# Supplementary material for: Selective Moonlighting Cell-Penetrating Peptides
Source: Pharmaceutics. 2021 Jul 22;13(8):1119. doi: 10.3390/pharmaceutics13081119 (PMC8400200; doi:10.3390/pharmaceutics13081119)
Supplement: Supplementary file 1 [file pharmaceutics-13-01119-s001.zip › FigureS1.pdf]

Supplemental Data for the work entitled “Selective Moonlighting Cell-Penetrating Peptides” by Rafael Morán-Torres, David A. Castillo González, Beatriz Aguilar Maldonado, Maria Luisa Durán-Pastén, Susana Castro-Obregon & Gabriel Del Rio

Figure S1. Original Western blots
A) Neprilysin

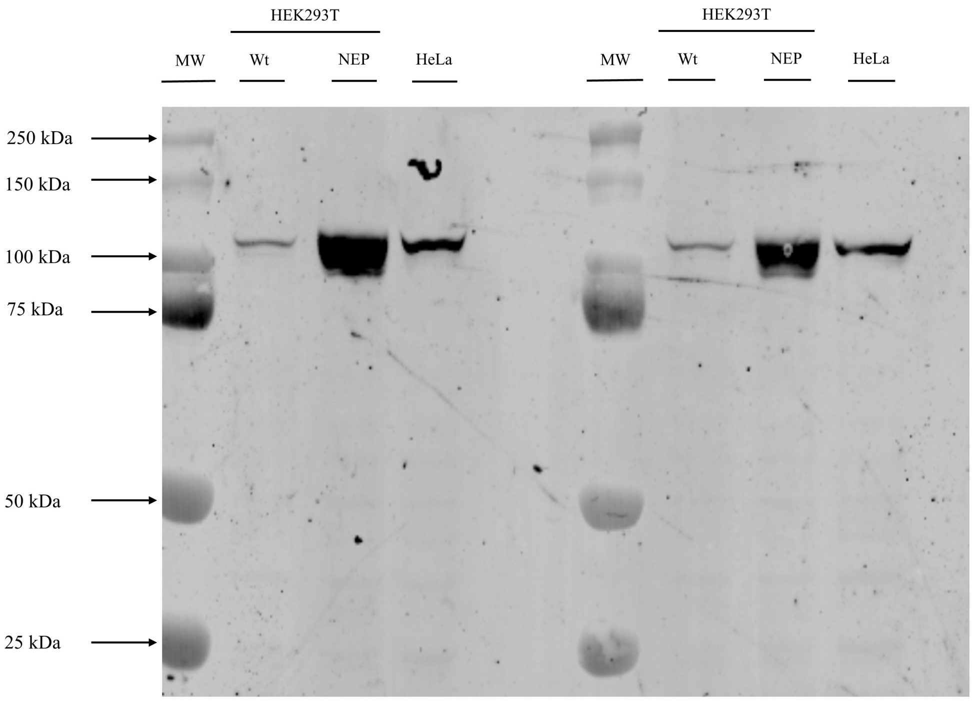

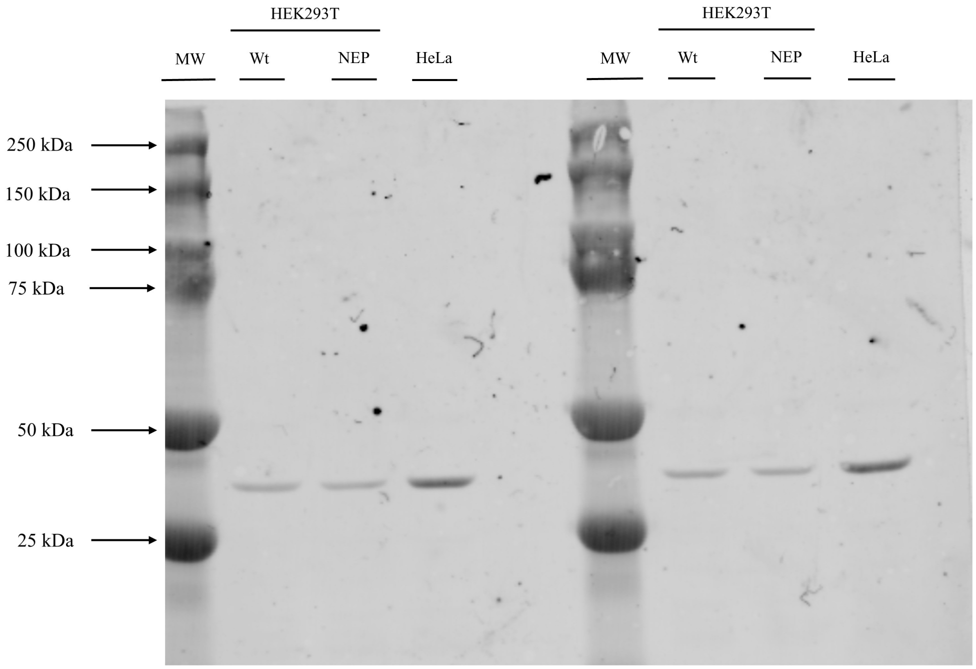

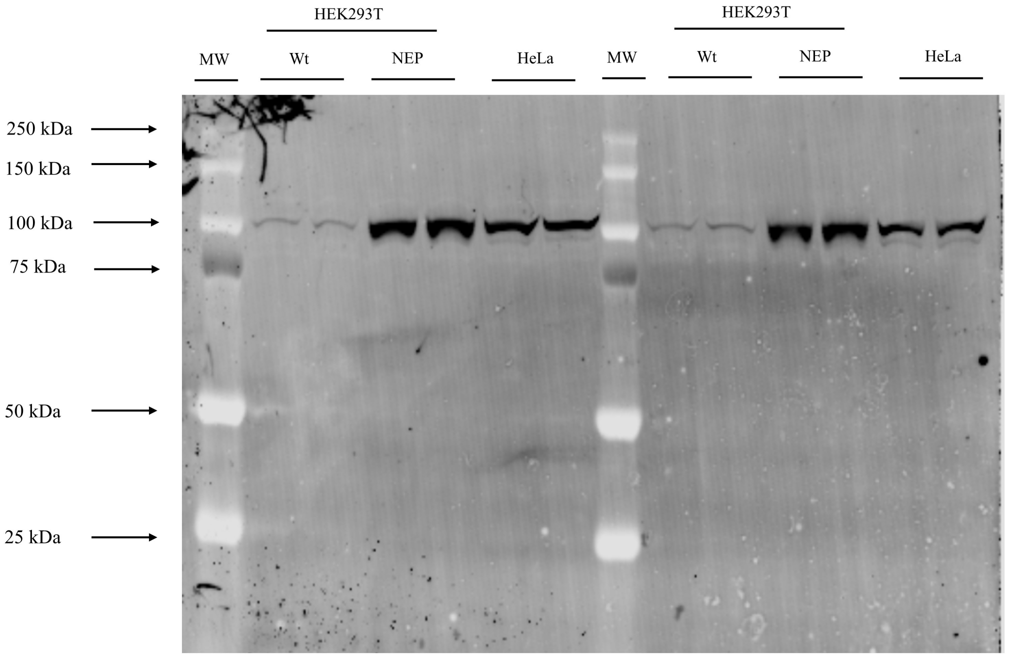
HEK293T
HEK293T
MW
Wt
NEP
HeLa
MW
Wt
NEP
HeLa
250 kDa
150 kDa
100 kDa
75 kDa
50 kDa
25 kDa

B) Actin

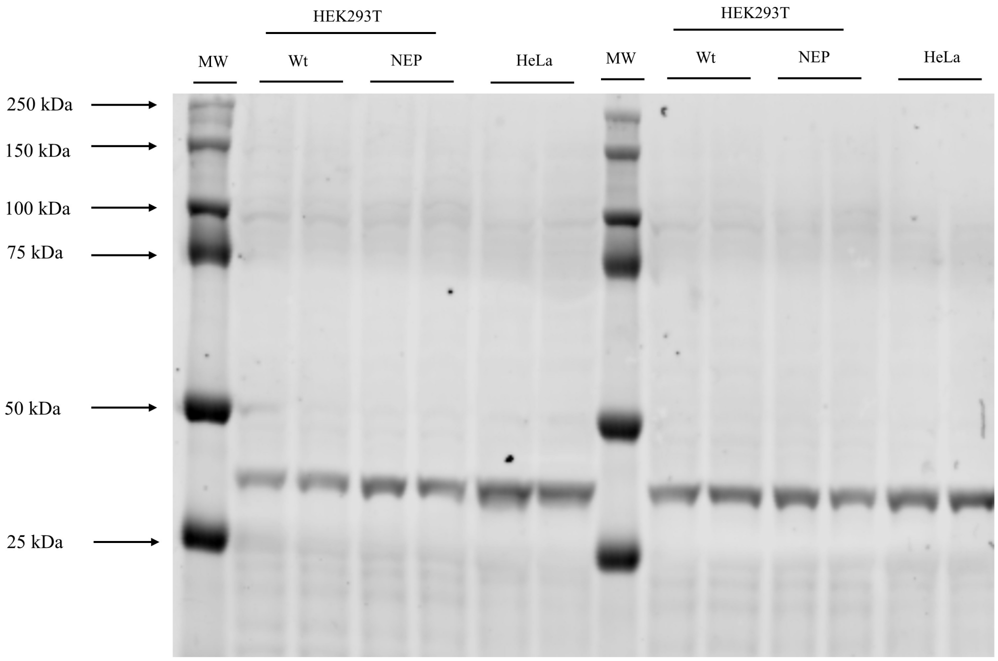
HEK293T
HEK293T
MW
Wt
NEP
HeLa
MW
Wt
NEP
HeLa
250 kDa
150 kDa
100 kDa
75 kDa
50 kDa
25 kDa

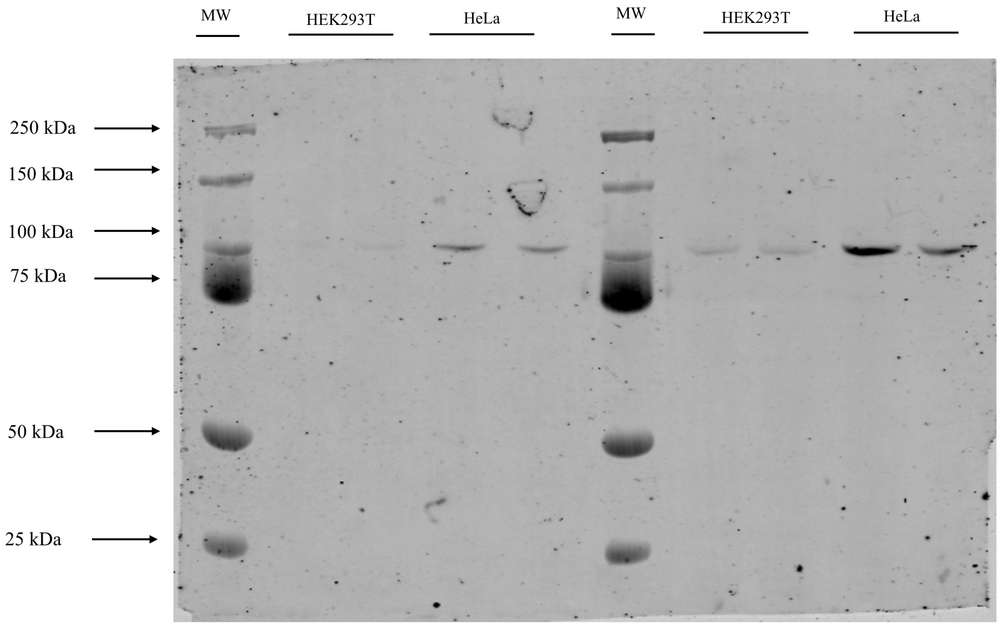

MW
HEK293T
HeLa
MW
HEK293T
HeLa
250 kDa
150 kDa
100 kDa
75 kDa
50 kDa
25 kDa

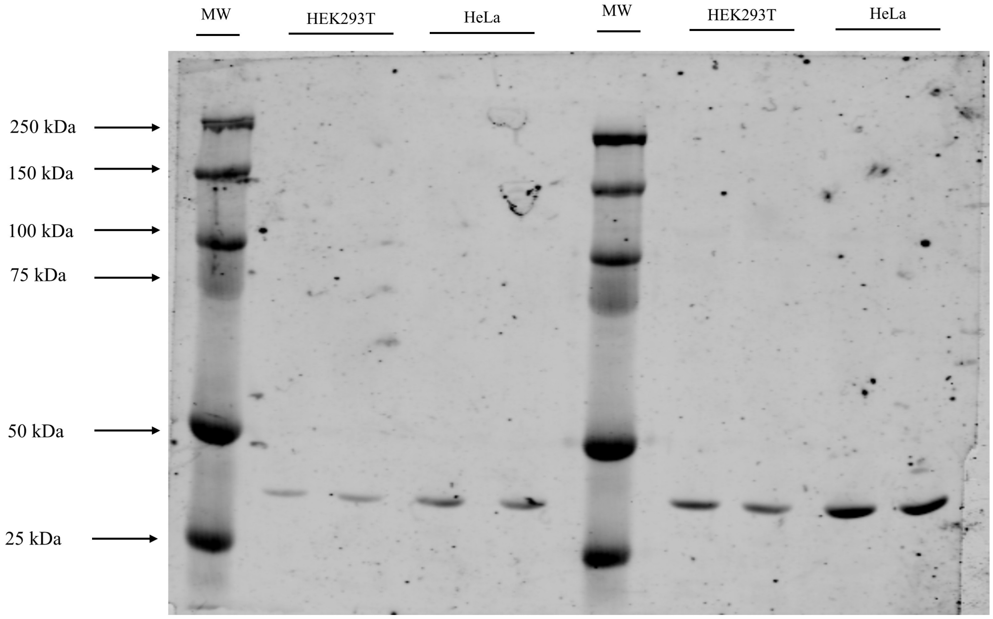

MW
HEK293T
HeLa
MW
HEK293T
HeLa
250 kDa
150 kDa
100 kDa
75 kDa
50 kDa
25 kDa
