# Supplementary material for: Selective Moonlighting Cell-Penetrating Peptides
Source: Pharmaceutics. 2021 Jul 22;13(8):1119. doi: 10.3390/pharmaceutics13081119 (PMC8400200; doi:10.3390/pharmaceutics13081119)

Supplemental Data for the work entitled “Selective Moonlighting Cell-Penetrating Peptides” by Rafael Morán-Torres, David A. Castillo González, Beatriz Aguilar Maldonado, Maria Luisa Durán-Pastén, Susana Castro-Obregon & Gabriel Del Rio

Figure S2. Image examples showing the internalization of activatable CPP.
A) TAMRA and DAPI staining for D-TatNepNoCPP peptide

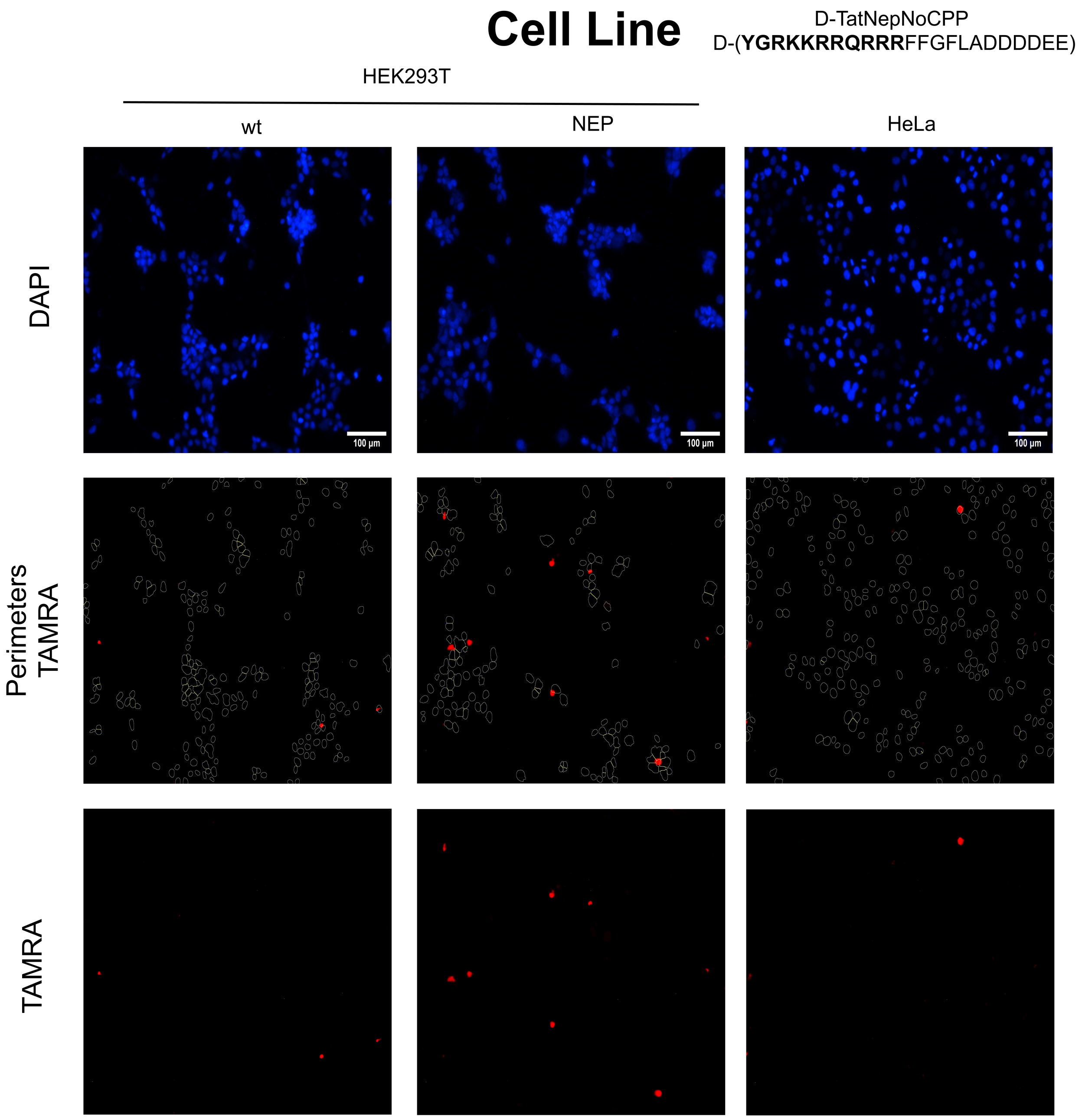

B) TAMRA and DAPI staining for TatNep peptide

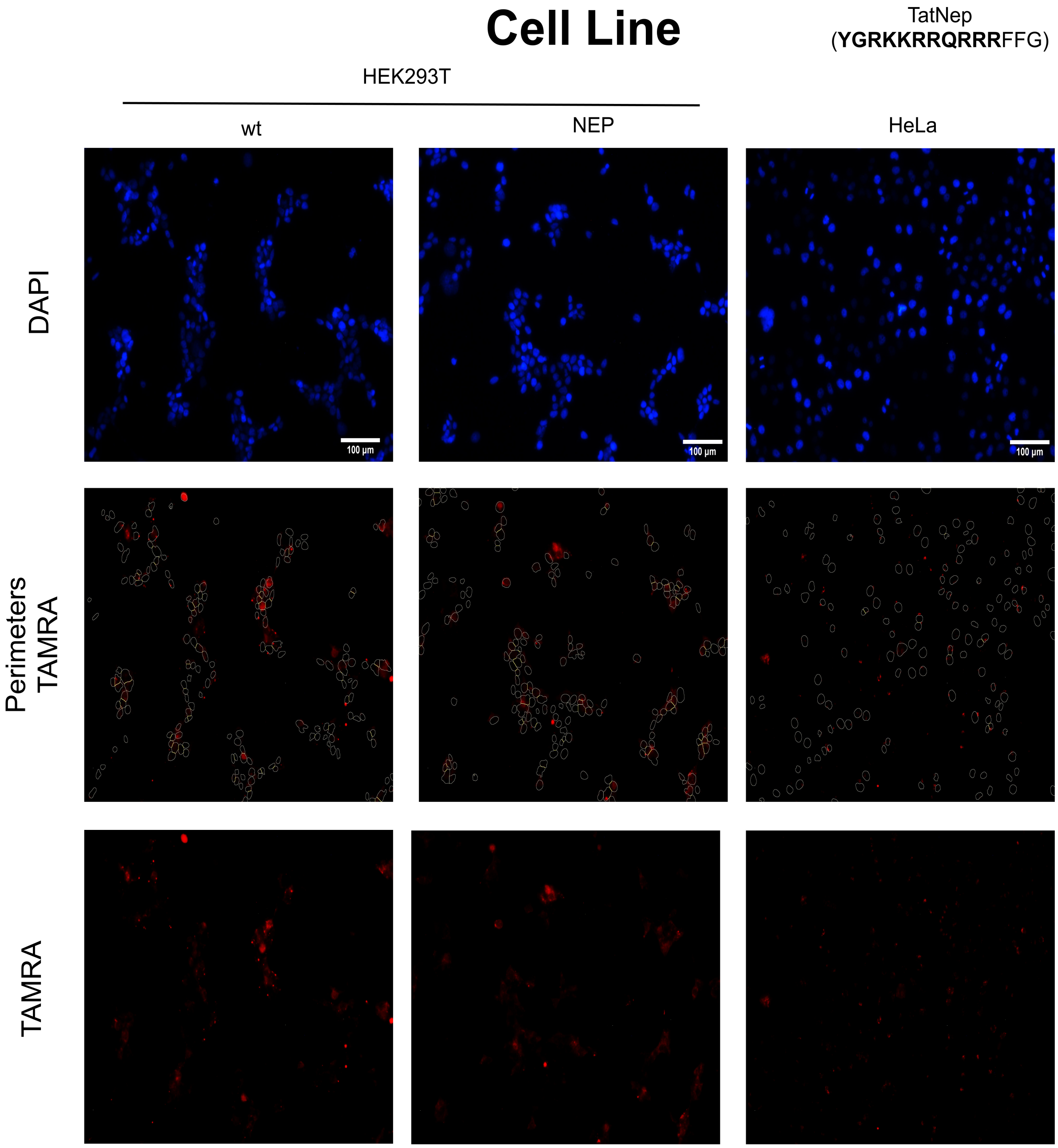

C) TAMRA and DAPI staining for TatNepNoCPP peptide

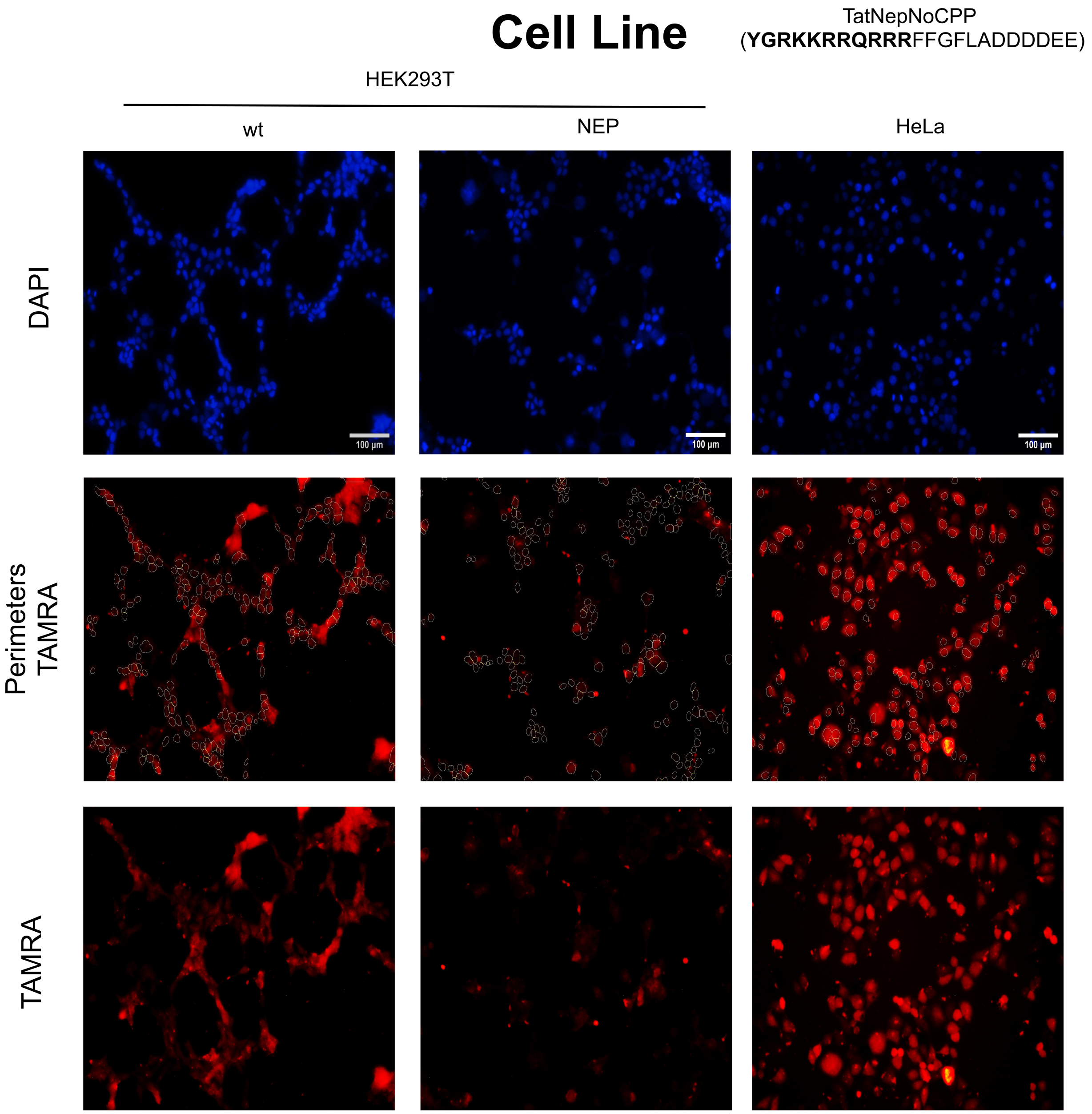

Supplement: Supplementary file 1 [file pharmaceutics-13-01119-s001.zip › FigureS2.pdf]
