# Supplementary material for: Selective Moonlighting Cell-Penetrating Peptides
Source: Pharmaceutics. 2021 Jul 22;13(8):1119. doi: 10.3390/pharmaceutics13081119 (PMC8400200; doi:10.3390/pharmaceutics13081119)
Supplement: Supplementary file 1 [file pharmaceutics-13-01119-s001.zip › FigureS3.pdf]

Supplemental Data for the work entitled “Selective Moonlighting Cell-Penetrating Peptides” by Rafael Morán-Torres, David A. Castillo González, Beatriz Aguilar Maldonado, Maria Luisa Durán-Pastén, Susana Castro-Obregon & Gabriel Del Rio

Figure S3. Cell viability of different cell lines exposed to activatable peptides.

A) HEK293T cells

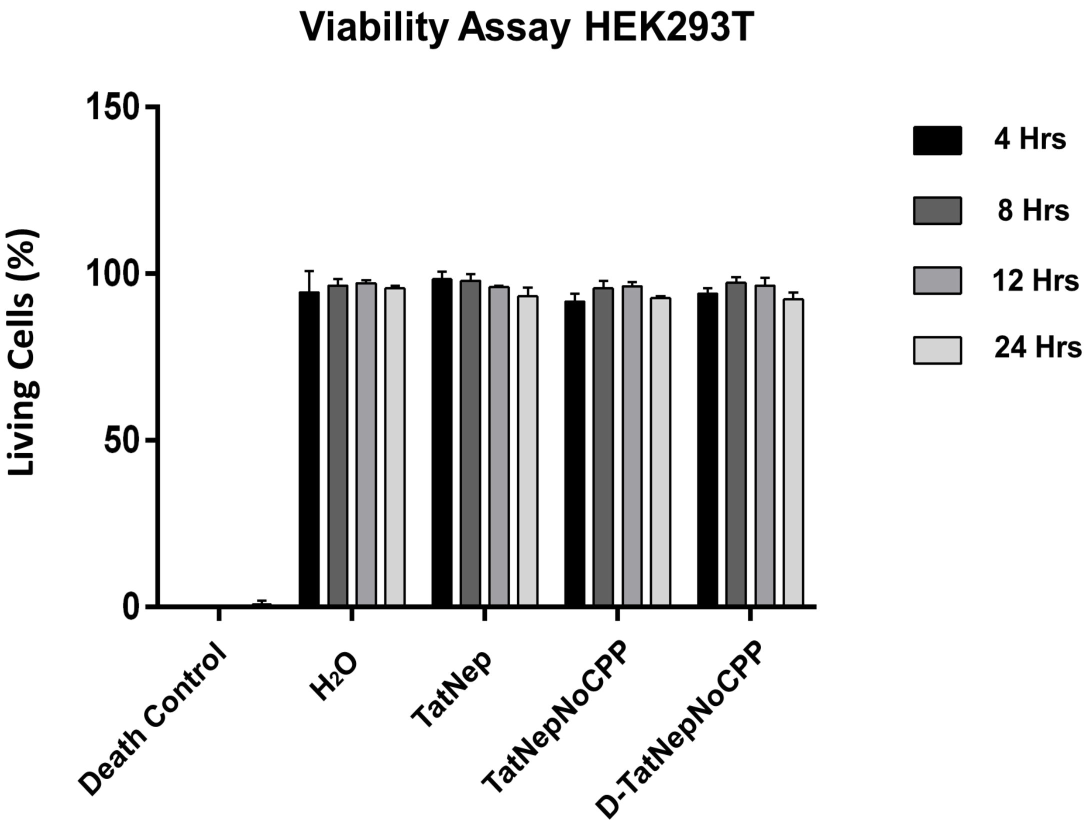

Three independent biological experiments were conducted for each condition and each period of time, three replicates were used in all cases. The plot presents the average of 9 data points and the bars show the mean +/- standard deviation.

B) HEK293T-NEP transfected cells

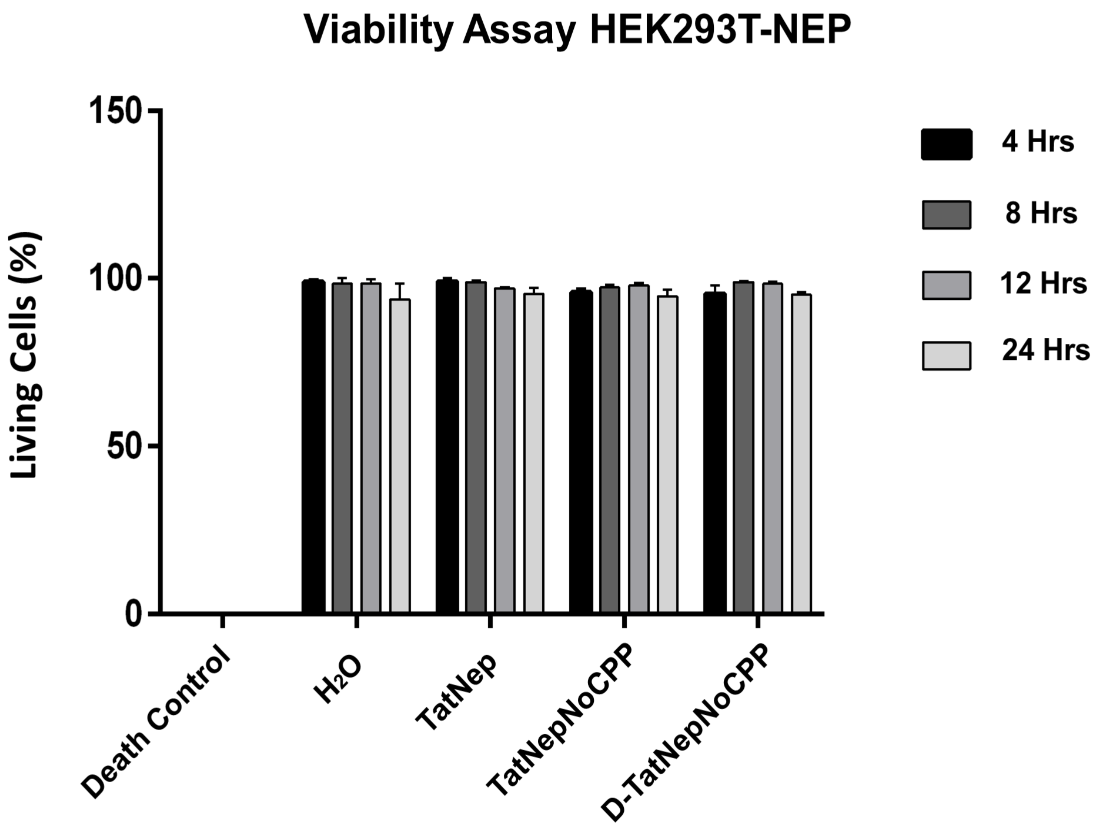

Three independent biological experiments were conducted for each condition and each period of time, three replicates were used in all cases. The plot presents the average of 9 data points and the bars show the mean +/- standard deviation.

C) HeLa cells

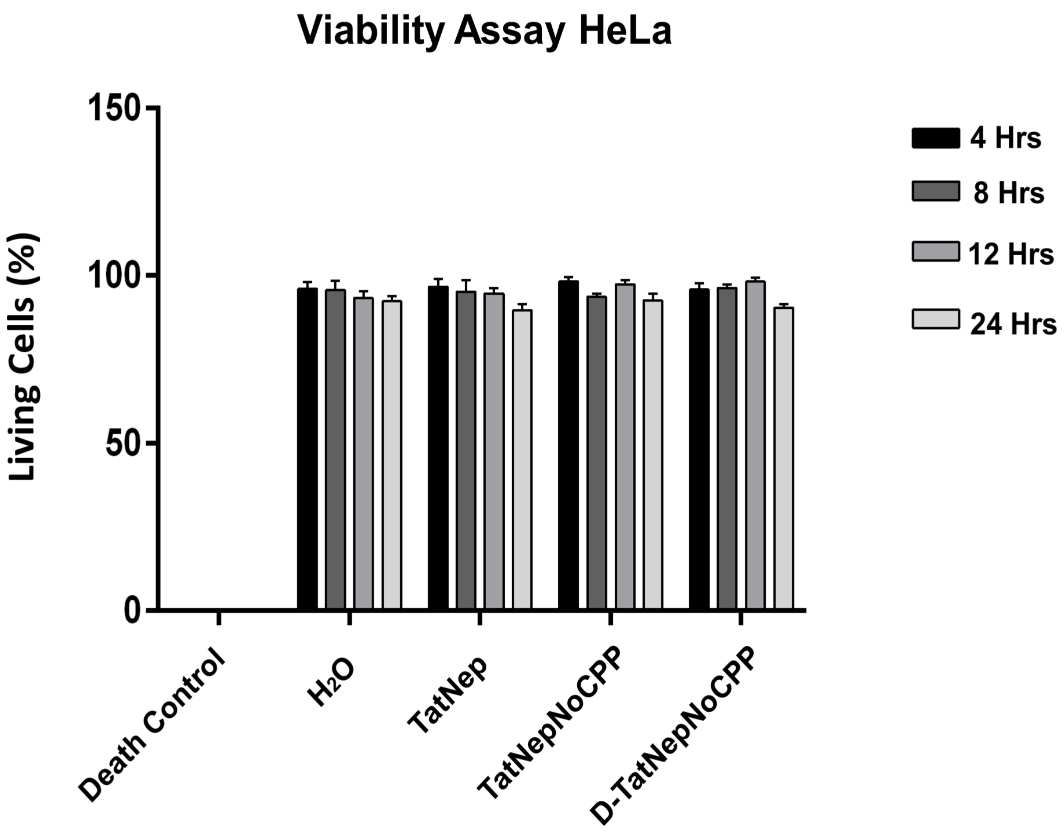

Three independent biological experiments were conducted for each condition and each period of time, three replicates were used in all cases. The plot presents the average of 9 data points and the bars show the mean +/- standard deviation.

D) Cell viability after 24 h of exposure to peptide solvents

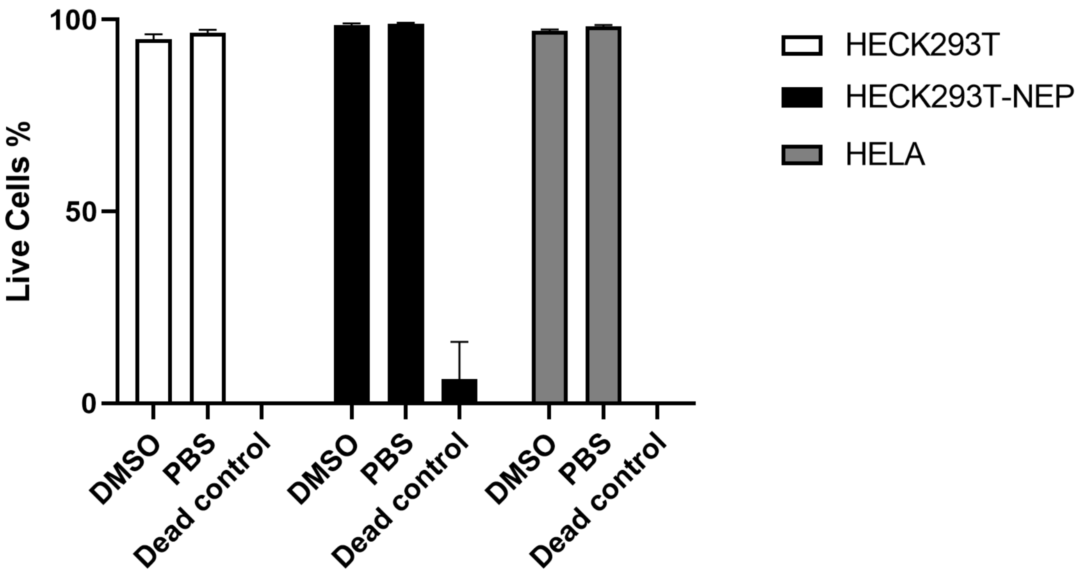

Three independent biological experiments were conducted for each condition and each period of time, three replicates were used in all cases. The plot presents the average of 9 data points and the bars show the mean +/- standard deviation.

E) Cell viability after 48 h of exposure to peptide solvents

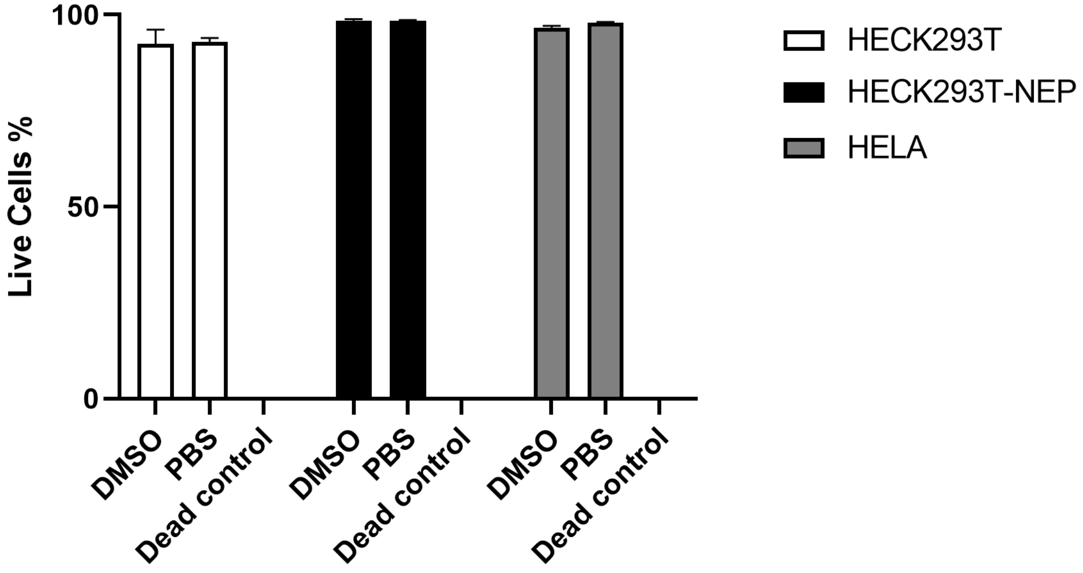

Three independent biological experiments were conducted for each condition and each period of time, three replicates were used in all cases. The plot presents the average of 9 data points and the bars show the mean +/- standard deviation.
