# Supplementary material for: Selective Moonlighting Cell-Penetrating Peptides
Source: Pharmaceutics. 2021 Jul 22;13(8):1119. doi: 10.3390/pharmaceutics13081119 (PMC8400200; doi:10.3390/pharmaceutics13081119)
Supplement: Supplementary file 1 [file pharmaceutics-13-01119-s001.zip › TableS1.pdf]

Supplemental Data for the work entitled “Selective Moonlighting Cell-Penetrating Peptides” by Rafael Morán-Torres, David A. Castillo González, Beatriz Aguilar Maldonado, Maria Luisa Durán-Pastén, Susana Castro-Obregon & Gabriel Del Rio

Table S1 A) Intensity recorded for peptide alpha-NLS-C on MATa and MATalpha cells. MATa recordings correspond with odd Time numbers, and MATalpha with even Time numbers.

Time,GroupByColor,Intensity
1,1,14247.875000
1,1,1647.575000
1,1,7098.816667
1,1,3319.325581
1,1,1779.362903
1,1,8738.350000
1,1,2171.104167
1,1,2083.425676
1,1,7217.899194
1,1,4002.988372
1,1,7882.542857
1,1,2563.000000
1,1,3030.750000
1,1,8553.037234
1,1,4426.178977
1,1,3087.338235
1,1,6593.800000
1,1,17387.866667
1,1,5166.166667
1,1,2083.000000
1,1,7057.202206
1,1,2469.600000
1,1,1589.066176
1,1,5495.152439
1,1,6506.109375
1,1,1523.029762
1,1,7239.500000
1,1,2060.246429
1,1,9361.081081
1,1,7108.888889
1,1,4756.570513
1,1,6631.312500
1,1,2418.431818
1,1,1698.750000
1,1,15519.732143
1,1,13709.125000
1,1,2266.425532
1,1,8009.590000
1,1,1837.378676
1,1,2055.694444
1,1,7484.750000
1,1,6357.156250
1,1,9456.275568
1,1,2205.179487
1,1,14153.219512
1,1,3626.600000
1,1,1898.365741
1,1,2238.217105
1,1,5597.926136
1,1,1853.882979
1,1,13698.312500
1,1,2240.994681
1,1,18279.532143
1,1,2010.230769
1,1,7494.174242
1,1,14093.000000
1,1,9568.451613
1,1,5507.676282
1,1,7975.857955
1,1,15793.387500
1,1,8315.262500
1,1,1791.955128

1,1,2716.000000
1,1,2511.788462
1,1,2115.669118
1,1,18740.725000
1,1,7565.316860
1,1,2182.948529
1,1,2178.980769
1,1,1924.482143
1,1,4255.060976
1,1,1828.598684
1,1,15769.325658
1,1,6934.450000
1,1,2352.076613
1,1,1962.346939
1,1,3099.323529
1,1,3375.968750
1,1,16948.528571
1,1,2487.714286
1,1,8101.250000
1,1,1649.692308
1,1,3810.353659
1,1,5397.992647
1,1,11325.978125
1,1,11758.815789
1,1,8961.423077
1,1,3387.784722
1,1,7485.086538
1,1,16766.761628
1,1,3439.562500
1,1,4049.743750
1,1,3429.527778
1,1,15182.105000
1,1,3645.558824
1,1,4362.048387
1,1,1525.479592
1,1,10279.500000
1,1,1505.016667
1,1,2446.052885
1,1,13189.223214
1,1,2479.167969
1,1,2838.462500
1,1,2386.992647
1,1,2698.398810
1,1,1863.036765
1,1,2541.793103
1,1,5660.738095
1,1,2155.243902
1,1,3724.071429
1,1,2738.336538
1,1,5394.413793
1,1,1690.993590
1,1,5073.522727
1,1,2786.322581
1,1,11848.821429
1,1,6748.758065
1,1,5199.956081
1,1,7713.282609
1,1,1606.326087
1,1,12209.250000
1,1,2579.758929
1,1,5943.546875
1,1,1556.950000
1,1,1602.912500
1,1,6483.154891
1,1,8184.753676
1,1,9320.048387

1,1,6400.496429
1,1,2829.000000
1,1,3592.735294
1,1,7163.120370
1,1,7679.971774
1,1,15572.229730
1,1,2650.788462
1,1,5094.794643
1,1,7783.705882
1,1,4963.363095
1,1,4547.048780
1,1,2126.925000
1,1,5642.546875
1,1,2265.073529
1,1,2598.375000
1,1,1644.189286
1,1,4123.141667
1,1,2588.918750
1,1,3916.935484
1,1,5871.685484
1,1,7760.155405
1,1,1646.593750
1,1,4918.659375
1,1,4584.518939
1,1,8084.775000
1,1,7928.187500
1,1,1628.550000
1,1,7036.445946
1,1,6762.682927
1,1,3981.372093
1,1,15185.098837
1,1,1724.363636
1,1,1936.337500
1,1,9547.418103
1,1,2545.613636
1,1,4999.650000
1,1,1683.535714
1,1,2051.167763
1,1,6563.525000
1,1,2591.846154
1,1,7586.195652
1,1,2238.055921
1,1,3527.174419
1,1,1903.822368
1,1,6981.868421
1,1,4049.722656
1,1,14347.187500
1,1,3098.727273
1,1,1798.634409
1,1,2784.369565
1,1,7170.892857
1,1,2010.441667
1,1,8118.254310
1,1,2154.667683
1,1,7196.090909
1,1,3528.519231
1,1,1666.350000
1,1,13189.934783
1,1,14167.958333
1,1,7860.440000
1,1,1506.055147
1,1,5375.500000
1,1,7949.525000
1,1,7938.545455
1,1,8818.605769
1,1,2510.529412

1,1,13335.193182
1,1,1715.756579
1,1,7507.000000
1,1,15408.147059
1,1,2220.401515
1,1,2255.233108
1,1,3183.364865
1,1,2436.703488
1,1,8040.500000
1,1,6285.892857
1,1,6222.300000
1,1,1708.881250
1,1,5492.093750
1,1,1709.115132
1,1,4334.910000
1,1,15052.585714
1,1,8140.263514
1,1,8385.421875
1,1,3333.500000
1,1,4767.818182
1,1,8177.114362
1,1,1848.570652
1,1,3181.138158
1,1,5553.488636
1,1,3712.875000
1,1,6430.046875
1,1,3583.411932
1,1,2756.982143
1,1,1644.068750
1,1,1821.263158
1,1,2298.598958
1,1,1508.105263
1,1,3540.586207
1,1,1574.600000
1,1,1583.085714
1,1,14157.966346
1,1,2206.252841
1,1,2139.404762
1,1,14504.222222
1,1,14509.187500
1,1,1847.635000
1,1,1724.500000
1,1,9104.667614
1,1,2331.573529
1,1,3389.948718
1,1,12521.742188
1,1,2755.824324
1,1,4740.135714
1,1,3059.428571
1,1,9496.490385
1,1,8009.655612
1,1,10157.640000
1,1,13692.295455
1,1,7186.995192
1,1,6489.350446
1,1,2560.020270
1,1,12070.375000
1,1,1852.765625
1,1,2228.464286
1,1,2412.445122
1,1,5051.075000
1,1,6216.365385
1,1,5640.125000
1,1,5379.715426
1,1,7821.125000
1,1,2197.333333

1,1,14235.151163
1,1,17983.600000
1,1,1595.835938
1,1,1532.397222
1,1,10717.786207
1,1,2026.475000
1,1,7426.512195
1,1,4179.545455
1,1,12756.187500
1,1,4566.628125
1,1,16494.468085
1,1,4570.883721
1,1,18125.261364
1,1,7753.525000
1,1,5747.000000
1,1,18868.250000
1,1,2470.160714
1,1,7666.788462
1,1,6171.063830
1,1,5344.000000
1,1,6452.525000
1,1,15938.178571
1,1,10413.750000
1,1,6471.395833
1,1,2632.644737
1,1,6995.160714
1,1,5592.605469
1,1,1684.904412
1,1,1931.155488
1,1,7833.730263
1,1,3270.772727
1,1,2739.437500
1,1,3216.850746
1,1,3895.862500
1,1,12697.042763
1,1,3353.343750
1,1,4994.722973
1,1,4216.952703
1,1,1923.463415
1,1,3400.750000
1,1,1993.070946
1,1,1553.460123
1,1,2084.144737
1,1,8015.577586
1,1,15908.150000
1,1,5014.947674
1,1,3145.488095
1,1,3632.250000
1,1,8191.926829
1,1,8927.944767
1,1,3113.892857
1,1,3499.869186
1,1,3120.552147
1,1,7782.056818
1,1,3921.197917
1,1,18904.880952
1,1,2317.375000
1,1,1602.300676
1,1,1716.209459
1,1,2051.903846
1,1,15765.642857
1,1,1859.503289
1,1,6561.811321
1,1,2336.500000
1,1,5587.433333
1,1,5088.523438

```
1,1,4654.217105
1,1,2947.500000
1,1,4322.290441
1,1,15977.188953
1,1,2042.000000
1,1,3711.866667
1,1,3977.400000
1,1,1506.040816
1,1,7338.948718
1,1,2986.636364
1,1,8185.150000
1,1,6859.340116
1,1,9644.891447
1,1,2694.568627
1,1,2638.771429
1,1,7010.461538
1,1,6356.320000
1,1,10922.535714
1,1,4731.503676
1,1,13509.980263
1,1,9815.966912
1,1,7924.444444
1,1,18896.961538
1,1,7475.098485
1,1,2867.203125
1,1,18134.426136
1,1,7755.169643
1,1,2278.634868
1,1,13926.643939
1,1,16215.357639
1,1,4812.522959
1,1,1767.600000
1,1,6605.640306
1,1,1637.125000
1,1,7296.724138
1,1,7031.338068
1,1,2082.823529
1,1,1578.416667
1,1,2774.153846
1,1,3037.263021
1,1,3669.852941
1,1,7110.166667
1,1,6875.454545
1,1,5341.637755
1,1,6555.894737
1,1,3096.399038
1,1,3955.921053
1,1,5677.744681
1,1,2155.947368
1,1,6174.064516
1,1,6821.262821
1,1,3625.421875
1,1,2813.850610
1,1,1697.380435
1,1,1596.036765
1,1,5469.664286
1,1,8829.125000
1,1,2913.887500
1,1,4341.322917
1,1,2645.461957
1,1,13612.829082
1,1,6849.406250
1,1,10508.000000
1,1,1853.185000
1,1,8644.057692
1,1,7840.733333
```

1,1,5976.357143
1,1,5305.163462
1,1,17093.102941
1,1,6984.277778
1,1,2926.783854
1,1,2085.643293
1,1,2114.000000
1,1,1569.508929
1,1,2112.218750
1,1,2941.170000
1,1,2648.480769
1,1,9376.225000
1,1,4939.400000
1,1,13160.148148
1,1,16881.750000
1,1,4911.153846
1,1,1537.109756
1,1,2036.380952
1,1,1678.392045
1,1,14299.598214
1,1,3511.704167
1,1,8066.265152
1,1,8175.710938
1,1,3313.716463
1,1,10544.240000
1,1,7974.000000
1,1,1717.024390
1,1,2110.700581
1,1,7458.156250
1,1,4232.130952
1,1,4285.858696
1,1,7401.600962
1,1,3739.669643
1,1,1906.310976
1,1,9062.474194
1,1,3456.625000
1,1,2304.300781
1,1,9161.882353
1,1,16086.671053
1,1,7412.554348
1,1,10977.822581
1,1,1606.964286
1,1,7934.602273
1,1,12740.555556
1,1,7753.937500
1,1,3945.692308
1,1,2961.863281
1,1,1998.875000
1,1,14669.500000
1,1,5543.958333
1,1,3609.650000
1,1,7896.442857
1,1,2321.614865
1,1,8551.000000
1,1,3773.150000
1,1,3248.562500
1,1,2008.721154
1,1,7282.711538
1,1,7856.592391
1,1,3459.062500
1,1,1777.780303
1,1,7575.671875
1,1,7012.875000
1,1,10133.673913
1,1,5522.607143
1,1,2062.772959

1,1,1788.000000
1,1,1820.041667
1,1,1802.888889
1,1,1756.000000
1,1,2027.820513
1,1,19454.243750
1,1,1737.877717
1,1,4572.375000
1,1,4177.171875
1,1,8534.343750
1,1,1926.050481
1,1,2374.968023
1,1,6793.217391
1,1,3111.214286
1,1,16716.129032
1,1,13619.944444
1,1,9105.993421
1,1,15305.450000
1,1,2186.201923
1,1,4088.976190
1,1,6196.946970
1,1,7956.211538
1,1,3284.500000
1,1,1558.442308
1,1,5527.569149
1,1,4135.669118
1,1,7058.343750
1,1,5524.709375
1,1,7749.055556
1,1,13344.988636
1,1,8139.817857
1,1,14218.872222
1,1,7476.816667
1,1,3282.359375
1,1,8638.076389
1,1,11349.704545
1,1,11294.812500
1,1,4389.381579
1,1,1721.583333
1,1,1678.229167
1,1,2159.581522
1,1,16664.825000
1,1,16648.122093
1,1,2003.476744
1,1,2304.329787
1,1,2624.100000
1,1,3370.477273
1,1,18365.923077
1,1,7211.928125
1,1,3541.357143
1,1,2755.262097
1,1,14774.940000
1,1,2548.298387
1,1,17307.690000
1,1,4378.750000
1,1,3668.608696
1,1,19253.078947
1,1,1600.793103
1,1,3881.362500
1,1,3053.306548
1,1,4744.750000
1,1,2410.394737
1,1,14977.006757
1,1,4654.578947
1,1,3611.130435
1,1,4042.333333

1,1,13233.907407
1,1,2327.177778
1,1,2247.551136
1,1,2737.887019
1,1,6937.380682
1,1,2268.875000
1,1,3010.318182
1,1,5538.812500
1,1,14116.234848
1,1,1697.053922
1,1,7619.955556
1,1,2599.261628
1,1,2273.100000
1,1,3318.804878
1,1,5694.875000
1,1,4580.444444
1,1,14315.125000
1,1,6192.190476
1,1,17200.663043
1,1,8064.798077
1,1,2949.676136
1,1,1555.411765
1,1,2362.692308
1,1,4487.523438
1,1,6966.147727
1,1,5402.025568
1,1,6039.375000
1,1,1793.144022
1,1,2453.920732
1,1,2657.948718
1,1,2081.963415
1,1,1930.083333
1,1,2237.800000
1,1,3521.176829
1,1,4355.295455
1,1,2838.226563
1,1,5336.902027
1,1,5349.281863
1,1,5430.872222
1,1,5085.727273
1,1,4863.681818
1,1,2096.975543
1,1,1741.800000
1,1,7481.894231
1,1,5305.512500
1,1,7711.209677
1,1,1770.908537
1,1,8891.363636
1,1,18282.107143
1,1,7476.607143
1,1,4240.252841
1,1,7681.000000
1,1,16507.844660
1,1,3696.303977
1,1,2721.768293
1,1,3787.848168
1,1,2092.458333
1,1,6153.428571
1,1,4904.062500
1,1,3781.356383
1,1,3767.357558
1,1,3441.750000
1,1,5393.381098
1,1,1819.630814
1,1,1597.892857
1,1,2222.302083

1,1,3489.188312
1,1,10452.512097
1,1,2655.247283
1,1,7789.090517
1,1,2104.466463
1,1,7837.645833
1,1,8225.733333
1,1,13044.875000
1,1,7137.166667
1,1,6456.924419
1,1,9311.854167
1,1,2702.803571
1,1,2044.444767
1,1,2212.564103
1,1,3342.954545
1,1,14548.680556
1,1,1862.906977
1,1,17845.625000
1,1,1574.605000
1,1,14650.253289
1,1,3584.046053
1,1,3311.703488
1,1,1767.321875
1,1,1705.175000
1,1,3696.075000
1,1,2531.437500
1,1,2703.520833
1,1,2581.608491
1,1,5230.170455
1,1,1719.115741
1,1,5640.400000
1,1,5357.785714
1,1,2881.352941
1,1,1935.446809
1,1,2433.121951
1,1,2212.416667
1,1,3853.285714
1,1,17423.568182
1,1,2876.480769
1,1,5098.540541
1,1,15884.996711
1,1,3228.664634
1,1,3153.053191
1,1,14168.607143
1,1,13531.222222
1,1,8419.526786
1,1,12486.750000
1,1,2875.302326
1,1,1685.966667
1,1,3014.846154
1,1,2243.416667
1,1,3233.875000
1,1,1762.591216
1,1,2491.488372
1,1,5070.113208
1,1,10635.250000
1,1,10206.559375
1,1,7730.661290
1,1,2110.615385
1,1,10123.381757
1,1,2539.187500
1,1,1750.625000
1,1,2008.229167
1,1,4218.178977
1,1,4752.622222
1,1,14687.875000

1,1,2194.833333
1,1,14991.125000
1,1,1810.991935
1,1,7644.327586
1,1,6233.137755
1,1,6312.761905
1,1,3042.810976
1,1,10991.867188
1,1,17997.050676
1,1,6438.880000
1,1,3541.712500
1,1,1672.768750
1,1,2769.000000
1,1,4412.182432
1,1,2561.532051
1,1,8434.358333
1,1,3163.833333
1,1,1727.698529
1,1,6724.898438
1,1,1679.778571
1,1,1816.483333
1,1,15081.593750
1,1,15837.106250
1,1,7699.529412
1,1,1973.750000
1,1,2092.048295
1,1,5523.812500
1,1,13987.180921
1,1,12510.819149
1,1,1551.585987
1,1,1550.552632
1,1,7039.338889
1,1,4784.325000
1,1,4133.768382
1,1,10012.043478
1,1,2343.217500
1,1,2452.162791
1,1,2279.140625
1,1,7972.865385
1,1,3456.156250
1,1,2204.882353
1,1,10891.109043
1,1,2191.325000
1,1,6916.145833
1,1,4958.531746
1,1,2130.544118
1,1,3622.590426
1,1,7919.900000
1,1,15389.544118
1,1,4748.787234
1,1,19661.250000
1,1,4057.995833
1,1,10676.587500
1,1,7827.315217
1,1,1633.486842
1,1,4052.500000
1,1,4575.316176
1,1,4442.223214
1,1,7736.630435
1,1,17941.323980
1,1,7685.931818
1,1,2863.937500
1,1,4940.209302
1,1,7345.338710
1,1,14766.729167
1,1,14811.625000

1,1,2448.576087
1,1,4947.854167
1,1,12302.987500
1,1,2505.583333
1,1,5518.428571
1,1,1717.079545
1,1,1919.229730
1,1,12055.500000
1,1,7454.568182
1,1,1919.601562
1,1,2049.889423
1,1,13583.400000
1,1,4238.393162
1,1,2165.881410
1,1,13622.201923
1,1,3933.406250
1,1,2155.000000
1,1,2514.534574
1,1,6234.607143
1,1,1888.301471
1,1,2243.435484
1,1,8085.528846
1,1,2395.482955
1,1,4931.812500
1,1,10334.288462
1,1,2195.826923
1,1,2017.500000
1,1,5146.239583
1,1,2496.082192
1,1,4865.142857
1,1,1835.405488
1,1,4057.125000
1,1,2113.461538
1,1,7332.159375
1,1,2261.311047
1,1,1570.258929
1,1,2417.119565
1,1,5712.356383
1,1,2168.950000
1,1,5530.996528
1,1,2699.593750
1,1,3269.875000
1,1,2184.311111
1,1,1903.675000
1,1,1673.354167
1,1,2815.541667
1,1,15770.636364
1,1,5084.321429
1,1,3959.545455
1,1,3964.682692
1,1,7279.402778
1,1,2984.525641
1,1,7647.000000
1,1,6547.394737
1,1,5392.475000
1,1,4305.700000
1,1,6387.120000
1,1,4499.125000
1,1,7242.000000
1,1,8630.789634
1,1,7100.159722
1,1,6291.948276
1,1,3179.262195
1,1,2280.992188
1,1,5535.250000
1,1,1566.315217

1,1,2544.713542
1,1,5884.617021
1,1,3206.947917
1,1,1941.392857
1,1,8660.397059
1,1,1634.215909
1,1,7260.060000
1,1,10211.359281
1,1,5020.580882
1,1,1820.377778
1,1,5946.625000
1,1,5517.359756
1,1,3114.737500
1,1,6079.803571
1,1,16326.911765
1,1,5470.767045
1,1,3893.734375
1,1,9396.313953
1,1,4879.017857
1,1,2393.467391
1,1,1871.159091
1,1,2691.451613
1,1,5400.636029
1,1,18151.677083
1,1,1761.642857
1,1,2175.245370
1,1,2410.000000
1,1,2035.148936
1,1,7787.725000
1,1,1637.615385
1,1,4250.883929
1,1,6722.782738
1,1,7260.267500
1,1,7984.480769
1,1,4903.155172
1,1,1626.689516
1,1,2922.382653
1,1,5915.801471
1,1,19340.166667
1,1,2450.420455
1,1,19381.767442
1,1,7964.844828
1,1,3513.609756
1,1,4576.593137
1,1,1655.045455
1,1,1523.532500
1,1,5423.947917
1,1,1571.380952
1,1,1666.750000
1,1,5541.206250
1,1,9892.733333
1,1,12815.357143
1,1,3294.960938
1,1,10808.259615
1,1,14196.693548
1,1,2124.414634
1,1,10056.750000
1,1,6311.972222
1,1,6717.846429
1,1,4292.513393
1,1,6606.247283
1,1,2853.723404
1,1,1920.869565
1,1,2226.414894
1,1,3881.903846
1,1,1751.381579

1,1,2163.357143
1,1,3328.800000
1,1,3224.800000
1,1,11350.411111
1,1,1903.676630
1,1,2429.748466
1,1,13722.140625
1,1,1795.554054
1,1,2215.517857
1,1,1590.958333
1,1,2853.726064
1,1,3020.090909
1,1,3434.160959
1,1,2126.280488
1,1,1900.496622
1,1,2013.538462
1,1,2997.676471
1,1,6539.125000
1,1,4148.794444
1,1,7030.500000
1,1,1858.330000
1,1,13534.274194
1,1,1810.982143
1,1,5338.333333
1,1,5425.486842
1,1,2895.279412
1,1,1972.900000
1,1,3325.216216
1,1,9620.852941
1,1,13891.925000
1,1,1625.054487
1,1,3978.944444
1,1,2305.160714
1,1,1841.951613
1,1,4408.712121
1,1,1815.625000
1,1,5036.535714
1,1,3701.720000
1,1,2395.034014
1,1,7233.257143
1,1,2077.769231
1,1,1977.611111
1,1,2324.400000
1,1,15404.709459
1,1,2232.989130
1,1,2336.336538
1,1,4396.357955
1,1,3787.375000
1,1,9836.406250
1,1,3952.891892
1,1,5088.181818
1,1,1860.431818
1,1,5136.529412
1,1,4205.255814
1,1,13167.444444
1,1,1771.458333
1,1,7956.750000
1,1,2960.884375
1,1,6417.995000
1,1,2207.000000
1,1,3524.679487
1,1,10523.503378
1,1,1752.359043
1,1,2437.441860
1,1,2326.625000
1,1,4590.527027

1,1,1720.543919
1,1,8527.826087
1,1,4389.625000
1,1,11264.500000
1,1,6883.625000
1,1,19616.833333
1,1,13735.548077
1,1,2276.393617
1,1,5743.418605
1,1,2569.695652
1,1,2667.684028
1,1,3600.478261
1,1,3582.000000
1,1,4081.750000
1,1,1825.020833
1,1,8192.433036
1,1,4202.933824
1,1,1900.500000
1,1,15081.416667
1,1,3985.221939
1,1,2416.687500
1,1,2771.375000
1,1,4352.839286
1,1,4843.776316
1,1,8764.850000
1,1,3788.379808
1,1,14158.790179
1,1,1848.900000
1,1,8979.210526
1,1,7247.992188
1,1,3125.859694
1,1,1956.048295
1,1,3324.889706
1,1,7784.937500
1,1,5876.609375
1,1,4844.381944
1,1,14485.404605
1,1,2762.277344
1,1,2819.812500
1,1,1617.862805
1,1,2907.792969
1,1,1751.025735
1,1,5175.327500
1,1,1677.078125
1,1,2036.804878
1,1,7298.518519
1,1,5103.281977
1,1,7039.075472
1,1,4411.760417
1,1,2672.635714
1,1,5733.292683
1,1,10364.518519
1,1,3990.000000
1,1,11524.531250
1,1,1695.198980
1,1,11339.502976
1,1,2060.250000
1,1,1565.781250
1,1,12806.526316
1,1,1613.982955
1,1,15861.259615
1,1,5429.267045
1,1,3630.007653
1,1,17579.837838
1,1,14258.333333
1,1,7126.375000

1,1,2951.229167
1,1,4086.244681
1,1,12672.031250
1,1,1507.375000
1,1,11249.940000
1,1,3418.000000
1,1,6300.411932
1,1,2386.554487
1,1,1910.883721
1,1,1609.719388
1,1,11540.375000
1,1,3121.502778
1,1,13189.645833
1,1,1845.131098
1,1,1531.218750
1,1,1652.613636
1,1,12609.767857
1,1,1984.655405
1,1,7728.666667
1,1,4255.652778
1,1,7123.500000
1,1,2255.500000
1,1,3391.783019
1,1,4050.476562
1,1,2472.052083
1,1,8117.958333
1,1,7583.000000
1,1,3148.600000
1,1,7787.851351
1,1,9378.727273
1,1,7937.232143
1,1,15754.083333
1,1,10917.160156
1,1,1862.765244
1,1,11199.411765
1,1,7563.344444
1,1,7923.246528
1,1,2733.982143
1,1,1658.461538
1,1,10506.092857
1,1,6180.725000
1,1,3556.025000
1,1,2628.372283
1,1,2576.386364
1,1,17997.069767
1,1,1822.900000
1,1,4031.221774
1,1,13303.212500
1,1,1637.367347
1,1,3590.075000
1,1,2571.321429
1,1,2137.585938
1,1,12398.764205
1,1,2418.860465
1,1,6949.131944
1,1,7110.257576
1,1,2261.552083
1,1,10051.011364
1,1,1636.750000
1,1,12771.200000
1,1,3698.026596
1,1,9238.968750
1,1,7276.100000
1,1,6206.343750
1,1,8369.408333
1,1,7257.951613

1,1,3271.250000
1,1,7883.769531
1,1,1820.420732
1,1,2741.492188
1,1,12359.717949
1,1,7746.200000
1,1,4305.875000
1,1,19168.779412
1,1,3288.576389
1,1,2889.628788
1,1,7958.531250
1,1,8877.746324
1,1,6400.583333
1,1,1521.000000
1,1,2789.256757
1,1,3062.397727
1,1,3049.292969
1,1,10617.125000
1,1,9836.390244
1,1,19915.421053
1,1,4825.875000
1,1,14741.506098
1,1,5355.869565
1,1,11655.409091
1,1,3212.881944
1,1,4436.872222
1,1,5878.220930
1,1,10576.592593
1,1,3429.607143
1,1,2619.703125
1,1,1839.210366
1,1,2192.677083
1,1,3611.250000
1,1,2559.750000
1,1,13142.314189
1,1,2779.857143
1,1,10728.336957
1,1,1988.380952
1,1,15219.000000
1,1,4547.053571
1,1,3740.218750
1,1,2079.651163
1,1,14338.571429
1,1,3302.132353
1,1,13387.517857
1,1,1501.568182
1,1,3767.687500
1,1,13609.000000
1,1,7417.472222
1,1,6895.721154
1,1,13420.506250
1,1,8786.143617
1,1,1509.000000
1,1,7277.479839
1,1,2240.275568
1,1,1985.058824
1,1,2520.329268
1,1,4033.635638
1,1,7576.000000
1,1,1667.062500
1,1,11225.936047
1,1,14902.418605
1,1,2298.769231
1,1,3632.869318
1,1,3551.984756
1,1,3821.410714

1,1,16597.127907
1,1,1681.413462
1,1,2919.983553
1,1,13257.942568
1,1,4615.939103
1,1,3774.012500
1,1,4874.533537
1,1,2222.125000
1,1,6033.147059
1,1,3202.023810
1,1,8596.083333
1,1,3910.526786
1,1,14805.985714
1,1,7807.388889
1,1,1507.000000
1,1,1604.475000
1,1,6020.128378
1,1,5256.000000
1,1,2121.962209
1,1,9967.916667
1,1,15385.405488
1,1,5477.394231
1,1,7689.285156
1,1,12008.218750
1,1,1827.303571
1,1,2544.003205
1,1,6785.580556
1,1,6669.685345
1,1,3673.277778
1,1,14354.825521
1,1,4565.303191
1,1,3024.031250
1,1,2602.202778
1,1,18747.348214
1,1,11095.437500
1,1,3666.970588
1,1,3502.906250
1,1,12770.472561
1,1,3336.412500
1,1,3383.350000
1,1,1921.985294
1,1,3408.867188
1,1,1628.561170
1,1,2273.835227
1,1,1721.238971
1,1,1935.021739
1,1,1691.772727
1,1,5467.218750
1,1,2460.578125
1,1,6745.710000
1,1,12561.733108
1,1,1933.982517
1,1,13369.777778
1,1,19119.654167
1,1,9475.043750
1,1,4998.041667
1,1,5452.851695
1,1,8089.212500
1,1,15446.521552
1,1,11713.000000
1,1,2478.522727
1,1,3570.719697
1,1,2012.250000
1,1,5178.510870
1,1,3841.913462
1,1,2817.348837

1,1,8044.790541
1,1,2115.783537
1,1,5267.615385
1,1,1826.401316
1,1,6484.302326
1,1,7816.707317
1,1,1501.146597
1,1,8998.944444
1,1,8237.401316
1,1,4451.857143
1,1,1920.580357
1,1,11937.285714
1,1,4618.812500
1,1,6600.263158
1,1,3440.357500
1,1,4069.012500
1,1,2195.762500
1,1,5706.716216
1,1,4251.798913
1,1,8398.244565
1,1,3381.283784
1,1,2183.596774
1,1,3133.395833
1,1,19995.265625
1,1,2573.043478
1,1,3629.923295
1,1,7950.666667
1,1,1595.059028
1,1,3289.539474
1,1,7048.736842
1,1,2274.867347
1,1,3119.136628
1,1,5985.255435
1,1,4246.101744
1,1,8024.975000
1,1,3370.986842
1,1,1976.916667
1,1,2408.028571
1,1,1529.956019
1,1,1563.460938
1,1,11500.696429
1,1,1855.500000
1,1,1614.127717
1,1,2267.181034
1,1,8204.228125
1,1,2585.387821
1,1,3097.815789
1,1,11000.066667
1,1,1942.615385
1,1,14215.390957
1,1,7400.115385
1,1,2569.956522
1,1,7598.207031
1,1,7878.537500
1,1,5350.298913
1,1,6458.796053
1,1,15223.000000
1,1,3299.222222
1,1,1670.588235
1,1,1527.212121
1,1,1705.090909
1,1,4168.246711
1,1,1885.085106
1,1,7206.825000
1,1,6675.083333
1,1,5401.459459

1,1,3829.671196
1,1,7102.980263
1,1,2597.570312
1,1,1979.875000
1,1,2289.883721
1,1,5756.153409
1,1,1890.388587
1,1,2852.250000
1,1,2553.260638
1,1,9789.988095
1,1,2837.750000
1,1,1714.621528
1,1,4574.640625
1,1,5474.516667
1,1,4172.144737
1,1,1596.944853
1,1,2300.303571
1,1,2411.951220
1,1,7066.906250
1,1,2201.875000
1,1,1907.383721
1,1,3131.642857
1,1,4477.195652
1,1,3773.644737
1,1,1572.800000
1,1,2341.404412
1,1,4147.097561
1,1,13660.204787
1,1,7762.546053
1,1,7374.909091
1,1,2426.980263
1,1,9919.867647
1,1,5227.714744
1,1,10757.523810
1,1,1572.076087
1,1,2229.517241
1,1,12303.120370
1,1,10308.913194
1,1,1690.400000
1,1,4700.277778
1,1,3383.011364
1,1,14399.977273
1,1,8607.920213
1,1,10072.369898
1,1,4170.236413
1,1,3559.061224
1,1,6735.312500
1,1,1895.558824
1,1,5644.687500
1,1,5598.765306
1,1,2059.952381
1,1,7841.008065
1,1,1504.528846
1,1,2193.120690
1,1,19045.952703
1,1,1898.441667
1,1,2025.348837
1,1,15125.997340
1,1,3897.993902
1,1,15591.000000
1,1,4917.469828
1,1,7055.218750
1,1,2851.763158
1,1,2062.408163
1,1,5210.750000
1,1,12841.703704

1,1,5785.180233
1,1,1853.935897
1,1,6600.425000
1,1,2372.845588
1,1,9037.465625
1,1,2984.090116
1,1,3336.710106
1,1,14574.897059
1,1,5438.496528
1,1,2538.869048
1,1,3926.096774
1,1,14301.506757
1,1,10578.961310
1,1,8664.237805
1,1,3092.682927
1,1,7509.470588
1,1,7229.441176
1,1,7374.519531
1,1,6963.327586
1,1,1553.381579
1,1,2162.864583
1,1,18987.796875
1,1,6448.363636
1,1,12414.728261
1,1,4028.412162
1,1,1972.517857
1,1,6272.990385
1,1,2861.810811
1,1,2626.414474
1,1,2229.764706
1,1,3384.620968
1,1,2571.093023
1,1,1595.708333
1,1,2611.480263
1,1,5076.955645
1,1,2543.267857
1,1,3289.829787
1,1,11458.289474
1,1,3804.335227
1,1,2942.247449
1,1,14817.041667
1,1,2264.647059
1,1,5464.407407
1,1,4848.500000
1,1,2676.266304
1,1,13322.416667
1,1,6760.923077
1,1,2002.889205
1,1,2744.461957
1,1,2415.810811
1,1,7816.777778
1,1,13196.418103
1,1,5881.533333
1,1,7023.960000
1,1,1832.895833
1,1,12089.120000
1,1,1751.396739
1,1,14027.750000
1,1,7397.163462
1,1,5172.378049
1,1,7866.519737
1,1,2175.971875
1,1,7283.828125
1,1,1690.125000
1,1,1668.290541
1,1,11007.100806

1,1,1662.470588
1,1,3561.057143
1,1,10415.162162
1,1,13313.548077
1,1,8200.573718
1,1,12081.788194
1,1,7490.578125
1,1,1626.357143
1,1,1510.708333
1,1,1867.928571
1,1,8325.602564
1,1,8118.875000
1,1,2445.242188
1,1,1537.884615
1,1,12319.705882
1,1,7701.056034
1,1,2885.562500
1,1,2212.407895
1,1,2754.807692
1,1,2697.836806
1,1,1654.600000
1,1,2205.810976
1,1,2205.214674
1,1,5712.500000
1,1,2449.726190
1,1,1725.790541
1,1,7306.411585
1,1,7260.428571
1,1,8544.781250
1,1,2583.914634
1,1,3879.359375
1,1,2460.910000
1,1,4147.882979
1,1,1601.750000
1,1,2448.366071
1,1,2191.576220
1,1,1699.312500
1,1,1639.744048
1,1,2806.990132
1,1,3351.179878
1,1,2235.620690
1,1,3080.869318
1,1,2778.356061
1,1,6899.263889
1,1,3033.257143
1,1,2101.791045
1,1,4414.386628
1,1,3611.310811
1,1,1990.160920
1,1,11243.007812
1,1,16956.508333
1,1,1758.558824
1,1,1961.698370
1,1,6155.000000
1,1,15517.125000
1,1,7073.333333
1,1,2958.008065
1,1,8859.734694
1,1,4240.464286
1,1,1634.470588
1,1,10813.977778
1,1,7870.823864
1,1,7120.729167
1,1,2568.240132
1,1,1898.331731
1,1,3886.831522

1,1,14299.718750
1,1,2221.053571
1,1,5362.552326
1,1,2873.731707
1,1,10333.029255
1,1,1637.955645
1,1,2224.707386
1,1,2145.187500
1,1,6835.289474
1,1,3565.557927
1,1,2911.708333
1,1,1615.592391
1,1,2236.687500
1,1,7602.103448
1,1,6563.912791
1,1,2717.007979
1,1,12238.181034
1,1,3132.591216
1,1,3070.000000
1,1,3458.672500
1,1,3732.704082
1,1,5512.907895
1,1,15091.728125
1,1,17905.284314
1,1,3496.563830
1,1,4659.056250
1,1,7494.125000
1,1,14095.414773
1,1,6622.000000
1,1,1804.437126
1,1,7142.833333
1,1,2419.117188
1,1,12075.884615
1,1,12261.915000
1,1,7419.629808
1,1,1867.437500
1,1,13656.157895
1,1,3554.028846
1,1,16520.637931
1,1,8235.692073
1,1,2195.201923
1,1,6338.000000
1,1,2469.259146
1,1,2617.914286
1,1,7548.298077
1,1,4245.466216
1,1,2047.923077
1,1,7488.437500
1,1,9220.703488
1,1,8225.663793
1,1,11940.670732
1,1,13184.229167
1,1,8972.633333
1,1,2113.453125
1,1,2391.711111
1,1,7329.625000
1,1,7845.080000
1,1,15145.600000
1,1,11599.911111
1,1,2055.991848
1,1,2283.500000
1,1,10982.406977
1,1,15894.454545
1,1,5862.255952
1,1,4038.106707
1,1,4004.500000

1,1,10499.199074
1,1,2516.363636
1,1,1784.213235
1,1,5036.545455
1,1,2808.058824
1,1,16842.320000
1,1,13158.684211
1,1,1552.846154
1,1,3602.985714
1,1,1679.826087
1,1,4915.850000
1,1,2787.363636
1,1,5222.075000
1,1,2290.062500
1,1,2984.563830
1,1,1721.539474
1,1,1698.240625
1,1,4857.618421
1,1,5170.005556
1,1,2210.443627
1,1,5294.391892
1,1,1523.975000
1,1,19163.152778
1,1,3058.401840
1,1,5944.595395
1,1,2110.872449
1,1,10472.400000
1,1,12656.125000
1,1,4432.125000
1,1,3984.884146
1,1,1563.789286
1,1,2875.058824
1,1,2986.465116
1,1,6456.047619
1,1,7179.647321
1,1,5750.351351
1,1,3732.536765
1,1,4839.044271
1,1,12108.500000
1,1,4422.336957
1,1,8120.685714
1,1,2776.750000
1,1,2401.000000
1,1,6449.500000
1,1,1587.823529
1,1,8014.243421
1,1,8052.477273
1,1,4504.414773
1,1,1658.654762
1,1,4197.281250
1,1,12295.187500
1,1,3759.712963
1,1,4108.544118
1,1,5349.265306
1,1,1592.867647
1,1,3496.107143
1,1,1701.585052
1,1,8005.640244
1,1,9908.166667
1,1,2616.388889
1,1,8810.044872
1,1,1995.182927
1,1,2468.757353
1,1,9047.854167
1,1,3574.365385
1,1,1529.690625

1,1,7997.454545
1,1,4804.417969
1,1,4209.837209
1,1,15407.500000
1,1,1701.500000
1,1,1699.187500
1,1,4731.987179
1,1,3637.182065
1,1,2100.368590
1,1,1664.074405
1,1,5333.500000
1,1,1601.279412
1,1,5278.342857
1,1,8206.918919
1,1,1731.008065
1,1,6221.923077
1,1,3574.348214
1,1,2359.375000
1,1,2630.000000
1,1,5897.687500
1,1,2732.918919
1,1,7015.218750
1,1,1696.500000
1,1,2964.675000
1,1,4658.452703
1,1,12312.104730
1,1,13164.085938
1,1,7645.156250
1,1,3228.059524
1,1,3075.091837
1,1,2817.852941
1,1,10826.133333
1,1,5690.675532
1,1,2243.940476
1,1,3871.947674
1,1,3756.859375
1,1,6347.562500
1,1,1750.160714
1,1,13067.300781
1,1,12178.340278
1,1,3659.950581
1,1,12211.845238
1,1,13572.511111
1,1,3451.127660
1,1,6801.712500
1,1,2087.668750
1,1,1545.411765
1,1,7990.367424
1,1,18573.570513
1,1,4243.600000
1,1,8079.765957
1,1,6963.091346
1,1,8364.071429
1,1,1829.888889
1,1,1701.836957
1,1,2658.666667
1,1,2060.375000
1,1,1619.557292
1,1,5302.657895
1,1,7801.375000
1,1,1522.222222
1,1,5018.593750
1,1,6281.375000
1,1,5402.483553
1,1,5532.846154
1,1,19787.908784

1,1,1563.142857
1,1,1559.399306
1,1,7200.072368
1,1,19456.261364
1,1,9968.156250
1,1,1571.354167
1,1,2887.882353
1,1,3219.959375
1,1,3841.806818
1,1,3230.808511
1,1,6836.142857
1,1,7996.103448
1,1,5574.426136
1,1,1563.166667
1,1,3775.267045
1,1,15013.614286
1,1,1739.800000
1,1,8755.091463
1,1,6298.605263
1,1,9169.109848
1,1,11262.141304
1,1,2214.025641
1,1,10664.730469
1,1,3024.276596
1,1,7434.375000
1,1,3941.392857
1,1,1892.317308
1,1,4336.863636
1,1,3941.166667
1,1,2069.364130
1,1,4833.450000
1,1,9070.952128
1,1,2778.559783
1,1,4668.691489
1,1,4494.168269
1,1,1883.714286
1,1,3957.000000
1,1,2860.205882
1,1,13385.258523
1,1,4391.847826
1,1,7281.661290
1,1,11532.783654
1,1,6811.945946
1,1,2882.384615
1,1,6916.408654
1,1,15227.189394
1,1,2443.000000
1,1,13179.521739
1,1,1749.184211
1,1,7582.932692
1,1,4115.853571
1,1,1971.312500
1,1,3606.477273
1,1,18067.162500
1,1,2118.309028
1,1,2477.200000
1,1,2644.716667
1,1,2559.681818
1,1,7130.054054
1,1,3852.683594
1,1,12830.923077
1,1,4102.375000
1,1,7837.375000
1,1,7768.271552
1,1,6644.000000
1,1,7621.208333

1,1,1781.435897
1,1,2206.604651
1,1,8056.520833
1,1,7197.690000
1,1,7321.386364
1,1,1788.934524
1,1,6923.573718
1,1,15958.470833
1,1,15938.073171
1,1,5073.098684
1,1,1583.796053
1,1,2274.437500
1,1,10504.250000
1,1,12552.861111
1,1,2847.229592
1,1,2252.425000
1,1,4458.500000
1,1,1900.363889
1,1,15242.990625
1,1,2633.704268
1,1,4848.372845
1,1,1556.325581
1,1,3506.200000
1,1,7060.127717
1,1,3690.367021
1,1,1778.923077
1,1,7460.405405
1,1,3600.732558
1,1,3154.088710
1,1,14187.045732
1,1,3498.325581
1,1,1559.831250
1,1,8816.750000
1,1,1589.233173
1,1,3199.798780
1,1,5271.695652
1,1,2851.000000
1,1,8086.791667
1,1,2776.733871
1,1,2214.645270
1,1,6985.531250
1,1,3000.317073
1,1,10631.636364
1,1,1760.897436
1,1,2758.360795
1,1,7574.250000
1,1,1565.000000
1,1,1668.107143
1,1,2316.198864
1,1,1677.475000
1,1,2019.125000
1,1,12320.909091
1,1,6737.972973
1,1,4013.308511
1,1,9313.432692
1,1,4212.588816
1,1,6396.804878
1,1,13254.527778
1,1,3516.174107
1,1,4424.048780
1,1,3446.636111
1,1,1661.508621
1,1,2282.866071
1,1,4247.616477
1,1,12325.824219
1,1,6783.875000

1,1,3355.607143
1,1,12464.000000
1,1,2628.483333
1,1,2562.034884
1,1,4612.675595
1,1,1575.400000
1,1,5302.500000
1,1,1585.291667
1,1,1950.296875
1,1,4593.100000
1,1,3049.736842
1,1,1821.765306
1,1,4511.424419
1,1,6542.277778
1,1,3757.256983
1,1,1626.020408
1,1,2460.799419
1,1,2230.728261
1,1,1594.500000
1,1,2990.705645
1,1,1976.500000
1,1,6926.481481
1,1,3149.869565
1,1,2082.492424
1,1,2200.546875
1,1,9165.875000
1,1,4146.052632
1,1,2787.640625
1,1,5928.911458
1,1,2208.701923
1,1,1630.865385
1,1,1921.769231
1,1,6167.593750
1,1,1554.522222
1,1,4300.741477
1,1,11810.875000
1,1,4759.906250
1,1,3383.707143
1,1,3727.428571
1,1,16002.657895
1,1,9770.340426
1,1,5515.166667
1,1,3845.438776
1,1,7317.461538
1,1,6879.773649
1,1,3165.383721
1,1,3102.715909
1,1,1615.368421
1,1,1613.000000
1,1,18072.277027
1,1,3810.319149
1,1,9912.251748
1,1,10238.628205
1,1,2441.219444
1,1,6455.242424
1,1,2678.659091
1,1,2891.556452
1,1,3436.822222
1,1,1603.352564
1,1,5331.324468
1,1,7498.078947
1,1,5859.181818
1,1,10630.809524
1,1,2888.054878
1,1,1783.127660
1,1,1971.605769

1,1,2732.950000
1,1,4875.933140
1,1,2619.587500
1,1,2918.852273
1,1,2148.230769
1,1,2433.375000
1,1,4223.571429
1,1,4456.960938
1,1,12580.687500
1,1,2304.567568
1,1,3450.310976
1,1,1569.000000
1,1,3076.000000
1,1,4942.500000
1,1,1974.406250
1,1,3888.868421
1,1,2356.773438
1,1,2026.174419
1,1,2109.594595
1,1,13400.878049
1,1,2576.583333
1,1,5096.946809
1,1,10074.780000
1,1,5551.043750
1,1,5213.541667
1,1,1532.725000
1,1,3858.302326
1,1,3244.620690
1,1,2108.471354
1,1,13230.305147
1,1,7821.115385
1,1,1634.235795
1,1,5847.303571
1,1,2060.653846
1,1,1832.413462
1,1,6254.444444
1,1,3973.423077
1,1,6625.045918
1,1,2497.887821
1,1,8190.481481
1,1,2568.242021
1,1,10152.437500
1,1,5144.344595
1,1,2528.355263
1,1,6669.046296
1,1,3315.665179
1,1,1677.954545
1,1,12869.207386
1,1,14605.289474
1,1,5481.445513
1,1,3951.281250
1,1,7032.325000
1,1,1562.377660
1,1,1950.347561
1,1,1762.753378
1,1,6876.300000
1,1,2433.565341
1,1,2055.382979
1,1,6517.655488
1,1,8897.596154
1,1,3250.669355
1,1,1735.166667
1,1,12426.097826
1,1,3779.375000
1,1,2203.942073
1,1,3371.413043

1,1,14059.217391
1,1,6760.982143
1,1,2960.915625
1,1,7883.843750
1,1,3759.852041
1,1,1774.544118
1,1,6520.000000
1,1,7490.941964
1,1,3392.363636
1,1,8285.208333
1,1,7866.625000
1,1,8907.306034
1,1,4529.554878
1,1,2390.466912
1,1,10231.350000
1,1,7476.113281
1,1,2213.409722
1,1,9748.599265
1,1,3780.659574
1,1,5682.710526
1,1,1993.043478
1,1,4876.794872
1,1,1766.052632
1,1,1949.614865
1,1,6628.890244
1,1,5770.202381
1,1,3775.300000
1,1,1922.533784
1,1,2496.261905
1,1,2103.429688
1,1,1518.714286
1,1,18662.595588
1,1,3127.831395
1,1,2650.638587
1,1,4419.743056
1,1,12860.078947
1,1,7681.739130
1,1,7373.750000
1,1,2633.007353
1,1,1809.814024
1,1,2757.015625
1,1,2608.843023
1,1,13568.391892
1,1,13414.500000
1,1,8112.625000
1,1,3693.473837
1,1,3132.625000
1,1,2523.013587
1,1,2038.207447
1,1,2607.668750
1,1,2728.413793
1,1,3146.500000
1,1,4066.943750
1,1,2202.070513
1,1,13979.871324
1,1,8630.206395
1,1,2997.245192
1,1,6568.500000
1,1,8046.009615
1,1,1528.921053
1,1,1848.538732
1,1,3134.846154
1,1,3423.414634
1,1,1704.500000
1,1,7021.750000
1,1,2687.992647

1,1,1506.000000
1,1,12930.244318
1,1,6239.271739
1,1,7828.274194
1,1,3260.212500
1,1,1795.864796
1,1,10464.593750
1,1,3364.762821
1,1,7926.906250
1,1,4807.663043
1,1,10268.151786
1,1,2560.616279
1,1,3269.337500
1,1,2529.857143
1,1,6816.486111
1,1,2633.008929
1,1,11549.677632
1,1,3326.215116
1,1,6035.761029
1,1,2081.789474
1,1,6670.116667
1,1,1785.371429
1,1,7556.312500
1,1,2331.479452
1,1,1873.988372
1,1,3412.687500
1,1,6977.267241
1,1,1781.332547
1,1,6823.500000
1,1,3011.542857
1,1,3048.790625
1,1,7084.756410
1,1,2101.182065
1,1,2309.430921
1,1,2167.444444
1,1,3159.964286
1,1,2724.375000
1,1,1794.467949
1,1,4356.168919
1,1,4649.595238
1,1,2223.868421
1,1,4898.896341
1,1,7604.900000
1,1,3444.450521
1,1,4272.861979
1,1,9353.884615
1,1,2161.307692
1,1,2319.990566
1,1,8729.052083
1,1,2554.207317
1,1,5569.450000
1,1,1672.946875
1,1,1669.227273
1,1,3937.418605
1,1,14355.253788
1,1,15892.934783
1,1,3084.255319
1,1,7151.055556
1,1,1662.604712
1,1,2601.279070
1,1,2055.203947
1,1,2049.284375
1,1,3409.616667
1,1,2211.229167
1,1,19712.450000
1,1,2176.500000

```
1,1,5401.043478
1,1,7940.915179
1,1,1733.052326
1,1,7848.534483
1,1,15786.216216
1,1,3460.807692
1,1,6785.179487
1,1,6709.976190
1,1,15527.875000
1,1,8654.777778
1,1,2698.642045
1,1,8127.100000
1,1,2483.946429
1,1,5432.284091
1,1,1750.270833
1,1,1691.101562
1,1,3059.566176
1,1,8045.013514
1,1,7976.182692
1,1,2137.560976
1,1,5305.697368
1,1,5577.372283
1,1,1868.944712
1,1,8844.902778
1,1,11446.101190
1,1,8303.666667
1,1,1811.278409
1,1,3135.817568
1,1,6033.951531
1,1,1720.415441
1,1,4155.641026
1,1,7961.625000
1,1,12609.902778
1,1,3832.875000
1,1,3159.960526
1,1,11404.000000
1,1,1517.944767
1,1,7923.479167
1,1,1935.375000
1,1,5630.947368
1,1,11926.121212
1,1,3679.277027
1,1,1581.589286
1,1,9040.487805
1,1,2938.545455
1,1,1953.063889
1,1,12091.692308
1,1,6674.073529
1,1,1725.190625
1,1,6504.121622
1,1,8464.343750
1,1,4159.893382
1,1,1959.391447
1,1,15008.375000
1,1,2961.841837
1,1,7712.200000
1,1,2762.281915
1,1,2130.660256
1,1,7710.333333
1,1,18919.329032
1,1,7766.671875
1,1,1943.318750
1,1,13279.850962
1,1,1661.050000
1,1,2315.870968
1,1,3801.322581
```

1,1,2243.875000
1,1,1842.598684
1,1,7942.843750
1,1,2668.201705
1,1,17131.503472
1,1,3784.137500
1,1,2435.397727
1,1,2888.529412
1,1,4974.941860
1,1,4719.948171
1,1,7827.553571
1,1,3234.821875
1,1,4779.714286
1,1,2679.142857
1,1,1630.863636
1,1,7583.165644
1,1,4250.000000
1,1,7667.942308
1,1,18985.721154
1,1,6954.208333
1,1,1699.763587
1,1,7034.500000
1,1,7091.663043
1,1,18431.614286
1,1,14855.357143
1,1,3103.029762
1,1,2491.261029
1,1,5797.299479
1,1,4175.625000
1,1,5400.636364
1,1,6114.741279
1,1,2849.127604
1,1,15173.364865
1,1,4095.615385
1,1,4316.551630
1,1,5714.550000
1,1,1544.901515
1,1,1656.477273
1,1,1661.346591
1,1,2630.121951
1,1,1601.890625
1,1,7067.458333
1,1,1516.526042
1,1,7122.670455
1,1,4188.699219
1,1,4186.051282
1,1,1867.250000
1,1,15474.310345
1,1,15771.382114
1,1,7149.811321
1,1,3429.357143
1,1,3207.595588
1,1,11482.807927
1,1,2453.088235
1,1,3262.437500
1,1,5311.852273
1,1,3353.078947
1,1,2660.290000
1,1,13790.601852
1,1,2703.731707
1,1,3727.000000
1,1,2075.645833
1,1,12764.250000
1,1,4376.242857
1,1,6182.113889
1,1,1957.273810

1,1,2200.825000
1,1,5551.316964
1,1,12475.500000
1,1,2231.364865
1,1,6573.554688
1,1,14376.500000
1,1,7998.066667
1,1,3156.508333
1,1,6903.732955
1,1,7637.326271
1,1,1641.969512
1,1,3139.035000
1,1,8425.250000
1,1,5793.888889
1,1,3818.091837
1,1,4120.805556
1,1,14261.819767
1,1,1699.380435
1,1,2728.257812
1,1,3195.025735
1,1,5513.375000
1,1,7505.799107
1,1,8636.643939
1,1,8183.406250
1,1,11577.958333
1,1,2688.234043
1,1,4626.853659
1,1,4040.979167
1,1,2219.304348
1,1,2628.717742
1,1,14388.990385
1,1,1851.677083
1,1,2386.514286
1,1,1886.276596
1,1,7071.290323
1,1,4640.602041
1,1,9481.038462
1,1,1742.983696
1,1,19410.392857
1,1,4216.250000
1,1,1648.100543
1,1,2210.012195
1,1,7496.456897
1,1,6313.881579
1,1,8307.923077
1,1,1609.367188
1,1,1764.089286
1,1,18707.555233
1,1,13233.846154
1,1,13190.493590
1,1,12483.024390
1,1,2106.614286
1,1,5712.214844
1,1,4123.280172
1,1,3890.171875
1,1,4009.642857
1,1,2250.882353
1,1,5320.182292
1,1,2489.360714
1,1,1544.740000
1,1,3825.043478
1,1,8314.568750
1,1,4428.312500
1,1,4606.756098
1,1,8281.996711
1,1,1828.033333

1,1,1683.071429
1,1,19552.052632
1,1,13495.259615
1,1,2451.940341
1,1,8178.975000
1,1,1578.820946
1,1,12326.833333
1,1,3806.078125
1,1,2309.500000
1,1,2739.257143
1,1,8764.410714
1,1,7687.000000
1,1,3341.002604
1,1,7777.000000
1,1,2285.760870
1,1,8015.400000
1,1,3086.304348
1,1,4166.661765
1,1,4279.463235
1,1,2331.072368
1,1,12145.850806
1,1,1749.862745
1,1,3202.407407
1,1,3362.868750
1,1,14488.913793
1,1,8104.645833
1,1,1678.500000
1,1,16596.781915
1,1,3840.777778
1,1,1624.179688
1,1,3077.915625
1,1,2186.666667
1,1,6450.250000
1,1,3669.772727
1,1,7880.000000
1,1,2907.650000
1,1,7386.396277
1,1,1896.485795
1,1,15145.339286
1,1,4267.897436
1,1,2309.711957
1,1,7962.375000
1,1,7386.411765
1,1,11158.259542
1,1,2656.875000
1,1,1754.074468
1,1,5965.406250
1,1,3457.406250
1,1,4192.921569
1,1,7320.473684
1,1,4287.807692
1,1,1843.565217
2,2,1603.059524
2,2,1621.750000
2,2,4186.922705
2,2,2768.114583
2,2,1635.520270
2,2,2779.511574
2,2,1772.914894
2,2,1663.916667
2,2,1553.375000
2,2,4121.882353
2,2,2046.875000
2,2,4607.122222
2,2,8835.569444
2,2,3808.125000

2,2,2362.467262
2,2,1962.255102
2,2,1631.839286
2,2,1547.000000
2,2,1622.242188
2,2,2498.851415
2,2,2778.875000
2,2,1980.814189
2,2,3613.285714
2,2,3555.002604
2,2,1526.425676
2,2,1699.037500
2,2,1710.642857
2,2,1961.049261
2,2,4229.428571
2,2,2700.905488
2,2,2450.500000
2,2,1711.711310
2,2,3887.153017
2,2,4324.720982
2,2,1519.428571
2,2,1866.929348
2,2,1527.781250
2,2,5242.754310
2,2,3497.880952
2,2,7331.687500
2,2,2431.955556
2,2,3396.685714
2,2,6756.757812
2,2,2472.778409
2,2,2458.932500
2,2,1707.088889
2,2,15104.867647
2,2,1925.700000
2,2,2089.547414
2,2,5246.125000
2,2,2678.825000
2,2,3732.479167
2,2,7608.411765
2,2,1713.955000
2,2,1640.292683
2,2,2211.514706
2,2,8707.765690
2,2,2490.008621
2,2,1668.064815
2,2,1723.642857
2,2,2625.461538
2,2,2394.583333
2,2,2695.229592
2,2,5394.217391
2,2,1654.853659
2,2,1776.893617
2,2,2214.175000
2,2,1911.200000
2,2,3630.673077
2,2,2739.474359
2,2,4755.457547
2,2,2249.831522
2,2,11046.227273
2,2,3489.857143
2,2,1807.938776
2,2,2989.035714
2,2,7104.464286
2,2,1899.975446
2,2,2820.011173
2,2,7255.245536

2,2,13754.568966
2,2,1967.567500
2,2,1610.108696
2,2,4303.780000
2,2,1981.152174
2,2,9194.840426
2,2,1849.692308
2,2,4404.500000
2,2,2216.048077
2,2,2604.725000
2,2,4833.530303
2,2,4240.161585
2,2,2494.220588
2,2,1730.105263
2,2,1709.839286
2,2,2259.576389
2,2,2324.943878
2,2,9181.583333
2,2,3560.333333
2,2,2067.964286
2,2,6255.375000
2,2,1637.997596
2,2,2020.861486
2,2,4847.640625
2,2,1775.112245
2,2,2000.340164
2,2,1699.238889
2,2,2954.500000
2,2,3332.117647
2,2,2054.660714
2,2,7318.250000
2,2,1873.549107
2,2,2068.239362
2,2,4449.692308
2,2,1946.208333
2,2,2329.365854
2,2,4066.473958
2,2,1856.659722
2,2,1862.395833
2,2,1802.586957
2,2,3475.703431
2,2,4825.387755
2,2,6050.614286
2,2,4221.633065
2,2,1534.741706
2,2,3203.645833
2,2,2793.872340
2,2,3140.655172
2,2,3225.145714
2,2,7515.015957
2,2,17926.825000
2,2,1750.853261
2,2,2111.341346
2,2,7799.400000
2,2,1678.073980
2,2,2597.095000
2,2,3542.461165
2,2,2179.163043
2,2,1628.008929
2,2,2128.300000
2,2,8742.590909
2,2,1706.175000
2,2,4661.532738
2,2,1593.075000
2,2,8058.716981
2,2,1815.292614

2,2,5472.334184
2,2,2053.562500
2,2,4951.529412
2,2,1828.055288
2,2,2144.869444
2,2,2275.682927
2,2,1886.412162
2,2,1723.101744
2,2,2144.894737
2,2,1577.841463
2,2,1945.794118
2,2,4948.953125
2,2,1741.250000
2,2,1686.840426
2,2,1735.234756
2,2,1812.666667
2,2,5515.250000
2,2,1749.588235
2,2,2264.324468
2,2,2593.125000
2,2,5086.634615
2,2,3644.750000
2,2,2435.697115
2,2,1558.359043
2,2,1689.255814
2,2,4410.904255
2,2,7543.500000
2,2,1637.926020
2,2,1737.433824
2,2,1626.741071
2,2,3569.948529
2,2,1644.676471
2,2,3251.156250
2,2,1603.500000
2,2,1607.096354
2,2,1891.666667
2,2,3739.041667
2,2,1724.398936
2,2,2594.785377
2,2,6333.650000
2,2,3152.311224
2,2,1521.476064
2,2,4161.583333
2,2,15226.500000
2,2,3160.932432
2,2,3352.754630
2,2,2430.452830
2,2,1567.354167
2,2,2604.531250
2,2,7764.950980
2,2,1988.067308
2,2,13915.375000
2,2,1861.270270
2,2,3404.875000
2,2,5544.775735
2,2,14134.217262
2,2,2258.058333
2,2,4562.811321
2,2,1610.401163
2,2,1542.438830
2,2,2219.117647
2,2,2825.404891
2,2,6416.125000
2,2,2757.444444
2,2,2557.668919
2,2,4275.375000

2,2,2464.458333
2,2,3629.487805
2,2,2331.080357
2,2,1852.712838
2,2,1597.326923
2,2,3245.061224
2,2,1696.805851
2,2,2683.379487
2,2,1832.994186
2,2,1843.619565
2,2,3639.027778
2,2,1943.566456
2,2,1783.773585
2,2,2839.875000
2,2,6274.811321
2,2,1722.372093
2,2,2084.800000
2,2,1811.263636
2,2,2355.645161
2,2,7707.159091
2,2,3726.662162
2,2,2319.783333
2,2,10902.550781
2,2,2406.500000
2,2,2712.430556
2,2,2994.761628
2,2,1555.101351
2,2,1610.125000
2,2,2218.663043
2,2,1620.801471
2,2,2207.531250
2,2,2201.553191
2,2,2117.483796
2,2,3755.995536
2,2,5492.016892
2,2,1844.351064
2,2,1662.000000
2,2,2668.040761
2,2,2729.250000
2,2,2040.410256
2,2,1613.057692
2,2,2073.482955
2,2,1866.305556
2,2,2104.712264
2,2,2261.220000
2,2,6484.861486
2,2,1691.890000
2,2,2446.800000
2,2,1844.142857
2,2,2514.460000
2,2,2648.608108
2,2,10821.476190
2,2,1719.365854
2,2,1583.175000
2,2,4308.321429
2,2,3313.609375
2,2,2056.568750
2,2,2585.554688
2,2,2237.762500
2,2,14293.066964
2,2,1819.042683
2,2,2222.227273
2,2,2787.250000
2,2,4280.125000
2,2,2171.468085
2,2,4750.267857

2,2,5055.354730
2,2,2494.857955
2,2,2483.616071
2,2,1766.058824
2,2,2452.618421
2,2,2807.113839
2,2,1867.011628
2,2,7366.801020
2,2,2362.684896
2,2,3615.535256
2,2,1987.121951
2,2,1707.750000
2,2,1505.920000
2,2,5160.785276
2,2,2002.383333
2,2,1815.864583
2,2,4015.697368
2,2,4841.453846
2,2,1539.955882
2,2,1917.577778
2,2,3070.578947
2,2,1537.821875
2,2,2265.108333
2,2,1514.045455
2,2,2214.722561
2,2,1874.395833
2,2,1808.718085
2,2,2704.563953
2,2,1853.598684
2,2,2690.157895
2,2,4309.058824
2,2,2115.345930
2,2,2019.714286
2,2,2314.865196
2,2,2270.235000
2,2,2036.480263
2,2,9312.713235
2,2,2525.644608
2,2,1551.796875
2,2,2295.767857
2,2,6697.520408
2,2,1720.434783
2,2,4805.365854
2,2,1515.863971
2,2,8191.485849
2,2,2344.885417
2,2,13101.266949
2,2,1858.479167
2,2,1718.276596
2,2,1683.528409
2,2,1895.813953
2,2,4185.638728
2,2,2184.422222
2,2,2737.375000
2,2,1777.009804
2,2,5236.285714
2,2,5838.048780
2,2,3993.566038
2,2,5823.704268
2,2,2677.898438
2,2,4142.342629
2,2,1812.104651
2,2,3105.022222
2,2,1778.625000
2,2,1677.609626
2,2,8583.000000

2,2,7003.261905
2,2,2054.675532
2,2,2188.600000
2,2,5313.663136
2,2,2113.906977
2,2,4884.500000
2,2,1643.623037
2,2,3535.794444
2,2,6192.627273
2,2,2112.767500
2,2,1990.441860
2,2,1849.225000
2,2,15692.389881
2,2,2872.702381
2,2,1785.486607
2,2,1778.868421
2,2,2240.875000
2,2,1555.825000
2,2,1665.187500
2,2,5634.636364
2,2,1901.685897
2,2,2466.450000
2,2,8126.900000
2,2,2175.871795
2,2,1738.980769
2,2,3500.826705
2,2,6535.583333
2,2,3247.380952
2,2,3208.872449
2,2,2153.973684
2,2,1998.310000
2,2,5005.357143
2,2,2213.958333
2,2,2753.142857
2,2,13742.358333
2,2,1856.160000
2,2,8744.081081
2,2,1731.567568
2,2,2367.157895
2,2,1522.293478
2,2,4263.868304
2,2,1518.117647
2,2,3009.157895
2,2,1619.356383
2,2,1517.145161
2,2,1503.182432
2,2,4322.336538
2,2,2550.519608
2,2,2892.454545
2,2,1532.673913
2,2,2151.025862
2,2,1701.576220
2,2,5365.165441
2,2,1763.215426
2,2,1948.188830
2,2,1781.705128
2,2,1568.189103
2,2,11004.687500
2,2,5397.157895
2,2,4619.089286
2,2,1693.285714
2,2,3792.963415
2,2,2080.351190
2,2,5860.534091
2,2,1542.257353
2,2,2585.578488

2,2,4020.882812
2,2,2150.361111
2,2,1918.683673
2,2,2092.195652
2,2,2114.921429
2,2,1511.519737
2,2,2440.943182
2,2,2239.694196
2,2,2559.057143
2,2,3634.348837
2,2,5297.307692
2,2,2373.461538
2,2,3826.994186
2,2,2613.137821
2,2,1920.532895
2,2,19628.824519
2,2,1773.403846
2,2,1650.272727
2,2,4980.492788
2,2,2784.035256
2,2,1610.892857
2,2,9951.582317
2,2,4119.982558
2,2,1909.609043
2,2,1572.543478
2,2,1546.040541
2,2,2412.405063
2,2,1556.536585
2,2,2136.708333
2,2,2489.429825
2,2,11730.586957
2,2,1681.840000
2,2,1955.455357
2,2,2048.686047
2,2,2507.741667
2,2,4655.802326
2,2,8374.063636
2,2,6446.021277
2,2,1625.764151
2,2,1972.340426
2,2,4466.601351
2,2,6610.288265
2,2,1830.127451
2,2,6315.927500
2,2,7682.131579
2,2,11796.382353
2,2,2616.413265
2,2,11537.327778
2,2,2404.861702
2,2,2021.468750
2,2,2223.420455
2,2,2748.524123
2,2,1791.335714
2,2,1810.838235
2,2,2063.953125
2,2,14727.497159
2,2,1987.485000
2,2,1527.521226
2,2,1600.214623
2,2,2594.351744
2,2,3734.875000
2,2,11406.894231
2,2,1544.037500
2,2,6202.078431
2,2,1600.330357
2,2,1535.871795

2,2,1623.182927
2,2,3132.066406
2,2,3744.957447
2,2,2250.768293
2,2,8192.534314
2,2,1657.391304
2,2,1608.785714
2,2,2373.390244
2,2,3062.835106
2,2,1833.096875
2,2,5183.776316
2,2,1640.593750
2,2,1853.636364
2,2,3539.080882
2,2,3019.529412
2,2,1995.076705
2,2,6097.718750
2,2,3230.312500
2,2,16874.647059
2,2,1606.445000
2,2,2909.750000
2,2,3830.476562
2,2,1875.132653
2,2,2097.527174
2,2,6484.285714
2,2,3908.918478
2,2,3830.505682
2,2,1914.312500
2,2,14984.880682
2,2,1745.666667
2,2,1933.088542
2,2,2344.603723
2,2,3575.108014
2,2,2352.731771
2,2,2457.125000
2,2,3556.600000
2,2,1530.104167
2,2,10501.361702
2,2,2669.921296
2,2,2246.875000
2,2,2095.415094
2,2,1719.902439
2,2,1611.584239
2,2,2558.702381
2,2,11277.560976
2,2,8627.481250
2,2,1905.922619
2,2,2155.783482
2,2,2328.795000
2,2,2170.337719
2,2,1582.533981
2,2,2331.926020
2,2,1849.572917
2,2,2991.200000
2,2,1563.010593
2,2,1510.840426
2,2,3544.655488
2,2,4991.565789
2,2,1562.000000
2,2,2360.165698
2,2,1576.232558
2,2,7406.611111
2,2,1672.195122
2,2,2367.570000
2,2,3004.679878
2,2,1592.592500

2,2,1617.051471
2,2,9376.480000
2,2,3498.323529
2,2,3859.260417
2,2,2044.071429
2,2,2661.679245
2,2,1518.890909
2,2,3398.500000
2,2,2921.808511
2,2,15926.579545
2,2,1547.226852
2,2,1971.715426
2,2,2055.988426
2,2,1665.535326
2,2,1687.443878
2,2,14159.928571
2,2,1549.985294
2,2,1615.035714
2,2,1774.170000
2,2,1936.245690
2,2,2878.175532
2,2,2520.416667
2,2,7512.850000
2,2,1554.087500
2,2,1992.262500
2,2,4183.938889
2,2,2235.619048
2,2,16669.452830
2,2,1634.621951
2,2,2540.170000
2,2,1657.703125
2,2,6915.982143
2,2,1546.000000
2,2,2600.588816
2,2,1844.795918
2,2,3937.721429
2,2,1974.362245
2,2,2386.096591
2,2,1603.182692
2,2,3169.339286
2,2,1579.090909
2,2,1943.372093
2,2,1790.605263
2,2,15236.467593
2,2,2958.243902
2,2,8026.460106
2,2,4991.073171
2,2,8745.437500
2,2,1530.200893
2,2,9324.009146
2,2,1598.591667
2,2,2673.775000
2,2,2351.012195
2,2,4243.463542
2,2,3053.212264
2,2,10229.386364
2,2,7399.500000
2,2,2008.270833
2,2,3051.345238
2,2,2005.228972
2,2,1789.368243
2,2,2003.200658
2,2,3698.211538
2,2,2673.187500
2,2,2400.968750
2,2,1557.960526

2,2,3802.875000
2,2,1796.127551
2,2,1935.002551
2,2,5612.250000
2,2,2695.923077
2,2,2414.996429
2,2,1523.000000
2,2,1557.025000
2,2,2190.410714
2,2,2388.093750
2,2,3535.875000
2,2,3074.411111
2,2,1850.835000
2,2,1576.252841
2,2,5700.994681
2,2,1976.792683
2,2,1788.660377
2,2,2553.468750
2,2,1657.258065
2,2,2624.377358
2,2,7028.680147
2,2,1617.489362
2,2,2999.990625
2,2,1681.875000
2,2,1988.785714
2,2,1807.750000
2,2,2720.805000
2,2,1731.562500
2,2,1943.075377
2,2,3387.875000
2,2,3942.109375
2,2,1534.617801
2,2,4927.071429
2,2,8397.875000
2,2,11813.882353
2,2,13161.018293
2,2,1929.352564
2,2,3774.468750
2,2,6688.127717
2,2,4659.439732
2,2,2359.982143
2,2,4734.468085
2,2,1824.572917
2,2,1779.532609
2,2,4217.402174
2,2,1879.442308
2,2,3000.500000
2,2,3584.903846
2,2,4497.968610
2,2,1985.950521
2,2,8734.263889
2,2,2103.440000
2,2,12973.395833
2,2,1506.000000
2,2,1615.430000
2,2,2526.585526
2,2,3682.872340
2,2,6311.155000
2,2,2900.242188
2,2,1533.311321
2,2,4364.857143
2,2,4654.932692
2,2,2788.461538
2,2,2380.523810
2,2,2146.648438
2,2,1597.661585

2,2,3881.000000
2,2,1608.261111
2,2,3093.482143
2,2,2045.583333
2,2,8145.443750
2,2,4885.025000
2,2,2326.694767
2,2,4083.000000
2,2,6324.367424
2,2,14377.441489
2,2,2091.000000
2,2,1821.770270
2,2,1540.437500
2,2,3429.010870
2,2,2746.093023
2,2,3096.500000
2,2,2982.775510
2,2,4925.700000
2,2,1671.652174
2,2,2182.407143
2,2,1624.891667
2,2,1966.387097
2,2,13797.593085
2,2,2483.619898
2,2,4228.773810
2,2,2166.970238
2,2,1826.457237
2,2,1930.551020
2,2,4209.596154
2,2,3088.307292
2,2,2187.171053
2,2,2082.312500
2,2,2063.250000
2,2,1655.345395
2,2,1593.102041
2,2,1995.318452
2,2,1954.355769
2,2,1893.928571
2,2,5838.041667
2,2,2751.275000
2,2,3381.206731
2,2,1667.515625
2,2,2000.533019
2,2,2140.665698
2,2,1816.640000
2,2,5084.604167
2,2,1619.492647
2,2,2353.710526
2,2,2681.096154
2,2,4767.622727
2,2,1870.317073
2,2,2660.476190
2,2,2123.146552
2,2,2723.494318
2,2,4507.347222
2,2,2417.181818
2,2,2184.480000
2,2,1820.047727
2,2,1533.263158
2,2,2468.364865
2,2,1658.153846
2,2,2764.512931
2,2,3914.844340
2,2,1623.580189
2,2,11891.289062
2,2,2423.888514

2,2,4723.405556
2,2,6661.125000
2,2,11024.727273
2,2,1808.859375
2,2,2872.625000
2,2,2278.812500
2,2,1709.730114
2,2,2639.398026
2,2,3201.059211
2,2,3186.948529
2,2,2518.191176
2,2,4011.881757
2,2,2504.660156
2,2,2347.847368
2,2,4579.494186
2,2,1787.686364
2,2,1921.223214
2,2,2696.056818
2,2,1724.593750
2,2,1817.813953
2,2,1681.568750
2,2,7852.942073
2,2,2684.336538
2,2,2645.479167
2,2,4003.838362
2,2,1798.036184
2,2,2034.716146
2,2,3730.555556
2,2,1536.102564
2,2,12818.753676
2,2,2364.858491
2,2,2632.308824
2,2,2799.339674
2,2,1644.915948
2,2,4280.233010
2,2,2495.593085
2,2,1953.486111
2,2,4036.763636
2,2,1921.533654
2,2,4579.337838
2,2,2121.424528
2,2,1676.167614
2,2,2581.511905
2,2,1696.564815
2,2,3113.187135
2,2,2191.442308
2,2,2617.121951
2,2,2422.652542
2,2,2072.657895
2,2,4269.520833
2,2,2205.095745
2,2,2138.767442
2,2,2846.938776
2,2,6300.500000
2,2,3658.650000
2,2,2340.642857
2,2,1523.013298
2,2,3259.407407
2,2,2287.650000
2,2,1912.117021
2,2,2058.928571
2,2,2004.650000
2,2,1558.815972
2,2,2296.644231
2,2,14087.058824
2,2,1506.605263

2,2,3101.711538
2,2,1604.375000
2,2,1689.122396
2,2,3736.530000
2,2,13855.136111
2,2,13818.727273
2,2,4127.404255
2,2,2182.453488
2,2,1514.660326
2,2,3017.619318
2,2,2172.152439
2,2,2557.152174
2,2,1770.566667
2,2,2755.482143
2,2,2966.142857
2,2,16334.794872
2,2,2177.285714
2,2,3173.342857
2,2,1536.942857
2,2,8419.250000
2,2,1625.640244
2,2,1916.630682
2,2,7180.528846
2,2,1595.789286
2,2,1602.232143
2,2,2314.602273
2,2,2792.457317
2,2,13314.032609
2,2,10459.639706
2,2,9531.222727
2,2,2618.094595
2,2,4553.957447
2,2,7179.553191
2,2,1805.688482
2,2,5986.156250
2,2,1715.733333
2,2,1735.804688
2,2,2209.044643
2,2,1860.929348
2,2,8259.441176
2,2,3155.076531
2,2,4200.437500
2,2,1635.614130
2,2,2491.617788
2,2,2083.451220
2,2,1615.482558
2,2,2421.726190
2,2,1554.295000
2,2,1504.884434
2,2,2245.836364
2,2,2140.649038
2,2,2367.384615
2,2,2361.900000
2,2,1839.812500
2,2,1533.840278
2,2,3965.105263
2,2,4281.948171
2,2,13664.571429
2,2,1896.750000
2,2,5501.478261
2,2,2212.700521
2,2,1831.750000
2,2,1511.393868
2,2,4898.317073
2,2,1660.960526
2,2,1710.058673

2,2,7554.962766
2,2,2150.234043
2,2,3662.323370
2,2,3453.400735
2,2,1534.102941
2,2,1538.153846
2,2,1727.125000
2,2,1589.470109
2,2,2323.600000
2,2,1630.491667
2,2,1681.526163
2,2,1975.681818
2,2,3177.312500
2,2,2163.110526
2,2,1647.335000
2,2,7392.698718
2,2,1636.487805
2,2,2410.469388
2,2,1657.761628
2,2,3811.934322
2,2,2972.331395
2,2,8363.500000
2,2,2469.250000
2,2,3547.250000
2,2,4053.758523
2,2,3123.066176
2,2,2964.214844
2,2,2446.784091
2,2,1601.949519
2,2,3759.668605
2,2,1825.676829
2,2,7118.201613
2,2,3301.097826
2,2,3607.250000
2,2,2209.103723
2,2,2687.000000
2,2,1825.566667
2,2,1724.858491
2,2,4028.408537
2,2,7500.578125
2,2,1716.889535
2,2,6831.562500
2,2,2802.254902
2,2,7868.997024
2,2,6381.593023
2,2,5959.140625
2,2,1582.428571
2,2,2022.455000
2,2,1648.068182
2,2,2110.298611
2,2,2318.724359
2,2,1814.884615
2,2,3024.160714
2,2,1536.409091
2,2,1657.882353
2,2,2888.247159
2,2,2043.589674
2,2,3972.097222
2,2,1676.070000
2,2,2093.221154
2,2,2483.031915
2,2,1717.678571
2,2,2511.411765
2,2,4318.052632
2,2,3518.964286
2,2,1612.004717

```
2,2,8943.375000
2,2,1854.500000
2,2,2497.171053
2,2,2028.916667
2,2,2141.547872
2,2,2031.290000
2,2,1982.692308
2,2,2221.955556
2,2,2089.809659
2,2,2060.648148
2,2,5675.422872
2,2,1856.389671
2,2,2398.333333
2,2,4715.954545
2,2,1989.922170
2,2,3188.580000
2,2,3477.988095
2,2,2246.347826
2,2,2718.319444
2,2,10273.674419
2,2,2225.789474
2,2,2650.232759
2,2,7869.730769
2,2,1613.000000
2,2,2409.400000
2,2,1638.424107
2,2,1650.798469
2,2,1993.656250
2,2,3049.000000
2,2,1985.929245
2,2,3287.100000
2,2,1864.048077
2,2,3861.297872
2,2,5589.795455
2,2,1797.641304
2,2,7163.059524
2,2,1882.994792
2,2,3525.555556
2,2,2595.282609
2,2,2708.125000
2,2,1998.026316
2,2,2202.473684
2,2,1510.776596
2,2,1545.500000
2,2,1574.544872
2,2,1658.808190
2,2,2180.747222
2,2,1619.375000
2,2,1681.311364
2,2,3340.138158
2,2,8648.481481
2,2,1630.939103
2,2,2717.105603
2,2,11504.125000
2,2,1725.642857
2,2,8355.353403
2,2,3362.480000
2,2,2332.577778
2,2,2419.081633
2,2,1511.142857
2,2,2682.434896
2,2,2551.409091
2,2,2289.588235
2,2,4307.153646
2,2,1770.627660
2,2,1569.100000
```

2,2,4069.338235
2,2,3123.922280
2,2,2507.058140
2,2,2323.743243
2,2,2411.488372
2,2,13805.225000
2,2,2285.156250
2,2,7469.622340
2,2,3544.261364
2,2,3771.260870
2,2,2022.090426
2,2,2301.142857
2,2,5230.241071
2,2,2468.740385
2,2,2076.441038
2,2,1592.336111
2,2,1544.257812
2,2,1583.268293
2,2,1567.187500
2,2,1773.250000
2,2,8168.107558
2,2,1828.323980
2,2,6730.553125
2,2,1518.987500
2,2,1756.941176
2,2,2323.493590
2,2,2055.894231
2,2,1870.423913
2,2,3604.375000
2,2,1592.341146
2,2,2481.345946
2,2,1910.391892
2,2,4020.959459
2,2,1862.901042
2,2,2166.960000
2,2,1612.764881
2,2,2264.710526
2,2,1593.945122
2,2,3407.570312
2,2,2231.799020
2,2,1584.444079
2,2,1790.375000
2,2,1677.640244
2,2,2740.305000
2,2,2474.523438
2,2,2091.058824
2,2,2070.857143
2,2,1850.201389
2,2,2231.797872
2,2,2527.514706
2,2,4654.185976
2,2,1823.110000
2,2,1626.178571
2,2,7377.073171
2,2,2184.048077
2,2,1754.342593
2,2,1560.406250
2,2,8079.807692
2,2,2018.160377
2,2,7323.066406
2,2,2047.867021
2,2,1529.017857
2,2,1587.000000
2,2,2312.380208
2,2,1736.928910
2,2,2616.444444

2,2,9670.125000
2,2,1517.375000
2,2,4760.000000
2,2,2318.883721
2,2,1865.470000
2,2,4326.661111
2,2,2789.433824
2,2,1637.098958
2,2,2902.028302
2,2,2872.605978
2,2,4941.442623
2,2,3615.169118
2,2,3660.096429
2,2,2492.104651
2,2,18442.820946
2,2,2854.180851
2,2,4834.069182
2,2,1518.034091
2,2,1525.783333
2,2,2779.131579
2,2,2136.822368
2,2,1585.305556
2,2,2510.201923
2,2,6937.125000
2,2,1577.922872
2,2,2025.848958
2,2,2997.777174
2,2,3329.250000
2,2,1833.443038
2,2,1781.162791
2,2,2219.400000
2,2,8743.385135
2,2,2066.736842
2,2,4167.225000
2,2,12515.083333
2,2,2291.594595
2,2,1806.021875
2,2,1827.579082
2,2,3146.265625
2,2,16254.216216
2,2,1923.731013
2,2,5246.985294
2,2,1636.317568
2,2,3614.608696
2,2,2114.627907
2,2,1652.166667
2,2,3953.132653
2,2,1569.391304
2,2,1855.625000
2,2,2421.221591
2,2,1870.331522
2,2,8243.067568
2,2,1642.529412
2,2,8810.318182
2,2,2325.555288
2,2,1836.881098
2,2,2004.822917
2,2,15399.812500
2,2,2656.062500
2,2,1980.189655
2,2,6043.820122
2,2,1717.729730
2,2,2186.275000
2,2,3557.614362
2,2,1730.042553
2,2,4053.164216

2,2,6644.175000
2,2,1593.320313
2,2,6691.650000
2,2,5843.489540
2,2,4457.706818
2,2,2967.835106
2,2,2131.679054
2,2,3019.000000
2,2,1635.925532
2,2,3109.440000
2,2,1572.394886
2,2,2814.487805
2,2,1960.239583
2,2,2982.305556
2,2,2379.069444
2,2,5203.890244
2,2,4952.962500
2,2,4403.208333
2,2,2948.760870
2,2,8018.733333
2,2,1744.146341
2,2,2037.860294
2,2,1801.840909
2,2,1548.250000
2,2,2041.750000
2,2,2938.024390
2,2,9072.578947
2,2,3042.290816
2,2,1519.892857
2,2,2577.323661
2,2,2971.595745
2,2,3901.500000
2,2,1538.055556
2,2,2833.795213
2,2,2243.615385
2,2,2611.540816
2,2,1557.634615
2,2,3145.048611
2,2,3297.930233
2,2,1509.408854
2,2,3972.294118
2,2,2587.750000
2,2,3046.686813
2,2,8414.687500
2,2,4158.208333
2,2,1654.941176
2,2,3278.680851
2,2,1969.687500
2,2,1588.221591
2,2,3647.419643
2,2,2098.602041
2,2,2995.618343
2,2,13269.636364
2,2,2372.226064
2,2,3389.648936
2,2,2854.940000
2,2,5023.048913
2,2,5262.650000
2,2,7022.232143
2,2,2059.332447
2,2,1541.296053
2,2,1947.702128
2,2,2042.647727
2,2,5778.652174
2,2,2102.228723
2,2,2959.137500

2,2,2267.061404
2,2,5178.213415
2,2,2851.180233
2,2,1857.142857
2,2,1975.622340
2,2,1974.637500
2,2,3456.613636
2,2,2744.268617
2,2,10728.969388
2,2,1681.220238
2,2,5845.515625
2,2,5103.035714
2,2,4906.981618
2,2,1864.304688
2,2,2101.844444
2,2,1549.550000
2,2,1563.190341
2,2,11435.687500
2,2,2341.134615
2,2,1828.842105
2,2,2598.761905
2,2,1593.988095
2,2,1743.228723
2,2,7083.286458
2,2,1745.125000
2,2,4436.291667
2,2,2193.243056
2,2,19748.104167
2,2,2380.033784
2,2,2722.437500
2,2,2376.000000
2,2,8224.257812
2,2,2813.595238
2,2,1507.532895
2,2,1780.482759
2,2,3492.600000
2,2,6775.549180
2,2,1976.462500
2,2,15386.833333
2,2,1503.863889
2,2,4000.816489
2,2,2014.903409
2,2,2076.109375
2,2,2079.950000
2,2,3086.862805
2,2,2329.355000
2,2,2273.943396
2,2,2068.473214
2,2,3620.190217
2,2,5246.891667
2,2,4190.976190
2,2,2473.735119
2,2,1957.152027
2,2,2140.000000
2,2,6703.541667
2,2,1540.160714
2,2,1573.151961
2,2,3533.469697
2,2,2687.267045
2,2,3576.803571
2,2,1756.972222
2,2,3953.580357
2,2,4663.565789
2,2,9865.021277
2,2,3669.841912
2,2,2256.222222

2,2,1634.202128
2,2,1710.078125
2,2,2048.882979
2,2,1968.724432
2,2,3534.721154
2,2,3070.559091
2,2,1667.250000
2,2,2006.237805
2,2,5299.311275
2,2,1900.250000
2,2,2120.944444
2,2,1662.948864
2,2,1661.034722
2,2,3650.273256
2,2,2283.925847
2,2,2198.986979
2,2,8416.598684
2,2,2347.619444
2,2,3064.065789
2,2,1777.585366
2,2,1523.922222
2,2,2137.428571
2,2,3570.636364
2,2,2746.590278
2,2,3224.442935
2,2,6512.357143
2,2,1739.954545
2,2,1852.478659
2,2,1842.956633
2,2,2472.954545
2,2,1923.934211
2,2,2918.914894
2,2,3569.333333
2,2,2999.872093
2,2,1867.468085
2,2,1536.375000
2,2,1531.230114
2,2,2966.043478
2,2,1528.568452
2,2,4905.956395
2,2,4758.210938
2,2,2983.358173
2,2,2912.255556
2,2,3781.260638
2,2,1652.235294
2,2,3387.546875
2,2,1756.875000
2,2,4137.990000
2,2,3692.740385
2,2,2353.556604
2,2,2018.323529
2,2,2827.263889
2,2,12592.519231
2,2,3981.120000
2,2,4252.564904
2,2,2632.395349
2,2,1711.182927
2,2,1925.641026
2,2,1633.200000
2,2,2295.816489
2,2,3900.288889
2,2,6167.425000
2,2,1708.540541
2,2,2262.273026
2,2,1606.356383
2,2,1545.099432

2,2,1885.673913
2,2,3435.232143
2,2,2122.051829
2,2,2710.879310
2,2,3288.840909
2,2,1638.042553
2,2,2031.097561
2,2,3672.556250
2,2,1776.312500
2,2,8367.002660
2,2,3288.732143
2,2,1884.338068
2,2,18828.064286
2,2,2052.779255
2,2,2576.830357
2,2,2682.637500
2,2,1659.369186
2,2,3042.400000
2,2,2009.250000
2,2,6905.433333
2,2,1954.921053
2,2,1632.750000
2,2,2620.333333
2,2,2226.500000
2,2,3528.725000
2,2,1515.990000
2,2,1727.450000
2,2,2136.186047
2,2,5220.105769
2,2,1869.854167
2,2,1882.961538
2,2,1504.000000
2,2,10012.877551
2,2,3098.867788
2,2,2596.584906
2,2,1637.158537
2,2,1625.826923
2,2,2310.538690
2,2,1604.857143
2,2,2244.634091
2,2,2350.937500
2,2,9082.620283
2,2,2147.196078
2,2,2761.761111
2,2,3260.466102
2,2,1693.369186
2,2,1950.692708
2,2,1838.340426
2,2,6259.437500
2,2,13293.830357
2,2,1811.062500
2,2,2699.450658
2,2,1595.984694
2,2,2769.254545
2,2,1644.632653
2,2,6010.872222
2,2,1638.300000
2,2,3374.653846
2,2,1760.229167
2,2,2359.207317
2,2,17572.625000
2,2,2700.243421
2,2,2839.895161
2,2,2151.448171
2,2,2970.473214
2,2,2801.097561

2,2,5482.795455
2,2,2172.750000
2,2,1667.125000
2,2,6024.870370
2,2,3902.275568
2,2,1640.410256
2,2,4330.166667
2,2,2001.469388
2,2,3935.826389
2,2,5145.906250
2,2,4697.014706
2,2,2078.265957
2,2,3392.706522
2,2,3188.440476
2,2,2219.572917
2,2,3022.717105
2,2,2073.867925
2,2,1647.880319
2,2,2640.314024
2,2,1503.198529
2,2,1972.600000
2,2,1691.198864
2,2,2135.427083
2,2,1710.214286
2,2,7081.133803
2,2,3008.302632
2,2,3096.727500
2,2,1999.352941
2,2,2183.707317
2,2,3152.994186
2,2,1639.677083
2,2,1598.523810
2,2,1668.750000
2,2,2282.559375
2,2,1859.750000
2,2,2091.207447
2,2,7976.851064
2,2,1650.413462
2,2,6610.517361
2,2,2248.567308
2,2,2177.400000
2,2,1782.843750
2,2,1843.633065
2,2,4971.184211
2,2,1911.362245
2,2,2490.160714
2,2,2624.223404
2,2,4948.305556
2,2,1658.604730
2,2,2276.644231
2,2,1649.645833
2,2,2585.062500
2,2,1680.759615
2,2,2225.427673
2,2,2966.637500
2,2,2067.675000
2,2,2532.352941
2,2,2873.312500
2,2,1823.529412
2,2,1941.546875
2,2,3538.714286
2,2,3209.887821
2,2,3001.959302
2,2,1572.872340
2,2,1970.500000
2,2,8564.960000

2,2,2366.408163
2,2,1919.188725
2,2,2291.005102
2,2,1870.852041
2,2,1697.165441
2,2,1914.552326
2,2,2261.880435
2,2,3021.636364
2,2,2636.246875
2,2,1726.500000
2,2,1668.027439
2,2,1731.427326
2,2,1800.534483
2,2,2271.666667
2,2,5364.600000
2,2,2429.884146
2,2,2278.759434
2,2,1807.771277
2,2,3748.365625
2,2,11634.890625
2,2,2060.570312
2,2,4830.433036
2,2,1573.463235
2,2,2453.475000
2,2,2690.139535
2,2,1743.740000
2,2,1680.755435
2,2,4007.860795
2,2,2877.755814
2,2,1984.759868
2,2,1533.105114
2,2,2836.517045
2,2,4487.368056
2,2,2030.600000
2,2,1652.775510
2,2,1984.137500
2,2,1964.428571
2,2,1768.666667
2,2,5942.365385
2,2,4594.841837
2,2,1980.024038
2,2,2383.517857
2,2,1874.015075
2,2,6697.052632
2,2,2076.276596
2,2,2696.638393
2,2,4226.716216
2,2,2561.444149
2,2,2009.140625
2,2,2952.666667
2,2,1566.733696
2,2,1572.678977
2,2,1886.428571
2,2,2150.163462
2,2,10587.730556
2,2,1815.515625
2,2,1506.959184
2,2,7967.325521
2,2,2147.117347
2,2,2238.638514
2,2,1794.833333
2,2,3408.255682
2,2,1545.914474
2,2,3161.477500
2,2,6378.513514
2,2,19933.795732

2,2,1942.392857
2,2,2236.160377
2,2,2419.180233
2,2,2743.295455
2,2,1682.545455
2,2,5900.151515
2,2,1653.615385
2,2,4362.384615
2,2,1623.948171
2,2,3542.856061
2,2,3666.616848
2,2,2847.187500
2,2,1691.295455
2,2,1623.994681
2,2,4516.732759
2,2,5073.837500
2,2,2417.826220
2,2,3334.367021
2,2,2107.898148
2,2,3333.000000
2,2,1693.250000
2,2,2161.402778
2,2,2817.627976
2,2,2240.238971
2,2,1925.821429
2,2,5300.547170
2,2,2290.069853
2,2,4126.834052
2,2,1935.837209
2,2,1583.533333
2,2,1651.372500
2,2,3841.156250
2,2,1710.135870
2,2,2158.848958
2,2,2919.179245
2,2,2347.724138
2,2,1637.580357
2,2,2620.764706
2,2,1507.951531
2,2,1883.750000
2,2,1678.226562
2,2,2553.470588
2,2,2998.002500
2,2,2156.313953
2,2,1947.932432
2,2,1687.731707
2,2,11007.875000
2,2,1663.875000
2,2,1991.462264
2,2,6587.514706
2,2,1757.234043
2,2,1553.134146
2,2,2606.602679
2,2,2160.108696
2,2,1725.184211
2,2,1723.118182
2,2,1584.230769
2,2,1763.705357
2,2,1535.256250
2,2,2414.877155
2,2,1997.187500
2,2,1499.578947
2,2,1670.641026
2,2,1647.333333
2,2,2371.622396
2,2,1795.750000

2,2,2295.946809
2,2,2387.750000
2,2,1687.869898
2,2,1722.413043
2,2,1588.841346
2,2,1560.949074
2,2,9091.647959
2,2,1795.402174
2,2,2756.252717
2,2,3548.414894
2,2,1684.714286
2,2,1550.454545
2,2,1807.550000
2,2,3931.178125
2,2,4307.347826
2,2,1878.451389
2,2,1966.090909
2,2,3551.786765
2,2,3777.240385
2,2,9824.982143
2,2,13229.822761
2,2,2362.093750
2,2,1893.332317
2,2,1503.206081
2,2,2573.339674
2,2,3869.571429
2,2,4851.644231
2,2,2510.294643
2,2,2278.385870
2,2,2329.807692
2,2,1992.570946
2,2,2375.242188
2,2,3777.871951
2,2,4400.326087
2,2,1526.884615
2,2,1545.090909
2,2,1908.544811
2,2,1559.975000
2,2,1989.333333
2,2,9249.236111
2,2,4221.524194
2,2,1691.990625
2,2,1556.713816
2,2,3771.943878
2,2,2294.280488
2,2,2330.209302
2,2,2311.500000
2,2,3436.910256
2,2,1654.590909
2,2,1721.500000
2,2,1945.690909
2,2,2143.743421
2,2,2065.739130
2,2,2288.378378
2,2,2039.727273
2,2,3570.900000
2,2,2839.711538
2,2,12208.570122
2,2,1756.698980
2,2,1765.903846
2,2,1796.489130
2,2,2917.720000
2,2,2232.655093
2,2,3447.333333
2,2,14125.779762
2,2,1863.166667

2,2,5126.363636
2,2,2072.531250
2,2,1849.600000
2,2,2595.660377
2,2,1599.593750
2,2,1510.250000
2,2,3551.663043
2,2,2259.875000
2,2,1556.627660
2,2,3975.760204
2,2,2221.850000
2,2,2353.375000
2,2,1507.625000
2,2,6733.416667
2,2,1622.318182
2,2,2353.375000
2,2,3892.433824
2,2,3339.633333
2,2,1512.516667
2,2,2032.187500
2,2,1829.183333
2,2,11237.750000
2,2,1504.220588
2,2,4095.040761
2,2,2035.765244
2,2,1615.459302
2,2,2230.781250
2,2,2361.012295
2,2,3291.629630
2,2,1807.011905
2,2,2738.641509
2,2,5913.546053
2,2,3187.427778
2,2,4231.466667
2,2,4080.729730
2,2,1870.489362
2,2,2973.400000
2,2,3072.542857
2,2,8135.909091
2,2,6418.076923
2,2,2336.487310
2,2,3121.958333
2,2,3715.559659
2,2,2272.357500
2,2,8153.857143
2,2,1682.776163
2,2,2876.468085
2,2,1566.750000
2,2,1519.747093
2,2,1512.568627
2,2,1785.044944
2,2,2465.153631
2,2,1583.055556
2,2,4265.303125
2,2,2448.089286
2,2,2459.691327
2,2,1588.648438
2,2,1871.835227
2,2,1894.906250
2,2,1827.781250
2,2,1618.591837
2,2,1808.184375
2,2,2889.952500
2,2,2921.221591
2,2,6097.808511
2,2,1717.409091

2,2,1737.370370
2,2,5092.455128
2,2,2687.678571
2,2,1712.612745
2,2,1864.433824
2,2,1577.809783
2,2,2209.828125
2,2,3361.871324
2,2,3532.116279
2,2,2832.428571
2,2,1581.115385
2,2,1823.222222
2,2,2505.848837
2,2,1559.681818
2,2,14245.204082
2,2,1938.072917
2,2,1842.826087
2,2,1854.835366
2,2,1768.855932
2,2,4507.500000
2,2,2401.750000
2,2,2270.804348
2,2,2385.687500
2,2,1614.000000
2,2,2001.062500
2,2,1680.500000
2,2,1616.997642
2,2,1872.815789
2,2,2979.826923
2,2,10023.081250
2,2,2227.812500
2,2,4367.662500
2,2,1828.622549
2,2,2481.739583
2,2,2509.125000
2,2,1531.231707
2,2,1703.313830
2,2,12340.875000
2,2,2648.167614
2,2,3322.787234
2,2,2189.838235
2,2,2027.642857
2,2,1983.360465
2,2,13221.371622
2,2,10900.984649
2,2,4269.933333
2,2,2464.684524
2,2,2608.118902
2,2,2585.857143
2,2,2687.040000
2,2,2546.018868
2,2,1910.035176
2,2,4417.833333
2,2,2660.179688
2,2,3200.000000
2,2,3297.871711
2,2,4155.842500
2,2,3399.049020
2,2,3938.358696
2,2,3301.577519
2,2,2361.250000
2,2,2723.203125
2,2,2514.421875
2,2,1999.922705
2,2,6781.300000
2,2,6798.441860

2,2,3766.687500
2,2,2781.982143
2,2,3892.813830
2,2,1597.445312
2,2,7710.310897
2,2,17590.243243
2,2,2156.798077
2,2,1509.960526
2,2,1620.520000
2,2,1934.928191
2,2,1564.789474
2,2,3270.500000
2,2,1523.647541
2,2,1572.752315
2,2,2304.044872
2,2,2702.340580
2,2,1636.871795
2,2,1968.900000
2,2,6976.156250
2,2,2979.511628
2,2,1565.093750
2,2,2484.520408
2,2,1568.656818
2,2,1930.125000
2,2,4515.429878
2,2,3488.358974
2,2,2872.728365
2,2,2764.138298
2,2,3930.017857
2,2,7446.586207
2,2,5397.240196
2,2,1615.858553
2,2,2414.857143
2,2,1777.878049
2,2,3538.117500
2,2,13657.000000
2,2,13369.372549
2,2,2125.595588
2,2,5262.396739
2,2,2696.666667
2,2,2715.040000
2,2,3157.255319
2,2,1697.630952
2,2,1955.375000
2,2,3395.203125
2,2,2902.028646
2,2,1750.450000
2,2,4633.120283
2,2,1823.453125
2,2,1537.755435
2,2,15640.400000
2,2,17993.763158
2,2,3604.875000
2,2,2192.504902
2,2,2580.419643
2,2,7680.314394
2,2,1590.367021
2,2,2767.488889
2,2,1515.897959
2,2,15744.560484
2,2,2808.285714
2,2,17898.694444
2,2,3921.707317
2,2,1601.000000
2,2,2082.040816
2,2,2079.895349

2,2,1814.102041
2,2,3657.847826
2,2,3195.811047
2,2,2242.842262
2,2,1528.845238
2,2,5568.986979
2,2,2325.230366
2,2,2265.000000
2,2,1502.932065
2,2,1654.697917
2,2,2488.279070
2,2,1686.078125
2,2,4280.265152
2,2,5337.834302
2,2,3604.415625
2,2,3003.875000
2,2,1809.562500
2,2,1516.312500
2,2,2847.769231
2,2,2023.076923
2,2,2259.410000
2,2,1653.255952
2,2,4445.578947
2,2,2538.625000
2,2,2446.051282
2,2,2078.250000
2,2,1923.339286
2,2,2818.116279
2,2,3136.000000
2,2,2131.285714
2,2,2489.016827
2,2,1739.339674
2,2,2391.683824
2,2,2553.500000
2,2,1822.437500
2,2,1612.656250
2,2,1898.415816
2,2,4113.827225
2,2,2816.019737
2,2,8534.361702
2,2,1535.965909
2,2,1877.483696
2,2,1743.244565
2,2,2643.471698
2,2,2077.439252
2,2,3149.177500
2,2,2287.321023
2,2,2670.268519
2,2,1548.050676
2,2,1989.370283
2,2,8032.505025
2,2,14546.434783
2,2,3863.113281
2,2,2266.380952
2,2,1666.565217
2,2,14108.533333
2,2,2144.555288
2,2,1976.125000
2,2,2912.380435
2,2,3159.657895
2,2,1529.529891
2,2,2596.941327
2,2,14169.537500
2,2,3002.013158
2,2,1523.901163
2,2,1583.707317

2,2,4510.793478
2,2,2374.519737
2,2,3348.787500
2,2,1678.283019
2,2,4408.382979
2,2,2407.441176
2,2,4551.000000
2,2,1629.642241
2,2,1699.700000
2,2,2886.333333
2,2,2267.095238
2,2,2060.333333
2,2,1711.046569
2,2,2773.250000
2,2,1608.196429
2,2,1667.504167
2,2,1865.261905
2,2,2313.100000
2,2,1532.811224
2,2,3911.843750
2,2,1754.162234
2,2,1600.350000
2,2,2978.932927
2,2,1713.536585
2,2,3449.178571
2,2,2630.927632
2,2,2010.335366
2,2,2493.035714
2,2,3750.750000
2,2,1778.138889
2,2,4718.355263
2,2,3100.500000
2,2,2265.107143
2,2,1659.099432
2,2,4988.220930
2,2,1855.442308
2,2,2600.562500
2,2,1671.082447
2,2,4047.850000
2,2,1574.276596
2,2,1534.743902
2,2,2113.152778
2,2,3633.113208
2,2,4452.752660
2,2,2066.205128
2,2,3367.000000
2,2,1991.235849
2,2,3377.750000
2,2,1774.887500
2,2,1770.861111
2,2,2056.025641
2,2,7688.395408
2,2,2938.438889
2,2,2523.057500
2,2,2374.446023
2,2,3281.358333
2,2,3066.822917
2,2,7346.608173
2,2,4593.562500
2,2,1701.555000
2,2,1746.845109
2,2,1890.530612
2,2,2798.955556
2,2,1929.742500
2,2,3764.787234
2,2,1986.781250

2,2,2284.890625
2,2,3980.189286
2,2,2337.285714
2,2,1719.000000
2,2,1638.757764
2,2,1881.368902
2,2,2425.755435
2,2,2317.141304
2,2,1843.406780
2,2,2652.436321
2,2,7171.440000
2,2,2543.204082
2,2,2308.550000
2,2,18577.196429
2,2,15199.826389
2,2,1725.857843
2,2,2016.436170
2,2,4134.350962
2,2,2807.872500
2,2,6942.937500
2,2,2291.212209
2,2,2877.512195
2,2,6752.620370
2,2,1531.298077
2,2,2186.558140
2,2,2900.371951
2,2,3158.257353
2,2,2500.858974
2,2,1581.348837
2,2,5059.826087
2,2,3860.764706
2,2,2801.915000
2,2,2082.625000
2,2,11297.402439
2,2,2230.515306
2,2,1555.002660
2,2,4353.726562
2,2,1786.663462
2,2,3492.075000
2,2,1691.407609
2,2,8237.547619
2,2,1946.526316
2,2,4743.839744
2,2,1501.787500
2,2,3599.038462
2,2,2558.300000
2,2,2481.801020
2,2,1747.161765
2,2,2849.235000
2,2,1597.317857
2,2,5333.398148
2,2,5057.144444
2,2,2211.243421
2,2,18549.679245
2,2,7127.302500
2,2,3508.924528
2,2,2568.910714
2,2,1873.062500
2,2,4788.510204
2,2,12288.444712
2,2,1860.625000
2,2,1931.207317
2,2,9132.003378
2,2,9362.517361
2,2,4761.755319
2,2,3274.078947

2,2,1759.062500
2,2,1780.679878
2,2,1554.883871
2,2,1678.410714
2,2,1887.521552
2,2,2855.946875
2,2,2068.652174
2,2,2234.065476
2,2,3493.039773
2,2,2690.636364
2,2,2867.580357
2,2,2102.138889
2,2,6230.600490
2,2,1711.680556
2,2,2399.642857
2,2,2237.294118
2,2,2136.127551
2,2,3569.327128
2,2,8101.395349
2,2,2820.298780
2,2,3167.590426
2,2,1723.687500
2,2,2694.500000
2,2,1533.857955
2,2,2819.463542
2,2,2944.812500
2,2,2873.357143
2,2,1502.530660
2,2,4519.066667
2,2,1656.521875
2,2,2244.330189
2,2,1740.658784
2,2,1583.147959
2,2,5447.304245
2,2,5034.965909
2,2,1736.673469
2,2,3053.875000
2,2,2026.159091
2,2,1558.764706
2,2,6582.829268
2,2,2836.535714
2,2,2533.470238
2,2,1679.351562
2,2,5330.551282
2,2,2567.877778
2,2,2235.246795
2,2,5950.427083
2,2,2722.531250
2,2,3906.529605
2,2,2650.133721
2,2,1887.585366
2,2,1902.601190
2,2,5542.768750
2,2,12334.990196
2,2,1945.414062
2,2,3482.903125
2,2,5632.574468
2,2,1747.761905
2,2,2433.877551
2,2,4983.614130
2,2,1997.449519
2,2,2062.174419
2,2,2071.992788
3,1,3074.189189
3,1,14221.070513
3,1,1616.517241

3,1,2906.228261
3,1,2188.875000
3,1,3816.896341
3,1,1834.147436
3,1,2327.970588
3,1,3062.103723
3,1,4260.729167
3,1,11309.989130
3,1,6439.057292
3,1,2175.356250
3,1,2379.506579
3,1,3344.020161
3,1,1840.770833
3,1,2499.091667
3,1,2815.578125
3,1,3860.009375
3,1,7581.320000
3,1,1970.393750
3,1,3219.961538
3,1,15782.125000
3,1,4705.821429
3,1,4656.702381
3,1,10077.033088
3,1,2790.000000
3,1,1605.975000
3,1,19476.256757
3,1,2675.682927
3,1,1677.051471
3,1,3114.463235
3,1,3713.629630
3,1,18349.331522
3,1,1869.766447
3,1,3783.855769
3,1,2747.941489
3,1,2078.441176
3,1,2710.258929
3,1,1593.546429
3,1,4103.919444
3,1,15231.500000
3,1,2171.333333
3,1,2039.000000
3,1,6668.908654
3,1,1717.521739
3,1,2810.046053
3,1,1705.588235
3,1,6782.300000
3,1,2131.723404
3,1,3242.944444
3,1,8144.000000
3,1,5970.427083
3,1,1510.705357
3,1,2582.571429
3,1,2020.728125
3,1,2410.988372
3,1,1609.760870
3,1,5097.579545
3,1,1820.794872
3,1,7019.000000
3,1,1570.633333
3,1,2245.291667
3,1,5488.263158
3,1,5614.478261
3,1,2092.108871
3,1,1682.728261
3,1,3546.070313
3,1,2636.869565

3,1,2903.578431
3,1,2140.127660
3,1,2862.266234
3,1,2396.692308
3,1,2276.932692
3,1,15142.666667
3,1,6829.420290
3,1,13068.226351
3,1,1724.250000
3,1,3239.380952
3,1,1518.270833
3,1,3423.768116
3,1,1846.649254
3,1,2448.194444
3,1,2730.704545
3,1,3484.284884
3,1,6993.487179
3,1,4328.296875
3,1,3584.661290
3,1,2101.732143
3,1,2604.209239
3,1,4650.595000
3,1,1858.264706
3,1,3526.256579
3,1,3346.021277
3,1,5164.740625
3,1,3857.923913
3,1,1785.560976
3,1,6698.417763
3,1,7444.722222
3,1,6441.184211
3,1,10226.128205
3,1,1783.106618
3,1,2344.487179
3,1,1779.470588
3,1,2684.434211
3,1,9021.550000
3,1,2386.325581
3,1,2010.757353
3,1,10464.540541
3,1,2608.511628
3,1,2112.250000
3,1,2910.857143
3,1,9670.940972
3,1,6795.133333
3,1,11766.437500
3,1,1982.625000
3,1,4870.934783
3,1,3415.370370
3,1,5882.000000
3,1,4487.742857
3,1,3382.278846
3,1,2933.404605
3,1,1662.230769
3,1,4921.534884
3,1,1904.141026
3,1,10811.363636
3,1,3506.720395
3,1,3160.380000
3,1,3766.382653
3,1,4367.850000
3,1,1746.036932
3,1,17006.631944
3,1,6055.979167
3,1,2225.564286
3,1,2004.400000

3,1,16778.966102
3,1,8220.328571
3,1,4952.441176
3,1,1967.984375
3,1,5887.041667
3,1,3363.147059
3,1,3054.942308
3,1,3255.366667
3,1,1683.400000
3,1,3787.517442
3,1,3163.574324
3,1,2935.074074
3,1,3236.297297
3,1,4617.802419
3,1,1997.000000
3,1,3836.000000
3,1,2570.388889
3,1,5979.225000
3,1,13464.766892
3,1,12150.483333
3,1,1958.256098
3,1,2644.390000
3,1,4226.272727
3,1,11239.319444
3,1,8200.643939
3,1,1955.036932
3,1,9097.353741
3,1,3038.743750
3,1,4062.202703
3,1,6479.618182
3,1,1605.325581
3,1,12233.380000
3,1,1718.055556
3,1,8351.902439
3,1,6451.923077
3,1,9726.829787
3,1,2944.400641
3,1,3956.972973
3,1,1846.220000
3,1,4854.239362
3,1,14713.301471
3,1,2234.721591
3,1,3506.670455
3,1,2158.285714
3,1,1671.857143
3,1,4415.935897
3,1,2177.432432
3,1,2143.945122
3,1,4276.500000
3,1,2424.291667
3,1,1776.308824
3,1,2733.098485
3,1,10762.522727
3,1,2174.725000
3,1,6373.228571
3,1,2011.054688
3,1,1940.875000
3,1,10092.687500
3,1,17183.841216
3,1,2136.326923
3,1,5239.270000
3,1,6287.550000
3,1,5089.424419
3,1,2873.104167
3,1,2251.815476
3,1,2063.567935

3,1,3237.312500
3,1,5194.695652
3,1,2531.206250
3,1,14635.674479
3,1,2090.204545
3,1,2127.000000
3,1,2677.664634
3,1,8897.406250
3,1,6654.573171
3,1,1995.807927
3,1,4615.166667
3,1,1917.877907
3,1,1779.425676
3,1,3458.046053
3,1,3389.443182
3,1,2947.920833
3,1,1885.454545
3,1,9566.378676
3,1,2716.243902
3,1,1949.307692
3,1,11484.058824
3,1,5166.101124
3,1,1518.780405
3,1,1979.877841
3,1,7451.390625
3,1,1727.305556
3,1,7939.805147
3,1,3910.205357
3,1,2047.159722
3,1,11336.262931
3,1,1763.825000
3,1,9562.216667
3,1,4859.714286
3,1,4311.500000
3,1,3187.953125
3,1,2615.028846
3,1,2568.328704
3,1,2647.315000
3,1,6178.209302
3,1,3033.500000
3,1,4054.500000
3,1,4918.659483
3,1,5310.933333
3,1,3193.911765
3,1,3789.527027
3,1,4783.487179
3,1,3876.564286
3,1,9929.450000
3,1,10449.826087
3,1,3151.392857
3,1,4674.263158
3,1,1988.888158
3,1,17370.236111
3,1,2529.507812
3,1,2941.321429
3,1,2603.758333
3,1,1891.036184
3,1,5147.859375
3,1,5325.572674
3,1,2331.112179
3,1,2146.147059
3,1,2778.335106
3,1,2365.266667
3,1,12630.071875
3,1,7327.368421
3,1,19526.973684

3,1,11773.705882
3,1,2752.343750
3,1,2629.250000
3,1,1515.580882
3,1,3120.505952
3,1,5777.625000
3,1,2461.015244
3,1,3683.615385
3,1,2733.214744
3,1,1768.784884
3,1,3523.596591
3,1,2117.696023
3,1,2625.600000
3,1,4116.880952
3,1,4961.326613
3,1,2267.234375
3,1,7747.050000
3,1,8394.721154
3,1,2175.928571
3,1,4251.644531
3,1,3478.192308
3,1,1913.809524
3,1,4023.223214
3,1,7490.593750
3,1,3266.646429
3,1,2139.760638
3,1,2906.008721
3,1,8436.827778
3,1,10746.422794
3,1,1558.265625
3,1,3059.279891
3,1,18130.323370
3,1,1927.914634
3,1,18127.539634
3,1,2081.756410
3,1,5775.562500
3,1,2417.325581
3,1,1686.343750
3,1,6819.939189
3,1,3935.164474
3,1,6395.284375
3,1,1620.135417
3,1,8431.000000
3,1,1938.173913
3,1,2460.744444
3,1,3293.750000
3,1,7761.062500
3,1,5106.497093
3,1,5187.125000
3,1,2499.312500
3,1,2103.365385
3,1,5836.250000
3,1,3139.183333
3,1,7158.500000
3,1,6079.666667
3,1,8714.720588
3,1,5731.092105
3,1,3050.152778
3,1,2811.779762
3,1,4043.372159
3,1,3956.340426
3,1,2813.273649
3,1,10905.538462
3,1,4521.460106
3,1,11181.013736
3,1,2825.705882

3,1,3578.496622
3,1,3298.947368
3,1,1576.193878
3,1,10893.022727
3,1,7980.580128
3,1,5578.132353
3,1,3124.000000
3,1,5336.443182
3,1,4833.851852
3,1,1509.031250
3,1,9538.162162
3,1,11584.500000
3,1,3831.269531
3,1,2179.725610
3,1,16366.550000
3,1,6887.148649
3,1,2306.888514
3,1,13460.285156
3,1,3157.625000
3,1,4357.570833
3,1,10503.504808
3,1,17383.717949
3,1,8387.461806
3,1,1932.020833
3,1,6175.633721
3,1,1719.856618
3,1,8825.494898
3,1,4102.333333
3,1,2085.900735
3,1,2162.992366
3,1,3763.678571
3,1,2430.157895
3,1,2317.454545
3,1,1829.500000
3,1,7211.687500
3,1,1628.016129
3,1,3392.191176
3,1,2092.195122
3,1,5015.263158
3,1,2704.750000
3,1,6837.625000
3,1,2928.110119
3,1,3979.916667
3,1,17062.515625
3,1,3253.857639
3,1,12846.528846
3,1,14470.377976
3,1,4526.663462
3,1,2211.524038
3,1,2339.103659
3,1,4038.365385
3,1,2925.500000
3,1,2156.461538
3,1,2115.923077
3,1,17797.805556
3,1,2045.659574
3,1,5231.000000
3,1,2701.023810
3,1,8685.769697
3,1,1727.821429
3,1,9856.534483
3,1,3745.027344
3,1,9890.343750
3,1,3781.235294
3,1,4434.562500
3,1,1562.671875

3,1,2973.744898
3,1,3135.012019
3,1,3616.907895
3,1,4567.959302
3,1,4134.662162
3,1,5161.982143
3,1,3593.096774
3,1,3313.600000
3,1,1651.228125
3,1,1519.363095
3,1,18675.209459
3,1,2276.385870
3,1,10029.434783
3,1,5087.628676
3,1,1919.157895
3,1,5612.806452
3,1,5815.350000
3,1,4524.640000
3,1,3247.195122
3,1,3493.595238
3,1,15391.860795
3,1,1875.257812
3,1,2932.066860
3,1,9391.913194
3,1,2613.959459
3,1,5994.522222
3,1,3370.503378
3,1,18489.424419
3,1,1716.831250
3,1,3386.866667
3,1,5391.102273
3,1,2546.513158
3,1,9160.012195
3,1,1577.339286
3,1,2311.382353
3,1,1763.029412
3,1,10893.695122
3,1,5738.318182
3,1,6508.041667
3,1,4273.834239
3,1,7278.136364
3,1,6165.244318
3,1,15979.461538
3,1,9411.358333
3,1,5666.002717
3,1,4961.195122
3,1,7112.909091
3,1,1583.958333
3,1,2545.559783
3,1,15468.964286
3,1,10624.500000
3,1,2886.543478
3,1,3341.690217
3,1,1924.800000
3,1,1659.818548
3,1,1889.698370
3,1,7519.801282
3,1,1796.583333
3,1,5367.100000
3,1,1716.511905
3,1,2814.562500
3,1,1606.669444
3,1,2378.500000
3,1,5751.687500
3,1,2637.210938
3,1,1507.683824

3,1,5261.841912
3,1,1643.085366
3,1,3672.787234
3,1,3723.875000
3,1,3324.028846
3,1,5228.750000
3,1,3781.194079
3,1,9037.213816
3,1,1784.375000
3,1,18097.815789
3,1,4995.368750
3,1,4283.804878
3,1,1610.715116
3,1,3255.741071
3,1,10226.125000
3,1,4570.250000
3,1,4640.000000
3,1,8770.192857
3,1,2834.963415
3,1,8168.666667
3,1,2035.476190
3,1,2166.183333
3,1,6220.997093
3,1,16049.431548
3,1,3617.534091
3,1,2726.981250
3,1,4236.469512
3,1,1556.325581
3,1,11679.344595
3,1,1924.848485
3,1,4705.900943
3,1,2156.309524
3,1,3163.558824
3,1,3760.172794
3,1,2415.121212
3,1,2637.475000
3,1,7012.268617
3,1,2346.411765
3,1,3093.220000
3,1,2287.670391
3,1,2460.750000
3,1,3644.329268
3,1,15994.570513
3,1,7325.475000
3,1,3065.237903
3,1,4178.266667
3,1,1785.058824
3,1,2677.903846
3,1,2047.196429
3,1,5489.272727
3,1,6527.582317
3,1,1539.596591
3,1,2165.780303
3,1,2302.272727
3,1,2484.253676
3,1,2046.467949
3,1,3797.104651
3,1,1517.166667
3,1,5611.416667
3,1,8020.715116
3,1,3546.250000
3,1,2594.424419
3,1,6289.676829
3,1,4454.678571
3,1,2978.385714
3,1,2863.107143

3,1,5355.217742
3,1,4849.500000
3,1,1528.041667
3,1,3341.065000
3,1,1820.852041
3,1,3747.636364
3,1,3247.125000
3,1,3920.302326
3,1,2180.264881
3,1,8552.594595
3,1,3528.807692
3,1,6578.528409
3,1,3179.473684
3,1,2317.803030
3,1,1962.294118
3,1,1792.650735
3,1,4045.714286
3,1,2351.265244
3,1,2938.448864
3,1,6199.089674
3,1,4230.617647
3,1,1927.736842
3,1,2265.255814
3,1,7806.697674
3,1,2541.975610
3,1,2115.925000
3,1,4190.825000
3,1,1800.000000
3,1,1537.825581
3,1,2761.617647
3,1,2971.514423
3,1,2830.000000
3,1,19476.547619
3,1,2870.236842
3,1,6980.288889
3,1,9748.187500
3,1,4767.021277
3,1,2944.640625
3,1,3525.113281
3,1,2685.500000
3,1,2762.625000
3,1,1502.446809
3,1,3667.444444
3,1,6502.808824
3,1,3713.707317
3,1,2186.535256
3,1,2425.451389
3,1,4254.397436
3,1,6666.106383
3,1,3168.238281
3,1,6805.500000
3,1,3711.159091
3,1,1544.738372
3,1,3670.460000
3,1,2475.040541
3,1,1701.107558
3,1,13344.109375
3,1,3230.125000
3,1,7853.584559
3,1,3022.657609
3,1,6346.856250
3,1,2159.503788
3,1,4176.337500
3,1,1524.035714
3,1,7912.416667
3,1,2543.133721

3,1,3932.506579
3,1,3996.116667
3,1,2160.625000
3,1,4081.500000
3,1,3446.073529
3,1,3719.937500
3,1,4035.659091
3,1,1815.750000
3,1,2220.221591
3,1,1954.675595
3,1,4833.625000
3,1,4333.237500
3,1,19347.375000
3,1,8757.953125
3,1,1844.476064
3,1,2526.970930
3,1,14537.867188
3,1,2930.000000
3,1,2512.917476
3,1,4370.031915
3,1,3028.714286
3,1,16120.583333
3,1,4529.750000
3,1,2945.197368
3,1,4049.266667
3,1,1509.076923
3,1,3108.263158
3,1,12487.000000
3,1,9164.323718
3,1,4580.791667
3,1,2988.689286
3,1,6850.392857
3,1,2645.468750
3,1,1557.514881
3,1,1864.353659
3,1,4892.625000
3,1,2352.800000
3,1,4213.159375
3,1,2533.184211
3,1,10179.687500
3,1,9306.396341
3,1,19076.834821
3,1,1726.941176
3,1,2093.838710
3,1,7543.908333
3,1,2442.359375
3,1,8507.537234
3,1,1837.588235
3,1,1640.125000
3,1,10344.166667
3,1,1771.000000
3,1,6110.340909
3,1,6650.034483
3,1,2383.958333
3,1,2686.847222
3,1,2160.805851
3,1,2380.750000
3,1,1967.081250
3,1,2956.460843
3,1,2207.726562
3,1,3802.285714
3,1,3847.823529
3,1,4242.578571
3,1,15542.689189
3,1,9000.554878
3,1,3259.152439

3,1,1846.109375
3,1,2138.693750
3,1,1659.401163
3,1,3261.782895
3,1,2057.383333
3,1,3744.250000
3,1,1848.068182
3,1,12110.261905
3,1,2601.789773
3,1,2615.026316
3,1,19286.294872
3,1,3361.250000
3,1,1963.524194
3,1,5180.977273
3,1,2214.347973
3,1,2872.978571
3,1,3722.073529
3,1,3136.666667
3,1,2476.132353
3,1,1616.695000
3,1,2135.896552
3,1,12585.452778
3,1,3103.479730
3,1,3267.029605
3,1,1846.737805
3,1,2053.842105
3,1,5979.823529
3,1,9680.111111
3,1,1989.960714
3,1,2715.183824
3,1,1736.642857
3,1,10005.875000
3,1,2270.081081
3,1,7646.444444
3,1,3329.883065
3,1,5508.078125
3,1,1802.905488
3,1,2470.958333
3,1,2142.788462
3,1,2642.375000
3,1,10000.234043
3,1,2625.346591
3,1,9932.017857
3,1,13035.791667
3,1,5939.347826
3,1,6322.742647
3,1,2918.273256
3,1,4646.809783
3,1,2856.354167
3,1,5549.711538
3,1,2204.288462
3,1,4063.331250
3,1,3216.242188
3,1,1866.458333
3,1,3655.157609
3,1,8794.718750
3,1,3425.716346
3,1,3001.000000
3,1,4746.836957
3,1,2016.730469
3,1,2586.213068
3,1,2753.157895
3,1,5194.022059
3,1,3952.837209
3,1,5721.463235
3,1,2425.254464

3,1,5212.113095
3,1,5516.856771
3,1,11510.280488
3,1,2095.182143
3,1,1770.909722
3,1,3589.521739
3,1,2415.225000
3,1,12382.125000
3,1,7526.718750
3,1,11715.886111
3,1,1572.713068
3,1,4249.649254
3,1,2134.294944
3,1,4689.541667
3,1,1670.291667
3,1,6637.155556
3,1,5597.250000
3,1,1995.026596
3,1,11101.272727
3,1,1892.604167
3,1,7031.846154
3,1,3749.335526
3,1,1598.513514
3,1,4055.625000
3,1,13407.304245
3,1,3646.068182
3,1,1646.065476
3,1,1642.989130
3,1,4244.256757
3,1,1718.800000
3,1,1599.781250
3,1,3151.807065
3,1,3207.125000
3,1,2287.322581
3,1,6526.175000
3,1,11937.543605
3,1,5164.139881
3,1,1883.806818
3,1,3687.296053
3,1,4680.875000
3,1,11166.190789
3,1,1669.098214
3,1,3972.633929
3,1,7394.000000
3,1,1607.898649
3,1,3010.402174
3,1,14493.515351
3,1,5218.016129
3,1,1521.809783
3,1,5681.529412
3,1,5125.437500
3,1,2928.953125
3,1,2838.760000
3,1,3028.443750
3,1,1550.262821
3,1,4803.433594
3,1,2032.500000
3,1,4289.092262
3,1,2269.832386
3,1,6221.734375
3,1,2749.205882
3,1,2142.951220
3,1,2819.712838
3,1,1511.951220
3,1,1517.863281
3,1,5413.202381

3,1,4816.625000
3,1,2173.000000
3,1,2509.875000
3,1,2204.828125
3,1,11232.890244
3,1,2548.369898
3,1,5499.534722
3,1,1980.869444
3,1,3157.687500
3,1,2159.200658
3,1,1598.882353
3,1,1714.775000
3,1,1742.809375
3,1,15663.783333
3,1,6781.307065
3,1,2809.414062
3,1,12510.553571
3,1,9686.687500
3,1,1556.666667
3,1,17717.701923
3,1,2310.019531
3,1,2824.444444
3,1,5194.666667
3,1,1669.673077
3,1,3595.723214
3,1,2785.239865
3,1,3294.866935
3,1,1663.208333
3,1,1681.682927
3,1,3333.189189
3,1,3395.597826
3,1,9593.392442
3,1,4459.347826
3,1,11858.542683
3,1,4294.421875
3,1,2351.300000
3,1,2054.982143
3,1,2507.005682
3,1,3907.875000
3,1,9883.578947
3,1,2145.247159
3,1,2729.346154
3,1,4071.291667
3,1,2434.190476
3,1,12125.193182
3,1,7527.085714
3,1,2321.758065
3,1,5942.348958
3,1,2221.114362
3,1,2762.750000
3,1,1745.635870
3,1,4661.655172
3,1,2358.243243
3,1,1541.072368
3,1,3378.261364
3,1,1768.750000
3,1,8468.787879
3,1,3548.871951
3,1,4144.452586
3,1,2387.000000
3,1,2900.461538
3,1,3122.214286
3,1,2138.620192
3,1,6519.103261
3,1,10423.073529
3,1,7451.872340

3,1,3108.106061
3,1,3507.402439
3,1,1678.973684
3,1,2517.136364
3,1,2982.833333
3,1,14228.000000
3,1,3676.500000
3,1,12871.062500
3,1,5179.160714
3,1,4808.939655
3,1,5388.545455
3,1,1649.000000
3,1,8213.863636
3,1,2814.625000
3,1,1509.252717
3,1,1565.727273
3,1,4446.156250
3,1,2254.885246
3,1,1520.031915
3,1,2936.000000
3,1,1872.434211
3,1,2678.687500
3,1,2083.896341
3,1,6562.532051
3,1,12548.540441
3,1,3571.653846
3,1,13784.926136
3,1,7675.260870
3,1,1902.214286
3,1,1902.842391
3,1,4516.722222
3,1,2674.242647
3,1,4747.767857
3,1,1933.325893
3,1,3542.181250
3,1,14071.671875
3,1,1651.234043
3,1,10563.937500
3,1,1902.979839
3,1,2468.772059
3,1,2594.915441
3,1,4064.288043
3,1,4048.243902
3,1,1521.348837
3,1,1592.769737
3,1,16929.201923
3,1,3252.306122
3,1,1680.107143
3,1,4527.364362
3,1,2354.468085
3,1,4064.604167
3,1,3222.762500
3,1,3441.664634
3,1,2719.183824
3,1,2209.924419
3,1,3797.297872
3,1,7610.171875
3,1,1974.136905
3,1,1608.513514
3,1,6422.347222
3,1,3164.797222
3,1,6737.355769
3,1,6283.512195
3,1,2575.863636
3,1,2644.721429
3,1,4868.223684

3,1,1648.000000
3,1,2061.164634
3,1,2129.272436
3,1,3398.457237
3,1,9936.000000
3,1,8529.445122
3,1,3060.371094
3,1,10229.382353
3,1,8206.648810
3,1,1889.625000
3,1,6379.513158
3,1,8113.682432
3,1,4711.149390
3,1,4416.780488
3,1,2464.548077
3,1,2964.669444
3,1,2296.250000
3,1,6804.500000
3,1,3176.760870
3,1,2925.769231
3,1,5047.937500
3,1,2988.339286
3,1,7364.586957
3,1,4651.625000
3,1,12788.463415
3,1,1722.448718
3,1,6034.472222
3,1,7007.403226
3,1,7151.324074
3,1,10987.831250
3,1,1725.829268
3,1,4226.096154
3,1,1938.727273
3,1,3602.903846
3,1,5961.100000
3,1,2023.102273
3,1,3544.271429
3,1,6690.312500
3,1,8188.017857
3,1,2028.703804
3,1,3470.400000
3,1,1872.767442
3,1,3027.272727
3,1,3060.659091
3,1,3023.829268
3,1,5554.058824
3,1,1718.661290
3,1,13259.768750
3,1,2920.540323
3,1,1535.638158
3,1,3418.297297
3,1,6364.681452
3,1,5506.945000
3,1,3614.571429
3,1,2377.962500
3,1,6010.921875
3,1,7866.523026
3,1,4305.490000
3,1,2341.630435
3,1,19035.783784
3,1,3558.394737
3,1,2729.179688
3,1,1513.918478
3,1,7638.340426
3,1,2689.109756
3,1,3663.141026

3,1,7141.815789
3,1,2730.048387
3,1,6839.855263
3,1,1776.152778
3,1,3027.449219
3,1,6500.402027
3,1,3574.028226
3,1,7399.000000
3,1,9676.170732
3,1,3376.325000
3,1,3780.227273
3,1,8891.810976
3,1,6227.365854
3,1,2381.604167
3,1,2406.718750
3,1,10067.664894
3,1,2339.605263
3,1,2696.308140
3,1,1813.158088
3,1,3987.378378
3,1,4907.401163
3,1,2435.544118
3,1,1864.361111
3,1,1988.633929
3,1,3258.158537
3,1,2918.397351
3,1,1530.347826
3,1,8256.688889
3,1,1585.863636
3,1,2082.708333
3,1,2047.857143
3,1,1651.537791
3,1,4169.762195
3,1,2384.578947
3,1,3980.916667
3,1,2276.973684
3,1,2418.614130
3,1,9517.464286
3,1,1767.608696
3,1,5223.907258
3,1,4264.598684
3,1,2166.599432
3,1,3282.321429
3,1,3002.140244
3,1,2166.632353
3,1,3136.946809
3,1,3422.452381
3,1,5449.968750
3,1,9791.588235
3,1,1897.821809
3,1,2284.312500
3,1,1811.964286
3,1,5167.455357
3,1,6804.695312
3,1,1708.790698
3,1,2999.648649
3,1,3482.951220
3,1,2301.336538
3,1,4656.227273
3,1,2894.051020
3,1,3412.250000
3,1,9198.697581
3,1,9192.197115
3,1,2347.275000
3,1,3638.668367
3,1,3254.158088

3,1,8500.232143
3,1,1693.508152
3,1,1963.540323
3,1,1647.870968
3,1,9634.836310
3,1,2541.325000
3,1,2279.684211
3,1,4821.728723
3,1,2126.208333
3,1,7095.705882
3,1,3167.819149
3,1,5107.560811
3,1,1880.687500
3,1,3087.986413
3,1,12471.671053
3,1,2284.486842
3,1,7453.548913
3,1,2167.571429
3,1,2749.200000
3,1,4441.298387
3,1,5164.661458
3,1,1542.911932
3,1,3464.992857
3,1,2514.321429
3,1,6138.041667
3,1,5057.091346
3,1,3278.419355
3,1,11213.500000
3,1,2206.440217
3,1,2894.750000
3,1,8344.344595
3,1,2059.685976
3,1,2693.878788
3,1,11992.881250
3,1,2494.875000
3,1,7647.683673
3,1,8053.570652
3,1,1630.450000
3,1,6640.531250
3,1,1934.153846
3,1,10742.062500
3,1,1657.127907
3,1,5237.733333
3,1,2079.833333
3,1,8533.500000
3,1,1675.176471
3,1,1771.964286
3,1,5014.378378
3,1,2447.586538
3,1,4329.878125
3,1,2500.653846
3,1,4484.166667
3,1,18650.750000
3,1,4028.095238
3,1,2911.807692
3,1,4984.220238
3,1,3454.766447
3,1,2124.179878
3,1,3509.325000
3,1,8941.420455
3,1,3243.959459
3,1,2661.895833
3,1,2251.080128
3,1,5529.153061
3,1,3187.919355
3,1,1939.621622

3,1,2654.154412
3,1,2966.173077
3,1,17366.055556
3,1,8220.182692
3,1,2740.000000
3,1,4659.613372
3,1,2228.546196
3,1,4437.902778
3,1,12767.585185
3,1,1954.166667
3,1,3215.750000
3,1,2343.375000
3,1,4142.142857
3,1,8607.759868
3,1,3938.339844
3,1,2238.125000
3,1,3706.113095
3,1,9117.622642
3,1,2956.334459
3,1,1759.857143
3,1,17040.600000
3,1,2761.552326
3,1,6206.625000
3,1,1719.500000
3,1,12411.266667
3,1,1554.010135
3,1,2338.818182
3,1,3810.769231
3,1,2753.436170
3,1,8678.770833
3,1,1901.467105
3,1,3138.870370
3,1,1534.589286
3,1,2049.661585
3,1,2709.714286
3,1,3320.858333
3,1,4120.062500
3,1,19689.114583
3,1,4538.633065
3,1,2724.304348
3,1,8722.384868
3,1,1973.537162
3,1,4306.402027
3,1,3726.943182
3,1,1969.676471
3,1,3809.255319
3,1,2138.080000
3,1,1859.943966
3,1,4883.416667
3,1,6958.990385
3,1,8210.034574
3,1,4229.079545
3,1,2280.166667
3,1,11092.274194
3,1,7502.525000
3,1,16805.000000
3,1,1717.698276
3,1,3757.765625
3,1,2007.095238
3,1,4297.495192
3,1,13793.241935
3,1,2303.375000
3,1,2374.968750
3,1,2633.065476
3,1,1843.375000
3,1,1712.588889

3,1,1790.635135
3,1,5313.764706
3,1,1619.007143
3,1,3489.743243
3,1,9866.000000
3,1,4264.361702
3,1,2686.639706
3,1,4792.571429
3,1,6306.260000
3,1,1839.425532
3,1,4174.621622
3,1,3925.645833
3,1,17746.448370
3,1,1786.770833
3,1,6883.711735
3,1,2031.658537
3,1,3446.153846
3,1,5901.732558
3,1,7302.552083
3,1,1715.891892
3,1,1886.512195
3,1,1699.231250
3,1,6807.284574
3,1,2717.707447
3,1,2517.312500
3,1,2894.327381
3,1,3150.617647
3,1,2561.656250
3,1,1578.000000
3,1,8573.271552
3,1,2432.418605
3,1,11572.555556
3,1,2378.500000
3,1,4405.170732
3,1,8625.377500
3,1,2053.027174
3,1,3255.723214
3,1,2138.549419
3,1,3829.781250
3,1,3328.910714
3,1,2645.890625
3,1,12796.607558
3,1,12579.258621
3,1,2344.159884
3,1,8273.047619
3,1,1818.978125
3,1,14426.835106
3,1,1647.604167
3,1,4848.833333
3,1,4525.134615
3,1,5000.676829
3,1,7329.290541
3,1,4367.097561
3,1,4393.656250
3,1,1907.470930
3,1,2582.653409
3,1,2588.315972
3,1,10578.326087
3,1,12232.237500
3,1,2324.711806
3,1,6120.743243
3,1,4856.238971
3,1,3568.595745
3,1,2218.005814
3,1,12109.277778
3,1,11035.425000

3,1,5353.185000
3,1,3557.350000
3,1,1918.138158
3,1,3599.750000
3,1,2837.750000
3,1,12822.993421
3,1,1625.133621
3,1,3612.052083
3,1,2072.146341
3,1,17632.900000
3,1,2515.432432
3,1,4483.092262
3,1,7378.597222
3,1,2376.583333
3,1,3028.851562
3,1,8540.669643
3,1,4733.319444
3,1,1654.650000
3,1,2457.622222
3,1,4264.977273
3,1,3023.666667
3,1,3157.331250
3,1,3364.988636
3,1,2174.378788
3,1,2353.367647
3,1,6062.725610
3,1,13374.553191
3,1,3708.386364
3,1,5356.771429
3,1,1710.555556
3,1,10607.102941
3,1,4142.409091
3,1,3891.000000
3,1,1865.093750
3,1,8092.650000
3,1,1607.813953
3,1,1636.462121
3,1,3593.868056
3,1,4131.432692
3,1,2350.860294
3,1,3534.500000
3,1,2271.829545
3,1,6401.595588
3,1,11488.980000
3,1,13010.969512
3,1,3557.522727
3,1,3977.882353
3,1,8637.970588
3,1,5601.117021
3,1,3430.157500
3,1,1669.384615
3,1,2977.523810
3,1,4500.580556
3,1,1966.444444
3,1,6529.468750
3,1,4217.439815
3,1,4146.695652
3,1,3649.115854
3,1,3202.076923
3,1,5526.125000
3,1,6571.263158
3,1,2140.000000
3,1,2145.205882
3,1,1595.217742
3,1,6486.736842
3,1,1909.755319

3,1,1856.177500
3,1,6175.571429
3,1,4205.839286
3,1,5237.696429
3,1,5561.812950
3,1,11127.867647
3,1,2740.639706
3,1,1539.791667
3,1,4329.436170
3,1,5817.747881
3,1,2304.287879
3,1,3836.647059
3,1,5441.375000
3,1,8420.471429
3,1,4674.087209
3,1,4279.715278
3,1,2898.051829
3,1,2206.750000
3,1,2236.809524
3,1,3712.043478
3,1,8129.815625
3,1,4783.941176
3,1,8264.674847
3,1,15657.675000
3,1,12833.607143
3,1,7132.270833
3,1,3557.839744
3,1,2536.169643
3,1,2142.082317
3,1,3790.309783
3,1,2171.146341
3,1,2036.133621
3,1,1529.313953
3,1,1692.206395
3,1,3510.767857
3,1,2854.343750
3,1,12461.000000
3,1,7271.384375
3,1,2125.527027
3,1,1596.069444
3,1,2678.772222
3,1,2713.312500
3,1,5684.420000
3,1,2180.779070
3,1,3810.270408
3,1,1797.625000
3,1,2055.306818
3,1,2997.050595
3,1,2537.517442
3,1,2190.169872
3,1,2344.305147
3,1,5023.381250
3,1,3522.750000
3,1,2292.529412
3,1,6451.500000
3,1,1775.645833
3,1,1741.147727
3,1,7521.853723
3,1,3427.714286
3,1,19669.698529
3,1,2995.605263
3,1,1931.955556
3,1,3039.552083
3,1,2703.347973
3,1,2349.401786
3,1,1862.590909

3,1,10453.246528
3,1,11746.353125
3,1,14283.178571
3,1,3211.775000
3,1,2002.218750
3,1,1685.375000
3,1,8039.992647
3,1,3028.323529
3,1,3493.762500
3,1,3476.900000
3,1,3774.415816
3,1,10700.750000
3,1,5345.682692
3,1,3836.866667
3,1,7455.486486
3,1,2709.958333
3,1,1930.435811
3,1,4052.900735
3,1,10755.676471
3,1,4317.647059
3,1,2176.448529
3,1,14095.758621
3,1,3247.500000
3,1,3427.477941
3,1,8814.799528
3,1,16780.571429
3,1,2127.856383
3,1,2943.568182
3,1,1620.326923
3,1,7012.864130
3,1,2995.571429
3,1,2562.936170
3,1,6345.633929
3,1,4777.810606
3,1,3184.244681
3,1,1563.947917
3,1,6242.753125
3,1,3567.000000
3,1,1639.071429
3,1,2895.014706
3,1,5090.423077
3,1,1894.472973
3,1,3220.755102
3,1,13270.485577
3,1,1529.005435
3,1,2082.454545
3,1,7867.041667
3,1,6652.051136
3,1,2346.814815
3,1,3833.574468
3,1,18294.382979
3,1,4089.904255
3,1,1736.722561
3,1,5113.692857
3,1,2192.475694
3,1,2520.632353
3,1,3133.062500
3,1,3220.177419
3,1,7149.181034
3,1,2713.937500
3,1,8186.640000
3,1,4820.779255
3,1,6423.525000
3,1,2112.384615
3,1,2764.218750
3,1,3723.956897

3,1,1551.560976
3,1,6819.125000
3,1,3543.468750
3,1,1732.671053
3,1,9742.860000
3,1,4655.892442
3,1,5123.475610
3,1,3061.125000
3,1,8802.165761
3,1,2627.233553
3,1,3505.310811
3,1,2358.212121
3,1,4136.276316
3,1,2479.152174
3,1,4733.000000
3,1,7791.396739
3,1,2255.806250
3,1,2844.440625
3,1,1569.581395
3,1,10621.430921
3,1,6183.463710
3,1,1903.195652
3,1,10590.495968
3,1,3401.357143
3,1,7712.800000
3,1,9688.285714
3,1,1698.736842
3,1,6156.868056
3,1,3276.097561
3,1,4902.864796
3,1,1700.180233
3,1,3974.525000
3,1,8176.945946
3,1,2796.337500
3,1,1738.271739
3,1,2491.073529
3,1,3058.881098
3,1,10548.643939
3,1,3445.132353
3,1,3330.685345
3,1,3413.037791
3,1,1913.480114
3,1,2810.250000
3,1,3473.457447
3,1,4106.650000
3,1,6195.739583
3,1,2447.158333
3,1,2259.327206
3,1,2840.807432
3,1,1612.780488
3,1,1562.022581
3,1,2163.687500
3,1,1674.159375
3,1,3441.155488
3,1,1916.423077
3,1,2744.644737
3,1,3713.596774
3,1,1603.000000
3,1,6418.000000
3,1,2346.020349
3,1,3511.770833
3,1,4110.750000
3,1,5209.000000
3,1,1541.696970
3,1,4689.510638
3,1,4066.641026

3,1,2443.117021
3,1,4300.650735
3,1,3622.307927
3,1,2352.806818
3,1,8478.726562
3,1,1503.213235
3,1,3248.058333
3,1,3084.926829
3,1,2572.915625
3,1,2071.531250
3,1,5291.793478
3,1,1702.946429
3,1,3715.400000
3,1,2055.645270
3,1,2377.877907
3,1,4023.677419
3,1,3559.810345
3,1,1994.712500
3,1,3348.250000
3,1,3606.511364
3,1,2262.750000
3,1,2900.269231
3,1,3856.660714
3,1,1501.057927
3,1,3329.511905
3,1,6170.130435
3,1,6196.453125
3,1,2450.666667
3,1,1853.089286
3,1,4677.244048
3,1,2487.957447
3,1,3211.915000
3,1,3571.940625
3,1,3543.304878
3,1,10694.743750
3,1,3641.720930
3,1,2367.666667
3,1,2065.595238
3,1,3874.454082
3,1,4741.808824
3,1,3773.375000
3,1,3471.087766
3,1,1544.079787
3,1,2082.153846
3,1,6753.771739
3,1,5988.255319
3,1,2634.384615
3,1,3698.341146
3,1,2431.921429
3,1,18099.730769
3,1,16447.004032
3,1,2112.125000
3,1,5141.955645
3,1,2764.892241
3,1,2493.848958
3,1,17761.625000
3,1,3729.260870
3,1,7416.000000
3,1,1500.597656
3,1,4708.800000
3,1,3674.046875
3,1,1738.750000
3,1,3638.866935
3,1,1793.161765
3,1,7843.716463
3,1,7507.200000

3,1,1630.476562
3,1,3492.698276
3,1,1615.335106
3,1,19545.465909
3,1,3161.157407
3,1,2082.980769
3,1,1565.345588
3,1,2014.601351
3,1,1979.602273
3,1,1840.416667
3,1,3918.675532
3,1,2917.575000
3,1,4168.468085
3,1,7754.273585
3,1,7206.529891
3,1,1613.000000
3,1,1744.234375
3,1,8183.781250
3,1,6814.296875
3,1,2184.083333
3,1,1883.519737
3,1,1636.394531
3,1,10472.203125
3,1,2966.707143
3,1,1617.462500
3,1,4564.976974
3,1,1906.709239
3,1,3172.425781
3,1,9992.256098
3,1,1644.764535
3,1,2526.647727
3,1,10959.402439
3,1,1519.602941
3,1,4430.963942
3,1,11813.668605
3,1,1773.428571
3,1,6566.743421
3,1,3264.875000
3,1,6779.921196
3,1,3012.025000
3,1,6484.850543
3,1,3219.916667
3,1,1801.500000
3,1,1507.002976
3,1,3719.355978
3,1,5093.116848
3,1,4806.229167
3,1,3830.160000
3,1,5528.050420
3,1,2770.940000
3,1,2123.200000
3,1,2748.318182
3,1,1921.592857
3,1,2357.636364
3,1,13501.973404
3,1,2641.610465
3,1,8360.588235
3,1,2488.117647
3,1,5850.656977
3,1,1919.153226
3,1,1885.104167
3,1,2080.942073
3,1,1901.763158
3,1,1559.839286
3,1,4635.518750
3,1,6135.500000

3,1,6115.203125
3,1,8094.081395
3,1,4177.309524
3,1,2410.444444
3,1,1914.815217
3,1,2828.097973
3,1,3320.006757
3,1,1554.086111
3,1,2345.673295
3,1,2029.675000
3,1,3220.968750
3,1,8147.769737
3,1,1699.538462
3,1,13054.087209
3,1,2877.590909
3,1,3206.171429
3,1,1540.343750
3,1,1684.500000
3,1,3496.916667
3,1,1745.454545
3,1,1734.426471
3,1,6077.616477
3,1,1596.744186
3,1,5040.014535
3,1,1594.565217
3,1,7054.304054
3,1,11095.394737
3,1,8494.057692
3,1,3557.533537
3,1,1837.440341
3,1,1805.282051
3,1,1567.668750
3,1,3159.205357
3,1,3301.395833
3,1,12038.697368
3,1,6592.803571
3,1,10668.416667
3,1,5089.308594
3,1,4216.294118
3,1,18471.237500
3,1,8088.547794
3,1,2198.185606
3,1,3993.195122
3,1,2126.225000
3,1,3249.244186
3,1,3283.237805
3,1,3896.558140
3,1,2809.389286
3,1,12409.121795
3,1,10183.057143
3,1,11451.375000
3,1,4738.009868
3,1,1821.166667
3,1,1687.600000
3,1,4557.965116
3,1,3707.068548
3,1,8087.681818
3,1,3335.080000
3,1,1550.280000
3,1,1950.598684
3,1,3350.762195
3,1,1739.903226
3,1,4365.202128
3,1,2707.885417
3,1,4209.064189
3,1,2283.431373

3,1,8147.783784
3,1,13123.863971
3,1,4777.826087
3,1,1936.854167
3,1,3432.287879
3,1,1604.231707
3,1,1752.250000
3,1,2429.837500
3,1,3359.674242
3,1,1822.232639
3,1,1930.271429
3,1,2006.669643
3,1,5966.725694
3,1,14457.325000
3,1,9878.711538
3,1,2261.681818
3,1,2879.296512
3,1,15159.541667
3,1,1763.000000
3,1,2891.975962
3,1,3505.375000
3,1,1857.606250
3,1,1778.912791
3,1,3591.620690
3,1,5939.120370
3,1,1591.610795
3,1,1636.077381
3,1,2105.255682
3,1,7110.194149
3,1,5063.915625
3,1,2353.117647
3,1,3104.763158
3,1,1852.579861
3,1,2896.663462
3,1,1516.290541
3,1,2176.091463
3,1,4698.911765
3,1,1690.003378
3,1,6050.566964
3,1,2299.009146
3,1,2446.719697
3,1,2648.253788
3,1,3110.825000
3,1,1599.539286
3,1,3671.125000
3,1,15245.233871
3,1,2357.260870
3,1,2588.120192
3,1,6212.217949
3,1,3622.891892
3,1,3969.202206
3,1,2178.069079
3,1,3889.845930
3,1,1948.371711
3,1,1537.789474
3,1,3410.413043
3,1,1560.390244
3,1,3281.290698
3,1,8881.847973
3,1,1752.146739
3,1,6849.181122
3,1,1850.343750
3,1,5432.970588
3,1,16664.346939
3,1,2510.024390
3,1,4576.321429

3,1,3720.538462
3,1,7424.307692
3,1,6433.434211
3,1,1798.005435
3,1,1771.021552
3,1,4126.486486
3,1,1564.292683
3,1,1577.137195
3,1,1871.146341
3,1,1523.518293
3,1,4030.713235
3,1,5637.427083
3,1,3677.427083
3,1,2000.375000
3,1,6182.083333
3,1,2186.191176
3,1,1742.169872
3,1,2816.849432
3,1,2150.398936
3,1,3884.679487
3,1,12746.895161
3,1,2635.692308
3,1,16812.253676
3,1,6893.625000
3,1,3077.941176
3,1,6778.622093
3,1,6022.354167
3,1,6893.514535
3,1,2852.859375
3,1,4648.314024
3,1,7408.170000
3,1,4313.596154
3,1,1994.000000
3,1,3723.545455
3,1,2007.639706
3,1,3360.339806
3,1,4704.375000
3,1,4091.354167
3,1,2670.635135
3,1,10385.259615
3,1,12702.961957
3,1,2094.680556
3,1,1989.736842
3,1,8732.638298
3,1,12125.637755
3,1,17092.277439
3,1,1850.736842
3,1,8849.147059
3,1,4169.669355
3,1,15317.783088
3,1,1663.656250
3,1,2237.718750
3,1,9704.923611
3,1,7023.250000
3,1,2595.022059
3,1,1649.282609
3,1,3934.650000
3,1,2202.051471
3,1,3507.922794
3,1,15919.597222
3,1,2341.884615
3,1,2391.893293
3,1,7233.750000
3,1,3057.363636
3,1,3331.371951
3,1,2687.195122

3,1,7759.750000
3,1,5684.480769
3,1,1801.080645
3,1,4394.843023
3,1,1577.739130
3,1,6111.355978
3,1,3737.089286
3,1,3282.973684
3,1,2620.272059
3,1,10776.187500
3,1,11387.997340
3,1,2226.230769
3,1,3729.149390
3,1,12837.846154
3,1,4052.106250
3,1,7084.442857
3,1,5702.052419
3,1,1561.190476
3,1,13930.571429
3,1,1817.122340
3,1,2026.636905
3,1,5945.195312
3,1,4044.669643
3,1,3169.503676
3,1,3998.457031
3,1,1579.888889
3,1,13797.580128
3,1,5591.508929
3,1,9239.223684
3,1,1709.161765
3,1,2294.295732
3,1,1762.941176
3,1,15807.625000
3,1,2695.802778
3,1,5706.666667
3,1,2736.660714
3,1,6913.346154
3,1,3670.716216
3,1,1664.517241
3,1,5425.928571
3,1,13822.150000
3,1,1750.666667
3,1,3744.810606
3,1,5873.406250
3,1,2019.544118
3,1,1993.466667
3,1,2997.918750
3,1,1968.937500
3,1,9763.550781
3,1,2398.314286
3,1,3507.846154
3,1,3080.747396
3,1,8311.703125
3,1,6517.750000
3,1,1828.428571
3,1,14455.050000
3,1,2355.602273
3,1,2181.113208
3,1,4498.423077
3,1,1956.794355
3,1,3633.231618
3,1,3086.482955
3,1,3494.662500
3,1,5103.923913
3,1,7646.895349
3,1,1857.802632

3,1,3856.761905
3,1,3115.857143
3,1,1620.986395
3,1,16495.576923
3,1,4855.943182
3,1,4496.343023
3,1,1598.992424
3,1,6616.765152
3,1,9590.110465
3,1,1821.392857
3,1,1745.640152
3,1,1693.778409
3,1,2608.608974
3,1,1696.142857
3,1,6235.914634
3,1,4610.687500
3,1,3314.681373
3,1,2501.375000
3,1,1753.508621
3,1,4721.250000
3,1,1604.736842
3,1,5273.750000
3,1,2389.011719
3,1,1507.133523
3,1,2854.233871
3,1,3816.111111
3,1,3055.903409
3,1,4335.551471
3,1,3885.297297
3,1,3137.250000
3,1,2542.066667
3,1,3201.826087
3,1,1910.918919
3,1,3017.387195
3,1,1613.281250
3,1,1578.244444
3,1,1576.682692
3,1,5114.333333
3,1,1768.008721
3,1,11458.483871
3,1,7907.735294
3,1,1528.250000
3,1,14213.934783
3,1,1544.384868
3,1,3821.759615
3,1,5785.431818
3,1,7425.250000
3,1,2537.462121
3,1,2139.750000
3,1,7894.700000
3,1,3034.993750
3,1,2827.464286
3,1,2991.500000
3,1,2988.625000
3,1,3198.043478
3,1,1529.250000
3,1,1717.101974
3,1,6475.564103
3,1,3557.084302
3,1,6090.969697
3,1,1562.672872
3,1,11227.414894
3,1,10232.487805
3,1,3981.485000
3,1,17922.962500
3,1,4127.965116

3,1,2646.203125
3,1,1915.906250
3,1,1842.762821
3,1,3989.813953
3,1,14923.046875
3,1,1639.407051
3,1,1561.878788
3,1,9365.923077
3,1,3838.875000
3,1,5065.991935
3,1,1852.755814
3,1,2037.566176
3,1,2935.314394
3,1,14541.475490
3,1,3470.725806
3,1,2124.918605
3,1,2366.263889
3,1,4965.708333
3,1,1727.897959
3,1,1968.168919
3,1,3385.798295
3,1,1893.234375
3,1,11789.273256
3,1,5290.167763
3,1,1823.904762
3,1,18828.666667
3,1,2409.567308
3,1,2459.893293
3,1,6252.272727
3,1,3183.067708
3,1,2126.318452
3,1,1761.508152
3,1,9608.425781
3,1,2582.329545
3,1,13435.872396
3,1,2482.750000
3,1,4692.757812
3,1,2955.535256
3,1,6802.650735
3,1,8655.514286
3,1,3582.013158
3,1,1751.672222
3,1,2166.878378
3,1,13725.909091
3,1,3405.608108
3,1,16866.412500
3,1,5035.250000
3,1,1715.831633
3,1,1702.328488
3,1,2026.091463
3,1,2804.407609
3,1,4035.887500
3,1,6356.215278
3,1,2250.561111
3,1,2326.939024
3,1,7042.333333
3,1,4244.678571
3,1,17329.459459
3,1,10748.300000
3,1,2073.486486
3,1,3366.950000
3,1,1809.464286
3,1,16752.765625
3,1,6626.414773
3,1,1944.069767
3,1,5386.053571

3,1,3711.724432
3,1,1665.312500
3,1,1772.125000
3,1,4745.725000
3,1,2023.134615
3,1,7134.820946
3,1,1795.755435
3,1,2391.100610
3,1,3220.531250
3,1,1597.743243
3,1,3056.811047
3,1,13354.118750
3,1,3978.666667
3,1,9866.500000
3,1,3906.942857
3,1,3145.233333
3,1,2653.884615
3,1,2078.750000
3,1,13506.689655
3,1,2142.157895
3,1,5179.824324
3,1,15047.750000
3,1,2110.560976
3,1,2937.966216
3,1,2338.411765
3,1,7789.050000
3,1,2408.328125
3,1,1617.500000
3,1,3049.798913
3,1,4692.794271
3,1,2761.253571
3,1,5946.296875
3,1,3321.946809
3,1,3454.163043
3,1,5094.727941
3,1,7103.513158
3,1,1770.213235
3,1,3865.511364
3,1,5011.392361
4,2,1555.350694
4,2,2210.730263
4,2,9856.416667
4,2,2084.571429
4,2,2186.632653
4,2,2620.101064
4,2,1864.718750
4,2,2033.287037
4,2,1541.821809
4,2,2506.855114
4,2,13282.711538
4,2,1566.838542
4,2,1669.487245
4,2,5493.913043
4,2,1690.315789
4,2,9399.738839
4,2,2375.285714
4,2,3387.413462
4,2,2337.553922
4,2,1738.695431
4,2,4465.055851
4,2,1805.833333
4,2,1624.497396
4,2,1826.000000
4,2,2252.815625
4,2,3060.274510
4,2,1517.601064

4,2,1516.016304
4,2,2377.358491
4,2,2945.034091
4,2,1602.846154
4,2,2027.500000
4,2,2862.941176
4,2,9185.800000
4,2,1676.262500
4,2,4208.092500
4,2,2021.819712
4,2,1771.955128
4,2,2734.577778
4,2,2298.545455
4,2,1874.040000
4,2,1613.952381
4,2,1850.358639
4,2,2238.177778
4,2,3471.400000
4,2,1502.104592
4,2,1945.869792
4,2,1992.967391
4,2,2639.366667
4,2,2696.254630
4,2,2006.366667
4,2,2737.700000
4,2,3508.259615
4,2,2111.750000
4,2,5216.459239
4,2,1505.054054
4,2,10777.987500
4,2,4079.957447
4,2,1695.836538
4,2,13461.765152
4,2,1769.061224
4,2,2064.230769
4,2,1788.852564
4,2,2781.198864
4,2,2985.066327
4,2,2982.243421
4,2,1916.946429
4,2,1987.983333
4,2,1984.628472
4,2,17626.589286
4,2,2113.676923
4,2,1975.264151
4,2,3051.073529
4,2,1918.068627
4,2,1874.882653
4,2,2530.041667
4,2,2650.000000
4,2,1542.326923
4,2,9149.430556
4,2,3163.941176
4,2,7513.402439
4,2,1849.335000
4,2,4413.823171
4,2,3355.750000
4,2,1562.141667
4,2,1917.273936
4,2,3177.875000
4,2,2450.362245
4,2,2176.000000
4,2,6119.250000
4,2,2399.361111
4,2,2269.814024
4,2,2002.397059

4,2,2601.808824
4,2,1601.979167
4,2,1647.418478
4,2,3939.083333
4,2,1568.137500
4,2,1774.683140
4,2,1699.370000
4,2,2619.372500
4,2,1570.476190
4,2,1818.343750
4,2,5522.212766
4,2,2228.326705
4,2,2160.439815
4,2,4699.765306
4,2,2035.000000
4,2,2051.490385
4,2,2138.690000
4,2,2377.840909
4,2,2949.370370
4,2,2837.316176
4,2,1884.850000
4,2,5650.634615
4,2,2041.437500
4,2,1572.311321
4,2,2017.750000
4,2,1734.418919
4,2,2313.050000
4,2,2050.916667
4,2,1773.789474
4,2,2579.250000
4,2,2039.425000
4,2,2018.678977
4,2,3998.548077
4,2,2191.264423
4,2,2541.599490
4,2,1567.080000
4,2,1692.712500
4,2,2777.821990
4,2,4043.586592
4,2,1775.000000
4,2,3074.890306
4,2,1674.431548
4,2,2786.752778
4,2,2211.050000
4,2,15442.270000
4,2,4228.231618
4,2,1581.587500
4,2,1962.912500
4,2,2236.531250
4,2,1824.229167
4,2,1974.591837
4,2,2828.412371
4,2,5626.550000
4,2,6550.437500
4,2,2431.375000
4,2,2108.090909
4,2,2298.062500
4,2,1573.183333
4,2,1965.276961
4,2,2411.134434
4,2,1675.579545
4,2,1647.357143
4,2,6779.779167
4,2,2649.393750
4,2,5366.101852
4,2,3319.816489

4,2,1862.264706
4,2,1935.053571
4,2,8075.225000
4,2,1888.527273
4,2,1935.140625
4,2,8032.814655
4,2,2225.920000
4,2,3517.178571
4,2,1812.243902
4,2,1988.760870
4,2,4271.866071
4,2,1631.038043
4,2,2163.598039
4,2,1554.923780
4,2,2471.331395
4,2,1523.166667
4,2,18540.421053
4,2,2662.400000
4,2,1877.350000
4,2,1575.164634
4,2,2179.000000
4,2,5318.235294
4,2,3072.888889
4,2,2592.242021
4,2,2693.732558
4,2,3662.728261
4,2,4915.638889
4,2,2750.750000
4,2,2051.227941
4,2,2907.093750
4,2,3071.364362
4,2,3788.427632
4,2,2211.641026
4,2,1509.252500
4,2,1704.689189
4,2,2734.695122
4,2,1642.781250
4,2,1782.850000
4,2,3152.065625
4,2,1925.278302
4,2,1861.846698
4,2,1529.073529
4,2,2312.041667
4,2,2836.841346
4,2,1605.573529
4,2,2605.691176
4,2,4873.100000
4,2,1573.800676
4,2,15184.117347
4,2,3311.000000
4,2,10564.666667
4,2,2211.847222
4,2,1744.125000
4,2,4242.559701
4,2,1513.775000
4,2,1990.333333
4,2,1922.771341
4,2,2345.075000
4,2,3069.396277
4,2,2085.375000
4,2,8651.828488
4,2,2443.828947
4,2,5695.840517
4,2,1507.982051
4,2,1513.429348
4,2,2248.148148

4,2,6964.387755
4,2,2279.025000
4,2,2291.085938
4,2,4548.159483
4,2,2376.428571
4,2,2222.771429
4,2,1900.449468
4,2,2053.832386
4,2,4514.336898
4,2,6205.700000
4,2,1975.839286
4,2,1782.900662
4,2,2628.080214
4,2,2593.036458
4,2,2923.974359
4,2,1942.769608
4,2,4717.515625
4,2,2553.247340
4,2,1789.936170
4,2,3788.116279
4,2,1533.504032
4,2,5253.207317
4,2,3137.579545
4,2,1693.838028
4,2,2784.875000
4,2,1821.727941
4,2,1607.690476
4,2,6278.436047
4,2,6224.064732
4,2,1521.375000
4,2,1728.414474
4,2,1895.000000
4,2,2775.750000
4,2,2958.844595
4,2,3178.829268
4,2,1961.347656
4,2,2003.845000
4,2,1938.546512
4,2,2607.069767
4,2,1757.745192
4,2,2731.375000
4,2,2313.018868
4,2,1966.791667
4,2,2461.062500
4,2,5430.042857
4,2,3511.588415
4,2,3549.406915
4,2,1627.364130
4,2,2534.902439
4,2,3123.340278
4,2,2724.042553
4,2,3800.437500
4,2,2439.578947
4,2,1791.875000
4,2,2024.645408
4,2,1499.703125
4,2,1880.510000
4,2,1702.054545
4,2,16818.609375
4,2,1755.750000
4,2,5490.630435
4,2,2570.687500
4,2,1618.756098
4,2,1678.170732
4,2,2353.500000
4,2,1908.195312

4,2,2026.552632
4,2,5416.798611
4,2,1526.605263
4,2,1634.870370
4,2,1891.234973
4,2,3200.936170
4,2,2151.015625
4,2,2680.507075
4,2,1807.056250
4,2,3455.150943
4,2,2035.081731
4,2,4881.048780
4,2,2427.982143
4,2,1556.174242
4,2,2457.450980
4,2,1527.000000
4,2,2026.966463
4,2,1788.365000
4,2,7281.322581
4,2,1757.111111
4,2,2597.510417
4,2,12876.842105
4,2,3225.546512
4,2,2009.889706
4,2,2422.285000
4,2,2329.032787
4,2,3643.052326
4,2,2633.511628
4,2,1578.000000
4,2,4369.048295
4,2,1866.003676
4,2,1640.734848
4,2,10326.757576
4,2,1957.423077
4,2,2523.981132
4,2,1556.070652
4,2,4781.675000
4,2,2684.182692
4,2,3050.806122
4,2,1900.453846
4,2,2121.377976
4,2,1947.535000
4,2,2221.166667
4,2,2849.047619
4,2,3039.899123
4,2,2333.276596
4,2,2156.833333
4,2,2010.432692
4,2,1602.141667
4,2,1626.875000
4,2,1816.445652
4,2,3079.475000
4,2,1636.684211
4,2,1786.471154
4,2,9066.848684
4,2,2385.756579
4,2,3652.600000
4,2,1613.348214
4,2,1609.898256
4,2,2137.776596
4,2,1732.222561
4,2,1671.442708
4,2,1602.741848
4,2,16687.796053
4,2,1971.872396
4,2,3575.085106

4,2,3700.095588
4,2,8011.794118
4,2,3727.719388
4,2,2269.602273
4,2,2044.267045
4,2,2691.862500
4,2,2175.908537
4,2,1566.187500
4,2,2913.428191
4,2,1798.500000
4,2,1685.750000
4,2,3367.804878
4,2,2037.193359
4,2,2687.494792
4,2,2132.831897
4,2,6522.614865
4,2,1665.807065
4,2,1669.634868
4,2,2950.548780
4,2,8633.601695
4,2,1947.276316
4,2,2409.171053
4,2,2315.492021
4,2,1819.666667
4,2,1811.861702
4,2,5252.433673
4,2,2183.053922
4,2,1984.319149
4,2,2049.736702
4,2,3386.622159
4,2,1516.923077
4,2,2040.312500
4,2,4765.769231
4,2,1621.975000
4,2,4089.932065
4,2,1635.974432
4,2,3846.182432
4,2,2584.375000
4,2,1614.832386
4,2,1823.517857
4,2,4559.738636
4,2,1587.866477
4,2,1683.122283
4,2,2925.132353
4,2,1900.576531
4,2,6162.107143
4,2,1608.233553
4,2,2558.789474
4,2,4128.063830
4,2,13228.215909
4,2,2669.319444
4,2,1660.356383
4,2,3567.704545
4,2,3472.918605
4,2,3839.747768
4,2,1774.122642
4,2,3409.046512
4,2,1836.600000
4,2,14318.527778
4,2,1727.427083
4,2,6157.319797
4,2,1824.150000
4,2,3510.090909
4,2,1780.750000
4,2,1842.625000
4,2,1704.359756

4,2,1499.608173
4,2,1795.448370
4,2,1688.991071
4,2,1731.340426
4,2,2386.595745
4,2,2980.125749
4,2,5732.506250
4,2,2715.515306
4,2,2378.904545
4,2,1625.067901
4,2,2247.941176
4,2,6513.410714
4,2,2248.918919
4,2,2201.168750
4,2,1731.814904
4,2,5437.476974
4,2,1617.440789
4,2,14542.250000
4,2,1797.014706
4,2,1893.186321
4,2,2559.481481
4,2,1739.346354
4,2,9297.906250
4,2,1630.631068
4,2,2059.915761
4,2,1701.754386
4,2,4627.141026
4,2,4084.462500
4,2,5213.250000
4,2,1637.947368
4,2,1966.250000
4,2,1504.770833
4,2,3006.840426
4,2,2488.284091
4,2,2238.451087
4,2,1859.239130
4,2,1501.721311
4,2,3191.368421
4,2,3756.333333
4,2,2516.214286
4,2,1804.634146
4,2,1518.621094
4,2,2293.009804
4,2,2466.104167
4,2,3829.190000
4,2,1506.834906
4,2,2215.275000
4,2,1820.105263
4,2,1626.648437
4,2,2521.212766
4,2,2527.128378
4,2,2290.882576
4,2,2345.234043
4,2,5719.461538
4,2,3886.645455
4,2,5208.981132
4,2,1557.361702
4,2,2397.520000
4,2,2945.301887
4,2,12333.058824
4,2,1786.934211
4,2,7655.274390
4,2,3117.540541
4,2,2253.939189
4,2,2542.058696
4,2,3023.840000

4,2,1622.211538
4,2,1911.270833
4,2,1934.218750
4,2,1835.127551
4,2,6090.322115
4,2,1695.083333
4,2,1709.039062
4,2,1819.228125
4,2,2543.795000
4,2,3218.108553
4,2,2975.250000
4,2,6619.097458
4,2,1853.528646
4,2,3133.032895
4,2,2997.169118
4,2,2236.312500
4,2,2214.454545
4,2,2490.295000
4,2,1516.215116
4,2,3243.020408
4,2,3419.404762
4,2,2290.583333
4,2,2869.391304
4,2,3183.447368
4,2,2048.569079
4,2,2206.205882
4,2,1572.992857
4,2,2297.110795
4,2,3021.032258
4,2,1551.297872
4,2,2301.282609
4,2,2763.070513
4,2,3485.704861
4,2,1853.247549
4,2,1819.296053
4,2,2383.428571
4,2,1893.333333
4,2,2470.828125
4,2,1912.829787
4,2,7104.292683
4,2,2841.062500
4,2,4521.307692
4,2,1690.285714
4,2,2578.381250
4,2,5872.788462
4,2,3626.777778
4,2,2316.562500
4,2,1539.039773
4,2,2606.723684
4,2,1512.318182
4,2,2126.785000
4,2,1547.592262
4,2,2285.789474
4,2,7872.404762
4,2,1783.285714
4,2,3963.048611
4,2,1649.013514
4,2,2305.860465
4,2,2280.086957
4,2,3005.382653
4,2,2339.351064
4,2,7155.793860
4,2,4475.875000
4,2,1819.966667
4,2,1869.397727
4,2,2124.306122

4,2,2393.010417
4,2,3018.037037
4,2,12409.416667
4,2,3369.540816
4,2,1848.724490
4,2,2169.073171
4,2,16406.275701
4,2,2224.023585
4,2,2066.336957
4,2,10923.302326
4,2,2150.895349
4,2,1562.250000
4,2,1656.595930
4,2,1927.656250
4,2,2103.445652
4,2,3390.236364
4,2,2132.881757
4,2,1894.958333
4,2,2812.383523
4,2,1525.625000
4,2,3157.821429
4,2,1620.654762
4,2,2539.755319
4,2,1833.166667
4,2,2017.195122
4,2,1735.375000
4,2,1624.454545
4,2,1505.312500
4,2,1691.223214
4,2,3021.011111
4,2,2536.450000
4,2,1631.210938
4,2,8393.598039
4,2,2475.482500
4,2,3466.451220
4,2,2124.000000
4,2,1728.765625
4,2,2407.409091
4,2,2809.680851
4,2,1597.346875
4,2,4360.935976
4,2,1619.703488
4,2,2707.914062
4,2,4989.571429
4,2,1598.400000
4,2,1535.948718
4,2,5211.795213
4,2,1870.000000
4,2,2614.422872
4,2,2037.295833
4,2,1593.279070
4,2,2062.157895
4,2,1501.138158
4,2,4326.947917
4,2,1597.276042
4,2,2154.201923
4,2,2261.080000
4,2,2138.294118
4,2,4153.666667
4,2,1586.125000
4,2,2146.774510
4,2,1829.174342
4,2,1983.281250
4,2,2955.604592
4,2,1573.285714
4,2,3446.705357

4,2,4073.725610
4,2,4393.768293
4,2,1851.898437
4,2,2354.530488
4,2,1690.637755
4,2,1517.463816
4,2,3057.803571
4,2,3920.213592
4,2,3017.250000
4,2,1682.625000
4,2,1670.980392
4,2,4611.785326
4,2,2167.664474
4,2,2295.216981
4,2,2566.137500
4,2,1814.792553
4,2,1933.485119
4,2,2127.673611
4,2,1678.085106
4,2,2287.341463
4,2,2926.454545
4,2,1961.047727
4,2,2373.089286
4,2,1552.806122
4,2,2287.098485
4,2,2776.187500
4,2,5738.944444
4,2,1636.834135
4,2,3410.343750
4,2,5138.330000
4,2,1608.037500
4,2,6813.449074
4,2,1767.002404
4,2,2794.166667
4,2,2993.610169
4,2,2694.161290
4,2,1634.509615
4,2,2118.665541
4,2,2635.242424
4,2,2176.541436
4,2,4534.462500
4,2,1882.773256
4,2,9368.215686
4,2,6127.991279
4,2,5374.195312
4,2,5347.227273
4,2,7505.713235
4,2,1998.002717
4,2,2008.734694
4,2,1987.461538
4,2,3845.377604
4,2,1909.020000
4,2,2556.812500
4,2,1754.056818
4,2,3550.404762
4,2,1522.921569
4,2,1590.712838
4,2,2121.539216
4,2,1872.060811
4,2,4765.989865
4,2,2511.818182
4,2,1774.216667
4,2,1616.398148
4,2,1947.027439
4,2,2379.507500
4,2,5198.155172

4,2,2225.012755
4,2,2178.518519
4,2,6882.425676
4,2,2941.792453
4,2,3155.216463
4,2,2654.375000
4,2,4029.744318
4,2,2579.380952
4,2,1592.500000
4,2,1834.481707
4,2,2277.932500
4,2,4123.405488
4,2,2313.034314
4,2,1989.880435
4,2,2076.535000
4,2,1623.324519
4,2,1706.116071
4,2,1802.515000
4,2,7557.050532
4,2,2458.714286
4,2,1716.431250
4,2,1528.585366
4,2,1873.223214
4,2,1694.317073
4,2,1825.723404
4,2,2231.625000
4,2,1663.218750
4,2,8267.971591
4,2,1691.344828
4,2,1732.600000
4,2,2671.294118
4,2,2409.750000
4,2,7166.599265
4,2,3433.635417
4,2,2669.907143
4,2,2409.225108
4,2,1904.358974
4,2,3155.000000
4,2,1610.747748
4,2,2660.405556
4,2,7365.000000
4,2,2388.142045
4,2,1720.250000
4,2,2333.812500
4,2,1596.075658
4,2,2123.722826
4,2,1861.188776
4,2,1695.986842
4,2,1668.887179
4,2,1629.612565
4,2,1919.833333
4,2,2412.372340
4,2,2016.330645
4,2,2946.521552
4,2,4203.250000
4,2,3415.747093
4,2,5327.715517
4,2,2001.441667
4,2,1505.215517
4,2,19481.058824
4,2,2166.414894
4,2,2969.030172
4,2,1738.355000
4,2,4333.052326
4,2,2072.844828
4,2,1909.511029

4,2,4743.438889
4,2,2037.000000
4,2,1988.769231
4,2,2897.906250
4,2,2588.500000
4,2,2838.527027
4,2,1755.681818
4,2,1564.971591
4,2,1764.680851
4,2,1552.781250
4,2,1899.406915
4,2,1741.150568
4,2,1824.990385
4,2,2264.730000
4,2,2504.788194
4,2,2660.764205
4,2,1781.145833
4,2,1598.706522
4,2,1833.781818
4,2,1941.530000
4,2,2973.721429
4,2,3372.182692
4,2,2007.904891
4,2,3764.271605
4,2,2012.361702
4,2,7044.992647
4,2,1612.372340
4,2,1775.039474
4,2,5059.078125
4,2,2473.103774
4,2,2274.783784
4,2,2646.943452
4,2,1503.077381
4,2,4733.285714
4,2,3040.512821
4,2,2744.957500
4,2,3425.823529
4,2,1841.237500
4,2,1800.911765
4,2,1990.115854
4,2,5119.687500
4,2,1700.747449
4,2,7654.655000
4,2,3042.607143
4,2,2075.437500
4,2,1788.687500
4,2,2910.363636
4,2,2089.250000
4,2,1758.835366
4,2,1700.526316
4,2,2085.949324
4,2,2909.428191
4,2,1622.220930
4,2,4381.338542
4,2,2639.546875
4,2,1800.350877
4,2,1548.613095
4,2,2440.517391
4,2,2771.128947
4,2,4177.544737
4,2,1732.459135
4,2,2012.183824
4,2,1551.594828
4,2,1958.571429
4,2,2621.036458
4,2,2569.625000

4,2,7232.285714
4,2,3758.276786
4,2,5749.897727
4,2,1925.298780
4,2,1523.730000
4,2,3563.677326
4,2,1562.442857
4,2,2024.441406
4,2,2664.985000
4,2,1718.304878
4,2,2895.782051
4,2,4429.820833
4,2,1642.078947
4,2,1895.593137
4,2,1961.630435
4,2,5134.727273
4,2,2163.778571
4,2,2259.722222
4,2,4433.041667
4,2,1891.027778
4,2,5837.168981
4,2,4098.238342
4,2,1524.477564
4,2,2020.338415
4,2,1930.164474
4,2,4481.781915
4,2,2463.617925
4,2,2837.111111
4,2,1769.522624
4,2,1921.159884
4,2,4279.784615
4,2,1821.843750
4,2,1637.438889
4,2,1536.500000
4,2,1583.928571
4,2,2834.825000
4,2,6810.828571
4,2,2306.395455
4,2,2972.962766
4,2,1499.668605
4,2,2211.285714
4,2,2177.222222
4,2,2019.233108
4,2,2259.597656
4,2,2154.570048
4,2,2045.254717
4,2,2373.731383
4,2,5677.659314
4,2,7833.102941
4,2,2464.255814
4,2,1729.167500
4,2,1772.255102
4,2,5297.307692
4,2,1966.416667
4,2,6887.655172
4,2,5741.398936
4,2,3648.000000
4,2,1585.366379
4,2,1655.343750
4,2,2538.171053
4,2,2422.393617
4,2,4077.325000
4,2,7871.036932
4,2,2324.500000
4,2,3378.044643
4,2,6098.485849

4,2,2229.903061
4,2,2985.259259
4,2,1770.343750
4,2,2374.976190
4,2,1911.007500
4,2,3356.800000
4,2,1600.082386
4,2,2395.587838
4,2,1798.175000
4,2,1758.575758
4,2,1501.671875
4,2,2827.740741
4,2,1744.810976
4,2,1578.125000
4,2,1954.765957
4,2,2837.083333
4,2,2806.668182
4,2,1627.857143
4,2,8251.422819
4,2,3048.167614
4,2,1684.971875
4,2,1782.293981
4,2,2613.462209
4,2,1622.339623
4,2,1952.719512
4,2,2260.977941
4,2,1574.791667
4,2,6268.218750
4,2,7521.906250
4,2,2097.072917
4,2,3242.370614
4,2,2085.620098
4,2,2220.157895
4,2,1887.193182
4,2,2344.740000
4,2,3903.464286
4,2,1978.980769
4,2,2325.194444
4,2,1722.375000
4,2,1667.256098
4,2,2319.500000
4,2,2327.953125
4,2,1574.639423
4,2,2465.585106
4,2,3898.089286
4,2,5106.875000
4,2,1715.375000
4,2,1808.481132
4,2,1520.937500
4,2,2089.125000
4,2,1882.750000
4,2,2201.079861
4,2,3922.072727
4,2,3669.312500
4,2,1705.869318
4,2,6298.055147
4,2,2007.962264
4,2,1656.296512
4,2,6661.896739
4,2,1936.909091
4,2,1639.650602
4,2,2886.247768
4,2,1964.652083
4,2,2141.972561
4,2,5373.783898
4,2,2847.812500

4,2,1989.457500
4,2,3586.440678
4,2,2214.687500
4,2,7517.451923
4,2,16002.261364
4,2,1920.189189
4,2,1516.000000
4,2,16255.212963
4,2,2115.307692
4,2,1751.777542
4,2,1719.293478
4,2,1555.695833
4,2,1523.909091
4,2,1821.799020
4,2,3068.000000
4,2,2003.647059
4,2,2101.142157
4,2,5068.788265
4,2,1782.500000
4,2,9451.138393
4,2,2278.765625
4,2,3029.561224
4,2,1605.406863
4,2,1574.546875
4,2,2433.617021
4,2,4656.306122
4,2,2330.285714
4,2,3949.321429
4,2,2359.743590
4,2,2836.062500
4,2,1537.687500
4,2,3304.843750
4,2,4230.866477
4,2,5600.288462
4,2,2247.708333
4,2,2427.206897
4,2,2815.187500
4,2,2260.630000
4,2,4755.875000
4,2,2042.303571
4,2,1798.785326
4,2,2079.648026
4,2,2652.538462
4,2,2315.962264
4,2,2501.255814
4,2,2619.828804
4,2,1545.889535
4,2,3817.528302
4,2,1663.361702
4,2,2038.826087
4,2,2108.055233
4,2,2379.625000
4,2,2011.151596
4,2,2340.040816
4,2,2396.735849
4,2,1642.086957
4,2,3757.615385
4,2,2493.947917
4,2,1856.647727
4,2,2694.209302
4,2,4114.148649
4,2,1575.634146
4,2,2752.707547
4,2,2451.375000
4,2,3154.404255
4,2,2722.413043

4,2,1660.747340
4,2,2215.966667
4,2,1836.830357
4,2,2079.053191
4,2,1849.970588
4,2,1714.575000
4,2,4137.732143
4,2,1754.877907
4,2,2535.423469
4,2,1577.800000
4,2,1924.601064
4,2,2551.378049
4,2,2130.381410
4,2,3987.958333
4,2,2137.150641
4,2,1868.632979
4,2,1714.570312
4,2,9421.071970
4,2,1600.298883
4,2,2123.893229
4,2,1659.586735
4,2,1716.800000
4,2,9449.611650
4,2,2553.348837
4,2,1582.615625
4,2,1875.791667
4,2,1737.594444
4,2,2515.655405
4,2,2357.422222
4,2,9397.647727
4,2,1735.600000
4,2,1994.916667
4,2,5147.812500
4,2,1883.566845
4,2,2060.846154
4,2,2814.166667
4,2,1861.423077
4,2,2253.647059
4,2,1886.659091
4,2,1511.875000
4,2,3602.220588
4,2,1895.684211
4,2,3378.819149
4,2,2979.322917
4,2,2225.135135
4,2,2187.718023
4,2,6504.020000
4,2,2118.569444
4,2,1523.000000
4,2,1616.058824
4,2,1690.520833
4,2,2222.535714
4,2,2345.122500
4,2,1879.356618
4,2,1845.627404
4,2,3684.943878
4,2,2081.476190
4,2,2162.157895
4,2,2605.625000
4,2,5994.533333
4,2,1828.819853
4,2,1520.387097
4,2,3488.402778
4,2,1851.406250
4,2,1636.535714
4,2,1745.311111

4,2,2413.500000
4,2,1805.916667
4,2,2969.888889
4,2,2514.914286
4,2,2830.616071
4,2,2295.924623
4,2,1673.680000
4,2,2143.898026
4,2,1915.847561
4,2,1787.961538
4,2,2005.325658
4,2,1867.750000
4,2,2610.717105
4,2,2101.507353
4,2,1703.421512
4,2,3320.400000
4,2,2263.204545
4,2,1555.860294
4,2,2170.359375
4,2,1810.326705
4,2,2711.149554
4,2,1872.630952
4,2,5103.993243
4,2,3007.984694
4,2,7983.272727
4,2,2526.431122
4,2,2198.155660
4,2,1499.792614
4,2,2299.536585
4,2,1799.652778
4,2,2072.277778
4,2,1795.689474
4,2,5825.464286
4,2,5606.552632
4,2,4762.640957
4,2,3206.119048
4,2,2890.901961
4,2,1837.910256
4,2,2104.365385
4,2,1854.447917
4,2,1817.065217
4,2,2678.872024
4,2,2545.534314
4,2,1817.625000
4,2,5861.673469
4,2,2355.276442
4,2,1702.636364
4,2,2615.105263
4,2,2310.051724
4,2,8435.444444
4,2,3695.785000
4,2,2335.897059
4,2,1550.954082
4,2,1643.724138
4,2,3272.361702
4,2,2319.144444
4,2,1541.290441
4,2,5061.038265
4,2,5515.512821
4,2,3474.344388
4,2,1528.540761
4,2,1786.162162
4,2,3542.187500
4,2,1584.189103
4,2,1807.980769
4,2,2538.118590

4,2,5071.680556
4,2,1939.458333
4,2,1920.000000
4,2,6831.050532
4,2,1499.437500
4,2,2678.941860
4,2,1716.104167
4,2,1885.437500
4,2,1687.301724
4,2,1531.925532
4,2,2079.936047
4,2,5741.454545
4,2,1600.716981
4,2,3877.025000
4,2,2401.745000
4,2,2942.627358
4,2,3022.310345
4,2,2342.593750
4,2,1904.222222
4,2,2860.500000
4,2,1808.447917
4,2,1802.115625
4,2,1658.708333
4,2,1776.556604
4,2,1735.500000
4,2,3334.348837
4,2,1539.347222
4,2,3147.731707
4,2,2726.413043
4,2,5092.068182
4,2,2898.809783
4,2,6280.319149
4,2,1931.675000
4,2,1628.648649
4,2,8518.136905
4,2,1736.500000
4,2,1563.546875
4,2,2049.276786
4,2,1735.937500
4,2,1637.891304
4,2,6105.885135
4,2,1932.471311
4,2,3766.806452
4,2,2460.593750
4,2,3731.327778
4,2,9130.750000
4,2,1533.255319
4,2,3418.965278
4,2,1767.477778
4,2,1789.663043
4,2,1708.177778
4,2,2738.923077
4,2,2358.197368
4,2,2350.776744
4,2,2079.480769
4,2,3102.915094
4,2,1882.588235
4,2,1976.557927
4,2,3244.048780
4,2,1791.342949
4,2,1704.821429
4,2,3203.068182
4,2,2146.138298
4,2,1903.000000
4,2,1685.893519
4,2,5839.898585

4,2,1637.538462
4,2,2553.455556
4,2,3236.412500
4,2,2991.220588
4,2,2511.965278
4,2,2879.875000
4,2,1778.005319
4,2,1578.367347
4,2,1534.156915
4,2,2981.227564
4,2,1586.347826
4,2,3814.500000
4,2,2108.011111
4,2,4455.901163
4,2,2834.951923
4,2,1688.500000
4,2,1696.198113
4,2,2096.438679
4,2,2255.957895
4,2,1865.502907
4,2,2823.107955
4,2,2449.155556
4,2,1651.382500
4,2,3529.278509
4,2,2778.557692
4,2,2152.729592
4,2,1617.995283
4,2,2335.464286
4,2,1682.456081
4,2,1563.855263
4,2,1504.940000
4,2,2269.820122
4,2,1637.902778
4,2,2459.604651
4,2,3748.502717
4,2,1929.404255
4,2,1602.930921
4,2,1728.276596
4,2,1754.000000
4,2,1949.598039
4,2,2391.687500
4,2,3067.670213
4,2,1514.792683
4,2,1924.969697
4,2,4384.430233
4,2,2668.444444
4,2,1982.657895
4,2,1694.579545
4,2,2887.491279
4,2,2412.991150
4,2,3072.898585
4,2,1577.350000
4,2,2504.196809
4,2,2118.285714
4,2,1596.250000
4,2,1593.719444
4,2,1843.335938
4,2,1840.242647
4,2,2685.933333
4,2,2062.525568
4,2,1943.574468
4,2,3991.090164
4,2,1634.416667
4,2,1960.799342
4,2,1738.443878
4,2,2606.736111

4,2,2291.861111
4,2,1824.803279
4,2,1618.056250
4,2,2383.941176
4,2,1918.130102
4,2,1837.442308
4,2,1707.544444
4,2,1801.930233
4,2,4678.011628
4,2,1920.497685
4,2,2953.165541
4,2,1671.295732
4,2,2433.025000
4,2,1977.825581
4,2,5709.235795
4,2,2091.031250
4,2,3971.462838
4,2,4200.580645
4,2,2236.050000
4,2,3936.207447
4,2,1499.901316
4,2,1977.293478
4,2,17732.835227
4,2,1631.585366
4,2,2914.285714
4,2,2962.494681
4,2,2038.233796
4,2,1806.830769
4,2,1705.625000
4,2,2420.818182
4,2,1796.800000
4,2,2180.800000
4,2,2400.334239
4,2,1614.918919
4,2,2452.177632
4,2,2269.281407
4,2,2373.329787
4,2,2460.541916
4,2,3101.579710
4,2,18841.979167
4,2,2444.465116
4,2,2003.578431
4,2,2482.895000
4,2,9291.680851
4,2,2194.322917
4,2,1689.934375
4,2,1530.093750
4,2,4617.800532
4,2,14781.337500
4,2,1551.960938
4,2,2978.400000
4,2,13832.557143
4,2,1815.712500
4,2,2522.346154
4,2,4983.937500
4,2,2103.812500
4,2,1556.466667
4,2,1515.296296
4,2,12652.776596
4,2,1730.666667
4,2,2374.500000
4,2,2058.416667
4,2,2045.000000
4,2,1820.566327
4,2,6971.442073
4,2,1930.802326

4,2,2062.217105
4,2,5366.628571
4,2,2379.085937
4,2,4139.119186
4,2,1559.285714
4,2,1980.615385
4,2,3764.946429
4,2,3363.819712
4,2,1630.460227
4,2,9928.612745
4,2,3707.250000
4,2,2762.218750
4,2,19405.113636
4,2,16036.218750
4,2,1960.666667
4,2,1608.462963
4,2,2281.238095
4,2,4808.148810
4,2,1913.178125
4,2,7879.051020
4,2,1501.135135
4,2,2608.826531
4,2,13460.195122
4,2,1724.165541
4,2,1971.458333
4,2,1505.333333
4,2,2314.853125
4,2,3937.460648
4,2,2357.000000
4,2,1811.000000
4,2,9728.314516
4,2,2066.242021
4,2,1763.000000
4,2,3338.798913
4,2,7843.680851
4,2,2322.062500
4,2,5677.027174
4,2,2004.806604
4,2,1871.760000
4,2,3726.540000
4,2,2699.244681
4,2,6384.370833
4,2,2844.018293
4,2,1970.102273
4,2,15437.470588
4,2,1585.803241
4,2,7997.109756
4,2,1965.886364
4,2,11786.388393
4,2,4459.488636
4,2,1911.829268
4,2,2533.022500
4,2,1537.215116
4,2,6705.156250
4,2,4758.977564
4,2,1639.194444
4,2,2234.938953
4,2,4874.306373
4,2,4976.218543
4,2,5658.981132
4,2,1759.109649
4,2,1883.996622
4,2,3112.580000
4,2,5724.407328
4,2,2053.796512
4,2,4681.908482

4,2,19749.402439
4,2,2659.737805
4,2,1922.100000
4,2,1663.241477
4,2,4096.046512
4,2,1715.270833
4,2,1609.687500
4,2,2718.723684
4,2,1943.325581
4,2,1925.163043
4,2,1508.537975
4,2,3744.245283
4,2,5063.976190
4,2,1698.552326
4,2,1626.875000
4,2,1860.906250
4,2,2658.395000
4,2,10924.165323
4,2,1731.827128
4,2,3116.833333
4,2,5446.357143
4,2,3150.554054
4,2,2615.363636
4,2,2681.346154
4,2,2638.414474
4,2,2068.439698
4,2,1643.375000
4,2,2838.031250
4,2,1629.605263
4,2,1577.127119
4,2,5090.784091
4,2,2119.818182
4,2,1721.672131
4,2,2118.932292
4,2,1923.078947
4,2,1749.462963
4,2,2147.918367
4,2,6140.800000
4,2,3242.122596
4,2,1549.038462
4,2,2810.704545
4,2,7381.818750
4,2,1640.051630
4,2,1858.537500
4,2,2088.046875
4,2,1790.015957
4,2,3355.187500
4,2,1843.407143
4,2,8060.064815
4,2,1691.280303
4,2,1805.272727
4,2,2316.355769
4,2,8633.397059
4,2,2831.767857
4,2,13123.708333
4,2,1602.662791
4,2,3323.187500
4,2,1594.037736
4,2,2747.295455
4,2,2248.985849
4,2,2393.222222
4,2,4642.375000
4,2,3495.296196
4,2,1745.087209
4,2,2239.229911
4,2,1929.522727

4,2,1720.645349
4,2,2108.735294
4,2,2174.725962
4,2,2883.775000
4,2,1825.166667
4,2,1584.022436
4,2,1579.527174
4,2,2438.271875
4,2,1766.485465
4,2,5649.157895
4,2,2648.045455
4,2,1883.166667
4,2,2742.011719
4,2,3820.892857
4,2,1686.687500
4,2,3169.941176
4,2,2128.000000
4,2,5310.604545
4,2,12597.719595
4,2,2490.279891
4,2,9825.642045
4,2,1920.902439
4,2,2734.677273
4,2,1987.113889
4,2,1499.678571
4,2,1610.464286
4,2,2034.769231
4,2,1847.011364
4,2,1806.866667
4,2,2099.468750
4,2,1657.894231
4,2,14103.632353
4,2,2115.071429
4,2,1969.268750
4,2,1568.190000
4,2,1604.817568
4,2,1751.713542
4,2,1592.964467
4,2,1564.560811
4,2,3048.673913
4,2,2029.951389
4,2,1683.127660
4,2,2587.489796
4,2,2050.517157
4,2,11768.866120
4,2,1934.304054
4,2,2687.000000
4,2,1703.812500
4,2,5757.800926
4,2,1808.589286
4,2,1543.571429
4,2,3025.340426
4,2,2389.272727
4,2,1638.361111
4,2,13970.425000
4,2,1740.684524
4,2,1557.848684
4,2,2271.963889
4,2,2794.625000
4,2,1517.340426
4,2,4924.732955
4,2,2009.358108
4,2,4046.878571
4,2,1713.137255
4,2,2164.146226
4,2,3496.810811

4,2,1886.840426
4,2,2375.409722
4,2,8056.927885
4,2,2987.716667
4,2,1998.875000
4,2,2754.593750
4,2,1533.263889
4,2,3129.743421
4,2,3245.902439
4,2,2858.690000
4,2,1507.465116
4,2,16829.143617
4,2,2238.857143
4,2,2079.084906
4,2,1674.235294
4,2,1641.450000
4,2,13306.828125
4,2,3003.089623
4,2,1692.585366
4,2,4783.512500
4,2,1928.324324
4,2,1855.597561
4,2,17903.604839
4,2,2690.267045
4,2,2100.635417
4,2,2289.596154
4,2,5118.072222
4,2,6015.738636
4,2,2559.006250
4,2,2072.173077
4,2,1595.345000
4,2,1504.964286
4,2,3789.875000
4,2,1542.409091
4,2,1877.675926
4,2,2407.575472
4,2,2736.963542
4,2,2171.732558
4,2,2396.574468
4,2,1668.918367
4,2,3897.074468
4,2,2471.571429
4,2,1523.750000
4,2,1683.743017
4,2,2261.945513
4,2,1951.569712
4,2,2671.924528
4,2,3775.054688
4,2,2684.476351
4,2,1794.434524
4,2,5002.967105
4,2,4734.176630
4,2,1791.055147
4,2,10075.242788
4,2,5311.337766
4,2,1970.809524
4,2,1827.180233
4,2,4705.044118
4,2,1915.100000
4,2,5315.159375
4,2,2077.562500
4,2,1537.184524
4,2,2893.706522
4,2,2747.926829
4,2,1864.250000
4,2,2928.572368

4,2,3400.989130
4,2,1834.935897
4,2,19678.552083
4,2,3322.747768
4,2,4437.149306
4,2,1748.846154
4,2,5175.365385
4,2,1784.447674
4,2,1748.725694
4,2,2214.517857
4,2,2558.190217
4,2,7477.932292
4,2,1954.418367
4,2,1788.776316
4,2,1653.816810
4,2,1563.840686
4,2,2167.510204
4,2,2962.546875
4,2,3134.809524
4,2,2984.650510
4,2,1615.573171
4,2,1509.550000
4,2,2031.395833
4,2,1571.739362
4,2,1920.173469
4,2,3838.702703
4,2,2489.433333
4,2,7704.119048
4,2,3624.723404
4,2,1575.947674
4,2,1651.433962
4,2,2138.028846
4,2,2134.450000
4,2,1529.552885
4,2,3797.036765
4,2,4294.028061
4,2,2644.043478
4,2,9260.535714
4,2,1544.100000
4,2,2435.432292
4,2,2246.468750
4,2,1886.154412
4,2,2596.625000
4,2,1577.878049
4,2,4026.678571
4,2,1509.075521
4,2,1740.774390
4,2,2201.906977
4,2,2848.420732
4,2,1704.235000
4,2,7035.707031
4,2,3067.530000
4,2,2109.358696
4,2,2036.020833
4,2,1695.268229
4,2,1905.360000
4,2,1638.462766
4,2,2190.640625
4,2,1707.664634
4,2,5521.595588
4,2,3209.997093
4,2,3741.312500
4,2,3313.784314
4,2,2303.486413
4,2,1878.750000
4,2,2038.560000

4,2,2784.564103
4,2,2532.691489
4,2,6819.812500
4,2,2903.225000
4,2,1927.565217
4,2,2027.490132
4,2,6018.324324
4,2,1726.285714
4,2,2960.012500
4,2,2393.635417
4,2,2488.656250
4,2,3833.458333
4,2,3022.402174
4,2,2984.184524
4,2,1666.259146
4,2,2001.743304
4,2,2348.548913
4,2,2101.278646
4,2,2926.219697
4,2,13105.355769
4,2,1542.349057
4,2,2253.375000
4,2,1702.535714
4,2,1764.824405
4,2,1978.379808
4,2,2469.764205
4,2,1849.125000
4,2,1932.410326
4,2,1850.569767
4,2,3088.000000
4,2,3416.260638
4,2,2017.106707
4,2,2579.202128
4,2,1559.046512
4,2,1991.443750
4,2,3469.459016
4,2,1550.187500
4,2,10186.434211
4,2,1954.653846
4,2,2205.327778
4,2,1738.529412
4,2,1939.212766
4,2,2958.156250
4,2,6384.658537
4,2,1572.714286
4,2,6930.673387
4,2,2350.959677
4,2,1588.218023
4,2,5568.817568
4,2,1560.077586
4,2,5378.301471
4,2,2897.650000
4,2,2188.714286
4,2,3355.342949
4,2,5843.641304
4,2,3735.940789
4,2,3564.404661
4,2,2978.216216
4,2,2869.038265
4,2,2708.447368
4,2,1629.490000
4,2,1954.811170
4,2,2646.989130
4,2,2144.204082
4,2,2570.965426
4,2,1734.447368

4,2,2345.427419
4,2,2547.500000
4,2,2244.608696
4,2,1557.194444
4,2,1672.392500
4,2,1698.046053
4,2,5992.172589
4,2,1610.352941
4,2,2433.088542
4,2,3178.737500
4,2,2901.681818
4,2,2959.514706
4,2,1715.305556
4,2,5531.897436
4,2,2344.802326
4,2,1639.264205
4,2,1938.000000
4,2,2799.679348
4,2,2808.826923
4,2,3657.451613
4,2,7045.523810
4,2,1748.298780
4,2,1728.854167
4,2,7036.760870
4,2,1546.207447
4,2,6173.883721
4,2,3599.377451
4,2,3677.471591
4,2,2141.049419
4,2,1552.580882
4,2,1718.800000
4,2,5274.631579
4,2,2404.148936
4,2,1644.812500
4,2,1810.182927
4,2,5533.650794
4,2,1511.568182
4,2,2400.415761
4,2,2587.480769
4,2,1740.195122
4,2,2835.622449
4,2,1918.767500
4,2,2926.744681
4,2,2010.653226
4,2,5768.562500
4,2,1499.485437
4,2,2000.552632
4,2,2815.632353
4,2,3469.630435
4,2,4108.037234
4,2,3355.548387
4,2,3475.455882
4,2,2635.135638
4,2,1632.071429
4,2,3697.706081
4,2,2946.305233
4,2,1507.744898
4,2,3823.428922
4,2,2228.335938
4,2,2336.835000
4,2,1657.310345
4,2,5010.636364
4,2,2204.866071
4,2,1944.859375
4,2,1812.118421
4,2,2666.744681

4,2,1521.312500
4,2,2696.458333
4,2,2213.825000
4,2,1902.085227
4,2,1981.078431
4,2,2057.698864
4,2,2173.396635
4,2,2564.541667
4,2,2694.631579
4,2,1730.062500
4,2,3262.811321
4,2,2232.771635
4,2,4750.617021
4,2,1507.733333
4,2,1665.237226
4,2,2475.320313
4,2,2533.007353
4,2,12671.492857
4,2,10594.500000
4,2,4820.016667
4,2,1722.090517
4,2,1762.766304
4,2,2986.000000
4,2,1667.375000
4,2,2613.921875
4,2,2825.958333
4,2,2228.156863
4,2,1696.229651
4,2,2550.666667
4,2,2267.693182
4,2,3424.103448
4,2,2576.914894
4,2,1770.069767
4,2,2355.102500
4,2,3790.290625
4,2,2255.301887
4,2,2389.471698
4,2,3837.130000
4,2,1803.134615
4,2,1789.229167
4,2,3366.296875
4,2,2336.000000
4,2,4656.959184
4,2,8023.686321
4,2,3785.986607
4,2,1593.525000
4,2,2353.375000
4,2,1577.321429
4,2,5302.833333
4,2,3862.669811
4,2,5104.330357
4,2,2104.077830
4,2,2708.754310
4,2,2100.627660
4,2,7102.437500
4,2,1731.345455
4,2,1695.027778
4,2,2693.739362
4,2,2489.546053
4,2,1726.208333
4,2,2700.672222
4,2,2205.541237
4,2,1510.062500
4,2,5283.869792
4,2,1574.642500
4,2,1885.166667

4,2,2897.219388
4,2,1689.739583
4,2,3230.664773
4,2,2361.428191
4,2,2087.657738
4,2,1892.659574
4,2,1511.636364
4,2,3867.408163
4,2,9985.321078
4,2,1526.659574
4,2,2003.024457
4,2,13022.445946
4,2,2302.607843
4,2,2229.960106
4,2,1526.842391
4,2,2574.430233
4,2,1847.132653
4,2,10489.531250
4,2,2300.622642
4,2,1948.769231
4,2,3605.762500
4,2,5048.804348
4,2,4116.912500
4,2,6208.891204
4,2,7200.119444
4,2,2382.524390
4,2,2296.116071
4,2,2102.882653
4,2,1824.175676
4,2,2245.046875
4,2,4244.375000
4,2,4772.944444
4,2,2230.053191
4,2,2226.612245
4,2,2833.103365
4,2,6990.634615
4,2,1537.562500
4,2,1863.375000
4,2,2097.173469
4,2,16592.304688
4,2,2882.350000
4,2,2738.770408
4,2,1620.669271
4,2,6943.750000
4,2,1744.107759
4,2,2159.038043
4,2,1994.091667
4,2,1626.176471
4,2,1517.426829
4,2,3040.881048
4,2,3627.625000
4,2,2508.333333
4,2,1626.393293
4,2,5862.648649
4,2,1566.585938
4,2,2211.111111
4,2,1768.343750
4,2,2906.650000
4,2,2971.666667
4,2,4098.437500
4,2,1582.011111
4,2,1723.718750
4,2,2262.780000
4,2,2132.726744
4,2,1806.275000
4,2,1966.088235

4,2,1889.396226
4,2,2118.075000
4,2,1640.054054
4,2,4118.730769
4,2,1734.421053
4,2,2118.727778
4,2,2267.364035
4,2,2898.055556
4,2,3696.150538
4,2,1629.640625
4,2,1991.130952
4,2,1569.833333
4,2,1657.000000
4,2,1767.701389
4,2,1668.593750
4,2,3411.000000
4,2,2075.387097
4,2,6182.765957
4,2,1542.593750
4,2,1727.011364
4,2,3035.164474
4,2,2177.101562
4,2,2626.542553
4,2,1802.500000
4,2,3828.112069
4,2,1617.641026
4,2,5066.697500
4,2,2722.235000
4,2,3536.807292
4,2,2651.265306
4,2,2246.152778
4,2,2053.531915
4,2,1553.932927
4,2,1864.213068
4,2,2719.823529
4,2,1602.596939
4,2,1822.706522
4,2,4077.675000
4,2,1563.909091
4,2,1549.674419
4,2,2231.468750
4,2,4749.391204
4,2,4185.070652
4,2,8287.035714
4,2,1765.649390
4,2,2909.079787
4,2,6895.065217
4,2,3605.783019
4,2,1551.512500
4,2,1808.260204
4,2,2524.850806
4,2,1623.535714
4,2,1553.298913
4,2,4345.894608
4,2,1578.382813
4,2,1913.851190
4,2,3822.196721
4,2,2526.704268
4,2,2978.892157
4,2,1662.816176
4,2,2970.545455
4,2,1690.927885
4,2,15901.705645
4,2,2186.877551
4,2,1550.100000
4,2,4169.568452

4,2,1672.897959
4,2,1909.817073
4,2,2010.558333
4,2,2017.365385
4,2,2323.244681
4,2,2198.112805
4,2,3375.807143
4,2,1762.465116
4,2,8068.734375
4,2,3544.379310
4,2,2121.925532
4,2,2242.595745
4,2,2035.127717
4,2,2093.419355
4,2,2030.000000
4,2,1980.195652
4,2,6215.960000
4,2,1839.320652
4,2,18528.413462
4,2,2703.933333
4,2,5313.000000
4,2,2016.067568
4,2,2044.945946
4,2,1870.463542
4,2,1760.570000
4,2,1756.571429
4,2,4680.160494
4,2,2065.247283
4,2,3708.563253
4,2,4222.754717
4,2,2210.922414
4,2,1695.615385
4,2,2504.826087
4,2,2973.856383
4,2,2194.903061
4,2,1840.895000
4,2,1604.308511
4,2,1949.554348
4,2,2599.596154
4,2,2650.400943
4,2,1558.913043
4,2,1571.340278
4,2,1639.780000
4,2,2301.593085
4,2,1927.482759
4,2,1974.073171
4,2,18472.767442
4,2,1600.509615
4,2,1882.085366
4,2,2681.293605
4,2,1960.500000
4,2,7143.352941
4,2,2715.750000
4,2,1562.569444
4,2,3993.700000
4,2,3938.210526
4,2,2481.424528
4,2,1807.705357
4,2,2143.604651
4,2,5200.915094
4,2,2398.390625
4,2,2785.875000
4,2,1533.669811
4,2,2913.897959
4,2,1531.696429
4,2,1508.823529

4,2,3015.239583
4,2,2138.804878
4,2,2588.120283
4,2,1977.007353
4,2,2927.785714
4,2,3183.340426
4,2,3565.237805
4,2,3235.062500
4,2,1561.600000
4,2,2046.608696
4,2,2457.973262
4,2,3262.517442
4,2,12889.775000
4,2,2866.877451
4,2,1577.392857
4,2,2175.175532
4,2,2325.365854
4,2,1563.576923
4,2,14227.385417
4,2,1934.900000
4,2,3803.712500
4,2,5567.857143
4,2,1519.615385
4,2,2157.900000
4,2,1522.250000
4,2,4067.165000
4,2,2212.139423
4,2,4039.750000
4,2,1654.149457
4,2,2729.978261
4,2,4271.666667
4,2,7077.065789
4,2,1799.281250
4,2,2059.982955
4,2,1846.207237
4,2,1835.584677
4,2,2019.639286
4,2,2923.012019
4,2,2161.502660
4,2,1908.308140
4,2,2663.108696
4,2,2072.218750
4,2,2712.323529
4,2,1814.546296
4,2,1774.073529
4,2,3664.769737
4,2,5884.514706
4,2,2412.875000
4,2,1620.958333
4,2,1646.929054
4,2,4336.448864
4,2,2122.211538
4,2,2337.112245
4,2,2008.279070
4,2,3816.454082
4,2,1746.375000
4,2,2370.920000
4,2,2314.597561
4,2,5114.200472
4,2,1718.812500
4,2,4036.227273
4,2,3457.037736
4,2,7318.979730
4,2,1599.971698
4,2,1600.650568
4,2,2454.880000

4,2,1866.734375
4,2,1780.967213
4,2,2419.875000
4,2,2839.732143
4,2,2563.703704
4,2,1839.238636
4,2,5457.306604
4,2,3283.821429
4,2,4478.808673
4,2,6063.600000
4,2,1580.396552
4,2,2504.886628
4,2,2420.712500
4,2,1566.255682
4,2,2452.393617
4,2,2174.992647
4,2,1744.891304
4,2,3138.052083
4,2,1794.145000
4,2,2357.982143
4,2,1757.441860
4,2,2097.361111
4,2,3204.600000
4,2,3500.245283
4,2,2086.107955
4,2,2281.957447
4,2,4027.502604
4,2,5462.225000
4,2,2019.029412
4,2,1580.514286
4,2,1885.971154
4,2,2073.826923
4,2,4114.222222
4,2,4558.847222
4,2,4017.750000
4,2,3157.837209
4,2,3840.701220
4,2,1816.713942
4,2,1596.212766
4,2,2047.398810
4,2,3782.579545
4,2,1704.997500
4,2,3123.018293
4,2,1555.040698
4,2,3854.547619
4,2,4414.645833
4,2,2920.167763
4,2,1687.850000
4,2,1499.696809
4,2,1948.768868
4,2,8537.420000
4,2,5464.263158
4,2,5213.550000
4,2,1527.342105
4,2,2393.130952
4,2,3303.150259
4,2,1656.857143
4,2,2162.955000
4,2,3911.547872
4,2,1567.087838
4,2,1900.729730
4,2,3920.101036
4,2,3120.265000
4,2,2328.367188
4,2,2832.829268
4,2,3237.079787

4,2,2028.361111
4,2,1563.214744
4,2,3999.607143
4,2,1675.050481
4,2,1900.563830
4,2,2083.143617
4,2,2651.805233
4,2,1668.085938
4,2,1595.643617
4,2,4597.671875
4,2,2692.560811
4,2,2052.924419
4,2,6553.642857
4,2,1521.269531
4,2,1926.287500
4,2,2226.875000
4,2,2085.810345
4,2,2901.333333
4,2,1788.648515
4,2,1587.739583
4,2,2177.432292
4,2,3937.958333
4,2,1700.357500
4,2,1778.080000
4,2,3049.057143
4,2,1520.858696
4,2,2586.160804
4,2,3189.300000
4,2,3078.280488
4,2,3963.419118
4,2,1923.165000
4,2,2416.578125
4,2,3070.447115
4,2,1714.581395
4,2,1709.677500
4,2,2038.797872
4,2,3240.961957
4,2,2104.445000
4,2,1896.089286
4,2,2129.605263
4,2,1638.701087
4,2,4538.448980
4,2,1818.325000
4,2,1578.322368
4,2,6848.125000
4,2,4471.736842
4,2,4618.500000
4,2,2940.203125
4,2,2268.337209
4,2,5134.508333
4,2,2085.913043
4,2,2139.784884
4,2,7065.594595
4,2,3608.866071
4,2,2130.985294
4,2,1915.237745
4,2,6542.497222
4,2,1867.018868
4,2,1650.906977
4,2,1762.451923
4,2,3517.855769
4,2,1813.408333
4,2,2729.818182
4,2,13682.147727
4,2,2833.290816
4,2,2829.893617

4,2,1507.429245
4,2,1597.945513
4,2,4942.068627
4,2,1576.928571
4,2,2191.742188
4,2,3139.375000
4,2,1592.175532
4,2,1610.139423
4,2,1835.575000
4,2,1921.059375
4,2,1857.590062
4,2,3804.052326
4,2,3249.710938
4,2,4937.118056
4,2,2398.404412
4,2,4500.532609
4,2,5528.698661
4,2,5918.619048
4,2,2591.493421
4,2,1782.268293
4,2,1912.534091
4,2,2148.806122
4,2,1913.924479
4,2,2045.145000
4,2,1613.704545
4,2,7634.326271
4,2,8333.930085
4,2,4216.275000
4,2,3787.858491
4,2,1659.542614
4,2,5583.090909
4,2,1696.754717
4,2,2397.514286
4,2,1815.976744
4,2,1953.393939
4,2,1904.000000
4,2,1518.784091
4,2,3673.063830
4,2,3651.676471
4,2,2373.902778
4,2,2164.734375
4,2,3174.119898
4,2,3528.370192
4,2,1723.639810
4,2,2313.294872
4,2,15032.285714
4,2,2262.581522
4,2,1799.956522
4,2,1989.727273
4,2,2340.454268
4,2,1562.425000
4,2,2170.098684
4,2,4794.340625
4,2,1824.005682
4,2,2389.756757
4,2,2382.848684
4,2,1592.892157
4,2,1949.768519
4,2,2622.174020
4,2,2068.615385
4,2,9326.778075
4,2,1977.979592
4,2,3438.923077
4,2,2694.711111
4,2,2363.020833
4,2,4956.278846

4,2,1640.011765
4,2,2216.325000
4,2,4002.658654
4,2,1588.787162
4,2,3044.644737
4,2,2453.917683
4,2,3045.055851
4,2,1562.029891
4,2,1603.825000
4,2,2920.521739
4,2,10002.184524
4,2,1651.810000
4,2,1903.479167
4,2,1984.580000
4,2,1716.000000
4,2,1857.401786
4,2,1652.015625
4,2,1959.414216
4,2,2061.250000
4,2,3526.100000
4,2,1698.727273
4,2,1707.783784
4,2,3441.800532
4,2,2078.843750
4,2,1939.152778
4,2,1706.761628
4,2,3708.727273
4,2,9371.500000
4,2,3376.178977
4,2,2920.171875
4,2,4690.440789
4,2,2709.281553
4,2,3010.333333
4,2,4490.182692
4,2,1576.994186
4,2,1693.763587
4,2,2245.304545
4,2,2712.571970
4,2,3327.146341
4,2,2237.935897
4,2,2410.250000
4,2,2658.961111
4,2,4324.652027
4,2,3204.541667
4,2,5810.095238
4,2,7491.869565
4,2,3555.992788
4,2,4154.621951
4,2,4841.601190
4,2,2821.187151
4,2,1709.142857
4,2,2349.968750
4,2,2712.891892
4,2,1879.247283
4,2,5044.745614
4,2,2049.448113
4,2,1701.110465
4,2,2161.625000
4,2,4577.114796
4,2,2681.558511
4,2,2631.574324
4,2,1815.354839
4,2,1773.852941
4,2,1557.625000
4,2,16216.453125
4,2,1776.845588

4,2,4675.978261
4,2,3747.122283
4,2,2452.166667
4,2,2575.512821
4,2,2325.542553
4,2,2034.156250
4,2,2448.817500
4,2,3951.305851
4,2,5450.667500
4,2,5885.256098
4,2,1537.753289
4,2,3117.048913
4,2,12267.922222
4,2,1859.285714
4,2,3167.921875
4,2,2643.605769
4,2,9147.174342
4,2,2433.684783
4,2,2829.880435
4,2,2052.845455
4,2,5403.000000
4,2,2322.914894
4,2,1695.722973
4,2,2688.398438
4,2,1963.750000
4,2,2790.441038
4,2,1679.375000
4,2,2475.375000
4,2,3955.395833
4,2,3478.263587
4,2,1669.610795
4,2,1639.668605
4,2,1554.250000
4,2,2132.491071
4,2,2143.105978
4,2,1608.000000
4,2,2130.964286
4,2,4223.386364
4,2,1814.703125
4,2,1571.483696
4,2,3158.431818
4,2,2029.289474
4,2,2088.567708
4,2,2964.087500
4,2,1915.539474
4,2,7698.114286
4,2,9271.145000
4,2,3112.075000
4,2,1957.701613
4,2,3301.856771
4,2,1818.357143
4,2,3629.094388
4,2,3863.918478
4,2,2169.372549
4,2,1907.964674
4,2,1850.312500
4,2,2338.555556
4,2,1525.865854
4,2,2237.809659
4,2,1664.836538
4,2,2476.354167
4,2,1906.819767
4,2,3410.835526
4,2,2645.211310
4,2,1611.445946
4,2,1519.928571

4,2,3195.867788
4,2,2151.133333
4,2,2318.400000
4,2,1637.858108
4,2,2270.435714
4,2,2531.118056
4,2,2442.993750
4,2,5007.025000
4,2,2076.117188
4,2,1729.128049
4,2,4432.375000
4,2,1546.133929
4,2,1925.327128
4,2,7950.095109
5,1,2338.812500
5,1,5555.642857
5,1,1872.122449
5,1,1845.355556
5,1,1568.951220
5,1,4494.795455
5,1,2993.950000
5,1,1771.323529
5,1,3102.445652
5,1,4430.877841
5,1,13116.658019
5,1,1713.538889
5,1,4780.375000
5,1,6038.678571
5,1,4694.088710
5,1,3147.949324
5,1,3313.777778
5,1,15451.340000
5,1,2410.342742
5,1,3348.362179
5,1,5993.027027
5,1,4279.721774
5,1,4792.464286
5,1,1934.208333
5,1,2555.213415
5,1,8566.637931
5,1,3325.617188
5,1,3836.772727
5,1,1621.122449
5,1,2088.000000
5,1,2810.575000
5,1,1804.336957
5,1,19085.781250
5,1,5661.868243
5,1,2435.980263
5,1,2579.487500
5,1,1770.427326
5,1,2317.163043
5,1,2379.328125
5,1,1763.779412
5,1,6195.980469
5,1,2438.057692
5,1,9709.642857
5,1,16038.360000
5,1,2450.403846
5,1,2859.536585
5,1,10269.125000
5,1,9173.401316
5,1,17959.367188
5,1,2654.546512
5,1,3007.375000
5,1,10709.887500

5,1,3853.311170
5,1,11845.367925
5,1,1612.914773
5,1,3596.460526
5,1,1977.083333
5,1,13893.384146
5,1,6149.246622
5,1,1850.222973
5,1,2016.859848
5,1,1532.771341
5,1,14442.739583
5,1,1966.777027
5,1,1874.633721
5,1,4541.875000
5,1,2952.139535
5,1,5413.413462
5,1,5442.450000
5,1,1582.000000
5,1,19485.034884
5,1,3390.769737
5,1,2598.104167
5,1,3412.063953
5,1,8921.111111
5,1,4165.125000
5,1,3599.057143
5,1,9012.946429
5,1,10924.500000
5,1,13004.692308
5,1,1819.303571
5,1,2969.922222
5,1,7090.627907
5,1,2931.246795
5,1,5601.293478
5,1,2192.057143
5,1,2763.000000
5,1,6782.619565
5,1,11678.307927
5,1,2157.536765
5,1,19282.161184
5,1,3899.507353
5,1,1545.966667
5,1,2949.980769
5,1,6139.807143
5,1,2237.500000
5,1,7537.613636
5,1,11109.671875
5,1,3362.348837
5,1,9757.813725
5,1,4130.164894
5,1,5184.891304
5,1,4723.246094
5,1,1697.733333
5,1,3899.145349
5,1,6397.219828
5,1,3008.650000
5,1,2207.450000
5,1,4835.093750
5,1,7367.916667
5,1,2330.000000
5,1,2786.139706
5,1,10013.887500
5,1,2208.826531
5,1,1659.068750
5,1,4365.840909
5,1,13883.766892
5,1,10029.255556

5,1,1520.625000
5,1,15394.204545
5,1,3315.371951
5,1,6956.911585
5,1,6697.615196
5,1,11594.191667
5,1,2982.923913
5,1,11461.000000
5,1,4063.500000
5,1,4734.235714
5,1,5958.134146
5,1,5706.647059
5,1,2569.193182
5,1,1677.434783
5,1,1822.718750
5,1,2819.021277
5,1,1627.387500
5,1,3636.157500
5,1,2439.437500
5,1,8297.054054
5,1,2631.355469
5,1,5107.850000
5,1,4666.565972
5,1,2451.900000
5,1,3099.903846
5,1,2309.268293
5,1,3433.225000
5,1,3880.600000
5,1,2824.194444
5,1,2013.197917
5,1,3396.951220
5,1,2066.566667
5,1,17377.250000
5,1,1947.250000
5,1,5032.104167
5,1,5304.080000
5,1,2174.930556
5,1,1674.351064
5,1,11364.461538
5,1,3933.500000
5,1,6823.769231
5,1,14273.717949
5,1,3402.090278
5,1,2504.730769
5,1,2265.658537
5,1,5898.775641
5,1,8078.667683
5,1,1509.750000
5,1,2696.895000
5,1,2022.914286
5,1,4807.243902
5,1,3368.250000
5,1,3765.128378
5,1,4713.855263
5,1,8832.350000
5,1,1600.500000
5,1,12958.403846
5,1,2773.550000
5,1,2273.953947
5,1,4257.310345
5,1,5489.134615
5,1,3844.980000
5,1,2070.711538
5,1,7761.372093
5,1,1604.136719
5,1,8486.116279

5,1,1734.914474
5,1,16332.674419
5,1,1739.318182
5,1,3586.477273
5,1,17726.000000
5,1,6812.099359
5,1,9420.519886
5,1,5929.782258
5,1,3379.800000
5,1,2354.639205
5,1,3914.732955
5,1,9404.307692
5,1,3026.500000
5,1,2027.794643
5,1,14556.426630
5,1,1553.952381
5,1,8653.521429
5,1,18226.888889
5,1,6417.433333
5,1,2678.522727
5,1,2554.453125
5,1,6353.950000
5,1,3504.729839
5,1,5315.477564
5,1,3282.325000
5,1,1830.539773
5,1,1738.843750
5,1,4228.733333
5,1,5213.440476
5,1,6031.439024
5,1,4448.494186
5,1,3466.564024
5,1,2779.411243
5,1,4007.411932
5,1,1984.692308
5,1,2402.048780
5,1,2470.603448
5,1,18259.250000
5,1,3512.357955
5,1,3366.509804
5,1,13654.569767
5,1,5873.409722
5,1,1546.562500
5,1,1711.551136
5,1,2861.497159
5,1,1700.513889
5,1,3759.725000
5,1,3142.610294
5,1,2223.292614
5,1,2050.500000
5,1,1888.131579
5,1,3671.276596
5,1,2479.401786
5,1,1656.381098
5,1,5269.228261
5,1,2585.829268
5,1,1657.833333
5,1,2474.116279
5,1,12331.679487
5,1,10951.422872
5,1,2060.375000
5,1,3523.909091
5,1,4765.881250
5,1,1507.277027
5,1,3129.475610
5,1,3920.618056

5,1,1955.704082
5,1,2376.734375
5,1,3615.777174
5,1,1593.592262
5,1,9686.026119
5,1,1685.532609
5,1,1756.046512
5,1,1893.609756
5,1,3850.382979
5,1,3242.061170
5,1,2544.646875
5,1,2637.146875
5,1,5211.743421
5,1,5282.312500
5,1,3247.375000
5,1,1993.667500
5,1,9172.994318
5,1,2912.697581
5,1,2331.614286
5,1,3086.628571
5,1,3555.655556
5,1,2927.109589
5,1,4015.084821
5,1,1988.750000
5,1,2035.250000
5,1,2773.034574
5,1,1506.922222
5,1,1975.619048
5,1,1551.327206
5,1,2168.181818
5,1,2349.450000
5,1,4738.512500
5,1,1503.250000
5,1,4251.803571
5,1,8490.657143
5,1,2477.211538
5,1,1716.250000
5,1,1876.869565
5,1,1821.908333
5,1,3750.019737
5,1,3165.629032
5,1,3831.250000
5,1,8061.454545
5,1,3147.296053
5,1,1699.476974
5,1,3027.181818
5,1,7347.984375
5,1,5036.064516
5,1,1940.375000
5,1,19079.773256
5,1,1626.575000
5,1,9324.864865
5,1,3251.090625
5,1,1527.550000
5,1,2372.907895
5,1,1697.738971
5,1,6436.326772
5,1,7539.903409
5,1,2767.261364
5,1,1909.750000
5,1,3584.161765
5,1,2956.861413
5,1,1675.857143
5,1,1698.881944
5,1,2770.255208
5,1,3493.763889

5,1,6116.777778
5,1,7429.179688
5,1,15950.520270
5,1,3254.122093
5,1,6555.125000
5,1,13674.500000
5,1,2309.848684
5,1,2192.912500
5,1,2335.728659
5,1,16580.213542
5,1,1838.695122
5,1,3545.028125
5,1,3226.337500
5,1,2792.000000
5,1,3863.400000
5,1,2935.750000
5,1,1893.363636
5,1,5048.875000
5,1,6267.241071
5,1,12189.683333
5,1,4060.755000
5,1,4171.117647
5,1,6744.531250
5,1,4995.214286
5,1,5623.671875
5,1,10063.000000
5,1,1524.708333
5,1,9929.804348
5,1,17961.409091
5,1,1573.377551
5,1,6028.198370
5,1,2746.227273
5,1,9969.800000
5,1,5382.476190
5,1,5396.138889
5,1,1660.466667
5,1,11579.514286
5,1,11714.825000
5,1,1583.316176
5,1,4171.000000
5,1,5934.151163
5,1,7292.531250
5,1,5466.976744
5,1,1879.386364
5,1,1615.357143
5,1,3234.286585
5,1,2578.536290
5,1,2121.162791
5,1,11824.829268
5,1,2310.491071
5,1,2106.216667
5,1,4410.420455
5,1,2176.200000
5,1,4380.431250
5,1,2955.965517
5,1,4783.759146
5,1,3312.773438
5,1,3780.780000
5,1,1511.325581
5,1,2661.780488
5,1,1835.658654
5,1,6119.530488
5,1,3884.000000
5,1,3128.437500
5,1,4527.370833
5,1,2135.972973

5,1,3987.060976
5,1,2758.526316
5,1,1674.725000
5,1,2248.540816
5,1,1596.865385
5,1,1960.062500
5,1,6219.444444
5,1,2205.625000
5,1,2611.278409
5,1,14998.916667
5,1,1735.139423
5,1,3160.112903
5,1,3839.196809
5,1,1687.350000
5,1,3842.034091
5,1,1523.686047
5,1,2081.893293
5,1,6516.213235
5,1,1536.143868
5,1,7740.470833
5,1,5653.101351
5,1,1531.333333
5,1,2026.474359
5,1,4784.666667
5,1,4190.037879
5,1,13805.917969
5,1,3115.341667
5,1,4460.413462
5,1,4211.000000
5,1,2085.166667
5,1,1663.740625
5,1,7456.886364
5,1,2693.606452
5,1,5360.835664
5,1,2373.325000
5,1,3063.352941
5,1,3524.077778
5,1,1977.137500
5,1,3787.527027
5,1,1875.150000
5,1,1595.207447
5,1,3296.833333
5,1,7663.612500
5,1,14174.303571
5,1,3445.263514
5,1,2639.057692
5,1,2246.695652
5,1,7792.887500
5,1,3561.934896
5,1,2854.625000
5,1,4743.949219
5,1,2067.941176
5,1,17215.781977
5,1,6655.616667
5,1,2013.456522
5,1,1733.781250
5,1,2522.653646
5,1,2214.000000
5,1,1639.966667
5,1,1970.490000
5,1,4383.467391
5,1,5985.181250
5,1,10832.638158
5,1,3790.189103
5,1,3875.676630
5,1,3856.609756

5,1,2808.562500
5,1,3249.360577
5,1,1500.462963
5,1,5118.738372
5,1,2050.050000
5,1,3352.390625
5,1,2493.361842
5,1,6203.791667
5,1,3550.476744
5,1,2624.663265
5,1,5099.931818
5,1,9798.527778
5,1,8517.078125
5,1,1882.880682
5,1,3829.411585
5,1,3732.588816
5,1,5794.200000
5,1,2138.666667
5,1,3940.692073
5,1,4408.317935
5,1,6381.125000
5,1,3375.513158
5,1,5228.591667
5,1,1528.500000
5,1,2193.480769
5,1,1974.950000
5,1,2378.335714
5,1,3365.277439
5,1,5116.903846
5,1,2368.892857
5,1,1533.325000
5,1,3892.192308
5,1,6794.119318
5,1,2761.708333
5,1,6814.000000
5,1,2493.295455
5,1,1596.500000
5,1,3096.972973
5,1,14474.000000
5,1,6861.865854
5,1,4997.869048
5,1,3430.046512
5,1,3524.176471
5,1,1828.079268
5,1,2090.570312
5,1,7050.146277
5,1,4288.156250
5,1,1713.483696
5,1,1594.900000
5,1,2276.125000
5,1,2342.115385
5,1,4140.051282
5,1,5997.666667
5,1,13695.536585
5,1,9617.777778
5,1,6604.642857
5,1,1969.952381
5,1,4138.279167
5,1,13868.750000
5,1,3901.814394
5,1,3844.122024
5,1,3444.703947
5,1,2190.828571
5,1,3815.520833
5,1,1558.267442
5,1,1692.362500

5,1,2619.945312
5,1,3471.159091
5,1,7556.447222
5,1,1788.685315
5,1,1855.291667
5,1,13659.460714
5,1,2784.860465
5,1,7244.160714
5,1,2994.647727
5,1,2685.890244
5,1,2526.589286
5,1,11961.000000
5,1,9035.564024
5,1,16507.995968
5,1,3686.021739
5,1,2038.000000
5,1,1525.633333
5,1,2184.200000
5,1,7637.500000
5,1,5632.236979
5,1,15340.472222
5,1,10109.588816
5,1,2187.834375
5,1,4469.656977
5,1,1857.000000
5,1,2896.491071
5,1,2634.257143
5,1,8680.085106
5,1,1597.125000
5,1,2563.927083
5,1,2392.418605
5,1,1823.760870
5,1,9393.038168
5,1,3150.953804
5,1,5772.340625
5,1,1553.012195
5,1,11690.664773
5,1,2545.083333
5,1,5149.816327
5,1,1839.860294
5,1,2050.422222
5,1,18595.738372
5,1,2748.250000
5,1,7161.181818
5,1,2369.390244
5,1,3703.583333
5,1,7229.071429
5,1,5988.687500
5,1,1534.012195
5,1,15465.173077
5,1,2087.992647
5,1,1736.641447
5,1,1997.030303
5,1,9589.750000
5,1,4358.675000
5,1,2164.431548
5,1,6438.467742
5,1,10399.668919
5,1,2097.922619
5,1,2378.928571
5,1,18660.650641
5,1,2751.250000
5,1,3276.343750
5,1,3572.948864
5,1,2579.250000
5,1,1588.500000

5,1,2884.419355
5,1,1755.052632
5,1,8099.490132
5,1,3119.535156
5,1,2137.391304
5,1,9290.593750
5,1,4662.500000
5,1,5223.771277
5,1,2207.555556
5,1,5348.692308
5,1,6447.000000
5,1,7979.229167
5,1,9554.481481
5,1,2292.750000
5,1,4624.423077
5,1,4010.798077
5,1,5134.471154
5,1,4289.049419
5,1,11539.833333
5,1,1861.918919
5,1,1756.333333
5,1,3112.779605
5,1,10749.406250
5,1,2992.214286
5,1,4385.121324
5,1,11041.026316
5,1,6921.393750
5,1,5115.255814
5,1,2300.500000
5,1,4447.378788
5,1,1948.411765
5,1,5250.794118
5,1,5129.012195
5,1,7266.774194
5,1,6889.372093
5,1,1702.750000
5,1,2057.195946
5,1,1781.285714
5,1,1887.808333
5,1,9377.474490
5,1,2464.033333
5,1,1849.486111
5,1,3332.277344
5,1,3516.168919
5,1,3103.636364
5,1,1867.730263
5,1,1916.950000
5,1,2032.400000
5,1,3048.102273
5,1,1666.150000
5,1,3354.137500
5,1,6406.022727
5,1,9614.600000
5,1,1908.453125
5,1,3385.252033
5,1,3558.210938
5,1,1620.662500
5,1,1530.250000
5,1,3382.500000
5,1,5466.625000
5,1,2859.411290
5,1,8761.166667
5,1,6330.388158
5,1,2900.752778
5,1,2026.217391
5,1,1981.310811

5,1,8987.669643
5,1,1904.425000
5,1,2364.941667
5,1,3331.000000
5,1,8178.100694
5,1,4501.050676
5,1,1598.437500
5,1,3335.806548
5,1,1960.707317
5,1,1881.713415
5,1,5089.058824
5,1,2208.928030
5,1,4331.130682
5,1,2273.564189
5,1,9297.888514
5,1,5581.323864
5,1,1740.864865
5,1,3610.050000
5,1,1747.060000
5,1,5429.258065
5,1,5027.156250
5,1,1531.060811
5,1,18920.953125
5,1,12522.545455
5,1,4015.142045
5,1,2464.104167
5,1,1760.162162
5,1,2507.590909
5,1,3664.789474
5,1,5232.968085
5,1,1761.147727
5,1,9009.025000
5,1,2835.742347
5,1,4491.725000
5,1,1539.737500
5,1,7125.229167
5,1,2053.961111
5,1,1635.978125
5,1,2353.642857
5,1,10764.480469
5,1,15419.840909
5,1,1560.967105
5,1,10703.811224
5,1,2108.378378
5,1,1647.191176
5,1,10078.331081
5,1,7944.821429
5,1,2150.215625
5,1,11246.352941
5,1,5049.439024
5,1,1818.636364
5,1,7402.481707
5,1,11417.091912
5,1,2370.351064
5,1,3483.486111
5,1,2164.157407
5,1,4974.875000
5,1,4493.707386
5,1,4335.750000
5,1,1609.111111
5,1,1883.500000
5,1,4719.717949
5,1,19654.388514
5,1,3328.502976
5,1,1585.000000
5,1,4019.121951

5,1,1639.721591
5,1,7350.238636
5,1,1752.710526
5,1,1722.180233
5,1,5073.715116
5,1,3438.122093
5,1,12469.485714
5,1,2559.918919
5,1,6210.996622
5,1,4379.000000
5,1,5999.114286
5,1,5195.204082
5,1,2255.180233
5,1,5218.015152
5,1,5368.250000
5,1,2012.170139
5,1,1700.548387
5,1,9274.555921
5,1,3281.920213
5,1,2456.231061
5,1,3938.093023
5,1,1577.956633
5,1,2177.191489
5,1,4917.550000
5,1,7224.925781
5,1,5716.220930
5,1,4466.833333
5,1,12022.277778
5,1,2867.882212
5,1,7836.432692
5,1,5606.580645
5,1,1988.752976
5,1,3139.083333
5,1,2634.989583
5,1,12539.971774
5,1,1722.102564
5,1,6001.777174
5,1,2001.359375
5,1,4735.438953
5,1,13384.041667
5,1,2175.166667
5,1,1559.717949
5,1,5415.242925
5,1,4485.250000
5,1,6882.346154
5,1,1592.250000
5,1,3331.250000
5,1,8397.367188
5,1,2413.515625
5,1,2709.511628
5,1,19519.954082
5,1,2429.554348
5,1,6229.635870
5,1,2171.312500
5,1,1975.070000
5,1,4954.750000
5,1,9147.264706
5,1,18155.259868
5,1,9099.229167
5,1,2326.213415
5,1,18476.642857
5,1,5530.750000
5,1,1962.767442
5,1,15122.500000
5,1,1851.484043
5,1,2471.347826

5,1,2309.490625
5,1,2965.625000
5,1,1864.630208
5,1,3412.267857
5,1,2073.675000
5,1,5293.625000
5,1,3022.689516
5,1,11144.145349
5,1,7902.073864
5,1,10679.326531
5,1,2783.519231
5,1,1886.357143
5,1,1780.643229
5,1,6483.369565
5,1,5497.734375
5,1,5090.545455
5,1,15535.511364
5,1,9416.559375
5,1,2260.770270
5,1,3604.666667
5,1,7147.630814
5,1,4991.956250
5,1,1652.312500
5,1,3386.481707
5,1,2379.551020
5,1,2929.726190
5,1,3213.533654
5,1,5256.384868
5,1,1582.850000
5,1,2466.286184
5,1,6356.513298
5,1,3020.109756
5,1,6000.601562
5,1,1764.790541
5,1,18563.581395
5,1,2690.786184
5,1,14411.875000
5,1,2909.733696
5,1,1931.048611
5,1,1548.446429
5,1,6851.218750
5,1,1538.408163
5,1,1551.895833
5,1,2009.766447
5,1,3331.769231
5,1,18928.294118
5,1,2789.724359
5,1,6936.455882
5,1,8070.366667
5,1,1523.683824
5,1,13577.856707
5,1,5883.357143
5,1,11883.313830
5,1,2023.350694
5,1,17109.953125
5,1,2284.892045
5,1,9712.073171
5,1,1528.030303
5,1,9412.682143
5,1,2338.509615
5,1,3219.918919
5,1,6761.979167
5,1,10859.880556
5,1,1905.856383
5,1,1844.054054
5,1,11872.288889

5,1,8410.935484
5,1,2169.609756
5,1,8039.190476
5,1,4676.200000
5,1,4643.337500
5,1,4546.670213
5,1,9161.299020
5,1,11204.135135
5,1,3181.244318
5,1,2005.297297
5,1,2488.607143
5,1,8918.421429
5,1,1675.500000
5,1,1860.085106
5,1,4663.570652
5,1,1675.510135
5,1,3023.348039
5,1,4984.395349
5,1,5308.130435
5,1,2319.463542
5,1,4237.505682
5,1,1955.878049
5,1,1736.579545
5,1,2111.358974
5,1,3999.250000
5,1,10522.071078
5,1,2071.478723
5,1,9625.430921
5,1,17035.812500
5,1,2269.039062
5,1,1569.051471
5,1,4712.610465
5,1,5274.666667
5,1,1639.460000
5,1,1734.310484
5,1,14233.312500
5,1,4593.640625
5,1,12079.500000
5,1,3557.720000
5,1,4051.446875
5,1,2266.062500
5,1,2198.068182
5,1,3334.000000
5,1,4224.951613
5,1,3532.017045
5,1,6978.317935
5,1,6927.496875
5,1,4880.771429
5,1,1881.676471
5,1,3671.250000
5,1,2285.890957
5,1,1781.944767
5,1,4577.475000
5,1,6328.714286
5,1,11972.891892
5,1,2465.615385
5,1,1582.917683
5,1,1561.211538
5,1,3242.833333
5,1,3014.559783
5,1,16181.725000
5,1,8562.893750
5,1,2747.633721
5,1,2088.578125
5,1,6923.615385
5,1,4039.738372

5,1,1747.954545
5,1,6370.467742
5,1,1510.865854
5,1,3343.760000
5,1,3366.803571
5,1,1808.488636
5,1,1761.156250
5,1,1932.684211
5,1,6626.723404
5,1,5837.225000
5,1,7733.544118
5,1,2250.868421
5,1,2276.328125
5,1,1974.045455
5,1,1730.388889
5,1,12138.533088
5,1,2360.037500
5,1,10674.944149
5,1,6464.135417
5,1,3849.425676
5,1,15877.812500
5,1,2610.823529
5,1,6749.803150
5,1,4989.729592
5,1,1601.967391
5,1,2277.395349
5,1,3249.411290
5,1,5610.292763
5,1,3712.988889
5,1,5031.406250
5,1,16469.910714
5,1,1812.708333
5,1,2352.590116
5,1,1890.621622
5,1,2166.227273
5,1,3677.338542
5,1,4956.737500
5,1,7015.681452
5,1,1509.836207
5,1,6166.060606
5,1,9509.795455
5,1,3921.614130
5,1,6525.887931
5,1,1902.317073
5,1,6239.664894
5,1,7428.319149
5,1,2754.683140
5,1,2360.077844
5,1,4686.872283
5,1,8017.392857
5,1,8943.573171
5,1,4999.950000
5,1,2608.512500
5,1,7437.956522
5,1,1500.647727
5,1,13135.581395
5,1,2520.279070
5,1,2082.218750
5,1,3211.445312
5,1,1863.333333
5,1,1650.604167
5,1,3381.368243
5,1,5656.265625
5,1,4438.344595
5,1,2939.880000
5,1,4271.875000

5,1,1737.886364
5,1,2190.687500
5,1,11335.527344
5,1,8402.422222
5,1,1830.450000
5,1,1500.222222
5,1,4052.520000
5,1,4511.375000
5,1,5515.442500
5,1,3468.336538
5,1,2035.560127
5,1,1750.000000
5,1,3438.814286
5,1,18025.520270
5,1,5722.937500
5,1,6851.125000
5,1,1586.521277
5,1,1562.526316
5,1,2304.293750
5,1,7421.616477
5,1,6049.589844
5,1,3390.650000
5,1,7199.964286
5,1,14098.201389
5,1,1946.861842
5,1,3279.127660
5,1,2774.375000
5,1,6129.388889
5,1,8239.750000
5,1,1683.217949
5,1,7396.906915
5,1,2358.757576
5,1,15830.926829
5,1,2727.736842
5,1,8612.720930
5,1,6705.541667
5,1,6502.451220
5,1,9711.825658
5,1,2649.963636
5,1,5427.812500
5,1,3452.661111
5,1,2102.720930
5,1,7746.812500
5,1,11366.266667
5,1,18316.430556
5,1,14040.148438
5,1,2482.808511
5,1,4356.386364
5,1,7637.627907
5,1,1538.333333
5,1,9105.112500
5,1,3852.388889
5,1,2460.756579
5,1,3359.335366
5,1,2280.000000
5,1,1782.182927
5,1,3243.664634
5,1,6502.170157
5,1,2272.871212
5,1,3761.337209
5,1,2316.419355
5,1,1526.375000
5,1,4256.324324
5,1,3497.209559
5,1,1786.750000
5,1,1841.433594

5,1,1745.494792
5,1,4748.686047
5,1,1617.954545
5,1,4031.934659
5,1,2005.564394
5,1,2163.137500
5,1,2296.350000
5,1,1509.242187
5,1,3392.250000
5,1,4056.988372
5,1,6956.411765
5,1,1868.517857
5,1,3884.400000
5,1,2687.279070
5,1,5119.482143
5,1,3516.468750
5,1,1581.800000
5,1,17231.081897
5,1,2238.968750
5,1,9673.135417
5,1,2886.007353
5,1,3246.333333
5,1,16741.562500
5,1,14739.625000
5,1,2103.260870
5,1,2649.809783
5,1,3472.009434
5,1,2962.113095
5,1,5840.250000
5,1,1535.688953
5,1,12209.044444
5,1,2744.266304
5,1,3406.202381
5,1,4189.320312
5,1,2148.157895
5,1,2218.569853
5,1,3476.990625
5,1,2285.051136
5,1,16225.308511
5,1,2045.446429
5,1,5942.642857
5,1,1990.273649
5,1,14842.072368
5,1,2951.643293
5,1,7705.543478
5,1,18005.178571
5,1,4136.381250
5,1,4765.000000
5,1,1993.110119
5,1,4517.750000
5,1,1998.400000
5,1,4232.806818
5,1,2562.147727
5,1,5163.000000
5,1,2167.294118
5,1,1976.535714
5,1,3757.648438
5,1,6776.011905
5,1,3229.250000
5,1,1585.987500
5,1,1651.933333
5,1,3670.539062
5,1,3122.125000
5,1,1859.785714
5,1,5787.750000
5,1,2611.051136

5,1,2862.625000
5,1,2959.491279
5,1,2686.243902
5,1,7039.073529
5,1,4373.312500
5,1,1911.534483
5,1,1962.470588
5,1,19772.353659
5,1,1780.117647
5,1,1663.640152
5,1,1856.277778
5,1,2791.753289
5,1,12325.140625
5,1,8762.096591
5,1,7746.473404
5,1,2858.032895
5,1,5028.360795
5,1,3686.250000
5,1,3553.218750
5,1,2835.300000
5,1,1956.239130
5,1,14706.949219
5,1,7483.075000
5,1,3207.559211
5,1,14513.500000
5,1,2107.000000
5,1,1541.832237
5,1,1514.802326
5,1,3586.155556
5,1,3349.291667
5,1,1810.221591
5,1,4651.041667
5,1,6187.750000
5,1,5169.429487
5,1,5241.500000
5,1,2389.223214
5,1,11336.215517
5,1,1648.320946
5,1,1578.552147
5,1,2027.000000
5,1,5075.269737
5,1,1948.375000
5,1,6131.232759
5,1,1729.635870
5,1,15110.202703
5,1,4203.583333
5,1,1815.069079
5,1,6283.895349
5,1,2970.768293
5,1,1681.143382
5,1,1511.219512
5,1,3554.071023
5,1,4353.973684
5,1,4956.840909
5,1,2480.437500
5,1,8825.277778
5,1,2247.940000
5,1,4899.834239
5,1,6101.271341
5,1,3289.766667
5,1,1710.170732
5,1,3662.000000
5,1,7256.055556
5,1,2387.356383
5,1,18016.619318
5,1,1983.871429

5,1,3967.611111
5,1,5935.745000
5,1,2237.733129
5,1,1566.827128
5,1,10233.975000
5,1,7802.868421
5,1,3313.583333
5,1,2320.010000
5,1,2295.641026
5,1,2844.880952
5,1,5133.410714
5,1,5743.431818
5,1,2989.032258
5,1,6008.573864
5,1,11676.216667
5,1,2702.812500
5,1,9481.389706
5,1,6291.692308
5,1,1597.550676
5,1,5350.060811
5,1,2667.230263
5,1,4128.581081
5,1,18138.432432
5,1,7150.064024
5,1,2187.111111
5,1,2871.417683
5,1,2188.369898
5,1,1828.205128
5,1,7012.750000
5,1,15381.722222
5,1,4054.718750
5,1,2963.964286
5,1,4527.573718
5,1,2282.250000
5,1,2489.222222
5,1,3477.714286
5,1,6953.952381
5,1,2279.238095
5,1,13425.325000
5,1,12356.654762
5,1,2244.272727
5,1,1688.393750
5,1,1930.399457
5,1,2460.551020
5,1,3767.205357
5,1,17147.736111
5,1,8222.000000
5,1,4300.269231
5,1,2115.767442
5,1,8152.329545
5,1,2791.562500
5,1,1617.172222
5,1,1686.051282
5,1,5448.488372
5,1,10067.227564
5,1,16635.392045
5,1,3325.610294
5,1,4013.454545
5,1,2536.759615
5,1,1650.244681
5,1,2298.406250
5,1,2497.195122
5,1,6414.477941
5,1,1549.772727
5,1,1616.400000
5,1,3950.375000

5,1,2018.571429
5,1,17719.672619
5,1,3802.381944
5,1,3802.514706
5,1,3593.903226
5,1,6341.088816
5,1,2773.651042
5,1,1971.238095
5,1,4850.243902
5,1,3570.941176
5,1,4926.375000
5,1,2199.335664
5,1,10931.062500
5,1,3037.281250
5,1,5984.708333
5,1,2108.307692
5,1,11105.804878
5,1,4597.000000
5,1,9142.756757
5,1,10900.043478
5,1,2738.734756
5,1,3784.578431
5,1,7125.967857
5,1,9675.161184
5,1,2857.701220
5,1,1536.260870
5,1,2674.738095
5,1,1657.494737
5,1,4440.804688
5,1,4485.567568
5,1,2181.193182
5,1,2063.250000
5,1,4808.022222
5,1,2279.662879
5,1,2935.142857
5,1,1573.189189
5,1,3151.000000
5,1,4970.206522
5,1,1639.300000
5,1,2989.312500
5,1,4121.695652
5,1,5312.315217
5,1,12763.700000
5,1,1564.416667
5,1,3398.015957
5,1,2777.741279
5,1,15970.511628
5,1,12468.140244
5,1,4024.346591
5,1,12790.903846
5,1,1601.704082
5,1,4388.125000
5,1,4167.683824
5,1,6378.175676
5,1,7385.500000
5,1,2068.822674
5,1,3032.978723
5,1,1614.735465
5,1,1661.865385
5,1,3217.297872
5,1,2329.226190
5,1,3468.467391
5,1,3467.163462
5,1,2688.728261
5,1,1678.115385
5,1,8043.870000

5,1,5366.957237
5,1,2730.975610
5,1,2279.781250
5,1,2633.232143
5,1,6093.562500
5,1,3424.851744
5,1,9651.614865
5,1,2556.437500
5,1,1919.312500
5,1,5883.701389
5,1,1776.922619
5,1,4071.409091
5,1,2297.317073
5,1,1587.112069
5,1,3420.243902
5,1,2336.223404
5,1,5763.692308
5,1,5267.455882
5,1,1974.738095
5,1,6820.818182
5,1,3915.545455
5,1,4352.575000
5,1,5483.902439
5,1,1945.750000
5,1,1676.134615
5,1,19886.079861
5,1,2620.304348
5,1,6379.855263
5,1,1715.972222
5,1,9880.279891
5,1,4131.602941
5,1,2887.377551
5,1,1940.013889
5,1,1829.035714
5,1,2431.941176
5,1,3210.321429
5,1,2449.709677
5,1,1691.164474
5,1,2484.488372
5,1,2294.459459
5,1,1905.230978
5,1,2793.412500
5,1,5091.209677
5,1,3809.195122
5,1,8696.685811
5,1,15526.846591
5,1,3980.825000
5,1,4220.082386
5,1,2583.484848
5,1,2463.235000
5,1,11054.882353
5,1,3644.538462
5,1,2957.461957
5,1,2556.687500
5,1,1703.913043
5,1,3233.138889
5,1,2731.064885
5,1,2624.744898
5,1,3790.112500
5,1,4604.750000
5,1,2816.919355
5,1,2592.192308
5,1,7094.646341
5,1,1917.302632
5,1,3436.741667
5,1,4124.315789

5,1,1855.869565
5,1,2214.252660
5,1,4790.333333
5,1,4789.515244
5,1,4266.462838
5,1,2511.615385
5,1,3258.674479
5,1,1514.531250
5,1,4713.967949
5,1,10289.578571
5,1,1601.450000
5,1,5174.638158
5,1,6109.130556
5,1,2569.467742
5,1,4286.841463
5,1,2155.794643
5,1,2002.540000
5,1,1624.713415
5,1,7247.756098
5,1,8686.300000
5,1,9808.791667
5,1,1544.114865
5,1,1525.871429
5,1,2448.727273
5,1,7370.250000
5,1,1839.848837
5,1,2378.419355
5,1,2411.040625
5,1,8239.648936
5,1,2034.158163
5,1,5631.427778
5,1,2908.335526
5,1,2561.394231
5,1,1857.590909
5,1,5235.934375
5,1,5496.398437
5,1,2400.878676
5,1,1836.562500
5,1,4073.415541
5,1,1957.132143
5,1,7232.307692
5,1,6930.485795
5,1,1864.443750
5,1,4082.175000
5,1,5134.718750
5,1,10847.000000
5,1,10150.420455
5,1,4245.325000
5,1,5205.901786
5,1,3142.820513
5,1,5435.937500
5,1,3941.132812
5,1,3596.478261
5,1,2062.000000
5,1,4154.888158
5,1,2128.204545
5,1,2463.177885
5,1,2526.900000
5,1,3171.429688
5,1,7895.254310
5,1,1875.460227
5,1,2615.722973
5,1,8840.863971
5,1,1953.590909
5,1,1678.730263
5,1,2308.044444

5,1,8739.119048
5,1,2138.473684
5,1,3009.807947
5,1,2301.229651
5,1,4956.500000
5,1,3124.975610
5,1,1514.389706
5,1,2371.792308
5,1,4363.372159
5,1,3796.375839
5,1,3203.223684
5,1,4271.250000
5,1,4118.053571
5,1,6746.088710
5,1,5772.714744
5,1,11259.405063
5,1,2551.375000
5,1,12394.961310
5,1,11384.597826
5,1,4189.647059
5,1,5733.791667
5,1,2317.717105
5,1,2355.070652
5,1,1599.406250
5,1,2603.000000
5,1,1553.553571
5,1,6794.156250
5,1,1875.400000
5,1,4819.145270
5,1,2263.618750
5,1,1853.394231
5,1,14632.869565
5,1,4699.746622
5,1,5056.250000
5,1,3527.500000
5,1,2907.559211
5,1,1604.470588
5,1,3905.355556
5,1,1545.785714
5,1,1746.565789
5,1,2382.536184
5,1,3109.828488
5,1,5551.670732
5,1,1651.883929
5,1,2308.811111
5,1,2058.688776
5,1,6121.000000
5,1,2083.358974
5,1,5457.967262
5,1,1686.244792
5,1,18528.401316
5,1,17045.386364
5,1,5451.469388
5,1,2895.803922
5,1,3593.000000
5,1,5053.863636
5,1,6763.493902
5,1,6820.865854
5,1,3425.458333
5,1,3179.755556
5,1,4076.388889
5,1,1543.166667
5,1,1944.036290
5,1,7753.156250
5,1,6397.327381
5,1,2668.358852

5,1,9805.581395
5,1,1906.875000
5,1,2832.853659
5,1,2355.454545
5,1,1679.321429
5,1,1669.982143
5,1,12002.164773
5,1,7523.776786
5,1,3613.878049
5,1,3292.892442
5,1,1536.558140
5,1,13089.337500
5,1,13251.000000
5,1,3267.977273
5,1,2329.972826
5,1,2557.283784
5,1,3614.813776
5,1,11750.476351
5,1,2659.020833
5,1,4216.772959
5,1,7027.507812
5,1,1682.000000
5,1,1546.361111
5,1,3685.438202
5,1,1867.116667
5,1,3473.044872
5,1,12468.545455
5,1,10042.055556
5,1,2262.226190
5,1,3020.392241
5,1,4236.850000
5,1,1908.816327
5,1,1552.765244
5,1,3027.065217
5,1,2364.377907
5,1,4111.688889
5,1,4764.500000
5,1,5788.035714
5,1,1610.861413
5,1,18289.180000
5,1,4670.855114
5,1,1777.948980
5,1,6445.727273
5,1,2304.400000
5,1,10861.529412
5,1,3010.516304
5,1,15516.721154
5,1,1736.173077
5,1,4918.393617
5,1,15227.154412
5,1,16741.058824
5,1,2525.444444
5,1,4090.526786
5,1,2877.170139
5,1,1968.486842
5,1,1972.216837
5,1,4032.048077
5,1,3623.819892
5,1,1678.631579
5,1,4492.363636
5,1,3177.594595
5,1,7191.903061
5,1,3094.615385
5,1,2829.331250
5,1,6411.442708
5,1,1573.585938

5,1,8572.412946
5,1,13541.666667
5,1,1745.690476
5,1,2675.811224
5,1,1946.850000
5,1,3252.573171
5,1,2638.451220
5,1,3589.976562
5,1,1896.758721
5,1,3160.400000
5,1,8723.818182
5,1,8144.510870
5,1,2763.050000
5,1,6699.950521
5,1,11741.272059
5,1,2060.415441
5,1,5207.910000
5,1,1652.676136
5,1,3372.736111
5,1,12955.611111
5,1,5696.083333
5,1,5878.440104
5,1,1649.937500
5,1,3911.166667
5,1,2183.779070
5,1,5263.242857
5,1,2781.000000
5,1,9180.937500
5,1,14392.537791
5,1,2366.250000
5,1,10517.669753
5,1,2701.125000
5,1,2269.790698
5,1,1950.847826
5,1,8934.476190
5,1,7469.140306
5,1,1929.207386
5,1,3726.344828
5,1,2119.148649
5,1,7276.786290
5,1,13132.000000
5,1,3064.607759
5,1,7541.978723
5,1,4417.284091
5,1,2825.523256
5,1,1533.392857
5,1,1987.219512
5,1,4754.714286
5,1,1706.100000
5,1,1889.166667
5,1,2821.602941
5,1,1774.152174
5,1,1863.541667
5,1,1893.689189
5,1,1553.943750
5,1,4887.590517
5,1,5928.398148
5,1,2533.439024
5,1,10438.166667
5,1,6618.290698
5,1,2542.976562
5,1,3988.593750
5,1,2129.529762
5,1,13479.941667
5,1,2631.461538
5,1,2554.301887

5,1,1794.394022
5,1,3726.948864
5,1,4180.963889
5,1,2898.676471
5,1,6662.297297
5,1,14238.600694
5,1,1910.142857
5,1,1767.273649
5,1,1592.437500
5,1,4662.150000
5,1,1591.410326
5,1,6352.939024
5,1,5029.525000
5,1,4166.201923
5,1,4668.354167
5,1,1788.562500
5,1,10138.192308
5,1,1509.054487
5,1,1889.017361
5,1,13793.231250
5,1,1857.657143
5,1,18881.206250
5,1,2523.500000
5,1,2188.271429
5,1,6393.170732
5,1,1568.531250
5,1,8169.476744
5,1,5112.807692
5,1,2947.661290
5,1,3076.886364
5,1,1541.409091
5,1,6680.468750
5,1,2229.802083
5,1,1724.250000
5,1,2124.960366
5,1,2166.954545
5,1,5225.263158
5,1,3015.580882
5,1,8402.673469
5,1,6076.576705
5,1,4937.875000
5,1,2624.928125
5,1,2997.500000
5,1,6459.100000
5,1,4464.465278
5,1,5357.592105
5,1,8928.704167
5,1,3376.548780
5,1,5127.423611
5,1,7712.413793
5,1,4926.138889
5,1,3238.571429
5,1,1698.409091
5,1,2497.720588
5,1,3764.377907
5,1,3743.900000
5,1,1865.489362
5,1,1938.962500
5,1,3733.783088
5,1,2650.314103
5,1,2337.660714
5,1,14819.250000
5,1,4800.053571
5,1,2064.000000
5,1,5258.643617
5,1,9245.657143

5,1,1575.490132
5,1,3909.733333
5,1,12017.785714
5,1,3872.087500
5,1,17996.839286
5,1,2008.063830
5,1,1815.250000
5,1,2485.107143
5,1,4218.959459
5,1,2037.928571
5,1,3046.866667
5,1,8928.562500
5,1,2139.937500
5,1,9651.521429
5,1,3392.614286
5,1,10022.677083
5,1,11453.589286
5,1,1982.508333
5,1,2507.902439
5,1,3773.235294
5,1,1528.349265
5,1,2407.216912
5,1,4129.062500
5,1,2331.934524
5,1,4070.815476
5,1,3528.944079
5,1,8355.711111
5,1,5007.292683
5,1,3488.311111
5,1,6879.011719
5,1,10362.133333
5,1,17257.710526
5,1,9883.469697
5,1,11360.037879
5,1,3837.230114
5,1,2662.946429
5,1,13377.475000
5,1,2202.790323
5,1,4399.327778
5,1,18875.888889
5,1,17903.453947
5,1,2254.250000
5,1,2895.275000
5,1,4755.446809
5,1,14291.092105
5,1,6837.168750
5,1,1701.339806
5,1,1638.207386
5,1,9950.843750
5,1,3532.005882
5,1,11396.676020
5,1,5289.888889
5,1,4206.295455
5,1,2772.971429
5,1,6950.025000
5,1,2658.869792
5,1,6035.231481
5,1,11762.763780
5,1,3383.062500
5,1,1562.843750
5,1,1938.200000
5,1,2917.821429
5,1,1673.330357
5,1,11377.930921
5,1,4965.338235
5,1,7395.369048

5,1,4445.916667
5,1,3836.468085
5,1,3474.210227
5,1,14151.430556
5,1,2296.953488
5,1,4607.750000
5,1,1965.697674
5,1,9653.941176
5,1,8095.320000
5,1,3563.539474
5,1,4693.953125
5,1,2053.263158
5,1,9003.192308
5,1,3497.350543
5,1,3918.537500
5,1,6881.225694
5,1,12803.810811
5,1,1997.169118
5,1,5200.494898
5,1,1585.095745
5,1,1695.217593
5,1,1503.610465
5,1,1804.485294
5,1,5383.441176
5,1,12247.488372
5,1,8775.273438
5,1,5480.599432
5,1,18662.875000
5,1,3139.569307
5,1,9888.171053
5,1,6989.267857
5,1,2117.557692
5,1,1591.781250
5,1,1993.272727
5,1,4401.881579
5,1,10538.500000
5,1,8816.041667
5,1,7666.214286
5,1,6039.140625
5,1,2470.000000
5,1,2630.291667
5,1,17624.142857
5,1,2344.767857
5,1,2020.595395
5,1,1626.839286
5,1,6946.441176
5,1,5058.704787
5,1,5359.984375
5,1,1856.776163
5,1,2678.744186
5,1,3129.125000
5,1,2299.696429
5,1,2297.132353
5,1,7928.250000
5,1,4936.888889
5,1,2424.139535
5,1,14927.187500
5,1,1522.559783
5,1,4893.712766
5,1,1690.531250
5,1,4962.442308
5,1,1925.262195
5,1,4956.038462
5,1,1720.044271
5,1,4189.057377
5,1,8504.960526

5,1,6754.586207
5,1,19784.587209
5,1,5266.435811
5,1,3418.796053
5,1,2941.682692
5,1,5637.734043
5,1,2310.139535
5,1,19991.453125
5,1,15552.375000
5,1,2809.285714
5,1,3609.551471
5,1,3774.520161
5,1,2131.781250
5,1,3262.350000
5,1,3602.850000
5,1,2192.031250
5,1,11635.837838
5,1,10813.080000
5,1,1796.420455
5,1,1770.131579
5,1,3001.267857
5,1,3796.800000
5,1,3014.079545
5,1,4123.435714
5,1,1566.178571
5,1,1539.432927
5,1,9932.697368
5,1,12491.812500
5,1,3438.576923
5,1,1891.437500
5,1,2015.750000
5,1,5256.611842
5,1,11179.510870
5,1,3368.575758
5,1,9257.265957
5,1,2183.125000
5,1,4343.039634
5,1,9639.669118
5,1,1792.781250
5,1,3193.990385
5,1,2083.727941
5,1,2322.515152
5,1,2104.425532
5,1,18246.189394
5,1,2241.250000
5,1,2147.550898
5,1,3808.419118
5,1,1569.444444
5,1,4428.178030
5,1,9203.789474
5,1,19131.222222
5,1,3378.593750
6,2,2839.760000
6,2,11924.240196
6,2,4640.619792
6,2,1515.750000
6,2,1978.090909
6,2,1675.218750
6,2,1788.369110
6,2,2559.013514
6,2,2137.588235
6,2,5126.041667
6,2,5166.406250
6,2,2194.040625
6,2,3665.386792
6,2,2274.078947

6,2,1519.241071
6,2,3401.541667
6,2,1746.182927
6,2,3046.783654
6,2,1714.562500
6,2,3480.607143
6,2,2528.625000
6,2,1895.861842
6,2,2088.152174
6,2,6029.178571
6,2,5598.135417
6,2,2414.265000
6,2,2332.572368
6,2,4380.718750
6,2,3069.377358
6,2,2608.393519
6,2,1713.357843
6,2,5021.326923
6,2,1706.314655
6,2,2910.440104
6,2,7064.273438
6,2,2004.034091
6,2,3136.620283
6,2,2844.312500
6,2,1794.912500
6,2,2653.455882
6,2,2588.274510
6,2,2046.634615
6,2,1595.080882
6,2,2537.591463
6,2,1546.303977
6,2,2032.614865
6,2,1511.267045
6,2,3841.757143
6,2,2907.660714
6,2,3226.383929
6,2,1584.235714
6,2,2927.946023
6,2,2973.388587
6,2,1641.286458
6,2,1637.550000
6,2,2143.586538
6,2,2546.375000
6,2,1747.277174
6,2,1804.435185
6,2,3364.957386
6,2,2725.586957
6,2,1602.252841
6,2,1946.406250
6,2,1942.827381
6,2,1673.507246
6,2,2536.278846
6,2,1534.984043
6,2,1651.397222
6,2,2278.000000
6,2,2205.539063
6,2,2930.103448
6,2,2595.925532
6,2,4517.647059
6,2,5134.816667
6,2,3317.627907
6,2,2463.530055
6,2,1551.645161
6,2,2689.375000
6,2,1569.880319
6,2,4698.363281

6,2,1617.747596
6,2,1576.077128
6,2,1589.637195
6,2,1607.937500
6,2,12797.723404
6,2,1653.860465
6,2,2378.648810
6,2,1594.600000
6,2,1506.000000
6,2,2237.480000
6,2,1732.301020
6,2,1723.008621
6,2,1771.962209
6,2,2083.658654
6,2,3130.168367
6,2,2148.857558
6,2,2033.387097
6,2,6993.266667
6,2,1559.301471
6,2,3082.079545
6,2,1623.579268
6,2,3326.752717
6,2,3484.478261
6,2,1750.125000
6,2,1507.953947
6,2,1951.437500
6,2,1655.934524
6,2,1877.146739
6,2,5188.840426
6,2,2438.914634
6,2,3006.441176
6,2,1990.208333
6,2,11717.867647
6,2,3498.263158
6,2,1786.900000
6,2,1879.938636
6,2,1536.734043
6,2,1609.065217
6,2,2378.948276
6,2,3755.100962
6,2,2097.377660
6,2,4022.234375
6,2,2433.718750
6,2,4101.761905
6,2,1572.026961
6,2,1836.147959
6,2,1794.395000
6,2,2406.279762
6,2,1677.837766
6,2,2718.142857
6,2,2973.750000
6,2,11914.757353
6,2,2182.486979
6,2,2895.581250
6,2,3162.312500
6,2,1837.345238
6,2,3584.517857
6,2,1810.395349
6,2,1636.534483
6,2,6497.411017
6,2,5534.000000
6,2,1603.656250
6,2,2368.914634
6,2,2268.962500
6,2,1971.558140
6,2,1807.131944

6,2,1611.977941
6,2,2016.357143
6,2,7493.172794
6,2,2980.337209
6,2,4241.155405
6,2,11436.291667
6,2,2415.820652
6,2,2810.495536
6,2,5330.691176
6,2,2904.500000
6,2,2057.884375
6,2,2334.369565
6,2,2618.689189
6,2,3259.972222
6,2,4125.500000
6,2,2542.780488
6,2,2347.934524
6,2,2942.244681
6,2,4921.160000
6,2,1612.919643
6,2,2705.583333
6,2,1907.572500
6,2,1608.849057
6,2,2222.075472
6,2,2718.096154
6,2,1898.070000
6,2,5392.469512
6,2,2207.198529
6,2,2821.225610
6,2,1735.875000
6,2,1640.864865
6,2,4875.358333
6,2,1779.571429
6,2,2116.580000
6,2,2186.810811
6,2,1719.445513
6,2,1883.636029
6,2,1834.212766
6,2,1661.813725
6,2,3629.480978
6,2,2048.596875
6,2,1952.205357
6,2,1835.848214
6,2,2185.543605
6,2,1542.641026
6,2,3123.220588
6,2,2542.860119
6,2,4471.375000
6,2,2347.000000
6,2,1552.237805
6,2,1707.846154
6,2,1521.440789
6,2,1706.011628
6,2,2133.454545
6,2,1692.269737
6,2,2018.437500
6,2,2239.067308
6,2,1885.736842
6,2,1679.458333
6,2,5261.377976
6,2,1626.098684
6,2,2263.218750
6,2,2753.302885
6,2,2083.781250
6,2,3313.862745
6,2,5582.588542

6,2,1717.126316
6,2,1912.492925
6,2,3198.800000
6,2,2651.281863
6,2,1802.304348
6,2,2367.558824
6,2,1652.000000
6,2,2096.363636
6,2,1879.593085
6,2,3107.899038
6,2,1887.540000
6,2,1900.294643
6,2,2107.585000
6,2,1608.891447
6,2,1638.677632
6,2,1872.809524
6,2,4564.230556
6,2,8696.878788
6,2,3078.038462
6,2,1657.265244
6,2,1536.106250
6,2,3617.676471
6,2,2305.241279
6,2,14654.808140
6,2,2185.190217
6,2,1978.222500
6,2,3055.410714
6,2,2484.622159
6,2,6882.787234
6,2,5708.040984
6,2,2510.361702
6,2,2160.108974
6,2,2723.127404
6,2,1687.958904
6,2,2026.532609
6,2,1740.567708
6,2,2578.475000
6,2,1605.734043
6,2,3529.755000
6,2,2085.161765
6,2,1620.216346
6,2,2387.309322
6,2,1570.588235
6,2,1623.852500
6,2,1612.805233
6,2,2238.632353
6,2,2378.403846
6,2,4956.756944
6,2,6117.389706
6,2,1593.910256
6,2,6849.797872
6,2,1745.542553
6,2,2125.226852
6,2,2980.415094
6,2,1608.231250
6,2,5947.077381
6,2,6292.980603
6,2,9524.848214
6,2,1512.552632
6,2,1649.578947
6,2,1790.955357
6,2,2080.680147
6,2,2754.390000
6,2,1663.312500
6,2,1636.107784
6,2,2108.666667

6,2,1504.329545
6,2,2034.700000
6,2,1995.622642
6,2,1831.722222
6,2,5398.935897
6,2,2023.076087
6,2,2599.411364
6,2,1863.891892
6,2,1763.625000
6,2,1666.696875
6,2,10065.357143
6,2,1977.061047
6,2,1763.931818
6,2,4000.335000
6,2,1839.000000
6,2,2890.750000
6,2,2616.362705
6,2,1609.551020
6,2,3512.093023
6,2,2339.872093
6,2,2186.793478
6,2,1542.514706
6,2,2595.190217
6,2,1544.281250
6,2,2312.486111
6,2,2651.750000
6,2,2026.450000
6,2,1935.096154
6,2,1710.489130
6,2,1787.875000
6,2,1513.975610
6,2,2801.570312
6,2,2240.516304
6,2,2093.738636
6,2,2026.625000
6,2,2619.465116
6,2,1887.725490
6,2,2376.607143
6,2,2009.180233
6,2,4427.016667
6,2,2193.947674
6,2,2871.706731
6,2,3819.714286
6,2,3689.625000
6,2,1686.867347
6,2,1839.604167
6,2,19333.098485
6,2,3207.365741
6,2,2829.308511
6,2,2299.967890
6,2,3439.261364
6,2,2422.368056
6,2,2501.521739
6,2,1876.833333
6,2,2106.792553
6,2,1908.489796
6,2,2424.361702
6,2,2591.953125
6,2,19421.803571
6,2,1800.799020
6,2,1872.955882
6,2,8939.266026
6,2,2249.764535
6,2,2262.485849
6,2,1732.058824
6,2,2275.950000

6,2,3188.072917
6,2,2032.058824
6,2,1811.560976
6,2,1800.275000
6,2,2753.904070
6,2,1979.767857
6,2,5021.490909
6,2,3000.728261
6,2,2054.109375
6,2,1983.859375
6,2,1898.028061
6,2,3604.273936
6,2,1521.617647
6,2,1533.641304
6,2,1544.922222
6,2,3135.948864
6,2,4370.850000
6,2,2024.837838
6,2,2115.000000
6,2,1685.726744
6,2,1714.691667
6,2,2420.236842
6,2,3032.774510
6,2,1501.658854
6,2,3944.336207
6,2,4467.276596
6,2,2094.166667
6,2,1827.000000
6,2,4293.067568
6,2,2472.215686
6,2,1922.684211
6,2,1799.187500
6,2,1524.037234
6,2,12711.142857
6,2,2078.500000
6,2,1566.313830
6,2,2211.327778
6,2,1694.786458
6,2,8045.655000
6,2,1550.645000
6,2,2271.069767
6,2,5375.256250
6,2,1541.978723
6,2,2164.461538
6,2,1757.233173
6,2,3995.609467
6,2,7253.687500
6,2,1729.528061
6,2,2974.250000
6,2,2826.125000
6,2,1515.500000
6,2,1935.087209
6,2,1755.564024
6,2,1855.252717
6,2,1583.061111
6,2,2267.365385
6,2,1667.080645
6,2,1636.497717
6,2,1502.843023
6,2,3385.047619
6,2,1507.512500
6,2,2056.263889
6,2,1701.219388
6,2,2660.101064
6,2,1624.698529
6,2,3919.174528

6,2,3052.925532
6,2,2595.553030
6,2,2329.500000
6,2,4416.687500
6,2,1638.848214
6,2,3625.761574
6,2,3640.639423
6,2,4739.163462
6,2,2362.410000
6,2,1606.808140
6,2,1651.438776
6,2,1703.000000
6,2,2352.825000
6,2,1658.797753
6,2,2733.333333
6,2,3467.028061
6,2,1520.235294
6,2,2518.346591
6,2,1771.244898
6,2,4885.728448
6,2,1576.120283
6,2,2396.375000
6,2,1779.156250
6,2,2636.721875
6,2,1521.897959
6,2,1979.268868
6,2,1950.000000
6,2,4348.919118
6,2,2351.140625
6,2,2160.767857
6,2,1805.045918
6,2,8494.480000
6,2,2001.381696
6,2,3378.836066
6,2,1816.634146
6,2,1933.894231
6,2,1501.692308
6,2,3337.212500
6,2,2694.267442
6,2,1527.250000
6,2,1599.814815
6,2,12766.443878
6,2,5419.527174
6,2,2207.468085
6,2,1627.054054
6,2,1619.937500
6,2,2070.985000
6,2,1681.177632
6,2,2639.009259
6,2,1976.978723
6,2,2001.020408
6,2,2258.500000
6,2,2300.060000
6,2,11682.256579
6,2,2212.288462
6,2,2611.079268
6,2,2352.425000
6,2,2201.250000
6,2,3943.728261
6,2,1901.926471
6,2,1739.402439
6,2,2054.941489
6,2,1721.212500
6,2,9378.917969
6,2,2081.500000
6,2,1965.300000

6,2,1975.872396
6,2,1567.710938
6,2,1944.566489
6,2,4444.782609
6,2,2428.337500
6,2,1938.438356
6,2,2126.215909
6,2,3818.678571
6,2,1506.500000
6,2,1846.178571
6,2,3850.275510
6,2,1933.312500
6,2,3199.625000
6,2,1990.946429
6,2,2618.228723
6,2,1597.279070
6,2,1697.797619
6,2,1790.622807
6,2,2656.068966
6,2,2982.175000
6,2,1714.500000
6,2,2115.820122
6,2,2037.545918
6,2,1690.152439
6,2,3273.892157
6,2,3748.992500
6,2,1680.665816
6,2,1512.394737
6,2,3642.805804
6,2,4438.008621
6,2,4003.431373
6,2,1696.844595
6,2,9603.354651
6,2,1938.597500
6,2,2051.957447
6,2,1617.234043
6,2,1940.000000
6,2,2631.807692
6,2,3459.631818
6,2,1675.112245
6,2,2187.300000
6,2,2059.142157
6,2,5253.077206
6,2,1502.505102
6,2,1927.339286
6,2,14168.450000
6,2,3270.041237
6,2,2612.981771
6,2,1986.188679
6,2,1652.977273
6,2,3423.000000
6,2,2227.316327
6,2,2219.362179
6,2,2657.886905
6,2,2266.326531
6,2,1678.644444
6,2,2078.666667
6,2,1579.443396
6,2,1923.085106
6,2,1795.732143
6,2,2175.132979
6,2,1731.398936
6,2,2932.926471
6,2,4163.154018
6,2,2441.500000
6,2,2433.492187

6,2,1772.556122
6,2,1731.634615
6,2,1752.742021
6,2,1513.250000
6,2,2419.133333
6,2,1575.375000
6,2,1729.553571
6,2,6138.083333
6,2,3418.718750
6,2,2418.475000
6,2,1662.382979
6,2,1633.550802
6,2,1772.361111
6,2,1757.645833
6,2,2484.906250
6,2,1875.268519
6,2,2912.187500
6,2,2282.860577
6,2,1679.835526
6,2,4236.428571
6,2,1504.607143
6,2,2790.578947
6,2,1566.656250
6,2,2363.250000
6,2,3049.424107
6,2,1802.116071
6,2,2840.096154
6,2,2722.185976
6,2,2559.412234
6,2,1604.437500
6,2,2797.123404
6,2,1747.507353
6,2,3072.375000
6,2,1688.888889
6,2,1669.781977
6,2,2702.510000
6,2,1967.140625
6,2,2394.782051
6,2,2557.787234
6,2,1737.808673
6,2,2658.000000
6,2,2173.004902
6,2,2202.735849
6,2,1983.790698
6,2,2108.915789
6,2,3733.814815
6,2,4481.437500
6,2,1600.875000
6,2,3815.954545
6,2,3675.723404
6,2,1978.961538
6,2,1564.692708
6,2,1842.404762
6,2,3495.019737
6,2,1722.654255
6,2,1852.662500
6,2,3315.781250
6,2,1591.357500
6,2,2111.453488
6,2,3982.000000
6,2,1961.159375
6,2,4612.777344
6,2,3548.226562
6,2,1618.611702
6,2,1773.625000
6,2,3672.500000

6,2,2596.237981
6,2,3779.500000
6,2,4998.067308
6,2,1991.408163
6,2,1533.474490
6,2,2758.677885
6,2,1531.877660
6,2,2102.102273
6,2,3303.937500
6,2,1821.075000
6,2,1571.897959
6,2,1922.495536
6,2,1774.500000
6,2,2106.742857
6,2,2812.660000
6,2,8668.175595
6,2,2314.581395
6,2,1999.357143
6,2,2449.500000
6,2,2065.907895
6,2,1719.000000
6,2,1697.918605
6,2,3048.691176
6,2,10764.632653
6,2,1745.204787
6,2,2401.486301
6,2,2390.232143
6,2,2056.076389
6,2,1979.743056
6,2,1536.500000
6,2,1776.823980
6,2,1693.596154
6,2,3142.921053
6,2,3141.382812
6,2,2132.130952
6,2,3315.527174
6,2,2625.800000
6,2,2315.730263
6,2,2024.088816
6,2,2345.650000
6,2,2383.554054
6,2,2849.541667
6,2,3735.737245
6,2,1827.156250
6,2,1814.600000
6,2,1857.824324
6,2,1754.500000
6,2,3057.348404
6,2,1512.367347
6,2,2291.544503
6,2,2436.393617
6,2,3805.790000
6,2,3549.646341
6,2,3558.245192
6,2,1955.450581
6,2,2973.957447
6,2,1622.390244
6,2,16803.817708
6,2,2715.175000
6,2,2936.416667
6,2,1633.800595
6,2,2621.845745
6,2,2271.755556
6,2,1822.562500
6,2,6290.772727
6,2,3554.470930

6,2,2828.980769
6,2,1909.171196
6,2,3223.983871
6,2,1725.147059
6,2,2721.065000
6,2,3908.048780
6,2,2102.927500
6,2,1815.290000
6,2,1509.448864
6,2,1824.890625
6,2,9123.807692
6,2,1815.750000
6,2,1685.750000
6,2,1823.193750
6,2,2478.053977
6,2,1562.072500
6,2,3764.222561
6,2,6250.145833
6,2,1876.593750
6,2,2173.337500
6,2,2045.192568
6,2,1663.024590
6,2,1800.866279
6,2,2559.707447
6,2,2748.765244
6,2,7519.925532
6,2,5407.173913
6,2,2252.636792
6,2,2535.500000
6,2,3212.732558
6,2,4011.895408
6,2,2337.958333
6,2,11136.915625
6,2,2785.196023
6,2,1897.513514
6,2,3052.661765
6,2,1638.918367
6,2,1886.064103
6,2,3363.162736
6,2,1500.695755
6,2,5485.695312
6,2,1883.330882
6,2,1906.054688
6,2,2258.517442
6,2,1866.800000
6,2,1804.673077
6,2,2675.526596
6,2,1674.402174
6,2,4598.097902
6,2,2799.297872
6,2,1992.500000
6,2,2243.960526
6,2,2723.406250
6,2,2622.752604
6,2,1751.293478
6,2,2658.500000
6,2,4611.150000
6,2,2619.700000
6,2,1615.691489
6,2,6330.970395
6,2,1696.029255
6,2,1574.045918
6,2,3451.538043
6,2,1989.000000
6,2,1708.833333
6,2,11622.048611

6,2,2620.863095
6,2,1798.080189
6,2,1701.090000
6,2,2553.306452
6,2,2728.868056
6,2,1794.288462
6,2,1686.439024
6,2,1982.628571
6,2,2251.470930
6,2,1852.333333
6,2,1559.250000
6,2,2192.877778
6,2,2821.723404
6,2,2452.902778
6,2,1726.189024
6,2,3728.593750
6,2,1709.675676
6,2,4391.750000
6,2,2056.211454
6,2,1648.673913
6,2,1897.528846
6,2,2457.500000
6,2,1743.725000
6,2,2834.944186
6,2,6046.057692
6,2,3229.000000
6,2,2796.937500
6,2,1694.052885
6,2,1808.502404
6,2,2183.237745
6,2,6729.891892
6,2,1829.336364
6,2,1985.797619
6,2,2183.300532
6,2,2520.790865
6,2,6378.360000
6,2,2440.039474
6,2,1552.095588
6,2,1505.505208
6,2,2069.583333
6,2,3508.057692
6,2,3301.098214
6,2,1576.862245
6,2,1716.020270
6,2,1849.783333
6,2,1761.460000
6,2,1628.143617
6,2,2271.906250
6,2,3093.116667
6,2,2383.944444
6,2,1664.902439
6,2,1777.300613
6,2,1800.770833
6,2,2557.317708
6,2,1562.355114
6,2,3185.693878
6,2,3396.734694
6,2,3563.187500
6,2,2110.272727
6,2,1826.526786
6,2,3098.473214
6,2,3154.942857
6,2,2028.220588
6,2,1931.551630
6,2,2249.000000
6,2,1958.734756

6,2,1845.359375
6,2,2477.778302
6,2,1811.848958
6,2,2516.519737
6,2,2090.785714
6,2,1942.632353
6,2,3543.554167
6,2,1870.432292
6,2,1576.369186
6,2,2191.735795
6,2,2356.115385
6,2,2518.382353
6,2,1509.777778
6,2,2011.044118
6,2,2036.416667
6,2,2367.025568
6,2,1933.129808
6,2,1715.800000
6,2,2827.918182
6,2,3528.611111
6,2,1999.199074
6,2,3868.458333
6,2,1606.135417
6,2,2516.166667
6,2,1715.958333
6,2,3533.706250
6,2,3676.755682
6,2,2904.500000
6,2,5400.185976
6,2,1598.760417
6,2,3582.575000
6,2,1533.397727
6,2,1600.837209
6,2,3242.148148
6,2,7019.764706
6,2,13152.755682
6,2,2508.125000
6,2,2364.105114
6,2,3361.455882
6,2,1684.380435
6,2,2131.973404
6,2,1815.430556
6,2,1764.143750
6,2,5845.947368
6,2,3058.899590
6,2,2757.339286
6,2,1795.500000
6,2,1702.425926
6,2,4398.480978
6,2,1959.090909
6,2,4733.694444
6,2,1579.383721
6,2,1586.116071
6,2,6375.762755
6,2,1837.753049
6,2,2062.861702
6,2,2719.393617
6,2,2664.675676
6,2,5276.133333
6,2,1539.566038
6,2,2761.000000
6,2,1549.274194
6,2,1785.477941
6,2,11957.357143
6,2,1595.106383
6,2,1715.375000

6,2,3766.651316
6,2,2521.868056
6,2,5734.023256
6,2,1595.852941
6,2,2194.159091
6,2,1523.583333
6,2,2085.531250
6,2,1685.910714
6,2,1745.240741
6,2,1570.470297
6,2,2085.554878
6,2,3220.617347
6,2,12571.875000
6,2,1557.389205
6,2,1705.628289
6,2,1951.006410
6,2,1570.454268
6,2,2019.416667
6,2,1911.132450
6,2,2856.073770
6,2,3964.914286
6,2,2291.909375
6,2,1515.159091
6,2,2136.729167
6,2,1773.000000
6,2,6292.309211
6,2,2157.640000
6,2,2916.772436
6,2,3331.691860
6,2,3085.333333
6,2,2291.425000
6,2,2561.725490
6,2,2199.472973
6,2,3167.169811
6,2,1532.122340
6,2,2847.051471
6,2,2653.717391
6,2,1566.946429
6,2,2081.894737
6,2,1653.531250
6,2,3646.469388
6,2,1531.568452
6,2,1859.489362
6,2,2535.686275
6,2,1526.531250
6,2,2506.013514
6,2,3245.656863
6,2,4275.350000
6,2,1516.437500
6,2,1501.534375
6,2,2106.846939
6,2,1746.238839
6,2,1527.250000
6,2,2056.589744
6,2,1978.200000
6,2,1741.212766
6,2,1815.250000
6,2,1540.797222
6,2,5337.393617
6,2,1674.838542
6,2,1586.543478
6,2,6803.878906
6,2,1532.852941
6,2,3279.243421
6,2,2506.343085
6,2,2403.604167

6,2,1879.305851
6,2,4107.963636
6,2,1846.755435
6,2,1724.780000
6,2,1646.539216
6,2,1679.325153
6,2,1993.687500
6,2,4078.559211
6,2,2785.909574
6,2,3293.341837
6,2,5919.068182
6,2,1721.735294
6,2,1515.512195
6,2,1515.401042
6,2,3007.194444
6,2,2018.408537
6,2,2997.171875
6,2,6640.193750
6,2,1683.356707
6,2,4158.941860
6,2,2377.690341
6,2,1789.020161
6,2,1841.935000
6,2,1912.500000
6,2,1668.100543
6,2,2073.882212
6,2,2546.957317
6,2,1846.367347
6,2,1548.789474
6,2,3358.640000
6,2,2277.276596
6,2,3853.496622
6,2,1655.958333
6,2,2291.403846
6,2,2401.515625
6,2,2111.622449
6,2,1604.557927
6,2,1751.000000
6,2,2310.250000
6,2,2715.394444
6,2,1566.022901
6,2,2550.455000
6,2,2011.565217
6,2,3993.244681
6,2,1779.612500
6,2,1599.790698
6,2,1952.932870
6,2,1512.353659
6,2,3122.173913
6,2,2206.761905
6,2,1781.285714
6,2,1528.169118
6,2,2041.762500
6,2,2413.056604
6,2,1575.325581
6,2,2986.770833
6,2,1658.966667
6,2,2847.383721
6,2,3128.606250
6,2,1614.666667
6,2,1694.820000
6,2,2327.602941
6,2,1866.818627
6,2,2400.187500
6,2,3157.836957
6,2,1667.361111

6,2,2201.285714
6,2,1794.090000
6,2,9246.108553
6,2,2036.519737
6,2,2582.703125
6,2,1617.272727
6,2,2224.246622
6,2,2588.948864
6,2,1963.000000
6,2,2339.923077
6,2,2903.725490
6,2,2026.618421
6,2,2047.714286
6,2,3028.260638
6,2,4588.651442
6,2,2005.943182
6,2,2476.170213
6,2,2355.052885
6,2,3524.343750
6,2,2449.956522
6,2,2701.670732
6,2,1605.925000
6,2,2103.045455
6,2,4339.737179
6,2,3097.301887
6,2,6160.875000
6,2,1775.760870
6,2,3276.070652
6,2,3125.456818
6,2,1591.416667
6,2,1882.479730
6,2,6988.737805
6,2,1627.400000
6,2,3135.225000
6,2,1632.010870
6,2,2277.835227
6,2,1810.823529
6,2,1526.468750
6,2,1556.975490
6,2,2507.023936
6,2,2334.617347
6,2,2643.980392
6,2,3835.398810
6,2,2467.833333
6,2,1765.674342
6,2,1595.860000
6,2,1961.476190
6,2,3150.034884
6,2,1852.116279
6,2,5795.261792
6,2,1672.976562
6,2,9831.360000
6,2,1731.836538
6,2,2343.283019
6,2,1611.345238
6,2,2922.452128
6,2,1608.664773
6,2,1822.601064
6,2,3138.415948
6,2,2335.463889
6,2,2324.413889
6,2,2537.568878
6,2,1544.012755
6,2,1865.500000
6,2,4507.386364
6,2,4529.125000

6,2,1929.590909
6,2,2889.934783
6,2,7707.250000
6,2,2563.815000
6,2,2263.994186
6,2,1634.612245
6,2,1601.300000
6,2,1637.646277
6,2,2169.588235
6,2,2461.285714
6,2,2093.017045
6,2,2379.215909
6,2,3572.832317
6,2,1983.209459
6,2,2464.105769
6,2,1877.206897
6,2,3264.539773
6,2,1739.935811
6,2,6291.087209
6,2,1519.777174
6,2,2747.792553
6,2,7226.331081
6,2,1615.307065
6,2,4709.960784
6,2,1795.384615
6,2,2492.500000
6,2,2225.081081
6,2,1933.856383
6,2,2843.170833
6,2,3096.076923
6,2,1608.492347
6,2,1925.281250
6,2,1592.988095
6,2,3004.366279
6,2,1732.163265
6,2,1872.273936
6,2,1799.495614
6,2,3056.569767
6,2,2198.668103
6,2,2449.742647
6,2,1625.011364
6,2,1619.843750
6,2,1581.217105
6,2,1594.916667
6,2,1703.816327
6,2,2020.265244
6,2,1830.500000
6,2,1716.372549
6,2,1698.187500
6,2,1524.429688
6,2,4556.162162
6,2,1683.125000
6,2,2051.273438
6,2,2598.121795
6,2,3071.471983
6,2,1619.726415
6,2,5085.779412
6,2,2030.973684
6,2,19131.133721
6,2,1725.750000
6,2,1980.400000
6,2,2020.595745
6,2,11400.854730
6,2,1620.359756
6,2,11740.648649
6,2,2584.717949

6,2,1759.798611
6,2,1736.661765
6,2,1785.033333
6,2,1993.519231
6,2,5470.511628
6,2,2498.147436
6,2,1573.415761
6,2,1926.563636
6,2,2487.974359
6,2,2317.885638
6,2,3977.194444
6,2,3051.437500
6,2,6143.877358
6,2,3632.399038
6,2,2793.784574
6,2,2603.393750
6,2,2088.805556
6,2,8320.824519
6,2,2523.250000
6,2,4206.127907
6,2,3059.061111
6,2,1642.720588
6,2,2918.967213
6,2,3100.211957
6,2,8902.083333
6,2,2295.365625
6,2,2460.815000
6,2,2315.860000
6,2,3733.981481
6,2,2133.196429
6,2,4883.800000
6,2,3231.521739
6,2,6269.644737
6,2,2514.600000
6,2,2089.823529
6,2,1686.938953
6,2,1558.458333
6,2,3090.320000
6,2,3142.881579
6,2,2213.160714
6,2,1687.425532
6,2,6738.557692
6,2,2322.458333
6,2,1538.697115
6,2,2040.519231
6,2,3960.451613
6,2,3090.396226
6,2,3761.181818
6,2,1573.559211
6,2,1593.560417
6,2,2468.262931
6,2,2557.611511
6,2,2115.425714
6,2,4188.313953
6,2,4013.027778
6,2,1919.336735
6,2,1499.775862
6,2,1912.480769
6,2,1790.157895
6,2,8892.969512
6,2,1618.577500
6,2,2555.911765
6,2,2388.106383
6,2,2390.770349
6,2,5573.875000
6,2,8353.134804

6,2,2370.000000
6,2,2573.315789
6,2,1608.336957
6,2,1541.837209
6,2,5170.437500
6,2,2456.859091
6,2,2866.730769
6,2,3056.232558
6,2,1520.687500
6,2,18538.190476
6,2,2668.133152
6,2,1928.176471
6,2,2014.400000
6,2,1537.012755
6,2,2274.431373
6,2,1612.243750
6,2,2559.380814
6,2,1506.625000
6,2,2744.168919
6,2,2504.101744
6,2,3401.358553
6,2,1690.384615
6,2,1865.520833
6,2,1584.810811
6,2,1975.760479
6,2,4373.709184
6,2,1512.480769
6,2,2502.430851
6,2,2416.235294
6,2,1740.477273
6,2,3343.087209
6,2,2722.220264
6,2,1650.205128
6,2,7210.330882
6,2,2713.900000
6,2,1934.851064
6,2,3008.261905
6,2,2143.110000
6,2,3788.055556
6,2,2905.618902
6,2,2045.448980
6,2,1563.941860
6,2,1904.182292
6,2,2688.071429
6,2,2654.159091
6,2,3185.780612
6,2,3681.040909
6,2,1881.589286
6,2,1666.315789
6,2,2314.453125
6,2,3789.096774
6,2,3368.263587
6,2,2028.375000
6,2,1786.938650
6,2,2288.394737
6,2,2093.600000
6,2,1569.635870
6,2,3032.668033
6,2,2255.804348
6,2,1779.230769
6,2,2402.232955
6,2,2659.244898
6,2,1623.549479
6,2,1596.743590
6,2,1915.312500
6,2,2362.335227

6,2,2631.622549
6,2,12634.593750
6,2,2543.617021
6,2,1683.594512
6,2,1735.840426
6,2,2331.535714
6,2,3225.718750
6,2,3709.875000
6,2,1770.562500
6,2,1541.346154
6,2,2494.375000
6,2,4416.484375
6,2,2105.029412
6,2,1808.042683
6,2,1572.226744
6,2,3768.150862
6,2,1686.435714
6,2,2039.460106
6,2,2051.612500
6,2,6999.181818
6,2,1680.016949
6,2,1702.020833
6,2,1929.855000
6,2,2788.622449
6,2,1760.083333
6,2,2018.444444
6,2,2418.272947
6,2,1654.118421
6,2,3109.266827
6,2,1504.368421
6,2,1799.021429
6,2,1521.655556
6,2,1609.772222
6,2,1976.797872
6,2,3127.532258
6,2,1864.016509
6,2,2595.750000
6,2,2255.785714
6,2,2378.367347
6,2,4684.592391
6,2,1628.343750
6,2,1545.040698
6,2,2640.658537
6,2,1953.722826
6,2,2005.470000
6,2,2297.160714
6,2,11802.043478
6,2,1738.420455
6,2,3808.591837
6,2,2907.392361
6,2,2433.154762
6,2,2473.551136
6,2,2130.247768
6,2,1580.500000
6,2,2268.447115
6,2,4564.273256
6,2,1973.212766
6,2,2405.357843
6,2,3167.950658
6,2,1597.919444
6,2,1617.763298
6,2,1776.763298
6,2,1662.725000
6,2,2150.742021
6,2,3266.838542
6,2,1585.051630

6,2,3173.608108
6,2,3011.139535
6,2,1977.316860
6,2,1662.744681
6,2,3229.962500
6,2,3003.482143
6,2,5169.473171
6,2,1752.437500
6,2,2310.923077
6,2,2049.000000
6,2,1976.686170
6,2,2399.658088
6,2,2248.573864
6,2,3842.395349
6,2,5338.466912
6,2,2059.492021
6,2,1831.526596
6,2,2362.581818
6,2,1742.272727
6,2,1894.656250
6,2,2189.640957
6,2,1648.494681
6,2,1694.763158
6,2,9712.352941
6,2,2856.366071
6,2,1544.026786
6,2,2121.333333
6,2,2894.182432
6,2,2725.215116
6,2,1781.532609
6,2,1935.836111
6,2,1626.321809
6,2,3555.729167
6,2,2329.803571
6,2,2958.427419
6,2,1954.000000
6,2,1663.434211
6,2,1991.355000
6,2,1949.588235
6,2,2515.304167
6,2,4589.042553
6,2,3077.952128
6,2,3100.080882
6,2,1919.829268
6,2,2538.235294
6,2,1611.446429
6,2,1861.776316
6,2,3874.097222
6,2,1873.870455
6,2,1639.444079
6,2,1815.178082
6,2,1586.246711
6,2,1681.551724
6,2,2054.958333
6,2,6410.492647
6,2,2149.225000
6,2,3052.687500
6,2,1635.125000
6,2,3610.268293
6,2,1856.278409
6,2,1675.301136
6,2,3062.889423
6,2,3769.451923
6,2,1575.549020
6,2,3134.553191
6,2,1743.314685

6,2,2696.666667
6,2,3159.962264
6,2,3401.791667
6,2,9226.090909
6,2,2328.980769
6,2,1583.484375
6,2,5826.359756
6,2,16441.404412
6,2,4289.801471
6,2,2219.415698
6,2,2564.589844
6,2,2026.604790
6,2,1691.869565
6,2,3328.803922
6,2,1904.402500
6,2,1687.083333
6,2,1887.642045
6,2,3159.133333
6,2,2730.675000
6,2,1935.877551
6,2,7163.378571
6,2,2130.222222
6,2,2200.459016
6,2,2028.593750
6,2,2939.292553
6,2,2682.270833
6,2,1500.475000
6,2,3276.515957
6,2,3521.768868
6,2,2805.536765
6,2,2545.151163
6,2,2630.457447
6,2,1686.368421
6,2,1733.111111
6,2,2032.973214
6,2,4623.217105
6,2,1585.336538
6,2,2306.197368
6,2,1974.500000
6,2,1623.866667
6,2,2762.935000
6,2,5105.750000
6,2,3018.237179
6,2,2171.768750
6,2,2651.891892
6,2,1701.636364
6,2,4201.951087
6,2,5703.225806
6,2,1626.540000
6,2,1893.594828
6,2,2032.947917
6,2,2928.040000
6,2,1512.913043
6,2,2701.400000
6,2,2036.200000
6,2,2398.296512
6,2,2126.765625
6,2,2687.588235
6,2,2001.687500
6,2,2461.383152
6,2,1535.265625
6,2,4468.430556
6,2,2747.540541
6,2,2616.235294
6,2,3381.066667
6,2,4097.277174

6,2,1869.121951
6,2,2465.058140
6,2,1773.680804
6,2,2191.468599
6,2,1762.190217
6,2,7176.034722
6,2,3063.825000
6,2,3146.818182
6,2,6554.029762
6,2,2206.649038
6,2,1762.316860
6,2,3677.362500
6,2,1866.746094
6,2,1504.551136
6,2,2361.163462
6,2,1576.278061
6,2,2277.450000
6,2,1609.281250
6,2,3405.156250
6,2,2524.159314
6,2,1740.780488
6,2,3626.622449
6,2,1548.497500
6,2,1897.950000
6,2,1662.019231
6,2,2147.515625
6,2,2351.214286
6,2,2822.645349
6,2,6489.816176
6,2,1589.742857
6,2,9894.694767
6,2,1600.472222
6,2,2354.625000
6,2,2592.427326
6,2,3285.866803
6,2,2569.400943
6,2,6012.319149
6,2,1991.476190
6,2,1680.392157
6,2,1559.073171
6,2,3793.200000
6,2,1679.489583
6,2,10631.000000
6,2,1913.382353
6,2,5081.864130
6,2,1752.571429
6,2,1692.472222
6,2,2038.193878
6,2,1737.304545
6,2,2281.578125
6,2,2275.534574
6,2,1608.953488
6,2,3903.676923
6,2,2287.163265
6,2,3894.430233
6,2,1769.604839
6,2,1614.000000
6,2,1599.716981
6,2,2545.859375
6,2,2891.235294
6,2,3470.083333
6,2,1640.754717
6,2,1571.522727
6,2,4955.551136
6,2,4516.231383
6,2,1623.370968

6,2,1804.697034
6,2,2679.984925
6,2,4023.555921
6,2,2994.782609
6,2,1650.202381
6,2,1579.764706
6,2,2916.714286
6,2,2948.466667
6,2,1740.814103
6,2,2462.015306
6,2,1691.010204
6,2,1530.953125
6,2,1769.093750
6,2,1565.000000
6,2,2166.915000
6,2,2914.896875
6,2,4492.795455
6,2,5370.183673
6,2,2406.597222
6,2,1580.914894
6,2,8332.777174
6,2,2252.580357
6,2,2190.095238
6,2,1840.660000
6,2,1779.500000
6,2,2797.032967
6,2,2272.812500
6,2,2094.382653
6,2,1640.005952
6,2,1819.244318
6,2,1541.000000
6,2,4076.937500
6,2,2264.683673
6,2,2617.144444
6,2,2422.625000
6,2,2139.459559
6,2,2258.653061
6,2,2155.074074
6,2,2451.050595
6,2,5088.847826
6,2,1910.291667
6,2,2494.833333
6,2,1597.264205
6,2,5748.062500
6,2,4684.497500
6,2,2020.605769
6,2,1577.282051
6,2,1783.000000
6,2,6167.573171
6,2,1604.993902
6,2,4269.714286
6,2,5935.466837
6,2,1738.137500
6,2,2633.410088
6,2,3624.627778
6,2,2604.571023
6,2,1986.018750
6,2,6642.165323
6,2,2991.100000
6,2,1940.713068
6,2,1674.392241
6,2,2079.736842
6,2,4521.144444
6,2,2388.959052
6,2,1840.000000
6,2,2117.972973

6,2,2084.054054
6,2,2077.597500
6,2,1637.166667
6,2,3019.333333
6,2,2560.541667
6,2,2174.000000
6,2,2139.264881
6,2,1778.953947
6,2,1757.893293
6,2,1718.727273
6,2,2274.882812
6,2,2135.277778
6,2,1556.799242
6,2,3062.075000
6,2,8566.238095
6,2,2239.895349
6,2,1525.854911
6,2,4131.117647
6,2,4743.428571
6,2,6088.738889
6,2,2017.163462
6,2,2643.614583
6,2,3728.470588
6,2,3416.011364
6,2,2091.056122
6,2,1998.629630
6,2,4352.479545
6,2,1748.068182
6,2,3203.875000
6,2,2059.357143
6,2,4790.358974
6,2,2045.316327
6,2,4101.650000
6,2,7533.511905
6,2,3743.300000
6,2,1956.650000
6,2,1786.446809
6,2,1812.529762
6,2,4511.197674
6,2,2230.625000
6,2,4128.812500
6,2,1556.311321
6,2,3672.410714
6,2,2695.793750
6,2,3783.500000
6,2,1625.897727
6,2,3828.480769
6,2,2232.473684
6,2,2297.168478
6,2,3603.463235
6,2,2234.475000
6,2,3593.171875
6,2,4351.000000
6,2,4952.326220
6,2,4676.203125
6,2,2364.911017
6,2,1850.681818
6,2,1594.348958
6,2,1960.787162
6,2,1654.152439
6,2,2759.184211
6,2,1948.081633
6,2,3164.602041
6,2,2962.169643
6,2,1603.458333
6,2,1662.287500

6,2,2007.666667
6,2,1710.436275
6,2,4009.256944
6,2,2634.257353
6,2,1545.957792
6,2,2772.071429
6,2,1917.268868
6,2,2454.014423
6,2,2915.800000
6,2,2840.735849
6,2,1763.659574
6,2,1631.625000
6,2,4563.184028
6,2,2056.140306
6,2,3605.177778
6,2,2788.792453
6,2,3209.286184
6,2,2506.164634
6,2,1952.814394
6,2,2456.832569
6,2,1677.953704
6,2,2636.868932
6,2,4071.942308
6,2,1692.053191
6,2,10606.938725
6,2,1558.944444
6,2,1702.750000
6,2,1956.812500
6,2,1516.862500
6,2,2737.840116
6,2,1624.750000
6,2,1644.921875
6,2,2563.822917
6,2,1634.534884
6,2,3036.599359
6,2,1914.139535
6,2,1654.690341
6,2,1840.909091
6,2,1505.023551
6,2,1526.953125
6,2,3131.515625
6,2,2451.693750
6,2,1615.122159
6,2,1901.593750
6,2,1917.367424
6,2,4683.560000
6,2,14081.136364
6,2,2333.183824
6,2,1841.507653
6,2,2350.889706
6,2,1513.375000
6,2,1523.181818
6,2,1628.668605
6,2,9661.652778
6,2,6334.295213
6,2,1702.052326
6,2,5957.818750
6,2,2595.765625
6,2,5506.303241
6,2,2768.050000
6,2,2448.125000
6,2,1530.266892
6,2,1951.125000
6,2,2692.054878
6,2,1580.325000
6,2,2499.729167

6,2,1728.975000
6,2,2034.830882
6,2,1885.961957
6,2,1650.137500
6,2,2544.713235
6,2,2119.363372
6,2,1908.326923
6,2,1543.329787
6,2,3058.613636
6,2,2981.763889
6,2,2260.821429
6,2,2791.441176
6,2,2386.291667
6,2,6550.711111
6,2,1644.512195
6,2,3423.711735
6,2,1955.058673
6,2,4982.806604
6,2,1542.421875
6,2,7278.425000
6,2,1942.692308
6,2,7396.833333
6,2,2011.511364
6,2,2049.192708
6,2,2016.820000
6,2,1718.974138
6,2,2574.611111
6,2,2406.938776
6,2,2661.894737
6,2,2197.324468
6,2,3185.203947
6,2,2414.273585
6,2,2654.000000
6,2,1780.100543
6,2,1695.750000
6,2,1508.857143
6,2,2473.102273
6,2,5215.087838
6,2,2813.340426
6,2,1524.300000
6,2,3137.081081
6,2,2809.090909
6,2,2696.525000
6,2,1920.776316
6,2,3715.704545
6,2,4481.063830
6,2,1734.755952
6,2,4001.149457
6,2,2034.211957
6,2,2001.574850
6,2,4650.438830
6,2,2049.283784
6,2,1592.789216
6,2,1973.730392
6,2,3435.928571
6,2,1637.466667
6,2,2725.242021
6,2,2127.204787
6,2,4506.465625
6,2,1868.047872
6,2,1796.614865
6,2,1750.234375
6,2,1597.154605
6,2,1747.211538
6,2,1760.925532
6,2,2276.791667

6,2,1809.470588
6,2,1728.703125
6,2,1585.024554
6,2,1940.307927
6,2,2216.087838
6,2,2408.949153
6,2,2278.775000
6,2,3757.625000
6,2,2735.368421
6,2,2179.575000
6,2,2249.223404
6,2,2033.604592
6,2,2621.673077
6,2,1501.842105
6,2,3253.139535
6,2,1841.500000
6,2,1720.952500
6,2,2721.208955
6,2,2760.613122
6,2,5437.562500
6,2,1510.145985
6,2,2144.902273
6,2,2143.435000
6,2,3338.225000
6,2,2071.125000
6,2,2077.812500
6,2,7478.637500
6,2,2476.815657
6,2,8258.718750
6,2,12967.704545
6,2,2095.006098
6,2,1603.660377
6,2,1799.384615
6,2,5151.674419
6,2,1596.512821
6,2,1611.540816
6,2,3036.148438
6,2,1922.815217
6,2,1501.125000
6,2,2799.955729
6,2,1686.833333
6,2,5227.458333
6,2,3266.071429
6,2,1635.119792
6,2,2468.019886
6,2,3047.919643
6,2,1877.060976
6,2,1570.820122
6,2,1533.428125
6,2,2442.378676
6,2,1931.855263
6,2,1536.360000
6,2,1729.875000
6,2,12843.666667
6,2,17382.583333
6,2,1726.140625
6,2,3694.102041
6,2,1499.388889
6,2,3848.046875
6,2,2406.709302
6,2,2026.872093
6,2,1655.591837
6,2,2115.947368
6,2,2726.387755
6,2,1751.333333
6,2,7419.775943

6,2,1561.420732
6,2,1669.911765
6,2,2374.910256
6,2,3051.557692
6,2,2083.155612
6,2,1602.659574
6,2,6826.212500
6,2,4465.400000
6,2,1533.703947
6,2,6757.432692
6,2,2416.176136
6,2,6449.232143
6,2,2676.270000
6,2,1902.992647
6,2,2072.875000
6,2,1641.591837
6,2,1549.147059
6,2,2201.573529
6,2,2326.815000
6,2,2553.926282
6,2,1547.118750
6,2,1592.455556
6,2,2112.698864
6,2,2253.930804
6,2,7269.333333
6,2,1606.525862
6,2,2113.175481
6,2,1921.290698
6,2,1549.388889
6,2,2397.352941
6,2,1685.272727
6,2,5154.352941
6,2,4488.195000
6,2,2224.703196
6,2,2505.883721
6,2,1634.960784
6,2,2009.611842
6,2,2760.791667
6,2,2015.191176
6,2,2610.527439
6,2,2403.184783
6,2,1920.311404
6,2,2420.163793
6,2,2093.640625
6,2,1505.662234
6,2,1787.375000
6,2,2218.604545
6,2,2490.553977
6,2,2037.990854
6,2,2109.625000
6,2,1976.889205
6,2,3310.865854
6,2,2894.771277
6,2,3743.509434
6,2,3207.066176
6,2,1841.039474
6,2,2860.134146
6,2,1638.500000
6,2,1722.486111
6,2,1528.824468
6,2,1537.673611
6,2,4112.895833
6,2,4230.402439
6,2,1550.460937
6,2,1789.677500
6,2,6476.185714

6,2,1626.293478
6,2,4785.414634
6,2,1774.481771
6,2,1525.904070
6,2,2960.359375
6,2,1939.911950
6,2,2720.658537
6,2,4421.725000
6,2,2246.377551
6,2,2223.540625
6,2,5740.511905
6,2,2290.683333
6,2,1959.532609
6,2,1801.144886
6,2,1774.549020
6,2,1639.414634
6,2,2176.640625
6,2,1739.540698
6,2,1606.461538
6,2,2177.402174
6,2,2568.607843
6,2,2872.980263
6,2,2495.000000
6,2,1904.570652
6,2,2159.247159
6,2,2128.063889
6,2,1681.197674
6,2,1629.304054
6,2,1931.274457
6,2,12984.019231
6,2,1763.652778
6,2,1528.145349
6,2,3184.003676
6,2,2924.157609
6,2,2478.558824
6,2,1812.098039
6,2,15816.417553
6,2,4251.972222
6,2,1731.739130
6,2,3455.526042
6,2,2283.259804
6,2,3480.037234
6,2,2700.423913
6,2,3456.346154
6,2,1731.625000
6,2,2917.195122
6,2,1982.058511
6,2,2476.583333
6,2,2849.684783
6,2,2273.136364
6,2,1791.202381
6,2,1758.186047
6,2,2772.105932
6,2,2185.330000
6,2,2305.734043
6,2,1647.316667
6,2,3400.450581
6,2,3090.925000
6,2,3966.391026
6,2,1558.721154
6,2,2920.060976
6,2,1688.488889
6,2,6510.000000
6,2,2587.806250
6,2,1789.467105
6,2,3340.416667

6,2,2244.601190
6,2,2509.763298
6,2,11065.038462
6,2,2094.712264
6,2,2872.201531
6,2,1838.166667
6,2,2120.315217
6,2,2552.900000
6,2,2203.036111
6,2,1791.268868
6,2,1866.940476
6,2,2646.867188
6,2,1721.732484
6,2,1649.143939
6,2,2197.333333
6,2,1959.369048
6,2,1590.190476
6,2,2500.927632
6,2,1682.031250
6,2,1540.408333
6,2,3929.666667
6,2,1517.750000
6,2,2101.088889
6,2,1952.895455
6,2,1872.421053
6,2,2688.868750
6,2,2426.625000
6,2,3848.240000
6,2,3888.823113
6,2,1591.494444
6,2,3949.377907
6,2,1552.236702
6,2,2493.864407
6,2,1533.068182
6,2,1645.791667
6,2,2164.136364
6,2,2252.725000
6,2,1622.316489
6,2,4013.846154
6,2,1645.775000
6,2,1696.769231
6,2,1556.417763
6,2,2034.976744
6,2,2973.021875
6,2,5843.958333
6,2,2350.299479
6,2,3999.065000
6,2,1640.230263
6,2,3363.187500
6,2,1897.416667
6,2,2328.451220
6,2,1888.268293
6,2,1662.908163
6,2,1650.687500
6,2,1763.454545
6,2,2347.894022
6,2,1659.867647
6,2,2754.893617
6,2,2024.666667
6,2,2620.232558
6,2,2906.117500
6,2,2955.414062
6,2,1571.011111
6,2,2083.236111
6,2,2623.653409
6,2,1700.811321

6,2,1800.587500
6,2,2201.313725
6,2,1607.075000
6,2,2311.096154
6,2,1515.988636
6,2,2284.809375
6,2,2428.315789
6,2,2826.339744
6,2,1547.132353
6,2,1778.728723
6,2,1524.525641
6,2,1677.094828
6,2,1842.228723
6,2,2142.546875
6,2,14586.578488
6,2,2739.377778
6,2,2701.384615
6,2,4142.038043
6,2,3111.553571
6,2,2619.547297
6,2,2425.568182
6,2,1741.790323
6,2,2252.477041
6,2,3145.500000
6,2,1773.910326
6,2,1580.848214
6,2,3136.353125
6,2,18970.500000
6,2,3590.995192
6,2,3846.015625
6,2,2141.212500
6,2,2242.978261
6,2,1995.119048
6,2,1561.286765
6,2,3251.728261
6,2,14667.785714
6,2,3006.275000
6,2,2086.684211
6,2,2750.809524
6,2,1760.743842
6,2,2192.707317
6,2,2056.368750
6,2,1507.750000
6,2,1766.048913
6,2,1634.466667
6,2,7549.500000
6,2,1776.576923
6,2,1708.070652
6,2,9488.303571
6,2,2529.625000
6,2,2023.804545
6,2,1893.107143
6,2,3831.260000
6,2,2099.807692
6,2,1792.390244
6,2,1541.368750
6,2,5890.322917
6,2,2319.979592
6,2,2107.414894
6,2,1991.382979
6,2,2204.868421
6,2,2192.489130
6,2,1587.187500
6,2,3733.743455
6,2,2790.069767
6,2,1579.161184

6,2,7135.022727
6,2,2845.847222
6,2,1733.400000
6,2,1912.794643
6,2,3500.083333
6,2,1821.812500
6,2,1630.271739
6,2,3488.595745
6,2,1934.467500
6,2,3940.191327
6,2,2833.500000
6,2,4190.333333
6,2,1876.500000
6,2,4257.511364
6,2,2956.410714
6,2,2115.017442
6,2,2666.210938
6,2,1903.611111
6,2,2229.680000
6,2,3921.555000
6,2,2156.670455
6,2,1511.837054
6,2,3355.605932
6,2,2189.987805
6,2,2444.958333
6,2,1802.862179
6,2,2029.684211
6,2,1537.792683
6,2,2211.250000
6,2,3761.322222
6,2,17501.915761
6,2,2347.720000
6,2,2900.489362
6,2,1532.463542
6,2,2963.475000
6,2,2373.225000
6,2,1911.377419
6,2,1940.867188
6,2,2137.270833
6,2,2030.187500
6,2,1938.000000
6,2,1801.164062
6,2,1525.158537
6,2,1544.143382
6,2,1823.436321
6,2,2291.271739
6,2,2139.034091
6,2,4887.380435
6,2,2146.916667
6,2,3073.894022
6,2,1753.929688
6,2,1861.250000
6,2,1943.796512
6,2,1848.100490
6,2,2461.869565
6,2,1574.026786
6,2,1609.172297
6,2,4239.861111
6,2,1879.153061
6,2,2687.375000
6,2,1744.794118
6,2,10582.638587
6,2,1509.100000
6,2,2494.583333
6,2,1630.153846
6,2,1771.181250

6,2,2026.940476
6,2,1739.613095
6,2,1745.293367
6,2,1814.368182
6,2,2703.666667
6,2,2372.440000
6,2,1503.233333
6,2,2796.343750
6,2,2712.125000
6,2,2286.147436
6,2,1817.111702
6,2,1916.964286
6,2,6612.843750
6,2,1783.476190
6,2,1716.912037
6,2,4933.365385
6,2,1645.184375
6,2,1567.546512
6,2,3135.287500
6,2,1552.108696
6,2,4246.604167
6,2,1506.440000
6,2,2341.085938
6,2,3194.640244
6,2,2054.714286
6,2,1528.950000
6,2,5393.755556
6,2,1514.510870
6,2,2177.104167
6,2,2272.630000
6,2,2984.769231
6,2,1967.119048
6,2,4376.540761
6,2,1932.856250
6,2,1544.812500
6,2,5009.171053
6,2,1569.853659
6,2,2932.507143
6,2,2094.921053
6,2,2185.950980
6,2,4981.500000
6,2,2008.315217
6,2,2184.061224
6,2,3437.192982
6,2,1743.171512
6,2,1600.633333
6,2,1695.307692
6,2,2041.517857
6,2,2783.559783
6,2,2996.160714
6,2,1599.535714
6,2,2769.230114
6,2,3226.529255
6,2,1571.049479
6,2,3247.659574
6,2,7286.452206
6,2,1573.718750
6,2,5766.031250
6,2,1963.733333
6,2,3127.107558
6,2,1751.476190
6,2,2024.343023
6,2,2328.036184
6,2,3831.801471
6,2,4564.515625
6,2,1515.529070

6,2,1753.380435
6,2,1787.162500
6,2,1536.875000
6,2,2678.615385
6,2,2758.071429
6,2,2253.416667
6,2,5587.897727
6,2,1891.487981
6,2,7884.489362
6,2,1772.485000
6,2,1536.985849
6,2,3774.150510
6,2,2581.850000
6,2,6169.693182
6,2,2116.895833
6,2,1501.413043
6,2,1801.813953
6,2,2114.960526
6,2,3010.489362
6,2,2619.496875
6,2,1851.500000
6,2,3021.812500
6,2,2212.497449
6,2,5639.017157
6,2,2194.385417
6,2,1888.823864
6,2,2776.048913
6,2,1604.924342
6,2,2241.505102
6,2,1542.420455
6,2,1770.029255
6,2,3150.527778
6,2,2196.306250
6,2,6416.331395
6,2,1943.635870
6,2,2008.375000
6,2,4256.138158
6,2,2315.232143
6,2,2638.906977
6,2,3508.338068
6,2,1975.794444
6,2,1866.607143
6,2,1653.119444
6,2,3479.390000
6,2,2138.451613
6,2,2385.952500
6,2,1535.716463
6,2,2179.583333
6,2,1850.293750
6,2,2008.484756
6,2,1559.369318
6,2,8292.205128
6,2,2747.484649
6,2,2756.500000
6,2,2688.952703
6,2,2073.783730
6,2,2634.144886
6,2,1517.583333
6,2,1730.606250
6,2,2312.589744
6,2,1840.652174
6,2,1521.456250
6,2,5033.356707
6,2,2482.500000
6,2,2426.685000
6,2,2710.568182

6,2,1595.590909
6,2,3163.375000
6,2,1552.735294
6,2,2638.625000
6,2,4255.089844
6,2,4068.166667
6,2,1556.500000
6,2,1624.061224
6,2,3553.198529
6,2,1663.493902
6,2,3074.587054
6,2,1584.916667
6,2,1716.368243
6,2,2287.617647
6,2,1597.136628
6,2,2605.062500
6,2,1870.625000
6,2,2639.665761
6,2,4169.507212
6,2,10204.325000
6,2,2015.677419
6,2,1639.491379
6,2,5836.120968
6,2,1512.577703
6,2,2129.494318
6,2,1702.520833
6,2,2984.895161
6,2,2655.268519
6,2,2483.804348
6,2,7212.104167
6,2,4452.436842
6,2,2543.500000
6,2,3232.203488
6,2,1525.881579
6,2,1696.942857
6,2,2013.571429
6,2,1877.935484
6,2,1797.000000
6,2,8291.590909
6,2,2011.106771
6,2,2002.941667
6,2,4249.125000
6,2,1772.780000
6,2,2173.451220
6,2,3097.621951
6,2,1585.209302
6,2,6379.696078
6,2,1627.488636
6,2,5061.401442
6,2,1591.375000
6,2,1711.642857
6,2,5561.551724
6,2,2275.089286
6,2,1692.043750
6,2,1937.894737
6,2,2433.822222
6,2,4705.860465
6,2,2400.285714
6,2,4295.593750
6,2,2448.420213
6,2,4565.041667
6,2,1986.952381
6,2,4046.689024
6,2,2869.016892
6,2,1765.750000
6,2,7847.156250

6,2,3379.868421
6,2,2504.508065
6,2,4914.573529
6,2,2323.384615
6,2,3227.615385
6,2,1699.505435
6,2,4467.323045
6,2,14203.412500
6,2,2700.045455
6,2,1889.972826
6,2,1750.503571
6,2,5659.214286
6,2,1799.695312
6,2,4963.833333
6,2,3689.098039
6,2,2690.068182
6,2,3074.392857
6,2,1640.763158
6,2,2403.970588
6,2,10252.103261
6,2,1512.500000
6,2,2756.254902
6,2,1635.143617
6,2,1877.860000
6,2,2447.448113
6,2,3920.646739
6,2,4754.787500
6,2,1978.502976
6,2,7706.000000
6,2,1518.662162
6,2,2366.857143
6,2,6657.816176
6,2,2093.594512
6,2,3665.542453
6,2,2366.317308
6,2,3584.213889
6,2,1572.257143
6,2,1511.476744
6,2,1886.153846
6,2,2172.156863
6,2,3103.210000
6,2,2389.005814
6,2,4167.158163
6,2,1899.843750
6,2,2210.571429
6,2,2743.539286
6,2,1776.587838
6,2,7329.421053
6,2,6732.963235
6,2,13509.826705
6,2,2118.393617
6,2,5495.564516
6,2,2577.381410
6,2,2614.382143
6,2,1938.243590
6,2,2826.167614
6,2,2003.209302
6,2,1877.268293
6,2,1729.562500
6,2,2505.881944
6,2,9236.453431
6,2,2601.402439
6,2,1558.296429
6,2,3169.397727
6,2,2014.927885
6,2,1988.220588

6,2,2540.750000
6,2,2634.063636
6,2,5236.250000
6,2,1752.404891
6,2,2582.585106
6,2,1908.473684
6,2,2341.675676
6,2,1616.644809
6,2,2295.813953
6,2,1650.120690
6,2,1588.857500
6,2,1947.708333
6,2,4200.714286
6,2,4695.416667
6,2,1570.825581
6,2,4435.048913
6,2,1692.526316
6,2,1575.045918
6,2,2795.011364
6,2,6180.562500
6,2,1505.037500
6,2,2324.170455
6,2,2389.875000
6,2,1633.638889
6,2,1704.175000
6,2,3213.924479
6,2,1864.800000
6,2,6058.286364
6,2,1775.855769
6,2,1577.046053
6,2,1624.659091
6,2,4680.625000
6,2,1778.202703
6,2,2898.776042
6,2,2916.953125
6,2,3589.304813
6,2,2378.690909
6,2,16726.000000
6,2,1733.218085
6,2,2122.585106
6,2,1783.545455
6,2,1520.449664
6,2,1559.653846
6,2,1827.744898
6,2,1611.565972
6,2,5860.127315
6,2,1660.687500
6,2,1581.269231
6,2,1978.047619
6,2,3930.719388
6,2,2406.136905
6,2,2309.193396
6,2,1925.286765
6,2,9398.500000
6,2,2284.537500
6,2,2328.627451
6,2,1682.382653
6,2,1591.332317
6,2,2593.113636
6,2,3945.418269
6,2,2342.793269
6,2,1514.368421
6,2,1604.750000
6,2,3328.256579
6,2,1536.515625
6,2,3271.857143

6,2,1581.054348
6,2,1565.364865
7,1,2835.982143
7,1,10420.432432
7,1,5836.878472
7,1,1556.166667
7,1,4731.589744
7,1,5025.829545
7,1,1570.104167
7,1,8142.952703
7,1,14112.769737
7,1,15458.864130
7,1,2900.700000
7,1,4141.755814
7,1,2456.761719
7,1,13456.425000
7,1,3690.832317
7,1,2938.434659
7,1,2458.026316
7,1,19106.696970
7,1,2517.314103
7,1,3174.526316
7,1,2493.407143
7,1,2744.138889
7,1,2883.581395
7,1,3236.587500
7,1,4698.404412
7,1,4456.671296
7,1,7248.789062
7,1,2952.250000
7,1,1740.619681
7,1,16908.469388
7,1,6228.151596
7,1,4815.921512
7,1,6773.931818
7,1,17070.364865
7,1,8364.084302
7,1,13044.364362
7,1,2021.639706
7,1,2729.281481
7,1,3165.582317
7,1,13579.114583
7,1,2434.015625
7,1,6527.687500
7,1,1833.758929
7,1,2140.414894
7,1,12186.205556
7,1,19184.252874
7,1,7201.208333
7,1,2031.156250
7,1,5475.944444
7,1,16721.125000
7,1,1770.269231
7,1,1632.042553
7,1,4715.443548
7,1,2186.622222
7,1,1563.642857
7,1,5449.152174
7,1,2610.500000
7,1,5239.735294
7,1,3391.777778
7,1,2299.875000
7,1,1863.607558
7,1,2255.054688
7,1,3364.000000
7,1,3333.186047

7,1,2906.250000
7,1,3131.604730
7,1,3090.348684
7,1,2682.141304
7,1,4412.355769
7,1,1672.178125
7,1,1955.750000
7,1,3321.944444
7,1,2359.218750
7,1,1937.975543
7,1,2388.772727
7,1,3539.184783
7,1,4033.332547
7,1,18596.666667
7,1,3966.166667
7,1,3072.724265
7,1,4904.576923
7,1,5591.015625
7,1,8265.490854
7,1,7025.666667
7,1,10598.638587
7,1,2420.116667
7,1,6275.441964
7,1,2175.375000
7,1,1597.833333
7,1,4669.360795
7,1,6555.785256
7,1,5274.182927
7,1,5776.492647
7,1,3229.519231
7,1,4067.852941
7,1,1550.612981
7,1,2453.170213
7,1,13170.038462
7,1,5123.463235
7,1,1920.954861
7,1,2144.547297
7,1,2629.287234
7,1,2610.995000
7,1,2015.808511
7,1,2033.333333
7,1,2822.375000
7,1,2673.400000
7,1,4334.747093
7,1,12459.906977
7,1,2237.312500
7,1,2839.561047
7,1,1814.331731
7,1,3031.333333
7,1,2458.826923
7,1,10293.956522
7,1,15291.000000
7,1,2535.217391
7,1,3482.734375
7,1,5576.962838
7,1,13842.818452
7,1,2236.937500
7,1,12337.622283
7,1,1796.000000
7,1,5126.770833
7,1,1507.453125
7,1,2430.227273
7,1,3316.862245
7,1,6224.894737
7,1,6450.732955
7,1,8019.115385

7,1,1658.234043
7,1,3640.750000
7,1,8215.565217
7,1,2658.525000
7,1,2195.782609
7,1,3656.430233
7,1,7921.784884
7,1,1797.163265
7,1,2357.416667
7,1,9666.250000
7,1,2479.450000
7,1,3977.924242
7,1,1878.025641
7,1,1543.038462
7,1,13817.787500
7,1,16791.798469
7,1,4754.804348
7,1,2304.272727
7,1,2670.975610
7,1,3293.204082
7,1,2964.961538
7,1,1595.466667
7,1,6233.652174
7,1,10535.462500
7,1,2419.485294
7,1,6410.875000
7,1,1564.750000
7,1,2711.943396
7,1,3080.767857
7,1,2048.740385
7,1,7659.000000
7,1,3186.918103
7,1,1923.176829
7,1,3034.808824
7,1,1685.103571
7,1,2819.481707
7,1,3125.975000
7,1,2149.079545
7,1,2930.699324
7,1,6739.484043
7,1,3339.685897
7,1,3056.932692
7,1,1931.838710
7,1,18611.292683
7,1,12276.366667
7,1,1822.390625
7,1,4545.627404
7,1,3706.000000
7,1,4227.180556
7,1,2829.172794
7,1,3935.321875
7,1,12288.392857
7,1,8180.960227
7,1,2898.096774
7,1,7315.697917
7,1,5164.384615
7,1,4015.250000
7,1,3686.514535
7,1,5906.400000
7,1,4382.352273
7,1,2664.200000
7,1,1728.375000
7,1,2498.724265
7,1,7340.285714
7,1,1865.903846
7,1,2015.187500

7,1,5867.907051
7,1,2320.153846
7,1,1670.841463
7,1,2008.172222
7,1,5742.475610
7,1,1735.281250
7,1,14665.529412
7,1,1708.944149
7,1,3182.145833
7,1,3387.512500
7,1,2489.424242
7,1,7297.287500
7,1,1721.682432
7,1,3992.525000
7,1,3750.537736
7,1,5498.155000
7,1,2950.429878
7,1,2474.733333
7,1,3182.820513
7,1,5107.502315
7,1,2202.644444
7,1,1686.678571
7,1,1803.250000
7,1,2122.750000
7,1,2005.750000
7,1,1755.088542
7,1,4737.066667
7,1,17518.403061
7,1,2736.854730
7,1,2365.565625
7,1,7188.970930
7,1,2250.228571
7,1,3789.288462
7,1,2211.813953
7,1,3933.488889
7,1,7499.007143
7,1,1565.666667
7,1,3421.496711
7,1,5610.612245
7,1,2447.030488
7,1,7167.000000
7,1,4185.005435
7,1,2000.276786
7,1,7827.184211
7,1,2697.993056
7,1,2672.776596
7,1,2009.300000
7,1,11029.529412
7,1,3406.000000
7,1,1724.842105
7,1,5434.741379
7,1,6062.250000
7,1,4451.175926
7,1,2669.571429
7,1,1625.620690
7,1,2876.621622
7,1,4170.928977
7,1,1534.625000
7,1,6297.375000
7,1,2170.967262
7,1,2359.796053
7,1,2903.538793
7,1,1565.493590
7,1,3117.018939
7,1,1630.058824
7,1,5941.442857

7,1,4558.897727
7,1,2984.346154
7,1,1949.389706
7,1,6193.615385
7,1,1701.713415
7,1,1960.071429
7,1,6717.293269
7,1,8016.101351
7,1,1731.241379
7,1,9360.428571
7,1,10088.176471
7,1,4652.000000
7,1,1555.237805
7,1,4208.414634
7,1,1767.519231
7,1,2301.553571
7,1,3210.022222
7,1,2675.441860
7,1,2142.750000
7,1,1633.529412
7,1,7905.914474
7,1,3423.269231
7,1,2858.835366
7,1,2538.239130
7,1,2474.247093
7,1,5168.049479
7,1,2639.052632
7,1,1754.067935
7,1,1749.169643
7,1,14875.272727
7,1,16068.550000
7,1,3220.605769
7,1,9949.117857
7,1,1717.451389
7,1,5326.217391
7,1,1664.540000
7,1,1630.360465
7,1,3965.454327
7,1,6226.037500
7,1,5295.350000
7,1,2892.246875
7,1,8962.125000
7,1,6857.428571
7,1,3325.562500
7,1,2953.176471
7,1,2239.994565
7,1,5850.832143
7,1,2500.939189
7,1,8724.469697
7,1,1995.648649
7,1,1925.000000
7,1,2454.959302
7,1,4259.477564
7,1,3511.055556
7,1,2219.414634
7,1,9536.333333
7,1,1971.738462
7,1,1819.934659
7,1,10654.180556
7,1,2446.000000
7,1,3859.517857
7,1,6327.000000
7,1,4659.500000
7,1,3580.446023
7,1,2538.744565
7,1,5585.625000

7,1,1640.435606
7,1,2426.146875
7,1,10561.200000
7,1,1970.677083
7,1,16871.743590
7,1,3691.875000
7,1,2294.354839
7,1,4356.142857
7,1,4596.950000
7,1,1663.982143
7,1,1827.875000
7,1,5363.101064
7,1,1647.451531
7,1,1686.154605
7,1,2551.522727
7,1,1505.026786
7,1,5821.678571
7,1,5401.752632
7,1,6260.703947
7,1,1680.044118
7,1,1633.268750
7,1,3143.404255
7,1,2238.067308
7,1,2365.871429
7,1,2068.615625
7,1,8598.209375
7,1,5497.562500
7,1,6691.821429
7,1,2490.194444
7,1,10308.295455
7,1,2475.846154
7,1,4869.627660
7,1,6123.000000
7,1,2941.333333
7,1,1809.312500
7,1,3185.053571
7,1,3735.425000
7,1,3194.666667
7,1,3276.960000
7,1,10687.684211
7,1,3148.375000
7,1,2489.125000
7,1,1954.521739
7,1,2698.005682
7,1,1586.928571
7,1,1842.213235
7,1,19780.423387
7,1,2243.625000
7,1,6075.538462
7,1,3733.588235
7,1,1523.000000
7,1,2888.562500
7,1,2701.677778
7,1,2400.542857
7,1,2367.217391
7,1,6962.986486
7,1,6968.823529
7,1,19273.958333
7,1,13719.743902
7,1,2294.494048
7,1,1722.224265
7,1,15348.175595
7,1,2569.133523
7,1,16290.257143
7,1,1546.425000
7,1,12957.250000

7,1,4342.888889
7,1,3543.731707
7,1,2129.127451
7,1,6437.790625
7,1,1684.071429
7,1,2949.576087
7,1,4051.757353
7,1,5437.500000
7,1,2270.981250
7,1,2144.382353
7,1,3747.946875
7,1,2178.725000
7,1,2697.000000
7,1,2122.259375
7,1,16399.666667
7,1,4251.093750
7,1,5963.692308
7,1,5972.146341
7,1,2474.947674
7,1,1593.321429
7,1,2160.436047
7,1,3266.656250
7,1,1583.218750
7,1,2814.714674
7,1,7810.910156
7,1,3288.500000
7,1,7308.310811
7,1,2292.746711
7,1,8420.161290
7,1,3303.064516
7,1,4043.517857
7,1,5444.416667
7,1,12005.405405
7,1,12879.896429
7,1,3270.414474
7,1,1950.398649
7,1,2413.116935
7,1,1944.023256
7,1,8193.902027
7,1,16378.236486
7,1,1871.454082
7,1,2916.188679
7,1,1810.884615
7,1,1958.700581
7,1,1962.722892
7,1,4030.981250
7,1,3881.357143
7,1,5970.250000
7,1,2269.277778
7,1,1905.909091
7,1,2400.694767
7,1,5625.569079
7,1,4325.513158
7,1,3025.786932
7,1,2733.833333
7,1,8073.785326
7,1,3897.875000
7,1,2356.597656
7,1,10157.195122
7,1,12101.443750
7,1,2847.456897
7,1,4606.482143
7,1,10192.091463
7,1,2434.002604
7,1,9476.193548
7,1,10336.230769

7,1,3128.961538
7,1,8230.500000
7,1,13620.906977
7,1,4789.665441
7,1,3158.641304
7,1,5464.553030
7,1,2811.273438
7,1,10526.350000
7,1,10350.921196
7,1,13513.571429
7,1,2243.370370
7,1,6679.406250
7,1,2051.058824
7,1,2185.355769
7,1,1608.471429
7,1,1885.434783
7,1,2095.431250
7,1,7781.886111
7,1,4925.625000
7,1,4486.547297
7,1,4803.201923
7,1,7108.649390
7,1,2723.250000
7,1,1994.017857
7,1,1560.880814
7,1,6304.470109
7,1,5062.075581
7,1,1807.802778
7,1,5108.437500
7,1,3515.945312
7,1,3358.687500
7,1,2656.015625
7,1,3150.057692
7,1,1724.125000
7,1,2262.678571
7,1,13051.046875
7,1,3104.285714
7,1,1965.612500
7,1,3281.291667
7,1,1505.216216
7,1,1692.619565
7,1,8097.253472
7,1,9352.944444
7,1,4122.260417
7,1,2097.791667
7,1,6887.272222
7,1,11741.710526
7,1,7199.159091
7,1,2532.260135
7,1,2253.750000
7,1,19987.842593
7,1,2367.195652
7,1,1549.750000
7,1,2059.250000
7,1,2954.194149
7,1,12625.746622
7,1,2919.298913
7,1,3266.468750
7,1,1515.683333
7,1,4921.936170
7,1,1901.434783
7,1,7116.000000
7,1,1989.150862
7,1,12595.850610
7,1,2748.454787
7,1,2914.252778

7,1,5343.989796
7,1,1715.228261
7,1,3642.875000
7,1,2014.106771
7,1,16439.974138
7,1,1749.673077
7,1,2651.530405
7,1,2085.691860
7,1,4642.297794
7,1,3563.744318
7,1,1936.722222
7,1,1846.962209
7,1,3741.181818
7,1,2599.375000
7,1,11372.945946
7,1,2049.761111
7,1,1690.500000
7,1,4393.535714
7,1,3747.093023
7,1,2888.510204
7,1,2124.727273
7,1,4330.764706
7,1,7999.243056
7,1,1571.819853
7,1,2595.270833
7,1,9717.625000
7,1,4089.818182
7,1,2205.000000
7,1,12418.225806
7,1,2471.833333
7,1,1723.420000
7,1,18502.203488
7,1,1695.969697
7,1,3435.142857
7,1,4041.125000
7,1,15438.465909
7,1,2768.967262
7,1,3021.909091
7,1,2899.476190
7,1,4230.562500
7,1,2760.758333
7,1,8128.562500
7,1,1876.791667
7,1,1805.896739
7,1,5001.428571
7,1,4387.958333
7,1,2774.628205
7,1,1839.118421
7,1,1504.993243
7,1,10102.106481
7,1,6644.608108
7,1,17859.614583
7,1,3685.962500
7,1,4651.857143
7,1,4571.980769
7,1,9172.352273
7,1,2183.256098
7,1,1697.310976
7,1,2772.213068
7,1,1628.688889
7,1,7794.744565
7,1,2689.212500
7,1,2042.812500
7,1,18289.290816
7,1,2059.300781
7,1,12984.545139

7,1,4424.363889
7,1,2272.491477
7,1,1955.659091
7,1,2638.030172
7,1,1892.578125
7,1,9097.571875
7,1,1738.482558
7,1,3998.804348
7,1,2250.968750
7,1,1702.454545
7,1,2060.068182
7,1,1563.916667
7,1,2951.187500
7,1,4084.451613
7,1,2921.931034
7,1,1850.110000
7,1,1500.976562
7,1,2766.461806
7,1,3243.950000
7,1,2673.875000
7,1,7393.727041
7,1,1638.883721
7,1,7013.468750
7,1,1500.720000
7,1,3886.830128
7,1,6505.710938
7,1,1984.356707
7,1,3861.092742
7,1,10099.629808
7,1,1976.795213
7,1,9893.388889
7,1,4905.736111
7,1,1897.335526
7,1,7873.675000
7,1,4460.312500
7,1,1580.961538
7,1,2214.776316
7,1,4066.137097
7,1,5936.384868
7,1,2136.238208
7,1,1742.214286
7,1,15883.911184
7,1,15531.356250
7,1,6133.136364
7,1,2481.661111
7,1,1585.991071
7,1,1633.955556
7,1,15251.062500
7,1,9247.744186
7,1,3734.048387
7,1,15870.169118
7,1,8600.113889
7,1,1542.463889
7,1,2732.937500
7,1,5216.973684
7,1,2276.694444
7,1,1657.552632
7,1,4708.870466
7,1,7438.380952
7,1,10564.817073
7,1,4519.160000
7,1,7557.920455
7,1,10275.032895
7,1,2448.205882
7,1,16404.933333
7,1,9964.413043

7,1,2970.538462
7,1,2441.228261
7,1,1859.689024
7,1,2824.444444
7,1,3974.687500
7,1,1520.191489
7,1,6582.948171
7,1,2338.441860
7,1,1875.850610
7,1,4756.821429
7,1,2533.278846
7,1,1884.566489
7,1,2835.380952
7,1,1829.375000
7,1,5878.925676
7,1,4711.083333
7,1,2325.829787
7,1,1644.524306
7,1,3242.924107
7,1,6177.189189
7,1,2190.570000
7,1,9192.653409
7,1,2811.184211
7,1,2747.523810
7,1,3173.493750
7,1,2635.677632
7,1,4661.735577
7,1,2461.315476
7,1,1709.167969
7,1,3815.647321
7,1,6655.347222
7,1,1511.394737
7,1,3604.441176
7,1,12532.560811
7,1,1595.793103
7,1,4170.634615
7,1,5997.896277
7,1,2142.812500
7,1,13536.500000
7,1,6722.000000
7,1,2804.489362
7,1,2369.000000
7,1,1813.768382
7,1,4216.790210
7,1,4286.954545
7,1,2791.127660
7,1,5134.944444
7,1,1915.391304
7,1,5277.100610
7,1,3705.478723
7,1,2311.795918
7,1,17244.750000
7,1,2313.719512
7,1,2515.500000
7,1,2097.234104
7,1,8080.916667
7,1,3435.987500
7,1,1930.384146
7,1,17482.813953
7,1,1650.359375
7,1,4438.154762
7,1,3086.692308
7,1,2558.485714
7,1,5105.026042
7,1,9345.200658
7,1,3603.804348

7,1,2307.500000
7,1,11934.369048
7,1,8505.970745
7,1,16062.634615
7,1,3173.071429
7,1,3363.181818
7,1,1778.905488
7,1,1994.297794
7,1,1911.005952
7,1,1568.218750
7,1,1769.285714
7,1,1833.125000
7,1,1816.612500
7,1,15089.723485
7,1,13480.538462
7,1,12778.865385
7,1,2176.793750
7,1,8256.982558
7,1,1687.567568
7,1,5367.608696
7,1,2633.416667
7,1,1886.398026
7,1,2877.509868
7,1,2435.841912
7,1,8527.500000
7,1,3528.520833
7,1,4240.625000
7,1,7957.375000
7,1,1798.041667
7,1,6286.335366
7,1,2091.192568
7,1,17270.632979
7,1,4532.031250
7,1,14921.726190
7,1,4297.069767
7,1,12161.548780
7,1,1776.613636
7,1,5404.279891
7,1,2870.383152
7,1,15524.075000
7,1,1568.541667
7,1,2291.568452
7,1,2110.960937
7,1,5681.639423
7,1,2126.500000
7,1,3482.010135
7,1,6127.418605
7,1,3011.766129
7,1,17158.500000
7,1,4225.375000
7,1,8241.032258
7,1,1518.714286
7,1,2618.666667
7,1,2185.230769
7,1,3456.066667
7,1,4923.178571
7,1,3039.097561
7,1,14316.892857
7,1,2006.119565
7,1,5948.200000
7,1,4516.610577
7,1,4901.756757
7,1,1617.505435
7,1,2407.200000
7,1,11832.231618
7,1,7231.000000

7,1,17155.480769
7,1,19409.162500
7,1,2270.252451
7,1,3828.295455
7,1,1532.714286
7,1,4292.832258
7,1,2298.159574
7,1,3856.875000
7,1,2029.121951
7,1,3542.531250
7,1,4712.122396
7,1,1564.406250
7,1,8261.356164
7,1,13604.702128
7,1,2676.804054
7,1,15908.704787
7,1,3806.738372
7,1,1935.250000
7,1,2293.428571
7,1,1533.446875
7,1,1701.031250
7,1,13577.540323
7,1,3150.222222
7,1,2544.852273
7,1,6084.463415
7,1,2399.872642
7,1,2552.980769
7,1,2473.800000
7,1,6876.250000
7,1,5908.571429
7,1,9690.485294
7,1,2748.206522
7,1,2452.077381
7,1,2660.035714
7,1,1790.503378
7,1,2074.537500
7,1,1683.983333
7,1,4879.666667
7,1,9222.612903
7,1,3345.212766
7,1,2256.864865
7,1,8471.763889
7,1,1698.703488
7,1,5713.607143
7,1,1943.625000
7,1,3775.416667
7,1,1894.347222
7,1,3199.500000
7,1,8347.304688
7,1,4154.181250
7,1,12347.590625
7,1,4420.016129
7,1,4393.050532
7,1,2704.000000
7,1,1755.401515
7,1,5100.615385
7,1,1654.709677
7,1,15611.256579
7,1,5523.919118
7,1,1977.521341
7,1,2468.656250
7,1,4329.127907
7,1,5921.594512
7,1,2920.653846
7,1,1509.769231
7,1,9183.604651

7,1,5547.136364
7,1,3527.164773
7,1,2305.782051
7,1,6783.355769
7,1,6152.072368
7,1,1645.562500
7,1,5338.823529
7,1,4137.164062
7,1,4282.645161
7,1,4885.400000
7,1,3658.941176
7,1,2042.322581
7,1,1601.410714
7,1,2092.880102
7,1,15860.608108
7,1,7603.511111
7,1,6006.831081
7,1,4674.940476
7,1,2199.734375
7,1,1591.990625
7,1,2442.773148
7,1,2848.011628
7,1,10154.926630
7,1,1904.416667
7,1,2765.276596
7,1,1924.982143
7,1,4409.772059
7,1,1632.269022
7,1,5862.520833
7,1,1918.027778
7,1,1568.865854
7,1,2525.067568
7,1,2024.526316
7,1,4212.760870
7,1,7325.725000
7,1,1619.130435
7,1,2729.269231
7,1,1507.691489
7,1,2728.850000
7,1,2084.763889
7,1,4525.838235
7,1,1649.889706
7,1,3127.478723
7,1,15855.100610
7,1,2454.772727
7,1,2792.037500
7,1,1608.977941
7,1,2852.928571
7,1,13806.375000
7,1,1983.727273
7,1,3777.078704
7,1,3384.534091
7,1,2882.133929
7,1,3373.461538
7,1,2512.252976
7,1,1941.050000
7,1,1885.011029
7,1,2470.660714
7,1,2039.163793
7,1,5011.878125
7,1,2755.335366
7,1,9176.140000
7,1,13271.454545
7,1,3428.529412
7,1,4892.967742
7,1,6984.541667

7,1,6165.379630
7,1,9463.446429
7,1,1558.530172
7,1,1776.994186
7,1,1919.140625
7,1,2633.293919
7,1,1821.657143
7,1,4578.015625
7,1,13124.500000
7,1,3582.453125
7,1,6385.076923
7,1,5046.000000
7,1,3114.512500
7,1,1647.212963
7,1,9356.500000
7,1,3325.534884
7,1,1704.808673
7,1,2457.794872
7,1,8347.239796
7,1,2101.074074
7,1,5696.500000
7,1,3282.119048
7,1,1772.877907
7,1,3837.420732
7,1,2342.353659
7,1,4955.274882
7,1,3741.625000
7,1,1512.134375
7,1,4482.319079
7,1,1554.720109
7,1,1991.625000
7,1,3064.331081
7,1,1872.024390
7,1,2850.371711
7,1,5930.627451
7,1,3243.107143
7,1,13255.142045
7,1,5344.631250
7,1,1963.266129
7,1,3711.300000
7,1,16448.756757
7,1,3020.887500
7,1,9514.281250
7,1,1729.169643
7,1,5960.875000
7,1,2922.523148
7,1,9136.835366
7,1,13807.792969
7,1,10943.518293
7,1,2186.220395
7,1,14786.583333
7,1,1941.645833
7,1,1887.439024
7,1,3042.568182
7,1,1666.216216
7,1,5814.835714
7,1,4005.494186
7,1,7156.661458
7,1,2253.113636
7,1,1873.824324
7,1,2684.104167
7,1,1835.537500
7,1,3471.678571
7,1,4098.576613
7,1,1852.024390
7,1,2865.000000

7,1,3105.330357
7,1,2435.310811
7,1,3424.125000
7,1,1608.426136
7,1,11062.684211
7,1,4280.634615
7,1,2542.562500
7,1,7201.765152
7,1,1995.611111
7,1,1897.036932
7,1,5271.660714
7,1,2029.488372
7,1,1499.666667
7,1,11047.616071
7,1,5174.427419
7,1,4801.045732
7,1,1519.600000
7,1,6956.756410
7,1,7891.830882
7,1,1961.798387
7,1,5239.750000
7,1,1795.617188
7,1,8923.030405
7,1,2087.231618
7,1,2280.357143
7,1,3666.250000
7,1,3630.328125
7,1,3887.230769
7,1,3875.857143
7,1,2195.090909
7,1,1531.505435
7,1,1947.029605
7,1,1574.392857
7,1,5810.730114
7,1,3963.102941
7,1,16120.083333
7,1,14582.894737
7,1,3521.711111
7,1,3950.390244
7,1,2061.080645
7,1,5554.232143
7,1,8701.392857
7,1,2194.216837
7,1,3020.317308
7,1,4163.548387
7,1,9354.916667
7,1,2276.906250
7,1,1773.367647
7,1,5075.651961
7,1,1881.789062
7,1,1697.294118
7,1,3392.100000
7,1,1749.468085
7,1,2043.962500
7,1,8587.327128
7,1,7886.895349
7,1,6176.250000
7,1,1747.207317
7,1,3178.341463
7,1,2753.779891
7,1,9546.137500
7,1,10088.200000
7,1,1616.937500
7,1,18727.633333
7,1,1568.065217
7,1,1550.028409

7,1,1921.707143
7,1,1602.090909
7,1,4476.963710
7,1,1909.663043
7,1,3044.961538
7,1,13251.000000
7,1,2013.344371
7,1,4298.614865
7,1,3573.913690
7,1,3292.387019
7,1,6115.385714
7,1,5967.939024
7,1,1520.000000
7,1,15429.750000
7,1,2303.517857
7,1,8274.346154
7,1,4897.692308
7,1,2427.291667
7,1,2671.576389
7,1,1626.823529
7,1,9710.191860
7,1,3531.000000
7,1,2448.718750
7,1,10534.887755
7,1,2539.250000
7,1,2895.909091
7,1,4943.348214
7,1,1611.800000
7,1,4611.858696
7,1,7207.549020
7,1,3198.351064
7,1,10298.416667
7,1,4314.980769
7,1,15968.303030
7,1,8389.608108
7,1,3836.055233
7,1,2578.333333
7,1,10690.308333
7,1,7937.228571
7,1,2578.909884
7,1,7098.439516
7,1,17557.928571
7,1,8929.625000
7,1,14181.058824
7,1,1897.625000
7,1,1952.562500
7,1,1608.237500
7,1,9908.838542
7,1,2271.459459
7,1,4346.773438
7,1,2840.692857
7,1,8498.375000
7,1,14850.882353
7,1,1551.215517
7,1,5848.625000
7,1,6360.250000
7,1,3187.803571
7,1,1771.769231
7,1,1957.160156
7,1,2144.312500
7,1,1601.500000
7,1,6254.250000
7,1,3248.700000
7,1,1723.306452
7,1,1734.968750
7,1,6255.132353

7,1,4872.161184
7,1,1882.757353
7,1,2208.905405
7,1,3053.576923
7,1,8666.046512
7,1,2150.779070
7,1,2215.095588
7,1,1720.809783
7,1,2431.472561
7,1,13112.652027
7,1,5914.893519
7,1,4830.225806
7,1,10786.326087
7,1,4206.900000
7,1,2198.652778
7,1,8702.375000
7,1,1806.038462
7,1,3954.038194
7,1,7757.000000
7,1,10641.427419
7,1,6510.363636
7,1,1680.195122
7,1,1921.120000
7,1,8027.120833
7,1,1499.573370
7,1,1994.637500
7,1,7683.016304
7,1,15991.935606
7,1,3796.236364
7,1,2907.487805
7,1,2333.857143
7,1,2613.163043
7,1,5598.073171
7,1,2325.941176
7,1,1757.619565
7,1,2881.297619
7,1,1747.380000
7,1,5672.982759
7,1,1725.111111
7,1,1599.608108
7,1,7436.442568
7,1,1875.984375
7,1,1966.078947
7,1,7424.778125
7,1,2300.328947
7,1,2352.382500
7,1,2281.259259
7,1,7092.607143
7,1,3160.297872
7,1,2858.391304
7,1,3150.846591
7,1,2064.253571
7,1,1910.520833
7,1,7734.395833
7,1,3617.735294
7,1,2878.985294
7,1,1890.409677
7,1,2094.239130
7,1,1722.250000
7,1,4333.684211
7,1,3419.466912
7,1,1588.299419
7,1,1632.777500
7,1,4332.400000
7,1,5255.321429
7,1,6087.406250

7,1,7694.105263
7,1,5556.642500
7,1,6252.628571
7,1,1764.328125
7,1,2492.600000
7,1,2130.386364
7,1,2818.689286
7,1,1647.250000
7,1,3481.431818
7,1,10397.851064
7,1,3592.300000
7,1,2104.096154
7,1,5139.222561
7,1,14124.085366
7,1,2138.619565
7,1,4638.660714
7,1,2370.472222
7,1,3603.906250
7,1,2905.127551
7,1,7884.111111
7,1,2280.059524
7,1,2368.272059
7,1,3419.714286
7,1,2283.036585
7,1,4433.918367
7,1,5610.950000
7,1,2672.253125
7,1,2467.479651
7,1,9945.386111
7,1,1525.750000
7,1,1964.736842
7,1,13105.707317
7,1,19760.192308
7,1,3189.630102
7,1,1538.276596
7,1,4976.518519
7,1,1685.032895
7,1,4847.666667
7,1,9410.275510
7,1,3214.826087
7,1,2640.117647
7,1,2543.988281
7,1,6508.699219
7,1,1529.312500
7,1,7473.781250
7,1,3064.347222
7,1,2802.364865
7,1,6227.948864
7,1,2281.828125
7,1,1831.612903
7,1,1906.666667
7,1,2113.426136
7,1,3579.229839
7,1,7162.142857
7,1,2699.625000
7,1,2322.464286
7,1,1953.416667
7,1,17070.687500
7,1,2318.611111
7,1,1585.500000
7,1,7626.216216
7,1,1945.360119
7,1,4440.851852
7,1,2915.250000
7,1,4274.468085
7,1,1557.531250

7,1,2125.333333
7,1,6278.750000
7,1,3991.403226
7,1,4595.611979
7,1,6391.048077
7,1,7840.802632
7,1,1583.372283
7,1,1999.395833
7,1,1998.378049
7,1,2990.059524
7,1,1559.878289
7,1,5866.868421
7,1,1863.678191
7,1,19065.602941
7,1,1560.788462
7,1,3580.715909
7,1,4416.437500
7,1,17813.756098
7,1,1690.352273
7,1,2759.617021
7,1,5557.266667
7,1,4751.178977
7,1,1709.744186
7,1,3614.625000
7,1,5239.895000
7,1,3033.863636
7,1,13412.650000
7,1,4940.089286
7,1,12384.121951
7,1,6140.500000
7,1,1500.714286
7,1,7499.197581
7,1,6149.189394
7,1,3312.328125
7,1,7423.000000
7,1,5964.666667
7,1,1854.857143
7,1,4110.176282
7,1,16047.365385
7,1,3898.933333
7,1,2181.700000
7,1,8540.576087
7,1,2783.122222
7,1,12704.757353
7,1,2564.125000
7,1,2798.802632
7,1,5220.120879
7,1,6135.578488
7,1,1860.800000
7,1,16997.303571
7,1,5855.096491
7,1,8670.721591
7,1,1766.617187
7,1,1832.346429
7,1,1522.942857
7,1,13529.564103
7,1,8161.083333
7,1,6850.125000
7,1,1977.847561
7,1,2319.130952
7,1,2938.175000
7,1,1763.159574
7,1,4998.852941
7,1,1548.375000
7,1,3990.575000
7,1,7979.937500

7,1,1789.280488
7,1,4297.837209
7,1,5238.779412
7,1,7659.012500
7,1,2251.625000
7,1,3692.699387
7,1,2761.847826
7,1,1949.166667
7,1,3803.992857
7,1,2670.593750
7,1,2332.500000
7,1,2668.833333
7,1,15545.333333
7,1,4095.857143
7,1,7183.594512
7,1,1628.144737
7,1,2614.100000
7,1,1893.713235
7,1,2630.083333
7,1,3708.630682
7,1,4561.325000
7,1,1662.961538
7,1,2303.017442
7,1,2840.117647
7,1,6769.032609
7,1,1827.483974
7,1,2711.469697
7,1,3985.425000
7,1,7358.687500
7,1,1548.083333
7,1,1822.161765
7,1,2607.022727
7,1,3806.684783
7,1,16404.702381
7,1,13741.707317
7,1,3949.338235
7,1,1607.783537
7,1,2245.713235
7,1,10197.451613
7,1,11916.732558
7,1,1897.750000
7,1,1673.559028
7,1,1940.907738
7,1,6341.875000
7,1,1540.852941
7,1,1767.480769
7,1,18561.466667
7,1,1871.772727
7,1,10562.902256
7,1,6454.052632
7,1,3157.602941
7,1,18157.335000
7,1,3978.074405
7,1,1669.269737
7,1,1629.085227
7,1,3277.914474
7,1,2569.100000
7,1,2071.033537
7,1,6494.671512
7,1,2507.155488
7,1,13948.325000
7,1,2214.593750
7,1,6733.683511
7,1,4023.679688
7,1,2100.830882
7,1,4078.228814

7,1,2803.975000
7,1,1559.566667
7,1,5395.086957
7,1,5388.611607
7,1,15973.600000
7,1,3862.605263
7,1,4333.375000
7,1,5936.208333
7,1,4710.310606
7,1,5759.250000
7,1,3496.733871
7,1,2490.678922
7,1,3243.966667
7,1,1532.763587
7,1,4533.597826
7,1,2299.450521
7,1,9227.217857
7,1,1869.880814
7,1,4690.218750
7,1,2949.923077
7,1,11607.132143
7,1,2023.338235
7,1,5969.111111
7,1,1955.294118
7,1,1509.861842
7,1,4906.581731
7,1,2212.990385
7,1,2844.000000
7,1,9536.600000
7,1,2862.187500
7,1,3434.402027
7,1,5836.270270
7,1,2044.750000
7,1,12079.756757
7,1,3247.120370
7,1,4569.070946
7,1,2288.307692
7,1,2589.234375
7,1,8018.466216
7,1,2542.819853
7,1,2136.607143
7,1,6072.763158
7,1,1729.080556
7,1,2582.000000
7,1,14777.155172
7,1,1928.687500
7,1,2048.048780
7,1,6150.369231
7,1,11937.920000
7,1,7119.625000
7,1,4233.283784
7,1,4358.333333
7,1,6963.342500
7,1,4284.643382
7,1,1715.057692
7,1,1709.304878
7,1,12442.125000
7,1,2600.037736
7,1,3219.535714
7,1,3054.465625
7,1,5347.012821
7,1,6652.030303
7,1,3550.445312
7,1,5206.042500
7,1,2934.460227
7,1,2648.500000

7,1,2421.587500
7,1,6868.237179
7,1,1517.397727
7,1,2635.427632
7,1,2255.000000
7,1,2208.231250
7,1,1867.277778
7,1,2142.333333
7,1,2176.789773
7,1,5287.180147
7,1,2630.207031
7,1,3044.732955
7,1,1784.562500
7,1,4717.234375
7,1,5130.030303
7,1,3721.652027
7,1,1520.266667
7,1,2356.372671
7,1,3573.671875
7,1,1553.539773
7,1,6933.000000
7,1,1725.072581
7,1,1857.400000
7,1,3221.019231
7,1,1863.960526
7,1,3261.799419
7,1,1891.958333
7,1,3065.655405
7,1,8205.215909
7,1,2746.473214
7,1,3690.575581
7,1,1749.919118
7,1,1812.145833
7,1,2149.980000
7,1,2306.411585
7,1,3993.705882
7,1,3502.923077
7,1,1981.085784
7,1,7084.408654
7,1,5113.213415
7,1,1774.485714
7,1,5682.612500
7,1,11487.383333
7,1,2783.216216
7,1,2977.442935
7,1,5013.694853
7,1,1881.128472
7,1,3136.553191
7,1,7026.687500
7,1,1528.231707
7,1,4815.462500
7,1,6984.108108
7,1,2783.319444
7,1,2589.401163
7,1,7909.691176
7,1,2813.911765
7,1,3950.906977
7,1,1986.257353
7,1,2747.531250
7,1,4827.306818
7,1,19014.125000
7,1,3928.923611
7,1,3798.833333
7,1,10046.973214
7,1,7671.978261
7,1,1860.562500

7,1,1518.055147
7,1,1508.366935
7,1,3437.220588
7,1,3830.114865
7,1,5028.463415
7,1,4983.960227
7,1,6344.993421
7,1,2487.310811
7,1,2670.176136
7,1,2379.289634
7,1,2468.629032
7,1,2155.323171
7,1,1811.395833
7,1,17883.525735
7,1,2284.903846
7,1,5259.340000
7,1,2785.416667
7,1,1819.356383
7,1,3940.507653
7,1,1611.000000
7,1,1602.162500
7,1,2728.200000
7,1,17252.750000
7,1,15224.621951
7,1,1703.422872
7,1,2530.413043
7,1,10099.346154
7,1,2031.410000
7,1,9220.142857
7,1,15176.615625
7,1,3652.523438
7,1,1642.264535
7,1,2804.595238
7,1,3706.371711
7,1,13958.369565
7,1,1740.769231
7,1,2309.718750
7,1,7445.443750
7,1,2433.005682
7,1,9332.906250
7,1,3856.225000
7,1,3763.825000
7,1,11129.625000
7,1,16342.926136
7,1,2311.437500
7,1,5862.425676
7,1,11555.000000
7,1,1949.983871
7,1,3107.416667
7,1,2588.413669
7,1,2342.562500
7,1,3930.616279
7,1,1861.000000
7,1,2033.280488
7,1,2243.125000
7,1,4556.561047
7,1,1970.604651
7,1,3884.538043
7,1,3290.559375
7,1,1857.066667
7,1,18006.918919
7,1,2456.801630
7,1,5685.236842
7,1,5373.000000
7,1,8306.323529
7,1,1909.058140

7,1,3289.109375
7,1,2708.973958
7,1,3429.929054
7,1,2226.170455
7,1,3034.830000
7,1,1526.086538
7,1,8643.512500
7,1,16246.127660
7,1,13150.794643
7,1,3464.812500
7,1,8412.000000
7,1,2403.356250
7,1,2005.500000
7,1,4403.583333
7,1,4069.383065
7,1,8620.125000
7,1,5717.013889
7,1,2276.406250
7,1,3666.977273
7,1,3605.968750
7,1,1513.702128
7,1,1526.191860
7,1,4406.125000
7,1,13206.678571
7,1,7383.636364
7,1,2654.460784
7,1,3321.321429
7,1,1973.000000
7,1,5874.564286
7,1,17984.650000
7,1,13460.334559
7,1,6428.785714
7,1,1591.574468
7,1,1890.841463
7,1,1763.458333
7,1,11617.543605
7,1,1506.800000
7,1,5142.500000
7,1,2852.780488
7,1,15191.246795
7,1,8347.761719
7,1,1634.875000
7,1,15095.909884
7,1,2070.906250
7,1,2180.488636
7,1,9376.714286
7,1,4165.760870
7,1,3057.642857
7,1,13087.288690
7,1,2544.237245
7,1,5850.732558
7,1,6429.250000
7,1,5650.236111
7,1,4388.332447
7,1,2124.274390
7,1,2028.254902
7,1,3132.042683
7,1,3665.850000
7,1,5761.515625
7,1,5298.111111
7,1,2351.372159
7,1,1721.130952
7,1,7746.334677
7,1,4714.911765
7,1,2871.644737
7,1,11423.541667

7,1,2933.071429
7,1,2138.055147
7,1,4977.291667
7,1,17469.028125
7,1,2404.703125
7,1,12984.356618
7,1,5636.004630
7,1,18338.037500
7,1,1698.993243
7,1,5343.587500
7,1,10092.435897
7,1,10669.682927
7,1,8582.000000
7,1,2615.133929
7,1,7389.072917
7,1,18719.598485
7,1,2689.750000
7,1,1956.500000
7,1,3406.714286
7,1,2118.985119
7,1,1792.812500
7,1,10482.045455
7,1,2174.289474
7,1,5569.987903
7,1,4980.858108
7,1,1786.364130
7,1,5624.890625
7,1,1751.729730
7,1,5063.211538
7,1,2557.231707
7,1,1724.676471
7,1,1969.187500
7,1,3107.312500
7,1,2117.367500
7,1,11521.750000
7,1,2988.296512
7,1,6263.935484
7,1,2374.616071
7,1,6813.789474
7,1,5689.597656
7,1,15926.815789
7,1,5958.888889
7,1,1517.505435
7,1,2788.125000
7,1,8453.715909
7,1,1540.337838
7,1,15959.780303
7,1,2171.750000
7,1,2744.258333
7,1,12181.778571
7,1,6981.939189
7,1,10523.131410
7,1,6693.787234
7,1,4757.713235
7,1,2466.800000
7,1,3880.071429
7,1,2080.250000
7,1,2098.734756
7,1,5674.563953
7,1,5919.500000
7,1,17733.894737
7,1,2732.715116
7,1,5205.935484
7,1,11303.395270
7,1,4124.583333
7,1,3643.882143

7,1,1567.602273
7,1,5282.187500
7,1,6959.464286
7,1,2487.143750
7,1,2445.031250
7,1,6357.016129
7,1,3284.205357
7,1,9149.388889
7,1,1656.155556
7,1,2546.093750
7,1,3929.980769
7,1,2244.272727
7,1,9963.500000
7,1,2037.162162
7,1,3718.565476
7,1,3876.117188
7,1,2055.802632
7,1,3778.483553
7,1,1847.048295
7,1,3131.235294
7,1,2111.712838
7,1,2966.479167
7,1,4837.121622
7,1,3018.492647
7,1,5297.527778
7,1,3761.365854
7,1,12357.653409
7,1,3031.760000
7,1,2149.788043
7,1,5765.384615
7,1,1938.890244
7,1,6854.147059
7,1,5301.312500
7,1,2416.467262
7,1,4395.810976
7,1,2482.090909
7,1,4392.846774
7,1,8268.588235
7,1,2384.472561
7,1,7235.717742
7,1,1509.680556
7,1,4365.100000
7,1,4804.948529
7,1,8822.510417
7,1,3717.400000
7,1,3565.221591
7,1,18679.666667
7,1,1506.211538
7,1,2482.762500
7,1,5893.042683
7,1,4846.672414
7,1,2376.850000
7,1,1939.250000
7,1,3677.176471
7,1,13309.788306
7,1,2127.846154
7,1,3528.572917
7,1,13377.647727
7,1,1573.312500
7,1,2017.581395
7,1,8021.850490
7,1,1713.153061
7,1,2378.390625
7,1,6003.743902
7,1,6376.378049
7,1,16578.566667

7,1,2717.067073
7,1,3349.191176
7,1,4401.522436
7,1,5677.875000
7,1,4165.183333
7,1,15562.841667
7,1,10753.678571
7,1,1995.497159
7,1,7006.178571
7,1,2055.893293
7,1,3365.207317
7,1,4840.100000
7,1,1626.571429
7,1,5013.281250
7,1,1585.509804
7,1,5235.702381
7,1,3051.625000
7,1,5321.594512
7,1,9999.228659
7,1,3667.272727
7,1,3429.715000
7,1,1900.207317
7,1,1741.279070
7,1,2264.216146
7,1,1530.317073
7,1,7791.939024
7,1,4124.833333
7,1,8053.750000
7,1,6825.182927
7,1,2247.612069
7,1,2586.184375
7,1,14322.000000
7,1,6357.125000
7,1,9386.755435
7,1,1983.725610
7,1,3070.609756
7,1,16951.184091
7,1,8412.803977
7,1,4237.484848
7,1,1695.351351
7,1,4067.934659
7,1,6743.065104
7,1,2354.891026
7,1,4976.282609
7,1,6361.320000
7,1,3323.791667
7,1,4908.090909
7,1,4561.250000
7,1,2384.648148
7,1,1540.583333
7,1,7348.518750
7,1,2989.938776
7,1,2641.527439
7,1,3344.477273
7,1,2939.340909
7,1,1509.490196
7,1,2985.666667
7,1,5778.861702
7,1,1959.402344
7,1,1985.939189
7,1,2250.259615
7,1,2218.937500
7,1,2018.582237
7,1,8748.162791
7,1,2206.202381
7,1,3518.875000

7,1,2993.460938
7,1,3250.211111
7,1,1560.023256
7,1,2667.635417
7,1,1754.934783
7,1,6220.648810
7,1,1567.585366
7,1,4644.416667
7,1,2305.865854
7,1,1633.928571
7,1,2547.884615
7,1,1694.153846
7,1,2798.234375
8,2,1888.666667
8,2,2259.533019
8,2,3831.075000
8,2,2146.000000
8,2,2507.277778
8,2,1996.078125
8,2,2264.918605
8,2,1843.485294
8,2,2394.034091
8,2,2148.270000
8,2,1902.187500
8,2,2166.362805
8,2,1521.225000
8,2,1948.940476
8,2,2198.679688
8,2,2304.915094
8,2,13322.715909
8,2,1499.734756
8,2,1861.539063
8,2,3324.317568
8,2,1610.539773
8,2,1706.953488
8,2,2215.076923
8,2,4711.083333
8,2,3669.213068
8,2,1540.064024
8,2,2789.311111
8,2,2016.020408
8,2,2153.262755
8,2,1653.668605
8,2,3475.875000
8,2,1548.462963
8,2,2047.731250
8,2,2312.125000
8,2,2043.098684
8,2,2725.199468
8,2,2152.545455
8,2,3108.620968
8,2,1704.352273
8,2,1699.520833
8,2,2534.303571
8,2,2521.209375
8,2,3167.867347
8,2,2264.760776
8,2,2800.000000
8,2,1528.139665
8,2,2193.125000
8,2,1581.693452
8,2,2129.014706
8,2,1776.965116
8,2,3191.901961
8,2,2196.403509
8,2,12983.453125

8,2,2127.122093
8,2,1700.730769
8,2,2599.640000
8,2,2797.294118
8,2,1784.000000
8,2,2068.711538
8,2,6505.700000
8,2,1618.697115
8,2,1797.842391
8,2,1738.797222
8,2,1818.333333
8,2,3196.851190
8,2,2427.202381
8,2,2854.853261
8,2,4398.765306
8,2,2135.926829
8,2,1654.117188
8,2,3516.609375
8,2,3508.395833
8,2,2113.996212
8,2,4521.768116
8,2,3427.328431
8,2,3192.823529
8,2,2343.630435
8,2,6514.903846
8,2,2031.700000
8,2,1534.000000
8,2,2080.069149
8,2,1997.627717
8,2,3054.029070
8,2,2627.109375
8,2,1502.429825
8,2,1899.807692
8,2,2884.910000
8,2,1757.788462
8,2,1777.951531
8,2,3817.369048
8,2,2248.184659
8,2,2323.534091
8,2,2336.323718
8,2,5301.026786
8,2,1778.416667
8,2,1793.625000
8,2,1846.541667
8,2,1889.838415
8,2,1661.809783
8,2,2593.581250
8,2,1710.982143
8,2,2426.316038
8,2,2704.583333
8,2,1638.689103
8,2,1705.679487
8,2,1679.680851
8,2,2490.130435
8,2,1727.176829
8,2,1891.562500
8,2,2125.335938
8,2,1653.875000
8,2,1894.238636
8,2,1511.871622
8,2,3116.050595
8,2,2223.543478
8,2,1557.511111
8,2,3592.638298
8,2,3432.600000
8,2,2336.613208

8,2,1565.337500
8,2,1961.166667
8,2,1966.500000
8,2,2236.081522
8,2,1704.281250
8,2,1969.952128
8,2,3944.486486
8,2,2227.904255
8,2,2163.923077
8,2,1832.790865
8,2,3351.538043
8,2,1808.341837
8,2,3624.409091
8,2,1688.950000
8,2,2976.820000
8,2,2027.625000
8,2,7451.288462
8,2,4397.532407
8,2,1693.212766
8,2,2905.851064
8,2,2808.394737
8,2,1893.569767
8,2,2384.846154
8,2,2263.952830
8,2,1580.175000
8,2,1647.320313
8,2,3246.787791
8,2,1659.250000
8,2,3443.563830
8,2,1831.521875
8,2,1796.578947
8,2,1575.159091
8,2,2347.846154
8,2,1819.083333
8,2,7425.458333
8,2,1855.200000
8,2,2156.416667
8,2,2037.990909
8,2,1896.777027
8,2,2835.670000
8,2,1839.171569
8,2,1881.060000
8,2,3297.565476
8,2,1908.533333
8,2,2712.711310
8,2,4686.750000
8,2,3750.391304
8,2,2058.197917
8,2,1515.956522
8,2,5417.113971
8,2,3752.846154
8,2,14705.000000
8,2,5017.724490
8,2,1589.192708
8,2,2259.287234
8,2,2622.075581
8,2,1598.889423
8,2,2330.103659
8,2,6642.598802
8,2,5543.811508
8,2,1781.265306
8,2,4226.058824
8,2,1654.579545
8,2,1610.775000
8,2,1564.900000
8,2,2479.799020

8,2,1525.479167
8,2,1699.437173
8,2,1571.181818
8,2,2551.235294
8,2,11639.977564
8,2,5131.555000
8,2,1867.385417
8,2,1563.900000
8,2,1768.040000
8,2,2213.085714
8,2,1656.926829
8,2,2036.307065
8,2,1711.077778
8,2,1895.312500
8,2,2855.350877
8,2,2146.250000
8,2,1994.686170
8,2,2075.232143
8,2,1914.208333
8,2,1716.894231
8,2,1608.400000
8,2,1517.744565
8,2,1563.375000
8,2,1725.148256
8,2,2161.847561
8,2,1566.717949
8,2,1929.668605
8,2,6410.784574
8,2,2232.750000
8,2,2665.650000
8,2,5406.230769
8,2,3432.000000
8,2,1814.331633
8,2,2397.197917
8,2,1992.722222
8,2,3083.595238
8,2,2821.540000
8,2,1745.292763
8,2,1533.665816
8,2,1831.115385
8,2,3068.802273
8,2,1707.750000
8,2,4893.409722
8,2,1678.777174
8,2,2673.781863
8,2,1860.461538
8,2,2942.202381
8,2,1738.191489
8,2,1905.964286
8,2,4325.551020
8,2,2302.567308
8,2,4455.093023
8,2,1900.154412
8,2,1663.174342
8,2,2402.386905
8,2,1740.045000
8,2,2533.257500
8,2,1646.048469
8,2,2292.169118
8,2,2450.210227
8,2,1856.771429
8,2,2505.584906
8,2,1726.575342
8,2,1794.815341
8,2,1511.083333
8,2,7281.795918

8,2,1828.670673
8,2,2871.461538
8,2,1664.803571
8,2,2099.801724
8,2,3814.311258
8,2,1937.088235
8,2,3924.563679
8,2,2158.000000
8,2,2395.950980
8,2,3451.511364
8,2,1975.343750
8,2,1786.519444
8,2,2501.775510
8,2,1871.272727
8,2,4796.553571
8,2,3281.404255
8,2,6958.598214
8,2,3853.828804
8,2,2011.121951
8,2,1868.096591
8,2,2213.380000
8,2,1732.339623
8,2,2614.511905
8,2,1627.569149
8,2,1608.152778
8,2,2030.607143
8,2,2792.892857
8,2,3752.118902
8,2,1586.589286
8,2,1574.257812
8,2,6983.735849
8,2,1779.524038
8,2,2348.238426
8,2,2750.904412
8,2,1737.822917
8,2,2045.465116
8,2,3776.829787
8,2,4227.462500
8,2,2653.258333
8,2,2114.447115
8,2,1761.453125
8,2,2689.307692
8,2,2855.537500
8,2,1864.394737
8,2,2106.607843
8,2,2012.312500
8,2,2091.313776
8,2,2792.387755
8,2,1521.250000
8,2,1724.375000
8,2,2145.860000
8,2,7062.000000
8,2,1564.610465
8,2,1719.079268
8,2,4708.981771
8,2,6648.079545
8,2,1828.125000
8,2,2285.636364
8,2,3523.890957
8,2,2590.625000
8,2,3170.461538
8,2,2838.800595
8,2,1795.310976
8,2,2477.323171
8,2,2372.500000
8,2,1633.304487

8,2,2502.881410
8,2,2189.569767
8,2,2503.750000
8,2,5191.204545
8,2,3685.250000
8,2,1799.421875
8,2,1579.189189
8,2,3122.000000
8,2,3265.059783
8,2,1595.691667
8,2,4409.854167
8,2,3034.490741
8,2,1589.000000
8,2,3121.272727
8,2,2027.390000
8,2,2449.062500
8,2,1790.406977
8,2,2320.910714
8,2,1931.792453
8,2,4056.800000
8,2,1695.088068
8,2,4198.375000
8,2,4687.036458
8,2,10529.442568
8,2,1839.789474
8,2,2950.494681
8,2,1768.147059
8,2,1563.760417
8,2,2288.060000
8,2,1582.463068
8,2,9149.335106
8,2,1533.000000
8,2,1776.184659
8,2,1500.371795
8,2,2354.673913
8,2,3371.735577
8,2,2655.560976
8,2,1554.325581
8,2,2464.307143
8,2,3046.997159
8,2,2417.875000
8,2,6104.122449
8,2,1771.744898
8,2,1612.208333
8,2,6028.119565
8,2,1834.795918
8,2,3411.078488
8,2,4771.539474
8,2,4119.604167
8,2,2364.984756
8,2,3001.745283
8,2,2750.486413
8,2,2129.090909
8,2,1597.943878
8,2,3507.500000
8,2,14082.235294
8,2,2489.288889
8,2,2314.558824
8,2,2877.134228
8,2,2112.990132
8,2,4620.849490
8,2,17889.000000
8,2,1655.945755
8,2,1814.031977
8,2,1652.712766
8,2,3680.561224

8,2,3861.722222
8,2,2222.746988
8,2,1802.471698
8,2,1665.697674
8,2,2073.528302
8,2,1650.187500
8,2,1527.827273
8,2,2066.632653
8,2,1983.969388
8,2,2660.315000
8,2,1800.303571
8,2,2037.212766
8,2,1564.565789
8,2,4839.810185
8,2,1728.750000
8,2,2366.666667
8,2,1521.857143
8,2,1692.820312
8,2,1811.000000
8,2,1630.562500
8,2,5484.978261
8,2,1518.680921
8,2,2725.907143
8,2,1649.279412
8,2,3170.927215
8,2,2887.685484
8,2,1774.197500
8,2,1836.829384
8,2,3943.769784
8,2,1744.132353
8,2,1681.858025
8,2,3923.147727
8,2,5597.182927
8,2,1637.279255
8,2,2304.264151
8,2,7821.218750
8,2,1910.354592
8,2,2051.100000
8,2,2735.277778
8,2,2168.211538
8,2,1622.940909
8,2,2108.026163
8,2,3206.906250
8,2,1858.591837
8,2,1903.423077
8,2,1765.742925
8,2,8140.052632
8,2,1639.215000
8,2,1785.823529
8,2,2140.000000
8,2,3478.654255
8,2,2054.301136
8,2,3451.025000
8,2,3260.043367
8,2,3145.574468
8,2,1547.597561
8,2,1577.512195
8,2,1551.500000
8,2,2732.757353
8,2,3604.017442
8,2,2460.831395
8,2,4753.269737
8,2,2156.993750
8,2,2716.709091
8,2,1656.706422
8,2,4351.593750

8,2,2498.142857
8,2,1928.102941
8,2,2112.531250
8,2,1948.202500
8,2,1604.546512
8,2,1893.554348
8,2,5126.509434
8,2,2708.584906
8,2,2876.138122
8,2,1830.846591
8,2,1952.900000
8,2,4412.611111
8,2,3429.919643
8,2,1829.213068
8,2,12280.382353
8,2,2996.272059
8,2,3573.505000
8,2,2186.380000
8,2,1906.317308
8,2,2231.888889
8,2,1515.381757
8,2,1780.631579
8,2,1711.928571
8,2,1506.192935
8,2,1840.062500
8,2,2125.718750
8,2,1516.095930
8,2,2627.491071
8,2,2538.803571
8,2,2993.792683
8,2,1531.738372
8,2,1509.236842
8,2,6907.717949
8,2,5514.212766
8,2,8661.827586
8,2,4722.411765
8,2,1639.734694
8,2,2912.868421
8,2,1621.679688
8,2,1931.940625
8,2,5112.590000
8,2,11432.023438
8,2,1682.250000
8,2,1902.597500
8,2,5828.283163
8,2,1950.568627
8,2,2341.526786
8,2,2599.541667
8,2,1945.302500
8,2,1604.642857
8,2,4323.990625
8,2,1524.466837
8,2,1703.833333
8,2,2535.836310
8,2,3888.013514
8,2,3411.731132
8,2,2450.596591
8,2,2243.593750
8,2,5226.294643
8,2,4899.729730
8,2,1526.353659
8,2,1575.387500
8,2,1946.414634
8,2,4265.955556
8,2,1666.205357
8,2,4357.300439

8,2,1999.857143
8,2,1889.700893
8,2,2241.550000
8,2,1879.767442
8,2,2403.665761
8,2,2070.316038
8,2,2584.758152
8,2,5912.800000
8,2,1790.310976
8,2,2566.437500
8,2,1649.611111
8,2,1644.780000
8,2,2826.197368
8,2,1872.487179
8,2,2609.480114
8,2,3761.182065
8,2,2152.780405
8,2,1589.818182
8,2,2938.955882
8,2,1603.714286
8,2,7809.510000
8,2,2727.671875
8,2,1625.861979
8,2,2052.447917
8,2,2418.437500
8,2,3126.974265
8,2,5020.143750
8,2,2210.500000
8,2,1788.726744
8,2,1774.312500
8,2,1777.875000
8,2,3221.879870
8,2,1787.378205
8,2,1785.676724
8,2,2615.847222
8,2,2248.562500
8,2,1895.336735
8,2,1565.037736
8,2,1725.621951
8,2,2176.034884
8,2,2063.950000
8,2,4150.620192
8,2,1743.604167
8,2,1911.841463
8,2,2791.750000
8,2,1604.772222
8,2,1648.279605
8,2,1605.595588
8,2,1927.397059
8,2,2107.543367
8,2,1545.687500
8,2,4314.750000
8,2,1774.342105
8,2,4510.083333
8,2,3145.838415
8,2,1636.568182
8,2,1632.588235
8,2,2185.536585
8,2,2353.531250
8,2,1723.902439
8,2,1599.250000
8,2,1610.100000
8,2,1860.565789
8,2,8928.963235
8,2,2133.745000
8,2,3497.729167

8,2,5569.264286
8,2,15261.250000
8,2,2371.590909
8,2,2043.864583
8,2,1707.961538
8,2,1946.068627
8,2,1928.098404
8,2,1744.826389
8,2,2027.064904
8,2,2774.812500
8,2,2268.492857
8,2,2351.304054
8,2,1615.071429
8,2,8750.214286
8,2,12502.296053
8,2,4799.000000
8,2,2133.869565
8,2,3351.265306
8,2,1503.851064
8,2,1870.720000
8,2,1519.311047
8,2,3424.543103
8,2,2609.107143
8,2,1574.100000
8,2,2419.812500
8,2,14327.513158
8,2,2934.060000
8,2,1832.328125
8,2,2388.986111
8,2,1817.220000
8,2,1540.795732
8,2,3506.670455
8,2,1559.383721
8,2,2719.358333
8,2,1615.917500
8,2,1557.121711
8,2,3386.514286
8,2,1706.015957
8,2,2805.986979
8,2,6273.855978
8,2,1533.070000
8,2,7324.095238
8,2,2035.801020
8,2,1951.854167
8,2,2804.153846
8,2,3874.905405
8,2,1785.676829
8,2,2187.497845
8,2,2485.347500
8,2,4018.437500
8,2,1708.822115
8,2,5447.372024
8,2,2601.711538
8,2,1915.258065
8,2,3060.571429
8,2,2787.542683
8,2,1583.687500
8,2,4113.666667
8,2,1633.079861
8,2,2105.160000
8,2,3399.906250
8,2,2601.795455
8,2,8079.500000
8,2,1681.817500
8,2,2272.186170
8,2,1604.919872

8,2,6051.290000
8,2,1845.662234
8,2,2171.081522
8,2,2147.750000
8,2,1655.859375
8,2,1863.809783
8,2,1794.485417
8,2,3952.334302
8,2,1834.725962
8,2,1611.836957
8,2,1979.719298
8,2,1846.608696
8,2,1607.583333
8,2,3262.000000
8,2,1948.375000
8,2,1672.715625
8,2,3270.194444
8,2,1740.350000
8,2,1521.463542
8,2,3102.714286
8,2,2133.976744
8,2,1639.122283
8,2,2542.571875
8,2,1884.804878
8,2,4088.270833
8,2,2063.144737
8,2,2006.557432
8,2,2992.082317
8,2,4244.191176
8,2,1916.676471
8,2,1991.381098
8,2,2242.081967
8,2,1624.760204
8,2,2450.038462
8,2,1730.283537
8,2,5034.250000
8,2,3487.142857
8,2,3711.045833
8,2,5848.652174
8,2,1590.744898
8,2,1557.691860
8,2,2271.186170
8,2,1643.677500
8,2,3030.571429
8,2,2003.598214
8,2,1653.397959
8,2,1531.392857
8,2,1750.524390
8,2,2377.687500
8,2,1683.385870
8,2,3514.365000
8,2,1594.945122
8,2,2281.558511
8,2,2799.610119
8,2,3425.347500
8,2,1610.252907
8,2,5424.750000
8,2,1679.683673
8,2,3218.986014
8,2,1745.962264
8,2,1590.072727
8,2,1528.760000
8,2,3564.950581
8,2,1745.854651
8,2,4140.704545
8,2,1793.750000

8,2,16153.472973
8,2,2452.416667
8,2,3309.990000
8,2,4058.169118
8,2,1891.673077
8,2,1691.000000
8,2,5202.840909
8,2,2507.234375
8,2,12781.882353
8,2,1889.153302
8,2,1804.195000
8,2,1529.891304
8,2,2498.447115
8,2,2558.500000
8,2,2847.604167
8,2,1752.321875
8,2,2840.733333
8,2,1514.016129
8,2,1928.936275
8,2,2774.480769
8,2,1801.882653
8,2,3068.129310
8,2,1928.671875
8,2,1720.758475
8,2,1668.444444
8,2,4743.842105
8,2,1684.888393
8,2,1594.892157
8,2,2789.601562
8,2,1515.614130
8,2,2402.090909
8,2,1641.545455
8,2,1642.338710
8,2,1705.348168
8,2,1704.000000
8,2,3640.671875
8,2,2785.426829
8,2,1942.597222
8,2,1805.909091
8,2,1908.318548
8,2,1583.741848
8,2,2578.042683
8,2,6661.015152
8,2,3410.500000
8,2,1773.897059
8,2,2753.767241
8,2,1620.895000
8,2,1579.500000
8,2,8861.196429
8,2,1898.316667
8,2,1859.264706
8,2,1840.388889
8,2,3902.506098
8,2,3564.000000
8,2,1766.067308
8,2,1571.040865
8,2,1627.152174
8,2,1745.510000
8,2,3581.691038
8,2,1540.440217
8,2,3849.411932
8,2,1778.718750
8,2,11051.059375
8,2,2144.483333
8,2,2818.530612
8,2,3830.162791

8,2,2382.130000
8,2,3036.494048
8,2,3028.946875
8,2,5779.517361
8,2,1887.000000
8,2,1515.260204
8,2,3760.791667
8,2,2397.000000
8,2,2267.821429
8,2,1587.551471
8,2,2662.030405
8,2,2070.081633
8,2,13591.399038
8,2,1913.165625
8,2,2009.500000
8,2,2659.125000
8,2,2664.791667
8,2,3765.271277
8,2,3944.694079
8,2,2036.546512
8,2,1507.572917
8,2,1821.726974
8,2,1663.000000
8,2,2589.232143
8,2,1577.645349
8,2,1503.939394
8,2,5753.892857
8,2,2078.320000
8,2,1771.645833
8,2,1719.470588
8,2,3717.235577
8,2,3130.642361
8,2,5363.725000
8,2,1916.763158
8,2,1770.350000
8,2,2032.739130
8,2,3496.773438
8,2,2369.250000
8,2,16612.153846
8,2,2503.495000
8,2,1503.454545
8,2,1756.437500
8,2,1789.256410
8,2,4921.666667
8,2,3556.630319
8,2,1737.479167
8,2,1626.953704
8,2,2059.881818
8,2,3137.565217
8,2,1742.288462
8,2,1628.823864
8,2,1500.105556
8,2,2285.309524
8,2,1789.250000
8,2,1944.870192
8,2,2119.152778
8,2,2466.994444
8,2,1828.734375
8,2,1940.857759
8,2,3720.560811
8,2,3311.919118
8,2,3308.972826
8,2,1635.716216
8,2,5383.500000
8,2,2337.138298
8,2,3794.500000

8,2,1793.636161
8,2,2418.507143
8,2,2034.739796
8,2,1606.154605
8,2,2142.266667
8,2,2738.038043
8,2,1591.363636
8,2,2005.130000
8,2,1954.397727
8,2,1614.387755
8,2,2347.790698
8,2,2589.755435
8,2,1556.289062
8,2,2248.795000
8,2,1718.061475
8,2,2720.308333
8,2,5026.601744
8,2,4374.125000
8,2,1984.841837
8,2,1832.984375
8,2,1538.540698
8,2,1657.921196
8,2,1979.744186
8,2,3256.431452
8,2,2910.496622
8,2,2471.030726
8,2,1923.926829
8,2,3513.591837
8,2,1744.008523
8,2,2330.200000
8,2,2217.928191
8,2,1785.383929
8,2,1611.607143
8,2,2789.406977
8,2,2651.430147
8,2,1980.035714
8,2,3010.357955
8,2,1767.522222
8,2,2010.000000
8,2,2742.806122
8,2,3152.375000
8,2,1606.537162
8,2,2164.027778
8,2,6417.202381
8,2,1870.083333
8,2,2705.885204
8,2,3033.730000
8,2,2050.091837
8,2,1513.655405
8,2,2399.717391
8,2,1865.968750
8,2,1557.907738
8,2,2626.630682
8,2,2288.399038
8,2,3395.260000
8,2,2393.880435
8,2,1508.809211
8,2,1745.731383
8,2,1834.000000
8,2,4259.146341
8,2,1694.994444
8,2,2566.988024
8,2,2000.679348
8,2,1636.609375
8,2,1950.161765
8,2,1746.080556

8,2,1908.166667
8,2,2063.316327
8,2,1706.538889
8,2,1615.500000
8,2,1743.960000
8,2,3379.427885
8,2,2874.773585
8,2,3568.310976
8,2,2201.704327
8,2,10853.410256
8,2,1597.523810
8,2,1857.411765
8,2,1523.888889
8,2,3355.709302
8,2,2638.641026
8,2,3272.895833
8,2,1517.205000
8,2,4454.937500
8,2,1662.323529
8,2,1713.895833
8,2,4870.480000
8,2,1792.156863
8,2,5423.707317
8,2,16001.381443
8,2,2002.875000
8,2,1720.847500
8,2,1705.593750
8,2,1553.026786
8,2,2728.678191
8,2,2565.942500
8,2,2360.386364
8,2,3566.543478
8,2,3016.703804
8,2,9063.133333
8,2,2576.882353
8,2,2618.272222
8,2,1503.924107
8,2,2739.394619
8,2,3047.031250
8,2,2232.375000
8,2,2013.602941
8,2,4426.562500
8,2,3569.562500
8,2,3354.040000
8,2,3565.601293
8,2,3344.648837
8,2,1618.329545
8,2,2404.691617
8,2,2481.805556
8,2,1523.406250
8,2,1771.266026
8,2,1515.571429
8,2,2009.977273
8,2,2788.860465
8,2,2260.857843
8,2,2954.988372
8,2,1638.265957
8,2,2000.022727
8,2,1835.228571
8,2,1751.410256
8,2,1797.636364
8,2,2056.326087
8,2,1650.567568
8,2,2069.205128
8,2,3147.328125
8,2,1794.884146

8,2,1989.296512
8,2,2432.326531
8,2,1931.386628
8,2,1769.173913
8,2,2234.281915
8,2,5802.075521
8,2,1847.005814
8,2,1940.391827
8,2,1588.845395
8,2,2242.630435
8,2,1865.570312
8,2,3605.932203
8,2,2111.235000
8,2,1616.460937
8,2,2275.984375
8,2,1990.424528
8,2,2331.800000
8,2,2229.162109
8,2,1961.915761
8,2,3139.005102
8,2,7882.550676
8,2,1940.000000
8,2,1944.550595
8,2,1591.368304
8,2,3245.117647
8,2,2673.984091
8,2,1736.239130
8,2,1909.750000
8,2,1647.815625
8,2,1806.058594
8,2,4981.117647
8,2,2189.456522
8,2,3070.671498
8,2,1547.489130
8,2,1577.500000
8,2,2400.607843
8,2,6909.513636
8,2,3840.500000
8,2,2387.500000
8,2,1518.918367
8,2,3440.139423
8,2,3069.647727
8,2,1620.144737
8,2,1719.045918
8,2,1616.868750
8,2,4338.694444
8,2,2046.100000
8,2,3264.920732
8,2,1632.288462
8,2,3254.382653
8,2,2711.262500
8,2,2345.781250
8,2,1805.809211
8,2,2116.744186
8,2,1750.208333
8,2,2116.135204
8,2,2951.946429
8,2,2100.202128
8,2,2772.923780
8,2,1955.562500
8,2,2641.321429
8,2,2230.811224
8,2,2911.701531
8,2,1902.875000
8,2,2227.218750
8,2,2778.073529

8,2,1809.142857
8,2,1552.383929
8,2,3506.647959
8,2,1546.133721
8,2,3668.414062
8,2,9444.789474
8,2,1756.478448
8,2,1825.286585
8,2,1974.721591
8,2,1855.812500
8,2,2522.020833
8,2,6773.479167
8,2,1840.058140
8,2,1811.074468
8,2,3500.196429
8,2,1826.980769
8,2,3981.910714
8,2,1526.940217
8,2,2026.803571
8,2,2626.460938
8,2,2953.100000
8,2,1891.079787
8,2,1609.194767
8,2,2029.882979
8,2,2274.842105
8,2,2937.363636
8,2,2546.750000
8,2,9797.391667
8,2,12678.000000
8,2,2785.757353
8,2,1890.918919
8,2,2572.213816
8,2,1873.202381
8,2,1795.076531
8,2,1720.391509
8,2,2779.703431
8,2,1838.325000
8,2,3965.605000
8,2,2033.004902
8,2,2037.625000
8,2,2214.717105
8,2,3674.430233
8,2,1777.875000
8,2,3289.303030
8,2,17355.100000
8,2,1764.515000
8,2,2375.573529
8,2,1652.629032
8,2,1652.875000
8,2,1996.347826
8,2,1667.482759
8,2,1886.000000
8,2,1538.680288
8,2,3402.375000
8,2,2329.522599
8,2,1512.075377
8,2,2084.661765
8,2,1700.042553
8,2,4632.546875
8,2,1510.080769
8,2,1769.132075
8,2,2434.857143
8,2,5575.977564
8,2,1827.165644
8,2,2089.517857
8,2,2217.210526

8,2,3586.065000
8,2,1579.555147
8,2,1776.585366
8,2,1921.856250
8,2,1648.429487
8,2,1782.914894
8,2,3347.821429
8,2,1863.937500
8,2,1970.403846
8,2,2089.006250
8,2,2523.122159
8,2,2953.718750
8,2,1864.887500
8,2,2070.616279
8,2,1951.199468
8,2,5258.600000
8,2,2126.232143
8,2,1978.265306
8,2,2228.342262
8,2,2490.960784
8,2,1670.698864
8,2,2590.693878
8,2,2627.866667
8,2,2461.575000
8,2,1827.232143
8,2,2565.491071
8,2,1683.136029
8,2,1511.615385
8,2,1804.702703
8,2,2339.202128
8,2,1672.353261
8,2,2291.390244
8,2,2613.979167
8,2,2654.960000
8,2,1542.627451
8,2,3956.646341
8,2,1649.594512
8,2,4488.665323
8,2,2259.413889
8,2,1992.605442
8,2,3152.000000
8,2,1660.922222
8,2,4689.010870
8,2,4091.771277
8,2,2803.500000
8,2,1715.527027
8,2,2958.828947
8,2,1929.140625
8,2,1553.635135
8,2,1644.569767
8,2,4645.964286
8,2,2674.865854
8,2,2385.427778
8,2,2129.698718
8,2,1573.415761
8,2,2696.750000
8,2,2536.637755
8,2,1559.361111
8,2,2038.566860
8,2,1657.887500
8,2,1589.357955
8,2,1625.063830
8,2,1568.906977
8,2,7067.883929
8,2,2186.460000
8,2,1542.690341

8,2,1858.407609
8,2,1598.471774
8,2,2155.500000
8,2,1978.434783
8,2,2208.281250
8,2,2509.194444
8,2,1556.023810
8,2,1760.109043
8,2,4042.058824
8,2,4068.397436
8,2,2063.339286
8,2,2118.041667
8,2,3222.804348
8,2,1913.408654
8,2,1987.857143
8,2,2733.021277
8,2,1667.692308
8,2,3015.247024
8,2,1600.229545
8,2,2671.478261
8,2,2261.803571
8,2,1554.185430
8,2,1566.913043
8,2,7921.877604
8,2,4026.062500
8,2,2019.135417
8,2,1576.342593
8,2,1876.437500
8,2,3457.500000
8,2,3179.719512
8,2,5871.400000
8,2,1566.301630
8,2,1971.810000
8,2,1621.735294
8,2,1589.579545
8,2,3934.041667
8,2,2700.750000
8,2,2129.025000
8,2,1691.888889
8,2,2610.110577
8,2,1882.534884
8,2,4068.290000
8,2,1688.533333
8,2,1691.087209
8,2,6743.191558
8,2,1899.019737
8,2,1512.686170
8,2,4590.526596
8,2,2054.414894
8,2,1789.863636
8,2,1657.078804
8,2,2195.810976
8,2,3136.766304
8,2,1665.390909
8,2,1690.215116
8,2,2360.505682
8,2,1524.488095
8,2,8678.032258
8,2,1818.064815
8,2,1689.900000
8,2,3932.371528
8,2,2154.500000
8,2,1888.027027
8,2,2166.789216
8,2,1504.420455
8,2,1697.881579

8,2,2819.051020
8,2,2483.047222
8,2,1507.410714
8,2,1752.631579
8,2,6679.047619
8,2,3087.236111
8,2,2158.221154
8,2,2223.114583
8,2,1574.806604
8,2,1881.285714
8,2,1946.238764
8,2,1858.933673
8,2,2443.263021
8,2,1856.677273
8,2,3157.305556
8,2,1702.726776
8,2,2795.162946
8,2,2004.627551
8,2,2017.339744
8,2,1803.093750
8,2,3467.790000
8,2,1812.905660
8,2,1747.916667
8,2,1942.500000
8,2,2768.363636
8,2,2128.511905
8,2,1640.753205
8,2,2529.086957
8,2,2222.964286
8,2,1528.250000
8,2,1652.346591
8,2,1501.269886
8,2,13416.707447
8,2,2191.142857
8,2,1728.862745
8,2,1550.150000
8,2,1586.553571
8,2,1649.959135
8,2,1949.351852
8,2,2923.598039
8,2,2557.456522
8,2,4716.500000
8,2,2339.923611
8,2,1939.454787
8,2,1817.736413
8,2,3758.777778
8,2,4506.910714
8,2,1645.588710
8,2,4533.815000
8,2,1890.097561
8,2,3298.961538
8,2,1661.100000
8,2,4824.029412
8,2,3136.716981
8,2,1666.211957
8,2,3584.214286
8,2,1529.280000
8,2,1545.250000
8,2,1534.812500
8,2,4097.115196
8,2,2995.955357
8,2,6118.090909
8,2,3625.939189
8,2,2003.289634
8,2,2075.263158
8,2,2959.564516

8,2,3449.428571
8,2,1804.922619
8,2,2268.430851
8,2,1773.294118
8,2,1682.019444
8,2,2008.891304
8,2,1897.885417
8,2,1507.231707
8,2,1575.250000
8,2,1508.230000
8,2,1863.459677
8,2,1696.317500
8,2,3437.617021
8,2,1505.520833
8,2,1757.102041
8,2,1859.726695
8,2,1787.047872
8,2,2751.440758
8,2,1644.418750
8,2,2198.375000
8,2,3262.636364
8,2,2413.017157
8,2,1558.805000
8,2,1540.837500
8,2,3250.117647
8,2,2892.823980
8,2,3967.949074
8,2,1786.090686
8,2,1642.198171
8,2,7254.058824
8,2,3192.125000
8,2,2262.213542
8,2,1792.285714
8,2,1689.640625
8,2,1680.708333
8,2,2651.546512
8,2,13008.450000
8,2,1630.201087
8,2,1646.035714
8,2,1722.031977
8,2,2632.128472
8,2,1567.608696
8,2,1764.500000
8,2,2046.800000
8,2,2754.446429
8,2,1886.588235
8,2,1577.164062
8,2,1732.798295
8,2,1646.510638
8,2,1922.420732
8,2,1680.106383
8,2,2022.236842
8,2,1513.560000
8,2,2859.487069
8,2,3525.378378
8,2,3303.375000
8,2,3092.428922
8,2,1977.118421
8,2,5014.857143
8,2,1880.297297
8,2,1734.287234
8,2,4103.250000
8,2,1524.293478
8,2,1934.872500
8,2,1694.589286
8,2,2575.532895

8,2,1567.731383
8,2,2013.954545
8,2,1540.410714
8,2,3431.252551
8,2,1844.799020
8,2,2335.617347
8,2,1696.924084
8,2,1651.083333
8,2,2728.360294
8,2,5760.083333
8,2,1507.416667
8,2,1973.690789
8,2,2908.065476
8,2,4652.361413
8,2,2095.062500
8,2,3567.643617
8,2,1778.166667
8,2,1946.194444
8,2,2140.857143
8,2,2388.375000
8,2,2125.575521
8,2,2090.285714
8,2,4086.000000
8,2,2772.601064
8,2,1785.740000
8,2,1966.532051
8,2,1652.250000
8,2,1582.052083
8,2,4447.718750
8,2,2317.442500
8,2,2946.397727
8,2,6471.462500
8,2,1966.693182
8,2,1788.375000
8,2,1818.372549
8,2,1987.691489
8,2,1897.610169
8,2,1702.461538
8,2,3807.396739
8,2,4316.744681
8,2,1806.775000
8,2,1571.714286
8,2,1864.583333
8,2,1869.886628
8,2,1827.130814
8,2,1759.797297
8,2,1579.735632
8,2,1550.660377
8,2,2151.702381
8,2,2219.279605
8,2,3747.166667
8,2,1833.089286
8,2,1918.292857
8,2,4932.027778
8,2,2416.765957
8,2,1507.033654
8,2,1588.343750
8,2,2367.471429
8,2,2090.821809
8,2,1655.613636
8,2,5013.588235
8,2,1719.985119
8,2,5258.556250
8,2,2409.386792
8,2,2201.365854
8,2,1824.482143

8,2,10637.375000
8,2,5182.448370
8,2,5075.688889
8,2,2019.464286
8,2,1994.366071
8,2,2084.142857
8,2,2336.210000
8,2,2319.150000
8,2,1634.587500
8,2,3384.702128
8,2,5049.290698
8,2,1966.714286
8,2,1598.872024
8,2,2818.802632
8,2,2006.666667
8,2,9136.301471
8,2,2872.543478
8,2,1976.375000
8,2,4173.989796
8,2,2718.481132
8,2,2007.607143
8,2,2738.500000
8,2,1942.953704
8,2,2532.686275
8,2,3132.300000
8,2,1740.750000
8,2,1695.116071
8,2,1674.318182
8,2,5141.089286
8,2,3788.630000
8,2,1874.336310
8,2,5828.397059
8,2,2538.630102
8,2,1655.098684
8,2,1508.628125
8,2,1631.142857
8,2,1936.893382
8,2,2411.593750
8,2,5532.690000
8,2,2126.368421
8,2,1637.500000
8,2,1574.722727
8,2,1829.888393
8,2,2619.035714
8,2,1973.313776
8,2,2171.104651
8,2,1914.625000
8,2,1913.026786
8,2,2851.764706
8,2,1554.911765
8,2,2058.104167
8,2,2191.143519
8,2,1511.487500
8,2,1637.576531
8,2,3509.673913
8,2,1802.041667
8,2,1815.834906
8,2,1556.421053
8,2,2008.676020
8,2,2123.053191
8,2,2055.302083
8,2,2551.975000
8,2,2145.089286
8,2,1614.714286
8,2,2377.208333
8,2,2291.987805

8,2,1581.140625
8,2,1556.508333
8,2,2258.287500
8,2,2387.897059
8,2,1662.125000
8,2,3292.079545
8,2,1671.580000
8,2,6236.975610
8,2,1516.344595
8,2,3739.421053
8,2,1668.263889
8,2,2959.927778
8,2,3219.508721
8,2,3043.307292
8,2,3692.957589
8,2,1798.523810
8,2,2047.643750
8,2,1769.375000
8,2,2055.393617
8,2,1619.153846
8,2,1702.461111
8,2,1665.852273
8,2,1512.488372
8,2,1680.465909
8,2,1665.321429
8,2,2172.620000
8,2,4544.831633
8,2,3998.461957
8,2,2111.413043
8,2,4920.150000
8,2,1711.589674
8,2,2665.875000
8,2,2702.816667
8,2,1914.392361
8,2,2075.054487
8,2,1664.195312
8,2,2726.013298
8,2,1688.169872
8,2,1826.176136
8,2,2766.648936
8,2,6598.335227
8,2,1979.718023
8,2,3755.662791
8,2,2060.519608
8,2,1659.069444
8,2,4290.460000
8,2,1638.217105
8,2,2032.791667
8,2,2226.781250
8,2,3508.545455
8,2,2499.127841
8,2,2001.612745
8,2,1667.728723
8,2,2396.220000
8,2,3048.716912
8,2,5369.119444
8,2,1663.457447
8,2,1972.230769
8,2,1678.562500
8,2,4576.571429
8,2,2638.372549
8,2,5934.184211
8,2,2102.136364
8,2,1818.141026
8,2,3643.743902
8,2,2879.276042

8,2,1923.843023
8,2,2456.137931
8,2,4186.321875
8,2,1869.021277
8,2,2270.120000
8,2,1631.184375
8,2,1599.554054
8,2,2019.985849
8,2,1720.510417
8,2,1639.071429
8,2,1563.029412
8,2,1517.846939
8,2,1651.291667
8,2,1721.375000
8,2,2985.433333
8,2,4019.000000
8,2,2646.371951
8,2,3107.864865
8,2,2853.072727
8,2,1872.112245
8,2,2970.327869
8,2,3726.936170
8,2,2712.250000
8,2,2068.390625
8,2,4885.953125
8,2,1977.240000
8,2,1582.000000
8,2,1684.686275
8,2,2107.002976
8,2,3121.666667
8,2,7579.375000
8,2,2221.494444
8,2,1694.134146
8,2,2757.500000
8,2,1594.875000
8,2,3744.067797
8,2,3381.871212
8,2,1662.555851
8,2,1695.734694
8,2,1502.230769
8,2,2760.888298
8,2,1608.810976
8,2,1529.341837
8,2,2927.647727
8,2,1614.765625
8,2,6471.288043
8,2,3184.272727
8,2,1605.557377
8,2,4045.130814
8,2,2210.563953
8,2,2160.821429
8,2,1552.109375
8,2,1789.435897
8,2,3285.579082
8,2,1679.656863
8,2,2177.083333
8,2,4098.028646
8,2,1895.863636
8,2,2896.553191
8,2,1601.750000
8,2,2284.664062
8,2,1579.524590
8,2,2278.818750
8,2,1690.766892
8,2,1725.132212
8,2,3737.858108

8,2,1550.141304
8,2,1824.454082
8,2,2158.869565
8,2,3381.632812
8,2,2199.377358
8,2,2918.002907
8,2,3605.720588
8,2,1516.725543
8,2,1625.000000
8,2,1636.261364
8,2,2230.034884
8,2,2383.465116
8,2,2501.083333
8,2,1915.433333
8,2,3072.614362
8,2,2403.205128
8,2,2014.489362
8,2,1743.307065
8,2,2113.579787
8,2,1503.975000
8,2,1935.266304
8,2,2153.042500
8,2,1726.077381
8,2,1802.500000
8,2,5474.687500
8,2,1823.573171
8,2,1723.431122
8,2,1774.583333
8,2,2808.454545
8,2,1545.677632
8,2,1523.511628
8,2,2692.117647
8,2,19993.995000
8,2,1759.789894
8,2,1731.231481
8,2,2428.063953
8,2,1892.825000
8,2,1594.715686
8,2,1695.257812
8,2,3250.892857
8,2,3509.122093
8,2,2029.202128
8,2,1581.919811
8,2,1499.255102
8,2,4591.095745
8,2,1657.546875
8,2,2163.592391
8,2,1989.465909
8,2,2500.797500
8,2,2121.317500
8,2,1900.681548
8,2,1688.441489
8,2,1608.972561
8,2,1606.206587
8,2,1812.621875
8,2,2158.948529
8,2,1908.562500
8,2,1523.649306
8,2,2691.485294
8,2,3192.242021
8,2,2551.312500
8,2,1992.236842
8,2,1744.479730
8,2,1914.182432
8,2,2744.982500
8,2,2395.041667

8,2,2106.021277
8,2,2258.700000
8,2,2277.202128
8,2,2170.363636
8,2,1815.889423
8,2,2399.434659
8,2,3255.138756
8,2,2340.272727
8,2,2339.923077
8,2,2112.100000
8,2,4457.285714
8,2,1517.514151
8,2,2310.625000
8,2,4249.528061
8,2,3191.636905
8,2,3046.419075
8,2,1750.928571
8,2,2369.022222
8,2,5246.063953
8,2,1635.638298
8,2,2278.936275
8,2,1582.486111
8,2,1572.500000
8,2,16064.000000
8,2,6964.046875
8,2,2229.290094
8,2,3844.692708
8,2,2192.276596
8,2,16867.121212
8,2,1511.625000
8,2,2006.371622
8,2,1615.034483
8,2,3798.468750
8,2,1702.733696
8,2,1861.409884
8,2,1692.181818
8,2,2596.982143
8,2,3620.017857
8,2,2931.833333
8,2,1949.117021
8,2,6230.968085
8,2,1994.366279
8,2,2986.211310
8,2,4973.630435
8,2,1600.104730
8,2,2192.318627
8,2,3252.538690
8,2,2538.265625
8,2,2390.009146
8,2,1567.280612
8,2,1617.303571
8,2,1544.325893
8,2,4850.054348
8,2,1858.625000
8,2,2086.250000
8,2,1890.734043
8,2,1928.636364
8,2,1514.603261
8,2,1731.918919
8,2,3156.877778
8,2,2646.656915
8,2,1988.767442
8,2,2286.333333
8,2,1834.959184
8,2,10570.459459
8,2,1972.250000

8,2,1539.519231
8,2,5128.392857
8,2,2572.086538
8,2,1904.687500
8,2,1734.026042
8,2,1665.857143
8,2,2869.915865
8,2,2127.920000
8,2,2356.500000
8,2,2698.850340
8,2,1597.312500
8,2,1764.557292
8,2,1902.300676
8,2,1535.050000
8,2,1820.067308
8,2,2978.583333
8,2,1642.219907
8,2,1529.146277
8,2,3111.647727
8,2,3259.131579
8,2,1580.500000
8,2,1725.775943
8,2,2309.437500
8,2,2405.505319
8,2,2483.994898
8,2,2685.284091
8,2,1820.992647
8,2,3187.976064
8,2,1643.451087
8,2,2830.600000
8,2,3133.835366
8,2,12998.223958
8,2,1765.232955
8,2,3409.668750
8,2,2969.814815
8,2,2072.679054
8,2,1971.913462
8,2,2211.337209
8,2,2197.094388
8,2,1613.380000
8,2,1648.250000
8,2,2233.322917
8,2,2032.680556
8,2,8355.387821
8,2,2082.270000
8,2,3658.822368
8,2,2763.411184
8,2,2548.446429
8,2,1916.851064
8,2,8124.439024
8,2,1833.177778
8,2,1845.193182
8,2,2049.271277
8,2,6600.238095
8,2,1765.203947
8,2,1522.619048
8,2,3152.122093
8,2,1980.000000
8,2,1648.000000
8,2,1761.208333
8,2,1766.923913
8,2,1707.709375
8,2,1664.333333
8,2,1585.065476
8,2,6530.951705
8,2,1739.760870

8,2,1714.039474
8,2,1634.757979
8,2,1744.352740
8,2,1524.072500
8,2,1653.007353
8,2,1901.031250
8,2,4910.668919
8,2,2675.328947
8,2,2391.625000
8,2,1780.480000
8,2,3854.282609
8,2,7959.013514
8,2,1761.312500
8,2,2252.127451
8,2,3206.960784
8,2,1624.092920
8,2,1518.458824
8,2,2693.035000
8,2,1770.393750
8,2,1889.404255
8,2,2033.833333
8,2,1612.723404
8,2,2147.162500
8,2,5499.703488
8,2,1673.464674
8,2,3010.312500
8,2,1659.575521
8,2,2179.423295
8,2,1755.726190
8,2,1501.406250
8,2,2398.053191
8,2,1649.093023
8,2,2415.309896
8,2,2196.740385
8,2,4005.928571
8,2,1642.721939
8,2,1684.617647
8,2,1595.836957
8,2,2270.702128
8,2,1585.187500
8,2,1715.423913
8,2,13701.687500
8,2,1635.798077
8,2,1934.161765
8,2,1959.576531
8,2,2625.744318
8,2,2007.625000
8,2,1593.357143
8,2,2467.136628
8,2,1515.826923
8,2,1961.160714
8,2,2798.687500
8,2,2313.500000
8,2,2450.230769
8,2,1519.625000
8,2,1548.295000
8,2,1992.208333
8,2,1846.085227
8,2,2422.163265
8,2,2226.200000
8,2,1859.051630
8,2,2195.974490
8,2,16779.929245
8,2,1616.454545
8,2,2464.517500
8,2,1613.018617

8,2,1566.323529
8,2,2052.455128
8,2,1763.633929
8,2,3135.333333
8,2,1591.918919
8,2,1835.603125
8,2,2858.792857
8,2,2825.367647
8,2,1650.329268
8,2,1831.385870
8,2,2001.287500
8,2,1691.059783
8,2,1536.171429
8,2,1810.601852
8,2,1741.625000
8,2,2631.336111
8,2,1565.820000
8,2,1639.586538
8,2,2023.117647
8,2,1902.980769
8,2,1888.712500
8,2,2047.853659
8,2,1587.914773
8,2,2655.881579
8,2,3843.877907
8,2,1932.166667
8,2,2315.083333
8,2,3689.882353
8,2,1731.187500
8,2,2488.651786
8,2,1796.489362
8,2,2526.270000
8,2,1628.412234
8,2,1638.312500
8,2,3246.048077
8,2,2578.161290
8,2,1754.607143
8,2,3497.857759
8,2,4431.058333
8,2,5085.928571
8,2,1573.818182
8,2,2521.053571
8,2,3714.493590
8,2,1519.960000
8,2,1675.252358
8,2,1874.216912
8,2,2172.273585
8,2,4932.435811
8,2,1680.364130
8,2,2599.343750
8,2,2615.407609
8,2,2169.927966
8,2,1629.215426
8,2,2112.269737
8,2,1633.697674
8,2,3032.108108
8,2,2684.269737
8,2,1687.574468
8,2,1870.883929
8,2,9347.533163
8,2,2489.310185
8,2,2414.631579
8,2,1990.771739
8,2,4145.464286
8,2,8294.931122
8,2,1837.642241

8,2,1643.026442
8,2,5892.534314
8,2,1517.476190
8,2,1514.303571
8,2,2451.808511
8,2,1501.039062
8,2,1555.966667
8,2,1674.684211
8,2,1693.152439
8,2,3223.057432
8,2,1895.636905
8,2,2951.879808
8,2,2000.948980
8,2,1825.316176
8,2,1694.849490
8,2,1587.142857
8,2,2881.923077
8,2,1513.250000
8,2,2002.414894
8,2,1995.062500
8,2,1515.045455
8,2,4028.180000
8,2,7813.500000
8,2,1990.777344
8,2,1784.278509
8,2,1609.597222
8,2,2931.926471
8,2,3731.997159
8,2,1741.584375
8,2,4266.146119
8,2,1506.517157
8,2,1643.277174
8,2,2187.551020
8,2,2144.940217
8,2,1591.675000
8,2,1807.173913
8,2,1959.346591
8,2,1560.548611
8,2,1541.517045
8,2,6531.390625
8,2,1613.733607
8,2,2401.474490
8,2,2174.463636
8,2,1691.521739
8,2,1677.064815
8,2,2259.180851
8,2,1894.350000
8,2,1687.282609
8,2,1512.459302
8,2,1775.818750
8,2,4759.169492
8,2,1778.973684
8,2,1994.698980
8,2,1503.685714
8,2,1752.940476
8,2,3095.822115
8,2,1575.052326
8,2,2297.155172
8,2,1532.628049
8,2,3782.230159
8,2,1579.812500
8,2,1833.032143
8,2,1728.808140
8,2,2120.893617
8,2,1844.881579
8,2,3344.805556

8,2,2566.039773
8,2,3379.216667
8,2,7170.842857
8,2,3900.014706
8,2,4747.816327
8,2,2031.716667
8,2,2160.041667
8,2,18155.941176
8,2,3243.598214
8,2,4756.899497
8,2,16608.469298
8,2,11606.885135
8,2,2073.000000
8,2,1871.227273
8,2,12318.534375
8,2,2635.714286
8,2,5720.967500
8,2,1640.215909
8,2,3050.592391
8,2,2114.102679
8,2,10341.702703
8,2,1605.000000
8,2,7540.156250
8,2,2220.056122
8,2,2123.894737
8,2,2093.971154
8,2,1874.302778
8,2,3590.769231
8,2,2757.235294
8,2,1794.636364
8,2,1853.510417
8,2,3729.489583
8,2,2710.812500
8,2,5188.158654
8,2,2001.000000
8,2,1597.777439
8,2,4530.028846
8,2,2026.120000
8,2,1532.929348
8,2,1795.666667
8,2,7045.805556
8,2,1586.122449
8,2,2339.875000
8,2,6033.838235
8,2,1779.744681
8,2,2082.033654
8,2,2093.208333
8,2,3060.728723
8,2,1563.965625
8,2,2543.956522
8,2,3513.809783
8,2,2336.482143
8,2,3231.031250
8,2,1728.910326
8,2,2511.158019
8,2,2000.007979
8,2,4320.595109
8,2,3176.375000
8,2,4919.375000
8,2,2522.622024
8,2,1941.063953
8,2,1592.419118
8,2,2313.403846
8,2,2156.181373
8,2,1862.022727
8,2,3318.921875

8,2,1511.228723
8,2,1993.053571
8,2,2877.000000
8,2,2209.266304
8,2,3685.684211
8,2,2864.690909
8,2,15536.222222
8,2,4012.260417
8,2,2327.176471
8,2,5389.833333
8,2,1675.895833
8,2,1525.166667
8,2,2493.040000
8,2,6256.222222
8,2,3158.294118
8,2,2903.478125
8,2,3909.786458
8,2,1566.200000
8,2,1629.875000
8,2,3013.847500
8,2,1855.300926
8,2,5212.666667
8,2,1872.842500
8,2,5805.789583
8,2,1593.428191
8,2,1642.533333
8,2,2475.475000
8,2,1619.889706
8,2,3411.118243
8,2,2349.276316
8,2,4412.790000
8,2,1878.266304
8,2,1763.666667
8,2,1936.928571
8,2,7208.664474
8,2,2070.707547
8,2,1733.720588
8,2,1904.333333
8,2,1610.403846
8,2,1563.298077
8,2,3191.750000
8,2,1790.625000
8,2,2563.989362
8,2,1840.134615
8,2,2525.400000
8,2,1854.087500
8,2,2021.291667
8,2,1762.034375
8,2,6566.463415
8,2,1582.105769
8,2,6280.403125
8,2,1773.136364
8,2,2728.037500
8,2,1581.750000
8,2,2213.549419
8,2,10907.031250
8,2,1985.060345
8,2,1717.500000
8,2,3421.820122
8,2,1817.219512
8,2,3809.075000
8,2,2079.041667
8,2,1787.687500
8,2,6298.744792
8,2,2439.771739
8,2,1730.770270

8,2,1579.046512
8,2,1843.140957
8,2,9558.863636
8,2,2571.958333
8,2,4903.544118
8,2,2459.302326
8,2,1999.940000
8,2,5469.050000
8,2,2694.213415
8,2,1844.314286
8,2,2474.695652
8,2,4012.640306
8,2,1694.176829
8,2,2279.357143
8,2,2722.562500
8,2,1686.957447
8,2,2213.208333
8,2,1819.306122
8,2,1950.724138
8,2,1990.924107
8,2,1524.166667
8,2,3337.000000
8,2,1978.340625
8,2,1537.725000
8,2,17455.821918
8,2,2109.692308
8,2,3458.296196
8,2,3608.567308
8,2,3967.187500
8,2,2256.625000
8,2,1517.192308
8,2,1594.741071
8,2,1540.280000
8,2,1932.596939
8,2,2334.384615
8,2,3060.335821
8,2,4379.211538
8,2,6035.625000
8,2,1504.882653
8,2,10653.527778
8,2,1578.125000
8,2,2274.568878
8,2,1625.750000
8,2,1950.789474
8,2,1625.970238
8,2,3267.887500
8,2,2297.028481
8,2,5679.174528
8,2,2008.051020
8,2,3203.096774
8,2,2095.600000
8,2,2313.904762
8,2,1702.075000
8,2,2223.625000
8,2,1705.455000
8,2,2075.758333
8,2,2364.521739
8,2,1941.712838
8,2,1728.805556
8,2,3951.625000
8,2,1597.327703
8,2,1942.281250
8,2,1837.256579
8,2,1620.446043
8,2,1705.887755
8,2,3189.351852

8,2,2893.352273
8,2,2659.796196
8,2,2032.733696
8,2,1505.250000
8,2,1973.543478
8,2,2132.265625
8,2,2031.772727
8,2,1948.798077
8,2,3603.000000
8,2,4076.173913
8,2,2167.340000
8,2,1779.500000
8,2,2351.902174
8,2,2241.053977
8,2,1786.165000
8,2,1704.200980
8,2,4918.510638
8,2,4502.187500
8,2,3244.875000
8,2,1545.057065
8,2,4278.984694
8,2,1693.468750
8,2,1528.083333
8,2,2591.059091
8,2,1653.164634
8,2,2014.166667
8,2,6407.333333
8,2,9446.090426
8,2,6160.140625
8,2,1588.385638
8,2,2804.605469
8,2,1850.500000
8,2,12423.125000
8,2,9673.409091
8,2,1784.683824
8,2,11448.197368
8,2,2397.005859
8,2,1586.000000
8,2,1634.227273
8,2,2495.448529
8,2,2845.220000
9,1,16672.384375
9,1,1696.906250
9,1,5434.941176
9,1,11801.268519
9,1,2372.033784
9,1,8211.310606
9,1,1686.666667
9,1,6621.347727
9,1,4322.465909
9,1,3451.813953
9,1,1510.170732
9,1,3059.923913
9,1,10299.323529
9,1,10566.033088
9,1,10110.010870
9,1,3275.587838
9,1,3025.818182
9,1,1699.885870
9,1,1835.278125
9,1,11919.465909
9,1,1568.746875
9,1,6413.352941
9,1,3413.916667
9,1,2802.908537
9,1,3137.177419

9,1,8193.262500
9,1,4197.829268
9,1,2846.120000
9,1,2399.189189
9,1,2023.503571
9,1,4054.835196
9,1,3416.283784
9,1,5172.875000
9,1,5278.472973
9,1,1720.291667
9,1,3182.444444
9,1,4460.857143
9,1,3047.260714
9,1,2441.378289
9,1,2953.712766
9,1,1617.348404
9,1,8412.086310
9,1,3224.636364
9,1,10591.184211
9,1,1848.178571
9,1,14088.275000
9,1,8352.898810
9,1,1680.609043
9,1,6457.779412
9,1,2182.264423
9,1,2116.359375
9,1,2111.503378
9,1,1597.975610
9,1,13131.977273
9,1,14223.892857
9,1,1785.005435
9,1,1923.198864
9,1,15974.117021
9,1,16703.837500
9,1,3079.000000
9,1,3210.744681
9,1,10832.500000
9,1,8951.365385
9,1,5490.225000
9,1,2249.233333
9,1,1795.784314
9,1,1582.127778
9,1,1508.134615
9,1,6420.443750
9,1,4636.838235
9,1,19588.353659
9,1,3705.665761
9,1,3070.433824
9,1,2277.744186
9,1,1954.675000
9,1,4308.592262
9,1,6187.000000
9,1,1890.475000
9,1,1793.276596
9,1,3838.781250
9,1,3330.439655
9,1,1759.784722
9,1,1927.750000
9,1,2272.252841
9,1,3180.346591
9,1,1905.268072
9,1,9581.226974
9,1,5468.470745
9,1,4639.611842
9,1,2022.083333
9,1,5649.633065

9,1,12550.549242
9,1,2145.057692
9,1,2436.693750
9,1,5400.435185
9,1,1936.250000
9,1,19230.500000
9,1,6933.785326
9,1,18611.750000
9,1,1812.661184
9,1,2377.521277
9,1,2681.352941
9,1,2989.239796
9,1,8791.095745
9,1,2159.634146
9,1,1656.002717
9,1,3735.181818
9,1,4864.544643
9,1,2592.297297
9,1,3972.187500
9,1,1811.822917
9,1,2760.272059
9,1,1756.216463
9,1,2627.806818
9,1,2186.500000
9,1,1663.305851
9,1,1504.178571
9,1,1988.536765
9,1,2307.911058
9,1,1555.086957
9,1,4336.011719
9,1,2572.959459
9,1,2743.280000
9,1,2867.183673
9,1,14120.316406
9,1,2383.656250
9,1,5115.885417
9,1,1703.039474
9,1,2564.228723
9,1,12035.977273
9,1,1840.050000
9,1,6217.805147
9,1,3492.140625
9,1,1658.858108
9,1,1587.632653
9,1,1958.382353
9,1,7038.314103
9,1,7165.527027
9,1,1631.190000
9,1,7789.539634
9,1,2633.288462
9,1,3619.227941
9,1,2899.589286
9,1,7775.454545
9,1,2446.157895
9,1,2889.000000
9,1,2027.617647
9,1,1812.205128
9,1,1779.473404
9,1,10783.884868
9,1,5255.708333
9,1,1987.976744
9,1,5379.854167
9,1,1779.694444
9,1,2337.142857
9,1,1697.132576
9,1,1815.568548

9,1,3456.700000
9,1,4010.353659
9,1,2465.894737
9,1,1536.532258
9,1,3426.968085
9,1,14037.456250
9,1,2366.285714
9,1,4219.451613
9,1,14580.437500
9,1,3527.079545
9,1,4035.779891
9,1,7904.979167
9,1,15153.481618
9,1,8213.258065
9,1,4395.500000
9,1,4105.551724
9,1,13796.851852
9,1,16921.996429
9,1,2683.775000
9,1,3177.066176
9,1,16383.863636
9,1,6762.274194
9,1,4508.738095
9,1,15161.666667
9,1,3126.531915
9,1,2249.698171
9,1,3352.225962
9,1,1766.645833
9,1,3103.070312
9,1,4110.459821
9,1,4498.041667
9,1,2733.921053
9,1,11132.267241
9,1,5538.340278
9,1,4749.588235
9,1,12079.928191
9,1,2503.140244
9,1,3335.421053
9,1,2964.119681
9,1,5648.675000
9,1,2793.279070
9,1,2865.096154
9,1,1560.277439
9,1,1571.108696
9,1,2304.000000
9,1,3280.500000
9,1,3808.078571
9,1,7374.385417
9,1,7452.755556
9,1,4048.148438
9,1,1787.730978
9,1,1807.753906
9,1,9894.630435
9,1,1814.518617
9,1,5541.783784
9,1,3855.522059
9,1,4084.142857
9,1,3167.765625
9,1,3097.190789
9,1,2267.406977
9,1,3166.506410
9,1,2753.560606
9,1,2574.042683
9,1,2368.133721
9,1,2296.383333
9,1,3841.103571

9,1,5227.317500
9,1,18048.453125
9,1,2038.159091
9,1,6117.818750
9,1,1735.129032
9,1,1657.453488
9,1,4788.813953
9,1,2682.218750
9,1,2179.496324
9,1,1886.066176
9,1,17286.125000
9,1,2039.477273
9,1,2278.365132
9,1,1687.000000
9,1,2216.342949
9,1,5290.666667
9,1,3058.140244
9,1,9879.117647
9,1,4281.328804
9,1,4345.013514
9,1,1757.012500
9,1,1643.131944
9,1,2724.674020
9,1,15888.333333
9,1,3066.611111
9,1,5085.051630
9,1,3075.797500
9,1,2705.592262
9,1,2220.550000
9,1,16455.175000
9,1,1812.128378
9,1,2314.424419
9,1,14741.274510
9,1,1681.244318
9,1,2173.242424
9,1,2615.881579
9,1,8021.244048
9,1,8785.144531
9,1,4285.593750
9,1,9956.973485
9,1,2867.732143
9,1,8191.663462
9,1,12844.833333
9,1,4382.016667
9,1,2492.500000
9,1,19714.468750
9,1,6505.344388
9,1,6458.541667
9,1,2360.143293
9,1,1664.600694
9,1,3233.302083
9,1,3456.722222
9,1,8284.444444
9,1,4578.234043
9,1,4891.375000
9,1,4899.487500
9,1,11771.723404
9,1,12370.250000
9,1,2069.985294
9,1,1679.129032
9,1,1843.693878
9,1,3217.375000
9,1,2075.121711
9,1,3347.360000
9,1,2542.892157
9,1,18322.525000

9,1,3177.103723
9,1,3517.425532
9,1,1637.687500
9,1,2084.213068
9,1,11226.138514
9,1,2272.090116
9,1,4607.967391
9,1,11748.378125
9,1,10130.303571
9,1,1878.263514
9,1,7088.194767
9,1,3269.857143
9,1,2170.784091
9,1,3909.315217
9,1,1857.494565
9,1,4924.963542
9,1,1512.132812
9,1,2076.267442
9,1,5051.864583
9,1,2660.871212
9,1,14440.500000
9,1,7968.083333
9,1,5860.010135
9,1,1608.116667
9,1,2432.000000
9,1,8542.000000
9,1,9688.808140
9,1,3580.285714
9,1,3630.442857
9,1,7556.762195
9,1,2262.472222
9,1,5815.558036
9,1,2111.981707
9,1,2400.308824
9,1,2408.875000
9,1,14625.893939
9,1,2423.750000
9,1,12583.596154
9,1,6311.284884
9,1,4009.600000
9,1,1900.777778
9,1,2789.078125
9,1,2405.956731
9,1,11958.092500
9,1,1837.151515
9,1,3863.540323
9,1,3473.683824
9,1,1753.941860
9,1,5598.418605
9,1,2107.054054
9,1,2970.393382
9,1,1570.641447
9,1,1898.338235
9,1,3652.450000
9,1,3904.367347
9,1,4863.915698
9,1,11998.836538
9,1,18034.416667
9,1,1807.622222
9,1,2378.893293
9,1,4987.994681
9,1,2455.693750
9,1,2299.085714
9,1,6807.401316
9,1,2403.111111
9,1,6285.871795

9,1,15434.906250
9,1,17654.791667
9,1,1614.524194
9,1,1999.578125
9,1,1779.437500
9,1,1561.111413
9,1,1562.687117
9,1,1759.625000
9,1,1810.895833
9,1,17771.258065
9,1,1550.080882
9,1,8545.571429
9,1,1906.681548
9,1,10279.994048
9,1,2226.673913
9,1,3303.951613
9,1,16219.780576
9,1,3283.457031
9,1,1654.595238
9,1,2543.118421
9,1,1875.768750
9,1,1599.085366
9,1,3337.729839
9,1,3396.727273
9,1,3184.913793
9,1,1891.034722
9,1,1683.611702
9,1,2624.648438
9,1,17373.928571
9,1,12680.375000
9,1,1917.886364
9,1,2601.542683
9,1,3306.425676
9,1,5524.276596
9,1,2537.538462
9,1,8391.366935
9,1,1551.715116
9,1,1743.270833
9,1,3901.638158
9,1,7182.300000
9,1,2385.919118
9,1,2551.606618
9,1,6710.293750
9,1,12058.464286
9,1,3581.855769
9,1,1531.927632
9,1,3126.256410
9,1,7767.129032
9,1,6112.904255
9,1,1859.774194
9,1,11635.406250
9,1,5942.361111
9,1,12266.985294
9,1,3078.630435
9,1,8966.055556
9,1,3800.182927
9,1,4533.500000
9,1,1554.409091
9,1,5357.946809
9,1,2317.647959
9,1,7566.404255
9,1,2932.939516
9,1,3885.026144
9,1,2494.142857
9,1,7930.897222
9,1,1733.815217

9,1,1638.967391
9,1,2129.222561
9,1,9912.953488
9,1,11171.096429
9,1,1980.375000
9,1,2832.791667
9,1,4948.821429
9,1,7699.435976
9,1,3356.803191
9,1,1881.952381
9,1,7288.607143
9,1,7176.050000
9,1,3576.619048
9,1,3046.846154
9,1,3351.125000
9,1,7138.095238
9,1,4816.768750
9,1,1669.045714
9,1,16587.687500
9,1,1545.200000
9,1,1871.776316
9,1,4367.845588
9,1,7048.218750
9,1,3998.632812
9,1,3134.418367
9,1,1543.725000
9,1,3060.287234
9,1,18973.145349
9,1,2595.388889
9,1,2801.789634
9,1,11066.750000
9,1,1816.227273
9,1,2355.568182
9,1,18331.706633
9,1,8620.191406
9,1,4847.719595
9,1,5583.259375
9,1,1920.691860
9,1,3104.979592
9,1,15672.500000
9,1,6086.064024
9,1,1753.675000
9,1,1966.591216
9,1,3732.101562
9,1,4118.084677
9,1,8274.766892
9,1,2604.706250
9,1,15494.322917
9,1,2238.211538
9,1,2978.560976
9,1,4255.788043
9,1,4106.915625
9,1,4561.255319
9,1,2652.159091
9,1,1555.190476
9,1,5369.500000
9,1,3937.989796
9,1,2911.672794
9,1,1525.644737
9,1,4075.312500
9,1,1527.100000
9,1,2020.310345
9,1,3213.046875
9,1,2828.285714
9,1,5695.250000
9,1,6873.687500

9,1,8767.602564
9,1,1810.229839
9,1,3171.320513
9,1,7247.539474
9,1,11818.923077
9,1,17456.625000
9,1,6570.210526
9,1,1623.081633
9,1,6110.431250
9,1,2858.290698
9,1,2240.825581
9,1,1884.599265
9,1,6719.750000
9,1,9491.209239
9,1,12487.419643
9,1,4314.849265
9,1,4594.090909
9,1,18090.869565
9,1,2832.687500
9,1,2499.656250
9,1,1860.698529
9,1,1602.546875
9,1,2010.921429
9,1,2643.169643
9,1,1738.839744
9,1,2093.493421
9,1,5201.647727
9,1,3995.959184
9,1,1997.804054
9,1,2006.421053
9,1,1558.291667
9,1,5133.062500
9,1,5246.724359
9,1,3761.829787
9,1,7267.700000
9,1,4033.489583
9,1,12601.729730
9,1,3289.625000
9,1,6370.800000
9,1,8380.516129
9,1,13803.607143
9,1,1542.548387
9,1,2383.243902
9,1,1733.068182
9,1,2141.997340
9,1,3499.002841
9,1,9003.958333
9,1,5733.918919
9,1,4186.983333
9,1,3326.848214
9,1,6649.197674
9,1,1616.562500
9,1,8120.181548
9,1,3534.696023
9,1,6519.722973
9,1,6266.404070
9,1,1818.720238
9,1,5166.562500
9,1,2491.190476
9,1,11237.264706
9,1,2565.817308
9,1,2142.585938
9,1,11330.991935
9,1,2542.069767
9,1,8012.500000
9,1,9674.843750

9,1,9669.122024
9,1,3938.853723
9,1,3208.119048
9,1,4141.040000
9,1,3905.000000
9,1,3290.034375
9,1,5274.720588
9,1,1855.062500
9,1,3506.486111
9,1,1883.916667
9,1,1512.838235
9,1,4573.193182
9,1,2047.953488
9,1,1951.689103
9,1,2136.915698
9,1,2406.116071
9,1,8978.996528
9,1,2128.052632
9,1,14327.000000
9,1,8248.548611
9,1,1911.326923
9,1,1520.808824
9,1,2308.137755
9,1,10414.852941
9,1,3494.041667
9,1,11483.291667
9,1,2119.701389
9,1,3191.561224
9,1,3265.905556
9,1,2811.595238
9,1,2479.196429
9,1,3392.131579
9,1,3838.828571
9,1,3006.677419
9,1,2240.987805
9,1,3681.509615
9,1,2692.215116
9,1,2197.031915
9,1,4678.104167
9,1,2204.494048
9,1,3352.768293
9,1,17027.955882
9,1,9024.502907
9,1,3596.882075
9,1,11303.272727
9,1,2073.541667
9,1,4256.819079
9,1,2685.514706
9,1,14113.843023
9,1,1779.196429
9,1,2794.933594
9,1,7099.035714
9,1,1637.617021
9,1,2104.500000
9,1,6565.825000
9,1,15167.819444
9,1,2005.960938
9,1,1775.821429
9,1,2328.414286
9,1,19238.266667
9,1,2064.234375
9,1,7171.475000
9,1,1987.325000
9,1,2388.400000
9,1,8553.298780
9,1,3990.447368

9,1,2321.875000
9,1,2054.562500
9,1,3612.783088
9,1,10058.155405
9,1,4689.065625
9,1,3059.734375
9,1,13024.450000
9,1,14146.253472
9,1,1512.939394
9,1,3404.945652
9,1,2113.391304
9,1,6844.346875
9,1,3520.380682
9,1,10053.410714
9,1,1960.000000
9,1,4400.514706
9,1,4784.100000
9,1,2312.088235
9,1,2817.975610
9,1,2706.944134
9,1,1731.424419
9,1,4720.851562
9,1,2630.170455
9,1,1622.382812
9,1,13248.557143
9,1,1794.564024
9,1,3144.863095
9,1,1517.507812
9,1,17923.678571
9,1,1832.818750
9,1,4617.177083
9,1,10923.698171
9,1,2813.267857
9,1,2333.009259
9,1,5978.000000
9,1,4442.718750
9,1,7839.125000
9,1,3051.076923
9,1,6563.583333
9,1,3808.307692
9,1,1731.045455
9,1,2245.368421
9,1,16811.671875
9,1,1891.617188
9,1,19676.625000
9,1,1790.288889
9,1,2924.677778
9,1,11400.430556
9,1,1533.704545
9,1,3531.973837
9,1,5260.769444
9,1,2868.888298
9,1,1503.038265
9,1,6942.367925
9,1,3926.080357
9,1,2585.680851
9,1,3668.095588
9,1,5785.375000
9,1,12730.269231
9,1,2120.758721
9,1,3586.625000
9,1,1930.178571
9,1,8752.281250
9,1,6380.974265
9,1,4784.136364
9,1,2412.167683

9,1,2083.142857
9,1,13231.891304
9,1,3259.515957
9,1,3503.214286
9,1,1965.400000
9,1,1867.647059
9,1,16670.480769
9,1,5084.881579
9,1,2549.681818
9,1,14419.674242
9,1,17808.335106
9,1,5433.589286
9,1,1658.264706
9,1,19530.000000
9,1,3159.360465
9,1,6468.223684
9,1,1569.075581
9,1,13085.913043
9,1,6962.941176
9,1,1563.267857
9,1,8711.225000
9,1,2312.250000
9,1,2712.114286
9,1,3981.458333
9,1,3171.193182
9,1,1718.250000
9,1,2071.280488
9,1,1567.831250
9,1,2857.750000
9,1,3457.294118
9,1,3976.436111
9,1,2021.161290
9,1,17148.906250
9,1,4074.773649
9,1,7401.593750
9,1,4669.419118
9,1,8561.864130
9,1,10457.543478
9,1,6007.442500
9,1,14350.640000
9,1,5258.275735
9,1,3985.567568
9,1,5805.365385
9,1,2505.390957
9,1,5900.333333
9,1,18004.520833
9,1,2817.037500
9,1,18621.000000
9,1,3492.441176
9,1,1942.979167
9,1,2720.621951
9,1,1672.053571
9,1,3347.342262
9,1,2531.144886
9,1,4441.255319
9,1,2071.470395
9,1,1718.067857
9,1,2120.081522
9,1,3652.512195
9,1,10223.023256
9,1,4500.476562
9,1,2483.056818
9,1,4065.000000
9,1,16161.736842
9,1,1703.298780
9,1,1869.507812

9,1,2564.010417
9,1,2387.000000
9,1,1672.888889
9,1,1816.662500
9,1,9672.502717
9,1,12704.357143
9,1,19185.336957
9,1,3610.274390
9,1,2941.526596
9,1,2446.300676
9,1,5872.468750
9,1,1543.284091
9,1,3231.042683
9,1,2645.044444
9,1,6990.076613
9,1,7667.503378
9,1,2323.902439
9,1,4086.722222
9,1,15344.793919
9,1,2964.003571
9,1,11966.765957
9,1,3401.972973
9,1,5381.333333
9,1,4027.125000
9,1,5360.570000
9,1,1799.932692
9,1,1637.334559
9,1,4847.946429
9,1,2177.803191
9,1,3670.875000
9,1,11268.378378
9,1,3494.362903
9,1,5007.000000
9,1,11524.455882
9,1,2294.504237
9,1,1807.060976
9,1,8652.009615
9,1,2186.228261
9,1,1593.372093
9,1,1996.315217
9,1,2033.519231
9,1,4850.473837
9,1,3588.920139
9,1,7389.602273
9,1,2206.125000
9,1,4416.828947
9,1,8719.394737
9,1,3225.433333
9,1,1994.765000
9,1,11142.390625
9,1,1770.852273
9,1,5807.264706
9,1,4123.148810
9,1,6507.629464
9,1,1551.187500
9,1,2044.917763
9,1,1838.917553
9,1,3051.474432
9,1,5143.755682
9,1,1797.273810
9,1,1524.888587
9,1,1989.559211
9,1,1823.700000
9,1,4785.770000
9,1,1566.713542
9,1,16717.266667

9,1,2535.168605
9,1,9139.383929
9,1,7498.339744
9,1,3702.649390
9,1,11261.671875
9,1,3295.908333
9,1,1990.804688
9,1,2151.841463
9,1,1663.500000
9,1,1606.654412
9,1,12867.031250
9,1,2080.093750
9,1,7701.125000
9,1,8187.000000
9,1,2503.951220
9,1,2951.371795
9,1,1918.076705
9,1,11947.111111
9,1,2099.365625
9,1,2153.317935
9,1,3192.987500
9,1,11669.887500
9,1,1689.816176
9,1,3591.500000
9,1,12046.608696
9,1,9347.839286
9,1,7994.700000
9,1,2711.915441
9,1,5153.106383
9,1,4938.006757
9,1,1598.238095
9,1,14935.888587
9,1,1921.948864
9,1,2169.811111
9,1,1834.727273
9,1,5464.821429
9,1,2583.361702
9,1,6835.053191
9,1,1696.769231
9,1,1789.109848
9,1,1554.000000
9,1,2350.978723
9,1,8722.864362
9,1,4365.574405
9,1,5075.725000
9,1,9925.500000
9,1,13685.975610
9,1,2787.336207
9,1,4923.760870
9,1,3072.886364
9,1,1981.338415
9,1,1840.055921
9,1,2542.674342
9,1,9214.875000
9,1,2173.943182
9,1,1747.000000
9,1,10102.658537
9,1,3978.975610
9,1,18364.147727
9,1,2376.546875
9,1,2354.461538
9,1,4356.020833
9,1,4082.402778
9,1,2139.582237
9,1,2295.312500
9,1,6951.596154

9,1,3617.684211
9,1,1615.862500
9,1,15367.818182
9,1,1623.020270
9,1,6512.083333
9,1,2254.283537
9,1,17748.000000
9,1,7389.003289
9,1,10441.900510
9,1,7632.282051
9,1,2897.000000
9,1,14196.802632
9,1,2076.489362
9,1,6414.670732
9,1,4630.272727
9,1,4033.803279
9,1,9902.682927
9,1,1580.176471
9,1,2758.207317
9,1,4603.078125
9,1,1745.242188
9,1,6556.241071
9,1,6891.769737
9,1,2551.916667
9,1,4200.979167
9,1,3273.054054
9,1,1588.923077
9,1,12995.230769
9,1,5169.690000
9,1,4066.134615
9,1,2108.440217
9,1,4163.447368
9,1,4258.438830
9,1,6621.308824
9,1,6819.086538
9,1,2755.134146
9,1,1838.445946
9,1,2835.184524
9,1,3069.642857
9,1,7646.853448
9,1,2610.382576
9,1,2832.650000
9,1,4112.000000
9,1,11846.958333
9,1,2944.575581
9,1,3272.783537
9,1,2833.589286
9,1,4970.261628
9,1,3924.028846
9,1,13003.519231
9,1,1993.070946
9,1,7014.210366
9,1,6636.553030
9,1,1834.165323
9,1,2279.840000
9,1,2065.171875
9,1,1685.500000
9,1,1679.851562
9,1,2727.916667
9,1,11593.375000
9,1,16114.913889
9,1,3564.099359
9,1,8765.335366
9,1,1776.422619
9,1,1921.358974
9,1,16507.111842

9,1,2465.939394
9,1,6441.406250
9,1,3452.878049
9,1,4496.593750
9,1,2812.793478
9,1,8160.659722
9,1,2382.568182
9,1,2675.294118
9,1,1707.367021
9,1,2099.500000
9,1,9792.166667
9,1,2383.330882
9,1,7668.512195
9,1,3298.140625
9,1,7680.222222
9,1,1765.227941
9,1,2443.595238
9,1,4715.583333
9,1,3138.910714
9,1,2052.897059
9,1,2266.175000
9,1,4767.686275
9,1,4442.767442
9,1,2661.422619
9,1,1639.964286
9,1,4349.727273
9,1,3969.902174
9,1,2385.031250
9,1,2324.630682
9,1,7093.795455
9,1,3794.022727
9,1,6484.064516
9,1,1556.195946
9,1,2148.310976
9,1,3664.775000
9,1,1961.682432
9,1,3644.741007
9,1,2770.401163
9,1,5236.178191
9,1,6452.250000
9,1,2687.440104
9,1,4452.808511
9,1,1512.541667
9,1,4654.933333
9,1,2393.977778
9,1,7358.210106
9,1,1697.553571
9,1,2726.764205
9,1,1984.908088
9,1,2222.800000
9,1,6173.852941
9,1,6589.355114
9,1,1502.886364
9,1,2334.044355
9,1,8690.359375
9,1,6911.526596
9,1,6920.593750
9,1,10364.791667
9,1,12381.500000
9,1,18254.000000
9,1,7029.580882
9,1,1992.038462
9,1,6412.433333
9,1,2600.466216
9,1,1511.571429
9,1,1802.666667

9,1,3886.839674
9,1,2290.763889
9,1,8471.662879
9,1,5002.425000
9,1,3541.877907
9,1,2862.578125
9,1,2092.244186
9,1,1651.453947
9,1,18945.242424
9,1,8866.375000
9,1,3299.330882
9,1,6700.051471
9,1,1732.651163
9,1,4613.563776
9,1,5178.426829
9,1,2981.917683
9,1,9971.563596
9,1,2681.322917
9,1,3863.500000
9,1,1722.585366
9,1,2567.658537
9,1,6079.010000
9,1,1583.268750
9,1,14129.647059
9,1,5063.228448
9,1,5311.515152
9,1,13166.650000
9,1,1887.820000
9,1,2238.625000
9,1,5954.774194
9,1,18137.039062
9,1,2794.000000
9,1,3041.650000
9,1,19821.666667
9,1,1552.396277
9,1,4482.419355
9,1,2424.484756
9,1,5124.586957
9,1,5971.635417
9,1,14213.545732
9,1,2207.573171
9,1,1914.888158
9,1,1737.222973
9,1,2395.297297
9,1,11517.816667
9,1,6721.502315
9,1,9545.939024
9,1,12694.115385
9,1,2209.841837
9,1,5240.200000
9,1,13055.409091
9,1,2399.750000
9,1,4133.396341
9,1,1656.183594
9,1,5072.348837
9,1,3752.650000
9,1,19234.659574
9,1,3279.756944
9,1,2003.810484
9,1,3122.000000
9,1,2298.274390
9,1,9583.903226
9,1,2841.677419
9,1,1754.302632
9,1,2001.947368
9,1,7179.941176

9,1,6406.153846
9,1,2285.744565
9,1,3110.228571
9,1,9196.413043
9,1,4146.346154
9,1,4760.412500
9,1,3922.006667
9,1,3007.562500
9,1,3455.906250
9,1,3256.054348
9,1,6697.477941
9,1,6279.285714
9,1,1868.322581
9,1,11972.962766
9,1,19885.250000
9,1,3627.204545
9,1,10053.375000
9,1,2757.647727
9,1,13379.205357
9,1,6613.270833
9,1,7545.353723
9,1,4843.402439
9,1,16893.833333
9,1,3834.250000
9,1,18197.716216
9,1,7262.500000
9,1,3036.602941
9,1,2443.520349
9,1,6852.583333
9,1,7232.944444
9,1,3996.230263
9,1,2027.250000
9,1,1636.182927
9,1,2866.404891
9,1,2100.404070
9,1,16119.136364
9,1,2352.478448
9,1,12331.898438
9,1,2130.585366
9,1,2014.269737
9,1,2313.012500
9,1,3001.743243
9,1,2713.116071
9,1,1629.522727
9,1,1753.707447
9,1,8623.262821
9,1,3858.500000
9,1,5879.808824
9,1,5189.540000
9,1,19478.572917
9,1,1746.254167
9,1,3162.489130
9,1,3886.932692
9,1,2183.277778
9,1,12377.958333
9,1,1630.562500
9,1,1871.904762
9,1,10633.787791
9,1,2583.529255
9,1,13287.914286
9,1,16690.800000
9,1,2677.613636
9,1,2780.487805
9,1,2268.772059
9,1,4172.875000
9,1,3436.263158

9,1,2481.517857
9,1,2172.554054
9,1,2219.850000
9,1,2963.016304
9,1,1612.186047
9,1,8525.576531
9,1,17456.062500
9,1,3759.986607
9,1,5110.725000
9,1,5145.023438
9,1,1771.347222
9,1,4914.966667
9,1,3154.062500
9,1,2041.166667
9,1,4578.018868
9,1,9330.922297
9,1,1620.000000
9,1,15621.179348
9,1,2504.508197
9,1,2086.862500
9,1,4235.226190
9,1,4473.794118
9,1,1990.305556
9,1,3131.413194
9,1,15018.125000
9,1,10291.625000
9,1,1864.321429
9,1,6509.122024
9,1,2429.566667
9,1,1501.698864
9,1,2318.488372
9,1,5733.279255
9,1,6175.674419
9,1,2826.670732
9,1,4210.335227
9,1,1894.632143
9,1,4094.099057
9,1,4860.940789
9,1,3980.580645
9,1,8828.369186
9,1,5592.021429
9,1,3450.233333
9,1,3517.602273
9,1,3690.888889
9,1,1712.640625
9,1,7354.560976
9,1,3869.179487
9,1,5387.217742
9,1,3185.154762
9,1,2112.072115
9,1,1891.114130
9,1,1648.345238
9,1,3951.846154
9,1,1893.634615
9,1,7375.173077
9,1,1635.264535
9,1,3929.034375
9,1,3515.331395
9,1,4834.621951
9,1,4136.755102
9,1,2178.731707
9,1,5756.904412
9,1,1729.718750
9,1,2052.562500
9,1,1764.366848
9,1,4399.977273

9,1,2310.712766
9,1,10110.794811
9,1,8279.217262
9,1,2066.601744
9,1,1819.193548
9,1,5418.720000
9,1,7492.890244
9,1,4346.189189
9,1,3604.312500
9,1,8065.125000
9,1,1583.292857
9,1,4526.533333
9,1,15791.887500
9,1,1864.481481
9,1,7053.459375
9,1,2523.407895
9,1,2808.672794
9,1,6889.875000
9,1,4146.354839
9,1,2707.526316
9,1,4255.177778
9,1,1806.854651
9,1,3345.520161
9,1,8195.472222
9,1,4510.518617
9,1,2200.697222
9,1,3848.045732
9,1,11805.640625
9,1,2015.137097
9,1,1811.171429
9,1,3447.735119
9,1,1671.917857
9,1,17632.586806
9,1,7838.566667
9,1,18593.533333
9,1,2944.831395
9,1,5606.648438
9,1,2525.194444
9,1,3213.821875
9,1,3066.368243
9,1,7274.833333
9,1,7982.395408
9,1,1875.170732
9,1,1898.356383
9,1,1617.674107
9,1,1584.204545
9,1,2946.636364
9,1,3621.166667
9,1,2878.638587
9,1,1795.444444
9,1,2311.164894
9,1,1638.057143
9,1,9760.638889
9,1,3619.321429
9,1,6670.283784
9,1,19328.250000
9,1,12007.371795
9,1,4992.177326
9,1,3831.500000
9,1,2370.500000
9,1,7549.289474
9,1,1870.666667
9,1,5678.150000
9,1,3497.650000
9,1,2563.353571
9,1,7578.595238

9,1,2255.486111
9,1,2389.833333
9,1,1908.913462
9,1,8249.330357
9,1,2455.645349
9,1,8387.232558
9,1,1601.951220
9,1,6255.910256
9,1,7562.421875
9,1,2405.718085
9,1,8387.572289
9,1,15035.835937
9,1,17769.628571
9,1,1998.000000
9,1,13160.384146
9,1,3606.161290
9,1,15673.073171
9,1,2404.065789
9,1,3515.416667
9,1,2241.600000
9,1,5395.312500
9,1,4090.928571
9,1,1504.278571
9,1,1944.272727
9,1,8173.857143
9,1,1888.458333
9,1,4211.437500
9,1,13561.125000
9,1,6167.270270
9,1,2675.082500
9,1,4064.446809
9,1,2790.681250
9,1,2664.951613
9,1,3032.350000
9,1,9131.568182
9,1,4499.810714
9,1,9700.976974
9,1,2827.750000
9,1,17982.473684
9,1,3012.085366
9,1,6975.317857
9,1,2115.507143
9,1,8533.741379
9,1,6650.625000
9,1,1816.700000
9,1,3332.550000
9,1,4249.791667
9,1,2336.987500
9,1,2531.820652
9,1,2257.153846
9,1,14719.117647
9,1,1504.722222
9,1,4214.899038
9,1,2683.953488
9,1,2763.211111
9,1,3878.692308
9,1,1723.516667
9,1,1600.861842
9,1,1852.193069
9,1,3362.400000
9,1,3679.128571
9,1,2083.654891
9,1,1809.838542
9,1,1817.050000
9,1,3348.818182
9,1,5696.228448

9,1,3660.628571
9,1,4378.552632
9,1,6432.773810
9,1,11652.437500
9,1,6977.625000
9,1,8033.875000
9,1,15790.756410
9,1,5106.558333
9,1,2112.357143
9,1,13651.663889
9,1,2104.942771
9,1,3262.687500
9,1,15852.025000
9,1,3101.888889
9,1,6511.428571
9,1,1624.250000
9,1,1860.263514
9,1,3144.365385
9,1,6123.245098
9,1,3851.250000
9,1,4795.646341
9,1,4998.250000
9,1,8349.681818
9,1,4338.728571
9,1,3765.079787
9,1,3824.875000
9,1,4534.706081
9,1,2974.695652
9,1,18744.750000
9,1,2124.700000
9,1,3892.673077
9,1,2006.257353
9,1,2979.019531
9,1,1863.372093
9,1,4481.536111
9,1,11993.406250
9,1,2780.063830
9,1,7089.037143
9,1,19657.837838
9,1,1785.341463
9,1,8484.733333
9,1,1858.005814
9,1,2317.000000
9,1,5612.991935
9,1,2060.575000
9,1,15788.758929
9,1,5330.909884
9,1,2807.756098
9,1,1876.617647
9,1,3386.846591
9,1,1689.342105
9,1,2336.339286
9,1,16544.652174
9,1,1577.500000
9,1,1518.793103
9,1,1815.544444
9,1,1915.423077
9,1,1877.772727
9,1,1595.500000
9,1,7635.700658
9,1,5894.604651
9,1,5022.614362
9,1,11913.697368
9,1,3719.967742
9,1,2925.613636
9,1,5081.250000

9,1,8788.837209
9,1,1811.784091
9,1,3196.379630
9,1,1604.928125
9,1,7716.159375
9,1,7013.437500
9,1,2456.506579
9,1,2332.907609
9,1,3187.161290
9,1,2987.716463
9,1,18139.107143
9,1,6256.779070
9,1,2485.750000
9,1,3846.934375
9,1,4463.937500
9,1,1653.936275
9,1,8919.247283
9,1,18171.982558
9,1,2235.058511
9,1,2301.651316
9,1,1971.593750
9,1,7186.410000
9,1,6564.257576
9,1,9168.648026
9,1,4247.380435
9,1,3776.372093
9,1,3467.125000
9,1,2374.617021
9,1,2802.611111
9,1,13546.573980
9,1,2888.850694
9,1,6149.796875
9,1,3106.965517
9,1,3988.931122
9,1,7126.400000
9,1,7541.727273
9,1,3363.588889
9,1,1728.338235
9,1,2637.539773
9,1,3010.723529
9,1,1907.177632
9,1,6294.151163
9,1,5899.607955
9,1,2771.725490
9,1,2349.741667
9,1,6349.215909
9,1,1520.317073
9,1,3904.756579
9,1,13492.564103
9,1,2487.802632
9,1,2074.210106
9,1,2251.784091
9,1,1720.821429
9,1,2565.590000
9,1,3480.097222
9,1,2289.853659
9,1,2085.471875
9,1,4217.522436
9,1,1808.588235
9,1,2079.034091
9,1,1875.651685
9,1,1933.784375
9,1,10657.119048
9,1,1859.517157
9,1,10031.173077
9,1,2133.868421

9,1,5108.312500
9,1,3305.950000
9,1,6631.064024
9,1,2432.847826
9,1,9270.531915
9,1,2688.372222
9,1,2087.329787
9,1,6621.314286
9,1,5193.198370
9,1,3191.397436
9,1,10542.546875
9,1,1995.609375
9,1,4745.110465
9,1,9485.667969
9,1,3684.867347
9,1,1717.556818
9,1,1762.996951
9,1,1620.456395
9,1,2596.375000
9,1,7892.677632
9,1,2550.750000
9,1,8990.394737
9,1,3904.049342
9,1,1939.854839
9,1,2071.863281
9,1,6737.084239
9,1,10448.929487
9,1,7741.022059
9,1,6987.428571
9,1,11390.910326
9,1,1652.654412
9,1,1709.786932
9,1,4941.286585
9,1,2696.923077
9,1,10437.432870
9,1,1813.343750
9,1,2090.676471
9,1,3846.313953
9,1,3367.800000
9,1,19770.517241
9,1,1701.437500
9,1,1983.090909
9,1,3122.773437
9,1,1614.816860
9,1,13040.142857
9,1,12637.087500
9,1,11674.738889
9,1,3186.517857
9,1,3449.297297
9,1,12952.500000
9,1,2943.118421
9,1,13682.443182
9,1,16408.406250
9,1,3747.125000
9,1,5638.010870
9,1,2460.387500
9,1,2187.218750
9,1,1551.777439
9,1,1583.442935
9,1,1650.146552
9,1,3102.027027
9,1,11465.338235
9,1,5602.625000
9,1,1921.099265
9,1,1968.978659
9,1,2362.604651

9,1,2063.370968
9,1,10002.571429
9,1,1571.805147
9,1,11438.953947
9,1,2675.355556
9,1,1798.343750
9,1,17283.680147
9,1,11471.808140
9,1,2519.000000
9,1,5390.500000
9,1,7276.969388
9,1,2003.234375
9,1,2874.287500
9,1,1543.709302
9,1,3852.534884
9,1,2027.829412
9,1,1866.919643
9,1,16910.875000
9,1,1931.437500
9,1,3122.358333
9,1,1887.561224
9,1,4996.061170
9,1,2977.250000
9,1,4954.150000
9,1,4924.843085
9,1,3273.000000
9,1,6576.250000
9,1,6605.497549
9,1,2343.836957
9,1,2387.882353
9,1,4009.898256
9,1,1631.304348
9,1,14069.520161
9,1,3580.828571
9,1,1765.975000
9,1,7189.888889
9,1,2547.635135
9,1,1680.668478
9,1,15509.000000
9,1,1611.261905
9,1,2797.576087
9,1,3216.222222
9,1,5627.291667
9,1,2234.828488
9,1,2879.755682
9,1,3262.187500
9,1,5142.658784
9,1,4967.408088
9,1,10726.272727
9,1,2726.069767
10,2,1934.250000
10,2,1859.338235
10,2,1852.470588
10,2,2094.134146
10,2,1604.375000
10,2,7101.704167
10,2,4066.242347
10,2,1658.168367
10,2,2156.692308
10,2,1500.118750
10,2,3016.224359
10,2,2483.795918
10,2,2831.404762
10,2,3266.696078
10,2,1820.848214
10,2,1563.403846

10,2,1879.896341
10,2,3101.256098
10,2,2510.437500
10,2,2497.807870
10,2,1744.528409
10,2,2084.947674
10,2,2235.756579
10,2,4518.753906
10,2,1845.142442
10,2,1602.093750
10,2,2738.365854
10,2,1999.265625
10,2,2283.272727
10,2,3877.353659
10,2,9206.506944
10,2,1725.675000
10,2,3403.022727
10,2,2908.585366
10,2,1894.708333
10,2,1857.021277
10,2,1948.004717
10,2,2081.923077
10,2,5321.094595
10,2,1714.847222
10,2,1838.576389
10,2,1602.950000
10,2,1914.115854
10,2,1635.316176
10,2,1816.108696
10,2,1761.768617
10,2,2031.436170
10,2,1859.945122
10,2,3830.089286
10,2,3166.346774
10,2,2457.238220
10,2,2753.316038
10,2,2023.065789
10,2,2015.336735
10,2,2776.581395
10,2,7769.492424
10,2,1666.781250
10,2,4408.186170
10,2,1915.589674
10,2,1534.460106
10,2,1994.437500
10,2,2874.859375
10,2,1907.571429
10,2,1826.658854
10,2,1570.264706
10,2,3750.525000
10,2,1766.775510
10,2,1613.305921
10,2,3892.809524
10,2,4966.263514
10,2,2232.226064
10,2,2090.687500
10,2,1620.015625
10,2,2407.444767
10,2,1736.692308
10,2,1875.185897
10,2,2020.521226
10,2,2616.909375
10,2,2074.847222
10,2,3088.708333
10,2,1598.707547
10,2,2254.540541

10,2,1662.505952
10,2,2278.747449
10,2,2310.250000
10,2,1901.506098
10,2,3826.153846
10,2,1836.250000
10,2,1713.537736
10,2,1725.379808
10,2,1526.975000
10,2,1739.567073
10,2,2468.181122
10,2,3172.442708
10,2,2056.869186
10,2,1530.807927
10,2,2816.066176
10,2,1576.902500
10,2,2192.653409
10,2,1796.017857
10,2,4347.952128
10,2,1876.457447
10,2,2205.101562
10,2,2021.068182
10,2,1521.347826
10,2,2178.268293
10,2,2094.612500
10,2,1586.487805
10,2,2259.711735
10,2,1666.937500
10,2,3134.000000
10,2,3019.862069
10,2,2883.107143
10,2,2687.434524
10,2,8572.523810
10,2,1681.404762
10,2,1539.256250
10,2,3505.625000
10,2,1545.875000
10,2,3171.166667
10,2,2732.130000
10,2,2079.750000
10,2,1932.768293
10,2,4071.571429
10,2,1554.875000
10,2,1860.522059
10,2,1651.255814
10,2,1627.657143
10,2,3056.503676
10,2,2151.264706
10,2,1560.596859
10,2,1757.025000
10,2,2059.107143
10,2,5462.177500
10,2,3879.698529
10,2,2284.024457
10,2,2343.650000
10,2,1720.776786
10,2,2521.020000
10,2,2176.565789
10,2,2404.645833
10,2,2264.330645
10,2,1587.400000
10,2,1764.812500
10,2,1741.843023
10,2,2693.862500
10,2,1590.975610
10,2,2154.076220

10,2,1831.134615
10,2,4637.776786
10,2,15317.866667
10,2,1605.500000
10,2,1614.764205
10,2,8269.310811
10,2,2587.026786
10,2,7159.039062
10,2,2423.508929
10,2,1776.613636
10,2,1842.400000
10,2,3255.790179
10,2,1816.312500
10,2,2703.750000
10,2,2163.632353
10,2,2137.136126
10,2,1690.904762
10,2,1775.601064
10,2,3351.938889
10,2,1877.771739
10,2,3609.994681
10,2,3038.456522
10,2,2031.756098
10,2,1808.678191
10,2,2056.400000
10,2,1873.062500
10,2,1847.464286
10,2,2765.234694
10,2,1654.665541
10,2,1878.756250
10,2,2271.181034
10,2,2578.251185
10,2,2072.584821
10,2,1520.211538
10,2,1594.535714
10,2,3602.642857
10,2,2002.681122
10,2,1878.250000
10,2,14804.812500
10,2,2246.120000
10,2,1650.395349
10,2,2719.864583
10,2,1708.168478
10,2,2052.326531
10,2,1725.650000
10,2,1527.193878
10,2,4056.098214
10,2,2679.608696
10,2,1935.234375
10,2,4063.002660
10,2,1681.818182
10,2,1846.561224
10,2,1952.064394
10,2,1827.984043
10,2,2835.541667
10,2,1613.428571
10,2,2568.679245
10,2,2334.744681
10,2,2756.208333
10,2,1844.684896
10,2,1540.380952
10,2,1646.710784
10,2,3051.015000
10,2,1558.881579
10,2,11741.197674
10,2,1919.250000

10,2,1605.848837
10,2,2515.528409
10,2,2148.466837
10,2,1697.348214
10,2,2098.200000
10,2,1654.750000
10,2,2063.785714
10,2,1800.039062
10,2,1957.608974
10,2,2675.895000
10,2,1544.105263
10,2,1969.553571
10,2,6390.515152
10,2,2160.731707
10,2,5514.305921
10,2,3684.740385
10,2,1909.662500
10,2,2043.757075
10,2,2204.705128
10,2,1612.000000
10,2,2027.208333
10,2,1784.335366
10,2,1893.176887
10,2,1754.568750
10,2,3266.272059
10,2,1518.156250
10,2,2834.750000
10,2,1862.037500
10,2,2092.625000
10,2,4110.712838
10,2,1623.878049
10,2,3475.835938
10,2,1691.443750
10,2,1514.653125
10,2,1529.796512
10,2,4880.703947
10,2,4344.048913
10,2,2170.767857
10,2,1639.625000
10,2,1588.914894
10,2,2304.500000
10,2,1994.875000
10,2,1945.367500
10,2,2782.980769
10,2,1728.285714
10,2,1798.256098
10,2,3358.119048
10,2,2480.990000
10,2,1697.235795
10,2,2592.057377
10,2,2515.384615
10,2,1554.728448
10,2,1818.293269
10,2,2002.833333
10,2,1773.143293
10,2,6520.312500
10,2,2705.871324
10,2,10704.669118
10,2,1621.232558
10,2,1557.116848
10,2,1885.600000
10,2,2012.718750
10,2,19770.500000
10,2,2467.805288
10,2,1867.450000
10,2,1760.306373

10,2,2829.348837
10,2,2468.333333
10,2,1813.801136
10,2,3232.906977
10,2,1542.041667
10,2,8053.346939
10,2,2103.771429
10,2,2601.594444
10,2,1755.222826
10,2,1674.943396
10,2,1505.175000
10,2,1568.743421
10,2,1801.687500
10,2,1923.820513
10,2,2029.000000
10,2,4667.875000
10,2,1692.207447
10,2,1693.448113
10,2,1992.583333
10,2,4585.250000
10,2,1803.456522
10,2,3284.795732
10,2,3597.034314
10,2,2456.117347
10,2,2513.035326
10,2,1611.000000
10,2,2416.008333
10,2,2151.975000
10,2,1608.262500
10,2,1575.442073
10,2,5646.062500
10,2,1820.375000
10,2,2400.861702
10,2,1950.281250
10,2,2050.145833
10,2,1513.673913
10,2,1788.743902
10,2,1548.903846
10,2,1771.459302
10,2,2459.260000
10,2,1680.869565
10,2,2243.216667
10,2,5598.384615
10,2,1691.291667
10,2,1962.473684
10,2,1734.819095
10,2,1588.984043
10,2,1772.039773
10,2,3364.062500
10,2,1746.807927
10,2,3259.500000
10,2,2615.452500
10,2,1950.397959
10,2,2348.941667
10,2,1923.838235
10,2,1504.043750
10,2,1821.059524
10,2,1543.922619
10,2,2280.143443
10,2,6624.199519
10,2,2992.234375
10,2,1867.567500
10,2,1700.200000
10,2,3437.460227
10,2,2208.320000
10,2,2905.057692

10,2,2484.750000
10,2,2017.782738
10,2,1826.785714
10,2,1661.329268
10,2,1545.755814
10,2,3493.054545
10,2,5398.044811
10,2,2122.421512
10,2,2010.500000
10,2,1755.042500
10,2,2798.284091
10,2,2289.083333
10,2,1619.395349
10,2,7956.015306
10,2,2386.734043
10,2,2361.198980
10,2,1701.600446
10,2,5835.466912
10,2,3140.875000
10,2,2634.867925
10,2,3703.639151
10,2,5472.750000
10,2,1522.750000
10,2,2005.126582
10,2,1505.125000
10,2,1899.437500
10,2,1613.062500
10,2,5009.784884
10,2,1559.780488
10,2,2520.404255
10,2,1588.519608
10,2,1966.767500
10,2,1510.806548
10,2,2078.767857
10,2,1618.039352
10,2,5804.437500
10,2,1653.613636
10,2,1593.903302
10,2,2979.467262
10,2,4726.829787
10,2,1620.906250
10,2,1567.545455
10,2,1534.080000
10,2,2319.312500
10,2,3009.762500
10,2,1686.200000
10,2,1861.333333
10,2,3069.953125
10,2,1883.195122
10,2,7960.723684
10,2,2416.850000
10,2,1940.952703
10,2,1824.250000
10,2,3334.858974
10,2,2083.640000
10,2,2766.732143
10,2,2092.682692
10,2,2511.767857
10,2,2396.283333
10,2,1741.088235
10,2,1924.011111
10,2,1876.793750
10,2,4142.545732
10,2,10392.272727
10,2,1800.951087
10,2,1505.130208

10,2,2043.837500
10,2,1615.703593
10,2,1713.029070
10,2,1975.907895
10,2,4539.728261
10,2,2159.381250
10,2,3569.744681
10,2,4791.814189
10,2,1506.615385
10,2,2181.125000
10,2,1966.293750
10,2,1865.389706
10,2,1850.169444
10,2,2175.674419
10,2,2068.800000
10,2,3233.956019
10,2,1768.812500
10,2,1644.027778
10,2,2299.666667
10,2,1561.631098
10,2,1780.875000
10,2,1628.133333
10,2,1967.625000
10,2,1528.094595
10,2,4106.211538
10,2,2309.768182
10,2,3435.273026
10,2,1955.526316
10,2,2716.960000
10,2,2541.081633
10,2,1869.700000
10,2,1938.243243
10,2,1854.674242
10,2,2118.590909
10,2,1585.702381
10,2,2156.175000
10,2,3626.336310
10,2,2228.345745
10,2,3229.993750
10,2,1614.548913
10,2,2952.096154
10,2,2302.315789
10,2,1896.041667
10,2,2171.198980
10,2,2463.250000
10,2,2913.135000
10,2,2133.427500
10,2,9486.275000
10,2,2652.615854
10,2,1587.173077
10,2,2797.600000
10,2,1631.047619
10,2,1868.125000
10,2,1820.726852
10,2,2908.082386
10,2,2382.848214
10,2,2007.744048
10,2,2395.273585
10,2,1623.794643
10,2,1999.585938
10,2,1590.025510
10,2,1974.755319
10,2,3304.265957
10,2,5639.111111
10,2,3944.968750
10,2,1553.951220

10,2,2041.530303
10,2,1699.714286
10,2,1946.880435
10,2,2202.250000
10,2,2656.653302
10,2,1542.437500
10,2,3383.128205
10,2,2842.317073
10,2,2029.562500
10,2,1535.612179
10,2,1842.610465
10,2,1915.829268
10,2,1519.187500
10,2,2246.234375
10,2,1591.475000
10,2,2047.845745
10,2,1782.000000
10,2,2573.585227
10,2,2669.687500
10,2,2579.470000
10,2,2507.558036
10,2,1723.219512
10,2,6217.941489
10,2,2794.632653
10,2,1648.162162
10,2,1935.390244
10,2,1517.337500
10,2,4271.166667
10,2,3070.950000
10,2,1562.913043
10,2,3123.155172
10,2,5933.119898
10,2,1574.321429
10,2,1745.867188
10,2,2025.215686
10,2,2660.590909
10,2,1522.560185
10,2,3892.562500
10,2,1735.990000
10,2,2568.268229
10,2,1533.738636
10,2,1938.459459
10,2,17673.441176
10,2,2886.489362
10,2,1707.204545
10,2,17195.157143
10,2,2154.573864
10,2,2146.216216
10,2,1647.923469
10,2,1667.114286
10,2,2042.744681
10,2,1779.613971
10,2,2313.784574
10,2,2096.292763
10,2,3624.219388
10,2,1892.665000
10,2,4319.722973
10,2,1926.408537
10,2,1513.655488
10,2,2554.167683
10,2,2355.226562
10,2,1582.331250
10,2,1857.500000
10,2,2621.640625
10,2,1750.033854
10,2,1924.875000

10,2,1605.274306
10,2,1528.442568
10,2,3194.446429
10,2,1975.894737
10,2,5564.404605
10,2,3493.161184
10,2,1523.632979
10,2,1706.133523
10,2,1736.250000
10,2,1701.618750
10,2,5721.647727
10,2,1992.080000
10,2,1609.231707
10,2,2416.357143
10,2,2258.437500
10,2,2340.273810
10,2,1611.960227
10,2,1921.188889
10,2,2271.906250
10,2,2083.413889
10,2,1826.604167
10,2,1995.916667
10,2,1534.627660
10,2,2440.346875
10,2,2164.712766
10,2,15818.692568
10,2,2612.334559
10,2,2592.032895
10,2,1730.273026
10,2,1974.502404
10,2,2941.941176
10,2,2801.566327
10,2,2570.472500
10,2,1972.093750
10,2,1616.010638
10,2,1642.875000
10,2,2265.478448
10,2,1726.985119
10,2,1743.906780
10,2,5363.312500
10,2,1606.729592
10,2,1932.016484
10,2,2896.987500
10,2,5020.031250
10,2,3115.533537
10,2,1546.500000
10,2,2714.330000
10,2,1863.973837
10,2,1518.329082
10,2,2147.771277
10,2,1591.977941
10,2,5924.000000
10,2,2097.226190
10,2,14966.558824
10,2,1510.213542
10,2,1516.062500
10,2,1568.555556
10,2,1891.754630
10,2,3710.490566
10,2,2496.938953
10,2,1535.949468
10,2,1737.372449
10,2,2022.778846
10,2,1887.078125
10,2,3475.238095
10,2,2045.250000

10,2,3038.695652
10,2,3634.344059
10,2,2363.294643
10,2,1509.005952
10,2,3400.652174
10,2,1878.264881
10,2,3085.445313
10,2,1549.449468
10,2,3166.718750
10,2,1971.304688
10,2,2133.658537
10,2,1679.541667
10,2,1611.823718
10,2,1612.934783
10,2,1668.150510
10,2,2351.495370
10,2,2784.508523
10,2,2358.880952
10,2,2010.569149
10,2,2205.227041
10,2,2015.000000
10,2,1637.107143
10,2,3560.069853
10,2,1574.356132
10,2,2357.095000
10,2,1626.850000
10,2,3692.732143
10,2,1513.583333
10,2,5318.675676
10,2,4024.057692
10,2,5344.725000
10,2,1730.980000
10,2,1810.688776
10,2,2094.803879
10,2,1604.508929
10,2,2552.110000
10,2,1541.392857
10,2,13235.054688
10,2,2729.803571
10,2,3666.257812
10,2,3192.367347
10,2,2178.328125
10,2,2441.426020
10,2,2169.375000
10,2,2337.666667
10,2,1731.437500
10,2,1609.601562
10,2,4052.014286
10,2,2495.138298
10,2,1634.625000
10,2,1572.270833
10,2,1813.551136
10,2,2630.340000
10,2,1586.625000
10,2,9797.486842
10,2,1656.381818
10,2,1626.473684
10,2,2201.915441
10,2,1603.700000
10,2,1718.188725
10,2,5258.170732
10,2,2617.335106
10,2,5083.884615
10,2,2250.712500
10,2,1670.642857
10,2,1860.183824

10,2,1799.366071
10,2,2156.295455
10,2,1706.565476
10,2,2274.693878
10,2,3624.818182
10,2,4047.354167
10,2,2009.964286
10,2,2998.421875
10,2,2649.125000
10,2,2182.803191
10,2,2595.454545
10,2,1832.872340
10,2,2781.350962
10,2,2068.416667
10,2,5865.041667
10,2,2284.776596
10,2,1547.117021
10,2,2944.059524
10,2,1820.116848
10,2,1866.475000
10,2,2524.556122
10,2,2288.337766
10,2,1719.968750
10,2,1759.100000
10,2,1596.260638
10,2,2716.833333
10,2,2242.841216
10,2,1748.329268
10,2,3699.317130
10,2,2125.089286
10,2,3473.811111
10,2,2749.819767
10,2,1922.719512
10,2,2982.578125
10,2,1734.816176
10,2,1877.336957
10,2,1946.557927
10,2,5527.739130
10,2,1548.801724
10,2,4340.523026
10,2,1903.372642
10,2,1574.480000
10,2,1763.742647
10,2,1572.379902
10,2,1562.142857
10,2,1598.187500
10,2,2157.278646
10,2,3169.500000
10,2,1763.988095
10,2,1874.464286
10,2,1808.107143
10,2,1901.925595
10,2,1977.681548
10,2,1777.858796
10,2,1951.142857
10,2,3534.032143
10,2,2349.825000
10,2,5036.122449
10,2,1851.340909
10,2,1995.428571
10,2,1891.862500
10,2,3461.481383
10,2,2013.270833
10,2,2908.803922
10,2,2165.375000
10,2,2726.426471

10,2,2539.404255
10,2,17884.875000
10,2,1681.109375
10,2,2301.984091
10,2,2199.075000
10,2,2097.035714
10,2,2143.522059
10,2,2286.412500
10,2,1721.770000
10,2,2701.047872
10,2,16905.750000
10,2,7336.194313
10,2,4089.937500
10,2,1888.651786
10,2,2544.609375
10,2,1805.789474
10,2,1833.553191
10,2,4791.389881
10,2,2161.232143
10,2,1798.860795
10,2,5997.085526
10,2,2532.331250
10,2,3216.693878
10,2,2311.729167
10,2,1797.957447
10,2,1525.605263
10,2,1590.096354
10,2,1568.121951
10,2,1826.620968
10,2,2395.258929
10,2,1693.446809
10,2,3273.348039
10,2,1846.285714
10,2,2254.707317
10,2,2178.091463
10,2,2822.382812
10,2,1560.799020
10,2,1742.500000
10,2,2068.125000
10,2,3757.950000
10,2,8428.311111
10,2,12023.636111
10,2,5530.000000
10,2,2242.005319
10,2,1823.250000
10,2,1751.030612
10,2,2081.833333
10,2,1685.885204
10,2,1510.333333
10,2,3087.030556
10,2,2375.541667
10,2,1880.707447
10,2,2136.166667
10,2,1545.642857
10,2,1798.636364
10,2,1700.366667
10,2,3061.375000
10,2,2083.640244
10,2,1934.890000
10,2,1504.891509
10,2,2909.171875
10,2,4421.076087
10,2,1811.441176
10,2,3176.825000
10,2,2754.500000
10,2,1873.463415

10,2,4792.340426
10,2,4671.064024
10,2,3201.500000
10,2,3396.842949
10,2,2967.080357
10,2,1647.677778
10,2,1585.530556
10,2,4183.723684
10,2,3694.046512
10,2,1743.351064
10,2,1871.169643
10,2,3706.302326
10,2,2880.250000
10,2,1638.110577
10,2,1722.310976
10,2,1747.788690
10,2,1759.665816
10,2,1583.679245
10,2,2035.378571
10,2,1700.164773
10,2,2471.894737
10,2,1797.976744
10,2,2473.580357
10,2,7605.230769
10,2,2679.156250
10,2,2066.971154
10,2,2698.035714
10,2,1504.378289
10,2,2123.187500
10,2,1503.587766
10,2,1657.860000
10,2,1652.687500
10,2,1740.698370
10,2,1503.894886
10,2,2460.953125
10,2,3300.179348
10,2,2080.146277
10,2,1558.139535
10,2,2016.272727
10,2,4954.125000
10,2,1826.093750
10,2,1654.897959
10,2,1519.767045
10,2,1740.378676
10,2,1989.562500
10,2,4345.975962
10,2,1903.867021
10,2,2395.614362
10,2,5634.181818
10,2,2101.426136
10,2,4004.242857
10,2,2065.486486
10,2,2219.041667
10,2,1569.414634
10,2,1954.977273
10,2,2431.583333
10,2,1868.187500
10,2,1625.211538
10,2,1607.597826
10,2,2052.535714
10,2,2173.353261
10,2,1575.634868
10,2,2560.185185
10,2,2150.210526
10,2,6222.433594
10,2,1676.827206

10,2,1707.909091
10,2,1675.942073
10,2,2173.641509
10,2,1662.700000
10,2,1898.853659
10,2,2092.400000
10,2,3475.935096
10,2,1511.352564
10,2,1613.564103
10,2,1503.826531
10,2,1677.836538
10,2,1742.505556
10,2,3932.078125
10,2,3455.140957
10,2,1576.953947
10,2,2874.237245
10,2,2603.233696
10,2,2062.372449
10,2,1708.000000
10,2,1569.708333
10,2,2747.309524
10,2,2003.614796
10,2,1879.594444
10,2,1753.012195
10,2,2039.295213
10,2,1548.355978
10,2,1571.571429
10,2,1590.016204
10,2,7063.875000
10,2,4621.388889
10,2,1912.781250
10,2,8077.678571
10,2,3291.852941
10,2,1582.082317
10,2,2239.370536
10,2,2215.848837
10,2,2179.243590
10,2,1585.691327
10,2,4894.046875
10,2,4212.282051
10,2,2160.794118
10,2,2243.050000
10,2,1563.666667
10,2,2092.378125
10,2,1581.851974
10,2,2537.914894
10,2,2070.138514
10,2,1787.000000
10,2,1521.875000
10,2,2490.224359
10,2,1613.571429
10,2,2846.495614
10,2,1818.586957
10,2,1890.479592
10,2,4121.480263
10,2,4511.956633
10,2,1548.339744
10,2,1502.005814
10,2,3088.631579
10,2,1857.587963
10,2,3380.332500
10,2,2181.140000
10,2,2700.276786
10,2,5297.286765
10,2,1838.833333
10,2,2001.246795

10,2,1781.192073
10,2,5525.698718
10,2,3469.385475
10,2,3018.601852
10,2,2314.678571
10,2,1499.351562
10,2,2306.211538
10,2,1574.116667
10,2,3381.500000
10,2,2044.207547
10,2,2361.358974
10,2,3114.063830
10,2,2314.573529
10,2,10684.733108
10,2,1503.122340
10,2,2584.310185
10,2,1799.994565
10,2,1745.558511
10,2,1995.809896
10,2,1778.828488
10,2,2038.227941
10,2,3170.692308
10,2,1742.698864
10,2,2018.750000
10,2,2701.653846
10,2,1821.022727
10,2,3139.110119
10,2,3403.244444
10,2,1804.668367
10,2,1988.382979
10,2,3170.693182
10,2,2272.091837
10,2,2903.767857
10,2,4266.927632
10,2,1557.538462
10,2,2430.943452
10,2,2795.047872
10,2,2671.201220
10,2,1665.127841
10,2,2660.948276
10,2,2019.542553
10,2,3525.400000
10,2,5933.955128
10,2,1670.031250
10,2,1549.886364
10,2,1574.500000
10,2,1602.000000
10,2,1647.156250
10,2,1756.247396
10,2,1884.484694
10,2,1783.371528
10,2,1594.956250
10,2,2227.957386
10,2,2273.819712
10,2,3425.432870
10,2,1553.100000
10,2,1805.875000
10,2,1724.009346
10,2,2660.891892
10,2,6705.089286
10,2,1738.470588
10,2,1551.804348
10,2,2681.770000
10,2,2528.482558
10,2,1980.085526
10,2,1676.587500

10,2,10589.975000
10,2,1988.981250
10,2,1528.656250
10,2,2353.804545
10,2,3601.622222
10,2,9747.134375
10,2,1690.072917
10,2,1784.863095
10,2,14297.975610
10,2,7790.500000
10,2,1688.808962
10,2,1955.540909
10,2,1958.197368
10,2,2043.588710
10,2,1763.562500
10,2,1859.143617
10,2,1818.810811
10,2,2200.695652
10,2,1759.712500
10,2,2057.586735
10,2,1691.931604
10,2,1859.079268
10,2,1967.187500
10,2,1667.272059
10,2,2159.163462
10,2,2165.207447
10,2,1629.213415
10,2,1623.593750
10,2,1636.593750
10,2,3271.064189
10,2,4294.500000
10,2,4510.589286
10,2,1681.339286
10,2,1865.325000
10,2,3605.534574
10,2,1756.866279
10,2,3215.303191
10,2,3944.261111
10,2,2132.107143
10,2,2408.530000
10,2,1645.925532
10,2,1637.583333
10,2,3171.000000
10,2,1568.831250
10,2,1616.452830
10,2,2681.962963
10,2,3471.418367
10,2,1667.079545
10,2,2652.224490
10,2,2068.187500
10,2,9980.835366
10,2,1681.405488
10,2,2612.000000
10,2,2022.854167
10,2,2995.800000
10,2,4966.961538
10,2,1746.450000
10,2,1943.000000
10,2,2863.940476
10,2,2327.393750
10,2,1936.215116
10,2,2460.529070
10,2,1663.718137
10,2,1936.542553
10,2,4783.620833
10,2,2205.410326

10,2,1962.484694
10,2,1978.280488
10,2,2329.211957
10,2,2009.627404
10,2,5801.285714
10,2,1662.030405
10,2,10420.728070
10,2,1608.280702
10,2,2105.375000
10,2,1662.397590
10,2,2272.339744
10,2,1732.970238
10,2,2084.559524
10,2,2331.127273
10,2,1545.247093
10,2,1983.770833
10,2,1533.437500
10,2,1738.625641
10,2,5364.555556
10,2,2461.500000
10,2,2107.490196
10,2,1756.941964
10,2,1639.161765
10,2,2268.429245
10,2,2927.705882
10,2,7687.789773
10,2,5764.130435
10,2,4497.116279
10,2,1668.714286
10,2,1557.341146
10,2,2255.097561
10,2,1923.213542
10,2,2974.500000
10,2,2436.179878
10,2,1891.923913
10,2,4852.456522
10,2,1828.071429
10,2,2673.118056
10,2,2122.995098
10,2,2553.950000
10,2,1751.125000
10,2,2505.073171
10,2,1536.628049
10,2,2018.724599
10,2,1651.130952
10,2,1671.395000
10,2,1706.730978
10,2,3859.177632
10,2,1711.318452
10,2,3601.238095
10,2,2140.125000
10,2,1922.214286
10,2,2375.420000
10,2,1617.000000
10,2,1852.805556
10,2,1577.794355
10,2,1509.594595
10,2,2896.500000
10,2,2097.857143
10,2,1692.375000
10,2,2778.000000
10,2,1712.299419
10,2,1931.936170
10,2,1772.418605
10,2,2637.153409
10,2,1817.229730

10,2,3109.250000
10,2,3838.923077
10,2,2446.346154
10,2,1804.035088
10,2,1668.000000
10,2,2965.494681
10,2,1660.436170
10,2,1808.200000
10,2,8640.839744
10,2,1899.740000
10,2,5332.057692
10,2,2116.622093
10,2,2002.139423
10,2,1574.812500
10,2,2804.294118
10,2,1698.110119
10,2,2096.833333
10,2,5782.216912
10,2,1703.132653
10,2,2071.393382
10,2,2536.335106
10,2,2030.925000
10,2,3208.007353
10,2,3188.500000
10,2,1740.145833
10,2,1609.463415
10,2,1691.073171
10,2,3934.473837
10,2,2133.829268
10,2,2617.275000
10,2,2108.025641
10,2,1608.698529
10,2,1605.490196
10,2,1606.455556
10,2,3706.784091
10,2,1500.505319
10,2,1716.632653
10,2,2651.617188
10,2,2253.200000
10,2,1565.556250
10,2,1864.463889
10,2,4994.452500
10,2,4117.781915
10,2,4182.200000
10,2,1743.422222
10,2,1737.965116
10,2,1754.000000
10,2,1806.250000
10,2,2825.722973
10,2,2068.895000
10,2,2602.637755
10,2,2956.375000
10,2,3126.716912
10,2,2037.213235
10,2,1516.733333
10,2,4029.000000
10,2,2367.441176
10,2,4100.357955
10,2,1515.365854
10,2,7887.436047
10,2,1782.931250
10,2,2183.416667
10,2,2369.983333
10,2,2677.602273
10,2,1564.772727
10,2,1538.523437

10,2,4102.780000
10,2,1689.714286
10,2,2657.657143
10,2,2194.000000
10,2,2102.272727
10,2,1961.365625
10,2,5180.301887
10,2,2525.000000
10,2,1707.760000
10,2,5411.257212
10,2,3122.052326
10,2,2537.932432
10,2,2131.885417
10,2,1516.721591
10,2,1546.984375
10,2,2161.302326
10,2,2605.292553
10,2,2217.401596
10,2,2067.940909
10,2,1668.964286
10,2,6872.830508
10,2,1523.678571
10,2,2521.784091
10,2,1812.321875
10,2,7568.390244
10,2,2014.257812
10,2,1979.250000
10,2,2131.347561
10,2,6791.590909
10,2,2148.552778
10,2,3709.375000
10,2,2092.250000
10,2,1527.875000
10,2,1567.750000
10,2,1942.552885
10,2,1818.149390
10,2,3281.473684
10,2,2359.545455
10,2,1834.621429
10,2,2304.770833
10,2,2669.339674
10,2,1592.686047
10,2,1759.062500
10,2,1574.718085
10,2,3237.087209
10,2,1582.094444
10,2,1597.292553
10,2,1507.337838
10,2,2120.206395
10,2,3313.935714
10,2,1731.573718
10,2,2619.483871
10,2,1584.633333
10,2,2194.486364
10,2,1842.324468
10,2,2034.934783
10,2,2808.425000
10,2,2241.310000
10,2,2572.687500
10,2,4590.313953
10,2,3017.805233
10,2,1515.687500
10,2,1780.577381
10,2,1792.816667
10,2,1523.016667
10,2,3194.375000

10,2,2624.058824
10,2,1575.182692
10,2,2158.052632
10,2,2887.097222
10,2,3384.472561
10,2,1770.941176
10,2,2393.234375
10,2,4525.087500
10,2,2157.946429
10,2,2341.961538
10,2,1514.448276
10,2,2610.108173
10,2,2379.958333
10,2,1686.196429
10,2,2475.092391
10,2,17333.530612
10,2,1562.107143
10,2,2093.325472
10,2,2165.985577
10,2,2404.182927
10,2,2367.791667
10,2,1613.821429
10,2,2436.338816
10,2,1552.877907
10,2,1515.637500
10,2,1560.451923
10,2,3283.543269
10,2,3136.529661
10,2,5850.919492
10,2,1636.392857
10,2,2004.169118
10,2,1787.474138
10,2,1732.428571
10,2,5324.952096
10,2,1572.743421
10,2,1556.138587
10,2,2142.380435
10,2,3597.951754
10,2,2415.863636
10,2,1685.962766
10,2,1532.794872
10,2,5081.154762
10,2,1669.583333
10,2,1502.994186
10,2,2208.444767
10,2,6145.314904
10,2,2663.391304
10,2,2353.433333
10,2,3401.744681
10,2,3163.447917
10,2,2467.336538
10,2,1855.325000
10,2,2243.903125
10,2,2116.028571
10,2,1645.052885
10,2,2903.743750
10,2,3339.000000
10,2,2262.781250
10,2,3073.270000
10,2,2183.390135
10,2,2086.989796
10,2,2232.898936
10,2,1535.950000
10,2,1776.614362
10,2,1840.071429
10,2,1556.258065

10,2,2150.447917
10,2,1540.709302
10,2,1502.648649
10,2,1790.458333
10,2,1549.127451
10,2,13665.487179
10,2,1691.000000
10,2,1636.184783
10,2,2106.609756
10,2,1664.157500
10,2,2326.900735
10,2,2500.612179
10,2,5229.894737
10,2,2579.869565
10,2,2002.070652
10,2,7152.037736
10,2,1727.740566
10,2,2121.493902
10,2,1853.942308
10,2,3098.093750
10,2,1634.122642
10,2,2086.658333
10,2,1918.728774
10,2,1665.190476
10,2,1783.148810
10,2,1536.657895
10,2,3568.642857
10,2,8911.545455
10,2,1628.777778
10,2,2557.525000
10,2,1967.794118
10,2,1579.365385
10,2,1527.209239
10,2,1953.625000
10,2,3932.654891
10,2,2045.357143
10,2,4073.398936
10,2,2712.734694
10,2,2025.175532
10,2,2815.510870
10,2,2512.650000
10,2,2787.416667
10,2,3512.023438
10,2,1511.231250
10,2,2674.341463
10,2,10526.657407
10,2,2621.797414
10,2,2125.214286
10,2,2965.166667
10,2,1937.298387
10,2,2055.829545
10,2,3646.125000
10,2,2712.395408
10,2,1993.622449
10,2,1668.817857
10,2,1653.563953
10,2,1913.578125
10,2,7996.011364
10,2,2024.823529
10,2,2361.000000
10,2,2000.375000
10,2,2170.687500
10,2,2382.992021
10,2,1977.195000
10,2,4300.572500
10,2,1776.463235

10,2,2169.679348
10,2,2019.025000
10,2,3095.982500
10,2,1618.871951
10,2,1941.117925
10,2,1647.182692
10,2,2123.735465
10,2,3312.200000
10,2,1839.294118
10,2,3488.774457
10,2,1622.259259
10,2,1701.500000
10,2,1948.361702
10,2,2318.688889
10,2,1544.813725
10,2,1989.285326
10,2,2568.000000
10,2,1926.675000
10,2,1658.024390
10,2,1614.416667
10,2,2067.733333
10,2,1514.902439
10,2,1954.833333
10,2,1526.637500
10,2,2470.750000
10,2,2031.757979
10,2,2292.318396
10,2,1558.986842
10,2,1668.591837
10,2,3017.852273
10,2,3113.067708
10,2,2541.419118
10,2,1985.295455
10,2,1507.850000
10,2,1613.000000
10,2,2536.798246
10,2,2848.531915
10,2,1701.601064
10,2,2082.779762
10,2,1959.020000
10,2,2642.189904
10,2,1508.192308
10,2,1920.571429
10,2,1625.454545
10,2,1735.628205
10,2,1824.546875
10,2,2586.455000
10,2,3071.465909
10,2,1649.113636
10,2,1723.722973
10,2,1618.575980
10,2,1653.127717
10,2,2503.670139
10,2,3906.953947
10,2,2251.141304
10,2,3322.175000
10,2,1655.500000
10,2,2303.404412
10,2,3145.735294
10,2,1568.250000
10,2,1531.153846
10,2,2292.375000
10,2,1857.676829
10,2,1618.290000
10,2,3914.258929
10,2,1918.330000

10,2,2733.170213
10,2,1949.020833
10,2,2319.000000
10,2,1976.589286
10,2,5585.892857
10,2,3381.120000
10,2,1880.578125
10,2,1879.500000
10,2,2394.710000
10,2,1604.420732
10,2,1657.859375
10,2,2301.000000
10,2,1617.915698
10,2,2594.380952
10,2,2076.371622
10,2,1993.409574
10,2,1924.957500
10,2,3776.042453
10,2,1805.788462
10,2,1646.768868
10,2,2027.872449
10,2,1790.332547
10,2,2297.000000
10,2,2354.099057
10,2,1563.475000
10,2,3499.193878
10,2,2192.230000
10,2,1609.195122
10,2,3517.694444
10,2,1619.048077
10,2,3073.500000
10,2,3588.619565
10,2,1757.385417
10,2,1834.267857
10,2,2478.253289
10,2,2042.077273
10,2,2074.902174
10,2,4858.170000
10,2,3050.426136
10,2,2389.382500
10,2,3479.466837
10,2,10330.086957
10,2,1737.411765
10,2,1715.195652
10,2,4262.470238
10,2,1812.671053
10,2,2166.630319
10,2,2221.550000
10,2,1743.409091
10,2,1633.566667
10,2,2032.200000
10,2,1616.551020
10,2,1629.333333
10,2,2683.408854
10,2,1944.237745
10,2,2534.520000
10,2,1564.250000
10,2,1604.900000
10,2,3356.559659
10,2,2210.375000
10,2,2073.980769
10,2,1550.526316
10,2,1513.602273
10,2,2310.468750
10,2,1681.919118
10,2,2038.770833

10,2,3550.011029
10,2,1829.602041
10,2,1664.975000
10,2,1924.889535
10,2,1743.052326
10,2,1746.032609
10,2,2315.032051
10,2,2143.755814
10,2,1742.180000
10,2,7681.030488
10,2,1841.954545
10,2,1727.127907
10,2,2183.890000
10,2,2234.413462
10,2,2019.272727
10,2,3229.350000
10,2,1607.650000
10,2,2296.456522
10,2,2822.861111
10,2,1685.796875
10,2,3312.272277
10,2,1521.469388
10,2,2445.140351
10,2,1774.720000
10,2,13315.772021
10,2,1808.033088
10,2,2481.928571
10,2,3770.818182
10,2,1610.090909
10,2,1798.389706
10,2,3646.980769
10,2,1755.590909
10,2,3185.946108
10,2,2881.767442
10,2,1646.783163
10,2,2175.833333
10,2,1567.160714
10,2,1712.864865
10,2,1589.964286
10,2,1825.671196
10,2,6512.891304
10,2,1892.718750
10,2,2058.529891
10,2,1602.781250
10,2,1499.810976
10,2,2024.387755
10,2,4333.642857
10,2,1756.030488
10,2,2027.744898
10,2,2239.885714
10,2,1856.937500
10,2,1677.125000
10,2,2381.700000
10,2,2473.583333
10,2,3445.896739
10,2,1631.282051
10,2,8003.420455
10,2,3346.045455
10,2,2567.526042
10,2,2016.778846
10,2,2011.687500
10,2,3641.723684
10,2,1569.261905
10,2,2295.288462
10,2,2721.014205
10,2,2452.445946

10,2,4505.510714
10,2,1781.829787
10,2,2286.880435
10,2,1537.458333
10,2,2146.244444
10,2,1565.718750
10,2,3743.820565
10,2,1880.875000
10,2,2131.218750
10,2,1553.307500
10,2,2431.239130
10,2,2470.462209
10,2,1989.456250
10,2,1856.591146
10,2,2999.939904
10,2,1506.968750
10,2,2245.964286
10,2,13136.583333
10,2,1872.794872
10,2,5429.466667
10,2,10527.315789
10,2,2649.458333
10,2,2009.220238
10,2,5230.512821
10,2,2551.681818
10,2,2066.191489
10,2,1503.109375
10,2,1723.224359
10,2,1858.400641
10,2,3560.844340
10,2,2065.687500
10,2,1510.500000
10,2,14405.321429
10,2,1718.797500
10,2,2417.771739
10,2,1677.036058
10,2,2299.907609
10,2,1566.948529
10,2,3987.221154
10,2,1818.321875
10,2,2176.157500
10,2,2723.564103
10,2,1938.413889
10,2,1744.006410
10,2,1528.103125
10,2,2227.541667
10,2,3709.580189
10,2,2244.808511
10,2,5354.430233
10,2,2458.669118
10,2,3182.610000
10,2,3294.140625
10,2,2484.382353
10,2,1891.179487
10,2,2557.354839
10,2,1611.247396
10,2,1583.177778
10,2,1579.415541
10,2,1562.964286
10,2,2014.409574
10,2,1523.760204
10,2,2637.187500
10,2,1649.932065
10,2,2023.173913
10,2,2766.570312
10,2,3946.052885

10,2,1898.104167
10,2,1568.625000
10,2,2105.478365
10,2,2384.467262
10,2,2402.677778
10,2,1569.528302
10,2,1547.830357
10,2,2293.732143
10,2,1900.406915
10,2,1555.389535
10,2,1509.764706
10,2,1957.384615
10,2,3798.510204
10,2,3458.648936
10,2,1592.726562
10,2,2735.794118
10,2,1872.429878
10,2,2029.995000
10,2,5006.527273
10,2,1845.500000
10,2,2770.029412
10,2,1636.972727
10,2,1676.222222
10,2,1654.000000
10,2,2478.240196
10,2,2040.807692
10,2,2268.054054
10,2,1713.349359
10,2,1564.181250
10,2,1697.648256
10,2,3004.428571
10,2,1614.256250
10,2,2695.000000
10,2,1573.783537
10,2,1611.531977
10,2,2040.923077
10,2,4559.211735
10,2,1953.071429
10,2,3316.845000
10,2,2039.487500
10,2,1541.812500
10,2,1961.358796
10,2,1799.065789
10,2,1695.130000
10,2,2504.951531
10,2,2561.361702
10,2,1724.763298
10,2,1818.852564
10,2,2933.175532
10,2,1915.161932
10,2,2223.895349
10,2,1511.885000
10,2,1728.487500
10,2,3475.729167
10,2,2171.120690
10,2,3258.803191
10,2,3519.451220
10,2,3100.886628
10,2,1969.117647
10,2,6030.852941
10,2,1506.091463
10,2,1707.769231
10,2,3037.721939
10,2,1933.803922
10,2,3157.363208
10,2,17129.131579

10,2,1816.555556
10,2,2737.026163
10,2,1722.696429
10,2,1862.014706
10,2,1984.885204
10,2,1749.320513
10,2,2977.014535
10,2,2104.752273
10,2,4042.724057
10,2,3204.108696
10,2,2238.967391
10,2,1981.222222
10,2,3350.865566
10,2,2197.500000
10,2,2599.854167
10,2,2449.734375
10,2,2193.300595
10,2,3081.341346
10,2,1678.015625
10,2,4142.042553
10,2,3493.162162
10,2,1806.685096
10,2,1673.535714
10,2,1890.782609
10,2,1768.666667
10,2,1680.648810
10,2,1523.500000
10,2,3320.260870
10,2,1505.000000
10,2,1561.625000
10,2,1835.397436
10,2,2032.723039
10,2,1859.625000
10,2,1649.062500
10,2,1927.364865
10,2,2000.369681
10,2,2327.355769
10,2,4157.600000
10,2,6781.222222
10,2,2170.747159
10,2,1646.226562
10,2,1576.309524
10,2,3473.829787
10,2,1704.725000
10,2,2480.277778
10,2,3692.494048
10,2,1967.844444
10,2,3173.585526
10,2,4009.218085
10,2,2116.966667
10,2,1875.638889
10,2,3305.900000
10,2,1918.046875
10,2,6140.948980
10,2,2738.174479
10,2,1720.535714
10,2,2589.543919
10,2,1631.453125
10,2,2352.137500
10,2,2992.500000
10,2,1555.428571
10,2,1671.727041
10,2,3289.388298
10,2,2894.341216
10,2,2401.117021
10,2,2381.173077

10,2,1608.458333
10,2,1692.228261
10,2,1737.857143
10,2,3052.459459
10,2,4553.129630
10,2,1768.064286
10,2,2423.392857
10,2,2367.361413
10,2,1571.720280
10,2,2144.000000
10,2,4760.680000
10,2,2802.433333
10,2,4716.803922
10,2,1621.943182
10,2,2688.345455
10,2,1994.312500
10,2,1790.385714
10,2,1525.204082
10,2,2207.353125
10,2,2779.890625
10,2,1523.680147
10,2,2155.570000
10,2,4102.188830
10,2,1538.410714
10,2,2157.507812
10,2,1693.805556
10,2,2249.655172
10,2,2278.671512
10,2,5540.104167
10,2,1615.229651
10,2,2783.580729
10,2,3281.547297
10,2,1846.913265
10,2,6391.795455
10,2,2538.570312
10,2,2318.158537
10,2,2853.902778
10,2,1591.965625
10,2,1773.523936
10,2,1891.720588
10,2,1928.340278
10,2,1641.125000
10,2,1532.250000
10,2,2635.191038
10,2,2995.638298
10,2,1523.142857
10,2,5932.892857
10,2,1621.875000
10,2,1692.067073
10,2,1550.287879
10,2,1980.053191
10,2,3086.120000
10,2,3798.240196
10,2,1544.216981
10,2,3044.513369
10,2,1793.521739
10,2,2063.741071
10,2,2860.705000
10,2,1982.697115
10,2,1912.995192
10,2,2402.211735
10,2,2199.436047
10,2,1516.815217
10,2,2387.621951
10,2,1624.040698
10,2,1619.005682

10,2,6230.409091
10,2,5331.077670
10,2,1580.211538
10,2,3012.137500
10,2,1846.653125
10,2,6147.636364
10,2,1539.417553
10,2,2118.571429
10,2,2173.926471
10,2,1932.968182
10,2,2585.500000
10,2,1905.200000
10,2,1687.750000
10,2,2026.952381
10,2,1781.093750
10,2,1693.867347
10,2,1936.243902
10,2,1899.433962
10,2,2544.000000
10,2,3127.451923
10,2,2519.731771
10,2,3096.393145
10,2,1604.695652
10,2,2331.699468
10,2,3880.843750
10,2,2000.006944
10,2,3035.913462
10,2,2299.138158
10,2,1651.010417
10,2,2327.066489
10,2,1851.012755
10,2,2947.920455
10,2,1638.524390
10,2,1577.300000
10,2,1666.790698
10,2,3124.663043
10,2,2439.296512
10,2,1884.648936
10,2,6500.196429
10,2,2794.203704
10,2,3969.044643
10,2,4360.135417
10,2,2325.742500
10,2,3021.000000
10,2,1932.607759
10,2,2745.625000
10,2,1984.431548
10,2,1576.568452
10,2,1847.329787
10,2,1615.333333
10,2,2225.660714
10,2,1502.562500
10,2,1791.240854
10,2,7442.756098
10,2,1567.925000
10,2,1784.000000
10,2,1586.551630
10,2,2048.902027
10,2,6931.950980
10,2,2980.558140
10,2,1835.398026
10,2,1700.889831
10,2,2588.961538
10,2,3067.164062
10,2,3181.142857
10,2,1646.921512

10,2,1594.571429
10,2,3701.708333
10,2,4504.671875
10,2,4966.678571
10,2,1848.045673
10,2,2919.889535
10,2,3168.000000
10,2,1727.747449
10,2,1976.276596
10,2,2259.584135
10,2,2698.326531
10,2,4025.973404
10,2,1513.062500
10,2,2031.204545
10,2,1762.680000
10,2,2035.045000
10,2,1996.601351
10,2,1657.413690
10,2,1953.323529
10,2,9008.205000
10,2,1518.572222
10,2,1537.367347
10,2,2290.445652
10,2,2096.714286
10,2,1757.534091
10,2,1716.352941
10,2,1935.663043
10,2,1867.684896
10,2,1749.031250
10,2,1533.451087
10,2,1637.920455
10,2,1552.795918
10,2,3377.875000
10,2,1809.711364
10,2,1704.267857
10,2,1804.367188
10,2,1500.297872
10,2,1820.993421
10,2,1884.848214
10,2,1731.915761
10,2,2029.166667
10,2,1820.288462
10,2,2511.367925
10,2,1553.989130
10,2,2000.398438
10,2,1561.759615
10,2,3374.414894
10,2,1938.369898
10,2,1955.340909
10,2,2094.782051
10,2,3001.015625
10,2,1598.400000
10,2,1987.554687
10,2,1921.693750
10,2,1671.480000
10,2,1781.596154
10,2,1529.104651
10,2,9759.527174
10,2,1619.746795
10,2,2238.328947
10,2,3443.531915
10,2,1706.525000
10,2,1572.375000
10,2,1774.097561
10,2,1513.096154
10,2,1694.046875

10,2,1617.685000
10,2,1638.572368
10,2,1714.143750
10,2,2489.161058
10,2,2699.205882
10,2,2894.000000
10,2,1793.457547
10,2,1674.633166
10,2,1560.750000
10,2,2037.889423
10,2,2229.110000
10,2,8678.992647
10,2,14033.774510
10,2,4505.321429
10,2,1672.402778
10,2,6850.608333
10,2,1859.396552
10,2,1532.613208
10,2,2572.468085
10,2,2541.590909
10,2,1881.150510
10,2,3264.163265
10,2,1674.440789
10,2,1785.391304
10,2,1873.988095
10,2,3083.593750
10,2,1855.055556
10,2,1932.358333
10,2,4293.435714
10,2,2081.902778
10,2,1731.572917
10,2,2043.593750
10,2,1699.135870
10,2,1547.437500
10,2,2898.972222
10,2,1549.726562
10,2,1680.685811
10,2,2175.567500
10,2,1558.625000
10,2,3323.605392
10,2,2052.951923
10,2,2945.018229
10,2,1796.046875
10,2,4025.010638
10,2,1691.122951
10,2,1988.500000
10,2,2675.369048
10,2,1516.697500
10,2,1821.546512
10,2,2442.125000
10,2,13881.823529
10,2,1931.884868
10,2,1931.918478
10,2,3439.680851
10,2,1592.505952
10,2,2051.640000
10,2,1604.511628
10,2,1615.156250
10,2,2194.255319
10,2,2203.666667
10,2,1596.976415
10,2,2276.188953
10,2,1694.577778
10,2,1561.482143
10,2,5068.307692
10,2,1636.068750

10,2,1799.950000
10,2,3157.750000
10,2,2478.481707
10,2,1865.784574
10,2,3247.925532
10,2,1670.859375
10,2,1849.700000
10,2,1603.512500
10,2,1532.913043
10,2,1908.250000
10,2,1826.858696
10,2,2710.150000
10,2,2316.250000
10,2,2108.400000
10,2,1810.090361
10,2,1655.861702
10,2,3734.886029
10,2,2994.428571
10,2,1802.950000
10,2,12087.680556
10,2,2130.911111
10,2,2758.300000
10,2,1688.554054
10,2,1541.367647
10,2,13400.489286
10,2,1815.923469
10,2,1984.239362
10,2,2277.011364
10,2,2443.512755
10,2,2495.512755
10,2,4330.305556
10,2,1524.600962
10,2,2304.593750
10,2,2067.191489
10,2,2040.875000
10,2,1510.061224
10,2,1545.221939
10,2,1600.769231
10,2,2065.240625
10,2,1540.790816
10,2,4744.683824
10,2,2657.366071
10,2,1926.011905
10,2,2127.640000
10,2,1954.428571
10,2,2082.142857
10,2,2225.494681
10,2,2499.524390
10,2,2369.867925
10,2,1975.073171
10,2,2039.470238
10,2,2004.743590
10,2,3871.404412
10,2,1790.641509
10,2,2103.098958
10,2,1590.309091
10,2,1777.738095
10,2,4093.391304
10,2,1901.475000
10,2,3941.872340
10,2,1886.686364
10,2,1971.771429
10,2,1766.348684
10,2,3019.428571
10,2,1973.103448
10,2,1591.500000

10,2,1877.352041
10,2,2617.627660
10,2,1966.800000
10,2,1680.164894
10,2,1754.829787
10,2,1643.738372
10,2,2572.410256
10,2,2229.850610
10,2,1595.500000
10,2,1535.216981
10,2,2070.272222
10,2,2488.738636
10,2,2396.288265
10,2,1512.018229
10,2,2367.717949
10,2,2718.631579
10,2,3678.677778
10,2,1523.928571
10,2,3536.002660
10,2,1710.000000
10,2,1637.550000
10,2,1550.687500
10,2,3932.306818
10,2,1777.062814
10,2,3098.319079
10,2,2833.279255
10,2,2571.777778
10,2,2918.239583
10,2,1602.298295
10,2,2053.682927
10,2,2155.345930
10,2,3670.218182
10,2,2849.800000
10,2,2253.613636
10,2,7107.125000
10,2,1735.307500
10,2,1774.914474
10,2,13374.006410
10,2,3056.840000
10,2,1961.912500
10,2,2748.323529
10,2,1542.335366
10,2,2192.807292
10,2,2088.290541
10,2,1548.029661
10,2,3765.000000
10,2,2160.610577
10,2,1708.848958
10,2,1821.574468
10,2,1752.682432
10,2,5807.565000
10,2,1501.336735
10,2,4681.883929
10,2,1749.220930
10,2,1796.000000
10,2,8105.121795
10,2,2518.720859
10,2,2307.305851
10,2,2524.136364
10,2,4451.956250
10,2,2635.505435
10,2,2919.864286
10,2,2249.500000
11,1,2202.781250
11,1,7196.878676
11,1,2022.960938

11,1,1599.125000
11,1,6535.483871
11,1,11298.857143
11,1,4600.312500
11,1,4783.545918
11,1,3372.159091
11,1,2012.160156
11,1,2505.011628
11,1,1734.375000
11,1,2226.000000
11,1,8717.898256
11,1,4336.400000
11,1,1747.050633
11,1,2673.689394
11,1,4084.000000
11,1,3823.397727
11,1,12602.977941
11,1,11083.182927
11,1,4319.593750
11,1,2475.095930
11,1,1538.365385
11,1,4370.187500
11,1,6852.138021
11,1,1610.487805
11,1,2117.826613
11,1,2109.250000
11,1,4807.351351
11,1,1908.690217
11,1,2407.607500
11,1,3322.947368
11,1,1585.960526
11,1,9568.210227
11,1,5951.657143
11,1,2781.156250
11,1,15512.452381
11,1,6575.125000
11,1,2851.809524
11,1,2273.159574
11,1,2074.628125
11,1,10041.914062
11,1,3543.301471
11,1,3134.575758
11,1,3124.000000
11,1,2004.277778
11,1,4371.928571
11,1,1740.890244
11,1,1947.456522
11,1,2675.785156
11,1,6159.796053
11,1,2730.126289
11,1,17646.895000
11,1,5639.647059
11,1,1728.400000
11,1,2276.193548
11,1,3561.392857
11,1,2888.694444
11,1,3753.925000
11,1,3724.360465
11,1,3433.898026
11,1,7007.281250
11,1,3056.429878
11,1,3525.175000
11,1,1618.941176
11,1,11312.913462
11,1,4455.803977
11,1,5848.250000

11,1,2145.587209
11,1,2448.705357
11,1,17776.843750
11,1,1900.168449
11,1,6854.170732
11,1,1667.487500
11,1,2013.642241
11,1,9853.037234
11,1,6237.043478
11,1,5166.036585
11,1,3235.948529
11,1,7215.282500
11,1,7707.215116
11,1,1648.578125
11,1,2371.375000
11,1,18718.470588
11,1,2501.775641
11,1,2897.333333
11,1,2068.088415
11,1,8645.448718
11,1,2353.756098
11,1,9655.841727
11,1,2183.428571
11,1,13821.347656
11,1,6353.557292
11,1,3427.812500
11,1,1976.534483
11,1,2574.029412
11,1,2576.366935
11,1,2199.382979
11,1,3009.145161
11,1,2509.500000
11,1,1808.452381
11,1,1547.727778
11,1,2247.625000
11,1,2995.075581
11,1,5417.340426
11,1,1855.560811
11,1,1705.454545
11,1,6601.005952
11,1,5431.216216
11,1,2157.378049
11,1,2745.250000
11,1,3534.322581
11,1,2616.306122
11,1,2339.852679
11,1,15310.522727
11,1,7874.186170
11,1,9621.423077
11,1,3782.318182
11,1,11723.958333
11,1,1993.750000
11,1,4273.857143
11,1,15787.942708
11,1,5278.213710
11,1,10269.157895
11,1,4605.500000
11,1,12029.834375
11,1,8002.615196
11,1,3278.600000
11,1,2532.875000
11,1,2445.519886
11,1,1889.285714
11,1,4381.010417
11,1,2272.214674
11,1,3912.012195

11,1,12067.287946
11,1,5151.616279
11,1,2597.314103
11,1,2028.666667
11,1,1940.625000
11,1,1874.647727
11,1,4213.267857
11,1,1905.821429
11,1,2046.833333
11,1,2068.250000
11,1,19275.508065
11,1,2638.491848
11,1,2780.205357
11,1,14454.819444
11,1,3715.780488
11,1,3917.608333
11,1,19808.230000
11,1,1939.007143
11,1,2789.145833
11,1,2573.864865
11,1,7931.235795
11,1,1570.852941
11,1,11152.705357
11,1,2009.871711
11,1,11344.800000
11,1,3692.161290
11,1,8514.180000
11,1,1524.375000
11,1,5872.375000
11,1,3296.697368
11,1,3354.076087
11,1,6942.507812
11,1,3150.821429
11,1,1975.833333
11,1,6442.000000
11,1,2524.131250
11,1,2021.306548
11,1,3388.281915
11,1,2424.012500
11,1,17287.243590
11,1,4144.705882
11,1,5786.906250
11,1,2435.028846
11,1,2972.660714
11,1,3005.334302
11,1,2258.246711
11,1,1937.481618
11,1,1600.090116
11,1,1749.093750
11,1,3062.853659
11,1,2337.218750
11,1,2220.125000
11,1,2572.285714
11,1,4605.886364
11,1,2298.585526
11,1,7113.484375
11,1,7811.250000
11,1,2114.057692
11,1,2488.705357
11,1,2140.031250
11,1,4314.690789
11,1,2452.558824
11,1,14680.578571
11,1,1642.842105
11,1,1808.155172
11,1,12213.714286

11,1,4041.768750
11,1,3476.812500
11,1,7819.354167
11,1,5934.109756
11,1,1933.700000
11,1,5628.156250
11,1,14260.892361
11,1,1900.713710
11,1,5851.106707
11,1,2148.363971
11,1,9400.690972
11,1,2442.946875
11,1,1843.908333
11,1,14481.234375
11,1,1567.191176
11,1,2118.967105
11,1,6067.586207
11,1,5182.426829
11,1,11737.301471
11,1,7731.535714
11,1,2211.230769
11,1,5840.558824
11,1,4307.444444
11,1,5343.040625
11,1,2420.554878
11,1,5917.340426
11,1,2064.625000
11,1,6883.105556
11,1,1674.231707
11,1,12323.293605
11,1,3506.409091
11,1,10891.648936
11,1,2339.300000
11,1,1594.796875
11,1,3580.908088
11,1,4744.555556
11,1,8981.075581
11,1,2270.300000
11,1,2495.690341
11,1,8489.134615
11,1,1530.160714
11,1,2636.593750
11,1,19899.180851
11,1,8262.625000
11,1,2622.600000
11,1,2035.800000
11,1,1544.687500
11,1,3447.195312
11,1,2307.290323
11,1,2878.847222
11,1,7985.208333
11,1,13714.423913
11,1,1686.428571
11,1,1540.974359
11,1,18534.288462
11,1,3329.296512
11,1,3175.524457
11,1,9421.125000
11,1,2952.375000
11,1,9612.281250
11,1,9656.916667
11,1,1720.532895
11,1,3460.734848
11,1,16345.755435
11,1,3746.138158
11,1,2629.056548

11,1,1506.723684
11,1,8961.581395
11,1,5044.658537
11,1,10993.250000
11,1,1530.738636
11,1,2324.187500
11,1,5409.830357
11,1,3771.433333
11,1,2135.085366
11,1,12978.725962
11,1,1534.258929
11,1,3802.329268
11,1,11079.495968
11,1,1606.441860
11,1,1976.412234
11,1,1718.976744
11,1,1601.531250
11,1,3827.387500
11,1,1542.071429
11,1,9686.180921
11,1,9940.664634
11,1,2541.327703
11,1,10074.825000
11,1,10657.333333
11,1,3296.767045
11,1,6706.937500
11,1,2226.484848
11,1,1588.828947
11,1,2597.884615
11,1,2515.982558
11,1,6754.272727
11,1,2520.709677
11,1,2072.000000
11,1,1814.750000
11,1,4065.675532
11,1,1519.949219
11,1,7669.371795
11,1,3082.837209
11,1,7909.439189
11,1,1994.694444
11,1,2486.197674
11,1,18928.666667
11,1,1943.737805
11,1,14711.500000
11,1,11342.048387
11,1,3957.147059
11,1,6164.860465
11,1,1723.878049
11,1,1639.358108
11,1,1555.666667
11,1,2031.815789
11,1,11119.567568
11,1,4173.497449
11,1,14011.011111
11,1,1973.732143
11,1,3809.562500
11,1,4035.255000
11,1,3442.886905
11,1,2783.006757
11,1,1570.740385
11,1,5648.202703
11,1,6316.315625
11,1,1555.516129
11,1,12676.758929
11,1,2008.712766
11,1,10073.168103

11,1,3537.500000
11,1,1627.198529
11,1,12027.117021
11,1,1837.928571
11,1,2888.596774
11,1,1622.172619
11,1,4536.000000
11,1,2863.172297
11,1,5716.696429
11,1,3735.798077
11,1,4128.452381
11,1,2785.217105
11,1,7257.163462
11,1,19183.535714
11,1,3496.392857
11,1,1737.551282
11,1,7210.178571
11,1,3746.371212
11,1,8853.105263
11,1,12457.386364
11,1,3927.291667
11,1,2365.691860
11,1,1677.425197
11,1,2542.875000
11,1,1967.920213
11,1,2328.505682
11,1,18325.855263
11,1,6513.461538
11,1,1630.092857
11,1,4061.392857
11,1,5411.500000
11,1,1949.812500
11,1,3555.208333
11,1,6507.191489
11,1,2585.142857
11,1,1837.781250
11,1,2501.562500
11,1,2848.212500
11,1,12927.962500
11,1,6228.097561
11,1,9310.937500
11,1,1830.421053
11,1,2264.832317
11,1,13436.178571
11,1,1506.277778
11,1,7523.816667
11,1,5240.074468
11,1,3183.142045
11,1,2581.850000
11,1,2449.323529
11,1,8217.209677
11,1,7502.000000
11,1,5613.979730
11,1,2722.468750
11,1,15233.607143
11,1,2448.625000
11,1,3850.259615
11,1,8450.859375
11,1,1817.857143
11,1,3200.230978
11,1,2650.900000
11,1,8098.127604
11,1,6478.158088
11,1,6634.593750
11,1,11020.163690
11,1,1673.500000

11,1,4858.608108
11,1,2406.426471
11,1,17941.750000
11,1,4073.537500
11,1,5128.747024
11,1,5254.600000
11,1,6714.267857
11,1,2527.701220
11,1,4607.842105
11,1,1576.255952
11,1,7190.336538
11,1,3427.536184
11,1,1705.929688
11,1,1723.858108
11,1,6661.589744
11,1,7817.555556
11,1,6825.006098
11,1,4209.336538
11,1,1758.644444
11,1,2535.282609
11,1,2975.470395
11,1,5675.709184
11,1,3488.664894
11,1,1738.231250
11,1,2360.000000
11,1,1673.289773
11,1,2894.164062
11,1,7361.184211
11,1,1501.946429
11,1,18770.978571
11,1,1758.976744
11,1,2035.666667
11,1,4324.040816
11,1,18981.800000
11,1,5594.520270
11,1,7085.769231
11,1,9972.611111
11,1,5734.500000
11,1,3252.426829
11,1,6622.055556
11,1,3284.512500
11,1,19169.796053
11,1,2024.309896
11,1,1992.726190
11,1,14873.210938
11,1,4782.198529
11,1,7738.699074
11,1,1584.378378
11,1,2908.817935
11,1,2181.116848
11,1,2245.671875
11,1,2237.855769
11,1,3082.125000
11,1,2015.890000
11,1,2393.094697
11,1,1925.298780
11,1,2677.594595
11,1,1690.198529
11,1,8768.927632
11,1,8375.798780
11,1,1533.732558
11,1,7540.666667
11,1,2936.704545
11,1,19821.677885
11,1,2267.000000
11,1,8429.523026

11,1,8590.238372
11,1,6648.894737
11,1,5479.648649
11,1,13613.927419
11,1,3633.160714
11,1,10225.625000
11,1,4617.369186
11,1,5244.500000
11,1,2366.268293
11,1,3039.906915
11,1,3051.362805
11,1,2415.718750
11,1,2979.683824
11,1,3664.285714
11,1,14420.954545
11,1,2356.493750
11,1,1781.131579
11,1,6840.199219
11,1,13755.200000
11,1,2430.796875
11,1,3748.062500
11,1,3215.722222
11,1,5917.722973
11,1,7386.375000
11,1,9820.854167
11,1,6948.518519
11,1,2948.583333
11,1,3377.120000
11,1,5253.487805
11,1,4528.818182
11,1,9295.554878
11,1,1918.348837
11,1,1807.038043
11,1,14749.226852
11,1,5580.726562
11,1,3426.967391
11,1,6695.761364
11,1,3361.061224
11,1,1717.362500
11,1,4946.208333
11,1,3019.939904
11,1,15866.934783
11,1,11205.872222
11,1,2731.643293
11,1,1802.215909
11,1,1546.181818
11,1,1593.779605
11,1,1499.111413
11,1,5945.932692
11,1,4909.571023
11,1,4689.431818
11,1,5703.406250
11,1,3250.030488
11,1,2770.489796
11,1,12022.529412
11,1,2307.855263
11,1,5060.851064
11,1,2437.298913
11,1,1540.666667
11,1,4405.875000
11,1,1511.738889
11,1,3159.485714
11,1,1843.065789
11,1,1905.129032
11,1,13105.715909
11,1,7426.941176

11,1,2491.902344
11,1,12456.886364
11,1,4512.062500
11,1,9403.757812
11,1,4526.404412
11,1,5143.500000
11,1,3433.833333
11,1,4937.191489
11,1,2137.593750
11,1,1846.976974
11,1,1858.170000
11,1,1898.052632
11,1,4775.750000
11,1,1887.540541
11,1,1621.669444
11,1,1856.595238
11,1,3524.801282
11,1,1615.157895
11,1,6816.675595
11,1,1990.733871
11,1,14688.669355
11,1,1613.487805
11,1,2990.937500
11,1,3749.739130
11,1,6793.477564
11,1,6786.214286
11,1,2736.382979
11,1,2099.619792
11,1,9425.846154
11,1,13788.536184
11,1,2063.894737
11,1,2766.880682
11,1,1664.700000
11,1,5385.429167
11,1,3947.220395
11,1,1706.878378
11,1,1960.730769
11,1,7953.909091
11,1,1835.841463
11,1,7506.676471
11,1,2716.500000
11,1,1640.829545
11,1,4510.340000
11,1,1826.940972
11,1,11131.130435
11,1,1958.450000
11,1,2500.375000
11,1,3600.027778
11,1,4001.984375
11,1,4150.047619
11,1,10826.553125
11,1,12405.419355
11,1,1551.052326
11,1,1965.069853
11,1,3563.478261
11,1,2834.281250
11,1,1547.149038
11,1,2542.606618
11,1,4250.689189
11,1,3975.333333
11,1,4583.337766
11,1,2735.902439
11,1,16001.500000
11,1,2734.510000
11,1,4013.072917
11,1,11767.098684

11,1,2719.183673
11,1,2665.493056
11,1,2551.763889
11,1,2366.222222
11,1,1666.410714
11,1,6565.103659
11,1,2691.459302
11,1,2972.680147
11,1,3346.483333
11,1,2498.250000
11,1,2570.533742
11,1,3729.023438
11,1,6399.875000
11,1,3106.250000
11,1,5583.555000
11,1,5145.114865
11,1,6696.457447
11,1,11560.079861
11,1,7095.845238
11,1,1562.835227
11,1,1609.790698
11,1,2828.993750
11,1,5013.222222
11,1,2482.150000
11,1,4050.736702
11,1,4365.828125
11,1,6500.094595
11,1,9382.869318
11,1,2494.785714
11,1,5604.187500
11,1,6122.669643
11,1,5941.986486
11,1,1630.954545
11,1,9398.333333
11,1,5431.201220
11,1,3589.806452
11,1,1552.465116
11,1,8893.750000
11,1,3994.074324
11,1,2282.812500
11,1,3311.619048
11,1,3988.821429
11,1,3191.250000
11,1,1598.294118
11,1,3206.554054
11,1,3873.833333
11,1,2007.652174
11,1,3281.094697
11,1,2183.294643
11,1,5558.871875
11,1,3543.146341
11,1,9675.019531
11,1,1749.085366
11,1,12828.087121
11,1,2651.665323
11,1,3582.705882
11,1,4265.843750
11,1,1626.562500
11,1,1739.885417
11,1,13517.000000
11,1,7343.794643
11,1,4424.864130
11,1,3234.685714
11,1,3342.793103
11,1,2246.324405
11,1,6428.197674

11,1,7371.625000
11,1,7002.525000
11,1,1771.018939
11,1,2154.702703
11,1,2331.621622
11,1,2870.676829
11,1,9450.923077
11,1,1941.166667
11,1,5659.817857
11,1,8735.000000
11,1,4290.784091
11,1,2300.000000
11,1,4641.937500
11,1,11675.250000
11,1,10329.644231
11,1,7584.800000
11,1,3394.399390
11,1,8357.700000
11,1,4981.141892
11,1,1881.188953
11,1,2198.970109
11,1,5454.058824
11,1,6566.454545
11,1,2465.127551
11,1,5436.552326
11,1,3484.000000
11,1,7774.506410
11,1,4362.270270
11,1,1540.991935
11,1,6861.060714
11,1,3814.952778
11,1,16064.000000
11,1,4947.800000
11,1,3087.510000
11,1,1831.325000
11,1,8575.550000
11,1,2126.604167
11,1,4398.800000
11,1,1642.600000
11,1,3535.783088
11,1,8731.461538
11,1,7155.197674
11,1,2483.103125
11,1,16378.901786
11,1,2586.076923
11,1,6061.786585
11,1,10167.073171
11,1,1701.127778
11,1,6824.483871
11,1,15415.347222
11,1,1598.699219
11,1,1541.681250
11,1,3600.889535
11,1,6217.906250
11,1,1510.303191
11,1,6105.134375
11,1,3469.304054
11,1,1790.556818
11,1,4716.487245
11,1,1914.826087
11,1,13736.562500
11,1,2874.875000
11,1,13962.810811
11,1,5726.877976
11,1,1876.536585
11,1,2394.162791

11,1,4479.054348
11,1,2687.404762
11,1,7936.187500
11,1,2742.427632
11,1,1961.092262
11,1,13633.013514
11,1,3826.188830
11,1,3944.568750
11,1,8989.525641
11,1,3285.584677
11,1,3674.642857
11,1,3896.592391
11,1,2333.476562
11,1,5448.608696
11,1,2781.200000
11,1,1806.156250
11,1,15470.101064
11,1,2428.505435
11,1,2324.078571
11,1,10652.605263
11,1,4330.862745
11,1,5201.525641
11,1,2154.640625
11,1,13978.346154
11,1,8840.333333
11,1,3326.812500
11,1,7944.798077
11,1,7610.073864
11,1,2252.560976
11,1,6329.906977
11,1,3470.875000
11,1,3998.808673
11,1,17481.851064
11,1,5749.202381
11,1,2155.650000
11,1,2145.765957
11,1,2138.666667
11,1,3439.769231
11,1,5740.973684
11,1,17314.045455
11,1,3773.131944
11,1,2725.560976
11,1,1568.142857
11,1,3763.076087
11,1,2971.926829
11,1,1792.062500
11,1,3569.877907
11,1,1759.618590
11,1,1903.357143
11,1,2611.300000
11,1,2433.953125
11,1,10105.341912
11,1,2838.560714
11,1,2197.204787
11,1,3579.409722
11,1,4519.315000
11,1,1577.416667
11,1,2648.926136
11,1,2685.608333
11,1,4923.281250
11,1,1831.618243
11,1,1745.900568
11,1,2082.240625
11,1,9620.287500
11,1,11780.597826
11,1,1853.363636

11,1,10468.320755
11,1,3371.005952
11,1,2488.665441
11,1,4301.450000
11,1,7001.225000
11,1,3610.798780
11,1,2758.761905
11,1,5487.500000
11,1,9221.097561
11,1,3787.794872
11,1,4690.458333
11,1,14603.573529
11,1,4735.916667
11,1,2323.006757
11,1,2137.485294
11,1,4775.544643
11,1,3604.520833
11,1,3828.517857
11,1,1764.051282
11,1,2184.451531
11,1,2096.043605
11,1,5763.722222
11,1,2867.171053
11,1,3442.679688
11,1,3624.780000
11,1,6776.877778
11,1,6898.980000
11,1,2368.496429
11,1,3972.636364
11,1,1659.031250
11,1,1811.542553
11,1,2620.942308
11,1,2584.981618
11,1,7990.707386
11,1,2696.394531
11,1,1610.000000
11,1,2250.329787
11,1,3082.415441
11,1,2272.571429
11,1,11304.113281
11,1,3046.960938
11,1,1628.571429
11,1,4649.461538
11,1,14704.339744
11,1,2422.444444
11,1,9888.125000
11,1,2049.316176
11,1,16269.640957
11,1,1512.454545
11,1,1616.646341
11,1,2906.250000
11,1,1642.940000
11,1,8349.371429
11,1,1816.503125
11,1,4436.488971
11,1,3722.096154
11,1,2137.292683
11,1,15441.229839
11,1,2475.411765
11,1,2399.023256
11,1,13805.413194
11,1,2318.607143
11,1,16786.108108
11,1,2074.963415
11,1,2358.875000
11,1,3823.320122

11,1,6082.517500
11,1,1788.739130
11,1,2681.709677
11,1,17450.072917
11,1,4975.115385
11,1,3664.075000
11,1,1506.459677
11,1,12440.152174
11,1,2993.769231
11,1,5120.241935
11,1,4292.087963
11,1,2792.764706
11,1,4382.870968
11,1,3765.489362
11,1,1720.732143
11,1,3070.586735
11,1,2646.214286
11,1,2528.408537
11,1,1877.487500
11,1,2803.500000
11,1,3225.576531
11,1,2226.950000
11,1,3033.162162
11,1,1798.635135
11,1,9119.583333
11,1,2010.355000
11,1,8494.419355
11,1,1749.343750
11,1,3344.787500
11,1,2879.250000
11,1,2771.391304
11,1,6589.795455
11,1,6443.524194
11,1,9175.388298
11,1,5668.648585
11,1,4158.045732
11,1,19354.230769
11,1,2031.949219
11,1,2142.426471
11,1,1846.612903
11,1,3993.800000
11,1,4611.044643
11,1,2644.617647
11,1,5071.116667
11,1,2783.021429
11,1,3345.660000
11,1,2627.194149
11,1,3426.390244
11,1,5028.179487
11,1,1768.929348
11,1,4466.385417
11,1,18645.095395
11,1,7941.706731
11,1,4034.541667
11,1,2389.303922
11,1,3802.409884
11,1,1748.687500
11,1,5173.687500
11,1,2837.083333
11,1,2106.469512
11,1,7441.224490
11,1,2036.832237
11,1,15605.525000
11,1,11838.014706
11,1,4258.808140
11,1,2166.961538

11,1,3101.261029
11,1,2019.792614
11,1,6981.004808
11,1,1630.199324
11,1,16308.196875
11,1,2128.226974
11,1,2743.736842
11,1,3096.130319
11,1,14524.953125
11,1,7364.128788
11,1,3876.723684
11,1,9115.648438
11,1,1914.766892
11,1,3534.500000
11,1,5814.750000
11,1,7064.831250
11,1,1609.480263
11,1,15819.578947
11,1,12400.052632
11,1,4555.654255
11,1,1910.179878
11,1,2176.118421
11,1,2069.580357
11,1,5453.485294
11,1,3382.833333
11,1,1927.235577
11,1,5625.816406
11,1,1889.546875
11,1,5522.389205
11,1,1791.621795
11,1,2742.132353
11,1,3172.604651
11,1,1964.815476
11,1,13783.580729
11,1,6193.352273
11,1,7747.500000
11,1,8308.187500
11,1,13067.460000
11,1,5331.637500
11,1,1660.156250
11,1,13047.250000
11,1,13620.145833
11,1,8318.676724
11,1,1626.762500
11,1,1962.340909
11,1,10149.168367
11,1,12631.015244
11,1,1666.321809
11,1,1947.108974
11,1,6520.601190
11,1,13051.500000
11,1,11328.500000
11,1,2575.363095
11,1,4655.587209
11,1,6797.436224
11,1,3657.155000
11,1,1543.632979
11,1,2126.973404
11,1,6544.728125
11,1,6612.271277
11,1,2305.263889
11,1,1567.250000
11,1,2306.867647
11,1,1762.166667
11,1,3083.679487
11,1,3966.613281

11,1,1762.206395
11,1,19291.000000
11,1,8245.510204
11,1,3032.044643
11,1,3791.002551
11,1,1695.842857
11,1,2352.506757
11,1,5270.704545
11,1,12437.019608
11,1,12938.335227
11,1,1623.880102
11,1,1840.280488
11,1,6290.695652
11,1,2320.073864
11,1,1538.896104
11,1,1559.772727
11,1,1551.848837
11,1,1659.642857
11,1,17466.353659
11,1,3175.885135
11,1,3078.375000
11,1,4383.275735
11,1,1960.605263
11,1,6066.100000
11,1,2297.554348
11,1,2324.090000
11,1,3308.068182
11,1,2081.607143
11,1,7774.387821
11,1,1686.602041
11,1,3276.569767
11,1,7917.454545
11,1,7258.354167
11,1,5970.864583
11,1,2042.246212
11,1,1895.150000
11,1,13533.930851
11,1,2460.109375
11,1,1916.148810
11,1,17819.875000
11,1,5913.201923
11,1,3665.750000
11,1,4170.953488
11,1,2357.133721
11,1,1871.677632
11,1,2048.000000
11,1,6091.881250
11,1,1541.483696
11,1,2298.344595
11,1,1509.019737
11,1,8512.532609
11,1,2860.446429
11,1,1569.405405
11,1,3264.837209
11,1,2442.916667
11,1,2092.789062
11,1,2503.135714
11,1,4091.463415
11,1,2488.083333
11,1,1727.467626
11,1,5573.538462
11,1,1591.136364
11,1,2257.576389
11,1,2344.058333
11,1,1698.442308
11,1,8327.279412

11,1,7929.257576
11,1,6666.740132
11,1,1522.222222
11,1,4357.750000
11,1,2250.719512
11,1,4011.631579
11,1,5420.771341
11,1,11869.000000
11,1,14947.264535
11,1,5778.600000
11,1,13296.452206
11,1,2016.041667
11,1,1577.583333
11,1,9481.329268
11,1,1552.021277
11,1,2961.900000
11,1,1707.000000
11,1,1677.651316
11,1,3445.450000
11,1,3343.660256
11,1,5479.454268
11,1,3055.437500
11,1,18210.666667
11,1,2917.308824
11,1,1500.319712
11,1,9663.451220
11,1,1613.062500
11,1,3206.244444
11,1,2335.133929
11,1,6805.765957
11,1,1506.541667
11,1,19394.341463
11,1,2569.841216
11,1,2490.275862
11,1,4414.184211
11,1,1997.800000
11,1,1986.831081
11,1,7107.437500
11,1,4255.553571
11,1,10681.231618
11,1,19775.941176
11,1,3549.050000
11,1,3741.943548
11,1,2244.381098
11,1,18463.015625
11,1,2778.474490
11,1,3875.150000
11,1,2948.188953
11,1,3324.714286
11,1,1622.009146
11,1,2899.733333
11,1,3229.475000
11,1,2169.299342
11,1,12903.985294
11,1,3387.131579
11,1,3854.343750
11,1,1671.674479
11,1,3617.230769
11,1,14638.447368
11,1,1844.515625
11,1,11245.181034
11,1,9005.318182
11,1,2356.362500
11,1,13483.375000
11,1,8857.391304
11,1,3462.255814

11,1,1702.812500
11,1,18826.070122
11,1,2462.875000
11,1,2697.455645
11,1,1589.189189
11,1,3107.125000
11,1,1549.303571
11,1,2589.200000
11,1,7624.588816
11,1,1579.979167
11,1,10960.500000
11,1,8023.775000
11,1,12608.111702
11,1,8274.274194
11,1,3810.209302
11,1,1934.895833
11,1,5921.315341
11,1,3071.897059
11,1,4079.031250
11,1,19774.062500
11,1,17446.573171
11,1,4444.288462
11,1,2005.073370
11,1,4259.984375
11,1,1670.483871
11,1,19137.241667
11,1,2658.789474
11,1,4602.500000
11,1,1627.380556
11,1,8347.500000
11,1,2184.830000
11,1,3078.950000
11,1,1677.916667
11,1,2044.312500
11,1,2034.536585
11,1,4385.941176
11,1,2136.869318
11,1,7665.996711
11,1,2288.115385
11,1,2972.770349
11,1,8182.277778
11,1,12771.244792
11,1,1721.849138
11,1,2783.262500
11,1,4957.476562
11,1,2588.911184
11,1,6491.567568
11,1,4603.011364
11,1,9605.142857
11,1,1790.007353
11,1,2469.041667
11,1,4991.388514
11,1,5066.250000
11,1,1523.000000
11,1,18679.621875
11,1,4297.679348
11,1,1902.543103
11,1,5199.446429
11,1,4180.138158
11,1,2235.944444
11,1,3042.805556
11,1,1542.000000
11,1,4846.150000
11,1,2254.455882
11,1,11356.196809
11,1,13916.993750

11,1,7516.173387
11,1,11499.971429
11,1,1848.443182
11,1,1884.881250
11,1,4329.515625
11,1,2298.750000
11,1,5860.681818
11,1,1825.659091
11,1,3125.122340
11,1,2214.731250
11,1,11007.062500
11,1,2277.116279
11,1,4291.546875
11,1,2989.673387
11,1,2597.460366
11,1,1932.970238
11,1,1982.871622
11,1,5510.772436
11,1,5842.594512
11,1,4462.256098
11,1,17794.500000
11,1,10197.735294
11,1,2360.547414
11,1,2346.125000
11,1,3263.897959
11,1,7480.252660
11,1,3523.946809
11,1,1525.941176
11,1,1795.548387
11,1,3153.738889
11,1,5316.150000
11,1,2137.987179
11,1,10000.463889
11,1,2866.891304
11,1,3032.306818
11,1,3840.805147
11,1,4954.225000
11,1,5692.093023
11,1,1838.750000
11,1,3840.639706
11,1,2539.400000
11,1,15474.741667
11,1,9215.777174
11,1,10620.706250
11,1,2928.738281
11,1,3600.294118
11,1,5022.718750
11,1,1798.561224
11,1,6969.428571
11,1,2833.022321
11,1,1542.262195
11,1,8909.332143
11,1,3058.593750
11,1,2924.755814
11,1,2518.689815
11,1,3150.600000
11,1,6903.179688
11,1,2125.982143
11,1,1540.020833
11,1,3121.461538
11,1,5374.568182
11,1,1714.898810
11,1,14650.250000
11,1,7751.217391
11,1,1993.835106
11,1,1918.921053

11,1,16606.785000
11,1,12254.604167
11,1,6161.575581
11,1,1671.083333
11,1,14312.589147
11,1,1984.121094
11,1,2804.187500
11,1,4363.946429
11,1,2658.842105
11,1,6950.114583
11,1,2324.983871
11,1,16348.627907
11,1,1668.875000
11,1,8961.728125
11,1,3317.428571
11,1,3004.877660
11,1,1994.823529
11,1,13918.361111
11,1,1719.500000
11,1,10487.394444
11,1,2840.456081
11,1,3627.753205
11,1,1614.668367
11,1,8698.944444
11,1,2674.252976
11,1,8409.760204
11,1,12860.156250
11,1,5262.392857
11,1,4285.875000
11,1,8270.086207
11,1,7020.692308
11,1,4024.469697
11,1,1690.028571
11,1,2188.194079
11,1,7028.497159
11,1,14957.843750
11,1,2705.607143
11,1,9583.369565
11,1,1726.655556
11,1,1750.173077
11,1,1709.945312
11,1,1553.913462
11,1,2187.941176
11,1,5676.608696
11,1,4092.437500
11,1,11369.675000
11,1,18076.375000
11,1,1530.989796
11,1,3744.211538
11,1,4920.666667
11,1,2218.900000
11,1,1628.771429
11,1,2557.291667
11,1,3445.368902
11,1,6891.556250
11,1,2524.522059
11,1,9004.015152
11,1,3270.322581
11,1,2162.187500
11,1,3462.446429
11,1,3053.120000
11,1,1897.088235
11,1,2839.898649
11,1,8583.468750
11,1,2270.883721
11,1,7253.269231

11,1,4070.885870
11,1,7223.250000
11,1,7623.894531
11,1,7500.010870
11,1,2640.611702
11,1,2414.063830
11,1,4527.058333
11,1,5943.531250
11,1,5422.525862
11,1,5772.151596
11,1,2328.676829
11,1,8658.190476
11,1,5293.045455
11,1,2020.060000
11,1,1711.500000
11,1,6031.426136
11,1,2067.956250
11,1,11304.106509
11,1,6675.853659
11,1,5823.488372
11,1,3583.684375
11,1,3177.791667
11,1,2430.545455
11,1,9387.194079
11,1,5673.655172
11,1,3983.826389
11,1,3917.741071
11,1,5881.175000
11,1,3709.157895
11,1,2610.494565
11,1,2376.500000
11,1,14043.935897
11,1,3674.829268
11,1,19345.625000
11,1,13008.153846
11,1,5714.146226
11,1,14287.987805
11,1,3186.450000
11,1,1792.046875
11,1,1983.125000
11,1,4331.147059
11,1,4621.914474
11,1,2714.250000
11,1,1652.687500
11,1,2654.720588
11,1,4039.977273
11,1,12929.853448
11,1,2514.983333
11,1,2752.801829
11,1,1841.343750
11,1,1799.964286
11,1,2219.944444
11,1,12665.953125
11,1,2109.791667
11,1,5671.159091
11,1,3545.756098
11,1,3395.285000
11,1,2229.029762
11,1,5766.333333
11,1,6996.750000
11,1,2205.864865
11,1,7370.929688
11,1,2996.666667
11,1,1520.713235
11,1,3024.125000
11,1,3381.687500

11,1,5435.891304
11,1,2619.294872
11,1,1650.442308
11,1,3585.455645
11,1,12788.633929
11,1,1926.914062
11,1,2803.326531
11,1,10604.562500
11,1,9101.260638
11,1,9339.773438
11,1,1686.375000
11,1,2057.062500
11,1,18271.250000
11,1,8466.982143
11,1,2911.742647
11,1,1508.921875
11,1,2949.524390
11,1,4701.502273
11,1,6740.615385
11,1,2071.428571
11,1,4316.823718
11,1,3676.833333
11,1,11573.842105
11,1,4284.666667
11,1,1731.548387
11,1,3154.904070
11,1,4832.137500
11,1,4746.625000
11,1,1700.653125
11,1,3441.080882
11,1,2728.093085
11,1,4343.675000
11,1,1880.500000
11,1,1677.929348
11,1,3286.110294
11,1,2024.870968
11,1,7656.219512
11,1,3979.875000
11,1,2609.065476
11,1,9887.674419
11,1,11337.525000
11,1,13840.750000
11,1,2875.308511
11,1,1940.062500
11,1,1713.730769
11,1,2140.970588
11,1,1862.250000
11,1,1955.230769
11,1,3331.320000
11,1,1606.109756
11,1,1986.708333
11,1,2628.390244
11,1,3442.177885
11,1,3387.945946
11,1,11572.573529
11,1,2076.787879
11,1,9734.429688
11,1,4754.736111
11,1,3921.173913
11,1,2239.000000
11,1,1976.666667
11,1,11198.532609
11,1,8069.423387
11,1,2321.102041
11,1,3428.333333
11,1,13908.279762

11,1,2924.903125
11,1,16698.368902
11,1,1655.272727
11,1,6356.594697
11,1,1502.942073
11,1,3138.162791
11,1,2101.116848
11,1,1849.832237
11,1,1833.375000
11,1,2963.169643
11,1,1610.969512
11,1,9243.077206
11,1,1697.533654
11,1,2092.136905
11,1,1704.156250
11,1,15457.850694
11,1,13305.221154
11,1,2937.732143
11,1,4156.370833
11,1,3350.894022
11,1,11490.261364
11,1,3605.597973
11,1,4096.568182
11,1,2908.058824
11,1,2128.500000
11,1,2691.270000
11,1,18204.991667
11,1,2431.725000
11,1,10937.964286
11,1,16466.513021
11,1,2189.952703
11,1,2668.117647
11,1,8824.000000
11,1,4955.000000
11,1,6413.558824
11,1,6735.682927
11,1,4910.711538
11,1,3758.991279
11,1,1887.183333
11,1,4282.021277
11,1,2173.347826
11,1,2951.297619
11,1,2504.750000
11,1,6052.807692
11,1,1863.909091
11,1,4529.233553
11,1,6731.270833
11,1,1521.844595
11,1,4254.758333
11,1,7052.000000
11,1,17572.769231
11,1,3638.230114
11,1,1789.527778
11,1,9494.736842
11,1,3804.310000
11,1,1766.042763
11,1,2763.925000
11,1,7206.091837
11,1,4931.319444
11,1,19251.225000
11,1,5343.782609
11,1,6454.071429
11,1,5760.230769
11,1,2260.632353
11,1,9991.863095
11,1,2028.666667

11,1,2986.322222
11,1,2728.965000
11,1,6174.379032
11,1,2924.877551
11,1,1617.234375
11,1,3286.078125
11,1,3415.078125
11,1,8250.663793
11,1,7835.250000
11,1,3713.666667
11,1,3897.475000
11,1,7813.241935
11,1,5315.837500
11,1,4190.625000
11,1,3180.836538
11,1,1704.855263
11,1,3914.928571
11,1,2033.654762
11,1,3080.125000
11,1,3996.272727
12,2,2648.668478
12,2,1970.585784
12,2,1537.531250
12,2,1615.281250
12,2,1588.708333
12,2,1511.812500
12,2,1561.553977
12,2,2341.500000
12,2,1606.953488
12,2,1979.490132
12,2,1701.412500
12,2,3297.243243
12,2,2423.935897
12,2,1711.554348
12,2,2488.739583
12,2,1538.000000
12,2,2021.500000
12,2,2305.934524
12,2,3476.968023
12,2,3572.109756
12,2,2994.320755
12,2,1808.010417
12,2,2474.672131
12,2,1677.950000
12,2,2096.660714
12,2,2194.111111
12,2,2097.545455
12,2,5556.585366
12,2,1968.715909
12,2,2110.804878
12,2,1595.201087
12,2,1527.581633
12,2,1754.852778
12,2,1623.375000
12,2,2912.358974
12,2,1561.608696
12,2,2658.833333
12,2,4696.062500
12,2,14197.415625
12,2,1533.779412
12,2,1713.000000
12,2,1940.550000
12,2,4456.371528
12,2,1802.797872
12,2,3749.264706
12,2,1525.846154

12,2,1870.956522
12,2,2667.000000
12,2,2200.125000
12,2,2098.480392
12,2,2251.533654
12,2,4521.576531
12,2,2989.968085
12,2,2336.625000
12,2,1656.293478
12,2,1901.385135
12,2,1617.500000
12,2,3202.468085
12,2,1898.270408
12,2,2171.189286
12,2,1845.372340
12,2,1825.728814
12,2,1973.711864
12,2,1707.589674
12,2,2008.680233
12,2,1564.975000
12,2,1508.865854
12,2,1897.644444
12,2,3272.691860
12,2,1766.900000
12,2,1534.331633
12,2,2034.824324
12,2,1817.205882
12,2,1853.065789
12,2,1778.219388
12,2,2611.927632
12,2,1598.994318
12,2,1798.785714
12,2,2552.062500
12,2,2359.521341
12,2,1837.343023
12,2,4471.837500
12,2,2453.177632
12,2,1627.093023
12,2,2086.600000
12,2,2143.666667
12,2,2445.422222
12,2,1512.200000
12,2,3214.517857
12,2,3407.923611
12,2,1847.466667
12,2,2058.480769
12,2,2120.051630
12,2,2011.226974
12,2,1704.727273
12,2,2046.523810
12,2,2500.750000
12,2,1996.368421
12,2,1574.930556
12,2,1631.132653
12,2,1507.839286
12,2,2469.017544
12,2,1524.625000
12,2,1538.496711
12,2,1660.606250
12,2,3478.445000
12,2,4061.208333
12,2,1722.653061
12,2,1664.035714
12,2,1552.500000
12,2,1918.573171
12,2,1513.655405

12,2,2903.978723
12,2,1743.545455
12,2,1786.403846
12,2,1714.125000
12,2,2185.655000
12,2,6611.666667
12,2,14117.107143
12,2,3251.375000
12,2,2270.555556
12,2,2172.615132
12,2,1526.803571
12,2,2361.125000
12,2,2299.543103
12,2,2624.500000
12,2,3242.750000
12,2,1556.368421
12,2,2818.576531
12,2,2184.469880
12,2,2060.829082
12,2,5001.326087
12,2,4851.993590
12,2,2329.142857
12,2,2551.365625
12,2,4746.929688
12,2,2745.695652
12,2,1610.437500
12,2,2794.674342
12,2,1736.303125
12,2,1602.433594
12,2,1588.100610
12,2,2068.811275
12,2,3620.016447
12,2,1798.446809
12,2,2344.241935
12,2,4229.157609
12,2,2670.377358
12,2,1718.440678
12,2,1596.294118
12,2,6526.314103
12,2,3098.200000
12,2,2403.550000
12,2,1726.209302
12,2,1526.062500
12,2,2517.625000
12,2,1573.880682
12,2,3474.076705
12,2,2100.888889
12,2,2824.666667
12,2,1787.634615
12,2,1822.040698
12,2,1589.093220
12,2,2676.670455
12,2,1623.973404
12,2,4560.510204
12,2,2146.739583
12,2,1972.024038
12,2,4006.195652
12,2,1624.642157
12,2,2753.272059
12,2,8340.842262
12,2,1574.253205
12,2,1643.981707
12,2,1992.593750
12,2,1932.588068
12,2,2052.500000
12,2,4732.615385

12,2,4478.458333
12,2,2243.831395
12,2,3806.625000
12,2,2031.298077
12,2,1792.282609
12,2,1767.393617
12,2,17716.431818
12,2,11081.357143
12,2,1975.238636
12,2,1631.083333
12,2,2067.020833
12,2,2110.976351
12,2,2436.612745
12,2,2193.235294
12,2,6646.518519
12,2,2434.715278
12,2,2802.524457
12,2,2210.554245
12,2,1773.269737
12,2,1778.875000
12,2,1788.897059
12,2,1622.026316
12,2,2213.916667
12,2,3727.297872
12,2,1535.954545
12,2,1905.485795
12,2,1661.196721
12,2,2157.000000
12,2,1593.218750
12,2,1649.545918
12,2,2960.518750
12,2,1934.627778
12,2,2190.082447
12,2,5749.355263
12,2,2914.863636
12,2,1966.802885
12,2,1631.128378
12,2,3374.062500
12,2,2081.517045
12,2,1809.513889
12,2,1816.062500
12,2,1594.914634
12,2,1520.327128
12,2,3841.649457
12,2,1515.377660
12,2,1694.787500
12,2,2055.250000
12,2,1501.040000
12,2,1828.425000
12,2,2855.801471
12,2,3084.944712
12,2,3199.750000
12,2,1890.878049
12,2,2950.194767
12,2,1921.330645
12,2,1946.100000
12,2,3009.323529
12,2,2421.526316
12,2,1642.347826
12,2,2781.375000
12,2,1906.906863
12,2,2381.908163
12,2,2466.147959
12,2,3839.725490
12,2,2248.625000
12,2,1702.755952

12,2,1788.554245
12,2,2401.300595
12,2,1627.657609
12,2,4997.727941
12,2,4292.492647
12,2,2295.230769
12,2,3062.204545
12,2,3201.192308
12,2,1971.727778
12,2,1825.321429
12,2,2519.600000
12,2,1561.183333
12,2,2920.937500
12,2,1585.190476
12,2,2065.810256
12,2,1966.605263
12,2,2831.400000
12,2,1611.250000
12,2,2861.234694
12,2,1628.536458
12,2,2318.303879
12,2,1844.137500
12,2,1953.308824
12,2,3987.836538
12,2,1831.806818
12,2,2031.738208
12,2,1908.613372
12,2,3714.000000
12,2,1634.911765
12,2,1569.083333
12,2,1827.981132
12,2,1762.929167
12,2,1687.500000
12,2,1508.600000
12,2,2198.539062
12,2,2797.138587
12,2,2062.050000
12,2,2267.478261
12,2,1917.318182
12,2,1613.395833
12,2,1741.187500
12,2,2259.296512
12,2,1800.750000
12,2,1666.520408
12,2,1842.933511
12,2,2265.269231
12,2,1686.895161
12,2,2584.425532
12,2,2865.144886
12,2,2099.237179
12,2,2436.203947
12,2,2864.905000
12,2,3709.000000
12,2,1671.580357
12,2,1756.620853
12,2,1845.050000
12,2,1941.934211
12,2,3431.083333
12,2,1601.860000
12,2,1819.197674
12,2,4493.250000
12,2,2294.230114
12,2,2234.339623
12,2,2167.169118
12,2,1641.154412
12,2,1607.731383

12,2,2213.117647
12,2,2133.386364
12,2,2688.250000
12,2,2910.296875
12,2,3003.571429
12,2,2052.658019
12,2,1840.657609
12,2,2313.372340
12,2,1519.707317
12,2,2979.935897
12,2,2426.875000
12,2,1582.792683
12,2,1964.489583
12,2,4323.510638
12,2,1987.330000
12,2,1621.582500
12,2,1743.806818
12,2,2618.135638
12,2,2745.130000
12,2,1519.972973
12,2,1807.127907
12,2,5693.292553
12,2,2144.844298
12,2,2320.520833
12,2,2108.642857
12,2,2634.121212
12,2,3227.724490
12,2,3916.382979
12,2,2172.619048
12,2,1771.573529
12,2,1732.800000
12,2,2625.232500
12,2,1814.740000
12,2,2354.779412
12,2,1526.889205
12,2,2181.000000
12,2,1885.081522
12,2,1562.037234
12,2,1884.534091
12,2,1975.635000
12,2,4318.824324
12,2,3464.968750
12,2,2297.226190
12,2,3186.625000
12,2,2702.259615
12,2,2215.866071
12,2,1815.211538
12,2,4172.011364
12,2,1558.747222
12,2,2935.931250
12,2,2461.843137
12,2,1680.880000
12,2,2919.378378
12,2,4319.656915
12,2,1876.728261
12,2,1841.340426
12,2,2224.396341
12,2,11373.633094
12,2,2416.250000
12,2,1715.718750
12,2,2118.322115
12,2,1693.398936
12,2,1733.988095
12,2,1697.040625
12,2,2702.069444
12,2,2073.235119

12,2,3610.295918
12,2,1909.006410
12,2,2295.318627
12,2,1626.046196
12,2,1616.088415
12,2,3476.853659
12,2,1616.546512
12,2,1642.128472
12,2,13779.392857
12,2,2960.908784
12,2,2693.760000
12,2,3485.639423
12,2,1525.052500
12,2,1520.836735
12,2,1559.168919
12,2,3010.224490
12,2,9654.881579
12,2,1500.838942
12,2,1546.257812
12,2,1734.435976
12,2,1741.130000
12,2,2586.421875
12,2,1662.206633
12,2,2090.023256
12,2,1618.975543
12,2,1628.414634
12,2,1745.438830
12,2,1684.461310
12,2,2372.955882
12,2,1878.798077
12,2,1786.102564
12,2,1771.372093
12,2,2298.990566
12,2,2284.135417
12,2,2026.287791
12,2,2006.160714
12,2,2500.813830
12,2,2761.416667
12,2,1551.000000
12,2,1528.916667
12,2,1814.861702
12,2,1636.555556
12,2,1893.200000
12,2,2408.772321
12,2,4726.414062
12,2,2023.252660
12,2,1690.639706
12,2,1557.552778
12,2,2147.875000
12,2,1693.184524
12,2,2255.072368
12,2,2160.392857
12,2,4672.383929
12,2,2072.650000
12,2,2683.177778
12,2,8645.368750
12,2,1559.000000
12,2,1986.408019
12,2,2302.338710
12,2,1757.500000
12,2,1987.320513
12,2,3212.050000
12,2,1560.000000
12,2,1675.033333
12,2,3059.096154
12,2,3198.400000

12,2,2415.666667
12,2,1787.878205
12,2,1941.689815
12,2,1679.294118
12,2,2121.200000
12,2,1514.002315
12,2,3777.426829
12,2,2732.868056
12,2,2548.975000
12,2,2335.046053
12,2,4983.260638
12,2,4445.670213
12,2,2704.904762
12,2,1662.817708
12,2,3180.596154
12,2,1906.840000
12,2,4388.942500
12,2,2558.485000
12,2,4224.235000
12,2,1643.432432
12,2,2821.818878
12,2,1564.333333
12,2,1582.761905
12,2,2193.673077
12,2,11592.213816
12,2,3554.703804
12,2,2159.285326
12,2,2772.859375
12,2,1668.517500
12,2,4588.666667
12,2,2370.718750
12,2,1563.750000
12,2,2083.002841
12,2,2013.000000
12,2,1560.031250
12,2,2067.635417
12,2,4379.687500
12,2,1655.743590
12,2,1607.291667
12,2,1500.942308
12,2,1527.910714
12,2,2421.125000
12,2,1945.005556
12,2,1525.000000
12,2,1923.380952
12,2,1660.255814
12,2,1609.662791
12,2,2219.111111
12,2,2361.870370
12,2,1594.348837
12,2,1642.736364
12,2,2500.733796
12,2,1564.198980
12,2,1772.718750
12,2,1540.266304
12,2,1974.029255
12,2,1800.360000
12,2,4860.543478
12,2,1647.222222
12,2,2173.528846
12,2,6822.059375
12,2,3049.108974
12,2,1710.879902
12,2,2086.346939
12,2,1638.767857
12,2,1834.083333

12,2,1664.470588
12,2,1860.125000
12,2,1783.459459
12,2,2270.328947
12,2,4069.931034
12,2,2253.937500
12,2,2061.974490
12,2,3068.772059
12,2,2079.258523
12,2,1683.390625
12,2,2684.971875
12,2,1528.414773
12,2,9807.386792
12,2,2075.410000
12,2,1652.491071
12,2,2636.510870
12,2,1719.957447
12,2,1532.875000
12,2,1866.000000
12,2,6049.081633
12,2,1833.835938
12,2,2060.922500
12,2,3277.000000
12,2,1540.959091
12,2,1769.544643
12,2,1529.096296
12,2,2351.744898
12,2,1610.859375
12,2,2311.309524
12,2,4215.015957
12,2,2955.400000
12,2,1797.375000
12,2,2437.007143
12,2,1611.609375
12,2,2039.638587
12,2,2807.190909
12,2,3251.636364
12,2,5501.000000
12,2,1876.750000
12,2,1588.225543
12,2,1552.875000
12,2,1704.635417
12,2,2638.634615
12,2,1845.566489
12,2,1501.579787
12,2,1606.218750
12,2,2300.941489
12,2,4161.093750
12,2,1542.945946
12,2,3666.035714
12,2,1745.473404
12,2,10028.505682
12,2,2167.072674
12,2,3302.062500
12,2,4742.918367
12,2,3494.181818
12,2,2032.663462
12,2,2155.716837
12,2,1749.240625
12,2,1664.958333
12,2,1582.750000
12,2,2007.882500
12,2,1932.883721
12,2,3026.767857
12,2,2342.526786
12,2,2103.556122

12,2,4247.033784
12,2,1706.073864
12,2,1727.695652
12,2,2599.231250
12,2,1775.487981
12,2,3764.583333
12,2,1879.351064
12,2,1520.784722
12,2,2926.472656
12,2,1736.911765
12,2,6550.993750
12,2,3026.000000
12,2,2481.819149
12,2,2160.762500
12,2,1911.265244
12,2,1503.080729
12,2,3427.669643
12,2,2544.894737
12,2,1777.231132
12,2,1722.287234
12,2,15311.107558
12,2,1697.948529
12,2,1718.617347
12,2,3381.640957
12,2,2203.526316
12,2,1706.764706
12,2,2511.205556
12,2,9671.342105
12,2,1908.810976
12,2,2287.807018
12,2,3415.099490
12,2,1678.615385
12,2,2400.560976
12,2,2296.400000
12,2,1685.937500
12,2,1553.585227
12,2,1630.029605
12,2,2256.000000
12,2,2449.326271
12,2,3475.837500
12,2,3384.169643
12,2,1657.410000
12,2,1683.325758
12,2,1568.160714
12,2,1852.832237
12,2,2859.687500
12,2,1906.730769
12,2,1588.848684
12,2,1682.750000
12,2,2209.788889
12,2,2592.812500
12,2,1906.645000
12,2,3305.052500
12,2,4108.367925
12,2,2649.557692
12,2,2981.847826
12,2,2073.511628
12,2,1847.000000
12,2,3629.091837
12,2,3250.218750
12,2,2532.354167
12,2,1758.132979
12,2,2355.265957
12,2,1817.983333
12,2,1718.567708
12,2,1583.946078

12,2,1808.774194
12,2,1625.076531
12,2,1639.723684
12,2,1590.641892
12,2,3540.319712
12,2,6983.022059
12,2,3041.177885
12,2,2329.460674
12,2,2101.855603
12,2,2055.477778
12,2,3846.301829
12,2,4242.066667
12,2,1647.843750
12,2,1749.156250
12,2,1644.240000
12,2,1970.825472
12,2,1879.000000
12,2,2433.927632
12,2,1936.714286
12,2,1748.181548
12,2,1815.074468
12,2,3344.055288
12,2,1621.956522
12,2,1593.414063
12,2,1514.319444
12,2,2596.263636
12,2,1826.971154
12,2,1610.514535
12,2,1784.892442
12,2,1652.769231
12,2,2132.355469
12,2,1654.627976
12,2,2070.695000
12,2,1684.649038
12,2,1847.625000
12,2,3294.669444
12,2,4732.247159
12,2,2800.568750
12,2,2240.732500
12,2,11092.577778
12,2,1510.020833
12,2,3664.459239
12,2,4292.863839
12,2,5171.276786
12,2,2095.946429
12,2,1591.892442
12,2,2804.250000
12,2,2083.442308
12,2,1906.355263
12,2,2999.809524
12,2,5358.772959
12,2,1654.612245
12,2,2233.519231
12,2,3046.189189
12,2,1802.750000
12,2,2537.162791
12,2,1876.038690
12,2,2468.762500
12,2,2626.106618
12,2,1569.430851
12,2,1753.392857
12,2,3851.179245
12,2,2512.762500
12,2,3880.636364
12,2,1883.944444
12,2,2610.659574

12,2,2414.857143
12,2,2881.318182
12,2,2126.375000
12,2,3526.044872
12,2,1648.366477
12,2,1711.806818
12,2,1512.865385
12,2,1659.404762
12,2,1879.959184
12,2,1914.625000
12,2,2054.354167
12,2,1500.056122
12,2,2243.553191
12,2,1748.116848
12,2,2401.042614
12,2,2274.776744
12,2,1584.576705
12,2,2287.010204
12,2,2207.293269
12,2,2181.000000
12,2,7190.801630
12,2,3501.005435
12,2,3247.020000
12,2,1614.043478
12,2,1838.428571
12,2,1567.799419
12,2,3797.136364
12,2,2755.111111
12,2,1991.537162
12,2,4255.596154
12,2,10710.465116
12,2,1797.130435
12,2,4199.039773
12,2,3619.135417
12,2,1542.294118
12,2,1705.640625
12,2,2579.878261
12,2,1895.866667
12,2,2718.080882
12,2,3956.625000
12,2,2104.928571
12,2,1579.735294
12,2,1520.164474
12,2,2144.064103
12,2,1871.204268
12,2,1782.327128
12,2,2057.030556
12,2,2148.263158
12,2,3287.519231
12,2,4079.402027
12,2,1998.306250
12,2,1904.968750
12,2,1767.095930
12,2,2798.850000
12,2,1575.046512
12,2,3527.801630
12,2,3106.675481
12,2,1930.211111
12,2,1586.864130
12,2,2402.920732
12,2,2095.092391
12,2,1665.303571
12,2,2289.321429
12,2,1703.653846
12,2,2909.416667
12,2,2032.500000

12,2,1795.101974
12,2,3441.851562
12,2,2582.478723
12,2,2065.315341
12,2,1673.526316
12,2,6605.364865
12,2,1511.855769
12,2,1949.075000
12,2,2651.281250
12,2,1683.956522
12,2,1763.161765
12,2,1607.470930
12,2,1837.073864
12,2,1917.864130
12,2,1744.916667
12,2,9161.369141
12,2,1675.505000
12,2,2772.105978
12,2,1961.643617
12,2,2548.802734
12,2,1839.360465
12,2,2455.548077
12,2,1571.920833
12,2,3656.517857
12,2,8143.910000
12,2,1792.893519
12,2,3793.592105
12,2,1568.319149
12,2,2066.510204
12,2,3046.297619
12,2,1720.468750
12,2,1578.389535
12,2,2404.337838
12,2,1627.642442
12,2,2108.418182
12,2,4118.625000
12,2,2404.152174
12,2,1714.264151
12,2,2636.696429
12,2,1930.422794
12,2,4700.421196
12,2,1873.279762
12,2,2317.668103
12,2,1756.383178
12,2,2106.777174
12,2,1760.556818
12,2,3252.000000
12,2,1682.682927
12,2,1994.070000
12,2,2466.240000
12,2,3150.827500
12,2,18399.222826
12,2,1513.788889
12,2,2852.431548
12,2,1741.482955
12,2,3965.068182
12,2,1953.839286
12,2,1692.802326
12,2,2575.125000
12,2,1652.467593
12,2,1711.696429
12,2,2039.777778
12,2,1730.039894
12,2,1739.324405
12,2,1934.824405
12,2,3552.500000

12,2,2201.062500
12,2,1741.764706
12,2,1575.039062
12,2,1987.865854
12,2,1569.970745
12,2,2543.907895
12,2,1666.030612
12,2,2091.065217
12,2,5230.216463
12,2,1631.864796
12,2,2050.989362
12,2,1647.428571
12,2,1547.158537
12,2,2042.849057
12,2,1536.602041
12,2,1516.984293
12,2,1936.813953
12,2,3136.000000
12,2,2689.225962
12,2,1866.767857
12,2,1714.991379
12,2,5163.513587
12,2,3092.426829
12,2,1558.304545
12,2,1800.372093
12,2,1814.419643
12,2,1929.651042
12,2,2785.294643
12,2,2086.142442
12,2,2476.226562
12,2,1957.304054
12,2,2106.169643
12,2,2174.197917
12,2,3477.627907
12,2,1767.159314
12,2,2984.104167
12,2,1752.147059
12,2,2744.772500
12,2,3241.539894
12,2,1638.770349
12,2,2562.880734
12,2,1790.148437
12,2,1872.070313
12,2,1667.963415
12,2,2087.700000
12,2,2008.084239
12,2,2205.901316
12,2,2277.375000
12,2,2604.570175
12,2,1796.184783
12,2,2500.383721
12,2,4966.286585
12,2,2360.300926
12,2,1621.585366
12,2,2479.398810
12,2,4540.756757
12,2,2057.065000
12,2,1685.468750
12,2,1513.951220
12,2,1864.531250
12,2,2104.429348
12,2,2707.643750
12,2,1592.660000
12,2,2010.662791
12,2,3345.259259
12,2,1703.411058

12,2,2529.706633
12,2,1826.750000
12,2,1677.032500
12,2,2310.058824
12,2,1584.668750
12,2,5951.659574
12,2,2050.766304
12,2,1520.553571
12,2,2506.100610
12,2,1552.875000
12,2,2303.943966
12,2,2041.791667
12,2,1734.891892
12,2,3575.203488
12,2,14333.979167
12,2,5998.053191
12,2,1746.790698
12,2,2235.250000
12,2,2348.920000
12,2,1526.057692
12,2,2516.329787
12,2,2841.940476
12,2,1818.453947
12,2,4185.722222
12,2,4201.308824
12,2,1690.085106
12,2,1667.857143
12,2,1905.159091
12,2,1757.825000
12,2,2056.573661
12,2,2499.008523
12,2,1624.298780
12,2,15848.077778
12,2,2384.923295
12,2,1533.047794
12,2,2399.750000
12,2,1958.871622
12,2,2287.375000
12,2,1683.192308
12,2,3076.682432
12,2,1619.046875
12,2,1676.357143
12,2,3318.279255
12,2,2207.710000
12,2,2180.000000
12,2,2071.848404
12,2,1673.041667
12,2,2658.651961
12,2,1516.919283
12,2,1722.722561
12,2,2770.260870
12,2,4550.925532
12,2,1699.812500
12,2,2102.665948
12,2,2453.037500
12,2,1768.726562
12,2,2909.328947
12,2,1899.185976
12,2,1931.198980
12,2,2333.250000
12,2,1536.342262
12,2,2091.345000
12,2,2918.806604
12,2,2784.260000
12,2,5406.625000
12,2,2907.489362

12,2,2409.440476
12,2,1506.416667
12,2,1813.200581
12,2,1537.092105
12,2,2420.392857
12,2,1848.290909
12,2,2172.730978
12,2,2053.540816
12,2,2000.200000
12,2,1954.104167
12,2,2615.053571
12,2,2883.165698
12,2,2070.836735
12,2,3278.551020
12,2,1935.737981
12,2,2109.094907
12,2,1542.314189
12,2,1928.032609
12,2,2823.625000
12,2,1669.140625
12,2,1673.104651
12,2,3015.372093
12,2,1503.064904
12,2,2333.355263
12,2,1775.132075
12,2,1521.875000
12,2,1903.709184
12,2,2734.186170
12,2,2306.467949
12,2,1958.625000
12,2,1589.882353
12,2,2647.888587
12,2,1506.705882
12,2,2323.729167
12,2,1773.677083
12,2,5260.933140
12,2,1786.418848
12,2,1824.302326
12,2,1969.247159
12,2,7733.459239
12,2,1579.027778
12,2,3335.472973
12,2,3352.764706
12,2,1724.578947
12,2,5913.090116
12,2,2070.326531
12,2,1596.000000
12,2,1560.770833
12,2,1699.460366
12,2,3437.416667
12,2,1999.590909
12,2,1781.950000
12,2,2018.214286
12,2,1719.845745
12,2,1540.119048
12,2,8590.468750
12,2,2120.610465
12,2,1502.892857
12,2,3303.702128
12,2,1583.262195
12,2,2269.274510
12,2,1804.355769
12,2,1870.413043
12,2,2040.958333
12,2,1607.518182
12,2,2862.546875

12,2,2002.178571
12,2,1504.763158
12,2,1656.636792
12,2,10679.162791
12,2,1608.833333
12,2,2662.632653
12,2,1528.718750
12,2,9498.154762
12,2,4873.400000
12,2,1588.357143
12,2,1595.146226
12,2,2427.917500
12,2,3818.921875
12,2,5157.324074
12,2,2997.571429
12,2,2744.000000
12,2,1879.950000
12,2,1864.035714
12,2,1966.072222
12,2,1730.817073
12,2,1745.548913
12,2,2479.183824
12,2,1950.595745
12,2,1749.871622
12,2,1816.297619
12,2,1631.000000
12,2,2647.158654
12,2,1900.662500
12,2,1913.662791
12,2,2488.531915
12,2,17601.529412
12,2,1512.848214
12,2,2067.963068
12,2,1917.500000
12,2,4655.193182
12,2,3652.755319
12,2,1838.652174
12,2,2117.407407
12,2,2183.696429
12,2,2779.428571
12,2,2747.280702
12,2,4420.367647
12,2,3558.619048
12,2,2378.523810
12,2,1653.862745
12,2,1591.794444
12,2,1528.010638
12,2,2232.517857
12,2,2387.703125
12,2,1648.200000
12,2,2493.660714
12,2,12040.207317
12,2,3098.255952
12,2,7474.159574
12,2,2238.494318
12,2,3209.219595
12,2,2837.583333
12,2,1546.517857
12,2,4770.564103
12,2,1654.148936
12,2,4019.002604
12,2,2633.571429
12,2,1544.907258
12,2,3660.107143
12,2,3482.946429
12,2,3626.109375

12,2,4005.551136
12,2,1553.615385
12,2,1601.372549
12,2,2040.896341
12,2,2564.693182
12,2,3209.000000
12,2,1860.588235
12,2,6888.200000
12,2,2030.500000
12,2,1558.400000
12,2,3776.875000
12,2,2163.878049
12,2,3310.704082
12,2,1499.146341
12,2,1614.318182
12,2,2306.890000
12,2,2554.846154
12,2,1919.300000
12,2,3132.167411
12,2,4449.807927
12,2,2309.730000
12,2,2682.048611
12,2,3713.948113
12,2,3610.512195
12,2,2393.250000
12,2,2650.461538
12,2,1671.632500
12,2,2760.452128
12,2,2022.316667
12,2,4923.500000
12,2,1661.530612
12,2,14016.594388
12,2,3355.520833
12,2,2040.131944
12,2,1513.149457
12,2,2121.658019
12,2,1614.284375
12,2,4140.759091
12,2,2332.790761
12,2,1522.720745
12,2,3662.846154
12,2,2727.318878
12,2,1848.906250
12,2,2665.020833
12,2,1937.466667
12,2,2675.388298
12,2,1659.122549
12,2,2500.121622
12,2,2187.000000
12,2,2438.609375
12,2,2873.796053
12,2,1577.291667
12,2,3165.972500
12,2,2537.666667
12,2,1973.281250
12,2,1813.175676
12,2,1591.534091
12,2,3217.103365
12,2,1766.648148
12,2,1738.541667
12,2,18288.058036
12,2,3548.671429
12,2,3092.058824
12,2,1501.020408
12,2,1566.772727
12,2,1671.855000

12,2,3174.904605
12,2,1833.960938
12,2,2160.277344
12,2,3408.481481
12,2,3030.617021
12,2,1556.968750
12,2,10417.750000
12,2,1980.904762
12,2,1778.541667
12,2,1988.900000
12,2,2774.875000
12,2,1774.523256
12,2,6120.541667
12,2,1543.743243
12,2,1676.053571
12,2,1867.714286
12,2,1541.107143
12,2,2443.664634
12,2,1702.349265
12,2,2995.671569
12,2,2729.622596
12,2,1538.237500
12,2,1620.837696
12,2,2266.000000
12,2,2298.359043
12,2,1531.187500
12,2,2028.125000
12,2,2204.285714
12,2,4693.533333
12,2,1768.567568
12,2,1742.455000
12,2,3801.530612
12,2,1584.750000
12,2,2971.176829
12,2,2268.416667
12,2,1854.912791
12,2,2073.923077
12,2,2073.651596
12,2,1928.564286
12,2,1561.130435
12,2,1534.091837
12,2,3891.475543
12,2,1854.086538
12,2,2265.218750
12,2,2761.768293
12,2,1682.071429
12,2,1747.909091
12,2,2833.941176
12,2,2134.573661
12,2,2340.232558
12,2,1533.101064
12,2,1951.714286
12,2,1581.341146
12,2,3079.612500
12,2,1690.414894
12,2,2339.692857
12,2,2019.719512
12,2,2792.783019
12,2,1523.380435
12,2,1512.253125
12,2,3909.023256
12,2,1557.333333
12,2,3039.510204
12,2,2103.735294
12,2,1581.732558
12,2,2868.375000

12,2,1660.698171
12,2,2347.033708
12,2,2361.453571
12,2,1565.139706
12,2,2002.041667
12,2,7917.298387
12,2,3300.098684
12,2,2376.500000
12,2,1602.308594
12,2,2956.529070
12,2,2363.861963
12,2,1852.500000
12,2,2883.850000
12,2,1707.972222
12,2,3540.556604
12,2,3062.782609
12,2,1765.460000
12,2,1534.808511
12,2,3490.403846
12,2,2496.250000
12,2,1533.977273
12,2,3714.851562
12,2,9392.283784
12,2,1526.057432
12,2,2790.125000
12,2,2122.734694
12,2,2169.480000
12,2,3214.666667
12,2,1879.630000
12,2,1559.937500
12,2,1883.121795
12,2,2101.897436
12,2,1728.210227
12,2,1720.560000
12,2,1801.539474
12,2,4727.080000
12,2,2291.237069
12,2,1810.093023
12,2,1517.019608
12,2,1525.931034
12,2,2680.986111
12,2,1799.953125
12,2,1640.469907
12,2,1510.968085
12,2,2913.708333
12,2,1887.349432
12,2,2041.076923
12,2,1877.900000
12,2,2716.011111
12,2,2609.427419
12,2,2111.609375
12,2,2094.285714
12,2,2548.451705
12,2,1973.949468
12,2,12318.285256
12,2,1771.714286
12,2,2465.233333
12,2,2776.000000
12,2,1659.689189
12,2,1900.375000
12,2,4295.506757
12,2,1949.441860
12,2,1921.140957
12,2,1955.010417
12,2,1715.892857
12,2,2872.551282

12,2,2146.437500
12,2,1919.375000
12,2,5392.298077
12,2,1506.272059
12,2,2081.232558
12,2,4140.043269
12,2,1564.222561
12,2,6522.843182
12,2,2708.444767
12,2,1564.328125
12,2,2157.423469
12,2,2016.234043
12,2,1619.792683
12,2,1719.262500
12,2,1862.875000
12,2,1756.152439
12,2,1502.000000
12,2,3873.575581
12,2,2806.934211
12,2,1889.386364
12,2,4386.189815
12,2,1932.922170
12,2,1840.585714
12,2,1819.428571
12,2,1952.024194
12,2,2731.002500
12,2,1799.475543
12,2,2501.636364
12,2,4891.581967
12,2,2418.800481
12,2,1627.820513
12,2,1510.910000
12,2,1785.203125
12,2,1925.117188
12,2,1746.960000
12,2,1579.907895
12,2,7718.000000
12,2,1889.000000
12,2,2800.122340
12,2,1612.367188
12,2,1665.687500
12,2,2266.644444
12,2,3458.978723
12,2,2319.678977
12,2,3458.148810
12,2,3586.359375
12,2,1523.729381
12,2,2475.970930
12,2,1815.111702
12,2,2636.000000
12,2,2245.129630
12,2,2987.250000
12,2,1677.028409
12,2,1961.765000
12,2,2429.489362
12,2,2062.481481
12,2,2661.742188
12,2,1585.618902
12,2,1680.829545
12,2,1801.300000
12,2,2103.291667
12,2,1551.073171
12,2,1634.722222
12,2,1766.301630
12,2,3397.431373
12,2,3470.187500

12,2,1871.955729
12,2,2632.487805
12,2,1647.343750
12,2,2479.980392
12,2,2534.360465
12,2,1518.481818
12,2,4720.275862
12,2,4290.792763
12,2,1708.338942
12,2,8310.571970
12,2,1780.262500
12,2,1632.669811
12,2,4512.910714
12,2,2701.500000
12,2,2048.381579
12,2,1750.833333
12,2,1980.684783
12,2,7031.776596
12,2,1624.610000
12,2,2448.827500
12,2,1850.778846
12,2,1787.218750
12,2,1705.570312
12,2,3047.154412
12,2,1989.642857
12,2,2003.627551
12,2,2052.692568
12,2,1912.867347
12,2,3239.562500
12,2,1766.069149
12,2,1806.096154
12,2,3385.372093
12,2,10885.030675
12,2,2897.019231
12,2,1765.428571
12,2,2031.636364
12,2,1744.909091
12,2,2797.704082
12,2,1647.910714
12,2,16007.253125
12,2,2633.250000
12,2,4061.701087
12,2,2507.250000
12,2,1678.668919
12,2,2124.458333
12,2,1581.520408
12,2,2394.586957
12,2,1650.231707
12,2,1913.181818
12,2,2729.415948
12,2,1520.065678
12,2,2141.337264
12,2,1968.351351
12,2,1755.510870
12,2,2724.698529
12,2,4801.093750
12,2,2077.422619
12,2,1569.666667
12,2,3200.196023
12,2,1692.861486
12,2,1572.281250
12,2,4242.937500
12,2,2111.606481
12,2,17496.423780
12,2,1688.935829
12,2,2722.952586

12,2,1858.026786
12,2,1951.744898
12,2,5032.321429
12,2,1553.010870
12,2,2791.625000
12,2,2180.530093
12,2,1647.906250
12,2,2480.372449
12,2,1516.834677
12,2,2184.937500
12,2,2134.178571
12,2,1555.348214
12,2,1764.385870
12,2,1956.924242
12,2,2688.071429
12,2,2345.434783
12,2,2153.140351
12,2,2172.652027
12,2,1707.450000
12,2,1698.522222
12,2,1948.844340
12,2,1631.675595
12,2,1680.540865
12,2,1931.023256
12,2,1719.109694
12,2,1722.500000
12,2,2384.160714
12,2,1590.557500
12,2,1987.519737
12,2,1818.380208
12,2,1865.000000
12,2,2038.027027
12,2,1694.375000
12,2,2142.502778
12,2,2635.740385
12,2,1795.141304
12,2,4890.326923
12,2,1815.322404
12,2,1693.029412
12,2,2028.692308
12,2,2926.992647
12,2,1526.656250
12,2,2505.722222
12,2,3691.478261
12,2,2391.737500
12,2,2537.724490
12,2,2747.400000
12,2,2226.785714
12,2,1502.607143
12,2,1850.027273
12,2,2987.083333
12,2,2205.839378
12,2,2893.690476
12,2,1526.836111
12,2,2838.808824
12,2,1897.212766
12,2,1585.963415
12,2,1751.625000
12,2,1874.005814
12,2,2475.088068
12,2,1784.113095
12,2,1744.328947
12,2,1584.960227
12,2,1528.867788
12,2,1719.922500
12,2,7985.559375

12,2,1520.100000
12,2,1576.726562
12,2,2421.545455
12,2,15183.792763
12,2,2126.691860
12,2,1509.264706
12,2,5187.821078
12,2,2577.000000
12,2,3080.500000
12,2,2882.026316
12,2,1505.562500
12,2,1604.081522
12,2,1722.763158
12,2,1789.670213
12,2,2035.155556
12,2,5250.656250
12,2,2708.548780
12,2,1600.583333
12,2,1505.310976
12,2,2400.045455
12,2,5877.209459
12,2,1913.009434
12,2,1576.730769
12,2,1881.550676
12,2,1972.732558
12,2,1926.817073
12,2,1650.267857
12,2,1640.790698
12,2,2028.444444
12,2,7207.861486
12,2,2688.133333
12,2,1564.448529
12,2,1631.375000
12,2,1740.762500
12,2,2767.793269
12,2,2326.272727
12,2,3335.534884
12,2,1983.308333
12,2,3003.845000
12,2,1932.581633
12,2,2544.912500
12,2,2059.985119
12,2,1533.794872
12,2,1942.622642
12,2,1627.328804
12,2,1535.768750
12,2,1797.250000
12,2,1633.061047
12,2,2127.653061
12,2,2584.125000
12,2,1839.396875
12,2,2174.625000
12,2,4685.490566
12,2,1642.756098
12,2,2649.189103
12,2,2600.262712
12,2,1654.482955
12,2,1907.344444
12,2,9009.132353
12,2,2080.555556
12,2,1520.134375
12,2,4029.811275
12,2,1510.950000
12,2,2016.973214
12,2,1585.828947
12,2,1516.612500

12,2,1516.712766
12,2,3745.422619
12,2,1835.041667
12,2,1597.828488
12,2,2231.715909
12,2,1670.569079
12,2,1611.700000
12,2,1676.232558
12,2,1637.075658
12,2,3703.000000
12,2,1562.959091
12,2,1699.673913
12,2,1523.350000
12,2,7732.218750
12,2,1826.944712
12,2,3684.625000
12,2,1860.966216
12,2,3683.285714
12,2,2102.416667
12,2,2231.793750
12,2,2357.384259
12,2,2191.899390
12,2,1636.625000
12,2,1868.651163
12,2,2039.928125
12,2,1518.523810
12,2,3046.166667
12,2,1805.235849
12,2,2415.125000
12,2,1664.009615
12,2,1748.829787
12,2,2336.438889
12,2,1747.140000
12,2,2499.107955
12,2,1826.575000
12,2,1687.154070
12,2,1992.425532
12,2,2333.306122
12,2,3420.000000
12,2,4519.106383
12,2,1608.535714
12,2,3111.113208
12,2,1811.529412
12,2,2324.169811
12,2,2418.900000
12,2,1535.589286
12,2,16049.107955
12,2,1676.449468
12,2,2338.902439
12,2,1627.718750
12,2,1554.250000
12,2,2323.692308
12,2,1779.500000
12,2,1631.958333
12,2,3298.796296
12,2,1714.285714
12,2,3018.144737
12,2,2832.650000
12,2,1988.447368
12,2,1888.574468
12,2,1910.152778
12,2,2437.723214
12,2,1565.243243
12,2,1852.489796
12,2,1552.860119
12,2,2232.166667

12,2,2745.789216
12,2,1668.607143
12,2,1526.238636
12,2,1752.632353
12,2,1993.950000
12,2,1505.916667
12,2,1580.416667
12,2,2515.150000
12,2,5979.428571
12,2,1523.543478
12,2,1779.649510
12,2,1658.952381
12,2,2465.647059
12,2,1614.356707
12,2,2107.238208
12,2,2860.666667
12,2,1809.971154
12,2,1518.500000
12,2,2090.910959
12,2,2171.957447
12,2,3635.636364
12,2,2368.053191
12,2,3383.720588
12,2,2305.106771
12,2,1690.932500
12,2,2006.800000
12,2,2731.799213
12,2,2408.535714
12,2,3591.932065
12,2,1594.317073
12,2,2182.056122
12,2,1768.573171
12,2,1920.563830
12,2,4330.825000
12,2,1946.275000
12,2,2507.693182
12,2,2919.658088
12,2,2989.223361
12,2,1597.909091
12,2,1813.351562
12,2,2405.539474
12,2,1509.036585
12,2,1800.521341
12,2,1965.285714
12,2,3644.500000
12,2,4741.558824
12,2,1776.490196
12,2,2559.472973
12,2,1601.963415
12,2,2059.547486
12,2,1734.500000
12,2,1854.428571
12,2,2955.990196
12,2,1587.473039
12,2,2188.331395
12,2,2162.677083
12,2,1583.227273
12,2,1590.020833
12,2,3058.234375
12,2,2067.479592
12,2,2517.412500
12,2,1713.589286
12,2,2887.647436
12,2,7275.305921
12,2,1586.336207
12,2,6719.400000

12,2,2970.251282
12,2,2271.614362
12,2,2782.963415
12,2,1691.509375
12,2,2400.000000
12,2,1545.765957
12,2,1674.145833
12,2,1986.780488
12,2,2994.800000
12,2,4758.050000
12,2,1824.236842
12,2,2364.054348
12,2,1771.263158
12,2,1798.305000
12,2,1540.259146
12,2,2829.574324
12,2,1658.604167
12,2,1504.362245
12,2,1638.700000
12,2,1611.015957
12,2,2568.392857
12,2,1590.193452
12,2,5467.861842
12,2,1908.375000
12,2,1705.862434
12,2,1658.567935
12,2,1518.644737
12,2,2409.846154
12,2,1827.277439
12,2,5084.500000
12,2,1597.740854
12,2,1549.339286
12,2,1508.375000
12,2,1574.350000
12,2,1572.518519
12,2,2700.000000
12,2,1678.063776
12,2,1585.172794
12,2,1788.968750
12,2,2272.070000
12,2,1809.567568
12,2,3131.134146
12,2,1793.250000
12,2,1969.250000
12,2,4770.391667
12,2,4012.436364
12,2,6335.583333
12,2,1846.328804
12,2,3553.129717
12,2,1621.585227
12,2,1990.892857
12,2,2965.042763
12,2,1619.555556
12,2,1567.085937
12,2,2320.950000
12,2,1548.035000
12,2,1759.390306
12,2,5591.395833
12,2,2015.436364
12,2,2144.204545
12,2,2318.490000
12,2,1546.909091
12,2,8635.200000
12,2,1684.540698
12,2,1882.187500
12,2,1696.320122

12,2,1847.718750
12,2,1834.831522
12,2,3359.437500
12,2,2612.200000
12,2,1606.075000
12,2,2732.759615
12,2,2046.230000
12,2,2991.705357
12,2,2124.054054
12,2,1879.118421
12,2,2817.239362
12,2,1820.378378
12,2,1917.433824
12,2,1798.975000
12,2,1881.684211
12,2,1795.567073
12,2,2147.360000
12,2,11452.060606
12,2,2162.794643
12,2,16360.976974
12,2,1905.956522
12,2,2691.043478
12,2,1877.956522
12,2,2540.648936
12,2,1826.245000
12,2,2542.142857
12,2,2001.448171
12,2,1726.272727
12,2,4650.840000
12,2,1597.909574
12,2,1590.903846
12,2,7533.892241
12,2,1944.818966
12,2,2091.703804
12,2,10831.929878
12,2,3154.423295
12,2,2076.833333
12,2,3699.576923
12,2,2211.880000
12,2,2573.865854
12,2,6697.930000
12,2,1712.000000
12,2,1527.684375
12,2,1791.189732
12,2,3189.346939
12,2,1940.833333
12,2,5484.875000
12,2,2703.143617
12,2,1891.656250
12,2,4147.003676
12,2,12752.819444
12,2,2099.965000
12,2,2757.666667
12,2,1771.155556
12,2,1565.002326
12,2,1571.875000
12,2,1743.750000
12,2,1558.485000
12,2,1719.586207
12,2,1781.369318
12,2,7412.111702
12,2,1506.304348
12,2,1912.656863
12,2,1826.250000
12,2,1517.627976
12,2,8698.577128

12,2,1649.750000
12,2,2942.178571
12,2,3700.584375
12,2,2094.880000
12,2,1550.208333
12,2,1630.068452
12,2,1514.943396
12,2,1665.666667
12,2,1603.429878
12,2,1876.110000
12,2,3690.625000
12,2,2201.990385
12,2,10706.755952
12,2,3532.750000
12,2,3564.425532
12,2,1553.843137
12,2,2256.450000
12,2,2841.702128
12,2,1966.470588
12,2,2559.205128
12,2,4606.326220
12,2,1773.445122
12,2,2866.275735
12,2,1526.300000
12,2,1743.929487
12,2,2637.230769
12,2,3101.300000
12,2,3633.347561
12,2,1909.293478
12,2,2827.901961
12,2,1991.146789
12,2,4092.500000
12,2,7934.652439
12,2,2061.475000
12,2,2430.254545
12,2,2028.036585
12,2,1711.199324
12,2,2562.020408
12,2,2920.322368
12,2,2494.023256
12,2,1948.015625
12,2,2711.719340
12,2,2101.986111
12,2,1670.285714
12,2,1943.111111
12,2,1604.500000
12,2,2252.312500
12,2,2034.936170
12,2,1709.050000
12,2,3868.881579
12,2,2902.061224
12,2,1549.571429
12,2,1890.583333
12,2,1506.965426
12,2,1583.747159
12,2,2617.315789
12,2,3056.157343
12,2,1829.622222
12,2,1545.129630
12,2,2516.437500
12,2,1662.375000
12,2,1738.896277
12,2,1986.599432
12,2,2617.732143
12,2,4229.104651
12,2,4636.250000

12,2,2691.453488
12,2,1606.662791
12,2,1589.986486
12,2,1934.000000
12,2,1786.840000
12,2,2540.437500
12,2,2269.580357
12,2,3925.112245
12,2,2562.949519
12,2,1667.883065
12,2,3257.335106
12,2,6266.816860
12,2,2489.196429
12,2,2327.250000
12,2,2136.291667
12,2,1525.653846
12,2,2141.446809
12,2,7343.815789
12,2,1747.119681
12,2,1840.029412
12,2,1601.239130
12,2,2403.797297
12,2,2099.214286
12,2,1896.574324
12,2,2580.732143
12,2,2347.866667
12,2,1557.744681
12,2,1921.841912
12,2,2887.100000
12,2,1718.572917
12,2,3368.883721
12,2,2958.235556
12,2,1575.875000
12,2,3989.240385
12,2,2129.773438
12,2,1898.339286
12,2,1848.086310
12,2,1540.622549
12,2,1509.214286
12,2,1586.317500
12,2,4574.257353
12,2,1592.065341
12,2,4943.205882
12,2,2329.833333
12,2,2123.005102
12,2,2110.315068
12,2,1884.866667
12,2,2020.941176
12,2,2051.823529
12,2,2502.073529
12,2,1653.885417
12,2,1837.255814
12,2,1623.531250
12,2,1582.796875
12,2,7139.451389
12,2,1941.303571
12,2,1893.058824
12,2,1573.509615
12,2,4256.100610
12,2,2363.903061
12,2,1558.875000
12,2,2013.850000
12,2,4980.268293
12,2,2046.541667
12,2,2598.143713
12,2,4010.195122

12,2,2965.656250
12,2,1695.438889
12,2,1574.445312
12,2,2033.677083
12,2,1611.039474
12,2,2361.872500
12,2,1550.892157
12,2,1525.115385
12,2,1579.500000
12,2,2663.561224
12,2,3640.362500
12,2,1935.132813
12,2,1554.729651
12,2,1514.678571
12,2,1814.235294
12,2,1643.250000
12,2,1712.500000
12,2,1947.786765
12,2,1829.283784
12,2,2060.180905
12,2,3142.562500
12,2,1499.092391
12,2,1759.107143
12,2,3983.857500
12,2,2059.520833
12,2,2415.714286
12,2,2786.214286
12,2,1500.500000
12,2,1581.076923
12,2,1716.912736
12,2,1543.692308
12,2,3148.415816
12,2,1635.250000
12,2,1621.000000
12,2,2404.476190
12,2,1819.402174
12,2,2187.250000
12,2,1948.596354
12,2,5522.950000
12,2,1910.000000
12,2,4132.518229
12,2,1774.000000
12,2,7022.357143
12,2,1596.111842
12,2,10818.105263
12,2,1907.166667
12,2,1619.584302
12,2,1599.680328
12,2,3558.103261
12,2,8331.303191
12,2,3113.176020
12,2,2402.228365
12,2,3024.596154
12,2,1836.063725
12,2,2317.719512
12,2,2354.913613
12,2,1538.000000
12,2,1769.686170
12,2,2262.420000
12,2,2406.812500
12,2,1751.114865
12,2,3937.808824
12,2,3765.830882
12,2,1599.550000
12,2,1551.971154
12,2,1811.315972

12,2,2115.422872
12,2,1639.294643
12,2,1650.612500
12,2,1797.710938
12,2,2089.458333
12,2,2400.283654
12,2,3458.328947
12,2,1785.910112
12,2,2821.694767
12,2,1620.630435
12,2,2866.274390
12,2,1736.589744
12,2,3822.398477
12,2,1561.228659
12,2,2971.800532
12,2,2363.178571
12,2,2131.092500
12,2,2289.415541
12,2,2597.636364
12,2,1839.385965
12,2,2600.893382
12,2,1690.000000
12,2,1938.638889
12,2,2906.156915
12,2,1655.696809
12,2,1713.727273
12,2,2307.847826
12,2,2961.011364
12,2,2005.075301
12,2,2106.180000
12,2,6957.640244
12,2,2023.891892
12,2,1962.920000
12,2,1503.664894
12,2,2365.493590
12,2,3276.419118
12,2,1731.385417
12,2,5615.880000
12,2,2074.437500
12,2,1636.058333
12,2,1707.239362
12,2,1499.903061
12,2,3821.571078
12,2,2009.012195
12,2,1752.178571
12,2,2766.991667
12,2,1798.103659
12,2,2283.387500
12,2,1627.660377
12,2,2287.347826
12,2,3213.785714
12,2,1945.068627
12,2,2843.566038
12,2,1768.433962
12,2,2206.809524
12,2,2131.148936
12,2,1506.014205
12,2,3944.338235
12,2,2311.352941
12,2,1499.085714
12,2,2530.041667
12,2,2400.053571
12,2,2105.130952
12,2,1935.037037
12,2,2846.063830
12,2,1954.336538

12,2,2449.017857
12,2,2170.053571
12,2,1624.619048
12,2,2359.889535
12,2,3217.093137
12,2,2506.472500
12,2,2300.110849
12,2,2478.180000
12,2,1606.000000
12,2,1808.928571
12,2,1967.086310
12,2,3130.750000
12,2,4853.459135
12,2,1788.000000
12,2,1680.294444
12,2,3628.910256
12,2,1798.375000
12,2,2095.297872
12,2,7182.086957
12,2,2321.959821
12,2,4373.563830
12,2,1539.101695
12,2,1755.687500
12,2,2096.291667
12,2,1637.763158
12,2,2775.984043
12,2,2218.957143
12,2,1780.195122
12,2,1657.878049
12,2,1794.704545
12,2,1567.536932
12,2,8350.632979
12,2,12103.383721
12,2,2868.725610
12,2,1644.030000
12,2,1607.469388
12,2,1948.880319
12,2,1706.528061
12,2,1596.719444
12,2,2349.421875
12,2,2828.311364
12,2,1617.565000
12,2,1632.613095
12,2,1710.317708
12,2,2212.612981
12,2,3567.062500
12,2,7589.136364
12,2,1566.148438
12,2,3163.375000
12,2,1502.125000
12,2,6275.357558
12,2,1984.461957
12,2,1692.888021
12,2,2561.140625
12,2,1542.689189
12,2,1509.576705
12,2,1505.156915
12,2,1944.945122
12,2,2104.456250
12,2,2025.493902
12,2,4053.309524
12,2,3057.022727
12,2,1809.492925
12,2,1869.412234
12,2,1696.245000
12,2,1575.695652

12,2,1625.221154
12,2,2685.885135
12,2,1542.044118
12,2,3317.285000
12,2,1583.219512
12,2,1502.633333
12,2,1825.390351
12,2,1789.718750
12,2,2995.154762
12,2,2211.852273
12,2,1985.625000
12,2,1923.909574
12,2,2126.904762
12,2,1607.015306
12,2,2406.611111
12,2,5153.805147
12,2,2202.645833
12,2,1891.440000
12,2,1513.446875
12,2,1511.232955
12,2,1548.581395
12,2,1542.432065
12,2,5855.997222
12,2,2021.661932
12,2,1535.994565
12,2,1508.416667
12,2,6796.188953
12,2,1779.701923
12,2,3917.666667
12,2,1614.829787
12,2,2421.438679
12,2,1899.825000
12,2,1695.139205
12,2,3494.956522
12,2,2766.525000
12,2,2066.508721
12,2,1867.943452
12,2,2186.534091
12,2,1552.470588
12,2,2356.704545
12,2,1658.440476
12,2,2510.240132
12,2,2654.024390
12,2,2032.938776
12,2,2065.571809
12,2,2606.578431
12,2,1609.905556
12,2,2075.079545
12,2,1595.046875
12,2,5528.241071
12,2,3238.100000
12,2,14069.598958
12,2,1785.408537
12,2,4115.039634
12,2,1897.769634
12,2,1536.875000
12,2,1878.096154
12,2,3210.298913
12,2,2134.828804
12,2,2997.581633
12,2,1764.395833
12,2,2911.449468
12,2,2766.889706
12,2,1697.062500
12,2,1579.646429
12,2,1884.121875

12,2,2159.923077
12,2,2839.086538
12,2,1565.068182
12,2,2072.269231
12,2,1552.516447
12,2,1518.565789
12,2,2869.423077
12,2,1646.463816
12,2,2254.375000
12,2,3637.740132
12,2,1896.478723
12,2,1629.247449
12,2,1612.102564
12,2,3567.785714
12,2,1596.324324
12,2,11531.334821
12,2,1635.101064
12,2,1767.640000
12,2,2211.767857
12,2,2059.625000
12,2,2967.392157
12,2,1525.747283
12,2,1584.455000
12,2,1773.615385
12,2,2564.120000
12,2,1961.000000
12,2,1977.904255
12,2,3938.650000
12,2,1924.752660
12,2,2955.428571
12,2,1759.121875
12,2,2313.587963
12,2,2116.226190
12,2,1750.984375
12,2,1829.625000
12,2,2144.636364
12,2,2414.448529
12,2,2327.433333
12,2,4093.543478
12,2,4342.145349
12,2,2132.737745
12,2,1852.078125
12,2,2338.803571
12,2,2093.900000
12,2,2186.750000
12,2,2166.301887
12,2,1880.792683
12,2,2312.595745
12,2,1643.113636
12,2,3183.221354
12,2,2134.275510
12,2,1565.340426
12,2,1540.394444
12,2,1690.459906
12,2,5506.144231
12,2,1542.228571
12,2,2106.054688
12,2,1613.780556
12,2,2602.619318
12,2,1615.785377
12,2,1668.323864
12,2,2152.618421
12,2,1920.884615
12,2,1506.696023
12,2,2178.208333
12,2,1533.240000

12,2,1985.162500
12,2,1547.882500
12,2,2875.000000
12,2,1564.760000
12,2,3421.602273
12,2,2847.037736
12,2,2559.566327
12,2,1541.263021
12,2,1873.400000
12,2,1975.394886
12,2,2505.144022
13,1,3817.828125
13,1,8303.230769
13,1,1913.993902
13,1,5313.277027
13,1,4525.733333
13,1,4215.910326
13,1,2900.854167
13,1,8143.722561
13,1,17958.000000
13,1,1888.072581
13,1,10748.017241
13,1,1545.127907
13,1,5871.464286
13,1,11883.638889
13,1,4803.190217
13,1,12406.964286
13,1,8151.372159
13,1,2123.524390
13,1,5572.526316
13,1,6798.387195
13,1,2317.538462
13,1,2074.869792
13,1,2254.758523
13,1,2813.500000
13,1,4120.333333
13,1,5833.410156
13,1,6407.854730
13,1,14061.122222
13,1,8223.000000
13,1,4284.101744
13,1,9575.121951
13,1,5302.651786
13,1,1927.742188
13,1,4951.370192
13,1,4276.740132
13,1,3627.044444
13,1,7808.838710
13,1,11640.056250
13,1,4538.801282
13,1,4187.535714
13,1,13168.833333
13,1,4084.681818
13,1,1984.540698
13,1,1592.159574
13,1,7759.066860
13,1,1941.303571
13,1,3500.695312
13,1,7003.360294
13,1,2795.771429
13,1,4271.266129
13,1,2159.382353
13,1,13185.979651
13,1,14369.428571
13,1,4398.352941
13,1,1818.242647

13,1,8017.242857
13,1,2615.134615
13,1,3865.877717
13,1,3778.525735
13,1,1838.209302
13,1,2248.565476
13,1,14040.385135
13,1,1829.888889
13,1,1960.810811
13,1,3782.661765
13,1,2394.436224
13,1,6159.741071
13,1,2209.000000
13,1,4705.875000
13,1,8051.380435
13,1,5135.993421
13,1,4471.812500
13,1,1526.975610
13,1,1766.225806
13,1,5778.729167
13,1,1685.572917
13,1,5340.156250
13,1,7457.000000
13,1,5021.162162
13,1,1727.683824
13,1,13730.655405
13,1,5616.625000
13,1,2443.831081
13,1,2732.894737
13,1,1708.563953
13,1,6740.312500
13,1,8512.321429
13,1,4885.010417
13,1,2470.091216
13,1,3859.441860
13,1,3601.142857
13,1,10287.578947
13,1,1932.134615
13,1,5569.244792
13,1,2975.509259
13,1,4318.761905
13,1,5582.112500
13,1,11475.538462
13,1,6680.800000
13,1,7409.670455
13,1,6692.336310
13,1,2454.846939
13,1,5786.324324
13,1,12546.347826
13,1,6626.642857
13,1,8973.867647
13,1,2022.446809
13,1,3520.640000
13,1,2271.320312
13,1,1948.073718
13,1,2943.523810
13,1,3730.702703
13,1,3375.395833
13,1,2219.821429
13,1,2026.762195
13,1,4012.496622
13,1,15002.340000
13,1,3676.375000
13,1,1728.611111
13,1,1638.625000
13,1,5515.894231

13,1,3167.674419
13,1,1595.371795
13,1,2266.963889
13,1,10220.731771
13,1,1719.505682
13,1,14101.260417
13,1,18364.556818
13,1,16940.648438
13,1,7793.000000
13,1,1746.759146
13,1,3923.977273
13,1,3850.761029
13,1,2287.564904
13,1,3916.937500
13,1,13831.844595
13,1,17665.466667
13,1,12716.621875
13,1,6937.312500
13,1,2614.829268
13,1,4985.231250
13,1,5334.914634
13,1,6659.000000
13,1,4342.514535
13,1,6794.470588
13,1,3955.214844
13,1,1812.333333
13,1,6213.359375
13,1,5400.667763
13,1,2463.944853
13,1,2157.631579
13,1,7885.076087
13,1,1893.279070
13,1,10285.405488
13,1,11921.242187
13,1,4368.250000
13,1,12207.428977
13,1,3555.196429
13,1,7771.250000
13,1,1786.094444
13,1,4534.346154
13,1,4362.090909
13,1,3918.097561
13,1,2836.958333
13,1,2585.333333
13,1,5409.151515
13,1,1790.866667
13,1,2490.937500
13,1,2269.980769
13,1,3670.902439
13,1,1654.762500
13,1,5938.378049
13,1,4805.937500
13,1,1761.000000
13,1,2639.764706
13,1,8357.172794
13,1,3335.617647
13,1,7823.781250
13,1,5168.613636
13,1,8124.363636
13,1,1750.441176
13,1,9352.892857
13,1,3523.581395
13,1,3688.125000
13,1,1634.971591
13,1,2866.951613
13,1,16012.000000

13,1,1861.941176
13,1,4343.500000
13,1,3697.293367
13,1,2354.478261
13,1,9326.425000
13,1,2271.295918
13,1,1605.176829
13,1,1727.217949
13,1,2136.250000
13,1,4690.241667
13,1,2740.600000
13,1,1859.454545
13,1,19947.700000
13,1,6479.948171
13,1,9563.312500
13,1,5573.279412
13,1,1786.359375
13,1,11395.736842
13,1,2268.035714
13,1,2734.716667
13,1,2632.750000
13,1,1913.524390
13,1,2201.160714
13,1,4668.901786
13,1,18284.941406
13,1,3414.926829
13,1,7919.385714
13,1,10941.383333
13,1,7919.000000
13,1,13026.315789
13,1,1858.676829
13,1,3718.676282
13,1,12367.644231
13,1,8767.423611
13,1,11791.744318
13,1,3828.723684
13,1,2131.431818
13,1,2962.107143
13,1,2556.428241
13,1,2245.222561
13,1,2868.346154
13,1,2358.594444
13,1,1604.275862
13,1,2506.000000
13,1,3685.750000
13,1,2664.077778
13,1,12619.500000
13,1,1912.142857
13,1,5340.076923
13,1,12374.476744
13,1,10065.702206
13,1,6449.188953
13,1,5869.109756
13,1,5645.000000
13,1,1961.875000
13,1,4837.945946
13,1,2705.267857
13,1,4567.274390
13,1,1734.281250
13,1,8591.888889
13,1,6660.608696
13,1,8109.816176
13,1,2467.444444
13,1,2026.487500
13,1,5284.190104
13,1,10079.049479

13,1,11782.121622
13,1,1511.616071
13,1,4686.487805
13,1,4215.840909
13,1,17513.250000
13,1,3210.712121
13,1,2805.500000
13,1,5385.853448
13,1,2854.503571
13,1,2023.602273
13,1,18215.943182
13,1,6004.164634
13,1,1750.022222
13,1,5608.720109
13,1,2761.915323
13,1,1758.796875
13,1,14250.666667
13,1,1748.714286
13,1,3213.163462
13,1,2004.632353
13,1,3239.644022
13,1,11359.008065
13,1,2511.791667
13,1,1879.781250
13,1,2190.887931
13,1,1567.653846
13,1,3996.000000
13,1,2774.023810
13,1,3294.681452
13,1,6401.500000
13,1,14065.315789
13,1,5229.400735
13,1,6257.750000
13,1,2073.987500
13,1,7605.567568
13,1,3145.647059
13,1,3325.706250
13,1,2988.018382
13,1,2403.175676
13,1,14467.107143
13,1,1764.258065
13,1,5641.288462
13,1,3936.718750
13,1,10464.113281
13,1,1814.894231
13,1,1640.145833
13,1,6999.291667
13,1,7860.781915
13,1,3493.359043
13,1,3110.041667
13,1,2771.636364
13,1,3603.920455
13,1,3489.802632
13,1,10176.487500
13,1,5830.000000
13,1,7856.457317
13,1,5254.258333
13,1,6874.664474
13,1,5979.923611
13,1,4763.940972
13,1,2358.609375
13,1,2047.315789
13,1,1709.424342
13,1,3691.250000
13,1,6059.659722
13,1,4167.132812

13,1,5411.525000
13,1,7688.871324
13,1,3008.978723
13,1,2710.500000
13,1,3656.994792
13,1,11619.226562
13,1,8104.666667
13,1,6309.300000
13,1,18091.953947
13,1,5569.102941
13,1,15099.857143
13,1,4298.198529
13,1,1906.800676
13,1,1693.875000
13,1,12125.325758
13,1,3288.733333
13,1,2807.863636
13,1,1943.500000
13,1,11205.212500
13,1,2174.456250
13,1,17595.691489
13,1,2149.567708
13,1,7297.705882
13,1,4803.618421
13,1,4555.906250
13,1,2713.919118
13,1,6880.920455
13,1,2890.875000
13,1,2655.591912
13,1,2342.784091
13,1,2897.019231
13,1,2818.642442
13,1,6096.565217
13,1,8429.416667
13,1,5238.734375
13,1,3040.719512
13,1,7914.340116
13,1,2675.015957
13,1,2614.069767
13,1,6692.187500
13,1,2011.894231
13,1,4526.855556
13,1,1678.534884
13,1,8648.794872
13,1,3052.941489
13,1,8346.235294
13,1,7959.910714
13,1,2229.040441
13,1,2326.396277
13,1,4148.625000
13,1,5205.904412
13,1,6187.783333
13,1,2431.900000
13,1,1581.731250
13,1,2392.709302
13,1,4332.717949
13,1,1671.666667
13,1,19065.096774
13,1,1954.486842
13,1,1509.482955
13,1,1533.235294
13,1,12947.478261
13,1,5772.116071
13,1,8395.200658
13,1,4678.215909
13,1,10299.364583

13,1,7574.137500
13,1,8173.090909
13,1,2709.243902
13,1,19446.548077
13,1,6005.875000
13,1,2059.750000
13,1,2953.656250
13,1,6243.579787
13,1,5605.829268
13,1,6098.176136
13,1,6897.653226
13,1,2375.808594
13,1,12742.267361
13,1,6183.469945
13,1,14370.250000
13,1,10580.772727
13,1,4214.485119
13,1,1901.784091
13,1,12416.956731
13,1,1609.691860
13,1,4561.534722
13,1,6054.965625
13,1,3404.743421
13,1,15441.679688
13,1,7285.801471
13,1,2495.708333
13,1,6118.880952
13,1,5479.880682
13,1,1723.343750
13,1,7950.689655
13,1,1513.619318
13,1,11867.380435
13,1,1928.909091
13,1,1689.194079
13,1,2734.906250
13,1,1687.725543
13,1,1870.125000
13,1,2733.272727
13,1,2149.337500
13,1,13064.358025
13,1,9313.416667
13,1,2411.099265
13,1,4544.200980
13,1,2750.035156
13,1,8777.856618
13,1,3188.356707
13,1,2859.257812
13,1,7553.453125
13,1,13008.125000
13,1,4355.959239
13,1,12499.432692
13,1,6132.253049
13,1,12308.027439
13,1,6257.852941
13,1,9459.619048
13,1,6885.187500
13,1,5974.000000
13,1,3539.214674
13,1,16911.947917
13,1,1902.421053
13,1,4540.781250
13,1,2721.513889
13,1,11195.887500
13,1,10461.691667
13,1,3880.141304
13,1,2125.816489

13,1,3596.000000
13,1,5273.146875
13,1,1778.268293
13,1,2462.128472
13,1,1836.864583
13,1,2120.365385
13,1,12603.782407
13,1,14018.071023
13,1,2871.823529
13,1,8956.392857
13,1,5127.461538
13,1,4454.073529
13,1,10835.971154
13,1,2322.802083
13,1,4267.709677
13,1,3729.692308
13,1,1688.662500
13,1,6309.250000
13,1,2241.166667
13,1,1802.386792
13,1,5074.113889
13,1,9675.477941
13,1,1721.099359
13,1,14069.838816
13,1,3284.062500
13,1,2129.682692
13,1,1875.946875
13,1,2898.289474
13,1,1935.970588
13,1,1961.740385
13,1,10187.273437
13,1,4438.361111
13,1,4433.197917
13,1,6204.682143
13,1,3294.812500
13,1,2255.650000
13,1,1560.206250
13,1,7531.703125
13,1,4386.987805
13,1,3779.259615
13,1,1956.089286
13,1,2263.613065
13,1,2521.402344
13,1,12577.935897
13,1,2866.205128
13,1,4046.052632
13,1,3121.805556
13,1,1748.093750
13,1,7061.708333
13,1,3158.805195
13,1,5432.500000
13,1,2571.914634
13,1,4554.000000
13,1,5272.350000
13,1,6210.298780
13,1,2991.853659
13,1,3916.992188
13,1,6693.279070
13,1,3167.067308
13,1,4801.772059
13,1,1682.176471
13,1,3270.187500
13,1,3369.780488
13,1,2163.807692
13,1,1538.105263
13,1,1528.100000

13,1,9219.375000
13,1,2463.400000
13,1,2051.375000
13,1,1604.000000
13,1,2678.589286
13,1,10295.989286
13,1,1977.391447
13,1,2925.363636
13,1,4364.705882
13,1,2821.319149
13,1,1663.767045
13,1,2002.640000
13,1,7915.253125
13,1,4208.893617
13,1,13038.095238
13,1,3344.464286
13,1,4174.058824
13,1,6730.035714
13,1,3134.909091
13,1,19698.769737
13,1,3591.303571
13,1,9300.538265
13,1,4523.871212
13,1,4600.066176
13,1,4390.104167
13,1,3464.914634
13,1,1597.257143
13,1,8997.562500
13,1,1932.039634
13,1,1562.243056
13,1,12400.919643
13,1,5263.761905
13,1,4166.517857
13,1,15667.138158
13,1,2735.350000
13,1,16515.875000
13,1,2984.699324
13,1,5341.342593
13,1,2149.196875
13,1,2464.102941
13,1,10571.825000
13,1,8001.146739
13,1,6812.432432
13,1,2703.670732
13,1,1516.600000
13,1,2053.542683
13,1,10811.020833
13,1,4148.453125
13,1,15392.950000
13,1,7255.928030
13,1,2281.088710
13,1,4003.441176
13,1,1663.985294
13,1,4813.750000
13,1,5373.346154
13,1,3421.661765
13,1,1844.743056
13,1,1917.380556
13,1,5732.723684
13,1,4149.103125
13,1,12540.914634
13,1,2065.108696
13,1,9893.047619
13,1,4742.348684
13,1,3900.802521
13,1,1664.230769

13,1,2206.897059
13,1,9291.312500
13,1,1871.744318
13,1,2334.666667
13,1,1916.789773
13,1,1609.864865
13,1,7775.802632
13,1,12518.214286
13,1,3257.725610
13,1,5406.500000
13,1,3372.993750
13,1,1504.593750
13,1,19639.932927
13,1,4655.127660
13,1,1808.714286
13,1,17274.897059
13,1,2846.714286
13,1,1617.465116
13,1,1962.668269
13,1,3007.913043
13,1,7901.701220
13,1,2177.378289
13,1,5706.364286
13,1,1566.538462
13,1,5253.266667
13,1,13560.460938
13,1,8295.643750
13,1,3465.575758
13,1,6053.125000
13,1,3309.538194
13,1,1611.661290
13,1,1690.217262
13,1,4093.000000
13,1,2909.750000
13,1,2527.367647
13,1,1769.119565
13,1,1540.590909
13,1,1761.058824
13,1,4451.023438
13,1,1769.179878
13,1,1828.994565
13,1,1944.317935
13,1,2641.494186
13,1,4953.928571
13,1,8846.697674
13,1,1845.261905
13,1,7357.340909
13,1,4268.952381
13,1,2595.028125
13,1,2709.195312
13,1,3656.560976
13,1,1556.653846
13,1,8786.659091
13,1,2849.388587
13,1,2409.671875
13,1,1520.157895
13,1,1560.336957
13,1,6522.709559
13,1,1941.419118
13,1,7282.480769
13,1,14064.025000
13,1,3618.893617
13,1,1919.984756
13,1,9012.500000
13,1,2626.533784
13,1,2707.044355

13,1,2516.714286
13,1,2519.575000
13,1,2528.625000
13,1,12994.500000
13,1,1582.319853
13,1,2431.840909
13,1,3138.040541
13,1,3074.575658
13,1,5306.972973
13,1,1582.766447
13,1,19841.398437
13,1,2788.734375
13,1,3021.211538
13,1,4100.325000
13,1,5260.741071
13,1,1978.306548
13,1,4384.855263
13,1,3313.500000
13,1,3355.428571
13,1,1991.833333
13,1,4649.576923
13,1,2793.514286
13,1,1776.803030
13,1,2798.100806
13,1,14174.133929
13,1,3968.915323
13,1,2289.625000
13,1,2141.616071
13,1,6182.000000
13,1,1765.318750
13,1,1834.358974
13,1,5243.196429
13,1,2787.133333
13,1,2594.443966
13,1,1708.468085
13,1,8637.000000
13,1,9125.125000
13,1,1935.573718
13,1,10767.436047
13,1,1598.375000
13,1,1919.625000
13,1,1658.538462
13,1,13741.390625
13,1,1761.145833
13,1,8156.750000
13,1,6012.181250
13,1,2592.636364
13,1,2918.128205
13,1,1908.926829
13,1,5981.479167
13,1,2065.187500
13,1,19712.869565
13,1,1577.588889
13,1,4447.375000
13,1,2344.718750
13,1,1727.616667
13,1,3107.655405
13,1,2695.151515
13,1,1714.437500
13,1,1572.737179
13,1,3481.455882
13,1,2628.929487
13,1,14799.282738
13,1,4004.216216
13,1,1562.340426
13,1,4019.857143

13,1,1950.288889
13,1,2439.187500
13,1,3871.304348
13,1,1667.500000
13,1,1874.700000
13,1,1947.455882
13,1,10220.263889
13,1,5245.653846
13,1,2847.450000
13,1,10159.833333
13,1,6792.308824
13,1,10434.437500
13,1,6650.557143
13,1,5220.300000
13,1,11059.892241
13,1,10161.176282
13,1,3981.200000
13,1,18139.262195
13,1,7090.545455
13,1,2711.441176
13,1,2100.806818
13,1,15641.790541
13,1,1821.242647
13,1,3015.945513
13,1,5047.250000
13,1,1766.229167
13,1,3113.312500
13,1,3088.909091
13,1,2251.809211
13,1,4972.937500
13,1,2558.379032
13,1,7212.399194
13,1,5988.786765
13,1,2385.000000
13,1,2750.054054
13,1,3736.750000
13,1,2475.802885
13,1,2554.804878
13,1,5862.812500
13,1,3015.719697
13,1,1732.353659
13,1,3532.630952
13,1,17124.226190
13,1,4657.162791
13,1,2981.165625
13,1,3985.419118
13,1,6912.202128
13,1,8614.344697
13,1,2616.891667
13,1,8009.140625
13,1,10487.172727
13,1,6537.476190
13,1,1562.125000
13,1,3449.957317
13,1,1780.308333
13,1,11213.312500
13,1,2227.159091
13,1,2960.414063
13,1,3123.193182
13,1,2350.925781
13,1,2870.000000
13,1,1679.217105
13,1,7897.470588
13,1,2009.797500
13,1,1945.724359
13,1,3164.786184

13,1,9386.980769
13,1,2545.356618
13,1,5544.945946
13,1,3249.056548
13,1,8047.362805
13,1,2012.643382
13,1,2465.181818
13,1,2723.289062
13,1,3680.858333
13,1,6907.868421
13,1,2643.173780
13,1,9168.980769
13,1,4751.969388
13,1,5301.194444
13,1,3627.757143
13,1,3822.125000
13,1,11127.902778
13,1,6065.883721
13,1,1580.833333
13,1,4409.875000
13,1,1806.208333
13,1,2965.707317
13,1,8031.633721
13,1,3324.511905
13,1,3774.695122
13,1,3440.432692
13,1,11270.285714
13,1,4030.862500
13,1,2293.976562
13,1,6385.718750
13,1,19624.666667
13,1,5196.047414
13,1,1763.184783
13,1,1709.862179
13,1,3615.811111
13,1,7311.714286
13,1,2574.473958
13,1,1797.651163
13,1,5144.690217
13,1,10490.050314
13,1,1629.310976
13,1,15402.825000
13,1,5705.393617
13,1,5882.500000
13,1,4599.131579
13,1,2864.368590
13,1,10585.047794
13,1,1650.500000
13,1,9919.968750
13,1,2639.000000
13,1,3357.350000
13,1,1782.265625
13,1,3548.939189
13,1,3313.634146
13,1,2126.829268
13,1,1986.833333
13,1,2126.617021
13,1,2858.972973
13,1,2075.000000
13,1,3040.619565
13,1,12830.372340
13,1,6240.707317
13,1,2086.785156
13,1,4777.101562
13,1,5203.285714
13,1,8833.900000

13,1,7538.593750
13,1,1507.358696
13,1,13553.921875
13,1,2048.128205
13,1,4865.615385
13,1,4150.020408
13,1,8364.909091
13,1,4526.153846
13,1,10306.400000
13,1,2715.206250
13,1,2093.929688
13,1,3533.493750
13,1,16913.410256
13,1,2791.375000
13,1,4480.409091
13,1,14017.687500
13,1,7355.852273
13,1,4033.250000
13,1,2942.252976
13,1,4596.000000
13,1,1843.768293
13,1,8110.281977
13,1,16065.875000
13,1,3152.071429
13,1,1519.650000
13,1,3082.078125
13,1,10552.281915
13,1,6367.409091
13,1,7207.106618
13,1,2158.333333
13,1,2665.508333
13,1,2619.244186
13,1,1572.347561
13,1,2279.117021
13,1,3218.402778
13,1,2218.841912
13,1,5504.261364
13,1,3029.692308
13,1,3480.763158
13,1,19487.384615
13,1,1586.062500
13,1,3559.404070
13,1,2473.863372
13,1,12877.328125
13,1,5823.621429
13,1,17642.588235
13,1,10604.234848
13,1,1567.125000
13,1,9117.864865
13,1,15500.425781
13,1,1615.219512
13,1,2410.875000
13,1,4256.897059
13,1,1809.641026
13,1,2264.937500
13,1,3429.686275
13,1,2105.150000
13,1,3530.227500
13,1,15153.338235
13,1,2656.394737
13,1,7155.208333
13,1,2354.875000
13,1,1645.958333
13,1,2599.377778
13,1,5112.728571
13,1,2609.092857

13,1,5859.750000
13,1,1674.166667
13,1,1713.510714
13,1,2278.463816
13,1,4827.750000
13,1,1569.558594
13,1,2500.606061
13,1,2250.031250
13,1,2536.158784
13,1,4849.794872
13,1,2734.265306
13,1,1518.390625
13,1,2730.233333
13,1,7386.367647
13,1,7304.458333
13,1,6448.090909
13,1,3230.807692
13,1,1675.208333
13,1,3546.305147
13,1,1875.095238
13,1,1663.747159
13,1,1856.704545
13,1,1754.297619
13,1,2141.286765
13,1,2667.545455
13,1,6298.846154
13,1,9947.775000
13,1,10221.944444
13,1,5022.302632
13,1,15423.946429
13,1,18391.867925
13,1,7386.750000
13,1,9415.268293
13,1,7853.000000
13,1,7089.524390
13,1,8925.410256
13,1,1713.135135
13,1,2654.241379
13,1,1656.792763
13,1,4287.925926
13,1,16389.687500
13,1,1907.050000
13,1,2259.297872
13,1,9744.727273
13,1,2059.687500
13,1,2007.430556
13,1,1692.656977
13,1,3186.203125
13,1,3066.347826
13,1,4451.464286
13,1,4969.742857
13,1,3615.538462
13,1,3709.045213
13,1,8346.310976
13,1,3608.000000
13,1,2854.937500
13,1,1975.382812
13,1,6156.588235
13,1,6153.916667
13,1,1807.015244
13,1,2081.872340
13,1,2465.975000
13,1,1569.127907
13,1,7506.500000
13,1,19559.406250
13,1,1781.316860

13,1,9702.392857
13,1,2669.465116
13,1,2275.142857
13,1,2121.810811
13,1,1649.185811
13,1,1577.372093
13,1,2059.022727
13,1,1816.375000
13,1,8703.169355
13,1,2805.868750
13,1,2554.521277
13,1,8988.406250
13,1,2566.909091
13,1,1509.110577
13,1,1682.022727
13,1,1826.017442
13,1,2037.358696
13,1,5890.435000
13,1,3416.247549
13,1,3196.711957
13,1,1995.256098
13,1,2760.979592
13,1,4135.237245
13,1,5860.716667
13,1,3200.082386
13,1,13708.335938
13,1,2111.746324
13,1,1504.672794
13,1,7462.904762
13,1,1813.312500
13,1,1580.762195
13,1,2435.887500
13,1,4157.991279
13,1,3068.295455
13,1,6635.694767
13,1,2069.625000
13,1,2520.500000
13,1,9189.504717
13,1,4542.486486
13,1,9280.952703
13,1,9978.473958
13,1,2524.798077
13,1,3268.976744
13,1,2982.553030
13,1,7667.612245
13,1,4413.279412
13,1,5016.928571
13,1,4834.492925
13,1,2415.333333
13,1,13091.959677
13,1,4046.648256
13,1,11769.364130
13,1,15564.323529
13,1,2134.243590
13,1,1679.489796
13,1,3060.750000
13,1,3175.761905
13,1,2140.652439
13,1,7482.386719
13,1,3787.825581
13,1,9786.875000
13,1,1758.031250
13,1,1501.392442
13,1,4251.671875
13,1,1740.051282
13,1,2289.583333

13,1,7966.547619
13,1,4254.484848
13,1,2360.625000
13,1,16310.026042
13,1,1833.475806
13,1,2676.800000
13,1,13367.211806
13,1,6654.937500
13,1,2926.334559
13,1,1504.625000
13,1,3444.108696
13,1,2010.973684
13,1,2207.046875
13,1,1526.142857
13,1,1717.039773
13,1,10609.815000
13,1,1943.447368
13,1,2907.958333
13,1,1737.571429
13,1,3200.808594
13,1,3667.988636
13,1,14427.475962
13,1,1697.208029
13,1,11629.789286
13,1,1663.360849
13,1,2283.744186
13,1,11491.769737
13,1,3342.993243
13,1,6734.938830
13,1,3366.581250
13,1,3706.111111
13,1,2340.605000
13,1,1513.938312
13,1,4509.267442
13,1,3283.234375
13,1,12887.900424
13,1,5558.052632
13,1,2481.150568
13,1,2361.265625
13,1,5136.627907
13,1,2327.179054
13,1,1704.408088
13,1,5580.515625
13,1,3008.005556
13,1,2435.181818
13,1,3174.128378
13,1,4586.102564
13,1,8304.250000
13,1,3397.176724
13,1,4053.799419
13,1,1594.846154
13,1,5799.986842
13,1,11583.664474
13,1,2199.397436
13,1,5790.477273
13,1,3221.031250
13,1,2386.658537
13,1,14691.386905
13,1,2058.765625
13,1,3092.445946
13,1,1901.513514
13,1,2596.125000
13,1,5904.809524
13,1,15206.201087
13,1,5521.576705
13,1,7199.051282

13,1,14968.834459
13,1,2081.487805
13,1,1824.602564
13,1,2458.063953
13,1,1518.464286
13,1,5625.206250
13,1,3584.494565
13,1,1934.948370
13,1,7799.942308
13,1,2321.750000
13,1,14796.500000
13,1,4442.870370
13,1,4331.312500
13,1,1735.776786
13,1,2960.324468
13,1,1972.000000
13,1,1566.596154
13,1,3779.500000
13,1,10391.875000
13,1,12626.062500
13,1,13824.052632
13,1,1785.125000
13,1,10239.796875
13,1,1721.272727
13,1,2296.081633
13,1,6474.177885
13,1,4278.214286
13,1,1800.195652
13,1,6516.127551
13,1,1933.000000
13,1,1998.092593
13,1,2071.024390
13,1,4088.375000
13,1,2572.279412
13,1,6188.841837
13,1,3795.800000
13,1,1733.586538
13,1,14137.368750
13,1,3676.357143
13,1,6898.677419
13,1,3415.514706
13,1,4471.720930
13,1,3117.062500
13,1,2035.316489
13,1,4915.781250
13,1,5505.000000
13,1,7491.823529
13,1,4618.441176
13,1,12534.396429
13,1,2133.500000
13,1,1703.900862
13,1,1708.914286
13,1,2474.078947
13,1,2390.432065
13,1,1722.712963
13,1,8617.875000
13,1,1921.690476
13,1,3887.587500
13,1,1999.909091
13,1,2489.955645
13,1,2610.467391
13,1,3579.552778
13,1,9232.544118
13,1,11307.612805
13,1,6423.502778
13,1,2719.750000

13,1,9107.728125
13,1,7514.632500
13,1,3634.512500
13,1,15484.641026
13,1,10011.960000
13,1,1687.927632
13,1,2420.521277
13,1,8022.671053
13,1,1868.000000
13,1,2455.913043
13,1,4858.112500
13,1,1552.345238
13,1,1578.901163
13,1,4504.178571
13,1,3917.500000
13,1,4082.750000
13,1,3144.307692
13,1,3257.162500
13,1,3196.720930
13,1,9571.089674
13,1,1809.218750
13,1,1615.489362
13,1,5468.642857
13,1,5241.000000
13,1,8434.617188
13,1,4518.308511
13,1,8041.219595
13,1,1745.308511
13,1,10390.744186
13,1,10903.401316
13,1,3394.375000
13,1,8825.650000
13,1,2036.668269
13,1,2252.095588
13,1,5758.410256
13,1,6884.750000
13,1,1792.500000
13,1,1572.258152
13,1,2328.684211
13,1,3062.203125
13,1,12208.901316
13,1,3613.173077
13,1,2131.729730
13,1,2152.529605
13,1,2185.666667
13,1,3531.577381
13,1,4842.764706
13,1,3245.589674
13,1,1763.456395
13,1,1831.806452
13,1,3161.875000
13,1,2945.853659
13,1,2265.341912
13,1,6760.175000
13,1,3282.429167
13,1,2568.968085
13,1,7052.428571
13,1,7396.572222
13,1,6277.318182
13,1,8214.180556
13,1,3128.964286
13,1,3251.816176
13,1,4387.388514
13,1,6224.385204
13,1,14508.464286
13,1,7356.625000

13,1,1535.466912
13,1,8483.250000
13,1,1879.585106
13,1,16628.183673
13,1,10889.398936
13,1,4925.562500
13,1,3170.963235
13,1,4600.540323
13,1,6313.884615
13,1,6224.177083
13,1,2942.931452
13,1,1598.843085
13,1,3052.567568
13,1,3304.976974
13,1,5879.510000
13,1,2312.859375
13,1,1573.096154
13,1,12244.375000
13,1,3243.220395
13,1,7536.706250
13,1,4739.684211
13,1,1684.335938
13,1,5924.056250
13,1,10466.229730
13,1,4012.698529
13,1,4082.990132
13,1,4275.275000
13,1,14388.678571
13,1,10473.583333
13,1,1859.416667
13,1,7216.534091
13,1,1978.861702
13,1,7885.007143
13,1,2510.806818
13,1,3389.229730
13,1,1940.193750
13,1,19364.823529
13,1,1781.625000
13,1,5772.786765
13,1,1703.545455
13,1,18358.000000
13,1,2058.683140
13,1,1958.750000
13,1,12919.781250
13,1,6485.453125
13,1,3611.312500
13,1,1802.019022
13,1,2113.214286
13,1,2761.404762
13,1,12016.125000
13,1,1508.156250
13,1,5367.851351
13,1,7925.544118
13,1,2978.551630
13,1,1975.083333
13,1,3018.000000
13,1,3868.925000
13,1,3697.816667
13,1,1996.887500
13,1,8286.950000
13,1,1881.357143
13,1,2346.728261
13,1,1763.182927
13,1,2016.527778
13,1,4165.711538
13,1,5478.294118

13,1,4279.627907
13,1,10276.464674
13,1,4011.201923
13,1,10443.175532
13,1,6811.166667
13,1,1665.166667
13,1,1638.236111
13,1,1613.300000
13,1,2287.250000
13,1,2391.532609
13,1,11574.333333
13,1,1571.562500
13,1,3207.250000
13,1,5070.352941
13,1,12262.722222
13,1,1503.903302
13,1,2774.541667
13,1,6085.015000
13,1,1909.593750
13,1,7493.754717
13,1,2057.162162
13,1,2392.000000
13,1,2879.190217
13,1,3250.921053
13,1,6160.390957
13,1,4776.182500
13,1,3721.711538
13,1,2514.365854
13,1,3962.576923
13,1,2948.671875
13,1,12576.212500
13,1,2256.663194
13,1,1688.363636
13,1,9211.300000
13,1,8014.850000
13,1,8049.168750
13,1,4402.190789
13,1,17854.805233
13,1,2843.538462
13,1,4452.169118
13,1,2690.432692
13,1,11274.822368
13,1,2771.871711
13,1,6656.394444
13,1,5229.371711
13,1,4980.857143
13,1,1538.086957
13,1,2824.333333
13,1,6946.000000
13,1,2381.163690
13,1,18912.967742
13,1,1675.036458
13,1,1571.779412
13,1,16704.993590
13,1,1872.730769
13,1,1990.763889
13,1,2226.125000
13,1,2761.923077
13,1,2052.966667
13,1,15978.954545
13,1,1845.156250
13,1,10994.971154
13,1,2715.255435
13,1,8394.457317
13,1,1556.091667
13,1,2106.019737

13,1,16883.148438
13,1,2416.041667
13,1,1551.097561
13,1,3821.405000
13,1,3921.097561
13,1,1706.912791
13,1,6680.831731
13,1,1524.178977
13,1,7778.720395
13,1,4062.935606
13,1,2817.246951
13,1,6467.528846
13,1,4082.975000
13,1,11796.086957
13,1,3756.546512
13,1,5027.000000
13,1,13507.933333
13,1,7435.967742
13,1,2317.981771
13,1,1851.352941
13,1,2961.650000
13,1,12627.119565
13,1,3514.269231
13,1,3178.220930
13,1,10006.882353
13,1,1768.767857
13,1,5405.931818
13,1,2469.308824
13,1,1917.083333
13,1,5963.618421
13,1,2114.821429
13,1,7874.176136
13,1,11229.912162
13,1,1912.371429
13,1,1663.515244
13,1,7964.059375
13,1,5694.200893
13,1,4709.237805
13,1,1821.666667
13,1,3393.095000
13,1,10586.278571
13,1,2533.206522
13,1,8246.886364
13,1,5253.828125
13,1,2023.312500
13,1,3815.000000
14,2,1551.740566
14,2,1563.263889
14,2,2263.175000
14,2,5718.800926
14,2,8417.250000
14,2,2234.659091
14,2,3326.000000
14,2,1692.587500
14,2,2068.416667
14,2,2518.505814
14,2,7986.112245
14,2,3583.021739
14,2,1584.996951
14,2,2612.225000
14,2,1762.890000
14,2,2340.767857
14,2,2020.941964
14,2,3380.054348
14,2,2231.983553
14,2,2741.468750

14,2,3223.545000
14,2,1642.926471
14,2,1679.587766
14,2,1869.537162
14,2,4129.222222
14,2,6150.025862
14,2,1696.772727
14,2,2738.882812
14,2,8436.497283
14,2,1894.750000
14,2,1791.973404
14,2,1605.700000
14,2,1859.445122
14,2,2173.669643
14,2,2806.090426
14,2,2176.552326
14,2,1529.195000
14,2,2640.053030
14,2,1628.574074
14,2,14780.903846
14,2,4145.625000
14,2,2913.805556
14,2,2016.015625
14,2,1610.753676
14,2,1637.273585
14,2,1988.812500
14,2,5870.135000
14,2,1833.867925
14,2,2453.760870
14,2,1656.419643
14,2,2081.482394
14,2,1644.448980
14,2,2014.770349
14,2,2653.190217
14,2,1980.264423
14,2,1523.260417
14,2,2642.735465
14,2,1528.416667
14,2,4246.597222
14,2,10054.296875
14,2,1639.516304
14,2,1543.947917
14,2,4547.732639
14,2,8150.320000
14,2,2273.896277
14,2,2597.347826
14,2,5415.610294
14,2,7105.531250
14,2,2311.479730
14,2,2278.852941
14,2,2023.337719
14,2,3429.173295
14,2,1714.338542
14,2,2308.190476
14,2,2376.308140
14,2,1822.567935
14,2,1599.688830
14,2,7561.239583
14,2,2852.718750
14,2,1737.406250
14,2,1500.500000
14,2,1638.205882
14,2,1648.533784
14,2,1642.078125
14,2,4490.385638
14,2,1683.316327

14,2,5978.441176
14,2,1514.066667
14,2,1595.781250
14,2,10611.243243
14,2,3818.201754
14,2,2999.018182
14,2,1587.446429
14,2,5214.057927
14,2,2405.235294
14,2,1908.273256
14,2,1958.031915
14,2,1760.545455
14,2,2195.390957
14,2,4277.252315
14,2,1594.339286
14,2,5266.711538
14,2,1752.299528
14,2,2117.659091
14,2,1505.861702
14,2,1654.191406
14,2,2304.657895
14,2,2163.510135
14,2,1989.682927
14,2,5088.884615
14,2,2687.559211
14,2,1539.527273
14,2,1753.430288
14,2,2568.399038
14,2,1583.000000
14,2,2172.081250
14,2,3845.972222
14,2,1744.500000
14,2,2257.507979
14,2,3380.767857
14,2,2653.075000
14,2,1645.677419
14,2,1970.350000
14,2,3243.000000
14,2,2405.617500
14,2,2079.565217
14,2,15053.650000
14,2,1582.244681
14,2,1670.031250
14,2,1566.089820
14,2,1527.818182
14,2,1653.844512
14,2,3176.188953
14,2,2900.944444
14,2,1846.362805
14,2,1604.072368
14,2,4421.520833
14,2,2139.285714
14,2,1864.036111
14,2,1715.980769
14,2,2935.292683
14,2,1500.163194
14,2,6365.900000
14,2,2144.292453
14,2,1579.657407
14,2,1577.730769
14,2,2533.546875
14,2,1994.791667
14,2,1770.656250
14,2,2652.487805
14,2,1509.782051
14,2,1739.802326

14,2,1860.155000
14,2,2265.046875
14,2,1730.826923
14,2,1960.688995
14,2,1985.728723
14,2,2173.628049
14,2,2532.245098
14,2,2836.819149
14,2,3147.782895
14,2,3092.793956
14,2,1675.912500
14,2,2250.300000
14,2,1534.000000
14,2,8719.803571
14,2,2034.500000
14,2,2485.377551
14,2,1829.025641
14,2,2705.142857
14,2,2087.966667
14,2,1519.522959
14,2,1755.687500
14,2,2590.296296
14,2,1509.250000
14,2,1856.326705
14,2,2832.353125
14,2,1782.729167
14,2,2895.735577
14,2,1563.807692
14,2,18764.800000
14,2,1782.190476
14,2,2563.106481
14,2,2205.615385
14,2,1557.726563
14,2,2372.837838
14,2,2671.221154
14,2,2757.238372
14,2,11153.864130
14,2,3495.360465
14,2,9136.243750
14,2,1917.700000
14,2,2049.461207
14,2,2964.514151
14,2,1824.538462
14,2,3673.547872
14,2,1753.880000
14,2,2281.226562
14,2,4022.036765
14,2,1715.291667
14,2,6592.656977
14,2,2076.185185
14,2,1559.622093
14,2,2699.087500
14,2,3022.368243
14,2,1522.312500
14,2,2148.883721
14,2,1768.868590
14,2,1569.211207
14,2,5051.823529
14,2,2047.437500
14,2,7188.806604
14,2,3210.218750
14,2,1621.312500
14,2,1585.938776
14,2,3101.542553
14,2,1638.766667
14,2,1662.000000

14,2,1567.563830
14,2,2980.927966
14,2,1562.000000
14,2,3259.808673
14,2,2983.170732
14,2,2050.940341
14,2,1684.463816
14,2,2039.710106
14,2,2478.977273
14,2,1706.320000
14,2,1715.930556
14,2,1647.683824
14,2,1782.225806
14,2,1530.981250
14,2,1951.391667
14,2,1630.061111
14,2,2571.328947
14,2,1580.437500
14,2,2981.925926
14,2,2211.500000
14,2,1698.360000
14,2,1533.023923
14,2,1917.333333
14,2,1549.086957
14,2,1952.656250
14,2,1746.720238
14,2,1510.791667
14,2,1841.068182
14,2,2730.750000
14,2,9441.975000
14,2,2295.775000
14,2,2267.095238
14,2,2669.140909
14,2,14530.625000
14,2,5401.897959
14,2,1747.606818
14,2,1767.010000
14,2,2022.713889
14,2,1607.000000
14,2,3270.588889
14,2,3489.387255
14,2,2138.432292
14,2,2791.920213
14,2,1951.578704
14,2,5259.426887
14,2,3749.937500
14,2,2534.081395
14,2,2055.404762
14,2,3412.476974
14,2,6092.201923
14,2,1617.409884
14,2,5075.352041
14,2,3066.812500
14,2,5588.989583
14,2,1588.014151
14,2,2814.905830
14,2,1500.633152
14,2,1535.925532
14,2,1803.974265
14,2,1917.197222
14,2,1709.808824
14,2,1814.793750
14,2,1950.434783
14,2,1760.716216
14,2,1549.414634
14,2,9853.853659

14,2,1499.719388
14,2,2267.555556
14,2,1817.957447
14,2,1979.846939
14,2,1533.596774
14,2,3194.468085
14,2,3659.520349
14,2,1509.832335
14,2,1568.696809
14,2,2262.513514
14,2,1790.872727
14,2,1773.692308
14,2,3098.565217
14,2,1747.986702
14,2,1869.639535
14,2,2222.787234
14,2,7489.866477
14,2,1844.778302
14,2,1527.978125
14,2,2025.421512
14,2,7397.813953
14,2,2410.851064
14,2,1937.320000
14,2,2708.160000
14,2,1789.845238
14,2,2917.310000
14,2,2473.466346
14,2,2957.109375
14,2,1838.833333
14,2,2766.356383
14,2,6118.038462
14,2,1547.524390
14,2,5550.253788
14,2,2676.093750
14,2,3181.903846
14,2,2494.250000
14,2,1774.216146
14,2,2542.505319
14,2,2332.585106
14,2,1780.631250
14,2,3620.576923
14,2,2236.505000
14,2,2754.089912
14,2,3704.095745
14,2,1641.077703
14,2,1802.647059
14,2,1717.939655
14,2,3645.615385
14,2,7820.625000
14,2,4725.851064
14,2,2365.469512
14,2,2540.519231
14,2,1569.838542
14,2,6017.761905
14,2,4752.675532
14,2,1735.299419
14,2,5645.404412
14,2,3806.718085
14,2,2899.020408
14,2,5214.035000
14,2,1531.487245
14,2,1507.445000
14,2,4306.354167
14,2,2788.281250
14,2,1805.711538
14,2,1504.580000

14,2,2033.464744
14,2,5486.197222
14,2,1746.684375
14,2,2926.441176
14,2,5101.518229
14,2,4485.900568
14,2,1689.921429
14,2,1663.457386
14,2,2231.861702
14,2,18639.678571
14,2,4144.288462
14,2,2158.470930
14,2,1939.161111
14,2,1651.628378
14,2,2210.265625
14,2,1555.010417
14,2,1730.083333
14,2,1654.209559
14,2,1718.680233
14,2,2093.250000
14,2,2512.910714
14,2,1647.760204
14,2,1592.875000
14,2,10020.034483
14,2,3034.812500
14,2,2577.414634
14,2,2418.359694
14,2,2649.013889
14,2,7847.686275
14,2,1915.491279
14,2,2319.205357
14,2,2770.724490
14,2,2135.905405
14,2,2116.125000
14,2,9848.000000
14,2,2611.870968
14,2,3557.279070
14,2,1581.440476
14,2,1858.759146
14,2,3838.110000
14,2,3462.105263
14,2,1899.950000
14,2,1799.588816
14,2,3423.452128
14,2,1770.825000
14,2,1562.772727
14,2,1634.894737
14,2,2868.203125
14,2,1790.218023
14,2,1597.738095
14,2,1529.535714
14,2,1524.294643
14,2,1894.828947
14,2,6692.178571
14,2,1980.615854
14,2,2783.810606
14,2,2572.673913
14,2,2582.485294
14,2,1701.366071
14,2,6590.385417
14,2,14704.543478
14,2,2855.619048
14,2,1860.031250
14,2,1724.000000
14,2,2902.063953
14,2,2170.730000

14,2,2889.200000
14,2,1981.648148
14,2,1656.043478
14,2,2725.909091
14,2,5609.239130
14,2,3026.479592
14,2,2034.978723
14,2,2665.704545
14,2,2146.194767
14,2,1578.878205
14,2,1739.905405
14,2,2118.717105
14,2,1845.888158
14,2,2500.508929
14,2,1527.768617
14,2,1567.554487
14,2,1520.326389
14,2,2075.750000
14,2,1521.714744
14,2,2752.180288
14,2,1590.707143
14,2,1847.446809
14,2,2968.363208
14,2,5680.041667
14,2,3361.870968
14,2,2295.208333
14,2,1589.786585
14,2,1734.606250
14,2,6142.862245
14,2,3919.064103
14,2,1883.907609
14,2,3373.406818
14,2,2389.350000
14,2,2303.530660
14,2,1975.235294
14,2,1548.558140
14,2,2039.697917
14,2,1757.294872
14,2,2267.750000
14,2,2835.345745
14,2,2279.389535
14,2,3009.856707
14,2,1620.163265
14,2,3943.053191
14,2,6325.843750
14,2,1785.638298
14,2,1897.266304
14,2,1864.756757
14,2,2943.888298
14,2,2614.444444
14,2,2276.500000
14,2,1826.586957
14,2,1912.135135
14,2,2396.787234
14,2,1871.202586
14,2,2071.703125
14,2,1709.571429
14,2,1506.755814
14,2,2188.854730
14,2,6836.125000
14,2,3229.535714
14,2,1786.453488
14,2,1948.727500
14,2,1659.673077
14,2,2436.544118
14,2,1708.640000

14,2,3894.454545
14,2,1526.166667
14,2,2553.470238
14,2,2085.122549
14,2,1527.953947
14,2,2663.151786
14,2,1525.916667
14,2,1919.530488
14,2,3356.947368
14,2,2264.063725
14,2,1615.764706
14,2,2692.693627
14,2,1818.375000
14,2,1654.466346
14,2,1727.908163
14,2,3857.599057
14,2,2725.232143
14,2,1904.542553
14,2,1553.195122
14,2,1840.702128
14,2,1688.250000
14,2,1563.989865
14,2,4014.525000
14,2,1942.478723
14,2,1950.172872
14,2,1504.250000
14,2,2474.758333
14,2,1638.818182
14,2,1556.743243
14,2,1891.110119
14,2,4291.291667
14,2,2128.183824
14,2,3181.945000
14,2,1982.420918
14,2,1842.798913
14,2,2893.877155
14,2,5788.897196
14,2,13376.500000
14,2,1686.691327
14,2,8517.972222
14,2,2214.195946
14,2,2290.220930
14,2,1893.069767
14,2,3116.198864
14,2,2015.370192
14,2,2354.584906
14,2,1798.674419
14,2,3320.943966
14,2,1553.238095
14,2,1529.400000
14,2,2246.272059
14,2,2063.500000
14,2,2567.681818
14,2,2104.422222
14,2,1810.800000
14,2,1581.421053
14,2,3625.600000
14,2,2329.187500
14,2,1690.386905
14,2,1558.390625
14,2,2686.991071
14,2,2396.367347
14,2,1581.418367
14,2,2933.750000
14,2,2648.128342
14,2,1718.916667

14,2,5758.381818
14,2,2825.540698
14,2,2188.840625
14,2,2489.721591
14,2,3004.562500
14,2,1621.020833
14,2,1585.906250
14,2,1582.604167
14,2,3677.361842
14,2,1885.212302
14,2,1823.756098
14,2,2740.222826
14,2,3969.635204
14,2,1656.107143
14,2,3731.235294
14,2,2825.142857
14,2,2048.760417
14,2,1834.809375
14,2,2162.338068
14,2,2094.500000
14,2,1748.746875
14,2,3663.750000
14,2,2643.882500
14,2,3653.437500
14,2,3194.270000
14,2,1991.108491
14,2,1801.885638
14,2,2274.964286
14,2,1803.223684
14,2,1995.593750
14,2,1852.730000
14,2,1631.437500
14,2,3176.375000
14,2,1663.886905
14,2,9769.714286
14,2,3329.677419
14,2,1882.445755
14,2,2863.192073
14,2,1530.828488
14,2,2279.061224
14,2,1618.946809
14,2,2007.620536
14,2,2089.554688
14,2,1894.791667
14,2,1873.476744
14,2,3242.475000
14,2,1505.229167
14,2,1624.979167
14,2,1591.581081
14,2,1603.500000
14,2,1994.720930
14,2,1562.596354
14,2,2054.051282
14,2,1578.734043
14,2,1750.686364
14,2,13730.727273
14,2,1721.324324
14,2,1559.164773
14,2,1726.765714
14,2,3649.556548
14,2,1810.263158
14,2,2136.692308
14,2,3163.820000
14,2,1980.380000
14,2,3383.210714
14,2,1817.244681

14,2,1512.372024
14,2,1683.063725
14,2,2087.070312
14,2,2350.583333
14,2,1863.304878
14,2,1555.086538
14,2,1662.446429
14,2,2242.775862
14,2,2344.395349
14,2,1527.000000
14,2,3651.437500
14,2,1950.328125
14,2,2709.000000
14,2,1956.963235
14,2,1581.940476
14,2,1764.166667
14,2,2292.205446
14,2,2276.833333
14,2,2036.576923
14,2,1943.345395
14,2,1708.204861
14,2,2662.697917
14,2,2921.883152
14,2,2329.858333
14,2,2658.697674
14,2,1673.902439
14,2,3368.664062
14,2,2275.088942
14,2,1974.369565
14,2,1924.500000
14,2,2025.785714
14,2,1835.090278
14,2,3310.829268
14,2,1609.495098
14,2,2538.548718
14,2,4437.445652
14,2,1623.773438
14,2,1976.520000
14,2,2389.018293
14,2,3135.916667
14,2,1693.827381
14,2,2741.572581
14,2,2089.138889
14,2,1653.606383
14,2,1858.291667
14,2,1500.709302
14,2,3060.508523
14,2,2996.166667
14,2,2728.880208
14,2,9030.166667
14,2,2060.404255
14,2,2248.455729
14,2,9044.312500
14,2,2513.808511
14,2,1567.961538
14,2,1648.676829
14,2,1855.510870
14,2,2241.104167
14,2,1542.039216
14,2,1930.543103
14,2,2144.036364
14,2,5849.869681
14,2,2706.179245
14,2,1714.523437
14,2,2614.308140
14,2,4393.539474

14,2,2439.339286
14,2,1663.072917
14,2,1766.270833
14,2,1570.644737
14,2,1886.349490
14,2,4210.783784
14,2,1582.265306
14,2,1521.122596
14,2,1609.023810
14,2,1847.252778
14,2,1503.750000
14,2,2708.194444
14,2,1568.325000
14,2,1600.125000
14,2,1906.527778
14,2,2703.286458
14,2,2496.500000
14,2,3056.012500
14,2,1631.520270
14,2,1644.559375
14,2,3654.760417
14,2,4531.523707
14,2,2972.916667
14,2,1569.106383
14,2,2762.433673
14,2,4306.836957
14,2,2044.214286
14,2,2645.007812
14,2,1535.820312
14,2,3297.312500
14,2,1499.011364
14,2,4061.256098
14,2,1939.635000
14,2,1509.072115
14,2,3441.743304
14,2,1734.411765
14,2,2015.410714
14,2,1546.664634
14,2,2540.599338
14,2,1969.000000
14,2,1511.912500
14,2,3098.132353
14,2,4177.745098
14,2,2787.755952
14,2,3650.283333
14,2,12386.000000
14,2,1743.169118
14,2,1754.972222
14,2,5163.529412
14,2,2060.291667
14,2,5555.227273
14,2,6460.739583
14,2,1771.227041
14,2,1958.317308
14,2,1852.157895
14,2,1659.227041
14,2,1618.280405
14,2,2961.319149
14,2,11915.982843
14,2,2014.388298
14,2,1638.239130
14,2,1578.548077
14,2,1519.218750
14,2,2727.162791
14,2,1672.000000
14,2,2769.375000

14,2,2155.625000
14,2,3994.341463
14,2,3118.167614
14,2,2480.481481
14,2,1512.333333
14,2,5327.812500
14,2,1861.115385
14,2,2859.960227
14,2,1572.625000
14,2,1653.189815
14,2,2549.468750
14,2,2152.235294
14,2,1802.051020
14,2,3108.039216
14,2,3253.747768
14,2,2212.512195
14,2,1873.720930
14,2,2859.089744
14,2,5004.200581
14,2,2572.655556
14,2,14120.473485
14,2,2908.704787
14,2,1837.135135
14,2,1797.106061
14,2,2463.870000
14,2,1938.344262
14,2,1838.875000
14,2,2941.110000
14,2,1579.444444
14,2,2938.808511
14,2,2463.260000
14,2,2882.489865
14,2,4491.185897
14,2,2729.659091
14,2,1507.056818
14,2,3267.428571
14,2,3239.687500
14,2,1559.089623
14,2,2405.960227
14,2,1722.290323
14,2,1978.960526
14,2,1582.918478
14,2,2319.638889
14,2,1712.697917
14,2,5014.727083
14,2,2591.843750
14,2,2325.689189
14,2,1621.875000
14,2,2296.872340
14,2,2370.354167
14,2,1887.088889
14,2,2529.110294
14,2,2099.063889
14,2,2666.770408
14,2,2025.410714
14,2,2172.750000
14,2,2180.178571
14,2,2066.860000
14,2,3337.804878
14,2,5085.864130
14,2,1996.142857
14,2,3139.860577
14,2,1578.048780
14,2,2841.652439
14,2,1534.615385
14,2,1995.323864

14,2,2392.442568
14,2,2561.989286
14,2,1545.500000
14,2,7687.293478
14,2,1653.216346
14,2,1702.877660
14,2,1942.375000
14,2,7935.222826
14,2,2175.078125
14,2,1753.333333
14,2,4890.858696
14,2,4086.535714
14,2,2389.750000
14,2,3098.148438
14,2,1537.200000
14,2,1827.481132
14,2,3144.463415
14,2,1932.250000
14,2,2056.338415
14,2,3370.967105
14,2,3353.209906
14,2,2069.380319
14,2,1848.367347
14,2,7145.590909
14,2,1619.520833
14,2,2100.080000
14,2,1803.867647
14,2,5337.637500
14,2,4311.857143
14,2,5101.231707
14,2,1947.036765
14,2,2006.175676
14,2,2080.319444
14,2,4134.437500
14,2,4303.320755
14,2,1790.521739
14,2,1791.387500
14,2,1811.554545
14,2,2046.490196
14,2,3809.970588
14,2,3198.659574
14,2,1520.521739
14,2,1901.574468
14,2,2539.375000
14,2,2530.413194
14,2,7496.583333
14,2,2856.957547
14,2,2240.232558
14,2,2033.874449
14,2,2499.632653
14,2,1681.406250
14,2,2940.553571
14,2,2025.447674
14,2,2743.716981
14,2,1518.308511
14,2,3802.012931
14,2,2142.527778
14,2,1519.931452
14,2,2293.489583
14,2,1686.142157
14,2,3229.779070
14,2,10210.892857
14,2,1737.147727
14,2,1565.144886
14,2,2459.334906
14,2,1736.537736

14,2,7267.269231
14,2,1844.640244
14,2,1944.281250
14,2,2208.759615
14,2,2385.596154
14,2,1801.000000
14,2,4660.467593
14,2,1624.218750
14,2,1636.186170
14,2,1594.071429
14,2,1933.617647
14,2,2319.215426
14,2,2128.616279
14,2,1739.085000
14,2,4137.003049
14,2,5498.866848
14,2,1958.936224
14,2,1550.716216
14,2,3876.505102
14,2,2574.472222
14,2,1518.156250
14,2,3281.415094
14,2,2269.627907
14,2,1597.512500
14,2,4041.865000
14,2,3919.769231
14,2,3315.120690
14,2,2842.045918
14,2,1622.328125
14,2,1648.750000
14,2,2139.365385
14,2,1633.226562
14,2,2056.684049
14,2,3814.736842
14,2,2282.197674
14,2,3767.165865
14,2,1949.061224
14,2,1986.231707
14,2,2859.916667
14,2,1781.411932
14,2,1844.750000
14,2,1603.816327
14,2,2331.980263
14,2,1624.709821
14,2,2028.406250
14,2,1907.346154
14,2,1666.606250
14,2,1784.569444
14,2,2643.597826
14,2,2585.552500
14,2,2267.886905
14,2,1918.386719
14,2,1751.221939
14,2,1533.913462
14,2,2572.416667
14,2,1766.490854
14,2,4598.603175
14,2,2001.909884
14,2,2389.895000
14,2,1567.937500
14,2,1595.535714
14,2,1865.101744
14,2,1538.170455
14,2,2430.375000
14,2,1501.911765
14,2,3052.378676

14,2,1976.291667
14,2,1680.070652
14,2,2726.686813
14,2,5100.480000
14,2,2184.105263
14,2,2449.750000
14,2,1513.836538
14,2,1670.447115
14,2,2295.875000
14,2,2583.221698
14,2,2013.908163
14,2,1801.462766
14,2,1565.131579
14,2,4298.316038
14,2,2669.050000
14,2,4216.819079
14,2,1582.211538
14,2,2294.938776
14,2,1659.942308
14,2,1738.859694
14,2,3893.522059
14,2,1615.500000
14,2,1818.902778
14,2,2400.944444
14,2,4463.795000
14,2,2306.136364
14,2,2296.605263
14,2,2765.783537
14,2,2376.993590
14,2,1858.485714
14,2,1817.655172
14,2,1540.416667
14,2,3884.163462
14,2,1708.430921
14,2,1684.387195
14,2,1572.708333
14,2,1569.609756
14,2,2319.463636
14,2,1735.377660
14,2,2215.292683
14,2,1718.017544
14,2,1922.555556
14,2,1818.611111
14,2,1594.975000
14,2,4823.777439
14,2,1603.996324
14,2,5133.380117
14,2,2264.021277
14,2,2006.402778
14,2,1630.825000
14,2,1648.214286
14,2,2637.177632
14,2,1821.900000
14,2,4453.477941
14,2,2843.470588
14,2,5214.589623
14,2,1576.029070
14,2,1692.527027
14,2,3612.005952
14,2,2464.020408
14,2,1827.875000
14,2,2040.971591
14,2,1586.087500
14,2,3717.330882
14,2,2509.412791
14,2,4886.196429

14,2,3389.459559
14,2,2525.543269
14,2,6692.424479
14,2,7055.723684
14,2,3278.844388
14,2,2613.022541
14,2,1943.915816
14,2,2221.250000
14,2,1695.886905
14,2,1715.948718
14,2,1704.024390
14,2,2125.576389
14,2,1634.161458
14,2,1547.807692
14,2,2667.416667
14,2,1721.435897
14,2,6373.432692
14,2,1814.545455
14,2,1516.542553
14,2,1608.704545
14,2,2041.889706
14,2,2515.544872
14,2,1834.734043
14,2,2506.427907
14,2,1979.090426
14,2,2031.031250
14,2,2392.541667
14,2,1736.472222
14,2,1553.927083
14,2,3217.636364
14,2,1699.566667
14,2,3756.250000
14,2,9195.000000
14,2,1718.602941
14,2,1750.687500
14,2,4191.729730
14,2,1575.319079
14,2,2187.500000
14,2,2596.917553
14,2,2465.901163
14,2,2157.076754
14,2,1639.833333
14,2,9088.111486
14,2,1501.255319
14,2,1991.750000
14,2,1766.556122
14,2,3229.340909
14,2,1622.014706
14,2,2977.240000
14,2,1869.895349
14,2,5824.617647
14,2,8587.120968
14,2,2050.720930
14,2,1789.432432
14,2,1885.250000
14,2,2310.546053
14,2,4064.788462
14,2,1508.809211
14,2,2153.460106
14,2,4558.250000
14,2,2479.755208
14,2,9286.333333
14,2,1763.130435
14,2,5701.951389
14,2,1549.008929
14,2,1724.530612

14,2,1535.605392
14,2,1853.750000
14,2,7349.655814
14,2,4749.020408
14,2,4048.986486
14,2,2360.847500
14,2,1827.572581
14,2,2131.769231
14,2,2163.293269
14,2,1544.107143
14,2,2325.888889
14,2,2714.612245
14,2,1543.250000
14,2,3052.916667
14,2,1714.784091
14,2,1707.146341
14,2,1776.867647
14,2,2367.531863
14,2,3406.153846
14,2,1795.009434
14,2,2210.714286
14,2,1649.744048
14,2,2396.500000
14,2,2683.490000
14,2,1856.653302
14,2,2203.132653
14,2,1813.516667
14,2,1584.247024
14,2,2024.584906
14,2,3153.660000
14,2,2153.699405
14,2,3409.405488
14,2,4084.702500
14,2,2748.202703
14,2,1776.682292
14,2,1869.112805
14,2,2329.043478
14,2,2767.114130
14,2,2064.367021
14,2,2311.538462
14,2,1525.986413
14,2,3244.307018
14,2,2187.770833
14,2,2716.079545
14,2,2102.905172
14,2,1710.500000
14,2,2266.487500
14,2,1712.421875
14,2,1992.625000
14,2,1801.217391
14,2,2853.250000
14,2,1795.724138
14,2,1598.412946
14,2,4950.071429
14,2,2158.952128
14,2,1914.678571
14,2,1772.406250
14,2,1506.385714
14,2,1574.668750
14,2,1714.234694
14,2,1516.669643
14,2,2577.485294
14,2,2820.437500
14,2,2561.816860
14,2,2108.921569
14,2,1604.840000

14,2,1975.009146
14,2,1788.340426
14,2,3087.028646
14,2,2237.031250
14,2,1884.511905
14,2,2114.718750
14,2,2030.075000
14,2,2271.558824
14,2,1675.982143
14,2,2602.872642
14,2,1516.295918
14,2,2395.914894
14,2,4344.633929
14,2,1855.826087
14,2,1999.700000
14,2,1940.741007
14,2,1506.882353
14,2,3620.377660
14,2,2328.570000
14,2,1501.091216
14,2,2103.652439
14,2,1745.582143
14,2,2513.763636
14,2,2207.411458
14,2,3304.811321
14,2,1935.790698
14,2,1795.000000
14,2,1813.319149
14,2,4205.423611
14,2,1784.425000
14,2,3231.250000
14,2,1702.948980
14,2,1623.247093
14,2,2144.489362
14,2,1515.758929
14,2,1634.116279
14,2,2175.263889
14,2,2323.703804
14,2,1896.914216
14,2,1640.247500
14,2,4194.755435
14,2,2898.416667
14,2,1573.684211
14,2,1534.063953
14,2,5536.793605
14,2,1742.753571
14,2,1820.606707
14,2,1510.005952
14,2,1936.883523
14,2,1809.554348
14,2,2028.272727
14,2,1907.505102
14,2,1542.174419
14,2,1633.557432
14,2,1603.767857
14,2,2984.210526
14,2,1515.625000
14,2,7297.465116
14,2,2617.520833
14,2,2266.920732
14,2,2258.301136
14,2,1681.062500
14,2,6435.333333
14,2,4216.375000
14,2,2419.567308
14,2,1858.733333

14,2,6392.206522
14,2,2789.900943
14,2,1971.788043
14,2,2622.884375
14,2,2501.562500
14,2,1526.465116
14,2,5444.307692
14,2,1569.375000
14,2,2210.156250
14,2,2929.866667
14,2,8558.350543
14,2,1580.533333
14,2,2317.697917
14,2,3344.934524
14,2,1690.934211
14,2,1548.883721
14,2,2054.835526
14,2,5831.020833
14,2,7138.843750
14,2,5736.398810
14,2,1505.517442
14,2,1754.494792
14,2,2110.101744
14,2,1825.872449
14,2,3510.924528
14,2,2067.745370
14,2,1943.643617
14,2,1927.759615
14,2,1690.600000
14,2,6874.593750
14,2,2260.945312
14,2,2196.750000
14,2,3110.923077
14,2,3104.750000
14,2,1657.945946
14,2,1749.477500
14,2,2370.804688
14,2,2331.281915
14,2,2367.117647
14,2,2515.127660
14,2,1687.764151
14,2,2020.425926
14,2,1795.512195
14,2,1721.208333
14,2,2189.717105
14,2,2314.826531
14,2,1730.432292
14,2,3292.256039
14,2,1789.407407
14,2,2204.250000
14,2,3084.912500
14,2,3052.726064
14,2,1551.122449
14,2,2154.327830
14,2,2084.662500
14,2,1566.230000
14,2,3447.727273
14,2,4578.300000
14,2,2448.156250
14,2,1666.217391
14,2,2162.550000
14,2,2979.055556
14,2,1503.988372
14,2,7984.697674
14,2,5808.432292
14,2,3322.459184

14,2,1583.994792
14,2,7683.145833
14,2,1606.520833
14,2,2412.943396
14,2,2148.394022
14,2,5677.041667
14,2,2367.360000
14,2,3169.696078
14,2,1682.000000
14,2,2430.640957
14,2,2541.701705
14,2,1603.505682
14,2,4951.404255
14,2,2983.923077
14,2,1544.276596
14,2,2756.377049
14,2,2772.397059
14,2,1586.330189
14,2,3652.816176
14,2,1853.804348
14,2,3418.119792
14,2,1565.504630
14,2,1848.828947
14,2,3074.775000
14,2,1582.019737
14,2,1807.750000
14,2,2554.892857
14,2,2068.325000
14,2,2324.354592
14,2,1824.172872
14,2,2220.500000
14,2,1581.588235
14,2,3447.331395
14,2,2940.564904
14,2,1944.546196
14,2,1792.321429
14,2,2247.551020
14,2,2140.562500
14,2,1596.075658
14,2,2191.157500
14,2,2608.189286
14,2,2091.750000
14,2,2152.766892
14,2,3947.151163
14,2,3232.825521
14,2,2024.636792
14,2,4155.339286
14,2,5264.730000
14,2,1763.191489
14,2,3655.970588
14,2,1660.888889
14,2,1552.750000
14,2,2743.232558
14,2,2138.552632
14,2,3462.930481
14,2,5850.217391
14,2,2441.300532
14,2,2182.081395
14,2,1835.740000
14,2,1738.954082
14,2,2004.022059
14,2,2784.651316
14,2,14488.152500
14,2,1513.520000
14,2,1618.036585
14,2,1927.083333

14,2,2152.733333
14,2,1964.502358
14,2,1530.558824
14,2,2810.719907
14,2,1505.660000
14,2,2964.187500
14,2,3889.859375
14,2,2974.210526
14,2,1591.210714
14,2,1631.900000
14,2,17487.240132
14,2,1751.953488
14,2,1732.980263
14,2,1684.882075
14,2,4540.412338
14,2,1731.044643
14,2,13253.455357
14,2,2086.713068
14,2,3604.055000
14,2,2825.098404
14,2,1858.174528
14,2,1558.222222
14,2,3569.405405
14,2,2026.745192
14,2,2316.400000
14,2,1751.931818
14,2,1539.080357
14,2,1746.418367
14,2,2753.822917
14,2,1709.946023
14,2,1953.847500
14,2,2189.750000
14,2,1725.976064
14,2,1908.045918
14,2,2508.212766
14,2,1754.952381
14,2,4213.716981
14,2,1590.525000
14,2,3394.347826
14,2,1897.473958
14,2,1917.918367
14,2,1505.809524
14,2,3264.784314
14,2,6624.040816
14,2,2859.744681
14,2,1828.173295
14,2,1510.216216
14,2,1775.794872
14,2,1875.705729
14,2,1601.250000
14,2,2141.683673
14,2,1968.083333
14,2,2048.961538
14,2,2052.277439
14,2,2112.510870
14,2,1507.027174
14,2,2136.036765
14,2,1679.801829
14,2,2589.018750
14,2,1510.147727
14,2,6527.879032
14,2,1768.265957
14,2,3700.188679
14,2,1920.104651
14,2,1550.665179
14,2,2723.035714

14,2,2876.857143
14,2,1919.580357
14,2,1569.804878
14,2,1692.283784
14,2,1649.091981
14,2,2146.459184
14,2,1581.906250
14,2,1717.693627
14,2,2234.609043
14,2,14526.427632
14,2,2068.603774
14,2,1515.258929
14,2,4019.702703
14,2,1624.357955
14,2,1994.728448
14,2,6304.405488
14,2,1742.280702
14,2,2431.174419
14,2,1910.617801
14,2,2097.328125
14,2,3168.805556
14,2,2670.723684
14,2,1636.835227
14,2,1703.511364
14,2,5775.897059
14,2,1519.190476
14,2,1562.854167
14,2,1556.952778
14,2,7706.806818
14,2,3962.490000
14,2,7502.727273
14,2,1816.316327
14,2,5871.705882
14,2,1688.000000
14,2,3330.419118
14,2,1788.496875
14,2,1752.200000
14,2,1634.758929
14,2,3194.911585
14,2,1565.256098
14,2,1714.109043
14,2,1568.636943
14,2,7331.056452
14,2,1857.600000
14,2,1688.193548
14,2,1991.500000
14,2,1722.897059
14,2,2247.339286
14,2,1710.888889
14,2,2086.258721
14,2,1610.573661
14,2,2733.960000
14,2,2025.630952
14,2,1634.575000
14,2,2408.771277
14,2,2352.000000
14,2,1721.076923
14,2,2068.421053
14,2,2019.187500
14,2,9101.956522
14,2,2462.101190
14,2,3344.705882
14,2,1635.800000
14,2,1707.352941
14,2,1788.481132
14,2,1957.053571

14,2,2522.758621
14,2,1988.800000
14,2,5700.858974
14,2,2087.707447
14,2,1609.842500
14,2,2071.250000
14,2,1756.667763
14,2,2612.164286
14,2,1597.290816
14,2,2833.933962
14,2,3946.761792
14,2,2307.583333
14,2,2633.841518
14,2,2231.046875
14,2,3134.967949
14,2,3547.988095
14,2,2003.113426
14,2,1599.812500
14,2,1589.478723
14,2,1895.431604
14,2,6675.919643
14,2,1530.861702
14,2,2560.946809
14,2,1886.160377
14,2,2061.195946
14,2,7842.912791
14,2,1996.733491
14,2,2092.439815
14,2,1974.329787
14,2,2061.836735
14,2,1833.215909
14,2,2712.862500
14,2,2389.149038
14,2,2737.915816
14,2,3254.710938
14,2,2007.252874
14,2,3462.250000
14,2,2259.162791
14,2,1744.154070
14,2,1633.147959
14,2,2160.510204
14,2,3448.307692
14,2,1536.000000
14,2,2131.531250
14,2,1751.461111
14,2,2088.808140
14,2,1877.463415
14,2,2808.691327
14,2,5710.940000
14,2,1721.170330
14,2,1893.788690
14,2,2178.018519
14,2,4344.219512
14,2,4481.614286
14,2,1922.385638
14,2,1952.084091
14,2,1799.439815
14,2,1669.053977
14,2,1656.497396
14,2,2420.180085
14,2,8058.640000
14,2,1884.201220
14,2,2919.750000
14,2,2087.000000
14,2,2004.500000
14,2,2475.817073

14,2,2226.035714
14,2,1931.925000
14,2,2071.104730
14,2,1637.040541
14,2,1780.433673
14,2,6358.538462
14,2,3757.375000
14,2,2076.824074
14,2,2088.700000
14,2,2412.807692
14,2,2107.777273
14,2,2721.625000
14,2,6038.974138
14,2,1837.846154
14,2,1531.098214
14,2,2220.293269
14,2,1501.937500
14,2,2325.044118
14,2,1542.159091
14,2,2236.353723
14,2,1744.434783
14,2,4666.403509
14,2,2405.833333
14,2,2245.937500
14,2,3358.000000
14,2,1596.541667
14,2,2294.767241
14,2,1984.235294
14,2,3425.984375
14,2,3394.438596
14,2,2068.327778
14,2,1706.598214
14,2,2026.039062
14,2,1585.581633
14,2,2006.361979
14,2,2593.442708
14,2,2085.562874
14,2,1708.321429
14,2,4033.003205
14,2,1571.383523
14,2,2801.278846
14,2,1997.982558
14,2,1996.525568
14,2,2414.238636
14,2,1528.833333
14,2,2667.950000
14,2,3314.981250
14,2,2660.233696
14,2,2854.311224
14,2,1885.316327
14,2,1688.538043
14,2,3389.834375
14,2,2592.980769
14,2,1844.267442
14,2,1755.352941
14,2,1512.640625
14,2,2553.750000
14,2,1874.745536
14,2,4030.683962
14,2,1605.160714
14,2,2126.852273
14,2,1902.185976
14,2,1656.154412
14,2,2018.662500
14,2,1657.630556
14,2,1850.384868

14,2,1501.314189
14,2,1609.636364
14,2,2158.720745
14,2,2828.558824
14,2,2215.153061
14,2,1960.677885
14,2,1773.312500
14,2,1893.456395
14,2,3562.500000
14,2,1702.102041
14,2,5109.977273
14,2,6152.227273
14,2,1680.310714
14,2,2333.716667
14,2,4058.062500
14,2,1710.529412
14,2,2027.368056
14,2,1844.362705
14,2,1803.107143
14,2,3177.987903
14,2,1515.800000
14,2,1752.911765
14,2,3621.451531
14,2,3423.868421
14,2,1541.900000
14,2,1555.300000
14,2,1963.227273
14,2,1818.031250
14,2,2166.940000
14,2,2144.848404
14,2,3924.365385
14,2,4182.310000
14,2,2496.425000
14,2,1588.215426
14,2,1619.951220
14,2,1663.558282
14,2,1879.205000
14,2,1956.442308
14,2,2467.340909
14,2,1718.401163
14,2,1536.842391
14,2,3670.069149
14,2,2602.140625
14,2,2814.616279
14,2,1541.178571
14,2,2471.394022
14,2,1658.769444
14,2,1812.847222
14,2,2333.912234
14,2,2403.180804
14,2,1577.703947
14,2,2320.593750
14,2,1614.553571
14,2,2328.144737
14,2,2894.717213
14,2,1819.571429
14,2,2405.116379
14,2,1951.792614
14,2,19178.916667
14,2,2374.163194
14,2,1666.791667
14,2,3057.445455
14,2,2768.759434
14,2,7261.351351
14,2,3921.364130
14,2,1568.417969

14,2,2688.090278
14,2,1858.483607
14,2,1571.405556
14,2,4815.394886
14,2,1846.069767
14,2,3048.463415
14,2,2776.075893
14,2,1609.000000
14,2,2625.461957
14,2,2644.135965
14,2,2529.312500
14,2,2836.911765
14,2,3348.546875
14,2,2702.620000
14,2,1533.420000
14,2,1704.196809
14,2,6204.930233
14,2,1647.828488
14,2,1535.207547
14,2,1505.963415
14,2,3929.371622
14,2,2243.389706
14,2,1846.076923
14,2,2004.880208
14,2,3460.982955
14,2,2765.581522
14,2,1502.500000
14,2,1655.443182
14,2,1943.478659
14,2,1819.341667
14,2,1852.861111
14,2,7149.928191
14,2,3084.250000
14,2,2608.375000
14,2,2089.238636
14,2,2538.286765
14,2,2580.968750
14,2,1523.163158
14,2,2645.672269
14,2,3522.146341
14,2,3232.407407
14,2,2270.600000
14,2,3541.272727
14,2,4104.482759
14,2,1542.050000
14,2,2859.813725
14,2,1587.215625
14,2,3058.484043
14,2,1819.632653
14,2,1599.244681
14,2,9715.855263
14,2,2328.913043
14,2,1660.804878
14,2,2340.494681
14,2,1987.387821
14,2,1686.323171
14,2,2725.820513
14,2,2314.750000
14,2,2180.034314
14,2,2201.352941
14,2,1986.600543
14,2,1653.946809
14,2,1943.968750
14,2,1991.444444
14,2,1996.397727
14,2,3078.476562

14,2,3468.900943
14,2,1529.419872
14,2,1576.900568
14,2,2393.941176
14,2,1822.182432
14,2,3124.706522
14,2,3932.045000
14,2,17483.593750
14,2,1657.500000
14,2,2801.736979
14,2,13477.839286
14,2,1617.558511
14,2,1717.047619
14,2,2183.791667
14,2,1606.812500
14,2,1874.518182
14,2,1542.647059
14,2,4828.805556
14,2,1656.230769
14,2,1514.918919
14,2,13014.857143
14,2,15470.075000
14,2,1917.718750
14,2,2446.983051
14,2,6602.074468
14,2,1708.724432
14,2,2261.950000
14,2,4771.211735
14,2,1850.482143
14,2,2199.795455
14,2,5788.062500
14,2,1636.750000
14,2,1793.289474
14,2,2588.283854
14,2,11276.142857
14,2,1652.559211
14,2,3258.638889
14,2,3447.513889
14,2,5406.891827
14,2,14777.021341
14,2,7164.424419
14,2,3942.191860
14,2,1692.785714
14,2,1580.585526
14,2,3837.166667
14,2,2066.647727
14,2,1725.007812
14,2,2287.067308
14,2,2560.058824
14,2,3316.734375
14,2,2020.548913
14,2,1961.665179
14,2,1575.609756
14,2,1781.850490
14,2,1809.853723
14,2,1515.533654
14,2,1547.697917
14,2,5616.966981
14,2,1902.522959
14,2,1916.409722
14,2,10879.375000
14,2,2487.166667
14,2,1733.265625
14,2,2020.725490
14,2,1641.201923
14,2,1752.250000

14,2,2351.687500
14,2,1525.218750
14,2,1560.000000
14,2,2007.100000
14,2,1512.000000
14,2,1579.147436
14,2,4397.454545
14,2,3383.135870
14,2,2064.595745
14,2,1976.791667
14,2,2520.000000
14,2,2249.375000
14,2,2250.218750
14,2,1965.382979
14,2,2130.005102
14,2,3042.560811
14,2,1704.888298
14,2,2195.692308
14,2,1635.308140
14,2,2162.558140
14,2,3151.105263
14,2,2286.281250
14,2,2228.596154
14,2,6058.537736
14,2,1570.794643
14,2,3108.544643
14,2,1813.829082
14,2,1792.490385
14,2,1786.052632
14,2,3364.605392
14,2,2283.058594
14,2,1555.060976
14,2,6062.156250
14,2,1815.926136
14,2,1901.666667
14,2,1972.000000
14,2,1969.312500
14,2,2233.174419
14,2,2002.188679
14,2,1799.235294
14,2,2666.670213
14,2,1554.383929
14,2,1568.212264
14,2,1542.369681
14,2,1741.250000
14,2,2567.148936
14,2,2495.038462
14,2,2661.979167
14,2,1954.596154
14,2,1742.087500
14,2,2533.766667
14,2,1892.383333
14,2,2895.739362
14,2,1798.453488
14,2,1964.461538
14,2,2907.454861
14,2,1995.625000
14,2,1715.935829
14,2,2471.104167
14,2,1622.197115
14,2,1639.727273
14,2,2700.224359
14,2,2170.522959
14,2,3455.298077
14,2,2010.700000
14,2,1549.817708

14,2,2118.901163
14,2,2602.000000
14,2,2974.853571
14,2,3504.039216
14,2,1838.607143
14,2,2685.343023
14,2,1679.217391
14,2,1898.500000
14,2,3455.191176
14,2,3351.478261
14,2,17527.686441
14,2,17993.051471
14,2,1512.166667
14,2,1585.303191
14,2,5058.250000
14,2,1556.005435
14,2,4951.607143
14,2,1745.760417
14,2,1655.139881
14,2,2024.476190
14,2,1618.875000
14,2,1658.761364
14,2,1916.312500
14,2,3081.630682
14,2,4036.961957
14,2,1776.035714
14,2,9694.020833
14,2,4981.460106
14,2,5974.750000
14,2,3637.863095
14,2,2547.733668
14,2,3078.837838
14,2,2451.596939
14,2,2587.661458
14,2,1621.053279
14,2,1762.000000
14,2,3123.400000
14,2,1886.000000
14,2,14076.631579
14,2,1774.875000
14,2,1733.612500
14,2,1928.906915
14,2,1588.768868
14,2,1645.840909
14,2,3856.644737
14,2,3968.050000
14,2,3021.856707
14,2,1842.984043
14,2,1551.938725
14,2,2287.573864
14,2,2030.062500
14,2,1572.576923
14,2,2745.755682
14,2,1529.633333
14,2,3976.018382
14,2,1747.968137
14,2,1539.117021
14,2,16401.864865
14,2,2993.281977
14,2,1655.639205
14,2,2909.400000
14,2,1588.435897
14,2,1607.785714
14,2,2207.615385
14,2,2045.177778
14,2,2697.935000

14,2,1802.815000
14,2,4980.432432
14,2,2541.926829
14,2,1944.450000
14,2,1771.625000
14,2,2487.304348
14,2,1573.048387
14,2,2131.459184
14,2,3032.537500
14,2,1674.523585
14,2,7574.000000
14,2,2471.042683
14,2,1536.500000
14,2,3250.166667
14,2,2909.113208
14,2,1663.594340
14,2,5048.195000
14,2,2127.882653
14,2,4149.641892
14,2,2138.571429
14,2,1753.250000
14,2,3128.435096
14,2,7226.710366
14,2,3134.867925
14,2,2276.583333
14,2,1695.375000
14,2,1758.187500
14,2,4896.750000
14,2,1860.394737
14,2,2014.500000
14,2,1711.162162
14,2,6008.718750
14,2,1662.116959
14,2,2449.800926
14,2,2489.548077
14,2,3165.741071
14,2,1602.377551
14,2,6097.860294
14,2,1554.936170
14,2,1534.410000
14,2,1829.970395
14,2,2696.071429
14,2,1825.230114
14,2,1939.985577
14,2,3296.670918
14,2,7490.138298
14,2,1592.142857
14,2,2021.109375
14,2,1918.765306
14,2,2223.823113
14,2,2721.369565
14,2,1737.208333
14,2,1669.507500
14,2,2315.000000
14,2,8213.389706
14,2,1548.829861
14,2,2509.488636
14,2,2159.144022
14,2,2110.080357
14,2,1989.140306
14,2,2044.485294
14,2,3456.015625
14,2,1720.773148
14,2,3152.540761
14,2,3863.931034
14,2,2633.416667

14,2,3321.087766
14,2,1783.794118
14,2,3035.648649
14,2,3201.869565
14,2,3524.607143
14,2,1568.665761
14,2,1758.648352
14,2,2133.050000
14,2,2452.734375
14,2,1813.833333
14,2,2589.275000
14,2,1658.617647
14,2,3309.325000
14,2,1963.745000
14,2,1656.024194
14,2,2557.447368
14,2,2160.325000
14,2,2831.330882
14,2,2053.228261
14,2,1674.238095
14,2,1861.302632
14,2,2160.544118
14,2,2262.132353
14,2,3637.639535
14,2,2073.687500
14,2,1944.866848
14,2,3043.157895
14,2,2693.600000
14,2,2298.008772
14,2,1711.925000
14,2,3264.595238
14,2,1499.671569
14,2,2283.227273
14,2,1818.846154
14,2,1963.898305
14,2,1858.425000
14,2,2005.981383
14,2,2965.105263
14,2,3079.930556
14,2,2300.800000
14,2,4203.814189
14,2,2801.375000
14,2,3423.621795
14,2,1539.849490
14,2,2421.614583
14,2,3630.435976
14,2,1734.877976
14,2,5514.584821
14,2,3424.744186
14,2,7676.218750
14,2,2221.780172
14,2,7808.113208
14,2,4226.653846
14,2,4962.322581
14,2,1847.842105
14,2,1634.230769
14,2,2018.531915
14,2,2454.471939
14,2,4652.250000
14,2,4593.863636
14,2,3431.484043
14,2,1696.923077
14,2,3565.823171
14,2,1777.600000
14,2,2032.875000
14,2,4275.701987

14,2,3103.726190
14,2,2038.500000
14,2,2142.795918
14,2,5223.151316
14,2,2112.338542
14,2,1856.701705
14,2,3007.794118
14,2,2105.625000
14,2,1516.004310
14,2,2949.449074
14,2,2183.829545
14,2,6686.000000
14,2,1982.552632
14,2,2738.212766
14,2,1679.975000
14,2,1758.760417
14,2,1670.840909
14,2,1654.387500
14,2,2638.213942
14,2,2879.543478
14,2,2940.767442
14,2,2177.365132
14,2,3963.544643
14,2,1975.125000
14,2,2642.349359
14,2,2893.417683
14,2,3080.098214
14,2,3453.409091
14,2,7529.827206
14,2,1658.177885
14,2,1692.200000
14,2,1771.964674
14,2,1677.425532
14,2,5695.539216
14,2,1872.609375
14,2,4578.007576
14,2,3215.942500
14,2,1533.093023
14,2,2557.800000
14,2,1795.250000
14,2,2125.979651
14,2,1959.415179
14,2,10657.474576
14,2,1920.986111
14,2,1806.346154
14,2,1875.853659
14,2,1568.725000
14,2,2267.541667
14,2,1682.864583
14,2,1977.391509
14,2,1680.969828
14,2,1572.558824
14,2,3133.787234
14,2,3155.595745
14,2,2890.754717
14,2,2950.437500
14,2,2162.029412
14,2,1947.914894
14,2,1887.880000
14,2,1619.255208
14,2,1983.851190
14,2,2235.581522
14,2,2121.054054
14,2,1942.364362
14,2,1888.684896
14,2,1921.893750

14,2,1561.147959
14,2,3251.433673
14,2,1698.406250
14,2,2930.412500
14,2,2413.756250
14,2,1557.985294
14,2,2009.075581
14,2,1626.413551
14,2,2829.657895
14,2,1665.366667
14,2,1542.184211
14,2,3102.093750
14,2,3449.746622
14,2,12685.746795
14,2,5092.772541
14,2,2206.470588
14,2,1809.897436
14,2,1533.445652
14,2,1576.621622
14,2,2979.842949
14,2,2351.078947
14,2,5677.982955
14,2,1528.040761
14,2,1892.892500
14,2,1561.711957
14,2,3741.044118
14,2,2075.511111
14,2,1837.369898
14,2,2353.333333
14,2,1954.596154
14,2,2060.263889
14,2,1562.269231
14,2,1928.812500
14,2,2200.333333
14,2,4101.384615
14,2,4970.833333
14,2,1596.574074
14,2,2493.632576
14,2,1876.296296
14,2,3541.872596
14,2,3472.247024
14,2,1860.747685
14,2,1537.704545
14,2,2010.866477
14,2,1835.102941
14,2,1607.687500
14,2,2073.398256
14,2,1595.494681
14,2,5897.000000
14,2,16356.380682
14,2,1546.058333
14,2,9690.416667
14,2,3030.062500
14,2,1529.190217
14,2,5180.970238
14,2,1922.882075
14,2,2333.218750
14,2,2250.432432
14,2,3116.916667
14,2,2962.716981
14,2,5261.690000
14,2,1506.903846
14,2,1966.710227
14,2,2228.768293
14,2,1506.000000
14,2,1504.208333

14,2,1848.963415
14,2,1951.358333
14,2,4314.653061
14,2,2201.177326
14,2,2326.090278
14,2,2475.833333
14,2,1692.464286
14,2,2555.965000
14,2,2232.290909
14,2,2022.854167
14,2,2105.100543
14,2,2250.747093
14,2,1713.909091
14,2,1641.857143
14,2,2494.400000
14,2,1560.962500
14,2,1906.809659
14,2,1776.080128
14,2,2650.450521
14,2,2250.619444
14,2,1803.720930
14,2,4486.982143
14,2,1663.697917
14,2,1815.500000
14,2,1586.518750
14,2,1564.931818
14,2,1687.777778
14,2,2290.110795
14,2,1731.437500
14,2,6975.774194
14,2,2039.750000
14,2,1965.276442
14,2,2067.487500
14,2,1523.260417
14,2,2657.169118
14,2,1915.544444
14,2,3834.526316
14,2,5478.387019
14,2,1668.066667
14,2,2805.507353
14,2,3632.323529
14,2,2380.090000
14,2,2338.875000
14,2,2001.254717
14,2,1657.351562
14,2,2439.118750
14,2,2272.208333
14,2,1864.627841
14,2,2439.492347
14,2,1632.558824
14,2,1684.390957
14,2,4755.385000
14,2,2585.061111
14,2,1570.217391
14,2,1552.604167
14,2,2209.307692
14,2,2486.043478
14,2,2524.166667
14,2,1524.632653
14,2,3306.600000
14,2,7714.456140
14,2,2084.922872
14,2,4528.209906
14,2,1976.646739
14,2,1956.833333
14,2,1862.968023

14,2,2928.074468
14,2,1550.140625
14,2,2430.058140
14,2,1600.912500
14,2,3267.623037
14,2,2063.111842
14,2,1815.738095
14,2,3253.715116
14,2,2315.166667
14,2,1504.707447
14,2,2063.674419
14,2,2957.328125
14,2,2752.108108
14,2,3751.023438
14,2,2302.160156
14,2,3261.050000
14,2,5288.381579
14,2,1720.264205
14,2,3533.003205
14,2,2191.539773
14,2,1807.893617
14,2,2673.632212
14,2,1698.212766
14,2,2079.769231
14,2,1580.925532
14,2,1708.918478
14,2,1661.634804
14,2,3114.240196
14,2,1721.389535
14,2,4161.666667
14,2,6797.556818
14,2,1674.196429
14,2,2487.000000
14,2,2095.656250
14,2,1666.203704
14,2,5625.652778
14,2,1736.561404
14,2,2461.127660
14,2,3469.413793
14,2,2596.079295
14,2,2215.018382
14,2,5545.416667
14,2,2508.705882
14,2,1915.923469
14,2,1505.159884
14,2,1761.236702
14,2,1645.112069
14,2,1604.808511
14,2,1854.069149
14,2,4479.583333
14,2,1623.156977
14,2,1560.289634
14,2,4556.886792
14,2,1530.404891
14,2,3782.044444
14,2,2434.029412
14,2,1589.395833
14,2,1859.364130
14,2,6579.527778
14,2,2923.408163
14,2,1677.967500
14,2,5534.080000
14,2,2318.090116
14,2,2228.759615
14,2,3069.774854
14,2,3388.273585

14,2,10280.663265
14,2,3549.611111
14,2,1766.859375
14,2,1549.766026
14,2,1506.187500
14,2,1514.841667
14,2,2563.571429
14,2,2277.906977
14,2,2125.189904
14,2,2670.917453
14,2,1753.208333
14,2,2186.320312
14,2,2438.293605
14,2,1889.769231
14,2,1523.809045
14,2,1929.551020
14,2,2067.773810
14,2,1803.788934
14,2,2601.750000
14,2,1799.175595
14,2,1529.519231
14,2,1694.750000
14,2,3162.891026
14,2,2091.416667
14,2,2840.084135
14,2,2263.655172
14,2,2038.000000
14,2,2455.843750
14,2,1914.292453
14,2,2915.735119
14,2,4817.230000
14,2,1897.750000
14,2,1603.217857
14,2,2105.431548
14,2,2224.078431
14,2,3533.926829
14,2,2159.203704
14,2,2792.461538
14,2,1514.400000
14,2,4229.937500
14,2,2862.646341
14,2,1605.378205
14,2,4258.226190
14,2,1712.094444
14,2,3808.395455
14,2,1591.672872
14,2,3053.667453
14,2,1505.062500
14,2,1538.269886
14,2,2695.340909
14,2,1936.013889
14,2,1921.275510
14,2,2432.788462
14,2,1574.616279
14,2,1551.924020
14,2,4210.834906
14,2,2836.226974
14,2,1744.903061
14,2,1553.439024
14,2,1693.702703
14,2,1770.147059
14,2,1523.591837
14,2,17932.996875
14,2,1565.979730
14,2,1875.802326
14,2,1635.225000

14,2,2122.318182
14,2,3001.692857
14,2,3538.072222
14,2,1573.177632
14,2,3078.925532
14,2,1808.142857
14,2,1744.205882
14,2,3278.839286
14,2,1544.357143
14,2,1827.752551
14,2,2108.675676
14,2,1974.228571
14,2,1981.862069
14,2,2474.922619
14,2,1536.250000
14,2,2461.707317
14,2,2569.178571
14,2,2812.801020
14,2,2075.948980
14,2,1840.310000
14,2,1940.720588
14,2,2511.090909
14,2,2123.957547
14,2,1916.639881
14,2,1752.919643
14,2,3724.093750
14,2,1620.480000
14,2,3663.752604
14,2,5608.888393
14,2,2096.850000
14,2,1972.122093
14,2,1694.292857
14,2,2362.093023
14,2,4671.276442
14,2,6144.752660
14,2,2008.922222
14,2,3512.866071
14,2,2273.424460
14,2,1884.292453
14,2,1790.558140
14,2,5039.530488
14,2,3545.521739
14,2,1754.952830
14,2,2025.932692
14,2,2938.437500
14,2,1947.616071
14,2,3917.416318
14,2,9477.048128
14,2,1594.092262
14,2,1518.625000
14,2,1717.769231
14,2,1784.695313
14,2,2815.218750
14,2,1686.065104
14,2,4660.556818
14,2,1617.650000
14,2,3963.042763
14,2,5108.162791
14,2,1558.605769
14,2,1687.806818
14,2,1883.110119
14,2,2351.294271
14,2,1809.883929
14,2,2111.700000
14,2,1950.988372
14,2,1589.365854

14,2,2198.729167
14,2,1946.473684
14,2,2528.686047
14,2,1907.632353
14,2,7126.108553
14,2,2181.200000
14,2,1664.622024
14,2,2878.098684
14,2,3440.500000
14,2,1965.936321
14,2,1891.893750
14,2,4643.125000
14,2,1528.455882
14,2,1594.858696
14,2,2048.452830
14,2,2188.375000
14,2,3187.903846
14,2,1948.255952
14,2,1658.764205
14,2,2135.400000
14,2,1608.652500
14,2,2365.909091
14,2,1718.178571
14,2,2786.977564
14,2,1645.546512
14,2,19379.768750
14,2,2307.625000
14,2,1773.227273
14,2,2913.414894
14,2,1537.852941
14,2,1505.425676
14,2,1779.372340
14,2,1680.587500
14,2,1895.823529
14,2,2528.342857
14,2,2570.307692
14,2,1738.897059
14,2,3150.794271
14,2,3007.712766
14,2,1948.173077
14,2,13841.860465
14,2,1651.997685
14,2,1625.857143
14,2,1577.818452
14,2,1796.975610
14,2,1565.785714
14,2,4228.625000
14,2,1773.963415
14,2,1551.000000
14,2,1546.949468
14,2,2311.894737
14,2,1574.353261
14,2,4387.107143
14,2,1708.704268
14,2,1510.921875
14,2,2028.705000
14,2,2674.461310
14,2,1695.923077
14,2,1576.135870
14,2,5325.406250
14,2,1712.714286
14,2,2134.272059
14,2,3576.613636
14,2,1909.733974
14,2,1729.307692
14,2,1816.000000

14,2,3897.965812
14,2,1825.536458
14,2,2280.829741
14,2,5579.483108
14,2,1849.545455
14,2,4040.571429
14,2,3008.979167
14,2,2169.173469
14,2,1846.225000
14,2,1517.361111
14,2,1979.521341
14,2,2086.297872
14,2,3661.301471
14,2,5477.203947
14,2,1571.731383
14,2,6439.273438
14,2,2553.635000
14,2,3317.216418
14,2,1562.108696
14,2,2974.353659
14,2,2532.464286
14,2,1937.353261
14,2,1721.560000
14,2,2215.682692
14,2,2153.500000
14,2,1876.706250
14,2,2078.855000
14,2,4012.448529
14,2,2362.820946
14,2,1606.198171
14,2,4593.468085
14,2,1510.633929
14,2,1919.546512
14,2,1701.243243
14,2,1775.752500
14,2,4211.767857
14,2,2302.828704
14,2,1733.625000
14,2,2444.612500
14,2,5974.387755
14,2,4908.000000
14,2,2805.573770
14,2,2283.781250
14,2,1957.312500
14,2,3591.340000
14,2,3764.483696
14,2,1698.181250
14,2,4384.875000
14,2,1844.403846
14,2,1920.653846
14,2,2263.917553
14,2,1675.000000
14,2,2966.187500
14,2,1727.902344
14,2,1727.254808
14,2,2483.286932
14,2,1983.085366
14,2,2551.875000
14,2,1507.772222
14,2,2082.098404
14,2,1544.723214
14,2,1876.000000
14,2,1765.437500
14,2,1756.082500
14,2,2008.737805
14,2,2515.638158

14,2,1524.822222
14,2,2728.747024
14,2,3342.601974
14,2,3816.937500
14,2,2219.029412
14,2,2186.500000
14,2,2041.953947
14,2,3383.712500
14,2,2553.045455
14,2,2533.528846
14,2,3963.437500
14,2,1547.210784
14,2,2114.966216
14,2,1731.151442
14,2,1798.500000
14,2,3094.705882
14,2,2354.456522
14,2,2049.115854
14,2,1657.608247
14,2,1861.865979
14,2,1580.186047
14,2,1748.456818
14,2,1876.156818
14,2,1708.676471
14,2,2119.503676
14,2,1673.433673
14,2,2287.318182
14,2,10977.526316
14,2,1685.875000
14,2,1802.342391
14,2,2071.240741
14,2,1845.255814
14,2,1661.000000
14,2,1816.452381
14,2,1791.404762
14,2,2789.234375
14,2,2344.995098
14,2,1577.968750
14,2,2268.645408
14,2,7964.138298
14,2,2855.363636
14,2,1935.978022
14,2,5266.201923
14,2,1681.920455
14,2,1833.326389
14,2,2091.182927
14,2,2003.761905
14,2,7942.723404
14,2,12625.681818
14,2,2199.864130
14,2,2478.447222
14,2,3843.848485
14,2,3145.217500
14,2,2527.356383
14,2,1669.854167
14,2,1558.560000
14,2,8615.058824
14,2,1571.978723
14,2,4329.406250
14,2,1633.941176
14,2,5531.985294
14,2,3153.074519
14,2,1746.817073
14,2,2516.881579
14,2,2684.737143
14,2,1508.105263

14,2,1662.062802
15,1,2240.075000
15,1,1774.191176
15,1,5361.421053
15,1,6550.850000
15,1,2002.857143
15,1,2085.483974
15,1,4462.371951
15,1,2427.733333
15,1,3900.848214
15,1,3527.911765
15,1,2238.793269
15,1,3089.316176
15,1,1621.750000
15,1,3846.739130
15,1,2888.970588
15,1,1845.087500
15,1,3245.562500
15,1,5109.853723
15,1,2549.320652
15,1,4818.870968
15,1,4492.701754
15,1,1812.246622
15,1,1886.457317
15,1,12216.013514
15,1,3387.046875
15,1,3016.319079
15,1,2022.344512
15,1,2001.419847
15,1,1909.130682
15,1,1698.755208
15,1,1892.100000
15,1,2933.718750
15,1,2974.761905
15,1,4908.790816
15,1,2040.666667
15,1,16347.875000
15,1,14193.364286
15,1,1800.222222
15,1,2578.062500
15,1,4286.333333
15,1,11351.886905
15,1,3259.155488
15,1,4246.828125
15,1,5300.153846
15,1,4885.750000
15,1,2583.227941
15,1,5053.500000
15,1,4961.840426
15,1,6644.687500
15,1,8981.500000
15,1,3130.996575
15,1,1846.933511
15,1,4525.176471
15,1,3309.600000
15,1,6499.468750
15,1,4241.780488
15,1,4938.377778
15,1,5423.191176
15,1,10190.116279
15,1,11099.819853
15,1,5646.056548
15,1,1812.822368
15,1,2342.725000
15,1,2548.469388
15,1,6647.885714

15,1,4420.317073
15,1,4531.663043
15,1,1598.208333
15,1,4217.320513
15,1,2173.407500
15,1,6278.513158
15,1,2470.602941
15,1,2895.236111
15,1,3584.625000
15,1,1632.155488
15,1,5843.390625
15,1,2184.225000
15,1,1507.066406
15,1,2370.565476
15,1,1540.472222
15,1,1645.287500
15,1,3274.125000
15,1,1561.867188
15,1,2568.010714
15,1,3739.181818
15,1,5851.475610
15,1,2256.361111
15,1,1640.437500
15,1,1873.617647
15,1,1618.894737
15,1,9826.964744
15,1,2318.317308
15,1,5822.952703
15,1,2270.745509
15,1,2840.194767
15,1,1805.642857
15,1,2046.222973
15,1,6867.973684
15,1,9985.395833
15,1,8481.086735
15,1,12245.270833
15,1,3845.250000
15,1,3176.317073
15,1,2145.401163
15,1,3645.584135
15,1,3335.500000
15,1,7632.659091
15,1,2961.650000
15,1,9644.049242
15,1,4169.384615
15,1,6126.839744
15,1,2994.092857
15,1,1578.965625
15,1,9779.107955
15,1,1624.279412
15,1,4321.573171
15,1,7027.596154
15,1,2278.205357
15,1,7623.303571
15,1,12159.647727
15,1,17778.596154
15,1,3678.060976
15,1,12441.705128
15,1,4320.509615
15,1,3485.985294
15,1,1668.147436
15,1,16895.548913
15,1,1521.375000
15,1,3801.127907
15,1,4034.789062
15,1,13362.250000

15,1,3250.534091
15,1,3262.847826
15,1,5495.769231
15,1,2028.684211
15,1,4865.500000
15,1,2057.000000
15,1,2853.690476
15,1,3021.823171
15,1,4651.045732
15,1,11848.093750
15,1,11908.020833
15,1,10282.320000
15,1,2288.625000
15,1,7616.695000
15,1,6291.800000
15,1,6418.170732
15,1,14614.714286
15,1,4953.769231
15,1,2722.520270
15,1,3453.375000
15,1,5903.842105
15,1,5141.163462
15,1,9285.909091
15,1,1974.034884
15,1,2816.281250
15,1,1924.237805
15,1,2593.500000
15,1,17694.750000
15,1,15023.324324
15,1,1669.776786
15,1,1893.500000
15,1,7569.800595
15,1,1951.161765
15,1,2654.943396
15,1,3059.877358
15,1,3702.244681
15,1,15026.963889
15,1,1730.930147
15,1,3190.098404
15,1,8642.324405
15,1,3898.562500
15,1,3491.022727
15,1,2324.595588
15,1,5375.230114
15,1,3706.250000
15,1,3909.095745
15,1,6715.884146
15,1,1636.100610
15,1,1599.128676
15,1,2352.255000
15,1,4830.302326
15,1,4519.055556
15,1,4100.348404
15,1,1906.800000
15,1,6951.000000
15,1,1647.459459
15,1,5181.300000
15,1,8658.687500
15,1,3731.663043
15,1,2471.926471
15,1,11927.435897
15,1,8634.434211
15,1,1543.148649
15,1,4567.982143
15,1,1851.096774
15,1,1712.111842

15,1,9045.025000
15,1,1733.994048
15,1,2116.075000
15,1,6825.023256
15,1,6578.453804
15,1,6720.786765
15,1,4442.886905
15,1,7680.155405
15,1,2442.861111
15,1,5239.292553
15,1,6023.332317
15,1,1539.069767
15,1,15401.454545
15,1,4977.023810
15,1,2845.701220
15,1,15478.571429
15,1,1646.402778
15,1,17037.187500
15,1,1563.815476
15,1,4468.500000
15,1,6742.696078
15,1,6827.101562
15,1,4996.500000
15,1,6486.160714
15,1,2913.544872
15,1,1953.709677
15,1,5277.142857
15,1,1829.392857
15,1,8392.615385
15,1,2333.013889
15,1,10694.163793
15,1,15223.034483
15,1,2405.400510
15,1,8952.645000
15,1,4394.639706
15,1,3879.518519
15,1,1510.614362
15,1,3712.772059
15,1,19630.209302
15,1,14012.467742
15,1,2669.613095
15,1,2228.145833
15,1,16332.195652
15,1,3125.035256
15,1,2412.304054
15,1,2653.705128
15,1,1713.238095
15,1,4202.833333
15,1,2527.858974
15,1,5764.528571
15,1,6825.621795
15,1,1627.725000
15,1,5251.500000
15,1,3614.384615
15,1,14295.095588
15,1,2780.378125
15,1,2165.857143
15,1,1552.823980
15,1,8580.531250
15,1,2309.000000
15,1,4034.934783
15,1,1546.600000
15,1,1631.865385
15,1,1559.258929
15,1,6409.705128
15,1,2576.567073

15,1,3058.015625
15,1,5103.720238
15,1,4231.031250
15,1,2638.761905
15,1,4598.750000
15,1,17343.562500
15,1,1713.162879
15,1,12466.414634
15,1,5647.000000
15,1,4080.500000
15,1,3573.864865
15,1,2670.878378
15,1,1517.928571
15,1,2212.861607
15,1,5016.913462
15,1,5862.156250
15,1,7805.510204
15,1,4064.150735
15,1,4131.951923
15,1,15524.500000
15,1,4655.135135
15,1,1517.357558
15,1,7109.468750
15,1,2104.323171
15,1,1926.125000
15,1,18646.878676
15,1,8311.838235
15,1,2001.000000
15,1,3505.487805
15,1,11520.358333
15,1,1819.918919
15,1,1623.991848
15,1,13125.078125
15,1,2117.511029
15,1,1858.816176
15,1,3944.976190
15,1,5051.012931
15,1,5727.262500
15,1,11927.402439
15,1,5126.077586
15,1,3065.864286
15,1,9810.763889
15,1,2001.150000
15,1,14261.083333
15,1,4428.512500
15,1,2097.466216
15,1,2506.125000
15,1,9415.401786
15,1,5156.039474
15,1,1762.156250
15,1,2386.482759
15,1,13707.906250
15,1,1816.887195
15,1,2724.220588
15,1,1521.864865
15,1,4432.196970
15,1,4597.642857
15,1,1548.901596
15,1,3717.085106
15,1,3808.756579
15,1,17016.230769
15,1,2526.666667
15,1,2224.500000
15,1,2731.959184
15,1,2354.781250
15,1,4323.542614

15,1,19788.750000
15,1,5745.669118
15,1,14547.437500
15,1,18435.926471
15,1,2869.616667
15,1,3027.554348
15,1,6836.865854
15,1,2504.844595
15,1,4763.622159
15,1,3197.968750
15,1,6391.310345
15,1,2252.421429
15,1,2153.586538
15,1,2340.937500
15,1,1764.102564
15,1,5702.092105
15,1,2147.670455
15,1,8431.229167
15,1,1620.471429
15,1,9546.725000
15,1,1905.577778
15,1,3473.274457
15,1,3681.625000
15,1,5461.758721
15,1,2682.034884
15,1,10176.182692
15,1,7571.956731
15,1,7401.500000
15,1,12133.666667
15,1,1581.437500
15,1,2440.658784
15,1,10410.347656
15,1,6937.280303
15,1,14922.238095
15,1,3023.220000
15,1,4128.104651
15,1,1584.795455
15,1,3421.309211
15,1,6848.947368
15,1,2229.679348
15,1,1971.571970
15,1,3465.750000
15,1,1907.695652
15,1,2892.215517
15,1,11672.382812
15,1,2438.320513
15,1,1771.312500
15,1,3025.972973
15,1,3382.375000
15,1,3775.522222
15,1,8872.554878
15,1,10018.913462
15,1,5988.263158
15,1,2281.000000
15,1,6145.453804
15,1,1748.473214
15,1,1778.163265
15,1,3604.940789
15,1,16781.500000
15,1,2789.303571
15,1,3088.243243
15,1,2621.857143
15,1,6348.160000
15,1,1743.375000
15,1,1984.170455
15,1,1864.675000

15,1,1717.735294
15,1,5106.735294
15,1,6018.843750
15,1,1705.018750
15,1,4478.154762
15,1,1956.969512
15,1,18343.821429
15,1,2684.940341
15,1,9460.878049
15,1,2736.913462
15,1,7261.279070
15,1,3340.000000
15,1,1871.539773
15,1,2424.416667
15,1,1847.031250
15,1,1727.781250
15,1,1965.833333
15,1,1654.256410
15,1,5653.333333
15,1,2398.882353
15,1,1729.875000
15,1,12067.008929
15,1,5435.345745
15,1,1504.279070
15,1,7064.978261
15,1,2755.828571
15,1,8845.340517
15,1,5533.468085
15,1,10481.391304
15,1,17602.000000
15,1,1720.057143
15,1,2204.829268
15,1,10091.500000
15,1,4987.250000
15,1,14509.536458
15,1,1776.420213
15,1,3235.923077
15,1,4630.700000
15,1,3766.509375
15,1,1804.239796
15,1,1947.388889
15,1,1932.428571
15,1,2457.864286
15,1,1854.893382
15,1,1899.985294
15,1,4070.151515
15,1,15481.815789
15,1,7598.276316
15,1,2074.632500
15,1,13747.540625
15,1,5102.863636
15,1,17731.260000
15,1,12077.435811
15,1,3540.800000
15,1,13484.262500
15,1,4816.962264
15,1,1706.540541
15,1,10687.228723
15,1,1838.898438
15,1,3715.254808
15,1,1843.898438
15,1,6016.722973
15,1,4320.352500
15,1,8074.930147
15,1,1587.316176
15,1,2826.322581

15,1,2787.683673
15,1,5052.235294
15,1,4653.286765
15,1,3294.147222
15,1,6586.062500
15,1,3572.042683
15,1,1844.264706
15,1,2083.766447
15,1,1767.500000
15,1,1874.088415
15,1,5779.842391
15,1,2621.400000
15,1,5375.000000
15,1,2122.548780
15,1,1955.536585
15,1,2204.436170
15,1,1989.437500
15,1,10121.000000
15,1,1588.787500
15,1,2622.050000
15,1,1795.250000
15,1,2029.078571
15,1,1925.967742
15,1,12079.875000
15,1,9226.205128
15,1,7652.200000
15,1,8251.351852
15,1,12643.968750
15,1,1599.323529
15,1,2095.542683
15,1,2101.234043
15,1,7568.826087
15,1,3069.160714
15,1,5221.666667
15,1,2036.925000
15,1,1951.078125
15,1,1759.921875
15,1,3671.985714
15,1,1630.437500
15,1,10097.523256
15,1,4345.888889
15,1,5347.743902
15,1,3984.761029
15,1,3667.250000
15,1,1596.548780
15,1,2974.875000
15,1,5334.128571
15,1,18118.882353
15,1,13154.715909
15,1,8846.585714
15,1,3439.650000
15,1,2111.000000
15,1,4684.875000
15,1,2832.730769
15,1,5112.330882
15,1,10024.344512
15,1,7056.324324
15,1,2512.723404
15,1,1966.250000
15,1,4827.713889
15,1,7436.685185
15,1,2515.164634
15,1,18680.600000
15,1,7252.089286
15,1,2278.111111
15,1,5935.000000

15,1,6127.692308
15,1,4135.466216
15,1,1946.568182
15,1,8301.658537
15,1,3572.216216
15,1,1567.029412
15,1,11686.804878
15,1,2937.769231
15,1,3017.125000
15,1,2178.400000
15,1,1607.955357
15,1,8351.708333
15,1,9017.464286
15,1,4251.281250
15,1,6162.618590
15,1,3300.212766
15,1,5388.059659
15,1,2208.898810
15,1,1549.622549
15,1,5498.979167
15,1,1740.140625
15,1,2014.274390
15,1,1802.984375
15,1,4113.627907
15,1,1594.135714
15,1,12978.941176
15,1,4968.896552
15,1,2708.408537
15,1,4179.803571
15,1,18056.223684
15,1,10446.948276
15,1,2491.494681
15,1,3040.968750
15,1,5845.513298
15,1,4597.750000
15,1,1711.761111
15,1,7215.221154
15,1,2260.048077
15,1,1507.476744
15,1,1541.385204
15,1,1843.568182
15,1,14902.690476
15,1,1906.342105
15,1,2607.466216
15,1,1610.423469
15,1,6511.785714
15,1,3186.625000
15,1,5416.886029
15,1,5629.599265
15,1,5476.500000
15,1,2774.571970
15,1,1523.680556
15,1,6300.754717
15,1,2536.218750
15,1,2161.641129
15,1,3295.011628
15,1,3284.465625
15,1,1646.357143
15,1,9353.361842
15,1,6177.046875
15,1,4782.717277
15,1,9970.600000
15,1,4657.500000
15,1,7035.723404
15,1,17049.907609
15,1,2669.800000

15,1,4773.875000
15,1,1903.517857
15,1,4077.028571
15,1,4334.229167
15,1,7229.687500
15,1,4852.923077
15,1,5770.467742
15,1,2523.069767
15,1,9707.948718
15,1,1553.236111
15,1,2460.692308
15,1,6326.772727
15,1,5856.636905
15,1,6074.907609
15,1,5412.538462
15,1,2479.954545
15,1,1668.487500
15,1,2814.750000
15,1,3563.166667
15,1,2317.756250
15,1,4547.574405
15,1,14858.333333
15,1,2435.794118
15,1,2204.562500
15,1,6568.871212
15,1,2485.782500
15,1,1688.715625
15,1,6751.250000
15,1,3097.331522
15,1,2314.209302
15,1,3403.470588
15,1,3512.794118
15,1,1560.743750
15,1,8829.000000
15,1,11886.903846
15,1,6527.784574
15,1,2604.200000
15,1,2463.579082
15,1,3271.365132
15,1,18762.829268
15,1,12446.137931
15,1,2757.523810
15,1,2988.317308
15,1,4387.891667
15,1,1880.287500
15,1,1996.961538
15,1,2402.384615
15,1,1764.490741
15,1,2742.672078
15,1,2660.212766
15,1,2574.571429
15,1,1823.083333
15,1,1986.872340
15,1,8509.161290
15,1,2022.769697
15,1,4331.053571
15,1,2207.265625
15,1,3206.656250
15,1,1866.000000
15,1,3064.201613
15,1,6229.502976
15,1,7923.246951
15,1,12231.142857
15,1,5271.513158
15,1,2540.322917
15,1,1523.750000

15,1,3006.250000
15,1,1595.750000
15,1,8821.164286
15,1,2738.906250
15,1,3072.161765
15,1,7672.562500
15,1,4254.221875
15,1,1757.419872
15,1,2775.690789
15,1,3724.384615
15,1,2096.263587
15,1,2019.603659
15,1,4138.793750
15,1,4217.606707
15,1,5031.722561
15,1,4677.130952
15,1,4472.769231
15,1,6802.180556
15,1,4186.625000
15,1,1666.414894
15,1,18864.173077
15,1,1939.414474
15,1,2131.271739
15,1,9046.618750
15,1,4205.600000
15,1,3335.708333
15,1,2337.700000
15,1,4060.773810
15,1,2684.133333
15,1,1879.125000
15,1,2147.182692
15,1,1645.705128
15,1,3204.693878
15,1,7533.918367
15,1,3742.396341
15,1,9496.483333
15,1,2530.708333
15,1,16868.470588
15,1,2341.596154
15,1,4670.926471
15,1,1881.962500
15,1,3726.391304
15,1,1973.575658
15,1,2250.145270
15,1,9341.512195
15,1,3270.500000
15,1,8444.750000
15,1,1845.668750
15,1,17935.303030
15,1,2829.800000
15,1,16197.125000
15,1,3834.921875
15,1,3121.788043
15,1,2115.790698
15,1,1501.972222
15,1,5682.833333
15,1,1595.060976
15,1,3567.343750
15,1,1602.000000
15,1,4369.657609
15,1,4128.775000
15,1,10409.444444
15,1,4057.870370
15,1,1981.323718
15,1,2463.195122
15,1,1795.000000

15,1,8557.182065
15,1,9320.833333
15,1,2558.083333
15,1,17818.137500
15,1,2991.216912
15,1,6146.307692
15,1,13631.815972
15,1,4694.651163
15,1,3431.243902
15,1,5543.659884
15,1,5295.022727
15,1,6631.625000
15,1,2845.081522
15,1,8435.713235
15,1,1806.936047
15,1,8650.525000
15,1,2191.612179
15,1,4259.774457
15,1,2868.144231
15,1,14513.252778
15,1,1601.310606
15,1,16522.900000
15,1,3222.154605
15,1,3777.573529
15,1,3158.488095
15,1,1745.301136
15,1,1717.511364
15,1,2970.583333
15,1,1790.539773
15,1,2522.253378
15,1,3319.226190
15,1,3849.756098
15,1,1550.221591
15,1,3494.215517
15,1,2850.500000
15,1,11739.083333
15,1,18967.333333
15,1,2003.187500
15,1,2774.050000
15,1,15453.928571
15,1,1880.090909
15,1,7745.824324
15,1,5245.958333
15,1,4234.714286
15,1,10120.642857
15,1,5328.813830
15,1,1831.292683
15,1,6614.491935
15,1,12703.282258
15,1,2007.391304
15,1,5824.036765
15,1,5188.517857
15,1,2630.097561
15,1,17132.416667
15,1,2283.106771
15,1,2505.081395
15,1,1725.142857
15,1,5427.272727
15,1,4904.580882
15,1,2515.625000
15,1,1715.710938
15,1,8421.027778
15,1,6992.256579
15,1,3139.270270
15,1,3020.000000
15,1,4891.172414

15,1,2264.250000
15,1,3630.948718
15,1,1614.335366
15,1,10178.750000
15,1,1946.186111
15,1,12312.148148
15,1,3280.718750
15,1,4517.928571
15,1,2073.590426
15,1,5837.500000
15,1,3750.236486
15,1,7889.691406
15,1,6979.760417
15,1,2082.708333
15,1,3157.573171
15,1,17636.302632
15,1,2581.933511
15,1,6411.913690
15,1,3850.260870
15,1,2381.235119
15,1,1786.795455
15,1,2182.125000
15,1,11413.986842
15,1,6394.597656
15,1,16798.076923
15,1,18181.629310
15,1,11118.378378
15,1,4203.252976
15,1,1803.541667
15,1,2859.350962
15,1,19723.630952
15,1,12131.928125
15,1,2147.782051
15,1,6182.700000
15,1,4869.835366
15,1,14381.967105
15,1,5914.992857
15,1,5979.384146
15,1,9591.331250
15,1,4941.822368
15,1,3642.985294
15,1,14134.479730
15,1,4573.922872
15,1,1996.194444
15,1,9527.682927
15,1,1547.938889
15,1,1602.516509
15,1,2157.384615
15,1,8564.841463
15,1,4390.750000
15,1,3703.184211
15,1,3606.875000
15,1,3093.992188
15,1,1620.500000
15,1,1914.803977
15,1,2845.321429
15,1,8849.159091
15,1,13898.807692
15,1,8963.625000
15,1,7607.782738
15,1,3398.166667
15,1,3092.500000
15,1,2429.393939
15,1,6995.077830
15,1,3030.021277
15,1,2741.706081

15,1,2052.958333
15,1,19765.548387
15,1,3248.025735
15,1,1523.403125
15,1,3944.011029
15,1,6287.326923
15,1,5335.005952
15,1,10762.072581
15,1,3443.152778
15,1,7259.700000
15,1,5382.512500
15,1,2216.362500
15,1,11023.733696
15,1,3699.934211
15,1,3692.297619
15,1,1560.615385
15,1,6023.823529
15,1,1779.282051
15,1,2723.125000
15,1,2783.768293
15,1,8552.090909
15,1,1529.937500
15,1,2121.906250
15,1,3613.000000
15,1,9706.087963
15,1,1651.250000
15,1,2118.839286
15,1,3186.923077
15,1,8306.792857
15,1,2856.105263
15,1,10095.395161
15,1,7745.755952
15,1,5422.215426
15,1,5441.444079
15,1,3829.756281
15,1,9766.382353
15,1,3923.200000
15,1,14005.712500
15,1,12765.479730
15,1,3840.809524
15,1,2029.345238
15,1,5049.284722
15,1,1661.335366
15,1,1835.443182
15,1,1698.897436
15,1,3543.321429
15,1,4430.707317
15,1,2081.264535
15,1,2048.000000
15,1,1512.103448
15,1,1914.770349
15,1,2303.000000
15,1,1749.825000
15,1,2301.234694
15,1,6616.928571
15,1,3471.666667
15,1,5930.003676
15,1,1775.127717
15,1,8952.101351
15,1,3372.794118
15,1,4746.571429
15,1,14136.277778
15,1,11101.500000
15,1,1851.807692
15,1,1889.628125
15,1,6690.952381

15,1,11614.403226
15,1,5481.921875
15,1,10311.902778
15,1,2282.781250
15,1,12127.307143
15,1,11709.909091
15,1,4689.906250
15,1,5895.650000
15,1,2361.622024
15,1,5673.428571
15,1,4036.132353
15,1,6699.539634
15,1,6864.770270
15,1,3237.109375
15,1,11053.872093
15,1,4934.085366
15,1,6926.102564
15,1,1729.846154
15,1,2848.500000
15,1,1648.974359
15,1,2136.631579
15,1,2401.959559
15,1,11808.312500
15,1,3822.125000
15,1,1821.483871
15,1,1930.939024
15,1,10646.517857
15,1,11318.428571
15,1,2803.364130
15,1,5682.784375
15,1,2593.307292
15,1,5053.242188
15,1,1512.602941
15,1,3680.121622
15,1,9442.052083
15,1,1889.067073
15,1,2842.961538
15,1,3920.723214
15,1,1890.128571
15,1,2224.861111
15,1,2897.256410
15,1,6695.318750
15,1,5599.807692
15,1,12724.636029
15,1,1597.470930
15,1,7241.470588
15,1,2827.138889
15,1,1628.837209
15,1,7382.727273
15,1,5602.390244
15,1,5552.318182
15,1,3195.758523
15,1,3703.218750
15,1,2763.720745
15,1,13673.428571
15,1,3371.714286
15,1,3236.077128
15,1,3295.058140
15,1,8288.910714
15,1,4497.258065
15,1,9147.274390
15,1,9379.542857
15,1,4384.286585
15,1,3270.295918
15,1,16367.187500
15,1,2479.268229

15,1,2752.227941
15,1,13370.845109
15,1,2853.741071
15,1,2896.825000
15,1,2554.717949
15,1,12807.495000
15,1,6405.857143
15,1,2293.957317
15,1,1781.500000
15,1,6100.385638
15,1,5800.809524
15,1,2209.432065
15,1,4798.643939
15,1,1909.071429
15,1,1662.534722
15,1,1956.758333
15,1,19001.463415
15,1,11615.319853
15,1,1554.225000
15,1,4139.344512
15,1,5550.000000
15,1,14878.452830
15,1,3972.000000
15,1,5023.316176
15,1,1834.357143
15,1,3235.162500
15,1,3377.551020
15,1,1981.574074
15,1,8730.237500
15,1,1838.404412
15,1,6430.009868
15,1,14009.160714
15,1,5859.032895
15,1,5041.146552
15,1,1805.166667
15,1,10275.197917
15,1,6314.325000
15,1,3491.002976
15,1,1501.189024
15,1,1567.338542
15,1,7057.259615
15,1,8577.500000
15,1,3743.023649
15,1,9892.606383
15,1,8393.141026
15,1,5724.323529
15,1,5753.823529
15,1,4177.202703
15,1,2273.843750
15,1,15984.297872
15,1,1611.723684
15,1,1650.959184
15,1,3545.760736
15,1,2401.951220
15,1,2331.205556
15,1,10334.345109
15,1,4604.448718
15,1,1891.269231
15,1,4936.406250
15,1,10982.236842
15,1,5326.425000
15,1,19534.925532
15,1,5459.350694
15,1,1630.234043
15,1,1588.216463
15,1,2235.827128

15,1,2662.052632
15,1,4873.000000
15,1,1550.597222
15,1,4624.203390
15,1,2661.246324
15,1,4341.598404
15,1,2008.666667
15,1,3593.414062
15,1,1503.410256
15,1,5234.271875
15,1,6180.152439
15,1,14035.878378
15,1,3995.854167
15,1,5922.221591
15,1,2759.550000
15,1,2671.128049
15,1,4344.884375
15,1,3944.219697
15,1,4545.171875
15,1,2264.109043
15,1,2180.371795
15,1,1970.021739
15,1,1608.588235
15,1,3509.500000
15,1,2174.750000
15,1,1506.000000
15,1,3299.333333
15,1,2726.150000
15,1,2812.298611
15,1,1552.973684
15,1,1868.083333
15,1,2200.954545
15,1,2042.306250
15,1,4019.494186
15,1,3771.212838
15,1,2809.091463
15,1,5383.709239
15,1,6935.611111
15,1,10766.272727
15,1,1822.125000
15,1,3708.782609
15,1,2561.511364
15,1,1561.450000
15,1,6402.094697
15,1,3835.427419
15,1,9898.812500
15,1,1670.418605
15,1,3206.923077
15,1,7769.546875
15,1,3194.372340
15,1,7251.043605
15,1,4048.062500
15,1,9079.750000
15,1,3592.458333
15,1,14894.707317
15,1,1967.306250
15,1,2422.833333
15,1,2179.320755
15,1,8848.488636
15,1,1730.064516
15,1,1556.708333
15,1,2456.494318
15,1,4440.953125
15,1,3675.698529
15,1,3359.468750
15,1,3887.644444

15,1,2694.689024
15,1,8739.793103
15,1,6241.455357
15,1,8874.878676
15,1,6588.627907
15,1,6421.066667
15,1,8159.621795
15,1,3870.943750
15,1,3821.213415
15,1,5456.447115
15,1,2037.323529
15,1,1787.349432
15,1,1784.453947
15,1,1651.000000
15,1,6618.000000
15,1,1560.771429
15,1,2031.791667
15,1,2346.509868
15,1,9831.696429
15,1,1600.750000
15,1,5398.911765
15,1,2843.597973
15,1,12077.272436
15,1,1878.432927
15,1,1795.928571
15,1,3535.543750
15,1,6706.041667
15,1,6442.000000
15,1,3966.185484
15,1,2550.212500
15,1,15861.416667
15,1,2629.243243
15,1,3916.750000
15,1,17263.471154
15,1,3342.759615
15,1,3177.578125
15,1,1761.305000
15,1,13335.842857
15,1,12763.496094
15,1,8581.600543
15,1,4090.106061
15,1,2628.268293
15,1,1845.083333
15,1,3153.115625
15,1,2118.377778
15,1,4326.726974
15,1,2792.945122
15,1,6171.595238
15,1,5810.113095
15,1,10391.977778
15,1,8787.835938
15,1,2020.333333
15,1,4561.010204
15,1,2938.809783
15,1,2447.880208
15,1,2343.937500
15,1,4539.060811
15,1,2955.770161
15,1,1794.350000
15,1,4196.773810
15,1,1905.045732
15,1,1874.337500
15,1,2060.364583
15,1,11484.779412
15,1,2783.677083
15,1,2477.035714

15,1,1991.558824
15,1,2514.552885
15,1,5495.290816
15,1,2226.125000
15,1,1921.330556
15,1,3051.521277
15,1,3202.400000
15,1,3515.416667
15,1,2873.250000
15,1,11371.747449
15,1,3859.461538
15,1,6316.632812
15,1,6948.670139
15,1,4569.352273
15,1,5075.895161
15,1,3715.605556
15,1,3082.592105
15,1,15754.727941
15,1,2149.909091
15,1,3288.073864
15,1,2068.530612
15,1,4751.780000
15,1,5986.421053
15,1,2127.801136
15,1,2012.464674
15,1,6773.180556
15,1,4482.329545
15,1,7951.941176
15,1,3024.428571
15,1,1715.664474
15,1,12600.682692
15,1,1880.397727
15,1,2547.704545
15,1,3159.000000
15,1,2372.368750
15,1,2044.183673
15,1,7430.058140
15,1,3442.640625
15,1,2288.412736
15,1,4920.841463
15,1,7923.280303
15,1,1694.697368
15,1,16610.055556
15,1,5943.697368
15,1,2682.970588
15,1,3566.875000
15,1,1684.593023
15,1,2598.108553
15,1,4918.102041
15,1,9857.404762
15,1,3981.964286
15,1,3167.609756
15,1,3719.988889
15,1,1533.000000
15,1,3399.986413
15,1,5084.631579
15,1,12683.166667
15,1,3245.437500
15,1,5298.285714
15,1,3160.541667
15,1,8506.829268
15,1,1806.395833
15,1,2342.364583
15,1,5766.240625
15,1,3140.397059
15,1,5767.671053

15,1,5638.000000
15,1,1996.128049
15,1,3397.911765
15,1,16452.968085
15,1,1625.833333
15,1,4561.714286
15,1,4590.592391
15,1,19393.058824
15,1,1917.331250
15,1,2391.293605
15,1,4241.355556
15,1,3200.334459
15,1,2641.021429
15,1,4976.169118
15,1,1688.657609
15,1,1618.246212
15,1,2153.867647
15,1,3072.011628
15,1,2953.517857
15,1,5878.301136
15,1,2684.295455
15,1,2268.875000
15,1,2402.785714
15,1,1868.015625
15,1,2514.928571
15,1,1773.019231
15,1,2202.925000
15,1,6615.628205
15,1,4596.703125
15,1,3743.953125
15,1,10046.246875
15,1,8689.161765
15,1,2459.900000
15,1,4299.218750
15,1,4935.625000
15,1,4854.614286
15,1,4890.104167
15,1,1673.521739
15,1,2273.428571
15,1,7762.500000
15,1,2490.181818
15,1,10956.500000
15,1,4882.464844
15,1,6756.589286
15,1,10458.375000
15,1,2629.803571
15,1,5404.200000
15,1,4927.990385
15,1,6614.785714
15,1,1894.125000
15,1,3244.833333
15,1,2759.982759
15,1,5264.757353
15,1,2290.752976
15,1,4519.173077
15,1,3782.814103
15,1,12998.358553
15,1,1939.515244
15,1,4896.855769
15,1,4066.524390
15,1,3234.428571
15,1,3051.596429
15,1,8396.410714
15,1,13639.732558
15,1,3688.595395
15,1,2661.308824

15,1,1777.847561
15,1,2588.926471
15,1,5516.267857
15,1,3471.406250
15,1,1914.888158
15,1,13393.191489
15,1,8053.714286
15,1,11295.576923
15,1,7034.040441
15,1,16783.477941
15,1,8445.343750
15,1,2162.750000
16,2,2721.502841
16,2,2128.769737
16,2,1749.875000
16,2,2547.596154
16,2,1537.893617
16,2,2671.690000
16,2,2875.678125
16,2,2291.238095
16,2,1910.867788
16,2,1873.601744
16,2,4743.250000
16,2,1968.737805
16,2,1591.412791
16,2,2934.175481
16,2,2335.012755
16,2,2198.710000
16,2,1751.429348
16,2,1589.564516
16,2,1873.613095
16,2,1798.400000
16,2,5023.440000
16,2,2944.125000
16,2,2189.444444
16,2,1865.597561
16,2,1629.600000
16,2,1700.435811
16,2,2076.820312
16,2,1925.934211
16,2,1691.008152
16,2,1716.000000
16,2,2363.477941
16,2,1739.828947
16,2,2779.729167
16,2,1685.225000
16,2,2072.621711
16,2,1552.210784
16,2,1604.394737
16,2,1940.843750
16,2,1813.635870
16,2,2619.192857
16,2,2682.534375
16,2,2414.050000
16,2,2062.488889
16,2,1767.145833
16,2,2449.350877
16,2,1547.350000
16,2,1789.769737
16,2,2774.771277
16,2,1566.500000
16,2,2229.331395
16,2,3640.575000
16,2,2172.812500
16,2,6090.735849
16,2,2269.000000

16,2,1749.936170
16,2,1661.533333
16,2,1957.816489
16,2,1566.395833
16,2,2148.079082
16,2,1681.478261
16,2,1945.312500
16,2,1829.825893
16,2,2532.250000
16,2,2034.521226
16,2,1632.317500
16,2,2939.925926
16,2,1737.710526
16,2,2554.632159
16,2,2282.028409
16,2,4809.959302
16,2,1579.119048
16,2,1658.938889
16,2,3522.511111
16,2,3378.168367
16,2,1966.382353
16,2,2322.080882
16,2,3349.794776
16,2,1941.521429
16,2,6079.165323
16,2,2721.875000
16,2,2002.015625
16,2,2068.561404
16,2,2004.612903
16,2,1557.778302
16,2,2311.295455
16,2,5134.416667
16,2,1547.351974
16,2,2947.270000
16,2,2290.852941
16,2,3162.548913
16,2,1783.744792
16,2,2155.732143
16,2,2497.093750
16,2,1591.875000
16,2,2243.583333
16,2,5270.842308
16,2,8001.464286
16,2,1724.434524
16,2,1604.709302
16,2,2011.833333
16,2,1754.500000
16,2,3638.690789
16,2,2999.796296
16,2,2514.015152
16,2,1675.795000
16,2,1824.533019
16,2,2644.090909
16,2,1806.147059
16,2,1585.750000
16,2,1738.845930
16,2,2625.177500
16,2,2555.627551
16,2,4882.954545
16,2,2877.437500
16,2,3218.951923
16,2,2142.845930
16,2,1606.960000
16,2,1538.361413
16,2,1894.367188
16,2,1824.761111

16,2,1866.031915
16,2,1715.250000
16,2,6940.625000
16,2,3892.596354
16,2,1980.234043
16,2,1505.808511
16,2,1888.560000
16,2,8125.750000
16,2,1840.400000
16,2,1978.362245
16,2,2180.760870
16,2,2552.850000
16,2,1537.916667
16,2,1950.340426
16,2,2293.250000
16,2,1797.413265
16,2,1962.027778
16,2,1727.779412
16,2,3436.208333
16,2,1625.473837
16,2,3149.815789
16,2,2763.800000
16,2,1715.769231
16,2,2210.301339
16,2,1616.766667
16,2,1998.581522
16,2,2302.250000
16,2,2377.684896
16,2,2104.662791
16,2,2302.686275
16,2,2031.363636
16,2,2123.192308
16,2,1706.836538
16,2,2250.166667
16,2,1562.312500
16,2,1891.796569
16,2,2745.369444
16,2,2523.293478
16,2,1561.163690
16,2,1554.659574
16,2,1971.000000
16,2,1628.093750
16,2,10543.801136
16,2,1875.924419
16,2,1798.950581
16,2,9525.819149
16,2,2079.681818
16,2,2225.680000
16,2,2487.618421
16,2,1991.530000
16,2,1528.046875
16,2,1790.489796
16,2,1804.562500
16,2,2033.875000
16,2,3796.941026
16,2,7856.829545
16,2,3819.500000
16,2,1882.538043
16,2,1538.000000
16,2,2050.454167
16,2,1678.782609
16,2,4802.885246
16,2,3564.660326
16,2,1660.333333
16,2,8589.375000
16,2,6836.116071

16,2,1989.868750
16,2,1606.702128
16,2,4261.696809
16,2,2761.312500
16,2,2084.140000
16,2,1750.723404
16,2,1843.350446
16,2,2452.875000
16,2,2014.129386
16,2,1574.347826
16,2,1558.347222
16,2,1760.882353
16,2,1947.333333
16,2,1959.170213
16,2,1848.333333
16,2,1810.115000
16,2,2585.088235
16,2,2305.015957
16,2,2115.669643
16,2,2647.186224
16,2,3378.179487
16,2,4173.375000
16,2,1693.713942
16,2,2258.851293
16,2,2547.075000
16,2,1895.654167
16,2,1695.847222
16,2,1562.564024
16,2,2784.944444
16,2,2054.187500
16,2,4528.800000
16,2,3771.162500
16,2,1523.307500
16,2,3909.491071
16,2,1748.390625
16,2,2233.610795
16,2,1748.609375
16,2,2173.500000
16,2,1575.118750
16,2,2145.229592
16,2,5718.447368
16,2,2064.404412
16,2,15721.295455
16,2,3806.319149
16,2,1635.089286
16,2,1645.022959
16,2,2503.500000
16,2,1840.500000
16,2,6865.832589
16,2,2094.083333
16,2,1875.375000
16,2,2572.650000
16,2,1646.053571
16,2,2505.669118
16,2,5097.621951
16,2,1936.000000
16,2,4227.167832
16,2,1921.808140
16,2,1636.402174
16,2,2160.387500
16,2,3732.656915
16,2,2020.826923
16,2,1771.993056
16,2,2004.583333
16,2,1620.638298
16,2,1669.461538

16,2,6999.230392
16,2,1787.928571
16,2,1531.705882
16,2,3580.010417
16,2,2004.644231
16,2,1516.400000
16,2,5852.145000
16,2,1502.908537
16,2,2077.093085
16,2,2632.166667
16,2,1823.204545
16,2,1818.571429
16,2,1544.682927
16,2,1624.573171
16,2,1543.594828
16,2,3246.668478
16,2,1629.293750
16,2,1675.916667
16,2,1779.101064
16,2,2222.915094
16,2,1814.404762
16,2,1664.597826
16,2,1646.093750
16,2,1719.071429
16,2,2245.310000
16,2,1606.890625
16,2,1539.500000
16,2,1988.274510
16,2,2203.108974
16,2,1890.059524
16,2,4792.025463
16,2,1994.787234
16,2,1801.787500
16,2,5414.106250
16,2,1917.046569
16,2,1859.979592
16,2,4485.184375
16,2,1876.250000
16,2,1568.729730
16,2,1580.840909
16,2,2826.411765
16,2,4921.576531
16,2,9812.328125
16,2,1929.421053
16,2,3807.115385
16,2,6497.172500
16,2,2835.539326
16,2,1677.750000
16,2,2351.509615
16,2,1908.413265
16,2,2187.625000
16,2,2859.962719
16,2,1773.814103
16,2,6941.625000
16,2,3018.593750
16,2,1769.397959
16,2,2078.788043
16,2,2487.698370
16,2,2788.084906
16,2,1856.581731
16,2,3374.229167
16,2,2332.980000
16,2,1877.631579
16,2,1898.106383
16,2,4535.026961
16,2,3332.618750

16,2,2681.267857
16,2,1871.675000
16,2,1862.364796
16,2,4398.146341
16,2,1894.841463
16,2,1574.562500
16,2,2252.250000
16,2,2027.966981
16,2,2517.225962
16,2,1678.882353
16,2,2254.562500
16,2,2889.725000
16,2,2938.671875
16,2,4951.993421
16,2,2081.448864
16,2,2344.980000
16,2,1574.642857
16,2,1581.022500
16,2,1714.798913
16,2,4773.157692
16,2,3288.277778
16,2,1852.755102
16,2,1920.764706
16,2,2556.428571
16,2,2011.661765
16,2,1658.143293
16,2,2337.572917
16,2,2061.824324
16,2,1791.928571
16,2,1536.676630
16,2,1934.000000
16,2,1589.275862
16,2,2479.250000
16,2,1954.635714
16,2,1766.562500
16,2,1963.173611
16,2,1812.852941
16,2,1613.726190
16,2,3489.852778
16,2,2377.105556
16,2,1604.113372
16,2,1652.892157
16,2,2792.711538
16,2,1748.690476
16,2,2259.600000
16,2,1905.410714
16,2,1872.954545
16,2,1709.388393
16,2,1660.618182
16,2,2790.808673
16,2,1526.206522
16,2,1541.740385
16,2,1551.944444
16,2,4881.941176
16,2,5731.368421
16,2,17008.285714
16,2,1882.813830
16,2,3804.450000
16,2,1563.185000
16,2,1525.878049
16,2,2835.596154
16,2,2480.437500
16,2,1789.578431
16,2,4137.648936
16,2,2959.096154
16,2,2042.687500

16,2,2650.250000
16,2,2000.145349
16,2,2751.296296
16,2,2310.409091
16,2,1612.260870
16,2,2263.090909
16,2,3970.500000
16,2,10649.515244
16,2,2105.231481
16,2,10956.792411
16,2,1518.238095
16,2,3214.333333
16,2,1646.141304
16,2,8677.880682
16,2,3695.617647
16,2,15434.973214
16,2,9655.595745
16,2,6814.454054
16,2,1524.494318
16,2,2630.287736
16,2,3433.645455
16,2,3957.957447
16,2,1698.581818
16,2,2596.804054
16,2,2377.147727
16,2,2585.872024
16,2,1665.831633
16,2,1508.614130
16,2,3629.347826
16,2,1891.394737
16,2,3559.238889
16,2,1830.914773
16,2,1605.812500
16,2,1759.138889
16,2,1731.640625
16,2,2078.506696
16,2,2154.901786
16,2,2766.132353
16,2,3485.892857
16,2,2240.937209
16,2,2489.801932
16,2,1905.434783
16,2,16708.454545
16,2,1935.917910
16,2,1637.651163
16,2,2551.125000
16,2,3169.163265
16,2,2299.840000
16,2,1695.208333
16,2,3959.368750
16,2,1761.566667
16,2,3649.170732
16,2,2167.000000
16,2,2023.825000
16,2,3866.128049
16,2,4120.622549
16,2,8376.016949
16,2,2051.404255
16,2,5545.250000
16,2,1637.604651
16,2,2889.815217
16,2,1602.464286
16,2,2273.006356
16,2,1562.950000
16,2,1778.214286
16,2,4510.956522

16,2,1553.000000
16,2,2242.902778
16,2,2512.948864
16,2,1551.122283
16,2,2310.875000
16,2,1653.545455
16,2,3199.364583
16,2,1758.302083
16,2,7757.255556
16,2,1709.800000
16,2,1573.408163
16,2,1862.872093
16,2,1979.728723
16,2,2190.442708
16,2,1587.392857
16,2,2582.494565
16,2,1959.562500
16,2,1605.000000
16,2,2365.968085
16,2,1710.900000
16,2,1709.076220
16,2,1973.000000
16,2,1719.883152
16,2,1524.038265
16,2,2167.846591
16,2,2706.742788
16,2,3189.017857
16,2,12716.742188
16,2,1591.597561
16,2,1621.026042
16,2,1588.492647
16,2,1623.750000
16,2,1839.452128
16,2,1876.372845
16,2,2142.869792
16,2,2033.609375
16,2,2141.919118
16,2,2992.563725
16,2,1554.500000
16,2,2589.447115
16,2,1854.326316
16,2,3383.596591
16,2,1790.209302
16,2,2078.717391
16,2,2094.470455
16,2,3005.351351
16,2,1593.528125
16,2,2425.377778
16,2,2264.454545
16,2,2074.961538
16,2,2733.760417
16,2,2197.388021
16,2,1661.075000
16,2,2548.051136
16,2,2754.122024
16,2,1656.428571
16,2,1661.813253
16,2,2845.687500
16,2,1911.591216
16,2,1939.750000
16,2,4155.166667
16,2,3094.217593
16,2,5809.554348
16,2,2093.500000
16,2,1621.937500
16,2,1862.413043

16,2,1706.045455
16,2,2787.255208
16,2,1527.648649
16,2,1823.573171
16,2,1750.541667
16,2,3048.575000
16,2,1955.280172
16,2,1799.909091
16,2,1953.800000
16,2,2507.397059
16,2,1964.243243
16,2,5718.213415
16,2,2061.166667
16,2,2000.605769
16,2,2787.000000
16,2,1503.226064
16,2,1915.000000
16,2,2414.395349
16,2,2903.709302
16,2,2662.408654
16,2,1861.262500
16,2,1589.510638
16,2,1968.357143
16,2,2598.098958
16,2,4426.782609
16,2,2441.211957
16,2,1989.651515
16,2,2421.234756
16,2,2267.955556
16,2,2666.777273
16,2,2291.783654
16,2,2551.565104
16,2,1980.147222
16,2,3485.456522
16,2,2586.855769
16,2,1589.358491
16,2,1840.050000
16,2,3127.000000
16,2,3165.766667
16,2,2006.559278
16,2,1971.881944
16,2,1599.885417
16,2,6173.463415
16,2,2696.525000
16,2,2944.772727
16,2,1543.563953
16,2,2938.333333
16,2,2298.266447
16,2,1646.000000
16,2,1689.615385
16,2,1717.487805
16,2,2807.083333
16,2,1550.143750
16,2,1528.375000
16,2,1933.954082
16,2,1751.148148
16,2,2134.680000
16,2,1962.804348
16,2,2075.933824
16,2,1579.908784
16,2,12755.172872
16,2,2439.043750
16,2,2596.617647
16,2,3875.169271
16,2,2126.840426
16,2,2344.042553

16,2,2080.533333
16,2,1965.830189
16,2,2245.812500
16,2,1660.544118
16,2,2114.710000
16,2,3489.960526
16,2,2487.304054
16,2,2243.692308
16,2,2257.963415
16,2,2645.489796
16,2,2603.152778
16,2,1815.423913
16,2,1630.848837
16,2,3534.037037
16,2,4073.557927
16,2,1772.565476
16,2,1982.328125
16,2,2176.801471
16,2,2608.846154
16,2,1710.971591
16,2,7125.247159
16,2,1847.134146
16,2,2461.400000
16,2,3823.764706
16,2,1794.836735
16,2,1790.138298
16,2,1835.528846
16,2,1905.097561
16,2,1608.116071
16,2,2335.831633
16,2,4975.862903
16,2,3229.846939
16,2,1858.342105
16,2,1688.950000
16,2,6561.910714
16,2,1979.665761
16,2,1554.000000
16,2,1711.104167
16,2,1788.738636
16,2,2895.783654
16,2,16490.388889
16,2,1910.795455
16,2,1636.074074
16,2,1947.702703
16,2,3043.258621
16,2,2406.012195
16,2,2243.106383
16,2,1974.375000
16,2,3549.709091
16,2,1755.309375
16,2,1590.731383
16,2,1830.986979
16,2,4393.619048
16,2,1899.169811
16,2,1514.902778
16,2,6901.565341
16,2,2711.928571
16,2,2348.504808
16,2,1618.564904
16,2,1505.147321
16,2,1808.348214
16,2,1996.078488
16,2,2609.977273
16,2,2036.837838
16,2,1686.950521
16,2,2294.731707

16,2,2182.025641
16,2,1524.687500
16,2,2801.151042
16,2,2959.520833
16,2,10632.002660
16,2,2391.000000
16,2,2698.151316
16,2,2968.412234
16,2,1960.252551
16,2,2029.696809
16,2,1589.795918
16,2,8638.921875
16,2,2094.290000
16,2,1781.088889
16,2,1697.534574
16,2,2208.430556
16,2,3532.760870
16,2,3053.850610
16,2,1589.330357
16,2,5775.551020
16,2,2266.468750
16,2,2088.800000
16,2,1723.988372
16,2,2720.079545
16,2,4443.377193
16,2,2354.128676
16,2,1828.860294
16,2,2348.127660
16,2,1702.153409
16,2,2114.140244
16,2,4188.232143
16,2,1708.940789
16,2,1698.036458
16,2,1817.300000
16,2,1880.132979
16,2,1508.838415
16,2,1791.500000
16,2,2216.687500
16,2,1506.018293
16,2,4817.984375
16,2,1627.204082
16,2,2934.375000
16,2,2669.139881
16,2,1654.718750
16,2,1958.769231
16,2,2064.034375
16,2,2030.363208
16,2,8283.716981
16,2,3532.225131
16,2,5586.179054
16,2,2275.469388
16,2,4238.444444
16,2,2301.062500
16,2,4017.837500
16,2,1523.888889
16,2,11304.943182
16,2,3390.903846
16,2,1546.921053
16,2,5885.145000
16,2,1752.813953
16,2,1526.767857
16,2,1769.988636
16,2,4851.525000
16,2,2970.010638
16,2,2039.062500
16,2,4593.857143

16,2,1976.790698
16,2,3021.750000
16,2,1987.884146
16,2,1957.250000
16,2,1505.250000
16,2,1588.782609
16,2,2628.750000
16,2,1596.740854
16,2,1551.152439
16,2,2312.218085
16,2,1597.388889
16,2,4248.968750
16,2,4906.404661
16,2,1574.928571
16,2,1523.331818
16,2,2513.438776
16,2,1653.070312
16,2,2369.533163
16,2,5011.844595
16,2,2478.375000
16,2,1689.530405
16,2,1734.262019
16,2,2670.638298
16,2,1933.411765
16,2,2763.156250
16,2,2897.365909
16,2,1607.857143
16,2,1598.315104
16,2,2131.352273
16,2,1978.685096
16,2,9215.087500
16,2,1883.625000
16,2,2569.385135
16,2,3607.953125
16,2,2238.875000
16,2,1932.375000
16,2,2662.005319
16,2,1723.128205
16,2,1607.823370
16,2,1784.921875
16,2,2025.496875
16,2,1932.500000
16,2,2264.539773
16,2,1648.609375
16,2,2937.066489
16,2,1639.676829
16,2,2201.254545
16,2,1969.890909
16,2,1702.829082
16,2,2375.459239
16,2,3601.434783
16,2,3237.942308
16,2,2176.741071
16,2,18208.267857
16,2,2984.318182
16,2,1600.261905
16,2,1637.460227
16,2,3483.393617
16,2,3729.257812
16,2,3920.422500
16,2,2940.669118
16,2,2402.837209
16,2,2327.331250
16,2,2424.503289
16,2,2622.946429
16,2,9939.118421

16,2,4441.542683
16,2,8862.516667
16,2,3119.836864
16,2,1752.806250
16,2,1513.275000
16,2,1732.071429
16,2,1713.410714
16,2,12193.768293
16,2,1688.163462
16,2,1732.894231
16,2,3297.106132
16,2,2090.406250
16,2,5958.983412
16,2,2003.000000
16,2,2705.542553
16,2,1780.944149
16,2,1628.712500
16,2,1639.456522
16,2,1792.172619
16,2,1724.268293
16,2,1639.261111
16,2,5636.002976
16,2,19300.073171
16,2,2936.325000
16,2,1709.317308
16,2,2194.608553
16,2,1673.494792
16,2,4404.585526
16,2,3325.222222
16,2,2626.183673
16,2,2867.902439
16,2,2619.709184
16,2,2132.507500
16,2,1820.423077
16,2,1684.861702
16,2,3567.062500
16,2,1540.125000
16,2,3328.131868
16,2,2546.472561
16,2,1663.625000
16,2,1522.482143
16,2,2100.210526
16,2,1749.031250
16,2,2606.559783
16,2,1651.200000
16,2,1967.071429
16,2,1863.479592
16,2,1671.500000
16,2,2851.733333
16,2,3779.517857
16,2,1530.843750
16,2,1709.923295
16,2,1534.220000
16,2,1857.772727
16,2,2296.773026
16,2,2681.000000
16,2,2191.964286
16,2,1507.015625
16,2,2457.800000
16,2,1571.743421
16,2,1664.500000
16,2,1501.392157
16,2,1804.782051
16,2,2814.125000
16,2,2355.214286
16,2,13605.480769

16,2,2443.946429
16,2,3237.125000
16,2,2757.651163
16,2,2677.883721
16,2,1768.089674
16,2,3818.827500
16,2,1887.352273
16,2,1865.283333
16,2,2463.834906
16,2,2778.840686
16,2,2545.179688
16,2,2201.833333
16,2,1805.556818
16,2,1916.211735
16,2,6823.372727
16,2,2794.309783
16,2,5361.489583
16,2,2286.830357
16,2,3269.865132
16,2,1894.525000
16,2,1726.312500
16,2,2142.853659
16,2,2492.430556
16,2,2396.765625
16,2,1688.625000
16,2,1604.508721
16,2,4231.329787
16,2,1616.750000
16,2,1513.114583
16,2,1523.785714
16,2,2713.368421
16,2,4792.140000
16,2,1524.424342
16,2,2797.616071
16,2,2107.627551
16,2,1848.437500
16,2,1709.993750
16,2,2843.161932
16,2,2608.959239
16,2,1939.901786
16,2,1546.301020
16,2,1920.056548
16,2,2909.323370
16,2,2240.871622
16,2,1630.925847
16,2,1703.976744
16,2,2321.215385
16,2,1721.405556
16,2,1620.618902
16,2,1531.600000
16,2,3966.691489
16,2,1893.825472
16,2,7678.551136
16,2,5004.605263
16,2,3159.051471
16,2,1560.234375
16,2,1629.267442
16,2,1689.648936
16,2,1661.490625
16,2,1619.735000
16,2,2139.035714
16,2,1930.595745
16,2,1536.741071
16,2,4110.445652
16,2,1944.183673
16,2,3201.862069

16,2,1605.750000
16,2,2391.314103
16,2,1692.637500
16,2,9377.160714
16,2,1758.985000
16,2,2539.208333
16,2,2292.416667
16,2,3885.282895
16,2,2082.344444
16,2,2288.476562
16,2,1537.383523
16,2,1502.447368
16,2,1540.186170
16,2,1981.000000
16,2,7254.326531
16,2,2038.726852
16,2,3595.330000
16,2,1717.804878
16,2,1541.548780
16,2,4241.551020
16,2,2992.978723
16,2,1903.176136
16,2,1847.892857
16,2,1517.565217
16,2,2462.500000
16,2,1720.780612
16,2,1621.279070
16,2,2244.878049
16,2,1748.808824
16,2,3124.510638
16,2,2217.255814
16,2,2084.734043
16,2,1641.477564
16,2,2679.882353
16,2,1906.477941
16,2,2695.253049
16,2,1770.222826
16,2,1789.241071
16,2,1871.442857
16,2,1578.000000
16,2,1755.510638
16,2,2710.533333
16,2,1763.171875
16,2,1789.464286
16,2,2822.303571
16,2,3229.052885
16,2,1931.594595
16,2,2135.956522
16,2,1872.987805
16,2,18118.527027
16,2,1616.670673
16,2,3002.672619
16,2,1970.657895
16,2,2648.823529
16,2,4720.500000
16,2,1716.336735
16,2,1708.938679
16,2,4287.295455
16,2,1916.512931
16,2,1619.972222
16,2,3552.975962
16,2,1872.546875
16,2,1728.723404
16,2,2028.950000
16,2,3242.463415
16,2,2027.301724

16,2,1803.234756
16,2,1717.343750
16,2,2075.132500
16,2,1510.788462
16,2,2268.616848
16,2,2151.300000
16,2,1895.612500
16,2,1551.540323
16,2,1531.153409
16,2,1611.495370
16,2,1836.523585
16,2,1956.303922
16,2,1845.696809
16,2,2636.020833
16,2,6023.011029
16,2,2198.687500
16,2,1816.333333
16,2,2250.420455
16,2,1922.000000
16,2,1827.173077
16,2,1997.196809
16,2,2099.990566
16,2,15283.512195
16,2,1904.023256
16,2,1970.877778
16,2,1586.571429
16,2,1583.951220
16,2,1560.364583
16,2,1535.920732
16,2,1536.097561
16,2,6712.807692
16,2,1520.865385
16,2,2939.463415
16,2,4413.074468
16,2,1691.822368
16,2,1643.701087
16,2,2322.478723
16,2,2841.888298
16,2,3285.534014
16,2,2239.502717
16,2,1505.500000
16,2,2367.257653
16,2,1539.630000
16,2,1520.627907
16,2,1652.711957
16,2,1784.640625
16,2,2431.627778
16,2,2074.979592
16,2,1814.812500
16,2,2120.277778
16,2,2193.727273
16,2,1681.488095
16,2,2576.500000
16,2,2369.423077
16,2,2375.513158
16,2,1506.578431
16,2,3042.901961
16,2,3018.000000
16,2,3087.821429
16,2,2671.250000
16,2,2688.140097
16,2,2054.702128
16,2,1604.687500
16,2,1544.750000
16,2,4129.607143
16,2,1785.033333

16,2,12491.534483
16,2,1644.932018
16,2,1986.333333
16,2,2932.339286
16,2,1545.985119
16,2,2864.333333
16,2,2310.493056
16,2,1564.250000
16,2,1687.855769
16,2,1625.432143
16,2,3120.921875
16,2,1721.437500
16,2,2147.162162
16,2,1868.335227
16,2,1695.131579
16,2,1571.500000
16,2,1784.073529
16,2,2250.959184
16,2,2066.398936
16,2,4945.625000
16,2,3019.388587
16,2,3452.265306
16,2,2098.606771
16,2,2221.317130
16,2,3957.313953
16,2,2508.800000
16,2,1718.229651
16,2,1942.329545
16,2,1603.989865
16,2,2104.400735
16,2,2030.000000
16,2,2780.480978
16,2,1567.300000
16,2,1666.122449
16,2,1526.058511
16,2,3470.702128
16,2,1649.421875
16,2,2593.568182
16,2,14144.989865
16,2,2052.646277
16,2,2042.220395
16,2,1968.481383
16,2,4838.147436
16,2,2012.272727
16,2,1653.127717
16,2,2525.975000
16,2,2266.726064
16,2,1949.285714
16,2,1605.561224
16,2,2702.384615
16,2,16480.912791
16,2,1559.455357
16,2,2843.611111
16,2,1829.750000
16,2,5569.701923
16,2,1544.242500
16,2,1775.857143
16,2,1609.323980
16,2,1715.555556
16,2,2019.446809
16,2,2160.712963
16,2,2001.833333
16,2,3481.428571
16,2,1950.421875
16,2,2383.869565
16,2,3174.131696

16,2,1897.117188
16,2,1660.816038
16,2,1890.485000
16,2,2883.159574
16,2,2445.354167
16,2,3401.284091
16,2,4837.394231
16,2,1751.877907
16,2,1558.406250
16,2,2474.280488
16,2,2821.318182
16,2,1816.648438
16,2,1660.463710
16,2,1939.510638
16,2,1656.198661
16,2,1995.640625
16,2,1501.679878
16,2,1868.253049
16,2,1884.958333
16,2,3621.331395
16,2,5709.533333
16,2,1697.125000
16,2,2402.893617
16,2,2726.541667
16,2,2049.287234
16,2,1698.776786
16,2,1662.727500
16,2,2261.700000
16,2,2820.739286
16,2,1832.610000
16,2,2078.013889
16,2,2525.544643
16,2,1719.039474
16,2,1544.063830
16,2,1788.783654
16,2,2216.571429
16,2,1896.163265
16,2,1538.883333
16,2,1525.450000
16,2,1932.765957
16,2,1618.448718
16,2,1796.234756
16,2,1802.500000
16,2,1570.250000
16,2,6187.535714
16,2,1519.037500
16,2,2590.607500
16,2,1853.682292
16,2,3643.391827
16,2,1681.957447
16,2,1615.830882
16,2,2326.887097
16,2,4440.375000
16,2,3962.809659
16,2,1548.418605
16,2,2841.483696
16,2,1632.306818
16,2,1625.963415
16,2,2152.789894
16,2,1958.561828
16,2,1992.640000
16,2,1600.004808
16,2,2192.204082
16,2,1846.395833
16,2,1793.019231
16,2,2182.906250

16,2,2635.085526
16,2,1730.617647
16,2,2562.512195
16,2,1650.573529
16,2,1646.460526
16,2,1517.937500
16,2,1642.428571
16,2,2132.798077
16,2,2845.137755
16,2,4131.086957
16,2,1746.687500
16,2,1512.789474
16,2,2854.665865
16,2,2349.112500
16,2,1808.643382
16,2,2416.000000
16,2,4299.583333
16,2,1921.134146
16,2,3545.019231
16,2,1582.262195
16,2,1878.899390
16,2,3173.505319
16,2,1849.518293
16,2,1507.586957
16,2,2444.035714
16,2,3177.011905
16,2,1974.957317
16,2,2151.487805
16,2,2456.660256
16,2,2012.782609
16,2,2462.339623
16,2,1583.596154
16,2,1943.052500
16,2,2547.272500
16,2,1865.407609
16,2,1533.512195
16,2,6623.050000
16,2,1522.935897
16,2,2306.120000
16,2,1502.462264
16,2,1712.500000
16,2,1712.923077
16,2,1547.545455
16,2,1741.020833
16,2,1522.416667
16,2,1845.547872
16,2,2792.574074
16,2,1526.529412
16,2,2426.891827
16,2,1990.051471
16,2,3458.048077
16,2,2289.766667
16,2,2661.900510
16,2,1887.180556
16,2,1816.513889
16,2,1825.534722
16,2,1548.500000
16,2,1654.750000
16,2,4741.218750
16,2,1710.767857
16,2,2716.090909
16,2,4449.613636
16,2,2876.961340
16,2,2818.710227
16,2,2612.611111
16,2,2141.371795

16,2,2930.362500
16,2,2174.241477
16,2,3052.240909
16,2,1564.720930
16,2,1920.908537
16,2,1552.096939
16,2,2521.479651
16,2,2145.212500
16,2,1580.352273
16,2,2133.082447
16,2,2361.583333
16,2,2344.910714
16,2,2100.577128
16,2,1523.021277
16,2,1642.116071
16,2,2921.948529
16,2,2315.464286
16,2,2666.484848
16,2,1733.120690
16,2,2062.750000
16,2,1797.275862
16,2,1606.586957
16,2,2681.900000
16,2,3121.651442
16,2,2715.151515
16,2,1843.105000
16,2,1570.044118
16,2,1699.407895
16,2,1634.923913
16,2,1599.197500
16,2,2090.719444
16,2,2135.313830
16,2,3293.607143
16,2,2627.385870
16,2,2700.171196
16,2,1663.101449
16,2,1731.059211
16,2,1993.044444
16,2,2202.444444
16,2,2782.805556
16,2,2280.350000
16,2,1782.042683
16,2,16113.812500
16,2,1765.603659
16,2,2120.838415
16,2,1748.638393
16,2,1733.950658
16,2,11977.090116
16,2,2413.985294
16,2,1857.000000
16,2,2479.510870
16,2,1649.690625
16,2,2017.752232
16,2,8615.046512
16,2,1639.900000
16,2,1758.043478
16,2,2562.000000
16,2,2166.392157
16,2,2321.322917
16,2,1809.583333
16,2,3166.119048
16,2,1787.184783
16,2,1798.815000
16,2,1591.191489
16,2,4809.757353
16,2,1559.811321

16,2,1505.901163
16,2,1986.323529
16,2,1612.732143
16,2,1606.865625
16,2,1557.643382
16,2,2016.315000
16,2,2289.730769
16,2,2362.884868
16,2,1819.887097
16,2,1791.170213
16,2,1617.583333
16,2,1615.515625
16,2,5008.778571
16,2,1661.382653
16,2,2864.868590
16,2,2107.325000
16,2,1900.834184
16,2,2006.386139
16,2,3286.987179
16,2,4107.714286
16,2,1698.800000
16,2,1922.463235
16,2,2123.750000
16,2,1977.500000
16,2,1690.909574
16,2,2066.347826
16,2,1851.391304
16,2,2412.787500
16,2,1532.465116
16,2,2344.757812
16,2,1819.955882
16,2,2026.542683
16,2,1539.721354
16,2,1509.360294
16,2,3111.351064
16,2,1622.817308
16,2,1537.477273
16,2,1830.804688
16,2,2015.570000
16,2,1580.300000
16,2,4473.105556
16,2,2787.625000
16,2,2795.545455
16,2,1655.162879
16,2,1918.615854
16,2,2271.021277
16,2,1499.576923
16,2,1611.639151
16,2,1661.930000
16,2,2227.760000
16,2,2139.067073
16,2,1646.610000
16,2,1599.083333
16,2,2926.597826
16,2,2649.619048
16,2,2243.411585
16,2,2762.617188
16,2,1688.042553
16,2,1827.195122
16,2,1603.308333
16,2,1502.718182
16,2,3899.279255
16,2,3470.007812
16,2,1608.345000
16,2,2868.187500
16,2,1865.352941

16,2,1721.547170
16,2,1924.319444
16,2,2667.226190
16,2,2299.365854
16,2,3788.978659
16,2,2336.410714
16,2,2454.660714
16,2,1745.690000
16,2,3119.478261
16,2,1775.119048
16,2,2099.793750
16,2,1775.716981
16,2,1596.576531
16,2,1772.111111
16,2,1966.818182
16,2,4775.866848
16,2,2584.821429
16,2,1574.750000
16,2,1511.978261
16,2,2345.474359
16,2,2110.058140
16,2,2521.687500
16,2,2161.883333
16,2,2305.109589
16,2,1731.292683
16,2,2404.250000
16,2,1969.750000
16,2,2264.361111
16,2,1611.781250
16,2,1829.103261
16,2,2741.500000
16,2,2502.750000
16,2,4295.663265
16,2,2061.256098
16,2,2027.311224
16,2,1820.709302
16,2,1700.073864
16,2,3690.141827
16,2,1690.717949
16,2,1700.944853
16,2,1883.076923
16,2,1991.500000
16,2,2078.364865
16,2,1871.903061
16,2,4237.813830
16,2,2236.097561
16,2,1708.619898
16,2,1993.877358
16,2,2801.384375
16,2,2096.600000
16,2,1638.812500
16,2,1740.521739
16,2,1868.045000
16,2,2461.579268
16,2,1986.390625
16,2,2869.937500
16,2,1951.600000
16,2,3236.227273
16,2,1850.750000
16,2,1661.307692
16,2,1526.554878
16,2,1959.798611
16,2,1895.000000
16,2,1530.260870
16,2,1761.150000
16,2,1985.792453

16,2,2059.798387
16,2,3069.500000
16,2,1931.339744
16,2,2834.829032
16,2,2660.698324
16,2,4794.002604
16,2,2025.250000
16,2,2287.635000
16,2,2183.760870
16,2,4457.840000
16,2,1666.750000
16,2,2367.122449
16,2,1677.065217
16,2,1954.610465
16,2,2445.475000
16,2,4081.058824
16,2,3840.883721
16,2,1749.790698
16,2,1763.076923
16,2,1573.617788
16,2,1875.431250
16,2,2033.454545
16,2,1770.696429
16,2,3056.234043
16,2,5462.138889
16,2,1692.220588
16,2,1796.544271
16,2,1554.959184
16,2,3417.344828
16,2,3235.858696
16,2,1677.958333
16,2,4290.267442
16,2,1707.136364
16,2,2457.267857
16,2,1924.125000
16,2,2718.571429
16,2,3298.390000
16,2,19736.365854
16,2,5582.017544
16,2,3735.375000
16,2,1633.805000
16,2,16162.697368
16,2,1933.271429
16,2,2576.936170
16,2,2063.019608
16,2,1540.988095
16,2,2319.712766
16,2,2208.313725
16,2,2111.855000
16,2,2092.886364
16,2,7646.687500
16,2,1564.770833
16,2,1996.200472
16,2,1771.977778
16,2,1877.000000
16,2,2355.532609
16,2,1521.125000
16,2,1558.521341
16,2,4550.435714
16,2,3034.841912
16,2,2860.690000
16,2,1723.195946
16,2,2524.875000
16,2,2195.431818
16,2,3106.225806
16,2,5047.847826

16,2,16181.837209
16,2,8432.430921
16,2,2026.800000
16,2,1785.597826
16,2,1530.653846
16,2,16278.723684
16,2,4049.620370
16,2,1906.872449
16,2,3071.800000
16,2,2334.625000
16,2,2592.820000
16,2,1759.392857
16,2,2537.903125
16,2,1979.609375
16,2,1534.500000
16,2,1595.544118
16,2,3240.838415
16,2,1792.440625
16,2,2916.934028
16,2,2190.690217
16,2,2174.230000
16,2,1767.250000
16,2,7730.125000
16,2,1820.280000
16,2,1533.181818
16,2,1701.892045
16,2,1640.042553
16,2,4054.895833
16,2,2915.200000
16,2,1562.268987
16,2,1736.000000
16,2,1915.650000
16,2,5931.741848
16,2,6439.572115
16,2,1874.428571
16,2,2512.305556
16,2,2207.125000
16,2,1827.580128
16,2,2269.488372
16,2,2450.022727
16,2,1775.050926
16,2,2632.966346
16,2,1693.617347
16,2,1733.920732
16,2,1925.617347
16,2,1566.958333
16,2,1501.613971
16,2,1578.742500
16,2,3492.739583
16,2,1717.317073
16,2,2107.054688
16,2,1802.459746
16,2,2092.546512
16,2,2419.548780
16,2,2086.208333
16,2,2971.983333
16,2,5081.831250
16,2,1988.739362
16,2,1779.241071
16,2,5220.201923
16,2,3659.324468
16,2,2189.741279
16,2,2622.688830
16,2,2214.804487
16,2,1912.492188
16,2,2166.560811

16,2,1659.547872
16,2,2015.714286
16,2,2872.820000
16,2,2432.967949
16,2,2197.904762
16,2,1641.571429
16,2,2471.263514
16,2,2187.083333
16,2,1738.077778
16,2,3490.250000
16,2,2036.250000
16,2,2374.744186
16,2,1685.197368
16,2,2302.530000
16,2,2650.171875
16,2,1674.840426
16,2,1987.632075
16,2,3462.014535
16,2,4223.473822
16,2,2046.650000
16,2,2636.583333
16,2,1905.133152
16,2,3544.732955
16,2,1775.586538
16,2,1633.602564
16,2,2027.705556
16,2,1564.251366
16,2,1997.647500
16,2,12995.328947
16,2,1874.000000
16,2,1536.828947
16,2,1606.307870
16,2,1669.992347
16,2,2943.282051
16,2,1787.622396
16,2,1546.742021
16,2,2462.250000
16,2,3379.521739
16,2,1647.234286
16,2,2019.146341
16,2,1901.621324
16,2,1660.586735
16,2,2471.950000
16,2,2249.553571
16,2,1741.210000
16,2,4327.910156
16,2,3638.529412
16,2,2072.938953
16,2,1600.406250
16,2,1601.375000
16,2,1667.172872
16,2,1740.070755
16,2,1972.781250
16,2,3437.576531
16,2,2533.353261
16,2,1818.411765
16,2,2927.291667
16,2,1521.752500
16,2,10868.261111
16,2,2154.598214
16,2,1699.163265
16,2,10505.969595
16,2,1850.795455
16,2,2006.310811
16,2,2084.500000
16,2,2276.756757

16,2,2392.307692
16,2,1612.962500
16,2,1969.088235
16,2,1940.500000
16,2,1692.537234
16,2,7555.583333
16,2,2819.872340
16,2,1551.562500
16,2,1580.579545
16,2,1563.239130
16,2,2464.308511
16,2,1756.737500
16,2,2921.000000
16,2,2354.804878
16,2,2414.122222
16,2,1611.800000
16,2,1802.765625
16,2,19153.439024
16,2,3041.892857
16,2,3473.760638
16,2,1733.585366
16,2,1939.003289
16,2,1949.320513
16,2,1516.000000
16,2,5241.291667
16,2,1529.039062
16,2,1957.351190
16,2,1638.298077
16,2,1758.500000
16,2,1847.613372
16,2,2602.003676
16,2,6275.675000
16,2,1964.225490
16,2,2073.296875
16,2,2187.739796
16,2,1500.250000
16,2,1866.821809
16,2,2196.466837
16,2,2985.955000
16,2,1628.401042
16,2,5881.426136
16,2,1809.780488
16,2,1850.203125
16,2,1771.954082
16,2,2174.357143
16,2,2011.973214
16,2,1886.642857
16,2,2272.000000
16,2,1964.681250
16,2,3055.086957
16,2,10301.774390
16,2,2820.864130
16,2,1580.103175
16,2,1538.562500
16,2,1885.090426
16,2,1675.808511
16,2,2131.955128
16,2,2101.500000
16,2,2440.816327
16,2,2312.889706
16,2,2716.586957
16,2,2230.400000
16,2,1550.429348
16,2,1718.837662
16,2,1653.204082
16,2,2243.722222

16,2,2592.078947
16,2,2164.360294
16,2,2016.308036
16,2,4586.211111
16,2,9083.982639
16,2,1975.253049
16,2,2285.320000
16,2,2627.819149
16,2,1614.225000
16,2,1553.160714
16,2,1680.236111
16,2,2159.200000
16,2,1596.875000
16,2,1628.193750
16,2,2974.527174
16,2,1537.850694
16,2,2214.947368
16,2,1609.689655
16,2,2528.464481
16,2,1590.740000
16,2,1663.271277
16,2,3184.407609
16,2,2289.703030
16,2,1555.397959
16,2,1993.611111
16,2,2856.781250
16,2,4498.770833
16,2,1668.043269
16,2,1542.770000
16,2,1572.230556
16,2,1691.448980
16,2,1761.557065
16,2,1537.437500
16,2,2453.890244
16,2,1593.260000
16,2,1637.808140
16,2,1992.738095
16,2,1967.132812
16,2,4259.125000
16,2,2001.636364
16,2,3723.434896
16,2,4436.227500
16,2,1627.439560
16,2,9587.974359
16,2,2169.983696
16,2,1521.022222
16,2,2110.645833
16,2,1929.047222
16,2,1929.538462
16,2,1917.044643
16,2,2618.201389
16,2,1773.277778
16,2,1965.153846
16,2,1714.000000
16,2,4531.264205
16,2,1561.731707
16,2,1529.319149
16,2,1738.266667
16,2,1815.117647
16,2,3068.187500
16,2,2947.539062
16,2,1680.829268
16,2,2229.237500
16,2,1638.817308
16,2,1859.190000
16,2,4063.200581

16,2,1664.478723
16,2,1524.708333
16,2,2620.976744
16,2,3739.666667
16,2,2528.522222
16,2,1605.294872
16,2,2355.662500
16,2,1520.533654
16,2,2534.125000
16,2,2401.771739
16,2,1659.388298
16,2,3099.110294
16,2,1663.125000
16,2,2383.853659
16,2,1917.540000
16,2,2214.901442
16,2,5686.515957
16,2,2836.795455
16,2,2470.165761
16,2,7071.304545
16,2,2905.375000
16,2,1908.760000
16,2,1604.022727
16,2,4556.858696
16,2,1905.260000
16,2,2272.000000
16,2,3929.346154
16,2,1611.486842
16,2,4407.006250
16,2,1683.223837
16,2,1938.056122
16,2,2100.718182
16,2,1843.538462
16,2,2802.565217
16,2,1669.709302
16,2,1937.833333
16,2,2057.334906
16,2,2163.423913
16,2,2612.857955
16,2,1521.897059
16,2,2144.449405
16,2,5666.509375
16,2,1555.821023
16,2,1558.428571
16,2,10586.785714
16,2,3885.041667
16,2,2138.367021
16,2,2534.279412
16,2,1910.273256
16,2,1538.076923
16,2,2545.263158
16,2,1924.183511
16,2,1952.285714
16,2,1554.237179
16,2,1803.855769
16,2,1742.115854
16,2,2874.068878
16,2,2081.033333
16,2,1848.000000
16,2,1730.797872
16,2,1563.004902
16,2,1855.156250
16,2,2697.606061
16,2,1608.282051
16,2,1549.457447
16,2,2648.994565

16,2,4140.812500
16,2,1705.166667
16,2,1761.100000
16,2,1822.006579
16,2,1648.416667
16,2,1756.222222
16,2,2448.696429
16,2,2465.318182
16,2,2914.788462
16,2,7977.396226
16,2,1885.461538
16,2,2167.831395
16,2,1863.892857
16,2,1825.791667
16,2,1639.020408
16,2,1741.416667
16,2,1518.075581
16,2,1766.607843
16,2,1809.141892
16,2,2059.214286
16,2,2051.420673
16,2,1588.069767
16,2,2662.361702
16,2,1659.104651
16,2,2775.000000
16,2,1827.330000
16,2,1649.268750
16,2,1993.198864
16,2,1677.752358
16,2,1841.843023
16,2,2886.820000
16,2,1544.636364
16,2,1622.731383
16,2,1823.831897
16,2,1833.717262
16,2,1646.883929
16,2,2539.000000
16,2,2427.785714
16,2,1499.750000
16,2,2435.440217
16,2,2102.500000
16,2,3855.224359
16,2,1688.331081
16,2,2914.526316
16,2,1694.266667
16,2,2391.119469
16,2,3216.020408
16,2,1636.893617
16,2,2383.238095
16,2,1824.661585
16,2,1794.989796
16,2,3000.203488
16,2,13267.571429
16,2,1546.772727
16,2,2972.562500
16,2,1735.977041
16,2,2499.500000
16,2,1851.583333
16,2,3226.024510
16,2,1907.744186
16,2,2162.400000
16,2,1862.938776
16,2,6319.081081
16,2,2960.197368
16,2,1602.202128
16,2,3639.654070

16,2,2180.000000
16,2,2205.776596
16,2,2620.289474
16,2,2165.128049
16,2,2345.000000
16,2,1820.045732
16,2,3147.203125
16,2,1921.521429
16,2,2257.561170
16,2,2741.930851
16,2,1903.877841
16,2,2304.666667
16,2,1567.505682
16,2,2038.375000
16,2,1505.421569
16,2,2009.557143
16,2,2984.312500
16,2,5732.550595
16,2,1799.000000
16,2,1855.705128
16,2,1802.654762
16,2,2014.762500
16,2,2100.144444
16,2,1693.357143
16,2,1706.155172
16,2,3619.300000
16,2,1813.171875
16,2,1706.954545
16,2,1995.333333
16,2,2540.696335
16,2,3234.654412
16,2,9535.778409
16,2,3209.000000
16,2,2053.634615
16,2,1754.000000
16,2,2401.353659
16,2,3039.903226
16,2,1871.587209
16,2,1902.128049
16,2,2409.327381
16,2,1641.079268
16,2,2488.103659
16,2,3117.914286
16,2,1961.721154
16,2,2733.924528
16,2,7729.910256
16,2,2531.883929
16,2,2502.788462
16,2,3479.994048
16,2,2063.070513
16,2,3289.630000
16,2,1536.631579
16,2,2356.714844
16,2,1848.712766
16,2,2042.600000
16,2,2906.854651
16,2,1776.845000
16,2,2742.303191
16,2,4683.767857
16,2,5713.461538
16,2,1602.075000
16,2,1618.220588
16,2,4265.354839
16,2,2253.453125
16,2,2262.632353
16,2,2153.798469

16,2,2139.539062
16,2,2010.936170
16,2,1507.055000
16,2,1525.581633
16,2,1818.250000
16,2,3266.730556
16,2,1555.250000
16,2,2206.500000
16,2,3616.687500
16,2,1629.664634
16,2,2760.010870
16,2,1769.966346
16,2,1816.121622
16,2,1992.125000
16,2,3402.583333
16,2,2044.823864
16,2,1718.798077
16,2,1569.156250
16,2,3507.260638
16,2,1863.024457
16,2,1708.882353
16,2,2388.862500
16,2,1548.947917
16,2,8509.912500
16,2,2779.800000
16,2,3545.091146
16,2,2261.814394
16,2,2747.031250
16,2,2097.256944
16,2,2079.975410
16,2,2429.054054
16,2,2040.792683
16,2,2077.837209
16,2,1706.957317
16,2,1695.333333
16,2,1987.978723
16,2,1510.487179
16,2,1556.800000
16,2,1633.012195
16,2,1780.625000
16,2,2789.058673
16,2,1919.474576
16,2,1730.848837
16,2,2475.454545
16,2,1895.235795
16,2,1507.357143
16,2,2869.839286
16,2,1683.484472
16,2,2667.234694
16,2,3115.245370
16,2,2110.058824
16,2,3173.844444
16,2,2187.554688
16,2,4575.075472
16,2,1776.281250
16,2,2033.525000
16,2,1727.761905
16,2,2269.851064
16,2,2104.852041
16,2,2409.500000
16,2,2811.272727
16,2,3928.171875
16,2,1589.076923
16,2,2097.211538
16,2,1656.015957
16,2,13185.782895

16,2,1539.328125
16,2,2132.611111
16,2,2162.151961
16,2,2258.625000
16,2,1783.200000
16,2,2298.187500
16,2,2156.046512
16,2,3920.892857
16,2,2740.008621
16,2,1507.088235
16,2,2348.191489
16,2,3790.351695
16,2,13462.551282
16,2,2264.017500
16,2,3924.507812
16,2,1881.884615
16,2,1780.346154
16,2,2375.955357
16,2,1771.808824
16,2,2140.919643
16,2,3377.089286
16,2,2491.625000
16,2,2916.127717
16,2,1941.307692
16,2,1627.900000
16,2,1607.076531
16,2,2809.583333
16,2,3395.606383
16,2,1540.802326
16,2,1713.797297
16,2,8193.678571
16,2,1992.593023
16,2,3819.896552
16,2,1578.944444
16,2,1686.919643
16,2,1507.906863
16,2,2426.500000
16,2,1725.645833
16,2,2047.000000
16,2,3497.750000
16,2,1769.112245
16,2,1556.637500
16,2,1827.416667
16,2,2613.100000
16,2,1925.119898
16,2,1638.377451
16,2,2280.671512
16,2,13389.263889
16,2,1657.435000
16,2,2417.168478
16,2,1534.080000
16,2,1712.235294
16,2,4706.880000
16,2,1554.446809
16,2,1538.094512
16,2,1622.906250
16,2,1804.763393
16,2,2040.208333
16,2,3039.392361
16,2,2171.506944
16,2,2171.137755
16,2,1830.518382
16,2,1765.358696
16,2,3574.485294
16,2,1606.208333
16,2,2396.962963

16,2,2142.473404
16,2,1903.046875
16,2,1499.710938
16,2,1650.917614
16,2,1707.323864
16,2,1738.852941
16,2,1913.125000
16,2,1825.201389
16,2,1747.518293
16,2,1576.257979
16,2,1815.593750
16,2,5079.250000
16,2,1677.570312
16,2,2121.948529
16,2,2121.652778
16,2,2980.239286
16,2,2206.675000
16,2,1709.782609
16,2,5395.862245
16,2,2592.066667
16,2,5730.985577
16,2,1746.000000
16,2,2166.363636
16,2,1499.696809
16,2,1712.785714
16,2,9139.579787
16,2,3103.973039
16,2,11468.575000
16,2,1702.159314
16,2,3589.970588
16,2,10037.301829
16,2,2371.771739
16,2,1920.465426
16,2,2073.659483
16,2,2101.487500
16,2,1754.384434
16,2,2280.591912
16,2,7700.430556
16,2,1598.337209
16,2,1949.130682
16,2,1682.140957
16,2,4230.002841
16,2,2271.571429
16,2,2505.268750
16,2,2225.066667
16,2,2247.916667
16,2,1863.406250
16,2,2448.000000
16,2,7237.250000
16,2,1717.952830
16,2,2330.476190
16,2,2407.792553
16,2,2889.772727
16,2,1561.561170
16,2,2947.129870
16,2,1582.216216
16,2,2734.401515
16,2,2519.665816
16,2,1606.528846
16,2,2892.790761
16,2,2705.856250
16,2,4380.000000
16,2,1922.697917
16,2,1577.466346
16,2,4433.105114
16,2,5078.604167

16,2,1567.666667
16,2,3514.979651
16,2,2074.044872
16,2,1588.732759
16,2,2088.017442
16,2,1619.073171
16,2,6513.037234
16,2,1576.531915
16,2,3047.500000
16,2,2541.500000
16,2,2401.164062
16,2,1723.259259
16,2,1582.394231
16,2,1827.807500
16,2,4209.454268
16,2,1618.529605
16,2,1856.771635
16,2,2715.535714
16,2,2601.454787
16,2,1648.180851
16,2,2153.260870
16,2,1500.682927
16,2,2234.647059
16,2,4511.333333
16,2,1794.476351
16,2,1512.197917
16,2,2453.347826
16,2,1719.500000
16,2,1564.225610
16,2,2464.371795
16,2,1934.500000
16,2,2059.009615
16,2,2819.929245
16,2,1874.283019
16,2,2067.948113
16,2,2001.239130
16,2,2387.576389
16,2,2512.095745
16,2,2231.913690
16,2,2644.553571
16,2,2140.114286
16,2,2046.500000
16,2,1594.201923
16,2,2296.578947
16,2,1909.119565
16,2,2294.238426
16,2,2219.367647
16,2,1892.925000
16,2,1666.134615
16,2,3453.557692
16,2,5010.281250
16,2,2004.875000
16,2,3726.273438
16,2,2251.375000
16,2,1516.286184
16,2,1501.114583
16,2,1551.050000
16,2,2563.785714
16,2,1643.250000
16,2,1516.191489
16,2,1630.336735
16,2,1935.383929
16,2,2738.230769
16,2,1823.758772
16,2,1853.621212
16,2,1735.050000

16,2,1648.946809
16,2,1647.531250
16,2,1665.796875
16,2,2988.899390
16,2,5407.691964
16,2,2031.130000
16,2,2429.201923
16,2,2558.009615
16,2,2113.614362
16,2,2283.136364
16,2,2405.916667
16,2,2000.292553
16,2,1512.064655
16,2,3485.130682
16,2,1573.250000
16,2,1938.218750
16,2,1577.431579
16,2,2987.120000
16,2,3969.500000
16,2,1846.766667
16,2,1743.432692
16,2,3296.913043
16,2,4482.937500
16,2,1587.896186
16,2,1683.775510
16,2,1864.784314
16,2,3276.011364
16,2,2194.238208
16,2,2548.447115
16,2,1657.056818
16,2,2535.880952
16,2,3719.825472
16,2,1993.000000
16,2,1873.584559
16,2,1895.497396
16,2,2101.250000
16,2,3337.510638
16,2,1885.638889
16,2,1598.781818
16,2,14046.944444
16,2,2111.435000
16,2,1571.122222
16,2,2953.531915
16,2,1585.325000
16,2,1922.368902
16,2,2955.478659
16,2,1767.813830
16,2,1592.465116
16,2,4935.144444
16,2,1811.439024
16,2,2539.290000
16,2,1756.713542
16,2,2688.525000
16,2,2734.273256
16,2,2239.600000
16,2,2182.189286
16,2,1573.285714
16,2,1529.651042
16,2,1775.100000
16,2,1951.820122
16,2,4241.631579
16,2,1506.714286
16,2,1827.429878
16,2,2453.150000
16,2,1623.947917
16,2,5472.541667

16,2,3654.916667
16,2,1735.839286
16,2,2346.847826
16,2,2136.840000
16,2,1614.007353
16,2,2033.200000
16,2,2913.390957
16,2,1724.000000
16,2,3223.644231
16,2,1698.833333
16,2,2440.925000
16,2,2104.672619
16,2,9244.250000
16,2,1897.664062
16,2,19998.702128
16,2,3253.088983
16,2,1816.872500
16,2,1837.622642
16,2,2729.346154
16,2,2686.022727
16,2,2067.031250
16,2,3131.862500
16,2,1520.728723
16,2,1988.299419
16,2,2383.384615
16,2,1603.193452
16,2,2316.474432
16,2,1706.410256
16,2,3418.441860
16,2,3193.968023
16,2,1659.615132
16,2,1687.953488
16,2,1683.987500
16,2,1776.458333
16,2,2032.603774
16,2,2546.419118
16,2,8314.250000
16,2,1590.796875
16,2,2034.547500
16,2,1761.250000
16,2,1511.345395
16,2,1579.388889
16,2,2342.415816
16,2,1871.700000
16,2,3243.375000
16,2,2161.250000
16,2,3660.558511
16,2,2769.241477
16,2,2351.830000
16,2,1678.470588
16,2,1935.817500
16,2,1678.714286
16,2,1539.875000
16,2,3721.372973
16,2,3171.500000
16,2,4337.371429
16,2,3261.601562
16,2,2108.487805
16,2,4980.015625
16,2,1521.596154
16,2,1563.339623
16,2,1687.209459
16,2,2582.457547
16,2,1771.855769
16,2,2338.074468
16,2,1794.545455

16,2,1508.500000
16,2,1565.156250
16,2,4349.731544
16,2,2052.697917
16,2,1751.570652
16,2,1506.300000
16,2,1719.375000
16,2,1794.685976
16,2,2862.059524
16,2,2885.204545
16,2,5111.544643
16,2,1868.250000
16,2,2578.032609
16,2,3422.781915
16,2,10658.685976
16,2,1783.339674
16,2,1573.633721
16,2,1573.383929
16,2,1506.023438
16,2,1991.357143
16,2,2365.813725
16,2,1704.587209
16,2,2700.638889
16,2,1899.170213
16,2,2342.625000
16,2,2563.043605
16,2,2304.557927
16,2,1520.755208
16,2,1521.681818
16,2,1785.914062
16,2,1733.962766
16,2,1527.593750
16,2,2384.923077
16,2,5190.639706
16,2,1526.435000
16,2,3511.418182
16,2,1640.041667
16,2,1832.021277
16,2,1591.671875
16,2,1858.375000
16,2,3951.909091
16,2,5280.214286
16,2,1947.957500
16,2,3184.446809
16,2,4444.350543
16,2,1727.000000
16,2,1528.000000
16,2,2281.354545
16,2,2661.250000
16,2,2122.652778
16,2,1504.577128
16,2,2096.820000
16,2,1588.089286
16,2,1548.564189
16,2,1767.765625
16,2,2546.524038
16,2,7364.010417
16,2,1505.416667
16,2,2987.572500
16,2,2268.276596
16,2,2680.847973
16,2,4206.434211
16,2,2896.896739
16,2,1542.955882
16,2,6471.428571
16,2,2014.486486

16,2,2331.606383
16,2,2055.177885
16,2,1818.292683
16,2,1956.563679
16,2,2366.944149
16,2,3048.729592
16,2,1949.500000
16,2,1655.051887
16,2,3100.789474
16,2,1651.891892
16,2,1971.840426
16,2,2645.947368
16,2,1802.792763
16,2,1858.273437
16,2,1741.360000
16,2,1837.600000
16,2,1782.132075
16,2,1607.250000
16,2,2002.870000
16,2,2463.191038
16,2,19407.109375
16,2,1636.075000
16,2,2291.459184
16,2,1651.732143
16,2,2715.991071
16,2,2338.570312
16,2,7632.220930
16,2,3437.441176
16,2,1734.981707
16,2,3356.092391
16,2,2069.015000
16,2,3451.989796
16,2,1987.082317
16,2,17658.307692
16,2,3728.809211
16,2,2558.059375
16,2,1527.062500
16,2,1767.346875
16,2,2650.979651
16,2,2344.275000
16,2,2557.994681
16,2,1630.675676
16,2,3285.850806
16,2,2218.927083
16,2,2257.850000
16,2,2419.500000
16,2,3597.328947
16,2,1525.170000
16,2,1711.102778
16,2,3041.181818
16,2,1545.241279
16,2,1696.750000
16,2,1818.484043
16,2,3885.734375
16,2,2423.534884
16,2,1591.021739
16,2,3283.972826
16,2,2129.000000
16,2,3200.803571
16,2,3231.628676
16,2,2969.988889
16,2,2521.340909
16,2,1641.939189
16,2,2117.325521
16,2,1621.130952
16,2,2491.150568

16,2,3498.708333
16,2,1997.966667
16,2,2107.372340
16,2,2553.426471
16,2,2149.681818
16,2,2836.238281
16,2,2246.619048
16,2,5399.722222
16,2,1521.115385
16,2,4316.976744
16,2,1511.204082
17,1,1748.446429
17,1,6087.775000
17,1,6493.116071
17,1,5700.878676
17,1,3206.240385
17,1,2559.524390
17,1,2955.511364
17,1,2376.646739
17,1,8150.943182
17,1,18762.595238
17,1,9448.021739
17,1,2357.880435
17,1,2354.333333
17,1,14446.184783
17,1,1671.244318
17,1,1564.920732
17,1,4854.130435
17,1,6470.279412
17,1,8401.142045
17,1,1766.071429
17,1,18464.520000
17,1,4800.362805
17,1,8342.410000
17,1,8058.937500
17,1,2884.182927
17,1,4144.226562
17,1,1519.958333
17,1,1891.719595
17,1,2303.185096
17,1,2439.250000
17,1,8778.333333
17,1,3037.301471
17,1,13541.250000
17,1,12730.473684
17,1,5430.500000
17,1,1678.000000
17,1,13474.375000
17,1,6866.625000
17,1,1778.134146
17,1,4314.134146
17,1,2371.414634
17,1,4381.622449
17,1,13774.043478
17,1,8401.625000
17,1,5902.556818
17,1,7944.930233
17,1,8883.186170
17,1,2468.718085
17,1,2307.010870
17,1,8446.729911
17,1,3240.211111
17,1,4214.160000
17,1,4025.421053
17,1,3042.637500
17,1,6878.826087

17,1,1894.481707
17,1,2980.765244
17,1,1839.662500
17,1,1587.966912
17,1,13151.369565
17,1,12332.491667
17,1,2557.958333
17,1,3327.235577
17,1,7743.169811
17,1,8693.544444
17,1,1861.119048
17,1,1733.641304
17,1,6749.276515
17,1,2937.950000
17,1,1680.442857
17,1,3013.266447
17,1,10427.096354
17,1,5066.000000
17,1,1910.125000
17,1,4879.521635
17,1,2808.527344
17,1,2214.903846
17,1,1831.987500
17,1,14059.086735
17,1,19619.546875
17,1,3832.746951
17,1,3260.583333
17,1,1975.555556
17,1,1847.108871
17,1,1943.062500
17,1,2951.080556
17,1,2333.656818
17,1,3336.206081
17,1,1818.562500
17,1,6229.496875
17,1,7271.906977
17,1,6250.104651
17,1,12720.145833
17,1,13070.596875
17,1,1775.136905
17,1,1988.326923
17,1,3903.945122
17,1,1884.127907
17,1,3614.031250
17,1,2242.226190
17,1,1587.800000
17,1,3500.140541
17,1,1671.583333
17,1,5285.695946
17,1,1730.262195
17,1,2214.671512
17,1,5225.627451
17,1,1529.375000
17,1,1723.131579
17,1,1585.054348
17,1,10411.517857
17,1,1951.847222
17,1,14853.715625
17,1,4121.250000
17,1,6169.270270
17,1,1906.900000
17,1,1583.735795
17,1,4847.310096
17,1,1949.951087
17,1,2273.253788
17,1,4638.785714

17,1,3771.025735
17,1,9133.615385
17,1,4888.086207
17,1,1946.111486
17,1,16538.536184
17,1,2008.106383
17,1,4496.386364
17,1,8571.588816
17,1,3540.743750
17,1,16839.315104
17,1,6131.958333
17,1,7965.230769
17,1,15498.951923
17,1,2055.439024
17,1,1728.791667
17,1,2577.666667
17,1,3439.071429
17,1,6676.800000
17,1,11974.986842
17,1,5486.117647
17,1,1711.064103
17,1,7942.111842
17,1,13382.320312
17,1,11550.625000
17,1,1585.048077
17,1,12949.500000
17,1,2219.589844
17,1,3821.610119
17,1,3874.187500
17,1,2741.500000
17,1,2659.104651
17,1,3102.116071
17,1,16808.812500
17,1,7750.698718
17,1,2982.731707
17,1,2370.243750
17,1,4070.750000
17,1,13080.312500
17,1,2337.307692
17,1,5941.750000
17,1,4950.035714
17,1,3901.649038
17,1,2922.127717
17,1,3484.662879
17,1,4364.298387
17,1,2236.522059
17,1,3409.179878
17,1,18645.886029
17,1,1763.544118
17,1,2509.640244
17,1,3524.050000
17,1,2567.779762
17,1,1886.647059
17,1,19407.980469
17,1,7501.954545
17,1,5642.875000
17,1,1811.344697
17,1,2685.000000
17,1,6184.942308
17,1,2729.021739
17,1,1716.350000
17,1,2100.916667
17,1,1670.187500
17,1,4245.892857
17,1,8734.489796
17,1,1775.871429

17,1,6767.975610
17,1,2765.432292
17,1,3879.187500
17,1,2421.250000
17,1,3611.869565
17,1,4470.380000
17,1,1612.047794
17,1,3627.875000
17,1,10513.750000
17,1,11616.625000
17,1,4400.730978
17,1,1587.755102
17,1,7484.073718
17,1,4147.872500
17,1,10587.236842
17,1,2860.973684
17,1,18937.948980
17,1,5315.470588
17,1,7997.662500
17,1,1576.725000
17,1,4118.466667
17,1,19752.163462
17,1,3588.063830
17,1,12975.869565
17,1,3600.687500
17,1,1644.482955
17,1,7074.805147
17,1,3537.361702
17,1,3634.729167
17,1,5082.858974
17,1,7030.740741
17,1,1506.390625
17,1,4419.845930
17,1,2371.242347
17,1,2820.750000
17,1,1946.841216
17,1,1506.007979
17,1,16283.279605
17,1,6518.093023
17,1,1904.312500
17,1,4577.758929
17,1,7160.819767
17,1,3179.687500
17,1,2276.472973
17,1,1897.085938
17,1,2853.987805
17,1,3038.973485
17,1,3882.411765
17,1,8610.546875
17,1,4411.236842
17,1,13717.180233
17,1,6174.966981
17,1,2569.285714
17,1,1961.111979
17,1,2554.608333
17,1,17894.194079
17,1,2141.300000
17,1,12256.867647
17,1,8042.992188
17,1,6915.637755
17,1,3327.443182
17,1,7662.315217
17,1,2686.141892
17,1,3536.629630
17,1,1872.694444
17,1,1977.959184

17,1,1640.857143
17,1,14988.256410
17,1,6542.102273
17,1,12959.187500
17,1,2296.764706
17,1,10339.401316
17,1,3458.091463
17,1,1545.660256
17,1,2015.786184
17,1,1533.054878
17,1,4206.183673
17,1,4647.227273
17,1,1819.844697
17,1,15796.125000
17,1,15335.724359
17,1,5918.841463
17,1,2310.780172
17,1,2257.106618
17,1,4103.442308
17,1,13760.390625
17,1,1815.796875
17,1,6779.994186
17,1,1978.685714
17,1,11821.729730
17,1,2271.634868
17,1,2246.527778
17,1,2125.544118
17,1,5006.500000
17,1,8175.971774
17,1,1658.329545
17,1,3154.953125
17,1,2971.640625
17,1,12093.833333
17,1,2093.600000
17,1,1992.404891
17,1,1845.091346
17,1,2946.794872
17,1,5722.750000
17,1,2087.641667
17,1,1560.232558
17,1,1969.744186
17,1,2739.031250
17,1,1605.292683
17,1,1751.920455
17,1,2250.681818
17,1,8465.830556
17,1,1786.037234
17,1,2263.367188
17,1,1775.190000
17,1,2742.500000
17,1,2146.243902
17,1,1579.560976
17,1,10324.666667
17,1,11021.967105
17,1,2212.413043
17,1,2587.500000
17,1,10808.939024
17,1,6808.574468
17,1,15389.250000
17,1,8164.904412
17,1,4695.146341
17,1,1847.936047
17,1,19226.464744
17,1,6661.764706
17,1,2085.053191
17,1,2013.097826

17,1,3116.448171
17,1,2964.029605
17,1,2507.572581
17,1,8690.000000
17,1,11377.791667
17,1,4062.478125
17,1,1825.205729
17,1,6257.804878
17,1,17793.000000
17,1,1719.757979
17,1,1730.242424
17,1,4795.604167
17,1,1913.812500
17,1,10234.430556
17,1,1906.519444
17,1,8531.906250
17,1,3414.478261
17,1,2149.864865
17,1,3224.402439
17,1,3095.564103
17,1,18011.271277
17,1,14462.659091
17,1,2626.106383
17,1,1597.307692
17,1,3015.413043
17,1,11169.375000
17,1,3281.353659
17,1,12948.000000
17,1,1663.931818
17,1,5908.383333
17,1,18403.514423
17,1,2214.894886
17,1,1633.122340
17,1,2629.263158
17,1,4589.064815
17,1,8985.750000
17,1,1852.616477
17,1,3053.025000
17,1,3250.037879
17,1,5984.222656
17,1,2469.834302
17,1,6454.869186
17,1,7275.916667
17,1,2985.000000
17,1,18058.889535
17,1,1694.344595
17,1,5339.146341
17,1,7535.250000
17,1,8262.727564
17,1,2389.375000
17,1,2918.636364
17,1,2526.970109
17,1,4602.028125
17,1,11707.145349
17,1,1664.993243
17,1,5460.727273
17,1,12010.965909
17,1,2132.425532
17,1,3724.821429
17,1,7127.596774
17,1,12943.564286
17,1,8841.855769
17,1,2535.932927
17,1,9944.191489
17,1,7435.734694
17,1,17166.852941

17,1,5751.789474
17,1,15950.312500
17,1,2424.411585
17,1,1519.746212
17,1,7955.000000
17,1,5330.654412
17,1,4323.383721
17,1,8661.017857
17,1,5115.492647
17,1,2345.382353
17,1,1752.456731
17,1,4677.383929
17,1,17303.651515
17,1,1683.541667
17,1,13184.151786
17,1,3361.303191
17,1,9539.269231
17,1,3047.187500
17,1,6978.277500
17,1,1609.546875
17,1,1756.708333
17,1,12842.718750
17,1,2111.608108
17,1,8687.480000
17,1,1679.088235
17,1,4028.916667
17,1,4651.230769
17,1,2736.215278
17,1,2623.515152
17,1,2883.750000
17,1,4004.437500
17,1,3858.048780
17,1,4256.524038
17,1,15575.307692
17,1,2424.928571
17,1,5392.258065
17,1,7805.329787
17,1,11033.375000
17,1,5008.262821
17,1,6830.500000
17,1,4526.951923
17,1,9297.135417
17,1,9045.483333
17,1,2601.388889
17,1,9566.397059
17,1,2761.962766
17,1,10199.284375
17,1,10727.062500
17,1,2352.841912
17,1,13908.420000
17,1,9751.875000
17,1,2655.312500
17,1,2656.426630
17,1,3121.708333
17,1,6095.156250
17,1,4226.229167
17,1,1611.087838
17,1,2304.482143
17,1,4611.185484
17,1,7307.565789
17,1,4409.164062
17,1,3100.277778
17,1,3686.256098
17,1,3241.468750
17,1,19394.450000
17,1,4851.419118

17,1,2316.319444
17,1,1649.524194
17,1,2826.367647
17,1,2055.502778
17,1,2718.547619
17,1,8378.525641
17,1,3115.346154
17,1,2967.945312
17,1,3878.821429
17,1,15315.250000
17,1,1665.000000
17,1,3274.488971
17,1,4873.426471
17,1,12314.576923
17,1,1732.328488
17,1,3872.937500
17,1,3952.178571
17,1,5442.500000
17,1,17872.966981
17,1,1903.219512
17,1,5014.018750
17,1,9149.104167
17,1,2169.054348
17,1,14432.000000
17,1,2579.738971
17,1,3670.206522
17,1,4225.260870
17,1,10390.768293
17,1,10790.908163
17,1,3154.796512
17,1,7021.898026
17,1,8323.080128
17,1,3609.285714
17,1,3758.585526
17,1,9444.553571
17,1,5075.207031
17,1,3710.765306
17,1,2625.121795
17,1,5552.859756
17,1,4418.162162
17,1,1875.042553
17,1,4820.488971
17,1,2886.190972
17,1,2757.492424
17,1,5203.303922
17,1,2249.136364
17,1,5596.050000
17,1,19626.174528
17,1,5794.477941
17,1,5793.037879
17,1,14714.378906
17,1,17129.687500
17,1,3946.714286
17,1,1957.474359
17,1,1646.102778
17,1,1825.608696
17,1,1983.260417
17,1,2037.875000
17,1,3857.347826
17,1,12907.114583
17,1,2689.125000
17,1,3354.909375
17,1,4629.282895
17,1,2189.472973
17,1,2271.978723
17,1,6838.153226

17,1,19072.750000
17,1,4435.000000
17,1,1778.664062
17,1,3496.263158
17,1,3370.446970
17,1,2703.921875
17,1,11984.502907
17,1,1643.717949
17,1,10263.005952
17,1,2042.942308
17,1,1855.545139
17,1,2785.200000
17,1,1903.059211
17,1,4356.458333
17,1,2284.485714
17,1,1720.221154
17,1,5337.260638
17,1,6630.502841
17,1,4401.384615
17,1,7753.037500
17,1,6821.714286
17,1,5190.201613
17,1,2193.914062
17,1,4188.966667
17,1,9261.214286
17,1,2248.375000
17,1,3082.024390
17,1,3166.875000
17,1,3683.557927
17,1,7488.562500
17,1,3539.512195
17,1,2463.285714
17,1,17714.500000
17,1,2031.789062
17,1,5100.175781
17,1,3918.509146
17,1,2618.868421
17,1,3765.906250
17,1,2415.615385
17,1,3337.826087
17,1,4911.024390
17,1,10534.541667
17,1,1681.500000
17,1,3889.800000
17,1,4000.442857
17,1,2328.423077
17,1,8119.200000
17,1,14029.663043
17,1,2374.593750
17,1,3858.096875
17,1,1910.181818
17,1,9215.663462
17,1,4143.625000
17,1,3139.851562
17,1,5335.069767
17,1,3467.649306
17,1,8370.578947
17,1,2487.125000
17,1,1946.452381
17,1,2918.363636
17,1,2664.115385
17,1,7280.920455
17,1,10914.722222
17,1,3037.017045
17,1,3435.386364
17,1,17880.646429

17,1,2941.526316
17,1,1739.255814
17,1,4201.676471
17,1,1517.975806
17,1,5077.370192
17,1,7366.509146
17,1,1666.000000
17,1,6338.808824
17,1,2751.089286
17,1,2638.769231
17,1,6934.551020
17,1,5226.700000
17,1,2143.619318
17,1,1777.378049
17,1,5240.458333
17,1,2524.836735
17,1,4647.790698
17,1,3931.898026
17,1,2018.872340
17,1,15825.447368
17,1,12813.446023
17,1,5691.026596
17,1,2330.968023
17,1,2097.421875
17,1,2304.784722
17,1,1942.000000
17,1,3031.276786
17,1,5084.083333
17,1,13660.284091
17,1,9655.586207
17,1,4560.819853
17,1,8425.933824
17,1,4375.273438
17,1,2675.800000
17,1,8046.157143
17,1,3425.776163
17,1,4885.625000
17,1,5216.224265
17,1,1799.593750
17,1,2660.236486
17,1,3156.090909
17,1,18837.097561
17,1,1697.796053
17,1,6048.962500
17,1,2283.135000
17,1,6767.522727
17,1,2368.338816
17,1,5380.730769
17,1,10778.731707
17,1,10379.461957
17,1,3443.935897
17,1,2172.431250
17,1,1910.600000
17,1,1830.888158
17,1,2633.666667
17,1,5701.589744
17,1,9954.088710
17,1,5647.354167
17,1,2916.731132
17,1,18705.870968
17,1,4795.916667
17,1,3131.714286
17,1,2921.627907
17,1,8176.315341
17,1,4375.150000
17,1,3228.987179

17,1,2840.666667
17,1,1761.993902
17,1,2589.005952
17,1,2406.465000
17,1,1727.866071
17,1,3488.932143
17,1,6676.147727
17,1,1522.015306
17,1,4991.846591
17,1,2531.396552
17,1,15158.410256
17,1,3770.677885
17,1,1941.394231
17,1,3829.657407
17,1,1716.187500
17,1,3123.634146
17,1,6670.762019
17,1,3517.575581
17,1,5118.525735
17,1,2490.450000
17,1,4890.203125
17,1,1754.937500
17,1,4736.053571
17,1,4417.358974
17,1,4938.734848
17,1,18205.052632
17,1,16096.461538
17,1,1804.363636
17,1,9421.315625
17,1,2712.765306
17,1,4549.989362
17,1,6166.964286
17,1,9696.881250
17,1,1981.224490
17,1,7563.867647
17,1,2352.791667
17,1,2686.625000
17,1,1796.116279
17,1,2205.580882
17,1,1785.878049
17,1,6095.166667
17,1,2944.191176
17,1,2275.072222
17,1,3311.148810
17,1,3081.983696
17,1,3546.756098
17,1,1883.750000
17,1,3418.909091
17,1,5746.088415
17,1,2240.437500
17,1,1878.671053
17,1,9656.980263
17,1,4073.000000
17,1,7526.923913
17,1,7153.180288
17,1,4048.191667
17,1,1895.868056
17,1,8418.003049
17,1,2091.336957
17,1,4236.986842
17,1,12114.110294
17,1,2790.421053
17,1,2301.678571
17,1,3906.671053
17,1,1951.848684
17,1,9925.000000

17,1,9664.679348
17,1,2686.111413
17,1,1842.500000
17,1,5077.508152
17,1,6667.410714
17,1,3937.447368
17,1,7592.425926
17,1,2437.325758
17,1,5516.363095
17,1,17223.587121
17,1,8204.923077
17,1,2015.875000
17,1,2841.720395
17,1,5307.520270
17,1,9992.361111
17,1,4478.795455
17,1,2907.000000
17,1,3793.076389
17,1,12628.160000
17,1,5786.628049
17,1,2307.454545
17,1,10228.772727
17,1,2691.316176
17,1,5879.882653
17,1,2302.343023
17,1,6401.180851
17,1,3925.852273
17,1,10075.228571
17,1,3423.500000
17,1,7312.294643
17,1,12382.639706
17,1,1847.384146
17,1,8903.506579
17,1,4191.581731
17,1,2119.250000
17,1,1626.773810
17,1,2166.277778
17,1,4590.241071
17,1,2567.942308
17,1,8207.083333
17,1,1917.019231
17,1,2579.333333
17,1,5540.884146
17,1,2167.500000
17,1,1730.458333
17,1,4100.657895
17,1,2265.446429
17,1,1686.861702
17,1,3809.790698
17,1,1677.365385
17,1,2097.306818
17,1,4912.184211
17,1,3894.236111
17,1,2358.337500
17,1,8588.127660
17,1,2204.988281
17,1,1847.609375
17,1,2516.575000
17,1,12065.595238
17,1,5466.719388
17,1,3268.668367
17,1,15135.372340
17,1,15056.795732
17,1,15995.375000
17,1,7681.513158
17,1,4285.170732

17,1,5065.766892
17,1,3350.602273
17,1,2999.196429
17,1,13275.711538
17,1,6360.351351
17,1,2123.728261
17,1,1550.702381
17,1,1732.575000
17,1,2139.987500
17,1,7270.600000
17,1,2172.434783
17,1,2126.148026
17,1,11093.762195
17,1,10502.875000
17,1,8150.465625
17,1,2371.000000
17,1,3623.495098
17,1,3203.119048
17,1,2750.197674
17,1,19173.972656
17,1,4424.121094
17,1,1890.065789
17,1,11656.242188
17,1,9519.285714
17,1,4655.609756
17,1,1772.904762
17,1,2147.375000
17,1,5532.617188
17,1,4714.771277
17,1,18685.935714
17,1,2542.337209
17,1,8756.358974
17,1,10700.409722
17,1,3588.691489
17,1,18165.000000
17,1,3407.560976
17,1,1854.431250
17,1,2901.000000
17,1,5967.409091
17,1,1862.360000
17,1,2087.867788
17,1,3640.687500
17,1,6791.451220
17,1,7656.179487
17,1,3694.839286
17,1,1559.015306
17,1,2068.593750
17,1,4635.284091
17,1,2626.500000
17,1,2151.243902
17,1,17378.234043
17,1,11280.416667
17,1,4278.275000
17,1,5450.243243
17,1,2047.550000
17,1,8092.785000
17,1,5308.637500
17,1,5240.392857
17,1,13438.269444
17,1,3311.375000
17,1,1598.203804
17,1,1561.500000
17,1,1865.760135
17,1,6810.915441
17,1,6332.725000
17,1,2922.500000

17,1,1924.924242
17,1,13942.640625
17,1,5689.796875
17,1,1748.461538
17,1,1893.408654
17,1,3161.885714
17,1,4556.646341
17,1,3894.400000
17,1,2525.875000
17,1,1803.854167
17,1,11019.632353
17,1,15181.974265
17,1,3482.298780
17,1,19785.503472
17,1,5465.464286
17,1,13635.418605
17,1,1748.256579
17,1,2494.803191
17,1,13211.120690
17,1,5757.000000
17,1,1534.385417
17,1,2442.439516
17,1,2975.829787
17,1,2876.812500
17,1,7031.938953
17,1,3354.321429
17,1,12464.733333
17,1,1678.725000
17,1,4096.458333
17,1,6469.904412
17,1,10441.381579
17,1,5857.487903
17,1,2059.212766
17,1,8316.135135
17,1,5422.345588
17,1,3698.744565
17,1,17736.125000
17,1,4897.935976
17,1,4951.902439
17,1,2841.357143
17,1,2955.191489
17,1,2971.625000
17,1,2163.069767
17,1,1624.353659
17,1,5178.475000
17,1,2621.633333
17,1,11107.780488
17,1,3830.878571
17,1,1532.937500
17,1,4017.190789
17,1,3121.285714
17,1,2694.000000
17,1,2529.958333
17,1,1783.790698
17,1,5215.900510
17,1,7569.914634
17,1,9484.319853
17,1,12775.652778
17,1,2286.943878
17,1,6856.475000
17,1,4183.409091
17,1,7323.174342
17,1,5671.636364
17,1,2414.875000
17,1,5477.600000
17,1,17797.150568

17,1,3500.788462
17,1,4531.847973
17,1,10954.500000
17,1,3841.584135
17,1,2395.592391
17,1,7890.956522
17,1,6140.939655
17,1,4813.588235
17,1,2109.875000
17,1,1968.204545
17,1,7778.311047
17,1,1831.843750
17,1,5685.419118
17,1,17807.894737
17,1,7119.272727
17,1,2709.973837
17,1,1744.076613
17,1,5936.083333
17,1,7794.602273
17,1,2494.823171
17,1,2909.531250
17,1,1758.850000
17,1,6013.602941
17,1,8759.211538
17,1,4615.801471
17,1,8975.000000
17,1,1706.742188
17,1,1760.750000
17,1,1728.743902
17,1,2663.394231
17,1,10345.625000
17,1,2187.937500
17,1,9697.575000
17,1,1554.800781
17,1,1833.152344
17,1,1539.301020
17,1,19650.613095
17,1,1794.075658
17,1,4350.456818
17,1,3069.366477
17,1,11765.781250
17,1,7459.678030
17,1,3983.450758
17,1,4851.166667
17,1,2295.375000
17,1,5862.753676
17,1,9820.689024
17,1,2192.425676
17,1,8937.119048
17,1,5605.646739
17,1,2847.264706
17,1,7482.601744
17,1,2832.062500
17,1,6076.486842
17,1,5004.287234
17,1,12427.171875
17,1,5749.333333
17,1,2235.437500
17,1,4847.298780
17,1,10405.315625
17,1,8834.818182
17,1,5627.852941
17,1,5718.476562
17,1,8065.756757
17,1,10651.137500
17,1,2022.375000

17,1,5261.823529
17,1,1914.290441
17,1,9771.000000
17,1,1665.488636
17,1,13137.298077
17,1,18320.678571
17,1,8388.044355
17,1,2060.756757
17,1,8583.410714
17,1,1954.864865
17,1,1547.375000
17,1,6976.497340
17,1,5812.411111
17,1,2048.625000
17,1,1546.276596
17,1,1590.142857
17,1,10386.615385
17,1,3404.651163
17,1,2951.802326
17,1,5573.759615
17,1,1928.310345
17,1,2626.300000
17,1,1588.605769
17,1,1835.825000
17,1,2405.320652
17,1,17622.875000
17,1,1825.369048
17,1,8410.985465
17,1,1968.425000
17,1,6651.269231
17,1,6965.421875
17,1,2614.631410
17,1,1709.597826
17,1,5397.937500
17,1,10492.088235
17,1,1895.831250
17,1,2345.662736
17,1,3419.264706
17,1,1589.953125
17,1,3196.580645
17,1,5309.187500
17,1,1691.500000
17,1,14683.105114
17,1,4157.894231
17,1,10656.705357
17,1,9895.642857
17,1,5421.143293
17,1,8768.232143
17,1,19137.886029
17,1,4348.818182
17,1,8448.880556
17,1,19216.666667
17,1,5095.733333
17,1,2150.625000
17,1,2042.325000
17,1,10410.228125
17,1,6327.625000
17,1,3746.597222
17,1,3735.717949
17,1,3166.252660
17,1,2786.250000
17,1,7114.068750
17,1,7773.323529
17,1,3701.015306
17,1,5774.727273
17,1,12572.594828

17,1,3366.826220
17,1,2677.583333
17,1,2564.875000
17,1,14688.980769
17,1,3005.649390
17,1,2893.916667
17,1,2598.029605
17,1,1923.782051
17,1,1519.770833
17,1,2948.608696
17,1,7663.302419
17,1,1919.060976
17,1,9441.678571
17,1,5687.803571
17,1,2594.812500
17,1,4616.589286
17,1,1932.218750
17,1,9692.692308
17,1,14013.022727
17,1,8494.634615
17,1,3887.338235
17,1,18455.297872
17,1,1730.475000
17,1,9614.884615
17,1,8073.884615
17,1,1539.106383
17,1,2581.693182
17,1,1727.750000
17,1,11578.750000
17,1,16097.421053
17,1,2687.133333
17,1,3599.268293
17,1,7297.144737
17,1,8437.267442
17,1,1834.985294
17,1,4619.187500
17,1,17238.089623
17,1,17482.875000
17,1,4176.306604
17,1,4428.375000
17,1,1721.448276
17,1,5037.466146
17,1,2723.000000
17,1,2463.264535
17,1,19321.767857
17,1,1822.174419
17,1,1836.569444
17,1,5146.018750
17,1,3625.710227
17,1,5404.255319
17,1,2695.017045
17,1,1942.416667
17,1,6559.392857
17,1,5151.795455
17,1,3048.611111
17,1,3144.777778
17,1,3733.479167
17,1,4136.897436
17,1,3090.943750
17,1,1688.484043
17,1,8442.145161
17,1,4386.912162
17,1,2649.202381
17,1,7476.592391
17,1,8283.698171
17,1,9918.070312

17,1,3840.451923
17,1,15499.475000
17,1,8949.862069
17,1,7062.513393
17,1,3296.125000
17,1,1916.125000
17,1,10228.712500
17,1,1720.828947
17,1,1988.256579
17,1,1602.413690
17,1,4450.954545
17,1,1549.460938
17,1,3337.419118
17,1,5836.425532
17,1,3973.152027
17,1,1783.755814
17,1,3014.157609
17,1,4738.500000
17,1,2014.307692
17,1,2565.018868
17,1,4370.597500
17,1,2705.346154
17,1,13464.411765
17,1,11671.792411
17,1,1620.353659
17,1,16317.551020
17,1,4533.375000
17,1,3017.285714
17,1,9291.840116
17,1,1715.884868
17,1,2150.125000
17,1,2695.707317
17,1,4713.043478
17,1,3851.398810
17,1,3215.158654
17,1,1781.466667
17,1,2642.453488
17,1,1997.818750
17,1,10851.439655
17,1,5946.562500
17,1,2682.080128
17,1,2901.264706
17,1,1934.833333
17,1,8823.138889
17,1,3685.853659
17,1,2192.500000
17,1,1598.838816
17,1,8811.696875
17,1,14292.565217
17,1,5273.250000
17,1,3842.888021
17,1,1599.187500
17,1,1551.184783
17,1,4954.333333
17,1,6003.875000
17,1,9241.581081
17,1,6686.090000
17,1,2799.952381
17,1,1964.031250
17,1,4753.779255
17,1,14252.867647
17,1,5112.000000
17,1,4075.000000
17,1,1506.365625
17,1,3850.875000
17,1,8107.695122

17,1,12184.475000
17,1,2021.839474
17,1,2031.655172
17,1,1933.968750
17,1,11014.897727
17,1,12739.329545
17,1,2519.487500
17,1,3498.783333
17,1,8691.170732
17,1,3230.773026
17,1,4206.128205
17,1,1591.875000
17,1,1945.267442
17,1,2710.281915
17,1,5174.900000
17,1,9053.514706
17,1,4931.477564
17,1,4135.636364
17,1,5639.000000
17,1,18222.437500
17,1,6221.245690
17,1,1633.244898
17,1,2367.771739
17,1,3949.391026
17,1,3971.344444
17,1,11110.371094
17,1,1787.665179
17,1,1620.150568
17,1,2173.335366
17,1,2197.337838
17,1,1970.920455
17,1,6514.413043
17,1,1740.872340
17,1,8532.539286
17,1,4500.136364
17,1,1592.258929
17,1,3058.422619
17,1,2655.558140
17,1,3944.926829
17,1,4402.824074
17,1,4685.553571
17,1,1686.579268
17,1,2466.231707
17,1,4166.011029
17,1,13000.059524
17,1,1510.250000
17,1,18606.678571
17,1,16303.261905
17,1,4794.095238
17,1,1973.561224
17,1,2115.948171
17,1,4315.634615
17,1,6149.590909
17,1,1515.197222
17,1,6414.330357
17,1,1548.390244
17,1,16642.493902
17,1,4118.521277
17,1,1968.729167
17,1,1544.291667
17,1,3617.460227
17,1,1607.649038
17,1,3491.854167
17,1,2359.209184
17,1,6797.500000
17,1,5302.085526

17,1,6115.825758
17,1,5604.954545
17,1,4200.200000
17,1,2494.615854
17,1,2686.794118
17,1,11314.219512
17,1,2241.684211
17,1,8263.660714
17,1,2454.572115
17,1,7966.700000
17,1,5712.156250
17,1,2168.563953
17,1,6534.349432
17,1,6927.918919
17,1,4645.595930
17,1,7442.951220
17,1,3652.195652
17,1,11508.462121
17,1,1511.058333
17,1,4802.050000
17,1,2118.562500
17,1,12248.324324
17,1,14855.792683
17,1,3651.202703
17,1,2446.085227
17,1,3458.250000
17,1,7597.205882
17,1,6423.127451
17,1,6493.513587
17,1,2024.057692
18,2,2655.860294
18,2,2449.682692
18,2,3447.152439
18,2,1733.734375
18,2,1531.549342
18,2,1750.584906
18,2,1609.905488
18,2,2196.285714
18,2,1949.712264
18,2,2854.616379
18,2,2275.940789
18,2,2250.562500
18,2,2859.924107
18,2,1662.016304
18,2,1562.339286
18,2,2098.399038
18,2,4104.296875
18,2,5565.603774
18,2,2004.208333
18,2,6533.117647
18,2,1975.390244
18,2,1896.085526
18,2,3300.309278
18,2,1673.962963
18,2,1951.375000
18,2,1746.319712
18,2,18125.458333
18,2,1851.538462
18,2,2429.934211
18,2,2928.057692
18,2,1587.389881
18,2,1651.395408
18,2,1536.132212
18,2,1965.278409
18,2,2104.796875
18,2,3630.890244

18,2,1582.762500
18,2,2929.354651
18,2,1514.656250
18,2,3444.558140
18,2,2087.960784
18,2,2071.900000
18,2,1613.493421
18,2,3006.195312
18,2,1940.109375
18,2,1635.929878
18,2,2052.136364
18,2,1990.979167
18,2,1568.625000
18,2,1513.558594
18,2,1942.725490
18,2,2490.177632
18,2,2452.762755
18,2,1876.026042
18,2,6179.576271
18,2,3246.439698
18,2,2366.422297
18,2,2005.319767
18,2,2099.750000
18,2,7514.388587
18,2,1947.328804
18,2,2231.052778
18,2,7405.146635
18,2,4825.622283
18,2,1625.068182
18,2,2937.408163
18,2,8268.147436
18,2,1640.556034
18,2,2153.875000
18,2,2144.771739
18,2,2314.815217
18,2,2520.666667
18,2,1601.887195
18,2,3472.222222
18,2,2256.142857
18,2,1771.163265
18,2,2011.988827
18,2,1646.337209
18,2,2900.418269
18,2,1938.981771
18,2,4325.815476
18,2,1588.411932
18,2,2336.335106
18,2,1689.547486
18,2,3799.473214
18,2,4395.761905
18,2,2039.266827
18,2,2223.309659
18,2,2597.354167
18,2,1903.426136
18,2,2290.771739
18,2,2696.906250
18,2,3375.000000
18,2,1874.793103
18,2,11148.752155
18,2,1831.180000
18,2,1779.244444
18,2,2525.691176
18,2,1938.781250
18,2,1511.777778
18,2,1546.944444
18,2,1642.925926

18,2,1543.970443
18,2,1916.500000
18,2,1555.409091
18,2,1837.556548
18,2,2644.250000
18,2,2818.493421
18,2,1642.483696
18,2,2697.746622
18,2,2293.539773
18,2,5562.216463
18,2,1935.750000
18,2,2037.750000
18,2,1833.541667
18,2,1606.857143
18,2,1609.176020
18,2,2137.118056
18,2,3188.897500
18,2,3835.800000
18,2,1631.927083
18,2,3104.375000
18,2,1775.406977
18,2,1962.457317
18,2,1506.827206
18,2,3032.122159
18,2,2309.962500
18,2,1580.244318
18,2,4911.578947
18,2,1952.208333
18,2,1602.137255
18,2,2382.744048
18,2,2863.396226
18,2,2335.301724
18,2,2481.137755
18,2,2306.469828
18,2,1941.984375
18,2,6849.078947
18,2,4094.557692
18,2,2517.415094
18,2,1560.491848
18,2,2724.266484
18,2,1574.058511
18,2,1667.354592
18,2,7105.169118
18,2,2619.428977
18,2,1896.067935
18,2,2658.505682
18,2,1982.406977
18,2,5018.911765
18,2,2251.988208
18,2,1612.869565
18,2,2656.553030
18,2,3819.818750
18,2,2477.827128
18,2,3040.206897
18,2,1732.551282
18,2,2939.780702
18,2,1535.678191
18,2,2391.175000
18,2,2384.734848
18,2,3402.918605
18,2,1515.112805
18,2,17857.135000
18,2,2556.250000
18,2,1604.744186
18,2,1602.105405
18,2,16978.596154

18,2,2455.808333
18,2,2721.357143
18,2,2117.816327
18,2,1567.382353
18,2,3401.520000
18,2,2455.317708
18,2,1523.389706
18,2,10466.560606
18,2,1763.756757
18,2,1659.953125
18,2,1663.135000
18,2,2964.524390
18,2,2478.352500
18,2,2996.460938
18,2,2981.500000
18,2,13460.351562
18,2,1728.811321
18,2,2781.988636
18,2,1604.812500
18,2,4118.034091
18,2,1534.000000
18,2,1520.957447
18,2,1780.000000
18,2,2801.582888
18,2,2450.675000
18,2,2483.676471
18,2,2493.578125
18,2,4294.344444
18,2,1734.250000
18,2,1825.378378
18,2,1679.541667
18,2,1563.187500
18,2,2297.057692
18,2,1726.577778
18,2,2734.112069
18,2,3981.886364
18,2,1899.666667
18,2,4555.482422
18,2,3770.325980
18,2,1556.283333
18,2,2118.976562
18,2,1815.860000
18,2,3445.803571
18,2,1965.434783
18,2,2462.793103
18,2,2994.823529
18,2,1615.727273
18,2,3421.236111
18,2,1598.451613
18,2,5472.290323
18,2,1581.666667
18,2,1649.650000
18,2,2675.188776
18,2,2820.867647
18,2,1535.539773
18,2,2137.000000
18,2,3090.141304
18,2,2170.011111
18,2,1854.660377
18,2,2829.988095
18,2,1809.857143
18,2,2497.052083
18,2,2428.940000
18,2,2433.163462
18,2,3568.889706
18,2,3086.885135

18,2,2388.540541
18,2,1627.875000
18,2,1622.637195
18,2,1850.231707
18,2,2179.702381
18,2,2326.370000
18,2,2050.460227
18,2,3822.689024
18,2,2557.878289
18,2,1582.121429
18,2,1686.546875
18,2,1549.200581
18,2,1909.664634
18,2,2122.896552
18,2,1916.350000
18,2,1823.240854
18,2,1765.513889
18,2,3783.599138
18,2,1919.551829
18,2,1809.659091
18,2,2321.265909
18,2,1945.054545
18,2,2673.351744
18,2,2998.732143
18,2,10249.057143
18,2,1822.207547
18,2,2943.826923
18,2,2150.163462
18,2,1859.173913
18,2,1957.937500
18,2,1725.140704
18,2,1820.818182
18,2,1813.235849
18,2,2543.025641
18,2,3178.214286
18,2,1499.450549
18,2,1872.619048
18,2,12563.115741
18,2,2384.196429
18,2,3763.822222
18,2,1916.023256
18,2,1615.200000
18,2,2032.414773
18,2,3477.806548
18,2,2023.298611
18,2,1836.352941
18,2,1762.983108
18,2,1529.924528
18,2,3027.590909
18,2,4587.638021
18,2,1547.916667
18,2,2941.657895
18,2,2000.905488
18,2,2255.429825
18,2,2468.421875
18,2,7050.006383
18,2,1584.250000
18,2,1610.908088
18,2,2789.948276
18,2,5077.232955
18,2,1504.354167
18,2,3173.401042
18,2,1539.125000
18,2,2646.465909
18,2,1683.849057
18,2,1917.666667

18,2,2292.925000
18,2,1860.500000
18,2,1806.094828
18,2,2202.649306
18,2,2885.951087
18,2,1955.037500
18,2,1626.451087
18,2,1972.500000
18,2,2146.796875
18,2,2399.840426
18,2,1803.250000
18,2,1587.212121
18,2,1686.255319
18,2,1945.017361
18,2,1547.750000
18,2,1833.600694
18,2,1692.808824
18,2,2733.395349
18,2,1704.071023
18,2,2068.650000
18,2,5087.386719
18,2,1540.019231
18,2,1628.423077
18,2,2591.034722
18,2,4615.068182
18,2,4699.950000
18,2,1859.625000
18,2,4202.981132
18,2,6316.843750
18,2,2528.970588
18,2,2925.509804
18,2,5018.981250
18,2,3178.875000
18,2,2140.353659
18,2,1981.058140
18,2,2413.247685
18,2,1841.443878
18,2,6012.942308
18,2,1830.050000
18,2,1658.631944
18,2,2272.460784
18,2,2059.347561
18,2,1532.125000
18,2,2129.718750
18,2,2141.618750
18,2,2872.857143
18,2,2047.583333
18,2,1593.646667
18,2,2173.359756
18,2,1579.627717
18,2,1802.688776
18,2,2255.490854
18,2,5650.069444
18,2,2409.681818
18,2,2710.907895
18,2,1865.453125
18,2,2094.675926
18,2,2688.585784
18,2,4927.508152
18,2,1528.706731
18,2,2594.527778
18,2,2885.885870
18,2,1966.114583
18,2,2403.616071
18,2,1808.367347
18,2,2622.820000

18,2,2045.228723
18,2,1568.327830
18,2,1742.730000
18,2,2658.336898
18,2,2829.148148
18,2,4000.575658
18,2,1514.466667
18,2,1903.912162
18,2,2251.870000
18,2,2018.130435
18,2,1833.653846
18,2,2681.316860
18,2,2251.115385
18,2,2559.863636
18,2,1501.732143
18,2,1868.894737
18,2,1735.332237
18,2,2496.361111
18,2,2690.000000
18,2,1693.755556
18,2,1564.330729
18,2,1591.145349
18,2,2029.431373
18,2,3559.948370
18,2,1622.200000
18,2,2281.000000
18,2,1991.812500
18,2,1686.442308
18,2,16217.269608
18,2,2432.181818
18,2,3823.957447
18,2,3391.977500
18,2,1696.525641
18,2,1655.671429
18,2,1612.770349
18,2,1852.886792
18,2,1519.827500
18,2,1602.013298
18,2,1811.690341
18,2,1810.591667
18,2,1834.062500
18,2,2152.823529
18,2,13023.734694
18,2,5410.476562
18,2,1519.337838
18,2,2787.274457
18,2,1556.000000
18,2,1784.272727
18,2,1703.102273
18,2,4152.196078
18,2,2950.156250
18,2,2455.020833
18,2,1958.878049
18,2,2023.886029
18,2,1847.264706
18,2,3768.280488
18,2,1598.358871
18,2,4076.846154
18,2,2339.163265
18,2,3236.275000
18,2,3769.378571
18,2,2807.000000
18,2,1740.553191
18,2,1859.970238
18,2,1733.037037
18,2,1729.141129

18,2,1618.481250
18,2,2972.102941
18,2,2010.197674
18,2,1613.194444
18,2,2889.250000
18,2,2098.190476
18,2,2410.823276
18,2,1554.529412
18,2,2017.348018
18,2,1822.868421
18,2,2115.583333
18,2,1991.656250
18,2,2013.673913
18,2,1559.875000
18,2,1833.381579
18,2,5639.244792
18,2,2726.964744
18,2,2044.525510
18,2,1511.219512
18,2,2770.577703
18,2,1521.060897
18,2,2835.571429
18,2,3404.743590
18,2,2576.500000
18,2,2244.891892
18,2,2465.036585
18,2,1825.601415
18,2,2249.735849
18,2,1709.507075
18,2,1884.365385
18,2,1674.342262
18,2,3073.489362
18,2,2237.856250
18,2,3383.471591
18,2,2284.334906
18,2,1506.029605
18,2,2286.271635
18,2,2017.908163
18,2,1765.446429
18,2,5611.583333
18,2,1846.314286
18,2,2442.731771
18,2,2621.343023
18,2,1964.752404
18,2,1583.418182
18,2,1859.767442
18,2,2376.646341
18,2,2392.500000
18,2,2073.062500
18,2,1669.903125
18,2,4760.187500
18,2,2551.989362
18,2,3297.000000
18,2,1868.931818
18,2,1539.795455
18,2,3152.380208
18,2,2762.592672
18,2,2510.692308
18,2,2276.358974
18,2,1619.025000
18,2,2444.522727
18,2,2518.762238
18,2,7286.648936
18,2,1646.204545
18,2,2168.592593
18,2,1841.655000

18,2,2095.736607
18,2,1607.489362
18,2,2437.618902
18,2,2165.969388
18,2,3656.522727
18,2,1609.000000
18,2,1801.117647
18,2,6018.222222
18,2,1650.321429
18,2,1509.677419
18,2,3717.500000
18,2,2769.672297
18,2,1505.796875
18,2,2770.285714
18,2,1891.346939
18,2,4532.651163
18,2,1607.090625
18,2,1969.330000
18,2,3749.118056
18,2,1969.104167
18,2,1725.436170
18,2,1549.156863
18,2,2141.289773
18,2,2451.015625
18,2,2296.645161
18,2,8567.616379
18,2,1543.178571
18,2,1609.653302
18,2,1789.608974
18,2,2464.018293
18,2,2095.427500
18,2,2652.543981
18,2,1844.419118
18,2,2167.250000
18,2,1522.285714
18,2,5685.614796
18,2,3294.791667
18,2,1565.687500
18,2,2051.296512
18,2,1620.833333
18,2,1544.442708
18,2,2108.750000
18,2,1989.160000
18,2,2665.308511
18,2,3922.960784
18,2,3014.556818
18,2,2045.700000
18,2,5094.735294
18,2,3065.355114
18,2,1830.625000
18,2,2539.448370
18,2,3287.507979
18,2,1593.303125
18,2,1609.491279
18,2,2254.169118
18,2,1533.175000
18,2,2399.579268
18,2,4745.195122
18,2,2047.568750
18,2,1912.280000
18,2,2537.428571
18,2,3079.000000
18,2,15764.843750
18,2,1767.131944
18,2,1528.057692
18,2,2539.614583

18,2,2631.679245
18,2,3482.242291
18,2,1710.656250
18,2,1563.820513
18,2,4114.285714
18,2,2176.780488
18,2,1731.716667
18,2,2425.299451
18,2,3560.054878
18,2,2183.000000
18,2,4346.303977
18,2,1656.668919
18,2,1722.756098
18,2,1607.286585
18,2,1521.679348
18,2,1741.235577
18,2,1564.525000
18,2,1511.372449
18,2,1571.817073
18,2,2476.294118
18,2,2243.755102
18,2,3265.183824
18,2,3635.625000
18,2,3210.510638
18,2,2394.375000
18,2,1626.657738
18,2,2582.287791
18,2,2595.254167
18,2,1606.212766
18,2,1556.765244
18,2,1982.805556
18,2,2166.632353
18,2,2327.780612
18,2,2050.250000
18,2,3135.804687
18,2,2011.636905
18,2,1991.978723
18,2,1577.244186
18,2,1618.528226
18,2,2561.704787
18,2,1609.285714
18,2,3172.565217
18,2,2159.595745
18,2,1716.349057
18,2,2229.979651
18,2,1685.289474
18,2,2319.267857
18,2,2425.140625
18,2,3602.791667
18,2,3897.980769
18,2,4060.009434
18,2,2304.453125
18,2,1574.806604
18,2,3567.073113
18,2,2663.972222
18,2,3953.526316
18,2,4188.130000
18,2,1512.382212
18,2,5449.028061
18,2,1731.017442
18,2,1888.976974
18,2,1557.487179
18,2,3392.345000
18,2,1612.800000
18,2,2707.679487
18,2,2199.809659

18,2,1619.277273
18,2,1814.341463
18,2,2128.625000
18,2,2503.483333
18,2,1643.529762
18,2,2141.073529
18,2,1913.034314
18,2,1857.085714
18,2,1573.177083
18,2,2199.701923
18,2,3154.455000
18,2,2083.755000
18,2,3134.203488
18,2,2560.285714
18,2,1961.538889
18,2,1551.312500
18,2,1704.034091
18,2,2359.533088
18,2,2236.051724
18,2,2271.913690
18,2,2476.002907
18,2,2332.457447
18,2,2959.239583
18,2,2056.170213
18,2,2034.967500
18,2,2438.648026
18,2,2332.393750
18,2,2555.715278
18,2,2202.000000
18,2,2278.037037
18,2,2699.271277
18,2,3052.389706
18,2,3133.646635
18,2,3878.195000
18,2,1780.315217
18,2,2456.562500
18,2,7002.458333
18,2,1804.500000
18,2,1538.260000
18,2,1996.904255
18,2,1743.148649
18,2,3611.424419
18,2,2757.512931
18,2,1797.011628
18,2,2333.252717
18,2,2566.235294
18,2,1568.364362
18,2,2686.725000
18,2,4932.203947
18,2,1500.267500
18,2,1596.028125
18,2,2842.593750
18,2,3234.558824
18,2,2779.908163
18,2,1804.025735
18,2,2138.638514
18,2,1933.207589
18,2,2813.033537
18,2,2169.187500
18,2,2922.877358
18,2,1631.510638
18,2,2175.731132
18,2,1693.695652
18,2,2240.354911
18,2,2721.776596
18,2,2752.176471

18,2,2232.977041
18,2,3854.781250
18,2,2745.821809
18,2,2080.040865
18,2,2503.075581
18,2,1840.091912
18,2,1827.926829
18,2,3072.459821
18,2,1896.413690
18,2,1739.108108
18,2,1943.290179
18,2,4615.750000
18,2,1899.589674
18,2,3298.397959
18,2,1904.530612
18,2,1612.298780
18,2,2248.669811
18,2,1876.275000
18,2,2744.798077
18,2,2198.052356
18,2,2847.350000
18,2,2001.475000
18,2,1861.692500
18,2,1541.806818
18,2,1939.152174
18,2,1605.000000
18,2,4401.187500
18,2,2014.152439
18,2,2368.345238
18,2,1573.335227
18,2,2324.280702
18,2,1942.638298
18,2,1783.600000
18,2,2801.785714
18,2,1806.555556
18,2,1598.962963
18,2,2360.242647
18,2,1906.569149
18,2,2420.292453
18,2,2188.000000
18,2,1568.928571
18,2,1817.354839
18,2,2861.587500
18,2,1837.615385
18,2,1767.647727
18,2,1846.110465
18,2,1533.476744
18,2,3846.048077
18,2,3110.122093
18,2,2277.825472
18,2,2042.423077
18,2,1925.829268
18,2,4522.413462
18,2,2837.070755
18,2,1947.335227
18,2,1900.406250
18,2,1981.425000
18,2,5468.895833
18,2,1504.250000
18,2,3406.852941
18,2,1855.461538
18,2,2736.855263
18,2,2011.433824
18,2,1584.431818
18,2,1513.594595
18,2,6883.229167

18,2,6292.391827
18,2,1782.723837
18,2,4057.923077
18,2,2408.458333
18,2,2003.823529
18,2,1504.502907
18,2,12920.133333
18,2,1777.329787
18,2,1937.808511
18,2,1921.520833
18,2,2132.021429
18,2,2046.031250
18,2,2031.791667
18,2,1600.740566
18,2,3487.440789
18,2,2028.383929
18,2,1533.963415
18,2,3364.161458
18,2,2757.019531
18,2,2334.602273
18,2,1594.882653
18,2,1512.462766
18,2,2191.872881
18,2,1713.502358
18,2,1883.729032
18,2,4026.718750
18,2,1539.311111
18,2,2005.037500
18,2,2513.566176
18,2,1942.455556
18,2,2869.583333
18,2,2115.452586
18,2,1502.318627
18,2,1808.000000
18,2,2527.904605
18,2,2153.767857
18,2,1583.576389
18,2,1867.141361
18,2,1610.923077
18,2,3853.442857
18,2,2761.500000
18,2,1933.318966
18,2,15534.833333
18,2,13309.709091
18,2,2415.930233
18,2,1565.500000
18,2,2888.301829
18,2,1771.274457
18,2,2020.478261
18,2,3315.089744
18,2,3468.821429
18,2,3323.825000
18,2,3532.598684
18,2,12468.950000
18,2,2234.247549
18,2,1664.453488
18,2,3023.285377
18,2,1502.878378
18,2,2679.888889
18,2,1651.790865
18,2,4649.790000
18,2,2143.734694
18,2,1720.009868
18,2,2539.585106
18,2,1563.959459
18,2,6714.274457

18,2,2694.612745
18,2,2710.585366
18,2,1657.992308
18,2,1553.700000
18,2,2451.865385
18,2,2244.127451
18,2,3269.928571
18,2,2989.160000
18,2,1952.260417
18,2,1714.287879
18,2,1864.988095
18,2,2631.895833
18,2,2781.443750
18,2,2721.544118
18,2,2721.125000
18,2,1816.136364
18,2,1555.732759
18,2,4190.769737
18,2,4992.562500
18,2,2031.500000
18,2,13949.730769
18,2,1850.750000
18,2,1546.445312
18,2,4477.105263
18,2,1624.200000
18,2,1835.858696
18,2,2870.800595
18,2,2188.962963
18,2,2445.941860
18,2,12562.451923
18,2,1768.049020
18,2,1587.901786
18,2,1507.257812
18,2,2490.216216
18,2,2841.747642
18,2,1534.260870
18,2,2151.541667
18,2,1781.459302
18,2,2249.510135
18,2,1966.785714
18,2,1830.133929
18,2,1975.000000
18,2,3588.672414
18,2,2122.349515
18,2,1656.670673
18,2,1550.737179
18,2,2201.680556
18,2,2102.768617
18,2,4174.400000
18,2,2505.279412
18,2,8488.173913
18,2,1830.958333
18,2,1506.804348
18,2,4659.810096
18,2,1707.945122
18,2,1898.790698
18,2,6230.886792
18,2,1771.858553
18,2,6058.530612
18,2,1906.263514
18,2,6301.971154
18,2,2338.483871
18,2,2235.430233
18,2,2105.058140
18,2,2302.307692
18,2,1639.280488

18,2,1753.526316
18,2,3584.524510
18,2,1814.786458
18,2,1705.943750
18,2,4180.322368
18,2,2445.895652
18,2,2355.215909
18,2,6905.141509
18,2,1805.957237
18,2,1921.163462
18,2,2306.480978
18,2,6520.311364
18,2,4760.972973
18,2,1572.863636
18,2,2037.000000
18,2,2263.405488
18,2,3676.854167
18,2,1651.743056
18,2,1654.082500
18,2,2579.576087
18,2,4255.970588
18,2,1996.686170
18,2,2595.139881
18,2,2782.316176
18,2,1833.840625
18,2,4436.370968
18,2,2034.599057
18,2,1576.240385
18,2,1858.165909
18,2,2845.000000
18,2,1565.265625
18,2,2572.210000
18,2,1713.834459
18,2,1574.909375
18,2,1746.454082
18,2,2276.854545
18,2,1685.679348
18,2,1865.760000
18,2,3283.704545
18,2,1615.646635
18,2,1632.360577
18,2,1590.729167
18,2,7109.687500
18,2,3458.609043
18,2,2546.276316
18,2,2309.708333
18,2,9656.544643
18,2,2114.545918
18,2,2445.037791
18,2,2013.203125
18,2,15726.425532
18,2,2787.181818
18,2,3050.933333
18,2,2742.300000
18,2,5262.681818
18,2,1637.603659
18,2,2433.573604
18,2,2433.937500
18,2,1888.550000
18,2,1853.676471
18,2,1730.833333
18,2,4242.750000
18,2,2018.833333
18,2,2648.700000
18,2,3442.317073
18,2,1987.647059

18,2,2259.712209
18,2,1705.000000
18,2,1728.892857
18,2,1526.937500
18,2,1714.206522
18,2,1677.167411
18,2,2583.815000
18,2,3185.937500
18,2,2455.333333
18,2,2019.119048
18,2,1563.721875
18,2,1503.208333
18,2,1569.670213
18,2,1508.750000
18,2,2885.180851
18,2,2320.220455
18,2,2063.865385
18,2,1499.026316
18,2,3598.090625
18,2,3365.041667
18,2,1560.428571
18,2,1562.180000
18,2,2348.383333
18,2,2733.161765
18,2,2591.506410
18,2,1974.094340
18,2,2888.662500
18,2,1512.836735
18,2,3120.829545
18,2,3900.137712
18,2,1507.084270
18,2,6385.166667
18,2,5304.530612
18,2,2793.654412
18,2,3145.534884
18,2,1966.781690
18,2,3197.542553
18,2,3098.283163
18,2,1917.036638
18,2,2091.465278
18,2,2107.605000
18,2,1687.700000
18,2,1929.973214
18,2,2451.469595
18,2,1943.355263
18,2,2783.065789
18,2,6829.500000
18,2,2339.914894
18,2,3458.750000
18,2,1537.107500
18,2,3173.971591
18,2,2104.500000
18,2,5360.493056
18,2,2252.926724
18,2,1513.551471
18,2,5366.101562
18,2,4512.076923
18,2,1812.578125
18,2,3887.252273
18,2,1937.128205
18,2,2013.375000
18,2,2727.445946
18,2,1703.132075
18,2,1776.938679
18,2,2991.880319
18,2,1963.153061

18,2,1977.692708
18,2,6342.170213
18,2,2477.642857
18,2,8773.062500
18,2,2010.854167
18,2,1563.808333
18,2,2074.914773
18,2,1926.187500
18,2,1939.468085
18,2,2736.060000
18,2,5261.202778
18,2,3861.787234
18,2,1931.250000
18,2,1523.618902
18,2,2591.437500
18,2,3051.380952
18,2,4126.923077
18,2,2189.515000
18,2,2332.551020
18,2,1896.955556
18,2,2235.700000
18,2,2503.185714
18,2,1502.031250
18,2,1743.928571
18,2,1656.800000
18,2,2672.000000
18,2,1755.882979
18,2,3676.476415
18,2,3244.421875
18,2,2536.861111
18,2,1717.750000
18,2,1858.455882
18,2,2524.462264
18,2,1941.347222
18,2,1889.976974
18,2,2658.083333
18,2,1852.759615
18,2,3161.340000
18,2,4914.175676
18,2,2827.000000
18,2,10608.315789
18,2,7463.383621
18,2,1788.043750
18,2,4915.952703
18,2,3049.890625
18,2,2956.270349
18,2,2315.761111
18,2,1975.918478
18,2,6014.831395
18,2,3555.076923
18,2,5401.607143
18,2,9089.575000
18,2,1775.733945
18,2,3993.204787
18,2,2464.429245
18,2,2224.881250
18,2,2470.262755
18,2,2891.842365
18,2,1734.496795
18,2,4473.765957
18,2,2433.069519
18,2,1985.482143
18,2,1695.811111
18,2,1649.880319
18,2,4048.180556
18,2,1770.850000

18,2,7778.506944
18,2,2382.401786
18,2,1810.570000
18,2,2583.673246
18,2,1520.378378
18,2,3787.723404
18,2,1695.200000
18,2,7086.292857
18,2,1965.900000
18,2,2654.966667
18,2,2886.476351
18,2,2155.875000
18,2,1781.058673
18,2,1643.882353
18,2,1738.926829
18,2,4310.810345
18,2,7531.306818
18,2,3011.714286
18,2,2620.500000
18,2,1609.290865
18,2,2274.085938
18,2,1546.467391
18,2,3099.437500
18,2,1800.216837
18,2,2946.832386
18,2,2593.010638
18,2,7352.617021
18,2,1721.703488
18,2,1611.490854
18,2,3399.607843
18,2,1727.000000
18,2,1766.077273
18,2,1870.959184
18,2,2079.060000
18,2,1585.423077
18,2,6487.802632
18,2,2204.692308
18,2,1587.146875
18,2,3283.153846
18,2,1854.541667
18,2,3342.594595
18,2,2251.062500
18,2,9152.704787
18,2,2996.843750
18,2,2369.410000
18,2,1799.475000
18,2,2505.306250
18,2,1591.974057
18,2,2210.689189
18,2,2354.583333
18,2,1876.260870
18,2,2107.159574
18,2,3130.220930
18,2,4817.159091
18,2,2242.984043
18,2,2273.617647
18,2,2233.615000
18,2,3897.930233
18,2,1685.401316
18,2,8131.165625
18,2,2804.255556
18,2,2830.052632
18,2,2734.404762
18,2,9302.942935
18,2,5348.750000
18,2,1557.279412

18,2,2103.470745
18,2,2594.360000
18,2,2647.838068
18,2,1738.609756
18,2,3553.471939
18,2,1850.558140
18,2,1588.733333
18,2,1977.587500
18,2,1843.529412
18,2,2032.107143
18,2,1854.655629
18,2,2289.952830
18,2,1768.949405
18,2,1541.153846
18,2,5378.200000
18,2,2239.128125
18,2,1880.750000
18,2,2051.048077
18,2,1707.046296
18,2,2738.880952
18,2,2392.767045
18,2,4337.250000
18,2,1826.058824
18,2,2422.882353
18,2,1613.062500
18,2,4394.207317
18,2,2032.581081
18,2,1617.858696
18,2,1820.750000
18,2,1526.393443
18,2,1956.000000
18,2,6375.810976
18,2,2063.461538
18,2,1627.441406
18,2,2922.265152
18,2,1766.375000
18,2,4838.930921
18,2,2682.429245
18,2,2438.156250
18,2,1742.567568
18,2,1898.892857
18,2,2307.478723
18,2,2676.704545
18,2,4201.883333
18,2,1551.163462
18,2,1931.013158
18,2,2191.349057
18,2,2293.800000
18,2,5713.812500
18,2,2381.355556
18,2,2160.593085
18,2,2095.538043
18,2,1663.224490
18,2,1973.435897
18,2,3005.944079
18,2,4447.903409
18,2,2719.340625
18,2,1990.256579
18,2,1747.858696
18,2,4485.245902
18,2,2148.033333
18,2,1813.801630
18,2,2198.846154
18,2,1978.463235
18,2,1760.625000
18,2,1854.228365

18,2,4449.187500
18,2,1908.390909
18,2,1866.500000
18,2,2639.810000
18,2,5956.926829
18,2,2146.963068
18,2,2229.510204
18,2,3382.197368
18,2,2693.437500
18,2,1696.803191
18,2,1863.250000
18,2,5689.702703
18,2,3093.463889
18,2,3569.720000
18,2,3181.689189
18,2,2234.881818
18,2,2375.767857
18,2,3655.344512
18,2,10724.000000
18,2,3149.177778
18,2,1574.837209
18,2,1518.045455
18,2,2198.509615
18,2,3309.000000
18,2,2271.405303
18,2,4977.479167
18,2,1611.547872
18,2,5571.827957
18,2,4750.325472
18,2,1576.326923
18,2,2647.910714
18,2,1732.800000
18,2,16101.820000
18,2,2127.750000
18,2,1775.178571
18,2,2780.160714
18,2,1794.638298
18,2,1734.226744
18,2,1549.557692
18,2,1907.923780
18,2,8153.328804
18,2,2996.554348
18,2,3205.226667
18,2,2092.089744
18,2,1542.638889
18,2,2426.205357
18,2,1758.680851
18,2,1630.988636
18,2,2598.158163
18,2,2351.000000
18,2,2280.154070
18,2,5796.986111
18,2,1882.562500
18,2,1754.769231
18,2,1510.825000
18,2,1501.824468
18,2,2295.169271
18,2,1857.025510
18,2,1799.196759
18,2,2997.250000
18,2,9331.276163
18,2,2590.414286
18,2,2137.869565
18,2,1746.212500
18,2,1886.177778
18,2,2608.703125

18,2,1821.201220
18,2,1780.718750
18,2,1751.240642
18,2,3651.478261
18,2,1884.000000
18,2,11727.907609
18,2,2248.323529
18,2,2044.657895
18,2,1795.963889
18,2,1677.285256
18,2,1748.875000
18,2,1654.016026
18,2,2127.544118
18,2,2111.717949
18,2,11964.000000
18,2,2117.844595
18,2,1800.776316
18,2,5914.593750
18,2,1728.371951
18,2,1975.736364
18,2,2785.158333
18,2,11910.843023
18,2,1747.035714
18,2,1774.120192
18,2,1509.453125
18,2,1546.380952
18,2,1714.192857
18,2,1538.572917
18,2,1784.967262
18,2,2659.994444
18,2,2404.456522
18,2,1792.163636
18,2,2359.319444
18,2,3657.937500
18,2,2519.314286
18,2,1687.875000
18,2,8610.595000
18,2,2240.721154
18,2,3031.366492
18,2,2524.164671
18,2,12483.893617
18,2,5245.105263
18,2,2029.166667
18,2,7088.172794
18,2,1618.264205
18,2,2275.122093
18,2,1625.092391
18,2,1525.875000
18,2,1755.792453
18,2,1649.633065
18,2,8735.750000
18,2,1696.333333
18,2,2004.053125
18,2,2335.997093
18,2,16735.291667
18,2,1531.000000
18,2,1591.119792
18,2,2993.922222
18,2,2971.023256
18,2,14179.333333
18,2,1518.801630
18,2,2912.305556
18,2,2014.885417
18,2,1802.044118
18,2,4452.566327
18,2,1653.433036

18,2,1738.627273
18,2,1807.867424
18,2,1781.750000
18,2,2904.242347
18,2,1738.265306
18,2,2183.828125
18,2,2202.189189
18,2,1655.870283
18,2,1704.157895
18,2,2695.116848
18,2,17035.618243
18,2,3655.318878
18,2,1653.700000
18,2,2660.936170
18,2,6857.815789
18,2,3156.520833
18,2,1893.113839
18,2,2306.502907
18,2,1773.621711
18,2,3490.464286
18,2,1546.697917
18,2,2138.439286
18,2,1689.114130
18,2,2665.684524
18,2,1813.506250
18,2,2337.206731
18,2,3446.381944
18,2,2686.270408
18,2,4863.045455
18,2,2127.875000
18,2,2433.528302
18,2,5378.826705
18,2,2281.901961
18,2,8561.342949
18,2,1588.109375
18,2,2225.946429
18,2,2132.282609
18,2,1502.968750
18,2,5496.163462
18,2,2795.984043
18,2,3006.772321
18,2,1556.959677
18,2,1675.209677
18,2,4547.215116
18,2,6322.750000
18,2,2522.423611
18,2,1946.169271
18,2,3315.897959
18,2,1859.863372
18,2,4524.910828
18,2,7965.562500
18,2,3614.828947
18,2,1931.452703
18,2,2540.656977
18,2,2376.004902
18,2,3441.500000
18,2,2740.573864
18,2,1878.119048
18,2,1739.343085
18,2,3424.220588
18,2,1620.461957
18,2,1837.847222
18,2,1523.041667
18,2,2156.702830
18,2,1513.187500
18,2,1976.165094

18,2,1624.750000
18,2,1879.991071
18,2,1615.908163
18,2,1914.735849
18,2,2471.938776
18,2,1712.061170
18,2,2306.901316
18,2,7594.745968
18,2,2173.877551
18,2,2068.162500
18,2,5402.700000
18,2,1625.302632
18,2,2163.350000
18,2,1530.038462
18,2,2001.653226
18,2,1851.708333
18,2,2092.500000
18,2,3918.481250
18,2,1735.510638
18,2,2490.568182
18,2,3229.716981
18,2,1918.897059
18,2,3163.679245
18,2,2026.430147
18,2,1542.298780
18,2,3549.956250
18,2,2116.584416
18,2,1686.207317
18,2,4575.227273
18,2,4966.313679
18,2,2394.000000
18,2,11342.461538
18,2,1890.095000
18,2,2806.307065
18,2,2588.145492
18,2,1995.538462
18,2,2079.736111
18,2,4511.305556
18,2,1921.698370
18,2,1861.842262
18,2,2139.360577
18,2,19397.693182
18,2,1698.915625
18,2,2274.151515
18,2,9956.375000
18,2,2150.306122
18,2,2474.731771
18,2,1912.011364
18,2,3824.229167
18,2,3098.535326
18,2,2278.555556
18,2,2022.906250
18,2,1555.059783
18,2,1520.095588
18,2,3051.409091
18,2,2583.049020
18,2,2427.897959
18,2,2383.740741
18,2,2118.250000
18,2,1738.235795
18,2,4398.625000
18,2,2661.265957
18,2,3075.428571
18,2,2625.681818
18,2,1528.919271
18,2,3882.444149

18,2,3968.061364
18,2,1697.845395
18,2,2135.517045
18,2,3174.085526
18,2,1903.736111
18,2,1955.307692
18,2,7716.431122
18,2,1788.606250
18,2,2272.914216
18,2,1939.647059
18,2,1609.479651
18,2,3263.043367
18,2,2241.945652
18,2,2235.571429
18,2,2215.593750
18,2,2331.151042
18,2,1571.375000
18,2,2230.625000
18,2,1558.077128
18,2,1730.855769
18,2,18456.721774
18,2,2746.948718
18,2,2931.472727
18,2,3394.180556
18,2,2478.390625
18,2,17637.000000
18,2,5121.848684
18,2,2504.218085
18,2,3935.921296
18,2,2298.937500
18,2,2978.682143
18,2,2581.931818
18,2,2315.652174
18,2,1812.625000
18,2,3419.250000
18,2,2035.435185
18,2,1726.468085
18,2,2704.541667
18,2,1952.859296
18,2,1540.250000
18,2,1716.862745
18,2,2813.711538
18,2,2192.142857
18,2,1698.360294
18,2,1792.159091
18,2,4807.456633
18,2,1837.375000
18,2,3110.406250
18,2,1930.934211
18,2,1537.328488
18,2,2053.232143
18,2,13624.615385
18,2,2367.394737
18,2,2387.234043
18,2,1870.000000
18,2,3175.553191
18,2,2108.780556
18,2,2202.313679
18,2,1651.159091
18,2,2714.375000
18,2,2503.285714
18,2,2827.500000
18,2,2427.673913
18,2,1661.933155
18,2,2225.687500
18,2,2320.500000

18,2,1544.375000
18,2,2584.948370
18,2,2618.000000
18,2,2202.060000
18,2,1686.305851
18,2,2730.465426
18,2,1582.342105
18,2,1597.731707
18,2,2251.667614
18,2,1971.084135
18,2,2302.294118
18,2,1693.877551
18,2,1556.500000
18,2,1953.323113
18,2,2170.056818
18,2,2254.757500
18,2,2135.750000
18,2,1836.941860
18,2,1715.555556
18,2,1658.464286
18,2,1507.671196
18,2,1971.505102
18,2,1936.093137
18,2,1944.567308
18,2,2349.495000
18,2,1992.210648
18,2,4800.916667
18,2,2763.664894
18,2,1525.845395
18,2,2983.744318
18,2,3435.319149
18,2,1650.304688
18,2,1695.113208
18,2,2470.938776
18,2,1564.278409
18,2,1886.114130
18,2,1681.607143
18,2,3997.352041
18,2,3507.927778
18,2,1680.500000
18,2,2157.484375
18,2,2577.027778
18,2,1961.600000
18,2,3620.980392
18,2,1510.433673
18,2,2712.812500
18,2,1797.955000
18,2,2432.703704
18,2,1656.337500
18,2,1584.441860
18,2,1584.875000
18,2,3727.808962
18,2,5291.101562
18,2,1553.214286
18,2,2960.860000
18,2,1884.531250
18,2,2624.690476
18,2,2793.189189
18,2,2478.020408
18,2,2083.200000
18,2,2094.575000
18,2,1789.518519
18,2,2001.513089
18,2,2139.519231
18,2,2397.458333
18,2,2965.708333

18,2,3171.235294
18,2,8937.851562
18,2,2411.900000
18,2,1652.710526
18,2,1640.821429
18,2,4508.605263
18,2,1692.916667
18,2,1636.214286
18,2,1731.514881
18,2,12238.000000
18,2,1546.759868
18,2,2012.081395
18,2,2614.830000
18,2,10910.199219
18,2,1892.218182
18,2,2622.348214
18,2,1620.886792
18,2,2345.424020
18,2,1543.861111
18,2,1978.606707
18,2,2225.260638
18,2,2374.790909
18,2,2524.091837
18,2,1522.783784
18,2,2108.439024
18,2,3234.740000
18,2,1823.686047
18,2,2072.579861
18,2,4275.340909
18,2,5959.833333
18,2,1898.250000
18,2,1693.666667
18,2,1509.025735
18,2,2826.468750
18,2,2919.535714
18,2,3611.115385
18,2,1743.195652
18,2,1626.359375
18,2,3760.653409
18,2,2081.693878
18,2,1698.445652
18,2,2487.133929
18,2,2470.345000
18,2,1548.727778
18,2,3265.455556
18,2,1885.653846
18,2,1636.627193
18,2,1920.120536
18,2,1867.716146
18,2,1545.304348
18,2,2713.616279
18,2,2362.051282
18,2,1605.400000
18,2,3472.372549
18,2,2555.593750
18,2,2233.552632
18,2,2308.957237
18,2,2424.482143
18,2,2219.581250
18,2,1675.222222
18,2,2777.000000
18,2,2283.770833
18,2,5475.472973
18,2,1880.000000
18,2,2251.823980
18,2,2989.186047

18,2,1583.117647
18,2,4564.788889
18,2,12763.269444
18,2,1591.551829
18,2,1853.171875
18,2,2031.583333
18,2,9136.457447
18,2,2259.921875
18,2,2079.875000
18,2,8512.703125
18,2,1668.233491
18,2,2890.534884
18,2,2890.357843
18,2,1551.963855
18,2,1739.675481
18,2,4256.175926
18,2,3029.880000
18,2,2587.867187
18,2,1684.274457
18,2,3305.892857
18,2,1502.718750
18,2,5051.694444
18,2,2475.290000
18,2,3737.559783
18,2,5519.723404
18,2,1641.000000
18,2,1713.433140
18,2,1696.500000
18,2,1870.003125
18,2,1530.692308
18,2,1726.505435
18,2,2025.708333
18,2,1966.142500
18,2,4771.133333
18,2,4909.077586
18,2,2280.889706
18,2,3999.652174
18,2,3075.511628
18,2,1961.595745
18,2,1637.549020
18,2,2249.196078
18,2,2533.049020
18,2,1983.414474
18,2,1993.262500
18,2,3820.669811
18,2,1918.811321
18,2,2729.678571
18,2,1722.123656
18,2,1771.819444
18,2,1951.331250
18,2,1758.542553
18,2,2318.280660
18,2,2556.602564
18,2,1530.217105
18,2,1674.635870
18,2,1681.728261
18,2,2014.472222
18,2,2065.164062
18,2,1790.343750
18,2,2189.127119
18,2,3212.834559
18,2,2094.767442
18,2,7492.558511
18,2,1866.952381
18,2,2150.088235
18,2,3269.316327

18,2,4391.240385
18,2,2278.700000
18,2,2890.618421
18,2,2004.617021
18,2,2288.216216
18,2,3051.433962
18,2,1761.920213
18,2,2688.160377
18,2,2222.112500
18,2,2049.880952
18,2,1501.590000
18,2,2452.250000
18,2,3949.777778
18,2,2074.265625
18,2,2239.033333
18,2,18095.294643
18,2,2429.395833
18,2,3745.664336
18,2,1987.083333
18,2,4410.298913
18,2,2027.944767
18,2,1617.047297
18,2,1913.000000
18,2,3752.100000
18,2,1791.380319
18,2,2967.662791
18,2,2096.693750
18,2,1642.877500
18,2,1877.279412
18,2,1798.298611
18,2,1582.257143
18,2,3809.453947
18,2,4250.515909
18,2,2252.168182
18,2,1668.339623
18,2,2623.891447
18,2,1735.711538
18,2,5025.946809
18,2,2298.546875
18,2,8278.762821
18,2,2148.653846
18,2,2715.591463
18,2,1772.500000
18,2,4249.312500
18,2,2349.161765
18,2,2661.706522
18,2,1642.524390
18,2,1670.968750
18,2,3061.359375
18,2,1717.407407
18,2,1738.737805
18,2,2431.627119
18,2,2199.861111
18,2,6213.100000
18,2,2441.383721
18,2,2133.388889
18,2,5442.058824
18,2,2038.097973
18,2,2186.853659
18,2,2588.011905
18,2,5582.432432
18,2,6080.551136
18,2,3529.845588
18,2,2595.455000
18,2,1556.214286
18,2,2476.651584

18,2,2080.259615
18,2,2755.750000
18,2,1525.692308
18,2,1985.812500
18,2,13836.100000
18,2,3866.393617
18,2,1801.091912
18,2,4974.509615
18,2,1505.847222
18,2,1678.423077
18,2,1566.878125
18,2,2406.375887
18,2,4825.147727
18,2,1525.009434
18,2,2056.587500
18,2,2614.566489
18,2,1669.479167
18,2,1730.081395
18,2,3787.076923
18,2,10923.197674
18,2,4654.921875
18,2,8829.169444
18,2,6000.103659
18,2,1977.779412
18,2,1518.284091
18,2,2031.150000
18,2,4618.670139
18,2,2772.950521
18,2,1604.000000
18,2,2203.156863
18,2,2069.913043
18,2,1583.511111
18,2,1965.026316
18,2,1616.607143
18,2,2130.322674
18,2,2548.965517
18,2,2491.513514
18,2,2238.744681
18,2,1919.048780
18,2,1916.815789
18,2,1851.387500
18,2,1850.100000
18,2,2683.424479
18,2,1526.600000
18,2,1573.219072
18,2,3202.000000
18,2,2546.706633
18,2,1546.042553
18,2,4483.934659
18,2,1598.696078
18,2,10724.695122
18,2,1536.250000
18,2,2639.516129
18,2,3281.108696
18,2,11963.951087
18,2,2361.042500
18,2,1805.690909
18,2,7740.923780
18,2,1506.437500
18,2,2094.063776
18,2,3611.265625
18,2,2457.021739
18,2,6493.707386
18,2,1746.937500
18,2,2071.106061
18,2,1762.770000

18,2,3004.730769
18,2,4667.222222
18,2,10024.233696
18,2,2001.142857
18,2,2094.171875
18,2,1705.854651
18,2,3052.954327
18,2,3490.641509
18,2,1536.403061
18,2,10742.923913
18,2,2002.708333
18,2,2206.800000
18,2,1847.695312
18,2,2248.764205
18,2,2426.370833
18,2,3403.365132
18,2,8982.367925
18,2,2622.925926
18,2,1978.424528
18,2,1518.809896
18,2,2580.176829
18,2,1914.265625
18,2,3569.361607
18,2,2359.495000
18,2,9839.287500
18,2,1588.463889
18,2,1788.977500
18,2,1582.776596
18,2,2013.551136
18,2,2552.119318
18,2,2530.463415
18,2,3639.799107
18,2,2187.641509
18,2,3540.828125
18,2,2194.204082
18,2,2436.739130
18,2,2352.220588
18,2,3300.800532
18,2,2051.907895
18,2,13832.327778
18,2,2114.312500
18,2,2034.959184
18,2,2060.519231
18,2,1573.475000
18,2,2028.534722
18,2,1751.425000
18,2,9804.508152
18,2,2051.205189
18,2,10299.089844
18,2,2572.666667
18,2,3973.475877
18,2,3949.009868
18,2,1955.207547
18,2,1677.198529
18,2,1844.530612
18,2,3649.700000
18,2,2046.180000
18,2,1746.197368
18,2,1566.853659
18,2,1656.440758
18,2,2251.331081
18,2,2405.415094
18,2,2269.500000
18,2,2039.343750
18,2,3143.606481
18,2,2287.803571

18,2,1901.744318
18,2,3812.529412
18,2,1718.591518
18,2,1794.414634
18,2,1648.046875
18,2,2135.803922
18,2,1850.792453
18,2,3655.538462
18,2,2103.385000
18,2,1762.500000
18,2,2713.926829
18,2,4590.699324
18,2,1534.862500
18,2,9103.372727
18,2,1924.714286
18,2,1771.697674
18,2,2078.975000
18,2,3831.025862
18,2,2531.525000
18,2,6203.651596
18,2,1771.017045
18,2,1884.423077
18,2,3191.208333
18,2,4540.596491
18,2,2569.653509
18,2,1909.473684
18,2,3329.375000
18,2,2834.146119
18,2,1633.829268
18,2,1892.763158
18,2,1502.803191
18,2,2661.110577
18,2,2508.108491
18,2,1531.548077
18,2,1893.589286
18,2,2244.756944
18,2,3145.278125
18,2,1991.250000
18,2,2421.956522
18,2,2065.656250
18,2,5562.094340
18,2,2873.388889
18,2,2990.357955
18,2,3962.097561
18,2,10801.556250
18,2,2284.689474
18,2,3147.326389
18,2,10229.348214
18,2,2474.320313
18,2,4199.655000
18,2,2397.237143
18,2,1823.823529
18,2,1652.422619
18,2,1819.170391
18,2,1522.602649
18,2,3532.441489
18,2,2444.793605
18,2,1761.215116
18,2,2534.891892
18,2,1546.146341
18,2,2525.287234
18,2,14269.226562
18,2,1908.062500
18,2,2235.555000
18,2,1875.037500
18,2,4333.247642

18,2,1727.818182
18,2,4324.027778
18,2,2050.360000
18,2,1663.576087
18,2,1923.983333
18,2,3420.817881
18,2,1606.891892
18,2,2096.897727
18,2,1509.695312
18,2,2230.084906
18,2,9009.826220
18,2,2218.396739
18,2,4579.000000
18,2,4580.548913
18,2,3730.496711
18,2,2037.933511
18,2,1697.320175
18,2,2317.187783
18,2,2645.937500
18,2,1798.787500
18,2,1568.996622
18,2,1569.348837
18,2,5722.797297
18,2,1729.064655
18,2,1606.904255
18,2,2613.263158
18,2,1788.855114
18,2,2194.189516
18,2,1654.527778
18,2,1769.372093
18,2,2131.336735
18,2,1937.043605
18,2,2434.514151
18,2,3140.140306
18,2,3301.152778
18,2,1913.566667
18,2,1595.677632
18,2,1992.343750
18,2,2885.375000
18,2,2020.088889
18,2,1517.000000
18,2,2222.468750
18,2,2023.297297
18,2,1771.913690
18,2,1996.774457
18,2,2574.769231
18,2,1626.395349
18,2,3890.223684
18,2,3961.875000
18,2,10584.133333
18,2,10665.511719
18,2,1940.729167
18,2,3373.826923
18,2,6663.627551
18,2,3426.032738
18,2,3290.465116
18,2,2175.968085
18,2,1596.170213
18,2,1982.127451
18,2,3123.955645
18,2,2435.645000
18,2,1502.239583
18,2,1706.761628
18,2,1917.357143
18,2,1885.101190
18,2,2621.527778

18,2,1991.250000
18,2,1915.329787
18,2,3606.138889
18,2,2072.018519
18,2,1982.932432
18,2,3481.177083
18,2,3352.636905
18,2,1541.348214
18,2,1673.625000
18,2,2505.005682
18,2,1885.200000
18,2,1603.104167
18,2,2014.845745
18,2,3098.681818
18,2,5920.122449
18,2,1736.723684
18,2,1852.635870
18,2,1502.666667
18,2,1849.349432
18,2,2405.906977
18,2,2648.047500
18,2,1662.276596
18,2,2083.781250
18,2,2986.986413
18,2,2515.403846
18,2,9530.405488
18,2,4714.794643
18,2,11914.083333
18,2,1778.000000
18,2,1765.070652
18,2,5753.172131
18,2,1587.461111
18,2,2022.416667
18,2,1701.677083
18,2,2424.720930
18,2,2600.310976
18,2,4008.203571
18,2,1599.762500
18,2,3829.221675
18,2,2326.968750
18,2,2299.600000
18,2,2132.039634
18,2,2056.669355
18,2,1597.204082
18,2,1865.453947
18,2,1663.562500
18,2,5221.201923
18,2,1541.039474
18,2,1584.016393
18,2,1657.482955
18,2,3118.629808
18,2,2263.750000
18,2,1697.051429
18,2,1724.326816
18,2,1937.796875
18,2,1579.358108
18,2,5714.155556
18,2,1705.208333
18,2,4571.388298
18,2,3151.193396
18,2,1512.180851
18,2,1827.925926
18,2,2937.812500
18,2,1629.679167
18,2,1585.317073
18,2,4023.337838

18,2,2117.772727
18,2,2198.886905
18,2,2656.409091
18,2,3513.190476
18,2,1768.000000
18,2,6067.574324
18,2,3271.627976
18,2,1770.753906
18,2,3720.989583
18,2,6225.000000
18,2,3299.757576
18,2,2971.583333
18,2,1848.380435
18,2,1775.879902
18,2,1566.926829
18,2,1742.281250
18,2,2613.801136
18,2,2465.309896
18,2,1713.002273
18,2,5077.372093
18,2,2057.947368
18,2,1531.062500
18,2,1499.695946
18,2,1635.166667
18,2,1559.765000
18,2,1571.406250
18,2,1802.400568
18,2,1593.683333
18,2,3937.250000
18,2,1645.027174
18,2,1567.354167
18,2,2084.169355
18,2,7569.512195
18,2,2561.413462
18,2,2383.202128
18,2,7027.726562
18,2,3955.055233
18,2,1983.400000
18,2,1606.196429
18,2,2542.689189
18,2,1851.719388
18,2,1561.101190
18,2,1673.045455
18,2,1646.338235
18,2,4514.458333
18,2,2230.650510
18,2,1758.420732
18,2,1827.875000
18,2,1925.348039
18,2,1737.967593
18,2,1775.893868
18,2,1803.454545
18,2,5009.115741
18,2,1939.669271
18,2,2022.663462
18,2,4203.044444
18,2,2249.405405
18,2,1646.566667
18,2,1802.362069
18,2,2783.058824
18,2,4295.274419
18,2,2411.987500
18,2,1730.933673
18,2,1604.725000
18,2,2092.759091
18,2,1558.458333

18,2,3292.620000
18,2,2192.637500
18,2,1609.528302
18,2,1787.375000
18,2,3000.073718
18,2,2039.363372
18,2,2150.951923
18,2,2579.852941
18,2,1634.104167
18,2,1789.986111
18,2,2030.523936
18,2,1604.257353
18,2,1656.914894
18,2,2402.879630
18,2,1564.450472
18,2,1537.377841
18,2,10563.243750
18,2,1855.730769
18,2,1828.492424
18,2,18353.824519
18,2,2430.669872
18,2,1540.443452
18,2,2344.054688
18,2,2243.741206
18,2,1552.910714
18,2,4974.368421
18,2,1534.562500
18,2,1794.864796
18,2,2714.875000
18,2,1700.746951
18,2,1857.500000
18,2,2241.125000
18,2,3455.787500
18,2,2467.287037
18,2,1663.375000
18,2,1528.684091
18,2,1907.474490
18,2,1620.880435
18,2,2228.615854
18,2,1885.418605
18,2,7729.959459
18,2,3161.243750
18,2,2385.095395
18,2,3242.793750
18,2,9189.163194
18,2,3409.125000
18,2,3254.293478
18,2,1540.295455
18,2,1749.632653
18,2,2217.607143
18,2,5804.609375
18,2,1798.411765
18,2,1894.434375
18,2,1828.387195
18,2,1723.086207
18,2,1938.971698
18,2,1594.453488
18,2,3959.547619
18,2,1815.316176
18,2,1520.156977
18,2,2023.394231
18,2,1594.867188
18,2,2720.005102
18,2,1710.385714
18,2,2461.087264
18,2,4241.707317

18,2,1516.407895
18,2,2263.476744
18,2,3013.635417
18,2,3742.431250
18,2,2694.009615
18,2,2294.526316
18,2,2278.277027
18,2,2624.625000
18,2,1613.478365
18,2,9942.428571
18,2,3773.007813
18,2,6333.261905
18,2,1801.092105
18,2,2039.782609
18,2,3982.824074
18,2,1502.250000
18,2,1879.883721
18,2,1826.091463
18,2,3564.710227
18,2,1962.863636
18,2,2094.768293
18,2,1658.150000
18,2,1499.151961
18,2,14848.125000
18,2,4520.919643
18,2,3944.487500
18,2,6575.697368
18,2,1563.192073
18,2,1637.208333
18,2,2166.691489
18,2,2849.084746
18,2,5263.332237
18,2,1825.250000
18,2,2952.390625
18,2,2364.617647
18,2,2439.557692
18,2,2352.866667
18,2,7057.101449
18,2,1792.312500
18,2,1888.630208
18,2,2496.278409
18,2,1721.853723
18,2,2069.395000
18,2,2047.187500
18,2,9046.950820
18,2,1594.197674
18,2,1687.489362
18,2,1865.320652
18,2,4221.223214
18,2,2870.430000
18,2,1552.182927
18,2,4653.000000
18,2,2189.375000
18,2,2429.635000
18,2,1980.895349
18,2,3889.250000
18,2,3214.228261
18,2,2308.679878
18,2,2646.365854
18,2,2909.693878
18,2,3722.375000
18,2,1973.270349
18,2,1761.526316
18,2,2849.457317
18,2,2250.263636
18,2,4227.468085

18,2,2448.736842
18,2,1564.953488
18,2,3600.900000
18,2,4543.287234
18,2,1542.725000
18,2,1552.406250
18,2,1830.709302
18,2,3073.798077
18,2,2645.348039
18,2,1827.415094
18,2,3362.937500
18,2,4175.272727
18,2,1726.893750
18,2,2283.244318
18,2,1660.033854
18,2,1838.208333
18,2,3384.625000
18,2,2103.308511
18,2,1747.562500
18,2,4887.961538
18,2,3037.392857
18,2,1587.308219
18,2,1961.286585
18,2,2060.320652
18,2,1873.089623
18,2,1655.347826
18,2,1962.725806
18,2,2401.313253
18,2,1834.328125
18,2,1530.697581
18,2,2164.872340
18,2,2392.600000
18,2,2814.666667
18,2,5557.585106
18,2,3448.080508
18,2,1590.335000
18,2,1537.462264
18,2,1562.822581
18,2,1971.121795
18,2,2262.654255
18,2,1511.093023
18,2,1639.153061
18,2,2069.765625
18,2,1697.576923
18,2,2171.223404
18,2,1600.844444
18,2,3160.325342
18,2,2415.390244
18,2,4462.233696
18,2,2195.273148
18,2,3107.148148
18,2,4253.046512
18,2,1840.346939
18,2,2290.085106
18,2,3488.500000
18,2,2792.049107
18,2,2704.872283
18,2,1880.362745
18,2,1922.826087
18,2,2763.795673
18,2,2237.742188
18,2,1919.993590
18,2,1734.442708
18,2,2521.120192
18,2,1504.494186
18,2,2015.147222

18,2,3249.683333
18,2,1602.725490
18,2,1578.250000
18,2,1582.666667
18,2,2005.945122
18,2,1886.837500
18,2,1977.042553
18,2,3014.192308
18,2,1677.000000
18,2,3124.159574
18,2,2116.087838
18,2,3996.875000
18,2,2166.794872
18,2,2447.671429
18,2,3157.732143
18,2,1665.147059
18,2,3106.219512
18,2,2548.715517
18,2,2191.592500
18,2,1501.337838
18,2,1599.294118
18,2,3418.928571
18,2,2330.095833
18,2,2650.723404
18,2,1654.724432
18,2,8810.229730
18,2,1995.554687
18,2,3453.302778
18,2,3048.572917
18,2,4481.566176
18,2,2799.521341
18,2,2226.250000
18,2,2639.234043
18,2,6110.313725
18,2,4639.065789
18,2,2025.491071
18,2,1734.021429
18,2,1672.883721
18,2,1568.500000
18,2,1542.002778
18,2,1846.445000
18,2,2013.222222
18,2,4067.220833
18,2,1571.163793
18,2,1832.282895
18,2,3072.750000
18,2,1548.487069
18,2,1915.866667
18,2,1947.050000
18,2,2228.258152
18,2,3851.970588
18,2,4241.250000
18,2,1877.424528
18,2,1568.000000
18,2,2095.720588
18,2,1619.928571
18,2,1670.885000
18,2,2693.565217
18,2,2438.883721
18,2,2166.360465
18,2,3858.484375
18,2,1759.042857
18,2,2091.800000
18,2,6363.332386
18,2,1771.173469
18,2,2222.809524

18,2,3227.116477
18,2,3535.574074
18,2,1707.819149
18,2,1955.547619
18,2,2391.750000
18,2,1560.934783
18,2,1961.607143
18,2,1502.770408
18,2,2065.397436
18,2,1522.104651
18,2,1649.359043
18,2,2052.577778
18,2,2459.669811
18,2,1869.605556
18,2,2880.002358
18,2,5094.375000
18,2,1505.256410
18,2,1576.769737
18,2,1725.584906
18,2,2609.718750
18,2,1861.848214
18,2,1536.186047
18,2,3863.500000
18,2,2029.032609
18,2,1961.987179
18,2,11008.000000
18,2,1597.869565
18,2,1503.830000
18,2,2907.305233
18,2,2646.083333
18,2,1692.571429
18,2,2118.022727
18,2,1502.000000
18,2,2436.233696
18,2,1757.937500
18,2,2413.281250
18,2,1612.895833
18,2,3069.460526
18,2,1597.076923
18,2,2750.606132
18,2,2409.107843
18,2,1700.210000
18,2,2025.358974
18,2,1637.763889
18,2,1800.680921
18,2,2217.236364
18,2,3531.161184
18,2,1912.077778
18,2,2128.164894
18,2,2184.108974
18,2,3853.100000
18,2,4179.053435
18,2,1856.864583
18,2,1848.477941
18,2,2068.369318
18,2,1552.728261
18,2,1909.737288
18,2,2937.481818
18,2,2888.552083
18,2,1690.843137
18,2,2460.390625
18,2,1550.724359
18,2,3100.084906
18,2,1584.500000
18,2,1885.600000
18,2,1505.313725

18,2,1672.500000
18,2,1954.402439
18,2,9015.073980
18,2,2732.953947
18,2,1942.250000
18,2,2273.412037
18,2,2752.063830
18,2,1535.426829
18,2,2810.768939
18,2,1501.787879
18,2,1538.957589
18,2,2102.188889
18,2,2163.617647
18,2,2053.331522
18,2,1547.494048
18,2,2681.973684
18,2,3472.756098
18,2,2081.872549
18,2,1642.701087
18,2,1722.056818
18,2,1665.392045
18,2,2270.177778
18,2,2112.666667
18,2,1722.468085
18,2,1559.403226
18,2,1500.119681
18,2,3953.596939
18,2,1883.577128
18,2,1709.358974
18,2,2218.719298
18,2,1619.622549
18,2,2376.798942
18,2,4001.914286
18,2,1671.538462
18,2,2658.015625
18,2,3414.223558
18,2,1526.110119
18,2,1700.013889
18,2,2304.343085
18,2,1858.155000
18,2,1505.405405
18,2,3200.438710
18,2,1867.559783
18,2,2190.177778
18,2,1867.083333
18,2,3809.500000
18,2,2485.438424
18,2,2071.339623
18,2,1804.483108
18,2,2880.351064
18,2,2546.821429
18,2,1942.977041
18,2,5618.741848
18,2,1718.625000
18,2,2285.440217
18,2,1841.500000
18,2,2382.273810
18,2,11694.731618
18,2,2258.715909
18,2,1593.027027
18,2,3808.435714
18,2,2720.534091
18,2,3387.675000
18,2,2353.482143
18,2,5712.000000
18,2,4177.022727

18,2,2266.107143
18,2,3037.796875
18,2,2540.854167
18,2,1991.804878
18,2,1689.078125
18,2,19475.921053
18,2,1575.493056
18,2,2000.375000
18,2,1539.689286
18,2,3609.344262
18,2,1675.084184
18,2,2025.369565
18,2,1675.000000
18,2,2243.722222
18,2,1753.388021
18,2,2812.457447
18,2,1505.250000
18,2,1692.507576
18,2,5681.997024
18,2,2961.969388
18,2,2035.802326
18,2,2193.522222
18,2,2392.482955
18,2,1639.515306
18,2,2323.470588
18,2,2650.778017
18,2,2601.127660
18,2,4429.375000
18,2,2117.293103
18,2,1918.267857
18,2,2624.819149
18,2,1780.301829
18,2,1642.607143
18,2,1989.566176
18,2,3083.729167
18,2,2020.000000
18,2,2738.955556
18,2,3337.916107
18,2,12045.875000
18,2,2136.397059
18,2,1726.894608
18,2,1765.390625
18,2,1760.243243
18,2,2317.409091
18,2,2761.800000
18,2,2290.400000
18,2,2146.419118
18,2,2431.435897
18,2,5957.378049
18,2,2250.875000
18,2,3082.538265
18,2,1999.650000
18,2,1928.250000
18,2,1816.281977
18,2,3266.025000
18,2,3979.531250
18,2,1885.082237
18,2,3320.575581
18,2,1605.250000
18,2,2142.716216
18,2,3588.276042
18,2,11112.181818
18,2,1626.644737
18,2,6857.349490
18,2,2869.571429
18,2,2387.750000

18,2,1871.798913
18,2,1838.593750
18,2,2065.916667
18,2,1575.948718
18,2,4525.905172
18,2,1547.044811
18,2,2156.141304
18,2,10758.668478
18,2,1571.875000
18,2,1628.421875
18,2,7171.675000
18,2,2855.666667
18,2,1579.073529
18,2,3992.562500
18,2,1825.404070
18,2,1634.397727
18,2,4291.522727
18,2,2544.904891
18,2,3268.710526
18,2,2225.836538
18,2,4878.232143
18,2,2487.526163
18,2,9401.016667
18,2,5308.880952
18,2,1671.767442
18,2,1909.088889
18,2,2232.558140
18,2,5660.961538
18,2,3344.691406
18,2,2795.234694
18,2,1500.515625
18,2,1527.431034
18,2,1550.095238
18,2,1988.785714
18,2,1756.908537
18,2,3786.162162
18,2,2121.194444
18,2,1765.616279
18,2,2021.520833
18,2,2521.095588
18,2,2632.150000
18,2,2049.210106
18,2,2165.863095
18,2,2164.152174
18,2,2381.854651
18,2,1772.260870
18,2,3764.996324
18,2,7151.984375
18,2,3533.532258
18,2,3991.725000
18,2,2060.357843
18,2,3353.679245
18,2,1653.397727
18,2,1706.300000
18,2,2299.650000
18,2,1999.918605
18,2,2561.700000
18,2,3079.832237
18,2,1598.878049
18,2,6233.750000
18,2,3098.347222
18,2,1502.412736
18,2,1506.090909
19,1,5524.115625
19,1,5908.378049
19,1,2779.481618

19,1,17329.475000
19,1,3479.330000
19,1,3927.717949
19,1,8216.164474
19,1,4105.821429
19,1,2232.497340
19,1,1620.500000
19,1,2903.920455
19,1,14642.744186
19,1,2888.471154
19,1,1899.173913
19,1,11817.340426
19,1,7190.314516
19,1,6197.681818
19,1,5351.450000
19,1,1902.916667
19,1,1502.104167
19,1,2946.595930
19,1,8140.125000
19,1,7951.746622
19,1,1744.863281
19,1,3251.400000
19,1,6173.940217
19,1,2889.395833
19,1,1892.992188
19,1,2075.525000
19,1,1701.458333
19,1,1522.744318
19,1,1541.208333
19,1,12062.858333
19,1,3974.841912
19,1,11950.994048
19,1,3852.440789
19,1,4081.270270
19,1,1742.091216
19,1,12596.600000
19,1,8299.666667
19,1,1534.983333
19,1,2557.188679
19,1,2338.744186
19,1,10015.600000
19,1,2605.460938
19,1,2876.010870
19,1,12476.181818
19,1,3512.475000
19,1,1787.931298
19,1,5919.062500
19,1,9052.437500
19,1,4848.275000
19,1,6443.418919
19,1,2470.222973
19,1,2901.281977
19,1,3260.926829
19,1,2845.153846
19,1,3980.234043
19,1,5673.000000
19,1,5001.781250
19,1,8571.812500
19,1,5335.904167
19,1,1994.122283
19,1,5089.333333
19,1,16015.430851
19,1,4080.975610
19,1,2551.986486
19,1,3522.375000
19,1,3723.763158

19,1,4640.750000
19,1,5457.583333
19,1,1541.676471
19,1,2061.953947
19,1,17087.803571
19,1,5798.738889
19,1,16371.662162
19,1,10647.208333
19,1,2704.047619
19,1,2140.750000
19,1,2308.439024
19,1,3523.130319
19,1,6001.456395
19,1,1656.472561
19,1,2141.877717
19,1,7111.302632
19,1,7452.693182
19,1,5131.560976
19,1,8908.678571
19,1,2993.034884
19,1,2315.375000
19,1,17841.409722
19,1,6203.972973
19,1,5679.550000
19,1,1712.016304
19,1,4622.761905
19,1,12593.500000
19,1,1594.080000
19,1,1881.750000
19,1,2384.310976
19,1,4325.648936
19,1,4744.115385
19,1,2036.046296
19,1,2593.789894
19,1,4455.547619
19,1,2700.425532
19,1,6704.812500
19,1,1531.555556
19,1,1709.615385
19,1,1593.995690
19,1,3469.937500
19,1,2047.500000
19,1,2049.451613
19,1,2870.856061
19,1,4369.750000
19,1,3855.400735
19,1,1555.023256
19,1,1635.961111
19,1,4391.194079
19,1,1639.378676
19,1,7097.875000
19,1,2952.816667
19,1,2838.760870
19,1,2145.130952
19,1,2540.839844
19,1,1897.090909
19,1,2713.971429
19,1,1577.076220
19,1,4085.195946
19,1,4136.318182
19,1,7391.145349
19,1,11242.040441
19,1,1581.187500
19,1,5225.500000
19,1,1751.326220
19,1,1565.704545

19,1,1710.390244
19,1,4904.093750
19,1,3443.500000
19,1,2497.423077
19,1,2125.704787
19,1,4667.234043
19,1,5306.157895
19,1,2261.097561
19,1,2513.157895
19,1,1719.515625
19,1,14335.392857
19,1,7864.403846
19,1,2330.580882
19,1,3379.166667
19,1,7543.750000
19,1,7320.869681
19,1,3101.605114
19,1,2190.333333
19,1,9925.964674
19,1,14748.060714
19,1,9201.925676
19,1,1770.525000
19,1,5477.518293
19,1,1537.564286
19,1,1708.437500
19,1,6146.393382
19,1,8563.687500
19,1,6545.622024
19,1,2112.383333
19,1,1627.576923
19,1,2272.681548
19,1,9601.945652
19,1,4317.253472
19,1,1607.219512
19,1,1951.400000
19,1,5320.197674
19,1,7066.733333
19,1,3917.640625
19,1,1730.598214
19,1,6356.430000
19,1,3389.740566
19,1,1542.053030
19,1,1771.512195
19,1,2022.515625
19,1,3478.982143
19,1,14841.993243
19,1,2440.920732
19,1,3769.055000
19,1,2593.343750
19,1,2398.615385
19,1,1839.921512
19,1,4664.916667
19,1,3269.171512
19,1,5026.020833
19,1,2286.537879
19,1,4128.083333
19,1,1589.230769
19,1,2765.236842
19,1,1529.266892
19,1,2553.868590
19,1,4855.000000
19,1,2383.632143
19,1,4987.162162
19,1,6202.630952
19,1,7304.172619
19,1,5876.775000

19,1,1792.791667
19,1,1723.392857
19,1,6870.884615
19,1,7683.709302
19,1,3458.310000
19,1,11506.685185
19,1,6082.143519
19,1,14350.221154
19,1,15952.826923
19,1,4971.115385
19,1,2856.586806
19,1,19067.538462
19,1,16206.296875
19,1,3113.115854
19,1,2711.555556
19,1,2482.834677
19,1,7039.487179
19,1,2687.681250
19,1,1737.775000
19,1,3230.000000
19,1,7858.954545
19,1,3230.071429
19,1,2215.478659
19,1,2675.050000
19,1,5487.725000
19,1,2307.059211
19,1,3462.700658
19,1,4600.519231
19,1,3634.475000
19,1,4431.936170
19,1,1983.076923
19,1,1854.060897
19,1,1971.152174
19,1,1900.061224
19,1,5626.750000
19,1,5061.391447
19,1,2976.280405
19,1,12433.990132
19,1,2071.500000
19,1,7627.343023
19,1,8843.585227
19,1,1876.779412
19,1,3631.447368
19,1,2945.051020
19,1,1866.977041
19,1,3630.255102
19,1,15293.287791
19,1,1741.620968
19,1,1845.208333
19,1,1945.500000
19,1,2281.885714
19,1,1607.987500
19,1,1777.964286
19,1,11576.832386
19,1,3498.625000
19,1,14319.846154
19,1,2067.884615
19,1,8259.293860
19,1,2409.677326
19,1,3643.832500
19,1,6723.031250
19,1,2869.880556
19,1,7678.166667
19,1,5791.454545
19,1,17070.206522
19,1,2049.062500

19,1,2871.223404
19,1,2338.163462
19,1,2148.395349
19,1,16988.033088
19,1,2483.725000
19,1,3832.900568
19,1,5256.849138
19,1,4186.689189
19,1,2922.645833
19,1,4836.333333
19,1,3002.684211
19,1,3224.634615
19,1,15336.274306
19,1,4976.622222
19,1,2357.812500
19,1,6406.483333
19,1,2484.629630
19,1,5839.005682
19,1,2125.303571
19,1,1668.615385
19,1,15695.418919
19,1,2203.818182
19,1,15753.993750
19,1,15718.572674
19,1,2941.863636
19,1,5342.206522
19,1,1556.555556
19,1,3042.996429
19,1,8155.366279
19,1,2120.716216
19,1,1515.833333
19,1,7124.195313
19,1,4976.181818
19,1,3564.661290
19,1,10923.767857
19,1,8343.250000
19,1,1634.609756
19,1,1656.416667
19,1,3355.878049
19,1,2231.421875
19,1,1958.405556
19,1,1624.264286
19,1,4896.000000
19,1,2971.268293
19,1,2041.872093
19,1,2319.947368
19,1,9121.404255
19,1,1615.196429
19,1,2984.984043
19,1,8468.829268
19,1,9087.416667
19,1,1747.630556
19,1,2212.816667
19,1,1978.921053
19,1,1554.246951
19,1,14455.820122
19,1,3190.094512
19,1,1788.000000
19,1,2334.960106
19,1,1654.734694
19,1,2067.439394
19,1,2449.534483
19,1,4673.164773
19,1,2267.250000
19,1,1735.125000
19,1,9966.464286

19,1,6441.309783
19,1,2336.333333
19,1,3756.500000
19,1,4458.740385
19,1,1845.625000
19,1,4530.522222
19,1,4195.079167
19,1,17112.835227
19,1,1518.705882
19,1,2462.125000
19,1,4397.000000
19,1,5828.579861
19,1,7112.125000
19,1,3420.847973
19,1,14125.253571
19,1,2764.356771
19,1,1538.930851
19,1,4015.775000
19,1,1711.618421
19,1,12938.000000
19,1,4179.658163
19,1,4686.195946
19,1,6703.911111
19,1,4493.455128
19,1,5306.179487
19,1,3777.587766
19,1,7126.007812
19,1,4061.511628
19,1,12784.313953
19,1,1996.862500
19,1,12678.888889
19,1,4678.548611
19,1,1619.355978
19,1,15219.919118
19,1,2907.407500
19,1,8327.266827
19,1,3978.714286
19,1,3272.494681
19,1,2098.767857
19,1,3319.193750
19,1,3237.687500
19,1,2942.555556
19,1,4322.893293
19,1,2085.282609
19,1,10344.639205
19,1,3467.900568
19,1,1724.307692
19,1,4747.250000
19,1,5317.200000
19,1,3852.888889
19,1,4454.239286
19,1,18497.038889
19,1,2458.611111
19,1,2143.105114
19,1,10006.329545
19,1,2741.145161
19,1,2191.647059
19,1,6356.265306
19,1,1811.853659
19,1,12446.676829
19,1,12552.230769
19,1,2129.906250
19,1,8332.785714
19,1,2773.218085
19,1,2692.750000
19,1,1770.377005

19,1,2164.096154
19,1,3564.692568
19,1,4425.867500
19,1,2482.151961
19,1,2148.953125
19,1,4472.337209
19,1,2342.591912
19,1,4970.571429
19,1,5324.136364
19,1,3406.860000
19,1,1881.000000
19,1,2604.762500
19,1,3654.052083
19,1,10548.142857
19,1,1928.723837
19,1,1617.840909
19,1,4930.173387
19,1,2926.941176
19,1,8694.452128
19,1,3495.485294
19,1,4871.343750
19,1,8611.655172
19,1,4064.106061
19,1,2158.545455
19,1,1782.102041
19,1,2380.755814
19,1,3445.062500
19,1,3274.222222
19,1,8051.452381
19,1,1663.258065
19,1,5095.285714
19,1,2308.648649
19,1,2318.568182
19,1,1707.038265
19,1,4329.229167
19,1,1891.268293
19,1,2675.671875
19,1,8223.937500
19,1,3148.217105
19,1,13324.764706
19,1,5764.937500
19,1,5522.500000
19,1,7176.465116
19,1,4707.068182
19,1,1596.000000
19,1,10707.355556
19,1,1612.704545
19,1,1806.441176
19,1,9691.754464
19,1,1570.756098
19,1,9518.601351
19,1,4486.812500
19,1,18658.791667
19,1,2842.674419
19,1,8543.517857
19,1,2548.202703
19,1,3939.418919
19,1,3665.230769
19,1,3208.086806
19,1,2655.952381
19,1,2268.187500
19,1,2978.588235
19,1,1883.072581
19,1,17096.972222
19,1,6408.288793
19,1,1526.648026

19,1,10909.500000
19,1,2306.009146
19,1,1868.496324
19,1,1570.171429
19,1,2636.675000
19,1,3175.879808
19,1,2845.666667
19,1,6215.448276
19,1,8262.589286
19,1,2925.172414
19,1,2604.432927
19,1,6303.272727
19,1,4516.955882
19,1,9104.893617
19,1,3867.578125
19,1,2479.058824
19,1,1761.583333
19,1,3468.914894
19,1,6544.610294
19,1,4554.000000
19,1,2058.911765
19,1,3334.868421
19,1,1684.159091
19,1,3971.849138
19,1,10055.018519
19,1,2009.905000
19,1,8093.989865
19,1,16927.161585
19,1,1553.000000
19,1,3613.113636
19,1,1627.933511
19,1,3030.937500
19,1,3551.500000
19,1,1593.991071
19,1,8707.531250
19,1,3249.625000
19,1,2108.005319
19,1,2854.298077
19,1,6265.014205
19,1,2798.988636
19,1,2064.638889
19,1,1506.219697
19,1,2848.110465
19,1,1781.925000
19,1,1921.486413
19,1,3311.918269
19,1,1772.055556
19,1,5329.750000
19,1,4859.343750
19,1,3993.393750
19,1,1710.726562
19,1,5619.531250
19,1,2305.883929
19,1,2220.677305
19,1,17880.206897
19,1,4147.976562
19,1,4020.169811
19,1,3780.090909
19,1,1530.600610
19,1,1950.026042
19,1,4995.798780
19,1,4793.936170
19,1,2888.125000
19,1,4738.109756
19,1,1666.676471
19,1,19525.136905

19,1,4068.738095
19,1,1669.650000
19,1,2390.755435
19,1,5283.601351
19,1,5649.391026
19,1,5102.060606
19,1,1684.892857
19,1,3164.314024
19,1,9336.800000
19,1,3636.727273
19,1,1645.656250
19,1,2457.975962
19,1,2279.617647
19,1,2390.305556
19,1,3060.293478
19,1,1984.817073
19,1,2597.516667
19,1,3267.884146
19,1,10782.937500
19,1,1920.045732
19,1,2108.521739
19,1,6505.250000
19,1,5259.482143
19,1,1629.512195
19,1,3943.000000
19,1,2703.017857
19,1,4768.842105
19,1,3761.811111
19,1,1764.958333
19,1,4779.750000
19,1,5978.346154
19,1,4295.833333
19,1,2216.767442
19,1,16858.662500
19,1,13133.950000
19,1,19222.505102
19,1,5589.692308
19,1,3798.808824
19,1,2375.000000
19,1,2166.422222
19,1,3448.950658
19,1,2964.597500
19,1,4076.020833
19,1,2867.166667
19,1,3850.887324
19,1,1512.172727
19,1,2868.675000
19,1,1648.211538
19,1,11781.761905
19,1,3705.707317
19,1,2609.660256
19,1,1893.104167
19,1,3556.538043
19,1,5076.136029
19,1,1842.487805
19,1,1844.927632
19,1,4106.863636
19,1,1553.586207
19,1,4484.989583
19,1,18114.400000
19,1,2008.941176
19,1,15622.830357
19,1,10963.880435
19,1,5230.500000
19,1,3808.192935
19,1,1617.846875

19,1,11935.039773
19,1,3794.530405
19,1,2558.363889
19,1,10820.554348
19,1,5000.736979
19,1,3380.940000
19,1,17658.750000
19,1,2322.918269
19,1,3507.826087
19,1,16701.831731
19,1,4215.254032
19,1,2589.397727
19,1,3483.000000
19,1,1852.269886
19,1,2958.681034
19,1,2337.113636
19,1,5622.882353
19,1,1682.777778
19,1,3384.581633
19,1,14993.826923
19,1,2276.477941
19,1,5624.809524
19,1,2315.287500
19,1,2240.845745
19,1,8199.909091
19,1,2964.617647
19,1,1612.200000
19,1,3493.682927
19,1,9105.125000
19,1,3287.668919
19,1,4780.304348
19,1,10435.785714
19,1,7470.486486
19,1,4980.157895
19,1,5109.694444
19,1,7740.043103
19,1,1504.660714
19,1,11259.857143
19,1,2460.000000
19,1,5367.820000
19,1,4975.062500
19,1,1930.328125
19,1,5053.416667
19,1,8447.308824
19,1,1711.302326
19,1,2655.289773
19,1,3920.640625
19,1,3833.745690
19,1,5498.303977
19,1,2129.895833
19,1,8955.594595
19,1,5455.191489
19,1,5578.985849
19,1,2323.194444
19,1,9669.417683
19,1,5613.000000
19,1,4181.040816
19,1,2089.763889
19,1,8092.741848
19,1,1767.134615
19,1,1885.865854
19,1,8043.934211
19,1,6354.436275
19,1,5531.731707
19,1,1517.997642
19,1,2771.000000

19,1,6901.625000
19,1,4213.520349
19,1,2248.120192
19,1,1989.847973
19,1,2316.000000
19,1,6329.055851
19,1,5616.500000
19,1,3022.570513
19,1,1885.579787
19,1,3902.214286
19,1,3960.458333
19,1,5125.387195
19,1,2363.736111
19,1,1971.111111
19,1,6951.923077
19,1,11643.458333
19,1,6334.993590
19,1,2538.726351
19,1,1899.590909
19,1,6554.733333
19,1,1718.528846
19,1,5820.247283
19,1,2363.325000
19,1,3352.516667
19,1,12232.481250
19,1,3823.028409
19,1,16250.283333
19,1,4716.625000
19,1,1868.552885
19,1,5323.813830
19,1,5571.041667
19,1,5864.093085
19,1,1734.787234
19,1,11897.833333
19,1,1901.125000
19,1,15155.262500
19,1,12357.127717
19,1,15688.541667
19,1,1546.000000
19,1,2298.280556
19,1,12108.295455
19,1,3927.660377
19,1,4907.952381
19,1,8578.888889
19,1,2756.642500
19,1,3261.151515
19,1,3283.619048
19,1,3581.247596
19,1,4170.663194
19,1,11137.524510
19,1,3509.979592
19,1,3206.162791
19,1,6221.234756
19,1,3161.629032
19,1,3439.000000
19,1,5396.208333
19,1,17652.076923
19,1,3701.805556
19,1,2912.991477
19,1,2189.177778
19,1,4335.305921
19,1,7397.692073
19,1,6229.790441
19,1,4404.038043
19,1,4521.854167
19,1,13027.214286

19,1,1677.261364
19,1,1949.307292
19,1,2126.600000
19,1,1600.714286
19,1,18027.766129
19,1,2146.797619
19,1,2004.453125
19,1,7710.000000
19,1,4397.736486
19,1,2702.454787
19,1,5307.808824
19,1,1865.416667
19,1,1823.721429
19,1,3401.638889
19,1,2562.455000
19,1,7343.875000
19,1,9477.511111
19,1,8620.584375
19,1,5642.238095
19,1,2485.375000
19,1,11585.255319
19,1,1622.000000
19,1,3651.562500
19,1,2041.446809
19,1,3321.600000
19,1,4142.940476
19,1,11622.688889
19,1,19201.923077
19,1,6099.617424
19,1,2154.859756
19,1,7365.034091
19,1,4651.540541
19,1,6714.323171
19,1,2394.707317
19,1,19126.077381
19,1,5386.625000
19,1,1975.547619
19,1,1815.878205
19,1,7352.063953
19,1,8762.942857
19,1,9232.851190
19,1,7470.812500
19,1,7372.941406
19,1,2259.562500
19,1,6449.283088
19,1,1975.182692
19,1,7299.265625
19,1,1986.011905
19,1,4182.617424
19,1,5428.875000
19,1,1544.517857
19,1,2722.392857
19,1,18788.731707
19,1,1923.317073
19,1,4485.218750
19,1,6333.158088
19,1,12209.113636
19,1,1622.955882
19,1,1887.310606
19,1,10494.933333
19,1,5495.568182
19,1,1539.104167
19,1,1574.934524
19,1,1551.285714
19,1,2918.280488
19,1,14605.942308

19,1,6616.799020
19,1,4720.765957
19,1,2169.317308
19,1,1775.965909
19,1,3124.933333
19,1,8718.922794
19,1,1949.397059
19,1,2330.361842
19,1,3493.340426
19,1,4390.547794
19,1,7538.406977
19,1,1792.000000
19,1,5160.843750
19,1,2789.456522
19,1,2164.428571
19,1,4377.641827
19,1,1824.756757
19,1,3686.727273
19,1,2030.714286
19,1,1979.180556
19,1,1916.767857
19,1,1691.404255
19,1,7037.802083
19,1,3733.910000
19,1,3411.666667
19,1,2970.895270
19,1,3501.640625
19,1,11993.833333
19,1,3575.657258
19,1,4355.170455
19,1,5568.316667
19,1,3371.767442
19,1,2738.192857
19,1,3576.160714
19,1,17170.638298
19,1,7335.833333
19,1,3453.010050
19,1,1885.382979
19,1,2395.940217
19,1,6799.703125
19,1,5928.349265
19,1,2852.184375
19,1,4144.599265
19,1,11974.750000
19,1,1834.860294
19,1,4908.250000
19,1,8132.994565
19,1,5791.850543
19,1,4493.092949
19,1,2357.153846
19,1,12228.186047
19,1,1961.902174
19,1,11007.617500
19,1,2793.678571
19,1,7782.125000
19,1,5548.398810
19,1,1872.646341
19,1,1723.523256
19,1,5798.442308
19,1,4928.628906
19,1,3001.166667
19,1,4451.870192
19,1,2778.812500
19,1,1631.855263
19,1,5730.000000
19,1,1768.000000

19,1,8891.732558
19,1,2677.232143
19,1,6300.455128
19,1,3433.758721
19,1,2111.475000
19,1,10963.243902
19,1,2139.679878
19,1,11706.567308
19,1,9545.142857
19,1,7615.625000
19,1,4052.918919
19,1,3126.443750
19,1,16347.125000
19,1,8581.039894
19,1,2102.023256
19,1,8312.339844
19,1,6096.601744
19,1,13259.233696
19,1,6557.143382
19,1,3939.824219
19,1,2450.000000
19,1,4610.657895
19,1,3972.777344
19,1,1730.390625
19,1,17665.809783
19,1,2592.375000
19,1,9057.830729
19,1,1846.115385
19,1,3311.545455
19,1,3040.014706
19,1,1502.381579
19,1,5759.639706
19,1,4441.250000
19,1,4717.920732
19,1,1970.482143
19,1,14108.158537
19,1,4001.535714
19,1,8275.593023
19,1,4655.950000
19,1,2180.000000
19,1,2438.184524
19,1,6790.844595
19,1,2659.815476
19,1,7009.926136
19,1,2354.320312
19,1,1547.891892
19,1,1877.823171
19,1,6455.391129
19,1,3088.142857
19,1,2333.954082
19,1,4459.883333
19,1,4142.990854
19,1,8545.244318
19,1,2496.480769
19,1,4487.877500
19,1,2260.659574
19,1,1691.335938
19,1,1910.575758
19,1,6451.379310
19,1,5744.625000
19,1,2005.576687
19,1,1676.674479
19,1,8584.775000
19,1,1875.871795
19,1,4939.381757
19,1,4261.313776

19,1,1849.000000
19,1,3149.909091
19,1,8559.892045
19,1,5785.716981
19,1,4636.728723
19,1,1579.697674
19,1,6910.558673
19,1,5402.204545
19,1,10219.713235
19,1,9257.843750
19,1,6934.256098
19,1,2329.264535
19,1,2396.176471
19,1,2731.776786
19,1,5112.090000
19,1,6225.392857
19,1,4184.700000
19,1,12732.830645
19,1,1594.062500
19,1,12896.000000
19,1,3705.913043
19,1,13127.067308
19,1,2216.336538
19,1,2057.777778
19,1,2511.555556
19,1,2356.928571
19,1,5198.070652
19,1,12854.326531
19,1,1660.240625
19,1,2618.281863
19,1,2849.309783
19,1,1886.543367
19,1,3756.692857
19,1,5581.464844
19,1,1604.869565
19,1,3937.200000
19,1,4507.659091
19,1,2114.939516
19,1,7710.146739
19,1,18260.880000
19,1,1930.815789
19,1,2641.440476
19,1,1618.214286
19,1,3164.128125
19,1,3123.140957
19,1,6019.364362
19,1,7705.527778
19,1,2335.492857
19,1,5035.875000
19,1,1600.934211
19,1,1894.648437
19,1,2172.265625
19,1,4051.097826
19,1,2664.097500
19,1,11277.013158
19,1,7696.906250
19,1,7632.088235
19,1,2453.355000
19,1,16591.527174
19,1,2158.714286
19,1,3339.187500
19,1,1795.341667
19,1,2645.242424
19,1,3766.375000
19,1,6290.267241
19,1,1531.578125

19,1,3196.988636
19,1,2193.886719
19,1,12644.692308
19,1,4253.314286
19,1,5540.063830
19,1,2002.433333
19,1,1642.134146
19,1,4527.906250
19,1,1685.981132
19,1,6018.405405
19,1,4276.682500
19,1,3474.860294
19,1,1614.545000
19,1,1621.982639
19,1,18814.692308
19,1,1913.550781
19,1,1720.642857
19,1,1575.786458
19,1,2105.695652
19,1,4789.888889
19,1,3365.494048
19,1,1752.687500
19,1,2326.132353
19,1,4365.500000
19,1,5948.933333
19,1,4449.107558
19,1,1725.152174
19,1,1821.633929
19,1,4730.306818
19,1,5272.048828
19,1,1615.013298
19,1,8099.153846
19,1,10814.402174
19,1,3512.097561
19,1,14195.666667
19,1,9123.906736
19,1,4430.814815
19,1,1654.868243
19,1,7868.845588
19,1,2045.769231
19,1,2327.213816
19,1,2423.065000
19,1,4105.500000
19,1,7087.302966
19,1,3636.068966
19,1,5927.269231
19,1,7300.285714
19,1,8162.809524
19,1,2261.789474
19,1,2727.000000
19,1,1536.625000
19,1,19730.281250
19,1,5093.320755
19,1,1992.125000
19,1,17169.974265
19,1,8417.642857
19,1,1529.737500
19,1,9286.466667
19,1,14642.789062
19,1,1792.000000
19,1,1891.107143
19,1,3603.395270
19,1,1900.432292
19,1,1674.448276
19,1,14322.468750
19,1,3377.187500

19,1,3287.097222
19,1,1606.080882
19,1,1518.856618
19,1,1782.250000
19,1,1786.209677
19,1,4734.559524
19,1,1798.039062
19,1,11702.500000
19,1,3948.977273
19,1,12996.315217
19,1,15529.429348
19,1,1525.625000
19,1,16948.036932
19,1,2157.750000
19,1,1949.909091
19,1,2023.861111
19,1,15388.354839
19,1,2448.767442
19,1,2189.750000
19,1,1950.800000
19,1,1725.694444
19,1,5253.448276
19,1,3967.493902
19,1,3188.078125
19,1,4125.948718
19,1,1916.875000
19,1,7601.460526
19,1,3371.234694
19,1,2346.675000
19,1,4298.125000
19,1,6481.027174
19,1,3041.914773
19,1,2155.076705
19,1,2266.622396
19,1,3873.653846
19,1,3031.538462
19,1,11018.031250
19,1,14491.250000
19,1,9602.585938
19,1,2185.761628
19,1,11944.375000
19,1,1548.861702
19,1,1761.947368
19,1,3126.689024
19,1,3069.433824
19,1,3272.250000
19,1,2903.079268
19,1,3855.099490
19,1,7915.468750
19,1,10023.222656
19,1,2411.220000
19,1,5575.677778
19,1,3208.636364
19,1,1829.814024
19,1,4173.204082
19,1,2706.590909
19,1,1662.176471
19,1,2595.823529
19,1,5156.031250
19,1,2646.650000
19,1,7930.108974
19,1,2458.279412
19,1,2850.837209
19,1,12362.545455
19,1,1827.803571
19,1,2223.495146

19,1,4313.559278
19,1,12877.503289
19,1,3273.781250
19,1,2315.621951
19,1,2631.212121
19,1,2520.212500
19,1,18609.640625
19,1,3335.375000
19,1,7631.166667
19,1,2090.942308
19,1,8513.392857
19,1,2930.045455
19,1,1991.756757
19,1,6577.590909
19,1,10977.056122
19,1,2283.814815
19,1,4512.116667
19,1,11469.288462
19,1,11851.655172
19,1,5350.000000
19,1,1858.900000
19,1,17356.397727
19,1,2000.390244
19,1,1646.154605
19,1,2048.451613
19,1,3165.609848
19,1,5646.315789
19,1,2245.719512
19,1,6543.851190
19,1,10075.594388
19,1,5889.579787
19,1,2322.881250
19,1,6120.772727
19,1,7371.340686
19,1,8912.141129
19,1,15486.974265
19,1,4134.473214
19,1,2847.570000
19,1,2298.885135
19,1,7797.009259
19,1,1925.948529
19,1,2075.042553
19,1,8417.875000
19,1,12256.475000
19,1,4777.400000
19,1,1855.684659
19,1,3660.654255
19,1,1582.816176
19,1,1972.825000
19,1,11852.104545
19,1,2984.923077
19,1,3554.953704
19,1,1548.587209
19,1,1830.941176
19,1,2106.132576
19,1,6665.096154
19,1,18898.675676
19,1,2684.263158
19,1,5172.189655
19,1,2866.317308
19,1,2548.948718
19,1,4800.518750
19,1,7432.554688
19,1,1505.671053
19,1,6178.750000
19,1,2706.190476

19,1,1514.916667
19,1,9645.164474
19,1,3551.731707
19,1,4423.017045
19,1,1828.992021
19,1,3751.934211
19,1,5045.928030
19,1,3673.198529
19,1,3389.214286
19,1,2511.616279
19,1,17758.625000
19,1,4412.729167
19,1,2028.607143
19,1,4473.460938
19,1,4553.552632
19,1,9174.264881
19,1,6777.390625
19,1,2738.000000
19,1,3487.125000
19,1,2834.453125
19,1,1740.904412
19,1,3990.581250
19,1,9246.236842
19,1,2547.171875
19,1,4782.487805
19,1,4232.833333
19,1,3885.281250
19,1,2608.891026
19,1,6267.155488
19,1,8787.106061
19,1,4634.946429
19,1,13268.967391
19,1,7579.500000
19,1,12300.812500
19,1,1865.796875
19,1,2725.950000
19,1,5297.629630
19,1,1535.696429
19,1,16083.139286
19,1,7120.100806
19,1,5063.365385
19,1,2941.875000
19,1,2070.425000
19,1,3573.852941
19,1,3588.952206
19,1,1923.631579
19,1,2501.926471
19,1,6974.865385
19,1,4051.632576
19,1,1608.207031
19,1,2399.478261
19,1,11020.625000
19,1,3902.901042
19,1,2346.162791
19,1,9145.222222
19,1,3803.945652
19,1,4920.454545
19,1,3406.649038
19,1,6525.260870
19,1,1984.555556
19,1,4751.132812
19,1,8522.363636
19,1,1848.597561
19,1,2048.712766
19,1,2903.895349
19,1,1888.051282

19,1,2369.926829
19,1,4306.491667
19,1,3335.625000
19,1,2505.661458
19,1,6590.265957
19,1,5968.600000
19,1,4727.304348
19,1,2216.000000
19,1,2227.893617
19,1,1506.029412
19,1,1979.759868
19,1,4846.267045
19,1,1544.172794
19,1,2627.836538
19,1,1762.750000
19,1,8444.612805
19,1,4605.080645
19,1,3992.036585
19,1,3340.115000
19,1,2512.967391
19,1,1770.070000
19,1,15938.945833
19,1,16716.947674
19,1,1850.319767
19,1,2504.914062
19,1,1843.194149
19,1,10878.352273
19,1,13950.848684
19,1,3453.970930
19,1,1857.173077
19,1,3654.333333
19,1,6033.292857
19,1,7600.954545
19,1,4720.115385
19,1,16989.893382
19,1,1828.357143
19,1,2354.578947
19,1,1843.463415
19,1,3749.738281
19,1,3146.375000
19,1,2542.931818
19,1,10269.763889
19,1,4474.705729
19,1,7878.115625
19,1,4715.815476
19,1,7104.422619
19,1,3964.517045
19,1,6638.093023
19,1,5389.121324
19,1,3367.307927
19,1,6163.145161
19,1,3164.160000
19,1,2390.843750
19,1,1707.619792
19,1,1733.701923
19,1,1636.972222
19,1,8835.294118
19,1,2231.656250
19,1,1810.839286
19,1,5347.830882
19,1,7357.359375
19,1,15193.882353
19,1,8126.978659
19,1,5975.238095
19,1,4322.242188
19,1,2597.857143

19,1,4510.802083
19,1,4313.306034
19,1,3150.566176
19,1,3534.559524
19,1,2808.836735
19,1,3458.937500
19,1,2236.458333
19,1,10569.323529
19,1,9893.388298
19,1,4394.573864
19,1,2440.220930
19,1,8473.194444
19,1,3458.378676
19,1,3353.679348
19,1,6830.918478
19,1,6138.862500
19,1,2777.553030
19,1,1797.761628
19,1,13344.619318
19,1,10260.986842
19,1,2159.453488
19,1,2773.652439
19,1,1698.428571
19,1,1554.181818
19,1,11049.713816
19,1,3424.595930
19,1,4007.440574
19,1,4403.796512
19,1,3389.328431
19,1,1865.212766
19,1,12076.642442
19,1,7423.655612
19,1,1532.562500
19,1,6038.888372
19,1,14940.474359
19,1,1792.637931
19,1,6291.449219
19,1,3073.265306
19,1,8951.000000
19,1,1797.854167
19,1,2649.017857
19,1,3241.609375
19,1,3176.463415
19,1,1899.781915
19,1,8223.388587
19,1,17603.177083
19,1,9729.551471
19,1,3545.418478
19,1,4073.096354
19,1,2377.301020
19,1,1715.621094
19,1,4388.682692
19,1,1628.454545
19,1,1730.451087
19,1,1714.941489
19,1,1860.850000
19,1,2206.073171
19,1,1951.222826
19,1,6859.048469
19,1,3828.541667
19,1,1902.361842
19,1,19225.758929
19,1,7040.917553
19,1,2791.343750
19,1,1813.826705
19,1,7745.186047

19,1,7878.969512
19,1,12944.323529
19,1,2118.550000
19,1,1764.227273
19,1,4385.961111
19,1,5572.284375
19,1,5705.843750
19,1,6752.223404
19,1,5826.163265
19,1,1732.218750
19,1,2142.698718
19,1,7379.158654
19,1,7877.071429
19,1,2536.676471
19,1,2177.177083
19,1,6202.820122
19,1,5792.616279
19,1,4129.418367
19,1,2260.093750
19,1,3496.132812
19,1,3821.854839
19,1,5469.166667
19,1,3288.333333
19,1,2993.362069
19,1,3593.081250
19,1,5398.210938
19,1,3676.929487
19,1,7285.883929
19,1,2012.524390
19,1,1956.351064
19,1,4290.083333
19,1,7319.284884
19,1,1914.504808
19,1,1831.530303
19,1,10577.854839
19,1,2555.538462
19,1,1523.750000
19,1,8129.257353
19,1,10813.415323
19,1,1879.306818
19,1,2540.068182
19,1,11799.215000
19,1,9484.454787
19,1,3305.586111
19,1,5457.172414
19,1,6348.077586
19,1,5196.823529
19,1,2408.833333
19,1,1665.991667
19,1,3724.913043
19,1,2478.232955
19,1,1967.312500
19,1,10893.947674
19,1,3665.609756
19,1,2210.562500
19,1,4281.178571
19,1,15493.108333
19,1,1601.111111
19,1,2665.260417
19,1,3743.894737
19,1,11306.142857
19,1,1914.800000
19,1,17239.284884
19,1,2218.867021
19,1,2434.352941
19,1,4738.363095

19,1,1609.583333
19,1,2732.589744
19,1,8898.790761
19,1,12992.936170
19,1,16708.320513
19,1,2234.707447
19,1,5082.741379
19,1,10145.815476
19,1,4248.000000
19,1,2342.283784
19,1,1555.980114
19,1,11075.059524
19,1,12842.200000
19,1,1856.628571
19,1,2903.350000
19,1,2288.592105
19,1,2955.317935
19,1,2570.619048
19,1,1628.120192
19,1,2431.991477
19,1,2820.736111
19,1,15987.420000
19,1,4639.729167
19,1,17779.578125
19,1,2204.743902
19,1,4079.788889
19,1,5062.100000
19,1,5537.801282
19,1,17262.936170
19,1,4132.625000
19,1,2356.585938
19,1,7923.063830
19,1,5875.846154
19,1,5339.732955
19,1,2962.396739
19,1,2328.892045
19,1,2495.521277
19,1,8885.385135
19,1,5455.500000
19,1,1985.503571
19,1,2322.558140
19,1,4922.529412
19,1,1784.794872
19,1,2176.897436
19,1,2330.898936
19,1,19373.785714
19,1,2037.894022
19,1,14718.194853
19,1,1908.740854
19,1,7216.425000
19,1,5023.740000
19,1,1937.367424
19,1,1537.387097
19,1,1902.837500
19,1,2557.233871
19,1,8581.563889
19,1,9194.164634
19,1,2543.038889
19,1,2431.872449
19,1,1661.940625
19,1,4110.898649
19,1,1640.433824
19,1,4538.058333
19,1,4352.848485
19,1,4087.425000
19,1,2039.652174

19,1,4600.266667
19,1,13944.805000
19,1,19065.720833
19,1,2552.350000
20,2,1975.127841
20,2,3511.206522
20,2,2738.893617
20,2,1702.189024
20,2,9596.483333
20,2,2205.072115
20,2,4936.095588
20,2,1978.511364
20,2,1630.850000
20,2,9016.465278
20,2,1727.655405
20,2,1869.812500
20,2,1697.700658
20,2,2022.447115
20,2,5884.923913
20,2,3928.719697
20,2,2447.465116
20,2,2303.014535
20,2,2631.965116
20,2,1982.115385
20,2,2473.601064
20,2,2448.654891
20,2,2742.302632
20,2,9499.848837
20,2,1680.300000
20,2,1806.587838
20,2,1572.015625
20,2,4153.500000
20,2,2022.202381
20,2,3415.218750
20,2,1926.639423
20,2,2185.115385
20,2,5864.368421
20,2,1869.925000
20,2,1935.441489
20,2,1695.844828
20,2,2495.526316
20,2,1685.147727
20,2,2833.743750
20,2,2343.268519
20,2,2189.750000
20,2,1501.359375
20,2,4685.161364
20,2,2119.464286
20,2,2738.000000
20,2,1645.122449
20,2,1905.022500
20,2,2858.054245
20,2,3147.727273
20,2,2732.420455
20,2,3333.232955
20,2,4592.841346
20,2,2863.844262
20,2,1519.321429
20,2,2393.902439
20,2,2212.565217
20,2,1803.595395
20,2,1799.915000
20,2,3406.775943
20,2,1830.270000
20,2,4250.209302
20,2,1977.468750

20,2,9856.687500
20,2,2273.750000
20,2,2525.338235
20,2,1744.147727
20,2,1582.625000
20,2,1516.590116
20,2,1918.738095
20,2,2613.500000
20,2,4681.843750
20,2,1958.864865
20,2,2136.930233
20,2,1619.732759
20,2,1794.666667
20,2,2741.747396
20,2,1936.433735
20,2,1696.218750
20,2,1816.281250
20,2,1909.571970
20,2,2378.022321
20,2,2114.667614
20,2,1558.477273
20,2,3582.691038
20,2,2608.920000
20,2,1649.448370
20,2,4246.296053
20,2,2851.160000
20,2,1717.869231
20,2,1853.911111
20,2,2370.446809
20,2,2138.005814
20,2,2203.512195
20,2,2086.656250
20,2,1663.177083
20,2,1540.819444
20,2,2182.303030
20,2,2058.110577
20,2,1912.392857
20,2,1909.994898
20,2,1983.933333
20,2,2153.184028
20,2,1548.457317
20,2,2863.703125
20,2,1869.477273
20,2,2743.459821
20,2,1863.528846
20,2,2265.355769
20,2,2867.833333
20,2,3875.643617
20,2,3025.197368
20,2,2665.387500
20,2,1643.707447
20,2,1685.928571
20,2,1966.642458
20,2,2644.530837
20,2,2087.266055
20,2,2548.008929
20,2,2684.434524
20,2,2046.648256
20,2,2053.732143
20,2,2206.271875
20,2,3862.450000
20,2,1548.069149
20,2,1575.312500
20,2,1730.904255
20,2,1681.574324
20,2,1777.617647

20,2,5287.375000
20,2,5978.375000
20,2,1529.187500
20,2,1699.097561
20,2,2397.888158
20,2,3056.897959
20,2,1638.542553
20,2,1650.636364
20,2,2344.333333
20,2,1819.382812
20,2,1747.388298
20,2,3457.130000
20,2,2051.012195
20,2,2461.817308
20,2,2539.787234
20,2,2958.727941
20,2,2464.230769
20,2,2957.511628
20,2,3416.470588
20,2,1717.744186
20,2,1954.377778
20,2,2254.233333
20,2,3095.812500
20,2,2880.381579
20,2,5753.566667
20,2,2201.569444
20,2,1713.750000
20,2,2694.050000
20,2,2206.716346
20,2,1613.051020
20,2,2136.000000
20,2,19102.581818
20,2,2480.581081
20,2,1995.605769
20,2,1632.237500
20,2,3848.678571
20,2,2866.953488
20,2,2919.429687
20,2,1810.500000
20,2,1554.968750
20,2,1904.021277
20,2,2121.740000
20,2,2089.933824
20,2,1652.140704
20,2,1709.771654
20,2,1797.837500
20,2,1580.840909
20,2,2640.595745
20,2,1687.319149
20,2,5448.016447
20,2,2701.638298
20,2,4871.384804
20,2,1503.317708
20,2,2006.648649
20,2,2203.984043
20,2,1933.455357
20,2,1628.058511
20,2,1641.282609
20,2,1538.902778
20,2,1730.302326
20,2,1635.367647
20,2,1516.400000
20,2,6569.397500
20,2,2912.785326
20,2,2716.093137
20,2,13532.571429

20,2,2145.000000
20,2,1532.156250
20,2,1516.815000
20,2,2446.143868
20,2,1716.796875
20,2,6247.585427
20,2,1920.712500
20,2,1859.595000
20,2,2102.693878
20,2,2093.600000
20,2,1621.670732
20,2,1893.435976
20,2,2254.447368
20,2,1862.782609
20,2,2413.595109
20,2,1887.761905
20,2,3237.911585
20,2,1886.375000
20,2,1829.245283
20,2,1828.901316
20,2,1620.091463
20,2,1625.820312
20,2,1570.715000
20,2,1988.770833
20,2,2912.434524
20,2,1650.243902
20,2,7516.984375
20,2,4838.875000
20,2,1620.000000
20,2,1530.567708
20,2,2801.083333
20,2,1555.823864
20,2,2610.556604
20,2,6534.941860
20,2,1767.140625
20,2,1504.634615
20,2,2061.492424
20,2,1879.266667
20,2,3706.666667
20,2,2017.732143
20,2,1700.466667
20,2,1643.703704
20,2,2378.199074
20,2,13849.542857
20,2,3890.250000
20,2,2709.985294
20,2,1840.432292
20,2,2605.305000
20,2,1661.428571
20,2,2085.283019
20,2,1610.108108
20,2,4265.967742
20,2,1945.445946
20,2,1695.986842
20,2,2089.163462
20,2,2004.449468
20,2,2700.875000
20,2,1877.857143
20,2,3377.953488
20,2,1695.802632
20,2,1629.203125
20,2,1716.365909
20,2,1768.000000
20,2,2800.163043
20,2,2043.502976
20,2,6930.208333

20,2,2827.756579
20,2,2153.542763
20,2,2759.443182
20,2,6922.125000
20,2,2657.329268
20,2,2805.410000
20,2,1731.737500
20,2,3217.540948
20,2,1828.107955
20,2,1585.625000
20,2,2011.062500
20,2,2519.529412
20,2,3285.549342
20,2,7598.047619
20,2,2113.834135
20,2,1652.810897
20,2,3372.365854
20,2,2428.830000
20,2,1837.250000
20,2,1617.547794
20,2,1533.275000
20,2,2814.467105
20,2,2436.632653
20,2,1826.677326
20,2,3523.023936
20,2,1960.062500
20,2,2450.534091
20,2,3177.705882
20,2,1668.468750
20,2,1547.170213
20,2,2375.733333
20,2,6133.956522
20,2,2044.187500
20,2,2954.673469
20,2,2388.307692
20,2,1772.694444
20,2,2551.168367
20,2,1584.529762
20,2,1570.240854
20,2,4712.418367
20,2,1577.736979
20,2,2301.204082
20,2,2040.503676
20,2,1738.044271
20,2,1527.474265
20,2,4378.276074
20,2,2623.333333
20,2,7342.821429
20,2,4557.067797
20,2,1633.083333
20,2,1650.174569
20,2,2223.179426
20,2,1543.095000
20,2,2429.028302
20,2,2385.315476
20,2,3124.928571
20,2,12519.617187
20,2,3803.584906
20,2,1563.913043
20,2,2663.696078
20,2,5426.875000
20,2,2792.239362
20,2,2927.648936
20,2,1503.276042
20,2,1621.770000
20,2,1533.391892

20,2,1734.714286
20,2,3760.978659
20,2,1790.446429
20,2,1508.546053
20,2,2836.941176
20,2,1818.250000
20,2,3678.673469
20,2,2827.774510
20,2,1659.866667
20,2,2849.071429
20,2,3013.652439
20,2,2299.642857
20,2,3463.250000
20,2,5209.250000
20,2,4622.500000
20,2,1820.397059
20,2,8070.097222
20,2,2049.497159
20,2,1745.218009
20,2,1930.779762
20,2,1811.168367
20,2,2533.325000
20,2,2377.733696
20,2,1758.470588
20,2,1969.911184
20,2,1564.035714
20,2,1770.699468
20,2,1616.845109
20,2,1723.668919
20,2,1654.610465
20,2,2528.078125
20,2,1909.351744
20,2,1601.329861
20,2,4016.025943
20,2,1788.128906
20,2,2068.041667
20,2,2129.500000
20,2,2045.431122
20,2,2050.750000
20,2,1592.848485
20,2,2367.117647
20,2,1657.905660
20,2,8553.653125
20,2,1941.562500
20,2,2561.228723
20,2,1622.779605
20,2,5323.933511
20,2,1846.198864
20,2,3180.311111
20,2,1704.163265
20,2,1903.009615
20,2,1683.284314
20,2,3344.600000
20,2,1907.210227
20,2,1589.345395
20,2,4226.750000
20,2,3187.531250
20,2,5116.736842
20,2,2109.469512
20,2,1550.486486
20,2,2838.000000
20,2,1641.055556
20,2,1922.317568
20,2,1765.756757
20,2,3594.284314
20,2,2900.371429

20,2,1760.084302
20,2,2599.505435
20,2,2221.541667
20,2,1991.269231
20,2,1673.413265
20,2,1638.463918
20,2,2152.950000
20,2,2883.225490
20,2,11786.296512
20,2,2212.552632
20,2,1628.125000
20,2,2243.725000
20,2,1770.835227
20,2,2400.980000
20,2,3296.436275
20,2,2714.072674
20,2,2877.000000
20,2,1545.156250
20,2,1566.687500
20,2,5079.217262
20,2,1693.688679
20,2,1650.003378
20,2,2401.625000
20,2,3449.191860
20,2,1798.178571
20,2,2579.027174
20,2,2858.872396
20,2,9841.434783
20,2,1874.569149
20,2,2615.457547
20,2,2203.492647
20,2,1934.436170
20,2,7991.784722
20,2,2089.245536
20,2,4184.851351
20,2,3137.280000
20,2,1903.747222
20,2,2098.316327
20,2,1874.205357
20,2,2173.233696
20,2,1523.950000
20,2,2300.750000
20,2,3725.476351
20,2,3257.125000
20,2,5181.518617
20,2,3378.877119
20,2,3151.785714
20,2,1874.778646
20,2,1784.358333
20,2,3833.933824
20,2,2733.312500
20,2,2714.774194
20,2,1696.462500
20,2,2388.505319
20,2,5393.218750
20,2,2019.959459
20,2,1784.607955
20,2,4162.821429
20,2,2826.174020
20,2,5327.447581
20,2,1938.678571
20,2,1747.489583
20,2,2095.831081
20,2,1808.420455
20,2,2481.689189
20,2,2576.640625

20,2,1801.345000
20,2,1679.311224
20,2,2569.000000
20,2,3602.682292
20,2,1811.387500
20,2,1794.967391
20,2,1572.062500
20,2,1678.852941
20,2,5047.824468
20,2,2545.000000
20,2,2421.035714
20,2,2836.310897
20,2,1613.133929
20,2,1519.971098
20,2,2276.264706
20,2,1642.861111
20,2,2305.424242
20,2,1660.403846
20,2,2406.434783
20,2,1509.826220
20,2,1816.853659
20,2,2581.841463
20,2,2460.432065
20,2,1581.682692
20,2,4502.217391
20,2,2925.452381
20,2,1715.887195
20,2,2185.467742
20,2,3659.291667
20,2,2547.600000
20,2,3569.612500
20,2,8107.037736
20,2,2312.593750
20,2,3846.722222
20,2,1586.070833
20,2,1504.693878
20,2,2592.736111
20,2,2271.891304
20,2,1768.687500
20,2,1860.075000
20,2,2322.116279
20,2,1640.646226
20,2,2339.244898
20,2,2053.037500
20,2,1710.162109
20,2,1799.255319
20,2,1704.000000
20,2,1775.383929
20,2,3902.566176
20,2,1510.509615
20,2,1806.282051
20,2,1557.179878
20,2,1723.298246
20,2,2623.724490
20,2,2387.000000
20,2,1751.247396
20,2,4487.369565
20,2,10534.914773
20,2,1541.275000
20,2,2010.302083
20,2,2982.500000
20,2,2284.913043
20,2,1691.230000
20,2,3902.939189
20,2,4172.975000
20,2,2170.241071

20,2,2054.597222
20,2,1749.805000
20,2,2427.437500
20,2,1735.568182
20,2,2471.414474
20,2,2109.466146
20,2,2254.156250
20,2,7143.083333
20,2,2635.818966
20,2,1931.786765
20,2,2211.506410
20,2,1512.109375
20,2,2125.759615
20,2,3426.288462
20,2,9372.000000
20,2,4064.310976
20,2,2941.727500
20,2,2116.344444
20,2,1599.072368
20,2,1584.319767
20,2,2644.824561
20,2,2096.690476
20,2,3408.240196
20,2,1727.724265
20,2,3209.932692
20,2,1881.618750
20,2,1667.558140
20,2,3389.254237
20,2,1572.591837
20,2,1928.481250
20,2,3085.923295
20,2,1748.551471
20,2,1802.834184
20,2,1522.537736
20,2,2424.465909
20,2,1613.535326
20,2,2065.858173
20,2,5510.451531
20,2,3259.750000
20,2,2199.645833
20,2,1582.428571
20,2,1927.700000
20,2,2621.718750
20,2,2951.155612
20,2,1674.875000
20,2,4674.440789
20,2,2004.544872
20,2,3924.500000
20,2,2416.109375
20,2,3191.371795
20,2,2302.723684
20,2,1887.820513
20,2,7736.888889
20,2,1538.480000
20,2,3517.735294
20,2,5563.384615
20,2,2271.725806
20,2,2366.877660
20,2,3312.750000
20,2,2092.244681
20,2,1699.705729
20,2,1885.592105
20,2,2579.415816
20,2,1975.414894
20,2,3989.222222
20,2,1719.112745

20,2,1587.136364
20,2,2161.441038
20,2,1815.476562
20,2,2201.647727
20,2,2581.260870
20,2,2394.111702
20,2,1789.427632
20,2,1992.852778
20,2,3033.659091
20,2,2092.500000
20,2,2896.714286
20,2,1576.877604
20,2,2586.360795
20,2,1558.947368
20,2,1743.976190
20,2,3399.936275
20,2,1579.160714
20,2,13851.173529
20,2,1535.477273
20,2,1879.181818
20,2,3123.975000
20,2,1682.493750
20,2,3606.706522
20,2,1959.236111
20,2,1598.926471
20,2,1577.625000
20,2,2361.479730
20,2,1653.153509
20,2,1800.832258
20,2,2798.417763
20,2,1641.127500
20,2,2072.750000
20,2,1926.021739
20,2,1589.375000
20,2,2438.736413
20,2,1563.864865
20,2,2206.835000
20,2,8177.342213
20,2,1751.571429
20,2,1609.431452
20,2,2248.600000
20,2,2045.451705
20,2,2485.135747
20,2,2391.993902
20,2,3169.710938
20,2,1516.365000
20,2,2614.490385
20,2,1801.480263
20,2,1734.190000
20,2,2140.437500
20,2,4253.680000
20,2,2501.419355
20,2,1810.732143
20,2,3050.362245
20,2,1719.000000
20,2,3196.588068
20,2,1553.148936
20,2,2122.800926
20,2,4702.429348
20,2,2344.218750
20,2,1804.069767
20,2,2044.961538
20,2,1877.083333
20,2,3905.250000
20,2,2572.000000
20,2,2548.633523

20,2,1690.147436
20,2,6616.802632
20,2,1644.847458
20,2,2087.799020
20,2,1644.877907
20,2,2478.746528
20,2,1931.497768
20,2,2527.572674
20,2,2288.536585
20,2,2693.105769
20,2,2016.200000
20,2,3540.004808
20,2,1504.744681
20,2,1733.714286
20,2,3708.456522
20,2,2176.548780
20,2,1786.788889
20,2,1636.357143
20,2,1614.815789
20,2,1710.144231
20,2,5393.550000
20,2,1893.232143
20,2,2691.851852
20,2,2551.134615
20,2,2082.051724
20,2,2437.302632
20,2,2107.705882
20,2,3491.769231
20,2,2817.421875
20,2,2137.919811
20,2,5918.896226
20,2,2051.195122
20,2,1680.139151
20,2,2086.890000
20,2,2091.026596
20,2,3112.975610
20,2,1504.959459
20,2,5039.643617
20,2,1629.423469
20,2,2185.415761
20,2,2070.093750
20,2,1504.039062
20,2,2325.966981
20,2,1990.844444
20,2,5142.408088
20,2,2819.403846
20,2,2252.281250
20,2,3509.536585
20,2,3814.500000
20,2,3143.117647
20,2,4829.875000
20,2,3341.495000
20,2,1721.303571
20,2,1632.112805
20,2,1862.015086
20,2,1648.513514
20,2,1582.000000
20,2,1915.636364
20,2,3613.434659
20,2,2058.702128
20,2,2348.750000
20,2,1635.050000
20,2,1781.947368
20,2,2270.750000
20,2,5480.121711
20,2,6434.500000

20,2,1723.511111
20,2,2850.111413
20,2,2287.345588
20,2,2195.187500
20,2,1755.758929
20,2,2293.719262
20,2,3553.375000
20,2,1560.820513
20,2,3521.610714
20,2,2106.783654
20,2,2233.500000
20,2,2946.651961
20,2,1567.826087
20,2,1745.857955
20,2,1784.542453
20,2,2699.378906
20,2,2452.847561
20,2,2325.475000
20,2,1629.047297
20,2,1563.093750
20,2,1860.616071
20,2,4566.125000
20,2,2628.150000
20,2,2253.258929
20,2,2037.921053
20,2,1714.070652
20,2,2869.230769
20,2,1791.867347
20,2,2292.500000
20,2,1832.595238
20,2,4527.866667
20,2,3011.493243
20,2,1623.424242
20,2,1540.600000
20,2,2536.794118
20,2,1917.764706
20,2,3203.342500
20,2,2904.060976
20,2,2076.950980
20,2,1562.111111
20,2,1923.183333
20,2,1651.750000
20,2,1502.375000
20,2,2788.696429
20,2,2385.912791
20,2,6922.389535
20,2,1772.860465
20,2,1561.028571
20,2,3413.394737
20,2,1791.602041
20,2,2431.840625
20,2,2001.211735
20,2,1852.133721
20,2,2454.600000
20,2,2589.700000
20,2,2793.500000
20,2,2954.262500
20,2,2616.102041
20,2,1869.414773
20,2,1605.045673
20,2,4007.203125
20,2,1566.807292
20,2,2125.545455
20,2,2365.192308
20,2,3995.203125
20,2,2223.906977

20,2,2513.787234
20,2,3032.220000
20,2,1681.312500
20,2,1681.139706
20,2,1702.851064
20,2,5203.481132
20,2,1566.153125
20,2,5953.508523
20,2,1990.057692
20,2,2771.079545
20,2,2137.127660
20,2,2695.388889
20,2,1942.500000
20,2,2337.555556
20,2,1507.750000
20,2,2069.459016
20,2,3411.205882
20,2,2035.500000
20,2,2103.875000
20,2,1970.789474
20,2,2514.241477
20,2,1697.416667
20,2,3260.671053
20,2,3213.312500
20,2,1996.018182
20,2,1949.236979
20,2,1645.560811
20,2,3318.574219
20,2,2736.115385
20,2,3364.382979
20,2,4060.383333
20,2,1724.453431
20,2,3539.750000
20,2,2080.616848
20,2,3396.794872
20,2,4323.682065
20,2,2709.507812
20,2,2149.513158
20,2,1750.687500
20,2,1639.425781
20,2,2396.434783
20,2,2634.941176
20,2,1591.847826
20,2,1525.275000
20,2,7834.750000
20,2,2479.938596
20,2,4113.906250
20,2,1514.259615
20,2,1657.062500
20,2,1747.107143
20,2,3280.039062
20,2,3040.265625
20,2,2829.375000
20,2,1738.803030
20,2,2752.230769
20,2,3039.861111
20,2,5380.953125
20,2,2229.119318
20,2,1915.266129
20,2,3442.176136
20,2,2346.882353
20,2,3694.625000
20,2,2175.441176
20,2,2110.068878
20,2,1602.638587
20,2,3535.882353

20,2,1808.931548
20,2,1578.989796
20,2,1629.923077
20,2,1630.750000
20,2,1558.914894
20,2,1509.571429
20,2,1746.080882
20,2,2576.031250
20,2,1681.636905
20,2,2324.779762
20,2,1751.913462
20,2,2093.983553
20,2,2241.474359
20,2,1850.307692
20,2,1570.078947
20,2,2136.986111
20,2,1659.944444
20,2,1712.819196
20,2,1986.494792
20,2,3719.813953
20,2,1709.527778
20,2,1524.312500
20,2,2626.125000
20,2,3048.722222
20,2,1603.097561
20,2,2862.900000
20,2,1684.858974
20,2,1761.000000
20,2,1514.546875
20,2,1882.076923
20,2,2177.860000
20,2,1564.908537
20,2,1892.883333
20,2,2361.290625
20,2,2020.652778
20,2,1732.133721
20,2,1661.166667
20,2,1583.128205
20,2,4952.487805
20,2,1755.340517
20,2,2097.181818
20,2,1749.291667
20,2,2081.979167
20,2,2452.159884
20,2,2435.664474
20,2,3999.012821
20,2,1598.154605
20,2,1529.812500
20,2,2044.355769
20,2,2374.305921
20,2,1658.094595
20,2,1509.514423
20,2,2143.576923
20,2,2693.956250
20,2,3148.284884
20,2,1507.285714
20,2,3329.312500
20,2,1580.500000
20,2,1558.625000
20,2,2217.553191
20,2,1521.086111
20,2,2196.303191
20,2,2348.863636
20,2,2169.800000
20,2,2076.590426
20,2,2544.928571

20,2,1609.515625
20,2,1714.930380
20,2,1935.573529
20,2,5596.813953
20,2,7593.909091
20,2,8735.725000
20,2,7212.794118
20,2,2311.280612
20,2,2783.343137
20,2,1541.742647
20,2,2165.827500
20,2,2522.250000
20,2,3311.176282
20,2,4576.220930
20,2,2622.910000
20,2,1745.364583
20,2,3286.302500
20,2,2026.344444
20,2,1943.147500
20,2,1971.586735
20,2,1662.917683
20,2,3420.420455
20,2,1988.000000
20,2,3895.736111
20,2,1885.400000
20,2,1751.500000
20,2,4239.729730
20,2,2130.157143
20,2,7887.481707
20,2,1742.847973
20,2,2145.568182
20,2,2642.307377
20,2,1928.459459
20,2,2618.535714
20,2,1613.264151
20,2,1949.724490
20,2,3490.882812
20,2,3364.032609
20,2,2858.176471
20,2,1856.673246
20,2,1835.461538
20,2,1504.837209
20,2,1632.575581
20,2,1515.946429
20,2,1931.590909
20,2,2244.891667
20,2,3049.968750
20,2,1591.256579
20,2,1950.858696
20,2,2317.622283
20,2,6575.881944
20,2,1546.705882
20,2,2931.500000
20,2,2694.468571
20,2,1572.676829
20,2,6101.757653
20,2,3959.059896
20,2,1988.622396
20,2,2162.413043
20,2,2779.941038
20,2,1682.750000
20,2,1527.511364
20,2,6331.440000
20,2,3028.387755
20,2,2132.750000
20,2,1601.700000

20,2,1506.132653
20,2,1609.996622
20,2,4592.687500
20,2,2837.026042
20,2,4211.953804
20,2,1699.156863
20,2,2479.178571
20,2,1551.925781
20,2,3319.978723
20,2,2347.471698
20,2,2451.303191
20,2,4120.250000
20,2,1971.000000
20,2,2294.570000
20,2,2248.000000
20,2,3241.353261
20,2,1545.356707
20,2,1613.336957
20,2,3278.760000
20,2,2069.523256
20,2,2431.808962
20,2,1713.722222
20,2,2122.824519
20,2,1958.088710
20,2,1732.024590
20,2,2377.560345
20,2,1926.413265
20,2,1567.613636
20,2,1966.432990
20,2,1889.820513
20,2,1815.598214
20,2,2314.944444
20,2,2926.278409
20,2,3887.630435
20,2,1714.312500
20,2,2349.420455
20,2,2157.171875
20,2,2407.320000
20,2,1840.642857
20,2,3466.860656
20,2,2121.267857
20,2,3468.062500
20,2,2035.288136
20,2,2759.414773
20,2,1747.025000
20,2,1505.500000
20,2,3320.086957
20,2,2252.650000
20,2,2117.573529
20,2,3209.331140
20,2,8661.082447
20,2,1556.946429
20,2,13399.295455
20,2,1965.656250
20,2,2499.184028
20,2,1690.877717
20,2,3075.591216
20,2,5579.125000
20,2,2447.742424
20,2,1536.828125
20,2,3846.517857
20,2,1512.475806
20,2,3753.814103
20,2,4887.502119
20,2,7336.285714
20,2,2196.699074

20,2,1689.912500
20,2,1739.802632
20,2,1720.252841
20,2,1532.365385
20,2,1713.579787
20,2,2583.208333
20,2,3659.454545
20,2,2471.150000
20,2,1598.156250
20,2,1601.734043
20,2,2000.821429
20,2,3601.950000
20,2,2495.838415
20,2,2159.929245
20,2,1750.715909
20,2,1543.882353
20,2,1878.955882
20,2,2540.690476
20,2,4262.766667
20,2,2391.963068
20,2,3959.865566
20,2,2765.727500
20,2,1891.968750
20,2,2197.281250
20,2,1718.214286
20,2,1534.003289
20,2,2617.780488
20,2,2954.625000
20,2,3105.431250
20,2,1681.377551
20,2,1874.914063
20,2,2641.583333
20,2,1745.904412
20,2,2593.875000
20,2,2430.100000
20,2,3421.018293
20,2,2019.106383
20,2,3232.537500
20,2,2090.550000
20,2,2401.166667
20,2,1767.250000
20,2,1896.804487
20,2,3491.933673
20,2,4519.568878
20,2,1703.221106
20,2,3027.636364
20,2,5878.068627
20,2,4806.377358
20,2,2388.857143
20,2,2546.110000
20,2,2154.045455
20,2,1788.923077
20,2,1847.797170
20,2,2081.921569
20,2,3111.958333
20,2,1955.485294
20,2,1737.978125
20,2,3006.456897
20,2,1591.199324
20,2,1621.497283
20,2,1626.452381
20,2,2826.000000
20,2,2848.593750
20,2,2500.544271
20,2,2607.893617
20,2,1812.480769

20,2,1663.471154
20,2,1868.768229
20,2,1986.462963
20,2,1833.586735
20,2,2136.323529
20,2,1887.684783
20,2,11593.878378
20,2,4506.194444
20,2,2014.115385
20,2,7388.297619
20,2,1711.659091
20,2,2143.722727
20,2,2704.046875
20,2,3091.209302
20,2,1696.846429
20,2,1747.569149
20,2,2005.162234
20,2,2075.833333
20,2,2050.533854
20,2,6104.591837
20,2,1774.280488
20,2,3274.032609
20,2,1734.412037
20,2,3837.681818
20,2,1965.125000
20,2,2358.143293
20,2,2585.021739
20,2,3917.495192
20,2,8450.041667
20,2,1694.047619
20,2,1644.097561
20,2,1767.309524
20,2,2624.938776
20,2,1636.420000
20,2,2106.892308
20,2,1722.401786
20,2,12520.553571
20,2,3687.500000
20,2,1836.647783
20,2,2254.557292
20,2,1746.345745
20,2,2025.448113
20,2,1828.383152
20,2,1513.855556
20,2,1538.570313
20,2,2348.146277
20,2,1669.355263
20,2,2693.328125
20,2,1591.843750
20,2,1746.938889
20,2,3459.338542
20,2,2261.997093
20,2,8038.605769
20,2,1897.037234
20,2,3141.368421
20,2,1944.822368
20,2,2374.176471
20,2,1938.625000
20,2,3933.502551
20,2,1998.788043
20,2,1718.378378
20,2,3836.323370
20,2,2371.053191
20,2,1839.634615
20,2,2404.981818
20,2,2074.385638

20,2,2795.517857
20,2,2307.846154
20,2,1810.074468
20,2,1928.940476
20,2,2270.672500
20,2,1510.014423
20,2,1883.217949
20,2,2934.491279
20,2,7230.341463
20,2,1499.828488
20,2,2102.156250
20,2,2162.854167
20,2,1550.714286
20,2,1508.454268
20,2,1732.022727
20,2,4858.512500
20,2,1817.549133
20,2,1840.408019
20,2,2324.836364
20,2,1892.160714
20,2,2054.444444
20,2,2435.579545
20,2,1769.112069
20,2,2562.071429
20,2,2277.259259
20,2,1573.878049
20,2,2008.140625
20,2,1935.558824
20,2,3183.669643
20,2,11373.688596
20,2,2297.025735
20,2,1863.989796
20,2,2192.625000
20,2,2810.891892
20,2,2492.823529
20,2,2888.450000
20,2,1603.598837
20,2,2857.067308
20,2,1853.910714
20,2,2123.673077
20,2,2027.536458
20,2,1655.471591
20,2,3107.889706
20,2,2910.952128
20,2,1778.430233
20,2,2174.845238
20,2,4305.243243
20,2,2281.112500
20,2,2204.766304
20,2,5946.042553
20,2,1605.425373
20,2,4237.430556
20,2,2050.040201
20,2,1867.449219
20,2,6798.000000
20,2,2425.144022
20,2,1538.208333
20,2,1888.050000
20,2,1834.184211
20,2,1639.222826
20,2,4014.210526
20,2,2335.590426
20,2,2082.895833
20,2,4547.875000
20,2,2104.686047
20,2,2124.514205

20,2,2289.848039
20,2,2832.670455
20,2,1618.226563
20,2,1910.780000
20,2,3442.076087
20,2,1776.267442
20,2,3433.398305
20,2,3100.700000
20,2,1515.705882
20,2,2224.676471
20,2,1692.300000
20,2,1855.375000
20,2,1578.535714
20,2,3282.775000
20,2,1790.406250
20,2,2156.809783
20,2,1674.996094
20,2,3165.553191
20,2,1870.085000
20,2,3508.184783
20,2,2614.558442
20,2,2624.206250
20,2,1500.000000
20,2,1774.222222
20,2,1560.951705
20,2,2909.853659
20,2,1596.250000
20,2,2325.285000
20,2,2739.756757
20,2,1853.580000
20,2,2197.357143
20,2,2416.800000
20,2,1678.076220
20,2,1538.125000
20,2,4447.775510
20,2,2620.421196
20,2,1990.991935
20,2,1511.250000
20,2,2788.475000
20,2,1995.526316
20,2,2493.886905
20,2,4453.950000
20,2,3494.345238
20,2,2532.785714
20,2,3347.159574
20,2,1815.375000
20,2,1930.017857
20,2,2932.875000
20,2,8294.600000
20,2,1603.825000
20,2,6534.187500
20,2,2001.975000
20,2,1748.987500
20,2,2427.000000
20,2,1551.812500
20,2,2283.087500
20,2,3125.564103
20,2,2595.117647
20,2,2343.088235
20,2,5144.962054
20,2,1670.338068
20,2,10832.934783
20,2,2383.816327
20,2,1910.097826
20,2,1669.307692
20,2,2081.970238

20,2,2653.494624
20,2,5301.532051
20,2,5057.567073
20,2,2181.555556
20,2,1517.607143
20,2,2255.899510
20,2,1937.928977
20,2,1862.928571
20,2,1636.882353
20,2,1626.158333
20,2,4161.980978
20,2,2768.875000
20,2,1541.933511
20,2,2447.400000
20,2,2299.170918
20,2,1528.512097
20,2,1689.675532
20,2,6503.750000
20,2,1806.016667
20,2,2021.968023
20,2,1855.491071
20,2,1703.958333
20,2,2916.011364
20,2,10817.937500
20,2,1701.145000
20,2,2110.220000
20,2,2064.625000
20,2,2694.487500
20,2,3839.980769
20,2,1805.625000
20,2,1650.290323
20,2,3627.880137
20,2,12312.469388
20,2,1845.543750
20,2,3305.187500
20,2,1690.821429
20,2,2837.750000
20,2,2448.020000
20,2,2241.734375
20,2,2362.950000
20,2,1840.289474
20,2,2444.199468
20,2,2559.320122
20,2,2887.342105
20,2,2733.793478
20,2,4022.625000
20,2,2432.000000
20,2,3616.120000
20,2,2372.274194
20,2,2095.304054
20,2,2053.375000
20,2,2809.656250
20,2,2760.616667
20,2,1793.592391
20,2,2558.880319
20,2,2204.968750
20,2,3009.400000
20,2,2001.129902
20,2,2427.093023
20,2,1961.795455
20,2,2641.142857
20,2,2793.297872
20,2,1929.243902
20,2,1544.464286
20,2,1513.671569
20,2,1612.019231

20,2,2103.125000
20,2,4845.165816
20,2,2737.980263
20,2,1628.478723
20,2,1735.627451
20,2,3657.500000
20,2,1537.444444
20,2,1737.500000
20,2,3142.209375
20,2,2216.125000
20,2,2712.060000
20,2,2894.159314
20,2,1842.486979
20,2,4052.934524
20,2,1591.912500
20,2,1536.761905
20,2,2727.601852
20,2,8265.844444
20,2,4365.351351
20,2,1675.134804
20,2,1682.900000
20,2,1575.600000
20,2,1544.125000
20,2,2090.833333
20,2,2489.760417
20,2,2320.826923
20,2,1797.478261
20,2,5224.740000
20,2,3509.372093
20,2,2512.034884
20,2,2024.803191
20,2,2990.255814
20,2,3550.315789
20,2,1572.541667
20,2,1657.904523
20,2,3391.099138
20,2,2002.765957
20,2,2348.927374
20,2,2436.336735
20,2,2371.069079
20,2,2893.056122
20,2,1810.527500
20,2,1569.352941
20,2,4067.309659
20,2,1530.022727
20,2,1545.659574
20,2,2335.442308
20,2,2133.634146
20,2,4085.398649
20,2,2004.086111
20,2,2623.875000
20,2,1521.460000
20,2,3074.076923
20,2,4903.455357
20,2,2136.397059
20,2,5384.778125
20,2,2723.914894
20,2,1655.798913
20,2,3090.053571
20,2,1606.700000
20,2,2001.077128
20,2,2147.683824
20,2,2207.787234
20,2,1686.978261
20,2,1834.007813
20,2,1794.927778

20,2,5567.221698
20,2,2222.584906
20,2,1555.040816
20,2,1610.378205
20,2,2600.970238
20,2,1892.326531
20,2,2674.000000
20,2,1525.187500
20,2,2089.192500
20,2,1613.295455
20,2,2389.484375
20,2,2268.020833
20,2,1838.805147
20,2,1531.682692
20,2,3288.333333
20,2,1626.238764
20,2,2259.375000
20,2,2030.448276
20,2,3371.165698
20,2,1776.740000
20,2,1976.073718
20,2,1989.541667
20,2,2117.528646
20,2,3388.562500
20,2,3013.229167
20,2,2323.027174
20,2,2549.769444
20,2,2368.865385
20,2,1504.353659
20,2,2118.756579
20,2,3430.425000
20,2,2657.940476
20,2,1693.367647
20,2,3030.662791
20,2,3106.095745
20,2,5385.125000
20,2,2551.659722
20,2,2147.264151
20,2,2597.911364
20,2,3173.292614
20,2,1508.965000
20,2,2498.000000
20,2,4059.350000
20,2,1501.219512
20,2,2212.512500
20,2,1803.926471
20,2,1643.136364
20,2,1917.989796
20,2,1660.593023
20,2,2111.750000
20,2,2180.125000
20,2,1653.338235
20,2,2260.187500
20,2,3268.264706
20,2,1631.787234
20,2,15587.780000
20,2,2854.787791
20,2,2174.014706
20,2,2397.873563
20,2,1924.702381
20,2,5342.881148
20,2,2102.325581
20,2,2733.845588
20,2,3613.075000
20,2,6062.718750
20,2,1642.147059

20,2,2475.922872
20,2,2543.236607
20,2,3389.264423
20,2,6050.084746
20,2,1658.329787
20,2,2016.634146
20,2,2885.377451
20,2,5177.816489
20,2,4562.791667
20,2,2388.572727
20,2,2225.242857
20,2,1655.786765
20,2,3722.469388
20,2,1802.029240
20,2,4043.115196
20,2,2342.804878
20,2,1756.875000
20,2,1801.436170
20,2,1499.821429
20,2,2184.493056
20,2,1985.740000
20,2,1911.346154
20,2,2031.343750
20,2,2558.652174
20,2,4018.246324
20,2,1634.986111
20,2,3712.166667
20,2,8026.021875
20,2,1898.040000
20,2,2714.581019
20,2,2704.887640
20,2,1866.180851
20,2,4006.939189
20,2,2642.492188
20,2,1597.500000
20,2,1543.900568
20,2,3068.930921
20,2,2599.843750
20,2,2065.294643
20,2,1598.100610
20,2,1794.801829
20,2,5376.300000
20,2,3243.609375
20,2,1801.810811
20,2,1889.584821
20,2,1736.482143
20,2,2565.776042
20,2,1897.315972
20,2,1573.508333
20,2,1997.023952
20,2,3294.493421
20,2,1625.127273
20,2,2291.754717
20,2,2483.022727
20,2,2539.010417
20,2,1799.800000
20,2,5366.944444
20,2,2265.000000
20,2,1540.244444
20,2,1687.796053
20,2,2137.875000
20,2,6561.081081
20,2,1803.820122
20,2,1743.935976
20,2,1749.346939
20,2,1551.762500

20,2,2882.044118
20,2,2295.175000
20,2,3582.958333
20,2,2092.461364
20,2,1759.215116
20,2,1879.513158
20,2,1745.826531
20,2,2538.853659
20,2,2097.151596
20,2,2130.115385
20,2,4064.357143
20,2,2518.447115
20,2,1787.904762
20,2,2297.255682
20,2,1602.600000
20,2,2205.846154
20,2,1567.732143
20,2,2185.268519
20,2,2483.512195
20,2,1812.712209
20,2,1806.925676
20,2,1812.975490
20,2,2068.939189
20,2,5320.698276
20,2,1531.021739
20,2,2773.687500
20,2,2227.878125
20,2,4614.615854
20,2,2595.178571
20,2,1507.138889
20,2,3432.664063
20,2,1525.542500
20,2,2869.103774
20,2,3643.571429
20,2,2911.614583
20,2,1626.089844
20,2,1601.366848
20,2,1874.786585
20,2,2878.000000
20,2,1742.416667
20,2,1767.962963
20,2,2392.109756
20,2,9007.635000
20,2,1590.724138
20,2,1926.760204
20,2,2025.250000
20,2,3798.331250
20,2,1615.051136
20,2,2335.815789
20,2,2515.972826
20,2,1558.227273
20,2,1695.666667
20,2,1855.664894
20,2,3376.579268
20,2,4725.375000
20,2,4995.247951
20,2,2007.800000
20,2,1803.307432
20,2,1760.965909
20,2,1989.602273
20,2,1840.828125
20,2,3381.294118
20,2,2909.630814
20,2,2885.307292
20,2,2070.155556
20,2,2139.978723

20,2,1595.629310
20,2,3650.272727
20,2,1647.394444
20,2,1500.987500
20,2,3941.273810
20,2,1707.316860
20,2,1845.434524
20,2,6085.926136
20,2,4204.795455
20,2,17970.696809
20,2,2870.387500
20,2,2483.635870
20,2,1558.129167
20,2,1866.446759
20,2,1802.757813
20,2,3348.066667
20,2,2190.802326
20,2,2513.961538
20,2,1740.444444
20,2,2968.236111
20,2,2227.511574
20,2,2365.570755
20,2,3794.869792
20,2,2429.640625
20,2,12930.571809
20,2,1884.801020
20,2,2241.743056
20,2,1829.505952
20,2,1890.095588
20,2,1672.750000
20,2,1811.800000
20,2,1945.303922
20,2,1562.519444
20,2,1902.861979
20,2,1846.990385
20,2,2499.096491
20,2,3397.117647
20,2,4006.210526
20,2,2648.904255
20,2,2076.147727
20,2,3297.460938
20,2,2078.875000
20,2,2448.618644
20,2,1954.122449
20,2,1933.119565
20,2,1827.910000
20,2,3963.664216
20,2,1834.382653
20,2,2002.175000
20,2,2461.625000
20,2,2419.010204
20,2,1570.118056
20,2,1564.973684
20,2,4043.227545
20,2,1612.694175
20,2,1914.803191
20,2,2107.183962
20,2,2434.803922
20,2,2771.884146
20,2,2183.882353
20,2,1899.133333
20,2,2274.235294
20,2,2108.000000
20,2,1834.255814
20,2,1647.771739
20,2,2123.370370

20,2,2086.980114
20,2,2124.181818
20,2,6418.666667
20,2,1834.651163
20,2,1518.250000
20,2,2932.093750
20,2,7114.125000
20,2,2574.313953
20,2,2923.333333
20,2,4452.077206
20,2,1832.804688
20,2,3257.647727
20,2,3133.576220
20,2,1648.442708
20,2,2123.455882
20,2,14769.163043
20,2,1909.791667
20,2,2665.716981
20,2,4151.005000
20,2,2133.289634
20,2,2862.277174
20,2,2334.788462
20,2,5796.545455
20,2,3621.969388
20,2,1753.092391
20,2,2005.970000
20,2,1750.750000
20,2,2029.322115
20,2,2348.312500
20,2,3013.388889
20,2,6201.581395
20,2,1680.915094
20,2,2082.332636
20,2,1616.111111
20,2,4539.117021
20,2,2240.744898
20,2,2651.790541
20,2,1981.244898
20,2,1904.737745
20,2,1570.808511
20,2,2646.372093
20,2,1639.697500
20,2,3458.923913
20,2,1541.275862
20,2,1825.541667
20,2,1615.818182
20,2,2046.850000
20,2,1825.576923
20,2,2197.323529
20,2,1759.680851
20,2,3458.734694
20,2,1869.345000
20,2,2482.405000
20,2,2166.158333
20,2,1998.122340
20,2,1719.548913
20,2,2424.389205
20,2,2198.093750
20,2,2038.551020
20,2,1611.012195
20,2,2899.790865
20,2,2575.850000
20,2,4527.695652
20,2,3904.970588
20,2,2108.812500
20,2,2116.750000

20,2,1769.934783
20,2,1589.875000
20,2,1901.052632
20,2,2189.755814
20,2,2402.120000
20,2,1589.367021
20,2,2916.345000
20,2,5039.656250
20,2,2796.108796
20,2,5412.352273
20,2,1771.202830
20,2,2415.109043
20,2,2475.000000
20,2,1572.380000
20,2,1867.255435
20,2,7510.203431
20,2,5096.125000
20,2,1594.013158
20,2,3660.884615
20,2,2247.786765
20,2,1700.394231
20,2,1932.109091
20,2,3102.945122
20,2,1506.062500
20,2,2019.152778
20,2,3889.187500
20,2,1696.328947
20,2,1805.000000
20,2,1558.576923
20,2,3088.260638
20,2,1792.997642
20,2,6392.875000
20,2,3522.429487
20,2,2135.302326
20,2,1652.934783
20,2,5362.142045
20,2,1622.510870
20,2,2465.131250
20,2,1811.244318
20,2,2532.018182
20,2,2078.375000
20,2,4819.663043
20,2,5915.127660
20,2,3612.925481
20,2,2325.557143
20,2,2741.505319
20,2,3269.331731
20,2,4924.511628
20,2,3464.108333
20,2,1952.550595
20,2,1532.971354
20,2,5676.320755
20,2,1560.010638
20,2,1815.336957
20,2,2460.677966
20,2,1941.125000
20,2,2475.050000
20,2,1646.017045
20,2,2895.409091
20,2,2863.220588
20,2,3392.434375
20,2,2296.908537
20,2,4095.105114
20,2,1888.018519
20,2,2271.285377
20,2,2294.348558

20,2,1523.082237
20,2,1784.062500
20,2,2369.009804
20,2,2542.712719
20,2,1613.078431
20,2,3684.042969
20,2,2188.175532
20,2,1935.793478
20,2,1544.025000
20,2,2709.524510
20,2,1783.409091
20,2,6459.000000
20,2,1564.750000
20,2,1685.828125
20,2,2417.607143
20,2,2051.485294
20,2,1847.810897
20,2,2439.006944
20,2,5797.500000
20,2,1675.041667
20,2,2002.328125
20,2,2370.360119
20,2,5094.698529
20,2,1753.625000
20,2,6224.993243
20,2,1562.907609
20,2,1550.391304
20,2,2138.250000
20,2,1990.408333
20,2,6215.256098
20,2,2014.412500
20,2,7582.000000
20,2,2015.739583
20,2,1562.683673
20,2,3573.756098
20,2,2487.535714
20,2,3504.021277
20,2,1884.986111
20,2,3806.000000
20,2,1593.024306
20,2,1518.679687
20,2,1632.461538
20,2,2253.618421
20,2,1604.600000
20,2,1578.214286
20,2,1595.611702
20,2,3268.750000
20,2,5244.485714
20,2,3014.587500
20,2,2242.208333
20,2,1742.305921
20,2,1528.489362
20,2,1578.297297
20,2,3151.583333
20,2,1683.975000
20,2,2004.800000
20,2,2878.938889
20,2,1816.013089
20,2,3002.650641
20,2,1836.590625
20,2,2624.218750
20,2,3601.562500
20,2,1966.191964
20,2,2481.173913
20,2,2371.517857
20,2,1972.741379

20,2,1982.650000
20,2,1596.833333
20,2,1640.375000
20,2,1826.276596
20,2,1529.635135
20,2,4160.433824
20,2,2110.000000
20,2,1625.392857
20,2,1928.493056
20,2,1633.961207
20,2,4238.562500
20,2,2848.973214
20,2,2391.270000
20,2,1660.473684
20,2,2250.833333
20,2,1747.491071
20,2,2544.400000
20,2,2140.254545
20,2,1712.053191
20,2,1846.931818
20,2,1799.657895
20,2,2109.470000
20,2,3066.666667
20,2,2187.400000
20,2,1707.661836
20,2,2186.250000
20,2,1555.786585
20,2,1704.493421
20,2,1931.642857
20,2,1640.359375
20,2,3004.040816
20,2,1711.461806
20,2,2887.393939
20,2,2575.087209
20,2,1828.514706
20,2,2128.901163
20,2,1845.325472
20,2,1511.617021
20,2,2365.264706
20,2,3803.080189
20,2,5012.790179
20,2,5335.997340
20,2,1627.000000
20,2,1567.809859
20,2,2212.630000
20,2,1617.509615
20,2,2159.758621
20,2,1620.631250
20,2,1691.636111
20,2,2915.992500
20,2,1653.750000
20,2,2129.598837
20,2,1758.932692
20,2,2380.735294
20,2,3169.100000
20,2,4587.769231
20,2,1701.952381
20,2,1739.743243
20,2,2547.512821
20,2,1636.585714
20,2,1518.959302
20,2,2190.844512
20,2,3097.856383
20,2,3478.637255
20,2,3014.495968
20,2,1586.481250

20,2,2681.425150
20,2,2630.945161
20,2,3682.500000
20,2,2002.583333
20,2,2361.397959
20,2,1782.183824
20,2,8132.568182
20,2,1666.673611
20,2,3138.513514
20,2,6763.668367
20,2,1533.381579
20,2,1661.476190
20,2,5028.828125
20,2,3020.333333
20,2,1607.917763
20,2,1938.655405
20,2,1630.991477
20,2,1855.566176
20,2,2689.666667
20,2,2367.125000
20,2,2603.470588
20,2,2596.153846
20,2,2538.963235
20,2,2066.414062
20,2,3759.200000
20,2,2839.241228
20,2,1746.884868
20,2,1689.337500
20,2,2500.173611
20,2,2918.758333
20,2,2304.803922
20,2,15914.750000
20,2,2357.408163
20,2,1588.018750
20,2,3647.739394
20,2,1549.500000
20,2,2008.058511
20,2,5953.412281
20,2,1826.593750
20,2,5000.506944
20,2,1754.361702
20,2,1956.823171
20,2,2443.144231
20,2,11141.662500
20,2,4615.312500
20,2,1856.051339
20,2,1996.805851
20,2,1916.857143
20,2,2678.509615
20,2,1565.454545
20,2,1687.000000
20,2,1559.086538
20,2,3428.031250
20,2,1781.295732
20,2,2220.025000
20,2,1779.870000
20,2,2329.956250
20,2,1534.333333
20,2,1520.911111
20,2,2023.163265
20,2,1762.712500
20,2,1945.285714
20,2,1865.062500
20,2,2672.138587
20,2,1855.102941
20,2,2308.312500

20,2,2336.391304
20,2,2377.518519
20,2,2335.555556
20,2,1779.310096
20,2,1944.625000
20,2,1541.659574
20,2,2419.036765
20,2,2295.233696
20,2,2244.078431
20,2,2273.728659
20,2,1530.905488
20,2,2276.461538
20,2,1514.653061
20,2,2489.802500
20,2,2137.528846
20,2,1827.053571
20,2,3054.908333
20,2,1792.578947
20,2,3238.687500
20,2,3394.699074
20,2,2704.122449
20,2,1577.715909
20,2,1536.000000
20,2,2272.320122
20,2,1809.041667
20,2,1757.000000
20,2,4058.759259
20,2,2715.068396
20,2,2162.714286
20,2,2095.441810
20,2,1964.875000
20,2,1612.671875
20,2,2683.901042
20,2,3502.506579
20,2,2717.634615
20,2,1956.547170
20,2,1642.750000
20,2,2984.782609
20,2,2052.074074
20,2,1696.354167
20,2,1928.398437
20,2,6293.379310
20,2,2889.530612
20,2,1729.033333
20,2,3153.799479
20,2,4158.967593
20,2,2991.551020
20,2,3463.909574
20,2,1833.560811
20,2,2196.611979
20,2,2525.763158
20,2,1533.269231
20,2,1623.825000
20,2,5017.187500
20,2,2083.435897
20,2,2262.034884
20,2,1978.570513
20,2,2583.042857
20,2,1667.601744
20,2,1948.370000
20,2,1908.666667
20,2,1799.012500
20,2,1893.489796
20,2,2474.794643
20,2,3077.570652
20,2,1554.792614

20,2,2055.108108
20,2,3070.423469
20,2,4701.168478
20,2,3611.937500
20,2,1849.704268
20,2,6122.660156
20,2,1605.488372
20,2,3079.500000
20,2,1980.015244
20,2,2404.541667
20,2,3401.036585
20,2,10337.019231
20,2,1674.875000
20,2,1777.559211
20,2,1679.090909
20,2,1677.028846
20,2,1651.638889
20,2,1968.785256
20,2,1976.414474
20,2,1656.958333
20,2,4831.397059
20,2,2355.397727
20,2,10742.500000
20,2,1897.370000
20,2,1558.945455
20,2,1750.500000
20,2,1793.875000
20,2,2885.135135
20,2,7408.706522
20,2,3582.640625
20,2,1822.703125
20,2,2156.000000
20,2,2116.148438
20,2,2360.658088
20,2,2001.218750
20,2,8811.613636
20,2,1879.850877
20,2,2971.062500
20,2,1789.666667
20,2,2297.187500
20,2,2781.207547
20,2,2343.676471
20,2,1513.937500
20,2,1565.780000
20,2,1717.459677
20,2,2636.400000
20,2,3896.518987
20,2,2446.087500
20,2,2691.718085
20,2,1622.544118
20,2,3089.407609
20,2,2611.297414
20,2,2356.038462
20,2,1548.072917
20,2,2460.495283
20,2,1644.333333
20,2,10272.004902
20,2,2886.848958
20,2,1529.643293
20,2,4703.509146
20,2,2145.982143
20,2,2034.517157
20,2,4557.025641
20,2,4151.129808
20,2,1613.895000
20,2,1577.891304

20,2,2541.714286
20,2,1843.625000
20,2,2609.877660
20,2,2430.310000
20,2,2782.543367
20,2,2485.459239
20,2,2641.706140
20,2,1802.116071
20,2,2206.145161
20,2,2057.893750
20,2,4683.741935
20,2,1745.223214
20,2,5518.029412
20,2,3989.906250
20,2,9196.750000
20,2,1657.777027
20,2,1796.461957
20,2,1848.108333
20,2,3180.516447
20,2,1723.881250
20,2,2429.102941
20,2,1565.444079
20,2,1528.104167
20,2,2857.542683
20,2,2504.446875
20,2,2437.326389
20,2,5463.890957
20,2,2370.046053
20,2,2076.016129
20,2,3436.617925
20,2,1915.884615
20,2,1842.172414
20,2,1705.915000
20,2,2079.187500
20,2,1722.805921
20,2,2092.086538
20,2,1526.875000
20,2,4175.292683
20,2,2077.695122
20,2,1737.500000
20,2,6235.375000
20,2,2470.691489
20,2,1788.843750
20,2,19841.281250
20,2,2026.250000
20,2,10521.052632
20,2,2517.897959
20,2,2876.027778
20,2,2693.000000
20,2,8552.292683
20,2,1584.384058
20,2,1811.045455
20,2,1562.708333
20,2,1915.002358
20,2,7984.261905
20,2,2064.835106
20,2,1861.345588
20,2,1630.734375
20,2,1536.235294
20,2,2229.704082
20,2,1622.034375
20,2,2257.445000
20,2,1947.500000
20,2,2281.319149
20,2,2142.166667
20,2,1869.514286

20,2,1927.757353
20,2,3184.609091
20,2,1524.294118
20,2,1969.511628
20,2,14265.817073
20,2,3057.187500
20,2,1615.846154
20,2,1726.346154
20,2,5818.648585
20,2,1964.000000
20,2,1858.002551
20,2,3251.010417
20,2,1667.633333
20,2,1867.395833
20,2,1499.524390
20,2,1723.875000
20,2,2428.762195
20,2,3004.259868
20,2,3606.007979
20,2,2225.100000
20,2,1886.207386
20,2,1731.692308
20,2,2500.578125
20,2,1707.537037
20,2,6094.300000
20,2,3662.761905
20,2,1620.062500
20,2,1913.750000
20,2,1691.333333
20,2,2877.444444
20,2,1999.982143
20,2,1847.942073
20,2,3178.730769
20,2,11448.277778
20,2,1607.134146
20,2,1712.000000
20,2,5202.312500
20,2,2006.884868
20,2,1687.100000
20,2,3325.692308
20,2,2640.610577
20,2,3008.639535
20,2,2340.294118
20,2,1929.837838
20,2,1610.048913
20,2,2297.331250
20,2,2678.839286
20,2,1710.602273
20,2,1670.825581
20,2,2886.209184
20,2,1515.707317
20,2,2121.275000
20,2,2106.787500
20,2,3139.360000
20,2,3767.925000
20,2,1766.214674
20,2,2284.115385
20,2,1537.604651
20,2,2940.507653
20,2,2127.089674
20,2,3741.080645
20,2,2622.326923
20,2,5909.073370
20,2,2547.443750
20,2,2172.042614
20,2,8002.803571

20,2,2497.007075
20,2,1835.871711
20,2,5407.620000
20,2,4709.418367
20,2,2326.649306
20,2,4080.861111
20,2,2145.690000
20,2,4926.152778
20,2,4144.800000
20,2,1649.667683
20,2,2040.245968
20,2,2303.258065
20,2,2242.000000
20,2,3336.842105
20,2,2046.976562
20,2,1765.625000
20,2,1757.203947
20,2,3758.463816
20,2,2589.566038
20,2,1985.066860
20,2,2679.343750
20,2,4203.525000
20,2,3272.968750
20,2,2337.125000
20,2,2924.450000
20,2,2095.976744
20,2,1561.255924
20,2,1925.237500
20,2,2905.750000
20,2,1552.714286
20,2,3892.795000
20,2,3501.962500
20,2,5062.125000
20,2,2750.000000
20,2,5477.812500
20,2,1704.979592
20,2,1857.661111
20,2,4196.042683
20,2,2071.355263
20,2,1659.420455
20,2,2406.740741
20,2,3238.083333
20,2,1770.828125
20,2,2659.955128
20,2,3098.187500
20,2,1968.755000
20,2,2033.684211
20,2,2290.718750
20,2,2328.645833
20,2,1741.404762
20,2,1559.851562
20,2,1532.538462
20,2,2290.523585
20,2,1881.700000
20,2,3102.556373
20,2,2190.680851
20,2,1573.975446
20,2,3232.694079
20,2,1957.139151
20,2,1837.031250
20,2,2304.922222
20,2,1870.439024
20,2,1704.371429
20,2,1796.880952
20,2,1713.295918
20,2,1822.160714

20,2,1504.973958
20,2,5061.985849
20,2,2176.642857
20,2,3772.540000
20,2,2149.699519
20,2,1663.216667
20,2,1646.062500
20,2,2466.750000
20,2,2948.669118
20,2,2474.875000
20,2,2118.079545
20,2,1545.000000
20,2,1694.477273
20,2,4866.889286
20,2,2140.977564
20,2,2329.305556
20,2,2032.166667
20,2,2561.420213
20,2,1733.772727
20,2,1720.963235
20,2,1816.317308
20,2,1870.119318
20,2,1848.912500
20,2,2107.893617
20,2,2950.714844
20,2,1929.833333
20,2,1622.562500
20,2,3457.166667
20,2,2013.980392
20,2,1547.955556
20,2,1680.859375
20,2,1501.982143
20,2,1781.038462
20,2,2074.450980
20,2,1588.939815
20,2,1614.907609
20,2,1522.640625
20,2,3159.269608
20,2,2781.892045
20,2,1718.489362
20,2,2788.431250
20,2,3722.601974
20,2,2258.397059
20,2,1656.437500
20,2,2297.320000
20,2,2461.719828
20,2,2344.849138
20,2,2322.851852
20,2,2002.368902
20,2,2550.346154
20,2,1780.958333
20,2,1642.392857
20,2,5200.100000
20,2,1737.250000
20,2,2475.840686
20,2,14756.338710
20,2,3758.928571
20,2,1615.756757
20,2,2513.503289
20,2,4554.288043
20,2,2482.619565
20,2,4891.530093
20,2,1808.200000
20,2,4440.586735
20,2,2473.674242
20,2,3094.521739

20,2,2363.972973
20,2,1500.985294
20,2,1953.128205
20,2,3227.078652
20,2,1981.369318
20,2,1599.365385
20,2,1504.000000
20,2,2188.137500
20,2,2187.577381
20,2,1707.791667
20,2,2399.962500
20,2,2502.000000
20,2,3464.465909
20,2,1689.457237
20,2,5903.383721
20,2,1543.439024
20,2,1901.724432
20,2,1593.134615
20,2,1688.412791
20,2,1513.875000
20,2,2654.227273
20,2,2298.195652
20,2,3092.000000
20,2,3076.788265
20,2,5813.574468
20,2,7751.540698
20,2,5695.227041
20,2,1873.226415
20,2,1651.775641
20,2,2568.250000
20,2,3805.000000
20,2,1842.785714
20,2,2902.333333
20,2,1515.032609
20,2,1536.487805
20,2,1594.691176
20,2,3031.757813
20,2,2263.081081
20,2,2531.051282
20,2,2448.554245
20,2,1897.412879
20,2,2359.465000
20,2,2098.473958
20,2,2012.227273
20,2,1527.143617
20,2,5732.227778
20,2,1810.073171
20,2,2629.410714
20,2,4595.586735
20,2,2368.284091
20,2,2021.150000
20,2,1553.842105
20,2,1653.141667
20,2,1510.454268
20,2,1561.400000
20,2,1626.378125
20,2,2103.215909
20,2,2072.145833
20,2,2356.412500
20,2,2213.239583
20,2,1503.035714
20,2,2226.000000
20,2,1605.957447
20,2,3722.260776
20,2,2319.012887
20,2,1521.000000

20,2,2764.107143
20,2,2105.250000
20,2,1560.504237
20,2,2739.264706
20,2,1861.549342
20,2,2378.846939
20,2,1670.818878
20,2,2093.800000
20,2,1763.968750
20,2,2548.451389
20,2,1889.375000
20,2,2433.312500
20,2,2454.000000
20,2,3455.320455
20,2,5354.250000
20,2,1987.035714
20,2,1502.730769
20,2,2275.662234
20,2,1726.731250
20,2,3004.090625
20,2,2493.267857
20,2,3889.271277
20,2,2706.414894
20,2,3686.076531
20,2,1536.976190
20,2,1715.447115
20,2,1594.179825
20,2,2748.511628
20,2,1534.153846
20,2,1742.670455
20,2,2674.806818
20,2,2461.750000
20,2,1624.200000
20,2,4488.127907
20,2,3644.781977
20,2,2081.615625
20,2,1884.595745
20,2,2200.360294
20,2,1802.133333
20,2,3012.960526
20,2,1968.957317
20,2,3486.152778
20,2,1595.547619
20,2,3066.973684
20,2,2674.187500
20,2,1640.770161
20,2,2384.961538
20,2,1668.260417
20,2,1695.835938
20,2,4971.454545
20,2,1891.750000
20,2,2408.687500
20,2,1784.701220
20,2,3282.671429
20,2,1995.882653
20,2,1574.707317
20,2,1824.039062
20,2,1718.377717
20,2,2728.777778
20,2,1541.352941
20,2,1687.868421
20,2,2510.571875
20,2,1635.000000
20,2,1918.041667
20,2,4601.526786
20,2,1891.822917

20,2,1703.325000
20,2,2591.819444
20,2,2654.577586
20,2,1638.848958
20,2,2074.958333
20,2,4122.896552
20,2,4755.158537
20,2,1553.291667
20,2,7058.992857
20,2,1584.042553
20,2,1512.147059
20,2,2097.262755
20,2,1655.708333
20,2,1505.635762
20,2,2063.262195
20,2,2892.971429
20,2,1530.386364
20,2,5146.221154
20,2,1941.017857
20,2,3021.666667
20,2,1949.194767
20,2,5463.750000
20,2,1945.587500
20,2,1854.515957
20,2,1546.656250
20,2,3529.039216
20,2,1823.632653
20,2,1589.200000
20,2,2382.489583
20,2,2009.848837
20,2,1804.282895
20,2,3443.280488
20,2,1620.588889
20,2,2658.846154
20,2,1503.047872
20,2,1584.617188
20,2,2139.766304
20,2,2344.723958
20,2,2024.250000
20,2,2576.446429
20,2,1506.023026
20,2,2163.844595
20,2,1559.279412
20,2,2869.566176
20,2,2437.045455
20,2,3860.472500
20,2,1885.291667
20,2,2219.427083
20,2,2557.902778
20,2,3961.885417
20,2,1634.340909
20,2,1592.434375
20,2,4072.416667
20,2,3821.400000
20,2,1796.900000
20,2,1514.950000
20,2,1688.954787
20,2,1545.871324
20,2,2308.838942
20,2,3655.478723
20,2,2344.820122
20,2,1588.187500
20,2,1530.083333
20,2,1720.205729
20,2,2002.478261
20,2,1642.551020

20,2,1857.500000
20,2,3475.181818
20,2,1521.575000
20,2,1958.188830
20,2,4003.000000
20,2,1834.362500
20,2,1537.744186
20,2,1658.261161
20,2,2986.966292
20,2,2214.220000
20,2,2009.859375
20,2,3191.309524
20,2,1883.707317
20,2,2779.230769
20,2,1667.500000
20,2,1566.083333
20,2,2516.479167
20,2,2016.681548
20,2,1602.174342
20,2,2543.825000
20,2,2011.377778
20,2,1672.352564
20,2,1950.330189
20,2,2390.150000
20,2,2689.553191
20,2,2860.010870
20,2,1919.435811
20,2,2412.740741
20,2,2382.560811
20,2,1960.166667
20,2,1645.513158
20,2,7619.187500
20,2,8031.523256
20,2,5040.000000
20,2,1771.607143
20,2,2673.517857
20,2,2441.928922
20,2,1752.148936
20,2,2086.500000
20,2,2095.348684
20,2,1791.913580
20,2,1622.822917
20,2,3463.766667
20,2,12366.527778
20,2,1610.208333
20,2,1976.090909
20,2,1862.211957
20,2,1946.419872
20,2,1651.488889
20,2,1678.773026
20,2,1662.350000
20,2,2121.021429
20,2,1918.037791
20,2,1864.625000
20,2,1672.274194
20,2,1863.687500
20,2,1821.704268
20,2,2461.593750
20,2,1617.452703
20,2,1623.569444
20,2,4302.075000
20,2,1550.937500
20,2,1684.930921
20,2,3005.664773
20,2,2016.348404
20,2,1754.914286

20,2,1558.375000
20,2,3228.054545
20,2,1677.750000
20,2,2672.989796
20,2,1812.900568
20,2,1627.946429
20,2,3313.492021
20,2,1768.906250
20,2,1686.690789
20,2,3067.367647
20,2,1897.888889
20,2,3343.058824
20,2,2445.240000
20,2,2144.617021
20,2,2290.941327
20,2,1759.263889
20,2,1607.530488
20,2,3637.039216
20,2,2621.750000
20,2,3457.875000
20,2,2357.208333
20,2,1613.994186
20,2,1556.330000
20,2,2015.281250
20,2,1767.960106
20,2,3086.202500
20,2,3138.364796
20,2,2088.141026
20,2,4044.531915
20,2,2450.978723
20,2,3671.712500
20,2,1612.010417
20,2,11765.367347
20,2,1570.666667
20,2,2021.946429
20,2,2636.677083
20,2,7178.666667
20,2,2401.823529
20,2,1975.734694
20,2,2040.226351
20,2,2603.947917
20,2,3015.257653
20,2,2427.000000
20,2,3045.500000
20,2,3765.663043
20,2,1947.227273
20,2,12726.510135
20,2,1695.730769
20,2,1511.125000
20,2,1538.188889
20,2,8262.526316
20,2,1602.531250
20,2,1832.414634
20,2,1581.890625
20,2,2755.750000
20,2,1921.143750
20,2,2139.753049
20,2,1696.645833
20,2,2480.906736
20,2,1582.500000
20,2,3470.917763
20,2,2312.601351
20,2,1644.789773
20,2,1657.823980
20,2,1544.625000
20,2,5779.818182

20,2,1727.784091
20,2,1708.937500
20,2,6678.403846
20,2,1585.791667
20,2,2224.070122
20,2,4732.034722
20,2,5072.055851
20,2,1635.190909
20,2,1881.032609
20,2,1865.544643
20,2,1649.857558
20,2,3256.675439
20,2,2751.256250
20,2,2284.500000
20,2,2723.350543
20,2,1519.530000
20,2,2551.042553
20,2,1537.750000
20,2,2058.125000
20,2,3218.921875
20,2,2164.625000
20,2,1843.398438
20,2,1733.625000
20,2,2234.140909
20,2,1732.855469
20,2,2188.500000
20,2,5901.528571
20,2,2728.957237
20,2,1855.055556
20,2,2398.609756
20,2,2805.600000
20,2,1935.133333
20,2,5488.210526
20,2,4965.968750
20,2,4335.125000
20,2,2910.988636
20,2,1516.765957
20,2,2441.817797
20,2,1977.375000
20,2,11596.266304
20,2,2807.051471
20,2,3789.500000
20,2,1924.693182
20,2,1749.063636
20,2,5464.525000
20,2,2376.484375
20,2,1503.914216
20,2,1687.488095
20,2,1673.446023
20,2,1616.358491
20,2,2718.507353
20,2,1972.455729
20,2,1923.610465
20,2,1878.125000
20,2,4337.815476
20,2,1695.287234
20,2,2403.222826
20,2,4877.170455
20,2,2355.772727
20,2,2247.534884
20,2,2327.428571
20,2,1501.360577
20,2,2934.900000
20,2,3051.230000
20,2,1754.232843
20,2,1702.323529

20,2,1600.181122
20,2,2685.776163
20,2,1858.522436
20,2,2397.414894
20,2,1563.627907
20,2,2400.608696
20,2,1672.588942
20,2,3106.323529
20,2,4616.044503
20,2,5079.485577
20,2,1841.937500
20,2,3350.355263
20,2,4675.104167
20,2,1968.208333
20,2,9134.156250
20,2,2409.300000
20,2,1499.914894
20,2,2100.010135
20,2,1983.145695
20,2,1975.153846
20,2,1567.055556
20,2,1746.755556
20,2,1652.725610
20,2,2498.647059
20,2,3657.487179
20,2,6674.263158
20,2,2288.793103
20,2,3764.244505
20,2,2426.065217
20,2,2970.627660
20,2,3096.172170
20,2,2670.901786
20,2,2153.250000
20,2,1665.845745
20,2,2835.512195
20,2,2593.651786
20,2,4146.521875
20,2,2253.480263
20,2,1504.213415
20,2,1565.690217
20,2,1590.437500
20,2,4139.023026
20,2,1751.537791
20,2,3369.875000
20,2,1769.960937
20,2,2638.986111
20,2,1640.705556
20,2,2335.590000
20,2,2132.315476
20,2,3813.044444
20,2,1653.500000
20,2,3502.190476
20,2,1913.897059
20,2,1978.867925
20,2,2103.216981
20,2,1943.125000
20,2,2138.937500
20,2,2043.415698
20,2,4152.821429
20,2,2763.701220
20,2,1983.812500
20,2,1634.293103
20,2,2238.671875
20,2,1614.836538
20,2,1617.793367
20,2,2028.493590

20,2,3377.289062
20,2,2894.446429
20,2,1563.000000
20,2,8015.025641
20,2,2078.744565
20,2,1866.090426
20,2,1795.437500
20,2,2162.716346
20,2,1881.000000
20,2,2749.359043
20,2,1902.602941
20,2,1565.116279
20,2,1879.260204
20,2,2169.109375
20,2,1622.921875
20,2,2261.892857
20,2,1573.052500
20,2,2517.606383
20,2,1729.494872
20,2,1741.610465
20,2,2627.137255
20,2,4680.455556
20,2,3230.500000
20,2,1761.377717
20,2,2027.283333
20,2,1944.441860
20,2,2086.562500
20,2,1962.515244
20,2,1608.000000
20,2,1859.322727
20,2,2277.421053
20,2,2079.360000
20,2,1501.354167
20,2,1637.312500
20,2,1683.188889
20,2,2395.333333
20,2,2189.333333
20,2,1915.785714
20,2,2910.818182
20,2,2042.464286
20,2,1703.583333
20,2,3791.627907
20,2,1573.728814
20,2,3379.699324
20,2,3640.607143
20,2,1960.793478
20,2,1708.200000
20,2,3592.000000
20,2,2026.176724
20,2,3312.387255
20,2,1851.705128
21,1,17880.975610
21,1,2950.285714
21,1,1499.243243
21,1,1909.780488
21,1,2380.125000
21,1,19579.574468
21,1,2033.722826
21,1,5623.536585
21,1,1995.426136
21,1,16399.872685
21,1,7168.923913
21,1,2084.861111
21,1,4162.078947
21,1,1733.357143
21,1,10183.736413

21,1,1552.420732
21,1,17309.000000
21,1,2387.172297
21,1,3697.253289
21,1,3759.078947
21,1,2303.900000
21,1,4605.167683
21,1,4556.403846
21,1,2896.526316
21,1,7594.236486
21,1,2037.929348
21,1,14196.731707
21,1,4088.813953
21,1,2542.959559
21,1,2890.718750
21,1,12644.000000
21,1,2107.705882
21,1,1723.333333
21,1,1724.791667
21,1,2616.267442
21,1,1623.005435
21,1,1551.664474
21,1,4258.472656
21,1,17000.185185
21,1,4170.923077
21,1,1872.801136
21,1,6283.060714
21,1,2947.677885
21,1,3543.310811
21,1,2315.273438
21,1,2206.810976
21,1,2279.685000
21,1,4313.632812
21,1,11154.085366
21,1,1577.147727
21,1,1506.095238
21,1,3983.303030
21,1,14174.186047
21,1,11557.161290
21,1,2450.421196
21,1,4857.754032
21,1,1836.097826
21,1,3396.242647
21,1,8854.625000
21,1,3978.500000
21,1,1874.250000
21,1,3271.651515
21,1,4877.769231
21,1,14290.424242
21,1,3364.512195
21,1,3708.216912
21,1,7700.435484
21,1,1884.100000
21,1,1527.089744
21,1,2934.653846
21,1,3859.055556
21,1,6363.653846
21,1,2385.571429
21,1,2446.403846
21,1,10813.875000
21,1,3386.318182
21,1,7785.558824
21,1,6417.986486
21,1,16398.602273
21,1,3695.569853
21,1,2044.183824

21,1,2137.230769
21,1,1950.113971
21,1,1522.242424
21,1,1749.538462
21,1,7464.286585
21,1,4946.381250
21,1,2073.716463
21,1,16784.900000
21,1,3348.250000
21,1,1702.554688
21,1,6589.937500
21,1,4795.071429
21,1,2873.182432
21,1,2094.114286
21,1,3728.000000
21,1,7241.775735
21,1,8425.319444
21,1,18663.256944
21,1,4031.000000
21,1,1763.226190
21,1,5659.809211
21,1,1619.150000
21,1,3096.037037
21,1,3103.072368
21,1,1657.762500
21,1,2019.091146
21,1,8941.190789
21,1,2007.774194
21,1,13988.976190
21,1,5596.803922
21,1,5410.444444
21,1,1836.678977
21,1,2944.427632
21,1,1927.278125
21,1,4640.026042
21,1,2257.714286
21,1,14504.523438
21,1,4298.728571
21,1,16951.321429
21,1,2469.923780
21,1,18451.556250
21,1,2450.381579
21,1,3805.279412
21,1,3273.136364
21,1,6368.040541
21,1,8070.646552
21,1,7550.610465
21,1,6217.678571
21,1,14622.111111
21,1,4888.500000
21,1,1676.840909
21,1,10506.606481
21,1,2166.110577
21,1,8761.266667
21,1,10193.250000
21,1,3511.977564
21,1,2760.372449
21,1,18307.465116
21,1,2237.000000
21,1,1642.329268
21,1,2552.738372
21,1,3266.875000
21,1,2557.363636
21,1,1879.768382
21,1,14274.863636
21,1,3771.250000

21,1,2884.951923
21,1,2993.090426
21,1,8425.163522
21,1,7077.797794
21,1,14227.421053
21,1,9280.187500
21,1,2588.090909
21,1,15167.105000
21,1,10756.921875
21,1,6575.806452
21,1,1512.889205
21,1,1756.583333
21,1,3102.291667
21,1,8198.318548
21,1,2248.040441
21,1,2608.785714
21,1,13463.301136
21,1,2075.750000
21,1,7325.244681
21,1,1847.852941
21,1,4408.744681
21,1,2119.380682
21,1,4870.766304
21,1,6355.536765
21,1,3665.286458
21,1,12297.270000
21,1,6967.637821
21,1,6676.878289
21,1,5484.486486
21,1,3169.463415
21,1,2383.065217
21,1,2324.812500
21,1,2016.454545
21,1,2927.477273
21,1,9319.886364
21,1,4472.115625
21,1,11725.897059
21,1,2154.035714
21,1,19564.189189
21,1,1885.183333
21,1,2330.181818
21,1,2761.383929
21,1,17187.800000
21,1,2385.445000
21,1,3422.368590
21,1,4438.531250
21,1,2921.714286
21,1,5127.996429
21,1,12738.000000
21,1,3303.545455
21,1,2616.130952
21,1,4030.534091
21,1,8994.770833
21,1,6765.266667
21,1,6200.765625
21,1,6858.915761
21,1,4639.369048
21,1,2107.363971
21,1,4702.125000
21,1,3983.459459
21,1,3905.347826
21,1,1649.687500
21,1,3953.690789
21,1,5500.656250
21,1,4527.704545
21,1,15733.312500

21,1,1976.000000
21,1,1720.570000
21,1,7416.725543
21,1,1516.125000
21,1,2250.360000
21,1,8011.846154
21,1,15858.684322
21,1,4409.039474
21,1,9989.500000
21,1,2067.061170
21,1,1503.358696
21,1,6561.838235
21,1,3239.585366
21,1,3746.441667
21,1,1587.068182
21,1,8025.666667
21,1,2286.300000
21,1,4913.500000
21,1,2150.052632
21,1,5081.500000
21,1,7866.882353
21,1,17676.139073
21,1,3105.673077
21,1,1871.011628
21,1,6999.696133
21,1,7811.888636
21,1,2169.945122
21,1,11735.857143
21,1,3545.608108
21,1,9091.132353
21,1,9462.773026
21,1,4746.456522
21,1,14197.147059
21,1,4318.342857
21,1,2345.638889
21,1,16505.107143
21,1,1785.791667
21,1,1659.436782
21,1,1773.655914
21,1,2360.875000
21,1,2494.000000
21,1,5658.733553
21,1,1566.832143
21,1,2383.957386
21,1,1691.761364
21,1,4975.486842
21,1,2751.937500
21,1,9937.937500
21,1,4442.458333
21,1,2394.170455
21,1,1912.169872
21,1,10380.111702
21,1,2642.208333
21,1,3601.636364
21,1,2268.135135
21,1,1686.941176
21,1,13164.138587
21,1,10923.975610
21,1,2281.411765
21,1,3162.222656
21,1,2215.904070
21,1,3348.127551
21,1,2001.065909
21,1,3534.500000
21,1,18109.071970
21,1,7187.088710

21,1,1526.604651
21,1,3405.732143
21,1,14478.846154
21,1,3156.777778
21,1,2053.083333
21,1,6971.375000
21,1,1665.648936
21,1,4466.673913
21,1,4317.628378
21,1,6750.632353
21,1,1594.628205
21,1,1822.375000
21,1,4673.315217
21,1,4700.446429
21,1,1516.118421
21,1,4274.388889
21,1,2380.676136
21,1,2992.529605
21,1,1593.813953
21,1,8335.375000
21,1,8152.173077
21,1,17130.416667
21,1,1874.993243
21,1,1545.209459
21,1,4463.164062
21,1,2863.318182
21,1,2304.906250
21,1,1499.580645
21,1,6908.790698
21,1,2358.632653
21,1,2826.949405
21,1,15520.613095
21,1,2582.083333
21,1,7109.411111
21,1,11206.781250
21,1,5573.815972
21,1,7773.718750
21,1,2444.613636
21,1,5276.978659
21,1,3981.718750
21,1,2418.215278
21,1,1707.375000
21,1,2184.282609
21,1,10284.205882
21,1,6418.103571
21,1,12819.730769
21,1,13649.382979
21,1,3509.986702
21,1,7560.242647
21,1,15027.222222
21,1,3849.988636
21,1,7252.212500
21,1,1963.803977
21,1,5660.229167
21,1,3499.184896
21,1,7842.329545
21,1,2504.437500
21,1,1655.936170
21,1,1710.945946
21,1,1920.111702
21,1,3381.060000
21,1,2167.210526
21,1,2128.113636
21,1,6487.571429
21,1,2893.931250
21,1,5510.287791

21,1,1791.769231
21,1,2803.253472
21,1,7541.548780
21,1,4146.170213
21,1,4400.050000
21,1,2268.284091
21,1,2129.875000
21,1,1540.659091
21,1,4163.272727
21,1,3013.937500
21,1,11239.375000
21,1,5560.615385
21,1,2760.698980
21,1,10815.437500
21,1,3857.500000
21,1,1566.173077
21,1,4414.131579
21,1,1732.106383
21,1,1585.779412
21,1,5544.472561
21,1,10826.244565
21,1,3739.245399
21,1,4056.116848
21,1,2202.343750
21,1,3925.100000
21,1,8199.732759
21,1,7095.539062
21,1,3690.349432
21,1,8886.172414
21,1,1856.218750
21,1,13113.278571
21,1,13871.677419
21,1,6116.392045
21,1,1940.750000
21,1,6218.406250
21,1,1797.209559
21,1,1754.692308
21,1,2260.546875
21,1,1628.377358
21,1,13518.750000
21,1,4070.692308
21,1,4246.314516
21,1,17831.401786
21,1,5147.239865
21,1,6545.935811
21,1,4208.058824
21,1,4665.703125
21,1,14964.689655
21,1,6165.927326
21,1,2951.410714
21,1,9482.955882
21,1,2052.328571
21,1,2621.913462
21,1,15003.236842
21,1,1995.187500
21,1,3805.035714
21,1,2587.068452
21,1,6226.096774
21,1,5775.513889
21,1,14210.416667
21,1,1711.280000
21,1,6713.682292
21,1,2938.657143
21,1,16897.757143
21,1,3355.437500
21,1,1908.972222

21,1,1902.000000
21,1,4060.994186
21,1,2705.919118
21,1,2815.291667
21,1,1537.933673
21,1,3113.097222
21,1,15458.937500
21,1,6244.000000
21,1,16298.637500
21,1,3495.425781
21,1,3763.923913
21,1,11926.909091
21,1,2406.326087
21,1,4790.110000
21,1,1884.813830
21,1,1753.672222
21,1,8759.024194
21,1,7125.553191
21,1,3142.041667
21,1,14941.975610
21,1,1536.875000
21,1,3340.090909
21,1,5836.578125
21,1,8303.564024
21,1,4826.645349
21,1,2099.559211
21,1,1761.290625
21,1,13011.837209
21,1,3087.182927
21,1,5147.887195
21,1,2795.000000
21,1,2794.218750
21,1,7287.632353
21,1,1670.736979
21,1,2055.090909
21,1,2724.139535
21,1,1796.983871
21,1,16004.719595
21,1,2380.019231
21,1,3980.775000
21,1,4225.829787
21,1,1924.955729
21,1,1715.636364
21,1,13481.323980
21,1,1521.029412
21,1,1627.187500
21,1,3576.443182
21,1,2585.038194
21,1,2361.289474
21,1,2418.030303
21,1,8946.820946
21,1,2359.070312
21,1,3865.989130
21,1,2290.375000
21,1,2499.379464
21,1,2321.726744
21,1,12773.519608
21,1,1617.066176
21,1,3511.661765
21,1,3411.028846
21,1,2965.995833
21,1,10504.285714
21,1,2563.215278
21,1,2454.588235
21,1,3317.715116
21,1,2445.762500

21,1,4160.871324
21,1,4005.578947
21,1,2160.610795
21,1,1819.953488
21,1,2577.982558
21,1,4446.200521
21,1,6294.928191
21,1,1613.709677
21,1,6088.116071
21,1,2263.300000
21,1,8252.500000
21,1,2261.071875
21,1,2260.336310
21,1,2604.543919
21,1,12404.838816
21,1,1775.541667
21,1,2973.062500
21,1,2468.352273
21,1,4387.552632
21,1,7785.632353
21,1,1837.868750
21,1,5356.721154
21,1,1968.384615
21,1,4006.586538
21,1,2007.493056
21,1,1812.027500
21,1,5461.454545
21,1,14570.304348
21,1,1507.742188
21,1,6846.977778
21,1,5867.144231
21,1,4477.395833
21,1,6184.861111
21,1,2947.855769
21,1,2889.742857
21,1,1568.338415
21,1,2112.585000
21,1,3603.455357
21,1,4007.817308
21,1,13589.361842
21,1,3972.125000
21,1,10797.000000
21,1,5282.715278
21,1,3107.617647
21,1,4887.955696
21,1,14997.311224
21,1,2232.698718
21,1,3835.484375
21,1,3359.725694
21,1,1930.379032
21,1,2661.288462
21,1,17983.765625
21,1,1517.000000
21,1,10131.479167
21,1,2112.937500
21,1,2504.425532
21,1,15166.892857
21,1,4355.066667
21,1,3723.840278
21,1,3589.000000
21,1,8210.320652
21,1,12974.000000
21,1,2072.745192
21,1,4234.125000
21,1,1577.857955
21,1,2635.545455

21,1,1633.600000
21,1,2744.108173
21,1,6204.154255
21,1,5046.178571
21,1,1707.372159
21,1,6611.761628
21,1,7236.901316
21,1,12794.058673
21,1,2768.557692
21,1,15479.193182
21,1,11653.758929
21,1,1538.022727
21,1,1747.824324
21,1,13800.544811
21,1,16193.434783
21,1,2308.839844
21,1,4504.878125
21,1,16095.825758
21,1,4000.082386
21,1,1732.260000
21,1,3778.027500
21,1,2985.954545
21,1,2284.885638
21,1,4842.062500
21,1,3497.750000
21,1,2336.490385
21,1,5270.593137
21,1,2552.700000
21,1,2970.088816
21,1,4374.093750
21,1,4041.107500
21,1,8567.782609
21,1,6122.562500
21,1,17006.877660
21,1,6249.901042
21,1,13592.956897
21,1,5317.444444
21,1,2843.138298
21,1,1987.432692
21,1,3577.600000
21,1,4063.250000
21,1,10224.500000
21,1,4009.454545
21,1,2073.913462
21,1,1551.288889
21,1,18884.458333
21,1,3260.800000
21,1,7654.184211
21,1,3629.894737
21,1,11845.988372
21,1,1574.487179
21,1,11280.465909
21,1,2572.087838
21,1,9389.256410
21,1,1827.403061
21,1,1718.205882
21,1,4268.692857
21,1,12789.080000
21,1,6269.000000
21,1,14907.750000
21,1,14873.477941
21,1,13345.044118
21,1,15591.752841
21,1,3337.679878
21,1,4518.427419
21,1,1620.833333

21,1,2585.904762
21,1,18241.094512
21,1,2566.852941
21,1,2459.405172
21,1,4458.033088
21,1,4406.801020
21,1,4095.593750
21,1,1659.750000
21,1,12464.223214
21,1,12116.031250
21,1,17627.750000
21,1,1577.472826
21,1,10629.637500
21,1,3947.066327
21,1,1993.779605
21,1,1517.419444
21,1,4224.812500
21,1,5987.423077
21,1,7715.229167
21,1,5845.092949
21,1,2060.058824
21,1,14028.456522
21,1,3402.312500
21,1,3837.717262
21,1,3275.516129
21,1,6067.963542
21,1,2613.729167
21,1,7466.807692
21,1,2020.812500
21,1,1901.292683
21,1,1899.741935
21,1,17120.543478
21,1,14507.848214
21,1,6022.867647
21,1,1703.105263
21,1,3218.560976
21,1,5237.400000
21,1,2634.279070
21,1,3925.236702
21,1,2404.308511
21,1,2582.054054
21,1,6563.027027
21,1,5481.709677
21,1,3797.526316
21,1,2121.100806
21,1,1637.761905
21,1,1696.157609
21,1,16933.263889
21,1,15621.976190
21,1,4882.556452
21,1,1738.030303
21,1,3739.800000
21,1,1934.950000
21,1,3515.664474
21,1,2423.613281
21,1,1724.764706
21,1,3112.470588
21,1,5003.583333
21,1,5335.000000
21,1,4092.295213
21,1,7698.125000
21,1,2107.762500
21,1,5293.875000
21,1,5803.632812
21,1,4796.016129
21,1,5424.441176

21,1,1811.884146
21,1,1504.105263
21,1,4739.444444
21,1,3490.635135
21,1,1685.250000
21,1,1815.202703
21,1,6256.420213
21,1,2666.750000
21,1,2949.455357
21,1,11369.770833
21,1,1594.000000
21,1,3332.881250
21,1,3310.468750
21,1,19957.339744
21,1,8434.375000
21,1,6512.822917
21,1,2068.250000
21,1,2928.953488
21,1,3380.777027
21,1,4316.875000
21,1,2026.474359
21,1,8485.158333
21,1,3112.571875
21,1,1508.114130
21,1,15257.406977
21,1,3436.095588
21,1,4656.464744
21,1,5178.462209
21,1,2653.661765
21,1,13510.979167
21,1,12541.986842
21,1,4052.705357
21,1,12919.250000
21,1,1749.643293
21,1,7003.000000
21,1,10797.040441
21,1,1962.250000
21,1,4099.801471
21,1,2090.171875
21,1,1583.766892
21,1,6020.335227
21,1,2751.952381
21,1,1575.160326
21,1,2081.417683
21,1,2865.384615
21,1,1729.631579
21,1,7273.701220
21,1,1856.643939
21,1,1695.090909
21,1,15433.085938
21,1,1935.166667
21,1,1826.842857
21,1,3835.687500
21,1,1799.198171
21,1,1772.926829
21,1,4409.878788
21,1,4649.106618
21,1,3620.307692
21,1,3665.500000
21,1,16456.871094
21,1,4274.323529
21,1,15311.056818
21,1,12307.323171
21,1,2554.509091
21,1,1650.852941
21,1,5665.839286

21,1,7555.288793
21,1,2135.925000
21,1,7751.162500
21,1,9393.375000
21,1,6171.420732
21,1,11295.012195
21,1,1790.003049
21,1,4198.932692
21,1,3390.787879
21,1,5342.544118
21,1,4275.025000
21,1,2137.160156
21,1,2757.723214
21,1,1598.679688
21,1,4241.893617
21,1,1990.939394
21,1,1560.947368
21,1,4508.083333
21,1,6137.295455
21,1,4760.625000
21,1,1783.495968
21,1,1654.038462
21,1,2243.317073
21,1,2434.572368
21,1,3243.309524
21,1,7040.006944
21,1,13334.932292
21,1,2359.921053
21,1,2656.066667
21,1,2961.141026
21,1,18678.444444
21,1,2479.662500
21,1,1952.423077
21,1,1763.200000
21,1,9705.090426
21,1,3468.366071
21,1,1505.593750
21,1,2932.174528
21,1,2082.413793
21,1,3952.674419
21,1,4468.965000
21,1,6367.180000
21,1,4230.888889
21,1,10647.493902
21,1,4125.075758
21,1,16866.660714
21,1,1727.016026
21,1,3574.575000
21,1,19614.588235
21,1,7211.404762
21,1,2574.867857
21,1,2166.000000
21,1,5661.933673
21,1,6401.181818
21,1,2386.734375
21,1,15248.583333
21,1,2711.600000
21,1,2916.410714
21,1,2512.109091
21,1,15470.133333
21,1,1721.284091
21,1,1615.413043
21,1,4961.912500
21,1,8214.000000
21,1,2090.234043
21,1,3657.321429

21,1,7437.384615
21,1,2372.390244
21,1,10835.115385
21,1,3104.312500
21,1,2143.087500
21,1,7529.458333
21,1,6795.012255
21,1,3058.605769
21,1,2435.302326
21,1,1576.923077
21,1,14694.038462
21,1,1866.337500
21,1,15067.125000
21,1,2733.210526
21,1,5281.666667
21,1,4181.285714
21,1,5503.078804
21,1,3633.263514
21,1,9609.200000
21,1,7263.745000
21,1,2972.600000
21,1,3583.216216
21,1,1916.341463
21,1,7098.892857
21,1,1964.375000
21,1,1768.737805
21,1,4863.488372
21,1,16669.838235
21,1,3498.617647
21,1,2401.944444
21,1,2317.714286
21,1,2746.142857
21,1,1704.702381
21,1,2731.496622
21,1,2731.878049
21,1,14731.250000
21,1,1915.081250
21,1,1755.574324
21,1,10705.000000
21,1,5094.448276
21,1,3928.382353
21,1,2022.000000
21,1,4858.708333
21,1,15774.457447
21,1,17223.555921
21,1,3333.242188
21,1,5865.800781
21,1,4182.011111
21,1,3492.062500
21,1,2151.055851
21,1,4098.228261
21,1,7378.771812
21,1,2827.343750
21,1,8301.180000
21,1,4838.131579
21,1,4046.987805
21,1,3717.465909
21,1,1556.025641
21,1,1866.880952
21,1,3639.103659
21,1,2945.625000
21,1,2868.719697
21,1,2078.872024
21,1,1691.990625
21,1,1767.285714
21,1,2244.702703

21,1,1513.266667
21,1,2207.745690
21,1,17869.323718
21,1,3246.960526
21,1,1591.980769
21,1,1522.735294
21,1,4224.959459
21,1,3706.986413
21,1,1739.497340
21,1,8091.556250
21,1,2086.428571
21,1,3583.823529
21,1,11229.416667
21,1,3155.910156
21,1,3342.319149
21,1,9723.602273
21,1,1687.201220
21,1,9749.062500
21,1,2941.875000
21,1,3243.829787
21,1,4221.846154
21,1,1836.538462
21,1,3491.343750
21,1,5971.500000
21,1,18520.789062
21,1,6457.178571
21,1,7713.487805
21,1,2500.898649
21,1,5981.099265
21,1,4234.395833
21,1,5558.317308
21,1,6499.718750
21,1,3081.964286
21,1,18422.558140
21,1,1517.612500
21,1,1787.221429
21,1,6456.973485
21,1,7092.562500
21,1,15724.692308
21,1,2262.058824
21,1,5104.172222
21,1,4360.214286
21,1,3325.060345
21,1,3056.663889
21,1,2104.187500
21,1,2160.422619
21,1,2449.531915
21,1,2234.769231
21,1,1559.243590
21,1,3560.897436
21,1,1984.545732
21,1,2890.702128
21,1,3037.107143
21,1,3697.500000
21,1,4715.428571
21,1,2685.894231
21,1,1651.453125
21,1,12721.596154
21,1,4641.543478
21,1,13922.211310
21,1,1551.128676
21,1,3632.613971
21,1,16056.500000
21,1,1752.647059
21,1,2112.500000
21,1,3161.408654

21,1,6456.428571
21,1,3467.185811
21,1,1873.646341
21,1,3919.557692
21,1,7160.621324
21,1,6195.849359
21,1,3363.375000
21,1,3933.378049
21,1,1726.055147
21,1,6427.243902
21,1,7170.080882
21,1,2907.700658
21,1,3797.030405
21,1,3888.000000
21,1,12595.405303
21,1,1517.807692
21,1,3058.725806
21,1,2283.005319
21,1,12506.681034
21,1,8283.442308
21,1,3059.857143
21,1,4270.454861
21,1,2905.732143
21,1,7784.261905
21,1,5012.534483
21,1,6090.636719
21,1,16894.300000
21,1,8343.000000
21,1,1766.405172
21,1,4910.390625
21,1,3289.625000
21,1,5693.865385
21,1,4858.388889
21,1,7501.459239
21,1,1669.971154
21,1,5914.550000
21,1,3473.765625
21,1,2849.000000
21,1,2469.193182
21,1,10002.906250
21,1,4957.923077
21,1,3723.433824
21,1,11186.500000
21,1,4238.363636
21,1,7347.768293
21,1,2038.750000
21,1,8167.220588
21,1,7017.950893
21,1,4722.545455
21,1,3347.651786
21,1,9297.250000
21,1,15496.334677
21,1,2227.858333
21,1,7113.121951
21,1,2028.647059
21,1,3254.470588
21,1,9733.988636
21,1,4028.646341
21,1,2036.209559
21,1,4995.820513
21,1,9700.718750
21,1,3535.012195
21,1,1521.109375
21,1,13817.734756
21,1,6298.727941
21,1,3997.029412

21,1,4040.906977
21,1,16200.698276
21,1,3604.630952
21,1,2842.153846
21,1,6890.963235
21,1,7098.262500
21,1,10693.992188
21,1,2213.757143
21,1,1923.231707
21,1,9206.892857
21,1,1576.357143
21,1,8923.481707
21,1,1613.740132
21,1,3131.444444
21,1,8061.220588
21,1,4434.321429
21,1,16170.791667
21,1,7673.875000
21,1,1892.968750
21,1,6181.591346
21,1,2540.529412
21,1,8352.634615
21,1,2970.000000
21,1,1942.977941
21,1,4558.454545
21,1,2947.470588
21,1,6048.195513
21,1,2842.794872
21,1,2013.058140
21,1,5411.891892
21,1,8038.764706
21,1,4166.337838
21,1,9712.020833
21,1,1562.446970
21,1,11633.525735
21,1,1677.000000
21,1,8511.388298
21,1,2866.931250
21,1,2742.087500
21,1,1537.687500
21,1,2769.764423
21,1,6632.312500
21,1,10912.504310
21,1,6056.507353
21,1,4203.913462
21,1,15249.468750
21,1,2189.833333
21,1,1757.415323
21,1,6770.325000
21,1,4240.487179
21,1,5193.730769
21,1,4197.303977
21,1,3391.985294
21,1,2854.263158
21,1,1548.678571
21,1,6778.396341
21,1,4532.055556
21,1,2410.339286
21,1,7641.000000
21,1,5737.338235
21,1,4267.256410
21,1,3221.962766
21,1,3328.496951
21,1,2951.421053
21,1,3381.033333
21,1,1633.622222

21,1,5117.696429
21,1,9641.932432
21,1,2456.397436
21,1,4451.166667
21,1,2986.679878
21,1,3627.250000
21,1,4470.755435
21,1,19968.939394
21,1,2413.853659
21,1,1556.147059
21,1,2404.761905
21,1,3434.012500
21,1,7958.736486
21,1,18192.450000
21,1,5812.967742
21,1,5146.000000
21,1,17487.840278
21,1,2095.870370
21,1,3882.423611
21,1,1854.855000
21,1,4490.316176
21,1,5628.886719
21,1,7931.031250
21,1,2250.042683
21,1,10018.203125
21,1,2176.625000
21,1,5661.353125
21,1,1799.145349
21,1,1502.479167
21,1,2777.451389
21,1,3612.375000
21,1,2990.330882
21,1,3544.835227
21,1,5966.585366
21,1,2902.967742
21,1,5237.037500
21,1,1704.472222
21,1,10219.964286
21,1,11377.682927
21,1,1832.297619
21,1,2194.589286
21,1,3210.160714
21,1,9055.639535
21,1,9871.229167
21,1,3059.384615
21,1,1750.027778
21,1,1704.547222
21,1,1821.704268
21,1,2191.804878
21,1,11892.000000
21,1,11118.907738
21,1,1752.625000
21,1,17796.717949
21,1,2912.110294
21,1,2500.473214
21,1,3931.090278
21,1,3088.388298
21,1,2247.141304
21,1,5626.724359
21,1,2376.953488
21,1,5702.913265
21,1,1622.010638
21,1,3087.107955
21,1,1732.596875
21,1,7196.977941
21,1,2300.000000

21,1,4751.252404
21,1,7418.438144
21,1,3193.358696
21,1,5221.196023
21,1,1984.666667
21,1,10905.000000
21,1,1828.632812
21,1,4436.117647
21,1,3047.082500
21,1,1952.787879
21,1,6150.651786
21,1,3726.937500
21,1,4460.484375
21,1,2924.865385
21,1,10289.625000
21,1,1689.694149
21,1,9380.931548
21,1,3404.293478
21,1,3858.250000
21,1,4067.521739
21,1,13349.750000
21,1,18850.000000
21,1,4692.913690
21,1,4312.833333
21,1,5951.532895
21,1,3060.510870
21,1,6694.209677
21,1,5015.731707
21,1,16107.402174
21,1,2545.848039
21,1,3749.734375
21,1,2396.510870
21,1,3316.425000
21,1,9775.867021
21,1,3827.675000
21,1,11627.812500
21,1,12272.175676
21,1,1840.720588
21,1,7200.000000
21,1,7225.793919
21,1,4460.900000
21,1,17565.000000
21,1,2784.866667
21,1,6314.947500
21,1,5648.750000
21,1,3351.575000
21,1,10332.567073
21,1,6718.823529
21,1,2684.678977
21,1,3073.393617
21,1,19500.075000
21,1,2646.250000
21,1,1977.750000
21,1,2773.886364
21,1,2410.472222
21,1,4037.437500
21,1,8696.964286
21,1,2231.669872
21,1,9564.820513
21,1,1718.753049
21,1,7196.610465
21,1,17451.273256
21,1,6720.546196
21,1,2558.211957
21,1,1812.959677
21,1,10578.076613

21,1,3811.550000
21,1,2016.641304
21,1,2237.774194
21,1,2878.203488
21,1,3344.093750
21,1,15180.652027
21,1,5890.821429
21,1,2180.828431
21,1,2824.892857
21,1,1747.470588
21,1,4775.546875
21,1,4913.367347
21,1,1713.601563
21,1,2340.619898
21,1,16227.705882
21,1,9844.120000
21,1,8225.187500
21,1,2528.519231
21,1,4829.467391
21,1,3292.176282
21,1,6898.112069
21,1,18585.899457
21,1,3091.527778
21,1,6129.461538
21,1,9740.771552
21,1,4844.673077
21,1,7747.229167
21,1,1812.561224
21,1,1713.875000
21,1,3867.523810
21,1,3980.057692
21,1,6044.359756
21,1,3645.434375
21,1,3430.421512
21,1,5709.339286
21,1,3883.519231
21,1,5823.200000
21,1,3877.729730
21,1,7413.258523
21,1,2088.984375
21,1,3215.284091
21,1,17583.051282
21,1,8138.764706
21,1,16369.997340
21,1,11691.560897
21,1,2512.008065
21,1,4742.109375
21,1,3040.675000
21,1,8126.073529
21,1,4807.833333
21,1,14629.887255
21,1,1541.985294
21,1,2234.863095
21,1,7668.014706
21,1,10006.857143
21,1,1857.176829
21,1,7800.024510
21,1,2692.810976
21,1,6497.614583
21,1,7317.642361
21,1,1591.149306
21,1,9112.567568
21,1,19106.000000
21,1,3361.727273
21,1,13930.923611
21,1,12080.958333

21,1,1879.388514
21,1,1983.916667
21,1,6119.645161
21,1,4342.375000
21,1,2476.750000
21,1,2368.066667
21,1,2407.750000
21,1,3845.250000
21,1,4878.859375
21,1,3778.359756
21,1,1934.896277
21,1,15613.127660
21,1,2668.059659
21,1,2874.380208
21,1,1829.137255
21,1,4362.547170
21,1,2258.691860
21,1,4682.500000
21,1,7006.025000
21,1,6958.604592
21,1,2597.145833
21,1,3482.219512
21,1,4416.504630
21,1,7618.702128
21,1,1874.234375
21,1,7320.953125
21,1,14713.894231
21,1,9315.914062
21,1,4163.020349
21,1,4714.192308
21,1,6665.219512
21,1,3682.916667
21,1,4566.851064
21,1,2542.540441
21,1,2195.559211
21,1,2244.896552
21,1,1826.209302
21,1,4902.572222
21,1,1536.000000
21,1,15042.746429
21,1,7357.069853
21,1,2342.550000
21,1,1520.815217
21,1,7860.977500
21,1,1711.318182
21,1,12713.622093
21,1,18630.009615
21,1,1802.205128
21,1,2472.461207
21,1,19329.345395
21,1,1817.175532
21,1,2145.731707
21,1,4748.664286
21,1,6379.819767
21,1,3751.496212
21,1,5984.232558
21,1,2308.984043
21,1,2040.846774
21,1,8095.666667
21,1,12971.179688
21,1,5212.145833
21,1,2027.838068
21,1,2720.233108
21,1,1807.966216
21,1,13444.139423
21,1,17061.128049

21,1,14167.730469
21,1,12059.580882
21,1,3179.054054
21,1,4335.072581
21,1,17405.708333
21,1,3822.450000
22,2,8515.612500
22,2,1691.115385
22,2,2027.730159
22,2,2154.866667
22,2,1551.437500
22,2,1534.255102
22,2,1579.448980
22,2,2981.643868
22,2,2956.391304
22,2,2533.107143
22,2,1975.484375
22,2,1535.886905
22,2,2858.565789
22,2,1860.295732
22,2,1540.062500
22,2,1745.364583
22,2,2330.363636
22,2,1869.140244
22,2,2396.298913
22,2,1542.470238
22,2,2754.061224
22,2,7145.085366
22,2,6682.605769
22,2,4458.370370
22,2,2264.029412
22,2,2046.625000
22,2,2041.410000
22,2,1555.421569
22,2,2243.318182
22,2,1638.510638
22,2,2542.471591
22,2,1646.338889
22,2,2282.602500
22,2,2216.083333
22,2,1828.655612
22,2,1689.562500
22,2,1630.795455
22,2,2144.517857
22,2,2585.291667
22,2,4445.419643
22,2,2409.041667
22,2,1504.158654
22,2,1782.316327
22,2,1726.935000
22,2,2460.377778
22,2,2205.622093
22,2,2337.142857
22,2,2975.556034
22,2,5502.079787
22,2,1968.695122
22,2,1799.045455
22,2,3969.480114
22,2,2793.846154
22,2,16579.951220
22,2,2463.781250
22,2,2352.264205
22,2,1701.333333
22,2,5002.786932
22,2,1698.651786
22,2,1543.331731

22,2,4189.011719
22,2,2034.178571
22,2,1926.990741
22,2,1782.380952
22,2,3757.985465
22,2,2103.324519
22,2,1748.895270
22,2,1827.106383
22,2,2043.475000
22,2,2052.520833
22,2,2552.480769
22,2,4359.757576
22,2,1517.513514
22,2,1590.707317
22,2,5932.433673
22,2,2110.971875
22,2,2723.780000
22,2,2536.707447
22,2,2052.443182
22,2,2177.072674
22,2,1660.768293
22,2,1837.920213
22,2,2096.725000
22,2,2283.162162
22,2,1575.884434
22,2,2834.150943
22,2,2105.436275
22,2,2019.755208
22,2,2665.245536
22,2,2120.742925
22,2,3013.931818
22,2,3069.325581
22,2,1773.191860
22,2,3570.538462
22,2,1784.954545
22,2,1707.675595
22,2,2152.804825
22,2,2314.280612
22,2,2081.797619
22,2,1653.927632
22,2,1544.323529
22,2,1779.555556
22,2,10471.515957
22,2,1834.456081
22,2,3167.406250
22,2,2206.058824
22,2,1755.392857
22,2,1621.982143
22,2,3043.858491
22,2,4784.075000
22,2,1556.243243
22,2,1559.132500
22,2,3206.269737
22,2,1811.356771
22,2,2614.750000
22,2,1567.801471
22,2,1816.139881
22,2,1929.940217
22,2,2068.383721
22,2,2457.969388
22,2,3236.329787
22,2,1906.621951
22,2,2870.685185
22,2,7226.617500
22,2,2179.000000
22,2,2163.199468

22,2,1723.691489
22,2,1529.166667
22,2,2231.280000
22,2,1603.335000
22,2,1541.326531
22,2,2190.332447
22,2,11644.448819
22,2,1844.506250
22,2,3483.750000
22,2,1515.317073
22,2,1520.972222
22,2,2049.411458
22,2,2304.230392
22,2,3206.750000
22,2,1615.929687
22,2,1584.773026
22,2,1671.923977
22,2,1671.058140
22,2,1573.780488
22,2,1592.842105
22,2,2045.181159
22,2,1586.997807
22,2,2979.482759
22,2,4165.144231
22,2,5857.769737
22,2,1516.329268
22,2,1768.000000
22,2,1916.787037
22,2,1626.177778
22,2,1720.012500
22,2,2543.400000
22,2,1795.885204
22,2,3277.476974
22,2,1576.422500
22,2,2155.620000
22,2,1633.802326
22,2,1944.704545
22,2,8304.627273
22,2,5438.221591
22,2,2941.198980
22,2,1516.000000
22,2,3178.591146
22,2,1769.491667
22,2,5407.215426
22,2,2125.458333
22,2,2810.211538
22,2,2376.065217
22,2,1516.700000
22,2,2123.925714
22,2,1750.670455
22,2,1785.357143
22,2,1828.080882
22,2,1593.828804
22,2,3177.836957
22,2,2023.472727
22,2,2299.923077
22,2,1755.633721
22,2,1742.128571
22,2,2811.618182
22,2,1919.022321
22,2,3249.944444
22,2,1767.333333
22,2,2014.724138
22,2,1723.260870
22,2,1671.127358
22,2,2695.547170

22,2,1763.339674
22,2,1627.656977
22,2,1915.953488
22,2,2050.875000
22,2,1613.911765
22,2,3102.798295
22,2,2642.307292
22,2,3297.487245
22,2,2741.823529
22,2,2246.233333
22,2,1805.651316
22,2,2384.052632
22,2,5360.517241
22,2,2617.250000
22,2,1726.825000
22,2,1746.352273
22,2,5200.808140
22,2,1635.333333
22,2,1503.577128
22,2,1903.150000
22,2,1744.877358
22,2,1612.990385
22,2,8432.618750
22,2,2532.766304
22,2,1972.316327
22,2,2888.661058
22,2,2319.625000
22,2,2003.881579
22,2,3366.105263
22,2,1722.459184
22,2,1910.679487
22,2,10386.601190
22,2,2487.625000
22,2,1697.235294
22,2,2189.101064
22,2,2050.720588
22,2,1838.080000
22,2,1544.805556
22,2,1827.187500
22,2,1592.990385
22,2,1683.941176
22,2,2583.294643
22,2,2318.425000
22,2,1541.803571
22,2,2626.785714
22,2,1659.492647
22,2,1811.000000
22,2,1538.125000
22,2,3694.088068
22,2,2721.375000
22,2,1548.023026
22,2,1640.695652
22,2,1824.983333
22,2,2413.695513
22,2,2914.073171
22,2,1920.308190
22,2,1839.125000
22,2,3314.520000
22,2,2344.971154
22,2,1786.058673
22,2,3130.983051
22,2,2393.035326
22,2,7564.431034
22,2,1651.604592
22,2,2012.598039
22,2,1690.173780

22,2,1747.072674
22,2,1677.108696
22,2,1531.146875
22,2,2854.734375
22,2,1813.312500
22,2,2766.000000
22,2,2819.000000
22,2,1619.870000
22,2,1706.705128
22,2,1881.142857
22,2,2425.285714
22,2,1584.190217
22,2,1945.312500
22,2,4836.281646
22,2,1575.508929
22,2,1563.458333
22,2,2835.016447
22,2,9282.174419
22,2,11864.698113
22,2,8882.622159
22,2,2076.400000
22,2,1887.325581
22,2,2803.094828
22,2,1810.426829
22,2,1659.714286
22,2,2538.583333
22,2,1881.102273
22,2,1584.333333
22,2,4764.327381
22,2,2652.740385
22,2,2721.580189
22,2,1506.377358
22,2,1772.175000
22,2,2363.165761
22,2,1659.500000
22,2,2073.500000
22,2,1668.165094
22,2,2153.837838
22,2,2422.454327
22,2,1499.875000
22,2,2824.272727
22,2,2268.154762
22,2,2191.455729
22,2,2123.605000
22,2,1598.187500
22,2,1779.125000
22,2,3073.353659
22,2,2856.714286
22,2,1658.890244
22,2,3383.036458
22,2,2189.472222
22,2,1817.381579
22,2,15662.422872
22,2,1536.245000
22,2,2474.591912
22,2,3627.433333
22,2,1689.497340
22,2,4458.100000
22,2,1939.660714
22,2,2708.460784
22,2,1572.450000
22,2,2389.187500
22,2,1802.771277
22,2,7020.720930
22,2,1514.077206
22,2,1620.452128

22,2,2026.486413
22,2,2007.484375
22,2,1851.981383
22,2,1708.043478
22,2,1983.827586
22,2,3671.723684
22,2,2049.440341
22,2,2216.177419
22,2,2029.235849
22,2,1623.175532
22,2,2259.547297
22,2,3788.770270
22,2,2185.093023
22,2,3145.453125
22,2,2005.995614
22,2,2022.780488
22,2,1520.196809
22,2,1615.846875
22,2,2739.593085
22,2,1701.906250
22,2,1911.573171
22,2,1693.432292
22,2,1940.875000
22,2,1895.362245
22,2,1600.510204
22,2,3778.346939
22,2,1616.260870
22,2,1621.888889
22,2,2629.532895
22,2,1594.350000
22,2,1499.910714
22,2,3022.966981
22,2,1818.500000
22,2,2282.545455
22,2,3922.562500
22,2,1663.472973
22,2,1755.900000
22,2,1562.906250
22,2,1522.474537
22,2,3074.464744
22,2,1641.727941
22,2,2658.202778
22,2,1556.180000
22,2,2275.700581
22,2,3141.822917
22,2,1839.409884
22,2,2799.200000
22,2,1574.614130
22,2,2331.467105
22,2,1959.414286
22,2,2404.304348
22,2,1616.940104
22,2,2008.553977
22,2,1850.122222
22,2,2868.935897
22,2,2009.128571
22,2,1887.875000
22,2,1719.578947
22,2,1574.872340
22,2,3306.864130
22,2,2195.078431
22,2,2131.414773
22,2,2066.200000
22,2,1788.448864
22,2,1707.054054
22,2,2458.180000

22,2,2215.955882
22,2,2723.050000
22,2,2136.870370
22,2,1722.815476
22,2,3387.419643
22,2,3579.489130
22,2,2340.678571
22,2,5033.755319
22,2,1771.531977
22,2,2055.576271
22,2,3544.865741
22,2,5596.872093
22,2,1951.636364
22,2,1899.263514
22,2,1601.636792
22,2,1574.221698
22,2,1803.162500
22,2,1674.525862
22,2,2258.645408
22,2,5026.858553
22,2,1608.908163
22,2,1641.125000
22,2,2594.875000
22,2,2351.596591
22,2,1523.300000
22,2,3435.240000
22,2,1608.513514
22,2,2101.423077
22,2,3205.149038
22,2,1516.562500
22,2,2322.364130
22,2,1783.912791
22,2,3485.403846
22,2,1690.266667
22,2,1725.477041
22,2,5043.622449
22,2,4607.380952
22,2,3578.090909
22,2,1719.742647
22,2,3066.115385
22,2,3125.363636
22,2,3174.166667
22,2,3037.172414
22,2,3257.044444
22,2,2056.359375
22,2,3405.913690
22,2,3080.659091
22,2,1588.937500
22,2,2413.432692
22,2,4883.553977
22,2,2258.439815
22,2,2324.937500
22,2,2627.056122
22,2,3590.407895
22,2,3988.553571
22,2,1979.821429
22,2,3515.732143
22,2,2319.767857
22,2,2606.406250
22,2,2325.702381
22,2,3150.437500
22,2,3088.429487
22,2,1527.484375
22,2,2013.787500
22,2,1648.267442
22,2,1698.878378

22,2,1742.866667
22,2,2984.540816
22,2,2874.026442
22,2,1858.358108
22,2,4416.367347
22,2,2236.050000
22,2,2319.913043
22,2,1901.198370
22,2,2175.775862
22,2,1654.872024
22,2,2701.024390
22,2,1819.125000
22,2,3059.150000
22,2,2500.431034
22,2,1642.968750
22,2,1766.487500
22,2,3856.750000
22,2,3970.780000
22,2,1882.000000
22,2,1587.906250
22,2,2345.038462
22,2,2820.602041
22,2,2405.169643
22,2,1741.836957
22,2,2550.500000
22,2,2032.692308
22,2,2106.000000
22,2,2220.581395
22,2,3890.971154
22,2,1578.654762
22,2,2344.238095
22,2,2282.652174
22,2,1985.910714
22,2,1879.710526
22,2,1797.658537
22,2,2182.902174
22,2,2189.717391
22,2,2504.631579
22,2,4231.309524
22,2,2398.412162
22,2,2739.875000
22,2,1742.427632
22,2,4146.446524
22,2,10239.521875
22,2,1808.250000
22,2,2783.052632
22,2,2124.625000
22,2,2097.259434
22,2,1548.037736
22,2,1618.000000
22,2,2645.660377
22,2,1920.529412
22,2,1652.972222
22,2,1877.593085
22,2,9416.370833
22,2,2248.142857
22,2,1570.744444
22,2,2055.558824
22,2,1513.073171
22,2,1594.984375
22,2,3405.719388
22,2,1540.425926
22,2,1662.000000
22,2,5866.938679
22,2,1831.000000
22,2,1862.788043

22,2,3295.778409
22,2,1603.381944
22,2,1584.769022
22,2,2538.730932
22,2,1654.771739
22,2,2219.375000
22,2,2320.416667
22,2,2230.875000
22,2,4508.224490
22,2,1617.602778
22,2,1702.375000
22,2,1846.407895
22,2,1740.125000
22,2,2103.812500
22,2,1519.687500
22,2,1569.570652
22,2,1750.704861
22,2,1677.283784
22,2,5342.239130
22,2,2024.375000
22,2,2522.355978
22,2,2497.333333
22,2,2074.497807
22,2,4267.353659
22,2,1893.476190
22,2,2410.200000
22,2,1804.788043
22,2,2428.050000
22,2,2558.787500
22,2,1732.781250
22,2,1565.163265
22,2,3562.153846
22,2,1794.062500
22,2,3340.662500
22,2,2183.307692
22,2,2206.399194
22,2,2531.594388
22,2,2816.173913
22,2,1544.264706
22,2,3159.875000
22,2,1503.000000
22,2,3542.467262
22,2,19372.500000
22,2,2848.366667
22,2,2534.064286
22,2,1870.363636
22,2,1894.171429
22,2,1725.243902
22,2,1739.000000
22,2,3215.689024
22,2,1636.827381
22,2,2127.563679
22,2,1819.738372
22,2,1951.073980
22,2,3087.764706
22,2,2214.588235
22,2,1594.905405
22,2,1615.715909
22,2,3419.460000
22,2,1723.071429
22,2,4062.479545
22,2,4347.500000
22,2,1564.076087
22,2,1675.182927
22,2,3335.227273
22,2,2545.312500

22,2,1673.717105
22,2,2216.312500
22,2,2049.533163
22,2,3130.180556
22,2,3037.360000
22,2,1852.139535
22,2,3421.245283
22,2,1903.250000
22,2,1504.804688
22,2,3254.772727
22,2,1903.400000
22,2,1540.380952
22,2,7185.318966
22,2,3421.494318
22,2,2331.351190
22,2,2247.862179
22,2,2368.933824
22,2,1559.815789
22,2,1887.086735
22,2,1501.742647
22,2,3377.250000
22,2,2285.837766
22,2,1965.829787
22,2,2362.037037
22,2,1611.813953
22,2,1750.760000
22,2,2287.653846
22,2,1737.695652
22,2,1814.125000
22,2,1632.347826
22,2,1599.447368
22,2,2553.187500
22,2,1706.000000
22,2,1563.326087
22,2,1523.558036
22,2,3550.336323
22,2,3788.367647
22,2,1653.943182
22,2,2151.448980
22,2,1905.806122
22,2,2321.507653
22,2,1978.073171
22,2,2461.052632
22,2,1562.807500
22,2,2086.042857
22,2,2147.850000
22,2,12262.125000
22,2,3632.386905
22,2,2356.545455
22,2,2681.435096
22,2,2123.505814
22,2,3172.187500
22,2,1509.820000
22,2,1615.769608
22,2,2484.166667
22,2,5553.819444
22,2,1818.096154
22,2,2237.446429
22,2,3285.710227
22,2,1712.837500
22,2,1800.000000
22,2,1728.916667
22,2,1839.921296
22,2,1528.404762
22,2,1499.321970
22,2,2564.919355

22,2,1914.587838
22,2,2222.263393
22,2,1968.538462
22,2,1626.112245
22,2,2247.492500
22,2,4617.694853
22,2,1762.866667
22,2,1525.718750
22,2,2762.733333
22,2,1936.000000
22,2,5204.823171
22,2,4565.736842
22,2,3163.093750
22,2,1679.313776
22,2,1691.594340
22,2,2129.193878
22,2,3261.601744
22,2,2228.914773
22,2,1699.750000
22,2,4632.908088
22,2,2637.210000
22,2,1623.823864
22,2,2243.839286
22,2,1673.108108
22,2,2299.440341
22,2,2000.997159
22,2,3238.200000
22,2,2521.218085
22,2,1717.866071
22,2,1506.152439
22,2,1543.571429
22,2,1636.187500
22,2,2773.665441
22,2,1606.712209
22,2,2849.994792
22,2,2123.696429
22,2,1515.313830
22,2,2757.125000
22,2,1976.824627
22,2,1825.490385
22,2,1935.666667
22,2,1649.333333
22,2,1751.418750
22,2,1545.505556
22,2,1859.450000
22,2,1871.151442
22,2,1726.122093
22,2,1534.019022
22,2,3175.141304
22,2,3160.484375
22,2,2067.595930
22,2,3916.809896
22,2,2108.551020
22,2,1704.437500
22,2,1525.707386
22,2,1707.000000
22,2,8429.736842
22,2,2578.857143
22,2,2172.256098
22,2,1720.649038
22,2,2953.316176
22,2,3098.606383
22,2,3103.734694
22,2,1745.825000
22,2,1550.413462
22,2,2008.594444

22,2,2253.186047
22,2,1711.535000
22,2,1796.294872
22,2,1810.832447
22,2,1821.038194
22,2,3060.539773
22,2,1946.519022
22,2,1519.319149
22,2,2034.644444
22,2,1985.502604
22,2,2148.061170
22,2,2590.226190
22,2,1840.315476
22,2,2193.789189
22,2,2124.807692
22,2,1704.744444
22,2,4220.330645
22,2,1851.139706
22,2,1507.495192
22,2,1803.858974
22,2,1891.202128
22,2,2840.922727
22,2,4369.078947
22,2,1839.683908
22,2,2070.047619
22,2,2405.477273
22,2,2995.387755
22,2,1943.783537
22,2,3934.538462
22,2,2981.820513
22,2,2750.063830
22,2,3660.085714
22,2,2065.811321
22,2,1668.453947
22,2,1761.685096
22,2,4963.611111
22,2,3694.200000
22,2,5150.065217
22,2,2518.017857
22,2,1891.977273
22,2,2182.991279
22,2,1522.750000
22,2,1721.118304
22,2,2331.836957
22,2,2942.640625
22,2,2042.702703
22,2,2284.043478
22,2,2034.818182
22,2,1560.117021
22,2,1541.212121
22,2,1925.284483
22,2,1636.375000
22,2,1633.896341
22,2,2387.800000
22,2,1842.937500
22,2,2547.125000
22,2,1785.180851
22,2,2043.583333
22,2,3194.634868
22,2,1870.037037
22,2,1563.416667
22,2,1790.000000
22,2,1651.086957
22,2,2302.613372
22,2,1562.393229
22,2,1511.550000

22,2,3389.734694
22,2,2357.708333
22,2,3229.256410
22,2,1918.625000
22,2,1842.959184
22,2,1881.394886
22,2,1534.191489
22,2,1549.818878
22,2,5088.125000
22,2,1561.272727
22,2,2141.684211
22,2,1538.565217
22,2,1751.225000
22,2,2230.757979
22,2,4867.855978
22,2,2162.500000
22,2,1735.299242
22,2,2064.254902
22,2,1887.357143
22,2,1560.692308
22,2,1660.000000
22,2,2850.750000
22,2,3021.529412
22,2,2700.782609
22,2,2406.468085
22,2,1917.855263
22,2,3353.837209
22,2,3137.160000
22,2,1539.041667
22,2,1677.327500
22,2,1971.517857
22,2,2876.703947
22,2,1726.229167
22,2,1812.149457
22,2,1929.687500
22,2,2740.060000
22,2,3482.220588
22,2,2456.340278
22,2,2148.731132
22,2,2245.236842
22,2,2795.942308
22,2,2462.782051
22,2,1594.797727
22,2,1783.975610
22,2,1499.120690
22,2,2868.571429
22,2,1500.222222
22,2,1827.297297
22,2,2265.200581
22,2,1751.989130
22,2,2625.959184
22,2,1655.196429
22,2,2247.875000
22,2,2017.425595
22,2,2437.369444
22,2,1741.484375
22,2,2019.441176
22,2,1502.214286
22,2,1613.441860
22,2,2209.564516
22,2,2608.488636
22,2,1874.281250
22,2,1820.180233
22,2,2146.397959
22,2,1910.315341
22,2,4115.471154

22,2,3196.622222
22,2,2658.843750
22,2,1943.014706
22,2,1999.375000
22,2,1854.661932
22,2,1571.896739
22,2,3123.832386
22,2,1954.996875
22,2,2242.518617
22,2,2413.027174
22,2,2065.256098
22,2,2645.454545
22,2,4796.920455
22,2,2326.369565
22,2,2409.000000
22,2,2482.824503
22,2,3151.813830
22,2,1754.026316
22,2,2111.654070
22,2,1659.199468
22,2,15976.062500
22,2,6062.840116
22,2,1774.243902
22,2,1520.241379
22,2,1913.785714
22,2,2901.562500
22,2,1690.543750
22,2,1724.464744
22,2,3373.776515
22,2,2254.192500
22,2,1580.500000
22,2,2139.020000
22,2,2177.552885
22,2,1619.833333
22,2,1683.666667
22,2,2091.095238
22,2,1905.041667
22,2,1719.781863
22,2,2651.094512
22,2,2074.945755
22,2,3265.169811
22,2,2157.758152
22,2,2132.887255
22,2,1769.572674
22,2,2761.970745
22,2,2656.565217
22,2,1503.437500
22,2,2352.165094
22,2,1720.692308
22,2,5031.821023
22,2,2607.848958
22,2,11968.421875
22,2,1586.356383
22,2,2112.337766
22,2,2862.958333
22,2,2993.441176
22,2,5841.100000
22,2,2496.956522
22,2,1655.085034
22,2,1588.864362
22,2,1878.556818
22,2,1616.803571
22,2,2517.862500
22,2,3739.586957
22,2,1785.872093
22,2,3288.250000

22,2,2483.750000
22,2,2949.298387
22,2,1926.459184
22,2,1540.722222
22,2,2020.668605
22,2,3162.967105
22,2,1987.772727
22,2,8631.442500
22,2,2280.397959
22,2,2077.875000
22,2,1850.561111
22,2,1586.176471
22,2,3477.679545
22,2,1817.823718
22,2,1739.255000
22,2,2047.882979
22,2,1857.601744
22,2,1691.839286
22,2,3361.289773
22,2,1560.165761
22,2,2133.071429
22,2,1601.081081
22,2,1879.125000
22,2,3393.145833
22,2,1880.520833
22,2,1712.840625
22,2,1750.470930
22,2,6267.290000
22,2,2095.369318
22,2,6602.686475
22,2,1998.590909
22,2,1503.714286
22,2,1695.206897
22,2,1756.100000
22,2,1841.750000
22,2,1665.750000
22,2,1971.125000
22,2,3935.500000
22,2,3364.000000
22,2,1548.390306
22,2,1940.338068
22,2,1657.619318
22,2,1751.250000
22,2,6844.581731
22,2,2500.079755
22,2,1583.788889
22,2,1779.982456
22,2,2275.041667
22,2,1519.379464
22,2,1540.000000
22,2,1549.880435
22,2,2322.101064
22,2,3153.406915
22,2,2101.357143
22,2,1567.128205
22,2,2114.311224
22,2,1991.571429
22,2,1577.712121
22,2,1546.771277
22,2,1975.030612
22,2,3760.687500
22,2,1783.242188
22,2,2372.250000
22,2,3228.503472
22,2,2318.970930
22,2,2425.827778

22,2,1499.524096
22,2,2715.360294
22,2,2343.760417
22,2,1609.180147
22,2,1518.856383
22,2,1872.928571
22,2,1861.488095
22,2,1521.750000
22,2,1651.033333
22,2,2404.329268
22,2,1961.593750
22,2,2420.469388
22,2,2451.036458
22,2,1626.192568
22,2,2035.951613
22,2,1651.490385
22,2,1689.000000
22,2,1796.156863
22,2,1739.082031
22,2,1822.000000
22,2,1909.809524
22,2,1560.354545
22,2,1671.164894
22,2,6264.016129
22,2,3764.777778
22,2,2414.254630
22,2,1948.307692
22,2,4003.857143
22,2,1880.780000
22,2,3807.908730
22,2,2486.610256
22,2,4020.724432
22,2,2658.165094
22,2,1941.652439
22,2,3166.850694
22,2,5167.161184
22,2,2639.432927
22,2,2163.262712
22,2,1613.078125
22,2,1695.836538
22,2,1636.500000
22,2,2279.995690
22,2,1972.387755
22,2,2189.110577
22,2,2231.460227
22,2,1722.062500
22,2,2065.625000
22,2,1652.087500
22,2,3853.461538
22,2,1570.578947
22,2,2586.250000
22,2,2330.625000
22,2,2231.000000
22,2,14489.992857
22,2,3894.161017
22,2,3941.673469
22,2,1862.911111
22,2,2825.966667
22,2,2007.808673
22,2,2597.797619
22,2,3028.976562
22,2,7704.937500
22,2,2517.829545
22,2,2668.000000
22,2,4355.481481
22,2,1986.875000

22,2,1642.677326
22,2,2839.119792
22,2,1705.845395
22,2,2266.957237
22,2,1850.483333
22,2,1784.492857
22,2,2998.720588
22,2,4674.423611
22,2,2575.229730
22,2,1735.822222
22,2,1717.444444
22,2,3149.764151
22,2,1581.445312
22,2,1847.401316
22,2,1775.199468
22,2,1861.825000
22,2,1868.032738
22,2,1986.712500
22,2,1714.107558
22,2,2187.425000
22,2,4457.433673
22,2,2601.183333
22,2,1898.783784
22,2,2824.830882
22,2,15704.693548
22,2,1554.819444
22,2,2015.448980
22,2,2169.567308
22,2,2129.398649
22,2,1596.355556
22,2,3392.321429
22,2,1528.333333
22,2,2197.079268
22,2,1547.088710
22,2,1554.446429
22,2,1913.558511
22,2,2191.415816
22,2,1523.534091
22,2,2081.482143
22,2,2009.422619
22,2,1818.500000
22,2,1509.085106
22,2,1591.174419
22,2,2903.990000
22,2,10384.263158
22,2,1596.993421
22,2,1778.000000
22,2,3565.454545
22,2,3536.221154
22,2,1802.133523
22,2,1864.340426
22,2,1957.500000
22,2,1604.377358
22,2,8387.970000
22,2,3051.295455
22,2,1551.392857
22,2,1574.668478
22,2,2840.400000
22,2,1505.500000
22,2,10685.140244
22,2,1703.489796
22,2,1611.950980
22,2,1665.000000
22,2,1607.936170
22,2,2483.345000
22,2,1515.809896

22,2,2703.533784
22,2,1970.736979
22,2,1501.750000
22,2,1727.452381
22,2,1735.355769
22,2,1504.145833
22,2,2353.655556
22,2,1570.166667
22,2,1707.680921
22,2,1646.783163
22,2,1511.807692
22,2,3564.485714
22,2,2792.625000
22,2,1521.700000
22,2,2380.774510
22,2,1666.025510
22,2,2042.660714
22,2,1761.608491
22,2,2207.650000
22,2,2192.107143
22,2,1574.809524
22,2,1963.232143
22,2,4073.659574
22,2,4475.090426
22,2,1925.806818
22,2,4796.522727
22,2,1595.162500
22,2,4647.000000
22,2,2803.796875
22,2,2271.610294
22,2,2383.450000
22,2,1730.000000
22,2,2654.531915
22,2,2150.854651
22,2,2014.794872
22,2,2698.420103
22,2,1765.807692
22,2,1642.750000
22,2,2088.314815
22,2,4346.552632
22,2,4425.146875
22,2,1806.400000
22,2,1515.755556
22,2,3085.103365
22,2,1722.171875
22,2,2409.587719
22,2,2073.250000
22,2,1766.687500
22,2,4049.548544
22,2,6050.227500
22,2,3322.726562
22,2,1867.244186
22,2,3105.604651
22,2,3028.768293
22,2,3471.636628
22,2,2718.133152
22,2,2025.577778
22,2,1750.142442
22,2,4583.314286
22,2,2161.113095
22,2,1776.200581
22,2,2425.164216
22,2,2401.599432
22,2,2486.680000
22,2,3097.655093
22,2,1685.285714

22,2,1914.083832
22,2,4104.596154
22,2,3207.680000
22,2,1600.648026
22,2,1889.128289
22,2,3273.346939
22,2,5970.518293
22,2,2531.660000
22,2,1886.305000
22,2,2238.222826
22,2,2639.561111
22,2,1765.708333
22,2,1889.125000
22,2,2117.777027
22,2,2301.054545
22,2,2918.720000
22,2,2582.523438
22,2,2068.258065
22,2,2727.101415
22,2,2504.042453
22,2,2325.127049
22,2,1777.978723
22,2,2952.290094
22,2,5816.996795
22,2,2091.009091
22,2,2894.000000
22,2,2124.961538
22,2,2813.221698
22,2,1784.750000
22,2,2485.225000
22,2,2027.179878
22,2,16691.101744
22,2,12840.541667
22,2,6272.095238
22,2,6968.892857
22,2,2390.875000
22,2,8546.468137
22,2,1549.353448
22,2,2159.545455
22,2,3677.736842
22,2,1556.937500
22,2,1766.109375
22,2,2231.625000
22,2,1984.765625
22,2,3270.828125
22,2,2758.317708
22,2,2782.282609
22,2,1975.506098
22,2,2659.466912
22,2,4260.010000
22,2,1586.905660
22,2,1712.125000
22,2,1981.328571
22,2,1997.528409
22,2,1756.515152
22,2,2722.175000
22,2,1566.332547
22,2,2220.301136
22,2,2859.744444
22,2,4271.800000
22,2,2528.315217
22,2,10827.018750
22,2,1814.105978
22,2,1554.068182
22,2,5658.231707
22,2,2324.280488

22,2,1621.077830
22,2,1792.771429
22,2,8965.976190
22,2,1642.944149
22,2,1710.000000
22,2,1956.943548
22,2,2724.585714
22,2,3147.910000
22,2,2480.079787
22,2,1740.913043
22,2,1695.340686
22,2,2589.901163
22,2,1722.406915
22,2,1963.727941
22,2,4635.810345
22,2,2591.473404
22,2,1529.107143
22,2,1647.093023
22,2,2182.905172
22,2,3992.179641
22,2,2577.825000
22,2,2053.978723
22,2,4571.800000
22,2,6238.051887
22,2,2785.550847
22,2,2234.391304
22,2,2594.957031
22,2,1646.178571
22,2,1782.166667
22,2,2453.673913
22,2,1837.000000
22,2,4491.953704
22,2,1759.120000
22,2,1504.625000
22,2,1532.625000
22,2,2896.125000
22,2,4280.046875
22,2,1715.396226
22,2,1505.036585
22,2,1524.142857
22,2,1619.039062
22,2,1809.801136
22,2,2280.855114
22,2,1644.853659
22,2,1593.439024
22,2,2699.496212
22,2,1537.263158
22,2,1685.705000
22,2,1660.187500
22,2,1904.463636
22,2,2078.625000
22,2,1986.940341
22,2,1659.772436
22,2,1631.787037
22,2,2319.708333
22,2,1564.142857
22,2,2546.425000
22,2,1933.954545
22,2,1518.843750
22,2,4784.960784
22,2,2015.703804
22,2,10604.658088
22,2,1890.744898
22,2,10985.777778
22,2,5763.391304
22,2,2167.063830

22,2,1966.615385
22,2,1915.707447
22,2,3839.194444
22,2,1790.937500
22,2,1854.063953
22,2,1841.690217
22,2,2036.000000
22,2,1577.338889
22,2,2627.696809
22,2,2027.118902
22,2,1733.853723
22,2,4270.316327
22,2,3532.400000
22,2,7253.911765
22,2,1709.724359
22,2,2463.205357
22,2,1608.920918
22,2,1678.950000
22,2,9934.471429
22,2,2518.710526
22,2,1504.914894
22,2,1505.820513
22,2,2672.606383
22,2,1673.723837
22,2,1748.800000
22,2,1820.095238
22,2,1762.418919
22,2,4688.828704
22,2,2675.164975
22,2,1621.968750
22,2,2819.941176
22,2,1930.120000
22,2,3013.203704
22,2,2488.333333
22,2,2068.776042
22,2,2446.032895
22,2,2104.176471
22,2,3391.220588
22,2,1582.669643
22,2,3610.567568
22,2,8024.989583
22,2,1768.670455
22,2,10831.975694
22,2,1752.304054
22,2,1802.688889
22,2,3469.904762
22,2,2260.825472
22,2,2008.630208
22,2,1584.363636
22,2,8351.513889
22,2,1733.007979
22,2,4667.926829
22,2,2228.213953
22,2,1926.736842
22,2,1765.794271
22,2,2314.812500
22,2,2233.094697
22,2,2364.631579
22,2,1618.132353
22,2,2018.366071
22,2,3419.627232
22,2,2730.384615
22,2,1666.774457
22,2,2138.279605
22,2,12365.600000
22,2,2101.097500

22,2,1525.025000
22,2,3592.783333
22,2,8593.110000
22,2,2773.435897
22,2,3534.598958
22,2,1571.363636
22,2,1958.210366
22,2,1864.359375
22,2,1893.458333
22,2,1823.290698
22,2,6547.255034
22,2,1901.750000
22,2,2340.204545
22,2,2242.682500
22,2,4805.250000
22,2,2088.476562
22,2,1543.081081
22,2,1591.356707
22,2,2239.712766
22,2,1708.750000
22,2,1501.000000
22,2,1681.495000
22,2,1998.331522
22,2,1911.284884
22,2,2737.363636
22,2,1742.693182
22,2,1501.875000
22,2,1962.078125
22,2,1706.265244
22,2,2251.733333
22,2,8181.260000
22,2,2812.160000
22,2,2386.701987
22,2,2778.531977
22,2,2621.660714
22,2,1612.005000
22,2,2347.418605
22,2,1531.724490
22,2,1748.162162
22,2,3016.312500
22,2,1709.311047
22,2,1924.308824
22,2,2007.163636
22,2,1874.083333
22,2,2840.670455
22,2,1777.857143
22,2,2279.006098
22,2,2035.988636
22,2,2276.666667
22,2,1915.737245
22,2,3343.173469
22,2,1622.000000
22,2,3000.520000
22,2,3519.396226
22,2,1822.700000
22,2,2465.297297
22,2,3161.232143
22,2,1961.585366
22,2,1813.632353
22,2,1574.657895
22,2,2549.615385
22,2,2506.062500
22,2,1783.950980
22,2,2415.091837
22,2,1790.807927
22,2,1889.500000

22,2,2114.250000
22,2,1917.632812
22,2,1614.808824
22,2,2569.213235
22,2,8144.071429
22,2,2153.308511
22,2,3348.073529
22,2,1683.632653
22,2,1604.575000
22,2,1961.601974
22,2,2607.040816
22,2,2276.513889
22,2,3071.745192
22,2,1517.397959
22,2,7053.689189
22,2,5917.154255
22,2,4142.156780
22,2,2112.005952
22,2,1905.792453
22,2,1519.605769
22,2,1509.630435
22,2,1807.559659
22,2,1745.833333
22,2,1654.785714
22,2,8412.078125
22,2,1926.183824
22,2,2173.279762
22,2,3385.703125
22,2,3232.015152
22,2,2072.159574
22,2,1918.976351
22,2,2333.857143
22,2,3184.713568
22,2,1800.038690
22,2,2229.669643
22,2,1788.900510
22,2,3364.865000
22,2,1710.027027
22,2,7573.342105
22,2,1710.833333
22,2,2010.010870
22,2,2878.877604
22,2,2255.643750
22,2,1728.378378
22,2,2536.763158
22,2,1741.850000
22,2,2113.090909
22,2,1697.012019
22,2,1709.797727
22,2,6022.480769
22,2,2946.859091
22,2,2814.986607
22,2,1833.302083
22,2,1994.145349
22,2,1836.545455
22,2,2387.187500
22,2,2417.738208
22,2,1804.752551
22,2,1678.755435
22,2,1529.225000
22,2,2127.261364
22,2,4775.287500
22,2,3138.068452
22,2,1657.063830
22,2,2775.989362
22,2,18029.331522

22,2,3397.833333
22,2,2305.534091
22,2,1520.396226
22,2,1552.241477
22,2,1570.604651
22,2,4859.906250
22,2,2028.573864
22,2,2121.926471
22,2,2591.229592
22,2,2814.156250
22,2,2079.125000
22,2,2150.512755
22,2,17683.039216
22,2,3141.450000
22,2,1825.717391
22,2,1939.900510
22,2,1548.028846
22,2,7293.176282
22,2,2401.009615
22,2,2755.141447
22,2,3690.781780
22,2,1884.233696
22,2,1547.427673
22,2,1777.732500
22,2,1857.630319
22,2,8425.083333
22,2,1636.568878
22,2,1623.500000
22,2,2931.817073
22,2,2094.979167
22,2,2023.745192
22,2,1818.364865
22,2,2124.837719
22,2,2174.223214
22,2,1628.712500
22,2,2708.015957
22,2,2032.303191
22,2,6082.470395
22,2,1852.576087
22,2,2155.200000
22,2,2352.932432
22,2,1762.456633
22,2,1667.794271
22,2,2313.275000
22,2,4250.510870
22,2,2931.983333
22,2,1980.104167
22,2,2014.588235
22,2,3408.529018
22,2,1860.664286
22,2,1591.228125
22,2,1588.750000
22,2,1818.769886
22,2,2659.222222
22,2,4179.326923
22,2,1565.580838
22,2,2054.639344
22,2,2606.574519
22,2,1818.203125
22,2,1787.860849
22,2,1591.625000
22,2,1605.092105
22,2,1583.535714
22,2,1534.657407
22,2,1620.722561
22,2,17894.044444

22,2,1847.106383
22,2,2573.399038
22,2,2795.440000
22,2,2812.536111
22,2,1513.507812
22,2,1572.023810
22,2,2189.467593
22,2,2366.785714
22,2,1947.381356
22,2,4176.762931
22,2,4413.602679
22,2,3627.981707
22,2,4761.877315
22,2,3654.000000
22,2,2372.210938
22,2,3130.370455
22,2,3862.433333
22,2,2039.934783
22,2,3585.687500
22,2,3218.825000
22,2,1831.263158
22,2,2250.316327
22,2,2510.744681
22,2,2377.116477
22,2,1589.584184
22,2,1569.471875
22,2,3264.500000
22,2,1987.833333
22,2,1850.845395
22,2,2489.397321
22,2,6754.270833
22,2,2272.000000
22,2,3091.348958
22,2,1676.882682
22,2,2763.018868
22,2,2988.500000
22,2,2391.756579
22,2,1535.958333
22,2,1714.316327
22,2,2864.929032
22,2,2074.804545
22,2,2058.200000
22,2,1880.033333
22,2,2233.543269
22,2,2445.847458
22,2,3004.344512
22,2,1976.119792
22,2,5048.400000
22,2,9394.237805
22,2,1537.465116
22,2,1499.695513
22,2,1705.744681
22,2,3328.162791
22,2,1516.484375
22,2,2467.343750
22,2,1806.481383
22,2,2653.607955
22,2,2579.276596
22,2,2828.010204
22,2,1530.350490
22,2,2432.250000
22,2,2609.375000
22,2,1818.139423
22,2,2083.190476
22,2,1589.343750
22,2,2318.586047

22,2,3554.333333
22,2,2705.454545
22,2,2412.451705
22,2,1879.250000
22,2,1563.656250
22,2,1992.535714
22,2,2340.422872
22,2,1541.342857
22,2,1598.300000
22,2,2342.500000
22,2,2244.048951
22,2,3205.072222
22,2,1702.294872
22,2,2126.608871
22,2,5279.437500
22,2,1990.279070
22,2,5870.047794
22,2,1509.428571
22,2,3062.960938
22,2,1502.232955
22,2,4419.671053
22,2,2099.323370
22,2,2325.448718
22,2,7195.544643
22,2,2609.659091
22,2,2142.062500
22,2,1632.206897
22,2,1928.204082
22,2,1755.875000
22,2,1541.910256
22,2,1778.312500
22,2,1511.400000
22,2,1558.588235
22,2,2015.793981
22,2,4240.245614
22,2,1582.857143
22,2,3387.750000
22,2,2410.750000
22,2,2502.867925
22,2,6512.971014
22,2,2879.208333
22,2,3059.923567
22,2,1865.000000
22,2,2405.310811
22,2,2298.632653
22,2,3314.062500
22,2,6637.897959
22,2,1736.659574
22,2,1502.132653
22,2,1732.500000
22,2,7420.065789
22,2,2019.130208
22,2,5248.312500
22,2,2291.073864
22,2,1750.923469
22,2,2543.940476
22,2,1533.446078
22,2,1510.391304
22,2,1609.600000
22,2,1546.258929
22,2,8045.804878
22,2,4985.933333
22,2,1785.875000
22,2,1520.071875
22,2,1910.997449
22,2,1529.125000

22,2,1922.143519
22,2,3517.311475
22,2,1889.791667
22,2,11048.114130
22,2,6003.291667
22,2,3925.279070
22,2,3632.131579
22,2,4416.584906
22,2,2217.622159
22,2,2017.098404
22,2,2217.161290
22,2,1781.441176
22,2,1810.690789
22,2,1712.002976
22,2,1920.406977
22,2,2047.662879
22,2,1544.544643
22,2,2214.250000
22,2,1600.764706
22,2,2017.069712
22,2,1763.375000
22,2,1973.349057
22,2,1882.837209
22,2,4360.364865
22,2,1642.677326
22,2,2585.476562
22,2,2669.697674
22,2,1865.307500
22,2,1541.369792
22,2,1560.560976
22,2,2378.246212
22,2,1773.346154
22,2,1586.791667
22,2,1940.926829
22,2,2466.062500
22,2,2961.288889
22,2,1513.058511
22,2,5540.739362
22,2,3722.076923
22,2,2578.000000
22,2,1890.650510
22,2,2006.026961
22,2,1941.707547
22,2,4182.803571
22,2,2362.986111
22,2,1769.171875
22,2,3790.000000
22,2,6066.485000
22,2,4885.174528
22,2,2502.979545
22,2,8585.436170
22,2,2277.000000
22,2,4662.457500
22,2,1616.389151
22,2,2322.783784
22,2,2524.073171
22,2,2366.443452
22,2,3810.803571
22,2,4123.208333
22,2,2076.905612
22,2,3823.035088
22,2,2021.433333
22,2,1519.151596
22,2,1660.588235
22,2,1998.921053
22,2,1622.263889

22,2,2059.672414
22,2,2254.427083
22,2,1735.676471
22,2,2397.879464
22,2,1822.671296
22,2,2017.553571
22,2,1829.845395
22,2,2416.024390
22,2,2131.333333
22,2,2005.134868
22,2,1581.666667
22,2,1586.511111
22,2,2771.255814
22,2,1908.400000
22,2,6378.839286
22,2,1806.434783
22,2,1663.938776
22,2,2151.635135
22,2,2356.333333
22,2,1499.611413
22,2,5191.050000
22,2,1545.700000
22,2,1664.544643
22,2,2188.423077
22,2,2015.150000
22,2,2142.562500
22,2,1937.926471
22,2,1500.153846
22,2,2344.462766
22,2,2112.922297
22,2,1810.892857
22,2,1754.479592
22,2,1953.531250
22,2,1530.076220
22,2,8549.851190
22,2,6672.665094
22,2,1876.421875
22,2,1783.345930
22,2,1560.925532
22,2,4083.881356
22,2,1770.944444
22,2,2268.031977
22,2,1558.346354
22,2,2120.553191
22,2,2479.523256
22,2,8363.275000
22,2,1716.555085
22,2,4750.483696
22,2,1801.977941
22,2,2556.637500
22,2,1774.538462
22,2,1499.428571
22,2,4114.326923
22,2,1666.621711
22,2,3831.939189
22,2,1983.582500
22,2,2338.223404
22,2,1819.787500
22,2,3022.928571
22,2,2896.042857
22,2,2932.864796
22,2,1830.500000
22,2,1507.281250
22,2,1699.510638
22,2,3467.283333
22,2,1915.437500

22,2,2541.164634
22,2,2793.000000
22,2,2085.733974
22,2,2181.038462
22,2,1835.625000
22,2,1666.707031
22,2,1854.673469
22,2,3065.040541
22,2,1833.355556
22,2,2921.027778
22,2,3660.607955
22,2,2062.125000
22,2,2549.929878
22,2,1740.568182
22,2,1824.412234
22,2,3192.338235
22,2,1601.708333
22,2,1874.428571
22,2,1685.253289
22,2,1817.553191
22,2,2085.752660
22,2,1544.812500
22,2,1846.313953
22,2,2751.989796
22,2,3048.337209
22,2,2174.313636
22,2,1541.007353
22,2,3441.545918
22,2,1975.456250
22,2,1847.573171
22,2,2100.509146
22,2,2689.722222
22,2,2109.671875
22,2,1518.383929
22,2,1783.000000
22,2,2231.250000
22,2,1507.125000
22,2,1679.179878
22,2,1952.729167
22,2,3496.888889
22,2,5013.160714
22,2,1973.577889
22,2,1504.681818
22,2,1873.447115
22,2,2285.548246
22,2,2884.055000
22,2,2192.038043
22,2,1796.250000
22,2,1606.430921
22,2,1521.180851
22,2,1924.043605
22,2,2613.661058
22,2,1638.756757
22,2,2160.226562
22,2,3612.839286
22,2,1912.454545
22,2,1509.103659
22,2,1620.396341
22,2,1589.042683
22,2,2133.197727
22,2,1649.771739
22,2,1654.218750
22,2,2766.065104
22,2,1813.821990
22,2,1843.583333
22,2,2483.287500

22,2,10269.724432
22,2,2877.937500
22,2,1975.350962
22,2,1541.671875
22,2,2674.718750
22,2,5730.556452
22,2,10647.775000
22,2,3111.027027
22,2,1725.824468
22,2,2038.863636
22,2,2479.942308
22,2,2287.016949
22,2,3441.073171
22,2,1589.110465
22,2,3031.269231
22,2,1685.162162
22,2,2166.890244
22,2,2893.392857
22,2,4229.250000
22,2,2204.752604
22,2,1966.250000
22,2,1942.454545
22,2,8533.570312
22,2,1655.459239
22,2,1618.769231
22,2,2008.909091
22,2,4425.966463
22,2,2083.756098
22,2,1745.285714
22,2,1868.113889
22,2,2180.522222
22,2,3341.428571
22,2,2048.975000
22,2,2390.676829
22,2,1962.207317
22,2,1614.004310
22,2,2747.933962
22,2,2605.461538
22,2,2254.714286
22,2,3515.444444
22,2,1563.372549
22,2,2415.889205
22,2,6155.954545
22,2,1624.898438
22,2,1616.100000
22,2,3168.493902
22,2,1838.547486
22,2,1830.125000
22,2,1920.892857
22,2,2013.687500
22,2,3847.359375
22,2,2187.898256
22,2,2300.237805
22,2,2153.141892
22,2,1571.029412
22,2,2095.333333
22,2,1855.206818
22,2,2007.455882
22,2,3179.028846
22,2,3183.137195
22,2,2365.956522
22,2,2137.566176
22,2,1735.631250
22,2,1951.650000
22,2,2529.502155
22,2,2360.705000

22,2,3210.294118
22,2,1772.477273
22,2,1702.120000
22,2,1816.710526
22,2,1671.096154
22,2,2360.560976
22,2,3312.474432
22,2,2694.875000
22,2,1633.142857
22,2,4665.664773
22,2,2062.559211
22,2,1684.213942
22,2,2971.636628
22,2,1913.613889
22,2,1884.029255
22,2,1766.050000
22,2,1750.343023
22,2,2177.409375
22,2,2319.950980
22,2,1698.784091
22,2,1553.786184
22,2,2078.096154
22,2,7064.117647
22,2,1879.666667
22,2,2650.856250
22,2,3117.831081
22,2,1590.191667
22,2,2888.007500
22,2,2066.333333
22,2,1510.210227
22,2,2555.787500
22,2,8681.460526
22,2,1836.037037
22,2,1533.688889
22,2,1584.777778
22,2,2303.562500
22,2,1507.168367
22,2,3116.016667
22,2,2770.072368
22,2,1852.728070
22,2,1914.182692
22,2,1603.000000
22,2,1792.916667
22,2,1899.500000
22,2,3482.209302
22,2,2191.468085
22,2,3432.487437
22,2,2292.125000
22,2,1876.678571
22,2,2753.111111
22,2,1774.591667
22,2,1972.302632
22,2,1872.722826
22,2,1964.487500
22,2,1609.393229
22,2,1641.310160
22,2,2093.693750
22,2,1633.845238
22,2,7065.611111
22,2,1899.500000
22,2,3435.221154
22,2,2373.000000
22,2,1939.782500
22,2,1918.304054
22,2,2022.548077
22,2,1776.035326

22,2,2511.500000
22,2,3440.514706
22,2,1685.488889
22,2,2170.617647
22,2,2605.331395
22,2,2136.528846
22,2,1572.052083
22,2,3207.696078
22,2,1715.415698
22,2,2421.116279
22,2,1795.038462
22,2,2832.075472
22,2,1634.054348
22,2,3513.410714
22,2,2042.000000
22,2,3610.505682
22,2,2336.451220
22,2,14893.502717
22,2,1888.854167
22,2,2024.709184
22,2,2168.816667
22,2,3566.913043
22,2,1636.723404
22,2,1730.625000
22,2,2009.575758
22,2,3633.969595
22,2,2241.178571
22,2,1788.195000
22,2,1531.242188
22,2,8002.816667
22,2,2230.742424
22,2,3324.456522
22,2,1891.164773
22,2,2363.021277
22,2,1660.925000
22,2,1885.246875
22,2,1696.630435
22,2,1645.000000
22,2,1641.978774
22,2,1914.621622
22,2,2454.243243
22,2,2337.916667
22,2,4504.670139
22,2,1747.901786
22,2,3902.610169
22,2,6355.897959
22,2,1546.390625
22,2,1691.837209
22,2,2040.510638
22,2,2427.970588
22,2,2216.813725
22,2,2032.893939
22,2,2458.392045
22,2,2666.165563
22,2,2902.781250
22,2,3639.551020
22,2,3567.161458
22,2,1956.144444
22,2,6490.755556
22,2,1837.607143
22,2,2438.500000
22,2,19070.062500
22,2,1753.682927
22,2,1901.595745
22,2,2317.931122
22,2,2545.225806

22,2,2652.421875
22,2,4157.729167
22,2,2708.733333
22,2,1514.000000
22,2,1731.954545
22,2,1866.365385
22,2,8614.031250
22,2,2273.046875
22,2,1908.233491
22,2,2411.310976
22,2,5343.223558
22,2,1526.562500
22,2,2342.866279
22,2,7057.775641
22,2,1783.352041
22,2,1635.187500
22,2,1773.294643
22,2,2195.409483
22,2,2216.500000
22,2,1724.392442
22,2,2069.829268
22,2,3364.816176
22,2,2739.104592
22,2,1723.441038
22,2,1588.541667
22,2,1609.837500
22,2,2533.597826
22,2,1525.779762
22,2,1630.598540
22,2,5645.868966
22,2,1687.375000
22,2,2014.833333
22,2,1505.988024
22,2,2192.400000
22,2,1957.013393
22,2,3218.718023
22,2,1863.035714
22,2,2265.466346
22,2,1923.466667
22,2,4297.444444
22,2,2076.560811
22,2,1713.293194
22,2,1549.744681
22,2,2720.125000
22,2,2677.712963
22,2,1849.161290
22,2,2956.315789
22,2,1536.508333
22,2,1508.165000
22,2,5178.985294
22,2,1891.736842
22,2,1569.532609
22,2,1899.100000
22,2,2473.837838
22,2,1654.259375
22,2,1954.058824
22,2,1825.611429
22,2,1995.775785
22,2,1847.516129
22,2,1900.367188
22,2,1510.789474
22,2,2652.877660
22,2,4309.877500
22,2,1755.666667
22,2,3408.214286
22,2,1646.592262

22,2,1733.928571
22,2,1952.750000
22,2,2064.564103
22,2,1785.375000
22,2,1807.429687
22,2,1509.795031
22,2,3119.067073
22,2,3762.045000
22,2,1552.612245
22,2,3224.393617
22,2,1843.584559
22,2,6876.345109
22,2,1886.858796
22,2,1522.513889
22,2,1757.069767
22,2,8055.621795
22,2,2422.534314
22,2,1829.375000
22,2,1904.585366
22,2,2084.825581
22,2,1976.540698
22,2,1509.782609
22,2,1849.882353
22,2,1752.048246
22,2,2070.662500
22,2,1996.490566
22,2,1860.933140
22,2,2799.107955
22,2,4517.450292
22,2,2304.625000
22,2,2632.046512
22,2,1802.885870
22,2,1927.219340
22,2,1597.352273
22,2,3015.687500
22,2,1978.410326
22,2,1887.169811
22,2,1669.867647
22,2,2203.618421
22,2,2249.272222
22,2,1862.771226
22,2,2499.505319
22,2,1625.064103
22,2,2998.209302
22,2,1692.039474
22,2,9335.493421
22,2,3350.885135
22,2,1983.485294
22,2,2401.863095
22,2,6044.018293
22,2,8944.215116
22,2,2011.187500
22,2,1798.502500
22,2,1706.397959
22,2,1726.014535
22,2,2862.394737
22,2,1643.665698
22,2,3250.562500
22,2,3511.622340
22,2,2477.232639
22,2,1857.554878
22,2,1649.145455
22,2,2547.385000
22,2,4400.982143
22,2,1543.275000
22,2,2122.267857

22,2,3430.121875
22,2,1905.476744
22,2,2743.695755
22,2,3802.214286
22,2,3913.083333
22,2,2273.037234
22,2,1758.579545
22,2,1908.191489
22,2,1512.190104
22,2,2769.401042
22,2,1565.926829
22,2,1672.300000
22,2,2907.370370
22,2,1589.403941
22,2,4874.283333
22,2,1996.851190
22,2,1589.437500
22,2,19702.714286
22,2,2618.416667
22,2,1532.129630
22,2,3119.539352
22,2,1718.921569
22,2,2067.632653
22,2,2037.702128
22,2,6199.005556
22,2,6655.487069
22,2,2118.450000
22,2,2072.261905
22,2,1535.305233
22,2,2566.965000
22,2,1667.375000
22,2,1572.357143
22,2,1783.400000
22,2,1717.206633
22,2,1927.496622
22,2,1661.226974
22,2,2760.256757
22,2,6397.127551
22,2,2099.289894
22,2,2462.646277
22,2,1849.806604
22,2,3367.165541
22,2,2185.828947
22,2,1896.687500
22,2,3595.500000
22,2,2026.845238
22,2,1863.937500
22,2,2257.071429
22,2,1648.733333
22,2,1941.950000
22,2,1619.000000
22,2,1512.768229
22,2,2467.650943
22,2,1645.223214
22,2,1605.393617
22,2,2633.151042
22,2,4029.668367
22,2,1547.843750
22,2,2085.944149
22,2,7904.286765
22,2,8405.941176
22,2,1589.765625
22,2,1802.703125
22,2,1979.959239
22,2,3007.485714
22,2,2049.547414

22,2,1646.833333
22,2,1582.351351
22,2,1546.027778
22,2,2757.219388
22,2,2151.323529
22,2,1713.333333
22,2,1532.740132
22,2,1633.204787
22,2,2036.844512
22,2,2223.355769
22,2,2691.250000
22,2,1821.732143
22,2,1751.053191
22,2,3904.250000
22,2,12866.934211
22,2,1650.023256
22,2,2077.279070
22,2,2651.375000
22,2,1512.200000
22,2,1820.337054
22,2,2560.168919
22,2,1615.032609
22,2,2317.985849
22,2,2611.922222
22,2,2270.122449
22,2,5076.414894
22,2,1591.505814
22,2,2179.152778
22,2,2944.817308
22,2,1943.394737
22,2,2714.397727
22,2,2116.550000
22,2,4088.944079
22,2,2508.729167
22,2,1898.210526
22,2,2009.352273
22,2,2084.489796
22,2,2434.242021
22,2,2882.643617
22,2,2348.413690
22,2,2141.875000
22,2,1535.418605
22,2,2424.194444
22,2,2643.343750
22,2,2432.125000
22,2,7401.500000
22,2,2563.409483
22,2,1591.348837
22,2,2020.533333
22,2,1777.500000
22,2,2805.778846
22,2,1975.709091
22,2,1635.183824
22,2,3148.765306
22,2,1889.881250
22,2,3227.096154
22,2,1623.665816
22,2,2173.278409
22,2,2570.959302
22,2,2460.780000
22,2,1575.192073
22,2,2062.259259
22,2,1663.651786
22,2,1651.378378
22,2,2199.097561
22,2,2031.642857

22,2,2982.882500
22,2,2178.158537
22,2,3856.246324
22,2,1523.408537
22,2,2142.676829
22,2,2381.307692
22,2,2521.190217
22,2,2300.750000
22,2,3086.203704
22,2,2395.489583
22,2,2916.655556
22,2,1931.825000
22,2,2276.857143
22,2,4997.175000
22,2,1561.822222
22,2,1608.074074
22,2,2236.690476
22,2,1715.935714
22,2,1932.875000
22,2,2372.504167
22,2,1975.755814
22,2,1977.705128
22,2,1623.333333
22,2,1639.656915
22,2,1866.666667
22,2,1752.577206
22,2,1852.411458
22,2,2247.832500
22,2,2350.707317
22,2,2258.892857
22,2,2317.708333
22,2,1608.184524
22,2,1604.185811
22,2,1614.000000
22,2,3431.631579
22,2,3781.920635
22,2,2205.226852
22,2,3189.679487
22,2,2877.858491
22,2,1947.103659
22,2,2144.150000
22,2,1589.724490
22,2,3034.666667
22,2,2037.456522
22,2,5111.258333
22,2,2284.497449
22,2,2402.072115
22,2,2431.019022
22,2,3084.925926
22,2,2309.125000
22,2,2618.680851
22,2,3234.218750
22,2,1628.525000
22,2,3492.312500
22,2,2229.125000
22,2,1755.490000
22,2,1554.375000
22,2,15342.500000
22,2,3433.212766
22,2,2776.864362
22,2,5127.590426
22,2,1674.420455
22,2,1506.391304
22,2,2100.786585
22,2,3704.912162
22,2,3156.924342

22,2,2128.417763
22,2,2326.512195
22,2,1937.000000
22,2,1547.296296
22,2,1516.517361
22,2,2169.375000
22,2,1515.474490
22,2,2496.700000
22,2,3003.351852
22,2,1556.640625
22,2,14505.761905
22,2,1619.081250
22,2,1768.594595
22,2,2588.609375
22,2,1728.156250
22,2,3346.125000
22,2,2325.063953
22,2,1517.669811
22,2,1956.142857
22,2,1571.806604
22,2,2287.473404
22,2,1571.961735
22,2,5371.325000
22,2,1952.014706
22,2,3488.981818
22,2,3065.276596
22,2,2247.180233
22,2,3120.468750
22,2,2026.554878
22,2,2692.902174
22,2,1689.069444
22,2,1597.356771
22,2,1755.086806
22,2,2374.700000
22,2,2504.474576
22,2,4320.978571
22,2,1679.375000
22,2,1705.954545
22,2,1681.000000
22,2,3985.051020
22,2,9733.974576
22,2,2239.590909
22,2,1914.380682
22,2,2963.732143
22,2,2517.402500
22,2,2961.180851
22,2,1801.828947
22,2,1531.243243
22,2,1589.393229
22,2,1637.472727
22,2,4704.186047
22,2,2528.436170
22,2,1808.100000
22,2,7611.657895
22,2,4065.433594
22,2,1854.236111
22,2,1540.723404
22,2,1604.868421
22,2,2655.518293
22,2,1591.716146
22,2,2186.900000
22,2,2218.324468
22,2,1654.228723
22,2,1522.436170
22,2,1811.300000
22,2,3065.585106

22,2,3521.747396
22,2,3735.102273
22,2,1700.178571
22,2,2734.375000
22,2,2203.864865
22,2,2651.000000
22,2,2655.545455
22,2,2216.214286
22,2,2121.771341
22,2,2234.850543
22,2,10954.275000
22,2,2436.057692
22,2,2855.129545
22,2,1851.080000
22,2,7393.699219
22,2,1966.245833
22,2,2074.059524
22,2,2712.400000
22,2,4241.145349
22,2,5026.431452
22,2,1540.777174
22,2,2253.129032
22,2,1769.400000
22,2,1503.364865
22,2,4688.750000
22,2,1624.860119
22,2,2246.341837
22,2,1514.776442
22,2,1609.010714
22,2,2260.358491
22,2,2984.951220
22,2,1507.583333
22,2,2126.808511
22,2,2972.947500
22,2,1809.209906
22,2,2106.147727
22,2,2060.592262
22,2,5703.625000
22,2,1521.407609
22,2,2024.268293
22,2,1836.707447
22,2,3352.972222
22,2,1669.666667
22,2,2125.375000
22,2,2872.782609
22,2,2367.125000
22,2,2491.326531
22,2,3489.769397
22,2,2204.596154
22,2,1753.121951
22,2,17958.625000
22,2,1983.750000
22,2,1959.506098
22,2,1793.771277
22,2,1864.450000
22,2,1723.768293
22,2,4504.071429
22,2,3952.105263
22,2,2038.568750
22,2,2236.604167
22,2,1569.657895
22,2,1823.676630
22,2,2174.093750
22,2,3110.369898
22,2,1787.514706
22,2,2533.598214

22,2,2694.421687
22,2,1968.024390
22,2,3485.032895
22,2,1584.681122
22,2,1844.451705
22,2,2915.566406
22,2,4209.562500
22,2,2682.127660
22,2,3072.378049
22,2,2086.191489
22,2,1955.058333
22,2,1525.279605
22,2,2174.925000
22,2,2737.816038
22,2,1730.007576
22,2,1840.358974
22,2,3007.958333
22,2,1736.929688
22,2,1543.089674
22,2,2715.111364
22,2,1671.237500
22,2,3520.921875
22,2,1587.039216
22,2,1555.100000
22,2,15391.478261
22,2,4018.737500
22,2,5242.872340
22,2,4628.640625
22,2,1553.167553
22,2,1997.500000
22,2,1735.648256
22,2,2692.882500
22,2,2357.000000
22,2,1561.014205
22,2,2904.825000
22,2,1923.416667
22,2,2011.018293
22,2,3091.154255
22,2,2082.439024
22,2,1635.492647
22,2,1783.717391
22,2,2702.889706
22,2,3742.004310
22,2,3052.062500
22,2,1829.392857
22,2,2770.621429
22,2,10572.375000
22,2,3948.329787
22,2,2754.686047
22,2,3186.897059
22,2,7002.588235
22,2,2459.709302
22,2,2137.571429
22,2,3214.153646
22,2,1860.000000
22,2,2190.958333
22,2,1710.775510
22,2,2316.770000
22,2,2735.239865
22,2,1569.200000
22,2,2345.049479
22,2,1764.177083
22,2,2316.675926
22,2,2735.241071
22,2,2412.563830
22,2,1622.196023

22,2,1538.041667
22,2,2416.101064
22,2,5732.250000
22,2,1552.358974
22,2,2688.338235
22,2,9368.836207
22,2,2198.676887
22,2,2171.654255
22,2,1585.562500
22,2,1504.684211
22,2,1678.267857
22,2,4073.316667
22,2,3775.875000
22,2,2117.000000
22,2,3284.336538
22,2,2349.345930
22,2,1562.760870
22,2,3601.508523
22,2,2053.043478
22,2,1571.918919
22,2,1572.673469
22,2,4428.857143
22,2,2261.166667
22,2,1897.961538
22,2,2545.526163
22,2,1744.890000
22,2,3631.398026
22,2,1751.715426
22,2,1889.303571
22,2,3265.610619
22,2,5433.320312
22,2,1566.222222
22,2,2248.353125
22,2,2007.093750
22,2,1836.515152
22,2,1669.775424
22,2,1619.650000
22,2,2612.850610
22,2,1911.986842
22,2,1697.025641
22,2,1653.070000
22,2,1724.813679
22,2,2683.177778
22,2,1590.614130
22,2,1982.757812
22,2,6999.613208
22,2,1571.711111
22,2,2696.740000
22,2,1823.558824
22,2,5966.520833
22,2,3929.011905
22,2,2062.266509
22,2,1580.153409
22,2,2052.000000
22,2,1852.753049
22,2,1880.586957
22,2,1648.948529
22,2,1711.752907
22,2,1860.437500
22,2,3028.059783
22,2,1721.500000
22,2,5576.115000
22,2,2559.453488
22,2,10054.269737
22,2,1534.635000
22,2,1757.984293

22,2,2409.030660
22,2,1592.500000
22,2,1942.589286
22,2,1956.235000
22,2,1999.654412
22,2,2830.876923
22,2,2459.548469
22,2,1828.875000
22,2,1602.223214
22,2,16495.012500
22,2,1534.815972
22,2,1619.531250
22,2,1531.121528
22,2,1544.812500
22,2,9587.059375
22,2,1838.210526
22,2,2114.161932
22,2,3629.375000
22,2,2456.360294
22,2,2179.443750
22,2,2289.789062
22,2,1742.854545
22,2,1906.930233
22,2,2233.636364
22,2,6402.402273
22,2,1663.011364
22,2,4245.054688
22,2,1634.955882
22,2,3036.272727
22,2,1683.462963
22,2,3634.740909
22,2,1609.172414
22,2,1588.187500
22,2,14455.294118
22,2,3007.848039
22,2,3322.890000
22,2,2501.097561
22,2,1660.937500
22,2,1920.357558
22,2,2072.867925
22,2,2347.574405
22,2,1586.128205
22,2,4210.723558
22,2,3512.442308
22,2,3275.245098
22,2,7928.878049
22,2,3908.721154
22,2,3908.938776
22,2,1591.125000
22,2,2903.691176
22,2,2376.576142
22,2,2825.625000
22,2,2337.957317
22,2,2828.780220
22,2,2824.812500
22,2,2392.383333
22,2,4037.467391
22,2,1664.445122
22,2,1952.584459
22,2,3006.245283
22,2,4064.937500
22,2,1999.236842
22,2,2246.343195
22,2,1704.300000
22,2,4744.144231
22,2,5836.003125

22,2,1949.775510
22,2,4067.352941
22,2,3171.448276
22,2,1786.531250
22,2,16730.513021
22,2,2119.677885
22,2,3544.405556
22,2,1888.892857
22,2,1535.162791
22,2,1627.536585
22,2,2435.022222
22,2,1660.428571
22,2,3461.494681
22,2,1778.040541
22,2,3282.865854
22,2,2147.520710
22,2,2619.375000
22,2,3581.771739
22,2,1714.555288
22,2,2618.784574
22,2,1731.488372
22,2,2595.500000
22,2,1738.184783
22,2,2692.521739
22,2,4365.000000
22,2,1554.181818
22,2,3255.540816
22,2,1760.394231
22,2,1642.759777
22,2,2826.285714
22,2,1552.514706
22,2,1561.839286
22,2,1849.677083
22,2,1541.023649
22,2,2186.086957
22,2,1698.312500
22,2,3235.216080
22,2,1891.703125
22,2,3768.932292
22,2,1878.364130
22,2,2102.064655
22,2,9283.666667
22,2,15867.600000
22,2,3616.337500
22,2,2326.180556
22,2,1952.663462
22,2,1653.449324
22,2,1501.606707
22,2,1688.162736
22,2,1569.569444
22,2,2939.085937
22,2,1557.851064
22,2,1858.713415
22,2,2134.425532
22,2,1785.650000
22,2,1652.333333
22,2,1761.500000
22,2,1705.163462
22,2,2376.697115
22,2,1782.274457
22,2,1938.804878
22,2,1805.210526
22,2,1676.291667
22,2,2359.188889
22,2,2351.395833
22,2,3080.169811

22,2,1635.200000
22,2,1685.183594
22,2,1754.427632
22,2,2028.059783
22,2,3705.210106
22,2,1920.500000
22,2,1731.903846
22,2,2402.776163
22,2,1668.255319
22,2,3152.428571
22,2,1688.714286
22,2,2040.125000
22,2,1973.512255
22,2,10114.996094
22,2,1871.000000
22,2,2460.473684
22,2,2385.569444
22,2,2382.426136
22,2,1943.218750
22,2,1567.269737
22,2,1833.644444
22,2,1920.053922
22,2,2238.795000
22,2,1626.300000
22,2,1940.158537
22,2,1566.403061
22,2,1773.584375
22,2,2566.783019
22,2,2994.384146
22,2,1714.040441
23,1,1514.395349
23,1,1721.753378
23,1,3487.348485
23,1,9032.608696
23,1,13544.383929
23,1,9237.953947
23,1,10083.090909
23,1,1777.228261
23,1,2695.868421
23,1,11581.335227
23,1,2753.302885
23,1,5726.742857
23,1,4717.022727
23,1,13895.480978
23,1,1895.115385
23,1,2825.107143
23,1,2058.363636
23,1,3058.848485
23,1,4282.093750
23,1,4988.458333
23,1,2679.169643
23,1,5354.107143
23,1,17753.320122
23,1,5460.432927
23,1,2675.312500
23,1,16722.571429
23,1,13515.306122
23,1,5688.333333
23,1,2716.496622
23,1,2127.125000
23,1,4866.500000
23,1,2030.750000
23,1,1527.560976
23,1,3174.950000
23,1,3634.807692
23,1,8709.303030

23,1,11631.000000
23,1,1626.267857
23,1,8092.319149
23,1,10008.642857
23,1,3253.107143
23,1,13992.976562
23,1,7483.610294
23,1,10280.797794
23,1,1588.585366
23,1,1694.142857
23,1,1689.727273
23,1,2110.916667
23,1,2644.939103
23,1,7200.764535
23,1,1506.557927
23,1,1621.839744
23,1,3070.857143
23,1,13747.369444
23,1,19575.607143
23,1,1509.315789
23,1,16426.354167
23,1,15005.546512
23,1,9189.777027
23,1,1569.396875
23,1,1581.505814
23,1,17156.592105
23,1,2176.298780
23,1,9532.828804
23,1,14124.301282
23,1,1835.460366
23,1,5391.900424
23,1,4328.267857
23,1,9392.996875
23,1,4782.666667
23,1,3669.494565
23,1,1539.000000
23,1,6104.022727
23,1,18096.550676
23,1,2678.907500
23,1,14569.583333
23,1,5303.031250
23,1,5724.714286
23,1,4095.125000
23,1,1894.454545
23,1,8025.418103
23,1,4091.253676
23,1,1612.901786
23,1,2783.025641
23,1,3075.461290
23,1,5959.418605
23,1,10855.145161
23,1,1669.125000
23,1,1540.125000
23,1,12872.433333
23,1,1588.031250
23,1,4336.505814
23,1,3518.686111
23,1,3169.314516
23,1,1990.900000
23,1,8806.346154
23,1,6686.500000
23,1,2031.041667
23,1,3569.176630
23,1,3167.031250
23,1,4647.433824
23,1,3350.919271

23,1,13248.336111
23,1,8407.015625
23,1,3551.404255
23,1,11401.256944
23,1,3001.979730
23,1,1747.015625
23,1,2383.000000
23,1,4496.620370
23,1,7039.059211
23,1,18969.224359
23,1,5132.541667
23,1,2496.930556
23,1,2093.344595
23,1,1850.085106
23,1,2345.884146
23,1,8961.408333
23,1,1948.611702
23,1,6553.477273
23,1,7276.579545
23,1,3329.280303
23,1,3151.805851
23,1,5989.082143
23,1,8617.336111
23,1,1766.008523
23,1,6124.958333
23,1,1538.530612
23,1,3760.875000
23,1,13295.545918
23,1,2944.282738
23,1,15174.794118
23,1,3335.954787
23,1,1568.782738
23,1,4064.055147
23,1,1605.116667
23,1,1787.203125
23,1,9013.553571
23,1,3858.763514
23,1,5118.997340
23,1,6501.780488
23,1,2440.955882
23,1,2630.023256
23,1,2419.958333
23,1,2288.345930
23,1,2169.508929
23,1,5741.500000
23,1,1959.881579
23,1,5072.182927
23,1,2009.075472
23,1,4584.324324
23,1,2714.145349
23,1,4487.958333
23,1,4175.500000
23,1,18113.869565
23,1,2882.542411
23,1,5791.016667
23,1,2607.872340
23,1,19112.384615
23,1,3857.030000
23,1,2830.486111
23,1,17526.756757
23,1,4191.694444
23,1,10424.915625
23,1,2382.250000
23,1,2200.856618
23,1,1957.500000
23,1,9286.980769

23,1,3525.322917
23,1,10832.456250
23,1,5062.833333
23,1,1824.835227
23,1,5962.968085
23,1,5092.552632
23,1,1642.708333
23,1,4033.777778
23,1,2085.875000
23,1,2761.607500
23,1,17116.759615
23,1,2599.834302
23,1,3190.240000
23,1,17725.532609
23,1,2415.150000
23,1,1997.600000
23,1,2379.680851
23,1,1560.032609
23,1,7992.601744
23,1,1835.829268
23,1,9320.677632
23,1,2529.996951
23,1,2210.808511
23,1,2432.630208
23,1,11095.412500
23,1,1808.695122
23,1,15850.351562
23,1,4391.462500
23,1,3048.600000
23,1,6628.625000
23,1,16833.166667
23,1,10285.402542
23,1,5519.766667
23,1,2837.940104
23,1,1550.062500
23,1,9655.104839
23,1,2241.187500
23,1,9096.409091
23,1,8934.166667
23,1,8969.327703
23,1,13675.684659
23,1,4910.630208
23,1,2615.755102
23,1,6543.815789
23,1,7208.207447
23,1,1768.628571
23,1,3953.153846
23,1,1631.802083
23,1,1711.865854
23,1,3740.937500
23,1,1723.024390
23,1,6255.250000
23,1,1663.815789
23,1,11673.515957
23,1,6343.063830
23,1,12519.960526
23,1,4329.502907
23,1,5131.125000
23,1,1858.750000
23,1,2999.351351
23,1,4816.726852
23,1,1921.464286
23,1,14961.937500
23,1,2456.491935
23,1,6225.156250
23,1,9675.448718

23,1,1747.882353
23,1,3253.906977
23,1,2599.337838
23,1,1534.414773
23,1,4121.423611
23,1,6189.909091
23,1,1991.791667
23,1,2515.295455
23,1,18818.954861
23,1,15677.632353
23,1,3329.906250
23,1,17143.125000
23,1,11724.987500
23,1,5842.647059
23,1,5397.255319
23,1,9637.868750
23,1,3638.630435
23,1,2112.260417
23,1,2340.149390
23,1,2724.827586
23,1,1733.952586
23,1,2125.428571
23,1,14315.263514
23,1,1849.500000
23,1,3554.622500
23,1,2440.464844
23,1,3435.539474
23,1,3937.750000
23,1,8453.760135
23,1,1553.131579
23,1,3685.112500
23,1,1552.375000
23,1,1642.961957
23,1,2195.793919
23,1,6380.034091
23,1,10979.666667
23,1,6927.937500
23,1,12448.744681
23,1,3965.448529
23,1,5741.012821
23,1,2686.243590
23,1,7238.333333
23,1,1739.966667
23,1,2014.062500
23,1,2846.421053
23,1,4010.886364
23,1,1570.664062
23,1,8320.794118
23,1,4250.736842
23,1,14488.729167
23,1,7722.892857
23,1,1939.823370
23,1,2052.609375
23,1,4074.943750
23,1,1666.753125
23,1,9920.906250
23,1,4685.375000
23,1,1536.714286
23,1,11762.375000
23,1,4476.935000
23,1,2811.684028
23,1,1996.242424
23,1,2922.062500
23,1,2711.887255
23,1,2089.250000
23,1,1557.684211

23,1,1986.997340
23,1,7632.425532
23,1,4332.426471
23,1,10217.700000
23,1,2909.586111
23,1,2425.642857
23,1,3717.923780
23,1,10338.978448
23,1,6115.810185
23,1,17742.384615
23,1,14228.429688
23,1,6814.035714
23,1,15460.963415
23,1,2197.112805
23,1,2999.213710
23,1,1708.232143
23,1,3406.900000
23,1,2553.651316
23,1,2825.103659
23,1,5182.121795
23,1,2909.478261
23,1,5058.797297
23,1,12505.271277
23,1,10780.070312
23,1,8895.125000
23,1,3794.272727
23,1,12093.333333
23,1,5048.159091
23,1,12739.584559
23,1,3607.485294
23,1,2125.218750
23,1,2213.753378
23,1,7401.437500
23,1,4277.493902
23,1,2689.538265
23,1,3544.145161
23,1,9472.363636
23,1,1706.250000
23,1,3309.189655
23,1,14684.953125
23,1,4378.580645
23,1,2553.412500
23,1,2974.082143
23,1,1828.400000
23,1,14138.531250
23,1,1567.886364
23,1,3010.600000
23,1,10726.466216
23,1,11533.905303
23,1,7781.947368
23,1,4554.500000
23,1,7445.200000
23,1,16024.181818
23,1,4106.136364
23,1,7103.483871
23,1,16072.153846
23,1,12088.419872
23,1,10406.341837
23,1,2591.812500
23,1,2347.238889
23,1,5072.800000
23,1,2559.320513
23,1,4865.523256
23,1,5075.812500
23,1,3099.293269
23,1,2496.650568

23,1,2566.965517
23,1,3654.265625
23,1,19517.585227
23,1,9831.416667
23,1,12700.095238
23,1,5246.892857
23,1,2641.329167
23,1,10341.641892
23,1,3357.625000
23,1,2996.167553
23,1,8076.340278
23,1,2312.774390
23,1,14713.566406
23,1,2771.852564
23,1,2479.571429
23,1,3703.573864
23,1,5216.032258
23,1,12003.092742
23,1,1925.193548
23,1,1775.990854
23,1,8379.905405
23,1,2317.141667
23,1,1907.459559
23,1,2091.395349
23,1,1710.739796
23,1,4589.909091
23,1,11999.839286
23,1,2640.687500
23,1,18452.648649
23,1,4427.018519
23,1,3143.218750
23,1,2091.638514
23,1,6951.823529
23,1,12722.912791
23,1,1746.487805
23,1,14598.830882
23,1,5379.609694
23,1,1914.128049
23,1,1685.144737
23,1,3396.012821
23,1,4190.029412
23,1,2948.121622
23,1,4121.963235
23,1,11281.884146
23,1,1650.584375
23,1,1902.597826
23,1,13261.943182
23,1,2184.985577
23,1,2018.069444
23,1,3338.772727
23,1,10231.782895
23,1,1584.500000
23,1,17941.279605
23,1,10757.910156
23,1,2095.960227
23,1,8516.304878
23,1,1916.230769
23,1,3280.088235
23,1,7004.780488
23,1,3956.996324
23,1,2286.045455
23,1,12938.844444
23,1,2451.192308
23,1,2892.659091
23,1,4198.113095
23,1,1889.993902

23,1,2622.554688
23,1,5864.818750
23,1,2341.522727
23,1,7085.512500
23,1,9839.343750
23,1,1792.891447
23,1,4769.416667
23,1,2908.113636
23,1,1516.760000
23,1,4132.446429
23,1,4255.136719
23,1,2728.538462
23,1,3205.648438
23,1,5806.166667
23,1,11620.556818
23,1,2931.650000
23,1,11668.992188
23,1,1511.975610
23,1,2711.941860
23,1,3126.695312
23,1,6748.294118
23,1,1693.000000
23,1,11738.051724
23,1,1516.600000
23,1,1851.764706
23,1,4735.692308
23,1,3707.179487
23,1,5377.916667
23,1,5606.942857
23,1,2957.151515
23,1,4801.316667
23,1,1892.786585
23,1,17208.960000
23,1,1815.814189
23,1,2761.654762
23,1,8320.625000
23,1,1853.666667
23,1,11855.371429
23,1,12647.916667
23,1,12175.232143
23,1,1657.750000
23,1,15346.237179
23,1,2080.423077
23,1,2956.922794
23,1,4364.081897
23,1,19889.823529
23,1,2268.845238
23,1,12391.229730
23,1,1676.205357
23,1,1516.410156
23,1,1538.860795
23,1,2040.647059
23,1,3456.620690
23,1,11003.152174
23,1,5104.190789
23,1,4421.615385
23,1,3199.114583
23,1,3529.500000
23,1,10527.256098
23,1,5502.482143
23,1,2440.650000
23,1,4677.006098
23,1,11712.738372
23,1,1562.125000
23,1,5439.985795
23,1,1779.463415

23,1,3652.191176
23,1,2566.284483
23,1,14206.105769
23,1,18427.909574
23,1,3147.857143
23,1,2497.763514
23,1,4795.218750
23,1,4541.818182
23,1,15262.285156
23,1,7747.588235
23,1,16105.022727
23,1,7930.093750
23,1,16251.089674
23,1,2562.500000
23,1,3764.048913
23,1,2157.187500
23,1,11959.452703
23,1,2799.700000
23,1,2161.678571
23,1,1767.168919
23,1,5613.738636
23,1,2173.421053
23,1,3601.267442
23,1,4890.153846
23,1,3007.115385
23,1,6472.071429
23,1,10334.118902
23,1,14706.865385
23,1,3379.455882
23,1,4441.333333
23,1,2209.532051
23,1,4212.371951
23,1,2713.232143
23,1,3504.035156
23,1,3401.363971
23,1,2189.767857
23,1,7701.012195
23,1,2011.375000
23,1,9396.105769
23,1,5010.846875
23,1,2039.429688
23,1,1821.532500
23,1,3209.489286
23,1,4566.414634
23,1,5600.400000
23,1,4115.666667
23,1,10846.238095
23,1,8268.205882
23,1,4355.319444
23,1,1949.229167
23,1,1874.862069
23,1,4312.419643
23,1,9337.154605
23,1,2311.458333
23,1,2499.695652
23,1,3264.935897
23,1,3537.379808
23,1,2831.337500
23,1,6626.307692
23,1,11450.911111
23,1,4537.069853
23,1,3505.816176
23,1,2016.334677
23,1,1914.890625
23,1,5537.782609
23,1,3722.675595

23,1,3578.306250
23,1,4141.483696
23,1,2014.871094
23,1,3774.835227
23,1,6468.100694
23,1,4315.534884
23,1,4466.829861
23,1,4545.833333
23,1,1880.128049
23,1,1635.516447
23,1,8832.837209
23,1,3362.281250
23,1,4363.045455
23,1,12469.634375
23,1,11517.517045
23,1,1585.333333
23,1,12807.094512
23,1,5316.395833
23,1,7508.340909
23,1,5351.281250
23,1,1974.785714
23,1,6241.209677
23,1,3283.872340
23,1,1965.735714
23,1,1821.608108
23,1,2482.900735
23,1,4767.000000
23,1,5379.753289
23,1,2944.500000
23,1,3056.694853
23,1,1826.255319
23,1,2221.065476
23,1,2731.750000
23,1,7263.357143
23,1,5259.290323
23,1,2473.847826
23,1,4491.576613
23,1,17990.028571
23,1,2964.653846
23,1,4592.923611
23,1,1973.279412
23,1,2804.475000
23,1,10382.365385
23,1,2444.255556
23,1,3889.553125
23,1,9334.984375
23,1,18404.952381
23,1,13296.421053
23,1,6358.297619
23,1,3329.425676
23,1,2098.650641
23,1,2295.854839
23,1,4311.121622
23,1,2209.236842
23,1,15361.417614
23,1,11112.455882
23,1,4741.713542
23,1,14261.225000
23,1,11842.044444
23,1,2120.898438
23,1,2467.783537
23,1,1972.150000
23,1,3212.256944
23,1,2268.690476
23,1,2819.314655
23,1,2239.389205

23,1,9250.416667
23,1,2374.096939
23,1,10658.528571
23,1,3534.467857
23,1,9287.006250
23,1,10986.058824
23,1,11942.818182
23,1,2397.710526
23,1,2081.125000
23,1,10463.025641
23,1,2942.840909
23,1,3543.775641
23,1,1512.910714
23,1,8346.697674
23,1,16846.058824
23,1,1783.687500
23,1,1792.708333
23,1,2855.033333
23,1,2328.073529
23,1,15720.375000
23,1,2272.175676
23,1,3733.000000
23,1,15840.250000
23,1,1617.319853
23,1,1615.823529
23,1,9238.648649
23,1,5744.084239
23,1,6508.743590
23,1,12102.476974
23,1,2011.525000
23,1,2727.418103
23,1,3228.019231
23,1,6896.122093
23,1,1884.307692
23,1,1522.296429
23,1,2088.910714
23,1,2413.551282
23,1,5367.598837
23,1,3872.445312
23,1,11451.883333
23,1,3330.482143
23,1,2087.488889
23,1,3032.402344
23,1,2614.144231
23,1,11982.875000
23,1,6923.445652
23,1,1665.540323
23,1,2087.250000
23,1,8748.165698
23,1,2193.701220
23,1,1644.423077
23,1,2946.577381
23,1,3745.494048
23,1,4848.000000
23,1,3180.365854
23,1,12795.883929
23,1,3999.578125
23,1,2463.854167
23,1,8977.625000
23,1,1674.833333
23,1,3185.400000
23,1,14945.082143
23,1,1892.994565
23,1,9971.639344
23,1,1734.200000
23,1,1904.946429

23,1,16062.711864
23,1,1557.113636
23,1,4357.198171
23,1,7394.833333
23,1,7271.116279
23,1,1862.375000
23,1,3945.529070
23,1,1821.285714
23,1,3184.527273
23,1,5166.287736
23,1,1627.888889
23,1,15587.589286
23,1,5893.717391
23,1,2853.569444
23,1,6174.656250
23,1,7401.239583
23,1,15956.269737
23,1,1522.875000
23,1,2447.833333
23,1,1995.238636
23,1,3543.008065
23,1,2831.729651
23,1,2744.614362
23,1,1629.375000
23,1,5502.171875
23,1,3342.680921
23,1,10785.607143
23,1,1974.500000
23,1,3075.503731
23,1,1781.000000
23,1,3355.333333
23,1,3430.338235
23,1,2365.026596
23,1,2217.296512
23,1,8450.486486
23,1,2416.230978
23,1,9155.294118
23,1,1953.867925
23,1,1745.460526
23,1,2484.180000
23,1,4849.325581
23,1,3914.459459
23,1,5969.520833
23,1,8159.806452
23,1,7600.800000
23,1,1618.000000
23,1,3526.457547
23,1,2270.270270
23,1,5591.961111
23,1,2351.150000
23,1,2557.477273
23,1,1840.535714
23,1,6950.273026
23,1,1743.402439
23,1,5330.243902
23,1,15158.500000
23,1,2088.768182
23,1,6436.025641
23,1,1994.666667
23,1,11109.515957
23,1,2519.947674
23,1,1754.051471
23,1,1969.568182
23,1,18900.455729
23,1,5430.894737
23,1,15184.419643

23,1,7674.160000
23,1,6409.171875
23,1,6363.413043
23,1,3760.312500
23,1,2007.039062
23,1,4396.014286
23,1,1911.910714
23,1,12886.093750
23,1,5450.544643
23,1,2384.640000
23,1,6954.666667
23,1,2495.972222
23,1,5285.136792
23,1,4298.276163
23,1,3595.216667
23,1,7522.203125
23,1,1603.534091
23,1,14622.510417
23,1,2317.657895
23,1,3783.102273
23,1,4348.815972
23,1,3410.500000
23,1,3099.944149
23,1,2714.701923
23,1,6864.055804
23,1,6853.461538
23,1,13196.019531
23,1,4827.146635
23,1,1638.841463
23,1,5111.096591
23,1,19447.510638
23,1,12624.142442
23,1,4635.453125
23,1,1816.369565
23,1,1582.534722
23,1,3579.358491
23,1,3246.173077
23,1,6992.125000
23,1,9735.882812
23,1,6016.057692
23,1,12120.835227
23,1,8784.214674
23,1,1794.604651
23,1,2214.668478
23,1,4797.958333
23,1,1624.187500
23,1,2180.462500
23,1,2829.755102
23,1,2165.100806
23,1,1780.802083
23,1,4068.520833
23,1,12601.625000
23,1,17179.403846
23,1,9109.048913
23,1,2498.916667
23,1,5561.691406
23,1,3721.840909
23,1,4573.607143
23,1,19963.723837
23,1,3407.468750
23,1,4645.336538
23,1,2138.238372
23,1,1707.000000
23,1,6169.695652
23,1,9530.600000
23,1,2199.534091

23,1,10575.468085
23,1,1628.125000
23,1,3814.224537
23,1,2426.350000
23,1,1696.294118
23,1,1719.133929
23,1,18418.625000
23,1,2465.700000
23,1,10485.842105
23,1,9123.152439
23,1,6601.250000
23,1,17427.509434
23,1,1572.628049
23,1,3529.645161
23,1,1765.125000
23,1,11663.041667
23,1,16452.950000
23,1,5255.951389
23,1,1789.450658
23,1,1769.503289
23,1,6723.256579
23,1,1941.923077
23,1,1803.334239
23,1,1713.468750
23,1,5026.936170
23,1,4562.537234
23,1,3287.125000
23,1,11899.156250
23,1,10560.425000
23,1,5119.557692
23,1,13974.678241
23,1,1946.902439
23,1,17517.899457
23,1,2253.454545
23,1,7574.209302
23,1,3372.516667
23,1,2412.027273
23,1,9414.862500
23,1,3939.385000
23,1,8271.589623
23,1,2962.492188
23,1,4968.043750
23,1,5688.973558
23,1,1523.523936
23,1,5337.347222
23,1,15813.842857
23,1,2895.992347
23,1,3395.521739
23,1,2302.351351
23,1,3383.835526
23,1,3469.409091
23,1,1986.845930
23,1,5734.835106
23,1,7082.137931
23,1,4601.210526
23,1,3103.236842
23,1,2915.000000
23,1,3062.750000
23,1,15146.020833
23,1,2728.083333
23,1,2387.657738
23,1,10594.358974
23,1,10484.432927
23,1,3032.363636
23,1,1568.547872
23,1,2395.183673

23,1,3897.559375
23,1,18804.253906
23,1,13954.707447
23,1,1595.187500
23,1,1520.246324
23,1,2900.600000
23,1,16165.381250
23,1,11179.187500
23,1,3599.763514
23,1,15721.072368
23,1,13626.148649
23,1,3109.098958
23,1,12547.750000
23,1,3447.161932
23,1,3873.375000
23,1,2663.146875
23,1,13253.500000
23,1,2281.087500
23,1,4419.054688
23,1,2639.125000
23,1,1885.333333
23,1,2756.682927
23,1,2868.218182
23,1,2952.862069
23,1,8527.243750
23,1,1537.785714
23,1,14725.905172
23,1,8067.890625
23,1,4536.790441
23,1,2087.675000
23,1,2531.340625
23,1,2250.395833
23,1,14622.691860
23,1,8754.781250
23,1,1691.384615
23,1,2715.220000
23,1,17029.035714
23,1,7659.500000
23,1,11203.517045
23,1,5866.420732
23,1,6598.357143
23,1,1991.500000
23,1,1929.480769
23,1,4945.710714
23,1,5363.875000
23,1,3275.072115
23,1,1702.103659
23,1,3259.371429
23,1,6467.102941
23,1,10808.387500
23,1,5221.500000
23,1,5696.403846
23,1,10777.889706
23,1,4316.823171
23,1,1575.500000
23,1,5889.556818
23,1,2164.632353
23,1,5477.766129
23,1,5867.757576
23,1,2333.168750
23,1,8473.250000
23,1,2307.273438
23,1,4237.589286
23,1,2432.402778
23,1,10621.448718
23,1,1878.162500

23,1,4579.618750
23,1,3194.000000
23,1,2279.433594
23,1,2747.510870
23,1,1707.074468
23,1,4125.562500
23,1,7215.589286
23,1,9211.723684
23,1,3093.178030
23,1,2036.433824
23,1,4708.017857
23,1,2295.207143
23,1,2118.215909
23,1,3172.804054
23,1,6573.901316
23,1,1558.250000
23,1,5831.635870
23,1,15394.971154
23,1,16155.634868
23,1,5292.637500
23,1,19667.341912
23,1,2874.413793
23,1,10974.939024
23,1,3351.058594
23,1,4741.687500
23,1,4123.292683
23,1,1677.095109
23,1,6389.250000
23,1,2053.125000
23,1,6776.396947
23,1,8368.304688
23,1,2827.537879
23,1,6188.544118
23,1,2299.250000
23,1,5016.257143
23,1,3864.717105
23,1,3324.718750
23,1,4601.650000
23,1,8361.209677
23,1,13552.513587
23,1,7603.931250
23,1,3546.413690
23,1,1717.875000
23,1,2834.000000
23,1,1556.125000
23,1,2053.926829
23,1,10096.939516
23,1,7069.515625
23,1,8467.192308
23,1,3463.982143
23,1,6340.361111
23,1,10427.666667
23,1,2825.631579
23,1,5530.847561
23,1,13597.322917
23,1,1910.718750
23,1,2203.416667
23,1,15455.657895
23,1,2576.666667
23,1,8373.462500
23,1,12721.790816
23,1,2947.753472
23,1,2249.981618
23,1,14718.468085
23,1,2320.772436
23,1,3229.755952

23,1,1907.838415
23,1,15871.323171
23,1,3812.352273
23,1,5656.621622
23,1,15841.830882
23,1,2471.750000
23,1,6326.531250
23,1,4507.852941
23,1,4197.385135
23,1,1938.197115
23,1,12371.906250
23,1,3033.000000
23,1,8382.198171
23,1,2930.384615
23,1,2325.178571
23,1,9211.551136
23,1,5809.368056
23,1,11429.081250
23,1,2319.857143
23,1,2322.357143
23,1,17132.136364
23,1,12124.809211
23,1,15484.633333
23,1,1754.344512
23,1,5201.009259
23,1,9753.000000
23,1,5271.835366
23,1,16175.362500
23,1,14691.798077
23,1,15338.973404
23,1,12394.681818
23,1,7128.535000
23,1,3502.277778
23,1,3029.101852
23,1,2166.556818
23,1,10950.705357
23,1,3059.067857
23,1,2471.088235
23,1,1789.854167
23,1,7001.000000
23,1,4118.642857
23,1,5670.198864
23,1,9898.970588
23,1,13681.711111
23,1,3837.946875
23,1,1536.744792
23,1,1546.384615
23,1,6541.705000
23,1,3680.533333
23,1,16641.607143
23,1,3265.968750
23,1,6395.461538
23,1,1982.964286
23,1,6266.916667
23,1,7010.714286
23,1,3018.948718
23,1,2310.647059
23,1,1584.386905
23,1,2326.845588
23,1,14018.832317
23,1,2682.835714
23,1,12549.898256
23,1,2646.451220
23,1,2310.970588
23,1,1848.949219
23,1,2202.115854

23,1,19648.708333
23,1,3327.525000
23,1,1682.644231
23,1,5586.291667
23,1,2891.974265
23,1,3335.055147
23,1,4597.000000
23,1,3537.037500
23,1,2516.000000
23,1,9425.332317
23,1,1531.904412
23,1,1570.062500
23,1,10656.625000
23,1,1968.125000
23,1,3142.838710
23,1,7766.647059
23,1,13788.903226
23,1,8137.208333
23,1,1676.185811
23,1,3047.959559
23,1,11587.973684
23,1,1939.500000
23,1,12255.463415
23,1,3592.545455
23,1,1826.833333
23,1,8956.036184
23,1,13530.812500
23,1,13441.812500
23,1,2627.320122
23,1,5472.432692
23,1,13859.817073
23,1,1611.930000
23,1,5323.381098
23,1,1596.446429
23,1,2634.500000
23,1,3293.833333
23,1,1653.351190
23,1,3411.833333
23,1,2075.243902
23,1,2879.625000
23,1,1906.297619
23,1,7891.375000
23,1,8250.902439
23,1,10659.878205
23,1,3388.073171
23,1,6491.838235
23,1,3022.911765
23,1,3494.692308
23,1,1930.673077
23,1,7712.770408
23,1,4026.290441
23,1,11766.080357
23,1,3017.059524
23,1,11013.303125
23,1,4890.880208
23,1,4684.363636
23,1,6801.847561
23,1,1861.843750
23,1,7830.897436
23,1,1558.940476
23,1,18880.440789
23,1,2391.750000
23,1,4485.690625
23,1,2793.625000
23,1,1500.720930
23,1,1929.576923

23,1,2127.779412
23,1,2119.926471
23,1,5416.062500
23,1,2903.341837
23,1,4008.324219
23,1,3213.775568
23,1,1610.192308
23,1,4843.578125
23,1,13311.861111
23,1,1934.563830
23,1,2284.204545
23,1,7210.276596
23,1,3323.455556
23,1,4798.727941
23,1,13749.202381
23,1,7939.472656
23,1,13496.057432
23,1,14515.625000
23,1,1593.085784
23,1,2902.640625
23,1,1870.912791
23,1,5394.761719
23,1,7013.840000
23,1,2291.923077
23,1,14462.243902
23,1,16536.857143
23,1,1593.853261
23,1,1534.971429
23,1,19148.628472
23,1,17979.250000
23,1,4090.211538
23,1,2634.271341
23,1,7399.540541
23,1,2227.119681
23,1,7662.661765
23,1,7945.554054
23,1,5383.735294
23,1,4409.529255
23,1,2738.011574
23,1,3937.663462
23,1,2946.683824
23,1,10875.500000
23,1,8912.041667
23,1,2721.218750
23,1,7171.829545
23,1,2330.143293
23,1,1703.609375
23,1,6159.180000
23,1,13348.288265
23,1,16966.535714
23,1,3063.605263
23,1,7438.106618
23,1,2208.529412
23,1,1783.536765
23,1,1502.367424
23,1,6629.017857
23,1,3094.200000
23,1,8748.814394
23,1,6180.781250
23,1,2984.808824
23,1,1595.759868
23,1,14855.481132
23,1,4204.500000
23,1,1510.355556
23,1,2864.842105
23,1,2769.352564

23,1,1560.761905
23,1,7517.093137
23,1,14732.767442
23,1,2351.146739
23,1,3882.079082
23,1,4607.625000
23,1,2160.410714
23,1,2118.286458
23,1,19664.535714
23,1,4720.241667
23,1,2038.125000
23,1,8052.989130
23,1,1797.695652
23,1,3311.777778
23,1,2237.476562
23,1,4161.947368
23,1,3220.599265
23,1,3822.669118
23,1,3316.244444
23,1,3407.891447
23,1,16561.970238
23,1,1651.692073
23,1,2376.336735
23,1,7950.135135
23,1,3165.260135
23,1,2739.913194
24,2,1775.150641
24,2,2672.102273
24,2,1759.055147
24,2,2555.019231
24,2,2028.439024
24,2,5127.383333
24,2,1875.067935
24,2,1949.684783
24,2,1571.670213
24,2,4980.785714
24,2,2015.911290
24,2,1651.509375
24,2,2559.365132
24,2,1679.971774
24,2,2506.058511
24,2,1524.446809
24,2,2007.675000
24,2,2319.245283
24,2,2646.941176
24,2,2069.195755
24,2,2572.842742
24,2,1852.400000
24,2,7359.578125
24,2,1934.825000
24,2,2453.577778
24,2,1856.875000
24,2,3376.709091
24,2,2287.875000
24,2,2758.551471
24,2,1572.800000
24,2,1642.730556
24,2,8364.363636
24,2,2084.702381
24,2,1558.125000
24,2,2131.852273
24,2,2078.150000
24,2,2478.807500
24,2,3757.297297
24,2,2277.446809
24,2,2777.571429

24,2,2158.329268
24,2,1730.136364
24,2,2462.325000
24,2,9271.033654
24,2,2034.340426
24,2,2828.195313
24,2,1587.523256
24,2,2386.922619
24,2,2587.960000
24,2,1750.592593
24,2,1600.031818
24,2,3431.846154
24,2,2759.783333
24,2,1544.710526
24,2,4178.921569
24,2,2015.491848
24,2,1643.957447
24,2,1853.957237
24,2,1537.055556
24,2,1958.978723
24,2,1523.769231
24,2,1716.085000
24,2,2493.384615
24,2,9573.642857
24,2,1737.521739
24,2,3180.712963
24,2,2632.451923
24,2,2159.020270
24,2,1554.758929
24,2,2424.111111
24,2,4659.032407
24,2,1724.536585
24,2,3092.747093
24,2,1861.814024
24,2,1610.447500
24,2,1589.500000
24,2,2122.411765
24,2,3437.322500
24,2,3972.276316
24,2,10127.687500
24,2,3763.955357
24,2,1837.953125
24,2,2287.940000
24,2,4217.000000
24,2,1499.391026
24,2,1572.965000
24,2,1885.302083
24,2,1599.511628
24,2,2763.505102
24,2,2005.470395
24,2,1966.252841
24,2,2196.278689
24,2,5080.270349
24,2,7721.454082
24,2,3174.305233
24,2,2520.076220
24,2,2111.202586
24,2,4033.720588
24,2,2391.000000
24,2,1675.688953
24,2,1513.148649
24,2,5099.941406
24,2,1566.444915
24,2,1657.430000
24,2,2044.633929
24,2,2927.945652

24,2,1571.988889
24,2,1631.277174
24,2,3673.900000
24,2,1698.560000
24,2,1901.260870
24,2,1724.692308
24,2,1834.263393
24,2,1870.576923
24,2,2092.535156
24,2,2188.244681
24,2,1543.725490
24,2,5712.975000
24,2,1983.909091
24,2,2168.638298
24,2,2334.181818
24,2,2782.722222
24,2,1706.680556
24,2,2139.690104
24,2,1971.285256
24,2,11188.801887
24,2,2630.394737
24,2,2759.366379
24,2,1887.660714
24,2,2145.645000
24,2,2006.800000
24,2,1592.239130
24,2,2010.788265
24,2,1691.584677
24,2,1517.221154
24,2,2363.069767
24,2,2085.140000
24,2,4038.952381
24,2,1752.651316
24,2,1519.308333
24,2,2062.285714
24,2,2311.757653
24,2,1936.896739
24,2,1630.750000
24,2,2003.892045
24,2,2097.220588
24,2,1502.864865
24,2,1824.646341
24,2,1853.941860
24,2,2239.852564
24,2,1526.324468
24,2,2209.634434
24,2,1986.113636
24,2,1706.042614
24,2,2295.207317
24,2,3580.527778
24,2,1602.480000
24,2,2694.115000
24,2,1576.664062
24,2,5992.264706
24,2,1689.932692
24,2,1526.977778
24,2,1995.050000
24,2,1969.880435
24,2,1809.275000
24,2,1538.761029
24,2,3910.308824
24,2,1569.675676
24,2,1617.705882
24,2,1533.600000
24,2,6163.027174
24,2,1692.875000

24,2,3035.341837
24,2,1589.528571
24,2,3015.236842
24,2,1758.750000
24,2,2775.017857
24,2,1888.113636
24,2,2023.333333
24,2,1522.860465
24,2,1999.021739
24,2,2316.631579
24,2,2011.377358
24,2,1525.754310
24,2,1862.798780
24,2,1960.729730
24,2,2304.041667
24,2,2227.901961
24,2,1791.846429
24,2,2102.836957
24,2,3609.323864
24,2,1698.855769
24,2,2075.323529
24,2,2420.093750
24,2,2491.520000
24,2,2308.892045
24,2,2628.297872
24,2,1588.149390
24,2,2691.275000
24,2,1749.096154
24,2,2786.904762
24,2,1757.181818
24,2,4695.937500
24,2,1524.490196
24,2,1825.000000
24,2,1755.039773
24,2,8885.896341
24,2,2031.819767
24,2,3993.235294
24,2,4070.031250
24,2,1607.520408
24,2,1591.517857
24,2,1623.041667
24,2,4012.062500
24,2,1599.000000
24,2,1813.144444
24,2,1932.020270
24,2,4609.275000
24,2,3824.454545
24,2,1660.922619
24,2,2461.243304
24,2,1826.000000
24,2,1587.757500
24,2,1615.704545
24,2,3305.653846
24,2,1899.425595
24,2,1541.717500
24,2,2303.950000
24,2,2515.200000
24,2,2078.976190
24,2,1499.944444
24,2,1736.988095
24,2,3471.076531
24,2,4372.054348
24,2,1720.006098
24,2,3228.710938
24,2,1536.750000
24,2,1868.607143

24,2,2053.010000
24,2,2339.680851
24,2,2915.779412
24,2,1532.664516
24,2,3389.866477
24,2,1644.723404
24,2,1975.109756
24,2,2475.056250
24,2,2914.682065
24,2,2673.234375
24,2,1796.393617
24,2,1563.118421
24,2,3313.683036
24,2,2212.875000
24,2,1822.585366
24,2,2885.855978
24,2,2616.200000
24,2,1520.500000
24,2,5719.709302
24,2,2362.326087
24,2,2234.714286
24,2,1612.608108
24,2,1652.616279
24,2,1623.872340
24,2,3497.915441
24,2,1568.951220
24,2,1657.113095
24,2,1650.731250
24,2,1788.157609
24,2,2623.413265
24,2,1985.640244
24,2,1758.616848
24,2,2235.618750
24,2,1609.173913
24,2,1655.083333
24,2,2275.588235
24,2,1804.987981
24,2,3052.098684
24,2,2800.382979
24,2,1823.375000
24,2,1738.051282
24,2,3843.750000
24,2,2890.572500
24,2,1577.750000
24,2,7459.297872
24,2,2036.365000
24,2,1900.896226
24,2,2858.884615
24,2,2778.854167
24,2,1499.400000
24,2,2125.882353
24,2,1638.244048
24,2,2093.429245
24,2,2069.851562
24,2,4539.567568
24,2,1599.372093
24,2,4405.773585
24,2,2111.247024
24,2,2449.530405
24,2,1604.081250
24,2,1562.393382
24,2,5085.010204
24,2,2317.441860
24,2,1901.743243
24,2,1630.357143
24,2,2284.462500

24,2,12515.375000
24,2,1521.111111
24,2,1975.386364
24,2,1796.619792
24,2,3681.738208
24,2,1742.529762
24,2,2166.926136
24,2,1979.354167
24,2,2444.725000
24,2,2426.620536
24,2,1706.603175
24,2,2114.722222
24,2,1834.665909
24,2,1886.806452
24,2,1683.687500
24,2,2590.531250
24,2,1740.080357
24,2,2281.409091
24,2,1812.843750
24,2,1577.672297
24,2,2232.637500
24,2,2109.986111
24,2,2140.744681
24,2,1663.750000
24,2,2144.980392
24,2,1621.301136
24,2,2419.813953
24,2,1708.000000
24,2,5170.750000
24,2,3318.985849
24,2,1811.476351
24,2,2646.526042
24,2,2567.239796
24,2,1676.735294
24,2,1725.656250
24,2,1943.664634
24,2,3309.709677
24,2,1991.875000
24,2,3505.721154
24,2,1946.083333
24,2,2057.480392
24,2,2882.291667
24,2,1825.651786
24,2,1827.333333
24,2,1855.888889
24,2,6333.076923
24,2,1858.888889
24,2,2245.297872
24,2,3094.000000
24,2,2466.233108
24,2,1758.533333
24,2,2806.700000
24,2,2306.087500
24,2,2627.073864
24,2,4462.409574
24,2,2401.796875
24,2,1682.329545
24,2,1500.432927
24,2,3344.813636
24,2,8653.210227
24,2,1836.575000
24,2,1777.803571
24,2,18039.741935
24,2,1564.969828
24,2,3318.100000
24,2,1730.255814

24,2,2478.469388
24,2,1767.500000
24,2,5532.855263
24,2,2904.187500
24,2,2315.250000
24,2,2289.400000
24,2,1852.526316
24,2,1793.574074
24,2,1545.343750
24,2,1507.682692
24,2,2163.828125
24,2,2235.797872
24,2,2443.575000
24,2,2836.833333
24,2,1769.208333
24,2,1524.997340
24,2,2095.957547
24,2,2420.491071
24,2,2157.607143
24,2,3311.348214
24,2,2946.104592
24,2,1614.544643
24,2,2296.528125
24,2,2417.644231
24,2,1832.568182
24,2,1566.937500
24,2,4738.308140
24,2,1782.859756
24,2,1551.410714
24,2,3232.265625
24,2,2409.442308
24,2,7644.404412
24,2,1570.419492
24,2,2831.540000
24,2,2530.071942
24,2,2804.450000
24,2,2475.104592
24,2,1880.986111
24,2,2857.414634
24,2,1566.746711
24,2,1888.363095
24,2,2798.432432
24,2,1590.613636
24,2,1604.294118
24,2,2219.000000
24,2,1656.295732
24,2,1546.658537
24,2,1850.017857
24,2,2894.696429
24,2,2678.582237
24,2,1792.500000
24,2,3721.063679
24,2,2095.003378
24,2,2892.919118
24,2,2282.450000
24,2,2088.201220
24,2,11012.192308
24,2,2474.679775
24,2,1910.085938
24,2,2485.576531
24,2,2115.559783
24,2,1874.065217
24,2,3199.958333
24,2,2281.384615
24,2,1659.255319
24,2,2344.776316

24,2,2124.951220
24,2,1885.864865
24,2,1561.678571
24,2,2371.375000
24,2,3054.833333
24,2,2441.655612
24,2,3263.702381
24,2,2255.896739
24,2,12208.000000
24,2,1718.954861
24,2,1765.497449
24,2,1515.723837
24,2,2564.871951
24,2,1729.117647
24,2,2335.036765
24,2,2320.766667
24,2,1529.828125
24,2,3451.073864
24,2,1994.010753
24,2,1527.020408
24,2,2052.255435
24,2,1953.613095
24,2,1757.800000
24,2,1634.397059
24,2,2497.653061
24,2,2659.297794
24,2,1959.752551
24,2,2056.011628
24,2,2564.265625
24,2,1753.842857
24,2,1655.081081
24,2,2253.886503
24,2,4255.007075
24,2,2018.940000
24,2,1846.942308
24,2,1708.619048
24,2,4809.681818
24,2,4340.750000
24,2,2168.602941
24,2,1754.846939
24,2,1776.360294
24,2,1542.700000
24,2,3240.688830
24,2,2374.444444
24,2,2031.777778
24,2,1807.526316
24,2,2143.975610
24,2,1576.145833
24,2,6478.544910
24,2,1519.714623
24,2,1977.279070
24,2,1524.023936
24,2,2493.507212
24,2,2022.336957
24,2,5071.953704
24,2,1742.047170
24,2,1756.764423
24,2,2129.750000
24,2,2485.855263
24,2,1789.884868
24,2,1648.702703
24,2,2195.000000
24,2,2271.460000
24,2,1613.537234
24,2,1662.114362
24,2,3203.580357

24,2,2594.494792
24,2,4247.957143
24,2,14885.821429
24,2,1674.018939
24,2,1608.967742
24,2,3548.932927
24,2,2515.701389
24,2,1811.222561
24,2,1742.245000
24,2,1663.705882
24,2,1959.896739
24,2,2040.878049
24,2,1621.222973
24,2,2018.478365
24,2,1528.583333
24,2,2012.000000
24,2,1938.489796
24,2,2333.591912
24,2,3494.794118
24,2,2425.377907
24,2,1795.922222
24,2,1563.909574
24,2,1903.142857
24,2,1837.829268
24,2,2377.602273
24,2,1909.956395
24,2,1700.228571
24,2,3631.415888
24,2,1556.398649
24,2,1678.635870
24,2,2652.941860
24,2,1805.720000
24,2,2748.321875
24,2,4304.625000
24,2,1572.784091
24,2,1510.605000
24,2,3975.666667
24,2,2026.390244
24,2,2732.797872
24,2,2859.475000
24,2,1655.236842
24,2,1629.290698
24,2,6209.225000
24,2,2074.772727
24,2,4337.252232
24,2,1716.854167
24,2,1689.197368
24,2,2144.256098
24,2,1579.616667
24,2,2136.666667
24,2,3229.420918
24,2,2131.966981
24,2,2026.774390
24,2,1597.166667
24,2,1509.448980
24,2,3204.007042
24,2,2002.200000
24,2,1793.731844
24,2,2829.105556
24,2,1711.875000
24,2,2902.637931
24,2,2557.617647
24,2,2003.060096
24,2,3842.065000
24,2,4950.031250
24,2,1992.256098

24,2,2376.237903
24,2,4291.937500
24,2,1970.707386
24,2,2195.136364
24,2,1658.261905
24,2,2176.182870
24,2,1996.297872
24,2,2305.423469
24,2,1718.762712
24,2,1499.583333
24,2,5337.916667
24,2,2325.834302
24,2,2847.633929
24,2,4063.102273
24,2,5250.798077
24,2,1698.057870
24,2,2089.030000
24,2,1643.878049
24,2,2481.069444
24,2,1826.346939
24,2,1650.954787
24,2,2997.750000
24,2,1920.772727
24,2,1715.389535
24,2,2442.250000
24,2,2496.580645
24,2,2033.426829
24,2,6020.052326
24,2,2027.752273
24,2,2222.546429
24,2,3672.811170
24,2,1856.677419
24,2,1796.477273
24,2,3185.063830
24,2,1897.558824
24,2,1694.395349
24,2,1679.522222
24,2,3664.163265
24,2,3544.125000
24,2,1809.702128
24,2,1747.577778
24,2,1774.200000
24,2,2032.119681
24,2,1771.577778
24,2,1558.181818
24,2,1881.360465
24,2,1606.687500
24,2,1775.825000
24,2,2004.048913
24,2,1866.728448
24,2,1967.395349
24,2,3653.562500
24,2,2900.204082
24,2,4134.853448
24,2,1877.892857
24,2,1646.590278
24,2,1587.008152
24,2,2069.132075
24,2,5177.299419
24,2,3516.634146
24,2,2816.762821
24,2,2002.750000
24,2,1667.272500
24,2,2943.251256
24,2,1966.390909
24,2,2268.171053

24,2,1773.108696
24,2,1694.515306
24,2,4598.331522
24,2,1500.144231
24,2,3359.380952
24,2,2269.691860
24,2,1817.601562
24,2,3040.200000
24,2,1711.277500
24,2,3182.421053
24,2,2124.202128
24,2,2243.820000
24,2,5259.333333
24,2,1549.186047
24,2,4159.931452
24,2,2138.204082
24,2,2606.397436
24,2,1939.948718
24,2,1726.709184
24,2,3587.321429
24,2,4109.062500
24,2,3393.101064
24,2,1824.941176
24,2,1852.085366
24,2,1712.849057
24,2,1691.517857
24,2,5701.060811
24,2,1689.407738
24,2,2527.636029
24,2,2220.098131
24,2,1850.105263
24,2,3229.076923
24,2,2121.091463
24,2,1700.079268
24,2,2726.840426
24,2,6823.763158
24,2,1951.231707
24,2,2299.095109
24,2,1670.693750
24,2,4451.875000
24,2,1968.142157
24,2,1645.531250
24,2,1741.662791
24,2,2592.487500
24,2,3131.348684
24,2,1623.682927
24,2,3888.625000
24,2,4760.235795
24,2,2278.555288
24,2,2313.525641
24,2,2147.156250
24,2,5489.852083
24,2,1665.602564
24,2,2286.913043
24,2,3543.622727
24,2,1662.062500
24,2,5313.714286
24,2,2048.892308
24,2,4133.886364
24,2,7040.532609
24,2,2010.224490
24,2,2729.480000
24,2,2964.739130
24,2,1748.487805
24,2,2850.121711
24,2,2021.350962

24,2,1663.846154
24,2,1904.851563
24,2,2800.600543
24,2,2204.254902
24,2,1544.875000
24,2,1995.727749
24,2,2588.524038
24,2,1678.993750
24,2,1874.610294
24,2,3061.888889
24,2,1901.446023
24,2,1851.317073
24,2,1713.255102
24,2,2065.163265
24,2,2028.750000
24,2,1641.254902
24,2,3675.946429
24,2,4867.559211
24,2,3045.048780
24,2,3037.851190
24,2,2222.644737
24,2,2352.224490
24,2,2992.016760
24,2,18600.656250
24,2,6049.863636
24,2,1628.000000
24,2,1638.442308
24,2,2112.614286
24,2,2544.312500
24,2,5846.577778
24,2,8880.588362
24,2,1898.098214
24,2,1768.825000
24,2,2810.092391
24,2,2287.000000
24,2,1719.419643
24,2,2201.484848
24,2,9558.277778
24,2,14534.526316
24,2,2587.441860
24,2,2765.250000
24,2,5273.016892
24,2,1589.375000
24,2,1586.400000
24,2,1570.190217
24,2,2299.000000
24,2,2247.775000
24,2,1521.979167
24,2,3474.900000
24,2,2304.625000
24,2,2822.680000
24,2,4087.579082
24,2,4652.823661
24,2,3623.109091
24,2,1537.214286
24,2,2553.491489
24,2,7580.642857
24,2,4240.951087
24,2,1641.619048
24,2,2172.967742
24,2,2998.878205
24,2,1987.604167
24,2,5467.865000
24,2,2569.352941
24,2,4088.260870
24,2,2662.146341

24,2,1690.224490
24,2,2353.557143
24,2,1717.361111
24,2,1562.852564
24,2,1706.630000
24,2,3844.357955
24,2,1599.602041
24,2,2743.562500
24,2,3358.850000
24,2,5927.291667
24,2,2849.778409
24,2,1732.740196
24,2,5811.818182
24,2,1834.685714
24,2,1718.484043
24,2,4033.698864
24,2,1700.320312
24,2,2363.366071
24,2,1976.802500
24,2,2411.226744
24,2,1814.071429
24,2,6562.491848
24,2,2305.165000
24,2,2713.006250
24,2,2467.625000
24,2,2075.368056
24,2,2094.190217
24,2,1692.480769
24,2,3667.935185
24,2,1660.500000
24,2,2434.585227
24,2,2321.163265
24,2,1599.509434
24,2,2641.571429
24,2,1797.413462
24,2,1690.130435
24,2,2351.565217
24,2,2089.571429
24,2,1645.696809
24,2,1569.835000
24,2,1749.121951
24,2,2579.935185
24,2,1873.762255
24,2,1530.451613
24,2,2524.500000
24,2,2535.625000
24,2,1872.680000
24,2,10866.208333
24,2,5113.691176
24,2,1975.092105
24,2,2890.104545
24,2,1985.114865
24,2,1887.000000
24,2,1594.196429
24,2,3298.490132
24,2,3424.400000
24,2,2157.125000
24,2,1874.317073
24,2,2373.403846
24,2,1607.125000
24,2,4279.500000
24,2,3737.364865
24,2,2029.757143
24,2,4305.085227
24,2,2859.651042
24,2,2013.307870

24,2,1757.678125
24,2,2217.184211
24,2,1617.263158
24,2,2430.110714
24,2,1771.642857
24,2,1775.275000
24,2,1917.676471
24,2,2386.850000
24,2,9783.657500
24,2,5361.789773
24,2,1863.310345
24,2,2444.963415
24,2,2133.895833
24,2,1528.250000
24,2,2561.258929
24,2,2687.915625
24,2,3560.727273
24,2,2294.562500
24,2,1804.683824
24,2,2886.701531
24,2,2690.530093
24,2,1701.179487
24,2,1944.228261
24,2,2168.088889
24,2,3253.030303
24,2,2056.734694
24,2,1807.202381
24,2,1637.487500
24,2,2380.602041
24,2,1980.882812
24,2,2325.335648
24,2,1989.800532
24,2,1970.203947
24,2,1539.968750
24,2,1575.920000
24,2,1906.665816
24,2,3181.450000
24,2,1740.520833
24,2,2216.339623
24,2,3538.817500
24,2,2190.253125
24,2,1570.925000
24,2,1599.342995
24,2,4387.095238
24,2,4031.243243
24,2,3449.177885
24,2,3709.060547
24,2,1631.271277
24,2,2473.657895
24,2,2390.432692
24,2,1912.769231
24,2,1804.548387
24,2,2616.250000
24,2,2144.150000
24,2,1872.351974
24,2,1989.500000
24,2,1707.309783
24,2,3396.489583
24,2,2644.589623
24,2,1755.533163
24,2,6432.726415
24,2,1872.464286
24,2,1877.341346
24,2,1932.461957
24,2,11454.055851
24,2,1518.720000

24,2,2144.928571
24,2,2544.940104
24,2,1984.367347
24,2,1862.110465
24,2,8764.894737
24,2,1931.596354
24,2,2631.000000
24,2,3019.589623
24,2,1621.604167
24,2,1873.713235
24,2,1577.092949
24,2,1512.333333
24,2,1848.495370
24,2,2616.556250
24,2,3017.032143
24,2,2183.519231
24,2,2472.730769
24,2,1667.133523
24,2,1565.514535
24,2,1751.778846
24,2,2642.047222
24,2,2338.125000
24,2,3264.615000
24,2,1945.381410
24,2,8845.920000
24,2,1766.593750
24,2,2025.277778
24,2,1725.562500
24,2,1918.878049
24,2,4528.494318
24,2,1527.051136
24,2,1558.071970
24,2,2270.867647
24,2,3295.849057
24,2,5637.290541
24,2,10730.771875
24,2,1748.193182
24,2,2040.681122
24,2,1651.788462
24,2,1676.771429
24,2,1595.369792
24,2,1995.983333
24,2,3422.105769
24,2,2388.420732
24,2,2516.560000
24,2,1699.307692
24,2,2403.762500
24,2,1896.846591
24,2,2783.112179
24,2,2259.778409
24,2,2276.600000
24,2,2782.365385
24,2,2226.052500
24,2,2403.481481
24,2,1873.750000
24,2,1739.877500
24,2,1935.750000
24,2,3478.761792
24,2,7332.395000
24,2,2300.269231
24,2,1924.017857
24,2,2668.395833
24,2,2310.708333
24,2,1597.909574
24,2,1537.536765
24,2,1739.031250

24,2,1962.631757
24,2,2338.478261
24,2,2231.631579
24,2,2033.750000
24,2,1896.528571
24,2,1920.625000
24,2,5441.766827
24,2,1988.046196
24,2,2452.661765
24,2,2994.770408
24,2,1504.063830
24,2,1882.519737
24,2,1594.063953
24,2,2180.301887
24,2,1629.926471
24,2,3762.410256
24,2,12384.823529
24,2,5353.904762
24,2,1763.850000
24,2,1516.590909
24,2,2080.300000
24,2,3544.333333
24,2,1608.315217
24,2,2085.672297
24,2,2236.802885
24,2,2917.246429
24,2,3122.948276
24,2,1856.875000
24,2,1738.932500
24,2,1602.435484
24,2,1581.000000
24,2,2272.500000
24,2,1540.568627
24,2,1511.015625
24,2,3373.088889
24,2,1757.187500
24,2,3198.348214
24,2,1605.152174
24,2,3876.450000
24,2,1986.056122
24,2,1730.633929
24,2,4536.368421
24,2,1930.190104
24,2,6677.083333
24,2,3514.625000
24,2,4704.433468
24,2,2440.598837
24,2,1978.529070
24,2,4834.080645
24,2,1544.388587
24,2,2174.024457
24,2,1802.496951
24,2,2157.375000
24,2,1931.092593
24,2,1883.876712
24,2,1858.663462
24,2,2583.641026
24,2,2012.935714
24,2,2629.312500
24,2,1583.461538
24,2,3403.656250
24,2,3731.420000
24,2,2588.300781
24,2,1656.117647
24,2,5623.256696
24,2,2386.560000

24,2,2002.521739
24,2,1804.515625
24,2,1881.092262
24,2,2217.264706
24,2,2430.826087
24,2,2426.154412
24,2,1927.350000
24,2,1822.800532
24,2,3418.420213
24,2,2080.144172
24,2,1524.944079
24,2,1916.405128
24,2,1716.329609
24,2,3174.666667
24,2,2888.163265
24,2,1811.098958
24,2,1785.589744
24,2,1838.002841
24,2,1812.590278
24,2,2345.222222
24,2,5887.424419
24,2,2035.198718
24,2,5266.017241
24,2,1656.120000
24,2,2041.000000
24,2,1819.608696
24,2,1646.464286
24,2,3830.837500
24,2,1548.588415
24,2,2569.388636
24,2,3114.045139
24,2,1853.888889
24,2,1684.553191
24,2,1721.812500
24,2,1867.445652
24,2,1771.202381
24,2,3586.437500
24,2,2411.500000
24,2,1678.500000
24,2,1797.407609
24,2,1502.627907
24,2,1854.807692
24,2,1740.136905
24,2,2241.857143
24,2,2877.628049
24,2,1978.467742
24,2,2175.369565
24,2,1874.630102
24,2,2015.410000
24,2,2264.722826
24,2,2043.997727
24,2,1511.714286
24,2,2978.602041
24,2,2899.292453
24,2,2000.440104
24,2,2642.487903
24,2,3464.125000
24,2,14167.018750
24,2,3567.851852
24,2,3090.867647
24,2,1682.244186
24,2,3936.923913
24,2,2569.647059
24,2,8351.671196
24,2,1791.000000
24,2,1717.130000

24,2,1739.542553
24,2,2342.391304
24,2,2994.616071
24,2,15938.718750
24,2,2567.101562
24,2,1760.476974
24,2,11864.580882
24,2,1723.464286
24,2,2213.530769
24,2,6840.081081
24,2,1705.504808
24,2,1505.034483
24,2,1538.500000
24,2,1672.593023
24,2,15946.312500
24,2,1913.739583
24,2,2296.085227
24,2,2357.104167
24,2,1734.101190
24,2,2386.796512
24,2,1554.628378
24,2,1993.480000
24,2,1530.560976
24,2,1623.517442
24,2,2159.750000
24,2,1500.586310
24,2,1995.735577
24,2,1865.153846
24,2,2269.201087
24,2,1808.218750
24,2,3006.708333
24,2,2048.100000
24,2,1609.098039
24,2,1564.807065
24,2,2046.039474
24,2,1897.500000
24,2,2099.829268
24,2,2812.227041
24,2,2094.607143
24,2,1812.750000
24,2,2185.447368
24,2,1874.279070
24,2,2754.561224
24,2,6683.276316
24,2,1608.652500
24,2,1963.590909
24,2,2239.027174
24,2,2578.727941
24,2,1710.254902
24,2,1923.289474
24,2,1717.350446
24,2,2197.194444
24,2,2151.678571
24,2,2950.844444
24,2,1965.375000
24,2,1506.761905
24,2,1921.311275
24,2,1657.583333
24,2,1847.821429
24,2,1568.706395
24,2,2593.720000
24,2,1660.621951
24,2,2097.007653
24,2,1862.772321
24,2,2148.122449
24,2,2917.071429

24,2,2871.161765
24,2,2446.530172
24,2,1815.767442
24,2,1548.625000
24,2,2168.750000
24,2,5423.687500
24,2,1996.707237
24,2,4824.328571
24,2,1841.658537
24,2,1649.561047
24,2,2026.366071
24,2,1765.000000
24,2,1603.786458
24,2,4218.234375
24,2,5068.191489
24,2,2710.419643
24,2,2355.848837
24,2,2223.660714
24,2,3443.905612
24,2,2785.618421
24,2,2139.684659
24,2,1901.428571
24,2,2805.000000
24,2,1529.295000
24,2,2544.050000
24,2,2062.143519
24,2,2796.902439
24,2,2542.750000
24,2,5151.200000
24,2,5049.340909
24,2,2198.098131
24,2,1775.882500
24,2,2259.734375
24,2,8103.885417
24,2,1934.505208
24,2,2643.750000
24,2,2278.466667
24,2,1872.058511
24,2,2914.296196
24,2,1557.410714
24,2,3331.846154
24,2,2683.621429
24,2,1530.393750
24,2,1923.250000
24,2,1539.937500
24,2,2051.538462
24,2,1992.981250
24,2,1966.882653
24,2,1625.242788
24,2,1717.681818
24,2,1536.619792
24,2,2441.090909
24,2,2606.280612
24,2,2227.878205
24,2,6946.762500
24,2,6760.043478
24,2,2560.898810
24,2,1608.529605
24,2,2049.456522
24,2,4586.733553
24,2,2556.778571
24,2,1865.326531
24,2,1503.625000
24,2,6003.163462
24,2,4813.766304
24,2,1767.925532

24,2,1858.865566
24,2,2454.357143
24,2,1661.242188
24,2,1621.179487
24,2,2098.020270
24,2,1883.040441
24,2,5595.012500
24,2,2839.954268
24,2,1777.127551
24,2,1970.212766
24,2,5443.072581
24,2,1605.828571
24,2,1745.142857
24,2,1805.646739
24,2,1953.889831
24,2,3249.410714
24,2,1796.686275
24,2,2385.439024
24,2,4067.264706
24,2,2485.875000
24,2,4780.000000
24,2,1837.227941
24,2,1654.250000
24,2,1735.430556
24,2,1719.121951
24,2,1576.918750
24,2,2398.571809
24,2,1619.161458
24,2,3863.666667
24,2,2116.520833
24,2,7608.043605
24,2,1591.337766
24,2,1786.000000
24,2,2264.035714
24,2,2600.593085
24,2,1713.997159
24,2,2252.543478
24,2,2249.013298
24,2,2805.852941
24,2,2058.650000
24,2,1776.497596
24,2,2034.964623
24,2,2659.132075
24,2,1503.471875
24,2,1823.731250
24,2,1701.817500
24,2,2274.577586
24,2,2385.960938
24,2,1499.135638
24,2,1513.950000
24,2,1683.662234
24,2,1729.510638
24,2,2822.282609
24,2,1948.510204
24,2,2175.200000
24,2,4811.911058
24,2,1933.922222
24,2,2470.993902
24,2,2408.916230
24,2,1958.705357
24,2,3140.808511
24,2,1746.218750
24,2,1637.959239
24,2,1948.819444
24,2,2351.663462
24,2,1625.356383

24,2,2638.333333
24,2,1609.325581
24,2,2741.618421
24,2,2015.069444
24,2,1845.785714
24,2,1838.446809
24,2,1582.209184
24,2,3338.593023
24,2,2363.875000
24,2,7225.010929
24,2,2238.782895
24,2,2487.406250
24,2,1778.174847
24,2,1675.333333
24,2,1963.375000
24,2,1726.762097
24,2,1673.230769
24,2,1730.796875
24,2,2067.654412
24,2,5117.319444
24,2,2063.348837
24,2,1563.518382
24,2,1827.253289
24,2,6082.135714
24,2,3079.341463
24,2,2004.867647
24,2,1537.562500
24,2,1717.120536
24,2,2569.464674
24,2,1847.850000
24,2,1810.000000
24,2,2596.205128
24,2,1550.817073
24,2,2252.125000
24,2,2245.709302
24,2,2905.709302
24,2,1524.710000
24,2,1670.843023
24,2,3750.465909
24,2,1522.861111
24,2,2151.856250
24,2,1823.054348
24,2,4149.461864
24,2,3409.556604
24,2,2296.760870
24,2,1792.777778
24,2,1622.303191
24,2,1589.793478
24,2,1772.073171
24,2,2371.204082
24,2,2010.703488
24,2,2387.078571
24,2,2117.726190
24,2,3958.911765
24,2,1766.757353
24,2,2064.564583
24,2,1717.800000
24,2,1532.986111
24,2,6730.711538
24,2,2321.594595
24,2,3540.070122
24,2,1695.466667
24,2,2170.696429
24,2,1641.392857
24,2,3691.562500
24,2,14508.707317

24,2,1956.627717
24,2,1504.137500
24,2,1616.084906
24,2,3371.750000
24,2,1662.916667
24,2,1716.483333
24,2,4035.473404
24,2,1588.505556
24,2,2512.087963
24,2,2494.583333
24,2,7362.996429
24,2,1807.689516
24,2,2226.989796
24,2,1654.883065
24,2,1655.151786
24,2,1821.629310
24,2,1632.402597
24,2,1930.057292
24,2,1604.629032
24,2,1560.294118
24,2,2876.162791
24,2,2810.687500
24,2,1510.792553
24,2,2529.312500
24,2,1742.782609
24,2,3497.517241
24,2,1635.482143
24,2,4143.897059
24,2,1810.607143
24,2,1836.096154
24,2,1595.333333
24,2,2218.000000
24,2,2367.997159
24,2,9467.773438
24,2,2332.130435
24,2,2953.764423
24,2,2036.900000
24,2,2067.634146
24,2,3691.685000
24,2,2574.206250
24,2,3549.676339
24,2,2167.196429
24,2,2040.208333
24,2,2451.154545
24,2,1941.647500
24,2,1760.804348
24,2,2953.900000
24,2,7782.268293
24,2,2885.666667
24,2,2546.448529
24,2,1666.062500
24,2,3077.372549
24,2,1762.779070
24,2,2011.100000
24,2,2076.508929
24,2,4159.467857
24,2,1872.743455
24,2,4825.588235
24,2,2435.527174
24,2,2217.237500
24,2,3207.826389
24,2,3876.573171
24,2,1558.800000
24,2,2144.483871
24,2,2552.092105
24,2,2842.319444

24,2,1869.487500
24,2,5223.019531
24,2,1637.875000
24,2,1746.375000
24,2,2473.224490
24,2,5595.310000
24,2,1947.192308
24,2,1797.080882
24,2,1659.125000
24,2,1838.932065
24,2,1800.494792
24,2,1943.181818
24,2,4886.559322
24,2,3725.336538
24,2,2214.278302
24,2,1606.460000
24,2,7864.487179
24,2,1773.731481
24,2,2624.707317
24,2,8486.390000
24,2,2847.066327
24,2,1593.459302
24,2,13936.127841
24,2,2495.166667
24,2,1571.125000
24,2,1578.844512
24,2,3087.428571
24,2,4891.676647
24,2,1926.942857
24,2,2493.855769
24,2,4164.949219
24,2,3017.912500
24,2,8118.625000
24,2,2203.799107
24,2,1709.500000
24,2,7324.416667
24,2,1638.932432
24,2,2569.812500
24,2,2138.927500
24,2,2127.700000
24,2,1604.333333
24,2,1763.120000
24,2,1650.519084
24,2,2520.375000
24,2,2950.609756
24,2,1623.942857
24,2,3230.937500
24,2,1824.186047
24,2,2887.476852
24,2,1727.906250
24,2,1718.078125
24,2,1728.826087
24,2,2186.541667
24,2,1938.714286
24,2,1516.521875
24,2,6390.244681
24,2,3786.972222
24,2,2068.847561
24,2,1767.039352
24,2,3115.463415
24,2,1891.296512
24,2,5322.735849
24,2,2566.300000
24,2,1527.414352
24,2,3177.630814
24,2,1975.525000

24,2,1870.966667
24,2,1998.632979
24,2,3088.142857
24,2,1662.674419
24,2,3773.864130
24,2,3779.211538
24,2,1615.580882
24,2,2815.492857
24,2,1593.437500
24,2,2121.617021
24,2,2697.250000
24,2,3103.508475
24,2,1999.864583
24,2,1633.737179
24,2,1820.816038
24,2,2165.320513
24,2,3521.029605
24,2,1554.211538
24,2,2006.038690
24,2,1812.456250
24,2,1598.045732
24,2,5403.048077
24,2,1771.086957
24,2,2058.223837
24,2,1676.622500
24,2,1600.960526
24,2,8484.803571
24,2,2468.597561
24,2,1980.038043
24,2,1605.870968
24,2,2219.227500
24,2,3058.154255
24,2,2163.813953
24,2,2194.185000
24,2,2417.857143
24,2,1511.316667
24,2,2491.687500
24,2,3234.607143
24,2,1759.811047
24,2,1712.294643
24,2,2210.250000
24,2,2021.456633
24,2,1716.312500
24,2,2697.776786
24,2,1537.097656
24,2,2585.142857
24,2,2329.336864
24,2,2595.971875
24,2,1625.679688
24,2,1669.817708
24,2,2067.779070
24,2,10485.921875
24,2,2024.977273
24,2,1978.338942
24,2,1773.118421
24,2,2107.911765
24,2,1775.349490
24,2,2678.085227
24,2,2839.021739
24,2,2690.225000
24,2,1805.244898
24,2,1551.296875
24,2,2932.526042
24,2,4381.473837
24,2,1575.275862
24,2,3731.708333

24,2,2018.925000
24,2,2356.375000
24,2,2984.734375
24,2,1516.655488
24,2,1967.910256
24,2,2757.511111
24,2,2019.038603
24,2,3245.000000
24,2,1859.727500
24,2,6380.977041
24,2,2846.914894
24,2,1715.277027
24,2,2543.444444
24,2,1963.869565
24,2,2802.647959
24,2,2302.278061
24,2,2471.375000
24,2,1918.750000
24,2,4703.315789
24,2,2940.593750
24,2,2133.820513
24,2,1835.542553
24,2,2238.577778
24,2,2840.470930
24,2,2366.525641
24,2,1936.747549
24,2,1509.305233
24,2,2125.298913
24,2,2910.636646
24,2,2483.342105
24,2,2223.596591
24,2,1612.472222
24,2,3578.993750
24,2,1819.000000
24,2,1940.565217
24,2,3326.012195
24,2,3397.600000
24,2,2741.190000
24,2,1931.857143
24,2,1671.475000
24,2,2820.952381
24,2,1599.279412
24,2,2361.731707
24,2,1558.125000
24,2,2005.460106
24,2,1936.883721
24,2,2280.975000
24,2,2430.238208
24,2,1764.386364
24,2,2564.334906
24,2,1563.882979
24,2,1549.932927
24,2,1831.489362
24,2,4644.933962
24,2,1651.610465
24,2,3377.903846
24,2,1849.300481
24,2,1702.927083
24,2,1862.000000
24,2,1711.396277
24,2,2606.878049
24,2,1550.750000
24,2,7424.558824
24,2,1631.500000
24,2,1558.587838
24,2,2300.491477

24,2,3184.619355
24,2,3178.699074
24,2,1582.395833
24,2,1679.753049
24,2,1642.772959
24,2,2731.682292
24,2,1501.780488
24,2,2799.875000
24,2,1504.137500
24,2,2121.687500
24,2,1661.776042
24,2,2775.593750
24,2,3106.095238
24,2,2331.019531
24,2,2007.395000
24,2,1613.946429
24,2,2810.615132
24,2,1576.128125
24,2,2017.563679
24,2,1744.006696
24,2,2178.829545
24,2,3619.420000
24,2,4628.040625
24,2,1764.241848
24,2,1756.333333
24,2,2543.596698
24,2,5332.845395
24,2,1567.728723
24,2,1667.333333
24,2,2515.135417
24,2,2033.740000
24,2,1995.893519
24,2,4886.922500
24,2,2968.170000
24,2,2083.456522
24,2,1675.196429
24,2,1860.335937
24,2,1861.308824
24,2,1862.633333
24,2,2079.204545
24,2,1707.826531
24,2,1741.071429
24,2,1622.116279
24,2,1910.534483
24,2,4379.929245
24,2,2058.279070
24,2,1834.738636
24,2,1667.677326
24,2,3253.573529
24,2,1617.054348
24,2,4014.246212
24,2,1743.446875
24,2,1640.787791
24,2,3093.333333
24,2,6628.605263
24,2,1938.895349
24,2,1959.277778
24,2,1670.000000
24,2,1521.012500
24,2,1530.586735
24,2,2075.000000
24,2,3130.634146
24,2,1629.978365
24,2,2879.213235
24,2,2081.500000
24,2,3951.269231

24,2,2606.897436
24,2,2646.114583
24,2,2017.571429
24,2,2297.947222
24,2,2688.310976
24,2,2574.152778
24,2,4332.511905
24,2,2243.282051
24,2,2683.750000
24,2,1829.813725
24,2,1683.000000
24,2,2441.494898
24,2,2611.294118
24,2,1856.882143
24,2,1805.663462
24,2,2053.727273
24,2,2126.285714
24,2,1950.589744
24,2,2522.625000
24,2,3232.355769
24,2,1790.141447
24,2,2030.850000
24,2,2042.625000
24,2,1945.255000
24,2,2805.411765
24,2,1514.969388
24,2,11520.156977
24,2,2309.583333
24,2,1929.861842
24,2,4960.038462
24,2,2154.221591
24,2,1690.166667
24,2,1542.730978
24,2,1755.672727
24,2,2822.756250
24,2,1502.041667
24,2,2188.606250
24,2,3796.107143
24,2,2138.000000
24,2,1515.593750
24,2,4504.333333
24,2,4029.924242
24,2,1851.625000
24,2,1891.856250
24,2,4137.085366
24,2,2332.325431
24,2,1798.491018
24,2,2247.087500
24,2,2095.287162
24,2,1837.505682
24,2,2961.622222
24,2,1605.088415
24,2,2006.335938
24,2,1533.384146
24,2,4044.755102
24,2,1933.265625
24,2,2262.313725
24,2,1764.160326
24,2,1860.857143
24,2,2664.658537
24,2,3082.060000
24,2,2229.184783
24,2,2389.134615
24,2,3310.392857
24,2,2103.279412
24,2,3911.793269

24,2,12062.865854
24,2,2023.731250
24,2,2482.159091
24,2,1755.209302
24,2,3498.350000
24,2,1845.814286
24,2,1962.729592
24,2,1737.892500
24,2,1634.050000
24,2,1967.463068
24,2,1587.026442
24,2,3151.317708
24,2,2530.969595
24,2,1618.263587
24,2,1786.259615
24,2,6165.485294
24,2,1765.590625
24,2,2855.087054
24,2,1798.165625
24,2,1580.138021
24,2,2198.263889
24,2,1870.737179
24,2,2534.673913
24,2,1690.232143
24,2,3669.107143
24,2,2336.490909
24,2,2156.571429
24,2,3262.521739
24,2,2053.678571
24,2,1839.750000
24,2,12174.525862
24,2,3689.142857
24,2,1885.375000
24,2,2234.569444
24,2,1983.000000
24,2,2369.145833
24,2,2209.000000
24,2,1735.675000
24,2,1690.067568
24,2,3100.000000
24,2,2706.439286
24,2,3099.836364
24,2,2133.198980
24,2,8991.382353
24,2,3859.000000
24,2,1549.190476
24,2,1921.897727
24,2,1704.010638
24,2,1869.699153
24,2,3215.637500
24,2,2007.016667
24,2,2536.005319
24,2,1776.828488
24,2,1866.023810
24,2,2341.665761
24,2,2043.158537
24,2,1507.413043
24,2,2330.052632
24,2,1882.934375
24,2,1560.468750
24,2,2376.974057
24,2,2209.000000
24,2,1862.583333
24,2,2682.027174
24,2,1540.879902
24,2,1549.095109

24,2,2598.170213
24,2,2807.590909
24,2,2239.823529
24,2,1647.351064
24,2,1621.710526
24,2,1554.911765
24,2,3065.202128
24,2,1661.448276
24,2,1679.607843
24,2,1859.712500
24,2,2363.500000
24,2,2403.695313
24,2,2374.942308
24,2,2393.300000
24,2,2037.455189
24,2,1621.796053
24,2,2111.534091
24,2,2013.401786
24,2,2571.835937
24,2,1850.578571
24,2,3131.870968
24,2,2426.014706
24,2,1726.939189
24,2,1686.304348
24,2,3043.921875
24,2,1674.488095
24,2,2039.050000
24,2,2592.056604
24,2,1806.537234
24,2,1773.480769
24,2,3891.018443
24,2,2733.727679
24,2,3981.076923
24,2,15118.325581
24,2,1827.724868
24,2,2018.972826
24,2,2136.928191
24,2,3609.175000
24,2,1932.977273
24,2,2440.953947
24,2,1682.211735
24,2,1867.591837
24,2,1960.750000
24,2,1661.928571
24,2,2262.834239
24,2,1944.892500
24,2,1928.597561
24,2,1604.833333
24,2,2556.315000
24,2,1550.193966
24,2,1843.833333
24,2,2010.091346
24,2,2013.013158
24,2,1530.684783
24,2,1562.714286
24,2,1593.108696
24,2,1586.000000
24,2,2119.922619
24,2,2546.070946
24,2,3562.959459
24,2,1697.055000
24,2,2560.000000
24,2,1733.702703
24,2,1878.737981
24,2,2001.970455
24,2,1742.858974

24,2,2313.768519
24,2,1764.932500
24,2,1599.665000
24,2,3603.345238
24,2,3134.406250
24,2,1761.222222
24,2,3833.292683
24,2,1584.687500
24,2,3631.622159
24,2,2509.562500
24,2,1743.628676
24,2,1819.726415
24,2,1719.818182
24,2,3086.758152
24,2,1518.757500
24,2,4590.143617
24,2,2136.185185
24,2,2859.500000
24,2,7546.100000
24,2,2826.447674
24,2,1711.095745
24,2,2087.439024
24,2,1948.829268
24,2,1979.210227
24,2,1846.573864
24,2,1925.182927
24,2,1568.525735
24,2,1714.045732
24,2,1618.027778
24,2,1582.226415
24,2,2602.302419
24,2,7230.356618
24,2,1514.500000
24,2,2554.785714
24,2,1929.980769
24,2,4067.656915
24,2,4250.514286
24,2,1796.793367
24,2,1595.208333
24,2,3341.150000
24,2,1703.935897
24,2,2185.744681
24,2,1798.832317
24,2,9709.593750
24,2,1536.465909
24,2,2136.375000
24,2,3375.046512
24,2,1654.782209
24,2,2398.978261
24,2,2850.211538
24,2,1698.641892
24,2,1647.487179
24,2,2989.406863
24,2,7030.040816
24,2,2374.683140
24,2,1586.940217
24,2,1873.262821
24,2,2783.901163
24,2,1619.315217
24,2,1913.036585
24,2,2096.881818
24,2,1515.571429
24,2,2258.434524
24,2,1799.256579
24,2,1749.233333
24,2,1657.368590

24,2,2135.000000
24,2,1968.783854
24,2,1575.541096
24,2,3730.234375
24,2,1710.550000
24,2,1681.268293
24,2,2142.079787
24,2,8336.778125
24,2,1795.450000
24,2,1962.142857
24,2,2619.303571
24,2,5237.060811
24,2,1705.810811
24,2,1811.852564
24,2,2132.428571
24,2,1657.363128
24,2,2929.922500
24,2,1602.763514
24,2,1778.052632
24,2,1620.189189
24,2,2079.647500
24,2,4388.981481
24,2,1500.391892
24,2,2161.680556
24,2,1529.760714
24,2,3384.750000
24,2,3373.713889
24,2,1653.411765
24,2,2365.628125
24,2,1877.351351
24,2,1899.317308
24,2,1609.830556
24,2,1673.668919
24,2,1518.236111
24,2,2479.352564
24,2,1657.377660
24,2,4185.385204
24,2,1920.312500
24,2,5112.113636
24,2,1887.879902
24,2,3010.281250
24,2,9801.000000
24,2,2095.714286
24,2,4603.434211
24,2,2568.941176
24,2,2160.269886
24,2,2268.145833
24,2,2276.300000
24,2,4929.194149
24,2,1829.491379
24,2,3261.300000
24,2,1959.469388
24,2,1767.476190
24,2,1837.441667
24,2,2623.848404
24,2,2551.792453
24,2,1887.066176
24,2,2207.102941
24,2,4410.840909
24,2,2833.545213
24,2,1979.313889
24,2,2217.312500
24,2,1949.500000
24,2,2697.292683
24,2,2888.695122
24,2,2233.901786

24,2,3030.090426
24,2,4514.038889
24,2,11014.750000
24,2,1503.406250
24,2,1521.900000
24,2,1908.377717
24,2,3050.520833
24,2,1654.921569
24,2,1783.257143
24,2,2070.523810
24,2,1623.737179
24,2,2403.850000
24,2,2003.970588
24,2,1704.000000
24,2,2923.744898
24,2,1569.011364
24,2,1658.468085
24,2,2502.627660
24,2,5622.654605
24,2,1909.202586
24,2,1784.330000
24,2,2933.656250
24,2,1787.721154
24,2,2416.863208
24,2,2259.365625
24,2,1736.350000
24,2,1726.334459
24,2,1804.372093
24,2,2047.957386
24,2,3082.510417
24,2,3392.153061
24,2,1842.244186
24,2,1799.173469
24,2,1826.830645
24,2,1593.674419
24,2,1928.572917
24,2,1669.609375
24,2,3404.684211
24,2,1564.547794
24,2,1542.478261
24,2,2481.291667
24,2,2066.514706
24,2,1716.404018
24,2,7267.534375
24,2,8332.000000
24,2,1951.182927
24,2,2387.742347
24,2,2151.121951
24,2,1507.616279
24,2,1719.692708
24,2,1614.053571
24,2,2024.685811
24,2,2643.021277
24,2,1825.615385
24,2,1576.443548
24,2,1683.250000
24,2,1509.025510
24,2,1510.833333
24,2,2291.775510
24,2,1644.186047
24,2,2391.895833
24,2,2652.000000
24,2,1833.697674
24,2,1684.023810
24,2,12061.916667
24,2,1611.784091

24,2,2213.420918
24,2,2034.979167
24,2,1844.875000
24,2,1992.531250
24,2,1927.568878
24,2,1579.601064
24,2,1762.475000
24,2,1869.890957
24,2,8997.209040
24,2,2157.588235
24,2,6561.122951
24,2,1753.562500
24,2,1568.115385
24,2,4506.789744
24,2,1508.558824
24,2,2825.471698
24,2,1912.734375
24,2,2304.143750
24,2,1536.587766
24,2,2081.635135
24,2,1725.618750
24,2,2558.865000
24,2,2300.317568
24,2,3026.293269
24,2,2354.980392
24,2,2148.653846
24,2,2052.976293
24,2,1802.137821
24,2,1605.940625
24,2,2443.166667
24,2,2318.207317
24,2,2702.435714
24,2,2297.921875
24,2,1786.434375
24,2,2976.770833
24,2,2350.723684
24,2,3082.826087
24,2,1788.625000
24,2,1873.500000
24,2,2921.117647
24,2,2087.964286
24,2,12961.984043
24,2,2896.250000
24,2,2040.315000
24,2,2093.195122
24,2,1600.580189
24,2,2937.800000
24,2,2034.790816
24,2,1512.138298
24,2,1684.600000
24,2,2336.583333
24,2,1508.900000
24,2,1815.125000
24,2,2142.518382
24,2,1515.253049
24,2,1700.450000
24,2,3180.200000
24,2,3972.190476
24,2,1758.662500
24,2,2489.416667
24,2,5633.944444
24,2,1989.513889
24,2,1550.525000
24,2,1502.736111
24,2,2692.365854
24,2,5737.566176

24,2,4527.560606
24,2,2684.597458
24,2,2938.937500
24,2,1804.157895
24,2,2892.637255
24,2,1803.980769
24,2,2125.519608
24,2,1705.272727
24,2,3195.942857
24,2,1682.700000
24,2,2652.794643
24,2,2808.127273
24,2,3025.750000
24,2,2740.961957
24,2,1639.934783
24,2,1683.011905
24,2,4184.465426
24,2,1663.632653
24,2,1501.308673
24,2,1625.011364
24,2,1684.615385
24,2,1575.117647
24,2,1592.114796
24,2,2451.834239
24,2,2908.267045
24,2,6256.135135
24,2,5096.795000
24,2,1884.247727
24,2,2028.913462
24,2,3133.388889
24,2,2128.638298
24,2,1717.525641
24,2,1783.120000
24,2,2324.919872
24,2,2458.390625
24,2,2048.477564
24,2,1587.468085
24,2,1810.035326
24,2,3339.625000
24,2,1509.750000
24,2,2347.714286
24,2,2267.854167
24,2,2376.840625
24,2,5242.411765
24,2,1950.327830
24,2,1715.773585
24,2,1568.851562
24,2,1648.459302
24,2,9211.521277
24,2,6004.612245
24,2,2574.418919
24,2,1799.532609
24,2,2591.615385
24,2,2276.796875
24,2,2017.974359
24,2,3868.268868
24,2,1695.333333
24,2,2847.752212
24,2,1909.436047
24,2,2760.723684
24,2,2309.158163
24,2,1615.070312
24,2,1748.222222
24,2,12177.025000
24,2,2031.812500
24,2,1766.211111

24,2,4487.149390
24,2,2470.170000
24,2,1510.730769
24,2,2045.095745
24,2,1547.062500
24,2,4575.295455
24,2,3701.538462
24,2,1730.585366
24,2,3181.962766
24,2,2896.240196
24,2,1554.955357
24,2,1589.111111
24,2,2791.250000
24,2,3297.006944
24,2,1517.388298
24,2,1573.674419
24,2,3333.939394
24,2,2156.763889
24,2,1846.181818
24,2,3787.224265
24,2,1569.511628
24,2,1738.320513
24,2,3326.915094
24,2,8797.683594
24,2,3261.243902
24,2,1736.770833
24,2,5246.239130
24,2,1789.766304
24,2,3901.065217
24,2,2353.084559
24,2,2195.271739
24,2,2436.901596
24,2,2204.306818
24,2,3506.708333
24,2,1574.921875
24,2,2037.304878
24,2,2123.758621
24,2,3446.286458
24,2,1630.933824
24,2,2217.854167
24,2,2525.912500
24,2,7281.763021
24,2,1850.375000
24,2,2222.167453
24,2,2130.470588
24,2,1817.232558
24,2,1804.363636
24,2,2176.285714
24,2,2293.875000
24,2,2391.915761
24,2,12828.489362
24,2,1554.191667
24,2,1532.689394
24,2,12578.480469
24,2,2173.528302
24,2,4793.656863
24,2,1767.317308
24,2,4392.537879
24,2,2182.848958
24,2,1619.136905
24,2,2469.132353
24,2,1542.473958
24,2,2115.666667
24,2,3907.842105
24,2,1567.987981
24,2,1683.372596

24,2,2615.241071
24,2,3079.365625
24,2,2457.597561
24,2,1854.836735
24,2,2367.390000
24,2,3715.683140
24,2,1587.955556
24,2,2026.897059
24,2,1738.000000
24,2,2263.508721
24,2,2454.790441
24,2,2986.714286
24,2,2068.730769
24,2,1798.349432
24,2,1732.661458
24,2,2522.922872
24,2,1710.778409
24,2,1794.050000
24,2,1692.055556
24,2,2618.352941
24,2,3592.105000
24,2,1570.166667
24,2,2102.243056
24,2,1726.630435
24,2,2104.225806
24,2,3612.500000
24,2,1783.620000
24,2,2266.396739
24,2,1593.743902
24,2,3880.927083
24,2,1953.493902
24,2,3311.586957
24,2,1747.070000
24,2,2507.461735
24,2,1804.412500
24,2,2931.828125
24,2,1637.750000
24,2,3162.568548
24,2,1866.756579
24,2,2442.904255
24,2,2641.681818
24,2,1523.812500
24,2,8804.350000
24,2,3291.367788
24,2,1995.222826
24,2,1621.875000
24,2,1571.191176
24,2,3848.755435
24,2,1569.703125
24,2,4179.172619
24,2,1596.089912
24,2,3346.656250
24,2,1502.676471
24,2,2226.562500
24,2,4489.500000
24,2,10390.166667
24,2,2105.423077
24,2,2838.984375
24,2,4244.856383
24,2,1579.928571
24,2,2929.465116
24,2,1516.000000
24,2,1678.050847
24,2,2087.477941
24,2,2706.555147
24,2,4039.582386

24,2,3879.008772
24,2,1865.062500
24,2,1975.922297
24,2,3018.603774
24,2,1503.701220
24,2,2308.397959
24,2,4488.342262
24,2,1512.975610
24,2,1504.406250
24,2,3114.166667
24,2,3014.782895
24,2,2163.150000
24,2,2169.416667
24,2,1670.833333
24,2,6146.250000
24,2,2434.543919
24,2,2321.659314
24,2,1932.521635
24,2,1824.104651
24,2,2102.351064
24,2,2832.218750
24,2,3419.891892
24,2,2029.040816
24,2,1738.197674
24,2,1855.857143
24,2,1523.081250
24,2,2143.136905
24,2,2925.000000
24,2,2184.142857
24,2,1745.292553
24,2,1596.548077
24,2,1557.156250
24,2,1779.117647
24,2,1677.666667
24,2,1797.536585
24,2,3533.357143
24,2,1523.933333
24,2,7047.506757
24,2,1562.402439
24,2,1577.852500
24,2,2080.937500
24,2,3161.612500
24,2,1719.994792
24,2,1584.571429
24,2,1838.142857
24,2,2079.909091
24,2,2127.844444
24,2,2209.725000
24,2,1542.708333
24,2,1569.096591
24,2,1531.027778
24,2,1667.739583
24,2,2130.036585
24,2,2031.315625
24,2,2191.636792
24,2,2593.698529
24,2,3011.778061
24,2,2033.006410
24,2,2466.032258
24,2,1936.054688
24,2,2025.157895
24,2,1977.429688
24,2,1505.914634
24,2,1860.634375
24,2,3490.593023
24,2,2499.807500

24,2,5600.640625
24,2,1582.841837
24,2,2699.300000
24,2,1984.886905
24,2,4227.496951
24,2,2055.659091
24,2,2235.346591
24,2,5036.355159
24,2,1606.840625
24,2,1840.500000
24,2,2383.080000
24,2,1920.184783
24,2,2304.087500
24,2,2167.250000
24,2,1534.922500
24,2,1858.640625
24,2,1563.921569
24,2,2858.771739
24,2,1964.375000
24,2,1852.375000
24,2,2007.027778
24,2,2819.688623
24,2,1652.029545
24,2,6435.805556
24,2,1545.242347
24,2,2161.658537
24,2,1796.953125
24,2,2519.951220
24,2,3113.500000
24,2,1854.237500
24,2,1679.785714
24,2,1709.410714
24,2,3631.039773
24,2,1902.180233
24,2,3193.736842
24,2,1634.984694
24,2,1751.450000
24,2,1861.018293
24,2,1717.812500
24,2,4379.440000
24,2,2633.599537
24,2,1676.271739
24,2,2054.375000
24,2,2232.147436
24,2,4004.731707
24,2,2983.521226
24,2,1604.777778
24,2,1958.818182
24,2,2096.067073
24,2,3232.975000
24,2,3223.000000
24,2,3048.613636
24,2,2438.500000
24,2,3733.733696
24,2,1546.941176
24,2,1762.756757
24,2,1753.851562
24,2,1552.444444
24,2,2106.469388
24,2,12707.277439
24,2,1639.576705
24,2,4567.295455
24,2,1596.168605
24,2,1970.585106
24,2,2110.600000
24,2,2521.500000

24,2,1823.356771
24,2,5125.151316
24,2,3381.200000
24,2,2240.364583
24,2,2469.505814
24,2,2476.460000
24,2,1506.238971
24,2,1709.648936
24,2,2293.710227
24,2,2708.500000
24,2,7927.270833
24,2,1822.900000
24,2,2464.209052
24,2,1540.875000
24,2,5873.897959
24,2,2127.170732
24,2,1515.913043
24,2,1829.409091
24,2,2234.114130
24,2,1590.723837
24,2,2307.333333
24,2,1912.857143
24,2,1988.000000
24,2,1786.848039
24,2,13382.375000
24,2,2480.375000
24,2,1712.227273
24,2,1587.625000
24,2,1706.541667
24,2,2339.147436
24,2,1528.500000
24,2,1704.193452
24,2,1706.336207
24,2,7740.246875
24,2,2109.120000
24,2,2866.457386
24,2,3100.555215
24,2,1554.995327
24,2,2299.059748
24,2,1611.234375
24,2,2062.992647
24,2,2351.000000
24,2,3951.406250
24,2,1889.567308
24,2,5266.161364
24,2,2180.133333
24,2,2539.349359
24,2,1695.450658
24,2,2181.901786
24,2,2033.377778
24,2,1597.306818
24,2,9563.390625
24,2,2272.738095
24,2,1669.535714
24,2,2017.586957
24,2,2340.069767
24,2,2525.630208
24,2,1533.955882
24,2,1577.590909
24,2,2252.627451
24,2,2353.909091
24,2,2157.610577
24,2,2485.573171
24,2,1598.270000
24,2,1591.272059
24,2,1603.445513

24,2,2641.916667
24,2,1943.170213
24,2,3677.798077
24,2,1864.073171
24,2,1958.175000
24,2,1906.750000
24,2,1547.950000
24,2,1579.125000
24,2,1695.548077
24,2,3495.707237
24,2,2039.056122
24,2,2457.750000
24,2,1546.384615
24,2,2295.837500
24,2,2496.150943
24,2,2958.937500
24,2,1598.851064
24,2,5480.092262
24,2,2184.291667
24,2,1865.159091
24,2,2659.661017
24,2,1643.250000
24,2,2053.021552
24,2,2535.365854
24,2,2875.665761
24,2,1776.380208
24,2,1562.267442
24,2,1799.541667
24,2,4044.418750
24,2,1713.986702
24,2,2347.871951
24,2,1967.367647
24,2,1630.750000
24,2,2792.644022
24,2,1506.142857
24,2,1771.433511
24,2,1543.300000
24,2,2190.421053
24,2,1942.500000
24,2,1879.704545
24,2,1793.266332
24,2,1603.865854
24,2,2018.566327
24,2,1836.086538
24,2,1529.403409
24,2,1821.204082
24,2,1872.593750
24,2,1888.595588
24,2,1801.326087
24,2,1537.529412
24,2,1562.696809
24,2,1519.909091
24,2,1698.786585
24,2,1777.127841
24,2,2236.060096
24,2,1978.772321
24,2,2295.811594
24,2,1686.250000
24,2,2328.798780
24,2,2336.162162
24,2,5147.799020
24,2,1695.657609
24,2,3564.950820
24,2,1681.127551
24,2,2120.375000
24,2,2014.451923

24,2,2485.173077
24,2,2341.576923
24,2,1537.806250
24,2,4926.565217
24,2,1855.608333
24,2,2637.127604
24,2,2288.366667
24,2,2760.500000
24,2,2894.879630
24,2,1719.562500
24,2,1704.750000
24,2,2797.943396
24,2,2137.465686
24,2,2800.390244
24,2,1709.735294
24,2,1819.035714
24,2,1802.108108
24,2,1809.797872
24,2,3856.000000
24,2,1760.043367
24,2,1702.646226
24,2,3422.678571
24,2,3405.330882
24,2,1725.290323
24,2,3704.569444
24,2,4865.840909
24,2,3388.691176
24,2,2641.696809
24,2,1821.039773
24,2,1928.638298
24,2,2134.976744
24,2,1932.218023
24,2,1804.523026
24,2,1880.586957
24,2,1722.916667
24,2,1857.635870
24,2,1666.350000
24,2,1736.603659
24,2,1835.478261
24,2,2811.859375
24,2,2416.631356
24,2,1937.843750
24,2,2375.793269
24,2,2032.237981
24,2,1703.047500
24,2,3662.533854
24,2,3651.394231
24,2,5586.366071
24,2,1962.894231
24,2,1510.671875
24,2,5689.684211
24,2,1660.300000
24,2,1814.086207
24,2,1650.231132
24,2,1885.482558
24,2,1632.678571
24,2,2311.997596
24,2,1655.000000
24,2,1511.357143
24,2,3590.352941
24,2,2882.669643
24,2,1774.250000
24,2,1772.276596
24,2,2316.714286
24,2,2762.421875
24,2,2068.964286

24,2,1955.720588
24,2,2411.160000
24,2,2509.074324
24,2,1833.648438
24,2,1539.200000
24,2,2088.650943
24,2,2947.595745
24,2,3667.366848
24,2,2860.842105
24,2,8842.710526
24,2,1932.239130
24,2,2510.300000
24,2,7145.171569
24,2,3352.418605
24,2,2540.276596
24,2,2952.256039
24,2,1724.925000
24,2,1654.406250
24,2,2692.555556
24,2,1615.416667
24,2,3033.905172
24,2,1904.500000
24,2,4279.877551
24,2,3572.717391
24,2,2369.834459
24,2,1660.173077
24,2,1980.407609
24,2,2321.069767
24,2,2125.400000
24,2,1692.666667
24,2,2217.307692
24,2,1767.311111
24,2,1717.013889
24,2,2880.142857
24,2,4406.570000
24,2,2103.840278
24,2,7848.125000
24,2,1953.446809
24,2,3490.342672
24,2,2363.738636
24,2,2946.494565
24,2,3121.052632
24,2,3858.707317
24,2,4018.683333
24,2,1631.706250
24,2,2118.140625
24,2,1835.017045
24,2,1731.989796
24,2,3976.616071
24,2,2396.375000
24,2,2189.306818
24,2,1656.564815
24,2,2162.586538
24,2,1779.310096
24,2,2085.357143
24,2,4321.015957
24,2,1687.677083
24,2,7310.578125
24,2,1969.660714
24,2,1562.529412
24,2,2228.500000
24,2,1582.918103
24,2,2336.345588
24,2,4552.583333
24,2,2661.857143
24,2,2201.254808

24,2,2814.031250
24,2,3872.457317
24,2,2285.493827
24,2,1773.323171
24,2,1707.166667
24,2,1753.084459
24,2,1744.083333
24,2,2945.292683
24,2,2926.033333
24,2,2552.046875
24,2,2088.608108
24,2,2115.000000
24,2,3116.030000
24,2,2156.937500
24,2,4031.848684
24,2,2446.536058
24,2,1805.363636
24,2,1547.480769
24,2,2852.595109
24,2,1557.000000
24,2,1777.505435
24,2,1510.966981
24,2,1666.261905
24,2,2857.812500
24,2,1931.735465
24,2,4140.173469
24,2,1960.200000
24,2,1854.633929
24,2,2876.800000
24,2,2576.251232
24,2,1870.581633
24,2,1500.500000
24,2,2578.250000
24,2,1937.619048
24,2,2123.635204
24,2,1545.166667
24,2,1941.395408
24,2,2080.534884
24,2,2988.391667
24,2,2186.666667
24,2,15683.666667
24,2,1544.425532
24,2,19132.355263
24,2,1754.489583
24,2,6248.058824
24,2,2655.085784
24,2,2217.136364
24,2,5321.398585
24,2,1909.618421
24,2,19743.040441
24,2,1581.140625
24,2,1908.523026
24,2,1917.125000
24,2,1787.482143
24,2,2001.953125
24,2,2771.307692
24,2,1737.756098
24,2,2924.506410
24,2,3471.068182
24,2,1502.825581
24,2,1532.666667
24,2,2429.062500
24,2,2228.945652
24,2,1610.105263
24,2,1630.872159
24,2,2858.214286

24,2,1727.880000
24,2,2527.416667
24,2,2677.497642
24,2,3094.132979
24,2,1724.851415
24,2,1682.239583
24,2,1598.990625
24,2,2150.829545
24,2,1927.937500
24,2,1618.152466
24,2,3142.700980
24,2,2345.769231
24,2,1715.003049
24,2,1551.218085
24,2,1592.605263
24,2,1644.967857
24,2,1967.722222
24,2,2674.454741
24,2,1501.035714
24,2,1945.771552
24,2,1681.528846
24,2,5343.437500
24,2,4461.242187
24,2,2737.172872
24,2,6508.187500
24,2,3653.201923
24,2,1839.198529
24,2,3271.938776
24,2,2185.368902
24,2,3469.227041
24,2,1580.619048
24,2,2143.105263
24,2,1769.644231
24,2,6466.012500
24,2,1702.324519
24,2,2413.341346
24,2,1556.714286
24,2,2590.111111
24,2,2227.884615
24,2,2658.205128
24,2,2007.198529
24,2,1786.581395
24,2,3350.026163
24,2,1824.411458
24,2,1512.613372
24,2,1632.772222
24,2,6398.385246
24,2,3489.127551
24,2,2076.728774
24,2,1686.880000
24,2,2083.500000
24,2,2401.321429
24,2,2194.707317
24,2,1684.769231
24,2,1634.908333
24,2,2514.146277
24,2,3077.109091
24,2,1779.410000
24,2,2428.581818
24,2,2293.359375
24,2,2751.197115
24,2,4600.138889
24,2,1737.558673
24,2,3560.804878
24,2,6154.781250
24,2,2040.406250

24,2,2063.186404
24,2,1958.660000
24,2,3916.347826
24,2,1878.323276
24,2,2915.924342
24,2,1765.897959
24,2,1504.516667
24,2,5271.224490
24,2,2046.860465
24,2,3999.056818
24,2,1932.772727
24,2,2413.593750
24,2,2084.360577
24,2,1499.969388
24,2,1580.272059
24,2,1921.354167
24,2,1819.166667
24,2,2385.982143
24,2,1597.801471
24,2,1839.877193
24,2,3411.292500
24,2,2044.681818
24,2,2038.042614
24,2,1499.714286
24,2,1509.607143
24,2,1533.058824
24,2,1880.744444
24,2,1999.916667
24,2,1546.376884
24,2,2404.945946
24,2,1999.375000
24,2,2499.902439
24,2,2443.691489
24,2,5454.215426
24,2,6229.052778
24,2,1570.700000
24,2,1914.108108
24,2,5086.328947
24,2,2540.000000
24,2,2323.595745
24,2,2111.625000
24,2,2297.888889
24,2,2412.452703
24,2,4243.517241
24,2,1641.315000
24,2,1611.197368
24,2,2174.333333
24,2,1766.482558
24,2,2750.340116
24,2,3650.238636
24,2,1938.392157
24,2,2372.990385
24,2,1822.361702
24,2,6882.702381
24,2,2125.743304
24,2,1536.505556
24,2,2249.451389
24,2,4066.089623
24,2,2352.204545
24,2,1801.057065
24,2,2655.035714
24,2,2209.772727
24,2,1781.680233
24,2,2226.782609
24,2,3319.942308
24,2,1499.059375

24,2,2116.019231
24,2,2930.260870
24,2,5348.093023
24,2,2629.717105
24,2,1607.675000
24,2,6613.307692
24,2,3385.500000
24,2,2458.083333
24,2,2254.416667
24,2,2233.215625
24,2,1671.404255
24,2,1854.576087
24,2,3164.884615
24,2,2711.185000
24,2,2049.530405
24,2,1573.333333
24,2,1703.872024
24,2,2590.464286
24,2,2929.867788
24,2,2879.218750
24,2,1808.305233
24,2,4157.540816
24,2,2267.668478
24,2,1585.906250
24,2,1612.761905
24,2,1980.244565
24,2,2347.437500
24,2,1504.349490
24,2,1832.947917
24,2,2898.769231
24,2,1701.444444
24,2,1886.976562
24,2,1945.161932
24,2,6123.166667
24,2,1539.585714
24,2,1709.451220
24,2,1595.519231
24,2,2378.722222
24,2,5761.627717
24,2,2764.386364
24,2,1820.516026
24,2,2753.326389
24,2,2031.000000
24,2,2158.189189
24,2,3903.288690
24,2,1676.353774
24,2,2216.034375
24,2,2348.341216
24,2,9061.648585
24,2,2390.150000
24,2,1756.303922
24,2,3972.945455
24,2,2605.018750
24,2,2222.178977
24,2,2210.642857
24,2,1700.547170
24,2,2241.281250
24,2,7803.391447
24,2,2100.596774
24,2,3635.000000
24,2,2087.418478
24,2,1722.316964
24,2,1709.846154
24,2,2836.674419
24,2,1905.783784
24,2,2910.522059

24,2,2669.278409
24,2,2396.937500
24,2,1618.950658
24,2,2748.524390
24,2,2063.970588
24,2,2199.219444
24,2,3001.955556
24,2,2397.223214
24,2,2093.754237
24,2,5511.613372
24,2,1950.095238
24,2,1776.291667
24,2,2605.712054
Table S1 B) Intensity recorded for peptide NLS-alpha-CE on MATa and MATalpha cells. MATa recordings correspond with odd Time numbers, and MATalpha with even Time numbers
Time,GroupByColor,Intensity
1,1,1676.875000
1,1,3710.336538
1,1,30623.134146
1,1,2598.895349
1,1,1888.300000
1,1,3021.195513
1,1,1584.239583
1,1,9160.490625
1,1,83743.326923
1,1,1618.116477
1,1,4466.380814
1,1,10945.911765
1,1,1525.451220
1,1,42763.820122
1,1,4395.460526
1,1,3379.822368
1,1,1572.208333
1,1,7716.968750
1,1,34489.689189
1,1,4490.738426
1,1,4236.010870
1,1,2013.947368
1,1,4285.689394
1,1,10259.875000
1,1,3084.826087
1,1,3000.625000
1,1,8298.184028
1,1,2931.800000
1,1,2915.000000
1,1,12215.052273
1,1,2183.478659
1,1,2355.292553
1,1,1564.476744
1,1,5775.648936
1,1,1761.770408
1,1,1678.750000
1,1,3516.937500
1,1,17391.648707
1,1,1694.960526
1,1,3536.617021
1,1,1689.000000
1,1,51235.333333
1,1,10072.181818
1,1,10188.718750
1,1,2991.750000
1,1,2037.226563
1,1,5751.229167
1,1,13869.789773
1,1,1917.142045

1,1,7003.802469
1,1,4909.672222
1,1,8408.326087
1,1,2151.292614
1,1,9931.201531
1,1,5452.000000
1,1,4243.744624
1,1,7161.930233
1,1,2732.375000
1,1,8552.418301
1,1,5518.765060
1,1,4418.408854
1,1,4830.384615
1,1,1994.260234
1,1,37735.505882
1,1,112283.737805
1,1,6831.315217
1,1,8919.697368
1,1,1558.919118
1,1,10172.562500
1,1,1727.809524
1,1,2020.082447
1,1,1727.197674
1,1,1672.367188
1,1,3797.511628
1,1,1571.191667
1,1,6886.130435
1,1,7211.431818
1,1,11029.545455
1,1,2719.960526
1,1,1506.727273
1,1,2411.952128
1,1,10078.863636
1,1,4307.508929
1,1,2252.266667
1,1,16984.000000
1,1,6831.840909
1,1,2727.604651
1,1,7930.381250
1,1,1934.585938
1,1,2301.500000
1,1,3916.057143
1,1,4556.945946
1,1,1905.416667
1,1,2591.555556
1,1,1524.997024
1,1,6584.934783
1,1,3198.060000
1,1,2836.312139
1,1,8342.600000
1,1,4504.910714
1,1,6047.875000
1,1,74946.100000
1,1,28061.229167
1,1,1615.730978
1,1,2630.358333
1,1,4620.145349
1,1,3422.788889
1,1,1890.532895
1,1,4005.085714
1,1,8255.106061
1,1,2009.547486
1,1,2583.946809
1,1,2166.766667
1,1,4279.633523
1,1,2478.927326

1,1,5391.787234
1,1,1954.149425
1,1,2541.250000
1,1,6313.176471
1,1,1620.085938
1,1,4176.157258
1,1,3062.659091
1,1,2963.384615
1,1,3896.875000
1,1,2803.062500
1,1,7447.920000
1,1,1694.494318
1,1,3132.611111
1,1,4113.070312
1,1,3553.670213
1,1,4277.364865
1,1,1923.875000
1,1,2069.855346
1,1,6505.775253
1,1,5579.515152
1,1,4496.273810
1,1,14378.544444
1,1,2571.593750
1,1,19936.468085
1,1,3239.900000
1,1,6045.523810
1,1,2799.386364
1,1,2615.229167
1,1,3927.205357
1,1,17267.179487
1,1,2879.338889
1,1,1769.942073
1,1,1748.471154
1,1,3646.062500
1,1,9031.919444
1,1,7276.508523
1,1,5080.682432
1,1,1668.428030
1,1,4543.937500
1,1,2169.987179
1,1,2920.836957
1,1,2999.966667
1,1,2177.884146
1,1,1780.791667
1,1,7485.846591
1,1,60324.460938
1,1,1696.562500
1,1,3600.609649
1,1,5397.349057
1,1,2034.584239
1,1,1716.817073
1,1,1639.135135
1,1,2397.735000
1,1,3941.634146
1,1,2249.174419
1,1,2260.851852
1,1,2533.794872
1,1,2801.469388
1,1,7736.462963
1,1,2261.712766
1,1,36852.521429
1,1,40570.703125
1,1,5012.153409
1,1,1559.875000
1,1,17554.785256
1,1,5078.717949

1,1,2130.719697
1,1,3597.604651
1,1,1596.882979
1,1,3271.804878
1,1,2654.853723
1,1,1946.981250
1,1,2187.301948
1,1,1784.192073
1,1,2688.822917
1,1,2640.173077
1,1,18911.094697
1,1,4062.970588
1,1,2204.285714
1,1,1919.460526
1,1,2618.314286
1,1,1762.910714
1,1,3654.266055
1,1,23131.657895
1,1,2323.976562
1,1,3443.946429
1,1,3302.610294
1,1,19008.500000
1,1,2319.788235
1,1,2748.238636
1,1,6158.171053
1,1,2917.250000
1,1,32700.184659
1,1,4217.182857
1,1,10321.323171
1,1,3108.670732
1,1,5295.781915
1,1,2355.801136
1,1,4905.975000
1,1,11865.108108
1,1,1595.664634
1,1,8243.950000
1,1,7681.965625
1,1,9586.854592
1,1,1765.670139
1,1,2531.750000
1,1,2476.923780
1,1,56311.663043
1,1,3366.720690
1,1,2360.484043
1,1,4263.006098
1,1,3723.650000
1,1,2797.500000
1,1,10118.087838
1,1,2358.100000
1,1,2511.771277
1,1,4061.850962
1,1,2332.088415
1,1,3514.312500
1,1,1598.235465
1,1,1849.673077
1,1,2873.204545
1,1,1966.242647
1,1,30619.471154
1,1,1530.634615
1,1,1780.780788
1,1,4042.604651
1,1,15146.762500
1,1,3155.768293
1,1,3334.892857
1,1,13379.861111
1,1,36668.300000

1,1,3578.200000
1,1,2527.650000
1,1,16763.850543
1,1,1629.788889
1,1,11774.578947
1,1,6031.277778
1,1,1648.545455
1,1,4425.457447
1,1,2182.415000
1,1,1742.185714
1,1,2021.863636
1,1,1871.465426
1,1,2720.854730
1,1,7503.320000
1,1,4845.143617
1,1,4369.415385
1,1,2686.414634
1,1,5449.892857
1,1,3710.500000
1,1,11832.159483
1,1,1598.913043
1,1,6040.094595
1,1,2176.906250
1,1,1975.217391
1,1,1758.888889
1,1,6440.756944
1,1,2114.643382
1,1,2817.012019
1,1,5163.883436
1,1,39961.236842
1,1,6012.170732
1,1,4728.000000
1,1,12270.710366
1,1,2004.678977
1,1,5005.686111
1,1,2026.996377
1,1,2440.932692
1,1,2583.915761
1,1,5963.602273
1,1,27233.318548
1,1,2121.558824
1,1,38904.818182
1,1,3048.687500
1,1,79388.460526
1,1,5978.588816
1,1,2553.000000
1,1,4898.262500
1,1,5718.200000
1,1,1709.316176
1,1,4885.425532
1,1,8009.000000
1,1,2234.965278
1,1,1877.930921
1,1,1672.693878
1,1,33249.559028
1,1,10112.181818
1,1,11626.577778
1,1,5930.840278
1,1,2721.324468
1,1,2537.266667
1,1,2728.928571
1,1,10198.135135
1,1,2118.692308
1,1,42081.660326
1,1,2265.402778
1,1,3167.397436

1,1,1576.490132
1,1,9938.276786
1,1,1829.864362
1,1,2557.560976
1,1,1523.779255
1,1,2155.670833
1,1,65799.385714
1,1,4471.352941
1,1,2008.827586
1,1,2346.105263
1,1,3521.000000
1,1,2610.977273
1,1,2112.511628
1,1,1950.817857
1,1,1713.784574
1,1,8702.857143
1,1,1631.898256
1,1,2500.710366
1,1,5287.121622
1,1,6171.143617
1,1,2302.958333
1,1,3439.541667
1,1,3541.548780
1,1,5119.460227
1,1,4712.923077
1,1,4105.458333
1,1,2457.578947
1,1,1844.333333
1,1,2041.610795
1,1,100597.909091
1,1,1892.855556
1,1,43261.325000
1,1,3628.604396
1,1,2792.000000
1,1,1540.979730
1,1,3319.782051
1,1,2531.393750
1,1,4681.224490
1,1,5808.593137
1,1,2170.247024
1,1,3720.395349
1,1,2825.419355
1,1,6961.343750
1,1,10392.219298
1,1,1650.743902
1,1,5893.875000
1,1,13074.125000
1,1,3074.706522
1,1,114372.043478
1,1,1517.937500
1,1,5827.060606
1,1,1552.957447
1,1,7592.578125
1,1,7052.541667
1,1,1545.081081
1,1,1736.133333
1,1,40707.821429
1,1,3185.398810
1,1,1557.771739
1,1,49513.333333
1,1,1781.107143
1,1,9579.442308
1,1,6327.688623
1,1,3390.107759
1,1,4413.851562
1,1,2012.090909

1,1,14055.893617
1,1,6603.012821
1,1,20087.950758
1,1,1972.239583
1,1,2366.244898
1,1,2342.439024
1,1,14254.600694
1,1,2159.714286
1,1,6022.237500
1,1,1654.078125
1,1,2990.720588
1,1,90112.697917
1,1,6685.383234
1,1,1782.371622
1,1,2984.746951
1,1,99060.532895
1,1,10614.544811
1,1,1598.050000
1,1,2874.035714
1,1,2058.616667
1,1,6909.685811
1,1,4395.764706
1,1,8438.308824
1,1,2142.110465
1,1,4043.101351
1,1,2086.514423
1,1,1925.194444
1,1,2951.275862
1,1,3822.500000
1,1,2878.122093
1,1,4637.500000
1,1,2002.965278
1,1,4073.886364
1,1,3478.912162
1,1,3768.359375
1,1,2595.189189
1,1,2012.836957
1,1,2842.444444
1,1,4167.427136
1,1,1668.684375
1,1,3234.071809
1,1,1804.103448
1,1,6259.125000
1,1,5432.652174
1,1,5131.309659
1,1,1998.412500
1,1,3252.714286
1,1,8663.000000
1,1,1824.378049
1,1,2091.768382
1,1,3098.208333
1,1,6051.450000
1,1,1645.613095
1,1,2062.207447
1,1,4490.071770
1,1,1560.393939
1,1,1925.441860
1,1,2597.750000
1,1,66752.666667
1,1,13401.982143
1,1,2924.890625
1,1,2873.182390
1,1,2682.157895
1,1,1583.534722
1,1,8543.765957
1,1,4785.235294

1,1,22541.014205
1,1,5582.113636
1,1,65176.019231
1,1,2043.664634
1,1,1619.673469
1,1,1656.022857
1,1,4294.670213
1,1,2017.083333
1,1,5968.288462
1,1,3715.901316
1,1,100087.144231
1,1,18514.772727
1,1,3146.031250
1,1,3099.642857
1,1,3811.705882
1,1,119801.729167
1,1,6072.054688
1,1,2976.266892
1,1,3997.138889
1,1,12272.043367
1,1,6084.744186
1,1,1501.142857
1,1,3076.755556
1,1,6238.531250
1,1,2658.936111
1,1,3493.329545
1,1,1552.380952
1,1,20120.909091
1,1,1853.657143
1,1,2494.263158
1,1,6130.668605
1,1,10252.913043
1,1,11070.298387
1,1,7265.047619
1,1,92875.335106
1,1,9247.926829
1,1,13049.975610
1,1,37033.875000
1,1,2151.883721
1,1,11614.226415
1,1,5288.103448
1,1,2992.250000
1,1,4065.083333
1,1,41872.937500
1,1,4989.057065
1,1,2159.969388
1,1,5581.921875
1,1,45600.566406
1,1,8659.151786
1,1,10030.223214
1,1,3696.592949
1,1,2057.977273
1,1,4085.304054
1,1,4006.607143
1,1,2527.896875
1,1,2960.430233
1,1,26572.875000
1,1,2374.545732
1,1,1793.596154
1,1,4381.704545
1,1,3466.729167
1,1,5871.125000
1,1,1714.888889
1,1,2976.851744
1,1,1603.460227
1,1,1825.817568

1,1,4156.187500
1,1,3962.451923
1,1,4014.500000
1,1,2796.280303
1,1,2391.344595
1,1,1761.012500
1,1,2088.657143
1,1,2069.778846
1,1,1545.171429
1,1,2725.316667
1,1,2651.000000
1,1,1838.058824
1,1,22257.248428
1,1,54703.946429
1,1,1553.113095
1,1,10873.443452
1,1,3717.271429
1,1,1714.923295
1,1,4095.108108
1,1,1682.185811
1,1,1866.029070
1,1,6011.515152
1,1,7338.045455
1,1,1885.091667
1,1,87514.723958
1,1,5436.247159
1,1,9062.100694
1,1,2977.812500
1,1,1570.361486
1,1,19344.591837
1,1,10580.175000
1,1,18305.243243
1,1,5241.076923
1,1,1674.851190
1,1,11350.462500
1,1,3965.423295
1,1,26247.900568
1,1,2351.238636
1,1,2023.968750
1,1,16062.469697
1,1,10614.395833
1,1,3464.091463
1,1,1510.568182
1,1,2688.925000
1,1,3825.628289
1,1,5997.138889
1,1,2578.888889
1,1,2337.781250
1,1,2654.946429
1,1,2044.170455
1,1,65429.974359
1,1,4913.956522
1,1,2412.045732
1,1,2564.062500
1,1,6997.321429
1,1,2779.341667
1,1,3258.684524
1,1,2667.375000
1,1,4677.750000
1,1,1800.865385
1,1,1834.822368
1,1,1672.088757
1,1,3600.021277
1,1,5108.825581
1,1,3660.490964
1,1,2031.608696

1,1,11450.250000
1,1,135978.088957
1,1,5240.911111
1,1,12905.550000
1,1,4207.440341
1,1,3608.986486
1,1,1937.148936
1,1,3703.384615
1,1,3071.267857
1,1,3761.125000
1,1,18633.844595
1,1,2366.180556
1,1,93156.944444
1,1,11265.378289
1,1,3551.867188
1,1,1668.611111
1,1,2821.005319
1,1,4666.093750
1,1,1829.250000
1,1,122064.587209
1,1,2566.169118
1,1,5524.361364
1,1,19329.236842
1,1,3301.048077
1,1,1935.500000
1,1,9182.812500
1,1,9823.921512
1,1,2587.272727
1,1,4070.590000
1,1,1854.750000
1,1,7533.397059
1,1,12583.324324
1,1,1660.102941
1,1,2563.923913
1,1,11478.181818
1,1,7005.160156
1,1,60848.144231
1,1,2275.575581
1,1,2732.098039
1,1,9221.711538
1,1,4824.057692
1,1,1868.010870
1,1,3432.913462
1,1,4324.869565
1,1,2592.695122
1,1,3696.234375
1,1,2139.025641
1,1,5878.125000
1,1,4145.641304
1,1,2571.136364
1,1,7253.729167
1,1,2219.022727
1,1,6841.201117
1,1,5905.838235
1,1,1569.500000
1,1,1694.250000
1,1,36981.150641
1,1,3103.333333
1,1,5087.093750
1,1,6885.806818
1,1,4933.406250
1,1,4114.125000
1,1,1730.121324
1,1,4736.625000
1,1,6372.450000
1,1,2148.416667

1,1,1580.785326
1,1,2182.201220
1,1,1720.689516
1,1,4273.757143
1,1,2043.594286
1,1,1600.692073
1,1,1833.466667
1,1,1982.051282
1,1,3076.652778
1,1,1941.605114
1,1,2632.036585
1,1,1854.413043
1,1,4384.791667
1,1,1665.280488
1,1,4648.659091
1,1,2381.038265
1,1,2553.382979
1,1,3716.218750
1,1,6656.861111
1,1,4224.130435
1,1,17076.316406
1,1,7180.695312
1,1,6199.558824
1,1,3707.750000
1,1,3551.058673
1,1,85457.538462
1,1,1521.108553
1,1,2220.125000
1,1,3894.666667
1,1,3111.887195
1,1,6461.669811
1,1,8020.091146
1,1,11088.434211
1,1,3661.342857
1,1,2319.615385
1,1,9012.988636
1,1,1603.851190
1,1,5712.538462
1,1,4941.000000
1,1,12947.980000
1,1,2615.473684
1,1,63807.945946
1,1,1677.011364
1,1,2162.048649
1,1,3629.763889
1,1,3931.212500
1,1,9182.295213
1,1,3063.079787
1,1,2727.936047
1,1,5301.813830
1,1,4413.722222
1,1,5846.705357
1,1,7320.625000
1,1,5663.657143
1,1,4076.758621
1,1,1780.056818
1,1,3441.635135
1,1,7174.365625
1,1,1956.896277
1,1,7871.445122
1,1,6214.933824
1,1,1814.416667
1,1,6385.031915
1,1,2260.064103
1,1,3602.717262
1,1,6116.659420

1,1,4976.750000
1,1,14417.268293
1,1,3338.411184
1,1,3297.192308
1,1,4352.333333
1,1,1823.000000
1,1,2864.889706
1,1,1557.987179
1,1,1674.842949
1,1,1904.023256
1,1,9709.913043
1,1,4947.378378
1,1,23505.192308
1,1,4305.266667
1,1,5904.966216
1,1,4210.329545
1,1,4497.615385
1,1,68260.268293
1,1,1682.727273
1,1,1964.000000
1,1,2077.826220
1,1,12664.000000
1,1,3333.011111
1,1,1926.695652
1,1,3759.586538
1,1,7412.688889
1,1,5069.718750
1,1,3311.314103
1,1,8087.302083
1,1,7211.500000
1,1,7581.840426
1,1,2230.807432
1,1,2985.000000
1,1,2182.424242
1,1,9325.000000
1,1,1616.361111
1,1,6222.173295
1,1,3839.625000
1,1,3389.609043
1,1,15606.404255
1,1,1611.487805
1,1,1519.647059
1,1,2985.239583
1,1,5479.267857
1,1,4679.714286
1,1,2861.628205
1,1,2119.086957
1,1,49330.304688
1,1,3186.285714
1,1,16319.028571
1,1,8735.062500
1,1,6769.100490
1,1,2721.206522
1,1,2994.736842
1,1,2046.744186
1,1,2440.352273
1,1,7046.307692
1,1,13537.864865
1,1,24866.500000
1,1,1673.187500
1,1,4285.942935
1,1,64525.063953
1,1,1946.847222
1,1,5067.385135
1,1,2059.770833
1,1,44576.135135

1,1,8215.937500
1,1,2694.837209
1,1,9931.461538
1,1,3725.508152
1,1,8884.722222
1,1,60978.022059
1,1,4670.315217
1,1,1653.026316
1,1,4283.000000
1,1,9780.625000
1,1,11366.545455
1,1,7538.983333
1,1,1701.901163
1,1,18131.086957
1,1,10277.170732
1,1,3375.217105
1,1,3411.920139
1,1,2700.505102
1,1,3683.229167
1,1,3108.445946
1,1,6748.755556
1,1,2684.132812
1,1,3578.375000
1,1,3743.720930
1,1,5709.108108
1,1,4933.825581
1,1,4665.590909
1,1,1770.225694
1,1,2580.724359
1,1,6645.557692
1,1,5313.846154
1,1,4618.181818
1,1,1730.581081
1,1,4115.000000
1,1,1809.175000
1,1,5693.960526
1,1,3099.182927
1,1,7831.207447
1,1,1958.222222
1,1,5863.125000
1,1,2129.229167
1,1,21364.096875
1,1,7476.270833
1,1,2874.500000
1,1,1631.195122
1,1,18604.736111
1,1,2292.365854
1,1,5577.984848
1,1,8511.753247
1,1,10695.235000
1,1,3433.162162
1,1,7360.013889
1,1,36314.164634
1,1,3358.187500
1,1,40983.926471
1,1,4346.390625
1,1,1841.904255
1,1,3745.750000
1,1,2342.179688
1,1,1522.375000
1,1,2353.906977
1,1,6187.604730
1,1,3487.541985
1,1,2002.785714
1,1,61093.475610
1,1,2560.703125

1,1,8586.463235
1,1,1599.430556
1,1,49929.008475
1,1,83086.000000
1,1,1912.371622
1,1,1719.943548
1,1,9887.287879
1,1,1748.260638
1,1,4737.196721
1,1,7929.503906
1,1,2966.785714
1,1,1596.163462
1,1,40471.642857
1,1,6680.189655
1,1,145734.262500
1,1,2166.025568
1,1,2791.098039
1,1,1656.824324
1,1,11438.661765
1,1,4601.743590
1,1,9253.328125
1,1,3995.896552
1,1,3220.742857
1,1,1726.368421
1,1,2053.988636
1,1,2479.185811
1,1,5204.605263
1,1,1797.556452
1,1,10133.133929
1,1,7668.093750
1,1,3363.487500
1,1,3473.116667
1,1,8783.101744
1,1,3468.909483
1,1,3391.965116
1,1,7776.318182
1,1,4514.816176
1,1,2497.658537
1,1,2998.142857
1,1,2043.384615
1,1,1599.965116
1,1,7087.659314
1,1,5484.852941
1,1,5617.500000
1,1,1741.539634
1,1,5259.775000
1,1,51700.785714
1,1,6236.892857
1,1,1647.165761
1,1,6536.538462
1,1,3146.527174
1,1,9256.596939
1,1,2577.803922
1,1,3247.943750
1,1,13474.493056
1,1,7204.509868
1,1,3464.480769
1,1,4512.151042
1,1,1876.116848
1,1,1830.622222
1,1,6298.375000
1,1,20558.333333
1,1,2150.275000
1,1,1588.134375
1,1,27025.122093
1,1,46200.140625

1,1,1803.210227
1,1,7407.388889
1,1,2416.937500
1,1,2556.996951
1,1,2702.164773
1,1,3373.534884
1,1,7743.197500
1,1,5443.000000
1,1,6011.059091
1,1,2324.527174
1,1,7569.200000
1,1,2550.234375
1,1,4566.579787
1,1,7336.025000
1,1,2607.157143
1,1,22575.307927
1,1,3008.079545
1,1,1634.403571
1,1,2304.900000
1,1,2708.784091
1,1,2007.281250
1,1,5886.877551
1,1,2954.631944
1,1,4454.142857
1,1,2799.971429
1,1,3297.576923
1,1,7453.029412
1,1,5956.465839
1,1,2211.351562
1,1,2946.296053
1,1,2307.390244
1,1,19442.407143
1,1,4308.130435
1,1,15078.727273
1,1,28761.378378
1,1,5477.785714
1,1,59290.758333
1,1,8947.173913
1,1,53533.654412
1,1,3852.092105
1,1,3543.560976
1,1,2057.805556
1,1,10364.450000
1,1,2830.875000
1,1,3175.048077
1,1,7132.993243
1,1,3761.253676
1,1,10015.887417
1,1,4927.769231
1,1,1522.000000
1,1,5686.879310
1,1,1770.173913
1,1,4446.085106
1,1,2480.500000
1,1,3775.142857
1,1,2977.282051
1,1,2143.290441
1,1,1701.255814
1,1,2411.731250
1,1,10262.168478
1,1,2567.260000
1,1,3897.635638
1,1,2773.952500
1,1,2346.245399
1,1,1579.739496
1,1,5842.739362

1,1,1812.097561
1,1,1511.781250
1,1,3154.525424
1,1,1508.472973
1,1,2665.891447
1,1,2622.337989
1,1,3249.552632
1,1,2215.354286
1,1,7338.265625
1,1,3353.320312
1,1,2103.902778
1,1,1916.382979
1,1,9109.632075
1,1,27813.411585
1,1,5476.446927
1,1,11407.421875
1,1,7451.085714
1,1,1955.769231
1,1,7087.125000
1,1,4408.898438
1,1,1835.673469
1,1,36501.257812
1,1,1705.053191
1,1,13751.500000
1,1,1656.562500
1,1,3429.662500
1,1,3663.040000
1,1,4177.655405
1,1,1925.794118
1,1,6806.412791
1,1,7375.687500
1,1,2893.199219
1,1,2594.543478
1,1,2546.437500
1,1,3820.578947
1,1,2140.801136
1,1,2136.491071
1,1,2600.413265
1,1,2522.552632
1,1,1865.894022
1,1,5226.881579
1,1,1512.795322
1,1,5959.146853
1,1,1742.593750
1,1,3924.343750
1,1,4044.524038
1,1,11508.135135
1,1,1787.671053
1,1,22212.934211
1,1,30765.334459
1,1,2291.222222
1,1,2004.350000
1,1,11381.715686
1,1,2383.389535
1,1,4646.476190
1,1,2793.432692
1,1,4343.307263
1,1,2979.667476
1,1,5367.769231
1,1,5603.601190
1,1,2311.390244
1,1,2510.738095
1,1,3247.310811
1,1,2573.650641
1,1,6736.078947
1,1,3888.459239

1,1,1885.096154
1,1,1957.799419
1,1,4446.613636
1,1,2572.173077
1,1,4424.576389
1,1,29884.826087
1,1,2381.450581
1,1,9752.066406
1,1,2672.033333
1,1,1566.040881
1,1,3801.869565
1,1,151383.578125
1,1,7017.381579
1,1,1762.111650
1,1,2265.250000
1,1,3394.425000
1,1,9092.369318
1,1,2070.227273
1,1,12155.400000
1,1,81675.360248
1,1,1941.900000
1,1,18658.807432
1,1,1882.110294
1,1,2310.521739
1,1,1872.951220
1,1,1553.974194
1,1,3353.882353
1,1,3621.220588
1,1,4594.500000
1,1,2903.906250
1,1,7795.756757
1,1,14545.871951
1,1,2094.635135
1,1,2575.031250
1,1,3772.375000
1,1,19988.280488
1,1,1634.921875
1,1,1872.086538
1,1,1755.014205
1,1,4733.025641
1,1,6277.278409
1,1,7821.812500
1,1,1678.652439
1,1,5848.147727
1,1,2450.187500
1,1,2511.054054
1,1,1501.781250
1,1,2020.375000
1,1,41335.359375
1,1,1596.949405
1,1,4146.639535
1,1,5308.011364
1,1,2047.111111
1,1,20264.511364
1,1,2739.000000
1,1,6288.969828
1,1,8528.157609
1,1,1675.479730
1,1,2080.583333
1,1,7865.250000
1,1,8938.156977
1,1,1569.269886
1,1,4422.218750
1,1,3777.506098
1,1,3710.159722
1,1,2491.914474

1,1,1813.128342
1,1,3201.764706
1,1,6360.819444
1,1,2918.395349
1,1,1522.152174
1,1,6253.762712
1,1,20707.888889
1,1,1884.446429
1,1,1591.857143
1,1,1536.906667
1,1,11270.133333
1,1,1926.250000
1,1,2890.571429
1,1,3844.857143
1,1,4129.479730
1,1,4425.920000
1,1,5534.500000
1,1,6115.529412
1,1,2453.707317
1,1,13541.327206
1,1,6975.455882
1,1,58774.400000
1,1,1728.304487
1,1,8117.641026
1,1,4011.068182
1,1,7615.500000
1,1,13940.736486
1,1,4159.038889
1,1,3613.402778
1,1,1980.000000
1,1,11781.000000
1,1,1988.210227
1,1,16942.176471
1,1,41600.513158
1,1,2147.866667
1,1,4907.207386
1,1,3744.453488
1,1,6409.323864
1,1,31701.625000
1,1,2119.917553
1,1,7270.544304
1,1,1653.040698
1,1,1676.710526
1,1,7697.125000
1,1,2783.794643
1,1,3603.850000
1,1,6686.651163
1,1,1931.567568
1,1,7982.333333
1,1,2408.380814
1,1,1597.109677
1,1,2983.573298
1,1,3264.836735
1,1,3225.342105
1,1,1906.987500
1,1,3279.857955
1,1,2127.026316
1,1,3984.671053
1,1,2598.355769
1,1,7547.734375
1,1,6476.831633
1,1,2548.118421
1,1,4382.434783
1,1,3214.954787
1,1,1908.017857
1,1,2352.393548

1,1,12466.746324
1,1,1595.290698
1,1,3325.265306
1,1,1581.544444
1,1,3286.257143
1,1,1988.818182
1,1,4201.071429
1,1,15209.381944
1,1,1991.608333
1,1,6164.664773
1,1,83250.577206
1,1,3717.335106
1,1,5270.786932
1,1,7273.305147
1,1,2906.544872
1,1,1897.173913
1,1,12559.012755
1,1,62076.653846
1,1,3885.342105
1,1,2147.528409
1,1,5611.390625
1,1,1939.202778
1,1,2960.230114
1,1,6580.368263
1,1,10432.952381
1,1,4968.407407
1,1,2161.234375
1,1,4481.621094
1,1,1687.025000
1,1,6284.705882
1,1,1563.125000
1,1,3226.568182
1,1,1540.792683
1,1,3333.063953
1,1,10593.687500
1,1,3569.625000
1,1,2764.067073
1,1,2936.676630
1,1,2481.692857
1,1,3519.103448
1,1,52286.208333
1,1,1783.809524
1,1,8667.444149
1,1,2443.250000
1,1,4285.229167
1,1,4392.581395
1,1,5693.000000
1,1,1859.122222
1,1,5795.950000
1,1,41424.058140
1,1,2821.073529
1,1,3139.139535
1,1,4360.276596
1,1,3948.138889
1,1,1909.919271
1,1,6935.375000
1,1,2286.318182
1,1,38088.190476
1,1,13371.687500
1,1,2597.365000
1,1,3336.904255
1,1,2075.390244
1,1,2941.086538
1,1,2134.000000
1,1,5456.583333
1,1,6352.904523

1,1,2349.244898
1,1,2323.304878
1,1,2830.988971
1,1,3741.091837
1,1,3926.444079
1,1,2236.487805
1,1,3691.317308
1,1,4219.142500
1,1,13398.341463
1,1,3229.785714
1,1,3662.241477
1,1,7468.771739
1,1,4283.750000
1,1,2374.934211
1,1,12520.052326
1,1,4103.400000
1,1,4355.000000
1,1,52556.053977
1,1,3366.209302
1,1,2309.010204
1,1,9900.092105
1,1,3722.598684
1,1,3797.478261
1,1,6618.147727
1,1,1998.199405
1,1,4926.977941
1,1,2750.353125
1,1,2034.097561
1,1,2016.227273
1,1,4531.554054
1,1,1694.837500
1,1,2085.272059
1,1,3666.722222
1,1,2839.956522
1,1,3609.858696
1,1,39759.650000
1,1,6113.178977
1,1,2438.336538
1,1,2496.244444
1,1,7944.581395
1,1,2511.750000
1,1,1815.775000
1,1,2024.770833
1,1,2524.066667
1,1,4459.486842
1,1,1744.631250
1,1,4926.246711
1,1,34582.368421
1,1,2620.383333
1,1,1892.600000
1,1,4567.717949
1,1,3158.597826
1,1,19413.605263
1,1,7036.714286
1,1,6922.992188
1,1,3816.734375
1,1,3545.888298
1,1,8933.860294
1,1,1903.096591
1,1,3368.852941
1,1,1979.631579
1,1,1512.482558
1,1,8331.908537
1,1,1580.144231
1,1,1637.404762
1,1,2590.627907

1,1,2840.908537
1,1,6850.437500
1,1,2867.854167
1,1,1843.278125
1,1,1941.486607
1,1,8577.663889
1,1,9657.068627
1,1,1858.000000
1,1,6295.276596
1,1,2775.875000
1,1,10877.510638
1,1,1963.340278
1,1,1644.069767
1,1,1572.923913
1,1,1534.750000
1,1,1568.878571
1,1,2317.650000
1,1,2938.764535
1,1,5433.114583
1,1,6097.378571
1,1,10492.359756
1,1,2589.468750
1,1,36058.025281
1,1,2353.383929
1,1,2827.571429
1,1,2249.255000
1,1,1887.756250
1,1,1634.082237
1,1,1894.466146
1,1,2280.054688
1,1,4578.352564
1,1,2729.135204
1,1,11691.419271
1,1,1636.733108
1,1,8695.916667
1,1,2182.281250
1,1,3492.869565
1,1,1786.951220
1,1,1955.568182
1,1,5254.117647
1,1,3032.305851
1,1,2776.067708
1,1,1885.500000
1,1,3904.687500
1,1,2407.008721
1,1,128963.328125
1,1,2643.319767
1,1,4415.610465
1,1,1967.467949
1,1,19392.203571
1,1,2226.895349
1,1,2159.253049
1,1,9475.737762
1,1,5774.567568
1,1,2343.398649
1,1,9132.918367
1,1,8181.108696
1,1,3483.888889
1,1,9977.078947
1,1,3381.479167
1,1,2399.153646
1,1,2542.851064
1,1,7434.170000
1,1,1564.986842
1,1,3449.427500
1,1,3498.218750

1,1,1700.483974
1,1,1758.937500
1,1,6438.195652
1,1,90853.096591
1,1,3367.642857
1,1,11157.551020
1,1,2509.983333
1,1,4803.236842
1,1,1521.277778
1,1,9609.083333
1,1,4631.951220
1,1,7967.121951
1,1,3745.923077
1,1,37748.145833
1,1,2467.500000
1,1,3089.582237
1,1,1702.791667
1,1,25349.039474
1,1,2683.057143
1,1,3097.047872
1,1,9273.955882
1,1,2799.038462
1,1,4767.885870
1,1,3697.107759
1,1,8873.750000
1,1,1569.500000
1,1,1917.185811
1,1,3508.454787
1,1,17556.191489
1,1,5000.273585
1,1,2220.466837
1,1,3290.342391
1,1,2993.351351
1,1,3285.758065
1,1,3943.871622
1,1,5405.793478
1,1,5913.333333
1,1,2387.612069
1,1,1706.757979
1,1,2412.070652
1,1,2206.681250
1,1,2611.960526
1,1,7321.849057
1,1,2051.333333
1,1,2086.601562
1,1,3719.189655
1,1,2372.363636
1,1,6145.527174
1,1,7847.811111
1,1,2474.000000
1,1,1898.364865
1,1,1699.475000
1,1,2374.619186
1,1,1920.545455
1,1,3002.228261
1,1,2852.000000
1,1,3762.166667
1,1,2405.220109
1,1,2313.069767
1,1,3839.662162
1,1,6812.941176
1,1,1891.861111
1,1,4966.291667
1,1,1775.204678
1,1,2281.608696
1,1,2856.218750

1,1,3416.715909
1,1,5530.332447
1,1,5037.360294
1,1,1966.911111
1,1,1672.030405
1,1,1562.738372
1,1,3486.909091
1,1,2762.113636
1,1,21217.714286
1,1,5488.032258
1,1,2095.442073
1,1,6739.449468
1,1,2386.894231
1,1,2597.894040
1,1,14426.530488
1,1,53911.315476
1,1,3085.653846
1,1,8719.062500
1,1,3798.535714
1,1,16158.597122
1,1,4168.048295
1,1,3342.701705
1,1,2623.414286
1,1,1506.943182
1,1,1852.093023
1,1,5175.894737
1,1,4262.363636
1,1,1869.328947
1,1,5354.948171
1,1,2446.195652
1,1,41975.243750
1,1,4157.558140
1,1,6352.307692
1,1,3637.250000
1,1,8872.023256
1,1,4945.393617
1,1,7837.195652
1,1,2245.105469
1,1,6158.338983
1,1,4969.756579
1,1,4574.427632
1,1,3060.116279
1,1,2978.045455
1,1,3759.964286
1,1,5513.230769
1,1,16461.645349
1,1,1877.841216
1,1,6661.890000
1,1,31836.154412
1,1,4432.695312
1,1,7700.062500
1,1,1523.539634
1,1,2233.400000
1,1,49290.857143
1,1,10374.500000
1,1,52955.055921
1,1,4992.561170
1,1,1669.958333
1,1,7374.052632
1,1,1769.904070
1,1,8396.636364
1,1,1739.861413
1,1,68256.706897
1,1,3722.268382
1,1,2627.610119
1,1,3117.613636

1,1,1665.758152
1,1,1925.414634
1,1,4203.553125
1,1,1613.227564
1,1,24848.886364
1,1,1829.500000
1,1,43892.611486
1,1,1521.421053
1,1,3015.013889
1,1,11836.268293
1,1,1876.594697
1,1,3677.944079
1,1,2224.251572
1,1,1852.837209
1,1,2355.046512
1,1,1950.858553
1,1,2169.411111
1,1,16860.645161
1,1,1914.615385
1,1,3955.668919
1,1,1642.969388
1,1,4073.260870
1,1,10572.281250
1,1,13433.410714
1,1,3750.023256
1,1,2901.884615
1,1,1649.942308
1,1,9609.875000
1,1,3569.893617
1,1,1703.555556
1,1,1546.512821
1,1,3160.067568
1,1,1983.455882
1,1,9226.275735
1,1,5773.382979
1,1,2585.500000
1,1,2593.112245
1,1,1594.435976
1,1,1780.485465
1,1,4688.638587
1,1,16520.482143
1,1,4580.851190
1,1,1679.580000
1,1,7079.885417
1,1,12925.480263
1,1,2914.315789
1,1,4667.421875
1,1,6247.222222
1,1,4161.121951
1,1,5553.085714
1,1,3895.011364
1,1,2724.533333
1,1,2066.125000
1,1,2674.973684
1,1,1581.500000
1,1,1785.390957
1,1,104803.520231
1,1,4940.084459
1,1,1558.120567
1,1,2310.728659
1,1,1849.097561
1,1,2158.500000
1,1,2869.109375
1,1,1652.687500
1,1,5645.533333
1,1,2321.538462

1,1,4606.800000
1,1,2064.888298
1,1,3603.257353
1,1,4418.500000
1,1,2144.181818
1,1,2534.297872
1,1,2092.075000
1,1,3092.226064
1,1,2197.919463
1,1,3819.489583
1,1,7564.872549
1,1,39053.229730
1,1,7308.480000
1,1,2329.144737
1,1,2245.247283
1,1,3220.437500
1,1,1761.081522
1,1,2325.842500
1,1,1870.594595
1,1,3111.892857
1,1,2175.371795
1,1,5943.451389
1,1,15029.017361
1,1,5337.568182
1,1,3996.083333
1,1,1892.319149
1,1,3770.394737
1,1,3196.948718
1,1,6692.988636
1,1,4064.215000
1,1,4160.228261
1,1,2812.009146
1,1,2178.250000
1,1,3724.354167
1,1,4059.811170
1,1,149268.258523
1,1,6183.633333
1,1,1574.106383
1,1,3829.104730
1,1,2435.900000
1,1,2362.819149
1,1,3734.712500
1,1,13046.025568
1,1,1887.818182
1,1,4812.925000
1,1,8744.739583
1,1,1695.989130
1,1,7410.720745
1,1,2126.840000
1,1,2388.871951
1,1,4670.375000
1,1,1965.833333
1,1,2685.800000
1,1,1882.053571
1,1,1621.619444
1,1,1677.028646
1,1,2492.718750
1,1,4508.416667
1,1,11068.814103
1,1,2835.909091
1,1,5716.780347
1,1,2813.834459
1,1,3047.977273
1,1,1587.933333
1,1,1653.166667
1,1,3138.254286

1,1,12648.542553
1,1,1783.732143
1,1,8488.100543
1,1,4746.091463
1,1,1560.937500
1,1,5155.221591
1,1,5406.488889
1,1,5796.475000
1,1,1978.793605
1,1,15582.971154
1,1,22112.521739
1,1,22811.348214
1,1,1872.500000
1,1,5342.850962
1,1,2887.198795
1,1,1642.615132
1,1,2707.786932
1,1,2843.851562
1,1,2424.802326
1,1,49907.911932
1,1,11926.015152
1,1,2210.776786
1,1,8186.431373
1,1,1923.222222
1,1,2443.125000
1,1,3539.564516
1,1,2029.452703
1,1,2831.719136
1,1,9147.580420
1,1,4952.964286
1,1,1648.200000
1,1,1742.977041
1,1,2335.809524
1,1,1661.209302
1,1,24002.250000
1,1,4647.296875
1,1,6674.197917
1,1,4558.741379
1,1,59418.000000
1,1,12684.645833
1,1,2011.904762
1,1,5157.274306
1,1,6658.241071
1,1,7006.930556
1,1,3449.101562
1,1,2700.666667
1,1,9994.315789
1,1,2441.241071
1,1,2277.541667
1,1,6816.421053
1,1,2034.366279
1,1,2230.750000
1,1,3109.684211
1,1,2006.255814
1,1,2628.145349
1,1,57901.625000
1,1,3746.966667
1,1,2974.425532
1,1,1522.615385
1,1,1688.349359
1,1,6202.966463
1,1,2608.530120
1,1,4687.595745
1,1,5226.007212
1,1,1681.410714
1,1,3049.326923

1,1,1529.687500
1,1,9063.456250
1,1,1716.587500
1,1,4332.477679
1,1,4080.038462
1,1,34999.020833
1,1,2170.609756
1,1,5730.613636
1,1,128628.946429
1,1,4561.166667
1,1,2400.634615
1,1,6916.487500
1,1,2579.065625
1,1,2214.729885
1,1,3138.522727
1,1,1889.161765
1,1,4195.651596
1,1,3496.777778
1,1,11400.097561
1,1,2511.750000
1,1,1575.687500
1,1,2610.500000
1,1,60277.113333
1,1,2337.354651
1,1,4023.940789
1,1,16640.312500
1,1,3697.134615
1,1,2078.232558
1,1,5132.375000
1,1,2002.355556
1,1,4878.036765
1,1,2637.035714
1,1,1962.880682
1,1,7509.500000
1,1,11177.250000
1,1,2321.820946
1,1,3350.571429
1,1,4481.700000
1,1,1915.687500
1,1,3536.049107
1,1,1957.000000
1,1,44678.230769
1,1,4156.829545
1,1,1805.718750
1,1,1718.312500
1,1,5693.810976
1,1,8240.704545
1,1,2856.500000
1,1,2077.073864
1,1,1794.836735
1,1,2647.100000
1,1,9353.892857
1,1,4229.881579
1,1,7377.285714
1,1,11237.938776
1,1,1732.750000
1,1,2156.576087
1,1,1532.983333
1,1,2732.052083
1,1,1570.875000
1,1,8946.777542
1,1,29941.379032
1,1,3005.979167
1,1,3651.250000
1,1,1735.942073
1,1,8157.144737

1,1,3136.160714
1,1,1905.900000
1,1,4078.744186
1,1,6234.425781
1,1,3610.477273
1,1,1527.384615
1,1,2115.406250
1,1,2448.668605
1,1,10557.660526
1,1,2922.772727
1,1,1877.554878
1,1,2664.321229
1,1,3104.946809
1,1,2526.518293
1,1,4120.428571
1,1,6523.225000
1,1,2740.178571
1,1,4683.834459
1,1,12868.720930
1,1,6831.259259
1,1,1741.683673
1,1,2598.625000
1,1,9798.219512
1,1,1977.390625
1,1,3208.318182
1,1,3267.203125
1,1,1954.175676
1,1,32128.397436
1,1,3767.650000
1,1,3922.929348
1,1,4802.314189
1,1,4730.265625
1,1,17879.538690
1,1,2146.535714
1,1,72037.621622
1,1,1589.312500
1,1,43103.182065
1,1,4951.333333
1,1,2248.243243
1,1,2939.319149
1,1,2159.095238
1,1,3399.534884
1,1,2101.378472
1,1,2197.918367
1,1,2173.937500
1,1,9853.429054
1,1,2190.764706
1,1,4309.141447
1,1,8728.527439
1,1,2151.604730
1,1,6332.750000
1,1,4162.081395
1,1,5969.965517
1,1,2358.055233
1,1,3150.425595
1,1,1585.391447
1,1,1892.045455
1,1,2075.692308
1,1,4478.633880
1,1,4189.922500
1,1,3709.740854
1,1,1875.833333
1,1,3661.824405
1,1,127442.729167
1,1,2768.792453
1,1,1896.850000

1,1,1812.478261
1,1,5409.327128
1,1,4710.941860
1,1,1959.834532
1,1,4084.096774
1,1,92502.796196
1,1,2405.891892
1,1,1529.326923
1,1,2288.625000
1,1,1641.203125
1,1,26942.759615
1,1,2607.346154
1,1,6683.382653
1,1,1515.589286
1,1,3131.011364
1,1,4651.625000
1,1,2959.642857
1,1,1797.521739
1,1,7942.055556
1,1,9645.863636
1,1,6511.938776
1,1,79191.824324
1,1,3670.194444
1,1,5140.900000
1,1,3064.603125
1,1,8049.363636
1,1,10739.067130
1,1,3598.737903
1,1,1742.650000
1,1,3252.300000
1,1,23400.987500
1,1,2327.621622
1,1,2284.090116
1,1,4461.041667
1,1,3556.350543
1,1,3005.920455
1,1,4177.367347
1,1,1606.203488
1,1,6072.841463
1,1,56592.033333
1,1,3316.333333
1,1,105167.800000
1,1,2350.431818
1,1,2111.892857
1,1,14928.380208
1,1,3056.103571
1,1,7251.437500
1,1,16648.655612
1,1,11671.051829
1,1,2696.091837
1,1,1724.936170
1,1,6971.625000
1,1,2939.114286
1,1,2176.803571
1,1,1705.233974
1,1,3377.920455
1,1,4388.086806
1,1,3182.432432
1,1,8048.466667
1,1,3418.569444
1,1,12245.000000
1,1,2647.494565
1,1,3339.571429
1,1,14371.867925
1,1,47008.729167
1,1,1648.875000

1,1,2325.270833
1,1,3609.647727
1,1,10955.546053
1,1,2313.000000
1,1,5893.028302
1,1,4825.147059
1,1,8390.800000
1,1,3275.767857
1,1,7407.750000
1,1,2671.903226
1,1,6362.613636
1,1,6883.893382
1,1,6024.571429
1,1,2056.209459
1,1,8355.421053
1,1,35031.826220
1,1,2255.011364
1,1,4790.227273
1,1,1680.976190
1,1,1530.723837
1,1,3845.660377
1,1,14053.390411
1,1,5352.054348
1,1,2239.315789
1,1,1502.000000
1,1,5042.583333
1,1,1916.583333
1,1,4970.733553
1,1,4531.375000
1,1,7396.856383
1,1,5336.625000
1,1,1730.630682
1,1,13210.425000
1,1,5745.200000
1,1,2366.244444
1,1,2077.230769
1,1,1958.581395
1,1,3292.560976
1,1,9203.508621
1,1,4819.382653
1,1,4977.434783
1,1,3705.525000
1,1,7596.472973
1,1,2520.366279
1,1,6247.352941
1,1,2090.959064
1,1,2230.454545
1,1,4428.094828
1,1,24057.450000
1,1,8005.505814
1,1,3554.860000
1,1,7625.200000
1,1,27004.022727
1,1,5898.603659
1,1,2515.368750
1,1,3104.627976
1,1,1679.897959
1,1,2503.346154
1,1,2373.775862
1,1,3047.803571
1,1,14526.810606
1,1,3231.963542
1,1,20601.397059
1,1,3028.519886
1,1,5023.687500
1,1,3099.428571

1,1,3345.895349
1,1,3290.183673
1,1,4796.720930
1,1,7681.609043
1,1,3047.343750
1,1,1674.029762
1,1,4828.062500
1,1,4552.898649
1,1,7341.191176
1,1,2263.125000
1,1,2233.567568
1,1,8505.389423
1,1,10283.293605
1,1,4121.146552
1,1,2011.555000
1,1,3491.190217
1,1,2091.936709
1,1,6104.477941
1,1,4061.083333
1,1,2536.651163
1,1,2223.878205
1,1,4465.233333
1,1,10706.906250
1,1,1703.318878
1,1,1955.151079
1,1,22553.464744
1,1,2829.298780
1,1,1567.366279
1,1,17257.775000
1,1,1590.410256
1,1,3227.500000
1,1,1695.460000
1,1,2544.166667
1,1,2522.000000
1,1,6599.446809
1,1,3669.267045
1,1,2030.345930
1,1,7545.537879
1,1,3750.551471
1,1,3850.017857
1,1,4550.000000
1,1,49027.776163
1,1,2809.351744
1,1,8034.608333
1,1,1903.423077
1,1,2784.932692
1,1,2297.727679
1,1,5464.851744
1,1,1813.514286
1,1,1647.160714
1,1,1921.261628
1,1,3462.141304
1,1,5702.027027
1,1,4542.400000
1,1,6020.618421
1,1,12505.665761
1,1,2451.307692
1,1,1919.345395
1,1,2082.844595
1,1,1994.684211
1,1,13335.309659
1,1,2491.375000
1,1,3673.448171
1,1,6913.089744
1,1,2356.507812
1,1,12576.255814

1,1,2893.700000
1,1,4722.691860
1,1,3192.709677
1,1,1582.571429
1,1,3149.016304
1,1,4192.734694
1,1,4209.451389
1,1,94485.292683
1,1,2131.789474
1,1,2379.000000
1,1,3092.864865
1,1,16588.187500
1,1,2539.527174
1,1,27389.416667
1,1,5507.222973
1,1,2870.759615
1,1,2202.845395
1,1,1936.580882
1,1,2155.777778
1,1,2651.958333
1,1,3442.720588
1,1,3666.767500
1,1,3239.900000
1,1,2501.500000
1,1,4891.480114
1,1,1747.444444
1,1,5572.118421
1,1,1799.882353
1,1,34760.560241
1,1,2134.097561
1,1,5764.833333
1,1,1924.701923
1,1,36992.636628
1,1,2176.866667
1,1,7839.525000
1,1,2824.517045
1,1,1787.884615
1,1,58768.893939
1,1,3610.491071
1,1,3168.096774
1,1,49052.000000
1,1,1550.175573
1,1,2344.953488
1,1,1896.388298
1,1,17049.686170
1,1,6929.188889
1,1,1643.431250
1,1,1540.084459
1,1,5662.041667
1,1,2179.238095
1,1,3800.906250
1,1,3412.527778
1,1,3610.666667
1,1,61515.301471
1,1,6006.136364
1,1,1855.901316
1,1,2350.865385
1,1,12958.757576
1,1,5041.307292
1,1,4187.816176
1,1,1717.846626
1,1,3451.500000
1,1,1588.432624
1,1,2081.614286
1,1,15501.780488
1,1,5199.116667

1,1,2275.647727
1,1,3459.367347
1,1,4625.336111
1,1,1869.722222
1,1,26255.036585
1,1,9864.250000
1,1,2223.828947
1,1,7318.750000
1,1,2789.008152
1,1,1668.343137
1,1,2229.189516
1,1,25655.763889
1,1,3236.954268
1,1,2056.224265
1,1,1606.625000
1,1,4954.441176
1,1,1714.788043
1,1,3762.068182
1,1,1795.108108
1,1,1521.288690
1,1,2192.583333
1,1,28228.875000
1,1,1886.563776
1,1,3595.236486
1,1,1805.750000
1,1,2975.816860
1,1,1509.206250
1,1,3565.581395
1,1,2349.458333
1,1,2593.398649
1,1,1741.333333
1,1,8271.003289
1,1,5014.438889
1,1,4068.211538
1,1,1776.333333
1,1,1566.589286
1,1,11756.025000
1,1,1900.281250
1,1,3241.755319
1,1,4982.500000
1,1,6573.312500
1,1,1986.437500
1,1,5155.057432
1,1,3580.615385
1,1,54036.625000
1,1,1508.500000
1,1,3608.753378
1,1,60543.937500
1,1,6158.891447
1,1,3434.235294
1,1,1582.975694
1,1,4633.169118
1,1,1624.840491
1,1,7156.736842
1,1,3663.137097
1,1,4006.612500
1,1,4592.300481
1,1,21512.815476
1,1,11607.954545
1,1,4116.953488
1,1,1657.836806
1,1,17300.866667
1,1,1762.375000
1,1,38909.959677
1,1,2719.640625
1,1,2444.607143

1,1,9822.400000
1,1,1544.388889
1,1,8870.523810
1,1,2523.853723
1,1,21493.250000
1,1,26515.169118
1,1,3038.894737
1,1,2951.551282
1,1,3481.407143
1,1,4159.290698
1,1,2695.531250
1,1,2664.465278
1,1,1596.179487
1,1,26886.040000
1,1,2547.293919
1,1,6819.272727
1,1,2042.914286
1,1,2005.013158
1,1,2250.000000
1,1,1613.861413
1,1,3473.599490
1,1,2544.566667
1,1,1838.444767
1,1,4246.443966
1,1,2154.825581
1,1,2840.100000
1,1,2586.266892
1,1,24324.300000
1,1,12649.607143
1,1,8045.359375
1,1,1822.022727
1,1,1628.067708
1,1,139115.011364
1,1,2817.666667
1,1,3296.714286
1,1,2537.598684
1,1,2104.890244
1,1,2424.500000
1,1,2490.187500
1,1,5412.797170
1,1,2680.833333
1,1,13455.575000
1,1,2503.850000
1,1,1658.597561
1,1,14836.384615
1,1,29782.950000
1,1,2357.531250
1,1,1620.536585
1,1,4896.788194
1,1,9322.023936
1,1,2414.709559
1,1,5046.323980
1,1,4165.209302
1,1,24869.336806
1,1,2199.933333
1,1,8238.913462
1,1,2064.235795
1,1,13932.516304
1,1,1548.108108
1,1,3776.543605
1,1,3121.000000
1,1,65637.000000
1,1,1560.781250
1,1,2062.292763
1,1,8567.496622
1,1,1767.095070

1,1,12939.048780
1,1,5117.732639
1,1,1764.801136
1,1,12977.017857
1,1,4449.299020
1,1,17856.506757
1,1,8625.562500
1,1,1516.204545
1,1,1581.641026
1,1,6163.117647
1,1,3890.060811
1,1,2784.807692
1,1,2248.754098
1,1,2277.562500
1,1,3688.391892
1,1,1948.626404
1,1,1821.106383
1,1,4136.392157
1,1,2573.867188
1,1,12286.125000
1,1,2499.692308
1,1,86201.353261
1,1,92147.365854
1,1,2374.000000
1,1,75668.741477
1,1,3454.822917
1,1,4633.572222
1,1,3702.432432
1,1,25722.775000
1,1,7409.806452
1,1,2304.642857
1,1,2840.896341
1,1,1841.982143
1,1,3109.375000
1,1,2580.875000
1,1,1696.891892
1,1,2625.939394
1,1,2001.325658
1,1,11617.122449
1,1,3965.625000
1,1,7960.625000
1,1,2634.000000
1,1,5460.293750
1,1,42235.000000
1,1,2236.451220
1,1,1782.597222
1,1,13003.059091
1,1,4340.459119
1,1,5333.021277
1,1,12649.177778
1,1,2254.833333
1,1,5103.442708
1,1,1727.955645
1,1,3247.646875
1,1,4371.210526
1,1,48877.250000
1,1,1900.388889
1,1,4998.633094
1,1,2534.500000
1,1,1919.911111
1,1,3021.473214
1,1,4832.167553
1,1,1865.135714
1,1,2283.764706
1,1,3730.677083
1,1,3039.267857

1,1,2898.390625
1,1,2617.900000
1,1,2552.625000
1,1,1965.059783
1,1,3575.730392
1,1,11650.167857
1,1,2817.292683
1,1,68739.750000
1,1,1923.796875
1,1,26067.000000
1,1,6954.645833
1,1,3766.445652
1,1,1834.571429
1,1,2487.812500
1,1,64802.962500
1,1,2508.872093
1,1,4479.186170
1,1,12001.288462
1,1,2293.875000
1,1,3845.062500
1,1,6136.753125
1,1,7200.959239
1,1,1688.738636
1,1,1934.394231
1,1,3101.817500
1,1,6111.201389
1,1,6512.597222
1,1,1642.397059
1,1,2327.625000
1,1,27295.341463
1,1,3217.437500
1,1,8981.413194
1,1,1818.750000
1,1,1670.416667
1,1,1515.215426
1,1,4736.564516
1,1,17831.943182
1,1,6398.966146
1,1,3331.442308
1,1,2471.744186
1,1,5625.267442
1,1,1651.954545
1,1,7749.181818
1,1,2894.682692
1,1,1888.254717
1,1,8954.615625
1,1,4670.886029
1,1,1508.512821
1,1,2515.606707
1,1,26337.122093
1,1,18473.693750
1,1,4720.946078
1,1,6117.884615
1,1,2130.500000
1,1,2149.800000
1,1,2252.125000
1,1,7675.482143
1,1,3895.844444
1,1,2268.935811
1,1,3786.000000
1,1,2272.020833
1,1,2102.617347
1,1,3013.210000
1,1,9565.631579
1,1,2291.950000
1,1,3199.484848

1,1,4649.642045
1,1,7465.113636
1,1,3609.572581
1,1,4538.059524
1,1,20922.601744
1,1,2998.478261
1,1,7026.978261
1,1,7343.958333
1,1,1657.500000
1,1,3196.515152
1,1,1820.743902
1,1,3455.163194
1,1,4354.558333
1,1,13377.839286
1,1,9265.964674
1,1,2273.079082
1,1,3307.886364
1,1,1506.304054
1,1,2035.993590
1,1,2071.076923
1,1,89411.443750
1,1,28709.283784
1,1,2087.670213
1,1,5799.714286
1,1,13745.808140
1,1,13814.100000
1,1,6730.125000
1,1,25334.875000
1,1,2210.850000
1,1,2169.580645
1,1,2616.653846
1,1,1837.167500
1,1,2113.920139
1,1,2218.265060
1,1,1862.343023
1,1,2043.434659
1,1,8241.090000
1,1,18584.218750
1,1,3837.762500
1,1,1669.244186
1,1,2046.798077
1,1,1712.837838
1,1,43312.330645
1,1,1594.384868
1,1,1920.212500
1,1,156517.725000
1,1,10620.602273
1,1,1698.821809
1,1,2829.039063
1,1,2044.666667
1,1,4053.285000
1,1,9343.755319
1,1,1822.445946
1,1,2608.985465
1,1,4890.312500
1,1,2689.755102
1,1,7060.642157
1,1,87577.562500
1,1,1794.029070
1,1,2358.750000
1,1,38157.130435
1,1,8966.601093
1,1,1574.891026
1,1,2451.432065
1,1,10155.411765
1,1,3090.347973

1,1,3102.733333
1,1,86583.933775
1,1,102185.626667
1,1,9187.069444
1,1,3429.393750
1,1,3164.452381
1,1,1888.231707
1,1,7688.260870
1,1,1715.693182
1,1,1767.000000
1,1,5244.896907
1,1,6199.714744
1,1,3448.420455
1,1,5912.511364
1,1,1508.595238
1,1,5933.587662
1,1,1642.134615
1,1,5101.519737
1,1,2664.835106
1,1,1715.000000
1,1,12452.205128
1,1,5500.571429
1,1,3880.807065
1,1,2128.595588
1,1,10778.994681
1,1,1917.085000
1,1,9883.883929
1,1,4664.439394
1,1,7495.561047
1,1,17516.634615
1,1,3990.855263
1,1,2309.951691
1,1,4223.595745
1,1,11437.083333
1,1,4084.296512
1,1,1674.910714
1,1,1984.661765
1,1,6188.692308
1,1,3447.441860
1,1,7458.427083
1,1,6042.012755
1,1,4268.778846
1,1,2728.409091
1,1,9539.269886
1,1,3067.414474
1,1,4022.562500
1,1,2554.125000
1,1,1735.863388
1,1,102165.676282
1,1,2289.326087
1,1,5823.326531
1,1,149750.550000
1,1,20324.083333
1,1,2275.097561
1,1,12796.399194
1,1,17563.081081
1,1,11136.952381
1,1,1949.035714
1,1,3690.965116
1,1,45880.628205
1,1,5505.575581
1,1,2220.908537
1,1,4240.915625
1,1,3103.071429
1,1,1607.152174
1,1,1967.968750

1,1,3005.294872
1,1,11036.076087
1,1,4406.734848
1,1,2649.002551
1,1,2794.625000
1,1,3567.380952
1,1,2708.724359
1,1,2321.056452
1,1,3759.074324
1,1,1957.980978
1,1,2681.800000
1,1,3194.600610
1,1,11349.630952
1,1,2074.363636
1,1,3193.090909
1,1,2911.139706
1,1,3618.797297
1,1,5392.687500
1,1,3352.392442
1,1,11402.625000
1,1,2065.402778
1,1,6229.523810
1,1,4469.944444
1,1,3924.189655
1,1,4912.400000
1,1,3277.788462
1,1,8397.446875
1,1,4762.649194
1,1,3956.429648
1,1,6355.046196
1,1,1907.165761
1,1,90457.316176
1,1,4181.263158
1,1,3611.255682
1,1,6136.282143
1,1,2474.261905
1,1,27119.381579
1,1,1865.233553
1,1,3256.056818
1,1,44703.671053
1,1,4704.653125
1,1,2326.581395
1,1,53271.811024
1,1,3785.485000
1,1,3101.934783
1,1,1866.437500
1,1,2056.708333
1,1,3337.413043
1,1,8550.407051
1,1,36287.884375
1,1,5389.305851
1,1,41433.661290
1,1,2902.250000
1,1,3331.019886
1,1,13528.615854
1,1,2130.425000
1,1,5417.328571
1,1,3569.804348
1,1,1911.781915
1,1,9281.941589
1,1,2797.125000
1,1,2535.620000
1,1,5921.959302
1,1,8540.812500
1,1,1600.134146
1,1,1545.300000

1,1,4266.382514
1,1,2562.834225
1,1,1553.354167
1,1,3032.601064
1,1,4306.075000
1,1,1903.149457
1,1,1713.883721
1,1,5529.720930
1,1,6549.295455
1,1,2824.166667
1,1,2575.333333
1,1,2208.341216
1,1,84897.852273
1,1,3796.295455
1,1,9676.152439
1,1,1959.140957
1,1,2705.272959
1,1,3547.159375
1,1,7030.128289
1,1,8012.486486
1,1,2326.264205
1,1,3058.166667
1,1,3157.681818
1,1,3036.418478
1,1,5093.799342
1,1,2051.039634
1,1,7920.550000
1,1,32128.406250
1,1,6165.027027
1,1,2261.777778
1,1,1585.800000
1,1,4688.272059
1,1,2725.908163
1,1,1499.924419
1,1,4005.103774
1,1,2103.125000
1,1,3648.333333
1,1,3395.250000
1,1,1925.466216
1,1,122179.893750
1,1,7616.736842
1,1,64096.789474
1,1,6936.894737
1,1,4788.673469
1,1,7263.133523
1,1,6168.255435
1,1,3556.870968
1,1,1931.730769
1,1,4841.771341
1,1,2713.068182
1,1,1539.617500
1,1,9580.564394
1,1,2589.972826
1,1,2569.070000
1,1,2538.949468
1,1,4418.716216
1,1,2175.727273
1,1,4152.950000
1,1,118677.625000
1,1,4338.178571
1,1,8053.401961
1,1,1828.279070
1,1,5556.332386
1,1,1580.258333
1,1,5101.722222
1,1,5083.771739

1,1,5883.555556
1,1,2404.529412
1,1,1935.279070
1,1,8343.770833
1,1,1908.513889
1,1,2352.051282
1,1,2327.130556
1,1,79926.470588
1,1,3680.762791
1,1,4760.500000
1,1,13209.447674
1,1,3401.426724
1,1,2285.155488
1,1,2897.394737
1,1,2940.884375
1,1,1524.887755
1,1,15940.364583
1,1,5334.616667
1,1,1799.878378
1,1,5255.318452
1,1,2762.020833
1,1,7489.827206
1,1,4191.750000
1,1,6776.404255
1,1,3070.739286
1,1,4279.428125
1,1,62344.808824
1,1,2762.769231
1,1,6700.209302
1,1,1803.771429
1,1,2955.293750
1,1,3745.421053
1,1,1642.788591
1,1,8980.963068
1,1,10968.372093
1,1,4428.375000
1,1,2079.750000
1,1,2436.010417
1,1,1632.921569
1,1,3857.573529
1,1,1670.159375
1,1,6182.805851
1,1,2051.150000
1,1,4270.008065
1,1,2228.366197
1,1,1759.568548
1,1,3685.775000
1,1,2161.085227
1,1,2605.053571
1,1,2398.466912
1,1,72484.719595
1,1,2207.761905
1,1,1622.073171
1,1,6366.696970
1,1,1870.955729
1,1,3548.880597
1,1,1998.986111
1,1,3170.434211
1,1,1858.411765
1,1,27183.588235
1,1,1688.976744
1,1,44892.710938
1,1,14364.750000
1,1,3454.250000
1,1,4468.021277
1,1,2903.622449

1,1,41146.040541
1,1,3393.335000
1,1,2665.000000
1,1,3209.912500
1,1,2414.000000
1,1,5544.175000
1,1,6326.795918
1,1,10548.068452
1,1,3552.554878
1,1,2168.425595
1,1,2469.938889
1,1,6116.019022
1,1,1588.012097
1,1,3490.878378
1,1,6500.917683
1,1,62477.381579
1,1,2516.117188
1,1,18435.562500
1,1,4542.087500
1,1,1915.981383
1,1,5590.233696
1,1,6394.344595
1,1,2279.984375
1,1,2528.148438
1,1,40746.809783
1,1,6819.521429
1,1,89264.702703
1,1,10597.666667
1,1,1696.692308
1,1,5844.017241
1,1,3043.069444
1,1,1945.046512
1,1,2980.050676
1,1,13698.722973
1,1,4608.314286
1,1,2280.833333
1,1,1735.551136
1,1,2896.375000
1,1,4383.100610
1,1,5212.364865
1,1,7082.425000
1,1,45290.634868
1,1,3565.428571
1,1,6654.636029
1,1,95083.196875
1,1,13002.150000
1,1,5400.446970
1,1,1942.198171
1,1,7044.908163
1,1,10321.496403
1,1,3416.481707
1,1,1797.750000
1,1,4465.523256
1,1,2056.622093
1,1,13056.820652
1,1,4020.991279
1,1,3469.875000
1,1,1805.531250
1,1,3851.886111
1,1,3151.462500
1,1,2089.588415
1,1,2163.500000
1,1,2680.088710
1,1,17660.571429
1,1,3038.125000
1,1,1561.482143

1,1,6172.206081
1,1,9763.435976
1,1,2087.310811
1,1,2720.857558
1,1,3715.595092
1,1,4189.746212
1,1,2420.404070
1,1,4663.043478
1,1,3584.304688
1,1,5911.994681
1,1,8474.824324
1,1,2331.348837
1,1,16841.990132
1,1,5937.277778
1,1,2311.750000
1,1,4466.388889
1,1,11803.772059
1,1,2507.447368
1,1,4489.222222
1,1,9904.375000
1,1,5773.221154
1,1,4788.341463
1,1,2851.799479
1,1,4873.870690
1,1,3347.539773
1,1,6273.588235
1,1,4289.500000
1,1,4255.586957
1,1,1574.256250
1,1,1656.911765
1,1,8125.929348
1,1,1726.793478
1,1,3038.557432
1,1,2666.786885
1,1,9196.381944
1,1,2596.756696
1,1,2704.128049
1,1,5061.012097
1,1,3362.453237
1,1,20036.079545
1,1,2253.230769
1,1,5326.517045
1,1,9420.392857
1,1,2122.343750
1,1,1662.108696
1,1,12992.005814
1,1,1933.085227
1,1,3463.241758
1,1,2896.893750
1,1,1798.007979
1,1,4535.326531
1,1,2262.027778
1,1,2887.734694
1,1,2182.000000
1,1,4489.322581
1,1,5776.800000
1,1,6142.187500
1,1,11679.605769
1,1,2168.766827
1,1,9103.861486
1,1,5951.494792
1,1,10018.864130
1,1,4139.959559
1,1,2676.879032
1,1,3618.794872
1,1,4600.875000

1,1,6577.875000
1,1,2471.932927
1,1,2181.047619
1,1,2392.553125
1,1,4958.122093
1,1,2105.900000
1,1,7957.062500
1,1,3882.028571
1,1,1857.250000
1,1,3073.051136
1,1,2136.372024
1,1,1545.866667
1,1,11726.853261
1,1,50991.891892
1,1,10235.370690
1,1,4211.497340
1,1,12985.644737
1,1,3698.750000
1,1,2483.125828
1,1,7097.892857
1,1,18643.114583
1,1,6628.392857
1,1,2575.970395
1,1,3687.465116
1,1,3621.368750
1,1,2194.280612
1,1,52227.434375
1,1,7307.031250
1,1,3511.109375
1,1,21584.348361
1,1,2154.833333
1,1,3959.539773
1,1,1877.920213
1,1,4819.335227
1,1,4451.863636
1,1,4515.388889
1,1,2983.703297
1,1,2577.951807
1,1,2447.849432
1,1,1983.312500
1,1,1680.494318
1,1,2804.875000
1,1,2449.392857
1,1,4004.575000
1,1,1593.887500
1,1,2286.533333
1,1,3510.483333
1,1,8246.019231
1,1,2283.736527
1,1,6947.625000
1,1,12171.000000
1,1,1685.403646
1,1,6180.509615
1,1,2406.664773
1,1,2189.653061
1,1,3387.952381
1,1,13775.981618
1,1,3947.763158
1,1,22721.176829
1,1,1787.505319
1,1,1780.415541
1,1,68870.698529
1,1,3051.794872
1,1,2401.348837
1,1,7678.098361
1,1,3457.941667

1,1,13523.500000
1,1,2067.370968
1,1,1781.473684
1,1,30870.172222
1,1,4170.826087
1,1,2153.567857
1,1,4782.476744
1,1,2068.210526
1,1,3935.463415
1,1,3190.337209
1,1,3953.140244
1,1,30170.028125
1,1,2281.411111
1,1,12819.500000
1,1,6442.161111
1,1,41847.835938
1,1,3754.038462
1,1,16795.955556
1,1,1717.778409
1,1,1639.617021
1,1,7643.772727
1,1,1878.486486
1,1,3434.305921
1,1,1689.062500
1,1,1529.566667
1,1,17948.212838
1,1,3405.400000
1,1,2578.950000
1,1,1917.472561
1,1,3261.908163
1,1,2309.632653
1,1,35917.770833
1,1,6540.237179
1,1,2305.267045
1,1,2077.600000
1,1,2077.110795
1,1,3271.406977
1,1,5669.470588
1,1,4778.837209
1,1,2354.835000
1,1,13592.984375
1,1,1647.408333
1,1,38590.539474
1,1,3821.546875
1,1,1538.000000
1,1,5012.281915
1,1,1977.011029
1,1,5778.916667
1,1,11465.566901
1,1,1992.927326
1,1,2697.519231
1,1,4473.120690
1,1,92456.069079
1,1,22094.395833
1,1,4566.766520
1,1,2496.475000
1,1,1926.756757
1,1,4596.305031
1,1,7003.013514
1,1,10861.172414
1,1,27716.957143
1,1,2169.184659
1,1,16395.395000
1,1,2529.161184
1,1,4355.243902
1,1,2282.896341

1,1,12789.729730
1,1,2237.283088
1,1,2613.915441
1,1,10030.358553
1,1,2010.741477
1,1,5465.951389
1,1,2533.057692
1,1,7375.935897
1,1,2578.423077
1,1,5510.687117
1,1,5560.400568
1,1,2996.750000
1,1,1637.477273
1,1,4923.571429
1,1,1682.423780
1,1,3726.141892
1,1,3821.950000
1,1,1687.375000
1,1,2283.875000
1,1,8167.111111
1,1,27379.368421
1,1,2965.472222
1,1,5581.455556
1,1,5339.641304
1,1,4664.888393
1,1,88385.886364
1,1,3891.846154
1,1,6341.030405
1,1,4433.264706
1,1,4133.081081
1,1,2034.582317
1,1,13942.242857
1,1,1932.079787
1,1,10152.625000
1,1,7778.546875
1,1,3078.683962
1,1,18783.035256
1,1,8784.600000
1,1,3702.930233
1,1,2582.615385
1,1,5570.937500
1,1,1579.465649
1,1,9313.983333
1,1,3876.636364
1,1,12891.385135
1,1,5314.418605
1,1,1812.200000
1,1,4433.343750
1,1,6095.937500
1,1,3708.206897
1,1,2152.776786
1,1,2934.551282
1,1,8095.664063
1,1,2194.641447
1,1,9372.855978
1,1,2321.533333
1,1,4117.105114
1,1,3452.250000
1,1,4395.548780
1,1,1725.568182
1,1,2981.136364
1,1,21524.014286
1,1,2361.527778
1,1,2717.517045
1,1,3243.168605
1,1,4276.730114

1,1,1986.973404
1,1,4412.846774
1,1,3587.798077
1,1,18396.500000
1,1,5675.715909
1,1,1705.065217
1,1,4315.022727
1,1,51909.017857
1,1,24375.290541
1,1,4520.644022
1,1,2939.281250
1,1,2506.469388
1,1,8912.468750
1,1,4116.971429
1,1,7141.641667
1,1,12511.657609
1,1,7842.525000
1,1,3793.297297
1,1,2468.255814
1,1,7201.317308
1,1,37476.856250
1,1,3460.296196
1,1,31087.616667
1,1,22816.318548
1,1,1718.933333
1,1,1779.679487
1,1,7959.261421
1,1,3818.551020
1,1,12025.437500
1,1,2823.911765
1,1,3420.435393
1,1,3136.250000
1,1,7048.697368
1,1,7274.150641
1,1,9875.240132
1,1,3196.152174
1,1,180422.863057
1,1,4677.708333
1,1,1603.540761
1,1,3983.578947
1,1,2428.452206
1,1,1500.448864
1,1,3595.090909
1,1,8639.687500
1,1,2275.662791
1,1,25308.663194
1,1,4134.766667
1,1,3881.396341
1,1,3827.151316
1,1,1541.738372
1,1,2125.835106
1,1,114887.000000
1,1,3429.502907
1,1,1798.646552
1,1,2562.659574
1,1,9250.479730
1,1,5832.390244
1,1,11929.577703
1,1,2150.105263
1,1,3820.128049
1,1,14214.904762
1,1,3222.729167
1,1,9769.500000
1,1,116295.354167
1,1,15160.099359
1,1,4612.229167

1,1,3060.587500
1,1,5016.503205
1,1,66871.607143
1,1,4127.756410
1,1,1768.633523
1,1,4019.179688
1,1,36829.218750
1,1,8733.867647
1,1,87868.196023
1,1,6857.052632
1,1,13706.703125
1,1,4363.341912
1,1,3702.304348
1,1,4317.417910
1,1,2114.916667
1,1,2342.136364
1,1,7127.820513
1,1,3194.190341
1,1,4184.618421
1,1,2564.515244
1,1,2307.618243
1,1,2115.529412
1,1,6116.981250
1,1,9783.015625
1,1,38972.984375
1,1,60637.934524
1,1,7905.823529
1,1,1938.125000
1,1,1987.659722
1,1,11475.186321
1,1,25011.109756
1,1,7646.289474
1,1,4193.236842
1,1,1666.061224
1,1,2759.755435
1,1,5435.951389
1,1,3183.621212
1,1,2308.613636
1,1,9366.007576
1,1,2074.020833
1,1,2997.396104
1,1,6075.562500
1,1,1633.673077
1,1,1673.459459
1,1,1578.581250
1,1,3710.000000
1,1,6958.453947
1,1,11103.493902
1,1,1855.896277
1,1,17560.046512
1,1,1717.805556
1,1,3598.617188
1,1,1500.600000
1,1,2633.203125
1,1,4209.477987
1,1,5698.014535
1,1,17189.191176
1,1,12209.365385
1,1,1555.765625
1,1,6278.172794
1,1,4634.581006
1,1,2484.986486
1,1,1631.079861
1,1,6761.088710
1,1,3868.000000
1,1,42814.787234

1,1,3503.005682
1,1,1507.462121
1,1,3254.802632
1,1,3886.394608
1,1,2418.110465
1,1,2133.045455
1,1,55318.106250
1,1,70210.093023
1,1,3450.958333
1,1,4586.571429
1,1,6523.712264
1,1,3260.013889
1,1,2186.520833
1,1,4272.285714
1,1,5826.587500
1,1,2442.503049
1,1,4005.541667
1,1,4628.032787
1,1,14721.678571
1,1,4229.900000
1,1,1985.770833
1,1,4702.117021
1,1,3010.727273
1,1,3630.392857
1,1,2403.638889
1,1,4115.279070
1,1,6179.000000
1,1,2245.437500
1,1,5578.533784
1,1,2255.250000
1,1,1891.000000
1,1,7092.465909
1,1,13252.481250
1,1,5850.384615
1,1,3881.500000
1,1,2018.615385
1,1,2595.279070
1,1,2171.956522
1,1,2017.021739
1,1,51500.771429
1,1,2991.683140
1,1,5564.752778
1,1,3070.357143
1,1,3185.461538
1,1,2526.895833
1,1,2784.278646
1,1,4733.413462
1,1,2982.826087
1,1,2740.383929
1,1,1728.458333
1,1,1922.534722
1,1,8666.000000
1,1,5453.886628
1,1,4691.864362
1,1,3621.996212
1,1,22832.981707
1,1,4937.470982
1,1,3350.369898
1,1,1764.798469
1,1,3059.108333
1,1,1675.390625
1,1,1543.007576
1,1,8889.277174
1,1,1929.531915
1,1,3461.281250
1,1,2081.756410

1,1,4570.644737
1,1,2296.778846
1,1,4039.062500
1,1,2296.682432
1,1,55068.093750
1,1,3617.264706
1,1,18649.713816
1,1,2051.014706
1,1,2671.156250
1,1,1669.612805
1,1,3364.282051
1,1,2212.885135
1,1,2521.934896
1,1,7715.726190
1,1,10458.622093
1,1,3236.714286
1,1,3954.954545
1,1,6813.838542
1,1,1733.848684
1,1,3503.928125
1,1,2298.489130
1,1,2905.052486
1,1,2756.234375
1,1,4145.750000
1,1,4506.915179
1,1,1642.919643
1,1,40481.868902
1,1,1902.540698
1,1,2005.320833
1,1,3739.304348
1,1,4476.429348
1,1,1806.684524
1,1,10908.940141
1,1,5435.617647
1,1,6115.179688
1,1,10571.267045
1,1,3081.107143
1,1,2901.293605
1,1,2028.720588
1,1,4851.312500
1,1,18849.793478
1,1,3790.477941
1,1,5100.450000
1,1,1937.397436
1,1,1915.864583
1,1,2049.312500
1,1,5172.792254
1,1,1699.437500
1,1,2293.833333
1,1,1895.106061
1,1,4192.211957
1,1,3468.703947
1,1,6933.450000
1,1,1827.083333
1,1,2075.256579
1,1,4743.572368
1,1,1893.046961
1,1,57649.641892
1,1,2040.623037
1,1,1986.081395
1,1,3302.816860
1,1,1550.365385
1,1,3403.400000
1,1,1840.548780
1,1,3117.461538
1,1,2585.931818

1,1,1688.733333
1,1,2227.241935
1,1,188515.977273
1,1,3698.928571
1,1,7667.971429
1,1,6229.777027
1,1,3952.129747
1,1,2401.917553
1,1,2545.028409
1,1,16175.142857
1,1,5644.826087
1,1,11538.215278
1,1,4901.541667
1,1,47221.133333
1,1,2418.341463
1,1,4922.045455
1,1,3836.955556
1,1,2311.300000
1,1,2370.930233
1,1,2748.187500
1,1,6088.015152
1,1,2455.966667
1,1,7142.750000
1,1,2331.121711
1,1,1660.151042
1,1,4238.396226
1,1,9573.339286
1,1,1850.888514
1,1,7155.885714
1,1,2940.986111
1,1,3342.197368
1,1,3271.350000
1,1,22600.329268
1,1,1966.875000
1,1,9535.976744
1,1,3508.312500
1,1,18288.945312
1,1,19301.606061
1,1,2598.015957
1,1,47239.911111
1,1,23923.916667
1,1,14609.438889
1,1,1766.033784
1,1,1855.648649
1,1,2179.250000
1,1,2420.053571
1,1,4434.375000
1,1,2162.801282
1,1,7257.836864
1,1,1771.419643
1,1,20033.697674
1,1,1860.218750
1,1,68047.480769
1,1,4817.437500
1,1,3247.888889
1,1,2510.716667
1,1,2117.500000
1,1,1850.217391
1,1,2009.820513
1,1,85780.500000
1,1,6719.317308
1,1,15001.524390
1,1,1507.563953
1,1,7089.678571
1,1,14729.860294
1,1,1700.725000

1,1,1606.496795
1,1,7688.764706
1,1,4209.276596
1,1,1609.628205
1,1,6089.382143
1,1,6152.835714
1,1,3730.770161
1,1,2655.125000
1,1,10784.048077
1,1,2941.786585
1,1,7246.588889
1,1,25385.829545
1,1,1615.361413
1,1,2791.898438
1,1,14602.350000
1,1,9944.762500
1,1,1551.341216
1,1,3613.326923
1,1,2463.725000
1,1,2989.411765
1,1,13903.581522
1,1,1569.778846
1,1,4829.750000
1,1,1613.654891
1,1,111322.578947
1,1,27175.937500
1,1,3663.735484
1,1,2476.965909
1,1,2311.688889
1,1,3306.648438
1,1,6577.442568
1,1,15617.720930
1,1,3703.717391
1,1,6649.903226
1,1,2120.821429
1,1,4122.906250
1,1,15365.936508
1,1,5624.242647
1,1,2074.434211
1,1,3373.910256
1,1,2103.723404
1,1,4085.017857
1,1,2515.631579
1,1,3378.108696
1,1,13305.595238
1,1,11833.655488
1,1,4615.602941
1,1,4163.549419
1,1,2370.258929
1,1,7313.250000
1,1,1660.662162
1,1,2356.808176
1,1,3423.923077
1,1,1752.331395
1,1,48352.575758
1,1,1587.287500
1,1,6806.425676
1,1,7807.689815
1,1,4806.047059
1,1,4959.777778
1,1,6440.663743
1,1,2289.817568
1,1,3328.357143
1,1,3860.892045
1,1,11472.375000
1,1,9725.125000

1,1,4232.361272
1,1,5148.048077
1,1,2577.767442
1,1,118956.818182
1,1,4232.000000
1,1,2183.950000
1,1,2134.434659
1,1,7745.678571
1,1,4392.041667
1,1,2345.333333
1,1,2508.256410
1,1,3650.784091
1,1,3900.030864
1,1,5049.434211
1,1,5314.439286
1,1,1793.214286
1,1,7645.284916
1,1,1969.244898
1,1,78026.692771
1,1,3552.750000
1,1,14320.284375
1,1,3293.930851
1,1,22963.354167
1,1,1603.726190
1,1,2122.480769
1,1,1526.648649
1,1,5306.000000
1,1,1844.203571
1,1,3660.540441
1,1,2217.409091
1,1,1827.308333
1,1,6825.310976
1,1,2251.744681
1,1,3911.690476
1,1,3014.225000
1,1,3413.783333
1,1,14925.634615
1,1,5407.564815
1,1,33704.432692
1,1,9836.500000
1,1,4017.822368
1,1,15591.963415
1,1,2018.621711
1,1,2034.495000
1,1,1662.500000
1,1,3000.734043
1,1,4758.010638
1,1,3152.335714
1,1,8576.951220
1,1,2958.879032
1,1,2110.000000
1,1,2000.240854
1,1,10956.988372
1,1,1617.430851
1,1,6484.033149
1,1,1739.125000
1,1,1906.182143
1,1,4810.925676
1,1,3901.003378
1,1,5417.067568
1,1,2848.785714
1,1,3969.593750
1,1,2019.312500
1,1,11055.134615
1,1,4221.666667
1,1,18630.187500

1,1,1651.630682
1,1,4726.025000
1,1,1523.980769
1,1,2214.806250
1,1,1729.134146
1,1,4851.139344
1,1,4089.652174
1,1,2341.374233
1,1,5997.446809
1,1,3516.149701
1,1,1518.500000
1,1,2394.652778
1,1,2511.936047
1,1,14888.877660
1,1,2234.750000
1,1,5046.854545
1,1,2684.909375
1,1,3260.612245
1,1,2456.232955
1,1,1816.369318
1,1,82533.040541
1,1,2054.314103
1,1,6096.192308
1,1,24244.661765
1,1,2458.304348
1,1,37160.302419
1,1,3510.576923
1,1,156936.328125
1,1,47593.372159
1,1,4630.179487
1,1,11599.017857
1,1,13987.357143
1,1,2394.388158
1,1,1568.070312
1,1,2025.467742
1,1,1791.636364
1,1,5325.071429
1,1,9128.652174
1,1,2680.026596
1,1,4129.823529
1,1,2024.302632
1,1,6815.633523
1,1,2851.028846
1,1,2440.493902
1,1,29151.012500
1,1,1673.611650
1,1,2350.000000
1,1,2140.694444
1,1,2389.714286
1,1,1734.144737
1,1,2336.884615
1,1,2107.466667
1,1,1538.308140
1,1,12886.121622
1,1,4280.950000
1,1,4421.082237
1,1,22383.613636
1,1,1977.110714
1,1,2460.135870
1,1,23370.444444
1,1,6924.336735
1,1,1592.464286
1,1,7376.086957
1,1,3924.976744
1,1,2885.198370
1,1,3573.562500

1,1,15810.885135
1,1,7421.166667
1,1,3025.656250
1,1,2077.558140
1,1,9171.896739
1,1,2564.339286
1,1,6426.267857
1,1,1841.294118
1,1,4978.000000
1,1,2268.620968
1,1,43455.327206
1,1,1511.986111
1,1,2282.086957
1,1,2013.795213
1,1,3599.390909
1,1,2331.051282
1,1,26517.482558
1,1,1647.313889
1,1,1903.472973
1,1,2601.811047
1,1,1963.148649
1,1,2368.706250
1,1,3129.500000
1,1,5038.288770
1,1,3771.500000
1,1,4268.525568
1,1,6837.307692
1,1,3250.179878
1,1,3731.524510
1,1,2419.802083
1,1,2812.750000
1,1,2248.086957
1,1,2041.270270
1,1,7736.468750
1,1,3596.280488
1,1,3192.959239
1,1,16850.775862
1,1,4655.792553
1,1,1676.164634
1,1,3517.585366
1,1,4115.821809
1,1,9986.785714
1,1,2740.596875
1,1,5977.237179
1,1,3328.836207
1,1,3951.232558
1,1,5738.862805
1,1,1703.150000
1,1,6690.756579
1,1,1524.685714
1,1,26098.171512
1,1,2494.216667
1,1,2287.830357
1,1,2899.993902
1,1,65716.422222
1,1,8181.500000
1,1,2438.038194
1,1,4485.529412
1,1,2039.768750
1,1,31989.237342
1,1,1915.778571
1,1,2574.735294
1,1,8433.861111
1,1,2210.250000
1,1,1667.027027
1,1,72433.096154

1,1,42087.986111
1,1,1783.583333
1,1,1811.782609
1,1,10039.500000
1,1,3653.114130
1,1,4335.750000
1,1,2031.257895
1,1,8335.078947
1,1,3223.070513
1,1,6327.642045
1,1,3076.102273
1,1,3790.702206
1,1,2064.287234
1,1,1926.696429
1,1,11745.330882
1,1,1907.210227
1,1,7331.600000
1,1,4658.566860
1,1,4192.635417
1,1,2584.384615
1,1,3060.971429
1,1,47118.475610
1,1,3102.292683
1,1,50981.815972
1,1,4143.392857
1,1,2189.959302
1,1,6338.588235
1,1,3422.519022
1,1,5714.673913
1,1,6616.071809
1,1,3319.068182
1,1,192460.320000
1,1,14489.964286
1,1,1739.625000
1,1,1768.586538
1,1,2457.677184
1,1,1533.335227
1,1,4057.647727
1,1,1643.500000
1,1,1709.409884
1,1,3936.279070
1,1,1655.600000
1,1,23378.046512
1,1,2824.812500
1,1,5249.277778
1,1,1919.200581
1,1,5100.312500
1,1,1659.804348
1,1,18798.214286
1,1,3964.678571
1,1,44952.666667
1,1,7445.500000
1,1,3106.179348
1,1,1812.456250
1,1,6056.552326
1,1,4068.345395
1,1,2860.875000
1,1,2040.029412
1,1,1997.715116
1,1,12302.213889
1,1,2267.882353
1,1,2091.135714
1,1,2190.733108
1,1,2384.420455
1,1,1515.948718
1,1,5524.135135

1,1,4007.391304
1,1,1500.325000
1,1,1917.060897
1,1,57233.446023
1,1,1790.466667
1,1,7621.482143
1,1,1626.500000
1,1,7163.907407
1,1,3244.985000
1,1,1792.979167
1,1,2250.038462
1,1,2103.898438
1,1,4350.059783
1,1,5276.214286
1,1,3630.770833
1,1,2904.683824
1,1,1651.240196
1,1,1510.250000
1,1,11763.413043
1,1,12836.195946
1,1,7065.725543
1,1,5020.166667
1,1,10622.678571
1,1,1500.562500
1,1,2421.000000
1,1,5116.003378
1,1,12761.040698
1,1,5936.872024
1,1,2403.471591
1,1,5297.137255
1,1,12276.690972
1,1,7671.875000
1,1,6638.400000
1,1,1535.150000
1,1,61995.950000
1,1,4294.588235
1,1,3429.000000
1,1,13625.621622
1,1,2982.489583
1,1,8029.904762
1,1,3499.608696
1,1,2497.051136
1,1,8930.357143
1,1,4266.383333
1,1,6397.217105
1,1,35456.164474
1,1,4579.265625
1,1,14319.853774
1,1,5525.041667
1,1,3163.000000
1,1,1889.291667
1,1,5076.917683
1,1,2549.323171
1,1,3847.012755
1,1,1574.492188
1,1,4237.121711
1,1,4770.333333
1,1,13219.348837
1,1,4075.597222
1,1,141833.492021
1,1,6393.093750
1,1,2109.311111
1,1,8757.121094
1,1,2033.228125
1,1,7648.272222
1,1,3110.000000

1,1,4087.484375
1,1,1593.326923
1,1,12004.477564
1,1,1556.791667
1,1,1834.732143
1,1,5113.963542
1,1,4204.777778
1,1,3924.500000
1,1,3396.657738
1,1,2016.973404
1,1,1983.660714
1,1,2211.065789
1,1,53036.323529
1,1,2506.270408
1,1,3423.000000
1,1,1782.988889
1,1,2405.520349
1,1,1925.627778
1,1,2473.000000
1,1,4713.256757
1,1,3637.325000
1,1,1859.742424
1,1,1660.471354
1,1,92155.544643
1,1,1801.594828
1,1,3780.166667
1,1,36403.255435
1,1,22027.128378
1,1,15911.117647
1,1,2475.375000
1,1,5919.726974
1,1,3490.250000
1,1,3477.666667
1,1,2466.809524
1,1,1882.023256
1,1,1708.428571
1,1,2059.253676
1,1,1524.350610
1,1,2450.032738
1,1,2472.307692
1,1,4708.900000
1,1,6357.675000
1,1,5411.489130
1,1,3731.159574
1,1,3619.636364
1,1,3161.281250
1,1,3059.333333
1,1,2609.008929
1,1,42003.000000
1,1,10261.733696
1,1,1720.182432
1,1,5349.783854
1,1,2045.036585
1,1,2779.505814
1,1,3895.909091
1,1,2636.389881
1,1,2571.458333
1,1,6063.044872
1,1,4732.709677
1,1,1501.029605
1,1,1748.400000
1,1,23058.738636
1,1,2664.333333
1,1,2245.509146
1,1,1893.585714
1,1,3991.138889

1,1,3716.190625
1,1,2427.738889
1,1,1690.616071
1,1,2786.482143
1,1,1633.527439
1,1,22937.721591
1,1,3314.960938
1,1,3130.538889
1,1,9320.536765
1,1,1974.500000
1,1,42943.714286
1,1,52361.381579
1,1,4278.140625
1,1,16135.568182
1,1,3541.445312
1,1,4313.090278
1,1,21312.816489
1,1,7074.847134
1,1,62768.416667
1,1,8168.093023
1,1,5191.731250
1,1,2818.855263
1,1,4027.837264
1,1,3597.333333
1,1,4370.307143
1,1,2699.846591
1,1,4771.017857
2,2,1668.465116
2,2,1948.058824
2,2,2506.548913
2,2,16408.241071
2,2,4129.072674
2,2,3029.085714
2,2,5687.636364
2,2,2735.042105
2,2,9976.083333
2,2,4824.486111
2,2,2180.594298
2,2,2060.205882
2,2,1860.562500
2,2,1516.828125
2,2,2703.000000
2,2,3202.164894
2,2,1895.621951
2,2,2163.315789
2,2,2391.736842
2,2,2107.666667
2,2,2475.256818
2,2,1769.756098
2,2,2089.901961
2,2,2127.319444
2,2,1519.038462
2,2,3061.872928
2,2,2181.371429
2,2,3673.500000
2,2,3561.844595
2,2,2239.923497
2,2,5643.031447
2,2,10000.957317
2,2,2454.857143
2,2,1559.846154
2,2,4899.785714
2,2,2157.453947
2,2,4627.555556
2,2,2385.280702
2,2,1927.269737

2,2,1886.509375
2,2,3560.520833
2,2,1816.967857
2,2,5275.946108
2,2,2611.719298
2,2,1584.621622
2,2,4096.807692
2,2,16341.976744
2,2,1805.544118
2,2,2853.936842
2,2,2862.522472
2,2,2187.005435
2,2,5104.222892
2,2,2174.540698
2,2,1911.520833
2,2,6237.693548
2,2,2296.967742
2,2,2897.646739
2,2,4964.750000
2,2,3215.255319
2,2,13667.265306
2,2,2763.827381
2,2,2648.442708
2,2,2794.291667
2,2,6218.187500
2,2,13987.580000
2,2,1653.147059
2,2,2789.826923
2,2,5796.900474
2,2,2490.200000
2,2,12596.000000
2,2,7003.878049
2,2,3004.752809
2,2,5753.317935
2,2,3834.520408
2,2,1534.031250
2,2,6242.676471
2,2,6580.697674
2,2,1642.072917
2,2,3376.468750
2,2,13484.300676
2,2,3023.314815
2,2,1775.604651
2,2,2659.113636
2,2,3499.803125
2,2,3313.767045
2,2,2126.459135
2,2,3125.083333
2,2,2040.921569
2,2,3759.083333
2,2,1669.173077
2,2,3732.942308
2,2,4500.521739
2,2,5329.913043
2,2,7812.668508
2,2,2553.921466
2,2,5886.985537
2,2,2761.539683
2,2,1702.143293
2,2,3038.887417
2,2,1851.668449
2,2,4053.258242
2,2,3095.920000
2,2,5301.755435
2,2,1643.857143
2,2,3704.447368

2,2,9121.944444
2,2,2305.787234
2,2,1901.490385
2,2,1785.915000
2,2,2415.215686
2,2,3956.552778
2,2,3396.000000
2,2,3089.879808
2,2,6976.102941
2,2,11336.401042
2,2,2377.500000
2,2,3662.308333
2,2,2684.750000
2,2,5487.385870
2,2,1650.569444
2,2,1554.817610
2,2,3614.860000
2,2,2417.500000
2,2,19713.526596
2,2,2835.892045
2,2,6480.969512
2,2,1540.344828
2,2,2707.387097
2,2,1519.300000
2,2,1724.418919
2,2,3893.142857
2,2,3147.245000
2,2,1665.744792
2,2,1727.404605
2,2,4415.250000
2,2,2696.381579
2,2,2283.352273
2,2,1580.677419
2,2,2342.833333
2,2,5108.571429
2,2,1896.714286
2,2,3013.993590
2,2,3116.838068
2,2,3318.608108
2,2,4122.487805
2,2,2781.813953
2,2,1750.000000
2,2,2887.445946
2,2,2019.221154
2,2,1694.414062
2,2,1520.688776
2,2,2439.997093
2,2,1576.125000
2,2,6197.078947
2,2,2686.398104
2,2,5772.834437
2,2,3438.250000
2,2,2335.734848
2,2,1597.408163
2,2,9868.161932
2,2,12814.087264
2,2,9827.641975
2,2,3590.841530
2,2,6733.259146
2,2,3516.554054
2,2,2022.992424
2,2,1702.557065
2,2,2283.790541
2,2,4259.750000
2,2,2159.128125
2,2,15170.342105

2,2,3728.817308
2,2,5290.287582
2,2,6736.882629
2,2,2885.785714
2,2,1960.777778
2,2,2097.712500
2,2,2706.288265
2,2,1803.968750
2,2,3969.295918
2,2,4932.270833
2,2,9740.851351
2,2,7090.171498
2,2,2467.931818
2,2,4938.341346
2,2,1969.818713
2,2,6118.042553
2,2,1671.456731
2,2,2018.020270
2,2,4291.682927
2,2,1585.148810
2,2,4885.209302
2,2,3028.916667
2,2,3569.640244
2,2,2328.428571
2,2,3775.055556
2,2,5142.666667
2,2,1848.071429
2,2,3369.828804
2,2,2749.067308
2,2,6365.146875
2,2,2524.987730
2,2,5430.536585
2,2,2042.914474
2,2,2674.572368
2,2,2451.515625
2,2,1730.650000
2,2,3146.872222
2,2,1760.054348
2,2,2258.506410
2,2,2834.988095
2,2,4836.750000
2,2,8620.872396
2,2,3578.692308
2,2,2813.100000
2,2,1763.615385
2,2,5822.100000
2,2,6302.153846
2,2,7908.547945
2,2,2021.462766
2,2,5882.551724
2,2,1580.767857
2,2,3069.236111
2,2,1652.034314
2,2,2870.658654
2,2,2042.959302
2,2,6059.040816
2,2,5133.408654
2,2,2084.182927
2,2,1574.603774
2,2,1620.718023
2,2,1580.532609
2,2,5593.375000
2,2,4490.347727
2,2,1505.543269
2,2,1521.057692
2,2,1590.485795

2,2,1990.651316
2,2,5216.016667
2,2,4331.737805
2,2,3990.781818
2,2,3212.458333
2,2,8787.201923
2,2,18469.552778
2,2,4113.562500
2,2,2494.420000
2,2,1900.340517
2,2,3946.571429
2,2,4361.152174
2,2,2476.959184
2,2,2800.860465
2,2,2724.649390
2,2,6647.349057
2,2,3110.047619
2,2,1580.046512
2,2,2126.370588
2,2,1622.653846
2,2,5779.542614
2,2,2929.548469
2,2,1785.140306
2,2,1621.300676
2,2,2301.521875
2,2,4226.702857
2,2,1634.735897
2,2,2097.748879
2,2,2334.755682
2,2,2245.225694
2,2,4057.128472
2,2,3262.678571
2,2,3676.500000
2,2,2557.732143
2,2,2537.895000
2,2,1702.010714
2,2,3419.000000
2,2,2779.802139
2,2,1595.631757
2,2,2436.225000
2,2,2162.102041
2,2,3584.337209
2,2,11455.334746
2,2,3070.914634
2,2,14720.784091
2,2,1799.909884
2,2,1565.598214
2,2,4863.896186
2,2,2276.365854
2,2,1941.744681
2,2,10068.653409
2,2,2867.681250
2,2,3150.095238
2,2,6692.794872
2,2,5506.594595
2,2,2802.834906
2,2,7222.441026
2,2,1510.138462
2,2,1536.380682
2,2,4066.084746
2,2,2078.250000
2,2,3049.618243
2,2,1721.631410
2,2,3328.703947
2,2,8693.012500
2,2,4131.086957

2,2,3201.630952
2,2,3340.892857
2,2,2421.348039
2,2,1693.418605
2,2,5093.410000
2,2,1577.627660
2,2,2546.726562
2,2,2688.980263
2,2,2100.698718
2,2,3073.033333
2,2,1912.951923
2,2,3234.740385
2,2,1594.451923
2,2,2965.731132
2,2,4591.304545
2,2,2199.013889
2,2,2988.706395
2,2,2701.060096
2,2,1512.505682
2,2,2435.605769
2,2,10588.708333
2,2,13608.973958
2,2,4594.250000
2,2,7130.875000
2,2,2009.225000
2,2,3327.112745
2,2,4209.628205
2,2,5371.440000
2,2,3555.354167
2,2,1581.100000
2,2,7587.196809
2,2,2809.966463
2,2,1628.692308
2,2,17611.048780
2,2,3557.538462
2,2,5044.166667
2,2,2020.825658
2,2,6200.161765
2,2,8042.485795
2,2,1522.603774
2,2,2236.095745
2,2,3212.479167
2,2,15424.263889
2,2,5905.146919
2,2,2561.990854
2,2,2133.000000
2,2,2414.519231
2,2,2152.812834
2,2,2035.187500
2,2,3676.297872
2,2,1751.378378
2,2,1758.183673
2,2,11294.052273
2,2,2262.590909
2,2,2892.127358
2,2,7094.000000
2,2,6565.035928
2,2,1786.031250
2,2,2693.932692
2,2,4057.306818
2,2,2377.460227
2,2,1950.105263
2,2,8007.759615
2,2,1691.809524
2,2,4604.418750
2,2,2123.837209

2,2,2792.961538
2,2,2054.230769
2,2,1686.040625
2,2,2483.384615
2,2,1802.947368
2,2,2352.994681
2,2,7807.916667
2,2,9610.137255
2,2,2826.600000
2,2,1670.600000
2,2,3720.522959
2,2,7864.784314
2,2,1888.181250
2,2,2009.100000
2,2,2355.684783
2,2,1834.846154
2,2,5502.905172
2,2,8487.589744
2,2,3655.663043
2,2,2388.975610
2,2,2233.720000
2,2,1721.934659
2,2,4214.115854
2,2,2646.250000
2,2,7265.672222
2,2,4324.066667
2,2,7733.457317
2,2,2306.312500
2,2,10504.666667
2,2,4327.036199
2,2,2748.312500
2,2,2507.339450
2,2,2563.000000
2,2,2142.243750
2,2,4887.569149
2,2,11377.006410
2,2,4130.808824
2,2,2331.146739
2,2,2550.558140
2,2,2521.486486
2,2,5000.656250
2,2,1659.809524
2,2,3382.531915
2,2,3197.291667
2,2,3090.605634
2,2,3439.361702
2,2,2110.697368
2,2,3422.416667
2,2,1840.671053
2,2,2326.712575
2,2,17489.103774
2,2,3614.859375
2,2,3202.049505
2,2,1840.878472
2,2,9067.290816
2,2,1782.449468
2,2,2828.385870
2,2,3943.880952
2,2,1746.766667
2,2,2939.492188
2,2,1876.602339
2,2,1503.000000
2,2,2001.555556
2,2,4225.718750
2,2,4709.993865
2,2,8838.314516

2,2,1965.053571
2,2,4036.286184
2,2,17477.515625
2,2,2780.429878
2,2,6037.154070
2,2,4504.517045
2,2,1915.774038
2,2,3498.615385
2,2,3609.756098
2,2,4306.703804
2,2,2050.748603
2,2,3103.205882
2,2,13647.208333
2,2,85032.611111
2,2,1652.209375
2,2,4915.720238
2,2,4305.535714
2,2,10003.893258
2,2,1841.331250
2,2,4898.890625
2,2,1524.587500
2,2,3823.882979
2,2,2402.125000
2,2,2505.557870
2,2,4327.346939
2,2,2303.167614
2,2,3013.384615
2,2,5263.950000
2,2,2634.615646
2,2,1960.711538
2,2,2059.777273
2,2,3930.272727
2,2,1840.241379
2,2,2692.096875
2,2,2448.000000
2,2,2090.472973
2,2,4838.914773
2,2,2557.307692
2,2,1641.687500
2,2,2426.712766
2,2,2307.452830
2,2,4121.956954
2,2,1544.800000
2,2,1744.148936
2,2,9925.780000
2,2,2497.986111
2,2,2614.348837
2,2,1929.500000
2,2,5014.528302
2,2,3525.706897
2,2,3761.164773
2,2,9959.494681
2,2,8542.078947
2,2,2056.312500
2,2,3217.627907
2,2,1665.093750
2,2,6149.041667
2,2,7652.437500
2,2,6394.112245
2,2,3862.125532
2,2,4634.791667
2,2,3709.934896
2,2,3291.168712
2,2,2133.500000
2,2,11747.489362
2,2,3695.812500

2,2,3005.367500
2,2,5393.368304
2,2,2736.165803
2,2,9138.672500
2,2,2400.192308
2,2,3088.467290
2,2,4827.856250
2,2,3333.602041
2,2,2132.024457
2,2,1560.533784
2,2,10255.475410
2,2,4443.386364
2,2,1608.983957
2,2,8324.943182
2,2,4304.925000
2,2,2989.487981
2,2,10593.419643
2,2,1662.896341
2,2,2399.382353
2,2,8790.505000
2,2,3611.764706
2,2,3358.952128
2,2,2903.985795
2,2,12004.879310
2,2,1833.893333
2,2,5377.372642
2,2,3041.250000
2,2,2662.980769
2,2,2202.576705
2,2,1762.820513
2,2,3921.454082
2,2,7840.401163
2,2,4665.830769
2,2,4018.554054
2,2,3689.863095
2,2,3100.379808
2,2,1699.219907
2,2,6175.510204
2,2,1518.786585
2,2,3034.812500
2,2,1736.296875
2,2,2589.142857
2,2,5110.822368
2,2,1712.413043
2,2,3035.761628
2,2,14276.483051
2,2,1945.892857
2,2,1920.437500
2,2,1901.161111
2,2,3183.705882
2,2,5859.500000
2,2,3501.925000
2,2,8993.750000
2,2,6501.776596
2,2,7265.671512
2,2,1618.489130
2,2,2402.875000
2,2,3236.934211
2,2,8246.301370
2,2,2176.008152
2,2,11453.900000
2,2,3195.940217
2,2,1911.229167
2,2,4612.480392
2,2,8286.798969
2,2,3047.341837

2,2,10398.270408
2,2,2631.762195
2,2,2440.096774
2,2,1634.853261
2,2,2862.777778
2,2,2066.617021
2,2,1500.908537
2,2,2542.639831
2,2,5506.394089
2,2,6051.751397
2,2,2425.068452
2,2,1716.259542
2,2,3610.578704
2,2,5531.062500
2,2,2777.653125
2,2,11750.469388
2,2,13618.090909
2,2,9129.145833
2,2,4215.048077
2,2,4775.730000
2,2,2192.037500
2,2,2305.005208
2,2,4420.117647
2,2,3305.085106
2,2,3739.600000
2,2,3658.700000
2,2,15561.718750
2,2,5728.441489
2,2,1585.056818
2,2,3510.967105
2,2,1751.553571
2,2,3042.031915
2,2,1990.553571
2,2,5761.877907
2,2,2623.600000
2,2,2657.795918
2,2,8222.372449
2,2,1523.344512
2,2,3972.142045
2,2,1816.594444
2,2,4481.696429
2,2,3301.714623
2,2,3781.457317
2,2,2008.047297
2,2,2338.822917
2,2,2927.750000
2,2,1573.443182
2,2,12614.612805
2,2,1548.396875
2,2,1523.436047
2,2,4958.105978
2,2,1901.903061
2,2,1666.798077
2,2,10364.200000
2,2,4543.656863
2,2,5601.640000
2,2,2263.050926
2,2,10427.425532
2,2,4910.277439
2,2,1735.443299
2,2,5979.284653
2,2,1983.252976
2,2,1997.478261
2,2,6909.240385
2,2,3812.049020
2,2,1773.166667

2,2,3555.886905
2,2,3927.750000
2,2,2602.890244
2,2,2999.287234
2,2,1824.818182
2,2,5688.502941
2,2,2095.591549
2,2,1666.042169
2,2,1637.229730
2,2,6446.625000
2,2,3131.625571
2,2,2455.350000
2,2,2040.300000
2,2,2585.319149
2,2,1568.755682
2,2,4125.315104
2,2,7641.369347
2,2,2093.214286
2,2,1544.975000
2,2,8313.484649
2,2,1696.230769
2,2,14300.985294
2,2,3812.083333
2,2,8295.700000
2,2,12305.204036
2,2,4813.196429
2,2,1650.812500
2,2,2840.266026
2,2,2186.655702
2,2,2518.452128
2,2,1557.062500
2,2,3233.232143
2,2,1653.052632
2,2,1906.695000
2,2,4713.226974
2,2,5164.723404
2,2,1672.377660
2,2,5041.255102
2,2,2552.763889
2,2,2424.403061
2,2,1600.075000
2,2,1573.414474
2,2,2815.068878
2,2,2318.974359
2,2,3940.727273
2,2,3983.578947
2,2,7423.050000
2,2,4480.750000
2,2,1555.628049
2,2,5090.824468
2,2,2271.844595
2,2,2618.468750
2,2,1673.408537
2,2,20042.652778
2,2,2521.562500
2,2,2591.308140
2,2,2189.872093
2,2,5743.963415
2,2,6009.610577
2,2,2943.731707
2,2,5754.531915
2,2,2297.600000
2,2,5829.419118
2,2,3625.533537
2,2,6084.377551
2,2,3227.817073

2,2,1779.325581
2,2,2621.875000
2,2,3276.931250
2,2,3971.762500
2,2,2040.533163
2,2,2342.175595
2,2,3845.812500
2,2,37065.678125
2,2,3173.104167
2,2,3346.495283
2,2,3418.330882
2,2,5645.433333
2,2,4263.363636
2,2,9799.147059
2,2,2242.732955
2,2,5245.590000
2,2,5692.052632
2,2,4486.000000
2,2,1970.163265
2,2,2750.640625
2,2,1899.045455
2,2,1900.937500
2,2,2837.250000
2,2,2575.133333
2,2,1572.408019
2,2,4292.070968
2,2,2034.661765
2,2,19882.209677
2,2,3336.949495
2,2,4088.750000
2,2,5345.090909
2,2,1694.500000
2,2,3078.750000
2,2,3920.362745
2,2,6033.464674
2,2,1598.586207
2,2,3421.207237
2,2,2886.462617
2,2,2393.937037
2,2,1605.525714
2,2,1793.019685
2,2,4954.982906
2,2,2502.428571
2,2,2164.493590
2,2,2934.218563
2,2,1728.097222
2,2,3237.440000
2,2,3133.787234
2,2,1728.000000
2,2,1736.261111
2,2,7472.997500
2,2,1727.610000
2,2,13252.695946
2,2,4596.480769
2,2,2724.152381
2,2,4238.228022
2,2,2675.186813
2,2,4333.275568
2,2,2062.181818
2,2,1758.078488
2,2,2646.883333
2,2,2047.497396
2,2,4990.712838
2,2,5034.076087
2,2,7235.404762
2,2,2728.011905

2,2,1707.365854
2,2,2881.284091
2,2,12931.948980
2,2,2718.473684
2,2,4798.671053
2,2,4794.175000
2,2,1587.656250
2,2,1558.110465
2,2,3003.036364
2,2,4387.857143
2,2,7623.353659
2,2,8363.751412
2,2,2433.462366
2,2,38041.696203
2,2,3473.372340
2,2,3085.479167
2,2,4019.446078
2,2,3459.715000
2,2,7331.105769
2,2,13676.993902
2,2,1685.311275
2,2,1532.470930
2,2,3087.428571
2,2,4503.009804
2,2,4541.803922
2,2,2903.255208
2,2,3886.405556
2,2,11858.558824
2,2,2814.489796
2,2,2222.459375
2,2,2424.729167
2,2,14879.939024
2,2,2147.643590
2,2,1547.945122
2,2,1541.750000
2,2,6641.798077
2,2,2438.086310
2,2,3846.312500
2,2,4019.354592
2,2,4169.406250
2,2,3455.782609
2,2,2175.130435
2,2,2584.666667
2,2,4731.090909
2,2,1668.230769
2,2,1961.671429
2,2,2711.615132
2,2,4002.677500
2,2,5127.680233
2,2,3317.285714
2,2,2658.583333
2,2,2913.649457
2,2,2144.739583
2,2,1693.058673
2,2,3672.829114
2,2,2890.139706
2,2,3314.842105
2,2,1628.014706
2,2,1835.677632
2,2,7174.224057
2,2,4234.978723
2,2,5662.750000
2,2,6985.770270
2,2,3324.109375
2,2,2314.988636
2,2,2024.117021

2,2,5092.567500
2,2,4316.377604
2,2,2401.763158
2,2,1931.069149
2,2,2652.914773
2,2,2291.133333
2,2,6760.191489
2,2,1782.762500
2,2,8060.789474
2,2,3979.134146
2,2,4846.223464
2,2,3707.962264
2,2,1545.562500
2,2,2304.634615
2,2,2280.750000
2,2,13434.822222
2,2,4631.513514
2,2,4121.245399
2,2,5818.538462
2,2,2046.443878
2,2,2471.016667
2,2,3225.016667
2,2,1797.808511
2,2,4211.068750
2,2,2551.500000
2,2,2057.609756
2,2,1919.389474
2,2,2342.955128
2,2,4340.763514
2,2,6497.095238
2,2,5017.828125
2,2,2223.866071
2,2,3786.333333
2,2,8214.031746
2,2,2742.096774
2,2,3597.571429
2,2,2591.149390
2,2,2049.899329
2,2,3542.755102
2,2,3125.000000
2,2,5351.142077
2,2,1849.125000
2,2,1892.039352
2,2,21705.416256
2,2,6502.510638
2,2,2442.267442
2,2,2348.811321
2,2,2223.296875
2,2,12408.208333
2,2,1964.292553
2,2,5858.933333
2,2,6068.232759
2,2,8003.305233
2,2,1765.520000
2,2,2765.021472
2,2,5325.424658
2,2,1821.295455
2,2,2876.000000
2,2,1987.480769
2,2,2722.175355
2,2,4600.850299
2,2,1823.500000
2,2,2865.848837
2,2,1560.034091
2,2,2433.979592
2,2,2860.542857

2,2,13773.687500
2,2,7940.978723
2,2,1898.515625
2,2,2325.283920
2,2,6214.597015
2,2,1615.017442
2,2,13423.117925
2,2,3014.469512
2,2,4384.082237
2,2,2837.509615
2,2,1750.000000
2,2,6628.511364
2,2,1605.222826
2,2,4021.412500
2,2,3581.087500
2,2,3246.527778
2,2,2868.306818
2,2,11114.442857
2,2,2277.630000
2,2,1528.009146
2,2,3387.319767
2,2,1517.898026
2,2,2161.234694
2,2,26518.809091
2,2,2023.191011
2,2,2000.572917
2,2,3032.215686
2,2,4096.705714
2,2,3308.122340
2,2,1722.628289
2,2,2265.350000
2,2,7112.805556
2,2,1831.547619
2,2,2698.534091
2,2,3727.184615
2,2,4242.269886
2,2,3245.773256
2,2,8130.469697
2,2,3651.786765
2,2,4801.371951
2,2,1690.328947
2,2,5371.750000
2,2,1664.000000
2,2,1758.050532
2,2,2594.195652
2,2,9878.134615
2,2,7258.160000
2,2,17078.692500
2,2,1830.181818
2,2,1954.187500
2,2,4557.441176
2,2,3888.937500
2,2,3379.061224
2,2,2132.810056
2,2,4570.914286
2,2,1544.420765
2,2,1548.053191
2,2,1548.079470
2,2,7627.254980
2,2,5765.852459
2,2,1852.615385
2,2,8289.591837
2,2,16658.035326
2,2,4175.395349
2,2,7971.531250
2,2,3291.507353

2,2,1790.784091
2,2,14245.677778
2,2,2213.844444
2,2,6126.500000
2,2,1845.701987
2,2,10618.705128
2,2,1548.125749
2,2,2644.049020
2,2,3602.382075
2,2,4163.287234
2,2,2045.769231
2,2,3299.488372
2,2,2319.238372
2,2,2454.807692
2,2,1512.000000
2,2,2712.911932
2,2,2631.105769
2,2,3874.755556
2,2,9203.275862
2,2,3772.244048
2,2,3470.951031
2,2,2959.785714
2,2,2279.726415
2,2,1522.663265
2,2,5224.151741
2,2,3037.286585
2,2,1609.838816
2,2,2112.509375
2,2,1901.625000
2,2,1792.786517
2,2,2731.962500
2,2,4568.046875
2,2,1621.273684
2,2,2006.407895
2,2,1930.843750
2,2,4862.905556
2,2,1960.173913
2,2,4903.508333
2,2,4322.043478
2,2,1713.968750
2,2,2755.975000
2,2,4081.023256
2,2,3047.896739
2,2,3516.074324
2,2,1985.049342
2,2,5066.264706
2,2,4144.061224
2,2,5201.500000
2,2,2043.075000
2,2,1728.775568
2,2,4072.572222
2,2,1501.947917
2,2,5996.559211
2,2,2524.258152
2,2,1596.250000
2,2,2891.680000
2,2,1903.726994
2,2,1970.966887
2,2,3344.452381
2,2,3123.467066
2,2,1732.800000
2,2,2732.074405
2,2,3646.396341
2,2,6188.279412
2,2,2549.311828
2,2,1842.862069

2,2,2262.339674
2,2,1885.851852
2,2,9195.117647
2,2,1931.875000
2,2,15874.227273
2,2,4230.966146
2,2,4765.833333
2,2,8413.000000
2,2,2911.989796
2,2,2418.102041
2,2,1708.125000
2,2,2246.756944
2,2,1848.711806
2,2,3831.986842
2,2,2853.290698
2,2,2506.490066
2,2,8301.872685
2,2,1794.358553
2,2,1742.666667
2,2,3977.088889
2,2,5570.709135
2,2,2276.051282
2,2,2043.367347
2,2,2021.000000
2,2,2233.530405
2,2,2508.775000
2,2,1617.542857
2,2,6736.456250
2,2,2581.727273
2,2,2397.068966
2,2,2332.073171
2,2,1657.401961
2,2,11676.715164
2,2,1851.325000
2,2,2693.805556
2,2,15793.521739
2,2,1800.699387
2,2,5644.222222
2,2,5461.396226
2,2,1894.968750
2,2,4976.000000
2,2,2169.387255
2,2,2589.926136
2,2,4161.567500
2,2,2857.215686
2,2,7038.789216
2,2,1813.527027
2,2,2144.500000
2,2,3518.416667
2,2,3426.484848
2,2,1778.569444
2,2,1639.254717
2,2,8406.842105
2,2,13863.489362
2,2,1561.375000
2,2,2772.900641
2,2,1570.388021
2,2,13848.713636
2,2,5662.080357
2,2,4195.781250
2,2,6550.952500
2,2,4555.805714
2,2,6911.863281
2,2,2769.647727
2,2,2199.227749
2,2,1665.511628

2,2,8664.466667
2,2,1556.869565
2,2,4784.583333
2,2,6157.085106
2,2,2751.515625
2,2,2623.571429
2,2,3522.031250
2,2,1520.947500
2,2,2462.825000
2,2,5522.061224
2,2,2835.808511
2,2,2788.230769
2,2,8370.574803
2,2,6484.275510
2,2,3552.288571
2,2,2376.937500
2,2,1698.607143
2,2,2026.318182
2,2,3746.003049
2,2,4296.514368
2,2,1752.500000
2,2,8189.275510
2,2,9489.524390
2,2,13756.630952
2,2,2408.200000
2,2,4240.425532
2,2,3989.781977
2,2,2037.561111
2,2,2149.195455
2,2,2622.250000
2,2,3597.789352
2,2,3034.000000
2,2,1925.784810
2,2,4899.107143
2,2,2904.792683
2,2,2270.484536
2,2,5486.798077
2,2,3824.877551
2,2,2703.633333
2,2,2616.595930
2,2,4165.209821
2,2,7847.426630
2,2,6533.189189
2,2,4984.952381
2,2,2057.739583
2,2,3059.465000
2,2,3641.130952
2,2,1841.141447
2,2,2086.250000
2,2,9114.375000
2,2,1594.343750
2,2,1978.411765
2,2,10317.058333
2,2,1793.375000
2,2,5044.764901
2,2,7206.365385
2,2,3838.709184
2,2,2876.649457
2,2,2130.678125
2,2,3095.000000
2,2,3807.428977
2,2,1853.350000
2,2,1780.696875
2,2,6826.086957
2,2,3336.114583
2,2,4079.098684

2,2,2676.055556
2,2,1721.067568
2,2,4466.386364
2,2,2997.647059
2,2,5149.258721
2,2,1514.461538
2,2,4268.918605
2,2,4191.281250
2,2,4356.020349
2,2,2287.127778
2,2,4212.250000
2,2,1703.487179
2,2,3098.403125
2,2,2045.622340
2,2,1717.029762
2,2,1704.480000
2,2,2148.600000
2,2,1610.477273
2,2,11542.522422
2,2,2944.850000
2,2,2109.423077
2,2,4459.836538
2,2,1513.425000
2,2,2217.067308
2,2,2371.627451
2,2,1645.620690
2,2,6624.605405
2,2,7494.183036
2,2,2325.394886
2,2,9831.270270
2,2,2107.009036
2,2,6003.800926
2,2,3678.750000
2,2,7701.449324
2,2,7761.806604
2,2,15001.090909
2,2,4845.968750
2,2,2477.293750
2,2,3204.744048
2,2,2615.774390
2,2,1777.890052
2,2,7839.914286
2,2,2186.639810
2,2,10266.366071
2,2,4345.882353
2,2,2420.898810
2,2,6300.921053
2,2,3065.687500
2,2,4001.581395
2,2,2417.977679
2,2,4885.817708
2,2,2228.250000
2,2,3812.790541
2,2,5915.676829
2,2,8832.118534
2,2,3984.475000
2,2,1740.884868
2,2,2395.895833
2,2,12083.867647
2,2,6694.333333
2,2,2279.472222
2,2,7045.470930
2,2,1745.669118
2,2,3673.043478
2,2,1836.978125
2,2,3332.000000

2,2,5250.356383
2,2,3146.128205
2,2,2706.860465
2,2,1831.594388
2,2,1525.740196
2,2,2727.869565
2,2,5036.400000
2,2,2290.415541
2,2,2757.314103
2,2,2096.187500
2,2,8296.470339
2,2,4130.750000
2,2,1832.518750
2,2,2101.988889
2,2,3867.818182
2,2,10138.976190
2,2,2419.305804
2,2,4780.365385
2,2,2345.265306
2,2,3603.250000
2,2,1998.292683
2,2,7484.250000
2,2,7482.284091
2,2,26310.245614
2,2,6612.708333
2,2,3520.309211
2,2,1900.735119
2,2,7892.577869
2,2,3996.997500
2,2,12477.182692
2,2,2208.756098
2,2,7057.075000
2,2,3146.961957
2,2,2266.472500
2,2,9802.681818
2,2,5232.500000
2,2,2422.322674
2,2,1627.652439
2,2,3138.652174
2,2,7890.191860
2,2,3108.562500
2,2,1586.997449
2,2,15718.598837
2,2,2317.047414
2,2,1711.965278
2,2,4228.796196
2,2,2425.718750
2,2,3457.941860
2,2,2371.793919
2,2,3375.795455
2,2,2156.431250
2,2,1743.616071
2,2,1684.817935
2,2,3679.156250
2,2,1844.468750
2,2,1750.661765
2,2,13352.566176
2,2,1644.582386
2,2,3188.324324
2,2,1806.275510
2,2,2159.710227
2,2,1614.207317
2,2,2033.250000
2,2,5394.287500
2,2,3009.944444
2,2,2414.535156

2,2,3479.042453
2,2,1992.212766
2,2,2655.541667
2,2,1983.697368
2,2,2760.250000
2,2,2534.670455
2,2,2656.255814
2,2,1721.302083
2,2,30038.000000
2,2,1504.128571
2,2,5538.776596
2,2,2778.360714
2,2,5916.530093
2,2,4618.051282
2,2,5690.650000
2,2,3211.402778
2,2,2569.115132
2,2,2730.215909
2,2,2709.245283
2,2,4307.395000
2,2,3348.414894
2,2,5268.859694
2,2,1633.425000
2,2,4275.006410
2,2,5746.153846
2,2,7190.463816
2,2,7743.586735
2,2,1607.164062
2,2,1976.145000
2,2,2015.000000
2,2,1929.256944
2,2,2424.912921
2,2,2525.045918
2,2,9575.715909
2,2,2172.081522
2,2,7071.709091
2,2,2718.364286
2,2,5580.779412
2,2,5312.835526
2,2,2036.675000
2,2,4492.375000
2,2,1535.426136
2,2,7215.144608
2,2,12752.370166
2,2,8200.048295
2,2,4664.715116
2,2,10180.857955
2,2,7848.500000
2,2,5212.809524
2,2,20782.445000
2,2,5100.724138
2,2,2097.228346
2,2,6950.143617
2,2,1971.968750
2,2,8964.059783
2,2,3655.076923
2,2,1632.840625
2,2,4156.435897
2,2,3811.961111
2,2,1628.786585
2,2,8527.437500
2,2,1676.048851
2,2,2218.708333
2,2,1803.950581
2,2,2041.982143
2,2,3486.744898

2,2,3425.090278
2,2,1572.646341
2,2,3653.508380
2,2,4076.174863
2,2,2505.312500
2,2,3296.818436
2,2,1994.553571
2,2,1688.519022
2,2,4096.905000
2,2,3257.052885
2,2,2168.333333
2,2,2132.988235
2,2,2491.673913
2,2,4822.594907
2,2,2315.611111
2,2,4447.704545
2,2,2309.535354
2,2,2381.107500
2,2,3662.979592
2,2,2061.154545
2,2,2992.854651
2,2,1815.185484
2,2,3996.028646
2,2,1666.955556
2,2,4557.133929
2,2,2525.849057
2,2,2778.469512
2,2,3206.468085
2,2,3989.463816
2,2,1502.243421
2,2,8016.807692
2,2,1939.356707
2,2,3577.793605
2,2,1732.644022
2,2,10042.663043
2,2,6121.492647
2,2,4492.764706
2,2,1560.096875
2,2,10275.041237
2,2,5620.288462
2,2,3575.059172
2,2,2960.786624
2,2,2652.326531
2,2,1930.176471
2,2,2524.516304
2,2,3973.280556
2,2,6426.810204
2,2,4686.339572
2,2,1718.000000
2,2,3978.560976
2,2,5705.048544
2,2,2473.702381
2,2,7426.953488
2,2,2474.832418
2,2,2749.893617
2,2,4204.351724
2,2,3974.640719
2,2,9536.179167
2,2,3285.500000
2,2,4822.732143
2,2,7842.585987
2,2,4169.731707
2,2,4277.114094
2,2,4323.605978
2,2,4921.513158
2,2,2835.780488

2,2,1892.672414
2,2,2151.577206
2,2,6394.160256
2,2,2523.520833
2,2,2092.600000
2,2,4153.214286
2,2,1526.656250
2,2,1797.767606
2,2,6828.193548
2,2,3892.974432
2,2,4830.890957
2,2,2875.471292
2,2,5517.471154
2,2,3864.084302
2,2,8305.235294
2,2,3996.750000
2,2,3040.643229
2,2,1819.533333
2,2,1765.955882
2,2,2191.453125
2,2,3869.531250
2,2,10863.763587
2,2,12036.017442
2,2,4731.951923
2,2,2314.460526
2,2,3872.085784
2,2,2085.549296
2,2,1946.937500
2,2,2081.812500
2,2,18473.056452
2,2,3321.092391
2,2,2385.363372
2,2,1851.261905
2,2,1630.116279
2,2,2368.790698
2,2,1537.736842
2,2,7546.607558
2,2,4220.304878
2,2,2469.407895
2,2,3663.673077
2,2,4207.990654
2,2,3082.111111
2,2,2683.861650
2,2,1920.179348
2,2,7361.215640
2,2,3850.192308
2,2,2484.154412
2,2,2936.727749
2,2,2944.805556
2,2,2274.778443
2,2,5509.813830
2,2,1659.111111
2,2,1758.887097
2,2,3199.108108
2,2,2030.444444
2,2,4828.795455
2,2,2637.226804
2,2,2238.221154
2,2,2323.972973
2,2,2198.392857
2,2,3664.640625
2,2,2819.812500
2,2,2976.948529
2,2,1600.276042
2,2,1615.652174
2,2,1939.555556

2,2,6038.031977
2,2,2598.020833
2,2,4897.915254
2,2,5248.388889
2,2,11756.432065
2,2,6440.659091
2,2,6542.125000
2,2,1538.058824
2,2,2007.236413
2,2,4392.325581
2,2,2077.663462
2,2,1502.266304
2,2,6095.392500
2,2,2134.369681
2,2,3280.636364
2,2,3374.467391
2,2,1617.507353
2,2,8808.500000
2,2,4182.076220
2,2,2964.735294
2,2,2314.013514
2,2,2471.357143
2,2,6396.250000
2,2,4131.691919
2,2,2791.535714
2,2,4088.277778
2,2,3812.671196
2,2,2287.851190
2,2,1709.801724
2,2,18517.931373
2,2,5465.927083
2,2,1641.075000
2,2,7218.324324
2,2,5871.054054
2,2,4826.299479
2,2,10784.285714
2,2,1850.115789
2,2,5119.490798
2,2,7982.317647
2,2,2817.525000
2,2,2656.814570
2,2,2032.222826
2,2,2455.090686
2,2,1525.303571
2,2,2562.250000
2,2,2995.793103
2,2,3386.167614
2,2,2206.666667
2,2,1524.853723
2,2,3680.513158
2,2,5614.612500
2,2,3498.119565
2,2,4075.947500
2,2,4024.687500
2,2,4035.706522
2,2,3935.460227
2,2,7903.026316
2,2,11439.875000
2,2,4083.095455
2,2,3636.300000
2,2,3284.643275
2,2,6454.272500
2,2,2857.153846
2,2,1595.510135
2,2,1588.882813
2,2,1517.983553

2,2,6713.178571
2,2,1966.148936
2,2,3607.961538
2,2,3726.201087
2,2,4466.826923
2,2,1826.138158
2,2,5554.391204
2,2,2680.166667
2,2,5475.451531
2,2,2410.406250
2,2,2192.453488
2,2,1532.258333
2,2,3289.040094
2,2,1814.826389
2,2,1712.847222
2,2,7730.753304
2,2,4361.019802
2,2,3238.198980
2,2,3585.071918
2,2,4057.017157
2,2,76737.756410
2,2,1767.203704
2,2,5706.182292
2,2,1669.416667
2,2,2211.331731
2,2,3302.741848
2,2,1823.173913
2,2,2302.388889
2,2,1751.892857
2,2,3746.297872
2,2,1816.317308
2,2,13127.971154
2,2,2238.303571
2,2,5538.375000
2,2,1914.953488
2,2,2181.583333
2,2,1622.135135
2,2,5192.714286
2,2,2149.782609
2,2,3968.988372
2,2,2065.000000
2,2,6501.891304
2,2,5314.218750
2,2,1754.320000
2,2,4231.641304
2,2,3946.565868
2,2,2791.859375
2,2,2790.804878
2,2,7821.808824
2,2,5914.125000
2,2,8737.193182
2,2,3804.385000
2,2,1826.731771
2,2,2230.913793
2,2,1512.333333
2,2,3473.326923
2,2,5574.250000
2,2,3068.109756
2,2,2419.494444
2,2,2416.394737
2,2,2198.875000
2,2,3694.544118
2,2,3371.648438
2,2,3145.832386
2,2,4409.081818
2,2,4716.500000

2,2,5634.305936
2,2,2499.673611
2,2,2101.395833
2,2,1794.116379
2,2,2695.979545
2,2,3913.618750
2,2,3037.360465
2,2,9174.556122
2,2,10554.949275
2,2,2297.025641
2,2,4547.282486
2,2,1892.664634
2,2,1957.514970
2,2,6338.952128
2,2,4169.633333
2,2,3971.389706
2,2,1499.135135
2,2,1530.651163
2,2,8207.741071
2,2,1878.875000
2,2,8470.704188
2,2,7778.717949
2,2,1658.521277
2,2,2665.433962
2,2,7872.397059
2,2,4699.096354
2,2,1614.795455
2,2,1776.585366
2,2,1837.875000
2,2,7842.730978
2,2,2076.390306
2,2,4314.775568
2,2,4520.250000
2,2,1979.750000
2,2,2136.152174
2,2,1646.625000
2,2,1786.934783
2,2,2112.052326
2,2,5924.331522
2,2,18301.887500
2,2,4418.955556
2,2,3298.550000
2,2,2645.675325
2,2,6043.450000
2,2,4175.535885
2,2,2389.550000
2,2,5521.550336
2,2,3078.602871
2,2,1766.031250
2,2,2204.375000
2,2,3229.041667
2,2,6936.546196
2,2,1860.136076
2,2,2284.520468
2,2,8534.656489
2,2,4505.599432
2,2,2257.409091
2,2,2536.370000
2,2,2152.487179
2,2,4320.509375
2,2,2486.807927
2,2,1684.284722
2,2,2594.526316
2,2,3616.077778
2,2,1534.545000
2,2,4600.784574

2,2,3574.850000
2,2,3565.381944
2,2,3371.766234
2,2,2819.929032
2,2,4908.468085
2,2,12030.217262
2,2,2171.121951
2,2,4551.500000
2,2,12100.729730
2,2,20813.263889
2,2,1968.894737
2,2,7765.037037
2,2,4571.273196
2,2,1818.368421
2,2,5519.315000
2,2,3737.608974
2,2,3898.918367
2,2,6618.989796
2,2,5936.931818
2,2,21858.717391
2,2,1764.695000
2,2,3399.842365
2,2,6563.529851
2,2,2835.147727
2,2,16819.617424
2,2,2596.375000
2,2,5095.306977
2,2,2447.552632
2,2,3547.736842
2,2,3894.750000
2,2,3733.843085
2,2,2329.840909
2,2,4333.250000
2,2,3193.005952
2,2,1749.866071
2,2,43713.735294
2,2,5174.385965
2,2,2196.090452
2,2,1868.083333
2,2,1790.833333
2,2,1681.493750
2,2,3055.312500
2,2,2049.500000
2,2,4426.489510
2,2,6235.153846
2,2,9820.314732
2,2,3215.198171
2,2,5349.087209
2,2,16042.238971
2,2,1615.183673
2,2,1673.118243
2,2,1712.420455
2,2,1739.812500
2,2,2158.135135
2,2,2333.644444
2,2,5122.753378
2,2,2939.833333
2,2,5417.236364
2,2,3066.196203
2,2,2230.969388
2,2,8769.776316
2,2,5336.250000
2,2,2196.166667
2,2,2766.085227
2,2,1731.712500
2,2,3030.250000

2,2,2980.806283
2,2,3498.074286
2,2,2643.778409
2,2,7733.312500
2,2,1549.955497
2,2,2635.205128
2,2,2688.307692
2,2,10879.180233
2,2,2235.410256
2,2,2292.371429
2,2,2997.609375
2,2,1519.908784
2,2,2864.166667
2,2,5896.250000
2,2,1919.532051
2,2,1936.675676
2,2,4260.670918
2,2,22605.132812
2,2,2560.909884
2,2,3290.891667
2,2,8557.515957
2,2,2278.072727
2,2,3863.944767
2,2,2470.641509
2,2,1691.398256
2,2,5621.794872
2,2,2486.300000
2,2,7201.089623
2,2,7147.250000
2,2,3617.250000
2,2,3230.961538
2,2,6595.519886
2,2,1639.545213
2,2,4626.279762
2,2,5222.267857
2,2,5597.637195
2,2,2657.210526
2,2,5187.782857
2,2,1508.267857
2,2,2222.850711
2,2,4361.448980
2,2,4401.604651
2,2,1511.400000
2,2,5277.877451
2,2,1940.380102
2,2,5046.240196
2,2,1635.090909
2,2,1962.076389
2,2,2880.263158
2,2,2808.333333
2,2,2631.461957
2,2,2472.172297
2,2,4397.130000
2,2,2373.164634
2,2,2132.098958
2,2,5086.607500
2,2,5228.978723
2,2,2956.760417
2,2,1915.380952
2,2,1639.585106
2,2,2382.875000
2,2,1902.250000
2,2,11676.484375
2,2,2436.815909
2,2,2525.854167
2,2,2780.447222

2,2,12638.570755
2,2,2937.364706
2,2,2340.936073
2,2,3798.450000
2,2,2768.103774
2,2,5777.107558
2,2,8052.925170
2,2,4239.846154
2,2,5713.328947
2,2,1582.523810
2,2,2281.738636
2,2,20363.170732
2,2,8044.902778
2,2,6982.914894
2,2,4122.302632
2,2,2466.994118
2,2,3755.729167
2,2,3696.677632
2,2,2397.586207
2,2,1695.353261
2,2,3649.823864
2,2,1864.701389
2,2,4492.011905
2,2,8393.731250
2,2,1739.415698
2,2,6447.627778
2,2,11516.480392
2,2,2099.394231
2,2,4517.014706
2,2,2636.348837
2,2,2755.752577
2,2,5311.875000
2,2,1653.525000
2,2,4876.552273
2,2,2685.070313
2,2,2214.215686
2,2,19763.035714
2,2,2634.944444
2,2,1855.830729
2,2,14095.333333
2,2,1537.085714
2,2,3184.666667
2,2,4570.641509
2,2,3723.872449
2,2,4862.260204
2,2,3488.890244
2,2,2905.802395
2,2,1835.000000
2,2,2663.116667
2,2,2001.857616
2,2,4150.097826
2,2,1729.776074
2,2,3108.226562
2,2,2305.202128
2,2,6552.000000
2,2,2927.176471
2,2,1597.642857
2,2,1594.785714
2,2,2477.437838
2,2,12295.394737
2,2,2481.010695
2,2,2347.908784
2,2,4397.625000
2,2,3182.500000
2,2,4852.518293
2,2,4572.276471

2,2,4348.872093
2,2,1577.946023
2,2,1558.882353
2,2,3521.869318
2,2,4083.621951
2,2,4665.972561
2,2,7699.235294
2,2,1869.526316
2,2,2203.972826
2,2,1898.595109
2,2,4002.121951
2,2,2611.581395
2,2,2777.375000
2,2,5509.580357
2,2,1543.739796
2,2,3281.168605
2,2,3580.391827
2,2,2085.142500
2,2,2209.729167
2,2,16067.539474
2,2,1675.743421
2,2,3730.914894
2,2,1752.176630
2,2,5968.352410
2,2,2048.363636
2,2,3456.370000
2,2,2861.000000
2,2,2559.096774
2,2,37133.068878
2,2,3721.538462
2,2,2138.332317
2,2,1562.636364
2,2,1931.519231
2,2,2592.693878
2,2,2949.211806
2,2,3323.942308
2,2,2538.107143
2,2,5565.312500
2,2,2266.510638
2,2,9951.927273
2,2,7634.202128
2,2,2121.209375
2,2,3152.559783
2,2,4095.443182
2,2,4999.992806
2,2,3802.634146
2,2,2421.328947
2,2,1640.142157
2,2,2757.350446
2,2,2194.686275
2,2,4515.015306
2,2,7296.379310
2,2,4134.392857
2,2,7432.192982
2,2,4621.735294
2,2,8763.370283
2,2,2747.831522
2,2,1921.000000
2,2,4005.060484
2,2,4154.584906
2,2,1705.625000
2,2,2080.309659
2,2,2544.900000
2,2,1857.315789
2,2,3580.204545
2,2,3074.761905

2,2,1837.718750
2,2,1774.810976
2,2,2329.843750
2,2,1862.071429
2,2,3022.788462
2,2,6393.719212
2,2,3897.968085
2,2,5225.371795
2,2,4917.425532
2,2,4156.852941
2,2,1773.259615
2,2,3935.261905
2,2,3808.607955
2,2,2308.048295
2,2,2194.825000
2,2,1973.686170
2,2,3577.684783
2,2,5663.352941
2,2,4765.446809
2,2,1887.162304
2,2,3992.305000
2,2,4571.339286
2,2,2843.222222
2,2,4071.981818
2,2,2879.323077
2,2,1551.803571
2,2,5003.222222
2,2,2128.500000
2,2,2082.483333
2,2,2471.207386
2,2,3145.269737
2,2,6021.226190
2,2,8395.395161
2,2,1773.458333
2,2,3420.681818
2,2,9430.750000
2,2,3399.919355
2,2,1544.500000
2,2,2379.133333
2,2,10486.799528
2,2,3082.892857
2,2,2063.595238
2,2,2582.304054
2,2,1673.783333
2,2,2284.206522
2,2,3948.680851
2,2,5808.468750
2,2,3452.566667
2,2,2047.006579
2,2,4401.816754
2,2,2421.541872
2,2,2142.412791
2,2,2949.696078
2,2,2392.500000
2,2,1716.865000
2,2,4895.675000
2,2,6243.484375
2,2,2057.580556
2,2,2198.926829
2,2,1747.272727
2,2,1723.800000
2,2,1559.473684
2,2,1685.443966
2,2,3762.953488
2,2,5711.166667
2,2,4113.301020

2,2,15731.136364
2,2,1947.878378
2,2,5057.101064
2,2,2255.222222
2,2,3674.297872
2,2,1929.298742
2,2,7806.936170
2,2,1690.094118
2,2,2850.930233
2,2,2945.885000
2,2,3380.020270
2,2,4526.187500
2,2,3218.408867
2,2,1602.750000
2,2,4463.006410
2,2,6759.237500
2,2,2935.767157
2,2,4992.363636
2,2,4006.135135
2,2,1545.353333
2,2,4017.454545
2,2,1907.850000
2,2,1636.755435
2,2,2079.425000
2,2,2410.000000
2,2,2629.540816
2,2,7321.044811
2,2,4140.604651
2,2,5221.789474
2,2,5645.908537
2,2,4275.084302
2,2,8937.000000
2,2,10911.357143
2,2,1746.608696
2,2,2156.673469
2,2,2246.000000
2,2,4441.431250
2,2,1762.444444
2,2,2802.037500
2,2,3573.342949
2,2,2875.534884
2,2,2185.312500
2,2,3691.216080
2,2,2074.408537
2,2,26295.418605
2,2,4071.533654
2,2,11563.191176
2,2,2450.184211
2,2,1699.225000
2,2,6671.513514
2,2,1653.000000
2,2,2189.454545
2,2,2460.795455
2,2,3059.000000
2,2,3132.063452
2,2,2046.279070
2,2,2638.308989
2,2,2023.197674
2,2,3179.875000
2,2,2078.846875
2,2,6724.217500
2,2,3089.136628
2,2,4520.592105
2,2,4474.957317
2,2,2462.266509
2,2,2814.251397

2,2,1856.000000
2,2,1678.864865
2,2,2721.862245
2,2,3432.102941
2,2,3047.112821
2,2,2172.125000
2,2,4115.192982
2,2,10230.777778
2,2,2994.512500
2,2,5269.897500
2,2,1562.608696
2,2,2118.600000
2,2,2063.157895
2,2,2323.677632
2,2,3256.175532
2,2,3914.930921
2,2,4199.750000
2,2,11050.534031
2,2,2290.003497
2,2,2563.145000
2,2,3792.979167
2,2,2660.174419
2,2,2306.301829
2,2,2490.326613
2,2,12319.574468
2,2,1638.664894
2,2,1793.201220
2,2,1840.455357
2,2,2096.180556
2,2,1626.600000
2,2,15321.473684
2,2,1751.269231
2,2,1649.050000
2,2,16388.000000
2,2,6616.375000
2,2,5705.481618
2,2,4481.466667
2,2,1613.815789
2,2,1522.340000
2,2,10763.320175
2,2,2199.200000
2,2,1678.487500
2,2,2448.090909
2,2,2978.650000
2,2,1817.779762
2,2,3724.625000
2,2,2022.851351
2,2,13063.960106
2,2,2071.128205
2,2,3899.934783
2,2,1984.145833
2,2,2252.506250
2,2,1774.000000
2,2,3038.801887
2,2,2805.645833
2,2,2088.040541
2,2,11728.322222
2,2,3472.175000
2,2,4942.644886
2,2,1543.470588
2,2,1675.367021
2,2,2568.173469
2,2,2092.812950
2,2,3170.051471
2,2,2128.292683
2,2,6803.423256

2,2,7391.020833
2,2,5003.348684
2,2,4189.000000
2,2,6886.525000
2,2,8832.875000
2,2,2493.615385
2,2,11172.385204
2,2,21867.771341
2,2,2825.363372
2,2,1859.140306
2,2,7449.000000
2,2,3543.219444
2,2,3454.130952
2,2,2321.489796
2,2,1511.512500
2,2,1607.854730
2,2,4592.344512
2,2,3217.695652
2,2,3003.986979
2,2,1692.988095
2,2,4458.184783
2,2,8849.425676
2,2,3352.029255
2,2,5387.459302
2,2,1509.531250
2,2,2743.296569
2,2,10582.695312
2,2,2722.729469
2,2,10703.036269
2,2,2578.751220
2,2,1721.266169
2,2,1625.916667
2,2,1649.085586
2,2,5384.098958
2,2,5253.144231
2,2,2074.640704
2,2,3384.413408
2,2,1965.237805
2,2,3007.497340
2,2,2465.921875
2,2,2556.440217
2,2,4221.031250
2,2,1620.593750
2,2,5291.012019
2,2,3749.429688
2,2,5393.250000
2,2,1547.516667
2,2,6314.103125
2,2,13680.128655
2,2,2615.118557
2,2,2267.545455
2,2,2634.659722
2,2,2501.100610
2,2,5273.391304
2,2,3076.405405
2,2,7605.341912
2,2,1617.146429
2,2,1558.831754
2,2,4761.338816
2,2,2921.729167
2,2,1986.884615
2,2,4833.828947
2,2,5147.024390
2,2,3840.682692
2,2,3889.718750
2,2,1539.214286

2,2,3064.321429
2,2,3192.073171
2,2,4215.201923
2,2,6834.740741
2,2,10269.463636
2,2,1527.918367
2,2,1568.432432
2,2,1855.492925
2,2,2202.812500
2,2,1542.180982
2,2,1641.591837
2,2,1901.150568
2,2,1933.828947
2,2,2256.250000
2,2,2337.912162
2,2,2836.731707
2,2,6781.929348
2,2,5154.920000
2,2,5116.661638
2,2,4137.816129
2,2,7042.411765
2,2,2711.607558
2,2,3144.243590
2,2,3966.393333
2,2,2287.881579
2,2,1584.425000
2,2,2134.438953
2,2,12486.309524
2,2,2198.510870
2,2,5297.781915
2,2,7177.061224
2,2,2053.186528
2,2,1542.445087
2,2,1777.857143
2,2,7381.053125
2,2,1837.229592
2,2,3065.318182
2,2,1681.833333
2,2,3086.840580
2,2,4129.383152
2,2,3999.512821
2,2,1854.880000
2,2,5195.000000
2,2,1816.421622
2,2,3000.972458
2,2,1712.356250
2,2,1593.786585
2,2,4518.132353
2,2,2064.770833
2,2,4731.020833
2,2,2705.869565
2,2,3512.918919
2,2,1866.000000
2,2,8152.551887
2,2,3798.692308
2,2,1861.642857
2,2,2162.000000
2,2,2319.611842
2,2,1922.074468
2,2,11787.648438
2,2,1838.333333
2,2,2652.550000
2,2,5649.627500
2,2,9825.949074
2,2,2167.908333
2,2,4457.775862

2,2,3672.139881
2,2,5967.059211
2,2,3509.586207
2,2,4107.302326
2,2,3124.827225
2,2,3359.987500
2,2,3871.828571
2,2,1628.450000
2,2,6327.550000
2,2,10410.345455
2,2,5523.288043
2,2,7058.244444
2,2,4337.431604
2,2,2978.766827
2,2,2466.250000
2,2,9006.263158
2,2,1970.185811
2,2,23712.100000
2,2,3882.365854
2,2,4872.180851
2,2,6792.353261
2,2,1589.579268
2,2,1590.285714
2,2,3864.179348
2,2,2301.937500
2,2,10010.095588
2,2,3095.100000
2,2,8048.022222
2,2,1506.637755
2,2,3737.347222
2,2,15249.527174
2,2,2612.680328
2,2,1837.351351
2,2,6585.101093
2,2,2193.212291
2,2,4848.920000
2,2,3215.691038
2,2,2455.430851
2,2,3422.774457
2,2,12542.272727
2,2,6311.761905
2,2,2020.500000
2,2,7756.403846
2,2,1772.617021
2,2,4463.087209
2,2,5261.966518
2,2,1920.731707
2,2,3307.508929
2,2,8915.864796
2,2,5053.987013
2,2,4855.000000
2,2,2267.621053
2,2,14332.348018
2,2,4635.271277
2,2,11843.748466
2,2,25841.412903
2,2,1578.796748
2,2,2652.476974
2,2,1509.937500
2,2,2830.207317
2,2,4326.468750
2,2,3324.291667
2,2,2366.981818
2,2,1697.750000
2,2,2326.097561
2,2,5151.872340

2,2,4113.523649
2,2,3341.600000
2,2,3364.036364
2,2,1619.137255
2,2,7860.304688
2,2,1630.835366
2,2,2477.909091
2,2,3529.878049
2,2,2813.657051
2,2,2119.566667
2,2,2968.371951
2,2,2587.416667
2,2,4469.437500
2,2,1544.738636
2,2,3077.526042
2,2,3226.604167
2,2,5510.525735
2,2,22881.615196
2,2,2294.821023
2,2,2267.798611
2,2,5001.823864
2,2,5574.474359
2,2,24304.428030
2,2,2498.687500
2,2,1734.261792
2,2,1721.520408
2,2,2803.848214
2,2,2487.481707
2,2,2065.384615
2,2,13015.279605
2,2,12890.941176
2,2,3007.167614
2,2,8353.166667
2,2,2488.045455
2,2,1601.000000
2,2,1811.267361
2,2,2377.638889
2,2,2182.478723
2,2,1843.588235
2,2,2900.481707
2,2,1908.650735
2,2,2349.365566
2,2,3936.883721
2,2,2222.669271
2,2,2443.323370
2,2,1989.833333
2,2,2292.255319
2,2,2914.398256
2,2,2231.528409
2,2,3648.100000
2,2,2557.771739
2,2,6093.625000
2,2,2874.778125
2,2,1923.475000
2,2,2046.583333
2,2,4164.013298
2,2,5263.390625
2,2,1885.659574
2,2,4496.100000
2,2,4161.166667
2,2,1816.297297
2,2,2069.078431
2,2,2433.656250
2,2,2761.016026
2,2,1980.639286
2,2,4947.632653

2,2,3074.828947
2,2,1755.013514
2,2,2413.057692
2,2,1597.185976
2,2,1575.564356
2,2,3317.467949
2,2,3782.615385
2,2,2116.854167
2,2,2864.948864
2,2,3096.940625
2,2,5208.216049
2,2,1768.890625
2,2,4502.443350
2,2,2291.855330
2,2,8541.381579
2,2,1698.244444
2,2,2964.437500
2,2,1798.807692
2,2,2949.280788
2,2,4864.220126
2,2,6886.659574
2,2,1848.250000
2,2,7317.000000
2,2,1931.835366
2,2,5044.311111
2,2,3523.652439
2,2,3169.437500
2,2,5311.895349
2,2,9380.892857
2,2,3183.666667
2,2,4493.687500
2,2,4425.100000
2,2,1927.000000
2,2,3984.956633
2,2,1867.412791
2,2,1709.983333
2,2,3810.000000
2,2,2407.941176
2,2,4279.330882
2,2,2519.375000
2,2,6788.295732
2,2,1963.524390
2,2,1777.625000
2,2,4680.411111
2,2,2012.450331
2,2,2576.481132
2,2,3742.500000
2,2,4799.566489
2,2,2460.210526
2,2,5315.659091
2,2,6040.750000
2,2,4149.440217
2,2,5928.272436
2,2,3604.089623
2,2,2877.663462
2,2,2402.666667
2,2,2846.468085
2,2,2593.695364
2,2,2205.371622
2,2,1929.500000
2,2,1809.802326
2,2,2256.849057
2,2,2359.800000
2,2,6709.058140
2,2,2089.055556
2,2,2104.794118

2,2,4032.428125
2,2,3334.676136
2,2,1519.145833
2,2,1513.162500
2,2,1603.480769
2,2,7372.537234
2,2,4415.917614
2,2,3731.965625
2,2,1519.542683
2,2,1982.663462
2,2,3276.302632
2,2,5179.988372
2,2,1571.721875
2,2,2863.500000
2,2,2063.364130
2,2,2544.125000
2,2,2978.750000
2,2,4836.500000
2,2,2073.238426
2,2,2939.561224
2,2,2470.775000
2,2,2951.459459
2,2,4377.446809
2,2,1669.019231
2,2,5136.456522
2,2,1785.551136
2,2,5089.504630
2,2,10627.574519
2,2,6135.333333
2,2,2832.244444
2,2,8540.983333
2,2,3120.382979
2,2,8098.866228
2,2,2505.817500
2,2,2122.101351
2,2,1874.885714
2,2,2388.666667
2,2,2403.250000
2,2,8072.361702
2,2,3853.902500
2,2,3934.500000
2,2,4419.346535
2,2,1654.545455
2,2,1933.848837
2,2,3456.961538
2,2,7785.651786
2,2,1563.308989
2,2,2356.903846
2,2,2463.679641
2,2,4541.376963
2,2,1910.104167
2,2,2552.975000
2,2,2135.363636
2,2,1637.208333
2,2,4951.279412
2,2,2731.529787
2,2,5677.535714
2,2,2234.857143
2,2,1505.710526
2,2,9241.436047
2,2,9506.647727
2,2,3466.663717
2,2,5147.847826
2,2,5808.042683
2,2,1528.726974
2,2,2969.835404

2,2,3677.767857
2,2,3306.160194
2,2,4206.637640
2,2,2876.500000
2,2,4501.411458
2,2,3329.298507
2,2,1555.829114
2,2,3613.493421
2,2,3851.611765
2,2,6951.490338
2,2,2047.290816
2,2,6564.186508
2,2,7807.627119
2,2,10765.562500
2,2,3778.106383
2,2,5968.438830
2,2,1860.857143
2,2,1655.184211
2,2,6955.662420
2,2,3372.645833
2,2,4314.297872
2,2,1985.068750
2,2,3050.937500
2,2,2449.840278
2,2,2234.750000
2,2,3727.255952
2,2,3309.789474
2,2,6207.787234
2,2,5027.390625
2,2,3060.150000
2,2,2376.466346
2,2,4284.252907
2,2,3402.614130
2,2,1629.375000
2,2,6716.002604
2,2,3660.240385
2,2,5286.000000
2,2,9617.786538
2,2,7479.692308
2,2,2560.411765
2,2,2644.000000
2,2,7108.959052
2,2,2952.521739
2,2,9708.621622
2,2,15802.209302
2,2,8100.500000
2,2,4123.288889
2,2,2249.407895
2,2,2270.108108
2,2,3843.500000
2,2,2539.665541
2,2,2121.380597
2,2,1694.073864
2,2,2795.848485
2,2,1970.557143
2,2,2171.521739
2,2,3957.141892
2,2,6348.818182
2,2,2541.636364
2,2,2044.990000
2,2,12803.203947
2,2,9295.130597
2,2,2191.322368
2,2,2043.117647
2,2,2088.854651
2,2,9424.400000

2,2,2294.882979
2,2,7998.451220
2,2,1854.083333
2,2,10085.961538
2,2,6352.829082
2,2,2389.137500
2,2,2151.485465
2,2,2750.487500
2,2,6231.418605
2,2,2878.095109
2,2,4594.405556
2,2,2940.083333
2,2,1938.909274
2,2,1693.306452
2,2,2869.197368
2,2,4071.494444
2,2,26844.772727
2,2,6294.395833
2,2,4549.812500
2,2,5010.385000
2,2,5764.754902
2,2,2653.981250
2,2,14387.573661
2,2,1588.959184
2,2,1689.457237
2,2,1522.780000
2,2,20711.129032
2,2,1812.745370
2,2,2302.282051
2,2,1749.866279
2,2,1567.229730
2,2,5829.666667
2,2,1641.250000
2,2,3098.795699
2,2,7497.489899
2,2,1926.806818
2,2,1636.533537
2,2,2839.353659
2,2,1778.360248
2,2,5846.847953
2,2,2694.698171
2,2,1635.892857
2,2,3801.620192
2,2,1662.750000
2,2,1589.043478
2,2,3509.586957
2,2,6007.127193
2,2,1817.533333
2,2,2826.846154
2,2,1633.012500
2,2,4090.830000
2,2,3503.569444
2,2,3049.229167
2,2,3249.493243
2,2,7403.000000
2,2,3857.821429
2,2,2476.543478
2,2,2282.691489
2,2,6778.338068
2,2,2415.875000
2,2,3734.054054
2,2,8895.867788
2,2,1541.829268
2,2,4864.150000
2,2,7937.192708
2,2,5868.125000

2,2,4922.730337
2,2,2313.186916
2,2,2112.943299
2,2,2212.178571
2,2,3078.354651
2,2,2443.787234
2,2,1787.300000
2,2,2129.461957
2,2,2133.520833
2,2,5769.814815
2,2,3479.839286
2,2,2044.530516
2,2,3096.670673
2,2,3409.350610
2,2,4211.918919
2,2,16062.010776
2,2,1781.351064
2,2,2103.453125
2,2,7951.489130
2,2,3687.828488
2,2,4009.021875
2,2,34060.099490
2,2,2972.265244
2,2,2091.483696
2,2,3677.320000
2,2,1622.666667
2,2,8043.447674
2,2,3499.687500
2,2,1579.227273
2,2,2168.229167
2,2,2776.775510
2,2,2273.857143
2,2,2640.983173
2,2,2849.663462
2,2,4201.937500
2,2,2639.573276
2,2,4735.844660
2,2,1633.864583
2,2,1893.232143
2,2,2628.843049
2,2,4389.478070
2,2,1783.400000
2,2,4759.833333
2,2,10955.422170
2,2,5071.316860
2,2,9184.062500
2,2,2037.507979
2,2,4270.777778
2,2,2093.982639
2,2,3409.473214
2,2,2544.250000
2,2,1659.692308
2,2,2772.062937
2,2,15603.259259
2,2,2215.163043
2,2,2069.575000
2,2,5728.438776
2,2,5102.945455
2,2,3180.914634
2,2,2922.893519
2,2,4187.343750
2,2,1533.503205
2,2,2820.925532
2,2,3942.468750
2,2,3110.774390
2,2,1864.932432

2,2,2414.020270
2,2,1735.127717
2,2,1624.102041
2,2,1912.102564
2,2,2262.731061
2,2,12978.805000
2,2,1606.471591
2,2,3096.794872
2,2,4082.184375
2,2,2115.133508
2,2,2635.379310
2,2,1977.877778
2,2,13199.882353
2,2,5199.666667
2,2,2788.764706
2,2,1811.399457
2,2,4774.243590
2,2,2069.000000
2,2,3014.566176
2,2,5214.139241
2,2,1670.615578
2,2,3435.211429
2,2,1635.500000
2,2,2032.000000
2,2,1882.062500
2,2,2431.312500
2,2,11868.426230
2,2,2207.840909
2,2,5980.859375
2,2,2337.994318
2,2,2815.562500
2,2,2622.443299
2,2,2059.375000
2,2,2407.968750
2,2,1770.777778
2,2,1927.347561
2,2,2901.840426
2,2,5767.812500
2,2,1701.523810
2,2,3554.270270
2,2,2843.256944
2,2,3979.729730
2,2,4534.681818
2,2,2617.000000
2,2,1787.468023
2,2,2512.520833
2,2,3128.125000
2,2,3569.689189
2,2,3258.975000
2,2,3320.702703
2,2,7694.761364
2,2,3980.143939
2,2,1920.550781
2,2,5096.125000
2,2,2220.800000
2,2,5062.162736
2,2,3460.142857
2,2,3438.358491
2,2,2575.437500
2,2,2216.142857
2,2,2069.566667
2,2,9449.181122
2,2,2713.132143
2,2,1610.932692
2,2,5301.764706
2,2,4110.775000

2,2,2942.541667
2,2,2227.486111
2,2,2266.369048
2,2,2891.314394
2,2,5410.229730
2,2,4538.473262
2,2,2687.632353
2,2,3563.574468
2,2,2364.189189
2,2,2016.312500
2,2,2576.994186
2,2,1861.594595
2,2,2513.969595
2,2,5724.031250
2,2,4248.472826
2,2,1652.343023
2,2,2701.171875
2,2,2882.508380
2,2,3203.527174
2,2,3768.054348
2,2,5479.109756
2,2,2336.340909
2,2,2822.078125
2,2,19089.352273
2,2,3005.250000
2,2,3266.666667
2,2,3242.314024
2,2,1750.256757
2,2,3463.804348
2,2,4260.848039
2,2,1699.894737
2,2,12990.165000
2,2,2663.464286
2,2,8079.384615
2,2,4063.055288
2,2,1641.500000
2,2,1672.956835
2,2,2303.444444
2,2,1522.423780
2,2,2015.895270
2,2,1740.628049
2,2,1608.279070
2,2,5066.851064
2,2,2134.741279
2,2,2241.832447
2,2,3424.021739
2,2,2827.835000
2,2,2555.716667
2,2,1962.641667
2,2,1540.750000
2,2,2633.916667
2,2,2305.088889
2,2,1977.386364
2,2,2369.875000
2,2,17310.382979
2,2,3837.035714
2,2,2497.308824
2,2,5673.167630
2,2,3541.312500
2,2,5029.051948
2,2,1695.991935
2,2,3576.280488
2,2,1998.349057
2,2,4481.416667
2,2,1592.326316
2,2,3241.689655

2,2,1517.922156
2,2,10657.064815
2,2,5004.180095
2,2,2255.005435
2,2,6338.700000
2,2,5500.554054
2,2,3530.335366
2,2,6316.103365
2,2,3282.290698
2,2,2149.935976
2,2,1790.517857
2,2,1736.156250
2,2,4408.937500
2,2,1634.674419
2,2,2960.100000
2,2,2063.916667
2,2,1754.286932
2,2,3360.315625
2,2,4195.075000
2,2,2399.055000
2,2,3803.656977
2,2,1764.532051
2,2,2702.091463
2,2,1979.000000
2,2,2502.035256
2,2,2378.167857
2,2,1883.781250
2,2,4536.823529
2,2,1500.479730
2,2,5096.487745
2,2,3746.128205
2,2,3793.687500
2,2,1883.750000
2,2,2093.295139
2,2,2038.493750
2,2,2571.255435
2,2,14672.156250
2,2,1523.130208
2,2,1633.912736
2,2,2806.210526
2,2,14699.625000
2,2,2710.034091
2,2,7041.426020
2,2,2242.666667
2,2,1890.812500
2,2,19506.559113
2,2,2164.644444
2,2,3176.865385
2,2,6046.592593
2,2,2124.000000
2,2,2494.988636
2,2,5436.584746
2,2,6233.347222
2,2,3267.837766
2,2,2916.301282
2,2,2192.790698
2,2,1714.135135
2,2,6535.779070
2,2,4100.355556
2,2,3123.596591
2,2,3224.750000
2,2,8986.539773
2,2,1735.000000
2,2,2482.813953
2,2,1939.387701
2,2,1515.367742

2,2,1730.033537
2,2,1700.936170
2,2,2386.587500
2,2,3560.877841
2,2,2360.134146
2,2,6510.005814
2,2,5353.558333
2,2,4169.166667
2,2,2183.225490
2,2,5205.865854
2,2,10244.112069
2,2,1610.596875
2,2,4646.944444
2,2,3401.256579
2,2,2466.972973
2,2,5227.264881
2,2,1713.787234
2,2,3084.279412
2,2,2749.347826
2,2,1644.614865
2,2,5109.948529
2,2,11349.372642
2,2,2580.133523
2,2,4328.262500
2,2,7999.875000
2,2,1884.888514
2,2,5299.500000
2,2,3298.234043
2,2,4796.927273
2,2,2762.133333
2,2,2279.864865
2,2,2933.147059
2,2,5928.789062
2,2,2212.837838
2,2,2263.772222
2,2,2871.133333
2,2,1923.428571
2,2,3143.228261
2,2,12914.276316
2,2,4836.104167
2,2,3277.489865
2,2,3189.884615
2,2,1874.644737
2,2,3639.312500
2,2,2644.403846
2,2,2084.741279
2,2,1739.302632
2,2,12481.401042
2,2,3140.700000
2,2,11675.752778
2,2,7356.138393
2,2,1776.437500
2,2,4208.968085
2,2,2273.777174
2,2,2520.609756
2,2,1912.976744
2,2,3220.515625
2,2,2401.542553
2,2,2666.061453
2,2,8888.866667
2,2,3377.757979
2,2,4391.666667
2,2,2034.696429
2,2,1604.026316
2,2,5427.984043
2,2,2596.770270

2,2,3099.842593
2,2,1866.092391
2,2,3510.594059
2,2,8384.292453
2,2,2904.101852
2,2,2429.780000
2,2,2120.368421
2,2,5915.989362
2,2,1781.866667
2,2,1764.467949
2,2,3994.540107
2,2,2709.567251
2,2,2239.399497
2,2,1511.500000
2,2,5167.526882
2,2,1791.539634
2,2,2700.336207
2,2,2249.007353
2,2,2106.733871
2,2,1623.844444
2,2,1853.009146
2,2,1565.458333
2,2,2704.780488
2,2,2055.584615
2,2,3530.602857
2,2,10844.646341
2,2,2822.000000
2,2,22359.798077
2,2,7927.225000
2,2,8957.005556
2,2,5489.750000
2,2,7112.218750
2,2,4079.087805
2,2,3887.627660
2,2,3646.470588
2,2,2137.698718
2,2,5668.344828
2,2,1587.454082
2,2,2386.250000
2,2,3404.136364
2,2,1922.705128
2,2,2411.302326
2,2,1693.784884
2,2,1609.240000
2,2,12388.500000
2,2,11005.750000
2,2,5298.714286
2,2,2051.018868
2,2,2215.500000
2,2,8947.500000
2,2,2448.945122
2,2,2556.356771
2,2,1948.284153
2,2,4172.244898
2,2,1692.920455
2,2,6532.612500
2,2,8437.406915
2,2,1848.142857
2,2,2575.886792
2,2,4716.456522
2,2,1733.895408
2,2,1776.951220
2,2,2043.868750
2,2,1973.600000
2,2,2932.716667
2,2,1568.159091

2,2,3613.250000
2,2,4640.382979
2,2,1730.887755
2,2,3789.315789
2,2,12248.093085
2,2,3404.056962
2,2,2149.161932
2,2,3435.840426
2,2,2178.343750
2,2,2682.600000
2,2,2151.625000
2,2,2698.948864
2,2,1541.027778
2,2,3658.555851
2,2,4605.648352
2,2,5147.546512
2,2,2072.960227
2,2,2149.423469
2,2,11200.982843
2,2,4068.095109
2,2,2262.251572
2,2,4977.083799
2,2,2778.444444
2,2,2395.547794
2,2,5574.108040
2,2,2271.909677
2,2,7771.333333
2,2,2136.275000
2,2,5677.265957
2,2,3229.087766
2,2,5600.870098
2,2,8200.139535
2,2,1590.283537
2,2,3441.910256
2,2,5811.539474
2,2,1805.891304
2,2,7061.041667
2,2,5697.883152
2,2,2002.978261
2,2,1531.815104
2,2,2197.833333
2,2,10018.936047
2,2,4262.625000
2,2,1713.231707
2,2,2263.097826
2,2,3919.362500
2,2,1519.005814
2,2,2811.500000
2,2,3644.038462
2,2,3610.365854
2,2,4513.132075
2,2,2153.239130
2,2,2780.736111
2,2,5721.752717
2,2,2437.241379
2,2,5014.347561
2,2,1504.763587
2,2,6059.443452
2,2,2069.214660
2,2,1623.192982
2,2,1657.621622
2,2,3636.581395
2,2,1654.531250
2,2,2629.500000
2,2,2552.717949
2,2,2390.676923

2,2,2683.573171
2,2,4503.247685
2,2,4320.425926
2,2,2483.324324
2,2,1838.104651
2,2,2123.572674
2,2,3217.261364
2,2,1943.895954
2,2,3124.171196
2,2,1693.734043
2,2,1936.451429
2,2,2438.621711
2,2,2533.322500
2,2,1550.488889
2,2,2166.166667
2,2,2076.128205
2,2,5784.312500
2,2,16848.875000
2,2,4470.000000
2,2,13149.836066
2,2,1883.069149
2,2,2467.750000
2,2,2723.801630
2,2,2858.237500
2,2,9270.186170
2,2,4011.957237
2,2,1841.869186
2,2,3361.909091
2,2,2807.850543
2,2,1810.913669
2,2,2668.341463
2,2,2246.605769
2,2,1665.800000
2,2,6609.296875
2,2,3896.000000
2,2,13218.765957
2,2,1997.821809
2,2,1730.562500
2,2,2987.164634
2,2,3762.250000
2,2,1935.618750
2,2,2253.218750
2,2,1703.258929
2,2,2705.530612
2,2,2517.572368
2,2,1972.730263
2,2,2190.526316
2,2,3843.166667
2,2,5979.861111
2,2,1864.557692
2,2,1991.822086
2,2,2448.196429
2,2,2224.776596
2,2,2296.539683
2,2,5762.250000
2,2,3438.741667
2,2,5749.569712
2,2,1594.338235
2,2,2135.291667
2,2,1948.794811
2,2,6784.583333
2,2,1578.189024
2,2,1555.534091
2,2,4026.808333
2,2,4997.030702
2,2,7029.093023

2,2,3606.945055
2,2,5750.845238
2,2,1520.928977
2,2,7912.846939
2,2,4709.929204
2,2,2099.690000
2,2,8810.285714
2,2,8830.290698
2,2,1637.027174
2,2,2112.130435
2,2,2733.000000
2,2,5450.312500
2,2,1875.244186
2,2,2008.694444
2,2,14628.229592
2,2,8817.290909
2,2,2434.437500
2,2,2088.917614
2,2,1829.456522
2,2,5719.616766
2,2,1847.243902
2,2,5867.084906
2,2,3773.235294
2,2,1836.111111
2,2,3788.595588
2,2,2041.519324
2,2,4198.209184
2,2,4402.875000
2,2,2660.989865
2,2,9866.567568
2,2,3158.707602
2,2,6763.812500
2,2,2159.250000
2,2,3875.192308
2,2,19926.085366
2,2,2293.390000
2,2,2622.478774
2,2,11255.485465
2,2,2416.545455
2,2,6531.257212
2,2,2610.911585
2,2,1682.355978
2,2,4720.510417
2,2,3278.265000
2,2,2761.115741
2,2,2594.049451
2,2,1602.094828
2,2,2763.117021
2,2,2011.988636
2,2,3789.991935
2,2,6332.627451
2,2,6637.150000
2,2,2328.520833
2,2,2584.425000
2,2,2504.209459
2,2,4256.902857
2,2,19446.977273
2,2,1823.287162
2,2,2514.182065
2,2,2704.901961
2,2,1961.714286
2,2,2536.687500
2,2,2979.317073
2,2,1687.942857
2,2,1555.781977
2,2,3050.309211

2,2,1662.100000
2,2,1946.500000
2,2,1916.575000
2,2,1706.209677
2,2,3823.445652
2,2,3826.888889
2,2,1681.153846
2,2,1809.319728
2,2,1935.414474
2,2,2298.892857
2,2,3972.306533
2,2,2716.722826
2,2,2245.802500
2,2,1790.902778
2,2,2884.275000
2,2,5033.526316
2,2,2509.670455
2,2,2183.295000
2,2,6501.646409
2,2,16195.517857
2,2,2484.178082
2,2,2503.400000
2,2,13597.447917
2,2,2034.754808
2,2,3506.741071
2,2,4603.160714
2,2,1780.309524
2,2,2805.797619
2,2,1502.784091
2,2,2018.617647
2,2,2722.533333
2,2,2228.436464
2,2,2052.735849
2,2,1665.251852
2,2,3302.399103
2,2,3323.899457
2,2,5086.151316
2,2,4666.806122
2,2,8648.613426
2,2,16550.916667
2,2,1868.666667
2,2,2397.240437
2,2,10340.072500
2,2,5787.811321
2,2,2426.971354
2,2,1935.785714
2,2,1502.230978
2,2,5156.357500
2,2,3657.884615
2,2,1812.933333
2,2,3522.400621
2,2,1773.270408
2,2,1508.635870
2,2,1879.747093
2,2,17088.231707
2,2,2848.981132
2,2,2548.890449
2,2,5422.063830
2,2,3475.500000
2,2,2634.972826
2,2,2121.866667
2,2,3315.122340
2,2,1727.289216
2,2,6532.542683
2,2,2089.926282
2,2,2826.625000

2,2,2242.698864
2,2,4573.681122
2,2,7780.658537
2,2,2647.503205
2,2,3751.166667
2,2,7078.329861
2,2,5500.810000
2,2,5105.615646
2,2,4740.935961
2,2,2802.505000
2,2,5019.372881
2,2,2997.822917
2,2,1587.791339
2,2,1718.937500
2,2,1691.150943
2,2,7400.113402
2,2,1569.148936
2,2,10668.723404
2,2,8279.323276
2,2,3758.654762
2,2,2515.250000
2,2,1818.437500
2,2,2470.918539
2,2,3409.853659
2,2,8108.294872
2,2,2174.437500
2,2,3380.612245
2,2,2913.053571
2,2,1980.303922
2,2,3485.295213
2,2,1952.000000
2,2,4688.846939
2,2,4266.860465
2,2,2962.071429
2,2,4818.484375
2,2,4757.500000
2,2,3081.267857
2,2,3575.342105
2,2,5553.603352
2,2,4878.652778
2,2,2575.670673
2,2,1885.951389
2,2,3503.053571
2,2,21268.268868
2,2,2553.697674
2,2,4172.862705
2,2,1841.625000
2,2,1958.384615
2,2,2268.445161
2,2,2819.297872
2,2,4875.040909
2,2,1561.272727
2,2,2755.147727
2,2,2881.583333
2,2,5339.836957
2,2,6711.958333
2,2,8791.452514
2,2,2531.968750
2,2,4783.174194
2,2,10265.386364
2,2,10979.920213
2,2,2095.166667
2,2,2451.943005
2,2,4350.923077
2,2,4157.220930
2,2,2038.091837

2,2,4306.874172
2,2,10269.625000
2,2,4514.687500
2,2,3225.634615
2,2,1662.078704
2,2,6178.710526
2,2,4229.584184
2,2,6320.654412
2,2,4335.586111
2,2,1708.966667
2,2,1553.837500
2,2,1752.975610
2,2,3447.000000
2,2,2586.117187
2,2,3081.948718
2,2,1733.250000
2,2,6051.977099
2,2,3702.465278
2,2,4046.473684
2,2,1624.442130
2,2,8796.278846
2,2,1543.386364
2,2,6242.819444
2,2,2005.154839
2,2,2490.334884
2,2,3577.555556
2,2,1576.777778
2,2,5158.099490
2,2,10050.475000
2,2,1538.319149
2,2,3264.886792
2,2,1814.378378
2,2,3118.147059
2,2,1676.805233
2,2,2657.743590
2,2,2726.833333
2,2,1922.252976
2,2,2885.198864
2,2,1667.723404
2,2,2718.790698
2,2,2903.302326
2,2,1687.787234
2,2,5031.303030
2,2,3492.825175
2,2,6279.470833
2,2,14366.371795
2,2,2493.125000
2,2,1702.875000
2,2,5141.235119
2,2,1811.258065
2,2,3253.721154
2,2,3586.407258
2,2,9177.230769
2,2,2238.584184
2,2,11159.792411
2,2,3746.243590
2,2,2470.644737
2,2,2002.390000
2,2,8652.295455
2,2,1824.746951
2,2,3583.685484
2,2,2449.562500
2,2,1712.722222
2,2,4737.643902
2,2,2429.537500
2,2,1657.779605

2,2,4552.437500
2,2,4154.203125
2,2,2460.592391
2,2,3800.296296
2,2,1646.370968
2,2,12622.135922
2,2,5952.105556
2,2,2305.085366
2,2,1660.244186
2,2,4190.247727
2,2,1517.630000
2,2,1593.306452
2,2,2841.760870
2,2,4369.371585
2,2,2703.346734
2,2,2318.289773
2,2,4778.781250
2,2,6962.750000
2,2,4360.178571
2,2,3154.233696
2,2,11892.782609
2,2,3358.060976
2,2,2642.081633
2,2,4331.189024
2,2,1522.118421
2,2,1877.024691
2,2,1974.961111
2,2,2958.320000
2,2,1991.092857
2,2,1937.244186
2,2,2825.226562
2,2,6633.779412
2,2,1629.168421
2,2,2511.959459
2,2,13268.812500
2,2,4551.846354
2,2,6722.750000
2,2,2396.862500
2,2,1736.779891
2,2,7659.263158
2,2,1779.022727
2,2,1691.416667
2,2,1561.892241
2,2,1500.224359
2,2,3617.423780
2,2,1821.426471
2,2,2352.100000
2,2,7324.695531
2,2,7811.500000
2,2,5312.986702
2,2,4480.879828
2,2,3216.828488
2,2,6590.959184
2,2,4212.325000
2,2,2546.774390
2,2,3432.948864
2,2,3314.225490
2,2,3061.142157
2,2,5375.610169
2,2,3988.474490
2,2,1690.269737
2,2,7021.742647
2,2,2530.015625
2,2,3343.336538
2,2,1873.895408
2,2,2632.486979

2,2,1868.292553
2,2,1564.880682
2,2,2049.288889
2,2,1542.593750
2,2,2279.123153
2,2,2446.762500
2,2,4243.478261
2,2,2448.955556
2,2,2741.200000
2,2,4927.512931
2,2,4268.551724
2,2,2267.423077
2,2,2228.828125
2,2,5305.663043
2,2,3139.334559
2,2,1902.035714
2,2,3719.933673
2,2,1705.679348
2,2,2459.788462
2,2,14718.446809
2,2,3554.815000
2,2,1748.342105
2,2,2043.967105
2,2,1664.384615
2,2,5956.653846
2,2,4204.910180
2,2,15962.274112
2,2,3946.209302
2,2,4082.890625
2,2,2449.369444
2,2,2234.306818
2,2,6303.429515
2,2,2244.163934
2,2,2113.401198
2,2,3303.169399
2,2,4719.368932
2,2,2272.875000
2,2,2470.322581
2,2,2358.056548
2,2,2371.769737
2,2,5452.542553
2,2,4933.406250
2,2,2806.829268
2,2,2798.437500
2,2,6119.425926
2,2,4153.700000
2,2,4998.393145
2,2,10101.107500
2,2,6393.333333
2,2,1856.583333
2,2,3652.113208
2,2,7791.390000
2,2,2077.051136
2,2,1682.939286
2,2,5494.301630
2,2,4351.551136
2,2,2345.812500
2,2,4931.452500
2,2,1724.978571
2,2,5526.443452
2,2,1711.621951
2,2,1995.417969
2,2,2320.181818
2,2,3472.336538
2,2,1544.490385
2,2,2471.290094

2,2,10181.963542
2,2,5254.506944
2,2,3159.829787
2,2,4878.453125
2,2,1619.139831
2,2,2504.752841
2,2,11152.225000
2,2,5146.923077
2,2,10002.405000
2,2,3127.664062
2,2,4909.502778
2,2,1516.618902
2,2,3806.423469
2,2,2809.116667
2,2,2349.303922
2,2,4869.542553
2,2,2991.661458
2,2,1580.439189
2,2,2169.257075
2,2,2351.875000
2,2,2924.435714
2,2,6621.918919
2,2,4410.983796
2,2,2751.625668
2,2,1850.268293
2,2,1716.910615
2,2,2578.958333
2,2,4737.650463
2,2,1509.953125
2,2,8079.543478
2,2,1514.625000
2,2,3528.593750
2,2,6583.420213
2,2,2022.106383
2,2,1963.478261
2,2,1599.562500
2,2,3352.780612
2,2,3350.380000
2,2,1656.380319
2,2,1908.400000
2,2,1562.449219
2,2,2479.311364
2,2,4924.650568
2,2,15022.188679
2,2,8537.954545
2,2,4766.385650
2,2,2690.175000
2,2,1964.724138
2,2,3034.744318
2,2,7080.298611
2,2,5654.539216
2,2,5471.280000
2,2,3957.458333
2,2,1590.141447
2,2,7914.479042
2,2,6746.767296
2,2,8335.384434
2,2,4703.000000
2,2,3588.521739
2,2,2594.300000
2,2,8990.793103
2,2,3037.344828
2,2,2294.861111
2,2,1810.817073
2,2,4357.583333
2,2,1824.333333

2,2,2067.372881
2,2,2093.828125
2,2,5492.875000
2,2,2288.250000
2,2,12646.068966
2,2,11959.570312
2,2,8628.831250
2,2,2012.861111
2,2,3988.288265
2,2,4061.000000
2,2,3177.963576
2,2,4242.702703
2,2,2012.604651
2,2,2399.318841
2,2,2572.544118
2,2,6220.010989
2,2,3406.483871
2,2,3887.922460
2,2,3685.764205
2,2,4608.325581
2,2,5427.560976
2,2,2062.276596
2,2,3740.007813
2,2,3979.268182
2,2,3777.277174
2,2,2196.333333
2,2,4597.407407
2,2,2884.580556
2,2,3094.245098
2,2,2077.014706
2,2,1755.931548
2,2,4272.496795
2,2,5199.768182
2,2,2862.141304
2,2,2788.686170
2,2,3395.812500
2,2,6824.000000
2,2,2161.098361
2,2,4838.818627
2,2,2468.066667
2,2,7817.371560
2,2,4161.763298
2,2,2005.102564
2,2,2873.919811
2,2,4259.641935
2,2,2042.458333
2,2,1989.282895
2,2,1501.062842
2,2,5093.000000
2,2,8267.055046
2,2,1537.707317
2,2,1688.533333
2,2,6227.560345
2,2,1554.970109
2,2,5195.669643
2,2,6456.650000
2,2,5694.984694
2,2,2474.827500
2,2,6642.302885
2,2,1557.488372
2,2,1519.246875
2,2,4402.500000
2,2,1785.638889
2,2,5505.613475
2,2,3059.570000
2,2,3413.052632

2,2,2188.052632
2,2,1889.524038
2,2,3258.776316
2,2,3221.962264
2,2,2887.875000
2,2,14500.812500
2,2,3018.909722
2,2,1612.093750
2,2,3282.097561
2,2,2577.941176
2,2,3336.088235
2,2,1925.566038
2,2,5178.203911
2,2,2126.581522
2,2,5192.173246
2,2,1501.550802
2,2,1918.289773
2,2,1990.935897
2,2,3314.641026
2,2,7358.606250
2,2,1880.835366
2,2,4058.222222
2,2,2825.500000
2,2,1587.581395
2,2,4842.846591
2,2,1508.230769
2,2,2411.485849
2,2,1769.179054
2,2,4441.896552
2,2,2160.961538
2,2,5257.346939
2,2,1831.694444
2,2,8673.863636
2,2,1583.144737
2,2,3539.545455
2,2,2255.631818
2,2,2421.841346
2,2,1827.151163
2,2,4673.398810
2,2,2768.979651
2,2,6711.172222
2,2,3867.054878
2,2,2494.491848
2,2,2530.805556
2,2,5575.024038
2,2,3791.040000
2,2,3527.286585
2,2,1854.500000
2,2,8998.666667
2,2,4899.163889
2,2,1872.628049
2,2,4117.976064
2,2,4030.818182
2,2,6804.179104
2,2,2568.000000
2,2,2472.750000
2,2,2178.979167
2,2,3504.776596
2,2,1682.812500
2,2,3135.437500
2,2,1551.043367
2,2,1523.840116
2,2,1810.015625
2,2,2310.201923
2,2,1792.936709
2,2,4418.237500

2,2,1840.815000
2,2,2446.803125
2,2,4421.062500
2,2,3228.459302
2,2,6008.228659
2,2,4136.744186
2,2,1607.046196
2,2,2251.533333
2,2,2095.271277
2,2,6836.165746
2,2,1954.483051
2,2,2556.815029
2,2,3286.821429
2,2,1579.848684
2,2,2858.902235
2,2,1602.390625
2,2,9614.668981
2,2,7326.563830
2,2,1751.000000
2,2,5199.752976
2,2,3496.500000
2,2,1549.200000
2,2,1581.678571
2,2,2440.472222
2,2,3359.116071
2,2,3440.666667
2,2,14210.268293
2,2,3145.470588
2,2,3171.441860
2,2,8777.183673
2,2,8560.750000
2,2,2483.347826
2,2,1877.540816
2,2,3761.148936
2,2,3695.625000
2,2,1527.927500
2,2,2447.558140
2,2,8735.333333
2,2,3181.605769
2,2,6238.500000
2,2,2276.353659
2,2,1845.305147
2,2,1798.065789
2,2,2819.293103
2,2,1706.474265
2,2,3237.296196
2,2,1586.778090
2,2,3565.991935
2,2,3939.660000
2,2,1734.054795
2,2,2237.796875
2,2,11034.187500
2,2,7195.895735
2,2,1938.906977
2,2,5705.625000
2,2,1983.000000
2,2,1792.480769
2,2,7458.175439
2,2,4519.540625
2,2,1570.017857
2,2,2524.427778
2,2,2974.832558
2,2,3781.880435
2,2,4699.350490
2,2,1887.978571
2,2,4679.646875

2,2,2856.069832
2,2,2065.308824
2,2,5680.930000
2,2,7241.535714
2,2,3559.305851
2,2,3313.664474
2,2,1796.365854
2,2,2963.031250
2,2,1631.921053
2,2,4930.045455
2,2,3649.357143
2,2,2422.489583
2,2,1526.145833
2,2,2837.688636
2,2,2039.708333
2,2,4156.760870
2,2,3379.052632
2,2,3672.079787
2,2,7324.316017
2,2,2339.137931
2,2,2135.917683
2,2,8505.039352
2,2,6922.069149
2,2,3157.514151
2,2,2290.307692
2,2,3864.275000
2,2,6657.142857
2,2,9508.430233
2,2,5569.340625
2,2,3754.167598
2,2,2655.496795
2,2,3311.724490
2,2,1941.250000
2,2,7405.000000
2,2,3755.065789
2,2,3154.500000
2,2,14297.352564
2,2,2201.835366
2,2,1715.693878
2,2,5941.525000
2,2,2991.619718
2,2,3686.131313
2,2,3030.357143
2,2,3465.117021
2,2,4750.310000
2,2,5017.903061
2,2,4040.154255
2,2,3321.983173
2,2,3243.019022
2,2,2139.192308
2,2,3630.245370
2,2,2952.500000
2,2,7083.212042
2,2,8847.000000
2,2,1651.403974
2,2,5631.983974
2,2,7201.625000
2,2,3166.162791
2,2,8127.643216
2,2,8962.138298
2,2,4677.591837
2,2,14406.188679
2,2,2143.800000
2,2,5462.852459
2,2,1963.142857
2,2,3461.746667

2,2,3253.485465
2,2,3305.583333
2,2,3528.575581
2,2,3112.779605
2,2,5174.040816
2,2,2382.173077
2,2,2180.247340
2,2,8302.736515
2,2,8670.960000
2,2,10710.139706
2,2,2297.950000
2,2,3771.463415
2,2,2066.416667
2,2,4610.672500
2,2,4209.324675
2,2,3047.576923
2,2,1817.477941
2,2,2503.750000
2,2,14169.785714
2,2,4275.945313
2,2,5116.713068
2,2,8073.913462
2,2,4602.857143
2,2,1529.587500
2,2,1792.031250
2,2,2222.081395
2,2,5425.000000
2,2,3185.328261
2,2,1764.276786
2,2,6269.414634
2,2,6410.346591
2,2,2088.794872
2,2,2524.864865
2,2,4288.211538
2,2,3794.666667
2,2,10594.887019
2,2,3182.721154
2,2,4849.850000
2,2,2655.484043
2,2,3656.210227
2,2,2592.464674
2,2,1624.988372
2,2,2606.225000
2,2,1657.831081
2,2,9820.835648
2,2,1559.000000
2,2,2675.089744
2,2,6703.339744
2,2,1622.337748
2,2,2103.593750
2,2,1689.846154
2,2,3035.500000
2,2,4214.023810
2,2,1928.505952
2,2,2245.105263
2,2,1597.632353
2,2,1683.023256
2,2,7182.333333
2,2,6872.656250
2,2,2816.968750
2,2,2138.635417
2,2,5406.459559
2,2,6029.048780
2,2,17574.400000
2,2,3287.461749
2,2,2256.191489

2,2,2443.334906
2,2,6666.687500
2,2,2026.554974
2,2,1528.273256
2,2,6182.895270
2,2,1724.358491
2,2,4384.960526
2,2,2060.467949
2,2,4987.539773
2,2,1611.586207
2,2,6573.609865
2,2,2553.010050
2,2,6369.967213
2,2,5276.538012
2,2,2754.073171
2,2,3043.974194
2,2,5755.653846
2,2,1877.496815
2,2,2906.609375
2,2,1980.368056
2,2,2437.200483
2,2,3709.767045
2,2,3893.729167
2,2,3533.439024
2,2,2289.981250
2,2,1896.857558
2,2,2356.579268
2,2,19014.132353
2,2,2713.868902
2,2,3674.625000
2,2,1824.146341
2,2,16151.148148
2,2,2934.826816
2,2,7115.778409
2,2,4677.164474
2,2,2755.593750
2,2,2329.630435
2,2,3300.952830
2,2,3044.984043
2,2,10529.129032
2,2,2224.994186
2,2,7503.571429
2,2,7882.419643
2,2,13682.714286
2,2,1722.861486
2,2,1614.277778
2,2,1722.600694
2,2,8694.268750
2,2,2759.156863
2,2,3424.132979
2,2,1621.269939
2,2,2360.072917
2,2,2525.347826
2,2,2199.750000
2,2,1833.828947
2,2,1914.307692
2,2,1608.000000
2,2,2621.661111
2,2,6630.795455
2,2,3136.877193
2,2,2067.341935
2,2,2302.238372
2,2,1991.333333
2,2,7111.040698
2,2,3317.229730
2,2,4086.755000

2,2,6803.143750
2,2,3487.973118
2,2,1755.600000
2,2,2474.711538
2,2,2329.519231
2,2,2595.677019
2,2,1914.256944
2,2,9048.919355
2,2,5136.242188
2,2,7677.675000
2,2,5039.384615
2,2,3201.906250
2,2,2237.871875
2,2,2439.605263
2,2,1571.529412
2,2,2591.674419
2,2,7058.071429
2,2,8103.732500
2,2,4552.549342
2,2,1908.877660
2,2,2185.441489
2,2,4806.549107
2,2,1825.666667
2,2,2503.026163
2,2,2655.685897
2,2,4102.588068
2,2,2155.562500
2,2,6205.654494
2,2,4218.052632
2,2,2294.139535
2,2,1967.897059
2,2,2558.895089
2,2,2706.552632
2,2,2748.691860
2,2,4959.682018
2,2,3286.156627
2,2,8064.764940
2,2,2354.260638
2,2,2183.541985
2,2,2029.490385
2,2,1903.707317
2,2,16906.564767
2,2,4942.391304
2,2,2242.218023
2,2,3721.817130
2,2,2304.093750
2,2,3763.657143
2,2,3509.869565
2,2,2624.556291
2,2,3678.757576
2,2,3809.065476
2,2,2366.307432
2,2,3108.394886
2,2,1685.658291
2,2,3570.591463
2,2,7535.250000
2,2,6766.331633
2,2,1687.375000
2,2,3883.350000
2,2,2957.687500
2,2,2445.333333
2,2,11004.031250
2,2,3428.000000
2,2,2276.700000
2,2,2120.396277
2,2,7972.179825

2,2,1624.381944
2,2,1849.000000
2,2,5428.928125
2,2,7743.130435
2,2,15345.790000
2,2,2048.192073
2,2,4805.335664
2,2,3798.948370
2,2,5141.229651
2,2,2085.759259
2,2,6978.412162
2,2,10052.843137
2,2,2766.296053
2,2,2813.311047
2,2,1837.737805
2,2,1603.255556
2,2,2275.500000
2,2,1580.175000
2,2,2114.946429
2,2,1833.977273
2,2,5810.996795
2,2,2892.677885
2,2,3444.875000
2,2,3461.110465
2,2,4416.573171
2,2,2454.227273
2,2,8035.703125
2,2,1513.211538
2,2,7298.091216
2,2,6257.690217
2,2,2733.892157
2,2,5946.264706
2,2,10343.032258
2,2,3995.058824
2,2,2522.981250
2,2,2491.225694
2,2,3217.702703
2,2,93743.870968
2,2,2801.380319
2,2,2932.818182
2,2,1599.004717
2,2,3008.918367
2,2,2820.575758
2,2,2125.627027
2,2,3420.300481
2,2,3916.689655
2,2,1954.952500
2,2,4049.299419
2,2,4355.073529
2,2,5232.582418
2,2,1855.830189
2,2,1778.967391
2,2,4373.425824
2,2,2222.076923
2,2,4384.819149
2,2,3668.303125
2,2,1962.835366
2,2,2313.123762
2,2,1658.408867
2,2,8745.495575
2,2,2017.333333
2,2,2345.595588
2,2,8488.167553
2,2,2753.857143
2,2,3194.584270
2,2,2626.773438

2,2,1726.682836
2,2,9347.836538
2,2,2146.944149
2,2,2090.640625
2,2,1952.362832
2,2,2443.135870
2,2,2373.600000
2,2,3220.627907
2,2,1708.714286
2,2,10656.875000
2,2,1734.512821
2,2,2742.967320
2,2,1731.062500
2,2,7187.625000
2,2,4244.774038
2,2,1941.331522
2,2,11909.657895
2,2,1952.160714
2,2,3848.705189
2,2,4358.115566
2,2,3579.566667
2,2,4176.801630
2,2,36233.307692
2,2,3356.022222
2,2,7194.407186
2,2,1834.179104
2,2,2640.096354
2,2,21369.237981
2,2,1900.500000
2,2,2766.090426
2,2,3861.339286
2,2,6555.194444
2,2,4973.760000
2,2,5640.534314
2,2,1619.021429
2,2,6108.846774
2,2,2126.750000
2,2,14526.273438
2,2,14980.894472
2,2,2195.601227
2,2,1848.292683
2,2,4739.167453
2,2,7765.285714
2,2,1594.200581
2,2,1886.791667
2,2,3743.050439
2,2,2796.055556
2,2,5745.588235
2,2,1531.153061
2,2,5193.818182
2,2,2862.822368
2,2,4759.137255
2,2,5733.005208
2,2,6938.914894
2,2,2850.803571
2,2,4716.318182
2,2,4836.908654
2,2,2035.612500
2,2,1939.186170
2,2,2358.643243
2,2,6772.138393
2,2,2303.012255
2,2,2613.569892
2,2,2202.936170
2,2,3375.091346
2,2,3656.993750

2,2,3366.798913
2,2,3231.985465
2,2,8429.883721
2,2,3591.426136
2,2,5830.257353
2,2,1795.184211
2,2,3169.184375
2,2,1603.583333
2,2,2939.031250
2,2,1513.270833
2,2,8509.177083
2,2,7221.900000
2,2,2348.720930
2,2,1657.955729
2,2,5328.516667
2,2,2186.906250
2,2,2484.051282
2,2,5423.188776
2,2,6652.238095
2,2,3688.694444
2,2,3366.882653
2,2,5535.639205
2,2,6587.040201
2,2,2900.222222
2,2,4239.388554
2,2,2751.625000
2,2,5028.287736
2,2,5128.890995
2,2,3580.387755
2,2,6106.000000
2,2,2739.421053
2,2,18075.923077
2,2,3422.666667
2,2,2145.409836
2,2,1741.506098
2,2,1667.425532
2,2,3380.195652
2,2,14116.035714
2,2,3287.413889
2,2,3480.269737
2,2,2174.508621
2,2,2063.375000
2,2,1601.562500
2,2,4451.097561
2,2,2042.358491
2,2,4160.830189
2,2,2956.000000
2,2,3074.297872
2,2,2371.875000
2,2,2478.435714
2,2,2047.587838
2,2,1788.275000
2,2,2858.431250
2,2,3293.722222
2,2,2064.218750
2,2,2650.118243
2,2,3510.540323
2,2,3107.755556
2,2,2504.250000
2,2,2586.000000
2,2,2051.782609
2,2,4030.400000
2,2,2306.731250
2,2,1587.057692
2,2,7626.857143
2,2,2333.038265

2,2,3175.328125
2,2,2901.381250
2,2,2207.417763
2,2,2998.115385
2,2,1587.166667
2,2,6953.277778
2,2,3004.288462
2,2,7099.057692
2,2,2844.970588
2,2,4178.130682
2,2,30217.675000
2,2,4303.400000
2,2,4114.056250
2,2,2233.784574
2,2,5577.104651
2,2,5861.290948
2,2,9865.781977
2,2,1607.733696
2,2,8323.649457
2,2,3970.750000
2,2,2353.803571
2,2,5189.855556
2,2,4990.552083
2,2,5551.312500
2,2,3231.278846
2,2,1898.515625
2,2,4212.092105
2,2,3012.233333
2,2,2982.361702
2,2,6072.787234
2,2,3392.189024
2,2,3629.093750
2,2,4813.346698
2,2,1519.718750
2,2,2046.390625
2,2,2058.213235
2,2,6339.298295
2,2,2097.710000
2,2,2357.018293
2,2,3897.297619
2,2,1786.934028
2,2,1584.959677
2,2,5033.074468
2,2,6488.867925
2,2,3447.243590
2,2,1984.460526
2,2,1664.036765
2,2,3292.553191
2,2,1898.637500
2,2,1634.472727
2,2,7018.274390
2,2,3201.235000
2,2,5174.875000
2,2,1536.500000
2,2,1989.636792
2,2,10367.300000
2,2,5661.250000
2,2,5218.200000
2,2,7812.555556
2,2,1911.326087
2,2,1833.950581
2,2,1772.878049
2,2,2171.011905
2,2,4454.806452
2,2,12526.266667
2,2,2112.818182

2,2,2637.447368
2,2,3678.534722
2,2,5509.632353
2,2,3032.135135
2,2,2274.473684
2,2,2279.176136
2,2,3506.122642
2,2,1684.443878
2,2,1687.854167
2,2,2208.915625
2,2,9989.736486
2,2,2843.328125
2,2,1816.431548
2,2,2152.095395
2,2,1831.779605
2,2,4437.943069
2,2,2070.959184
2,2,1655.000000
2,2,2062.454545
2,2,1635.225806
2,2,3991.840426
2,2,1983.882653
2,2,1687.456633
2,2,3169.787500
2,2,1599.365854
2,2,1640.125000
2,2,5734.796875
2,2,8033.571121
2,2,3674.985294
2,2,2205.633721
2,2,1512.666667
2,2,5961.230769
2,2,1998.759868
2,2,6111.812500
2,2,1579.333333
2,2,1619.582888
2,2,2925.671875
2,2,1576.732143
2,2,21060.791667
2,2,5971.413462
2,2,5049.902778
2,2,2623.337209
2,2,10698.025000
2,2,7239.584135
2,2,3631.000000
2,2,2428.416667
2,2,1791.447368
2,2,3176.681818
2,2,1725.928977
2,2,1639.467391
2,2,1636.036585
2,2,14151.235577
2,2,1693.887195
2,2,2549.433140
2,2,2428.859756
2,2,6736.095930
2,2,2141.181818
2,2,5525.574519
2,2,1957.759091
2,2,5915.436275
2,2,3776.072500
2,2,4719.555233
2,2,3230.616667
2,2,1937.619048
2,2,2435.065217
2,2,3553.715000

2,2,1845.777174
2,2,1740.950000
2,2,12660.225000
2,2,6856.962264
2,2,10117.388601
2,2,9933.700893
2,2,7654.778378
2,2,3660.135922
2,2,4028.677596
2,2,1723.213287
2,2,2313.338129
2,2,2153.619318
2,2,1843.398649
2,2,2225.545455
2,2,2488.080556
2,2,2971.760000
2,2,2394.078125
2,2,1612.230114
2,2,1862.228261
2,2,1953.015625
2,2,2504.456522
2,2,3511.767442
2,2,1738.516304
2,2,2194.777778
2,2,2303.427632
2,2,3278.553191
2,2,2082.540541
2,2,4615.844828
2,2,1648.614286
2,2,6000.188953
2,2,1663.000000
2,2,1879.306373
2,2,1817.325000
2,2,9645.200000
2,2,2749.094595
2,2,2175.258294
2,2,3082.235000
2,2,4551.310976
2,2,4427.847973
2,2,1653.470930
2,2,20918.911765
2,2,6905.027778
2,2,4122.625000
2,2,3601.134615
2,2,2912.105556
2,2,3539.852941
2,2,4190.965517
2,2,2082.899457
2,2,4904.583333
2,2,5010.127660
2,2,7245.906250
2,2,4751.769608
2,2,2511.470588
2,2,1933.440789
2,2,5973.675497
2,2,2374.512500
2,2,1759.583333
2,2,7706.810185
2,2,2046.718085
2,2,3253.954082
2,2,2235.258278
2,2,6431.298246
2,2,3289.104651
2,2,6505.264423
2,2,11939.214286
2,2,1948.326705

2,2,2161.621711
2,2,5904.559748
2,2,17905.025140
2,2,3104.679012
2,2,2998.596774
2,2,5661.666667
2,2,1837.494565
2,2,2689.437500
2,2,2614.038889
2,2,2014.920455
2,2,1585.948980
2,2,1746.853659
2,2,3034.531915
2,2,15972.937500
2,2,7965.991453
2,2,1726.666667
2,2,2595.111842
2,2,2752.927885
2,2,2350.250000
2,2,2463.743421
2,2,7057.317500
2,2,1796.600000
2,2,3384.839109
2,2,1716.085366
2,2,1976.066489
2,2,6083.488152
2,2,1590.875000
2,2,9924.430556
2,2,5767.986979
2,2,3714.534884
2,2,7254.178571
2,2,6011.169492
2,2,5401.716346
2,2,3439.520833
2,2,5735.068182
2,2,3459.835938
2,2,2427.673469
2,2,3989.784091
2,2,2522.694767
2,2,4337.307432
2,2,3275.835106
2,2,1936.023026
2,2,3459.742188
2,2,4420.397959
2,2,4989.813953
2,2,2043.762500
2,2,6686.161184
2,2,28897.958333
2,2,1725.361842
2,2,2191.776042
2,2,2689.530488
2,2,2076.520000
2,2,3029.803571
2,2,2721.390625
2,2,1881.988439
2,2,1824.500000
2,2,2129.283871
2,2,4466.343590
2,2,1700.558140
2,2,4998.363636
2,2,1720.783784
2,2,3221.096059
2,2,6272.612565
2,2,5958.000000
2,2,2939.061111
2,2,1609.634615

2,2,2020.754545
2,2,3829.112500
2,2,4619.461538
2,2,1698.051471
2,2,1550.395683
2,2,5857.814978
2,2,3290.084302
2,2,6628.880952
2,2,3037.829327
2,2,2029.894040
2,2,2906.825000
2,2,1785.141892
2,2,2075.711538
2,2,2192.039474
2,2,2906.666667
2,2,2066.660377
2,2,3388.807692
2,2,9022.197222
2,2,6815.425676
2,2,4636.323529
2,2,2294.536932
2,2,3413.666667
2,2,2819.390244
2,2,4999.148936
2,2,12111.714286
2,2,2169.666667
2,2,1987.105263
2,2,4397.090000
2,2,1983.921569
2,2,2725.296053
2,2,3759.815217
2,2,1571.931818
2,2,2018.789474
2,2,5269.333333
2,2,2686.058140
2,2,1511.107692
2,2,4028.300000
2,2,1616.333333
2,2,2258.188679
2,2,3351.903846
2,2,3857.000000
2,2,9703.500000
2,2,1580.005682
2,2,12030.890351
2,2,2797.472362
2,2,7887.053476
2,2,1946.051163
2,2,1607.036458
2,2,2647.780000
2,2,5242.292553
2,2,4471.076531
2,2,1618.154762
2,2,2670.218750
2,2,5410.154930
2,2,37993.739130
2,2,4744.433962
2,2,2077.394231
2,2,2444.187192
2,2,3186.415625
2,2,2641.632353
2,2,2595.755102
2,2,8697.388393
2,2,5619.821429
2,2,4707.544271
2,2,10308.929245
2,2,3670.733108

2,2,2172.437126
2,2,2302.460227
2,2,1515.986301
2,2,4387.794286
2,2,1772.136364
2,2,1632.206395
2,2,5294.666667
2,2,1644.420673
2,2,3211.178571
2,2,1843.480000
2,2,1524.234043
2,2,5494.579741
2,2,10151.795290
2,2,1702.736842
2,2,14037.068421
2,2,3682.660494
2,2,4835.852941
2,2,5862.080000
2,2,4317.731707
2,2,12849.684426
2,2,2497.302326
2,2,1791.592262
2,2,4207.871951
2,2,2325.400510
2,2,3735.923913
2,2,5962.158163
2,2,2569.329167
2,2,2544.747283
2,2,2935.197727
2,2,2839.194444
2,2,11269.150000
2,2,17531.385714
2,2,8694.054348
2,2,1578.628205
2,2,3851.994536
2,2,1499.152174
2,2,4080.463415
2,2,3024.964286
2,2,33540.950000
2,2,7552.732558
2,2,1542.923077
2,2,2616.367299
2,2,1985.420530
2,2,2173.453488
2,2,3425.928571
2,2,5896.025510
2,2,3246.693750
2,2,2107.980000
2,2,9432.833333
2,2,1543.968750
2,2,4139.407407
2,2,2343.050000
2,2,2028.091837
2,2,2897.869565
2,2,3817.333333
2,2,1761.955729
2,2,2242.296196
2,2,11342.600000
2,2,4564.077093
2,2,6559.282609
2,2,12283.875000
2,2,6842.463415
2,2,2952.114130
2,2,2060.391304
2,2,5994.421525
2,2,2898.661765

2,2,2680.542553
2,2,3918.931818
2,2,7967.686235
2,2,1840.387755
2,2,2017.810256
2,2,3356.825658
2,2,1963.437500
2,2,2099.976331
2,2,2051.200581
2,2,1900.250000
2,2,1875.856383
2,2,1657.622500
2,2,4923.203804
2,2,1565.476440
2,2,5855.189944
2,2,2288.304348
2,2,2108.072115
2,2,2559.755814
2,2,3740.439103
2,2,2004.644737
2,2,1527.750000
2,2,3078.827381
2,2,3422.125000
2,2,1833.503125
2,2,1982.648438
2,2,4276.097222
2,2,1759.837209
2,2,3687.000000
2,2,3069.102564
2,2,2310.758621
2,2,2195.593750
2,2,10664.845982
2,2,2099.334586
2,2,14724.546703
2,2,3025.558252
2,2,4483.000000
2,2,1646.000000
2,2,3793.687500
2,2,1636.813953
2,2,2831.734597
2,2,4258.766447
2,2,5161.754545
2,2,10776.812834
2,2,5550.176471
2,2,3386.000000
2,2,24813.818653
2,2,3545.158416
2,2,2881.709459
2,2,2280.909091
2,2,5663.595745
2,2,2068.824324
2,2,2927.787500
2,2,6746.189655
2,2,7356.367347
2,2,3800.278409
2,2,3801.069767
2,2,1980.316327
2,2,6192.773585
2,2,2715.970000
2,2,2046.382812
2,2,1924.670455
2,2,1688.812500
2,2,3059.665541
2,2,3570.536765
2,2,9592.944444
2,2,5769.663043

2,2,1499.212766
2,2,3180.205128
2,2,2539.735795
2,2,4687.437500
2,2,1908.436111
2,2,6060.706383
2,2,5807.358333
2,2,7291.852500
2,2,4397.861111
2,2,1993.583333
2,2,1712.897959
2,2,1523.673913
2,2,3123.328125
2,2,2312.665816
2,2,2204.321429
2,2,6242.973214
2,2,5807.370192
2,2,2968.150000
2,2,5839.476293
2,2,3589.574850
2,2,4122.584475
2,2,2024.617021
2,2,6601.785000
2,2,3082.812500
2,2,20355.723214
2,2,2462.966463
2,2,2225.558824
2,2,6160.500000
2,2,1576.918919
2,2,23005.372549
2,2,2500.045455
2,2,1549.927673
2,2,2163.564103
2,2,1767.261364
2,2,7828.165909
2,2,1724.156977
2,2,2327.261628
2,2,3487.685897
2,2,2812.963942
2,2,3084.246622
2,2,1886.306122
2,2,3102.706522
2,2,1740.027174
2,2,4183.078804
2,2,2230.484043
2,2,3410.516304
2,2,3045.553571
2,2,1994.395833
2,2,2259.500000
2,2,15041.049180
2,2,3117.039062
2,2,3861.294643
2,2,12871.255208
2,2,2051.037791
2,2,5416.829082
2,2,2240.750000
2,2,6288.825658
2,2,1690.522727
2,2,4077.763158
2,2,2125.000000
2,2,1606.000000
2,2,2672.791667
2,2,1947.063953
2,2,2454.806452
2,2,2187.906250
2,2,3220.585106

2,2,6062.479167
2,2,3734.565217
2,2,5705.151163
2,2,3251.636364
2,2,3732.636364
2,2,3102.857143
2,2,2604.477778
2,2,2495.804348
2,2,2440.585106
2,2,2474.088816
2,2,4988.719388
2,2,3911.113426
2,2,4065.477528
2,2,3120.725000
2,2,3748.925532
2,2,12291.314286
2,2,1936.114362
2,2,4365.275000
2,2,3503.780612
2,2,4463.062500
2,2,3258.976048
2,2,3776.214286
2,2,3037.829787
2,2,4646.511628
2,2,2166.789474
2,2,2937.739011
2,2,1532.128049
2,2,2413.632143
2,2,3224.416667
2,2,24810.209677
2,2,1770.950000
2,2,1778.758929
2,2,2247.239583
2,2,3125.707317
2,2,33256.717949
2,2,11841.552273
2,2,2573.600000
2,2,2775.768156
2,2,2000.928571
2,2,2330.125000
2,2,1980.088889
2,2,2002.962766
2,2,6417.597561
2,2,4451.957627
2,2,8156.909091
2,2,1908.599359
2,2,3367.786765
2,2,5349.693548
2,2,1829.160000
2,2,8527.379717
2,2,1751.333333
2,2,2825.045872
2,2,5299.000000
2,2,23223.530612
2,2,2231.500000
2,2,1737.750000
2,2,2371.239130
2,2,8716.675481
2,2,6702.722222
2,2,4001.659091
2,2,6022.275000
2,2,3917.594241
2,2,1642.600000
2,2,2564.845109
2,2,8397.380319
2,2,1791.177419

2,2,2002.360294
2,2,9650.930952
2,2,2034.201923
2,2,3155.835227
2,2,4760.173469
2,2,12324.704082
2,2,1602.546053
2,2,2744.922727
2,2,5612.269231
2,2,9382.168367
2,2,2790.173913
2,2,8039.406977
2,2,7089.937500
2,2,2513.857143
2,2,2920.565789
2,2,3453.361111
2,2,8576.262931
2,2,1617.187500
2,2,2628.555556
2,2,4042.982759
2,2,2219.831081
2,2,4372.431818
2,2,5593.709135
2,2,3388.062500
2,2,3386.803030
2,2,2884.235294
2,2,1936.500000
2,2,2315.195312
2,2,2341.710106
2,2,6191.040816
2,2,3419.953125
2,2,48246.500000
2,2,5188.809211
2,2,2614.953488
2,2,4248.105263
2,2,2264.210526
2,2,3038.855263
2,2,2315.603448
2,2,1965.708609
2,2,4796.704545
2,2,2582.763889
2,2,4332.620087
2,2,2418.843750
2,2,1836.386243
2,2,2635.281250
2,2,2104.170455
2,2,1601.715116
2,2,1514.506757
2,2,6985.963415
2,2,2801.250000
2,2,2034.602941
2,2,4668.830556
2,2,2081.519231
2,2,2365.700000
2,2,1860.714286
2,2,3321.230769
2,2,7001.946429
2,2,1684.794271
2,2,4093.070064
2,2,1658.358974
2,2,1675.261765
2,2,4399.840000
2,2,2370.615385
2,2,6216.530172
2,2,3426.023256
2,2,1990.682353

2,2,5059.607143
2,2,3949.325301
2,2,1835.739130
2,2,3057.962500
2,2,2840.398268
2,2,12796.428571
2,2,4177.252907
2,2,3199.402174
2,2,2391.585227
2,2,7524.553571
2,2,9790.127451
2,2,2474.600000
2,2,9994.343373
2,2,3160.519886
2,2,7305.000000
2,2,1499.464674
2,2,3280.291667
2,2,4012.102273
2,2,2567.423469
2,2,2561.923077
2,2,2196.951087
2,2,1862.819079
2,2,3164.420765
2,2,3801.571429
2,2,2298.689655
2,2,2151.789286
2,2,1597.145833
2,2,5162.556818
2,2,1540.724359
2,2,5049.646429
2,2,5481.908654
2,2,1518.947368
2,2,6977.267544
2,2,9937.219512
2,2,9467.310096
2,2,4085.905000
2,2,3903.070652
2,2,4088.100962
2,2,2737.125000
2,2,2055.807692
2,2,6511.023649
2,2,2200.190476
2,2,1628.442935
2,2,1504.932927
2,2,3546.760870
2,2,2037.596154
2,2,3440.895833
2,2,6307.453488
2,2,1771.952500
2,2,1519.853659
2,2,3999.125000
2,2,4398.988372
2,2,1975.978610
2,2,5233.956522
2,2,1815.961538
2,2,1860.934783
2,2,2919.898148
2,2,2056.093750
2,2,32444.519022
2,2,1657.878049
2,2,3951.812500
2,2,17966.301370
2,2,3474.305556
2,2,3589.625000
2,2,7441.968750
2,2,3683.408854

2,2,2184.857143
2,2,2287.279605
2,2,3426.250000
2,2,5153.651961
2,2,1521.456250
2,2,2073.942857
2,2,2386.108911
2,2,1735.687097
2,2,1777.039634
2,2,3008.609756
2,2,2022.956522
2,2,1774.163462
2,2,1939.167683
2,2,8296.440341
2,2,2051.604651
2,2,1524.153571
2,2,2981.812500
2,2,2384.869444
2,2,5883.687500
2,2,2815.409091
2,2,1900.190476
2,2,1858.875000
2,2,2740.635417
2,2,11749.922170
2,2,4373.041667
2,2,1701.012500
2,2,2874.821429
2,2,2309.640625
2,2,5758.500000
2,2,5547.330645
2,2,3532.798658
2,2,1593.433333
2,2,7454.950000
2,2,1743.903846
2,2,3095.884615
2,2,1790.422500
2,2,3481.210526
2,2,2762.625000
2,2,5960.727545
2,2,5017.089744
2,2,5726.411765
2,2,2499.845238
2,2,1617.531250
2,2,2095.596154
2,2,1782.197674
2,2,2815.877143
2,2,2321.812821
2,2,7827.865385
2,2,3086.000000
2,2,1583.764706
2,2,1879.300000
2,2,2048.052632
2,2,2071.588068
2,2,2525.756757
2,2,3236.928571
2,2,5284.573864
2,2,2028.679070
2,2,5811.937500
2,2,4014.440789
2,2,2354.485294
2,2,2550.363208
2,2,1942.324324
2,2,4731.250000
2,2,16157.025510
2,2,1701.312500
2,2,4909.766990

2,2,2784.614943
2,2,2821.711538
2,2,30144.435583
2,2,2981.375000
2,2,1546.723214
2,2,3949.118280
2,2,1972.813131
2,2,1656.000000
2,2,5689.666667
2,2,4190.360000
2,2,1842.558140
2,2,1836.422414
2,2,2399.144444
2,2,5236.444444
2,2,1544.243902
2,2,4387.943750
2,2,3296.351190
2,2,9791.040761
2,2,4493.021739
2,2,1760.583333
2,2,8260.333333
2,2,2033.136628
2,2,6845.719340
2,2,7647.650000
2,2,4277.025000
2,2,1965.020833
2,2,1655.704787
2,2,3103.578947
2,2,1703.107143
2,2,2985.096774
2,2,3005.965116
2,2,4450.659524
2,2,3913.267773
2,2,3260.068720
2,2,1864.574324
2,2,6761.579545
2,2,2352.809783
2,2,5086.729651
2,2,2757.437500
2,2,3460.762500
2,2,1751.406977
2,2,1711.840278
2,2,3228.636364
2,2,1889.992647
2,2,1557.142857
2,2,10626.394231
2,2,2737.676724
2,2,3301.756696
2,2,6205.775510
2,2,1624.513158
2,2,2257.541667
2,2,3951.944079
2,2,1876.512500
2,2,2033.334559
2,2,6300.692308
2,2,12414.944444
2,2,1725.231707
2,2,7577.452778
2,2,3440.826923
2,2,7542.953704
2,2,1971.703704
2,2,2707.500000
2,2,1923.711538
2,2,7283.688889
2,2,2336.511628
2,2,1524.378049

2,2,9375.224490
2,2,11459.488636
2,2,14729.804348
2,2,1521.401130
2,2,3135.060606
2,2,5034.887931
2,2,10910.088235
2,2,1644.950000
2,2,1899.672794
2,2,2146.161585
2,2,3169.440625
2,2,3892.920732
2,2,1995.708543
2,2,4742.225610
2,2,2029.072500
2,2,2603.254386
2,2,2396.885135
2,2,1615.969849
2,2,4188.167665
2,2,1803.434375
2,2,3071.771277
2,2,6591.296774
2,2,3781.269231
2,2,4982.663462
2,2,3462.000000
2,2,1538.783537
2,2,1627.358696
2,2,3532.409091
2,2,8041.536458
2,2,1594.880597
2,2,4816.719340
2,2,2425.413043
2,2,2915.186047
2,2,2605.968750
2,2,1767.203947
2,2,1623.230978
2,2,5814.095238
2,2,3193.095588
2,2,4649.739130
2,2,1795.700000
2,2,3836.175481
2,2,3912.500000
2,2,13394.276316
2,2,3238.756098
2,2,5241.118902
2,2,8255.456250
2,2,1999.426829
2,2,2693.528634
2,2,1848.901042
2,2,1815.842857
2,2,2309.778646
2,2,1614.496644
2,2,2340.598404
2,2,1934.412500
2,2,9970.701087
2,2,4067.071875
2,2,2583.832386
2,2,16008.683824
2,2,2270.918605
2,2,6331.652500
2,2,2912.517442
2,2,7902.829787
2,2,5647.853365
2,2,3620.010000
2,2,7148.666667
2,2,1828.277778

2,2,2582.103352
2,2,2711.009615
2,2,4777.562500
2,2,8290.875000
2,2,1724.362069
2,2,1705.093750
2,2,5108.507812
2,2,2817.743750
2,2,1660.862745
2,2,15693.500000
2,2,2332.411111
2,2,1631.914773
2,2,11466.953571
2,2,2063.177083
2,2,1913.535211
2,2,2730.860465
2,2,3014.405000
2,2,7590.880435
2,2,3654.673077
2,2,2353.959677
2,2,2142.405063
2,2,2141.333333
2,2,3233.507853
2,2,1784.622500
2,2,1590.777439
3,1,14164.562500
3,1,4000.576087
3,1,39280.637195
3,1,3647.697368
3,1,2194.910180
3,1,5292.042553
3,1,1575.931548
3,1,188886.902439
3,1,2924.375000
3,1,3411.515152
3,1,2645.848958
3,1,2849.555556
3,1,6091.934524
3,1,7776.769737
3,1,4559.935484
3,1,3252.085366
3,1,2494.989362
3,1,1642.886364
3,1,1768.928571
3,1,5426.741071
3,1,8573.176020
3,1,8797.698529
3,1,3234.852679
3,1,2405.863971
3,1,12678.215278
3,1,6651.091667
3,1,1640.110294
3,1,7506.406250
3,1,2657.755952
3,1,1897.170213
3,1,6736.470588
3,1,5518.014706
3,1,4778.600000
3,1,3173.176471
3,1,8329.178571
3,1,1607.431818
3,1,3043.913462
3,1,1997.230114
3,1,3762.766129
3,1,7209.269531
3,1,170015.375000

3,1,170078.554878
3,1,3415.900000
3,1,1561.414773
3,1,1517.666667
3,1,16598.000000
3,1,4158.041667
3,1,5492.141509
3,1,4683.370968
3,1,54933.648649
3,1,2841.941176
3,1,3236.500000
3,1,1548.948718
3,1,2678.069444
3,1,2395.942308
3,1,3607.400000
3,1,21966.171875
3,1,10795.272727
3,1,3912.952128
3,1,9362.841346
3,1,2572.370536
3,1,1715.996795
3,1,186548.903846
3,1,4334.138889
3,1,27753.584459
3,1,4846.585526
3,1,1543.925000
3,1,3234.775000
3,1,2817.140244
3,1,3963.920455
3,1,69219.750000
3,1,3551.883929
3,1,1641.192308
3,1,6297.705882
3,1,6682.617021
3,1,3466.979167
3,1,3369.487245
3,1,1722.500000
3,1,2124.143229
3,1,10046.776515
3,1,3927.900943
3,1,3505.625000
3,1,1745.840909
3,1,1927.445312
3,1,10717.607143
3,1,1887.598837
3,1,2918.414634
3,1,9203.443396
3,1,25648.440476
3,1,1578.692308
3,1,1704.796196
3,1,4523.645455
3,1,1677.156250
3,1,2929.603448
3,1,2802.054054
3,1,8213.880814
3,1,2448.152439
3,1,1878.434659
3,1,1833.750000
3,1,3665.375000
3,1,14618.950000
3,1,4112.923077
3,1,5790.139706
3,1,161799.826923
3,1,60742.874251
3,1,7499.984925
3,1,6760.250000

3,1,3237.557692
3,1,2914.625000
3,1,3844.130208
3,1,2019.429348
3,1,2080.401042
3,1,1861.815341
3,1,30868.303125
3,1,4749.404412
3,1,62071.068966
3,1,16639.135417
3,1,2301.092105
3,1,3096.475000
3,1,1828.000000
3,1,2042.296875
3,1,1610.250000
3,1,2258.727273
3,1,2126.333333
3,1,79063.731250
3,1,4376.739130
3,1,6274.428571
3,1,1911.219512
3,1,3862.992647
3,1,4486.560606
3,1,20439.033784
3,1,1625.309524
3,1,6871.372283
3,1,5698.346591
3,1,1587.452206
3,1,3032.255682
3,1,2487.509804
3,1,2228.275000
3,1,2570.334906
3,1,6818.162791
3,1,11522.750000
3,1,12962.136364
3,1,3675.297794
3,1,4426.884146
3,1,11159.321429
3,1,2753.878378
3,1,1927.675000
3,1,9710.743590
3,1,12039.000000
3,1,2154.187500
3,1,4394.500000
3,1,2650.962500
3,1,1537.000000
3,1,1607.450000
3,1,6194.000000
3,1,2044.595588
3,1,2588.500000
3,1,5542.046875
3,1,1543.145000
3,1,3110.863636
3,1,1740.306250
3,1,2031.926829
3,1,43808.821429
3,1,3448.009259
3,1,2211.125000
3,1,4023.244898
3,1,2288.622222
3,1,5778.709459
3,1,2133.836957
3,1,2134.280488
3,1,1866.257143
3,1,48344.534884
3,1,1830.333333

3,1,22940.905405
3,1,9680.209459
3,1,2098.723214
3,1,3659.598485
3,1,6264.566667
3,1,4862.844340
3,1,2463.566667
3,1,2644.134868
3,1,2428.281250
3,1,12012.677835
3,1,2089.089286
3,1,2927.647727
3,1,6415.099138
3,1,2847.953297
3,1,1724.505556
3,1,36315.045732
3,1,75351.990625
3,1,3100.775000
3,1,3764.641304
3,1,10917.066327
3,1,2662.441176
3,1,1677.988095
3,1,4631.370098
3,1,2769.487805
3,1,5626.455696
3,1,2756.710938
3,1,1991.910326
3,1,2678.701923
3,1,1524.600000
3,1,2005.269231
3,1,1547.946023
3,1,25941.340909
3,1,1696.914634
3,1,1701.913462
3,1,5069.782609
3,1,3161.671053
3,1,2341.613636
3,1,2360.551630
3,1,5205.000000
3,1,2507.533784
3,1,1551.039474
3,1,13832.000000
3,1,3198.333333
3,1,4116.666667
3,1,2460.221154
3,1,2290.448980
3,1,4973.933140
3,1,10760.378049
3,1,11659.011364
3,1,2326.325581
3,1,4034.072115
3,1,3188.833333
3,1,2964.125000
3,1,4162.446809
3,1,1617.900524
3,1,12956.502242
3,1,2083.620690
3,1,2420.394737
3,1,1643.899281
3,1,1666.576389
3,1,1588.890909
3,1,3009.378676
3,1,3568.827778
3,1,4264.625000
3,1,1616.562500
3,1,1848.882812

3,1,7367.083333
3,1,14052.936047
3,1,3297.348958
3,1,1926.340278
3,1,2000.550000
3,1,2397.916667
3,1,21208.500000
3,1,3149.091837
3,1,2169.525000
3,1,4143.683673
3,1,1531.458333
3,1,11068.717593
3,1,2589.666667
3,1,2501.106383
3,1,9019.187500
3,1,3327.452500
3,1,2318.389535
3,1,5947.725610
3,1,11519.778646
3,1,3530.736842
3,1,2216.699468
3,1,14407.000000
3,1,5751.895833
3,1,4296.000000
3,1,25024.770833
3,1,3430.776515
3,1,6518.485000
3,1,2394.238095
3,1,2030.384615
3,1,4177.964286
3,1,3328.392045
3,1,19077.934783
3,1,7425.893750
3,1,5185.886364
3,1,1647.515625
3,1,6025.621429
3,1,2059.102564
3,1,168308.866071
3,1,1573.926630
3,1,4086.852941
3,1,14384.927632
3,1,3528.352273
3,1,10765.344595
3,1,2914.017857
3,1,3449.513021
3,1,1757.571429
3,1,4350.615385
3,1,5619.625000
3,1,1802.735099
3,1,1925.046512
3,1,2550.244318
3,1,3350.474490
3,1,2231.619792
3,1,3273.285000
3,1,3233.478261
3,1,6232.921429
3,1,2625.042553
3,1,10893.815476
3,1,2052.395349
3,1,2637.798611
3,1,3130.923077
3,1,20695.640244
3,1,2559.269231
3,1,5103.717277
3,1,2216.613636
3,1,2379.916667

3,1,2887.571429
3,1,3467.800926
3,1,7050.904545
3,1,17089.025943
3,1,7224.206522
3,1,2896.222222
3,1,4706.781250
3,1,4114.333333
3,1,27591.000000
3,1,5152.000000
3,1,1630.524306
3,1,19783.546512
3,1,1830.452381
3,1,2386.488372
3,1,6301.814815
3,1,7342.601852
3,1,3962.722826
3,1,1971.012500
3,1,2293.942308
3,1,2824.268750
3,1,3242.468750
3,1,14085.243056
3,1,1792.048780
3,1,16189.788462
3,1,3018.145833
3,1,1529.348837
3,1,4058.875000
3,1,4359.103723
3,1,1712.661111
3,1,3067.230469
3,1,10194.666667
3,1,2958.933824
3,1,7783.864865
3,1,164389.140625
3,1,2733.100000
3,1,14465.812500
3,1,3859.231061
3,1,1984.664634
3,1,1861.552083
3,1,6471.095000
3,1,1519.077778
3,1,1775.956522
3,1,2736.427326
3,1,2364.628676
3,1,1703.750000
3,1,2662.666667
3,1,67197.318966
3,1,2284.725000
3,1,5551.500000
3,1,2800.066667
3,1,4604.880208
3,1,6980.531250
3,1,1926.357143
3,1,1639.093750
3,1,37330.510204
3,1,5616.309659
3,1,4021.389535
3,1,3539.951923
3,1,1764.807143
3,1,1881.550000
3,1,1926.570312
3,1,1739.834239
3,1,4838.203431
3,1,7264.202128
3,1,5318.916667
3,1,2481.975000

3,1,2973.739286
3,1,11407.569149
3,1,2463.425000
3,1,5176.094595
3,1,4883.500000
3,1,1885.848214
3,1,3966.262255
3,1,2918.670455
3,1,3050.867647
3,1,2681.520833
3,1,22309.709302
3,1,7636.818182
3,1,5790.309659
3,1,1627.848214
3,1,3819.717391
3,1,1666.786765
3,1,23697.722656
3,1,6311.483696
3,1,1872.016304
3,1,2337.603571
3,1,64709.845000
3,1,3755.418103
3,1,2579.209302
3,1,2097.937500
3,1,3753.557927
3,1,2153.208333
3,1,120442.631410
3,1,1893.269737
3,1,2044.285714
3,1,91648.590909
3,1,101935.300000
3,1,3312.930168
3,1,13796.981707
3,1,1826.000000
3,1,2211.875000
3,1,2319.090426
3,1,1749.384211
3,1,5465.375000
3,1,2309.658537
3,1,2247.978261
3,1,3220.800000
3,1,3313.017857
3,1,3345.812500
3,1,1744.602941
3,1,96544.064286
3,1,1779.305000
3,1,4193.259434
3,1,1653.903409
3,1,2439.659722
3,1,28672.418605
3,1,2952.340000
3,1,1528.445946
3,1,1763.142857
3,1,1690.296512
3,1,2964.200000
3,1,3408.033784
3,1,2808.687500
3,1,1532.875000
3,1,4655.713415
3,1,4134.211111
3,1,2576.387755
3,1,7734.063889
3,1,8006.610119
3,1,2708.285714
3,1,8076.250000
3,1,4382.242188

3,1,3682.338235
3,1,3754.275735
3,1,1952.562500
3,1,15905.375000
3,1,10661.350000
3,1,4177.506757
3,1,8888.728261
3,1,2355.351190
3,1,3348.243243
3,1,3071.461310
3,1,2760.567073
3,1,7152.522321
3,1,11270.846591
3,1,24266.674658
3,1,4503.256757
3,1,2049.375000
3,1,2062.154412
3,1,1708.420213
3,1,8779.575000
3,1,1527.036364
3,1,4645.272222
3,1,3014.657258
3,1,11871.812500
3,1,5545.704225
3,1,3194.406250
3,1,1693.983108
3,1,2161.477273
3,1,6090.430233
3,1,1650.494186
3,1,16493.645833
3,1,2568.852273
3,1,2480.108696
3,1,6078.921053
3,1,2172.566176
3,1,16628.484375
3,1,1749.416667
3,1,1738.013514
3,1,15979.804878
3,1,2494.353125
3,1,8186.781250
3,1,5775.410377
3,1,4300.455128
3,1,3293.710938
3,1,2447.381944
3,1,2882.487179
3,1,90220.802632
3,1,1665.125000
3,1,2782.812500
3,1,7055.257812
3,1,1598.156250
3,1,2736.510638
3,1,6785.256757
3,1,1652.087209
3,1,2116.544643
3,1,2421.953488
3,1,4617.654070
3,1,2049.625000
3,1,7699.972222
3,1,10566.392857
3,1,4139.656250
3,1,1899.530612
3,1,19558.977500
3,1,5067.200000
3,1,8301.035714
3,1,2936.127778
3,1,2377.875000

3,1,8719.165441
3,1,2778.983333
3,1,3975.750000
3,1,2765.533333
3,1,3055.422222
3,1,2362.684211
3,1,100164.062500
3,1,2599.689189
3,1,4741.078125
3,1,3562.068182
3,1,18133.007812
3,1,3934.404762
3,1,3349.636905
3,1,6969.279605
3,1,2480.657051
3,1,4258.154255
3,1,1524.875000
3,1,3574.303191
3,1,4154.452381
3,1,5588.735000
3,1,4008.986111
3,1,7186.856132
3,1,1540.410256
3,1,102009.720109
3,1,5530.111111
3,1,2998.677632
3,1,2324.828947
3,1,5900.875000
3,1,2052.128378
3,1,5897.104839
3,1,1557.127660
3,1,15758.754310
3,1,1857.532895
3,1,2186.611111
3,1,4047.666667
3,1,2718.604167
3,1,61233.528846
3,1,1591.403846
3,1,4291.589674
3,1,1590.479167
3,1,1647.964286
3,1,1735.519022
3,1,10485.884615
3,1,5641.989865
3,1,2489.548913
3,1,3548.636364
3,1,1848.565217
3,1,1995.312500
3,1,1532.285714
3,1,11774.570833
3,1,7898.852500
3,1,5308.602041
3,1,2131.666667
3,1,2946.435096
3,1,7631.769231
3,1,12208.875000
3,1,17334.050000
3,1,3651.234043
3,1,2754.079787
3,1,1929.964286
3,1,1539.520833
3,1,1668.424419
3,1,6725.518293
3,1,6606.695000
3,1,12466.153226
3,1,5763.817568

3,1,4222.087500
3,1,3238.031250
3,1,54725.333333
3,1,2589.468750
3,1,9536.195652
3,1,2123.271739
3,1,2040.651163
3,1,5843.978261
3,1,1800.026316
3,1,7635.707500
3,1,2603.587838
3,1,1988.098837
3,1,23990.687500
3,1,3968.869565
3,1,4960.250000
3,1,8087.644531
3,1,4112.107955
3,1,57410.384146
3,1,1807.707547
3,1,1658.450000
3,1,3620.678571
3,1,3453.022727
3,1,1854.622159
3,1,2366.385870
3,1,20453.822917
3,1,2255.761905
3,1,2611.008929
3,1,1853.851190
3,1,1920.701220
3,1,2103.784091
3,1,2077.468750
3,1,2692.342105
3,1,1854.906250
3,1,33171.901316
3,1,1771.858696
3,1,3234.956522
3,1,19757.583333
3,1,1657.402778
3,1,3118.485714
3,1,7294.219512
3,1,1726.275641
3,1,103955.836806
3,1,2724.428571
3,1,3655.406915
3,1,22407.166667
3,1,4581.290541
3,1,5627.896341
3,1,2265.021739
3,1,5386.606132
3,1,2024.006944
3,1,1792.250000
3,1,2153.706522
3,1,62374.230769
3,1,3173.414773
3,1,2876.966216
3,1,16094.454787
3,1,7093.090909
3,1,1957.200000
3,1,4076.500000
3,1,3858.477564
3,1,3762.867347
3,1,3587.145833
3,1,22771.552632
3,1,1555.000000
3,1,3796.541667
3,1,6898.012500

3,1,1648.005952
3,1,6417.263158
3,1,1975.256410
3,1,2595.697581
3,1,7768.628205
3,1,4690.553191
3,1,4169.769231
3,1,1516.408333
3,1,2775.387097
3,1,3994.583333
3,1,2436.168605
3,1,1592.973684
3,1,144212.983108
3,1,3941.274390
3,1,3847.630952
3,1,35304.377358
3,1,5852.833333
3,1,2016.411184
3,1,3440.784722
3,1,2613.746875
3,1,1781.000000
3,1,2845.706587
3,1,2577.520833
3,1,1724.729651
3,1,1556.125000
3,1,5302.277344
3,1,41345.625000
3,1,2569.003125
3,1,4292.518293
3,1,3781.913043
3,1,4002.975610
3,1,2271.546429
3,1,2174.478261
3,1,7903.886029
3,1,2439.000000
3,1,1828.542683
3,1,2076.280000
3,1,8114.500000
3,1,16108.127778
3,1,1650.400000
3,1,2229.896875
3,1,2073.341463
3,1,1801.142857
3,1,1652.257576
3,1,1641.978261
3,1,3538.890625
3,1,6216.871951
3,1,2176.771739
3,1,1560.444444
3,1,3340.176630
3,1,8675.395833
3,1,1785.645349
3,1,26747.312500
3,1,3224.842391
3,1,16538.189189
3,1,12779.857143
3,1,2852.333333
3,1,7403.536765
3,1,1573.962209
3,1,3733.500000
3,1,6611.208333
3,1,4024.500000
3,1,2599.644444
3,1,5601.392045
3,1,11220.558511
3,1,1960.750000

3,1,2148.108333
3,1,3255.500000
3,1,12558.134615
3,1,1627.377907
3,1,2107.668605
3,1,63848.164634
3,1,3251.549342
3,1,2548.424419
3,1,2194.121875
3,1,3377.732955
3,1,2437.609756
3,1,4848.142857
3,1,2754.494048
3,1,1928.909091
3,1,1672.631944
3,1,3178.467742
3,1,10050.575000
3,1,2494.462500
3,1,10109.425781
3,1,2947.891304
3,1,3383.857143
3,1,1885.057927
3,1,5466.062500
3,1,6683.328125
3,1,4634.383333
3,1,2566.151515
3,1,1768.857143
3,1,1523.500000
3,1,1547.232558
3,1,14831.112245
3,1,32999.691406
3,1,11172.537234
3,1,30929.676471
3,1,2185.358974
3,1,6046.048780
3,1,14848.143939
3,1,18259.265957
3,1,11822.111111
3,1,1516.692308
3,1,2874.621094
3,1,25440.690789
3,1,3120.168605
3,1,8033.651596
3,1,9181.416667
3,1,3395.000000
3,1,16046.324324
3,1,2385.227273
3,1,2185.848837
3,1,6725.852273
3,1,7418.833333
3,1,3003.750000
3,1,1625.837209
3,1,10192.828125
3,1,7106.714286
3,1,162228.306818
3,1,1746.890244
3,1,1735.871795
3,1,12680.291667
3,1,2588.294118
3,1,4407.925000
3,1,2521.000000
3,1,3225.539062
3,1,2675.503906
3,1,1602.188953
3,1,1830.208333
3,1,2372.018382

3,1,2674.482759
3,1,1543.154762
3,1,1781.750000
3,1,1880.690972
3,1,12024.414474
3,1,3102.889423
3,1,18099.564103
3,1,3551.432692
3,1,2830.176471
3,1,2069.183140
3,1,3949.676136
3,1,25535.293919
3,1,4481.676471
3,1,1704.868421
3,1,2609.166667
3,1,122760.833333
3,1,129414.687500
3,1,1707.731707
3,1,21610.756757
3,1,8958.833333
3,1,2645.011364
3,1,1949.939024
3,1,2125.500000
3,1,1777.718750
3,1,8863.695513
3,1,1532.242857
3,1,24223.536585
3,1,89089.417614
3,1,57344.409722
3,1,2845.241935
3,1,16201.000000
3,1,1659.040323
3,1,5627.109375
3,1,1987.500000
3,1,15863.448529
3,1,15387.974265
3,1,1598.571429
3,1,2749.833333
3,1,7657.610577
3,1,1568.982955
3,1,3731.861111
3,1,17652.386029
3,1,2870.389706
3,1,1572.256579
3,1,113251.068493
3,1,6226.678571
3,1,3699.087766
3,1,45021.348684
3,1,2662.505435
3,1,2202.263889
3,1,1592.281977
3,1,7623.837838
3,1,110909.597561
3,1,11786.500000
3,1,2612.609375
3,1,4266.741071
3,1,6769.750000
3,1,1609.858974
3,1,36799.937500
3,1,83091.000000
3,1,1762.359375
3,1,1537.220930
3,1,1971.902439
3,1,2671.398438
3,1,2065.290541
3,1,3072.151163

3,1,2575.481707
3,1,5819.306250
3,1,2026.500000
3,1,2487.047619
3,1,7793.735294
3,1,8584.657258
3,1,2183.473684
3,1,2753.550000
3,1,3614.222222
3,1,2344.239796
3,1,1643.395349
3,1,11325.948529
3,1,6862.860465
3,1,1692.693750
3,1,5861.666667
3,1,5060.217742
3,1,3523.250000
3,1,3412.235294
3,1,1981.000000
3,1,2136.357143
3,1,9418.967742
3,1,5347.927419
3,1,2305.617647
3,1,1517.425000
3,1,1892.200000
3,1,2597.645833
3,1,62534.467105
3,1,19442.954545
3,1,7246.857143
3,1,4746.211538
3,1,2253.562500
3,1,2564.847561
3,1,2975.031250
3,1,2601.226744
3,1,5894.723485
3,1,1556.198171
3,1,1695.382353
3,1,2692.330189
3,1,8369.663462
3,1,1541.000000
3,1,7412.448718
3,1,4184.166667
3,1,1956.191667
3,1,2100.020833
3,1,5773.109756
3,1,3002.890625
3,1,1951.864362
3,1,4239.869318
3,1,3310.776042
3,1,3078.598684
3,1,4103.552326
3,1,1571.244792
3,1,1915.375000
3,1,4205.278571
3,1,1546.267857
3,1,1593.973684
3,1,2113.462838
3,1,68463.319149
3,1,3098.182292
3,1,2181.147727
3,1,2384.702381
3,1,7561.312500
3,1,2860.812500
3,1,2446.781250
3,1,2130.214286
3,1,1839.222222

3,1,2985.277228
3,1,1555.621622
3,1,1784.543605
3,1,12839.785714
3,1,1505.266667
3,1,6181.145833
3,1,2070.865385
3,1,1642.202128
3,1,2982.000000
3,1,4153.198864
3,1,4362.648438
3,1,3066.482955
3,1,2778.625000
3,1,1675.500000
3,1,2470.583333
3,1,3522.750000
3,1,2769.171875
3,1,8305.858974
3,1,17283.266304
3,1,2201.384615
3,1,2255.445652
3,1,20385.833333
3,1,22013.062500
3,1,23102.565789
3,1,18937.054054
3,1,2097.509375
3,1,2061.821429
3,1,1769.619318
3,1,22206.515625
3,1,3270.215116
3,1,78576.024390
3,1,1518.228723
3,1,1604.883929
3,1,2681.110119
3,1,23536.078947
3,1,3904.260870
3,1,3597.234043
3,1,1892.675676
3,1,2690.548780
3,1,1574.350000
3,1,2223.288043
3,1,3523.714286
3,1,2272.774194
3,1,2010.533333
3,1,1640.930851
3,1,3882.707317
3,1,2818.600000
3,1,86880.310976
3,1,7483.368750
3,1,18407.353261
3,1,2448.319767
3,1,1842.057692
3,1,22057.891892
3,1,2806.469697
3,1,32676.578947
3,1,2162.586207
3,1,3788.558824
3,1,93656.554054
3,1,6414.926829
3,1,90560.645833
3,1,1607.180851
3,1,14088.384146
3,1,1878.527778
3,1,21368.182065
3,1,1529.829268
3,1,2217.525000

3,1,3693.203125
3,1,12127.279255
3,1,14345.542683
3,1,2004.569767
3,1,6653.979167
3,1,5272.325000
3,1,8678.674419
3,1,2858.600000
3,1,2878.440476
3,1,9703.279762
3,1,19279.750000
3,1,1755.977941
3,1,2591.315217
3,1,2441.544872
3,1,2248.231250
3,1,32152.470238
3,1,2875.562500
3,1,4626.335938
3,1,18976.431818
3,1,8997.057692
3,1,12126.558824
3,1,2716.121212
3,1,1653.706250
3,1,1927.426471
3,1,4570.065625
3,1,7071.375000
3,1,13506.697917
3,1,2778.934783
3,1,2401.812500
3,1,1555.148026
3,1,2012.845238
3,1,4448.375000
3,1,3150.188830
3,1,1632.838235
3,1,1793.916667
3,1,1865.125000
3,1,2470.425532
3,1,2588.500000
3,1,1778.766447
3,1,2534.018382
3,1,1595.285714
3,1,2482.051471
3,1,3898.587500
3,1,2350.537500
3,1,2922.159091
3,1,3465.064024
3,1,1575.941176
3,1,4883.268293
3,1,1648.580357
3,1,17124.385714
3,1,15358.857143
3,1,2468.693878
3,1,4014.034314
3,1,1509.044355
3,1,4223.468750
3,1,83971.875000
3,1,3947.943182
3,1,2292.283088
3,1,2867.750000
3,1,2980.258065
3,1,3781.587209
3,1,1761.738372
3,1,11424.861842
3,1,6835.750000
3,1,2817.073529
3,1,2601.000000

3,1,16039.389535
3,1,2182.666667
3,1,1887.187500
3,1,6576.067500
3,1,3044.086111
3,1,4256.445313
3,1,3258.884146
3,1,1681.279070
3,1,2225.436170
3,1,2311.689189
3,1,1637.288194
3,1,26734.012195
3,1,2759.583333
3,1,1788.714286
3,1,3814.391026
3,1,2206.847826
3,1,102889.812500
3,1,4100.970000
3,1,18223.009868
3,1,17436.311111
3,1,1503.812500
3,1,4374.141892
3,1,7597.923077
3,1,8480.191860
3,1,4265.934211
3,1,3560.151163
3,1,5331.125000
3,1,4717.562500
3,1,16300.952830
3,1,2147.328125
3,1,5025.977273
3,1,9182.494898
3,1,30758.058511
3,1,9406.103261
3,1,26507.931034
3,1,15767.061224
3,1,2985.704545
3,1,2640.075000
3,1,3285.387500
3,1,5094.321429
3,1,3326.994048
3,1,3286.497500
3,1,6583.078125
3,1,9262.149390
3,1,5150.348837
3,1,3598.782609
3,1,178610.327869
3,1,5760.557927
3,1,11571.449405
3,1,2568.370098
3,1,45480.786458
3,1,2217.666667
3,1,2629.695122
3,1,1948.536184
3,1,6743.270408
3,1,25906.316406
3,1,4009.622340
3,1,6648.928191
3,1,4126.044872
3,1,22907.488889
3,1,3773.423469
3,1,2187.127660
3,1,6562.558333
3,1,9401.043269
3,1,14307.062500
3,1,2703.619048

3,1,1743.542553
3,1,1508.757576
3,1,2583.783333
3,1,1634.740196
3,1,1632.105114
3,1,2408.333333
3,1,8725.991279
3,1,2255.089844
3,1,2554.010417
3,1,11156.062500
3,1,3796.809659
3,1,4701.000000
3,1,3777.619792
3,1,2359.788043
3,1,2606.166667
3,1,25091.415094
3,1,3530.322368
3,1,2115.740196
3,1,1937.956395
3,1,3590.153409
3,1,3035.732143
3,1,1986.193717
3,1,4544.346354
3,1,7274.414634
3,1,2646.456250
3,1,14330.814516
3,1,7972.497024
3,1,8033.857843
3,1,5470.268293
3,1,4572.813830
3,1,2643.680851
3,1,1546.340426
3,1,3590.955357
3,1,8756.883929
3,1,3053.680556
3,1,4083.222561
3,1,14495.419118
3,1,1780.807692
3,1,1992.750000
3,1,2089.148649
3,1,2129.981818
3,1,1748.437500
3,1,2731.664286
3,1,8147.589286
3,1,2725.674419
3,1,2246.537500
3,1,3347.941176
3,1,1937.170732
3,1,2833.858333
3,1,8719.398256
3,1,3189.088068
3,1,7000.315476
3,1,3200.219512
3,1,6577.825000
3,1,1608.434211
3,1,6212.194767
3,1,2904.741071
3,1,3309.080882
3,1,1744.530612
3,1,34565.991071
3,1,2418.479592
3,1,8023.817308
3,1,3535.117925
3,1,24134.500000
3,1,67629.804878
3,1,2893.925532

3,1,2845.557927
3,1,5408.638158
3,1,22410.104167
3,1,7977.638889
3,1,3246.901163
3,1,3764.718750
3,1,6250.022727
3,1,1555.551829
3,1,5613.179487
3,1,2970.791667
3,1,3924.257812
3,1,2539.058594
3,1,4664.121951
3,1,2602.753086
3,1,4335.050000
3,1,43739.000000
3,1,15160.595238
3,1,1566.631757
3,1,3668.913462
3,1,1599.247283
3,1,2530.076923
3,1,5139.595238
3,1,1633.403846
3,1,55710.600610
3,1,1559.764151
3,1,2294.000000
3,1,2686.881720
3,1,4485.142857
3,1,5574.302083
3,1,2197.705128
3,1,1757.338235
3,1,1919.107955
3,1,5406.772727
3,1,1784.926471
3,1,4107.608696
3,1,7691.887195
3,1,10009.682927
3,1,6299.312500
3,1,5082.217391
3,1,3184.062500
3,1,5040.225694
3,1,1700.300532
3,1,2639.938679
3,1,2035.971591
3,1,3294.183824
3,1,1755.695652
3,1,5458.200000
3,1,4214.678191
3,1,3611.940217
3,1,20689.228571
3,1,2288.296875
3,1,9530.726190
3,1,1813.086538
3,1,3056.265625
3,1,2222.850610
3,1,3358.765625
3,1,8190.300532
3,1,11314.462185
3,1,1686.878788
3,1,2896.750000
3,1,4435.222826
3,1,6384.203947
3,1,56574.216216
3,1,1700.296296
3,1,3963.113636
3,1,2122.903646

3,1,7752.333333
3,1,21742.169872
3,1,3199.000000
3,1,1876.380319
3,1,1719.105114
3,1,1686.649390
3,1,2075.662162
3,1,1730.866667
3,1,4534.105769
3,1,1546.877358
3,1,23269.859375
3,1,2305.366279
3,1,5758.006098
3,1,4645.679245
3,1,2233.795213
3,1,3048.360294
3,1,1549.726744
3,1,2768.255814
3,1,1947.213415
3,1,2781.645349
3,1,3169.936275
3,1,1923.470238
3,1,1932.510638
3,1,6877.204545
3,1,2515.913462
3,1,1926.575758
3,1,2478.238372
3,1,1680.657895
3,1,2535.611111
3,1,3833.071429
3,1,5096.503676
3,1,2579.366667
3,1,5843.146552
3,1,5050.428571
3,1,4890.294872
3,1,3381.625000
3,1,3890.210526
3,1,20829.798780
3,1,2544.968750
3,1,2639.772222
3,1,5964.428571
3,1,7273.456522
3,1,2217.007353
3,1,9270.053571
3,1,81814.346939
3,1,12246.596591
3,1,7184.743750
3,1,2294.674419
3,1,2220.149457
3,1,3682.628049
3,1,4108.350543
3,1,2870.670000
3,1,2894.608696
3,1,1973.647059
3,1,1722.637500
3,1,1530.597826
3,1,2724.261029
3,1,4989.101852
3,1,15920.456522
3,1,2661.443750
3,1,9369.065789
3,1,22759.014151
3,1,1731.794643
3,1,3704.142857
3,1,1587.420732
3,1,2391.266892

3,1,2170.656250
3,1,18746.366667
3,1,2952.825000
3,1,2017.487500
3,1,3945.581250
3,1,7439.588235
3,1,8017.458333
3,1,5568.000000
3,1,4702.035714
3,1,1876.590116
3,1,1546.007979
3,1,6801.490909
3,1,12140.785714
3,1,2149.411585
3,1,6546.278846
3,1,2668.346354
3,1,6132.775862
3,1,2121.536184
3,1,1551.184211
3,1,5398.046875
3,1,15945.257353
3,1,4286.636364
3,1,2414.533333
3,1,5051.412791
3,1,10696.181818
3,1,1881.930851
3,1,1602.602941
3,1,12543.260870
3,1,2957.461538
3,1,29769.696429
3,1,2454.635135
3,1,3177.857143
3,1,32196.443452
3,1,2451.885204
3,1,1506.693878
3,1,60825.333333
3,1,4846.548913
3,1,2410.720109
3,1,5164.678977
3,1,10541.494186
3,1,2370.437500
3,1,1719.428571
3,1,2298.548780
3,1,1530.931250
3,1,17131.479167
3,1,1986.388889
3,1,6142.157609
3,1,2671.590000
3,1,1728.403846
3,1,2778.306818
3,1,2670.100000
3,1,4952.187500
3,1,6484.582317
3,1,21376.113372
3,1,2868.150000
3,1,2011.044118
3,1,2418.598214
3,1,4363.473214
3,1,4418.131757
3,1,7964.820513
3,1,2494.113636
3,1,35939.750000
3,1,13522.743056
3,1,2093.573171
3,1,25435.750000
3,1,2795.000000

3,1,103893.208333
3,1,1692.726744
3,1,2079.297619
3,1,5403.059524
3,1,19022.638158
3,1,9037.385870
3,1,1562.594595
3,1,3015.500000
3,1,16200.735294
3,1,3546.837838
3,1,3504.488971
3,1,23953.584302
3,1,1888.178571
3,1,1900.231707
3,1,2854.779070
3,1,2816.200000
3,1,2378.875000
3,1,77400.027027
3,1,13167.605000
3,1,2668.000000
3,1,1965.920455
3,1,4089.900000
3,1,23856.846154
3,1,4222.038043
3,1,6221.037500
3,1,37228.850000
3,1,4870.222222
3,1,3437.903846
3,1,4586.730769
3,1,2090.000000
3,1,4061.244186
3,1,5955.642857
3,1,9221.181818
3,1,5393.250000
3,1,6076.537500
3,1,13700.285714
3,1,1667.666667
3,1,3570.678571
3,1,14344.011364
3,1,5092.970588
3,1,3848.175532
3,1,2405.170213
3,1,2251.352941
3,1,3974.685897
3,1,25412.184211
3,1,1737.540865
3,1,8195.250000
3,1,3851.347222
3,1,1556.517857
3,1,31606.268293
3,1,1632.676282
3,1,3985.318182
3,1,2734.480769
3,1,2576.386364
3,1,47548.826923
3,1,76845.837209
3,1,47843.491071
3,1,3603.106383
3,1,2464.206522
3,1,2180.386029
3,1,3423.882353
3,1,3003.746988
3,1,87124.184375
3,1,2988.325000
3,1,5017.486111
3,1,6467.768293

3,1,1760.948864
3,1,2307.605114
3,1,3839.461538
3,1,6286.840909
3,1,45249.733333
3,1,11217.777778
3,1,2091.989130
3,1,2645.723684
3,1,18878.250000
3,1,2010.800000
3,1,1524.048780
3,1,1866.116477
3,1,2078.065625
3,1,5244.837121
3,1,1806.948171
3,1,5290.351351
3,1,4867.046875
3,1,1763.469388
3,1,5013.013889
3,1,5305.144231
3,1,148832.814189
3,1,7896.494048
3,1,12375.687500
3,1,2005.837838
3,1,61133.951923
3,1,1801.343750
3,1,4666.155556
3,1,9427.628125
3,1,16456.260135
3,1,3516.479167
3,1,3065.478448
3,1,4183.865385
3,1,4462.616279
3,1,1552.025641
3,1,2100.187500
3,1,4677.838710
3,1,8547.723684
3,1,27296.265385
3,1,1725.982143
3,1,190061.084459
3,1,2985.162234
3,1,9297.267296
3,1,3200.710938
3,1,2223.731707
3,1,1553.229167
3,1,2424.875000
3,1,4887.416667
3,1,1985.487179
3,1,4537.384615
3,1,2448.750000
3,1,1617.916667
3,1,1803.608333
3,1,35007.106383
3,1,1783.786164
3,1,2580.275000
3,1,1634.515244
3,1,4151.709677
3,1,3786.551020
3,1,4015.144231
3,1,1978.712500
3,1,2394.172414
3,1,3685.613636
3,1,4353.577703
3,1,2999.125000
3,1,24464.266667
3,1,2840.205882

3,1,9380.321429
3,1,3446.033784
3,1,2297.969466
3,1,34438.011905
3,1,1510.392857
3,1,1840.424419
3,1,1983.375000
3,1,2266.963415
3,1,5085.741935
3,1,1989.664474
3,1,63711.000000
3,1,5997.616935
3,1,1569.937500
3,1,7144.937500
3,1,5466.878049
3,1,2815.211538
3,1,3258.800000
3,1,3451.595745
3,1,8012.081081
3,1,3915.683824
3,1,2705.541667
3,1,7370.257812
3,1,12647.484848
3,1,2121.268293
3,1,27034.103261
3,1,2657.417763
3,1,6166.774194
3,1,2006.362805
3,1,14138.019231
3,1,1666.794118
3,1,6609.715909
3,1,12980.235294
3,1,3031.437500
3,1,16508.669355
3,1,1798.131250
3,1,33412.143836
3,1,8754.665323
3,1,1976.317073
3,1,2837.260417
3,1,4156.028846
3,1,2867.212500
3,1,2063.946429
3,1,5473.062500
3,1,15120.838710
3,1,13633.454545
3,1,6354.018750
3,1,2247.308824
3,1,8004.033333
3,1,5652.125000
3,1,13958.625000
3,1,4211.408333
3,1,1674.175532
3,1,2323.139706
3,1,7245.320513
3,1,8461.511719
3,1,2459.196429
3,1,23218.006098
3,1,1729.355263
3,1,9593.256410
3,1,1547.701389
3,1,2669.090000
3,1,2673.887097
3,1,6239.604167
3,1,6194.496711
3,1,3818.735294
3,1,2083.411458

3,1,1503.884259
3,1,5405.343750
3,1,4340.500000
3,1,1671.114286
3,1,3517.786458
3,1,3983.416667
3,1,1897.035714
3,1,1973.950000
3,1,4944.515152
3,1,2857.375000
3,1,11430.344444
3,1,2794.521739
3,1,135429.559524
3,1,1579.007692
3,1,2037.696429
3,1,1524.744318
3,1,5133.048077
3,1,2574.634615
3,1,1519.173913
3,1,6428.744681
3,1,1589.823529
3,1,1884.142857
3,1,1690.829787
3,1,1754.163462
3,1,1709.437500
3,1,3972.815476
3,1,2888.271341
3,1,25687.651163
3,1,1517.750000
3,1,2842.850610
3,1,3816.457317
3,1,3042.741935
3,1,2609.000000
3,1,2311.946970
3,1,2773.871094
3,1,2237.041667
3,1,1723.365385
3,1,2296.312500
3,1,1913.576087
3,1,2853.131148
3,1,6329.019737
3,1,1637.258152
3,1,4150.100543
3,1,3690.857143
3,1,1735.812500
3,1,22732.438776
3,1,4087.032407
3,1,12207.102273
3,1,4541.645161
3,1,5170.630435
3,1,5937.546053
3,1,7287.128049
3,1,5955.728261
3,1,2465.520161
3,1,3268.329545
3,1,17809.000000
3,1,18755.825000
3,1,8267.700000
3,1,1930.428571
3,1,6443.961039
3,1,2309.982558
3,1,1919.809896
3,1,3038.883929
3,1,5656.180556
3,1,17902.157895
3,1,2223.059524

3,1,14948.763514
3,1,1764.654891
3,1,67780.687500
3,1,2615.531250
3,1,3902.172297
3,1,2836.375000
3,1,14309.523810
3,1,2262.744681
3,1,2813.415625
3,1,3020.236413
3,1,1788.380952
3,1,2743.125000
3,1,20386.406250
3,1,4804.953125
3,1,1521.417857
3,1,5571.000000
3,1,3001.719697
3,1,2711.229167
3,1,2909.226190
3,1,2508.375000
3,1,3353.978261
3,1,4324.527778
3,1,2047.527778
3,1,24883.290441
3,1,7488.637500
3,1,44804.156250
3,1,5511.148438
3,1,4002.250000
3,1,1761.651786
3,1,6646.075000
3,1,6705.194853
3,1,1716.042553
3,1,3432.696970
3,1,158321.779070
3,1,5781.357143
3,1,8581.335366
3,1,42162.724359
3,1,4211.942073
3,1,17213.563725
3,1,3051.250000
3,1,4051.898649
3,1,2337.352273
3,1,2102.714286
3,1,15066.093750
3,1,2932.710526
3,1,1989.381757
3,1,1786.265625
3,1,2301.804878
3,1,1974.344697
3,1,1689.534091
3,1,3079.150000
3,1,1537.262500
3,1,2054.050595
3,1,3005.513587
3,1,2997.285714
3,1,2029.792683
3,1,3217.854167
3,1,38616.125000
3,1,2756.882353
3,1,2127.118902
3,1,3506.047222
3,1,8792.464844
3,1,3168.888889
3,1,15037.031250
3,1,2805.785714
3,1,8079.081395

3,1,2018.378676
3,1,2398.033784
3,1,9648.580645
3,1,1722.500000
3,1,99932.734375
3,1,16912.230769
3,1,1822.381410
3,1,3155.877660
3,1,1608.791667
3,1,1812.054054
3,1,1889.924242
3,1,3384.361413
3,1,1717.818182
3,1,1923.750000
3,1,36020.517361
3,1,1644.619792
3,1,1776.889535
3,1,2708.241935
3,1,2142.922222
3,1,1519.863095
3,1,1534.111111
3,1,1705.187500
3,1,5079.814103
3,1,2568.475610
3,1,2647.923077
3,1,10252.707237
3,1,6135.668919
3,1,10813.090909
3,1,1855.444444
3,1,17534.760000
3,1,1849.195122
3,1,2073.542857
3,1,4184.942308
3,1,42735.701613
3,1,3219.448529
3,1,21572.404255
3,1,2258.764706
3,1,1741.946429
3,1,21443.415541
3,1,14652.221154
3,1,4806.553030
3,1,11241.391304
3,1,3621.047619
3,1,5063.125000
3,1,1976.888514
3,1,13067.652439
3,1,2503.176829
3,1,3251.650000
3,1,3531.710937
3,1,2452.625000
3,1,1826.042614
3,1,2844.444444
3,1,1767.756410
3,1,2195.361702
3,1,3667.209677
3,1,3517.058824
3,1,15430.082143
3,1,1722.058140
3,1,86713.322368
3,1,1784.195946
3,1,3770.594388
3,1,1787.524194
3,1,1632.333333
3,1,2720.596774
3,1,3174.265625
3,1,5531.694444

3,1,6051.575000
3,1,7779.666667
3,1,1976.635714
3,1,1675.656977
3,1,2356.239130
3,1,3741.500000
3,1,2717.767857
3,1,3079.617187
3,1,3244.730114
3,1,3610.418750
3,1,10871.215426
3,1,5746.383721
3,1,6065.187500
3,1,3050.980000
3,1,3122.315789
3,1,6357.266667
3,1,2525.958333
3,1,4284.252778
3,1,2974.915000
3,1,2283.142857
3,1,2555.122642
3,1,41503.613971
3,1,4780.261111
3,1,1589.705882
3,1,9289.109756
3,1,1702.365385
3,1,99658.333333
3,1,1530.700980
3,1,4308.201220
3,1,17544.833333
3,1,9119.537736
3,1,3595.015625
3,1,1601.660256
3,1,2983.849246
3,1,1892.425000
3,1,1697.833333
3,1,1808.643382
3,1,1605.545455
3,1,2393.776536
3,1,2092.567839
3,1,3307.184783
3,1,3269.125000
3,1,1620.388889
3,1,10151.986842
3,1,3970.291667
3,1,1902.347826
3,1,22521.750000
3,1,2423.395349
3,1,1719.611111
3,1,19061.835227
3,1,9213.659091
3,1,3047.919118
3,1,2098.517857
3,1,2037.425676
3,1,5855.034091
3,1,3448.662500
3,1,5926.847222
3,1,2608.892045
3,1,3010.182927
3,1,7083.625000
3,1,3047.211538
3,1,47643.062500
3,1,1792.257576
3,1,12178.764706
3,1,3866.122222
3,1,5669.366935

3,1,2599.284091
3,1,16920.555921
3,1,2328.833333
3,1,1752.465909
3,1,2811.982143
3,1,4840.011905
3,1,3511.400000
3,1,36114.272059
3,1,7670.125000
3,1,15360.196721
3,1,1689.593750
3,1,2434.352941
3,1,5126.522059
3,1,12289.180556
3,1,5349.691176
3,1,23497.863636
3,1,2191.868056
3,1,1673.343750
3,1,4314.614130
3,1,5323.191860
3,1,1731.802083
3,1,3511.245000
3,1,11968.657407
3,1,9198.893939
3,1,3906.929348
3,1,142623.364583
3,1,14479.975000
3,1,1900.136364
3,1,1744.500000
3,1,1926.317708
3,1,7182.400000
3,1,2287.000000
3,1,4885.540373
3,1,3924.638743
3,1,7906.470588
3,1,2043.983553
3,1,1809.842105
3,1,3043.154891
3,1,10646.977941
3,1,4599.897059
3,1,1906.250000
3,1,1882.420455
3,1,26665.562500
3,1,3609.645833
3,1,46313.951299
3,1,8816.946429
3,1,4203.000000
3,1,4840.669271
3,1,4094.761628
3,1,33232.687500
3,1,1704.907258
3,1,2757.884146
3,1,1897.145833
3,1,1976.037736
3,1,1557.696970
3,1,5955.380682
3,1,6647.534091
3,1,3919.656977
3,1,16114.520833
3,1,78320.050000
3,1,4647.239865
3,1,4277.371429
3,1,4771.760000
3,1,3231.146635
3,1,2107.666667
3,1,2297.729592

3,1,2037.702381
3,1,6075.425532
3,1,3794.908163
3,1,3683.388889
3,1,7815.841146
3,1,4806.814189
3,1,9671.570652
3,1,1598.822917
3,1,2936.345588
3,1,2708.945312
3,1,2091.660714
3,1,7495.534091
3,1,10728.797794
3,1,4263.000000
3,1,2302.958861
3,1,50879.848404
3,1,1611.949324
3,1,2980.312500
3,1,9007.666667
3,1,3341.842105
3,1,3268.842742
3,1,8596.520000
3,1,6016.175847
3,1,7385.325000
3,1,2693.509615
3,1,6972.630208
3,1,2045.015464
3,1,1844.342742
3,1,2450.676829
3,1,6497.604167
3,1,7987.884615
3,1,1897.984043
3,1,5641.000000
3,1,8831.312500
3,1,3139.153846
3,1,1730.979275
3,1,19006.713415
3,1,4345.648936
3,1,20911.093750
3,1,2261.535714
3,1,1973.817857
3,1,4208.700535
3,1,2002.208609
3,1,2154.809524
3,1,1709.761628
3,1,2071.590000
3,1,7502.975806
3,1,4442.827586
3,1,3278.932292
3,1,3523.423913
3,1,6714.896739
3,1,2290.025862
3,1,11090.343750
3,1,1810.933333
3,1,56534.035714
3,1,2148.316860
3,1,11394.357558
3,1,39224.900000
3,1,5432.052632
3,1,3550.071429
3,1,4305.633333
3,1,47781.692308
3,1,2668.768617
3,1,1741.194767
3,1,2327.666667
3,1,15061.580000

3,1,3196.875000
3,1,2227.312500
3,1,1753.352500
3,1,7052.005000
3,1,9711.090909
3,1,3194.500000
3,1,10193.526316
3,1,7473.333333
3,1,1670.028571
3,1,1637.701087
3,1,9368.961735
3,1,6778.857143
3,1,6599.675926
3,1,1732.839744
3,1,10790.395833
3,1,7954.210526
3,1,7415.854167
3,1,1605.500000
3,1,26807.426282
3,1,20530.957317
3,1,1500.666667
3,1,1894.528409
3,1,2423.714286
3,1,2580.625000
3,1,1870.476190
3,1,3334.854167
3,1,6078.162500
3,1,2238.083333
3,1,1673.240385
3,1,4055.125000
3,1,11470.178571
3,1,4040.669540
3,1,1891.808824
3,1,1614.583333
3,1,1822.844737
3,1,3692.639881
3,1,3779.671610
3,1,15031.246429
3,1,2667.756757
3,1,6283.910714
3,1,1873.986413
3,1,1802.090909
3,1,7145.262019
3,1,6815.507812
3,1,2180.431452
3,1,2799.397849
3,1,4192.604167
3,1,2784.584239
3,1,4238.896635
3,1,2574.388889
3,1,4950.459091
3,1,2081.546053
3,1,1636.843750
3,1,2208.348837
3,1,6839.086957
3,1,1759.194444
3,1,5109.090000
3,1,5143.840426
3,1,3133.190625
3,1,2694.394737
3,1,2752.840909
3,1,10359.193750
3,1,27115.228571
3,1,1663.164634
3,1,22156.750000
3,1,13866.306818

3,1,2311.105114
3,1,4188.583770
3,1,2069.000000
3,1,4350.372642
3,1,1959.625000
3,1,3649.743590
3,1,1758.381250
3,1,126959.817568
3,1,1940.707317
3,1,1743.846154
3,1,3536.991667
3,1,6185.673729
3,1,8289.750000
3,1,1683.161290
3,1,5338.976562
3,1,1789.464286
3,1,7673.833333
3,1,4548.208333
3,1,2373.579787
3,1,2233.372159
3,1,87345.702273
3,1,11409.600000
3,1,30413.056818
3,1,15693.529018
3,1,3708.203947
3,1,3048.305851
3,1,6209.966667
3,1,1585.136364
3,1,88907.506098
3,1,18820.435897
3,1,1995.828125
3,1,6071.250000
3,1,1640.115854
3,1,2912.166667
3,1,3625.757353
3,1,1767.372340
3,1,2569.567073
3,1,15245.755952
3,1,1871.162500
3,1,2476.000000
3,1,24819.175676
3,1,2149.011029
3,1,3500.706250
3,1,3364.134146
3,1,1785.065789
3,1,7279.329787
3,1,1674.881944
3,1,3569.000000
3,1,2778.316667
3,1,2013.681818
3,1,2644.006757
3,1,1586.115854
3,1,2708.642442
3,1,1661.757812
3,1,11818.280000
3,1,1562.000000
3,1,2713.210714
3,1,5446.000000
3,1,8906.554878
3,1,1846.129032
3,1,2465.906977
3,1,8541.000000
3,1,1763.255814
3,1,3063.831250
3,1,3457.880814
3,1,3553.343750

3,1,1907.261628
3,1,3605.250000
3,1,12133.000000
3,1,5085.727273
3,1,17818.829861
3,1,1757.088889
3,1,1989.465116
3,1,1770.892045
3,1,2348.560976
3,1,3853.700000
3,1,2909.204167
3,1,1763.788194
3,1,2435.578125
3,1,2249.468750
3,1,1991.739130
3,1,10338.045455
3,1,2518.045455
3,1,3571.500000
3,1,19780.375000
3,1,2260.133621
3,1,2384.981818
3,1,2668.283333
3,1,2807.041667
3,1,2623.890244
3,1,2186.808511
3,1,2537.252717
3,1,3600.785326
3,1,87039.915541
3,1,2338.849138
3,1,10642.214286
3,1,8414.445946
3,1,1500.337838
3,1,1639.788889
3,1,46428.750000
3,1,2004.776786
3,1,5089.902344
3,1,2048.500000
3,1,1716.280488
3,1,2749.100000
3,1,1552.082317
3,1,1918.316327
3,1,9034.375000
3,1,28391.989583
3,1,1553.421512
3,1,11988.571429
3,1,1584.142857
3,1,5085.214286
3,1,7101.281915
3,1,2778.808511
3,1,6637.588235
3,1,1828.989130
3,1,2687.906250
3,1,3089.093750
3,1,5264.375000
3,1,1905.138889
3,1,2134.819853
3,1,8644.538194
3,1,2529.235294
3,1,1787.667553
3,1,15273.875000
3,1,12215.656250
3,1,5146.428571
3,1,1919.615385
3,1,3004.047619
3,1,28661.638889
3,1,7031.600000

3,1,37181.977941
3,1,1846.517045
3,1,6794.052632
3,1,2008.205128
3,1,2311.937500
3,1,1595.341912
3,1,2125.277778
3,1,3111.290323
3,1,5323.586207
3,1,1972.366071
3,1,2548.508621
3,1,60653.260417
3,1,3445.115385
3,1,7469.381944
3,1,108596.440476
3,1,4355.000000
3,1,1632.734694
3,1,2087.924419
3,1,9940.330882
3,1,17758.839286
3,1,1753.831522
3,1,16675.302326
3,1,7002.312500
3,1,1512.628289
3,1,2229.825581
3,1,3853.448370
3,1,4869.379310
3,1,8951.500000
3,1,2825.680000
3,1,3456.250000
3,1,2557.552419
3,1,1662.338889
3,1,1898.773026
3,1,3900.521552
3,1,49327.068627
3,1,4271.281977
3,1,1641.387821
3,1,1737.130208
3,1,3439.875000
3,1,10292.525000
3,1,1530.630682
3,1,4643.480263
3,1,14593.032895
3,1,3329.226562
3,1,3095.893750
3,1,13092.997549
3,1,3143.758333
3,1,6092.714286
3,1,2114.682927
3,1,3072.028846
3,1,2155.596429
3,1,13171.923611
3,1,2631.403226
3,1,6029.931452
3,1,4043.194079
3,1,38819.951705
3,1,175732.957317
3,1,2094.278125
3,1,21750.133333
3,1,2751.345000
3,1,77271.029412
3,1,3560.192308
3,1,1706.068182
3,1,2311.430851
3,1,11859.496350
3,1,9053.750000

3,1,5173.579545
3,1,1859.697368
3,1,2033.666667
3,1,1788.494318
3,1,1935.682927
3,1,3140.275641
3,1,6767.608553
3,1,3461.647059
3,1,8440.012931
3,1,4106.542500
3,1,2670.607843
3,1,2981.821429
3,1,2572.335366
3,1,11050.515152
3,1,58754.161290
3,1,5599.914286
3,1,3518.522436
3,1,2054.868421
3,1,2271.046875
3,1,2295.154286
3,1,111649.444444
3,1,37740.470588
3,1,1882.460106
3,1,23755.392857
3,1,3076.576705
3,1,6410.739286
3,1,1851.492857
3,1,17483.714286
3,1,1602.364286
3,1,3957.928977
3,1,18669.418919
3,1,5636.607500
3,1,19372.763158
3,1,2437.581731
3,1,9444.133721
3,1,17200.500000
3,1,2124.874016
3,1,4807.352564
3,1,2442.550676
3,1,2699.259615
3,1,32099.000000
3,1,11142.868421
3,1,3906.750000
3,1,2554.224057
3,1,3517.314286
3,1,1605.523810
3,1,1688.980000
3,1,1532.675000
3,1,1656.168605
3,1,7534.057692
3,1,2954.747500
3,1,1787.614865
3,1,1944.261905
3,1,4078.769231
3,1,2492.993421
3,1,9446.540094
3,1,1531.434659
3,1,1724.790698
3,1,3096.508523
3,1,6617.489130
3,1,2338.326923
3,1,2783.250000
3,1,2148.562500
3,1,4560.324519
3,1,1692.023256
3,1,1610.618243

3,1,3374.906250
3,1,88491.218750
3,1,1854.710526
3,1,3778.456522
3,1,2591.400000
3,1,4309.712209
3,1,1723.777778
3,1,2047.307692
3,1,2515.747807
3,1,3651.840000
3,1,2285.638889
3,1,35446.150735
3,1,3963.979167
3,1,1893.765957
3,1,20197.578947
3,1,3641.062500
3,1,6240.125000
3,1,5705.733333
3,1,7401.073529
3,1,3839.918367
3,1,6023.462766
3,1,3990.265957
3,1,6332.538462
3,1,7320.116935
3,1,3742.187500
3,1,1764.729730
3,1,1936.354167
3,1,2330.720395
3,1,5367.809524
3,1,4859.458333
3,1,2086.026316
3,1,2930.527778
3,1,7363.378049
3,1,9353.390306
3,1,8271.630435
3,1,3462.532143
3,1,4484.047794
3,1,1606.711538
3,1,2437.500000
3,1,5245.401316
3,1,7566.901515
3,1,2284.280000
3,1,1596.644444
3,1,2618.697674
3,1,3083.843750
3,1,4559.475000
3,1,12296.268617
3,1,1943.421875
3,1,1711.233696
3,1,9574.285714
3,1,1853.107955
3,1,2102.485119
3,1,2995.250000
3,1,3923.315476
3,1,2002.619318
3,1,34065.585366
3,1,28022.505882
3,1,30823.793103
3,1,10227.052632
3,1,2952.028571
3,1,4017.426630
3,1,2995.537500
3,1,1796.010204
3,1,10297.075472
3,1,4603.108333
3,1,5887.143617

3,1,2645.402778
3,1,5699.200521
3,1,2020.882812
3,1,5934.654762
3,1,1617.896341
3,1,45447.051471
3,1,3320.714286
3,1,7098.808036
3,1,1992.263393
3,1,9788.425000
3,1,8701.000000
3,1,8596.308824
3,1,4716.486607
3,1,2715.750000
3,1,3069.471591
3,1,2790.479167
3,1,7993.982143
3,1,2772.830882
3,1,1700.790000
3,1,12676.786585
3,1,1642.060714
3,1,4362.502674
3,1,1845.156977
3,1,2110.929348
3,1,3592.550000
3,1,4358.100000
3,1,2177.793605
3,1,3687.973684
3,1,1742.821809
3,1,56248.130435
3,1,23902.351351
3,1,1751.293478
3,1,6516.875000
3,1,24122.424242
3,1,1728.375000
3,1,4320.798077
3,1,1551.872159
3,1,5004.565476
3,1,2822.295732
3,1,8357.901316
3,1,50094.838068
3,1,3122.142857
3,1,1607.331250
3,1,1568.093750
3,1,2272.179878
3,1,1817.539773
3,1,1583.377717
3,1,3569.833333
3,1,6372.976190
3,1,15181.448529
3,1,3378.000000
3,1,27847.787500
3,1,2204.614362
3,1,2264.365000
3,1,1785.948718
3,1,1746.609694
3,1,2406.750000
3,1,1626.965116
3,1,2499.000000
3,1,5837.420673
3,1,2251.741279
3,1,2010.489583
3,1,51379.916667
3,1,89456.765957
3,1,2610.207317
3,1,2170.744444

3,1,7413.452830
3,1,3213.429487
3,1,2067.535714
3,1,2125.500000
3,1,5686.178571
3,1,3945.051471
3,1,2280.158854
3,1,9114.021226
3,1,30925.380952
3,1,5151.343750
3,1,2432.000000
3,1,1878.695652
3,1,196468.633721
3,1,2632.232558
3,1,2592.226562
3,1,2348.480769
3,1,4298.782738
3,1,3391.380556
3,1,16353.246711
3,1,69503.798077
3,1,2635.544118
3,1,21958.233553
3,1,3826.770000
3,1,1796.820946
3,1,1588.728659
3,1,3417.078947
3,1,3535.375000
3,1,2109.576087
3,1,8473.804245
3,1,1669.797872
3,1,2607.538860
3,1,3022.884848
3,1,72211.285714
3,1,3102.054878
3,1,2861.024390
3,1,3963.141304
3,1,6508.755556
3,1,2203.500000
3,1,2195.416667
3,1,2307.572917
3,1,2236.105769
3,1,2162.255319
3,1,1705.877551
3,1,33470.884615
3,1,1560.095238
3,1,2352.125000
3,1,1714.343750
3,1,169891.254545
3,1,1606.391026
3,1,2970.108974
3,1,2800.500000
3,1,12215.208333
3,1,22951.730263
3,1,2077.714286
3,1,16749.510638
3,1,4421.758523
3,1,7892.439286
3,1,1936.137755
3,1,1533.357955
3,1,1737.340426
3,1,20481.616667
3,1,2768.274390
3,1,4918.947917
3,1,2830.444444
3,1,14086.392157
3,1,1528.564103

3,1,4059.976190
3,1,20025.322368
3,1,1851.028409
3,1,1765.875000
3,1,4613.191860
3,1,1960.697368
3,1,4816.389706
3,1,1874.294872
3,1,1912.572368
3,1,1744.731707
3,1,2550.125000
3,1,3474.403226
3,1,2070.428571
3,1,2113.276316
3,1,1790.062500
3,1,2685.541667
3,1,6657.231481
3,1,2243.972973
3,1,1573.401163
3,1,3454.529703
3,1,47207.968750
3,1,5426.500000
3,1,5969.687500
3,1,3392.875000
3,1,1727.994565
3,1,7484.932143
3,1,14974.033333
3,1,3675.138587
3,1,3751.000000
3,1,3603.937500
3,1,1527.000000
3,1,3158.437500
3,1,1640.446023
3,1,3237.300000
3,1,10608.390625
3,1,1715.232955
3,1,7884.616667
3,1,2918.292683
3,1,4568.130435
3,1,10135.800000
3,1,1569.500000
3,1,3946.613636
3,1,1816.916667
3,1,7727.800000
3,1,1875.568182
3,1,13210.820946
3,1,1604.763158
3,1,1669.756579
3,1,4351.702703
3,1,2088.918605
3,1,1875.094595
3,1,3611.329545
3,1,5059.401042
3,1,1868.139535
3,1,21790.294872
3,1,2737.428571
3,1,17132.173077
3,1,1680.581395
3,1,6314.531250
3,1,2473.146875
3,1,1598.438889
3,1,4497.875000
3,1,1939.325521
3,1,2206.764535
3,1,4383.619186
3,1,74221.625000

3,1,4665.191667
3,1,1559.666667
3,1,2566.411932
3,1,6514.334239
3,1,7385.357143
3,1,35027.431250
3,1,1567.766304
3,1,4078.644444
3,1,2378.641892
3,1,1521.282143
3,1,2007.804878
3,1,2871.750000
3,1,6317.994186
3,1,4987.875000
3,1,1675.196429
3,1,1549.750000
3,1,2611.684524
3,1,74140.171429
3,1,1958.869565
3,1,2423.918919
3,1,1772.404255
3,1,2074.190000
3,1,13273.556818
3,1,10809.039216
3,1,8708.306034
3,1,1871.323370
3,1,2265.125000
3,1,14921.300000
3,1,2254.861702
3,1,1556.450758
3,1,1831.216667
3,1,5037.333333
3,1,2169.654762
3,1,2188.673913
3,1,2162.778125
3,1,48233.413462
3,1,1681.187500
3,1,9032.118421
3,1,2199.431818
3,1,124221.434211
3,1,8178.254032
3,1,3693.500000
3,1,11294.636364
3,1,1524.810000
3,1,6417.062500
3,1,2614.193548
3,1,9365.170213
3,1,1906.712500
3,1,10204.297297
3,1,6087.845109
3,1,4583.600000
3,1,12164.106618
3,1,8056.621875
3,1,3112.625000
3,1,5252.787879
3,1,8874.515625
3,1,1600.845588
3,1,1957.477941
3,1,13672.657895
3,1,3189.265152
3,1,3519.121324
3,1,3558.400000
3,1,8471.641026
3,1,109029.859756
3,1,1512.058824
3,1,12965.171875

3,1,1765.907895
3,1,1666.371622
3,1,2049.023256
3,1,1755.739130
3,1,1610.037162
3,1,3302.025641
3,1,1902.182432
3,1,2709.679878
3,1,2173.541667
3,1,1550.339844
3,1,4477.086957
3,1,6826.720930
3,1,7553.186047
3,1,2924.947674
3,1,15116.024194
3,1,2107.903846
3,1,3087.683333
3,1,2721.668919
3,1,1711.384615
3,1,4743.986413
3,1,3159.516667
3,1,2001.720395
3,1,58896.566038
3,1,8811.706522
3,1,1629.377907
3,1,2022.111111
3,1,3530.914634
3,1,8019.422222
3,1,2713.756579
3,1,7301.417763
3,1,1563.404762
3,1,36715.450000
3,1,2750.632576
3,1,11390.971292
3,1,1629.105263
3,1,2136.111111
3,1,2503.732143
3,1,1719.134146
3,1,1601.231707
3,1,8017.187500
3,1,1527.830556
3,1,36461.636364
3,1,12859.384868
3,1,1754.026316
3,1,5140.358108
3,1,4157.734375
3,1,1893.074405
3,1,2510.794643
3,1,19571.073171
3,1,4051.420000
3,1,11517.973684
3,1,5212.428571
3,1,1992.096591
3,1,3882.000000
3,1,1959.655660
3,1,4574.833333
3,1,2166.500000
3,1,2771.155172
3,1,13107.695312
3,1,1696.500000
3,1,9012.993750
3,1,3438.435606
3,1,4321.946429
3,1,3917.923780
3,1,7004.195122
3,1,1621.559211

3,1,130398.000000
3,1,4964.145161
3,1,8660.847561
3,1,51739.216912
3,1,1839.625000
3,1,1905.290698
3,1,2095.328125
3,1,1704.554688
3,1,4570.634146
3,1,10679.075521
3,1,48649.347222
3,1,3316.954545
3,1,5377.814516
3,1,4523.384615
3,1,3816.128378
3,1,4328.288043
3,1,4677.482143
3,1,7029.086111
3,1,1712.317073
3,1,3294.080357
3,1,1737.993902
3,1,3850.088542
3,1,9209.500000
3,1,1763.368421
3,1,17111.423387
3,1,1536.194853
3,1,1612.153846
3,1,1542.877551
3,1,25959.250000
3,1,1911.621429
3,1,1661.793750
3,1,2554.262500
3,1,2616.477941
3,1,1698.142857
3,1,3106.562500
3,1,22292.745968
3,1,4283.284091
3,1,1814.866667
3,1,11648.604839
3,1,9187.869318
3,1,118606.375000
3,1,7786.458333
3,1,1581.448718
3,1,1678.217391
3,1,5189.506024
3,1,16969.568182
3,1,40684.108108
3,1,3850.067308
3,1,2538.470930
3,1,1685.923077
3,1,1648.840116
3,1,1562.000000
3,1,1958.508065
3,1,1823.745342
3,1,2377.841463
3,1,4511.269231
3,1,18576.869318
3,1,10283.366071
3,1,106489.973039
3,1,1692.414062
3,1,47488.258929
3,1,2652.456250
3,1,3610.547297
3,1,1941.232143
3,1,10576.651786
3,1,2789.600000

3,1,10917.397059
3,1,2239.949405
3,1,6057.141304
3,1,2062.355263
3,1,4339.217391
3,1,1978.587838
3,1,5332.703125
3,1,2672.047619
3,1,8379.571970
3,1,12606.362637
3,1,2521.929245
3,1,7711.186441
3,1,2280.378378
3,1,1844.994318
3,1,1585.534884
3,1,2498.375000
3,1,2660.174419
3,1,34016.091463
3,1,3350.553571
3,1,9840.687500
3,1,1598.255814
3,1,7540.239583
3,1,3930.902778
3,1,1597.160000
3,1,1504.207317
3,1,13927.573529
3,1,21015.551020
3,1,3989.343750
3,1,15768.531250
3,1,2355.235714
3,1,2077.720238
3,1,2140.561224
3,1,93172.342105
3,1,3017.200000
3,1,31009.580357
3,1,2827.187500
3,1,3691.810976
3,1,1818.430556
3,1,2759.732558
3,1,1970.906250
3,1,51200.750000
3,1,3032.468750
3,1,2883.297170
3,1,3223.577586
3,1,9879.790698
3,1,6632.785714
3,1,1733.856707
3,1,1581.284483
3,1,1895.480769
3,1,5681.458333
3,1,3116.468085
3,1,9878.647959
3,1,55143.531250
3,1,2778.279605
3,1,3410.960784
3,1,7342.120690
3,1,1779.797500
3,1,4877.978723
3,1,3593.710106
3,1,21189.743421
3,1,5300.095238
3,1,7229.670213
3,1,3498.752315
3,1,3298.481250
3,1,5913.861111
3,1,3177.812500

3,1,41262.875000
3,1,3976.020833
3,1,10320.195652
3,1,1740.827586
3,1,3512.504630
3,1,3515.754902
3,1,2697.335526
3,1,2036.323864
3,1,2862.268519
3,1,5758.035714
3,1,1879.277027
3,1,2251.818627
3,1,1664.014286
3,1,14869.557692
3,1,5473.482143
3,1,5470.750000
3,1,7914.448276
3,1,2867.397436
3,1,2375.513889
3,1,3857.115854
3,1,2432.346154
3,1,11493.482955
3,1,1646.735294
3,1,2152.021739
3,1,5669.391667
3,1,1990.937500
3,1,3520.086806
3,1,3139.488208
3,1,31224.689320
3,1,3446.145833
3,1,3386.500000
3,1,1558.028409
3,1,2139.655093
3,1,43470.085308
3,1,1801.818750
3,1,2880.138889
3,1,65435.086957
3,1,8902.852273
3,1,4289.078488
3,1,1990.160714
3,1,1781.293103
3,1,5665.122159
3,1,50154.743056
3,1,4232.000000
3,1,3341.770000
3,1,5324.391304
3,1,4306.971429
3,1,11777.881579
3,1,1697.640244
3,1,15995.835526
3,1,37801.025000
3,1,3467.979167
3,1,2175.224490
3,1,2604.441176
3,1,2072.386364
3,1,9614.766447
3,1,3112.750000
3,1,12339.789474
3,1,16194.923077
3,1,9670.288462
3,1,2653.937500
3,1,3292.836735
3,1,2333.055556
3,1,10257.221591
3,1,2447.711735
3,1,3845.797872

3,1,1572.592105
3,1,1635.072289
3,1,1689.031250
3,1,1804.497159
3,1,5282.312500
3,1,1907.462766
3,1,3296.785714
3,1,8703.377358
3,1,1767.472826
3,1,5172.404412
3,1,3216.234375
3,1,19073.375000
3,1,2693.632353
3,1,6041.312500
3,1,1887.658537
3,1,2347.333333
3,1,2428.295455
3,1,2108.105263
3,1,13442.489130
3,1,3290.431818
3,1,1552.139706
3,1,2232.444767
3,1,1814.741379
3,1,4816.127717
3,1,2343.565217
3,1,2273.411058
3,1,1586.103261
3,1,1729.161585
3,1,25343.805233
3,1,3175.136364
3,1,1609.161585
3,1,4864.375000
3,1,4182.831081
3,1,3006.000000
3,1,35317.733108
3,1,6535.750000
3,1,2322.035714
3,1,8858.981250
3,1,1579.892045
3,1,1907.096154
3,1,2031.576705
3,1,3257.517857
3,1,2150.863636
3,1,2017.450000
3,1,2068.033163
3,1,1933.962500
3,1,4199.900000
3,1,3161.952381
3,1,3566.442073
3,1,5271.093750
3,1,13204.710106
3,1,2938.349359
3,1,5646.645833
3,1,10040.420000
3,1,2571.380682
3,1,5674.556122
3,1,2687.491071
3,1,2325.500000
3,1,2876.697917
3,1,2848.250000
3,1,3932.772727
3,1,5799.721925
3,1,2723.612500
3,1,2588.014205
3,1,25501.408163
3,1,5381.552632

3,1,5279.867647
3,1,17760.776042
3,1,1945.323718
3,1,2129.388889
3,1,3541.534884
3,1,35503.700980
3,1,102106.435268
3,1,148910.788462
3,1,1737.660714
3,1,8910.183673
3,1,3069.482143
3,1,89301.380435
3,1,3056.500000
3,1,1754.296296
3,1,2190.454545
3,1,24086.031250
3,1,14826.625000
3,1,3102.278409
3,1,4888.946429
3,1,2510.348485
3,1,4587.468750
3,1,1748.443396
3,1,1511.538462
3,1,1636.081395
3,1,9007.734375
3,1,5982.038889
3,1,1852.187500
3,1,3790.871622
3,1,4169.729730
3,1,1678.572917
3,1,2955.171875
3,1,2312.500000
3,1,1724.500000
3,1,2081.667553
3,1,7961.204082
3,1,2841.493421
3,1,15031.500000
3,1,5345.823529
3,1,1556.550000
3,1,8271.978261
3,1,1899.540816
3,1,29857.956522
3,1,2421.883721
3,1,2397.084848
3,1,9336.188596
3,1,3296.037037
3,1,2296.950521
3,1,1776.053571
3,1,55864.187500
3,1,3850.600000
3,1,4308.513158
3,1,2826.630952
3,1,2259.790698
3,1,1549.098837
3,1,1731.957317
3,1,3320.671875
3,1,5187.750000
3,1,3195.250000
3,1,2854.664286
3,1,8055.271739
3,1,3139.146739
3,1,4742.679487
3,1,1924.153846
3,1,2666.965909
3,1,2632.060976
3,1,1552.332500

3,1,1541.750000
3,1,5436.153302
3,1,8833.229730
3,1,24768.923077
3,1,10510.242424
3,1,1499.478723
3,1,1765.781250
3,1,4160.882979
3,1,5131.953125
3,1,17234.291667
3,1,2959.218750
3,1,1820.262097
3,1,2011.947368
3,1,2108.910714
3,1,11118.930556
3,1,15853.875000
3,1,1507.710526
3,1,2099.967391
3,1,1708.041899
3,1,4711.464844
3,1,5184.703804
3,1,3177.959184
3,1,8718.118421
3,1,1598.095395
3,1,20251.937500
3,1,2322.631579
3,1,3166.705882
3,1,2109.628571
3,1,2068.797794
3,1,3297.451531
3,1,9354.006329
3,1,15182.853659
3,1,1750.360119
3,1,4240.975610
3,1,12066.344595
3,1,27389.625000
3,1,1852.169271
3,1,2581.920455
3,1,2022.510870
3,1,2241.448276
3,1,1691.787162
3,1,2471.347561
3,1,3356.317073
3,1,4029.680921
3,1,2495.804688
3,1,23791.593750
3,1,5121.085938
3,1,4902.051136
3,1,2857.558511
3,1,2668.222561
3,1,3399.842105
3,1,1504.934524
3,1,57303.032051
3,1,2470.189189
3,1,6007.109756
3,1,8511.769231
3,1,14194.089744
3,1,6717.142857
3,1,1954.766667
3,1,1501.463415
3,1,1972.100000
3,1,5923.530303
3,1,13090.303571
3,1,5434.288043
3,1,1789.505814
3,1,1611.600000

3,1,1502.165000
3,1,3745.586538
3,1,31710.236111
3,1,4813.352041
3,1,5405.081081
3,1,2111.295918
3,1,3260.600000
3,1,4065.500000
3,1,12902.000000
3,1,4272.857143
3,1,1807.511905
3,1,4367.858108
3,1,9939.390625
3,1,15016.073529
3,1,2816.000000
3,1,1785.256098
3,1,1541.939189
3,1,4654.285714
3,1,1513.488281
3,1,1871.978261
3,1,3171.448276
3,1,1612.111111
3,1,7598.875000
3,1,17495.074586
3,1,9352.478659
3,1,6218.571023
3,1,74254.016026
3,1,4585.413043
3,1,2875.189189
3,1,25507.523256
3,1,7701.705128
3,1,2950.258929
3,1,13441.917969
3,1,6204.437500
3,1,7806.773707
3,1,2460.043605
3,1,1559.244318
3,1,2167.424419
3,1,8277.291667
3,1,8615.909884
3,1,2301.530303
3,1,6565.324324
3,1,2550.588710
3,1,2302.891892
3,1,3725.716518
3,1,2959.075758
3,1,1670.778481
3,1,2633.804878
3,1,2250.125000
3,1,2664.442568
3,1,86606.221429
3,1,8775.335526
3,1,4742.220745
3,1,1953.880435
3,1,12275.083333
3,1,1668.895349
3,1,1644.061224
3,1,2243.443182
3,1,1688.271277
3,1,41603.632812
3,1,1532.671875
3,1,2115.666667
3,1,15456.329268
3,1,1847.832317
3,1,2226.130102
3,1,1617.554487

3,1,2576.675676
3,1,2753.152344
3,1,3265.085106
3,1,1984.584559
3,1,3658.008065
3,1,1674.339286
3,1,1748.375000
3,1,2265.440789
3,1,4670.772727
3,1,3557.892045
3,1,5810.540698
3,1,29549.166667
3,1,2466.700599
3,1,3598.642857
3,1,9128.843750
3,1,1611.875000
3,1,3825.788462
3,1,2402.732143
3,1,1540.967949
3,1,3828.419444
3,1,1803.031250
3,1,2310.045455
3,1,1820.215116
3,1,25852.223485
3,1,1804.250000
3,1,1704.362805
3,1,2156.519231
3,1,4669.888298
3,1,2935.733333
3,1,3629.881944
3,1,2138.452381
3,1,3173.312500
3,1,14803.232143
3,1,2068.205357
3,1,1692.256410
3,1,1636.259615
3,1,2682.219828
3,1,1736.395349
3,1,15495.891304
3,1,3421.458101
3,1,2514.241935
3,1,12683.925676
3,1,37362.038043
3,1,2302.321429
3,1,4335.371622
3,1,82722.130435
3,1,13975.363636
3,1,3128.145349
3,1,2691.788194
3,1,4959.753125
3,1,14207.982143
3,1,3862.000000
3,1,2413.655488
3,1,10360.511111
3,1,6220.835938
3,1,5602.218750
3,1,15549.952206
3,1,5349.801829
3,1,7902.282258
3,1,6919.458333
3,1,6550.284884
3,1,1595.837838
3,1,35613.974265
3,1,3895.175000
3,1,1673.736979
3,1,3618.195652

3,1,7177.966912
3,1,1927.987500
3,1,2777.673077
3,1,1944.000000
3,1,1529.085937
3,1,9593.600000
3,1,2387.625000
3,1,2119.887097
3,1,2682.484848
3,1,4556.822115
3,1,15918.290625
3,1,1742.000000
3,1,2696.340909
3,1,3369.594388
3,1,1521.654762
3,1,2604.765625
3,1,10553.300000
3,1,1704.461538
3,1,3997.510870
3,1,1675.137500
3,1,2551.031250
3,1,1637.083333
3,1,3724.304878
3,1,6627.750000
3,1,2415.252717
3,1,2926.718750
3,1,5592.734043
3,1,29287.454545
3,1,2847.315476
3,1,5026.315625
3,1,4684.387500
3,1,3684.516667
3,1,2693.754167
3,1,3799.500000
3,1,13442.984043
3,1,1923.469388
3,1,15916.594595
3,1,14091.413043
3,1,2615.907895
3,1,2299.394886
3,1,2638.469595
3,1,2531.973214
3,1,2340.658537
3,1,1569.000000
3,1,1682.039474
3,1,2913.698529
3,1,7239.523684
3,1,2805.000000
3,1,2245.589286
3,1,1691.660714
3,1,1548.304687
3,1,9361.823529
3,1,14145.371622
3,1,2820.583333
3,1,2611.613636
3,1,8622.800000
3,1,4216.595930
3,1,2155.181818
3,1,4088.024390
3,1,4326.401163
3,1,4609.147436
3,1,3752.500000
3,1,3556.340625
3,1,6833.245968
3,1,7083.750000
3,1,5292.692308

3,1,8791.925595
3,1,1923.500000
3,1,6952.985294
3,1,3318.136364
3,1,3379.217391
3,1,1854.985294
3,1,1517.215909
3,1,12252.763158
3,1,2372.168919
3,1,4691.080357
3,1,2447.957143
3,1,3094.939655
3,1,3247.312500
3,1,1512.431818
3,1,2346.500000
3,1,28927.890244
3,1,2999.954545
3,1,1550.570946
3,1,40007.204082
3,1,3060.270270
3,1,1819.098684
3,1,3734.000000
3,1,1727.000000
3,1,8760.465909
3,1,2111.578947
3,1,2505.666667
3,1,2335.027027
3,1,28529.724138
3,1,11729.500000
3,1,2928.527027
3,1,5470.726190
3,1,3633.983974
3,1,3168.737179
3,1,1727.640625
3,1,2596.952381
3,1,2905.500000
3,1,3700.664062
3,1,5264.457447
3,1,1892.326923
3,1,1981.779762
3,1,1803.949324
3,1,3970.508065
3,1,1993.960938
3,1,5478.144737
3,1,6476.889706
3,1,1580.917553
3,1,1751.562500
3,1,2956.644022
3,1,9336.063636
3,1,6720.007812
3,1,9685.791667
3,1,3662.609375
3,1,2596.353261
3,1,5943.285714
3,1,2076.795455
3,1,1957.250000
3,1,3998.562500
3,1,5410.182432
3,1,3111.943548
3,1,2441.125000
3,1,4649.465116
3,1,2438.051829
3,1,1774.176471
3,1,2403.610714
3,1,4592.086957
3,1,8503.714286

3,1,2684.875000
3,1,8709.897059
3,1,3889.765625
3,1,2008.446429
3,1,2090.781250
3,1,2971.125000
3,1,4397.583333
3,1,2106.104651
3,1,8477.835366
3,1,3628.666667
3,1,2182.589286
3,1,7205.151786
3,1,11066.095588
3,1,2371.974359
3,1,1704.250000
3,1,4189.890625
3,1,1606.333333
3,1,5668.927778
3,1,1880.567308
3,1,1826.659091
3,1,1994.134615
3,1,6276.692308
3,1,72523.886364
3,1,7997.175595
3,1,5140.250000
3,1,1564.850000
3,1,21964.012195
3,1,1853.906250
3,1,12748.000000
3,1,1850.786585
3,1,1806.665625
3,1,2081.841667
3,1,21462.710526
3,1,3569.116279
3,1,1560.238971
3,1,1786.397222
3,1,2300.909375
3,1,4167.179878
3,1,2330.510638
3,1,9808.014706
3,1,1530.066667
3,1,4793.117188
3,1,2713.779070
3,1,1735.233333
3,1,2783.847561
3,1,2480.095588
3,1,1725.929348
3,1,2192.666667
3,1,1633.867500
3,1,2887.310000
3,1,3861.975694
3,1,2068.448980
3,1,2157.575000
3,1,23892.989130
3,1,2611.323171
3,1,2222.611842
3,1,8243.279255
3,1,2087.750000
3,1,2902.312500
3,1,3195.833333
3,1,2246.981061
3,1,2514.943750
3,1,2771.700000
3,1,2461.302326
3,1,1810.096939
3,1,2416.358108

3,1,1900.255000
3,1,1648.663043
3,1,2301.888889
3,1,2002.258152
3,1,8268.489362
3,1,9618.863281
3,1,1975.978261
3,1,8639.119444
3,1,3184.564103
3,1,6845.101064
3,1,1912.473684
3,1,1575.672872
3,1,62446.103448
3,1,2781.720395
3,1,4543.076087
3,1,1773.554167
3,1,12716.000000
3,1,4370.166667
3,1,11943.560000
3,1,2693.437500
3,1,2052.281250
3,1,3562.368243
3,1,3264.055556
3,1,1781.350000
3,1,19566.200000
3,1,12415.794872
3,1,68210.218750
3,1,3124.725490
3,1,5001.695122
3,1,5229.328125
3,1,2384.620192
3,1,2815.336957
3,1,4933.821429
3,1,2090.275000
3,1,3184.130682
3,1,2515.419872
3,1,2479.400000
3,1,12274.500000
3,1,1594.789474
3,1,1603.669118
3,1,1709.604167
3,1,26714.430851
3,1,4362.698661
3,1,1505.334975
3,1,2071.545455
3,1,3794.509434
3,1,1554.609375
3,1,1516.355978
3,1,2178.166667
3,1,2201.546875
3,1,111711.025735
3,1,2695.115385
3,1,25488.919643
3,1,1874.875000
3,1,19374.663793
3,1,6019.164706
3,1,7102.371795
3,1,1920.036111
3,1,17718.331395
3,1,14453.946429
3,1,1950.157895
3,1,6554.177632
3,1,5480.650000
3,1,1558.250000
3,1,2266.865385
3,1,2689.408163

3,1,14874.813953
3,1,3645.884259
3,1,2474.655000
3,1,2110.400000
3,1,4354.125000
3,1,19250.433333
3,1,10361.709559
3,1,7515.765625
3,1,14639.527273
3,1,4469.218750
3,1,22240.372340
3,1,2571.000000
3,1,9783.562500
3,1,3824.685811
3,1,2618.471875
3,1,6349.536932
3,1,1927.713415
3,1,3836.388889
3,1,2667.645349
3,1,3848.890000
3,1,16101.359375
3,1,6478.750000
3,1,1955.750000
3,1,1939.244186
3,1,2700.122449
3,1,1612.631579
3,1,4694.183673
3,1,24701.445946
3,1,1551.659091
3,1,2642.592391
3,1,2204.382353
3,1,5430.054487
3,1,3093.233553
3,1,5843.867647
3,1,1806.807927
3,1,6511.415625
3,1,7629.625000
3,1,2270.329787
3,1,4042.986702
3,1,6394.349265
3,1,1863.768293
3,1,3528.541667
3,1,2155.764706
3,1,117055.041667
3,1,2055.250000
3,1,10253.041667
3,1,23404.500000
3,1,1541.383333
3,1,7958.552239
3,1,4668.351064
3,1,114738.285326
3,1,11648.531250
3,1,2381.776163
3,1,8135.146341
3,1,1773.774510
3,1,1622.293605
3,1,1829.857955
3,1,22947.093750
3,1,1710.818182
3,1,2063.800000
3,1,1880.094828
3,1,19166.695312
3,1,5805.989362
3,1,3957.959184
3,1,3721.592391
3,1,2857.975543

3,1,4038.209091
3,1,1647.821970
3,1,6468.278689
3,1,11395.977987
3,1,24963.804469
3,1,3100.218750
3,1,4627.911392
3,1,1562.662500
3,1,17479.565789
3,1,70951.887755
3,1,6227.028409
3,1,28806.633523
3,1,10294.030702
3,1,1649.714286
3,1,2018.500000
3,1,2655.375000
3,1,18276.732955
3,1,6794.102273
3,1,43908.653846
3,1,3169.743056
3,1,6348.235294
3,1,5864.857143
3,1,9931.936170
3,1,11352.020270
3,1,1970.518293
3,1,7396.750000
3,1,10446.083333
3,1,3833.418605
3,1,2070.844444
3,1,1859.813953
3,1,34302.215909
3,1,6672.709302
3,1,1548.985294
3,1,3695.143293
3,1,2306.163265
3,1,2807.083333
3,1,2251.609756
3,1,7056.793478
3,1,6750.264706
3,1,1766.155556
3,1,4673.220588
3,1,2909.134804
3,1,4164.350000
3,1,7450.714286
3,1,8852.752660
3,1,1508.157500
3,1,11343.905405
3,1,3522.159091
3,1,5390.666667
3,1,3262.187500
3,1,5664.200000
3,1,1822.636111
3,1,3755.029762
3,1,2452.840000
3,1,8682.662234
3,1,2129.234756
3,1,3019.595930
3,1,5752.937500
3,1,22865.589286
3,1,2232.169903
3,1,4591.045455
3,1,4070.819149
3,1,3556.866667
3,1,3077.677778
3,1,1582.550481
3,1,37578.714286

3,1,12260.032787
3,1,101050.548780
3,1,2318.076087
3,1,1603.921512
3,1,5808.031447
3,1,1670.629630
3,1,32620.572368
3,1,88983.557692
3,1,3494.195652
3,1,3888.461111
3,1,4715.663462
3,1,2586.375000
3,1,8421.008772
3,1,10429.379310
3,1,3119.192708
3,1,8293.271277
3,1,1518.825000
3,1,5226.907258
3,1,4315.737500
3,1,16793.338816
3,1,2897.890625
3,1,25302.959677
3,1,5536.943182
3,1,3525.666667
3,1,14394.838235
3,1,3014.369792
3,1,7822.113636
3,1,1901.116279
3,1,2688.109756
3,1,2415.010081
3,1,7537.578629
3,1,2963.183333
3,1,3569.442708
3,1,3761.166667
3,1,4515.018868
3,1,37584.727273
3,1,3113.240000
3,1,7355.612500
3,1,1621.108696
3,1,2122.033784
3,1,1571.181034
3,1,1820.562500
3,1,3345.405488
3,1,3484.210227
3,1,4387.131410
3,1,14622.306818
3,1,1750.000000
3,1,21244.202703
3,1,3457.605263
3,1,24155.185976
3,1,4153.437500
3,1,5055.656250
3,1,2632.416667
3,1,5147.481771
3,1,1660.312500
3,1,2358.535714
3,1,68692.170732
3,1,8259.000000
3,1,4870.659091
3,1,57787.139706
3,1,26325.442308
3,1,2886.215686
3,1,10701.139706
3,1,2482.773936
3,1,4820.136364
3,1,1697.234043

3,1,3867.545732
3,1,3547.546512
3,1,1645.812500
3,1,7382.000000
3,1,2680.480769
4,2,6892.320652
4,2,2089.869565
4,2,1686.707500
4,2,3249.757353
4,2,2452.678977
4,2,2392.609091
4,2,5436.945783
4,2,1777.920103
4,2,3822.359375
4,2,2316.016304
4,2,2036.949468
4,2,7196.296875
4,2,6517.532143
4,2,4275.400000
4,2,1809.031250
4,2,1597.275000
4,2,1726.476562
4,2,1737.120455
4,2,9608.012500
4,2,2244.434375
4,2,4752.726562
4,2,2011.000000
4,2,3122.008152
4,2,7005.142857
4,2,5003.739130
4,2,9133.500000
4,2,2279.968750
4,2,4833.250000
4,2,3146.182692
4,2,5886.982143
4,2,1839.692568
4,2,2214.475000
4,2,11556.713068
4,2,3473.570000
4,2,4365.491018
4,2,2267.642458
4,2,2801.821429
4,2,2904.164286
4,2,3969.178571
4,2,3583.916667
4,2,3120.827128
4,2,3951.706395
4,2,2515.557018
4,2,7263.838710
4,2,1662.721088
4,2,4429.761905
4,2,1857.405405
4,2,2159.752358
4,2,2988.297872
4,2,1574.421053
4,2,4699.918367
4,2,2166.505000
4,2,4771.852941
4,2,1600.000000
4,2,5619.212054
4,2,2784.573529
4,2,4407.411765
4,2,1710.959184
4,2,5410.940341
4,2,4576.212264
4,2,2228.609756

4,2,2412.726974
4,2,3263.054688
4,2,2295.645714
4,2,2218.636364
4,2,1681.550000
4,2,3779.517045
4,2,3778.157500
4,2,1824.962963
4,2,14675.052632
4,2,1932.000000
4,2,3956.197674
4,2,17697.456522
4,2,1680.707865
4,2,3993.973837
4,2,3957.712838
4,2,2258.770833
4,2,2010.741071
4,2,8614.395000
4,2,3434.170455
4,2,1902.833333
4,2,2751.916667
4,2,1824.537500
4,2,4071.259868
4,2,5210.075000
4,2,2533.500000
4,2,10463.353448
4,2,4272.841346
4,2,8332.380208
4,2,12992.982500
4,2,4239.718137
4,2,10291.309211
4,2,3765.950000
4,2,4570.215909
4,2,4928.382353
4,2,7263.338942
4,2,3141.910256
4,2,9898.398437
4,2,3905.755102
4,2,4676.417614
4,2,4153.649390
4,2,4077.562500
4,2,3942.830882
4,2,3493.062500
4,2,6029.705882
4,2,5133.518939
4,2,2687.882979
4,2,2892.433333
4,2,2321.387500
4,2,8065.750000
4,2,1662.144886
4,2,2102.958333
4,2,1835.665761
4,2,7414.125000
4,2,1512.387755
4,2,1541.000000
4,2,2210.668750
4,2,1647.365385
4,2,6585.285714
4,2,6513.285714
4,2,3785.895238
4,2,2988.080556
4,2,7102.644608
4,2,15944.895833
4,2,1907.446809
4,2,1974.603659
4,2,1903.872340

4,2,1611.220238
4,2,5255.416667
4,2,2473.812500
4,2,4158.059880
4,2,5349.754717
4,2,8303.046053
4,2,2284.667763
4,2,2014.364865
4,2,10104.984375
4,2,1747.571970
4,2,2508.160000
4,2,2902.613095
4,2,6510.937173
4,2,1819.795455
4,2,3840.154412
4,2,8780.019231
4,2,4223.825000
4,2,1729.381250
4,2,3766.053191
4,2,13515.812500
4,2,3253.043605
4,2,2850.477941
4,2,7017.904762
4,2,2571.031250
4,2,4360.112500
4,2,2375.840278
4,2,4893.383333
4,2,2452.560209
4,2,2887.058824
4,2,4613.461538
4,2,4049.344920
4,2,1960.686047
4,2,2456.769737
4,2,2580.607143
4,2,2133.842105
4,2,2751.494186
4,2,8829.456897
4,2,3683.907216
4,2,3496.211957
4,2,1606.781250
4,2,1909.776316
4,2,2922.692308
4,2,2239.935268
4,2,1761.155405
4,2,1706.100000
4,2,1653.216216
4,2,2577.702381
4,2,1717.098039
4,2,2550.627778
4,2,3317.302326
4,2,2225.529070
4,2,8360.875000
4,2,1740.437500
4,2,2547.968750
4,2,2482.425000
4,2,4931.200000
4,2,3824.387195
4,2,1877.105978
4,2,4875.343750
4,2,7423.965116
4,2,1683.900735
4,2,7955.214286
4,2,6887.918103
4,2,4604.307692
4,2,1506.868056
4,2,7425.666667

4,2,3439.500000
4,2,3398.423077
4,2,1619.732558
4,2,1940.222222
4,2,2202.593750
4,2,1851.010638
4,2,3191.317857
4,2,2690.645833
4,2,2043.147059
4,2,6404.562500
4,2,3411.445455
4,2,3781.599057
4,2,1986.065217
4,2,4181.921053
4,2,1965.910256
4,2,1841.400943
4,2,4055.000000
4,2,2890.452586
4,2,1926.925676
4,2,3124.654135
4,2,10770.170000
4,2,1875.538462
4,2,1538.081522
4,2,2141.875000
4,2,5203.425532
4,2,3272.574561
4,2,5749.359756
4,2,1581.000000
4,2,6182.187500
4,2,2397.327500
4,2,3699.807432
4,2,3054.237805
4,2,5048.625000
4,2,2007.537234
4,2,9167.809524
4,2,2728.483173
4,2,3359.250000
4,2,6344.051630
4,2,8625.013453
4,2,4260.500000
4,2,25887.611111
4,2,2050.125000
4,2,1683.230769
4,2,1909.821875
4,2,1815.131579
4,2,7661.166667
4,2,2005.770270
4,2,1503.044118
4,2,4125.040816
4,2,3926.396739
4,2,4017.625000
4,2,2982.161932
4,2,11308.608333
4,2,3522.307927
4,2,2132.500000
4,2,1903.586207
4,2,5403.095238
4,2,2278.318750
4,2,2771.729730
4,2,5123.255102
4,2,3013.496622
4,2,3463.037791
4,2,2309.837838
4,2,1655.937173
4,2,3593.428571
4,2,2293.689024

4,2,6183.296875
4,2,7387.764706
4,2,2847.285714
4,2,10073.970968
4,2,9195.000000
4,2,2361.741848
4,2,4220.864796
4,2,1980.343750
4,2,1788.664894
4,2,3036.898649
4,2,3399.864130
4,2,1544.724684
4,2,15803.115385
4,2,3899.177273
4,2,50830.500000
4,2,3221.972222
4,2,2666.903409
4,2,2101.825581
4,2,3210.613095
4,2,2711.462428
4,2,4178.535714
4,2,8050.472973
4,2,2652.007692
4,2,6067.111702
4,2,6842.454545
4,2,2743.176471
4,2,1885.475000
4,2,3091.022959
4,2,17120.747396
4,2,5068.165094
4,2,2555.285714
4,2,2693.375000
4,2,2454.773256
4,2,1623.187500
4,2,2351.100694
4,2,2673.883929
4,2,2082.540441
4,2,2479.553571
4,2,2170.040948
4,2,2441.188725
4,2,5074.187204
4,2,9812.120079
4,2,1991.406863
4,2,2656.530000
4,2,1709.763158
4,2,3235.844388
4,2,2814.104651
4,2,5980.078804
4,2,2457.803922
4,2,2048.529101
4,2,1714.660000
4,2,2348.083333
4,2,1668.068182
4,2,5544.125000
4,2,5929.777778
4,2,1901.153302
4,2,2002.261364
4,2,2045.309028
4,2,6520.502660
4,2,4341.953125
4,2,1752.016026
4,2,1668.193750
4,2,1700.361111
4,2,3286.171875
4,2,2457.924419
4,2,3139.453704

4,2,2619.232143
4,2,3479.669643
4,2,2961.469595
4,2,2682.273585
4,2,1874.980769
4,2,2110.953125
4,2,1632.238636
4,2,4854.269231
4,2,2109.321429
4,2,5709.817308
4,2,1916.423077
4,2,2049.125000
4,2,28635.024390
4,2,2813.875000
4,2,2582.255000
4,2,4546.029940
4,2,5801.875000
4,2,2119.369318
4,2,3059.861702
4,2,3115.865385
4,2,5403.719340
4,2,1500.222222
4,2,7280.490566
4,2,2317.283537
4,2,3476.754902
4,2,2241.854167
4,2,5257.565476
4,2,3436.937500
4,2,1734.773437
4,2,3677.475000
4,2,3671.307018
4,2,1583.959302
4,2,5711.418605
4,2,2384.346154
4,2,2438.682927
4,2,1642.006849
4,2,3025.083333
4,2,5133.631757
4,2,8645.461111
4,2,2381.283163
4,2,12776.157895
4,2,3530.500000
4,2,2033.406250
4,2,3239.953804
4,2,1679.252660
4,2,3033.902174
4,2,3647.114035
4,2,10972.295455
4,2,1882.162500
4,2,1683.645390
4,2,5618.652778
4,2,3082.236111
4,2,1680.090909
4,2,3868.000000
4,2,10442.154412
4,2,7679.038462
4,2,1835.010417
4,2,2516.887821
4,2,1507.964286
4,2,2346.094595
4,2,1910.451923
4,2,8347.909091
4,2,3569.907609
4,2,3189.400000
4,2,3207.750000
4,2,3595.637755

4,2,3414.686364
4,2,1572.118750
4,2,1869.887324
4,2,1641.222222
4,2,11314.786932
4,2,3177.523438
4,2,1667.148810
4,2,7288.000000
4,2,2711.818750
4,2,2007.857955
4,2,5864.492424
4,2,1582.958333
4,2,2459.524390
4,2,3103.170000
4,2,1957.821429
4,2,1761.065476
4,2,3514.850000
4,2,2376.166667
4,2,9764.861111
4,2,2829.365854
4,2,2305.154070
4,2,3035.553571
4,2,3148.489796
4,2,1588.686047
4,2,2268.061111
4,2,1938.337838
4,2,8311.078125
4,2,2499.515625
4,2,3870.325820
4,2,2624.357143
4,2,2686.115132
4,2,6612.565341
4,2,1787.750000
4,2,5090.062500
4,2,4841.961538
4,2,2583.232143
4,2,1617.027174
4,2,2110.935811
4,2,2368.860465
4,2,4165.413265
4,2,22406.465517
4,2,4989.217105
4,2,4333.421053
4,2,2003.982143
4,2,2916.567073
4,2,4581.325000
4,2,2786.662162
4,2,4105.474359
4,2,1887.744318
4,2,4506.961538
4,2,1991.875000
4,2,3287.356707
4,2,20673.995283
4,2,3976.877500
4,2,3194.733333
4,2,2203.343750
4,2,6290.359375
4,2,9282.940000
4,2,10791.804167
4,2,5540.709790
4,2,1602.836478
4,2,1986.873418
4,2,2664.494624
4,2,1873.439024
4,2,2804.051887
4,2,5340.982143

4,2,2899.784314
4,2,6772.932065
4,2,1528.092949
4,2,3857.470588
4,2,10678.136842
4,2,2641.128049
4,2,7919.255319
4,2,1556.166667
4,2,4212.156250
4,2,6345.855769
4,2,2118.425532
4,2,2845.747768
4,2,1991.625000
4,2,4463.780000
4,2,2289.885417
4,2,2528.691489
4,2,1819.010870
4,2,1817.600000
4,2,2687.031250
4,2,1988.348214
4,2,3790.544872
4,2,2183.989583
4,2,2027.073864
4,2,1681.680921
4,2,2910.475000
4,2,4077.403409
4,2,4720.554187
4,2,1956.714286
4,2,1901.520833
4,2,8267.887500
4,2,4073.312500
4,2,1768.631579
4,2,11110.938144
4,2,6315.451807
4,2,4127.437126
4,2,4827.639474
4,2,1868.807895
4,2,2241.861111
4,2,2306.810976
4,2,3098.350000
4,2,3652.500000
4,2,2221.000000
4,2,1536.393939
4,2,3838.232558
4,2,2083.367089
4,2,2216.366848
4,2,1838.150000
4,2,3598.744444
4,2,1578.365854
4,2,3006.914474
4,2,5395.783019
4,2,4132.750000
4,2,4464.428082
4,2,1984.539474
4,2,3648.857143
4,2,15985.729167
4,2,1714.000000
4,2,1619.682432
4,2,5800.088235
4,2,2299.241935
4,2,3582.231707
4,2,16461.425532
4,2,3820.114583
4,2,2557.625000
4,2,1612.606250
4,2,7975.041667

4,2,4981.934896
4,2,2227.125000
4,2,5477.850000
4,2,11990.318548
4,2,2444.894231
4,2,2463.281250
4,2,1690.345238
4,2,2709.625000
4,2,2628.048077
4,2,4631.675676
4,2,1857.497222
4,2,2186.527778
4,2,2931.572674
4,2,5487.617647
4,2,2053.269231
4,2,23393.785714
4,2,8123.406250
4,2,2567.724359
4,2,2784.425000
4,2,8322.388889
4,2,3242.062500
4,2,7156.029412
4,2,3503.875000
4,2,5698.775000
4,2,2605.382979
4,2,2738.502660
4,2,10131.379913
4,2,2147.996403
4,2,7512.737805
4,2,8745.625000
4,2,2346.728571
4,2,1551.851351
4,2,2335.750000
4,2,5083.331658
4,2,4340.342466
4,2,3136.757310
4,2,2044.342105
4,2,1512.292857
4,2,3537.309091
4,2,1789.652439
4,2,1568.807692
4,2,2612.044118
4,2,1863.062500
4,2,2103.790323
4,2,2036.423913
4,2,1769.288043
4,2,8547.372093
4,2,1829.990741
4,2,1553.526163
4,2,7795.962838
4,2,3983.269886
4,2,3596.185714
4,2,4281.672131
4,2,5629.311111
4,2,1535.504902
4,2,3959.196429
4,2,2147.773585
4,2,1846.799342
4,2,7705.671779
4,2,15074.427885
4,2,1937.268293
4,2,16600.973958
4,2,1684.269231
4,2,10987.250000
4,2,2300.505814
4,2,9026.946429

4,2,7665.013158
4,2,4751.957447
4,2,1947.625000
4,2,1771.336735
4,2,1564.697674
4,2,5014.371901
4,2,2019.990000
4,2,2397.561224
4,2,3678.601351
4,2,3593.976190
4,2,2792.425532
4,2,3013.886111
4,2,4072.619565
4,2,2500.918750
4,2,4919.640957
4,2,1617.035714
4,2,2070.562500
4,2,6644.825980
4,2,1609.409091
4,2,3352.844444
4,2,4612.607143
4,2,3120.969388
4,2,1513.620690
4,2,5940.653061
4,2,2658.285326
4,2,3871.246875
4,2,3735.864796
4,2,4477.840000
4,2,1936.156250
4,2,2746.700000
4,2,4774.100543
4,2,3377.844595
4,2,1591.846154
4,2,5311.780556
4,2,1790.049479
4,2,1596.114286
4,2,3007.525000
4,2,2269.706731
4,2,10513.812500
4,2,2757.764706
4,2,4432.857143
4,2,4502.707447
4,2,1671.450000
4,2,2745.414474
4,2,1600.911111
4,2,5026.294872
4,2,5699.875000
4,2,1930.813830
4,2,3803.500000
4,2,3485.048295
4,2,3276.651442
4,2,2662.157407
4,2,4262.648936
4,2,1539.091216
4,2,4495.375000
4,2,4005.816327
4,2,3056.966867
4,2,1500.746269
4,2,2676.046512
4,2,1726.569767
4,2,4006.920213
4,2,1919.094444
4,2,14843.700000
4,2,1852.353659
4,2,1707.194767
4,2,1950.925000

4,2,6937.507812
4,2,3208.080882
4,2,8483.886364
4,2,4260.564189
4,2,4484.139423
4,2,2399.739583
4,2,3898.832500
4,2,5302.787234
4,2,4469.790698
4,2,6592.000000
4,2,12878.744898
4,2,4455.674757
4,2,1611.513514
4,2,1707.848837
4,2,4788.606383
4,2,4472.002212
4,2,4156.107558
4,2,1969.881579
4,2,10063.496711
4,2,2564.031915
4,2,1547.500000
4,2,2130.289286
4,2,2608.479651
4,2,2105.733108
4,2,1668.801020
4,2,19000.497768
4,2,1719.465909
4,2,1525.429878
4,2,3027.900000
4,2,4274.093023
4,2,1769.484694
4,2,2821.100559
4,2,2014.225806
4,2,1532.506173
4,2,17001.877358
4,2,3075.729508
4,2,3237.920213
4,2,3214.026786
4,2,1732.336957
4,2,6760.590426
4,2,3804.245902
4,2,1676.390625
4,2,1794.706897
4,2,2868.702128
4,2,3166.280488
4,2,3827.384615
4,2,5220.549419
4,2,6506.750000
4,2,3528.997283
4,2,10369.769231
4,2,5942.569079
4,2,14673.000000
4,2,2195.090116
4,2,5237.633929
4,2,3692.739796
4,2,2520.836806
4,2,1509.558140
4,2,6082.571429
4,2,9257.229167
4,2,9222.451220
4,2,6263.216981
4,2,3167.400000
4,2,1925.642857
4,2,1605.560976
4,2,3347.431818
4,2,10827.618677

4,2,12352.552326
4,2,2529.461538
4,2,3466.822086
4,2,2677.725610
4,2,1838.522222
4,2,10691.358974
4,2,3680.661765
4,2,2135.989362
4,2,6566.583333
4,2,3147.142857
4,2,2773.533654
4,2,2479.039634
4,2,5826.716837
4,2,3217.922794
4,2,2836.078125
4,2,9696.806122
4,2,2683.312500
4,2,1522.000000
4,2,2522.320513
4,2,3385.234043
4,2,4802.850000
4,2,3501.560811
4,2,5444.380435
4,2,2807.500000
4,2,1688.619681
4,2,1848.651163
4,2,4570.101351
4,2,2114.250000
4,2,3533.916667
4,2,5199.142857
4,2,4803.028646
4,2,1839.924342
4,2,2255.597500
4,2,3502.583333
4,2,3923.264516
4,2,2182.714286
4,2,1595.254054
4,2,1765.657143
4,2,5595.565104
4,2,7936.000000
4,2,1863.630208
4,2,4215.063830
4,2,2220.125000
4,2,2797.734043
4,2,5691.689815
4,2,3572.940397
4,2,2706.424342
4,2,1563.516026
4,2,1801.493590
4,2,3515.568182
4,2,1540.164474
4,2,2414.175000
4,2,4883.378289
4,2,8887.584184
4,2,3129.867647
4,2,2636.315789
4,2,2031.742424
4,2,2698.796460
4,2,3877.136598
4,2,4249.881188
4,2,5318.683511
4,2,12932.455882
4,2,4929.777778
4,2,9287.660377
4,2,1887.552083
4,2,8641.160377

4,2,2731.042254
4,2,9977.211268
4,2,2257.236111
4,2,3887.413265
4,2,3664.551020
4,2,1531.750000
4,2,1763.798780
4,2,11170.235294
4,2,3514.125000
4,2,2442.397959
4,2,6687.997596
4,2,1527.464286
4,2,3222.071429
4,2,6205.178771
4,2,4521.760000
4,2,4938.500000
4,2,1616.863281
4,2,1646.233108
4,2,1614.898438
4,2,3843.000000
4,2,3912.750000
4,2,3988.176829
4,2,6575.583333
4,2,13041.380814
4,2,4138.797297
4,2,4087.020833
4,2,5099.921875
4,2,4255.777027
4,2,3001.517857
4,2,1796.804054
4,2,2571.725000
4,2,3008.609375
4,2,5358.852941
4,2,2399.845339
4,2,1742.263473
4,2,2807.857955
4,2,4296.617647
4,2,2016.488372
4,2,5204.410000
4,2,1674.292500
4,2,2203.308824
4,2,2001.887195
4,2,1950.488636
4,2,1613.842391
4,2,2655.812500
4,2,5773.142012
4,2,3704.055556
4,2,2624.822222
4,2,1938.917553
4,2,8275.653846
4,2,5999.570946
4,2,5068.419811
4,2,2778.639706
4,2,1891.454301
4,2,3651.000000
4,2,3721.840000
4,2,1959.892157
4,2,3281.460227
4,2,2539.453125
4,2,2287.790698
4,2,2411.000000
4,2,5738.241026
4,2,3385.085890
4,2,4410.169591
4,2,2542.965909
4,2,8486.620853

4,2,2454.412791
4,2,4067.300000
4,2,2071.537500
4,2,2283.677632
4,2,10685.617021
4,2,3782.375000
4,2,1629.581395
4,2,1960.750000
4,2,1706.511628
4,2,7152.234375
4,2,1702.560897
4,2,2258.797170
4,2,2398.829545
4,2,2594.167553
4,2,4696.571429
4,2,9471.525000
4,2,1719.000000
4,2,5884.223958
4,2,1500.000000
4,2,1564.055000
4,2,1799.702830
4,2,3223.401163
4,2,11043.214286
4,2,1875.664062
4,2,8011.097561
4,2,2783.918367
4,2,1843.854167
4,2,10658.255208
4,2,2716.802326
4,2,1702.005435
4,2,9732.212766
4,2,9321.884470
4,2,1625.450000
4,2,3618.000000
4,2,1714.139205
4,2,8458.908654
4,2,1982.656250
4,2,3501.594828
4,2,4805.726190
4,2,2297.039634
4,2,2680.195122
4,2,4623.480769
4,2,1985.040541
4,2,2236.079327
4,2,2129.352778
4,2,2073.274194
4,2,1805.572222
4,2,4373.710106
4,2,2148.100000
4,2,1679.496875
4,2,2036.125000
4,2,2111.589286
4,2,3627.923077
4,2,4125.093023
4,2,2358.743590
4,2,2063.229947
4,2,4709.125000
4,2,1737.406250
4,2,2533.117647
4,2,6837.858173
4,2,4970.823529
4,2,3043.780405
4,2,3145.329082
4,2,4837.983796
4,2,11712.579268
4,2,3062.774390

4,2,1584.480676
4,2,3554.901639
4,2,1670.780612
4,2,2966.217391
4,2,1953.000000
4,2,1780.431548
4,2,4156.414894
4,2,1678.125000
4,2,2975.750000
4,2,24171.894231
4,2,10332.947115
4,2,2136.406250
4,2,1523.188679
4,2,3851.416667
4,2,11687.641827
4,2,1781.595628
4,2,13061.122396
4,2,4003.157895
4,2,5683.784884
4,2,3636.375000
4,2,4026.871711
4,2,4825.234043
4,2,14901.387500
4,2,3600.552356
4,2,4813.766509
4,2,2771.242424
4,2,2454.469512
4,2,3321.348315
4,2,2595.724432
4,2,2432.022727
4,2,11312.834302
4,2,2347.000000
4,2,6686.781250
4,2,9540.367188
4,2,4714.792857
4,2,1788.086538
4,2,2706.888889
4,2,7616.157738
4,2,1838.895408
4,2,3031.145270
4,2,2166.638298
4,2,9297.932292
4,2,2426.948370
4,2,2640.785714
4,2,3328.004902
4,2,3521.519886
4,2,4645.775641
4,2,4574.710000
4,2,23931.315000
4,2,1982.487069
4,2,2849.795918
4,2,3229.590909
4,2,2806.596591
4,2,2269.292763
4,2,1754.750000
4,2,3678.526946
4,2,2769.018868
4,2,2414.711111
4,2,5879.469388
4,2,1750.895349
4,2,6807.000000
4,2,1573.675000
4,2,24985.585938
4,2,2526.000000
4,2,2163.401316
4,2,17631.204082

4,2,10716.094298
4,2,5225.529412
4,2,8323.000000
4,2,1798.268750
4,2,2053.440476
4,2,3074.670455
4,2,6234.043478
4,2,11926.019231
4,2,8096.941860
4,2,2948.571429
4,2,3013.401316
4,2,3815.914894
4,2,2547.875000
4,2,5310.046512
4,2,2501.861842
4,2,19262.971154
4,2,6090.750000
4,2,5360.298387
4,2,2286.490566
4,2,2122.541667
4,2,2047.279070
4,2,1879.083333
4,2,3078.571023
4,2,1813.437126
4,2,1883.375000
4,2,4891.898936
4,2,1513.883721
4,2,1972.900000
4,2,2969.332447
4,2,3820.733696
4,2,8352.438830
4,2,1970.090426
4,2,2128.000000
4,2,2564.329609
4,2,3492.598684
4,2,7634.800000
4,2,5235.787879
4,2,4092.915000
4,2,5013.888889
4,2,2666.189573
4,2,3238.490566
4,2,3683.000000
4,2,3291.904255
4,2,6306.904070
4,2,5452.377907
4,2,2384.000000
4,2,1982.161932
4,2,5434.812500
4,2,4754.882353
4,2,3505.494565
4,2,5664.153646
4,2,2022.351190
4,2,8601.008523
4,2,5062.659574
4,2,2013.275000
4,2,3393.044444
4,2,6870.565104
4,2,1660.473684
4,2,1875.250000
4,2,1629.890728
4,2,3919.401786
4,2,1516.209770
4,2,1512.294118
4,2,3024.086957
4,2,1898.634615
4,2,2139.000000

4,2,1964.337500
4,2,2814.117647
4,2,2042.500000
4,2,2798.700472
4,2,3888.840909
4,2,9277.875000
4,2,1606.639881
4,2,11049.201613
4,2,1921.628289
4,2,1953.937500
4,2,2172.880952
4,2,3129.906977
4,2,8962.438679
4,2,4002.732143
4,2,10069.194231
4,2,1560.107143
4,2,1836.028571
4,2,4280.041916
4,2,2481.695122
4,2,8655.993056
4,2,3637.464674
4,2,2154.425000
4,2,3531.788889
4,2,4057.018868
4,2,2557.732143
4,2,1639.097973
4,2,2347.686275
4,2,1766.000000
4,2,4101.515625
4,2,4135.407143
4,2,2861.099490
4,2,2999.062500
4,2,1709.886598
4,2,2707.989011
4,2,4895.280000
4,2,6427.995000
4,2,7524.576923
4,2,3007.256250
4,2,4280.419811
4,2,3571.142857
4,2,5376.482143
4,2,2198.085526
4,2,2420.118750
4,2,1568.875000
4,2,6414.016026
4,2,2725.372549
4,2,2690.241477
4,2,11612.538636
4,2,2805.168919
4,2,2932.949468
4,2,2486.128205
4,2,9092.023438
4,2,3232.041667
4,2,1689.611628
4,2,6453.613636
4,2,2768.150000
4,2,4682.867925
4,2,3042.147436
4,2,4651.590625
4,2,4271.041667
4,2,2557.843137
4,2,2301.596154
4,2,4169.750000
4,2,2568.701087
4,2,7251.183824
4,2,3374.461538

4,2,5909.109005
4,2,13066.885417
4,2,2122.145349
4,2,1713.750000
4,2,5179.633803
4,2,3671.758389
4,2,1876.714286
4,2,1536.967532
4,2,2285.301887
4,2,2095.267857
4,2,1749.318878
4,2,3152.425000
4,2,1505.230769
4,2,1888.691489
4,2,3685.802326
4,2,1741.945755
4,2,8426.204082
4,2,4051.125000
4,2,3220.280000
4,2,7356.583333
4,2,5255.993056
4,2,2278.122159
4,2,4581.398026
4,2,3580.872685
4,2,1743.657895
4,2,4705.519231
4,2,1663.047872
4,2,5408.422111
4,2,2127.634615
4,2,8222.808140
4,2,2261.794118
4,2,2360.834395
4,2,3807.130208
4,2,1618.169444
4,2,2882.754902
4,2,2446.666667
4,2,2704.701754
4,2,2327.663194
4,2,2889.052083
4,2,2758.918129
4,2,3127.188725
4,2,2568.113208
4,2,3356.000000
4,2,1672.430233
4,2,2849.412500
4,2,1530.443182
4,2,6286.732456
4,2,1513.516026
4,2,4775.054688
4,2,3178.435897
4,2,2665.617021
4,2,4061.685185
4,2,2396.455000
4,2,2255.472973
4,2,1955.296196
4,2,3101.800532
4,2,3094.125000
4,2,2525.144737
4,2,1731.500000
4,2,7976.969388
4,2,2553.000000
4,2,8188.321429
4,2,1866.935897
4,2,4523.341837
4,2,4058.396739
4,2,3497.294872

4,2,2550.271739
4,2,4070.519022
4,2,4403.826087
4,2,2243.257212
4,2,1743.665625
4,2,1958.962209
4,2,1685.584135
4,2,5583.335570
4,2,1701.344828
4,2,4206.402439
4,2,17670.195652
4,2,1638.675676
4,2,2929.881250
4,2,4216.093750
4,2,3315.562500
4,2,2587.000000
4,2,2364.828125
4,2,6253.393617
4,2,1920.620000
4,2,10837.809524
4,2,1734.125000
4,2,2542.522727
4,2,5162.462500
4,2,1533.281250
4,2,2039.817073
4,2,2600.625000
4,2,4473.767857
4,2,2878.250000
4,2,1551.097222
4,2,17353.164502
4,2,5118.391061
4,2,4472.385870
4,2,2116.387500
4,2,1592.479592
4,2,2796.826087
4,2,1984.371429
4,2,6809.392694
4,2,10644.366120
4,2,5423.522727
4,2,1508.494565
4,2,3035.746875
4,2,13586.875000
4,2,6393.325581
4,2,15527.107143
4,2,2189.272727
4,2,1592.777778
4,2,1678.333333
4,2,2991.899457
4,2,1876.394231
4,2,6890.844340
4,2,1759.986842
4,2,1532.560976
4,2,5719.963362
4,2,2664.697674
4,2,3035.950000
4,2,1627.409722
4,2,8822.818182
4,2,3744.886364
4,2,2996.882812
4,2,3150.114362
4,2,2165.351064
4,2,3445.156250
4,2,1652.314103
4,2,19354.491071
4,2,2664.000000
4,2,4193.263636

4,2,3901.506466
4,2,1900.587500
4,2,10617.362745
4,2,7696.375000
4,2,3167.705882
4,2,4609.214286
4,2,1874.329861
4,2,6437.082317
4,2,1610.100000
4,2,1621.300000
4,2,3723.976744
4,2,3454.023649
4,2,9137.312500
4,2,8632.988636
4,2,4760.025641
4,2,3035.634146
4,2,1622.755319
4,2,5555.164894
4,2,2135.944444
4,2,2434.657051
4,2,2518.604294
4,2,2567.797872
4,2,2129.731707
4,2,3740.182500
4,2,2456.662500
4,2,4622.641304
4,2,1586.570652
4,2,3094.050000
4,2,6105.068750
4,2,1509.000000
4,2,2439.788636
4,2,2167.858209
4,2,1706.294872
4,2,1831.297297
4,2,1532.422222
4,2,2820.138365
4,2,2664.673077
4,2,5976.500000
4,2,4776.093023
4,2,1707.897959
4,2,1723.113889
4,2,3195.565217
4,2,11716.670455
4,2,1944.835366
4,2,1572.414365
4,2,2132.843023
4,2,1832.041667
4,2,6451.941935
4,2,2477.250000
4,2,3938.750000
4,2,3002.513889
4,2,5769.500000
4,2,2290.427083
4,2,3160.019231
4,2,3655.100000
4,2,3765.059783
4,2,4170.634615
4,2,1835.106383
4,2,2198.177632
4,2,2811.125000
4,2,5177.151786
4,2,2701.263158
4,2,5043.883152
4,2,1901.000000
4,2,1695.914634
4,2,2305.179775

4,2,3107.791667
4,2,6098.533537
4,2,3491.908602
4,2,2219.886667
4,2,4675.411058
4,2,2673.304795
4,2,7157.414474
4,2,1556.771605
4,2,4469.375000
4,2,4886.033333
4,2,3826.542553
4,2,3751.860119
4,2,2363.417647
4,2,1595.714286
4,2,5766.245098
4,2,2075.211538
4,2,4972.344595
4,2,3225.720238
4,2,3204.910326
4,2,1851.067568
4,2,3388.377907
4,2,2376.384615
4,2,1786.168919
4,2,2969.217949
4,2,2955.652174
4,2,2263.281437
4,2,7757.166667
4,2,9228.897436
4,2,19634.335443
4,2,7033.078125
4,2,1778.500000
4,2,3793.810096
4,2,3246.750000
4,2,2658.605769
4,2,1663.521277
4,2,2441.187500
4,2,8228.380319
4,2,1590.461957
4,2,1989.414894
4,2,3562.775641
4,2,2710.365385
4,2,2310.763158
4,2,1535.897436
4,2,2382.063830
4,2,3443.425000
4,2,1986.442568
4,2,2298.250000
4,2,5197.237500
4,2,3839.000000
4,2,3080.347826
4,2,3787.057018
4,2,1822.068878
4,2,5414.460526
4,2,4242.188953
4,2,5967.679487
4,2,2617.300676
4,2,1815.341216
4,2,7557.485294
4,2,4530.112745
4,2,12193.342672
4,2,2829.076531
4,2,11835.057692
4,2,1857.371875
4,2,3511.210084
4,2,2010.463235
4,2,5777.305785

4,2,2320.184524
4,2,3106.653846
4,2,2203.600000
4,2,10225.954082
4,2,3156.278409
4,2,8568.677778
4,2,4606.250000
4,2,3150.000000
4,2,2222.996711
4,2,2562.881579
4,2,2108.544910
4,2,2568.954268
4,2,8419.000000
4,2,15088.631579
4,2,2875.561275
4,2,5333.178571
4,2,2575.968750
4,2,2853.777778
4,2,11174.318182
4,2,2140.571429
4,2,6532.934524
4,2,4376.802469
4,2,1860.505155
4,2,2085.470588
4,2,2972.923295
4,2,1690.242021
4,2,1756.982143
4,2,2538.571429
4,2,4863.323529
4,2,3514.925000
4,2,4615.234043
4,2,3504.194444
4,2,1979.683511
4,2,5423.928571
4,2,2282.918478
4,2,1923.285714
4,2,4239.922222
4,2,3852.825000
4,2,3246.923913
4,2,2939.843137
4,2,2192.696429
4,2,1765.666667
4,2,3641.785000
4,2,4630.437500
4,2,3340.981250
4,2,1506.114865
4,2,4855.765957
4,2,1943.530405
4,2,2388.858553
4,2,3262.186047
4,2,1859.719697
4,2,8177.532895
4,2,3463.867647
4,2,33002.836735
4,2,2349.609948
4,2,2642.965278
4,2,2614.779070
4,2,11788.042308
4,2,1774.364796
4,2,2496.790816
4,2,7897.485000
4,2,2903.357143
4,2,6440.983108
4,2,3109.377778
4,2,3668.254167
4,2,9609.919118

4,2,4871.811224
4,2,1817.604730
4,2,2743.617647
4,2,1979.071429
4,2,6258.971698
4,2,1526.542553
4,2,3540.666667
4,2,3035.540816
4,2,2672.893617
4,2,2731.568182
4,2,2624.437500
4,2,2486.509804
4,2,4437.988636
4,2,5297.000000
4,2,2579.733333
4,2,1881.593750
4,2,1659.125000
4,2,6036.211111
4,2,4755.975610
4,2,3583.250000
4,2,1892.611413
4,2,3086.608696
4,2,1627.601064
4,2,1672.943750
4,2,2794.000000
4,2,2624.468137
4,2,5600.464286
4,2,1844.608333
4,2,9488.877907
4,2,1815.551829
4,2,3883.305556
4,2,2691.357143
4,2,2488.156250
4,2,3946.237500
4,2,1778.375000
4,2,2013.661932
4,2,3284.367925
4,2,1838.750000
4,2,2003.364865
4,2,1633.981707
4,2,2624.174419
4,2,1636.729592
4,2,1499.420732
4,2,2238.295732
4,2,2365.913043
4,2,2899.487179
4,2,2612.898810
4,2,5289.950000
4,2,4681.406250
4,2,1790.181250
4,2,1973.218750
4,2,2026.980769
4,2,4008.500000
4,2,5288.321429
4,2,1743.686111
4,2,4994.000000
4,2,1532.312500
4,2,2417.960938
4,2,3833.490385
4,2,2505.727273
4,2,4716.066667
4,2,4874.687500
4,2,2010.342500
4,2,2970.027174
4,2,2040.762195
4,2,2500.553191

4,2,2236.428571
4,2,1816.393293
4,2,1590.472222
4,2,3815.331633
4,2,1962.235294
4,2,3584.619565
4,2,4643.513514
4,2,2236.730769
4,2,2938.687500
4,2,2438.692308
4,2,1764.500000
4,2,6863.307692
4,2,1526.608553
4,2,1733.802885
4,2,13793.948171
4,2,2833.904891
4,2,10032.904412
4,2,2105.947368
4,2,1870.283333
4,2,9609.432500
4,2,1629.642857
4,2,2471.191919
4,2,8624.286585
4,2,3464.670732
4,2,2774.727273
4,2,2034.732143
4,2,2559.294118
4,2,1938.814516
4,2,2626.696429
4,2,8464.262019
4,2,1623.959459
4,2,1862.615132
4,2,13724.954545
4,2,3358.625000
4,2,2000.421053
4,2,1768.357143
4,2,1558.033333
4,2,6923.718750
4,2,4563.250000
4,2,1613.664634
4,2,1645.291667
4,2,1706.434211
4,2,2631.830189
4,2,2409.395833
4,2,6363.969512
4,2,2376.289894
4,2,1615.701220
4,2,3118.841667
4,2,7280.140777
4,2,2726.772727
4,2,2377.367347
4,2,3018.662791
4,2,3905.152174
4,2,3824.945312
4,2,5149.418848
4,2,2967.537879
4,2,2705.086758
4,2,1548.880795
4,2,4655.081395
4,2,1725.719512
4,2,2106.212500
4,2,3124.431250
4,2,8428.431818
4,2,1630.840909
4,2,2537.268293
4,2,6392.768293

4,2,2246.444444
4,2,1790.950000
4,2,3027.910377
4,2,3186.317073
4,2,3282.363636
4,2,2567.875000
4,2,7719.772727
4,2,11444.880435
4,2,102682.914110
4,2,1712.360294
4,2,6547.223485
4,2,1575.966667
4,2,5227.360465
4,2,10082.422170
4,2,3160.784722
4,2,6328.866667
4,2,4812.962766
4,2,6998.604651
4,2,2271.923077
4,2,2454.644886
4,2,1566.550000
4,2,2113.826087
4,2,2311.330049
4,2,2960.035714
4,2,1575.000000
4,2,3791.442623
4,2,4585.184211
4,2,2721.407051
4,2,2775.421053
4,2,2154.710526
4,2,4150.654054
4,2,1754.972678
4,2,1775.663265
4,2,9177.495192
4,2,1849.087209
4,2,1728.285714
4,2,1513.423077
4,2,15783.375000
4,2,2522.843750
4,2,5213.244681
4,2,8917.012755
4,2,2051.118421
4,2,4499.551887
4,2,1991.637097
4,2,3432.520349
4,2,1990.247423
4,2,1513.642857
4,2,3245.089744
4,2,6467.012195
4,2,6592.660377
4,2,9949.659389
4,2,2669.082474
4,2,2339.266667
4,2,1978.584337
4,2,2087.677083
4,2,3507.397727
4,2,2352.038462
4,2,2059.033816
4,2,3425.186275
4,2,2528.336538
4,2,2037.125000
4,2,3315.574074
4,2,1910.763158
4,2,2084.547872
4,2,24793.172897
4,2,2730.000000

4,2,3773.884868
4,2,2892.054688
4,2,6609.541667
4,2,4228.378378
4,2,2861.994565
4,2,1669.669643
4,2,3720.186275
4,2,1683.363095
4,2,2677.488426
4,2,3631.420455
4,2,1808.324405
4,2,1999.021875
4,2,13412.668605
4,2,1613.875000
4,2,3085.850543
4,2,6073.048077
4,2,2004.039216
4,2,2143.895349
4,2,3628.428571
4,2,2740.567308
4,2,4979.750000
4,2,4587.950000
4,2,7023.063953
4,2,5165.372340
4,2,4487.578125
4,2,2979.481818
4,2,4624.424342
4,2,8598.605263
4,2,2413.333333
4,2,3734.275000
4,2,2047.200000
4,2,2023.150754
4,2,1505.576389
4,2,5365.884615
4,2,4007.782051
4,2,4133.661765
4,2,2401.494565
4,2,1678.277500
4,2,1548.875000
4,2,2556.901163
4,2,6528.000000
4,2,1697.426339
4,2,3090.612245
4,2,2637.813636
4,2,2749.908163
4,2,8465.397059
4,2,2327.046875
4,2,1595.179348
4,2,3353.000000
4,2,4739.787500
4,2,5834.375000
4,2,6797.858696
4,2,6991.700000
4,2,3483.923077
4,2,2988.726563
4,2,2632.076705
4,2,1758.791667
4,2,9951.852941
4,2,3642.847826
4,2,2602.959459
4,2,4854.931548
4,2,11467.566502
4,2,5465.032086
4,2,1942.164773
4,2,2025.283251
4,2,1603.325581

4,2,2452.921182
4,2,1632.952381
4,2,6910.723214
4,2,6781.391304
4,2,2436.325000
4,2,3710.428571
4,2,1889.727941
4,2,5832.636364
4,2,2570.899457
4,2,2635.722561
4,2,5204.872449
4,2,2427.422222
4,2,4695.362500
4,2,2058.111702
4,2,3287.497093
4,2,2632.914894
4,2,10406.270270
4,2,3876.250000
4,2,1792.429688
4,2,2022.540816
4,2,1732.509375
4,2,3428.794643
4,2,3034.817308
4,2,3950.759259
4,2,2266.782895
4,2,6169.077419
4,2,2198.125000
4,2,3569.006098
4,2,1593.822222
4,2,3007.528846
4,2,1813.383152
4,2,2113.259615
4,2,8766.081522
4,2,6154.734043
4,2,29288.294479
4,2,5374.295455
4,2,7334.229592
4,2,2300.178571
4,2,4243.750000
4,2,4700.108571
4,2,1794.608040
4,2,4865.761905
4,2,14527.181818
4,2,8405.700000
4,2,1908.203209
4,2,2110.562500
4,2,3699.495000
4,2,1552.293919
4,2,3285.944444
4,2,5415.983766
4,2,1554.081395
4,2,3257.157895
4,2,3080.465116
4,2,2290.825000
4,2,2843.617021
4,2,3542.055288
4,2,4624.408163
4,2,4489.511628
4,2,2311.924419
4,2,2193.964912
4,2,3898.911058
4,2,5361.937500
4,2,9287.780488
4,2,3027.297222
4,2,2726.682432
4,2,5227.669118

4,2,7649.089286
4,2,3861.538462
4,2,2063.336538
4,2,5203.616279
4,2,1580.158854
4,2,1545.107558
4,2,8494.282051
4,2,3500.521739
4,2,3158.547297
4,2,4749.472826
4,2,2080.010204
4,2,2299.453390
4,2,3305.591837
4,2,2219.398649
4,2,2153.602679
4,2,2401.309375
4,2,3820.361702
4,2,2164.453125
4,2,2425.705882
4,2,7544.330882
4,2,2613.060976
4,2,2283.128205
4,2,1722.875000
4,2,2140.425532
4,2,1554.615385
4,2,3126.843750
4,2,2030.700000
4,2,6883.142857
4,2,4801.216837
4,2,3062.090000
4,2,4194.441489
4,2,1798.682927
4,2,1867.239286
4,2,1842.508721
4,2,3014.410256
4,2,10278.808333
4,2,1959.656442
4,2,6885.702128
4,2,2726.333333
4,2,1906.702128
4,2,2639.871795
4,2,3054.315789
4,2,3517.152174
4,2,1942.858553
4,2,1636.666667
4,2,3762.467391
4,2,3373.737179
4,2,3144.622596
4,2,1794.721088
4,2,4359.441860
4,2,2600.298077
4,2,6953.796512
4,2,11901.375000
4,2,2031.490625
4,2,7498.753555
4,2,3091.872340
4,2,2926.284884
4,2,1556.616580
4,2,2249.040761
4,2,2004.808333
4,2,2856.135638
4,2,2121.631579
4,2,1611.919355
4,2,3142.095745
4,2,4430.433511
4,2,2495.437500

4,2,5656.602041
4,2,2285.310976
4,2,1907.940828
4,2,1766.750000
4,2,2926.424569
4,2,8616.027273
4,2,3080.880240
4,2,1657.392442
4,2,6586.586957
4,2,6117.179245
4,2,1821.978610
4,2,4633.683544
4,2,1703.909091
4,2,4759.512712
4,2,2840.021277
4,2,1983.192308
4,2,2584.017045
4,2,2002.596591
4,2,2232.632979
4,2,1954.707237
4,2,3785.535256
4,2,4774.679245
4,2,1543.638554
4,2,1984.030822
4,2,2591.030000
4,2,3284.643519
4,2,5656.548780
4,2,2294.092593
4,2,3675.024510
4,2,1962.465625
4,2,5914.631579
4,2,3708.916667
4,2,2908.162162
4,2,2227.180556
4,2,11643.593750
4,2,2249.502500
4,2,1743.502907
4,2,3158.864865
4,2,2182.875000
4,2,2205.095745
4,2,3321.500000
4,2,2388.502907
4,2,7617.843750
4,2,5019.546296
4,2,6133.878049
4,2,1525.862500
4,2,5771.851744
4,2,1665.770349
4,2,2479.884615
4,2,1791.187500
4,2,2832.166667
4,2,3454.083333
4,2,2146.313725
4,2,2162.811224
4,2,1540.275862
4,2,3932.477778
4,2,1540.195652
4,2,2566.090909
4,2,3480.294872
4,2,12007.962025
4,2,1844.375000
4,2,1519.923077
4,2,2555.530405
4,2,1559.538462
4,2,6934.202830
4,2,1793.241379

4,2,3157.478261
4,2,6592.008333
4,2,2161.600000
4,2,2723.625000
4,2,1715.600000
4,2,1908.211310
4,2,2528.524510
4,2,3628.581522
4,2,7270.340000
4,2,4703.666667
4,2,3052.119565
4,2,10752.024390
4,2,1731.273256
4,2,2542.442568
4,2,4030.943182
4,2,5043.459239
4,2,4551.519231
4,2,3353.259434
4,2,4077.884868
4,2,2574.500000
4,2,7460.037234
4,2,1611.578125
4,2,1865.186321
4,2,1720.422297
4,2,1791.500000
4,2,1963.822917
4,2,16019.328431
4,2,1766.206522
4,2,3039.153846
4,2,2046.337500
4,2,4563.102273
4,2,2215.409091
4,2,3454.670455
4,2,11565.178571
4,2,4115.222222
4,2,1533.750000
4,2,2079.468750
4,2,1984.437500
4,2,4922.886364
4,2,7253.653646
4,2,11759.500000
4,2,6121.961538
4,2,1616.340625
4,2,1818.848837
4,2,2816.652174
4,2,7447.116352
4,2,1583.134375
4,2,2032.557377
4,2,2618.407407
4,2,6907.564732
4,2,8949.877358
4,2,1870.393750
4,2,1755.634615
4,2,1575.000000
4,2,1624.574468
4,2,4354.742021
4,2,4833.286184
4,2,4883.366071
4,2,1969.867647
4,2,3716.741477
4,2,1865.847727
4,2,2835.524390
4,2,2767.964912
4,2,23068.141256
4,2,4539.933036
4,2,6655.975610

4,2,6294.652027
4,2,5591.696809
4,2,4542.396341
4,2,1702.058511
4,2,2334.728261
4,2,3406.743590
4,2,1872.058511
4,2,1749.162162
4,2,2447.127660
4,2,2905.881517
4,2,1748.488372
4,2,1553.735294
4,2,2650.074468
4,2,3460.369863
4,2,1933.511628
4,2,2839.460432
4,2,2605.400000
4,2,2372.022059
4,2,4670.676471
4,2,2148.132979
4,2,2702.015625
4,2,12520.340426
4,2,2186.342391
4,2,1719.425000
4,2,2089.800000
4,2,6257.108333
4,2,6958.776596
4,2,2994.621795
4,2,2361.625000
4,2,6864.671053
4,2,1798.405405
4,2,2374.109694
4,2,7902.883721
4,2,2137.250000
4,2,2254.119186
4,2,2568.812500
4,2,1985.047297
4,2,2216.786458
4,2,2031.308511
4,2,3025.163462
4,2,3992.316038
4,2,4528.344595
4,2,2494.116279
4,2,1532.043919
4,2,3854.042683
4,2,8731.031250
4,2,2598.870000
4,2,3617.634868
4,2,3016.320755
4,2,4346.066176
4,2,10194.936170
4,2,3132.555556
4,2,1641.942446
4,2,5584.122754
4,2,5618.331551
4,2,2362.472561
4,2,2764.486842
4,2,2092.976190
4,2,2429.516304
4,2,2571.115385
4,2,1634.776316
4,2,1961.551136
4,2,2818.664773
4,2,1987.256098
4,2,6290.941520
4,2,2491.770161

4,2,4707.691542
4,2,1671.881579
4,2,5320.458763
4,2,3236.338028
4,2,2662.937500
4,2,6308.588235
4,2,9571.108108
4,2,1732.164474
4,2,4899.299020
4,2,2363.581081
4,2,1610.200000
4,2,2170.389535
4,2,7407.506579
4,2,2346.884615
4,2,2364.048077
4,2,6710.562500
4,2,3032.151042
4,2,3808.390244
4,2,3189.583333
4,2,4860.388889
4,2,3916.646226
4,2,4495.530405
4,2,1824.692308
4,2,6190.730769
4,2,2044.005556
4,2,1609.093750
4,2,3249.785000
4,2,1767.475610
4,2,3333.508772
4,2,2626.845109
4,2,1829.959184
4,2,2473.585106
4,2,3040.217593
4,2,4383.992021
4,2,1600.507143
4,2,1849.390625
4,2,1713.152778
4,2,1579.190476
4,2,2847.329787
4,2,2931.198864
4,2,1752.684211
4,2,1595.426471
4,2,7681.549020
4,2,2350.410714
4,2,8955.839912
4,2,4529.802326
4,2,1639.434211
4,2,2358.853659
4,2,2439.462766
4,2,3366.898936
4,2,7916.403125
4,2,2820.390244
4,2,3953.521930
4,2,5893.031915
4,2,15803.696429
4,2,7015.642045
4,2,4690.452830
4,2,4132.656915
4,2,2487.590909
4,2,2643.660526
4,2,1537.836538
4,2,3865.750000
4,2,3257.110656
4,2,1607.625000
4,2,3885.338415
4,2,3141.478723

4,2,1807.729730
4,2,2557.514881
4,2,5567.409091
4,2,4630.354592
4,2,1955.502841
4,2,3651.375000
4,2,3663.325000
4,2,4934.040984
4,2,9688.669811
4,2,2304.832237
4,2,4033.383333
4,2,14527.360577
4,2,4431.997340
4,2,6101.446429
4,2,3141.916667
4,2,1882.461538
4,2,1726.829545
4,2,2309.750000
4,2,8396.500000
4,2,1644.250000
4,2,5612.423077
4,2,1971.040541
4,2,1843.086538
4,2,6001.072115
4,2,2556.581395
4,2,2189.944444
4,2,7143.687500
4,2,1704.931034
4,2,2883.650943
4,2,3648.769737
4,2,1911.631579
4,2,2223.791667
4,2,1571.411765
4,2,1830.425532
4,2,14378.971875
4,2,1714.411765
4,2,4190.437500
4,2,3047.511628
4,2,2912.212838
4,2,2847.128049
4,2,4190.989362
4,2,3515.416667
4,2,2322.435976
4,2,2982.577381
4,2,1656.500000
4,2,3121.625000
4,2,5938.441176
4,2,1608.601351
4,2,1878.912500
4,2,15916.969388
4,2,2400.243056
4,2,1903.560811
4,2,2815.531250
4,2,6994.341667
4,2,4186.577778
4,2,3746.320225
4,2,30361.610465
4,2,2136.652174
4,2,2317.909574
4,2,4501.956019
4,2,4009.487805
4,2,2074.900000
4,2,2104.698864
4,2,5235.272727
4,2,1633.487179
4,2,2477.027273

4,2,1875.275000
4,2,1622.976744
4,2,2008.557377
4,2,1940.350000
4,2,2205.567308
4,2,2096.523256
4,2,2977.263889
4,2,7057.237500
4,2,3172.479167
4,2,3339.191860
4,2,5773.529412
4,2,8606.189024
4,2,1550.100000
4,2,1588.246914
4,2,3130.698113
4,2,1940.775568
4,2,9267.000000
4,2,2510.772727
4,2,1842.378289
4,2,1786.289634
4,2,5368.291667
4,2,2593.461538
4,2,3707.079618
4,2,12734.365517
4,2,3499.692308
4,2,2139.654762
4,2,3052.597222
4,2,2250.625000
4,2,11333.136111
4,2,1829.594595
4,2,4349.304545
4,2,2090.444444
4,2,1892.615385
4,2,3149.391304
4,2,2069.481818
4,2,2919.237745
4,2,7604.131466
4,2,2449.007143
4,2,9072.865854
4,2,1548.800000
4,2,10126.030660
4,2,1959.905172
4,2,2988.149038
4,2,1566.423611
4,2,2798.802885
4,2,2103.500000
4,2,29837.919444
4,2,12108.559659
4,2,1625.388889
4,2,4546.625749
4,2,2487.272947
4,2,4631.336538
4,2,2157.384615
4,2,3435.713450
4,2,4599.063636
4,2,18511.400000
4,2,2676.933333
4,2,3940.358491
4,2,1736.219388
4,2,2059.093023
4,2,1892.833333
4,2,2060.694712
4,2,5551.100000
4,2,6915.134259
4,2,3047.020408
4,2,1853.809524

4,2,2001.739362
4,2,4872.412500
4,2,8905.083333
4,2,3813.200000
4,2,3648.612745
4,2,1961.100000
4,2,4596.673913
4,2,3617.114865
4,2,9398.605000
4,2,3550.871795
4,2,1975.970588
4,2,2073.723684
4,2,8487.682432
4,2,3151.952381
4,2,5236.118750
4,2,13389.761905
4,2,2866.475543
4,2,6461.750000
4,2,1832.296512
4,2,2092.000000
4,2,2029.800000
4,2,2779.138158
4,2,2143.406250
4,2,8541.877551
4,2,4385.183824
4,2,1691.145833
4,2,3989.175000
4,2,2066.736486
4,2,2666.906977
4,2,1667.281496
4,2,9752.625000
4,2,2003.330882
4,2,1550.106383
4,2,3261.574713
4,2,3694.000000
4,2,1521.908163
4,2,2378.219101
4,2,2286.285714
4,2,1551.488372
4,2,5645.685897
4,2,5019.914062
4,2,4677.500000
4,2,2401.941860
4,2,1846.869048
4,2,1875.153409
4,2,2153.879808
4,2,3072.062500
4,2,1883.594595
4,2,3156.404040
4,2,1761.412500
4,2,4114.437500
4,2,1796.993750
4,2,2378.250000
4,2,1858.500000
4,2,1718.727273
4,2,2442.875000
4,2,5364.895833
4,2,2005.255319
4,2,1532.101974
4,2,5642.450000
4,2,3826.805556
4,2,2536.063830
4,2,11448.643357
4,2,2451.945000
4,2,2153.943182
4,2,8986.846875

4,2,2744.856250
4,2,2597.644444
4,2,5041.128205
4,2,1570.628125
4,2,6570.698864
4,2,2531.625000
4,2,2029.638889
4,2,3412.779412
4,2,2054.960227
4,2,3032.994681
4,2,4064.510000
4,2,3493.575000
4,2,4224.075000
4,2,7600.027027
4,2,2873.945946
4,2,7073.864198
4,2,1537.820261
4,2,1724.533784
4,2,5283.346154
4,2,3198.951087
4,2,3603.761905
4,2,2206.750000
4,2,2899.540881
4,2,1949.494681
4,2,2021.266332
4,2,8792.237885
4,2,2031.264398
4,2,2221.875000
4,2,5775.477564
4,2,2688.864078
4,2,2070.453488
4,2,2154.400000
4,2,16047.389151
4,2,1782.663462
4,2,1573.815789
4,2,3139.098522
4,2,25111.663462
4,2,1546.644231
4,2,2147.614362
4,2,3909.161290
4,2,4955.974843
4,2,2291.594512
4,2,1698.297297
4,2,13139.700000
4,2,10013.712500
4,2,2582.529412
4,2,1745.281250
4,2,7593.785714
4,2,3349.049479
4,2,5680.500000
4,2,2448.108974
4,2,1844.161765
4,2,1851.386792
4,2,2422.980769
4,2,2639.250000
4,2,2112.163043
4,2,4010.538690
4,2,3495.500000
4,2,2129.094340
4,2,1619.085526
4,2,2490.780000
4,2,1906.688889
4,2,5719.338415
4,2,2475.082011
4,2,1671.596154
4,2,1759.031250

4,2,1985.810976
4,2,3324.406915
4,2,3745.840426
4,2,2750.230769
4,2,2309.892857
4,2,1889.750000
4,2,3926.937500
4,2,3763.173077
4,2,2287.237179
4,2,2128.057432
4,2,2158.040698
4,2,1531.518939
4,2,6162.621622
4,2,3367.269737
4,2,2416.793605
4,2,3839.166667
4,2,4744.542714
4,2,2710.696721
4,2,1710.355769
4,2,1876.933673
4,2,5306.640625
4,2,1656.112500
4,2,4160.381250
4,2,3856.178571
4,2,2613.218137
4,2,3228.547170
4,2,7040.586310
4,2,5215.445652
4,2,2319.788462
4,2,2554.790698
4,2,3291.997596
4,2,6419.340909
4,2,2172.556818
4,2,2517.843750
4,2,2802.491477
4,2,2940.453125
4,2,2279.782609
4,2,3205.347826
4,2,1853.987903
4,2,5490.804020
4,2,2058.888889
4,2,5882.960227
4,2,11918.977273
4,2,2953.307692
4,2,3342.330000
4,2,6492.116667
4,2,3362.145833
4,2,2291.500000
4,2,3118.718750
4,2,4080.453488
4,2,1717.911765
4,2,1841.500000
4,2,2791.459459
4,2,1703.444149
4,2,2846.613636
4,2,3918.996951
4,2,6318.500000
4,2,3106.121429
4,2,2498.602273
4,2,10573.847826
4,2,2276.375000
4,2,4033.737500
4,2,1503.982143
4,2,3100.961538
4,2,2552.107143
4,2,4906.636792

4,2,2721.797222
4,2,15288.295918
4,2,2395.625000
4,2,4088.343750
4,2,2977.200549
4,2,1528.956897
4,2,2511.645833
4,2,2815.750000
4,2,2066.500000
4,2,1789.823980
4,2,1698.838235
4,2,2737.850000
4,2,1873.493056
4,2,53174.000000
4,2,2989.687500
4,2,4197.047297
4,2,2743.441860
4,2,1565.000000
4,2,2248.115385
4,2,2139.845395
4,2,3919.333333
4,2,1911.597087
4,2,1651.225000
4,2,9922.018293
4,2,2304.827869
4,2,2768.011364
4,2,2714.854545
4,2,2165.071429
4,2,3162.740196
4,2,7441.950000
4,2,15103.175439
4,2,2337.222222
4,2,4488.038043
4,2,2684.250000
4,2,1978.992021
4,2,2312.889205
4,2,2001.144231
4,2,3163.520833
4,2,2719.593596
4,2,2540.743961
4,2,2768.968750
4,2,6963.607500
4,2,11285.200000
4,2,1733.923077
4,2,4783.760000
4,2,2644.306250
4,2,3911.687500
4,2,1575.137255
4,2,4103.775000
4,2,2995.054878
4,2,3396.806122
4,2,2942.928571
4,2,3269.291667
4,2,4368.315217
4,2,5240.040323
4,2,2833.636905
4,2,1736.372340
4,2,9642.067073
4,2,40699.625000
4,2,7489.178571
4,2,1919.159091
4,2,2720.745370
4,2,2851.879545
4,2,4034.707237
4,2,3883.492188
4,2,8782.515625

4,2,3314.467391
4,2,3444.178571
4,2,2176.134146
4,2,2361.304348
4,2,2390.070652
4,2,1813.515152
4,2,1902.125000
4,2,2260.640000
4,2,3240.341463
4,2,4261.555556
4,2,2713.663265
4,2,2069.872093
4,2,5408.695652
4,2,10528.011905
4,2,1732.821429
4,2,2499.883495
4,2,3256.776442
4,2,2034.954545
4,2,1809.000000
4,2,11263.375000
4,2,4374.020408
4,2,1882.000000
4,2,10124.891667
4,2,1764.471591
4,2,1537.105263
4,2,13597.657143
4,2,1765.000000
4,2,5128.477273
4,2,1950.793750
4,2,2554.135417
4,2,1968.064103
4,2,3336.130682
4,2,1861.152174
4,2,2793.725694
4,2,1875.163462
4,2,2953.900000
4,2,3129.574468
4,2,7751.805233
4,2,2177.864865
4,2,4708.920000
4,2,2251.250000
4,2,3457.069767
4,2,3215.865854
4,2,2579.525000
4,2,5627.642857
4,2,1648.494898
4,2,10106.000000
4,2,2771.587838
4,2,2978.121429
4,2,1574.539474
4,2,5995.750000
4,2,1579.116667
4,2,7534.173913
4,2,9851.294118
4,2,4026.222222
4,2,13186.889423
4,2,4254.822917
4,2,3557.994898
4,2,1964.000000
4,2,4509.117021
4,2,1622.534884
4,2,1506.277344
4,2,3496.213542
4,2,5950.707237
4,2,1563.638587
4,2,4908.528846

4,2,3025.835227
4,2,4049.965625
4,2,3137.660377
4,2,2336.889535
4,2,1709.693878
4,2,6822.278689
4,2,4609.560606
4,2,3621.760000
4,2,54831.325658
4,2,7877.081731
4,2,12013.807692
4,2,11841.271277
4,2,4316.034574
4,2,3279.187500
4,2,6387.430851
4,2,1810.529412
4,2,2521.637681
4,2,2406.119565
4,2,4827.406250
4,2,3168.708333
4,2,3274.100000
4,2,2327.761364
4,2,1768.722973
4,2,1570.637195
4,2,1625.658537
4,2,2462.325581
4,2,5562.557692
4,2,2004.467500
4,2,1896.878049
4,2,3339.888889
4,2,9068.174419
4,2,2541.654661
4,2,2399.875000
4,2,3773.582938
4,2,1767.725490
4,2,9531.232955
4,2,13753.522346
4,2,4722.330189
4,2,2891.656250
4,2,1976.918478
4,2,7312.703804
4,2,1541.848214
4,2,1650.019651
4,2,1779.450581
4,2,16242.288043
4,2,2060.058333
4,2,4700.680851
4,2,1519.668269
4,2,2243.906977
4,2,1897.525000
4,2,1724.261364
4,2,1747.000000
4,2,2065.357143
4,2,1790.377358
4,2,3049.719512
4,2,2261.685714
4,2,1655.878049
4,2,3614.418367
4,2,3120.330882
4,2,2988.100543
4,2,2792.619565
4,2,9153.335000
4,2,3636.245098
4,2,4307.173077
4,2,2372.593750
4,2,2461.163889

4,2,2940.943182
4,2,2972.076923
4,2,2767.863889
4,2,3988.988636
4,2,2206.750000
4,2,4194.500000
4,2,1774.517442
4,2,2342.961538
4,2,8146.363636
4,2,2819.401596
4,2,3747.194712
4,2,1996.462963
4,2,1910.297101
4,2,2481.825000
4,2,2047.587766
4,2,2054.000000
4,2,3621.794118
4,2,2516.448980
4,2,3413.189024
4,2,1526.055851
4,2,1571.128205
4,2,2383.381098
4,2,3732.736413
4,2,4306.937500
4,2,6346.368902
4,2,2372.887931
4,2,1519.215909
4,2,4600.500000
4,2,2162.401042
4,2,5622.108173
4,2,5805.575472
4,2,1800.750000
4,2,1638.674419
4,2,1839.653846
4,2,3071.673684
4,2,1543.668605
4,2,3171.072222
4,2,1625.250000
4,2,2089.556548
4,2,2964.420455
4,2,2372.434783
4,2,2133.011905
4,2,3806.020000
4,2,2611.659091
4,2,2798.512195
4,2,3104.739286
4,2,1680.215580
4,2,2619.173780
4,2,2756.888889
4,2,3116.671875
4,2,1651.060976
4,2,2878.352273
4,2,4699.933190
4,2,4830.645161
4,2,4836.928879
4,2,1832.692308
4,2,4245.660000
4,2,2010.790055
4,2,2894.982234
4,2,7488.877551
4,2,8000.405556
4,2,2642.520349
4,2,6195.190789
4,2,5027.668750
4,2,3411.117647
4,2,1607.198529

4,2,7272.894022
4,2,2933.897959
4,2,14757.939815
4,2,1556.090909
4,2,6612.413462
4,2,1805.000000
4,2,5338.701531
4,2,7914.931034
4,2,2422.017857
4,2,1904.259146
4,2,12204.560345
4,2,3053.419872
4,2,1598.908537
4,2,3336.512500
4,2,1595.465909
4,2,10100.851923
4,2,2199.841667
4,2,2212.520000
4,2,2701.625000
4,2,1561.562500
4,2,4365.020408
4,2,1550.318182
4,2,5483.589385
4,2,10930.056604
4,2,2247.000000
4,2,5292.807870
4,2,3513.862500
4,2,1704.235294
4,2,8369.286364
4,2,1912.505208
4,2,7491.157895
4,2,2969.529412
4,2,2087.142857
4,2,10524.421875
4,2,2842.602564
4,2,5268.318182
4,2,2150.778846
4,2,1775.812500
4,2,7804.265625
4,2,8556.217391
4,2,4693.014535
4,2,2507.166667
4,2,1675.804020
4,2,3689.698370
4,2,6617.125000
4,2,6040.448980
4,2,4088.315625
4,2,2579.920000
4,2,1964.559748
4,2,3417.547619
4,2,1772.644737
4,2,3385.086420
4,2,2223.575269
4,2,7014.976744
4,2,2435.419872
4,2,2314.520349
4,2,1796.494898
4,2,3019.051724
4,2,2113.085938
4,2,4354.113839
4,2,1662.920000
4,2,4433.629412
4,2,2257.813218
4,2,4928.372727
4,2,2719.915541
4,2,7183.347826

4,2,4679.887500
4,2,26471.687500
4,2,1503.532258
4,2,4320.994318
4,2,11960.228723
4,2,2441.674888
4,2,8427.981735
4,2,2918.372449
4,2,2143.166667
4,2,1571.505435
4,2,2371.817308
4,2,1877.191176
4,2,25572.214674
4,2,1602.096939
4,2,3237.634146
4,2,2442.937500
4,2,3128.931818
4,2,5481.091216
4,2,6028.460227
4,2,15722.890625
4,2,3451.512500
4,2,2871.653846
4,2,2074.937500
4,2,1593.890625
4,2,1546.366848
4,2,2224.278302
4,2,1792.847826
4,2,6173.946429
4,2,9197.415625
4,2,1864.567568
4,2,1716.029661
4,2,2496.705882
4,2,11188.430233
4,2,2065.554878
4,2,4144.769231
4,2,2044.213235
4,2,7784.718182
4,2,2407.214286
4,2,1712.291667
4,2,6908.278894
4,2,2957.674419
4,2,3031.750000
4,2,3526.196429
4,2,2301.952381
4,2,7862.112676
4,2,3673.774194
4,2,1606.763158
4,2,7021.353659
4,2,5480.505682
4,2,1626.934783
4,2,3997.493056
4,2,1841.488636
4,2,1537.860465
4,2,4842.252717
4,2,1523.923077
4,2,15259.230769
4,2,1822.625000
4,2,2397.198370
4,2,4148.784722
4,2,3305.312500
4,2,14873.937500
4,2,2604.975610
4,2,2325.618750
4,2,2679.837838
4,2,2515.175000
4,2,4698.036765

4,2,3641.105128
4,2,5446.300000
4,2,2447.926471
4,2,2412.380952
4,2,1640.426829
4,2,3095.312500
4,2,2014.960000
4,2,4941.529412
4,2,2497.187500
4,2,4586.411184
4,2,2994.803347
4,2,3260.208333
4,2,1650.685897
4,2,2727.000000
4,2,2443.142857
4,2,3836.883721
4,2,4302.500000
4,2,2308.244681
4,2,3514.968085
4,2,1927.285714
4,2,6135.096154
4,2,6077.454545
4,2,5217.012255
4,2,2062.512195
4,2,3954.211538
4,2,1799.951220
4,2,4939.968750
4,2,2243.327778
4,2,1712.553191
4,2,6140.722826
4,2,1986.647577
4,2,2198.475000
4,2,2564.000000
4,2,1616.527027
4,2,4282.791667
4,2,4553.771429
4,2,3152.687500
4,2,5625.586806
4,2,2467.142857
4,2,2223.564103
4,2,2266.888889
4,2,2345.284375
4,2,6376.388514
4,2,3361.500000
4,2,1941.833333
4,2,1726.375000
4,2,1914.088235
4,2,21116.162879
4,2,2270.490196
4,2,3625.384615
4,2,1811.027027
4,2,1534.347222
4,2,3833.292614
4,2,2333.538462
4,2,2094.782895
4,2,2890.846591
4,2,3959.635417
4,2,4974.187500
4,2,5699.698171
4,2,1619.115854
4,2,2857.701923
4,2,2412.084239
4,2,1798.205128
4,2,7564.446429
4,2,6925.582627
4,2,2700.558659

4,2,1785.578125
4,2,4924.762255
4,2,6267.900000
4,2,3500.942308
4,2,2925.949324
4,2,6466.644737
4,2,4669.604651
4,2,5115.437500
4,2,2014.868852
4,2,5610.743316
4,2,2800.951220
4,2,2693.494681
4,2,7293.442308
4,2,2308.050000
4,2,1606.935484
4,2,1598.702128
4,2,5671.762500
4,2,1767.083799
4,2,3795.788043
4,2,2064.372340
4,2,2645.163265
4,2,5722.276382
4,2,16372.346939
4,2,5083.709135
4,2,3994.743590
4,2,2288.304878
4,2,2516.938889
4,2,4269.207547
4,2,9531.870629
4,2,1960.459375
4,2,3077.938889
4,2,2560.619186
4,2,9598.377604
4,2,5529.803571
4,2,1550.366279
4,2,3002.282895
4,2,3495.720000
4,2,3967.574468
4,2,10126.491379
4,2,8283.843373
4,2,1568.846154
4,2,5552.531646
4,2,4045.839286
4,2,2099.500000
4,2,1959.896739
4,2,1893.545455
4,2,4543.961538
4,2,3094.666667
4,2,2272.384868
4,2,2890.705729
4,2,1509.119565
4,2,1941.500000
4,2,2749.127660
4,2,1680.625000
4,2,2606.365132
4,2,1936.113971
4,2,1822.579909
4,2,1678.771300
4,2,1743.346154
4,2,2630.625000
4,2,6772.255814
4,2,2005.773810
4,2,2100.109756
4,2,1928.926829
4,2,4834.438462
4,2,2176.996622

4,2,1684.371429
4,2,2417.235294
4,2,3619.741071
4,2,2812.130000
4,2,1625.021739
4,2,11022.393617
4,2,4747.086957
4,2,3303.358696
4,2,8494.242188
4,2,4971.083333
4,2,4067.900000
4,2,2167.147222
4,2,3778.000000
4,2,1809.750000
4,2,2065.107143
4,2,7978.877551
4,2,1535.900568
4,2,26901.555556
4,2,4764.345109
4,2,1949.818182
4,2,7617.875000
4,2,2522.927907
4,2,2530.438776
4,2,3392.327731
4,2,3204.421053
4,2,2734.102649
4,2,2422.034884
4,2,2196.996875
4,2,2524.758621
4,2,1845.595395
4,2,5867.689024
4,2,4760.902439
4,2,3552.015385
4,2,6118.000000
4,2,4442.502841
4,2,1606.404412
4,2,6923.022727
4,2,2793.367347
4,2,2079.457447
4,2,3959.071429
4,2,2429.681818
4,2,5262.594340
4,2,2036.882212
4,2,9648.271930
4,2,1928.744395
4,2,2296.809783
4,2,9264.000000
4,2,2976.519231
4,2,2384.771739
4,2,12963.000000
4,2,2659.843750
4,2,3103.753125
4,2,3858.065789
4,2,1517.238636
4,2,2258.250000
4,2,25152.383721
4,2,3983.664062
4,2,2273.227273
4,2,1759.666667
4,2,1617.825000
4,2,1715.836310
4,2,2139.635220
4,2,5623.031429
4,2,6816.955556
4,2,4406.612245
4,2,3864.154930

4,2,2780.756098
4,2,2461.157895
4,2,6414.075000
4,2,2912.625000
4,2,3201.562500
4,2,1917.928571
4,2,1760.742347
4,2,2054.379310
4,2,1966.965116
4,2,1970.219895
4,2,1715.874251
4,2,4289.848168
4,2,5122.677500
4,2,2023.299528
4,2,2663.453488
4,2,1609.793605
4,2,3375.500000
4,2,2247.305556
4,2,7626.253049
4,2,4233.334677
4,2,2428.230769
4,2,1870.231250
4,2,3171.856707
4,2,3678.406250
4,2,2168.581395
4,2,12110.422222
4,2,3080.553571
4,2,3063.590909
4,2,1644.193750
4,2,2950.075521
4,2,3826.243243
4,2,6279.955414
4,2,8806.698565
4,2,5412.440476
4,2,2774.161585
4,2,1737.298780
4,2,3434.047619
4,2,2759.445312
4,2,2977.649306
4,2,2385.171875
4,2,4339.476974
4,2,1575.610465
4,2,4934.412037
4,2,8130.425595
4,2,6722.696078
4,2,4882.691489
4,2,2150.823370
4,2,2895.719907
4,2,5810.818750
4,2,5456.461538
4,2,1679.052778
4,2,1774.384615
4,2,2270.375000
4,2,1777.266667
4,2,3691.097561
4,2,4681.997222
4,2,8509.448529
4,2,2961.000000
4,2,7938.144886
4,2,2064.229167
4,2,1654.253788
4,2,2068.524038
4,2,1689.838710
4,2,1785.683333
4,2,4126.193548
4,2,3527.669951

4,2,1634.375000
4,2,3256.285714
4,2,2989.551282
4,2,3329.269886
4,2,4943.283854
4,2,4262.361486
4,2,3177.258929
4,2,4467.755459
4,2,2515.847826
4,2,8990.285714
4,2,1587.500000
4,2,3611.000000
4,2,7808.821078
4,2,2202.863372
4,2,1653.053977
4,2,2295.544776
4,2,1905.974359
4,2,32261.983173
4,2,3123.208333
4,2,1981.300000
4,2,3856.976744
4,2,1547.000000
4,2,2207.226471
4,2,3577.539062
4,2,1658.019531
4,2,4761.479545
4,2,9359.333333
4,2,3781.112805
4,2,2966.700658
4,2,3510.534091
4,2,2827.034091
4,2,5185.142857
4,2,5355.140244
4,2,2087.364286
4,2,5205.868421
4,2,1697.860465
4,2,4925.785714
4,2,10930.890625
4,2,3800.795918
4,2,4915.574468
4,2,9367.319444
4,2,1832.260000
4,2,16543.023013
4,2,1959.093750
4,2,2323.532609
4,2,3061.361702
4,2,7098.753125
4,2,2087.921875
4,2,2522.510417
4,2,2179.568750
4,2,2977.588235
4,2,4486.871429
4,2,2950.514423
4,2,2806.701087
4,2,10148.016949
4,2,4649.655738
4,2,2162.677143
4,2,3881.956522
4,2,2576.679245
4,2,1653.333333
4,2,4571.969388
4,2,2847.411765
4,2,2052.729560
4,2,1751.000000
4,2,9139.116848
4,2,8862.823718

4,2,2286.906250
4,2,1658.708333
4,2,2738.948718
4,2,2236.777027
4,2,1692.212766
4,2,4792.788194
4,2,6302.101852
4,2,7493.478261
4,2,5744.990625
4,2,3024.250000
4,2,4774.654891
4,2,2847.884615
4,2,1562.225806
4,2,3301.056818
4,2,1737.494444
4,2,3060.447368
4,2,6340.329268
4,2,3735.416667
4,2,3117.140625
4,2,1746.165000
4,2,3286.531250
4,2,3247.387500
4,2,2340.108974
4,2,1571.028571
4,2,3606.869565
4,2,4119.396040
4,2,8701.441176
4,2,2524.141509
4,2,2904.793478
4,2,9877.598837
4,2,6254.367788
4,2,1759.833333
4,2,1678.415625
4,2,4333.695000
4,2,2312.844595
4,2,2001.925000
4,2,7320.906383
4,2,2626.625000
4,2,1533.577586
4,2,6669.103659
4,2,3116.500000
4,2,11093.590909
4,2,5197.656977
4,2,4499.968750
4,2,6747.833333
4,2,2207.279412
4,2,2842.011364
4,2,3756.327703
4,2,2388.444444
4,2,3150.276316
4,2,2167.000000
4,2,2285.616279
4,2,3293.531250
4,2,1763.361486
4,2,3145.329787
4,2,8314.953125
4,2,1536.728659
4,2,3966.423913
4,2,1572.041667
4,2,1951.653409
4,2,2470.320000
4,2,2485.232143
4,2,1659.973684
4,2,3138.371795
4,2,2205.300000
4,2,2057.617021

4,2,6346.329146
4,2,3526.077778
4,2,3001.021277
4,2,2541.250000
4,2,3011.717791
4,2,2473.214286
4,2,3431.223214
4,2,2638.562500
4,2,5526.400510
4,2,6992.993056
4,2,17666.447368
4,2,3257.687500
4,2,9644.517045
4,2,5881.166667
4,2,2833.686275
4,2,2070.385638
4,2,1667.208333
4,2,1875.153846
4,2,9739.804054
4,2,2167.109551
4,2,6228.943396
4,2,3838.068878
4,2,2981.791667
4,2,2076.256757
4,2,3761.359375
4,2,2191.769231
4,2,6856.750000
4,2,3630.069767
4,2,3073.102941
4,2,3862.002551
4,2,1952.769231
4,2,2327.588542
4,2,3939.306122
4,2,4836.968750
4,2,2014.565574
4,2,4284.614130
4,2,1870.069231
4,2,2057.010417
4,2,6294.777778
4,2,2177.256944
4,2,2582.070000
4,2,3183.150000
4,2,1709.209375
4,2,1593.687500
4,2,3985.782738
4,2,1726.607143
4,2,2771.524390
4,2,10023.760870
4,2,2545.335366
4,2,3424.527778
4,2,2354.642500
4,2,2491.024390
4,2,1788.837500
4,2,4049.023256
4,2,11609.954787
4,2,2206.000000
4,2,1560.525140
4,2,1683.137725
4,2,2463.405405
4,2,2312.000000
4,2,4529.529412
4,2,2892.339623
4,2,2225.225000
4,2,2675.397727
4,2,3616.500000
4,2,3119.432692

4,2,2533.840909
4,2,2212.535714
4,2,5861.933333
4,2,2596.343750
4,2,1936.508721
4,2,2181.176887
4,2,3285.127778
4,2,1666.330000
4,2,9131.377907
4,2,7103.735119
4,2,2125.062500
4,2,1842.488889
4,2,7517.224359
4,2,4874.606250
4,2,2219.791667
4,2,1855.309783
4,2,1714.641026
4,2,2554.502358
4,2,2181.810096
4,2,2192.870000
4,2,5549.469388
4,2,4745.571429
4,2,5367.152632
4,2,2668.120603
4,2,4437.342105
4,2,2142.768750
4,2,1577.703488
4,2,2539.225000
4,2,1929.750000
4,2,17274.936441
4,2,5252.857143
4,2,2813.272727
4,2,2634.845455
4,2,12492.778481
4,2,3206.534759
4,2,2282.459302
4,2,2660.598684
4,2,1728.100000
4,2,2306.078947
4,2,4621.125000
4,2,12593.769231
4,2,2050.952128
4,2,9643.647959
4,2,3815.665000
4,2,3296.187500
4,2,1695.437500
4,2,2349.391304
4,2,3672.967391
4,2,1499.078125
4,2,5451.824121
4,2,3038.312500
4,2,4065.882500
4,2,2705.923404
4,2,2471.634259
4,2,1977.391720
4,2,2107.520833
4,2,2228.464286
4,2,2115.708333
4,2,8362.454545
4,2,1816.540000
4,2,2123.714286
4,2,1742.634868
4,2,2540.721591
4,2,3033.755208
4,2,5685.157143
4,2,7448.298077

4,2,2853.720930
4,2,2866.576220
4,2,9997.000000
4,2,4639.610063
4,2,13526.500000
4,2,4292.805556
4,2,5612.583333
4,2,3439.961538
4,2,3047.687151
4,2,2608.545455
4,2,1681.324468
4,2,4459.739362
4,2,12726.826923
4,2,3318.153005
4,2,2031.461905
4,2,2451.807692
4,2,3912.166667
4,2,2164.218137
4,2,5064.543182
4,2,2084.523256
4,2,10890.687500
4,2,1795.527027
4,2,3760.109756
4,2,1875.009615
4,2,1765.905612
4,2,1821.982955
4,2,9557.011628
4,2,1777.234375
4,2,1564.813830
4,2,2185.253125
4,2,1652.179687
4,2,3470.563830
4,2,2063.186047
4,2,2183.402778
4,2,3166.425000
4,2,3508.159091
4,2,4108.157407
4,2,4362.481481
4,2,4145.906250
4,2,6326.369565
4,2,2678.902439
4,2,1657.333333
4,2,2019.216216
4,2,1655.962500
4,2,4245.971591
4,2,1882.250000
4,2,1776.401042
4,2,1787.034946
4,2,3192.000000
4,2,5011.085714
4,2,1900.745283
4,2,1694.979730
4,2,1676.615000
4,2,1684.513514
4,2,1998.466216
4,2,2949.000000
4,2,9085.181250
4,2,1858.169643
4,2,9024.707500
4,2,3640.250000
4,2,1586.812500
4,2,4190.301471
4,2,1900.894737
4,2,9848.133721
4,2,1989.305000
4,2,4362.166667

4,2,3177.843750
4,2,2929.787500
4,2,3224.250000
4,2,2824.218750
4,2,6239.925532
4,2,2312.763587
4,2,2264.098684
4,2,2154.295918
4,2,2600.665441
4,2,3105.826923
4,2,1642.477778
4,2,3053.250000
4,2,6566.320000
4,2,2426.463816
4,2,3661.655405
4,2,1841.333333
4,2,3313.750000
4,2,3402.220930
4,2,5354.613095
4,2,2538.134868
4,2,3999.236111
4,2,2265.368243
4,2,4497.362559
4,2,1630.495098
4,2,2042.663462
4,2,2068.095109
4,2,3185.357558
4,2,2665.518868
4,2,1579.705128
4,2,3229.923077
4,2,5047.288945
4,2,1598.065000
4,2,2392.994318
4,2,6844.523438
4,2,2856.090580
4,2,2142.569048
4,2,4170.621875
4,2,2320.390244
4,2,2255.603261
4,2,2844.288462
4,2,3800.980978
4,2,3380.239796
4,2,3742.893293
4,2,1860.422111
4,2,2585.243316
4,2,3273.459459
4,2,1871.364286
4,2,6644.116279
4,2,1981.138365
4,2,5998.690789
4,2,4706.641026
4,2,3428.829545
4,2,5023.595000
4,2,3223.669643
4,2,3417.451754
4,2,8014.320513
4,2,6290.019231
4,2,3089.051136
4,2,1936.346591
4,2,2039.720588
4,2,2590.035714
4,2,1699.804054
4,2,2722.729167
4,2,1787.767857
4,2,2584.593750
4,2,1537.457386

4,2,3442.764423
4,2,2178.290000
4,2,5459.988095
4,2,1781.196970
4,2,1910.194444
4,2,2693.790698
4,2,8868.372549
4,2,5770.590909
4,2,10861.745098
4,2,2594.490196
4,2,1790.160714
4,2,3574.187500
4,2,1599.472222
4,2,19345.432990
4,2,1813.393939
4,2,1895.425000
4,2,2587.494186
4,2,1804.194444
4,2,19069.300000
4,2,5135.482143
4,2,2629.263158
4,2,1723.161290
4,2,2179.653125
4,2,6511.557927
4,2,2212.434783
4,2,4967.882353
4,2,2640.596591
4,2,4854.822917
4,2,3570.450000
4,2,29714.197115
4,2,7793.489362
4,2,1542.546512
4,2,4009.054687
4,2,4066.727273
4,2,2682.428571
4,2,5520.000000
4,2,2481.247619
4,2,1806.937500
4,2,1540.243590
4,2,4040.658163
4,2,1561.250000
4,2,2048.230769
4,2,1951.011364
4,2,3799.171053
4,2,2324.238889
4,2,2122.392670
4,2,1551.900000
4,2,2067.347826
4,2,5851.972973
4,2,16573.148241
4,2,2731.520833
4,2,1926.083333
4,2,1844.555556
4,2,1910.816327
4,2,2444.109677
4,2,2125.913907
4,2,2891.245000
4,2,4723.853659
4,2,6828.609091
4,2,2355.000000
4,2,14566.439024
4,2,1719.473684
4,2,2028.200000
4,2,7656.670082
4,2,1995.428571
4,2,5416.175000

4,2,3718.118902
4,2,1751.435897
4,2,2447.959799
4,2,4754.061728
4,2,1657.924837
4,2,15592.076923
4,2,4018.258929
4,2,9231.372340
4,2,10443.850000
4,2,1574.600000
4,2,1555.851955
4,2,2319.366667
4,2,2471.750000
4,2,4344.462500
4,2,3330.000000
4,2,2663.203608
4,2,5304.221154
4,2,2267.250000
4,2,2702.699405
4,2,1565.153846
4,2,5484.593373
4,2,4866.694737
4,2,2148.650000
4,2,2685.637143
4,2,3016.056604
4,2,1775.545455
4,2,1919.303571
4,2,5207.390000
4,2,3225.393617
4,2,2206.892857
4,2,1532.960526
4,2,3922.986842
4,2,2445.740385
4,2,2694.104167
4,2,1985.993243
4,2,8563.062500
4,2,1663.625000
4,2,3146.628289
4,2,4800.857500
4,2,3792.372222
4,2,6195.863095
4,2,6390.198864
4,2,1803.591837
4,2,2219.555556
4,2,2362.346154
4,2,7879.142857
4,2,2184.019022
4,2,33763.702899
4,2,1927.125000
4,2,4354.514151
4,2,5478.667969
4,2,2360.956897
4,2,2143.671875
4,2,5871.170000
4,2,6648.786765
4,2,6307.830357
4,2,2651.000000
4,2,1791.031250
4,2,4575.394886
4,2,4564.884615
4,2,2921.676136
4,2,4783.093458
4,2,2275.908784
4,2,1621.244318
4,2,2572.500000
4,2,50719.700000

4,2,9537.625000
4,2,1612.743902
4,2,1631.070652
4,2,2092.755952
4,2,2415.937500
4,2,1761.387821
4,2,2123.138158
4,2,54056.062500
4,2,5936.625000
4,2,2844.592262
4,2,1651.140625
4,2,4931.695513
4,2,1968.500000
4,2,1829.800676
4,2,2817.288690
4,2,2774.496711
4,2,1517.444444
4,2,2643.017857
4,2,1501.552632
4,2,3363.833333
4,2,17695.696970
4,2,2176.312500
4,2,5763.729167
4,2,1921.921875
4,2,1646.310811
4,2,3847.798077
4,2,3245.116279
4,2,2941.160714
4,2,1698.758523
4,2,6686.691176
4,2,2222.363889
4,2,1893.524390
4,2,20943.313776
4,2,3995.581897
4,2,1931.815789
4,2,2234.689024
4,2,13752.974359
4,2,2192.609756
4,2,3817.820513
4,2,4083.875000
4,2,7114.840000
4,2,4180.372642
4,2,3352.534884
4,2,9119.086538
4,2,2174.737903
4,2,2583.000000
4,2,1572.312500
4,2,6349.534091
4,2,4653.500000
4,2,1957.297872
4,2,7253.615000
4,2,4814.127358
4,2,1976.358974
4,2,2963.175676
4,2,3289.295455
4,2,3281.460000
4,2,1542.083333
4,2,3485.716216
4,2,1851.692073
4,2,2627.018293
4,2,2467.347561
4,2,1661.270833
4,2,4748.744898
4,2,5970.581395
4,2,7809.808140
4,2,3525.323353

4,2,5957.479167
4,2,4265.306122
4,2,3480.872222
4,2,3049.820175
4,2,3358.305556
4,2,1557.777778
4,2,1704.205882
4,2,2365.053922
4,2,2000.027778
4,2,5246.515000
4,2,4973.362500
4,2,2264.109756
4,2,1838.875000
4,2,5296.910714
4,2,1707.926471
4,2,2759.641104
4,2,6232.923913
4,2,3962.191489
4,2,1713.000000
4,2,3104.916667
4,2,2185.100543
4,2,3332.400000
4,2,1616.265363
4,2,1559.522059
4,2,5004.434375
4,2,2667.250000
4,2,1920.422297
4,2,2129.142105
4,2,2672.729167
4,2,4762.402439
4,2,4005.293478
4,2,1983.889706
4,2,4598.054054
4,2,1833.014045
4,2,2140.012821
4,2,3186.486702
4,2,98087.655405
4,2,1867.510135
4,2,10236.319149
4,2,7743.350000
4,2,4855.413043
4,2,1616.721429
4,2,1611.423913
4,2,4353.166667
4,2,6603.068750
4,2,1908.930000
4,2,4188.671429
4,2,3026.602564
4,2,2755.035714
4,2,1611.162500
4,2,3066.391509
4,2,1591.804878
4,2,4141.617647
4,2,3131.920455
4,2,3259.477273
4,2,3267.703125
4,2,2799.555921
4,2,4546.408163
4,2,5446.612805
4,2,7821.817073
4,2,2704.333333
4,2,4590.582386
4,2,1769.230769
4,2,4200.575000
4,2,4272.320513
4,2,2621.698113

4,2,5788.489583
4,2,3824.597826
4,2,1808.666667
4,2,1605.454082
4,2,1732.697368
4,2,3413.875000
4,2,4197.154028
4,2,2666.647727
4,2,2800.104167
4,2,2710.453488
4,2,1589.189189
4,2,2264.003472
4,2,4031.479592
4,2,1511.281250
4,2,2909.915698
4,2,2866.170213
4,2,1668.548851
4,2,4949.617647
4,2,3569.107143
4,2,1717.650000
4,2,1768.122340
4,2,3868.199519
4,2,1689.125000
4,2,6742.333333
4,2,3880.341667
4,2,2479.520833
4,2,1793.088889
4,2,7683.067568
4,2,5547.300481
4,2,4131.752778
4,2,3601.906250
4,2,1753.000000
4,2,5865.734694
4,2,2676.448113
4,2,2623.804487
4,2,5347.308962
4,2,2952.912162
4,2,2960.636719
4,2,5484.767016
4,2,3223.562500
4,2,1795.562500
4,2,2816.834437
4,2,4412.918750
4,2,2153.223837
4,2,1780.375000
4,2,11440.936170
4,2,1565.375000
4,2,2590.342857
4,2,2741.328125
4,2,1946.347826
4,2,1562.215278
4,2,8124.250000
4,2,3736.070000
4,2,5856.951705
4,2,6468.916667
4,2,5000.329545
4,2,3255.697917
4,2,2798.193989
4,2,3368.153846
4,2,3294.031250
4,2,2701.730392
4,2,2970.284091
4,2,2228.595588
4,2,5743.853659
4,2,4444.186047
4,2,1631.692308

4,2,2731.679687
4,2,1863.484375
4,2,4426.763393
4,2,2014.785714
4,2,2087.057065
4,2,1823.951220
4,2,4931.178977
4,2,2638.105263
4,2,2620.850000
4,2,2318.953488
4,2,1623.773256
4,2,2370.794444
4,2,12745.917683
4,2,3596.982143
4,2,8060.493590
4,2,1691.443627
4,2,2711.562500
4,2,2434.575581
4,2,3001.875000
4,2,4108.360849
4,2,13150.121429
4,2,3830.166667
4,2,1769.514286
4,2,2947.585366
4,2,2359.192308
4,2,3211.553571
4,2,3001.968023
4,2,2578.000000
4,2,2460.684211
4,2,4871.154255
4,2,2272.914352
4,2,2477.342857
4,2,2617.857143
4,2,2334.410494
4,2,6944.744898
4,2,3233.000000
4,2,2950.664063
4,2,5061.800595
4,2,2181.324324
4,2,1553.700000
4,2,1875.687500
4,2,1620.494898
4,2,1697.197674
4,2,2358.983333
4,2,4411.382353
4,2,1557.250000
4,2,3048.109375
4,2,1539.073864
4,2,3094.125000
4,2,1903.497222
4,2,2427.073034
4,2,13108.888889
4,2,3286.341463
4,2,2635.846154
4,2,13087.200000
4,2,2912.846154
4,2,3686.800000
4,2,2703.647436
4,2,3862.717262
4,2,2720.677083
4,2,2480.935484
4,2,4972.282500
4,2,4038.886574
4,2,5947.994318
4,2,1963.119048
4,2,1712.800000

4,2,1740.036184
4,2,2812.259459
4,2,29579.416667
4,2,3390.488095
4,2,4175.666667
4,2,2228.198830
4,2,7189.131737
4,2,1869.837662
4,2,3878.827778
4,2,9246.161765
4,2,1578.923077
4,2,2852.182927
4,2,5957.622596
4,2,5666.945122
4,2,6327.298077
4,2,2210.735294
4,2,2520.067568
4,2,4704.762500
4,2,4008.785714
4,2,2407.625000
4,2,1820.825521
4,2,3330.775000
4,2,2946.204082
4,2,6784.214286
4,2,5061.916667
4,2,4214.812500
4,2,3930.883929
4,2,2060.612500
4,2,3950.883721
4,2,3618.995283
4,2,2045.541667
4,2,4315.576705
4,2,1596.692568
4,2,3679.125000
4,2,1623.705882
4,2,1516.853659
4,2,2901.600000
4,2,2295.660377
4,2,1522.488636
4,2,1569.425150
4,2,1585.400000
4,2,7580.730337
4,2,4367.714286
4,2,3039.725000
4,2,1722.936170
4,2,1741.625000
4,2,5998.173077
4,2,1747.798077
4,2,3811.571429
4,2,2408.542453
4,2,1971.018229
4,2,4024.027027
4,2,3529.411765
4,2,4554.924528
4,2,3543.393082
4,2,1701.705128
4,2,3913.120000
4,2,2390.185976
4,2,1938.291667
4,2,3367.838415
4,2,1705.302885
4,2,3460.627500
4,2,4029.187500
4,2,1751.800000
4,2,1606.839286
4,2,1855.016304

4,2,4012.472973
4,2,2741.000000
4,2,4236.543367
4,2,1553.957237
4,2,3230.804348
4,2,3308.162162
4,2,1699.296053
4,2,3650.367347
4,2,1804.566176
4,2,2848.928571
4,2,6758.768085
4,2,2035.581395
4,2,1620.505952
4,2,1526.000000
4,2,2494.043478
4,2,2241.894022
4,2,2569.563953
4,2,2060.722973
4,2,3200.900735
4,2,5651.208333
4,2,2449.523810
4,2,5300.148148
4,2,8893.194444
4,2,10224.537698
4,2,1715.848214
4,2,2295.639205
4,2,1879.113095
4,2,1784.701923
4,2,1843.061224
4,2,3049.052500
4,2,7283.777778
4,2,1782.070122
4,2,2695.878788
4,2,4917.428241
4,2,2068.542683
4,2,3297.334302
4,2,2900.700000
4,2,3360.515625
4,2,2745.042683
4,2,2157.101744
4,2,2164.060000
4,2,2480.710526
4,2,4742.791667
4,2,11298.159091
4,2,4496.701220
4,2,1980.695946
4,2,2688.245370
4,2,2208.233333
4,2,8187.393617
4,2,1844.993421
4,2,3541.775943
4,2,1504.853659
4,2,2783.132500
4,2,1954.159091
4,2,2051.820513
4,2,3661.812500
4,2,5014.076923
4,2,1537.570122
4,2,10886.170213
4,2,3299.602941
4,2,6479.949074
4,2,3110.555556
4,2,5149.596939
4,2,1903.346154
4,2,1994.343750
4,2,2746.497449

4,2,2157.800000
4,2,3168.185897
4,2,1851.536585
4,2,3495.487179
4,2,4677.112500
4,2,11750.539216
4,2,7059.581897
4,2,4978.282051
4,2,1677.607143
4,2,2202.811111
4,2,2508.383523
4,2,1846.416667
4,2,2545.990196
4,2,2381.357143
4,2,4138.375000
4,2,13008.160256
4,2,2069.200980
4,2,2487.514286
4,2,4774.280405
4,2,2547.920213
4,2,2472.864583
4,2,3457.853723
4,2,5652.940217
4,2,4310.673077
4,2,2903.980392
4,2,1954.849057
4,2,3784.250000
4,2,8136.937500
4,2,2105.791667
4,2,2715.620000
4,2,3023.655172
4,2,9298.300000
4,2,2800.352941
4,2,4793.909091
4,2,2120.664634
4,2,2346.179687
4,2,2015.171296
4,2,2664.094595
4,2,3117.594737
4,2,2196.333333
4,2,2417.625000
4,2,5786.975379
4,2,2488.467105
4,2,3367.166667
4,2,2059.338235
4,2,2275.315000
4,2,2308.502273
4,2,1687.700000
4,2,6020.683962
4,2,13853.137500
4,2,3052.343750
4,2,1771.982143
4,2,2170.046429
4,2,5028.921569
4,2,3336.941176
4,2,2867.015625
4,2,3149.213636
4,2,8387.892045
4,2,4691.353261
4,2,3605.517241
4,2,2177.875000
4,2,2218.511364
4,2,2070.761364
4,2,3074.041667
4,2,5522.548387
4,2,1542.304348

4,2,4701.187500
4,2,4143.393617
4,2,2494.138514
4,2,4154.589286
4,2,2265.857639
4,2,2202.609043
4,2,1771.185000
4,2,2998.666667
4,2,5197.449074
4,2,3173.189474
4,2,2096.500000
4,2,2229.025000
4,2,3071.404255
4,2,2223.674312
4,2,3117.404762
4,2,1559.357143
4,2,8092.883721
4,2,4478.513889
4,2,2885.122222
4,2,2558.096591
4,2,2409.237245
4,2,2712.294118
4,2,4896.153846
4,2,3423.476510
4,2,5368.088235
4,2,5280.387755
4,2,1972.328125
4,2,2054.296482
4,2,3416.812500
4,2,2864.233974
4,2,3200.940789
4,2,2719.845238
4,2,6276.011364
4,2,3045.984043
4,2,5266.341463
4,2,1731.978261
4,2,2268.008523
4,2,5142.854167
4,2,7025.000000
4,2,2819.510135
4,2,1610.096429
4,2,2517.166667
4,2,4596.861111
4,2,1948.511628
4,2,1550.067073
4,2,1742.643750
4,2,2663.213415
4,2,3216.522059
4,2,2294.200000
4,2,8110.546875
4,2,2823.181818
4,2,1867.166667
4,2,2506.534091
4,2,2137.438776
4,2,2678.138889
4,2,2367.354369
4,2,1886.158824
4,2,1596.050000
4,2,2656.719595
4,2,2492.085648
4,2,3576.425000
4,2,6311.851351
4,2,3552.671429
4,2,8245.734375
4,2,2835.510417
4,2,2351.545455

4,2,3479.837054
4,2,3990.291429
4,2,1986.559659
4,2,5177.671429
4,2,2810.170918
4,2,1529.785714
4,2,1701.552083
4,2,2193.750000
4,2,1685.905941
4,2,4267.524390
4,2,2023.154605
4,2,1748.919118
4,2,31533.525424
4,2,4945.023256
4,2,5057.375000
4,2,1532.824468
4,2,1978.418605
4,2,1710.750000
4,2,5110.277027
4,2,1738.633333
4,2,3912.372093
4,2,2264.040761
4,2,2240.295732
4,2,2733.189189
4,2,2331.500000
4,2,3216.535714
4,2,6011.875000
4,2,5822.231061
4,2,5032.997159
4,2,2938.925926
4,2,3305.097561
4,2,2126.697368
4,2,3642.088889
4,2,2107.705556
4,2,3887.880000
4,2,2537.870968
4,2,1732.750000
4,2,1894.637500
4,2,6476.184211
4,2,5776.736842
4,2,3254.307692
4,2,2534.153846
4,2,1712.101351
4,2,2991.400000
4,2,1706.301887
4,2,2793.269022
4,2,1513.472561
4,2,1874.342857
4,2,1526.538462
4,2,3223.764706
4,2,2560.662791
4,2,2099.173913
4,2,4132.765714
4,2,4813.006452
4,2,2625.022059
4,2,2334.442308
4,2,2600.548295
4,2,2604.916667
4,2,2690.312500
4,2,2329.165698
4,2,4913.125000
4,2,7384.990000
4,2,2780.833333
4,2,16310.312500
4,2,3498.863839
4,2,6411.537037

4,2,1559.818182
4,2,1623.279070
4,2,4425.166667
4,2,5544.409375
4,2,1553.000000
4,2,3649.969512
4,2,9438.070000
4,2,3700.065789
4,2,5509.440341
4,2,2103.502119
4,2,2015.168919
4,2,3178.393750
4,2,1630.437500
4,2,2908.277439
4,2,3465.812500
4,2,1873.506410
4,2,3129.738636
4,2,4405.166667
4,2,2338.924419
4,2,6971.382682
4,2,4309.911765
4,2,3291.254545
4,2,3429.729730
4,2,2316.250000
4,2,1843.818182
4,2,4562.912500
4,2,1614.770270
4,2,4961.155556
4,2,1577.681122
4,2,1580.459302
4,2,3202.842500
4,2,1816.340278
4,2,6306.057065
4,2,2693.801471
4,2,2560.805556
4,2,3115.300000
4,2,1638.625000
4,2,2639.702381
4,2,2060.022222
4,2,2201.918269
4,2,4589.883333
4,2,3305.969231
4,2,3221.568750
4,2,16740.698529
4,2,2390.260870
4,2,8775.596354
4,2,2455.478261
4,2,1740.810976
4,2,11500.363636
4,2,2205.073529
4,2,4233.668367
4,2,3457.409091
4,2,2954.739130
4,2,1760.795732
4,2,3991.043367
4,2,4662.781250
4,2,1624.093023
4,2,2315.000000
4,2,1587.443396
4,2,8336.665957
4,2,7461.218750
4,2,3062.403941
4,2,1933.517986
4,2,11123.720588
4,2,2201.262069
4,2,3154.685083

4,2,2283.564865
4,2,2536.937500
4,2,1929.385870
4,2,3186.426471
4,2,1772.355978
4,2,2371.565657
4,2,2520.458101
4,2,4736.613636
4,2,1535.161184
4,2,3474.489362
4,2,4746.237705
4,2,2898.958763
4,2,13636.522727
4,2,1991.336538
4,2,1842.669951
4,2,2938.000000
4,2,1988.021739
4,2,11652.002874
4,2,3778.536585
4,2,1526.060417
4,2,3397.916667
4,2,1730.331081
4,2,1789.079268
4,2,5735.391892
4,2,2564.478261
4,2,6265.200000
4,2,8876.437500
4,2,2007.642045
4,2,2693.727273
4,2,4966.255000
4,2,2824.315000
4,2,2803.316327
4,2,8829.007812
4,2,2503.932692
4,2,1734.925000
4,2,3662.408163
4,2,4260.768293
4,2,3037.122340
4,2,3550.571429
4,2,1707.202381
4,2,5597.897436
4,2,4560.305000
4,2,5322.962264
4,2,2078.268293
4,2,1577.326087
4,2,2169.147059
4,2,2943.051471
4,2,3592.562500
4,2,1895.190789
4,2,4942.716667
4,2,1544.951220
4,2,3453.535714
4,2,1823.615385
4,2,3023.584906
4,2,5409.969512
4,2,1730.000000
4,2,2330.067073
4,2,3562.424623
4,2,1666.232258
4,2,1979.782857
4,2,4672.865854
4,2,7383.542601
4,2,6408.417857
4,2,3036.945946
4,2,5822.053571
4,2,2927.219388

4,2,3160.382653
4,2,1503.625000
4,2,4717.457447
4,2,3040.241477
4,2,3326.238636
4,2,4311.748571
4,2,3294.415698
4,2,1762.740933
4,2,2037.858553
4,2,8540.453947
4,2,2110.881250
4,2,3234.559172
4,2,3024.956395
4,2,2573.351064
4,2,2590.000000
4,2,3482.958333
4,2,2627.828125
4,2,1543.633721
4,2,1689.616071
4,2,3838.717949
4,2,2100.496711
4,2,4327.059783
4,2,1797.568182
4,2,2005.588235
4,2,2471.604294
4,2,4645.965426
4,2,2420.933333
4,2,6601.170455
4,2,2045.904762
4,2,3082.345000
4,2,3123.479730
4,2,4564.375000
4,2,3860.020349
4,2,3583.722973
4,2,1994.442500
4,2,1511.959184
4,2,1621.242500
4,2,2943.981707
4,2,3344.198113
4,2,1678.500000
4,2,3474.714286
4,2,17846.902344
4,2,10077.837004
4,2,1723.538674
4,2,3331.879747
4,2,1785.736413
4,2,2068.941860
4,2,7607.339623
4,2,3262.819079
4,2,15385.745614
4,2,2491.485795
4,2,3332.320000
4,2,1555.699324
4,2,2027.767442
4,2,10911.831395
4,2,1681.485119
4,2,2869.257653
4,2,2720.468085
4,2,6019.945946
4,2,1790.250000
4,2,9161.296296
4,2,4328.170455
4,2,1521.683333
4,2,4771.851562
4,2,2475.516304
4,2,2426.230114

4,2,6646.546875
4,2,1962.529070
4,2,3881.743590
4,2,2459.454545
4,2,9829.837264
4,2,2364.978261
4,2,2533.815789
4,2,1622.179856
4,2,3003.005682
4,2,18698.650000
4,2,2173.659574
4,2,12761.903226
4,2,5547.538462
4,2,12893.465909
4,2,1618.138158
4,2,1948.740132
4,2,1723.907787
4,2,3131.000000
4,2,5691.195000
4,2,2353.217391
4,2,1654.833333
4,2,2427.786111
4,2,3151.904255
4,2,2519.844512
4,2,3014.709459
4,2,2581.500000
4,2,3961.375000
4,2,4610.481383
4,2,8294.954082
4,2,3595.194079
4,2,2936.900000
4,2,1538.099432
4,2,15034.895833
4,2,3916.944444
4,2,2277.476064
4,2,2140.662162
4,2,1661.087432
4,2,4806.881818
4,2,1875.347826
4,2,1731.094512
4,2,5307.104167
4,2,3474.677885
4,2,1833.442708
4,2,5145.227273
4,2,4303.396277
4,2,2481.926136
4,2,2796.350000
4,2,2994.285714
4,2,3331.776316
4,2,3793.078488
4,2,2598.755435
4,2,1514.375000
4,2,2002.488372
4,2,1582.025000
4,2,3519.000000
4,2,2595.428571
4,2,2685.292453
4,2,5402.758523
4,2,3561.881098
4,2,2226.647059
4,2,3277.788889
4,2,1780.818182
4,2,2039.844595
4,2,2542.572864
4,2,4940.125000
4,2,7845.298077

4,2,3414.926136
4,2,4958.683333
4,2,2500.000000
4,2,5126.717489
4,2,5872.317073
4,2,3749.520000
4,2,3218.579787
4,2,4267.639535
4,2,3179.125000
4,2,1874.385714
4,2,2785.802500
4,2,6413.072917
4,2,1987.106771
4,2,3828.000000
4,2,4796.946809
4,2,5470.090909
4,2,1889.152439
4,2,1939.238636
4,2,1843.766447
4,2,2001.989583
4,2,1790.055556
4,2,2697.790761
4,2,6154.482500
4,2,1769.738636
4,2,1736.493902
4,2,2388.450000
4,2,2735.644231
4,2,2123.421053
4,2,9475.106250
4,2,1775.785714
4,2,11439.531250
4,2,20597.781250
4,2,1772.583333
4,2,10075.585000
4,2,2917.718750
4,2,2769.418919
4,2,1521.585366
4,2,1968.035714
4,2,2164.765000
4,2,7156.146341
4,2,14298.901786
4,2,2641.181818
4,2,1844.773810
4,2,1764.425714
4,2,3245.125000
4,2,1937.333333
4,2,1712.368421
4,2,39546.584416
4,2,3970.674757
4,2,2903.274390
4,2,4901.984375
4,2,1595.237179
4,2,7811.788793
4,2,3420.350000
4,2,2736.323529
4,2,3165.586957
4,2,3732.331633
4,2,3915.238220
4,2,1922.468023
4,2,14464.201923
4,2,5850.039683
4,2,1814.617021
4,2,2825.218750
4,2,2745.803922
4,2,3751.583333
4,2,2845.405405

4,2,4466.450000
4,2,10038.332317
4,2,2910.230366
4,2,1679.160156
4,2,2614.017949
4,2,2134.176923
4,2,8717.884615
4,2,2646.347826
4,2,2424.770833
4,2,1939.031250
4,2,4309.250000
4,2,1559.027778
4,2,1546.981707
4,2,2003.381250
4,2,1517.696809
4,2,6835.640449
4,2,2809.466667
4,2,2341.039683
4,2,5436.559322
4,2,6366.875000
4,2,3853.125000
4,2,2397.000000
4,2,3314.850000
4,2,4865.000000
4,2,3336.527778
4,2,6772.240741
4,2,2679.986842
4,2,2174.255319
4,2,2065.125000
4,2,5270.244565
4,2,1522.267974
4,2,2966.836806
4,2,3256.456376
4,2,1911.760563
4,2,3558.479167
4,2,1856.459459
4,2,5505.571429
4,2,2772.061224
4,2,7023.862179
4,2,1542.780612
4,2,3351.171875
4,2,2783.307692
4,2,1889.166667
4,2,2171.500000
4,2,3571.262500
4,2,2410.491803
4,2,7734.000000
4,2,3762.271875
4,2,3439.323671
4,2,3128.693069
4,2,1673.032468
4,2,2983.945652
4,2,1792.193182
4,2,5450.419492
4,2,14807.093750
4,2,2340.700000
4,2,4071.166667
4,2,6004.675532
4,2,2679.180556
4,2,2179.533333
4,2,2787.982143
4,2,3644.813953
4,2,2763.792411
4,2,1892.750000
4,2,2384.958333
4,2,3159.586538

4,2,2017.987805
4,2,4662.127907
4,2,2882.188889
4,2,1582.000000
4,2,2802.125000
4,2,2421.277439
4,2,10399.278409
4,2,6842.019231
4,2,6642.541667
4,2,14374.557692
4,2,10749.452500
4,2,2955.355769
4,2,3414.804878
4,2,3793.043750
4,2,1802.680000
4,2,2091.769231
4,2,1708.181319
4,2,10725.875000
4,2,8891.500000
4,2,2098.935000
4,2,7147.702703
4,2,1837.090909
4,2,2511.083333
4,2,2885.107955
4,2,2014.768293
4,2,2046.020349
4,2,2082.842105
4,2,2276.751337
4,2,10057.444444
4,2,5835.292929
4,2,3064.904412
4,2,1909.288690
4,2,2165.936111
4,2,6459.796875
4,2,4757.497283
4,2,2608.891667
4,2,2556.250000
4,2,1679.647727
4,2,3002.183333
4,2,1553.567010
4,2,3350.414226
4,2,4595.223837
4,2,5123.612245
4,2,5459.189904
4,2,56984.861111
4,2,10660.534884
4,2,3329.767857
4,2,2022.643293
4,2,1948.125000
4,2,3552.490741
4,2,1610.600000
4,2,1610.952381
4,2,1826.700000
4,2,7898.800926
4,2,10363.066667
4,2,2909.380000
4,2,2034.903846
4,2,6458.223558
4,2,15556.446429
4,2,2332.480114
4,2,2628.571809
4,2,145294.733696
4,2,6197.938889
4,2,3528.550676
4,2,2151.720183
4,2,4303.600000

4,2,5079.513298
4,2,4678.942308
4,2,6970.187500
4,2,3119.349057
4,2,3757.604592
4,2,2011.500000
4,2,3375.865625
4,2,2176.235119
4,2,3027.125000
4,2,1995.817797
4,2,8044.755208
4,2,7194.138298
4,2,1695.433824
4,2,2243.037736
4,2,5119.328125
4,2,5915.783333
4,2,6782.552632
4,2,4229.398649
4,2,1742.727273
4,2,11447.304167
4,2,5351.227941
4,2,3371.319149
4,2,1567.155405
4,2,4412.261905
4,2,2364.285714
4,2,7423.335526
4,2,4204.937500
4,2,5885.718750
4,2,4239.891892
4,2,4215.985294
4,2,7029.146341
4,2,1676.720930
4,2,3971.400000
4,2,3351.451923
4,2,6542.930851
4,2,2857.069767
4,2,5053.843750
4,2,2760.840000
4,2,1754.260638
4,2,3193.853571
4,2,2829.135000
4,2,2341.750000
4,2,3168.644231
4,2,1723.210526
4,2,2385.328125
4,2,2035.881944
4,2,11901.200000
4,2,1623.348684
4,2,19584.764706
4,2,1828.714286
4,2,3075.522222
4,2,2615.879581
4,2,3401.967391
4,2,10897.105263
4,2,5547.612245
4,2,1539.877778
4,2,3785.611111
4,2,5810.201389
4,2,2228.660714
4,2,6176.619318
4,2,1735.641026
4,2,1715.213235
4,2,5281.142857
4,2,1544.000000
4,2,3300.866667
4,2,3296.898438

4,2,7491.112500
4,2,2067.666667
4,2,2211.044218
4,2,9415.550000
4,2,1845.179487
4,2,2988.100000
4,2,2130.098160
4,2,2122.838816
4,2,1616.636364
4,2,1580.585937
4,2,1591.026316
4,2,2006.666667
4,2,3830.785714
4,2,4979.616279
4,2,1817.868056
4,2,4575.463068
4,2,1844.714744
4,2,4269.885714
4,2,2666.090909
4,2,3576.093750
4,2,2068.100000
4,2,4425.260593
4,2,3570.775568
4,2,2034.959302
4,2,3105.760000
4,2,2529.983696
4,2,13368.041667
4,2,2072.621622
4,2,2288.776596
4,2,3488.330000
4,2,3995.961326
4,2,1628.047739
4,2,2201.690955
4,2,3134.547170
4,2,3765.516509
4,2,4661.670732
4,2,1928.030303
4,2,2812.428571
4,2,2644.660377
4,2,3757.446328
4,2,4607.660714
4,2,2662.759434
4,2,3101.192308
4,2,3226.764706
4,2,2038.342105
4,2,5484.423611
4,2,2133.877551
4,2,2324.178571
4,2,2969.849359
4,2,2118.600000
4,2,3059.682692
4,2,1592.975610
4,2,6419.289474
4,2,3728.091346
4,2,3202.678571
4,2,5353.951923
4,2,4184.852071
4,2,8313.695652
4,2,1939.776042
4,2,2065.057692
4,2,4308.680000
4,2,1549.066176
4,2,2863.500000
4,2,5143.112903
4,2,2561.593085
4,2,3447.906250

4,2,4412.303279
4,2,1965.428571
4,2,2665.547872
4,2,3687.500000
4,2,2822.117647
4,2,2005.451149
4,2,3009.905263
4,2,5121.002841
4,2,2564.037975
4,2,13152.902174
4,2,8046.368421
4,2,3769.445000
4,2,7575.057292
4,2,3696.375000
4,2,6340.683140
4,2,2951.728261
4,2,5392.511628
4,2,1752.285714
4,2,2916.850000
4,2,3408.967949
4,2,10896.981132
4,2,1853.010050
4,2,7802.440217
4,2,4118.823077
4,2,2495.562500
4,2,1581.706633
4,2,2365.386364
4,2,2019.369565
4,2,2309.015625
4,2,2742.570000
4,2,7211.427632
4,2,5803.969388
4,2,1829.144578
4,2,2128.453125
4,2,5714.857955
4,2,3490.472222
4,2,2405.509615
4,2,11250.500000
4,2,2190.000000
4,2,5770.911765
4,2,6210.975000
4,2,2648.248889
4,2,6743.421053
4,2,11329.776596
4,2,1695.945946
4,2,2740.323171
4,2,3522.230769
4,2,2144.015306
4,2,2336.321023
4,2,1895.655172
4,2,2888.250000
4,2,3661.990385
4,2,2150.845000
4,2,3426.802326
4,2,3815.017442
4,2,1757.260090
4,2,5928.079268
4,2,1633.750000
4,2,6799.097561
4,2,6865.244898
4,2,1499.843750
4,2,1804.295455
4,2,4597.778061
4,2,5092.000000
4,2,1859.182927
4,2,12247.777778

4,2,4494.162162
4,2,1740.912500
4,2,2424.107143
4,2,13993.411932
4,2,1842.882353
4,2,1671.250000
4,2,2371.862069
4,2,1549.661111
4,2,2094.968750
4,2,1875.256410
4,2,14523.569915
4,2,5541.619565
4,2,4352.619048
4,2,1910.062500
4,2,6699.107143
4,2,2919.598765
4,2,2336.913043
4,2,5826.914474
4,2,3819.953052
4,2,1956.972222
4,2,2477.147059
4,2,2355.193717
4,2,2545.976744
4,2,2091.958333
4,2,2850.096154
4,2,12569.366822
4,2,14959.990625
4,2,7423.560811
4,2,3052.040094
4,2,1977.101974
4,2,4457.285000
4,2,2869.986486
4,2,2573.852564
4,2,1684.400000
4,2,1608.629630
4,2,1518.230263
4,2,17326.949153
4,2,1541.546053
4,2,5345.843137
4,2,3319.449735
4,2,4953.615385
4,2,2347.958333
4,2,3915.547619
4,2,2855.600000
4,2,3946.551020
4,2,3842.318182
4,2,1757.476562
4,2,6129.270000
4,2,2442.540541
4,2,2584.436047
4,2,1755.173077
4,2,1641.416667
4,2,2084.502500
4,2,2032.902778
4,2,1763.221875
4,2,2869.040000
4,2,4438.305556
4,2,1566.153846
4,2,1556.158537
4,2,4127.064394
4,2,2318.032895
4,2,3334.250000
4,2,1923.166667
4,2,3128.129902
4,2,1851.200000
4,2,2332.773437

4,2,5416.057500
4,2,4877.540094
4,2,2210.250000
4,2,2310.944079
4,2,4069.560976
4,2,3336.458333
4,2,4534.315789
4,2,3417.791667
4,2,4122.740132
4,2,1562.632353
4,2,3213.601562
4,2,2847.643750
4,2,2285.472561
4,2,2152.495000
4,2,3085.616477
4,2,1551.187500
4,2,5148.531915
4,2,8254.474490
4,2,26763.375000
4,2,7378.625000
4,2,5349.532609
4,2,2267.062500
4,2,2547.583333
4,2,2999.436275
4,2,4696.005208
4,2,3060.400000
4,2,2868.444785
4,2,10075.397436
4,2,1524.100962
4,2,1636.401786
4,2,2699.562500
4,2,4854.187500
4,2,5268.537791
4,2,5455.809524
4,2,3710.277778
4,2,1728.276074
4,2,5038.670000
4,2,1791.489130
4,2,2882.329843
4,2,6993.835526
4,2,2612.143590
4,2,7104.764423
4,2,5117.795455
4,2,2148.516588
4,2,2557.083333
4,2,2158.018617
4,2,15542.677632
4,2,4187.283333
4,2,2508.762500
4,2,4278.697674
4,2,2930.644444
4,2,3729.663462
4,2,4659.281250
4,2,4154.619318
4,2,2062.979592
4,2,2700.847222
4,2,4794.276596
4,2,19461.864130
4,2,2624.333333
4,2,3424.730114
4,2,1821.617647
4,2,1661.764706
4,2,2627.667683
4,2,2875.400000
4,2,1542.038462
4,2,4126.648026

4,2,7401.915179
4,2,4156.755556
4,2,4053.878205
4,2,2109.875000
4,2,4542.752451
4,2,5198.536585
4,2,2323.622517
4,2,1921.166667
4,2,3507.500000
4,2,6689.979532
4,2,5390.625000
4,2,24403.402542
4,2,1734.333333
4,2,2540.542683
4,2,3854.865741
4,2,4639.765625
4,2,3557.791667
4,2,1579.819444
4,2,2101.839286
4,2,14426.700581
4,2,3273.161137
4,2,6728.517964
4,2,15994.245509
4,2,4494.000000
4,2,3355.904255
4,2,3632.428571
4,2,1535.723214
4,2,2144.569712
4,2,1899.044199
4,2,4467.228723
4,2,1746.743056
4,2,2527.944444
4,2,3822.871429
4,2,1664.546429
4,2,2162.600000
4,2,1530.338816
4,2,16800.346154
4,2,2150.730769
4,2,5768.362500
4,2,3642.616848
4,2,2426.120000
4,2,5025.609756
4,2,7538.534884
4,2,3552.500000
4,2,2478.511111
4,2,4783.067308
4,2,2719.807692
4,2,2327.728723
4,2,2098.597826
4,2,5098.577778
4,2,3833.356061
4,2,2736.850000
4,2,2167.469136
4,2,3594.221910
4,2,3660.367925
4,2,7534.336207
4,2,2666.000000
4,2,11855.000000
4,2,2792.607143
4,2,2234.139423
4,2,11102.714286
4,2,6640.756098
4,2,1894.309783
4,2,9412.883152
4,2,5238.869565
4,2,6206.037500

4,2,3457.320122
4,2,3976.705189
4,2,8796.351064
4,2,9932.663462
4,2,1565.750000
4,2,2277.709459
4,2,1904.447368
4,2,5023.333333
4,2,5876.328125
4,2,1501.954545
4,2,1946.350000
4,2,3977.134615
4,2,2386.707547
4,2,2349.218750
4,2,1732.895408
4,2,3208.371711
4,2,2641.125000
4,2,4890.423077
4,2,2605.775641
4,2,1817.125000
4,2,2363.020000
4,2,1955.920455
4,2,10300.200000
4,2,4754.780612
4,2,1964.960938
4,2,37509.806452
4,2,1658.738636
4,2,5003.500000
4,2,2868.400000
4,2,1812.447917
4,2,4179.972826
4,2,5833.468531
4,2,6330.349359
4,2,3849.244318
4,2,11485.343750
4,2,3756.976744
4,2,2662.439103
4,2,2043.125683
4,2,7830.602823
4,2,4413.350000
4,2,1581.153846
4,2,3911.422222
4,2,4302.713983
4,2,3121.310976
4,2,4243.954268
4,2,2830.556373
4,2,3903.625000
4,2,6000.820652
4,2,2842.122222
4,2,8458.207317
4,2,1903.548611
4,2,1543.241477
4,2,7030.093023
4,2,6617.117647
4,2,2111.344444
4,2,2945.144886
4,2,1543.027027
4,2,1556.122995
4,2,1631.391667
4,2,6080.625000
4,2,2419.130682
4,2,6788.692308
4,2,8471.712500
4,2,3391.317130
4,2,2452.861842
4,2,4137.872642

4,2,11753.000000
4,2,6820.823718
4,2,1606.000000
4,2,3354.241477
4,2,2619.840000
4,2,9259.120763
4,2,2364.564103
4,2,1852.264516
4,2,5830.271930
4,2,4982.861111
4,2,4256.439024
4,2,5876.158333
4,2,2900.741071
4,2,3486.274510
4,2,7796.817857
4,2,6583.561404
4,2,4605.127907
4,2,3477.520833
4,2,4419.447115
4,2,3051.910000
4,2,2462.405405
4,2,6311.500000
4,2,8211.495098
4,2,6487.783019
4,2,3946.906250
4,2,4790.262500
4,2,2082.671569
4,2,2665.958140
4,2,5513.312500
4,2,2072.250000
4,2,3655.071023
4,2,3434.700000
4,2,2023.846154
4,2,1520.937500
4,2,3659.760870
4,2,7143.080882
4,2,2969.415179
4,2,1613.325472
4,2,10455.865741
4,2,19086.375000
4,2,5557.519886
4,2,2560.581522
4,2,2226.560976
4,2,3547.086735
4,2,3265.662162
5,1,2337.562500
5,1,1595.952381
5,1,2876.394231
5,1,3415.357500
5,1,1528.658537
5,1,5658.048077
5,1,3092.411585
5,1,4440.322674
5,1,5263.000000
5,1,46122.240132
5,1,2443.307692
5,1,11898.628378
5,1,5605.937500
5,1,1968.520349
5,1,2734.071429
5,1,4974.763889
5,1,3016.846591
5,1,2289.716837
5,1,2386.444444
5,1,7208.818182
5,1,2588.948718

5,1,3511.875000
5,1,1684.502551
5,1,3188.533333
5,1,8993.968750
5,1,11702.489130
5,1,34805.551282
5,1,1804.010870
5,1,6894.079545
5,1,3936.957447
5,1,1641.625000
5,1,6175.365132
5,1,3140.921053
5,1,1954.875000
5,1,5726.143293
5,1,3723.977564
5,1,2667.548077
5,1,1634.171875
5,1,1608.131410
5,1,8385.500000
5,1,2286.696023
5,1,11610.808511
5,1,2298.589286
5,1,1773.279070
5,1,2148.529412
5,1,2534.250000
5,1,27144.918750
5,1,6565.872093
5,1,13534.996875
5,1,6888.934211
5,1,16323.397436
5,1,8260.122449
5,1,8610.402174
5,1,2915.808140
5,1,8872.666667
5,1,3001.194767
5,1,2068.664062
5,1,17056.300439
5,1,38906.869048
5,1,7288.479167
5,1,36022.921053
5,1,14382.350000
5,1,1790.135638
5,1,5024.303030
5,1,5374.033333
5,1,3505.747340
5,1,15934.659884
5,1,10352.360577
5,1,145455.660256
5,1,1852.899457
5,1,13041.646277
5,1,3174.139535
5,1,4111.341912
5,1,3583.925926
5,1,2033.994318
5,1,2445.914474
5,1,18387.044271
5,1,176569.025000
5,1,5041.755814
5,1,8226.082500
5,1,2957.506250
5,1,9442.674419
5,1,1753.079268
5,1,6490.473684
5,1,6205.657500
5,1,3483.296053
5,1,5319.356481

5,1,9887.021341
5,1,1525.329861
5,1,1767.948718
5,1,24476.102041
5,1,3537.622642
5,1,2641.718750
5,1,1696.194149
5,1,7283.366667
5,1,1895.052083
5,1,4547.797297
5,1,41875.573529
5,1,4042.625000
5,1,1563.087629
5,1,3956.347826
5,1,2764.792969
5,1,2623.758721
5,1,2359.875000
5,1,1788.854651
5,1,17928.850000
5,1,1573.125000
5,1,2799.579545
5,1,2608.328947
5,1,1708.158537
5,1,1994.621324
5,1,3318.091463
5,1,4074.615854
5,1,64389.258721
5,1,6446.581731
5,1,5092.752551
5,1,2901.035714
5,1,1870.402500
5,1,8641.750000
5,1,2068.450000
5,1,2278.261792
5,1,2535.500000
5,1,3187.492647
5,1,4621.540865
5,1,27402.727273
5,1,71721.440625
5,1,2691.050000
5,1,6485.889706
5,1,2086.054878
5,1,1900.561111
5,1,25428.661290
5,1,7090.325000
5,1,2536.900000
5,1,4675.094444
5,1,171967.243056
5,1,1525.412791
5,1,76610.075000
5,1,6234.175676
5,1,1707.902439
5,1,4197.118750
5,1,2805.414286
5,1,7850.910714
5,1,56084.507143
5,1,7883.600000
5,1,2588.796875
5,1,2668.321429
5,1,5256.414286
5,1,1513.519231
5,1,35411.759615
5,1,35724.315789
5,1,9524.130435
5,1,3673.433333
5,1,2090.656250

5,1,5803.000000
5,1,3209.000000
5,1,5902.135135
5,1,5513.804245
5,1,4449.386364
5,1,1877.210938
5,1,1618.916667
5,1,96633.965278
5,1,1939.631250
5,1,1674.209302
5,1,48168.437500
5,1,1678.705882
5,1,15078.125000
5,1,2957.581250
5,1,4135.866667
5,1,3222.572368
5,1,121670.025641
5,1,8853.344828
5,1,3856.302083
5,1,1793.153846
5,1,17038.920732
5,1,2044.686111
5,1,2790.892857
5,1,41012.607143
5,1,3449.061224
5,1,2068.283088
5,1,7019.093750
5,1,4165.957317
5,1,1801.000000
5,1,3007.180000
5,1,4227.557927
5,1,3294.649457
5,1,5142.593750
5,1,5176.973684
5,1,1516.386364
5,1,1676.619048
5,1,12887.729167
5,1,3031.335526
5,1,2669.000000
5,1,3227.175000
5,1,3793.065476
5,1,1602.893750
5,1,6786.824219
5,1,7457.000000
5,1,2580.589744
5,1,1894.406250
5,1,1887.583333
5,1,61359.736842
5,1,2000.421875
5,1,24499.951087
5,1,6257.607500
5,1,2338.819767
5,1,12242.776256
5,1,112884.565341
5,1,1922.228723
5,1,7899.746528
5,1,21425.250000
5,1,3024.272277
5,1,4815.937500
5,1,2191.666667
5,1,2891.566667
5,1,4979.705882
5,1,4291.989583
5,1,8002.482759
5,1,1915.000000
5,1,1583.857639

5,1,52978.641026
5,1,3494.048780
5,1,5431.333333
5,1,14523.828125
5,1,6754.898438
5,1,57223.646875
5,1,1587.091463
5,1,151140.447368
5,1,1639.125000
5,1,2304.833333
5,1,21154.465278
5,1,2294.722500
5,1,1517.171875
5,1,1843.281915
5,1,3620.843750
5,1,7264.400000
5,1,1939.818966
5,1,2586.896341
5,1,5326.758333
5,1,1678.496324
5,1,2924.915761
5,1,3577.558824
5,1,2293.604651
5,1,4358.887500
5,1,4788.529167
5,1,2066.750000
5,1,2727.187500
5,1,2207.779412
5,1,71350.840278
5,1,32288.080645
5,1,1572.541667
5,1,37222.233871
5,1,25560.935811
5,1,1757.869565
5,1,2781.714286
5,1,58585.855263
5,1,33277.125000
5,1,3333.619565
5,1,3053.590909
5,1,2132.488372
5,1,1536.550000
5,1,4580.500000
5,1,1935.416667
5,1,25902.038462
5,1,2693.266667
5,1,9616.189103
5,1,1838.409722
5,1,1588.813559
5,1,2554.681034
5,1,2209.660714
5,1,2573.822674
5,1,2128.896739
5,1,3321.162234
5,1,1636.308824
5,1,2973.187500
5,1,17266.494318
5,1,1646.500000
5,1,3573.318182
5,1,6269.860465
5,1,14111.348837
5,1,2062.732558
5,1,3987.459184
5,1,4991.114286
5,1,5026.838235
5,1,145024.313776
5,1,4502.948718

5,1,181172.585227
5,1,35501.000000
5,1,6454.971429
5,1,125485.573529
5,1,2221.341837
5,1,2148.487179
5,1,1547.422619
5,1,2799.291667
5,1,33162.928977
5,1,85854.118243
5,1,2173.116667
5,1,2004.700000
5,1,2148.567568
5,1,3305.763514
5,1,11147.777174
5,1,4758.326087
5,1,38563.571429
5,1,5378.750000
5,1,1719.045213
5,1,6695.510870
5,1,148307.371795
5,1,6224.888889
5,1,1499.984375
5,1,1862.189189
5,1,1719.244792
5,1,85630.850694
5,1,2527.453125
5,1,2051.978261
5,1,8269.141026
5,1,2412.216912
5,1,8663.000000
5,1,2652.552419
5,1,1829.697222
5,1,2945.718750
5,1,7369.632653
5,1,31867.729730
5,1,12080.970238
5,1,2326.890244
5,1,6875.860294
5,1,2049.083333
5,1,3427.190625
5,1,1550.562500
5,1,4578.065476
5,1,2496.478659
5,1,1672.239583
5,1,1915.180851
5,1,77343.500000
5,1,8416.613636
5,1,31757.111842
5,1,1949.574468
5,1,1520.941860
5,1,3688.979167
5,1,5025.268750
5,1,2419.608696
5,1,2981.062500
5,1,2710.225806
5,1,1571.628571
5,1,2224.875000
5,1,2865.582317
5,1,5415.059524
5,1,4465.454545
5,1,1640.727273
5,1,2601.750000
5,1,1607.125000
5,1,1956.510417
5,1,8051.500000

5,1,1885.975000
5,1,33516.812500
5,1,1686.546512
5,1,7560.549342
5,1,71145.000000
5,1,2705.750000
5,1,10677.619048
5,1,3224.372340
5,1,1861.574468
5,1,1570.266667
5,1,11460.084239
5,1,5425.585366
5,1,15049.324324
5,1,7937.662162
5,1,2165.500000
5,1,1758.976744
5,1,7706.062500
5,1,7826.268293
5,1,63867.420455
5,1,4931.665761
5,1,6627.531250
5,1,2137.910714
5,1,3311.920732
5,1,1504.289474
5,1,6492.705882
5,1,2295.562827
5,1,1648.358108
5,1,2041.153409
5,1,4031.830882
5,1,2273.380952
5,1,3094.092593
5,1,2860.812500
5,1,3220.911585
5,1,4332.914634
5,1,37598.000000
5,1,3077.846591
5,1,1726.675000
5,1,7733.395349
5,1,2082.360714
5,1,2204.558333
5,1,4979.500000
5,1,1749.797619
5,1,1754.418750
5,1,1701.479651
5,1,2289.954545
5,1,2429.073171
5,1,2554.010870
5,1,19624.662162
5,1,32423.851064
5,1,5244.571429
5,1,1924.694444
5,1,1623.271739
5,1,18055.460526
5,1,2156.324324
5,1,31898.170732
5,1,2615.141892
5,1,1515.884615
5,1,2121.688525
5,1,11439.608696
5,1,24609.422619
5,1,13040.588816
5,1,2622.005882
5,1,2838.132812
5,1,3422.062500
5,1,2269.559211
5,1,1960.585366

5,1,7075.519231
5,1,18537.923913
5,1,4794.746711
5,1,3054.875000
5,1,5979.895161
5,1,1637.871795
5,1,2946.398437
5,1,4844.800000
5,1,16109.967105
5,1,2817.321023
5,1,48555.636364
5,1,3273.714286
5,1,2434.150000
5,1,5528.344828
5,1,2233.125000
5,1,2004.994186
5,1,27508.333333
5,1,11817.000000
5,1,2491.208333
5,1,1515.391304
5,1,5235.437500
5,1,1550.665441
5,1,4272.267857
5,1,3459.280702
5,1,2732.000000
5,1,3733.329268
5,1,1657.750000
5,1,11033.304878
5,1,8712.625000
5,1,4936.103723
5,1,8177.471154
5,1,1901.718750
5,1,4900.578125
5,1,2186.700758
5,1,82886.929487
5,1,4060.294521
5,1,4344.477273
5,1,28806.401163
5,1,13521.940217
5,1,5295.541667
5,1,1735.477273
5,1,5235.275510
5,1,37010.492424
5,1,1632.412500
5,1,1705.013298
5,1,8533.020161
5,1,6221.375000
5,1,1662.617188
5,1,3052.744898
5,1,1739.578125
5,1,1901.137500
5,1,2058.426829
5,1,3717.683824
5,1,3221.000000
5,1,2408.271341
5,1,1925.348684
5,1,6145.863971
5,1,1677.769531
5,1,2361.400000
5,1,10763.454861
5,1,5203.152174
5,1,2962.823864
5,1,1567.060976
5,1,7061.178571
5,1,5593.428571
5,1,18259.437500

5,1,1499.483333
5,1,1917.125000
5,1,2888.732143
5,1,3519.500000
5,1,5374.166667
5,1,2563.731618
5,1,3299.615385
5,1,1515.740132
5,1,4111.797727
5,1,4199.272436
5,1,3207.875000
5,1,1954.500000
5,1,1563.250000
5,1,4966.663462
5,1,5695.622222
5,1,2065.716463
5,1,1739.024390
5,1,60300.585938
5,1,6368.190217
5,1,2276.961735
5,1,55865.765625
5,1,8254.306250
5,1,2651.285714
5,1,5771.881250
5,1,3294.129808
5,1,2714.638298
5,1,9031.800000
5,1,3003.221154
5,1,1520.166667
5,1,4929.546196
5,1,2183.500000
5,1,1720.426829
5,1,1679.476064
5,1,1873.625000
5,1,2792.955882
5,1,2324.184211
5,1,1765.701087
5,1,7436.500000
5,1,4612.352941
5,1,12230.245614
5,1,1616.561047
5,1,1842.756250
5,1,1557.927500
5,1,4242.912500
5,1,19667.058824
5,1,12542.673913
5,1,8901.000000
5,1,8115.993644
5,1,3539.869565
5,1,36198.625000
5,1,1775.228261
5,1,128104.036111
5,1,16098.044118
5,1,14458.652344
5,1,1725.441176
5,1,3773.562500
5,1,6933.853125
5,1,2645.897727
5,1,2496.583333
5,1,2615.262755
5,1,1980.571429
5,1,28792.051282
5,1,1690.617949
5,1,1925.084239
5,1,5347.625000
5,1,198839.733871

5,1,1512.805233
5,1,1643.025000
5,1,4262.707317
5,1,3292.375000
5,1,2832.058824
5,1,8343.459559
5,1,9020.250000
5,1,1703.061047
5,1,3145.343023
5,1,1585.689189
5,1,5803.173913
5,1,2167.167553
5,1,6067.838415
5,1,1523.142857
5,1,1938.763021
5,1,26402.236111
5,1,48739.318750
5,1,3189.500000
5,1,2515.726744
5,1,3505.900000
5,1,3693.633333
5,1,39133.326087
5,1,1770.681250
5,1,3278.605263
5,1,18878.178571
5,1,1903.705128
5,1,2594.551829
5,1,1555.410714
5,1,1593.033333
5,1,2825.895833
5,1,3655.181818
5,1,1984.199405
5,1,3825.615385
5,1,1610.573529
5,1,6991.939394
5,1,8026.837209
5,1,1955.000000
5,1,9933.955729
5,1,2588.231383
5,1,22534.014706
5,1,2592.493056
5,1,2219.789062
5,1,5199.836957
5,1,14305.733333
5,1,1599.756098
5,1,1676.641026
5,1,1935.465116
5,1,14606.568750
5,1,1584.540541
5,1,2913.180272
5,1,2374.500000
5,1,4107.000000
5,1,2673.500000
5,1,28232.508065
5,1,2571.677632
5,1,3320.625000
5,1,8897.224138
5,1,3760.890625
5,1,1755.914634
5,1,2608.744565
5,1,11650.375000
5,1,29821.705882
5,1,75688.509615
5,1,2971.420732
5,1,5256.371951
5,1,1971.585366

5,1,1840.785714
5,1,5831.083333
5,1,5719.207317
5,1,3414.416667
5,1,1698.548913
5,1,3216.878289
5,1,46091.224265
5,1,3809.562500
5,1,8117.364286
5,1,1953.086957
5,1,2394.875000
5,1,4834.925595
5,1,55561.000000
5,1,3992.375000
5,1,2724.187500
5,1,2655.246324
5,1,2893.412500
5,1,1657.681818
5,1,3119.627660
5,1,16613.875000
5,1,9280.607500
5,1,1974.991848
5,1,23519.380682
5,1,6709.343750
5,1,1727.519737
5,1,37990.552083
5,1,2366.310714
5,1,6260.085106
5,1,3615.877660
5,1,2088.110294
5,1,5984.129630
5,1,63104.903061
5,1,1550.866667
5,1,135697.778846
5,1,1522.715625
5,1,3195.200000
5,1,1960.669271
5,1,4713.637500
5,1,2663.795732
5,1,1842.024390
5,1,2966.458333
5,1,8377.690476
5,1,5087.731707
5,1,14541.543750
5,1,6288.101064
5,1,1768.284722
5,1,4793.043269
5,1,4281.656250
5,1,3014.360465
5,1,21782.487069
5,1,1525.313953
5,1,6606.209302
5,1,4362.195513
5,1,2447.021739
5,1,1704.660714
5,1,2314.537162
5,1,24534.187500
5,1,6889.032895
5,1,3001.571429
5,1,1900.571429
5,1,5241.000000
5,1,2558.348485
5,1,9065.297170
5,1,1512.962500
5,1,1965.857500
5,1,2547.425676

5,1,4222.994048
5,1,2770.111111
5,1,7908.716867
5,1,120836.147436
5,1,2411.884868
5,1,1835.414062
5,1,4421.000000
5,1,95507.400000
5,1,1676.333333
5,1,1835.000000
5,1,1902.681818
5,1,8135.854839
5,1,3330.045918
5,1,139430.487805
5,1,1806.072368
5,1,2961.997340
5,1,2912.000000
5,1,3405.777174
5,1,6492.190476
5,1,1888.750000
5,1,11969.071429
5,1,1769.984043
5,1,18575.997024
5,1,2861.434211
5,1,2819.111111
5,1,1666.783784
5,1,3428.157609
5,1,9423.186224
5,1,2680.494186
5,1,2323.888889
5,1,3292.479839
5,1,2873.897059
5,1,1964.048077
5,1,2402.389535
5,1,2405.550000
5,1,10691.592742
5,1,21128.817308
5,1,1799.344444
5,1,8068.850000
5,1,4670.588235
5,1,1820.353261
5,1,6110.096875
5,1,21333.505319
5,1,2246.547619
5,1,2389.641667
5,1,1585.793478
5,1,2067.579545
5,1,3408.117188
5,1,2389.714286
5,1,1971.031250
5,1,4387.483871
5,1,17572.681818
5,1,1529.634146
5,1,17111.207317
5,1,2152.200000
5,1,1748.162791
5,1,3605.765000
5,1,2488.105469
5,1,27463.569444
5,1,3262.372093
5,1,1990.227273
5,1,2871.625000
5,1,1570.000000
5,1,1676.115854
5,1,45985.937500
5,1,7729.061170

5,1,9639.800000
5,1,2156.821970
5,1,3640.846774
5,1,3714.428571
5,1,2559.565217
5,1,9596.810976
5,1,21854.640000
5,1,6195.486111
5,1,3423.756098
5,1,2446.753472
5,1,2161.153125
5,1,2702.909091
5,1,5176.906250
5,1,9678.500000
5,1,4262.444444
5,1,6943.785714
5,1,2193.986111
5,1,4596.973958
5,1,5397.483333
5,1,8595.116279
5,1,2544.130814
5,1,4749.942073
5,1,24700.621951
5,1,23901.677419
5,1,5815.125000
5,1,21018.268293
5,1,5665.721354
5,1,2240.780488
5,1,1694.959302
5,1,13949.233333
5,1,1812.820755
5,1,26156.185714
5,1,3578.125000
5,1,2711.878788
5,1,1603.925676
5,1,1839.304878
5,1,6826.255435
5,1,6262.750000
5,1,2411.357558
5,1,2157.360294
5,1,47839.535326
5,1,2927.460938
5,1,2652.628834
5,1,3276.131250
5,1,17721.086538
5,1,3178.500000
5,1,1532.875000
5,1,6275.984375
5,1,9774.680851
5,1,2971.776316
5,1,2587.052632
5,1,6987.957386
5,1,4643.818182
5,1,4830.991071
5,1,1955.435714
5,1,2038.182927
5,1,1583.439189
5,1,6789.162500
5,1,2292.200000
5,1,3400.096429
5,1,19404.444079
5,1,2034.200000
5,1,3599.614130
5,1,7082.000000
5,1,5420.089286
5,1,2300.540541

5,1,2868.173913
5,1,5087.247093
5,1,1884.769737
5,1,1915.941176
5,1,7229.848214
5,1,1780.712766
5,1,36406.411111
5,1,3600.882075
5,1,118598.232558
5,1,3586.414894
5,1,1748.817073
5,1,1610.034884
5,1,4537.372685
5,1,5485.987069
5,1,2977.416667
5,1,8222.750000
5,1,2623.927083
5,1,3646.642857
5,1,24871.250000
5,1,18734.900000
5,1,52116.400000
5,1,3711.419811
5,1,3133.600000
5,1,2876.447917
5,1,1738.458333
5,1,39532.855978
5,1,1623.302326
5,1,100386.487179
5,1,2061.375000
5,1,15768.500000
5,1,3069.500000
5,1,1571.541667
5,1,7056.165323
5,1,4072.342742
5,1,5982.702128
5,1,2483.790698
5,1,1762.244048
5,1,2281.031250
5,1,1853.404412
5,1,19504.169872
5,1,6897.551282
5,1,3055.457143
5,1,2303.035714
5,1,1662.522059
5,1,2490.295455
5,1,29908.763514
5,1,1713.527778
5,1,5445.117857
5,1,86129.085714
5,1,2677.928571
5,1,1966.265957
5,1,1826.280488
5,1,4011.275641
5,1,6444.151316
5,1,95141.000000
5,1,95085.390244
5,1,5424.187500
5,1,2191.562500
5,1,1689.281250
5,1,3436.700000
5,1,15730.000000
5,1,4375.968750
5,1,2173.044444
5,1,1766.428571
5,1,17440.054487
5,1,1928.429348

5,1,2417.791667
5,1,33527.807692
5,1,1524.225610
5,1,1887.208333
5,1,3177.425000
5,1,1562.586957
5,1,1855.363636
5,1,1563.000000
5,1,4558.260870
5,1,3560.284314
5,1,4239.078818
5,1,16322.428571
5,1,3000.865854
5,1,1764.764331
5,1,8771.504630
5,1,27748.572368
5,1,1989.679245
5,1,5079.664773
5,1,2353.473684
5,1,3584.604651
5,1,2330.278571
5,1,12822.420455
5,1,2037.260000
5,1,2701.000000
5,1,22660.442073
5,1,10468.809524
5,1,2113.500000
5,1,51993.557292
5,1,7010.602273
5,1,6181.761905
5,1,2825.595745
5,1,1836.085526
5,1,4875.537500
5,1,4666.076220
5,1,2696.011111
5,1,1887.478261
5,1,7244.912791
5,1,2270.812500
5,1,6416.400000
5,1,13860.451705
5,1,17422.089286
5,1,8998.583333
5,1,2244.000000
5,1,52153.707143
5,1,1808.512195
5,1,38041.838710
5,1,2097.562500
5,1,1764.339286
5,1,3528.437500
5,1,9726.157895
5,1,1796.750000
5,1,15317.951220
5,1,6401.035000
5,1,2540.664773
5,1,60820.158537
5,1,2385.602941
5,1,1623.718750
5,1,5213.669643
5,1,76781.576923
5,1,7952.358696
5,1,2655.520833
5,1,4327.744186
5,1,13735.611111
5,1,2339.344262
5,1,1624.180147
5,1,7776.178571

5,1,33555.475610
5,1,31198.423611
5,1,1853.937500
5,1,49114.506410
5,1,10121.315789
5,1,3499.421053
5,1,2135.071429
5,1,1551.801829
5,1,3850.375000
5,1,1525.988636
5,1,2619.468750
5,1,12049.300000
5,1,2047.475000
5,1,4995.110294
5,1,6988.920455
5,1,7469.821429
5,1,2104.027778
5,1,50224.422222
5,1,3517.756098
5,1,2066.549242
5,1,3691.075000
5,1,2609.718750
5,1,1580.763889
5,1,2259.693182
5,1,1633.635135
5,1,1686.191327
5,1,1798.803571
5,1,1735.043478
5,1,3236.853261
5,1,6821.779412
5,1,2731.750000
5,1,8195.238372
5,1,1859.544118
5,1,9359.551724
5,1,2096.505682
5,1,2161.033088
5,1,4219.171429
5,1,1982.889535
5,1,5726.625000
5,1,1577.941860
5,1,1723.363636
5,1,4296.718750
5,1,2646.630435
5,1,9774.600000
5,1,12279.041667
5,1,57176.691667
5,1,7632.243902
5,1,16502.576389
5,1,26765.861963
5,1,5528.777778
5,1,2425.978723
5,1,46496.517361
5,1,4383.455882
5,1,53830.906977
5,1,11061.659574
5,1,3450.272727
5,1,2160.173295
5,1,4241.000000
5,1,3943.205357
5,1,2494.062500
5,1,68346.315217
5,1,1757.911111
5,1,1567.406250
5,1,2322.258523
5,1,1878.275641
5,1,31698.275000

5,1,3738.756757
5,1,4547.222826
5,1,3883.048077
5,1,1991.898810
5,1,10815.468750
5,1,4058.488372
5,1,165667.937500
5,1,11045.239224
5,1,1577.952381
5,1,2081.945000
5,1,3354.482143
5,1,33477.321429
5,1,9127.961538
5,1,28089.322674
5,1,9024.333333
5,1,18501.531250
5,1,7302.925676
5,1,10759.598837
5,1,3706.108974
5,1,23096.280405
5,1,1745.522727
5,1,2367.997642
5,1,1671.692568
5,1,121137.378378
5,1,2694.750000
5,1,2921.440625
5,1,91155.125000
5,1,1911.509146
5,1,2182.181250
5,1,3756.236364
5,1,3208.812500
5,1,6902.005814
5,1,62622.750000
5,1,4359.872549
5,1,3571.375000
5,1,2888.128906
5,1,2320.701220
5,1,7090.916667
5,1,2479.815789
5,1,4353.189189
5,1,3238.906977
5,1,12360.869565
5,1,22846.133929
5,1,6602.915441
5,1,45379.129032
5,1,1501.820946
5,1,2362.437500
5,1,3210.282895
5,1,1993.208333
5,1,3966.627976
5,1,23115.503571
5,1,11863.595000
5,1,2590.159722
5,1,5097.843085
5,1,2919.265625
5,1,30546.345238
5,1,2346.552326
5,1,16482.403571
5,1,2199.500000
5,1,3924.953947
5,1,1600.606383
5,1,2326.870000
5,1,2916.632353
5,1,2510.387931
5,1,46596.111842
5,1,2387.622222

5,1,41671.529412
5,1,44149.806250
5,1,1780.790323
5,1,2002.269737
5,1,15808.697674
5,1,4043.487500
5,1,5902.181818
5,1,1664.729167
5,1,1908.365854
5,1,1912.500000
5,1,4557.812500
5,1,2322.875000
5,1,4566.755556
5,1,5485.002660
5,1,1603.000000
5,1,1903.318182
5,1,12826.427083
5,1,23041.319853
5,1,2897.027027
5,1,3378.125000
5,1,4541.452381
5,1,20246.180147
5,1,10046.861111
5,1,2444.372222
5,1,6664.960227
5,1,9772.139423
5,1,1913.365854
5,1,8008.542079
5,1,12547.331250
5,1,2606.121711
5,1,2599.686047
5,1,123892.320513
5,1,3325.604651
5,1,9777.438953
5,1,13577.375000
5,1,4016.733333
5,1,13207.614865
5,1,2315.450000
5,1,31449.048780
5,1,1809.133523
5,1,2385.280488
5,1,3477.838542
5,1,1909.393939
5,1,18352.705882
5,1,86031.250000
5,1,7833.527027
5,1,49629.666667
5,1,2140.803571
5,1,8142.442073
5,1,2385.262500
5,1,4000.311111
5,1,20637.455882
5,1,13345.571429
5,1,8119.153302
5,1,2000.911765
5,1,161132.283784
5,1,3175.307692
5,1,1561.000000
5,1,2218.088710
5,1,1975.857143
5,1,4412.985294
5,1,2473.877660
5,1,1620.300000
5,1,4686.547619
5,1,60412.540441
5,1,3835.837766

5,1,1663.060976
5,1,33141.320000
5,1,8257.600000
5,1,2263.645833
5,1,6329.307692
5,1,26879.503289
5,1,5052.518750
5,1,2599.221154
5,1,5129.693182
5,1,24239.144886
5,1,1568.392857
5,1,3532.564286
5,1,2263.110465
5,1,63720.187500
5,1,1986.956522
5,1,16446.634146
5,1,3895.916667
5,1,3639.396341
5,1,13147.055921
5,1,6080.679487
5,1,2681.476190
5,1,1826.602500
5,1,2569.684211
5,1,5453.576705
5,1,4122.562500
5,1,2202.500000
5,1,18690.148026
5,1,7024.246429
5,1,1534.692308
5,1,36187.264706
5,1,4854.365625
5,1,1775.848404
5,1,27466.843750
5,1,2078.272727
5,1,1726.550000
5,1,1779.125000
5,1,2507.000000
5,1,3291.562500
5,1,25411.460366
5,1,28100.768382
5,1,90524.466216
5,1,6234.654891
5,1,106847.331395
5,1,6902.809375
5,1,7087.363636
5,1,11393.592593
5,1,18356.125000
5,1,1585.647436
5,1,1712.878571
5,1,4323.216837
5,1,9622.439103
5,1,23313.153409
5,1,6345.742188
5,1,3481.920918
5,1,12271.648026
5,1,2804.540698
5,1,1616.960227
5,1,4967.317308
5,1,2322.923077
5,1,2631.219595
5,1,18641.083333
5,1,1569.010135
5,1,1586.396739
5,1,3532.479167
5,1,3918.907738
5,1,13769.560606

5,1,1858.547872
5,1,2714.807500
5,1,2387.882353
5,1,2349.512821
5,1,3599.475490
5,1,1741.176471
5,1,39782.393750
5,1,6416.161290
5,1,36776.875000
5,1,1605.222222
5,1,9458.636792
5,1,7380.229730
5,1,1846.704545
5,1,1674.054054
5,1,1502.409574
5,1,11323.488636
5,1,6557.066667
5,1,2018.200000
5,1,1547.875000
5,1,4865.816327
5,1,3259.775000
5,1,3689.698529
5,1,2841.744681
5,1,3266.750000
5,1,4105.509615
5,1,2345.553571
5,1,1732.093750
5,1,3786.000000
5,1,2664.743590
5,1,4706.459459
5,1,6609.083333
5,1,4395.390244
5,1,1665.573529
5,1,1594.067568
5,1,2102.300000
5,1,2570.916667
5,1,20706.511628
5,1,1711.981383
5,1,2338.416667
5,1,6613.454545
5,1,3068.379032
5,1,3783.893617
5,1,12078.270833
5,1,2236.352941
5,1,2988.742857
5,1,2167.666667
5,1,3798.564024
5,1,1667.102273
5,1,2024.665698
5,1,34994.714286
5,1,7002.170000
5,1,3923.201923
5,1,18452.276596
5,1,4461.392857
5,1,1618.882353
5,1,2375.329787
5,1,1591.656250
5,1,2054.781250
5,1,32940.763889
5,1,13559.666667
5,1,13923.720000
5,1,2491.157738
5,1,2188.697581
5,1,3205.200000
5,1,1718.774390
5,1,1866.500000

5,1,5978.977778
5,1,21265.305556
5,1,8416.029412
5,1,2489.025000
5,1,2745.282609
5,1,6162.475610
5,1,1548.354651
5,1,4027.370732
5,1,2325.347561
5,1,3682.071429
5,1,6368.334375
5,1,2330.913043
5,1,1884.333333
5,1,12627.172794
5,1,10753.502451
5,1,5311.724138
5,1,10095.711538
5,1,12038.207317
5,1,1838.250000
5,1,1517.792763
5,1,1550.407143
5,1,1928.326923
5,1,1894.839286
5,1,5609.410000
5,1,2271.101190
5,1,115644.125000
5,1,8028.509615
5,1,1781.680851
5,1,4654.755319
5,1,2446.720588
5,1,1570.853659
5,1,32661.109375
5,1,6874.158854
5,1,2490.848214
5,1,5283.645833
5,1,1764.757353
5,1,2722.562500
5,1,45838.384615
5,1,3239.933333
5,1,6895.687500
5,1,3225.648649
5,1,3833.970395
5,1,2155.750000
5,1,1968.228723
5,1,18176.869681
5,1,12379.707407
5,1,12474.158537
5,1,3646.208333
5,1,4964.615625
5,1,4698.439024
5,1,3125.035714
5,1,2387.777778
5,1,27302.013514
5,1,2402.414062
5,1,43302.381250
5,1,2857.323718
5,1,2269.317308
5,1,6030.128049
5,1,2430.687500
5,1,1917.973214
5,1,1590.392045
5,1,1627.734375
5,1,33254.459459
5,1,2280.297872
5,1,84653.411765
5,1,1500.372340

5,1,1736.881098
5,1,14387.925595
5,1,3570.153846
5,1,50681.496429
5,1,1667.227273
5,1,2322.770588
5,1,5562.111111
5,1,2699.609375
5,1,3402.706395
5,1,6081.621212
5,1,4414.602273
5,1,48319.555147
5,1,2041.610465
5,1,9459.957031
5,1,2744.837963
5,1,4482.919118
5,1,2398.116071
5,1,1562.825000
5,1,6390.301020
5,1,2442.951923
5,1,2942.928571
5,1,2063.880952
5,1,5898.500000
5,1,1525.560000
5,1,13506.260000
5,1,10432.909091
5,1,1602.250000
5,1,7575.375000
5,1,4301.430147
5,1,2515.447917
5,1,6811.573171
5,1,2080.731250
5,1,1628.378788
5,1,2956.573529
5,1,4214.210526
5,1,16880.306250
5,1,2880.467391
5,1,39659.385714
5,1,30848.220238
5,1,2524.042969
5,1,1860.256579
5,1,1531.622396
5,1,13888.137500
5,1,1540.421569
5,1,1595.483696
5,1,4663.223404
5,1,4392.000000
5,1,17507.342857
5,1,2260.835784
5,1,3001.114286
5,1,1584.114583
5,1,11110.679054
5,1,3364.139706
5,1,4519.675978
5,1,1609.200000
5,1,5435.741071
5,1,1915.224432
5,1,4416.143382
5,1,11195.416667
5,1,2776.875000
5,1,4931.607143
5,1,4798.049479
5,1,6868.149457
5,1,6056.977273
5,1,2556.252551
5,1,3853.812500

5,1,7124.044118
5,1,4699.653409
5,1,2672.350962
5,1,1552.452381
5,1,2934.625000
5,1,2169.812500
5,1,4594.000000
5,1,14139.403846
5,1,155872.545455
5,1,12763.043478
5,1,3318.106618
5,1,4258.198864
5,1,1608.583333
5,1,18773.093023
5,1,1698.131757
5,1,8971.615385
5,1,7088.169355
5,1,3519.375000
5,1,6840.011628
5,1,2010.666667
5,1,2490.796875
5,1,2844.050000
5,1,2827.500000
5,1,1952.684896
5,1,4769.750000
5,1,3726.307292
5,1,5346.429487
5,1,2067.837209
5,1,19686.536765
5,1,8056.000000
5,1,3889.300000
5,1,2782.059211
5,1,1770.818396
5,1,1581.714286
5,1,41729.914474
5,1,1908.796053
5,1,2329.874332
5,1,3034.148148
5,1,1976.348837
5,1,3270.705357
5,1,2677.687500
5,1,5393.662879
5,1,4260.066667
5,1,1839.607143
5,1,2266.717262
5,1,4126.616279
5,1,2116.537500
5,1,1791.107143
5,1,3212.531250
5,1,1946.926282
5,1,9336.995536
5,1,103271.173913
5,1,2297.875000
5,1,2108.200000
5,1,10071.670139
5,1,3240.920455
5,1,5391.398649
5,1,2694.596774
5,1,12209.577206
5,1,33313.388158
5,1,2837.743902
5,1,3554.351744
5,1,2721.941176
5,1,20818.437500
5,1,2857.632353
5,1,4750.833333

5,1,1645.029070
5,1,1711.603448
5,1,4075.250000
5,1,1626.179348
5,1,1619.870000
5,1,57574.617424
5,1,1787.107143
5,1,2876.740854
5,1,2899.470588
5,1,2165.114286
5,1,1772.291667
5,1,9581.363636
5,1,8933.203125
5,1,2688.135638
5,1,7523.603774
5,1,2686.914773
5,1,1922.635135
5,1,2088.683453
5,1,4327.564024
5,1,5259.015957
5,1,2588.237500
5,1,4942.494186
5,1,5179.250000
5,1,1803.144231
5,1,48052.787500
5,1,1875.925000
5,1,2996.612179
5,1,14175.835938
5,1,8291.402174
5,1,58707.175676
5,1,1687.292683
5,1,2056.487805
5,1,2242.942308
5,1,1558.576923
5,1,4046.997340
5,1,2713.022727
5,1,3040.966667
5,1,5379.809524
5,1,53671.312500
5,1,1671.957447
5,1,7293.847826
5,1,2562.964844
5,1,3449.645349
5,1,4394.696429
5,1,6453.539773
5,1,1587.503472
5,1,21830.684211
5,1,5244.355978
5,1,2276.487179
5,1,1519.986111
5,1,2362.479167
5,1,10064.985577
5,1,2212.930233
5,1,3179.653409
5,1,8593.459459
5,1,2909.159091
5,1,91486.516129
5,1,6466.717391
5,1,1673.963415
5,1,11835.567935
5,1,7657.388298
5,1,13647.800000
5,1,195631.302326
5,1,1829.528125
5,1,6045.408854
5,1,7263.860294

5,1,4340.552326
5,1,9908.119792
5,1,9571.640909
5,1,2698.163462
5,1,2748.406977
5,1,4728.891304
5,1,21384.064516
5,1,1723.564024
5,1,9341.582317
5,1,3063.920732
5,1,7155.530303
5,1,1555.000000
5,1,43196.250000
5,1,10203.440104
5,1,3075.656250
5,1,24122.685714
5,1,3468.794872
5,1,33882.171429
5,1,10941.806034
5,1,3230.833333
5,1,2910.345109
5,1,2943.750000
5,1,4764.478261
5,1,1638.753472
5,1,1789.621622
5,1,1507.750000
5,1,2167.712500
5,1,3446.200000
5,1,10106.387755
5,1,3896.375000
5,1,1949.453488
5,1,1947.000000
5,1,44363.808824
5,1,18498.134146
5,1,6391.150000
5,1,33717.513514
5,1,4048.339844
5,1,1523.158163
5,1,1666.789474
5,1,1913.797297
5,1,5973.635135
5,1,89404.705882
5,1,5704.342857
5,1,41773.759146
5,1,3546.303571
5,1,3534.424107
5,1,2735.685976
5,1,8375.963415
5,1,2698.041667
5,1,11731.984375
5,1,1796.750000
5,1,7912.638514
5,1,2797.911765
5,1,4902.131098
5,1,1779.689103
5,1,3569.636364
5,1,8467.414474
5,1,2341.557927
5,1,3547.616667
5,1,6096.530303
5,1,11966.515625
5,1,3704.500000
5,1,4208.347561
5,1,1741.625000
5,1,2856.983696
5,1,5201.396226

5,1,3670.791667
5,1,3240.272727
5,1,2795.503472
5,1,2139.911765
5,1,44343.190476
5,1,6527.692708
5,1,15001.125000
5,1,11797.505682
5,1,1613.625000
5,1,1778.199324
5,1,24958.419872
5,1,25672.848485
5,1,2198.155556
5,1,1755.500000
5,1,8097.875000
5,1,4725.714286
5,1,2311.889535
5,1,4914.369318
5,1,25576.057143
5,1,3883.061170
5,1,3672.060606
5,1,1591.500000
5,1,4322.208333
5,1,2366.333333
5,1,2416.978723
5,1,2592.250000
5,1,2189.406250
5,1,5842.175781
5,1,4309.625000
5,1,4634.779412
5,1,28281.607143
5,1,2071.875000
5,1,22945.220588
5,1,12155.243243
5,1,4348.205882
5,1,99085.406250
5,1,2839.966463
5,1,3014.688889
5,1,13307.260870
5,1,2217.875000
5,1,3856.616667
5,1,1543.403646
5,1,2761.341216
5,1,23135.000000
5,1,1762.657068
5,1,1829.878788
5,1,1804.744898
5,1,22155.250000
5,1,6605.782500
5,1,2231.103659
5,1,4260.344828
5,1,1602.825000
5,1,2710.000000
5,1,2004.339286
5,1,26262.364583
5,1,7227.215625
5,1,5739.670213
5,1,3518.292614
5,1,13910.868421
5,1,2511.587838
5,1,2417.213235
5,1,3942.062500
5,1,2060.925676
5,1,3535.886364
5,1,5362.465686
5,1,18402.968023

5,1,2547.301630
5,1,3145.578947
5,1,61181.394886
5,1,12121.875000
5,1,84965.540741
5,1,3018.342857
5,1,2399.565476
5,1,1731.697674
5,1,10038.521277
5,1,9856.562500
5,1,11083.625000
5,1,5164.250000
5,1,7164.340278
5,1,1517.148256
5,1,25996.500000
5,1,1641.725610
5,1,14380.597222
5,1,2248.855556
5,1,1922.475610
5,1,15985.188953
5,1,2458.161290
5,1,2487.807692
5,1,23757.909091
5,1,4611.691964
5,1,4487.166667
5,1,2664.937500
5,1,8107.750000
5,1,2550.346354
5,1,2077.232558
5,1,4594.635135
5,1,7461.438889
5,1,1677.643617
5,1,1518.228261
5,1,1944.444444
5,1,6191.436170
5,1,13308.428571
5,1,2339.843750
5,1,74092.625000
5,1,1546.045455
5,1,2305.246622
5,1,1547.515152
5,1,78485.930556
5,1,3075.333333
5,1,3945.104430
5,1,6660.178571
5,1,11989.065217
5,1,1566.107143
5,1,1560.550000
5,1,71108.315789
5,1,5224.396875
5,1,4727.964286
5,1,2469.687500
5,1,4013.848837
5,1,1872.538462
5,1,3333.896739
5,1,2662.947115
5,1,40225.686047
5,1,70385.055000
5,1,1604.412791
5,1,6598.176471
5,1,25491.176471
5,1,2431.097561
5,1,13321.826923
5,1,43389.437500
5,1,2420.244898
5,1,2340.290698

5,1,27343.437500
5,1,2751.362069
5,1,2855.116667
5,1,2939.105263
5,1,7337.743304
5,1,2056.611111
5,1,1640.540541
5,1,40640.710227
5,1,4606.306250
5,1,4504.688889
5,1,3019.782609
5,1,1881.875000
5,1,2975.642857
5,1,2456.228571
5,1,1809.147727
5,1,3703.534091
5,1,2804.281250
5,1,2100.200000
5,1,10354.002976
5,1,2170.875000
5,1,1764.143617
5,1,1633.510638
5,1,2687.250000
5,1,3142.974490
5,1,5580.250000
5,1,9428.222826
5,1,1643.409091
5,1,1609.755556
5,1,113173.823980
5,1,5124.291667
5,1,2789.202128
5,1,4129.563953
5,1,1653.648026
5,1,2031.103659
5,1,1789.910326
5,1,124422.161765
5,1,4796.524390
5,1,136203.948718
5,1,1866.090909
5,1,3379.125000
5,1,2118.480769
5,1,2407.252551
5,1,2383.546875
5,1,191192.133333
5,1,1863.521341
5,1,40579.125000
5,1,6722.191406
5,1,2854.488889
5,1,1987.791667
5,1,2702.784375
5,1,3626.524590
5,1,4980.811429
5,1,20828.151316
5,1,1599.198953
5,1,3889.912791
5,1,1571.337209
5,1,2192.666667
5,1,6873.468085
5,1,169712.221591
5,1,2344.843023
5,1,2451.736111
5,1,2978.145833
5,1,5839.620690
5,1,2940.184783
5,1,5293.375000
5,1,4072.850000

5,1,7355.674419
5,1,66017.276316
5,1,13032.347826
5,1,9173.351064
5,1,2218.071429
5,1,2748.000000
5,1,1536.333333
5,1,3136.418605
5,1,14643.180556
5,1,7538.669118
5,1,4251.537791
5,1,22310.100694
5,1,4072.290698
5,1,3143.451087
5,1,2198.986111
5,1,1507.180556
5,1,8708.294118
5,1,2626.305233
5,1,1554.997093
5,1,1664.656250
5,1,7131.531250
5,1,1646.307692
5,1,7705.714286
5,1,3508.241848
5,1,2738.882353
5,1,96089.076389
5,1,3174.859375
5,1,7300.935484
5,1,1809.616279
5,1,1801.200000
5,1,4582.934211
5,1,2435.904040
5,1,3530.597222
5,1,2592.830357
5,1,3898.735294
5,1,3150.032051
5,1,2955.541667
5,1,1631.586806
5,1,1948.551020
5,1,1962.531250
5,1,1951.758721
5,1,29663.708333
5,1,3608.600575
5,1,7694.310526
5,1,4087.943182
5,1,6227.000000
5,1,23449.441860
5,1,3189.367188
5,1,2680.375000
5,1,1703.601351
5,1,2068.817073
5,1,2552.106707
5,1,7745.004032
5,1,10195.695946
5,1,10688.009615
5,1,9398.839286
5,1,3004.684524
5,1,3103.361702
5,1,8300.031250
5,1,34196.805556
5,1,3496.250000
5,1,5036.250000
5,1,2525.679688
5,1,1638.448276
5,1,3785.642857
5,1,2238.586111

5,1,1687.378049
5,1,7156.264881
5,1,1669.576087
5,1,35145.300000
5,1,2173.000000
5,1,1513.545455
5,1,1647.222222
5,1,2665.408537
5,1,2903.851562
5,1,3176.567308
5,1,2037.642857
5,1,3251.816489
5,1,2438.814103
5,1,2199.912162
5,1,8929.671196
5,1,2794.534574
5,1,2630.451389
5,1,2257.818452
5,1,1991.827586
5,1,11524.791667
5,1,6194.698718
5,1,1573.667683
5,1,3922.453125
5,1,49678.958333
5,1,2622.166667
5,1,39792.307432
5,1,7776.002551
5,1,28895.041667
5,1,48157.512931
5,1,1917.780488
5,1,2394.247549
5,1,12249.428571
5,1,2900.497449
5,1,2109.104167
5,1,5171.064103
5,1,2096.335106
5,1,3267.119318
5,1,6389.682927
5,1,45203.578571
5,1,2393.437500
5,1,4584.829545
5,1,4270.346591
5,1,28043.073864
5,1,4803.600000
5,1,85695.073370
5,1,17772.698529
5,1,8039.225694
5,1,2569.565476
5,1,1709.412234
5,1,1668.508523
5,1,2570.516393
5,1,122910.734375
5,1,75013.016667
5,1,3867.656250
5,1,1914.622222
5,1,4567.053191
5,1,13309.881356
5,1,5366.307692
5,1,1710.683333
5,1,4016.375000
5,1,2479.723404
5,1,3708.145833
5,1,2250.785256
5,1,7006.658537
5,1,27491.796875
5,1,7730.423077

5,1,1744.611111
5,1,2232.771277
5,1,1706.331250
5,1,2288.959184
5,1,1504.113636
5,1,1817.133523
5,1,1921.283784
5,1,3367.944444
5,1,11222.737903
5,1,89038.038462
5,1,2165.354167
5,1,3167.182692
5,1,1713.886364
5,1,6741.110465
5,1,14385.083333
5,1,1782.333333
5,1,4387.220000
5,1,1549.583333
5,1,85972.333333
5,1,3334.511628
5,1,3490.973684
5,1,30374.763889
5,1,2694.462121
5,1,2500.666667
5,1,1587.957447
5,1,14994.421429
5,1,28510.500000
5,1,5073.333333
5,1,6706.593750
5,1,33596.743056
5,1,3349.793478
5,1,1934.832386
5,1,3339.531863
5,1,4637.285714
5,1,61763.090278
5,1,32815.042763
5,1,1728.290698
5,1,2163.566667
5,1,1674.559211
5,1,2765.394531
5,1,2435.471154
5,1,1710.894444
5,1,2630.788462
5,1,2674.920455
5,1,1552.487500
5,1,2038.513889
5,1,2755.835526
5,1,14345.295455
5,1,1910.323980
5,1,2453.071809
5,1,99547.953488
5,1,2209.732955
5,1,3208.432927
5,1,10993.226994
5,1,14495.440299
5,1,62214.682432
5,1,9111.625000
5,1,2794.390244
5,1,3618.406250
5,1,8513.428571
5,1,1966.125000
5,1,3114.238971
5,1,16919.192857
5,1,2325.065217
5,1,43765.939024
5,1,4136.576220

5,1,3653.052326
5,1,5077.920455
5,1,2266.500000
5,1,1786.066860
5,1,6340.340278
5,1,2645.720000
5,1,2444.387006
5,1,1995.440341
5,1,2672.238372
5,1,1638.950000
5,1,4213.750000
5,1,4346.076220
5,1,4428.625000
5,1,2713.912500
5,1,3293.313953
5,1,14308.433511
5,1,3973.858333
5,1,1569.804878
5,1,11988.222656
5,1,1630.697674
5,1,2410.970000
5,1,2292.196023
5,1,2894.250000
5,1,3089.464286
5,1,2728.558824
5,1,28180.687500
5,1,3507.931818
5,1,85928.422297
5,1,2086.923077
5,1,6361.250000
5,1,4783.678571
5,1,22465.636364
5,1,2956.348837
5,1,20336.700000
5,1,13213.443182
5,1,66877.375000
5,1,2727.259868
5,1,84335.713415
5,1,22840.377976
5,1,2034.500000
5,1,1633.750000
5,1,1977.887195
5,1,1654.027439
5,1,3080.069767
5,1,14859.168478
5,1,3142.403846
5,1,21499.720238
5,1,5126.250000
5,1,4650.592391
5,1,2471.037500
5,1,4342.028061
5,1,31236.500000
5,1,5002.017442
5,1,7663.687500
5,1,5412.878788
5,1,2829.883721
5,1,3172.559524
5,1,3540.333333
5,1,2931.048780
5,1,4944.804348
5,1,2038.204545
5,1,1710.835227
5,1,2783.625000
5,1,31441.250000
5,1,2184.135870
5,1,16009.237903

5,1,2446.020833
5,1,2389.500000
5,1,2163.050000
5,1,1761.841463
5,1,1505.188889
5,1,18639.450000
5,1,3309.772727
5,1,5675.026163
5,1,37195.132812
5,1,10662.113971
5,1,1946.144578
5,1,40289.806250
5,1,15998.412500
5,1,4273.836735
5,1,2090.956522
5,1,1729.441667
5,1,2004.479592
5,1,2299.581395
5,1,1615.497093
5,1,2179.785714
5,1,1830.944444
5,1,13983.875000
5,1,1785.466667
5,1,7160.869898
5,1,2924.563725
5,1,6422.730769
5,1,13324.333333
5,1,3055.275000
5,1,1693.562500
5,1,3617.567073
5,1,24263.148810
5,1,1670.588235
5,1,1612.062500
5,1,1977.247449
5,1,74998.371875
5,1,3893.800000
5,1,32741.235294
5,1,1657.317935
5,1,2251.921875
5,1,1727.500000
5,1,7191.846774
5,1,3734.387755
5,1,3875.959302
5,1,1821.886364
5,1,1682.437500
5,1,3800.625000
5,1,1732.528846
5,1,3784.928571
5,1,39078.347973
5,1,5213.090909
5,1,4910.295213
5,1,2932.851744
5,1,2790.056995
5,1,9855.333333
5,1,53813.932692
5,1,1607.342105
5,1,1694.270833
5,1,3723.159884
5,1,1958.343750
5,1,34209.119186
5,1,15763.422297
5,1,9829.122093
5,1,8016.055921
5,1,5195.402439
5,1,23414.067073
5,1,2627.483696

5,1,10985.381579
5,1,6652.781250
5,1,2325.160156
5,1,13313.093750
5,1,1995.642857
5,1,4971.057692
5,1,3020.461957
5,1,4958.649457
5,1,21252.696429
5,1,2430.410714
5,1,4521.500000
5,1,1835.571429
5,1,1767.506757
5,1,7857.584906
5,1,3445.289634
5,1,3471.500000
5,1,2174.681818
5,1,6164.206818
5,1,1685.278846
5,1,2595.916667
5,1,38058.363095
5,1,1509.000000
5,1,29815.054688
5,1,2017.700000
5,1,2633.354167
5,1,1539.004032
5,1,2494.787234
5,1,1945.250000
5,1,2117.804878
5,1,4346.415698
5,1,3060.119048
5,1,2162.497396
5,1,4034.225490
5,1,2664.750000
5,1,1884.891447
5,1,23968.214286
5,1,12366.715426
5,1,9958.180233
5,1,3457.390244
5,1,3142.407143
5,1,17984.853448
5,1,2101.666667
5,1,48905.200000
5,1,29838.878289
5,1,3074.800000
5,1,1917.197917
5,1,4994.591837
5,1,5728.240132
5,1,2272.134615
5,1,2629.943182
5,1,1643.497340
5,1,3755.019022
5,1,3223.408088
5,1,3181.696203
5,1,3529.731707
5,1,10655.480769
5,1,38989.142045
5,1,4637.586310
5,1,1997.523256
5,1,3633.458333
5,1,11822.423387
5,1,3321.872222
5,1,2099.446429
5,1,1905.272436
5,1,1684.681818
5,1,1690.030405

5,1,3727.680000
5,1,58539.885714
5,1,2161.939516
5,1,2339.763158
5,1,4500.875000
5,1,18017.767857
5,1,31600.891129
5,1,2980.062500
5,1,4683.812500
5,1,11256.070312
5,1,98234.120690
5,1,5435.266129
5,1,1513.500000
5,1,45231.181818
5,1,1559.062500
5,1,2721.083333
5,1,13243.267857
5,1,3739.332447
5,1,2604.781250
5,1,8721.877049
5,1,6030.615854
5,1,4504.581522
5,1,51333.250000
5,1,1855.798611
5,1,8429.033333
5,1,3694.581522
5,1,6516.750000
5,1,22606.057870
5,1,7696.804688
5,1,1610.419118
5,1,6247.062500
5,1,1930.139286
5,1,4941.660714
5,1,9135.830556
5,1,84619.290541
5,1,2332.181818
5,1,5776.551136
5,1,2616.652174
5,1,12120.696023
5,1,7569.806818
5,1,5741.977500
5,1,27783.245283
5,1,1714.312500
5,1,2384.904412
5,1,4653.687500
5,1,4060.016304
5,1,4940.188679
5,1,5576.066327
5,1,3388.883721
5,1,4330.117021
5,1,1855.867647
5,1,1614.411765
5,1,47009.513514
5,1,1986.884146
5,1,3863.423077
5,1,7284.065789
5,1,1613.974359
5,1,3164.590426
5,1,4194.261307
5,1,3374.972222
5,1,2634.636364
5,1,1895.005319
5,1,3282.982955
5,1,12193.392442
5,1,1688.158854
5,1,6515.900000

5,1,6485.235294
5,1,3250.157895
5,1,8201.300000
5,1,1645.595588
5,1,38102.522059
5,1,9732.073529
5,1,53088.144886
5,1,2921.209184
5,1,4584.827586
5,1,4304.702128
5,1,15925.333333
5,1,104983.981481
5,1,2385.008197
5,1,1640.070312
5,1,1501.788462
5,1,17188.677778
5,1,2803.304348
5,1,2012.861702
5,1,3712.997283
5,1,1975.166667
5,1,37075.301471
5,1,1886.875000
5,1,2093.722656
5,1,9451.078947
5,1,2989.788462
5,1,13027.113208
5,1,2427.343750
5,1,2226.436047
5,1,12699.136364
5,1,3813.755814
5,1,9192.258333
5,1,2604.433036
5,1,1587.414474
5,1,2417.995098
5,1,3475.423469
5,1,4860.834459
5,1,26805.868750
5,1,2196.133333
5,1,3115.097561
5,1,2558.021277
5,1,3507.980769
5,1,2526.631579
5,1,4305.972222
5,1,24447.980769
5,1,1849.781250
5,1,20831.970930
5,1,2472.851190
5,1,1608.000000
5,1,2795.066667
5,1,2105.743750
5,1,3496.063830
5,1,2780.505208
5,1,6367.475000
5,1,16509.359043
5,1,11862.375000
5,1,26272.419643
5,1,9249.337838
5,1,18418.105263
5,1,3082.464286
5,1,4983.718750
5,1,20410.107955
5,1,1741.174051
5,1,5266.078125
5,1,4388.571429
5,1,21972.280488
5,1,23578.317073

5,1,3400.044118
5,1,3063.571429
5,1,1593.628289
5,1,2905.653846
5,1,2281.659091
5,1,2319.166667
5,1,1642.284884
5,1,3269.619048
5,1,16589.730114
5,1,4011.730769
5,1,11034.886792
5,1,18068.448718
5,1,6444.966667
5,1,2089.895833
5,1,1614.517857
5,1,41885.771341
5,1,3231.000000
5,1,7702.160326
5,1,8437.647059
5,1,1734.755814
5,1,3111.094595
5,1,3887.196429
5,1,6225.950000
5,1,3757.093750
5,1,1778.088068
5,1,3203.334459
5,1,2226.212766
5,1,8559.779762
5,1,2068.382979
5,1,1628.085106
5,1,24742.932692
5,1,1558.282051
5,1,2455.000000
5,1,2773.861111
5,1,1965.099490
5,1,15687.174419
5,1,10602.347727
5,1,4079.134228
5,1,60402.056604
5,1,3892.164062
5,1,3263.965625
5,1,12268.155172
5,1,103213.133333
5,1,1597.851351
5,1,3068.436170
5,1,16144.937500
5,1,1539.145833
5,1,2010.942308
5,1,2483.800000
5,1,2079.650568
5,1,70615.102564
5,1,4260.250000
5,1,6905.027439
5,1,2072.019022
5,1,1927.356383
5,1,2276.508721
5,1,6159.431280
5,1,12916.310976
5,1,2264.095930
5,1,59614.990000
5,1,2598.973404
5,1,21723.960938
5,1,31638.545455
5,1,2094.956522
5,1,2794.807692
5,1,2326.440000

5,1,1559.336957
5,1,1938.333333
5,1,7543.468750
5,1,8303.567073
5,1,3037.567857
5,1,2944.800000
5,1,128635.138636
5,1,2869.389831
5,1,1891.869792
5,1,2000.074468
5,1,4090.179487
5,1,1548.933333
5,1,3171.673077
5,1,2032.835526
5,1,5265.932432
5,1,15420.401163
5,1,5517.852761
5,1,5301.996795
5,1,5926.375000
5,1,2332.500000
5,1,2162.725806
5,1,59063.267442
5,1,1769.762195
5,1,32870.615854
5,1,6416.644531
5,1,14083.466667
5,1,1574.232759
5,1,3223.285714
5,1,2859.698529
5,1,3516.558333
5,1,7471.595745
5,1,33747.000000
5,1,1516.820513
5,1,28798.957143
5,1,2276.787879
5,1,2316.760000
5,1,12144.599265
5,1,2021.309028
5,1,2526.171875
5,1,2604.481250
5,1,4333.758929
5,1,1516.162234
5,1,23677.711538
5,1,2237.032895
5,1,23028.402439
5,1,1724.157895
5,1,1966.494444
5,1,27471.095000
5,1,4658.340517
5,1,3674.618421
5,1,1525.473684
5,1,2090.020000
5,1,1707.185811
5,1,3076.171512
5,1,40336.454545
5,1,10764.718750
5,1,2183.712500
5,1,4859.972222
5,1,2018.820312
5,1,3288.658333
5,1,16815.266447
5,1,2792.310185
5,1,1499.070946
5,1,3710.869231
5,1,9299.375000
5,1,2390.544444

5,1,1948.914634
5,1,3750.000000
5,1,2124.400000
5,1,5008.839286
5,1,1716.559524
5,1,2056.457386
5,1,33942.579545
5,1,4809.585000
5,1,56381.375000
5,1,1856.343750
5,1,10966.411765
5,1,80116.742647
5,1,22696.510417
5,1,2155.796053
5,1,3664.869565
5,1,3006.864286
5,1,2549.572917
5,1,2644.750000
5,1,3905.224490
5,1,3753.341463
5,1,5148.550000
5,1,4344.153846
5,1,2567.647059
5,1,4100.875000
5,1,7394.540179
5,1,2189.160326
5,1,1557.130000
5,1,2376.600000
5,1,1584.009375
5,1,2822.671053
5,1,1545.589674
5,1,9569.068182
5,1,1981.062500
5,1,2451.000000
5,1,2710.497283
5,1,1701.422297
5,1,99296.664474
5,1,12187.058824
5,1,5526.447674
5,1,137799.819444
5,1,2100.188953
5,1,2976.384615
5,1,11627.391509
5,1,1548.039634
5,1,3582.540541
5,1,2208.750000
5,1,1637.911290
5,1,1580.668367
5,1,8820.802326
5,1,2035.459459
5,1,25472.435897
5,1,2168.267857
5,1,7837.685096
5,1,2445.334459
5,1,2756.342391
5,1,1982.951087
5,1,4210.762500
5,1,1783.509615
5,1,1518.260638
5,1,100079.486111
5,1,2129.543919
5,1,2412.500000
5,1,9390.830128
5,1,6402.801471
5,1,3982.971591
5,1,105576.270270

5,1,9182.875000
5,1,3829.810000
5,1,2988.156977
5,1,1517.482143
5,1,8151.320312
5,1,2036.425532
5,1,4345.116848
5,1,2662.056604
5,1,3772.817708
5,1,1928.563830
5,1,2772.692568
5,1,3243.483553
5,1,29327.078947
5,1,3943.028409
5,1,1846.396875
5,1,61645.477564
5,1,2156.586957
5,1,2414.479167
5,1,2418.428125
5,1,4069.972973
5,1,1712.800000
5,1,4023.977778
5,1,1658.068182
5,1,45515.903846
5,1,1525.812500
5,1,3722.809524
5,1,2060.285714
5,1,5532.106383
5,1,1606.207317
5,1,5100.964286
5,1,15019.386719
5,1,1532.250000
5,1,1670.730114
5,1,1726.550000
5,1,4877.693182
5,1,2482.565476
5,1,1760.318878
5,1,20221.043478
5,1,1891.821429
5,1,1620.218750
5,1,2638.024390
5,1,4126.313953
5,1,6186.459459
5,1,22734.750000
5,1,1882.979167
5,1,68475.940789
5,1,1672.390000
5,1,1610.402439
5,1,2768.297619
5,1,6265.609375
5,1,2106.000000
5,1,1587.625000
5,1,11533.880952
5,1,1594.744186
5,1,1964.483974
5,1,1849.562500
5,1,5904.926829
5,1,1738.824405
5,1,2129.658537
5,1,2007.333333
5,1,1812.223958
5,1,14434.552778
5,1,3836.125000
5,1,2374.187500
5,1,1764.271739
5,1,16218.067568

5,1,2127.267045
5,1,48059.537879
5,1,2464.297872
5,1,1904.854651
5,1,1537.146341
5,1,1530.310976
5,1,1578.522059
5,1,3563.102564
5,1,4016.530612
5,1,22917.558511
5,1,3259.977778
5,1,32642.486842
5,1,9514.021739
5,1,1706.613636
5,1,5457.371795
5,1,3119.534091
5,1,8096.605469
5,1,1913.608696
5,1,2510.750000
5,1,2088.690789
5,1,10787.687500
5,1,9037.316667
5,1,1523.826316
5,1,1715.727273
5,1,3086.763158
5,1,2475.755814
5,1,4300.779070
5,1,1966.723214
5,1,2661.858696
5,1,20865.000000
5,1,2278.521739
5,1,3100.437500
5,1,2675.013393
5,1,15788.841463
5,1,1975.756098
5,1,2949.410714
5,1,3596.701087
5,1,4548.319149
5,1,113836.310484
5,1,2300.955556
5,1,5851.375000
5,1,21949.358696
5,1,2914.890625
5,1,2256.900000
5,1,2670.375000
5,1,4986.562500
5,1,2140.750000
5,1,2625.972067
5,1,2800.441964
5,1,45683.320312
5,1,3278.261364
5,1,2189.552083
5,1,2131.768293
5,1,2573.578125
5,1,1506.255682
5,1,3648.800000
5,1,2044.371094
5,1,1555.517045
5,1,1562.210714
5,1,2850.500000
5,1,64872.868902
5,1,1576.944444
5,1,3216.256757
5,1,60347.281250
5,1,1753.633721
5,1,1632.526786

```
5,1,53300.133929
5,1,3527.500000
5,1,2125.911585
5,1,40321.453125
5,1,19864.843750
5,1,9277.051136
5,1,3344.189189
5,1,4764.836735
5,1,2364.416667
5,1,5544.833333
5,1,2084.673780
5,1,2788.222826
5,1,7510.232558
5,1,6112.557292
5,1,1946.905488
5,1,3226.129808
5,1,1562.139535
5,1,2025.722973
5,1,2066.317073
5,1,1614.513158
5,1,3001.586592
5,1,1624.364865
5,1,5807.190217
5,1,25330.592593
5,1,1700.901316
5,1,17808.343750
5,1,1624.406977
5,1,4084.878049
5,1,3030.444767
5,1,1854.569149
5,1,2532.151316
5,1,2838.944444
5,1,2372.965426
5,1,141226.496711
5,1,3117.916667
5,1,4254.093750
5,1,4524.891129
5,1,3489.186047
5,1,4591.101064
5,1,2458.950000
5,1,1665.588542
5,1,13785.000000
5,1,5147.378676
5,1,1684.361702
5,1,1813.191489
5,1,2258.237500
5,1,32286.297297
5,1,2560.000000
5,1,2534.666667
5,1,10398.000000
5,1,3766.761628
5,1,3337.461957
5,1,6028.257653
5,1,1724.410714
5,1,8180.302326
5,1,134679.800000
5,1,13387.921053
5,1,1776.909091
5,1,2667.625000
5,1,5300.989583
5,1,40852.387255
5,1,13433.023438
5,1,1583.913462
5,1,26636.179487
5,1,1854.692308
5,1,7146.847826
```

5,1,3065.807692
5,1,39718.634615
5,1,101917.500000
5,1,2205.429054
5,1,2639.546429
5,1,2013.604651
5,1,1677.406915
5,1,4998.171875
5,1,1697.060606
5,1,4433.031250
5,1,4526.210526
5,1,2568.476190
5,1,1778.800000
5,1,2636.812500
5,1,2352.625000
5,1,3712.253472
5,1,2455.214286
5,1,2429.558824
5,1,1970.403846
5,1,2832.000000
5,1,13061.360465
5,1,1539.170213
5,1,63572.513514
5,1,41618.785714
5,1,3614.930556
5,1,2695.216463
5,1,3257.789773
5,1,23692.993421
5,1,1682.309211
5,1,3393.010417
5,1,4876.227273
5,1,38718.079268
5,1,2174.783537
5,1,43104.563953
5,1,4492.872396
5,1,2162.200000
5,1,2402.378788
5,1,2449.478261
5,1,1773.660714
5,1,5361.166667
5,1,1741.344595
5,1,1649.579268
5,1,1671.135870
5,1,4514.060714
5,1,3672.813953
5,1,1554.315104
5,1,18570.143750
5,1,3587.250000
5,1,14334.200000
5,1,2438.550000
5,1,3474.819767
5,1,2378.750000
5,1,4960.375000
5,1,1778.323529
5,1,3081.500000
5,1,2066.633333
5,1,17823.555556
5,1,30814.854167
5,1,39506.723214
5,1,1734.897959
5,1,85216.240385
5,1,4350.343085
5,1,28446.533163
5,1,2015.117647
5,1,1993.891892
5,1,1621.803191

5,1,1989.691176
5,1,8009.000000
5,1,174997.646739
5,1,1688.804094
5,1,10081.496622
5,1,5519.057927
5,1,2584.473684
5,1,25840.062500
5,1,2750.864796
5,1,3129.571429
5,1,2789.914894
5,1,2185.153846
5,1,3122.678571
5,1,2071.657738
5,1,1582.921053
5,1,5170.000000
5,1,3526.750000
5,1,4866.055556
5,1,2872.025000
5,1,2944.807692
5,1,1785.060976
5,1,5952.958333
5,1,3626.625000
5,1,15192.042453
5,1,47804.439189
5,1,6837.465909
5,1,2558.010870
5,1,3083.625000
5,1,4734.243902
5,1,16716.517241
5,1,1883.428571
5,1,2991.675676
5,1,2798.000000
5,1,2730.068966
5,1,2143.030556
5,1,2644.677632
5,1,3084.139535
5,1,3324.132979
5,1,1687.321429
5,1,1986.859375
5,1,2424.905405
5,1,3925.396341
5,1,25347.073529
5,1,14857.218750
5,1,3923.076087
5,1,1955.029762
5,1,8249.500000
5,1,1501.854167
5,1,8660.704082
5,1,1833.715625
5,1,6075.581395
5,1,1588.523256
5,1,5884.142857
5,1,14494.727273
5,1,5620.276786
5,1,3242.159722
5,1,2117.833333
5,1,1569.986842
5,1,3496.279891
5,1,1843.980263
5,1,7898.562500
5,1,32755.717857
5,1,2268.103261
5,1,1998.545455
5,1,3912.060811
5,1,4055.150000

5,1,5219.071809
5,1,2914.809524
5,1,1741.209302
5,1,3764.908088
5,1,1681.942308
5,1,3035.578947
5,1,12983.375000
5,1,5782.187500
5,1,1560.662500
5,1,49938.548611
5,1,13358.625000
5,1,3424.000000
5,1,1851.510714
5,1,4299.533333
5,1,7920.488764
5,1,1990.783854
5,1,44889.603261
5,1,13429.791667
5,1,2620.673077
5,1,4849.578125
5,1,1873.609375
5,1,3075.496795
5,1,1942.399306
5,1,1801.875000
5,1,3696.034722
5,1,2803.109375
5,1,5248.272727
5,1,9668.023026
5,1,5580.635870
5,1,3814.702128
5,1,2860.395833
5,1,5875.406250
5,1,19509.664062
5,1,1900.608696
5,1,2873.406977
5,1,6233.959375
5,1,5236.548077
5,1,1843.333333
5,1,11051.500000
5,1,2047.722826
5,1,1808.000000
5,1,25951.937500
5,1,3620.375000
5,1,2058.539773
5,1,3737.295455
5,1,2194.617021
5,1,13216.200000
5,1,4212.326087
5,1,2626.595238
5,1,3088.654605
5,1,37640.835714
5,1,44944.680556
5,1,1676.774194
5,1,2728.574468
5,1,1841.531250
5,1,2855.429688
5,1,2032.666667
5,1,5788.252551
5,1,4742.913386
5,1,3562.394737
5,1,3768.897222
5,1,1604.000000
5,1,53015.463415
5,1,3119.620000
5,1,22863.299419
5,1,11041.618902

5,1,64958.790698
5,1,3617.665698
5,1,2287.750000
5,1,1618.810811
5,1,2063.325758
5,1,2089.692308
5,1,1513.725694
5,1,2249.006944
5,1,8965.250000
5,1,1637.154070
5,1,1532.716667
5,1,3521.443182
5,1,2231.557500
5,1,2739.767857
5,1,17671.088235
5,1,5509.833333
5,1,2067.436111
5,1,2135.946429
5,1,89311.552326
5,1,2550.904762
5,1,1705.830357
5,1,11058.662500
5,1,15963.298246
5,1,27413.540625
5,1,3548.616477
5,1,1527.268293
5,1,3038.516667
5,1,5699.744681
5,1,44079.705882
5,1,3163.555556
5,1,2497.684524
5,1,134196.677632
5,1,22595.057500
5,1,56246.775000
5,1,32105.552419
5,1,2311.137500
5,1,1946.605978
5,1,5791.912791
5,1,1738.395833
5,1,3677.522222
5,1,1930.005435
5,1,1797.191489
5,1,32702.410714
5,1,59136.599265
5,1,2500.914634
5,1,2461.800000
5,1,4013.000000
5,1,31915.327703
5,1,22511.250000
5,1,1536.671429
5,1,2092.143868
5,1,2510.503049
5,1,4042.048387
5,1,3483.125000
5,1,3598.668750
5,1,4998.500000
5,1,2306.875000
5,1,8122.426829
5,1,61262.500000
5,1,2507.688889
5,1,2018.605114
5,1,78543.589744
5,1,4085.737500
5,1,2801.940476
5,1,3739.234694
5,1,4382.514205

5,1,1836.583333
5,1,7559.890625
5,1,58406.239286
5,1,48302.833333
5,1,2543.994444
5,1,2205.785714
5,1,1625.750000
5,1,3309.550781
5,1,2644.312500
5,1,1695.137195
5,1,1581.333333
5,1,2339.333333
5,1,5365.884615
5,1,2530.553571
5,1,2668.548077
5,1,3732.944444
5,1,9005.875000
5,1,123969.772222
5,1,11875.851351
5,1,3951.762500
5,1,102691.044872
5,1,2125.675532
5,1,4199.986486
5,1,2067.700000
5,1,2125.164773
5,1,4745.687500
5,1,36563.485714
5,1,3228.375000
5,1,3178.146875
5,1,2659.000000
5,1,1524.850000
5,1,1870.325581
5,1,11961.494792
5,1,2468.897727
5,1,3877.791045
5,1,6020.246988
5,1,6869.536585
5,1,3840.400000
5,1,10086.532738
5,1,2287.125000
5,1,3795.755102
5,1,8589.277778
5,1,3028.316129
5,1,1743.262295
5,1,2366.888889
5,1,22734.181818
5,1,8014.139205
5,1,2188.414894
5,1,2482.360294
5,1,15232.526316
5,1,4454.352273
5,1,3339.714286
5,1,3957.289773
5,1,2979.556548
5,1,24999.875000
5,1,20863.150000
5,1,3434.350000
5,1,3324.898810
5,1,2917.278409
5,1,6251.416667
5,1,5967.812500
5,1,2098.238636
5,1,1557.804878
5,1,2216.807927
5,1,2162.627907
5,1,1740.302326

5,1,1742.041667
5,1,1940.553977
5,1,1802.487805
5,1,2107.734694
5,1,2246.250000
5,1,3780.408537
5,1,1725.637500
5,1,2382.177778
5,1,8783.187500
5,1,2447.732639
5,1,5169.122093
5,1,5275.515625
5,1,4063.125000
5,1,1669.113372
5,1,2066.195652
6,2,3173.208092
6,2,2439.982955
6,2,1979.385787
6,2,1500.250000
6,2,4370.323864
6,2,1970.087838
6,2,1775.839286
6,2,7528.229592
6,2,5419.343750
6,2,4606.229592
6,2,1554.358553
6,2,2479.226190
6,2,3855.031977
6,2,4125.750000
6,2,1547.871711
6,2,5027.181818
6,2,7455.943182
6,2,2414.111111
6,2,2192.550000
6,2,5383.916667
6,2,3049.140625
6,2,1971.250000
6,2,2494.789474
6,2,4545.136719
6,2,3338.510204
6,2,2113.576355
6,2,1964.359375
6,2,8357.220109
6,2,3354.711864
6,2,2663.070988
6,2,2762.366279
6,2,10250.871728
6,2,4773.133333
6,2,4254.472222
6,2,4047.698630
6,2,2493.345238
6,2,3026.535714
6,2,6434.625000
6,2,2127.163793
6,2,9137.080882
6,2,11513.811364
6,2,15251.782857
6,2,6299.355769
6,2,6427.142857
6,2,2428.178363
6,2,7376.325714
6,2,1973.122995
6,2,5376.555556
6,2,3404.510870
6,2,2057.670918
6,2,1859.292614

6,2,8851.197917
6,2,1829.841463
6,2,2343.880795
6,2,3959.164063
6,2,2479.053977
6,2,11082.051502
6,2,2743.430556
6,2,11146.997093
6,2,1610.009146
6,2,11157.293033
6,2,1753.437500
6,2,4208.600000
6,2,1741.701923
6,2,3652.460106
6,2,5256.894737
6,2,1722.531250
6,2,8830.923729
6,2,6402.833333
6,2,4077.609043
6,2,9988.142857
6,2,6729.592391
6,2,5791.051282
6,2,8409.800000
6,2,6396.336957
6,2,3245.861111
6,2,1757.166667
6,2,5307.044444
6,2,6154.383721
6,2,2424.744681
6,2,1961.195122
6,2,3954.392857
6,2,2161.259615
6,2,2787.750000
6,2,3901.548913
6,2,2929.609756
6,2,4953.955497
6,2,1544.736842
6,2,3360.470588
6,2,3118.580729
6,2,2668.848214
6,2,7801.783784
6,2,3730.512195
6,2,2420.222222
6,2,8158.500000
6,2,1742.865000
6,2,2535.295918
6,2,7952.693627
6,2,2502.365000
6,2,2439.108696
6,2,17572.345109
6,2,2205.453947
6,2,3304.960000
6,2,2135.580000
6,2,5346.476744
6,2,3670.422222
6,2,1843.346591
6,2,3208.634146
6,2,7226.965116
6,2,1831.928571
6,2,2930.911290
6,2,2121.788462
6,2,10087.276923
6,2,3249.697917
6,2,3822.500000
6,2,1984.923077
6,2,2219.980769

6,2,4646.682566
6,2,3680.115385
6,2,1916.242188
6,2,2114.833333
6,2,2309.204787
6,2,6411.059211
6,2,1674.325000
6,2,2579.228723
6,2,2078.233108
6,2,2121.632353
6,2,13759.261905
6,2,1962.659218
6,2,2921.849057
6,2,2191.347328
6,2,3109.058824
6,2,4631.893750
6,2,3473.414216
6,2,4285.821429
6,2,3664.187500
6,2,5545.316860
6,2,4570.875000
6,2,5618.670732
6,2,1768.675676
6,2,1701.000000
6,2,3476.544118
6,2,1918.742647
6,2,2902.210714
6,2,2566.173077
6,2,3972.043750
6,2,1824.990385
6,2,7426.666667
6,2,3161.297872
6,2,3774.536290
6,2,2099.217105
6,2,3770.299479
6,2,2935.013587
6,2,3427.828125
6,2,2805.512931
6,2,4672.473684
6,2,4499.953488
6,2,14560.195122
6,2,7098.267361
6,2,2403.312500
6,2,1572.795213
6,2,13345.465116
6,2,2157.578125
6,2,3995.038835
6,2,1862.146341
6,2,2256.151515
6,2,2556.910714
6,2,5321.093750
6,2,1723.328947
6,2,2250.136612
6,2,2207.071429
6,2,2088.774038
6,2,9025.627358
6,2,3138.300000
6,2,5889.074766
6,2,5959.500000
6,2,4148.484848
6,2,6186.421875
6,2,3203.980296
6,2,1851.383333
6,2,2255.690217
6,2,2795.185897
6,2,5766.019737

6,2,9270.250000
6,2,1502.642361
6,2,4254.154255
6,2,7023.964674
6,2,1747.215116
6,2,5012.588889
6,2,4717.362745
6,2,1580.112245
6,2,1772.834239
6,2,2695.544944
6,2,4220.895349
6,2,4560.522727
6,2,2619.850543
6,2,7570.354839
6,2,10397.608108
6,2,1942.701923
6,2,7709.017442
6,2,11294.622596
6,2,1713.375000
6,2,2251.195000
6,2,2097.503125
6,2,6390.204082
6,2,9777.755814
6,2,2970.048913
6,2,2789.250000
6,2,1811.171429
6,2,2033.932927
6,2,2953.718750
6,2,2769.883152
6,2,3389.432432
6,2,4726.390625
6,2,7469.810096
6,2,4075.324324
6,2,1914.564286
6,2,2466.445000
6,2,19187.812500
6,2,2599.851064
6,2,6032.720238
6,2,1531.240385
6,2,2406.291667
6,2,3751.891960
6,2,3259.851064
6,2,2479.447581
6,2,2190.878378
6,2,1811.765957
6,2,1686.929487
6,2,4505.350000
6,2,4477.055556
6,2,2133.289773
6,2,3145.415385
6,2,6702.096939
6,2,2072.111486
6,2,1817.234756
6,2,1714.252660
6,2,2377.177083
6,2,2232.764706
6,2,1792.866279
6,2,7938.815789
6,2,2171.863636
6,2,3428.117647
6,2,2141.796512
6,2,7123.224599
6,2,6418.259777
6,2,2260.085937
6,2,4309.838816
6,2,3692.354167

6,2,3203.111111
6,2,3945.357639
6,2,5947.797500
6,2,4009.317568
6,2,2364.153846
6,2,4121.635135
6,2,2773.073980
6,2,9891.785714
6,2,1943.155405
6,2,3665.681250
6,2,1622.532895
6,2,21540.291667
6,2,2974.320122
6,2,8778.961538
6,2,2089.859375
6,2,4116.050000
6,2,1623.732558
6,2,1618.428571
6,2,2411.404255
6,2,6330.134615
6,2,2035.933333
6,2,1998.422222
6,2,1665.625000
6,2,1577.236486
6,2,1592.615385
6,2,2018.988636
6,2,2417.406250
6,2,10432.840000
6,2,2212.627907
6,2,2654.933735
6,2,3052.765306
6,2,2075.383495
6,2,4486.479730
6,2,3337.289340
6,2,64467.990385
6,2,2343.405093
6,2,1511.845000
6,2,3814.331395
6,2,2380.595745
6,2,1695.788462
6,2,2648.384615
6,2,5383.928191
6,2,1787.000000
6,2,2728.914773
6,2,3762.904605
6,2,7255.697917
6,2,4325.776316
6,2,3904.714286
6,2,5790.552083
6,2,3491.912500
6,2,2557.170455
6,2,1804.603261
6,2,5678.307692
6,2,1905.183140
6,2,4141.122517
6,2,2012.668605
6,2,1954.492147
6,2,1794.040541
6,2,3278.156250
6,2,2459.263158
6,2,2673.606481
6,2,1847.520833
6,2,3182.725694
6,2,1531.720745
6,2,1734.093023
6,2,5444.911111

6,2,4752.437853
6,2,2845.211538
6,2,1504.761905
6,2,4064.812500
6,2,9095.986486
6,2,30370.413462
6,2,3553.997685
6,2,1839.255435
6,2,6112.258475
6,2,2553.625000
6,2,1776.938776
6,2,1850.083333
6,2,5630.138158
6,2,4525.371324
6,2,2480.842105
6,2,1879.517857
6,2,3508.148649
6,2,2207.355556
6,2,2135.405405
6,2,4155.431373
6,2,2434.875000
6,2,1636.735000
6,2,4068.497222
6,2,4909.270408
6,2,2785.291667
6,2,3630.747093
6,2,1720.875000
6,2,1648.386029
6,2,2689.366667
6,2,7548.901070
6,2,2497.764286
6,2,3977.191489
6,2,5240.553191
6,2,2325.093023
6,2,2710.306818
6,2,8735.065678
6,2,1785.963415
6,2,9594.363889
6,2,11958.906780
6,2,2517.667614
6,2,4769.585938
6,2,2181.825000
6,2,2675.476744
6,2,2279.087264
6,2,5562.120370
6,2,2125.190341
6,2,16659.488636
6,2,1827.389286
6,2,1680.555556
6,2,2471.831897
6,2,2108.000000
6,2,2579.500000
6,2,5078.650000
6,2,1807.368421
6,2,5598.041667
6,2,2210.230978
6,2,2403.024390
6,2,2292.907895
6,2,2291.278846
6,2,3394.337209
6,2,2071.658537
6,2,1712.063187
6,2,2036.391447
6,2,4206.750000
6,2,1910.673077
6,2,9323.308824

6,2,4575.625000
6,2,9608.134868
6,2,2105.823529
6,2,8134.118750
6,2,1525.653846
6,2,1512.560000
6,2,2141.041667
6,2,4073.435897
6,2,2940.605978
6,2,1663.418605
6,2,4110.722727
6,2,10700.425926
6,2,2950.490909
6,2,1847.659091
6,2,10201.620370
6,2,7495.632353
6,2,1981.970588
6,2,2580.400000
6,2,11323.101695
6,2,1853.377907
6,2,2758.578125
6,2,7847.551020
6,2,2914.046512
6,2,3659.490385
6,2,2539.405660
6,2,2551.384615
6,2,3758.135870
6,2,2400.404891
6,2,5620.322581
6,2,2237.394737
6,2,8453.335616
6,2,3898.112981
6,2,1628.845395
6,2,2232.466667
6,2,2247.945652
6,2,2002.625000
6,2,2883.281250
6,2,2859.831019
6,2,1979.533784
6,2,4858.781250
6,2,5022.764706
6,2,2320.512821
6,2,2000.580645
6,2,2961.333333
6,2,2692.750000
6,2,8833.466346
6,2,12616.613426
6,2,2251.531915
6,2,2771.669118
6,2,4738.569444
6,2,4867.521739
6,2,3713.750000
6,2,2675.022436
6,2,9373.710000
6,2,2279.500000
6,2,5884.014205
6,2,2519.828125
6,2,7972.012500
6,2,3392.615385
6,2,3979.801630
6,2,1791.454545
6,2,1663.607143
6,2,2550.000000
6,2,1679.181250
6,2,2260.617647
6,2,3535.325000

6,2,3382.581522
6,2,3043.771875
6,2,2657.217105
6,2,2381.953125
6,2,3661.011364
6,2,1948.046429
6,2,2715.446809
6,2,5383.043478
6,2,3350.211921
6,2,2984.223684
6,2,1607.147436
6,2,3472.306122
6,2,1651.215278
6,2,2897.888889
6,2,1510.276316
6,2,4405.251462
6,2,13739.153191
6,2,7985.669872
6,2,1675.000000
6,2,2484.428571
6,2,1932.605769
6,2,2255.398438
6,2,1609.074324
6,2,5151.375000
6,2,4253.724138
6,2,8807.375000
6,2,3088.828125
6,2,2844.521053
6,2,8542.571809
6,2,15930.608974
6,2,3321.067708
6,2,6216.529412
6,2,3085.824324
6,2,6461.296053
6,2,3622.050000
6,2,2717.559524
6,2,2919.461187
6,2,1531.475000
6,2,1639.692308
6,2,3911.357868
6,2,1689.651934
6,2,5822.945455
6,2,1593.535714
6,2,11703.682692
6,2,3764.872159
6,2,2988.292763
6,2,5887.771739
6,2,2121.368902
6,2,3648.050000
6,2,2071.912281
6,2,3981.361702
6,2,4682.910714
6,2,7287.403846
6,2,4803.000000
6,2,2860.350446
6,2,2912.727612
6,2,10085.000000
6,2,3293.542553
6,2,2300.871795
6,2,8284.032738
6,2,1532.808824
6,2,2495.116438
6,2,4448.197115
6,2,2332.166667
6,2,5482.137195
6,2,1660.625000

6,2,4897.842500
6,2,1579.069079
6,2,2243.573446
6,2,1865.238372
6,2,3092.578947
6,2,6211.214286
6,2,1538.550000
6,2,11873.171196
6,2,2463.602273
6,2,11774.921875
6,2,1737.541667
6,2,3973.875000
6,2,2072.810811
6,2,2990.358553
6,2,2447.053571
6,2,6135.019231
6,2,3400.604167
6,2,2881.514535
6,2,5435.454023
6,2,1649.493976
6,2,1704.813953
6,2,2887.000000
6,2,5989.377404
6,2,3479.781250
6,2,1592.193548
6,2,2169.645161
6,2,7482.718750
6,2,2054.692308
6,2,2833.562500
6,2,2579.571809
6,2,1646.500000
6,2,12734.717593
6,2,2519.010000
6,2,1576.821429
6,2,2630.872340
6,2,4313.719388
6,2,2045.630952
6,2,3254.842857
6,2,4823.476415
6,2,2721.054054
6,2,1913.458065
6,2,6199.461538
6,2,2843.000000
6,2,6163.018519
6,2,4095.696602
6,2,2319.647727
6,2,2460.278125
6,2,1580.218750
6,2,1969.173077
6,2,6083.935976
6,2,1618.100559
6,2,3308.187166
6,2,1812.854592
6,2,6769.029412
6,2,1571.618902
6,2,2270.354167
6,2,3601.823529
6,2,2708.806122
6,2,2248.921053
6,2,6978.176991
6,2,2012.863636
6,2,1746.000000
6,2,1513.461957
6,2,3209.609375
6,2,2431.007653
6,2,3261.824000

6,2,2930.412162
6,2,7306.358974
6,2,8112.062500
6,2,3971.250000
6,2,2347.326923
6,2,2190.750000
6,2,1589.323034
6,2,1631.777778
6,2,2058.988372
6,2,9731.185185
6,2,1865.000000
6,2,1556.472973
6,2,2488.916667
6,2,4300.340000
6,2,6099.216346
6,2,2582.451389
6,2,2601.335000
6,2,3428.503289
6,2,2581.543981
6,2,2959.209135
6,2,1750.894886
6,2,2328.649289
6,2,8858.848485
6,2,6065.902778
6,2,13344.860795
6,2,1615.857143
6,2,3483.774038
6,2,7348.363636
6,2,1567.157895
6,2,1862.473684
6,2,6060.783333
6,2,8801.779070
6,2,14553.863636
6,2,1892.211538
6,2,6508.421875
6,2,2824.206977
6,2,2635.113497
6,2,1965.000000
6,2,2161.047170
6,2,1850.089286
6,2,2788.965000
6,2,4647.000000
6,2,3263.779255
6,2,2430.461538
6,2,4685.612500
6,2,1665.344340
6,2,2838.750000
6,2,2634.628641
6,2,2393.337766
6,2,3754.534884
6,2,2250.267857
6,2,4497.831522
6,2,5749.714912
6,2,2276.521341
6,2,6308.683673
6,2,3225.820122
6,2,1980.907609
6,2,4516.508929
6,2,3940.839286
6,2,3591.807692
6,2,1888.625000
6,2,5964.446429
6,2,15619.335227
6,2,1967.182432
6,2,1727.688889
6,2,1922.468750

6,2,5579.614583
6,2,3786.994792
6,2,1870.930233
6,2,3956.440104
6,2,1584.186047
6,2,1921.784884
6,2,3286.301136
6,2,1878.172619
6,2,2946.565972
6,2,1744.042683
6,2,2778.362745
6,2,2083.673913
6,2,1998.939024
6,2,4232.000000
6,2,9807.082447
6,2,2044.517857
6,2,2234.041667
6,2,1741.953804
6,2,3741.381579
6,2,1530.461538
6,2,1769.919271
6,2,2846.818182
6,2,5248.141026
6,2,6479.862745
6,2,3830.904762
6,2,2324.461735
6,2,1630.061111
6,2,4809.200000
6,2,8255.155405
6,2,3272.962963
6,2,1667.263514
6,2,4065.651163
6,2,4686.296875
6,2,12322.192308
6,2,1683.564103
6,2,5976.866667
6,2,1713.796875
6,2,2720.571429
6,2,2195.579787
6,2,1780.500000
6,2,3521.164773
6,2,1830.407895
6,2,1641.708333
6,2,6990.976562
6,2,2815.314433
6,2,3572.100000
6,2,4694.021341
6,2,3516.630952
6,2,3049.209239
6,2,4219.344907
6,2,4947.608911
6,2,2419.766304
6,2,2392.500000
6,2,2678.375000
6,2,1788.951087
6,2,2117.205000
6,2,2386.294643
6,2,2872.902439
6,2,2552.414894
6,2,1556.351351
6,2,3282.031250
6,2,4188.833333
6,2,6969.773585
6,2,3966.057927
6,2,3233.576923
6,2,2283.044118

6,2,2773.734043
6,2,3243.465909
6,2,6115.946429
6,2,1920.625000
6,2,4470.802632
6,2,2154.461957
6,2,4270.336957
6,2,2824.975410
6,2,2751.053977
6,2,2064.368421
6,2,1649.125000
6,2,5399.694915
6,2,2697.158333
6,2,1608.159091
6,2,2946.592857
6,2,1518.964286
6,2,7582.038462
6,2,2405.411932
6,2,9031.323718
6,2,3901.354651
6,2,9534.256818
6,2,5776.339744
6,2,3897.811047
6,2,3089.867647
6,2,3803.923469
6,2,6185.596698
6,2,3872.757075
6,2,1683.868750
6,2,1610.527778
6,2,2319.481013
6,2,1699.461538
6,2,2329.612745
6,2,6491.062500
6,2,2209.057692
6,2,5888.993750
6,2,1512.187500
6,2,19012.791667
6,2,2172.076923
6,2,1775.250000
6,2,2252.524590
6,2,1709.713450
6,2,1811.218023
6,2,2599.670455
6,2,8553.129808
6,2,2040.211538
6,2,4340.457547
6,2,13976.797297
6,2,2929.704082
6,2,4604.451220
6,2,2537.977500
6,2,2195.022222
6,2,3813.542553
6,2,4290.846154
6,2,1966.615854
6,2,1721.872093
6,2,4144.505682
6,2,4179.771429
6,2,3789.375000
6,2,2353.280612
6,2,1526.196429
6,2,1630.197368
6,2,7327.654605
6,2,9630.142857
6,2,4054.571875
6,2,2503.341463
6,2,1531.136905

6,2,2283.121951
6,2,5252.825000
6,2,1917.834459
6,2,3203.481132
6,2,3442.986364
6,2,2102.013889
6,2,2056.255102
6,2,15948.783333
6,2,2298.644231
6,2,1691.934375
6,2,2983.707447
6,2,1634.214286
6,2,7748.940341
6,2,2199.393229
6,2,2752.068182
6,2,11848.039474
6,2,5785.000000
6,2,2129.213816
6,2,2244.819444
6,2,2113.958333
6,2,3811.021277
6,2,2703.878378
6,2,6185.329268
6,2,3844.321429
6,2,3863.872340
6,2,3079.192308
6,2,7798.564103
6,2,1602.250000
6,2,1578.286624
6,2,2401.306034
6,2,8145.541667
6,2,2304.540000
6,2,1662.316860
6,2,3611.594595
6,2,1590.621622
6,2,2129.298295
6,2,4472.834091
6,2,3640.555556
6,2,2607.218750
6,2,1959.000000
6,2,14789.136628
6,2,12025.788043
6,2,1896.091146
6,2,1695.668605
6,2,2276.236413
6,2,2127.964286
6,2,1684.732143
6,2,16736.711538
6,2,2819.637500
6,2,1768.013158
6,2,5399.695513
6,2,2084.506944
6,2,2525.250000
6,2,3311.125000
6,2,1717.086538
6,2,2399.923913
6,2,4496.542553
6,2,5498.002717
6,2,6835.000000
6,2,2738.548077
6,2,2292.850000
6,2,8597.675000
6,2,2189.720930
6,2,1885.192708
6,2,4534.414634
6,2,3852.500000

6,2,1884.139073
6,2,3940.853723
6,2,1585.908163
6,2,2234.453125
6,2,1611.044643
6,2,5842.641667
6,2,27084.437500
6,2,2208.082386
6,2,3796.234375
6,2,4412.034759
6,2,2781.306122
6,2,8577.652174
6,2,4749.290698
6,2,1671.864130
6,2,3181.829268
6,2,1801.992500
6,2,3248.250000
6,2,18036.196023
6,2,4286.562500
6,2,2158.703125
6,2,1600.207317
6,2,1735.831019
6,2,1740.665816
6,2,8677.837500
6,2,1744.519737
6,2,2202.976190
6,2,4288.297872
6,2,1906.078947
6,2,2941.270270
6,2,73063.333333
6,2,1644.250000
6,2,1940.926829
6,2,2341.937500
6,2,3659.942308
6,2,6514.150000
6,2,1692.821656
6,2,1935.321053
6,2,1996.056818
6,2,1685.128571
6,2,1513.011905
6,2,3933.645833
6,2,5710.136364
6,2,2436.705882
6,2,1891.029412
6,2,1524.651163
6,2,8264.515625
6,2,2420.766026
6,2,3354.383333
6,2,3754.366071
6,2,1857.875000
6,2,2518.358974
6,2,2113.694444
6,2,7504.118644
6,2,1519.836957
6,2,1535.250000
6,2,1580.871622
6,2,2346.002404
6,2,1961.557927
6,2,1601.781250
6,2,3621.596591
6,2,1793.682065
6,2,1809.733813
6,2,1545.833333
6,2,8079.262821
6,2,12546.051887
6,2,2925.572581

6,2,2067.767045
6,2,2804.985294
6,2,1944.277439
6,2,2423.782609
6,2,3768.670213
6,2,3674.646341
6,2,2914.285714
6,2,2654.808824
6,2,1798.532895
6,2,1604.743590
6,2,3825.600000
6,2,2031.611111
6,2,4250.718220
6,2,2606.721154
6,2,3465.567308
6,2,3034.529070
6,2,1874.989583
6,2,2740.802469
6,2,2518.925532
6,2,4884.259615
6,2,2350.729167
6,2,2082.093750
6,2,3912.180921
6,2,2342.048077
6,2,5657.573171
6,2,2189.919118
6,2,7382.861702
6,2,1669.962766
6,2,1769.555233
6,2,4461.528846
6,2,1628.281437
6,2,2161.250000
6,2,4384.724432
6,2,2290.884615
6,2,2373.502778
6,2,3435.756098
6,2,3042.172680
6,2,9729.775000
6,2,2086.575000
6,2,2947.635135
6,2,3237.961538
6,2,1616.887755
6,2,6142.441860
6,2,7575.929825
6,2,2711.931373
6,2,1551.307692
6,2,4217.306250
6,2,1772.469388
6,2,8863.000000
6,2,6353.942073
6,2,2965.850000
6,2,3316.004630
6,2,3322.058594
6,2,11711.195652
6,2,1887.400000
6,2,1884.250000
6,2,4873.636029
6,2,1609.403125
6,2,2698.000000
6,2,4012.322581
6,2,3429.750000
6,2,2239.250000
6,2,2745.668103
6,2,11969.317073
6,2,4014.407143
6,2,2076.371951

6,2,3962.542683
6,2,3329.148936
6,2,1583.284574
6,2,2919.837838
6,2,1941.940625
6,2,3220.533537
6,2,1561.057927
6,2,2211.086957
6,2,5232.026163
6,2,1808.279255
6,2,1994.287500
6,2,3437.703947
6,2,2379.370787
6,2,2729.895349
6,2,2196.190625
6,2,1914.261905
6,2,3147.178571
6,2,2888.601064
6,2,3065.754902
6,2,3168.978125
6,2,3930.590164
6,2,3319.061224
6,2,1921.360795
6,2,1879.746795
6,2,1903.715640
6,2,3826.550000
6,2,1827.250000
6,2,7123.904762
6,2,8838.520833
6,2,3181.265909
6,2,2446.564103
6,2,3906.750000
6,2,5192.538462
6,2,1678.777027
6,2,8031.296875
6,2,5128.802817
6,2,3588.313953
6,2,2817.517442
6,2,1760.383721
6,2,3993.256757
6,2,2598.351974
6,2,2986.179487
6,2,2080.846429
6,2,3198.289474
6,2,4826.557065
6,2,1820.875000
6,2,1838.411932
6,2,5026.229167
6,2,3165.750000
6,2,3518.031250
6,2,5410.181818
6,2,4963.005952
6,2,4306.829268
6,2,2535.914062
6,2,4270.919192
6,2,4033.469444
6,2,3105.880435
6,2,25616.857759
6,2,1763.750000
6,2,1540.744186
6,2,5723.005525
6,2,1796.519022
6,2,2139.887500
6,2,1838.633721
6,2,2273.800000
6,2,10213.117647

6,2,3120.209459
6,2,4285.095000
6,2,3055.192308
6,2,3665.115854
6,2,5932.750000
6,2,1934.872093
6,2,7352.316860
6,2,1612.092105
6,2,2109.189815
6,2,2106.978125
6,2,2771.500000
6,2,5635.952500
6,2,1790.800000
6,2,1781.565217
6,2,6019.000000
6,2,2323.743902
6,2,2712.959302
6,2,3244.960227
6,2,2258.277273
6,2,2028.417969
6,2,5926.119048
6,2,1865.866477
6,2,26517.005208
6,2,2243.971429
6,2,4955.082857
6,2,2770.185185
6,2,7249.400000
6,2,3408.644231
6,2,2272.320755
6,2,1605.181548
6,2,2644.402174
6,2,2765.188725
6,2,2314.767045
6,2,4568.891304
6,2,10513.653846
6,2,2774.908088
6,2,13203.580189
6,2,3908.065972
6,2,5418.034091
6,2,7944.870536
6,2,2374.914634
6,2,1576.692308
6,2,4389.908163
6,2,1833.563830
6,2,3050.016447
6,2,1602.162791
6,2,2238.097674
6,2,2530.927807
6,2,3448.337423
6,2,11305.585938
6,2,4422.747283
6,2,4256.561224
6,2,7377.605769
6,2,3375.612245
6,2,8022.988636
6,2,4220.411765
6,2,2615.861364
6,2,3541.260204
6,2,1621.631250
6,2,1755.698864
6,2,2195.975000
6,2,4982.572864
6,2,2407.205357
6,2,1896.883234
6,2,2235.112069
6,2,12853.367647

6,2,6701.872340
6,2,5255.039216
6,2,3452.467391
6,2,3184.235714
6,2,3335.045455
6,2,10987.717949
6,2,2529.744444
6,2,2356.666667
6,2,1509.000000
6,2,6399.579787
6,2,1816.573864
6,2,7306.986702
6,2,1724.225806
6,2,5011.434783
6,2,2281.494186
6,2,2404.777778
6,2,6157.365741
6,2,11428.696970
6,2,2373.847973
6,2,9083.734694
6,2,2754.322222
6,2,15248.616279
6,2,2521.966667
6,2,3777.102649
6,2,11049.175781
6,2,3039.352041
6,2,2392.267857
6,2,3129.897500
6,2,1618.112805
6,2,1581.849727
6,2,5238.000000
6,2,1773.673077
6,2,1915.310976
6,2,2448.093750
6,2,8825.683333
6,2,5785.278509
6,2,1632.319079
6,2,10093.021053
6,2,2763.875000
6,2,1662.083333
6,2,2110.944444
6,2,6573.648936
6,2,3260.285714
6,2,2141.872024
6,2,2087.659375
6,2,2638.975000
6,2,3406.494186
6,2,1870.448276
6,2,2150.641711
6,2,1588.460227
6,2,3055.062500
6,2,8711.697143
6,2,4596.455497
6,2,11580.782609
6,2,1648.885714
6,2,5428.653846
6,2,1593.386364
6,2,7496.406863
6,2,11590.000000
6,2,6962.665254
6,2,4772.590909
6,2,2156.406250
6,2,2908.191406
6,2,1715.571429
6,2,2970.958333
6,2,2393.250000

6,2,4063.528061
6,2,1644.845238
6,2,1731.494536
6,2,1600.356481
6,2,3587.255435
6,2,2084.321429
6,2,4728.113095
6,2,4079.811275
6,2,3351.377778
6,2,2169.291667
6,2,1672.832447
6,2,5098.846154
6,2,8934.327273
6,2,2802.284314
6,2,2081.418919
6,2,2132.087500
6,2,4637.490909
6,2,5309.923077
6,2,3665.561111
6,2,14579.898936
6,2,2104.768473
6,2,2145.550000
6,2,1943.575000
6,2,4803.284722
6,2,9810.082317
6,2,4013.762500
6,2,1750.116848
6,2,2014.145349
6,2,4592.111111
6,2,1765.450000
6,2,2292.637725
6,2,1535.816327
6,2,1638.743421
6,2,13967.423077
6,2,3008.790541
6,2,4658.432692
6,2,2654.664773
6,2,3861.760870
6,2,4141.919643
6,2,3605.880435
6,2,1540.500000
6,2,1768.422222
6,2,1713.942308
6,2,3143.531250
6,2,2403.207447
6,2,1603.372093
6,2,5492.375000
6,2,2195.230769
6,2,7392.300000
6,2,5127.332237
6,2,3108.645349
6,2,2825.012195
6,2,1575.081081
6,2,5247.802326
6,2,1530.581081
6,2,1846.985294
6,2,1866.674342
6,2,1535.976562
6,2,3668.590909
6,2,1800.484756
6,2,3004.031250
6,2,7008.265306
6,2,1903.582386
6,2,1558.875000
6,2,40894.265537
6,2,6229.971154

6,2,4109.078125
6,2,1808.333333
6,2,3861.631250
6,2,1866.988636
6,2,6057.122449
6,2,2173.421053
6,2,2417.118056
6,2,2264.151316
6,2,2065.639752
6,2,1510.153571
6,2,2085.328125
6,2,22110.416667
6,2,2392.795918
6,2,3208.014205
6,2,2702.714286
6,2,3992.433333
6,2,2148.375000
6,2,3466.059211
6,2,4515.000000
6,2,1560.489130
6,2,3633.139535
6,2,4163.670455
6,2,1569.041667
6,2,3653.315789
6,2,1774.452514
6,2,6283.189904
6,2,5258.768750
6,2,7443.442708
6,2,3284.008523
6,2,3605.530556
6,2,5845.125000
6,2,3409.103659
6,2,1517.060606
6,2,6233.712500
6,2,2362.865854
6,2,3918.975000
6,2,16953.890909
6,2,2694.518750
6,2,1947.143617
6,2,2969.333333
6,2,1812.494318
6,2,4287.119565
6,2,5254.554348
6,2,1670.750000
6,2,4437.715909
6,2,2509.310897
6,2,12097.789474
6,2,8209.382979
6,2,2388.182320
6,2,3473.865772
6,2,3714.448113
6,2,5235.419118
6,2,3626.367347
6,2,3279.551020
6,2,4182.969325
6,2,2293.471698
6,2,3852.316327
6,2,2679.237745
6,2,2918.776163
6,2,5012.579787
6,2,1732.354730
6,2,4354.003289
6,2,2400.325000
6,2,6815.494681
6,2,1611.063830
6,2,2127.625000

6,2,13582.747727
6,2,6490.233333
6,2,4116.478261
6,2,3407.619048
6,2,4658.739130
6,2,2393.560976
6,2,2739.621528
6,2,2503.234091
6,2,9154.969231
6,2,4505.945122
6,2,2348.470588
6,2,1558.511628
6,2,4347.209135
6,2,2509.591837
6,2,1699.395210
6,2,6971.216981
6,2,5574.057692
6,2,12446.738636
6,2,2277.013333
6,2,1506.920139
6,2,1983.261111
6,2,2674.560811
6,2,1954.631579
6,2,1913.538462
6,2,2032.859756
6,2,1924.927374
6,2,1725.007812
6,2,4411.173780
6,2,2516.988636
6,2,2629.867021
6,2,2544.960714
6,2,7880.275281
6,2,10723.224390
6,2,2148.532847
6,2,5685.993750
6,2,8729.194712
6,2,1710.567073
6,2,5017.384804
6,2,1649.176630
6,2,8749.580311
6,2,2190.643939
6,2,1735.658228
6,2,2360.475000
6,2,9225.190476
6,2,1821.400000
6,2,1608.513514
6,2,1993.038265
6,2,2675.875000
6,2,2551.667614
6,2,4444.500000
6,2,8456.625000
6,2,5625.042553
6,2,2012.543478
6,2,1864.418750
6,2,3337.434524
6,2,4781.516827
6,2,2811.102273
6,2,7227.678571
6,2,2734.105263
6,2,7536.339286
6,2,2918.549479
6,2,1680.528846
6,2,3768.641509
6,2,12047.041667
6,2,1831.666667
6,2,8239.558824

6,2,1689.281046
6,2,4849.902174
6,2,3052.518293
6,2,1676.558140
6,2,2556.307870
6,2,2939.540865
6,2,2204.022222
6,2,2272.815972
6,2,2418.850000
6,2,1671.969388
6,2,3824.977723
6,2,3116.702128
6,2,2485.470000
6,2,1568.148649
6,2,6932.278409
6,2,2143.881579
6,2,7490.752747
6,2,2009.845588
6,2,1857.046196
6,2,1512.625000
6,2,5982.516667
6,2,3158.619048
6,2,3532.913636
6,2,2507.153846
6,2,3315.914474
6,2,1825.288265
6,2,4934.149351
6,2,1811.825000
6,2,3587.854430
6,2,3532.725146
6,2,6597.844828
6,2,4357.696809
6,2,3274.621287
6,2,2371.327320
6,2,9588.777778
6,2,2004.900000
6,2,4469.984043
6,2,3527.900000
6,2,3611.634146
6,2,1601.000000
6,2,3495.440217
6,2,4583.861111
6,2,1661.116959
6,2,2558.291667
6,2,1961.187500
6,2,1625.211111
6,2,3847.877778
6,2,2745.427632
6,2,8293.951389
6,2,1894.666667
6,2,4154.783784
6,2,2315.936170
6,2,2983.407143
6,2,4049.705556
6,2,4034.933333
6,2,3014.986486
6,2,3085.428571
6,2,2826.414634
6,2,2304.833333
6,2,2953.078947
6,2,3320.831633
6,2,6637.730556
6,2,2510.823529
6,2,3147.035714
6,2,1909.777778
6,2,3877.763006

6,2,2935.730570
6,2,4352.730994
6,2,3697.776744
6,2,3855.284884
6,2,2254.843023
6,2,2369.631098
6,2,4718.243243
6,2,1925.660000
6,2,19023.882353
6,2,1621.048780
6,2,3797.359375
6,2,13144.777778
6,2,2669.772727
6,2,1907.658228
6,2,2585.572917
6,2,7516.562500
6,2,5866.992347
6,2,2898.468085
6,2,1674.125000
6,2,1676.750000
6,2,3461.154891
6,2,22595.914062
6,2,2345.279762
6,2,4841.781250
6,2,17215.330508
6,2,3705.920673
6,2,4050.230769
6,2,2224.309896
6,2,2217.698864
6,2,3109.414894
6,2,9044.973958
6,2,5111.480000
6,2,1871.981675
6,2,6345.250000
6,2,1945.230769
6,2,1742.160000
6,2,2484.994898
6,2,1718.260870
6,2,3648.441176
6,2,2963.353125
6,2,1575.823864
6,2,1875.695122
6,2,3459.508929
6,2,10738.206452
6,2,1780.080275
6,2,2695.153061
6,2,8985.228125
6,2,4522.625000
6,2,1589.505556
6,2,3297.860465
6,2,3326.691860
6,2,3443.972826
6,2,1820.646277
6,2,5960.439024
6,2,4418.247222
6,2,1789.714286
6,2,13832.416667
6,2,1742.020833
6,2,2606.486842
6,2,1696.250000
6,2,1622.791667
6,2,2120.677711
6,2,2937.600610
6,2,2017.000000
6,2,2182.279070
6,2,3133.696809

6,2,3645.125000
6,2,2645.363208
6,2,1792.069767
6,2,8125.511905
6,2,4503.140957
6,2,2079.883929
6,2,1764.275000
6,2,2945.392045
6,2,2142.589286
6,2,2259.862745
6,2,1755.808511
6,2,1817.224359
6,2,1652.200000
6,2,2149.510417
6,2,1980.877778
6,2,2560.957895
6,2,4710.825000
6,2,2358.000000
6,2,4745.089286
6,2,1568.956522
6,2,6003.062500
6,2,4696.750000
6,2,5765.990741
6,2,3229.007075
6,2,7457.510204
6,2,1658.171875
6,2,1814.397959
6,2,2951.819079
6,2,2115.653646
6,2,2031.130769
6,2,2424.658537
6,2,2908.125000
6,2,2722.826087
6,2,3277.737500
6,2,2099.326531
6,2,1596.357143
6,2,10790.893617
6,2,2058.141892
6,2,3411.625000
6,2,7340.346591
6,2,6792.025862
6,2,2261.000000
6,2,1987.676630
6,2,1900.375000
6,2,3403.576923
6,2,5827.724790
6,2,4074.673077
6,2,2042.521429
6,2,2864.262821
6,2,9276.721154
6,2,25484.611111
6,2,7350.547872
6,2,12504.697917
6,2,5375.865000
6,2,3929.586111
6,2,4152.462500
6,2,1555.958084
6,2,1783.445652
6,2,2064.859375
6,2,9288.303977
6,2,4521.036842
6,2,2081.630814
6,2,2722.040865
6,2,2859.708333
6,2,6898.841667
6,2,1503.750000

6,2,2687.224490
6,2,2837.128049
6,2,8551.671171
6,2,1686.351064
6,2,2038.409574
6,2,1836.768707
6,2,17604.373950
6,2,14413.006098
6,2,8346.083770
6,2,7562.054688
6,2,4465.558974
6,2,4928.287500
6,2,7021.705000
6,2,3369.704545
6,2,2237.578212
6,2,1810.947368
6,2,2415.625000
6,2,5573.529412
6,2,3721.420455
6,2,12969.447222
6,2,2502.755000
6,2,3422.625000
6,2,1939.959459
6,2,4245.833333
6,2,3744.946429
6,2,2543.750000
6,2,2890.500000
6,2,3441.042683
6,2,2729.875000
6,2,2312.052632
6,2,3976.025641
6,2,2636.385870
6,2,4497.052632
6,2,1551.500000
6,2,2085.343750
6,2,1635.222222
6,2,6949.420213
6,2,2308.976744
6,2,5043.041667
6,2,7231.345588
6,2,16419.921053
6,2,1952.158654
6,2,1993.197917
6,2,9221.032609
6,2,5573.706395
6,2,11852.345930
6,2,1542.693182
6,2,1528.750000
6,2,2169.625000
6,2,2716.193878
6,2,14943.234043
6,2,1964.468750
6,2,7141.392857
6,2,2399.700000
6,2,2676.807692
6,2,4065.812500
6,2,2662.082386
6,2,2563.569767
6,2,1813.670455
6,2,2451.375000
6,2,3627.858333
6,2,3078.439189
6,2,2137.918033
6,2,4212.354167
6,2,6266.347368
6,2,2308.899371

6,2,2065.333333
6,2,16064.500000
6,2,2104.210526
6,2,2492.105769
6,2,7743.040201
6,2,4252.201389
6,2,1951.055556
6,2,2489.902439
6,2,3212.875000
6,2,2479.204082
6,2,6420.680556
6,2,1592.576531
6,2,2235.802632
6,2,4889.750000
6,2,1858.014535
6,2,2177.872159
6,2,4243.567073
6,2,2447.259868
6,2,2603.102273
6,2,2457.754386
6,2,3254.028205
6,2,3649.750000
6,2,6260.235000
6,2,1657.000000
6,2,5316.564286
6,2,1860.336735
6,2,1499.618590
6,2,1718.205357
6,2,1931.983871
6,2,1707.641509
6,2,1618.721591
6,2,3431.512821
6,2,2877.898810
6,2,1567.246951
6,2,8302.949761
6,2,2390.021739
6,2,1653.212121
6,2,2766.839888
6,2,7484.333333
6,2,3467.237245
6,2,2152.047297
6,2,6869.636364
6,2,10340.648148
6,2,8742.000000
6,2,1751.806250
6,2,3882.750000
6,2,5600.077670
6,2,2613.900510
6,2,1799.076923
6,2,3203.343750
6,2,2552.790698
6,2,7152.194805
6,2,2706.128049
6,2,1504.801282
6,2,3832.039216
6,2,1774.714286
6,2,13416.528409
6,2,1958.628205
6,2,3235.519231
6,2,1957.088235
6,2,2523.802632
6,2,1595.893939
6,2,2407.979592
6,2,1532.566667
6,2,3536.062500
6,2,1611.051282

6,2,1939.377551
6,2,1796.560976
6,2,2846.200581
6,2,4196.455882
6,2,1547.625000
6,2,2279.511628
6,2,1913.260417
6,2,1556.138158
6,2,2865.434783
6,2,2863.221939
6,2,1955.046053
6,2,3520.318919
6,2,1504.646907
6,2,2273.831325
6,2,1670.260000
6,2,7889.089286
6,2,2385.493213
6,2,6067.382353
6,2,2495.441489
6,2,1608.270349
6,2,3426.163717
6,2,1774.818182
6,2,5023.707317
6,2,1523.529605
6,2,2783.333333
6,2,2444.367188
6,2,3670.138889
6,2,2789.272222
6,2,5094.857143
6,2,13884.919643
6,2,3236.022727
6,2,8450.580569
6,2,6297.866667
6,2,2195.428571
6,2,2355.541667
6,2,1993.569444
6,2,1667.761628
6,2,1751.168478
6,2,2176.756579
6,2,1857.781250
6,2,1621.418919
6,2,2792.333333
6,2,5080.046053
6,2,4084.221939
6,2,10624.861111
6,2,3078.078125
6,2,2102.589286
6,2,6165.278226
6,2,4668.404255
6,2,6841.930693
6,2,2677.372093
6,2,2273.412791
6,2,4884.477273
6,2,7372.164894
6,2,4547.261905
6,2,5169.638889
6,2,3428.187500
6,2,2795.590909
6,2,5483.955556
6,2,2844.571429
6,2,2903.935000
6,2,5356.563830
6,2,4258.285714
6,2,2741.000000
6,2,2632.250000
6,2,6039.027778

6,2,1522.005319
6,2,2131.048780
6,2,1796.025568
6,2,2847.761364
6,2,2949.625000
6,2,3823.660377
6,2,1608.702703
6,2,2456.840909
6,2,2608.588235
6,2,1699.872159
6,2,1677.823864
6,2,2436.574468
6,2,1652.516304
6,2,4662.976562
6,2,2331.461957
6,2,2950.125000
6,2,4018.316176
6,2,2056.632653
6,2,2796.392857
6,2,2340.416667
6,2,2900.117647
6,2,3206.517647
6,2,2912.222222
6,2,5685.343318
6,2,1934.421053
6,2,21712.634518
6,2,3539.459184
6,2,2455.996528
6,2,6237.048780
6,2,3958.801724
6,2,2631.820513
6,2,3268.321078
6,2,3469.377451
6,2,4005.662234
6,2,12424.196429
6,2,6676.237745
6,2,13323.234375
6,2,1896.216463
6,2,2797.132653
6,2,3646.489796
6,2,11754.341667
6,2,1505.000000
6,2,8037.500000
6,2,2845.756098
6,2,1766.406250
6,2,4512.916667
6,2,7303.348837
6,2,2714.700000
6,2,3474.421053
6,2,11508.872642
6,2,2857.875000
6,2,1546.752976
6,2,6239.368421
6,2,3521.772059
6,2,1956.441860
6,2,2617.993902
6,2,1579.278846
6,2,3137.875000
6,2,2281.138393
6,2,3594.059896
6,2,4882.500000
6,2,2054.954545
6,2,1821.980769
6,2,2001.250000
6,2,6279.777778
6,2,1518.728261

6,2,6214.823529
6,2,3458.704268
6,2,7990.018868
6,2,3492.833333
6,2,2640.031250
6,2,1835.888158
6,2,4204.451705
6,2,3728.450000
6,2,5001.888889
6,2,2681.163934
6,2,1983.035714
6,2,3019.618357
6,2,2428.262570
6,2,1675.384615
6,2,1519.545918
6,2,2268.900000
6,2,2290.412791
6,2,3491.769397
6,2,1766.524306
6,2,1539.844444
6,2,4337.681250
6,2,7638.172500
6,2,4993.243902
6,2,2639.761905
6,2,8316.278846
6,2,1597.625000
6,2,13012.440000
6,2,2280.162304
6,2,7385.200000
6,2,2526.261364
6,2,3035.893519
6,2,2096.139535
6,2,2063.963889
6,2,3287.535088
6,2,2252.372549
6,2,3665.840116
6,2,1919.076923
6,2,1651.365385
6,2,3398.906250
6,2,1709.296053
6,2,10279.129310
6,2,1806.000000
6,2,2404.075472
6,2,2480.250000
6,2,1703.061224
6,2,5237.641447
6,2,1880.395349
6,2,4394.607955
6,2,3478.208791
6,2,2786.885246
6,2,6205.166667
6,2,1530.434211
6,2,6394.859043
6,2,1817.741477
6,2,4260.506579
6,2,2874.546875
6,2,3331.737179
6,2,1957.029605
6,2,2382.735465
6,2,2033.186047
6,2,2411.559524
6,2,3963.790179
6,2,2615.280488
6,2,2663.335106
6,2,2049.846983
6,2,9684.943548

6,2,4548.921053
6,2,2197.511364
6,2,8584.970000
6,2,6651.500000
6,2,2638.678571
6,2,2729.437500
6,2,1545.305389
6,2,3908.521472
6,2,2248.750000
6,2,2856.171875
6,2,1587.068750
6,2,1679.293367
6,2,19665.712963
6,2,5935.655172
6,2,2123.960526
6,2,2322.848404
6,2,2416.230769
6,2,3650.313953
6,2,2302.348684
6,2,8466.189873
6,2,1679.615196
6,2,1906.910326
6,2,11481.417683
6,2,1504.753425
6,2,2177.569767
6,2,1835.323864
6,2,4348.055000
6,2,3018.741860
6,2,1699.544118
6,2,3439.125000
6,2,12807.180556
6,2,1722.923077
6,2,8844.977273
6,2,2219.342857
6,2,2290.395833
6,2,1640.809896
6,2,7801.218137
6,2,1560.837209
6,2,4498.286585
6,2,1566.152500
6,2,2989.902439
6,2,2219.695652
6,2,5337.380952
6,2,8053.804054
6,2,2714.327778
6,2,7069.272321
6,2,1548.500000
6,2,6290.088889
6,2,3696.481707
6,2,9629.976744
6,2,6397.006757
6,2,2722.075000
6,2,6859.622378
6,2,2162.972826
6,2,1567.000000
6,2,2519.558511
6,2,3308.885246
6,2,2386.836957
6,2,6934.000000
6,2,3215.605769
6,2,1617.177778
6,2,1890.150000
6,2,2360.250000
6,2,1526.625000
6,2,3713.227273
6,2,6373.333333

6,2,1981.783505
6,2,3114.992647
6,2,3171.658537
6,2,13383.098958
6,2,2160.615196
6,2,2324.357143
6,2,3526.500000
6,2,4306.780612
6,2,3146.301630
6,2,2853.368421
6,2,1764.322674
6,2,1591.411458
6,2,4080.702586
6,2,1530.607143
6,2,5374.442797
6,2,7704.909091
6,2,2333.593548
6,2,3799.464286
6,2,3321.349359
6,2,1669.117021
6,2,1514.482143
6,2,3140.034574
6,2,4248.200000
6,2,2921.232143
6,2,3016.803571
6,2,1526.542683
6,2,2554.575000
6,2,4812.905612
6,2,2243.196429
6,2,2797.655367
6,2,3176.857143
6,2,5951.950000
6,2,1684.042683
6,2,3846.247253
6,2,2282.195313
6,2,1866.585034
6,2,2154.812500
6,2,1878.666667
6,2,7845.021390
6,2,11129.319767
6,2,4473.184659
6,2,1544.375000
6,2,6887.377841
6,2,1521.947368
6,2,5345.466837
6,2,1670.171053
6,2,1567.812500
6,2,6382.750000
6,2,5520.936170
6,2,2133.329082
6,2,2862.384868
6,2,4929.082386
6,2,1645.715116
6,2,2577.773810
6,2,2544.271226
6,2,4342.237179
6,2,4672.398256
6,2,1592.421053
6,2,3825.569620
6,2,2558.125000
6,2,18715.300000
6,2,2977.500000
6,2,3586.673913
6,2,3256.448980
6,2,5244.502717
6,2,2230.541667

6,2,2135.022222
6,2,2342.552326
6,2,2786.006579
6,2,3250.565217
6,2,7122.406863
6,2,3598.411765
6,2,5300.189024
6,2,2025.431818
6,2,2745.565789
6,2,8138.562500
6,2,3491.696429
6,2,1683.961538
6,2,1672.005814
6,2,2232.769737
6,2,4052.361702
6,2,1832.292683
6,2,2350.111702
6,2,2135.219512
6,2,1872.827381
6,2,2390.966346
6,2,2021.903846
6,2,3976.071429
6,2,3961.635870
6,2,1852.812500
6,2,1758.228723
6,2,6374.795732
6,2,2145.514881
6,2,1933.113402
6,2,4292.009662
6,2,2849.945402
6,2,7418.052632
6,2,2440.206044
6,2,3299.066667
6,2,5220.834302
6,2,1715.733108
6,2,3445.429487
6,2,1676.708333
6,2,3057.479651
6,2,4710.275000
6,2,8071.166667
6,2,6661.615385
6,2,1716.704082
6,2,2306.900000
6,2,2240.185792
6,2,2173.767857
6,2,4674.145833
6,2,2760.479651
6,2,1666.539063
6,2,2665.661765
6,2,14184.112245
6,2,4599.027027
6,2,1548.472222
6,2,1574.720109
6,2,2763.076923
6,2,2896.529255
6,2,3417.646341
6,2,2842.216867
6,2,1873.281250
6,2,2474.973684
6,2,5736.717143
6,2,1582.550802
6,2,2497.754491
6,2,2783.630682
6,2,4571.564516
6,2,1811.084459
6,2,2163.107143

6,2,9305.244444
6,2,3551.051020
6,2,8610.377419
6,2,1611.530093
6,2,1508.792683
6,2,1780.007812
6,2,1963.859375
6,2,3877.486111
6,2,5240.701087
6,2,1809.042500
6,2,1593.468085
6,2,1881.047904
6,2,2330.387701
6,2,2759.694079
6,2,5315.333333
6,2,2624.109043
6,2,3131.387324
6,2,1998.733333
6,2,2859.046512
6,2,3512.620370
6,2,8886.247222
6,2,6221.556886
6,2,1707.592105
6,2,1782.722222
6,2,1952.812500
6,2,6168.527174
6,2,2238.959459
6,2,1946.428125
6,2,1928.755208
6,2,3924.737981
6,2,2563.657895
6,2,14520.260000
6,2,1906.486486
6,2,2600.642857
6,2,3464.035714
6,2,6206.744792
6,2,2375.812500
6,2,3073.267857
6,2,2880.804348
6,2,4477.364286
6,2,2730.441176
6,2,2726.667553
6,2,7520.131250
6,2,2374.711538
6,2,1705.241758
6,2,2078.733333
6,2,17941.234375
6,2,2892.694444
6,2,3446.535714
6,2,16589.105263
6,2,2134.532895
6,2,2321.327586
6,2,10569.593137
6,2,1672.975000
6,2,1821.233108
6,2,4510.927136
6,2,2486.090000
6,2,3556.972826
6,2,1740.828125
6,2,9758.329545
6,2,2110.016304
6,2,1568.475610
6,2,3481.284091
6,2,3983.725490
6,2,1615.016304
6,2,1522.647059

6,2,1942.175000
6,2,3999.253289
6,2,1711.852740
6,2,4718.781818
6,2,10069.225000
6,2,19185.080000
6,2,4065.070000
6,2,2595.898936
6,2,1653.880000
6,2,9245.000000
6,2,2157.900000
6,2,2070.142857
6,2,4538.817935
6,2,1507.608974
6,2,3313.640244
6,2,3631.288889
6,2,1949.372340
6,2,5401.843750
6,2,4533.912791
6,2,2137.804878
6,2,9576.810000
6,2,1936.826531
6,2,7996.118280
6,2,2569.000000
6,2,4699.728022
6,2,5070.747475
6,2,1782.345930
6,2,1792.027027
6,2,1758.193396
6,2,6811.678571
6,2,32150.880952
6,2,3207.597222
6,2,1896.266667
6,2,1655.521739
6,2,2192.076389
6,2,4334.044643
6,2,2807.753289
6,2,2284.955000
6,2,8908.580189
6,2,3383.746622
6,2,3402.516667
6,2,1820.984694
6,2,2382.480447
6,2,3727.500000
6,2,8021.178571
6,2,3885.648810
6,2,10107.821429
6,2,2129.734375
6,2,1878.369318
6,2,5093.683140
6,2,2983.573864
6,2,2485.176282
6,2,6040.514706
6,2,1667.825000
6,2,2334.243902
6,2,5918.448276
6,2,2932.512195
6,2,2297.273936
6,2,4388.177778
6,2,1600.869565
6,2,3633.916667
6,2,2109.444444
6,2,4261.527273
6,2,4159.292614
6,2,2055.377404
6,2,2614.076923

6,2,1597.915698
6,2,5742.046784
6,2,3881.110849
6,2,1710.820513
6,2,7030.659091
6,2,5030.675676
6,2,6548.910615
6,2,2931.389908
6,2,3155.736111
6,2,3017.808140
6,2,4175.730088
6,2,2159.544304
6,2,10316.288462
6,2,2895.900000
6,2,3587.207143
6,2,2434.186047
6,2,7064.782738
6,2,6975.987864
6,2,1973.266892
6,2,5063.898148
6,2,4090.019737
6,2,5137.250000
6,2,3748.916667
6,2,6042.250000
6,2,5301.436224
6,2,4366.000000
6,2,2419.395349
6,2,2702.485000
6,2,1519.404762
6,2,2452.350543
6,2,3345.600000
6,2,1951.930851
6,2,5435.114130
6,2,1779.000000
6,2,3556.500000
6,2,1575.560811
6,2,5274.351064
6,2,1526.208333
6,2,1713.904891
6,2,4192.226562
6,2,7031.514851
6,2,2449.565789
6,2,11137.764706
6,2,4384.678571
6,2,2583.085106
6,2,5364.953125
6,2,2051.240854
6,2,1673.553191
6,2,1710.843137
6,2,2014.792056
6,2,3038.347087
6,2,3124.967391
6,2,7136.146552
6,2,2330.445122
6,2,6971.151042
6,2,13696.904167
6,2,1947.682065
6,2,2310.666667
6,2,3240.083333
6,2,1992.483696
6,2,16107.870283
6,2,4627.500000
6,2,1833.684896
6,2,4840.127604
6,2,4415.000000
6,2,2685.215686

6,2,3088.128713
6,2,2286.915385
6,2,2441.426316
6,2,6786.581395
6,2,2381.826531
6,2,8429.065789
6,2,3089.750000
6,2,2913.710714
6,2,9081.075758
6,2,2060.203947
6,2,5510.025000
6,2,3808.054688
6,2,2042.985577
6,2,2530.607143
6,2,5525.389205
6,2,1576.250000
6,2,3302.966146
6,2,4312.377660
6,2,3876.812500
6,2,4302.152174
6,2,4102.519231
6,2,2927.483333
6,2,10523.797872
6,2,14075.625000
6,2,3047.038462
6,2,5544.057692
6,2,7855.529412
6,2,1519.097561
6,2,2253.308901
6,2,2035.606218
6,2,2374.546875
6,2,2374.460227
6,2,1831.154762
6,2,1748.000000
6,2,11506.897368
6,2,2053.823529
6,2,4860.714286
6,2,4091.585227
6,2,7694.921053
6,2,3398.223958
6,2,2919.954082
6,2,3238.079545
6,2,3325.281250
6,2,2812.524390
6,2,6343.709302
6,2,3881.754464
6,2,9136.021875
6,2,2870.176471
6,2,5466.473404
6,2,2732.250000
6,2,3163.019231
6,2,3836.733696
6,2,1718.856383
6,2,1599.189189
6,2,4589.494681
6,2,3985.127660
6,2,1601.173653
6,2,2186.357639
6,2,11633.931250
6,2,2536.421053
6,2,2876.666667
6,2,5786.870968
6,2,3172.674528
6,2,3065.841216
6,2,34178.205128
6,2,2341.509615

6,2,2988.354839
6,2,1919.042857
6,2,2201.952381
6,2,1893.463816
6,2,7709.480114
6,2,1828.953488
6,2,1947.215116
6,2,3458.514286
6,2,1515.356383
6,2,1844.682432
6,2,1803.530612
6,2,5839.062500
6,2,4873.472222
6,2,2407.500000
6,2,8515.272727
6,2,5434.972973
6,2,2036.893617
6,2,4999.816327
6,2,5272.442708
6,2,1597.882353
6,2,2303.875000
6,2,3024.017241
6,2,2763.011364
6,2,2377.008523
6,2,5244.957589
6,2,4581.395349
6,2,4443.257812
6,2,2642.746795
6,2,5271.106383
6,2,4210.387500
6,2,1796.061224
6,2,1743.737931
6,2,2975.339286
6,2,2139.953488
6,2,4469.005208
6,2,15280.796296
6,2,5958.722222
6,2,1773.712500
6,2,2529.525000
6,2,4605.400000
6,2,1656.739130
6,2,5200.755556
6,2,9645.002660
6,2,10242.736702
6,2,3465.872340
6,2,11527.083333
6,2,2047.861111
6,2,2065.731383
6,2,4455.540541
6,2,1612.578947
6,2,1512.488889
6,2,2472.910377
6,2,2862.888298
6,2,5014.423780
6,2,5007.852041
6,2,4991.277174
6,2,6399.833333
6,2,1561.920673
6,2,2696.358974
6,2,2092.594595
6,2,1885.654762
6,2,2093.808511
6,2,4133.922619
6,2,2188.796196
6,2,1523.008152
6,2,4165.714286

6,2,2346.675000
6,2,2065.759259
6,2,3525.151832
6,2,7226.810056
6,2,3405.616667
6,2,6272.605556
6,2,1716.930556
6,2,2372.541667
6,2,43333.882591
6,2,1920.300000
6,2,4551.312500
6,2,2986.705882
6,2,3724.020833
6,2,3397.750000
6,2,3724.692308
6,2,3022.594444
6,2,5813.152439
6,2,2336.560000
6,2,2460.204545
6,2,4387.045732
6,2,3958.895349
6,2,2121.648256
6,2,10003.156250
6,2,2599.882979
6,2,1564.318182
6,2,2868.759375
6,2,2836.950000
6,2,2068.780488
6,2,5114.054545
6,2,1642.128289
6,2,1552.367347
6,2,2242.615909
6,2,6632.506579
6,2,2085.993377
6,2,3569.084302
6,2,3645.863426
6,2,1830.622093
6,2,5580.744681
6,2,5818.171875
6,2,4470.646226
6,2,12993.382979
6,2,6266.795745
6,2,14701.000000
6,2,2363.671196
6,2,1574.035256
6,2,1566.950000
6,2,7629.200000
6,2,2614.849265
6,2,1543.628049
6,2,2466.535000
6,2,2621.500000
6,2,14605.153846
6,2,3937.587766
6,2,1660.816327
6,2,2519.295082
6,2,4513.109948
6,2,7273.459184
6,2,4271.300000
6,2,8175.048077
6,2,1833.370000
6,2,5028.779605
6,2,2487.006250
6,2,2452.453947
6,2,26292.009146
6,2,2771.653846
6,2,3617.982143

6,2,2001.612500
6,2,2936.590909
6,2,5522.059896
6,2,2898.786458
6,2,1838.743902
6,2,2591.390306
6,2,4540.925926
6,2,5872.087500
6,2,2853.278571
6,2,4592.675000
6,2,3653.588235
6,2,2046.075000
6,2,4516.951220
6,2,12861.490798
6,2,6080.882353
6,2,4649.400000
6,2,2870.332447
6,2,17958.235465
6,2,5387.420732
6,2,2858.581522
6,2,2463.263514
6,2,3855.244565
6,2,3103.221557
6,2,3421.573529
6,2,1547.241379
6,2,1984.926829
6,2,1696.855263
6,2,6413.854167
6,2,3844.416667
6,2,8260.015625
6,2,3582.279070
6,2,2023.602273
6,2,1671.989796
6,2,1674.400000
6,2,1538.231618
6,2,1564.766667
6,2,2362.297297
6,2,2650.500000
6,2,1656.886364
6,2,2014.228448
6,2,3683.829787
6,2,1791.192982
6,2,3924.417178
6,2,2022.239130
6,2,8459.200000
6,2,2610.444444
6,2,1862.964674
6,2,3508.741667
6,2,5132.888889
6,2,1790.718750
6,2,8474.247222
6,2,1712.171875
6,2,2179.304348
6,2,14635.500000
6,2,4461.259375
6,2,3322.793367
6,2,3806.576705
6,2,3585.317568
6,2,2784.569149
6,2,2257.696429
6,2,9484.640625
6,2,4870.821023
6,2,2158.548077
6,2,3094.552486
6,2,1989.750000
6,2,2361.739362

6,2,3485.812500
6,2,2821.048611
6,2,5148.062500
6,2,3822.006250
6,2,4228.750000
6,2,4913.887500
6,2,2029.601266
6,2,1635.758929
6,2,8148.600000
6,2,3250.062500
6,2,2485.688889
6,2,3029.200000
6,2,4329.473214
6,2,3340.622093
6,2,1841.578947
6,2,2178.418750
6,2,2359.980676
6,2,2092.375000
6,2,7196.083333
6,2,1685.015625
6,2,4016.263587
6,2,3330.320652
6,2,2013.768072
6,2,2258.106509
6,2,2098.528409
6,2,3678.500000
6,2,2107.134146
6,2,1884.857143
6,2,3307.805921
6,2,3416.773585
6,2,1842.309211
6,2,1697.776596
6,2,3625.795918
6,2,4999.194853
6,2,6188.894737
6,2,5621.079268
6,2,5169.200000
6,2,14061.243421
6,2,1902.108108
6,2,4528.700000
6,2,4270.080000
6,2,4136.543478
6,2,2664.208333
6,2,3271.386905
6,2,1605.743243
6,2,7239.284375
6,2,4465.619048
6,2,3571.062500
6,2,7100.015000
6,2,2325.536585
6,2,3213.159091
6,2,6498.478723
6,2,1695.783019
6,2,2537.344828
6,2,2344.382353
6,2,8413.062500
6,2,2205.698276
6,2,3804.204545
6,2,10161.237500
6,2,2159.820225
6,2,2096.167401
6,2,2887.911765
6,2,2391.525000
6,2,9607.382979
6,2,7498.694805
6,2,2953.406250

6,2,4089.375000
6,2,4977.028846
6,2,2033.214286
6,2,3185.280405
6,2,2569.855263
6,2,2075.583333
6,2,1631.491979
6,2,22401.796117
6,2,2726.570588
6,2,1591.552778
6,2,2260.375000
6,2,2102.659091
6,2,9076.625000
6,2,4114.196429
6,2,5801.732143
6,2,3425.239130
6,2,3595.018519
6,2,3149.702128
6,2,2098.195313
6,2,3869.945455
6,2,5199.585938
6,2,2006.580556
6,2,2023.017045
6,2,1814.625000
6,2,8589.250000
6,2,1690.009868
6,2,1794.031977
6,2,1572.282895
6,2,2723.487179
6,2,1721.198529
6,2,3022.821429
6,2,2820.163793
6,2,1534.489362
6,2,3700.150000
6,2,3228.978723
6,2,2817.702381
6,2,1934.368421
6,2,2425.101064
6,2,7019.348837
6,2,2475.988095
6,2,4065.602410
6,2,4478.787500
6,2,2614.181818
6,2,1721.893617
6,2,1602.204545
6,2,1890.559659
6,2,1638.289474
6,2,2909.388889
6,2,2827.565217
6,2,3596.686321
6,2,4387.220455
6,2,1604.315789
6,2,3513.680556
6,2,2238.789773
6,2,5772.714286
6,2,1995.342105
6,2,3919.789634
6,2,3367.319712
6,2,7053.231383
6,2,6558.648649
6,2,5056.148148
6,2,2451.321809
6,2,1726.108974
6,2,1957.730000
6,2,2884.040541
6,2,5202.668919

6,2,1710.750000
6,2,2468.057895
6,2,3601.838816
6,2,2627.621429
6,2,3076.442308
6,2,1617.872093
6,2,2425.502778
6,2,2495.541667
6,2,3562.864865
6,2,1951.378472
6,2,3194.804054
6,2,7090.962500
6,2,5263.585938
6,2,1832.840909
6,2,2776.875000
6,2,3006.957447
6,2,6315.872340
6,2,4819.173295
6,2,2420.028571
6,2,1509.062500
6,2,1682.671429
6,2,12074.178571
6,2,3585.292453
6,2,11443.584184
6,2,3183.321429
6,2,8364.511364
6,2,1550.270408
6,2,5902.833333
6,2,3777.603352
6,2,1506.019737
6,2,2634.056250
6,2,3128.348039
6,2,3329.600000
6,2,2924.372549
6,2,3020.531250
6,2,5420.700000
6,2,1755.472973
6,2,3932.798077
6,2,2754.253205
6,2,2164.125000
6,2,1529.538462
6,2,3102.150000
6,2,3774.720745
6,2,4047.483871
6,2,5402.679144
6,2,1683.368182
6,2,1617.567073
6,2,26644.500000
6,2,2527.221698
6,2,10153.787500
6,2,2573.608553
6,2,4348.479167
6,2,2413.500000
6,2,1783.684659
6,2,4062.500000
6,2,1626.760135
6,2,2191.448171
6,2,1517.502907
6,2,7136.487179
6,2,2550.785714
6,2,32866.349432
6,2,7812.810056
6,2,3226.513514
6,2,3438.500000
6,2,2677.302083
6,2,4253.270270

6,2,2117.771739
6,2,2736.171053
6,2,1884.080000
6,2,2551.987179
6,2,1842.164773
6,2,7942.120000
6,2,2735.714286
6,2,3538.682065
6,2,3165.346939
6,2,3025.631696
6,2,7685.614130
6,2,3422.843023
6,2,177181.134286
6,2,8181.288462
6,2,3213.847222
6,2,6608.661364
6,2,2804.418919
6,2,3214.672297
6,2,2158.628472
6,2,4405.111111
6,2,5492.854651
6,2,4140.166667
6,2,6314.392045
6,2,4285.588816
6,2,1976.878049
6,2,1516.461538
6,2,1510.795455
6,2,2952.913043
6,2,2456.070707
6,2,2304.247525
6,2,6894.787129
6,2,4030.027778
6,2,2481.959091
6,2,2242.511364
6,2,12470.864780
6,2,5298.748889
6,2,6751.483516
6,2,1812.007353
6,2,1521.548913
6,2,7947.627604
6,2,3629.210227
6,2,2178.436275
6,2,2692.133333
6,2,3041.776786
6,2,10974.387097
6,2,1734.710366
6,2,1713.329268
6,2,7790.271739
6,2,2801.330357
6,2,2180.520270
6,2,3593.622222
6,2,13367.508696
6,2,1565.421053
6,2,7112.104938
6,2,1737.179487
6,2,1524.033333
6,2,5457.170732
6,2,1762.730769
6,2,4772.906250
6,2,2306.953804
6,2,3379.760000
6,2,1844.255682
6,2,23762.250000
6,2,1811.748603
6,2,2547.105263
6,2,8754.331522

6,2,7272.975000
6,2,2450.494898
6,2,9992.678571
6,2,5536.647222
6,2,6387.500000
6,2,2320.979532
6,2,2535.854167
6,2,2315.423077
6,2,2895.613636
6,2,2320.548913
6,2,3779.218750
6,2,2948.744076
6,2,5249.817568
6,2,2576.200000
6,2,2566.325581
6,2,14207.693878
6,2,1572.263587
6,2,8385.236842
6,2,13361.264151
6,2,2344.857143
6,2,3412.648936
6,2,3866.241477
6,2,6814.828125
6,2,4730.774457
6,2,4380.272727
6,2,1772.304878
6,2,2037.517361
6,2,4524.976064
6,2,2133.125000
6,2,3430.333333
6,2,1524.678571
6,2,2578.304878
6,2,2656.075000
6,2,2072.000000
6,2,15244.309406
6,2,5424.840909
6,2,6695.101351
6,2,1837.680851
6,2,1772.800000
6,2,4239.866667
6,2,3477.008929
6,2,1524.404255
6,2,2831.721311
6,2,1510.635638
6,2,5125.648256
6,2,5549.298387
6,2,4802.210884
6,2,1769.977941
6,2,6792.850000
6,2,5115.183908
6,2,1944.758523
6,2,4511.817568
6,2,8868.022727
6,2,2412.163462
6,2,1951.211538
6,2,2564.411111
6,2,4677.115385
6,2,2894.055851
6,2,2739.274390
6,2,3695.235795
6,2,3837.455556
6,2,1597.631579
6,2,4925.614865
6,2,1890.118421
6,2,1722.418981
6,2,7653.259615

6,2,4292.312500
6,2,9945.607143
6,2,1645.068182
6,2,3871.871324
6,2,11434.823529
6,2,2865.201117
6,2,8240.634615
6,2,2360.733766
6,2,3307.197674
6,2,2549.740506
6,2,1771.054054
6,2,5487.319149
6,2,3065.987805
6,2,1756.809524
6,2,2443.593750
6,2,2152.166667
6,2,6411.491071
6,2,3852.279255
6,2,9443.794872
6,2,1638.133333
6,2,2328.981250
6,2,2231.192568
6,2,1957.400602
6,2,3106.086538
6,2,2290.179688
6,2,2018.195531
6,2,12734.221311
6,2,5871.212766
6,2,3249.369186
6,2,3466.137500
6,2,2253.750000
6,2,2642.201087
6,2,7317.851852
6,2,2818.347826
6,2,2910.481250
6,2,1772.298780
6,2,7019.959677
6,2,2124.625000
6,2,1911.638158
6,2,2592.135638
6,2,3567.338235
6,2,3419.338235
6,2,1556.837838
6,2,2302.600000
6,2,18195.734756
6,2,2728.364706
6,2,3114.187500
6,2,4213.244898
6,2,1697.000000
6,2,2152.908163
6,2,2497.360000
6,2,3315.943182
6,2,2511.632353
6,2,2136.607143
6,2,4653.589674
6,2,2196.555556
6,2,2626.097765
6,2,4886.778443
6,2,8194.578804
6,2,2420.236842
6,2,2637.926829
6,2,4691.513158
6,2,4279.602778
6,2,2037.022222
6,2,2566.369318
6,2,5343.678571

6,2,2956.339744
6,2,2160.375000
6,2,1802.395833
6,2,12735.375000
6,2,4724.335526
6,2,1743.132812
6,2,2315.346154
6,2,2376.211735
6,2,3826.798913
6,2,5696.416667
6,2,8388.571875
6,2,1568.994186
6,2,12056.689286
6,2,1967.900000
6,2,4246.125000
6,2,1971.529762
6,2,2372.526042
6,2,2181.784091
6,2,2842.717391
6,2,6277.960106
6,2,4483.426471
6,2,4426.781250
6,2,3039.097826
6,2,8112.145833
6,2,3526.702381
6,2,3285.695652
6,2,2050.795918
6,2,4002.500000
6,2,9921.508929
6,2,3088.680556
6,2,1697.300948
6,2,20398.648649
6,2,5398.761905
6,2,1645.687500
6,2,2449.609375
6,2,3577.840000
6,2,1978.189103
6,2,2574.935484
6,2,1659.700000
6,2,2160.591195
6,2,12801.698718
6,2,3268.875000
6,2,4416.375000
6,2,4072.951220
6,2,1997.070122
6,2,3113.767500
6,2,3322.231818
6,2,6085.972727
6,2,7154.675000
6,2,1651.576687
6,2,2179.437500
6,2,2675.711538
6,2,4812.163043
6,2,9593.221354
6,2,2920.696429
6,2,3995.686047
6,2,1733.708333
6,2,1622.380000
6,2,9050.033333
6,2,2701.564024
6,2,2375.055556
6,2,5333.539634
6,2,7298.500000
6,2,5325.361979
6,2,4329.040541
6,2,19208.552966

6,2,6310.000000
6,2,3921.112245
6,2,1992.577500
6,2,1895.500000
6,2,11301.503067
6,2,1994.500000
6,2,2494.670732
6,2,2051.304348
6,2,7907.468750
6,2,2189.916667
6,2,2751.288194
6,2,13281.380597
6,2,4557.219512
6,2,1953.127907
6,2,2562.800000
6,2,3950.767857
6,2,2770.285000
6,2,3567.813830
6,2,2392.643750
6,2,1814.346154
6,2,3245.830357
6,2,2000.875000
6,2,1878.682119
6,2,6485.227273
6,2,34297.625000
6,2,1810.726190
6,2,1752.888158
6,2,2252.513514
6,2,1752.427632
6,2,2505.105263
6,2,2078.931818
6,2,3456.720238
6,2,8223.176136
6,2,3659.083333
6,2,12413.478774
6,2,2703.687500
6,2,1968.293478
6,2,1897.605769
6,2,13301.648936
6,2,1716.625000
6,2,3248.114796
6,2,2077.671512
6,2,1564.750000
6,2,4410.980769
6,2,2891.500000
6,2,1643.141892
6,2,2999.440217
6,2,3072.617318
6,2,3734.156250
6,2,1880.951087
6,2,3339.225000
6,2,1632.465909
6,2,2186.421053
6,2,3095.698020
6,2,1842.477528
6,2,2432.905556
6,2,7493.794811
6,2,5786.656863
6,2,3333.157609
6,2,4275.535294
6,2,4480.482558
6,2,3083.492188
6,2,2076.476190
6,2,3210.041667
6,2,3760.770833
6,2,4900.742515

6,2,2458.150685
6,2,2453.782051
6,2,1783.763158
6,2,3608.733108
6,2,1773.582500
6,2,1642.278846
6,2,4492.565789
6,2,3101.658537
6,2,2000.484375
6,2,8571.437500
6,2,5009.095890
6,2,1987.443182
6,2,3874.895480
6,2,8023.390625
6,2,2623.280105
6,2,6148.464286
6,2,1667.750000
6,2,5230.510000
6,2,1993.803977
6,2,3165.393293
6,2,5486.140625
6,2,1924.755682
6,2,7872.648936
6,2,5385.597222
6,2,2728.159236
6,2,2231.206250
6,2,2672.646875
6,2,2006.385965
6,2,2476.757576
6,2,3504.420455
6,2,2226.500000
6,2,6758.994792
6,2,2172.337079
6,2,5671.919786
6,2,3544.601485
6,2,3494.317500
6,2,2898.377778
6,2,3373.000000
6,2,3935.602041
6,2,1892.898438
6,2,4108.240385
6,2,1563.561047
6,2,3464.710938
6,2,3940.407143
6,2,6329.687500
6,2,6431.612805
6,2,1934.174419
6,2,1766.037791
6,2,2138.087302
6,2,3833.230769
6,2,7611.585366
6,2,2603.047619
6,2,1637.431373
6,2,2144.395349
6,2,5888.250000
6,2,3878.421053
6,2,4617.500000
6,2,1969.846939
6,2,2202.689024
6,2,2129.476351
6,2,1621.130208
6,2,1987.513514
6,2,1511.505263
6,2,3134.168421
6,2,5588.963542
6,2,3815.760000

6,2,3112.545455
6,2,3329.227564
6,2,3344.361111
6,2,4026.601351
6,2,2682.597500
6,2,2005.304878
6,2,2016.155000
6,2,1744.812500
6,2,2325.675676
6,2,1973.732143
6,2,1665.924479
6,2,2475.262821
6,2,6097.913043
6,2,3918.682927
6,2,1660.676829
6,2,3932.182927
6,2,6297.303125
6,2,6931.441667
6,2,1779.000000
6,2,2402.104167
6,2,4593.506944
6,2,1636.846875
6,2,2046.644737
6,2,2316.500000
6,2,2643.300781
6,2,2078.096939
6,2,7628.850000
6,2,2148.868902
6,2,4204.675676
6,2,5524.504717
6,2,2078.122845
6,2,1990.695455
6,2,7653.839286
6,2,2509.642857
6,2,12127.000000
6,2,8443.443396
6,2,2887.551282
6,2,5667.671512
6,2,2184.191489
6,2,2477.029557
6,2,2623.051829
6,2,3476.072500
6,2,5097.633333
6,2,1540.731250
6,2,3733.302326
6,2,2477.130000
6,2,4375.061538
6,2,2518.300000
6,2,3705.063953
6,2,1879.625000
6,2,3840.513158
6,2,2218.877778
6,2,4183.979167
6,2,2313.580645
6,2,7597.250000
6,2,1971.870968
6,2,1563.719178
6,2,4216.943878
6,2,9603.648649
6,2,5317.553571
6,2,2440.425000
6,2,4327.702128
6,2,4638.928191
6,2,2866.418367
6,2,2732.899441
6,2,2796.139037

6,2,2194.744681
6,2,2267.302139
6,2,2498.670732
6,2,1917.541860
6,2,5535.743902
6,2,6595.442500
6,2,3215.439024
6,2,2424.107639
6,2,5049.654440
6,2,1773.312500
6,2,3300.494186
6,2,4594.014151
6,2,3916.488636
6,2,1916.847059
6,2,1814.821429
6,2,4596.806452
6,2,16584.361111
6,2,2259.192857
6,2,2445.433140
6,2,1693.861842
6,2,2793.462500
6,2,2488.941176
6,2,2984.750000
6,2,2954.024390
6,2,5025.081250
6,2,9389.375000
6,2,4010.816860
6,2,4123.041667
6,2,6528.435484
6,2,2950.121622
6,2,2498.888889
6,2,3681.047619
6,2,2273.937500
6,2,1904.796875
6,2,4509.383721
6,2,1642.209375
6,2,4987.821429
6,2,1609.595930
6,2,4429.273438
6,2,2051.048077
6,2,1961.255102
6,2,1697.798780
6,2,4916.010000
6,2,5003.904891
6,2,2620.604167
6,2,1844.585366
6,2,2123.202454
6,2,3052.806604
6,2,2269.143750
6,2,3724.451705
6,2,2446.500000
6,2,4278.096591
6,2,2017.134146
6,2,4871.814286
6,2,2740.186047
6,2,2138.145349
6,2,4840.371951
6,2,3289.892857
6,2,1683.891304
6,2,3904.473558
6,2,69835.721429
6,2,6548.371795
6,2,6993.931818
6,2,6421.781977
6,2,5455.027027
6,2,2323.831633

6,2,2766.229167
6,2,1833.976190
6,2,11113.136364
6,2,1529.105263
6,2,4856.616071
6,2,2065.441489
6,2,2151.700000
6,2,4484.062500
6,2,3633.312500
6,2,5367.653846
6,2,1511.073864
6,2,3051.091216
6,2,1989.738372
6,2,5349.977941
6,2,2015.679487
6,2,2004.331250
6,2,2612.209375
6,2,1641.924419
6,2,1651.896226
6,2,1720.707547
6,2,5042.023256
6,2,6217.417553
6,2,3228.862069
6,2,4224.815789
6,2,2719.228571
6,2,11687.575758
6,2,1834.547297
6,2,1823.000000
6,2,4008.061798
6,2,1635.640244
6,2,2333.996528
6,2,1790.217391
6,2,2363.663462
6,2,1924.934524
6,2,7952.087500
6,2,3018.362500
6,2,12918.196262
6,2,3736.123457
6,2,4102.666667
6,2,2069.961735
6,2,2156.250000
6,2,2816.256579
6,2,14298.875000
6,2,2412.350000
6,2,2036.735714
6,2,12264.741206
6,2,3440.596154
6,2,3595.212264
6,2,1574.241667
6,2,5746.985849
6,2,3455.692308
6,2,2323.067308
6,2,3878.990385
6,2,9161.526042
6,2,5298.300481
6,2,2432.037500
6,2,2785.071429
6,2,7393.307692
6,2,2115.421875
6,2,3972.235294
6,2,6099.949324
6,2,11822.557377
6,2,4245.090909
6,2,3041.646341
6,2,1912.705128
6,2,1690.619048

6,2,1792.524390
6,2,2031.806818
6,2,3701.500000
6,2,2634.216667
6,2,3037.835227
6,2,1545.792453
6,2,2127.560976
6,2,1719.596154
6,2,1925.519022
6,2,2256.000000
6,2,7269.362069
6,2,2462.009868
6,2,7513.000000
6,2,15240.946023
6,2,1796.207831
6,2,2122.500000
6,2,12933.925466
6,2,3643.148438
6,2,2080.179104
6,2,4060.532051
6,2,2790.283784
6,2,1687.218750
6,2,5672.765957
6,2,6021.184322
6,2,1733.125000
6,2,1755.300000
6,2,3760.544271
6,2,2335.718750
6,2,6553.580882
6,2,2921.968750
6,2,10046.976562
6,2,4882.866667
6,2,6276.307692
6,2,3429.686111
6,2,1539.493865
6,2,1903.070513
6,2,2396.386740
6,2,2588.275000
6,2,1550.408163
6,2,1904.521875
6,2,3574.666667
6,2,2493.404255
6,2,1521.231707
6,2,5443.459184
6,2,4678.358974
6,2,3389.442308
6,2,4346.732143
6,2,6871.678879
6,2,4697.884804
6,2,8191.625000
6,2,5127.037500
6,2,1753.010135
6,2,8754.323529
6,2,1829.890625
6,2,2403.837500
6,2,3008.737500
6,2,3425.875000
6,2,5168.500000
6,2,2206.471591
6,2,6051.683333
6,2,4096.336538
6,2,5784.435897
6,2,3234.412234
6,2,14650.644444
6,2,1976.178571
6,2,1690.511364

6,2,4825.469828
6,2,1899.982955
6,2,1921.125000
6,2,3284.926829
6,2,3773.531250
6,2,3180.970109
6,2,5527.468750
6,2,1909.727273
6,2,2573.184211
6,2,1543.406250
6,2,16482.384615
6,2,3506.213235
6,2,5503.458333
6,2,3276.098214
6,2,1742.154412
6,2,4713.593407
6,2,4747.329545
6,2,7755.284314
6,2,7644.122093
6,2,12699.263889
6,2,6878.883721
6,2,2712.367347
6,2,1878.357143
6,2,2229.642442
6,2,5315.573370
6,2,1504.047486
6,2,9534.505814
6,2,4691.308511
6,2,2416.125000
6,2,6506.125000
6,2,5993.093023
6,2,3548.970238
6,2,3865.000000
6,2,2535.786885
6,2,2237.705882
6,2,2350.750000
6,2,1749.086957
6,2,3826.081633
6,2,4538.437500
6,2,3391.018293
6,2,4956.465000
6,2,4397.597826
6,2,4973.535714
6,2,3377.906250
6,2,1752.625000
6,2,5984.596154
6,2,1539.432292
6,2,10645.543367
6,2,2795.894231
6,2,6424.240000
6,2,1753.982558
6,2,3514.890244
6,2,2794.422222
6,2,20540.003472
6,2,4228.181818
6,2,2304.246711
6,2,4870.700000
6,2,3000.282895
6,2,2389.921053
6,2,3214.000000
6,2,4832.666667
6,2,2076.842262
6,2,1808.859375
6,2,7469.666667
6,2,3540.693182
6,2,5748.000000

6,2,2286.394022
6,2,3251.183333
6,2,2389.765625
6,2,5510.117188
6,2,3459.799020
6,2,2384.298780
6,2,2749.256579
6,2,5121.422872
6,2,6867.517045
6,2,3993.953125
6,2,6968.176887
6,2,2801.500000
6,2,1976.723404
6,2,8297.776536
6,2,2121.114796
6,2,1703.004630
6,2,2734.035000
6,2,1585.805556
6,2,1622.442073
6,2,1992.425595
6,2,2688.741848
6,2,6698.389535
6,2,1890.839286
6,2,2693.652174
6,2,1656.370098
6,2,11948.642857
6,2,3765.329545
6,2,4083.677632
6,2,3247.837500
6,2,4688.811047
6,2,1629.273936
6,2,2505.344828
6,2,2161.411765
6,2,5732.752841
6,2,2398.927885
6,2,12576.878049
6,2,4938.416667
6,2,1542.707500
6,2,2188.082317
6,2,6434.731844
6,2,2298.751479
6,2,2247.523810
6,2,3472.656915
6,2,1876.541872
6,2,2273.214286
6,2,2005.444444
6,2,6416.930233
6,2,2471.226744
6,2,4720.568182
6,2,6841.781818
6,2,4192.532895
6,2,4145.416667
6,2,4914.397727
6,2,2272.021739
6,2,1733.323529
6,2,2270.326087
6,2,1635.151163
6,2,2637.454545
6,2,2188.500000
6,2,2983.076087
6,2,1578.209239
6,2,2400.977273
6,2,1895.166667
6,2,9284.254464
6,2,2695.000000
6,2,2722.486911

6,2,4077.357955
6,2,1533.658163
6,2,5072.750000
6,2,2080.528125
6,2,2731.053571
6,2,4245.690000
6,2,2306.526786
6,2,2142.058140
6,2,7053.450000
6,2,2165.682292
6,2,11336.322917
6,2,1658.252841
6,2,2652.308333
6,2,1646.045455
6,2,7623.704545
6,2,2123.093750
6,2,1780.738636
6,2,9052.090909
6,2,14263.775000
6,2,2775.708333
6,2,2166.728261
6,2,8521.750000
6,2,2915.153846
6,2,1953.375000
6,2,6517.477500
6,2,1635.602778
6,2,3411.907407
6,2,9194.513889
6,2,2160.581522
6,2,2482.863372
6,2,5013.055556
6,2,7581.293750
6,2,2698.473684
6,2,5244.256757
6,2,12437.330189
6,2,1780.630952
6,2,2673.630682
6,2,2555.034091
6,2,5450.190909
6,2,3176.562500
6,2,2842.325000
6,2,6282.189474
6,2,4015.415865
6,2,2494.832335
6,2,7293.630137
6,2,2477.784314
6,2,1550.575581
6,2,2468.720238
6,2,1944.256579
6,2,3461.200581
6,2,5865.500000
6,2,2066.993976
6,2,2239.943925
6,2,2707.687500
6,2,2999.212500
6,2,2160.473214
6,2,4334.170732
6,2,1755.489362
6,2,2242.651163
6,2,1823.670455
6,2,4223.012821
6,2,3665.641304
6,2,2296.380952
6,2,1680.471774
6,2,2279.007812
6,2,2101.131098

6,2,1674.432432
6,2,2368.632979
6,2,1651.620253
6,2,2883.948276
6,2,2014.984043
6,2,3990.547297
6,2,1646.225962
6,2,2646.064103
6,2,3484.436224
6,2,1661.909091
6,2,13602.654255
6,2,7158.750000
6,2,3168.750000
6,2,3068.296875
6,2,2690.882979
6,2,9432.127500
6,2,2045.546053
6,2,1683.671875
6,2,3692.665094
6,2,4229.747396
6,2,1641.713068
6,2,1902.692857
6,2,3004.365385
6,2,1667.782738
6,2,1573.332237
6,2,2265.739362
6,2,2106.297546
6,2,1839.354167
6,2,3992.046196
6,2,1764.887500
6,2,1897.698171
6,2,3075.010417
6,2,3850.695122
6,2,9250.946429
6,2,7870.702128
6,2,2111.767442
6,2,4253.307692
6,2,7306.562500
6,2,12249.737805
6,2,2952.048077
6,2,5172.888636
6,2,2345.281250
6,2,6857.913043
6,2,2526.643258
6,2,2404.333333
6,2,2139.289474
6,2,3206.812500
6,2,2886.324324
6,2,4983.742647
6,2,4217.017045
6,2,2829.134146
6,2,3646.227273
6,2,9750.183673
6,2,10299.473684
6,2,8422.000000
6,2,3316.528409
6,2,9943.722222
6,2,1617.887931
6,2,1771.229730
6,2,4384.607914
6,2,2426.405488
6,2,5673.617021
6,2,3213.617647
6,2,1994.252604
6,2,1917.888298
6,2,5418.894737

6,2,2896.111111
6,2,1567.769231
6,2,3331.218750
6,2,3332.250000
6,2,7291.402542
6,2,1511.777778
6,2,4026.497549
6,2,10064.358491
6,2,2785.832447
6,2,2855.129464
6,2,7169.592391
6,2,2080.208333
6,2,1542.479730
6,2,4115.309524
6,2,3222.505263
6,2,6861.250000
6,2,2678.604651
6,2,6513.735294
6,2,1814.200000
6,2,2208.778409
6,2,3358.512821
6,2,3734.326923
6,2,6009.400000
6,2,4648.670673
6,2,2255.710227
6,2,5859.627907
6,2,2029.011628
6,2,2093.000000
6,2,4992.483696
6,2,4987.220588
6,2,2263.133333
6,2,11924.010526
6,2,4936.555556
6,2,1780.785714
6,2,1596.230769
6,2,1703.250000
6,2,6571.015625
6,2,4250.208333
6,2,4013.974432
6,2,2596.092105
6,2,2639.113208
6,2,4931.632812
6,2,2166.461538
6,2,4586.357143
6,2,5211.857143
6,2,1700.048077
6,2,1633.220588
6,2,4269.967391
6,2,1731.952273
6,2,4293.384615
6,2,15081.384615
6,2,4802.224390
6,2,2066.190217
6,2,1656.388889
6,2,5237.055556
6,2,1759.601351
6,2,1869.597222
6,2,2018.000000
6,2,4900.175000
6,2,2361.266304
6,2,5565.812500
6,2,3946.900000
6,2,3886.723837
6,2,4757.994898
6,2,3358.013158
6,2,3704.531250

6,2,2388.104651
6,2,1672.307692
6,2,3892.476974
6,2,1852.384615
6,2,1998.684783
6,2,1718.641026
6,2,2219.767857
6,2,2540.107143
6,2,2748.823810
6,2,1584.678571
6,2,2689.160256
6,2,1660.768868
6,2,2458.002778
6,2,3607.500000
6,2,2724.942177
6,2,2214.452500
6,2,2909.366279
6,2,1520.824324
6,2,7191.233333
6,2,1616.929936
6,2,13884.819149
6,2,3529.023256
6,2,4701.000000
6,2,4554.478659
6,2,9948.242925
6,2,1651.842105
6,2,13929.890957
6,2,2270.658019
6,2,4776.245098
6,2,1718.826087
6,2,4605.383929
6,2,2539.532609
6,2,1544.709459
6,2,5694.818182
6,2,2928.021739
6,2,3849.250000
6,2,2471.009868
6,2,1508.590426
6,2,2251.045455
6,2,4124.553191
6,2,12901.880208
6,2,1575.125000
6,2,3653.100000
6,2,6648.750000
6,2,1692.239264
6,2,3662.222222
6,2,2264.305851
6,2,6976.363636
6,2,1724.318452
6,2,1622.142857
6,2,14334.902439
6,2,3159.024457
6,2,3133.137931
6,2,4608.459906
6,2,2678.837209
6,2,4131.235119
6,2,1643.828571
6,2,2576.089286
6,2,7180.490625
6,2,2529.625000
6,2,7615.153846
6,2,2337.257485
6,2,2110.976064
6,2,1650.571429
6,2,4443.523305
6,2,3685.500000

6,2,2062.555851
6,2,3171.040323
6,2,5158.304688
6,2,2291.833333
6,2,1742.844444
6,2,1709.794286
6,2,1695.723837
6,2,18996.337500
6,2,3537.715909
6,2,4713.375839
6,2,3551.472561
6,2,2561.150000
6,2,7749.562500
6,2,8098.455128
6,2,2024.458333
6,2,2194.838068
6,2,6818.087500
6,2,2887.500000
6,2,3116.420455
6,2,1734.344444
6,2,1510.795455
6,2,3731.215909
6,2,2697.850877
6,2,2954.470109
6,2,3977.886364
6,2,1538.338710
6,2,5268.168269
6,2,2315.520833
6,2,2730.430233
6,2,6149.714286
6,2,3654.161290
6,2,1976.907895
6,2,1854.200000
6,2,1610.115385
6,2,2006.583333
6,2,2295.575000
6,2,2338.278302
6,2,6127.782609
6,2,2804.312500
6,2,5389.315789
6,2,5975.640704
6,2,2807.731250
6,2,1779.762570
6,2,16006.366667
6,2,1926.301630
6,2,3091.060096
6,2,2293.400000
6,2,14296.141667
6,2,1987.601064
6,2,1614.714286
6,2,4393.821429
6,2,2504.728643
6,2,2956.165094
6,2,2334.537500
6,2,14120.078740
6,2,4452.698324
6,2,9407.650510
6,2,5362.420455
6,2,2267.089109
6,2,10294.944444
6,2,4002.425000
6,2,3394.524390
6,2,1686.215232
6,2,5419.513514
6,2,14821.022727
6,2,5143.275000

6,2,8101.480000
6,2,1661.054054
6,2,2168.148936
6,2,2104.236486
6,2,1703.758929
6,2,2163.668269
6,2,4160.411111
6,2,3128.096939
6,2,2325.766667
6,2,1710.061798
6,2,2024.280488
6,2,2676.750000
6,2,6309.004098
6,2,1596.578616
6,2,4372.483696
6,2,4364.500000
6,2,15915.518868
6,2,1789.971591
6,2,3577.505051
6,2,4711.912791
6,2,1742.682432
6,2,4347.928571
6,2,20775.871429
6,2,1734.471698
6,2,4522.642857
6,2,4386.075000
6,2,4050.666667
6,2,3103.161458
6,2,2304.359375
6,2,2480.766010
6,2,2951.890957
6,2,1998.204545
6,2,8188.090909
6,2,3217.861111
6,2,16850.928879
6,2,3565.089286
6,2,2265.020833
6,2,3400.560000
6,2,4003.208333
6,2,3069.641026
6,2,2772.000000
6,2,1840.430556
6,2,2588.521739
6,2,3622.630435
6,2,1996.384615
6,2,2465.284091
6,2,4308.630435
6,2,2777.651852
6,2,1525.861702
6,2,14717.942308
6,2,14800.762255
6,2,2444.134146
6,2,1967.948370
6,2,1654.511628
6,2,1857.046512
6,2,2094.574468
6,2,1626.197727
6,2,7737.397338
6,2,18292.285714
6,2,3244.160000
6,2,3808.403141
6,2,2666.961957
6,2,3924.472222
6,2,4461.934783
6,2,2382.809028
6,2,4310.964744

6,2,7439.285714
6,2,7782.458333
6,2,1631.777778
6,2,6927.945876
6,2,1689.174419
6,2,2408.214286
6,2,1918.839286
6,2,2384.968750
6,2,11159.652027
6,2,6274.156250
6,2,5133.843750
6,2,3637.828571
6,2,2391.343750
6,2,8316.750000
6,2,2000.164557
6,2,4399.070312
6,2,2641.489796
6,2,15553.347826
6,2,1883.344444
6,2,3153.645349
6,2,1857.556604
6,2,4451.375000
6,2,1880.729839
6,2,3771.155488
6,2,5651.298507
6,2,1750.416667
6,2,2892.684783
6,2,2606.107843
6,2,3539.310606
6,2,2285.398649
6,2,5438.050439
6,2,4205.648936
6,2,1687.209581
6,2,2302.043750
6,2,3326.472222
6,2,2270.953125
6,2,7294.920455
6,2,7343.112903
6,2,2273.583333
6,2,17660.392857
6,2,3334.890625
6,2,2490.750000
6,2,2125.403846
6,2,1510.438889
6,2,21383.089286
6,2,5575.959239
6,2,11242.812500
6,2,2887.801282
6,2,1797.649194
6,2,1656.494792
6,2,6321.567708
6,2,4582.052632
6,2,2238.330189
6,2,3722.860795
6,2,1735.750000
6,2,1806.131696
6,2,1821.435714
6,2,2138.428571
6,2,2229.821429
6,2,5981.850000
6,2,7717.605263
6,2,2468.043478
6,2,4259.937500
6,2,5342.566964
6,2,3348.000000
6,2,4106.081731

6,2,4609.192308
6,2,4728.355114
6,2,1592.397661
6,2,4668.011628
6,2,6032.084906
6,2,2297.321429
6,2,2895.800000
6,2,3258.531250
6,2,5811.979730
6,2,1986.662088
6,2,3084.500000
6,2,6292.404891
6,2,2430.334459
6,2,6056.162562
6,2,5436.196721
6,2,2087.492147
6,2,1589.054878
6,2,2315.506944
6,2,1904.210526
6,2,3591.013966
6,2,1728.842105
6,2,1558.829268
6,2,6173.540000
6,2,6418.319767
6,2,9298.840116
6,2,3281.819915
6,2,2598.115854
6,2,3710.744792
6,2,7471.086538
6,2,4228.625000
6,2,2989.392857
6,2,6627.343023
6,2,1503.519231
6,2,2977.564516
6,2,7526.639205
6,2,3694.051282
6,2,4963.098639
6,2,1983.070513
6,2,2037.040000
6,2,2175.900552
6,2,2767.525641
6,2,3044.038217
6,2,2085.899329
6,2,13350.538462
6,2,2607.847500
6,2,1876.638298
6,2,5156.673913
6,2,3897.687500
6,2,4655.900000
6,2,2978.541667
6,2,20104.262712
6,2,2626.545455
6,2,2435.857143
6,2,3953.458333
6,2,3947.956284
6,2,3642.754808
6,2,2011.902542
6,2,4424.225962
6,2,4171.225000
6,2,38559.895833
6,2,2288.126316
6,2,3834.288660
6,2,2429.417553
6,2,10250.437500
6,2,1874.845395
6,2,10053.259259

6,2,31152.709877
6,2,2496.569444
6,2,2569.073171
6,2,2240.185714
6,2,4239.555556
6,2,1507.882812
6,2,1688.661765
6,2,1855.461538
6,2,4866.671875
6,2,5137.601744
6,2,3560.816327
6,2,2227.734043
6,2,2719.858333
6,2,2758.985795
6,2,5395.527094
6,2,2071.408537
6,2,2967.370690
6,2,7758.920000
6,2,1816.474490
6,2,1801.266892
6,2,5051.673913
6,2,6537.649038
6,2,4434.441860
6,2,4970.523810
6,2,1578.375000
6,2,4628.377551
6,2,3349.414894
6,2,2872.083333
6,2,3822.397059
6,2,1547.875000
6,2,1527.858434
6,2,2787.983553
6,2,3629.427885
6,2,4435.259615
6,2,3399.585903
6,2,1754.718023
6,2,3524.134615
6,2,5829.746875
6,2,1893.768293
6,2,1896.966667
6,2,2589.827815
6,2,2735.064103
6,2,2600.428571
6,2,3977.067308
6,2,1620.828947
6,2,2265.938830
6,2,7823.645833
6,2,1559.012500
6,2,1892.848684
6,2,4413.522222
6,2,4372.425000
6,2,1775.775862
6,2,9300.027778
6,2,5869.097561
6,2,2216.818182
6,2,2934.400000
6,2,2774.098837
6,2,1702.768293
6,2,2466.194444
6,2,2140.127551
6,2,4915.305328
6,2,3880.400000
6,2,1817.910714
6,2,2009.878205
6,2,1811.907895
6,2,2063.281250

6,2,1549.914634
6,2,1683.597826
6,2,8717.027778
6,2,5611.101974
6,2,2071.736842
6,2,2824.500000
6,2,2236.777439
6,2,4598.802632
6,2,2157.860294
6,2,3990.680851
6,2,1884.350000
6,2,2024.761574
6,2,5935.750000
6,2,1507.369565
6,2,2998.384615
6,2,4712.864130
6,2,5012.165761
6,2,2425.625000
6,2,2866.320000
6,2,1921.686275
6,2,4338.360000
6,2,3819.323171
6,2,2245.088889
6,2,2029.450000
6,2,6412.397059
6,2,4219.363636
6,2,1777.931373
6,2,4564.298295
6,2,2164.753012
6,2,8243.534091
6,2,1570.625000
6,2,1663.481250
6,2,2356.756098
6,2,2919.842105
6,2,5280.825000
6,2,2793.559375
6,2,1839.965000
6,2,2203.459302
6,2,31130.884146
6,2,7347.346154
6,2,1612.472603
6,2,1875.053191
6,2,3184.710000
6,2,6572.278646
6,2,1680.658163
6,2,1823.602041
6,2,6412.068452
6,2,1532.421053
6,2,1698.329268
6,2,5833.411290
6,2,3430.013889
6,2,1591.685000
6,2,1630.851974
6,2,7030.727273
6,2,5288.136364
6,2,3376.308673
6,2,2029.857143
6,2,2556.632812
6,2,1588.675676
6,2,4575.015625
6,2,10952.610465
6,2,4186.288945
6,2,4632.812500
6,2,3244.651042
6,2,4766.966667
6,2,1599.410256

6,2,7871.641447
6,2,1744.866667
6,2,2423.324561
6,2,5817.891429
6,2,2531.358639
6,2,6291.630000
6,2,7820.217262
6,2,2122.054348
6,2,1709.004854
6,2,1658.392857
6,2,5854.312500
6,2,2927.890625
6,2,2317.487245
6,2,1944.538043
6,2,3762.296512
6,2,16247.071429
6,2,8798.234375
6,2,7127.115000
6,2,1765.617021
6,2,1956.653061
6,2,3523.594286
6,2,2141.710526
6,2,2627.620000
6,2,2792.240741
6,2,1572.940217
6,2,2003.253049
6,2,3926.046875
6,2,2039.916667
6,2,1632.785714
6,2,2177.204082
6,2,13371.421053
6,2,3569.106618
6,2,1817.234375
6,2,2503.855769
6,2,6023.463415
6,2,11586.539216
6,2,3989.988095
6,2,2374.141304
6,2,5055.125000
6,2,2710.250000
6,2,1530.551471
6,2,1934.973214
6,2,3779.685096
6,2,1991.902174
6,2,2741.107143
6,2,4353.800781
6,2,2582.848837
6,2,2036.101852
6,2,2551.074866
6,2,5978.819079
6,2,7010.858696
6,2,2464.595238
6,2,1672.056373
6,2,1922.723837
6,2,1884.923913
6,2,161580.512755
6,2,1972.895833
6,2,2129.785714
6,2,2481.359043
6,2,3260.548780
6,2,1984.973822
6,2,1974.891626
6,2,2001.139706
6,2,2760.363636
6,2,1606.312500
6,2,4545.705882

6,2,2207.821429
6,2,2243.681250
6,2,3117.625000
6,2,3344.673469
6,2,5708.330882
6,2,1708.200000
6,2,1964.744681
6,2,1717.601744
6,2,1507.825000
6,2,1654.462963
6,2,1696.424699
6,2,3028.358696
6,2,7673.767241
6,2,3580.814815
6,2,3499.716667
6,2,1776.946809
6,2,2550.060976
6,2,23948.238636
6,2,3751.075000
6,2,2521.687500
6,2,4610.162736
6,2,3404.847500
6,2,2785.278846
6,2,3435.493902
6,2,2945.320652
6,2,3809.728365
6,2,8412.526316
6,2,1569.220930
6,2,3249.357143
6,2,5408.400000
6,2,2807.310811
6,2,2706.558824
6,2,3585.773585
6,2,3223.400000
6,2,1852.463068
6,2,2537.480769
6,2,1536.058511
6,2,3816.142857
6,2,1600.418919
6,2,2361.654255
6,2,2746.044444
6,2,2011.066845
6,2,3622.970000
6,2,3275.611429
6,2,2244.362500
6,2,2296.634615
6,2,5962.086310
6,2,2379.847561
6,2,1519.715625
6,2,2809.000000
6,2,3001.250000
6,2,4802.869318
6,2,2135.658537
6,2,1591.177419
6,2,4794.752907
6,2,2277.658537
6,2,1610.637500
6,2,3016.959184
6,2,6692.281250
6,2,2235.496875
6,2,1746.370536
6,2,3719.076923
6,2,6107.046875
6,2,5371.715426
6,2,15723.684211
6,2,3619.958333

6,2,13579.197674
6,2,4246.110465
6,2,3224.900641
6,2,8871.252451
6,2,4171.739583
6,2,2074.756250
6,2,2139.415663
6,2,1894.178082
6,2,3627.744681
6,2,1662.849432
6,2,1793.022346
6,2,11102.850000
6,2,6055.035928
6,2,2514.850000
6,2,6621.575758
6,2,1736.224432
6,2,5242.710366
6,2,1530.454787
6,2,2575.243902
6,2,1712.347222
6,2,2069.055046
6,2,5042.684896
6,2,3751.759615
6,2,2449.267857
6,2,2885.343750
6,2,4266.496429
6,2,6115.546512
6,2,2442.038462
6,2,3586.620000
6,2,1740.330189
6,2,2870.500000
6,2,6220.591463
6,2,2048.250000
6,2,1640.863095
6,2,1720.627907
6,2,2349.535484
6,2,3720.323370
6,2,3650.811224
6,2,7033.217105
6,2,1774.409722
6,2,2154.742188
6,2,9829.301587
6,2,1777.951389
6,2,4967.518692
6,2,5018.696429
6,2,2949.566489
6,2,2937.720238
6,2,2859.337209
6,2,3063.827586
6,2,6031.052632
6,2,6884.408333
6,2,13749.614407
6,2,1552.557692
6,2,3756.061047
6,2,1745.515625
6,2,5673.333333
6,2,3347.785714
6,2,6195.229358
6,2,196635.837004
6,2,5571.895210
6,2,2992.785714
6,2,2412.000000
6,2,6869.665541
6,2,3318.718750
6,2,3136.454545
6,2,2479.671429

6,2,1968.550595
6,2,2813.510000
6,2,2213.445000
6,2,7312.173913
6,2,1854.388158
6,2,2287.103352
6,2,2744.706806
6,2,11496.034091
6,2,2286.491667
6,2,2426.900510
6,2,2202.928571
6,2,5094.608333
6,2,2939.671875
6,2,2404.678571
6,2,1990.812500
6,2,6082.860000
6,2,2540.367021
6,2,5309.614362
6,2,2385.882653
6,2,4942.703125
6,2,2783.005102
6,2,1702.244318
6,2,1901.286164
6,2,3603.994318
6,2,3008.111702
6,2,3266.918919
6,2,4349.372500
6,2,2994.975610
6,2,7122.513158
6,2,15210.500000
6,2,2643.155556
6,2,4112.733333
6,2,5397.791667
6,2,4743.536585
6,2,2854.545455
6,2,2863.411111
6,2,3809.625000
6,2,9581.605128
6,2,4300.176887
6,2,1747.238636
6,2,1988.587500
6,2,5562.639535
6,2,9199.906780
6,2,4120.337500
6,2,20080.260870
6,2,3306.875000
6,2,5549.400000
6,2,2237.954545
6,2,1713.572222
6,2,34972.687500
6,2,2816.443396
6,2,3174.656915
6,2,2606.140000
6,2,5663.329787
6,2,2116.076923
6,2,4908.406250
6,2,4600.801630
6,2,20771.632653
6,2,4612.789474
6,2,2153.419643
6,2,4922.132850
6,2,4102.241722
6,2,5429.750965
6,2,1984.875000
6,2,5564.117647
6,2,1592.236486

6,2,1561.821429
6,2,2003.191176
6,2,13584.333333
6,2,5459.771635
6,2,4528.872093
6,2,1723.089286
6,2,3942.987805
6,2,28381.782407
6,2,1811.812500
6,2,5202.114865
6,2,1695.050000
6,2,2085.082418
6,2,1967.415541
6,2,1666.583333
6,2,5699.607143
6,2,2072.710526
6,2,1721.679245
6,2,5886.880435
6,2,10030.809211
6,2,2791.433824
6,2,1713.382212
6,2,2411.013889
6,2,3271.895833
6,2,3138.696429
6,2,2895.112981
6,2,2324.972222
6,2,1547.913889
6,2,7382.139205
6,2,1886.352941
6,2,2702.468023
6,2,2937.375000
6,2,4181.451220
6,2,3855.991477
6,2,2079.728261
6,2,1702.741379
6,2,2017.657895
6,2,1758.888889
6,2,2338.288889
6,2,4634.397222
6,2,5319.022436
6,2,3120.265000
6,2,1635.988372
6,2,4946.514706
6,2,7771.268750
6,2,6486.717105
6,2,2109.378378
6,2,3907.516304
6,2,3500.279412
6,2,2240.341837
6,2,2854.697674
6,2,2080.769608
6,2,2780.666667
6,2,2482.090426
6,2,7526.531250
6,2,3841.271739
6,2,2270.880952
6,2,4131.207317
6,2,2265.137755
6,2,4366.819672
6,2,4674.733974
6,2,6882.926020
6,2,4815.875000
6,2,8072.710366
6,2,8889.560000
6,2,2057.140244
6,2,3281.711111

6,2,3066.419355
6,2,22706.803571
6,2,2582.349206
6,2,4731.798611
6,2,1595.923077
6,2,2666.514205
6,2,4215.589286
6,2,5663.625000
6,2,5290.333333
6,2,3628.718750
6,2,1533.197368
6,2,12226.659091
6,2,2138.974432
6,2,3564.310241
6,2,8748.305921
6,2,2145.075000
6,2,5297.465116
6,2,5748.811170
6,2,5877.828571
6,2,2922.330189
6,2,2729.244565
6,2,5934.700000
6,2,2071.825000
6,2,1617.186047
6,2,2934.974359
6,2,2087.928571
6,2,1554.557065
6,2,3281.130435
6,2,2069.593023
6,2,2408.000000
6,2,1831.390625
6,2,1890.463415
6,2,5159.191176
6,2,14227.013514
6,2,2303.506250
6,2,15632.775943
6,2,2417.937500
6,2,1676.400000
6,2,4726.616279
6,2,1811.352273
6,2,1618.375000
6,2,5607.638587
6,2,1532.470745
6,2,2274.552500
6,2,1593.603261
6,2,1686.750000
6,2,11382.341040
6,2,3486.886364
6,2,7900.177083
6,2,6050.531915
6,2,2527.883152
6,2,2101.856250
6,2,3138.500000
6,2,1901.978261
6,2,11908.391304
6,2,4393.424419
6,2,2748.295213
6,2,5207.750000
6,2,2452.078947
6,2,2106.969388
6,2,2798.991071
6,2,6297.843750
6,2,2031.606383
6,2,6986.530612
6,2,1717.830357
6,2,7287.156250

6,2,1780.222222
6,2,4566.856818
6,2,7696.648585
6,2,4127.962500
6,2,4242.545918
6,2,2519.261364
6,2,5416.722500
6,2,4355.500000
6,2,13519.313953
6,2,1735.255814
6,2,1855.233173
6,2,3725.632979
6,2,1593.093750
6,2,6117.107143
6,2,2680.804688
6,2,4834.648148
6,2,2509.515625
6,2,2173.806548
6,2,3044.381250
6,2,2568.846154
6,2,6211.975000
6,2,2351.492647
6,2,1625.261628
6,2,4192.000000
6,2,2301.325581
6,2,1930.983696
6,2,2641.720930
6,2,11444.875000
6,2,2730.470745
6,2,2315.170213
6,2,11558.113636
6,2,2304.650000
6,2,2210.760000
6,2,1604.926471
6,2,2995.945312
6,2,1725.117021
6,2,5688.140625
6,2,2162.315385
6,2,2762.717742
6,2,3429.267045
6,2,3266.552885
6,2,3699.353723
6,2,10156.468750
6,2,7534.703125
6,2,3567.288043
6,2,2017.577703
6,2,1799.523936
6,2,2521.033654
6,2,2425.372093
6,2,3636.346821
6,2,2176.565000
6,2,5598.601974
6,2,1554.941176
6,2,7525.214286
6,2,3987.773256
6,2,6578.040000
6,2,7176.500000
6,2,10606.378205
6,2,1769.325000
6,2,2997.032609
6,2,3201.641827
6,2,3430.582589
6,2,3412.300000
6,2,1685.198980
6,2,4579.943182
6,2,4210.452830

6,2,16572.429448
6,2,1615.000000
6,2,2212.527027
6,2,2298.790698
6,2,3064.881944
6,2,6844.986702
6,2,2169.910326
6,2,1659.692623
6,2,6546.470588
6,2,2212.250000
6,2,1592.795918
6,2,2375.177778
6,2,1612.632353
6,2,1705.395349
6,2,2121.166667
6,2,1901.205556
6,2,3345.022727
6,2,1667.171875
6,2,3071.590909
6,2,2155.617647
6,2,3536.118644
6,2,1583.551471
6,2,2869.908854
6,2,8866.000000
6,2,2902.427632
6,2,2239.703125
6,2,6488.021186
6,2,2794.605263
6,2,1878.222222
6,2,8169.275000
6,2,4679.441860
6,2,8463.765957
6,2,5050.800000
6,2,1963.645833
6,2,1696.000000
6,2,2930.010638
6,2,1666.535714
6,2,2151.800000
6,2,1659.193182
6,2,1914.298077
6,2,1672.811224
6,2,2524.425532
6,2,4648.250000
6,2,1906.918605
6,2,2778.674419
6,2,2181.000000
6,2,2752.394444
6,2,4331.048077
6,2,6856.738372
6,2,2146.577381
6,2,3609.678571
6,2,1501.447917
6,2,3660.103659
6,2,2782.016892
6,2,1522.648256
6,2,1904.395349
6,2,13080.440909
6,2,4175.392045
6,2,2412.473404
6,2,4563.550000
6,2,2653.764706
6,2,3105.945000
6,2,3350.596591
6,2,1959.269608
6,2,3563.540179
6,2,1876.483871

6,2,2002.558140
6,2,7483.906250
6,2,2297.316489
6,2,5757.107143
6,2,3946.763636
6,2,3969.734756
6,2,3062.897260
6,2,5537.783333
6,2,3940.424569
6,2,1580.718750
6,2,4940.029762
6,2,2349.301676
6,2,2463.351351
6,2,3142.625000
6,2,1777.404762
6,2,2649.972527
6,2,8260.196581
6,2,3026.841837
6,2,1519.573113
6,2,4517.359694
6,2,2814.075000
6,2,3691.165094
6,2,1949.606061
6,2,1939.585714
6,2,2487.448864
6,2,6758.117021
6,2,1774.729730
6,2,3006.710526
6,2,3408.314103
6,2,5958.984756
6,2,2392.125000
6,2,3357.755814
6,2,3633.500000
6,2,2679.122449
6,2,2388.960000
6,2,6199.252232
6,2,3758.679245
6,2,2182.224490
6,2,3852.132979
6,2,3089.000000
6,2,3596.809524
6,2,3790.371257
6,2,2003.767045
6,2,7985.255435
6,2,5286.283784
6,2,1957.477273
6,2,2134.250000
6,2,7698.044118
6,2,2113.761628
6,2,10493.902778
6,2,2257.795455
6,2,2245.836683
6,2,1701.994413
6,2,6560.166667
6,2,5966.104651
6,2,4036.500000
6,2,1909.338889
6,2,2733.369565
6,2,3686.816327
6,2,1911.538462
6,2,2396.703125
6,2,1591.464286
6,2,9098.833333
6,2,2249.523952
6,2,5128.322500
6,2,2153.551020

6,2,1672.286184
6,2,2436.773543
6,2,4958.259434
6,2,1578.851064
6,2,2393.004310
6,2,4592.446721
6,2,2787.532895
6,2,4516.489130
6,2,2999.727273
6,2,2747.370629
6,2,189856.340314
6,2,2072.354430
6,2,4087.370482
6,2,2178.500000
6,2,5355.893617
6,2,3207.791444
6,2,3603.600000
6,2,10632.914474
6,2,2191.576220
6,2,2005.085106
6,2,2444.878049
6,2,6758.125000
6,2,2105.414474
6,2,1623.000000
6,2,2254.218085
6,2,1800.181818
6,2,3268.350000
6,2,9428.464286
6,2,3638.190000
6,2,9061.439024
6,2,2774.304245
6,2,1548.360000
6,2,4249.812500
6,2,2202.636364
6,2,4793.730769
6,2,7505.034884
6,2,5216.677083
6,2,5497.727564
6,2,10282.081967
6,2,1788.755556
6,2,10279.000000
6,2,3001.666667
6,2,4305.651163
6,2,3540.640244
6,2,4641.314904
6,2,1919.000000
6,2,4531.600000
6,2,3821.925000
6,2,1810.821429
6,2,1741.000000
6,2,10871.016129
6,2,3649.817308
6,2,2148.838235
6,2,1534.851064
6,2,2010.636029
6,2,4005.385135
6,2,6404.134199
6,2,2722.125000
6,2,8483.818182
6,2,1589.810811
6,2,8115.722826
6,2,4786.333333
6,2,6143.173913
6,2,1811.782609
6,2,1516.230114
6,2,3537.877604

6,2,12666.125000
6,2,3865.500000
6,2,4815.024194
6,2,1521.492462
6,2,1579.000000
6,2,8183.328125
6,2,1815.564189
6,2,1890.000000
6,2,2043.315000
6,2,2523.850649
6,2,6148.727778
6,2,4290.857143
6,2,3644.981707
6,2,3557.994220
6,2,1562.435294
6,2,4360.464497
6,2,5145.181818
6,2,2454.343750
6,2,3018.197500
6,2,2902.019022
6,2,2790.601744
6,2,4022.606771
6,2,2329.473404
6,2,4495.310976
6,2,1668.714286
6,2,18266.314103
6,2,10032.953488
6,2,2537.716667
6,2,2788.087379
6,2,2650.413978
6,2,3111.444444
6,2,1981.484375
6,2,2689.171512
6,2,145226.468750
6,2,2777.981707
6,2,2904.611111
6,2,2093.318627
6,2,2549.320513
6,2,4096.673267
6,2,2840.384146
6,2,2019.021739
6,2,1598.642857
6,2,2614.387500
6,2,2466.589286
6,2,2171.902778
6,2,1836.906250
6,2,6473.853659
6,2,1977.953488
6,2,1649.088816
6,2,7679.320565
6,2,3954.881250
6,2,3416.307692
6,2,5047.534375
6,2,3579.395000
6,2,4540.122222
6,2,2891.877778
6,2,1539.333333
6,2,7825.367021
6,2,2322.750000
6,2,5050.598684
6,2,5655.655738
6,2,2643.850785
6,2,2442.376623
6,2,2640.006757
6,2,8690.333333
6,2,4936.014881

6,2,4799.500000
6,2,2194.163265
6,2,1811.123116
6,2,3171.219595
6,2,2861.974684
6,2,3505.896875
6,2,7962.785714
6,2,14682.125000
6,2,6103.268868
6,2,7299.425000
6,2,1904.608696
6,2,5651.897959
6,2,4179.516892
6,2,4454.532895
6,2,3163.209239
6,2,1746.701087
6,2,2373.450000
6,2,8332.135135
6,2,4966.569767
6,2,2589.100000
6,2,3088.669271
6,2,1815.486631
6,2,1524.686275
6,2,2117.632258
6,2,1893.500000
6,2,3292.579545
6,2,2068.245283
6,2,2705.000000
6,2,3420.357143
6,2,2314.607143
6,2,1546.665025
6,2,1726.582237
6,2,1844.000000
6,2,4658.134831
6,2,3328.968750
6,2,2018.666667
6,2,16713.732955
6,2,3062.000000
6,2,1779.246622
6,2,2021.146875
6,2,1980.471591
6,2,3160.000000
6,2,2712.349432
6,2,2971.736979
6,2,5552.270833
6,2,5957.835366
6,2,1611.205882
6,2,5093.375000
6,2,2356.362500
6,2,2986.410714
6,2,2612.297872
6,2,2637.597826
6,2,6198.411111
6,2,2027.831250
6,2,1864.093750
6,2,5034.591463
6,2,2954.221154
6,2,5774.101351
6,2,1625.000000
6,2,2062.166667
6,2,2977.062500
6,2,1925.446078
6,2,6388.425000
6,2,5475.535714
6,2,1843.482843
6,2,14135.125000

6,2,4292.650000
6,2,4258.796875
6,2,3461.122340
6,2,4166.309211
6,2,1537.500000
6,2,2605.820513
6,2,2208.220000
6,2,1691.645349
6,2,1938.552500
6,2,5510.592105
6,2,3015.772500
6,2,2121.870000
6,2,8555.000000
6,2,4014.557692
6,2,5661.053571
6,2,4579.285714
6,2,2908.916667
6,2,2291.453571
6,2,6270.561170
6,2,5854.547368
6,2,3110.137755
6,2,3194.250000
6,2,2811.025000
6,2,3640.134615
6,2,2421.828125
6,2,1865.187500
6,2,2043.122093
6,2,3564.644737
6,2,8067.505376
6,2,1530.743243
6,2,13962.333333
6,2,1785.714286
6,2,1950.294118
6,2,2014.289062
6,2,8838.588785
6,2,2404.185897
6,2,1854.652778
6,2,4002.980978
6,2,17392.883721
6,2,3651.527933
6,2,2203.975000
6,2,1824.755814
6,2,3719.000000
6,2,5067.878049
6,2,2287.003205
6,2,1525.435897
6,2,2376.265306
6,2,2335.019704
6,2,1726.845178
6,2,1980.000000
6,2,2579.960784
6,2,2109.319853
6,2,1721.288462
6,2,3929.740385
6,2,5564.218750
6,2,3498.006579
6,2,4137.800000
6,2,1586.107143
6,2,5020.071429
6,2,2148.000000
6,2,2040.639706
6,2,2540.714674
6,2,2023.034483
6,2,1894.447500
6,2,3321.681250
6,2,3593.682065

6,2,10912.075472
6,2,3942.925000
6,2,2768.368902
6,2,1533.311111
6,2,3839.347561
6,2,3105.235714
6,2,14260.330357
6,2,2090.125000
6,2,13720.184783
6,2,5340.308511
6,2,2597.543478
6,2,3005.522599
6,2,3098.558480
6,2,1896.250000
6,2,3152.889706
6,2,4365.970745
6,2,5527.951220
6,2,1618.074405
6,2,2228.965909
6,2,1590.842105
6,2,5801.254902
6,2,1638.022727
6,2,1926.053922
6,2,5024.101351
6,2,3015.200000
6,2,1542.735484
6,2,1633.744681
6,2,2254.184783
6,2,5086.115854
6,2,4128.000000
6,2,1610.049383
6,2,2454.110390
6,2,2084.519444
6,2,3156.027907
6,2,1557.000000
6,2,2644.000000
6,2,2470.806818
6,2,2431.218750
6,2,1598.467391
6,2,1796.409091
6,2,1590.550439
6,2,5416.850962
6,2,4551.263514
6,2,7214.302198
6,2,8695.418848
6,2,3721.807692
6,2,3784.363426
6,2,2680.416667
6,2,6878.200000
6,2,4081.002551
6,2,3901.270440
6,2,4911.028061
6,2,1806.259146
6,2,2648.835106
6,2,3167.558673
6,2,4158.549528
6,2,6986.833333
6,2,5669.355263
6,2,3782.111702
6,2,6849.312500
6,2,3987.567982
6,2,3171.794271
6,2,39014.403302
6,2,4115.762500
6,2,3052.375000
6,2,2117.546512

6,2,2220.644022
6,2,5240.679688
6,2,1523.921569
6,2,10129.265086
6,2,3253.542683
6,2,4127.851351
6,2,2413.026163
6,2,42572.826389
6,2,8146.720000
6,2,1718.593750
6,2,1847.100000
6,2,4607.051020
6,2,6391.516827
6,2,2058.328125
6,2,3512.894444
6,2,7771.924528
6,2,11028.500000
6,2,2186.228125
6,2,7943.984694
6,2,2570.860119
6,2,4047.798913
6,2,3588.285714
6,2,5333.122222
6,2,2272.645408
6,2,5193.361702
6,2,2549.957447
6,2,3284.262195
6,2,3038.352564
6,2,22480.907216
6,2,3520.968586
6,2,4940.092965
6,2,1658.538462
6,2,9438.728571
6,2,1637.371711
6,2,1977.500000
6,2,3276.391667
6,2,2538.687500
6,2,1639.255682
6,2,8355.956633
6,2,1770.621849
6,2,5154.128049
6,2,2064.433673
6,2,7974.760417
6,2,8435.742187
6,2,2256.392857
6,2,5087.740196
6,2,5333.414773
6,2,1812.000000
6,2,6858.108491
6,2,2240.466216
6,2,3764.083333
6,2,2185.478365
6,2,1636.362500
6,2,3155.936170
6,2,2456.368421
6,2,4044.292683
6,2,1586.902439
6,2,19940.064516
6,2,2329.784884
6,2,8082.316176
6,2,3130.119318
6,2,9899.802326
6,2,11408.206061
6,2,2438.490625
6,2,6804.894737
6,2,6003.925000

6,2,2004.562500
6,2,1510.857143
6,2,1672.388298
6,2,1508.661458
6,2,5594.450000
6,2,3035.522727
6,2,10565.037791
6,2,1607.411111
6,2,3191.788889
6,2,12453.457627
6,2,1616.341346
6,2,4965.948864
6,2,8120.964912
6,2,1509.642857
6,2,2306.324324
6,2,5840.225000
6,2,1517.782609
6,2,5599.370536
6,2,2105.747283
6,2,2709.106707
6,2,2151.712500
6,2,18114.187500
6,2,1835.282895
6,2,2437.869186
6,2,4374.040323
6,2,2577.618056
6,2,2019.971154
6,2,1948.095000
6,2,5569.652439
6,2,2827.298611
6,2,1523.250000
6,2,3014.390625
6,2,18016.833333
6,2,3451.015000
6,2,3508.250000
6,2,1564.360577
6,2,2646.650000
6,2,1859.420455
6,2,1725.348684
6,2,2160.105114
6,2,3749.625000
6,2,3227.345000
6,2,3125.063218
6,2,2018.940120
6,2,2782.571429
6,2,5101.471591
6,2,1778.466667
6,2,1965.482143
6,2,2015.294118
6,2,1631.678571
6,2,4759.014113
6,2,4088.434783
6,2,2768.793478
6,2,6916.637500
6,2,3000.857143
6,2,1997.283784
6,2,1629.853659
6,2,3143.674020
6,2,1636.500000
6,2,2408.625000
6,2,1814.553571
6,2,1678.694149
6,2,2720.375000
6,2,3082.908537
6,2,2404.857143
6,2,5860.385776

6,2,2443.015306
6,2,1516.710526
6,2,14266.704082
6,2,4918.250000
6,2,3794.113372
6,2,3805.474432
6,2,5490.185096
6,2,1559.600000
6,2,2086.210784
6,2,6848.514286
6,2,2854.259375
6,2,1700.610795
6,2,2664.372549
6,2,5081.179688
6,2,4581.473333
6,2,4668.250000
6,2,1793.704082
6,2,2068.250000
6,2,2717.122222
6,2,15151.595000
6,2,7823.568627
6,2,2194.100000
6,2,3260.404018
6,2,2424.767857
6,2,2113.989362
6,2,6170.359043
6,2,2688.077703
6,2,1590.592105
6,2,5718.368056
6,2,5041.584270
6,2,1537.126437
7,1,1650.914894
7,1,1958.006410
7,1,52019.546196
7,1,4301.438679
7,1,82181.923077
7,1,23111.908088
7,1,18598.350543
7,1,25035.669118
7,1,8646.176136
7,1,23568.844444
7,1,3021.500000
7,1,41547.625000
7,1,2547.953488
7,1,14341.702206
7,1,2638.691860
7,1,99638.434211
7,1,87460.000000
7,1,11024.632353
7,1,38561.912500
7,1,4583.333333
7,1,1760.133333
7,1,2226.250000
7,1,2314.675000
7,1,3069.576923
7,1,1749.361702
7,1,3281.329861
7,1,5619.457447
7,1,4556.893293
7,1,1788.075000
7,1,4756.571429
7,1,3393.340909
7,1,2645.565789
7,1,11581.864865
7,1,2187.649390
7,1,2136.071809

7,1,3712.338816
7,1,1504.511364
7,1,1997.048295
7,1,20028.812500
7,1,1577.750000
7,1,2150.878378
7,1,3215.607955
7,1,2509.117647
7,1,1710.542683
7,1,2495.855556
7,1,2384.508333
7,1,2070.362245
7,1,1661.089286
7,1,2510.128049
7,1,1783.700000
7,1,6063.703488
7,1,21833.394531
7,1,44498.631579
7,1,2175.372549
7,1,1669.500000
7,1,7033.598684
7,1,1730.325581
7,1,131685.263298
7,1,1511.652174
7,1,7653.635870
7,1,30258.394231
7,1,2192.217391
7,1,1878.068182
7,1,43569.094595
7,1,6549.477778
7,1,2260.483871
7,1,1945.375000
7,1,10050.406977
7,1,5915.083333
7,1,4773.275000
7,1,10576.023256
7,1,2088.222561
7,1,4718.531250
7,1,3130.333333
7,1,20573.750000
7,1,2177.197674
7,1,1954.292500
7,1,4302.769231
7,1,2373.432432
7,1,1876.700521
7,1,67156.390625
7,1,2276.705882
7,1,43777.000000
7,1,13112.239264
7,1,47100.742188
7,1,5488.358491
7,1,12942.976190
7,1,2946.557927
7,1,1737.137755
7,1,59278.846154
7,1,11262.325581
7,1,2190.500000
7,1,1900.651163
7,1,4988.944882
7,1,3818.348958
7,1,14347.729730
7,1,1539.611111
7,1,47265.539474
7,1,2587.658537
7,1,6928.172872
7,1,2628.480000

7,1,2182.048780
7,1,21025.976351
7,1,58651.790000
7,1,3611.076389
7,1,2970.709239
7,1,1680.033854
7,1,39787.250000
7,1,16574.192308
7,1,3284.679245
7,1,20446.562500
7,1,2619.847222
7,1,2738.267857
7,1,54791.707143
7,1,1714.438830
7,1,1898.366071
7,1,19175.548913
7,1,1538.590909
7,1,1516.532609
7,1,1582.716912
7,1,6393.648438
7,1,39628.336806
7,1,4076.875000
7,1,3434.553125
7,1,1784.651163
7,1,10692.368421
7,1,2507.312500
7,1,2127.464286
7,1,1979.470930
7,1,2841.522727
7,1,21549.508929
7,1,3933.086207
7,1,3060.837838
7,1,2165.410000
7,1,2815.595000
7,1,8381.857143
7,1,30695.484375
7,1,5043.533784
7,1,3531.333333
7,1,2631.062500
7,1,2696.006250
7,1,12655.086538
7,1,6658.512136
7,1,6939.023810
7,1,26261.060897
7,1,6964.428571
7,1,4465.304054
7,1,2261.222222
7,1,20804.769231
7,1,1589.486111
7,1,2286.494565
7,1,2736.019231
7,1,3614.805825
7,1,2968.761111
7,1,37503.567568
7,1,41456.302326
7,1,3522.596875
7,1,2855.191489
7,1,7217.333333
7,1,181404.262500
7,1,133626.750000
7,1,1560.454545
7,1,2693.296196
7,1,4442.653846
7,1,1679.553977
7,1,1888.927083
7,1,46255.396429

7,1,12152.114362
7,1,54518.006757
7,1,1868.024390
7,1,6388.226415
7,1,2288.160714
7,1,1922.255208
7,1,1620.717105
7,1,69397.296053
7,1,8651.220109
7,1,3083.289062
7,1,3772.200000
7,1,8602.250000
7,1,14754.144231
7,1,58710.621324
7,1,29071.666667
7,1,4897.343023
7,1,4995.821429
7,1,2955.825758
7,1,5122.505814
7,1,3477.300676
7,1,3285.762195
7,1,19752.897059
7,1,66116.687500
7,1,51571.185714
7,1,3410.863636
7,1,4199.692073
7,1,1785.691860
7,1,5688.049419
7,1,2430.462838
7,1,2591.666667
7,1,1916.403125
7,1,2158.609375
7,1,1780.774390
7,1,2176.000000
7,1,8014.560811
7,1,12004.851351
7,1,3036.609375
7,1,1532.750000
7,1,8254.341463
7,1,1624.181818
7,1,1755.250000
7,1,117729.765957
7,1,3279.256757
7,1,2502.358108
7,1,3837.690476
7,1,41862.375000
7,1,26464.669643
7,1,1809.723837
7,1,45513.742424
7,1,1593.253205
7,1,133688.773256
7,1,1937.319149
7,1,2416.833333
7,1,1876.590278
7,1,1771.191489
7,1,3586.956522
7,1,50252.565625
7,1,1798.270408
7,1,44999.844595
7,1,45306.200000
7,1,2101.479730
7,1,14450.870968
7,1,1587.982955
7,1,3531.500000
7,1,1535.125000
7,1,2315.281250

7,1,61323.418103
7,1,8665.111486
7,1,15443.632812
7,1,1737.403226
7,1,1994.636364
7,1,1977.741477
7,1,57532.916667
7,1,2385.456522
7,1,13364.068966
7,1,1857.338415
7,1,3610.845745
7,1,2157.462500
7,1,3524.401786
7,1,35102.023810
7,1,2795.000000
7,1,7648.352679
7,1,4925.846154
7,1,2048.325581
7,1,1787.053125
7,1,5557.454545
7,1,2496.083333
7,1,39899.418367
7,1,36714.225000
7,1,2256.494681
7,1,22341.722892
7,1,1790.880319
7,1,12163.500000
7,1,2884.827381
7,1,2859.325581
7,1,1977.928571
7,1,2147.105769
7,1,103759.179487
7,1,175809.675000
7,1,19991.220588
7,1,1821.200000
7,1,2732.391667
7,1,44503.000000
7,1,38432.071875
7,1,1642.259868
7,1,2636.250000
7,1,9539.700000
7,1,3546.176471
7,1,3445.230000
7,1,2478.408163
7,1,5763.684211
7,1,10822.680000
7,1,96691.968750
7,1,4265.993243
7,1,1977.790000
7,1,118856.382979
7,1,2237.386628
7,1,65123.597561
7,1,2720.769022
7,1,1651.168605
7,1,2855.601064
7,1,3697.986486
7,1,2671.909574
7,1,11344.983871
7,1,5600.000000
7,1,55694.000000
7,1,4871.996429
7,1,16004.385135
7,1,5064.500000
7,1,19531.750000
7,1,59165.466667
7,1,2215.667763

7,1,1799.647727
7,1,2565.120000
7,1,1979.677326
7,1,7336.633152
7,1,2522.090625
7,1,1915.341667
7,1,2519.902344
7,1,11009.325581
7,1,41815.838235
7,1,15673.062500
7,1,4221.581250
7,1,8253.235000
7,1,64976.766304
7,1,97114.847561
7,1,21651.396825
7,1,4238.185714
7,1,70067.397436
7,1,1691.732558
7,1,55932.922297
7,1,2671.545455
7,1,1602.909091
7,1,74384.356250
7,1,2292.614286
7,1,2445.415541
7,1,5673.444444
7,1,2943.210938
7,1,2320.265957
7,1,90352.125000
7,1,1763.937500
7,1,6467.914474
7,1,1692.252604
7,1,4674.232143
7,1,3272.813830
7,1,1768.045455
7,1,3756.025000
7,1,29612.100000
7,1,2255.994186
7,1,129738.098837
7,1,13264.357955
7,1,5603.020349
7,1,1818.933333
7,1,5196.105769
7,1,1677.910714
7,1,5254.242347
7,1,3013.579268
7,1,1922.659574
7,1,11080.221154
7,1,2384.684091
7,1,2556.045139
7,1,1744.522727
7,1,2354.125000
7,1,5956.385638
7,1,1802.761364
7,1,1823.448718
7,1,1835.352941
7,1,3125.914894
7,1,1800.661184
7,1,42730.000000
7,1,1531.230769
7,1,44856.476562
7,1,80948.187500
7,1,18648.806452
7,1,2716.968750
7,1,2680.081522
7,1,17720.565341
7,1,19564.914773

7,1,1625.257143
7,1,3432.196429
7,1,2386.939103
7,1,91707.493243
7,1,17866.648649
7,1,16214.875000
7,1,3275.765625
7,1,4039.017857
7,1,1620.152174
7,1,3449.335784
7,1,3894.448171
7,1,1940.886905
7,1,134790.708333
7,1,42716.000000
7,1,3976.098214
7,1,9121.880952
7,1,38034.150735
7,1,1696.518229
7,1,2963.110294
7,1,1561.200000
7,1,2231.210714
7,1,18065.521739
7,1,5689.893617
7,1,32969.656977
7,1,49270.743902
7,1,2336.656250
7,1,42156.815341
7,1,26389.312500
7,1,35884.282609
7,1,2001.869565
7,1,3228.000000
7,1,2008.056250
7,1,42614.530488
7,1,2206.619565
7,1,5406.405405
7,1,1524.419118
7,1,1848.000000
7,1,23603.591912
7,1,12039.062500
7,1,2985.540541
7,1,50514.491071
7,1,16013.214286
7,1,2393.704545
7,1,5435.088235
7,1,53042.600000
7,1,2682.440789
7,1,14506.928571
7,1,2976.238636
7,1,3206.621212
7,1,21985.851064
7,1,2443.650735
7,1,1829.411111
7,1,5593.875000
7,1,3828.163462
7,1,51161.585938
7,1,6915.625000
7,1,3206.699405
7,1,9153.466346
7,1,3605.526316
7,1,29933.736842
7,1,1897.875000
7,1,1983.184028
7,1,1594.025000
7,1,15552.217391
7,1,2131.091667
7,1,2148.033333

7,1,2021.511905
7,1,4381.208333
7,1,2540.046512
7,1,8363.033784
7,1,1560.940789
7,1,105579.017857
7,1,1974.400000
7,1,2083.304348
7,1,1550.922222
7,1,111487.255319
7,1,2863.006250
7,1,2080.546875
7,1,2369.500000
7,1,1886.500000
7,1,1721.822674
7,1,10119.469697
7,1,2466.945000
7,1,7924.988971
7,1,28962.849265
7,1,5663.277439
7,1,34352.781250
7,1,14583.208333
7,1,1728.166667
7,1,4083.093750
7,1,2198.875000
7,1,1512.166667
7,1,10049.902439
7,1,2744.296053
7,1,1892.159722
7,1,1940.288462
7,1,10216.907609
7,1,2404.212500
7,1,2533.687500
7,1,3135.174419
7,1,1545.065625
7,1,10152.571429
7,1,36792.003289
7,1,85507.687500
7,1,1942.704787
7,1,3767.763889
7,1,7513.095238
7,1,83622.679878
7,1,5467.063291
7,1,3384.833333
7,1,3504.341463
7,1,4800.595000
7,1,76858.552326
7,1,3553.950000
7,1,1611.162162
7,1,2099.127907
7,1,50846.097561
7,1,5212.264881
7,1,4687.571429
7,1,3092.736111
7,1,1685.994681
7,1,73700.914062
7,1,3778.210366
7,1,1683.478261
7,1,2312.125000
7,1,4824.968750
7,1,41642.316860
7,1,43148.860465
7,1,3471.250000
7,1,3671.987500
7,1,1739.262821
7,1,2050.240000

7,1,89666.992424
7,1,3026.454082
7,1,18427.222222
7,1,16227.411932
7,1,2461.942073
7,1,1704.436224
7,1,38624.766447
7,1,1569.446809
7,1,1812.630682
7,1,23507.235795
7,1,79057.250000
7,1,52254.834559
7,1,14223.488636
7,1,17080.928571
7,1,1702.591667
7,1,1844.660714
7,1,2107.674419
7,1,35097.500000
7,1,1661.543919
7,1,2712.205357
7,1,80554.200000
7,1,55347.638889
7,1,1793.250000
7,1,32337.709677
7,1,1590.000000
7,1,6425.666667
7,1,1897.685976
7,1,38711.331250
7,1,1732.500000
7,1,58069.150000
7,1,63448.835938
7,1,4156.426829
7,1,2523.642857
7,1,5336.720109
7,1,32626.407895
7,1,11819.125000
7,1,11001.666667
7,1,6841.646552
7,1,1951.404762
7,1,1875.871795
7,1,43546.312500
7,1,3494.410714
7,1,10509.767857
7,1,3067.528571
7,1,3109.500000
7,1,3314.394444
7,1,1970.103659
7,1,19030.175000
7,1,2811.627660
7,1,8783.765244
7,1,2097.920918
7,1,30786.272727
7,1,2083.672619
7,1,5162.417500
7,1,1640.986702
7,1,2519.666667
7,1,2582.798780
7,1,4450.580882
7,1,4130.417553
7,1,42098.558594
7,1,1703.616667
7,1,5926.500000
7,1,1944.414894
7,1,3942.070946
7,1,1912.682927
7,1,2823.922619

7,1,4058.729730
7,1,5815.750000
7,1,44268.492647
7,1,2162.839286
7,1,1808.486413
7,1,13692.019737
7,1,19585.529762
7,1,24530.664062
7,1,14276.121622
7,1,2329.241667
7,1,2122.783784
7,1,3658.765957
7,1,4745.950000
7,1,18402.606771
7,1,3078.307692
7,1,43398.570423
7,1,13073.183333
7,1,5695.768293
7,1,4255.307692
7,1,1525.059375
7,1,70423.834302
7,1,18023.500000
7,1,1681.857143
7,1,2546.076923
7,1,2671.125000
7,1,1609.736842
7,1,8608.142857
7,1,49737.297619
7,1,4978.422872
7,1,34168.171875
7,1,2710.971429
7,1,4453.234043
7,1,2107.078947
7,1,2512.798077
7,1,22555.250000
7,1,27098.455128
7,1,1537.013158
7,1,41180.445876
7,1,4727.439024
7,1,1499.709184
7,1,2284.976190
7,1,136751.658163
7,1,4945.925000
7,1,1629.857955
7,1,2193.804878
7,1,3219.542945
7,1,1551.741477
7,1,13873.071429
7,1,1781.000000
7,1,33587.846154
7,1,3074.975610
7,1,1533.533784
7,1,3338.933333
7,1,3506.785714
7,1,3661.171875
7,1,10204.347222
7,1,1842.347826
7,1,58384.772436
7,1,18367.917500
7,1,4794.350000
7,1,29960.000000
7,1,2050.377604
7,1,119019.270833
7,1,2199.768293
7,1,2131.792683
7,1,2870.531250

7,1,1762.865385
7,1,2817.380435
7,1,2378.910714
7,1,2452.750000
7,1,3733.821429
7,1,9381.333333
7,1,13642.770270
7,1,2482.539062
7,1,4198.800000
7,1,4028.105263
7,1,6133.725543
7,1,2014.250000
7,1,12009.774194
7,1,1891.658537
7,1,2656.632212
7,1,3397.425000
7,1,2514.917500
7,1,3203.839674
7,1,4220.781250
7,1,4710.480769
7,1,1954.250000
7,1,2012.659091
7,1,1824.058511
7,1,1882.136905
7,1,1795.654605
7,1,1948.604592
7,1,3134.686047
7,1,9254.525641
7,1,2424.939024
7,1,1943.667553
7,1,1854.278846
7,1,41779.857143
7,1,4406.777439
7,1,28685.066667
7,1,2470.174419
7,1,3808.875000
7,1,2358.548611
7,1,2998.647222
7,1,3846.622222
7,1,2465.588889
7,1,5277.726562
7,1,1717.793548
7,1,1692.175676
7,1,3231.666667
7,1,4139.802326
7,1,1888.425676
7,1,3281.768750
7,1,11340.529891
7,1,23048.591463
7,1,1566.670455
7,1,13838.017857
7,1,1833.845238
7,1,4694.000000
7,1,3819.000000
7,1,2576.408537
7,1,42065.674479
7,1,60077.833333
7,1,64738.514151
7,1,2606.635417
7,1,2481.111111
7,1,3083.187500
7,1,19422.304688
7,1,2190.659574
7,1,4201.814208
7,1,2688.440000
7,1,1614.290541

7,1,1712.181818
7,1,3432.119565
7,1,1619.410256
7,1,97111.796053
7,1,4995.574324
7,1,3950.095588
7,1,2463.428571
7,1,41657.996324
7,1,2315.877358
7,1,1582.794643
7,1,2298.588235
7,1,3014.352941
7,1,5261.750000
7,1,7603.133929
7,1,2194.489362
7,1,59608.138889
7,1,4054.272727
7,1,1642.882353
7,1,4051.826389
7,1,34975.052885
7,1,2860.931818
7,1,1961.073171
7,1,3327.585366
7,1,3184.595000
7,1,42276.852941
7,1,5818.093023
7,1,9816.360294
7,1,1879.209375
7,1,10402.636364
7,1,1855.031250
7,1,1675.652778
7,1,44857.763514
7,1,1630.610119
7,1,20111.791667
7,1,5038.852941
7,1,1504.839286
7,1,7876.383721
7,1,1837.858333
7,1,16102.079545
7,1,2436.020270
7,1,19803.659574
7,1,95046.114865
7,1,1891.853125
7,1,2051.229651
7,1,34807.250000
7,1,2526.781250
7,1,83688.686275
7,1,45029.201220
7,1,36492.500000
7,1,1558.408163
7,1,1867.500000
7,1,51013.666667
7,1,2608.848404
7,1,3796.564286
7,1,4888.216216
7,1,3095.811111
7,1,1525.250000
7,1,1973.437500
7,1,43010.078125
7,1,10001.947674
7,1,2059.330882
7,1,4356.531250
7,1,7278.286458
7,1,5188.287234
7,1,46677.782051
7,1,2220.066667

7,1,91158.115132
7,1,2872.775000
7,1,5192.823171
7,1,8080.917411
7,1,4106.875000
7,1,3322.571429
7,1,14726.235294
7,1,36708.992857
7,1,14972.325581
7,1,16034.850000
7,1,23544.375000
7,1,40494.618902
7,1,2083.950000
7,1,1977.600000
7,1,2193.714674
7,1,1746.073718
7,1,2574.642500
7,1,2544.050000
7,1,1798.186224
7,1,1838.904070
7,1,28487.950581
7,1,1763.115132
7,1,9524.342857
7,1,2410.546053
7,1,2112.500000
7,1,96427.594595
7,1,2323.766447
7,1,1935.142857
7,1,1890.959559
7,1,1777.195122
7,1,1674.922222
7,1,3323.177778
7,1,24038.625000
7,1,2837.864865
7,1,31787.384615
7,1,2066.299342
7,1,4069.468750
7,1,55857.764706
7,1,1589.630435
7,1,1738.717143
7,1,48944.393939
7,1,19286.120968
7,1,25023.660714
7,1,1974.270349
7,1,196136.309659
7,1,22918.148438
7,1,4260.649123
7,1,2106.351190
7,1,50549.658784
7,1,4674.531250
7,1,53167.350000
7,1,14523.840000
7,1,13712.906250
7,1,3824.581395
7,1,4909.051630
7,1,2145.733333
7,1,1562.080729
7,1,1958.068182
7,1,16754.571429
7,1,3166.923497
7,1,1730.789634
7,1,13076.370690
7,1,4459.589286
7,1,72135.893750
7,1,1924.185976
7,1,6301.530488

7,1,1512.958333
7,1,4380.787500
7,1,4150.425532
7,1,5001.404255
7,1,3662.142857
7,1,192441.790816
7,1,65408.316176
7,1,6780.148936
7,1,5453.806122
7,1,78004.028571
7,1,4543.491667
7,1,4280.959459
7,1,2696.081633
7,1,5232.687500
7,1,6213.991228
7,1,42859.268293
7,1,2476.148438
7,1,3307.543750
7,1,2619.404412
7,1,6130.741935
7,1,4140.363636
7,1,2171.291667
7,1,2569.423077
7,1,5253.576087
7,1,3063.159574
7,1,121048.555556
7,1,3650.889286
7,1,22885.218750
7,1,1998.162162
7,1,3248.323529
7,1,1671.217105
7,1,2187.733333
7,1,16170.744681
7,1,7454.537234
7,1,2236.142857
7,1,2529.317073
7,1,5000.027027
7,1,2382.186047
7,1,34712.462500
7,1,7201.411932
7,1,2470.330128
7,1,4784.333333
7,1,4147.233696
7,1,1993.543750
7,1,4746.815217
7,1,2591.988095
7,1,1591.348684
7,1,1683.685714
7,1,1909.750000
7,1,1826.779070
7,1,45700.941176
7,1,4481.285714
7,1,1867.765957
7,1,1624.918367
7,1,1911.365591
7,1,5420.505319
7,1,3611.753378
7,1,9235.270408
7,1,80131.912500
7,1,5691.997093
7,1,2770.034884
7,1,2121.000000
7,1,2641.911765
7,1,2113.003472
7,1,2684.765957
7,1,2215.545732

7,1,5473.583333
7,1,2464.637755
7,1,1927.508721
7,1,2131.916667
7,1,1719.836111
7,1,2040.633333
7,1,52085.450000
7,1,37517.983974
7,1,14727.692308
7,1,3892.915698
7,1,4900.000000
7,1,70333.398810
7,1,37157.980263
7,1,25623.875000
7,1,6537.340426
7,1,3621.013298
7,1,1806.361111
7,1,2448.883523
7,1,1615.611111
7,1,5533.883721
7,1,1814.529762
7,1,3123.018293
7,1,7161.400000
7,1,1728.553977
7,1,16458.851415
7,1,4843.551136
7,1,24306.448276
7,1,2048.438776
7,1,6506.953804
7,1,5704.418478
7,1,1832.789062
7,1,99224.438953
7,1,63926.909091
7,1,1932.400641
7,1,38001.320312
7,1,26198.056250
7,1,12100.420455
7,1,1872.375000
7,1,31347.170455
7,1,84955.391026
7,1,2218.653846
7,1,1517.916667
7,1,2311.950893
7,1,3608.017857
7,1,2727.431548
7,1,45859.493421
7,1,7915.768939
7,1,2170.809375
7,1,3857.160714
7,1,1646.067568
7,1,13897.166667
7,1,39803.697368
7,1,8262.886792
7,1,3351.075000
7,1,2272.965909
7,1,1545.493421
7,1,2283.609756
7,1,2028.934896
7,1,3048.928571
7,1,11945.170732
7,1,4000.303191
7,1,75701.183333
7,1,2049.493902
7,1,6437.153846
7,1,40571.801020
7,1,1573.968085

7,1,5971.358108
7,1,1878.127778
7,1,2410.854730
7,1,1779.761905
7,1,2650.600000
7,1,27989.852273
7,1,27205.325758
7,1,1642.508929
7,1,1621.059783
7,1,33628.305556
7,1,4400.382979
7,1,6834.820652
7,1,2743.523437
7,1,1690.415094
7,1,3100.716463
7,1,1531.328947
7,1,24404.851562
7,1,2122.678571
7,1,6848.420732
7,1,1864.907895
7,1,1701.744186
7,1,2146.514706
7,1,4328.830882
7,1,1564.625000
7,1,2996.114362
7,1,8068.602041
7,1,8128.384375
7,1,2182.635870
7,1,1521.312500
7,1,1690.894737
7,1,58224.586207
7,1,2934.730769
7,1,2692.580357
7,1,20697.880952
7,1,2540.050000
7,1,2291.497093
7,1,4311.820312
7,1,16799.235294
7,1,1819.444444
7,1,6809.372549
7,1,5760.883929
7,1,34819.325000
7,1,4283.037500
7,1,2837.833333
7,1,5070.600000
7,1,4730.299107
7,1,5346.208333
7,1,34297.352941
7,1,1873.767442
7,1,1709.578125
7,1,5801.453947
7,1,2629.789474
7,1,4417.666667
7,1,2669.611702
7,1,1615.843750
7,1,3440.677326
7,1,3759.521739
7,1,2028.852273
7,1,27187.285714
7,1,1683.947368
7,1,2166.786184
7,1,2804.000000
7,1,2977.666667
7,1,24533.500000
7,1,6665.829082
7,1,1920.447917

7,1,2183.251366
7,1,2936.201087
7,1,1748.219512
7,1,4475.333333
7,1,1548.655488
7,1,72752.412162
7,1,1950.790625
7,1,3217.521739
7,1,2917.216216
7,1,19874.194444
7,1,25671.387097
7,1,2718.206081
7,1,5546.471875
7,1,6114.979592
7,1,1763.878788
7,1,3468.855769
7,1,1912.275000
7,1,2580.752907
7,1,1794.727273
7,1,5495.535000
7,1,3167.875000
7,1,1810.380682
7,1,28110.378378
7,1,19081.512097
7,1,1751.423077
7,1,2314.540541
7,1,26640.875000
7,1,1720.200000
7,1,1674.165625
7,1,1771.000000
7,1,7858.348039
7,1,41010.618056
7,1,2685.053191
7,1,20989.800000
7,1,4191.118902
7,1,67994.898649
7,1,6254.776316
7,1,53305.250000
7,1,11881.833333
7,1,1688.482955
7,1,2007.114130
7,1,12743.000000
7,1,3624.875000
7,1,26436.802083
7,1,4553.308036
7,1,21861.934783
7,1,7842.442308
7,1,2616.946875
7,1,2406.783333
7,1,6806.710526
7,1,1930.625000
7,1,7983.889706
7,1,6386.044444
7,1,41094.130952
7,1,4215.945122
7,1,70940.582237
7,1,34413.865854
7,1,44687.083333
7,1,2336.348837
7,1,4118.515625
7,1,34964.789474
7,1,2307.082143
7,1,117210.468750
7,1,4696.694444
7,1,38692.750000
7,1,38619.916667

7,1,2510.446809
7,1,7351.257653
7,1,1599.157738
7,1,2638.469388
7,1,3050.842105
7,1,1688.256098
7,1,3556.918605
7,1,1938.200000
7,1,5810.935185
7,1,1626.125000
7,1,80052.328947
7,1,3028.323529
7,1,1735.430556
7,1,75612.250000
7,1,1835.475000
7,1,6965.917500
7,1,1981.725000
7,1,113463.552326
7,1,7774.443182
7,1,1729.163265
7,1,3318.727778
7,1,5109.117347
7,1,35473.887500
7,1,45754.558824
7,1,15669.600000
7,1,68192.461111
7,1,1564.341463
7,1,10215.806034
7,1,7056.151316
7,1,1582.843085
7,1,30531.550781
7,1,4013.408163
7,1,91462.042553
7,1,4181.786932
7,1,4974.151786
7,1,3355.208333
7,1,1584.387500
7,1,6905.991477
7,1,6923.548913
7,1,6861.571023
7,1,94540.383065
7,1,67474.631579
7,1,1988.650641
7,1,20397.507874
7,1,35283.404412
7,1,2572.514706
7,1,42430.884615
7,1,4366.213415
7,1,22397.033333
7,1,2068.117647
7,1,6492.523026
7,1,2497.618902
7,1,2256.000000
7,1,34488.135870
7,1,4516.097500
7,1,67408.675497
7,1,33981.352273
7,1,3111.721354
7,1,48372.157143
7,1,34290.270270
7,1,2185.456522
7,1,18246.618243
7,1,27965.829268
7,1,3044.219880
7,1,4345.611842
7,1,2600.782609

7,1,9929.612903
7,1,36249.216216
7,1,83398.562500
7,1,3201.788462
7,1,2234.340116
7,1,2233.410000
7,1,24276.437500
7,1,3233.934783
7,1,2548.587838
7,1,29324.854167
7,1,3872.487805
7,1,4088.907738
7,1,6298.304878
7,1,10729.943548
7,1,1508.825806
7,1,9517.272727
7,1,6590.600000
7,1,5619.247368
7,1,1883.714286
7,1,3434.673469
7,1,5166.255682
7,1,1509.928571
7,1,121160.898810
7,1,3275.086957
7,1,1616.805851
7,1,2385.017045
7,1,2557.523585
7,1,2461.607143
7,1,19587.335938
7,1,3963.323529
7,1,38113.065217
7,1,12661.261364
7,1,16243.019886
7,1,2880.367347
7,1,7089.276316
7,1,7773.081522
7,1,3389.127907
7,1,4999.656250
7,1,2296.418919
7,1,2055.454545
7,1,7716.905405
7,1,3315.910714
7,1,1887.850000
7,1,5319.678571
7,1,8995.511111
7,1,1938.590909
7,1,13774.651316
7,1,5333.404891
7,1,15134.875000
7,1,107522.403226
7,1,2973.951613
7,1,138647.456522
7,1,4776.694444
7,1,12406.284314
7,1,11553.653846
7,1,89544.905405
7,1,3609.880000
7,1,3408.446809
7,1,2088.243902
7,1,3981.375000
7,1,1986.232143
7,1,2548.600000
7,1,5857.735795
7,1,41077.300000
7,1,7031.878378
7,1,13343.303191

7,1,3803.000000
7,1,73872.679487
7,1,4402.056604
7,1,14617.832317
7,1,12449.371951
7,1,23841.576271
7,1,2194.135135
7,1,2449.683333
7,1,14356.260417
7,1,1993.915698
7,1,1993.697368
7,1,15288.000000
7,1,10141.395349
7,1,2398.000000
7,1,5193.143750
7,1,63996.110795
7,1,3092.897500
7,1,25151.793750
7,1,2814.500000
7,1,18046.081395
7,1,2391.000000
7,1,12658.852679
7,1,3459.486111
7,1,3509.062500
7,1,1662.948864
7,1,1729.702128
7,1,3941.520408
7,1,4945.705128
7,1,2736.142857
7,1,2823.600000
7,1,2225.711111
7,1,1899.928191
7,1,15079.812500
7,1,3618.095808
7,1,3768.948718
7,1,18474.871711
7,1,6966.290698
7,1,6395.896341
7,1,3876.424242
7,1,13440.059659
7,1,17804.351190
7,1,2883.958333
7,1,3299.243902
7,1,2147.107143
7,1,13494.043367
7,1,2488.156250
7,1,2346.549451
7,1,4129.046512
7,1,1684.181818
7,1,1673.289773
7,1,5327.973684
7,1,1908.800000
7,1,9559.500000
7,1,2546.771930
7,1,24221.812500
7,1,11068.847222
7,1,1747.181818
7,1,17577.371324
7,1,3550.857143
7,1,27242.243243
7,1,5497.625000
7,1,1597.031250
7,1,10738.656250
7,1,2747.195652
7,1,2122.529412
7,1,35468.798295

7,1,2257.183511
7,1,1567.666667
7,1,12133.612069
7,1,1864.454082
7,1,12937.650000
7,1,6274.202128
7,1,2574.000000
7,1,70358.190476
7,1,2064.058511
7,1,5103.350000
7,1,3984.173780
7,1,19764.142857
7,1,2119.651786
7,1,5815.521739
7,1,54272.636364
7,1,49404.333333
7,1,13477.253676
7,1,3318.978261
7,1,3168.945122
7,1,2267.258065
7,1,1781.060897
7,1,2218.606707
7,1,1571.045455
7,1,2092.225191
7,1,31354.302326
7,1,2608.780405
7,1,2190.856250
7,1,3383.096154
7,1,1575.946809
7,1,1516.226562
7,1,1611.238281
7,1,2343.200000
7,1,3366.564024
7,1,13946.455128
7,1,2319.207547
7,1,3753.015152
7,1,30488.139706
7,1,1505.539474
7,1,17899.000000
7,1,3501.000000
7,1,8080.412162
7,1,1819.447222
7,1,1506.791667
7,1,3688.045918
7,1,1617.230769
7,1,2546.625000
7,1,3227.041667
7,1,1597.072917
7,1,77618.217857
7,1,1559.213415
7,1,31953.236842
7,1,3753.579268
7,1,5396.088235
7,1,10620.913043
7,1,3827.296875
7,1,7268.536765
7,1,5122.906250
7,1,16010.625000
7,1,1590.390625
7,1,3285.818182
7,1,2300.943548
7,1,3775.218750
7,1,3162.298913
7,1,2714.318182
7,1,94867.791667
7,1,8091.168919

7,1,10626.234091
7,1,1528.183824
7,1,37553.209302
7,1,6269.929348
7,1,5450.527778
7,1,5985.613636
7,1,2857.239865
7,1,3544.000000
7,1,3533.120968
7,1,4969.362245
7,1,7400.953947
7,1,2146.125000
7,1,23944.696970
7,1,20585.352273
7,1,1501.687500
7,1,3486.562500
7,1,6995.602041
7,1,6194.760870
7,1,2008.610577
7,1,2135.576087
7,1,1813.282895
7,1,4561.390625
7,1,2782.558140
7,1,43965.000000
7,1,7191.964674
7,1,2561.857143
7,1,2703.505814
7,1,6424.611842
7,1,6966.148026
7,1,1961.455882
7,1,1502.758721
7,1,2926.201613
7,1,1829.298780
7,1,2310.392857
7,1,1520.947115
7,1,3077.298077
7,1,30951.197368
7,1,15333.614583
7,1,1850.177778
7,1,2313.306122
7,1,5896.437500
7,1,1685.975000
7,1,2481.690476
7,1,3556.669811
7,1,1505.976190
7,1,8173.227941
7,1,61552.858553
7,1,87006.647727
7,1,1680.543478
7,1,5438.716667
7,1,16088.913907
7,1,1935.690476
7,1,106579.075000
7,1,1633.700758
7,1,2777.153846
7,1,45284.216216
7,1,1603.478261
7,1,3314.933333
7,1,6675.201220
7,1,2179.068182
7,1,29741.531250
7,1,4909.570946
7,1,16117.793103
7,1,16245.892857
7,1,2147.806122
7,1,14198.151515

7,1,1842.054054
7,1,3579.812500
7,1,1735.813953
7,1,5688.527439
7,1,1886.200000
7,1,3131.691667
7,1,2222.060811
7,1,1677.880319
7,1,7558.147959
7,1,2623.125000
7,1,3214.897727
7,1,11469.878788
7,1,4152.200000
7,1,1597.250000
7,1,2945.513298
7,1,3932.175595
7,1,1745.410714
7,1,2952.094595
7,1,1957.000000
7,1,1629.375000
7,1,23424.487903
7,1,2107.202703
7,1,2354.581522
7,1,12809.202381
7,1,1973.169643
7,1,7039.058824
7,1,1595.488372
7,1,1974.100000
7,1,1513.333333
7,1,2034.382812
7,1,18317.500000
7,1,1753.687500
7,1,5582.396552
7,1,3204.292683
7,1,1765.146277
7,1,76663.130952
7,1,11354.911111
7,1,1684.734848
7,1,2272.000000
7,1,87281.000000
7,1,5450.186111
7,1,64513.000000
7,1,26387.851351
7,1,2553.500000
7,1,3598.794355
7,1,1546.992424
7,1,148823.095238
7,1,35103.692308
7,1,4962.583333
7,1,65393.622159
7,1,1849.702857
7,1,3348.009868
7,1,2232.048077
7,1,9805.979167
7,1,2039.127717
7,1,2582.290000
7,1,1783.117647
7,1,15781.691489
7,1,82201.616477
7,1,1918.173780
7,1,31393.946429
7,1,2522.534884
7,1,47195.812500
7,1,1662.585000
7,1,4074.011194
7,1,1718.322581

7,1,43266.943452
7,1,10049.858974
7,1,1849.708333
7,1,24194.392857
7,1,23065.353659
7,1,6074.387195
7,1,4779.189189
7,1,6089.110795
7,1,3850.485119
7,1,1861.028302
7,1,1825.333333
7,1,1558.719512
7,1,50994.750000
7,1,3170.696429
7,1,3246.093750
7,1,35398.027027
7,1,2453.079545
7,1,12607.263158
7,1,1575.643617
7,1,4885.885417
7,1,2706.328947
7,1,2407.445946
7,1,3232.899390
7,1,11256.833333
7,1,1667.350962
7,1,28730.907609
7,1,1820.125000
7,1,42676.406250
7,1,31883.527027
7,1,3518.740000
7,1,2049.869186
7,1,5694.229167
7,1,1584.731771
7,1,3798.600000
7,1,63795.847826
7,1,2000.891892
7,1,1937.508929
7,1,2886.176829
7,1,2632.423077
7,1,2604.944767
7,1,3884.900510
7,1,99182.821429
7,1,2122.140000
7,1,8502.173469
7,1,5098.634615
7,1,2097.292683
7,1,52329.812500
7,1,100015.967391
7,1,2343.724432
7,1,35156.050000
7,1,33906.270833
7,1,87969.493421
7,1,2033.187500
7,1,5337.029412
7,1,62374.369186
7,1,1799.302419
7,1,1541.000000
7,1,1681.080128
7,1,3019.007143
7,1,2394.875000
7,1,2715.390625
7,1,2069.250000
7,1,1591.040625
7,1,3696.359375
7,1,111961.820513
7,1,56067.958333

7,1,2660.800000
7,1,7171.703947
7,1,16010.826923
7,1,3112.845109
7,1,108347.464286
7,1,2579.778125
7,1,2089.372093
7,1,4857.113372
7,1,2674.800000
7,1,14648.296053
7,1,2300.378049
7,1,2008.473214
7,1,22671.718085
7,1,4168.621429
7,1,1575.829268
7,1,4225.187500
7,1,6099.747396
7,1,11584.738372
7,1,3210.862805
7,1,20000.386719
7,1,2301.941176
7,1,1848.036585
7,1,6924.382353
7,1,1525.200000
7,1,162704.000000
7,1,2934.669118
7,1,4272.533333
7,1,49825.774834
7,1,57183.909375
7,1,80491.448718
7,1,1545.272727
7,1,8802.611842
7,1,3189.518519
7,1,1944.488636
7,1,1536.125000
7,1,2091.472222
7,1,2516.023810
7,1,2408.000000
7,1,1612.269231
7,1,7231.984848
7,1,2420.091216
7,1,6855.848485
7,1,3037.070652
7,1,14534.988889
7,1,15958.951220
7,1,5623.630435
7,1,1588.872093
7,1,8157.644231
7,1,10951.916667
7,1,4180.659091
7,1,2370.083333
7,1,3346.647959
7,1,1809.096591
7,1,1805.819149
7,1,4839.872340
7,1,4405.359375
7,1,1799.939394
7,1,38399.878289
7,1,1520.577778
7,1,57604.750000
7,1,12700.750000
7,1,2023.396341
7,1,2083.302632
7,1,1804.599010
7,1,1721.402174
7,1,2528.011719

7,1,12636.978261
7,1,2546.189024
7,1,10350.735000
7,1,2725.481675
7,1,144096.670455
7,1,5251.947368
7,1,7490.151786
7,1,2465.000000
7,1,5839.517857
7,1,1756.634146
7,1,110514.700000
7,1,1907.829787
7,1,3305.939394
7,1,1558.308140
7,1,2375.277778
7,1,51204.125000
7,1,13294.534091
7,1,24789.833333
7,1,1664.121212
7,1,1930.512821
7,1,34119.783784
7,1,2819.500000
7,1,1832.141304
7,1,1621.544643
7,1,2674.456731
7,1,5017.875000
7,1,4209.166667
7,1,1796.048295
7,1,2392.666667
7,1,1840.480769
7,1,3787.290816
7,1,5858.663043
7,1,8266.515625
7,1,4996.694079
7,1,1515.862069
7,1,1823.437500
7,1,2009.263158
7,1,2077.902439
7,1,1806.416667
7,1,1526.676136
7,1,17869.408088
7,1,1768.500000
7,1,2494.083333
7,1,4088.440789
7,1,42591.547297
7,1,36916.781250
7,1,4261.281250
7,1,3134.560976
7,1,4012.101064
7,1,43037.920455
7,1,1703.451220
7,1,2505.285326
7,1,3703.875000
7,1,2889.514881
7,1,6792.433333
7,1,2170.179688
7,1,4137.235294
7,1,9078.500000
7,1,9163.187500
7,1,2115.206250
7,1,3439.358586
7,1,8243.898810
7,1,18054.600000
7,1,10352.600000
7,1,90273.895270
7,1,3456.551630

7,1,9923.437500
7,1,3998.474432
7,1,2302.291667
7,1,33459.329861
7,1,1653.651163
7,1,3126.275000
7,1,2684.616279
7,1,1706.062500
7,1,135940.658537
7,1,5640.119186
7,1,26248.357955
7,1,2008.136364
7,1,3250.785714
7,1,117997.705000
7,1,2601.198718
7,1,25339.200758
7,1,2567.833333
7,1,2802.151163
7,1,2127.335714
7,1,49172.128788
7,1,3256.549419
7,1,3652.385870
7,1,20468.612069
7,1,2161.382353
7,1,2188.750000
7,1,4748.875000
7,1,2379.294118
7,1,2034.214286
7,1,2377.570312
7,1,3899.641304
7,1,83993.432692
7,1,14206.000000
7,1,4793.531088
7,1,18852.906250
7,1,43464.062500
7,1,1774.227273
7,1,1794.153846
7,1,1743.127907
7,1,1515.460227
7,1,1792.478261
7,1,1730.658537
7,1,1736.367347
7,1,1791.264535
7,1,4968.687500
7,1,84927.736842
7,1,2609.977778
7,1,1581.986842
7,1,4562.418919
7,1,2697.200000
7,1,4831.511905
7,1,7795.389706
7,1,4988.951923
7,1,3966.318750
7,1,9420.416667
7,1,1946.475543
7,1,2184.593750
7,1,1870.729167
7,1,2776.125000
7,1,3427.790698
7,1,2350.521739
7,1,11778.837500
7,1,2317.397059
7,1,18565.898305
7,1,17729.739130
7,1,20754.293269
7,1,4279.232955

7,1,2597.938889
7,1,3627.315476
7,1,2208.853659
7,1,7429.227941
7,1,72344.578947
7,1,2033.712500
7,1,3766.243902
7,1,3679.875000
7,1,2053.778846
7,1,8670.085106
7,1,2479.174419
7,1,1903.465116
7,1,3771.932432
7,1,2738.651786
7,1,31640.475694
7,1,34904.059659
7,1,1985.259615
7,1,2725.421512
7,1,13998.523438
7,1,71539.263889
7,1,4009.070312
7,1,4856.130435
7,1,2340.514706
7,1,1542.934524
7,1,2420.391304
7,1,2781.940789
7,1,90386.270833
7,1,2986.200000
7,1,2136.068182
7,1,2798.755814
7,1,3624.296875
7,1,3359.931138
7,1,7044.360000
7,1,1754.804878
7,1,3685.407609
7,1,5770.470930
7,1,2004.438953
7,1,2602.408163
7,1,1600.243902
7,1,5232.333333
7,1,38663.694444
7,1,2090.558594
7,1,7592.254902
7,1,85794.025568
7,1,1898.923077
7,1,2112.534247
7,1,32983.099315
7,1,4467.325521
7,1,2297.250000
7,1,2483.795455
7,1,1931.990196
7,1,84151.265957
7,1,3580.815104
7,1,3132.735000
7,1,2421.681818
7,1,12802.786996
7,1,2217.478261
7,1,12120.567568
7,1,2448.141304
7,1,5220.612805
7,1,58072.086538
7,1,1782.132979
7,1,6006.530660
7,1,1539.073171
7,1,1919.630435
7,1,3255.769231

7,1,4203.112500
7,1,2519.409375
7,1,1726.553571
7,1,100589.689189
7,1,7324.482143
7,1,8368.156977
7,1,2468.021739
7,1,2404.088415
7,1,1805.450000
7,1,2615.832500
7,1,4024.964286
7,1,1945.250000
7,1,42389.831081
7,1,5712.292553
7,1,10101.791667
7,1,12429.441463
7,1,13881.427419
7,1,84524.069079
7,1,60909.994318
7,1,43583.005435
7,1,44867.934211
7,1,1834.407609
7,1,1513.075843
7,1,4206.064516
7,1,1503.250000
7,1,3548.625000
7,1,6220.375000
7,1,8412.350694
7,1,3900.511111
7,1,2160.975806
7,1,1815.250000
7,1,3759.622222
7,1,2122.125000
7,1,36457.375000
7,1,32370.600000
7,1,2209.348958
7,1,23666.482993
7,1,1906.948980
7,1,7450.540179
7,1,1557.666667
7,1,3727.278409
7,1,16635.866667
7,1,2555.396739
7,1,7570.500000
7,1,32071.461111
7,1,1781.631944
7,1,8468.509146
7,1,5432.987245
7,1,7734.915698
7,1,2257.255435
7,1,30759.554348
7,1,4813.483871
7,1,15353.222656
7,1,2953.437500
7,1,3717.252660
7,1,12587.250000
7,1,50987.506757
7,1,2336.983696
7,1,3685.161765
7,1,23682.875000
7,1,23137.579082
7,1,1848.562500
7,1,3591.083333
7,1,23854.709302
7,1,2245.029412
7,1,4674.038889

7,1,3228.062500
7,1,7554.847222
7,1,4323.427083
7,1,3324.892857
7,1,2547.295455
7,1,15351.803571
7,1,1810.934524
7,1,1873.937198
7,1,1684.404891
7,1,1716.110294
7,1,3530.062500
7,1,7157.032609
7,1,1569.830357
7,1,10584.724138
7,1,1725.820513
7,1,7702.960227
7,1,3411.583333
7,1,1716.843750
7,1,21598.765625
7,1,2229.275463
7,1,4011.193452
7,1,3112.846154
7,1,1665.326705
7,1,2679.508721
7,1,65571.510870
7,1,12985.113636
7,1,3753.046512
7,1,1630.964286
7,1,2346.647727
7,1,1666.887195
7,1,50538.875000
7,1,7259.137500
7,1,1703.758333
7,1,57645.244186
7,1,2869.576923
7,1,2277.600000
7,1,3305.475610
7,1,2744.395349
7,1,1690.621711
7,1,2427.560976
7,1,32657.500000
7,1,1940.008929
7,1,9625.752841
7,1,3238.261111
7,1,4256.763889
7,1,86247.034483
7,1,2276.924342
7,1,2573.842105
7,1,72344.678571
7,1,2150.178571
7,1,1969.000000
7,1,2911.664740
7,1,2461.730769
7,1,5310.238372
7,1,2500.865741
7,1,4568.375000
7,1,15003.750000
7,1,2008.742647
7,1,21201.015625
7,1,2472.800000
7,1,1779.698529
7,1,4805.335938
7,1,2087.377907
7,1,39439.134328
7,1,3086.884868
7,1,2025.764286

7,1,2578.558511
7,1,1594.750000
7,1,7099.758152
7,1,2693.482759
7,1,2175.888298
7,1,1656.812500
7,1,2949.401961
7,1,10720.515625
7,1,16694.611486
7,1,3541.630556
7,1,2117.409091
7,1,1666.142857
7,1,2475.111111
7,1,103830.285714
7,1,2034.056818
7,1,2169.800000
7,1,3898.868243
7,1,53951.588608
7,1,1981.009868
7,1,10401.973684
7,1,6559.837838
7,1,89308.833333
7,1,59432.916667
7,1,71960.218023
7,1,1558.250000
7,1,9057.775000
7,1,4712.500000
7,1,1714.219697
7,1,1749.151316
7,1,1699.578571
7,1,21751.817568
7,1,2051.519886
7,1,1555.024194
7,1,63281.261628
7,1,2259.563889
7,1,1735.133333
7,1,2015.382576
7,1,3213.625000
7,1,2298.410326
7,1,3176.316667
7,1,2641.365385
7,1,62515.187500
7,1,2022.105556
7,1,49165.100000
7,1,56605.135135
7,1,1665.598214
7,1,30084.571429
7,1,3129.392157
7,1,61555.453488
7,1,2993.969512
7,1,1699.100000
7,1,17990.000000
7,1,1582.282051
7,1,2799.428571
7,1,40470.285714
7,1,79233.782051
7,1,5391.495000
7,1,2789.529412
7,1,2351.544444
7,1,1942.230769
7,1,3958.625000
7,1,59992.326923
7,1,36928.142857
7,1,3002.095238
7,1,41081.506410
7,1,1572.756250

7,1,1861.322034
7,1,9697.785714
7,1,2103.261905
7,1,1678.766447
7,1,5476.110000
7,1,3281.284091
7,1,40889.375000
7,1,3523.675000
7,1,1964.146875
7,1,4225.859375
7,1,15574.389881
7,1,1529.524457
7,1,1788.833333
7,1,4009.780488
7,1,3937.197222
7,1,3681.765306
7,1,1680.718750
7,1,1685.850000
7,1,3759.138889
7,1,14381.540441
7,1,1518.170732
7,1,6400.994048
7,1,1564.329787
7,1,5989.700000
7,1,7831.113971
7,1,4088.703947
7,1,10323.741071
7,1,30350.715827
7,1,1949.565217
7,1,5350.712121
7,1,2845.234375
7,1,3766.134615
7,1,14810.542553
7,1,41640.090909
7,1,2367.207317
7,1,2206.161290
7,1,1626.026316
7,1,9589.130435
7,1,2402.616071
7,1,5293.744444
7,1,1657.551020
7,1,2202.809524
7,1,2328.594512
7,1,35323.887500
7,1,3028.303191
7,1,1614.045213
7,1,5874.325000
7,1,3072.335106
7,1,32060.106618
7,1,2118.986413
7,1,2680.867925
7,1,3146.614130
7,1,3683.300000
7,1,130188.834184
7,1,4552.703488
7,1,42483.585366
7,1,2616.423077
7,1,1893.348684
7,1,16730.286765
7,1,2185.925000
7,1,1836.320122
7,1,104754.750000
7,1,2882.293919
7,1,51816.857143
7,1,3639.204082
7,1,21482.271277

7,1,117523.166667
7,1,2545.500000
7,1,6743.914894
7,1,2260.594286
7,1,1564.293919
7,1,1847.739583
7,1,57155.138158
7,1,23500.319444
7,1,5772.935897
7,1,2092.565217
7,1,12709.713710
7,1,6347.057692
7,1,1867.697674
7,1,87720.730769
7,1,16617.500000
7,1,1707.867188
7,1,3925.786585
7,1,81428.812500
7,1,2669.823529
7,1,4705.391026
7,1,2612.889706
7,1,20245.573171
7,1,3075.186047
7,1,2462.428571
7,1,4829.551282
7,1,25604.919355
7,1,2833.378289
7,1,19785.816860
7,1,5012.088235
7,1,2594.190217
7,1,1786.785714
7,1,6822.416667
7,1,2970.042553
7,1,3906.894444
7,1,2025.416667
7,1,2520.804054
7,1,2065.256250
7,1,6619.145833
7,1,93983.687500
7,1,17842.958333
7,1,2209.736702
7,1,3937.880952
7,1,4985.597826
7,1,57111.107143
7,1,5563.319149
7,1,1541.750000
7,1,4613.997283
7,1,18484.511111
7,1,76011.261905
7,1,11016.580645
7,1,10068.775000
7,1,1515.750000
7,1,63825.250000
7,1,1842.250000
7,1,44771.315476
7,1,3491.781250
7,1,30557.428571
7,1,3359.456522
7,1,1683.440341
7,1,7850.340909
7,1,121399.500000
7,1,3570.062500
7,1,38662.500000
7,1,6644.906250
7,1,33981.275943
7,1,1537.144737

7,1,1867.238636
7,1,20116.154412
7,1,2362.291667
7,1,11789.530172
7,1,9068.581395
7,1,6515.540698
7,1,2512.703488
7,1,3385.336735
7,1,1644.693182
7,1,5845.980769
7,1,2189.621711
7,1,2110.796196
7,1,2326.520000
7,1,4392.271341
7,1,42446.964286
7,1,2649.406250
7,1,1514.016667
7,1,2715.486111
7,1,1506.523810
7,1,1528.375000
7,1,2750.861702
7,1,4138.579268
7,1,34655.541667
7,1,2005.075000
7,1,1600.576220
7,1,2252.345238
7,1,131584.465909
7,1,3614.132143
7,1,1530.005682
7,1,2732.780556
7,1,22952.450000
7,1,11850.125000
7,1,69058.866279
7,1,61477.510000
7,1,1525.485714
7,1,2339.611486
7,1,1556.098404
7,1,6791.791667
7,1,2175.800000
7,1,2747.000000
7,1,6406.787234
7,1,4316.864865
7,1,33829.088710
7,1,1984.585106
7,1,11871.579082
7,1,66135.232558
7,1,2558.234043
7,1,2654.725806
7,1,2868.630102
7,1,7464.236842
7,1,5483.039894
7,1,2352.614583
7,1,74719.053125
7,1,2031.905405
7,1,1894.945946
7,1,7036.584559
7,1,2688.500000
7,1,2748.581395
7,1,5072.610465
7,1,32342.204918
7,1,24288.670732
7,1,60975.904412
7,1,1511.213068
7,1,1816.307927
7,1,64251.142857
7,1,54512.180556

7,1,2293.314655
7,1,3308.673913
7,1,1882.115385
7,1,175906.743750
7,1,10450.078125
7,1,1655.526786
7,1,1614.593750
7,1,19706.336207
7,1,1733.963415
7,1,2007.340426
7,1,68880.390625
7,1,5180.823529
7,1,1668.489796
7,1,24797.687500
7,1,3131.860577
7,1,2790.895349
7,1,11522.275000
7,1,2044.723837
7,1,1936.295455
7,1,1526.233553
7,1,11469.769022
7,1,2252.875000
7,1,1798.750000
7,1,25844.926471
7,1,2443.263889
7,1,28779.866935
7,1,5218.981481
7,1,63000.897436
7,1,43206.314516
7,1,4267.915625
7,1,4645.616071
7,1,82561.101190
7,1,54930.171053
7,1,2010.991848
7,1,1721.000000
7,1,44898.750000
7,1,2044.475000
7,1,2468.290761
7,1,1505.245000
7,1,83813.500000
7,1,2983.789773
7,1,2687.612981
7,1,75648.986111
7,1,1799.368421
7,1,6918.005319
7,1,7244.437500
7,1,1889.277778
7,1,8469.638298
7,1,31211.458333
7,1,1827.582386
7,1,2485.730263
7,1,1861.411765
7,1,1725.045455
7,1,2129.878378
7,1,2103.775000
7,1,2217.348837
7,1,3462.704861
7,1,56407.011364
7,1,51592.554054
7,1,2231.522727
7,1,1582.195122
7,1,2502.954286
7,1,6717.744186
7,1,1829.801630
7,1,3169.074074
7,1,2520.103774

7,1,2982.347561
7,1,31900.065341
7,1,32991.227053
7,1,2956.667969
7,1,11968.937500
7,1,1628.468750
7,1,8933.625000
7,1,2506.548913
7,1,1648.372159
7,1,1922.121951
7,1,2453.255319
7,1,1991.398649
7,1,39073.993243
7,1,2306.214286
7,1,1629.507812
7,1,1688.846939
7,1,11877.617647
7,1,5013.452381
7,1,5154.923913
7,1,3458.128049
7,1,1655.971154
7,1,13487.343023
7,1,2168.464286
7,1,9077.829861
7,1,58197.662162
7,1,36354.197368
7,1,1547.138889
7,1,1723.166667
7,1,6543.910000
7,1,10639.600000
7,1,67937.756098
7,1,1552.928571
7,1,7398.304348
7,1,1726.967105
7,1,6356.531250
7,1,1675.062500
7,1,4804.615385
7,1,2612.732500
7,1,4305.418919
7,1,2806.027778
7,1,1969.750000
7,1,3156.157609
7,1,3543.654762
7,1,4578.101744
7,1,1715.250000
7,1,1777.453704
7,1,2156.890625
7,1,1520.287234
7,1,2896.358108
7,1,5990.390625
7,1,13829.614362
7,1,3615.875000
7,1,11239.518382
7,1,7536.323529
7,1,5773.000000
7,1,27385.572581
7,1,2729.009375
7,1,3403.191489
7,1,1582.694149
7,1,46527.742188
7,1,5230.544643
7,1,40220.173077
7,1,9017.218750
7,1,2657.826613
7,1,44751.071429
7,1,2235.647727

7,1,15242.470588
7,1,1742.543750
7,1,87117.795455
7,1,2077.534884
7,1,2981.209239
7,1,8091.275000
7,1,93682.520833
7,1,6528.219512
7,1,1698.239521
7,1,2515.634868
7,1,1526.763158
7,1,3200.483696
7,1,1565.638889
7,1,1502.505208
7,1,5153.065625
7,1,2586.511628
7,1,97820.585526
7,1,2748.477273
7,1,1773.951087
7,1,53078.712838
7,1,1900.706250
7,1,1669.761628
7,1,2122.442857
7,1,2804.642157
7,1,1746.305556
7,1,8133.732143
7,1,42622.885135
7,1,5022.375000
7,1,3406.512658
7,1,2485.354167
7,1,2785.409091
7,1,23159.750000
7,1,1720.425000
7,1,3463.970238
7,1,7059.375000
7,1,4255.709302
7,1,1628.059783
7,1,2346.414634
7,1,13577.979592
7,1,2639.920213
7,1,79886.731250
7,1,6061.466837
7,1,17449.593750
7,1,1716.250000
7,1,31653.094595
7,1,3913.336735
7,1,81739.426282
7,1,53485.365385
7,1,4411.338415
7,1,29703.666667
7,1,3097.864865
7,1,1889.776163
7,1,197312.910326
7,1,3646.186170
7,1,2867.485000
7,1,3332.363636
7,1,1569.265957
7,1,85705.115385
7,1,2346.582386
7,1,2357.019231
7,1,7288.860119
7,1,3313.194196
7,1,12414.662791
7,1,1527.795918
7,1,18455.217949
7,1,1795.875000

7,1,90018.282609
7,1,1995.448980
7,1,25616.159091
7,1,1668.000000
7,1,6984.468750
7,1,2618.975610
7,1,1887.562500
7,1,3865.247283
7,1,3737.218750
7,1,2323.988636
7,1,1890.166667
7,1,3705.269231
7,1,1647.152439
7,1,2175.259146
7,1,5544.051020
7,1,2175.861486
7,1,2953.281250
7,1,1537.396277
7,1,2018.316667
7,1,6147.642857
7,1,1852.113636
7,1,3123.739865
7,1,36400.648026
7,1,29245.482143
7,1,1988.867021
7,1,25809.727941
7,1,7715.065000
7,1,1630.353261
7,1,67120.482143
7,1,3151.300000
7,1,7624.011364
7,1,8388.603659
7,1,3616.875000
7,1,3312.710106
7,1,2761.920000
7,1,34766.268382
7,1,1663.708333
7,1,2067.443452
7,1,2543.739130
7,1,44504.076923
7,1,2256.488636
7,1,1545.402778
7,1,9340.829545
7,1,2073.838235
7,1,2166.378289
7,1,2583.945652
7,1,157484.703488
7,1,2123.454545
7,1,3134.658537
7,1,34678.517241
7,1,4052.166667
7,1,3035.416667
7,1,11733.829545
7,1,2946.913043
7,1,2858.785714
7,1,3531.108696
7,1,1693.116071
7,1,4151.741071
7,1,3225.584302
7,1,39489.285714
7,1,3295.406250
7,1,2635.630435
7,1,2012.050000
7,1,4118.898438
7,1,3998.420455
7,1,18342.625000

7,1,10268.692308
7,1,17796.563830
7,1,46377.243902
7,1,1944.720930
7,1,3931.891892
7,1,1957.765625
7,1,1979.007353
7,1,2895.384615
7,1,1504.910714
7,1,1612.401163
7,1,3231.238095
7,1,6829.940476
7,1,5759.281250
7,1,1569.554688
7,1,1629.850000
7,1,1909.403226
7,1,2023.541667
7,1,4180.333333
7,1,3022.111111
7,1,3259.950521
7,1,15137.951705
7,1,20439.000000
7,1,55854.487500
7,1,4559.328947
7,1,9899.864796
7,1,2395.534884
7,1,46640.325658
7,1,2310.000000
7,1,8674.403846
7,1,96153.575000
7,1,1836.935606
7,1,1707.538462
7,1,2077.991071
7,1,2161.473404
7,1,8992.248571
7,1,153226.706522
7,1,8005.039062
7,1,3157.395270
7,1,2370.488372
7,1,1500.000000
7,1,1772.372449
7,1,5507.369565
7,1,2576.812500
7,1,2619.253289
7,1,2368.041667
7,1,2003.342105
7,1,15048.518293
7,1,9955.022727
7,1,2264.000000
7,1,1521.549479
7,1,16080.077381
7,1,1795.515625
7,1,1568.903061
7,1,36323.856618
7,1,4847.835294
7,1,2216.750000
7,1,5575.469274
7,1,19820.500000
7,1,4004.795455
7,1,15801.000000
7,1,2602.700000
7,1,3622.148936
7,1,1525.279412
7,1,9632.188312
7,1,2111.987179
7,1,17690.794118

7,1,3046.131410
7,1,30589.987805
7,1,29214.014706
7,1,4402.541667
7,1,1865.725610
7,1,4555.574074
7,1,13509.345109
7,1,2375.250000
7,1,4429.964286
7,1,2309.929487
7,1,9660.383152
7,1,2520.053571
7,1,43475.970588
7,1,6502.500000
7,1,2193.447368
7,1,57512.414634
7,1,8043.500000
7,1,21812.000000
7,1,2148.307692
7,1,4522.556548
7,1,4889.522222
7,1,2948.278443
7,1,3735.457143
7,1,3295.000000
7,1,4630.080128
7,1,67925.852273
7,1,4680.212209
7,1,4918.142857
7,1,34269.658537
7,1,1737.193182
7,1,1727.167683
7,1,2106.813830
7,1,5261.899306
7,1,1883.404255
7,1,3390.102564
7,1,2854.009615
7,1,38720.590909
7,1,47147.625000
7,1,1511.580000
7,1,7400.513514
7,1,52028.273973
7,1,35261.215625
7,1,2082.965278
7,1,2658.104651
7,1,19632.181250
7,1,4031.584677
7,1,3416.515957
7,1,2884.711111
7,1,29586.650000
7,1,113077.413043
7,1,35554.859375
7,1,1624.752907
7,1,4096.602041
7,1,9443.888158
7,1,2709.921053
7,1,7360.482843
7,1,60485.450000
7,1,2721.081250
7,1,18551.290000
7,1,2775.770833
7,1,3908.185811
7,1,5112.615385
7,1,3866.164286
7,1,17510.224265
7,1,3353.395349
7,1,1959.750000

7,1,4531.428571
7,1,10970.346154
7,1,75546.184375
7,1,1923.772436
7,1,6075.217391
7,1,2982.539062
7,1,20383.612179
7,1,4593.447500
7,1,16945.000000
7,1,1535.346154
7,1,1861.829268
7,1,1644.268293
7,1,2047.511905
7,1,5460.696429
7,1,6051.750000
7,1,1519.581081
7,1,5038.282609
7,1,1647.609195
7,1,3325.299242
7,1,1968.650000
7,1,4638.385417
7,1,12620.590909
7,1,3427.571429
7,1,1857.405405
7,1,1763.059783
7,1,2568.250000
7,1,9095.826087
7,1,1953.488095
7,1,1808.808511
7,1,40173.143939
7,1,58164.898649
7,1,1646.952500
7,1,72984.422794
7,1,1633.682432
7,1,16810.700000
7,1,1910.413462
7,1,3218.150000
7,1,1517.906977
7,1,2971.781250
7,1,4410.968750
7,1,3477.593750
7,1,30064.734375
7,1,2558.977941
7,1,5084.959184
7,1,2670.295732
7,1,30546.109375
7,1,20716.203125
7,1,2168.021739
7,1,140025.753927
7,1,25504.424419
7,1,15692.699219
7,1,2397.819767
7,1,6365.215116
7,1,1614.763587
7,1,3756.368421
7,1,1518.844937
7,1,13143.575758
7,1,1844.679054
7,1,3673.725490
7,1,3531.500000
7,1,4807.371951
7,1,2544.229167
7,1,4365.039773
7,1,2525.162234
7,1,4917.925000
7,1,1581.397727

7,1,3159.148936
7,1,34748.419355
7,1,17557.109375
7,1,31393.797619
7,1,2684.488372
7,1,5811.481061
7,1,3655.284091
7,1,2274.110000
7,1,1592.575000
7,1,2387.477564
7,1,3367.836735
7,1,12263.773810
7,1,2600.807500
7,1,3183.453125
7,1,2963.154255
7,1,1704.675000
7,1,2416.178571
8,2,2150.127660
8,2,2033.927083
8,2,7232.916667
8,2,6350.361979
8,2,1602.700521
8,2,5703.838816
8,2,1557.381250
8,2,3015.363095
8,2,5239.993421
8,2,2617.942308
8,2,1686.848837
8,2,2030.606707
8,2,3989.247788
8,2,1717.785714
8,2,2803.363636
8,2,2057.835366
8,2,8204.685393
8,2,3590.765244
8,2,3726.960000
8,2,1738.559524
8,2,1972.267045
8,2,4664.761111
8,2,2039.560976
8,2,2587.310897
8,2,7517.802083
8,2,3860.552632
8,2,2648.934211
8,2,2010.452880
8,2,7147.147321
8,2,3804.418269
8,2,1956.578818
8,2,1893.886364
8,2,2111.478261
8,2,1761.163522
8,2,2991.293478
8,2,4205.875000
8,2,6143.529891
8,2,3365.575000
8,2,2561.684211
8,2,2560.031915
8,2,3165.335714
8,2,3742.433036
8,2,2974.541667
8,2,3547.110000
8,2,1939.851351
8,2,3426.392857
8,2,2652.255952
8,2,3211.984375
8,2,2009.393617

8,2,2601.303977
8,2,1704.094595
8,2,2863.093750
8,2,2198.494048
8,2,4248.708333
8,2,9242.005000
8,2,1644.184211
8,2,5580.067568
8,2,2968.583333
8,2,2603.071429
8,2,14196.562500
8,2,1718.529412
8,2,2354.282609
8,2,3823.102273
8,2,6250.819672
8,2,2267.873786
8,2,5218.413793
8,2,1904.898876
8,2,5574.154545
8,2,3718.562500
8,2,1812.162921
8,2,6545.937500
8,2,1536.843373
8,2,3650.544271
8,2,2041.214286
8,2,5939.154255
8,2,3394.875000
8,2,7797.967391
8,2,2221.027778
8,2,1633.941176
8,2,1933.470588
8,2,7591.653333
8,2,1975.796875
8,2,3786.148810
8,2,2330.097222
8,2,12730.706818
8,2,3269.095395
8,2,11759.883721
8,2,1510.150000
8,2,2973.828947
8,2,1641.729167
8,2,3050.304878
8,2,1774.110429
8,2,6961.727679
8,2,2854.375000
8,2,2776.369444
8,2,12956.366228
8,2,5027.847727
8,2,2392.711538
8,2,4636.798295
8,2,4752.527027
8,2,4536.781977
8,2,3249.173469
8,2,6097.570312
8,2,11810.135417
8,2,4696.067708
8,2,2110.769022
8,2,1904.117021
8,2,1934.355263
8,2,1609.609375
8,2,3410.408163
8,2,2135.401163
8,2,2389.250000
8,2,9187.701389
8,2,1965.927632
8,2,21519.261792

8,2,2152.454545
8,2,3777.625000
8,2,1536.667553
8,2,3658.371951
8,2,3669.528409
8,2,3064.500000
8,2,2955.600000
8,2,3315.323718
8,2,1585.050000
8,2,3238.096154
8,2,2570.750000
8,2,6991.690104
8,2,2044.690104
8,2,2821.854167
8,2,6377.250000
8,2,8295.493274
8,2,6068.449664
8,2,3152.146277
8,2,2224.404494
8,2,4464.790698
8,2,2224.638298
8,2,2368.331933
8,2,2524.767442
8,2,4232.787879
8,2,3365.085526
8,2,6806.444444
8,2,2029.578431
8,2,3498.309524
8,2,2542.068750
8,2,3226.750000
8,2,14453.807175
8,2,1904.336735
8,2,4789.975000
8,2,4411.980769
8,2,4988.693878
8,2,4545.563889
8,2,1711.665698
8,2,4164.478774
8,2,7506.959184
8,2,1751.562500
8,2,3061.079545
8,2,1869.400943
8,2,5979.949519
8,2,2834.695652
8,2,3014.306548
8,2,4376.957317
8,2,4864.720930
8,2,1551.433333
8,2,3272.000000
8,2,5107.929688
8,2,2208.418605
8,2,2325.409091
8,2,2388.846154
8,2,2556.320000
8,2,2802.025000
8,2,4134.416667
8,2,2771.300000
8,2,4353.784722
8,2,7172.849359
8,2,1843.982143
8,2,3863.584302
8,2,4132.160714
8,2,3513.534091
8,2,2385.648649
8,2,2119.775510
8,2,1755.315789

8,2,2224.261364
8,2,5532.269886
8,2,2418.975000
8,2,2790.194444
8,2,6440.552500
8,2,8651.385135
8,2,1819.887500
8,2,6693.491935
8,2,6524.765152
8,2,1972.773810
8,2,3012.602273
8,2,1686.539007
8,2,2408.720109
8,2,1851.465116
8,2,6392.583333
8,2,2303.331395
8,2,2215.202778
8,2,1519.166667
8,2,2474.600000
8,2,10399.576271
8,2,3285.152027
8,2,2147.027778
8,2,1745.522727
8,2,2432.581633
8,2,4144.717262
8,2,2755.700000
8,2,5436.025126
8,2,3272.628289
8,2,1533.677632
8,2,5998.029412
8,2,2037.666667
8,2,5900.394886
8,2,8021.357143
8,2,6267.000000
8,2,3616.696133
8,2,1950.475962
8,2,2100.682927
8,2,2314.718232
8,2,1738.561181
8,2,2687.942308
8,2,4734.975806
8,2,2313.040698
8,2,1713.795455
8,2,5155.910377
8,2,1552.592105
8,2,4449.241071
8,2,4210.054054
8,2,1757.190476
8,2,1648.365922
8,2,4944.581395
8,2,2214.181548
8,2,3681.435000
8,2,3337.057692
8,2,3017.390244
8,2,2004.633333
8,2,6660.824074
8,2,1640.390244
8,2,5399.383721
8,2,3627.531915
8,2,2113.040865
8,2,2189.132353
8,2,1998.205882
8,2,2142.246988
8,2,2180.500000
8,2,2833.508929
8,2,1524.333333

8,2,54187.850610
8,2,3637.086957
8,2,4911.463415
8,2,7196.553571
8,2,9280.810547
8,2,2369.433962
8,2,4732.856707
8,2,1554.154412
8,2,1539.744048
8,2,1793.082317
8,2,3356.722826
8,2,4258.255319
8,2,8778.006098
8,2,1660.932692
8,2,5336.177273
8,2,4028.686047
8,2,3550.868750
8,2,2968.875000
8,2,8717.081250
8,2,2852.883721
8,2,3299.442935
8,2,10659.147727
8,2,6110.480769
8,2,10096.928571
8,2,1622.750000
8,2,3759.015152
8,2,8351.044872
8,2,3050.609091
8,2,2450.367647
8,2,6285.042553
8,2,2810.000000
8,2,2070.333333
8,2,1568.221591
8,2,4673.696429
8,2,1845.670854
8,2,1679.192308
8,2,2270.357955
8,2,1883.470833
8,2,4006.474359
8,2,6369.713942
8,2,2266.101852
8,2,1634.577500
8,2,5753.572222
8,2,2411.979592
8,2,4886.276730
8,2,2563.505814
8,2,3690.094340
8,2,2243.855114
8,2,1576.559211
8,2,1550.118750
8,2,1500.404605
8,2,1537.112500
8,2,1934.200000
8,2,6239.721429
8,2,2330.230114
8,2,5303.471939
8,2,2323.070988
8,2,6075.062500
8,2,2093.109375
8,2,4734.888298
8,2,6906.790816
8,2,1584.576159
8,2,7917.111111
8,2,1825.383721
8,2,2995.280303
8,2,2459.298429

8,2,5558.432065
8,2,3188.050532
8,2,3418.460938
8,2,1796.471354
8,2,3657.980392
8,2,13207.218750
8,2,1819.562500
8,2,3251.120000
8,2,2053.000000
8,2,5959.428571
8,2,3975.978723
8,2,4974.461538
8,2,4495.173913
8,2,4842.974359
8,2,1956.173611
8,2,5105.476190
8,2,4755.591667
8,2,4347.054348
8,2,2052.000000
8,2,3606.230769
8,2,3016.693878
8,2,6671.771429
8,2,3183.409091
8,2,1585.279070
8,2,2074.977778
8,2,1607.295732
8,2,21999.474576
8,2,2702.243902
8,2,1543.558333
8,2,1989.816327
8,2,1673.554348
8,2,2832.075000
8,2,1771.382212
8,2,7525.945161
8,2,2840.600000
8,2,3619.612903
8,2,1764.005000
8,2,9124.125000
8,2,2333.256757
8,2,1542.110429
8,2,8127.972603
8,2,3308.329787
8,2,20944.000000
8,2,8040.005682
8,2,1647.781437
8,2,3345.612245
8,2,2530.897727
8,2,1668.877841
8,2,4806.610294
8,2,3765.240909
8,2,2160.567308
8,2,1562.343750
8,2,7037.911458
8,2,1774.787736
8,2,4710.822917
8,2,2418.225694
8,2,5567.085106
8,2,3140.637500
8,2,4493.861111
8,2,5455.664894
8,2,2805.615385
8,2,4105.620000
8,2,45111.604027
8,2,3252.024823
8,2,2795.854167
8,2,13021.589286

8,2,2088.050000
8,2,2895.625000
8,2,3518.773707
8,2,6836.714286
8,2,1669.853723
8,2,3793.441489
8,2,1596.775000
8,2,1652.810714
8,2,1836.075000
8,2,1664.551020
8,2,2408.093023
8,2,3627.457447
8,2,1573.186224
8,2,2585.614865
8,2,2653.937500
8,2,9948.677966
8,2,1670.675000
8,2,5891.340909
8,2,6299.000000
8,2,3256.241848
8,2,2752.967391
8,2,8868.188462
8,2,3364.250000
8,2,2468.403846
8,2,1654.217742
8,2,3039.435897
8,2,2698.526316
8,2,4485.365269
8,2,3680.810811
8,2,2869.544118
8,2,2548.592593
8,2,2371.898810
8,2,4119.968750
8,2,3941.325000
8,2,2064.000000
8,2,3751.995370
8,2,2685.053571
8,2,10642.333333
8,2,3853.072222
8,2,2632.965909
8,2,1827.886364
8,2,1855.094340
8,2,1780.006410
8,2,4563.250000
8,2,1785.857143
8,2,2816.271930
8,2,4872.964286
8,2,5159.826087
8,2,14116.222222
8,2,1961.833333
8,2,4324.417749
8,2,4682.833333
8,2,1962.536458
8,2,4797.105263
8,2,2448.851190
8,2,3570.053191
8,2,3619.000000
8,2,9086.637681
8,2,3561.825472
8,2,1802.797872
8,2,2143.377246
8,2,3479.380814
8,2,4080.691860
8,2,1853.630814
8,2,1775.354592
8,2,3112.209135

8,2,1926.805233
8,2,2349.235577
8,2,3696.475000
8,2,4208.625000
8,2,2468.661765
8,2,4234.642857
8,2,1931.050000
8,2,1705.037234
8,2,4173.994318
8,2,5037.875000
8,2,1898.090278
8,2,3928.075000
8,2,3829.055288
8,2,1549.297872
8,2,2672.354271
8,2,3773.850962
8,2,2060.629630
8,2,1801.137931
8,2,1842.755319
8,2,1549.076923
8,2,1772.265625
8,2,1727.000000
8,2,5773.206522
8,2,4038.363636
8,2,1582.590909
8,2,1920.580000
8,2,1984.601852
8,2,1869.935714
8,2,1972.541667
8,2,5094.054217
8,2,3050.972093
8,2,18125.837209
8,2,2971.781977
8,2,1960.304348
8,2,3826.233333
8,2,1507.818878
8,2,5796.948864
8,2,1988.541667
8,2,2391.584375
8,2,2785.087838
8,2,2147.126667
8,2,2301.650000
8,2,2697.106250
8,2,9755.500000
8,2,2777.540323
8,2,2644.572917
8,2,1676.777778
8,2,5113.255319
8,2,5134.875000
8,2,6174.390000
8,2,3109.865000
8,2,2858.875000
8,2,2701.437500
8,2,2752.521472
8,2,4608.000000
8,2,3950.612500
8,2,2709.162791
8,2,2975.636364
8,2,5566.000000
8,2,3393.528846
8,2,4591.555556
8,2,12998.580000
8,2,6521.958333
8,2,9778.062500
8,2,2046.700000
8,2,2455.750000

8,2,2602.262195
8,2,3620.882353
8,2,6020.827830
8,2,4648.275510
8,2,1992.869898
8,2,2989.715426
8,2,10715.013298
8,2,3710.091146
8,2,2078.922156
8,2,2080.461957
8,2,1562.712500
8,2,1797.113636
8,2,4151.484375
8,2,1652.053571
8,2,3605.603659
8,2,6826.426630
8,2,9463.703125
8,2,4156.000000
8,2,2559.648026
8,2,2170.593137
8,2,4665.500000
8,2,4404.177326
8,2,5991.750000
8,2,3622.591133
8,2,1700.524272
8,2,3319.959302
8,2,3713.566265
8,2,2375.809375
8,2,3281.700000
8,2,4472.361809
8,2,8404.790816
8,2,1531.596859
8,2,4171.210526
8,2,2537.780488
8,2,1528.688953
8,2,4110.666667
8,2,1614.057143
8,2,5729.782609
8,2,5682.960784
8,2,2557.656250
8,2,1503.156627
8,2,3367.178125
8,2,1856.190000
8,2,2929.950000
8,2,2060.125000
8,2,2344.517857
8,2,4828.230556
8,2,1554.208333
8,2,3357.770000
8,2,4816.790323
8,2,1754.600000
8,2,6338.250000
8,2,3873.081081
8,2,6247.980978
8,2,4445.898707
8,2,6919.319767
8,2,5916.848214
8,2,4202.631579
8,2,3156.853659
8,2,6462.890547
8,2,2192.993421
8,2,12700.352381
8,2,2506.000000
8,2,6829.142857
8,2,1615.615385
8,2,4251.752358

8,2,3432.341837
8,2,1505.592105
8,2,8268.579861
8,2,2091.301471
8,2,2395.783019
8,2,5594.678571
8,2,2867.767500
8,2,17530.947917
8,2,3008.522059
8,2,1966.500000
8,2,3766.566489
8,2,8474.083682
8,2,11174.475936
8,2,12751.380682
8,2,2456.617021
8,2,8139.420732
8,2,7156.493776
8,2,2126.324324
8,2,2077.412500
8,2,3056.500000
8,2,2775.193627
8,2,3176.259615
8,2,5349.634146
8,2,2118.804878
8,2,3022.000000
8,2,5255.691489
8,2,2834.733333
8,2,2905.030612
8,2,1648.644444
8,2,1908.708333
8,2,2483.593750
8,2,1742.964912
8,2,10865.555000
8,2,1824.988372
8,2,8367.392442
8,2,4564.348837
8,2,4566.625000
8,2,12496.663265
8,2,2936.823864
8,2,18222.788043
8,2,1987.250000
8,2,1547.140000
8,2,2529.666667
8,2,2260.724490
8,2,7764.904762
8,2,2129.818750
8,2,9160.085000
8,2,3079.640244
8,2,4412.488208
8,2,1772.575000
8,2,11116.201389
8,2,10182.377698
8,2,2267.836957
8,2,3049.850610
8,2,4298.289894
8,2,2932.225000
8,2,7491.611702
8,2,5683.230114
8,2,2121.798780
8,2,3673.541667
8,2,2067.780488
8,2,1953.317073
8,2,1648.555556
8,2,1600.362245
8,2,5975.465909
8,2,5521.232227

8,2,4314.109649
8,2,4608.121951
8,2,3676.727273
8,2,4590.640625
8,2,3089.778125
8,2,1989.272727
8,2,1978.217391
8,2,6979.842105
8,2,3897.225806
8,2,2703.230769
8,2,1620.906250
8,2,5801.054054
8,2,5645.619469
8,2,2142.065217
8,2,5645.859375
8,2,2553.918675
8,2,2882.784753
8,2,5818.397059
8,2,9708.125000
8,2,16096.686047
8,2,2001.579545
8,2,2133.350000
8,2,4451.600000
8,2,8697.194444
8,2,1588.409091
8,2,3889.380435
8,2,3661.333333
8,2,3300.656250
8,2,3494.615854
8,2,7622.668675
8,2,4169.185841
8,2,5043.383495
8,2,2539.257353
8,2,2266.402439
8,2,2580.500000
8,2,2365.159375
8,2,2276.418750
8,2,7545.964844
8,2,1864.250000
8,2,3133.800000
8,2,2189.793478
8,2,1949.564286
8,2,1846.351163
8,2,7551.504566
8,2,6441.718750
8,2,1571.709821
8,2,30374.608553
8,2,1520.267045
8,2,5571.855769
8,2,3496.226190
8,2,3587.644737
8,2,3964.619444
8,2,5124.364583
8,2,2571.600000
8,2,1561.263889
8,2,6891.569444
8,2,4727.325581
8,2,4581.880952
8,2,1854.731707
8,2,1843.980769
8,2,1695.401786
8,2,1728.893617
8,2,7103.944444
8,2,1981.326220
8,2,2349.784091
8,2,3002.459459

8,2,1562.840625
8,2,3468.057065
8,2,1882.358974
8,2,2679.178977
8,2,1503.250000
8,2,4526.909091
8,2,7907.930851
8,2,10358.044643
8,2,1778.130319
8,2,3451.666667
8,2,6698.971154
8,2,1653.093750
8,2,19910.038462
8,2,6179.238938
8,2,8888.267241
8,2,6199.384615
8,2,3637.097561
8,2,4509.921512
8,2,1978.005000
8,2,11116.186047
8,2,2109.277778
8,2,2074.675000
8,2,6972.470238
8,2,2759.282051
8,2,8050.654255
8,2,3649.439815
8,2,2658.100000
8,2,7477.507463
8,2,5717.670732
8,2,1526.154255
8,2,3163.916667
8,2,2860.769886
8,2,3442.387500
8,2,2452.845361
8,2,2681.434483
8,2,16622.777778
8,2,2615.479167
8,2,3854.174863
8,2,5047.629808
8,2,1880.620536
8,2,4159.300000
8,2,1916.203125
8,2,13586.013889
8,2,1871.971154
8,2,2444.898936
8,2,8924.085106
8,2,15857.056410
8,2,2528.338235
8,2,2230.010929
8,2,4783.166667
8,2,1976.393229
8,2,1609.315789
8,2,5216.794643
8,2,1499.243590
8,2,1824.125000
8,2,1578.150000
8,2,2097.165761
8,2,4754.860577
8,2,2753.028571
8,2,5325.000000
8,2,4115.677273
8,2,5650.480000
8,2,6441.301676
8,2,3809.006818
8,2,1982.733333
8,2,2395.867347

8,2,6226.511628
8,2,4818.109375
8,2,2393.215909
8,2,3720.424020
8,2,3253.954545
8,2,2256.875000
8,2,4103.299419
8,2,2750.750000
8,2,2979.970109
8,2,11332.603365
8,2,4039.468137
8,2,2659.973404
8,2,3310.914439
8,2,4580.956731
8,2,5241.694444
8,2,1552.454787
8,2,3680.093750
8,2,7474.475000
8,2,3159.050000
8,2,3279.015625
8,2,14399.000000
8,2,1760.893617
8,2,2035.271739
8,2,2284.288462
8,2,3230.875000
8,2,2300.654762
8,2,1597.266667
8,2,2091.190625
8,2,2378.953125
8,2,9260.201923
8,2,1749.948864
8,2,3161.682692
8,2,9453.777174
8,2,2659.625000
8,2,2241.191860
8,2,4205.992021
8,2,4511.625000
8,2,3221.841216
8,2,1927.750000
8,2,3290.684896
8,2,1842.393939
8,2,1632.598039
8,2,3001.517442
8,2,2168.503378
8,2,6829.015000
8,2,3834.170213
8,2,1917.151163
8,2,2389.885638
8,2,4234.510204
8,2,3861.692308
8,2,1906.600000
8,2,3814.865385
8,2,8124.642857
8,2,3179.375000
8,2,2487.768293
8,2,2457.045455
8,2,1935.871711
8,2,2318.831731
8,2,9661.455882
8,2,1532.039062
8,2,4228.125000
8,2,2649.829545
8,2,2561.562500
8,2,3352.468085
8,2,2814.884615
8,2,2334.097561

8,2,5677.745614
8,2,6105.678571
8,2,10773.015873
8,2,3386.421053
8,2,3564.959459
8,2,2490.196429
8,2,1504.386364
8,2,6939.455128
8,2,10245.239583
8,2,3520.586538
8,2,2001.562500
8,2,2675.062500
8,2,2560.526882
8,2,1859.041667
8,2,2678.519886
8,2,3258.914894
8,2,4401.375000
8,2,1890.838068
8,2,3262.851064
8,2,1733.052326
8,2,3373.466667
8,2,6540.200000
8,2,4082.113636
8,2,1759.098837
8,2,1862.542553
8,2,5615.031250
8,2,1660.985795
8,2,4557.000000
8,2,1524.160714
8,2,6069.425150
8,2,2682.473214
8,2,3941.272727
8,2,2021.950000
8,2,7090.826705
8,2,3067.652174
8,2,1780.174419
8,2,2570.630000
8,2,1542.268939
8,2,6200.009434
8,2,6321.843750
8,2,2002.332386
8,2,8503.000000
8,2,2946.450000
8,2,2095.777174
8,2,1865.733333
8,2,1813.936170
8,2,2525.319149
8,2,4276.762295
8,2,19739.152027
8,2,2900.203488
8,2,1704.176471
8,2,1729.485714
8,2,6610.179688
8,2,4263.842391
8,2,2218.627660
8,2,4281.495192
8,2,2090.057692
8,2,4924.444444
8,2,1650.290625
8,2,4211.013514
8,2,22482.750000
8,2,4905.219828
8,2,4592.559524
8,2,5787.987245
8,2,2551.825000
8,2,1505.485577

8,2,6299.000000
8,2,1613.517442
8,2,1710.666667
8,2,1785.009259
8,2,2322.483333
8,2,1860.836478
8,2,2385.130435
8,2,1519.558442
8,2,1819.799383
8,2,3305.368715
8,2,3662.668919
8,2,1566.461538
8,2,2254.185714
8,2,2354.028409
8,2,20110.962264
8,2,17761.775000
8,2,1669.293750
8,2,10880.557927
8,2,60817.529412
8,2,2286.046875
8,2,2726.634615
8,2,2746.313636
8,2,3866.595745
8,2,11707.354651
8,2,3438.452128
8,2,2026.500000
8,2,8447.855263
8,2,2384.984043
8,2,1834.625000
8,2,3314.650862
8,2,3135.070312
8,2,4685.437500
8,2,2056.718750
8,2,2957.765625
8,2,3903.635135
8,2,10086.397727
8,2,3334.652174
8,2,4958.334091
8,2,2638.884146
8,2,1943.336538
8,2,2617.834239
8,2,10311.734146
8,2,3689.945946
8,2,3964.510638
8,2,2479.806452
8,2,3524.200000
8,2,2265.849057
8,2,1700.046875
8,2,2598.375000
8,2,5267.592391
8,2,2256.142857
8,2,1826.119565
8,2,2211.872340
8,2,3279.285714
8,2,10201.475000
8,2,3609.000000
8,2,7964.451271
8,2,1900.625000
8,2,7516.625000
8,2,2387.111702
8,2,2542.250000
8,2,3529.297872
8,2,2383.733696
8,2,3954.750000
8,2,2098.015625
8,2,4478.586207

8,2,1553.650000
8,2,3203.021552
8,2,11890.978571
8,2,3493.812500
8,2,12593.867187
8,2,2603.056818
8,2,2056.029762
8,2,8567.552632
8,2,1806.075000
8,2,3394.337209
8,2,1795.178571
8,2,9968.375000
8,2,3210.656250
8,2,6914.326087
8,2,9805.254545
8,2,2872.562500
8,2,3033.402439
8,2,1902.341463
8,2,5294.200000
8,2,11617.207547
8,2,4605.072115
8,2,2412.416667
8,2,2379.885638
8,2,1876.484043
8,2,3017.106481
8,2,3885.445652
8,2,2691.509375
8,2,3160.930851
8,2,5787.902098
8,2,2486.781250
8,2,1630.184091
8,2,1576.017442
8,2,1912.592105
8,2,3471.416667
8,2,4207.340909
8,2,2329.341346
8,2,2398.214286
8,2,2985.505882
8,2,3709.800000
8,2,2631.186047
8,2,5001.000000
8,2,2922.149289
8,2,3701.522222
8,2,1701.537234
8,2,4204.887821
8,2,7218.960526
8,2,2291.532051
8,2,1697.680000
8,2,2734.766304
8,2,1743.968553
8,2,1836.062500
8,2,2969.000000
8,2,2755.639175
8,2,2923.201613
8,2,5770.201087
8,2,6420.765000
8,2,6070.922872
8,2,3941.250000
8,2,2666.206522
8,2,2238.791667
8,2,4454.897727
8,2,3909.185185
8,2,15298.894737
8,2,2632.946602
8,2,1812.342105
8,2,1527.892473

8,2,1514.375000
8,2,3160.916667
8,2,4337.139151
8,2,2981.142857
8,2,1638.542763
8,2,1788.073529
8,2,5371.215686
8,2,2781.750000
8,2,1848.420455
8,2,1892.736111
8,2,2487.465116
8,2,9204.875000
8,2,2508.941176
8,2,1521.527174
8,2,4346.447917
8,2,3930.798295
8,2,1736.266447
8,2,4396.367021
8,2,1617.446809
8,2,2254.896277
8,2,2106.250000
8,2,4311.541667
8,2,2738.400000
8,2,1910.416667
8,2,4003.871429
8,2,8239.495192
8,2,2637.050000
8,2,7220.532609
8,2,6390.713542
8,2,2415.400000
8,2,1953.180000
8,2,2132.305000
8,2,5142.756579
8,2,2144.937500
8,2,3484.448864
8,2,3488.294118
8,2,11661.500000
8,2,4160.147436
8,2,2824.470000
8,2,9561.718085
8,2,1804.666667
8,2,5902.863636
8,2,7825.175000
8,2,40634.500000
8,2,2173.011628
8,2,6555.414894
8,2,9045.864865
8,2,2009.795213
8,2,2291.166667
8,2,7072.916667
8,2,1855.158537
8,2,2668.875000
8,2,9919.733696
8,2,2043.353659
8,2,2098.621711
8,2,13266.425000
8,2,2896.931034
8,2,2836.392157
8,2,3673.338542
8,2,3532.741935
8,2,5398.843137
8,2,5505.752778
8,2,55579.670213
8,2,3203.769444
8,2,4751.182266
8,2,2643.984375

8,2,6917.605000
8,2,2512.995614
8,2,4960.318750
8,2,3193.454082
8,2,1927.200431
8,2,2957.632653
8,2,9748.456522
8,2,4680.543919
8,2,5974.312500
8,2,9677.777500
8,2,4888.191327
8,2,3025.231481
8,2,2025.100000
8,2,5456.729167
8,2,13264.500000
8,2,2654.978723
8,2,2721.970588
8,2,1985.465116
8,2,4995.115385
8,2,2167.897727
8,2,1918.284091
8,2,2118.572368
8,2,4548.062500
8,2,2425.588235
8,2,1970.823171
8,2,1879.640625
8,2,3907.590116
8,2,2062.152778
8,2,2665.389423
8,2,3547.941860
8,2,8159.361635
8,2,1661.125000
8,2,1864.334459
8,2,7923.750000
8,2,4709.483516
8,2,1924.625000
8,2,4346.040816
8,2,6517.125000
8,2,13437.933333
8,2,2375.143293
8,2,3306.357500
8,2,1878.945312
8,2,2788.531250
8,2,3775.562500
8,2,8795.570312
8,2,5943.981132
8,2,2003.966667
8,2,1795.375000
8,2,15228.893939
8,2,1521.540541
8,2,4469.297872
8,2,2516.333333
8,2,1529.564024
8,2,5333.436321
8,2,7682.622222
8,2,5623.653061
8,2,2339.177083
8,2,4604.588415
8,2,4894.076923
8,2,6584.192308
8,2,10658.770349
8,2,4306.457386
8,2,2329.125000
8,2,2421.307692
8,2,2772.500000
8,2,1575.242647

8,2,1661.531250
8,2,4919.055556
8,2,8048.812500
8,2,3273.538462
8,2,4443.560000
8,2,2863.936275
8,2,1781.450000
8,2,7443.792135
8,2,6176.372449
8,2,2760.524038
8,2,2746.086957
8,2,1647.441860
8,2,73943.698980
8,2,3555.000000
8,2,39834.279070
8,2,3225.625000
8,2,2542.744048
8,2,1825.187500
8,2,5232.115741
8,2,2298.733333
8,2,4420.795455
8,2,2859.750000
8,2,4379.619681
8,2,2778.718750
8,2,6690.464286
8,2,2902.520270
8,2,2965.152174
8,2,3839.287162
8,2,3471.906250
8,2,7163.463235
8,2,4126.025510
8,2,3789.236364
8,2,1766.851064
8,2,4525.252193
8,2,7077.940789
8,2,8031.267045
8,2,1640.718750
8,2,1719.901961
8,2,3767.237500
8,2,1929.812500
8,2,1535.290541
8,2,5633.818182
8,2,2113.041667
8,2,2870.061224
8,2,8082.539474
8,2,7367.250000
8,2,5593.331633
8,2,2538.574468
8,2,5778.673913
8,2,1684.443590
8,2,6786.540284
8,2,5598.744681
8,2,9438.115385
8,2,2869.360577
8,2,1631.300000
8,2,2494.762376
8,2,3559.145455
8,2,1774.238095
8,2,1850.227273
8,2,3800.046875
8,2,24842.460366
8,2,2123.812500
8,2,3088.535714
8,2,3925.116071
8,2,9311.293839
8,2,6149.272727

8,2,2245.480769
8,2,22275.707317
8,2,12122.080000
8,2,9548.243902
8,2,7975.075000
8,2,10964.580645
8,2,1570.482955
8,2,2003.005319
8,2,3521.654762
8,2,7503.047414
8,2,2346.901042
8,2,2277.578947
8,2,5675.916667
8,2,1770.604651
8,2,23824.668103
8,2,9313.313889
8,2,5475.345745
8,2,3782.571429
8,2,3293.143750
8,2,13106.778061
8,2,3524.028846
8,2,5410.018182
8,2,2603.213235
8,2,4718.021739
8,2,2566.398256
8,2,4527.183511
8,2,8155.680000
8,2,1767.812500
8,2,2263.785714
8,2,6596.627907
8,2,1639.951220
8,2,10307.034188
8,2,2815.791209
8,2,6049.285714
8,2,5727.160526
8,2,6314.574713
8,2,1747.647059
8,2,9160.411765
8,2,1811.090909
8,2,3868.010204
8,2,1842.383721
8,2,4490.138889
8,2,3850.413043
8,2,2428.125000
8,2,1787.268293
8,2,3015.197802
8,2,2008.206667
8,2,3522.007389
8,2,3729.897727
8,2,7410.250000
8,2,2596.908482
8,2,4618.343750
8,2,1578.244444
8,2,6877.934524
8,2,2074.687500
8,2,2343.555556
8,2,2807.562500
8,2,1801.375000
8,2,4217.937500
8,2,2557.316176
8,2,2936.541667
8,2,6400.598485
8,2,3369.428571
8,2,1902.421875
8,2,4425.988372
8,2,6816.345588

8,2,14088.655405
8,2,1719.602041
8,2,5801.334821
8,2,2360.326923
8,2,5958.587500
8,2,3185.887755
8,2,1695.395833
8,2,2136.484375
8,2,3752.750000
8,2,8628.103261
8,2,2102.882653
8,2,4705.127072
8,2,1551.583333
8,2,4544.114754
8,2,4554.000000
8,2,4994.194444
8,2,2263.506757
8,2,2144.190000
8,2,4239.692568
8,2,2413.592213
8,2,5363.402439
8,2,2286.480000
8,2,2064.840426
8,2,4245.500000
8,2,1644.182692
8,2,2702.483871
8,2,7774.941489
8,2,1683.941489
8,2,3214.614583
8,2,7332.153846
8,2,6096.676471
8,2,16140.250000
8,2,2459.187500
8,2,4104.655556
8,2,11998.524457
8,2,32148.659091
8,2,5975.593023
8,2,5230.868421
8,2,14769.921875
8,2,2509.250000
8,2,4218.824074
8,2,2158.847682
8,2,2189.048780
8,2,2189.044444
8,2,1565.936170
8,2,5851.562500
8,2,5428.142857
8,2,2883.583333
8,2,26625.046512
8,2,2539.875000
8,2,1965.137500
8,2,9084.048295
8,2,3372.262500
8,2,1848.338235
8,2,2110.450000
8,2,3554.327778
8,2,2502.740909
8,2,4182.948980
8,2,2526.993750
8,2,2922.914773
8,2,2338.330556
8,2,3508.469298
8,2,1682.242857
8,2,3260.172297
8,2,4323.964706
8,2,2045.226667

8,2,3198.347826
8,2,1529.833333
8,2,2371.228261
8,2,2283.750000
8,2,12057.720745
8,2,11171.375000
8,2,13469.766990
8,2,4077.358491
8,2,2863.816176
8,2,7331.451531
8,2,14454.179245
8,2,1549.830357
8,2,1983.600000
8,2,1553.569767
8,2,2015.761628
8,2,2824.293750
8,2,4433.661932
8,2,2745.463415
8,2,27283.138889
8,2,2798.877717
8,2,5217.197674
8,2,1895.000000
8,2,2582.125000
8,2,2113.200581
8,2,2574.947674
8,2,1999.724359
8,2,1762.269430
8,2,1749.743902
8,2,2313.625000
8,2,7064.627193
8,2,1878.750000
8,2,1901.804054
8,2,5126.990741
8,2,17384.412791
8,2,3510.668571
8,2,1717.398256
8,2,4897.199519
8,2,3009.139881
8,2,2471.146341
8,2,1756.405000
8,2,3528.628571
8,2,5841.617788
8,2,1665.512195
8,2,2319.165865
8,2,3279.412791
8,2,2587.950000
8,2,4703.378049
8,2,2819.088068
8,2,2890.448980
8,2,2306.032051
8,2,2783.179688
8,2,6253.750000
8,2,5443.693627
8,2,3036.220238
8,2,7825.178977
8,2,1847.875000
8,2,2645.604651
8,2,5411.333333
8,2,12840.757576
8,2,4794.000000
8,2,2495.842105
8,2,4531.425532
8,2,5093.125000
8,2,2750.343750
8,2,1792.638889
8,2,3427.732955

8,2,3698.142857
8,2,6455.953125
8,2,6546.219388
8,2,1906.428571
8,2,2085.201705
8,2,1587.250000
8,2,1991.259375
8,2,2453.675532
8,2,2471.492021
8,2,2091.833333
8,2,2119.005814
8,2,1540.913265
8,2,2498.000000
8,2,10132.625000
8,2,4509.573171
8,2,2079.089744
8,2,8874.115196
8,2,1680.950581
8,2,2085.266667
8,2,3302.264535
8,2,1803.333333
8,2,1859.250000
8,2,2838.250000
8,2,3067.902985
8,2,6483.233173
8,2,3612.775000
8,2,7810.216667
8,2,3178.279070
8,2,4789.309783
8,2,4028.300000
8,2,9722.951220
8,2,1520.011628
8,2,21515.600000
8,2,2805.107527
8,2,2781.810345
8,2,6600.066832
8,2,3851.183673
8,2,7931.750000
8,2,2566.302083
8,2,1725.659091
8,2,2697.755102
8,2,1577.894886
8,2,2744.091667
8,2,3164.890625
8,2,1754.518072
8,2,1778.166667
8,2,3093.595238
8,2,5867.313679
8,2,2944.818713
8,2,1812.338462
8,2,1525.353723
8,2,2515.926829
8,2,1599.761628
8,2,5333.843023
8,2,1739.270270
8,2,1651.701087
8,2,2277.350610
8,2,1983.347826
8,2,1648.931818
8,2,6914.716981
8,2,3217.250000
8,2,1527.028846
8,2,7387.527027
8,2,8115.967213
8,2,1620.000000
8,2,2123.785714

8,2,2255.841667
8,2,2544.447222
8,2,2483.598404
8,2,2182.201087
8,2,3948.928571
8,2,1625.341216
8,2,1661.606132
8,2,2467.293478
8,2,1963.336310
8,2,2375.904255
8,2,3932.754717
8,2,1721.580357
8,2,1503.906250
8,2,4542.043478
8,2,2235.659091
8,2,2872.906250
8,2,2077.701923
8,2,3360.872093
8,2,2299.942708
8,2,4654.510204
8,2,7791.285714
8,2,1677.595745
8,2,3925.400000
8,2,2141.538462
8,2,6725.244681
8,2,1540.665698
8,2,2105.810897
8,2,4793.130435
8,2,2815.000000
8,2,6263.933333
8,2,3291.548387
8,2,2556.048913
8,2,1707.761364
8,2,12148.593750
8,2,9128.269068
8,2,29290.358696
8,2,3531.107143
8,2,3071.466837
8,2,3573.843750
8,2,15274.683962
8,2,5746.796569
8,2,9160.693182
8,2,3168.806818
8,2,3609.597561
8,2,5323.762295
8,2,1530.945122
8,2,2386.406250
8,2,6825.397727
8,2,2623.815789
8,2,3357.099359
8,2,4911.002841
8,2,4203.877551
8,2,7175.750000
8,2,4039.238095
8,2,2205.806818
8,2,8110.857143
8,2,5107.760638
8,2,8557.184783
8,2,1645.552632
8,2,1843.062500
8,2,5317.033333
8,2,8999.744186
8,2,3559.631579
8,2,4718.282609
8,2,5293.230769
8,2,9550.703125

8,2,3869.274457
8,2,4840.423228
8,2,1805.000000
8,2,3296.521739
8,2,14922.846154
8,2,10264.858407
8,2,6985.609375
8,2,1886.560976
8,2,2427.869048
8,2,3311.596875
8,2,10132.000000
8,2,2031.750000
8,2,9308.505525
8,2,2026.095238
8,2,5050.219512
8,2,2190.076087
8,2,1578.069079
8,2,3169.666667
8,2,1791.406915
8,2,2905.392857
8,2,5208.535000
8,2,1738.515152
8,2,5282.173913
8,2,2175.395349
8,2,3769.244681
8,2,6326.139706
8,2,6369.355392
8,2,2295.692308
8,2,1722.171053
8,2,2623.223881
8,2,2537.525862
8,2,2390.334459
8,2,1633.171196
8,2,2737.939655
8,2,1935.138889
8,2,1805.287234
8,2,1783.098837
8,2,1978.627604
8,2,5086.223684
8,2,7749.816176
8,2,5945.528302
8,2,2829.951220
8,2,2288.586957
8,2,3312.068750
8,2,4145.476744
8,2,3578.145349
8,2,5749.913265
8,2,2480.266667
8,2,8035.189732
8,2,5497.200000
8,2,3046.834459
8,2,2640.495098
8,2,1972.454787
8,2,2464.554054
8,2,2091.658537
8,2,4149.973684
8,2,2038.480556
8,2,1724.332237
8,2,4001.403125
8,2,9691.548077
8,2,2230.541667
8,2,6741.064286
8,2,2140.521739
8,2,2307.363636
8,2,4349.079327
8,2,2104.615000

8,2,2182.776786
8,2,4313.691489
8,2,2742.601064
8,2,5387.540816
8,2,4554.000000
8,2,3433.813253
8,2,5145.567633
8,2,2417.839623
8,2,2376.685000
8,2,1641.087209
8,2,2838.679054
8,2,2221.554688
8,2,4523.651786
8,2,5223.632653
8,2,1914.916667
8,2,4486.333333
8,2,3068.609375
8,2,2181.958333
8,2,7009.867788
8,2,4339.271739
8,2,1576.500000
8,2,1630.412500
8,2,1796.145833
8,2,3592.468023
8,2,2283.166667
8,2,6518.093220
8,2,2177.940789
8,2,2920.529255
8,2,2516.230556
8,2,6492.747807
8,2,4111.759615
8,2,7109.041667
8,2,2512.140306
8,2,2723.920455
8,2,7737.020000
8,2,3600.068182
8,2,2671.148438
8,2,4178.348485
8,2,2412.363636
8,2,3794.651163
8,2,2975.671053
8,2,3706.505435
8,2,2809.520000
8,2,2004.795455
8,2,3232.260870
8,2,7688.718750
8,2,2185.625000
8,2,1617.692308
8,2,1905.792683
8,2,5598.968023
8,2,11891.945312
8,2,4368.125000
8,2,6370.475610
8,2,11658.693182
8,2,23639.653846
8,2,12494.470199
8,2,1977.750000
8,2,4549.531250
8,2,1716.485714
8,2,1563.265625
8,2,4062.010695
8,2,3690.068627
8,2,2949.225490
8,2,1945.487500
8,2,1525.428571
8,2,6572.715426

8,2,4114.562500
8,2,3500.468750
8,2,2978.414634
8,2,3066.384615
8,2,2347.459459
8,2,2160.375000
8,2,1680.136905
8,2,1701.500000
8,2,2288.142857
8,2,6135.174419
8,2,5386.524390
8,2,2788.666667
8,2,1787.293478
8,2,10200.834375
8,2,1959.416667
8,2,6746.674419
8,2,14469.696429
8,2,2797.671875
8,2,3430.909884
8,2,2653.181548
8,2,3038.739583
8,2,1773.494444
8,2,2619.406452
8,2,3040.799107
8,2,7668.246795
8,2,23250.964844
8,2,3714.550000
8,2,2658.339286
8,2,2109.179348
8,2,2745.863636
8,2,3215.052632
8,2,5247.769231
8,2,2181.083832
8,2,1648.938424
8,2,3395.850000
8,2,4776.137255
8,2,3004.022727
8,2,2479.610577
8,2,5871.423529
8,2,2309.750000
8,2,1550.740566
8,2,12700.187500
8,2,1677.280488
8,2,5847.279070
8,2,2431.229167
8,2,3633.317073
8,2,1978.320652
8,2,3066.532609
8,2,1980.598837
8,2,1657.204861
8,2,4257.271429
8,2,9401.704955
8,2,1617.160448
8,2,3913.268072
8,2,3254.476064
8,2,4759.305000
8,2,4107.166667
8,2,6178.096154
8,2,2323.640000
8,2,2602.957317
8,2,4028.551136
8,2,34848.537879
8,2,4836.813953
8,2,2617.143750
8,2,3364.937500
8,2,3674.630814

8,2,2218.989796
8,2,2714.744565
8,2,2155.520000
8,2,4073.859155
8,2,9932.666667
8,2,7543.045455
8,2,2918.395833
8,2,2860.725000
8,2,6274.721649
8,2,2434.761290
8,2,2661.155405
8,2,4565.510000
8,2,3680.344444
8,2,6279.064904
8,2,6031.898438
8,2,4540.616279
8,2,1957.411765
8,2,16474.814480
8,2,6352.852941
8,2,7245.774510
8,2,1538.702830
8,2,4959.724537
8,2,1806.184492
8,2,2433.841912
8,2,4554.523936
8,2,4054.469388
8,2,3012.692935
8,2,5363.931319
8,2,6333.142857
8,2,2454.864130
8,2,6008.714286
8,2,10949.500000
8,2,3845.087500
8,2,10570.470588
8,2,1888.197917
8,2,7204.196721
8,2,1768.985294
8,2,1880.402778
8,2,2602.008523
8,2,4350.725490
8,2,3361.310345
8,2,2896.347682
8,2,1709.295455
8,2,5760.851744
8,2,1618.308824
8,2,3407.274194
8,2,2102.396277
8,2,9460.662088
8,2,3519.840909
8,2,4555.313776
8,2,2231.192857
8,2,4029.571429
8,2,4674.680365
8,2,6079.200000
8,2,10583.704545
8,2,1521.264423
8,2,6733.905660
8,2,3671.365132
8,2,7779.103723
8,2,2917.087838
8,2,25194.422727
8,2,1582.875000
8,2,6666.894231
8,2,2370.651163
8,2,2118.095745
8,2,5682.086957

8,2,2334.500000
8,2,1842.494565
8,2,3773.921196
8,2,15285.730088
8,2,2999.538462
8,2,5842.166667
8,2,2067.717822
8,2,3446.806604
8,2,15588.289634
8,2,3037.741848
8,2,1973.937500
8,2,1673.396875
8,2,9804.384375
8,2,5342.352941
8,2,2040.107143
8,2,2139.628205
8,2,7149.494048
8,2,2429.410256
8,2,13569.105263
8,2,2700.892857
8,2,2039.646739
8,2,2580.570652
8,2,2067.898810
8,2,4614.000000
8,2,7400.723214
8,2,6812.387019
8,2,5887.779070
8,2,6051.459906
8,2,1530.678571
8,2,1549.922794
8,2,3719.666667
8,2,3893.159763
8,2,2089.002146
8,2,5619.448529
8,2,2232.386905
8,2,1885.084906
8,2,2423.113426
8,2,1703.512195
8,2,4324.081250
8,2,9337.344697
8,2,1511.686170
8,2,3852.375000
8,2,2724.208333
8,2,1968.317073
8,2,1872.941176
8,2,4103.701923
8,2,1641.434783
8,2,4441.280556
8,2,3639.807692
8,2,7253.754717
8,2,1840.460227
8,2,5118.673611
8,2,10685.472500
8,2,7678.957447
8,2,3728.160000
8,2,2602.158371
8,2,1514.480769
8,2,2769.765957
8,2,2444.410714
8,2,3021.771429
8,2,8236.452586
8,2,1905.414634
8,2,1570.485714
8,2,2683.600000
8,2,2221.883929
8,2,1968.446875

8,2,3904.790816
8,2,1689.007979
8,2,2351.704545
8,2,2158.212500
8,2,5516.009868
8,2,1532.950000
8,2,2161.868590
8,2,3277.250000
8,2,4433.774457
8,2,2686.100000
8,2,1621.182927
8,2,1564.208333
8,2,3283.000000
8,2,3009.500000
8,2,5658.409091
8,2,13736.485294
8,2,2375.918367
8,2,1593.757143
8,2,5791.872449
8,2,2492.848485
8,2,3286.947917
8,2,2946.693750
8,2,2438.450000
8,2,2009.856618
8,2,2648.225000
8,2,2641.385135
8,2,4047.971591
8,2,5387.523936
8,2,1693.500000
8,2,2669.381098
8,2,3133.810000
8,2,9215.725806
8,2,3117.207447
8,2,2202.808824
8,2,2418.401914
8,2,4526.831633
8,2,2512.176136
8,2,1788.321429
8,2,3134.968750
8,2,8507.724432
8,2,3392.480769
8,2,2881.716981
8,2,3532.870787
8,2,2344.544737
8,2,1503.014368
8,2,6697.101351
8,2,3404.612903
8,2,1734.230769
8,2,3070.972222
8,2,3751.051402
8,2,1575.834375
8,2,1827.420000
8,2,2102.015000
8,2,6054.652174
8,2,2483.611842
8,2,3312.108974
8,2,1864.347826
8,2,2425.056872
8,2,2116.137755
8,2,2458.253521
8,2,4938.970588
8,2,11250.949074
8,2,1881.260638
8,2,4399.150735
8,2,1760.727273
8,2,2150.368902

8,2,6497.700000
8,2,10856.117021
8,2,2421.635000
8,2,2024.581522
8,2,2156.000000
8,2,2152.714286
8,2,2477.316038
8,2,2767.089286
8,2,4313.154762
8,2,27479.432065
8,2,7059.771277
8,2,4191.625000
8,2,7256.187500
8,2,2022.542553
8,2,7620.864130
8,2,4253.099359
8,2,1979.000000
8,2,1843.340426
8,2,6561.346698
8,2,1743.505682
8,2,3234.093750
8,2,2600.789474
8,2,2112.095092
8,2,1888.761905
8,2,6985.593023
8,2,2878.406977
8,2,2095.694767
8,2,1630.271341
8,2,2380.150685
8,2,2521.325000
8,2,2557.549242
8,2,4093.591837
8,2,3534.234043
8,2,7325.250000
8,2,2120.022727
8,2,1647.246951
8,2,1708.342105
8,2,2069.170213
8,2,7276.315217
8,2,3454.788043
8,2,1652.369565
8,2,1561.087766
8,2,2100.829268
8,2,1869.539474
8,2,9991.495000
8,2,2060.647059
8,2,3836.062500
8,2,10442.886792
8,2,2583.875000
8,2,3847.255556
8,2,2726.731250
8,2,2339.844340
8,2,5722.503125
8,2,2186.437500
8,2,3159.194079
8,2,2067.750000
8,2,1523.330645
8,2,2074.972500
8,2,1631.146341
8,2,3461.526786
8,2,4094.451220
8,2,31819.410714
8,2,1625.801471
8,2,4837.250000
8,2,7865.222543
8,2,4720.310606

8,2,4581.642105
8,2,2987.759259
8,2,1922.568452
8,2,2125.711207
8,2,9287.472826
8,2,27562.304878
8,2,7343.425926
8,2,1724.960000
8,2,1734.842105
8,2,2049.292969
8,2,1731.504808
8,2,2097.259434
8,2,1941.839286
8,2,2389.216216
8,2,2161.011628
8,2,4647.636364
8,2,3042.118056
8,2,4477.250000
8,2,2734.736486
8,2,2106.235000
8,2,6295.097222
8,2,6607.709497
8,2,5836.769531
8,2,8064.558140
8,2,1683.416667
8,2,5364.476923
8,2,2191.621622
8,2,3486.692308
8,2,6042.932692
8,2,4948.888889
8,2,1867.984375
8,2,2707.875000
8,2,2059.291667
8,2,3563.595395
8,2,3451.476415
8,2,3410.782895
8,2,2984.956522
8,2,2474.250000
8,2,9839.694149
8,2,5687.373171
8,2,1979.286486
8,2,2240.475524
8,2,4019.416667
8,2,2427.669643
8,2,4018.193717
8,2,7298.318681
8,2,8162.546358
8,2,2953.492925
8,2,1912.312500
8,2,3107.938725
8,2,2304.361702
8,2,2246.455882
8,2,8042.051282
8,2,3455.065574
8,2,3339.528302
8,2,1994.148148
8,2,3785.497423
8,2,5452.502551
8,2,4779.833333
8,2,6496.860465
8,2,6187.097222
8,2,2711.958333
8,2,1555.197917
8,2,1570.112500
8,2,1919.290909
8,2,4687.700000

8,2,2273.317308
8,2,1902.608696
8,2,15147.402439
8,2,9770.000000
8,2,2128.915865
8,2,7359.625000
8,2,2291.023256
8,2,3383.545918
8,2,1666.164474
8,2,1985.564024
8,2,1599.402174
8,2,2401.537791
8,2,2293.428125
8,2,2387.005435
8,2,1823.427632
8,2,3423.849138
8,2,2176.333333
8,2,2769.189189
8,2,2956.478723
8,2,5008.177778
8,2,2010.459135
8,2,2623.478723
8,2,3998.723558
8,2,1585.736842
8,2,2407.323171
8,2,3709.679878
8,2,2392.797619
8,2,2519.243902
8,2,2866.617021
8,2,7472.925000
8,2,7049.542553
8,2,2510.551887
8,2,1746.604730
8,2,1598.625000
8,2,2769.902778
8,2,3616.312500
8,2,3040.937500
8,2,5171.611111
8,2,1637.685714
8,2,1837.088068
8,2,2320.829268
8,2,3523.875000
8,2,16700.801020
8,2,4247.970000
8,2,3905.563953
8,2,5762.321429
8,2,1572.156250
8,2,5051.000000
8,2,2498.658784
8,2,2731.369318
8,2,12662.703488
8,2,2590.900000
8,2,8563.154676
8,2,2420.268229
8,2,3304.000000
8,2,1867.000000
8,2,4587.450000
8,2,12836.744681
8,2,1777.276596
8,2,6722.650000
8,2,4002.830645
8,2,3279.279070
8,2,3288.392045
8,2,5970.733333
8,2,2092.031250
8,2,2135.918605

8,2,1687.695122
8,2,3554.850610
8,2,3004.920213
8,2,2051.057692
8,2,6216.416667
8,2,7481.078261
8,2,4709.627660
8,2,2933.055556
8,2,2062.579268
8,2,3060.234694
8,2,4689.406250
8,2,2974.900000
8,2,2082.682927
8,2,2275.716667
8,2,2212.654545
8,2,4270.223214
8,2,1786.282051
8,2,1695.786111
8,2,3739.101770
8,2,3087.691489
8,2,2470.026786
8,2,6371.281250
8,2,3223.531250
8,2,1734.534884
8,2,4853.572368
8,2,2798.383152
8,2,1826.076220
8,2,2579.607143
8,2,1826.801242
8,2,2670.000000
8,2,2167.361842
8,2,4661.897436
8,2,1560.250000
8,2,17164.920000
8,2,4793.000000
8,2,9703.887755
8,2,5269.909091
8,2,1668.803571
8,2,2477.540541
8,2,5418.540541
8,2,6335.244681
8,2,1659.585938
8,2,1859.740000
8,2,1878.628571
8,2,2820.989130
8,2,3191.529801
8,2,2076.757500
8,2,6391.489130
8,2,1614.636364
8,2,2218.505952
8,2,2103.800000
8,2,1921.583333
8,2,5298.801587
8,2,1823.390244
8,2,3442.380952
8,2,1607.305921
8,2,7231.450000
8,2,1645.247191
8,2,6861.990385
8,2,3199.187500
8,2,2296.631250
8,2,1576.576923
8,2,3154.900000
8,2,5274.500000
8,2,6916.202128
8,2,1903.972973

8,2,4556.072115
8,2,3596.166667
8,2,1735.997159
8,2,2687.976190
8,2,2950.245283
8,2,3197.964286
8,2,3567.782051
8,2,5693.832386
8,2,1604.576923
8,2,2121.017766
8,2,4655.833333
8,2,1600.939024
8,2,4409.047500
8,2,1630.734375
8,2,3361.103774
8,2,22802.162162
8,2,2641.991848
8,2,1669.733333
8,2,1950.435000
8,2,3014.514286
8,2,2340.777778
8,2,2337.513298
8,2,1680.532609
8,2,1894.255814
8,2,25488.130208
8,2,2183.304348
8,2,1674.838068
8,2,2105.000000
8,2,2242.333333
8,2,1911.189189
8,2,1727.468750
8,2,2945.447500
8,2,6217.484375
8,2,2136.444444
8,2,2120.000000
8,2,2081.587500
8,2,4224.474057
8,2,3645.160000
8,2,2248.861111
8,2,5633.944444
8,2,1610.715000
8,2,1990.674419
8,2,5856.021277
8,2,6355.712575
8,2,3715.546512
8,2,4337.080189
8,2,2173.952128
8,2,6753.070000
8,2,2753.288462
8,2,2577.440000
8,2,2999.613636
8,2,2829.127660
8,2,1895.387195
8,2,4369.602679
8,2,2623.941860
8,2,6748.879630
8,2,8327.000000
8,2,4445.857143
8,2,4022.244253
8,2,1735.459459
8,2,1573.176471
8,2,2947.848684
8,2,2124.361702
8,2,1933.587500
8,2,1956.170732
8,2,2478.166667

8,2,1939.050000
8,2,3539.750000
8,2,4091.833333
8,2,2256.942308
8,2,4809.089286
8,2,7287.515625
8,2,2438.555556
8,2,1792.231707
8,2,2600.909091
8,2,10607.073770
8,2,3196.747596
8,2,2214.136126
8,2,4908.050898
8,2,3444.171196
8,2,8477.975000
8,2,1763.019231
8,2,5525.212500
8,2,8904.287500
8,2,2384.616279
8,2,1645.576923
8,2,6089.174419
8,2,2249.882353
8,2,6814.687500
8,2,3146.682292
8,2,4136.875000
8,2,1865.873418
8,2,4787.506329
8,2,6233.760638
8,2,2046.668269
8,2,2238.642361
8,2,1649.087413
8,2,1767.200581
8,2,2891.658537
8,2,1919.272455
8,2,21190.530220
8,2,4428.469274
8,2,2711.531250
8,2,4212.222222
8,2,5090.952381
8,2,2305.703125
8,2,1685.592593
8,2,2396.243902
8,2,4592.937500
8,2,15558.442308
8,2,1763.729299
8,2,1785.693299
8,2,2656.500000
8,2,3307.081731
8,2,1812.357143
8,2,12306.843750
8,2,2087.173913
8,2,1770.310811
8,2,1713.848214
8,2,4771.071429
8,2,2804.421875
8,2,4211.197674
8,2,2166.565789
8,2,2369.267241
8,2,2998.468750
8,2,3231.380952
8,2,1727.769444
8,2,1661.357558
8,2,4809.560000
8,2,2072.808511
8,2,3532.561404
8,2,4244.916667

8,2,7803.827128
8,2,1872.573171
8,2,1522.945312
8,2,3521.791667
8,2,2364.506757
8,2,5364.566327
8,2,2512.035714
8,2,9357.594444
8,2,1583.795918
8,2,5599.207729
8,2,1907.097727
8,2,2450.375000
8,2,10977.957447
8,2,1584.152439
8,2,3136.894737
8,2,3397.092262
8,2,1690.835526
8,2,3506.096154
8,2,12468.306818
8,2,4468.493421
8,2,4053.337209
8,2,18783.455882
8,2,6143.203125
8,2,13031.346939
8,2,4443.075581
8,2,1882.000000
8,2,2550.015000
8,2,1696.555921
8,2,4246.384615
8,2,1955.904255
8,2,3127.246231
8,2,4931.263889
8,2,5626.321429
8,2,2599.000000
8,2,1952.500000
8,2,4739.180851
8,2,4336.466667
8,2,2925.036111
8,2,3625.558140
8,2,1900.022500
8,2,2003.092949
8,2,2145.326220
8,2,2000.212766
8,2,2059.826087
8,2,2610.928977
8,2,2203.473837
8,2,2830.093750
8,2,1789.927632
8,2,3370.556250
8,2,2780.540816
8,2,2527.350000
8,2,1999.394737
8,2,3845.100000
8,2,1626.071856
8,2,2314.320946
8,2,3184.628676
8,2,3997.454545
8,2,1635.219512
8,2,3528.900000
8,2,2647.928571
8,2,2011.069307
8,2,4226.329268
8,2,1671.345946
8,2,6308.266839
8,2,3539.060976
8,2,8008.833333

8,2,1928.410256
8,2,1536.670732
8,2,1930.218750
8,2,2487.962766
8,2,2910.658088
8,2,1719.777778
8,2,5841.244681
8,2,2297.200000
8,2,3067.210526
8,2,2924.021341
8,2,2381.153646
8,2,2188.710526
8,2,2944.622093
8,2,9047.225000
8,2,2905.750000
8,2,1752.687500
8,2,1735.076087
8,2,4250.950000
8,2,4702.890086
8,2,1544.321608
8,2,11311.520408
8,2,3237.860000
8,2,4359.446927
8,2,3223.318182
8,2,1579.433673
8,2,2056.743243
8,2,2760.457143
8,2,9576.627907
8,2,3576.123153
8,2,2339.915205
8,2,2519.784091
8,2,2027.451613
8,2,2532.711957
8,2,1985.770408
8,2,2293.120000
8,2,3052.434211
8,2,2778.108974
8,2,2616.281250
8,2,2081.563830
8,2,2607.420213
8,2,4000.982500
8,2,1774.250000
8,2,2949.005814
8,2,2493.843182
8,2,3376.470588
8,2,4693.586538
8,2,4990.968182
8,2,4236.611111
8,2,3669.169811
8,2,2233.883721
8,2,2423.032895
8,2,2951.783163
8,2,2479.122283
8,2,2812.306818
8,2,5128.100000
8,2,3521.000000
8,2,3673.557292
8,2,1927.109375
8,2,3292.277027
8,2,1965.123711
8,2,8296.162500
8,2,1724.788043
8,2,3815.237113
8,2,3093.216518
8,2,2469.308140
8,2,5703.342105

8,2,5601.716981
8,2,2420.514706
8,2,1760.522222
8,2,2298.718182
8,2,1572.425000
8,2,2277.585366
8,2,2892.424419
8,2,4992.064189
8,2,3013.445714
8,2,4979.500000
8,2,1627.476923
8,2,2408.194767
8,2,3079.985714
8,2,1947.128205
8,2,2553.540984
8,2,2134.092949
8,2,3185.063679
8,2,7932.979167
8,2,2799.436224
8,2,4298.214286
8,2,1770.425481
8,2,3988.120690
8,2,1726.800000
8,2,1718.687500
8,2,2580.690789
8,2,1726.000000
8,2,2401.202778
8,2,4564.611111
8,2,2070.400510
8,2,2364.414634
8,2,5839.390625
8,2,2210.195652
8,2,2572.315789
8,2,2643.388889
8,2,5048.310096
8,2,3030.650424
8,2,8862.005319
8,2,1975.390244
8,2,2191.000000
8,2,3233.666667
8,2,14550.418919
8,2,2759.452703
8,2,2656.115183
8,2,1601.337838
8,2,4479.812500
8,2,1938.192308
8,2,1943.215909
8,2,2934.778125
8,2,3638.693182
8,2,13402.211207
8,2,4238.867647
8,2,2101.715116
8,2,2461.796053
8,2,2126.758621
8,2,5431.466667
8,2,8823.240385
8,2,2288.625000
8,2,4760.174479
8,2,3227.785714
8,2,2546.272727
8,2,5247.454268
8,2,1772.462500
8,2,1881.118750
8,2,1602.187500
8,2,6326.828704
8,2,1935.090909

8,2,2113.265957
8,2,3169.202703
8,2,4045.134615
8,2,2375.740000
8,2,4699.223404
8,2,3462.588957
8,2,1771.793194
8,2,1812.488636
8,2,2220.834302
8,2,5727.443878
8,2,2339.777143
8,2,9196.489362
8,2,1528.952830
8,2,1658.362500
8,2,2244.565625
8,2,6509.368952
8,2,6406.184375
8,2,2780.421053
8,2,3823.000000
8,2,6415.328125
8,2,2361.528846
8,2,3372.160256
8,2,1773.546053
8,2,3342.225000
8,2,2625.333333
8,2,15265.342105
8,2,1732.506173
8,2,3544.600000
8,2,3335.750000
8,2,2325.691489
8,2,1664.837838
8,2,1523.479730
8,2,1963.375000
8,2,1848.453883
8,2,5940.315476
8,2,3777.625000
8,2,3742.476190
8,2,3560.105263
8,2,2938.713889
8,2,7943.096154
8,2,7825.009615
8,2,3012.701087
8,2,4783.700617
8,2,2388.162791
8,2,4164.010870
8,2,3694.107558
8,2,1791.853659
8,2,3952.384146
8,2,2612.387195
8,2,1874.979167
8,2,4191.640244
8,2,2300.287162
8,2,1903.506098
8,2,2863.385417
8,2,2353.837963
8,2,2110.347826
8,2,2904.450000
8,2,3189.593750
8,2,4202.186813
8,2,6963.759162
8,2,11638.880682
8,2,2074.418478
8,2,31325.292683
8,2,5634.101562
8,2,4097.463235
8,2,1828.402778

8,2,1728.659091
8,2,9201.650000
8,2,4716.000000
8,2,2626.263736
8,2,1636.300000
8,2,1851.918269
8,2,3280.208333
8,2,3617.614130
8,2,2114.173611
8,2,1980.821053
8,2,2672.842105
8,2,3748.604167
8,2,4740.536313
8,2,4843.985294
8,2,7115.448276
8,2,3310.804348
8,2,2518.451389
8,2,2459.340625
8,2,1628.255952
8,2,1732.678571
8,2,3706.916667
8,2,1822.432432
8,2,1692.473958
8,2,2235.588889
8,2,1881.327778
8,2,2745.825658
8,2,3283.000000
8,2,3965.941327
8,2,6495.760000
8,2,2062.356061
8,2,2163.237805
8,2,4098.875000
8,2,1520.448980
8,2,1805.687500
8,2,3463.514706
8,2,2603.696875
8,2,2622.170732
8,2,4076.250000
8,2,2914.031250
8,2,1575.125000
8,2,3062.502660
8,2,5615.961538
8,2,4348.138249
8,2,4781.506250
8,2,3527.012500
8,2,2569.756410
8,2,7165.180556
8,2,4691.712209
8,2,4331.062500
8,2,1933.075893
8,2,3062.493750
8,2,3786.221154
8,2,3382.168103
8,2,2106.132812
8,2,1906.443627
8,2,4677.625000
8,2,3319.030612
8,2,2049.125000
8,2,1726.522222
8,2,2390.195946
8,2,2901.925000
8,2,6618.722222
8,2,1992.600000
8,2,1728.842767
8,2,10069.111111
8,2,4118.497674

8,2,4011.138298
8,2,1536.454802
8,2,7643.221622
8,2,2672.727273
8,2,2083.666667
8,2,3942.742021
8,2,1893.700000
8,2,1667.708333
8,2,1749.750000
8,2,5086.906977
8,2,1865.153846
8,2,3059.315789
8,2,3569.232143
8,2,2349.067568
8,2,1672.020833
8,2,3821.172414
8,2,2468.621622
8,2,2052.401786
8,2,1872.158537
8,2,6295.944196
8,2,1994.789062
8,2,2287.380682
8,2,2514.300847
8,2,2346.987342
8,2,4719.974359
8,2,8193.510417
8,2,5016.329167
8,2,1720.675676
8,2,4702.547619
8,2,3469.840909
8,2,1660.718750
8,2,4783.697674
8,2,5133.551020
8,2,2732.510000
8,2,4797.659375
8,2,7595.118750
8,2,8008.491379
8,2,2675.002841
8,2,2780.338983
8,2,2209.972561
8,2,5405.584135
8,2,4782.531646
8,2,3066.005682
8,2,4152.107143
8,2,2948.662234
8,2,3202.651596
8,2,1743.926020
8,2,2545.743590
8,2,5119.880319
8,2,1745.045455
8,2,1669.883333
8,2,1746.947368
8,2,5151.677130
8,2,7334.418129
8,2,2905.478723
8,2,2411.340909
8,2,3286.333333
8,2,3804.380000
8,2,6037.074074
8,2,2434.641892
8,2,2886.625000
8,2,2951.258929
8,2,3469.918605
8,2,3952.328125
8,2,1846.872159
8,2,5228.303571

8,2,3772.265625
8,2,2391.894737
8,2,7621.919118
8,2,6796.839286
8,2,1870.750000
8,2,5875.192708
8,2,6443.720930
8,2,2956.640000
8,2,5380.618090
8,2,1876.322581
8,2,3451.460938
8,2,15789.117925
8,2,3996.579545
8,2,1911.767857
8,2,3166.448113
8,2,8029.717391
8,2,1922.218750
8,2,5384.793103
8,2,7398.385000
8,2,5610.360577
8,2,1556.571023
8,2,9614.434524
8,2,24818.428571
8,2,1525.939024
8,2,6608.716216
8,2,4417.614286
8,2,2312.555556
8,2,20218.540625
8,2,2874.109694
8,2,9145.773585
8,2,2975.458333
8,2,3366.437500
8,2,2171.843023
8,2,4460.452381
8,2,7368.693878
8,2,3218.880814
8,2,3410.728125
8,2,2337.775785
8,2,2532.786932
8,2,3443.412791
8,2,2121.397436
8,2,3519.216216
8,2,8963.600000
8,2,2827.688830
8,2,5130.131250
8,2,2197.846354
8,2,4810.760417
8,2,3059.301136
8,2,7699.416667
8,2,2506.815104
8,2,2123.055851
8,2,2787.025641
8,2,6543.439103
8,2,6953.859375
8,2,3219.928571
8,2,2324.317708
8,2,6437.932927
8,2,3492.936170
8,2,2736.489362
8,2,1611.052083
8,2,3447.892857
8,2,2325.266667
8,2,3930.615000
8,2,1602.300000
8,2,2746.149390
8,2,3716.970588

8,2,4222.829787
8,2,4150.352941
8,2,10004.300000
8,2,4389.925000
8,2,3604.756410
8,2,6961.675926
8,2,1570.850000
8,2,1511.544643
8,2,1830.913043
8,2,6691.919643
8,2,10330.640625
8,2,2752.789216
8,2,3657.483240
8,2,2056.703125
8,2,30461.017442
8,2,1579.864865
8,2,1767.872283
8,2,5653.675000
8,2,4298.448529
8,2,1587.562500
8,2,1867.195755
8,2,1546.160000
8,2,4232.283784
8,2,4515.514423
8,2,9667.943182
8,2,2101.914894
8,2,2605.190341
8,2,4741.294444
8,2,8022.474537
8,2,1623.527778
8,2,4556.250000
8,2,11847.966346
8,2,3935.993056
8,2,4412.745763
8,2,1566.324324
8,2,1798.602564
8,2,3700.308511
8,2,2014.136364
8,2,3363.883333
8,2,1673.757653
8,2,1674.238095
8,2,2620.014706
8,2,2145.398438
8,2,6420.524064
8,2,3239.483146
8,2,3776.982759
8,2,5474.977273
8,2,2343.770701
8,2,6849.611399
8,2,6586.765244
8,2,1846.483173
8,2,2501.666667
8,2,12521.901786
8,2,3742.875000
8,2,5147.913889
8,2,2651.745536
8,2,1872.643382
8,2,7980.111111
8,2,2922.250000
8,2,4152.191176
8,2,1507.225962
8,2,5594.275000
8,2,2200.475490
8,2,3176.000000
8,2,4383.828125
8,2,1780.196429

8,2,3054.907609
8,2,4098.986486
8,2,2166.848485
8,2,1798.394886
8,2,1683.161184
8,2,3014.903409
8,2,1613.736842
8,2,4137.803571
8,2,2086.454819
8,2,5066.488095
8,2,1584.913690
8,2,2001.333333
8,2,1740.371622
8,2,2354.338889
8,2,2316.325000
8,2,1534.125000
8,2,5839.483696
8,2,3655.150568
8,2,3894.500000
8,2,3689.698020
8,2,2753.911111
8,2,1532.253378
8,2,1706.375000
8,2,3083.612500
8,2,4210.202128
8,2,2664.276382
8,2,3881.545455
8,2,18245.389121
8,2,3087.177083
8,2,1643.567073
8,2,15958.765432
8,2,3933.272727
8,2,4681.585366
8,2,3818.128788
8,2,4319.250000
8,2,2691.087766
8,2,1584.875000
8,2,4283.807500
8,2,2155.177419
8,2,2904.937500
8,2,3757.383065
8,2,4374.010417
8,2,2966.571429
8,2,4797.727778
8,2,10501.188679
8,2,3455.250000
8,2,10357.796875
8,2,2957.256250
8,2,10337.125000
8,2,9593.364754
8,2,4716.111842
8,2,2410.636364
8,2,8901.084239
8,2,2199.461957
8,2,2354.384615
8,2,3128.225000
8,2,2902.000000
8,2,10879.559091
8,2,1736.634868
8,2,8730.000000
8,2,2135.653846
8,2,2565.484375
8,2,4742.815789
8,2,1973.312500
8,2,3922.586310
8,2,1717.283422

8,2,3029.965969
8,2,2741.448529
8,2,2136.078488
8,2,4738.015625
8,2,3410.165761
8,2,5853.521277
8,2,4750.480769
8,2,6375.704545
8,2,8143.047619
8,2,1808.837500
8,2,12489.052885
8,2,1769.009434
8,2,9755.520408
8,2,3639.952381
8,2,3646.009615
8,2,3551.241279
8,2,5260.526639
8,2,11944.351190
8,2,4209.186047
8,2,1897.025641
8,2,6341.583333
8,2,9141.187500
8,2,2131.370861
8,2,2190.258065
8,2,2968.282051
8,2,2309.463235
8,2,4478.244565
8,2,9002.596154
8,2,2555.382812
8,2,1652.464286
8,2,2430.992188
8,2,3963.485294
8,2,3382.803571
8,2,3992.292683
8,2,2456.519231
8,2,3101.573529
8,2,3010.880952
8,2,5968.714286
8,2,1864.380952
8,2,1686.680556
8,2,4926.381818
8,2,4027.712766
8,2,13792.693966
8,2,5884.050000
8,2,4675.151596
8,2,2990.142857
8,2,2634.519737
8,2,66060.125000
8,2,2846.595745
8,2,1999.000000
8,2,2799.384615
8,2,3311.810185
8,2,3590.603723
8,2,2393.117647
8,2,2620.117857
8,2,3014.826087
8,2,1985.081633
8,2,7565.090909
8,2,2869.777778
8,2,2828.331250
8,2,2769.888889
8,2,2459.578125
8,2,2117.966146
8,2,3896.640244
8,2,3944.684211
8,2,2689.566489

8,2,2760.576923
8,2,2462.363636
8,2,1520.063679
8,2,3352.658088
8,2,7165.302326
8,2,3045.962766
8,2,4638.951923
8,2,1961.575000
8,2,2565.114035
8,2,1904.113636
8,2,4056.312500
8,2,4458.610795
8,2,1950.032895
8,2,4947.711538
8,2,3451.272059
8,2,3273.181818
8,2,7849.390244
8,2,9019.642857
8,2,2153.955000
8,2,1766.125000
8,2,2230.814024
8,2,1761.683544
8,2,2369.771739
8,2,1927.428571
8,2,2819.204188
8,2,6449.430000
8,2,2455.737179
8,2,7729.454545
8,2,2458.250000
8,2,4844.300000
8,2,6253.078431
8,2,1859.064516
8,2,5883.341463
8,2,3476.688776
8,2,4975.500000
8,2,4385.750000
8,2,2028.535256
8,2,2094.688889
8,2,1871.320000
8,2,3737.732143
8,2,6976.577128
8,2,2542.006993
8,2,2875.978610
8,2,4960.194149
8,2,2880.785714
8,2,4447.762948
8,2,1742.951220
8,2,178431.683333
8,2,7365.458333
8,2,5877.507212
8,2,2340.902500
8,2,3084.957447
8,2,5503.934783
8,2,1568.492647
8,2,4256.691558
8,2,6904.929577
8,2,2472.157895
8,2,3231.272059
8,2,3220.631868
8,2,1910.377358
8,2,4012.521552
8,2,2526.593220
8,2,4016.111111
8,2,4043.415865
8,2,1768.869565
8,2,4246.366477

8,2,4438.448980
8,2,3393.416667
8,2,13802.170732
8,2,2445.174342
8,2,2010.914062
8,2,4822.562500
8,2,2375.328125
8,2,4353.878049
8,2,4050.312500
8,2,6846.054348
8,2,4180.473684
8,2,2171.366337
8,2,3881.643519
8,2,2169.133721
8,2,2061.000000
8,2,3001.378378
8,2,1655.550532
8,2,2678.975610
8,2,2722.267361
8,2,1968.140625
8,2,6122.156667
8,2,8584.696970
8,2,1677.408537
8,2,5653.215625
8,2,8277.000000
8,2,2440.706522
8,2,2887.631098
8,2,2345.227273
8,2,2430.331522
8,2,3874.922222
8,2,2620.428571
8,2,3722.500000
8,2,1744.538265
8,2,3208.095109
8,2,1885.428571
8,2,1501.291667
8,2,16582.125000
8,2,2912.889706
8,2,7562.678571
8,2,2050.071023
8,2,1665.208333
8,2,1645.432432
8,2,2277.955882
8,2,6315.750000
8,2,2837.120968
8,2,1658.093750
8,2,1531.118421
8,2,2485.416667
8,2,9077.093085
8,2,3394.548611
8,2,3212.018382
8,2,2024.900000
8,2,5187.639706
8,2,4968.115196
8,2,10162.328488
8,2,1570.577703
8,2,2043.122283
8,2,2542.352941
8,2,2000.945122
8,2,3139.730769
8,2,4133.828431
8,2,3298.641509
8,2,2262.465116
8,2,1519.635135
8,2,2662.861842
8,2,4310.950980

8,2,2636.460938
8,2,1644.062500
8,2,2192.762500
8,2,3238.390625
8,2,3549.990385
8,2,5860.026596
8,2,3225.066667
8,2,3468.871951
8,2,1679.756757
8,2,3795.476190
8,2,10061.672872
8,2,1961.718137
8,2,3627.035714
8,2,2571.046632
8,2,3361.836957
8,2,3559.543353
8,2,1935.279070
8,2,1970.915625
8,2,3837.000000
8,2,1950.618750
8,2,9883.337017
8,2,4739.885621
8,2,2444.333333
8,2,1808.700000
8,2,21539.568182
8,2,3460.240000
8,2,2258.312500
8,2,5395.500000
8,2,4334.042781
8,2,10302.915730
8,2,1677.945205
8,2,2354.051282
8,2,3624.242188
8,2,6163.452830
8,2,3986.440789
8,2,1640.907407
8,2,2313.319079
8,2,5259.840686
8,2,3560.206250
8,2,3679.381466
8,2,2057.893229
8,2,5664.597087
8,2,3664.164352
8,2,2332.882812
8,2,4475.406250
8,2,1712.353659
8,2,4157.909091
8,2,2626.125000
8,2,5000.984043
8,2,4570.519231
8,2,3367.365132
8,2,2724.707921
8,2,6952.893939
8,2,1926.846995
8,2,11245.103448
8,2,2977.432065
8,2,4876.026316
8,2,1651.000000
8,2,1811.722513
8,2,2663.153846
8,2,1572.104430
8,2,2195.785326
8,2,3199.294118
8,2,1870.832317
8,2,4152.395210
8,2,3886.824074

8,2,1739.305288
8,2,4169.666667
8,2,2495.482955
8,2,1592.793103
8,2,9647.723214
8,2,3168.289634
8,2,1785.747283
8,2,2220.239796
8,2,2316.512500
8,2,7537.268293
8,2,9025.538462
8,2,1839.103659
8,2,9434.053977
8,2,6490.516304
8,2,6749.556452
8,2,4932.500000
8,2,2323.917763
8,2,1780.776744
8,2,6044.488571
8,2,1898.312500
8,2,4012.733796
8,2,4460.163690
8,2,2973.952381
8,2,2616.250000
8,2,2374.631579
8,2,3293.684524
8,2,5829.632979
8,2,3126.620536
8,2,1574.977273
8,2,1823.391753
8,2,1744.621212
8,2,1522.236842
8,2,2360.404255
8,2,3826.383562
8,2,9255.143617
8,2,3259.189189
8,2,9745.000000
8,2,3881.416667
8,2,1726.250000
8,2,2363.616279
8,2,2099.983553
8,2,1554.395833
8,2,2599.826087
8,2,8024.548077
8,2,2188.565217
8,2,11357.793269
8,2,2808.121622
8,2,3926.256098
8,2,4167.535714
8,2,2211.200000
8,2,2367.906977
8,2,7217.944444
8,2,3358.103571
8,2,1850.597222
8,2,3102.081967
8,2,4526.875000
8,2,3694.838710
8,2,3164.795812
8,2,1589.981595
8,2,3668.659639
8,2,3535.529412
8,2,4545.327778
8,2,9892.216837
8,2,1731.655172
8,2,8782.760000
8,2,5046.833333

8,2,2678.458333
8,2,2080.587838
8,2,2062.908537
8,2,3385.075758
8,2,3719.237245
8,2,2649.750000
8,2,2434.756757
8,2,5607.915698
8,2,3116.487745
8,2,1944.944444
8,2,2140.065000
8,2,4355.980769
8,2,1666.391667
8,2,6445.181818
8,2,1741.354545
8,2,3455.123762
8,2,2317.722222
8,2,1518.509804
8,2,4414.000000
8,2,2230.070513
8,2,1858.320946
8,2,2106.000000
8,2,1502.720930
8,2,1585.337500
8,2,4762.869565
8,2,4154.415730
8,2,6367.600000
8,2,1526.636364
8,2,10725.446429
8,2,2177.823529
8,2,3021.184783
8,2,3479.281609
8,2,3431.922414
8,2,3750.125000
8,2,3510.591837
8,2,2169.625000
8,2,3508.346774
8,2,29524.262755
8,2,11297.500000
8,2,1843.595395
8,2,9357.688889
8,2,2380.423077
8,2,7693.683333
8,2,1651.305233
8,2,10434.327869
8,2,1536.842105
8,2,1843.079268
8,2,2064.857988
8,2,6093.843750
8,2,5714.750000
8,2,2393.716346
8,2,9673.363636
8,2,4947.971354
8,2,4684.506726
8,2,7949.107143
8,2,4782.578947
8,2,2281.000000
8,2,3587.714674
8,2,4520.654545
8,2,3039.633745
8,2,3604.223214
8,2,7337.013158
8,2,2030.894231
8,2,5354.425000
8,2,13886.462264
8,2,3130.093750

8,2,10570.606707
8,2,2621.243902
8,2,7285.134146
8,2,13087.416667
8,2,3651.139535
8,2,2634.291667
8,2,2316.500000
8,2,1611.441860
8,2,3791.166667
8,2,7778.000000
8,2,2036.337963
8,2,2265.456731
8,2,3818.096774
8,2,3249.357143
8,2,2283.985507
8,2,7164.877232
8,2,3848.505319
8,2,1501.671875
8,2,7537.635417
8,2,49315.032051
8,2,2339.218750
8,2,1917.928571
8,2,1989.161932
8,2,1578.141129
8,2,2908.445946
8,2,1913.621622
8,2,3067.568627
8,2,5359.263158
8,2,5388.336364
8,2,2467.902344
8,2,3368.750000
8,2,4765.030303
8,2,2452.309524
8,2,11402.550971
8,2,3158.933333
8,2,3975.080460
8,2,3120.376884
8,2,1888.812500
8,2,3401.774869
8,2,3409.850000
8,2,1933.786842
8,2,1934.958333
8,2,1793.911932
8,2,1987.363861
8,2,1679.347826
8,2,3801.818878
8,2,2039.159375
8,2,1965.093750
8,2,10866.353774
8,2,1810.190217
8,2,1663.782051
8,2,2889.625000
8,2,3885.229167
8,2,1915.828804
8,2,6145.329268
8,2,4886.157895
8,2,1821.886076
8,2,9405.512821
8,2,10398.551913
8,2,2284.500000
8,2,5941.779891
8,2,3935.042683
8,2,3053.715116
8,2,5549.857143
8,2,5344.317935
8,2,2387.790625

8,2,2321.554348
8,2,1716.175000
8,2,3226.184896
8,2,1764.937500
8,2,2136.105263
8,2,1682.666667
8,2,2747.333333
8,2,1513.210366
8,2,3013.421053
8,2,2192.491071
8,2,4952.959184
8,2,3426.468750
8,2,2085.255682
8,2,18258.965909
8,2,11018.880000
8,2,12144.928191
8,2,2898.147727
8,2,1874.487805
8,2,2337.000000
8,2,2936.066176
8,2,2799.976190
8,2,6472.538306
8,2,4223.156250
8,2,1891.541667
8,2,1577.818182
8,2,3683.086538
8,2,1947.633152
8,2,5569.391304
8,2,1520.875000
8,2,1839.612500
8,2,21445.355769
8,2,3300.328804
8,2,1840.945122
8,2,3239.967949
8,2,2830.997093
8,2,2457.712766
8,2,1645.562500
8,2,11217.038462
8,2,3803.725000
8,2,2058.644608
8,2,1532.195946
8,2,2556.666667
8,2,14701.500000
8,2,2668.020833
8,2,2370.828390
8,2,8209.041667
8,2,13695.100000
8,2,1738.476744
8,2,2963.263889
8,2,2926.862319
8,2,3228.312500
8,2,4235.450000
8,2,1726.740741
8,2,2013.100000
8,2,4095.861111
8,2,2884.635870
8,2,1884.570122
8,2,2185.312500
8,2,3574.656250
8,2,3893.385246
8,2,6054.926829
8,2,1765.728261
8,2,4482.173469
8,2,3928.145833
8,2,1899.023438
8,2,4075.255319

8,2,2510.837500
8,2,7650.707317
8,2,4293.423077
8,2,14220.475862
8,2,2360.247642
8,2,2757.952273
8,2,6404.269022
8,2,3358.442308
8,2,6096.463415
8,2,6200.542553
8,2,3690.462500
8,2,9688.541667
8,2,4568.750000
8,2,1646.229167
8,2,2452.928191
8,2,5768.529412
8,2,1567.633523
8,2,1996.558140
8,2,4048.051020
8,2,12998.947727
8,2,2706.789474
8,2,12701.925000
8,2,1686.314010
8,2,1503.161765
8,2,2769.836538
8,2,2833.492462
8,2,4723.666667
8,2,2148.750000
8,2,36141.811170
8,2,1629.646875
8,2,2782.750000
8,2,2975.940104
8,2,2249.448276
8,2,3431.045918
8,2,3168.000000
8,2,2328.353659
8,2,4060.111702
8,2,2890.990741
8,2,11486.500000
8,2,2178.812102
8,2,5983.500000
8,2,3276.083333
8,2,2597.526316
8,2,4692.343750
8,2,1957.103226
8,2,2279.971698
8,2,3400.307927
8,2,2573.924528
8,2,3891.818750
8,2,2626.785714
8,2,1503.675532
8,2,2865.688830
8,2,2488.074468
8,2,4973.545000
8,2,2245.166667
8,2,3312.968750
8,2,4037.875000
8,2,2043.083333
8,2,1666.715909
8,2,1659.461538
8,2,3708.108696
8,2,2540.250000
8,2,2726.200000
8,2,1783.315789
8,2,4335.433594
8,2,1753.818182

8,2,4002.798077
8,2,3027.341837
8,2,2009.783537
8,2,5482.154255
8,2,4053.525000
8,2,4831.985849
8,2,5031.620000
8,2,2754.937500
8,2,4904.000000
8,2,13128.750000
8,2,4106.413043
8,2,1957.492347
8,2,1838.388889
8,2,3565.630952
8,2,5887.250000
8,2,1959.404605
8,2,3261.520833
8,2,7856.512195
8,2,1622.871094
8,2,5328.595745
8,2,1567.239437
8,2,12602.904661
8,2,4927.500000
8,2,2195.395833
8,2,1802.972222
8,2,14271.645270
8,2,4829.669683
8,2,5512.238342
8,2,14792.142857
8,2,1791.212766
8,2,4141.210177
8,2,2241.448087
8,2,2028.618590
8,2,2745.178771
8,2,1952.722826
8,2,3732.939189
8,2,4095.843750
8,2,2060.062500
8,2,3571.659091
8,2,5496.426471
8,2,3477.090206
8,2,3155.224490
8,2,4012.585714
8,2,3806.891447
8,2,2307.833333
8,2,3935.939891
8,2,2822.931818
8,2,2620.642857
8,2,2030.142857
8,2,12178.187500
8,2,2993.195652
8,2,1622.995370
8,2,1662.036585
8,2,2051.871795
8,2,5064.421569
8,2,3538.979167
8,2,2919.318750
8,2,5848.680233
8,2,2403.304054
8,2,2510.585714
8,2,1550.246951
8,2,5360.062500
8,2,3882.323864
8,2,4616.558824
8,2,2190.725000
8,2,3572.298913

8,2,4442.859043
8,2,3442.730978
8,2,1939.955357
8,2,8946.562500
8,2,15127.192073
8,2,2780.454545
8,2,23034.788043
8,2,3195.179775
8,2,4405.482143
8,2,8299.343750
8,2,1803.695312
8,2,2809.959375
8,2,1808.192308
8,2,1714.842105
8,2,4930.801508
8,2,1824.412162
8,2,2351.181818
8,2,7279.776119
8,2,3313.531250
8,2,2518.652174
8,2,4461.732558
8,2,2009.789062
8,2,1895.543919
8,2,6125.912500
8,2,1627.641667
8,2,7864.162500
8,2,2296.038690
8,2,3685.250000
8,2,2923.077778
8,2,1961.285714
8,2,4220.915179
8,2,4488.559524
8,2,6321.233333
8,2,3926.009804
8,2,4044.944767
8,2,3546.157407
8,2,7741.782609
8,2,2553.290541
8,2,5443.406250
8,2,1585.243902
8,2,2133.921053
8,2,28514.139535
8,2,1656.723684
8,2,4521.573171
8,2,6784.400000
8,2,11207.796875
8,2,3331.789773
8,2,2343.500000
8,2,1828.180147
8,2,2062.375000
8,2,2196.515957
8,2,1836.229730
8,2,1963.235294
8,2,10411.325581
8,2,5817.100000
8,2,4655.688679
8,2,6565.439024
8,2,7280.657609
8,2,7378.913043
8,2,2049.648438
8,2,2409.721591
8,2,6652.785714
8,2,6308.151442
8,2,5384.687500
8,2,2040.736842
8,2,5960.035377

8,2,5682.416667
8,2,5429.368750
8,2,1982.736702
8,2,1807.935714
8,2,3768.166667
8,2,9551.125000
8,2,13665.482759
8,2,1912.097561
8,2,3548.600000
8,2,8650.680000
8,2,2072.244444
8,2,3525.048387
8,2,2237.343750
8,2,1562.636364
8,2,5372.004167
8,2,1678.512195
8,2,7104.676471
8,2,4162.045226
8,2,5428.977273
8,2,1585.486239
8,2,11040.293478
8,2,1833.021277
8,2,2793.513298
8,2,1735.786585
8,2,2237.293478
8,2,4038.433333
8,2,4424.375000
8,2,2115.713816
8,2,4312.954545
8,2,4225.658537
8,2,6007.255814
8,2,4073.766026
8,2,1709.861111
8,2,5058.896552
8,2,7806.240000
8,2,7611.091270
8,2,3755.639896
8,2,2409.520833
8,2,1689.555556
8,2,148876.916667
8,2,4541.994048
8,2,1916.329670
8,2,1898.665663
8,2,1715.095238
8,2,4367.000000
8,2,4600.040000
8,2,2250.057692
8,2,1506.283163
8,2,2740.176471
8,2,3470.276442
8,2,1816.651316
8,2,14054.128889
8,2,2736.061224
8,2,1793.271875
8,2,3731.380682
8,2,2310.279070
8,2,4595.051613
8,2,3248.018229
8,2,3742.195000
8,2,7129.668605
8,2,2030.960227
8,2,1614.365385
8,2,5759.670732
8,2,3826.030488
8,2,3769.285714
8,2,4994.595982

8,2,2348.275000
8,2,4933.791667
8,2,11349.241379
8,2,1511.630435
8,2,3030.028302
8,2,3712.431122
8,2,4499.312500
8,2,2202.985294
8,2,1836.356707
8,2,2438.000000
8,2,2294.445652
8,2,1713.177083
8,2,4617.662500
8,2,3542.780488
8,2,1509.206250
8,2,1853.166667
8,2,2302.913462
8,2,1934.095808
8,2,5228.233173
8,2,2139.694853
8,2,4106.821429
8,2,3942.735294
8,2,2715.216346
8,2,1980.677083
8,2,2750.781646
8,2,69089.276786
8,2,7742.703488
8,2,9430.875000
8,2,4199.357923
8,2,2562.574324
8,2,6490.444444
8,2,3381.654255
8,2,1634.357843
8,2,5512.472561
8,2,14078.162791
8,2,3254.666667
8,2,3171.980000
8,2,7551.140244
8,2,1975.729651
8,2,4075.306122
8,2,2969.644022
8,2,3268.118902
8,2,6697.346939
8,2,1787.152174
8,2,3710.375000
8,2,2696.590686
8,2,10318.655612
8,2,2303.013889
8,2,1792.552632
8,2,1594.750000
8,2,3080.366667
8,2,2794.468085
8,2,7844.000000
8,2,2592.666667
8,2,3647.273438
8,2,3002.395270
8,2,2137.861111
8,2,2755.284722
8,2,7120.736842
8,2,1695.670213
8,2,8832.222222
8,2,13128.070175
8,2,1697.758152
8,2,1684.673780
8,2,4081.806818
8,2,1793.372024

8,2,2325.360606
8,2,1751.569079
8,2,3522.750000
8,2,4848.753788
8,2,2479.385135
8,2,1794.828571
8,2,7873.239130
8,2,1734.569444
8,2,2871.083333
8,2,5347.597222
8,2,2328.592965
8,2,2697.569149
8,2,7330.943878
8,2,3620.111702
8,2,3664.428571
8,2,3629.333333
8,2,1607.256757
8,2,5680.469388
8,2,2807.215000
8,2,6612.872642
8,2,2316.536458
8,2,2234.841146
8,2,1720.750000
8,2,1824.196875
8,2,9042.708333
8,2,1725.375000
8,2,2438.544554
8,2,2474.083333
8,2,1947.037500
8,2,4244.725000
8,2,1693.472067
8,2,2326.437500
8,2,2335.975000
8,2,13737.745536
8,2,2746.373457
8,2,1806.375000
8,2,2908.887500
8,2,10051.137500
8,2,1995.400000
8,2,2324.042339
8,2,4728.666667
8,2,3078.916667
8,2,1755.508929
8,2,2275.439516
8,2,2879.955357
8,2,8090.461735
8,2,4827.186047
8,2,2261.875000
8,2,3688.336310
8,2,1685.540000
8,2,1846.446809
8,2,3834.455000
8,2,3844.844444
8,2,1740.921053
8,2,2389.891304
8,2,3921.580729
8,2,1867.433594
8,2,2122.818182
8,2,2320.841667
8,2,6189.826087
8,2,3296.262195
8,2,1927.242188
8,2,1724.296089
8,2,11368.516393
8,2,3622.887500
8,2,2222.062500

8,2,2382.866279
8,2,1896.502907
8,2,4232.439904
8,2,1749.152778
8,2,2161.163462
8,2,6612.648438
8,2,2437.448370
8,2,2958.537791
8,2,1807.057377
8,2,6153.934555
8,2,1730.104651
8,2,1590.177632
8,2,2450.615854
8,2,2145.725000
8,2,2825.775000
8,2,1598.378125
8,2,3513.490000
8,2,4077.578125
8,2,1607.857955
8,2,5609.647959
8,2,2033.700000
8,2,1795.871345
8,2,1867.319892
8,2,8726.000000
8,2,6740.994737
8,2,2657.076923
8,2,30454.410714
8,2,4777.875000
8,2,4091.567500
8,2,8942.546875
8,2,6557.160256
8,2,1585.250000
8,2,24296.951923
8,2,3824.290323
8,2,7485.500000
8,2,6604.755814
8,2,17061.212195
8,2,1718.400000
8,2,4015.362245
8,2,11540.165179
8,2,2229.097561
8,2,1569.865132
8,2,6861.796875
8,2,9111.642500
8,2,9428.941667
8,2,1800.996711
8,2,2458.077586
8,2,3221.355769
8,2,6258.861842
8,2,1941.845238
8,2,3783.709239
8,2,1584.250000
8,2,2553.160714
8,2,3270.070652
8,2,8231.108696
8,2,5050.259434
8,2,1895.615385
8,2,2163.192073
8,2,4714.010417
8,2,1977.535377
8,2,2273.755952
8,2,2980.868056
8,2,1919.489362
8,2,1613.875000
8,2,2239.012755
8,2,2210.114583

8,2,1596.844595
8,2,4200.988839
8,2,2217.075000
8,2,2925.800000
8,2,1617.955882
8,2,3265.178241
8,2,4642.948718
8,2,1624.500000
8,2,1802.128205
8,2,12128.149390
8,2,2363.000000
8,2,2477.128378
8,2,15292.067308
8,2,9879.863636
8,2,2610.045455
8,2,2120.562500
8,2,2814.788889
8,2,2754.833333
8,2,3991.523256
8,2,9813.294444
8,2,11329.328846
8,2,7966.725000
8,2,3298.912234
8,2,1752.790698
8,2,1681.818182
8,2,1537.645270
8,2,2792.406250
8,2,9861.630859
8,2,3619.510695
8,2,2817.579909
8,2,4858.434524
8,2,7881.573864
8,2,5512.250000
8,2,2122.947368
8,2,6040.554054
8,2,2954.757576
8,2,5267.648515
8,2,17855.983240
8,2,4295.546512
8,2,2836.353125
8,2,2070.386364
8,2,2680.412234
8,2,1632.525568
8,2,4888.000000
8,2,1895.750000
8,2,5185.106383
8,2,7695.228723
8,2,1902.175676
8,2,1732.984925
8,2,7889.010695
8,2,2872.472500
8,2,2322.552486
8,2,5783.027027
8,2,2283.234043
8,2,7495.285714
8,2,2970.375000
8,2,2272.250000
8,2,1911.000000
8,2,4315.250000
8,2,4320.590116
8,2,13874.206522
8,2,2180.622222
8,2,2172.923077
8,2,1773.114130
8,2,6586.250000
8,2,30238.546512

8,2,3221.478261
8,2,1784.295139
8,2,2851.323718
8,2,2051.297872
8,2,10748.470588
8,2,5591.133621
8,2,2424.554878
8,2,1652.469388
8,2,5587.766667
8,2,3745.392857
8,2,1605.947674
8,2,3932.032258
8,2,2455.843750
8,2,2306.100000
8,2,1560.843750
8,2,3574.338583
8,2,1949.756757
8,2,6224.125000
8,2,3523.253378
8,2,4412.572917
8,2,8195.425532
8,2,7801.235294
8,2,3321.684783
8,2,1790.725446
8,2,4308.043103
8,2,3115.458333
8,2,2476.582237
8,2,1631.891892
8,2,1570.300000
8,2,2131.109756
8,2,2142.478261
8,2,5712.491848
8,2,2255.762821
8,2,3858.671875
8,2,3527.221698
8,2,4271.891892
8,2,1619.250000
8,2,2415.458333
8,2,3112.299528
8,2,2701.591667
8,2,2407.054878
8,2,2830.762019
8,2,3282.054348
8,2,4016.769231
8,2,2545.641892
8,2,2767.244318
8,2,3656.940000
8,2,2354.575000
8,2,6572.285714
8,2,2656.437500
8,2,13534.290000
8,2,1883.780405
8,2,4219.290640
8,2,4020.456731
8,2,2573.562500
8,2,4617.093750
8,2,1577.659884
8,2,1585.250000
8,2,5082.345455
8,2,4465.935811
8,2,6101.804348
8,2,32152.372222
8,2,12257.769231
8,2,2244.210843
8,2,8131.083333
8,2,2942.289062

8,2,4284.573980
8,2,2498.780660
8,2,4687.915698
8,2,5131.711111
8,2,6018.855556
8,2,16173.212766
8,2,13408.658163
8,2,1544.333333
8,2,1565.055233
8,2,1736.337079
8,2,3644.541667
8,2,2432.765957
8,2,2246.031250
8,2,2487.581395
8,2,2077.405488
8,2,6512.430556
8,2,1689.134615
8,2,2675.648649
8,2,6325.041667
8,2,2703.280000
8,2,4618.375000
8,2,3614.315789
8,2,1959.243750
8,2,2024.059896
8,2,3150.166667
8,2,4818.573171
8,2,4207.790323
8,2,6141.341667
8,2,5655.546875
8,2,2498.316176
8,2,1715.076389
8,2,4279.400000
8,2,1941.705882
8,2,1890.627604
8,2,4762.937500
8,2,5984.655488
8,2,2023.904255
8,2,2203.114286
8,2,2180.406977
8,2,2140.335664
8,2,3024.281250
8,2,2004.297297
8,2,2027.886905
8,2,6080.174528
8,2,4204.132212
8,2,2215.187500
8,2,1927.697674
8,2,4436.468750
8,2,1723.946023
8,2,8783.262821
8,2,8972.781250
8,2,2509.967391
8,2,1743.594697
8,2,2759.462500
8,2,4334.776596
8,2,2853.300000
8,2,2561.074468
8,2,12727.562500
8,2,4600.000000
8,2,2230.451087
8,2,5572.222222
8,2,1543.875000
8,2,1633.833333
8,2,2713.558511
8,2,1734.201087
8,2,3337.051471

8,2,6046.647500
8,2,1655.894737
8,2,1601.450000
8,2,10418.436019
8,2,3758.698565
8,2,4411.272727
8,2,12956.665816
8,2,3204.262195
8,2,2371.436047
8,2,2474.750000
8,2,6052.222222
8,2,1875.502451
8,2,6026.898585
8,2,3008.742424
8,2,2324.800000
8,2,4100.431818
8,2,2713.031863
8,2,8476.973684
8,2,5342.500000
8,2,2540.919444
8,2,2371.834184
8,2,1995.420732
8,2,1529.541667
8,2,11382.817073
8,2,7134.560811
8,2,2003.319149
8,2,2191.317130
8,2,2055.866071
8,2,3285.614796
8,2,4947.173077
8,2,4009.921875
8,2,1569.178571
8,2,1777.401515
8,2,5909.872159
8,2,1520.176020
8,2,4380.930288
8,2,2320.386364
8,2,7000.041667
8,2,2707.421053
8,2,5925.950893
8,2,3687.930233
8,2,3221.730000
8,2,6383.000000
8,2,7965.578125
8,2,1616.295732
8,2,1751.490625
8,2,3550.823529
8,2,1872.312500
8,2,1544.117647
8,2,5017.720000
8,2,2117.810976
8,2,6789.986979
8,2,4294.506410
8,2,19426.454918
8,2,2216.780488
8,2,2777.642857
8,2,2998.985577
8,2,3744.772727
8,2,3640.243421
8,2,7813.869898
8,2,5042.687500
8,2,1672.250000
8,2,2009.722973
8,2,1959.366071
8,2,24614.713415
8,2,4552.115385

8,2,3621.573171
8,2,10653.260870
8,2,3470.187500
8,2,2710.987500
8,2,1616.680556
8,2,2349.996622
8,2,5162.862069
8,2,3110.327815
8,2,1688.691099
8,2,2672.000000
8,2,1912.451923
8,2,2759.675000
8,2,10845.937500
8,2,3817.750000
8,2,1786.449405
8,2,4210.268750
8,2,2288.250000
8,2,6558.858173
8,2,1574.718750
8,2,5598.902778
8,2,2031.892857
8,2,1694.923611
8,2,2753.932692
8,2,2640.170213
8,2,2883.003906
8,2,1661.211538
8,2,1662.864362
8,2,1747.363874
8,2,4846.734694
8,2,2225.467105
8,2,3854.750000
8,2,2359.796196
8,2,2835.310811
8,2,9125.243697
8,2,4820.630332
8,2,1703.019737
8,2,7394.812500
8,2,9054.750000
8,2,6540.173913
8,2,11975.968000
8,2,2257.052419
8,2,2462.972222
8,2,2758.493421
8,2,5308.819767
8,2,2631.862245
8,2,3986.706522
8,2,1791.250000
8,2,3671.250000
8,2,5043.481283
8,2,2669.553571
8,2,2073.181818
8,2,1772.269231
8,2,5840.516447
8,2,2651.392857
8,2,3849.115000
8,2,5435.285714
8,2,4924.020000
8,2,4697.673913
8,2,1701.176020
8,2,2461.172222
8,2,2036.371875
8,2,1855.764706
8,2,3172.839844
8,2,1842.189189
8,2,1779.884393
8,2,3324.456522

8,2,2572.250000
8,2,3389.930851
8,2,5970.361607
8,2,4258.066327
8,2,2674.006250
8,2,4071.947368
8,2,2023.829787
8,2,3823.484375
8,2,1573.127660
8,2,2205.644444
8,2,1781.151042
8,2,5681.225877
8,2,1684.671875
8,2,2811.718750
8,2,4540.125000
8,2,2749.703125
8,2,6071.812500
8,2,3243.856250
8,2,5192.916667
8,2,4793.525000
8,2,2934.384615
8,2,4125.937500
8,2,7728.881579
8,2,1583.597222
8,2,1596.788043
8,2,3483.526316
8,2,2727.286184
8,2,3588.123288
8,2,2011.418367
8,2,3680.375000
8,2,2878.392157
8,2,3866.704433
8,2,4159.288462
8,2,1643.150000
8,2,4497.718391
8,2,3396.695122
8,2,3578.909091
8,2,2009.643750
8,2,3586.873239
8,2,2612.942308
8,2,4897.640000
8,2,3591.777778
8,2,2090.863636
8,2,8017.000000
8,2,4265.020833
8,2,10162.758929
8,2,4167.030488
8,2,1721.045455
8,2,2052.555921
8,2,2011.717262
8,2,3743.577778
8,2,3190.982955
8,2,1534.338415
8,2,2017.214286
8,2,5899.703125
8,2,5762.913043
8,2,1732.657143
8,2,3124.721519
8,2,2912.333333
8,2,4398.650000
8,2,4189.529255
8,2,3009.975490
8,2,4089.812500
8,2,2586.865385
8,2,4544.232558
8,2,4221.111842

8,2,1820.446429
8,2,1921.585714
8,2,8371.409884
8,2,3759.333333
8,2,5069.734375
8,2,9685.246154
8,2,7766.500000
8,2,2713.041667
8,2,5284.590164
8,2,3563.215000
8,2,2502.468750
8,2,4377.144330
8,2,3106.916667
8,2,2370.533654
8,2,11010.450000
8,2,2464.895408
8,2,3793.250000
8,2,7346.044118
8,2,4520.677083
8,2,1561.391892
8,2,2194.908088
8,2,2374.605263
8,2,9557.531977
8,2,2539.803571
8,2,1666.148026
8,2,9821.171053
8,2,3680.946875
8,2,2021.198324
8,2,4423.833333
8,2,1631.557692
8,2,2067.279070
8,2,4226.220395
8,2,3870.687500
8,2,1902.482143
8,2,1972.815625
8,2,5946.230769
8,2,3758.928571
8,2,1902.604592
8,2,1743.159091
8,2,6031.000000
8,2,7307.125000
8,2,4927.161058
8,2,4890.043750
8,2,2222.153061
8,2,1540.367347
8,2,3425.049342
8,2,2395.442308
8,2,3545.563889
8,2,1757.408163
8,2,2977.200472
8,2,6565.785714
8,2,3658.195652
8,2,6924.125000
8,2,1693.064024
8,2,2320.668478
8,2,12203.769231
8,2,3271.664894
8,2,7998.704545
8,2,2216.644444
8,2,2746.815217
8,2,1812.342105
8,2,20932.640110
8,2,1853.786932
8,2,9980.935484
8,2,2372.298077
8,2,7245.688830

8,2,6135.440678
8,2,1859.250000
8,2,1932.485000
8,2,3171.250000
8,2,1894.183333
8,2,2347.265306
8,2,3212.508671
8,2,2565.021277
8,2,3098.000000
8,2,2005.198171
8,2,3909.850000
8,2,2267.000000
8,2,2249.914894
8,2,5136.333333
8,2,5505.058824
8,2,1749.433511
8,2,7953.956522
8,2,3122.121622
8,2,2726.629310
8,2,1658.296875
8,2,7514.320175
8,2,2245.307692
8,2,2069.750000
8,2,1761.617801
8,2,3243.217949
8,2,4988.508772
8,2,4414.056818
8,2,7105.250000
8,2,2239.437500
8,2,7169.991848
8,2,1561.224359
8,2,5309.230769
8,2,3317.899457
8,2,8625.026596
8,2,2006.395480
8,2,3476.672414
8,2,3535.193548
8,2,4638.457447
8,2,2964.197368
8,2,5425.116477
8,2,5470.926136
8,2,2033.909314
8,2,7810.085366
8,2,1754.264706
8,2,2199.005952
8,2,22094.450472
8,2,5334.484649
8,2,3716.815217
8,2,1945.052885
8,2,1934.445122
8,2,2026.480392
8,2,3641.711538
8,2,7694.062500
8,2,5755.857143
8,2,3365.000000
8,2,7901.463542
8,2,2202.008333
8,2,2344.032609
8,2,4929.250000
8,2,1992.576923
8,2,2031.075658
8,2,5847.375000
8,2,4718.020349
8,2,6548.590909
8,2,3427.265625
8,2,1504.337209

8,2,2609.489474
8,2,1895.393750
8,2,2886.779605
8,2,1592.846154
8,2,1822.750000
8,2,1749.170139
8,2,2930.768750
8,2,5695.207547
8,2,1574.137500
8,2,7537.015625
8,2,3350.267544
8,2,2639.653061
8,2,1874.437500
8,2,19897.256579
8,2,47136.432143
8,2,7628.012500
8,2,2424.062500
8,2,4228.164894
8,2,8714.616071
8,2,22521.048780
8,2,8570.000000
8,2,2499.190476
8,2,5385.622449
8,2,5465.181818
8,2,7284.586735
8,2,2628.542553
8,2,2074.844595
8,2,2421.837209
8,2,4603.063830
8,2,5368.302326
8,2,4950.991848
8,2,2054.031250
8,2,2604.970443
8,2,1549.397790
8,2,2531.493333
8,2,1654.232955
8,2,3104.658163
8,2,2999.061224
8,2,1535.486842
8,2,3761.450000
8,2,1835.862500
8,2,2560.590909
8,2,2932.160156
8,2,2402.366071
8,2,1697.187500
8,2,7672.137755
8,2,3024.271739
8,2,3934.204082
8,2,10069.411458
8,2,2558.291667
8,2,1667.986413
8,2,2280.411765
8,2,6068.995690
8,2,3081.643617
8,2,2043.261628
8,2,2370.616071
8,2,2198.894231
8,2,4834.846939
8,2,1885.673469
8,2,3916.970395
8,2,2699.387500
8,2,1828.142487
8,2,3390.758065
8,2,2059.489130
8,2,2204.232143
8,2,3028.130319

8,2,3640.630556
8,2,2245.361842
8,2,1822.250000
8,2,4378.163043
8,2,2019.782051
8,2,2142.945946
8,2,4807.930233
8,2,6900.027778
8,2,1817.115196
8,2,1776.960526
8,2,3493.586486
8,2,9584.931641
8,2,3260.468750
8,2,3531.462264
8,2,1999.605469
8,2,2596.414773
8,2,1514.273810
8,2,2390.526316
8,2,1960.743902
8,2,2728.400966
8,2,1501.107143
8,2,2370.545455
8,2,3005.699324
8,2,6414.581395
8,2,2973.291667
8,2,1816.437500
8,2,3627.941406
8,2,5680.056818
8,2,1696.360000
8,2,3160.734694
8,2,2842.181818
8,2,6432.687500
8,2,1797.175676
8,2,2057.728125
8,2,2032.340426
8,2,4450.305556
8,2,4851.000000
8,2,7343.555556
8,2,4096.875000
8,2,3904.333333
8,2,2288.223214
8,2,3779.762195
8,2,2868.368056
8,2,2298.555556
8,2,4974.404412
8,2,3288.114583
8,2,2279.393519
8,2,8010.558824
8,2,1943.555556
8,2,2381.074219
8,2,2022.250000
8,2,5457.572816
8,2,7764.971014
8,2,32293.671196
8,2,2469.614583
8,2,5899.192308
8,2,3240.831731
8,2,1727.284884
8,2,1962.950820
8,2,1612.105263
8,2,5990.194712
8,2,1610.750000
8,2,2956.575000
8,2,2993.452128
8,2,1864.745370
8,2,5027.840278

8,2,4353.551724
8,2,2509.765625
8,2,7834.178363
8,2,1684.078947
8,2,2411.898204
8,2,3679.532164
8,2,1708.592500
8,2,1502.041667
8,2,1607.125000
8,2,2688.870000
8,2,2634.248649
8,2,3056.246512
8,2,2364.111413
8,2,3351.463415
8,2,2460.552632
8,2,4273.625000
8,2,4803.500000
8,2,4912.287234
8,2,11989.860465
8,2,1502.587500
8,2,3369.598837
8,2,4899.092965
8,2,2170.480263
8,2,9613.727564
8,2,5380.915789
8,2,9366.157500
8,2,3504.540816
8,2,7001.400000
8,2,2188.218750
8,2,14813.217391
8,2,1875.421512
8,2,1957.594595
8,2,1890.561224
8,2,2152.886364
8,2,1653.278846
8,2,2958.660000
8,2,1834.750000
8,2,2681.895349
8,2,6662.177778
8,2,1839.500000
8,2,2012.350000
8,2,3918.212209
8,2,4594.064286
8,2,2343.721875
8,2,1555.244681
8,2,20646.321429
8,2,3502.682143
8,2,4129.750000
8,2,2430.634868
8,2,3204.673295
8,2,1925.188889
8,2,3379.593750
8,2,5812.699074
8,2,2274.679487
8,2,11250.750000
8,2,2815.648936
8,2,6487.333333
8,2,4306.071429
8,2,4022.460526
8,2,2750.218750
8,2,1724.601351
8,2,2091.957317
8,2,9915.625000
8,2,11209.614035
8,2,3181.102941
8,2,5270.187500

8,2,2814.901163
8,2,7714.186275
8,2,3923.223684
8,2,2537.881356
8,2,1750.969512
8,2,1632.468750
8,2,18268.857143
8,2,9668.260465
8,2,2543.029891
8,2,3889.188976
8,2,8532.909091
8,2,1621.355263
8,2,2389.092179
8,2,6845.265957
8,2,3713.792553
8,2,2723.079787
8,2,2069.507212
8,2,3155.343750
8,2,6460.429348
8,2,1557.776730
8,2,1837.634615
8,2,2242.386628
8,2,2365.162500
8,2,3770.488636
8,2,11921.583333
8,2,2132.565217
8,2,2802.955556
8,2,1581.273810
8,2,3376.425532
8,2,4075.007109
8,2,2379.906250
8,2,3707.578125
8,2,3196.354167
8,2,1746.744792
8,2,12863.396341
8,2,2511.994565
8,2,5350.894737
8,2,7151.062500
8,2,8937.305556
8,2,1625.214286
8,2,5845.346154
8,2,3025.190217
8,2,8294.402439
8,2,11760.754464
8,2,2174.000000
8,2,1898.865854
8,2,1903.310127
8,2,3864.781915
8,2,11402.729167
8,2,3958.234756
8,2,2070.160000
8,2,1711.900000
8,2,10140.274590
8,2,1897.531250
8,2,10521.788462
8,2,9700.872340
8,2,5394.909722
8,2,6145.741848
8,2,5033.300000
8,2,4065.861111
8,2,2273.194767
8,2,4192.350000
8,2,1526.003378
8,2,1576.475000
8,2,2182.581633
8,2,1614.242515

8,2,3775.219595
8,2,4748.222222
8,2,2010.127778
8,2,6226.734694
8,2,2685.028846
8,2,2615.541667
8,2,2638.462366
8,2,1734.104167
8,2,17497.446809
8,2,5254.272727
8,2,12123.217391
8,2,2317.803977
8,2,4110.541667
8,2,3452.235294
8,2,1771.674051
8,2,5665.647727
8,2,3992.300000
8,2,5484.168750
8,2,2455.036585
8,2,10779.666667
8,2,2498.193182
8,2,1724.312500
8,2,1930.810811
8,2,2152.444737
8,2,3065.402439
8,2,4462.891304
8,2,3148.771739
8,2,5876.237245
8,2,2046.669643
8,2,2098.511628
8,2,1860.196429
8,2,2620.013889
8,2,9228.840909
8,2,4480.723404
8,2,1534.222826
8,2,6200.973684
8,2,2186.750000
8,2,3314.994898
8,2,4059.360465
8,2,21616.490132
8,2,2127.703125
8,2,1841.847222
8,2,1807.125000
8,2,2017.294872
8,2,2401.687500
8,2,2362.570000
8,2,3887.673077
8,2,3773.593750
8,2,1868.375000
8,2,1617.666667
8,2,3919.111111
8,2,2925.865132
8,2,1579.500000
8,2,10952.435897
8,2,9726.833333
8,2,1698.663265
8,2,2967.000000
8,2,3209.500000
8,2,5444.813084
8,2,2088.866667
8,2,6517.926829
8,2,5670.875000
8,2,1645.753571
8,2,3208.441327
8,2,2911.991667
8,2,5223.973684

8,2,11938.907609
8,2,2904.605263
8,2,2688.312500
8,2,2707.775510
8,2,4531.777778
8,2,2141.753049
8,2,3764.477273
8,2,2729.650000
8,2,3922.063830
8,2,1880.250000
8,2,1550.176829
8,2,4397.968750
8,2,1509.956250
8,2,3991.401316
8,2,3874.847500
8,2,5011.889831
8,2,2027.210526
8,2,1716.150000
8,2,6638.900510
8,2,7572.137255
8,2,2398.592391
8,2,1538.447674
8,2,2797.589744
8,2,2467.053571
8,2,1531.209184
8,2,2637.360465
8,2,3304.969388
8,2,3299.919598
8,2,4608.187500
8,2,3023.076923
8,2,8018.045455
8,2,4316.000000
8,2,2663.916667
8,2,7162.720395
8,2,2029.596939
8,2,9148.050000
8,2,3472.958333
8,2,1977.184524
8,2,2977.593750
8,2,3911.507812
8,2,2468.506250
8,2,1629.171053
8,2,1926.975610
8,2,4289.895000
8,2,9152.842105
8,2,3660.525000
8,2,1527.928571
8,2,1548.125000
8,2,2603.032609
8,2,5694.475000
8,2,2484.695946
8,2,1805.616379
8,2,3409.220238
8,2,3188.702830
8,2,3555.224490
8,2,1664.750000
8,2,3325.875000
8,2,6661.303922
8,2,2151.362903
8,2,3126.074324
8,2,2060.636364
8,2,6797.375000
8,2,2399.650000
8,2,4643.643617
8,2,4773.445946
8,2,2525.646259

8,2,12348.387097
8,2,2409.729787
8,2,3323.214286
8,2,1561.529255
8,2,2561.724359
8,2,1987.223684
8,2,3009.750000
8,2,1772.989362
8,2,9939.594828
8,2,1793.849693
8,2,2384.043243
8,2,13288.478873
8,2,1678.150000
8,2,3183.137255
8,2,1782.897436
8,2,6911.355769
8,2,2065.648810
8,2,5425.941176
8,2,6371.250000
8,2,3501.044271
8,2,2368.300000
8,2,1736.741071
8,2,5355.967593
8,2,8416.783898
8,2,1833.750000
8,2,2083.528846
8,2,6667.139535
8,2,2883.217391
8,2,6999.885870
8,2,3540.034091
8,2,2550.250000
8,2,2411.151163
8,2,4564.918103
8,2,1875.274011
8,2,5704.872642
8,2,4407.329268
8,2,2749.208333
8,2,8527.087838
8,2,2627.340000
8,2,13707.702128
8,2,10979.121622
8,2,6088.420732
8,2,2790.671053
8,2,2404.700000
8,2,2012.393548
8,2,1928.039894
8,2,7213.706897
8,2,1703.926471
8,2,4239.989130
8,2,6887.825000
8,2,1783.333333
8,2,1706.146341
8,2,1620.956731
8,2,9589.226415
8,2,3170.837500
8,2,11248.315000
8,2,6927.020202
8,2,3378.073864
8,2,5957.840426
8,2,3527.966480
8,2,10728.337900
8,2,7665.206897
8,2,3390.511029
8,2,2707.788265
8,2,8103.557065
8,2,2430.367500

8,2,2007.907407
8,2,7177.085366
8,2,3107.283163
8,2,1852.842949
8,2,2056.958333
8,2,2832.285714
8,2,2016.709821
8,2,4760.434524
8,2,2002.750000
8,2,12703.277778
8,2,4576.550532
8,2,5739.180233
8,2,1734.486111
8,2,8977.970430
8,2,1723.000000
8,2,2580.911290
8,2,3474.533163
8,2,2174.743243
8,2,1543.511628
8,2,2599.902439
8,2,2346.551887
8,2,2140.486111
8,2,4196.250000
8,2,1552.273438
8,2,1520.745763
8,2,3234.765957
8,2,4337.847222
8,2,3693.947368
8,2,3043.024194
8,2,4559.062500
8,2,9432.774123
8,2,1869.276596
8,2,1537.508152
8,2,2245.730263
8,2,2481.212963
8,2,4425.120000
8,2,1871.283784
8,2,1934.476744
8,2,4339.277778
8,2,6237.613636
8,2,4665.514881
8,2,4677.000000
8,2,3485.539634
8,2,1809.612981
8,2,1941.631098
8,2,3855.500000
8,2,1967.511905
8,2,1517.320652
8,2,6115.191489
8,2,12436.011976
8,2,2859.428125
8,2,1774.243243
8,2,1616.285714
8,2,8407.304878
8,2,11768.444444
8,2,6501.781250
8,2,22310.509615
8,2,2668.481132
8,2,2373.560096
8,2,4399.567039
8,2,1511.173611
8,2,4844.786127
8,2,8936.446629
8,2,3621.073333
8,2,2119.754601
8,2,2404.950739

8,2,5224.976852
8,2,2721.326923
8,2,10152.675000
8,2,4866.914773
8,2,2240.750000
8,2,3088.383152
8,2,2678.750000
8,2,5021.129717
8,2,7920.899457
8,2,4430.350299
8,2,6101.201258
8,2,2789.073171
8,2,3870.428571
8,2,3363.717391
8,2,2697.733333
8,2,5590.539062
8,2,5854.090909
8,2,1637.563830
8,2,1886.303191
8,2,14856.701220
8,2,1997.300000
8,2,1670.701220
8,2,3370.000000
8,2,2166.632812
8,2,4792.737805
8,2,4748.725000
8,2,2764.578035
8,2,2214.852349
8,2,3317.668394
8,2,5368.703704
8,2,14983.159274
8,2,1867.571429
8,2,8180.112069
8,2,3770.895833
8,2,8042.744048
8,2,1982.973684
8,2,1975.009146
8,2,6349.400000
8,2,1506.862500
8,2,5519.478723
8,2,1790.802632
8,2,1691.927152
8,2,2317.472222
8,2,5652.300613
8,2,2370.590909
8,2,5542.625000
8,2,1838.090452
8,2,1709.364130
8,2,2481.218750
8,2,12040.714286
8,2,2343.109043
8,2,2947.987179
8,2,11021.934524
8,2,3027.122093
8,2,2640.514151
8,2,20906.038462
8,2,6154.382812
8,2,4631.867647
8,2,8615.475000
8,2,2634.786585
8,2,3387.089286
8,2,1606.093750
8,2,1687.237805
8,2,6642.625000
8,2,7111.689655
8,2,1553.048780

8,2,1659.360190
8,2,3758.415865
8,2,2353.821429
8,2,5654.389535
8,2,1745.408333
8,2,3249.112981
8,2,2397.996429
8,2,4435.350000
8,2,3057.133333
8,2,1558.451220
8,2,7662.660326
8,2,2416.838235
8,2,20379.558511
8,2,6195.711735
8,2,10917.692308
8,2,6953.805851
8,2,1864.756410
8,2,2655.673077
8,2,4179.961735
8,2,1816.978723
8,2,6028.615385
8,2,5580.825000
8,2,3604.833333
8,2,4025.177326
8,2,4146.337838
8,2,2471.901163
8,2,12330.253333
8,2,2787.000000
8,2,1674.195652
8,2,4239.597222
8,2,2022.908333
8,2,4448.058824
8,2,11275.039474
8,2,3678.819149
8,2,1522.513889
8,2,2036.885135
8,2,2552.264205
8,2,3330.708333
8,2,45873.340385
8,2,5559.686047
8,2,3976.675676
8,2,6338.076923
8,2,3829.318750
8,2,2713.964286
8,2,13292.247642
8,2,3679.437500
8,2,2045.202703
8,2,2891.075000
8,2,3451.630682
8,2,4167.611732
8,2,2857.975610
8,2,7116.432203
8,2,4535.250000
8,2,2208.500000
8,2,1539.494898
8,2,4597.877778
8,2,1923.193750
8,2,2539.375000
8,2,2224.353261
8,2,1872.983553
8,2,1571.849359
8,2,12058.738839
8,2,5039.477273
8,2,4808.016393
8,2,3049.919271
8,2,6974.171569

8,2,1601.089674
8,2,1807.590426
8,2,5290.909574
8,2,3840.267045
8,2,3266.261538
8,2,1697.977941
8,2,4379.758454
8,2,8371.986486
8,2,7611.443182
8,2,4430.204787
8,2,2061.321429
8,2,3788.669355
8,2,8165.275641
8,2,1683.318182
8,2,1861.070755
8,2,6222.087649
8,2,4676.575000
8,2,12508.250000
8,2,6159.572674
8,2,3156.857513
8,2,13229.220408
8,2,2273.657360
8,2,4877.305085
8,2,6173.678571
8,2,3330.448276
8,2,14976.287234
8,2,3326.521739
8,2,4261.759615
8,2,2042.845455
8,2,2860.922222
8,2,1910.645833
8,2,1503.583333
8,2,2753.627778
8,2,3040.024390
8,2,3753.928571
8,2,2398.551724
8,2,3664.375000
8,2,5121.968085
8,2,1639.178571
8,2,3068.973837
8,2,2825.437500
8,2,5803.111111
8,2,6682.137019
8,2,10292.308824
8,2,2461.857143
8,2,2557.820312
8,2,3073.289063
8,2,1629.075581
8,2,2343.687500
8,2,1512.676630
8,2,3924.767442
8,2,2454.375000
8,2,1679.166667
8,2,2465.280405
8,2,8452.120690
8,2,1746.864865
8,2,7123.250000
8,2,2922.022222
8,2,2775.255814
8,2,1669.437500
8,2,4378.967593
8,2,5875.625000
8,2,2278.390625
8,2,3358.511905
8,2,5410.336538
8,2,6822.780488

8,2,2201.598039
8,2,1932.000000
8,2,8150.476562
8,2,6369.214286
8,2,4560.041667
8,2,7644.438725
8,2,5744.910448
8,2,1900.980263
8,2,3161.232558
8,2,2256.465116
8,2,3950.850962
8,2,3869.125000
8,2,2751.967949
8,2,4114.800000
8,2,6245.567308
8,2,9095.240625
8,2,6280.235294
8,2,6181.490741
8,2,8675.804124
8,2,9042.391566
8,2,3690.945000
8,2,1834.265000
8,2,4856.941176
8,2,2000.102041
8,2,2823.312500
8,2,3535.579545
8,2,2579.636905
8,2,2559.027027
8,2,5878.473404
8,2,5833.762500
8,2,2930.467105
8,2,3075.291667
8,2,3632.000000
8,2,2374.911932
8,2,2121.527778
8,2,3251.868421
8,2,12450.291667
8,2,2013.588710
8,2,2426.766892
8,2,16704.888889
8,2,3401.507212
8,2,2785.832589
8,2,6658.902778
8,2,11958.581019
8,2,3663.134615
8,2,2397.470588
8,2,1675.583333
8,2,2305.319372
8,2,3457.219512
8,2,2981.059524
8,2,2480.146341
8,2,5806.461735
8,2,3509.687500
8,2,2362.152439
8,2,1651.278125
8,2,1854.574519
8,2,4699.250000
8,2,4802.500000
8,2,2999.465116
8,2,17533.795673
8,2,7228.500000
8,2,2308.580000
8,2,3530.035000
8,2,3010.477564
8,2,3320.337662
8,2,7601.520349

8,2,2583.175532
8,2,3305.937500
8,2,1766.789216
8,2,2277.500000
8,2,4192.875000
8,2,2255.570652
8,2,2355.728774
8,2,3455.181818
8,2,4890.112069
8,2,9629.756757
8,2,2164.961538
8,2,2263.750000
8,2,8321.625000
8,2,3460.416667
8,2,5304.210526
8,2,3340.673469
8,2,4750.696078
8,2,4339.732143
8,2,1676.448864
8,2,12462.103814
8,2,3743.317568
8,2,2260.816667
8,2,1627.815508
8,2,5095.339286
8,2,2467.743590
8,2,7202.439024
8,2,5698.066845
8,2,4751.937173
8,2,5230.502404
8,2,9348.937500
8,2,2496.500000
8,2,6187.611111
8,2,7827.011628
8,2,2248.630435
8,2,2331.424419
8,2,1611.243094
8,2,2941.222222
8,2,5469.111111
8,2,4672.421429
8,2,3395.900000
8,2,1910.268229
8,2,3276.666667
8,2,2426.691489
8,2,3841.827586
8,2,3532.166667
8,2,1613.850000
8,2,2111.835526
8,2,3439.687500
8,2,3847.569697
8,2,1841.525000
8,2,3419.755435
8,2,1639.115385
8,2,5743.718750
8,2,6590.009091
8,2,4412.962500
8,2,2387.915000
8,2,3742.255319
8,2,2938.658163
8,2,1546.955357
8,2,6740.789474
8,2,2980.512821
8,2,2604.125000
8,2,4232.585635
8,2,3432.447368
8,2,2803.290761
8,2,2670.453125

8,2,3859.000000
8,2,2251.528409
8,2,1798.074074
8,2,3851.716981
8,2,1800.040698
8,2,2994.968750
8,2,1781.260000
8,2,2306.951923
8,2,3519.184896
8,2,3100.260204
8,2,7538.744681
8,2,1639.080000
8,2,5401.632500
8,2,2761.261628
8,2,3681.452778
8,2,10378.095930
8,2,3148.335106
8,2,1738.327869
8,2,2144.037162
8,2,2829.528090
8,2,2276.365385
8,2,2710.088710
8,2,7678.187500
8,2,2013.275362
8,2,1925.000000
8,2,15500.564103
8,2,7317.250000
8,2,2882.546875
8,2,1750.178571
8,2,2702.187500
8,2,3089.852941
8,2,3125.250000
8,2,1766.430851
8,2,3350.104651
8,2,2894.692308
8,2,2684.888587
8,2,4281.659574
8,2,1734.887500
8,2,3192.071429
8,2,2205.083333
8,2,2773.122222
8,2,2416.481132
8,2,1667.794872
8,2,4685.750000
8,2,1930.643750
8,2,2354.627976
8,2,2797.682692
8,2,1594.542969
8,2,2177.250000
8,2,1822.600000
8,2,1796.375000
8,2,7697.535377
8,2,2595.530405
8,2,24373.541667
8,2,5189.785714
8,2,1707.553191
8,2,8493.325000
8,2,7575.301020
8,2,5550.593750
8,2,3210.727273
8,2,3885.300000
8,2,3107.696429
8,2,4206.695122
8,2,1555.119565
8,2,3345.401163
8,2,2409.541667

8,2,5850.702128
8,2,3916.062500
8,2,2312.542553
8,2,4126.725610
8,2,1673.150000
8,2,6246.217105
8,2,1523.139535
8,2,2154.515306
8,2,5097.736842
8,2,8947.755814
8,2,1847.142857
8,2,2694.607955
8,2,2175.585227
8,2,11180.756250
8,2,2572.684211
8,2,6247.931373
8,2,1720.127660
8,2,6097.121622
8,2,10182.613208
8,2,2441.200000
8,2,1670.905660
8,2,5932.925000
8,2,23141.431818
8,2,3085.250000
8,2,4276.324468
8,2,3645.145833
8,2,3471.188830
8,2,1745.395349
8,2,1635.875000
8,2,4034.115385
8,2,24607.720000
8,2,11410.622500
8,2,2218.664286
8,2,8548.230769
8,2,2694.125000
8,2,2217.250000
8,2,10298.079082
8,2,2888.282051
8,2,2007.045455
8,2,11909.293919
8,2,1807.187097
8,2,1530.950000
8,2,6263.252907
8,2,1730.682692
8,2,1959.608108
8,2,2455.742038
8,2,4680.450000
8,2,3921.625000
8,2,5432.600000
8,2,3940.800000
8,2,9696.043478
8,2,3644.817308
8,2,2014.250000
8,2,3485.368304
8,2,9132.010417
8,2,1553.482759
8,2,4670.588068
8,2,6604.132353
8,2,2651.333333
8,2,5302.581818
8,2,2756.829268
8,2,3917.183962
8,2,4533.585106
8,2,2905.318750
8,2,1942.666667
8,2,4755.265306

8,2,8824.389881
8,2,2300.482143
8,2,12640.055288
8,2,3177.785256
8,2,2518.430921
8,2,2216.827500
8,2,10508.776786
8,2,5042.493750
8,2,3034.059524
8,2,1776.422297
8,2,5884.425926
8,2,1797.744792
8,2,5259.361702
8,2,4220.089674
8,2,1700.134615
8,2,8069.883152
8,2,7311.152273
8,2,2057.524306
8,2,7136.247093
8,2,10241.019737
8,2,2278.779412
8,2,4592.062500
8,2,3229.845238
8,2,3895.678571
8,2,2957.820755
8,2,6825.783784
8,2,2987.791667
8,2,3962.266667
8,2,5155.531915
8,2,1678.091837
8,2,8032.072034
8,2,3461.444444
8,2,2051.814815
8,2,4107.600000
8,2,1517.792857
8,2,5448.312500
8,2,3160.932927
8,2,2377.463542
8,2,3087.884615
8,2,2349.066667
8,2,1826.641447
8,2,3676.742857
8,2,5211.868243
8,2,3760.083333
8,2,13800.267857
8,2,2211.238095
8,2,2290.125000
8,2,5552.640625
8,2,5457.308824
8,2,1917.963816
9,1,2603.482639
9,1,3743.250000
9,1,2960.804878
9,1,34206.356061
9,1,11085.859756
9,1,5672.583333
9,1,2168.487179
9,1,2903.500000
9,1,1991.889535
9,1,2109.463415
9,1,140363.631579
9,1,1501.935606
9,1,2506.250000
9,1,64270.270833
9,1,1824.694444
9,1,1665.950000

9,1,2219.113636
9,1,7105.625000
9,1,42934.378049
9,1,1542.761364
9,1,2741.040441
9,1,4994.625000
9,1,1984.622222
9,1,4189.375000
9,1,8358.034722
9,1,60410.805556
9,1,1555.816176
9,1,1727.051471
9,1,2457.958333
9,1,1582.494048
9,1,2666.225806
9,1,55495.213542
9,1,1523.977273
9,1,7493.502660
9,1,3732.614865
9,1,1501.297872
9,1,17875.004902
9,1,1646.507143
9,1,2600.569149
9,1,1503.938776
9,1,2549.757576
9,1,2517.630682
9,1,2690.007576
9,1,1608.897436
9,1,105054.093023
9,1,1592.625000
9,1,1731.087500
9,1,1550.600000
9,1,4970.507812
9,1,1909.302326
9,1,22944.285714
9,1,66448.707317
9,1,29383.385417
9,1,26876.617424
9,1,2401.047619
9,1,1603.128378
9,1,61873.076923
9,1,64875.351744
9,1,1712.854167
9,1,39867.517857
9,1,52148.117925
9,1,78413.058511
9,1,55442.888889
9,1,19593.146341
9,1,1908.062500
9,1,3577.597561
9,1,1778.522436
9,1,6637.191667
9,1,32880.015625
9,1,1997.407051
9,1,7142.377907
9,1,77711.937500
9,1,9156.090909
9,1,1897.300000
9,1,1800.963235
9,1,1544.334375
9,1,113331.341463
9,1,3355.937500
9,1,44157.227941
9,1,3731.735465
9,1,1631.843750
9,1,8574.917969

9,1,92056.250000
9,1,2864.830000
9,1,3667.591837
9,1,2692.042857
9,1,7220.012195
9,1,15191.166667
9,1,2713.383929
9,1,1747.366279
9,1,38560.110294
9,1,43473.239865
9,1,3948.036765
9,1,2084.312500
9,1,4480.515625
9,1,1756.428571
9,1,21809.775641
9,1,7771.921053
9,1,1910.192308
9,1,2286.696429
9,1,2066.517045
9,1,24218.318966
9,1,100652.400000
9,1,2033.188889
9,1,3662.153846
9,1,2027.585366
9,1,2390.406250
9,1,5216.715116
9,1,24998.750000
9,1,51665.212121
9,1,16545.246875
9,1,101986.155488
9,1,1701.732143
9,1,1710.321429
9,1,3544.200581
9,1,5447.900000
9,1,4522.800000
9,1,1845.156977
9,1,2945.669643
9,1,5170.295000
9,1,2007.304348
9,1,60306.065789
9,1,43862.500000
9,1,2354.111413
9,1,1585.052326
9,1,7393.332317
9,1,2245.361111
9,1,2284.505814
9,1,1714.719512
9,1,2760.521341
9,1,21969.477273
9,1,77420.685811
9,1,4414.656977
9,1,17727.639286
9,1,8737.912162
9,1,2860.021739
9,1,1977.278846
9,1,12922.750000
9,1,2901.622222
9,1,32379.531250
9,1,60659.182692
9,1,4573.860465
9,1,3142.307292
9,1,2298.510417
9,1,1537.544643
9,1,11332.153846
9,1,2759.181818
9,1,11598.977273

9,1,2514.779070
9,1,4075.371795
9,1,2789.258523
9,1,22049.198052
9,1,4416.494186
9,1,6289.774038
9,1,2679.817308
9,1,1969.785714
9,1,1976.242188
9,1,5065.093750
9,1,4339.750000
9,1,46498.923387
9,1,2702.039062
9,1,7558.737500
9,1,6734.423780
9,1,2143.456395
9,1,2513.860795
9,1,7821.307692
9,1,9140.507653
9,1,4411.454545
9,1,1901.598837
9,1,16911.628571
9,1,2252.568750
9,1,3680.686047
9,1,4197.487805
9,1,1630.218750
9,1,4199.278646
9,1,4972.500000
9,1,3091.277027
9,1,1817.528846
9,1,1886.000000
9,1,66579.036184
9,1,5830.333333
9,1,25751.391892
9,1,41016.083333
9,1,9666.056548
9,1,12268.500000
9,1,1499.451220
9,1,1653.687500
9,1,5992.750000
9,1,2958.542763
9,1,18989.757353
9,1,7661.862500
9,1,28411.342857
9,1,1873.000000
9,1,85705.939024
9,1,2286.065476
9,1,1591.621951
9,1,9795.450000
9,1,5894.680556
9,1,1579.311111
9,1,1521.457317
9,1,1613.946809
9,1,14465.800000
9,1,1572.000000
9,1,4140.747222
9,1,2446.300000
9,1,1640.585366
9,1,1766.262097
9,1,6588.775000
9,1,1944.197917
9,1,71040.355769
9,1,8517.309524
9,1,1684.645833
9,1,36763.275000
9,1,1617.143750

9,1,1789.834239
9,1,40721.227778
9,1,1863.076923
9,1,183481.161765
9,1,4077.264706
9,1,3455.072222
9,1,2721.917614
9,1,9210.400000
9,1,2273.204861
9,1,8181.764535
9,1,19681.109375
9,1,1573.702128
9,1,1652.500000
9,1,3723.765152
9,1,70650.022059
9,1,1659.511111
9,1,1568.524390
9,1,64580.385000
9,1,1895.238636
9,1,12203.331658
9,1,1913.726974
9,1,119325.551136
9,1,8117.772727
9,1,2501.727273
9,1,4437.523438
9,1,6765.514535
9,1,11225.736842
9,1,33392.607143
9,1,2620.380682
9,1,15134.450000
9,1,2362.088542
9,1,46397.137500
9,1,3187.764535
9,1,3301.293750
9,1,1786.934783
9,1,31641.018750
9,1,1781.829268
9,1,2699.883721
9,1,1521.997222
9,1,1546.500000
9,1,2452.947917
9,1,5079.697500
9,1,5026.897727
9,1,1806.801136
9,1,30301.934211
9,1,1827.403846
9,1,53092.334677
9,1,1950.331126
9,1,2604.702128
9,1,78803.753049
9,1,1645.891304
9,1,10200.773936
9,1,2044.208333
9,1,1530.891667
9,1,1631.443182
9,1,2434.935897
9,1,40808.250000
9,1,1641.304348
9,1,1789.609756
9,1,100334.432927
9,1,2865.625000
9,1,24812.657051
9,1,2384.926829
9,1,9774.140625
9,1,1602.847826
9,1,3079.367021

9,1,42062.972973
9,1,5504.015625
9,1,2387.385870
9,1,31625.518750
9,1,31958.804054
9,1,2881.639706
9,1,3196.391026
9,1,2461.471429
9,1,4880.486111
9,1,5361.721429
9,1,1928.932292
9,1,43717.214674
9,1,2075.815476
9,1,2148.869565
9,1,3109.228873
9,1,2910.790541
9,1,1935.457447
9,1,2043.038690
9,1,2551.198529
9,1,2005.901515
9,1,4234.583333
9,1,99196.013514
9,1,2655.815476
9,1,2227.023256
9,1,2264.112500
9,1,2562.500000
9,1,1588.027778
9,1,8081.429688
9,1,37178.693878
9,1,1735.175000
9,1,2501.024390
9,1,4122.804878
9,1,2115.155405
9,1,1867.835106
9,1,1803.940104
9,1,64834.769231
9,1,2977.133333
9,1,54222.157051
9,1,4373.814815
9,1,8537.524390
9,1,4872.560811
9,1,1925.179487
9,1,2502.775862
9,1,117823.013158
9,1,4490.459302
9,1,63058.253676
9,1,2700.662791
9,1,3662.091837
9,1,116539.281250
9,1,1711.500000
9,1,1653.090909
9,1,2113.908537
9,1,24719.600000
9,1,2406.282738
9,1,1812.343023
9,1,13519.027174
9,1,2091.231618
9,1,30688.142857
9,1,2685.845238
9,1,2302.500000
9,1,1668.127660
9,1,27767.012195
9,1,2170.250000
9,1,122530.150000
9,1,7033.260417
9,1,4234.094340

9,1,4585.240000
9,1,6210.428571
9,1,1967.184524
9,1,139235.961538
9,1,117822.256098
9,1,1937.922619
9,1,1872.194079
9,1,3132.300000
9,1,51032.500000
9,1,3285.857143
9,1,1801.275000
9,1,2629.486486
9,1,35166.250000
9,1,2189.130952
9,1,4446.000000
9,1,5002.991667
9,1,10765.654255
9,1,1531.000000
9,1,2736.548295
9,1,9254.709677
9,1,56221.599359
9,1,72250.779070
9,1,3147.649306
9,1,2592.979730
9,1,75442.097561
9,1,24074.902174
9,1,59573.960526
9,1,1826.776316
9,1,31239.000000
9,1,3500.198113
9,1,183538.500000
9,1,2598.756098
9,1,1618.488636
9,1,80555.082386
9,1,1912.490132
9,1,3678.030000
9,1,75338.187500
9,1,7268.872159
9,1,81559.833333
9,1,2648.486413
9,1,2015.851744
9,1,22479.652174
9,1,1739.785714
9,1,1882.227273
9,1,4528.828125
9,1,10222.139205
9,1,6482.319444
9,1,33418.996212
9,1,3046.200000
9,1,3106.325581
9,1,25638.807692
9,1,5558.861842
9,1,19311.636364
9,1,1551.407051
9,1,2379.900000
9,1,8488.042857
9,1,2111.666667
9,1,1716.250000
9,1,2514.224138
9,1,1806.314024
9,1,3503.920732
9,1,3793.781915
9,1,3180.471698
9,1,4166.875000
9,1,3169.711111
9,1,2715.572222

9,1,3261.676630
9,1,1734.175000
9,1,21229.161458
9,1,1894.125000
9,1,24958.361842
9,1,8779.043478
9,1,2657.799419
9,1,2428.115132
9,1,47362.006250
9,1,91506.250000
9,1,75025.875000
9,1,11735.140625
9,1,8898.268293
9,1,2300.327381
9,1,5465.867647
9,1,38469.976744
9,1,35329.000000
9,1,30322.041667
9,1,5834.666667
9,1,3120.775000
9,1,43867.000000
9,1,1956.901596
9,1,2570.109375
9,1,32782.043605
9,1,128838.344907
9,1,1879.557927
9,1,47641.807692
9,1,2946.023256
9,1,1552.360465
9,1,6795.625000
9,1,3728.750000
9,1,35538.526316
9,1,6235.300000
9,1,69714.847973
9,1,2078.767857
9,1,11162.258152
9,1,1963.762195
9,1,1596.654070
9,1,6805.526163
9,1,3963.330645
9,1,135504.759146
9,1,1532.649306
9,1,2084.622024
9,1,3126.201087
9,1,1749.058140
9,1,13608.771429
9,1,2114.804348
9,1,1643.241477
9,1,1673.721154
9,1,2513.056250
9,1,1712.461538
9,1,36805.830882
9,1,2269.506579
9,1,2886.488889
9,1,1567.865385
9,1,1551.792857
9,1,63751.962838
9,1,58283.625000
9,1,1708.000000
9,1,1743.425000
9,1,2616.192568
9,1,48806.265625
9,1,3735.154255
9,1,20793.250000
9,1,13920.140909
9,1,28154.453947

9,1,2257.733333
9,1,3468.901163
9,1,4065.975610
9,1,1575.119318
9,1,2309.493421
9,1,2247.101064
9,1,35003.878049
9,1,2756.236702
9,1,1925.131944
9,1,50077.122340
9,1,9087.473684
9,1,2897.352941
9,1,2823.088235
9,1,1899.928571
9,1,53649.769531
9,1,1506.964286
9,1,8358.510870
9,1,4749.662946
9,1,54255.125000
9,1,18113.636111
9,1,31825.965517
9,1,1880.971154
9,1,1720.377551
9,1,45591.986111
9,1,2638.717391
9,1,1923.722826
9,1,69392.305556
9,1,2031.104651
9,1,1562.678977
9,1,3503.211735
9,1,1567.250000
9,1,9729.914062
9,1,21636.523810
9,1,3022.346429
9,1,2668.128205
9,1,4354.173913
9,1,28796.161585
9,1,1650.170213
9,1,58304.019531
9,1,5812.703125
9,1,62926.204268
9,1,46882.486111
9,1,2403.198113
9,1,51764.907895
9,1,17089.182927
9,1,36137.278571
9,1,1742.321429
9,1,10070.120098
9,1,4550.331731
9,1,2368.330000
9,1,7073.803571
9,1,1981.806548
9,1,40494.138889
9,1,2127.116279
9,1,2421.484375
9,1,2086.223404
9,1,1558.907895
9,1,2224.469512
9,1,3226.895833
9,1,2272.534375
9,1,36568.960526
9,1,1927.736486
9,1,39820.709677
9,1,4090.298077
9,1,1954.740741
9,1,2418.900000

9,1,3732.907143
9,1,29735.679293
9,1,120150.951220
9,1,2509.841216
9,1,2690.398649
9,1,11011.180000
9,1,2329.288690
9,1,1858.000000
9,1,2298.021341
9,1,9667.871622
9,1,70828.186047
9,1,2708.096154
9,1,24933.548077
9,1,1784.508929
9,1,2944.808140
9,1,86606.489865
9,1,8749.596774
9,1,1516.959302
9,1,2932.857143
9,1,4001.562500
9,1,13729.448171
9,1,2062.483333
9,1,40125.375000
9,1,80234.295732
9,1,4909.176471
9,1,42933.888158
9,1,39647.042553
9,1,5739.888889
9,1,3476.285714
9,1,1608.883721
9,1,1735.038462
9,1,2556.540541
9,1,1667.541667
9,1,1542.017857
9,1,63289.666667
9,1,57018.345395
9,1,3381.316176
9,1,9936.285156
9,1,38316.803125
9,1,2056.119048
9,1,2104.423077
9,1,1636.908854
9,1,8255.085106
9,1,1567.885714
9,1,5569.244681
9,1,2969.959184
9,1,1707.062500
9,1,29927.235795
9,1,28982.712963
9,1,8273.757576
9,1,2056.062500
9,1,3168.235294
9,1,1516.172297
9,1,45015.884615
9,1,1764.168750
9,1,39664.858108
9,1,1729.850000
9,1,2092.405000
9,1,4938.260776
9,1,1585.276316
9,1,5462.987500
9,1,2311.505000
9,1,1958.914634
9,1,111225.847826
9,1,2019.757576
9,1,4590.904762

9,1,1638.581633
9,1,2608.203804
9,1,4384.839286
9,1,1916.489796
9,1,39036.607143
9,1,3321.232143
9,1,20155.442073
9,1,2350.812500
9,1,3751.011905
9,1,39477.954545
9,1,2993.312500
9,1,10093.460938
9,1,23683.525000
9,1,4438.236111
9,1,2060.485294
9,1,34096.287500
9,1,1778.800595
9,1,24932.659091
9,1,17979.000000
9,1,3589.209559
9,1,4490.769737
9,1,61577.079545
9,1,26486.562500
9,1,1642.250000
9,1,2751.424342
9,1,4156.784884
9,1,118345.696970
9,1,1535.715686
9,1,2416.089286
9,1,9779.487069
9,1,71791.500000
9,1,2074.304348
9,1,8501.625000
9,1,1585.297872
9,1,2726.725000
9,1,2458.500000
9,1,3059.865854
9,1,2330.228261
9,1,9108.577381
9,1,1671.906977
9,1,2863.264706
9,1,2200.061170
9,1,155711.907500
9,1,6261.837500
9,1,5394.162500
9,1,1502.263158
9,1,41840.050000
9,1,8787.229167
9,1,1509.500000
9,1,2895.267857
9,1,1671.048780
9,1,1596.763514
9,1,3131.926829
9,1,3644.380102
9,1,6677.857143
9,1,2500.435811
9,1,7403.612245
9,1,2900.234043
9,1,102866.772059
9,1,84660.826087
9,1,16452.930556
9,1,3557.202703
9,1,2826.187500
9,1,3770.424342
9,1,58467.043605
9,1,45300.450000

9,1,1872.095238
9,1,1871.701923
9,1,3408.973684
9,1,2336.227273
9,1,1580.543750
9,1,5570.004808
9,1,4379.260870
9,1,38520.118590
9,1,82954.562500
9,1,5782.392157
9,1,3853.575000
9,1,1803.891304
9,1,3019.589744
9,1,4152.643750
9,1,23622.076923
9,1,1811.325000
9,1,11793.413793
9,1,4452.118590
9,1,3735.885714
9,1,1760.600000
9,1,47231.845000
9,1,7880.343750
9,1,1867.420732
9,1,1953.562500
9,1,5358.550000
9,1,35988.638158
9,1,38227.827206
9,1,16896.954861
9,1,23850.484848
9,1,2674.963889
9,1,2917.286458
9,1,1548.850000
9,1,2591.237179
9,1,20330.422222
9,1,15598.692708
9,1,2295.558824
9,1,61500.281250
9,1,1511.287500
9,1,2354.566489
9,1,1900.681818
9,1,1660.768293
9,1,17540.031915
9,1,62871.358974
9,1,33821.152027
9,1,1948.166667
9,1,3145.652778
9,1,44780.795455
9,1,1613.796875
9,1,1886.310811
9,1,3086.943182
9,1,2006.297872
9,1,1840.845395
9,1,1838.404255
9,1,12053.780488
9,1,40857.183673
9,1,1692.085366
9,1,1526.764706
9,1,2646.858974
9,1,1530.026042
9,1,1950.415541
9,1,2802.647436
9,1,2621.833333
9,1,3525.125000
9,1,12463.739130
9,1,1863.255682
9,1,37438.208333

9,1,1694.650000
9,1,1767.622396
9,1,4143.621951
9,1,1518.387755
9,1,1815.263298
9,1,1705.132979
9,1,4678.084906
9,1,70310.956250
9,1,3459.802632
9,1,1926.884615
9,1,1814.513158
9,1,1641.050676
9,1,106978.230769
9,1,1884.436111
9,1,2513.386628
9,1,1578.769737
9,1,89317.604167
9,1,3554.736413
9,1,12150.364583
9,1,110605.611111
9,1,37639.384146
9,1,48628.812500
9,1,1583.963636
9,1,2011.820122
9,1,2053.828125
9,1,6535.816860
9,1,3305.445122
9,1,1519.993902
9,1,8807.708333
9,1,21636.968750
9,1,1554.422619
9,1,3116.500000
9,1,8970.133929
9,1,2167.144737
9,1,2620.583333
9,1,3812.006250
9,1,2619.660714
9,1,50436.590909
9,1,2985.402778
9,1,3674.589744
9,1,8791.939024
9,1,85551.554054
9,1,1851.212766
9,1,6389.083333
9,1,1936.765244
9,1,1625.465625
9,1,75500.956250
9,1,4040.156250
9,1,1557.136364
9,1,14749.483333
9,1,6546.220745
9,1,3030.262195
9,1,2254.458333
9,1,4178.875000
9,1,4836.036765
9,1,1610.860294
9,1,1815.529412
9,1,2259.723404
9,1,11214.977273
9,1,17236.204082
9,1,26002.500000
9,1,90014.000000
9,1,32012.549020
9,1,1787.641304
9,1,7636.839286
9,1,1692.316667

9,1,5706.843750
9,1,27836.587500
9,1,59337.758621
9,1,55909.176829
9,1,9407.612903
9,1,3202.625000
9,1,17183.644231
9,1,2764.922619
9,1,1715.817708
9,1,1504.208333
9,1,32881.120192
9,1,1978.753676
9,1,2591.585526
9,1,1878.291667
9,1,5829.500000
9,1,3133.448370
9,1,3460.428571
9,1,3508.944444
9,1,6014.538462
9,1,1603.225610
9,1,1832.887500
9,1,3965.609375
9,1,55791.659091
9,1,4665.404255
9,1,2796.325581
9,1,3282.390244
9,1,1503.205357
9,1,3185.642857
9,1,2277.439189
9,1,136916.824468
9,1,73955.822857
9,1,6990.284722
9,1,1707.743902
9,1,3110.250000
9,1,55832.780488
9,1,2972.708333
9,1,1651.744565
9,1,1673.045455
9,1,133962.571023
9,1,4045.201923
9,1,88467.661765
9,1,42623.964744
9,1,5682.821429
9,1,1895.741935
9,1,16287.088235
9,1,47913.843750
9,1,6372.444444
9,1,8516.684783
9,1,3279.834302
9,1,1938.739286
9,1,172250.633333
9,1,69146.292683
9,1,61552.567568
9,1,64088.319079
9,1,1565.777778
9,1,36257.973837
9,1,85089.065625
9,1,4645.050000
9,1,46650.819444
9,1,35394.516026
9,1,20597.897959
9,1,1984.690476
9,1,6394.786184
9,1,1501.692308
9,1,4545.872340
9,1,1521.687500

```
9,1,2440.828125
9,1,5167.932065
9,1,5967.963710
9,1,1648.428571
9,1,47051.625000
9,1,2955.326923
9,1,3947.450581
9,1,2972.995000
9,1,1602.416667
9,1,3205.732558
9,1,69576.937500
9,1,2005.228571
9,1,58226.752907
9,1,2815.000000
9,1,4041.246951
9,1,3230.194853
9,1,2541.200000
9,1,9896.493421
9,1,18075.812500
9,1,2657.538462
9,1,56803.780405
9,1,7288.608696
9,1,29615.302419
9,1,8603.400000
9,1,25167.868421
9,1,3131.500000
9,1,1552.839744
9,1,53056.891667
9,1,16504.218750
9,1,2641.765625
9,1,3072.060000
9,1,2008.410000
9,1,2507.782609
9,1,49854.459559
9,1,14273.944444
9,1,2981.270270
9,1,19258.375000
9,1,2156.112903
9,1,2324.765000
9,1,3013.014535
9,1,40163.846354
9,1,4299.125000
9,1,39778.871212
9,1,4943.871429
9,1,51009.890625
9,1,11478.829268
9,1,3031.605000
9,1,1752.703571
9,1,4840.723684
9,1,5497.860465
9,1,53077.777778
9,1,1788.780488
9,1,2861.413265
9,1,22126.830000
9,1,2018.448980
9,1,1849.250000
9,1,18376.328000
9,1,3739.763975
9,1,1667.531250
9,1,10049.246324
9,1,1719.500000
9,1,2309.918129
9,1,2319.125000
9,1,2923.574405
9,1,1590.138889
9,1,3697.598404
```

9,1,2576.625000
9,1,6025.285714
9,1,13899.860465
9,1,56238.404762
9,1,2352.769231
9,1,2861.777027
9,1,2781.533537
9,1,103629.155556
9,1,4636.132212
9,1,2056.472826
9,1,3598.750000
9,1,2671.814103
9,1,1724.472222
9,1,2417.600000
9,1,16023.286290
9,1,86826.461538
9,1,7669.147959
9,1,113360.062500
9,1,1710.713415
9,1,44033.362903
9,1,5705.047170
9,1,1672.901515
9,1,2780.985465
9,1,2208.573864
9,1,1966.357143
9,1,1547.171196
9,1,1518.653125
9,1,1703.225610
9,1,6478.203488
9,1,1855.328125
9,1,4326.981132
9,1,16440.286290
9,1,5175.690000
9,1,107751.166667
9,1,3489.125000
9,1,2633.858333
9,1,2799.944444
9,1,1760.237500
9,1,3932.726190
9,1,3801.388889
9,1,3624.142857
9,1,3965.968085
9,1,2287.046875
9,1,2827.790698
9,1,1871.572222
9,1,6525.975000
9,1,5776.533088
9,1,43028.475000
9,1,1885.192568
9,1,25238.983871
9,1,1634.130208
9,1,3407.243243
9,1,155221.122093
9,1,5081.655556
9,1,6964.573864
9,1,2236.265625
9,1,1551.665541
9,1,3833.549020
9,1,7485.196809
9,1,71037.810811
9,1,1573.351064
9,1,1936.358696
9,1,3064.182927
9,1,50507.791667
9,1,1607.200521
9,1,4330.830882

9,1,2292.388889
9,1,3829.666667
9,1,1791.408333
9,1,7578.535714
9,1,51742.005814
9,1,7076.214286
9,1,1981.371094
9,1,30580.516129
9,1,2685.444444
9,1,3684.923913
9,1,3053.360849
9,1,1865.937500
9,1,1882.011628
9,1,1673.125000
9,1,10204.378049
9,1,6514.097222
9,1,2605.319767
9,1,7240.627660
9,1,84902.177083
9,1,1605.500000
9,1,7106.822222
9,1,3202.384615
9,1,6868.375000
9,1,30148.828125
9,1,36688.065789
9,1,7509.339286
9,1,18663.047297
9,1,1770.641667
9,1,5391.898256
9,1,102160.330357
9,1,58382.038462
9,1,31066.371324
9,1,4523.985577
9,1,1517.720930
9,1,3230.789474
9,1,49849.179688
9,1,12137.700000
9,1,2556.942073
9,1,1546.353261
9,1,3073.252604
9,1,1566.131579
9,1,13046.096774
9,1,6289.125000
9,1,2645.268229
9,1,7468.845930
9,1,2470.338235
9,1,1796.577778
9,1,139694.151163
9,1,3628.821429
9,1,37455.000000
9,1,1993.539634
9,1,9997.169643
9,1,47478.020833
9,1,16899.772959
9,1,1669.500000
9,1,1522.412791
9,1,140252.350000
9,1,2543.541667
9,1,2280.315789
9,1,1897.198864
9,1,3894.083333
9,1,3621.437500
9,1,9433.388889
9,1,33240.650000
9,1,4708.720238
9,1,2521.585938

9,1,52354.875000
9,1,38580.926136
9,1,61157.795139
9,1,2546.089286
9,1,4135.461957
9,1,81936.826531
9,1,3589.534884
9,1,93275.692308
9,1,4156.191489
9,1,11717.195122
9,1,1989.262500
9,1,1579.384615
9,1,1838.306818
9,1,4713.869792
9,1,1832.000000
9,1,34295.293919
9,1,3280.480769
9,1,1737.836735
9,1,10507.976562
9,1,1705.538462
9,1,56563.000000
9,1,11888.909091
9,1,3674.875000
9,1,6670.394737
9,1,1593.604651
9,1,3905.443182
9,1,3261.166667
9,1,1507.824468
9,1,8632.582317
9,1,2514.391447
9,1,2141.698718
9,1,18144.131944
9,1,6519.666667
9,1,32840.825658
9,1,2218.274390
9,1,2689.280488
9,1,5429.428571
9,1,69069.833333
9,1,3606.363095
9,1,85413.930921
9,1,9751.437500
9,1,29478.567073
9,1,2031.000000
9,1,1790.125000
9,1,4245.983696
9,1,4179.350000
9,1,2226.565341
9,1,32052.846154
9,1,3380.910714
9,1,4022.904762
9,1,2405.550000
9,1,2390.848214
9,1,1992.128049
9,1,11733.613372
9,1,150415.300000
9,1,1804.625000
9,1,1647.022222
9,1,45981.252874
9,1,3245.730769
9,1,22388.525568
9,1,3826.377451
9,1,3365.087209
9,1,1528.152778
9,1,6229.212766
9,1,1769.082447
9,1,178839.295000

9,1,3440.746622
9,1,4312.737805
9,1,2616.000000
9,1,1937.818182
9,1,1643.456395
9,1,3820.979651
9,1,2128.666667
9,1,43343.800000
9,1,2136.832237
9,1,5517.486486
9,1,2153.574468
9,1,2801.324468
9,1,7239.693878
9,1,3987.112805
9,1,9855.200000
9,1,75247.550000
9,1,1535.189286
9,1,2011.926471
9,1,1841.024306
9,1,2871.662234
9,1,4425.000000
9,1,47330.322368
9,1,104190.802083
9,1,75243.003289
9,1,34602.421875
9,1,4701.963415
9,1,3754.272436
9,1,3984.440476
9,1,4126.250000
9,1,1529.847222
9,1,4071.822222
9,1,3183.500000
9,1,2656.369863
9,1,8415.237500
9,1,5102.911765
9,1,4458.820122
9,1,159554.253125
9,1,1547.019444
9,1,3844.795918
9,1,71936.912791
9,1,5379.703488
9,1,1984.357143
9,1,55612.596429
9,1,56132.980263
9,1,3367.277027
9,1,9861.460526
9,1,16574.265152
9,1,2552.028846
9,1,2606.566667
9,1,91031.441667
9,1,11939.390306
9,1,116631.847826
9,1,118278.759615
9,1,2048.446541
9,1,4680.764706
9,1,3434.609756
9,1,1818.828125
9,1,3065.250000
9,1,57422.009868
9,1,2444.343750
9,1,139043.744318
9,1,17127.988636
9,1,6558.821429
9,1,1736.011628
9,1,41779.447368
9,1,6192.302326

9,1,2214.073171
9,1,5020.100000
9,1,13766.641304
9,1,57235.392405
9,1,3714.600610
9,1,3899.890244
9,1,73115.562500
9,1,1688.682292
9,1,2136.359848
9,1,3023.741071
9,1,2099.668605
9,1,12346.306818
9,1,3357.956250
9,1,7892.587500
9,1,2649.600000
9,1,3742.833333
9,1,3438.700000
9,1,107437.885870
9,1,2527.136364
9,1,177018.348039
9,1,148008.476190
9,1,42388.250000
9,1,21161.181818
9,1,5787.750000
9,1,2420.713415
9,1,2306.967949
9,1,3625.110465
9,1,73756.833333
9,1,3190.541667
9,1,1590.627604
9,1,3706.447674
9,1,2976.125000
9,1,1682.975610
9,1,2848.300000
9,1,3604.023810
9,1,7156.723039
9,1,1987.420455
9,1,83854.692308
9,1,4216.625000
9,1,1876.523148
9,1,4262.621951
9,1,2534.140625
9,1,3466.806818
9,1,61469.681818
9,1,2817.785714
9,1,2709.946429
9,1,3220.867347
9,1,2188.630556
9,1,1691.203804
9,1,1796.815789
9,1,2008.145455
9,1,1845.021875
9,1,33883.636111
9,1,56504.190789
9,1,110062.461538
9,1,4966.877660
9,1,32756.000000
9,1,19332.612500
9,1,2582.554054
9,1,1620.239796
9,1,5945.476190
9,1,2043.683824
9,1,44062.719595
9,1,1674.666667
9,1,1910.279070
9,1,14230.728261

9,1,57417.866279
9,1,1704.812500
9,1,1511.184211
9,1,5113.353261
9,1,2109.894737
9,1,100172.156250
9,1,56421.047297
9,1,1579.497093
9,1,1963.152406
9,1,1765.782738
9,1,1593.227273
9,1,19362.125000
9,1,138195.050000
9,1,2103.593750
9,1,3112.480769
9,1,77369.972222
9,1,1932.041667
9,1,1999.630435
9,1,2223.053977
9,1,7719.864130
9,1,6160.668367
9,1,9681.651786
9,1,52275.250000
9,1,3041.785714
9,1,1731.397059
9,1,2410.786585
9,1,1937.214286
9,1,3017.211111
9,1,2751.218750
9,1,5519.462209
9,1,32424.573529
9,1,1801.647727
9,1,58760.056250
9,1,84884.178571
9,1,1743.695312
9,1,1570.302632
9,1,1924.837209
9,1,58474.303571
9,1,4513.700000
9,1,2154.989796
9,1,2768.041667
9,1,55605.267361
9,1,2383.185976
9,1,79871.418919
9,1,26188.145833
9,1,1644.842105
9,1,1513.231707
9,1,4579.665441
9,1,2303.640000
9,1,1560.424242
9,1,2470.411765
9,1,16478.875000
9,1,72524.784574
9,1,2867.000000
9,1,6822.863281
9,1,25795.371429
9,1,1574.487805
9,1,4127.716981
9,1,1757.550532
9,1,153743.319149
9,1,2376.253289
9,1,9054.187500
9,1,2464.469512
9,1,2084.545455
9,1,70106.215909
9,1,4697.424479

9,1,4988.782258
9,1,2066.168919
9,1,2304.939394
9,1,1950.002976
9,1,21508.333333
9,1,18426.112500
9,1,85589.617857
9,1,6772.041667
9,1,3508.456633
9,1,1645.283784
9,1,9813.573171
9,1,40381.354839
9,1,4456.533333
9,1,53238.000000
9,1,44845.268293
9,1,22706.333333
9,1,2206.090909
9,1,2186.060811
9,1,2376.816860
9,1,1863.111979
9,1,1521.903061
9,1,16853.098404
9,1,2539.578125
9,1,1515.690789
9,1,2548.782051
9,1,1906.228365
9,1,3076.268617
9,1,5535.575581
9,1,1579.567073
9,1,49123.000000
9,1,46763.109756
9,1,3644.208333
9,1,1613.684375
9,1,66255.844444
9,1,1671.222222
9,1,28316.136364
9,1,2111.880682
9,1,2691.928571
9,1,59339.497283
9,1,4438.133523
9,1,8804.201923
9,1,1693.500000
9,1,1607.744186
9,1,52548.146739
9,1,2049.388889
9,1,2614.166667
9,1,51610.522727
9,1,2164.414062
9,1,43911.586957
9,1,69117.584559
9,1,2230.031250
9,1,5977.744681
9,1,51803.054878
9,1,4689.539474
9,1,42958.000000
9,1,3443.176471
9,1,2001.103723
9,1,1626.567308
9,1,5617.714286
9,1,71260.810811
9,1,39843.392857
9,1,1932.250000
9,1,6552.850000
9,1,46935.875000
9,1,87293.290541
9,1,3337.414773

9,1,2946.314815
9,1,20269.216912
9,1,103706.500000
9,1,1921.806122
9,1,36801.181250
9,1,1996.409091
9,1,1589.407051
9,1,42399.041667
9,1,12224.904762
9,1,3249.366848
9,1,4099.486842
9,1,2268.226190
9,1,2159.452381
9,1,1547.389535
9,1,1976.583333
9,1,12602.513514
9,1,6425.480769
9,1,49905.976562
9,1,54329.480769
9,1,1683.750000
9,1,10505.640625
9,1,7951.042683
9,1,2867.644737
9,1,5316.857143
9,1,26464.053030
9,1,2309.207386
9,1,6461.642857
9,1,1785.691860
9,1,29296.391304
9,1,2391.628571
9,1,66853.214286
9,1,3359.289474
9,1,1621.545455
9,1,1791.298913
9,1,7545.203947
9,1,3665.317073
9,1,2879.395833
9,1,4257.411765
9,1,14127.331395
9,1,76454.003378
9,1,1597.026786
9,1,3261.345455
9,1,6153.543478
9,1,3974.651515
9,1,53913.549342
9,1,2473.184659
9,1,2279.625000
9,1,2443.548611
9,1,2116.990566
9,1,57970.551282
9,1,87878.238636
9,1,7872.500000
9,1,3433.029586
9,1,4678.086538
9,1,11219.384868
9,1,1631.525000
9,1,6058.426136
9,1,71804.672872
9,1,24302.670455
9,1,2848.226351
9,1,54121.000000
9,1,5735.485000
9,1,6161.050781
9,1,94752.393939
9,1,1592.558824
9,1,4387.700000

9,1,4052.566176
9,1,2230.613636
9,1,86716.802632
9,1,16905.500000
9,1,1790.833333
9,1,1761.000000
9,1,70956.250000
9,1,2737.925000
9,1,1791.388889
9,1,1725.750000
9,1,1857.428571
9,1,10824.157609
9,1,3234.315789
9,1,12644.545918
9,1,1637.300000
9,1,2321.607143
9,1,1611.814024
9,1,1729.674419
9,1,7063.193182
9,1,6731.989796
9,1,3048.186047
9,1,2607.884615
9,1,6631.616379
9,1,4155.899390
9,1,2355.994792
9,1,20050.625000
9,1,4999.288462
9,1,2460.420455
9,1,3789.567857
9,1,30664.639706
9,1,44215.953947
9,1,46357.025735
9,1,9610.141892
9,1,2034.955357
9,1,2966.137500
9,1,1936.802326
9,1,16220.972222
9,1,3304.627907
9,1,1527.470588
9,1,64206.319767
9,1,4954.280172
9,1,36703.720588
9,1,2513.363636
9,1,3330.195000
9,1,2172.500000
9,1,5762.117647
9,1,53690.052632
9,1,32567.514706
9,1,3166.469444
9,1,15598.862903
9,1,50041.222826
9,1,26727.414634
9,1,1785.462500
9,1,3956.877551
9,1,1802.224265
9,1,3354.355978
9,1,4679.453125
9,1,63569.534884
9,1,1781.000000
9,1,3692.789634
9,1,4813.714286
9,1,7237.100000
9,1,121419.277344
9,1,1858.693182
9,1,1627.364796
9,1,2301.637755

9,1,56545.375000
9,1,1940.397436
9,1,1926.086310
9,1,110184.361842
9,1,1646.750000
9,1,10696.457143
9,1,2136.257353
9,1,3348.477273
9,1,2445.314136
9,1,99040.789634
9,1,1557.400000
9,1,2956.166667
9,1,21575.564024
9,1,14291.159091
9,1,2797.416667
9,1,27513.818750
9,1,2019.558511
9,1,48852.359375
9,1,4603.071429
9,1,9948.111413
9,1,9220.437500
9,1,1891.125000
9,1,11665.350962
9,1,4293.541667
9,1,2206.333333
9,1,31079.953125
9,1,4370.104651
9,1,88147.062500
9,1,7985.762500
9,1,2519.164474
9,1,14009.817308
9,1,100886.825000
9,1,5869.327778
9,1,6106.029412
9,1,5714.762500
9,1,4798.493902
9,1,84683.977941
9,1,3590.587209
9,1,1769.788889
9,1,4621.710784
9,1,5222.770833
9,1,1657.191489
9,1,2672.753125
9,1,79110.340909
9,1,2506.720109
9,1,3403.007812
9,1,98713.562500
9,1,2022.900000
9,1,4908.734375
9,1,2656.102500
9,1,2163.042553
9,1,44826.842857
9,1,1675.653061
9,1,2877.333333
9,1,26835.609375
9,1,3360.500000
9,1,1557.033537
9,1,6146.727273
9,1,1686.888298
9,1,4744.128205
9,1,2451.187500
9,1,13019.239362
9,1,86494.809211
9,1,1628.040698
9,1,15103.022727
9,1,40709.354651

9,1,7146.031250
9,1,1935.500000
9,1,33739.007812
9,1,5058.776316
9,1,3459.791667
9,1,4326.916667
9,1,72936.500000
9,1,1698.828804
9,1,27231.833333
9,1,2372.291667
9,1,1760.870192
9,1,18656.516129
9,1,37458.760204
9,1,76822.196429
9,1,3473.841667
9,1,4182.071429
9,1,7379.575581
9,1,12998.669355
9,1,2093.292683
9,1,23857.190476
9,1,3564.551282
9,1,4861.809211
9,1,1823.788462
9,1,33143.191176
9,1,2613.128125
9,1,15702.065476
9,1,7666.923611
9,1,1837.961538
9,1,1654.000000
9,1,2476.043478
9,1,1815.422619
9,1,2835.542857
9,1,7819.500000
9,1,32357.378906
9,1,5151.933333
9,1,83728.693750
9,1,1747.686047
9,1,2481.452128
9,1,1985.149390
9,1,28464.285714
9,1,13244.183594
9,1,2930.375000
9,1,2324.872642
9,1,35230.942857
9,1,6509.331633
9,1,1694.109043
9,1,63744.375000
9,1,73335.805556
9,1,2128.896341
9,1,1716.988889
9,1,7740.191176
9,1,3744.291667
9,1,34136.517857
9,1,1737.068182
9,1,48975.225000
9,1,2709.291667
9,1,1850.750000
9,1,24833.618182
9,1,13612.996324
9,1,1679.604167
9,1,1757.720000
9,1,1545.738372
9,1,120237.127907
9,1,24130.500000
9,1,40315.589744
9,1,4248.634434

9,1,2736.006757
9,1,146652.744898
9,1,76273.179878
9,1,3164.585366
9,1,76952.231707
9,1,138684.423077
9,1,3753.746711
9,1,2010.387500
9,1,4567.500000
9,1,2486.637500
9,1,1806.137500
9,1,1689.742347
9,1,1582.354730
9,1,6073.375000
9,1,70370.577778
9,1,3965.117647
9,1,2253.602941
9,1,38945.417969
9,1,11310.506410
9,1,4254.222222
9,1,32028.405405
9,1,81769.346154
9,1,1993.714286
9,1,5153.859375
9,1,3232.714286
9,1,15661.909375
9,1,3231.312500
9,1,8001.453125
9,1,3263.087766
9,1,4186.976064
9,1,4317.296296
9,1,2837.321429
9,1,17778.529412
9,1,4620.019608
9,1,2489.279070
9,1,50842.207031
9,1,3256.588235
9,1,1977.815217
9,1,33624.675676
9,1,4401.846154
9,1,1764.196970
9,1,12642.343023
9,1,14855.790541
9,1,2623.443750
9,1,78127.916667
9,1,54179.536765
9,1,140819.821429
9,1,1728.961538
9,1,111260.556818
9,1,1514.430851
9,1,3342.906977
9,1,13381.631098
9,1,1547.297872
9,1,1628.541667
9,1,4485.489362
9,1,1857.317308
9,1,2059.953488
9,1,126145.011364
9,1,1682.958333
9,1,3874.145349
9,1,14917.268293
9,1,9366.787791
9,1,1802.142857
9,1,108077.000000
9,1,2369.448370
9,1,11083.506410

9,1,33311.731250
9,1,62442.456250
9,1,1891.380952
9,1,11985.946429
9,1,1702.826220
9,1,2750.369565
9,1,1591.678125
9,1,13161.355072
9,1,2549.402344
9,1,1676.632500
9,1,1696.392442
9,1,23083.393617
9,1,1733.733696
9,1,90106.770833
9,1,2602.789474
9,1,2585.112805
9,1,3722.302326
9,1,1979.512500
9,1,7087.930233
9,1,68070.406977
9,1,1787.732558
9,1,2099.360714
9,1,2055.093750
9,1,2031.700000
9,1,2007.535714
9,1,3223.347561
9,1,5654.530612
9,1,1499.812500
9,1,146482.613095
9,1,6072.843750
9,1,67883.596429
9,1,1854.114130
9,1,2931.266667
9,1,1620.750000
9,1,44422.523437
9,1,6142.080357
9,1,37810.724832
9,1,110063.823529
9,1,3942.442308
9,1,2018.661585
9,1,22786.423387
9,1,2979.391447
9,1,176440.583333
9,1,2275.954787
9,1,2063.630435
9,1,62152.347561
9,1,7233.965278
9,1,2900.615385
9,1,134150.030612
9,1,4242.294118
9,1,151842.301136
9,1,2311.154762
9,1,1866.696875
9,1,50277.819767
9,1,23866.536585
9,1,1627.269444
9,1,3822.055556
9,1,31258.485294
9,1,4831.329268
9,1,24501.778409
9,1,1913.268293
9,1,7782.850000
9,1,55201.250000
9,1,61633.409091
9,1,2079.197674
9,1,3477.425000

9,1,7998.342391
9,1,13745.992647
9,1,11005.303571
9,1,83144.000000
9,1,2709.875000
9,1,1708.225000
9,1,9333.828947
9,1,9511.636943
9,1,1547.875000
9,1,1738.977273
9,1,2764.537234
9,1,6405.353659
9,1,2760.460526
9,1,2711.219512
9,1,31952.389706
9,1,134960.479651
9,1,44703.308824
9,1,15279.863636
9,1,5587.601351
9,1,1821.977273
9,1,1813.080882
9,1,1761.430000
9,1,7987.227273
9,1,1631.571429
9,1,1503.791667
9,1,2087.607143
9,1,2300.833333
9,1,3631.368421
9,1,1849.246795
9,1,94275.398649
9,1,63404.605469
9,1,6393.962500
9,1,2360.990385
9,1,55692.217391
9,1,2638.947368
9,1,4179.340909
9,1,106045.325000
9,1,66972.333333
9,1,3786.350000
9,1,13885.428571
9,1,8841.416667
9,1,2500.853261
9,1,70729.506098
9,1,65536.710938
9,1,25501.405000
9,1,2745.592105
9,1,1745.320000
9,1,2404.635638
9,1,105011.875000
9,1,47215.616477
9,1,1908.580000
9,1,39466.209459
9,1,4989.286364
9,1,2140.531250
9,1,33475.411458
9,1,4419.214286
9,1,11660.551282
9,1,4543.510638
9,1,4739.294872
9,1,11961.828571
9,1,2073.375000
9,1,15185.702500
9,1,3840.989583
9,1,2116.477273
9,1,2962.146341
9,1,56776.431818

9,1,9171.122024
9,1,2515.785714
9,1,3213.743243
9,1,52947.756579
9,1,6065.773438
9,1,23518.035256
9,1,2047.628378
9,1,67333.843750
9,1,63874.553571
9,1,2130.430921
9,1,82083.684524
9,1,2851.634146
9,1,2338.601064
9,1,2117.006579
9,1,3405.312500
9,1,1597.208333
9,1,1668.724490
9,1,5918.932692
9,1,15159.742925
9,1,1655.908088
9,1,2605.225000
9,1,9212.731707
9,1,2618.553191
9,1,6450.909483
9,1,3802.471774
9,1,3842.410714
9,1,2285.295732
9,1,6357.625000
9,1,4530.529412
9,1,5421.450521
9,1,1832.242647
9,1,1859.455882
9,1,14852.090909
9,1,44017.595930
9,1,48559.921053
9,1,3495.530405
9,1,22537.282258
9,1,9634.697674
9,1,1639.743056
9,1,16521.194149
9,1,13131.423611
9,1,1539.173780
9,1,2352.458333
9,1,4844.500000
9,1,3355.106250
9,1,1843.688889
9,1,2384.687500
9,1,2871.160714
9,1,2168.920455
9,1,86141.043605
9,1,16381.894231
9,1,6311.734694
9,1,1778.229167
9,1,4955.531250
9,1,32478.035714
9,1,64414.055921
9,1,60581.532051
9,1,3036.978723
9,1,6468.500000
9,1,16422.554167
9,1,6533.515086
9,1,26979.479167
9,1,1852.959239
9,1,1720.053125
9,1,23379.447368
9,1,2116.772727

9,1,50455.562500
9,1,3582.628289
9,1,46678.179487
9,1,3592.141892
9,1,96769.346154
9,1,1859.994792
9,1,3728.125000
9,1,43168.722222
9,1,3832.171875
9,1,72419.265244
9,1,100290.000000
9,1,6765.255319
9,1,8634.306604
9,1,1730.708333
9,1,26963.480769
9,1,3742.170732
9,1,78020.118421
9,1,11871.807292
9,1,13064.571429
9,1,10468.279412
9,1,2545.437500
9,1,1836.557500
9,1,2158.250000
9,1,3565.793750
9,1,3206.187500
9,1,1729.637500
9,1,4329.275510
9,1,3365.618421
9,1,4561.869565
9,1,1786.500000
9,1,51478.692308
9,1,2062.267442
9,1,11019.301508
9,1,149141.625000
9,1,5713.245614
9,1,60510.937500
9,1,10183.105556
9,1,1508.264205
9,1,1756.572674
9,1,1501.416667
9,1,1654.554054
9,1,8713.494048
9,1,57362.628049
9,1,2335.750000
9,1,3473.363636
9,1,26847.041667
9,1,61284.526316
9,1,63200.086538
9,1,3205.718750
9,1,2509.904762
9,1,1916.734756
9,1,7225.437500
9,1,2055.360119
9,1,4596.932292
9,1,2814.398649
9,1,74336.082500
9,1,2398.717391
9,1,12504.697917
9,1,10092.772959
9,1,4905.563830
9,1,2211.600000
9,1,8923.102273
9,1,2746.400000
9,1,1784.062500
9,1,1808.595238
9,1,1596.880682

9,1,2514.871951
9,1,2061.395833
9,1,4843.355556
9,1,8301.103659
9,1,123162.277778
9,1,41286.658537
9,1,9283.083333
9,1,2833.515625
9,1,2549.611702
9,1,1758.964744
9,1,14113.470833
9,1,1584.090909
9,1,1964.068182
9,1,1958.780405
9,1,5836.437500
9,1,5426.614130
9,1,2590.302632
9,1,4875.910326
9,1,81866.662791
9,1,4663.366667
9,1,6729.275568
9,1,8624.731707
9,1,9604.991477
9,1,3463.216346
9,1,1528.250000
9,1,1705.192935
9,1,2040.583333
9,1,1863.597561
9,1,2907.000000
9,1,47856.363636
9,1,4676.500000
9,1,33942.616935
9,1,3292.520408
9,1,2822.202703
9,1,2420.418919
9,1,2846.698864
9,1,1644.611111
9,1,1569.220930
9,1,10080.145833
9,1,65111.888158
9,1,1666.772436
9,1,10079.329545
9,1,2794.829268
9,1,24795.320312
9,1,1969.812500
9,1,26313.284091
9,1,9908.486413
9,1,22246.514423
9,1,3660.573171
9,1,43733.152778
9,1,2144.930556
9,1,6139.652174
9,1,4865.140625
9,1,2528.684783
9,1,59821.769737
9,1,1791.630208
9,1,86044.500000
9,1,32603.250000
9,1,5898.467262
9,1,152749.338068
9,1,11833.950000
9,1,1887.109756
9,1,3660.477273
9,1,20592.878788
9,1,67919.072222
9,1,3208.750000

9,1,2050.421875
9,1,191240.263158
9,1,64503.195946
9,1,2652.925781
9,1,1927.521277
9,1,79218.250000
9,1,37207.789474
9,1,44887.256410
9,1,3915.529762
9,1,4572.640625
9,1,1814.097561
9,1,3561.802778
9,1,53785.565789
9,1,26143.618590
9,1,2595.180085
9,1,1546.435811
9,1,39189.652778
9,1,12439.798077
9,1,1879.269737
9,1,1654.055556
9,1,2201.265306
9,1,27473.488971
9,1,44022.989130
9,1,4578.375000
9,1,2153.872093
9,1,1988.985294
9,1,3059.752907
9,1,2026.764205
9,1,1519.715278
9,1,7895.975610
9,1,4364.343750
9,1,1593.025641
9,1,2076.997024
9,1,13444.353261
9,1,4075.000000
9,1,1695.808333
9,1,2165.426829
9,1,5462.047297
9,1,1572.818182
9,1,2245.276596
9,1,4131.439759
9,1,1626.046875
9,1,9800.128205
9,1,2235.444444
9,1,45968.045455
9,1,4162.237500
9,1,1708.950000
9,1,2699.750000
9,1,3486.523437
9,1,5252.445355
9,1,2587.084459
9,1,62334.934211
9,1,13710.149457
9,1,1898.056548
9,1,1511.130435
9,1,3070.546875
9,1,4554.779891
9,1,2829.989130
9,1,3064.359375
9,1,7337.398810
9,1,2050.656250
9,1,44031.428571
9,1,3273.255435
9,1,3606.676829
9,1,3329.274390
9,1,25863.470588

9,1,1600.711111
9,1,2005.212766
9,1,104646.233108
9,1,1604.053571
9,1,2234.270833
9,1,3350.312500
9,1,2231.930556
9,1,40076.176471
9,1,2855.162791
9,1,2959.300000
9,1,30774.241379
9,1,1705.898936
9,1,14543.125000
9,1,46542.720588
9,1,45466.295139
9,1,2064.609756
9,1,4610.910714
9,1,1867.318182
9,1,43663.927083
9,1,58946.666667
9,1,7516.452703
9,1,1505.625000
9,1,32592.779070
9,1,3992.715909
9,1,1997.300532
9,1,3893.529070
9,1,3901.375000
9,1,139309.313953
9,1,2158.178571
9,1,65147.038462
9,1,1518.219512
9,1,2236.829545
9,1,8296.731250
9,1,11476.773437
9,1,1674.462766
9,1,154559.150000
9,1,4676.928571
9,1,5075.380682
9,1,2449.985294
9,1,2197.910714
9,1,12162.708333
9,1,1586.457143
9,1,1633.750000
9,1,1572.803191
9,1,1814.152174
9,1,5205.763158
9,1,31855.230769
9,1,3809.933333
9,1,4918.800000
9,1,1614.196078
9,1,3000.738095
9,1,64353.536585
9,1,51899.292683
9,1,33307.769531
9,1,1833.138889
9,1,1660.558140
9,1,7921.306604
9,1,2274.653846
9,1,1770.200000
9,1,25255.285714
9,1,6418.992188
9,1,2513.111702
9,1,2055.737500
9,1,1516.285714
9,1,2429.557432
9,1,3566.277027

9,1,1727.180233
9,1,1841.073171
9,1,1770.534091
9,1,2504.250000
9,1,3070.989583
9,1,66115.875000
9,1,72501.109375
9,1,6779.191810
9,1,1829.340686
9,1,2484.807432
9,1,21191.871528
9,1,3824.600000
9,1,5964.196809
9,1,36382.093023
9,1,12445.212121
9,1,12854.130556
9,1,51399.040541
9,1,3883.789474
9,1,1504.985294
9,1,4469.595745
9,1,4820.303571
9,1,3252.125000
9,1,1919.116279
9,1,3207.390625
9,1,49673.072368
9,1,5672.848214
9,1,2245.083333
9,1,2948.987179
9,1,57667.957143
9,1,20163.441176
9,1,16155.345000
9,1,2204.448171
9,1,5148.356250
9,1,2649.862245
9,1,2651.173913
9,1,2263.743902
9,1,1546.513587
9,1,57804.321429
9,1,2394.144231
9,1,1503.798295
9,1,14435.682692
9,1,3895.516667
9,1,119631.980769
9,1,4039.405405
9,1,55902.303571
9,1,3731.945122
9,1,7999.906250
9,1,3454.361963
9,1,12105.200000
9,1,3978.255556
9,1,3528.640625
9,1,2760.545455
9,1,15364.581522
9,1,1674.882353
9,1,40355.256757
9,1,96050.074405
9,1,58230.963816
9,1,1980.359756
9,1,4694.273438
9,1,1530.905556
9,1,3737.799479
9,1,13013.724265
9,1,1973.975610
9,1,1909.214286
9,1,77145.604167
9,1,1739.020833

9,1,36038.077206
9,1,2370.409091
9,1,8339.718023
9,1,1734.093023
9,1,130331.633721
9,1,13028.863636
9,1,1872.058824
9,1,2525.076923
9,1,1773.304878
9,1,3337.994898
9,1,5390.342262
9,1,5346.281250
9,1,1843.915865
9,1,2392.393750
9,1,1729.285714
9,1,111607.957317
9,1,3036.937500
9,1,2059.000000
9,1,42950.527027
9,1,4431.297872
9,1,6958.158784
9,1,130061.007812
9,1,1689.059375
9,1,65809.250000
9,1,23062.375000
9,1,2585.176471
9,1,14209.092949
9,1,1772.030488
9,1,2139.193548
9,1,112579.350000
9,1,1926.383152
9,1,5038.812500
9,1,34581.664062
9,1,1932.825000
9,1,26912.078125
9,1,1817.826087
9,1,1775.261029
9,1,3310.470588
9,1,3123.779891
9,1,20296.500000
9,1,2186.556250
9,1,3066.049419
9,1,3559.574324
9,1,14400.211207
9,1,1646.511628
9,1,2015.063725
9,1,1746.050000
9,1,1886.121951
9,1,4220.315476
9,1,5194.593750
9,1,18502.213068
9,1,2316.127660
9,1,119598.607143
9,1,2347.107558
9,1,15296.838235
9,1,1695.592391
9,1,1523.225806
9,1,10602.925000
9,1,2039.442935
9,1,4338.307692
9,1,1969.931818
9,1,2088.915698
9,1,23448.650000
9,1,78155.625000
9,1,32952.119048
9,1,2776.243421

9,1,64471.609375
9,1,2496.546875
9,1,72335.651961
9,1,4020.453125
9,1,1640.578947
9,1,2214.357143
9,1,9590.768293
9,1,56765.795455
9,1,6587.217391
9,1,1757.364362
9,1,4486.280488
9,1,1767.636364
9,1,7812.617021
9,1,2170.774390
9,1,2647.734756
9,1,1577.538462
9,1,6815.226744
9,1,2696.826087
9,1,5656.650000
9,1,106494.179487
9,1,3408.949074
9,1,1596.701923
9,1,2000.562500
9,1,1518.711957
9,1,6655.561441
9,1,1624.225000
9,1,2907.900000
9,1,2840.286585
9,1,2653.323864
9,1,11538.906250
9,1,2405.826220
9,1,21884.457386
9,1,38009.523256
9,1,4540.533333
9,1,4362.264706
9,1,50813.073529
9,1,2642.149510
9,1,2708.328488
9,1,1781.645270
9,1,26936.116279
9,1,2573.000000
9,1,19475.147059
9,1,38642.818182
9,1,2833.828571
9,1,1559.375000
9,1,38595.872093
9,1,1786.183824
9,1,3189.562500
9,1,3488.231707
9,1,2150.000000
9,1,32589.580357
9,1,28183.278846
9,1,2361.053977
9,1,1544.464286
9,1,2157.049419
9,1,2523.000000
9,1,6844.250000
9,1,5774.328571
9,1,2642.606250
9,1,13956.475610
9,1,1534.968750
9,1,6834.000000
9,1,4170.041667
9,1,67165.631579
9,1,106574.718750
9,1,7652.595588

9,1,1856.139535
9,1,2325.199405
9,1,23439.500000
9,1,1986.017442
9,1,2284.036765
9,1,5491.865854
9,1,1697.387500
9,1,7657.127778
9,1,69430.164474
9,1,1851.019737
9,1,1569.443750
9,1,5475.250000
9,1,1877.315217
9,1,2524.727273
9,1,57445.753472
9,1,119066.792683
9,1,6174.917453
9,1,2118.678571
9,1,24986.937500
9,1,16395.519886
9,1,3776.697917
9,1,2163.866279
9,1,52863.961806
9,1,1609.655556
9,1,3777.189024
9,1,59645.440000
9,1,8885.102041
9,1,2613.076923
9,1,1529.111486
9,1,2881.500000
9,1,2315.000000
9,1,19625.818750
9,1,79018.746951
9,1,3986.463235
9,1,1729.750000
9,1,8482.945122
9,1,1924.548611
9,1,43362.931818
9,1,2155.477273
9,1,41388.758721
9,1,2564.595238
9,1,2734.668478
9,1,1713.585366
9,1,1733.835366
9,1,2626.937500
9,1,2736.441860
9,1,2084.194444
9,1,5416.005208
9,1,8039.872340
9,1,8442.010417
9,1,2078.750000
9,1,1987.071429
9,1,2736.761364
9,1,2219.000000
9,1,1896.519737
9,1,5563.821429
9,1,4909.317073
9,1,52931.575000
9,1,2296.000000
9,1,1928.797297
9,1,2235.843750
9,1,5555.772727
9,1,1596.902778
9,1,14111.261364
9,1,4779.944444
9,1,2350.596591

9,1,5802.375000
9,1,2324.429348
9,1,1770.193182
9,1,3013.346154
9,1,32665.440789
9,1,53364.637427
9,1,1933.226351
9,1,5873.333333
9,1,2196.918367
9,1,44933.230769
9,1,4166.016304
9,1,1594.903846
9,1,47234.685714
9,1,5740.674419
9,1,2798.338889
9,1,27460.250000
9,1,4916.242647
9,1,1745.845745
9,1,13384.628049
9,1,2751.528409
9,1,4143.308594
9,1,1607.093750
9,1,1937.514881
9,1,1801.892857
9,1,4496.848684
9,1,3177.078571
9,1,1879.077206
9,1,2681.550000
9,1,3633.899390
9,1,60630.907051
9,1,6530.268617
9,1,3990.363636
9,1,2106.430233
9,1,24695.729839
9,1,3291.821429
9,1,1660.250000
9,1,2929.833333
9,1,1602.057143
9,1,2121.575000
9,1,4833.325521
9,1,106154.256250
9,1,2014.689024
9,1,6310.687500
9,1,24298.648649
9,1,4498.369048
9,1,8486.660714
9,1,1825.375000
9,1,2429.562500
9,1,2032.874396
9,1,1939.396341
9,1,2180.609375
9,1,2800.205696
9,1,9913.739362
9,1,2042.687500
9,1,2193.384615
9,1,1912.759146
9,1,1730.042105
9,1,9023.105263
9,1,81133.597973
9,1,4385.395270
9,1,2171.261905
9,1,3263.547794
9,1,1525.377976
9,1,3313.142857
9,1,64534.606250
9,1,1968.617647

9,1,91875.556250
9,1,4212.509615
9,1,1538.266667
9,1,129286.406977
9,1,5182.777778
9,1,1541.091146
9,1,2365.045000
9,1,99901.250000
9,1,1669.684524
9,1,17824.000000
9,1,4276.645833
9,1,9523.435484
9,1,2007.200000
9,1,2086.000000
9,1,1944.823529
9,1,7960.842105
9,1,8028.950658
9,1,3451.029762
9,1,22801.565217
9,1,6918.500000
9,1,1623.428571
9,1,1762.842949
9,1,5905.923077
9,1,91225.786184
9,1,51386.960227
9,1,5847.739796
9,1,3770.325658
9,1,2809.763587
9,1,5257.279070
9,1,27038.611413
9,1,14721.020833
9,1,1626.984375
9,1,1997.675000
9,1,2023.867647
9,1,3200.680851
9,1,2501.664062
9,1,2050.048295
9,1,11522.050532
9,1,77488.107143
9,1,2085.263587
9,1,4730.076531
9,1,7599.333333
9,1,23972.625000
9,1,90292.630682
9,1,3285.900000
9,1,1764.506944
9,1,2038.022727
9,1,2521.614865
9,1,55759.461538
9,1,18399.937500
9,1,1746.821429
9,1,1905.418605
9,1,7214.922619
9,1,5087.153846
9,1,1636.470930
9,1,62938.210714
9,1,12858.300000
9,1,3267.916667
9,1,1866.586957
9,1,1826.642857
9,1,7050.820513
9,1,54218.834459
9,1,192800.035714
9,1,1717.755556
9,1,1917.378788
9,1,4877.588235

9,1,2962.516892
9,1,1993.937500
10,2,10419.055556
10,2,2842.343750
10,2,1742.170918
10,2,3600.500000
10,2,4948.954082
10,2,1999.081081
10,2,1621.513393
10,2,3498.142857
10,2,3099.348837
10,2,3679.303571
10,2,2023.399194
10,2,2336.106383
10,2,2751.416667
10,2,3468.407895
10,2,2346.325581
10,2,7779.095930
10,2,1619.777778
10,2,2460.093750
10,2,1600.738095
10,2,1874.816038
10,2,1539.750000
10,2,11076.000000
10,2,2119.558511
10,2,2198.760870
10,2,6246.702703
10,2,1512.409574
10,2,1926.132979
10,2,9039.755102
10,2,4794.898936
10,2,2372.688830
10,2,5594.689815
10,2,4195.116071
10,2,2520.689655
10,2,3148.410256
10,2,5293.015625
10,2,1647.625000
10,2,1711.671053
10,2,1963.454861
10,2,2251.557377
10,2,6245.891213
10,2,1788.750000
10,2,6018.950000
10,2,3685.572674
10,2,1683.000000
10,2,2702.732794
10,2,2183.500000
10,2,9905.762755
10,2,1645.475000
10,2,5172.426020
10,2,11864.268293
10,2,5733.062500
10,2,3253.700000
10,2,4107.838542
10,2,2261.180272
10,2,4213.440559
10,2,13568.337037
10,2,5746.425000
10,2,6022.385000
10,2,3205.954545
10,2,1696.640625
10,2,7533.000000
10,2,1664.131579
10,2,1966.305233
10,2,3080.750000

10,2,1673.444149
10,2,2960.308140
10,2,4030.357955
10,2,4198.555556
10,2,5124.484375
10,2,3087.137500
10,2,4438.091743
10,2,3470.642857
10,2,3648.928571
10,2,2724.697500
10,2,3145.496711
10,2,2810.088235
10,2,3143.237069
10,2,3070.026786
10,2,3708.795918
10,2,1946.117647
10,2,3810.213415
10,2,3457.181122
10,2,6050.743182
10,2,1798.986486
10,2,2547.036585
10,2,2751.921569
10,2,2054.432500
10,2,6445.005952
10,2,3610.750000
10,2,2322.316038
10,2,1595.916667
10,2,3898.019231
10,2,2859.015152
10,2,1842.163690
10,2,5878.615909
10,2,1971.610119
10,2,1584.937500
10,2,1993.886792
10,2,3482.125000
10,2,2155.352941
10,2,2664.493750
10,2,1805.616667
10,2,7707.582031
10,2,5917.895349
10,2,2807.757353
10,2,1605.477778
10,2,1569.200000
10,2,1612.796053
10,2,2117.108333
10,2,3657.138514
10,2,1577.988372
10,2,5805.692708
10,2,4610.521635
10,2,3924.109375
10,2,12948.796875
10,2,3266.687500
10,2,3865.250000
10,2,12695.757653
10,2,1871.091146
10,2,2598.056818
10,2,1881.538462
10,2,3706.163265
10,2,4882.888158
10,2,5317.218750
10,2,1972.727778
10,2,5608.359375
10,2,2575.204082
10,2,4031.212500
10,2,11139.743590
10,2,1628.770115

10,2,2642.568966
10,2,4575.840000
10,2,2243.181250
10,2,4581.767442
10,2,4934.800000
10,2,9442.420732
10,2,2349.784091
10,2,5305.734694
10,2,6400.460938
10,2,6640.969907
10,2,2009.055000
10,2,5146.108108
10,2,17473.391304
10,2,1618.154762
10,2,1562.153846
10,2,2838.870098
10,2,2838.646341
10,2,3001.515625
10,2,3612.456395
10,2,8528.437500
10,2,6097.266667
10,2,6612.743056
10,2,3752.321429
10,2,4016.633929
10,2,10048.849359
10,2,1725.022222
10,2,6838.137097
10,2,8729.831250
10,2,1801.696809
10,2,2194.187500
10,2,5438.488372
10,2,2186.019126
10,2,1836.541667
10,2,3233.579545
10,2,4199.000000
10,2,4465.637681
10,2,2695.656250
10,2,2602.552147
10,2,4783.467391
10,2,3911.280612
10,2,2747.102273
10,2,3864.800000
10,2,8071.466837
10,2,2614.340426
10,2,10556.000000
10,2,3522.976190
10,2,2379.822222
10,2,33910.430233
10,2,6532.562500
10,2,4221.772059
10,2,4148.765625
10,2,1589.297619
10,2,2741.060000
10,2,4289.759868
10,2,2934.406977
10,2,2275.736842
10,2,9556.328740
10,2,1566.173077
10,2,1974.586957
10,2,2342.720000
10,2,3354.756757
10,2,2826.388889
10,2,1522.512500
10,2,5889.240385
10,2,4967.453804
10,2,12400.614130

10,2,7266.600000
10,2,4212.080357
10,2,1589.774510
10,2,2081.027778
10,2,2996.272727
10,2,2791.052083
10,2,4853.193548
10,2,3839.966981
10,2,3624.861925
10,2,2960.636364
10,2,4505.644444
10,2,4436.756757
10,2,2496.750000
10,2,3683.992718
10,2,6403.681818
10,2,1784.637755
10,2,1679.525000
10,2,1654.200000
10,2,2484.521429
10,2,5935.753125
10,2,4116.380000
10,2,3204.833333
10,2,3141.005435
10,2,2090.131579
10,2,9521.416667
10,2,1899.511719
10,2,10057.531915
10,2,7978.095238
10,2,2159.693878
10,2,1695.039216
10,2,2281.105114
10,2,3309.093750
10,2,16815.875000
10,2,8163.727273
10,2,4467.125000
10,2,2356.714286
10,2,3117.923077
10,2,3299.973404
10,2,8308.158416
10,2,1839.513158
10,2,3438.677885
10,2,2279.817935
10,2,2782.894737
10,2,3976.812500
10,2,3023.507500
10,2,2077.605114
10,2,4245.119318
10,2,10114.745000
10,2,2659.416667
10,2,4724.081633
10,2,7269.127907
10,2,5002.571429
10,2,2594.839286
10,2,2641.983333
10,2,2678.615385
10,2,1586.676316
10,2,3824.005155
10,2,1857.500000
10,2,1968.114583
10,2,5834.592593
10,2,4249.737864
10,2,2367.730769
10,2,2195.150000
10,2,4004.538462
10,2,2333.128125
10,2,1538.937500

10,2,11284.453125
10,2,6800.407500
10,2,1739.652778
10,2,3670.975543
10,2,3721.224490
10,2,2334.489796
10,2,1942.250000
10,2,2002.326923
10,2,10500.173077
10,2,2558.573529
10,2,1613.737500
10,2,1690.403846
10,2,1709.868644
10,2,1930.563830
10,2,2110.000000
10,2,4050.238095
10,2,4545.487705
10,2,17150.000000
10,2,1584.977273
10,2,2327.246711
10,2,11408.976562
10,2,5491.436364
10,2,5864.093750
10,2,2785.482639
10,2,7172.875000
10,2,2163.043269
10,2,4012.459302
10,2,3489.367021
10,2,2585.850000
10,2,9309.230769
10,2,7368.109756
10,2,1816.562500
10,2,2055.676991
10,2,13022.092391
10,2,1834.268750
10,2,3103.315789
10,2,2213.609756
10,2,12414.659639
10,2,3576.606383
10,2,2006.139706
10,2,4074.068750
10,2,3867.047872
10,2,2021.699507
10,2,3123.232558
10,2,1513.263298
10,2,2431.921788
10,2,14787.011364
10,2,12061.033333
10,2,1886.208333
10,2,1626.256250
10,2,2116.638158
10,2,3441.575581
10,2,7507.033019
10,2,1676.825581
10,2,3285.722826
10,2,3230.348837
10,2,2550.164062
10,2,1818.552326
10,2,1859.773810
10,2,6926.473684
10,2,5301.997093
10,2,6802.650943
10,2,2946.604167
10,2,3906.937500
10,2,3240.842105
10,2,1782.230000

10,2,3382.991848
10,2,2204.629310
10,2,5290.181818
10,2,3358.769231
10,2,10290.281250
10,2,1733.220238
10,2,1664.948980
10,2,1735.007812
10,2,2272.674419
10,2,4295.460000
10,2,3699.880000
10,2,2153.220109
10,2,4449.433333
10,2,2278.733108
10,2,2520.834375
10,2,4654.573171
10,2,1834.975610
10,2,5764.405405
10,2,1582.681250
10,2,6891.162791
10,2,2770.953125
10,2,4196.893617
10,2,1794.906250
10,2,2406.220930
10,2,1782.500000
10,2,9448.562500
10,2,3876.500000
10,2,4081.375000
10,2,2093.012821
10,2,2538.838235
10,2,1911.269737
10,2,4412.606707
10,2,7777.566667
10,2,3177.423077
10,2,14681.970060
10,2,6208.919118
10,2,5614.596774
10,2,1811.130000
10,2,1978.178771
10,2,2087.167614
10,2,3009.272727
10,2,4681.166667
10,2,5864.299020
10,2,4290.701357
10,2,1968.304878
10,2,4795.562500
10,2,2449.223958
10,2,2230.093750
10,2,2221.000000
10,2,2912.814815
10,2,5143.349490
10,2,1550.416667
10,2,2761.056452
10,2,1803.941176
10,2,1995.093750
10,2,3335.000000
10,2,5436.450549
10,2,2798.727273
10,2,4268.528302
10,2,1575.243056
10,2,1981.875000
10,2,7625.299020
10,2,1975.153846
10,2,6095.913462
10,2,5771.429448
10,2,3218.117647

10,2,6128.994565
10,2,1550.730978
10,2,1607.345930
10,2,2516.859375
10,2,1574.682692
10,2,4149.437500
10,2,5457.294872
10,2,2261.000000
10,2,8569.682927
10,2,2446.976744
10,2,4452.968085
10,2,4016.347345
10,2,1663.752688
10,2,11927.059783
10,2,8323.294271
10,2,2501.509091
10,2,1540.375000
10,2,1667.483553
10,2,49852.764423
10,2,1896.130435
10,2,2274.744048
10,2,2711.044118
10,2,2028.333333
10,2,2270.097561
10,2,2073.565000
10,2,11512.444444
10,2,2893.918478
10,2,1842.239583
10,2,2930.788194
10,2,1890.159884
10,2,1579.921875
10,2,1551.572674
10,2,2168.318750
10,2,1755.291667
10,2,3060.200000
10,2,1937.441860
10,2,3675.250000
10,2,12527.204787
10,2,4557.787234
10,2,5443.027174
10,2,5095.950000
10,2,1587.588235
10,2,2299.227679
10,2,19940.082447
10,2,2102.218750
10,2,10892.666667
10,2,6843.570175
10,2,4522.392857
10,2,1772.517857
10,2,6665.618056
10,2,2894.005319
10,2,1831.312500
10,2,7732.380682
10,2,2451.375000
10,2,4051.570312
10,2,2018.640000
10,2,6956.956522
10,2,9205.820000
10,2,7619.437500
10,2,1835.583333
10,2,5742.250000
10,2,61953.972222
10,2,3121.761905
10,2,2134.075000
10,2,1598.888889
10,2,1748.685714

10,2,8248.420833
10,2,3863.100543
10,2,1771.415094
10,2,4036.892857
10,2,5316.344444
10,2,3799.214286
10,2,3263.678571
10,2,5885.692308
10,2,2564.349206
10,2,4667.509091
10,2,7021.546569
10,2,1958.922222
10,2,2127.468750
10,2,2103.812500
10,2,7446.367150
10,2,1714.787234
10,2,2973.480769
10,2,1601.201923
10,2,4352.374269
10,2,2635.500000
10,2,1874.575000
10,2,2686.437500
10,2,2340.193750
10,2,18371.428571
10,2,3378.657609
10,2,4751.418033
10,2,8670.505319
10,2,11932.040201
10,2,16568.758333
10,2,5362.470588
10,2,1665.446429
10,2,2475.215909
10,2,3575.905000
10,2,7609.187500
10,2,4785.685714
10,2,2945.771739
10,2,2090.050000
10,2,2054.084821
10,2,1573.276596
10,2,2107.129310
10,2,5293.625000
10,2,3321.614362
10,2,2643.668605
10,2,1559.801630
10,2,3432.182692
10,2,4602.897727
10,2,1586.524390
10,2,5458.622642
10,2,1559.133333
10,2,4929.125000
10,2,5515.687500
10,2,9356.185864
10,2,2265.512500
10,2,3457.284722
10,2,13403.270408
10,2,4424.415816
10,2,2202.477564
10,2,5225.114130
10,2,4249.252358
10,2,1554.840426
10,2,1887.414634
10,2,2332.121951
10,2,7248.732484
10,2,2187.744565
10,2,2498.310345
10,2,2455.023256

10,2,5911.127907
10,2,2064.750000
10,2,2281.212871
10,2,3998.250000
10,2,2225.750000
10,2,2508.160714
10,2,5116.336066
10,2,1751.317568
10,2,7726.743243
10,2,42131.357143
10,2,2682.760638
10,2,2826.662281
10,2,3499.052500
10,2,8695.517857
10,2,5658.674528
10,2,5589.872340
10,2,2962.177500
10,2,2239.500000
10,2,4803.836364
10,2,1793.316667
10,2,2115.153846
10,2,3427.440000
10,2,3573.368902
10,2,1626.416667
10,2,2805.994444
10,2,8725.883772
10,2,2777.333333
10,2,4025.535000
10,2,2495.444444
10,2,6314.198113
10,2,7600.026316
10,2,1549.187500
10,2,6834.960526
10,2,7572.677966
10,2,15960.558333
10,2,2377.225000
10,2,1997.529762
10,2,1918.285714
10,2,3499.833333
10,2,5473.161290
10,2,4516.759146
10,2,3542.538462
10,2,5843.156915
10,2,1950.716346
10,2,1922.414894
10,2,1928.664773
10,2,10055.908163
10,2,24963.115854
10,2,36658.781250
10,2,10158.108491
10,2,7466.350000
10,2,4830.922500
10,2,1942.221649
10,2,4640.142857
10,2,2075.486842
10,2,2576.250000
10,2,2703.228814
10,2,2068.906250
10,2,2979.767857
10,2,1503.666667
10,2,2007.571429
10,2,1898.891447
10,2,2428.781250
10,2,2616.670139
10,2,2506.986486
10,2,2195.343750

10,2,1925.144737
10,2,5694.244565
10,2,1861.851064
10,2,3132.843085
10,2,3561.912037
10,2,2706.318182
10,2,1689.331395
10,2,3095.390244
10,2,2610.912234
10,2,2372.093750
10,2,2653.813725
10,2,4891.400000
10,2,3220.129630
10,2,3441.807292
10,2,2029.673469
10,2,5179.433673
10,2,8853.725806
10,2,2121.753378
10,2,2660.805556
10,2,1986.868243
10,2,7696.414062
10,2,5619.703488
10,2,1606.584906
10,2,2146.684211
10,2,1842.560345
10,2,3952.547170
10,2,3465.068182
10,2,1938.586538
10,2,1961.612500
10,2,1566.508523
10,2,10143.740000
10,2,1845.000000
10,2,5770.106742
10,2,8557.109649
10,2,3565.331522
10,2,2469.828947
10,2,1521.792105
10,2,1601.030612
10,2,7250.226744
10,2,5153.903125
10,2,1776.000000
10,2,6902.548913
10,2,1542.968750
10,2,2362.312500
10,2,1840.053571
10,2,22610.250000
10,2,5761.755319
10,2,9694.690909
10,2,3013.356383
10,2,2340.882075
10,2,1602.150000
10,2,3413.627232
10,2,1797.857143
10,2,2265.863636
10,2,3258.125000
10,2,2562.654891
10,2,2631.465116
10,2,6607.180851
10,2,2121.417266
10,2,1963.819712
10,2,1543.374150
10,2,2490.875000
10,2,1890.000000
10,2,2253.920455
10,2,1874.948113
10,2,10223.863636

10,2,1825.838542
10,2,2750.740741
10,2,2178.319079
10,2,3951.446429
10,2,2349.200658
10,2,1595.782609
10,2,15844.136364
10,2,10020.659091
10,2,1732.752809
10,2,7341.402542
10,2,2061.388889
10,2,21098.351351
10,2,4845.961538
10,2,2420.488372
10,2,1957.663462
10,2,2259.090278
10,2,3433.512195
10,2,9011.635593
10,2,3478.732143
10,2,3159.337209
10,2,3990.062500
10,2,2750.528409
10,2,6652.216667
10,2,3302.382979
10,2,1637.988095
10,2,5890.666667
10,2,2751.105000
10,2,4151.088415
10,2,2695.000000
10,2,2443.459052
10,2,5431.315789
10,2,8919.340000
10,2,3654.326531
10,2,4881.615385
10,2,4012.242009
10,2,3399.786585
10,2,3300.313725
10,2,3583.493750
10,2,5412.331897
10,2,1787.728723
10,2,4474.851190
10,2,4111.556548
10,2,6369.100000
10,2,5864.627841
10,2,2263.968085
10,2,1600.416667
10,2,3681.631757
10,2,3950.865000
10,2,4908.024038
10,2,3923.394737
10,2,1891.567568
10,2,1926.247093
10,2,1539.404255
10,2,2061.924419
10,2,1846.000000
10,2,5459.630000
10,2,1618.165000
10,2,1851.165000
10,2,4089.961538
10,2,3139.775281
10,2,5652.944444
10,2,2685.292683
10,2,3233.196429
10,2,1893.095745
10,2,3986.388889
10,2,3608.740385

10,2,3131.085366
10,2,1861.959239
10,2,2411.312500
10,2,2270.401786
10,2,7711.375000
10,2,5203.144231
10,2,4891.900763
10,2,1935.585366
10,2,6376.375000
10,2,8533.767544
10,2,1519.826923
10,2,13409.420213
10,2,2223.594512
10,2,1535.943182
10,2,2766.217391
10,2,1785.562500
10,2,1943.632653
10,2,4289.118421
10,2,4541.186170
10,2,3641.485294
10,2,2432.385417
10,2,5970.375000
10,2,2695.037500
10,2,2254.468750
10,2,4467.390957
10,2,1593.447368
10,2,10291.302083
10,2,2795.666667
10,2,1746.750000
10,2,1526.058824
10,2,3202.050279
10,2,5531.804348
10,2,4393.050000
10,2,1762.682927
10,2,1730.879310
10,2,2636.265625
10,2,5372.866071
10,2,7311.485294
10,2,6466.890244
10,2,5013.661017
10,2,6081.075000
10,2,1706.550000
10,2,2584.735849
10,2,2962.057927
10,2,5632.692308
10,2,1701.954545
10,2,1905.228571
10,2,2299.000000
10,2,3003.712209
10,2,5625.437500
10,2,1666.581633
10,2,4515.289216
10,2,2334.039062
10,2,3099.444196
10,2,4425.158537
10,2,1881.571875
10,2,1816.111702
10,2,5724.455882
10,2,8045.046875
10,2,2371.635135
10,2,4752.316327
10,2,2797.284211
10,2,2343.341463
10,2,1624.128205
10,2,2124.437500
10,2,1665.636364

10,2,3412.915094
10,2,2957.162791
10,2,2921.375000
10,2,2532.000000
10,2,4451.556122
10,2,2004.505814
10,2,1675.487179
10,2,2081.598214
10,2,3352.273438
10,2,5366.235795
10,2,3241.375000
10,2,2438.312500
10,2,1726.281250
10,2,1820.312500
10,2,6196.641304
10,2,1696.786585
10,2,4303.154639
10,2,7536.320388
10,2,1838.915663
10,2,7364.101485
10,2,8976.360000
10,2,1822.038235
10,2,5923.742857
10,2,1563.310000
10,2,2545.534884
10,2,5817.159524
10,2,3879.160714
10,2,1501.333333
10,2,1544.434211
10,2,4586.309028
10,2,3576.385204
10,2,1635.000000
10,2,4751.094595
10,2,11521.915761
10,2,1659.142857
10,2,2591.046875
10,2,3535.460000
10,2,1756.664706
10,2,2075.076923
10,2,3839.421196
10,2,3088.547872
10,2,2210.235294
10,2,1931.521875
10,2,9772.337209
10,2,5079.108333
10,2,3213.215909
10,2,1638.451220
10,2,2425.377778
10,2,3653.150943
10,2,7247.993056
10,2,2605.786585
10,2,6868.500000
10,2,4696.066964
10,2,1638.531250
10,2,2339.974490
10,2,1917.656522
10,2,2245.900000
10,2,2184.223214
10,2,2799.375000
10,2,6581.119792
10,2,6071.494186
10,2,3530.613281
10,2,3214.098039
10,2,2605.994186
10,2,7791.948980
10,2,6020.963542

10,2,1753.677083
10,2,1888.083333
10,2,2815.713235
10,2,6274.442130
10,2,5917.125000
10,2,3175.841146
10,2,6411.500000
10,2,16231.253521
10,2,2804.722222
10,2,6620.755435
10,2,5389.132812
10,2,10287.333333
10,2,10734.500000
10,2,6111.145833
10,2,6090.677632
10,2,1961.381579
10,2,2015.776536
10,2,3381.848684
10,2,2298.163366
10,2,2702.556701
10,2,3752.364865
10,2,3346.125000
10,2,1803.037500
10,2,1586.691667
10,2,1657.684783
10,2,6578.368421
10,2,5527.464286
10,2,2471.080000
10,2,2890.480769
10,2,2928.658654
10,2,2069.762195
10,2,7118.916667
10,2,4883.670673
10,2,1875.442308
10,2,3608.851852
10,2,3996.752273
10,2,3836.698171
10,2,3705.159091
10,2,3725.000000
10,2,2333.245283
10,2,2347.017045
10,2,3593.261111
10,2,6178.275862
10,2,1600.302326
10,2,5386.668269
10,2,5168.020408
10,2,2550.144737
10,2,3907.805556
10,2,2727.790698
10,2,2809.486111
10,2,8268.285714
10,2,1908.803571
10,2,3813.440217
10,2,2244.548387
10,2,19359.857143
10,2,1952.188776
10,2,1890.734375
10,2,5938.010050
10,2,2182.747283
10,2,1708.720000
10,2,1678.750000
10,2,4573.211207
10,2,3791.750000
10,2,2379.485465
10,2,3603.190000
10,2,1667.793103

10,2,5777.425532
10,2,2898.976351
10,2,1978.553030
10,2,3658.000000
10,2,2108.350000
10,2,2585.318182
10,2,11190.543919
10,2,3694.569853
10,2,2847.303191
10,2,2654.892216
10,2,2858.026042
10,2,2868.653846
10,2,1993.388298
10,2,4364.457143
10,2,1533.546218
10,2,3416.777778
10,2,1802.000000
10,2,3457.553922
10,2,2410.054348
10,2,2114.911765
10,2,4391.312500
10,2,4373.606771
10,2,2131.602941
10,2,2432.594444
10,2,8734.475610
10,2,6323.483871
10,2,5078.238372
10,2,2402.887500
10,2,14268.923913
10,2,1897.807065
10,2,1576.524390
10,2,3994.039773
10,2,2064.025510
10,2,2156.558824
10,2,12683.770833
10,2,2222.045455
10,2,1738.682927
10,2,2740.575658
10,2,10960.234848
10,2,3851.513158
10,2,4581.817708
10,2,2640.750000
10,2,2908.350962
10,2,2496.131250
10,2,3280.986486
10,2,1736.323171
10,2,1696.367347
10,2,1679.636364
10,2,1822.275510
10,2,2575.322500
10,2,4611.922414
10,2,2604.141304
10,2,1666.052326
10,2,5382.512755
10,2,3933.386935
10,2,1789.028409
10,2,3094.500000
10,2,1510.816176
10,2,1941.288462
10,2,7809.745283
10,2,1928.335106
10,2,3254.306122
10,2,5306.956000
10,2,3739.458333
10,2,7539.667373
10,2,5104.146739

10,2,8914.825243
10,2,3343.931034
10,2,1739.885417
10,2,4901.073298
10,2,1546.190341
10,2,3748.500000
10,2,15763.137255
10,2,5024.500000
10,2,2802.132979
10,2,2915.765306
10,2,1872.596154
10,2,1970.829787
10,2,1963.945578
10,2,3555.645729
10,2,6363.456522
10,2,2267.371094
10,2,3339.246795
10,2,3533.862981
10,2,2599.000000
10,2,2548.693182
10,2,3806.342105
10,2,4008.520408
10,2,6540.725000
10,2,2108.875000
10,2,1596.625000
10,2,10710.265306
10,2,1944.946429
10,2,3295.377232
10,2,5599.447674
10,2,3355.012821
10,2,3210.976415
10,2,2664.810811
10,2,6514.520349
10,2,4398.761905
10,2,1671.416667
10,2,3167.354167
10,2,2105.995614
10,2,2367.767442
10,2,2933.493590
10,2,2256.100000
10,2,3947.134615
10,2,1720.862500
10,2,5763.166667
10,2,2965.289474
10,2,3050.733173
10,2,3540.679245
10,2,1551.295455
10,2,2749.800000
10,2,3693.687500
10,2,2776.340782
10,2,1499.676829
10,2,2110.386503
10,2,6280.921466
10,2,1771.801546
10,2,5834.343750
10,2,2154.515625
10,2,1869.720000
10,2,2748.471698
10,2,2913.521739
10,2,1853.469101
10,2,2614.646040
10,2,3197.046512
10,2,7395.507895
10,2,4525.410714
10,2,3469.556818
10,2,1678.259804

10,2,3261.727273
10,2,1592.398256
10,2,7364.055556
10,2,3153.387755
10,2,1541.937500
10,2,2770.754902
10,2,2784.762755
10,2,3154.485437
10,2,3890.197674
10,2,3469.194444
10,2,2371.273256
10,2,6951.579235
10,2,2954.912234
10,2,1634.678125
10,2,3974.139205
10,2,1938.107595
10,2,2580.522843
10,2,1756.095238
10,2,2058.437500
10,2,2234.744681
10,2,1979.576923
10,2,7877.168367
10,2,2793.038674
10,2,2171.902027
10,2,2238.814815
10,2,10233.154762
10,2,4439.295213
10,2,5986.551020
10,2,5930.500000
10,2,1918.871622
10,2,9185.453488
10,2,11859.105263
10,2,4587.141827
10,2,2868.863372
10,2,1634.778846
10,2,3222.645833
10,2,5111.490385
10,2,7454.781250
10,2,8098.377778
10,2,3036.703390
10,2,11028.605932
10,2,1737.414894
10,2,1833.963542
10,2,1888.702381
10,2,1856.631098
10,2,3618.208333
10,2,2279.987437
10,2,5488.304813
10,2,1685.939189
10,2,7259.455128
10,2,7785.466346
10,2,4609.391026
10,2,2300.408784
10,2,2274.000000
10,2,3421.084746
10,2,2138.911538
10,2,1996.464286
10,2,7224.856481
10,2,4844.000000
10,2,5000.465753
10,2,2200.505495
10,2,4412.662921
10,2,1684.500000
10,2,1504.255682
10,2,5759.081140
10,2,6853.324074

10,2,2273.642857
10,2,2466.131579
10,2,2565.294643
10,2,6242.938596
10,2,2106.780105
10,2,3460.364583
10,2,9247.053241
10,2,1756.644737
10,2,4586.294118
10,2,5781.831081
10,2,3303.078125
10,2,1506.250000
10,2,7802.312500
10,2,5008.014706
10,2,1557.714286
10,2,1776.859375
10,2,2207.579545
10,2,4172.169643
10,2,3076.238208
10,2,1621.855978
10,2,3403.703488
10,2,1708.305233
10,2,5248.474057
10,2,2755.815217
10,2,1504.500000
10,2,6301.850000
10,2,2903.078125
10,2,1822.918367
10,2,1653.913462
10,2,3952.500000
10,2,2368.726744
10,2,4532.470745
10,2,1869.062500
10,2,11964.031915
10,2,1928.875000
10,2,1913.625000
10,2,2598.453125
10,2,2004.072674
10,2,4612.826087
10,2,3039.559748
10,2,1806.702703
10,2,4711.894231
10,2,7174.914894
10,2,2563.026316
10,2,6667.108696
10,2,1775.882353
10,2,2652.270000
10,2,4733.607500
10,2,1511.081761
10,2,1735.843137
10,2,3884.054455
10,2,1908.005435
10,2,5080.869565
10,2,3238.036585
10,2,1669.692308
10,2,2699.276316
10,2,3553.285714
10,2,1571.356707
10,2,1503.327703
10,2,7897.454545
10,2,4352.655000
10,2,10744.386364
10,2,2031.269939
10,2,3018.363636
10,2,3420.978723
10,2,4915.189286

10,2,2085.575581
10,2,1701.890244
10,2,2833.312500
10,2,13474.851064
10,2,4642.973684
10,2,3461.875000
10,2,1527.102941
10,2,5263.593750
10,2,4343.694444
10,2,2685.858974
10,2,1808.187500
10,2,1732.750000
10,2,1712.045918
10,2,4791.939189
10,2,1585.412162
10,2,2011.739837
10,2,2672.451807
10,2,51771.510204
10,2,5395.956221
10,2,2048.114094
10,2,1556.175000
10,2,3574.144737
10,2,4057.680556
10,2,2426.400000
10,2,5625.148585
10,2,4073.364407
10,2,2721.447917
10,2,3272.028125
10,2,10558.486842
10,2,2209.267045
10,2,3211.441860
10,2,2408.500000
10,2,1673.186047
10,2,3068.427083
10,2,2284.157143
10,2,4183.801653
10,2,3099.076923
10,2,4664.226519
10,2,28110.041667
10,2,2218.870192
10,2,2222.375000
10,2,4576.443396
10,2,3702.448980
10,2,4105.205882
10,2,3223.276442
10,2,1524.733333
10,2,39395.196429
10,2,2173.534884
10,2,1995.384615
10,2,2420.888158
10,2,3209.483871
10,2,3433.380208
10,2,2589.556604
10,2,1661.765957
10,2,1877.310897
10,2,3670.833333
10,2,2122.181818
10,2,2507.036017
10,2,4168.368056
10,2,3301.976190
10,2,2061.205882
10,2,2292.836957
10,2,7394.842105
10,2,3321.779070
10,2,1851.551546
10,2,2016.192708

10,2,2796.338889
10,2,6018.653061
10,2,2336.933333
10,2,8212.000000
10,2,5301.246445
10,2,7309.694444
10,2,1877.191176
10,2,4701.826087
10,2,63653.377976
10,2,2225.696809
10,2,7351.500000
10,2,3705.273256
10,2,14197.043269
10,2,9558.809524
10,2,2386.276316
10,2,3235.226190
10,2,1733.231707
10,2,2420.680851
10,2,2821.825000
10,2,1621.512821
10,2,3057.643750
10,2,2273.100000
10,2,3315.328571
10,2,14328.300000
10,2,1511.567308
10,2,2184.125000
10,2,10352.144022
10,2,2591.539062
10,2,2507.113636
10,2,1619.482558
10,2,4505.825000
10,2,4543.875000
10,2,2318.681818
10,2,4240.601974
10,2,4021.636364
10,2,1636.857143
10,2,1761.058824
10,2,1924.756410
10,2,1861.592391
10,2,4262.722222
10,2,19583.989796
10,2,2507.550000
10,2,4845.756757
10,2,1718.023256
10,2,8119.000000
10,2,8215.529255
10,2,3439.259146
10,2,1760.000000
10,2,2972.689394
10,2,2236.439024
10,2,4766.197727
10,2,22318.060000
10,2,3161.723039
10,2,2515.990000
10,2,6243.950000
10,2,1835.777439
10,2,1865.237500
10,2,5542.040541
10,2,2355.541667
10,2,1753.136628
10,2,8894.618421
10,2,3876.796875
10,2,4022.311111
10,2,1659.770833
10,2,3849.493421
10,2,2645.250000

10,2,6213.428571
10,2,3868.591463
10,2,6721.919492
10,2,6440.354730
10,2,3530.606383
10,2,5413.241379
10,2,3022.558824
10,2,3108.775000
10,2,7316.578947
10,2,1752.745690
10,2,4353.909091
10,2,15050.031250
10,2,8967.016667
10,2,2856.750000
10,2,2642.878378
10,2,3985.000000
10,2,5189.019608
10,2,5349.554348
10,2,2103.795732
10,2,1782.000000
10,2,3665.250000
10,2,2769.830189
10,2,1966.033333
10,2,3549.398936
10,2,4139.714286
10,2,4177.927326
10,2,2810.514388
10,2,16347.369792
10,2,3383.345455
10,2,7972.777778
10,2,1799.750000
10,2,3404.270349
10,2,5421.465116
10,2,2786.196682
10,2,1603.336538
10,2,2095.466463
10,2,1853.906863
10,2,2591.146341
10,2,1781.831395
10,2,2319.020408
10,2,1782.250000
10,2,2162.937500
10,2,1853.044355
10,2,38926.403061
10,2,2198.803571
10,2,6575.508621
10,2,1763.979167
10,2,1913.076220
10,2,7432.695652
10,2,2177.625000
10,2,2898.020270
10,2,4830.987981
10,2,3124.385870
10,2,5480.015625
10,2,3099.132812
10,2,3079.105263
10,2,5332.387500
10,2,1651.478261
10,2,1778.379902
10,2,1833.696429
10,2,2490.345238
10,2,1873.197917
10,2,6921.338816
10,2,2271.535326
10,2,3633.170213
10,2,2336.960000

10,2,6824.961538
10,2,1698.833333
10,2,1563.688889
10,2,2488.891827
10,2,2544.127660
10,2,2468.809783
10,2,2449.413043
10,2,1562.092857
10,2,1611.967262
10,2,6622.468750
10,2,10327.851064
10,2,3266.644444
10,2,8841.102273
10,2,3160.715164
10,2,1980.750000
10,2,4734.323370
10,2,2913.137500
10,2,6797.964286
10,2,2032.000000
10,2,2483.857143
10,2,4129.601415
10,2,5288.526316
10,2,3144.553191
10,2,3932.992021
10,2,4150.256410
10,2,2547.775000
10,2,2753.180921
10,2,3556.151042
10,2,9664.357143
10,2,1730.145833
10,2,3602.792453
10,2,3263.715909
10,2,3173.505319
10,2,7010.808962
10,2,3806.950000
10,2,13284.598214
10,2,5934.173913
10,2,1892.019231
10,2,2446.462264
10,2,2220.500000
10,2,11850.322115
10,2,3702.015625
10,2,5103.502717
10,2,2522.651042
10,2,1825.800000
10,2,5638.519397
10,2,1727.157895
10,2,1756.653061
10,2,1894.936321
10,2,4713.333333
10,2,17625.782609
10,2,2932.256250
10,2,3337.990000
10,2,1647.937500
10,2,7771.050403
10,2,4481.787234
10,2,5091.119565
10,2,1783.795918
10,2,2331.391304
10,2,2337.935000
10,2,6956.845455
10,2,5152.604651
10,2,14004.440000
10,2,2493.824818
10,2,2022.968750
10,2,7416.771875

10,2,1964.166667
10,2,3141.128125
10,2,2303.916230
10,2,1706.761290
10,2,1794.666667
10,2,2297.578947
10,2,6685.051724
10,2,1661.493750
10,2,9916.910256
10,2,2505.064904
10,2,2064.343750
10,2,3983.278351
10,2,5167.634615
10,2,2165.858491
10,2,3693.000000
10,2,5487.219212
10,2,1931.600000
10,2,3801.578947
10,2,1868.861486
10,2,4984.333333
10,2,3039.800000
10,2,3825.786932
10,2,2476.196429
10,2,2984.494318
10,2,1596.403846
10,2,2649.776042
10,2,5466.384615
10,2,4623.997585
10,2,8978.974432
10,2,2287.811927
10,2,3618.571429
10,2,1988.550000
10,2,5326.100000
10,2,3398.990385
10,2,1753.680921
10,2,8121.391304
10,2,2588.380319
10,2,7650.890000
10,2,2726.235294
10,2,1718.636905
10,2,4274.890244
10,2,4124.537234
10,2,1567.944444
10,2,6042.065217
10,2,7352.576613
10,2,2955.865625
10,2,5079.444444
10,2,2600.900000
10,2,3676.064516
10,2,3934.618590
10,2,14555.000000
10,2,1900.236111
10,2,2768.500000
10,2,5347.846591
10,2,2268.142857
10,2,3367.508475
10,2,10513.184524
10,2,1517.763889
10,2,8597.917614
10,2,1547.575000
10,2,2424.276316
10,2,5166.877273
10,2,4416.637255
10,2,3638.278226
10,2,3902.744048
10,2,2580.812500

10,2,9898.223214
10,2,10680.003049
10,2,2296.150000
10,2,2063.572917
10,2,3241.804545
10,2,4840.695652
10,2,3103.265152
10,2,2506.406250
10,2,2298.789474
10,2,2435.363095
10,2,1600.450000
10,2,3823.812500
10,2,2140.661765
10,2,6581.424342
10,2,4657.532258
10,2,1573.988372
10,2,13172.289216
10,2,5931.370833
10,2,2338.791667
10,2,1841.733333
10,2,4835.881773
10,2,2916.217391
10,2,1574.071429
10,2,1973.923077
10,2,1904.215000
10,2,2139.018293
10,2,2585.562500
10,2,2045.750000
10,2,7283.906250
10,2,5518.852273
10,2,1731.272727
10,2,6746.435000
10,2,1671.886905
10,2,1551.557432
10,2,1539.750000
10,2,1663.469388
10,2,2481.322115
10,2,4377.037736
10,2,2829.879032
10,2,2035.020833
10,2,2290.195652
10,2,3136.369318
10,2,2333.875000
10,2,7652.953804
10,2,2845.893617
10,2,16050.847826
10,2,2839.934211
10,2,1767.554878
10,2,2738.533333
10,2,2439.076923
10,2,3264.520000
10,2,2929.162281
10,2,1971.330645
10,2,2540.419643
10,2,2519.918750
10,2,2134.542857
10,2,5246.589744
10,2,3918.487421
10,2,3651.702778
10,2,1904.122340
10,2,8436.353261
10,2,5200.627451
10,2,1548.284574
10,2,2829.500000
10,2,4354.769912
10,2,2595.989011

10,2,1799.643750
10,2,1549.089744
10,2,2589.728261
10,2,1875.318548
10,2,1534.653846
10,2,3098.360000
10,2,3589.804348
10,2,9275.895833
10,2,98705.610795
10,2,9458.971154
10,2,37294.397059
10,2,4318.294872
10,2,32645.207547
10,2,4722.500000
10,2,2383.644444
10,2,1726.180851
10,2,2280.055556
10,2,2709.934211
10,2,3152.596154
10,2,2212.262755
10,2,2174.034091
10,2,5920.341667
10,2,60191.580556
10,2,1951.875000
10,2,4493.198980
10,2,3003.859375
10,2,2576.527778
10,2,2514.500000
10,2,7306.962963
10,2,1867.149457
10,2,11686.817073
10,2,3622.744444
10,2,1847.875000
10,2,3325.096774
10,2,6004.633929
10,2,1514.567568
10,2,5876.308036
10,2,2240.068493
10,2,2289.689815
10,2,3834.500000
10,2,3903.264205
10,2,1900.112500
10,2,1846.600610
10,2,1605.220395
10,2,3729.714286
10,2,2293.312500
10,2,2091.905000
10,2,4143.250000
10,2,2033.008523
10,2,11830.384422
10,2,3945.666667
10,2,8380.150754
10,2,1611.153846
10,2,3023.403846
10,2,1542.531250
10,2,5919.477778
10,2,3502.333333
10,2,2069.332237
10,2,1604.375000
10,2,16715.007812
10,2,11541.678010
10,2,4225.879397
10,2,1993.467949
10,2,1938.200000
10,2,3670.350000
10,2,1777.127660

10,2,33720.695946
10,2,2427.058511
10,2,3920.966837
10,2,2580.445652
10,2,11446.745000
10,2,2905.586957
10,2,2168.367647
10,2,22201.617021
10,2,1499.230263
10,2,8101.114583
10,2,2508.348214
10,2,1895.800595
10,2,4475.340909
10,2,3173.287500
10,2,3210.842105
10,2,1625.385870
10,2,1735.673469
10,2,2363.727273
10,2,2536.388889
10,2,1860.468750
10,2,4309.581395
10,2,3262.300000
10,2,2405.483146
10,2,5050.088710
10,2,2037.892405
10,2,1848.464286
10,2,3719.111111
10,2,2201.550000
10,2,3415.678571
10,2,5157.600000
10,2,1680.250000
10,2,1898.400000
10,2,2040.430000
10,2,5309.952381
10,2,2988.244444
10,2,6105.574468
10,2,2510.093023
10,2,4036.804020
10,2,6908.344262
10,2,2088.122222
10,2,1616.714844
10,2,1715.319048
10,2,4985.086111
10,2,3552.268229
10,2,2747.615854
10,2,3295.894737
10,2,1981.333333
10,2,5423.044811
10,2,7414.918367
10,2,4636.250000
10,2,2478.781250
10,2,2209.872340
10,2,2388.081395
10,2,2910.350000
10,2,4160.307692
10,2,5137.956633
10,2,2247.613372
10,2,1501.586207
10,2,1858.500000
10,2,4946.125874
10,2,2263.909375
10,2,4046.154255
10,2,9773.447368
10,2,1675.616279
10,2,6802.035242
10,2,5471.553922

10,2,1525.955882
10,2,8398.911458
10,2,6747.448864
10,2,3024.447917
10,2,3446.750000
10,2,3358.335106
10,2,2437.833333
10,2,5075.488889
10,2,5226.167683
10,2,1955.875000
10,2,1727.906250
10,2,1746.833333
10,2,1614.297619
10,2,1574.298913
10,2,3891.081633
10,2,5667.746795
10,2,4594.523026
10,2,2648.040000
10,2,1557.255319
10,2,2880.848558
10,2,8058.119565
10,2,3761.810811
10,2,2009.576389
10,2,2069.156250
10,2,16722.368852
10,2,3999.747951
10,2,11356.604478
10,2,2538.241667
10,2,1848.670213
10,2,3764.044444
10,2,1948.364865
10,2,1642.442857
10,2,1528.160714
10,2,2307.227273
10,2,2877.719512
10,2,2053.044643
10,2,1791.546875
10,2,4426.605263
10,2,2583.285714
10,2,3567.487500
10,2,2043.699405
10,2,1774.447581
10,2,1903.628049
10,2,1575.455357
10,2,1920.000000
10,2,5274.611111
10,2,3143.014706
10,2,2733.924419
10,2,12575.181818
10,2,3487.152174
10,2,10038.610577
10,2,6354.918033
10,2,8311.073864
10,2,2464.739362
10,2,3894.581522
10,2,4548.510638
10,2,36693.846154
10,2,3089.333333
10,2,3110.467391
10,2,5035.487805
10,2,2922.548077
10,2,5628.509804
10,2,4609.203947
10,2,3223.220238
10,2,3008.833333
10,2,2104.614213

10,2,8758.676887
10,2,1540.787037
10,2,1795.940000
10,2,1800.304348
10,2,1863.464286
10,2,2098.964286
10,2,10888.653509
10,2,4884.834135
10,2,2063.729167
10,2,3911.864865
10,2,1757.350490
10,2,4491.452703
10,2,1653.220000
10,2,3043.865385
10,2,3650.379781
10,2,5485.092500
10,2,5317.594340
10,2,1843.002907
10,2,1724.068182
10,2,3216.490854
10,2,4697.103261
10,2,3219.765306
10,2,2160.244444
10,2,9051.241206
10,2,2859.869565
10,2,6773.193182
10,2,4831.298780
10,2,2453.936224
10,2,1747.900000
10,2,19200.730469
10,2,1612.969512
10,2,20202.739286
10,2,2239.246875
10,2,3890.864865
10,2,2367.128205
10,2,2214.843750
10,2,12284.508772
10,2,2615.735294
10,2,2439.957547
10,2,3353.760000
10,2,5440.426471
10,2,1675.277778
10,2,3411.786290
10,2,5530.719298
10,2,2982.130000
10,2,1501.621622
10,2,3211.846154
10,2,2045.451613
10,2,1536.288043
10,2,1659.394737
10,2,1847.453125
10,2,3269.943182
10,2,6180.718750
10,2,3179.598958
10,2,1642.852273
10,2,2414.868750
10,2,42099.452830
10,2,1540.937500
10,2,4818.134146
10,2,15570.714286
10,2,2027.849057
10,2,5534.714286
10,2,4876.593750
10,2,1629.218750
10,2,2198.627551
10,2,3413.633721

10,2,3012.806250
10,2,11705.786290
10,2,8046.650000
10,2,1723.588235
10,2,5803.300000
10,2,1574.591837
10,2,2489.464286
10,2,3118.625000
10,2,3253.651042
10,2,1559.539634
10,2,3035.481707
10,2,1863.666667
10,2,4844.627907
10,2,1578.804878
10,2,2270.369898
10,2,1863.419492
10,2,3457.369792
10,2,7250.707386
10,2,1670.521277
10,2,7800.143617
10,2,2919.887755
10,2,1788.534722
10,2,2902.250000
10,2,3148.500000
10,2,3575.913136
10,2,1512.134146
10,2,1653.685393
10,2,5898.528302
10,2,9529.634615
10,2,4213.477273
10,2,4557.980000
10,2,3309.765625
10,2,3910.375000
10,2,1521.634146
10,2,2820.648148
10,2,11650.050000
10,2,8988.074561
10,2,7510.323980
10,2,1813.593750
10,2,3640.352500
10,2,8482.978723
10,2,7208.312500
10,2,6245.978723
10,2,2759.225000
10,2,1600.130952
10,2,3261.661058
10,2,2382.103659
10,2,1642.212500
10,2,2844.668605
10,2,2718.839181
10,2,2097.750000
10,2,2808.784431
10,2,2385.331126
10,2,2173.687500
10,2,1585.827128
10,2,1721.176471
10,2,3901.222222
10,2,2466.625000
10,2,2521.883721
10,2,4376.441964
10,2,8147.414634
10,2,2430.939189
10,2,22133.195513
10,2,4426.684426
10,2,2078.459239
10,2,3155.187500

10,2,13997.304688
10,2,2123.295918
10,2,5165.982143
10,2,2346.666667
10,2,1553.980263
10,2,2445.191489
10,2,2687.238372
10,2,5554.392157
10,2,3559.004237
10,2,2000.614583
10,2,1566.250000
10,2,1988.298077
10,2,8332.125000
10,2,2164.328947
10,2,8153.071749
10,2,9328.611413
10,2,4787.282895
10,2,3249.154762
10,2,4836.750000
10,2,3187.704545
10,2,9516.583333
10,2,1843.750000
10,2,1526.320513
10,2,2562.285714
10,2,2842.035326
10,2,2705.375000
10,2,7097.256579
10,2,4619.811321
10,2,7829.051020
10,2,3454.742647
10,2,13699.670000
10,2,2027.847561
10,2,1747.021277
10,2,2820.851064
10,2,13114.900000
10,2,1625.989796
10,2,1740.729167
10,2,7294.006410
10,2,5165.663462
10,2,6252.284091
10,2,5793.620192
10,2,5898.706395
10,2,15929.707589
10,2,5665.052083
10,2,1500.744318
10,2,15711.973214
10,2,3344.739130
10,2,2060.168605
10,2,1747.179487
10,2,2451.433333
10,2,5137.292683
10,2,8978.589552
10,2,6921.488889
10,2,1729.826923
10,2,3791.627717
10,2,2349.517442
10,2,1590.939024
10,2,5475.696023
10,2,1814.800000
10,2,3330.203704
10,2,5819.769231
10,2,4594.076220
10,2,2548.247312
10,2,3557.878125
10,2,4422.339286
10,2,2696.093023

10,2,5587.725000
10,2,1547.130000
10,2,2353.755952
10,2,4544.412234
10,2,2909.604167
10,2,14735.525000
10,2,4906.000000
10,2,1774.987805
10,2,3692.393617
10,2,4501.085938
10,2,4300.875000
10,2,5880.033163
10,2,1585.622093
10,2,2250.928571
10,2,1738.101064
10,2,3501.638158
10,2,1639.565789
10,2,1811.697917
10,2,5021.653061
10,2,5291.304878
10,2,9261.803279
10,2,1564.271739
10,2,5449.790123
10,2,1820.410714
10,2,2420.597938
10,2,12302.509615
10,2,7646.594262
10,2,1797.508621
10,2,11528.164352
10,2,3491.991477
10,2,3691.750000
10,2,8178.709524
10,2,29273.756757
10,2,2169.371795
10,2,3288.798077
10,2,3214.833333
10,2,11355.946429
10,2,1804.975543
10,2,3492.837500
10,2,7998.125000
10,2,1880.784884
10,2,4725.687500
10,2,3069.750000
10,2,2518.518519
10,2,1687.892857
10,2,1701.655000
10,2,4174.225000
10,2,13093.140244
10,2,3603.266447
10,2,4974.395833
10,2,2854.318627
10,2,2662.694444
10,2,2322.665865
10,2,1769.670000
10,2,4456.406863
10,2,1845.837748
10,2,2116.511574
10,2,6136.339147
10,2,2279.707500
10,2,2603.859375
10,2,17327.445122
10,2,3122.080357
10,2,2615.839286
10,2,2384.312500
10,2,1662.108974
10,2,2631.648438

10,2,3365.422111
10,2,21914.194079
10,2,65984.270440
10,2,1729.738462
10,2,2013.946429
10,2,3170.517241
10,2,9701.722826
10,2,2096.727273
10,2,1588.880435
10,2,2610.465909
10,2,2898.159722
10,2,3329.273936
10,2,7865.063559
10,2,3009.854730
10,2,1764.314136
10,2,1781.827778
10,2,1743.111413
10,2,2378.777174
10,2,1738.037037
10,2,1564.191011
10,2,4037.238693
10,2,2507.470109
10,2,9624.982143
10,2,3126.551887
10,2,1780.125000
10,2,2223.993902
10,2,2046.595238
10,2,6651.818627
10,2,2248.405405
10,2,2184.176020
10,2,1672.598592
10,2,2171.726415
10,2,13723.641204
10,2,2133.989130
10,2,1718.187500
10,2,1713.075000
10,2,3576.068750
10,2,2634.675000
10,2,2136.860577
10,2,3634.063830
10,2,1883.755435
10,2,2697.516447
10,2,18734.807692
10,2,6118.772959
10,2,2797.534884
10,2,2709.947500
10,2,2590.773649
10,2,1719.778646
10,2,3654.894022
10,2,2528.918367
10,2,1912.900000
10,2,2584.750000
10,2,7140.892045
10,2,10971.519737
10,2,5884.317708
10,2,2949.234043
10,2,3175.931373
10,2,2550.665541
10,2,2477.375000
10,2,5192.086957
10,2,6635.900000
10,2,2572.080000
10,2,8738.162791
10,2,3181.982143
10,2,2301.409375
10,2,12538.403846

10,2,8661.031250
10,2,2031.145349
10,2,5707.546875
10,2,5045.274390
10,2,1601.725000
10,2,3973.668750
10,2,4125.905612
10,2,3825.027439
10,2,2389.022959
10,2,2501.510870
10,2,1770.339744
10,2,3097.562500
10,2,2040.418367
10,2,7377.194149
10,2,2125.107143
10,2,5470.592500
10,2,3032.000000
10,2,1754.612903
10,2,2172.000000
10,2,2821.187500
10,2,8979.688679
10,2,3199.191860
10,2,2353.579268
10,2,2905.535088
10,2,3653.168367
10,2,8425.281977
10,2,6337.964286
10,2,27138.250000
10,2,3205.233333
10,2,3082.091667
10,2,2196.767857
10,2,1780.043750
10,2,1880.395833
10,2,2512.608696
10,2,1606.538043
10,2,2436.835000
10,2,3214.833333
10,2,4367.160714
10,2,1850.708661
10,2,1803.725000
10,2,1757.210526
10,2,1567.786290
10,2,4936.923077
10,2,2070.804688
10,2,4886.692708
10,2,8880.567708
10,2,3259.840000
10,2,2052.371875
10,2,2661.787179
10,2,2065.013393
10,2,4483.351351
10,2,4257.500000
10,2,1516.170213
10,2,4284.230603
10,2,2689.750000
10,2,2458.493056
10,2,1511.265625
10,2,6456.027273
10,2,23973.291667
10,2,2697.000000
10,2,2853.526316
10,2,4275.670213
10,2,3365.702381
10,2,5944.880435
10,2,2651.063830
10,2,5916.063492

10,2,6240.977612
10,2,7232.314286
10,2,2607.919872
10,2,4225.400000
10,2,2687.274038
10,2,1829.556931
10,2,2380.696023
10,2,2030.741071
10,2,10362.551471
10,2,7507.344828
10,2,1828.026316
10,2,15952.957547
10,2,7471.406977
10,2,4450.725962
10,2,2804.750000
10,2,8217.035714
10,2,1574.772727
10,2,1681.450000
10,2,4336.673295
10,2,14920.943396
10,2,1832.114286
10,2,1688.361111
10,2,6296.352941
10,2,2562.612245
10,2,3301.022222
10,2,2372.029255
10,2,2461.628378
10,2,3644.783784
10,2,8305.046610
10,2,2943.323529
10,2,1508.791667
10,2,1604.250000
10,2,2837.207317
10,2,3111.437500
10,2,1916.089286
10,2,5510.347500
10,2,2864.078804
10,2,33848.718750
10,2,7948.064103
10,2,2049.000000
10,2,14586.625000
10,2,2104.217172
10,2,5992.250000
10,2,1657.035714
10,2,2992.560000
10,2,2598.551887
10,2,4545.703125
10,2,2154.807692
10,2,1574.037736
10,2,4541.134615
10,2,7874.433036
10,2,1527.950658
10,2,2321.461538
10,2,5065.759615
10,2,2336.690104
10,2,2938.898810
10,2,1740.857143
10,2,3961.168269
10,2,5728.481818
10,2,4612.401316
10,2,2019.416667
10,2,3777.903846
10,2,1928.666667
10,2,2219.241279
10,2,1882.842857
10,2,2435.763514

10,2,1609.093750
10,2,13678.500000
10,2,3683.789216
10,2,4528.554348
10,2,5846.840278
10,2,3158.437500
10,2,2624.756098
10,2,3174.722222
10,2,9772.946429
10,2,4338.530806
10,2,2293.566667
10,2,3578.125000
10,2,6639.416667
10,2,1544.131737
10,2,1500.111111
10,2,5167.662500
10,2,3750.653846
10,2,7552.535714
10,2,2794.698864
10,2,2065.611111
10,2,2197.602041
10,2,2105.333333
10,2,1795.791667
10,2,1674.500000
10,2,4667.039063
10,2,2545.897727
10,2,2103.279255
10,2,6501.290909
10,2,2839.621429
10,2,2112.787234
10,2,3087.428571
10,2,6365.460106
10,2,3899.637500
10,2,11052.859375
10,2,1962.122283
10,2,14445.478469
10,2,2515.073171
10,2,3071.471591
10,2,6065.065574
10,2,2313.905213
10,2,3027.163043
10,2,5506.705357
10,2,4519.929348
10,2,6623.576923
10,2,5222.500000
10,2,1499.261364
10,2,6623.582500
10,2,1864.825000
10,2,2422.650888
10,2,3737.388060
10,2,2410.905660
10,2,1911.357558
10,2,1975.609756
10,2,6767.914894
10,2,4008.590909
10,2,2736.090909
10,2,5538.794872
10,2,1835.812500
10,2,1716.975000
10,2,2833.848837
10,2,2477.597973
10,2,3437.125000
10,2,2095.500000
10,2,2209.160000
10,2,3234.997449
10,2,5823.031250

10,2,2333.132075
10,2,3294.664474
10,2,4917.943182
10,2,2604.462500
10,2,4059.122222
10,2,7610.840000
10,2,5034.445312
10,2,1764.202703
10,2,5479.413043
10,2,3086.046610
10,2,3273.231250
10,2,3847.176471
10,2,7985.117188
10,2,6922.309783
10,2,11456.622222
10,2,2448.890957
10,2,2213.108333
10,2,3171.300000
10,2,1647.617021
10,2,2648.518293
10,2,3008.187192
10,2,1732.398810
10,2,2810.750000
10,2,5878.540816
10,2,2110.750000
10,2,2777.250000
10,2,11343.666667
10,2,1951.657143
10,2,2445.896341
10,2,5299.798883
10,2,2495.557692
10,2,7503.333333
10,2,9899.363636
10,2,2499.901042
10,2,3202.024390
10,2,5406.355769
10,2,4817.400000
10,2,2932.048780
10,2,1933.604651
10,2,9001.000000
10,2,1606.109375
10,2,11994.333333
10,2,3624.173469
10,2,1847.350000
10,2,3138.685096
10,2,1635.318182
10,2,3846.723404
10,2,1559.707317
10,2,5612.101124
10,2,15170.517857
10,2,6238.473913
10,2,5527.359375
10,2,10888.715909
10,2,1758.609756
10,2,1545.851064
10,2,9628.697674
10,2,1507.250000
10,2,22405.234848
10,2,1593.862805
10,2,2552.666667
10,2,2823.212121
10,2,5614.450000
10,2,2099.883929
10,2,2508.833333
10,2,5611.121622
10,2,2035.709459

10,2,5653.378378
10,2,4293.808140
10,2,1957.933962
10,2,3390.950000
10,2,3882.763889
10,2,1730.841463
10,2,2264.526042
10,2,4731.550000
10,2,2924.390957
10,2,2242.315217
10,2,9881.555556
10,2,3596.283784
10,2,2392.765957
10,2,3013.332447
10,2,15381.673913
10,2,2006.786585
10,2,5414.432432
10,2,5872.440789
10,2,9944.903302
10,2,2362.962264
10,2,2327.271277
10,2,4046.512500
10,2,3097.141447
10,2,18474.656250
10,2,4977.787234
10,2,2775.258523
10,2,5864.661017
10,2,9054.965409
10,2,5086.507212
10,2,1752.583333
10,2,15524.743590
10,2,1755.281818
10,2,3765.763158
10,2,2568.750000
10,2,1596.131098
10,2,2784.538889
10,2,5067.342391
10,2,4941.538889
10,2,2465.516892
10,2,2427.181159
10,2,2091.212766
10,2,3342.045732
10,2,7621.428879
10,2,2562.505556
10,2,1530.948980
10,2,1686.148438
10,2,6254.258929
10,2,3442.175000
10,2,2121.997283
10,2,9667.765152
10,2,2599.119565
10,2,2401.565217
10,2,1872.851064
10,2,2674.298469
10,2,5489.022831
10,2,4379.236486
10,2,1828.404762
10,2,4585.206349
10,2,5682.485294
10,2,5696.854167
10,2,3010.964286
10,2,4061.742857
10,2,2260.025641
10,2,1794.045455
10,2,1940.067308
10,2,2211.822222

10,2,2360.517045
10,2,2458.750000
10,2,2933.335000
10,2,1976.152778
10,2,6557.590000
10,2,7399.088942
10,2,3857.853774
10,2,1692.600000
10,2,3875.308673
10,2,4342.369565
10,2,3323.826087
10,2,1767.026786
10,2,11911.590909
10,2,4956.725000
10,2,1832.917614
10,2,2021.468750
10,2,3054.712500
10,2,2975.451923
10,2,7978.000000
10,2,4666.800000
10,2,2296.986702
10,2,4430.895833
10,2,2818.710526
10,2,2452.511364
10,2,1622.343023
10,2,2628.734375
10,2,2091.192935
10,2,3513.166667
10,2,4008.530093
10,2,1774.525000
10,2,2246.875000
10,2,4637.338235
10,2,4090.244444
10,2,2986.545455
10,2,2625.748571
10,2,1900.312500
10,2,2935.205357
10,2,9082.814286
10,2,3568.026316
10,2,5855.676471
10,2,1848.351744
10,2,2995.974490
10,2,2753.200000
10,2,1918.632653
10,2,1761.453488
10,2,2246.625000
10,2,2407.693750
10,2,13658.842857
10,2,4337.500000
10,2,3495.363057
10,2,5845.322034
10,2,7190.395349
10,2,4450.069767
10,2,2156.083333
10,2,6432.925481
10,2,2477.923077
10,2,4556.421569
10,2,5812.658537
10,2,6682.428977
10,2,3437.467391
10,2,3931.080882
10,2,3365.772059
10,2,4658.995283
10,2,1551.489796
10,2,2413.302326
10,2,3470.313953

10,2,2132.009259
10,2,2018.558824
10,2,3449.165094
10,2,1950.647959
10,2,3012.000000
10,2,1905.428571
10,2,14544.965116
10,2,4359.378049
10,2,2474.743590
10,2,1906.502874
10,2,2336.129412
10,2,4531.000000
10,2,2434.240854
10,2,1673.733696
10,2,2228.428571
10,2,4667.347826
10,2,9498.466667
10,2,2285.103261
10,2,3897.259434
10,2,5613.747596
10,2,1880.402273
10,2,3980.437500
10,2,3059.654255
10,2,2146.375000
10,2,1735.011905
10,2,7353.591837
10,2,2129.994186
10,2,5898.375000
10,2,5841.907609
10,2,2076.045977
10,2,2386.697368
10,2,28603.738636
10,2,1665.333333
10,2,2098.033333
10,2,4956.066327
10,2,6351.601852
10,2,1950.430233
10,2,3378.259615
10,2,1858.870370
10,2,7500.083333
10,2,2015.826923
10,2,2642.674419
10,2,8406.143750
10,2,4377.916667
10,2,2159.377358
10,2,3600.575472
10,2,2045.185811
10,2,6882.535176
10,2,4732.450000
10,2,10127.311475
10,2,4853.153846
10,2,8428.688679
10,2,2871.875000
10,2,2623.000000
10,2,1759.486842
10,2,8255.225490
10,2,9930.616071
10,2,3702.412162
10,2,2638.931373
10,2,1528.202703
10,2,5567.742857
10,2,6540.426471
10,2,2602.000000
10,2,1695.451087
10,2,77024.468750
10,2,1776.142012

10,2,7708.320675
10,2,10907.765086
10,2,12946.687500
10,2,4805.500000
10,2,2140.579787
10,2,1613.776596
10,2,2386.847561
10,2,1931.458333
10,2,6852.489796
10,2,7517.550000
10,2,3570.322581
10,2,2901.985294
10,2,2072.000000
10,2,5103.771277
10,2,5000.293103
10,2,2305.487805
10,2,5620.358209
10,2,4022.360577
10,2,2454.638514
10,2,1705.267857
10,2,3829.202500
10,2,1516.069767
10,2,1638.215278
10,2,4234.276316
10,2,3370.106383
10,2,3578.730769
10,2,2394.829268
10,2,5024.471366
10,2,3969.734756
10,2,1858.836066
10,2,2171.955307
10,2,6662.949721
10,2,2951.545894
10,2,8485.102564
10,2,7670.041451
10,2,2504.950980
10,2,5420.107143
10,2,2642.368421
10,2,2182.396226
10,2,2125.889286
10,2,3624.399457
10,2,2521.780612
10,2,2580.461310
10,2,2928.391667
10,2,4170.436170
10,2,1802.729730
10,2,1606.781915
10,2,3027.206081
10,2,1908.426136
10,2,4796.012821
10,2,1531.937500
10,2,9955.664894
10,2,1626.538462
10,2,9712.776596
10,2,1730.101399
10,2,1691.500000
10,2,2966.562500
10,2,2407.288660
10,2,3885.422500
10,2,2632.176101
10,2,1704.955128
10,2,4754.922170
10,2,7996.636364
10,2,2445.257143
10,2,2692.478723
10,2,2408.204380

10,2,2496.000000
10,2,6747.281250
10,2,4149.155556
10,2,2592.361413
10,2,3867.012500
10,2,5998.926887
10,2,5299.211111
10,2,4096.090000
10,2,3252.025000
10,2,2223.115625
10,2,2578.622500
10,2,2937.637931
10,2,2018.500000
10,2,3177.582386
10,2,2025.782609
10,2,5863.439189
10,2,1604.502717
10,2,3058.729730
10,2,2218.036585
10,2,4967.011905
10,2,1788.035533
10,2,6567.563636
10,2,2691.434286
10,2,4755.110577
10,2,2494.155689
10,2,1681.058480
10,2,2101.968750
10,2,2070.012195
10,2,4493.935000
10,2,5257.996711
10,2,4626.677885
10,2,14398.933824
10,2,1605.668605
10,2,1709.836538
10,2,5474.034884
10,2,3311.447368
10,2,2312.494318
10,2,1626.454545
10,2,11895.612179
10,2,2734.763889
10,2,2834.726695
10,2,3747.094512
10,2,1907.517361
10,2,2086.986842
10,2,4030.096591
10,2,1971.239583
10,2,5301.285714
10,2,3588.694444
10,2,2121.277027
10,2,2091.156250
10,2,4639.268750
10,2,1661.510204
10,2,2090.732143
10,2,2997.206250
10,2,3899.415000
10,2,2074.526316
10,2,1505.758929
10,2,3261.612179
10,2,4287.088889
10,2,2350.437500
10,2,2633.198718
10,2,2044.923077
10,2,5075.171053
10,2,5054.482143
10,2,4039.375000
10,2,5828.803571

10,2,5012.116279
10,2,4381.454106
10,2,4559.916256
10,2,2908.523316
10,2,1831.375000
10,2,5206.550000
10,2,2670.072500
10,2,2022.010417
10,2,1913.774390
10,2,5197.833333
10,2,10808.041667
10,2,2287.092391
10,2,3560.000000
10,2,1593.532895
10,2,4423.393939
10,2,12376.125000
10,2,8796.058824
10,2,1556.800000
10,2,2249.385417
10,2,3896.591837
10,2,2358.119565
10,2,2416.487805
10,2,6372.861650
10,2,1772.636364
10,2,3581.728261
10,2,3748.397727
10,2,1898.198324
10,2,18048.136364
10,2,6474.609442
10,2,1918.300676
10,2,2743.064904
10,2,9530.444444
10,2,5330.714286
10,2,1638.656250
10,2,27445.767857
10,2,2031.953125
10,2,1685.994505
10,2,3110.304487
10,2,5074.378049
10,2,2397.869565
10,2,3367.398438
10,2,3382.494792
10,2,1937.894444
10,2,1827.735849
10,2,1788.012195
10,2,2467.062500
10,2,10033.691964
10,2,1653.550000
10,2,1766.097561
10,2,4212.100410
10,2,3252.694149
10,2,2320.319149
10,2,5651.038265
10,2,2098.880435
10,2,2967.559211
10,2,2519.002475
10,2,4675.770270
10,2,1689.578947
10,2,4207.576220
10,2,6027.055288
10,2,11537.512755
10,2,2281.454545
10,2,5306.923077
10,2,2592.411765
10,2,3493.522727
10,2,15777.714286

10,2,3845.178571
10,2,14105.875000
10,2,1911.586735
10,2,3852.321429
10,2,8556.562500
10,2,2189.914894
10,2,4807.187500
10,2,3867.656250
10,2,3119.205882
10,2,2407.028646
10,2,6330.166667
10,2,7607.357143
10,2,1940.475000
10,2,7150.964286
10,2,1510.935484
10,2,3388.660377
10,2,4494.788043
10,2,5151.151163
10,2,3137.133333
10,2,5022.102273
10,2,3139.078125
10,2,7163.991279
10,2,4721.340426
10,2,10086.877660
10,2,1704.760000
10,2,3694.515625
10,2,1992.292683
10,2,7829.894444
10,2,3712.287234
10,2,1585.600000
10,2,6233.723404
10,2,3475.988971
10,2,2224.775000
10,2,3776.233491
10,2,2664.572917
10,2,54820.092949
10,2,2905.011111
10,2,5799.708333
10,2,1554.292683
10,2,2366.807927
10,2,2589.004739
10,2,3478.867188
10,2,1746.614362
10,2,1792.987805
10,2,7294.666667
10,2,2886.412500
10,2,3011.673780
10,2,6249.209302
10,2,2707.375000
10,2,9137.343750
10,2,3243.128205
10,2,1762.571023
10,2,2717.819853
10,2,2984.728723
10,2,3485.747222
10,2,2787.880000
10,2,7933.202703
10,2,9588.010714
10,2,1928.902439
10,2,2747.565000
10,2,2408.528409
10,2,5889.795918
10,2,3937.420732
10,2,12200.619318
10,2,3638.862500
10,2,1621.000000

10,2,1813.250000
10,2,6619.415842
10,2,3366.044872
10,2,40282.127404
10,2,9454.375000
10,2,1596.735714
10,2,1557.378378
10,2,2699.103261
10,2,5924.731250
10,2,1687.488304
10,2,1784.405405
10,2,1824.861607
10,2,1565.845745
10,2,5793.060976
10,2,2120.814286
10,2,2364.691176
10,2,2976.625000
10,2,2747.630952
10,2,2045.750000
10,2,3195.375000
10,2,2657.961538
10,2,3704.088942
10,2,4899.418605
10,2,1777.361111
10,2,3387.833333
10,2,9687.888889
10,2,4275.636792
10,2,5863.405488
10,2,3357.658333
10,2,1812.500000
10,2,2760.613260
10,2,2806.538860
10,2,8580.791829
10,2,4456.363636
10,2,3245.788462
10,2,4619.365854
10,2,1758.103723
10,2,3683.818750
10,2,2947.125000
10,2,5143.343137
10,2,2669.760000
10,2,6940.821875
10,2,3080.607143
10,2,3317.574468
10,2,1844.092105
10,2,4119.872340
10,2,2323.066667
10,2,1979.455357
10,2,8640.750000
10,2,2152.870968
10,2,3652.302326
10,2,1654.841216
10,2,5212.402174
10,2,1574.465909
10,2,1624.104651
10,2,7978.600000
10,2,3299.850000
10,2,3113.399194
10,2,1802.322368
10,2,1530.842105
10,2,1553.664634
10,2,1960.277778
10,2,1705.312500
10,2,1852.887417
10,2,2285.500000
10,2,3090.053571

10,2,5530.682692
10,2,2711.447222
10,2,4760.211957
10,2,4564.712500
10,2,4404.205128
10,2,4251.334375
10,2,4911.137821
10,2,1849.947368
10,2,3123.568182
10,2,2429.710784
10,2,3609.629630
10,2,1988.742857
10,2,2145.642857
10,2,3042.704082
10,2,1939.611111
10,2,2554.302885
10,2,4374.177778
10,2,2969.600543
10,2,2810.191860
10,2,7082.755435
10,2,7161.000000
10,2,3296.678571
10,2,2749.972222
10,2,3836.804878
10,2,1711.568750
10,2,6553.037234
10,2,3040.680851
10,2,1647.862500
10,2,1540.529255
10,2,2251.777027
10,2,1681.082237
10,2,1690.403846
10,2,2466.397959
10,2,1938.682292
10,2,9706.343750
10,2,10819.781818
10,2,2000.950893
10,2,2543.997159
10,2,2709.105000
10,2,2562.108374
10,2,4624.796407
10,2,6948.517442
10,2,6954.322581
10,2,3540.416667
10,2,2965.419118
10,2,11435.656977
10,2,2750.588889
10,2,10963.571429
10,2,6972.840426
10,2,2066.500000
10,2,5312.464286
10,2,2323.388889
10,2,5824.937500
10,2,9713.101562
10,2,1823.351064
10,2,1529.253472
10,2,1831.451389
10,2,2518.054054
10,2,2482.076923
10,2,2190.037736
10,2,1932.522727
10,2,3506.430288
10,2,22491.585366
10,2,1857.914062
10,2,1929.904412
10,2,2591.454545

10,2,3921.280660
10,2,3416.305000
10,2,3156.507576
10,2,2343.553299
10,2,1967.367021
10,2,2395.925287
10,2,4691.043478
10,2,10802.402116
10,2,20424.987069
10,2,6248.573864
10,2,3776.477273
10,2,5608.539216
10,2,2468.858696
10,2,4017.993056
10,2,1990.723958
10,2,3105.837209
10,2,1756.180556
10,2,1778.721154
10,2,3558.839286
10,2,4267.250000
10,2,2170.410714
10,2,4162.867925
10,2,1501.211957
10,2,1594.896552
10,2,2020.532609
10,2,1937.957219
10,2,3173.536649
10,2,3464.830189
10,2,3791.031250
10,2,11110.946429
10,2,1518.312500
10,2,13858.543269
10,2,2005.465116
10,2,10737.756757
10,2,7175.821429
10,2,2629.562500
10,2,10440.881250
10,2,7120.512821
10,2,2034.764706
10,2,29021.216216
10,2,2457.181034
10,2,10424.518293
10,2,3646.154070
10,2,17053.829268
10,2,10749.291667
10,2,1838.900862
10,2,1553.954286
10,2,3515.566667
10,2,1696.746711
10,2,2050.315476
10,2,2128.059211
10,2,1920.163043
10,2,7431.908333
10,2,3291.971154
10,2,3366.948052
10,2,6109.403125
10,2,2472.084337
10,2,2462.125000
10,2,1750.099490
10,2,3087.152778
10,2,2733.625000
10,2,2467.111111
10,2,2821.726190
10,2,8576.826923
10,2,1744.086735
10,2,3307.265625

10,2,1656.848837
10,2,2150.000000
10,2,2250.875000
10,2,2406.792553
10,2,9462.857843
10,2,8108.493590
10,2,8118.777778
10,2,1959.566327
10,2,1845.604592
10,2,2853.927885
10,2,2643.086592
10,2,2326.005208
10,2,2219.172775
10,2,41754.797222
10,2,2062.878641
10,2,5512.666667
10,2,4477.303571
10,2,2049.385135
10,2,1726.554054
10,2,2622.706250
10,2,20182.939024
10,2,2705.697368
10,2,1722.272727
10,2,2340.666667
10,2,2153.057143
10,2,13389.502907
10,2,4604.562500
10,2,9953.327273
10,2,2445.450980
10,2,3718.834239
10,2,2157.159574
10,2,2067.220430
10,2,2695.892857
10,2,4446.796875
10,2,5157.636364
10,2,4487.412500
10,2,2365.016026
10,2,6652.509146
10,2,2382.693182
10,2,6936.958333
10,2,1549.383929
10,2,2450.483696
10,2,3092.536885
10,2,1608.782051
10,2,4443.055556
10,2,4455.428571
10,2,6063.695122
10,2,7387.181818
10,2,1536.809211
10,2,2216.680628
10,2,3152.742021
10,2,4220.011628
10,2,2233.500000
10,2,3279.915541
10,2,1933.692308
10,2,2195.816667
10,2,2486.875000
10,2,1878.492647
10,2,1510.348684
10,2,4619.227273
10,2,6066.928571
10,2,3974.763158
10,2,3133.118750
10,2,2483.619048
10,2,3904.096591
10,2,7287.727273

10,2,3120.218750
10,2,2683.113636
10,2,2015.850000
10,2,5497.916667
10,2,1500.763158
10,2,3334.853659
10,2,4020.675000
10,2,3980.889535
10,2,7622.140000
10,2,4266.096354
10,2,1668.510417
10,2,4565.301136
10,2,1764.291667
10,2,3223.983051
10,2,4237.627660
10,2,2578.750000
10,2,4305.352941
10,2,2955.421233
10,2,4100.585227
10,2,2012.698113
10,2,2094.968750
10,2,93333.272500
10,2,4756.240132
10,2,1581.073171
10,2,1714.356383
10,2,2390.850000
10,2,1808.430348
10,2,3570.772059
10,2,3665.475000
10,2,2338.767857
10,2,3707.176471
10,2,1955.750000
10,2,2504.117021
10,2,2913.600000
10,2,4194.979592
10,2,3172.357143
10,2,4682.963235
10,2,1744.662500
10,2,2684.482143
10,2,1869.822034
10,2,2879.047619
10,2,8187.150000
10,2,2921.000000
10,2,4074.375000
10,2,1820.192308
10,2,1507.692308
10,2,2407.500000
10,2,3314.018182
10,2,1871.607955
10,2,1661.791925
10,2,3172.761364
10,2,7507.182500
10,2,5105.669811
10,2,2869.526316
10,2,3043.205128
10,2,4282.739362
10,2,2055.565217
10,2,9104.734597
10,2,1985.012712
10,2,5316.919872
10,2,2217.358974
10,2,2629.138021
10,2,2490.965909
10,2,1671.742857
10,2,1624.468750
10,2,24299.437500

10,2,2898.181548
10,2,2476.875000
10,2,1606.390625
10,2,5963.097561
10,2,4992.024390
10,2,1684.776163
10,2,2468.160256
10,2,6565.540000
10,2,3352.552632
10,2,4198.750000
10,2,18060.000000
10,2,7347.538462
10,2,2279.095745
10,2,7158.933673
10,2,2558.805147
10,2,3545.270270
10,2,9872.623106
10,2,3876.331250
10,2,3194.510870
10,2,3720.823171
10,2,1798.531915
10,2,1519.533784
10,2,2091.575000
10,2,3153.912736
10,2,2801.421875
10,2,1948.057065
10,2,6143.445455
10,2,3123.962500
10,2,1533.558511
10,2,6373.262821
10,2,2331.594595
10,2,8655.002451
10,2,3122.773256
10,2,3015.727273
10,2,3843.312500
10,2,3652.310811
10,2,2990.333333
10,2,7141.782895
10,2,2386.255814
10,2,4831.708333
10,2,1592.208333
10,2,14942.948980
10,2,3638.531250
10,2,2203.769886
10,2,2170.968750
10,2,1679.812500
10,2,5067.762500
10,2,2341.243902
10,2,4495.279891
10,2,2041.477778
10,2,1554.531250
10,2,2240.457447
10,2,4334.643750
10,2,34537.589744
10,2,1592.664286
10,2,5799.957447
10,2,2624.628788
10,2,2675.741848
10,2,1735.020349
10,2,4226.934783
10,2,6721.371212
10,2,4270.955000
10,2,1915.212766
10,2,3368.431373
10,2,1828.622222
10,2,2843.579787

10,2,2447.422170
10,2,9370.882353
10,2,6958.240854
10,2,1947.189655
10,2,1711.197917
10,2,1617.378289
10,2,1555.757812
10,2,1713.000000
10,2,1853.182143
10,2,4361.666667
10,2,2503.550000
10,2,3192.715686
10,2,8131.288889
10,2,2390.382353
10,2,20106.811224
10,2,1589.104167
10,2,1525.447368
10,2,2127.026316
10,2,6960.697674
10,2,2705.305288
10,2,5042.396739
10,2,1693.494792
10,2,1610.411458
10,2,3659.687500
10,2,2750.740385
10,2,1575.551282
10,2,3553.964286
10,2,41914.375000
10,2,4241.548077
10,2,2526.808673
10,2,1791.836538
10,2,5422.684659
10,2,4795.513587
10,2,3088.625000
10,2,8960.432432
10,2,4399.625000
10,2,7556.733333
10,2,4906.103125
10,2,2041.666667
10,2,1640.769231
10,2,3065.760000
10,2,2549.156250
10,2,5261.214286
10,2,4260.938830
10,2,9096.014151
10,2,4748.000000
10,2,3028.135922
10,2,2615.100000
10,2,4797.755102
10,2,8991.425847
10,2,4749.583333
10,2,1655.685897
10,2,2094.697674
10,2,2268.956395
10,2,1976.980978
10,2,2006.512500
10,2,7059.229730
10,2,2798.789474
10,2,2625.021277
10,2,2511.214286
10,2,2661.895349
10,2,3535.208333
10,2,3858.232955
10,2,6203.886905
10,2,4244.800000
10,2,4062.428571

10,2,3377.340116
10,2,2110.788043
10,2,2147.441327
10,2,2875.384615
10,2,5755.816667
10,2,4333.012931
10,2,6902.677778
10,2,2188.121622
10,2,2053.461538
10,2,2054.169355
10,2,3863.041667
10,2,2182.404605
10,2,1719.846386
10,2,2470.888889
10,2,8771.769231
10,2,1817.565217
10,2,1603.750000
10,2,4504.754808
10,2,1768.621622
10,2,1693.353448
10,2,6800.377358
10,2,6170.076923
10,2,2303.965909
10,2,3335.052632
10,2,5106.625000
10,2,4816.463415
10,2,2324.527094
10,2,1531.416667
10,2,8136.072464
10,2,1545.746324
10,2,2572.804348
10,2,2266.000000
10,2,1641.425000
10,2,5501.851351
10,2,9966.351759
10,2,1639.216346
10,2,2112.935976
10,2,9521.384615
10,2,2643.792683
10,2,1676.100000
10,2,3957.109375
10,2,4643.406250
10,2,1914.968750
10,2,2299.180851
10,2,3194.257143
10,2,1736.974265
10,2,6175.280788
10,2,6416.153846
10,2,12895.930233
10,2,3372.503049
10,2,1528.926829
10,2,3093.567568
10,2,6927.695652
10,2,2781.341463
10,2,15341.509709
10,2,5912.868421
10,2,2031.987805
10,2,17970.232143
10,2,4223.723684
10,2,2453.736842
10,2,4930.340909
10,2,5772.947115
10,2,5907.625000
10,2,2258.244898
10,2,1706.230769
10,2,3005.275000

10,2,3123.520000
10,2,2905.482143
10,2,2188.931818
10,2,9371.036585
10,2,2860.037500
10,2,1887.343750
10,2,11380.388158
10,2,3747.410000
10,2,20253.232558
10,2,2709.090406
10,2,7494.913043
10,2,4861.963235
10,2,4004.152344
10,2,3323.333333
10,2,4595.405556
10,2,4173.166667
10,2,1659.928571
10,2,5885.433824
10,2,4734.278061
10,2,1522.028846
10,2,1545.973684
10,2,1541.026316
10,2,3487.772727
10,2,2697.493902
10,2,2378.072222
10,2,4102.151961
10,2,2909.016026
10,2,6913.065476
10,2,2076.309659
10,2,4063.325000
10,2,2807.066667
10,2,2825.026042
10,2,3511.430000
10,2,5886.960227
10,2,6663.105000
10,2,4248.551829
10,2,2905.450292
10,2,3690.962766
10,2,8998.495614
10,2,1892.411765
10,2,5025.031250
10,2,7341.505682
10,2,61611.535714
10,2,5298.875000
10,2,2417.600000
10,2,3399.677885
10,2,3440.529412
10,2,8688.063953
10,2,9644.901163
10,2,2589.331250
10,2,2983.872549
10,2,2362.228659
10,2,3770.500000
10,2,5124.175926
10,2,5753.639053
10,2,4200.230769
10,2,3403.619318
10,2,1534.394231
10,2,3960.015957
10,2,3211.672414
10,2,5342.793478
10,2,7209.096698
10,2,4229.544355
10,2,3781.508065
10,2,2918.381410
10,2,2998.804688

10,2,2259.000000
10,2,1533.627854
10,2,1710.889881
10,2,3885.323171
10,2,2002.520408
10,2,8038.781250
10,2,3254.604592
10,2,3269.002358
10,2,7755.631579
10,2,4332.242424
10,2,1753.875000
10,2,4509.122549
10,2,4802.534884
10,2,4541.187500
10,2,2681.489796
10,2,1826.000000
10,2,9671.709302
10,2,5713.703488
10,2,4935.307692
10,2,2506.825472
10,2,1607.850299
10,2,2637.663265
10,2,3688.112500
10,2,2380.484848
10,2,1588.875000
10,2,3245.325581
10,2,8916.055556
10,2,25037.387195
10,2,1612.646739
10,2,5462.717949
10,2,1856.747283
10,2,2027.176471
10,2,3401.218750
10,2,4675.713592
10,2,5161.782258
10,2,5879.325980
10,2,1797.191176
10,2,1578.283854
10,2,1896.280000
10,2,3981.732558
10,2,1996.470588
10,2,1582.561111
10,2,6154.803571
10,2,2700.207500
10,2,3142.206250
10,2,2493.461538
10,2,2198.702703
10,2,1592.717949
10,2,2545.297794
10,2,1919.428191
10,2,1785.244898
10,2,2572.921569
10,2,3777.352941
10,2,3640.055556
10,2,3809.097826
10,2,3194.603723
10,2,3647.394231
10,2,11177.458333
10,2,2882.859375
10,2,2075.320513
10,2,2472.050000
10,2,2666.611979
10,2,2463.510638
10,2,2977.015625
10,2,9868.238739
10,2,4263.453488

10,2,2154.745192
10,2,7437.945000
10,2,4611.255952
10,2,4777.440000
10,2,6815.153846
10,2,2166.925676
10,2,10942.064103
10,2,2699.156977
10,2,1962.552083
10,2,1609.514423
10,2,4033.666667
10,2,2397.328829
10,2,4559.423077
10,2,1592.587838
10,2,2281.274359
10,2,1673.174359
10,2,10151.217593
10,2,11699.744565
10,2,1892.409884
10,2,3014.769231
10,2,2558.846154
10,2,3749.736111
10,2,2174.346154
10,2,5945.770833
10,2,1685.953947
10,2,4429.045455
10,2,3614.920213
10,2,4333.798469
10,2,10499.931818
10,2,7130.622857
10,2,4632.695652
10,2,3269.303571
10,2,4529.458333
10,2,3242.940476
10,2,4402.083333
10,2,3642.164286
10,2,5564.062500
10,2,1577.201220
10,2,2331.250000
10,2,4586.117647
10,2,2035.000000
10,2,7352.107143
10,2,3767.201531
10,2,1710.567073
10,2,1538.134868
10,2,1978.060897
10,2,1729.275000
10,2,4300.625000
10,2,1611.857843
10,2,3214.365591
10,2,2164.963415
10,2,12563.912621
10,2,2713.795918
10,2,2969.637405
10,2,2169.472222
10,2,6088.011628
10,2,2970.877660
10,2,10340.333333
10,2,2628.871795
10,2,2248.802083
10,2,4443.150000
10,2,2729.463415
10,2,4231.156250
10,2,2059.428571
10,2,2005.423077
10,2,8831.655303

10,2,2519.921875
10,2,1566.284884
10,2,5200.689655
10,2,4534.026163
10,2,1783.755556
10,2,1602.200000
10,2,1719.529070
10,2,2215.854167
10,2,4648.307692
10,2,8087.617021
10,2,1721.774038
10,2,1522.981707
10,2,1785.646875
10,2,4684.165625
10,2,2295.647059
10,2,3071.006579
10,2,5894.572727
10,2,2124.530612
10,2,7522.637500
10,2,2994.940625
10,2,16077.407738
10,2,2338.543750
10,2,5615.244186
10,2,1647.125000
10,2,2959.000000
10,2,1560.783333
10,2,2620.040541
10,2,3596.410377
10,2,8271.996154
10,2,3630.272727
10,2,4172.502463
10,2,2933.979651
10,2,6798.375000
10,2,1988.755814
10,2,4432.809045
10,2,1528.260417
10,2,2225.776316
10,2,13059.709459
10,2,12610.652174
10,2,1909.197917
10,2,1672.636364
10,2,1790.215116
10,2,3529.516949
10,2,2914.000000
10,2,6828.222561
10,2,2070.964286
10,2,8098.530612
10,2,1822.715278
10,2,2069.537162
10,2,2619.650000
10,2,2280.375000
10,2,4158.606383
10,2,2155.000000
10,2,4898.014706
10,2,3071.000000
10,2,7676.304688
10,2,2303.908805
10,2,4751.254902
10,2,9998.212500
10,2,3362.053571
10,2,1724.232143
10,2,1832.625000
10,2,1984.571429
10,2,5220.735294
10,2,4429.091837
10,2,5169.185185

10,2,2752.449438
10,2,4485.782738
10,2,3920.026596
10,2,2039.068182
10,2,7099.263889
10,2,12330.914062
10,2,1514.258721
10,2,5730.288934
10,2,2145.586957
10,2,2261.806452
10,2,2493.884831
10,2,3023.150376
10,2,2975.845109
10,2,2100.683824
10,2,2864.729167
10,2,1871.317073
10,2,2311.289216
10,2,2547.738889
10,2,7143.265306
10,2,1566.411017
10,2,2692.500000
10,2,1984.820122
10,2,2864.770833
10,2,1965.719512
10,2,2750.518750
10,2,1717.600000
10,2,4752.906863
10,2,1750.395349
10,2,1655.613636
10,2,2420.777027
10,2,4228.549180
10,2,2849.064904
10,2,4344.364286
10,2,3574.275000
10,2,3868.900000
10,2,3447.773810
10,2,1546.664894
10,2,2350.686111
10,2,8473.026442
10,2,1527.609756
10,2,2645.841530
10,2,1892.200000
10,2,2616.412234
10,2,2061.154605
10,2,4316.475694
10,2,1606.358491
10,2,1568.375000
10,2,1611.151163
10,2,6179.450000
10,2,3330.346591
10,2,2091.271739
10,2,9027.926630
10,2,1597.067568
10,2,4606.090909
10,2,2871.759615
10,2,2944.641447
10,2,2224.487805
10,2,3813.803125
10,2,3100.804348
10,2,23346.000000
10,2,5376.000000
10,2,5009.788043
10,2,8916.277778
10,2,2796.566327
10,2,1965.987805
10,2,1737.909091

10,2,2436.360795
10,2,2228.170886
10,2,1734.930000
10,2,2801.957547
10,2,3220.008523
10,2,2301.345000
10,2,2016.806452
10,2,3806.757282
10,2,2107.536932
10,2,1842.629630
10,2,5115.656566
10,2,1798.489583
10,2,1741.711538
10,2,3363.351064
10,2,4331.309375
10,2,5812.250000
10,2,5827.130682
10,2,1603.824468
10,2,1724.415000
10,2,1777.005682
10,2,4762.048469
10,2,1652.300000
10,2,6641.163265
10,2,3269.191489
10,2,3442.254717
10,2,4699.447917
10,2,3539.115000
10,2,3518.075472
10,2,2368.840426
10,2,12901.406250
10,2,2471.730263
10,2,2662.750000
10,2,2988.348958
10,2,2928.225000
10,2,1652.490909
10,2,4627.020270
10,2,2179.005988
10,2,2454.502404
10,2,1816.875000
10,2,1916.915094
10,2,4445.533333
10,2,2719.425532
10,2,2599.393519
10,2,1661.444853
10,2,6392.649123
10,2,2181.429032
10,2,1738.519886
10,2,6029.296512
10,2,4272.439024
10,2,4172.500000
10,2,2243.643939
10,2,2781.743421
10,2,1516.512500
10,2,3221.635417
10,2,16296.097826
10,2,9046.278409
10,2,1990.375000
10,2,3018.485915
10,2,2171.090000
10,2,13101.103774
10,2,3987.958333
10,2,2127.000000
10,2,2335.050676
10,2,4203.843023
10,2,2314.679688
10,2,1711.162879

10,2,2751.701754
10,2,2322.952830
10,2,1504.072917
10,2,5169.482759
10,2,9256.367188
10,2,10123.862500
10,2,5531.181818
10,2,3082.150235
10,2,7918.628205
10,2,1644.763889
10,2,4218.200000
10,2,3668.285714
10,2,2412.322034
10,2,3053.789474
10,2,2352.185000
10,2,3215.622340
10,2,2596.321429
10,2,9601.330645
10,2,2197.500000
10,2,1579.364706
10,2,5180.602941
10,2,2437.076923
10,2,2140.132979
10,2,2280.035256
10,2,13421.800000
10,2,1508.910714
10,2,2848.375000
10,2,3590.635417
10,2,22164.116071
10,2,4183.779070
10,2,2728.172619
10,2,1722.000000
10,2,2998.802326
10,2,5164.486842
10,2,8190.926829
10,2,2830.667683
10,2,8241.478261
10,2,2244.375000
10,2,3505.516892
10,2,1907.071429
10,2,2404.722222
10,2,3008.435000
10,2,10035.900641
10,2,4353.705882
10,2,8034.565476
10,2,2209.545213
10,2,3477.017361
10,2,2641.718750
10,2,5243.965909
10,2,6492.468750
10,2,3461.515625
10,2,10838.580838
10,2,2292.396277
10,2,2980.872449
10,2,1948.391892
10,2,4146.750000
10,2,2728.540865
10,2,2815.742857
10,2,1935.458333
10,2,4061.172222
10,2,1970.045455
10,2,2210.835227
10,2,2852.441327
10,2,3757.656250
10,2,7017.411765
10,2,2274.458333

10,2,1737.485294
10,2,1733.494318
10,2,2086.000000
10,2,3189.083333
10,2,2659.621212
10,2,1624.861111
10,2,2164.739583
10,2,2004.292763
10,2,3194.066860
10,2,1616.387755
10,2,3898.073171
10,2,8887.210526
10,2,1924.632353
10,2,2061.559211
10,2,8108.004717
10,2,2743.213415
10,2,2325.884615
10,2,5861.159574
10,2,2084.437500
10,2,2270.323529
10,2,1869.497093
10,2,1880.700000
10,2,10682.114362
10,2,2739.900000
10,2,2820.500000
10,2,2711.466960
10,2,2100.660000
10,2,3138.510870
10,2,6638.398773
10,2,2103.993590
10,2,3499.867647
10,2,6036.041667
10,2,2192.640000
10,2,7758.432432
10,2,19659.016026
10,2,2780.553571
10,2,3504.745455
10,2,4768.128571
10,2,8312.290816
10,2,3625.585938
10,2,2683.862745
10,2,18490.326923
10,2,4928.147783
10,2,3461.110294
10,2,8101.867647
10,2,2337.034884
10,2,8310.179348
10,2,2441.587500
10,2,2287.135638
10,2,10278.827128
10,2,5019.324324
10,2,2820.021341
10,2,19335.437500
10,2,3747.854167
10,2,3119.137019
10,2,8814.666667
10,2,2635.125000
10,2,5291.931818
10,2,1567.217949
10,2,2409.923295
10,2,1631.883152
10,2,3814.325000
10,2,1862.355263
10,2,2638.537736
10,2,1732.955556
10,2,3994.479651

10,2,2626.382979
10,2,2567.291667
10,2,1939.250000
10,2,6534.744318
10,2,2806.035326
10,2,1830.250000
10,2,9568.281250
10,2,2671.666667
10,2,2563.478261
10,2,2083.596154
10,2,1811.300000
10,2,1852.028125
10,2,3655.750000
10,2,1820.892045
10,2,2806.959302
10,2,2907.481707
10,2,1918.941176
10,2,2221.866071
10,2,2753.552083
10,2,1871.421875
10,2,1753.805556
10,2,2143.440476
10,2,3339.854167
10,2,9436.187500
10,2,5129.720930
10,2,5564.440000
10,2,13733.208333
10,2,4244.567073
10,2,1587.306548
10,2,2821.118056
10,2,2070.580645
10,2,3379.873239
10,2,4135.863208
10,2,3292.639535
10,2,2878.553571
10,2,1981.385000
10,2,5279.936321
10,2,1904.012195
10,2,2177.100000
10,2,2314.056818
10,2,3753.539855
10,2,2152.003049
10,2,1592.371711
10,2,4359.790576
10,2,1600.308824
10,2,2293.320755
10,2,2429.812500
10,2,2918.279070
10,2,2870.531915
10,2,4903.592262
10,2,3877.432692
10,2,12065.583333
10,2,2392.108108
10,2,6093.570513
10,2,14500.579268
10,2,4208.842105
10,2,2557.591837
10,2,3459.578378
10,2,2180.250000
10,2,1625.085366
10,2,2036.755102
10,2,2548.575000
10,2,2750.826087
10,2,4761.989130
10,2,8018.355978
10,2,4778.997500

10,2,42143.428571
10,2,4509.015957
10,2,8192.785326
10,2,1880.411111
10,2,1671.518519
10,2,16205.346429
10,2,7491.425595
10,2,2631.842391
10,2,4570.461538
10,2,3945.238636
10,2,8845.765625
10,2,10132.259615
10,2,4983.932432
10,2,4511.642045
10,2,1800.073171
10,2,5480.785714
10,2,1792.562500
10,2,6069.344444
10,2,2459.587500
10,2,1803.215426
10,2,3715.578125
10,2,1791.423841
10,2,2257.010204
10,2,6138.382500
10,2,2593.459239
10,2,1562.259777
10,2,3711.023438
10,2,3900.302326
10,2,2045.252551
10,2,2099.549342
10,2,6174.629630
10,2,4635.971698
10,2,6379.011905
10,2,4985.346591
10,2,3849.717949
10,2,2179.557292
10,2,4447.128205
10,2,2536.000000
10,2,5636.509434
10,2,2462.418478
10,2,2218.289216
10,2,2805.062500
10,2,2343.276074
10,2,19504.000000
10,2,2986.341146
10,2,7544.216667
10,2,3207.183333
10,2,2416.010204
10,2,1745.423913
10,2,4511.839286
10,2,2722.158784
10,2,4752.971698
10,2,2557.102273
10,2,2450.453431
10,2,6525.250000
10,2,2008.704082
10,2,10261.873016
10,2,2583.921875
10,2,6728.016447
10,2,3244.563889
10,2,6189.400735
10,2,4647.229167
10,2,2062.469512
10,2,6308.473958
10,2,2099.300000
10,2,1841.985119

10,2,3388.267045
10,2,4016.770270
10,2,5291.532895
10,2,3269.026316
10,2,4471.375000
10,2,2051.594595
10,2,2985.185897
10,2,4259.133333
10,2,3931.454545
10,2,14374.550000
10,2,3315.440789
10,2,2936.912037
10,2,7807.644737
10,2,1783.960366
10,2,2996.432515
10,2,3230.168919
10,2,4788.095000
10,2,2417.823529
10,2,25004.125000
10,2,1709.276596
10,2,4290.654891
10,2,3020.627778
10,2,4339.907051
10,2,2278.415625
10,2,2763.836735
10,2,2641.217391
10,2,1969.847826
10,2,2710.500000
10,2,3041.727273
10,2,9050.347222
10,2,2232.031915
10,2,3045.187500
10,2,3385.797170
10,2,2279.039062
10,2,7505.076923
10,2,3011.932065
10,2,13976.633621
10,2,4413.358333
10,2,3161.904762
10,2,3600.131944
10,2,5926.856250
10,2,2806.487685
10,2,7136.323529
10,2,1915.113636
10,2,2738.171875
10,2,2304.153846
10,2,6091.688776
10,2,2044.058511
10,2,10658.171371
10,2,2817.833333
10,2,4744.981707
10,2,3819.987179
10,2,1859.570946
10,2,2773.653061
10,2,3256.907143
10,2,6986.558140
10,2,2990.182927
10,2,3295.050000
10,2,18869.633621
10,2,5767.068396
10,2,7064.437500
10,2,7803.384615
10,2,1738.343750
10,2,2761.000000
10,2,2650.071875
10,2,2371.481818

10,2,3127.987805
10,2,4037.428571
10,2,4336.562500
10,2,2552.816667
10,2,10654.860000
10,2,1580.294118
10,2,2369.406250
10,2,1660.625000
10,2,1809.425532
10,2,2384.363636
10,2,3433.725000
10,2,1579.198718
10,2,13471.500000
10,2,2386.372340
10,2,11834.400735
10,2,1819.545455
10,2,2105.528846
10,2,1899.572500
10,2,2691.603261
10,2,7670.063830
10,2,5864.750000
10,2,2724.978659
10,2,7040.458333
10,2,29925.645833
10,2,2049.602564
10,2,2799.589674
10,2,4035.695652
10,2,1714.300000
10,2,2268.142157
10,2,2047.236111
10,2,4689.292683
10,2,3662.308511
10,2,1812.332386
10,2,4335.522059
10,2,3130.971698
10,2,1528.005814
10,2,5334.346154
10,2,1644.454545
10,2,1717.032258
10,2,1801.024194
10,2,5717.445652
10,2,2939.361111
10,2,3362.846154
10,2,1930.878049
10,2,1525.263889
10,2,2105.929907
10,2,2909.866279
10,2,2869.510870
10,2,1733.459459
10,2,3022.981595
10,2,2323.502857
10,2,2884.050000
10,2,4668.400000
10,2,7491.385321
10,2,2107.250000
10,2,1795.036932
10,2,3815.245000
10,2,1689.354167
10,2,1612.750000
10,2,1695.889205
10,2,1589.595238
10,2,4817.429487
10,2,8777.802083
10,2,10855.948113
10,2,3747.887500
10,2,2140.545455

10,2,7504.000000
10,2,11146.586538
10,2,4981.267442
10,2,5043.125000
10,2,4560.402174
10,2,2038.117021
10,2,8686.158784
10,2,2108.343750
10,2,3405.673797
10,2,4812.452703
10,2,3760.005435
10,2,1734.420765
10,2,7446.752717
10,2,3626.773585
10,2,3494.826220
10,2,6194.851064
10,2,1731.390244
10,2,6125.565789
10,2,2061.040625
10,2,1846.138158
10,2,2164.200000
10,2,3822.083333
10,2,10225.875000
10,2,4991.411058
10,2,2159.006098
10,2,4769.080000
10,2,8810.206731
10,2,3689.777778
10,2,5010.878049
10,2,4759.021739
10,2,3995.057143
10,2,2501.906250
10,2,4375.767442
10,2,1562.604651
10,2,1596.702128
10,2,5908.781250
10,2,1545.011236
10,2,1512.725000
10,2,1650.755682
10,2,8732.927885
10,2,6327.621212
10,2,5049.383929
10,2,1927.867347
10,2,2865.476974
10,2,3635.537037
10,2,8791.482500
10,2,1989.312500
10,2,3682.052632
10,2,1523.383523
10,2,3331.812500
10,2,4363.957317
10,2,12825.741935
10,2,4430.607143
10,2,2874.942708
10,2,2439.297872
10,2,5452.360887
10,2,5193.227273
10,2,2063.044586
10,2,2953.439024
10,2,3324.905325
10,2,1736.831081
10,2,2782.447674
10,2,3953.857143
10,2,3066.857143
10,2,17700.622596
10,2,2924.321429

10,2,3119.071429
10,2,8512.932692
10,2,3883.142857
10,2,7105.315789
10,2,2864.051163
10,2,6156.188889
10,2,2208.714286
10,2,1644.362069
10,2,5353.338415
10,2,1841.000000
10,2,2321.593750
10,2,2201.440000
10,2,3140.971429
10,2,6499.604651
10,2,1814.255319
10,2,1717.533654
10,2,1871.230769
10,2,4395.978947
10,2,4577.534653
10,2,6336.179688
10,2,1584.090278
10,2,6903.480978
10,2,1624.188679
10,2,3154.976351
10,2,4445.452128
10,2,1812.242424
10,2,6581.715426
10,2,1982.672872
10,2,4153.621795
10,2,3415.664804
10,2,2044.977528
10,2,62352.287037
10,2,2368.380952
10,2,4551.133721
10,2,8676.918750
10,2,2059.869565
10,2,3296.372340
10,2,4026.945652
10,2,1641.436170
10,2,3599.031250
10,2,1636.366477
10,2,1528.093220
10,2,3440.995763
10,2,1737.187500
10,2,14500.085106
10,2,1794.043750
10,2,1865.470588
10,2,3541.715909
10,2,6300.346939
10,2,2542.656250
10,2,1533.625000
10,2,2278.569079
10,2,2752.728125
10,2,3614.204678
10,2,3975.250000
10,2,11025.335366
10,2,2195.910000
10,2,6926.109290
10,2,2081.951872
10,2,2391.591837
10,2,1807.578125
10,2,4381.244048
10,2,2885.710938
10,2,3315.382979
10,2,7282.601744
10,2,4285.277778

10,2,5084.870536
10,2,3143.688571
10,2,5836.240909
10,2,1832.000000
10,2,1900.916667
10,2,3524.571809
10,2,35995.406250
10,2,1708.575758
10,2,8472.895833
10,2,1607.250000
10,2,2350.078125
10,2,7613.791667
10,2,1608.915584
10,2,4827.592593
10,2,7982.120690
10,2,6831.269461
10,2,2005.718085
10,2,1891.305556
10,2,2601.790698
10,2,3476.904891
10,2,28805.555921
10,2,10370.033784
10,2,4442.676471
10,2,1752.718085
10,2,3395.907407
10,2,1892.933333
10,2,6270.167614
10,2,4405.358491
10,2,2514.184659
10,2,1941.991477
10,2,2897.795455
10,2,6249.711538
10,2,2433.193548
10,2,6679.566176
10,2,4631.605263
10,2,7962.857143
10,2,2585.000000
10,2,1619.400000
10,2,7916.670732
10,2,2984.970455
10,2,2148.425595
10,2,4188.583333
10,2,7691.000000
10,2,4189.088889
10,2,5414.948113
10,2,2570.214552
10,2,8415.974265
10,2,2928.860849
10,2,12949.839286
10,2,2227.553571
10,2,1954.437500
10,2,2949.556818
10,2,1967.700000
10,2,2439.804054
10,2,4964.170455
10,2,1996.664474
10,2,3947.968750
10,2,1828.208333
10,2,6325.100000
10,2,5281.402778
10,2,3805.174020
10,2,7250.645833
10,2,3945.261628
10,2,5981.227477
10,2,4706.136364
10,2,2002.990909

10,2,7078.110256
10,2,1610.834532
10,2,2570.545226
10,2,2619.942029
10,2,2447.887195
10,2,6752.457447
10,2,1748.150000
10,2,6681.038889
10,2,2693.215909
10,2,2057.643229
10,2,1917.067568
10,2,9434.083333
10,2,4766.663636
10,2,4063.006098
10,2,2503.401042
10,2,1800.592949
10,2,2488.880829
10,2,3688.470779
10,2,2256.775641
10,2,10785.369792
10,2,1990.562500
10,2,7350.850877
10,2,2063.517045
10,2,2086.840000
10,2,28502.435714
10,2,1718.875000
10,2,5555.000000
10,2,1595.095238
10,2,5289.727273
10,2,2135.148649
10,2,3621.700000
10,2,2244.480263
10,2,4478.203125
10,2,3600.210938
10,2,3446.094595
10,2,6584.672500
10,2,2952.233333
10,2,2158.673295
10,2,18568.000000
10,2,5283.467391
10,2,3076.671429
10,2,3189.861111
10,2,1650.994186
10,2,4521.070175
10,2,2283.500000
10,2,5022.729167
10,2,3303.568182
10,2,2497.614362
10,2,3053.157895
10,2,1868.033784
10,2,2523.029070
10,2,2584.965278
10,2,5857.827660
10,2,5120.954106
10,2,3304.110256
10,2,2881.115385
10,2,1608.000000
10,2,3208.845588
10,2,1591.031250
10,2,2396.270270
10,2,2149.050000
10,2,3869.480769
10,2,8165.375000
10,2,10735.262755
10,2,2496.146341
10,2,5062.171875

10,2,1576.156863
10,2,6735.223684
10,2,3345.549020
10,2,4334.637755
10,2,2305.823171
10,2,7723.328704
10,2,10329.938547
10,2,2703.902778
10,2,2317.469697
10,2,9572.039683
10,2,1532.326705
10,2,17902.500000
10,2,6482.738636
10,2,2521.034483
10,2,2815.756250
10,2,1976.364583
10,2,1548.492857
10,2,6326.871795
10,2,4905.696429
10,2,4606.619681
10,2,3120.891304
10,2,1520.031250
10,2,8623.970109
10,2,2210.676829
10,2,2885.250000
10,2,1951.715000
10,2,5670.268382
10,2,12912.000000
10,2,11549.905738
10,2,3011.000000
10,2,4407.524390
10,2,1505.423913
10,2,2458.335227
10,2,17525.170213
10,2,3382.781250
10,2,2399.316176
10,2,1532.114583
10,2,1677.465000
10,2,2143.838235
10,2,1966.836735
10,2,4773.828125
10,2,2146.980198
10,2,4156.206161
10,2,4884.214286
10,2,1934.648810
10,2,5221.110000
10,2,7980.627049
10,2,4106.967500
10,2,4642.948864
10,2,3788.510638
10,2,4673.752809
10,2,2250.968023
10,2,2792.628049
10,2,9416.593085
10,2,4388.000000
10,2,4487.302632
10,2,1939.100000
10,2,6170.211921
10,2,8036.658654
10,2,3601.702381
10,2,1986.819079
10,2,2140.176282
10,2,2083.432432
10,2,4750.867021
10,2,3126.855263
10,2,1633.743750

10,2,9891.932292
10,2,13603.819853
10,2,12936.490566
10,2,5748.162234
10,2,2111.257576
10,2,2502.000000
10,2,2876.462766
10,2,2566.790816
10,2,7848.100000
10,2,1728.404412
10,2,3555.500000
10,2,2808.883978
10,2,7459.339286
10,2,2334.783333
10,2,5157.880952
10,2,1987.467949
10,2,2345.279255
10,2,4067.725962
10,2,2256.062500
10,2,2341.684211
10,2,1864.967391
10,2,1774.142857
10,2,3969.037500
10,2,6511.632653
10,2,1683.090278
10,2,3191.717391
10,2,2111.098837
10,2,1814.068063
10,2,3802.840426
10,2,3539.128378
10,2,5966.417500
10,2,1885.045455
10,2,2047.921053
10,2,2043.000000
10,2,1879.562500
10,2,2568.832143
10,2,3744.622222
10,2,4070.136792
10,2,3134.926471
10,2,2120.789474
10,2,3583.028736
10,2,1771.328571
10,2,1742.155405
10,2,1676.500000
10,2,4839.494318
10,2,4290.350000
10,2,3704.236111
10,2,3400.102679
10,2,3460.375000
10,2,3980.513514
10,2,3256.500000
10,2,1802.046875
10,2,4315.410714
10,2,7490.325581
10,2,2297.635638
10,2,1803.384615
10,2,2757.421875
10,2,32059.629310
10,2,1792.684659
10,2,6651.258333
10,2,1961.806122
10,2,4845.273438
10,2,4361.163043
10,2,2006.348404
10,2,21737.052632
10,2,3622.219512

10,2,6578.850000
10,2,4513.574324
10,2,2494.304054
10,2,1499.437173
10,2,1680.019126
10,2,3796.091346
10,2,5064.290625
10,2,5200.300000
10,2,5030.829787
10,2,2107.380952
10,2,1530.173077
10,2,1874.687500
10,2,14229.855769
10,2,12364.422872
10,2,1598.315625
10,2,6399.477273
10,2,11315.049107
10,2,2764.195652
10,2,1573.000000
10,2,1710.845745
10,2,2164.803571
10,2,2114.080000
10,2,1769.386029
10,2,3041.071429
10,2,2093.022727
10,2,5208.044492
10,2,3349.738095
10,2,3708.141509
10,2,4673.750000
10,2,2083.464286
10,2,2439.500000
10,2,8493.323171
10,2,3133.753676
10,2,4254.312500
10,2,2314.486842
10,2,1684.606383
10,2,3411.381579
10,2,1641.852941
10,2,2280.227273
10,2,2471.600000
10,2,3464.906977
10,2,2551.163462
10,2,2236.250000
10,2,1878.133333
10,2,4882.269608
10,2,7040.392857
10,2,1738.414894
10,2,2596.776786
10,2,5717.250000
10,2,3143.375000
10,2,2918.735577
10,2,2296.956522
10,2,4054.470588
10,2,5350.000000
10,2,4034.295732
10,2,2899.407407
10,2,5817.535714
10,2,1815.041667
10,2,3631.808824
10,2,3013.032258
10,2,2547.672414
10,2,1939.770833
10,2,2209.148438
10,2,6349.605469
10,2,6197.000000
10,2,1500.205128

10,2,2360.697674
10,2,2426.236842
10,2,2285.523810
10,2,10709.367647
10,2,1830.951220
10,2,3161.243590
10,2,2475.754808
10,2,14601.140625
10,2,3014.841146
10,2,2463.921875
10,2,2354.577703
10,2,1734.863636
10,2,2612.271429
10,2,3495.669355
10,2,6180.000000
10,2,8812.605978
10,2,1616.285714
10,2,3454.074468
10,2,5226.366848
10,2,4829.762500
10,2,1549.828125
10,2,2436.475962
10,2,3014.240132
10,2,2487.897436
10,2,2065.816667
10,2,1745.666667
10,2,2780.608696
10,2,1669.325581
10,2,4567.464286
10,2,6364.187500
10,2,4084.089286
10,2,6288.666667
10,2,1534.431818
10,2,5815.590909
10,2,2976.250000
10,2,2360.977273
10,2,2114.764151
10,2,4747.515000
10,2,1583.827778
10,2,8993.233696
10,2,3176.416667
10,2,2636.229730
10,2,2317.722222
10,2,2230.959184
10,2,1867.040000
10,2,3164.971875
10,2,1955.796407
10,2,2214.105263
10,2,2360.299479
10,2,3076.052356
10,2,1675.464286
10,2,4094.806122
10,2,2718.885204
10,2,3056.718750
10,2,6508.074468
10,2,3275.547170
10,2,11575.614407
10,2,1901.200000
10,2,3177.985795
10,2,2657.402439
10,2,2420.571429
10,2,1565.568807
10,2,2696.469072
10,2,1983.750000
10,2,4150.631250
10,2,20760.979167

10,2,1804.534091
10,2,14225.744898
10,2,3386.900000
10,2,1625.500000
10,2,2668.117021
10,2,4449.325581
10,2,3132.743243
10,2,5940.056818
10,2,3667.644737
10,2,2520.178571
10,2,2231.262295
10,2,5900.277439
10,2,3732.881944
10,2,1974.566327
10,2,6382.440000
10,2,3310.335196
10,2,9893.036313
10,2,2507.731818
10,2,22682.659314
10,2,1559.700000
10,2,4525.266667
10,2,2757.880000
10,2,3688.618590
10,2,2661.593750
10,2,4547.182692
10,2,1881.606771
10,2,4359.536765
10,2,5967.500000
10,2,1606.571429
10,2,3890.130435
10,2,2467.472222
10,2,2883.187500
10,2,1972.807870
10,2,1588.559524
10,2,2535.750000
10,2,2577.218750
10,2,5695.030000
10,2,1653.708333
10,2,5252.064815
10,2,2883.193182
10,2,2638.125000
10,2,5986.825980
10,2,1516.836957
10,2,6605.238095
10,2,1627.614865
10,2,5742.950581
10,2,6045.281250
10,2,3104.662500
10,2,9248.767442
10,2,6159.000000
10,2,1921.666667
10,2,6391.608108
10,2,7058.187500
10,2,4590.142857
10,2,1774.192500
10,2,4345.169811
10,2,3398.631579
10,2,2664.321429
10,2,2694.988372
10,2,9202.750000
10,2,3299.198529
10,2,2063.717391
10,2,3573.300000
10,2,6730.849057
10,2,4600.044444
10,2,1641.326705

10,2,1820.252976
10,2,1549.926829
10,2,5215.480392
10,2,3730.612069
10,2,4078.184211
10,2,3808.019608
10,2,3436.432927
10,2,6298.475000
10,2,4104.379310
10,2,4195.635417
10,2,3172.213542
10,2,4801.441860
10,2,3740.190341
10,2,15061.614583
10,2,2443.243243
10,2,4428.365741
10,2,3459.296875
10,2,6610.392857
10,2,2461.862500
10,2,3232.360465
10,2,1770.843023
10,2,2282.489362
10,2,3889.977778
10,2,1806.311224
10,2,2111.648936
10,2,4872.871795
10,2,5207.756944
10,2,1629.144578
10,2,1558.622340
10,2,2461.634146
10,2,1880.153226
10,2,3158.809524
10,2,2723.947115
10,2,25808.050000
10,2,1929.931818
10,2,3508.652632
10,2,11526.801887
10,2,5614.394737
10,2,2433.277778
10,2,4305.982143
10,2,3656.311170
10,2,1851.467949
10,2,2407.552083
10,2,1669.875000
10,2,2876.950000
10,2,1815.500000
10,2,1985.576923
10,2,3658.094340
10,2,2167.650568
10,2,7529.701422
10,2,4398.260417
10,2,2575.383721
10,2,2125.791667
10,2,2050.039773
10,2,1694.825581
10,2,3266.627451
10,2,1956.141304
10,2,3239.437500
10,2,3679.671053
10,2,3482.651786
10,2,2871.119565
10,2,4869.648810
10,2,1976.930380
10,2,12381.916667
10,2,1947.191176
10,2,2547.862500

10,2,6169.277778
10,2,1810.759868
10,2,1521.027344
10,2,4759.731250
10,2,1779.090909
10,2,2254.034483
10,2,2725.158537
10,2,2054.591146
10,2,4393.611979
10,2,3324.111111
10,2,7225.736842
10,2,14313.669118
10,2,2953.331731
10,2,4248.678571
10,2,2954.202614
10,2,3470.294643
10,2,1866.400000
10,2,1939.043478
10,2,2692.146429
10,2,4842.613636
10,2,1965.323529
10,2,13350.308057
10,2,4323.216080
10,2,3225.681373
10,2,2814.200000
10,2,1707.592105
10,2,1661.125000
10,2,5078.710526
10,2,1632.041667
10,2,3140.593750
10,2,5543.154545
10,2,5625.945122
10,2,2441.906863
10,2,2488.045455
10,2,1744.780488
10,2,2310.822500
10,2,2316.321875
10,2,7916.875000
10,2,4241.635135
10,2,5770.701220
10,2,1537.675595
10,2,2111.460227
10,2,3938.875000
10,2,3077.434783
10,2,1673.552632
10,2,1642.866667
10,2,23416.187500
10,2,3184.636364
10,2,1810.301887
10,2,1522.469697
10,2,5171.715426
10,2,2313.238636
10,2,2278.519886
10,2,3497.633929
10,2,3474.787736
10,2,7272.908537
10,2,4452.450000
10,2,5799.979651
10,2,2067.471591
10,2,2534.695652
10,2,5840.682927
10,2,4399.000000
10,2,4569.987805
10,2,1589.600000
10,2,7075.240000
10,2,2998.237013

10,2,6663.488095
10,2,1986.775510
10,2,3126.684783
10,2,1758.298077
10,2,4587.406250
10,2,3721.761905
10,2,21766.522059
10,2,7655.609649
10,2,2433.321429
10,2,37136.486111
10,2,4488.943966
10,2,4498.797727
10,2,1724.704268
10,2,1822.707317
10,2,12661.739796
10,2,3515.111702
10,2,4423.518293
10,2,1592.547297
10,2,3505.061224
10,2,2800.200000
10,2,1507.709239
10,2,1955.547222
10,2,3969.263298
10,2,2851.730263
10,2,4116.702128
10,2,2898.305851
10,2,3000.005435
10,2,1742.628205
10,2,2735.000000
10,2,1520.604651
10,2,4942.136364
10,2,1592.939103
10,2,3584.212500
10,2,2245.781250
10,2,3733.134146
10,2,1916.285714
10,2,5937.086735
10,2,1523.533537
10,2,8123.956522
10,2,3670.053191
10,2,2708.597826
10,2,3192.255556
10,2,2838.434783
10,2,1750.072368
10,2,7421.073661
10,2,3534.284091
10,2,5918.321429
10,2,5041.138298
10,2,13594.428571
10,2,4134.581395
10,2,3014.861413
10,2,5273.854167
10,2,6552.239583
10,2,3049.906250
10,2,7028.185000
10,2,15616.076531
10,2,5270.658163
10,2,3281.000000
10,2,7622.255869
10,2,1549.071429
10,2,2519.826087
10,2,2264.446429
10,2,3159.493056
10,2,2199.467262
10,2,1702.277027
10,2,6845.869198

10,2,2501.928571
10,2,3492.539474
10,2,2974.658537
10,2,11488.437500
10,2,2141.263514
10,2,5714.225000
10,2,2831.619681
10,2,2228.038462
10,2,2022.267361
10,2,4492.068452
10,2,2740.601190
10,2,4728.230392
10,2,6205.098039
10,2,2740.293478
10,2,2738.781977
10,2,13512.533482
10,2,5156.829268
10,2,3744.906250
10,2,1702.600000
10,2,2154.185096
10,2,19866.539216
10,2,5521.173913
10,2,3333.422170
10,2,6449.444444
10,2,2494.881579
10,2,2585.913636
10,2,2130.757212
10,2,3157.328704
10,2,1655.096154
10,2,8839.520000
10,2,9343.071023
10,2,2274.208092
10,2,3944.406915
10,2,2165.144737
10,2,1535.616071
10,2,1791.621875
10,2,2260.679487
10,2,1757.324324
10,2,1707.000000
10,2,2670.326531
10,2,7391.891892
10,2,4265.938272
10,2,1576.014286
10,2,2681.719101
10,2,3762.680000
10,2,4095.600000
10,2,3348.366071
10,2,1836.833333
10,2,4642.671875
10,2,1884.162234
10,2,1551.519608
10,2,2455.393617
10,2,2790.444444
10,2,3217.574468
10,2,28056.705729
10,2,6791.053571
10,2,2114.957447
10,2,3938.056180
10,2,1940.022727
10,2,3670.918605
10,2,3472.924419
10,2,4784.638889
10,2,5661.695000
10,2,1963.153846
10,2,1500.135870
10,2,2812.450000

10,2,5972.914634
10,2,4408.346875
10,2,1616.156250
10,2,2615.914773
10,2,2318.664474
10,2,1516.104651
10,2,2793.428571
10,2,10352.801471
10,2,1531.969388
10,2,3831.008721
10,2,2157.079787
10,2,2498.733333
10,2,2055.642857
10,2,2059.656250
10,2,11236.576087
10,2,2197.360759
10,2,3562.800000
10,2,1560.215686
10,2,2438.199074
10,2,1877.255319
10,2,4921.111364
10,2,3977.890625
10,2,3556.695000
10,2,1555.212500
10,2,4762.796196
10,2,5576.298165
10,2,3635.283019
10,2,3202.500000
10,2,2252.847826
10,2,1877.706081
10,2,4358.390625
10,2,3907.604167
10,2,3730.211538
10,2,3353.053571
10,2,2569.552326
10,2,1544.107500
10,2,1905.528302
10,2,3875.296875
10,2,6498.800532
10,2,8359.010638
10,2,1773.402500
10,2,3895.833333
10,2,1805.508065
10,2,1738.979911
10,2,4478.785714
10,2,4256.668478
10,2,2143.584375
10,2,7485.641026
10,2,3437.962766
10,2,2909.791667
10,2,9158.830882
10,2,2215.023256
10,2,3042.157895
10,2,2704.622995
10,2,4591.481283
10,2,8452.705357
10,2,2283.666667
10,2,6183.500000
10,2,4257.843023
10,2,3287.980263
10,2,1519.437500
10,2,2039.265625
10,2,1762.002778
10,2,1761.370000
10,2,3066.375000
10,2,4298.548611

10,2,2045.934211
10,2,1502.400000
10,2,28359.399441
10,2,3600.678191
10,2,1940.521552
10,2,2822.271186
10,2,4298.474432
10,2,3649.125000
10,2,2463.625000
10,2,4833.391304
10,2,4239.977564
10,2,3313.773585
10,2,3591.175000
10,2,2780.823864
10,2,1798.352941
10,2,2328.655556
10,2,2692.917683
10,2,3246.509615
10,2,1987.562500
10,2,5048.847826
10,2,2843.556604
10,2,18318.781250
10,2,2081.533537
10,2,4247.750000
10,2,1548.628205
10,2,4056.250000
10,2,1692.647541
10,2,2491.457031
10,2,34854.109375
10,2,2002.060976
10,2,32354.928571
10,2,5439.191489
10,2,14590.395349
10,2,2267.451220
10,2,1523.056452
10,2,1666.450521
10,2,1799.521277
10,2,2392.085000
10,2,3218.045455
10,2,7999.832447
10,2,1587.276163
10,2,4235.138158
10,2,2282.073171
10,2,3860.391304
10,2,5061.333333
10,2,3199.625000
10,2,6633.520408
10,2,1956.900510
10,2,2864.017442
10,2,10908.932927
10,2,2319.164062
10,2,1922.375000
10,2,6402.046053
10,2,78824.250000
10,2,5165.976744
10,2,2925.411458
10,2,12491.417219
10,2,2075.208333
10,2,4482.201531
10,2,1956.500000
10,2,2412.201923
10,2,2825.762195
10,2,2078.609467
10,2,1870.450980
10,2,6687.912500
10,2,1567.679688

10,2,4912.921053
10,2,1799.194444
10,2,2539.500000
10,2,2288.155556
10,2,6292.090909
10,2,4708.318182
10,2,3671.015625
10,2,2034.006757
10,2,4730.351293
10,2,9707.525862
10,2,3325.427184
10,2,2585.625000
10,2,3868.750000
10,2,1953.605263
10,2,3005.261628
10,2,2082.317308
10,2,1733.823529
10,2,6841.568421
10,2,2317.923077
11,1,1795.785714
11,1,58275.798013
11,1,1902.272727
11,1,1719.205882
11,1,41169.500000
11,1,1995.009615
11,1,32262.875000
11,1,20031.021277
11,1,1613.365385
11,1,3629.063953
11,1,44690.604167
11,1,4134.180851
11,1,9027.378676
11,1,1507.062500
11,1,2201.085938
11,1,1645.714286
11,1,32369.143617
11,1,3530.195122
11,1,75969.894737
11,1,53764.942308
11,1,1852.583333
11,1,16721.347222
11,1,6652.702128
11,1,7814.770349
11,1,1870.346591
11,1,42082.270833
11,1,1732.094595
11,1,29091.300781
11,1,4015.520833
11,1,1838.477941
11,1,23023.307692
11,1,5837.276786
11,1,1820.172414
11,1,60138.067308
11,1,155011.700000
11,1,3725.679348
11,1,19711.917614
11,1,7724.267442
11,1,17137.535714
11,1,2104.544643
11,1,84226.125000
11,1,3489.735294
11,1,2710.031250
11,1,1844.279070
11,1,1570.756098
11,1,87312.000000
11,1,64123.638158

11,1,2046.627778
11,1,1601.271739
11,1,19046.750000
11,1,9878.846154
11,1,4149.459302
11,1,3369.695652
11,1,11658.151786
11,1,2843.210000
11,1,103766.116071
11,1,1569.000000
11,1,3975.904762
11,1,17683.316667
11,1,4057.891026
11,1,2654.447368
11,1,1963.697368
11,1,16727.132143
11,1,3167.175000
11,1,30142.565789
11,1,1594.096774
11,1,1943.377778
11,1,3158.036184
11,1,21848.500000
11,1,86292.000000
11,1,2591.402174
11,1,4692.951705
11,1,98884.300000
11,1,2207.843750
11,1,90991.635135
11,1,21658.157143
11,1,1938.287500
11,1,2160.614286
11,1,10346.391447
11,1,2746.044643
11,1,120071.600000
11,1,3206.646875
11,1,1702.278061
11,1,2577.604651
11,1,2315.200000
11,1,2548.392045
11,1,2803.106383
11,1,1877.593023
11,1,2498.493750
11,1,4532.960938
11,1,1581.750000
11,1,85307.500000
11,1,92888.675000
11,1,1607.466463
11,1,7322.625000
11,1,8558.000000
11,1,58249.397727
11,1,3593.333333
11,1,3971.175000
11,1,117271.000000
11,1,1502.475610
11,1,1916.673077
11,1,47095.687500
11,1,16384.125000
11,1,12186.166667
11,1,1840.759615
11,1,128740.853659
11,1,1863.943548
11,1,2749.385870
11,1,7058.000000
11,1,1638.861111
11,1,44786.358974
11,1,2153.804054

11,1,4275.470588
11,1,43764.042857
11,1,3998.744681
11,1,1723.872340
11,1,1797.888158
11,1,8464.714286
11,1,1534.531250
11,1,3064.234848
11,1,19405.747024
11,1,5443.256944
11,1,4349.990196
11,1,5844.961039
11,1,26939.496875
11,1,2406.202206
11,1,3531.366667
11,1,11419.718750
11,1,4029.434783
11,1,50769.618750
11,1,1609.551020
11,1,80469.043478
11,1,1630.961538
11,1,1934.911458
11,1,82410.845745
11,1,39119.004310
11,1,2975.341463
11,1,3934.000000
11,1,2281.632812
11,1,1930.552083
11,1,2627.845238
11,1,65761.592105
11,1,3331.556886
11,1,21413.979592
11,1,1938.316860
11,1,2201.194444
11,1,2230.155556
11,1,63934.907895
11,1,76677.493750
11,1,2570.420732
11,1,1770.740132
11,1,44154.414474
11,1,41998.825000
11,1,52431.487179
11,1,1843.788462
11,1,36117.916667
11,1,11250.714286
11,1,1532.900000
11,1,12054.630208
11,1,2109.695122
11,1,16591.843023
11,1,1628.850000
11,1,2515.897727
11,1,4764.486111
11,1,9920.993750
11,1,10699.744186
11,1,6242.675000
11,1,2048.867021
11,1,2116.694853
11,1,76140.785714
11,1,5147.727778
11,1,22978.437500
11,1,51024.354167
11,1,7345.414634
11,1,1895.966981
11,1,2623.984375
11,1,1571.904255
11,1,2415.451389

11,1,21880.336957
11,1,3964.325472
11,1,1600.377604
11,1,61285.540441
11,1,5371.960227
11,1,3659.655556
11,1,7159.431818
11,1,1960.290816
11,1,98705.181818
11,1,30628.217105
11,1,4330.906250
11,1,1679.362500
11,1,4274.458333
11,1,2447.380682
11,1,81754.086806
11,1,15497.770833
11,1,1543.590909
11,1,4271.631410
11,1,79991.539773
11,1,3238.062500
11,1,2628.788690
11,1,2412.261628
11,1,15984.500000
11,1,2021.369565
11,1,1904.477273
11,1,5256.822581
11,1,2504.750000
11,1,2046.250000
11,1,6284.482143
11,1,1612.075000
11,1,1649.469444
11,1,20592.030612
11,1,2292.478723
11,1,58349.445946
11,1,47068.062500
11,1,130608.666667
11,1,1795.239130
11,1,2139.634615
11,1,11327.375000
11,1,4463.026163
11,1,45062.593750
11,1,1837.074324
11,1,2629.322917
11,1,6082.077778
11,1,2354.468085
11,1,48055.318750
11,1,2109.284091
11,1,1650.407895
11,1,69752.346154
11,1,3279.593750
11,1,119119.339744
11,1,49234.981061
11,1,47680.609375
11,1,10924.722826
11,1,35537.322581
11,1,2968.979167
11,1,1981.041667
11,1,5329.057692
11,1,1896.448980
11,1,2337.945122
11,1,53123.440972
11,1,46948.656250
11,1,17767.317073
11,1,1683.868263
11,1,2164.695122
11,1,2188.034884

11,1,6968.337838
11,1,105575.539062
11,1,31329.002976
11,1,51265.055556
11,1,11454.307065
11,1,6039.597222
11,1,3711.023256
11,1,5051.277778
11,1,1759.308824
11,1,7638.886364
11,1,2425.302632
11,1,1676.394531
11,1,2540.997222
11,1,1623.410156
11,1,2101.406250
11,1,37239.460526
11,1,12650.617647
11,1,3040.375000
11,1,89054.087500
11,1,5621.023256
11,1,34600.850543
11,1,1500.642442
11,1,69748.789474
11,1,2268.553191
11,1,2837.121711
11,1,52186.269231
11,1,39713.121324
11,1,3115.975543
11,1,3283.343085
11,1,2190.142857
11,1,2113.488372
11,1,29614.137500
11,1,2300.861111
11,1,1624.279070
11,1,3568.852941
11,1,1597.190476
11,1,1927.000000
11,1,59033.539634
11,1,29043.209559
11,1,2733.292969
11,1,3312.138587
11,1,196310.032895
11,1,2086.093023
11,1,1701.319444
11,1,55852.256410
11,1,17538.813953
11,1,3719.484375
11,1,1671.219512
11,1,29926.854167
11,1,1749.987179
11,1,11283.446429
11,1,3768.534574
11,1,72119.641892
11,1,58916.000000
11,1,59713.048780
11,1,3905.488636
11,1,5855.574405
11,1,2759.287500
11,1,22402.414216
11,1,3710.587209
11,1,13136.109043
11,1,79924.363636
11,1,11579.909091
11,1,1815.788462
11,1,23965.755000
11,1,3402.000000

11,1,9023.342857
11,1,6721.443878
11,1,2432.465909
11,1,33100.153061
11,1,48966.250000
11,1,56836.604651
11,1,3583.682927
11,1,64616.426471
11,1,34551.756757
11,1,4331.025568
11,1,52916.930233
11,1,73698.604651
11,1,22718.603774
11,1,4056.263298
11,1,5008.909091
11,1,15379.186047
11,1,2305.750000
11,1,84039.176136
11,1,3781.250000
11,1,1627.694853
11,1,1586.435897
11,1,6827.631579
11,1,24865.275000
11,1,1730.000000
11,1,1848.212500
11,1,3122.969697
11,1,3797.633929
11,1,1744.507143
11,1,98927.059375
11,1,1785.811111
11,1,31779.054348
11,1,2112.477273
11,1,1861.291667
11,1,1580.112500
11,1,5848.924242
11,1,4911.734375
11,1,126137.146341
11,1,81489.451220
11,1,52290.942308
11,1,17232.198718
11,1,29682.312500
11,1,1792.718750
11,1,31262.600000
11,1,1644.040441
11,1,1696.006250
11,1,2140.261111
11,1,2110.678571
11,1,11498.399038
11,1,5672.507353
11,1,2022.233696
11,1,1778.936047
11,1,9917.343750
11,1,1803.166667
11,1,1514.195946
11,1,141639.649510
11,1,20742.308824
11,1,1620.488095
11,1,3760.311047
11,1,62278.601351
11,1,53623.378472
11,1,20854.735294
11,1,2167.468750
11,1,2737.169643
11,1,116456.407895
11,1,1772.107143
11,1,1599.550000

11,1,1725.909483
11,1,14960.940217
11,1,3985.000000
11,1,39710.522727
11,1,7575.731383
11,1,3249.700000
11,1,4465.045455
11,1,7026.344388
11,1,6488.610294
11,1,2384.500000
11,1,1980.244186
11,1,5512.446429
11,1,1957.656977
11,1,6843.741477
11,1,2095.687500
11,1,1566.812500
11,1,19456.605263
11,1,63505.817568
11,1,5245.506098
11,1,7017.839286
11,1,3790.662879
11,1,2690.631250
11,1,59008.715278
11,1,2974.364796
11,1,88002.296875
11,1,2221.287791
11,1,5925.000000
11,1,38663.771429
11,1,1590.809211
11,1,3847.575658
11,1,6544.568452
11,1,32575.043103
11,1,3368.125000
11,1,3467.625000
11,1,43908.900000
11,1,2411.821429
11,1,4089.860294
11,1,34707.905405
11,1,78311.117857
11,1,4341.210227
11,1,2890.073171
11,1,3097.258721
11,1,3075.284574
11,1,1585.204545
11,1,54350.465909
11,1,1906.596154
11,1,6059.375000
11,1,25399.875000
11,1,1771.761905
11,1,54442.000000
11,1,2388.916667
11,1,1587.069149
11,1,3015.482955
11,1,1635.589286
11,1,14074.686275
11,1,7919.482558
11,1,1702.055556
11,1,19529.400000
11,1,1564.418605
11,1,2079.285714
11,1,5875.608696
11,1,27168.202586
11,1,5045.235119
11,1,1978.534091
11,1,1516.663265
11,1,1603.025862

11,1,2455.272727
11,1,6555.778061
11,1,115934.615385
11,1,2094.375000
11,1,2785.776596
11,1,4147.921875
11,1,2659.805851
11,1,3506.930233
11,1,46408.314103
11,1,2789.200000
11,1,2108.590909
11,1,3305.560976
11,1,1554.437500
11,1,1599.602564
11,1,2404.967949
11,1,54955.045455
11,1,3292.072222
11,1,135248.052326
11,1,2617.180921
11,1,1613.137195
11,1,2417.620000
11,1,1522.628205
11,1,8152.932377
11,1,17839.433511
11,1,17224.477500
11,1,1814.543478
11,1,39001.406250
11,1,1551.768750
11,1,2983.434783
11,1,6683.111842
11,1,46789.533784
11,1,58619.000000
11,1,4132.973214
11,1,92112.980263
11,1,82351.607143
11,1,2125.723837
11,1,2288.812500
11,1,2320.575758
11,1,3190.375000
11,1,5226.410000
11,1,1766.256757
11,1,8356.683673
11,1,3998.232558
11,1,32820.929688
11,1,10674.543478
11,1,2100.419643
11,1,5869.172222
11,1,38739.622093
11,1,39260.347826
11,1,1600.891026
11,1,54898.596429
11,1,1719.086957
11,1,15232.862500
11,1,80691.764706
11,1,1983.616071
11,1,3373.210938
11,1,1776.529412
11,1,32747.741279
11,1,43795.363281
11,1,1627.944444
11,1,35609.838710
11,1,3013.732558
11,1,48253.522436
11,1,3770.125000
11,1,2584.500000
11,1,6441.375000

11,1,32783.158537
11,1,7412.552632
11,1,5406.122449
11,1,1510.140957
11,1,16225.462500
11,1,1692.500000
11,1,2178.767442
11,1,1526.065217
11,1,2868.468750
11,1,29420.149038
11,1,6574.397436
11,1,2074.812500
11,1,6535.250000
11,1,3191.953804
11,1,1786.912791
11,1,48717.166667
11,1,3058.233533
11,1,3389.628571
11,1,74843.357639
11,1,3010.343750
11,1,1704.215686
11,1,23054.050000
11,1,3189.000000
11,1,4479.375000
11,1,7683.540625
11,1,3092.170000
11,1,1882.610465
11,1,26893.558824
11,1,1517.197674
11,1,5738.348837
11,1,2058.162500
11,1,39227.839286
11,1,3316.984375
11,1,3793.673077
11,1,8068.500000
11,1,8145.186047
11,1,3797.207386
11,1,1759.441489
11,1,8637.354839
11,1,5093.205128
11,1,14340.326797
11,1,11112.721591
11,1,2160.825000
11,1,3244.540625
11,1,3451.351351
11,1,2592.575658
11,1,2589.978723
11,1,16813.389706
11,1,104622.509615
11,1,1551.109375
11,1,93359.615385
11,1,2705.647059
11,1,3940.348837
11,1,70050.559524
11,1,2360.690789
11,1,4051.695652
11,1,2601.000000
11,1,12764.400000
11,1,7630.127660
11,1,1813.500000
11,1,83356.429054
11,1,2807.420732
11,1,2202.289474
11,1,1567.142045
11,1,1713.178571
11,1,7364.715909

11,1,4344.381579
11,1,1525.593750
11,1,3441.948529
11,1,1545.477273
11,1,2625.328125
11,1,3808.090909
11,1,6279.102041
11,1,23683.057692
11,1,2217.648936
11,1,13935.588235
11,1,1956.750000
11,1,2052.307692
11,1,1643.359375
11,1,1548.906977
11,1,1661.580357
11,1,3687.040816
11,1,2362.312500
11,1,10878.039474
11,1,28650.935714
11,1,62990.408537
11,1,1624.756757
11,1,3978.980978
11,1,4216.336538
11,1,109250.608974
11,1,1656.851064
11,1,2577.567073
11,1,22180.798077
11,1,48832.831395
11,1,3409.637500
11,1,4577.657407
11,1,16917.545455
11,1,3407.653846
11,1,2730.440000
11,1,2329.658784
11,1,2261.570513
11,1,4006.017857
11,1,1507.162234
11,1,61738.662162
11,1,2567.357143
11,1,3333.031915
11,1,2665.075521
11,1,3971.556250
11,1,75139.590909
11,1,1636.611111
11,1,20580.625000
11,1,2973.676471
11,1,4173.730769
11,1,25311.086957
11,1,2563.156977
11,1,1564.067935
11,1,4001.809524
11,1,28949.180556
11,1,1596.788690
11,1,4096.662791
11,1,44679.841463
11,1,4772.338542
11,1,5637.738636
11,1,31723.356618
11,1,2356.503125
11,1,70436.666667
11,1,1695.468085
11,1,2689.781250
11,1,2357.787234
11,1,57544.287037
11,1,34715.065789
11,1,177420.192073

11,1,2065.576923
11,1,2929.266667
11,1,2357.423913
11,1,3160.029891
11,1,30251.439655
11,1,76255.682692
11,1,4068.023810
11,1,3403.659091
11,1,90467.236111
11,1,2102.978261
11,1,114538.188889
11,1,132820.582500
11,1,2356.773438
11,1,3794.505319
11,1,13395.750000
11,1,24853.578947
11,1,2708.855978
11,1,31681.415541
11,1,1642.009868
11,1,66289.250000
11,1,196837.773585
11,1,2822.500000
11,1,4354.669492
11,1,4320.488889
11,1,4629.529070
11,1,26197.647059
11,1,55222.103070
11,1,42265.242647
11,1,2016.045455
11,1,1558.551282
11,1,70797.000000
11,1,42136.385135
11,1,4682.641447
11,1,16985.918605
11,1,5854.649510
11,1,16428.076087
11,1,74029.093750
11,1,2242.804878
11,1,3109.422619
11,1,3029.323529
11,1,5999.150000
11,1,2008.561224
11,1,6042.088235
11,1,83998.522059
11,1,2051.697674
11,1,1664.013514
11,1,1539.000000
11,1,67288.384615
11,1,75699.246622
11,1,2522.500000
11,1,3015.614362
11,1,4398.962500
11,1,3126.272727
11,1,39399.692308
11,1,5756.005814
11,1,1960.868421
11,1,2233.733333
11,1,2034.207447
11,1,4319.937500
11,1,2074.656250
11,1,4829.571429
11,1,1896.837209
11,1,1807.022222
11,1,79847.571429
11,1,4312.727273
11,1,53093.503125

11,1,3946.362500
11,1,70437.110465
11,1,43825.871795
11,1,4970.559783
11,1,2726.250000
11,1,4221.292969
11,1,126084.701705
11,1,1632.080000
11,1,48558.743590
11,1,5546.348485
11,1,26601.682432
11,1,122400.240854
11,1,1876.756944
11,1,2061.896552
11,1,32432.457143
11,1,7220.560000
11,1,1774.833333
11,1,4320.650000
11,1,13025.096875
11,1,2028.964286
11,1,5818.125000
11,1,6039.750000
11,1,2995.504032
11,1,13546.155000
11,1,1984.375000
11,1,98318.250000
11,1,4467.003125
11,1,1521.601852
11,1,1686.878125
11,1,29073.871795
11,1,8804.142857
11,1,1805.570000
11,1,52271.128049
11,1,6412.048780
11,1,2277.843750
11,1,1974.138889
11,1,94647.283019
11,1,5524.000000
11,1,1699.530000
11,1,1554.976190
11,1,2683.400000
11,1,88268.712838
11,1,22048.727941
11,1,1522.937500
11,1,9801.750000
11,1,44190.200000
11,1,3274.783088
11,1,6232.774390
11,1,62600.588235
11,1,94756.394231
11,1,2060.158088
11,1,1973.794872
11,1,72585.378378
11,1,22553.257353
11,1,1627.782143
11,1,3717.232143
11,1,1896.125000
11,1,1552.866477
11,1,1501.916667
11,1,2412.446809
11,1,1543.875000
11,1,2592.494681
11,1,25847.200000
11,1,6526.205882
11,1,1847.569079
11,1,4824.639535

11,1,1513.987179
11,1,62560.750000
11,1,2917.554878
11,1,1797.956395
11,1,2376.955882
11,1,1559.319079
11,1,142369.783537
11,1,1697.350610
11,1,13336.454545
11,1,2371.377604
11,1,2370.795455
11,1,3039.323529
11,1,80893.488889
11,1,5416.238636
11,1,1971.704545
11,1,4066.154255
11,1,24424.969512
11,1,3298.913043
11,1,93775.352273
11,1,14369.069767
11,1,2574.500000
11,1,18468.412500
11,1,54442.344595
11,1,2569.943750
11,1,28643.003676
11,1,35334.375000
11,1,3670.169872
11,1,49681.656250
11,1,7139.147727
11,1,9767.932927
11,1,1810.133333
11,1,1991.818182
11,1,59033.210526
11,1,1875.219512
11,1,3114.442857
11,1,2688.937500
11,1,2372.350649
11,1,2511.496795
11,1,2339.585227
11,1,3586.559659
11,1,8165.441489
11,1,2481.364286
11,1,2453.459239
11,1,21999.952206
11,1,73410.152027
11,1,3138.412234
11,1,1850.386364
11,1,23567.668605
11,1,1815.000000
11,1,3321.975000
11,1,28326.040698
11,1,7870.238372
11,1,1907.053977
11,1,2603.615385
11,1,61088.500000
11,1,14502.773810
11,1,9267.437500
11,1,7178.756757
11,1,3441.634146
11,1,3453.290698
11,1,3071.775641
11,1,1544.875000
11,1,4085.964744
11,1,59159.730263
11,1,2774.395349
11,1,8264.473404

11,1,2004.315789
11,1,1584.391304
11,1,3652.144737
11,1,1749.500000
11,1,2058.029762
11,1,4573.846154
11,1,1678.184375
11,1,1937.810811
11,1,1755.268750
11,1,3561.406977
11,1,1537.382653
11,1,3895.875000
11,1,2943.044271
11,1,1943.521341
11,1,1872.022727
11,1,7966.934375
11,1,1730.357955
11,1,10404.851190
11,1,1874.000000
11,1,36520.101351
11,1,5017.435714
11,1,3037.900000
11,1,1670.492021
11,1,2452.131757
11,1,10765.107143
11,1,17888.517857
11,1,1818.062500
11,1,1537.000000
11,1,4637.196809
11,1,5645.915625
11,1,2427.375000
11,1,9046.728125
11,1,16324.758621
11,1,165154.186047
11,1,5699.522727
11,1,28472.676471
11,1,4454.928571
11,1,3703.685714
11,1,2862.496711
11,1,34870.780405
11,1,2440.603448
11,1,56856.545455
11,1,6339.685000
11,1,1834.078947
11,1,99095.713415
11,1,144054.865854
11,1,100139.076923
11,1,53737.562500
11,1,153161.000000
11,1,51649.421053
11,1,73018.094595
11,1,6277.032258
11,1,4058.170732
11,1,2077.591912
11,1,47289.000000
11,1,5346.213235
11,1,2479.145833
11,1,1769.485119
11,1,68397.403846
11,1,63517.000000
11,1,28544.079545
11,1,26755.093750
11,1,1763.014535
11,1,1529.807432
11,1,96305.755682
11,1,4470.946429

11,1,4684.204545
11,1,1555.145833
11,1,7370.456522
11,1,19372.216216
11,1,14009.375000
11,1,96454.405405
11,1,4962.331395
11,1,17440.156250
11,1,2624.912791
11,1,2541.848039
11,1,1598.578125
11,1,59337.746711
11,1,2635.575000
11,1,2245.571429
11,1,3075.572222
11,1,6229.841463
11,1,1838.062500
11,1,2904.837121
11,1,19436.368421
11,1,24057.986111
11,1,196480.250000
11,1,33600.761364
11,1,1734.875000
11,1,2785.516129
11,1,5503.342105
11,1,20438.625000
11,1,1583.125000
11,1,4550.892857
11,1,2399.400000
11,1,4703.541667
11,1,1627.302632
11,1,1794.400000
11,1,2358.198171
11,1,4834.831395
11,1,2360.669444
11,1,6239.224432
11,1,7628.500000
11,1,2864.643293
11,1,36913.726974
11,1,68122.851351
11,1,41170.503676
11,1,1639.690625
11,1,6196.565217
11,1,1540.822917
11,1,1883.984375
11,1,35745.533088
11,1,10182.320513
11,1,2076.256410
11,1,25844.421053
11,1,60458.230769
11,1,118488.979167
11,1,4365.946429
11,1,151368.585526
11,1,4231.036585
11,1,1766.708333
11,1,64050.578947
11,1,4002.818182
11,1,1898.269737
11,1,4172.294643
11,1,4447.864865
11,1,7106.666667
11,1,3795.117647
11,1,2367.853659
11,1,56157.027027
11,1,3111.260638
11,1,3225.210227

11,1,5144.966667
11,1,1985.230769
11,1,1848.627778
11,1,8568.360119
11,1,137701.808140
11,1,2842.688889
11,1,1540.496795
11,1,65481.000000
11,1,1909.259434
11,1,11358.463235
11,1,4767.062500
11,1,1974.278846
11,1,2603.755556
11,1,2908.100000
11,1,74796.197368
11,1,1662.081633
11,1,12683.361842
11,1,72051.425595
11,1,2042.143229
11,1,1581.371429
11,1,1815.936170
11,1,3662.743590
11,1,1602.651163
11,1,155121.127315
11,1,7264.981250
11,1,125014.219512
11,1,68078.575581
11,1,1519.884146
11,1,6261.830556
11,1,7294.904762
11,1,1819.591837
11,1,1652.169118
11,1,3254.564286
11,1,9708.406977
11,1,4023.869565
11,1,1537.625000
11,1,7924.218750
11,1,3684.933333
11,1,1601.921196
11,1,2603.482955
11,1,16632.793210
11,1,76130.108974
11,1,1653.969697
11,1,53194.657895
11,1,8336.718750
11,1,9895.700000
11,1,2496.500000
11,1,2412.729730
11,1,126632.062500
11,1,26439.594595
11,1,18617.717391
11,1,2286.825581
11,1,24955.670732
11,1,17517.425926
11,1,3039.820000
11,1,22710.586538
11,1,3431.880814
11,1,3845.828125
11,1,8501.111111
11,1,1771.309783
11,1,5194.500000
11,1,8432.257812
11,1,3027.706250
11,1,115038.223404
11,1,113220.445652
11,1,5719.803571

11,1,114075.862745
11,1,1555.875000
11,1,9361.541667
11,1,2009.000000
11,1,54915.143382
11,1,3507.916667
11,1,2577.447368
11,1,2796.232143
11,1,49545.291667
11,1,88729.478261
11,1,12792.479167
11,1,5257.223404
11,1,2872.863636
11,1,10495.848837
11,1,1572.555851
11,1,3705.290698
11,1,66740.131579
11,1,178743.015625
11,1,97029.194444
11,1,7980.408019
11,1,4531.144737
11,1,1941.195652
11,1,1875.795918
11,1,2269.230769
11,1,77136.097561
11,1,94347.398810
11,1,4092.857143
11,1,59320.425532
11,1,79875.398810
11,1,44940.666667
11,1,3468.095238
11,1,1514.166667
11,1,2004.750000
11,1,75373.851974
11,1,2964.062500
11,1,5107.258929
11,1,48962.657895
11,1,4369.994792
11,1,1777.165625
11,1,1530.538462
11,1,2595.933140
11,1,3497.155488
11,1,8930.336207
11,1,19153.426829
11,1,24189.157609
11,1,2459.974359
11,1,6441.234043
11,1,28374.086538
11,1,55262.657895
11,1,2139.531915
11,1,2288.736842
11,1,9913.317073
11,1,21585.359375
11,1,2104.059524
11,1,2268.608553
11,1,4386.402439
11,1,92774.109375
11,1,1639.725000
11,1,3303.205000
11,1,1522.471154
11,1,1828.963415
11,1,1507.750000
11,1,3139.411765
11,1,1501.426136
11,1,49905.666667
11,1,75070.850000

11,1,32538.324324
11,1,3112.108696
11,1,1966.581395
11,1,1843.255435
11,1,1705.968750
11,1,2882.718750
11,1,1935.571429
11,1,2627.907895
11,1,1913.644886
11,1,3610.760417
11,1,4173.318182
11,1,51964.073620
11,1,12101.217105
11,1,2613.176829
11,1,1876.585937
11,1,5696.203947
11,1,2655.821970
11,1,12912.563830
11,1,58583.060811
11,1,2612.142857
11,1,44181.156250
11,1,29562.686047
11,1,1578.132653
11,1,1713.843085
11,1,1617.762500
11,1,12861.588889
11,1,20080.846154
11,1,1866.067568
11,1,66832.291667
11,1,149297.750000
11,1,133164.909091
11,1,1567.880952
11,1,51024.659722
11,1,2782.358491
11,1,30474.406250
11,1,4312.750000
11,1,81701.698718
11,1,1771.250000
11,1,2526.468085
11,1,10376.764706
11,1,5465.883152
11,1,27255.712121
11,1,1925.276042
11,1,42529.203125
11,1,3015.577500
11,1,19458.394737
11,1,96851.993902
11,1,1859.703125
11,1,2448.357955
11,1,3988.384615
11,1,2103.219512
11,1,4710.440476
11,1,1949.242347
11,1,6504.250000
11,1,1805.134615
11,1,13367.870968
11,1,2824.521739
11,1,3272.700000
11,1,1500.953125
11,1,4685.462838
11,1,1503.562500
11,1,56401.818182
11,1,3342.980978
11,1,4468.111111
11,1,84896.057692
11,1,14861.215909

11,1,1835.963889
11,1,12316.930233
11,1,2538.795918
11,1,1732.756579
11,1,1843.394231
11,1,9616.941176
11,1,3677.272059
11,1,9446.344444
11,1,4683.821809
11,1,94431.923913
11,1,14442.682143
11,1,2925.595238
11,1,143449.145833
11,1,1940.046875
11,1,4685.903409
11,1,3632.884615
11,1,2836.136628
11,1,1668.500000
11,1,1836.811170
11,1,1633.687500
11,1,7208.541667
11,1,4328.218750
11,1,2987.730769
11,1,5069.304054
11,1,3630.617647
11,1,4879.478448
11,1,57741.032258
11,1,1510.215190
11,1,1737.807692
11,1,78704.842105
11,1,2232.613636
11,1,2798.779255
11,1,2303.164634
11,1,57147.886364
11,1,120245.219697
11,1,2172.114286
11,1,4653.270349
11,1,5038.394737
11,1,5274.312500
11,1,2450.195652
11,1,2958.605263
11,1,7348.687500
11,1,8798.162500
11,1,3026.728723
11,1,4613.002660
11,1,7368.118243
11,1,49774.750000
11,1,90865.432927
11,1,2253.100000
11,1,1645.990000
11,1,38224.491667
11,1,48008.733333
11,1,9818.008333
11,1,1655.987805
11,1,1712.984756
11,1,2984.957447
11,1,1617.728261
11,1,33961.600000
11,1,1595.163462
11,1,12577.458333
11,1,12037.739362
11,1,2172.250000
11,1,72595.702703
11,1,1710.191489
11,1,2098.976190
11,1,4968.562500

11,1,1601.128125
11,1,3552.345745
11,1,72598.786184
11,1,81828.661932
11,1,27917.264706
11,1,1502.432927
11,1,24723.128378
11,1,3147.137195
11,1,4194.298295
11,1,1746.050000
11,1,2418.445652
11,1,105771.087838
11,1,34721.714286
11,1,57446.780488
11,1,2121.567308
11,1,6343.404070
11,1,1943.288690
11,1,4546.490741
11,1,35306.205882
11,1,2369.085106
11,1,15120.038462
11,1,2334.098684
11,1,30275.225000
11,1,14109.564655
11,1,2664.351351
11,1,1741.660377
11,1,4500.535714
11,1,20010.100000
11,1,4456.915761
11,1,8311.235294
11,1,22740.851064
11,1,29237.263158
11,1,13496.227273
11,1,188707.223214
11,1,2161.256579
11,1,2443.859375
11,1,33463.860465
11,1,2149.975000
11,1,1507.022727
11,1,2076.173913
11,1,5667.443627
11,1,4004.052632
11,1,7457.747159
11,1,4289.479167
11,1,1829.633333
11,1,148564.329545
11,1,4117.762500
11,1,4766.577778
11,1,59399.782895
11,1,5008.963415
11,1,2323.618421
11,1,96146.072368
11,1,5689.439024
11,1,1569.486413
11,1,64655.117647
11,1,36604.523438
11,1,1543.294355
11,1,2993.861413
11,1,6012.000000
11,1,3841.000000
11,1,1783.014286
11,1,1545.658163
11,1,1823.186047
11,1,19915.750000
11,1,1803.638298
11,1,3367.000000

11,1,7409.317308
11,1,1686.911111
11,1,1548.558824
11,1,6366.207317
11,1,2136.241071
11,1,5094.218750
11,1,88741.384615
11,1,1761.071856
11,1,8888.250000
11,1,2197.128049
11,1,2610.244186
11,1,29820.875000
11,1,3081.677632
11,1,5422.670455
11,1,7789.289474
11,1,2590.574468
11,1,2100.775000
11,1,46055.600000
11,1,3002.250000
11,1,1640.850962
11,1,21580.210526
11,1,4476.664634
11,1,5526.664062
11,1,4997.597222
11,1,48772.470588
11,1,23402.142857
11,1,11969.212500
11,1,1737.589744
11,1,48151.016447
11,1,2512.772727
11,1,2681.523810
11,1,2425.230769
11,1,4343.048780
11,1,9839.308511
11,1,4330.043478
11,1,6569.460227
11,1,2752.188953
11,1,2886.926471
11,1,66042.746528
11,1,6374.269737
11,1,37660.850000
11,1,133354.496951
11,1,3147.338235
11,1,4615.540909
11,1,110623.432692
11,1,3242.631944
11,1,1524.024390
11,1,15688.421875
11,1,2002.648438
11,1,9222.945000
11,1,2039.565476
11,1,83404.845000
11,1,2056.541667
11,1,13599.090909
11,1,9572.995000
11,1,4468.395833
11,1,32045.562500
11,1,2203.500000
11,1,11310.975610
11,1,19741.250000
11,1,3573.642442
11,1,116607.465116
11,1,8656.164062
11,1,3657.883929
11,1,7891.152439
11,1,1740.369898

11,1,2489.343750
11,1,22652.044118
11,1,110672.097561
11,1,2607.965909
11,1,4348.609756
11,1,175281.006098
11,1,2996.923077
11,1,2789.125000
11,1,172450.750000
11,1,5251.243421
11,1,1760.284722
11,1,3432.250000
11,1,153339.419643
11,1,3238.496324
11,1,7826.567708
11,1,2875.996875
11,1,1637.597500
11,1,4055.298780
11,1,2006.266447
11,1,1619.903226
11,1,1825.344512
11,1,1775.000000
11,1,16385.637500
11,1,1895.110465
11,1,1709.942308
11,1,2465.601190
11,1,2184.696970
11,1,44613.117647
11,1,57865.589744
11,1,18487.784091
11,1,2294.002717
11,1,1738.663043
11,1,73904.570652
11,1,2198.142045
11,1,4618.307292
11,1,2028.218750
11,1,25353.613636
11,1,3135.375000
11,1,3586.125000
11,1,2726.132353
11,1,1743.265244
11,1,34683.150000
11,1,7252.800000
11,1,3862.947368
11,1,2689.416667
11,1,2449.359375
11,1,4471.125000
11,1,2032.593750
11,1,65148.694767
11,1,10844.756579
11,1,1795.194767
11,1,1830.583333
11,1,3835.187500
11,1,2611.312500
11,1,1998.065104
11,1,2010.673913
11,1,2675.660714
11,1,81394.886628
11,1,48030.472222
11,1,1641.800000
11,1,2670.542553
11,1,1506.340426
11,1,3290.483696
11,1,6933.089744
11,1,73883.343023
11,1,102206.282609

11,1,2126.085366
11,1,2717.166667
11,1,5092.957317
11,1,4114.552632
11,1,7140.695122
11,1,2401.955882
11,1,17177.764205
11,1,3137.540541
11,1,113309.980645
11,1,2873.474359
11,1,19652.609375
11,1,4448.408163
11,1,3558.323529
11,1,101989.551282
11,1,1687.000000
11,1,8023.073171
11,1,4015.962963
11,1,37438.973404
11,1,1848.250000
11,1,1754.500000
11,1,39160.750000
11,1,41320.062500
11,1,2758.354839
11,1,1503.206250
11,1,3123.346774
11,1,7943.816327
11,1,1549.408333
11,1,1987.875000
11,1,16158.674342
11,1,1546.930556
11,1,14658.781250
11,1,6436.244444
11,1,112106.500000
11,1,60397.442073
11,1,8744.032895
11,1,10705.674020
11,1,23494.358108
11,1,28354.025568
11,1,4760.247340
11,1,3403.875000
11,1,1578.306122
11,1,4376.346154
11,1,1941.076923
11,1,1729.642857
11,1,8128.843750
11,1,89773.687500
11,1,3959.285714
11,1,46172.680921
11,1,1758.300000
11,1,5352.390957
11,1,148622.875000
11,1,1809.358553
11,1,66357.288194
11,1,2520.442073
11,1,3670.912500
11,1,4305.806818
11,1,2238.276786
11,1,44684.941176
11,1,1748.698864
11,1,1850.977273
11,1,1659.018229
11,1,1553.117647
11,1,2386.901163
11,1,32488.955645
11,1,10803.607558
11,1,33834.953488

11,1,2756.762500
11,1,1725.388298
11,1,2774.183824
11,1,2518.562500
11,1,3059.191489
11,1,1569.170213
11,1,1732.977778
11,1,11404.366071
11,1,2465.869318
11,1,1804.426724
11,1,3387.104651
11,1,25031.773810
11,1,144977.681818
11,1,2229.343750
11,1,2878.750000
11,1,1758.643293
11,1,2176.255435
11,1,21765.798913
11,1,2022.909722
11,1,2288.278409
11,1,4226.370000
11,1,1639.577703
11,1,39824.479730
11,1,42942.859756
11,1,2616.771277
11,1,170757.937500
11,1,1689.968750
11,1,1652.642857
11,1,189406.891304
11,1,4786.119565
11,1,2280.697674
11,1,2468.782609
11,1,2016.227273
11,1,2532.902174
11,1,1929.888889
11,1,93195.594595
11,1,2668.073718
11,1,1613.448529
11,1,34459.967105
11,1,31487.960938
11,1,33968.821875
11,1,4841.803977
11,1,48860.482143
11,1,2049.928571
11,1,54489.391447
11,1,5780.740000
11,1,5434.452128
11,1,2514.250000
11,1,5352.566667
11,1,2173.034884
11,1,8280.661932
11,1,2051.326923
11,1,12975.279605
11,1,3678.591667
11,1,3606.904762
11,1,14207.552632
11,1,3892.730769
11,1,51352.667763
11,1,146971.666667
11,1,30258.000000
11,1,3400.447222
11,1,3801.386364
11,1,8552.489583
11,1,41634.750000
11,1,3084.125000
11,1,2544.133523

11,1,2437.145833
11,1,1959.408784
11,1,24209.227273
11,1,59161.182432
11,1,14614.978659
11,1,1820.257353
11,1,6169.000000
11,1,1668.387500
11,1,51052.054054
11,1,1937.525000
11,1,88229.519231
11,1,10299.238636
11,1,22008.758523
11,1,2737.029762
11,1,69084.185714
11,1,3962.378378
11,1,5090.685976
11,1,1600.231707
11,1,2399.495000
11,1,1799.914894
11,1,2888.497283
11,1,16111.901042
11,1,1882.135135
11,1,7778.560976
11,1,3636.484375
11,1,1554.933824
11,1,1661.368243
11,1,5773.299342
11,1,5347.883333
11,1,2100.516304
11,1,29229.555556
11,1,5642.345238
11,1,3170.541667
11,1,2283.984043
11,1,2155.760870
11,1,1501.302326
11,1,2434.640625
11,1,3292.353125
11,1,23010.066176
11,1,1936.204268
11,1,6498.200000
11,1,5612.219512
11,1,7209.344512
11,1,5529.900000
11,1,1527.182927
11,1,1763.350000
11,1,5364.125000
11,1,1550.005208
11,1,54092.090116
11,1,28675.197368
11,1,1527.305556
11,1,10916.741477
11,1,1882.807692
11,1,58826.795455
11,1,53611.316176
11,1,5867.750000
11,1,51173.317073
11,1,8602.619048
11,1,8231.408537
11,1,4205.031250
11,1,2418.983553
11,1,4337.045455
11,1,5818.684896
11,1,3700.214674
11,1,4817.406250
11,1,2382.528409

11,1,3996.265625
11,1,102836.081250
11,1,4658.297297
11,1,15253.604651
11,1,50964.275000
11,1,1579.643750
11,1,1515.713235
11,1,15435.013587
11,1,2286.000000
11,1,64616.867021
11,1,111693.150000
11,1,3175.795918
11,1,2644.306818
11,1,2501.027778
11,1,5455.269737
11,1,78996.416667
11,1,72852.281250
11,1,1596.300676
11,1,2963.897059
11,1,71851.552083
11,1,77160.138158
11,1,4270.250000
11,1,3089.000000
11,1,62242.402778
11,1,11999.000000
11,1,5171.872093
11,1,56092.558824
11,1,2245.875000
11,1,1919.428571
11,1,3008.600000
11,1,2446.731707
11,1,2037.608974
11,1,26162.092105
11,1,2084.125000
11,1,2186.271341
11,1,130676.675000
11,1,9982.709184
11,1,2324.443750
11,1,27423.647059
11,1,72058.544872
11,1,2110.367347
11,1,6066.743421
11,1,1938.960227
11,1,1609.441860
11,1,2943.115132
11,1,2910.393939
11,1,74199.563830
11,1,1667.166667
11,1,1800.427083
11,1,2058.960526
11,1,2757.494318
11,1,7531.267857
11,1,4552.201389
11,1,77445.810811
11,1,55852.315789
11,1,74382.875000
11,1,123894.062500
11,1,5145.520833
11,1,12224.432927
11,1,10100.000000
11,1,69089.852941
11,1,1518.274390
11,1,4398.100000
11,1,51750.119681
11,1,2569.187500
11,1,57349.733871

11,1,1511.545139
11,1,2719.193182
11,1,90202.405405
11,1,2305.583333
11,1,4409.017442
11,1,4981.057692
11,1,2125.337209
11,1,1979.133929
11,1,1749.184783
11,1,7317.350610
11,1,38471.949405
11,1,38110.324324
11,1,2833.743902
11,1,1527.232143
11,1,4964.134615
11,1,2422.465909
11,1,4251.448529
11,1,44781.205357
11,1,27156.568966
11,1,4955.669811
11,1,1526.758929
11,1,5323.562500
11,1,18077.937500
11,1,3391.355556
11,1,49748.753906
11,1,1759.823529
11,1,1531.721429
11,1,1658.317308
11,1,71628.737805
11,1,58589.517241
11,1,1713.893293
11,1,2386.811111
11,1,2051.133721
11,1,2231.250000
11,1,40988.434211
11,1,1586.821023
11,1,4341.166667
11,1,3974.255319
11,1,22877.686047
11,1,46461.858108
11,1,1996.494565
11,1,3113.360294
11,1,1688.144444
11,1,1604.192073
11,1,3891.702703
11,1,6111.796875
11,1,50682.625000
11,1,56905.570946
11,1,1873.948718
11,1,1732.595395
11,1,2543.517442
11,1,2372.794872
11,1,22807.745370
11,1,1898.159574
11,1,6391.306122
11,1,52022.568182
11,1,4231.257353
11,1,105540.326087
11,1,29848.200000
11,1,2450.542553
11,1,2267.303977
11,1,2325.033333
11,1,1748.234043
11,1,1768.780488
11,1,3631.437500
11,1,31555.610294

11,1,1792.404762
11,1,8281.467105
11,1,34796.193182
11,1,3304.346154
11,1,1664.939024
11,1,5636.148936
11,1,18379.134868
11,1,3889.027174
11,1,5680.186170
11,1,2188.611111
11,1,142445.185976
11,1,1799.953804
11,1,20404.670455
11,1,7818.986842
11,1,29127.377907
11,1,2463.752294
11,1,8520.676724
11,1,2318.872449
11,1,2274.189024
11,1,4984.536932
11,1,2993.140625
11,1,52266.692308
11,1,1913.250000
11,1,69049.472561
11,1,2088.165761
11,1,2780.775000
11,1,2059.558333
11,1,1576.577778
11,1,4023.625000
11,1,6777.900000
11,1,2716.604651
11,1,2803.500000
11,1,2750.485000
11,1,4444.844444
11,1,2036.813953
11,1,3152.540625
11,1,82084.483333
11,1,1617.000000
11,1,2291.628049
11,1,40463.341667
11,1,2056.970745
11,1,1740.048295
11,1,15315.496094
11,1,1519.551282
11,1,26110.605469
11,1,1774.402778
11,1,2605.023256
11,1,23422.262500
11,1,1659.631579
11,1,1539.045455
11,1,10844.965116
11,1,13417.178571
11,1,3315.168605
11,1,5353.628289
11,1,8831.561170
11,1,1596.619565
11,1,3764.035714
11,1,4902.666667
11,1,1692.285714
11,1,35950.705882
11,1,18744.687500
11,1,1965.644737
11,1,1587.923780
11,1,1782.437500
11,1,32538.619186
11,1,1775.208333

11,1,2598.378205
11,1,2359.668919
11,1,1955.923497
11,1,1981.293750
11,1,43747.303571
11,1,1602.395349
11,1,127459.923077
11,1,2165.062500
11,1,1752.237805
11,1,35848.721591
11,1,3141.488636
11,1,3475.351190
11,1,2093.893617
11,1,1721.542683
11,1,2154.132353
11,1,4067.733696
11,1,18887.234043
11,1,4810.195652
11,1,4324.408333
11,1,4470.586538
11,1,2487.267857
11,1,1662.298611
11,1,4669.936170
11,1,4640.691176
11,1,3325.855556
11,1,6298.075000
11,1,5742.631410
11,1,1895.315789
11,1,4335.950000
11,1,2537.600610
11,1,6459.307692
11,1,4733.541667
11,1,1686.250000
11,1,1815.717391
11,1,35447.571429
11,1,2371.389881
11,1,4108.875000
11,1,3965.500000
11,1,1625.887821
11,1,56777.064286
11,1,1678.464286
11,1,2196.358553
11,1,37359.350000
11,1,9850.645833
11,1,2116.273026
11,1,2001.625000
11,1,4860.852564
11,1,2896.169444
11,1,4796.932065
11,1,93402.138889
11,1,7743.611111
11,1,96448.437500
11,1,1819.326220
11,1,2894.115385
11,1,5178.582031
11,1,69820.299342
11,1,24040.757353
11,1,7235.897727
11,1,26594.697279
11,1,3082.896296
11,1,9468.722222
11,1,2505.458333
11,1,14043.662791
11,1,1977.095238
11,1,2590.427419
11,1,19744.831522

11,1,7520.527778
11,1,1797.627660
11,1,1681.573529
11,1,1640.371429
11,1,2129.685000
11,1,24072.243902
11,1,1609.137019
11,1,11868.104730
11,1,3517.121951
11,1,19283.375000
11,1,1613.836538
11,1,6435.333333
11,1,37580.927632
11,1,34796.714286
11,1,2048.418605
11,1,3971.671053
11,1,3344.218750
11,1,88396.953488
11,1,4308.791667
11,1,2142.898936
11,1,1647.723039
11,1,5937.465625
11,1,29698.725806
11,1,8405.762755
11,1,8856.761905
11,1,31997.986842
11,1,4160.965278
11,1,5073.128289
11,1,3280.475806
11,1,1555.500000
11,1,27639.200000
11,1,6563.641892
11,1,2265.647059
11,1,4372.727273
11,1,1751.662162
11,1,80443.085526
11,1,2775.050000
11,1,19931.400000
11,1,5676.317073
11,1,1579.125000
11,1,6198.575581
11,1,2071.465116
11,1,3602.410256
11,1,3264.750000
11,1,13802.459821
11,1,2538.393293
11,1,2884.269737
11,1,14912.286458
11,1,1567.702128
11,1,1875.548611
11,1,3593.375000
11,1,1948.205128
11,1,26901.259868
11,1,2467.900000
11,1,2261.875000
11,1,33914.550000
11,1,17944.125000
11,1,1961.562500
11,1,52057.125000
11,1,2123.611111
11,1,62699.694079
11,1,1604.118421
11,1,67961.201220
11,1,36714.851351
11,1,4651.451389
11,1,3215.139423

11,1,15407.358696
11,1,1624.250000
11,1,3218.511111
11,1,29246.348214
11,1,61990.420213
11,1,61677.555556
11,1,1538.337500
11,1,1520.936170
11,1,2993.627660
11,1,2480.363636
11,1,1929.443182
11,1,2105.833333
11,1,88792.785714
11,1,59464.564103
11,1,2590.404891
11,1,3567.285714
11,1,23228.844633
11,1,6153.111486
11,1,6182.111111
11,1,2457.284884
11,1,4912.277174
11,1,34946.705729
11,1,51056.739286
11,1,5581.079545
11,1,2109.125000
11,1,85144.901316
11,1,40946.419643
11,1,3268.557292
11,1,7477.675595
11,1,2620.046875
11,1,59549.720588
11,1,19892.043605
11,1,1709.000000
11,1,2006.425000
11,1,5333.000000
11,1,62034.055556
11,1,5360.305147
11,1,107235.091463
11,1,2078.493243
11,1,65000.618902
11,1,4014.800000
11,1,6996.558673
11,1,1916.462209
11,1,28987.259615
11,1,1765.098214
11,1,2969.761364
11,1,48857.454545
11,1,36119.909091
11,1,43463.254808
11,1,2831.812500
11,1,1674.289894
11,1,6960.957237
11,1,1561.450000
11,1,1592.975610
11,1,12656.839286
11,1,12874.912500
11,1,4099.364130
11,1,12702.301829
11,1,2354.372093
11,1,88745.354167
11,1,4805.443878
11,1,1641.357143
11,1,41524.096257
11,1,2150.591837
11,1,50255.551471
11,1,2207.000000

11,1,9360.358108
11,1,1891.340909
11,1,2455.728723
11,1,29155.083333
11,1,1519.377604
11,1,2580.416667
11,1,1553.357143
11,1,2007.193182
11,1,4366.195000
11,1,65241.913690
11,1,19843.108108
11,1,1896.530488
11,1,3986.168367
11,1,9722.554687
11,1,5307.135714
11,1,4002.464286
11,1,4741.742647
11,1,1752.029070
11,1,100898.836111
11,1,13100.508621
11,1,4162.759615
11,1,1617.406250
11,1,45218.059211
11,1,2940.451220
11,1,2246.043478
11,1,2325.609756
11,1,2844.907143
11,1,1887.078431
11,1,85219.157895
11,1,2164.500000
11,1,160311.209302
11,1,2241.054054
11,1,44809.515244
11,1,4307.349359
11,1,3883.036932
11,1,45221.228571
11,1,36206.734375
11,1,1884.657895
11,1,79752.628049
11,1,31690.023256
11,1,45143.318182
11,1,25074.104651
11,1,95874.414634
11,1,16511.428571
11,1,4514.100000
11,1,1570.102041
11,1,1987.351064
11,1,25650.816406
11,1,2399.392442
11,1,6732.461538
11,1,7646.500000
11,1,3634.869048
11,1,2688.028571
11,1,3423.969512
11,1,1947.613889
11,1,1712.306548
11,1,2295.572674
11,1,1678.581250
11,1,53616.118421
11,1,11855.397727
11,1,29347.250000
11,1,1962.743421
11,1,8943.961538
11,1,7162.147059
11,1,3903.569149
11,1,5271.864583

11,1,29310.918367
11,1,127975.444767
11,1,2738.446023
11,1,1686.828947
11,1,2151.471354
11,1,1897.234375
11,1,91682.168605
11,1,9671.507813
11,1,2038.285088
11,1,2811.617188
11,1,105923.084459
11,1,3678.588710
11,1,3516.029412
11,1,62073.242021
11,1,31382.954545
11,1,3332.802326
11,1,1874.891026
11,1,4250.199405
11,1,2733.290625
11,1,2723.488372
11,1,3501.058140
11,1,59096.418605
11,1,1543.175532
11,1,45907.129032
11,1,72733.817308
11,1,136384.729167
11,1,4249.407738
11,1,6201.062500
11,1,2115.502907
11,1,1772.057432
11,1,131180.350610
11,1,2299.857143
11,1,3381.090909
11,1,1975.031250
11,1,19793.675000
11,1,2003.871094
11,1,1909.672619
11,1,64606.019231
11,1,1587.833333
11,1,15769.263889
11,1,1784.457143
11,1,2570.000000
11,1,17175.642857
11,1,4075.289773
11,1,6200.244792
11,1,2176.022727
11,1,2803.060811
11,1,4281.382353
11,1,2192.884868
11,1,9422.854167
11,1,1676.216216
11,1,1910.069767
11,1,71488.104839
11,1,3134.170732
11,1,28697.107143
11,1,2162.063953
11,1,2662.732558
11,1,2273.871795
11,1,154040.973485
11,1,1860.554348
11,1,20286.468085
11,1,2496.589286
11,1,3262.298077
11,1,68776.750000
11,1,6070.985795
11,1,2302.922619

11,1,62991.378472
11,1,6885.354651
11,1,71615.346154
11,1,2371.000000
11,1,1715.673780
11,1,1763.149457
11,1,3466.116477
11,1,1772.338816
11,1,30647.837838
11,1,24971.205128
11,1,2230.183824
11,1,1861.144886
11,1,3122.367188
11,1,1803.253676
11,1,3860.709559
11,1,1817.162234
11,1,3605.425532
11,1,1559.882353
11,1,2780.303030
11,1,3861.807065
11,1,2101.011628
11,1,2176.634615
11,1,2447.500000
11,1,4982.394531
11,1,4914.567568
11,1,1957.622093
11,1,6696.989286
11,1,1727.005952
11,1,3127.636792
11,1,61079.114286
11,1,73721.125000
11,1,69755.783333
11,1,90113.186047
11,1,1992.352941
11,1,3336.906977
11,1,3108.750000
11,1,13622.730769
11,1,54219.925000
11,1,13546.025000
11,1,1506.437500
11,1,3540.225694
11,1,2059.508929
11,1,2146.702128
11,1,18583.645833
11,1,8743.975610
11,1,96075.012500
11,1,22545.764706
11,1,3808.583333
11,1,3016.175000
11,1,3538.255952
11,1,2967.092391
11,1,2986.110294
11,1,3143.724265
11,1,140554.160256
11,1,4279.265957
11,1,6879.462766
11,1,1698.892500
11,1,2177.462209
11,1,5342.503571
11,1,9461.008333
11,1,21269.156250
11,1,2389.192308
11,1,6924.680851
11,1,1825.376884
11,1,1776.500000
11,1,1985.990854

11,1,46692.545732
11,1,75795.274390
11,1,1575.762500
11,1,2534.905405
11,1,1533.092105
11,1,1891.746711
11,1,1705.826531
11,1,1982.719512
11,1,1682.250000
11,1,1887.426471
11,1,9326.862500
11,1,2355.505814
11,1,1916.756757
11,1,1737.441489
11,1,59324.146341
11,1,16998.488095
11,1,8582.022727
11,1,43808.470588
11,1,2169.357143
11,1,24438.168750
11,1,3356.397059
11,1,1873.330645
11,1,47305.976744
11,1,3919.555556
11,1,8878.956522
11,1,1779.826705
11,1,70731.217949
11,1,84864.500000
11,1,4043.680851
11,1,1861.420213
11,1,4725.933333
11,1,2123.356250
11,1,1732.834559
11,1,2511.062500
11,1,1612.432692
11,1,1574.794444
11,1,2433.247283
11,1,39804.604651
11,1,171531.750000
11,1,2693.565217
11,1,2940.686170
11,1,30838.708333
11,1,30304.636905
11,1,6303.519886
11,1,3381.201220
11,1,68889.192308
11,1,94239.682927
11,1,17584.250000
11,1,10396.468085
11,1,1554.657143
11,1,2044.214286
11,1,2222.991667
11,1,10311.029762
11,1,1683.791667
11,1,1546.485795
11,1,2042.242647
11,1,4090.122396
11,1,1931.461538
11,1,72617.514493
11,1,23600.015625
11,1,5681.614796
11,1,3937.656250
11,1,4054.585366
11,1,39601.875000
11,1,30055.153846
11,1,4381.932515

11,1,5501.680556
11,1,1942.952381
11,1,2387.352941
11,1,12174.752315
11,1,5075.781250
11,1,1847.604651
11,1,1678.400000
11,1,11981.437500
11,1,3307.400000
11,1,6411.180000
11,1,9289.625000
11,1,42004.112500
11,1,44745.500000
11,1,1512.370000
11,1,2090.791667
11,1,18360.887755
11,1,55167.586538
11,1,89502.821429
11,1,6522.143750
11,1,31825.250000
11,1,3804.914773
11,1,3263.250000
11,1,97342.953488
11,1,51048.875000
11,1,1887.668750
11,1,2234.239362
11,1,38496.613497
11,1,71578.791667
11,1,4301.207317
11,1,66481.137195
11,1,2158.447222
11,1,5298.006098
11,1,4392.888889
11,1,168736.378125
11,1,5681.046875
11,1,76530.571429
11,1,48997.814394
11,1,2681.909091
11,1,114531.539634
11,1,6663.716216
11,1,1543.843750
11,1,157158.006098
11,1,56282.220395
11,1,2988.048246
11,1,35809.596154
11,1,37485.625000
11,1,4759.041667
11,1,56503.774306
11,1,11665.426471
11,1,2630.466667
11,1,1785.380682
11,1,6254.100000
11,1,23682.031250
11,1,3266.949405
11,1,6193.577381
11,1,3317.562500
11,1,3021.437500
11,1,1771.910256
11,1,65432.846154
11,1,10073.808824
11,1,3178.000000
11,1,2779.366667
11,1,11115.843023
11,1,7472.867647
11,1,1729.560000
11,1,4165.366667

11,1,1743.781915
11,1,2129.531250
11,1,55374.291667
11,1,15185.491071
11,1,2361.536585
11,1,1706.918919
11,1,87546.781250
11,1,5408.527778
11,1,1502.043478
11,1,1693.534091
11,1,2060.440789
11,1,2977.253049
11,1,2970.500000
11,1,1571.538462
11,1,1606.705882
11,1,2191.590426
11,1,29050.975000
11,1,1678.652174
11,1,2219.732558
11,1,18961.820513
11,1,70862.792553
11,1,11253.636364
11,1,1804.234375
11,1,8930.173077
11,1,1976.854167
11,1,9286.750000
11,1,1978.838235
11,1,1887.095930
11,1,1795.513298
11,1,2094.550000
11,1,1947.318750
11,1,2685.133721
11,1,2306.562500
11,1,4319.959559
11,1,2058.333333
11,1,8494.718750
11,1,3932.116279
11,1,64633.537162
11,1,2095.448370
11,1,2871.239130
11,1,2473.079545
11,1,3861.622642
11,1,2286.256410
11,1,2435.058824
11,1,68765.100000
11,1,24748.532895
11,1,1733.704545
11,1,2232.936224
11,1,21981.250000
11,1,2212.927632
11,1,1964.696970
11,1,5679.250000
11,1,5312.212766
12,2,6376.710784
12,2,32823.900510
12,2,7873.836364
12,2,2061.562500
12,2,2940.827381
12,2,5858.450000
12,2,4715.069444
12,2,2642.230769
12,2,4646.708333
12,2,3878.334184
12,2,4676.573171
12,2,5877.113095
12,2,1677.691176

12,2,1870.200000
12,2,3140.166667
12,2,7099.145833
12,2,1867.850000
12,2,1671.988636
12,2,6895.098558
12,2,1608.340909
12,2,2956.833333
12,2,7174.087079
12,2,1594.212500
12,2,5935.471591
12,2,1954.279018
12,2,8544.760563
12,2,1656.851190
12,2,3785.406250
12,2,3438.977612
12,2,3893.843137
12,2,1526.756098
12,2,2173.034722
12,2,8422.574468
12,2,14223.850000
12,2,2119.536458
12,2,7325.875000
12,2,2204.166667
12,2,2788.268293
12,2,3787.968750
12,2,4188.653846
12,2,1691.653061
12,2,1948.991667
12,2,2455.462209
12,2,1881.125000
12,2,3118.465909
12,2,1575.915816
12,2,1856.953947
12,2,1582.377500
12,2,4705.346154
12,2,4999.274306
12,2,1911.435897
12,2,1709.666667
12,2,6271.332500
12,2,3312.964674
12,2,1552.125000
12,2,3902.807692
12,2,1920.187500
12,2,1664.000000
12,2,1521.968750
12,2,1644.067308
12,2,4111.461538
12,2,3356.256831
12,2,1798.903846
12,2,2538.371212
12,2,2317.975610
12,2,1823.772727
12,2,11223.275568
12,2,39152.044444
12,2,5276.427083
12,2,1804.057143
12,2,1994.936111
12,2,5588.656250
12,2,2698.434783
12,2,6780.533333
12,2,45170.507576
12,2,1597.223684
12,2,4440.976190
12,2,2222.258152
12,2,2422.608974

12,2,2063.771277
12,2,10762.888889
12,2,3185.875000
12,2,1793.119048
12,2,1806.000000
12,2,3206.704861
12,2,3556.931034
12,2,3286.530556
12,2,2864.204545
12,2,1953.196809
12,2,3168.916667
12,2,2367.187500
12,2,1664.500000
12,2,1642.136364
12,2,10644.240000
12,2,3282.545455
12,2,1850.646341
12,2,3474.583333
12,2,1588.577703
12,2,4869.521635
12,2,6793.112745
12,2,3567.790541
12,2,5038.325000
12,2,1639.156863
12,2,2468.683511
12,2,1909.982993
12,2,1771.191489
12,2,3803.500000
12,2,8057.485119
12,2,1780.534031
12,2,3312.508772
12,2,2293.529412
12,2,3714.142857
12,2,8297.196429
12,2,6361.875000
12,2,1565.293919
12,2,2673.579545
12,2,4984.141304
12,2,4058.393868
12,2,1906.485294
12,2,2300.500000
12,2,1566.911765
12,2,1546.694712
12,2,1811.633508
12,2,2597.357143
12,2,9891.525714
12,2,1499.395000
12,2,5722.947368
12,2,1963.804348
12,2,3726.510870
12,2,2378.710714
12,2,16478.847222
12,2,4681.478261
12,2,2460.943182
12,2,3308.488636
12,2,4410.081633
12,2,2002.500000
12,2,1601.943182
12,2,5131.178571
12,2,2345.100000
12,2,7414.541667
12,2,9306.261029
12,2,3098.984694
12,2,1623.090278
12,2,6447.381579
12,2,8250.310345

12,2,2509.267500
12,2,4208.662162
12,2,14575.663043
12,2,1798.734756
12,2,3835.776042
12,2,3573.602273
12,2,3468.446429
12,2,2194.157609
12,2,2043.138889
12,2,8567.193396
12,2,2842.441860
12,2,2955.617788
12,2,5822.489362
12,2,2625.000000
12,2,5625.331522
12,2,15254.125000
12,2,132957.375000
12,2,4796.843137
12,2,2048.160377
12,2,3782.747159
12,2,2304.634146
12,2,2728.633621
12,2,4927.945946
12,2,4605.159091
12,2,21780.150000
12,2,2179.101562
12,2,2431.830729
12,2,1956.019231
12,2,2474.994186
12,2,3213.516588
12,2,1552.192308
12,2,2286.016667
12,2,1607.006757
12,2,3948.995455
12,2,11529.091146
12,2,2373.250000
12,2,2447.222222
12,2,7928.000000
12,2,1552.756757
12,2,2596.718750
12,2,4615.535714
12,2,2733.111702
12,2,1737.829384
12,2,4523.916667
12,2,4457.258929
12,2,2389.191667
12,2,1512.949405
12,2,6895.714286
12,2,1803.097500
12,2,1917.578804
12,2,4948.546875
12,2,3529.122449
12,2,2550.510204
12,2,1736.525000
12,2,3305.763889
12,2,6016.776596
12,2,2388.951923
12,2,1980.440341
12,2,11163.423913
12,2,2724.964286
12,2,2158.255208
12,2,1646.063953
12,2,2513.402439
12,2,2497.846939
12,2,1998.182432
12,2,2541.875000

12,2,3710.313725
12,2,5732.772959
12,2,2089.653571
12,2,5192.963235
12,2,1999.840361
12,2,2160.507979
12,2,2791.396875
12,2,2319.937500
12,2,1775.750000
12,2,2409.263736
12,2,4825.941667
12,2,3425.865385
12,2,3878.185022
12,2,1795.260870
12,2,2227.363636
12,2,9513.390873
12,2,2284.399038
12,2,1815.680233
12,2,2336.537234
12,2,4060.767773
12,2,8093.639706
12,2,2565.812903
12,2,2963.440217
12,2,1977.625000
12,2,4713.552083
12,2,1666.754902
12,2,9627.781250
12,2,3154.610577
12,2,2409.562500
12,2,2846.000000
12,2,2459.507853
12,2,2371.638393
12,2,2669.394958
12,2,9805.353659
12,2,3074.725191
12,2,3781.271341
12,2,1854.779255
12,2,1885.588710
12,2,4935.337719
12,2,14959.619565
12,2,1681.436170
12,2,1618.925532
12,2,10029.583333
12,2,3082.835526
12,2,4543.653846
12,2,5133.386574
12,2,1976.243243
12,2,3299.916667
12,2,1741.919872
12,2,3073.091463
12,2,4921.772500
12,2,2513.275568
12,2,2484.680851
12,2,13269.395161
12,2,1773.392500
12,2,2156.400000
12,2,5561.367188
12,2,6836.680851
12,2,1890.925000
12,2,3043.901316
12,2,3551.089286
12,2,19190.950000
12,2,6072.529412
12,2,2087.583333
12,2,10160.502717
12,2,2437.306818

12,2,6605.553241
12,2,2593.968137
12,2,3058.887755
12,2,9706.020833
12,2,9905.586207
12,2,8898.176630
12,2,1782.860465
12,2,2339.772436
12,2,60653.637019
12,2,3594.837500
12,2,7608.100000
12,2,2099.375000
12,2,2045.970588
12,2,3456.575000
12,2,2520.668750
12,2,2203.825000
12,2,2284.851852
12,2,1708.470588
12,2,3532.866667
12,2,2834.252660
12,2,2710.545000
12,2,3875.000000
12,2,5585.477273
12,2,1784.255319
12,2,4026.328125
12,2,5652.275000
12,2,5605.133929
12,2,2577.296053
12,2,3842.545455
12,2,1785.265086
12,2,2263.226415
12,2,3192.364583
12,2,3435.826923
12,2,8095.622159
12,2,2875.625000
12,2,1591.947581
12,2,3174.084906
12,2,2809.272109
12,2,2363.957447
12,2,2757.167763
12,2,5622.384615
12,2,2659.603261
12,2,4923.204545
12,2,4363.435976
12,2,1555.176136
12,2,1590.596154
12,2,1618.589286
12,2,1955.584184
12,2,3770.324561
12,2,3662.131707
12,2,7581.290503
12,2,1535.500000
12,2,4294.547619
12,2,3909.994681
12,2,2203.796512
12,2,1679.952778
12,2,2549.086735
12,2,2944.524554
12,2,2216.006098
12,2,1521.525641
12,2,1665.314516
12,2,1968.613636
12,2,11591.075581
12,2,1653.479730
12,2,5102.406250
12,2,10329.451389

12,2,3295.959184
12,2,4590.851351
12,2,2307.576389
12,2,3002.707386
12,2,9566.647500
12,2,1613.725000
12,2,1909.042683
12,2,4055.752688
12,2,2276.969512
12,2,4300.372222
12,2,2978.184615
12,2,7447.890244
12,2,6354.260000
12,2,1591.515306
12,2,2088.615000
12,2,1979.483333
12,2,2001.120690
12,2,8917.521930
12,2,1643.734043
12,2,4146.595109
12,2,5975.191489
12,2,3121.980392
12,2,2181.845930
12,2,2868.342105
12,2,1563.902439
12,2,1536.171512
12,2,4186.000000
12,2,2416.371795
12,2,1953.250000
12,2,24369.204545
12,2,3746.865741
12,2,2027.716518
12,2,2129.096591
12,2,1693.113372
12,2,4462.539062
12,2,1740.483333
12,2,3819.889205
12,2,2134.068750
12,2,2989.009259
12,2,1500.079787
12,2,1811.773585
12,2,2358.508772
12,2,2874.426829
12,2,4352.619898
12,2,2199.387931
12,2,4508.130952
12,2,4395.976563
12,2,2393.333333
12,2,28826.250000
12,2,2575.166667
12,2,3204.058333
12,2,1739.500000
12,2,1517.403846
12,2,1672.948864
12,2,2870.921875
12,2,3085.352273
12,2,2728.500000
12,2,1586.560000
12,2,3609.081250
12,2,5248.589286
12,2,2989.864583
12,2,3200.396739
12,2,4063.702830
12,2,13665.256098
12,2,2132.500000
12,2,4181.867347

12,2,1983.285714
12,2,4067.666667
12,2,2313.889535
12,2,31128.777778
12,2,1525.468750
12,2,2443.411765
12,2,1542.064516
12,2,4924.938953
12,2,5975.497340
12,2,3112.855114
12,2,3372.587766
12,2,4759.079787
12,2,1631.821429
12,2,3501.533854
12,2,8954.925000
12,2,6306.033019
12,2,4559.186404
12,2,1904.221875
12,2,1881.397959
12,2,4415.016393
12,2,3173.418605
12,2,1534.125000
12,2,3528.594388
12,2,7411.709184
12,2,2099.671875
12,2,4876.616279
12,2,3712.228261
12,2,1937.552632
12,2,1768.822917
12,2,1596.802632
12,2,3765.468750
12,2,2139.253695
12,2,9659.139344
12,2,7540.552863
12,2,1548.079295
12,2,9878.390625
12,2,1631.011628
12,2,4369.040000
12,2,2415.312500
12,2,1543.195652
12,2,2826.729167
12,2,1680.292553
12,2,1849.448370
12,2,17645.846154
12,2,1814.572917
12,2,5479.250000
12,2,2408.177083
12,2,1573.906250
12,2,1616.390244
12,2,4871.218750
12,2,5818.070652
12,2,6602.826271
12,2,2198.916667
12,2,1736.503125
12,2,2857.383721
12,2,1543.250000
12,2,2102.656250
12,2,7455.276316
12,2,8493.772059
12,2,2230.843750
12,2,6637.086538
12,2,2175.500000
12,2,2186.602941
12,2,1756.386364
12,2,2517.135638
12,2,1943.517442

12,2,3325.588235
12,2,1845.526210
12,2,10931.462963
12,2,2185.072917
12,2,1822.939394
12,2,1818.359756
12,2,1953.904255
12,2,1949.719512
12,2,2798.125000
12,2,2981.119186
12,2,1632.424479
12,2,3561.105263
12,2,1521.694444
12,2,1959.114583
12,2,4030.176829
12,2,3498.478261
12,2,5175.031250
12,2,5439.271277
12,2,6653.054348
12,2,3315.738636
12,2,5051.798780
12,2,4578.607955
12,2,3484.503049
12,2,5102.644886
12,2,3403.446809
12,2,1895.162791
12,2,1871.875000
12,2,2576.867925
12,2,2059.710937
12,2,4341.247449
12,2,3436.158333
12,2,3352.679348
12,2,1527.320122
12,2,4081.903061
12,2,2111.375000
12,2,4997.568452
12,2,4816.038265
12,2,23955.159091
12,2,1812.750000
12,2,1595.243750
12,2,2881.605882
12,2,4419.085938
12,2,3889.159091
12,2,1683.730769
12,2,2303.189427
12,2,2666.673469
12,2,1967.427632
12,2,5618.752315
12,2,4820.241477
12,2,2444.558824
12,2,3217.433511
12,2,7192.384868
12,2,2243.038043
12,2,9150.437500
12,2,6885.525735
12,2,2594.593023
12,2,2025.581731
12,2,2474.834559
12,2,1843.381250
12,2,1594.164062
12,2,1623.025641
12,2,3251.129630
12,2,1733.837264
12,2,45691.930804
12,2,1978.450000
12,2,10787.500000

12,2,2077.887500
12,2,2236.756757
12,2,1538.792500
12,2,2061.162791
12,2,1618.821429
12,2,2777.046053
12,2,4193.332386
12,2,3405.255319
12,2,2139.094697
12,2,1537.250000
12,2,1530.666667
12,2,3615.727778
12,2,6386.527778
12,2,4747.941667
12,2,6435.663043
12,2,2665.581081
12,2,3592.954545
12,2,4757.578804
12,2,2530.128378
12,2,2592.788889
12,2,4740.027439
12,2,2479.625000
12,2,16487.847973
12,2,4425.210000
12,2,11071.888158
12,2,3217.122549
12,2,1717.231771
12,2,1571.642045
12,2,3144.150000
12,2,5477.836538
12,2,1802.857143
12,2,2199.033088
12,2,1645.677632
12,2,4733.548295
12,2,6491.209016
12,2,10248.103261
12,2,3055.024457
12,2,2284.131579
12,2,1952.950000
12,2,2524.440104
12,2,4410.521739
12,2,4039.233974
12,2,1696.887755
12,2,7693.109756
12,2,2514.048951
12,2,3538.902778
12,2,1616.404523
12,2,4052.815789
12,2,1761.703488
12,2,1536.800000
12,2,1931.727041
12,2,5868.953488
12,2,2412.092500
12,2,3885.306122
12,2,1804.161932
12,2,2102.970745
12,2,1756.940217
12,2,3559.920000
12,2,1753.402778
12,2,2883.801282
12,2,5212.101974
12,2,11659.454861
12,2,4843.671875
12,2,20641.913889
12,2,2436.127660
12,2,2042.062500

12,2,1731.911111
12,2,2007.631579
12,2,2996.671569
12,2,6870.500000
12,2,16020.863839
12,2,2567.388889
12,2,2056.093750
12,2,3699.290698
12,2,3198.903846
12,2,8284.276786
12,2,3441.189815
12,2,3666.422222
12,2,1978.872396
12,2,1812.927083
12,2,2264.184896
12,2,2512.226562
12,2,5205.025000
12,2,4469.210106
12,2,6843.600000
12,2,19325.075980
12,2,2070.043478
12,2,7381.545213
12,2,5710.607143
12,2,2151.512019
12,2,5412.566860
12,2,3042.362805
12,2,3003.230769
12,2,1835.857143
12,2,2288.937500
12,2,1954.983108
12,2,5991.072368
12,2,2990.930851
12,2,2441.628049
12,2,3170.608696
12,2,2961.000000
12,2,5918.155488
12,2,5456.437500
12,2,1681.521739
12,2,2691.945312
12,2,9575.866379
12,2,2754.619048
12,2,1850.069149
12,2,3957.272727
12,2,2018.405405
12,2,1548.250000
12,2,4650.274096
12,2,2431.250000
12,2,2566.734375
12,2,2514.200000
12,2,4302.540816
12,2,2433.414062
12,2,2409.879808
12,2,4686.533333
12,2,1751.153061
12,2,2953.025000
12,2,2402.840000
12,2,3042.423077
12,2,9667.068182
12,2,8296.750000
12,2,5915.993243
12,2,6633.735849
12,2,4911.706522
12,2,1725.240000
12,2,2339.511364
12,2,2145.844444
12,2,7996.500000

12,2,11810.250000
12,2,2093.776316
12,2,4938.919598
12,2,5561.735577
12,2,2106.737500
12,2,2619.239362
12,2,5341.134146
12,2,5434.214844
12,2,1564.464286
12,2,3102.618421
12,2,4525.430921
12,2,3311.764706
12,2,1901.232500
12,2,3645.630682
12,2,2582.652439
12,2,2007.979508
12,2,2758.000000
12,2,1684.260204
12,2,2813.836364
12,2,1823.419872
12,2,3306.039216
12,2,1720.954545
12,2,2379.494186
12,2,7023.114583
12,2,1865.736486
12,2,1551.941176
12,2,2045.890625
12,2,1536.422872
12,2,4795.020468
12,2,3376.009479
12,2,2872.750000
12,2,2388.066667
12,2,1575.687500
12,2,1599.342391
12,2,2675.287500
12,2,26580.426396
12,2,1832.539474
12,2,5059.056122
12,2,2915.083333
12,2,4821.328431
12,2,33421.866279
12,2,13303.589286
12,2,1687.375000
12,2,1903.750000
12,2,1759.259375
12,2,3375.666667
12,2,4461.127358
12,2,2339.758333
12,2,2511.931319
12,2,47205.642045
12,2,2088.351351
12,2,3384.648649
12,2,4303.162500
12,2,3755.250000
12,2,2159.741071
12,2,1840.484694
12,2,3174.592105
12,2,20326.466667
12,2,6016.964286
12,2,2740.955882
12,2,1892.211538
12,2,1748.638889
12,2,2227.322500
12,2,4665.653409
12,2,4160.966667
12,2,3759.965714

12,2,5008.254902
12,2,1950.852273
12,2,2729.182927
12,2,1815.188679
12,2,7448.759494
12,2,1590.585106
12,2,3831.787234
12,2,3283.311224
12,2,13565.435096
12,2,3594.653846
12,2,1862.422222
12,2,2392.588235
12,2,1583.125000
12,2,6905.062500
12,2,1603.115385
12,2,1614.432432
12,2,2497.690789
12,2,6982.906977
12,2,2055.261628
12,2,4442.000000
12,2,7719.470588
12,2,12002.584375
12,2,5521.169811
12,2,4561.690909
12,2,1505.106132
12,2,4931.454545
12,2,3214.223404
12,2,3518.958333
12,2,2253.000000
12,2,2901.687500
12,2,3186.526316
12,2,2942.250000
12,2,3372.400000
12,2,3058.433962
12,2,10700.209302
12,2,3259.187500
12,2,1832.702703
12,2,16693.836735
12,2,1668.335714
12,2,13268.349537
12,2,3129.570000
12,2,4700.135870
12,2,2501.361702
12,2,1788.755319
12,2,4436.081019
12,2,11471.840909
12,2,2368.200000
12,2,11717.318182
12,2,5791.151316
12,2,1888.083333
12,2,3698.670918
12,2,2081.440000
12,2,5576.380368
12,2,1633.534091
12,2,3986.979651
12,2,1537.713415
12,2,1659.000000
12,2,5582.638889
12,2,1694.678922
12,2,7794.315000
12,2,11760.294271
12,2,1726.860465
12,2,4943.294118
12,2,1635.285714
12,2,4194.631579
12,2,3301.720930

12,2,2838.298469
12,2,6103.904545
12,2,1658.727273
12,2,18251.884615
12,2,6292.768293
12,2,2920.891667
12,2,2801.656250
12,2,2728.155556
12,2,1630.432432
12,2,3359.695312
12,2,1729.187500
12,2,1815.800676
12,2,7962.139205
12,2,2022.000000
12,2,6151.880000
12,2,3837.625000
12,2,1869.289062
12,2,2151.562500
12,2,1506.665698
12,2,2561.639151
12,2,4171.312500
12,2,2158.027027
12,2,2295.787500
12,2,5867.536585
12,2,5947.950000
12,2,3445.696429
12,2,3142.241379
12,2,2004.640244
12,2,2843.337500
12,2,1945.484756
12,2,1629.594595
12,2,6808.317935
12,2,2110.270833
12,2,9767.425532
12,2,2811.565217
12,2,4127.774510
12,2,1659.689904
12,2,8111.419271
12,2,3514.000000
12,2,2267.535714
12,2,5120.944767
12,2,2386.774390
12,2,3552.022059
12,2,3476.250000
12,2,2869.250000
12,2,8158.687500
12,2,21086.178082
12,2,3898.027322
12,2,5442.846939
12,2,8337.183962
12,2,4559.304878
12,2,6262.718085
12,2,2196.644737
12,2,2513.619444
12,2,1787.382022
12,2,6256.480000
12,2,2644.740741
12,2,1514.979167
12,2,2712.869565
12,2,6208.900000
12,2,2273.757353
12,2,2065.729167
12,2,3699.658537
12,2,2065.750000
12,2,5972.617647
12,2,3127.788043

12,2,1622.283784
12,2,3005.442708
12,2,9359.137500
12,2,3750.625000
12,2,2250.115854
12,2,2737.273585
12,2,2775.109756
12,2,2973.182432
12,2,3380.486364
12,2,3762.565217
12,2,3489.500000
12,2,10007.097561
12,2,3978.836735
12,2,1625.000000
12,2,1655.937500
12,2,3103.833333
12,2,2459.218750
12,2,1581.103659
12,2,8607.000000
12,2,3764.571429
12,2,2109.692308
12,2,2651.283654
12,2,1915.110577
12,2,2206.346154
12,2,27557.568182
12,2,2414.000000
12,2,3021.281250
12,2,1697.976064
12,2,2034.725000
12,2,4322.750000
12,2,2670.822917
12,2,2116.571429
12,2,1972.859756
12,2,1610.851562
12,2,1512.951220
12,2,12573.657895
12,2,1592.756579
12,2,1597.945175
12,2,1933.193069
12,2,1537.131944
12,2,4127.056604
12,2,2015.974359
12,2,1660.224599
12,2,2916.568182
12,2,3382.986111
12,2,3282.511792
12,2,4091.010204
12,2,1851.500000
12,2,3077.329268
12,2,5521.220833
12,2,2826.579545
12,2,19305.090517
12,2,2918.018750
12,2,12041.000000
12,2,1969.137255
12,2,8226.525000
12,2,2605.830882
12,2,3598.000000
12,2,6818.607143
12,2,3783.118421
12,2,2086.539062
12,2,3528.750000
12,2,4505.300000
12,2,5531.375000
12,2,7675.817568
12,2,16759.678879

12,2,3629.631944
12,2,2906.747500
12,2,5905.101093
12,2,4511.981481
12,2,3136.420455
12,2,3997.000000
12,2,5063.850610
12,2,4285.042553
12,2,2274.335366
12,2,2978.477778
12,2,9080.670455
12,2,2817.853261
12,2,2375.154412
12,2,4053.966837
12,2,2211.521552
12,2,1668.940789
12,2,2461.982143
12,2,1725.591346
12,2,4310.968085
12,2,1778.572500
12,2,8681.168539
12,2,1790.000000
12,2,4247.493243
12,2,2141.177083
12,2,10798.072848
12,2,2411.016760
12,2,2334.051282
12,2,2020.035714
12,2,1747.300000
12,2,4801.425532
12,2,6442.953488
12,2,8708.323864
12,2,1548.405000
12,2,2103.250000
12,2,4898.496855
12,2,6035.959459
12,2,5846.418750
12,2,1554.608696
12,2,4000.880435
12,2,4059.884615
12,2,9242.752907
12,2,1656.701531
12,2,5421.744681
12,2,4249.591346
12,2,3942.000000
12,2,1785.573529
12,2,1801.903061
12,2,2049.771739
12,2,1813.598837
12,2,9973.490566
12,2,5489.127907
12,2,3678.250000
12,2,3936.427083
12,2,3842.988636
12,2,1706.602041
12,2,3249.454545
12,2,3308.619318
12,2,1897.475000
12,2,5042.415865
12,2,1889.508152
12,2,1612.456250
12,2,4883.444444
12,2,3942.875000
12,2,3770.974138
12,2,1612.214286
12,2,13889.758721

12,2,3131.713542
12,2,2228.143617
12,2,5697.096939
12,2,1504.945513
12,2,5209.160714
12,2,3605.739362
12,2,1899.384259
12,2,5942.701389
12,2,2829.851852
12,2,2280.511364
12,2,3692.973958
12,2,1775.437500
12,2,2628.323353
12,2,4121.153061
12,2,4753.150862
12,2,2458.817610
12,2,12355.955357
12,2,2636.586310
12,2,3322.416667
12,2,33524.958333
12,2,3121.090909
12,2,3820.500000
12,2,2250.475446
12,2,4306.820000
12,2,2589.964286
12,2,1883.489130
12,2,1668.270370
12,2,5114.272727
12,2,4532.134434
12,2,3140.847222
12,2,5839.803571
12,2,5908.440909
12,2,11688.799528
12,2,5500.600000
12,2,19421.909091
12,2,3351.489583
12,2,10531.648256
12,2,2545.539773
12,2,2306.657258
12,2,4584.288265
12,2,2563.627907
12,2,4813.983108
12,2,2841.756579
12,2,4641.738372
12,2,2321.375000
12,2,2839.027778
12,2,1499.052941
12,2,2471.500000
12,2,3174.956731
12,2,7642.863636
12,2,4792.775510
12,2,1696.236842
12,2,2510.560976
12,2,3275.104545
12,2,2322.858491
12,2,4112.652439
12,2,1631.500000
12,2,1699.608696
12,2,2014.293269
12,2,3720.429448
12,2,1923.986842
12,2,7264.413043
12,2,2289.610714
12,2,2062.078947
12,2,2796.840909
12,2,3054.179487

12,2,4022.564815
12,2,2342.914894
12,2,1536.352941
12,2,9075.450000
12,2,4861.931250
12,2,5821.220238
12,2,1565.703488
12,2,4658.610465
12,2,12462.204082
12,2,2066.244792
12,2,2441.583333
12,2,3665.551136
12,2,1930.300000
12,2,11046.234375
12,2,1593.334184
12,2,2734.664773
12,2,7452.910326
12,2,3761.750000
12,2,3734.041667
12,2,1757.784091
12,2,5889.666667
12,2,2720.227848
12,2,2280.093023
12,2,4539.448529
12,2,2414.585938
12,2,2933.790761
12,2,3330.809045
12,2,2204.857143
12,2,1521.493243
12,2,6542.608696
12,2,4780.750000
12,2,4480.479592
12,2,4825.507979
12,2,5839.170732
12,2,2072.454082
12,2,6625.121795
12,2,2416.133333
12,2,4020.730114
12,2,5897.125000
12,2,5429.037037
12,2,3683.350000
12,2,1764.750000
12,2,3837.037500
12,2,1525.726562
12,2,10579.708333
12,2,2213.576923
12,2,5518.500000
12,2,8395.831395
12,2,5879.925481
12,2,1796.902439
12,2,3286.618750
12,2,3530.724432
12,2,4355.136364
12,2,3028.352041
12,2,1990.129032
12,2,2903.634615
12,2,2813.130208
12,2,3899.044271
12,2,5103.349359
12,2,2375.151316
12,2,1869.773585
12,2,5429.454545
12,2,4960.143216
12,2,5337.714286
12,2,2551.588235
12,2,2695.630682

12,2,4427.512500
12,2,2737.593750
12,2,2704.307432
12,2,4462.177632
12,2,3075.558511
12,2,1704.030000
12,2,2110.019231
12,2,9077.448980
12,2,1538.702128
12,2,1987.758152
12,2,16605.353659
12,2,2385.480000
12,2,3134.744444
12,2,5943.000000
12,2,2880.071429
12,2,2697.755682
12,2,1611.255102
12,2,3323.666667
12,2,2671.479167
12,2,7923.214286
12,2,2802.427083
12,2,5435.258929
12,2,3025.694149
12,2,9914.125000
12,2,2104.125000
12,2,2183.246622
12,2,5438.625000
12,2,2322.562500
12,2,1702.789286
12,2,1550.722826
12,2,2786.540761
12,2,3703.488372
12,2,4023.206395
12,2,5307.081395
12,2,1886.200000
12,2,2437.511628
12,2,8213.380556
12,2,3543.061224
12,2,1621.076923
12,2,2144.289773
12,2,1723.564189
12,2,3073.344340
12,2,4323.769022
12,2,1634.881579
12,2,1647.500000
12,2,2926.015625
12,2,5641.078431
12,2,1554.108108
12,2,12766.090909
12,2,2068.857143
12,2,4646.900000
12,2,2196.855263
12,2,4225.970238
12,2,2646.132653
12,2,5585.833333
12,2,5804.711538
12,2,3014.639423
12,2,2575.296053
12,2,1603.291667
12,2,2325.989583
12,2,2148.698630
12,2,2324.326923
12,2,6659.702381
12,2,2116.446809
12,2,1839.390625
12,2,5787.736979

12,2,1953.611111
12,2,2115.157500
12,2,3010.071429
12,2,1581.100000
12,2,3283.165000
12,2,25212.064189
12,2,2463.994186
12,2,1626.625000
12,2,4673.055000
12,2,1606.721354
12,2,3637.578947
12,2,3517.804348
12,2,3659.403846
12,2,3715.238636
12,2,2558.907609
12,2,3619.435897
12,2,4733.000000
12,2,3814.293839
12,2,49470.051471
12,2,6705.100000
12,2,3698.473684
12,2,3687.000000
12,2,1745.273256
12,2,13547.176829
12,2,4159.437500
12,2,4052.051471
12,2,1705.335227
12,2,1562.948529
12,2,1907.867925
12,2,2322.054054
12,2,1576.823718
12,2,3556.018405
12,2,3817.744444
12,2,22497.537879
12,2,2133.770270
12,2,1873.090278
12,2,6709.406250
12,2,2139.493902
12,2,6393.770000
12,2,1646.232558
12,2,4184.511111
12,2,1999.697917
12,2,1972.279070
12,2,1727.208333
12,2,6661.650000
12,2,2281.398936
12,2,5057.000000
12,2,6178.471591
12,2,1607.646552
12,2,3370.937500
12,2,1949.433824
12,2,6361.709821
12,2,1906.305556
12,2,5225.719388
12,2,4952.063776
12,2,6195.980000
12,2,8844.125000
12,2,2642.934028
12,2,1523.375000
12,2,3298.625000
12,2,1741.500000
12,2,4725.447115
12,2,2050.276042
12,2,12781.845395
12,2,4290.666667
12,2,8187.232558

12,2,1814.583333
12,2,2595.678125
12,2,5902.875000
12,2,3074.886076
12,2,1826.341463
12,2,6950.171875
12,2,1667.287037
12,2,3847.761364
12,2,1630.138889
12,2,9084.645833
12,2,2709.750000
12,2,3256.011111
12,2,4826.643145
12,2,2663.445652
12,2,1840.312500
12,2,2755.153846
12,2,8343.489583
12,2,2803.911765
12,2,1669.562500
12,2,2698.677083
12,2,6315.632653
12,2,1973.563953
12,2,3487.388889
12,2,3262.590909
12,2,2138.539894
12,2,4002.780000
12,2,6751.622642
12,2,1772.214286
12,2,6347.743750
12,2,1686.500000
12,2,5286.242647
12,2,8423.651316
12,2,2133.125000
12,2,5205.384615
12,2,2843.601351
12,2,2222.171875
12,2,6019.705189
12,2,6116.387755
12,2,2118.255556
12,2,1944.666667
12,2,3149.882979
12,2,10100.159483
12,2,3360.638889
12,2,2155.691667
12,2,1784.421875
12,2,2300.911111
12,2,17310.291667
12,2,5118.529070
12,2,3081.566667
12,2,5516.689655
12,2,3330.093023
12,2,5081.002976
12,2,1656.731707
12,2,9426.160000
12,2,7328.413462
12,2,1717.333333
12,2,2425.515000
12,2,7506.546875
12,2,1901.845982
12,2,4439.000000
12,2,5039.310897
12,2,1877.510870
12,2,4684.223837
12,2,1914.996875
12,2,1982.850000
12,2,1768.488372

12,2,2262.188202
12,2,1722.535714
12,2,8602.096774
12,2,1788.601852
12,2,1976.125000
12,2,2379.675000
12,2,1962.651961
12,2,3706.000000
12,2,4500.100000
12,2,2024.348837
12,2,3344.039634
12,2,3634.562500
12,2,6401.725610
12,2,2473.893617
12,2,10059.157895
12,2,4763.101852
12,2,3414.687500
12,2,3129.724432
12,2,5299.155556
12,2,4012.275000
12,2,2216.184211
12,2,2618.286058
12,2,4397.464286
12,2,2214.125000
12,2,3460.218750
12,2,3458.179688
12,2,4794.732143
12,2,1756.348837
12,2,1816.571429
12,2,2851.414474
12,2,1617.155556
12,2,1648.842105
12,2,2815.160714
12,2,2116.534091
12,2,5110.896552
12,2,6045.479167
12,2,4073.828704
12,2,3310.789634
12,2,6272.760417
12,2,2649.138889
12,2,5007.609375
12,2,1850.240625
12,2,2492.947917
12,2,2485.023256
12,2,5833.319444
12,2,4296.405405
12,2,3629.163636
12,2,7243.756944
12,2,1928.634731
12,2,4929.375000
12,2,3002.948370
12,2,4544.739362
12,2,3604.488636
12,2,1624.812500
12,2,2867.573171
12,2,3287.035088
12,2,3139.223529
12,2,2666.023649
12,2,1852.520833
12,2,21069.141667
12,2,2110.187500
12,2,1600.650000
12,2,4729.615854
12,2,2626.017442
12,2,3468.938596
12,2,2003.800000

12,2,3193.833333
12,2,2354.348837
12,2,6016.523256
12,2,2612.067935
12,2,3246.636364
12,2,1607.290909
12,2,2449.079545
12,2,2260.345930
12,2,3069.872340
12,2,2367.520833
12,2,4533.887255
12,2,3189.095745
12,2,5158.500000
12,2,3853.967391
12,2,2560.927326
12,2,19031.129310
12,2,1603.075000
12,2,4418.712575
12,2,1932.851190
12,2,2385.453125
12,2,6935.835366
12,2,4494.645408
12,2,1965.412234
12,2,5025.341667
12,2,23420.071429
12,2,3543.347727
12,2,3552.258065
12,2,4467.928571
12,2,28111.314286
12,2,6423.616477
12,2,1958.250000
12,2,10385.062500
12,2,1551.121951
12,2,13675.723684
12,2,4471.625000
12,2,3891.312500
12,2,3207.042553
12,2,3447.750000
12,2,2851.625000
12,2,2663.415761
12,2,3663.915789
12,2,4869.806122
12,2,2370.734694
12,2,3415.201220
12,2,2244.562500
12,2,3038.831731
12,2,57098.621134
12,2,4596.663462
12,2,4463.351293
12,2,2198.062500
12,2,1507.000000
12,2,1571.414062
12,2,4956.093750
12,2,1835.705128
12,2,64559.795918
12,2,2610.833333
12,2,3813.978774
12,2,4523.953125
12,2,3525.455128
12,2,2198.846698
12,2,8184.555556
12,2,1642.810811
12,2,3965.580000
12,2,1592.335106
12,2,3631.738095
12,2,3423.937500

12,2,4999.568966
12,2,3411.933511
12,2,4985.968610
12,2,2721.663934
12,2,7595.368421
12,2,2113.052326
12,2,9849.687500
12,2,2722.897959
12,2,1729.928125
12,2,8547.487500
12,2,7477.797814
12,2,2168.053892
12,2,1589.690789
12,2,7128.440217
12,2,2776.151042
12,2,3581.750000
12,2,4906.711538
12,2,1637.686047
12,2,3002.644737
12,2,21194.866667
12,2,7689.781915
12,2,2425.410256
12,2,1967.450980
12,2,2723.245283
12,2,1787.369186
12,2,1594.076923
12,2,1749.428571
12,2,2484.750000
12,2,6285.092593
12,2,1597.559211
12,2,1720.384146
12,2,3990.147059
12,2,12620.235849
12,2,4902.788793
12,2,2450.611364
12,2,1668.424528
12,2,7768.291667
12,2,2955.870968
12,2,2244.155172
12,2,4067.431937
12,2,4717.348958
12,2,3246.721154
12,2,3159.375000
12,2,1509.402778
12,2,2311.804487
12,2,2863.175926
12,2,3315.502242
12,2,2025.076923
12,2,6441.235656
12,2,1801.343750
12,2,2086.200000
12,2,5154.193182
12,2,5478.055556
12,2,9304.769643
12,2,7984.600000
12,2,2601.639535
12,2,1625.270531
12,2,2562.776730
12,2,1547.720930
12,2,2493.102941
12,2,3102.542500
12,2,1604.900000
12,2,1505.065000
12,2,3967.529070
12,2,1811.964115
12,2,3334.787234

12,2,4706.370283
12,2,2553.070312
12,2,4710.954545
12,2,2178.534884
12,2,3783.510870
12,2,3871.235000
12,2,2136.867925
12,2,2119.595455
12,2,2421.200000
12,2,2394.319079
12,2,4122.238095
12,2,5408.309211
12,2,4273.130814
12,2,1521.412500
12,2,2119.782443
12,2,1665.794872
12,2,2151.650000
12,2,4291.647059
12,2,8863.332461
12,2,5461.268473
12,2,4246.685714
12,2,2240.481707
12,2,21837.250000
12,2,1510.312500
12,2,6925.300000
12,2,2239.886628
12,2,4060.820312
12,2,1873.592391
12,2,2104.488372
12,2,4918.413265
12,2,3670.896341
12,2,4154.478723
12,2,2149.220109
12,2,13417.586207
12,2,5787.063636
12,2,1760.864865
12,2,5427.428571
12,2,8938.034091
12,2,6225.549419
12,2,1984.979167
12,2,3160.857143
12,2,7751.874251
12,2,4005.385417
12,2,2646.114130
12,2,6953.026042
12,2,1882.084135
12,2,2105.763587
12,2,8784.785714
12,2,2081.243421
12,2,3693.156250
12,2,2148.951087
12,2,2524.739362
12,2,13894.885204
12,2,2720.908654
12,2,3942.032609
12,2,7671.611413
12,2,2223.285714
12,2,2337.297297
12,2,2647.845109
12,2,2830.159091
12,2,3834.403125
12,2,4179.487981
12,2,1623.214286
12,2,2308.826389
12,2,3454.361111
12,2,2340.384615

12,2,2065.418605
12,2,1659.517857
12,2,1773.432143
12,2,3837.416667
12,2,4078.170732
12,2,2005.930556
12,2,4414.986111
12,2,1993.549479
12,2,2454.025641
12,2,7226.953975
12,2,4024.950739
12,2,1698.500000
12,2,4736.250000
12,2,1650.484694
12,2,1784.729730
12,2,2024.977778
12,2,9154.292453
12,2,3046.095395
12,2,1661.200000
12,2,2181.375000
12,2,5346.406250
12,2,1814.985119
12,2,38016.451613
12,2,5126.584302
12,2,1736.545455
12,2,2650.267857
12,2,2025.150735
12,2,2101.640000
12,2,2847.470588
12,2,3744.705882
12,2,1528.020833
12,2,3393.802885
12,2,4513.349265
12,2,2082.364583
12,2,4498.562500
12,2,1992.187500
12,2,1818.792398
12,2,3799.843750
12,2,1654.638889
12,2,2576.181034
12,2,1877.545455
12,2,1518.421053
12,2,8383.583333
12,2,1691.690000
12,2,22377.386574
12,2,4533.949640
12,2,3180.281915
12,2,2378.456522
12,2,2180.204545
12,2,3016.900000
12,2,1775.932927
12,2,1894.002841
12,2,2208.500000
12,2,54660.430851
12,2,4571.687500
12,2,2355.333333
12,2,1741.197368
12,2,12287.201878
12,2,6190.052910
12,2,1841.450000
12,2,10444.106855
12,2,2560.283537
12,2,14509.032407
12,2,1953.027778
12,2,11494.576923
12,2,7943.914063

12,2,1793.638889
12,2,6919.700000
12,2,2402.571429
12,2,6241.209302
12,2,6966.586538
12,2,1754.988372
12,2,1881.435000
12,2,3735.233333
12,2,2158.058594
12,2,5695.264423
12,2,3768.348684
12,2,4366.887255
12,2,1861.890909
12,2,3463.062500
12,2,3781.500000
12,2,7219.796569
12,2,2388.606383
12,2,2897.303125
12,2,2277.930000
12,2,1852.695000
12,2,2125.727273
12,2,3409.329268
12,2,3028.787500
12,2,1971.255814
12,2,6883.641393
12,2,2760.457447
12,2,3578.164474
12,2,3542.159574
12,2,3209.478723
12,2,7690.267442
12,2,4148.569149
12,2,2355.486842
12,2,8706.086957
12,2,1749.687500
12,2,7268.216463
12,2,4038.738095
12,2,9298.545455
12,2,2471.900943
12,2,2220.110119
12,2,4572.341981
12,2,2455.812500
12,2,1865.890625
12,2,3744.275510
12,2,2694.712500
12,2,13365.579545
12,2,1784.182927
12,2,11611.872642
12,2,2896.560976
12,2,7688.002273
12,2,12760.365546
12,2,6642.315217
12,2,3948.942708
12,2,1547.250000
12,2,4822.971698
12,2,3657.750000
12,2,2129.231771
12,2,2508.720930
12,2,3544.823529
12,2,2079.350000
12,2,2717.969388
12,2,1771.791667
12,2,3355.774390
12,2,18241.256579
12,2,4766.691327
12,2,8582.750000
12,2,2033.682432

12,2,1738.056650
12,2,5219.919643
12,2,1597.260417
12,2,1780.089888
12,2,2626.942935
12,2,2332.869048
12,2,3882.639151
12,2,1572.395408
12,2,1598.343750
12,2,3515.023437
12,2,1616.250000
12,2,11720.533898
12,2,5675.852273
12,2,4569.250000
12,2,5282.598214
12,2,8490.422581
12,2,1874.715278
12,2,2177.743421
12,2,1539.465116
12,2,4329.099490
12,2,4032.859375
12,2,2208.313953
12,2,2917.297872
12,2,2837.676136
12,2,2264.750000
12,2,2024.982143
12,2,2658.172222
12,2,1749.232143
12,2,1744.872340
12,2,3268.915698
12,2,2818.750000
12,2,3113.000000
12,2,1619.011628
12,2,2009.212500
12,2,6338.169811
12,2,1810.722826
12,2,23470.292969
12,2,4465.276316
12,2,11562.848404
12,2,2676.944444
12,2,1935.260870
12,2,2264.130814
12,2,2365.957746
12,2,3338.500000
12,2,2204.222826
12,2,1693.363095
12,2,3477.851064
12,2,2220.831522
12,2,3467.656250
12,2,4952.000000
12,2,1692.762500
12,2,2382.613636
12,2,15332.334459
12,2,3128.948980
12,2,4414.863636
12,2,2563.250000
12,2,8125.000000
12,2,3520.148649
12,2,4058.173469
12,2,11261.673077
12,2,9489.785714
12,2,2844.605978
12,2,2159.227273
12,2,1783.213542
12,2,2232.267045
12,2,10715.863636

12,2,2164.912500
12,2,1898.131579
12,2,10290.560185
12,2,4152.279070
12,2,2053.906977
12,2,5399.659091
12,2,2056.756250
12,2,12859.959091
12,2,3398.513587
12,2,3161.055233
12,2,4256.488889
12,2,5936.142857
12,2,3397.455556
12,2,5194.128472
12,2,6268.747500
12,2,9814.460000
12,2,2394.250000
12,2,5495.621528
12,2,2055.769231
12,2,3046.932203
12,2,3555.541667
12,2,3372.528409
12,2,1521.785714
12,2,7084.187500
12,2,2594.167553
12,2,2570.018293
12,2,5100.336538
12,2,2657.533654
12,2,2769.950000
12,2,3857.923077
12,2,2674.288889
12,2,11709.915094
12,2,4182.026786
12,2,1968.384615
12,2,5720.025641
12,2,1615.258621
12,2,8094.542683
12,2,1834.164634
12,2,2852.489865
12,2,2309.588068
12,2,1629.047619
12,2,3224.125000
12,2,1825.309783
12,2,2114.911932
12,2,8236.191489
12,2,1717.704545
12,2,1738.040323
12,2,3505.385417
12,2,3609.678571
12,2,9985.646930
12,2,12889.000000
12,2,4200.078947
12,2,1545.628125
12,2,3494.048913
12,2,4080.825000
12,2,4722.854167
12,2,3654.247449
12,2,2954.260870
12,2,4714.307692
12,2,2473.700000
12,2,5218.734694
12,2,3731.495098
12,2,2425.346154
12,2,2614.864516
12,2,6692.275000
12,2,9014.146341

12,2,2804.088889
12,2,4038.816176
12,2,3435.537500
12,2,4158.000000
12,2,1500.173913
12,2,2752.023256
12,2,3441.849057
12,2,6731.696875
12,2,5675.190972
12,2,2536.930000
12,2,3112.329082
12,2,4581.348837
12,2,2839.598039
12,2,2290.830729
12,2,3728.555556
12,2,1605.642857
12,2,55466.953431
12,2,1665.022727
12,2,1560.648649
12,2,1866.364130
12,2,1632.369444
12,2,6355.562500
12,2,9600.257322
12,2,2544.184783
12,2,5125.562500
12,2,2747.936170
12,2,1645.916667
12,2,1738.048611
12,2,4601.016447
12,2,1769.103448
12,2,2429.909574
12,2,1893.522500
12,2,1710.006098
12,2,2953.414062
12,2,3323.062500
12,2,2287.928571
12,2,1600.179878
12,2,3042.398649
12,2,9979.611111
12,2,6693.210000
12,2,1859.010135
12,2,9491.433014
12,2,3572.515152
12,2,2340.037037
12,2,2702.200000
12,2,3933.644737
12,2,2051.980769
12,2,5551.971154
12,2,4892.075000
12,2,4406.500000
12,2,1633.023810
12,2,2007.269231
12,2,3086.571023
12,2,1760.677083
12,2,9887.804020
12,2,2527.720109
12,2,2261.625000
12,2,1588.333333
12,2,4672.750000
12,2,2226.923913
12,2,8614.397959
12,2,4886.265625
12,2,2752.252451
12,2,8690.514706
12,2,1610.936170
12,2,4800.503289

12,2,3195.384146
12,2,15580.182927
12,2,5081.078804
12,2,3271.083333
12,2,1636.080000
12,2,2332.559091
12,2,12368.187500
12,2,2510.084302
12,2,2804.956667
12,2,3077.451613
12,2,2182.500000
12,2,1963.067308
12,2,1650.300000
12,2,3793.625000
12,2,3775.000000
12,2,2424.586957
12,2,4999.428571
12,2,1530.678571
12,2,1680.449468
12,2,3921.177500
12,2,1974.068750
12,2,2540.306250
12,2,5793.801724
12,2,2530.860795
12,2,4744.972826
12,2,14738.851563
12,2,2202.739583
12,2,2324.064815
12,2,2360.843750
12,2,1881.893617
12,2,2184.307927
12,2,4409.702586
12,2,4794.219340
12,2,3434.970395
12,2,6777.058962
12,2,3299.330357
12,2,2556.650000
12,2,5419.031250
12,2,5185.770833
12,2,3156.617647
12,2,1806.743902
12,2,2655.902439
12,2,2109.369318
12,2,1761.223404
12,2,4670.450000
12,2,2470.734375
12,2,5294.915254
12,2,7009.756098
12,2,9269.322368
12,2,3378.300000
12,2,2641.836735
12,2,2409.795918
12,2,1774.500000
12,2,1937.971875
12,2,1571.014535
12,2,1848.794118
12,2,2632.440476
12,2,4765.666667
12,2,5676.207447
12,2,7195.542135
12,2,1975.250000
12,2,6850.857759
12,2,2358.641892
12,2,2722.792553
12,2,4809.712042
12,2,2805.977833

12,2,6394.807292
12,2,2699.966837
12,2,1909.541667
12,2,4469.013158
12,2,2441.647059
12,2,7052.845238
12,2,5230.861486
12,2,1609.152778
12,2,1911.642857
12,2,2301.816038
12,2,2627.160000
12,2,3562.375000
12,2,5965.565217
12,2,4374.509375
12,2,3287.125000
12,2,1514.452830
12,2,1934.595745
12,2,1849.357955
12,2,3794.284884
12,2,2122.000000
12,2,4707.599432
12,2,7257.593750
12,2,5163.625000
12,2,1720.000000
12,2,1788.587097
12,2,1556.212500
12,2,1766.076220
12,2,2577.067308
12,2,3866.673295
12,2,2018.005556
12,2,3507.148936
12,2,2012.495370
12,2,3271.502907
12,2,1526.100610
12,2,2469.546392
12,2,2570.862745
12,2,2146.728723
12,2,1922.897959
12,2,2623.274390
12,2,1675.611364
12,2,4120.169872
12,2,1610.788265
12,2,1725.414634
12,2,2773.263889
12,2,4681.014706
12,2,1525.552632
12,2,3860.575658
12,2,2813.583333
12,2,2529.125000
12,2,3247.592391
12,2,9702.641447
12,2,2156.500000
12,2,3085.220745
12,2,5164.866279
12,2,4504.910714
12,2,3313.240566
12,2,13750.346939
12,2,6130.184783
12,2,2091.105769
12,2,2263.135135
12,2,8341.987448
12,2,2228.547500
12,2,2501.206250
12,2,3057.408163
12,2,3981.675000
12,2,1954.382813

12,2,3949.362500
12,2,3233.768519
12,2,2238.153846
12,2,3035.953488
12,2,2416.250000
12,2,4072.757653
12,2,1922.263158
12,2,2029.583333
12,2,3318.216463
12,2,4096.095238
12,2,2892.023256
12,2,10553.727273
12,2,8687.362745
12,2,4248.440217
12,2,3275.680556
12,2,4608.132353
12,2,2302.141304
12,2,2302.198171
12,2,4998.930851
12,2,3845.000000
12,2,1725.406250
12,2,4435.111111
12,2,2148.724359
12,2,2835.139535
12,2,4113.000000
12,2,1529.373239
12,2,4888.346939
12,2,2456.130952
12,2,2669.496875
12,2,1842.272727
12,2,5040.018182
12,2,1769.132653
12,2,2115.625000
12,2,4501.086538
12,2,2638.828125
12,2,3400.895833
12,2,1614.633523
12,2,2048.080556
12,2,2630.818182
12,2,4336.118902
12,2,1575.169643
12,2,3119.227041
12,2,4559.650000
12,2,3937.535714
12,2,1511.303191
12,2,2062.667683
12,2,2241.964286
12,2,2344.928571
12,2,1509.391827
12,2,2448.703704
12,2,5089.043011
12,2,2007.022059
12,2,2815.372093
12,2,1527.658537
12,2,3085.790323
12,2,2389.029412
12,2,2271.373984
12,2,4441.983696
12,2,2416.000000
12,2,81011.918605
12,2,1786.152027
12,2,7670.025510
12,2,5322.918919
12,2,1633.518868
12,2,3291.325000
12,2,4112.593750

12,2,2592.122449
12,2,2236.090426
12,2,2264.522222
12,2,1666.311224
12,2,2388.394737
12,2,5107.365000
12,2,4262.081395
12,2,9962.166667
12,2,1926.790698
12,2,2335.733333
12,2,2832.302326
12,2,1778.835227
12,2,10018.304545
12,2,1808.305000
12,2,3691.266892
12,2,2984.877828
12,2,1654.375000
12,2,3210.571429
12,2,3405.468750
12,2,2929.359375
12,2,2238.071429
12,2,2614.757979
12,2,2486.736842
12,2,10512.925000
12,2,3652.882979
12,2,2227.943396
12,2,5106.619632
12,2,3186.511111
12,2,4836.651163
12,2,2852.489362
12,2,6928.684896
12,2,1516.661585
12,2,3361.222500
12,2,12454.265625
12,2,3466.231771
12,2,4884.573370
12,2,2189.836735
12,2,1978.076531
12,2,3328.650000
12,2,4081.432927
12,2,2161.691489
12,2,5939.270408
12,2,1992.613636
12,2,3386.990625
12,2,1512.462209
12,2,3088.056122
12,2,5878.468750
12,2,4602.277778
12,2,3395.051282
12,2,1744.802083
12,2,5158.434211
12,2,9047.197115
12,2,2371.535714
12,2,4513.100000
12,2,14084.625000
12,2,2279.545732
12,2,1693.963235
12,2,1798.394231
12,2,4922.297872
12,2,1915.654070
12,2,2296.960227
12,2,2312.306548
12,2,16349.992857
12,2,3325.365854
12,2,1988.428571
12,2,2846.947368

12,2,3555.217391
12,2,2045.022727
12,2,3854.671512
12,2,1602.048295
12,2,1902.483108
12,2,3956.426471
12,2,11344.782500
12,2,8014.333333
12,2,2100.770833
12,2,1739.840909
12,2,2804.669811
12,2,5880.852941
12,2,4479.439103
12,2,3518.804348
12,2,5612.281250
12,2,1578.821429
12,2,2007.275641
12,2,4640.769231
12,2,2635.627841
12,2,1525.826816
12,2,6813.990385
12,2,3905.159722
12,2,4262.111979
12,2,3231.658654
12,2,5680.587500
12,2,1824.349057
12,2,2092.339286
12,2,18941.888889
12,2,3738.845238
12,2,18426.560976
12,2,3516.692073
12,2,3044.879717
12,2,6422.281250
12,2,72187.111111
12,2,2164.843137
12,2,2892.753571
12,2,2072.632353
12,2,6652.352941
12,2,1793.833333
12,2,9331.910000
12,2,2818.057692
12,2,1532.320513
12,2,2436.962209
12,2,1696.604167
12,2,3494.783333
12,2,6218.345000
12,2,2619.647059
12,2,1904.750000
12,2,2642.718750
12,2,2228.670455
12,2,3062.537500
12,2,2248.817500
12,2,3585.649123
12,2,2528.036458
12,2,2148.604167
12,2,4105.308511
12,2,3649.118644
12,2,3336.595506
12,2,11914.043478
12,2,1838.756098
12,2,1633.718750
12,2,6306.030000
12,2,2336.579082
12,2,1950.100962
12,2,3560.125000
12,2,2427.905000

12,2,4773.381579
12,2,6353.924528
12,2,2375.312500
12,2,6150.071875
12,2,2363.945946
12,2,1890.475000
12,2,1911.970109
12,2,4317.026316
12,2,6920.891393
12,2,3364.556122
12,2,2749.792683
12,2,2377.505102
12,2,1557.483108
12,2,3879.369186
12,2,3871.319444
12,2,2433.936047
12,2,4851.512500
12,2,6868.833333
12,2,3894.955357
12,2,3834.296053
12,2,2120.705882
12,2,2603.366071
12,2,2719.993421
12,2,10382.418367
12,2,4462.690000
12,2,6443.817857
12,2,3514.900000
12,2,5598.538043
12,2,3587.571429
12,2,2764.256250
12,2,2607.510593
12,2,1531.210526
12,2,3146.571429
12,2,2694.200000
12,2,2975.329787
12,2,4672.678571
12,2,4438.500000
12,2,1613.925532
12,2,2673.120690
12,2,1500.317073
12,2,3069.225166
12,2,6003.723404
12,2,3296.692500
12,2,19234.261364
12,2,2351.558824
12,2,2458.558140
12,2,3063.506410
12,2,9355.000000
12,2,2668.472561
12,2,1536.101974
12,2,3962.187500
12,2,1875.826087
12,2,8169.785714
12,2,5043.450000
12,2,5701.110795
12,2,3164.239796
12,2,3308.678125
12,2,5256.053191
12,2,4666.804878
12,2,2680.750000
12,2,1785.665625
12,2,2029.081081
12,2,5469.864583
12,2,10986.073171
12,2,7776.557692
12,2,2186.750000

12,2,1644.877907
12,2,9010.773585
12,2,30220.807143
12,2,1537.258152
12,2,5090.901163
12,2,1966.630102
12,2,2412.881579
12,2,4624.355556
12,2,1860.757353
12,2,3687.135417
12,2,5284.687500
12,2,5487.613372
12,2,3801.967949
12,2,1617.500000
12,2,5440.215426
12,2,2628.000000
12,2,10937.976744
12,2,1948.245000
12,2,4038.630137
12,2,1745.172414
12,2,3336.511905
12,2,1789.612245
12,2,1567.562500
12,2,1504.445946
12,2,1666.265152
12,2,4237.525000
12,2,2570.083333
12,2,3262.932927
12,2,2828.665541
12,2,8287.964623
12,2,5739.516667
12,2,1592.523256
12,2,3137.322115
12,2,2369.054245
12,2,1721.627358
12,2,10765.930851
12,2,2484.358289
12,2,6762.090909
12,2,1886.788043
12,2,2251.465909
12,2,2220.346875
12,2,2355.909091
12,2,5484.402439
12,2,1688.533537
12,2,6713.951923
12,2,4242.179348
12,2,8309.566667
12,2,1618.422222
12,2,3013.559659
12,2,1802.804348
12,2,1712.671875
12,2,4987.555233
12,2,1565.317935
12,2,6109.125000
12,2,1611.543860
12,2,2616.480263
12,2,4037.924171
12,2,1590.692737
12,2,1548.804688
12,2,5676.062500
12,2,1841.416667
12,2,4331.050481
12,2,5976.441406
12,2,1661.668605
12,2,6345.732759
12,2,2252.282609

12,2,2329.145833
12,2,1557.000000
12,2,2939.658537
12,2,6519.276442
12,2,8073.587209
12,2,2791.791667
12,2,2155.000000
12,2,3799.106707
12,2,4195.692073
12,2,2067.402174
12,2,20968.205882
12,2,2927.362745
12,2,5374.611413
12,2,3946.000000
12,2,2001.796569
12,2,2653.804348
12,2,10647.241071
12,2,5062.015000
12,2,3710.415205
12,2,2912.557143
12,2,4070.365854
12,2,1883.098684
12,2,1862.071429
12,2,1499.294872
12,2,2373.684524
12,2,3325.975610
12,2,1803.450000
12,2,8356.636364
12,2,3902.312500
12,2,6003.214286
12,2,2725.255102
12,2,3071.355556
12,2,2406.486486
12,2,9018.183511
12,2,2658.190341
12,2,1928.028846
12,2,2199.000000
12,2,1894.659884
12,2,2574.713415
12,2,1620.255208
12,2,4999.239130
12,2,2836.296053
12,2,5149.018750
12,2,2927.155405
12,2,2613.000000
12,2,2411.527500
12,2,9685.255319
12,2,2599.136364
12,2,2774.857143
12,2,4289.562500
12,2,5056.428977
12,2,7103.071429
12,2,4002.356771
12,2,4054.848039
12,2,2391.756944
12,2,2610.383117
12,2,2451.217391
12,2,1662.085714
12,2,2373.400000
12,2,1611.432432
12,2,2197.458333
12,2,2065.174342
12,2,2151.294872
12,2,6147.737500
12,2,2091.069672
12,2,2423.108108

12,2,3061.627500
12,2,2017.883721
12,2,2077.949468
12,2,2024.585000
12,2,3204.062500
12,2,6279.602041
12,2,3681.130435
12,2,2826.192308
12,2,2339.282609
12,2,1830.787037
12,2,8983.439024
12,2,3274.958333
12,2,4438.444134
12,2,6247.425481
12,2,4555.538462
12,2,2877.703947
12,2,7405.437500
12,2,1658.511364
12,2,3118.513514
12,2,5560.225962
12,2,2954.543750
12,2,2710.595238
12,2,3996.979592
12,2,2875.468137
12,2,3815.408416
12,2,1674.682927
12,2,2550.875000
12,2,7191.644231
12,2,3058.552632
12,2,3757.270408
12,2,1784.069149
12,2,2539.250000
12,2,1825.125000
12,2,2745.089286
12,2,3019.172872
12,2,2156.948571
12,2,1817.300000
12,2,3248.369681
12,2,1516.093750
12,2,2064.750000
12,2,2962.255000
12,2,2715.393750
12,2,1845.500000
12,2,2342.347826
12,2,4574.333333
12,2,6056.166667
12,2,14800.772059
12,2,4913.307692
12,2,1697.297619
12,2,4267.865741
12,2,2361.518293
12,2,2790.776316
12,2,2402.687500
12,2,17696.951705
12,2,2058.081081
12,2,3966.979592
12,2,1884.475610
12,2,15542.225000
12,2,2250.720109
12,2,3540.511364
12,2,6294.416667
12,2,1943.351351
12,2,1760.067568
12,2,3358.902174
12,2,16138.490385
12,2,2099.013158

12,2,3836.633333
12,2,2253.315789
12,2,3540.330357
12,2,2030.875000
12,2,1669.538462
12,2,3197.027778
12,2,3132.968750
12,2,3031.808824
12,2,1814.255814
12,2,2304.707317
12,2,3281.881250
12,2,2444.549342
12,2,4301.640000
12,2,7120.500000
12,2,3745.601064
12,2,1512.308824
12,2,15064.000000
12,2,5202.555556
12,2,1591.968750
12,2,8724.534884
12,2,4798.800633
12,2,4149.533333
12,2,2140.500000
12,2,5004.343750
12,2,7044.228261
12,2,2307.518293
12,2,3520.033816
12,2,20571.802817
12,2,5747.372340
12,2,4411.062500
12,2,1799.606383
12,2,2547.369318
12,2,5383.000000
12,2,2069.139535
12,2,1529.152174
12,2,2771.116848
12,2,1981.403509
12,2,6114.290761
12,2,2979.812500
12,2,6498.405405
12,2,2045.079268
12,2,51582.821429
12,2,1995.955556
12,2,6273.322368
12,2,4287.185096
12,2,2017.429348
12,2,3299.167553
12,2,1906.894737
12,2,1950.651163
12,2,1719.666667
12,2,1838.146277
12,2,1858.348039
12,2,2452.833333
12,2,6421.304688
12,2,3052.052632
12,2,4681.678899
12,2,3325.651786
12,2,2468.527174
12,2,6163.876838
12,2,2366.568182
12,2,2139.054348
12,2,3125.518750
12,2,3621.666667
12,2,1506.031250
12,2,2028.388889
12,2,3832.363636

12,2,3021.750000
12,2,8852.305556
12,2,7853.750000
12,2,2931.560976
12,2,2898.571023
12,2,2520.750000
12,2,1954.682692
12,2,5082.360465
12,2,1853.375000
12,2,5796.891026
12,2,5952.750000
12,2,4157.933962
12,2,2260.127907
12,2,5907.916667
12,2,4595.022222
12,2,1845.866071
12,2,87980.161765
12,2,4186.671875
12,2,2706.755319
12,2,3437.276596
12,2,2999.125000
12,2,6458.184783
12,2,4544.048913
12,2,3174.757075
12,2,2249.296053
12,2,2241.277778
12,2,3192.711957
12,2,3217.478261
12,2,3302.500000
12,2,3919.414894
12,2,2151.440000
12,2,1573.600000
12,2,4898.821429
12,2,3405.187500
12,2,3970.482143
12,2,1659.880814
12,2,2783.390625
12,2,3833.673516
12,2,2691.105128
12,2,3980.122283
12,2,1637.298780
12,2,2514.394022
12,2,5088.765957
12,2,2685.923913
12,2,3595.538265
12,2,7402.517857
12,2,1963.890306
12,2,1838.000000
12,2,4232.566964
12,2,1902.250000
12,2,2210.586957
12,2,1698.322674
12,2,5098.771341
12,2,12453.837500
12,2,3531.519737
12,2,5881.579545
12,2,2158.788889
12,2,3325.718750
12,2,6366.784375
12,2,2295.638021
12,2,2037.482412
12,2,1556.665829
12,2,3514.155556
12,2,1730.533784
12,2,3567.875000
12,2,13391.133333

12,2,4285.440217
12,2,15581.647436
12,2,2724.637755
12,2,5883.255814
12,2,23596.712042
12,2,2518.285714
12,2,1766.150000
12,2,2757.900000
12,2,7125.310000
12,2,2444.070755
12,2,3680.840909
12,2,3435.810000
12,2,1553.019231
12,2,3567.592391
12,2,11577.271277
12,2,3425.476440
12,2,2235.823034
12,2,3684.095000
12,2,3824.830357
12,2,2253.272059
12,2,2173.116848
12,2,25151.950617
12,2,4074.520000
12,2,1676.112245
12,2,1850.300595
12,2,3805.833333
12,2,26629.170732
12,2,3806.906250
12,2,2751.883152
12,2,2178.547297
12,2,4408.744681
12,2,2258.910256
12,2,3336.966667
12,2,3581.823529
12,2,2472.942073
12,2,1778.042614
12,2,1631.500000
12,2,7843.612583
12,2,4090.105263
12,2,2415.187500
12,2,1674.572917
12,2,16148.516204
12,2,2639.934211
12,2,1939.007212
12,2,2425.000000
12,2,2392.218750
12,2,1533.528302
12,2,10109.216667
12,2,3341.000000
12,2,4792.634804
12,2,7825.372222
12,2,5097.294643
12,2,3365.338235
12,2,2871.632653
12,2,2021.412791
12,2,1683.946023
12,2,1696.880000
12,2,2881.157895
12,2,2148.467105
12,2,2165.500000
12,2,10157.095238
12,2,3652.983796
12,2,6874.962963
12,2,1715.375000
12,2,1970.614130
12,2,4232.103723

12,2,1961.872449
12,2,4018.349432
12,2,6700.419271
12,2,2152.714286
12,2,6549.505208
12,2,2767.650000
12,2,1702.625000
12,2,14763.587500
12,2,2174.487500
12,2,2720.136364
12,2,1994.473684
12,2,5733.636364
12,2,2062.647059
12,2,3269.343137
12,2,6116.972826
12,2,1547.574468
12,2,1542.869565
12,2,3966.562500
12,2,1585.826087
12,2,2451.802326
12,2,3539.833333
12,2,4323.204545
12,2,7178.638889
12,2,1936.919811
12,2,2541.764706
12,2,1520.540816
12,2,2437.840278
12,2,1876.585938
12,2,13798.500000
12,2,1702.601974
12,2,2456.297619
12,2,2019.480769
12,2,8870.083333
12,2,1673.480000
12,2,2639.475000
12,2,8214.702703
12,2,1666.852941
12,2,2187.916667
12,2,9114.384434
12,2,2684.551282
12,2,2870.662162
12,2,4756.750000
12,2,2021.225000
12,2,2805.532609
12,2,2794.047619
12,2,2280.220930
12,2,2098.939394
12,2,3055.362500
12,2,2297.274510
12,2,3451.662162
12,2,2086.482143
12,2,8508.008671
12,2,4832.489583
12,2,2197.940217
12,2,2873.357143
12,2,4485.121951
12,2,1747.292553
12,2,4919.551020
12,2,3494.370968
12,2,4847.964286
12,2,2221.500000
12,2,2045.312500
12,2,2869.378049
12,2,3383.908537
12,2,2432.707386
12,2,2808.037500

12,2,2862.381579
12,2,2597.397727
12,2,18916.047222
12,2,2170.281250
12,2,3037.021739
12,2,2313.893617
12,2,3240.220588
12,2,1568.684524
12,2,1882.718750
12,2,3485.798077
12,2,1548.804348
12,2,2506.802326
12,2,14812.037234
12,2,9831.896341
12,2,2463.315217
12,2,2770.000000
12,2,2928.611111
12,2,3353.355978
12,2,2194.250000
12,2,2920.882812
12,2,3070.312500
12,2,1614.240000
12,2,1514.346154
12,2,1690.432432
12,2,3478.715426
12,2,6749.500000
12,2,2808.817568
12,2,1921.012500
12,2,1715.880319
12,2,4717.500000
12,2,2394.143229
12,2,1841.750000
12,2,7125.561404
12,2,4422.650000
12,2,1831.830688
12,2,9222.945000
12,2,2920.729592
12,2,2179.383721
12,2,2000.917614
12,2,7968.687500
12,2,1775.328125
12,2,4915.296296
12,2,1662.703125
12,2,2747.647059
12,2,3604.200980
12,2,6770.750000
12,2,2729.804878
12,2,8965.923469
12,2,2942.378205
12,2,14969.587629
12,2,10490.002500
12,2,5235.384868
12,2,5193.160714
12,2,7966.244048
12,2,2359.697368
12,2,2574.408163
12,2,2226.807947
12,2,1865.950000
12,2,8729.903061
12,2,2210.212766
12,2,1987.625000
12,2,5063.163265
12,2,2311.377551
12,2,5171.665761
12,2,2224.243421
12,2,2284.820755

12,2,1661.935897
12,2,1737.705556
12,2,2168.931373
12,2,5071.100962
12,2,2312.101744
12,2,8508.385204
12,2,2689.773810
12,2,6630.481707
12,2,2841.470000
12,2,4777.705882
12,2,2091.551136
12,2,1506.589844
12,2,2538.056548
12,2,2731.833333
12,2,1777.122222
12,2,2164.213816
12,2,6786.205882
12,2,8344.377358
12,2,1959.066176
12,2,1501.734043
12,2,1820.408163
12,2,1643.512755
12,2,1694.500000
12,2,2016.895349
12,2,5877.436364
12,2,1562.812500
12,2,2673.638889
12,2,2207.441038
12,2,4439.579327
12,2,4582.330189
12,2,2448.250000
12,2,2023.548387
12,2,4641.952206
12,2,1713.920000
12,2,3837.942308
12,2,10126.977273
12,2,1678.455000
12,2,2142.023256
12,2,1835.000000
12,2,5429.305389
12,2,4522.608796
12,2,11322.221719
12,2,4447.050000
12,2,4119.595745
12,2,3326.736979
12,2,10052.500000
12,2,5766.466667
12,2,2755.548295
12,2,2102.340278
12,2,3490.970930
12,2,6750.555288
12,2,40294.395270
12,2,2983.732558
12,2,17322.915761
12,2,6449.080128
12,2,2307.511628
12,2,1842.960784
12,2,1683.653846
12,2,1802.592949
12,2,3843.611486
12,2,2134.643750
12,2,2777.333333
12,2,7432.802083
12,2,7126.500000
12,2,2419.127717
12,2,2774.149390

12,2,2818.092593
12,2,2177.156716
12,2,1919.803571
12,2,2532.983696
12,2,4303.611842
12,2,5841.794643
12,2,7009.244444
12,2,2533.536364
12,2,2465.421875
12,2,3406.875000
12,2,2789.512195
12,2,1703.812500
12,2,5509.458333
12,2,3557.080000
12,2,2253.464286
12,2,3887.810345
12,2,1796.063636
12,2,1936.687500
12,2,3022.435897
12,2,8116.857143
12,2,5004.848214
12,2,1917.090686
12,2,2271.847826
12,2,9864.252907
12,2,4696.686047
12,2,10140.032407
12,2,2590.394608
12,2,2953.625000
12,2,3362.331081
12,2,1537.273810
12,2,2644.500000
12,2,2521.125000
12,2,5183.452381
12,2,2107.863971
12,2,1776.632979
12,2,67204.666667
12,2,4680.601563
12,2,3139.735119
12,2,6351.187500
12,2,1958.600000
12,2,5211.131818
12,2,3929.321229
12,2,4635.659091
12,2,1815.222222
12,2,3249.680851
12,2,3094.786184
12,2,1625.540541
12,2,9821.563776
12,2,1619.218750
12,2,4720.913462
12,2,2442.187500
12,2,3358.404762
12,2,4890.555556
12,2,4700.717949
12,2,5064.893617
12,2,2451.626506
12,2,5211.383178
12,2,2892.827586
12,2,10461.281250
12,2,1705.160714
12,2,2090.739362
12,2,2927.992500
12,2,2879.342262
12,2,1993.000000
12,2,2659.833333
12,2,2415.918919

12,2,2204.482143
12,2,1576.521739
12,2,4378.850000
12,2,4369.618590
12,2,3777.180556
12,2,3701.677500
12,2,2327.532051
12,2,1548.006579
12,2,5720.424107
12,2,1594.280556
12,2,7631.676339
12,2,3014.192073
12,2,5374.611979
12,2,5002.869565
12,2,5011.955307
12,2,4151.947368
12,2,2397.470930
12,2,4059.019886
12,2,1826.630952
12,2,10875.034483
12,2,1609.338235
12,2,7389.585366
12,2,2736.674419
12,2,1647.427500
12,2,1904.602459
12,2,6728.441860
12,2,13625.528846
12,2,7049.460366
12,2,1774.521429
12,2,1675.015707
12,2,1867.379310
12,2,9545.239130
12,2,2063.606818
12,2,6425.048913
12,2,1766.500000
12,2,8694.698718
12,2,5097.299465
12,2,8025.890625
12,2,3970.416667
12,2,3993.062500
12,2,2103.981061
12,2,1526.785256
12,2,3225.183511
12,2,10169.510000
12,2,1599.262195
12,2,2979.257979
12,2,3311.218750
12,2,3701.836735
12,2,3853.562500
12,2,1540.384615
12,2,6082.019608
12,2,4875.750000
12,2,3350.244898
12,2,2596.568627
12,2,2035.259146
12,2,4355.000000
12,2,1890.750000
12,2,1925.765625
12,2,2691.849432
12,2,2127.858491
12,2,2885.107143
12,2,7691.231132
12,2,3407.093750
12,2,2379.981250
12,2,5301.444444
12,2,8119.494792

12,2,4574.639535
12,2,2207.016667
12,2,5288.602273
12,2,3154.162234
12,2,4162.831731
12,2,2989.500000
12,2,4180.687500
12,2,1902.031250
12,2,3007.654676
12,2,1614.299020
12,2,1661.177083
12,2,7081.079545
12,2,2129.908654
12,2,2016.360795
12,2,1815.586538
12,2,1951.048780
12,2,3922.989583
12,2,7222.800000
12,2,2531.645161
12,2,2122.532143
12,2,24503.250000
12,2,1892.816327
12,2,5531.809179
12,2,1569.208556
12,2,2238.911765
12,2,3365.000000
12,2,1571.204545
12,2,3347.193182
12,2,4991.782609
12,2,2875.458333
12,2,1977.545455
12,2,11981.937500
12,2,2407.825658
12,2,2734.315789
12,2,4519.271825
12,2,2207.914286
12,2,3788.958333
12,2,1556.900641
12,2,1567.960000
12,2,27069.788136
12,2,5327.368421
12,2,12595.057203
12,2,1733.046512
12,2,2920.955357
12,2,7762.200000
12,2,2378.282609
12,2,3191.864583
12,2,1937.174528
12,2,8684.540541
12,2,6257.219355
12,2,1591.454545
12,2,5895.105263
12,2,4176.759146
12,2,3618.125000
12,2,4392.375000
12,2,8522.958333
12,2,2986.390625
12,2,2996.688953
12,2,3572.616279
12,2,2367.622340
12,2,3128.880682
12,2,2459.085366
12,2,3019.468750
12,2,5763.754386
12,2,5104.777778
12,2,2827.005814

12,2,10143.942786
12,2,1583.691327
12,2,8376.460526
12,2,2763.895833
12,2,3766.693182
12,2,3109.453125
12,2,2617.901316
12,2,4917.321212
12,2,8537.526316
12,2,2628.409091
12,2,1801.835227
12,2,3231.125000
12,2,1698.439904
12,2,5476.300000
12,2,2595.750000
12,2,7128.695000
12,2,9108.955497
12,2,4510.700000
12,2,37574.495000
12,2,4502.181818
12,2,8802.132075
12,2,3868.535714
12,2,1701.414894
12,2,3314.836538
12,2,6331.577540
12,2,2634.089286
12,2,4938.641509
12,2,1985.391204
12,2,6863.750000
12,2,4125.084239
12,2,6366.968750
12,2,2312.953125
12,2,1632.369565
12,2,6506.000000
12,2,1760.469388
12,2,31077.445312
12,2,3847.714286
12,2,2475.172872
12,2,2992.232558
12,2,11874.574468
12,2,2891.812500
12,2,2109.536585
12,2,1752.389163
12,2,6717.061224
12,2,1970.002604
12,2,2012.714286
12,2,3946.146552
12,2,2980.912736
12,2,3199.752660
12,2,1888.435976
12,2,4150.872727
12,2,4921.365000
12,2,1953.305000
12,2,6245.252500
12,2,1928.329545
12,2,4142.402439
12,2,6792.700000
12,2,7836.000000
12,2,5796.963235
12,2,28535.139535
12,2,1799.645270
12,2,3392.312865
12,2,1582.453125
12,2,5292.875000
12,2,4012.307927
12,2,2933.636364

12,2,1913.193182
12,2,6291.428030
12,2,1877.521277
12,2,3313.128049
12,2,4689.222222
12,2,4244.641026
12,2,2692.455357
12,2,5706.947368
12,2,2587.808333
12,2,3541.146341
12,2,2513.375000
12,2,9118.031250
12,2,1562.328947
12,2,2014.107143
12,2,8482.954545
12,2,4824.970000
12,2,4534.076220
12,2,3517.202128
12,2,2419.976190
12,2,2264.188679
12,2,2926.916667
12,2,4037.012195
12,2,2256.500000
12,2,1859.916667
12,2,2976.653846
12,2,2621.906250
12,2,2590.994186
12,2,1533.409722
12,2,5842.080000
12,2,1603.213636
12,2,3371.334646
12,2,1546.405405
12,2,3724.067308
12,2,3919.500000
12,2,1613.888889
12,2,3022.500000
12,2,1982.586957
12,2,4570.015957
12,2,1889.272727
12,2,2376.222561
12,2,10498.774390
12,2,1543.805556
12,2,2084.452703
12,2,2838.454787
12,2,3930.981250
12,2,2818.714286
12,2,2895.381579
12,2,10689.333333
12,2,6330.976744
12,2,63399.446541
12,2,1945.709184
12,2,2101.818182
12,2,3512.311111
12,2,5910.347826
12,2,1773.847222
12,2,2080.434028
12,2,3036.463889
12,2,2200.326389
12,2,2958.332317
12,2,2176.574468
12,2,3141.083333
12,2,2124.910000
12,2,6080.596154
12,2,13645.142857
12,2,1852.107143
12,2,5059.438776

12,2,1743.772727
12,2,4487.323232
12,2,5753.287190
12,2,7796.847826
12,2,4695.871951
12,2,4337.178571
12,2,6986.832447
12,2,2177.783069
12,2,16869.500000
12,2,2635.720588
12,2,2538.205882
12,2,1934.239130
12,2,2869.769737
12,2,7003.845745
12,2,2806.433824
12,2,1814.113636
12,2,2663.333333
12,2,2786.416667
12,2,1549.852564
12,2,3332.062500
12,2,1759.350490
12,2,2859.865854
12,2,2014.072222
12,2,2474.300000
12,2,2283.205128
12,2,2082.195000
12,2,6157.636364
12,2,4990.199405
12,2,18146.010000
12,2,2788.750000
12,2,2784.729167
12,2,2661.344444
12,2,4926.582447
12,2,4969.901961
12,2,3571.744186
12,2,2196.950000
12,2,2434.702128
12,2,1541.655556
12,2,4531.609043
12,2,2959.029891
12,2,2819.451389
12,2,3079.625000
12,2,3246.383929
12,2,1943.911111
12,2,1961.736842
12,2,6265.400000
12,2,5922.052083
12,2,5460.476744
12,2,4570.785714
12,2,2194.777174
12,2,2827.313679
12,2,2732.916667
12,2,4544.636364
12,2,1526.750000
12,2,4270.433333
12,2,1517.625000
12,2,7586.755208
12,2,1951.048828
12,2,2400.781250
12,2,2148.179487
12,2,2946.732143
12,2,2396.520408
12,2,5664.528061
12,2,2144.819712
12,2,5284.983696
12,2,1796.223684

12,2,80517.000000
12,2,4326.027174
12,2,1540.267442
12,2,2201.557292
12,2,6968.625000
12,2,1553.384868
12,2,2202.023256
12,2,3080.321256
12,2,8165.680851
12,2,1899.864130
12,2,1745.536111
12,2,7497.529255
12,2,51140.739865
12,2,10208.780488
12,2,2729.047872
12,2,2525.750000
12,2,18652.298246
12,2,2210.794271
12,2,4854.722222
12,2,7654.125000
12,2,6687.319149
12,2,1802.015707
12,2,1836.492188
12,2,2860.579268
12,2,1933.020833
12,2,29076.896226
12,2,2576.698113
12,2,1673.000000
12,2,1692.767442
12,2,3418.568750
12,2,2644.800000
12,2,2243.438953
12,2,4653.224490
12,2,6944.679245
12,2,1526.169811
12,2,3114.531250
12,2,2870.250000
12,2,1705.000000
12,2,45372.892045
12,2,4969.833333
12,2,2610.302857
12,2,1742.642857
12,2,8113.524038
12,2,6528.952273
12,2,3989.500000
12,2,1635.171875
12,2,1995.163690
12,2,3780.890000
12,2,2334.360000
12,2,3637.888587
12,2,1776.095745
12,2,8022.747283
12,2,5214.404255
12,2,6400.461957
12,2,2121.960784
12,2,3744.777778
12,2,1535.031250
12,2,2959.598039
12,2,4693.653846
12,2,5873.710938
12,2,5684.340278
12,2,10661.013393
12,2,4813.918750
12,2,2136.846154
12,2,8015.531977
12,2,5038.742063

12,2,3143.627451
12,2,8602.471698
12,2,2190.627660
12,2,2538.280488
12,2,8767.583333
12,2,4233.875000
12,2,4842.775000
12,2,2581.000000
12,2,11118.806011
12,2,1796.713415
12,2,2100.365385
12,2,4258.151316
12,2,5575.875000
12,2,2231.543605
12,2,2845.079545
12,2,3968.951220
12,2,2932.732955
12,2,2100.266827
12,2,2144.201613
12,2,3548.604651
12,2,1608.164773
12,2,2271.646739
12,2,3147.096154
12,2,1554.431818
12,2,2133.381250
12,2,4226.012626
12,2,2257.754808
12,2,2536.028571
12,2,1562.325000
12,2,2713.391204
12,2,4537.673077
12,2,8062.368421
12,2,4353.883721
12,2,2789.045455
12,2,11843.500000
12,2,2830.471591
12,2,3485.710843
12,2,16186.844444
12,2,3782.395349
12,2,5254.222917
12,2,2460.835938
12,2,14926.156977
12,2,2717.287162
12,2,1751.006944
12,2,2693.410714
12,2,3101.260246
12,2,2294.923780
12,2,7781.072115
12,2,4628.012195
12,2,1859.435897
12,2,2223.055556
12,2,14818.197044
12,2,1809.458333
12,2,2007.011364
12,2,4902.875000
12,2,6425.738636
12,2,2487.971154
12,2,2058.296296
12,2,3990.320652
12,2,5500.024390
12,2,2525.781250
12,2,2200.475352
12,2,6446.450980
12,2,1769.277778
12,2,1794.642442
12,2,7090.452830

12,2,2421.312500
12,2,4545.357143
12,2,4377.611842
12,2,1546.674419
12,2,18418.250000
12,2,21828.250000
12,2,2003.169811
12,2,4257.244444
12,2,3337.259459
12,2,4250.535714
12,2,3854.187500
12,2,11768.139706
12,2,5472.326923
12,2,3713.496429
12,2,2550.321429
12,2,1574.444444
12,2,12147.869565
12,2,13739.462719
12,2,1772.614035
12,2,6425.516588
12,2,9098.372093
12,2,2867.710106
12,2,8750.480978
12,2,2341.540865
12,2,5421.679688
12,2,4193.741477
12,2,4529.190476
12,2,10329.062500
12,2,2834.289062
12,2,2533.558140
12,2,2268.258523
12,2,2031.335000
12,2,4131.076923
12,2,3232.650000
12,2,3892.408537
12,2,3897.047619
12,2,4465.348837
12,2,4238.239865
12,2,1696.396739
12,2,7384.666667
12,2,1777.437500
12,2,4348.903226
12,2,2879.750000
12,2,1586.632075
12,2,2387.468085
12,2,1620.714623
12,2,4683.218085
12,2,3877.192935
12,2,18507.092025
12,2,3527.236111
12,2,5760.653061
12,2,1888.179878
12,2,4548.826087
12,2,2204.712500
12,2,1804.705882
12,2,1542.086207
12,2,7795.562500
12,2,3905.896277
12,2,3818.302632
12,2,6864.170732
12,2,1920.011364
12,2,2971.768868
12,2,2813.057692
12,2,3581.385417
12,2,1777.414894
12,2,3780.525943

12,2,1793.771635
12,2,9769.187500
12,2,1608.922619
12,2,4296.747500
12,2,3932.695652
12,2,9960.760870
12,2,2903.733333
12,2,1956.685811
12,2,9470.695312
12,2,1965.278846
12,2,2391.650000
12,2,1692.977941
12,2,3333.410714
12,2,3009.853571
12,2,2311.561224
12,2,3967.471591
12,2,3456.625000
12,2,3226.288690
12,2,6549.609694
12,2,1578.603774
12,2,2775.636364
12,2,1958.818182
12,2,1753.000000
12,2,4597.612500
12,2,1689.428571
12,2,1971.173295
12,2,6378.454787
12,2,4409.388889
12,2,4659.000000
12,2,26399.710648
12,2,2080.342262
12,2,1650.519231
12,2,1514.275000
12,2,4535.713115
12,2,4852.857558
12,2,4298.897959
12,2,4067.210106
12,2,1933.003289
12,2,7856.814189
12,2,2861.054054
12,2,6737.488372
12,2,6677.437500
12,2,4558.926887
12,2,1778.060897
12,2,6378.785714
12,2,1584.430147
12,2,8906.468085
12,2,1599.812500
12,2,2950.692857
12,2,1736.593750
12,2,17006.562500
12,2,1826.744186
12,2,2675.351351
12,2,2028.138298
12,2,2343.098404
12,2,8583.109091
12,2,3505.828947
12,2,5958.645833
12,2,3629.087121
12,2,2137.196429
12,2,2743.721875
12,2,6767.284091
12,2,2081.140625
12,2,2128.587766
12,2,1749.688889
12,2,2038.782609

12,2,9067.529412
12,2,3737.223958
12,2,2687.276596
12,2,2462.227273
12,2,1736.974359
12,2,11350.586957
12,2,2675.640625
12,2,2406.817073
12,2,5651.113636
12,2,2224.095000
12,2,2327.150000
12,2,2419.893182
12,2,6271.797500
12,2,2423.569444
12,2,3371.783251
12,2,2155.461538
12,2,2729.979592
12,2,9022.295259
12,2,3687.204082
12,2,1723.819672
12,2,6443.875000
12,2,4941.750000
12,2,58579.346939
12,2,4191.198980
12,2,3208.705357
12,2,6131.260204
12,2,2133.292614
12,2,3381.051020
12,2,4895.142857
12,2,1606.906250
12,2,2073.205556
12,2,4253.750000
12,2,6151.213636
12,2,2895.340426
12,2,1973.377907
12,2,3983.816860
12,2,2933.187500
12,2,1594.620192
12,2,2411.386792
12,2,12007.734091
12,2,3396.820755
12,2,5779.765957
12,2,3093.879167
12,2,6253.333333
12,2,3099.536290
12,2,2135.992857
12,2,1697.407583
12,2,1511.600000
12,2,1771.666667
12,2,7569.933673
12,2,2350.694149
12,2,1726.495098
12,2,2727.731707
12,2,8798.490066
12,2,1753.783019
12,2,4742.972561
12,2,1991.400000
12,2,10256.250000
12,2,1893.686170
12,2,2947.000000
12,2,13876.272917
12,2,2274.439024
12,2,4970.022727
12,2,4396.871711
12,2,2309.240000
12,2,2392.532738

12,2,2612.275000
12,2,5045.125000
12,2,5008.336111
12,2,2958.133621
12,2,1777.333333
12,2,2094.765000
12,2,2153.083333
12,2,3445.919811
12,2,5068.454545
12,2,5663.355263
12,2,1985.596154
12,2,4852.971154
12,2,2051.454545
12,2,2636.170732
12,2,1768.471154
12,2,4271.732843
12,2,3378.591216
12,2,1527.467262
12,2,1744.474432
12,2,2152.310345
12,2,1918.791667
12,2,14087.556818
12,2,6373.938776
12,2,2975.628289
12,2,6505.625000
12,2,3696.658163
12,2,3223.217391
12,2,4100.875000
12,2,8122.077206
12,2,1659.978261
12,2,2504.164062
12,2,2686.367188
12,2,3198.309091
12,2,2485.071429
12,2,1863.401163
12,2,3090.476190
12,2,4476.619048
12,2,5935.940299
12,2,2447.527027
12,2,4315.113208
12,2,2670.000000
12,2,2137.885870
12,2,3474.718750
12,2,2400.504386
12,2,2133.568047
12,2,4097.156250
12,2,6822.081081
12,2,3024.437500
12,2,1706.406250
12,2,3724.750000
12,2,5263.765306
12,2,2694.020833
12,2,2686.660000
12,2,2627.812500
12,2,3997.959184
12,2,2489.346939
12,2,1513.760870
12,2,3842.865854
12,2,1667.665000
12,2,148179.913462
12,2,2854.942308
12,2,4431.680000
12,2,6827.379717
12,2,2158.043750
12,2,3608.511905
12,2,7460.463054

12,2,2506.287879
12,2,4178.200000
12,2,8074.771127
12,2,4302.227273
12,2,2362.187500
12,2,1832.739130
12,2,3071.245536
12,2,3706.185629
12,2,16431.365724
12,2,2959.372093
12,2,1924.000000
12,2,2104.434783
12,2,2682.106707
12,2,4949.888889
12,2,5573.247881
12,2,5993.728723
12,2,3627.303279
12,2,1511.809659
12,2,6179.200000
12,2,7460.758523
12,2,2451.806818
12,2,1622.250000
12,2,2880.361111
12,2,5246.735714
12,2,7740.611111
12,2,1598.116279
12,2,4795.996988
12,2,1628.062500
12,2,1911.273810
12,2,6553.175926
12,2,1898.743421
12,2,7074.067935
12,2,5178.285714
12,2,1963.914894
12,2,6530.239362
12,2,1899.713235
12,2,1716.027907
12,2,2401.388158
12,2,5356.729730
12,2,4872.671512
12,2,1682.648438
12,2,5258.425000
12,2,3480.565217
12,2,6336.234375
12,2,1716.583333
12,2,13888.928571
12,2,2826.109375
12,2,2900.250000
12,2,3253.547170
12,2,2029.099057
12,2,1865.505682
12,2,13905.429268
12,2,2535.356383
12,2,10778.222222
12,2,4674.917553
12,2,1544.357143
12,2,2162.297619
12,2,2255.488636
12,2,2091.479592
12,2,4465.763889
12,2,3220.643617
12,2,1970.943182
12,2,3182.962963
12,2,1825.969697
12,2,2821.512500
12,2,3148.854167

12,2,4211.345930
12,2,5067.500000
12,2,4947.791667
12,2,2315.935897
12,2,2343.709302
12,2,12034.111111
12,2,1645.329327
12,2,3890.480469
12,2,2815.485849
12,2,2254.105769
12,2,1688.804878
12,2,2662.389744
12,2,1613.871069
12,2,6480.701087
12,2,3707.285714
12,2,2972.902778
12,2,2707.765625
12,2,4042.500000
12,2,1738.956250
12,2,2242.790541
12,2,4781.714286
12,2,1812.260000
12,2,1946.259615
12,2,2138.757500
12,2,7137.593750
12,2,2068.901961
12,2,2987.991935
12,2,4150.452128
12,2,3312.554878
12,2,2263.138393
12,2,1987.446429
12,2,4808.274390
12,2,2055.280000
12,2,3876.833333
12,2,6526.677083
12,2,3189.076923
12,2,1639.589286
12,2,3532.750000
12,2,2568.687500
12,2,5161.125000
12,2,1809.545455
12,2,2402.061224
12,2,4035.394231
12,2,1896.690909
12,2,6095.112245
12,2,6573.298246
12,2,4275.830189
12,2,1645.260766
12,2,9989.174342
12,2,4349.441489
12,2,8990.989754
12,2,1704.546053
12,2,1919.833333
12,2,6860.050000
12,2,2196.244186
12,2,5616.015000
12,2,4190.583333
12,2,3213.497024
12,2,2573.276596
12,2,2249.100000
12,2,3546.750000
12,2,4406.860577
12,2,2976.531250
12,2,3200.445122
12,2,2762.680328
12,2,2705.497159

12,2,1592.391061
12,2,6600.476190
12,2,6205.083333
12,2,7873.299180
12,2,3133.204082
12,2,3967.205556
12,2,11469.747500
12,2,8316.232143
12,2,2225.647059
12,2,4526.040816
12,2,7755.051471
12,2,4463.803191
12,2,2856.427500
12,2,2576.522727
12,2,4108.962766
12,2,3419.944444
12,2,7064.938830
12,2,3300.125000
12,2,1681.723837
12,2,4159.159574
12,2,3602.166667
12,2,4712.173469
12,2,1523.585165
12,2,1732.366667
12,2,1875.413889
12,2,5854.243902
12,2,2851.769231
12,2,3853.708333
12,2,8847.634615
12,2,2969.964286
12,2,2583.609375
12,2,3495.735294
12,2,2011.869565
12,2,4092.695652
12,2,2792.849057
12,2,2322.245455
12,2,1706.462209
12,2,2976.510638
12,2,2156.932432
12,2,9746.559211
12,2,2877.739362
12,2,1569.183673
12,2,6587.833333
12,2,1745.196429
12,2,1562.050000
12,2,3110.451220
12,2,2279.428571
12,2,4091.607143
12,2,6216.338983
12,2,3429.804348
12,2,4401.925000
12,2,3625.556818
12,2,2595.415541
12,2,4983.461538
12,2,3699.964286
12,2,6005.511236
12,2,2829.270588
12,2,7157.266854
12,2,1629.385714
12,2,4018.554545
12,2,1976.888021
12,2,6595.982857
12,2,2299.090116
12,2,5330.877778
12,2,4247.420082
12,2,1953.805000

12,2,6162.507853
12,2,4437.620192
12,2,2055.224359
12,2,2267.925676
12,2,2025.974138
12,2,2319.823529
12,2,9066.239796
12,2,1504.013158
12,2,11025.278049
12,2,16252.627273
12,2,2463.729839
12,2,1877.678571
12,2,1911.989362
12,2,2280.758333
12,2,3625.835938
12,2,5517.880000
12,2,4335.085366
12,2,1732.352273
12,2,12582.578947
12,2,2195.500000
12,2,3329.142045
12,2,3871.451220
12,2,5989.244681
12,2,2626.800000
12,2,1642.250000
12,2,4807.367347
12,2,2377.739130
12,2,2703.078125
12,2,4431.982843
12,2,2358.025000
12,2,2948.173077
12,2,3116.463068
12,2,2807.040625
12,2,2668.456395
12,2,1958.427500
12,2,7786.038462
12,2,1935.636719
12,2,7779.358974
12,2,6423.410714
12,2,2221.273148
12,2,5074.963415
12,2,3721.003289
12,2,6654.577830
12,2,2657.473837
12,2,3077.050000
12,2,5022.550000
12,2,1612.141304
12,2,2464.562500
12,2,1661.687500
12,2,4454.719388
12,2,2427.056122
12,2,2385.459677
12,2,5384.184524
12,2,1548.875000
12,2,2922.111111
12,2,4601.000000
12,2,5720.315625
12,2,5432.609375
12,2,1728.000000
12,2,1565.100000
12,2,1576.681818
12,2,20185.817857
12,2,3384.536585
12,2,6297.820312
12,2,1790.044118
12,2,2256.649510

12,2,8063.486842
12,2,1864.026042
12,2,4741.529412
12,2,5931.041667
12,2,1850.669444
12,2,1874.508721
12,2,2062.946108
12,2,7779.790541
12,2,3813.826923
12,2,2316.206395
12,2,5033.928571
12,2,1616.815217
12,2,8812.228516
12,2,13121.858586
12,2,3567.233333
12,2,7860.500000
12,2,2094.162500
12,2,2648.807692
12,2,3010.428571
12,2,4863.608333
12,2,2384.991071
12,2,1765.508721
12,2,2094.700000
12,2,1683.050000
12,2,2718.650000
12,2,2666.664773
12,2,6051.717500
12,2,7663.833333
12,2,2867.000000
12,2,5256.595109
12,2,3282.560109
12,2,6385.559375
12,2,4977.691964
12,2,3223.500000
12,2,3230.065972
12,2,7143.892241
12,2,22257.016447
12,2,4582.201220
12,2,2872.517857
12,2,2886.603365
12,2,4975.395349
12,2,1537.000000
12,2,3498.877717
12,2,4607.773707
12,2,7495.600000
12,2,2668.804348
12,2,1912.210938
12,2,3654.469512
12,2,2072.697222
12,2,2843.348684
12,2,7782.781250
12,2,6074.818627
12,2,1635.714674
12,2,1617.000000
12,2,1796.627604
12,2,3866.187500
12,2,2240.799242
12,2,5817.737500
12,2,3423.783582
12,2,5188.744681
12,2,6875.326923
12,2,5016.468137
12,2,5180.725410
12,2,8588.546875
12,2,3466.372549
12,2,1704.816327

12,2,2943.398256
12,2,3020.806250
12,2,1566.823718
12,2,2068.674419
12,2,2080.954545
12,2,2687.892241
12,2,4015.948113
12,2,5404.188492
12,2,3667.078125
12,2,3489.881250
12,2,3306.488208
12,2,6697.572115
12,2,5660.691667
12,2,2824.737500
12,2,1854.527778
12,2,2649.156250
12,2,4684.854167
12,2,5321.523256
12,2,3361.316129
12,2,2952.703608
12,2,2316.393868
12,2,2579.666667
12,2,1665.722826
12,2,1852.250000
12,2,2062.500000
12,2,1817.220395
12,2,18433.280488
12,2,1640.038194
12,2,1671.906250
12,2,5068.329787
12,2,4731.567073
12,2,3345.833333
12,2,1670.114583
12,2,83214.226064
12,2,2859.277778
12,2,3049.367021
12,2,4944.166667
12,2,8967.571429
12,2,5273.428571
12,2,2364.625000
12,2,1825.032895
12,2,1547.187500
12,2,1923.693878
12,2,2432.030612
12,2,6429.272727
12,2,2290.197222
12,2,3910.129032
12,2,6592.713542
12,2,3278.591837
12,2,4860.652174
12,2,1924.974057
12,2,3526.018519
12,2,1927.138889
12,2,2648.988208
12,2,4357.977273
12,2,1756.239796
12,2,3174.088068
12,2,2214.913043
12,2,2324.600000
12,2,2136.896739
12,2,7430.100000
12,2,5698.692308
12,2,1934.941176
12,2,6042.673575
12,2,4730.869822
12,2,5587.305851

12,2,1858.666667
12,2,1809.526786
12,2,12294.118421
12,2,1808.425000
12,2,2699.744898
12,2,2143.329843
12,2,3433.027027
12,2,5263.467391
12,2,1506.875000
12,2,5471.948113
12,2,2118.157143
12,2,6016.613208
12,2,1730.376963
12,2,2611.036585
12,2,2368.744681
12,2,1692.620098
12,2,2407.666667
12,2,3866.984375
12,2,1660.017500
12,2,1838.118421
12,2,2343.880435
12,2,3657.826923
12,2,1991.004717
12,2,7764.403846
12,2,1552.515957
12,2,4109.445175
12,2,6980.104167
12,2,3266.911885
12,2,1768.125000
12,2,2035.183036
12,2,1804.428571
12,2,2300.635870
12,2,4414.355932
12,2,3270.494681
12,2,4905.464674
12,2,3221.205556
12,2,2512.852273
12,2,4958.187500
12,2,8986.912281
12,2,1525.180000
12,2,2986.167453
12,2,1667.574324
12,2,2070.832386
12,2,3864.185897
12,2,2318.544444
12,2,4960.919643
12,2,6255.200000
12,2,4036.416279
12,2,3766.500000
12,2,3924.141667
12,2,3437.957447
12,2,2433.084906
12,2,3222.188830
12,2,2071.550000
12,2,2160.050000
12,2,3002.203125
12,2,1605.747159
12,2,2228.488372
12,2,4282.137755
12,2,3254.468750
12,2,10281.802083
12,2,5867.540541
12,2,3308.704819
12,2,2176.120000
12,2,5409.364865
12,2,1936.500000

12,2,3701.385638
12,2,3463.166667
12,2,2916.015244
12,2,6617.265957
12,2,3367.928191
12,2,2459.068878
12,2,3937.298611
12,2,6477.310160
12,2,3803.848404
12,2,5607.057692
12,2,1621.648810
12,2,1792.675000
12,2,2345.830508
12,2,3990.000000
12,2,5207.000000
12,2,3142.235294
12,2,2266.857143
12,2,1892.359694
12,2,6437.979730
12,2,3379.300481
12,2,17226.072368
12,2,63966.000000
12,2,1655.690104
12,2,1847.835648
12,2,5640.568182
12,2,2133.972826
12,2,3326.053571
12,2,4622.152174
12,2,1967.000000
12,2,3917.416667
12,2,4037.417614
12,2,2296.500000
12,2,3679.050000
12,2,2915.590625
12,2,4772.467949
12,2,6049.480000
12,2,7200.577869
12,2,4208.000000
12,2,3152.695000
12,2,2692.036585
12,2,1539.645833
12,2,3113.865385
12,2,1830.574468
12,2,3906.240132
12,2,4685.645000
12,2,1840.595109
12,2,3544.496528
12,2,1998.327128
12,2,6252.802632
12,2,3605.523529
12,2,4619.500000
12,2,2663.423295
12,2,3972.497093
12,2,1902.891026
12,2,3694.397436
12,2,2054.410256
12,2,7703.099432
12,2,3393.969298
12,2,2270.812500
12,2,4512.382979
12,2,5047.720930
12,2,41924.988889
12,2,1508.025943
12,2,1925.392500
12,2,3625.750000
12,2,5698.956522

12,2,2235.387255
12,2,2158.250000
12,2,2670.553191
12,2,13185.904167
12,2,1761.906250
12,2,2398.286111
12,2,3050.303191
12,2,4567.345930
12,2,4174.766667
12,2,2470.518750
12,2,4597.458333
12,2,1548.260204
12,2,2233.076923
12,2,1807.351351
12,2,1848.215686
12,2,1556.889610
12,2,12563.578947
12,2,2511.109375
12,2,2422.468085
12,2,2171.297203
12,2,1839.893048
12,2,1973.603365
12,2,2132.420455
12,2,2209.800481
12,2,6799.022222
12,2,1972.207865
12,2,2246.334507
12,2,3978.312500
12,2,1759.308824
12,2,5052.325581
12,2,2765.621711
12,2,2189.795918
12,2,4986.953125
12,2,8918.583333
12,2,2110.416667
12,2,6445.336735
12,2,3344.119565
12,2,2305.480263
12,2,1798.723404
12,2,2930.250000
12,2,5984.610465
12,2,2899.966667
12,2,1596.548387
12,2,7453.466667
12,2,6816.969444
12,2,1838.804878
12,2,1694.483553
12,2,3056.600000
12,2,11692.642857
12,2,1857.200000
12,2,6041.928571
12,2,3271.692568
12,2,1763.972973
12,2,2017.314189
12,2,12978.726415
12,2,8321.750000
12,2,1686.625000
12,2,7159.859296
12,2,10247.521739
12,2,4198.056604
12,2,3615.920213
12,2,1538.076923
12,2,12545.900943
12,2,11603.529412
12,2,7712.312500
12,2,1866.173295

12,2,1894.523585
12,2,12143.671875
12,2,2268.955556
12,2,3410.081395
12,2,11344.555556
12,2,4829.882979
12,2,5336.802326
12,2,3147.041667
12,2,6007.203488
12,2,4575.281780
12,2,1508.154762
12,2,1847.000000
12,2,2718.569853
12,2,1814.942935
12,2,2851.777778
12,2,2486.485294
12,2,2923.117925
12,2,2793.047222
12,2,3284.173913
12,2,8529.268293
12,2,2137.980392
12,2,2362.110294
12,2,3145.691176
12,2,2425.319149
12,2,2022.722222
12,2,1880.843750
12,2,2715.395408
12,2,3758.894022
12,2,2667.603723
12,2,2972.113636
12,2,2308.932692
12,2,1784.054878
12,2,2125.375000
12,2,1680.311558
12,2,1735.467105
12,2,3423.729787
12,2,4698.045161
12,2,6586.520000
12,2,4547.076531
12,2,1598.588235
12,2,3353.662162
12,2,9147.411765
12,2,2430.380597
12,2,7982.649123
12,2,3960.500000
12,2,4005.951613
12,2,3754.367647
12,2,2923.104167
12,2,1549.625000
12,2,2194.260870
12,2,3801.412791
12,2,3642.195122
12,2,2311.250000
12,2,3424.818182
12,2,2309.410714
12,2,2333.691327
12,2,2265.996324
12,2,1751.500000
12,2,2347.631579
12,2,3495.737805
12,2,1738.164773
12,2,8573.927273
12,2,5057.787791
12,2,1981.178571
12,2,4987.083721
12,2,6976.934673

12,2,4900.468085
12,2,3647.848214
12,2,1906.263158
12,2,3040.250000
12,2,1742.441176
12,2,2056.065217
12,2,1657.366071
12,2,1976.750000
12,2,2086.138298
12,2,1733.196078
12,2,2594.583333
12,2,2960.500000
12,2,1684.013587
12,2,3413.455729
12,2,3334.513158
12,2,2966.651515
12,2,1829.416667
12,2,4895.566489
12,2,2805.152439
12,2,2898.272059
12,2,4124.277778
12,2,1683.838415
12,2,1737.712766
12,2,5109.795455
12,2,1763.833333
12,2,1577.932432
12,2,7046.640306
12,2,4440.574468
12,2,5150.522222
12,2,2455.527027
12,2,5035.802885
12,2,1756.081897
12,2,19560.565022
12,2,2237.608696
12,2,2059.163462
12,2,1958.836538
12,2,2863.127660
12,2,4368.769397
12,2,11952.625000
12,2,3271.510204
12,2,1643.562500
12,2,2338.235294
12,2,1774.365591
12,2,1979.843750
12,2,1629.783654
12,2,7683.875000
12,2,2646.218750
12,2,2489.304348
12,2,1828.565341
12,2,2777.644231
12,2,4664.121429
12,2,1563.514286
12,2,2107.818182
12,2,2728.388889
12,2,4539.176471
12,2,2952.894737
12,2,2722.125000
12,2,45726.727273
12,2,14752.437500
12,2,1799.952703
12,2,2086.621622
12,2,4755.827160
12,2,13581.024306
12,2,2521.166667
12,2,2223.100610
12,2,1619.000000

12,2,3040.051887
12,2,2710.478814
12,2,4372.413462
12,2,5981.645833
12,2,1562.687500
12,2,1676.000000
12,2,2956.958333
12,2,5651.903061
12,2,5178.341837
12,2,3921.130859
12,2,3835.875000
12,2,2844.176471
12,2,9638.464286
12,2,2226.477273
12,2,2266.331522
12,2,2051.765625
12,2,2402.365000
12,2,5043.726064
12,2,4809.720588
12,2,5272.681818
12,2,2822.867188
12,2,2234.404605
12,2,1619.096591
12,2,5533.974490
12,2,2149.670455
12,2,5049.462500
12,2,5424.321429
12,2,3455.154762
12,2,4037.000000
12,2,14515.585106
12,2,5162.170213
12,2,2276.783537
12,2,5009.401163
12,2,5943.709821
12,2,5314.225000
12,2,2772.971429
12,2,6234.652439
12,2,1747.500000
12,2,4127.076355
12,2,7001.252604
12,2,3616.862069
12,2,2045.648649
12,2,5195.875000
12,2,4465.558824
12,2,1986.944444
12,2,8648.828804
12,2,2623.157895
12,2,8882.315789
12,2,4269.669271
12,2,4145.094444
12,2,1611.850000
12,2,5573.986607
12,2,3516.984615
12,2,1878.478723
12,2,1633.666667
12,2,6558.842593
12,2,2403.850694
12,2,2626.586957
12,2,4829.658537
12,2,2144.094512
12,2,1983.078804
12,2,2387.068182
12,2,6415.567708
12,2,4734.472500
12,2,2451.642857
12,2,3080.098558

```
12,2,12814.959184
12,2,1679.500000
12,2,5569.611111
12,2,1929.410377
12,2,1549.821429
12,2,1986.819149
12,2,6877.370370
12,2,1576.800481
12,2,6047.652439
12,2,8707.100000
12,2,8887.634146
12,2,18095.452703
12,2,4903.725000
12,2,3042.921875
12,2,3892.715517
12,2,2481.595588
12,2,2164.178125
12,2,2241.048780
12,2,5646.220238
12,2,8389.769886
12,2,2661.582447
12,2,2180.961538
12,2,1769.658537
12,2,2120.895833
12,2,9106.419872
12,2,1659.137255
12,2,1997.736413
12,2,37855.625000
12,2,5711.008929
12,2,3376.325581
12,2,4391.390000
12,2,2328.173077
12,2,4766.659574
12,2,2324.875000
12,2,5997.125000
12,2,3033.762295
12,2,1755.696429
12,2,3087.030000
12,2,3663.364641
12,2,2881.265306
12,2,6790.771739
12,2,2064.828947
12,2,2887.457447
12,2,3506.379630
12,2,1541.250000
12,2,1663.762500
12,2,2982.500000
12,2,1642.073171
12,2,7066.743842
12,2,6347.377976
12,2,1901.689655
12,2,2474.651163
12,2,10222.656250
12,2,4510.237143
12,2,9434.194313
12,2,5205.977011
12,2,1640.221429
12,2,3071.125000
12,2,1920.630682
12,2,5923.478571
12,2,1506.576271
12,2,7590.250000
12,2,2132.296875
12,2,1757.833333
12,2,2660.094828
12,2,5869.703125
```

12,2,1872.660000
12,2,1718.692130
12,2,10950.052083
12,2,5062.349432
12,2,2185.918367
12,2,4180.948052
12,2,3302.595092
12,2,1528.005025
12,2,6485.785714
12,2,8914.181122
12,2,2107.707547
12,2,5207.226415
12,2,4322.206395
12,2,2072.879237
12,2,4233.351562
12,2,3154.086538
12,2,1977.961538
12,2,2513.579268
12,2,1572.031447
12,2,2063.417553
12,2,3136.571429
12,2,3317.142857
12,2,1904.685976
12,2,1628.055556
12,2,2667.552083
12,2,8304.010638
12,2,2188.897849
12,2,3963.794444
12,2,3253.505319
12,2,21762.714286
12,2,6686.229167
12,2,2254.571078
12,2,4126.560209
12,2,2244.500000
12,2,1713.771930
12,2,2328.429688
12,2,2413.216216
12,2,5975.062500
12,2,2582.675926
12,2,17408.764205
12,2,6414.250000
12,2,1835.125000
12,2,2993.971939
12,2,1981.219388
12,2,1787.211538
12,2,3451.869565
12,2,1537.147436
12,2,2257.993056
12,2,2734.318966
12,2,3872.721154
12,2,7333.750000
12,2,3572.641304
12,2,2517.470930
12,2,6568.428571
12,2,7796.377778
12,2,3238.250000
12,2,1578.602273
12,2,1591.368421
12,2,3780.586538
12,2,2233.841667
12,2,2803.329670
12,2,3846.199367
12,2,3134.822368
12,2,2926.851190
12,2,33722.146341
12,2,2174.979167

12,2,7762.437500
12,2,1965.530405
12,2,5207.222727
12,2,1511.410256
12,2,4219.688053
12,2,5603.967033
12,2,11203.529126
12,2,3073.627907
12,2,2969.218750
12,2,1785.179487
12,2,2010.307065
12,2,2989.698980
12,2,2024.686275
12,2,1513.952128
12,2,2523.136364
12,2,2603.000000
12,2,7844.574405
12,2,4180.541667
12,2,14040.942308
12,2,3431.448864
12,2,2040.807500
12,2,2416.334375
12,2,7955.527174
12,2,4172.357143
12,2,1607.795918
12,2,5257.053030
12,2,2244.088235
12,2,4503.483796
12,2,2658.734694
12,2,3538.284091
12,2,2850.144444
12,2,4974.345000
12,2,2406.524510
12,2,2043.477941
12,2,5553.760204
12,2,4876.659884
12,2,5199.516667
12,2,2722.734375
12,2,6265.888889
12,2,12321.110169
12,2,1688.666667
12,2,5249.409091
12,2,2083.552083
12,2,2462.890710
12,2,3608.534884
12,2,1772.416667
12,2,7899.696429
12,2,38855.176471
12,2,5189.803125
12,2,2781.778125
12,2,1749.781250
12,2,1839.990385
12,2,1876.191176
12,2,2721.125000
12,2,2720.625000
12,2,3592.948864
12,2,9510.421196
12,2,2615.695364
12,2,2344.625000
12,2,2322.729167
12,2,6231.687500
12,2,4738.102041
12,2,2629.350610
12,2,4400.652439
12,2,12535.500000
12,2,1569.843023

12,2,3130.832599
12,2,1778.193878
12,2,2293.435897
12,2,2547.029412
12,2,14722.432292
12,2,8261.582386
12,2,3817.526163
12,2,1529.365385
12,2,9555.519231
12,2,3372.168478
12,2,3156.488095
12,2,2873.391026
12,2,1584.832143
12,2,2626.959091
12,2,10148.000000
12,2,2155.500000
12,2,2365.979651
12,2,1676.596939
12,2,2705.000000
12,2,4678.125000
12,2,2596.763021
12,2,2679.861111
12,2,3952.760204
12,2,10734.573171
12,2,3898.850267
12,2,3218.544118
12,2,14555.018868
12,2,1598.595745
12,2,2404.164865
12,2,4434.703704
12,2,3671.167614
12,2,5281.571429
12,2,2225.522222
12,2,2691.053571
12,2,3618.630952
12,2,2684.641304
12,2,6503.886364
12,2,2512.814286
12,2,5083.300847
12,2,1901.410714
12,2,1703.090116
12,2,2213.872500
12,2,1624.636364
12,2,5328.007812
12,2,2899.000000
12,2,4539.268519
12,2,5194.207317
12,2,2914.010000
12,2,3653.127551
12,2,5323.878378
12,2,4780.150000
12,2,5335.625000
12,2,3858.350000
12,2,8373.436364
12,2,2268.800000
12,2,1785.767857
12,2,2372.981132
12,2,1706.774390
12,2,1929.112903
12,2,1514.322368
12,2,2681.336957
12,2,5209.850000
12,2,16063.073171
12,2,2801.262821
12,2,2637.135593
12,2,7287.991667

12,2,8422.669643
12,2,2016.560000
12,2,3586.763514
12,2,1552.400568
12,2,8348.851351
12,2,1769.194767
12,2,3186.195652
12,2,3188.272059
12,2,37012.562500
12,2,3605.860465
12,2,2650.797872
12,2,2811.590116
12,2,1918.173913
12,2,2772.517500
12,2,2036.375000
12,2,5582.579268
12,2,1501.862179
12,2,2293.190000
12,2,13577.541667
12,2,2329.956522
12,2,4585.297170
12,2,1831.291667
12,2,2296.861446
12,2,1563.300000
12,2,3647.118590
12,2,4134.744318
12,2,3533.695312
12,2,1964.030612
12,2,4154.130682
12,2,6158.629717
12,2,4771.750000
12,2,5869.100000
12,2,2576.666667
12,2,2362.636364
12,2,2580.573171
12,2,1753.797794
12,2,17121.433824
12,2,2145.061224
12,2,3953.294118
12,2,5413.827500
12,2,3255.730769
12,2,2965.281250
12,2,1673.708333
12,2,13668.220930
12,2,4071.625000
12,2,2700.312500
12,2,2562.678977
12,2,2080.760870
12,2,2102.776042
12,2,3301.776596
12,2,1651.925000
12,2,12944.359694
12,2,2279.468750
12,2,7688.661017
12,2,4861.797753
12,2,5273.155172
12,2,1699.831395
12,2,2493.947368
12,2,2338.567568
12,2,2369.101562
12,2,2695.750000
12,2,3464.552778
12,2,4615.026786
12,2,12424.195122
12,2,6646.125000
12,2,8700.312500

12,2,1504.479167
12,2,2449.278846
12,2,4291.140625
12,2,2749.535484
12,2,2210.139535
12,2,6824.390135
12,2,2599.210526
12,2,7082.632653
12,2,1616.715000
12,2,2050.377551
12,2,3487.365854
12,2,1709.232143
12,2,1961.027027
12,2,2935.102273
12,2,2348.372727
12,2,5035.676829
12,2,2376.514706
12,2,2127.487179
12,2,2665.175000
12,2,1729.209375
12,2,2465.318627
12,2,3287.250000
12,2,2754.525510
12,2,4060.255952
12,2,2442.467593
12,2,2368.516667
12,2,3521.750000
12,2,1621.990385
12,2,7469.858696
12,2,1701.426136
12,2,5852.781250
13,1,5039.279070
13,1,103548.700000
13,1,54748.333333
13,1,39161.575658
13,1,1588.909091
13,1,55226.363636
13,1,70775.168605
13,1,1774.982955
13,1,15505.223684
13,1,1898.946809
13,1,4106.942568
13,1,26164.916667
13,1,28490.669118
13,1,1509.336806
13,1,1527.993056
13,1,37034.347222
13,1,2211.952381
13,1,13831.307692
13,1,53876.747093
13,1,1773.206250
13,1,2532.300000
13,1,1668.776596
13,1,48266.926471
13,1,1596.032500
13,1,6971.570513
13,1,30054.666667
13,1,17596.791667
13,1,2008.292683
13,1,1764.904762
13,1,151005.977273
13,1,6029.040698
13,1,14919.187500
13,1,56375.079268
13,1,3975.824405
13,1,93109.732639

13,1,1993.350000
13,1,142341.346591
13,1,9660.568548
13,1,1736.105978
13,1,3376.437500
13,1,2292.625000
13,1,114839.465116
13,1,10781.701923
13,1,54181.404255
13,1,6244.647059
13,1,12893.333333
13,1,1799.947674
13,1,1510.275000
13,1,1883.093750
13,1,1671.426282
13,1,3023.964623
13,1,4916.111111
13,1,33761.181818
13,1,2321.331395
13,1,31540.390625
13,1,1630.580556
13,1,1684.142857
13,1,1901.243902
13,1,84221.729730
13,1,1701.345930
13,1,1555.134375
13,1,1965.472222
13,1,73997.933333
13,1,76302.786585
13,1,75392.202703
13,1,4464.696809
13,1,19717.046875
13,1,10841.576923
13,1,64571.866071
13,1,4883.813953
13,1,1887.288889
13,1,1739.461957
13,1,48452.777027
13,1,1819.625000
13,1,93555.768293
13,1,3998.409722
13,1,5453.625000
13,1,5582.380435
13,1,10490.810976
13,1,4000.879808
13,1,1802.212121
13,1,8305.668478
13,1,96213.065789
13,1,1513.331081
13,1,4377.574324
13,1,1554.846939
13,1,26980.697674
13,1,96853.642442
13,1,21183.161585
13,1,111487.891026
13,1,5941.686170
13,1,31385.189189
13,1,9024.392857
13,1,2150.803571
13,1,2087.457317
13,1,1705.233553
13,1,3089.583333
13,1,2221.858553
13,1,1541.828125
13,1,1647.025510
13,1,4238.150000

13,1,23518.411765
13,1,12184.902439
13,1,1695.991071
13,1,105468.756944
13,1,21935.847222
13,1,3673.666667
13,1,112497.369318
13,1,2122.412500
13,1,4018.400641
13,1,8982.595588
13,1,47564.100000
13,1,88889.921053
13,1,4421.361111
13,1,64848.243421
13,1,3933.062500
13,1,4040.741071
13,1,37161.093750
13,1,5370.354730
13,1,1753.459459
13,1,5190.613636
13,1,5366.535714
13,1,1676.714286
13,1,77445.282051
13,1,1860.606250
13,1,11324.156250
13,1,71890.365385
13,1,1537.065625
13,1,2411.378205
13,1,69188.092105
13,1,2151.148936
13,1,3109.750000
13,1,1834.272059
13,1,5412.469388
13,1,2110.687500
13,1,184536.631285
13,1,45755.871429
13,1,59611.904412
13,1,1619.885417
13,1,7035.886628
13,1,2326.546053
13,1,2259.634146
13,1,20700.618421
13,1,62427.269531
13,1,1682.843750
13,1,2710.565789
13,1,19972.317935
13,1,29701.000000
13,1,4606.200000
13,1,141896.685976
13,1,63585.400568
13,1,16040.787234
13,1,1550.625000
13,1,4155.416667
13,1,2679.195122
13,1,39937.182692
13,1,2373.687500
13,1,1733.667763
13,1,2192.184211
13,1,1667.148936
13,1,151450.381579
13,1,1587.678571
13,1,3102.047619
13,1,6430.773810
13,1,71386.000000
13,1,6509.153846
13,1,4023.507812

13,1,11111.510870
13,1,27137.978261
13,1,1837.115385
13,1,4048.655612
13,1,166765.424419
13,1,1906.000000
13,1,1868.044271
13,1,34940.532051
13,1,3670.784091
13,1,72689.013514
13,1,3695.286932
13,1,46962.690789
13,1,1876.615385
13,1,2917.257143
13,1,34294.613636
13,1,1626.857143
13,1,2114.677778
13,1,79685.769886
13,1,1713.780405
13,1,3093.851064
13,1,7689.744898
13,1,46961.802083
13,1,2739.489362
13,1,24080.750000
13,1,31541.853261
13,1,2491.677778
13,1,3602.000000
13,1,53303.710526
13,1,44792.062500
13,1,39998.507353
13,1,2395.890625
13,1,1515.615385
13,1,1533.122159
13,1,46289.414286
13,1,10137.593750
13,1,1935.423469
13,1,11057.734756
13,1,55074.617857
13,1,167611.130952
13,1,1892.813953
13,1,1585.039894
13,1,1761.361413
13,1,8092.366667
13,1,1559.250000
13,1,3601.053571
13,1,1630.466463
13,1,4436.878205
13,1,5651.518868
13,1,70810.513514
13,1,16699.862500
13,1,2274.807692
13,1,21342.187500
13,1,24826.631579
13,1,3915.866279
13,1,55372.719697
13,1,8217.035714
13,1,3922.250000
13,1,2194.987179
13,1,1800.542553
13,1,2511.492188
13,1,1581.213415
13,1,5130.488372
13,1,1617.062500
13,1,2060.993902
13,1,2948.195122
13,1,47250.360465

13,1,6919.796053
13,1,5830.721154
13,1,100347.767857
13,1,18889.222826
13,1,2558.589674
13,1,3941.893293
13,1,40427.680921
13,1,1899.092391
13,1,41682.210714
13,1,1594.714286
13,1,43444.542969
13,1,103476.560976
13,1,20216.070312
13,1,1913.423077
13,1,12281.488636
13,1,11971.898649
13,1,1584.319767
13,1,10934.180851
13,1,44855.057143
13,1,2065.000000
13,1,1876.790541
13,1,4787.520833
13,1,1732.445513
13,1,1747.381250
13,1,2121.943182
13,1,4785.095588
13,1,3531.583333
13,1,46654.179054
13,1,1529.666667
13,1,65399.192308
13,1,1914.430233
13,1,2536.684375
13,1,110475.607143
13,1,1569.567073
13,1,6062.134375
13,1,24970.257576
13,1,50160.780488
13,1,5899.056250
13,1,9074.190476
13,1,3440.301471
13,1,1576.613636
13,1,4590.904255
13,1,62830.799242
13,1,44276.764706
13,1,6091.395833
13,1,47435.901163
13,1,71088.941176
13,1,1596.867925
13,1,31479.404412
13,1,22016.912162
13,1,171225.529891
13,1,98042.584302
13,1,3237.108696
13,1,8108.071023
13,1,1923.678125
13,1,3077.125000
13,1,4018.968750
13,1,1961.244318
13,1,24901.508929
13,1,2668.607143
13,1,1686.452206
13,1,49558.827206
13,1,3757.460366
13,1,1612.375000
13,1,79995.528409
13,1,3046.845745

13,1,56960.407143
13,1,5987.731383
13,1,1642.175532
13,1,32903.862903
13,1,1547.683333
13,1,37630.602941
13,1,2072.684524
13,1,1978.971591
13,1,41272.800000
13,1,25994.514706
13,1,2141.695946
13,1,4340.790625
13,1,1570.435897
13,1,4365.851190
13,1,17236.736702
13,1,5159.267857
13,1,4007.369565
13,1,4618.704268
13,1,3044.018293
13,1,2146.025000
13,1,103460.553030
13,1,171801.681818
13,1,1510.819767
13,1,1596.139535
13,1,13317.270833
13,1,2830.820000
13,1,1866.980114
13,1,1509.358696
13,1,1586.602273
13,1,1955.650000
13,1,2295.840426
13,1,1592.635417
13,1,4606.168750
13,1,6310.944444
13,1,14900.000000
13,1,1672.345588
13,1,3559.581395
13,1,1836.139706
13,1,1537.880435
13,1,56757.035156
13,1,1874.839286
13,1,1585.115854
13,1,14119.382813
13,1,2698.987500
13,1,3410.903846
13,1,2060.378125
13,1,19359.346939
13,1,1591.117647
13,1,25623.187500
13,1,1739.894022
13,1,64632.226351
13,1,43620.621795
13,1,5835.888158
13,1,3062.812500
13,1,36521.384615
13,1,1688.775000
13,1,5668.300000
13,1,5361.434783
13,1,1722.106707
13,1,2733.245000
13,1,2836.283654
13,1,39128.690789
13,1,3468.986702
13,1,1857.511111
13,1,32045.850000
13,1,59872.600000

13,1,7140.613636
13,1,52232.500000
13,1,2405.647500
13,1,1847.978723
13,1,2066.984043
13,1,5214.031250
13,1,142526.133929
13,1,4036.500000
13,1,7677.144886
13,1,190571.867021
13,1,1700.038690
13,1,1637.538462
13,1,166993.196809
13,1,109431.005814
13,1,103824.085366
13,1,4555.676471
13,1,40818.696970
13,1,3315.755952
13,1,1786.757143
13,1,2685.730769
13,1,2766.283088
13,1,1964.283784
13,1,42679.932432
13,1,4566.702128
13,1,8811.578488
13,1,54696.671053
13,1,79410.823718
13,1,12117.236413
13,1,2901.403846
13,1,38548.375000
13,1,2456.345930
13,1,1502.178571
13,1,1664.405405
13,1,2148.489286
13,1,3509.450000
13,1,1897.440000
13,1,13720.032258
13,1,69858.518750
13,1,1917.079787
13,1,96603.782051
13,1,2907.190058
13,1,76673.472826
13,1,2147.025000
13,1,1826.000000
13,1,5526.647436
13,1,22613.856061
13,1,26076.835366
13,1,1861.931250
13,1,18592.011628
13,1,45257.769231
13,1,2294.875000
13,1,9495.266304
13,1,2297.776316
13,1,47638.964286
13,1,3908.035714
13,1,2654.421053
13,1,3623.955128
13,1,3466.162791
13,1,1951.157895
13,1,1578.329545
13,1,3477.811111
13,1,2752.140625
13,1,49071.234756
13,1,3101.846154
13,1,1768.321429
13,1,29941.460526

13,1,1584.180851
13,1,1748.732759
13,1,1562.240385
13,1,2145.602941
13,1,8916.232143
13,1,2461.003289
13,1,1637.741935
13,1,107139.000000
13,1,1702.307692
13,1,19340.820513
13,1,4882.176471
13,1,25891.679878
13,1,2063.707317
13,1,14432.250000
13,1,20570.562500
13,1,40002.000000
13,1,133918.515244
13,1,113394.420732
13,1,4522.871795
13,1,2613.030612
13,1,1623.222222
13,1,20353.242857
13,1,1779.213415
13,1,44302.166667
13,1,29429.593750
13,1,15455.846154
13,1,28201.375000
13,1,1651.428571
13,1,1890.952128
13,1,15615.744681
13,1,11749.404255
13,1,4563.250000
13,1,162803.678571
13,1,12613.890625
13,1,57609.432432
13,1,1626.227941
13,1,91905.350000
13,1,2794.800000
13,1,6293.250000
13,1,4454.895833
13,1,11589.214286
13,1,66815.184783
13,1,40266.166667
13,1,1890.903409
13,1,3581.810976
13,1,19750.439189
13,1,2901.554878
13,1,4227.395833
13,1,1832.853659
13,1,126382.026316
13,1,4700.345238
13,1,2440.690909
13,1,1708.634615
13,1,1546.803571
13,1,2945.836735
13,1,1558.741477
13,1,37112.272727
13,1,2269.239362
13,1,54095.700000
13,1,10816.980469
13,1,2296.418605
13,1,46081.593750
13,1,16809.238889
13,1,2809.000000
13,1,2408.199324
13,1,2776.083333

13,1,10475.654412
13,1,1784.154891
13,1,147554.557065
13,1,2031.984375
13,1,114602.000000
13,1,1868.279412
13,1,1931.060606
13,1,2903.746711
13,1,78134.253472
13,1,1797.095745
13,1,2342.690476
13,1,1642.392045
13,1,2971.907738
13,1,1847.825581
13,1,2298.854167
13,1,4088.195946
13,1,43181.327586
13,1,74314.112500
13,1,7116.355556
13,1,179139.300000
13,1,72349.609756
13,1,11165.989583
13,1,3265.752976
13,1,33583.718750
13,1,59421.736842
13,1,2090.578125
13,1,93337.520270
13,1,2068.652439
13,1,5505.601064
13,1,5568.284091
13,1,1875.412791
13,1,72617.062500
13,1,36267.682692
13,1,151269.930921
13,1,6941.294872
13,1,56982.743902
13,1,10465.728448
13,1,92588.959375
13,1,8209.125000
13,1,125558.572368
13,1,29357.750000
13,1,35676.446429
13,1,4689.107558
13,1,20948.464286
13,1,1598.298780
13,1,2842.700000
13,1,128218.653846
13,1,119786.330556
13,1,2390.733696
13,1,2200.725000
13,1,3171.388889
13,1,3022.000000
13,1,44892.852941
13,1,4184.651316
13,1,1652.247549
13,1,112660.539474
13,1,32661.243243
13,1,7802.187500
13,1,2460.483696
13,1,2046.000000
13,1,1937.318182
13,1,2633.576923
13,1,116160.700000
13,1,38588.218750
13,1,2475.377451
13,1,20067.500000

13,1,6106.687500
13,1,51798.829268
13,1,1503.812500
13,1,9261.708333
13,1,9325.138889
13,1,1669.925676
13,1,43636.704545
13,1,1859.544271
13,1,1955.568878
13,1,59250.297297
13,1,29260.151163
13,1,8161.551724
13,1,1646.331395
13,1,3453.944444
13,1,2145.468085
13,1,1893.490625
13,1,2158.073864
13,1,1727.919643
13,1,3963.144231
13,1,13836.533333
13,1,42134.714286
13,1,1953.319149
13,1,1794.714286
13,1,2041.464286
13,1,1845.179878
13,1,2284.941176
13,1,2913.159091
13,1,5616.333333
13,1,83684.142157
13,1,2523.984375
13,1,11227.443182
13,1,4457.734914
13,1,2109.069149
13,1,94388.825000
13,1,1548.744186
13,1,30245.189189
13,1,5255.200000
13,1,1989.658537
13,1,14112.906250
13,1,2360.829545
13,1,2432.064103
13,1,78675.707143
13,1,3272.595745
13,1,9807.585000
13,1,2695.133929
13,1,36200.690789
13,1,5081.054688
13,1,1878.555556
13,1,1880.333333
13,1,12972.631818
13,1,30061.266667
13,1,1643.046512
13,1,4604.952778
13,1,1636.402439
13,1,2089.886029
13,1,53450.524457
13,1,42865.682927
13,1,2010.927273
13,1,2345.819444
13,1,4941.256410
13,1,1586.881250
13,1,2531.552632
13,1,51619.959559
13,1,19273.850000
13,1,1736.518293
13,1,10865.928571

13,1,40957.204268
13,1,2727.121429
13,1,1745.219828
13,1,1620.949324
13,1,82652.369565
13,1,4612.578947
13,1,1516.443548
13,1,32825.837838
13,1,47519.666667
13,1,4407.244565
13,1,10914.276316
13,1,1767.987179
13,1,53929.761905
13,1,40090.383333
13,1,50223.926471
13,1,6337.676020
13,1,18409.208333
13,1,14991.777778
13,1,51051.181818
13,1,56559.644737
13,1,3200.091216
13,1,10814.040541
13,1,2468.277174
13,1,31542.875000
13,1,2818.242857
13,1,1820.014286
13,1,171812.264423
13,1,28500.538462
13,1,22733.920000
13,1,10241.031250
13,1,6321.217949
13,1,1636.460227
13,1,1972.153846
13,1,4191.862069
13,1,2442.129032
13,1,1932.201220
13,1,3284.886364
13,1,3063.063830
13,1,4591.154255
13,1,2354.531915
13,1,2591.677632
13,1,5414.703125
13,1,7966.786585
13,1,5913.507353
13,1,2012.756098
13,1,3498.230114
13,1,2369.916667
13,1,2136.635870
13,1,100336.779412
13,1,2863.812500
13,1,2714.000000
13,1,1651.040698
13,1,1845.371951
13,1,38726.816327
13,1,155050.294118
13,1,3658.326923
13,1,1813.013514
13,1,9337.785714
13,1,6724.298913
13,1,5535.526316
13,1,6186.701087
13,1,36838.250000
13,1,55060.352941
13,1,37528.852941
13,1,6613.858491
13,1,2523.000000

13,1,27307.148649
13,1,2611.357143
13,1,73889.021429
13,1,8298.038043
13,1,65209.240625
13,1,2014.938953
13,1,1640.503049
13,1,6191.440625
13,1,12050.162791
13,1,2069.085714
13,1,40071.357143
13,1,1958.142857
13,1,19926.132075
13,1,2852.946429
13,1,2822.109375
13,1,78550.255000
13,1,1630.732143
13,1,2614.678030
13,1,35917.958333
13,1,1528.615854
13,1,57602.424419
13,1,124771.791667
13,1,67750.270833
13,1,1870.363636
13,1,1644.439024
13,1,10888.656250
13,1,51028.076923
13,1,2305.811111
13,1,2859.098214
13,1,1877.196429
13,1,1572.468085
13,1,3025.465909
13,1,2759.937500
13,1,1526.476064
13,1,100198.928571
13,1,2295.456395
13,1,113164.892857
13,1,3212.625000
13,1,6979.878378
13,1,1873.676471
13,1,148841.252500
13,1,42291.656250
13,1,1684.358974
13,1,7472.541667
13,1,3868.961538
13,1,2400.488095
13,1,53567.125000
13,1,80890.984375
13,1,15402.713235
13,1,2586.500000
13,1,9696.476064
13,1,4907.428571
13,1,52976.102041
13,1,125796.980556
13,1,10379.976190
13,1,1805.533333
13,1,2127.664773
13,1,2022.062500
13,1,74154.391447
13,1,1760.295918
13,1,86284.714286
13,1,1583.860119
13,1,135336.093750
13,1,35995.185484
13,1,1792.559091
13,1,5295.941176

13,1,1958.303191
13,1,3151.668605
13,1,48908.317935
13,1,2054.580357
13,1,49688.529412
13,1,168039.046584
13,1,26962.966912
13,1,80790.250000
13,1,1644.583333
13,1,6825.947500
13,1,4253.555556
13,1,15958.514706
13,1,2575.800000
13,1,5181.076923
13,1,1654.441860
13,1,2276.000000
13,1,1658.986413
13,1,1839.700000
13,1,4005.812500
13,1,52201.655303
13,1,1591.000000
13,1,50959.714286
13,1,2896.758333
13,1,10812.808333
13,1,2593.577778
13,1,5089.270833
13,1,1925.666667
13,1,2839.556548
13,1,9236.000000
13,1,1953.776316
13,1,2148.250000
13,1,2612.669444
13,1,7702.059524
13,1,2363.266667
13,1,8640.090047
13,1,18775.083333
13,1,2678.697674
13,1,1695.418478
13,1,178053.907407
13,1,34344.090426
13,1,40219.590909
13,1,2926.486486
13,1,1643.515244
13,1,127254.585000
13,1,3095.806250
13,1,28246.878472
13,1,34503.617647
13,1,82591.652027
13,1,7206.917614
13,1,22443.660000
13,1,2611.853659
13,1,1555.869048
13,1,1747.500000
13,1,14236.273438
13,1,56048.200000
13,1,58348.824324
13,1,5068.909091
13,1,2714.886364
13,1,1646.144737
13,1,3047.765625
13,1,1540.935606
13,1,2203.176471
13,1,4482.468750
13,1,2574.963235
13,1,3520.798077
13,1,1642.875000

13,1,12094.000000
13,1,1586.230769
13,1,1834.100000
13,1,114817.394737
13,1,4072.951220
13,1,47313.798611
13,1,3550.404762
13,1,93392.528302
13,1,1964.416667
13,1,3043.877604
13,1,25240.559748
13,1,29729.274390
13,1,69161.552326
13,1,2155.739362
13,1,2005.714286
13,1,4973.989865
13,1,1721.190476
13,1,35071.337500
13,1,64983.888889
13,1,2793.235294
13,1,3875.882353
13,1,5651.896875
13,1,3415.448529
13,1,2743.609043
13,1,25293.532258
13,1,5408.218750
13,1,2304.465116
13,1,47079.121429
13,1,88195.318750
13,1,2756.712766
13,1,45746.132812
13,1,1780.983333
13,1,4693.982143
13,1,4784.000000
13,1,1775.048913
13,1,1615.641447
13,1,3210.882353
13,1,1515.930233
13,1,107592.586111
13,1,1922.687500
13,1,1887.818182
13,1,141366.558824
13,1,6747.875000
13,1,42070.039062
13,1,140396.506579
13,1,2460.891667
13,1,6217.235294
13,1,2065.721591
13,1,4902.977273
13,1,7422.545732
13,1,1627.410714
13,1,10303.985294
13,1,1924.361111
13,1,156382.168605
13,1,7136.000000
13,1,1791.421053
13,1,7088.326923
13,1,2061.928571
13,1,1649.565789
13,1,1863.991279
13,1,1512.094444
13,1,1993.342105
13,1,1750.702703
13,1,50764.333333
13,1,2626.159091
13,1,6119.875000

13,1,48222.000000
13,1,175948.662736
13,1,2095.259615
13,1,39331.488372
13,1,3168.816667
13,1,3193.051471
13,1,1516.881098
13,1,1661.658537
13,1,1638.360714
13,1,18862.380952
13,1,2606.544118
13,1,46485.284091
13,1,18462.117647
13,1,19309.559028
13,1,1727.513514
13,1,30825.302500
13,1,35529.724359
13,1,5912.287234
13,1,39716.940972
13,1,1575.366667
13,1,1586.100000
13,1,1606.600000
13,1,1740.562500
13,1,121525.157051
13,1,43877.826389
13,1,2034.918919
13,1,1594.031250
13,1,45476.205882
13,1,58202.779891
13,1,10217.613372
13,1,39271.539474
13,1,1521.013158
13,1,4727.756410
13,1,91372.729730
13,1,2683.400000
13,1,103057.505814
13,1,59691.900000
13,1,88378.572917
13,1,21968.982639
13,1,2199.230556
13,1,5394.815476
13,1,18402.943396
13,1,2498.744186
13,1,17235.102273
13,1,6825.730769
13,1,6864.535714
13,1,3510.446429
13,1,1573.922619
13,1,3069.720588
13,1,3046.500000
13,1,54618.158088
13,1,2962.633929
13,1,1591.439024
13,1,2235.711538
13,1,2285.151786
13,1,38723.675781
13,1,24051.625000
13,1,11324.650735
13,1,22314.812500
13,1,2046.176282
13,1,36246.276442
13,1,4819.860119
13,1,1601.162791
13,1,53334.255319
13,1,4533.523936
13,1,7062.883721

13,1,39921.263158
13,1,8843.973684
13,1,6131.502500
13,1,1560.153374
13,1,52541.179348
13,1,11386.977273
13,1,1737.671512
13,1,2366.500000
13,1,1708.538462
13,1,45259.493421
13,1,14553.768293
13,1,1515.503125
13,1,3331.031250
13,1,6846.485119
13,1,6429.651786
13,1,19301.085366
13,1,2127.969444
13,1,1692.318182
13,1,113053.960526
13,1,3998.000000
13,1,6305.298780
13,1,56086.571429
13,1,4862.117188
13,1,6149.828947
13,1,5773.487805
13,1,16967.470588
13,1,2315.660000
13,1,2001.375000
13,1,2336.787791
13,1,4178.892857
13,1,15735.861413
13,1,3978.250000
13,1,3835.431250
13,1,168531.480769
13,1,6581.627404
13,1,15175.291667
13,1,91753.204545
13,1,23551.800000
13,1,100085.472973
13,1,3062.422222
13,1,6645.964286
13,1,1556.536585
13,1,12638.902439
13,1,6492.700000
13,1,2086.402439
13,1,6354.228261
13,1,1795.227941
13,1,1978.966463
13,1,4403.099432
13,1,1516.262195
13,1,37278.288462
13,1,1788.932432
13,1,1996.528646
13,1,1605.250000
13,1,7746.500000
13,1,1603.442568
13,1,1793.017500
13,1,2045.227273
13,1,3474.232143
13,1,73353.609848
13,1,3418.010638
13,1,2081.957447
13,1,27934.593750
13,1,67932.858553
13,1,1957.552632
13,1,1530.500000

13,1,65309.510135
13,1,51701.628378
13,1,3291.595930
13,1,5990.550781
13,1,36609.476351
13,1,3091.869681
13,1,78467.510417
13,1,4093.218023
13,1,1737.823718
13,1,1749.875000
13,1,12860.166667
13,1,1662.213235
13,1,2772.142857
13,1,113029.416667
13,1,7072.818396
13,1,55669.040323
13,1,10449.388889
13,1,49565.202381
13,1,3688.380952
13,1,2681.312500
13,1,7600.000000
13,1,1903.261111
13,1,15530.080357
13,1,4034.187500
13,1,15172.125000
13,1,65686.067568
13,1,1668.867188
13,1,11204.147727
13,1,36049.235294
13,1,1907.368243
13,1,69910.229167
13,1,34485.250000
13,1,144167.848837
13,1,2496.951087
13,1,2092.000000
13,1,12216.076923
13,1,16133.317073
13,1,3096.487179
13,1,6181.431250
13,1,1821.750000
13,1,1594.181818
13,1,1651.192708
13,1,4175.705729
13,1,2281.000000
13,1,18578.085938
13,1,19324.625000
13,1,78101.771341
13,1,1924.145833
13,1,19531.317073
13,1,17975.722222
13,1,1556.125000
13,1,1519.625000
13,1,4888.137500
13,1,2106.562500
13,1,4283.750000
13,1,2932.304348
13,1,13857.230000
13,1,1666.850000
13,1,101011.500000
13,1,55438.218750
13,1,1762.989130
13,1,3416.432692
13,1,103972.410256
13,1,4514.983696
13,1,1764.833333
13,1,4265.483871

13,1,2135.554878
13,1,2705.594595
13,1,3233.394231
13,1,1645.635135
13,1,3253.207237
13,1,2744.634615
13,1,45281.000000
13,1,51882.761029
13,1,7506.934783
13,1,2656.687500
13,1,1689.185811
13,1,6719.866667
13,1,6451.754717
13,1,2162.993750
13,1,1545.411765
13,1,2208.308824
13,1,19777.566489
13,1,68583.080645
13,1,37784.840625
13,1,1581.297619
13,1,44372.968750
13,1,133133.168478
13,1,5321.161765
13,1,6302.062500
13,1,1667.750000
13,1,9709.186047
13,1,3000.743902
13,1,2627.102941
13,1,8983.243590
13,1,20424.000000
13,1,28043.535156
13,1,2648.613636
13,1,8314.574324
13,1,1614.794643
13,1,98223.200658
13,1,4701.500000
13,1,5031.051136
13,1,3094.071429
13,1,2378.446429
13,1,22678.700000
13,1,3037.690476
13,1,2508.425000
13,1,62941.155488
13,1,118676.880952
13,1,2725.142442
13,1,30247.171875
13,1,3712.040323
13,1,1601.278571
13,1,1563.691176
13,1,147910.468750
13,1,3053.358696
13,1,1595.837838
13,1,20031.993750
13,1,1595.625000
13,1,1932.205882
13,1,83229.491667
13,1,14008.224432
13,1,5064.035714
13,1,5815.003676
13,1,49076.427632
13,1,9525.416667
13,1,83871.491071
13,1,145640.819149
13,1,10457.666667
13,1,2184.973404
13,1,4018.718085

13,1,6271.112500
13,1,2068.982143
13,1,31445.054054
13,1,1922.461957
13,1,39051.185811
13,1,34380.743421
13,1,91114.201389
13,1,62137.181818
13,1,5457.310976
13,1,60028.817568
13,1,54773.951613
13,1,2351.750000
13,1,5359.157143
13,1,8311.737805
13,1,19483.164634
13,1,74573.211806
13,1,50789.328571
13,1,1631.503030
13,1,2300.139535
13,1,3048.358333
13,1,54456.746528
13,1,39172.795455
13,1,36998.750000
13,1,2097.746212
13,1,1499.655556
13,1,1707.418478
13,1,121151.878125
13,1,77178.182692
13,1,2317.425595
13,1,1534.225610
13,1,2053.648649
13,1,1963.356618
13,1,4077.532738
13,1,52578.957143
13,1,3241.812500
13,1,58274.709184
13,1,13865.418605
13,1,118674.340426
13,1,3794.593085
13,1,62067.799020
13,1,2499.195652
13,1,13019.785714
13,1,2113.151961
13,1,47175.477273
13,1,6557.000000
13,1,2327.222222
13,1,50529.310897
13,1,6271.790625
13,1,97609.487805
13,1,4824.170732
13,1,159131.076923
13,1,2034.842949
13,1,3677.878472
13,1,84280.000000
13,1,45329.083333
13,1,4530.885135
13,1,2964.825000
13,1,1686.974359
13,1,3257.200000
13,1,83249.712766
13,1,2724.901163
13,1,15362.597826
13,1,1516.209459
13,1,3324.238806
13,1,10296.493590
13,1,1824.636792

13,1,1863.744792
13,1,7516.445122
13,1,60007.622222
13,1,6314.203125
13,1,7726.952381
13,1,1639.631579
13,1,3225.450000
13,1,1540.066667
13,1,68213.461538
13,1,41785.886029
13,1,2448.875000
13,1,2528.184783
13,1,2360.706395
13,1,2671.548913
13,1,51992.282609
13,1,36360.935484
13,1,6153.842105
13,1,54763.000000
13,1,3252.250000
13,1,54308.406250
13,1,1671.881579
13,1,95470.000000
13,1,9398.225000
13,1,1825.904070
13,1,3483.595745
13,1,3148.505814
13,1,3741.632653
13,1,1629.569444
13,1,2547.254902
13,1,2197.793269
13,1,41730.993421
13,1,3934.975543
13,1,149236.122642
13,1,2128.203125
13,1,1849.924342
13,1,3316.202206
13,1,1648.375000
13,1,55544.996429
13,1,1712.331081
13,1,2625.319149
13,1,9405.711538
13,1,4951.664062
13,1,72243.483108
13,1,1677.972826
13,1,5003.428571
13,1,3571.066038
13,1,69918.423077
13,1,1785.634146
13,1,1581.366071
13,1,78054.872340
13,1,1713.263514
13,1,2881.988281
13,1,7117.020833
13,1,2121.239583
13,1,3168.291667
13,1,5315.157895
13,1,89453.562500
13,1,1975.521277
13,1,160910.452381
13,1,1506.000000
13,1,5880.270833
13,1,3883.195455
13,1,2304.750000
13,1,1505.904762
13,1,2078.843750
13,1,153763.630435

13,1,2089.833333
13,1,1575.601744
13,1,1556.022222
13,1,5301.767857
13,1,1828.682065
13,1,3709.093750
13,1,2434.223214
13,1,5318.023810
13,1,2009.587209
13,1,2075.462500
13,1,6066.250000
13,1,1660.133929
13,1,80363.648026
13,1,166446.326087
13,1,11656.736413
13,1,17532.314189
13,1,8732.591667
13,1,28763.302083
13,1,8255.300000
13,1,3249.038690
13,1,15987.529891
13,1,1791.020833
13,1,58984.444444
13,1,85648.537791
13,1,104342.526596
13,1,152049.139423
13,1,1642.181818
13,1,2248.622093
13,1,143513.820122
13,1,9636.510870
13,1,29258.632143
13,1,6330.172222
13,1,133130.464286
13,1,107730.828488
13,1,7709.250000
13,1,1589.463415
13,1,2196.652174
13,1,2297.709302
13,1,12682.083333
13,1,21931.525568
13,1,1629.107143
13,1,1854.502778
13,1,46926.591837
13,1,3677.722561
13,1,5157.371951
13,1,22242.677419
13,1,3035.277027
13,1,4894.625000
13,1,2271.097561
13,1,76473.157143
13,1,8671.297619
13,1,5508.881944
13,1,39229.933594
13,1,1720.645349
13,1,2215.341667
13,1,56577.608108
13,1,2333.928571
13,1,2700.106707
13,1,1810.797619
13,1,1778.482955
13,1,2456.062500
13,1,1877.763158
13,1,10323.466667
13,1,2281.829545
13,1,3844.673913
13,1,2075.900000

13,1,37927.986486
13,1,3149.800000
13,1,45539.867647
13,1,1690.365854
13,1,1556.949468
13,1,12326.652174
13,1,47808.500000
13,1,2436.506944
13,1,3894.857143
13,1,75349.765957
13,1,2018.843750
13,1,18956.653571
13,1,190943.203125
13,1,1768.298780
13,1,4638.367021
13,1,4517.384146
13,1,14338.604167
13,1,2948.058594
13,1,3699.616848
13,1,1629.026316
13,1,1748.333333
13,1,1524.369048
13,1,57376.980000
13,1,2036.206522
13,1,37412.647059
13,1,1751.264706
13,1,33696.160156
13,1,30678.425000
13,1,2440.023437
13,1,1803.109756
13,1,14911.738095
13,1,1569.834239
13,1,3387.434211
13,1,16692.191667
13,1,4458.410714
13,1,2789.035714
13,1,33134.646277
13,1,19415.980263
13,1,45103.101852
13,1,1864.987805
13,1,62534.270270
13,1,136720.969388
13,1,1999.282738
13,1,7646.765625
13,1,16264.359375
13,1,31760.729730
13,1,5721.312500
13,1,155590.611111
13,1,93351.552500
13,1,4152.570122
13,1,2591.806548
13,1,1731.250000
13,1,7188.744048
13,1,20059.200000
13,1,42470.416667
13,1,2075.439189
13,1,2902.483173
13,1,2758.558511
13,1,9857.122093
13,1,11994.723485
13,1,1635.630952
13,1,2269.526316
13,1,2228.223404
13,1,16791.944853
13,1,1593.496622
13,1,1579.489130

13,1,8309.643939
13,1,13927.138298
13,1,1724.044872
13,1,97796.845238
13,1,2571.780488
13,1,27570.511364
13,1,32430.906250
13,1,15775.155405
13,1,2882.207317
13,1,1996.243243
13,1,69266.868421
13,1,134125.943452
13,1,5599.076613
13,1,97932.743902
13,1,66485.009868
13,1,3649.125000
13,1,3731.170213
13,1,7644.136111
13,1,166142.774194
13,1,3943.863636
13,1,46150.164062
13,1,2251.567568
13,1,11263.246094
13,1,7069.168919
13,1,2748.750000
13,1,7474.240000
13,1,96491.406250
13,1,2787.608108
13,1,1503.636364
13,1,10087.138158
13,1,27812.129032
13,1,1688.000000
13,1,134122.533333
13,1,61341.323529
13,1,6492.837838
13,1,2053.962500
13,1,3060.288690
13,1,66355.500000
13,1,39350.666667
13,1,122924.536585
13,1,10968.718085
13,1,117920.090000
13,1,31480.000000
13,1,1559.500000
13,1,5911.712500
13,1,6850.461310
13,1,1729.234694
13,1,2917.366279
13,1,1860.500000
13,1,56062.771429
13,1,1550.791667
13,1,23088.118421
13,1,136776.250000
13,1,73656.184211
13,1,2105.147059
13,1,1597.986111
13,1,2417.852564
13,1,2995.333333
13,1,1734.710366
13,1,1809.994898
13,1,5337.963415
13,1,2781.678571
13,1,1943.393617
13,1,1932.399390
13,1,1857.875000
13,1,81452.000000

13,1,7593.890957
13,1,2389.526316
13,1,35962.220588
13,1,2229.559028
13,1,61976.022727
13,1,84807.125000
13,1,1752.281250
13,1,33151.604651
13,1,28550.375000
13,1,3763.627907
13,1,4156.025000
13,1,1684.118750
13,1,41971.327703
13,1,3157.234375
13,1,41577.496795
13,1,1712.865385
13,1,1562.012500
13,1,30842.818182
13,1,1895.108696
13,1,2088.880000
13,1,38806.014286
13,1,2163.642857
13,1,3041.923077
13,1,7623.752841
13,1,106574.439024
13,1,14251.000000
13,1,50243.176471
13,1,1669.833333
13,1,3219.260870
13,1,2025.122093
13,1,4489.200000
13,1,68977.790000
13,1,46554.275000
13,1,3175.359756
13,1,2417.625000
13,1,142173.713816
13,1,20398.389286
13,1,42816.614865
13,1,33613.687500
13,1,1588.266304
13,1,3320.214286
13,1,155315.000000
13,1,2856.393750
13,1,1615.116667
13,1,1507.412162
13,1,1515.718023
13,1,3665.288889
13,1,88519.468750
13,1,32851.275000
13,1,7845.159574
13,1,3755.000000
13,1,38765.432143
13,1,2313.457447
13,1,3749.040698
13,1,2580.519444
13,1,1798.367347
13,1,2480.346154
13,1,2363.291667
13,1,61013.980769
13,1,13805.358974
13,1,71178.264423
13,1,8673.346154
13,1,15454.889535
13,1,12687.532051
13,1,1647.318452
13,1,6209.282051

13,1,3463.372093
13,1,1845.073864
13,1,1942.640625
13,1,4949.000000
13,1,5871.699324
13,1,1627.663462
13,1,1969.837500
13,1,11159.747396
13,1,2220.212500
13,1,15298.963235
13,1,3070.176471
13,1,2363.900000
13,1,1990.614865
13,1,2701.616279
13,1,2978.191667
13,1,4294.075581
13,1,1536.031250
13,1,3106.400000
13,1,113086.140957
13,1,6798.444444
13,1,62530.673611
13,1,4222.687500
13,1,18440.583333
13,1,55022.581081
13,1,21029.697368
13,1,18282.564189
13,1,2315.160714
13,1,1624.864130
13,1,8127.631579
13,1,3052.838542
13,1,10687.569444
13,1,121106.711538
13,1,3383.700000
13,1,42694.769231
13,1,5195.800000
13,1,1668.778571
13,1,11984.600000
13,1,54565.020833
13,1,2868.256250
13,1,4293.800000
13,1,74302.834459
13,1,31390.430000
13,1,44357.768382
13,1,2512.717949
13,1,2012.782738
13,1,5510.771277
13,1,2901.136905
13,1,2422.425532
13,1,1553.232955
13,1,1707.658537
13,1,105195.750000
13,1,35679.695652
13,1,17285.604651
13,1,165896.533333
13,1,7005.039634
13,1,7167.953804
13,1,38281.693750
13,1,3406.641447
13,1,1587.630435
13,1,1646.087121
13,1,5656.760870
13,1,2607.276596
13,1,1512.687500
13,1,53680.456081
13,1,1937.848214
13,1,1968.162500

13,1,5537.152778
13,1,2504.031250
13,1,1638.615385
13,1,1617.156250
13,1,94351.050000
13,1,1677.515625
13,1,2243.062500
13,1,8190.916667
13,1,2636.166667
13,1,2220.562500
13,1,83804.911765
13,1,1519.868243
13,1,13484.288889
13,1,73831.281250
13,1,1537.871795
13,1,1954.583333
13,1,2243.989583
13,1,2058.931250
13,1,4741.731707
13,1,90501.676471
13,1,7725.795455
13,1,33557.833333
13,1,2961.884375
13,1,4663.327206
13,1,86428.526316
13,1,4404.279070
13,1,1697.082317
13,1,33710.102273
13,1,51063.093750
13,1,133549.089286
13,1,1564.989583
13,1,8784.655556
13,1,6918.656250
13,1,3328.982143
13,1,22093.383721
13,1,1580.218085
13,1,3265.521277
13,1,14253.750000
13,1,47125.860119
13,1,1621.478125
13,1,2824.241935
13,1,15096.125000
13,1,3008.104730
13,1,2181.750000
13,1,3433.267857
13,1,2969.967742
13,1,2048.871795
13,1,97905.858974
13,1,1501.973684
13,1,2777.532738
13,1,8274.609756
13,1,3126.389535
13,1,2337.529412
13,1,4508.265625
13,1,2087.437500
13,1,4310.647059
13,1,3770.773256
13,1,4049.317073
13,1,4479.548295
13,1,1674.297297
13,1,17314.204545
13,1,6047.875000
13,1,2126.656250
13,1,1802.217949
13,1,8949.106061
13,1,2737.875000

13,1,27676.863636
13,1,1582.102041
13,1,4147.088235
13,1,1840.048780
13,1,78057.684211
13,1,24527.397059
13,1,3876.690000
13,1,71614.413793
13,1,2097.414634
13,1,2250.975610
13,1,60297.125000
13,1,3946.625000
13,1,15913.210526
13,1,3568.764706
13,1,3165.361702
13,1,3204.352564
13,1,1850.756579
13,1,2022.253289
13,1,3370.678571
13,1,45397.520833
13,1,6423.105000
13,1,104222.831081
13,1,2224.766667
13,1,8208.239583
13,1,6404.591346
13,1,2875.709302
13,1,185352.220930
13,1,19372.116071
13,1,7515.736413
13,1,7159.812500
13,1,2557.901316
13,1,3751.403409
13,1,12914.500000
13,1,1619.544872
13,1,126209.275000
13,1,3605.026786
13,1,4012.916667
13,1,3067.875000
13,1,2396.636111
13,1,2064.803571
13,1,70190.564103
13,1,1961.083333
13,1,10862.093023
13,1,2137.005682
13,1,42595.528571
13,1,2028.113208
13,1,2796.184211
13,1,2351.205645
13,1,2923.277439
13,1,44248.882812
13,1,2012.537500
13,1,2120.551471
13,1,4061.555851
13,1,94758.619898
13,1,5916.437500
13,1,11853.300000
13,1,2220.569149
13,1,1545.830882
13,1,5550.313953
13,1,139161.613095
13,1,3704.527778
13,1,31512.580645
13,1,54968.750000
13,1,5085.871622
13,1,2738.039894
13,1,1594.672619

13,1,38344.255952
13,1,1830.818452
13,1,17165.307692
13,1,4216.219697
13,1,1614.547794
13,1,1648.831325
13,1,4475.268293
13,1,96369.767857
13,1,4063.843750
13,1,3055.393939
13,1,2265.409091
13,1,5396.348837
13,1,3190.321875
13,1,65047.082317
13,1,1507.554348
13,1,27501.435000
13,1,2430.255814
13,1,33817.500000
13,1,19428.918478
13,1,2058.535714
13,1,2534.951705
13,1,3626.050000
13,1,22072.026786
13,1,12552.872222
13,1,2344.625000
13,1,40346.094595
13,1,12736.062500
13,1,3782.250000
13,1,136140.859756
13,1,32490.720745
13,1,6090.687500
13,1,5190.685976
13,1,1565.530488
13,1,114726.510870
13,1,7205.057692
13,1,3192.168605
13,1,4774.792683
13,1,29409.255319
13,1,8005.452381
13,1,5833.511628
13,1,2225.473404
13,1,2290.030488
13,1,2250.165000
13,1,113678.035714
13,1,4323.427778
13,1,71046.409722
13,1,2034.250000
13,1,2025.368421
13,1,9048.112805
13,1,14481.744681
13,1,2013.801282
13,1,3110.557692
13,1,58871.000000
13,1,1875.051020
13,1,2753.533537
13,1,6041.820000
13,1,6016.520833
13,1,123206.976974
13,1,39587.250000
13,1,7262.763514
13,1,3440.150000
13,1,1650.656250
13,1,3024.488372
13,1,2643.761905
13,1,1672.662500
13,1,2475.957447

13,1,33013.664474
13,1,35582.348485
13,1,2287.859375
13,1,2448.709302
13,1,34339.228125
13,1,2622.634146
13,1,25954.695122
13,1,49114.058824
13,1,2378.187500
13,1,5654.009375
13,1,2674.731250
13,1,29435.365385
13,1,4377.295455
13,1,1740.236111
13,1,63119.668478
13,1,8519.657895
13,1,1538.645455
13,1,2676.230769
13,1,2878.181818
13,1,7193.235714
13,1,68883.267361
13,1,7134.808824
13,1,5004.216912
13,1,28304.721154
13,1,1702.253378
13,1,3063.437500
13,1,3356.827381
13,1,92834.666667
13,1,3427.250000
13,1,1637.042553
13,1,2471.232143
13,1,57672.714286
13,1,4637.654762
13,1,4592.914474
13,1,196978.784091
13,1,24901.221429
13,1,1753.739796
13,1,10712.010638
13,1,100450.660714
13,1,1506.910326
13,1,1884.000000
13,1,3242.617647
13,1,3365.094595
13,1,2849.675000
13,1,2956.628378
13,1,2394.177632
13,1,2432.755814
13,1,18022.821429
13,1,5502.769737
13,1,107338.967105
13,1,4120.243902
13,1,3824.115385
13,1,53030.023438
13,1,5696.886905
13,1,23205.219595
13,1,167131.664634
13,1,2651.666667
13,1,7274.984375
13,1,4283.005952
13,1,5762.347826
13,1,7229.625000
13,1,1778.962838
13,1,1771.225610
13,1,1563.428571
13,1,62750.471875
13,1,34128.428030

13,1,7802.875000
13,1,1558.500000
13,1,2741.693750
13,1,1809.798913
13,1,3278.162791
13,1,11966.500000
13,1,191375.127976
13,1,1755.400000
13,1,11242.704327
13,1,25800.409091
13,1,58323.267442
13,1,2372.207317
13,1,3494.933333
13,1,8547.288889
13,1,7381.950000
13,1,4149.456522
13,1,2120.904762
13,1,9751.406250
13,1,1814.666667
13,1,2266.428571
13,1,12215.468750
13,1,34136.700658
13,1,6579.427419
13,1,2885.743902
13,1,47880.866279
13,1,2330.416667
13,1,9339.923077
13,1,1844.285714
13,1,3408.260638
13,1,76252.907895
13,1,2485.079545
13,1,1641.553191
13,1,2828.486842
13,1,1795.842857
13,1,7336.964286
13,1,8643.882353
13,1,5367.452128
13,1,4367.065972
13,1,162116.463068
13,1,2054.661585
13,1,70948.136364
13,1,1532.587500
13,1,1809.977654
13,1,7119.083333
13,1,64592.854730
13,1,10932.906250
13,1,1718.966146
13,1,1603.081081
13,1,3321.568627
13,1,4669.007353
13,1,1783.795455
13,1,1596.760204
13,1,2370.207031
13,1,8267.642857
13,1,96511.081250
13,1,1644.479651
13,1,4879.168750
13,1,2487.457143
13,1,1791.696429
13,1,7431.750000
13,1,4438.992647
13,1,2027.600000
13,1,1835.328125
13,1,5592.948370
13,1,2277.821970
13,1,2940.761364

13,1,1606.349490
13,1,51289.750000
13,1,10342.467593
13,1,98300.578947
13,1,4285.239362
13,1,2129.357143
13,1,1786.081522
13,1,2354.133929
13,1,5300.970588
13,1,4470.125000
13,1,1911.596154
13,1,2990.117647
13,1,2692.013514
13,1,141077.890244
13,1,1970.791667
13,1,9380.456897
13,1,73322.559896
13,1,1646.453488
13,1,9086.435484
13,1,49373.566406
13,1,81104.375000
13,1,2150.382143
13,1,159335.558140
13,1,1638.894231
13,1,2206.641509
13,1,37438.246324
13,1,5925.604167
13,1,19718.105263
13,1,21082.420213
13,1,1828.824324
13,1,4344.910256
13,1,1508.527174
13,1,2543.700658
13,1,27076.121711
13,1,10756.877660
13,1,5700.657609
13,1,3577.546875
13,1,90875.081081
13,1,2256.639706
13,1,3374.375000
13,1,57314.175000
13,1,126105.068750
13,1,22191.082500
13,1,2791.127717
13,1,1922.892857
13,1,1817.235465
13,1,43142.641447
13,1,28169.300000
13,1,2388.352941
13,1,98452.219512
13,1,2190.666667
13,1,65916.568182
13,1,5361.907051
13,1,31064.433333
13,1,2390.240385
13,1,2455.652439
13,1,3448.333333
13,1,4359.563953
13,1,2663.038462
13,1,81523.367647
13,1,61356.169872
13,1,1562.831633
13,1,1999.607143
13,1,2009.625000
13,1,2417.026042
13,1,13394.775000

13,1,2267.616279
13,1,136990.666667
13,1,2087.336806
13,1,9455.046512
13,1,47352.083333
13,1,1573.718750
13,1,1969.963415
13,1,4120.428571
13,1,5890.345588
13,1,2443.483871
13,1,1508.050000
13,1,4270.645833
13,1,7805.854167
13,1,5059.891892
13,1,13518.800000
13,1,2462.853659
13,1,12385.552419
13,1,3177.053125
13,1,3018.803571
13,1,1935.339286
13,1,2148.705882
13,1,36212.967105
13,1,1898.707447
13,1,41579.992647
13,1,40255.685714
13,1,2439.350000
13,1,18878.945312
13,1,51145.338235
13,1,14795.270833
13,1,1675.127660
13,1,1838.679054
13,1,1801.562500
13,1,3076.015244
13,1,1500.250000
13,1,1790.788043
13,1,1652.119048
13,1,77948.024390
13,1,2274.604167
13,1,11878.278846
13,1,9102.278846
13,1,2733.542683
13,1,4475.187500
13,1,5444.320000
13,1,2117.894737
13,1,39910.420732
13,1,15367.714286
13,1,36810.007353
13,1,1774.327586
13,1,1835.191489
13,1,63589.675676
13,1,2019.314103
13,1,2596.105263
13,1,1975.425595
13,1,82110.435714
13,1,52982.798611
13,1,1810.562500
13,1,1663.615385
13,1,3595.760870
13,1,60072.488372
13,1,2875.750000
13,1,1642.244186
13,1,2197.989130
13,1,4097.602041
13,1,7022.732143
13,1,6010.625000
13,1,2072.657609

13,1,1519.600000
13,1,41149.791667
13,1,2195.666667
13,1,17759.826087
13,1,2879.062500
13,1,8125.125000
13,1,10442.925000
13,1,5165.823529
13,1,5641.797619
13,1,65294.907051
13,1,3740.641026
13,1,40844.531250
13,1,134475.133333
13,1,7173.777439
13,1,32884.093750
13,1,120812.785714
13,1,3183.000000
13,1,1997.781250
13,1,5306.390000
13,1,23189.811170
14,2,3238.763889
14,2,2825.023256
14,2,2042.993421
14,2,3464.067708
14,2,3134.476562
14,2,3125.606383
14,2,2742.156977
14,2,1830.951220
14,2,2213.510417
14,2,3347.132075
14,2,1513.687500
14,2,2767.862245
14,2,2381.652439
14,2,1703.713542
14,2,1615.647727
14,2,23824.737500
14,2,5495.803571
14,2,3197.724490
14,2,3048.564103
14,2,3887.000000
14,2,2217.435897
14,2,30971.065217
14,2,2720.121711
14,2,14255.687500
14,2,4409.216667
14,2,4747.315625
14,2,1766.673684
14,2,1590.338028
14,2,4924.150000
14,2,4074.327778
14,2,1506.587209
14,2,2149.721154
14,2,2438.382353
14,2,3395.665179
14,2,1525.319079
14,2,6930.994565
14,2,9587.391304
14,2,2293.428571
14,2,1979.000000
14,2,7449.000000
14,2,2400.500000
14,2,23505.005435
14,2,4036.289773
14,2,1732.875000
14,2,2630.000000
14,2,4062.440789

14,2,3244.460000
14,2,3873.364583
14,2,1737.225000
14,2,1805.130435
14,2,3190.074468
14,2,1679.964286
14,2,3021.659574
14,2,1955.497093
14,2,2714.362832
14,2,4903.888889
14,2,2933.730263
14,2,2772.896875
14,2,3588.729167
14,2,2628.982558
14,2,10731.114796
14,2,2944.600000
14,2,3622.738372
14,2,2414.083333
14,2,3914.813559
14,2,9013.213115
14,2,2978.114583
14,2,16547.725410
14,2,2787.950581
14,2,5367.764535
14,2,2709.543269
14,2,3171.451220
14,2,10100.598039
14,2,3902.745455
14,2,4901.735294
14,2,4177.694149
14,2,3908.242647
14,2,2997.535714
14,2,2364.375000
14,2,3201.085227
14,2,4953.638298
14,2,9089.000000
14,2,5105.976852
14,2,2959.722222
14,2,4626.230769
14,2,4010.908537
14,2,6756.418919
14,2,7076.259259
14,2,3717.000000
14,2,3618.333333
14,2,1618.011111
14,2,17980.224490
14,2,1693.976744
14,2,2153.085227
14,2,3543.120000
14,2,1685.375000
14,2,2815.797872
14,2,1819.105978
14,2,2123.350000
14,2,6012.153846
14,2,1907.943878
14,2,2095.235849
14,2,4644.435185
14,2,5157.930556
14,2,5819.502262
14,2,2301.397790
14,2,2532.836957
14,2,2452.916667
14,2,1567.877907
14,2,2406.375000
14,2,1611.597500
14,2,1543.695122

14,2,1883.618590
14,2,4660.818182
14,2,6809.029762
14,2,2932.693878
14,2,8053.112360
14,2,5989.070652
14,2,2548.948171
14,2,2294.250000
14,2,1617.229008
14,2,8086.962312
14,2,11547.551913
14,2,5167.454286
14,2,4211.903226
14,2,9119.702128
14,2,4380.556818
14,2,2868.541667
14,2,4331.892655
14,2,2207.926380
14,2,4568.813665
14,2,2120.842105
14,2,2396.391129
14,2,1718.890244
14,2,6138.741477
14,2,1990.412500
14,2,5085.125000
14,2,2210.625000
14,2,2807.238095
14,2,2201.172414
14,2,1865.634868
14,2,1915.550000
14,2,6566.950000
14,2,4756.247312
14,2,1604.758242
14,2,1843.378676
14,2,4682.077869
14,2,1773.661458
14,2,11436.322368
14,2,1772.410377
14,2,2844.323980
14,2,1948.886905
14,2,2706.923077
14,2,8009.453988
14,2,4861.750000
14,2,2608.884615
14,2,20073.033898
14,2,9309.687500
14,2,10327.453488
14,2,2452.629808
14,2,1633.393519
14,2,7211.793860
14,2,5945.863014
14,2,3161.723464
14,2,1568.484848
14,2,1518.675595
14,2,9994.330357
14,2,4326.218750
14,2,4087.200000
14,2,3978.285714
14,2,1634.332278
14,2,3539.435897
14,2,2326.437500
14,2,7538.469512
14,2,1529.543605
14,2,4267.511628
14,2,5575.000000
14,2,1988.640000

14,2,5353.460106
14,2,2619.910714
14,2,4524.797872
14,2,2019.934783
14,2,3131.687500
14,2,8242.166667
14,2,2843.083333
14,2,17756.344595
14,2,4398.776442
14,2,21342.031250
14,2,8047.645833
14,2,2195.761364
14,2,1600.800000
14,2,1887.750000
14,2,6422.546875
14,2,1774.412791
14,2,4961.691860
14,2,4152.787234
14,2,2133.483974
14,2,1756.187500
14,2,3588.875000
14,2,3239.312500
14,2,1671.801471
14,2,1835.457237
14,2,1745.031250
14,2,8183.750000
14,2,4146.842105
14,2,5141.913043
14,2,9132.271186
14,2,1880.875000
14,2,1999.581395
14,2,3935.186170
14,2,2384.130890
14,2,4229.715000
14,2,2247.993421
14,2,4821.375000
14,2,1569.163265
14,2,2420.120000
14,2,5915.438830
14,2,2038.594512
14,2,1513.979592
14,2,6598.553191
14,2,3628.055556
14,2,2929.873786
14,2,2618.647059
14,2,5762.893868
14,2,2041.716463
14,2,4956.486842
14,2,1834.549342
14,2,3487.125000
14,2,2507.888158
14,2,2494.634146
14,2,3165.250000
14,2,2393.845745
14,2,4532.243421
14,2,1863.410256
14,2,1716.255319
14,2,4323.924528
14,2,1530.086538
14,2,4499.238281
14,2,3503.780660
14,2,2562.606250
14,2,2521.450000
14,2,2039.478261
14,2,1782.361486
14,2,1976.931373

14,2,3699.617021
14,2,3376.208333
14,2,2815.824324
14,2,1529.986111
14,2,3199.215116
14,2,10098.980769
14,2,3084.275862
14,2,1793.378125
14,2,2358.500000
14,2,2348.335526
14,2,2213.636364
14,2,4613.602041
14,2,5970.445755
14,2,4071.220588
14,2,2663.160000
14,2,4059.233553
14,2,2329.063830
14,2,3427.915000
14,2,2095.815789
14,2,3159.687500
14,2,8564.344340
14,2,10002.890625
14,2,3698.409091
14,2,5070.074324
14,2,14894.254545
14,2,10373.239796
14,2,1653.051020
14,2,6085.640000
14,2,1687.222826
14,2,1536.829341
14,2,2772.453125
14,2,4542.375000
14,2,5925.504630
14,2,2650.200581
14,2,5218.041667
14,2,5695.078125
14,2,1951.750000
14,2,9951.316964
14,2,3104.000000
14,2,1998.789894
14,2,8164.382353
14,2,2142.259259
14,2,10576.795918
14,2,2962.966667
14,2,2823.845238
14,2,2734.400000
14,2,1738.902778
14,2,5389.500000
14,2,1884.716981
14,2,1857.200000
14,2,1777.353448
14,2,7799.340909
14,2,1899.866279
14,2,7430.335938
14,2,2074.688679
14,2,3131.875000
14,2,7533.645000
14,2,3727.861413
14,2,2141.636905
14,2,1756.642857
14,2,1696.000000
14,2,3326.146875
14,2,2389.159091
14,2,8647.125000
14,2,4249.679054
14,2,4342.148387

14,2,4693.603659
14,2,2516.812500
14,2,20065.215278
14,2,1682.575581
14,2,2017.323529
14,2,7371.566225
14,2,1749.232143
14,2,1965.154412
14,2,2337.222222
14,2,3095.737952
14,2,2474.163265
14,2,2480.811364
14,2,4444.765625
14,2,6608.437500
14,2,1862.912500
14,2,1787.320423
14,2,3303.404412
14,2,4260.832547
14,2,3598.857558
14,2,5278.656250
14,2,15015.622396
14,2,4395.889222
14,2,2767.488281
14,2,1996.666667
14,2,5917.500000
14,2,2398.975610
14,2,1754.952381
14,2,4681.294118
14,2,2241.558140
14,2,3851.900000
14,2,1590.788043
14,2,2969.160714
14,2,4255.897321
14,2,5654.599576
14,2,3528.750000
14,2,13946.067568
14,2,6204.881410
14,2,3512.023256
14,2,2165.589744
14,2,1748.164062
14,2,2431.125000
14,2,2941.918750
14,2,1645.132143
14,2,1761.428571
14,2,1625.901163
14,2,2286.493750
14,2,2359.507812
14,2,6173.050000
14,2,2480.045455
14,2,1548.127717
14,2,12266.309091
14,2,2665.938492
14,2,6204.375000
14,2,1978.260000
14,2,3057.846154
14,2,3743.881944
14,2,5971.865000
14,2,5402.312500
14,2,2306.336538
14,2,2259.343750
14,2,1857.456731
14,2,2112.352500
14,2,1926.500000
14,2,2673.659091
14,2,9354.823864
14,2,3763.057292

14,2,10595.968182
14,2,4787.470588
14,2,2142.641667
14,2,2382.070122
14,2,7976.217949
14,2,3957.599359
14,2,4402.595238
14,2,3200.642857
14,2,4306.219388
14,2,5482.368243
14,2,2034.288889
14,2,6348.372093
14,2,1744.193878
14,2,1580.274390
14,2,2180.405405
14,2,2796.470930
14,2,2301.464286
14,2,157124.252404
14,2,1625.500000
14,2,1619.524457
14,2,1835.632500
14,2,2377.617647
14,2,2579.125000
14,2,5424.500000
14,2,2720.216463
14,2,5780.065476
14,2,2141.418750
14,2,3285.133929
14,2,3485.438953
14,2,2460.792453
14,2,3698.615741
14,2,2650.677083
14,2,1924.943627
14,2,2502.619444
14,2,2245.346154
14,2,5122.520833
14,2,10367.500000
14,2,2492.923469
14,2,15025.868421
14,2,4707.062500
14,2,2179.375000
14,2,2020.365854
14,2,1833.690476
14,2,3603.771028
14,2,2472.250000
14,2,3531.094340
14,2,3371.318452
14,2,7450.906250
14,2,3439.630000
14,2,2717.240000
14,2,2822.081633
14,2,1603.600000
14,2,1584.625000
14,2,4569.680851
14,2,2962.637255
14,2,6095.166667
14,2,2587.450658
14,2,6802.929825
14,2,3258.142857
14,2,2194.160714
14,2,3404.132353
14,2,2207.910256
14,2,2734.221939
14,2,2692.592105
14,2,3029.574468
14,2,7111.358491

14,2,1592.975806
14,2,10463.612245
14,2,2538.031977
14,2,2372.319767
14,2,5317.675676
14,2,3200.913043
14,2,7451.882979
14,2,3992.482143
14,2,1750.065217
14,2,2288.655172
14,2,5915.867187
14,2,13457.303309
14,2,1517.648810
14,2,2858.402778
14,2,4612.110795
14,2,1917.500000
14,2,5285.949074
14,2,1756.250000
14,2,3186.169118
14,2,5215.476744
14,2,2887.463415
14,2,1978.829787
14,2,7814.261628
14,2,2972.138158
14,2,1499.466912
14,2,15943.916667
14,2,26354.682927
14,2,2994.125000
14,2,2922.400000
14,2,3340.343023
14,2,2226.666667
14,2,3843.309211
14,2,1750.400000
14,2,3012.930556
14,2,3473.000000
14,2,2320.957746
14,2,3722.064935
14,2,1502.022727
14,2,1547.325581
14,2,1972.812500
14,2,1959.276042
14,2,4886.968750
14,2,1935.654605
14,2,5111.368056
14,2,1512.331081
14,2,5537.767442
14,2,6753.450000
14,2,2384.314103
14,2,3831.062500
14,2,1717.185714
14,2,2036.575000
14,2,5496.625000
14,2,2795.596354
14,2,2125.733333
14,2,4448.610465
14,2,10082.828571
14,2,20654.458333
14,2,12618.829545
14,2,2106.022500
14,2,10202.905000
14,2,2790.292683
14,2,8816.836538
14,2,4227.644886
14,2,2146.656593
14,2,4455.128205
14,2,3626.858974

14,2,3170.538889
14,2,2566.875000
14,2,5480.296875
14,2,2246.214286
14,2,2893.952703
14,2,1524.234043
14,2,1886.575758
14,2,1850.000000
14,2,3355.385870
14,2,1723.783784
14,2,2132.397959
14,2,2574.906250
14,2,1913.760000
14,2,2161.487500
14,2,5347.734694
14,2,1723.125000
14,2,8891.416667
14,2,5357.855114
14,2,1754.256410
14,2,1695.895833
14,2,3600.243750
14,2,8867.041667
14,2,2453.871795
14,2,10186.542763
14,2,1524.235000
14,2,2707.057432
14,2,2580.200658
14,2,3337.272727
14,2,3707.720000
14,2,1725.818182
14,2,2369.453804
14,2,5529.120253
14,2,2661.768617
14,2,1518.060000
14,2,1832.701087
14,2,1827.750000
14,2,4243.588415
14,2,4430.574219
14,2,3351.252778
14,2,6254.217949
14,2,1788.666667
14,2,2124.472727
14,2,1614.977041
14,2,3070.779070
14,2,14572.681818
14,2,2534.853814
14,2,2280.722222
14,2,2681.627660
14,2,2091.261628
14,2,10933.527778
14,2,3144.534884
14,2,4717.839286
14,2,2530.459459
14,2,2037.304545
14,2,2831.002660
14,2,1910.280488
14,2,4687.562500
14,2,1720.434783
14,2,2753.375000
14,2,2578.033333
14,2,2168.089623
14,2,1995.401099
14,2,5046.465278
14,2,4160.735294
14,2,3461.874251
14,2,3027.607955

14,2,6234.580189
14,2,1755.225610
14,2,5055.600000
14,2,3869.173077
14,2,1663.117021
14,2,4429.659314
14,2,2335.467949
14,2,2624.083333
14,2,3223.186441
14,2,7218.766892
14,2,3539.556250
14,2,1887.653061
14,2,8871.844595
14,2,10483.184091
14,2,3391.821429
14,2,4987.546512
14,2,1749.145833
14,2,1737.975000
14,2,2749.297414
14,2,2302.187500
14,2,5040.063830
14,2,7632.866667
14,2,2047.819712
14,2,2619.863436
14,2,2807.367021
14,2,4899.273543
14,2,1923.666667
14,2,4399.012195
14,2,1681.698529
14,2,2968.428191
14,2,4597.609091
14,2,1651.378289
14,2,2274.604167
14,2,10093.093750
14,2,1733.680851
14,2,1995.875000
14,2,2931.713115
14,2,6642.747253
14,2,4447.500000
14,2,1855.727273
14,2,2333.130435
14,2,4544.145349
14,2,1970.223837
14,2,1817.428571
14,2,2491.540000
14,2,9061.428571
14,2,5831.769231
14,2,1891.558140
14,2,1673.833333
14,2,3970.454545
14,2,3934.602941
14,2,1590.695652
14,2,5063.625000
14,2,1842.578947
14,2,2906.878049
14,2,2224.205882
14,2,8742.727273
14,2,7583.281250
14,2,5954.787037
14,2,2532.937500
14,2,4080.333333
14,2,1567.372727
14,2,4962.578947
14,2,3801.000000
14,2,1703.945513
14,2,2171.328431

14,2,4946.069079
14,2,1804.765244
14,2,3430.000000
14,2,3118.583333
14,2,2287.804348
14,2,2315.345455
14,2,6913.413194
14,2,3581.500000
14,2,3401.315789
14,2,4072.772436
14,2,1808.433511
14,2,6619.269231
14,2,1869.852740
14,2,1609.568182
14,2,2046.884375
14,2,2759.750000
14,2,2311.386364
14,2,8961.899038
14,2,4393.020408
14,2,4030.336066
14,2,1780.766892
14,2,1642.418478
14,2,3077.105263
14,2,2768.843750
14,2,17380.430851
14,2,2145.388889
14,2,2160.855769
14,2,1569.417614
14,2,2124.390244
14,2,4191.440000
14,2,4225.838710
14,2,2753.227778
14,2,2162.880682
14,2,1915.275000
14,2,2738.055556
14,2,8022.570312
14,2,6218.639535
14,2,4986.743056
14,2,5464.735294
14,2,2282.270408
14,2,5008.553571
14,2,3586.512987
14,2,1540.617500
14,2,1724.683140
14,2,2332.054348
14,2,2819.616071
14,2,3832.795213
14,2,3286.900000
14,2,7524.204082
14,2,1975.368421
14,2,1549.553571
14,2,8355.398438
14,2,6848.167553
14,2,3343.480000
14,2,1643.616477
14,2,5983.821429
14,2,10082.402439
14,2,1961.000000
14,2,1510.039474
14,2,2588.319079
14,2,2430.236111
14,2,1781.425000
14,2,2851.416667
14,2,7140.875000
14,2,1972.763298
14,2,3222.923077

14,2,4295.460526
14,2,2393.271277
14,2,3573.825581
14,2,3285.465116
14,2,4254.879902
14,2,6073.273438
14,2,1652.155405
14,2,2871.106383
14,2,6598.671429
14,2,3454.826705
14,2,1670.355978
14,2,3992.812500
14,2,2945.394444
14,2,2496.230263
14,2,3576.725000
14,2,1910.652174
14,2,4064.181818
14,2,4797.372093
14,2,5662.916667
14,2,3811.333333
14,2,4411.750000
14,2,3560.476563
14,2,1710.203125
14,2,10693.986842
14,2,2853.855346
14,2,3030.069915
14,2,2126.160714
14,2,3246.860795
14,2,2697.010417
14,2,5100.707386
14,2,4112.943396
14,2,2242.345982
14,2,1904.845745
14,2,16781.327128
14,2,3235.710526
14,2,6425.863636
14,2,1507.860465
14,2,3067.547619
14,2,3582.259259
14,2,4992.896226
14,2,1548.565068
14,2,4806.666667
14,2,1878.727273
14,2,2495.292683
14,2,5474.041667
14,2,12229.250000
14,2,3106.972973
14,2,4127.033898
14,2,5085.071429
14,2,4367.438776
14,2,2121.581152
14,2,1633.566667
14,2,5093.204082
14,2,19968.490385
14,2,3023.085000
14,2,2507.087209
14,2,1977.279070
14,2,2831.057500
14,2,5613.820122
14,2,2754.033333
14,2,3397.787791
14,2,1756.434783
14,2,11600.060870
14,2,2226.335526
14,2,2033.302326
14,2,2634.524038

14,2,8170.112245
14,2,6769.805804
14,2,2496.156250
14,2,3559.756098
14,2,5557.090000
14,2,3255.805556
14,2,2069.937500
14,2,11136.343023
14,2,2661.383929
14,2,4046.400000
14,2,3446.845745
14,2,1810.756579
14,2,4260.097222
14,2,3946.836066
14,2,22204.752809
14,2,2511.488095
14,2,1656.308824
14,2,3090.995327
14,2,5767.134615
14,2,2615.062500
14,2,9385.586957
14,2,2533.709239
14,2,12850.834239
14,2,7076.640000
14,2,6108.829268
14,2,3213.320000
14,2,2359.892857
14,2,6080.350962
14,2,1883.928571
14,2,1834.083333
14,2,5127.894022
14,2,2830.273936
14,2,2826.898936
14,2,1682.198171
14,2,3334.593085
14,2,1635.000000
14,2,2646.256818
14,2,2992.194712
14,2,4724.706522
14,2,2231.184783
14,2,6970.938679
14,2,8381.656250
14,2,1884.477041
14,2,1780.704545
14,2,1688.981250
14,2,3872.493902
14,2,2600.517857
14,2,4669.081250
14,2,2159.950521
14,2,1637.013514
14,2,4083.990000
14,2,3976.401163
14,2,2039.411765
14,2,2348.040000
14,2,4638.362745
14,2,3298.590000
14,2,39776.266304
14,2,1858.850000
14,2,1709.452381
14,2,2397.000000
14,2,2714.984043
14,2,3144.121324
14,2,2334.712766
14,2,1879.781250
14,2,3347.311275
14,2,1854.187500

14,2,1717.014286
14,2,1642.494652
14,2,3135.718750
14,2,2638.090909
14,2,2954.055556
14,2,2256.158163
14,2,3688.627232
14,2,6061.768382
14,2,4102.698171
14,2,6054.755102
14,2,5399.173913
14,2,3102.250000
14,2,5358.351351
14,2,4280.000000
14,2,2517.494318
14,2,1680.941176
14,2,1557.190000
14,2,1829.029762
14,2,6255.491848
14,2,1760.157895
14,2,46735.303571
14,2,5901.852564
14,2,1530.445946
14,2,4108.742788
14,2,6763.395408
14,2,1906.812500
14,2,2743.867647
14,2,3732.687500
14,2,8170.784884
14,2,11385.000000
14,2,4339.142857
14,2,3508.409091
14,2,5472.020000
14,2,1794.455319
14,2,4176.477612
14,2,1938.375000
14,2,1906.969512
14,2,16282.884615
14,2,5031.026042
14,2,4066.245000
14,2,1683.362500
14,2,1941.723684
14,2,2543.353659
14,2,1586.893750
14,2,3335.473404
14,2,1718.233333
14,2,2667.652720
14,2,3415.846154
14,2,6787.318182
14,2,3262.666667
14,2,11429.809417
14,2,7360.431138
14,2,6787.887255
14,2,4773.527273
14,2,3390.653846
14,2,3383.105978
14,2,1846.647059
14,2,1766.000000
14,2,2789.750000
14,2,3127.119898
14,2,2486.841667
14,2,2418.517857
14,2,5104.072266
14,2,2931.974138
14,2,1612.604651
14,2,2195.419811

14,2,2647.222826
14,2,3007.554688
14,2,5415.029412
14,2,5716.041667
14,2,2333.853774
14,2,6025.544118
14,2,2650.280992
14,2,1781.880000
14,2,4142.592920
14,2,2649.493902
14,2,2590.411765
14,2,2403.180000
14,2,7796.628205
14,2,5792.962963
14,2,1534.733696
14,2,2450.184783
14,2,3597.886364
14,2,1992.276316
14,2,4844.477273
14,2,2421.437500
14,2,3075.278409
14,2,2958.500000
14,2,2285.121528
14,2,2146.625000
14,2,5074.548077
14,2,8679.994565
14,2,6102.584302
14,2,3393.560000
14,2,4858.140957
14,2,4416.671875
14,2,17345.172131
14,2,2413.320513
14,2,8556.907051
14,2,1621.571429
14,2,2970.838235
14,2,5236.750000
14,2,1906.621429
14,2,4654.325581
14,2,7241.611111
14,2,8637.770833
14,2,3885.917453
14,2,2465.937500
14,2,2084.230769
14,2,1668.226974
14,2,10713.687500
14,2,2240.341667
14,2,10693.099138
14,2,4622.065934
14,2,2755.969697
14,2,1744.562500
14,2,1767.589286
14,2,1911.010870
14,2,4946.224490
14,2,4117.941860
14,2,4094.467949
14,2,2006.000000
14,2,2095.750000
14,2,1751.596939
14,2,1737.799419
14,2,2472.620000
14,2,1542.312500
14,2,2497.739130
14,2,8931.670000
14,2,2795.017500
14,2,1758.638514
14,2,2016.565625

14,2,2721.509804
14,2,1802.512500
14,2,5320.567500
14,2,1774.111842
14,2,6323.500000
14,2,1808.333333
14,2,2349.579268
14,2,1851.234146
14,2,4321.478873
14,2,1876.657895
14,2,4835.031250
14,2,1663.829268
14,2,2638.002564
14,2,5464.945833
14,2,1788.781250
14,2,9246.777778
14,2,3728.060897
14,2,2014.675000
14,2,2881.012195
14,2,3238.778846
14,2,2176.930000
14,2,2282.481771
14,2,1733.268817
14,2,3385.544872
14,2,1773.964744
14,2,6539.055556
14,2,1949.737805
14,2,2709.861111
14,2,5308.108696
14,2,2364.263021
14,2,36513.346154
14,2,1537.540816
14,2,1695.125000
14,2,8604.219298
14,2,3554.533537
14,2,1954.783333
14,2,1582.470109
14,2,3581.047368
14,2,7232.352740
14,2,1679.322581
14,2,2130.492647
14,2,3879.363636
14,2,3045.013889
14,2,1875.583333
14,2,3191.176630
14,2,1768.668874
14,2,4430.560976
14,2,19853.039062
14,2,5481.554348
14,2,2131.200000
14,2,23047.860465
14,2,2423.750000
14,2,1984.980978
14,2,2371.647500
14,2,2275.806034
14,2,1840.041667
14,2,2977.956522
14,2,3087.727273
14,2,3274.367021
14,2,7988.737805
14,2,1568.798780
14,2,1710.320000
14,2,3207.750000
14,2,4448.774809
14,2,2293.771341
14,2,2826.866667

14,2,2079.657500
14,2,1661.182432
14,2,3934.781863
14,2,2104.952381
14,2,2885.774390
14,2,1686.803665
14,2,3067.354651
14,2,1851.656250
14,2,6556.125000
14,2,1632.136364
14,2,2246.400000
14,2,4686.116279
14,2,1932.312500
14,2,2600.166667
14,2,2666.536458
14,2,1668.851190
14,2,1912.122093
14,2,4617.200000
14,2,2952.659091
14,2,4729.731707
14,2,5175.971154
14,2,1859.908784
14,2,1955.080357
14,2,4646.500000
14,2,1789.652778
14,2,3651.363636
14,2,2307.504902
14,2,3376.104730
14,2,3108.145161
14,2,2436.289773
14,2,1820.789634
14,2,3426.208333
14,2,2122.723404
14,2,3145.291667
14,2,4214.231707
14,2,2743.857143
14,2,2547.790698
14,2,1518.479452
14,2,7174.525000
14,2,6560.410959
14,2,2274.650000
14,2,4234.677632
14,2,1514.825000
14,2,2998.700000
14,2,1509.000000
14,2,2316.400000
14,2,3101.377451
14,2,3248.875000
14,2,1889.339286
14,2,4141.665865
14,2,3869.920530
14,2,2740.382979
14,2,8008.691964
14,2,4453.102273
14,2,1666.289474
14,2,16315.933333
14,2,13364.125000
14,2,2039.708333
14,2,19436.791667
14,2,3340.379121
14,2,1558.630952
14,2,10543.583333
14,2,3970.606061
14,2,8037.102941
14,2,12119.673469
14,2,4280.296875

14,2,38831.005435
14,2,11231.656250
14,2,2820.363636
14,2,4279.865079
14,2,1886.909884
14,2,3193.764706
14,2,7434.485714
14,2,5007.795000
14,2,2909.300595
14,2,1653.825000
14,2,3253.971591
14,2,3191.490000
14,2,2075.386792
14,2,4368.147059
14,2,6781.627273
14,2,5787.511628
14,2,16309.080508
14,2,2844.067308
14,2,12140.267442
14,2,3146.579167
14,2,3714.327586
14,2,3219.547170
14,2,4177.461538
14,2,3894.289474
14,2,37335.692308
14,2,1583.384375
14,2,2409.366071
14,2,1580.829545
14,2,3222.343750
14,2,3581.473684
14,2,3260.133333
14,2,2992.172414
14,2,2466.559091
14,2,3401.976563
14,2,4468.088235
14,2,3764.693627
14,2,1838.925532
14,2,12593.867424
14,2,2583.750000
14,2,1866.769231
14,2,1547.953488
14,2,4042.097222
14,2,3695.524336
14,2,7157.630435
14,2,4654.095238
14,2,50946.705882
14,2,3053.788043
14,2,1759.835366
14,2,9055.744681
14,2,6786.899390
14,2,3114.440341
14,2,8604.158163
14,2,1921.350000
14,2,2442.331081
14,2,1682.258152
14,2,3929.769231
14,2,8766.390957
14,2,3224.379630
14,2,2918.230769
14,2,3409.415761
14,2,2320.470109
14,2,3304.725694
14,2,6247.357500
14,2,3898.646226
14,2,4070.168367
14,2,2598.043478

14,2,20274.679688
14,2,4998.958333
14,2,8399.420732
14,2,6781.837121
14,2,2225.281250
14,2,1957.366667
14,2,2162.690341
14,2,1854.255319
14,2,4956.489362
14,2,13855.568396
14,2,2809.338235
14,2,7643.500000
14,2,8033.069444
14,2,3165.290698
14,2,2784.725000
14,2,2696.977273
14,2,1769.037500
14,2,4161.765957
14,2,2693.711538
14,2,9661.944444
14,2,4289.178571
14,2,3536.156863
14,2,2893.187500
14,2,5731.915179
14,2,4153.625000
14,2,6636.417112
14,2,4398.317073
14,2,3644.750000
14,2,1509.765625
14,2,10758.130435
14,2,3454.085227
14,2,3865.264706
14,2,7927.583333
14,2,2097.671053
14,2,8216.827225
14,2,4454.085366
14,2,2634.038043
14,2,5162.923077
14,2,4386.511364
14,2,1976.360294
14,2,2039.817857
14,2,6407.196809
14,2,4338.025140
14,2,2171.648649
14,2,2159.979167
14,2,3662.237245
14,2,4401.333333
14,2,68889.570313
14,2,12910.597656
14,2,2104.281915
14,2,2129.475000
14,2,1774.076531
14,2,3179.937500
14,2,2790.019231
14,2,1756.050000
14,2,1538.031250
14,2,3344.868421
14,2,2278.138889
14,2,1618.000000
14,2,3598.238208
14,2,1933.926829
14,2,10145.825758
14,2,2028.423469
14,2,6254.122881
14,2,1583.117647
14,2,3842.825000

14,2,2492.497024
14,2,1540.269231
14,2,1726.750000
14,2,7126.625000
14,2,3335.339286
14,2,1838.826531
14,2,3225.519231
14,2,2176.586957
14,2,1614.066667
14,2,10441.308140
14,2,4825.260638
14,2,8595.300532
14,2,3914.428571
14,2,2145.212500
14,2,1531.823529
14,2,2262.990385
14,2,1714.213415
14,2,2025.186275
14,2,9309.172872
14,2,1804.182266
14,2,2674.100559
14,2,8895.397959
14,2,2815.265403
14,2,1790.277778
14,2,2207.707447
14,2,2040.722222
14,2,5412.534722
14,2,2240.108108
14,2,3286.209459
14,2,2600.312500
14,2,3178.143229
14,2,2671.261364
14,2,4928.180556
14,2,4578.141892
14,2,1913.133929
14,2,3564.773026
14,2,2751.720930
14,2,2200.812500
14,2,6528.656250
14,2,3429.529070
14,2,4071.479452
14,2,3987.641509
14,2,2431.776316
14,2,8263.149123
14,2,13802.183140
14,2,2842.875000
14,2,2424.074468
14,2,1701.776316
14,2,1820.010989
14,2,1545.096491
14,2,3621.850000
14,2,3447.781250
14,2,1967.539474
14,2,5609.607143
14,2,2094.977273
14,2,1602.062500
14,2,3197.375000
14,2,5467.845109
14,2,1504.956522
14,2,2101.971591
14,2,3964.642857
14,2,2662.989362
14,2,1941.387500
14,2,4773.579545
14,2,12572.962500
14,2,1501.608108

14,2,1502.682432
14,2,3092.903846
14,2,25423.647959
14,2,2730.691860
14,2,2869.428571
14,2,2435.380208
14,2,4975.701705
14,2,2633.562500
14,2,4181.545213
14,2,2249.982143
14,2,14061.714286
14,2,3240.500000
14,2,1502.658537
14,2,7098.000000
14,2,5414.382576
14,2,17688.600000
14,2,2831.869565
14,2,2799.965517
14,2,1503.402439
14,2,3049.972222
14,2,2660.946429
14,2,1519.310811
14,2,9734.199519
14,2,2843.209184
14,2,1707.296875
14,2,7524.125000
14,2,2132.284314
14,2,2270.978571
14,2,4941.350000
14,2,2331.079082
14,2,2489.209302
14,2,2796.240566
14,2,2621.324324
14,2,1894.843750
14,2,6396.062500
14,2,2094.755952
14,2,2164.048295
14,2,2527.874459
14,2,1998.739362
14,2,2382.509259
14,2,2276.833333
14,2,4154.678571
14,2,4103.818182
14,2,2179.712766
14,2,6601.904255
14,2,2313.411765
14,2,6879.500000
14,2,2027.531915
14,2,1763.000000
14,2,1775.959302
14,2,4951.729592
14,2,3571.583333
14,2,9984.940341
14,2,1667.773585
14,2,3659.933511
14,2,7866.157895
14,2,3260.131250
14,2,3145.052083
14,2,2148.053191
14,2,1653.672414
14,2,6097.965909
14,2,6785.827586
14,2,1902.420732
14,2,3211.664286
14,2,1559.234043
14,2,1520.791262

14,2,1991.425824
14,2,2754.316327
14,2,1662.219512
14,2,2715.716667
14,2,2073.234375
14,2,1608.285326
14,2,3263.543103
14,2,7318.346154
14,2,42444.900000
14,2,4924.454545
14,2,4384.911932
14,2,4602.716346
14,2,3163.750000
14,2,4762.796196
14,2,1860.113372
14,2,4057.402985
14,2,25605.810811
14,2,8286.375000
14,2,4670.505682
14,2,1753.784884
14,2,3110.620098
14,2,1940.768868
14,2,7534.451923
14,2,1725.854730
14,2,2592.083333
14,2,2987.516447
14,2,3280.125000
14,2,3561.362069
14,2,2249.861702
14,2,2859.495000
14,2,2574.050000
14,2,1952.365385
14,2,4302.093750
14,2,2818.000000
14,2,6563.021277
14,2,3354.445652
14,2,2570.875000
14,2,4198.833333
14,2,2044.805556
14,2,4635.685185
14,2,3269.750000
14,2,6982.866071
14,2,3596.223404
14,2,9469.765625
14,2,1687.258929
14,2,2109.417857
14,2,2681.417857
14,2,1794.218750
14,2,2809.861111
14,2,7783.458333
14,2,2836.567073
14,2,5262.580189
14,2,4408.683511
14,2,1745.840909
14,2,11407.494048
14,2,3283.106383
14,2,2157.763889
14,2,3825.426829
14,2,6314.810000
14,2,4408.104167
14,2,6592.063559
14,2,4184.212500
14,2,2041.146341
14,2,4232.407895
14,2,2165.047904
14,2,4592.200000

14,2,3258.240000
14,2,1801.838068
14,2,2676.243902
14,2,1808.959375
14,2,3759.800000
14,2,4234.388298
14,2,2835.264286
14,2,6506.702500
14,2,2261.666667
14,2,3457.101064
14,2,9127.750000
14,2,2650.584071
14,2,2626.338542
14,2,3771.305000
14,2,2092.576923
14,2,3494.825581
14,2,5086.975962
14,2,1500.567568
14,2,1783.630952
14,2,3433.477143
14,2,4248.548387
14,2,5649.688889
14,2,8232.527778
14,2,2988.297297
14,2,1681.933333
14,2,2937.435484
14,2,7558.813559
14,2,4500.337209
14,2,1612.402778
14,2,3738.940625
14,2,1935.035000
14,2,1856.177083
14,2,13050.465278
14,2,3528.869186
14,2,3470.617021
14,2,1669.960366
14,2,5140.052632
14,2,2009.125000
14,2,4334.943750
14,2,1915.636364
14,2,8329.547872
14,2,3079.446154
14,2,1969.651163
14,2,5295.706395
14,2,5533.828947
14,2,2241.037037
14,2,2307.707317
14,2,2224.555556
14,2,1628.902098
14,2,5577.000000
14,2,2659.737624
14,2,3213.971014
14,2,4123.625000
14,2,4189.303571
14,2,6321.121951
14,2,7372.333333
14,2,1699.132353
14,2,7747.010204
14,2,3634.678322
14,2,8170.833333
14,2,1819.175000
14,2,5752.644737
14,2,2466.055215
14,2,1648.129032
14,2,4437.836066
14,2,6166.982379

14,2,1687.069444
14,2,6239.431818
14,2,5611.068396
14,2,3149.750000
14,2,1781.701220
14,2,9096.308824
14,2,14769.466981
14,2,1775.295455
14,2,10810.598958
14,2,6366.125000
14,2,33650.100000
14,2,3053.578431
14,2,6833.235795
14,2,3694.304878
14,2,1878.182432
14,2,6611.744565
14,2,19747.225000
14,2,3764.773148
14,2,8867.828488
14,2,1657.189189
14,2,2738.752294
14,2,4010.653846
14,2,2561.500000
14,2,1857.351562
14,2,2623.351064
14,2,1683.289894
14,2,4670.038636
14,2,1706.645349
14,2,4422.882812
14,2,4745.924479
14,2,2085.105978
14,2,2767.500000
14,2,2154.908854
14,2,6304.186224
14,2,6619.259259
14,2,2368.672515
14,2,1732.058140
14,2,1514.217105
14,2,2357.034884
14,2,2924.822917
14,2,1615.693750
14,2,2523.570423
14,2,14025.730769
14,2,2130.796875
14,2,2447.696970
14,2,14880.476415
14,2,4911.195312
14,2,1921.830000
14,2,2692.346154
14,2,2509.836957
14,2,1756.451613
14,2,1504.117647
14,2,4804.153061
14,2,2659.236111
14,2,6741.970930
14,2,5921.416667
14,2,2394.379310
14,2,7056.800000
14,2,4681.878378
14,2,3938.415525
14,2,3579.495614
14,2,3292.903846
14,2,11389.288889
14,2,5706.531977
14,2,2778.075000
14,2,2975.595745

14,2,9832.529891
14,2,1808.817961
14,2,2405.204545
14,2,9393.287234
14,2,2750.633721
14,2,2682.300000
14,2,2766.441176
14,2,2227.627660
14,2,1785.852273
14,2,2012.375000
14,2,2205.648810
14,2,5714.478261
14,2,2695.750000
14,2,4017.343750
14,2,2020.302632
14,2,3507.970000
14,2,1783.716667
14,2,7902.411585
14,2,2120.183962
14,2,1926.600000
14,2,2988.878378
14,2,3692.145089
14,2,7034.000000
14,2,2496.533854
14,2,1648.148810
14,2,7116.544643
14,2,4097.227564
14,2,2382.234375
14,2,9645.485795
14,2,7589.903846
14,2,3393.375000
14,2,3055.264151
14,2,4510.162037
14,2,2688.125000
14,2,7128.500000
14,2,2408.000000
14,2,1730.914894
14,2,3116.056818
14,2,1847.776316
14,2,8048.375000
14,2,3614.475000
14,2,2357.950980
14,2,2044.445513
14,2,3186.381757
14,2,2042.945652
14,2,1533.040816
14,2,1760.909091
14,2,3220.958333
14,2,2244.219595
14,2,3061.528302
14,2,2596.288462
14,2,5241.571429
14,2,3537.641304
14,2,1603.050000
14,2,6195.687500
14,2,2730.541667
14,2,1637.030660
14,2,2911.236702
14,2,3598.000000
14,2,7843.163636
14,2,5415.453125
14,2,4940.314815
14,2,1868.618056
14,2,2231.866667
14,2,3835.811321
14,2,1530.750000

14,2,5376.687500
14,2,2622.098485
14,2,1620.807453
14,2,3784.428571
14,2,1849.627451
14,2,1746.412162
14,2,3655.923077
14,2,2852.026316
14,2,3513.095930
14,2,2213.818182
14,2,1684.868421
14,2,5398.800000
14,2,1660.714286
14,2,1991.390374
14,2,1788.200000
14,2,2237.520930
14,2,2634.573770
14,2,2654.963235
14,2,1633.000000
14,2,2041.631944
14,2,4642.012821
14,2,4309.782609
14,2,13948.747449
14,2,4452.487805
14,2,3930.174569
14,2,1548.353659
14,2,4115.012195
14,2,2217.786585
14,2,5815.235000
14,2,9237.366667
14,2,6102.857143
14,2,7486.044444
14,2,2937.848837
14,2,11155.544503
14,2,1516.818841
14,2,7651.875000
14,2,1690.010204
14,2,2392.927632
14,2,2385.356250
14,2,5888.627660
14,2,2570.739865
14,2,3792.477273
14,2,15441.125000
14,2,4191.300000
14,2,2276.581395
14,2,7811.884434
14,2,6065.419271
14,2,4081.863636
14,2,3471.323864
14,2,2659.482143
14,2,8001.761628
14,2,32406.986413
14,2,4921.884146
14,2,2232.125000
14,2,46156.258929
14,2,3153.937500
14,2,1657.944149
14,2,5513.469444
14,2,3806.285714
14,2,7402.422414
14,2,2637.172619
14,2,4295.128289
14,2,3462.733607
14,2,4708.088942
14,2,7087.513889
14,2,1585.000000

14,2,6787.333333
14,2,39920.566810
14,2,1515.655172
14,2,10601.549479
14,2,3578.406250
14,2,6581.851485
14,2,4932.892857
14,2,2099.133929
14,2,1815.010638
14,2,1967.397727
14,2,2922.142857
14,2,4475.864796
14,2,2316.426724
14,2,3793.169444
14,2,4460.847222
14,2,6382.976744
14,2,2920.250000
14,2,2269.110465
14,2,4726.442308
14,2,1854.928977
14,2,1546.111842
14,2,7234.559278
14,2,5323.617021
14,2,1815.690909
14,2,43034.565217
14,2,2549.993056
14,2,4668.352941
14,2,1805.404762
14,2,6376.135135
14,2,3349.866279
14,2,3085.557500
14,2,1736.894737
14,2,3075.852778
14,2,4827.600000
14,2,1616.500000
14,2,3233.954787
14,2,1606.564103
14,2,4019.753571
14,2,2697.321429
14,2,2873.500000
14,2,1646.081731
14,2,2058.035714
14,2,3181.161765
14,2,1743.776316
14,2,4704.303571
14,2,7467.882143
14,2,2380.926829
14,2,2050.687500
14,2,2166.928571
14,2,1874.368557
14,2,5204.519355
14,2,2728.717391
14,2,5570.651786
14,2,1939.950000
14,2,2428.323171
14,2,1873.836957
14,2,2354.115385
14,2,1556.500000
14,2,3309.074324
14,2,3010.678571
14,2,3141.168605
14,2,1998.567073
14,2,1579.048780
14,2,3770.239130
14,2,5102.246324
14,2,1935.855263

14,2,3304.350877
14,2,3567.417763
14,2,2923.314655
14,2,1879.297872
14,2,1733.140306
14,2,2279.292683
14,2,2363.562500
14,2,2687.415816
14,2,3021.187500
14,2,1983.135204
14,2,4066.559211
14,2,5866.450000
14,2,4414.454268
14,2,2775.815217
14,2,8228.666667
14,2,3641.502551
14,2,2676.098837
14,2,2066.854167
14,2,5768.948529
14,2,6336.922018
14,2,3096.062500
14,2,35642.222727
14,2,5618.114865
14,2,4518.075581
14,2,6190.058594
14,2,2144.621528
14,2,3843.875000
14,2,2285.441667
14,2,7336.430769
14,2,18317.599537
14,2,2221.500000
14,2,2980.344828
14,2,2377.277778
14,2,4254.062500
14,2,4950.937500
14,2,4596.848958
14,2,1672.872549
14,2,2119.000000
14,2,3942.131579
14,2,7959.666667
14,2,7549.991935
14,2,10553.994898
14,2,1970.306122
14,2,11579.756098
14,2,3538.273810
14,2,6969.884615
14,2,4921.995370
14,2,4703.731481
14,2,9337.771226
14,2,4635.759375
14,2,10799.368952
14,2,4584.642361
14,2,2532.412791
14,2,3924.111111
14,2,8061.504717
14,2,2488.281250
14,2,4367.641026
14,2,1737.416667
14,2,7045.436047
14,2,2560.298077
14,2,7611.466216
14,2,2117.695455
14,2,3476.391892
14,2,13644.675439
14,2,2825.272727
14,2,7215.906250

14,2,2330.649390
14,2,1655.442308
14,2,2117.706250
14,2,4633.717391
14,2,4377.941176
14,2,3191.857143
14,2,7380.245455
14,2,5748.264151
14,2,2730.009615
14,2,1859.312500
14,2,2321.242188
14,2,11021.223214
14,2,2530.149289
14,2,2190.556250
14,2,1990.202703
14,2,3273.721154
14,2,5155.997222
14,2,10617.395833
14,2,2036.040323
14,2,3651.006098
14,2,2280.038043
14,2,1599.619048
14,2,2855.396739
14,2,1836.886628
14,2,2296.304878
14,2,6081.812500
14,2,4569.814189
14,2,4126.293269
14,2,5363.481557
14,2,3796.857143
14,2,2290.500000
14,2,3290.745370
14,2,3707.095238
14,2,4718.880102
14,2,4348.470745
14,2,5655.361111
14,2,2417.445122
14,2,1599.400000
14,2,4391.210227
14,2,2374.187500
14,2,2029.000000
14,2,1977.231132
14,2,4655.813889
14,2,1938.752315
14,2,3007.800847
14,2,5579.408824
14,2,1500.931034
14,2,4024.071429
14,2,2333.455556
14,2,2239.762500
14,2,6374.495098
14,2,2565.000000
14,2,1746.390625
14,2,4956.750000
14,2,5863.175000
14,2,1653.291667
14,2,4037.168605
14,2,1569.806373
14,2,26196.531915
14,2,2851.095238
14,2,4158.023256
14,2,5946.459135
14,2,1660.071429
14,2,3295.989583
14,2,6122.384615
14,2,3925.148438

14,2,3449.769231
14,2,2485.327778
14,2,15119.714286
14,2,1936.510638
14,2,2585.393939
14,2,1718.576087
14,2,6008.360000
14,2,1549.711538
14,2,2378.837500
14,2,1514.986842
14,2,4727.527778
14,2,11156.656250
14,2,1754.849265
14,2,2181.028571
14,2,4253.538462
14,2,11014.282609
14,2,4934.125000
14,2,6711.709677
14,2,4439.008929
14,2,3477.175000
14,2,1742.907143
14,2,4552.931034
14,2,4394.949519
14,2,5565.647059
14,2,2381.051020
14,2,1542.968750
14,2,2198.227500
14,2,4031.510638
14,2,3255.490566
14,2,8434.265625
14,2,2038.684783
14,2,1531.048469
14,2,9522.059783
14,2,3507.409483
14,2,51756.344340
14,2,9520.765625
14,2,1708.750000
14,2,4487.426230
14,2,1878.593750
14,2,3169.232500
14,2,1883.832143
14,2,5277.050000
14,2,2247.920455
14,2,2225.554688
14,2,2546.258152
14,2,7116.926020
14,2,5233.638514
14,2,4759.012295
14,2,10157.000000
14,2,2882.000000
14,2,5790.191176
14,2,2004.342105
14,2,2163.220833
14,2,2819.814394
14,2,4012.355556
14,2,2875.513298
14,2,2202.926282
14,2,4306.026163
14,2,1927.375000
14,2,5566.197917
14,2,4447.281250
14,2,1853.625000
14,2,1810.113208
14,2,3674.585366
14,2,2128.379310
14,2,5229.389706

14,2,2340.958333
14,2,9349.089286
14,2,1911.914474
14,2,19161.860465
14,2,2377.778226
14,2,3479.535377
14,2,2726.250000
14,2,2392.021930
14,2,1832.100000
14,2,5009.460366
14,2,2477.735849
14,2,4316.333333
14,2,2892.609375
14,2,15640.500000
14,2,2716.558824
14,2,5274.070122
14,2,4480.519737
14,2,2155.654762
14,2,2725.510204
14,2,1793.278125
14,2,2297.135417
14,2,5573.178977
14,2,3408.411765
14,2,2830.694444
14,2,18471.803191
14,2,4022.979167
14,2,2784.502841
14,2,1884.439462
14,2,1996.598837
14,2,2608.612903
14,2,1533.638889
14,2,3025.888889
14,2,1875.012019
14,2,4771.095930
14,2,4032.545455
14,2,10459.114068
14,2,1564.078947
14,2,3825.296512
14,2,4951.091518
14,2,6361.555556
14,2,2004.714286
14,2,1845.477778
14,2,2035.367347
14,2,1824.804054
14,2,1871.989130
14,2,3250.822500
14,2,1694.857955
14,2,1712.916667
14,2,3380.983957
14,2,1830.579787
14,2,3646.187500
14,2,2911.846591
14,2,2169.407895
14,2,5578.670918
14,2,2813.400000
14,2,1766.968750
14,2,1942.053125
14,2,3729.333333
14,2,2530.458333
14,2,5311.485294
14,2,1913.446809
14,2,4509.018868
14,2,3836.745000
14,2,2875.888889
14,2,9035.096939
14,2,3408.230769

14,2,2359.090909
14,2,2502.700000
14,2,5544.245098
14,2,2959.887755
14,2,2142.477941
14,2,9820.776163
14,2,3568.085938
14,2,1560.415385
14,2,3095.741935
14,2,6519.145408
14,2,4923.160326
14,2,3366.377717
14,2,11601.972222
14,2,2820.034574
14,2,2453.080556
14,2,8581.545455
14,2,1685.005952
14,2,1827.600000
14,2,3505.279762
14,2,4188.155405
14,2,2728.051282
14,2,5782.125000
14,2,3681.722772
14,2,2483.804878
14,2,6321.636771
14,2,2421.457547
14,2,1705.038043
14,2,2492.280405
14,2,2646.289062
14,2,8650.807692
14,2,2011.094444
14,2,65601.585938
14,2,3454.128205
14,2,2292.057143
14,2,2935.617021
14,2,2748.875000
14,2,1614.085366
14,2,1983.013158
14,2,1681.710526
14,2,2075.555000
14,2,5488.662736
14,2,3687.658537
14,2,4438.566406
14,2,3171.181250
14,2,1602.048913
14,2,2143.268293
14,2,18140.269231
14,2,22963.687500
14,2,3048.266667
14,2,2604.000000
14,2,1782.227778
14,2,2693.404255
14,2,3059.541667
14,2,5794.117021
14,2,4147.625000
14,2,2150.988636
14,2,2066.696078
14,2,1676.511111
14,2,3944.872727
14,2,2335.500000
14,2,1526.903846
14,2,1909.529412
14,2,8112.049180
14,2,8739.558824
14,2,2185.750000
14,2,6247.352941

14,2,6081.191860
14,2,6176.393868
14,2,3915.942308
14,2,3603.602273
14,2,9289.376812
14,2,3474.222973
14,2,1955.125000
14,2,4317.282051
14,2,3394.250000
14,2,4855.779412
14,2,2925.176136
14,2,2887.702128
14,2,2090.615385
14,2,2360.059880
14,2,5731.381818
14,2,7857.710106
14,2,2569.328125
14,2,1974.189024
14,2,1820.890625
14,2,2299.489130
14,2,3813.618590
14,2,3175.125000
14,2,45235.678571
14,2,2259.000000
14,2,2073.320000
14,2,3900.265625
14,2,4284.289216
14,2,7062.625000
14,2,3254.200521
14,2,1931.097826
14,2,1575.875000
14,2,3441.809524
14,2,7741.500000
14,2,2628.281250
14,2,1609.335526
14,2,1810.799401
14,2,2298.922500
14,2,7528.000000
14,2,1862.885417
14,2,3689.481707
14,2,11924.021277
14,2,4449.563636
14,2,4390.911111
14,2,2208.011364
14,2,5678.127660
14,2,4458.125000
14,2,1895.359375
14,2,4113.283019
14,2,1966.087838
14,2,5533.627451
14,2,2064.341216
14,2,4862.263158
14,2,4112.576923
14,2,4537.125000
14,2,3118.346154
14,2,1813.128571
14,2,4504.426087
14,2,4867.853659
14,2,1994.635417
14,2,15694.714286
14,2,4927.427419
14,2,2289.826923
14,2,1655.569444
14,2,3660.894472
14,2,4953.264151
14,2,16105.292969

14,2,3124.588235
14,2,2359.510638
14,2,6792.933333
14,2,1887.016667
14,2,5738.687500
14,2,1601.526042
14,2,5142.434524
14,2,1893.216146
14,2,3053.046512
14,2,18418.222222
14,2,1905.166667
14,2,5177.960938
14,2,2731.405405
14,2,2337.211538
14,2,1731.744565
14,2,10958.916667
14,2,2661.910714
14,2,6856.250000
14,2,2741.653846
14,2,3407.972727
14,2,3394.338235
14,2,3495.452381
14,2,2213.926136
14,2,6675.161458
14,2,4116.750000
14,2,7396.445000
14,2,1659.488372
14,2,17043.127451
14,2,3869.354839
14,2,2098.471698
14,2,3166.421296
14,2,3175.962766
14,2,1757.122449
14,2,1804.460000
14,2,4515.687500
14,2,4454.166667
14,2,5315.012821
14,2,2658.134615
14,2,1881.250000
14,2,10227.085106
14,2,1508.709459
14,2,1815.018182
14,2,6322.112069
14,2,4672.610390
14,2,2405.133721
14,2,2078.772222
14,2,1700.744681
14,2,1500.653226
14,2,1997.416667
14,2,4667.409884
14,2,10823.144860
14,2,6129.656250
14,2,2261.897059
14,2,3476.726064
14,2,1642.189189
14,2,3573.780347
14,2,1988.773585
14,2,5373.535714
14,2,3334.994286
14,2,3751.292614
14,2,7036.302083
14,2,3617.560345
14,2,2981.598958
14,2,10408.536765
14,2,2072.755208
14,2,4009.546875

14,2,6978.083333
14,2,2128.815341
14,2,9231.513514
14,2,10365.788043
14,2,2625.221311
14,2,5872.372596
14,2,1700.026316
14,2,3951.530612
14,2,1656.878788
14,2,5604.278646
14,2,4760.738462
14,2,2029.452500
14,2,3281.647500
14,2,1816.528571
14,2,7210.466667
14,2,6723.543478
14,2,4829.346591
14,2,1763.500000
14,2,4087.169643
14,2,5779.275000
14,2,3021.645161
14,2,3677.542683
14,2,2290.448529
14,2,2951.931818
14,2,3466.406977
14,2,3617.674419
14,2,2026.981481
14,2,2769.692308
14,2,5881.885714
14,2,5861.801325
14,2,3967.935897
14,2,2027.085890
14,2,2718.659686
14,2,8795.609375
14,2,2042.509804
14,2,1687.164062
14,2,1652.500000
14,2,3613.349138
14,2,3239.470588
14,2,2241.802083
14,2,3579.333333
14,2,5357.754808
14,2,7745.306250
14,2,3508.250000
14,2,1689.015625
14,2,9087.937500
14,2,2906.888889
14,2,3801.640625
14,2,5173.059375
14,2,19190.269608
14,2,2249.216749
14,2,5304.304348
14,2,2172.437500
14,2,1936.287234
14,2,2230.916667
14,2,7075.677083
14,2,7667.125000
14,2,3428.139344
14,2,1859.292857
14,2,17400.941860
14,2,2122.673469
14,2,1983.595745
14,2,2301.030303
14,2,3025.976064
14,2,1997.169811
14,2,2532.739130

14,2,1709.837209
14,2,4887.454327
14,2,3094.432927
14,2,2460.771341
14,2,1528.833333
14,2,4343.795213
14,2,4672.914286
14,2,6902.389151
14,2,1520.780488
14,2,2025.573864
14,2,3050.364780
14,2,2530.875000
14,2,1871.750000
14,2,1813.527174
14,2,4341.850000
14,2,9249.637097
14,2,2864.364796
14,2,3257.722222
14,2,4518.732143
14,2,2744.338542
14,2,5947.259804
14,2,2987.037500
14,2,6071.080000
14,2,2621.156250
14,2,2213.884146
14,2,3235.133621
14,2,10489.737500
14,2,1534.875000
14,2,4220.732143
14,2,2781.342105
14,2,4211.428571
14,2,3172.971698
14,2,5191.398396
14,2,1950.666667
14,2,7828.721774
14,2,1642.128049
14,2,4816.973684
14,2,4216.904762
14,2,4075.515625
14,2,1668.602273
14,2,6009.500000
14,2,1596.852273
14,2,5394.459375
14,2,4863.933333
14,2,2031.446809
14,2,2207.363636
14,2,2046.098214
14,2,4869.446429
14,2,8312.077206
14,2,2767.509434
14,2,3790.046512
14,2,4757.150000
14,2,3790.915625
14,2,2726.616667
14,2,1540.645833
14,2,4352.352041
14,2,4892.041667
14,2,1899.565217
14,2,2492.036458
14,2,1552.401442
14,2,3442.235000
14,2,3647.880435
14,2,5752.846154
14,2,5774.142857
14,2,2105.943548
14,2,1676.343750

14,2,3000.707317
14,2,4562.850000
14,2,2451.285714
14,2,6537.750000
14,2,13685.527273
14,2,2100.663690
14,2,11533.214286
14,2,1969.530660
14,2,1796.877551
14,2,5378.513636
14,2,6943.738095
14,2,3829.114286
14,2,8158.000000
14,2,25817.395556
14,2,1798.428571
14,2,1524.022222
14,2,1557.955556
14,2,9218.083333
14,2,4093.480769
14,2,2348.683333
14,2,1982.571429
14,2,6836.539841
14,2,2326.586957
14,2,13450.307692
14,2,3586.986486
14,2,3320.845000
14,2,9915.363636
14,2,6511.951220
14,2,2637.842105
14,2,2150.886364
14,2,2303.467391
14,2,2548.815625
14,2,3538.534722
14,2,2411.867257
14,2,2395.319149
14,2,2333.817416
14,2,1896.890909
14,2,2285.621622
14,2,4810.657895
14,2,2050.585106
14,2,2298.062500
14,2,1729.329268
14,2,6967.407051
14,2,4874.904255
14,2,5027.666667
14,2,1837.687500
14,2,4486.628289
14,2,1659.125000
14,2,3031.418367
14,2,6075.320000
14,2,5920.463415
14,2,1726.440476
14,2,6605.734375
14,2,4066.939655
14,2,2598.341463
14,2,4961.796296
14,2,2212.832402
14,2,1705.754839
14,2,3854.770833
14,2,1511.816667
14,2,2158.557692
14,2,1507.569444
14,2,2808.625000
14,2,6994.095930
14,2,1549.354167
14,2,2601.946809

14,2,3054.920000
14,2,2239.264151
14,2,2185.683333
14,2,3122.622449
14,2,1709.103365
14,2,2768.275000
14,2,3132.815217
14,2,2458.156915
14,2,3108.777778
14,2,1987.714286
14,2,6637.595588
14,2,8136.689024
14,2,1988.315000
14,2,7020.086957
14,2,6630.272727
14,2,1527.002841
14,2,8832.223214
14,2,1862.328125
14,2,2974.659091
14,2,2721.046196
14,2,2062.333333
14,2,5031.831140
14,2,2002.800000
14,2,15111.833333
14,2,2070.381757
14,2,4977.690476
14,2,3140.775943
14,2,2140.826087
14,2,15525.415865
14,2,2142.467213
14,2,38697.929688
14,2,3394.933824
14,2,2351.372024
14,2,7478.367347
14,2,3097.635135
14,2,3319.492188
14,2,4402.806818
14,2,1804.963889
14,2,2224.777027
14,2,2149.937500
14,2,7716.413043
14,2,1864.446429
14,2,2169.518229
14,2,6355.906716
14,2,14202.508368
14,2,1816.826087
14,2,1559.166667
14,2,12473.718750
14,2,3880.579327
14,2,3491.237245
14,2,4218.393617
14,2,4636.548387
14,2,1616.145833
14,2,3701.086735
14,2,7005.563725
14,2,4526.875000
14,2,2303.465909
14,2,4701.885776
14,2,3638.701705
14,2,2163.160714
14,2,3542.283019
14,2,1768.839744
14,2,1813.593750
14,2,2012.184524
14,2,1598.085526
14,2,4088.659193

14,2,1844.268817
14,2,19566.923913
14,2,1564.663462
14,2,2496.610169
14,2,1613.062500
14,2,2093.207090
14,2,3470.075000
14,2,2732.843023
14,2,1927.307692
14,2,1618.460366
14,2,1506.676829
14,2,5518.340909
14,2,2333.100000
14,2,3731.289773
14,2,7092.639344
14,2,2888.517500
14,2,1524.924528
14,2,2703.750000
14,2,8121.852941
14,2,1503.974359
14,2,2075.608939
14,2,6472.700980
14,2,5299.766509
14,2,1973.614583
14,2,2908.095930
14,2,7301.151316
14,2,7243.872222
14,2,2912.212766
14,2,1506.363636
14,2,4835.475000
14,2,10091.948529
14,2,4105.544444
14,2,1660.858696
14,2,2128.031977
14,2,1510.188830
14,2,4299.547619
14,2,2336.592391
14,2,3826.013587
14,2,2405.896429
14,2,2230.875000
14,2,11598.892857
14,2,2581.755814
14,2,1733.895408
14,2,6541.119048
14,2,2837.523810
14,2,8411.568548
14,2,2911.943878
14,2,1608.000000
14,2,2614.056604
14,2,1810.986842
14,2,2361.172775
14,2,3365.397436
14,2,5361.752551
14,2,3501.448171
14,2,5273.980000
14,2,3538.410326
14,2,6088.800847
14,2,4654.949324
14,2,3207.077703
14,2,3698.382979
14,2,3033.551829
14,2,4251.949468
14,2,5967.367347
14,2,2672.307692
14,2,2182.448980
14,2,4801.968750

14,2,2653.452830
14,2,2415.120000
14,2,3467.639535
14,2,3520.754237
14,2,5581.210526
14,2,5803.692308
14,2,1951.330000
14,2,3462.714286
14,2,6286.875000
14,2,9173.450000
14,2,2749.389535
14,2,1721.918367
14,2,6840.211957
14,2,1872.510204
14,2,1911.096774
14,2,6070.486607
14,2,2278.677778
14,2,1675.487805
14,2,1746.590909
14,2,14365.840426
14,2,2542.078571
14,2,10950.659420
14,2,2804.638298
14,2,2300.294355
14,2,2850.782051
14,2,2646.513889
14,2,3777.208333
14,2,3244.038043
14,2,2892.636364
14,2,1961.450000
14,2,3819.780172
14,2,2702.941176
14,2,2328.247093
14,2,3309.591981
14,2,1847.461111
14,2,3580.906250
14,2,3008.804636
14,2,2478.685606
14,2,4974.568878
14,2,8291.210784
14,2,6018.360795
14,2,3448.504098
14,2,1702.401316
14,2,6693.303279
14,2,2768.660714
14,2,3399.575000
14,2,1822.078125
14,2,1803.851064
14,2,2470.825472
14,2,1987.610795
14,2,1560.214286
14,2,2572.736364
14,2,3484.172775
14,2,2492.067039
14,2,1810.000000
14,2,2020.157895
14,2,2616.333333
14,2,8518.943005
14,2,2207.554913
14,2,2162.170732
14,2,2583.294944
14,2,4335.845588
14,2,16762.729167
14,2,3897.625000
14,2,8450.000000
14,2,3319.430000

14,2,5158.612981
14,2,6887.069767
14,2,5624.967949
14,2,1812.371795
14,2,1641.670000
14,2,1635.062500
14,2,2177.600000
14,2,8558.433824
14,2,1672.000000
14,2,3503.425000
14,2,22698.875000
14,2,3211.857143
14,2,3022.768750
14,2,3046.532609
14,2,8403.404255
14,2,3574.055085
14,2,4044.036058
14,2,9923.719907
14,2,1879.193182
14,2,2252.481567
14,2,4587.876000
14,2,8048.224359
14,2,4227.414063
14,2,2005.378378
14,2,2076.025000
14,2,8487.133333
14,2,5207.831081
14,2,15305.961538
14,2,4230.511905
14,2,4710.325581
14,2,3355.013514
14,2,4688.785714
14,2,4018.930233
14,2,8363.437500
14,2,1515.450000
14,2,2988.938776
14,2,3350.236111
14,2,1971.834906
14,2,1510.921296
14,2,3067.218750
14,2,7085.324324
14,2,6206.196429
14,2,16511.750000
14,2,3610.139423
14,2,2314.625000
14,2,1990.460526
14,2,7616.171875
14,2,3679.449468
14,2,3109.000000
14,2,2113.421384
14,2,1783.744565
14,2,9832.696429
14,2,2525.548387
14,2,2880.518229
14,2,1868.688830
14,2,3978.344444
14,2,10400.718750
14,2,4524.910714
14,2,2146.560345
14,2,2899.604167
14,2,9766.520833
14,2,1808.580000
14,2,1570.986842
14,2,1907.008523
14,2,7727.182692
14,2,15458.140625

14,2,4859.002551
14,2,3279.963942
14,2,2233.875000
14,2,9294.367925
14,2,1511.428962
14,2,5181.346939
14,2,3916.040000
14,2,1586.250000
14,2,7492.176829
14,2,1522.204545
14,2,8398.133333
14,2,6511.953125
14,2,2278.783333
14,2,2557.725000
14,2,1717.701183
14,2,4968.669492
14,2,3975.802239
14,2,2548.521739
14,2,4283.478261
14,2,4994.312500
14,2,135956.771277
14,2,1637.147959
14,2,7953.274390
14,2,2727.475000
14,2,4433.811047
14,2,2167.957317
14,2,6251.023256
14,2,2251.276786
14,2,2017.458333
14,2,9067.805085
14,2,4582.513158
14,2,1941.500000
14,2,1542.446809
14,2,8994.704545
14,2,7789.325301
14,2,2602.563380
14,2,5600.586111
14,2,9911.479167
14,2,2045.494681
14,2,2708.788194
14,2,2444.566667
14,2,2948.292763
14,2,1636.409091
14,2,1904.250000
14,2,10572.892857
14,2,3020.937500
14,2,1964.318182
14,2,4300.666667
14,2,2690.050000
14,2,2799.923077
14,2,7635.095588
14,2,4454.097368
14,2,11541.650190
14,2,4073.244444
14,2,5764.556122
14,2,1885.397959
14,2,4972.268519
14,2,2156.585227
14,2,2452.214286
14,2,2058.104651
14,2,1790.364130
14,2,12236.422619
14,2,1774.275510
14,2,7703.750000
14,2,13220.310976
14,2,4366.860577

14,2,5058.645349
14,2,2001.169355
14,2,3234.500000
14,2,10586.160714
14,2,4117.235465
14,2,1964.417453
14,2,2884.720745
14,2,15384.820755
14,2,8029.882353
14,2,2905.764423
14,2,7460.908163
14,2,4292.825000
14,2,2171.538462
14,2,2097.842857
14,2,1708.632653
14,2,2469.489130
14,2,2885.849265
14,2,4473.564246
14,2,1722.089286
14,2,3172.000000
14,2,9649.052500
14,2,1962.944444
14,2,9879.600000
14,2,2213.294811
14,2,3645.401163
14,2,6207.470588
14,2,2318.416667
14,2,3044.837500
14,2,3846.528646
14,2,13202.833333
14,2,5639.875000
14,2,3139.222826
14,2,6242.732143
14,2,3156.404018
14,2,2372.170732
14,2,10201.950521
14,2,2533.000000
14,2,1583.744681
14,2,1646.347973
14,2,1705.682927
14,2,2993.600000
14,2,2666.681122
14,2,2240.293269
14,2,5917.176020
14,2,2071.922297
14,2,2677.036199
14,2,6364.157895
14,2,2642.133333
14,2,8243.932292
14,2,3098.340278
14,2,3254.582386
14,2,22305.500000
14,2,1861.781250
14,2,1663.520000
14,2,2795.814583
14,2,4049.091346
14,2,2752.812500
14,2,2764.670732
14,2,1626.415541
14,2,3893.075000
14,2,2426.304348
14,2,6953.864583
14,2,2643.668919
14,2,1549.102273
14,2,2949.325472
14,2,4382.832317

14,2,3082.205189
14,2,2378.663690
14,2,14888.934211
14,2,1589.851190
14,2,2784.804348
14,2,1920.500000
14,2,4091.447917
14,2,2129.291667
14,2,15977.710000
14,2,3209.108696
14,2,4898.000000
14,2,4959.199519
14,2,3413.473404
14,2,2328.521739
14,2,10880.908854
14,2,2647.552083
14,2,2059.407895
14,2,1583.843750
14,2,3999.052764
14,2,12002.597826
14,2,2588.939394
14,2,154645.036765
14,2,2780.688889
14,2,2690.255814
14,2,5634.588068
14,2,2963.595238
14,2,3306.816176
14,2,1558.457143
14,2,9354.159836
14,2,3092.527778
14,2,2010.125000
14,2,1723.971154
14,2,2244.808333
14,2,5928.447368
14,2,2376.880102
14,2,2498.250000
14,2,2132.702206
14,2,8092.571429
14,2,28582.395210
14,2,1894.902439
14,2,1745.948529
14,2,2313.948718
14,2,7154.754902
14,2,1514.272727
14,2,2886.022222
14,2,2672.960000
14,2,2003.148148
14,2,2773.204787
14,2,1939.849138
14,2,3057.228448
14,2,1973.963889
14,2,7759.462025
14,2,2567.297030
14,2,3827.869565
14,2,12892.738971
14,2,4672.253394
14,2,3562.828947
14,2,4139.187500
14,2,2288.250000
14,2,2807.880435
14,2,5550.710227
14,2,2992.695000
14,2,12261.768868
14,2,2224.457447
14,2,4520.209459
14,2,1817.250000

14,2,3342.113208
14,2,5486.718593
14,2,2081.543590
14,2,8632.924528
14,2,1814.846154
14,2,2351.683333
14,2,2101.920213
14,2,1598.757576
14,2,1870.558140
14,2,3131.058140
14,2,1622.512195
14,2,6673.833333
14,2,1639.625000
14,2,5799.228659
14,2,1581.822368
14,2,174032.289474
14,2,2422.006944
14,2,2370.304054
14,2,1950.462500
14,2,2029.027027
14,2,1843.267500
14,2,4042.168182
14,2,1898.423077
14,2,1949.700000
14,2,13445.394531
14,2,2423.078341
14,2,10445.541176
14,2,2522.414286
14,2,2063.693750
14,2,7301.116279
14,2,1520.139286
14,2,1857.098404
14,2,3385.762712
14,2,1704.253049
14,2,1538.621951
14,2,4269.866492
14,2,1799.069767
14,2,3517.360577
14,2,1649.282895
14,2,10026.857143
14,2,4252.218182
14,2,2537.989362
14,2,2396.967742
14,2,3835.540761
14,2,1763.244898
14,2,2018.162162
14,2,3656.452830
14,2,1651.352041
14,2,6931.171610
14,2,5307.370370
14,2,3110.970745
14,2,11576.395161
14,2,2270.548780
14,2,3016.024390
14,2,1902.540541
14,2,3472.000000
14,2,6844.068182
14,2,2203.547872
14,2,14290.037500
14,2,3093.912736
14,2,1838.007143
14,2,1668.886228
14,2,2476.237805
14,2,3312.500000
14,2,1746.382022
14,2,2571.781250

14,2,3054.079787
14,2,10172.000000
14,2,2300.771429
14,2,2278.536585
14,2,3667.500000
14,2,3485.566860
14,2,5192.343750
14,2,2721.325581
14,2,1570.854545
14,2,2697.111111
14,2,1620.518750
14,2,1539.401042
14,2,2676.272727
14,2,6229.367925
14,2,2054.907489
14,2,1590.792683
14,2,2058.750000
14,2,4596.024510
14,2,1985.333333
14,2,8100.300000
14,2,2103.127273
14,2,1814.146341
14,2,1939.989796
14,2,9760.913043
14,2,3457.234375
14,2,3182.380000
14,2,2300.658537
14,2,1626.156250
14,2,6141.938776
14,2,4176.226744
14,2,1918.557292
14,2,3483.553191
14,2,1789.536184
14,2,13722.865854
14,2,8385.006098
14,2,10636.333333
14,2,2191.500000
14,2,2004.728261
14,2,2930.400000
14,2,2069.818182
14,2,7149.826087
14,2,5757.953488
14,2,1824.635870
14,2,7120.312500
14,2,2185.082317
14,2,2331.971154
14,2,3063.550926
14,2,2795.482143
14,2,5285.591463
14,2,7884.851562
14,2,2995.811170
14,2,1749.300000
14,2,1626.919643
14,2,1635.637500
14,2,3241.211538
14,2,1526.812500
14,2,6219.117647
14,2,4481.666667
14,2,2984.152439
14,2,2212.030660
14,2,1980.946809
14,2,3823.666667
14,2,2003.622596
14,2,55996.121324
14,2,3594.773026
14,2,3355.463235

14,2,2977.047872
14,2,1938.333333
14,2,10914.544811
14,2,10875.192308
14,2,12359.662791
14,2,1896.181818
14,2,1982.676630
14,2,3474.423077
14,2,2139.741477
14,2,1925.488095
14,2,2652.008929
14,2,1500.936170
14,2,2062.971429
14,2,2218.114754
14,2,2619.662577
14,2,3618.850000
14,2,3655.006410
14,2,1654.544271
14,2,3323.024194
14,2,1795.377717
14,2,1591.540441
14,2,6090.305556
14,2,5155.747283
14,2,2991.185976
14,2,9611.085000
14,2,3919.883929
14,2,1515.319149
14,2,18462.294118
14,2,1551.172872
14,2,2660.921429
14,2,2025.086957
14,2,5573.675676
14,2,11480.018868
14,2,55953.111702
14,2,9139.905660
14,2,1949.333333
14,2,3136.176471
14,2,1570.281250
14,2,3462.358491
14,2,2495.292553
14,2,5260.918605
14,2,3837.033333
14,2,3630.710227
14,2,15176.466837
14,2,3016.770833
14,2,1777.872222
14,2,1571.116667
14,2,1551.172500
14,2,2598.829545
14,2,1584.169872
14,2,4969.350000
14,2,2660.670455
14,2,2274.891304
14,2,4513.095238
14,2,3206.947917
14,2,1771.746875
14,2,1682.000000
14,2,11226.588517
14,2,6137.625000
14,2,4872.966667
14,2,2279.783537
14,2,3096.084239
14,2,7256.279412
14,2,2069.388889
14,2,6116.434783
14,2,2134.400000

14,2,1785.114286
14,2,3076.215686
14,2,6829.531977
14,2,2922.625000
14,2,4855.528302
14,2,3120.742647
14,2,1851.458333
14,2,8170.368750
14,2,2843.029851
14,2,5605.120763
14,2,4048.000000
14,2,6051.089506
14,2,36604.448052
14,2,5064.159722
14,2,6668.397143
14,2,13744.607143
14,2,2359.780488
14,2,2187.440476
14,2,2809.951220
14,2,10049.500000
14,2,4384.828125
14,2,5173.744318
14,2,1564.524390
14,2,5663.937500
14,2,4523.437500
14,2,2408.750000
14,2,3474.420139
14,2,5173.391667
14,2,5585.780000
14,2,2954.122159
14,2,2245.265152
14,2,5045.674419
14,2,1575.961806
14,2,3903.812500
14,2,2082.997449
14,2,22082.957447
14,2,1620.196429
14,2,2944.897436
14,2,1903.280405
14,2,2057.585000
14,2,10564.376623
14,2,2811.242105
14,2,1665.000000
14,2,1639.142857
14,2,2398.166667
14,2,2608.974359
14,2,6307.992857
14,2,2847.948529
14,2,2177.000000
14,2,4406.920000
14,2,7523.461538
14,2,2665.197368
14,2,2025.537037
14,2,2321.224490
14,2,1668.440217
14,2,3220.742021
14,2,5897.695312
14,2,1537.570455
14,2,1671.044271
14,2,1786.609694
14,2,2587.257075
14,2,3032.790323
14,2,10755.003049
14,2,6109.916667
14,2,2780.617021
14,2,1872.350000

14,2,9444.258333
14,2,1503.093023
14,2,2181.067935
14,2,4509.374359
14,2,5046.814570
14,2,7321.085714
14,2,3777.902439
14,2,1602.181818
14,2,6102.234694
14,2,3574.450000
14,2,2587.258152
14,2,3693.230263
14,2,1525.255814
14,2,1886.354167
14,2,5459.936111
14,2,4102.400000
14,2,3773.500000
14,2,2148.500000
14,2,1510.369565
14,2,6322.896739
14,2,2798.458333
14,2,3208.809783
14,2,8858.313830
14,2,9831.424107
14,2,6180.935268
14,2,6914.511628
14,2,4575.207447
14,2,1546.467949
14,2,3429.178125
14,2,5513.663043
14,2,9867.183824
14,2,5672.812500
14,2,6062.553571
14,2,4607.500000
14,2,2605.220930
14,2,2941.400000
14,2,5235.326531
14,2,2493.409483
14,2,2323.012500
14,2,49959.088889
14,2,3110.347826
14,2,7932.813830
14,2,3937.189904
14,2,4685.368056
14,2,2003.357143
14,2,2231.186170
14,2,4843.633721
14,2,5817.028846
14,2,1634.610465
14,2,7290.706731
14,2,1735.538462
14,2,5266.604167
14,2,2927.400000
14,2,1901.151786
14,2,4915.461538
14,2,4612.045918
14,2,3782.242424
14,2,4726.242647
14,2,5032.000000
14,2,4600.150000
14,2,8723.982456
14,2,2085.641026
14,2,2104.468750
14,2,3329.277500
14,2,2073.530612
14,2,2870.782609

14,2,2503.372093
14,2,2328.469512
14,2,9923.659375
14,2,2368.806818
14,2,9057.111111
14,2,2877.851064
14,2,3144.714286
14,2,2107.083333
14,2,7382.668605
14,2,2405.125000
14,2,5828.973214
14,2,1916.943750
14,2,1509.964286
14,2,16728.463889
14,2,1737.524457
14,2,3649.288462
14,2,2912.710227
14,2,3520.187500
14,2,1650.548780
14,2,3523.014423
14,2,2373.500000
14,2,2035.201087
14,2,2395.228571
14,2,2002.200000
14,2,4058.766447
14,2,2015.061111
14,2,1935.242500
14,2,2275.523649
14,2,2018.227273
14,2,7726.647500
14,2,6054.539894
14,2,2789.366071
14,2,1840.700000
14,2,2589.598837
14,2,18629.429688
14,2,6097.143443
14,2,2775.908537
14,2,6801.090909
14,2,1699.923077
14,2,1967.481771
14,2,2947.421053
14,2,1677.983696
14,2,4026.887097
14,2,3365.291667
14,2,1990.396104
14,2,2176.241206
14,2,1755.390244
14,2,1955.415094
14,2,8669.773585
14,2,1658.227273
14,2,3861.906250
14,2,2432.000000
14,2,2445.417553
14,2,9873.500000
14,2,2220.750000
14,2,1663.517241
14,2,6647.400000
14,2,2907.940476
14,2,1765.485549
14,2,4718.614583
14,2,3398.917683
14,2,1520.067308
14,2,5553.808594
14,2,1994.702128
14,2,2075.887500
14,2,4946.911765

14,2,5601.395833
14,2,2147.076923
14,2,6714.986842
14,2,4969.010638
14,2,5539.688073
14,2,2152.528846
14,2,1608.930233
14,2,4818.434896
14,2,1807.060268
14,2,8935.377551
14,2,1555.945946
14,2,1996.060096
14,2,6699.075472
14,2,1895.310526
14,2,3049.573298
14,2,4381.736842
14,2,3492.594595
14,2,4116.777778
14,2,23171.500000
14,2,3920.125000
14,2,3708.174020
14,2,1899.457143
14,2,2957.505952
14,2,1980.676471
14,2,1901.114130
14,2,5779.412500
14,2,2611.021277
14,2,16394.826389
14,2,9505.651163
14,2,4769.228814
14,2,1635.264205
14,2,6846.377193
14,2,6215.567839
14,2,1932.672297
14,2,4630.927885
14,2,7777.162791
14,2,4308.452830
14,2,1581.625000
14,2,2807.690000
14,2,10459.307229
14,2,3861.835366
14,2,2142.500000
14,2,13269.420455
14,2,3480.114583
14,2,5826.039604
14,2,3067.138554
14,2,2804.036765
14,2,2105.210227
14,2,7747.583333
14,2,4328.540698
14,2,7608.469828
14,2,1565.166667
14,2,3059.803571
14,2,6123.460000
14,2,3557.250000
14,2,5649.182692
14,2,4178.300000
14,2,1978.119565
14,2,1897.223684
14,2,6279.077586
14,2,15266.104839
14,2,2836.957447
14,2,8061.062500
14,2,3333.703125
14,2,2715.912791
14,2,4628.966981

14,2,2826.555556
14,2,2802.157609
14,2,3723.284091
14,2,2920.625000
14,2,3564.674419
14,2,7976.995098
14,2,2382.086111
14,2,9495.161765
14,2,1755.310526
14,2,4481.189189
14,2,1817.033163
14,2,4481.295000
14,2,4050.636029
14,2,2710.201531
14,2,5028.467105
14,2,7596.954545
14,2,1886.720395
14,2,4812.832237
14,2,3575.122340
14,2,3841.680556
14,2,3344.153409
14,2,1671.357143
14,2,2126.364583
14,2,1830.823980
14,2,2320.412791
14,2,4119.321429
14,2,9050.541667
14,2,2350.821429
14,2,6128.868243
14,2,2576.652174
14,2,1768.944079
14,2,3288.503472
14,2,3555.480769
14,2,4924.946429
14,2,5518.959302
14,2,2268.170732
14,2,2287.103571
14,2,4010.023026
14,2,2616.677419
14,2,2945.761364
14,2,3031.340000
14,2,3252.341463
14,2,1614.005848
14,2,2756.656977
14,2,5974.183673
14,2,2523.805000
14,2,2679.217391
14,2,6451.910714
14,2,2559.487500
14,2,11317.494186
14,2,2247.577381
14,2,3276.452128
14,2,20289.139535
14,2,2683.189189
14,2,3956.926829
14,2,2023.106132
14,2,5581.481707
14,2,2707.692568
14,2,1550.850610
14,2,1643.609375
14,2,1525.372093
14,2,4106.796053
14,2,4779.851351
14,2,2434.418605
14,2,1830.173469
14,2,3793.625000

14,2,8179.000000
14,2,1871.987500
14,2,3275.341463
14,2,2583.281977
14,2,1507.536585
14,2,1660.661111
14,2,4866.395349
14,2,3970.185567
14,2,2817.984375
14,2,4136.250000
14,2,2828.130682
14,2,6983.781250
14,2,2658.416667
14,2,2854.369444
14,2,1662.872024
14,2,3237.019022
14,2,1884.572864
14,2,2258.630000
14,2,5623.182692
14,2,7430.338983
14,2,2088.179245
14,2,3203.000000
14,2,1850.928571
14,2,1716.223837
14,2,3096.218750
14,2,1661.109756
14,2,1799.240000
14,2,1890.750000
14,2,2594.750000
14,2,82264.923077
14,2,1511.733696
14,2,4761.349138
14,2,6371.145000
14,2,2257.060185
14,2,1563.032258
14,2,4712.792818
14,2,6581.015625
14,2,1794.391304
14,2,2687.021277
14,2,5765.140000
14,2,2711.137500
14,2,2654.738095
14,2,2536.510204
14,2,6109.226190
14,2,2973.979592
14,2,2189.038462
14,2,3579.700581
14,2,4343.765306
14,2,2560.205882
14,2,3196.375000
14,2,1742.433140
14,2,2682.650000
14,2,2038.655488
14,2,8891.860577
14,2,2998.461538
14,2,5504.600000
14,2,3646.220588
14,2,2824.380952
14,2,4194.539062
14,2,1989.400000
14,2,1661.482143
14,2,1680.170213
14,2,4582.922414
14,2,6151.427885
14,2,3673.375000
14,2,1663.203125

14,2,8146.714286
14,2,1645.447368
14,2,2639.617347
14,2,3043.550000
14,2,1946.464286
14,2,2240.364130
14,2,4390.291667
14,2,5881.455882
14,2,2026.638158
14,2,2176.795918
14,2,3069.427778
14,2,1603.340426
14,2,1710.851064
14,2,10695.287234
14,2,4086.241071
14,2,2920.458333
14,2,6277.674419
14,2,11771.895000
14,2,7923.473684
14,2,19060.104508
14,2,2505.916667
14,2,1725.813830
14,2,3768.071429
14,2,2526.329787
14,2,1626.775000
14,2,6265.562500
14,2,7789.735465
14,2,10293.551020
14,2,3177.097765
14,2,2754.519886
14,2,1774.682500
14,2,4432.753049
14,2,4801.755208
14,2,2665.351351
14,2,2388.282895
14,2,1852.777778
14,2,8763.711111
14,2,3388.989362
14,2,7505.189024
14,2,2534.764706
14,2,2978.980556
14,2,2530.887097
14,2,8307.312500
14,2,10651.995098
14,2,7120.760417
14,2,1821.328125
14,2,2108.738636
14,2,4932.312500
14,2,2053.085106
14,2,3827.290541
14,2,2044.982955
14,2,3610.655488
14,2,9505.406250
14,2,1834.442308
14,2,1735.613095
14,2,4155.811170
14,2,1754.561702
14,2,4216.605769
14,2,2565.931280
14,2,9782.534483
14,2,1843.000000
14,2,1656.702586
14,2,5392.777778
14,2,2351.468085
14,2,5043.542683
14,2,1735.089286

14,2,3526.734694
14,2,4720.182692
14,2,7743.370000
14,2,1737.710526
14,2,5011.150862
14,2,15256.951923
14,2,1539.391509
14,2,4144.968750
14,2,3398.473404
14,2,2466.625000
14,2,2026.187500
14,2,5565.014706
14,2,1806.734375
14,2,2079.235294
14,2,1836.369565
14,2,4677.500000
14,2,4679.980769
14,2,2142.470588
14,2,1552.037500
14,2,8367.436170
14,2,1781.867647
14,2,3016.441860
14,2,6714.928571
14,2,6946.000000
14,2,2565.295652
14,2,2015.695312
14,2,3196.591837
14,2,5109.553571
14,2,2654.420000
14,2,1659.806452
14,2,4507.529891
14,2,4343.764286
14,2,1994.538265
14,2,2040.511628
14,2,3733.588235
14,2,3718.256098
14,2,3002.778846
14,2,5530.655488
14,2,1571.602041
14,2,1597.216981
14,2,3901.947917
14,2,19570.986111
14,2,6157.734043
14,2,3572.291667
14,2,5539.717391
14,2,5145.486111
14,2,7100.812500
14,2,5589.258475
14,2,1737.565789
14,2,2031.312500
14,2,4997.288462
14,2,2339.426829
14,2,6358.732323
14,2,5506.938830
14,2,2832.029891
14,2,3805.450000
14,2,5922.486111
14,2,5347.786058
14,2,2930.099432
14,2,3544.673469
14,2,2082.250000
14,2,3327.666667
14,2,47813.576613
14,2,2305.375000
14,2,2409.670732
14,2,2139.122340

14,2,3675.483553
14,2,1597.401596
14,2,2516.839779
14,2,4471.465278
14,2,1512.727273
14,2,2056.352941
14,2,2399.375000
14,2,3506.265306
14,2,2013.380435
14,2,7462.949468
14,2,3195.431818
14,2,1819.318182
14,2,4676.147321
14,2,1716.863636
14,2,2010.429878
14,2,1641.180000
14,2,2999.515625
14,2,4102.210784
14,2,3743.335366
14,2,1812.500000
14,2,2333.717105
14,2,2625.182203
14,2,4496.857143
14,2,5473.677686
14,2,1809.265823
14,2,14350.884615
14,2,5189.078947
14,2,8036.000000
14,2,3650.579268
14,2,1545.434783
14,2,5088.651596
14,2,1825.696809
14,2,1739.410326
14,2,5168.125000
14,2,14364.776316
14,2,7827.447368
14,2,7066.440678
14,2,1818.913462
14,2,14213.063953
14,2,4063.628906
14,2,3937.423645
14,2,2507.698675
14,2,6556.577957
14,2,2249.921429
14,2,2673.250000
14,2,2165.418605
14,2,3404.600000
14,2,1569.672794
14,2,2015.173077
14,2,2119.843750
14,2,2670.500000
14,2,1985.950980
14,2,2731.739583
14,2,1704.625000
14,2,2507.953488
14,2,1670.964674
14,2,2029.763889
14,2,1541.988636
14,2,33262.658537
14,2,3767.941176
14,2,6091.125000
14,2,5281.656250
14,2,2138.057692
14,2,1912.521739
14,2,2368.921053
14,2,2176.716216

14,2,2699.529070
14,2,7337.348214
14,2,3597.000000
14,2,5074.028846
14,2,2126.317073
14,2,3357.328488
14,2,2602.236842
14,2,2551.980263
14,2,2288.715686
14,2,8522.368421
14,2,2561.960000
14,2,1991.266667
14,2,3755.812500
14,2,2743.227273
14,2,6972.903226
14,2,4777.625000
14,2,1801.638889
14,2,10392.132653
14,2,1929.389535
14,2,1785.687500
14,2,8310.285714
14,2,3823.364130
14,2,2012.451220
14,2,9384.041667
14,2,4738.418750
14,2,4971.992188
14,2,4490.383721
14,2,2607.648649
14,2,1523.810897
14,2,2291.138298
14,2,2420.151163
14,2,1816.236842
14,2,2160.789474
14,2,2180.812500
14,2,9436.071429
14,2,3898.375000
14,2,3254.875000
14,2,3667.091518
14,2,1678.250000
14,2,3394.000000
14,2,3803.673077
14,2,3043.723837
14,2,8221.258242
14,2,2887.624224
14,2,1819.212838
14,2,7618.721154
14,2,3451.119681
14,2,3130.875000
14,2,1836.416667
14,2,7624.000000
14,2,4237.398649
14,2,8410.912500
14,2,4623.371795
14,2,2345.081081
14,2,2107.025000
14,2,1637.067568
14,2,3529.494681
14,2,1602.875000
14,2,4052.100000
14,2,1921.179487
14,2,5374.663462
14,2,1668.421053
14,2,11883.853846
14,2,38495.071429
14,2,1758.666667
14,2,8827.718750

14,2,2039.908088
14,2,4089.000000
14,2,1648.500000
14,2,4445.051630
14,2,1757.437500
14,2,3910.812500
14,2,3839.800000
14,2,2460.894737
14,2,2793.116071
14,2,1608.076087
14,2,7600.511628
14,2,1607.531250
14,2,2128.103774
14,2,3588.146341
14,2,6798.260870
14,2,5057.054054
14,2,3466.514706
14,2,1992.700000
14,2,4603.320312
14,2,3193.567130
14,2,1573.121711
14,2,2212.394886
14,2,3485.250000
14,2,3612.133562
14,2,5726.294643
14,2,2703.108974
14,2,2519.683036
14,2,1630.548780
14,2,1586.250000
14,2,2463.567500
14,2,3046.569444
14,2,1650.352941
14,2,1816.789474
14,2,2847.761628
14,2,3078.994764
14,2,6163.989362
14,2,9115.910714
14,2,1836.044355
14,2,1579.848684
14,2,5340.937500
14,2,2074.009804
14,2,2470.901596
14,2,61008.292683
14,2,2942.895000
14,2,3476.792683
14,2,3269.125000
14,2,4260.772727
14,2,2041.791667
14,2,2313.375000
14,2,2738.621622
14,2,2212.807500
14,2,1634.797619
14,2,2550.578488
14,2,3251.913136
14,2,4034.272500
14,2,2265.384615
14,2,3478.812500
14,2,2326.425000
14,2,3045.595745
14,2,2000.217391
14,2,3281.872449
14,2,1737.122159
14,2,3816.909375
14,2,3658.383721
14,2,2028.037736
14,2,1851.700000

14,2,1889.148148
14,2,1785.821429
14,2,3115.428571
14,2,4166.175676
14,2,6781.328125
14,2,2866.208333
14,2,5099.000000
14,2,7942.921053
14,2,1653.961806
14,2,2766.351064
14,2,2643.263298
14,2,3817.554348
14,2,4600.820000
14,2,3356.068182
14,2,2791.772727
14,2,2648.561224
14,2,3070.202381
14,2,4359.985714
14,2,3583.067935
14,2,3206.739583
14,2,1547.144608
14,2,3036.790698
14,2,1799.866071
14,2,4522.914773
14,2,38296.096591
14,2,5393.076923
14,2,3716.561224
14,2,1982.322034
14,2,3876.392405
14,2,1912.170455
14,2,5415.154412
14,2,3492.196429
14,2,9366.930233
14,2,1944.169271
14,2,6620.026316
14,2,11475.323171
14,2,1722.243750
14,2,4021.167553
14,2,4765.900000
14,2,2515.875000
14,2,8206.692308
14,2,4086.000000
14,2,1977.889286
14,2,5736.071121
14,2,1589.774554
14,2,3835.170455
14,2,2458.209677
14,2,5377.772059
14,2,4193.177326
14,2,1832.702381
14,2,5924.744186
14,2,3605.231481
14,2,1712.018868
14,2,2465.500000
14,2,2690.789474
14,2,2356.005208
14,2,1734.125000
14,2,2416.359375
14,2,10529.031250
14,2,8329.775974
14,2,1536.125000
14,2,1640.675676
14,2,5015.720930
14,2,1561.169811
14,2,1539.720588
14,2,1982.835366

14,2,2127.195652
14,2,6194.792453
14,2,1552.631098
14,2,2299.334184
14,2,4493.909722
14,2,9922.884615
14,2,4237.516304
14,2,10560.283654
14,2,2150.785714
14,2,2350.538462
14,2,2948.947368
14,2,3943.666667
14,2,1553.150000
14,2,3313.470930
14,2,4371.500000
14,2,8341.505556
14,2,4507.949074
14,2,4208.459302
14,2,3752.843085
14,2,2534.845000
14,2,2726.810345
14,2,2835.453125
14,2,4183.125000
14,2,1663.616279
14,2,9443.438776
14,2,12386.477679
14,2,1772.702500
14,2,1951.966667
14,2,5474.801020
14,2,2763.536585
14,2,1836.511111
14,2,7318.000000
14,2,1540.158163
14,2,3463.160326
14,2,10784.740586
14,2,3589.490066
14,2,3072.041667
14,2,2511.368750
14,2,1817.218750
14,2,2461.132979
14,2,3253.850000
14,2,3508.061798
14,2,2458.575342
14,2,3319.971910
14,2,2026.946429
14,2,9120.337500
14,2,1949.526316
14,2,2184.625000
14,2,4872.976562
14,2,1703.536458
14,2,3922.307292
14,2,2359.940909
14,2,3891.285714
14,2,2763.783654
14,2,6113.668269
14,2,2544.755814
14,2,2885.610000
14,2,4144.000000
14,2,3011.726744
14,2,2506.753049
14,2,7481.619444
14,2,2550.210526
14,2,8733.288462
14,2,1801.391667
14,2,2648.910714
14,2,4177.611111

14,2,4402.048193
14,2,4905.051724
14,2,10498.282051
14,2,1582.784722
14,2,1801.625000
14,2,2661.774510
14,2,4479.062500
14,2,1793.738095
14,2,1675.045455
14,2,3225.951087
14,2,4198.562500
14,2,1511.411765
14,2,2687.914352
14,2,3342.528689
14,2,2208.566667
14,2,4407.960526
14,2,15289.300000
14,2,1967.900524
14,2,1686.203390
14,2,6340.311224
14,2,6440.165909
14,2,2052.896552
14,2,2936.486842
14,2,6706.608696
14,2,5694.342949
14,2,6230.987179
14,2,6804.160714
14,2,1672.488372
14,2,3367.650000
14,2,6497.585366
14,2,2400.634615
14,2,3462.023256
14,2,3174.064516
14,2,6477.353261
14,2,2130.150000
14,2,2830.817857
14,2,2847.007979
14,2,4329.295918
14,2,2428.915541
14,2,7345.157895
14,2,2222.418919
14,2,1759.773305
14,2,4007.513966
14,2,3079.976744
14,2,2211.550000
14,2,6830.543478
14,2,2233.670000
14,2,10730.057692
14,2,18983.477941
14,2,2075.247159
14,2,4142.711538
14,2,7370.488372
14,2,4192.263158
14,2,1857.545673
14,2,5570.322222
14,2,1919.644737
14,2,3005.981818
14,2,1854.812500
14,2,3615.428571
14,2,3705.804878
14,2,1869.048780
14,2,1556.455882
14,2,1837.947674
14,2,2318.250000
14,2,5006.940217
14,2,1514.151316

14,2,8294.142857
14,2,4624.713542
14,2,2092.791667
14,2,1783.277778
14,2,1637.793478
14,2,2548.153061
14,2,3808.190476
14,2,24147.096154
14,2,2808.092262
14,2,3788.341216
14,2,2482.538462
14,2,1849.444444
14,2,2402.293269
14,2,2550.484043
14,2,2285.750000
14,2,2136.826705
14,2,2606.939189
14,2,4511.790179
14,2,8237.276596
14,2,3703.203125
14,2,3534.008152
14,2,3441.487805
14,2,7771.773810
14,2,2783.509615
14,2,2355.319149
14,2,8645.687500
14,2,2679.772727
14,2,1680.810811
14,2,2466.513298
14,2,4621.744444
14,2,1968.816327
14,2,2471.798611
14,2,6924.524664
14,2,15770.341463
14,2,3726.382212
14,2,10825.843137
14,2,2108.935185
14,2,2068.773256
14,2,2954.834239
14,2,8081.875000
14,2,1684.459239
14,2,12361.881944
14,2,3485.745455
14,2,2904.875000
14,2,2350.191327
14,2,3526.050000
14,2,6367.047500
14,2,3553.013514
14,2,1665.834302
14,2,1579.731771
14,2,2311.539062
14,2,3511.773026
14,2,6022.677632
14,2,3731.303125
14,2,6949.990000
14,2,1802.486111
14,2,2264.033784
14,2,2041.526316
14,2,3955.164634
14,2,2534.989362
14,2,2441.882353
14,2,3122.905405
14,2,1907.377841
14,2,11645.615909
14,2,3010.767442
14,2,4136.250000

14,2,2202.024390
14,2,2680.016447
14,2,11777.825000
14,2,2696.095109
14,2,2475.990385
14,2,2165.946429
14,2,6925.599490
14,2,2088.421875
14,2,3616.787234
14,2,3699.827703
14,2,4502.545732
14,2,2874.593750
14,2,5264.645349
14,2,2056.948864
14,2,4118.945652
14,2,2602.703947
14,2,3439.698718
14,2,2323.015625
14,2,1890.774390
14,2,2644.962500
14,2,2663.238095
14,2,3179.274038
14,2,1720.993902
14,2,9172.490909
14,2,3011.259146
14,2,5087.946429
14,2,5690.405660
14,2,2681.540984
14,2,4677.903846
14,2,3997.638298
14,2,2911.030556
14,2,1519.809211
14,2,1661.369565
14,2,2536.692308
14,2,5510.059375
14,2,1733.345982
14,2,4472.522059
14,2,5156.637195
14,2,3302.454545
14,2,1591.828947
14,2,1560.156977
14,2,3019.918367
14,2,5852.743056
14,2,6284.830357
14,2,11106.244898
14,2,11602.292683
14,2,5100.261364
14,2,4640.342857
14,2,2784.277778
14,2,22045.595588
14,2,5016.947368
14,2,2523.039634
14,2,4400.875000
14,2,1551.372222
14,2,3543.182692
14,2,2442.019231
14,2,2099.182292
14,2,2682.333333
14,2,1865.159091
14,2,1815.289474
14,2,1570.937500
14,2,4599.912500
14,2,2016.689904
14,2,3387.958333
14,2,2549.224359
14,2,1731.425676

14,2,1996.260000
14,2,3789.557692
14,2,1533.994186
14,2,2334.063953
14,2,2457.254545
14,2,6278.596939
14,2,10799.909091
14,2,1663.837500
14,2,6597.981707
14,2,22708.243421
14,2,3228.269231
14,2,7254.426829
14,2,4818.171053
14,2,45599.848684
14,2,3404.233333
14,2,1594.420000
14,2,2417.130102
14,2,1613.275641
14,2,3917.263780
14,2,1877.037736
14,2,2356.039474
14,2,1967.759146
14,2,4144.688679
14,2,7053.102564
14,2,8953.141667
14,2,15510.155844
14,2,4912.072674
14,2,3605.833333
14,2,1968.396739
14,2,2338.727500
14,2,2412.710526
14,2,4088.397959
14,2,2515.377841
14,2,3075.928571
14,2,6554.750000
14,2,2676.523810
14,2,4427.042614
14,2,4244.735795
14,2,2507.529412
14,2,2523.554054
14,2,6685.400000
14,2,8305.438725
14,2,11031.375000
14,2,4126.844828
14,2,2577.221591
14,2,1626.685484
14,2,6862.520833
14,2,4540.680233
14,2,5427.854651
14,2,1821.061224
14,2,3245.273810
14,2,10467.965426
14,2,2163.628378
14,2,3025.125000
14,2,2022.729730
14,2,10909.539877
14,2,7301.750000
14,2,1606.788043
14,2,9327.117647
14,2,1556.053030
14,2,3810.276042
14,2,1818.193750
14,2,3675.000000
14,2,5112.494792
14,2,100295.550000
14,2,1637.163265

14,2,1905.000000
14,2,1752.338068
14,2,2428.583333
14,2,1686.142857
14,2,1664.222973
14,2,3569.281250
14,2,1913.263587
14,2,2896.189024
14,2,1587.285714
14,2,4001.367500
14,2,6949.013158
14,2,4036.168478
14,2,2517.537879
14,2,4724.948980
14,2,2689.700000
14,2,1825.692857
14,2,8025.039062
14,2,6133.819767
14,2,4814.307692
14,2,1797.522727
14,2,1543.714660
14,2,1666.394022
14,2,6065.602941
14,2,2820.201087
14,2,3810.250000
14,2,1869.036458
14,2,7562.765909
14,2,2863.118421
14,2,2434.250000
14,2,2034.571429
14,2,3619.212766
14,2,4674.601190
14,2,1536.086538
14,2,1766.787791
14,2,10809.894737
14,2,2507.333333
14,2,4700.560976
14,2,1891.864865
14,2,5792.605114
14,2,1771.500000
14,2,2687.643617
14,2,4869.965517
14,2,4298.773936
14,2,3015.040816
14,2,9567.642500
14,2,3037.800000
14,2,1836.085000
14,2,1524.289634
14,2,4556.375000
14,2,9336.176471
14,2,3207.717105
14,2,3263.000000
14,2,1815.916667
14,2,8929.041667
14,2,3039.145078
14,2,1749.375000
14,2,18696.121951
14,2,1902.922619
14,2,4900.545455
14,2,1916.000000
14,2,1865.339286
14,2,1567.375000
14,2,1600.654762
14,2,5377.791667
14,2,1666.475000
14,2,11320.575000

14,2,2308.630208
14,2,2516.900000
14,2,2575.844444
14,2,3403.737500
14,2,5325.273438
14,2,29808.950000
14,2,1747.546875
14,2,2324.666667
14,2,3948.937500
14,2,2260.086957
14,2,1777.276163
14,2,2066.854167
14,2,3317.057692
14,2,1653.617647
14,2,7933.758333
14,2,3089.972826
14,2,3707.270270
14,2,1944.675676
14,2,2850.673913
14,2,1790.902500
14,2,2154.356383
14,2,1955.365625
14,2,3071.461538
14,2,1604.521875
14,2,7124.235119
14,2,2238.551136
14,2,9342.727273
14,2,30584.685714
14,2,2087.089744
14,2,9076.153409
14,2,3730.346939
14,2,2787.712766
14,2,2168.625000
14,2,2900.666667
14,2,4082.687500
14,2,3262.058824
14,2,3230.622449
14,2,2638.294444
14,2,3617.175000
14,2,6047.707447
14,2,17022.730769
14,2,3696.000000
14,2,1876.577778
14,2,2104.000000
14,2,3228.340782
14,2,2370.401163
14,2,5364.520349
14,2,3203.225000
14,2,1736.204082
14,2,2585.563776
14,2,1650.000000
14,2,1572.375000
14,2,1851.647059
14,2,4414.895349
14,2,4483.947500
14,2,1709.844595
14,2,7219.138889
14,2,1856.872093
14,2,1677.750000
14,2,5431.638498
14,2,9989.127389
14,2,3094.900000
14,2,6107.214286
14,2,2740.625000
14,2,3844.785000
14,2,2617.724490

14,2,2082.489362
14,2,2176.659884
14,2,3088.527174
14,2,4740.905405
14,2,1507.307692
14,2,3729.430000
14,2,2767.750000
14,2,3819.937500
14,2,2049.742021
14,2,2486.818182
14,2,3674.625000
14,2,2449.882353
14,2,2444.909091
14,2,10143.017045
14,2,1901.554839
14,2,2019.065476
14,2,2181.750000
14,2,1872.560000
14,2,8171.291045
14,2,4371.710843
14,2,2104.651832
14,2,2963.500000
14,2,4614.995098
14,2,1762.764535
14,2,3818.515625
14,2,3506.091463
14,2,1731.674107
14,2,3798.356771
14,2,2511.000000
14,2,1715.107143
14,2,3405.130319
14,2,2523.134804
14,2,2541.324324
14,2,1925.413043
14,2,4418.409884
14,2,1997.526316
14,2,1811.513158
14,2,11068.366477
14,2,2336.938953
14,2,2734.318182
14,2,3232.962121
14,2,3744.750000
14,2,4734.914352
14,2,1688.264423
14,2,10443.311321
14,2,1769.183140
14,2,12412.919643
14,2,6275.926471
14,2,2872.296875
14,2,1871.238095
14,2,3032.625000
14,2,6548.388298
14,2,2439.544118
14,2,2595.949324
14,2,1893.052632
14,2,1544.195652
14,2,7379.463235
14,2,3907.166667
14,2,3456.386574
14,2,37281.287162
14,2,1642.335000
14,2,4132.744444
14,2,7284.352941
14,2,4914.718750
14,2,4135.000000
14,2,3548.773585

14,2,1629.775000
14,2,3361.250000
14,2,3298.929348
14,2,1750.937500
14,2,2406.106250
14,2,5726.836735
14,2,2417.515625
14,2,2565.809659
14,2,3920.037383
14,2,3680.658537
14,2,2026.097561
14,2,2016.908537
14,2,5711.160714
14,2,2758.847826
14,2,4764.000000
14,2,3149.116279
14,2,2134.968750
14,2,1774.230769
14,2,2244.366279
14,2,2910.774390
14,2,6675.921875
14,2,2266.292683
14,2,2726.904070
14,2,35490.979167
14,2,1996.673295
14,2,1771.514706
14,2,5187.829787
14,2,3113.522059
14,2,3231.136628
14,2,2285.558824
14,2,1878.917453
14,2,7282.530612
14,2,13392.797500
14,2,14581.945813
14,2,2176.002976
14,2,2500.699519
14,2,4147.405660
14,2,12221.102273
14,2,8693.107143
14,2,2449.914634
14,2,2284.283784
14,2,1735.533333
14,2,1741.246795
14,2,4785.926020
14,2,2652.854167
14,2,10251.434426
14,2,5093.340625
14,2,2826.024390
14,2,3118.455357
14,2,3680.087766
14,2,4119.639205
14,2,2464.361702
14,2,5569.714286
14,2,2846.614130
14,2,2615.847826
14,2,1550.554878
14,2,4830.632979
14,2,9057.750000
14,2,7797.154070
14,2,3724.216667
14,2,1564.278646
14,2,1527.913265
14,2,8642.886792
14,2,1803.888889
14,2,2763.128205
14,2,3085.375000

14,2,5876.303571
14,2,1563.415761
14,2,3067.380435
14,2,2098.797872
14,2,1715.634146
14,2,9720.220395
14,2,4571.370968
14,2,2487.086957
14,2,4177.588542
14,2,2719.359375
14,2,2175.569079
14,2,2527.435714
14,2,2105.058824
14,2,2704.636029
14,2,5427.400943
14,2,2641.125000
14,2,2024.245283
14,2,1902.679245
14,2,4871.802632
14,2,3074.358333
14,2,1523.485294
14,2,1751.041667
14,2,1696.437500
14,2,1723.095745
14,2,3812.054054
14,2,2501.296053
14,2,5528.841981
14,2,2457.348214
14,2,2317.600000
14,2,5648.962054
14,2,3120.965517
14,2,3016.393617
14,2,6335.927326
14,2,3483.725000
14,2,5254.983491
14,2,4863.115183
14,2,2931.777778
14,2,3535.646277
14,2,23051.867347
14,2,8240.944444
14,2,13211.581395
14,2,2186.378472
14,2,2340.775000
14,2,2218.322115
14,2,1549.130952
14,2,1652.771341
14,2,2150.769231
14,2,2094.861111
14,2,1779.772959
14,2,3827.631250
14,2,2871.600000
14,2,1499.891892
14,2,3706.518072
14,2,2047.275510
14,2,8030.902913
14,2,2224.384375
14,2,7826.764535
14,2,3180.610465
14,2,4111.656250
14,2,12628.866667
14,2,2098.133621
14,2,5156.351562
14,2,5045.734043
14,2,2191.449468
14,2,2119.283784
14,2,12725.302419

14,2,2187.375000
14,2,28334.542614
14,2,6833.000000
14,2,11037.276596
14,2,3793.102679
14,2,2363.952778
14,2,7152.578125
14,2,13395.677273
14,2,2187.787500
14,2,1960.250000
14,2,1629.581699
14,2,2743.656250
14,2,4036.232955
14,2,4695.912500
14,2,1681.576531
14,2,3003.432927
14,2,4463.363426
14,2,5364.826087
14,2,2140.725000
14,2,2480.815972
14,2,4204.508523
14,2,3232.295918
14,2,1997.587209
14,2,4466.459459
14,2,1628.746622
14,2,1543.215116
14,2,2662.685976
14,2,5490.144366
14,2,6354.288462
14,2,2065.625000
14,2,2812.236842
14,2,2046.607143
14,2,2255.349490
14,2,4073.578571
14,2,1542.372449
14,2,2504.073529
14,2,6664.074074
14,2,2358.100000
14,2,1620.671429
14,2,6182.541667
14,2,2137.576220
14,2,1880.495000
14,2,4187.550000
14,2,2554.944767
14,2,11489.847458
14,2,1579.818182
14,2,1929.340000
14,2,2134.546875
14,2,3262.500000
14,2,1631.641026
14,2,2137.512195
14,2,8309.174419
14,2,1960.666667
14,2,2182.125000
14,2,3882.333333
14,2,2906.470588
14,2,3665.884615
14,2,12471.467105
14,2,10375.774457
14,2,1939.078125
14,2,6227.818627
14,2,2174.315789
14,2,12314.798828
14,2,5650.259259
14,2,4987.375000
14,2,4356.025641

14,2,1907.687500
14,2,2064.651786
14,2,2735.225000
14,2,3790.032051
14,2,1524.250000
14,2,1613.168478
14,2,1991.309524
14,2,2436.315217
14,2,3312.591837
14,2,1594.354167
14,2,1790.390244
14,2,1640.701389
14,2,5466.627273
14,2,2047.750000
14,2,4698.181122
14,2,3456.155242
14,2,2058.250000
14,2,2473.537791
14,2,8117.875000
14,2,15664.971311
14,2,4129.000000
14,2,3700.734043
14,2,9752.946078
14,2,3707.952830
14,2,3392.709677
14,2,2063.152500
14,2,29694.715686
14,2,1904.792857
14,2,3615.115909
14,2,3572.929348
14,2,3524.274457
14,2,3007.045455
14,2,3788.138158
14,2,20128.410714
14,2,1779.750000
14,2,2847.555556
14,2,1536.040625
14,2,2599.700000
14,2,1642.742347
14,2,3339.520833
14,2,2052.163690
14,2,2046.236842
14,2,7044.311224
14,2,4141.062500
14,2,1640.789062
14,2,18264.837264
14,2,6075.139706
14,2,1957.976744
14,2,3799.833333
14,2,4019.121622
14,2,2845.702128
14,2,2108.964744
14,2,9244.875000
14,2,1600.080645
14,2,1506.051136
14,2,2724.517442
14,2,1827.796512
14,2,6007.416667
14,2,3575.295775
14,2,4511.369792
14,2,3475.071429
14,2,1851.968750
14,2,1579.534375
14,2,1575.087209
14,2,1777.536585
14,2,2241.853261

14,2,3036.455729
14,2,7359.921053
14,2,1540.408537
14,2,1514.195946
14,2,3779.400641
14,2,2052.959559
14,2,11553.155556
14,2,2937.000000
14,2,2770.131250
14,2,2664.875000
14,2,3830.780488
14,2,1901.791667
14,2,3047.206522
14,2,6035.430288
14,2,1840.250000
14,2,2184.963068
14,2,2925.119048
14,2,4618.204082
14,2,1724.986111
14,2,1637.025641
14,2,16313.715909
14,2,2170.600000
14,2,5790.461538
14,2,3206.372093
14,2,1902.661932
14,2,7890.738095
14,2,1890.506098
14,2,8466.234043
14,2,2514.134146
14,2,10822.621951
14,2,3369.781553
14,2,1856.271845
14,2,1786.225694
14,2,9411.841463
14,2,9984.674569
14,2,10336.214015
14,2,5860.777778
14,2,3293.772727
14,2,3452.614286
14,2,10338.526316
14,2,1929.917553
14,2,1666.517500
14,2,2677.972973
14,2,5220.067073
14,2,28943.150568
14,2,2044.744444
14,2,2907.184524
14,2,2091.154412
14,2,10836.380000
14,2,5848.571429
14,2,3586.000000
14,2,6872.377358
14,2,29221.480392
14,2,2358.000000
14,2,3688.753769
14,2,8413.038462
14,2,11251.828488
14,2,2127.666667
14,2,8884.494898
14,2,2811.994681
14,2,4254.308511
14,2,1916.500000
14,2,4070.677778
14,2,2157.987500
14,2,3694.869565
14,2,1950.208333

14,2,35022.062500
14,2,2704.607143
14,2,3210.186047
14,2,1865.131250
14,2,4034.095455
14,2,2492.374332
14,2,3100.384615
14,2,4515.153846
14,2,8567.136905
14,2,3136.000000
14,2,2732.354545
14,2,3125.880000
14,2,2134.836538
14,2,2084.442308
14,2,5436.509615
14,2,11612.505952
14,2,9873.111111
14,2,12022.459135
14,2,1895.161111
14,2,4910.979592
14,2,1901.074468
14,2,7008.387755
14,2,4038.111111
14,2,2807.477273
14,2,1546.646739
14,2,2306.427083
14,2,2300.072674
14,2,10810.442308
14,2,1893.231707
14,2,2482.800926
14,2,2574.427083
14,2,2714.930851
14,2,12143.942308
14,2,4098.366972
14,2,7396.814516
14,2,4841.909639
14,2,1855.800000
14,2,2950.428571
14,2,2045.767442
14,2,3103.108491
14,2,3514.152174
14,2,6222.363372
14,2,1499.551630
14,2,1907.948529
14,2,2203.284574
14,2,5687.555556
14,2,1814.055000
14,2,7781.994565
14,2,4534.564394
14,2,3310.384868
14,2,5790.460938
14,2,5066.625000
14,2,3388.156250
14,2,2501.156250
14,2,2508.755102
14,2,6369.044393
14,2,8419.177083
14,2,3692.812500
14,2,4917.740385
14,2,10880.645833
14,2,3067.282609
14,2,6646.819079
14,2,2358.682692
14,2,2486.342262
14,2,2807.468750
14,2,1607.313953

14,2,3875.320000
14,2,2346.008721
14,2,4622.258929
14,2,5775.015306
14,2,1837.486842
14,2,1628.246711
14,2,1621.614130
14,2,2986.002193
14,2,1747.635638
14,2,3691.487179
14,2,7487.209239
14,2,2124.763158
14,2,5285.232143
14,2,2768.592391
14,2,3538.500000
14,2,1686.756098
14,2,1727.096154
14,2,1786.663043
14,2,1939.815789
14,2,2078.185000
14,2,1981.250000
14,2,1501.920732
14,2,1743.921875
14,2,2446.902778
14,2,1909.443182
14,2,2897.420455
14,2,7316.238971
14,2,3360.977778
14,2,37635.174603
14,2,1726.432065
14,2,10802.650000
14,2,22481.409091
14,2,1660.270349
14,2,4197.354167
14,2,2034.164062
14,2,4535.750000
14,2,2395.875000
14,2,3486.018817
14,2,2311.318182
14,2,3163.137195
14,2,1946.064189
14,2,4247.071429
14,2,5790.146341
14,2,3323.239035
14,2,1676.963415
14,2,2891.762195
14,2,2633.011905
14,2,2532.510204
14,2,2526.756098
14,2,3266.691176
14,2,1562.780405
14,2,1819.377500
14,2,8685.609272
14,2,2270.015625
14,2,1814.964674
14,2,4571.393293
14,2,3907.784314
14,2,2469.743421
14,2,1817.600000
14,2,2591.437500
14,2,23071.798077
14,2,1539.589286
14,2,6644.947917
14,2,2245.656250
14,2,5511.806122
14,2,2836.078125

14,2,1666.250000
14,2,9571.593750
14,2,2328.709375
14,2,2100.993243
14,2,1800.375000
14,2,3749.517442
14,2,2694.346154
14,2,2997.165000
14,2,3875.240854
14,2,3141.000000
14,2,2644.338542
14,2,1726.841667
14,2,2665.619681
14,2,3051.000000
14,2,1774.414894
14,2,1579.388889
14,2,3650.292683
14,2,2251.371324
14,2,2174.938776
14,2,2651.075000
14,2,3375.205128
14,2,16493.796610
14,2,1822.737500
14,2,2498.872340
14,2,2434.762500
14,2,5238.816327
14,2,1754.500000
14,2,3844.872340
14,2,3179.000000
14,2,5141.479730
14,2,3503.639535
14,2,4754.075658
14,2,6607.883333
14,2,4896.989362
14,2,1853.239130
14,2,2203.642857
14,2,1516.799419
14,2,1664.298077
14,2,6570.958333
14,2,8306.291667
14,2,2233.441489
14,2,3109.250000
14,2,1818.597222
14,2,1972.974138
14,2,4419.214286
14,2,5174.087766
14,2,1508.116379
14,2,1602.930556
14,2,3760.792857
14,2,2856.757485
14,2,2220.472362
14,2,3293.413242
14,2,1829.258621
14,2,2762.750000
14,2,2975.500000
14,2,3092.390244
14,2,1630.026316
14,2,2772.365854
14,2,1522.333333
14,2,3218.522727
14,2,2920.000000
14,2,2299.250000
14,2,1713.477273
14,2,2843.171123
14,2,1976.200000
14,2,6094.508982

14,2,2619.506579
14,2,3999.750000
14,2,5593.560976
14,2,23356.704545
14,2,4262.189024
14,2,3131.758333
14,2,5503.491935
14,2,2887.961111
14,2,1903.137255
14,2,2052.729592
14,2,7162.362500
14,2,3997.857143
14,2,4951.250000
14,2,9358.634615
14,2,3017.881098
14,2,10311.280702
14,2,5670.645833
14,2,3073.095109
14,2,4733.100000
14,2,2513.571023
14,2,3762.539286
14,2,1566.750000
14,2,5826.809896
14,2,9961.814516
14,2,1775.966346
14,2,4306.532500
14,2,1511.857143
14,2,5314.068862
14,2,4978.492188
14,2,10056.666667
14,2,3183.183333
14,2,2486.635870
14,2,5288.727273
14,2,8238.068966
14,2,3294.567935
14,2,3322.082524
14,2,2189.775000
14,2,5050.454545
14,2,7478.194444
14,2,3071.298913
14,2,3537.337500
14,2,6189.076923
14,2,2539.177778
14,2,2802.736842
14,2,3958.820652
14,2,35674.711538
14,2,2270.833333
14,2,2618.375000
14,2,3056.549479
14,2,1780.750000
14,2,3197.688776
14,2,13946.305556
14,2,9353.258929
14,2,3454.148936
14,2,3919.224490
14,2,2607.070755
14,2,2058.164634
14,2,1916.312500
14,2,1813.648256
14,2,4813.945122
14,2,2550.474093
14,2,19167.000000
14,2,7807.362245
14,2,2200.608696
14,2,2179.483516
14,2,3304.069712

14,2,1900.593023
14,2,1970.527027
14,2,3209.015244
14,2,6791.355556
14,2,2202.804878
14,2,6713.777174
14,2,18946.584302
14,2,2288.527027
14,2,1593.294737
14,2,1750.387097
14,2,1542.138393
14,2,5694.780000
14,2,2913.782609
14,2,4388.500000
14,2,1590.581081
14,2,5331.333333
14,2,6980.359375
14,2,3212.063725
14,2,2079.552885
14,2,1539.277778
14,2,7922.403846
14,2,2609.307692
14,2,1661.090625
14,2,7338.244681
14,2,3552.243902
14,2,8396.450000
14,2,4571.319149
14,2,4370.735849
14,2,4107.148241
14,2,1643.564103
14,2,1608.281915
14,2,7996.405172
14,2,1710.234043
14,2,4620.522167
14,2,6623.823171
14,2,8157.654412
14,2,5224.379310
14,2,1949.645161
14,2,1855.598837
14,2,2019.572115
14,2,4224.187500
14,2,1626.970745
14,2,2313.370000
14,2,1782.570513
14,2,4878.721591
14,2,2566.483333
14,2,2015.610294
14,2,2682.291667
14,2,5611.560000
14,2,1552.538462
14,2,2568.135417
14,2,1614.405000
14,2,1655.840686
14,2,5528.553191
14,2,1750.282143
14,2,2008.618421
14,2,6815.833333
14,2,2043.062500
14,2,1913.757979
14,2,6433.339286
14,2,1932.479167
14,2,1542.000000
14,2,2606.988636
14,2,19601.319149
14,2,7073.745763
14,2,3673.742857

14,2,3193.779070
14,2,2019.669355
14,2,4206.201923
14,2,3144.829268
14,2,3502.433333
14,2,3620.617021
14,2,5690.522222
14,2,6506.257653
14,2,1966.625000
14,2,2518.473404
14,2,3874.307292
14,2,8945.292763
14,2,7431.960366
14,2,2121.965909
14,2,1644.958333
14,2,2729.846939
14,2,2030.750000
14,2,4771.978571
14,2,1559.057927
14,2,1805.562500
14,2,2139.413462
14,2,4934.936170
14,2,8388.820000
14,2,4764.250000
14,2,5654.900000
14,2,2421.236842
14,2,2087.125000
14,2,2880.238095
14,2,2833.736702
14,2,1850.000000
14,2,4113.819444
14,2,4232.569444
14,2,18034.555556
14,2,59892.964286
14,2,3372.500000
14,2,7176.375000
14,2,2332.531250
14,2,2965.662791
14,2,1680.289474
14,2,1534.600000
14,2,2196.328704
14,2,16128.024038
14,2,2288.635870
14,2,1594.671875
14,2,1922.609375
14,2,5804.502404
14,2,6321.914474
14,2,1863.637500
14,2,1747.940625
14,2,1530.506410
14,2,6884.528646
14,2,1520.951220
14,2,1816.767442
14,2,2669.298077
15,1,2394.625000
15,1,58362.497159
15,1,6252.520833
15,1,2090.156250
15,1,1504.208333
15,1,1563.726744
15,1,53367.602273
15,1,4485.600000
15,1,1528.459559
15,1,10774.609756
15,1,1725.444853
15,1,25144.739865

15,1,3455.465116
15,1,4200.394886
15,1,2575.388889
15,1,48504.689024
15,1,1577.252976
15,1,42950.972973
15,1,2098.571429
15,1,2895.563830
15,1,3498.516667
15,1,1944.578125
15,1,1834.744186
15,1,2668.556250
15,1,15208.941176
15,1,2039.211310
15,1,1673.455882
15,1,51098.059211
15,1,7798.192308
15,1,90229.578125
15,1,2447.603659
15,1,53058.789474
15,1,1633.555147
15,1,13362.713816
15,1,1744.019444
15,1,1513.668605
15,1,4198.367857
15,1,2385.839286
15,1,10515.081818
15,1,4388.750000
15,1,23024.255435
15,1,175682.946809
15,1,13621.855769
15,1,12084.701087
15,1,2332.218023
15,1,4176.487805
15,1,2751.500000
15,1,126940.282051
15,1,15800.640625
15,1,40261.708333
15,1,1688.979592
15,1,92683.680233
15,1,1617.523026
15,1,144626.686047
15,1,47047.484375
15,1,31311.918605
15,1,29377.687500
15,1,2848.067708
15,1,3475.086957
15,1,2542.549419
15,1,3168.701220
15,1,81067.285714
15,1,2081.602273
15,1,4118.000000
15,1,1811.625000
15,1,128402.628049
15,1,79605.476190
15,1,2462.343750
15,1,8922.763158
15,1,1899.050595
15,1,5837.275000
15,1,1799.698864
15,1,2406.372340
15,1,89376.038690
15,1,1731.295732
15,1,50880.692857
15,1,3905.034722
15,1,2777.187500

15,1,27376.384615
15,1,8539.477564
15,1,89929.682927
15,1,49148.314024
15,1,1737.923077
15,1,1659.278846
15,1,1602.186047
15,1,91697.975000
15,1,2126.584375
15,1,1531.468750
15,1,1980.146341
15,1,1649.321429
15,1,2313.701087
15,1,29979.980769
15,1,38730.321429
15,1,1830.122222
15,1,1963.784314
15,1,11624.371711
15,1,38989.272727
15,1,25893.142857
15,1,3174.086957
15,1,66093.015152
15,1,1651.912234
15,1,1661.010870
15,1,4981.778409
15,1,2079.923077
15,1,107184.837209
15,1,3092.512500
15,1,9870.914894
15,1,1975.136364
15,1,1769.558824
15,1,90366.168605
15,1,2499.637500
15,1,4016.622449
15,1,1832.794643
15,1,36668.132353
15,1,2277.564024
15,1,9786.675000
15,1,1607.475543
15,1,14486.279762
15,1,4489.845930
15,1,1538.524457
15,1,3311.114130
15,1,14897.016667
15,1,81974.125000
15,1,3025.300000
15,1,1948.216912
15,1,1803.600000
15,1,1999.012195
15,1,122806.368902
15,1,61018.105769
15,1,1999.272727
15,1,2781.207237
15,1,2084.805556
15,1,2023.154070
15,1,149921.231383
15,1,4867.750000
15,1,1733.062500
15,1,3394.110795
15,1,105110.851064
15,1,34095.159091
15,1,7628.625000
15,1,1524.570000
15,1,51550.691176
15,1,4929.125000
15,1,2872.435345

15,1,9180.846154
15,1,2622.500000
15,1,1813.684211
15,1,87803.993750
15,1,3585.441176
15,1,2232.398438
15,1,3587.674419
15,1,25312.459459
15,1,3874.428571
15,1,1695.104730
15,1,13974.636364
15,1,2924.361111
15,1,3876.864583
15,1,3554.513889
15,1,23990.733333
15,1,4225.433140
15,1,4424.731707
15,1,1908.330882
15,1,2092.192308
15,1,7362.568627
15,1,4413.483974
15,1,4565.050000
15,1,3909.567308
15,1,2081.138298
15,1,30594.947917
15,1,1571.437500
15,1,2069.617647
15,1,4342.858108
15,1,1533.136628
15,1,2078.043919
15,1,7488.052083
15,1,92246.167763
15,1,2101.669118
15,1,1838.150000
15,1,1520.519231
15,1,3561.600000
15,1,22251.450000
15,1,43311.421875
15,1,6092.937500
15,1,1776.895833
15,1,9021.476744
15,1,15928.006410
15,1,2344.979167
15,1,63064.904762
15,1,1672.279412
15,1,3093.314103
15,1,6684.713710
15,1,7988.463415
15,1,7194.652174
15,1,11782.387931
15,1,3058.032258
15,1,10029.971154
15,1,110522.400000
15,1,13453.235795
15,1,63374.925000
15,1,1507.010870
15,1,17455.495000
15,1,36310.625000
15,1,4851.812500
15,1,3702.791667
15,1,63069.217857
15,1,135139.684375
15,1,1779.558140
15,1,61498.041667
15,1,1633.143293
15,1,4146.760638

15,1,3351.671875
15,1,129390.619318
15,1,1514.035714
15,1,2012.921875
15,1,13164.375000
15,1,1528.093750
15,1,1893.511111
15,1,1606.000000
15,1,1846.574324
15,1,3377.108974
15,1,135594.250000
15,1,3382.926829
15,1,3483.890625
15,1,1666.525000
15,1,8539.871324
15,1,194379.175532
15,1,94682.172794
15,1,3081.484694
15,1,7619.801282
15,1,1705.529412
15,1,2782.232558
15,1,2554.375000
15,1,6623.454545
15,1,6300.250000
15,1,49522.917857
15,1,1829.815909
15,1,1664.729167
15,1,72800.456250
15,1,98453.500000
15,1,1822.674020
15,1,1534.853261
15,1,6196.061224
15,1,41735.562500
15,1,3716.843137
15,1,2610.201613
15,1,78903.519737
15,1,103631.093750
15,1,8383.800000
15,1,1513.666667
15,1,1834.408333
15,1,195827.000000
15,1,2646.041667
15,1,15361.500000
15,1,1855.213068
15,1,57334.128472
15,1,2793.818750
15,1,4510.195513
15,1,1887.850000
15,1,7808.900000
15,1,2546.750000
15,1,3540.156863
15,1,1780.944444
15,1,2674.071875
15,1,2908.895161
15,1,5822.589286
15,1,14071.486111
15,1,2326.176630
15,1,92104.118750
15,1,2100.854651
15,1,1815.973214
15,1,2935.793103
15,1,6874.125000
15,1,103808.259434
15,1,8234.244186
15,1,3275.645833
15,1,28687.791667

15,1,1654.956395
15,1,2618.205556
15,1,21553.535714
15,1,3691.077128
15,1,6586.200000
15,1,49055.843750
15,1,12962.615385
15,1,5652.964286
15,1,2231.382143
15,1,4480.732558
15,1,7392.384615
15,1,6736.785714
15,1,93173.997093
15,1,66279.925926
15,1,19713.125000
15,1,55685.464286
15,1,4814.036585
15,1,69325.222973
15,1,20912.294643
15,1,1582.425676
15,1,81408.682065
15,1,5068.208333
15,1,1563.029762
15,1,2633.642857
15,1,37818.281250
15,1,1975.446809
15,1,66047.847222
15,1,6311.826087
15,1,100730.945312
15,1,18137.197917
15,1,1541.000000
15,1,4237.095238
15,1,98406.479592
15,1,6486.467105
15,1,77896.500000
15,1,2671.369048
15,1,2638.457317
15,1,3702.362805
15,1,1501.798387
15,1,3535.671196
15,1,1699.431818
15,1,3819.521739
15,1,36668.844828
15,1,8539.238636
15,1,185317.050595
15,1,2249.897436
15,1,1525.113636
15,1,51491.651961
15,1,59227.615385
15,1,149072.922222
15,1,5865.421053
15,1,7450.336735
15,1,59874.897059
15,1,3122.008523
15,1,2908.842105
15,1,1879.847826
15,1,1722.045455
15,1,82626.119186
15,1,4452.205556
15,1,43962.302326
15,1,2647.392857
15,1,17415.562500
15,1,38881.958333
15,1,6973.470588
15,1,3698.600000
15,1,2570.461538

15,1,38905.392857
15,1,4678.875000
15,1,1580.500000
15,1,2061.098684
15,1,10381.389535
15,1,59071.071429
15,1,2403.559211
15,1,3011.313725
15,1,105494.125000
15,1,22843.058824
15,1,109688.072674
15,1,6264.830357
15,1,12277.000000
15,1,25406.312500
15,1,1658.416667
15,1,3109.362500
15,1,43506.860465
15,1,12082.344828
15,1,197513.658654
15,1,2379.083333
15,1,36041.138158
15,1,157531.169444
15,1,1802.000000
15,1,7421.179487
15,1,10493.756579
15,1,1519.313953
15,1,2103.196970
15,1,39017.250000
15,1,4704.708333
15,1,31821.973485
15,1,24050.870968
15,1,4769.031250
15,1,90545.296875
15,1,73364.987500
15,1,1835.375000
15,1,5415.076923
15,1,38858.446429
15,1,3447.250000
15,1,33551.037162
15,1,58418.815217
15,1,2546.418605
15,1,57577.221154
15,1,32491.821875
15,1,1514.395833
15,1,1737.706250
15,1,1605.059524
15,1,23127.494565
15,1,1628.500000
15,1,56306.945946
15,1,4425.771635
15,1,28959.625000
15,1,2759.196809
15,1,2868.863636
15,1,5876.031250
15,1,2501.881944
15,1,2091.288043
15,1,86271.045455
15,1,1507.655612
15,1,43350.342857
15,1,2639.285714
15,1,48262.113636
15,1,1710.138889
15,1,2952.823171
15,1,6505.704545
15,1,2249.951220
15,1,1600.927083

15,1,3495.969388
15,1,1646.461310
15,1,3338.130435
15,1,6966.500000
15,1,2772.408482
15,1,2122.657738
15,1,1921.160714
15,1,1755.503788
15,1,3002.324324
15,1,11416.690476
15,1,3175.954545
15,1,1997.000000
15,1,1531.415816
15,1,1811.513514
15,1,20959.415625
15,1,2489.076705
15,1,1772.659091
15,1,9813.323718
15,1,1539.729167
15,1,187070.910326
15,1,1817.046512
15,1,105060.519231
15,1,103085.428571
15,1,1898.911765
15,1,1640.013889
15,1,1900.334559
15,1,8309.414634
15,1,151357.209302
15,1,3508.452703
15,1,3363.649194
15,1,8112.517857
15,1,3826.006579
15,1,1510.921875
15,1,2430.695652
15,1,58853.875000
15,1,2311.687500
15,1,2747.727273
15,1,16810.818548
15,1,11353.357143
15,1,2167.954082
15,1,3386.687500
15,1,1526.141667
15,1,11104.459559
15,1,4712.500000
15,1,2631.477941
15,1,58853.664773
15,1,34635.898438
15,1,3860.718750
15,1,1559.608696
15,1,2935.339674
15,1,15903.375000
15,1,51364.006250
15,1,40860.400000
15,1,1606.275000
15,1,18572.932927
15,1,3070.782895
15,1,38871.512097
15,1,97611.000000
15,1,27267.441176
15,1,5916.656250
15,1,3280.809524
15,1,30149.791667
15,1,4792.057927
15,1,9061.621875
15,1,124738.367347
15,1,2335.413043

15,1,2039.450000
15,1,2668.730114
15,1,2753.511628
15,1,2080.860294
15,1,9016.763158
15,1,21945.546875
15,1,1582.277778
15,1,28382.179688
15,1,1946.153846
15,1,190880.039634
15,1,2857.989796
15,1,1558.386364
15,1,44824.382576
15,1,79633.036585
15,1,2119.468085
15,1,2972.378049
15,1,1962.800000
15,1,6906.823529
15,1,1736.579545
15,1,2629.025000
15,1,140428.015625
15,1,19369.929487
15,1,13503.392500
15,1,8902.686170
15,1,24490.585938
15,1,1861.243243
15,1,2918.869565
15,1,7998.225446
15,1,1604.265957
15,1,28739.000000
15,1,6376.142045
15,1,29323.523026
15,1,2124.401786
15,1,31297.121622
15,1,160875.883721
15,1,9853.579861
15,1,96806.479167
15,1,1814.863636
15,1,6574.894737
15,1,9464.611111
15,1,4361.125000
15,1,1649.218750
15,1,4789.336735
15,1,2029.604167
15,1,2468.955556
15,1,199144.261364
15,1,1908.476064
15,1,10477.952703
15,1,76305.319853
15,1,1509.261905
15,1,12538.976190
15,1,9764.100000
15,1,2824.701705
15,1,6436.475000
15,1,192719.977273
15,1,1954.844444
15,1,3003.461957
15,1,2909.026596
15,1,1578.964286
15,1,1963.724432
15,1,34985.398026
15,1,23304.255814
15,1,1649.934783
15,1,8540.558824
15,1,4843.500000
15,1,1513.364130

15,1,59464.621212
15,1,2444.320122
15,1,7200.773810
15,1,116983.038462
15,1,4779.698370
15,1,193286.107558
15,1,38723.887195
15,1,3072.219512
15,1,34628.712121
15,1,1668.157895
15,1,5921.826087
15,1,14266.968750
15,1,55448.385135
15,1,75575.053571
15,1,17079.750000
15,1,2334.019444
15,1,2398.133929
15,1,1931.341463
15,1,33339.025641
15,1,2335.494318
15,1,1650.348214
15,1,2023.718750
15,1,81724.625000
15,1,28837.660714
15,1,42080.166667
15,1,57654.475000
15,1,1684.062500
15,1,1958.918919
15,1,2085.418269
15,1,23747.250000
15,1,2142.000000
15,1,4173.644022
15,1,9202.600610
15,1,25155.448276
15,1,82015.045918
15,1,2453.053191
15,1,20575.237500
15,1,40654.121212
15,1,4991.284722
15,1,3339.043750
15,1,1800.200000
15,1,3593.458333
15,1,1631.101190
15,1,56427.308824
15,1,1765.523810
15,1,3298.838068
15,1,42162.119444
15,1,19440.196970
15,1,20782.756250
15,1,3041.933673
15,1,1921.881944
15,1,80752.757353
15,1,1775.548077
15,1,65302.290761
15,1,13170.906250
15,1,3117.645000
15,1,4475.106383
15,1,2248.085106
15,1,1609.762195
15,1,43124.815789
15,1,3291.971154
15,1,2098.583333
15,1,3669.517045
15,1,1589.037234
15,1,1638.333333
15,1,11066.608871

15,1,26730.972656
15,1,113659.562500
15,1,3267.965909
15,1,1665.744444
15,1,2332.725490
15,1,1621.843023
15,1,3395.906250
15,1,2572.888889
15,1,7402.406977
15,1,27378.647436
15,1,1700.375000
15,1,32109.108108
15,1,52443.656250
15,1,2039.446023
15,1,41982.796512
15,1,80825.113636
15,1,9105.875000
15,1,1617.267857
15,1,4907.303571
15,1,1527.244318
15,1,120845.801282
15,1,4342.729730
15,1,5494.928571
15,1,1744.762195
15,1,20814.011628
15,1,60331.048780
15,1,7356.589744
15,1,2281.940000
15,1,1520.220109
15,1,2182.154762
15,1,11662.630682
15,1,3365.057432
15,1,1543.850000
15,1,3858.622340
15,1,3617.663889
15,1,187778.375000
15,1,14004.683140
15,1,46702.069079
15,1,2489.789773
15,1,99838.391304
15,1,29497.583333
15,1,2384.117647
15,1,3414.062500
15,1,83941.461538
15,1,5113.833333
15,1,3179.700980
15,1,12734.863095
15,1,34542.043478
15,1,1561.750000
15,1,87032.223684
15,1,1536.958333
15,1,198922.578947
15,1,1802.898438
15,1,78411.000000
15,1,64205.114286
15,1,2100.058594
15,1,2462.433962
15,1,1504.804878
15,1,1540.024457
15,1,152581.451613
15,1,1861.058140
15,1,16606.771739
15,1,65876.312500
15,1,2054.821023
15,1,3118.881250
15,1,82368.838816

15,1,2304.100000
15,1,129350.826087
15,1,64307.930556
15,1,5253.988095
15,1,12467.243421
15,1,1513.074219
15,1,58406.500000
15,1,4562.496933
15,1,30773.750000
15,1,13775.458333
15,1,2048.753472
15,1,2428.881250
15,1,49437.378571
15,1,1856.231383
15,1,3175.806452
15,1,42885.190972
15,1,1533.695652
15,1,10446.987500
15,1,133704.703125
15,1,1773.943182
15,1,3881.062500
15,1,1603.625000
15,1,1927.955357
15,1,5134.854651
15,1,2962.735465
15,1,72363.857143
15,1,3604.769231
15,1,2039.338235
15,1,94467.632653
15,1,45888.802083
15,1,48951.250000
15,1,22335.043605
15,1,3243.578947
15,1,13820.701087
15,1,1821.432927
15,1,2120.000000
15,1,2134.645161
15,1,69230.639535
15,1,2592.491279
15,1,53230.990741
15,1,3043.500000
15,1,3213.000000
15,1,4981.882353
15,1,1598.166667
15,1,1647.904762
15,1,179413.466463
15,1,2508.410714
15,1,1711.460938
15,1,2059.808511
15,1,54757.787879
15,1,59154.695652
15,1,3339.687500
15,1,100329.018382
15,1,2926.410256
15,1,30983.993421
15,1,8091.755814
15,1,61643.007653
15,1,1537.055556
15,1,1500.465116
15,1,2440.562500
15,1,2360.940217
15,1,30887.700000
15,1,80816.913462
15,1,88115.546053
15,1,13036.009259
15,1,1512.950000

15,1,92838.581081
15,1,6232.585938
15,1,15055.625000
15,1,37355.738281
15,1,2485.991667
15,1,19289.006944
15,1,58085.763158
15,1,37163.281250
15,1,2825.250000
15,1,8127.314189
15,1,4861.017964
15,1,33176.931818
15,1,2804.213415
15,1,12802.475000
15,1,3404.505814
15,1,2041.910714
15,1,46107.200000
15,1,43422.507812
15,1,5442.641026
15,1,2398.784375
15,1,5821.738372
15,1,1817.120000
15,1,24516.141892
15,1,2986.326531
15,1,5752.614130
15,1,1620.271739
15,1,9075.934783
15,1,1903.880435
15,1,1789.670732
15,1,1734.594512
15,1,2205.614583
15,1,20030.027174
15,1,3487.707317
15,1,55223.562500
15,1,29108.436275
15,1,23622.154605
15,1,1913.070312
15,1,126237.255556
15,1,1915.508523
15,1,5780.446809
15,1,1636.878472
15,1,4357.136364
15,1,6146.766447
15,1,1628.738971
15,1,3144.573370
15,1,49328.547619
15,1,5383.875000
15,1,2393.656250
15,1,23596.247024
15,1,59687.132353
15,1,93504.608108
15,1,44857.310976
15,1,14695.094388
15,1,120085.536585
15,1,4162.000000
15,1,4029.153061
15,1,1746.250000
15,1,2008.071429
15,1,142564.702128
15,1,22765.323276
15,1,63418.555556
15,1,89632.885417
15,1,2250.568750
15,1,183191.500000
15,1,10261.352564
15,1,2288.860465

15,1,1904.350000
15,1,2714.107143
15,1,3117.450000
15,1,2365.360000
15,1,101979.403061
15,1,1567.948980
15,1,7614.039634
15,1,1511.526042
15,1,1544.312500
15,1,2797.583333
15,1,64122.782895
15,1,68154.014286
15,1,11461.470930
15,1,5682.072368
15,1,1612.146341
15,1,3639.666667
15,1,50563.000000
15,1,1690.066860
15,1,2390.781250
15,1,4106.265957
15,1,1683.945946
15,1,1595.111413
15,1,1907.250000
15,1,36961.622995
15,1,3098.317708
15,1,48987.823718
15,1,20905.507813
15,1,2592.954545
15,1,43796.371429
15,1,7500.033854
15,1,61258.037791
15,1,3037.046053
15,1,3586.952128
15,1,157967.866667
15,1,3788.572917
15,1,3364.041667
15,1,1924.600000
15,1,1608.800000
15,1,2015.230769
15,1,7281.625000
15,1,1906.899390
15,1,6431.691176
15,1,3127.333333
15,1,1951.760135
15,1,12577.557692
15,1,5169.226190
15,1,3038.563953
15,1,64309.878378
15,1,117328.209375
15,1,4782.265000
15,1,1702.433333
15,1,2372.171875
15,1,2232.219512
15,1,103043.214286
15,1,5880.333333
15,1,56753.598485
15,1,17713.975000
15,1,46358.279070
15,1,2187.708333
15,1,18432.812500
15,1,1724.255319
15,1,6795.065217
15,1,2063.437500
15,1,10540.000000
15,1,6247.193182
15,1,1603.828125

15,1,1535.521341
15,1,115948.865625
15,1,4265.743590
15,1,19304.692308
15,1,5141.766304
15,1,2764.894737
15,1,1792.696429
15,1,3163.404255
15,1,25936.712121
15,1,77196.695312
15,1,85627.595238
15,1,2136.894444
15,1,1499.246951
15,1,2704.128205
15,1,3270.133523
15,1,1625.217949
15,1,13900.500000
15,1,1542.851351
15,1,1680.500000
15,1,60882.891667
15,1,7514.505952
15,1,1511.276596
15,1,1532.234756
15,1,3630.512500
15,1,35541.602941
15,1,10581.800000
15,1,3481.204268
15,1,3315.837209
15,1,1964.077381
15,1,1742.083333
15,1,3470.472973
15,1,5354.065217
15,1,37783.267857
15,1,2337.114583
15,1,5025.476190
15,1,13527.521739
15,1,1677.272959
15,1,2160.556818
15,1,66711.416667
15,1,150158.882500
15,1,6800.396875
15,1,3290.157895
15,1,4241.529412
15,1,110403.770833
15,1,2505.159091
15,1,6586.187500
15,1,3502.923077
15,1,90319.181818
15,1,1645.349359
15,1,7970.676630
15,1,16575.825658
15,1,1915.750000
15,1,6991.893617
15,1,2019.980263
15,1,5007.333333
15,1,3056.529762
15,1,72923.244444
15,1,75320.081395
15,1,3159.111111
15,1,53871.525735
15,1,1587.500000
15,1,1773.857143
15,1,1766.086310
15,1,3477.291667
15,1,5810.634146
15,1,3809.675532

15,1,1786.000000
15,1,2503.647222
15,1,10402.140244
15,1,80485.812500
15,1,52396.323529
15,1,1914.785714
15,1,15869.584239
15,1,139847.050000
15,1,1684.187500
15,1,2236.800000
15,1,3512.538889
15,1,1553.982143
15,1,26899.778409
15,1,6350.377660
15,1,76770.040541
15,1,41718.068548
15,1,67698.023649
15,1,2771.992647
15,1,4340.666667
15,1,68993.638889
15,1,54691.214286
15,1,2000.687500
15,1,1998.877660
15,1,45327.489583
15,1,24945.651786
15,1,113877.069767
15,1,19985.896341
15,1,1631.511111
15,1,5577.627976
15,1,1934.838415
15,1,151111.427500
15,1,160839.081081
15,1,8216.023256
15,1,10071.003049
15,1,2533.400000
15,1,2241.091216
15,1,1960.980769
15,1,69274.013889
15,1,1802.764706
15,1,1538.945652
15,1,66960.259375
15,1,57748.458333
15,1,11317.921875
15,1,3774.050898
15,1,57686.666667
15,1,1866.578125
15,1,4955.232143
15,1,1882.993421
15,1,72221.950000
15,1,21980.854592
15,1,1576.130682
15,1,69123.424242
15,1,18527.547297
15,1,1759.195652
15,1,1932.933673
15,1,3534.969512
15,1,10605.552632
15,1,7848.409091
15,1,25142.242424
15,1,5742.367788
15,1,2843.603125
15,1,2896.729730
15,1,4756.532609
15,1,1996.669643
15,1,1522.360465
15,1,2369.116071

15,1,1798.687500
15,1,6809.463542
15,1,22361.340909
15,1,1643.262500
15,1,1900.111111
15,1,8474.079268
15,1,11600.950000
15,1,119840.992021
15,1,37052.261905
15,1,1613.619681
15,1,38236.294643
15,1,12690.641026
15,1,7619.250000
15,1,84750.357143
15,1,14476.047297
15,1,4938.944444
15,1,54482.253676
15,1,5356.108696
15,1,1654.189024
15,1,2024.692708
15,1,7036.750000
15,1,3240.832447
15,1,3940.391667
15,1,59398.141892
15,1,2080.875000
15,1,4711.553191
15,1,6332.208333
15,1,72682.961310
15,1,7665.519737
15,1,38000.921053
15,1,1680.802326
15,1,2724.588816
15,1,4928.580882
15,1,5135.903846
15,1,60067.256757
15,1,3492.891892
15,1,7455.321429
15,1,7844.500000
15,1,2899.350000
15,1,4644.992647
15,1,2427.725000
15,1,29217.600000
15,1,87558.086957
15,1,41761.125000
15,1,4091.932432
15,1,1920.551136
15,1,131572.115854
15,1,30147.454268
15,1,5570.089286
15,1,6528.695000
15,1,47831.246951
15,1,3492.307692
15,1,17834.894022
15,1,2386.312500
15,1,18083.666667
15,1,2302.864865
15,1,4658.106383
15,1,1540.442857
15,1,2096.000000
15,1,2394.775000
15,1,5246.976744
15,1,51980.347561
15,1,16648.821429
15,1,2356.239130
15,1,1614.702128
15,1,2487.000000

15,1,2108.607143
15,1,178695.986842
15,1,1552.140625
15,1,1959.755556
15,1,64375.337748
15,1,59585.523256
15,1,5606.183333
15,1,2619.428571
15,1,2104.485714
15,1,4563.133333
15,1,1784.838816
15,1,24880.078125
15,1,4442.520270
15,1,3031.230263
15,1,1897.667683
15,1,70589.268293
15,1,6371.554348
15,1,61052.540541
15,1,1679.057065
15,1,3896.142857
15,1,3951.842105
15,1,3253.076923
15,1,7669.617188
15,1,8729.806818
15,1,2292.686111
15,1,6485.192073
15,1,2879.588235
15,1,20009.000000
15,1,2362.961538
15,1,77772.456250
15,1,11047.703571
15,1,1771.401163
15,1,10763.250000
15,1,2495.750000
15,1,2074.454545
15,1,35440.527344
15,1,6765.718750
15,1,186881.148936
15,1,3222.224359
15,1,1765.500000
15,1,29968.816667
15,1,87699.903846
15,1,11694.409091
15,1,2864.463415
15,1,1993.858108
15,1,2529.639535
15,1,1695.391447
15,1,3175.086957
15,1,7395.445946
15,1,66599.659091
15,1,42802.742647
15,1,152959.701705
15,1,51443.470930
15,1,133500.460526
15,1,2579.232558
15,1,27544.272727
15,1,61984.155488
15,1,2468.325581
15,1,3143.166667
15,1,2135.836806
15,1,3156.163462
15,1,56384.017857
15,1,73577.852273
15,1,104330.142857
15,1,2105.529891
15,1,1701.250000

15,1,11595.581395
15,1,5181.750000
15,1,1990.660714
15,1,3864.616071
15,1,30627.621212
15,1,5984.090909
15,1,1969.696078
15,1,147985.428571
15,1,3433.085000
15,1,50549.590909
15,1,1618.967391
15,1,1621.320197
15,1,4396.016667
15,1,1751.571429
15,1,2166.694767
15,1,27189.258065
15,1,73885.812500
15,1,4244.218750
15,1,8707.080357
15,1,4162.923469
15,1,1652.916667
15,1,6029.109043
15,1,195454.604651
15,1,3204.290865
15,1,1833.511905
15,1,10060.250000
15,1,8988.914894
15,1,1829.366667
15,1,2374.385417
15,1,8930.119048
15,1,117547.631250
15,1,5636.371429
15,1,2254.608696
15,1,1655.192935
15,1,153559.412234
15,1,4087.300000
15,1,26584.660714
15,1,171189.988095
15,1,159254.428571
15,1,121901.884615
15,1,21184.673469
15,1,1632.207317
15,1,171395.487805
15,1,3882.375000
15,1,15234.893750
15,1,1547.317073
15,1,15947.664894
15,1,3595.662791
15,1,4947.970588
15,1,47434.143750
15,1,1543.150000
15,1,28966.133929
15,1,2430.195122
15,1,1557.857143
15,1,75939.416667
15,1,3026.190476
15,1,163968.024390
15,1,147354.122093
15,1,7199.281250
15,1,2992.615854
15,1,13429.897959
15,1,8872.390244
15,1,4795.546512
15,1,2153.783784
15,1,1708.448980
15,1,108212.323864

15,1,2092.953947
15,1,7689.295455
15,1,2444.788043
15,1,23936.157895
15,1,1924.265625
15,1,7841.708333
15,1,2075.708333
15,1,1975.388298
15,1,65122.337500
15,1,1539.125000
15,1,5302.494681
15,1,1867.583333
15,1,21678.119318
15,1,1728.074468
15,1,21479.828125
15,1,133458.000000
15,1,2692.277778
15,1,2426.850000
15,1,3251.969697
15,1,6272.341667
15,1,1970.427083
15,1,89749.250000
15,1,116776.578125
15,1,45607.866279
15,1,1832.805556
15,1,1753.851064
15,1,5905.142857
15,1,1700.722561
15,1,7261.662500
15,1,4661.837766
15,1,39809.246528
15,1,1898.850000
15,1,148493.020833
15,1,1818.036765
15,1,2396.771429
15,1,114814.195122
15,1,1753.447917
15,1,8535.504310
15,1,92768.122159
15,1,3232.270833
15,1,1552.175532
15,1,31352.114286
15,1,63174.233871
15,1,22405.138393
15,1,1585.293478
15,1,2711.401515
15,1,162734.515244
15,1,6988.918478
15,1,6037.195652
15,1,55843.392045
15,1,43308.015625
15,1,2174.900735
15,1,5643.352941
15,1,47334.687500
15,1,1670.063830
15,1,3983.243243
15,1,2021.384615
15,1,1857.325000
15,1,8824.252404
15,1,4263.333333
15,1,3375.685976
15,1,22047.689024
15,1,4871.511628
15,1,2362.022436
15,1,95304.023810
15,1,2013.481707

15,1,1740.690476
15,1,3035.970588
15,1,3656.847561
15,1,33599.933824
15,1,41736.381579
15,1,9263.725000
15,1,2455.525000
15,1,2634.335526
15,1,7885.125000
15,1,1621.318878
15,1,124085.968750
15,1,1503.361413
15,1,2116.401786
15,1,1535.700658
15,1,149244.181548
15,1,58564.222973
15,1,17925.300000
15,1,2101.437500
15,1,1792.047619
15,1,71221.941176
15,1,1945.818182
15,1,66132.072917
15,1,2031.652174
15,1,2517.623494
15,1,1776.547619
15,1,13942.354651
15,1,6795.619318
15,1,17295.769231
15,1,2123.437500
15,1,55625.125000
15,1,10267.756098
15,1,1607.731618
15,1,2325.131579
15,1,3490.142857
15,1,1557.230263
15,1,1549.146341
15,1,113801.365854
15,1,14412.847826
15,1,5298.967105
15,1,95650.000000
15,1,44403.080882
15,1,36769.000000
15,1,1539.704082
15,1,4053.882353
15,1,6406.090909
15,1,2753.020833
15,1,4067.000000
15,1,14785.769737
15,1,4885.103261
15,1,53462.987179
15,1,196266.296875
15,1,7891.446429
15,1,2366.528846
15,1,2604.416667
15,1,8464.043478
15,1,4440.941667
15,1,1889.689189
15,1,2827.963415
15,1,39843.434211
15,1,3143.403846
15,1,1805.409091
15,1,4941.968750
15,1,2310.531250
15,1,1738.468085
15,1,1548.381579
15,1,3066.187500

15,1,2756.000000
15,1,143602.212766
15,1,7873.579268
15,1,1505.337209
15,1,43102.750000
15,1,3772.146341
15,1,6195.234043
15,1,50087.646875
15,1,5528.359375
15,1,2141.354651
15,1,81154.404762
15,1,2768.613372
15,1,2766.767857
15,1,1657.375000
15,1,5361.691489
15,1,2054.400000
15,1,9041.140625
15,1,4449.600000
15,1,7108.625000
15,1,2902.607143
15,1,13890.158088
15,1,2492.013158
15,1,1696.142857
15,1,2419.303571
15,1,91040.006757
15,1,3906.511628
15,1,5825.285714
15,1,45471.980263
15,1,2519.302632
15,1,17791.752907
15,1,2074.125000
15,1,1819.903846
15,1,2334.536765
15,1,2948.763889
15,1,3980.900641
15,1,7088.750000
15,1,76993.383721
15,1,64061.868421
15,1,51589.341463
15,1,1534.222222
15,1,2976.881868
15,1,30004.837500
15,1,10255.375000
15,1,43083.651316
15,1,1701.428125
15,1,2538.140957
15,1,193074.090909
15,1,19363.666667
15,1,3026.618182
15,1,2184.704787
15,1,51846.730263
15,1,102782.073864
15,1,64410.555921
15,1,4563.607143
15,1,1539.361702
15,1,7824.048077
15,1,5671.062500
15,1,32340.750000
15,1,2442.880682
15,1,2430.651163
15,1,1858.976744
15,1,4844.009146
15,1,18039.083333
15,1,31605.753906
15,1,1671.650000
15,1,4495.904412

15,1,5414.351351
15,1,3246.041667
15,1,2887.437500
15,1,2857.835526
15,1,3277.076923
15,1,1898.231707
15,1,8422.488636
15,1,4789.714286
15,1,5802.958333
15,1,5428.744681
15,1,2818.457143
15,1,18517.536364
15,1,132529.337838
15,1,2180.409574
15,1,75173.364865
15,1,3309.489796
15,1,1842.529412
15,1,2180.028846
15,1,77449.388514
15,1,2020.041667
15,1,45633.750000
15,1,1598.105769
15,1,34338.053571
15,1,10318.756944
15,1,33167.492188
15,1,161534.597826
15,1,109860.790541
15,1,6532.409884
15,1,1551.860795
15,1,1973.167614
15,1,1540.381250
15,1,1534.309211
15,1,2130.500000
15,1,1836.125000
15,1,1989.607143
15,1,3555.941860
15,1,2126.190476
15,1,37835.878378
15,1,1617.000000
15,1,1887.565789
15,1,4527.178571
15,1,1963.888889
15,1,11499.125000
15,1,5452.136364
15,1,4460.046875
15,1,3461.786458
15,1,3364.174242
15,1,11635.189189
15,1,1700.934028
15,1,2825.519022
15,1,8016.255435
15,1,2726.933333
15,1,2054.712500
15,1,1616.710526
15,1,3417.125000
15,1,1979.000000
15,1,60510.487179
15,1,29340.169643
15,1,11363.620000
15,1,42655.515152
15,1,192209.592262
15,1,36954.247826
15,1,39486.042857
15,1,4289.491477
15,1,2705.548780
15,1,24302.983871

15,1,71478.364583
15,1,2025.990000
15,1,2836.975000
15,1,2983.367188
15,1,7479.761364
15,1,1518.510417
15,1,2582.266667
15,1,19889.068182
15,1,2410.934211
15,1,8002.199324
15,1,2262.526786
15,1,1654.000000
15,1,80390.907895
15,1,4420.272059
15,1,6195.413690
15,1,1737.772727
15,1,1540.909722
15,1,4216.809524
15,1,1964.692308
15,1,37728.354839
15,1,28721.657895
15,1,3640.723404
15,1,3822.571429
15,1,1502.892857
15,1,25609.204082
15,1,57078.911765
15,1,2205.491071
15,1,78720.235294
15,1,1857.147059
15,1,1532.555556
15,1,2179.947917
15,1,2550.556250
15,1,35404.557143
15,1,7146.081250
15,1,2726.879310
15,1,3766.717105
15,1,2379.841463
15,1,78113.331395
15,1,2886.815476
15,1,1793.931122
15,1,104017.837500
15,1,14303.286932
15,1,1993.053571
15,1,7490.286585
15,1,4015.425532
15,1,122278.679688
15,1,2822.078804
15,1,2171.480769
15,1,8813.477941
15,1,1971.838415
15,1,107531.638889
15,1,1734.162791
15,1,1726.265625
15,1,1763.800000
15,1,23685.664474
15,1,31052.080645
15,1,66506.421875
15,1,52772.294444
15,1,2945.930556
15,1,7553.444444
15,1,5000.000000
15,1,1650.125000
15,1,1993.603659
15,1,82919.605769
15,1,153027.000000
15,1,2859.721875

15,1,37817.062500
15,1,62416.650641
15,1,5507.505556
15,1,10886.321429
15,1,10504.771552
15,1,4770.965625
15,1,4099.464744
15,1,2063.297619
15,1,1948.541667
15,1,2093.000000
15,1,5506.400000
15,1,2516.541667
15,1,24775.266667
15,1,41660.963068
15,1,3490.139423
15,1,42012.875000
15,1,145616.259615
15,1,2718.580556
15,1,5641.926829
15,1,6677.625000
15,1,1748.845588
15,1,1885.384615
15,1,1552.500000
15,1,4899.833333
15,1,7667.566667
15,1,3470.928571
15,1,4818.724490
15,1,2059.457447
15,1,2936.581395
15,1,1614.600000
15,1,1875.597973
15,1,42395.500000
15,1,40531.289062
15,1,4761.902439
15,1,34168.803571
15,1,4050.924242
15,1,2573.618421
15,1,4078.833333
15,1,2086.532143
15,1,25261.417763
15,1,25022.594595
15,1,3454.214286
15,1,2272.421429
15,1,10924.208333
15,1,135932.612179
15,1,4840.709239
15,1,2414.500000
15,1,1504.699468
15,1,5458.355828
15,1,1960.756757
15,1,81408.600000
15,1,1580.536111
15,1,14081.877500
15,1,4575.723404
15,1,1896.222222
15,1,1771.975610
15,1,1992.075581
15,1,9921.044118
15,1,2409.500000
15,1,5130.246324
15,1,3324.064103
15,1,1592.447368
15,1,73474.322368
15,1,106647.255319
15,1,1676.846154
15,1,2513.020833

15,1,5104.000000
15,1,5576.276163
15,1,1991.130556
15,1,2416.184524
15,1,132756.875000
15,1,2499.669643
15,1,1595.534884
15,1,166548.754464
15,1,3698.771739
15,1,29300.411765
15,1,1618.893939
15,1,4502.297619
15,1,1823.133333
15,1,1863.917683
15,1,8539.294444
15,1,148241.547619
15,1,2279.651786
15,1,137111.315789
15,1,63056.625000
15,1,5029.233173
15,1,9150.467262
15,1,99381.243056
15,1,14540.963415
15,1,2570.459459
15,1,5859.990566
15,1,3676.145833
15,1,29953.017045
15,1,3797.950000
15,1,3815.880952
15,1,1572.918919
15,1,10343.812500
15,1,4257.213415
15,1,12637.157895
15,1,179717.839286
15,1,1552.680921
15,1,1665.460938
15,1,9505.582237
15,1,166652.287500
15,1,4217.286585
15,1,1660.812500
15,1,2434.562500
15,1,27532.000000
15,1,3591.053571
15,1,6119.828125
15,1,71840.889706
15,1,2335.911765
15,1,1536.642857
15,1,11478.048295
15,1,4497.328947
15,1,76956.125828
15,1,1885.898256
15,1,52610.850806
15,1,1861.400000
15,1,1681.845588
15,1,8040.393750
15,1,5688.647059
15,1,1538.209302
15,1,2640.926630
15,1,55608.989865
15,1,2162.529412
15,1,2183.240000
15,1,1858.143750
15,1,104646.840625
15,1,6592.660714
15,1,44910.166667
15,1,7207.153846

15,1,42931.158333
15,1,3214.925000
15,1,7373.443182
15,1,3552.281250
15,1,2127.083333
15,1,46199.189024
15,1,45557.385000
15,1,70587.817308
15,1,2141.153846
15,1,4249.750000
15,1,1956.676471
15,1,12465.000000
15,1,10696.536585
15,1,2607.086957
15,1,1701.831522
15,1,3881.171053
15,1,23955.112903
15,1,4133.079545
15,1,8385.949405
15,1,5950.937500
15,1,2263.315217
15,1,30387.250000
15,1,4069.500000
15,1,1763.182927
15,1,60824.820513
15,1,1599.011364
15,1,1603.333333
15,1,20601.121622
15,1,16602.669643
15,1,1994.781250
15,1,1729.143229
15,1,1791.658537
15,1,2951.358696
15,1,1961.343023
15,1,1519.257353
15,1,3556.484375
15,1,1986.847826
15,1,3776.026163
15,1,5658.775000
15,1,4578.585366
15,1,10481.898256
15,1,1779.418478
15,1,106396.365854
15,1,83334.060976
15,1,2472.877976
15,1,2403.375000
15,1,3662.516129
15,1,3747.208333
15,1,66748.666667
15,1,5399.104592
15,1,1601.081395
15,1,2048.375000
15,1,17998.000000
15,1,2355.866477
15,1,7633.068452
15,1,1571.808140
15,1,1620.211806
15,1,46426.851562
15,1,14687.868421
15,1,4427.211735
15,1,1785.071429
15,1,27084.147059
15,1,4471.948529
15,1,78219.729167
15,1,134460.893293
15,1,1833.372483

15,1,3695.173469
15,1,109863.121951
15,1,61728.697368
15,1,14132.410714
15,1,1587.129032
15,1,6466.225000
15,1,37717.690789
15,1,2322.173077
15,1,60821.490854
15,1,4459.714286
15,1,2097.297297
15,1,3699.002604
15,1,17436.665816
15,1,1559.569444
15,1,3828.904070
15,1,2761.535714
15,1,4083.579327
15,1,2903.300676
15,1,1687.000000
15,1,73271.312500
15,1,8845.813953
15,1,1631.040761
15,1,2313.231618
15,1,7705.714286
15,1,1593.106250
15,1,10732.978723
15,1,14636.543478
15,1,8579.559524
15,1,1798.570652
15,1,16685.750000
15,1,129217.135417
15,1,6272.403226
15,1,2809.347826
15,1,2442.937500
15,1,1563.968750
15,1,2499.906977
15,1,1567.932927
15,1,82819.795455
15,1,18832.520833
15,1,4612.488889
15,1,69863.625000
15,1,2559.442308
15,1,171368.937500
15,1,2664.926630
15,1,2280.812500
15,1,1616.250000
15,1,175555.508721
15,1,156463.000000
15,1,1584.322368
15,1,6771.151316
15,1,2373.486486
15,1,2529.012195
15,1,2609.326220
15,1,62982.033537
15,1,1675.284884
15,1,3699.416667
15,1,114518.250000
15,1,3188.146875
15,1,1572.937500
15,1,1578.752976
15,1,4254.842949
15,1,35901.934783
15,1,4433.957447
15,1,48711.253049
15,1,1768.125000
15,1,59116.278571

15,1,2749.125000
15,1,2373.662791
15,1,17112.229167
15,1,1819.776596
15,1,94388.493750
15,1,1924.159574
15,1,3017.411765
15,1,1572.108108
15,1,61723.723684
15,1,139308.005814
15,1,2875.508929
15,1,15780.470588
15,1,18324.229167
15,1,7004.775000
15,1,3258.555147
15,1,1523.439024
15,1,50370.109375
15,1,1947.441860
15,1,74524.191489
15,1,1708.884375
15,1,32007.932432
15,1,48065.437500
15,1,2771.226190
15,1,2889.202206
15,1,3009.706081
15,1,5397.342105
15,1,6427.468085
15,1,5229.848837
15,1,11706.534884
15,1,12205.827381
15,1,71170.136029
15,1,22034.835714
15,1,1943.238636
15,1,5376.711111
15,1,3362.583333
15,1,164388.500000
15,1,14122.903125
15,1,3993.388889
15,1,86438.042553
15,1,38591.959559
15,1,34346.333333
15,1,173853.328947
15,1,5489.382143
15,1,18972.193182
15,1,4351.402174
15,1,141486.464286
15,1,1634.625000
15,1,1829.270833
15,1,19700.904762
15,1,161801.671875
15,1,45720.895833
15,1,3073.298780
15,1,4324.625000
15,1,1770.271739
15,1,1631.020000
15,1,4460.093750
15,1,79170.788690
15,1,1944.844512
15,1,1664.813889
15,1,72983.750000
15,1,4258.000000
15,1,30067.072581
15,1,1692.000000
15,1,2330.589286
15,1,1676.437500
15,1,44326.109091

15,1,6019.487179
15,1,70134.746622
15,1,1944.750000
15,1,1538.269531
15,1,11681.539474
15,1,1659.492347
15,1,3194.099265
15,1,24469.133333
15,1,4953.346154
15,1,1530.064103
15,1,1996.991667
15,1,122155.833333
15,1,60942.136364
15,1,1692.345745
15,1,2718.622222
15,1,2746.946429
15,1,3362.670213
15,1,61748.672297
15,1,5370.440217
15,1,115474.000000
15,1,45697.250000
15,1,32750.769231
15,1,53444.272727
15,1,154730.732955
15,1,35872.818182
15,1,12693.930851
15,1,3064.081522
15,1,3947.857143
15,1,3342.588816
15,1,93474.656250
15,1,3090.594512
15,1,1624.775568
15,1,7381.958333
15,1,12068.105263
15,1,2202.885135
15,1,111895.213889
15,1,55269.292683
15,1,48244.763889
15,1,84340.755952
15,1,44306.088235
15,1,8438.133152
15,1,132276.489130
15,1,78128.625000
15,1,1797.307692
15,1,6708.056122
15,1,5581.150000
15,1,50198.290541
15,1,2553.045455
15,1,4640.610294
15,1,51290.235294
15,1,2911.354167
15,1,17123.419118
15,1,10287.612245
15,1,1670.946023
15,1,1641.173295
15,1,9559.250000
15,1,11875.425000
15,1,12274.733333
15,1,1854.432927
15,1,4412.025568
15,1,32781.733333
15,1,45748.227564
15,1,48635.767857
15,1,1648.490741
15,1,2904.800000
15,1,2607.720930

15,1,2214.621951
15,1,6547.658537
15,1,8321.750000
15,1,2527.899390
15,1,151949.011905
15,1,6397.828571
15,1,4354.117347
15,1,2681.264535
15,1,22429.775735
15,1,4358.693452
15,1,7328.638298
15,1,5068.375000
15,1,3040.895349
15,1,132805.000000
15,1,2084.794118
15,1,2125.973958
15,1,10993.714286
15,1,61432.910256
15,1,52660.108108
15,1,49904.152439
15,1,1785.625000
15,1,31560.733871
15,1,73559.153846
15,1,114575.479167
15,1,47333.763514
15,1,115377.146429
15,1,1647.576923
15,1,1700.634615
15,1,14565.297297
15,1,43734.662234
15,1,1618.228261
15,1,12478.737245
15,1,3367.878378
15,1,8059.713415
15,1,23963.660714
15,1,1711.436111
15,1,32514.641026
15,1,4270.521739
15,1,1714.070312
15,1,39170.424242
15,1,1710.157051
15,1,2155.192308
15,1,17568.057692
15,1,60160.795918
15,1,19490.904412
15,1,16395.450000
15,1,3124.460000
15,1,5537.947917
15,1,1694.263158
15,1,20899.421053
15,1,1692.797872
15,1,19347.024390
15,1,1596.641892
15,1,1605.395349
15,1,126530.130208
15,1,53338.786765
15,1,5986.750000
15,1,131473.425000
15,1,11207.587264
15,1,64892.409722
15,1,4720.813953
15,1,2420.125000
15,1,69405.514706
15,1,15551.420732
15,1,120763.480114
15,1,2745.666667

15,1,2998.562500
15,1,1962.758929
15,1,1667.428125
15,1,10747.503676
15,1,1802.575000
15,1,34374.666667
15,1,6420.739130
15,1,5158.478261
15,1,1883.690476
15,1,11024.922222
15,1,2766.965625
15,1,1543.046512
15,1,4011.750000
15,1,47495.231707
15,1,1807.921053
15,1,2799.473958
15,1,3176.129032
15,1,14349.244681
15,1,3366.250000
15,1,1672.478659
15,1,25324.349359
15,1,8757.419643
15,1,3689.391667
15,1,5724.390244
15,1,4662.289062
15,1,1883.875000
15,1,10272.127660
15,1,3131.508427
15,1,1757.425000
15,1,2818.891892
15,1,8022.762195
15,1,2311.341216
15,1,1745.925000
15,1,1733.718750
15,1,87877.837838
15,1,59140.897436
15,1,1748.750000
15,1,2751.785714
15,1,23447.321809
15,1,2066.071875
15,1,3728.849432
15,1,56462.789474
15,1,3163.805233
15,1,14915.736842
15,1,107340.052632
15,1,3679.628049
15,1,2361.405556
15,1,2047.371711
15,1,64085.835526
15,1,4263.488636
15,1,2212.785714
15,1,2366.769231
15,1,147671.722222
15,1,8182.580882
15,1,26458.824324
15,1,3364.683036
15,1,1659.909091
15,1,2015.372093
15,1,5719.627907
15,1,3242.054054
15,1,52686.277778
15,1,1565.150000
15,1,2019.875000
15,1,1723.170732
15,1,29769.159091
15,1,6856.856383

15,1,2171.671512
15,1,11706.500000
15,1,1515.705882
15,1,2443.300000
15,1,2243.428571
15,1,3618.902439
15,1,1607.671875
15,1,40189.832143
15,1,1666.306250
15,1,41550.209559
15,1,3636.823529
15,1,82294.945652
15,1,7291.279255
15,1,5926.638298
15,1,1924.602941
15,1,1820.182143
15,1,53088.705882
15,1,2538.051136
15,1,1702.791667
15,1,1651.127660
15,1,1712.588889
15,1,47873.500000
15,1,9898.000000
15,1,8120.978261
15,1,4795.744565
15,1,46975.080882
15,1,2375.752404
15,1,5163.734375
15,1,2360.812500
15,1,1942.923077
15,1,6126.242188
15,1,54928.585938
15,1,2018.941489
15,1,18018.857143
15,1,91527.533088
15,1,2206.127404
15,1,1804.443878
15,1,2341.585106
15,1,101727.500000
15,1,10310.353125
15,1,43504.338235
15,1,1839.038462
15,1,1931.611979
15,1,33333.301282
15,1,106641.519231
15,1,1643.011111
15,1,1982.723214
15,1,75542.195122
15,1,4081.426630
15,1,3541.931818
15,1,62082.081633
15,1,1893.725000
15,1,1704.176630
15,1,1980.312500
15,1,2731.880952
15,1,6144.138298
15,1,17244.315789
15,1,1944.375000
15,1,2873.500000
15,1,13610.500000
15,1,1553.019231
15,1,66489.076389
15,1,1502.750000
15,1,2870.214286
15,1,1686.857143
15,1,10516.274510

15,1,23758.433333
15,1,4387.961111
15,1,89935.000000
15,1,2042.875000
15,1,39006.078125
15,1,9736.865854
15,1,7959.173077
15,1,8588.220745
15,1,54003.335714
15,1,1809.905405
15,1,2170.954787
15,1,1637.423611
15,1,21045.514286
15,1,2124.600000
15,1,4678.296512
15,1,1641.409091
15,1,29158.891892
15,1,1982.573980
15,1,2139.982143
15,1,31266.182692
15,1,4154.243902
15,1,2234.893617
15,1,59721.348214
15,1,1952.598214
15,1,48639.743243
15,1,1787.066327
15,1,50777.125000
15,1,2576.687500
15,1,1558.737903
15,1,1866.545455
15,1,1783.784722
15,1,1688.146341
15,1,44998.928571
15,1,65038.493590
15,1,1842.696023
15,1,2483.712766
15,1,21331.504545
15,1,1777.058824
15,1,21844.187500
15,1,1753.375000
15,1,20732.859375
15,1,3461.647059
15,1,4675.852941
15,1,1577.812500
15,1,3666.786111
15,1,1681.608871
15,1,43523.788462
15,1,11990.863636
15,1,1575.889881
15,1,10953.502907
15,1,1879.361486
15,1,1663.252551
15,1,1558.650000
15,1,7151.943878
15,1,10318.190476
15,1,3978.300532
15,1,6946.675532
15,1,7895.816667
15,1,1651.706522
16,2,2721.502841
16,2,2128.769737
16,2,1749.875000
16,2,2547.596154
16,2,1537.893617
16,2,45207.087500
16,2,2671.690000

16,2,2875.678125
16,2,2291.238095
16,2,1910.867788
16,2,1873.601744
16,2,4743.250000
16,2,1968.737805
16,2,1591.412791
16,2,2934.175481
16,2,2335.012755
16,2,2198.710000
16,2,1751.429348
16,2,1589.564516
16,2,1873.613095
16,2,1798.400000
16,2,5023.440000
16,2,2944.125000
16,2,2189.444444
16,2,1865.597561
16,2,1629.600000
16,2,1700.435811
16,2,2076.820312
16,2,30140.706522
16,2,1925.934211
16,2,1691.008152
16,2,1716.000000
16,2,2363.477941
16,2,1739.828947
16,2,2779.729167
16,2,1685.225000
16,2,2072.621711
16,2,1552.210784
16,2,1604.394737
16,2,1940.843750
16,2,1813.635870
16,2,2619.192857
16,2,2682.534375
16,2,2414.050000
16,2,2062.488889
16,2,1767.145833
16,2,63634.000000
16,2,2449.350877
16,2,1547.350000
16,2,1789.769737
16,2,2774.771277
16,2,1566.500000
16,2,2229.331395
16,2,3640.575000
16,2,2172.812500
16,2,6090.735849
16,2,2269.000000
16,2,1749.936170
16,2,1661.533333
16,2,1957.816489
16,2,1566.395833
16,2,2148.079082
16,2,1681.478261
16,2,1945.312500
16,2,1829.825893
16,2,2532.250000
16,2,2034.521226
16,2,1632.317500
16,2,2939.925926
16,2,1737.710526
16,2,2554.632159
16,2,2282.028409
16,2,4809.959302

16,2,1579.119048
16,2,1658.938889
16,2,3522.511111
16,2,3378.168367
16,2,1966.382353
16,2,2322.080882
16,2,3349.794776
16,2,1941.521429
16,2,6079.165323
16,2,2721.875000
16,2,2002.015625
16,2,2068.561404
16,2,2004.612903
16,2,1557.778302
16,2,2311.295455
16,2,5134.416667
16,2,1547.351974
16,2,2947.270000
16,2,2290.852941
16,2,3162.548913
16,2,1783.744792
16,2,2155.732143
16,2,2497.093750
16,2,1591.875000
16,2,2243.583333
16,2,5270.842308
16,2,8001.464286
16,2,1724.434524
16,2,1604.709302
16,2,2011.833333
16,2,1754.500000
16,2,3638.690789
16,2,2999.796296
16,2,2514.015152
16,2,1675.795000
16,2,1824.533019
16,2,2644.090909
16,2,1806.147059
16,2,1585.750000
16,2,1738.845930
16,2,2625.177500
16,2,2555.627551
16,2,4882.954545
16,2,2877.437500
16,2,3218.951923
16,2,2142.845930
16,2,1606.960000
16,2,1538.361413
16,2,1894.367188
16,2,1824.761111
16,2,1866.031915
16,2,1715.250000
16,2,6940.625000
16,2,3892.596354
16,2,1980.234043
16,2,1505.808511
16,2,1888.560000
16,2,8125.750000
16,2,1840.400000
16,2,1978.362245
16,2,2180.760870
16,2,2552.850000
16,2,1537.916667
16,2,1950.340426
16,2,2293.250000
16,2,1797.413265

16,2,1962.027778
16,2,1727.779412
16,2,3436.208333
16,2,1625.473837
16,2,3149.815789
16,2,2763.800000
16,2,1715.769231
16,2,2210.301339
16,2,1616.766667
16,2,1998.581522
16,2,2302.250000
16,2,2377.684896
16,2,2104.662791
16,2,2302.686275
16,2,2031.363636
16,2,2123.192308
16,2,1706.836538
16,2,2250.166667
16,2,1562.312500
16,2,1891.796569
16,2,2745.369444
16,2,2523.293478
16,2,1561.163690
16,2,1554.659574
16,2,1971.000000
16,2,1628.093750
16,2,10543.801136
16,2,1875.924419
16,2,1798.950581
16,2,9525.819149
16,2,2079.681818
16,2,2225.680000
16,2,2487.618421
16,2,1991.530000
16,2,1528.046875
16,2,1790.489796
16,2,1804.562500
16,2,2033.875000
16,2,3796.941026
16,2,7856.829545
16,2,3819.500000
16,2,1882.538043
16,2,1538.000000
16,2,2050.454167
16,2,1678.782609
16,2,4802.885246
16,2,3564.660326
16,2,1660.333333
16,2,8589.375000
16,2,6836.116071
16,2,1989.868750
16,2,1606.702128
16,2,4261.696809
16,2,2761.312500
16,2,2084.140000
16,2,1750.723404
16,2,1843.350446
16,2,2452.875000
16,2,2014.129386
16,2,1574.347826
16,2,1558.347222
16,2,1760.882353
16,2,1947.333333
16,2,1959.170213
16,2,1848.333333
16,2,1810.115000

16,2,2585.088235
16,2,2305.015957
16,2,2115.669643
16,2,2647.186224
16,2,3378.179487
16,2,4173.375000
16,2,1693.713942
16,2,2258.851293
16,2,28258.315789
16,2,2547.075000
16,2,1895.654167
16,2,1695.847222
16,2,1562.564024
16,2,2784.944444
16,2,2054.187500
16,2,4528.800000
16,2,3771.162500
16,2,1523.307500
16,2,3909.491071
16,2,1748.390625
16,2,2233.610795
16,2,1748.609375
16,2,2173.500000
16,2,1575.118750
16,2,2145.229592
16,2,5718.447368
16,2,2064.404412
16,2,15721.295455
16,2,3806.319149
16,2,1635.089286
16,2,1645.022959
16,2,2503.500000
16,2,1840.500000
16,2,6865.832589
16,2,2094.083333
16,2,1875.375000
16,2,2572.650000
16,2,1646.053571
16,2,2505.669118
16,2,5097.621951
16,2,1936.000000
16,2,4227.167832
16,2,1921.808140
16,2,1636.402174
16,2,2160.387500
16,2,3732.656915
16,2,2020.826923
16,2,1771.993056
16,2,2004.583333
16,2,1620.638298
16,2,1669.461538
16,2,6999.230392
16,2,1787.928571
16,2,1531.705882
16,2,3580.010417
16,2,2004.644231
16,2,1516.400000
16,2,5852.145000
16,2,1502.908537
16,2,2077.093085
16,2,2632.166667
16,2,1823.204545
16,2,1818.571429
16,2,1544.682927
16,2,1624.573171
16,2,1543.594828

16,2,3246.668478
16,2,1629.293750
16,2,1675.916667
16,2,1779.101064
16,2,2222.915094
16,2,1814.404762
16,2,1664.597826
16,2,1646.093750
16,2,1719.071429
16,2,2245.310000
16,2,40396.157738
16,2,1606.890625
16,2,1539.500000
16,2,1988.274510
16,2,2203.108974
16,2,1890.059524
16,2,4792.025463
16,2,1994.787234
16,2,1801.787500
16,2,5414.106250
16,2,1917.046569
16,2,1859.979592
16,2,4485.184375
16,2,1876.250000
16,2,1568.729730
16,2,1580.840909
16,2,2826.411765
16,2,4921.576531
16,2,9812.328125
16,2,1929.421053
16,2,3807.115385
16,2,6497.172500
16,2,2835.539326
16,2,1677.750000
16,2,2351.509615
16,2,1908.413265
16,2,2187.625000
16,2,2859.962719
16,2,1773.814103
16,2,6941.625000
16,2,3018.593750
16,2,1769.397959
16,2,2078.788043
16,2,2487.698370
16,2,2788.084906
16,2,1856.581731
16,2,3374.229167
16,2,2332.980000
16,2,1877.631579
16,2,1898.106383
16,2,4535.026961
16,2,3332.618750
16,2,2681.267857
16,2,1871.675000
16,2,1862.364796
16,2,4398.146341
16,2,1894.841463
16,2,1574.562500
16,2,2252.250000
16,2,2027.966981
16,2,2517.225962
16,2,1678.882353
16,2,2254.562500
16,2,2889.725000
16,2,2938.671875
16,2,4951.993421

16,2,2081.448864
16,2,2344.980000
16,2,1574.642857
16,2,1581.022500
16,2,1714.798913
16,2,4773.157692
16,2,3288.277778
16,2,1852.755102
16,2,1920.764706
16,2,2556.428571
16,2,2011.661765
16,2,1658.143293
16,2,2337.572917
16,2,2061.824324
16,2,1791.928571
16,2,1536.676630
16,2,1934.000000
16,2,1589.275862
16,2,2479.250000
16,2,1954.635714
16,2,1766.562500
16,2,1963.173611
16,2,1812.852941
16,2,1613.726190
16,2,3489.852778
16,2,2377.105556
16,2,1604.113372
16,2,1652.892157
16,2,2792.711538
16,2,1748.690476
16,2,2259.600000
16,2,1905.410714
16,2,1872.954545
16,2,1709.388393
16,2,1660.618182
16,2,2790.808673
16,2,1526.206522
16,2,1541.740385
16,2,1551.944444
16,2,4881.941176
16,2,5731.368421
16,2,17008.285714
16,2,1882.813830
16,2,3804.450000
16,2,1563.185000
16,2,1525.878049
16,2,2835.596154
16,2,2480.437500
16,2,1789.578431
16,2,4137.648936
16,2,2959.096154
16,2,2042.687500
16,2,2650.250000
16,2,2000.145349
16,2,2751.296296
16,2,2310.409091
16,2,1612.260870
16,2,2263.090909
16,2,3970.500000
16,2,10649.515244
16,2,2105.231481
16,2,10956.792411
16,2,1518.238095
16,2,3214.333333
16,2,1646.141304
16,2,8677.880682

16,2,3695.617647
16,2,15434.973214
16,2,9655.595745
16,2,6814.454054
16,2,1524.494318
16,2,2630.287736
16,2,3433.645455
16,2,3957.957447
16,2,1698.581818
16,2,2596.804054
16,2,2377.147727
16,2,2585.872024
16,2,1665.831633
16,2,1508.614130
16,2,3629.347826
16,2,1891.394737
16,2,3559.238889
16,2,1830.914773
16,2,1605.812500
16,2,1759.138889
16,2,1731.640625
16,2,2078.506696
16,2,2154.901786
16,2,2766.132353
16,2,3485.892857
16,2,2240.937209
16,2,2489.801932
16,2,1905.434783
16,2,16708.454545
16,2,1935.917910
16,2,1637.651163
16,2,55585.594828
16,2,2551.125000
16,2,3169.163265
16,2,2299.840000
16,2,1695.208333
16,2,3959.368750
16,2,1761.566667
16,2,3649.170732
16,2,2167.000000
16,2,2023.825000
16,2,39081.635870
16,2,3866.128049
16,2,4120.622549
16,2,8376.016949
16,2,2051.404255
16,2,5545.250000
16,2,1637.604651
16,2,2889.815217
16,2,1602.464286
16,2,2273.006356
16,2,1562.950000
16,2,1778.214286
16,2,4510.956522
16,2,1553.000000
16,2,2242.902778
16,2,2512.948864
16,2,1551.122283
16,2,2310.875000
16,2,1653.545455
16,2,3199.364583
16,2,1758.302083
16,2,7757.255556
16,2,1709.800000
16,2,1573.408163
16,2,1862.872093

16,2,1979.728723
16,2,2190.442708
16,2,1587.392857
16,2,2582.494565
16,2,1959.562500
16,2,1605.000000
16,2,2365.968085
16,2,1710.900000
16,2,78186.098039
16,2,1709.076220
16,2,1973.000000
16,2,1719.883152
16,2,1524.038265
16,2,2167.846591
16,2,2706.742788
16,2,3189.017857
16,2,12716.742188
16,2,1591.597561
16,2,1621.026042
16,2,1588.492647
16,2,1623.750000
16,2,1839.452128
16,2,1876.372845
16,2,2142.869792
16,2,2033.609375
16,2,2141.919118
16,2,2992.563725
16,2,1554.500000
16,2,2589.447115
16,2,1854.326316
16,2,3383.596591
16,2,1790.209302
16,2,2078.717391
16,2,2094.470455
16,2,3005.351351
16,2,1593.528125
16,2,2425.377778
16,2,2264.454545
16,2,2074.961538
16,2,2733.760417
16,2,2197.388021
16,2,1661.075000
16,2,2548.051136
16,2,2754.122024
16,2,1656.428571
16,2,1661.813253
16,2,2845.687500
16,2,1911.591216
16,2,1939.750000
16,2,4155.166667
16,2,3094.217593
16,2,5809.554348
16,2,2093.500000
16,2,1621.937500
16,2,1862.413043
16,2,1706.045455
16,2,2787.255208
16,2,1527.648649
16,2,1823.573171
16,2,1750.541667
16,2,3048.575000
16,2,1955.280172
16,2,1799.909091
16,2,1953.800000
16,2,2507.397059
16,2,45495.437500

16,2,1964.243243
16,2,5718.213415
16,2,2061.166667
16,2,2000.605769
16,2,2787.000000
16,2,1503.226064
16,2,1915.000000
16,2,2414.395349
16,2,2903.709302
16,2,2662.408654
16,2,1861.262500
16,2,1589.510638
16,2,1968.357143
16,2,2598.098958
16,2,4426.782609
16,2,2441.211957
16,2,1989.651515
16,2,2421.234756
16,2,2267.955556
16,2,78855.164474
16,2,2666.777273
16,2,2291.783654
16,2,2551.565104
16,2,1980.147222
16,2,3485.456522
16,2,2586.855769
16,2,1589.358491
16,2,1840.050000
16,2,25630.500000
16,2,3127.000000
16,2,3165.766667
16,2,2006.559278
16,2,1971.881944
16,2,1599.885417
16,2,6173.463415
16,2,2696.525000
16,2,2944.772727
16,2,1543.563953
16,2,2938.333333
16,2,2298.266447
16,2,1646.000000
16,2,1689.615385
16,2,1717.487805
16,2,2807.083333
16,2,1550.143750
16,2,1528.375000
16,2,1933.954082
16,2,1751.148148
16,2,2134.680000
16,2,1962.804348
16,2,2075.933824
16,2,1579.908784
16,2,12755.172872
16,2,2439.043750
16,2,2596.617647
16,2,3875.169271
16,2,2126.840426
16,2,2344.042553
16,2,2080.533333
16,2,1965.830189
16,2,2245.812500
16,2,1660.544118
16,2,2114.710000
16,2,3489.960526
16,2,2487.304054
16,2,2243.692308

16,2,2257.963415
16,2,2645.489796
16,2,2603.152778
16,2,1815.423913
16,2,1630.848837
16,2,3534.037037
16,2,4073.557927
16,2,1772.565476
16,2,1982.328125
16,2,2176.801471
16,2,2608.846154
16,2,1710.971591
16,2,7125.247159
16,2,1847.134146
16,2,152229.184896
16,2,2461.400000
16,2,3823.764706
16,2,1794.836735
16,2,1790.138298
16,2,1835.528846
16,2,1905.097561
16,2,1608.116071
16,2,2335.831633
16,2,4975.862903
16,2,3229.846939
16,2,1858.342105
16,2,1688.950000
16,2,6561.910714
16,2,1979.665761
16,2,1554.000000
16,2,1711.104167
16,2,1788.738636
16,2,2895.783654
16,2,16490.388889
16,2,1910.795455
16,2,1636.074074
16,2,1947.702703
16,2,3043.258621
16,2,2406.012195
16,2,2243.106383
16,2,1974.375000
16,2,3549.709091
16,2,1755.309375
16,2,1590.731383
16,2,1830.986979
16,2,4393.619048
16,2,1899.169811
16,2,1514.902778
16,2,6901.565341
16,2,2711.928571
16,2,2348.504808
16,2,1618.564904
16,2,1505.147321
16,2,1808.348214
16,2,1996.078488
16,2,2609.977273
16,2,2036.837838
16,2,1686.950521
16,2,2294.731707
16,2,2182.025641
16,2,1524.687500
16,2,2801.151042
16,2,2959.520833
16,2,10632.002660
16,2,2391.000000
16,2,2698.151316

16,2,2968.412234
16,2,1960.252551
16,2,2029.696809
16,2,1589.795918
16,2,8638.921875
16,2,2094.290000
16,2,1781.088889
16,2,1697.534574
16,2,2208.430556
16,2,3532.760870
16,2,3053.850610
16,2,1589.330357
16,2,5775.551020
16,2,2266.468750
16,2,2088.800000
16,2,1723.988372
16,2,2720.079545
16,2,4443.377193
16,2,2354.128676
16,2,1828.860294
16,2,2348.127660
16,2,1702.153409
16,2,2114.140244
16,2,64354.918367
16,2,97076.541667
16,2,4188.232143
16,2,1708.940789
16,2,1698.036458
16,2,1817.300000
16,2,1880.132979
16,2,1508.838415
16,2,1791.500000
16,2,2216.687500
16,2,1506.018293
16,2,4817.984375
16,2,1627.204082
16,2,2934.375000
16,2,2669.139881
16,2,1654.718750
16,2,1958.769231
16,2,2064.034375
16,2,2030.363208
16,2,8283.716981
16,2,3532.225131
16,2,5586.179054
16,2,2275.469388
16,2,4238.444444
16,2,2301.062500
16,2,4017.837500
16,2,1523.888889
16,2,11304.943182
16,2,3390.903846
16,2,1546.921053
16,2,5885.145000
16,2,1752.813953
16,2,1526.767857
16,2,1769.988636
16,2,4851.525000
16,2,2970.010638
16,2,2039.062500
16,2,4593.857143
16,2,1976.790698
16,2,3021.750000
16,2,1987.884146
16,2,1957.250000
16,2,1505.250000

16,2,1588.782609
16,2,2628.750000
16,2,1596.740854
16,2,1551.152439
16,2,2312.218085
16,2,1597.388889
16,2,4248.968750
16,2,4906.404661
16,2,1574.928571
16,2,1523.331818
16,2,2513.438776
16,2,1653.070312
16,2,2369.533163
16,2,5011.844595
16,2,2478.375000
16,2,1689.530405
16,2,1734.262019
16,2,2670.638298
16,2,1933.411765
16,2,2763.156250
16,2,2897.365909
16,2,1607.857143
16,2,1598.315104
16,2,2131.352273
16,2,1978.685096
16,2,9215.087500
16,2,1883.625000
16,2,2569.385135
16,2,3607.953125
16,2,2238.875000
16,2,1932.375000
16,2,2662.005319
16,2,1723.128205
16,2,1607.823370
16,2,1784.921875
16,2,2025.496875
16,2,1932.500000
16,2,2264.539773
16,2,1648.609375
16,2,2937.066489
16,2,1639.676829
16,2,2201.254545
16,2,1969.890909
16,2,1702.829082
16,2,2375.459239
16,2,3601.434783
16,2,3237.942308
16,2,2176.741071
16,2,18208.267857
16,2,2984.318182
16,2,24120.833333
16,2,1600.261905
16,2,1637.460227
16,2,3483.393617
16,2,3729.257812
16,2,3920.422500
16,2,2940.669118
16,2,2402.837209
16,2,2327.331250
16,2,2424.503289
16,2,2622.946429
16,2,9939.118421
16,2,4441.542683
16,2,8862.516667
16,2,3119.836864
16,2,1752.806250

16,2,1513.275000
16,2,1732.071429
16,2,1713.410714
16,2,12193.768293
16,2,55400.280000
16,2,1688.163462
16,2,1732.894231
16,2,3297.106132
16,2,2090.406250
16,2,5958.983412
16,2,2003.000000
16,2,2705.542553
16,2,1780.944149
16,2,1628.712500
16,2,1639.456522
16,2,1792.172619
16,2,1724.268293
16,2,75590.031250
16,2,68183.712054
16,2,1639.261111
16,2,27688.930921
16,2,5636.002976
16,2,19300.073171
16,2,2936.325000
16,2,1709.317308
16,2,2194.608553
16,2,1673.494792
16,2,4404.585526
16,2,3325.222222
16,2,2626.183673
16,2,2867.902439
16,2,2619.709184
16,2,2132.507500
16,2,1820.423077
16,2,1684.861702
16,2,3567.062500
16,2,1540.125000
16,2,3328.131868
16,2,2546.472561
16,2,1663.625000
16,2,1522.482143
16,2,2100.210526
16,2,1749.031250
16,2,2606.559783
16,2,1651.200000
16,2,1967.071429
16,2,1863.479592
16,2,1671.500000
16,2,2851.733333
16,2,3779.517857
16,2,1530.843750
16,2,1709.923295
16,2,1534.220000
16,2,1857.772727
16,2,2296.773026
16,2,2681.000000
16,2,2191.964286
16,2,1507.015625
16,2,2457.800000
16,2,1571.743421
16,2,1664.500000
16,2,1501.392157
16,2,1804.782051
16,2,2814.125000
16,2,2355.214286
16,2,13605.480769

16,2,2443.946429
16,2,97281.375000
16,2,3237.125000
16,2,2757.651163
16,2,2677.883721
16,2,1768.089674
16,2,3818.827500
16,2,1887.352273
16,2,1865.283333
16,2,2463.834906
16,2,2778.840686
16,2,2545.179688
16,2,2201.833333
16,2,1805.556818
16,2,1916.211735
16,2,6823.372727
16,2,2794.309783
16,2,5361.489583
16,2,2286.830357
16,2,3269.865132
16,2,1894.525000
16,2,1726.312500
16,2,2142.853659
16,2,2492.430556
16,2,2396.765625
16,2,1688.625000
16,2,1604.508721
16,2,4231.329787
16,2,1616.750000
16,2,1513.114583
16,2,1523.785714
16,2,2713.368421
16,2,4792.140000
16,2,1524.424342
16,2,2797.616071
16,2,2107.627551
16,2,1848.437500
16,2,1709.993750
16,2,2843.161932
16,2,2608.959239
16,2,1939.901786
16,2,1546.301020
16,2,1920.056548
16,2,25840.024390
16,2,2909.323370
16,2,2240.871622
16,2,1630.925847
16,2,1703.976744
16,2,2321.215385
16,2,1721.405556
16,2,1620.618902
16,2,1531.600000
16,2,3966.691489
16,2,1893.825472
16,2,7678.551136
16,2,5004.605263
16,2,3159.051471
16,2,1560.234375
16,2,1629.267442
16,2,1689.648936
16,2,1661.490625
16,2,1619.735000
16,2,2139.035714
16,2,1930.595745
16,2,1536.741071
16,2,4110.445652

16,2,1944.183673
16,2,39833.171875
16,2,3201.862069
16,2,1605.750000
16,2,2391.314103
16,2,1692.637500
16,2,9377.160714
16,2,1758.985000
16,2,2539.208333
16,2,2292.416667
16,2,3885.282895
16,2,2082.344444
16,2,2288.476562
16,2,1537.383523
16,2,1502.447368
16,2,1540.186170
16,2,1981.000000
16,2,7254.326531
16,2,2038.726852
16,2,3595.330000
16,2,1717.804878
16,2,1541.548780
16,2,4241.551020
16,2,2992.978723
16,2,1903.176136
16,2,1847.892857
16,2,1517.565217
16,2,2462.500000
16,2,1720.780612
16,2,1621.279070
16,2,2244.878049
16,2,1748.808824
16,2,3124.510638
16,2,2217.255814
16,2,2084.734043
16,2,1641.477564
16,2,2679.882353
16,2,1906.477941
16,2,24440.036458
16,2,2695.253049
16,2,1770.222826
16,2,1789.241071
16,2,1871.442857
16,2,1578.000000
16,2,1755.510638
16,2,2710.533333
16,2,1763.171875
16,2,1789.464286
16,2,2822.303571
16,2,3229.052885
16,2,1931.594595
16,2,2135.956522
16,2,1872.987805
16,2,18118.527027
16,2,1616.670673
16,2,3002.672619
16,2,1970.657895
16,2,2648.823529
16,2,4720.500000
16,2,1716.336735
16,2,1708.938679
16,2,4287.295455
16,2,1916.512931
16,2,1619.972222
16,2,3552.975962
16,2,1872.546875

16,2,1728.723404
16,2,2028.950000
16,2,3242.463415
16,2,2027.301724
16,2,1803.234756
16,2,1717.343750
16,2,2075.132500
16,2,1510.788462
16,2,2268.616848
16,2,2151.300000
16,2,1895.612500
16,2,1551.540323
16,2,1531.153409
16,2,1611.495370
16,2,1836.523585
16,2,1956.303922
16,2,1845.696809
16,2,2636.020833
16,2,6023.011029
16,2,2198.687500
16,2,1816.333333
16,2,2250.420455
16,2,1922.000000
16,2,1827.173077
16,2,1997.196809
16,2,2099.990566
16,2,15283.512195
16,2,1904.023256
16,2,1970.877778
16,2,1586.571429
16,2,1583.951220
16,2,1560.364583
16,2,1535.920732
16,2,1536.097561
16,2,6712.807692
16,2,1520.865385
16,2,2939.463415
16,2,4413.074468
16,2,1691.822368
16,2,1643.701087
16,2,2322.478723
16,2,2841.888298
16,2,3285.534014
16,2,2239.502717
16,2,1505.500000
16,2,2367.257653
16,2,1539.630000
16,2,1520.627907
16,2,1652.711957
16,2,1784.640625
16,2,2431.627778
16,2,2074.979592
16,2,1814.812500
16,2,2120.277778
16,2,2193.727273
16,2,1681.488095
16,2,2576.500000
16,2,2369.423077
16,2,2375.513158
16,2,1506.578431
16,2,3042.901961
16,2,3018.000000
16,2,3087.821429
16,2,2671.250000
16,2,2688.140097
16,2,2054.702128

16,2,1604.687500
16,2,1544.750000
16,2,4129.607143
16,2,1785.033333
16,2,12491.534483
16,2,1644.932018
16,2,1986.333333
16,2,2932.339286
16,2,1545.985119
16,2,2864.333333
16,2,2310.493056
16,2,1564.250000
16,2,1687.855769
16,2,1625.432143
16,2,3120.921875
16,2,1721.437500
16,2,2147.162162
16,2,1868.335227
16,2,1695.131579
16,2,1571.500000
16,2,1784.073529
16,2,2250.959184
16,2,2066.398936
16,2,4945.625000
16,2,3019.388587
16,2,3452.265306
16,2,2098.606771
16,2,2221.317130
16,2,3957.313953
16,2,2508.800000
16,2,1718.229651
16,2,1942.329545
16,2,1603.989865
16,2,2104.400735
16,2,2030.000000
16,2,2780.480978
16,2,1567.300000
16,2,1666.122449
16,2,34115.198718
16,2,1526.058511
16,2,3470.702128
16,2,1649.421875
16,2,2593.568182
16,2,14144.989865
16,2,2052.646277
16,2,2042.220395
16,2,1968.481383
16,2,4838.147436
16,2,2012.272727
16,2,1653.127717
16,2,2525.975000
16,2,2266.726064
16,2,1949.285714
16,2,1605.561224
16,2,2702.384615
16,2,16480.912791
16,2,57517.464286
16,2,1559.455357
16,2,2843.611111
16,2,1829.750000
16,2,5569.701923
16,2,1544.242500
16,2,1775.857143
16,2,1609.323980
16,2,1715.555556
16,2,2019.446809

16,2,2160.712963
16,2,2001.833333
16,2,3481.428571
16,2,1950.421875
16,2,2383.869565
16,2,3174.131696
16,2,1897.117188
16,2,1660.816038
16,2,102228.107143
16,2,1890.485000
16,2,2883.159574
16,2,2445.354167
16,2,3401.284091
16,2,4837.394231
16,2,1751.877907
16,2,1558.406250
16,2,2474.280488
16,2,2821.318182
16,2,1816.648438
16,2,1660.463710
16,2,1939.510638
16,2,1656.198661
16,2,1995.640625
16,2,1501.679878
16,2,1868.253049
16,2,174290.767442
16,2,1884.958333
16,2,3621.331395
16,2,5709.533333
16,2,1697.125000
16,2,2402.893617
16,2,2726.541667
16,2,2049.287234
16,2,1698.776786
16,2,1662.727500
16,2,2261.700000
16,2,2820.739286
16,2,1832.610000
16,2,2078.013889
16,2,46031.789062
16,2,2525.544643
16,2,1719.039474
16,2,1544.063830
16,2,1788.783654
16,2,2216.571429
16,2,1896.163265
16,2,1538.883333
16,2,1525.450000
16,2,1932.765957
16,2,1618.448718
16,2,1796.234756
16,2,1802.500000
16,2,1570.250000
16,2,6187.535714
16,2,1519.037500
16,2,2590.607500
16,2,1853.682292
16,2,3643.391827
16,2,1681.957447
16,2,1615.830882
16,2,2326.887097
16,2,88857.356250
16,2,4440.375000
16,2,3962.809659
16,2,1548.418605
16,2,2841.483696

16,2,1632.306818
16,2,1625.963415
16,2,2152.789894
16,2,1958.561828
16,2,1992.640000
16,2,1600.004808
16,2,2192.204082
16,2,1846.395833
16,2,1793.019231
16,2,2182.906250
16,2,2635.085526
16,2,1730.617647
16,2,65923.201923
16,2,2562.512195
16,2,1650.573529
16,2,1646.460526
16,2,1517.937500
16,2,1642.428571
16,2,2132.798077
16,2,2845.137755
16,2,4131.086957
16,2,1746.687500
16,2,1512.789474
16,2,2854.665865
16,2,2349.112500
16,2,1808.643382
16,2,2416.000000
16,2,4299.583333
16,2,30589.704545
16,2,1921.134146
16,2,3545.019231
16,2,1582.262195
16,2,1878.899390
16,2,3173.505319
16,2,1849.518293
16,2,1507.586957
16,2,2444.035714
16,2,3177.011905
16,2,1974.957317
16,2,2151.487805
16,2,2456.660256
16,2,2012.782609
16,2,2462.339623
16,2,41056.031250
16,2,1583.596154
16,2,1943.052500
16,2,2547.272500
16,2,1865.407609
16,2,1533.512195
16,2,6623.050000
16,2,1522.935897
16,2,2306.120000
16,2,1502.462264
16,2,37268.000000
16,2,1712.500000
16,2,1712.923077
16,2,1547.545455
16,2,1741.020833
16,2,1522.416667
16,2,1845.547872
16,2,2792.574074
16,2,1526.529412
16,2,2426.891827
16,2,1990.051471
16,2,3458.048077
16,2,2289.766667

16,2,2661.900510
16,2,1887.180556
16,2,1816.513889
16,2,1825.534722
16,2,1548.500000
16,2,1654.750000
16,2,4741.218750
16,2,1710.767857
16,2,2716.090909
16,2,4449.613636
16,2,2876.961340
16,2,2818.710227
16,2,2612.611111
16,2,2141.371795
16,2,2930.362500
16,2,2174.241477
16,2,3052.240909
16,2,1564.720930
16,2,38799.764706
16,2,1920.908537
16,2,1552.096939
16,2,2521.479651
16,2,2145.212500
16,2,1580.352273
16,2,2133.082447
16,2,2361.583333
16,2,2344.910714
16,2,2100.577128
16,2,1523.021277
16,2,1642.116071
16,2,2921.948529
16,2,2315.464286
16,2,2666.484848
16,2,1733.120690
16,2,2062.750000
16,2,1797.275862
16,2,1606.586957
16,2,2681.900000
16,2,3121.651442
16,2,2715.151515
16,2,1843.105000
16,2,1570.044118
16,2,1699.407895
16,2,1634.923913
16,2,1599.197500
16,2,2090.719444
16,2,2135.313830
16,2,3293.607143
16,2,2627.385870
16,2,2700.171196
16,2,1663.101449
16,2,1731.059211
16,2,1993.044444
16,2,2202.444444
16,2,2782.805556
16,2,2280.350000
16,2,1782.042683
16,2,16113.812500
16,2,1765.603659
16,2,2120.838415
16,2,1748.638393
16,2,1733.950658
16,2,11977.090116
16,2,2413.985294
16,2,1857.000000
16,2,2479.510870

16,2,1649.690625
16,2,2017.752232
16,2,8615.046512
16,2,1639.900000
16,2,1758.043478
16,2,2562.000000
16,2,2166.392157
16,2,34687.790816
16,2,2321.322917
16,2,1809.583333
16,2,3166.119048
16,2,1787.184783
16,2,1798.815000
16,2,1591.191489
16,2,4809.757353
16,2,1559.811321
16,2,1505.901163
16,2,1986.323529
16,2,1612.732143
16,2,1606.865625
16,2,1557.643382
16,2,2016.315000
16,2,2289.730769
16,2,2362.884868
16,2,1819.887097
16,2,1791.170213
16,2,1617.583333
16,2,1615.515625
16,2,5008.778571
16,2,1661.382653
16,2,2864.868590
16,2,2107.325000
16,2,1900.834184
16,2,2006.386139
16,2,3286.987179
16,2,4107.714286
16,2,1698.800000
16,2,1922.463235
16,2,2123.750000
16,2,1977.500000
16,2,1690.909574
16,2,30728.863636
16,2,2066.347826
16,2,1851.391304
16,2,2412.787500
16,2,1532.465116
16,2,2344.757812
16,2,1819.955882
16,2,2026.542683
16,2,1539.721354
16,2,1509.360294
16,2,3111.351064
16,2,1622.817308
16,2,1537.477273
16,2,1830.804688
16,2,2015.570000
16,2,1580.300000
16,2,4473.105556
16,2,2787.625000
16,2,2795.545455
16,2,1655.162879
16,2,1918.615854
16,2,2271.021277
16,2,1499.576923
16,2,1611.639151
16,2,1661.930000

16,2,2227.760000
16,2,2139.067073
16,2,1646.610000
16,2,1599.083333
16,2,2926.597826
16,2,2649.619048
16,2,2243.411585
16,2,2762.617188
16,2,1688.042553
16,2,1827.195122
16,2,36759.460938
16,2,1603.308333
16,2,1502.718182
16,2,3899.279255
16,2,3470.007812
16,2,1608.345000
16,2,2868.187500
16,2,1865.352941
16,2,1721.547170
16,2,1924.319444
16,2,2667.226190
16,2,2299.365854
16,2,3788.978659
16,2,2336.410714
16,2,2454.660714
16,2,1745.690000
16,2,3119.478261
16,2,1775.119048
16,2,2099.793750
16,2,1775.716981
16,2,1596.576531
16,2,1772.111111
16,2,1966.818182
16,2,4775.866848
16,2,2584.821429
16,2,1574.750000
16,2,1511.978261
16,2,2345.474359
16,2,2110.058140
16,2,2521.687500
16,2,2161.883333
16,2,2305.109589
16,2,1731.292683
16,2,2404.250000
16,2,1969.750000
16,2,2264.361111
16,2,1611.781250
16,2,1829.103261
16,2,2741.500000
16,2,2502.750000
16,2,4295.663265
16,2,2061.256098
16,2,2027.311224
16,2,1820.709302
16,2,1700.073864
16,2,3690.141827
16,2,1690.717949
16,2,1700.944853
16,2,1883.076923
16,2,1991.500000
16,2,2078.364865
16,2,32138.625000
16,2,1871.903061
16,2,4237.813830
16,2,2236.097561
16,2,1708.619898

16,2,1993.877358
16,2,2801.384375
16,2,2096.600000
16,2,1638.812500
16,2,1740.521739
16,2,1868.045000
16,2,2461.579268
16,2,1986.390625
16,2,2869.937500
16,2,1951.600000
16,2,3236.227273
16,2,1850.750000
16,2,1661.307692
16,2,1526.554878
16,2,1959.798611
16,2,1895.000000
16,2,1530.260870
16,2,1761.150000
16,2,1985.792453
16,2,2059.798387
16,2,3069.500000
16,2,1931.339744
16,2,2834.829032
16,2,158515.360000
16,2,2660.698324
16,2,4794.002604
16,2,2025.250000
16,2,2287.635000
16,2,2183.760870
16,2,4457.840000
16,2,1666.750000
16,2,2367.122449
16,2,1677.065217
16,2,1954.610465
16,2,2445.475000
16,2,4081.058824
16,2,3840.883721
16,2,1749.790698
16,2,1763.076923
16,2,1573.617788
16,2,1875.431250
16,2,2033.454545
16,2,1770.696429
16,2,3056.234043
16,2,5462.138889
16,2,1692.220588
16,2,1796.544271
16,2,1554.959184
16,2,3417.344828
16,2,3235.858696
16,2,1677.958333
16,2,4290.267442
16,2,1707.136364
16,2,2457.267857
16,2,1924.125000
16,2,2718.571429
16,2,3298.390000
16,2,19736.365854
16,2,5582.017544
16,2,3735.375000
16,2,1633.805000
16,2,16162.697368
16,2,1933.271429
16,2,2576.936170
16,2,38790.769737
16,2,47312.376289

16,2,2063.019608
16,2,1540.988095
16,2,2319.712766
16,2,2208.313725
16,2,2111.855000
16,2,2092.886364
16,2,7646.687500
16,2,1564.770833
16,2,1996.200472
16,2,1771.977778
16,2,1877.000000
16,2,2355.532609
16,2,1521.125000
16,2,1558.521341
16,2,4550.435714
16,2,3034.841912
16,2,2860.690000
16,2,1723.195946
16,2,2524.875000
16,2,2195.431818
16,2,3106.225806
16,2,50762.378571
16,2,5047.847826
16,2,16181.837209
16,2,8432.430921
16,2,2026.800000
16,2,1785.597826
16,2,1530.653846
16,2,35351.750000
16,2,16278.723684
16,2,4049.620370
16,2,1906.872449
16,2,3071.800000
16,2,31759.852941
16,2,2334.625000
16,2,2592.820000
16,2,1759.392857
16,2,2537.903125
16,2,1979.609375
16,2,1534.500000
16,2,1595.544118
16,2,3240.838415
16,2,1792.440625
16,2,2916.934028
16,2,2190.690217
16,2,2174.230000
16,2,1767.250000
16,2,7730.125000
16,2,1820.280000
16,2,1533.181818
16,2,1701.892045
16,2,1640.042553
16,2,4054.895833
16,2,2915.200000
16,2,1562.268987
16,2,1736.000000
16,2,1915.650000
16,2,5931.741848
16,2,6439.572115
16,2,1874.428571
16,2,2512.305556
16,2,2207.125000
16,2,1827.580128
16,2,2269.488372
16,2,2450.022727
16,2,1775.050926

16,2,2632.966346
16,2,1693.617347
16,2,1733.920732
16,2,1925.617347
16,2,1566.958333
16,2,1501.613971
16,2,1578.742500
16,2,3492.739583
16,2,1717.317073
16,2,2107.054688
16,2,1802.459746
16,2,2092.546512
16,2,2419.548780
16,2,2086.208333
16,2,2971.983333
16,2,5081.831250
16,2,1988.739362
16,2,1779.241071
16,2,5220.201923
16,2,3659.324468
16,2,2189.741279
16,2,2622.688830
16,2,2214.804487
16,2,1912.492188
16,2,2166.560811
16,2,1659.547872
16,2,2015.714286
16,2,2872.820000
16,2,2432.967949
16,2,2197.904762
16,2,1641.571429
16,2,2471.263514
16,2,2187.083333
16,2,24638.300000
16,2,1738.077778
16,2,3490.250000
16,2,2036.250000
16,2,2374.744186
16,2,1685.197368
16,2,2302.530000
16,2,2650.171875
16,2,1674.840426
16,2,1987.632075
16,2,3462.014535
16,2,4223.473822
16,2,2046.650000
16,2,2636.583333
16,2,1905.133152
16,2,3544.732955
16,2,1775.586538
16,2,1633.602564
16,2,2027.705556
16,2,1564.251366
16,2,1997.647500
16,2,12995.328947
16,2,1874.000000
16,2,1536.828947
16,2,1606.307870
16,2,1669.992347
16,2,2943.282051
16,2,1787.622396
16,2,1546.742021
16,2,2462.250000
16,2,3379.521739
16,2,1647.234286
16,2,2019.146341

16,2,1901.621324
16,2,28377.393293
16,2,1660.586735
16,2,2471.950000
16,2,2249.553571
16,2,1741.210000
16,2,4327.910156
16,2,3638.529412
16,2,2072.938953
16,2,1600.406250
16,2,1601.375000
16,2,1667.172872
16,2,1740.070755
16,2,1972.781250
16,2,65636.207237
16,2,3437.576531
16,2,2533.353261
16,2,1818.411765
16,2,2927.291667
16,2,1521.752500
16,2,10868.261111
16,2,2154.598214
16,2,1699.163265
16,2,10505.969595
16,2,1850.795455
16,2,2006.310811
16,2,2084.500000
16,2,2276.756757
16,2,2392.307692
16,2,1612.962500
16,2,1969.088235
16,2,1940.500000
16,2,1692.537234
16,2,7555.583333
16,2,2819.872340
16,2,1551.562500
16,2,1580.579545
16,2,1563.239130
16,2,2464.308511
16,2,1756.737500
16,2,2921.000000
16,2,2354.804878
16,2,2414.122222
16,2,1611.800000
16,2,1802.765625
16,2,41551.968750
16,2,19153.439024
16,2,3041.892857
16,2,3473.760638
16,2,1733.585366
16,2,1939.003289
16,2,1949.320513
16,2,1516.000000
16,2,5241.291667
16,2,1529.039062
16,2,1957.351190
16,2,1638.298077
16,2,1758.500000
16,2,1847.613372
16,2,2602.003676
16,2,6275.675000
16,2,1964.225490
16,2,2073.296875
16,2,2187.739796
16,2,1500.250000
16,2,1866.821809

16,2,2196.466837
16,2,2985.955000
16,2,1628.401042
16,2,5881.426136
16,2,1809.780488
16,2,1850.203125
16,2,1771.954082
16,2,2174.357143
16,2,2011.973214
16,2,1886.642857
16,2,2272.000000
16,2,1964.681250
16,2,3055.086957
16,2,10301.774390
16,2,2820.864130
16,2,1580.103175
16,2,1538.562500
16,2,40222.235294
16,2,1885.090426
16,2,1675.808511
16,2,2131.955128
16,2,2101.500000
16,2,2440.816327
16,2,2312.889706
16,2,2716.586957
16,2,2230.400000
16,2,1550.429348
16,2,1718.837662
16,2,1653.204082
16,2,2243.722222
16,2,2592.078947
16,2,2164.360294
16,2,2016.308036
16,2,4586.211111
16,2,9083.982639
16,2,1975.253049
16,2,2285.320000
16,2,96346.670213
16,2,2627.819149
16,2,156596.660000
16,2,1614.225000
16,2,1553.160714
16,2,1680.236111
16,2,2159.200000
16,2,1596.875000
16,2,1628.193750
16,2,2974.527174
16,2,1537.850694
16,2,2214.947368
16,2,1609.689655
16,2,2528.464481
16,2,1590.740000
16,2,1663.271277
16,2,3184.407609
16,2,2289.703030
16,2,1555.397959
16,2,1993.611111
16,2,2856.781250
16,2,4498.770833
16,2,1668.043269
16,2,1542.770000
16,2,1572.230556
16,2,1691.448980
16,2,1761.557065
16,2,1537.437500
16,2,2453.890244

16,2,1593.260000
16,2,1637.808140
16,2,1992.738095
16,2,30556.797872
16,2,1967.132812
16,2,4259.125000
16,2,28307.775862
16,2,2001.636364
16,2,3723.434896
16,2,4436.227500
16,2,1627.439560
16,2,9587.974359
16,2,2169.983696
16,2,1521.022222
16,2,2110.645833
16,2,1929.047222
16,2,1929.538462
16,2,1917.044643
16,2,2618.201389
16,2,1773.277778
16,2,1965.153846
16,2,1714.000000
16,2,4531.264205
16,2,1561.731707
16,2,1529.319149
16,2,1738.266667
16,2,1815.117647
16,2,3068.187500
16,2,2947.539062
16,2,1680.829268
16,2,2229.237500
16,2,1638.817308
16,2,1859.190000
16,2,4063.200581
16,2,1664.478723
16,2,1524.708333
16,2,2620.976744
16,2,3739.666667
16,2,2528.522222
16,2,1605.294872
16,2,2355.662500
16,2,1520.533654
16,2,2534.125000
16,2,2401.771739
16,2,1659.388298
16,2,3099.110294
16,2,1663.125000
16,2,2383.853659
16,2,1917.540000
16,2,2214.901442
16,2,5686.515957
16,2,2836.795455
16,2,2470.165761
16,2,7071.304545
16,2,2905.375000
16,2,1908.760000
16,2,1604.022727
16,2,4556.858696
16,2,1905.260000
16,2,2272.000000
16,2,3929.346154
16,2,1611.486842
16,2,4407.006250
16,2,1683.223837
16,2,41872.394886
16,2,1938.056122

16,2,2100.718182
16,2,1843.538462
16,2,2802.565217
16,2,1669.709302
16,2,1937.833333
16,2,2057.334906
16,2,2163.423913
16,2,2612.857955
16,2,1521.897059
16,2,2144.449405
16,2,5666.509375
16,2,1555.821023
16,2,1558.428571
16,2,10586.785714
16,2,3885.041667
16,2,2138.367021
16,2,2534.279412
16,2,1910.273256
16,2,1538.076923
16,2,2545.263158
16,2,1924.183511
16,2,1952.285714
16,2,1554.237179
16,2,1803.855769
16,2,1742.115854
16,2,2874.068878
16,2,2081.033333
16,2,1848.000000
16,2,1730.797872
16,2,1563.004902
16,2,1855.156250
16,2,2697.606061
16,2,1608.282051
16,2,1549.457447
16,2,2648.994565
16,2,4140.812500
16,2,1705.166667
16,2,1761.100000
16,2,1822.006579
16,2,1648.416667
16,2,1756.222222
16,2,2448.696429
16,2,2465.318182
16,2,2914.788462
16,2,7977.396226
16,2,1885.461538
16,2,2167.831395
16,2,1863.892857
16,2,1825.791667
16,2,1639.020408
16,2,1741.416667
16,2,1518.075581
16,2,1766.607843
16,2,1809.141892
16,2,2059.214286
16,2,2051.420673
16,2,1588.069767
16,2,2662.361702
16,2,1659.104651
16,2,2775.000000
16,2,1827.330000
16,2,1649.268750
16,2,1993.198864
16,2,1677.752358
16,2,1841.843023
16,2,2886.820000

16,2,1544.636364
16,2,1622.731383
16,2,1823.831897
16,2,1833.717262
16,2,1646.883929
16,2,2539.000000
16,2,2427.785714
16,2,1499.750000
16,2,2435.440217
16,2,2102.500000
16,2,3855.224359
16,2,1688.331081
16,2,2914.526316
16,2,1694.266667
16,2,2391.119469
16,2,3216.020408
16,2,1636.893617
16,2,2383.238095
16,2,1824.661585
16,2,1794.989796
16,2,128803.804688
16,2,3000.203488
16,2,13267.571429
16,2,1546.772727
16,2,2972.562500
16,2,1735.977041
16,2,2499.500000
16,2,1851.583333
16,2,3226.024510
16,2,1907.744186
16,2,2162.400000
16,2,1862.938776
16,2,6319.081081
16,2,2960.197368
16,2,1602.202128
16,2,3639.654070
16,2,2180.000000
16,2,2205.776596
16,2,2620.289474
16,2,2165.128049
16,2,46002.071795
16,2,2345.000000
16,2,1820.045732
16,2,3147.203125
16,2,1921.521429
16,2,2257.561170
16,2,2741.930851
16,2,1903.877841
16,2,2304.666667
16,2,1567.505682
16,2,2038.375000
16,2,1505.421569
16,2,2009.557143
16,2,2984.312500
16,2,5732.550595
16,2,1799.000000
16,2,1855.705128
16,2,1802.654762
16,2,2014.762500
16,2,2100.144444
16,2,37549.010870
16,2,1693.357143
16,2,1706.155172
16,2,3619.300000
16,2,1813.171875
16,2,1706.954545

16,2,1995.333333
16,2,2540.696335
16,2,3234.654412
16,2,9535.778409
16,2,3209.000000
16,2,2053.634615
16,2,1754.000000
16,2,2401.353659
16,2,3039.903226
16,2,1871.587209
16,2,1902.128049
16,2,2409.327381
16,2,1641.079268
16,2,2488.103659
16,2,3117.914286
16,2,1961.721154
16,2,2733.924528
16,2,7729.910256
16,2,2531.883929
16,2,2502.788462
16,2,3479.994048
16,2,2063.070513
16,2,3289.630000
16,2,1536.631579
16,2,2356.714844
16,2,1848.712766
16,2,2042.600000
16,2,2906.854651
16,2,1776.845000
16,2,2742.303191
16,2,4683.767857
16,2,5713.461538
16,2,1602.075000
16,2,1618.220588
16,2,4265.354839
16,2,2253.453125
16,2,2262.632353
16,2,2153.798469
16,2,2139.539062
16,2,2010.936170
16,2,1507.055000
16,2,1525.581633
16,2,1818.250000
16,2,3266.730556
16,2,1555.250000
16,2,2206.500000
16,2,3616.687500
16,2,1629.664634
16,2,2760.010870
16,2,1769.966346
16,2,1816.121622
16,2,1992.125000
16,2,3402.583333
16,2,2044.823864
16,2,1718.798077
16,2,1569.156250
16,2,3507.260638
16,2,1863.024457
16,2,1708.882353
16,2,2388.862500
16,2,1548.947917
16,2,8509.912500
16,2,2779.800000
16,2,3545.091146
16,2,2261.814394
16,2,2747.031250

16,2,2097.256944
16,2,2079.975410
16,2,2429.054054
16,2,2040.792683
16,2,2077.837209
16,2,1706.957317
16,2,1695.333333
16,2,1987.978723
16,2,1510.487179
16,2,1556.800000
16,2,1633.012195
16,2,1780.625000
16,2,2789.058673
16,2,1919.474576
16,2,1730.848837
16,2,2475.454545
16,2,1895.235795
16,2,1507.357143
16,2,2869.839286
16,2,1683.484472
16,2,2667.234694
16,2,3115.245370
16,2,2110.058824
16,2,3173.844444
16,2,2187.554688
16,2,4575.075472
16,2,1776.281250
16,2,2033.525000
16,2,1727.761905
16,2,2269.851064
16,2,2104.852041
16,2,2409.500000
16,2,2811.272727
16,2,3928.171875
16,2,1589.076923
16,2,27943.784926
16,2,2097.211538
16,2,1656.015957
16,2,13185.782895
16,2,1539.328125
16,2,2132.611111
16,2,2162.151961
16,2,2258.625000
16,2,73906.526316
16,2,1783.200000
16,2,2298.187500
16,2,2156.046512
16,2,3920.892857
16,2,2740.008621
16,2,1507.088235
16,2,2348.191489
16,2,3790.351695
16,2,13462.551282
16,2,2264.017500
16,2,3924.507812
16,2,1881.884615
16,2,1780.346154
16,2,34023.881757
16,2,2375.955357
16,2,1771.808824
16,2,2140.919643
16,2,3377.089286
16,2,2491.625000
16,2,2916.127717
16,2,1941.307692
16,2,1627.900000

16,2,1607.076531
16,2,2809.583333
16,2,3395.606383
16,2,1540.802326
16,2,1713.797297
16,2,8193.678571
16,2,1992.593023
16,2,3819.896552
16,2,1578.944444
16,2,1686.919643
16,2,1507.906863
16,2,2426.500000
16,2,24225.461538
16,2,1725.645833
16,2,2047.000000
16,2,3497.750000
16,2,1769.112245
16,2,1556.637500
16,2,1827.416667
16,2,2613.100000
16,2,1925.119898
16,2,1638.377451
16,2,2280.671512
16,2,13389.263889
16,2,1657.435000
16,2,2417.168478
16,2,1534.080000
16,2,1712.235294
16,2,4706.880000
16,2,1554.446809
16,2,1538.094512
16,2,1622.906250
16,2,1804.763393
16,2,2040.208333
16,2,3039.392361
16,2,2171.506944
16,2,2171.137755
16,2,1830.518382
16,2,1765.358696
16,2,3574.485294
16,2,1606.208333
16,2,2396.962963
16,2,2142.473404
16,2,1903.046875
16,2,1499.710938
16,2,1650.917614
16,2,1707.323864
16,2,1738.852941
16,2,1913.125000
16,2,1825.201389
16,2,1747.518293
16,2,1576.257979
16,2,1815.593750
16,2,5079.250000
16,2,1677.570312
16,2,2121.948529
16,2,2121.652778
16,2,2980.239286
16,2,2206.675000
16,2,1709.782609
16,2,5395.862245
16,2,2592.066667
16,2,5730.985577
16,2,1746.000000
16,2,2166.363636
16,2,1499.696809

16,2,56978.683140
16,2,1712.785714
16,2,9139.579787
16,2,3103.973039
16,2,11468.575000
16,2,1702.159314
16,2,3589.970588
16,2,10037.301829
16,2,2371.771739
16,2,1920.465426
16,2,2073.659483
16,2,2101.487500
16,2,1754.384434
16,2,2280.591912
16,2,7700.430556
16,2,1598.337209
16,2,1949.130682
16,2,55143.818182
16,2,1682.140957
16,2,4230.002841
16,2,2271.571429
16,2,2505.268750
16,2,2225.066667
16,2,2247.916667
16,2,1863.406250
16,2,2448.000000
16,2,7237.250000
16,2,1717.952830
16,2,2330.476190
16,2,2407.792553
16,2,2889.772727
16,2,1561.561170
16,2,2947.129870
16,2,1582.216216
16,2,2734.401515
16,2,2519.665816
16,2,1606.528846
16,2,2892.790761
16,2,2705.856250
16,2,4380.000000
16,2,1922.697917
16,2,1577.466346
16,2,4433.105114
16,2,5078.604167
16,2,1567.666667
16,2,112632.177273
16,2,3514.979651
16,2,2074.044872
16,2,1588.732759
16,2,2088.017442
16,2,1619.073171
16,2,6513.037234
16,2,1576.531915
16,2,3047.500000
16,2,2541.500000
16,2,2401.164062
16,2,1723.259259
16,2,1582.394231
16,2,1827.807500
16,2,4209.454268
16,2,1618.529605
16,2,1856.771635
16,2,2715.535714
16,2,2601.454787
16,2,1648.180851
16,2,2153.260870

16,2,1500.682927
16,2,2234.647059
16,2,4511.333333
16,2,1794.476351
16,2,1512.197917
16,2,2453.347826
16,2,1719.500000
16,2,1564.225610
16,2,2464.371795
16,2,1934.500000
16,2,2059.009615
16,2,2819.929245
16,2,1874.283019
16,2,2067.948113
16,2,2001.239130
16,2,2387.576389
16,2,2512.095745
16,2,2231.913690
16,2,2644.553571
16,2,2140.114286
16,2,2046.500000
16,2,1594.201923
16,2,2296.578947
16,2,1909.119565
16,2,2294.238426
16,2,2219.367647
16,2,1892.925000
16,2,1666.134615
16,2,3453.557692
16,2,5010.281250
16,2,2004.875000
16,2,3726.273438
16,2,2251.375000
16,2,1516.286184
16,2,1501.114583
16,2,1551.050000
16,2,2563.785714
16,2,1643.250000
16,2,1516.191489
16,2,1630.336735
16,2,1935.383929
16,2,2738.230769
16,2,1823.758772
16,2,1853.621212
16,2,1735.050000
16,2,1648.946809
16,2,1647.531250
16,2,1665.796875
16,2,2988.899390
16,2,5407.691964
16,2,2031.130000
16,2,53807.270270
16,2,2429.201923
16,2,2558.009615
16,2,2113.614362
16,2,2283.136364
16,2,2405.916667
16,2,2000.292553
16,2,1512.064655
16,2,3485.130682
16,2,1573.250000
16,2,1938.218750
16,2,1577.431579
16,2,2987.120000
16,2,3969.500000
16,2,1846.766667

16,2,1743.432692
16,2,3296.913043
16,2,4482.937500
16,2,1587.896186
16,2,1683.775510
16,2,1864.784314
16,2,3276.011364
16,2,2194.238208
16,2,2548.447115
16,2,1657.056818
16,2,2535.880952
16,2,3719.825472
16,2,1993.000000
16,2,1873.584559
16,2,1895.497396
16,2,2101.250000
16,2,47804.000000
16,2,3337.510638
16,2,1885.638889
16,2,1598.781818
16,2,14046.944444
16,2,2111.435000
16,2,1571.122222
16,2,2953.531915
16,2,1585.325000
16,2,1922.368902
16,2,2955.478659
16,2,1767.813830
16,2,1592.465116
16,2,4935.144444
16,2,1811.439024
16,2,2539.290000
16,2,1756.713542
16,2,129590.861111
16,2,2688.525000
16,2,2734.273256
16,2,2239.600000
16,2,2182.189286
16,2,1573.285714
16,2,1529.651042
16,2,1775.100000
16,2,1951.820122
16,2,4241.631579
16,2,1506.714286
16,2,1827.429878
16,2,2453.150000
16,2,1623.947917
16,2,5472.541667
16,2,3654.916667
16,2,1735.839286
16,2,2346.847826
16,2,2136.840000
16,2,1614.007353
16,2,2033.200000
16,2,2913.390957
16,2,1724.000000
16,2,3223.644231
16,2,1698.833333
16,2,2440.925000
16,2,2104.672619
16,2,9244.250000
16,2,1897.664062
16,2,19998.702128
16,2,3253.088983
16,2,1816.872500
16,2,1837.622642

16,2,2729.346154
16,2,2686.022727
16,2,2067.031250
16,2,3131.862500
16,2,1520.728723
16,2,1988.299419
16,2,2383.384615
16,2,34093.453571
16,2,1603.193452
16,2,2316.474432
16,2,1706.410256
16,2,3418.441860
16,2,3193.968023
16,2,57534.101695
16,2,1659.615132
16,2,1687.953488
16,2,1683.987500
16,2,1776.458333
16,2,2032.603774
16,2,2546.419118
16,2,8314.250000
16,2,1590.796875
16,2,2034.547500
16,2,1761.250000
16,2,1511.345395
16,2,1579.388889
16,2,2342.415816
16,2,1871.700000
16,2,3243.375000
16,2,2161.250000
16,2,3660.558511
16,2,2769.241477
16,2,2351.830000
16,2,1678.470588
16,2,1935.817500
16,2,1678.714286
16,2,1539.875000
16,2,3721.372973
16,2,3171.500000
16,2,4337.371429
16,2,3261.601562
16,2,2108.487805
16,2,4980.015625
16,2,1521.596154
16,2,1563.339623
16,2,1687.209459
16,2,2582.457547
16,2,1771.855769
16,2,2338.074468
16,2,1794.545455
16,2,1508.500000
16,2,1565.156250
16,2,4349.731544
16,2,2052.697917
16,2,1751.570652
16,2,1506.300000
16,2,1719.375000
16,2,1794.685976
16,2,2862.059524
16,2,2885.204545
16,2,5111.544643
16,2,1868.250000
16,2,2578.032609
16,2,3422.781915
16,2,10658.685976
16,2,1783.339674

16,2,36124.978571
16,2,1573.633721
16,2,1573.383929
16,2,1506.023438
16,2,1991.357143
16,2,2365.813725
16,2,1704.587209
16,2,2700.638889
16,2,1899.170213
16,2,2342.625000
16,2,2563.043605
16,2,2304.557927
16,2,1520.755208
16,2,1521.681818
16,2,1785.914062
16,2,1733.962766
16,2,1527.593750
16,2,2384.923077
16,2,5190.639706
16,2,1526.435000
16,2,3511.418182
16,2,1640.041667
16,2,1832.021277
16,2,1591.671875
16,2,1858.375000
16,2,3951.909091
16,2,5280.214286
16,2,1947.957500
16,2,3184.446809
16,2,4444.350543
16,2,1727.000000
16,2,1528.000000
16,2,2281.354545
16,2,2661.250000
16,2,2122.652778
16,2,1504.577128
16,2,2096.820000
16,2,1588.089286
16,2,1548.564189
16,2,1767.765625
16,2,2546.524038
16,2,7364.010417
16,2,1505.416667
16,2,2987.572500
16,2,2268.276596
16,2,2680.847973
16,2,4206.434211
16,2,2896.896739
16,2,1542.955882
16,2,6471.428571
16,2,2014.486486
16,2,2331.606383
16,2,2055.177885
16,2,1818.292683
16,2,1956.563679
16,2,2366.944149
16,2,3048.729592
16,2,1949.500000
16,2,1655.051887
16,2,3100.789474
16,2,1651.891892
16,2,1971.840426
16,2,2645.947368
16,2,1802.792763
16,2,1858.273437
16,2,1741.360000

16,2,1837.600000
16,2,1782.132075
16,2,1607.250000
16,2,2002.870000
16,2,2463.191038
16,2,19407.109375
16,2,1636.075000
16,2,2291.459184
16,2,1651.732143
16,2,2715.991071
16,2,2338.570312
16,2,7632.220930
16,2,3437.441176
16,2,130393.714286
16,2,1734.981707
16,2,3356.092391
16,2,2069.015000
16,2,49111.580882
16,2,3451.989796
16,2,1987.082317
16,2,17658.307692
16,2,3728.809211
16,2,2558.059375
16,2,1527.062500
16,2,1767.346875
16,2,2650.979651
16,2,2344.275000
16,2,2557.994681
16,2,1630.675676
16,2,3285.850806
16,2,2218.927083
16,2,2257.850000
16,2,2419.500000
16,2,3597.328947
16,2,1525.170000
16,2,1711.102778
16,2,3041.181818
16,2,1545.241279
16,2,1696.750000
16,2,1818.484043
16,2,3885.734375
16,2,2423.534884
16,2,1591.021739
16,2,3283.972826
16,2,2129.000000
16,2,3200.803571
16,2,3231.628676
16,2,2969.988889
16,2,2521.340909
16,2,1641.939189
16,2,2117.325521
16,2,1621.130952
16,2,2491.150568
16,2,3498.708333
16,2,1997.966667
16,2,2107.372340
16,2,2553.426471
16,2,2149.681818
16,2,2836.238281
16,2,2246.619048
16,2,5399.722222
16,2,1521.115385
16,2,4316.976744
16,2,1511.204082
17,1,1545.720588
17,1,42686.041667

17,1,36665.531250
17,1,15938.875000
17,1,55510.246324
17,1,82263.103125
17,1,134633.181818
17,1,1818.808333
17,1,4757.531915
17,1,1555.511364
17,1,3208.860795
17,1,2970.458333
17,1,6837.849432
17,1,59214.646739
17,1,9190.061047
17,1,105938.854651
17,1,1842.727941
17,1,1993.575000
17,1,15105.547619
17,1,3158.765625
17,1,4972.375000
17,1,4886.812500
17,1,1669.590909
17,1,1568.000000
17,1,1647.562500
17,1,1530.625000
17,1,3600.770833
17,1,37140.375000
17,1,2528.226190
17,1,3546.283333
17,1,1715.030405
17,1,93494.733974
17,1,1847.037234
17,1,2155.753571
17,1,1515.200000
17,1,1560.792763
17,1,1541.882979
17,1,2960.714286
17,1,1999.865385
17,1,1604.392442
17,1,1809.285714
17,1,1657.355556
17,1,1595.812500
17,1,6277.659091
17,1,102731.425000
17,1,4331.423387
17,1,4576.954082
17,1,1528.630682
17,1,42651.710526
17,1,1790.076923
17,1,3584.103723
17,1,1998.278409
17,1,29980.862069
17,1,2948.693452
17,1,6649.859375
17,1,3920.997449
17,1,4508.538462
17,1,2744.936275
17,1,2246.246875
17,1,1850.291667
17,1,2745.386364
17,1,1561.965909
17,1,25187.125000
17,1,89407.604167
17,1,10964.567568
17,1,1901.883721
17,1,2029.728261
17,1,1926.232558

17,1,2228.309524
17,1,42500.576087
17,1,9109.011905
17,1,40265.307692
17,1,2991.679688
17,1,128028.017442
17,1,1568.259146
17,1,99341.488372
17,1,27178.204545
17,1,6967.187500
17,1,6795.313953
17,1,4078.090909
17,1,6054.901316
17,1,1782.375000
17,1,4429.640625
17,1,35802.157895
17,1,14960.835526
17,1,4228.994681
17,1,1870.401786
17,1,8863.341912
17,1,1920.445652
17,1,17768.707317
17,1,9453.090426
17,1,1614.031250
17,1,15137.649194
17,1,2748.797794
17,1,27818.600000
17,1,1747.959302
17,1,83342.866279
17,1,17298.912791
17,1,1956.829545
17,1,1988.947222
17,1,1669.109756
17,1,2087.656977
17,1,2274.423077
17,1,1710.090909
17,1,65916.690789
17,1,5707.694149
17,1,50201.535714
17,1,5127.976064
17,1,1733.932692
17,1,2082.496951
17,1,2998.729167
17,1,10052.437500
17,1,1682.523810
17,1,60632.880682
17,1,1964.334197
17,1,3750.812500
17,1,5372.250000
17,1,6148.140244
17,1,96417.928571
17,1,1753.239865
17,1,2859.166667
17,1,62142.445122
17,1,4170.345238
17,1,95139.965909
17,1,1624.552083
17,1,2372.000000
17,1,10928.244444
17,1,86920.531250
17,1,1817.319149
17,1,1711.550000
17,1,5384.429688
17,1,110048.959459
17,1,1915.830882
17,1,117421.500000

17,1,2124.021875
17,1,1610.418605
17,1,45248.731707
17,1,60389.698370
17,1,2189.771341
17,1,45368.544872
17,1,45883.750000
17,1,2489.512195
17,1,4054.393617
17,1,1537.727273
17,1,1594.010135
17,1,1628.673295
17,1,1551.690789
17,1,2414.794872
17,1,116019.739583
17,1,3386.125000
17,1,1585.238636
17,1,2053.011111
17,1,68054.000000
17,1,1964.140625
17,1,2955.234756
17,1,1653.304348
17,1,1722.985795
17,1,1557.923913
17,1,2165.563953
17,1,2565.450000
17,1,3610.956522
17,1,20550.531915
17,1,24086.681250
17,1,1612.132353
17,1,152562.155660
17,1,2564.481383
17,1,2996.119565
17,1,2322.625000
17,1,3236.673469
17,1,129432.136364
17,1,12125.841463
17,1,73788.244898
17,1,29372.041667
17,1,142644.440217
17,1,1890.864583
17,1,129726.118590
17,1,7844.833333
17,1,41997.080882
17,1,3282.318750
17,1,2967.050000
17,1,2166.046512
17,1,1542.079861
17,1,1631.027174
17,1,4916.536585
17,1,78169.562500
17,1,1695.500000
17,1,56661.928571
17,1,18852.505208
17,1,5581.244898
17,1,2342.750000
17,1,1608.307692
17,1,1683.250000
17,1,3607.319149
17,1,4200.864130
17,1,2419.023256
17,1,3568.898256
17,1,3822.225000
17,1,93277.980000
17,1,6806.521739
17,1,1796.200000

17,1,2140.812500
17,1,125948.100000
17,1,2172.595395
17,1,2022.375000
17,1,1615.553571
17,1,2019.256098
17,1,27951.833333
17,1,1737.160714
17,1,37214.209677
17,1,1738.916667
17,1,21949.888889
17,1,1546.715909
17,1,2526.714286
17,1,36545.064189
17,1,2011.812500
17,1,2471.845588
17,1,52502.333333
17,1,40011.179687
17,1,2223.315789
17,1,2011.291667
17,1,2340.662791
17,1,2143.470930
17,1,7868.921053
17,1,1860.114583
17,1,124898.936111
17,1,1580.284884
17,1,101274.166667
17,1,4107.837500
17,1,2409.069767
17,1,1859.673469
17,1,1626.000000
17,1,3496.677632
17,1,11930.468085
17,1,1739.702381
17,1,6250.771341
17,1,2724.681818
17,1,49119.269231
17,1,2158.717391
17,1,174266.328125
17,1,1632.859756
17,1,3905.136628
17,1,2764.000000
17,1,151115.826923
17,1,8583.314189
17,1,3123.392857
17,1,5266.791667
17,1,65159.601974
17,1,2346.437500
17,1,8249.800000
17,1,10158.443878
17,1,2588.918919
17,1,1584.800000
17,1,1866.791667
17,1,2842.588235
17,1,83544.538462
17,1,2872.023256
17,1,1761.805233
17,1,7202.778571
17,1,64843.054054
17,1,8991.352941
17,1,3060.000000
17,1,2732.062500
17,1,23969.910256
17,1,1919.590116
17,1,70250.365854
17,1,2084.115385

17,1,29812.567073
17,1,1589.608696
17,1,23652.245968
17,1,115703.912500
17,1,4048.178571
17,1,113151.000000
17,1,1863.721154
17,1,1614.562500
17,1,1911.403846
17,1,5288.148810
17,1,1688.750000
17,1,88500.141892
17,1,5629.195122
17,1,1948.375000
17,1,1584.489362
17,1,2106.292614
17,1,115157.256250
17,1,1724.186047
17,1,5454.351064
17,1,8162.213235
17,1,1526.877976
17,1,62882.570946
17,1,61328.477941
17,1,2456.016667
17,1,1755.611111
17,1,29580.081081
17,1,1704.612500
17,1,4418.842262
17,1,2067.109756
17,1,9254.270833
17,1,44288.681818
17,1,5397.428191
17,1,11742.708333
17,1,2514.279762
17,1,114000.265306
17,1,1635.000000
17,1,2078.625000
17,1,15223.250000
17,1,102648.917763
17,1,2118.375000
17,1,3257.082237
17,1,142677.875000
17,1,2108.100000
17,1,2198.005952
17,1,4064.423313
17,1,3794.771341
17,1,117333.976190
17,1,5029.060976
17,1,3229.117021
17,1,90687.600000
17,1,58616.506250
17,1,1528.353659
17,1,5880.660000
17,1,39628.492647
17,1,2177.958333
17,1,116344.239865
17,1,2359.929487
17,1,17192.428571
17,1,9441.613208
17,1,2047.142857
17,1,2818.416667
17,1,2602.936170
17,1,5323.839286
17,1,1714.755102
17,1,118316.400000
17,1,2489.602273

17,1,2533.728125
17,1,2613.880952
17,1,122252.038043
17,1,12777.157895
17,1,1539.105263
17,1,2627.020000
17,1,9031.130952
17,1,1958.163889
17,1,22934.353659
17,1,4939.500000
17,1,3300.130435
17,1,142661.210526
17,1,27476.236111
17,1,5290.658163
17,1,1560.902174
17,1,1502.200000
17,1,4804.330000
17,1,1610.151786
17,1,22011.500000
17,1,1707.317073
17,1,156120.094512
17,1,46396.119318
17,1,157084.000000
17,1,4671.339286
17,1,24909.659722
17,1,192563.788889
17,1,1616.107955
17,1,8741.228261
17,1,1551.464286
17,1,1574.890306
17,1,1908.515625
17,1,54313.713235
17,1,2972.240385
17,1,114137.755208
17,1,1653.243902
17,1,1847.150000
17,1,3713.902439
17,1,2793.908537
17,1,1516.324324
17,1,15518.545732
17,1,57408.260638
17,1,1678.270270
17,1,1614.184783
17,1,40566.988971
17,1,1780.283088
17,1,3371.656250
17,1,3895.310811
17,1,3479.128676
17,1,3299.067073
17,1,1745.600000
17,1,125471.053571
17,1,1978.071429
17,1,10131.339286
17,1,3446.637755
17,1,41735.710526
17,1,1540.820652
17,1,107866.098039
17,1,1835.582317
17,1,8293.375000
17,1,5415.357143
17,1,2076.464286
17,1,1806.844444
17,1,3738.625000
17,1,55085.428030
17,1,20124.239130
17,1,3155.221939

17,1,53280.942073
17,1,1672.478261
17,1,2064.762821
17,1,2186.697368
17,1,1679.571429
17,1,2307.493056
17,1,44693.318182
17,1,1639.418605
17,1,2575.185976
17,1,2648.046512
17,1,3896.581250
17,1,5201.000000
17,1,2458.371711
17,1,1627.772727
17,1,2651.558824
17,1,31400.541667
17,1,5682.700000
17,1,2381.064189
17,1,16054.857143
17,1,1614.464286
17,1,4146.204545
17,1,27933.641026
17,1,123239.397222
17,1,3520.702128
17,1,69975.604167
17,1,42445.279070
17,1,8728.750000
17,1,2256.867647
17,1,111363.750000
17,1,2029.477941
17,1,4545.500000
17,1,33152.020000
17,1,3836.488095
17,1,153286.944444
17,1,1930.630952
17,1,3229.387821
17,1,1591.000000
17,1,38161.925000
17,1,1627.287234
17,1,89200.779762
17,1,29026.551724
17,1,1975.531915
17,1,7380.729730
17,1,112850.832237
17,1,1520.392157
17,1,1646.283784
17,1,191759.883929
17,1,7364.654762
17,1,1624.880952
17,1,1758.993548
17,1,85343.057692
17,1,2329.090909
17,1,5663.662162
17,1,44856.813953
17,1,50539.010135
17,1,16146.275000
17,1,1724.916667
17,1,84804.590000
17,1,75112.461538
17,1,1965.402027
17,1,96431.744444
17,1,2654.041667
17,1,1536.000000
17,1,84529.447917
17,1,5810.933511
17,1,4297.117647

17,1,3177.487805
17,1,1506.730978
17,1,2171.031250
17,1,2292.750000
17,1,2265.118590
17,1,1528.916667
17,1,2032.513158
17,1,133213.583333
17,1,1567.460938
17,1,80202.916667
17,1,1845.350000
17,1,2616.968750
17,1,1580.416667
17,1,5195.939024
17,1,2434.941176
17,1,5737.355556
17,1,2288.608696
17,1,2456.095745
17,1,3200.338235
17,1,1651.557143
17,1,8621.166667
17,1,2003.300000
17,1,36815.935811
17,1,2610.763889
17,1,2215.560976
17,1,32038.187500
17,1,2688.333333
17,1,7468.770833
17,1,1594.009146
17,1,85162.875000
17,1,2281.375000
17,1,26220.802326
17,1,1554.218085
17,1,12821.085938
17,1,29112.684211
17,1,3327.143750
17,1,3332.493750
17,1,3590.370000
17,1,158573.348837
17,1,2748.137500
17,1,12422.291667
17,1,4204.880952
17,1,36484.575758
17,1,2770.255435
17,1,2349.452381
17,1,106402.614865
17,1,55317.975610
17,1,1575.866279
17,1,1522.296875
17,1,42707.216216
17,1,2753.459302
17,1,2453.868590
17,1,4428.812500
17,1,2725.437500
17,1,39859.587209
17,1,4359.348837
17,1,1739.125000
17,1,2133.094595
17,1,14846.503289
17,1,2744.145270
17,1,64212.702703
17,1,5229.009868
17,1,35834.787234
17,1,3733.000000
17,1,2913.954545
17,1,3735.652174

17,1,4714.750000
17,1,4001.550000
17,1,107052.169118
17,1,1598.839286
17,1,1579.384615
17,1,1744.017442
17,1,5823.187500
17,1,1739.103448
17,1,1590.410714
17,1,108226.720238
17,1,1549.646875
17,1,47864.068182
17,1,1689.913043
17,1,43434.317073
17,1,3471.858871
17,1,3500.872222
17,1,1838.119681
17,1,151270.649457
17,1,164510.044118
17,1,1880.100000
17,1,4934.138889
17,1,1824.953125
17,1,1837.431034
17,1,88695.603723
17,1,52651.116667
17,1,1601.926829
17,1,2996.434211
17,1,1924.603659
17,1,3491.062500
17,1,33264.446875
17,1,89964.217742
17,1,3310.505952
17,1,76469.154255
17,1,1810.156250
17,1,2630.818182
17,1,1804.051829
17,1,4364.210227
17,1,18364.380952
17,1,1867.175000
17,1,2487.788462
17,1,4653.423913
17,1,9998.369565
17,1,7049.375000
17,1,1966.888889
17,1,131309.336957
17,1,1506.383152
17,1,8792.506250
17,1,4116.234375
17,1,2756.553571
17,1,36377.226562
17,1,76901.812500
17,1,4913.552632
17,1,43727.750000
17,1,27604.308140
17,1,16800.637255
17,1,5164.269231
17,1,4546.806818
17,1,1956.151163
17,1,10187.346939
17,1,91529.116071
17,1,2006.110294
17,1,97363.642857
17,1,43156.671875
17,1,161362.500000
17,1,2045.622222
17,1,9276.566327

17,1,2523.175676
17,1,12658.400000
17,1,1695.154070
17,1,2062.500000
17,1,13373.285714
17,1,2896.888889
17,1,1590.962766
17,1,18393.658333
17,1,5190.341912
17,1,1623.562500
17,1,161391.813953
17,1,49926.322368
17,1,85280.805556
17,1,3587.681122
17,1,113443.644231
17,1,3227.852941
17,1,63809.000000
17,1,194461.750000
17,1,4777.674419
17,1,4477.875000
17,1,1824.875000
17,1,2776.350000
17,1,3334.836735
17,1,7214.810811
17,1,2307.833333
17,1,1719.865854
17,1,2110.711111
17,1,92812.812500
17,1,41693.943182
17,1,2050.500000
17,1,1593.818182
17,1,3567.000000
17,1,8146.909091
17,1,8407.937500
17,1,3234.051282
17,1,2179.026786
17,1,4278.750000
17,1,46890.394444
17,1,6675.271739
17,1,3259.893229
17,1,17535.010000
17,1,3020.265625
17,1,4095.428571
17,1,4515.866071
17,1,23086.183333
17,1,182978.127273
17,1,1790.457143
17,1,2221.841463
17,1,1995.380952
17,1,2149.037500
17,1,1897.207317
17,1,1526.951220
17,1,2468.442308
17,1,3571.830882
17,1,5974.318750
17,1,49197.036765
17,1,2298.818182
17,1,2706.692308
17,1,2367.724359
17,1,3792.842391
17,1,187441.340000
17,1,2270.406250
17,1,23941.963235
17,1,3349.281915
17,1,2443.000000
17,1,3034.731707

17,1,2979.240909
17,1,5524.893617
17,1,18948.044643
17,1,1612.593750
17,1,137813.762500
17,1,3200.833333
17,1,5168.078125
17,1,2418.735294
17,1,1924.071429
17,1,2889.250000
17,1,175858.604651
17,1,1515.212766
17,1,4651.794872
17,1,57420.371951
17,1,123243.593750
17,1,3705.017045
17,1,3942.987805
17,1,1540.333333
17,1,34783.705882
17,1,2229.312500
17,1,59597.000000
17,1,1748.930921
17,1,56904.839286
17,1,2809.292683
17,1,4685.459302
17,1,1525.701531
17,1,74378.761364
17,1,1804.074405
17,1,2742.905405
17,1,179053.026042
17,1,2205.641026
17,1,3568.218085
17,1,11183.024038
17,1,77547.889706
17,1,16809.800000
17,1,1674.013158
17,1,1523.511628
17,1,2624.743902
17,1,1657.500000
17,1,3505.546512
17,1,126684.440789
17,1,4569.234043
17,1,23491.309524
17,1,37239.391892
17,1,3492.400000
17,1,5104.569767
17,1,14638.083333
17,1,1816.019608
17,1,21923.809524
17,1,5192.510135
17,1,52350.800000
17,1,1816.006250
17,1,5799.295455
17,1,38773.283784
17,1,4407.000000
17,1,10793.342949
17,1,5548.843750
17,1,3354.296512
17,1,14375.534884
17,1,1804.653846
17,1,1588.553191
17,1,71592.791667
17,1,2766.125000
17,1,11245.633333
17,1,3063.901786
17,1,2062.378378

17,1,12967.566667
17,1,5531.681818
17,1,3018.282895
17,1,1746.052083
17,1,185835.472826
17,1,2469.545455
17,1,133048.825000
17,1,1720.045213
17,1,3001.880000
17,1,2070.125000
17,1,1542.630435
17,1,4020.286765
17,1,16442.131410
17,1,11246.536585
17,1,3717.071875
17,1,51924.750000
17,1,3807.777778
17,1,2861.166667
17,1,20193.133065
17,1,11478.383333
17,1,1586.395349
17,1,16043.990385
17,1,3092.780405
17,1,172465.375000
17,1,3262.121622
17,1,1796.441860
17,1,22357.087121
17,1,1559.853659
17,1,1540.828571
17,1,1670.171512
17,1,13970.903061
17,1,3069.925000
17,1,64989.482143
17,1,4789.638889
17,1,5938.987500
17,1,2358.720588
17,1,11325.022222
17,1,9558.479730
17,1,1564.489130
17,1,2839.601744
17,1,73350.590909
17,1,76489.571429
17,1,5041.378049
17,1,2189.553571
17,1,1551.874251
17,1,4615.488372
17,1,1847.891892
17,1,1991.750000
17,1,1598.823864
17,1,3728.076389
17,1,40645.046154
17,1,7630.940476
17,1,1824.500000
17,1,4423.875000
17,1,17106.934211
17,1,2848.165698
17,1,2474.615385
17,1,5865.666667
17,1,3421.104730
17,1,12939.919118
17,1,3236.163265
17,1,155898.670455
17,1,1751.330882
17,1,84693.987500
17,1,79099.365854
17,1,8426.347826

17,1,5952.000000
17,1,110125.312500
17,1,86587.000000
17,1,4645.307692
17,1,2789.922872
17,1,1723.557692
17,1,34394.740196
17,1,4354.595588
17,1,32701.529412
17,1,5817.625000
17,1,1951.566667
17,1,1573.363636
17,1,1652.391667
17,1,3548.457386
17,1,1743.457386
17,1,83989.246711
17,1,3486.240000
17,1,6895.823529
17,1,71918.503676
17,1,2347.845109
17,1,1594.003676
17,1,2955.338235
17,1,1796.803977
17,1,105084.250000
17,1,1596.611702
17,1,3302.047297
17,1,2284.331081
17,1,2660.641509
17,1,2164.903125
17,1,1859.510638
17,1,4842.742857
17,1,5526.392857
17,1,41745.871212
17,1,5233.970109
17,1,1775.652174
17,1,1767.925000
17,1,2201.928977
17,1,4557.584459
17,1,3478.148936
17,1,1729.940789
17,1,1592.945946
17,1,199438.034091
17,1,2957.866848
17,1,14455.380814
17,1,47593.363636
17,1,2189.988372
17,1,1687.101974
17,1,162773.781915
17,1,44041.808511
17,1,1648.132353
17,1,27441.137931
17,1,159421.975610
17,1,2541.627907
17,1,81975.941489
17,1,1617.233108
17,1,1606.333333
17,1,1571.723214
17,1,56113.267857
17,1,1545.239583
17,1,6605.002907
17,1,2387.978723
17,1,14528.218750
17,1,3180.009615
17,1,1668.227941
17,1,10518.211538
17,1,1656.511905

17,1,5236.781250
17,1,3942.725610
17,1,2241.812500
17,1,7800.630435
17,1,2324.695122
17,1,3844.407895
17,1,2004.398649
17,1,3463.000000
17,1,31337.352941
17,1,2168.238636
17,1,1790.512195
17,1,2041.009615
17,1,10922.814189
17,1,50898.989865
17,1,1563.032738
17,1,1668.585366
17,1,2008.333333
17,1,5153.024390
17,1,1683.069767
17,1,1657.325000
17,1,6535.713415
17,1,17073.432432
17,1,30559.260638
17,1,41884.113636
17,1,10828.172619
17,1,1708.151316
17,1,1687.355978
17,1,2142.736486
17,1,6791.059524
17,1,2829.761905
17,1,3705.679487
17,1,2372.768293
17,1,1503.566176
17,1,3431.526316
17,1,1826.144737
17,1,83150.442857
17,1,4560.646341
17,1,2685.833333
17,1,1756.430233
17,1,2688.500000
17,1,2302.850000
17,1,1561.083333
17,1,3664.567500
17,1,4847.000000
17,1,81393.458333
17,1,4017.571429
17,1,3097.250000
17,1,58387.606061
17,1,2032.169118
17,1,2646.716216
17,1,3761.363281
17,1,1645.017857
17,1,2539.811321
17,1,11789.484756
17,1,1527.944444
17,1,4795.643617
17,1,4619.833333
17,1,1842.668750
17,1,1740.961538
17,1,53091.054878
17,1,1857.667553
17,1,2326.233173
17,1,1893.368421
17,1,2308.860119
17,1,5660.930147
17,1,101178.487179

17,1,3989.642857
17,1,2362.000000
17,1,69763.781250
17,1,3543.982558
17,1,36428.059783
17,1,1598.652174
17,1,2236.042969
17,1,64151.515244
17,1,29907.812500
17,1,2775.993243
17,1,71705.943878
17,1,32158.792857
17,1,3400.460366
17,1,4252.576923
17,1,3349.062500
17,1,3694.526596
17,1,43928.221154
17,1,57167.589744
17,1,2022.187500
17,1,6043.069079
17,1,5624.857143
17,1,57645.687500
17,1,68500.225000
17,1,2055.937500
17,1,1571.810484
17,1,2336.000000
17,1,5668.914634
17,1,2305.024306
17,1,1743.250000
17,1,1750.678571
17,1,1612.341463
17,1,2104.875000
17,1,79061.384615
17,1,8761.138514
17,1,3376.323529
17,1,185708.213415
17,1,3718.236413
17,1,73029.638889
17,1,3745.312500
17,1,88436.418750
17,1,12324.491071
17,1,2984.304878
17,1,2029.763889
17,1,62610.934783
17,1,99756.375000
17,1,3830.903846
17,1,3470.155405
17,1,2734.022727
17,1,7562.950000
17,1,4330.227273
17,1,11417.800000
17,1,25942.325000
17,1,1978.486486
17,1,3421.875000
17,1,2054.178191
17,1,34182.530000
17,1,12175.819444
17,1,22431.750000
17,1,23428.274306
17,1,1664.312500
17,1,1578.619792
17,1,145280.380952
17,1,7259.639706
17,1,8882.941860
17,1,7317.670455
17,1,2402.574468

17,1,4085.835938
17,1,45711.468750
17,1,2534.487805
17,1,3496.071429
17,1,2390.591463
17,1,1701.720000
17,1,11992.595238
17,1,33666.265957
17,1,1693.006410
17,1,11435.767442
17,1,1611.297794
17,1,2379.173077
17,1,1612.831250
17,1,11410.632576
17,1,6458.292614
17,1,2506.024390
17,1,1769.075758
17,1,2132.447115
17,1,12243.928571
17,1,20313.425676
17,1,68803.222222
17,1,2765.603659
17,1,19611.080357
17,1,58188.975610
17,1,159526.307692
17,1,13905.458333
17,1,1952.709239
17,1,1562.697115
17,1,77228.039634
17,1,11340.571429
17,1,5545.308824
17,1,43076.807432
17,1,5017.202381
17,1,2880.968750
17,1,2439.965625
17,1,5614.625000
17,1,2541.331633
17,1,59855.714286
17,1,7622.892157
17,1,83933.404762
17,1,1915.396739
17,1,145585.215625
17,1,2887.947581
17,1,2204.920673
17,1,7846.209677
17,1,2782.855769
17,1,79490.919872
17,1,44873.808594
17,1,5771.894737
17,1,53039.975000
17,1,18557.550000
17,1,4487.910156
17,1,2480.877778
17,1,2550.470930
17,1,139992.000000
17,1,152843.875000
17,1,1604.934524
17,1,2563.836735
17,1,1872.215909
17,1,39356.162162
17,1,3636.526786
17,1,35657.600000
17,1,1974.065476
17,1,2035.654412
17,1,99920.229730
17,1,4268.363971

17,1,5014.774457
17,1,2538.666667
17,1,10257.040816
17,1,71538.292553
17,1,1654.489362
17,1,4056.390625
17,1,144044.307692
17,1,154744.083333
17,1,48252.631250
17,1,1572.666667
17,1,2598.583333
17,1,4196.000000
17,1,24678.898256
17,1,82134.600000
17,1,86774.333333
17,1,2177.769886
17,1,2298.562500
17,1,1681.812500
17,1,1760.000000
17,1,82715.285714
17,1,2988.187500
17,1,1881.795918
17,1,1756.194444
17,1,157925.538462
17,1,4330.938596
17,1,1546.292857
17,1,1682.031915
17,1,130893.961538
17,1,16927.294643
17,1,1638.227273
17,1,4183.465909
17,1,2384.500000
17,1,1723.184896
17,1,1533.583333
17,1,4944.597973
17,1,4103.581395
17,1,6586.109375
17,1,1618.435714
17,1,3175.915761
17,1,154245.293750
17,1,117800.481618
17,1,42354.321429
17,1,2933.806250
17,1,1696.215426
17,1,64588.703947
17,1,3785.958333
17,1,64514.200000
17,1,16705.834270
17,1,1932.769444
17,1,12403.468750
17,1,55054.628571
17,1,5232.505682
17,1,1802.085106
17,1,73366.575658
17,1,4894.678571
17,1,6256.135135
17,1,1865.855263
17,1,90209.779412
17,1,2008.435811
17,1,4471.750000
17,1,3602.595588
17,1,1925.350000
17,1,2396.933333
17,1,1880.750000
17,1,56006.567708
17,1,2567.176471

17,1,113434.263158
17,1,26881.292683
17,1,4185.400000
17,1,10090.203947
17,1,2417.090909
17,1,2010.077381
17,1,62392.154545
17,1,25581.729167
17,1,10726.454545
17,1,2621.365132
17,1,1583.794118
17,1,10378.045213
17,1,115758.036585
17,1,2126.875000
17,1,2674.500000
17,1,145650.243902
17,1,33692.536585
17,1,2187.135135
17,1,2092.500000
17,1,1502.135417
17,1,55324.437500
17,1,1610.029412
17,1,116478.810976
17,1,3048.020833
17,1,3348.103261
17,1,2296.686224
17,1,1573.923077
17,1,1725.347973
17,1,91808.666667
17,1,1748.739130
17,1,3150.714286
17,1,82215.719512
17,1,179353.125000
17,1,10687.446809
17,1,110354.282609
17,1,1882.497340
17,1,61331.162162
17,1,184305.613095
17,1,8182.587209
17,1,2701.597826
17,1,2074.921875
17,1,8785.895349
17,1,58045.916667
17,1,22360.921875
17,1,4658.357143
17,1,61245.468750
17,1,2047.382143
17,1,3580.411765
17,1,51051.978659
17,1,2612.727564
17,1,2131.954545
17,1,1913.270833
17,1,2078.140625
17,1,2136.375000
17,1,54105.975610
17,1,1934.773256
17,1,10388.019397
17,1,1739.456522
17,1,75036.243243
17,1,103513.218750
17,1,35240.000000
17,1,21678.832031
17,1,45517.007812
17,1,4654.939024
17,1,90360.856383
17,1,7506.190476

17,1,7282.153846
17,1,2396.790698
17,1,1742.875000
17,1,1630.453488
17,1,68757.456250
17,1,4174.759146
17,1,26812.326923
17,1,73214.325658
17,1,3009.344444
17,1,14463.151163
17,1,6645.350000
17,1,3511.330556
17,1,1628.024390
17,1,85281.592949
17,1,1664.700000
17,1,2830.250000
17,1,48155.000000
17,1,4371.843023
17,1,34258.858108
17,1,1638.517857
17,1,3134.804348
17,1,122293.828125
17,1,58117.440972
17,1,129093.588889
17,1,1540.183673
17,1,9825.178344
17,1,1614.201087
17,1,89588.757143
17,1,1951.232558
17,1,2081.512821
17,1,3579.074074
17,1,6683.265152
17,1,111550.990385
17,1,22929.414474
17,1,87084.297619
17,1,3919.991848
17,1,23254.451705
17,1,175094.675000
17,1,5913.142857
17,1,1670.210106
17,1,42006.722222
17,1,2658.517857
17,1,1772.579268
17,1,1664.521739
17,1,8204.500000
17,1,28961.504032
17,1,3455.951087
17,1,166937.383152
17,1,1834.176136
17,1,3509.088710
17,1,3009.549419
17,1,2003.809524
17,1,36212.000000
17,1,1499.611111
17,1,2585.796875
17,1,16868.657895
17,1,60404.454545
17,1,126003.500000
17,1,2003.916667
17,1,12634.744565
17,1,3464.991071
17,1,1870.937500
17,1,47083.824324
17,1,2025.458333
17,1,62926.814286
17,1,6206.747449

17,1,1598.770833
17,1,21464.500000
17,1,23226.146552
17,1,1505.710106
17,1,1637.662500
17,1,3329.357143
17,1,1845.014205
17,1,26440.341216
17,1,1780.571429
17,1,11556.325000
17,1,20994.273148
17,1,3919.194444
17,1,4185.801724
17,1,2203.470588
17,1,2554.070652
17,1,1821.286765
17,1,1740.989130
17,1,1764.000000
17,1,2384.872283
17,1,5333.266667
17,1,61697.496622
17,1,14149.428977
17,1,100963.934783
17,1,51512.160256
17,1,10347.250000
17,1,1681.392857
17,1,4399.400000
17,1,8587.529605
17,1,1507.423077
17,1,2047.635135
17,1,17282.000000
17,1,2148.562500
17,1,2724.233333
17,1,3818.517857
17,1,2010.818750
17,1,72880.639706
17,1,1783.647059
17,1,5931.600000
17,1,29754.250000
17,1,2727.656250
17,1,1993.069853
17,1,1663.175000
17,1,70969.407895
17,1,1500.026786
17,1,3994.546875
17,1,84373.794118
17,1,2001.829861
17,1,2630.952381
17,1,3692.976563
17,1,4046.660714
17,1,2589.928571
17,1,1499.500000
17,1,1572.036585
17,1,2204.054688
17,1,114260.696429
17,1,100701.383721
17,1,169205.243902
17,1,15454.733333
17,1,3935.416667
17,1,1864.461111
17,1,1590.788462
17,1,1700.136905
17,1,6974.187500
17,1,25194.447368
17,1,2700.828947
17,1,2233.244792

17,1,34438.459677
17,1,43189.700000
17,1,66473.363636
17,1,1604.000000
17,1,3091.240385
17,1,1610.375000
17,1,15429.065789
17,1,3878.187500
17,1,92718.270270
17,1,88735.125000
17,1,140649.875000
17,1,2935.228571
17,1,2007.032609
17,1,1704.092105
17,1,1899.272222
17,1,1506.600000
17,1,7607.485294
17,1,26237.209302
17,1,37622.500000
17,1,2106.820513
17,1,2694.410714
17,1,3804.151163
17,1,2233.941176
17,1,13968.476190
17,1,14195.044118
17,1,3812.652778
17,1,5156.763889
17,1,1534.532258
17,1,9265.080000
17,1,1668.125000
17,1,22763.974359
17,1,73199.256757
17,1,1617.265625
17,1,4480.307692
17,1,2545.133333
17,1,15325.586538
17,1,13556.428571
17,1,2274.513889
17,1,3054.355556
17,1,1530.952381
17,1,1665.692308
17,1,4983.108974
17,1,2558.875000
17,1,63893.181818
17,1,125645.634146
17,1,63205.000000
17,1,1831.562500
17,1,8479.537791
17,1,1934.802632
17,1,145203.716667
17,1,5035.403226
17,1,1713.718085
17,1,62459.842105
17,1,2178.453488
17,1,1604.028571
17,1,7996.100000
17,1,1797.613971
17,1,2766.148936
17,1,1780.000000
17,1,3341.515152
17,1,5305.747222
17,1,3654.947368
17,1,2661.635870
17,1,50685.062500
17,1,79229.970930
17,1,6085.919643

17,1,38522.098485
17,1,82079.220588
17,1,1949.888298
17,1,2214.796053
17,1,3031.404070
17,1,13097.040698
17,1,2660.078125
17,1,2439.416667
17,1,2427.138889
17,1,3695.453947
17,1,50588.022059
17,1,2990.240385
17,1,1780.589286
17,1,2276.529070
17,1,1582.942073
17,1,3395.271277
17,1,4049.086957
17,1,3779.451389
17,1,8685.093023
17,1,4466.207447
17,1,44465.912879
17,1,41404.845395
17,1,73553.833333
17,1,3210.606250
17,1,1708.313889
17,1,2495.284574
17,1,162709.615385
17,1,1809.657738
17,1,95975.817308
17,1,123597.783854
17,1,2077.675595
17,1,109794.810000
17,1,150376.418750
17,1,5056.812500
17,1,33077.531250
17,1,1653.320122
17,1,6653.611111
17,1,2151.055556
17,1,48411.679054
17,1,119891.192308
17,1,58238.486842
17,1,136305.285714
17,1,3931.606771
17,1,128773.116279
17,1,1632.843750
17,1,9221.750000
17,1,1676.845588
17,1,5911.360465
17,1,9340.005319
17,1,1701.291429
17,1,6426.428571
17,1,5481.222561
17,1,158874.650000
17,1,1517.607143
17,1,9782.750000
17,1,18158.633333
17,1,45923.384375
17,1,2665.292763
17,1,1587.671875
17,1,1557.875000
17,1,93486.951220
17,1,3036.000000
17,1,8713.791667
17,1,2691.687500
17,1,3235.250000
17,1,1771.813889

17,1,1668.470588
17,1,43069.750000
17,1,49731.171053
17,1,1648.567308
17,1,2211.393750
17,1,2379.482143
17,1,2130.388889
17,1,89011.800000
17,1,1842.000000
17,1,11738.593750
17,1,4826.644737
17,1,166884.909884
17,1,3458.409091
17,1,1538.458333
17,1,4445.183333
17,1,3575.212500
17,1,163287.734043
17,1,58218.006944
17,1,2325.975000
17,1,1544.970588
17,1,1664.500000
17,1,2001.854167
17,1,1636.317073
17,1,2181.318750
17,1,1515.734375
17,1,1967.615000
17,1,48769.012195
17,1,2781.187500
17,1,2507.703947
17,1,1865.617647
17,1,1586.698171
17,1,4874.488636
17,1,2295.388514
17,1,40722.698171
17,1,2529.954545
17,1,5445.101351
17,1,4190.166667
17,1,1993.000000
17,1,2187.049419
17,1,1535.250000
17,1,2095.014205
17,1,52161.098485
17,1,45883.461538
17,1,52437.318182
17,1,2306.683333
17,1,1764.000000
17,1,3421.298295
17,1,1625.028226
17,1,11199.812500
17,1,2317.512821
17,1,2011.882979
17,1,4488.243902
17,1,2863.755682
17,1,3106.400000
17,1,2398.862500
17,1,1945.263158
17,1,23045.416667
17,1,1607.384615
17,1,20137.297222
17,1,1959.178571
17,1,23568.250000
17,1,59828.284091
17,1,2332.250000
17,1,147587.240000
17,1,2613.750000
17,1,54849.218750

17,1,127439.662791
17,1,1818.380952
17,1,41605.780488
17,1,129702.846354
17,1,2740.546875
17,1,3453.250000
17,1,1727.461538
17,1,2076.025641
17,1,1880.104167
17,1,2568.529412
17,1,3548.544872
17,1,4060.826087
17,1,1739.852941
17,1,58186.705357
17,1,2112.875000
17,1,150855.256410
17,1,2082.461538
17,1,2701.734375
17,1,5655.979167
17,1,2247.691176
17,1,1586.032051
17,1,10237.902439
17,1,149653.418605
17,1,5824.523026
17,1,4669.568182
17,1,1868.931548
17,1,1568.688889
17,1,1653.022059
17,1,1923.116071
17,1,5207.920732
17,1,1653.192708
17,1,1677.212209
17,1,2374.250000
17,1,3634.500000
17,1,2435.500000
17,1,159490.142045
17,1,9565.460938
17,1,2271.091463
17,1,1876.277439
17,1,19269.397059
17,1,1756.573864
17,1,1932.500000
17,1,1729.832317
17,1,2607.363636
17,1,1684.822368
17,1,84095.276316
17,1,1684.633333
17,1,3821.203125
17,1,2295.666667
17,1,61623.976744
17,1,33352.083333
17,1,8920.781250
17,1,103835.388158
17,1,1619.452206
17,1,1746.562500
17,1,2105.818452
17,1,1579.476744
17,1,5846.729730
17,1,47014.181818
17,1,1710.142857
17,1,5892.817708
17,1,3813.014706
17,1,1822.023810
17,1,35093.296429
17,1,8747.571429
17,1,1771.186224

17,1,2963.403409
17,1,87079.283784
17,1,1506.536585
17,1,60982.562500
17,1,86791.851351
17,1,8073.946809
17,1,27905.398649
17,1,3759.580882
17,1,7127.505952
17,1,10540.359375
17,1,1563.153409
17,1,1817.744681
17,1,6472.410714
17,1,5501.330882
17,1,1592.760204
17,1,1576.864865
17,1,49341.653846
17,1,3062.660714
17,1,8106.645833
17,1,4809.415094
17,1,1890.750000
17,1,1586.911184
17,1,3412.307927
17,1,93966.976744
17,1,2035.618421
17,1,2014.963235
17,1,3801.801471
17,1,1590.764881
17,1,124696.272727
17,1,41095.784091
17,1,50161.819149
17,1,1930.937500
17,1,9290.058140
17,1,2364.073171
17,1,5663.634615
17,1,71362.635417
17,1,5383.657143
17,1,3810.125000
17,1,1911.600000
17,1,63857.927632
17,1,3683.071429
17,1,2115.675595
17,1,3539.965426
17,1,3874.642857
17,1,1828.473214
17,1,1734.281250
17,1,88473.880208
17,1,6821.674419
17,1,71898.378378
17,1,1773.015625
17,1,10752.419118
17,1,3294.630814
17,1,7680.750000
17,1,7338.656250
17,1,3345.189024
17,1,39530.973958
17,1,4611.310714
17,1,1693.478659
17,1,21982.527778
17,1,91830.674342
17,1,1502.469388
17,1,1738.861413
17,1,5199.884615
17,1,4175.188776
17,1,3432.850000
17,1,3215.076531

17,1,1947.965625
17,1,3899.285714
17,1,3469.356383
17,1,1515.234375
17,1,2051.448980
17,1,1665.654762
17,1,17250.500000
17,1,4563.188953
17,1,1711.125000
17,1,46460.013889
17,1,4039.583333
17,1,2983.957447
17,1,3234.930851
17,1,3537.197917
17,1,125641.538462
17,1,1599.656250
17,1,13960.488095
17,1,33964.339286
17,1,110888.356771
17,1,18051.816327
17,1,106254.525568
17,1,8310.650000
17,1,2268.733333
17,1,2733.000000
17,1,5978.091463
17,1,1811.638889
17,1,90942.677632
17,1,2047.265625
17,1,28308.321429
17,1,4918.882353
17,1,101714.328947
17,1,7711.192308
17,1,97534.177632
17,1,3195.865854
17,1,6267.929348
17,1,99303.009615
17,1,45902.625000
17,1,30133.018519
17,1,6541.308140
17,1,1695.709302
17,1,2726.926136
17,1,93848.702703
17,1,3708.293750
17,1,2002.981250
17,1,2344.612805
17,1,5367.782609
17,1,1644.562500
17,1,31987.242857
17,1,1608.026786
17,1,70420.175595
17,1,15390.436224
17,1,1746.796875
17,1,5090.190217
17,1,6775.266447
17,1,4966.871324
17,1,8038.715000
17,1,9156.280488
17,1,1524.787234
17,1,2413.816667
17,1,2654.648438
17,1,59245.228571
17,1,56360.695312
17,1,1918.975610
17,1,1607.527439
17,1,8763.365854
17,1,3542.082237

17,1,2173.593750
17,1,6663.414474
17,1,1688.575000
17,1,1809.380952
17,1,1842.267500
17,1,1918.701923
17,1,3377.460366
17,1,108604.650568
17,1,2668.750000
17,1,63521.416667
17,1,1770.306122
17,1,5264.949324
17,1,80361.544444
17,1,67740.750000
17,1,1525.087209
17,1,62407.095238
17,1,1802.388514
17,1,7437.000000
17,1,2044.092391
17,1,4640.696429
17,1,5274.405488
17,1,4246.628788
17,1,2507.071429
17,1,64092.969697
17,1,1740.815217
17,1,11076.250000
17,1,13623.393617
17,1,40988.540541
17,1,155538.916667
17,1,1819.000000
17,1,51363.135714
17,1,51748.696970
17,1,28248.120833
17,1,20419.916667
17,1,1693.304487
17,1,1530.105556
17,1,5005.026316
17,1,2219.219512
17,1,2833.341463
17,1,38127.375000
17,1,194424.772727
17,1,1763.884146
17,1,31319.702206
17,1,18751.828125
17,1,32095.416667
17,1,20324.571429
17,1,6749.510204
17,1,1544.165541
17,1,147044.143750
17,1,83070.909091
17,1,6660.656250
17,1,1909.995192
17,1,8788.537162
17,1,2585.562500
17,1,4816.103723
17,1,1838.894737
17,1,5007.550000
17,1,1522.250000
17,1,1658.380952
17,1,1564.783163
17,1,1579.892857
17,1,74784.789474
17,1,1917.383929
17,1,1609.571429
17,1,108408.828125
17,1,2536.750000

17,1,2763.328947
17,1,1558.375000
17,1,1603.368590
17,1,1633.303571
17,1,61805.449324
17,1,71756.650735
17,1,7545.926136
17,1,7283.523810
17,1,7048.138889
17,1,2377.112745
17,1,15870.362500
17,1,1575.539894
17,1,7056.159375
17,1,3372.678571
17,1,1538.933333
17,1,1740.375000
17,1,27609.500000
17,1,2475.326923
17,1,62781.135135
17,1,2332.542969
17,1,1678.875000
17,1,3596.311170
17,1,2314.968750
17,1,17579.971875
17,1,3614.562500
17,1,6235.170213
17,1,3840.316406
17,1,1986.750000
17,1,1500.511628
17,1,81270.270833
17,1,4787.951923
17,1,4234.487179
17,1,3307.375000
17,1,1707.400000
17,1,25701.071970
17,1,2809.000000
17,1,2533.130102
17,1,1551.931818
17,1,35729.769231
17,1,16353.895833
17,1,1744.416667
17,1,17979.791667
17,1,6125.762500
17,1,4080.689103
17,1,2360.611842
17,1,9427.934426
17,1,9790.153409
17,1,1883.820312
17,1,3283.571429
17,1,8049.884615
17,1,2111.164894
17,1,3601.640625
17,1,1726.178571
17,1,4370.312500
17,1,2700.250000
17,1,1881.061111
17,1,2677.978723
17,1,19236.058824
17,1,2675.157895
17,1,3650.750000
17,1,3824.718750
17,1,2303.917683
17,1,1553.084559
17,1,1877.250000
17,1,61495.220588
17,1,56041.058824

17,1,1598.066667
17,1,1753.393750
17,1,23215.525735
17,1,1901.607143
17,1,55702.000000
17,1,33598.814516
17,1,1607.532895
17,1,1539.548913
17,1,58453.168919
17,1,1826.677632
17,1,1844.066667
17,1,4332.246951
17,1,4059.103659
17,1,69459.698529
17,1,54739.287162
17,1,1990.547872
17,1,2647.091667
17,1,102056.423077
17,1,1782.542553
17,1,3541.452381
17,1,2537.407895
17,1,1507.000000
17,1,2131.079268
17,1,2235.444079
17,1,1709.244898
17,1,5048.625000
17,1,11505.130319
17,1,4585.489583
17,1,26623.750000
17,1,2232.222222
17,1,2019.742857
17,1,1737.572115
17,1,1860.040000
17,1,74656.087838
17,1,4055.242500
17,1,2065.875000
17,1,4461.333333
17,1,4218.231250
17,1,16723.138158
17,1,1502.424242
17,1,176853.906977
17,1,2962.204861
17,1,2461.315789
17,1,1817.000000
17,1,23627.029605
17,1,86754.569444
17,1,40290.243243
17,1,1979.892045
17,1,4253.218750
17,1,4125.071429
17,1,6054.829268
17,1,36306.264535
17,1,1570.390625
17,1,1547.628125
17,1,2227.875000
17,1,108010.000000
17,1,2432.046875
17,1,3791.565789
17,1,21731.297619
17,1,3202.395270
17,1,32764.112500
17,1,2103.364286
17,1,1589.765244
17,1,51058.019531
17,1,2005.530612
17,1,5877.153846

17,1,8030.500000
17,1,3199.621711
17,1,1526.895833
17,1,172209.514706
17,1,3164.590426
17,1,87987.768293
17,1,1836.318182
17,1,2152.710106
17,1,3930.375000
17,1,3121.457447
17,1,13220.760638
17,1,87230.871795
17,1,2072.010638
17,1,2656.182143
17,1,33363.625000
17,1,6708.555233
17,1,33384.481618
17,1,4801.986486
17,1,76405.344340
17,1,5359.437500
17,1,1842.939189
17,1,3349.779412
17,1,1514.356383
17,1,1508.673469
17,1,43599.815476
17,1,6276.428571
17,1,1791.959459
17,1,81580.362500
17,1,3618.651163
17,1,1636.506579
17,1,2171.200000
17,1,9205.682292
17,1,21803.318182
17,1,1549.750000
17,1,2203.475000
17,1,47927.525735
17,1,9195.500000
17,1,40937.231707
17,1,2427.137097
17,1,3142.775510
17,1,1503.074324
17,1,3789.557692
17,1,46042.621622
17,1,1526.601562
17,1,1723.357143
17,1,41480.260135
17,1,18236.023810
17,1,180555.250000
17,1,1567.183511
17,1,2146.452381
17,1,8377.024390
17,1,56423.726974
17,1,146875.150000
17,1,5079.500000
17,1,3684.062500
17,1,2357.170732
17,1,4238.671196
17,1,1608.088889
17,1,1627.989796
17,1,2718.522222
17,1,60828.262500
17,1,4318.207317
17,1,50322.558824
17,1,29192.231618
17,1,2039.535714
17,1,1887.297872

17,1,127863.937500
17,1,2824.988095
17,1,54214.640244
17,1,1721.844444
17,1,1771.985714
17,1,17203.572581
17,1,8218.442708
17,1,62594.284375
17,1,4013.776786
17,1,4070.116667
17,1,1783.892857
17,1,4432.500000
17,1,1892.591837
17,1,42991.000000
17,1,6180.718750
17,1,121484.656250
17,1,8667.852273
17,1,1510.500000
17,1,3930.326087
17,1,3574.764706
17,1,1705.341463
17,1,27461.493421
17,1,32137.525000
17,1,1518.636905
17,1,1516.090909
17,1,25309.641026
17,1,1680.693452
17,1,1531.861842
17,1,3536.953488
17,1,2496.037500
17,1,2657.717949
17,1,172084.450000
17,1,17613.271341
17,1,116072.426829
17,1,34621.042857
17,1,21502.777778
17,1,1804.953488
17,1,187910.186441
17,1,7264.041667
17,1,3752.184211
17,1,2487.000000
17,1,3545.861413
17,1,2726.744898
17,1,3746.438776
17,1,1573.324468
17,1,69981.349432
17,1,43878.189286
17,1,1819.243902
18,2,4796.763598
18,2,3455.658537
18,2,1530.622222
18,2,2950.821429
18,2,4237.211538
18,2,8938.139535
18,2,2584.950581
18,2,6862.562500
18,2,1556.591398
18,2,3122.432692
18,2,20252.323529
18,2,3188.180556
18,2,1650.705128
18,2,4878.206250
18,2,6290.484043
18,2,4191.298077
18,2,3677.223776
18,2,1757.825521

```
18,2,3866.805714
18,2,4715.250000
18,2,2547.040698
18,2,4337.000000
18,2,20104.736979
18,2,1515.865854
18,2,7872.400000
18,2,4368.427326
18,2,2584.175000
18,2,2630.463362
18,2,2127.500000
18,2,3119.127717
18,2,1856.439024
18,2,8473.559091
18,2,5103.789474
18,2,2539.958333
18,2,1872.877358
18,2,3103.406915
18,2,3337.076923
18,2,1637.750000
18,2,2558.437500
18,2,2353.068396
18,2,3666.015625
18,2,3985.191327
18,2,3255.573171
18,2,31062.893293
18,2,2916.384615
18,2,4129.125000
18,2,2025.316964
18,2,2951.573171
18,2,4236.870192
18,2,2027.858108
18,2,15759.990741
18,2,33464.057500
18,2,1659.326923
18,2,7535.253731
18,2,4219.438725
18,2,4994.291667
18,2,1821.478261
18,2,2260.785714
18,2,2945.790698
18,2,3871.520000
18,2,1759.086957
18,2,5436.841667
18,2,2056.152778
18,2,6284.471698
18,2,1865.364865
18,2,4276.212766
18,2,1535.765625
18,2,2765.392857
18,2,6179.505208
18,2,2930.081818
18,2,2508.793478
18,2,11694.899038
18,2,2728.512195
18,2,2621.078431
18,2,2686.754545
18,2,3787.243902
18,2,2258.861111
18,2,2766.101562
18,2,7707.083333
18,2,1575.996429
18,2,3618.066327
18,2,1512.954545
18,2,1739.319149
18,2,1755.480769
```

18,2,9761.434783
18,2,2171.296875
18,2,2039.500000
18,2,2291.285714
18,2,15338.446809
18,2,1883.142857
18,2,5497.343750
18,2,1630.364583
18,2,1585.347561
18,2,4429.038462
18,2,1509.602941
18,2,1899.871795
18,2,2960.916667
18,2,2702.913462
18,2,4388.765957
18,2,1722.066667
18,2,1983.648810
18,2,2623.236111
18,2,6190.734043
18,2,1547.081250
18,2,12138.510204
18,2,6875.231771
18,2,6214.385135
18,2,3607.714286
18,2,2345.139241
18,2,6504.117647
18,2,10344.491228
18,2,16725.250000
18,2,5070.000000
18,2,3684.279412
18,2,3558.180328
18,2,2160.279661
18,2,3080.928571
18,2,3670.950000
18,2,1912.057065
18,2,5968.367021
18,2,3554.566327
18,2,2759.563776
18,2,1609.538043
18,2,7154.814371
18,2,2033.046875
18,2,6634.614525
18,2,1531.706522
18,2,2084.965517
18,2,2549.818182
18,2,2468.838235
18,2,4971.351744
18,2,3687.174528
18,2,2334.386905
18,2,1965.065972
18,2,2804.643293
18,2,1891.312500
18,2,1554.416667
18,2,1827.079787
18,2,3912.437500
18,2,1868.392045
18,2,3087.203125
18,2,1778.260135
18,2,2502.105556
18,2,8914.831081
18,2,21439.204082
18,2,2793.241379
18,2,6225.366379
18,2,1801.283019
18,2,1503.140625
18,2,1750.034091

18,2,6221.781250
18,2,5384.837838
18,2,2397.118056
18,2,4330.422043
18,2,2466.318182
18,2,1926.127551
18,2,2750.535714
18,2,5915.121951
18,2,7292.447674
18,2,2864.411458
18,2,5454.900000
18,2,5847.553571
18,2,2107.277439
18,2,5038.686275
18,2,2164.950000
18,2,2276.714286
18,2,2175.170732
18,2,4395.278509
18,2,6179.392857
18,2,3135.622396
18,2,2161.205729
18,2,4183.993750
18,2,3923.715426
18,2,9452.525568
18,2,1667.928571
18,2,9089.906250
18,2,7279.372727
18,2,6037.014881
18,2,20279.905738
18,2,4534.076087
18,2,4388.267157
18,2,3373.527027
18,2,1797.878049
18,2,13365.435000
18,2,1710.358696
18,2,2666.982558
18,2,3196.210526
18,2,7562.230769
18,2,6096.000000
18,2,10052.592233
18,2,5042.279412
18,2,4335.711957
18,2,3068.080851
18,2,2046.428571
18,2,3080.252688
18,2,2228.236842
18,2,4060.125000
18,2,6408.000000
18,2,2214.214286
18,2,1654.752336
18,2,42734.365471
18,2,1692.000000
18,2,3452.302419
18,2,2469.853571
18,2,1680.000000
18,2,3001.469512
18,2,6193.226974
18,2,2864.663043
18,2,14642.684211
18,2,1523.965116
18,2,2147.354167
18,2,2622.869565
18,2,1550.666667
18,2,2520.970000
18,2,3186.589674
18,2,4291.558140

18,2,2470.377778
18,2,1946.343750
18,2,1818.858553
18,2,1784.320313
18,2,7436.693878
18,2,2963.995283
18,2,3539.088889
18,2,3494.774390
18,2,6606.872340
18,2,2374.020349
18,2,1748.750000
18,2,5111.881579
18,2,6519.716216
18,2,2511.980769
18,2,2360.086735
18,2,3363.531915
18,2,3523.490741
18,2,3322.519231
18,2,1919.000000
18,2,3506.821429
18,2,1899.675676
18,2,1643.312500
18,2,9019.000000
18,2,3375.860465
18,2,12619.868852
18,2,1956.161058
18,2,3307.554878
18,2,2229.956835
18,2,5145.105263
18,2,4319.565217
18,2,1685.875000
18,2,1953.666667
18,2,1533.614130
18,2,2282.650000
18,2,3025.203488
18,2,2935.517857
18,2,2060.448980
18,2,1560.875000
18,2,3495.444079
18,2,1576.676471
18,2,5470.000000
18,2,3494.417453
18,2,13793.804878
18,2,3380.976562
18,2,2069.291667
18,2,3880.194444
18,2,2337.577778
18,2,13740.158019
18,2,1760.698718
18,2,2399.432432
18,2,8126.462963
18,2,5128.282051
18,2,5362.156250
18,2,3545.720000
18,2,2147.464286
18,2,3030.652174
18,2,3385.115385
18,2,3253.850962
18,2,5029.750000
18,2,2038.009615
18,2,6331.923913
18,2,1838.377551
18,2,1501.859375
18,2,2306.794643
18,2,8178.915698
18,2,2309.737805

18,2,4916.051887
18,2,1746.852941
18,2,13308.611607
18,2,6638.523810
18,2,4837.079787
18,2,5544.437500
18,2,2037.046512
18,2,1837.720588
18,2,1984.435811
18,2,2160.775000
18,2,4489.538462
18,2,5448.833333
18,2,4265.744048
18,2,2593.847222
18,2,2504.466216
18,2,2460.040541
18,2,9791.240566
18,2,2754.593750
18,2,2490.741071
18,2,1969.976562
18,2,9535.768041
18,2,2101.139241
18,2,2359.923387
18,2,2765.250000
18,2,1862.917763
18,2,2849.000000
18,2,2980.212291
18,2,7560.995192
18,2,1722.937500
18,2,4170.961538
18,2,3182.333333
18,2,1651.550000
18,2,2844.372222
18,2,1661.715278
18,2,1821.062500
18,2,4222.882813
18,2,1991.389706
18,2,2853.798077
18,2,2526.098039
18,2,3589.806122
18,2,9044.135638
18,2,2286.090909
18,2,3982.889706
18,2,4206.880814
18,2,2246.715232
18,2,5674.965909
18,2,2267.163265
18,2,2231.288889
18,2,21100.187500
18,2,2603.633333
18,2,1711.345395
18,2,1993.085366
18,2,2262.465608
18,2,15797.555556
18,2,4861.346939
18,2,2458.856250
18,2,7940.339286
18,2,2131.025000
18,2,5252.083333
18,2,2018.794872
18,2,2847.506579
18,2,10916.264706
18,2,1623.270270
18,2,3692.573661
18,2,1559.875000
18,2,1714.531818

18,2,4000.281250
18,2,2245.869565
18,2,1797.292614
18,2,3279.100000
18,2,4931.400000
18,2,3393.428571
18,2,7707.061475
18,2,3140.918367
18,2,4973.540541
18,2,2306.746951
18,2,3205.448276
18,2,6197.296196
18,2,7244.481618
18,2,6853.388889
18,2,6272.153846
18,2,7963.670455
18,2,2593.591837
18,2,1959.363636
18,2,3841.421053
18,2,2159.796875
18,2,6179.428571
18,2,1612.265625
18,2,1933.700000
18,2,2560.390244
18,2,4325.095000
18,2,19989.725543
18,2,4101.470588
18,2,1625.955128
18,2,6869.078431
18,2,3090.571429
18,2,14437.044118
18,2,1972.651163
18,2,2122.406452
18,2,1707.607143
18,2,3605.875000
18,2,7764.452830
18,2,4966.044118
18,2,7199.693182
18,2,1833.390000
18,2,1562.330882
18,2,3603.309896
18,2,3513.764706
18,2,6172.909091
18,2,6932.525281
18,2,2709.972222
18,2,52499.770833
18,2,1981.035714
18,2,1555.290625
18,2,3334.824324
18,2,23446.622642
18,2,6938.443750
18,2,1613.809524
18,2,2424.722222
18,2,5723.569767
18,2,8756.958333
18,2,3428.817708
18,2,3329.975000
18,2,1940.791667
18,2,2701.614865
18,2,2086.111842
18,2,10594.442308
18,2,1802.750000
18,2,3106.989130
18,2,2358.502717
18,2,3187.525000
18,2,4864.278846

18,2,3470.804878
18,2,2640.125000
18,2,1574.975610
18,2,4616.456522
18,2,5202.000000
18,2,1528.890625
18,2,3486.244898
18,2,8219.937500
18,2,3241.513021
18,2,9332.101961
18,2,6773.914692
18,2,6630.632500
18,2,6558.772727
18,2,5011.864286
18,2,1619.750000
18,2,12811.656250
18,2,3051.738208
18,2,1598.357143
18,2,1811.747664
18,2,1749.017857
18,2,1788.914894
18,2,2779.031915
18,2,3936.000000
18,2,1690.967949
18,2,22852.724138
18,2,2010.268868
18,2,2040.125000
18,2,4611.413265
18,2,1842.311321
18,2,1906.437500
18,2,2098.179348
18,2,1845.795455
18,2,2165.581731
18,2,5049.927632
18,2,4717.764286
18,2,4475.005556
18,2,2029.232759
18,2,6824.377660
18,2,4534.648026
18,2,2166.041667
18,2,1684.540698
18,2,1607.116279
18,2,14749.833333
18,2,3610.678571
18,2,1974.103448
18,2,5357.347826
18,2,1915.457055
18,2,5756.318182
18,2,2626.170000
18,2,2736.250000
18,2,4558.697674
18,2,3251.512195
18,2,5071.090909
18,2,2355.538462
18,2,2502.052632
18,2,11374.716418
18,2,3036.130208
18,2,4397.625000
18,2,3097.012821
18,2,3520.643293
18,2,1633.657609
18,2,1867.029762
18,2,4544.075000
18,2,7465.592105
18,2,3550.854651
18,2,3103.025000

18,2,5337.806818
18,2,1594.078488
18,2,4514.060096
18,2,5001.300000
18,2,3261.489130
18,2,10758.243182
18,2,3129.442073
18,2,1801.937500
18,2,1568.157895
18,2,2476.197368
18,2,9898.319149
18,2,3016.972222
18,2,2435.086735
18,2,1722.520270
18,2,2049.557500
18,2,5476.915761
18,2,3095.051630
18,2,2471.650568
18,2,2205.382812
18,2,4217.375000
18,2,1956.166667
18,2,3049.375000
18,2,5308.311111
18,2,2186.477273
18,2,2211.415094
18,2,5760.452830
18,2,5023.493590
18,2,4985.845000
18,2,3833.296296
18,2,1810.434783
18,2,12152.603261
18,2,3518.075000
18,2,10834.818182
18,2,2453.156863
18,2,1971.463415
18,2,3957.227941
18,2,1758.355114
18,2,4855.076923
18,2,2145.975610
18,2,2771.392857
18,2,1984.767857
18,2,1609.137500
18,2,1603.272727
18,2,2547.021277
18,2,4154.837264
18,2,2857.950000
18,2,2007.196023
18,2,2390.515909
18,2,2682.845109
18,2,4478.769231
18,2,2332.395833
18,2,2991.723958
18,2,2498.012821
18,2,1656.034091
18,2,4209.446429
18,2,5960.109589
18,2,1758.894737
18,2,2136.993243
18,2,1916.100446
18,2,2577.232500
18,2,2072.850962
18,2,5134.622159
18,2,3800.066038
18,2,1590.507653
18,2,2983.264398
18,2,5868.468750

18,2,5813.051471
18,2,7098.250000
18,2,4399.572917
18,2,5147.739130
18,2,1679.280488
18,2,2056.954545
18,2,2774.250000
18,2,3754.000000
18,2,6093.071429
18,2,3176.478261
18,2,8415.782051
18,2,3864.430556
18,2,2588.575472
18,2,1979.833333
18,2,2054.803571
18,2,4648.625000
18,2,2101.943182
18,2,2957.551887
18,2,6548.136905
18,2,1980.085526
18,2,1641.000000
18,2,3325.938889
18,2,3552.611111
18,2,1778.178571
18,2,1981.545918
18,2,9463.382979
18,2,6688.728571
18,2,2976.111842
18,2,2169.800000
18,2,2264.069444
18,2,5878.013393
18,2,2525.180233
18,2,2168.898026
18,2,2550.521341
18,2,2801.295918
18,2,20107.200000
18,2,3998.107143
18,2,2164.512821
18,2,3482.797872
18,2,1880.200000
18,2,6373.166667
18,2,1704.606061
18,2,2161.615854
18,2,1650.187500
18,2,4561.625000
18,2,2911.750000
18,2,1557.592105
18,2,1631.994048
18,2,2395.767045
18,2,1872.875000
18,2,2975.357143
18,2,5505.182432
18,2,4298.101064
18,2,3805.140884
18,2,2606.000000
18,2,1823.781250
18,2,6606.011111
18,2,5015.467593
18,2,4158.173077
18,2,4021.458333
18,2,3544.426136
18,2,7668.462121
18,2,3496.011628
18,2,2266.680851
18,2,3982.397727
18,2,1986.765625

18,2,8800.519022
18,2,1588.932500
18,2,2939.308962
18,2,2582.839674
18,2,4022.252315
18,2,4667.089286
18,2,1699.198675
18,2,3011.833333
18,2,4878.153670
18,2,1639.349138
18,2,1631.171875
18,2,1921.358696
18,2,4571.558140
18,2,8041.465517
18,2,2536.065625
18,2,1576.600610
18,2,5452.529018
18,2,6338.648649
18,2,2482.090909
18,2,1793.448529
18,2,3515.119565
18,2,3034.793478
18,2,5841.625000
18,2,11126.771186
18,2,2107.264205
18,2,6150.394737
18,2,1653.027027
18,2,3294.116667
18,2,4715.576923
18,2,3139.541667
18,2,3597.695652
18,2,5504.175000
18,2,2590.560000
18,2,1596.291667
18,2,2915.043478
18,2,4111.171598
18,2,1956.355556
18,2,5418.597222
18,2,5083.881048
18,2,4878.276316
18,2,2896.352778
18,2,4572.000000
18,2,2202.173729
18,2,4327.205882
18,2,2264.470930
18,2,1724.837838
18,2,9926.443182
18,2,12080.250000
18,2,3268.333333
18,2,2524.552885
18,2,3421.043750
18,2,2978.615000
18,2,2566.500000
18,2,14056.182796
18,2,7032.509804
18,2,2601.348837
18,2,2083.784091
18,2,4366.919598
18,2,11184.275000
18,2,1709.158163
18,2,3871.302326
18,2,2447.147059
18,2,1780.064286
18,2,1985.523305
18,2,6597.382979
18,2,2393.792553

18,2,3729.025000
18,2,3407.036649
18,2,6495.763285
18,2,4019.043269
18,2,1651.463415
18,2,1625.145000
18,2,2818.100000
18,2,10434.150000
18,2,1666.781250
18,2,2315.861111
18,2,59889.188889
18,2,4298.735000
18,2,5810.919811
18,2,2414.312500
18,2,2698.920168
18,2,1524.228365
18,2,4388.600000
18,2,1752.320000
18,2,5013.130952
18,2,3052.840625
18,2,2407.886364
18,2,1682.553571
18,2,2088.586538
18,2,1991.438559
18,2,1560.953488
18,2,14254.168605
18,2,4918.125000
18,2,2813.715909
18,2,10606.875000
18,2,1830.802778
18,2,2754.571875
18,2,2006.480263
18,2,4769.701923
18,2,5338.500000
18,2,2527.104167
18,2,1781.923913
18,2,2138.157895
18,2,2301.428571
18,2,13647.170000
18,2,2932.106383
18,2,2065.966667
18,2,4073.575000
18,2,3585.530612
18,2,9802.422222
18,2,10591.147727
18,2,3138.757353
18,2,8401.457447
18,2,3243.851064
18,2,5259.363372
18,2,8979.446023
18,2,4039.340426
18,2,2587.697674
18,2,2679.597500
18,2,1719.667614
18,2,1603.176136
18,2,17017.125000
18,2,2199.957317
18,2,2314.191406
18,2,3532.629630
18,2,2333.000000
18,2,5980.754237
18,2,4007.302632
18,2,5465.111111
18,2,2370.810811
18,2,1555.391304
18,2,3452.933333

18,2,8145.808140
18,2,2619.735849
18,2,1725.960366
18,2,2084.835938
18,2,6168.455357
18,2,3676.986364
18,2,1679.062500
18,2,1913.031250
18,2,17667.427419
18,2,2081.564103
18,2,2464.398438
18,2,2898.292683
18,2,2639.904412
18,2,2897.598214
18,2,5110.750000
18,2,2543.375000
18,2,2139.864865
18,2,1728.631579
18,2,2330.284884
18,2,13789.537736
18,2,1671.468750
18,2,2177.994681
18,2,9055.920000
18,2,10347.400000
18,2,1583.640909
18,2,6440.852564
18,2,1568.708333
18,2,5851.613636
18,2,4472.901042
18,2,15612.081633
18,2,44242.428571
18,2,1725.945652
18,2,2771.430851
18,2,2513.400641
18,2,2146.406250
18,2,1574.050000
18,2,2287.500000
18,2,1626.125000
18,2,19017.388889
18,2,4928.059524
18,2,1581.250000
18,2,3405.333333
18,2,3167.502778
18,2,1816.647059
18,2,2970.120370
18,2,3790.234091
18,2,4689.709375
18,2,2941.409836
18,2,2890.628571
18,2,3377.211364
18,2,2358.445455
18,2,3591.566489
18,2,2264.828125
18,2,2981.954545
18,2,2841.913690
18,2,1610.602941
18,2,9184.148438
18,2,2876.777778
18,2,5012.831522
18,2,8247.454787
18,2,1615.217514
18,2,1724.093750
18,2,3068.875000
18,2,2466.218023
18,2,2590.298077
18,2,1865.912195

18,2,10151.242647
18,2,1803.100694
18,2,2198.272727
18,2,1535.485795
18,2,9141.703704
18,2,2262.430000
18,2,1642.846154
18,2,10093.126437
18,2,4490.785276
18,2,56417.660377
18,2,1506.053571
18,2,2257.375000
18,2,2593.372340
18,2,1676.007075
18,2,2482.120000
18,2,3879.886861
18,2,2817.709375
18,2,6172.619318
18,2,85930.769231
18,2,1864.006466
18,2,4216.538462
18,2,2560.862500
18,2,1652.625000
18,2,3206.994792
18,2,1778.117500
18,2,5352.240132
18,2,6663.753049
18,2,1876.057927
18,2,1596.422222
18,2,5453.367647
18,2,1621.435811
18,2,1566.256098
18,2,2443.458333
18,2,2031.709302
18,2,2586.244792
18,2,3068.517857
18,2,1767.224490
18,2,1515.452206
18,2,2293.529070
18,2,4960.625000
18,2,1534.739362
18,2,1931.434783
18,2,1576.025000
18,2,1772.224359
18,2,6218.697115
18,2,2757.304348
18,2,2656.750000
18,2,3256.900000
18,2,1618.413462
18,2,3438.367647
18,2,1966.913043
18,2,8309.301508
18,2,6141.138889
18,2,1992.009091
18,2,6738.000000
18,2,6989.401961
18,2,1690.438953
18,2,3776.807692
18,2,4728.318182
18,2,1852.842949
18,2,1571.975000
18,2,22585.875000
18,2,1783.666667
18,2,1732.239130
18,2,7337.006250
18,2,6063.250000

18,2,2161.640000
18,2,2328.196121
18,2,2270.820513
18,2,3516.731383
18,2,3864.858757
18,2,2036.348958
18,2,6859.880515
18,2,1964.182927
18,2,2957.710526
18,2,24635.963942
18,2,2718.545732
18,2,1634.677083
18,2,1952.155405
18,2,2937.192308
18,2,1872.725806
18,2,2624.700000
18,2,1813.973837
18,2,1993.596354
18,2,4653.447917
18,2,1739.009852
18,2,1796.425532
18,2,3791.464623
18,2,4412.125000
18,2,2049.500000
18,2,1864.187500
18,2,2211.970588
18,2,4966.813953
18,2,2158.984043
18,2,4210.740234
18,2,5374.430233
18,2,2267.000000
18,2,2295.156250
18,2,3246.238839
18,2,2040.073171
18,2,2933.881579
18,2,1628.303571
18,2,2365.604651
18,2,19787.500000
18,2,61478.744186
18,2,4230.804348
18,2,3690.576923
18,2,3346.490196
18,2,1828.798077
18,2,5061.803571
18,2,2037.371711
18,2,2216.385417
18,2,2646.846154
18,2,1660.782051
18,2,1713.560976
18,2,6643.353125
18,2,3638.997396
18,2,17931.421053
18,2,5191.904255
18,2,10020.521739
18,2,12734.430233
18,2,3087.000000
18,2,2976.546875
18,2,1856.314024
18,2,28775.237981
18,2,13739.884615
18,2,2221.161290
18,2,1632.183962
18,2,2128.845455
18,2,14227.740741
18,2,3866.673469
18,2,1840.750000

18,2,1968.816038
18,2,2242.869565
18,2,1625.750000
18,2,7086.643750
18,2,2823.743421
18,2,1594.684211
18,2,11332.169643
18,2,11455.579268
18,2,2938.239286
18,2,6726.603774
18,2,1627.925532
18,2,1634.459459
18,2,4541.000000
18,2,2134.978261
18,2,1621.130435
18,2,2745.620690
18,2,1643.882353
18,2,8538.520833
18,2,3048.478261
18,2,2234.402174
18,2,2278.313725
18,2,2000.230769
18,2,3160.744048
18,2,1766.034014
18,2,2543.500000
18,2,3540.788934
18,2,3114.255000
18,2,4522.154762
18,2,1636.238636
18,2,12619.597222
18,2,4141.034091
18,2,35276.650000
18,2,3467.681250
18,2,4489.675000
18,2,3805.125000
18,2,3236.012195
18,2,1634.818182
18,2,1504.100000
18,2,2002.222222
18,2,2811.220238
18,2,1594.333333
18,2,1912.043478
18,2,44269.093750
18,2,5336.287791
18,2,6018.203488
18,2,2200.724359
18,2,6019.807065
18,2,2224.350543
18,2,2653.000000
18,2,4582.343750
18,2,5217.051724
18,2,2223.383333
18,2,1917.122396
18,2,2663.050926
18,2,1957.283784
18,2,1507.125000
18,2,15332.646739
18,2,10228.240385
18,2,9038.484756
18,2,4917.721875
18,2,2431.820652
18,2,1562.845000
18,2,3080.959375
18,2,6809.625000
18,2,3271.300532
18,2,2968.583333

18,2,7128.351064
18,2,1589.921875
18,2,1860.125000
18,2,2041.270531
18,2,2477.182500
18,2,2635.875000
18,2,1958.278125
18,2,1569.589623
18,2,3243.398649
18,2,7102.060976
18,2,5188.108696
18,2,1519.574468
18,2,2385.062500
18,2,2651.777273
18,2,3257.478261
18,2,1936.244681
18,2,16587.910326
18,2,4571.112245
18,2,3613.512195
18,2,1505.478261
18,2,2054.543367
18,2,3457.316406
18,2,2565.127778
18,2,3661.585227
18,2,2394.720745
18,2,2150.951220
18,2,2091.903571
18,2,2146.906250
18,2,6525.312500
18,2,1686.515625
18,2,1716.875000
18,2,2369.132979
18,2,2415.026316
18,2,1658.916667
18,2,1850.790698
18,2,4306.250000
18,2,2544.264535
18,2,5121.907895
18,2,7978.914894
18,2,3959.243902
18,2,2419.923469
18,2,2261.176471
18,2,3832.323144
18,2,2448.792683
18,2,2761.333333
18,2,10281.611111
18,2,5959.197368
18,2,2045.894022
18,2,6206.878049
18,2,1661.653226
18,2,1585.923077
18,2,7569.756250
18,2,1611.875000
18,2,2054.957500
18,2,1718.536946
18,2,3988.042553
18,2,6664.807692
18,2,3168.979167
18,2,5807.896739
18,2,2918.571429
18,2,1718.153846
18,2,1521.653846
18,2,3173.431818
18,2,1655.689732
18,2,10888.791667
18,2,10962.214286

18,2,2187.341463
18,2,3010.075893
18,2,1855.625000
18,2,3428.881696
18,2,2377.994186
18,2,1566.583333
18,2,2777.533333
18,2,2244.227273
18,2,4317.585366
18,2,3518.025641
18,2,1938.291667
18,2,2168.720588
18,2,2400.505435
18,2,5739.382653
18,2,4167.489362
18,2,1896.438776
18,2,2202.375000
18,2,3462.397727
18,2,1910.509868
18,2,4512.662234
18,2,1665.300000
18,2,6992.777027
18,2,4511.771186
18,2,1810.137019
18,2,7002.000000
18,2,3374.350610
18,2,11700.625000
18,2,1503.975610
18,2,2692.719388
18,2,2357.585714
18,2,2835.447917
18,2,3573.200658
18,2,7758.469828
18,2,1849.767442
18,2,4937.472222
18,2,1556.438953
18,2,2744.811475
18,2,2432.076503
18,2,5397.679245
18,2,2339.489437
18,2,4511.632812
18,2,2307.161017
18,2,3094.732558
18,2,1707.859375
18,2,3101.958333
18,2,2007.267544
18,2,5067.420213
18,2,6980.698718
18,2,10767.745098
18,2,2739.662500
18,2,3044.997283
18,2,1871.208333
18,2,4728.100610
18,2,3426.836957
18,2,2520.419271
18,2,4995.336957
18,2,4054.816327
18,2,1878.904762
18,2,7226.420732
18,2,6491.554878
18,2,3296.918478
18,2,1665.000000
18,2,2143.060714
18,2,24872.466667
18,2,5533.333333
18,2,7895.810096

18,2,6716.500000
18,2,1806.688889
18,2,2141.410256
18,2,3157.434783
18,2,2646.785714
18,2,8552.105263
18,2,2152.241848
18,2,3721.062500
18,2,5955.061321
18,2,2554.088235
18,2,2321.864130
18,2,6852.841837
18,2,2029.190476
18,2,2605.375000
18,2,1871.278090
18,2,2506.872093
18,2,3086.655405
18,2,5299.500000
18,2,1764.045455
18,2,2858.489583
18,2,5153.469595
18,2,1709.846154
18,2,3911.507812
18,2,3631.244898
18,2,2277.550676
18,2,1805.733974
18,2,2751.402778
18,2,4461.500000
18,2,2128.787879
18,2,4562.275000
18,2,8772.859375
18,2,4051.386364
18,2,1864.500000
18,2,2280.337079
18,2,3593.225000
18,2,2320.250000
18,2,1581.500000
18,2,10188.511364
18,2,2465.280000
18,2,2450.525000
18,2,2659.229167
18,2,2469.015625
18,2,3422.909091
18,2,2567.076923
18,2,2359.268229
18,2,2107.210526
18,2,4056.023810
18,2,2114.276923
18,2,2005.587766
18,2,7866.662736
18,2,1504.958333
18,2,3520.166667
18,2,2359.975000
18,2,31431.370455
18,2,4883.928191
18,2,3654.975610
18,2,1537.545455
18,2,2292.727273
18,2,3750.813953
18,2,2653.291667
18,2,1973.319767
18,2,1903.528846
18,2,31845.633721
18,2,2295.668269
18,2,2512.875000
18,2,4347.836957

18,2,1520.040000
18,2,8579.071429
18,2,2414.301471
18,2,2959.790909
18,2,2157.253571
18,2,4278.583333
18,2,2555.326923
18,2,2032.244048
18,2,2602.165000
18,2,1772.066667
18,2,2726.381098
18,2,2815.223837
18,2,1697.297619
18,2,6497.906977
18,2,2503.208333
18,2,1823.354167
18,2,5865.257143
18,2,3850.985119
18,2,1995.700000
18,2,4995.538462
18,2,2709.980663
18,2,5098.987805
18,2,9252.242424
18,2,4954.523256
18,2,3187.168750
18,2,1778.272455
18,2,3396.034884
18,2,1660.552083
18,2,4070.978528
18,2,3161.298077
18,2,2252.992347
18,2,2055.319444
18,2,2660.362805
18,2,1516.200000
18,2,2046.332386
18,2,5220.868056
18,2,3195.488372
18,2,18881.954545
18,2,19666.992188
18,2,4030.980263
18,2,4300.954545
18,2,3364.142857
18,2,14698.517442
18,2,2045.500000
18,2,2062.265909
18,2,8066.151163
18,2,4060.168605
18,2,2295.826220
18,2,3055.000000
18,2,2205.484043
18,2,3793.132184
18,2,2035.556604
18,2,2303.997093
18,2,6920.588235
18,2,3499.369048
18,2,2096.061170
18,2,5154.166667
18,2,1715.184211
18,2,2461.625000
18,2,2000.807927
18,2,4206.212766
18,2,2253.910714
18,2,2044.933824
18,2,1630.664773
18,2,1598.250000
18,2,2075.848837

18,2,1569.005208
18,2,1614.487500
18,2,10596.739583
18,2,2322.486842
18,2,3298.000000
18,2,1662.017857
18,2,4287.426471
18,2,7175.550595
18,2,2091.408088
18,2,1502.444444
18,2,4072.479167
18,2,11078.018182
18,2,2796.384146
18,2,2623.494565
18,2,1913.576531
18,2,3278.728448
18,2,3060.030612
18,2,10549.203125
18,2,1503.403226
18,2,5263.025641
18,2,4572.244792
18,2,8598.711111
18,2,3203.750000
18,2,2425.515957
18,2,1503.250000
18,2,4824.955128
18,2,2208.975610
18,2,8358.705357
18,2,11378.987245
18,2,2926.500000
18,2,2398.279070
18,2,4454.171875
18,2,3428.737805
18,2,7462.891026
18,2,1941.265957
18,2,2324.173077
18,2,5616.265306
18,2,4406.790816
18,2,3626.526596
18,2,5420.295455
18,2,2230.625000
18,2,1706.569444
18,2,1997.583333
18,2,9222.676829
18,2,3356.634259
18,2,2480.982558
18,2,4970.586364
18,2,2435.175481
18,2,1702.695652
18,2,4661.895833
18,2,4936.058824
18,2,3650.428571
18,2,2052.967742
18,2,2578.022177
18,2,5004.988636
18,2,2309.515625
18,2,2415.011628
18,2,1738.300000
18,2,1742.469512
18,2,5046.825581
18,2,2275.562500
18,2,3235.104651
18,2,7731.030000
18,2,15849.351744
18,2,2060.865385
18,2,5596.812500

18,2,1544.268293
18,2,2476.853659
18,2,47071.978723
18,2,7393.058824
18,2,15219.500000
18,2,2148.125000
18,2,1872.137500
18,2,4138.540323
18,2,3725.698925
18,2,5528.314917
18,2,7539.592025
18,2,1970.302326
18,2,2144.750000
18,2,1776.470588
18,2,1693.643293
18,2,1797.210784
18,2,3057.917763
18,2,1844.875000
18,2,8400.083333
18,2,1887.362245
18,2,2501.752660
18,2,5680.242647
18,2,1896.632979
18,2,1819.153509
18,2,3419.386792
18,2,1500.462838
18,2,1914.918079
18,2,2930.208333
18,2,2504.000000
18,2,2912.962766
18,2,2334.925000
18,2,4100.400000
18,2,4068.217105
18,2,3063.021277
18,2,5540.789634
18,2,2035.825658
18,2,2128.625000
18,2,3185.609375
18,2,2047.329545
18,2,5465.437500
18,2,2057.234043
18,2,3788.269444
18,2,2006.093750
18,2,3484.000000
18,2,1959.078125
18,2,5347.828125
18,2,2180.287037
18,2,3534.800000
18,2,2292.187500
18,2,1682.345745
18,2,5361.512821
18,2,6076.283721
18,2,1921.525714
18,2,1881.972973
18,2,6733.750000
18,2,4840.775000
18,2,2417.506944
18,2,2632.833333
18,2,4154.793269
18,2,3696.172619
18,2,1629.550000
18,2,4826.200000
18,2,3757.537500
18,2,2193.786458
18,2,10088.522727
18,2,2087.175781

18,2,2589.336207
18,2,3367.322917
18,2,6191.780105
18,2,6496.619792
18,2,5716.093750
18,2,3388.475000
18,2,10230.781250
18,2,1570.170000
18,2,25688.000000
18,2,1747.250000
18,2,1723.773438
18,2,4749.875000
18,2,2351.369048
18,2,6126.886364
18,2,1569.020833
18,2,5656.743902
18,2,6216.111364
18,2,5729.357143
18,2,3480.351064
18,2,8563.630000
18,2,3288.269231
18,2,13714.670103
18,2,5432.897436
18,2,3783.200000
18,2,2848.969388
18,2,5867.128125
18,2,14752.990741
18,2,3367.005319
18,2,33758.853659
18,2,5936.505435
18,2,3263.500000
18,2,2845.804487
18,2,2059.456250
18,2,4186.937500
18,2,1917.252976
18,2,2620.250000
18,2,8364.637500
18,2,24596.505814
18,2,3153.747283
18,2,3005.785340
18,2,1822.572222
18,2,2709.002674
18,2,1816.070312
18,2,3527.483051
18,2,1511.875000
18,2,3471.375000
18,2,3318.759615
18,2,2402.000000
18,2,1919.615385
18,2,9229.203846
18,2,2904.877551
18,2,1874.853723
18,2,3142.640625
18,2,3451.462500
18,2,3491.473837
18,2,3646.737981
18,2,5681.964286
18,2,2390.000000
18,2,1780.000000
18,2,6538.961957
18,2,1784.812500
18,2,7634.444149
18,2,1770.462500
18,2,5824.580189
18,2,6640.965909
18,2,2122.154255

18,2,2714.471354
18,2,1792.303571
18,2,3232.305556
18,2,3763.305233
18,2,1862.140000
18,2,2508.534091
18,2,5542.909091
18,2,1550.398649
18,2,2933.850000
18,2,2059.375000
18,2,5306.797872
18,2,3529.000000
18,2,2952.524390
18,2,1607.237500
18,2,3812.269231
18,2,4523.242188
18,2,6147.511628
18,2,4476.577381
18,2,3385.000000
18,2,4845.627604
18,2,10332.218750
18,2,9086.352679
18,2,1891.787440
18,2,2801.950000
18,2,3338.793478
18,2,1803.187500
18,2,1800.120000
18,2,2925.409091
18,2,2598.061321
18,2,7655.053571
18,2,2721.875000
18,2,2249.042135
18,2,1753.961207
18,2,9915.933962
18,2,1662.595745
18,2,12045.015086
18,2,3063.223404
18,2,4517.646186
18,2,8977.000000
18,2,2785.755102
18,2,2446.367500
18,2,11223.324074
18,2,3078.076923
18,2,2646.834302
18,2,4322.310000
18,2,2282.265957
18,2,13296.578488
18,2,2907.473214
18,2,3047.000000
18,2,3306.842105
18,2,1875.973958
18,2,12765.541667
18,2,3365.500000
18,2,1837.902439
18,2,3437.255814
18,2,1657.438953
18,2,3013.986413
18,2,1864.853261
18,2,2693.511364
18,2,2994.861111
18,2,2790.786111
18,2,2780.452381
18,2,10152.066667
18,2,1988.742500
18,2,1791.365385
18,2,4720.460526

18,2,2364.642857
18,2,3464.701923
18,2,9421.041667
18,2,5549.819079
18,2,6453.921053
18,2,4921.342593
18,2,2152.950920
18,2,1536.304348
18,2,3818.418605
18,2,1578.267857
18,2,3659.333333
18,2,2424.771341
18,2,2578.133333
18,2,2885.465909
18,2,2350.392045
18,2,1650.725000
18,2,5130.964286
18,2,7343.880208
18,2,3282.625000
18,2,9116.767442
18,2,4423.329082
18,2,4921.685185
18,2,2086.730769
18,2,4998.901961
18,2,5237.979866
18,2,3987.664634
18,2,2168.451613
18,2,4936.765306
18,2,4741.932065
18,2,3193.491935
18,2,8447.604167
18,2,5365.502358
18,2,1886.857143
18,2,4031.182292
18,2,2485.686170
18,2,2216.857955
18,2,3372.860465
18,2,5958.775510
18,2,9939.880952
18,2,1523.597561
18,2,4561.903125
18,2,1821.260870
18,2,2576.250000
18,2,2073.723404
18,2,13279.210227
18,2,8333.450000
18,2,9888.226744
18,2,2243.534884
18,2,8718.574866
18,2,3173.451923
18,2,4609.286585
18,2,5423.019231
18,2,2041.524390
18,2,1730.767296
18,2,1588.102041
18,2,3830.000000
18,2,5177.542683
18,2,5777.689815
18,2,3451.832317
18,2,6854.698980
18,2,4872.941489
18,2,3193.000000
18,2,2689.381944
18,2,2037.962209
18,2,2722.333333
18,2,5945.902439

18,2,2205.687500
18,2,5345.217949
18,2,3228.458647
18,2,1560.277027
18,2,1598.761111
18,2,4352.480392
18,2,4242.352273
18,2,8691.786885
18,2,3914.276786
18,2,3243.976415
18,2,4427.500000
18,2,2614.867925
18,2,1706.437500
18,2,1653.930233
18,2,6512.453571
18,2,2363.851562
18,2,2356.905172
18,2,2048.375000
18,2,12208.630208
18,2,2605.058140
18,2,2121.184524
18,2,13218.319149
18,2,3971.174419
18,2,2839.543860
18,2,4559.244681
18,2,3514.080357
18,2,2553.250000
18,2,2400.364865
18,2,8358.634615
18,2,1732.648936
18,2,2099.011111
18,2,1576.541667
18,2,3137.210227
18,2,2799.118590
18,2,4955.895833
18,2,2508.031250
18,2,2331.934783
18,2,2382.093750
18,2,2433.018293
18,2,2219.515625
18,2,4187.517857
18,2,4185.752660
18,2,3493.956250
18,2,2471.214286
18,2,2949.925481
18,2,3988.959184
18,2,6125.187500
18,2,2498.062500
18,2,8024.337500
18,2,8982.547297
18,2,2958.283019
18,2,2513.062500
18,2,2611.252155
18,2,5924.273438
18,2,6343.525510
18,2,2821.351190
18,2,17772.096939
18,2,11113.510274
18,2,2054.104651
18,2,1992.634146
18,2,1878.827128
18,2,3677.671875
18,2,32246.573171
18,2,2453.250000
18,2,4819.282609
18,2,2226.705357

18,2,2843.938596
18,2,1555.951923
18,2,6647.766667
18,2,2528.798913
18,2,1702.750000
18,2,1824.121212
18,2,8066.000000
18,2,2353.305921
18,2,6593.072222
18,2,3366.000000
18,2,2011.458333
18,2,1809.384615
18,2,4293.688679
18,2,3830.384615
18,2,2921.299419
18,2,6077.300439
18,2,2476.329082
18,2,1887.035714
18,2,2765.279412
18,2,2440.204787
18,2,6739.875000
18,2,5106.000000
18,2,4977.884615
18,2,2949.700000
18,2,1519.651042
18,2,1856.250000
18,2,3021.954545
18,2,3087.305556
18,2,3123.425000
18,2,3465.434524
18,2,1750.855000
18,2,2273.949468
18,2,2914.511574
18,2,4371.898256
18,2,2380.134615
18,2,1702.584906
18,2,9463.453804
18,2,5071.735294
18,2,6026.888889
18,2,1581.076923
18,2,1592.611111
18,2,2768.500000
18,2,1708.352564
18,2,5162.045455
18,2,5350.860465
18,2,3053.773438
18,2,5215.000000
18,2,4346.312500
18,2,4606.068750
18,2,6556.905660
18,2,3457.865854
18,2,3753.617347
18,2,2137.331361
18,2,3990.575000
18,2,2272.357639
18,2,3291.888889
18,2,2406.273256
18,2,2545.518293
18,2,1975.298469
18,2,4198.487179
18,2,14009.507576
18,2,3183.657143
18,2,3497.810096
18,2,9980.137931
18,2,4825.409091
18,2,2368.117021

18,2,7398.714286
18,2,4278.113889
18,2,4776.226190
18,2,3489.138158
18,2,2679.560897
18,2,4454.100000
18,2,1617.869318
18,2,2442.578947
18,2,3534.938776
18,2,9304.558333
18,2,1579.609375
18,2,3559.744565
18,2,3011.019231
18,2,4420.692308
18,2,2397.993421
18,2,1675.377358
18,2,4947.934211
18,2,6325.733491
18,2,1617.571429
18,2,1583.200000
18,2,3836.880435
18,2,1874.694118
18,2,1813.219512
18,2,1524.516304
18,2,1844.845361
18,2,3694.225962
18,2,1900.746835
18,2,4700.404070
18,2,4559.029070
18,2,2859.911585
18,2,22979.650000
18,2,1966.472727
18,2,2647.677885
18,2,2150.225000
18,2,5312.349057
18,2,1568.166667
18,2,8402.370130
18,2,2489.893519
18,2,2992.071429
18,2,11912.017045
18,2,2691.342857
18,2,1994.656250
18,2,1574.216912
18,2,3348.565789
18,2,4188.000000
18,2,2326.909091
18,2,1790.818182
18,2,2186.326923
18,2,6467.195513
18,2,4942.712264
18,2,1522.925532
18,2,2085.966981
18,2,3592.906250
18,2,6402.492347
18,2,2262.305556
18,2,1882.950000
18,2,1985.153017
18,2,2466.288043
18,2,2650.892857
18,2,2316.418605
18,2,1753.812500
18,2,2131.217391
18,2,9215.357759
18,2,1938.847826
18,2,2014.764706
18,2,1600.435714

18,2,3613.750000
18,2,2507.611979
18,2,3811.312500
18,2,1813.105263
18,2,3257.276596
18,2,2198.306452
18,2,31716.536585
18,2,19820.916667
18,2,4503.212500
18,2,1531.444444
18,2,3065.531250
18,2,5104.450472
18,2,3658.891304
18,2,2901.170455
18,2,2457.533333
18,2,7123.375000
18,2,3273.263298
18,2,2578.354167
18,2,7292.627907
18,2,1630.598214
18,2,2764.595000
18,2,2175.680000
18,2,3093.029412
18,2,1791.838235
18,2,2932.510638
18,2,26464.941489
18,2,2251.263158
18,2,2032.233696
18,2,2094.395349
18,2,5617.744444
18,2,20722.905303
18,2,3586.157407
18,2,1700.065217
18,2,4719.230769
18,2,1981.510638
18,2,1747.062500
18,2,9227.403846
18,2,4244.666667
18,2,6627.832237
18,2,2157.000000
18,2,4201.329897
18,2,2134.555556
18,2,3399.937500
18,2,7827.400000
18,2,5597.775862
18,2,1893.290541
18,2,1925.026163
18,2,5441.067308
18,2,2943.125000
18,2,2856.783784
18,2,2285.210938
18,2,4549.390625
18,2,6303.141026
18,2,2330.142045
18,2,1538.875000
18,2,1667.578488
18,2,3390.558140
18,2,2591.750000
18,2,2428.437500
18,2,4833.000000
18,2,4599.941667
18,2,7628.060976
18,2,2896.333333
18,2,1790.607143
18,2,2329.819767
18,2,1827.698795

18,2,2608.835526
18,2,5144.816327
18,2,2064.639706
18,2,9585.638298
18,2,1669.666667
18,2,2622.000000
18,2,2757.610169
18,2,3344.108696
18,2,3036.972500
18,2,2659.568182
18,2,2002.913462
18,2,5113.823077
18,2,1507.250000
18,2,2270.792683
18,2,2282.065000
18,2,2556.413690
18,2,6194.577703
18,2,4212.955357
18,2,2104.000000
18,2,8554.423684
18,2,2483.639881
18,2,6598.650000
18,2,3526.032051
18,2,6801.559375
18,2,2782.187500
18,2,5289.021277
18,2,4207.585227
18,2,3154.985714
18,2,3060.788462
18,2,1925.189904
18,2,11838.698113
18,2,1653.165625
18,2,1919.510714
18,2,3861.111111
18,2,21004.559783
18,2,4197.921875
18,2,7278.943452
18,2,2120.486486
18,2,1954.145270
18,2,1742.190789
18,2,2720.555556
18,2,4507.590909
18,2,8931.139151
18,2,15955.431373
18,2,4342.151685
18,2,1586.260000
18,2,2208.995370
18,2,13427.875000
18,2,4528.831250
18,2,2700.436111
18,2,1676.802198
18,2,6537.951807
18,2,1991.817568
18,2,2387.377778
18,2,17507.947917
18,2,2496.891304
18,2,2371.467391
18,2,1523.234043
18,2,2913.291667
18,2,2884.600000
18,2,2656.800000
18,2,2464.493421
18,2,3019.589286
18,2,3707.970930
18,2,4843.576923
18,2,3651.993506

18,2,3667.053125
18,2,2478.652027
18,2,3725.463855
18,2,2572.318750
18,2,2790.517857
18,2,2345.361486
18,2,3029.093023
18,2,2249.093750
18,2,2632.622093
18,2,4319.456140
18,2,6561.141304
18,2,5287.036885
18,2,8100.047500
18,2,2838.038462
18,2,1586.875000
18,2,13781.666667
18,2,1507.736111
18,2,1826.480000
18,2,2217.384615
18,2,2840.977778
18,2,3396.711538
18,2,2763.851190
18,2,4400.398936
18,2,4281.732558
18,2,2150.511905
18,2,4392.349359
18,2,1680.233813
18,2,2436.405556
18,2,2067.956522
18,2,5614.186893
18,2,2048.561798
18,2,6133.890625
18,2,4359.533019
18,2,2254.320513
18,2,1644.198171
18,2,8481.090909
18,2,4351.864583
18,2,4751.901198
18,2,2491.419355
18,2,12438.817844
18,2,6987.081395
18,2,2941.125000
18,2,1984.418605
18,2,1887.955357
18,2,1808.742347
18,2,5051.761111
18,2,3993.672170
18,2,3127.621429
18,2,7220.911111
18,2,2924.630435
18,2,2437.005208
18,2,2895.237805
18,2,2046.521341
18,2,1624.825000
18,2,2277.896875
18,2,3440.193182
18,2,2497.523026
18,2,10992.250000
18,2,8799.920000
18,2,2349.313830
18,2,1673.042857
18,2,1965.612500
18,2,2449.051471
18,2,3050.000000
18,2,4554.636364
18,2,4453.459184

18,2,2086.772727
18,2,4965.776442
18,2,4730.220930
18,2,1859.255682
18,2,2659.769231
18,2,5209.678977
18,2,6588.072917
18,2,1858.638037
18,2,2900.609756
18,2,2146.955000
18,2,2205.533333
18,2,3705.477273
18,2,1744.611842
18,2,4876.812500
18,2,5262.000000
18,2,6365.983871
18,2,29660.312500
18,2,2594.798611
18,2,1756.640000
18,2,11662.571429
18,2,1951.866279
18,2,2846.367925
18,2,2796.575581
18,2,5039.265625
18,2,1721.125000
18,2,2854.000000
18,2,2919.149038
18,2,5782.738889
18,2,1751.555556
18,2,3190.916667
18,2,1847.197674
18,2,2979.348214
18,2,3179.400000
18,2,2374.165698
18,2,1737.893617
18,2,2351.452128
18,2,5973.945946
18,2,3095.693750
18,2,6864.365854
18,2,2804.846354
18,2,2960.281250
18,2,1830.097458
18,2,2616.102041
18,2,11280.219444
18,2,14989.000000
18,2,1731.728659
18,2,2706.690594
18,2,2196.946429
18,2,3585.886364
18,2,11673.448661
18,2,2916.889423
18,2,2580.420213
18,2,4640.843085
18,2,2897.954787
18,2,3472.539216
18,2,27692.841463
18,2,2451.076220
18,2,1860.466146
18,2,1992.167857
18,2,6061.510638
18,2,4087.020455
18,2,3678.750000
18,2,3067.708333
18,2,2494.710106
18,2,10330.179878
18,2,1974.049080

18,2,2297.794118
18,2,1728.406863
18,2,1720.511905
18,2,4861.280000
18,2,2571.419271
18,2,9750.785714
18,2,2700.328125
18,2,4315.609375
18,2,1699.638514
18,2,1682.627660
18,2,2494.898601
18,2,1983.000000
18,2,1781.302326
18,2,1718.671875
18,2,4391.072674
18,2,6636.283019
18,2,1520.224359
18,2,2382.726064
18,2,6319.184783
18,2,3527.419643
18,2,2304.336957
18,2,2542.128571
18,2,2437.011364
18,2,1991.394231
18,2,3302.705882
18,2,1993.546875
18,2,1750.383929
18,2,2367.447368
18,2,10815.909357
18,2,1830.735484
18,2,2141.914474
18,2,4724.538462
18,2,11796.500000
18,2,1972.332447
18,2,1653.301282
18,2,1793.692308
18,2,2065.336538
18,2,15034.455882
18,2,5115.278409
18,2,1639.163043
18,2,10213.929348
18,2,2335.657609
18,2,5631.786585
18,2,28843.674419
18,2,1523.326389
18,2,2470.774194
18,2,3302.149123
18,2,6882.600000
18,2,3383.669811
18,2,12614.000000
18,2,1967.560000
18,2,3109.837209
18,2,2324.127778
18,2,4158.000000
18,2,2630.441176
18,2,7163.338235
18,2,3704.150000
18,2,2947.060811
18,2,2052.727273
18,2,4517.131410
18,2,2606.641892
18,2,2551.375000
18,2,1931.638298
18,2,1538.532738
18,2,2070.827225
18,2,14212.399103

18,2,2229.128342
18,2,2701.117647
18,2,2082.418367
18,2,1920.065217
18,2,4972.287037
18,2,6832.129808
18,2,5522.681818
18,2,3233.416667
18,2,2348.105000
18,2,1951.036364
18,2,5256.588542
18,2,7482.063830
18,2,2849.724432
18,2,6910.818182
18,2,1502.441860
18,2,3587.653061
18,2,2190.627273
18,2,3196.211538
18,2,2153.000000
18,2,2147.289773
18,2,4414.538462
18,2,2179.231707
18,2,6474.212500
18,2,1640.857500
18,2,3819.192308
18,2,3005.181818
18,2,4106.871795
18,2,2107.853659
18,2,1555.384375
18,2,5874.672348
18,2,1989.100000
18,2,1752.183333
18,2,4308.671569
18,2,2477.190217
18,2,2764.207589
18,2,31785.871795
18,2,2666.513298
18,2,2385.263889
18,2,1872.174107
18,2,1529.227778
18,2,1627.471591
18,2,7933.432927
18,2,3758.407051
18,2,4743.625000
18,2,8005.575000
18,2,6884.534574
18,2,4799.950581
18,2,3761.000000
18,2,7006.916667
18,2,1514.310811
18,2,1899.734694
18,2,2277.285068
18,2,7217.922619
18,2,60030.108696
18,2,3375.134146
18,2,6802.822500
18,2,1835.964286
18,2,1619.065476
18,2,2981.235577
18,2,4377.707317
18,2,5339.676020
18,2,5173.407407
18,2,5945.047619
18,2,9955.020000
18,2,7926.868421
18,2,2051.645833

18,2,2220.594340
18,2,4793.034091
18,2,4347.076531
18,2,1553.944444
18,2,4074.484043
18,2,12105.531250
18,2,6324.634615
18,2,3035.800000
18,2,2755.325000
18,2,1631.481884
18,2,4298.375000
18,2,1533.287234
18,2,2154.725000
18,2,2946.523256
18,2,3787.364865
18,2,5785.887019
18,2,30139.791667
18,2,1598.269231
18,2,5692.200581
18,2,3669.019231
18,2,4159.278689
18,2,5702.510000
18,2,2397.680851
18,2,2288.300000
18,2,2514.548387
18,2,2048.450000
18,2,2318.607143
18,2,2016.319767
18,2,11552.279070
18,2,6565.720000
18,2,18282.167401
18,2,2434.125000
18,2,1703.627778
18,2,1994.423077
18,2,2197.377778
18,2,2323.416667
18,2,6678.756098
18,2,1953.155000
18,2,2337.552083
18,2,9675.300000
18,2,6827.585366
18,2,6386.850000
18,2,2207.869318
18,2,4858.266304
18,2,3315.118421
18,2,4676.125000
18,2,7696.250000
18,2,5259.640000
18,2,1798.983784
18,2,2027.000000
18,2,1912.752551
18,2,2043.961538
18,2,9304.087963
18,2,1707.562500
18,2,4537.062500
18,2,4307.868132
18,2,2453.889423
18,2,1685.760000
18,2,1570.700000
18,2,5247.500000
18,2,13662.241279
18,2,5705.897959
18,2,3344.178571
18,2,6443.531915
18,2,2597.924419
18,2,4080.684834

18,2,1978.393229
18,2,2837.802083
18,2,2546.160000
18,2,4150.298469
18,2,2952.437500
18,2,2498.066038
18,2,3016.630208
18,2,9647.500000
18,2,2889.512195
18,2,3447.196429
18,2,25226.298246
18,2,2993.467391
18,2,1593.670213
18,2,2516.189904
18,2,17122.272059
18,2,1499.557229
18,2,1548.327869
18,2,1542.386792
18,2,2262.743243
18,2,2261.256466
18,2,2696.766667
18,2,2427.068750
18,2,5784.376344
18,2,3689.398256
18,2,4899.707627
18,2,2079.361842
18,2,3951.945312
18,2,1606.106383
18,2,4237.000000
18,2,3502.201923
18,2,1678.119318
18,2,2231.102941
18,2,7327.672414
18,2,4762.500000
18,2,2283.050000
18,2,7829.212500
18,2,2700.314024
18,2,1553.959184
18,2,4008.151442
18,2,2155.525000
18,2,5507.145408
18,2,2772.291667
18,2,1512.625000
18,2,2775.039216
18,2,9438.437500
18,2,2329.168478
18,2,1502.027439
18,2,3965.302778
18,2,2135.274390
18,2,4108.912109
18,2,4120.538462
18,2,1538.337500
18,2,2289.348837
18,2,1724.111702
18,2,2210.506944
18,2,11778.147500
18,2,5884.530864
18,2,1682.076923
18,2,2883.365196
18,2,3007.539063
18,2,2175.430556
18,2,3652.000000
18,2,2954.625000
18,2,3073.131313
18,2,9709.210526
18,2,9488.556122

18,2,2239.500000
18,2,4754.347826
18,2,3681.494792
18,2,2067.813889
18,2,2277.710227
18,2,4234.022059
18,2,3397.359375
18,2,2219.611842
18,2,4025.125000
18,2,1651.363636
18,2,9808.573370
18,2,2288.405405
18,2,1973.535714
18,2,6087.375000
18,2,6877.557432
18,2,1762.492647
18,2,1615.934783
18,2,1695.380952
18,2,2311.671875
18,2,2482.428977
18,2,4004.283482
18,2,3004.631944
18,2,2100.347305
18,2,1574.125000
18,2,5812.250000
18,2,2288.835227
18,2,6631.821429
18,2,3926.511364
18,2,8870.333333
18,2,6290.462500
18,2,4351.333333
18,2,4240.882653
18,2,7434.148936
18,2,1900.531250
18,2,2198.851429
18,2,1629.500000
18,2,1511.855978
18,2,7331.406250
18,2,1503.153614
18,2,6077.315789
18,2,4040.036585
18,2,5496.225000
18,2,5994.812500
18,2,3753.739130
18,2,5859.864583
18,2,5126.081250
18,2,2130.840116
18,2,3758.109375
18,2,2221.893617
18,2,1803.406250
18,2,1508.769737
18,2,3340.753571
18,2,2033.276163
18,2,5861.585227
18,2,3931.213415
18,2,1972.838235
18,2,75751.500000
18,2,1614.285714
18,2,1904.472603
18,2,3135.491018
18,2,1783.173913
18,2,1909.881410
18,2,2135.080189
18,2,3197.182692
18,2,3686.651042
18,2,1766.027778

18,2,2717.872024
18,2,3608.346154
18,2,28920.593750
18,2,7511.706731
18,2,4246.332317
18,2,2602.647959
18,2,2037.849490
18,2,4558.933333
18,2,6144.534091
18,2,2179.075658
18,2,3263.908854
18,2,2442.357143
18,2,2291.025510
18,2,6733.888889
18,2,3239.913194
18,2,2983.369048
18,2,5518.037037
18,2,2554.972826
18,2,1869.888889
18,2,2451.339744
18,2,2042.203125
18,2,2150.968750
18,2,11889.496622
18,2,3222.604651
18,2,7750.323671
18,2,1640.726351
18,2,3104.554974
18,2,9492.471354
18,2,3570.389205
18,2,2672.969512
18,2,4296.735849
18,2,1578.030556
18,2,7643.586957
18,2,6015.617188
18,2,3367.407895
18,2,3792.443750
18,2,1613.238636
18,2,6078.394231
18,2,5318.000000
18,2,1503.000000
18,2,5001.750000
18,2,2254.364130
18,2,1626.250000
18,2,5815.609375
18,2,3105.586538
18,2,12523.020270
18,2,1506.246711
18,2,1842.197368
18,2,3380.708333
18,2,19660.750000
18,2,2062.052326
18,2,2144.244444
18,2,1510.666667
18,2,2370.071429
18,2,3578.673913
18,2,4851.166667
18,2,7212.583333
18,2,1609.525568
18,2,5551.745455
18,2,3966.484043
18,2,3377.872093
18,2,3182.125000
18,2,4072.750000
18,2,1564.000000
18,2,1703.302083
18,2,2211.148438

18,2,1590.638298
18,2,5402.572368
18,2,3737.222222
18,2,2963.217105
18,2,3298.500000
18,2,7579.187500
18,2,2267.875000
18,2,1537.167598
18,2,4145.472727
18,2,3128.074689
18,2,4080.723404
18,2,3022.446429
18,2,12198.030364
18,2,3941.532609
18,2,4154.160714
18,2,2222.838542
18,2,2256.880682
18,2,1582.510135
18,2,2326.643750
18,2,4915.467391
18,2,3418.519737
18,2,2080.150000
18,2,4871.420833
18,2,3596.442308
18,2,4325.353191
18,2,6404.573770
18,2,1754.693182
18,2,8289.448171
18,2,10172.179878
18,2,13502.048611
18,2,1527.101351
18,2,6097.340000
18,2,5215.000000
18,2,1519.875000
18,2,1556.952830
18,2,2303.170732
18,2,4048.543147
18,2,1627.590426
18,2,7498.709091
18,2,4920.985294
18,2,2357.863208
18,2,2217.926136
18,2,2637.481132
18,2,8106.338542
18,2,2447.180921
18,2,7259.281250
18,2,5338.468137
18,2,2257.857143
18,2,4513.511905
18,2,1697.539474
18,2,1521.708333
18,2,2421.000000
18,2,3786.711538
18,2,4960.371951
18,2,2148.072115
18,2,3335.750000
18,2,3226.291667
18,2,5976.475610
18,2,3650.397727
18,2,2285.697917
18,2,3792.000000
18,2,6188.740000
18,2,1840.875000
18,2,3017.985294
18,2,1644.613158
18,2,5052.205128

18,2,3075.450980
18,2,2389.281977
18,2,4988.987500
18,2,2745.191489
18,2,7367.759434
18,2,1912.750000
18,2,2137.701087
18,2,2438.000000
18,2,3946.429245
18,2,2482.435897
18,2,2334.150735
18,2,4289.659091
18,2,1714.937500
18,2,1534.820312
18,2,3099.414634
18,2,5512.500000
18,2,5235.545000
18,2,1586.914894
18,2,3344.703488
18,2,1864.319149
18,2,2328.679688
18,2,9318.840625
18,2,4089.671875
18,2,2230.780405
18,2,6039.493056
18,2,1797.263158
18,2,2334.000000
18,2,3564.131579
18,2,10237.222222
18,2,2559.957317
18,2,3929.915698
18,2,3667.489796
18,2,2185.295455
18,2,1894.829787
18,2,58270.357955
18,2,3892.940476
18,2,5360.556818
18,2,10542.142857
18,2,4243.523649
18,2,2778.785714
18,2,4532.866279
18,2,2068.573171
18,2,4567.673295
18,2,5961.622222
18,2,3462.308140
18,2,6550.278409
18,2,3170.565972
18,2,3949.046569
18,2,2054.853365
18,2,3514.398148
18,2,2139.868421
18,2,2999.723404
18,2,1633.510000
18,2,3654.656250
18,2,8650.220395
18,2,2317.812500
18,2,12303.136905
18,2,1605.667857
18,2,2871.392442
18,2,1762.313953
18,2,30505.380952
18,2,3822.986486
18,2,2406.832386
18,2,8981.490566
18,2,2141.358333
18,2,3094.764205

18,2,53287.881579
18,2,1988.027174
18,2,2227.600543
18,2,6349.562500
18,2,2348.583333
18,2,20156.337500
18,2,2356.394737
18,2,1530.857143
18,2,2080.036111
18,2,1660.611111
18,2,6845.356250
18,2,6743.031250
18,2,4280.989583
18,2,7873.956250
18,2,2294.089286
18,2,3423.104167
18,2,6874.666667
18,2,14624.704545
18,2,1925.627907
18,2,1592.313953
18,2,2729.952663
18,2,7115.190323
18,2,2276.833333
18,2,1727.166667
18,2,1761.883721
18,2,4509.175214
18,2,6114.446429
18,2,2207.081081
18,2,3799.433824
18,2,5530.605263
18,2,2125.326220
18,2,1746.542857
18,2,3858.091837
18,2,7974.882979
18,2,3870.550000
18,2,1565.252841
18,2,11701.796053
18,2,4119.886010
18,2,3945.750000
18,2,2859.000000
18,2,8186.447368
18,2,2282.724432
18,2,2899.483333
18,2,4649.314732
18,2,1721.571023
18,2,1630.892857
18,2,6033.754717
18,2,3993.729592
18,2,2227.500000
18,2,3162.000000
18,2,2241.826531
18,2,3630.742500
18,2,3177.289773
18,2,4418.471939
18,2,1769.000000
18,2,7608.416667
18,2,8631.665761
18,2,1538.402500
18,2,1939.148649
18,2,1573.720109
18,2,4315.163265
18,2,1860.798611
18,2,1820.390957
18,2,1843.523560
18,2,1661.714286
18,2,2607.557692

18,2,4097.896277
18,2,1975.632353
18,2,1814.560606
18,2,2187.625000
18,2,1507.560811
18,2,1933.413043
18,2,5355.579545
18,2,4090.526596
18,2,5102.968750
18,2,1843.447917
18,2,1781.544444
18,2,5828.676829
18,2,4209.760000
18,2,1994.820513
18,2,1966.434783
18,2,4862.538462
18,2,2316.954545
18,2,2082.666667
18,2,4225.672297
18,2,3623.750000
18,2,4611.600000
18,2,2679.802083
18,2,2276.521739
18,2,3894.234043
18,2,1524.160714
18,2,4648.070122
18,2,2574.444223
18,2,2555.431818
18,2,4201.633523
18,2,4751.237805
18,2,3755.859375
18,2,3859.689024
18,2,3366.488636
18,2,5733.500000
18,2,1871.256250
18,2,2986.510870
18,2,4710.333333
18,2,19760.173611
18,2,3765.429878
18,2,2058.916667
18,2,3044.738095
18,2,5742.038462
18,2,6558.172414
18,2,2235.443182
18,2,1563.486486
18,2,19786.055147
18,2,2180.357143
18,2,5751.629630
18,2,2622.125000
18,2,1582.809375
18,2,1623.495192
18,2,2458.602941
18,2,1547.085366
18,2,1528.401316
18,2,2534.669355
18,2,3991.476562
18,2,1511.203125
18,2,7534.326923
18,2,7007.477273
18,2,3772.812500
18,2,11228.281250
18,2,5756.463542
18,2,4002.295455
18,2,3728.400000
18,2,1885.218750
18,2,1806.000000

18,2,3482.145833
18,2,6566.489796
18,2,3001.791667
18,2,3411.765957
18,2,8430.071429
18,2,3701.666667
18,2,3953.232877
18,2,2688.625000
18,2,2470.616848
18,2,87244.263473
18,2,4194.096429
18,2,13039.759162
18,2,3191.500000
18,2,6033.475000
18,2,3107.686047
18,2,2942.743750
18,2,2113.278846
18,2,3190.011364
18,2,1612.192308
18,2,4446.301887
18,2,3517.400000
18,2,2074.068750
18,2,4668.975610
18,2,2314.970000
18,2,2142.708333
18,2,2666.763021
18,2,5585.315789
18,2,3324.857143
18,2,7647.388889
18,2,3604.075658
18,2,3130.411765
18,2,3012.800000
18,2,2789.687500
18,2,2470.460000
18,2,3770.451389
18,2,7152.739583
18,2,1595.775862
18,2,5134.409091
18,2,5234.855769
18,2,1595.768750
18,2,2284.188976
18,2,1873.377778
18,2,7558.942308
18,2,3700.711538
18,2,1739.212766
18,2,1648.989362
18,2,8962.856618
18,2,1820.725806
18,2,3015.502841
18,2,2715.987500
18,2,3229.846154
18,2,3960.600000
18,2,2806.071875
18,2,1696.428571
18,2,4543.875000
18,2,3104.156863
18,2,2211.367647
18,2,2636.938953
18,2,1826.307692
18,2,5533.209497
18,2,2792.777027
18,2,6463.755208
18,2,15415.581395
18,2,48166.276786
18,2,2269.206522
18,2,5383.174342

18,2,1828.939516
18,2,2569.255319
18,2,4054.430851
18,2,2700.658163
18,2,1622.346154
18,2,1558.993243
18,2,2914.738372
18,2,1783.946429
18,2,4547.361702
18,2,5935.792135
18,2,24902.275000
18,2,2506.500000
18,2,1978.597826
18,2,1995.625000
18,2,5657.908854
18,2,4779.333333
18,2,3053.559524
18,2,2453.833333
18,2,2119.953488
18,2,6055.833333
18,2,5175.444444
18,2,3435.416667
18,2,2858.022727
18,2,10793.223684
18,2,1779.750000
18,2,11029.125000
18,2,1666.000000
18,2,1636.240223
18,2,1675.000000
18,2,6494.542763
18,2,4337.823171
18,2,2315.366667
18,2,2123.093750
18,2,5537.602941
18,2,1542.855769
18,2,3446.557292
18,2,1629.903846
18,2,3443.462209
18,2,3972.700000
18,2,1716.551282
18,2,3425.823529
18,2,2208.884146
18,2,12976.495902
18,2,4652.078431
18,2,42489.000000
18,2,2167.363095
18,2,6645.301887
18,2,1812.049261
18,2,1995.130000
18,2,1527.674342
18,2,2423.196429
18,2,1603.895349
18,2,3281.642857
18,2,7953.395349
18,2,1615.500000
18,2,2864.371711
18,2,1986.600000
18,2,4647.625000
18,2,2715.025000
18,2,1945.137405
18,2,1520.846875
18,2,2795.665698
18,2,2391.083333
18,2,3836.550000
18,2,2134.940104
18,2,11927.716981

18,2,1837.817073
18,2,2648.815287
18,2,2896.266667
18,2,7047.992500
18,2,1930.380952
18,2,3082.288889
18,2,6638.743590
18,2,1949.600000
18,2,1839.282895
18,2,3089.404255
18,2,4752.676471
18,2,2792.145833
18,2,2094.384615
18,2,2796.110465
18,2,2134.576923
18,2,1529.203704
18,2,5377.428571
18,2,1541.842105
18,2,2430.500000
18,2,12336.505000
18,2,1534.132075
18,2,1837.656250
18,2,3693.941489
18,2,3023.026163
18,2,6146.125000
18,2,2314.517857
18,2,4432.326705
18,2,2659.137755
18,2,2617.866279
18,2,3617.690000
18,2,2465.594340
18,2,3964.694444
18,2,1709.127717
18,2,1613.442308
18,2,5781.655093
18,2,2422.887195
18,2,7860.750000
18,2,1932.023936
18,2,3167.900000
18,2,2407.541667
18,2,3279.229508
18,2,2475.010000
18,2,1819.846154
18,2,10387.406250
18,2,2254.187500
18,2,1801.000000
18,2,7012.071429
18,2,5663.551630
18,2,2786.382979
18,2,5455.755102
18,2,4724.436047
18,2,1740.230769
18,2,2047.887500
18,2,2175.471591
18,2,2478.806122
18,2,1562.244898
18,2,1775.207031
18,2,1777.625000
18,2,4957.492188
18,2,6241.658333
18,2,7259.778125
18,2,1971.668449
18,2,6353.101093
18,2,1988.756831
18,2,1545.989529
18,2,1780.428571

18,2,13258.791667
18,2,7817.958333
18,2,6564.410714
18,2,5007.687500
18,2,4373.152778
18,2,7850.500000
18,2,3535.714286
18,2,7229.527638
18,2,3910.843750
18,2,2582.352273
18,2,1932.098837
18,2,1894.241379
18,2,43539.312500
18,2,2013.250000
18,2,1768.262500
18,2,1807.691176
18,2,2230.869792
18,2,70258.266827
18,2,4477.944444
18,2,7909.043182
18,2,6210.660428
18,2,1565.380682
18,2,2075.994681
18,2,2907.489583
18,2,1761.813953
18,2,1913.000000
18,2,3331.592105
18,2,3569.512931
18,2,7319.186275
18,2,7039.730769
18,2,1717.461538
18,2,9215.768293
18,2,2784.370192
18,2,2860.507075
18,2,5999.428571
18,2,1793.989130
18,2,4822.357143
18,2,3546.625000
18,2,1718.267045
18,2,4051.298077
18,2,4479.540984
18,2,1947.875000
18,2,2494.944149
18,2,2186.539474
18,2,1892.885417
18,2,2043.287791
18,2,1748.708333
18,2,2700.550000
18,2,1579.335227
18,2,4429.862150
18,2,8131.929487
18,2,2811.907609
18,2,2005.766304
18,2,2055.875000
18,2,4947.395833
18,2,26146.545139
18,2,2712.515625
18,2,5070.547170
18,2,5373.755102
18,2,3195.819444
18,2,1763.346875
18,2,4644.685268
18,2,3841.851562
18,2,3596.207447
18,2,1576.960000
18,2,3487.364583

18,2,1642.043605
18,2,2074.890625
18,2,1837.907609
18,2,6113.630000
18,2,8780.875000
18,2,1673.676136
18,2,3178.282738
18,2,2437.250000
18,2,1756.722826
18,2,2325.878788
18,2,6745.817708
18,2,70396.625000
18,2,5320.885417
18,2,2130.768293
18,2,3539.687500
18,2,3157.910000
18,2,2266.540000
18,2,1599.551282
18,2,3260.925926
18,2,9486.213068
18,2,3293.069767
18,2,3617.195000
18,2,2031.093458
18,2,2234.291667
18,2,2337.795918
18,2,3074.671053
18,2,2453.877976
18,2,2558.489583
18,2,1751.803125
18,2,9867.250000
18,2,2143.642857
18,2,4919.437500
18,2,2990.869444
18,2,6266.689189
18,2,2096.313830
18,2,1810.509615
18,2,2564.457447
18,2,3846.634146
18,2,5886.270833
18,2,5080.890244
18,2,2683.793750
18,2,3689.682927
18,2,1627.260714
18,2,2745.653061
18,2,2299.957317
18,2,2988.121951
18,2,3579.911458
18,2,2273.551887
18,2,4356.750000
18,2,2128.561224
18,2,2592.509615
18,2,2067.263158
18,2,1651.200000
18,2,2930.327586
18,2,4012.108696
18,2,4707.727941
18,2,4901.590909
18,2,2671.650602
18,2,2623.200000
18,2,3432.550000
18,2,5558.910714
18,2,5517.893868
18,2,13878.818750
18,2,2858.698113
18,2,3169.187500
18,2,1705.595238

18,2,7639.590625
18,2,1781.522959
18,2,20236.600000
18,2,2221.538690
18,2,1770.592105
18,2,1765.696759
18,2,3844.243902
18,2,3679.875000
18,2,2071.176471
18,2,7279.948529
18,2,1529.360577
18,2,4952.048469
18,2,1567.700000
18,2,6605.665948
18,2,1701.320513
18,2,2542.433962
18,2,4484.458333
18,2,2439.798611
18,2,2577.398936
18,2,2666.429545
18,2,2514.903409
18,2,5281.310345
18,2,1702.244565
18,2,5268.369565
18,2,1767.380435
18,2,2239.781250
18,2,3141.144737
18,2,5123.980263
18,2,2401.442308
18,2,1615.565217
18,2,2328.063830
18,2,8616.000000
18,2,4650.533854
18,2,5676.185811
18,2,1935.600000
18,2,2500.771277
18,2,4008.185185
18,2,1707.693182
18,2,3344.162791
18,2,7722.477273
18,2,2110.217949
18,2,2638.875000
18,2,5059.407895
18,2,9175.815476
18,2,1806.100000
18,2,5683.237245
18,2,3432.750000
18,2,1687.776961
18,2,4695.625000
18,2,3682.361702
18,2,1691.954545
18,2,4845.279070
18,2,9330.714286
18,2,4124.301136
18,2,2227.979592
18,2,2515.561047
18,2,6558.361702
18,2,5346.847222
18,2,3670.452830
18,2,4082.617021
18,2,6023.545455
18,2,5589.250000
18,2,7039.163265
18,2,4048.223958
18,2,9718.656250
18,2,3184.045213

18,2,2963.500000
18,2,4771.260870
18,2,2404.638298
18,2,4285.206250
18,2,3446.986842
18,2,2300.095395
18,2,4031.202830
18,2,2185.664773
18,2,1737.571429
18,2,7663.097561
18,2,3875.977273
18,2,1707.033784
18,2,2219.774510
18,2,7677.000000
18,2,7433.135294
18,2,3213.471910
18,2,4103.235294
18,2,3936.021739
18,2,4942.526316
18,2,2125.648438
18,2,7595.019737
18,2,1562.326923
18,2,4342.365854
18,2,13250.562500
18,2,8659.603659
18,2,2701.758929
18,2,2014.898148
18,2,3806.458333
18,2,11353.654545
18,2,3176.800000
18,2,2976.548780
18,2,7597.053571
18,2,2512.107955
18,2,8772.595395
18,2,2469.260870
18,2,4502.583333
18,2,2422.566667
18,2,1729.464286
18,2,3332.704741
18,2,1845.050000
18,2,3944.192308
18,2,1964.117647
18,2,1768.000000
18,2,3387.659091
18,2,2432.000000
18,2,12030.700000
18,2,2333.576923
18,2,1824.153846
18,2,8318.113208
18,2,2732.437500
18,2,1639.875000
18,2,4944.032609
18,2,2020.655000
18,2,10363.775401
18,2,3277.500000
18,2,2090.662162
18,2,1566.202381
18,2,5711.846154
18,2,20697.673913
18,2,6574.584746
18,2,6779.348837
18,2,6161.000000
18,2,3036.312500
18,2,3976.068396
18,2,2525.297872
18,2,7102.617021

18,2,2879.086538
18,2,3833.362245
18,2,26965.070513
18,2,2397.250000
18,2,2452.518868
18,2,2117.333333
18,2,1861.625000
18,2,3037.818182
18,2,1957.795000
18,2,2480.209302
18,2,1578.731383
18,2,2089.724490
18,2,2547.812500
18,2,4103.026316
18,2,2457.607143
18,2,1933.988372
18,2,3094.483974
18,2,7569.211957
18,2,3258.848039
18,2,2896.383333
18,2,2379.391892
18,2,2148.750000
18,2,3032.313433
18,2,2456.812500
18,2,6654.177500
18,2,5413.320388
18,2,3385.539157
18,2,3058.671569
18,2,2814.447368
18,2,19760.771930
18,2,2176.700000
18,2,1698.641026
18,2,4251.375000
18,2,1577.000000
18,2,3672.720000
18,2,2291.357143
18,2,2425.101190
18,2,2706.962264
18,2,2007.445161
18,2,1984.750000
18,2,5604.764398
18,2,2314.307692
18,2,4563.125000
18,2,7944.384615
18,2,2817.408537
18,2,5005.148936
18,2,2020.533854
18,2,4013.708333
18,2,6922.143939
18,2,12308.237500
18,2,3332.231707
18,2,2913.821078
18,2,3691.738636
18,2,2768.175000
18,2,2818.386364
18,2,3885.388298
18,2,3399.942708
18,2,6771.304878
18,2,2767.855469
18,2,1586.589286
18,2,4114.910377
18,2,2274.921875
18,2,2442.237069
18,2,2395.840909
18,2,3539.840909
18,2,17564.705729

18,2,2466.565217
18,2,7915.654545
18,2,4523.223404
18,2,4004.384615
18,2,2366.357558
18,2,3114.292683
18,2,2952.541667
18,2,1679.743421
18,2,1760.418919
18,2,2658.240000
18,2,1779.087500
18,2,1836.036364
18,2,3283.261628
18,2,3439.937500
18,2,2768.534884
18,2,13768.005952
18,2,1557.810811
18,2,2528.068182
18,2,2811.273649
18,2,2763.794872
18,2,21054.677083
18,2,1965.776042
18,2,5927.157068
18,2,1837.932927
18,2,3052.805556
18,2,3457.153846
18,2,4218.000000
18,2,4609.739130
18,2,2207.413043
18,2,3226.958333
18,2,1842.683962
18,2,2803.529412
18,2,2431.209302
18,2,4129.905405
18,2,2642.597826
18,2,4907.158537
18,2,4264.823529
18,2,7802.104592
18,2,2092.900735
18,2,1887.889881
18,2,6284.959459
18,2,1655.377778
18,2,4805.326471
18,2,2935.765957
18,2,2481.713816
18,2,4325.188953
18,2,16006.473684
18,2,3813.047872
18,2,1511.529605
18,2,2047.640625
18,2,3494.391960
18,2,2272.409574
18,2,1727.228125
18,2,1554.653846
18,2,1581.113372
18,2,3711.333333
18,2,5478.711538
18,2,9879.904661
18,2,2656.094118
18,2,1645.726190
18,2,2396.302632
18,2,2185.090909
18,2,10730.612245
18,2,19465.657143
18,2,3442.494624
18,2,1840.911585

18,2,2596.732558
18,2,2116.090909
18,2,2162.404605
18,2,1628.679878
18,2,3889.762500
18,2,6051.112805
18,2,2229.234043
18,2,3726.486911
18,2,2470.914634
18,2,13551.378049
18,2,2635.727273
18,2,3086.175000
18,2,1503.375000
18,2,4116.315789
18,2,1614.262755
18,2,2559.565341
18,2,16794.285714
18,2,4299.412791
18,2,3845.000000
18,2,4100.395349
18,2,47444.848837
18,2,3809.078431
18,2,13555.939655
18,2,14604.619048
18,2,3502.194444
18,2,5079.322917
18,2,4062.000000
18,2,6170.928571
18,2,1508.666667
18,2,2435.437500
18,2,3282.657895
18,2,1594.062500
18,2,1815.806452
18,2,3507.000000
18,2,2479.032895
18,2,1701.909375
18,2,3144.932292
18,2,1999.553571
18,2,7844.542553
18,2,4189.872500
18,2,1950.041667
18,2,3630.355769
18,2,1798.806548
18,2,6672.145833
18,2,3898.012195
18,2,2205.672619
18,2,16294.575000
18,2,4344.337264
18,2,2932.507653
18,2,2071.937500
18,2,2833.958333
18,2,1801.762821
18,2,9994.440678
18,2,3964.785256
18,2,1795.895349
18,2,2718.219444
18,2,5317.362805
18,2,3855.202703
18,2,4418.871795
18,2,1585.177632
18,2,4378.970930
18,2,4186.482955
18,2,4486.475000
18,2,1730.256944
18,2,4043.510204
18,2,4657.156863

18,2,2367.236413
18,2,24120.634146
18,2,1700.500000
18,2,1836.364583
18,2,7317.409091
18,2,1778.401786
18,2,1562.736607
18,2,5556.672414
18,2,1951.355556
18,2,15724.205357
18,2,3265.750000
18,2,9630.169540
18,2,1693.350000
18,2,3336.250000
18,2,2803.273438
18,2,4757.590909
18,2,5049.437500
18,2,3560.285000
18,2,2727.017857
18,2,2118.917614
18,2,3211.770408
18,2,2223.186047
18,2,1519.532895
18,2,2445.989583
18,2,9282.442623
18,2,3536.170213
18,2,1562.668750
18,2,1785.221154
18,2,5120.666667
18,2,1537.888889
18,2,1808.833333
18,2,7023.127451
18,2,6963.663462
18,2,1628.151515
18,2,2013.460938
18,2,3218.843750
18,2,6012.611702
18,2,3417.905660
18,2,4959.320755
18,2,7831.768293
18,2,2780.970238
18,2,18695.057143
18,2,6903.431818
18,2,1893.692308
18,2,46969.285714
18,2,1598.755208
18,2,2891.166667
18,2,3603.252660
18,2,24704.297619
18,2,3978.315789
18,2,3723.744898
18,2,6070.378676
18,2,3164.128676
18,2,6674.714286
18,2,5499.351351
18,2,1964.178808
18,2,1768.250000
18,2,1558.061170
18,2,11980.093750
18,2,3230.565217
18,2,5313.607143
18,2,4903.145833
18,2,2283.513158
18,2,1502.906250
18,2,1702.232143
18,2,2039.222222

18,2,2954.064189
18,2,20803.698529
18,2,2256.680233
18,2,1522.818182
18,2,13305.205000
18,2,1827.338889
18,2,2364.552632
18,2,1599.744681
18,2,2851.653226
18,2,2409.516304
18,2,2226.298077
18,2,3766.789286
18,2,2779.750000
18,2,1862.500000
18,2,5172.897059
18,2,2793.741379
18,2,5915.015625
18,2,4333.183673
18,2,7983.168478
18,2,3633.809524
18,2,3547.366071
18,2,5224.890052
18,2,2083.028796
18,2,2182.678947
18,2,2473.801546
18,2,2031.709302
18,2,5298.381188
18,2,1850.785714
18,2,4342.040000
18,2,1773.833333
18,2,6036.584375
18,2,2549.593923
18,2,3618.902439
18,2,5956.548913
18,2,2946.060000
18,2,2698.423077
18,2,1617.500000
18,2,3690.359375
18,2,3167.051829
18,2,2031.348780
18,2,44510.100000
18,2,1812.028571
18,2,2261.486364
18,2,4583.255319
18,2,15766.470588
18,2,2057.194444
18,2,1693.413265
18,2,1839.368421
18,2,3653.691489
18,2,3416.300000
18,2,6516.818878
18,2,1589.716216
18,2,2002.180851
18,2,4428.821429
18,2,2057.094595
18,2,2754.719512
18,2,3579.371728
18,2,7012.461957
18,2,6788.239130
18,2,10744.117021
18,2,2942.000000
18,2,1928.937500
18,2,1853.570513
18,2,1612.843750
18,2,1600.680000
18,2,6073.393617

18,2,1968.000000
18,2,4654.144231
18,2,1886.235577
18,2,2552.330000
18,2,4121.303571
18,2,2617.648936
18,2,2361.671429
18,2,10930.760638
18,2,41834.025641
18,2,2271.350962
18,2,3326.814815
18,2,4210.067073
18,2,1729.656250
18,2,2871.698113
18,2,2745.972973
18,2,1838.213636
18,2,3923.061170
18,2,4059.293367
18,2,2458.143617
18,2,2086.098404
18,2,2416.382653
18,2,3409.299419
18,2,2445.779412
18,2,4836.823171
18,2,2316.840625
18,2,2785.023256
18,2,7984.661184
18,2,5194.301136
18,2,2182.007979
18,2,1993.112500
18,2,1835.115385
18,2,2209.535714
18,2,2103.916667
18,2,1576.468750
18,2,5536.447674
18,2,127476.126582
18,2,7259.665025
18,2,10396.375000
18,2,3189.420000
18,2,1743.938596
18,2,1745.019022
18,2,1604.571429
18,2,1733.009259
18,2,3907.127778
18,2,2633.361842
18,2,11262.869565
18,2,1842.774390
18,2,5860.368421
18,2,2974.211538
18,2,3624.250000
18,2,1936.956522
18,2,2432.718750
18,2,5954.825000
18,2,6057.261628
18,2,2663.335938
18,2,6025.563679
18,2,12894.459184
18,2,5861.950658
18,2,2680.839286
18,2,4118.433962
18,2,3469.744792
18,2,3170.144737
18,2,2091.941532
18,2,2927.544444
18,2,2205.527778
18,2,1882.093750

18,2,3281.111702
18,2,1937.171512
18,2,2607.487805
18,2,2796.093750
18,2,1927.392857
18,2,33630.866397
18,2,1663.428571
18,2,6385.563830
18,2,2398.017045
18,2,4082.219512
18,2,1950.985119
18,2,3785.453488
18,2,15294.576271
18,2,2071.269231
18,2,3528.650000
18,2,2141.127841
18,2,3793.875000
18,2,11021.826087
18,2,1742.752907
18,2,3273.182692
18,2,2257.582781
18,2,2026.525943
18,2,4253.641026
18,2,4391.908537
18,2,3373.473404
18,2,1678.247283
18,2,4907.132653
18,2,3859.016304
18,2,5361.580882
18,2,2360.371711
18,2,2352.757225
18,2,1827.475410
18,2,4407.666667
18,2,3806.125000
18,2,2813.934524
18,2,1929.324324
18,2,4354.729167
18,2,2721.910714
18,2,2549.500000
18,2,4753.000000
18,2,3479.465426
18,2,1650.031250
18,2,32882.425000
18,2,2004.000000
18,2,4076.000000
18,2,4518.636628
18,2,3765.807692
18,2,2964.206897
18,2,3075.444767
18,2,4487.244048
18,2,4480.473684
18,2,10034.283537
18,2,9355.042857
18,2,4835.203571
18,2,1740.625000
18,2,4737.934466
18,2,3498.930233
18,2,6551.012500
18,2,2165.426829
18,2,1511.090909
18,2,2584.618902
18,2,5384.416667
18,2,4975.661765
18,2,3134.807229
18,2,2421.801887
18,2,7051.997487

18,2,1895.895408
18,2,5754.754717
18,2,5429.727273
18,2,3053.574074
18,2,4853.227273
18,2,8141.006098
18,2,5437.500000
18,2,1697.146341
18,2,8198.187500
18,2,5441.167539
18,2,2447.486486
18,2,1726.409574
18,2,6218.320312
18,2,4077.861111
18,2,6139.187500
18,2,1610.310811
18,2,2084.817073
18,2,3418.289474
18,2,1933.545455
18,2,6022.125000
18,2,4619.734043
18,2,4881.957055
18,2,1676.870690
18,2,3310.533537
18,2,4928.100000
18,2,5487.031977
18,2,3658.234536
18,2,6137.577381
18,2,2147.180556
18,2,4204.325000
18,2,11514.860169
18,2,2098.723214
18,2,2340.250000
18,2,12372.045455
18,2,3114.615385
18,2,2552.136905
18,2,1973.393617
18,2,2925.256281
18,2,1663.018293
18,2,3874.000000
18,2,2016.461538
18,2,1745.844156
18,2,1632.108974
18,2,1632.239130
18,2,2421.500000
18,2,1524.750000
18,2,5435.165698
18,2,4378.778226
18,2,3109.915761
18,2,3101.500000
18,2,2070.495192
18,2,1639.539773
18,2,3115.141026
18,2,2179.187500
18,2,7364.344828
18,2,4484.703125
18,2,1717.775568
18,2,4385.788043
18,2,2273.858696
18,2,2325.750000
18,2,2180.254902
18,2,5799.030000
18,2,1829.375000
18,2,1824.493243
18,2,19849.666667
18,2,3079.240000

18,2,11047.625000
18,2,2574.619565
18,2,1871.048701
18,2,7653.000000
18,2,6478.371875
18,2,2392.611607
18,2,4017.224057
18,2,1648.852564
18,2,11910.906977
18,2,4372.829787
18,2,2819.425532
18,2,2348.502604
18,2,14873.449519
18,2,3057.893617
18,2,3621.612805
18,2,14588.523026
18,2,2287.825000
18,2,4437.714286
18,2,2828.076087
18,2,4112.451087
18,2,1586.100000
18,2,2133.533333
18,2,1981.854015
18,2,2256.250000
18,2,3596.600610
18,2,1552.781250
18,2,1501.405405
18,2,3602.785714
18,2,1601.750000
18,2,2250.680556
18,2,8530.377778
18,2,1993.342949
18,2,2393.993750
18,2,4699.826923
18,2,1518.880435
18,2,4058.808511
18,2,2646.896875
18,2,5269.487500
18,2,3150.631579
18,2,5604.531250
18,2,2307.550000
18,2,2390.500000
18,2,4123.170732
18,2,2835.701923
18,2,2989.568878
18,2,2409.519608
18,2,1627.258929
18,2,8167.673469
18,2,2062.604839
18,2,1831.692308
18,2,1739.037162
18,2,1588.204268
18,2,12100.465116
18,2,1512.965278
18,2,31115.666667
18,2,2221.988636
18,2,1956.515957
18,2,3618.365385
18,2,2135.544872
18,2,10439.086957
18,2,1578.250000
18,2,5134.200000
18,2,5731.236842
18,2,1549.154255
18,2,2055.612069
18,2,2958.269231

18,2,1780.250000
18,2,1918.303125
18,2,2339.213068
18,2,3857.420455
18,2,2138.360000
18,2,6264.790698
18,2,2138.833333
18,2,3905.419872
18,2,10873.949074
18,2,5057.954545
18,2,7031.500000
18,2,6274.756579
18,2,1711.353723
18,2,8232.066860
18,2,5196.259259
18,2,2304.369565
18,2,2829.775000
18,2,18317.583333
18,2,4378.166667
18,2,1779.025806
18,2,11443.706250
18,2,2541.281250
18,2,3007.420455
18,2,17022.470588
18,2,1966.970588
18,2,3724.776536
18,2,2230.729592
18,2,7466.702703
18,2,1516.392442
18,2,2053.250000
18,2,1548.989130
18,2,1568.645833
18,2,2202.000000
18,2,2009.890000
18,2,2424.758333
18,2,5227.171875
18,2,11339.952381
18,2,3091.738636
18,2,1733.426136
18,2,2666.391304
18,2,1520.183333
18,2,5108.978448
18,2,7909.500000
18,2,4790.510638
18,2,1930.952703
18,2,3506.337054
18,2,8108.109375
18,2,4080.621622
18,2,7742.959016
18,2,4602.324468
18,2,6636.289062
18,2,5545.756757
18,2,2230.080729
18,2,1907.230769
18,2,4230.855769
18,2,8898.115854
18,2,1584.225694
18,2,3128.460106
18,2,9238.049479
18,2,4784.930851
18,2,6027.500000
18,2,2120.727564
18,2,7622.157895
18,2,2893.465116
18,2,1576.176471
18,2,1865.830000

18,2,3775.666667
18,2,5184.788265
18,2,3836.709677
18,2,5108.100000
18,2,2851.683673
18,2,4267.294479
18,2,2333.417763
18,2,2146.377841
18,2,1750.625000
18,2,8571.375000
18,2,1948.334302
18,2,6981.710106
18,2,9899.641129
18,2,1810.555556
18,2,2259.283784
18,2,9380.311111
18,2,4138.381818
18,2,1865.744186
18,2,2816.265000
18,2,2697.784091
18,2,1724.585000
18,2,1657.532051
18,2,2036.835526
18,2,12126.650943
18,2,5613.959091
18,2,2648.521875
18,2,1977.676471
18,2,4275.469274
18,2,3890.000000
18,2,75125.425000
18,2,6533.982558
18,2,2611.889205
18,2,2714.092105
18,2,3409.377841
18,2,2949.562112
18,2,1518.328704
18,2,9432.600000
18,2,2317.121795
18,2,3154.353774
18,2,15958.527778
18,2,5790.482955
18,2,1903.393868
18,2,2686.114130
18,2,2828.000000
18,2,2195.731707
18,2,4085.852500
18,2,27066.296196
18,2,5094.314189
18,2,1590.333333
18,2,4251.569444
18,2,6571.000000
18,2,5512.261364
18,2,6177.359375
18,2,2077.635204
18,2,4712.090909
18,2,3182.165254
18,2,10779.304348
18,2,1678.430851
18,2,8765.207237
18,2,2892.460000
18,2,2608.215426
18,2,4054.831395
18,2,24792.360825
18,2,7304.030660
18,2,4726.086111
18,2,5196.056548

18,2,7206.604167
18,2,2422.236842
18,2,5893.447368
18,2,2995.181818
18,2,2552.647059
18,2,8398.251337
18,2,12001.888889
18,2,1594.185185
18,2,7417.977564
18,2,2825.655488
18,2,3078.924107
18,2,9465.855072
18,2,1779.367742
18,2,12225.847222
18,2,2413.862500
18,2,7290.301047
18,2,1966.527439
18,2,2550.250000
18,2,1908.495192
18,2,3563.615385
18,2,2125.259804
18,2,3880.057692
18,2,2500.882353
18,2,1564.940217
18,2,1516.343558
18,2,1736.743750
18,2,2394.347222
18,2,5793.823980
18,2,2265.875000
18,2,1850.812500
18,2,2702.179348
18,2,1910.687500
18,2,5389.824561
18,2,4474.274882
18,2,1618.470968
18,2,2444.139535
18,2,6115.518519
18,2,4977.966851
18,2,1502.305851
18,2,6536.597826
18,2,2946.000000
18,2,2417.034884
18,2,2110.791667
18,2,3955.333333
18,2,3117.511628
18,2,7114.952128
18,2,2099.073718
18,2,9159.062500
18,2,2812.586364
18,2,11177.538889
18,2,5722.461538
18,2,2250.612745
18,2,6553.727273
18,2,2815.122283
18,2,5402.812500
18,2,7486.725490
18,2,1678.700000
18,2,7865.143657
18,2,1547.010870
18,2,4676.479911
18,2,1508.636364
18,2,2378.093750
18,2,1831.885000
18,2,10535.566176
18,2,6449.750000
18,2,1950.293103

18,2,2663.204082
18,2,11691.599265
18,2,1658.086957
18,2,2443.836066
18,2,2388.561644
18,2,1580.926136
18,2,3337.357143
18,2,6716.744681
18,2,2191.789474
18,2,2610.500000
18,2,3747.118421
18,2,3846.581395
18,2,2240.363636
18,2,4995.587629
18,2,1930.206250
18,2,4340.532895
18,2,8414.281250
18,2,15969.846939
18,2,3468.333333
18,2,3355.781250
18,2,2430.875000
18,2,2388.837838
18,2,1785.687500
18,2,195416.554054
18,2,8019.272727
18,2,2020.642157
18,2,1515.056452
18,2,1685.830128
18,2,1863.389423
18,2,2399.442308
18,2,10318.428241
18,2,1743.625000
18,2,1942.750000
18,2,1977.177778
18,2,1534.606383
18,2,1614.595238
18,2,3623.571429
18,2,1991.506757
18,2,6651.607843
18,2,2281.460227
18,2,2225.567073
18,2,7913.048913
18,2,4189.318878
18,2,1944.584302
18,2,1890.629464
18,2,2312.660326
18,2,2901.291667
18,2,3579.585000
18,2,5894.836735
18,2,2393.132653
18,2,5516.400000
18,2,2396.529412
18,2,3242.932432
18,2,5167.489796
18,2,1721.000000
18,2,4344.523936
18,2,1648.325581
18,2,1735.617021
18,2,1902.166667
18,2,5879.280000
18,2,35712.637500
18,2,5754.494681
18,2,2537.081081
18,2,2454.427778
18,2,1720.794872
18,2,2942.082447

18,2,5198.980978
18,2,3347.159091
18,2,1607.050595
18,2,2139.470238
18,2,2802.324324
18,2,3150.571429
18,2,2503.131579
18,2,2272.628289
18,2,15091.722222
18,2,5565.355263
18,2,3653.465426
18,2,3137.110000
18,2,2682.733553
18,2,1854.615000
18,2,9407.094595
18,2,5954.863415
18,2,2070.942935
18,2,5031.620283
18,2,3475.391447
18,2,1841.392857
18,2,6127.069079
18,2,12235.290625
18,2,1528.500000
18,2,2139.021277
18,2,9497.837302
18,2,2092.182927
18,2,3551.727273
18,2,4262.619792
18,2,10407.582589
18,2,3225.875000
18,2,8857.316964
18,2,2347.388889
18,2,2094.975000
18,2,2848.493750
18,2,2264.004808
18,2,6059.355769
18,2,2498.000000
18,2,3166.022727
18,2,2388.243902
18,2,1506.180645
18,2,4232.421875
18,2,2428.200000
18,2,3569.523810
18,2,4372.375000
18,2,2236.737500
18,2,9885.629630
18,2,3437.518519
18,2,3035.000000
18,2,2170.980556
18,2,2011.287037
18,2,1550.290323
18,2,2595.350000
18,2,1797.744186
18,2,5529.168367
18,2,6135.469512
18,2,1875.306122
18,2,5549.594340
18,2,4352.164251
18,2,2193.062500
18,2,3017.669643
18,2,2232.148936
18,2,1632.366848
18,2,3341.194444
18,2,6608.984375
18,2,4358.750000
18,2,3916.736842

18,2,1841.116438
18,2,2267.908854
18,2,3556.086735
18,2,1709.019231
18,2,1763.037500
18,2,2559.460526
18,2,2312.782979
18,2,2116.307692
18,2,1519.936047
18,2,2296.217143
18,2,11359.016892
18,2,4197.456818
18,2,5452.250000
18,2,2300.170000
18,2,1513.561170
18,2,2997.670455
18,2,1717.819767
18,2,5183.861979
18,2,2270.784314
18,2,2456.234694
18,2,4989.892857
18,2,4169.055556
18,2,16420.227273
18,2,6549.274457
18,2,6544.796569
18,2,1926.115646
18,2,4282.607143
18,2,2212.632353
18,2,4625.277027
18,2,1953.783784
18,2,1699.139881
18,2,2243.621622
18,2,6244.923469
18,2,2093.455556
18,2,3572.449324
18,2,2322.763006
18,2,2004.723404
18,2,16763.911765
18,2,5635.830357
18,2,2295.988304
18,2,2507.595092
18,2,1811.848958
18,2,2413.764706
18,2,4412.312500
18,2,2358.218023
18,2,2217.097222
18,2,1518.655488
18,2,2625.352564
18,2,1762.617647
18,2,3416.000000
18,2,6061.829268
18,2,4754.070968
18,2,5424.183673
18,2,3427.198113
18,2,3133.429487
18,2,13429.484091
18,2,3597.995370
18,2,6094.234043
18,2,3573.493056
18,2,2118.297872
18,2,3892.435484
18,2,3697.350962
18,2,3802.309028
18,2,4062.145833
18,2,1741.246622
18,2,2107.416667

18,2,3613.419643
18,2,3350.277778
18,2,3214.931818
18,2,4149.880435
18,2,2072.557927
18,2,1827.762821
18,2,2766.197115
18,2,2783.026316
18,2,3903.188679
18,2,2474.414634
18,2,2301.093750
18,2,3361.794872
18,2,2054.323529
18,2,2983.405530
18,2,2056.618919
18,2,1901.562500
18,2,3662.438830
18,2,2767.666667
18,2,2359.677326
18,2,3886.541667
18,2,1524.246622
18,2,2309.355769
18,2,1539.654891
18,2,1775.235577
18,2,2155.217105
18,2,1644.525000
18,2,3469.136364
18,2,4335.697674
18,2,2529.567857
18,2,5987.549342
18,2,5632.500000
18,2,3849.409574
18,2,2525.888889
18,2,5441.673469
18,2,1769.500000
18,2,1905.529412
18,2,1860.621951
18,2,2075.944444
18,2,1790.127660
18,2,1940.286458
18,2,2672.234043
18,2,5522.255208
18,2,1995.861702
18,2,3126.222222
18,2,3585.000000
18,2,1805.810811
18,2,2822.444175
18,2,8611.344444
18,2,1892.243750
18,2,2931.590909
18,2,2071.838542
18,2,3340.525000
18,2,5028.689103
18,2,32502.420455
18,2,3120.728448
18,2,1868.116279
18,2,3718.968750
18,2,2443.575000
18,2,4429.702128
18,2,1855.153409
18,2,1680.799342
18,2,3329.395833
18,2,5361.687500
18,2,2190.500000
18,2,2420.875000
18,2,2623.431373

18,2,4688.332237
18,2,4298.812500
18,2,1705.882979
18,2,7173.332447
18,2,2942.333333
18,2,3167.700000
18,2,2327.516304
18,2,2364.526596
18,2,1852.418182
18,2,2405.800000
18,2,7470.411058
18,2,1671.769231
18,2,2541.866667
18,2,2237.725543
18,2,4104.375000
18,2,2004.319372
18,2,4604.804469
18,2,1513.343750
18,2,1604.875000
18,2,1728.431818
18,2,1586.466019
18,2,12621.385593
18,2,2820.843750
18,2,3821.986047
18,2,2209.323171
18,2,4551.997596
18,2,16969.423077
18,2,1803.130435
18,2,4895.812500
18,2,2230.222973
18,2,5110.120000
18,2,4796.867647
18,2,1724.428571
18,2,2827.750000
18,2,2416.714286
18,2,1985.396186
18,2,20958.759259
18,2,3424.655000
18,2,4092.546053
18,2,3126.291667
18,2,1979.200000
18,2,3466.514535
18,2,4293.989950
18,2,5736.065217
18,2,2306.900000
18,2,1546.662791
18,2,1545.052326
18,2,9305.388889
18,2,3948.282738
18,2,9157.285000
18,2,10100.461538
18,2,7897.808673
18,2,1791.554688
18,2,4853.181818
18,2,3792.488372
18,2,2098.357843
18,2,2779.360577
18,2,1614.986842
18,2,1989.671875
18,2,6013.977778
18,2,5024.750000
18,2,2114.426136
18,2,3594.230769
18,2,15148.573980
18,2,7180.044118
18,2,1645.000000

18,2,2899.083916
18,2,37949.607143
18,2,9128.953846
18,2,34979.482143
18,2,2537.888889
18,2,5276.049419
18,2,2522.200000
18,2,4503.910714
18,2,2419.140000
18,2,3447.957447
18,2,4836.030769
18,2,2563.446429
18,2,63969.543367
18,2,1854.444444
18,2,6898.875000
18,2,2318.943182
18,2,2530.230769
18,2,3516.074468
18,2,1850.912162
18,2,7422.831633
18,2,2262.880814
18,2,8519.600000
18,2,3429.828125
18,2,5048.220588
18,2,1536.197917
18,2,1676.916667
18,2,4141.645270
18,2,2436.071429
18,2,2291.657143
18,2,2285.181250
18,2,2123.837766
18,2,1501.838710
18,2,2031.459459
18,2,3756.878049
18,2,2630.877551
18,2,1621.191667
18,2,2013.942073
18,2,5847.980978
18,2,3216.937500
18,2,22897.937500
18,2,2408.317308
18,2,2829.666667
18,2,9419.013158
18,2,2190.882143
18,2,2647.057203
18,2,2401.509375
18,2,4762.677778
18,2,2065.078125
18,2,2452.787037
18,2,2054.125000
18,2,2187.760870
18,2,1755.880000
18,2,3802.204861
18,2,10410.333333
18,2,3031.366667
18,2,1945.555556
18,2,7881.394231
18,2,9829.000000
18,2,5500.675000
18,2,4400.525510
18,2,1725.158854
18,2,1844.628378
18,2,1554.125000
18,2,1789.142857
18,2,2329.696429
18,2,2219.250000

18,2,6036.941176
18,2,4705.544271
18,2,1941.696875
18,2,6039.945513
18,2,2397.117347
18,2,2368.658537
18,2,3727.000000
18,2,10067.173077
18,2,1863.614130
18,2,2618.191860
18,2,7018.000000
18,2,8115.783088
18,2,1558.807692
18,2,1962.695312
18,2,1801.337838
18,2,6913.030769
18,2,3861.276163
18,2,7044.322816
18,2,4632.808511
18,2,2076.265625
18,2,2503.705000
18,2,10587.448370
18,2,2295.956250
18,2,4991.625000
18,2,5296.000000
18,2,1546.321429
18,2,5641.533784
18,2,2925.341463
18,2,1507.006098
18,2,5699.752717
18,2,1792.897959
18,2,4935.250000
18,2,4501.608974
18,2,1825.914063
18,2,2589.910714
18,2,19798.837500
18,2,2141.756579
18,2,2181.277778
18,2,7439.000000
18,2,2229.243902
18,2,12729.131579
18,2,2959.808511
18,2,4025.600000
18,2,3343.247222
18,2,2145.555556
18,2,5532.362981
18,2,2523.703125
18,2,3212.722222
18,2,3203.705128
18,2,1590.065789
18,2,1801.200000
18,2,1783.750000
18,2,1598.116477
18,2,22015.455357
18,2,2874.011628
18,2,6470.681818
18,2,1683.195652
18,2,3698.554878
18,2,3045.971429
18,2,2575.222222
18,2,3647.500000
18,2,2776.093750
18,2,2933.443878
18,2,2882.162162
18,2,1542.506579
18,2,3847.054878

18,2,6901.359649
18,2,1875.477500
18,2,8348.250000
18,2,1690.609694
18,2,2230.287879
18,2,3150.746324
18,2,9948.611111
18,2,3046.166667
18,2,4438.857143
18,2,2981.285714
18,2,2238.250000
18,2,1566.352273
18,2,1744.273936
18,2,7529.465517
18,2,1571.682051
18,2,5722.975000
18,2,1872.106383
18,2,3644.265000
18,2,1562.122340
18,2,5019.716667
18,2,3878.649038
18,2,2549.347561
18,2,1561.910714
18,2,4662.750000
18,2,2346.446809
18,2,3822.000000
18,2,2765.903409
18,2,6478.040000
18,2,1813.337838
18,2,1946.105263
18,2,25601.102941
18,2,4158.145089
18,2,1936.434783
18,2,5910.220339
18,2,3721.763889
18,2,1699.375000
18,2,2143.225904
18,2,2357.714286
18,2,5487.875000
18,2,3749.366667
18,2,5240.004630
18,2,2378.072115
18,2,1734.988889
18,2,4677.297500
18,2,5875.833333
18,2,4692.273438
18,2,3214.829787
18,2,8774.182065
18,2,3690.034483
18,2,2964.473684
18,2,7238.524390
18,2,2359.330357
18,2,2144.422872
18,2,8693.738636
18,2,3423.125000
18,2,4861.817708
18,2,1539.789474
18,2,4979.500000
18,2,7478.519737
18,2,9498.457447
18,2,1864.960000
18,2,3382.208333
18,2,2191.363636
18,2,1575.377907
18,2,12427.213415
18,2,4167.210526

18,2,1532.406250
18,2,1702.308511
18,2,2524.218137
18,2,3313.211538
18,2,1830.885714
18,2,10587.783333
18,2,2948.966216
18,2,3960.843750
18,2,3262.546512
18,2,5618.397727
18,2,1803.660326
18,2,5567.517857
18,2,1552.075000
18,2,4171.418367
18,2,1649.786232
18,2,1560.651316
18,2,4592.061224
18,2,3363.551020
18,2,2013.375000
18,2,3487.345000
18,2,2327.048780
18,2,1929.500000
18,2,1974.093750
18,2,3157.835000
18,2,5582.175000
18,2,2788.772059
18,2,3226.437500
18,2,4860.360656
18,2,3057.000000
18,2,2928.625000
18,2,2540.691176
18,2,6259.776786
18,2,44663.702703
18,2,1743.953947
18,2,5859.127841
18,2,3892.092391
18,2,2309.285714
18,2,2061.406250
18,2,1540.684211
18,2,2425.625000
18,2,4140.592105
18,2,3032.019737
18,2,3463.240854
18,2,2055.579082
18,2,3884.663043
18,2,2666.802326
18,2,2121.429688
18,2,1980.293860
18,2,6239.900000
18,2,5230.634146
18,2,1762.643750
18,2,2235.270833
18,2,3272.000000
18,2,6109.138021
18,2,3277.925000
18,2,3789.762755
18,2,2470.693627
18,2,1629.981132
18,2,1590.568182
18,2,2817.687500
18,2,2119.239583
18,2,1546.997283
18,2,4079.773810
18,2,4149.583333
18,2,2872.172297
18,2,1668.826923

18,2,2096.625000
18,2,5888.434211
18,2,2931.418182
18,2,2942.292553
18,2,16691.487179
18,2,1707.452703
18,2,2884.603723
18,2,2024.829787
18,2,2627.666667
18,2,2090.795455
18,2,2624.375000
18,2,4722.021739
18,2,4994.712766
18,2,2405.546512
18,2,4721.416667
18,2,1878.725000
18,2,1629.780000
18,2,1649.858333
18,2,6172.384615
18,2,1958.141667
18,2,7148.973837
18,2,4357.618750
18,2,11503.419118
18,2,1600.250000
18,2,3109.170213
18,2,4166.531250
18,2,2587.761628
18,2,3189.329268
18,2,3779.573171
18,2,2949.724551
18,2,6022.611842
18,2,1537.476190
18,2,3319.555556
18,2,24801.400474
18,2,1621.406250
18,2,12138.596154
18,2,9940.194313
18,2,1913.143617
18,2,2589.695652
18,2,2318.984375
18,2,2561.430233
18,2,1541.513889
18,2,5677.319712
18,2,6920.449102
18,2,4320.693750
18,2,4069.721311
18,2,2989.069767
18,2,2339.104911
18,2,5307.432432
18,2,2469.348214
18,2,7011.232558
18,2,7526.088889
18,2,3830.000000
18,2,3011.875000
18,2,1569.553571
18,2,2315.830000
18,2,21941.000000
18,2,2091.531915
18,2,3710.694444
18,2,2586.000000
18,2,8178.169271
18,2,2733.875000
18,2,3462.056962
18,2,2648.625000
18,2,1827.260870
18,2,2054.825000

18,2,1524.570652
18,2,1869.229167
18,2,2851.120536
18,2,2679.928571
18,2,5816.838415
18,2,3920.595745
18,2,2062.766892
18,2,4577.421875
18,2,6341.000000
18,2,1788.298611
18,2,2080.500000
18,2,5193.706522
18,2,10995.775000
18,2,5478.598404
18,2,2329.647059
18,2,5936.723684
18,2,9456.595238
18,2,2654.923841
18,2,2514.530000
18,2,2560.312500
18,2,2378.062500
18,2,2317.710227
18,2,1693.125000
18,2,1598.000000
18,2,9764.056250
18,2,3126.914474
18,2,1805.625000
18,2,2415.677778
18,2,2515.562500
18,2,1708.687500
18,2,5950.919444
18,2,6211.465909
18,2,17664.988372
18,2,2035.381579
18,2,4363.727273
18,2,3747.978261
18,2,1857.864130
18,2,3995.461538
18,2,8352.156250
18,2,2639.134615
18,2,2891.882653
18,2,1922.319444
18,2,1933.802632
18,2,1514.075472
18,2,1851.047619
18,2,2129.540541
18,2,1590.638889
18,2,2702.244681
18,2,3884.426020
18,2,4753.106742
18,2,2276.702128
18,2,3625.230769
18,2,11039.290816
18,2,2287.052326
18,2,5495.976415
18,2,2447.223684
18,2,4047.000000
18,2,1744.112500
18,2,9889.437500
18,2,6623.632500
18,2,1766.838710
18,2,3174.734973
18,2,3237.842105
18,2,3237.625000
18,2,1630.600000
18,2,1525.775000

18,2,10348.388889
18,2,5906.828488
18,2,1541.488636
18,2,4235.771341
18,2,1517.717857
18,2,4959.484375
18,2,1818.017857
18,2,3581.685714
18,2,4543.913043
18,2,1546.829268
18,2,1577.563953
18,2,2964.615000
18,2,1906.692308
18,2,3844.250000
18,2,3000.303922
18,2,1761.703125
18,2,6211.658784
18,2,2005.593750
18,2,15021.800000
18,2,5114.892361
18,2,7368.702128
18,2,1669.487805
18,2,1947.969907
18,2,1623.676829
18,2,6621.093750
18,2,6961.678571
18,2,2411.727941
18,2,2624.000000
18,2,3764.050000
18,2,2285.023026
18,2,3471.622222
18,2,2266.400000
18,2,10137.721698
18,2,6317.933511
18,2,2439.485577
18,2,1618.256579
18,2,7406.416667
18,2,4347.263889
18,2,1964.275641
18,2,4564.215686
18,2,7009.860000
18,2,3686.470588
18,2,2319.615385
18,2,1680.628205
18,2,1506.857143
18,2,2363.410714
18,2,2992.666667
18,2,2493.343750
18,2,2388.542553
18,2,6745.443878
18,2,1874.358108
18,2,1687.319149
18,2,12986.707500
18,2,1641.105263
18,2,1511.559783
18,2,13488.357143
18,2,1620.972656
18,2,8910.847826
18,2,2522.714286
18,2,3820.983553
18,2,2860.080729
18,2,4497.560096
18,2,1591.009146
18,2,4292.250000
18,2,2536.040816
18,2,1765.275000

18,2,3176.068627
18,2,2298.656250
18,2,1735.325000
18,2,4476.825000
18,2,5381.467033
18,2,26036.408451
18,2,2186.826754
18,2,15120.050847
18,2,11190.420213
18,2,3216.250000
18,2,2795.450000
18,2,2834.146277
18,2,4358.361650
18,2,2778.016509
18,2,2150.521635
18,2,7211.739130
18,2,3190.809211
18,2,1669.000000
18,2,2688.475000
18,2,6899.506757
18,2,2169.958333
18,2,3203.538462
18,2,7116.336957
18,2,58529.304545
18,2,6821.682692
18,2,7675.346939
18,2,2156.610119
18,2,2232.218023
18,2,1724.590909
18,2,3120.078125
18,2,6251.907609
18,2,2060.603571
18,2,2506.235795
18,2,3054.625000
18,2,4310.625000
18,2,6516.818182
18,2,2120.785714
18,2,2879.906250
18,2,2533.632979
18,2,1840.900000
18,2,2589.358974
18,2,2052.280488
18,2,3847.650000
18,2,2577.917614
18,2,3129.152778
18,2,7977.451220
18,2,3121.288462
18,2,8460.684659
18,2,1706.417763
18,2,2591.684659
18,2,4693.747340
18,2,4303.192073
18,2,2558.525510
18,2,2058.619792
18,2,2670.976415
18,2,2182.518868
18,2,3854.446429
18,2,2415.796512
18,2,2876.513514
18,2,2525.487500
18,2,4207.500000
18,2,6627.891509
18,2,3513.805556
18,2,1865.618902
18,2,1716.103125
18,2,2250.166667

18,2,3730.625000
18,2,2670.625000
18,2,2234.833333
18,2,1932.352273
18,2,3860.563636
18,2,9374.302326
18,2,4299.411364
18,2,3461.250000
18,2,8822.988636
18,2,3351.194444
18,2,7833.784314
18,2,1943.972222
18,2,8923.725490
18,2,1990.541667
18,2,2009.000000
18,2,3520.900000
18,2,1694.130000
18,2,2348.665625
18,2,2026.116279
18,2,1577.691489
18,2,1852.328125
18,2,1689.988636
18,2,1576.118421
18,2,3827.462500
18,2,13440.959302
18,2,3952.856250
18,2,5423.984375
18,2,2115.020408
18,2,13962.948077
18,2,3361.500000
18,2,3556.947674
18,2,2592.835106
18,2,3440.514881
18,2,2683.084746
18,2,5343.703431
18,2,2387.990385
18,2,12096.650000
18,2,2010.423077
18,2,1824.689189
18,2,4770.972222
18,2,2307.892857
18,2,1731.677165
18,2,2757.962766
18,2,5792.033854
18,2,6334.356383
18,2,2090.327778
18,2,1549.681548
18,2,4074.703488
18,2,6109.342742
18,2,3479.312500
18,2,2467.417614
18,2,2842.872159
18,2,2266.750000
18,2,7586.644608
18,2,3889.875000
18,2,6303.216912
18,2,6962.076923
18,2,19367.023438
18,2,1771.574405
18,2,2707.206522
18,2,2932.677083
18,2,1677.437500
18,2,3738.980000
18,2,7915.800000
18,2,2566.098214
18,2,1915.686047

18,2,1588.770833
18,2,1894.398585
18,2,1718.705000
18,2,1993.727273
18,2,10199.605556
18,2,2842.536585
18,2,10882.161364
18,2,4978.781250
18,2,3066.847222
18,2,3176.468750
18,2,2739.495192
18,2,16303.985795
18,2,3030.163677
18,2,1735.350000
18,2,1950.714844
18,2,3994.257143
18,2,2661.493056
18,2,3068.611872
18,2,2841.381643
18,2,2018.643275
18,2,8446.642157
18,2,20945.974359
18,2,3419.961538
18,2,1815.877841
18,2,2595.012500
18,2,6744.330882
18,2,1756.383721
18,2,1744.025641
18,2,3169.617500
18,2,2215.020000
18,2,1890.972222
18,2,1934.440000
18,2,7662.062500
18,2,4435.227273
18,2,1877.447368
18,2,3154.694712
18,2,3672.265625
18,2,1898.464744
18,2,2072.517857
18,2,4904.285714
18,2,1993.000000
18,2,5151.187500
18,2,2621.191489
18,2,3558.554688
18,2,5851.375000
18,2,2597.500000
18,2,2902.705000
18,2,1555.975962
18,2,6029.988636
18,2,4080.188776
18,2,2794.511628
18,2,5478.312500
18,2,6380.802885
18,2,2201.392857
18,2,1829.010417
18,2,1945.161111
18,2,3672.325581
18,2,2833.414634
18,2,4012.982143
18,2,3088.635204
18,2,3890.454545
18,2,4435.250000
18,2,5900.237500
18,2,2594.163690
18,2,2699.743902
18,2,5940.869565

18,2,1784.425532
18,2,2483.173780
18,2,13573.454545
18,2,1571.589674
18,2,2530.730769
18,2,4009.787500
18,2,1806.215000
18,2,3227.397351
18,2,5196.921429
18,2,5784.211957
18,2,2386.937500
18,2,5874.500000
18,2,6434.892857
18,2,7871.732143
18,2,2147.922619
18,2,4316.208333
18,2,2987.148936
18,2,4629.205128
18,2,2453.833333
18,2,6690.250000
18,2,2342.398810
18,2,1790.901639
18,2,2107.858553
18,2,1972.526442
18,2,9356.118182
18,2,2963.752907
18,2,3345.848404
18,2,1652.941176
18,2,9913.750000
19,1,2204.287500
19,1,2911.829268
19,1,2341.793605
19,1,2719.866667
19,1,2611.520833
19,1,61173.831081
19,1,2221.423077
19,1,140863.048780
19,1,135568.696429
19,1,7333.444149
19,1,4627.250000
19,1,34243.421053
19,1,1676.226190
19,1,6506.712500
19,1,6912.029762
19,1,2653.260135
19,1,124603.800000
19,1,6176.871795
19,1,2292.458333
19,1,184179.047619
19,1,1724.000000
19,1,2029.012821
19,1,1833.000000
19,1,2674.450000
19,1,2815.076389
19,1,3343.008929
19,1,1958.076531
19,1,2157.250000
19,1,3852.358108
19,1,2723.971154
19,1,1617.369048
19,1,3340.310811
19,1,1922.480769
19,1,6088.447368
19,1,1671.384615
19,1,5643.681818
19,1,72726.000000

19,1,7667.949405
19,1,17914.985119
19,1,1841.979167
19,1,1791.388158
19,1,10702.812500
19,1,23470.363372
19,1,30208.875000
19,1,4307.054054
19,1,19574.592391
19,1,18138.637500
19,1,1569.344512
19,1,61532.218085
19,1,1658.656250
19,1,13092.700000
19,1,112204.625000
19,1,2113.585366
19,1,197777.534884
19,1,69304.309211
19,1,2590.363636
19,1,11864.118750
19,1,3826.747283
19,1,37551.625000
19,1,1803.407609
19,1,1899.779255
19,1,1702.457317
19,1,1530.142045
19,1,12880.055233
19,1,2380.727273
19,1,7081.558824
19,1,1730.676471
19,1,3176.669872
19,1,1624.803977
19,1,2521.080000
19,1,1504.271739
19,1,9396.579545
19,1,1791.608974
19,1,1558.184783
19,1,3509.619565
19,1,22154.390625
19,1,1828.116883
19,1,1669.051282
19,1,6185.633721
19,1,68194.541667
19,1,1872.339286
19,1,44030.865854
19,1,5471.527273
19,1,11939.711957
19,1,4748.921429
19,1,31686.015152
19,1,5060.250000
19,1,19633.742857
19,1,2137.658333
19,1,61548.271277
19,1,1539.726562
19,1,2422.976744
19,1,14763.206897
19,1,32185.306034
19,1,1817.392045
19,1,2629.875000
19,1,13237.752907
19,1,78269.635417
19,1,2146.902174
19,1,1585.830882
19,1,4705.633721
19,1,5140.203947
19,1,2477.425676

19,1,2058.272321
19,1,2599.284091
19,1,41433.868056
19,1,2515.966216
19,1,53380.277027
19,1,5315.633929
19,1,12831.493243
19,1,5219.808824
19,1,25602.487179
19,1,2185.361702
19,1,1612.488636
19,1,1857.789894
19,1,1569.835106
19,1,1622.225000
19,1,2689.211538
19,1,2884.462121
19,1,28915.324324
19,1,3063.921053
19,1,17362.247283
19,1,3077.561047
19,1,4937.377778
19,1,2715.607143
19,1,1879.886364
19,1,9106.187500
19,1,3190.608108
19,1,105606.000000
19,1,8134.255435
19,1,16381.583333
19,1,7645.235849
19,1,1983.339674
19,1,1847.087209
19,1,1584.339286
19,1,161446.440217
19,1,2007.281250
19,1,15865.375000
19,1,4725.125000
19,1,1771.750000
19,1,95665.852941
19,1,46181.174342
19,1,34963.503571
19,1,62501.508929
19,1,4759.219512
19,1,1538.150000
19,1,16242.509868
19,1,2484.777778
19,1,2600.113208
19,1,1561.187500
19,1,40690.680147
19,1,88398.691489
19,1,1528.975000
19,1,10691.532258
19,1,1701.250000
19,1,37704.152439
19,1,2018.597561
19,1,119689.416667
19,1,16247.954545
19,1,1752.296875
19,1,1836.823529
19,1,158990.500000
19,1,4453.095588
19,1,1508.450000
19,1,2461.081522
19,1,2883.196429
19,1,2370.750000
19,1,15165.565217
19,1,6564.649123

19,1,1506.413462
19,1,2797.250000
19,1,85383.855000
19,1,7704.128049
19,1,20684.291667
19,1,1735.141304
19,1,4626.819444
19,1,32763.943878
19,1,2407.600000
19,1,4009.921875
19,1,10185.978659
19,1,2966.378049
19,1,31371.836538
19,1,48950.614286
19,1,2074.247024
19,1,5501.018617
19,1,1523.777500
19,1,4229.092105
19,1,3912.122340
19,1,48914.041667
19,1,136521.080128
19,1,2778.785714
19,1,3789.600000
19,1,89379.101351
19,1,4291.301136
19,1,37424.215116
19,1,3157.654412
19,1,11539.837209
19,1,5953.036585
19,1,15686.862805
19,1,1734.576923
19,1,2754.707447
19,1,10551.500000
19,1,2929.148810
19,1,70285.300000
19,1,2482.521739
19,1,4228.023256
19,1,4752.744565
19,1,5489.266667
19,1,1578.538462
19,1,2389.631579
19,1,2500.359375
19,1,1911.475694
19,1,2600.341463
19,1,13340.517241
19,1,1915.079545
19,1,2098.825000
19,1,15283.049342
19,1,2928.106383
19,1,20454.807927
19,1,1784.032609
19,1,2509.314286
19,1,1578.778409
19,1,2915.938462
19,1,3411.696429
19,1,1768.448276
19,1,2233.222973
19,1,1904.611979
19,1,2725.882979
19,1,197613.646875
19,1,9008.380814
19,1,8298.345238
19,1,2657.229167
19,1,2807.320946
19,1,54859.460526
19,1,18452.608108

19,1,22024.472222
19,1,3279.308824
19,1,1677.062500
19,1,7294.500000
19,1,3008.500000
19,1,1571.312500
19,1,21669.064189
19,1,1988.839394
19,1,19287.595395
19,1,2481.383333
19,1,1707.782051
19,1,102651.572917
19,1,1936.833333
19,1,3147.125000
19,1,80779.069444
19,1,50310.397059
19,1,1544.319444
19,1,1679.079268
19,1,35500.774510
19,1,23298.625000
19,1,2653.234043
19,1,3662.658333
19,1,1717.621324
19,1,4260.105263
19,1,12896.851351
19,1,108877.389535
19,1,3914.655556
19,1,80618.548780
19,1,8736.718750
19,1,1904.505682
19,1,4269.960938
19,1,3383.074468
19,1,33117.961538
19,1,6383.724359
19,1,4612.555556
19,1,1609.989583
19,1,4980.935976
19,1,4949.015625
19,1,1749.000000
19,1,2891.559524
19,1,12249.080882
19,1,1936.937500
19,1,111246.778571
19,1,38453.023026
19,1,125168.461538
19,1,1577.804878
19,1,2367.750000
19,1,47600.914286
19,1,3495.545455
19,1,4271.000000
19,1,1846.031250
19,1,46345.328571
19,1,1627.411111
19,1,2152.250000
19,1,8754.972222
19,1,2959.526786
19,1,2218.369048
19,1,1655.885204
19,1,1673.732143
19,1,3115.887500
19,1,3309.718750
19,1,1659.158537
19,1,9122.272436
19,1,14748.104167
19,1,7087.250000
19,1,1703.710526

19,1,2406.625000
19,1,5201.230263
19,1,1802.821875
19,1,2512.423780
19,1,26004.180851
19,1,4287.191860
19,1,4930.068182
19,1,6110.312500
19,1,2991.075581
19,1,1673.375000
19,1,1572.156915
19,1,1773.720109
19,1,22454.023810
19,1,2131.195122
19,1,1837.029412
19,1,3844.272727
19,1,5241.700000
19,1,1580.186047
19,1,1732.987805
19,1,41830.118750
19,1,1605.333333
19,1,5839.482955
19,1,3245.750000
19,1,1634.080882
19,1,2550.023256
19,1,2745.864286
19,1,1994.140625
19,1,3954.662500
19,1,1811.511628
19,1,1562.450658
19,1,5149.930000
19,1,136535.730769
19,1,5876.750000
19,1,6660.753125
19,1,3586.265152
19,1,2117.496212
19,1,2531.986413
19,1,1884.750000
19,1,6712.089674
19,1,2079.122159
19,1,4002.764706
19,1,4322.985714
19,1,2772.208333
19,1,1747.000000
19,1,1593.443750
19,1,6870.205882
19,1,1538.039634
19,1,1685.937500
19,1,4813.069853
19,1,3522.414815
19,1,4964.542683
19,1,1795.470588
19,1,2173.051020
19,1,52292.445122
19,1,1663.608696
19,1,1597.338542
19,1,2105.305556
19,1,2769.389881
19,1,1727.063953
19,1,1709.551829
19,1,8162.512821
19,1,2144.880814
19,1,2915.566667
19,1,9256.500000
19,1,13957.290698
19,1,49441.281250

19,1,1748.394231
19,1,2072.400000
19,1,2091.328125
19,1,105127.346154
19,1,1499.731707
19,1,78363.700000
19,1,2365.861842
19,1,4301.798077
19,1,2169.319767
19,1,1514.571429
19,1,1809.239583
19,1,94940.451220
19,1,95889.431818
19,1,29834.903226
19,1,2341.630208
19,1,19717.288462
19,1,14800.294118
19,1,6546.529605
19,1,81556.949468
19,1,2892.798780
19,1,1781.980000
19,1,2796.563830
19,1,1977.058140
19,1,5599.085938
19,1,1766.326923
19,1,3688.162791
19,1,2459.085714
19,1,68870.014286
19,1,1528.039634
19,1,1739.593750
19,1,4943.104167
19,1,3333.589286
19,1,1605.973404
19,1,3740.527397
19,1,98896.036765
19,1,72587.439024
19,1,3889.079268
19,1,1649.239362
19,1,9071.920918
19,1,8824.093750
19,1,1514.976744
19,1,1500.880682
19,1,67584.894231
19,1,1568.000000
19,1,2036.625000
19,1,1625.671875
19,1,1922.608333
19,1,56744.607143
19,1,2678.250000
19,1,5592.479167
19,1,3277.762195
19,1,4184.811111
19,1,3075.307927
19,1,2595.692073
19,1,2420.000000
19,1,2598.833333
19,1,2704.520833
19,1,1503.939024
19,1,2063.483974
19,1,37731.625000
19,1,26085.125000
19,1,6123.448718
19,1,2871.718750
19,1,35521.015625
19,1,1701.781250
19,1,49402.926471

19,1,3464.683333
19,1,6167.722826
19,1,71340.371875
19,1,2031.622340
19,1,10670.726562
19,1,2757.256579
19,1,3379.550000
19,1,1508.857143
19,1,7240.029762
19,1,5748.226415
19,1,1783.330882
19,1,129137.000000
19,1,7850.772059
19,1,2126.015038
19,1,178498.871951
19,1,4225.453125
19,1,17771.237805
19,1,177609.408333
19,1,3350.070122
19,1,61074.769737
19,1,29090.125000
19,1,28890.842105
19,1,75667.986111
19,1,194838.511364
19,1,8752.279070
19,1,5986.661585
19,1,3087.518182
19,1,3541.281250
19,1,40053.906250
19,1,1712.075000
19,1,1930.583333
19,1,1638.822368
19,1,1602.115385
19,1,1717.472826
19,1,3399.827206
19,1,3053.750000
19,1,2703.548913
19,1,7373.118902
19,1,3524.735119
19,1,50199.172297
19,1,7058.822368
19,1,3061.320000
19,1,2357.288889
19,1,1685.651786
19,1,2044.743902
19,1,2787.447368
19,1,6060.220930
19,1,2476.986486
19,1,19323.042763
19,1,6717.858108
19,1,1919.494681
19,1,2923.625000
19,1,18462.271739
19,1,41391.858974
19,1,17458.834375
19,1,6752.055556
19,1,14532.144444
19,1,5915.697674
19,1,198472.904605
19,1,5357.571023
19,1,62872.062500
19,1,2571.546053
19,1,6385.142857
19,1,63886.807143
19,1,1573.111413
19,1,114709.000000

19,1,2335.113971
19,1,1614.842391
19,1,3540.954545
19,1,8602.290541
19,1,2165.264706
19,1,5398.592949
19,1,1618.286585
19,1,40594.746094
19,1,3570.836310
19,1,1539.892857
19,1,1681.923077
19,1,7194.632979
19,1,1571.719512
19,1,19989.794643
19,1,168462.272727
19,1,2869.048913
19,1,2211.270833
19,1,5206.500000
19,1,7954.809524
19,1,2378.368750
19,1,2490.537946
19,1,7235.261111
19,1,2977.738372
19,1,1773.923077
19,1,2267.220238
19,1,1521.641667
19,1,1990.894737
19,1,102049.734375
19,1,18641.224551
19,1,4005.514706
19,1,1564.823529
19,1,3046.365854
19,1,4758.755814
19,1,1841.266447
19,1,1619.465625
19,1,1665.279070
19,1,2532.815789
19,1,6084.325658
19,1,36541.066327
19,1,1632.588542
19,1,47424.732955
19,1,5351.904412
19,1,2010.071429
19,1,134652.113372
19,1,4896.882353
19,1,1891.666667
19,1,1793.923780
19,1,2623.333333
19,1,56631.769231
19,1,3272.792683
19,1,164445.028409
19,1,39070.006579
19,1,4763.972222
19,1,2191.204082
19,1,5162.911765
19,1,72099.761029
19,1,11191.500000
19,1,2243.300595
19,1,3312.209302
19,1,1576.743750
19,1,2677.271277
19,1,1753.725000
19,1,1926.573529
19,1,2146.215426
19,1,3325.125000
19,1,4630.317073

19,1,49309.090278
19,1,123295.395833
19,1,2191.435897
19,1,3174.033537
19,1,1871.541667
19,1,2597.966463
19,1,136568.812500
19,1,4989.570652
19,1,1811.134146
19,1,2242.244681
19,1,120810.787500
19,1,42010.513889
19,1,4181.000000
19,1,2470.190476
19,1,9094.353659
19,1,1602.416667
19,1,19165.635714
19,1,2140.647059
19,1,1536.769231
19,1,2122.851852
19,1,3621.487805
19,1,2792.258929
19,1,69902.265625
19,1,5706.208333
19,1,44511.358108
19,1,1673.869048
19,1,3275.538043
19,1,4197.088710
19,1,22911.581081
19,1,3583.071429
19,1,4101.634615
19,1,2151.250000
19,1,1858.042553
19,1,1884.455556
19,1,31544.533088
19,1,1776.913265
19,1,5573.540441
19,1,90524.789474
19,1,2503.994792
19,1,3450.250000
19,1,3237.247024
19,1,4773.910326
19,1,5710.208333
19,1,1663.673913
19,1,1775.125000
19,1,2364.257353
19,1,2953.326923
19,1,1583.128049
19,1,2591.976190
19,1,3392.344444
19,1,4841.181818
19,1,4363.284091
19,1,4017.200000
19,1,21807.609375
19,1,66767.339744
19,1,77053.555556
19,1,30547.460526
19,1,3862.668750
19,1,1747.089286
19,1,2473.347222
19,1,18795.267442
19,1,2835.884615
19,1,1586.895408
19,1,1819.437500
19,1,1702.773810
19,1,42324.625000

19,1,2435.365854
19,1,1808.174419
19,1,27444.128205
19,1,3750.404762
19,1,4074.807692
19,1,1946.729651
19,1,2511.563953
19,1,114517.288889
19,1,1586.939024
19,1,2736.391304
19,1,121879.339623
19,1,5735.049479
19,1,1821.800000
19,1,15133.337500
19,1,16046.388889
19,1,1661.843750
19,1,1962.984043
19,1,3502.275000
19,1,48966.477273
19,1,3362.234375
19,1,1606.368421
19,1,28674.125000
19,1,1911.906915
19,1,2682.234848
19,1,2023.857143
19,1,26087.473684
19,1,10463.400000
19,1,1776.965116
19,1,5005.828947
19,1,2588.695652
19,1,26424.453488
19,1,1548.330357
19,1,2628.435096
19,1,6850.630952
19,1,3229.190476
19,1,4835.625000
19,1,1534.983553
19,1,13391.377907
19,1,2316.672794
19,1,3367.159574
19,1,2989.511905
19,1,3960.023256
19,1,1953.846591
19,1,24040.201923
19,1,3717.417553
19,1,2495.458333
19,1,3086.007979
19,1,3660.926829
19,1,5025.931818
19,1,29202.702128
19,1,3875.542683
19,1,7378.929348
19,1,3161.401596
19,1,1884.455882
19,1,2060.665816
19,1,41145.931034
19,1,2206.204545
19,1,6422.140625
19,1,18787.530612
19,1,75875.215909
19,1,170994.303030
19,1,15302.708333
19,1,1582.674419
19,1,2975.028571
19,1,3323.250000
19,1,16038.402174

19,1,4332.764706
19,1,2227.400000
19,1,2144.603659
19,1,1817.643293
19,1,1847.383929
19,1,34331.831250
19,1,35073.166667
19,1,1582.696875
19,1,24056.347561
19,1,1583.000000
19,1,4293.823529
19,1,2597.750000
19,1,160899.599432
19,1,104751.730769
19,1,2527.181818
19,1,48449.242857
19,1,101772.940217
19,1,6275.417857
19,1,68855.571429
19,1,25804.853659
19,1,1624.705729
19,1,19479.005208
19,1,1633.322222
19,1,2646.027027
19,1,50142.062500
19,1,7731.673913
19,1,1568.133929
19,1,59163.932692
19,1,6157.290441
19,1,2021.318182
19,1,3137.871951
19,1,10349.484694
19,1,7376.714286
19,1,106070.307692
19,1,1953.014706
19,1,1519.486413
19,1,4199.066176
19,1,9759.714286
19,1,1886.136029
19,1,2571.537879
19,1,80572.551829
19,1,12544.239865
19,1,2722.085366
19,1,124076.492188
19,1,1622.625000
19,1,24174.921875
19,1,2878.428571
19,1,59841.047619
19,1,2492.488095
19,1,14128.090909
19,1,19475.637500
19,1,2181.767857
19,1,2354.641304
19,1,21732.671429
19,1,1697.439024
19,1,1516.317073
19,1,21333.304688
19,1,3601.432065
19,1,101121.544872
19,1,2952.957237
19,1,49488.472222
19,1,2228.236486
19,1,3690.636905
19,1,1770.222222
19,1,2224.648936
19,1,1639.472222

19,1,1511.780488
19,1,17658.187500
19,1,165062.375000
19,1,6361.892045
19,1,6695.755556
19,1,3436.331522
19,1,2967.678125
19,1,1866.229651
19,1,14872.902439
19,1,3206.961310
19,1,69828.657143
19,1,2093.732558
19,1,1877.107143
19,1,51175.421053
19,1,1988.410256
19,1,43158.750000
19,1,3233.869048
19,1,2398.000000
19,1,167476.101064
19,1,3865.263158
19,1,127796.243421
19,1,15127.638889
19,1,2399.858696
19,1,3089.554348
19,1,3116.666667
19,1,1746.082317
19,1,1668.573718
19,1,1656.294118
19,1,27902.388889
19,1,167443.090000
19,1,7791.009804
19,1,145368.141667
19,1,3937.532544
19,1,1569.388158
19,1,75314.179487
19,1,2632.039063
19,1,10180.250000
19,1,130146.935976
19,1,2105.232143
19,1,1628.446429
19,1,9643.500000
19,1,3912.057432
19,1,132942.263158
19,1,36547.199219
19,1,41577.596875
19,1,2356.228261
19,1,1527.213542
19,1,4692.585366
19,1,58019.336538
19,1,7804.383333
19,1,2047.168605
19,1,2683.544118
19,1,1664.712209
19,1,7227.285156
19,1,2006.725000
19,1,57767.988636
19,1,5064.212264
19,1,72852.932927
19,1,4973.033088
19,1,5387.459459
19,1,1790.698864
19,1,91557.108108
19,1,110314.188889
19,1,4000.315625
19,1,3488.319149
19,1,1844.895833

19,1,41988.746875
19,1,2563.202381
19,1,13334.437500
19,1,2690.330357
19,1,113836.750000
19,1,23222.607843
19,1,2381.464286
19,1,1954.500000
19,1,1955.750000
19,1,1836.534091
19,1,71669.000000
19,1,8694.976974
19,1,2938.285714
19,1,156246.574468
19,1,2072.022222
19,1,1536.845109
19,1,70656.659722
19,1,42566.750000
19,1,52915.121622
19,1,138377.903409
19,1,1964.375000
19,1,2222.763298
19,1,12646.860294
19,1,6154.513158
19,1,149040.638889
19,1,2600.603125
19,1,2433.008065
19,1,3149.060241
19,1,1684.937500
19,1,3098.023256
19,1,46778.190476
19,1,39969.984375
19,1,2308.113095
19,1,1635.216216
19,1,2712.125000
19,1,15783.750000
19,1,1580.770349
19,1,1605.063953
19,1,84701.871795
19,1,1543.230769
19,1,2509.616477
19,1,12136.391304
19,1,52370.493902
19,1,2606.702206
19,1,90124.946429
19,1,1979.541667
19,1,144124.384615
19,1,1802.571429
19,1,1732.562500
19,1,23542.993243
19,1,1911.014286
19,1,2475.720745
19,1,8158.500000
19,1,86788.768293
19,1,1637.711538
19,1,2804.336957
19,1,5537.368056
19,1,1572.545732
19,1,147664.513158
19,1,28461.350000
19,1,6033.456522
19,1,5541.270833
19,1,9005.494048
19,1,62100.989865
19,1,1801.375000
19,1,172856.011628

19,1,92057.434211
19,1,89589.486486
19,1,1758.241848
19,1,9579.500000
19,1,25276.419643
19,1,57193.912162
19,1,5027.423077
19,1,16154.850000
19,1,77200.035714
19,1,3904.942771
19,1,3514.488372
19,1,2870.423469
19,1,11107.944444
19,1,1769.420455
19,1,30509.736111
19,1,8993.047619
19,1,2772.857143
19,1,1621.799342
19,1,69026.226190
19,1,2758.500000
19,1,2009.363636
19,1,3154.130102
19,1,4045.151163
19,1,1976.792683
19,1,3738.762500
19,1,6417.552632
19,1,2411.687500
19,1,1748.450000
19,1,78562.038194
19,1,1942.375000
19,1,2176.750000
19,1,1579.279070
19,1,1502.000000
19,1,4411.500000
19,1,3843.872222
19,1,9161.600000
19,1,1539.044118
19,1,1955.741071
19,1,1886.250000
19,1,3546.576705
19,1,16169.050000
19,1,78952.222222
19,1,1801.795213
19,1,1798.955357
19,1,1719.366667
19,1,1513.631579
19,1,2576.607143
19,1,2394.715909
19,1,3000.923077
19,1,1769.301136
19,1,14803.548780
19,1,1537.625000
19,1,11297.756944
19,1,1656.653061
19,1,77944.312500
19,1,1799.435976
19,1,177914.755814
19,1,1500.250000
19,1,2310.806548
19,1,22443.000000
19,1,3328.402439
19,1,34830.121429
19,1,4332.872283
19,1,1731.934375
19,1,1717.855000
19,1,2635.625000

19,1,46612.177083
19,1,3356.203947
19,1,22152.131098
19,1,1875.206522
19,1,133873.593750
19,1,8291.562500
19,1,1953.259615
19,1,2553.920455
19,1,66896.250000
19,1,9100.892045
19,1,2252.878205
19,1,53788.088235
19,1,2193.346591
19,1,1656.053571
19,1,145982.750000
19,1,1803.453804
19,1,8645.197368
19,1,1690.515625
19,1,9132.812500
19,1,10736.392857
19,1,1624.783537
19,1,3967.829268
19,1,108640.086957
19,1,2674.856250
19,1,1835.969697
19,1,2650.554054
19,1,171709.419811
19,1,1517.115385
19,1,3180.025000
19,1,2262.445946
19,1,26199.713068
19,1,1581.850543
19,1,2156.451923
19,1,6178.459559
19,1,7052.462500
19,1,2159.439024
19,1,12017.597561
19,1,1538.329545
19,1,15282.132143
19,1,44923.000000
19,1,12436.219512
19,1,2578.596591
19,1,2741.669872
19,1,5678.218750
19,1,1669.270408
19,1,2165.885135
19,1,2777.888889
19,1,1741.055921
19,1,1974.695122
19,1,2685.460227
19,1,4579.428571
19,1,3314.428571
19,1,5783.128049
19,1,120961.086806
19,1,103339.312500
19,1,47235.046429
19,1,60169.214286
19,1,27096.898148
19,1,101533.929487
19,1,1760.618590
19,1,1573.200000
19,1,1618.834459
19,1,7171.835106
19,1,21192.158333
19,1,15008.832386
19,1,2977.310345

19,1,1937.027778
19,1,1512.427632
19,1,2245.773256
19,1,2021.182692
19,1,53423.385417
19,1,2080.218023
19,1,3005.571429
19,1,13384.206522
19,1,2743.737245
19,1,3282.384615
19,1,7418.946429
19,1,14918.159884
19,1,15762.526786
19,1,1832.568182
19,1,84196.404762
19,1,2075.985465
19,1,22681.750000
19,1,58609.054878
19,1,3321.631410
19,1,150584.389881
19,1,1573.298611
19,1,12293.578125
19,1,1672.638298
19,1,1822.729651
19,1,2658.455128
19,1,1909.833333
19,1,1934.842262
19,1,2300.375000
19,1,1726.426136
19,1,10285.358974
19,1,3995.187500
19,1,2778.761628
19,1,72719.893939
19,1,2575.883929
19,1,10902.888889
19,1,37822.000000
19,1,2091.375000
19,1,152199.471154
19,1,16881.659574
19,1,89921.986755
19,1,2076.719512
19,1,3012.162500
19,1,2438.878049
19,1,11276.218023
19,1,4054.553191
19,1,75406.394737
19,1,1853.229167
19,1,39414.687500
19,1,40001.367188
19,1,2727.977778
19,1,2076.818182
19,1,182708.276786
19,1,11454.167614
19,1,11269.025510
19,1,35108.516892
19,1,3263.607143
19,1,57081.541667
19,1,41532.357143
19,1,2486.671053
19,1,37783.896552
19,1,2325.269231
19,1,2557.416667
19,1,24123.711310
19,1,4083.970930
19,1,171213.264205
19,1,1925.097656

19,1,28450.125000
19,1,2436.461538
19,1,1721.000000
19,1,34925.132812
19,1,1691.176282
19,1,3730.934211
19,1,21240.109375
19,1,1717.666667
19,1,2165.703125
19,1,1880.281250
19,1,24956.991935
19,1,1761.279070
19,1,1534.355769
19,1,1602.279762
19,1,1890.851744
19,1,60600.784091
19,1,12300.723404
19,1,1968.444444
19,1,2266.387755
19,1,2324.364865
19,1,1917.500000
19,1,101625.714286
19,1,61128.908784
19,1,1616.339286
19,1,2185.138889
19,1,2034.092949
19,1,52280.968750
19,1,4963.280660
19,1,15180.347826
19,1,2289.250000
19,1,2301.598837
19,1,26487.352941
19,1,7241.355000
19,1,1759.845588
19,1,2090.093750
19,1,50532.024390
19,1,31844.250000
19,1,1539.959302
19,1,1599.784091
19,1,197618.416667
19,1,3302.808511
19,1,1630.483333
19,1,1816.109375
19,1,2208.885000
19,1,24876.250000
19,1,167181.012195
19,1,6364.116071
19,1,1689.195652
19,1,19293.593750
19,1,3130.109043
19,1,7592.850000
19,1,1672.800000
19,1,3895.530488
19,1,7698.359375
19,1,1827.750000
19,1,4024.465426
19,1,2076.300000
19,1,31476.565789
19,1,2009.550676
19,1,5601.589286
19,1,1776.466667
19,1,27009.551136
19,1,7426.000000
19,1,19912.000000
19,1,119613.653846
19,1,6203.972222

19,1,1575.321429
19,1,119940.309524
19,1,20498.805556
19,1,2164.287500
19,1,2110.600000
19,1,3797.466667
19,1,1557.522059
19,1,4580.872340
19,1,98034.557143
19,1,76519.312500
19,1,1648.928571
19,1,2502.369048
19,1,1593.437500
19,1,1755.470588
19,1,38956.581633
19,1,5450.341463
19,1,2010.740385
19,1,1638.795455
19,1,1929.570312
19,1,60924.821970
19,1,1521.731707
19,1,1848.360465
19,1,121617.458333
19,1,1512.882979
19,1,4783.580882
19,1,44518.369186
19,1,1594.064024
19,1,2372.015152
19,1,1661.444444
19,1,17748.377717
19,1,5849.586310
19,1,11416.911765
19,1,2336.811224
19,1,3921.978261
19,1,28513.660256
19,1,1919.619792
19,1,1829.333333
19,1,36221.816860
19,1,11255.607143
19,1,3195.050000
19,1,2485.562500
19,1,95382.778846
19,1,2636.515625
19,1,1796.692935
19,1,40689.435811
19,1,46433.637097
19,1,2150.409091
19,1,2141.737179
19,1,69530.851351
19,1,65963.766667
19,1,1542.065217
19,1,1967.612805
19,1,10913.142857
19,1,17347.576531
19,1,12669.600000
19,1,115402.000000
19,1,44632.701923
19,1,3071.428571
19,1,1589.421053
19,1,4467.008721
19,1,93034.406250
19,1,2087.904605
19,1,2231.952128
19,1,6398.714286
19,1,3069.858974
19,1,15330.187500

19,1,2288.796875
19,1,9477.204545
19,1,2549.866279
19,1,141275.945946
19,1,7584.987500
19,1,12105.085106
19,1,1576.375000
19,1,2020.447917
19,1,3171.220745
19,1,1636.177083
19,1,15325.790179
19,1,7619.375000
19,1,1762.863636
19,1,155407.142857
19,1,1533.046875
19,1,109053.312500
19,1,64409.772727
19,1,3228.049419
19,1,2724.191176
19,1,1588.454545
19,1,6268.333333
19,1,6080.536585
19,1,2387.228261
19,1,1888.215000
19,1,53425.875000
19,1,2461.966216
19,1,112394.959375
19,1,2338.163043
19,1,2053.732955
19,1,1684.216837
19,1,5189.713068
19,1,1556.731707
19,1,1905.988095
19,1,1562.593750
19,1,5233.844595
19,1,147689.613095
19,1,12714.476744
19,1,2280.500000
19,1,76936.860000
19,1,1580.187500
19,1,6374.985465
19,1,150317.116477
19,1,2374.486842
19,1,140079.282895
19,1,2969.892442
19,1,5206.924242
19,1,100302.820122
19,1,2252.726351
19,1,15941.774834
19,1,1563.316489
19,1,1555.462500
19,1,73333.237805
19,1,1584.121951
19,1,2501.366667
19,1,40655.389706
19,1,3025.740625
19,1,15663.421875
19,1,8631.692708
19,1,1822.312500
19,1,5048.658537
19,1,1505.636905
19,1,5109.841463
19,1,1891.804348
19,1,3735.969444
19,1,1702.627907
19,1,1871.083333

19,1,5053.531915
19,1,1561.899457
19,1,142679.375000
19,1,113245.850000
19,1,2104.604167
19,1,1643.510638
19,1,59494.953125
19,1,1538.017857
19,1,1730.166667
19,1,4520.625000
19,1,5516.714286
19,1,51046.744565
19,1,3296.854167
19,1,2416.275000
19,1,2517.408333
19,1,43372.907258
19,1,1632.628205
19,1,2201.925000
19,1,8533.945652
19,1,188291.066667
19,1,4624.625000
19,1,39969.300000
19,1,119097.406250
19,1,1987.669118
19,1,5042.560976
19,1,1870.090426
19,1,19811.000000
19,1,53821.996094
19,1,1504.152778
19,1,7614.625000
19,1,196214.509434
19,1,1891.913043
19,1,19558.994444
19,1,2152.538043
19,1,17305.473404
19,1,3389.659218
19,1,4016.433155
19,1,2902.500000
19,1,1515.140152
19,1,8185.333333
19,1,2958.625000
19,1,96421.611111
19,1,2335.521739
19,1,29583.965278
19,1,144153.727273
19,1,1585.255319
19,1,1659.383333
19,1,199586.507500
19,1,2175.648810
19,1,119285.542614
19,1,121159.506250
19,1,2891.204268
19,1,77208.412500
19,1,5424.577778
19,1,188413.029891
19,1,10492.116477
19,1,1552.642857
19,1,1903.853261
19,1,74147.250000
19,1,1573.130952
19,1,1589.667598
19,1,153326.910714
19,1,17068.286585
19,1,1737.010870
19,1,3349.414062
19,1,2138.651163

19,1,11800.166667
19,1,5928.476744
19,1,1722.229167
19,1,2508.888889
19,1,12509.233333
19,1,1786.312500
19,1,2158.844595
19,1,2481.530612
19,1,2009.866071
19,1,38845.360294
19,1,14220.994186
19,1,33195.203947
19,1,2818.772222
19,1,7872.360119
19,1,41510.166667
19,1,2766.394231
19,1,7555.281250
19,1,1605.500000
19,1,2750.209302
19,1,6006.122449
19,1,2105.639881
19,1,1700.000000
19,1,78041.798780
19,1,4498.994048
19,1,2167.012195
19,1,2890.100000
19,1,185695.097561
19,1,3508.925000
19,1,2177.904762
19,1,155132.480978
19,1,1513.734043
19,1,43314.534884
19,1,18990.832317
19,1,1937.493750
19,1,1797.288889
19,1,4488.565789
19,1,2471.901786
19,1,3278.488889
19,1,4618.814103
19,1,2084.615385
19,1,18699.200000
19,1,2500.082317
19,1,7206.809524
19,1,1998.361702
19,1,1528.444444
19,1,50725.840278
19,1,2001.642857
19,1,1885.878613
19,1,1631.045213
19,1,63451.653846
19,1,1591.685976
19,1,50013.125000
19,1,1751.333333
19,1,2140.833333
19,1,73903.460714
[truncated: 1,522,597 more chars]
