# Supplementary material for: Selective Moonlighting Cell-Penetrating Peptides
Source: Pharmaceutics. 2021 Jul 22;13(8):1119. doi: 10.3390/pharmaceutics13081119 (PMC8400200; doi:10.3390/pharmaceutics13081119)
Supplement: Supplementary file 1 [file pharmaceutics-13-01119-s001.zip › TableS2.pdf]

Supplemental Data for the work entitled “Selective Moonlighting Cell-Penetrating Peptides” by Rafael Morán-Torres, David A. Castillo González, Beatriz Aguilar Maldonado, Maria Luisa Durán-Pastén, Susana Castro-Obregon & Gabriel Del Rio

Table S2 A) Intensity recorded for peptide alpha-NLS-C on MATa and MATalpha cells. MATa recordings correspond with odd Time numbers, and MATalpha with even Time numbers
Time,GroupByColor,Intensity
1,1,11121.2857142857
1,1,9039.8467741936
1,1,5807.2222222223
1,1,6719.3735955056
1,1,17616.0192307693
1,1,17693.6607142858
1,1,5628.9156976744
1,1,5640.5
1,1,9106.8954545454
1,1,11427.2321428572
1,1,14737.9999999999
1,1,19642.234375
1,1,7502
1,1,7009.9811320756
1,1,5177.8781250001
1,1,8541.9855769231
1,1,17606.5512820511
1,1,19613.4615384616
1,1,5262.6576086957
1,1,7803.7872340427
1,1,9519.0212765959
1,1,15654.1684782608
1,1,5744.6500000001
1,1,11451.6306306307
1,1,15963.3858695651
1,1,12627.7619047617
1,1,10290.6466346156
1,1,6169.4703389831
1,1,5445.4166666666
1,1,5606.203488372
1,1,6776.7529069769
1,1,9684.4267782426
1,1,7691.1540697675
1,1,8190.9335106383
1,1,8232.8826530613
1,1,6401.6458333334
1,1,9154.9166666666
1,1,5694.8614130434
1,1,5885.0592105265
1,1,8397.0921052632
1,1,8951.5833333333
1,1,16145.7499999998
1,1,5381.75
1,1,15697.2560975608
1,1,5641.0330188679
1,1,6510.0000000001
1,1,16948.1614583332
1,1,7950.0539772729
1,1,7750.475
1,1,7601.4137931034
1,1,5695.0184426231
1,1,9947.4600000002
1,1,8374.0664893618
1,1,7966.6675000001
1,1,6687.5539772729
1,1,5206.3421052632
1,1,5269
1,1,5385.7692307693
1,1,5758.9166666665
1,1,6973.3909090909
1,1,10016.5096153847
1,1,8859.5238095238

1,1,10499.9523809525
1,1,17929.4525000001
1,1,7519.6041666666
1,1,5718.5714285714
1,1,6357.8780487805
1,1,6449.6923076924
1,1,5517.75
1,1,5353.8392857143
1,1,15642.6153846155
1,1,14920.5735294118
1,1,5790.0555555556
1,1,5567.3687500001
1,1,5133.2105263158
1,1,5114.7774390244
1,1,6908.037037037
1,1,6794.8262711866
1,1,12099.3953488371
1,1,13094.53125
1,1,6780.6861702128
2,2,5428.6818181818
2,2,5900.2021276595
2,2,6675.6627906975
2,2,7125.4934210526
2,2,5288.0222222221
2,2,7286.3068181817
2,2,6158.5
2,2,5701.1250000001
2,2,9876.202380952
2,2,5493.8555555555
2,2,8253.9085365852
2,2,5788.5955882352
2,2,7339.5333333335
2,2,6852.5348837209
2,2,5236.2255434784
2,2,5027.9107142857
2,2,10484.4999999999
2,2,5746.3174999999
2,2,6716.8333333333
2,2,7435.277173913
2,2,5304.3333333333
2,2,6085.8260869565
2,2,5303.35
2,2,7173.6891891892
2,2,10667.4305555554
2,2,6287.7583333334
2,2,8047.0288461539
2,2,5687.3365384616
2,2,5297.9500000001
2,2,13008.4732142855
2,2,8180.2071428571
2,2,6281.1463414635
2,2,7405.4857142857
2,2,5848.0909090911
2,2,5019.3272058824
2,2,7666.5841836735
2,2,5480.3344594594
2,2,5264.0738636363
3,1,5825.6493902437
3,1,7239.9000000002
3,1,16140.5
3,1,5770.3375
3,1,5068.7325581395
3,1,7045.1555555556
3,1,8779.5977653632
3,1,6217
3,1,13406.3714285715

3,1,8071.2836538462
3,1,15095.2999999997
3,1,5887.4772727272
4,2,6796.8333333334
4,2,5095.8443396226
4,2,6283.4375
4,2,7713.8571428572
4,2,7641.9078947368
4,2,7021.4802631576
4,2,7909.2195121952
4,2,7774.4886363637
4,2,12650.5064102564
4,2,6372.3333333333
4,2,7751.3333333334
4,2,5284.8372093023
4,2,6635.3482758621
4,2,5189.9736842105
4,2,6874.7729591838
5,1,16161.5147058825
5,1,15912.588235294
5,1,5634.1081081081
5,1,5176.8894230769
5,1,14254.6015625
5,1,5693.1363636364
5,1,9306.3125000001
6,2,5949.8902439025
6,2,5505.659574468
6,2,5708.7763157895
6,2,5838.1249999999
6,2,5371.3653846154
6,2,5536.9505813953
6,2,5028.5390625
7,1,5106.375
7,1,5113.023255814
7,1,5298.3372641509
7,1,6761.1428571428
7,1,7300.0588235293
7,1,6413.4274193547
7,1,5376.5104166667
7,1,6833.4194915256
7,1,11455.4634146342
7,1,10172.5862068965
8,2,5832.7926136364
8,2,10090.7881355932
8,2,5153.861111111
8,2,8663.5999999999
8,2,10385.8562500001
8,2,6017.4583333333
8,2,7536.75
8,2,8633.9510869564
8,2,5919.6857142857
8,2,6080.6824324324
8,2,5962.0024509805
8,2,7526.2670454546
8,2,11568.8604651161
9,1,12505.7619047618
9,1,18732.2941176471
9,1,9065.0000000001
9,1,5275.0625
9,1,5031.6576086957
9,1,7923.7971698115
9,1,6036.1891891892
9,1,10455.8139534885
9,1,5855.6339285714
9,1,13076.7774390242
9,1,5538.75

9,1,7002.4375
9,1,19333.6249999999
9,1,6045.1136363636
9,1,7629.8749999999
9,1,5376.125
9,1,17264.1775000002
9,1,10882.8578431374
9,1,6906.09375
9,1,5621.9713541667
9,1,5088.9249999998
9,1,5380.5750000001
9,1,5185.3571428571
9,1,12171.1279761904
9,1,10877.8333333335
9,1,6557.2884615385
9,1,6553.1535714285
9,1,10433.2228260869
9,1,8344.4967948718
9,1,6102.3292682928
9,1,7692.7499999999
9,1,15997.9618055556
9,1,5090.9285714286
9,1,6223.9705882354
9,1,6370.5000000001
9,1,6689.5833333332
9,1,5454.9044117647
9,1,19714.5833333334
9,1,10638.5846153843
9,1,5099.5151515151
9,1,5372.85
9,1,5380.2391304347
9,1,5576.2500000002
9,1,16264.5540540539
10,2,7049.4880952381
10,2,6976.9873188407
10,2,10404.00625
10,2,6187.857142857
10,2,5274.4155405405
10,2,5945.6249999998
10,2,6229.1139705882
10,2,6823.9024390243
10,2,5358.3461538461
10,2,5173.7619047619
10,2,5658.9318181817
10,2,10650.5108695654
10,2,6609.8452380953
10,2,8171.3750000001
10,2,5713.4642857144
10,2,5985.0951086957
10,2,6813.7213541667
10,2,6673.4411764707
10,2,8402.7499999999
10,2,6976.4406779659
10,2,6480.181372549
10,2,5882.5918367348
10,2,5055.9705882353
10,2,7940.6006097561
10,2,7787.5714285711
10,2,5709.8571428572
10,2,5234.4308510636
10,2,5016.726744186
10,2,6118.425
10,2,5093.9054054055
11,1,12300.8537234041
11,1,17372.0849999999
11,1,5265.9545454545

11,1,11152.1874999998
11,1,5958.8571428571
11,1,5285.625
11,1,6210.3676470588
11,1,9375.4540816326
11,1,5381.2313829787
11,1,5924.5612244897
11,1,5504.28
11,1,14664.840909091
11,1,17798.7272727274
11,1,17119.4735576923
11,1,6814.9797297297
11,1,6598.4444444445
11,1,9900.7440476189
11,1,5846.3718749999
11,1,5141.3928571428
11,1,7089.8965517241
11,1,7224.1999999999
11,1,6688.0919117648
11,1,5696.019736842
11,1,5448.35
11,1,6917.8285714286
11,1,5231.1815789474
11,1,11013.8981481482
11,1,5126.9767441861
11,1,15168.4545454547
11,1,5506.6544117647
11,1,12094.0150000001
11,1,19945.956395349
11,1,15418.9387755101
11,1,14404.256818182
11,1,14834.9545454539
11,1,10192.2102272728
11,1,9210.6785714285
11,1,5064.9583333333
11,1,9068.8571428571
12,2,5860.6000000001
12,2,7796.7624999999
12,2,6531.2340425532
12,2,5396.5416666666
12,2,5287.9479166667
12,2,5459.511627907
12,2,8363.1057692308
12,2,6399.5029761905
12,2,5707.2692307693
12,2,5625.8465909091
12,2,5162.2580645161
12,2,5252.3
12,2,6196.7467948717
12,2,7683.9907407408
12,2,5082.9642857144
12,2,5994.375
12,2,5939.3602941177
12,2,6856.5609756099
12,2,6278.4772727273
12,2,6200.2884615385
12,2,5903.7218750001
12,2,5054.875
13,1,5330.6611842104
13,1,5958.5224999998
13,1,5193.8315217392
13,1,9059.7083333333
13,1,16070.1200000001
13,1,11057.0952380951
13,1,6440.798611111
13,1,12160.2222222223

13,1,5032.7291666667
13,1,18908.9825581394
13,1,5510.5625
13,1,5242.3030303031
13,1,13835.5625000001
13,1,18632.6666666666
13,1,16967.5364583333
13,1,5224.0238095239
13,1,19052.7722222222
13,1,8093.3537735849
13,1,17411.3413461538
13,1,5207.5324074075
13,1,15625.201923077
13,1,14968.7500000002
13,1,5018.1470588235
13,1,6664.080882353
14,2,7556.328125
14,2,7858.6071428572
14,2,5445.25
14,2,9556.2819148936
14,2,8545.2105263157
14,2,5811.5476190476
14,2,5172.5625000002
14,2,5261.515625
14,2,5017.8421052631
14,2,5710.955357143
14,2,8455.9102564103
14,2,7093.6818181819
14,2,6618.0535714285
14,2,5526.25
14,2,6153.05
14,2,6328.4575000001
14,2,7812.4852941176
15,1,15546.5478723406
15,1,6927.975
15,1,5665.7375
15,1,5861.3000000001
15,1,5966.4852941177
15,1,5404.2974137932
15,1,11861.8014705884
15,1,6080.9816176471
15,1,6066.893939394
15,1,12652.44
15,1,5316.4114583334
15,1,6958.5484693878
15,1,8431.2105263158
15,1,11473.2575757575
15,1,19014.051724138
15,1,6228.0357142858
15,1,5423.1858108109
16,2,5732.8835227273
16,2,5910.4111111112
16,2,6285.3944444444
16,2,7896.9476744185
16,2,5757.36
16,2,7375.3928571428
16,2,5343.8239795919
17,1,16629.2840909092
17,1,5790.75
17,1,10627.0212765956
17,1,15129.125
17,1,10754.1818181816
17,1,6737.975
17,1,16591.9360465117
17,1,13253.2259615383
17,1,14255.5588235295

17,1,13581.9695121954
17,1,9785.5094339621
17,1,15321.2958333333
17,1,10826.6621621622
17,1,15504.4595588236
17,1,5788.059375
17,1,15470.8881578946
17,1,5817.2340425533
17,1,17727.2692307693
17,1,5524.875
17,1,10032.9500000001
17,1,11037.25
17,1,7026.3676470588
17,1,12737.5374999999
17,1,13581.6999999999
17,1,19603.5568181819
17,1,14003.1707317072
17,1,10036.3750000002
17,1,19047.6875
17,1,14770.2083333337
17,1,11670.5572916666
17,1,5484.2117346939
17,1,9654.4324324324
17,1,16771
17,1,10606.1120689656
17,1,17433.721153846
17,1,15884.5
17,1,5398.190625
17,1,11485.5878378378
17,1,19049.2567567567
17,1,16314.3693181816
17,1,6832.7556818181
17,1,17847.3993055555
17,1,7328.195945946
17,1,5465.875
17,1,9344.6352040816
17,1,16157.5
17,1,16072.375
17,1,11578.625
17,1,8098.7928571428
17,1,5144.9333333333
17,1,15201.9921875
17,1,10336.6369047618
17,1,6568.8749999999
17,1,5525.9386363636
17,1,16494.419117647
17,1,16330.3658536587
17,1,9201.4144736843
17,1,6601.5999999999
17,1,5912.8295454544
18,2,6129.5520833333
18,2,5054.4761904762
18,2,6193.9583333334
18,2,5286.8562499999
18,2,6715.1805555556
18,2,7014.7715517242
18,2,5542.4967105263
18,2,6299.1439393939
18,2,6211.8684210527
18,2,9376.5499999999
18,2,7653.4714285715
18,2,5908.3250000001
18,2,7231.0588235295
18,2,5677.5520833334
18,2,13517.9539473686
18,2,7572.8878205128

18,2,5165.1049107144
18,2,6127.8411458334
18,2,5756.6309523811
18,2,5444.1853448276
18,2,6087.5426829269
18,2,6652.5482954546
18,2,5141.231707317
18,2,5500.7500000001
18,2,5004.8989361703
19,1,9680.25
19,1,5334.3846153846
19,1,15118.7307692308
19,1,5860.9510869564
19,1,6115.75
19,1,14307.6571428571
19,1,12838.0285714286
19,1,7187.0714285715
19,1,5709.4245283019
19,1,16450.8482142859
19,1,5554.375
19,1,16036.875
19,1,18977.2567567566
19,1,18321.8333333334
19,1,7610.1276595744
19,1,10568.0384615384
19,1,8582.5
19,1,7068.9166666667
19,1,15075.15625
19,1,14420.3076923078
19,1,19756.2022058825
19,1,12445.1451612904
19,1,14695.8269230766
19,1,15022.2095588237
19,1,10024.3825757576
19,1,5510.6458333333
19,1,10125.20754717
19,1,5043.83984375
19,1,18072.4594594595
19,1,13390.3630952381
19,1,17751.6216216215
19,1,5877.2091836735
19,1,10239.25
19,1,16263.811111111
19,1,16788.4324324324
19,1,5140.5454545452
19,1,18530.2312500002
19,1,6497.1524390244
19,1,13506.1470588234
19,1,7141.3405172413
19,1,17839.5914634144
19,1,8970.5312499999
19,1,7891.131097561
19,1,5130.3869047618
19,1,14077.2132352941
19,1,8593.5147058824
19,1,18234.7297297298
19,1,5098.3443396227
19,1,19426.2007575759
19,1,18560.8262195123
19,1,6517.1250000001
19,1,7548.7794117648
19,1,9104.625
19,1,11666.75
19,1,17799.867647059
19,1,13931.25
19,1,10698.7727272727

19,1,18097.4155405407
19,1,17934.4921875
20,2,8454.9758064517
20,2,8011.5975609757
20,2,5856.3970588236
20,2,7724.5384615385
20,2,5021.6148648648
20,2,7466.884375
20,2,5329.2279411765
20,2,6622.5657894737
20,2,5526.8467741936
20,2,7033.6666666666
20,2,10119.4448529411
20,2,5603.9444444445
20,2,7445.7884615385
20,2,5190.7647058823
20,2,9225.01171875
20,2,9792.1015625
20,2,6658.8388157894
20,2,6621.6470588235
20,2,9649.741935484
20,2,7163.2214285714
20,2,7225.1507352941
20,2,7774.4615384616
20,2,8561.75
20,2,6678.3378378379
20,2,5256.9276315789
20,2,5888.3552631579
20,2,6617.6631944444
20,2,9740.4886363637
20,2,8193.2352941176
20,2,6731.8235294117
20,2,7121.8124999999
21,1,11995.4743589744
21,1,10540.747340425
21,1,14281.9659090909
21,1,14428.8333333334
21,1,14736.7708333331
21,1,8336.1416666667
21,1,15133.884868421
21,1,17634.6532258066
21,1,6596.8557692308
21,1,12077.3602941176
21,1,15779.403409091
21,1,5538.1627906975
21,1,6005.62890625
21,1,5774.4547872341
21,1,9674.1707317071
21,1,14856.6250000001
21,1,11050.3039772724
21,1,5787.8125
21,1,7400.9117647058
21,1,13590
21,1,17149.4072580646
21,1,9322.947368421
21,1,5367.01953125
21,1,6297.136904762
21,1,9877.5914634148
21,1,7166.1875
21,1,18256.6585365852
21,1,7619.6829268291
21,1,6416.8076923078
21,1,15471.9285714286
21,1,6226.75625
21,1,16055.1818181817
21,1,19612.7894736843

21,1,16084.1572580645
21,1,7125.9375
21,1,9515.1904761904
21,1,7857.7620967741
21,1,19377.3967391304
21,1,9188.2678571429
21,1,5086.6379310345
21,1,11330.8333333332
21,1,10993.572368421
21,1,16567.7941176472
21,1,10825.1190476192
21,1,18699.2651515151
21,1,10793.1785714286
21,1,9367.1682692307
21,1,5362.2420212766
21,1,10371.2794117647
21,1,14193.400735294
21,1,8244.5731707317
21,1,14765.1250000001
21,1,5092.2867647059
21,1,12627.0735294117
21,1,11551.640625
21,1,18357.5168918922
21,1,12864.51171875
21,1,19218.9479166666
21,1,5992.2604166667
22,2,5662.3658536586
22,2,5443.0531914893
22,2,8022.6999999999
22,2,7369.0810810812
22,2,5465.7822580645
22,2,5255.2526595745
22,2,5186.2797619049
22,2,5859.9761904762
22,2,6325.0073529411
22,2,5435.6686046512
22,2,8498.7692307692
22,2,5456.3175675676
22,2,5101.2058823529
22,2,6805.5192307692
23,1,9178.1515151515
23,1,5434.8
23,1,6284.0681818182
23,1,5993.6666666665
23,1,11802.6465517242
23,1,13461.0201612904
23,1,7544.6838235294
23,1,17078.7647058823
23,1,8671.6509433962
23,1,9409.4166666664
23,1,12208.3092105264
23,1,6475.744047619
23,1,18085.484848485
23,1,9775.2812499998
23,1,19738.9999999999
23,1,17536.7499999998
23,1,19513.5493421054
23,1,19327.1644736843
23,1,18808.3781249999
23,1,11215.1428571428
23,1,8749.7880434783
23,1,17003.3157894738
23,1,12493.3749999999
23,1,14336.4047619049
23,1,17863.783333333
23,1,11018.8382352942

23,1,19880.784722222
23,1,16786.1750000001
23,1,13699.2419354839
23,1,8973.2083333332
23,1,18912.7348484847
23,1,12601.7272727272
23,1,11897.5750000001
23,1,18852.6451612903
23,1,15617.4999999999
23,1,5366.2857142857
23,1,17697.7426470585
23,1,15430.3823529411
23,1,6186.2553191489
23,1,14048.705882353
23,1,10196.5865384615
23,1,14650.3166666667
24,2,5060.609375
24,2,7209.3333333333
24,2,6858.1727941177
24,2,7032.3541666666
24,2,5191.7261904763
24,2,5118.14
24,2,5306.6875
24,2,7247.9487179488
24,2,7356.6442307692
24,2,5170.3378378379
24,2,6293.4448529411
24,2,5015.8333333333
24,2,5861.2340425532
24,2,7447.3194444444
Table S2 B) Intensity recorded for peptide NLS-alpha-CE on MATa and MATalpha cells. MATa recordings correspond with odd Time numbers, and MATalpha with even Time numbers
Time,GroupByColor,Intensity
1,1,8551.1333333333
1,1,9080.0460526315
1,1,8079.4517045453
1,1,6021.4114583335
1,1,15971.1627906973
1,1,8825.3000000001
1,1,5710.9651162792
1,1,8360.120614035
1,1,19925.6130952382
1,1,5994.7820512821
1,1,9492.1774999998
1,1,5950.5813953489
1,1,12517.5263157894
1,1,19936.6929347827
1,1,5505.6999999999
1,1,5887.6413043478
1,1,8834.2863636365
1,1,6103.5847457627
1,1,5311.8723404256
1,1,7388.4249999999
1,1,12373.0036764705
1,1,5708.475
1,1,5531.2159090909
1,1,5243.2500000001
1,1,5416.8974358974
1,1,5246.75
1,1,6055.8505434783
1,1,5778.9431818181
1,1,5326.0600961539
1,1,8230.8837209302
1,1,7807.5897435897
1,1,5213.3061224491

1,1,5903.4635416667
1,1,6467.0570652175
1,1,9757.5816326531
1,1,12450.4298245613
1,1,10243.17
1,1,6049.3406249999
1,1,6556.641025641
1,1,5088.3046875
1,1,10507.740909091
1,1,5969.9725
1,1,7829.9999999999
1,1,7317.6875
1,1,5297.8275862069
1,1,9413.3425925926
1,1,6309.8537234041
1,1,7215.9565217392
1,1,6112.3055555556
1,1,5386.2272727273
1,1,6323.8641304347
1,1,8862.731707317
1,1,9306.5555555556
1,1,6595.303030303
1,1,12131.320945946
1,1,13362.3452380952
1,1,11071.9895833334
1,1,9865.0164473683
1,1,8756.9781249999
1,1,8692.3809523811
1,1,5916.7754491018
1,1,5045.7
1,1,5533.7959183672
1,1,8792.5425531914
1,1,7332.3124999999
1,1,11285.195945946
1,1,6822.6666666666
1,1,16364.63671875
1,1,7118.8437500001
1,1,6210.25
1,1,9278.1960227273
1,1,7177.5625
1,1,5320.2965116279
1,1,11319.3884297521
1,1,5197.625
1,1,7270.4000000001
1,1,10159.2820512817
1,1,5021.0081967214
1,1,17603.6217105264
1,1,7128.7285714285
1,1,6187.5000000002
1,1,7745.5031249999
1,1,15684.5909090908
1,1,5741.8333333334
1,1,10951.3333333335
1,1,10294.1750000001
1,1,7517.032894737
1,1,6353.8885869567
1,1,5471.7
1,1,10670.95
1,1,6684.5106382978
1,1,7641.40625
1,1,5813.5660377359
1,1,6932.5972222223
1,1,10720.3749999999
1,1,5049.7579787234
1,1,7614.681818182
1,1,7319.5190677966

1,1,11563.9006410256
1,1,5271.47
1,1,13779.6833333332
1,1,7087.5396341464
1,1,5927.3111111111
1,1,12350.8794642857
1,1,5583.5666666665
1,1,10952.4809782607
1,1,5287.7264150943
1,1,5420
1,1,5047.0641891892
1,1,8145.8125
1,1,11604.0090909089
1,1,5091.3380681818
1,1,6536.5957446808
1,1,5921.8111702128
1,1,17513.6874999995
1,1,6252.3516949152
1,1,7690.1428571428
1,1,9226.5138888889
1,1,7797.7395833334
1,1,6824.441860465
1,1,7858.2745098038
1,1,7812.6785714285
1,1,9980.0884146342
1,1,9454.8233695652
1,1,7758.4732142858
1,1,5954.3749999999
1,1,5236.4594594595
1,1,5448.4875000001
1,1,6418.7674418605
1,1,9888.4097222224
1,1,16075.4732142856
1,1,13024.6999999998
1,1,7322.7093750001
1,1,11809.3333333334
1,1,15066.6382978721
1,1,6125.5625000001
1,1,7899.1488095238
1,1,15906.5049019606
1,1,8652.7159090911
1,1,16560.0238095239
1,1,6133.1489361703
1,1,9077.9192708333
1,1,10019.3642857143
1,1,5460.0499999998
1,1,6192.0681818181
1,1,12556.1071428571
1,1,11697.7738095237
1,1,6804.4438775507
1,1,17269.4821428569
1,1,18444.8014705885
1,1,17772.4857142858
1,1,6114.169871795
1,1,8262.045138889
1,1,6377.8680555555
1,1,10295.4000000002
1,1,6243.8260869565
1,1,11380.9791666667
1,1,5831.9451219513
1,1,6125
1,1,9444.5625
1,1,18861.0372340425
1,1,5830.2107843137
1,1,5421.8377659574
1,1,5970.296875

1,1,5072.4847560974
1,1,7304.1951219512
1,1,6246.4000000001
1,1,8741.5
1,1,8052.6335227273
1,1,5072.5656250001
1,1,5898.8382352941
1,1,14896.3815789476
1,1,7757.1145833333
1,1,5441.2978723404
1,1,5117.25
1,1,10314.0957446808
1,1,6709.6249999998
1,1,8404.9187499998
1,1,13569.8367346943
1,1,5933.8297872339
1,1,5919.3333333332
1,1,11491.7375000002
1,1,16198.2118055554
1,1,15264.7586206897
1,1,7194.4134615386
1,1,7146.5375000002
1,1,19248.0000000001
1,1,7986.21875
1,1,5086.0585106382
1,1,6313.3404255318
1,1,7251.7441860466
1,1,8530.8333333333
1,1,5815.6842105264
1,1,9212.5549999999
1,1,6508.825
1,1,10533.3392857143
1,1,5363.4329268293
1,1,5798.4166666665
1,1,15213.0691489358
1,1,10479.5116279072
1,1,5058.0174418604
1,1,5234.0512820513
1,1,6711.9464285715
1,1,10012.3782051283
2,2,5525.1874999998
2,2,5923.3055555555
2,2,5183.5365853658
2,2,8332.9318181819
2,2,8278.3676470589
2,2,6054.6666666669
2,2,5502.4274193549
2,2,11775.7727272726
2,2,5447.0729166666
2,2,8496.9968749999
2,2,5520.0263157895
2,2,5015.2043269231
2,2,10452.4891304348
2,2,5185.3083333334
2,2,5768.6023391812
2,2,5372.5914634146
2,2,5401.8963414634
2,2,5656.9107142856
2,2,9242.3387096774
2,2,8520.5760869568
2,2,5434.0065789474
2,2,6244.005988024
2,2,8494.1398809524
2,2,5282.0363636363
2,2,7215.5525
2,2,6882.0188679246

2,2,8034.5649038461
2,2,5498.1162790698
2,2,6306.9387755103
2,2,6289.554054054
2,2,6912.2430555556
2,2,5252.2499999999
2,2,9638.7232142858
2,2,7727.2222222223
2,2,6268.5555555555
2,2,5285.3962264151
2,2,8064.7659574468
2,2,6565.484375
2,2,6177.892857143
2,2,8170.4375000001
2,2,6884.4652777777
2,2,5386.2675000001
2,2,10120.1413043477
2,2,8366.8555555555
2,2,5532.4999999999
2,2,7555.1764705881
2,2,8056.4763513514
2,2,8509.1578947368
2,2,6019.8205128204
2,2,5938.7039473684
2,2,6648.0094339625
2,2,5941.359375
2,2,6746.9427083334
2,2,5141.2130681819
2,2,6235.3783783784
2,2,6611.3181818183
2,2,5887.32
2,2,8906.6587837838
2,2,6390.0391566265
2,2,7232.8333333334
2,2,6796.5
2,2,9766.0581395347
2,2,5269.5833333333
2,2,7713.9038461538
2,2,5375.5319148935
2,2,7019.5971074381
2,2,5655.59375
2,2,5094.5135135135
2,2,5081.6136363637
2,2,7030.590909091
2,2,6298.3125
2,2,5693.4999999999
2,2,5801.5128205128
2,2,8838.8962264151
2,2,11898.5
2,2,5301.9821428572
2,2,6160.0306122449
2,2,5343.9591836734
2,2,5306.3255813953
2,2,6734.2660256409
2,2,5757.8018292683
2,2,5687.7241379311
2,2,5515.8991228071
2,2,7368.4390243902
2,2,5319.3368644068
2,2,7102.1818181817
2,2,6405.1914893618
2,2,5513.6611842106
2,2,8597.8510638297
2,2,8527
2,2,6354.5843750001
2,2,5568.2926829269

2,2,8429.6375
2,2,5047.6692708334
2,2,7761.125
2,2,7826.8803191491
2,2,5065.8484042553
2,2,5418.8288043479
2,2,7559.3125000001
2,2,5284.8653846153
2,2,5636.2162162162
2,2,6178.3214285714
2,2,6108.09
2,2,7646.34375
2,2,6442.1346153847
2,2,6301.9558823529
2,2,10557.1875
2,2,5636.2604166667
2,2,5144.7361111111
2,2,5301.3175675676
2,2,5637.5337837838
2,2,8410.0714285713
2,2,7513.3793103448
2,2,7565.6515957447
2,2,5365.7732558138
2,2,5021.7708333336
2,2,5880.1794871796
2,2,6627.6611111112
2,2,5181.6625
2,2,7871.0625
3,1,18328.9200913242
3,1,5353.7870370371
3,1,7332.9375
3,1,6584.5405405407
3,1,5470.6972222223
3,1,13427.1335227274
3,1,8799.8430232558
3,1,10153.2180094788
3,1,5430.8596938776
3,1,9463.8181818181
3,1,6133.1666666666
3,1,5542.75
3,1,7983.9679487179
3,1,13189.130208333
3,1,13212.3977272727
3,1,8202.8076923077
3,1,6739.6250000001
3,1,8738.0454545453
3,1,5006.6976744187
3,1,9140.25
3,1,7349.5729166667
3,1,7755.585526316
3,1,6079.471590909
3,1,5303.7379032258
3,1,8692.9401197604
3,1,10321.78125
3,1,5003.846875
3,1,5264.1395348837
3,1,5700.2058823529
3,1,7448.4097222221
3,1,5802.2642045454
3,1,9954.1764705883
3,1,5803.1902173913
3,1,5268.0208333333
3,1,8818.7735849056
3,1,5220.85
3,1,5204.4431818183
3,1,6124.6151315788

3,1,5307.0518518519
3,1,5514.0500000002
3,1,5124
3,1,7309.0188679246
3,1,6891.3604651162
3,1,6752.0804020101
3,1,8055
3,1,7556.5740740742
3,1,7147.717948718
3,1,19396.8662790699
3,1,6696.8574999999
3,1,12598.1402439024
3,1,6477.2453703705
3,1,6232.8363636365
3,1,8144.6177884616
3,1,5881.1791044775
3,1,6513.2631578948
3,1,8052.4351851852
3,1,16759.820121951
3,1,13396.5601851847
3,1,16649.0762711866
3,1,7123.8061224489
3,1,14116.7109375
3,1,5916.2272727273
3,1,5243
3,1,7885.3181818181
3,1,5528.1644736841
3,1,5620.846590909
3,1,8783.9134615384
3,1,12361.1357142858
3,1,8074.2432432432
3,1,5444.693877551
3,1,6816.4
3,1,6136.90625
3,1,5402.7317073171
3,1,5615.9864864864
3,1,6100.7843137253
3,1,14353.5000000007
3,1,5530.2135416668
3,1,5684.5638297872
3,1,7553.956521739
3,1,5059.2173913044
3,1,5319.1739130434
3,1,8667.1415094341
3,1,9039.4078947367
3,1,17117.4774999999
3,1,13637.6702127659
3,1,6245.2682926828
3,1,7587.5333333334
3,1,5156.875
3,1,10613.75
3,1,5126.9875
3,1,9553.4871794873
3,1,10581.0828877007
3,1,14808.84375
3,1,5724.3695652173
3,1,7302.0731707316
3,1,7166.5326086956
3,1,6948.2916666667
3,1,12748.652173913
3,1,7266.1406249999
3,1,5099.2916666668
3,1,8286.1647727273
3,1,6634.6160714285
3,1,17862.9558823529
3,1,5940.2674418605

3,1,7957.869047619
3,1,5698.8897058823
3,1,9489.3333333334
3,1,6882.4893617022
3,1,9018.8837209301
3,1,5051.2763157895
3,1,19941.7222222223
3,1,9373.0585106385
3,1,5963.1310975609
3,1,19779.5562913906
3,1,19177.625
3,1,9512.6136363636
3,1,6515.2115384615
3,1,11054.5131578947
3,1,6119.2994186046
3,1,18716.6646341464
3,1,6599.2426470587
3,1,5462.6015625
3,1,12503.078125
3,1,9378
3,1,12395.7053571429
3,1,10547.0460526316
3,1,6031
3,1,7645.6489361703
3,1,5462.0697674418
3,1,9265.9999999999
3,1,6710.4583333334
3,1,7399.5
3,1,5970.5771276596
3,1,6525.7403846154
3,1,6697.7837837838
3,1,6808.8671875001
3,1,9421.359375
3,1,14652.984375
3,1,5310.8055555555
3,1,6251.90625
3,1,7705.6482558141
3,1,5226.5159574468
3,1,7421.4621212121
3,1,6619.5433673471
3,1,5651.28125
3,1,7815.8285714285
3,1,18511.625000001
3,1,5400.1193181817
3,1,7103.2000000001
3,1,5851.9204545454
3,1,5602.0104166666
3,1,11172.0534979421
3,1,5804.7499999999
3,1,9478
3,1,15933.1399999999
3,1,6020.8445121951
3,1,10150.0539772733
3,1,6330.8780487804
3,1,9560.8529411766
3,1,6722.0487804878
3,1,7978.3031250001
3,1,5479.9829545455
3,1,5327.3333333334
3,1,6751.5454545456
3,1,5242.8085106383
3,1,13715.031818182
3,1,7112.6341463416
3,1,5171.637755102
3,1,6130.9870689656
3,1,6640.7142857143

3,1,7735.7670454546
3,1,6509.0585106385
3,1,5090.9756097561
3,1,17014.1646341464
3,1,6311.3475
3,1,6696.0081521738
3,1,7501.8662790696
3,1,9500.828125
3,1,9191.553977273
3,1,6555.4656250001
3,1,6564.94375
3,1,9954.9818181817
3,1,6034.4296875
3,1,16867.6041666664
3,1,11685.328125
3,1,5916.2524509803
3,1,9914.4764150946
3,1,12349.0555555554
3,1,6680.5211864407
3,1,6363.0697674419
3,1,6909.011627907
3,1,8871.2897727272
3,1,6426.7784090908
3,1,13378.3522727276
3,1,5846.1696428572
3,1,5702.6588541666
3,1,7204.5396341465
3,1,8002.2333333333
3,1,6953.8125
3,1,5439.7774999999
3,1,5063.6947674418
3,1,7335.6329787234
3,1,8493.5387931035
3,1,16647.8106060605
3,1,7606.1914893618
3,1,8563.1707317073
3,1,5525.7764423077
3,1,11585.9027777778
3,1,5528.62
3,1,10282.5927419354
3,1,14030.5937499999
3,1,17512.6999999998
3,1,6284.0236486486
3,1,7497.8110465117
3,1,12278.1634615386
3,1,12333.1802884614
3,1,6368.3016304347
3,1,6713.9000000001
3,1,13842.4734042553
3,1,14330.2631578947
3,1,5551.8409090908
3,1,7491.9542682925
3,1,9670.4550898203
3,1,6576.0681818184
3,1,8349.7364130434
3,1,8752.4075000002
3,1,7519.0178571428
3,1,6296.4814814815
3,1,11919.46875
3,1,5042.5
3,1,9308.875
3,1,7229
4,2,5694.7727272726
4,2,8723.9268292684
4,2,6487.4893617023
4,2,8398.9206349205

4,2,5045.1770833333
4,2,5115.8809523808
4,2,9062.8571428574
4,2,7968.1500000001
4,2,6112.4437499999
4,2,5944
4,2,8784.3695652174
4,2,10032.2804878048
4,2,6807.3658536586
4,2,8047.1824324325
4,2,9351.5869565216
4,2,5385.0274390243
4,2,5913.9523809525
4,2,6629.4767441861
4,2,8757.2835365852
4,2,5049.4166666668
4,2,10351.3472222225
4,2,6857.5340909092
4,2,5811.6923076924
4,2,6201.780612245
4,2,9838.4325
4,2,5585.5670731707
4,2,5734.8295454546
4,2,11614.0762195123
4,2,7313.1428571427
4,2,6317.3070175439
4,2,9672.9744897959
4,2,5588.7015306122
4,2,6487.6935483871
4,2,5648.0548245614
4,2,8372.8295454545
4,2,5220.01875
4,2,6557.0638297874
4,2,7107.8372093024
4,2,9991.2325581395
4,2,7667.9166666666
4,2,6382.1178571428
4,2,7683.4102564102
4,2,5747.6388888889
4,2,8340.9772727273
4,2,6924.3529411765
4,2,14831.3333333333
4,2,7367.3468750001
4,2,11247.0892857143
4,2,10687.7625000001
4,2,7080.7023809524
4,2,7859.25
4,2,7135.8214285714
4,2,6856.625
4,2,7542.75
4,2,9938.0178571429
4,2,7850.4714285715
4,2,5877.9147727272
4,2,11211.375
4,2,5961.2593750002
4,2,5028.6136363637
4,2,5790.625
4,2,7497.9215116278
4,2,5635.1524390244
4,2,6079.4375
4,2,8440.953125
4,2,8547.2696078432
4,2,5409.383152174
4,2,7838.1891891892
4,2,7260.6494565216
4,2,10873.7281553398

4,2,9838.7142857143
4,2,6951.7105263158
4,2,10016.1463414633
4,2,9847.6404494384
4,2,6972.2558139534
4,2,9030.1319444443
4,2,10022.2275000001
4,2,5295.1776315789
4,2,5059.1197916667
4,2,5968.1143617022
4,2,12462.4318181819
4,2,5837.6067708332
4,2,9524.5370370369
4,2,5238.0096153847
4,2,7256.463888889
4,2,5161.607142857
4,2,7046.3255813954
4,2,7779.6656249999
4,2,6758.0425531915
4,2,6028.3678571429
4,2,6863.7872340427
4,2,5651.5
4,2,8149.5512820512
4,2,7300.24
4,2,6063.8125
4,2,5758.4693877551
4,2,5854.0217391304
4,2,5042.8682432433
4,2,6197
4,2,5010.4651162791
4,2,7642.8
4,2,7442.6283783782
4,2,8123.0518292684
4,2,5156.328125
4,2,7613.7333333334
4,2,10037.5955882354
4,2,5219.654109589
4,2,5439.2378048781
4,2,5385.1538461539
4,2,5513.8749999999
4,2,6917.1160714286
4,2,9260.0432692309
4,2,5789.0937500001
4,2,7975.328125
4,2,9331.7763157895
4,2,6166.9308510638
4,2,7038.4103260871
4,2,5139.469387755
4,2,10862.1448863636
4,2,8073.5749999999
4,2,7131.3333333332
4,2,5162.9999999999
4,2,5484.9099999999
4,2,5533.7897727272
4,2,7562.9673366834
4,2,6145.3028846153
4,2,5678.0255681818
4,2,5523.552419355
4,2,5507.3
4,2,5331.5928961748
4,2,6233.3529411764
4,2,5395.4230769231
4,2,6147.5833333334
4,2,5773.0445544555
4,2,6355.5754716981
4,2,7006.6586538461

4,2,5753.3970588235
4,2,7875.0749999999
4,2,6214.0714285714
4,2,8614.5
4,2,5171.2443181818
5,1,8644.84375
5,1,7316.3231707316
5,1,5620.955
5,1,5310.2437499999
5,1,15367.6462264151
5,1,6398.625
5,1,6385.671875
5,1,9935.201530612
5,1,10620.8611111111
5,1,14203.7419354839
5,1,5804.7819148937
5,1,10028.98125
5,1,7255.0833333335
5,1,17710.1538461537
5,1,14484.2209302327
5,1,9175.0343750001
5,1,17760.8785714287
5,1,7063.1555555556
5,1,6486.423076923
5,1,8928.7067307691
5,1,6262.4100877193
5,1,5523.8055555556
5,1,7338.2865853658
5,1,5088.4302325581
5,1,6437.7743055555
5,1,6622.5
5,1,6425.2200000001
5,1,7283.034883721
5,1,6266.0885416666
5,1,18626.4687499995
5,1,5115.7222222223
5,1,6100.9147727275
5,1,5828.1510416665
5,1,11874.828125
5,1,5067.3782894737
5,1,9978.559090909
5,1,8828.1517857144
5,1,11879.39453125
5,1,5152.751231527
5,1,18581.558139535
5,1,10935.7599999998
5,1,5687.3343023256
5,1,6124.7857142857
5,1,6925.3833333334
5,1,5905.4933333333
5,1,8991.5930851063
5,1,7656.935546875
5,1,12232.2400000003
5,1,9995.375
5,1,5080.7258064516
5,1,10604.8977272727
5,1,5772.1744791669
5,1,6707.3679245283
5,1,8228.515625
5,1,5875.909090909
5,1,8822.9875
5,1,6252.818181818
5,1,7913.3653846153
5,1,8503.9583333334
5,1,6133.1666666666
5,1,5198.9782608695

5,1,14773.5714285715
5,1,6099.4361702127
5,1,6594.2500000001
5,1,5054.9467592594
5,1,7642.25
5,1,5676.7358490566
5,1,5152.6981132076
5,1,8168.5683962264
5,1,6814.5333333332
5,1,7004.0641025641
5,1,9155.4146341462
5,1,13354.2264150945
5,1,5125.5000000001
5,1,6816.78125
5,1,5371.4951923078
5,1,6980.2631578947
5,1,10965.4972826089
5,1,6946.7886904762
5,1,6942.3125000001
5,1,5384.159090909
5,1,10331.6441717791
5,1,5086.4309210527
5,1,5364.8906249999
5,1,8581.5624999999
5,1,6993.6725
5,1,13552.8125000001
5,1,5616.7828947368
5,1,10392.1796407185
5,1,8933.4166666667
5,1,14751.9062499997
5,1,8064.0683962263
5,1,11742.8875000001
5,1,10823.3648648647
5,1,5409.3372641512
5,1,7606.0555555554
5,1,7408.9010416668
5,1,6121.0641025641
5,1,5751.0588235294
5,1,7058.9024390243
5,1,5987.6424418604
5,1,7009.2882653062
5,1,6802.0555555556
5,1,13237.875
5,1,9240.8750000001
5,1,18556.1960227274
5,1,5792.2972972973
5,1,6461.6250000002
5,1,6346.4068627451
5,1,9619
5,1,10424.4999999998
5,1,6712.84375
5,1,5702.25
5,1,10276.677631579
5,1,6269.2579787233
5,1,5216.5304347825
5,1,17394.56875
5,1,15005.03125
5,1,5206
5,1,9881.2000000001
5,1,7034.6542553192
5,1,7773.328125
5,1,5035.75
5,1,5195.8000000001
5,1,6054.2562499999
5,1,9306.8097826085
5,1,7821.345

5,1,8367.8449999999
5,1,7892.9318181818
5,1,17535.8809523811
5,1,5340.6875
5,1,7153.5135869565
5,1,6252.0000000001
5,1,6987.1607142856
5,1,11756.4081632653
5,1,11743.2765151513
5,1,6566.4107142857
5,1,5323.7111111112
5,1,10669.930327869
5,1,5421.0304878049
5,1,5230.1097560976
5,1,15641.5384615386
5,1,9453.8695652174
5,1,7581.9739583332
5,1,10668.3374999998
5,1,16995.9044117647
5,1,19764.0723684211
5,1,10827.0348837211
5,1,5137.1222222222
5,1,14734.1567796606
5,1,10424.1377551021
5,1,5343.875
5,1,15507.0426829269
5,1,16790.6357758623
5,1,14943.4802631577
5,1,6526.5106382977
5,1,5210.2295918366
5,1,10606.947368421
5,1,7721.506097561
5,1,5948.3839285714
5,1,19679.8399999999
5,1,19244.0737179486
5,1,11255.7616279072
5,1,7702.4941860466
5,1,6067.8070175439
5,1,18055.3285714286
5,1,6470.1020408163
5,1,8380.1274509804
5,1,10195.25
5,1,12076.2282608695
5,1,7173.7553191488
5,1,10140.3790322583
5,1,8196.4476744186
5,1,5052.8547297297
5,1,5196.4711538461
5,1,17984.9891304349
5,1,7456.4134615384
5,1,5197.1777777777
5,1,13669.3696808511
5,1,6143.5192307692
5,1,8316.6511627906
5,1,12682.0880681817
5,1,7417.1108490565
5,1,10595.4575
5,1,5078.2058823529
5,1,6102.7111111112
5,1,12451.5
5,1,7955.1630434785
5,1,17086.7719780218
5,1,5262.0803571428
5,1,10005.3414634146
5,1,9058.3333333331
5,1,6905.0188679244

5,1,7624.9499999998
5,1,5165.2500000001
5,1,8887.5967741936
5,1,18340.9817073169
5,1,6555.2239583332
5,1,6881.0759803922
5,1,11511.3783783785
5,1,5368.2291666668
5,1,15144.4692982453
5,1,5530.4767441861
5,1,6084.2173913045
5,1,6163.3693181818
5,1,8882.1078431372
5,1,7112.6931818182
5,1,9954.9999999998
5,1,7735.5833333332
5,1,5362.987745098
5,1,5619.3841463416
5,1,5726.1217105262
6,2,9009.3939393939
6,2,6032.7727272728
6,2,8069.5425531914
6,2,6000.4901960785
6,2,8434.1944444446
6,2,5093.5788043479
6,2,5180.2647058824
6,2,5009.9325
6,2,7914.625
6,2,6798.0457317073
6,2,5693.2244318181
6,2,5101.4749999999
6,2,7781.2500000002
6,2,6663.6607142858
6,2,8090.6562500002
6,2,5499.3520408164
6,2,7643.5255681817
6,2,5443.5051020408
6,2,5630.5512820513
6,2,5455.9196428569
6,2,5225.3451086956
6,2,6311.6413043478
6,2,10195.2840909092
6,2,6460.2819148937
6,2,5637.8795454546
6,2,7820.4761904761
6,2,5247.1397058824
6,2,5841.3935185185
6,2,8351.8260869565
6,2,5497.9157608697
6,2,10700.0222222224
6,2,7596.9347826087
6,2,6068.8333333332
6,2,6346.7435897436
6,2,5382.5
6,2,7787.015625
6,2,10823.9148936169
6,2,6292.1447368421
6,2,7081.6013513513
6,2,6498.268292683
6,2,5352.429245283
6,2,5154.75
6,2,7553.8191489362
6,2,5583.6107954546
6,2,10789.1441717791
6,2,5878.7180851065
6,2,7321.0352564103

6,2,7745.0102040818
6,2,5184.1219512197
6,2,6520.1940789474
6,2,5591.4893617022
6,2,5085.861111111
6,2,9763.9414893617
6,2,8152
6,2,8353.9285714286
6,2,8234.5555555557
6,2,8217.0853658538
6,2,12561.3953488371
6,2,11841.3750000001
6,2,5391.168478261
6,2,5917.8486842105
6,2,5209.8016304348
6,2,5032.8461538461
6,2,10463.3848039221
6,2,5755.2146226415
6,2,5534.0851063829
6,2,5858.3643617022
6,2,5527.2142857143
6,2,8033.8359375
6,2,5924.1136363638
6,2,5447.3999999999
6,2,7094.28
6,2,5845.875
6,2,9048.5178571428
6,2,6581.3125
6,2,5633.2670454546
6,2,9380.1785714286
6,2,8372.2119565217
6,2,6882.1812500001
6,2,8244.5789473684
6,2,7417.6991869918
6,2,8132.2717391305
6,2,6198.1622340425
6,2,5104.930232558
6,2,5268.7980769231
6,2,5140.8575581394
6,2,7072.465
6,2,6627.9702380953
6,2,7827.9277777777
6,2,7164.7435897436
6,2,5325.1312499999
6,2,5440.53125
6,2,9011.0863636363
6,2,5833.5
6,2,6170.472972973
6,2,6436.4158163264
6,2,6472.375
6,2,5019.7653061225
6,2,5548.9545454544
6,2,5443.3552631579
6,2,5366.7749999999
6,2,9643.9960317459
6,2,7296.703125
6,2,5508.1078431373
6,2,6362.96
6,2,9422.3749999999
6,2,7227.0611702128
6,2,6231.6361111109
6,2,5062.8039215686
6,2,6998.4177631579
6,2,8026.8365384616
6,2,5913.5
6,2,5685

6,2,5905.9199999999
6,2,8353.7743902439
6,2,9202.5552325581
6,2,5003.5216346154
6,2,6426.7890625001
6,2,7234.6947674419
6,2,9576.7663043479
6,2,8645.0526315789
6,2,5707.5416666667
6,2,9397.6590909091
6,2,7011.3368055556
6,2,6330.2329545454
6,2,7361.1000000001
6,2,5229.90625
6,2,7895.804054054
7,1,5228.4851190477
7,1,6915.7256097561
7,1,6765.6829268293
7,1,10081.41875
7,1,13534.3821989529
7,1,5241.6258503402
7,1,9308.4360465115
7,1,5180.1219512196
7,1,5313.7926829269
7,1,12815.2222222221
7,1,6598.0285714287
7,1,10301.1447368419
7,1,6133.0612244898
7,1,15581.5199999999
7,1,10751.0427631579
7,1,8789.2660098521
7,1,14979.6702127659
7,1,6672.48125
7,1,5678.0238095238
7,1,6250.1647727273
7,1,7612.7750000002
7,1,5439.40625
7,1,5515.8716216216
7,1,7907.804054054
7,1,5007.3
7,1,7833.7173913043
7,1,9698.4999999999
7,1,13208.0611111114
7,1,9792.59375
7,1,12944.2094594595
7,1,6900.4871794871
7,1,12248.25
7,1,7094.4763513513
7,1,9435.5349999999
7,1,6887.2139423077
7,1,6480.4705882354
7,1,11800.1333333333
7,1,7435.0249999999
7,1,11000.255952381
7,1,15778.5624999999
7,1,5211
7,1,7093.1595744679
7,1,5296.7194444444
7,1,7588.9605263158
7,1,8813
7,1,5854.8571428571
7,1,8152.0000000001
7,1,12716.3522727272
7,1,7812.4425675675
7,1,10679.4488636363
7,1,7628.3225806452

7,1,5700.9636363635
7,1,6391.8333333333
7,1,8235.9086538462
7,1,5424.5593750001
7,1,8867.4750000001
7,1,5746.46875
7,1,5319.4740566038
7,1,5091.2421875
7,1,5718.3263157896
7,1,5718.2348484849
7,1,7306.4512195122
7,1,5174.9414893618
7,1,18480.0769230768
7,1,7936.642857143
7,1,7270.2286585366
7,1,6109.8671875
7,1,5850.1548913044
7,1,5213.0729166666
7,1,11878.4255319147
7,1,5956.9840425531
7,1,13610.3846153845
7,1,6531.5808823527
7,1,5260.2543859649
7,1,6207.0833333332
7,1,9029.8039215686
7,1,7248.5984848484
7,1,6846.9107142858
7,1,5851.5
7,1,9884.8922413793
7,1,6458.8083333334
7,1,6621.1851851851
7,1,13382.5729166671
7,1,12292.9148936169
7,1,5189.6041666666
7,1,12782.1081081081
7,1,7457.8575581397
7,1,5159.4925
7,1,18409.9925373137
7,1,5890
7,1,12919.8085106383
7,1,6034.2619047619
7,1,7857.2224576271
7,1,5211.4999999999
7,1,9372.5000000002
7,1,6309.0465116279
7,1,6774.5862068965
7,1,16772.7200000001
7,1,9119.1481481483
7,1,8177.7833333333
7,1,5851.84375
7,1,5543.2833333333
7,1,6094.1328125
7,1,6413.4166666666
7,1,5743.6036585366
7,1,19520.5698324018
7,1,5080.205357143
7,1,8861.6666666667
7,1,7994.8600000001
7,1,6097.5670731707
7,1,7539.2250000001
7,1,18615.1470588235
7,1,9360.1331967214
7,1,7176.5361842104
7,1,14692.9005681818
7,1,12067.2347560976
7,1,13263.2350746273

7,1,6876.9673913044
7,1,5037.78125
7,1,6495.0869565218
7,1,15396.1117021273
7,1,15927.71875
7,1,5578.6036585366
7,1,7034.234375
7,1,6682.9807692309
7,1,7031.875
7,1,11083.9107142859
7,1,5280.3333333336
7,1,5154.684659091
7,1,5064.1203703704
7,1,10426.1583333336
7,1,11375
7,1,5947.1027777778
7,1,5180.6916666666
7,1,9637.9464285712
7,1,8418.8837209304
7,1,18024.0000000001
7,1,10211.5613207545
7,1,10757.7419354837
7,1,5124.1176470587
7,1,14739.5841836733
7,1,9823.773255814
7,1,8003.8378378378
7,1,8658.5357142857
7,1,5230.1580882353
7,1,9399.5212765957
7,1,8915.3662790696
7,1,6641.09
7,1,6744.5511363636
7,1,5260.95
7,1,6532.3990147783
7,1,7090.0000000001
7,1,7316
7,1,5833.6825
7,1,8082.2321428572
7,1,5460.1029411766
7,1,5475.4361702129
7,1,8638.5760869565
7,1,15351.2564102563
7,1,9022.0937500001
7,1,6432.8190789472
7,1,12268.5914634146
7,1,7134.9348958331
7,1,7088.3557692309
7,1,6640.2931034482
7,1,5215.396226415
7,1,18336.4354838711
7,1,6914.7021276596
7,1,10250.6542553192
7,1,17657.9083333334
7,1,13497.9210526314
7,1,9613.0409090911
7,1,7289.8048780487
7,1,9941.3414634146
7,1,11064.3680555555
7,1,8951.5409090909
7,1,7961.5462962962
7,1,9328.5732758622
7,1,7241.4944852942
7,1,6346.8706896553
7,1,5244.3679245284
7,1,15034.7959183671
7,1,5246.875

7,1,5158.6545454546
7,1,7415.1328125
7,1,5308.84
7,1,5448.4555555555
7,1,5962.5297029702
7,1,15214.1637931036
7,1,5553.2838983051
7,1,9900.5813953489
7,1,5782.3500000001
7,1,10848.6648936169
7,1,7378.1482558139
7,1,13914.9782608699
7,1,5532.0999999999
7,1,12455.857142857
7,1,14231.3214285712
7,1,6801.5353260869
7,1,7825.8430851065
7,1,8086.2872340426
7,1,5735.3031914893
7,1,5631.9791666667
7,1,7325.7333333332
7,1,5183.6875
7,1,8348.9799999999
7,1,10246.0187969925
7,1,6120.6420454545
7,1,10331.3203125
7,1,8813.4413793104
7,1,18062.1764705881
7,1,5394.1666666665
7,1,18500.0833333333
7,1,5255.325
7,1,5273.7154255319
7,1,5903.8536585367
7,1,6174.2916666667
7,1,8248.8653846153
7,1,5211.0597826088
7,1,5753.7784090909
7,1,19801.9375
7,1,5726.55078125
7,1,6168.1994047618
7,1,7749.6328125
7,1,6829.0816326532
7,1,5115.2222222222
7,1,9126.0398936171
7,1,5211.2073170732
7,1,7303.8128342246
7,1,5726.1198979591
7,1,7270
7,1,6494.4754098363
7,1,7941.4692982456
7,1,11703.6337209303
7,1,16772.75
7,1,11467.0574324323
7,1,15545.5833333335
7,1,6181.7250000001
7,1,5463.875
7,1,5109.9880952381
7,1,5185.2236842105
7,1,16247.1088709677
7,1,11832.2500000002
7,1,9329.0093749999
7,1,7407.9908536585
7,1,6187.2874999999
7,1,5055.0000000001
7,1,9889
7,1,15654.2727272728

7,1,13093.6968749999
7,1,6475
8,2,7177.075
8,2,7146.4625
8,2,8066.650862069
8,2,5397.9999999999
8,2,11038.7205882354
8,2,5258.152173913
8,2,7575.1874999999
8,2,9170.0047169813
8,2,7800.6710526316
8,2,9008.2702702703
8,2,5765.4883720932
8,2,6935.0131578948
8,2,7684.3384146339
8,2,7604.4166666666
8,2,6938.9797297298
8,2,7233.1442307692
8,2,5148.3475
8,2,7003.6979166668
8,2,6891.0714285715
8,2,5477.3804347825
8,2,5975.7403846154
8,2,6395.6923076923
8,2,7865.7777777777
8,2,5446.1517857143
8,2,5861.0408163266
8,2,5166.125
8,2,6325.6341463415
8,2,8939.6335227272
8,2,5524.1886792454
8,2,5898
8,2,9169.4473684211
8,2,8582.2368421053
8,2,6465.5000000001
8,2,5152.342105263
8,2,5657.7734375
8,2,5302.6428571428
8,2,5508.9111111113
8,2,6303.4555555554
8,2,10976.7279411766
8,2,5376.4375
8,2,5975.3125
8,2,5941.47265625
8,2,9862.4222222222
8,2,9449.0689655172
8,2,7327.4627659576
8,2,5155.28125
8,2,5928.1416666667
8,2,11488.3124999996
8,2,7822.6570512821
8,2,5809.4375
8,2,6537.5387931033
8,2,11008.1973684212
8,2,6614.2857142857
8,2,7721.6249999998
8,2,5039.8421052631
8,2,6007.7573529412
8,2,8598.4929245286
8,2,5079.1825396825
8,2,8315.4821428572
8,2,6521.3529411764
8,2,5400.9389534884
8,2,10105.6083333336
8,2,8462.5777027028
8,2,8738.2512315272

8,2,7336.6296296296
8,2,7129.5731707316
8,2,5609.8529411766
8,2,5350.2390350878
8,2,8011.6306818181
8,2,7313.5058139534
8,2,5213.0178571429
8,2,6479.2845744682
8,2,6287.4722222222
8,2,6182.4583333334
8,2,7126.8092105264
8,2,7837.0773809523
8,2,5327.5714285713
8,2,7214.2276785714
8,2,10423.8928571428
8,2,6584.1447368419
8,2,7684.4375
8,2,5258.3333333335
8,2,9517.3229166669
8,2,9935.46875
8,2,8710.9787234041
8,2,9303.7499999998
8,2,9859.6749999999
8,2,9645.9885714288
8,2,5233.2
8,2,10332.0661764708
8,2,5037.2038043478
8,2,5835.0923913042
8,2,5131.2340425532
8,2,8627.0142857142
8,2,9609.3181818181
8,2,5317.2886904762
8,2,6389.2093023255
8,2,6653.6170212766
8,2,5381.1333333335
8,2,5796.5600000001
8,2,8744.9285714286
8,2,5001.65625
8,2,5450.1071428572
8,2,5720.7039473685
8,2,6196.1590909093
8,2,9120.9388888889
8,2,5443.5656250001
8,2,6920.4843749999
8,2,5063.8936170212
8,2,8128.1097560977
8,2,12454.1249999998
3,1,5186.9349999999
9,1,5873.5706521741
9,1,13653.0000000002
9,1,7278.3921568626
9,1,5349.6564417177
9,1,7638.7398373984
9,1,5452.1127450981
9,1,9626.8534482759
9,1,5422.8095238095
9,1,8440.0769230769
9,1,5780.5288461539
9,1,6365
9,1,16416.55
9,1,9557.2056451613
9,1,12851.4000000001
9,1,9210.3406862744
9,1,11001.8111111111
9,1,6660.3937499999
9,1,7690.6280487806

9,1,14780.3194444447
9,1,9212.1796875
9,1,8214.1301020408
9,1,5671.552631579
9,1,5301.5663265306
9,1,7436.8173076924
9,1,11993.4146341463
9,1,5585.7824074074
9,1,6453.2299107141
9,1,8093.7824074074
9,1,5368.2592592593
9,1,7267.4119318181
9,1,10895.6041666668
9,1,8636.9107142856
9,1,6359.2777777777
9,1,7651.7812500002
9,1,5654.8081395349
9,1,5149.8837209302
9,1,6739.4107142857
9,1,8288.9622641507
9,1,5016.6219512195
9,1,7179.3535714286
9,1,7601.0755813956
9,1,5073.7407407407
9,1,6330.1666666668
9,1,8759.1875
9,1,8451.5948275861
9,1,8142.5294117648
9,1,12554.078125
9,1,6081.6343750001
9,1,13968.5505952381
9,1,5777.5714285717
9,1,7232.2905405405
9,1,16399.42
9,1,17450.4594594595
9,1,19575.6611842104
9,1,5108
9,1,16980.295138889
9,1,10000.457236842
9,1,5865.725
9,1,11064.296875
9,1,5108.8444444444
9,1,5198.7692307693
9,1,7177.5493421052
9,1,7600.8478260869
9,1,9394.1636904762
9,1,6880.4825581395
9,1,6827.8928571428
9,1,7478.441860465
9,1,6418.9540816326
9,1,7244.7297297297
9,1,7745.0738636363
9,1,6924.4725609757
9,1,5895.6875
9,1,6398.7034883722
9,1,5330.703125
9,1,8622.8382352938
9,1,7023.1187499998
9,1,12336.6052631579
9,1,7967.8088235294
9,1,7018.5599999998
9,1,18351.3730769228
9,1,13827.4681372551
9,1,6109.672413793
9,1,6642.3414634146
9,1,11867.6750000002

9,1,7211.4509803922
9,1,13994.2678571429
9,1,6424.7908163267
9,1,11091.2999999999
9,1,8709.4447674418
9,1,6093.8666666666
9,1,7094.9743589743
9,1,7112.1818181818
9,1,8233.7608695653
9,1,6572.462264151
9,1,12308.1840277777
9,1,5609.4854651163
9,1,8545.1818181819
9,1,11572.216216216
9,1,5966.3170731707
9,1,5495.4693877551
9,1,6799.5159574467
9,1,6816.7647058824
9,1,13547.116883117
9,1,14351.4756097564
9,1,6307.3307291667
9,1,14187.6249999999
9,1,5193.9508196721
9,1,7516.859375
9,1,7839.9107142858
9,1,11884.9527027027
9,1,7508.3181818183
9,1,5266.3806818181
9,1,6143.0084745763
9,1,5059.2433035714
9,1,5735.0125
9,1,6736.4479166666
9,1,5958.6000000001
9,1,11014.3846153843
9,1,5582.3179824561
9,1,8040.6499999999
9,1,6080.2261904762
9,1,8068.6818181819
9,1,5743.2831632654
9,1,6610
9,1,8477.7974999999
9,1,6367.0056818181
9,1,5609.5833333333
9,1,6722.0307017544
9,1,15423.4687499998
9,1,6656.9772727275
9,1,7433.1428571427
9,1,9986.7954545455
9,1,6674.8828125
9,1,7299.9407894737
9,1,14862.9671052632
9,1,5744.2291666667
9,1,5536.4767441859
9,1,6388.090116279
9,1,5028.3841463414
9,1,8052.5749999999
9,1,9861.875
9,1,10996.5482456138
9,1,6658.3409090909
9,1,10190.2412790695
9,1,5807.3541666666
9,1,16342.0520833336
9,1,9224.6413043478
9,1,8584.5454545453
9,1,6826.1063829787
9,1,5434.9972826088

9,1,13344.5336538458
9,1,7939.8628048781
9,1,14834.5797872341
9,1,8593.7678571429
9,1,6905.125
9,1,8460.2377049181
9,1,5505.8048780488
9,1,10654.1944444446
9,1,5701.5000000002
9,1,7330.7828947366
9,1,5078.5047169812
9,1,10712.8065476194
9,1,9470.1875
9,1,13997.1319444443
9,1,9026.9933035715
9,1,6249.6785714285
9,1,7803.7672413794
9,1,10080.7526595746
9,1,9144.7272727274
9,1,11319.552631579
9,1,5483.0833333332
9,1,13761.1634615385
9,1,14807.9756097561
9,1,14986.7065217392
9,1,6978.7388888887
9,1,8784.4999999999
9,1,7285.4391891892
9,1,8439.125
9,1,6017.38
9,1,5337.4210526316
9,1,14294.2321428567
9,1,6506.447368421
9,1,8375.6281249999
9,1,7805.5815217392
9,1,8529.1029411765
9,1,6607.9360465117
9,1,7383.3349056604
9,1,7748.5957446807
9,1,5512.3546511628
9,1,6719.8139534883
9,1,5167.8600000001
9,1,10279.7933673469
9,1,5818.5664893616
9,1,12592.597826087
9,1,5229.6534090909
9,1,15465.8374999999
9,1,5635.05
9,1,5119.2138157894
9,1,17218.3295454541
9,1,8649.6297169813
9,1,6249.1363636364
9,1,5801.923076923
9,1,15706.456521739
9,1,5745.6646341464
9,1,14697.1196808509
9,1,11551.5029069767
9,1,5481.9017857144
9,1,5718.5
9,1,7080.2750000001
9,1,5398.4701086957
9,1,7379.2282608695
9,1,6309.1730769229
9,1,11911.5055555554
9,1,5744.7391304348
9,1,10329.4375
9,1,6429.6293103447

9,1,5807
9,1,5418.5877659574
9,1,16430.5661764705
9,1,5010.7142857142
9,1,17471.8546511627
9,1,5918.328125
9,1,5828.8555555556
9,1,10466.026442308
9,1,10367.1473214283
9,1,5192.5102040816
9,1,6023.2790697674
10,2,6994.9204545455
10,2,8273.9642857142
10,2,5229.058510638
10,2,7103.0625
10,2,6673.8688524592
10,2,11396.96484375
10,2,5998.0433673469
10,2,9022.9000000003
10,2,8408.7325581391
10,2,8248.0847457625
10,2,8185.0190217392
10,2,7613.8142857141
10,2,8293.3513513513
10,2,5421.4888888889
10,2,9068.4591836735
10,2,13321.4583333335
10,2,6975.9655172414
10,2,5134.3010204081
10,2,5342.0717592594
10,2,5207.3068181818
10,2,7053.1889534883
10,2,5457.7708333332
10,2,5163.5928143713
10,2,8151.2432432432
10,2,5562.6363636364
10,2,8565.3506097562
10,2,6955.9036458335
10,2,7273.3043478262
10,2,7664.4047619048
10,2,6966.1918604649
10,2,6889.7621951219
10,2,5574.206632653
10,2,6469.4609375
10,2,5143.554347826
10,2,6824.9487179487
10,2,11028.5374999999
10,2,5397.8260869566
10,2,5523.1162790698
10,2,5154.9999999999
10,2,5893.2410714286
10,2,8686.3103448276
10,2,9117.6326530614
10,2,5980.9734042553
10,2,9460.1153846153
10,2,6611.3227848101
10,2,7105.96875
10,2,6859.8333333332
10,2,7082.8090452262
10,2,13108.0583333334
10,2,7942.5846774196
10,2,5986.8392857143
10,2,5780.948275862
10,2,5267.5581395348
10,2,5691.1614583332
10,2,7853.7906976744

10,2,8826.640350877
10,2,5054.9846938776
10,2,5500.305
10,2,5032.8717105262
10,2,6504.5500000001
10,2,6799.3181818182
10,2,6873.0833333334
10,2,6672.2042682924
10,2,5254.5
10,2,5103.7837837839
10,2,5462.4507575759
10,2,9187.3804347826
10,2,8756.3265306122
10,2,9415.0353260871
10,2,5392.8928571429
10,2,6319.625
10,2,5800.153409091
10,2,5586.5285714286
10,2,6170.9138888889
10,2,7604.0000000002
10,2,9324.5555555557
10,2,8138.0288461538
10,2,11276.4634146341
10,2,9933.8125
10,2,6880
10,2,5282.1538461538
10,2,7941.85
10,2,6763.0058139534
10,2,6379.1108870966
10,2,6519.4680851066
10,2,5933.2666666666
10,2,9382.0625
10,2,6185.6820175438
10,2,5096.7926136364
10,2,6663
10,2,6790.0675675676
10,2,7128.5163043478
10,2,5323.265625
10,2,6389.237244898
10,2,10255.6010638297
10,2,5380.7933673469
10,2,5399.8101851851
10,2,8391.1333333333
10,2,6877.6634615386
10,2,6513.1198979592
10,2,8050.8902439026
10,2,9491.9519230769
10,2,8791.0531914892
10,2,5409.9389534884
10,2,6119.7927631579
10,2,5978.7620192307
10,2,5768.5170454546
10,2,7849
10,2,5921.59375
10,2,6303.75
10,2,5080.1851851852
10,2,6842.3682432434
11,1,7507.0957446809
11,1,7074.0454545453
11,1,6057.94
11,1,11916.6118421052
11,1,5428.5483870967
11,1,5270.5833333333
11,1,6187.1775000001
11,1,7129.6530612246
11,1,17975.2000000001

11,1,7083
11,1,6804.9583333332
11,1,6830.7994186046
11,1,5395.6610576923
11,1,6080.8571428571
11,1,5535.6078431373
11,1,19659.1745689657
11,1,9386.6757990869
11,1,9679.9816513762
11,1,9132.0094339622
11,1,6883.25
11,1,8074.0153846152
11,1,7122.6420454546
11,1,10249.8888888891
11,1,5884.8000000001
11,1,7109.5259433963
11,1,7718.7946428572
11,1,7396.4817708335
11,1,7860.8695652174
11,1,15300.0599999998
11,1,5818.4755434783
11,1,9967.5641891892
11,1,5548.2329545454
11,1,5341.5326086956
11,1,5534.9557291667
11,1,18732.5692307687
11,1,5724.6744186046
11,1,8689.9655172414
11,1,6866.1985294118
11,1,8159.7391304348
11,1,7772.1500000001
11,1,8498.7543859649
11,1,6625.8
11,1,11251.6556603773
11,1,5020.6586538461
11,1,8038.6250000001
11,1,13064.1706730765
11,1,6245.2788461538
11,1,6792.8952702702
11,1,13723.833333333
11,1,6600.2102272727
11,1,12647.9999999999
11,1,16996.4444444442
11,1,13473.9107142857
11,1,5993.4975
11,1,15678.0588235295
11,1,8825.2458100557
11,1,6220.9999999999
11,1,7642.6419491525
11,1,6699.8617021277
11,1,5067.6956521738
11,1,5314.0526315791
11,1,10551.0285714285
11,1,15942.6250000002
11,1,8786.4130434784
11,1,6400.5875000001
11,1,5020.721875
11,1,8911.2942708333
11,1,10240.0196078432
11,1,9043.2558139534
11,1,5653.1431818182
11,1,17319.7075471701
11,1,8074.4042553191
11,1,8104.7202380951
11,1,7745.736111111
11,1,6013.1875000001

11,1,6078.5888888888
11,1,18745.6282051281
11,1,16382.7714285715
11,1,9067.8292682925
11,1,8713.4418604652
11,1,7125.9864864864
11,1,5212.1698113208
11,1,6011.21875
11,1,8206.5319148934
11,1,6776.1755319148
11,1,7933.3349056604
11,1,10996.3571428574
11,1,9964.6153846153
11,1,16168.2903225806
11,1,7532.4888888888
11,1,15709.1412037039
11,1,12170.7410714285
11,1,12504.6179245282
11,1,6373.25
11,1,6930.2200000001
11,1,5239.9517045455
11,1,12208.9912790699
11,1,7249.4076086955
11,1,7229.8587962965
11,1,6915.2727272727
11,1,5776.256097561
11,1,9888.446969697
11,1,8595.4396551725
11,1,13169.8168103447
11,1,11539.46875
11,1,5623.988888889
11,1,6391.7723214284
11,1,6803.8375
11,1,5385.3854166666
11,1,5077.1005434782
11,1,8471.3505434784
11,1,5051.6755319149
11,1,19421.923076923
11,1,6318.1630434784
11,1,7632.3
11,1,5996.8387096775
11,1,6340.0640243903
11,1,5768.7201086957
11,1,12932.6406249998
11,1,5897.5326086957
11,1,9504.4479166669
11,1,6914.3474999998
11,1,13448.8273809523
11,1,5812.55
11,1,5032.0306122449
11,1,5450.4517045455
11,1,12592.375
11,1,5438.2211538463
11,1,6714.9186046512
11,1,14154.3428571428
11,1,6879.585
11,1,15174.1999999995
11,1,10711.5499999999
11,1,8157.2999999998
11,1,5033.3985849057
11,1,10538.4062499999
11,1,8382.6783919599
11,1,6218.9763779528
11,1,5260.4294871794
11,1,5776.0613207548
11,1,6034.2280701756

11,1,9356.9565217394
11,1,5688.3835227273
11,1,5518.5700483091
11,1,6162.8333333334
11,1,5085.3113207547
11,1,5236.3833333334
11,1,6248.1323529411
11,1,5546.1249999999
11,1,6418.4468085107
11,1,9434.6177884615
11,1,7664.9694444445
11,1,13964.8235294118
11,1,8090.5652173913
11,1,5733.7244897959
11,1,5241.1047297297
11,1,5345.2440476189
11,1,6603.2173913044
11,1,6474.7823275861
11,1,14251.1976744184
11,1,6086.5436046511
11,1,5206.238095238
11,1,16823.294117647
11,1,9692.5573770491
11,1,5623.7453703702
11,1,5908.0400000001
11,1,8784.640625
11,1,11125.4583333331
11,1,6566.8389830509
11,1,12643.4897959185
11,1,6203.141304348
11,1,7086.2857142857
11,1,7958.5227272726
11,1,8295.3430232557
11,1,7207.3999999999
11,1,12254.9796511626
11,1,8642.3283898305
11,1,5399.25
11,1,7884.0263157895
11,1,16762.6341463416
11,1,13652.4811320759
11,1,6779.1937499999
11,1,7340.100490196
11,1,9011.3000000001
11,1,6702.20625
11,1,5032.1304347826
11,1,17118.8895348837
11,1,5059.7444444444
11,1,13424.53125
11,1,5636.0000000001
11,1,8577.5043103448
11,1,17045.7325581395
11,1,11264.6323529411
11,1,17615.6985294116
11,1,6986.641509434
11,1,6484.7499999999
11,1,18909.0581395349
11,1,5852.7916666666
11,1,14354.5000000001
11,1,18879.2374999994
11,1,5162.1363636362
11,1,7228.5367647059
11,1,10794.6312499998
11,1,9114.0994897962
11,1,18888.8846153847
11,1,8403.5851063829
11,1,6885.9407894736

11,1,5211.1793478261
11,1,7652.2105263158
11,1,5453.7777777779
11,1,5508.945652174
11,1,12532.3977272726
11,1,11391.260869565
11,1,12048.4411764707
11,1,5568.9272727273
11,1,7816.4147727272
11,1,18985.507653061
11,1,7291.8333333332
11,1,19419.4249999999
11,1,8826.03125
11,1,15549.6450000001
11,1,6594.0833333332
11,1,5341.9760638297
11,1,11455.451923077
11,1,9262.1444444443
11,1,9684.9147727275
11,1,6947.2916666665
11,1,7249.9454545455
11,1,10453.0404411763
11,1,6732.7826086957
11,1,6051
11,1,5667.2098214286
11,1,6625.6027397261
11,1,16134.6428571428
11,1,7071.7441860465
11,1,14236.6640625
11,1,6326.9513888887
11,1,7257.7115384616
11,1,12840.7405660379
11,1,9237.6249999999
11,1,10318.1587837837
11,1,7567.9393939393
11,1,6682.9007352941
11,1,6528.7004716982
11,1,6048.0119047618
11,1,16784.7499999998
11,1,8199.6296296296
11,1,7421.4333333334
11,1,9050
11,1,14852.7948717948
11,1,6114.175
11,1,17726.9895833333
11,1,14130.203125
11,1,5210.6219512195
11,1,5368.125
12,2,6001.3513513513
12,2,6069.8750000001
12,2,9956.5
12,2,6076.1947674418
12,2,5024.1764705883
12,2,5184.1875
12,2,8226.3519736844
12,2,12416.890625
12,2,6054.7619047619
12,2,7271.0822368422
12,2,9284.5657894738
12,2,10480.4386792455
12,2,6318.2134146342
12,2,5645.2265625001
12,2,6527.815
12,2,5034.3583333333
12,2,7505.68359375
12,2,5720.474137931

12,2,7318.8611111111
12,2,5305.3505154639
12,2,8630.9976851852
12,2,7508.1874999999
12,2,5834.6388888888
12,2,11005.7596153846
12,2,7371.6875
12,2,6008.9333333332
12,2,6625.095744681
12,2,5511.3525000001
12,2,7284.3885869566
12,2,6055.6118421052
12,2,5223.6590909091
12,2,7142.1642156862
12,2,6252.9642857143
12,2,5135.3951612903
12,2,5030.1071428571
12,2,5425.1572580645
12,2,5765.0714285712
12,2,5775.8090909091
12,2,5964.525510204
12,2,6360.3684210526
12,2,5075.2352941177
12,2,5646.0026041666
12,2,7802.715116279
12,2,8850.8962264152
12,2,5455.625
12,2,6211.2420212765
12,2,7974.7861111111
12,2,5710.4431818181
12,2,6611.5600961538
12,2,6616.4607142858
12,2,5812.8048780487
12,2,7111.7638888889
12,2,7815.2558139533
12,2,11841.0625
12,2,7815.265957447
12,2,5313.7794117648
12,2,5112.8888888889
12,2,6510.238372093
12,2,7267.9444444443
12,2,8522.8363636365
12,2,5657.5570175438
12,2,11405.6057692308
12,2,7085.58
12,2,5649.641509434
12,2,10900.8500000001
12,2,8016.1097560975
12,2,5690.1761363637
12,2,6955.6169354837
12,2,9591.3194444444
12,2,8260.8837209302
12,2,5903.0104166666
12,2,6346.9038461539
12,2,6993.6071428571
12,2,6944.7155172415
12,2,5876.6896551725
12,2,6992.3000000001
12,2,5706.0878378379
12,2,5070.3333333334
12,2,5747.6607142858
12,2,5822.796875
12,2,6482.7999999999
12,2,11280.3551136363
12,2,6602.4431818182
12,2,9474.5931818183

12,2,7007.068548387
12,2,5348.6249999999
12,2,6687.1586538461
12,2,5912.1086956521
12,2,9497.8397435896
12,2,5450.25
12,2,9377.9130434783
12,2,5465.5384615384
12,2,6669.5489130434
12,2,6871.6041666668
12,2,11129.8014705883
12,2,6339.3829787233
12,2,5535.2473404255
12,2,8073.5128205128
12,2,8192.2440476191
12,2,5255.6150000003
12,2,9238.9127906978
12,2,6212.625
12,2,5219.2727272727
12,2,7390.3776595746
12,2,6281.0384615386
12,2,11415.1250000001
12,2,6343.2585227272
12,2,7700.3722222223
12,2,6500.6595744681
12,2,5849.1310975611
12,2,5274.3676470588
12,2,9457.4342105264
12,2,5657.4088983051
12,2,7041.2162162163
12,2,5060.4468085107
12,2,5313.6637931034
12,2,7286.765625
12,2,5360.9583333334
12,2,7859.6527777776
12,2,5684.5489130435
12,2,5995.1515151515
12,2,9943.5491803276
12,2,7486.6666666669
12,2,5602.138888889
12,2,5408.5288461538
12,2,9684.5330188678
12,2,7207.375
12,2,5027.7352941175
12,2,5478.1302083332
12,2,5801.1217948717
12,2,8526.8549999998
12,2,6559.7625
12,2,5354.6542553192
12,2,7549.2756410257
12,2,7261.7792553192
12,2,5008.6212121212
13,1,7096
13,1,5999.3333333335
13,1,5116.4627659575
13,1,8170
13,1,6976.1756756756
13,1,5395.7840909091
13,1,5374.6875000001
13,1,5156.9204545454
13,1,5926.7017543858
13,1,9419.8777173915
13,1,6350.2309782608
13,1,9536.6904761906
13,1,6433.9528301885
13,1,8767.9448529414

13,1,16322.7355769231
13,1,7887.4499999998
13,1,11129.4583333332
13,1,9961.9662162164
13,1,6224.9812500001
13,1,6157.8418367347
13,1,6129.328488372
13,1,16391.599264706
13,1,14471.4136363633
13,1,6078.5068181819
13,1,10502.0625
13,1,6442.4074074073
13,1,6805.3035714286
13,1,6757.3170731707
13,1,7276.8374999998
13,1,19648.1141304348
13,1,5218.5178571429
13,1,5414.5142857144
13,1,5725.75
13,1,6369.3909574468
13,1,8832.5454545456
13,1,5010.3546511628
13,1,5212.3374999999
13,1,5164.9411764706
13,1,9157.5625
13,1,7241.8293269231
13,1,6524.4414893616
13,1,16100.2000000004
13,1,9146.8944444445
13,1,6895.9593023255
13,1,9109.9429347826
13,1,5703.9464285714
13,1,5756.2
13,1,5920.4787234043
13,1,5814.6493055557
13,1,5850.4795918368
13,1,7731.2666666665
13,1,14001.2907608694
13,1,5100.090909091
13,1,9181.0795454545
13,1,5514.6018518519
13,1,5746.2394067797
13,1,6433.8205128205
13,1,6973.1538461538
13,1,5707.90625
13,1,7021.9121621621
13,1,13899.352272727
13,1,5785.2978723402
13,1,5265.75
13,1,6291.8777777778
13,1,6671.4510869565
13,1,6252.3451086956
13,1,10085.2530864198
13,1,5694.58984375
13,1,10375.4390243906
13,1,6409.9714285715
13,1,5861.2916666668
13,1,5890.7692307692
13,1,7991.6547619048
13,1,7632.3203125
13,1,18222.1875
13,1,5713.6773255814
13,1,5510.6743421053
13,1,16524.3469387758
13,1,6634.2962962965
13,1,15297.7500000001

13,1,13624.3032407409
13,1,10001.75
13,1,8214.2472222221
13,1,5659.731707317
13,1,6478.1770833334
13,1,5411.9672619046
13,1,17968.8593750002
13,1,9460.8780487806
13,1,8734.9599056604
13,1,8843.1590909092
13,1,8074.0661764707
13,1,9646.883064516
13,1,13631.2543859652
13,1,7673.2870370371
13,1,7357.4027777779
13,1,6342.68
13,1,16033.5477707009
13,1,5188.0135135136
13,1,5751.5851063831
13,1,7177.2526595744
13,1,5112.32
13,1,9158.3729838709
13,1,6905.4228723406
13,1,6786.25
13,1,7183
13,1,11672.7986111112
13,1,6282.3725490198
13,1,11164.7853773583
13,1,10340.5588235294
13,1,5011.7045454545
13,1,11804.5306122447
13,1,6705.7234042554
13,1,6766.2105263158
13,1,6231.5535714286
13,1,9621.0000000001
13,1,8027.3141025642
13,1,7080.0000000001
13,1,17306.3641304348
13,1,12869.9743589743
13,1,5012.375
13,1,19363.7116564417
13,1,8650.6861702127
13,1,5542.9024390244
13,1,5708.2638888888
13,1,7033.4142857142
13,1,12838.0707547172
13,1,5490.09375
13,1,6456.5
13,1,5234.1521739131
13,1,5077.7187500001
13,1,5530.525
13,1,5135.2034313727
13,1,15095.8586956519
13,1,7052.6785714286
13,1,11158.098360656
13,1,6784.5357142857
13,1,5244.2329545455
13,1,8170.3478260871
13,1,7692.9566326529
13,1,12566.8820754717
13,1,5974.3999999999
13,1,6002.2037914693
13,1,17857.108108108
13,1,7020.8066037736
13,1,6780.2263513513
13,1,6853.914893617

13,1,7679
13,1,6549.0204081634
13,1,16707.7250000001
13,1,7809.3194444444
13,1,5763.2173913046
13,1,7093.7146739131
13,1,7093.5625
13,1,5345.2804878049
13,1,6525.7948717949
13,1,5218.8461538461
13,1,7050.2127659575
13,1,5527.7307692308
13,1,8391.511627907
13,1,8371.085106383
13,1,6111.0058139535
13,1,6454.2750000001
13,1,9491.5853658536
13,1,7494.1674528302
13,1,5286.1656976744
13,1,19665.6129807697
13,1,5141.71484375
13,1,5067.90625
13,1,5543.2556818182
13,1,6881.2928571429
13,1,5595.1944444444
13,1,5148.5714285714
13,1,5092.7424242424
13,1,6476.4852941178
13,1,5052.2500000001
13,1,5807.3255813953
13,1,6745.1890243904
13,1,8484.9934210526
13,1,5308.6328125
13,1,6354.1640625
13,1,8006.9976303314
13,1,6460.5568181818
13,1,7687.3454545454
13,1,6209.3947368421
13,1,6406.638888889
13,1,12691.3384146337
13,1,5717.7090517242
13,1,7079.7451923077
13,1,7014.2880434782
13,1,8608.875
13,1,15692.6340909092
13,1,10298.8912037034
13,1,5199.9663461537
13,1,8783.2727272727
13,1,5223.43125
13,1,5802.0409090909
13,1,9272.4000000002
13,1,6268.2613636364
13,1,10813.9282407408
13,1,6092.625
13,1,19946.631465517
13,1,5426.8736263736
13,1,5967.4305555555
13,1,5368.2249999999
13,1,6388.2875000001
13,1,19348.4090909092
13,1,5843.5535714285
13,1,8033.83125
13,1,5193.3318965517
13,1,7263.25
13,1,5345.75
13,1,5367.4166666667

13,1,6182.2717391304
13,1,9366.0408163265
13,1,5763.96875
13,1,6522.0060975609
13,1,5542.5759162302
13,1,5718.224489796
13,1,5618.55
13,1,6270.5094339622
13,1,5734.6886792452
13,1,6362.6116071428
13,1,13038.9857954546
13,1,9215.9056603774
13,1,8167.5833333335
13,1,10377.822222222
13,1,7976.7857142858
13,1,7423.380952381
13,1,10409.9695121951
13,1,5037.0104166667
13,1,7694.8477272726
13,1,13282.0909090907
13,1,7928.5133928573
13,1,5486.53125
13,1,11279.3999999997
13,1,6219.4285714286
13,1,10453.8062499998
13,1,8582.4007352941
13,1,5139.765625
13,1,5367.7037037036
14,2,12329.8402777778
14,2,10799.3414634143
14,2,5091.2941176471
14,2,8234.5489130434
14,2,6827.7448979592
14,2,8231.423076923
14,2,11224.0528846153
14,2,9430.0238095237
14,2,9084.875
14,2,6678.5033783784
14,2,7585.5
14,2,8415.634375
14,2,5435.9331395349
14,2,5991.4399999999
14,2,7014.5
14,2,7996.5142857142
14,2,8947.4324324325
14,2,6347.8382352942
14,2,7263.7927631578
14,2,6623.0833333333
14,2,8270.8571428571
14,2,5984.2083333334
14,2,5187.08984375
14,2,8268.431451613
14,2,9539.5348837206
14,2,8859.8073770491
14,2,5897.8
14,2,5939.9605263157
14,2,5573.5503144654
14,2,5035.9264705883
14,2,9364.6859756098
14,2,6609.671875
14,2,6837.3292682926
14,2,8707.125
14,2,6746.4358974358
14,2,7482.9738372093
14,2,6663.4034090906
14,2,7932.5187499999

14,2,9661.3550000001
14,2,5426.9107142858
14,2,8074.2403846155
14,2,5402.9593749999
14,2,5461.625
14,2,6395.2102272726
14,2,7759.6153846155
14,2,5383.8
14,2,6018.0755208334
14,2,6915.4244186047
14,2,7796.2721518986
14,2,8169.8166666667
14,2,6431.0539772726
14,2,5187.2942307693
14,2,7246.7325581395
14,2,5944.453125
14,2,5303.9999999999
14,2,7568.4941860464
14,2,10455.40625
14,2,7214.625
14,2,5600.1666666668
14,2,10174.0714285716
14,2,6389.5689655172
14,2,7493.5333333334
14,2,6508.48
14,2,7818.9821428571
14,2,6093.9285714286
14,2,9424.4828431372
14,2,5846.9264705883
14,2,7536.9268292683
14,2,8327.3357142858
14,2,5645
14,2,9006.0416666667
14,2,6012.2596153847
14,2,5919.4375
14,2,5221.8604651164
14,2,6954.9513888889
14,2,8851.5555555556
14,2,8542.5416666667
14,2,5421.222972973
14,2,8018.8369565217
14,2,5820.7954545454
14,2,5236.5913461537
14,2,5409.8260869563
14,2,5583.6666666667
14,2,5054.1105769231
14,2,7504.0045454547
14,2,5441.9291044776
14,2,6629.7105263158
14,2,5131.1621621622
14,2,5443.9396551725
14,2,5303.9847715736
14,2,6558.5
14,2,5380.2865853658
14,2,5633.0638297872
14,2,6375.1666666666
14,2,5017.7959183675
14,2,5815.1122448979
14,2,6192.2884615387
14,2,9050.95
14,2,6067.1275510203
14,2,6538.1934523809
14,2,6256.243902439
14,2,7604.5367647059
14,2,5749.1861702127
14,2,5022.463888889

14,2,5183.7195121952
14,2,6500.5000000001
14,2,5244.65625
15,1,6067.8170731706
15,1,8438.4840425533
15,1,5014.9146341464
15,1,5599.7383720931
15,1,7674.6071428572
15,1,13382.8717948716
15,1,7369.78125
15,1,5700.4681818181
15,1,5700.4050925926
15,1,10081.5775862064
15,1,6873.2010869566
15,1,17848.5966981135
15,1,8275.9772727272
15,1,11567.4107142858
15,1,5892.2635135135
15,1,10646.2812500001
15,1,5557.5399999999
15,1,6470.1935483869
15,1,10226.2875000001
15,1,14732.7267441858
15,1,5264.6602564103
15,1,6432.9482758622
15,1,7015.1195652172
15,1,12555.2276785715
15,1,6577.8275862069
15,1,6600.8031249999
15,1,6523.9285714285
15,1,14293.3829787233
15,1,6140.6222826087
15,1,8553.328125
15,1,5917.6025641025
15,1,15029.4861111113
15,1,5404.2780748663
15,1,6739.1388888888
15,1,9603.7067307691
15,1,5555.3859649122
15,1,7267.340116279
15,1,9295.2045454545
15,1,6802.552631579
15,1,10460.1249999999
15,1,12266.7692307691
15,1,16916.0524999998
15,1,6094.4948979592
15,1,5378.1046511628
15,1,9507.4782608695
15,1,7212.95625
15,1,5423.2987804878
15,1,11026.8479729729
15,1,11088.2324999998
15,1,6889.7795454547
15,1,5492.8750000001
15,1,16006.2125000001
15,1,9753.1875000001
15,1,10075.1041666666
15,1,7257.15625
15,1,5691.0288461538
15,1,10619.1081081081
15,1,6757.197368421
15,1,17997.9428571428
15,1,7580.9545454545
15,1,5204.8725490196
15,1,17331.7025862071
15,1,14705.7788461543

15,1,13806.3541666665
15,1,9309.5771276598
15,1,7151.1644736842
15,1,7072.625
15,1,5499.6346153845
15,1,5587.4255319149
15,1,6877.5
15,1,5397.0714285712
15,1,8138.9467213116
15,1,13951.1071428569
15,1,5174.3085106383
15,1,5540.0432692307
15,1,9244.8375000001
15,1,6781.4605263158
15,1,8601.8472222224
15,1,6254.0637755102
15,1,7496.2164634147
15,1,8331.1546052632
15,1,6714.2641509433
15,1,5915.8505434782
15,1,5553.244680851
15,1,12466.7234848484
15,1,6784.3970588235
15,1,16195.0532786888
15,1,5912.4402173913
15,1,6779.4361111111
15,1,18543.2083333333
15,1,8247.5892857143
15,1,5148.8273809524
15,1,7431.4261363637
15,1,10210.1756756757
15,1,6405.7169811321
15,1,10811.0601851852
15,1,5275.0865384615
15,1,9424.3333333333
15,1,9646.170212766
15,1,5966.0445859871
15,1,14361.2678571428
15,1,9050.8958333333
15,1,8705.0499999998
15,1,7677.1297169812
15,1,5280.1739130435
15,1,5283.9470338983
15,1,7757.5250000001
15,1,7661.5714285715
15,1,5132.0816326531
15,1,5273.375
15,1,5747.3618421053
15,1,5989.3416666667
15,1,7097.2870370369
15,1,5507.6134020619
15,1,16202.3124999998
15,1,6066.8244680851
15,1,10501.0792682925
15,1,11342.8000000001
15,1,10901.5476190477
15,1,5712.6875
15,1,11715.6530612246
15,1,5436.1081081081
15,1,8337.7632978723
15,1,12108.1428571431
15,1,7092.3720930233
15,1,5044.6402439025
15,1,16307.0364583335
15,1,5706.7500000001
15,1,5066.375

15,1,8761.0322580646
15,1,9418.7307692307
15,1,11157.3414634145
15,1,5403.784090909
15,1,9548.2926136365
15,1,7693.5051020408
15,1,6603.4237804875
15,1,5170.6556603777
15,1,13685.8636363635
15,1,6072.375
15,1,8864.4835526315
15,1,7735.5654761906
15,1,6313.9176829268
15,1,5434.3909574468
15,1,5503.0872093024
15,1,6608.7526595745
15,1,9172.861111111
15,1,6249.9932432433
15,1,5741.5
15,1,7311.9727272727
15,1,12671.1312500001
15,1,6027.34375
15,1,6967.6890243904
15,1,16684.3157894736
15,1,7081.6683673471
15,1,5804.8605769232
15,1,6161.0178571428
15,1,6005.8779069766
15,1,7344.6432926831
15,1,5410.9453125
15,1,8356.7297297298
15,1,12428.7361111112
15,1,10533.346153846
15,1,8646.4464285714
15,1,6067.4127358493
15,1,7305
15,1,12654.9999999999
15,1,7390.9473684209
15,1,17092.4127906976
15,1,7667.7222222223
15,1,12863.5480769231
15,1,11595.4114583337
15,1,19943.6875
15,1,19224.3090909089
15,1,5385.4468085105
15,1,15912.6764705883
15,1,11474.1696428572
15,1,10733.9333333337
15,1,9812.0133928572
15,1,16486.1923076925
15,1,7906.3356481482
15,1,6708.955882353
15,1,7120.5781249999
15,1,11118.1653846156
15,1,5621.347972973
15,1,7717.5454545455
15,1,5655.1875
15,1,17963.0789473687
15,1,6486.1006097561
15,1,16021.046875
15,1,7589.3369565215
15,1,6284.0265957447
15,1,10974.8292682924
15,1,5741.2103960396
15,1,5622.2380952382
15,1,8234.3020833332

15,1,5299.3125
15,1,8579.9423076923
15,1,7717.171875
15,1,5052.2307692307
15,1,10296.9444444445
15,1,5540.6904761904
15,1,8694.2906976742
15,1,5301.8431372549
15,1,9153.2093749998
15,1,7394.3214285713
15,1,6429.625
15,1,5014.5964285714
15,1,9949.0775862067
15,1,5082.3777777778
15,1,6183.5
15,1,9618.6470588236
15,1,6469.3194444444
15,1,5677.595
15,1,5697.7613636363
15,1,5557.0298913043
15,1,5065.4857142857
15,1,7389.53125
15,1,6994.5673076923
15,1,9103.25
15,1,8233.3368055557
15,1,10423.225490196
15,1,9873.6428571427
15,1,15409.7380952381
15,1,7226.1489361703
15,1,7933.6666666667
15,1,6488.7333333334
15,1,7891.5597826086
15,1,14624.2358490567
15,1,14278.3392857145
15,1,8015.9090909092
15,1,6327.4635416665
15,1,7138.804245283
15,1,5491.7578125
15,1,5331.3199999999
15,1,5022.3333333332
15,1,5994.7674418604
15,1,11096.0666666668
15,1,14925.6704545453
15,1,5693.3723404256
15,1,9835.0749999999
15,1,14610.8221153845
15,1,12808.1707317076
15,1,8115.8031914894
15,1,6144.7142857143
16,2,7478.1219512194
16,2,5116.1191860465
16,2,10982.980263158
16,2,7244.5625
16,2,6262.0909090909
16,2,7262.5416666667
16,2,5834.65
16,2,5113.1999999999
16,2,5284.6903409091
16,2,8236.4999999999
16,2,5846.6491228069
16,2,6133.8636363636
16,2,5407.5681818182
16,2,5236.3928571429
16,2,5366.0769230769
16,2,5117.6675
16,2,7172.455

16,2,9340.4687500001
16,2,5578.8303571428
16,2,6073.6630434783
16,2,6684.6000000001
16,2,5947.7659574468
16,2,6942.2616279072
16,2,5480.3365384617
16,2,7164.8375
16,2,8342.146067416
16,2,7488.546875
16,2,5246.8146551725
16,2,5557.4042553191
16,2,7448.2499999999
16,2,6576.4140625
16,2,5572.2236842106
16,2,5178
16,2,5019.9791666667
16,2,5307.2291666669
16,2,8172.0092592594
16,2,5397.9999999999
16,2,5528.5
16,2,5447.6
16,2,7113.6437500002
16,2,9215.1416666667
16,2,8129.9821428573
16,2,5789.7478813559
16,2,5088.0319767443
16,2,5934.205882353
16,2,7110.3125
16,2,8965.7367021277
16,2,8321.0543478259
16,2,6856.8000000001
16,2,9676.9937499999
16,2,7726.0232558139
16,2,8326.143292683
16,2,6680.6171875
16,2,8445.4182692311
16,2,5254.9259259259
16,2,5160.6139705883
16,2,5840.0528846155
16,2,5662.1586538462
16,2,5833.3125
16,2,7155.7720588237
16,2,6169.1595744682
16,2,6384.2173913044
16,2,5336.3072916667
16,2,5653.2180851064
16,2,8068.2222222223
16,2,10051.8177966102
16,2,8389.0882352941
16,2,8082.8235294118
16,2,6710.4074074074
16,2,8590.1914893617
16,2,7380.33125
16,2,13284.3522727272
16,2,5715.4795918369
16,2,6583.6157407409
16,2,7784.2999999999
16,2,5951.0677083333
16,2,6887.130952381
16,2,5804.2093023256
16,2,6927.9813829787
16,2,8604.8175675675
16,2,5842.9910714285
16,2,8441.4962121212
16,2,5026.4523809523

16,2,7752.0125
16,2,6426.40625
16,2,8421.8125
16,2,6006.119047619
16,2,5060.6000000002
16,2,5345.5526315789
16,2,6909.2627551021
16,2,8841.7261904762
16,2,7327.1711956522
16,2,11429.3392857144
16,2,8508.519736842
16,2,5899.6337209301
16,2,7220.3541666666
16,2,8891.224537037
16,2,5270.6923076922
16,2,6027.75
16,2,7059.6861702129
16,2,7205.7142857143
16,2,6569.0568181818
16,2,5496.37109375
16,2,7996.5500000001
16,2,6689.4085365852
16,2,6324.3299180325
17,1,8257.0324999999
17,1,6191.3452380953
17,1,6855.5855263157
17,1,10596.6025641025
17,1,6049.8693181818
17,1,18726.7099999996
17,1,5061.8909574468
17,1,6951.4423076923
17,1,16583.5025125628
17,1,15576.2
17,1,7772.2263513514
17,1,7656.5059523811
17,1,14610.5227272726
17,1,5949.35
17,1,6228.03125
17,1,5436.9857142856
17,1,8096.7401315791
17,1,9260.8750000001
17,1,15310.7725409835
17,1,6933.1627906977
17,1,5904.3
17,1,11279.170212766
17,1,6801.5471698111
17,1,8771.283018868
17,1,16264.3819444444
17,1,10937.8656716417
17,1,7453.3877551021
17,1,6445.4909090907
17,1,10074.511627907
17,1,5512.4402515724
17,1,6180.7888888889
17,1,17594.5047169812
17,1,7494.0290178571
17,1,5200.2536764705
17,1,6611.7578125
17,1,7212.3680555555
17,1,6653.3624999999
17,1,6565.5736196319
17,1,6622.0106382978
17,1,5173.6306818182
17,1,7893.4270833333
17,1,5622.0833333333
17,1,5901.4679487179

17,1,6126.5851063829
17,1,5253.125
17,1,12147.6510416667
17,1,15121.5197368424
17,1,13153.109090909
17,1,5699.5089285713
17,1,9653.5204081633
17,1,5876.3415178572
17,1,8004.5459183675
17,1,5766.3333333333
17,1,16357.6927083334
17,1,5982.375
17,1,10445.6875
17,1,6498.3465909092
17,1,15199.0199999997
17,1,5777.6304347827
17,1,5073.1687499998
17,1,5599.195652174
17,1,10916.5625
17,1,6122.625
17,1,7365.5230263158
17,1,13815.8837209303
17,1,13609.5572916666
17,1,11187.945652174
17,1,5095.2105263158
17,1,10525.0965909091
17,1,10321.3210227273
17,1,5901.3564814814
17,1,9004.625
17,1,5913.4573170731
17,1,6764.8175675676
17,1,6406.3166666667
17,1,18153.9148936171
17,1,7122.8947368421
17,1,7961.607142857
17,1,17811.2440476194
17,1,6887.7663043478
17,1,12969.703125
17,1,7534.1630434782
17,1,10893.1612903227
17,1,6601.9926470589
17,1,9190.7692307693
17,1,18054.4438775511
17,1,13402.3499999998
17,1,6084.2578125
17,1,6803.0209580839
17,1,8056.3624999998
17,1,6080.5697674418
17,1,10793.8421052632
17,1,6020.7202380954
17,1,5495.7499999999
17,1,16038.5948275861
17,1,5743.7125000002
17,1,8362.5921052631
17,1,6882.1052631579
17,1,6502.75
17,1,12691.265625
17,1,5838.5755813953
17,1,5455.8023255814
17,1,6116.230392157
17,1,8208.4531249999
17,1,13142.0612244896
17,1,5885.6451612904
17,1,6197.1863207547
17,1,5400.9593023256
17,1,8879.2083333334

17,1,6535.4326923077
17,1,6701.0833333334
17,1,5477.9021739131
17,1,9994.7213541669
17,1,6929.6489361701
17,1,10198.1931818181
17,1,18482.8210227276
17,1,9704.4285714285
17,1,5730.8070652173
17,1,9876.5277777778
17,1,5913.3052325583
17,1,8634.5
17,1,10549.9166666666
17,1,5630.4857954547
17,1,11571.9363636363
17,1,5681.826923077
17,1,6704.85106383
17,1,6108.3750000001
17,1,11222.7954545456
17,1,7635.0000000001
17,1,14183.4923469385
17,1,12181.125
17,1,8818
17,1,5270.8780487805
17,1,7969.4947916666
17,1,6493.2163461539
17,1,12622.1041666667
17,1,10928.6489361701
17,1,11783.6987179487
17,1,18713.8532608696
17,1,7479.0750000001
17,1,7596.6356382977
17,1,13015.9695121952
17,1,16006.4974489795
17,1,7056.5880681819
17,1,5599.8092105263
17,1,8028
17,1,8683.265625
17,1,7131.4651162791
17,1,8169.0212765957
17,1,9394.0930232559
17,1,8712.6527777777
17,1,5524.2333333334
17,1,8498.8718749999
17,1,5416.1590909092
17,1,12084.3555555557
17,1,5815.3125
17,1,7595.0625000001
17,1,5025.7469512195
17,1,5526.3333333333
17,1,6206.7027777778
17,1,6395.3199152543
17,1,9723.5047169811
17,1,7050.4521276596
17,1,9276.7413793104
17,1,16535.7142857143
17,1,5792.794117647
17,1,6919.5322580645
17,1,6572.5677083332
17,1,13769.7526041669
17,1,7170.34375
17,1,6263.318181818
17,1,7540.7720588236
17,1,6583.7
17,1,13499.8030303027
17,1,7728.8292682927

17,1,7219.230263158
17,1,5905.8005319149
17,1,9576.6392857142
17,1,9954.3773584906
17,1,7703.4975
17,1,6708.3125
17,1,5480.8277027027
17,1,10072.7477678572
17,1,8592.1145833333
17,1,7929.3950000001
17,1,9073.0433673468
17,1,7075.2647058823
17,1,7782.3617021277
17,1,5985.9825000002
17,1,17846.6654411766
17,1,8054.6874999999
17,1,8961.875
17,1,5026.660326087
17,1,6184.5285714286
17,1,12082.2500000001
17,1,11234.057065217
17,1,7897.9017857143
17,1,5520.4634146341
17,1,12005.9407894739
17,1,5426.5400000001
17,1,8925.5756578947
17,1,14722.8666666667
17,1,5952.4592391305
17,1,5707.2812500001
17,1,7783.4352941175
17,1,12850.0999999999
17,1,8388.8843749999
17,1,5546.1363636361
17,1,9628.1354166668
17,1,11459.2526595745
17,1,7910.7844827588
17,1,7727.9941860465
17,1,10488.1388888888
17,1,14837.6704545456
17,1,5027.9642857143
17,1,6723.7565789473
17,1,16324.5460526317
17,1,7924.5812499999
17,1,9077.4672619048
17,1,8915.0074626867
17,1,6153.9375000001
17,1,5530.1104651162
17,1,5676.7272727272
17,1,8127.0217391304
17,1,5106.0250000001
17,1,10059.5304878048
18,2,5345.1500000001
18,2,5131.0923913044
18,2,5722.076923077
18,2,11659.4147727272
18,2,6304.1951219512
18,2,6855.113372093
18,2,5880.7407407406
18,2,6402.5999999999
18,2,8163.274390244
18,2,10337.6904761906
18,2,6487.9375000001
18,2,5963.1385869566
18,2,5884.3823529412
18,2,6441.4594594595
18,2,7362.7241379306

18,2,7108.9635416668
18,2,8785.0906250001
18,2,5479.572368421
18,2,7718.0851063829
18,2,9007.15625
18,2,8896.6875
18,2,9069.6857142857
18,2,5832.15
18,2,6707.6201923076
18,2,6089.6714285714
18,2,7051.25
18,2,6519.1890243903
18,2,5639.2298387097
18,2,5106.5434782609
18,2,6287.6
18,2,8266.0208333333
18,2,7567.8125
18,2,9004.8321428572
18,2,8977.7526881719
18,2,5953.1756756757
18,2,6695.2155963305
18,2,10425.0324074074
18,2,6039.875
18,2,6297.6875
18,2,5025.3571428571
18,2,7970.0887096774
18,2,6956.7045454545
18,2,5627.2302631579
18,2,7274.6981132076
18,2,6512.4521276597
18,2,6511.1397058825
18,2,6185.4090909092
18,2,10324.8529411765
18,2,9914.2146226417
18,2,7684.5795454545
18,2,8985.0110294118
18,2,8664.8072916667
18,2,6928.4761904763
18,2,12437.1666666666
18,2,5592.4000000001
18,2,7471.777173913
18,2,5684.3513513514
18,2,6007.9750000001
18,2,9156.4
18,2,6480.4852941175
18,2,12299.912878788
18,2,7803.0863095237
18,2,8756.786764706
18,2,8225.1067073171
18,2,7277.7209302325
18,2,5044.7446808509
18,2,8658.2794117646
18,2,6794.3187500002
18,2,5480.6486486486
18,2,5481.9968750001
18,2,5796.909574468
18,2,8568.1490384613
18,2,5979.7613636364
18,2,8533.5573770492
18,2,8256.878125
18,2,8513.8290816325
18,2,6856.9940476191
18,2,6301.4312499999
18,2,5318.21484375
18,2,6354.1250000001
18,2,5965.3111702128

18,2,5177.6083333333
18,2,6051.875
18,2,5730.7634730539
18,2,8046.8796296296
18,2,6287.3673469388
18,2,9616.6489361702
18,2,6902.1730769231
18,2,9583.8190789474
18,2,7250.5590909093
18,2,5096.5290697674
18,2,5169.9111842106
18,2,7073.5108695652
18,2,6069.0743243244
18,2,8470.8627450981
18,2,9725.9315476192
18,2,6036.3671874999
18,2,5753.7642045455
18,2,7866.6017441862
18,2,5720.472972973
18,2,5447.75
18,2,6851.6
18,2,12131.4583333334
18,2,6228.3851351351
18,2,8668.9945945944
18,2,5724.6375
18,2,6269.5599999998
19,1,8160.9375000001
19,1,7094.7039473685
19,1,5671.2083333334
19,1,7435.8265306122
19,1,12470.6979166665
19,1,7856.7364864865
19,1,5976.7804878049
19,1,5531.575
19,1,10956.6531250001
19,1,5101.1277777779
19,1,6302.6480263158
19,1,5560.3564814816
19,1,15649.3894230768
19,1,5892
19,1,15608.276785714
19,1,6361.3443877552
19,1,6184.4709302327
19,1,7084.1982758622
19,1,5732.8488372094
19,1,6176.1118421052
19,1,5074.4182692308
19,1,6578.2965116277
19,1,14287.9545454546
19,1,9185.487804878
19,1,6571.7663043479
19,1,5061.0026595745
19,1,8964.78125
19,1,5978.8511235955
19,1,5916.7111111113
19,1,5656.7255434783
19,1,6188.6093750001
19,1,6853.9583333334
19,1,6143.25
19,1,5621.4846938774
19,1,7928.5
19,1,8205.7613636364
19,1,10658.2194444445
19,1,6978.9648241208
19,1,5109.265625
19,1,9775.4825581395

19,1,9427.2819148936
19,1,16986.7619047618
19,1,5252.9359756099
19,1,5647.2704081633
19,1,11608.0227272729
19,1,5363.6479591835
19,1,5948.3173076924
19,1,5201
19,1,14217.8333333332
19,1,7124.6397058823
19,1,5253.2750000001
19,1,8965.905612245
19,1,19746
19,1,8717.588235294
19,1,6984.5624999998
19,1,5019.6983695652
19,1,5583.5909090909
19,1,7182.9246031745
19,1,5483.886627907
19,1,8622.0294117647
19,1,6865.4358974361
19,1,5046.6875
19,1,6261.9375
19,1,9889.5
19,1,17259.13125
19,1,6023.8214285716
19,1,5846.8676470588
19,1,8621.2916666667
19,1,7925.345
19,1,13160.2244897959
19,1,5005.9542682927
19,1,5339.5174418605
19,1,6703.3367346937
19,1,8297.8571428571
19,1,5091.2391304348
19,1,5455.9244186048
19,1,6910.3388157896
19,1,10427.4756097561
19,1,8651.5217391305
19,1,7363.1158536586
19,1,7479.4047619047
19,1,7110.0098039215
19,1,6099.3125
19,1,11147.3224999996
19,1,5770.1875
19,1,8986.8975000001
19,1,6344.9655172413
19,1,6798.8295454546
19,1,7397.5859375
19,1,7270.6446078431
19,1,6802.0624999997
19,1,13530.5781249998
19,1,12840.9270833333
19,1,6503.4868421051
19,1,12712.7222222222
19,1,8593.6554054054
19,1,9246.9512195122
19,1,5208.1818181819
19,1,6009.7109375
19,1,5040.2441860466
19,1,8185.25
19,1,12673.731707317
19,1,6042.6333333333
19,1,7520.6141304347
19,1,5604.355263158
19,1,6118.9166666667

19,1,6017.6983695653
19,1,5230.5172413793
19,1,15469.5918367348
19,1,9295.6216216214
19,1,5024.9649122807
19,1,6684.15
19,1,19844.9805555556
19,1,5072.05
19,1,13661.1051136364
19,1,9641.2500000001
19,1,5034.4136904763
19,1,12499
19,1,6602.6845238095
19,1,15528.5543478263
19,1,7746.375
19,1,9775.1143617024
19,1,5544.9642857142
19,1,5465.2659574468
19,1,7466.7840909091
19,1,11380.4196428572
19,1,8207.7039473683
19,1,17689.137755102
19,1,8197.15625
19,1,6506.0208333334
19,1,6047.265625
19,1,7932.20625
19,1,6196.3714285715
19,1,5345.2884615385
19,1,9395.4305555555
19,1,10128.4
19,1,9470.9642857145
19,1,7335
19,1,5072.6916666667
19,1,13057.3382352942
19,1,7670.73
19,1,8419.0472222223
19,1,6890.1979166667
19,1,7750.4770408164
19,1,5173.3359375
19,1,5410.8951612902
19,1,8298.3506097561
19,1,8461
19,1,6396.5104166667
19,1,19764.2053571427
19,1,19262.6750000001
19,1,7278.090909091
19,1,13430.8004032261
19,1,5614.465
19,1,6030.9505813955
19,1,7468.7196969697
19,1,6359.7745098036
19,1,6346.5517241379
19,1,5615.8333333334
19,1,6103.1953125
19,1,9333.3372641508
19,1,5028.6875000001
19,1,11663.0182926827
19,1,16677.9090909089
19,1,5444.7896341463
19,1,5825.9122807019
19,1,7435.3281249999
19,1,5540.5416666667
19,1,9546.4308510638
19,1,8814.3488372092
19,1,7568.8125
19,1,8190.7115384616

19,1,6482.5523255813
19,1,6224.1041666668
19,1,5034.6308139535
19,1,5131.6666666666
19,1,10743.6598837206
19,1,15844.6382978721
19,1,9368.258064516
19,1,8203.6170212766
19,1,5089.5945945946
19,1,6382.7613636363
19,1,16174.0250000004
19,1,6346.6086956521
19,1,13226.9659090908
19,1,10933.1947674417
19,1,18815.5795454547
19,1,5343.2395833334
19,1,5344.8453947368
19,1,7500.5
19,1,10622.9479166667
19,1,6958.6346153846
19,1,6463.4311224489
19,1,5647.5833333333
19,1,11695.5066964284
19,1,5751.7499999999
19,1,5490.4230769232
19,1,6817.0227272728
19,1,9826.5
19,1,7087.1527777778
19,1,5804.2644230771
19,1,5638.0744680851
19,1,8698.081395349
19,1,7335.8721590911
19,1,8013.2500000001
19,1,10977.5290697677
19,1,8577.8854166668
19,1,7280.4617346938
19,1,7995.4361702126
19,1,11537.6153846152
19,1,7640.7548076923
19,1,5884.6399999999
19,1,18178.6666666664
19,1,9664.6415094339
19,1,7551.7571428571
19,1,12598.4507575757
19,1,7743.2194444445
19,1,5816.4722222223
19,1,17745.3333333332
20,2,6343.1363636364
20,2,6701.3513513512
20,2,7753.2238372093
20,2,10668.0274390241
20,2,9504.6875
20,2,6941.0147058821
20,2,6752.0810810811
20,2,11384.6276595745
20,2,9579.1428571429
20,2,5861.9017857142
20,2,8531.2250000002
20,2,8543.4999999999
20,2,8652.8372093025
20,2,9329.6041666667
20,2,5301.7575757576
20,2,10573.5250000002
20,2,6833.421875
20,2,7228.6273584906
20,2,6647.4642857143

20,2,5637.1632653061
20,2,6824.8231707318
20,2,11068.552631579
20,2,5442.175
20,2,8824.1029411764
20,2,8624.0199999998
20,2,6560.0757575757
20,2,5690.1750000001
20,2,5012.8170731707
20,2,6020.21
20,2,6737.2010869565
20,2,7865.2441860466
20,2,5063.2732558139
20,2,5636.8636363636
20,2,8344.6634615383
20,2,7368.2857142857
20,2,5264.8048780488
20,2,6459.5763888889
20,2,8345.9047619048
20,2,8190.7692307692
20,2,5287.9333333334
20,2,6207.8250000001
20,2,5114.7529069768
20,2,5670.3292682927
20,2,6591.9021739131
20,2,5373.5325
20,2,5412.140350877
20,2,5839.8810975612
20,2,6216.9069767441
20,2,7333.8260869565
20,2,7285.4042553192
20,2,7893.3628048781
20,2,5483.4273255815
20,2,8876.592105263
20,2,5481.9204545455
20,2,5344.5546875
20,2,9007.1524390242
20,2,6387.7790697674
20,2,5329.1875
20,2,8319.0892857142
20,2,5405.8611111112
20,2,6305.3414634147
20,2,5843.2708333335
20,2,6192.0460526316
20,2,7671.3706896551
20,2,5866.8867924527
20,2,10117.8222222223
20,2,10334.8000000002
20,2,5199.3076923078
20,2,7658.4125000001
20,2,5568.4573170733
20,2,6198.9011627908
20,2,6589.0477941176
20,2,5428.2083333335
20,2,5344.5148809524
20,2,6058.2352941177
20,2,8994.1403061224
20,2,7698.4239130435
20,2,5856.7013888891
20,2,7273.8786764706
20,2,7926.4999999998
20,2,6326.171875
20,2,6167.2916666666
20,2,6626.7727272726
20,2,6349.4431818181
20,2,5939.6458333332

20,2,10218.3205128205
20,2,5328.6448863635
20,2,5059.4899999999
20,2,5529.6999999998
20,2,5350.1931818181
20,2,8326.0606060605
20,2,6608.552631579
20,2,8284.5099999999
20,2,5045.28125
20,2,6928.3201219512
20,2,7809.6057692307
20,2,7412.125
20,2,6103.0771276596
20,2,5576.625
20,2,5490.4375
20,2,5692.5000000001
20,2,6814.0909090908
20,2,5015.108974359
20,2,10070.3658536586
20,2,7852.6686046512
20,2,6163.8749999999
20,2,7269.1352201258
20,2,6125.0944444443
20,2,5899.1428571428
20,2,5856.3321428572
20,2,6254.338235294
20,2,5885.8461538462
20,2,6756.9425675676
20,2,7579.7777777779
20,2,9203.7272727273
20,2,5620.74
20,2,5293.7142857142
20,2,5026.2528409091
20,2,7567.7134146341
20,2,6942.2305555558
20,2,6366.7406250001
20,2,7034.0000000001
20,2,9954.7687499999
20,2,5187.7234042553
20,2,7207.5928571429
20,2,6620.435897436
20,2,6902.3478260869
20,2,5822.5576923077
20,2,5009.6988636363
20,2,6555.7000000001
21,1,11237
21,1,5049.6875
21,1,9572.3058510635
21,1,8211.7195121951
21,1,5544.8835978837
21,1,6707.6492146597
21,1,8302.237244898
21,1,7283.75
21,1,5093.0581395348
21,1,15898.8680555552
21,1,7771.2727272729
21,1,10937.4342105264
21,1,5671.735
21,1,12411.918269231
21,1,7228.7743902439
21,1,6376.5
21,1,10022.015981735
21,1,8614.8499999999
21,1,5210.0434782609
21,1,5607.44140625
21,1,10516.3909090908

21,1,8201.45
21,1,18666.3076923079
21,1,7785.9128787879
21,1,7124.7448979591
21,1,5572.8265306124
21,1,7085.9767441861
21,1,19084.7625000002
21,1,8789.0540540541
21,1,6352.4755434782
21,1,8160.7117346938
21,1,6863.875
21,1,8088.5
21,1,7124.2916666666
21,1,15771.4285714285
21,1,7779.5
21,1,13978.0416666666
21,1,11305.18627451
21,1,7407.9250000001
21,1,8986.3942307692
21,1,5798.7727272728
21,1,7450.779296875
21,1,5685.673076923
21,1,7231.765957447
21,1,5519.126984127
21,1,6039.3125
21,1,18059.6744186046
21,1,6675.3048780488
21,1,11743.5
21,1,5037.9619565218
21,1,5634.5930232559
21,1,12227.9249999999
21,1,5054.165625
21,1,6653.4878048781
21,1,6226.9488636363
21,1,6644.625
21,1,5504.7109375
21,1,5711.4487179488
21,1,5404.1303191489
21,1,5785.125
21,1,10000.2012195121
21,1,7595.8571428572
21,1,8700.4594594594
21,1,6884.75
21,1,12791.0306603774
21,1,5138.3333333332
21,1,13642.9255319144
21,1,5017.445945946
21,1,10235.8958333334
21,1,8644.2598684211
21,1,5572.3749999999
21,1,16376.1467391306
21,1,18463.5
21,1,13888.2818181814
21,1,18990.0232558138
21,1,6905.3947368422
21,1,6101.1754385967
21,1,12618.2499999998
21,1,7145.2073170731
21,1,6975.5538461538
21,1,8521
21,1,5638.1493902439
21,1,5519.6585365854
21,1,6080.0243055555
21,1,12765.8149999999
21,1,7408.6153846153
21,1,7789.0657894737

21,1,7665.2173913043
21,1,6305.3916666667
21,1,12789.2305555556
21,1,9530.9166666667
21,1,5873.59375
21,1,8934.4310344827
21,1,6033.8776595746
21,1,5034.326923077
21,1,8276.6219512195
21,1,8262.7
21,1,8494.7045454544
21,1,6691.4042553191
21,1,17568.5384615384
21,1,6308.761904762
21,1,7748.3333333333
21,1,6340.59375
21,1,8180.9880952382
21,1,10066.6764705882
21,1,5480.3024193548
21,1,6240.3597560977
21,1,5515.9500000001
21,1,18780.238095238
21,1,5761.119680851
21,1,5997.3977272727
21,1,14704.2352941178
21,1,6273.8203124999
21,1,5336.0000000001
21,1,8194.9763513513
21,1,12448.8723404256
21,1,18525.7412790697
21,1,11880.0977272727
21,1,5102.32421875
21,1,8536.0196078431
21,1,13651.6046511628
21,1,9200.1388888889
21,1,7770.5999999999
21,1,8668.6861702129
21,1,7272.7872340426
21,1,16686.7569444443
21,1,8936.7732558139
21,1,11886.8343749997
21,1,18323.6785714287
21,1,5417.5952380952
21,1,8522.0208333333
21,1,5149.3974358974
21,1,9529.2282608696
21,1,8579.1522727273
21,1,7606.5919811319
21,1,10213.1379310345
21,1,16522.6155778894
21,1,7173.4875000001
21,1,5478.3255813954
21,1,15029.358695652
21,1,5025.8374999999
21,1,10652.6835106384
21,1,8199.0571428571
21,1,9583.0104166667
21,1,19735.5277777779
21,1,15285.4583333334
21,1,6660.7548076924
21,1,11101.3928571428
21,1,5782.125
21,1,9421.9090909091
21,1,7056.5151515154
21,1,17522.9722222224
21,1,5777.4335106384

21,1,11713.9423076919
21,1,5389.98125
21,1,6652.8749999999
21,1,9884.7547169812
21,1,10515.1046511628
21,1,6092.8515625002
21,1,9233.027027027
21,1,5082.2352941177
21,1,6964.2222222222
21,1,12135.6521739132
21,1,16311.59375
21,1,5088.7009803921
21,1,10012.6486486487
21,1,5410.6125
21,1,5527.2058823529
21,1,5535.0700000001
21,1,5918
21,1,6702.2022727272
21,1,8665.2749999997
21,1,5256.0598958332
21,1,17128.8833333336
21,1,9777.659375
21,1,6525.5
21,1,7396.3459302326
21,1,5258.03125
21,1,16919.3601694915
21,1,6368.9722222223
21,1,5724.3181818181
21,1,8181.25
21,1,6399.896226415
21,1,18400.18
21,1,8308.6216216217
21,1,9545.0510204082
21,1,15133.5714285713
21,1,6675.1020408164
22,2,7433.3787878788
22,2,8225.9062499999
22,2,5884.4522058823
22,2,5454.375
22,2,5053.552631579
22,2,8658.9807692307
22,2,8441.7613636363
22,2,5389.4407894737
22,2,5516.3256578948
22,2,5674.5487804878
22,2,5916.7954545454
22,2,8419
22,2,7455.5853658535
22,2,6104.5197368421
22,2,9220.8625000001
22,2,5355.125
22,2,6693.1375
22,2,6726.2499999999
22,2,6043.2499999999
22,2,5146.0178571429
22,2,6168.046875
22,2,7971.625
22,2,6998.858974359
22,2,7962.5208333334
22,2,6450.4666666666
22,2,7369.3026315789
22,2,5242.6166666667
22,2,8985.53125
22,2,6056.1481481481
22,2,5265.9
22,2,9870.165625

22,2,5818.3088235294
22,2,6324.7916666668
22,2,5766.6341463414
22,2,5623.7886904762
22,2,6336.1005434782
22,2,6138.8076923077
22,2,6869.2166666667
22,2,5698.7016129031
22,2,8305.2690217391
22,2,6644.1363636363
22,2,5485.7978723405
22,2,7177.0372340425
22,2,6534.1011904762
22,2,5099.8125
22,2,7711.7092391306
22,2,6588.5625
22,2,6898.5784883719
22,2,6186.8940217393
22,2,10177.1315789475
22,2,6121.3671875
22,2,6040.2305555555
22,2,8370.0771276596
22,2,8390.0803571428
22,2,9447.0172413792
22,2,5795.3392857142
22,2,6270.4879807693
22,2,11423.3557692307
22,2,6824.1047297296
22,2,10574.2678571429
22,2,5304.5052083332
22,2,5167.65625
22,2,7373.9759615384
22,2,12589.1317567568
22,2,6375.8536585365
22,2,5766.5425531915
22,2,6911.7261904762
22,2,8430.6646341464
22,2,10419.7000000002
22,2,7110.6693548387
22,2,5775.29
22,2,6578.8727272728
22,2,9443.4281914893
22,2,5055.6551724137
22,2,7325.8974358974
22,2,5716.4438775511
22,2,10533.5975609755
22,2,7521.3109756097
22,2,9436.4947916666
22,2,8272.8918918919
22,2,6372.6465517242
22,2,6008.25625
22,2,8692.6
22,2,5715.8676470588
22,2,7245.1941489361
22,2,6551.0857142857
22,2,5657.4891304348
22,2,6479.5
22,2,10648.8378378379
22,2,7877.8970588236
22,2,8974.8648648648
22,2,13012.5887096774
22,2,7681.7407407408
22,2,5616.4460227274
22,2,6838.0510204083
22,2,6340.0673076923
22,2,7263.9418604652

22,2,7044.2058823528
23,1,5602.0697674418
23,1,6054.2119565218
23,1,5816.3829787235
23,1,8634.3125
23,1,6290.4411764706
23,1,5157.7380952382
23,1,8362.1779661018
23,1,5738.1581632653
23,1,10459.3673469388
23,1,7932.1184210528
23,1,6486.7045454546
23,1,5116.6607142858
23,1,5953.9632352942
23,1,5535.96484375
23,1,5150.6547619048
23,1,6154.2755681818
23,1,7605.625
23,1,5527.4374999999
23,1,5427.8088235294
23,1,8309.4292452828
23,1,5680.4886363638
23,1,7040.3952702704
23,1,5426.25
23,1,6902.7083333333
23,1,5395.780172414
23,1,15266.2678571428
23,1,8009.6449999999
23,1,5845.8163265306
23,1,9177.55
23,1,7645.7378048781
23,1,17366.0961538459
23,1,5484.78125
23,1,5323.8125
23,1,7433.4081632653
23,1,18889.8725490196
23,1,7234.9519230768
23,1,5842.181818182
23,1,8566.431372549
23,1,5766.1170212766
23,1,7653.1617647059
23,1,14080.1063829789
23,1,8434.9227272727
23,1,10388.340909091
23,1,5720.3392857143
23,1,5936.875
23,1,14002.046875
23,1,10862.6964285712
23,1,19845.3782051282
23,1,6787.6250000001
23,1,10004.2664473686
23,1,7051.76875
23,1,12169.71875
23,1,9584.010638298
23,1,5557.1079545455
23,1,17170.5254629629
23,1,10021.0425531919
23,1,6499.6842105262
23,1,10020.445945946
23,1,17893.25
23,1,5794.2142857142
23,1,5614.2589285715
23,1,5198.5348837209
23,1,8470.7205882354
23,1,5226.3810975609
23,1,16728.8500000002

23,1,6705.4898648648
23,1,9858.0663265307
23,1,5123.16875
23,1,5724.2972972974
23,1,9254.513888889
23,1,5020.7000000001
23,1,15759.2683823532
23,1,11968.7999999999
23,1,19520.3505154636
23,1,5370.0833333334
23,1,9639.3579545454
23,1,8657.7325581395
23,1,14992.3421052632
23,1,17346.375
23,1,5986.3697916665
23,1,10226.3187500001
23,1,5494.9302325581
23,1,12153.3255813952
23,1,6860.4390243903
23,1,7579.8958333334
23,1,12486.2570754717
23,1,9605.528846154
23,1,5586.1726190478
23,1,6636.3723404256
23,1,11698.9330357143
23,1,8497.275
23,1,8437.8958333333
23,1,5550.3233695653
23,1,6172.6481481481
23,1,5530.1785714286
23,1,5395.125
23,1,6153.0096153847
23,1,8308.5178571428
23,1,5305.2239583334
23,1,5663.5777777779
23,1,10573.3068181817
23,1,5085.5000000001
23,1,7049.3011363636
23,1,11150.1079545454
23,1,8304.357142857
23,1,11171.8125000001
23,1,5766.1281249999
23,1,11719.4456521741
23,1,7490.0760869565
23,1,9684.7395833332
23,1,9963.5333333333
23,1,7053.7831632654
23,1,7659.9763513513
23,1,10468.2291666668
23,1,14141.9117647062
23,1,12703.1315789472
23,1,6212.375
23,1,19650.3275862071
23,1,7690.2155172413
23,1,7152.8097826087
23,1,16522
23,1,9204.1626506025
23,1,5961.75
23,1,6126.7289719625
23,1,12021.7031250001
23,1,5729.0500000001
23,1,14067.0170454543
23,1,12033.1718749997
23,1,5322.9438775511
23,1,7769.3292682926
23,1,6274.8999999999

23,1,5495.6923076923
23,1,7793.9802631579
23,1,19887.9189189188
23,1,7159.5882352942
23,1,5453.195754717
23,1,5762.5322580646
23,1,17354.625
23,1,6187.6428571429
24,2,6747.5994318183
24,2,6591.0033783783
24,2,6432.8125
24,2,5807.8461538463
24,2,9111.0227272727
24,2,5358.9361702128
24,2,6089.1505681819
24,2,5266.6875
24,2,5999.9047619047
24,2,5034.8014705882
24,2,9955.3932291667
24,2,11542.25
24,2,8348.3016304348
24,2,6356.8656249999
24,2,9180.8235294118
24,2,5430.6933962264
24,2,7776.8869047619
24,2,8193.1538461538
24,2,6292.4285714285
24,2,5013.625
24,2,6675.6599999999
24,2,6148.2678571429
24,2,7195.3125
24,2,6810.2826086957
24,2,6016.8250000001
24,2,5919.3079268293
24,2,8904.6642857143
24,2,5409.0640243902
24,2,5232.2239583334
24,2,9385.5299999999
24,2,10111.4749999999
24,2,6596.4030612245
24,2,8989.2977941176
24,2,8012.1354166668
24,2,6410.5272727273
24,2,5536.765625
24,2,7669.1987179488
24,2,5849.2075471698
24,2,7258.8953488372
24,2,8615.3913043478
24,2,5149.85
24,2,5422.5000000001
24,2,7979.8833333331
24,2,7653.3478260869
24,2,6542.1486486488
24,2,6079.5961538463
24,2,9391.5357142857
24,2,5882.6176470588
24,2,6580.3563829787
24,2,10113.7645348837
24,2,5883.2413793104
24,2,5131.9838709677
24,2,5760.25
24,2,8567.6732954547
24,2,5050.0670731708
24,2,7157.9107142857
24,2,11730.7195121952
24,2,8152.8461538461

24,2,5125.1999999998
24,2,5906.7407407408
24,2,6146.5238095238
24,2,6525.7378048782
24,2,6268.9156626505
24,2,6241.587837838
24,2,7974.765625
24,2,5119.909090909
24,2,5544.2151162791
24,2,6976.0545454545
24,2,6365.5921052632
24,2,6367.6428571429
24,2,5923.7111111111
24,2,10109.2987804879
24,2,5997.670212766
24,2,5219.3048780488
24,2,8141.1619318181
24,2,7354.6768292683
24,2,6489.0930232558
24,2,5528.7022058824
24,2,5616.1346153846
24,2,9045.9166666668
24,2,7606.5535714286
24,2,5307.9285714284
24,2,6283.8
Table S2 C) Intensity recorded for peptide chimera on MATa and MATalpha cells. MATa recordings correspond with odd Time numbers, and MATalpha with even Time numbers
Time,GroupByColor,Intensity
1,1,18611.1547619048
1,1,12613.5
1,1,7917.4289215685
1,1,5611.2678571428
1,1,14934.4754901958
1,1,12711.611111111
1,1,12208.3823529411
1,1,8625.090425532
1,1,5716.8263888889
1,1,5926.7739361703
1,1,6331.8380681816
1,1,6324.5572916667
1,1,5820.6
1,1,5654.0555555555
1,1,6478.6170212767
1,1,13772.2697368421
1,1,6323.1388888888
1,1,9389.4545454546
1,1,8660.3181818181
1,1,7392.6122448978
1,1,5255.8243243244
1,1,6181.3511904761
1,1,10489.0531249999
1,1,16275.4017857144
1,1,5099.7336956522
1,1,7724.21
1,1,12466.7122093022
1,1,10975.2687499999
1,1,6853.3696808509
1,1,9667.2589285714
1,1,15936.1413043477
1,1,8443.8256578947
1,1,9118.3
1,1,7168.215909091
1,1,6729.6906779662
1,1,11840.5364583335
1,1,5282.2980769231

1,1,6096.7678571429
1,1,9829.2307692308
1,1,6005.6486486488
1,1,5501.5243902438
1,1,6324.1249999997
1,1,7415.2083333333
1,1,8112.3958333333
1,1,13041.875
1,1,9539.3823529412
1,1,5215.8382352941
1,1,11958.0163934423
1,1,16890.0523255818
1,1,5444.4711538461
1,1,7024.3125
1,1,7414.0431034482
1,1,5439.08125
1,1,6979.625
1,1,5398.543478261
1,1,7798.5540540539
1,1,19168.4513888888
1,1,6166.993902439
1,1,17180.2
1,1,5731.6585365854
1,1,10900.5872093024
1,1,5365.8511904762
1,1,5038.2965116278
1,1,13973.9230769232
1,1,8513.4147727274
1,1,19968.5232558143
1,1,16738.036764706
1,1,5974.8803191488
1,1,9542.5640243903
1,1,8931.3882978723
1,1,6979.4339622642
1,1,5958.8617021276
1,1,11715.1904761904
1,1,9358.384615385
1,1,7970.3585526314
1,1,13385.6666666667
1,1,9589.5738636364
1,1,5066.8191489362
1,1,5319.9812499999
1,1,6159.8541666666
1,1,7693.7635135135
1,1,5753.3750000001
1,1,7200.3829787234
1,1,11589.3333333332
1,1,18365.1279069771
1,1,14701.5499999997
1,1,14179.1607142857
1,1,5476.268292683
1,1,7465.2367021277
1,1,14768.3783783786
1,1,5226.3109756097
1,1,13184.004032258
1,1,5913.3700000001
1,1,8324.9545454545
1,1,11595.9934210529
1,1,7334.125
1,1,5513.0978260869
1,1,19216.5197368421
1,1,11301.127777778
1,1,11933.1052631578
1,1,6957.34375
1,1,7520.9390243903
1,1,18251.9889705882

1,1,5697.0290697676
1,1,9632.2583333335
1,1,5363.2068965518
1,1,14847.7027777777
1,1,14877.6249999999
1,1,7765.2040816326
1,1,5730.75
1,1,6600.6193181818
1,1,8951.3836206897
1,1,8512.84765625
1,1,6390.2857142857
1,1,18848.8981481482
1,1,12980.2968749999
1,1,7964.4625
1,1,5674.7272727272
1,1,8291
1,1,19841.55
1,1,6030.39375
1,1,11698.03
1,1,5357.8445121951
1,1,7567.5312499999
1,1,5587.1768292683
1,1,10522.9411764706
1,1,10888.3509615384
1,1,8730.6538461539
1,1,15818.7209302325
1,1,5558.2207446809
1,1,14022.0625000002
1,1,19083.9767441861
1,1,6010.2062499999
1,1,19193.038888889
1,1,14060.6413043478
1,1,6067.5869565218
1,1,6884.5111111111
1,1,12707.8499999999
1,1,5564.5694444445
1,1,12011.3716216216
1,1,8725.5625
1,1,15462.2727272726
1,1,6907.816037736
1,1,6965.6013513514
1,1,6436.2573529411
1,1,19179.305555556
1,1,12904.6477272728
1,1,6416.1063829788
1,1,10675.0238095237
1,1,18626.9999999998
1,1,5246.3522727272
1,1,9440.4107142858
1,1,7692.7674418604
1,1,8872.2926136362
1,1,8181.0673076924
1,1,13034.4999999999
1,1,9182.8720238095
1,1,8377.3451086956
1,1,5449.9714285716
1,1,8548.147260274
1,1,5097.6694444445
1,1,13473.278846154
1,1,6049.5182926827
1,1,6969.6428571428
1,1,7485.5575000001
1,1,15668.5117924525
1,1,11919.358108108
1,1,10051.453125
1,1,9189.9534883721

1,1,5253.2738095238
1,1,15103.1276595744
1,1,13558.4926470589
1,1,8551.5625
1,1,18208.3048780488
1,1,8989.3799999997
1,1,8591.2374999999
1,1,5901.3775510203
1,1,11120.5069444442
1,1,8899.6470588235
1,1,14065.4663461539
1,1,16640.8140243903
1,1,5866.9182692307
1,1,11061.7321428577
1,1,7332.4772727272
1,1,17197.4605263155
1,1,5578.9725
1,1,5974.6358695653
1,1,7506.7169811321
1,1,13426.3164556962
1,1,11590.4302325582
1,1,7852.2884615385
1,1,13896.1363636365
1,1,14292.2692307693
1,1,8234.5401785716
1,1,5303.2234042553
1,1,9235.892857143
1,1,7098.4711538461
1,1,10576.5285714287
1,1,7465.1714285714
1,1,17288.5217391307
1,1,5137.2916666667
1,1,6341.5542168675
1,1,13555.1348684214
1,1,16017.6521739131
1,1,12929.08
1,1,5230.5000000002
1,1,18086.5729166673
1,1,5601.9829545455
1,1,10716.5624999997
2,2,7383
2,2,5415.2249999999
2,2,6193.784375
2,2,7351.9836956521
2,2,5726.8310810811
2,2,7691.1624999999
2,2,7323.6378205128
2,2,6875.7799999999
2,2,6334.9673913043
2,2,7201.0000000003
2,2,8193.0681818182
2,2,7526.0135135135
2,2,7662.0892857144
2,2,6513.2558139535
2,2,8318.4476744186
2,2,9634.9811320755
2,2,5502.0833333333
2,2,6500.3313953489
2,2,6145.5113636364
2,2,6669.4357142856
2,2,5369.6222222223
2,2,8273.8125
2,2,5243.3170731707
2,2,8274.5853658537
2,2,5123.9085365854
2,2,8932.8107142858

2,2,6370.2608695652
2,2,5305.2250000001
2,2,6367.0743243243
2,2,10597.4053030304
2,2,8838.5816326533
2,2,6497.5625
2,2,5961.6511627907
2,2,8080.9930555556
2,2,5003.7434210525
2,2,11579.2466216217
2,2,5318.0737179487
2,2,6009.9632352942
2,2,5927.7826086956
2,2,6675.5597826085
2,2,7747.4883720931
2,2,7321.9787234042
2,2,7191.7591463415
2,2,5644.7993421053
2,2,7925.3267045455
2,2,6354.3958333334
2,2,7642.4719387756
2,2,7912.4722222223
2,2,6148.5937500002
2,2,8397.2608695651
2,2,6184.15234375
2,2,8804.9560810811
2,2,7554.0000000001
2,2,5220.6395348837
2,2,9317.3009259259
2,2,6553.4044117646
2,2,5589.3048780487
2,2,5493.3152173912
2,2,5648.9331395351
2,2,5000.7016129033
2,2,7386.7
2,2,7387.5340909091
2,2,6539.7209302325
2,2,6394.5625
2,2,8109.9119318181
2,2,9698.7847222223
2,2,9256
2,2,6615.3
2,2,6749.8541666666
2,2,5462.6785714285
2,2,5469.9999999999
2,2,5159
2,2,6114.9281914894
2,2,6350.0052083331
2,2,7407.9558823529
2,2,6111.1275510204
2,2,6758.1999999999
2,2,7378.4387254902
2,2,7073.1029411765
2,2,6082.4499999999
2,2,5713
2,2,5412.1785714286
2,2,6657.2023809523
2,2,8583.2500000001
2,2,5046.6216216215
2,2,6544.8404255319
2,2,6085.6888888888
2,2,8234.7972972973
2,2,9292.987804878
2,2,7487.1011904762
2,2,5647.8221153846
2,2,6635.765625

2,2,6859.7045454546
2,2,5541.7916666666
2,2,5067.4519230769
2,2,5236.75
2,2,5227.8484848485
2,2,8955.284090909
2,2,8534.9593749999
2,2,5082.9
2,2,6771.8024193547
2,2,7590.9035714286
2,2,5729.24
2,2,8075.8011363636
2,2,5612.2604166667
2,2,6342.8416666667
2,2,5019.1220238095
2,2,5440.831521739
2,2,6872.2738095238
2,2,7144.4042553192
2,2,6340.3125
2,2,6046.8958333333
2,2,5756.4375
2,2,6427.2162162163
3,1,5825.6493902437
3,1,7239.9000000002
3,1,16140.5
3,1,5770.3375
3,1,5068.7325581395
3,1,7045.1555555556
3,1,8779.5977653632
3,1,6217
3,1,13406.3714285715
3,1,8071.2836538462
3,1,15095.2999999997
3,1,5887.4772727272
4,2,6796.8333333334
4,2,5095.8443396226
4,2,6283.4375
4,2,7713.8571428572
4,2,7641.9078947368
4,2,7021.4802631576
4,2,7909.2195121952
4,2,7774.4886363637
4,2,12650.5064102564
4,2,6372.3333333333
4,2,7751.3333333334
4,2,5284.8372093023
4,2,6635.3482758621
4,2,5189.9736842105
4,2,6874.7729591838
5,1,14447.9285714286
5,1,5277.2796052631
5,1,12577.1923076925
5,1,5463.45
5,1,7255.011627907
5,1,9746.3846153846
5,1,5131.6516393443
5,1,6088.3605769232
5,1,7067.3721590909
5,1,16153.5897435898
5,1,8216.1395348838
5,1,9191.7142857143
5,1,14690.9414893619
5,1,10386.1032608695
5,1,10202.9732142858
5,1,9509.7838541667
5,1,6896.0131578947

5,1,6923.2000000001
5,1,7278.0589622643
5,1,6911.4013157895
5,1,6978.8620689654
5,1,18651.6999999999
5,1,11378.542763158
5,1,10318
5,1,6360.8139534883
5,1,5112.5384615385
5,1,18944.605263159
5,1,7342.0197368422
5,1,5566.4722222223
5,1,11799.3650000001
5,1,8796.5223214284
5,1,5641.8416666666
5,1,5440.7349999999
5,1,5597.8577586207
5,1,6663.41875
5,1,6438.1666666667
5,1,5917
5,1,5524.0668604651
5,1,13873.8442622951
5,1,10965.6088709678
5,1,5086.931372549
5,1,5890.6385135137
5,1,9883.9000000001
5,1,7514.9100000001
5,1,5344.6796875
5,1,10327
5,1,8610.2343749999
5,1,7491.0833333333
5,1,7755.8869047616
5,1,6638.3154761905
5,1,8040.6494565217
5,1,5846.7291666667
5,1,11721.6521739132
5,1,6443.3058510637
5,1,8262.8658536586
5,1,6872.8494318182
5,1,11501.7277777776
5,1,14279.5500000001
5,1,6158.17
5,1,16205.052777778
5,1,17157.1874999994
5,1,18807.2861111117
5,1,7736.871527778
5,1,6378.5000000001
5,1,6372.7307692308
5,1,14331.638888889
5,1,5876.661764706
5,1,5615.9926470588
5,1,6054.15
5,1,10026.4444444444
5,1,11385.9534883722
5,1,6647.6744791666
5,1,5367.2053571431
5,1,12316.0625
5,1,6178.7211538462
5,1,9430.0701219511
5,1,6593.7202380952
5,1,7702.0188679245
5,1,5804.6170212766
5,1,5703.5945945946
5,1,7510.546875
5,1,6323.8181818182
5,1,5104.4659090909

5,1,9688.4910714285
5,1,14410.9062500003
5,1,6481.3297872341
5,1,17302.9868421049
5,1,5007.8697916666
5,1,14303
5,1,5296.8443396226
5,1,7487.0833333332
5,1,14230.6576086955
5,1,5085.4880952381
5,1,9364.3482142857
5,1,7269.25
5,1,16300.5625000003
5,1,6477.035
5,1,12715.9375
5,1,11559.7375
5,1,7925.2631578947
5,1,5985.666666667
5,1,8596.4999999998
5,1,5317.5
5,1,8367.5416666667
5,1,5128.8461538462
5,1,5456.46875
5,1,5595.365131579
5,1,8826.84375
5,1,7058.0416666667
5,1,5866.3553921567
5,1,5632.8897058822
5,1,12673.6521739131
5,1,6770.1710526315
5,1,13233.9812499995
5,1,12230.2232142858
5,1,5752.6666666668
5,1,5242.3072916666
5,1,5511.5235849056
5,1,5700.9176136365
5,1,14029.6335227272
5,1,11585.9642857143
5,1,10529.6666666667
5,1,7965.0813953489
5,1,5800.0833333335
5,1,5919.5652173914
5,1,7202.375
5,1,8635.267857143
5,1,7736.4187499998
5,1,10041.8678571426
5,1,5802.9910714286
5,1,5102.6820652173
5,1,8667.371875
5,1,12203.22265625
5,1,8729.9264705883
5,1,16662.8541666667
5,1,5676.1689189188
5,1,6163.5131578947
5,1,11627.0187499999
5,1,11521.130952381
5,1,5634.131097561
5,1,8032.9019607844
5,1,18850.8624999998
5,1,12552.6119791669
5,1,6649.8452380953
5,1,6078.5555555557
5,1,9013.7835365854
5,1,7697.7790697674
5,1,6245.375
5,1,9199.4359756098

5,1,5973.0178571429
5,1,14346.8510638295
5,1,6147.4871794872
5,1,9088.15625
5,1,8955.0054347831
5,1,19635.6953125
5,1,7589.3284883722
5,1,6871.9071428572
5,1,5902.7432432432
5,1,5540.2865853658
5,1,6374.8802083335
5,1,11537.401960784
5,1,5955.6547619048
5,1,9902.0568181819
5,1,6369.5
5,1,7208.5
5,1,5394.875
5,1,5942.3375
5,1,5453.4871794872
5,1,5501.2872340426
5,1,14427.4891304346
5,1,8751.235576923
5,1,7769.7179487179
5,1,11998.5166666666
5,1,7513.9847560976
5,1,5140.9222972973
5,1,6455.0178571428
5,1,12851.2962962961
5,1,10100.3984374999
5,1,8920.2727272728
5,1,14500.0813953489
5,1,14745.75
5,1,8231.875
5,1,5654.8676470588
5,1,5853.3233532934
5,1,18054.9722222223
5,1,7459.3645833334
5,1,13847.456140351
5,1,7097.3333333335
5,1,6752.2828947368
5,1,9413.0714285713
5,1,8248.375
5,1,14355.2965686279
5,1,9869.28125
5,1,6424.9107142856
5,1,5101.375
5,1,6999.3365384615
5,1,13885.6666666666
5,1,11642.9479166667
5,1,7016.8372093023
5,1,6099.3319327731
5,1,14275.3076923079
5,1,14005.5882352941
5,1,9992.9333333336
5,1,17042
5,1,18297.1874999994
5,1,14482.149390244
5,1,5697.9878048781
5,1,17581.9562499996
5,1,12027.51953125
5,1,5047.9375
5,1,9377.0874999998
5,1,7176.0561224489
5,1,6114.4
5,1,5015.890625
5,1,6809.4705882352

5,1,12816.0790816326
5,1,18224.4519230768
5,1,5609.1046511628
5,1,12226.0795454544
5,1,10612.9074074075
6,2,6375.2134146342
6,2,7341.3615384615
6,2,8842.3012820512
6,2,7937.3999999999
6,2,12872.4090909087
6,2,11537.9281914894
6,2,16595.9166666667
6,2,7360.505319149
6,2,7224.625
6,2,10171.8928571428
6,2,6187.2105263157
6,2,5625.0487804878
6,2,11973.6482558142
6,2,5705.5156250001
6,2,7240.4861111111
6,2,5389.53125
6,2,5148.5543478261
6,2,8492
6,2,6058.2166666667
6,2,7323.872881356
6,2,6756.6
6,2,6703.2635135135
6,2,6154.7000000002
6,2,6656.1279069768
6,2,7255.3190789474
6,2,5035.5921052633
6,2,6193.9772727272
6,2,9259.4222972973
6,2,5658.8669354838
6,2,8942.25
6,2,7373.1500000001
6,2,12072.4117647059
6,2,9118.9347826087
6,2,7450.1114130434
6,2,6814.4732142858
6,2,7460.3387096774
6,2,6393.5
6,2,8241.972972973
6,2,5379.1737804879
6,2,9961.3437500001
6,2,5311.6536885247
6,2,5725.3989361703
6,2,5271.2848837209
6,2,6549.9857142858
6,2,9207.5848214287
6,2,8635.5
6,2,5656.2987804877
6,2,10852.0975609757
6,2,5650.3994565218
6,2,5202.8310810811
6,2,8190.1219512195
6,2,7740.3653846153
6,2,5806.7708333334
6,2,6071.0548780487
6,2,5611.8092105264
6,2,6042.8722826088
6,2,5517.4127906978
6,2,8435.9090909091
6,2,7579.618902439
6,2,6838.1444444444
6,2,7905.9298780487

6,2,6000.9464285715
6,2,6363.0151515151
6,2,6959.4166666668
6,2,5914.7142857141
6,2,5913.538888889
6,2,6365.7072368421
6,2,7269.7718749999
6,2,5395.5306122449
6,2,5741.3284883721
6,2,8403.3970588237
6,2,5604.6452702703
6,2,9320.222826087
6,2,5325.3074324324
6,2,8946.6071428575
6,2,8924.5131578947
6,2,7202.0625000002
6,2,10544.972222222
6,2,5881.3877551021
6,2,9785.7857142858
6,2,6610.7010869564
6,2,7045.1071428571
6,2,8486.541984733
6,2,5156.5220588236
6,2,7144.6666666667
6,2,7513.2857142858
6,2,7735.8571428572
6,2,7498.8237179488
6,2,10949.65
6,2,5113.4444444444
6,2,6116.8888888888
6,2,7971.1968085105
6,2,14224.2962962963
6,2,5695.5472222222
6,2,5140.2272727272
6,2,9477.0548780488
6,2,7337.0249999999
6,2,6133.5965909091
6,2,5247.3628048781
6,2,6630.898255814
6,2,6311.25
6,2,5083.5102040813
6,2,10912.1858108108
6,2,6786.7368421051
6,2,10315.722826087
7,1,15918.71875
7,1,6778.4
7,1,9865.5588235296
7,1,5864.75
7,1,5217.2857142859
7,1,19814.7142857141
7,1,5313.3986486486
7,1,8924.4230769231
7,1,5353.3658536585
7,1,12537.1428571428
7,1,11779.8629032259
7,1,5159.5930851065
7,1,7970.35
7,1,10507.7093749999
7,1,7174.1875
7,1,18577.4702380953
7,1,5017.1707317073
7,1,8459.4358974359
7,1,8619.6428571431
7,1,13459.2596153844
7,1,18206.8942307692
7,1,9759.921875

7,1,13053.2649999995
7,1,17266.4444444445
7,1,17752.4285714288
7,1,17724.5166666667
7,1,6965.0468750001
7,1,7528.3878205127
7,1,6649.9345238096
7,1,5592.1890243901
7,1,5697.4730392157
7,1,7665.0408163264
7,1,6926.5539772726
7,1,5477.6857142858
7,1,9525.8611111112
7,1,10072.5520833334
7,1,11724.6911764705
7,1,11320.7564102562
7,1,12486.6388888885
7,1,5331.4097222221
7,1,6437.5524193548
7,1,12585.6363636363
7,1,12032.9906250001
7,1,19230.0231481482
7,1,8733.2806603775
7,1,5072.4593023256
7,1,7144.2549019607
7,1,12324.7449999999
7,1,7758.2391304348
7,1,8969.5738636363
7,1,11729.9779411764
7,1,18049.1646341462
7,1,15907.5609756097
7,1,10861.6250000003
7,1,7055.9673913043
7,1,8324.3658536586
7,1,5483.962962963
7,1,15950.5625
7,1,6160.6682692309
7,1,9067.7562499999
7,1,12006.0568181818
7,1,5189.1874999999
7,1,6807.5
7,1,7084.9705882352
7,1,13000
7,1,16353.5480769233
7,1,18957.6838235295
7,1,9302.3244047619
7,1,7979.5337837839
7,1,6360.5999999998
7,1,8862.1333333334
7,1,16275.9605263158
7,1,18263.9310344828
7,1,7184.9662162163
7,1,7266.8578431372
7,1,13319.7331081081
7,1,5730.6902173912
7,1,11159.8081395347
7,1,5316.1222222221
7,1,17016.5
7,1,5882.0265957447
7,1,9645.6890243902
7,1,17402.5943396224
7,1,8656.9389534884
7,1,6281.4006410258
7,1,6960.55
7,1,19195.4423076927
7,1,15905.0625

7,1,5574.9047619047
7,1,12963.5270270271
7,1,5150.3
7,1,10569.0075757576
7,1,13136.7403846155
7,1,8700.8937500001
7,1,8425
7,1,14473.5789473685
7,1,14970.9879032259
7,1,8483.5972222223
7,1,8083.8430232559
7,1,5478.8717948718
7,1,7025.5526315791
7,1,12007.9347826087
7,1,10167.2819148935
7,1,10902.9239130432
7,1,5798.3815789474
7,1,6300.7440476191
7,1,16917.55625
7,1,19258.2708333336
7,1,6391.3295454545
7,1,13484.2580645161
7,1,8083.6
7,1,15846.8457446814
7,1,5044.428125
7,1,5173.7809523809
7,1,7255.6698113209
7,1,8330.8090277777
7,1,12507.0851063833
7,1,13139.4999999999
7,1,7144.2916666667
7,1,19346.9310344827
7,1,9281.8837719296
7,1,8115.3833333334
7,1,10127.3750000002
7,1,10445.6315789473
7,1,11632.1540697675
7,1,5232.6666666666
7,1,9398.4090909092
7,1,5991.375
7,1,5474.4798387097
7,1,6851.7949999999
7,1,6347.4250000001
7,1,5007.3170731707
7,1,6737.2892156862
7,1,7159.4542682927
7,1,5786.0666666667
7,1,5324.8783783783
7,1,6838.6463414636
7,1,5113.6
7,1,8597.9207317073
7,1,6947.875
7,1,7153.1785714288
7,1,13822.6845238092
7,1,6588.8779069768
7,1,14942.3191489362
7,1,5603.4523809523
7,1,6041.1627906978
7,1,19590.879464286
7,1,9209.2
7,1,9930.7613636365
7,1,19973.8648648646
7,1,5750.9404761904
7,1,14630.4715909093
7,1,13390.60546875
7,1,6374.1971428571

7,1,18501.4308510639
7,1,6125.6187500001
7,1,11292.5972222221
7,1,10275.1947674418
7,1,5967.7946428571
7,1,6389.9013157896
7,1,7305.9671052631
7,1,13021.511904762
7,1,9749.6521739134
7,1,5419.5666666666
7,1,15720.3928571428
7,1,15174.90625
7,1,5117.8522727274
7,1,11716.1833333334
7,1,13466.9361111113
7,1,9376.4999999999
7,1,7952.5853658537
7,1,17631.6052631578
7,1,10104.8676470587
7,1,5841.8837209302
7,1,5183.3166666667
7,1,12110.4166666666
7,1,5854.875
7,1,10770.25
7,1,16495.7774390243
7,1,11566
7,1,8134.0571428571
7,1,14715.0478723405
7,1,12895.6321428572
7,1,7592.9166666666
7,1,7725.8333333332
7,1,5034.1666666665
7,1,6862.2019230771
7,1,10848.6644736842
7,1,5910.612244898
7,1,6997.779255319
7,1,10503.1829268295
7,1,19772.5563725478
7,1,5927.8776595745
7,1,5402.1843750002
7,1,9718.2702702703
7,1,13269.8388157894
7,1,5538.3125
8,2,7505.2173913044
8,2,5148.0833333333
8,2,6445.03125
8,2,5405.0399999998
8,2,8230.6975806451
8,2,5053.1750000001
8,2,5728.125
8,2,5231.7325581396
8,2,6498.3953488373
8,2,5820.9999999999
8,2,6426.9081632653
8,2,7188.0930232559
8,2,5050.2763157894
8,2,6232.8437499999
8,2,5619.7142857143
8,2,6153.9878048781
8,2,6954.7906976744
8,2,5419.3461538462
8,2,9380.9802631582
8,2,6542.0663265307
8,2,5994.4799107144
8,2,5538.295
8,2,9453.1655405406

8,2,8897.4
8,2,6794.3177083336
8,2,5436.0465116279
8,2,8428.1500000001
8,2,7990.1442307693
8,2,7020.1505102041
8,2,7622.7286585365
8,2,5627.8970588235
8,2,7688.8515625
8,2,7116.9999999999
8,2,6073.3813559322
8,2,6506.3292682927
8,2,7702.2931818182
8,2,5410.409090909
8,2,5390.1785714285
8,2,9414.7781249997
8,2,5538.6904761905
8,2,9289.0130208333
8,2,5774.6344339622
8,2,9718.0592105263
8,2,6745.25
8,2,5172.1995614036
8,2,5535.7307692308
8,2,5196.887195122
8,2,14093.029891304
8,2,12364.653846154
8,2,8286.5849056603
8,2,8608.1380208333
8,2,6854.4158163266
8,2,5279.8055555555
8,2,6655.6666666667
8,2,7071.8676470588
8,2,5147.1428571429
8,2,9457.5875
8,2,5912.2959183674
8,2,6376.9070512821
8,2,5651.1277173913
8,2,5537.78125
8,2,6895.137755102
8,2,5250.4107142858
8,2,6275.7282608695
8,2,9188.9475000003
8,2,6555
8,2,6945.0975609756
8,2,7791.085106383
8,2,5164
8,2,7683.1323529413
8,2,5195.9999999999
8,2,5208.3693181817
8,2,7162.2124999999
8,2,5758.5978260868
8,2,5154.1607142857
8,2,10866.3000000001
8,2,5741.8541666667
8,2,8481.3704545455
8,2,8204.909090909
8,2,7313.0625
8,2,6461.8636363637
8,2,5202.1739130435
8,2,5544
8,2,8227.5813953489
8,2,8144.8333333333
8,2,7009.6250000001
8,2,8177.5773809524
8,2,6090.3823529412
8,2,6052.6282894737

8,2,10655.9309210526
8,2,5865.6162790698
8,2,5278
8,2,6004.2162162163
8,2,8038.5739795917
8,2,11758.0972222224
8,2,9647.3999999999
8,2,8651.8190789473
8,2,12938.9268292683
8,2,5935.25
8,2,7860.0869565216
8,2,5266.828125
8,2,5744.0795454546
8,2,5297.20703125
8,2,9358.0000000001
8,2,9459.8148148152
8,2,6751.8493589744
8,2,6414.6818181819
8,2,5554.765
8,2,6936.972972973
8,2,6770.3885135135
8,2,6151.5855263158
8,2,7012
8,2,12165.4411764706
8,2,5162.2195121951
8,2,7154.7216981133
8,2,8272.3409090909
8,2,6523.018292683
8,2,5151.5067567568
9,1,17640.5859375
9,1,9015.5000000001
9,1,12037.375
9,1,12953.3841463414
9,1,10470.5121951217
9,1,10296.1761363638
9,1,10224.715425532
9,1,7133.375
9,1,6375.4878048782
9,1,5714.9040697675
9,1,16174.5760869567
9,1,7276.5994318182
9,1,5866.1162790698
9,1,5048.2777777777
9,1,10013.1914893616
9,1,12781.9521276596
9,1,7258.13
9,1,7397.2678571429
9,1,5062.9797297297
9,1,6076.3365384616
9,1,11722.5
9,1,17542.7616279069
9,1,15860.6959459459
9,1,11294.1538461539
9,1,8416.3450704224
9,1,9360.1923076923
9,1,16173.8947368422
9,1,5141.0851063831
9,1,16332.821875
9,1,13867.5
9,1,7302.8536585366
9,1,16843.1900000003
9,1,11732.4967948719
9,1,13632.8177966098
9,1,7432.7761627907
9,1,5520.1630434783
9,1,16307.2499999997

9,1,11930.2105263157
9,1,10876.0462962962
9,1,9737.0408163267
9,1,8065.8499999999
9,1,12996.4111842103
9,1,6729.3750000001
9,1,13013.1418918919
9,1,11821.7282608691
9,1,12759.0588235294
9,1,19057.96875
9,1,9802.2250000001
9,1,7319.2942708334
9,1,7527.6684782608
9,1,6608.6176470588
9,1,5978.7142857144
9,1,11871.2105263159
9,1,5593.5
9,1,10157.7560975612
9,1,7108.9088541668
9,1,16789.8269230767
9,1,14302.3437500002
9,1,6301.7173913043
9,1,5539.8457446808
9,1,7675.5259433961
9,1,7546.9651162791
9,1,6887.8139534883
9,1,12688.8292682924
9,1,9969.0340909091
9,1,19844.9333333334
9,1,5230.7331081081
9,1,6085.925925926
9,1,5231.4786585366
9,1,12309.1308139535
9,1,14135.5714285715
9,1,8926.2053571428
9,1,5188.3396226416
9,1,6819.2010869565
9,1,15090.2368421054
9,1,6628.1798780488
9,1,13323.226744186
9,1,5800.0255681818
9,1,7979.7952127661
9,1,8230.4662162162
9,1,5359.5897435897
9,1,13525.4264705887
9,1,14072.5357142859
9,1,14249.1447368419
9,1,7416.46875
9,1,8747.84375
9,1,6894.1802325581
9,1,13323.1707317071
9,1,6253.8823529412
9,1,5343.8902439025
9,1,6537.8982558139
9,1,9914.1764705883
9,1,17636.2499999998
9,1,5785.4340277778
9,1,8532.2325581395
9,1,12386.1223404254
9,1,7127.2613636362
9,1,5644.1197916665
9,1,8685.4883720931
9,1,8000.0609756096
9,1,14830.103448276
9,1,6042.1909722222
9,1,13732.0407407407

9,1,7988.5476190475
9,1,10991.9918478256
9,1,7581.8382352941
9,1,8144.3475609757
9,1,6153.9080882352
9,1,6369.8214285715
9,1,5734.5625
9,1,5026.2173913043
9,1,14978.599264706
9,1,5264.2916666666
9,1,6169.4999999999
9,1,12611.6527777777
9,1,18159.0384615386
9,1,9492.7499999999
9,1,6809.0106382979
9,1,19745.0738636367
9,1,9310.412735849
9,1,9950.3333333334
9,1,5539.2872340425
9,1,5259.2840909089
9,1,17463.954787234
9,1,5288.7620192306
9,1,19854.5625
9,1,10638.5274390245
9,1,9548.2393617024
9,1,5336.3725490196
9,1,6492.5784313726
9,1,7090.5342741935
9,1,7802.0722222225
9,1,7783.4519230768
9,1,5665.0606060606
9,1,5111.3421052632
9,1,5152.1666666666
9,1,15846.3096590908
9,1,19889.857142857
9,1,19105.3695652173
9,1,6706.215909091
9,1,5353.6875000001
9,1,10920.4244186046
9,1,16514.5053191488
9,1,5218.9110169492
9,1,5947.8181818183
9,1,7866.1781914894
9,1,14354.769230769
9,1,13605.7925531916
9,1,7521.829787234
9,1,9846.706521739
9,1,7641.4289772727
9,1,9700.0833333337
9,1,8556.3333333336
9,1,8785.6829268292
9,1,5176.4613095238
9,1,5216.203125
9,1,8169.1111111112
9,1,6817.75
9,1,6934.4875000001
9,1,18506.0645161291
9,1,14870.9189189188
9,1,7904.4390243903
9,1,17497.0110294117
9,1,8656.2641509432
9,1,5361.0714285714
9,1,5245.8445121951
9,1,18346.4000000004
9,1,13540.4702380956
9,1,17233.29296875

9,1,11224.1428571426
9,1,6767.5959821427
9,1,11678.3235294118
9,1,7069.9069148938
9,1,13671.9560810808
9,1,9307.3939393939
9,1,5358.1521739131
9,1,9288.4375
9,1,6195.1004901961
9,1,10304.2410714288
9,1,5600.4969512196
9,1,8096.2500000002
9,1,8330.657142857
9,1,9844.60546875
9,1,13327.3829787236
9,1,19946.2780612248
9,1,5900.3
9,1,9774.6451612903
9,1,14363.5375
9,1,5436.4627659574
9,1,11632.5263157892
9,1,8714.6168478263
9,1,11908.3499999999
9,1,5263.6702127659
9,1,16863.7750000001
9,1,8055.2357142857
9,1,6435.6037234041
9,1,13615.84375
9,1,9921.4945652174
9,1,13768.1796874999
9,1,6251.0203488371
9,1,6331.6075581395
9,1,8776.8111111111
9,1,10350.0994318184
9,1,8353
9,1,8953.8571428574
9,1,14248.1209677418
9,1,5462.2307692307
9,1,13453.2051282049
9,1,8090.2670454545
9,1,6348.1158536585
9,1,7471.6086956521
9,1,19077.9849999998
9,1,7929.11875
9,1,9286.6889534883
9,1,6634.5
9,1,14743.0363636364
9,1,6088.7619047618
9,1,7823.6104651163
9,1,7605.25
9,1,8521.37
9,1,18209.1944444444
9,1,12234.9627659573
9,1,15463.8858695651
9,1,6670.5244565218
10,2,5297.5483870968
10,2,5292.9680851064
10,2,9510.7717391304
10,2,6134.125
10,2,5222.4871794872
10,2,11591.2999999998
10,2,5823.375
10,2,5691.1174242425
10,2,5721.0338983051
10,2,5934
10,2,7608.7250000001

10,2,5307.6931818182
10,2,7072.8722222222
10,2,5010.5707547171
10,2,5593.1809210526
10,2,6098.7592592593
10,2,5881.2887931035
10,2,11519.465116279
10,2,5002.0434782609
10,2,5181.6000000002
10,2,7959.1
10,2,5719.5480769231
10,2,5403.8006756757
10,2,5789.075
10,2,5053.5789473685
10,2,6841.5182926829
10,2,7353.0724999999
10,2,8723.5849056604
10,2,11465.1940789473
10,2,5314.2238372093
10,2,7024.3750000001
10,2,7198.0877659574
10,2,6245.5239361703
10,2,6774.5116279069
10,2,6885.2877906977
10,2,5674.0377358491
10,2,5064.0243902439
10,2,7597.921875
10,2,5041.1973684211
10,2,7247.2275641025
10,2,5861.7727272727
10,2,7435.0525000002
10,2,9883.0714285715
10,2,5656.7500000002
10,2,7264.0454545454
10,2,9804.5694444446
10,2,6789.1743421053
10,2,6220.5551470588
10,2,5352.5217391304
10,2,10061.9055555558
10,2,7791
10,2,6228.1886792452
10,2,6935.7142857144
10,2,6012.5
10,2,10930.9642857142
10,2,5522.7337278106
10,2,9277.3541666667
10,2,5056.8534482759
10,2,5013.5
10,2,11101.8
10,2,5980.7261904761
10,2,5838.4361702127
10,2,8696.4338235294
10,2,7350.5256410256
10,2,5172.73
10,2,5012.0030487806
10,2,5419.6280487805
10,2,7177.0437500002
10,2,5427.9895833333
10,2,5867.3421052633
10,2,5346.8472222222
10,2,5204.7916666667
10,2,7973.4166666666
10,2,5138.6875
10,2,8169.15
10,2,10554.9555555559
10,2,7866.2648809526

10,2,5278.6190476191
10,2,8887.5833333334
10,2,6658.9226190476
10,2,5597.2678571429
10,2,5539.6964285714
10,2,8754.7446808511
10,2,12360.0576923073
10,2,5090.8333333334
10,2,7068.9011627905
10,2,5784.0833333331
10,2,6040.8947368421
10,2,5017.7307692308
10,2,6839.0857142857
10,2,5191.0443548387
10,2,7218.4444444444
10,2,9181.2976190476
10,2,13037.2205882355
10,2,5229.8677884615
10,2,5524.693181818
10,2,5768.9553072625
10,2,7875.465116279
10,2,6354.84375
10,2,6993.2321428571
10,2,6396.1202830189
10,2,9895.4264705884
10,2,7073.9795918366
10,2,5517.483974359
10,2,7385.0705128206
10,2,8171.3414634146
10,2,6432.4184782609
10,2,5935.5408163265
10,2,9689.3005952381
10,2,11249.5737179488
10,2,5646.0357142857
10,2,10762.0047169811
10,2,10359.2625000001
10,2,7409.4796511627
10,2,6240.6781914894
10,2,9888.0681818183
10,2,6722.8684210527
10,2,9848.8372093024
10,2,5917.8263888888
10,2,6021.1802325583
10,2,7876.6249999999
10,2,6355.6785714286
10,2,5331.3670212764
11,1,10924.6774999998
11,1,7756.5714285714
11,1,6076.6363636364
11,1,9760.9040697676
11,1,8403.6086956523
11,1,6002.7941176471
11,1,9289.2432432433
11,1,14394.2941176469
11,1,13519.9166666665
11,1,11012.9400000001
11,1,5785.0361842105
11,1,5998.25
11,1,5274.8472222223
11,1,9978.0535714285
11,1,9706.5813953488
11,1,5288.265
11,1,6161.5333333333
11,1,14609.4883720929
11,1,19361.322580645
11,1,5987.6785714285

11,1,6141.6860465117
11,1,9611.8882978725
11,1,7513.9437500001
11,1,9020.1
11,1,17739.1875000001
11,1,5731.268292683
11,1,9906.081632653
11,1,7748
11,1,5077.9500000001
11,1,5959.5546875
11,1,5679.3482142857
11,1,6588.8308823529
11,1,5247.529661017
11,1,6013.9261363637
11,1,7250.4972826087
11,1,11583.4499999999
11,1,5518.2857142857
11,1,6137.7142857142
11,1,5850
11,1,5232.9999999999
11,1,5365.6335227272
11,1,9365.0302419354
11,1,13722.5034722224
11,1,13497.6474999999
11,1,8467.2958333334
11,1,10543.898305085
11,1,5308.5
11,1,7231.3374999999
11,1,8296.7476851852
11,1,16881.2317073172
11,1,6352.201388889
11,1,7355.8693181818
11,1,19863.7222222222
11,1,8175.6016949154
11,1,14242.6627906977
11,1,7526.1737804879
11,1,10724.079787234
11,1,18297.0416666668
11,1,13766.5416666669
11,1,9366.6249999999
11,1,5725.5478723405
11,1,5358.8500000001
11,1,19289.9617346937
11,1,7884.2765957447
11,1,12377.3125000002
11,1,6927.3645833333
11,1,19069.8359375
11,1,5862.984375
11,1,11989.0937500002
11,1,9172.4893617021
11,1,5261.6585365854
11,1,16519.481707317
11,1,9744.0427631579
11,1,11097.4390243901
11,1,5887.4687499999
11,1,7298.3701923078
11,1,7185.0480769231
11,1,5204.0608108108
11,1,7535.8888888891
11,1,10894.5892857142
11,1,5289.9969512195
11,1,6122.3571428573
11,1,5804.0546875
11,1,18220.9664634147
11,1,5364.4285714286
11,1,7803.615131579

11,1,5619.2083333334
11,1,6073.3333333333
11,1,8946.2594339622
11,1,5827.7125
11,1,8454.9166666667
11,1,7419.3414634146
11,1,18516.8269230774
11,1,11582.3958333331
11,1,5955.8333333333
11,1,17860.6776315796
11,1,7817.8190789475
11,1,18568.8695652172
11,1,5065.7978723404
11,1,10184.3
11,1,6223.7812500001
11,1,19262.0833333334
11,1,11407.6463414633
11,1,7788.6517857144
11,1,5567.3293269231
11,1,14720.5789473684
11,1,6888.982142857
11,1,15098.7867647059
11,1,5063.2797619048
11,1,12908.6676136363
11,1,11261.7965116278
11,1,5820.9523809524
11,1,17183.6889534885
11,1,9190.5714285715
11,1,5801.19375
11,1,17220.512195122
11,1,10112.3658536585
11,1,18830.6530612246
11,1,6986.7960526316
11,1,5882.0406249999
11,1,18297.7567567569
11,1,7175.9791666668
11,1,7718.205357143
11,1,9188.4244186046
11,1,5851.6346153846
11,1,10433.9487179488
11,1,7281.0480769231
11,1,19456.2134146342
11,1,7129.9322916667
11,1,5910.8550000001
11,1,14637.5116279069
11,1,7039.4204545455
11,1,7422.2625000001
11,1,8055.1346153844
11,1,6182.5477941176
11,1,6732.9302325579
11,1,7026.6764705883
11,1,7498.25
11,1,5833.671875
11,1,5966.35
11,1,5238.6017441862
11,1,7287.8414634146
11,1,6404.2596153846
11,1,12659.0384615385
11,1,5973.3986486487
11,1,5871.5535714286
11,1,8575.1306818182
11,1,8219.6354166667
11,1,15379.9230769233
11,1,16829.1083333333
11,1,8650.2649999997
11,1,7353.9999999998

11,1,5487.6282051282
11,1,18388.5460526313
11,1,6716.6744186046
11,1,11488.6875000002
11,1,6363.4270833333
11,1,6480.024390244
11,1,16682.2692307694
11,1,9528.6515957448
11,1,7196.2142857145
11,1,6767.7556818182
11,1,18163.9695945946
11,1,5467.8109756098
11,1,12950.8815789476
11,1,7693.1462765956
11,1,15667.6249999997
11,1,19476.2837837838
11,1,15017.5735294118
11,1,13353.6838235293
11,1,5290.881097561
11,1,5985.3207547169
11,1,11606.1187499997
11,1,9789.7857142859
11,1,6214.4285714284
11,1,9780.7249999999
11,1,5566.6511627907
11,1,16568.8947368422
11,1,13258.7819767443
11,1,18833.4166666667
11,1,11331.75
11,1,5148.2127659575
11,1,6479.8125
11,1,5107.1520270271
11,1,13836.02734375
11,1,19180.6649999997
11,1,13425.9615384615
11,1,5691.7333333332
11,1,9266.1538461539
11,1,5154.7470238095
11,1,11264.5514705882
11,1,10353.595238095
11,1,5860.1369047619
11,1,11669.494565217
11,1,8453.5238095239
11,1,15225.2898936172
11,1,8330.5943396226
11,1,7639.5686274509
11,1,6281.4920634921
11,1,12415.3953488373
11,1,7158.798076923
11,1,5069.4088541666
11,1,15690.6666666665
11,1,9430.8970588236
11,1,10202.9062499997
11,1,7603.7625
11,1,8225.1588541666
11,1,15529.0280612245
11,1,14841.8367346936
11,1,9887.868902439
11,1,5765.8333333334
11,1,5885.8171296297
11,1,5871.8048780488
11,1,5678.6808510637
11,1,6493.7674418604
11,1,10654.7187500001
11,1,10294.6079545454
11,1,18709.0657894737

11,1,5137.8604651161
11,1,6393.2727272728
12,2,5797.7470930233
12,2,6545.9821428571
12,2,5673.4838709677
12,2,9425.0263157894
12,2,5768.3822115385
12,2,5961.6216216216
12,2,6560.3488372093
12,2,10366.1249999998
12,2,6469.5234375
12,2,6716.3108974359
12,2,8096.755952381
12,2,6514.7765151516
12,2,10178.975609756
12,2,9320.2724358976
12,2,5136.443452381
12,2,6775.4320652173
12,2,5526.9999999999
12,2,7815.9797297296
12,2,5061.0425531915
12,2,7490.4444444444
12,2,6701.9615384616
12,2,5133.3140243902
12,2,8673.2500000003
12,2,11599.2954545455
12,2,6532.6681034483
12,2,6802.6704545454
12,2,8693.5381355933
12,2,7377.8837209302
12,2,6770.0166666667
12,2,8256.4941860466
12,2,7157.2857142857
12,2,7852.8245192307
12,2,5621.5405405406
12,2,5495.8571428573
12,2,6830.8181818181
12,2,8676.7326388889
12,2,8558.1250000001
12,2,10885.9017857143
12,2,6313.4146341464
12,2,7407.6354166668
12,2,5900.3068181818
12,2,5639.1102941176
12,2,5963.2833333333
12,2,6429.1617647059
12,2,5463.9540816326
12,2,9333.1600000003
12,2,6221.4979166665
12,2,6320.2386363637
12,2,6055.4744318183
12,2,7204.1323529412
12,2,7876.9044117647
12,2,5171.3658536585
12,2,7135.4736842105
12,2,5592.83203125
12,2,9748.7848837208
12,2,5328.2720588235
12,2,6919.4666666667
12,2,10795.8532608695
12,2,6543.1291666666
12,2,11417.3494318182
12,2,7925.221590909
12,2,9670.421875
12,2,7253.3636363637
12,2,7173.7321428571

12,2,7126.5714285715
12,2,5218.6022727272
12,2,5941.9880952381
12,2,12614.605263158
12,2,5439.5303030303
12,2,5892.4054054054
12,2,5279.1805555555
12,2,6300.3157894736
12,2,5731.2523584907
12,2,5414.03125
12,2,7244.9166666668
12,2,7587.4285714285
12,2,9565.6428571427
12,2,5363.826923077
12,2,5529.9966216216
12,2,11527.0319148938
12,2,11063.1428571429
12,2,6461.5128205128
12,2,8012.205882353
12,2,6559.3524999999
12,2,6860.1700000001
12,2,8279.6540697675
12,2,5658.0111111112
12,2,10868.90625
12,2,7726.4821428571
12,2,6054.6666666668
12,2,8217.5386904762
12,2,6278.7647058824
12,2,5541.3823529414
12,2,5208.7333333333
12,2,6990.0294117648
12,2,10745.5036764707
12,2,5955.6666666666
12,2,9068.9230769232
12,2,5207.5992647058
12,2,6746.1488095238
12,2,8875.2925531917
12,2,5132.8125
12,2,8211.3333333334
12,2,5586.9423076923
12,2,7141.2822580645
13,1,17973.1499999999
13,1,8056.1597222223
13,1,6113.2777777778
13,1,13157.1888297872
13,1,9971.6704545455
13,1,5845.03125
13,1,9297.8125
13,1,11742.1569148936
13,1,16620.7203947368
13,1,9774.3804347824
13,1,9921.838362069
13,1,12378.75
13,1,9165.3897058823
13,1,5356.3514150943
13,1,7139.9014423077
13,1,9750.2784090909
13,1,8844.4255319151
13,1,9006.1171875
13,1,11214.9318181819
13,1,5628.1486486489
13,1,17345.7529761906
13,1,9938.2536764706
13,1,19287.7826086958
13,1,19312.746794872
13,1,6702.4343749999

13,1,8568.9908536585
13,1,9649.6538461539
13,1,5752.5892857143
13,1,7773.35
13,1,7267
13,1,13128.8947368421
13,1,11115.3670212769
13,1,7426.2419354839
13,1,17463.3494318182
13,1,16267.1101190476
13,1,13296.3529411765
13,1,5962.8749999999
13,1,5557.1933962264
13,1,7071.6219512195
13,1,9147.4285714286
13,1,8515.0130208333
13,1,14513.8624999998
13,1,14550.1691176475
13,1,15886.0568181819
13,1,7831.671875
13,1,14897.2207446809
13,1,10146.0723684208
13,1,5578.5857142857
13,1,6727.576923077
13,1,6175.5234375
13,1,12483.7165178574
13,1,12907.8589743591
13,1,13117.6410256411
13,1,6638.5
13,1,16037.7028301886
13,1,6148.6173469388
13,1,5762.2205882353
13,1,7637.8173076924
13,1,7851.078125
13,1,7464.3400000002
13,1,6091.0552325582
13,1,9847.875
13,1,9130.3070652174
13,1,5207.9222972973
13,1,11575.8666666668
13,1,9394
13,1,10339.0290697674
13,1,6261.1666666666
13,1,9428.1666666667
13,1,6856.5884146344
13,1,5053.4517045455
13,1,10107.2348484845
13,1,5518.8916666667
13,1,7441.4940476191
13,1,7272.8235294118
13,1,8720.0961538462
13,1,5692.2272727273
13,1,10953.641025641
13,1,6704.1125000001
13,1,6334.544117647
13,1,7516.9117647058
13,1,6062.16875
13,1,10996.9006410257
13,1,9273.4468085107
13,1,12518.5555555554
13,1,5043.3352272728
13,1,5684.5612244898
13,1,9836.625
13,1,17096.5642857142
13,1,14950.3256578948
13,1,16909.6808510637

13,1,6352.113372093
13,1,5245.9934210525
13,1,7815.3776041667
13,1,18454.0261627908
13,1,14351.659090909
13,1,13303.0239361705
13,1,6530.9285714285
13,1,10462.1781249999
13,1,10606.7386363634
13,1,10466.5476190477
13,1,13385.7500000001
13,1,10723.2105263158
13,1,16724.5483870965
13,1,7979.4468085108
13,1,19705.87890625
13,1,9491.81875
13,1,16961.8511904765
13,1,7492.2941176472
13,1,9584.3750000001
13,1,7264.0288461538
13,1,8045.7138157894
13,1,7589.3355263158
13,1,7097.6730769232
13,1,8142.2095588235
13,1,10847.7368421055
13,1,5994.2727272727
13,1,16204.9913793104
13,1,13211.4999999998
13,1,12238.1745283016
13,1,5038.39375
13,1,19024.8225806452
13,1,6484.6794871795
13,1,6750.5238095237
13,1,6545.6923076922
13,1,17801.217391304
13,1,18118.4673913044
13,1,5673.9545454545
13,1,12822.2142857142
13,1,5992.2016129034
13,1,8631.1458333333
13,1,17038.4375
13,1,5105.1875
13,1,11519.36
13,1,5756.966981132
13,1,13205.0769230771
13,1,5255.8537735848
13,1,7655.234375
13,1,5434.177631579
13,1,5760.9032258065
13,1,7436.783898305
13,1,15021.0142857142
13,1,9541.0076530613
13,1,5410.4481707316
13,1,6829.2875
13,1,5028.4523809523
13,1,5149.6583333333
13,1,10648.1666666666
13,1,6333.3125000001
13,1,14967.4180327867
13,1,9651.8719512195
13,1,12381.5161290322
13,1,5970.9027777777
13,1,5827.8750000001
13,1,13081.1773255815
13,1,10505.9508928568
13,1,15116.0303030304

13,1,9851.1818181818
13,1,9898.7037037037
13,1,10878.5384615383
13,1,12632.7894736841
13,1,9555.4044117647
13,1,8955.6785714286
13,1,9047.15
13,1,7941.4305555555
13,1,5043.918918919
13,1,19648.5749999999
13,1,6151.369047619
13,1,5045.8478260869
13,1,8945.9540816326
13,1,5645.302631579
13,1,18232.0142857144
14,2,9411.8139534884
14,2,7987.8804347826
14,2,6872.1718750001
14,2,8464.9312499999
14,2,8623.0921052632
14,2,6308.875
14,2,7858.9769736841
14,2,6283.5945121951
14,2,7789.2565789474
14,2,6290.6764705882
14,2,7268.2222222222
14,2,12591.534090909
14,2,7040.3333333332
14,2,11013.2285714284
14,2,7178.9756097561
14,2,8497.0978260869
14,2,9476.4473684209
14,2,7362.9891304348
14,2,6398.5657894738
14,2,5823.4375
14,2,6842.2857142858
14,2,7181.6923076924
14,2,6744.7039473685
14,2,8042.1170212766
14,2,6574.516025641
14,2,8494.3365384616
14,2,5288.2894736842
14,2,6681.6346153846
14,2,6084.3870967742
14,2,6783.4261363636
14,2,11203.216666667
14,2,7072.9093749999
14,2,6900.2302631581
14,2,5859.9177631579
14,2,8825.4573170731
14,2,6425.8571428572
14,2,5836.1730769232
14,2,11939.2749999999
14,2,5408.8666666666
14,2,5402.8310810811
14,2,10869.8392857146
14,2,5980.7419354839
14,2,6073.1993243244
14,2,7266.765625
14,2,5309.2784090908
14,2,8534.2500000001
14,2,10523.506097561
14,2,5006.3611111111
14,2,10485.4705882354
14,2,8679.4331395348
14,2,10463.6111111112

14,2,10926.8823529412
14,2,5111.5654761905
14,2,8495.0640243902
14,2,7935.1120689655
14,2,9212.6453488374
14,2,6011.5535714286
14,2,8534.0641891892
14,2,6697.1136363637
14,2,5869.2279411765
14,2,6364.109375
14,2,6325.1293103449
14,2,6831.2149122806
14,2,12318.6830357144
14,2,12548.582317073
14,2,8459.0243902439
14,2,8343.15
14,2,5430.6032608696
14,2,5537.8049999998
14,2,7169.3913043477
14,2,7433.8602941176
14,2,12535.6335227271
14,2,6429.7192982456
14,2,5204.7474489796
14,2,9980.9087837837
14,2,8376.856707317
14,2,6934.1304347826
14,2,6677
14,2,5605.75
14,2,5894.7352941176
14,2,11133.6057692307
14,2,6432.2765957448
14,2,9661.6433823529
14,2,6490.7692307692
14,2,6561.7741935483
14,2,5561.3660714286
14,2,7052.9310344827
14,2,10921.6470588236
14,2,5016.5964285715
14,2,6310.1175
14,2,5063.8140243903
14,2,9885.1219512193
15,1,5115.4545454546
15,1,10351.1213235292
15,1,5984.5535714285
15,1,17001.5357142856
15,1,18788.6341463416
15,1,12510.2629310344
15,1,6412.6842105263
15,1,14883.0454545456
15,1,13849.9285714288
15,1,5611.1777777777
15,1,11935.1190476189
15,1,13913.8142857142
15,1,6170.2777777778
15,1,16011.3125
15,1,16895.5806451613
15,1,13780.9375
15,1,6189.93125
15,1,14165.4807692309
15,1,11937.0218253966
15,1,12296.2812500001
15,1,5366.7907608696
15,1,7748.8571428571
15,1,15866.9729729729
15,1,6409.512195122
15,1,5263.1515957447

15,1,6590.6249999998
15,1,5560.0624999998
15,1,13858.2192982453
15,1,5951.5432692307
15,1,11157.5823170734
15,1,5194.3658536585
15,1,9104.078125
15,1,17521.96875
15,1,5424.7291666667
15,1,7558.9149999999
15,1,6070.1715116281
15,1,18106.0058139536
15,1,18718.8750000001
15,1,12453.9622093024
15,1,14211.0657894738
15,1,9916.09375
15,1,11454.1927083333
15,1,13324.1212121212
15,1,18455.2664473684
15,1,12656.5328947369
15,1,12305
15,1,6779.5454545454
15,1,6069.7872340425
15,1,11174.2897727277
15,1,13551.6800000001
15,1,7608.14
15,1,11429.9375
15,1,6606.514423077
15,1,5472.1075581395
15,1,9695.7205882351
15,1,5812.3546511628
15,1,9758.1923076923
15,1,10241.2647058821
15,1,7428.0767045455
15,1,5884.5818181817
15,1,10532.0972222222
15,1,8111.3666666667
15,1,15523.2111111109
15,1,5139.5384615385
15,1,12286.0261627906
15,1,14861.9999999999
15,1,12803.0913461538
15,1,19050.1774193549
15,1,11842.1484375
15,1,8758.96875
15,1,5440.4204545454
15,1,10862.09375
15,1,12188.2149999996
15,1,8000.8181818181
15,1,16077.2056451613
15,1,13738.697368421
15,1,13333.8083333334
15,1,8542.18877551
15,1,7817.0312500002
15,1,5438.44140625
15,1,9612.3892045454
15,1,11375.0714285712
15,1,8101.0833333334
15,1,7481.0416666668
15,1,13444.2439024394
15,1,11861.5
15,1,5576.1554054054
15,1,5856.2164634146
15,1,19454.875
15,1,5261.0625
15,1,11739.6301020406

15,1,9935.375
15,1,12880.875
15,1,5938.2653061224
15,1,8931.0294117646
15,1,9816.5
15,1,11136.6587837837
15,1,8054.6280487807
15,1,7778.1647727272
15,1,7633.5773809524
15,1,6713.1361111111
15,1,5523.4256756756
15,1,8562.125
15,1,9443.6223958333
15,1,7142.6293103449
15,1,10909.8214285715
15,1,13588.9572368422
15,1,15822.6041666666
15,1,6824.1958333334
15,1,7367.7692307692
15,1,5167.6909090909
15,1,16637.3295454545
15,1,14408.0517241379
15,1,9237.9210526316
15,1,19395.9230769231
15,1,6196
15,1,7725.8333333334
15,1,6312.3828125001
15,1,10658
15,1,5731.3857142857
15,1,16855.1666666666
15,1,7382.175595238
15,1,17905.4392857141
15,1,6235.6392045454
15,1,15969.5653409095
15,1,13083.2682926828
15,1,10740.8469387756
15,1,5889.5771276596
15,1,11355.0178571427
15,1,8881.6025
15,1,10429.3333333334
15,1,6550.8723404255
15,1,11724.7022058823
15,1,17835.125
15,1,9510.375
15,1,17973.8557692306
15,1,10779.3823529411
15,1,6596.2205882353
15,1,15109.3026315789
15,1,13817.6976744188
15,1,6703.049689441
15,1,12180.7678571428
15,1,12463.7403846152
15,1,6944.1312500001
15,1,5471.9034090909
15,1,17376.96875
15,1,6072.0135135136
15,1,14846.7291666668
15,1,11411.2836538461
15,1,8017.2727272727
15,1,8044.71875
15,1,12793.4870689655
15,1,13537.1666666666
15,1,14416.9433962266
15,1,8633.7666666667
15,1,6327.7022058823
15,1,6626.4210526316

15,1,5733.4451219512
15,1,8697.5
15,1,6116.2195121951
15,1,6051.2093023256
15,1,5133.7282608696
15,1,5766.8444444446
15,1,18054.0454545455
15,1,6301.2790697674
15,1,9572.0476190475
15,1,8330.2606382979
15,1,9720.7573529411
15,1,14692.356060606
15,1,5764.1385135135
15,1,13559.909090909
15,1,12838.6764705886
15,1,6261.5128205128
15,1,7193.1282051282
15,1,19697.046875
15,1,9138.9431818181
15,1,5204.1474358974
15,1,5098.6479591837
15,1,19886.5375000002
15,1,9831.4852941179
15,1,6392.6249999999
15,1,18194.5454545455
16,2,5367.1714285714
16,2,6448.2500000001
16,2,5237.3333333334
16,2,8151.0365853659
16,2,10560.8235294119
16,2,5813.3310810811
16,2,6874.4324324324
16,2,7716.2837837837
16,2,8750.1047297297
16,2,7381.9756097561
16,2,5974.4177631579
16,2,10107.8421052631
16,2,9113.0961538462
16,2,5726.0927419355
16,2,5456.8235294118
16,2,5021.7407407407
16,2,7247.8888888889
16,2,5156.6972222222
16,2,5914.4551282052
16,2,5007.5
16,2,5205.4966216216
16,2,6931.0526315789
16,2,5726.8333333332
16,2,6101.9324324324
16,2,6590.6118421053
16,2,5284.0902777778
16,2,5456.2794117647
16,2,7532.7890625
16,2,5605.1730769231
16,2,7067.95
16,2,8270.6785714285
16,2,6835.3696808511
16,2,12033.6691176472
16,2,5971.6666666666
16,2,6172.1004901961
16,2,6407.1702127658
16,2,5994.4893617021
16,2,6167.5919117647
16,2,8203.3839285714
16,2,6690.22
16,2,8358.3839285714

16,2,6125.75
16,2,7273.5224358973
16,2,5350.0865384615
16,2,5824.8963414635
16,2,5544.710106383
16,2,5282.5999999999
16,2,9657.0608108107
16,2,5398.320754717
16,2,5329.7763157895
16,2,6691.78125
16,2,8397.4545454546
16,2,5083.8445121951
16,2,7341.4042553191
16,2,10980.6458333334
16,2,6817.3722222222
16,2,6517.7357954547
16,2,9233.7
16,2,6212.1029411765
16,2,6035.3392857143
16,2,6210.8244680851
16,2,8209.63125
16,2,9509.625
16,2,7494.0666666667
16,2,6409.0148809524
16,2,5023.6822916666
16,2,10519.9583333333
16,2,6806.2980769231
16,2,6721.0555555554
16,2,8559.8920454545
16,2,11880.6158536587
16,2,7437.0735294116
16,2,5563.9078947368
16,2,6396.1130952381
16,2,6267.6836734693
16,2,11060.0888157895
16,2,5466.1627358491
16,2,7600.75
16,2,9371.3585526315
16,2,8037.3714285714
16,2,9148.7195121951
16,2,7553.6730769231
16,2,5216.5092592593
16,2,6535.2826086958
16,2,6512
16,2,6130.7058823529
16,2,5498.4329268293
16,2,6652.8378378378
16,2,5839.2307692308
16,2,5949.7703488372
16,2,8049.0192307692
16,2,9902.2865853659
16,2,6824.013157895
16,2,8797.0968750001
16,2,10117.1666666667
16,2,7658.4000000001
16,2,9997.4903846154
16,2,9022.0083333333
16,2,5900.5326086956
16,2,6855.0404411765
16,2,7541.3355263158
16,2,6396.7032258064
16,2,5052.8529411765
16,2,5484.8026315789
16,2,7748.5294117647
17,1,13153.892857143
17,1,16159.0769230767

17,1,6925.7211538461
17,1,5160.6909090909
17,1,9231.3059210525
17,1,10237.7647058823
17,1,5760.2884615385
17,1,8325.2007575756
17,1,16012.7916666664
17,1,5401.8970588236
17,1,18182.7499999999
17,1,8648.6875
17,1,16964.8823529411
17,1,13975.8863636364
17,1,18715.9473684208
17,1,7607.1686046511
17,1,10185.9649122806
17,1,5734.5535714285
17,1,5352.2445652175
17,1,12841.0735294118
17,1,6075.4007352941
17,1,5193.8579545454
17,1,19685.8684210522
17,1,11337.1874999999
17,1,5209.2536764706
17,1,5109.4375000001
17,1,10455.0823170732
17,1,9010.1951219512
17,1,19835.8343750002
17,1,13405.8617021276
17,1,7185.8525000001
17,1,6401.46875
17,1,16850.7589285713
17,1,18974.2218750001
17,1,9464.9848484848
17,1,8152.9320652174
17,1,18171.9249999999
17,1,12747.7529069765
17,1,6583.6071428572
17,1,6804.5000000001
17,1,8948.9375
17,1,5023.5500000001
17,1,9196.5138888888
17,1,7579.641025641
17,1,7640.0243902439
17,1,5527.130952381
17,1,8690.8703703704
17,1,14056.5310880829
17,1,17491.5089285715
17,1,8882.125
17,1,5627.9574468085
17,1,6387.5675675675
17,1,7053.9464285715
17,1,6082.6190476191
17,1,7763.5294117648
17,1,10478.0081521736
17,1,16948.4431818183
17,1,8774.3016304348
17,1,12688.044642857
17,1,8589.9285714286
17,1,5716.6756756757
17,1,7469.2401960785
17,1,14646.0000000004
17,1,14983.6249999997
17,1,13573.203125
17,1,5893.6423611111
17,1,19284.7051282051
17,1,5578.8958333333

17,1,10522.7685185185
17,1,19580.6764705885
17,1,11545.6457142857
17,1,15051.9962121212
17,1,6364.7173913044
17,1,9831.9411764707
17,1,5062.9761904761
17,1,6757.4803921569
17,1,5752.0000000001
17,1,9543.3081395348
17,1,10028.0500000001
17,1,5657.7926829269
17,1,9356.0795454546
17,1,7885.1475
17,1,5186.4901315789
17,1,7202.2819148937
17,1,13176.7976190471
17,1,13296.3547297297
17,1,6769.0000000001
17,1,17055.847222222
17,1,15168.6574999996
17,1,5481.0853658536
17,1,6120.6114130436
17,1,8841.9519230769
17,1,7061.8125000001
17,1,12694.9166666668
17,1,5830.9273255813
17,1,11000
17,1,6596.0961538462
17,1,10462.0416666668
17,1,9026.78
17,1,16290.857638889
17,1,15462.7105263158
17,1,6056.0510204082
17,1,19596.9999999998
17,1,19476.5500000004
17,1,11942.445945946
17,1,16665.6666666667
17,1,11125.9583333336
17,1,8923.5152439024
17,1,14116.2430555556
17,1,16431.8095238097
17,1,16690.8676470588
17,1,5478.1940789472
17,1,8878.3749999999
17,1,7438.1482558139
17,1,11045.6903409092
17,1,13790.0000000003
17,1,6706.9736842105
17,1,8500.1802325582
17,1,5272.0425531916
17,1,10947.15625
17,1,5384.8472222221
17,1,5917.0955882352
17,1,16740.5608108106
17,1,7267.3426573426
17,1,10581.2703488374
17,1,6733.6029411765
17,1,8039.862244898
17,1,8406.7432432434
17,1,10818.1015625
17,1,6740.6341463416
17,1,5719.7243589744
17,1,13562.9329268295
17,1,6241.6666666668
17,1,11414.9725609756

17,1,5883.8284883722
17,1,16423.9166666665
17,1,5731.8048780488
17,1,9521.3421052631
17,1,5866.7171052632
17,1,10960.6666666668
17,1,6824.8653846156
17,1,7182.0322580645
17,1,13279.4291666663
17,1,15940.8500000001
17,1,7899.0657894736
17,1,13569.1790540541
17,1,7074.0091463415
17,1,5366.005952381
17,1,7194.7535714286
17,1,12524.2312500001
17,1,7073.875
17,1,6691.9318181819
17,1,5978.0639534884
18,2,5302.2777777777
18,2,5676.9375000001
18,2,11492.0657894737
18,2,5421.0434782609
18,2,6988.8841463414
18,2,14140.9302325583
18,2,6910.1630434783
18,2,9781.1
18,2,9996.6627906976
18,2,7734.2439024391
18,2,10711.0697674417
18,2,10442.1749999999
18,2,5447.9743589743
18,2,5986.1726190475
18,2,5675.1612903226
18,2,8373.668478261
18,2,8737.8928571428
18,2,7388.6923076923
18,2,7582.3999999999
18,2,6833.3333333333
18,2,8854.75
18,2,8550.3552631579
18,2,9249.9114583333
18,2,6728.8571428572
18,2,5205.2072368421
18,2,9092.5772058824
18,2,11553.96875
18,2,9505.309375
18,2,9872.0681818181
18,2,5727.3717948718
18,2,5289.5465116279
18,2,5483.5
18,2,7924.75
18,2,5097.7083333334
18,2,5119.8467741936
18,2,5415.0523255814
18,2,7115.1634615384
18,2,10686.2364864864
18,2,6442.6636904762
18,2,6802.2857142856
18,2,7837.9625
18,2,11812.1547619047
18,2,5307.3774038462
18,2,8288.9935897436
18,2,8126.3579545454
18,2,5281.3600000001
18,2,5783.4736842107

18,2,5542.2113095238
18,2,7021.8852040817
18,2,6773.2647058824
18,2,8829.8571428572
18,2,9193.28125
18,2,5457.9821428572
18,2,11449.042613636
18,2,5285.0119047619
18,2,7319.9268292684
18,2,5126.8493589744
18,2,6688.875
18,2,5113.7420212766
18,2,7650.3475609756
18,2,5483.1403061223
18,2,5518.3194444444
18,2,9393.9111111112
18,2,6993.1538461539
18,2,8793.875
18,2,6493.7083333333
18,2,6182.75
18,2,5961.7000000001
18,2,8827.4340277777
18,2,8254.5357142857
18,2,5731.4973958334
18,2,6582.8076923077
18,2,8736.2983870968
18,2,8154.5625000001
18,2,13468.1057692308
18,2,6241.8352272726
18,2,6354.9338235295
18,2,5736.5457317073
18,2,6132.0535714286
18,2,5911.7692307692
18,2,5415.1449999999
18,2,5946.4736842105
18,2,5865.3846153847
19,1,5330.5865384615
19,1,10093.8687499999
19,1,7285.4567307693
19,1,7524.169642857
19,1,6650.8823529411
19,1,5774.8173076923
19,1,13916.3142857143
19,1,13843.0972222225
19,1,17305.2926829268
19,1,6232.8720930233
19,1,5912.1627906977
19,1,11932.6875
19,1,10739.875
19,1,9573.4661458334
19,1,14883.8749999999
19,1,11302.5673076922
19,1,13328.4742647057
19,1,5935.0397727274
19,1,5598.8699999999
19,1,6195.7318181818
19,1,10119.8238636362
19,1,5860.25
19,1,11059.843137255
19,1,7043.3557692308
19,1,5050.3571428572
19,1,5983.8579545454
19,1,11682.6875
19,1,12611.9448529411
19,1,14100.382142857
19,1,9571.1892857143

19,1,7326.2916666668
19,1,7733.2388888888
19,1,5476.7325581396
19,1,8554.0625
19,1,12000.2051282051
19,1,6839.9934210526
19,1,7067.9716981131
19,1,13857.196428571
19,1,5326.4000000001
19,1,14948.8919491525
19,1,15365.0343749998
19,1,17561.2875
19,1,13868.875
19,1,18167.40625
19,1,10289.0000000001
19,1,17365.5506756756
19,1,19198.4459459459
19,1,11479.0723684209
19,1,17222.8284883721
19,1,9508.6785714287
19,1,5668.7414772728
19,1,19586.75
19,1,11393.4329268295
19,1,13318.578125
19,1,5671.8255813952
19,1,19897.081521739
19,1,8337.9574468087
19,1,15373.1219512193
19,1,5470.82
19,1,6843.1726190476
19,1,5261.5999999999
19,1,13063.2968749999
19,1,10172.674418605
19,1,12052.0897435896
19,1,11478.296875
19,1,13166.8392857143
19,1,13760.7340909094
19,1,16422.3156250001
19,1,5392.4181818181
19,1,6012.8214285714
19,1,8214.9285714285
19,1,5179.2
19,1,7498.65625
19,1,10740.2678571424
19,1,13401.0681818182
19,1,5858.914893617
19,1,12112.1153846154
19,1,6456.1794871795
19,1,17423.6603773581
19,1,5569
19,1,7764.275
19,1,9997.4687499997
19,1,11783.1607142858
19,1,7915.5902777778
19,1,5538.6223958334
19,1,5338.4583333334
19,1,7862.9948979591
19,1,8913.2645348836
19,1,18926.3333333332
19,1,8344.9817073172
19,1,8589.9357142859
19,1,5339.4772727272
19,1,6267.6902173913
19,1,16566.0135135136
19,1,5357.0000000001
19,1,9321.4375

19,1,6895.4481707316
19,1,7752.3837209302
19,1,11203.4095744679
19,1,14480.9351851852
19,1,6795.8400000001
19,1,7518.7219387752
19,1,12166.2604166664
19,1,5715.9117647059
19,1,5563.25
19,1,13917.2019230769
19,1,6117.9505208334
19,1,8105.5297619046
19,1,5634
19,1,18735.4651162792
19,1,5449.1607142857
19,1,5715.4318181817
19,1,5608.2172619048
19,1,8666.0681818183
19,1,8787.0961538462
19,1,13402.7500000001
19,1,8795.2558139533
19,1,6440.4795918367
19,1,7895.875
19,1,6930.046875
19,1,18231.8749999999
19,1,12704.2606382977
19,1,18844.6911764704
19,1,6480.7990196078
19,1,8948.7142857143
19,1,9642.0729166666
19,1,9213.9144736842
19,1,5340
19,1,13891.05625
19,1,15124.0265957443
19,1,14827.6499999996
19,1,6753.8359375
19,1,9396.631818182
19,1,12032.2042682924
19,1,16799.1033653845
19,1,17923.2549019606
19,1,7726.900862069
19,1,5549.4245283019
19,1,19168.4999999999
19,1,11556.8384146341
19,1,13955.9054054053
19,1,6015.2333333332
19,1,11226.9800000001
19,1,5281.215625
19,1,15012.09375
19,1,13158.0773809524
19,1,12773.1121794872
19,1,7269.6486486485
19,1,5034.25
19,1,8130.1121794872
19,1,7356.03125
19,1,8535.775510204
19,1,5764.2828947369
19,1,11255.8316326534
19,1,13337.6973684211
19,1,8272.9736842106
19,1,5599.4175000001
19,1,8763.7023809523
19,1,6366.0638888889
19,1,11262.1086956522
19,1,6126.3928571428
19,1,7221.5542452827

19,1,5439.9345238096
19,1,10602.2934782609
19,1,7150.703125
19,1,12862.28515625
19,1,5345.2166666667
19,1,5126.8445945946
19,1,16484.1322115386
19,1,7814.1346153846
19,1,6241.25
19,1,15816.7980769231
19,1,5953.8181818181
19,1,18830.8645833333
19,1,12518.75
19,1,15961.1276041669
19,1,7572.2821428572
19,1,13659.9166666667
19,1,14316.4423076926
19,1,9826.0344827586
19,1,12383.5283018871
19,1,6212.6198979592
19,1,10330.5588235295
19,1,8975.8666666668
19,1,6011.5357142859
19,1,5273.8691860466
19,1,13477.5518292684
19,1,8689.7995283019
19,1,13768.2931034484
19,1,9373.4186046512
19,1,9138.2828947369
19,1,9791.2916666662
19,1,6784.1904761905
19,1,15194.9404761905
19,1,6909.6666666666
19,1,15674.4137931034
19,1,7974.2156250001
19,1,5418.1994047619
19,1,12025.4389534883
19,1,9132.4146341463
19,1,5248.8566176469
19,1,12998.2105263159
19,1,7173.3141025642
19,1,10504.6442307689
19,1,9673.3461538462
19,1,9969.0625
19,1,9756.0803571425
19,1,12075.2608695654
19,1,18762.5306122447
19,1,12029.4625000003
19,1,9250.1249999999
19,1,6889.25
19,1,5187.8720930234
19,1,8445.6517857142
19,1,5848.1829268295
19,1,14436.5500000002
19,1,9144.3482142856
19,1,6499.2259615387
19,1,8371.8538135593
19,1,10635.2321428572
19,1,16307.8472222225
19,1,8673.3809523809
19,1,5860.1348684211
19,1,17161.7142857141
19,1,10602.2177419353
19,1,8723.3529411765
19,1,7940.5166666667
19,1,7111.7279411765

19,1,6899.4677419354
19,1,9816.361111111
20,2,5424.5535714286
20,2,6493.8600000001
20,2,6200.7648809525
20,2,6481.7826086956
20,2,9434.2894736842
20,2,5270.1621621621
20,2,5353.5319148935
20,2,7561.6976744187
20,2,6787.9275000001
20,2,6028.1818181819
20,2,6445.4460784314
20,2,5256.5096153846
20,2,7867.3352272728
20,2,6442.1923076924
20,2,6158.5909090909
20,2,6436.89375
20,2,5465.6666666667
20,2,9097.3782051283
20,2,9146.3263473053
20,2,11634.955882353
20,2,6310.5
20,2,9125.7388888889
20,2,5019.4166666667
20,2,7127.6037735849
20,2,8772.125
20,2,9091.6022727272
20,2,11137.2583333334
20,2,8978.625
20,2,6802.0930232558
20,2,6688.3597560975
20,2,8815.2838427948
20,2,8892.8723404255
20,2,7557.5714285714
20,2,7951.4722222223
20,2,5108.13
20,2,6150.6666666666
20,2,5680.5528846154
20,2,6510.8365384616
20,2,7875.3993902439
20,2,6348.675595238
20,2,5939.515625
20,2,5937.9666666667
20,2,7131.6923076924
20,2,11613.0081521738
20,2,8010.65
20,2,7625.4285714285
20,2,5248.81875
20,2,7350.6428571428
20,2,6040.4893617021
20,2,7265.5406976744
20,2,6488.5737179487
20,2,6825.5182926828
20,2,5508.9735576923
20,2,7489.5984848484
20,2,7408.5137931035
20,2,10248.6437500001
20,2,6005.8546511628
20,2,8131.3679245282
20,2,9850.6902173913
20,2,9007.2916666667
20,2,7822.4038461539
20,2,11950.9056122448
20,2,5251.0982142857
20,2,6573.625

20,2,5926.4886363637
20,2,5392.8897058825
20,2,5338.2989130434
20,2,5051.3062499998
20,2,8070.0760869566
20,2,5077.0186170213
20,2,5598.0833333334
20,2,10047.5738916256
20,2,9235.9840425532
20,2,6903.75
20,2,9012.25
20,2,8729.5164473685
20,2,6410.6323529412
20,2,5542.2045454546
20,2,5724.0526315789
20,2,5686.1136363638
20,2,6515.2927631578
20,2,6636.4021739132
20,2,7828.9293478262
20,2,10438.140625
20,2,5532.625
20,2,6222.7621951221
20,2,11202.2234042552
20,2,5067.7708333331
20,2,11030.8333333332
20,2,5672.6015625
20,2,5069.4034090909
20,2,6031.4732142857
20,2,8640.3719512194
20,2,5984.1055555555
20,2,11910.6666666669
20,2,8124.6675531915
20,2,5604.7668918919
23,1,6733.125
23,1,16789.1499999998
23,1,18720.5366379316
23,1,13894.4017857143
23,1,6903.5085227274
23,1,6678.2200000001
23,1,5391.518292683
23,1,18828.0611111115
23,1,6102.4245283019
23,1,16752.7200000002
23,1,6665.2075471701
23,1,6069.1755319149
23,1,6376.1162790697
23,1,7128.8823529412
23,1,9993.1388888889
23,1,19787.010135135
23,1,9666.9583333332
23,1,11759.0558510638
23,1,9289.6666666667
23,1,6379.295
23,1,5055.6875
23,1,6958.4285714286
23,1,7375.5192307691
23,1,18247.5255681819
23,1,8827.2666666668
23,1,6225.4593023258
23,1,8804.6341463414
23,1,18540.5089820359
23,1,13783.0326086961
23,1,5415.9939024389
23,1,8635.3088235294
23,1,5290.2101063829
23,1,6604.4

23,1,15739.0625000001
23,1,17798.0892857142
23,1,5410.3035714286
23,1,15561.8035714285
23,1,10683.2250000001
23,1,7606.6189024389
23,1,8275.9281914894
23,1,10782.4875000002
23,1,5716.4903846155
23,1,7675.2173913043
23,1,5194.6439393939
23,1,8580.5357142858
23,1,16682.9861111114
23,1,9219.951219512
23,1,7360.7403846154
23,1,6401.7499999999
23,1,8153.7692307693
23,1,18558.544642857
23,1,5651.3571428572
23,1,19037.046511628
23,1,6514.4583333334
23,1,14026.6315789474
23,1,9449.3125
23,1,5545.5535714287
23,1,5102.1125
23,1,6186.2500000001
23,1,8363.3402777776
23,1,8220.3999999999
23,1,6913.6956521739
23,1,13098.2767857145
23,1,7267.6226851852
23,1,9341.36
23,1,5358.1666666665
23,1,5158.6381578949
23,1,18305.5337837837
23,1,6704.6071428569
23,1,17562.4799999998
23,1,10627.9375000001
23,1,5207.6656976743
23,1,6451.4634146342
23,1,5460.9522058823
23,1,5718.9277777776
23,1,17700.4871794871
23,1,8602.5000000001
23,1,8526.4305555555
23,1,6792.3749999999
23,1,16082.4744897958
23,1,9244.125
23,1,6955.5595238095
23,1,7657.7329545455
23,1,5594.4836956521
23,1,11556.1130952381
23,1,5785.9186046513
23,1,12352.9459459457
23,1,10894.1344696971
23,1,5595.4642857143
23,1,17507.9324324325
23,1,12573.5089285713
23,1,15902.2692307692
23,1,5176.1320754716
23,1,9694.450892857
23,1,11752.0599999999
23,1,7696.9903846154
23,1,7757.4042553193
23,1,8228.8406862745
23,1,5004.675

23,1,19975.1666666668
23,1,6591.029661017
23,1,8890.8177083334
23,1,6238.03
23,1,12250.7999999999
23,1,8254.534375
23,1,11344.75
23,1,6771.5930232558
23,1,16531.5462962964
23,1,5950.9493243244
23,1,6302.0452586205
23,1,5062.0135135135
23,1,15860.8629032259
23,1,12080.9285714286
23,1,18412.3371212121
23,1,15136.8151041667
23,1,5353.4779411765
23,1,8435.0025510204
23,1,11734.6354166663
23,1,7648.3414634146
23,1,8893.90625
23,1,5450.25
23,1,8063.8382352943
23,1,5624.5204081632
23,1,5935.0909090909
23,1,19211.4535714285
23,1,9351.875
23,1,17638.5694444441
23,1,8999.640625
23,1,12121.0281249998
23,1,10007.5789473685
23,1,7141.8632075473
23,1,7422.0493421052
23,1,12650.1602564104
23,1,15045.8815789473
23,1,17983.8857142857
23,1,13428.8055555557
23,1,8251.9034090908
23,1,5911.75
23,1,8788.9166666667
23,1,15033.8780487805
23,1,7978.2279411765
23,1,15492.5769230768
23,1,8736.25
23,1,6647.2682926828
23,1,16280.0749999997
23,1,19559.8455882352
23,1,7380.6029411765
23,1,14231.2604166667
23,1,6550.32
23,1,18615.9032258066
23,1,13369.4451219511
23,1,17654.2368421051
23,1,14082.1666666664
23,1,16798.2499999999
23,1,10162.2499999999
23,1,7726.159090909
23,1,18666.6530612243
23,1,5864.3397435898
23,1,15653.125
23,1,7425.0135135135
23,1,15371.0937499998
23,1,5659.875
23,1,9877.3333333334
23,1,9083.7410714284
23,1,18293.1944444442

23,1,6681.3863636364
23,1,14510.4899999999
23,1,6415.3488372094
23,1,5912.8717948719
23,1,13250.2196969697
23,1,5990.4243421052
23,1,17931.6989795919
23,1,5126.929054054
23,1,12319.0319148936
23,1,6476.2663043478
23,1,14832.9642857146
23,1,5035.8804347826
23,1,15103.0855263157
23,1,16719.875
23,1,15768.2034883719
23,1,7898.4553571428
23,1,19954
23,1,5941.3962765958
23,1,16594.7999999998
23,1,5084.8375
23,1,12452.3125
23,1,13578.0961538463
23,1,14337.3333333334
23,1,5119.5721153846
23,1,19394.8622448978
23,1,5158.5189393939
23,1,9238.1595744681
23,1,7771.5416666666
23,1,5191.3541666667
23,1,12078.8601694916
23,1,13853.375
23,1,10094.6577380953
23,1,6425.8750000001
23,1,5544.7975
23,1,5631.3399999999
23,1,15286.575892857
23,1,10527.975806452
23,1,5244.3414634146
24,2,6962.8947368422
24,2,7218.6923076922
24,2,6451.3422619047
24,2,6910.2837837838
24,2,5069.697368421
24,2,5653.0853658535
24,2,6161.2960526316
24,2,5397.9230769231
24,2,11000.5984848485
24,2,5045.9583333335
24,2,5846.2195945945
24,2,6771.9594594595
24,2,6492.1162790697
24,2,6904.0333333332
24,2,11219.8716216216
24,2,9604.703125
24,2,8198.3083333333
24,2,6390
24,2,7488.8414634145
24,2,5961.6425
24,2,5906.5833333334
24,2,7714.5320512821
24,2,5952.3863636363
24,2,8451.0178571429
24,2,8210.9787234043
24,2,6271.1533018869
24,2,6767.5108695652
24,2,6639.3491379311

24,2,5292.4155405405
24,2,5566.0224358975
24,2,7678.9375000002
24,2,11161.2115384616
24,2,5603.515625
24,2,5207.2734375
24,2,10310.9017857142
24,2,5151.703125
24,2,6446.5000000001
24,2,8642.5454545454
24,2,7974.7908163267
24,2,6048.125
24,2,7048.7171052632
24,2,8638.015625
24,2,7973.4358974359
24,2,5178.07
24,2,6652.5347222224
24,2,6361.5882352942
24,2,5578.6727272728
24,2,9335.8772321431
24,2,5251.9298780488
24,2,5777.9268292683
24,2,10768.8243243243
24,2,5376.0663265306
24,2,6363.4615384616
24,2,10578.8633720934
24,2,6455.3199999999
24,2,5450.59375
24,2,5658.7864583333
24,2,8864.7946428573
24,2,5135.75
24,2,5795.9807692308
24,2,11214.2169811321
24,2,5586.4607843137
24,2,8218.25
24,2,5424.3809523809
24,2,8809.709090909
24,2,5368.9943181819
24,2,8044.2741935485
24,2,7710.0431034483
24,2,6099.1121794871
24,2,7103.155
24,2,5224.4100000001
24,2,5394.3936170213
24,2,6963.6093749999
24,2,6906.93125
24,2,7606.4199999999
24,2,10286.4840425531
24,2,8038.1351351351
24,2,6787.2307692307
24,2,6597.2
24,2,5087.0227272727
21,1,5049.6875
21,1,9572.3058510635
21,1,8211.7195121951
21,1,5544.8835978837
21,1,6707.6492146597
21,1,8302.237244898
21,1,7283.75
21,1,15898.8680555552
21,1,10937.4342105264
21,1,12411.918269231
21,1,6376.5
21,1,10022.015981735
21,1,5210.0434782609
21,1,5607.44140625

21,1,10516.3909090908
21,1,18666.3076923079
21,1,7785.9128787879
21,1,7124.7448979591
21,1,5572.8265306124
21,1,7085.9767441861
21,1,19084.7625000002
21,1,8789.0540540541
21,1,8160.7117346938
21,1,8088.5
21,1,7124.2916666666
21,1,15771.4285714285
21,1,13978.0416666666
21,1,11305.18627451
21,1,7407.9250000001
21,1,8986.3942307692
21,1,5798.7727272728
21,1,7450.779296875
21,1,5685.673076923
21,1,7231.765957447
21,1,5519.126984127
21,1,6039.3125
21,1,18059.6744186046
21,1,6675.3048780488
21,1,11743.5
21,1,5037.9619565218
21,1,5634.5930232559
21,1,12227.9249999999
21,1,5054.165625
21,1,6653.4878048781
21,1,6226.9488636363
21,1,6644.625
21,1,5504.7109375
21,1,5711.4487179488
21,1,5404.1303191489
21,1,5785.125
21,1,10000.2012195121
21,1,7595.8571428572
21,1,8700.4594594594
21,1,6884.75
21,1,12791.0306603774
21,1,5138.3333333332
21,1,13642.9255319144
21,1,5017.445945946
21,1,10235.8958333334
21,1,8644.2598684211
21,1,5572.3749999999
21,1,16376.1467391306
21,1,18463.5
21,1,13888.2818181814
21,1,18990.0232558138
21,1,6905.3947368422
21,1,6101.1754385967
21,1,12618.2499999998
21,1,7145.2073170731
21,1,6975.5538461538
21,1,8521
21,1,5638.1493902439
21,1,5519.6585365854
21,1,6080.0243055555
21,1,12765.8149999999
21,1,7408.6153846153
21,1,7789.0657894737
21,1,7665.2173913043
21,1,6305.3916666667
21,1,12789.2305555556

21,1,9530.9166666667
21,1,5873.59375
21,1,8934.4310344827
21,1,6033.8776595746
21,1,5034.326923077
21,1,8276.6219512195
21,1,8262.7
21,1,8494.7045454544
21,1,6691.4042553191
21,1,17568.5384615384
21,1,6308.761904762
21,1,7748.3333333333
21,1,6340.59375
21,1,8180.9880952382
21,1,10066.6764705882
21,1,5480.3024193548
21,1,6240.3597560977
21,1,5515.9500000001
21,1,18780.238095238
21,1,5761.119680851
21,1,5997.3977272727
21,1,14704.2352941178
21,1,6273.8203124999
21,1,5336.0000000001
21,1,8194.9763513513
21,1,12448.8723404256
21,1,18525.7412790697
21,1,11880.0977272727
21,1,5102.32421875
21,1,8536.0196078431
21,1,13651.6046511628
21,1,9200.1388888889
21,1,7770.5999999999
21,1,8668.6861702129
21,1,7272.7872340426
21,1,16686.7569444443
21,1,8936.7732558139
21,1,11886.8343749997
21,1,18323.6785714287
21,1,5417.5952380952
21,1,8522.0208333333
21,1,5149.3974358974
21,1,9529.2282608696
21,1,8579.1522727273
21,1,7606.5919811319
21,1,10213.1379310345
21,1,16522.6155778894
21,1,7173.4875000001
21,1,5478.3255813954
21,1,15029.358695652
21,1,5025.8374999999
21,1,10652.6835106384
21,1,8199.0571428571
21,1,9583.0104166667
21,1,19735.5277777779
21,1,15285.4583333334
21,1,6660.7548076924
21,1,11101.3928571428
21,1,5782.125
21,1,9421.9090909091
21,1,7056.5151515154
21,1,17522.9722222224
21,1,5777.4335106384
21,1,11713.9423076919
21,1,5389.98125
21,1,6652.8749999999

21,1,9884.7547169812
21,1,10515.1046511628
21,1,6092.8515625002
21,1,9233.027027027
21,1,5082.2352941177
21,1,6964.2222222222
21,1,12135.6521739132
21,1,16311.59375
21,1,5088.7009803921
21,1,10012.6486486487
21,1,5410.6125
21,1,5527.2058823529
21,1,5535.0700000001
21,1,5918
21,1,6702.2022727272
21,1,8665.2749999997
21,1,5256.0598958332
21,1,17128.8833333336
21,1,9777.659375
21,1,6525.5
21,1,7396.3459302326
21,1,5258.03125
21,1,16919.3601694915
21,1,6368.9722222223
21,1,5724.3181818181
21,1,8181.25
21,1,6399.896226415
21,1,18400.18
21,1,8308.6216216217
21,1,9545.0510204082
21,1,15133.5714285713
21,1,6675.1020408164
22,2,7433.3787878788
22,2,8225.9062499999
22,2,5884.4522058823
22,2,5454.375
22,2,5053.552631579
22,2,8658.9807692307
22,2,8441.7613636363
22,2,5389.4407894737
22,2,5516.3256578948
22,2,5674.5487804878
22,2,5916.7954545454
22,2,8419
22,2,7455.5853658535
22,2,6104.5197368421
22,2,9220.8625000001
22,2,5355.125
22,2,6693.1375
22,2,6726.2499999999
22,2,6043.2499999999
22,2,5146.0178571429
22,2,6168.046875
22,2,7971.625
22,2,6998.858974359
22,2,7962.5208333334
22,2,6450.4666666666
22,2,7369.3026315789
22,2,5242.6166666667
22,2,8985.53125
22,2,6056.1481481481
22,2,5265.9
22,2,9870.165625
22,2,5818.3088235294
22,2,6324.7916666668
22,2,5766.6341463414

22,2,5623.7886904762
22,2,6336.1005434782
22,2,6138.8076923077
22,2,6869.2166666667
22,2,5698.7016129031
22,2,8305.2690217391
22,2,6644.1363636363
22,2,5485.7978723405
22,2,7177.0372340425
22,2,6534.1011904762
22,2,5099.8125
22,2,7711.7092391306
22,2,6588.5625
22,2,6898.5784883719
22,2,6186.8940217393
22,2,10177.1315789475
22,2,6121.3671875
22,2,6040.2305555555
22,2,8370.0771276596
22,2,8390.0803571428
22,2,9447.0172413792
22,2,5795.3392857142
22,2,6270.4879807693
22,2,11423.3557692307
22,2,6824.1047297296
22,2,10574.2678571429
22,2,5304.5052083332
22,2,5167.65625
22,2,7373.9759615384
22,2,12589.1317567568
22,2,6375.8536585365
22,2,5766.5425531915
22,2,6911.7261904762
22,2,8430.6646341464
22,2,10419.7000000002
22,2,7110.6693548387
22,2,5775.29
22,2,6578.8727272728
22,2,9443.4281914893
22,2,5055.6551724137
22,2,7325.8974358974
22,2,5716.4438775511
22,2,10533.5975609755
22,2,7521.3109756097
22,2,9436.4947916666
22,2,8272.8918918919
22,2,6372.6465517242
22,2,6008.25625
22,2,8692.6
22,2,5715.8676470588
22,2,7245.1941489361
22,2,6551.0857142857
22,2,5657.4891304348
22,2,6479.5
22,2,10648.8378378379
22,2,7877.8970588236
22,2,8974.8648648648
22,2,13012.5887096774
22,2,7681.7407407408
22,2,5616.4460227274
22,2,6838.0510204083
22,2,6340.0673076923
22,2,7263.9418604652
22,2,7044.2058823528
