# Supplementary material for: Selective Moonlighting Cell-Penetrating Peptides
Source: Pharmaceutics. 2021 Jul 22;13(8):1119. doi: 10.3390/pharmaceutics13081119 (PMC8400200; doi:10.3390/pharmaceutics13081119)
Supplement: Supplementary file 1 [file pharmaceutics-13-01119-s001.zip › TableS3.pdf]

Supplemental Data for the work entitled "Selective Moonlighting Cell-Penetrating Peptides" by Rafael Morán-Torres, David A. Castillo González, Beatriz Aguilar Maldonado, Maria Luisa Durán-Pastén, Susana Castro-Obregon & Gabriel Del Rio

Table S3. Median values of normalized fluorescence intensity for activatable peptides

		Peptide	
**Peptide**	**Cell line**	**TatNep**	**TatNepNoCPP**
**TatNep**	HEK293T		2.8
	HEK293T-NEP		2.4
	HeLa		3.7
**TatNepNoCPP**	HEK293T	2.4	
	HEK293T-NEP	1.6	
	HeLa	3.5	
**D-TatNepNoCPP**	HEK293T	2.8	7.1
	HEK293T-NEP	1.6	2.7
	HeLa	1.9	7.0
