# Supplementary material for: Selective Moonlighting Cell-Penetrating Peptides
Source: Pharmaceutics. 2021 Jul 22;13(8):1119. doi: 10.3390/pharmaceutics13081119 (PMC8400200; doi:10.3390/pharmaceutics13081119)
Supplement: Supplementary file 1 [file pharmaceutics-13-01119-s001.zip › TableS4.pdf]

Supplemental Data for the work entitled “Selective Moonlighting Cell-Penetrating Peptides” by Rafael Morán-Torres, David A. Castillo González, Beatriz Aguilar Maldonado, Maria Luisa Durán-Pastén, Susana Castro-Obregon & Gabriel Del Rio

Table S4. Fluorescence intensity data measuring the internalization of activatable CPP in mammalian cells.

HEK293_10_p1,HeLa_10_p1,HEK293_5_p1,HeLa_5_p1,HEK293_1_p1,HeLa_1_p1,HEK293_10_p2,HeLa_10_p2,HEK293_5_p2,HeLa_5_p2,HEK293_1_p2,HeLa_1_p2,HEK293_10_p4,HeLa_10_p4,HEK293_5_p4,HeLa_5_p4,HEK293_1_p4,HeLa_1_p4

9.737,8.153,4.689,8.462,3.93,3.116,31.784,39.225,24.748,47.146,3.718,9.127,11.847,18.006,5.098,7.548,3.196,17.064
5.226,9.27,5.137,10.143,4.289,3.59,36.927,9.923,15.417,47.533,5.448,8.603,9.57,17.187,7.883,8.2,1.585,5.495
5.974,9.366,2.972,15.857,2.284,7.582,28.209,65.356,30.343,18.476,6.818,2.331,7.949,9.515,20.174,18.66,6.023,10.99
8.157,7.367,7.445,7.354,1.211,9.509,43.636,40.097,14.835,46.861,5.293,8.34,12.82,21.783,9.54,37.313,3.395,7.959
7.483,11.227,2.456,16.271,2.943,2.354,15.899,11.526,27.445,23.924,3.138,8.156,7.641,11.855,8.928,6.953,5.574,4.079
17.198,17.264,5.911,17.58,3.299,4.533,19.77,6.043,22.194,44.164,3.433,4.473,11.514,14.38,8.013,17.202,2.241,8.773
11.518,8.423,3.972,7.123,3.445,5.431,13.652,46.347,24.071,30.425,5.753,10.416,8.598,20.224,8.714,5.525,2.396,10.16
10.816,15.067,4.456,12.314,1.907,4.451,24.594,23.97,24.292,24.471,5.714,9.377,16.177,12.104,8.542,6.997,3.155,7.883
5.939,7.884,9.148,10.015,4.819,4.345,28.592,21.571,21.274,44.727,4.872,9.295,20.095,10.636,5.113,6.228,2.309,4.183
9.519,9.38,6.946,24.844,2.633,4.061,31.75,66.149,16.434,41.58,5.147,22.466,3.22,11.132,8.569,25.759,2.819,8.289
13.721,8.455,4.628,8.652,1.339,6.96,29.275,30.514,16.253,32.746,2.142,7.448,7.873,13.722,9.406,2.993,5.123,6.994
26.795,13.048,3.261,9.54,3.293,6.154,22.516,57.686,19.104,12.493,5.592,8.087,3.822,23.485,9.575,8.888,2.96,11.638
8.181,5.806,4.455,6.238,2.409,9.195,37.289,29.402,14.413,23.594,3.435,5.755,3.161,11.248,2.359,16.994,1.831,5.497
5.664,12.911,2.075,4.496,2.718,5.279,38.58,40.893,25.952,11.61,4.245,7.675,8.037,3.245,5.718,3.789,2.631,12.375
8.147,4.884,3.128,10.11,2.932,7.693,37.136,50.338,20.287,36.374,5.77,6.256,11.021,7.962,6.528,21.587,2.696,8.61
6.283,12.914,8.506,6.988,3.63,2.879,10.098,28.427,18.608,39.087,4.022,9.263,10.589,32.871,4.202,12.723,1.663,14.971
8.57,9.178,6.33,8.369,3.651,3.867,20.989,53.384,35.271,36.771,5.49,4.664,6.378,28.595,7.453,13.205,1.684,13.611
5.852,8.927,5.833,9.551,3.599,1.138,12.042,28.63,10.627,9.658,4.274,7.734,18.858,15.624,12.588,22.327,2.612,5.853
9.4,4.917,6.724,7.713,2.51,7.97,28.112,66.012,11.41,16.272,9.413,7.253,2.789,14.933,8.489,5.388,3.958,4.814
11.523,11.61,3.531,12.196,4.207,4.374,27.304,62.978,7.091,57.018,4.301,11.093,5.831,15.899,8.427,10.218,2.02,14.839
16.046,16.898,3.104,8.183,1.408,4.024,26.789,46.968,18.156,25.635,7.548,8.565,7.085,21.661,12.361,5.486,2.102,4.199
7.634,9.414,9.48,3.876,2.838,5.474,29.413,40.777,14.168,41.697,4.183,12.611,4.617,16.506,4.218,5.852,2.097,14.786
8.881,10.186,4.478,16.765,1.704,4.959,34.858,5.324,16.143,36.477,3.398,14.063,11.195,19.509,9.887,20.399,3.352,23.017
14.404,17.942,7.564,16.659,3.094,2.24,20.275,43.967,29.661,11.23,9.458,4.941,11.078,16.108,2.824,20.936,3.583,7.159
7.025,6.353,4.88,12.091,2.383,4.863,27.126,32.063,10.021,8.606,3.384,10.218,6.485,13.115,9.522,12.648,1.315,9.123
9.683,6.725,2.422,11.736,1.761,7.845,43.306,32.947,46.229,26.245,8.505,5.388,8.103,6.844,10.677,14.101,4.26,5.494
19.819,7.58,5.953,10.299,5.395,1.96,43.825,43.082,39.811,22.346,3.804,5.779,6.177,6.52,5.708,16.316,2.686,14.253
16.072,8.378,3.532,7.786,1.509,6.66,20.762,30.403,52.103,11.363,2.955,9.48,6.88,14.388,10.175,17.053,2.236,6.163
8.778,9.582,5.205,9.965,1.849,1.412,32.585,43.121,15.95,20.597,8.793,5.817,10.149,16.439,13.514,4.351,4.05,12.492
5.95,9.94,2.212,16.212,1.287,4.206,30.339,67.68,25.97,56.679,2.587,12.489,14.83

3,8.008,7.49,11.933,2.696,7.441
15.802,11.018,3.996,6.872,3.056,3.946,40.265,6.988,18.352,38.295,2.846,5.843,8.
574,8.932,2.162,13.26,3.864,11.053
11.693,12.281,4.785,20.675,1.579,2.872,69.688,5.718,13.92,50.333,6.057,4.923,11
.512,27.889,16.12,27.439,4.219,16.999
9.112,9.782,2.253,8.859,2.493,6.944,20.172,42.03,17.695,53.083,9.104,6.308,5.33
5,10.153,9.76,14.23,2.382,8.582
12.398,8.734,7.327,12.85,3.832,9.899,21.392,14.121,20.444,41.036,4.392,6.238,5.
93,15.318,7.374,9.169,1.998,11.879
19.324,8.764,4.127,12.114,3.501,5.144,21.402,54.632,22.289,28.122,5.889,14.223,
6.514,3.782,6.179,7.701,4.12,16.614
10.367,8.468,2.8,11.101,2.457,9.169,33.113,39.553,6.392,39.983,8.574,11.61,4.88
3,11.652,11.031,4.518,2.15,4.851
9.587,8.752,5.518,8.256,1.96,7.486,20.652,19.604,24.754,14.315,4.169,13.154,9.8
62,7.63,3.993,9.059,2.799,9.385
10.638,16.986,5.56,7.892,5.598,4.002,25.167,47.15,21.959,60.749,14.267,5.801,5.
931,6.832,9.228,24.13,2.571,12.052
5.649,16.104,3.834,10.405,2.587,5.35,28.098,51.143,21.676,61.601,5.262,6.719,10
.433,31.706,10.736,5.614,3.946,8.359
14.008,8.474,5.704,6.603,1.437,3.566,29.145,56.742,13.498,31.365,3.775,4.777,6.
979,13.769,6.986,17.72,1.895,14.064
4.206,24.122,7.971,10.338,2.473,5.721,55.64,9.675,11.001,26.762,4.916,7.78,11.4
23,11.921,4.144,18.476,2.592,10.357
8.133,5.699,4.567,4.251,2.937,4.096,34.711,56.652,11.916,62.799,3.549,10.988,4.
632,10.787,8.147,9.737,1.809,17.17
19.852,10.077,3.014,25.092,3.208,3.566,17.01,63.375,14.947,8.56,11.207,6.082,10
.978,9.409,4.905,6.893,2.734,7.127
13.57,9.156,2.668,10.787,3.652,1.937,21.426,42.834,10.849,25.102,5.568,5.798,13
.444,11.391,7.852,16.035,2.449,5.484
8.027,8.713,1.921,72.563,2.18,5.344,13.014,59.262,19.646,14.779,7.181,5.688,13.
998,12.434,8.232,5.208,3.414,10.948
8.397,26.725,8.015,7.337,3.297,3.56,28.175,67.502,27.635,57.274,7.434,12.911,23
.848,12.879,6.322,20.859,4.16,9.493
11.543,8.641,4.576,6.69,1.751,5.996,12.315,41.509,19.489,50.946,9.527,6.852,14.
294,16.218,10.353,7.625,3.23,5.525
37.181,12.042,2.76,7.092,2.176,4.132,23.915,29.506,32.538,56.785,3.796,6.051,9.
454,11.836,9.479,18.975,3.134,18.161
5.679,5.849,7.104,5.486,2.947,1.607,36.065,31.318,25.291,50.796,3.907,16.212,13
.261,5.525,10.875,16.283,1.469,3.993
12.924,9.66,2.166,11.838,2.477,4.8,13.23,63.725,24.249,45.391,3.299,6.507,9.788
,13.422,8.624,7.326,2.117,13.733
8.261,12.826,4.969,9.429,1.541,5.049,39.246,11.429,33.061,26.778,9.475,6.242,9.
368,20.084,7.955,20.318,3.901,4.442
7.167,7.962,10.349,6.534,2.857,5.344,29.852,45.101,18.738,7.548,4.152,13.783,8.
384,19.635,3.967,24.5,2.744,20.887
12.701,10.705,3.991,6.46,3.465,8.231,40.118,6.353,16.067,17.926,6.692,11.661,7.
882,23.988,3.599,9.396,3.609,6.043
14.307,6.573,2.869,13.731,2.26,3.503,34.888,41.639,11.625,10.045,4.009,7.589,8.
602,19.769,4.312,9.675,1.95,14.198
13.93,16.134,3.121,12.629,1.544,8.172,28.94,65.198,8.309,9.606,3.122,6.104,11.9
38,13.557,10.727,5.267,3.835,16.648
15.239,7.298,4.516,7.836,3.925,8.999,24.632,7.883,15.839,8.062,5.803,8.802,9.40
3,15.871,11.889,9.126,6.046,9.881
5.392,11.183,3.87,15.107,1.917,5.602,24.754,8.91,25.416,47.069,3.751,8.872,14.4
15,12.575,5.823,17.758,2.203,14.435
31.394,9.727,4.714,13.433,2.838,7.978,28.343,44.327,12.925,45.38,4.5,8.256,5.75
7,18.399,4.972,6.174,1.689,17.202
13.643,7.294,4.976,11.325,2.422,4.153,16.544,45.764,18.188,58.412,7.037,5.504,1
0.206,22.491,5.174,8.421,1.462,3.955
8.621,11.73,5.059,8.56,8.043,2.783,28.12,46.276,14.203,29.582,2.502,11.111,7.20
8,13.048,8.12,19.692,3.738,3.919
12.304,10.609,4.044,10.286,3.283,3.318,41.848,54.529,20.496,62.496,7.947,10.242
,5.97,10.208,6.713,13.231,1.414,10.672
6.068,13.205,12.319,8.57,3.893,4.284,24.706,9.217,27.255,64.687,3.935,4.496,11.

921,11.69,9.282,8.306,3.05,23.747
5.564,5.108,5.236,4.995,4.342,3.243,24.686,59.872,15.748,31.797,2.644,10.352,16
.609,19.375,8.27,8.91,2.758,23.564
5.135,16.918,3.737,5.533,3.102,5.696,44.152,62.706,39.527,19.823,6.452,7.045,10
.9,14.332,4.003,7.872,1.643,8.465
10.861,12.967,8.254,5.539,1.343,3.834,20.478,8.736,20.63,26.065,11.432,9.356,8.
637,18.631,4.063,14.986,3.267,9.701
40.614,5.376,2.946,11.727,3.029,9.198,25.1,58.441,18.581,16.659,7.841,8.524,10.
519,10.838,11.701,35.063,2.873,20.544
13.558,10.057,3.32,10.482,3.263,5.068,45.369,49.732,25.851,9.263,2.867,11.557,8
.446,8.48,8.857,8.291,1.831,12.493
6.629,11.297,5.931,9.015,2.806,4.652,23.046,38.873,21.233,27.382,7.393,3.397,10
.483,16.667,3.532,19.676,1.247,9.329
9.121,7.997,3.335,10.697,3.368,4.332,40.642,58.44,21.701,10.218,3.027,7.125,11.
014,10.455,5.608,8.124,2.043,24.219
25.832,5.533,3.744,10.289,3.503,4.108,57.153,33.429,25.278,36.512,5.54,9.984,6.
059,17.526,11.635,4.114,1.684,11.42
9.195,18.422,3.363,6.997,3.676,3.775,37.759,65.414,14.049,12.629,6.027,6.93,7.9
77,12.219,7.534,14.082,2.765,12.091
7.431,7.883,3.819,14.782,1.797,2.946,25.071,37.747,19.102,16.283,6.569,12.035,1
4.039,16.055,8.248,10.12,2.11,13.298
8.19,23.504,3.849,9.683,1.956,7.13,32.309,48.959,11.495,16.218,9.238,7.852,10.3
01,11.55,9.383,9.431,2.837,10.592
7.424,8.723,5.231,12.198,1.303,3.737,25.162,33.375,18.564,29.31,5.171,11.544,7.
033,21.215,6.957,23.164,2.878,5.324
10.276,5.953,4.602,13.476,1.951,3.682,17.613,44.727,27.882,25.236,6.009,9.994,7
.012,10.61,12.083,18.991,1.134,7.389
14.549,5.388,5.352,8.123,3.291,4.493,19.347,5.799,21.617,17.75,3.625,8.403,9.94
3,10.772,3.455,15.842,4.324,11.267
13.649,8.678,2.064,8.2,1.52,7.401,30.716,52.979,33.952,22.83,4.218,11.189,8.99,
13.419,9.718,3.297,1.926,2.594
8.233,12.312,2.57,14.867,1.49,10.176,22.993,29.016,19.234,50.268,5.391,24.844,1
2.894,13.54,5.596,9.217,2.551,16.784
7.138,5.797,4.605,17.952,3.552,5.315,26.696,3.962,14.596,41.387,5.152,7.294,10.
383,14.791,6.032,13.927,2.221,7.653
11.152,11.554,3.307,4.085,3.825,4.437,25.461,51.462,39.45,35.022,5.806,6.462,9.
972,6.802,8.784,7.605,4.358,16.212
5.786,10.042,5.557,9.102,1.627,2.159,28.056,17.592,6.22,12.298,5.109,5.123,6.76
2,91.317,9.435,8.922,2.676,6.567
8.448,19.622,3.216,10.271,2.006,7.955,23.483,54.965,38.586,35.418,9.043,8.446,2
6.86,9.902,8.11,2.459,1.52,18.236
7.825,12.499,4.349,8.75,2.57,1.516,30.276,37.929,7.254,43.783,4.184,6.409,6.054
,6.548,7.973,11.363,3.19,22.715
10.089,6.96,4.353,12.996,2.201,5.173,33.56,7.778,19.246,48.831,4.967,10.875,10.
166,10.221,9.844,23.863,1.887,9.875
7.06,6.606,4.886,7.675,6.085,6.27,27.411,23.151,13.505,6.104,4.668,6.727,11.933
,13.669,11.929,21.34,1.947,3.102
8.329,13.8,4.745,10.603,1.553,5.186,19.386,19.622,13.987,38.832,4.21,5.66,20.23
7,12.264,5.969,23.6,2.88,6.782
10.162,7.147,2.445,4.415,1.061,6.69,20.082,32.139,54.996,55.585,2.599,9.919,3.8
11,5.195,10.468,7.157,4.188,19.967
4.047,16.166,3.149,9.017,3.075,2.199,6.38,60.717,22.845,45.69,6.997,6.556,2.446
,7.62,6.129,14.115,2.423,9.658
9.432,11.55,4.043,23.002,1.741,4.079,21.849,19.662,21.009,56.743,3.37,6.985,6.8
27,11.427,10.064,8.437,1.824,10.091
8.947,7.537,3.939,5.45,2.178,7.619,40.463,44.564,15.219,42.649,4.137,6.221,8.61
8,3.151,4.006,17.415,3.485,1.991
6.416,8.105,13.174,12.731,2.036,4.664,11.942,40.725,25.934,5.388,3.428,6.944,4.
623,8.832,12.879,17.484,2.528,3.628
4.901,31.461,1.725,9.727,2.341,8.462,12.815,57.262,13.867,33.793,6.621,6.071,10
.645,18.221,11.458,6.268,3.032,11.817
6.621,3.82,3.029,8.373,3.74,4.634,36.388,66.828,44.097,9.705,3.559,10.54,10.283
,17.31,7.711,3.343,3.184,13.276
41.12,29.497,2.77,5.243,1.274,5.586,32.188,62.186,17.94,35.632,8.074,6.048,12.4

86,15.304,3.019,14.059,4.943,6.618
10.801,8.606,3.362,13.048,3.679,6.981,35.435,30.869,19.111,23.017,8.612,10.118,
4.051,26.716,9.219,10.289,2.351,11.073
17.206,9.923,4.5,11.27,1.895,9.97,8.74,36.305,17.898,8.48,3.189,13.295,13.636,1
5.324,11.616,38.724,1.162,10.078
5.981,9.895,3.749,8.178,1.743,6.888,30.115,31.463,27.248,41.321,8.25,4.253,4.88
1,23.017,6.563,5.278,3.808,4.516
9.6,8.727,2.443,10.045,3.813,1.697,32.154,11.363,4.88,48.658,3.714,6.914,12.532
,9.377,8.698,8.925,1.852,6.714
6.367,11.073,3.382,4.242,3.377,2.567,27.758,36.163,26.505,44.559,4.102,10.294,1
1.787,5.519,9.783,18.745,2.777,8.966
13.509,5.082,4.554,15.871,1.732,5.275,27.16,38.277,26.729,29.533,4.684,5.994,10
.631,12.516,7.547,7.864,1.833,10.073
10.982,17.029,4.232,18.422,3.071,5.02,26.038,55.172,19.525,50.714,10.14,12.539,
10.02,13.843,8.24,4.198,2.892,9.723
13.811,8.66,2.961,5.812,3.201,4.01,35.109,63.335,30.285,58.729,7.33,4.044,9.365
,10.698,6.417,15.732,2.863,5.799
5.822,7.465,2.691,16.535,6.176,1.776,53.532,56.439,16.487,56.212,3.777,19.622,7
.422,16.916,9.405,14.887,2.001,3.923
69.096,6.031,3.242,15.339,2.691,3.388,14.252,44.109,20.449,63.074,3.437,8.439,8
.879,25.192,15.869,7.001,2.425,7.204
12.38,25.057,4.286,7.964,1.248,5.92,57.996,44.265,12.328,17.741,4.785,4.66,6.20
7,17.326,17.76,12.196,1.944,8.315
25.318,10.142,5.975,5.278,2.96,3.468,31.189,7.476,35.121,8.736,7.592,8.34,5.585
,8.899,12.507,12.374,3.667,9.533
8.466,17.109,6.495,29.497,5.105,7.055,21.16,57.956,16.954,35.444,5.032,7.914,8.
141,10.561,7.12,24.844,1.277,3.333
5.61,5.82,3.771,20.306,2.917,4.797,23.611,31.517,15.973,63.476,4.629,7.884,18.5
29,5.839,10.408,26.275,1.802,4.486
9.492,100.205,3.836,6.983,2.233,4.479,16.534,54.934,19.777,37.679,5.258,13.856,
9.763,14.356,7.943,15.067,1.489,20.543
10.874,15.899,2.17,29.398,5.613,7.111,23.253,61.822,21.708,46.808,3.769,5.103,5
.967,20.805,11.593,23.017,3.873,12.341
15.556,5.291,9.465,16.035,3.169,12.634,40.079,8.455,19.91,13.144,5.545,8.824,7.
34,14.479,9.034,7.226,3.722,13.261
30.139,8.149,2.718,10.685,1.822,5.339,47.252,8.764,13.197,53.429,9.354,3.708,9.
717,15.474,5.361,17.529,3.589,8.629
21.58,13.433,2.776,23.017,3.327,5.078,42.026,41.554,12.267,32.635,3.58,13.294,9
.513,12.099,3.859,5.02,4.275,11.853
8.582,15.955,4.807,53.674,2.315,6.674,40.896,50.535,25.984,60.603,2.752,7.393,6
.413,10.228,3.607,23.342,1.963,7.741
54.406,10.289,5.515,6.042,2.928,6.549,28.64,31.215,63.675,7.588,5.335,2.612,11.
062,12.175,6.648,23.647,1.997,10.965
32.811,12.618,4.532,16.283,2.419,7.244,19.046,60.678,21.231,30.93,5.055,61.911,
9.488,17.96,7.335,6.238,3.567,11.819
5.222,9.419,3.712,8.386,1.58,2.766,33.809,40.277,33.129,13.455,6.235,7.192,6.71
9,11.026,5.191,14.171,2.006,16.837
9.111,5.259,1.968,4.802,2.827,7.447,30.767,39.018,19.545,43.627,4.089,6.444,15.
038,12.309,14.494,15.916,2.261,10.876
10.117,9.083,3.58,8.315,4.374,3.831,17.833,17.222,29.061,9.217,3.665,9.941,13.9
71,15.516,7.61,16.731,3.076,2.445
95.022,14.096,6.337,6.764,3.615,3.317,18.581,15.476,20.781,35.956,3.27,9.129,14
.332,11.16,11.272,20.896,1.985,7.08
6.087,5.997,5.539,8.176,1.682,5.644,34.101,43.591,11.816,52.336,7.024,6.749,11.
476,9.778,5.838,7.557,2.942,7.55
23.552,12.738,4.079,9.043,2.928,4.583,38.272,59.486,29.899,35.35,3.059,6.161,3.
394,12.629,12.377,7.302,2.084,9.576
13.243,9.996,3.937,6.594,2.385,3.275,33.889,39.945,2.556,41.33,4.401,16.104,10.
465,17.268,5.568,27.089,2.104,16.765
7.93,10.045,3.788,11.487,2.184,4.148,28.4,47.509,20.578,9.494,4.578,7.417,8.32,
11.996,7.528,5.208,1.975,8.06
9.318,21.858,3.498,16.134,2.2,4.971,32.37,42.465,59.673,23.564,4.563,8.337,8.76
4,14.156,7.318,23.551,1.45,2.4
7.542,5.199,3.229,10.804,2.618,5.626,13.17,37.302,15.705,17.534,4.412,13.032,8.

339,13.564,3.386,16.396,3.654,14.982
8.269,13.847,2.372,6.78,2.209,1.452,30.793,47.32,28.812,39.082,3.413,8.736,8.731,26.541,20.467,14.121,3.513,12.204
3.51,11.61,6.076,13.055,8.265,4.191,28.996,8.092,21.852,108.338,4.822,11.325,24.747,13.011,12.813,24.598,3.58,4.859
11.731,4.079,4.798,9.279,2.518,5.273,16.508,10.091,11.551,64.182,2.294,11.61,2.551,6.359,10.95,15.187,1.503,10.603
6.551,7.711,3.208,7.543,4.124,4.06,33.113,33.854,17.558,6.45,3.503,5.991,7.847,8.683,11.718,10.965,2.253,8.947
6.919,10.948,5.192,9.658,3.186,11.824,17.874,36.77,20.705,36.013,2.958,11.297,5.176,18.09,5.521,8.159,5.219,11.414
8.541,5.687,4.438,3.897,2.597,5.912,34.103,53.089,37.561,18.613,2.801,5.846,4.964,12.953,11.384,10.796,1.666,6.44
24.756,7.604,4.461,18.894,2.683,5.142,26.49,44.302,30.307,17.952,4.568,12.996,3.054,7.514,10.122,5.108,3.124,12.339
13.618,18.613,3.481,12.106,2.342,3.332,30.192,43.708,8.211,57.301,6.652,6.149,5.822,24.084,5.532,12.698,1.626,12.629
9.098,92.554,4.56,7.05,2.738,5.958,27.842,54.918,45.42,16.104,4.991,8.197,10.914,11.768,9.741,3.862,2.897,6.433
8.875,7.667,1.407,15.067,1.81,4.381,32.631,33.743,26.803,40.131,5.707,9.542,8.182,8.103,7.822,15.316,1.53,6.808
6.614,16.283,8.968,23.988,1.926,3.243,35.29,58.215,22.763,51.782,4.555,10.965,10.398,11.008,3.543,5.894,2.215,8.092
8.596,2.616,6.277,5.915,3.401,7.289,50.247,24.209,17.054,36.058,3.181,7.269,12.761,9.606,4.553,5.594,1.94,15.057
10.212,8.367,3.532,9.652,2.464,6.666,18.4,40.861,34.408,13.473,2.08,14.34,11.985,21.21,11.592,10.504,3.144,10.068
5.247,18.35,2.694,5.108,4.288,5.157,20.427,30.517,28.191,42.91,4.015,4.185,9.779,13.518,9.821,6.987,2.787,7.124
8.259,6.043,4.027,5.398,2.91,3.958,32.751,47.619,34.865,13.205,3.826,24.219,12.936,13.07,5.395,6.018,1.216,7.548
9.753,8.91,6.457,11.064,1.687,3.849,19.042,28.525,19.012,12.571,6.332,14.191,7.763,3.672,7.559,8.916,1.779,6.598
23.029,9.889,5.215,9.263,1.924,4.982,22.073,53.493,29.927,25.93,2.595,12.483,7.715,20.133,11.296,6.622,5.297,4.513
12.244,6.355,4.268,16.218,2.914,8.98,32.693,11.325,34.709,31.576,5.812,8.313,4.791,25.243,7.037,6.493,1.834,15.606
9.82,4.715,5.58,7.647,3.547,1.426,17.839,23.019,32.312,10.965,7.007,9.169,8.327,13.255,4.485,8.508,3.948,17.222
8.965,10.603,7.436,12.249,1.77,5.347,10.061,58.97,22.435,31.496,8.24,4.814,6.219,13.642,9.661,13.037,2.616,27.089
7.102,4.422,3.636,13.368,1.493,7.514,44.939,45.421,3.895,12.309,4.686,10.87,5.166,11.834,4.413,3.943,3.364,1.756
8.462,10.281,4.421,10.363,3.95,9.157,18.808,48.149,25.568,13.927,6.143,5.194,3.397,11.14,6.756,18.613,1.355,6.326
17.042,80.873,4.595,5.628,3.292,6.835,28.283,20.887,23.722,9.015,5.856,7.548,6.642,12.938,20.253,12.708,1.873,15.768
7.955,14.833,3.264,12.053,2.065,2.226,31.737,46.724,29.64,50.664,5.107,10.377,5.829,7.234,11.664,19.622,2.05,8.65
7.716,6.067,5.956,6.016,2.132,5.107,22.12,33.885,27.946,33.776,3.555,2.722,8.303,11.837,1.98,38.758,2.155,8.91
5.279,12.217,4.047,13.788,3.105,4.077,54.787,31.881,23.14,43.628,5.634,5.426,2.78,13.114,8.623,14.419,1.427,12.117
6.612,8.345,8.314,9.648,3.341,2.885,17.953,51.825,34.715,7.092,3.085,3.977,8.535,13.897,12.767,10.978,1.866,2.591
27.326,12.112,6.129,7.729,2.369,5.893,19.894,52.708,27.949,26.139,5.829,10.289,2.602,11.952,3.269,3.153,2.952,15.732
5.91,3.292,4.127,5.131,3.666,2.39,31.86,65.398,14.39,35.122,2.892,16.105,5.476,108.26,5.691,10.493,2.371,5.278
9.934,6.975,3.402,19.194,3.233,1.303,30.132,42.939,15.533,42.667,8.293,10.271,9.488,17.309,7.195,6.342,3.431,16.607
13.632,14.237,5.74,7.299,2.853,2.968,24.325,64.157,8.675,54.784,3.79,4.78,10.234,20.927,10.401,11.817,2.086,14.823
39.082,26.456,3.756,10.184,3.983,3.024,20.447,35.511,15.223,16.167,3.833,6.471,

14.578,6.891,9.586,6.676,1.114,4.242
11.693,5.951,1.586,12.341,1.764,3.804,12.591,68.357,14.751,32.184,3.963,4.422,5.951,11.365,13.393,12.911,2.305,6.297
21.75,10.251,5.851,13.613,3.375,3.857,34.038,61.711,22.503,12.489,5.324,6.648,9.83,10.313,9.923,20.887,1.354,15.044
17.112,5.718,3.458,4.516,2.315,6.547,17.978,13.647,21.413,49.343,3.839,8.429,5.437,10.171,11.798,19.668,1.372,8.208
9.291,7.625,4.944,6.253,1.764,5.813,28.931,42.369,39.962,44.73,4.795,9.978,13.053,18.86,7.763,4.516,1.782,6.56
8.043,6.45,4.353,5.657,2.001,9.489,24.493,6.444,21.857,18.131,3.481,6.439,5.972,9.01,8.592,9.623,1.632,9.726
16.959,4.171,2.01,7.715,2.115,8.06,24.919,10.358,27.132,19.906,3.621,8.539,8.594,16.153,4.946,4.223,3.268,6.446
14.306,9.551,2.482,13.783,3.524,1.378,28.619,61.669,14.258,49.929,4.247,4.104,13.15,16.927,9.039,18.705,1.336,12.755
25.167,6.311,6.224,8.878,2.867,2.091,52.786,63.054,7.269,41.408,4.244,8.61,4.443,7.63,8.039,11.053,6.683,17.81
6.28,12.901,3.333,5.799,2.562,2.323,24.899,40.015,14.949,42.229,5.386,8.611,4.823,7.608,17.844,8.48,4.156,10.668
3.402,8.502,3.909,9.527,2.073,3.42,27.078,44.872,15.918,6.043,4.98,7.84,4.392,17.16,13.048,8.61,2.246,8.606
14.644,12.024,5.389,7.928,3.036,6.804,34.408,61.564,26.761,37.094,4.616,20.397,9.822,12.662,14.331,18.71,3.488,16.025
7.383,64.913,4.26,8.37,1.364,4.526,39.519,56.163,19.958,52.825,4.194,16.659,7.757,4.085,8.437,19.052,3.443,15.433
11.872,4.584,5.44,2.909,1.349,7.738,16.339,51.824,6.681,5.486,4.769,7.818,8.963,14.243,7.372,5.718,2.519,15.476
11.261,6.873,5.154,13.183,5.06,5.631,31.262,67.296,20.989,33.485,10.009,12.313,12.546,6.768,4.101,25.222,3.733,3.154
8.854,8.549,3.247,9.511,2.466,3.922,27.843,45.622,30.953,66.691,4.095,8.061,8.463,16.191,8.872,4.49,2.803,8.736
10.085,8.423,1.669,8.61,2.173,2.865,10.517,60.835,7.503,56.161,8.128,8.673,5.747,11.552,8.38,17.502,3.209,4.189
6.564,10.923,4.545,9.175,2.182,4.085,30.145,50.426,32.998,36.029,7.501,10.121,7.673,18.666,8.25,4.075,2.405,9.027
6.836,11.039,5.877,5.921,1.645,5.15,42.219,31.967,10.949,30.91,4.126,6.477,13.541,7.903,6.305,5.533,2.483,4.684
13.566,7.765,3.665,8.156,3.467,5.574,29.569,12.465,38.239,51.228,4.003,5.712,4.737,19.383,7.079,6.33,1.974,12.747
13.151,5.961,3.253,8.82,2.119,7.166,28.705,30.198,24.519,33.243,3.556,13.262,6.058,9.463,6.662,40.457,0.968,13.171
27.645,18.705,3.511,12.298,4.474,10.911,20.057,21.874,29.242,10.184,4.845,8.91,9.115,10.524,9.824,8.256,3.46,16.008
8.238,13.511,3.506,11.585,2.225,6.111,53.214,63.828,24.315,23.281,2.817,7.317,5.73,16.565,10.056,5.831,3.068,10.978
16.259,12.83,5.028,7.179,2.005,5.226,26.631,53.472,17.411,13.433,5.75,8.373,6.951,15.87,10.353,4.524,1.687,4.036
6.051,10.665,6.231,18.613,2.113,3.774,39.947,47.297,24.067,58.981,3.504,9.606,4.014,11.704,10.714,17.205,1.875,16.832
14.369,9.217,4.172,9.445,2.809,2.832,55.036,66.041,29.966,42.233,2.66,9.439,4.378,14.665,9.737,16.104,1.425,20.275
7.87,6.716,4.657,8.217,2.612,5.817,42.229,47.575,27.109,59.339,3.495,14.327,7.812,11.974,8.457,16.731,1.68,5.81
6.846,13.927,2.24,11.059,2.665,5.048,39.255,71.393,22.378,36.087,4.04,11.681,7.323,6.4,4.353,6.104,1.871,11.422
13.442,8.48,3.711,10.362,3.915,1.737,28.583,8.777,16.081,36.763,14.683,6.575,9.527,11.988,6.933,26.041,1.92,7.038
11.184,6.337,5.178,7.37,1.733,4.554,27.676,7.017,18.006,63.248,2.516,7.411,10.5,18.12,8.253,13.444,1.621,2.511
16.645,14.828,3.778,9.341,2.69,4.208,22.972,8.399,32.927,15.509,2.184,8.718,14.531,11.615,8.707,12.054,4.268,4.795
13.377,14.803,4.99,13.473,1.35,3.954,21.959,24.219,13.122,50.07,5.811,10.48,6.378,7.715,8.132,21.77,1.74,14.121
5.869,9.07,4.419,18.236,2.419,1.4,54.018,40.182,29.708,33.19,11.168,5.79,12.373

,13.123,8.791,13.613,1.704,11.068
9.178,4.437,5.362,7.254,1.535,6.766,19.931,49.333,27.748,5.799,3.486,7.26,7.139
,21.013,3.31,8.685,2.555,15.68
11.568,13.119,4.644,12.489,1.994,2.859,38.154,43.345,17.319,4.085,4.87,5.065,7.
835,12.513,6.278,10.358,3.878,6.667
5.295,66.858,2.899,10.099,2.255,6.122,36.698,41.289,24.679,42.516,7.811,41.312,
11.468,12.708,4.41,8.716,2.889,7.123
9.732,4.242,4.998,15.732,1.719,4.596,15.664,53.213,10.964,36.03,5.493,9.937,7.2
83,18.209,10.035,6.444,2.739,12.298
15.22,5.836,6.484,9.758,2.561,8.151,55.984,15.017,15.397,67.821,6.467,4.267,11.
865,17.202,9.765,5.605,2.586,4.153
14.258,8.145,7.841,6.444,3.241,3.766,18.899,7.326,27.104,68.582,6.176,5.751,16.
454,2.704,2.712,13.224,1.572,9.815
18.99,7.092,4.623,12.848,3.098,4.425,16.447,59.457,23.756,13.912,3.836,8.295,8.
816,9.902,12.819,9.593,1.661,2.562
16.589,9.986,3.263,19.604,2.284,4.027,13.359,17.202,5.051,39.182,7.807,3.639,5.
355,11.999,11.423,9.739,2.371,17.745
23.54,49.782,3.133,7.783,2.452,2.982,20.626,44.648,25.144,61.311,4.043,22.937,1
0.734,9.209,8.583,12.914,4.279,12.804
12.668,6.937,3.078,9.718,1.487,7.64,26.862,57.942,27.256,61.562,1.851,4.308,14.
912,14.154,7.857,21.606,3.843,13.901
11.963,11.912,2.856,9.826,3.153,16.707,33.538,47.861,10.228,39.912,4.164,13.502
,10.734,11.429,9.359,16.495,4.883,18.914
12.106,6.763,3.739,8.865,1.321,5.822,18.687,72.302,23.3,20.364,5.327,7.595,12.1
49,8.156,3.373,4.666,3.064,8.556
7.522,5.792,5.165,1.987,1.497,6.826,40.65,56.994,28.772,18.853,7.501,9.224,8.68
,15.46,7.761,14.484,5.575,7.884
12.434,8.852,3.479,10.561,1.42,1.436,39.882,50.581,17.116,36.603,3.808,9.07,10.
12,15.021,6.492,21.68,2.501,3.974
39.317,31.448,1.456,16.999,4.562,5.662,34.107,38.508,13.534,29.383,5.455,10.681
,11.191,7.017,6.192,23.212,3.605,14.099
15.62,7.044,11.693,23.564,2.786,4.081,16.76,63.247,25.676,51.056,2.891,5.799,19
.646,12.091,6.397,4.181,6.289,13.692
5.846,5.342,4.229,8.628,4.256,4.322,12.279,49.106,14.493,15.682,5.422,3.445,10.
06,11.253,7.549,23.03,3.126,13.983
8.653,7.325,4.367,7.562,3.674,4.65,20.167,32.547,25.454,37.097,4.388,7.061,7.12
8,9,8.154,11.338,4.04,26.456
15.989,5.736,5.607,10.126,3.293,5.484,24.961,48.301,25.662,49.306,5.271,10.978,
11.8,14.498,9.951,28.732,1.884,24.886
10.11,7.883,2.326,13.12,3.978,6.466,24.95,45.325,20.422,40.688,5.301,5.817,5.71
9,15.722,10.256,10.091,1.536,8.806
18.785,9.106,4.737,12.455,3.394,3.771,27.564,62.279,26.994,8.455,5.737,18.236,7
.34,12.745,4.061,12.493,2.08,10.557
10.829,9.705,3.592,11.559,1.671,3.707,38.282,43.968,25.28,59.193,4.707,18.131,1
1.324,18.728,6.897,21.013,1.875,8.921
5.339,10.744,9.995,20.85,2.14,3.723,52.435,54.561,15.363,41.153,5.892,7.972,9.3
55,12.078,7.444,14.084,2.12,11.448
8.822,12.123,2.706,10.644,4.558,9.828,16.174,11.093,29.124,36.205,4.945,9.261,8
.394,10.598,9.25,13.621,2.082,25.626
10.695,23.486,2.76,8.714,1.422,2.087,27.673,10.965,20.471,51.619,6.63,4.032,6.6
81,11.652,6.417,3.692,2.12,6.667
9,8.457,11.434,17.534,2.918,4.725,28.83,44.952,18.356,10.704,2.755,10.821,17.25
4,8.068,7.589,21.253,3.554,10.002
9.178,9.423,3.632,9.573,2.638,3.978,39.559,19.225,27.096,12.281,4.607,5.855,9.5
37,16.901,10.832,16.684,1.857,7.775
37.561,16.696,2.86,8.691,4.353,5.8,36.03,56.424,18.629,63.348,3.114,12.161,4.44
5,14.357,10.144,11.711,4.173,13.876
11.396,3.753,4.228,9.152,1.949,2.633,22.84,43.365,18.262,35.628,5.021,11.323,4.
704,14.059,4.849,4.338,2.21,5.742
26.835,7.919,4.886,8.45,2.44,4.431,18.002,47.704,32.885,10.978,5.921,9.652,13.9
11,12.289,8.005,18.823,1.682,6.45
6.717,8.256,2.572,14.223,2.748,5.412,11.42,62.393,17.205,11.817,4.385,7.752,14.
59,7.434,8.133,9.82,2.163,11.579
6.201,16.212,6.819,13.19,1.754,5.175,25.225,8.373,13.426,54.89,2.92,7.303,10.24

2,9.987,9.113,4.318,4.036,21.027
8.371,4.724,3.355,11.817,1.731,5.388,22.713,35.1,22.836,58.453,5.207,27.089,8.364,13.767,15.43,12.996,2.199,15.067
5.284,4.411,5.684,7.835,3.124,1.876,15.937,68.031,13.285,27.641,6.484,9.346,10.638,5.388,9.191,9.575,1.784,9.015
17.437,8.717,2.35,10.923,2.369,3.772,14.38,6.872,13.316,43.914,4.124,9.701,7.512,27.114,5.123,12.705,6.461,8.559
21.725,9.732,4.831,11.448,3.425,4.132,35.643,27.705,17.648,41.783,4.473,11.248,5.86,17.466,6.882,8.955,2.417,8.495
4.308,4.846,8.812,11.131,2.976,2.975,31.37,59.233,17.009,12.196,5.889,9.235,3.728,10.757,6.737,6.206,2.304,6.788
5.875,6.1,4.281,10.652,2.319,4.219,42.077,28.804,36.744,11.484,4.675,5.91,10.781,10.406,9.464,21.858,3.043,7.853
9.084,19.198,4.232,7.036,3.416,4.692,17.602,59.451,28.832,18.593,3.341,3.814,4.033,13.17,9.568,8.577,3.373,10.066
6.105,6.968,3.867,13.054,2.954,3.871,25.725,37.231,29.324,16.731,4.23,5.615,7.45,12.788,6.196,11.448,2.195,11.484
13.652,4.176,3.319,9.585,2.474,4.995,20.019,31.967,16.053,24.844,7.002,5.224,3.104,11.194,6.454,18.964,5.476,14.069
16.592,5.947,3.684,12.601,3.127,5.028,48.575,43.941,12.425,11.499,3.454,7.022,4.63,9.471,26.739,23.051,2.131,7.149
10.082,6.87,4.51,8.205,3.201,3.681,29.247,55.802,9.046,54.052,4.443,4.461,8.15,12.692,9.631,5.933,1.413,3.489
12.148,4.496,4.26,7.326,1.944,3.312,57.755,12.914,26.319,36.125,5.891,6.852,9.315,11.41,2.931,7.943,1.628,16.551
19.081,6.229,5.348,30.898,3.608,5.135,34.414,9.705,26.671,19.739,6.14,9.796,2.435,22.685,7.582,25.285,1.895,11.429
7.024,11.05,2.885,8.334,3.988,6.373,20.727,44.33,29.86,18.422,7.802,6.915,10.273,16.273,3.514,3.923,1.987,12.194
12.094,9.038,4.816,11.526,3.258,5.802,15.213,32.857,16.876,14.121,6.158,3.489,5.035,4.255,8.313,12.589,3.421,13.135
16.378,4.553,4.443,11.61,5.135,4.985,26.633,41.153,18.235,25.737,5.617,3.782,7.696,13.902,9.922,6.661,2.37,14.672
36.55,8.989,7.903,4.759,4.084,4.424,36.254,32.369,32.108,19.306,3.496,1.838,11.156,7.348,8.152,8.198,2.32,21.358
10.801,10.773,3.463,11.093,1.868,3.675,33.83,55.67,14.352,39.971,3.848,5.072,12.673,12.281,7.604,8.851,1.47,11.61
10.385,11.325,3.528,9.139,2.712,2.894,21.409,25.327,29.978,62.333,3.946,4.73,8.899,8.571,12.559,6.543,4.092,11.689
8.081,7.072,4.592,11.297,4.478,3.894,20.547,48.868,25.852,24.709,5.677,8.961,12.257,17.173,7.603,6.978,2.814,7.757
8.299,7.897,4.312,9.27,2.593,32.795,16.712,63.77,18.026,51.353,3.49,7.429,6.592,9.911,11.339,20.732,2.131,8.584
10.065,7.959,2.645,12.281,1.771,5.669,28.784,60.959,5.792,21.188,5.925,13.144,11.694,5.693,7.67,12.281,3.187,9.768
6.253,24.638,4.446,5.79,1.391,9.748,40.013,24.548,20.08,34.127,4.053,8.792,6.515,7.608,11.249,3.471,2.004,5.452
15.603,5.522,3.72,9.288,9.523,6.23,30.442,56.353,14.745,16.765,5.38,3.343,4.457,10.804,9.067,4.83,1.541,13.927
12.375,19.604,5.623,8.777,3.598,5.354,24.53,49.851,10.408,17.222,4.853,4.985,9.997,6.879,9.441,23.961,2.074,4.549
36.403,17.421,5.81,4.976,1.952,1.651,22.056,8.2,22.821,6.788,5.203,14.21,9.835,25.615,6.408,6.374,2.686,12.052
10.542,8.147,4.362,7.588,3.252,4.123,43.106,8.56,20.46,41.74,3.631,5.79,12.189,22.145,10.775,6.35,4.503,12.794
12.626,18.42,7.069,28.122,2.615,2.815,25.78,71.572,41.315,43.265,7.577,9.089,6.821,12.001,13.536,23.758,2.468,8.056
12.268,8.578,2.87,6,2.274,3.851,27.143,45.497,18.415,53.107,4.504,8.545,9.041,12.325,9.307,6.048,2.647,7.326
9.855,8.606,2.279,6.445,3.405,5.37,51.894,4.242,13.76,55.23,5.317,12.091,9.856,7.883,1.772,8.913,2.358,5.949
7.857,12.199,4.271,8.606,2.598,6.336,24.623,45.36,17.117,43.862,5.215,7.623,9.433,9.753,4.583,8.612,2.494,49.782
6.554,10.521,5.723,8.292,3.432,3.282,22.429,58.865,22.751,8.61,4.675,12.666,16.

784,12.624,10.065,12.489,1.888,5.388
10.356,7.326,3.508,10.268,3.338,3.999,36.165,65.591,10.683,51.604,5.183,7.777,1
3.481,8.767,9.834,13.667,2.702,14.181
7.375,19.034,6.927,9.986,3.063,4.958,16.727,59.218,9.029,40.583,5.636,8.824,8.1
57,26.605,8.058,11.499,2.494,5.893
7.16,11,3.038,10.14,4.977,8.45,23.641,51.402,26.728,8.315,4.664,10.565,8.299,19
.123,9.358,10.948,1.992,10.089
85.243,18.561,4.332,10.768,2.456,4.41,26.405,55.599,11.23,42.127,5.848,6.043,10
.74,17.916,2.212,20.026,1.859,20.675
9.198,30.83,5.35,8.53,4.803,6.819,52.846,46.812,40.13,44.158,3.798,8.56,5.569,1
2.312,12.506,14.781,1.951,7.536
6.331,5.37,3.837,9.72,3.358,4.206,41.244,36.635,22.635,16.986,4.95,9.279,3.958,
9.234,9.439,17.952,1.988,15.29
7.443,11.719,3.668,13.506,2.216,3.878,33.492,31.118,19.38,16.307,7.128,6.713,4.
487,21.361,7.379,22.596,1.76,16.207
7.561,9.962,6.006,9.54,3.387,2.231,60.798,35.182,24.419,33.05,8.254,11.709,6.89
4,26.279,7.136,15.878,2.587,16.951
7.008,8.216,8.753,7.723,3.558,2.678,24.58,50.785,15.818,47.191,6.516,5.894,7.01
8,6.419,2.049,55.468,3.763,5.747
44.143,9.675,3.643,7.963,3.724,11.518,38.468,56.998,24.508,33.632,5.756,9.523,5
.345,16.614,9.248,28.122,3.193,10.85
9.678,9.663,4.94,12.967,3.584,5.019,23.528,40.757,19.025,31.939,3.338,5.661,6.5
06,14.937,14.169,8.874,4.461,13.497
18.054,9.761,5.536,7.536,2.81,3.911,27.396,31.641,26.682,47.298,7.959,6.692,5.2
87,8.786,9.14,6.968,3.28,4.43
6.665,15.857,6.88,6.04,3.851,3.956,31.849,49.542,17.844,33.51,4.372,3.196,6.737
,10.791,7.659,11.937,1.837,10.504
12.056,11.448,5.144,9.547,2.48,6.861,45.764,62.567,33.765,35.649,8.205,17.952,9
.234,12.631,7.068,9.015,2.695,3.465
7.669,12.047,4.312,7.671,2.221,4.95,35.796,54.989,18.006,40.947,5.305,7.379,11.
321,18.217,5.696,7.351,3.396,10.912
10.739,16.218,4.791,7.031,2.857,3.94,29.716,61.599,17.851,37.462,12.375,6.988,1
9.892,5.501,9.712,11.975,3.03,5.61
14.408,5.278,14.778,7.741,5.407,6.658,23.153,35.144,20.104,47.613,6.611,9.658,4
.716,8.256,7.263,14.755,3.658,9.872
11.023,4.516,3.886,5.712,3.227,6.362,34.519,13.433,22.82,35.308,3.519,6.405,12.
753,9.006,5.203,12.492,2.497,2.663
9.183,6.897,8.737,10.22,3.568,6.165,42.019,27.742,28.863,51.849,4.283,7.218,4.7
9,7.455,5.044,14.779,5.834,18.613
5.872,12.112,4.165,5.358,1.512,3.221,26.045,51.517,27.495,56.131,4.089,6.659,9.
851,12.431,6.314,8.56,1.623,5.485
39.83,11.713,6.62,5.718,2.766,3.555,28.54,47.227,11.384,35.486,5.517,5.439,10.4
38,33.28,6.05,17.112,1.693,10.109
16.968,8.036,4.86,9.283,3.119,10.404,14.518,27.498,7.362,33.824,5.728,7.306,9.8
96,5.949,7.047,4.859,2.792,14.372
7.052,12.354,8.235,7.143,1.887,4.02,25.115,15.273,20.123,43.069,4.486,7.681,14.
614,16.283,9.906,6.086,14.831,9.003
6.936,8.883,3.986,12.58,2.347,56.758,18.542,52.065,16.638,5.525,3.782,9.467,3.6
6,8.907,5.457,19.736,1.996,14.059
11.883,7.03,2.24,13.348,2.836,4.138,9.849,37.103,26.516,36.378,4.946,11.839,5.4
72,6.104,12.701,18.92,1.968,4.487
10.553,18.131,11.731,9.418,2.323,3.648,35.322,47.467,15.192,47.154,4.807,9.702,
3.604,2.933,7.37,18.026,1.376,9.475
10.963,11.756,5.812,4.215,1.852,8.266,23.252,9.493,13.937,8.777,3.255,26.211,9.
003,7.379,4.197,15.476,1.778,4.724
10.973,17.373,3.022,9.606,2.97,4.272,32.292,51.539,26.722,13.783,9.091,10.53,19
.586,42.571,11.887,13.783,2.521,3.799
19.178,9.136,4.246,11.383,4.447,3.767,26.6,15.732,19.237,10.923,5.482,6.768,4.3
03,10.525,11.226,19.053,2.134,6.034
11.216,15.275,3.225,11.61,3.063,5.378,29.925,15.353,36.704,33.581,2.891,7.536,1
1.382,6.808,7.777,5.403,1.72,12.662
6.326,15.882,6.674,15.899,4.055,5.294,39.576,53.261,19.226,43.971,2.932,4.637,6
.558,12.211,6.024,8.81,2.336,9.217
10.865,6.22,3.424,7.773,2.123,1.5,11.804,11.61,25.702,58.401,2.611,8.718,3.573,

20.098,5.191,7.178,3.567,11.61
17.437,10.353,3.403,10.796,2.74,6.367,52.602,13.714,27.741,29.043,9.583,2.439,1
2.23,9.546,6.536,5.978,2.002,17.952
11.687,4.029,3.424,26.279,2.184,3.951,29.849,57.067,21.443,32.376,4.596,15.857,
4.659,7.757,11.063,20.14,3.423,14.484
6.211,11.002,3.006,6.882,6.436,3.698,17.13,36.213,12.156,20.887,8.031,4.073,10.
271,10.271,7.325,23.649,1.18,5.79
6.236,7.72,3.465,7.451,2.143,4.647,20.043,21.255,22.306,38.086,4.912,11.337,5.3
85,19.569,6.123,6.95,2.918,11.032
12.385,7.126,2.612,5.616,6.049,2.99,46.093,42.293,23.594,45.977,2.988,11.1,8.95
6,19.222,7.095,20.675,2.379,16.667
12.785,7.515,3.453,13.724,3.447,1.353,17.842,65.896,20.249,19.604,4.489,8.044,1
0.046,16.911,11.535,17.672,2.099,6.872
9.351,4.962,2.82,9.883,2.331,6.255,12.433,41.67,7.519,26.657,4.297,8.604,9.281,
19.33,7.468,13.183,1.787,6.653
7.114,11.195,3.677,16.912,1.607,4.583,34.458,60.834,10.985,26.583,4.789,7.9,5.6
06,12.835,2.881,7.383,2.819,5.712
26.233,12.315,3.682,11.404,3.534,4.975,19.17,48.941,14.113,15.732,2.543,5.981,8
.556,12.996,6.639,5.097,2.208,10.12
9.003,21.124,2.206,9.455,3.402,3.092,20.691,65.406,21.02,61.294,4.168,6.059,8.3
65,10.411,8.805,13.433,2.367,10.362
7.534,3.923,5.324,16.872,2.168,4.2,23.057,69.393,17.994,20.306,4.456,18.476,7.7
7,24.165,18.202,15.3,1.867,7.429
14.028,12.298,3.887,11.713,2.515,5.245,24.251,65.309,15.772,34.962,5.323,19.448
,7.086,10.978,3.026,11.325,2.877,8.82
11.446,6.189,5.279,8.603,2.413,6.678,38.281,27.089,13.9,16.058,4.039,12.914,4.8
3,6.49,4.63,10.787,1.503,7.092
16.546,13.134,3.346,9.68,3.745,3.942,34.092,63.699,28.254,52.944,5.867,10.141,9
.487,19.512,6.625,16.206,2.494,10.152
7.554,7.267,4.575,8.714,1.915,5.766,24.368,29.499,20.792,28.33,2.248,12.544,8.8
36,10.87,10.256,17.102,2.177,12.465
19.491,11.145,3.065,20.873,10.953,4.031,37.479,22.835,20.825,8.91,4.608,12.492,
13.589,12.912,9.538,7.115,1.42,9.94
8.895,9.932,3.668,6.998,2.615,9.205,38.839,39.568,17.068,16.212,5.248,4.671,8.7
11,18.221,10.099,31.423,2.125,8.56
11.812,6.048,6.793,6.529,2.232,1.617,11.222,53.152,26.932,15.107,3.389,8.02,11.
692,10.439,4.855,6.213,2.16,9.872
15.434,23.444,3.999,6.725,3.318,5.319,15.092,59.167,25.712,28.413,4.947,13.714,
5.792,11.479,7.423,4.947,3.132,4.649
5.556,9.287,6.9,10.12,2.204,4.291,31.271,24.137,32.117,52.88,4.445,8.565,4.881,
7.171,7.711,10.923,2.031,16.224
7.701,13.32,2.261,6.45,4.405,2.849,17.216,49.067,30.189,38.329,6.077,2.191,9.24
6,14.444,8.197,15.104,3.505,9.038
11.457,9.569,2.235,9.803,3.974,3.626,18.868,27.422,15.296,24.725,13.124,8.49,6.
508,7.078,9.146,4.479,3.103,17.534
63.375,9.773,3.542,8.698,4.14,7.568,18.043,33.698,15.073,5.79,4.263,10.787,4.68
,20.934,31.391,15.88,2.128,3.824
17.444,11.379,5.091,6.11,3.292,5.338,8.019,12.629,15.53,43.196,7.547,11.658,11.
668,13.443,2.543,6.413,3.394,13.473
12.255,16.731,8.036,16.182,3.196,1.679,22.45,69.525,35.15,48.168,2.786,8.588,5.
538,12.295,7.12,20.108,3.118,10.787
11.438,10.938,3.624,5.925,1.523,5.904,14.592,46.17,15.126,5.305,4.065,9.324,7.9
,11.233,21.071,23.504,4.373,8.306
6.757,8.582,3.834,10.055,2.795,5.95,31.204,56.117,32.406,38.073,9.715,5.33,6.10
6,6.953,2.817,6.38,3.857,6.019
19.875,6.521,4.377,23.504,4.145,5.819,11.332,48.669,20.768,30.554,4.949,8.03,10
.916,13.015,3.239,6.71,3.063,4.27
7.187,14.121,4.626,8.044,1.165,7.96,37.797,48.633,22.062,18.625,8.331,3.048,6.3
8,6.69,4.135,13.907,1.743,16.689
23.953,10.218,4.439,5.278,2.601,6.462,28.378,55.539,14.555,15.476,6.083,6.825,4
.463,16.593,10.118,12.593,7.028,15.447
9.6,15.732,2.185,12.563,1.388,3.734,28.758,55.682,23.28,32.096,4.944,6.997,7.34
5,10.971,6.709,11.705,2.361,5.67
13.226,6.444,10.443,7.38,2.406,6.361,37.797,15.857,25.763,39.192,3.615,7.991,5.

73,10.12,8.945,26.279,3.653,10.271
31.848,14.928,4.306,5.525,1.775,6.721,29.307,23.977,18.391,9.758,3.009,5.22,9.3
16,11.61,6.765,12.139,1.86,20.301
22.557,3.624,3.605,11.499,1.665,14.724,17.862,55.029,13.093,43.038,8.728,9.525,
5.161,2.788,6.277,8.82,3.48,9.075
14.708,12.632,8.693,4.663,4.77,4.197,34.977,5.712,14.226,4.333,2.914,3.974,28.4
15,6.353,13.482,16.986,3.079,12.522
60.58,11.429,5.455,10.473,1.842,4.65,28.61,64.581,17.146,11.073,5.167,6.127,14.
864,13.101,6.653,11.093,2.332,10.796
14.96,10.787,5.016,4.939,2.375,4.666,22.578,42.402,16.92,10.603,4.367,10.141,12
.741,13.01,6.075,6.816,1.739,18.178
7.57,5.899,4.635,7.556,3.158,5.14,16.007,7.959,5.53,62.044,5.914,3.169,7.191,16
.743,8.647,16.07,2.788,6.71
17.786,5.828,7.181,8.961,2.814,6.691,25.614,16.999,27.091,12.465,4.077,15.407,4
.708,20.85,6.521,16.999,1.76,16.104
15.763,15.107,5.712,8.764,2.302,7.162,53.186,57.52,22.246,51.853,2.597,8.82,8.6
86,9.402,11.482,10.271,2.327,16.876
12.432,10.735,3.594,12.421,1.42,5.918,58.472,43.598,26.3,13.298,7.281,5.071,7.4
55,4.5,6.789,48.452,1.904,15.107
16.392,7.259,3.321,20.045,3.132,5.043,26.626,31.836,11.404,103.341,2.523,6.252,
5.446,17.083,8.99,6.948,2.921,3.734
7.149,9.567,2.634,8.198,1.914,5.805,32.665,67.048,35.35,16.134,6.831,15.899,4.3
94,6.968,2.495,13.491,2.459,19.034
17.374,15.088,6.583,8.098,4.529,4.114,32.218,46.449,18.828,15.92,4.662,3.081,3.
987,10.845,8.35,9.715,3.169,12.421
6.277,8.88,5.203,14.056,2.674,8.973,27.908,34.115,15.776,6.048,1.954,3.731,14.7
9,7.851,7.385,13.822,2.164,7.941
6.072,4.502,6.322,12.49,3.158,6.322,17.813,46.166,21.859,24.881,2.894,9.66,14.0
21,10.156,8.203,7.536,1.623,19.282
26.51,14.779,2.122,8.741,3.033,4.327,22.617,55.055,11.633,7.883,4.827,28.122,9.
233,14.913,8.53,6.046,1.459,3.653
18.281,5.799,8.523,5.949,1.959,6.885,22.707,46.842,38.556,11.145,3.324,14.059,7
.598,4.618,9.341,14.688,4.083,10.266
91.054,5.467,3.567,18.319,1.521,4.506,23.389,64.998,11.934,48.153,4.919,11.429,
5.205,13.185,9.247,16.668,2.501,6.444
11.645,18.79,6.472,11.471,3.061,4.934,13.13,7.588,25.558,5.278,7.363,10.6,11.43
7,11.499,6.038,7.883,1.531,23.988
7.406,7.177,3.494,84.411,5.758,4.106,36.26,8.156,11.799,51.202,3.904,6.752,15.1
16,17.451,9.817,4.462,3.627,10.258
10.319,11.354,1.562,9.234,4.732,5.529,28.486,40.452,20.813,19.531,6.564,6.181,1
3.291,11.119,8.64,11.867,1.319,13.714
10.272,15.476,5.431,9.547,3.07,2.829,26.157,50.506,38.93,43.943,6.358,7.294,8.8
28,12.775,11.497,9.933,2.274,16.431
10.589,6.626,3.66,13.714,2.008,2.199,29.905,34.914,33.94,53.352,7.659,7.452,5.6
28,4.454,11.054,28.098,4.011,13.749
9.695,7.375,5.304,10.114,3.175,4.36,34.802,27.005,13.932,33.143,2.922,8.773,6.6
39,14.248,16.058,22.892,5.797,3.212
8.627,7.486,10.487,7.923,3.911,4.661,28.14,38.222,10.293,46.155,4.822,4.96,5.91
7,23.43,9.112,20.747,2.575,14.092
17.041,6.364,6.207,17.202,2.804,4.465,62.326,59.885,18.68,24.828,10.851,8.439,3
.966,10.489,5.031,6.202,1.583,12.402
7.026,9.207,2.768,6.675,3.606,6.553,28.973,34.704,8.045,49.07,4.563,12.556,12.3
95,12.04,9.605,4.716,2.693,9.675
7.556,11.363,5.672,7.017,2.747,4.224,16.983,40.068,7.92,6.444,3.505,6.031,3.018
,16.749,6.484,10.345,2.006,3.845
6.485,7.13,7.284,8.051,2.221,4.403,30.41,63.098,19.762,38.596,8.119,9.339,10.30
3,12.914,9.231,14.975,3.855,14.511
8.191,5.132,4.542,12.83,3.695,3.318,29.795,56.898,21.773,33.04,2.398,7.095,18.5
73,24.756,9.478,11.565,2.12,5.983
20.52,9.512,4.174,6.811,3.499,9.329,42.237,27.674,21.61,32.225,6.453,11.526,5.9
12,34.388,2.553,19.568,1.572,12.473
31.454,11.305,5.474,6.549,2.528,4.242,25.976,10.978,19.75,22.34,3.383,9.149,12.
854,8.324,9.119,16.454,1.701,9.283
49.13,7.004,3.023,6.104,2.713,4.173,24.826,57.162,34.976,56.617,2.726,8.272,16.

732,20.03,8.573,17.863,2.377,12.983
9.914,12.89,2.507,9.909,2.732,6.36,62.579,33.77,7.895,50.146,2.789,12.421,11.62
9,7.813,13.376,19.94,2.769,7.731
14.746,16.75,11.562,8.351,2.693,6.045,23.755,18.422,14.642,51.61,3.979,9.491,11
.131,9.358,3.644,4.882,3.883,6.048
18.819,12.107,1.725,8.275,2.67,4.233,39.095,7.092,14.572,29.418,2.109,7.855,7.8
91,15.008,13.897,10.639,29.575,5.467
8.963,8.445,3.374,11.383,3.115,2.868,56.022,76.785,18.516,30.678,4.651,10.53,6.
328,19.499,9.048,10.73,1.991,8.638
21.934,9.193,4.318,9.217,3.16,5.726,17.387,31.129,23.21,50.105,6.06,7.62,12.28,
12.888,6.664,5.051,1.651,13.273
8.33,7.526,3.851,2.813,5.865,4.356,21.331,5.358,18.971,21.868,3.511,8.254,24.63
9,19.209,9.31,26.844,3.295,10.869
72.317,24.844,2.29,12.309,3.422,6.732,29.384,13.048,18.708,12.117,4.279,10.809,
5.279,11.379,9.163,23.764,3.676,3.191
9.681,13.783,5.113,8.48,2.488,5.621,12.959,73.228,13.879,62.434,7.458,5.456,9.6
,17.812,4.501,23.97,3.413,5.221
4.571,7.022,1.527,8.533,2.006,3.789,18.847,46.04,16.828,46.852,6.038,5.519,12.2
22,3.377,6.976,16.212,3.441,9.606
11.269,4.821,3.37,8.91,4.045,6.441,33.963,53.81,12.802,18.573,8.458,9.215,3.922
,2.509,6.404,24.307,1.82,10.159
5.021,8.399,4.112,9.675,3.078,4.793,13.817,63.74,32.909,46.785,2.707,11.363,5.0
37,9.127,7.561,7.298,1.785,15.141
6.17,7.704,4.353,12.492,4.05,1.108,34.463,37.733,41.383,23.275,5.867,1.431,12.0
21,7.696,8.926,7.433,2.399,5.26
13.904,11.093,6.732,12.955,2.302,3.045,21.196,54.717,10.421,19.429,2.882,11.71,
6.682,15.237,7.907,26.456,2.16,8.777
7.127,4.457,6.215,16.07,8.294,2.99,42.735,54.186,10.655,34.016,4.676,5.905,7.69
8,9.525,8.481,8.571,3.43,14.01
5.115,8.672,2.974,10.85,1.6,4.667,29.597,54.034,14.044,44.969,2.243,13.639,8.33
8,13.56,5.885,16.235,2.635,8.164
7.57,19.194,3.866,10.745,1.987,4.06,32.807,54.156,30.13,12.402,4.258,6.569,4.80
5,24.89,12.337,13.714,1.483,13.892
48.332,4.192,5.372,10.358,1.957,2.149,22.355,47.95,21.009,44.005,1.98,9.124,7.0
28,11.78,8.838,5.324,2.304,5.571
25.255,12.421,3.552,23.97,3.012,3.98,18.146,48.955,35.726,32.295,5.357,6.084,5.
906,10.091,12.873,16.125,2.072,8.988
8.486,11.519,12.979,18.594,1.886,4.302,18.046,42.302,20.464,39.02,5.873,5.753,7
.714,12.689,6.146,9.493,3.362,10.054
27.165,5.542,4.721,10.002,2.217,2.928,32.415,33.568,32.723,15.272,3.038,9.632,6
.261,18.682,5.865,17.576,2.309,13.781
12.75,20.792,1.944,12.689,2.804,2.881,42.466,66.457,16.523,40.034,8.965,8.421,1
1.857,30.265,2.902,12.446,4.253,6.9
7.584,10.701,4.402,5.316,4.977,6.114,33.244,42.171,32.728,5.324,7.339,8.764,5.3
49,14.575,2.187,4.129,3.482,5.949
12.214,12.117,8.429,9.275,3.65,2.732,28.32,30.213,22.664,24.205,5.229,11.484,13
.989,7.326,8.439,11.145,1.377,13.205
12.328,16.487,3.77,6.493,2.102,4.786,16.036,53.168,41.99,27.067,4.747,3.919,11.
932,8.505,6.714,17.315,4.002,8.527
9.372,6.951,4.366,10.34,2.573,4.554,25.975,51.123,10.536,8.373,4.618,16.667,11.
245,9.217,5.314,22.145,3.203,6.407
29.307,7.638,1.414,7.539,2.724,3.871,8.494,25.854,19.603,24.263,8.536,2.558,9.8
49,10.695,6.45,17.924,2.385,13.904
12.308,4.872,5.837,6.968,6.452,9.855,12.255,66.933,24.677,26.456,5.84,7.998,14.
917,2.9,6.864,6.143,5.296,6.812
5.548,8.347,2.803,14.967,3.119,3.116,21.805,67.582,19.651,33.272,2.402,9.531,7.
699,25.035,10.785,17.743,1.648,7.778
8.019,8.536,3.466,11.868,1.726,3.226,33.274,69.969,6.219,13.714,4.154,8.619,10.
391,16.443,8.206,17.954,3.324,8.803
13.445,2.853,7.191,11.031,2.152,2.958,16.184,50.487,17.2,44.074,3.811,8.815,6.9
79,4.987,13.591,16.183,2.582,19.622
17.667,6.859,2.279,8.399,1.394,7.024,22.489,48.038,14.112,54.795,5.11,14.132,12
.303,14.601,2.092,19.034,4.335,4.915
14.829,10.549,2.578,6.833,3.483,5.845,37.648,61.074,30.637,30.688,3.832,2.49,3.

966,5.565,9.517,4.966,25.691,7.122
30.716,10.67,4.179,19.692,3.895,8.045,26.267,40.987,11.847,14.099,5.531,11.021,
13.52,6.435,13.73,4.186,2.589,9.294
6.795,6.815,4.004,5.761,2.356,5.578,24.662,58.704,19.121,36.863,9.538,12.117,16
.93,27.089,4.771,5.033,1.429,4.207
11.061,9.28,2.767,6.47,3.008,5.45,30.493,59.818,10.863,45.339,7.185,11.499,13.0
97,10.288,5.217,20.821,3.404,4.743
8.976,12.996,4.258,6.714,4.026,7.853,22.818,48.563,29.189,25.209,4.145,8.088,14
.43,14.496,8.36,13.464,1.69,8.166
15.707,8.113,4.285,11.429,2.77,6.152,8.354,47.711,31.366,8.105,4.447,9.778,13.6
46,14.854,8.121,20.207,2.894,7.806
13.875,4.317,2.834,9.501,2.899,6.181,21.409,31.523,10.772,8.092,6.393,20.675,12
.329,14.558,7.069,22.355,3.573,4.771
6.396,20.849,4.531,14.121,2.628,2.872,22.62,7.536,14.694,26.322,3.537,5.718,8.4
17,11.178,5.521,28.741,1.977,17.255
9.499,8.262,3.675,7.959,4.186,5.447,61.624,60.941,24.857,30.483,2.773,6.088,12.
658,23.504,9.997,16.765,1.894,5.179
15.514,10.804,2.596,8.75,3.355,6.904,68.869,32.832,28.526,53.592,4.33,6.095,9.4
51,10.641,4.492,18.961,2.51,8.371
10.49,13.888,3.905,16.731,5.897,4.443,14.555,21.92,41.584,9.455,7.588,4.181,3.1
33,9.214,7.937,5.018,1.414,5.943
7.949,4.467,6.928,8.683,1.802,5.372,6.847,56.912,24.934,34.371,4.572,7.682,11.8
28,31.161,9.889,4.213,2.758,7.629
63.1,10.271,3.372,11.644,2.86,10.34,16.55,23.376,11.588,9.234,9.514,15.732,9.93
8,15.636,12.367,5.17,1.509,4.532
22.536,17.952,3.947,19.622,3.064,6.749,27.842,44.8,23.026,7.536,5.837,10.095,10
.976,10.928,6.882,17.97,3.931,4.947
20.366,6.893,4.004,6.587,3.212,2.697,47.906,49.621,24.595,49.988,5.675,10.514,9
.844,17.114,6.385,12.87,3.077,14.779
10.511,4.776,3.193,7.476,2.223,3.958,33.789,35.916,12.257,7.778,6.13,10.948,11.
674,13.082,4.444,19.032,1.294,17.9
30.302,13.442,5.911,4.333,2.205,5.229,13.468,39.234,21.406,37.007,6.31,10.972,4
.371,10.272,7.78,10.917,2.488,15.144
15.125,9.015,4.2,5.324,1.639,5.005,23.135,62.681,8.805,43.419,4.869,4.955,7.756
,22.197,6.63,17.974,2.013,2.443
21.661,10.978,7.329,7.75,2.829,5.99,25.848,51.383,11.819,54.792,5.132,5.138,4.5
4,26.456,7.875,5.137,4.417,5.876
20.223,28.817,6.332,9.814,1.816,11.441,23.529,47.114,9.407,9.586,4.521,12.38,3.
603,3.073,9.448,66.957,2.174,2.24
31.187,11.479,11.804,37.465,7.782,11.73,12.071,22.382,9.806,2.553,5.772,3.28,4.
856,4.473,13.387,3.779,2.016,1.319
6.886,19.628,8.595,20,4.143,16.511,9.706,39.099,10.414,10.748,7.412,7.983,11.43
7,7.558,9.073,20.589,1.396,3.484
20.137,17.354,7.795,34.58,7.004,11.183,13.002,35.578,8.863,8.982,3.024,8.598,10
.311,3.302,9.526,3.549,1.725,1.57
15.141,16.735,10.354,13.188,5.078,9.394,23.235,9.034,9.042,2.754,2.825,5.111,10
.221,1.902,7.073,20.736,1.467,1.859
19.537,14.387,3.784,16.018,6.341,8.792,6.055,33.675,7.628,5.24,4.755,9.114,10.8
47,12.783,4.183,2.486,4.003,7.314
9.823,15.443,2.332,26.777,7.64,7.19,13.983,17.808,6.847,8.629,10.417,10.349,3.3
71,28.657,2.668,8.774,1.605,4.894
24.985,23.957,8.411,8.829,3.337,5.323,25.708,13.557,11.488,15.741,10.999,7.61,1
6.176,20.987,5.15,11.867,1.508,1.387
10.146,14.979,2.133,1.16,10.978,6.657,20.214,16.349,12.758,10.717,4.261,4.979,1
3.086,4.226,6.977,2.702,2.197,1.937
23.131,27.492,10.255,23.389,2.861,2.203,11.442,25.659,10.314,6.103,2.766,12.627
,7.585,5.542,11.787,8.66,1.52,1.3
10.83,10.265,6.818,29.127,6.678,11.369,25.406,47.106,9.612,33.23,3.239,12.677,2
.657,7.274,9.646,4.365,1.413,1.288
14.253,7.601,5.897,14.485,6.515,14.107,10.977,2.589,12.556,14.904,2.636,7.223,8
.535,2.338,10.031,8.021,1.651,1.279
13.403,17.54,10.739,16.067,5.383,16.796,17.108,12.877,6.541,4.675,2.581,60.094,
10.992,1.484,5.967,6.843,4.456,1.773
18.866,17.953,2.931,4.818,3.367,7.43,24.363,9.976,8.353,22.223,3.407,4.537,5.39

4,2.51,10.656,23.961,2.703,3.658
30.988,25.48,7.366,33.733,8.439,12.116,10.872,32.654,6.652,21.68,2.947,7.697,5.015,2.658,13.6,22.145,3.405,1.728
13.193,34.808,10.721,34.636,3.62,16.641,46.286,20.244,21.474,13.99,3.527,7.174,6.826,11.938,8.605,20.291,4.717,3.305
13.704,2.14,9.611,34.712,6.753,15.317,30.772,22.829,8.891,21.84,2.962,4.908,5.198,3.707,5.293,13.444,2.566,2.081
10.674,15.101,5.24,24.278,2.779,31.604,12.463,8.186,9.786,3.151,2.949,10.379,19.493,5.156,3.882,22.622,1.668,1.226
29.855,19.649,6.704,7.901,7.693,7.17,7.022,27.439,5.695,9.495,7.908,10.595,3.355,5.826,10.994,17.346,1.532,1.806
11.516,52.373,10.997,11.046,4.6,8.051,28.582,22.296,19.955,30.931,4.539,4.197,9.98,4.223,16.634,3.73,2.294,1.74
7.299,22.555,8.941,31.718,5.086,11.705,11.809,24.322,11.882,16.861,7.046,2.051,9.568,6.463,10.583,21.34,2.601,1.91
12.135,20.883,5.648,22.121,3.261,13.358,16.346,44.094,14.782,25.226,3.491,3.195,4.414,18.972,1.691,6.533,1.906,1.296
21.476,15.333,5.896,2.797,5.542,8.088,8.372,34.299,27.047,14.872,1.463,12.337,10.52,4.225,11.492,12.268,1.573,4.215
13.908,6.147,3.925,4.148,3.166,17.657,19.696,15.888,11.008,16.875,6.689,7.309,4.384,15.356,2.274,18.77,1.829,9.529
16.187,23.293,9.702,7.465,7.235,10.849,37.512,24.999,13.98,4.496,2.551,4.59,1.917,1.786,9.849,9.578,1.681,1.941
29.922,22.595,10.228,16.404,8.528,9.503,26.405,54.177,43.425,37.821,4.101,8.309,8.503,6.452,6.508,22.805,2.678,4.49
22.333,6.663,3.181,50.982,4.838,15.095,11.519,26.594,18.079,9.248,4.838,6.133,11.216,13.434,12.101,26.76,1.427,1.57
29.802,50.554,4.928,15.865,4.005,10.655,13.554,4.369,9.81,13.168,3.471,9.021,19.877,4.514,8.096,14.087,1.759,10.965
18.68,38.211,5.89,15.728,4.809,29.26,14.839,49.286,15.071,6.571,4.482,11.873,10.642,12.885,12.693,20.026,1.632,5.019
24.108,27.023,6.254,21.152,3.839,38.74,17.337,22.837,6.177,32.703,3.74,6.205,3.386,2.82,3.997,4.607,2.494,2.131
15.876,20.604,8.83,16.444,6.681,11.672,16.641,19.583,9.194,10.799,4.845,5.151,14.944,2.582,4.387,3.327,1.959,2.057
12.489,45.945,10.374,106.498,4.654,17.778,30.897,54.281,8.61,4.159,2.738,4.241,9.414,7.488,4.564,14.154,1.38,1.849
15.34,6.048,11.522,20.394,3.458,10.717,17.224,17.123,27.096,37.832,4.142,8.187,13.961,5.736,5.145,5.146,1.874,2.81
6.631,15.502,4.54,47.935,5.656,6.207,10.426,30.186,8.288,15.93,3.177,6.104,9.553,5.402,3.442,25.033,2.228,1.954
17.851,14.223,3.623,10.907,3.348,12.553,22.232,3.513,11.725,15.117,3.548,5.221,13.28,2.93,6.539,6.046,2.135,2.059
12.664,13.241,11.451,13.734,4.275,39.959,33.715,32.309,7.552,13.039,5.427,4.772,7.888,22.592,9.695,31.808,1.503,1.493
12.5,42.44,14.049,29.808,3.945,18.597,9.793,25.258,11.12,12.112,4.744,3.163,11.499,3.944,7.805,12.051,2.809,1.519
27.809,8.886,7.377,12.142,5.713,13.246,11.413,25.114,10.671,17.854,8.264,3.079,11.921,5.949,9.676,8.716,1.548,2.091
20.94,35.815,4.615,24.592,3.867,10.142,7.855,15.256,4.03,2.345,3.063,6.5,3.897,2.096,6.783,17.952,1.212,5.804
62.812,22.473,2.702,7.063,3.741,24.242,12.199,44.759,9.596,18.188,3.368,4.684,7.79,3.995,5.785,14.192,2.885,9.951
8.967,13.129,6.21,7.17,3.233,1.635,26.925,7.238,11.409,14.039,3.641,4.63,9.4,4.273,4.695,24.204,1.27,2.638
23.187,5.081,5.777,10.991,2.974,29.731,8.309,71.273,12.888,47.629,4.662,4.266,12.977,1.419,13.186,4.978,1.546,2.309
9.928,19.826,4.191,8.017,14.919,15.84,36.683,15.405,6.858,10.842,5.35,8.091,10.916,3.041,5.7,1.48,1.241,4.549
33.877,58.503,3.526,18.756,5.598,16.033,26.008,33.433,8.644,4.315,1.529,2.934,4.063,2.817,2.716,29.385,2.417,6.208
16.111,47.571,3.341,34.974,3.771,16.35,21.541,29.669,6.677,6.834,4.823,5.389,6.069,7.454,2.457,20.238,5.903,1.676
11.066,4.39,4.749,9.84,3.937,14.187,30.171,23.409,13.995,16.475,4.355,7.745,18.

568,6.503,4.525,18.223,1.864,0.912
8.897,51.928,4.3,20.552,5.259,17.004,18.562,24.214,3.607,13.419,3.689,2.082,3.2
93,3.163,6.442,1.134,4.097,1.211
8.935,37.751,9.452,16.765,3.846,11.912,13.389,11.491,9.958,17.985,5.357,7.416,4
.611,74.3,1.601,11.552,2.992,1.537
19.358,7.51,2.913,15.9,8.837,12.337,11.541,51.224,9.323,11.147,4.081,4.443,6.76
3,3.41,10.215,12.891,1.448,1.652
19.106,8.123,7.082,49.934,4.394,7.236,14.717,21.839,10.461,12.26,2.389,1.382,8.
447,4.091,2.171,11.096,2.289,11.531
16.405,33.031,5.105,25.714,4.826,11.19,12.91,24.217,12.636,10.38,9.78,6.829,10.
238,11.957,3.835,4.502,1.413,1.279
19.634,88.995,7.627,11.617,4.103,23.13,15.899,14.398,12.786,2.698,8.422,2.51,6.
163,10.706,13.313,12.038,2.613,12.982
32.819,39.413,8.088,11.16,2.407,13.535,29.016,69.952,15.026,2.192,2.743,2.627,1
2.23,7.765,5.296,14.631,2.282,2.957
33.453,47.059,7.694,29.67,4.166,15.987,17.355,17.559,8.045,32.266,4.867,11.179,
8.33,2.911,5.601,31.513,2.388,1.347
16.083,27.465,4.226,19.96,7.297,11.225,22.815,2.668,8.086,9.571,3.986,12.772,2.
82,3.281,4.998,16.519,3.334,1.179
30.631,17.283,6.263,11.834,4.705,10.789,17.04,3.707,11.579,4.448,4.058,8.689,13
.524,8.506,4.67,19.717,1.42,2.182
46.683,42.523,9.111,12.103,4.186,8.924,24.633,25.928,11.136,7.055,3.516,7.328,5
.931,4.283,2.533,1.766,1.791,6.179
19.448,25.12,10.327,22.519,6.11,15.363,14.942,33.058,9.533,15.774,2.411,5.807,2
.451,4.999,3.748,8.196,4.948,0.876
80.245,23.824,11.14,26.27,6.565,46.175,9.828,3.315,25.139,8.635,3.992,5.489,3.7
36,14.719,2.074,9.051,1.46,4.072
9.989,25.291,11.405,24.2,12.486,17.812,18.831,5.36,19.102,2.738,3.47,1.666,8.85
7,2.77,9.779,11.046,2.263,1.222
11.651,18.304,6.934,84.979,4.986,10.955,33.867,15.171,9.241,3.569,3.618,5.54,3.
355,6.992,2.944,16.702,2.836,1.348
13.037,10.559,5.913,16.019,3.545,16.902,14.485,28.998,8.451,13.156,5.733,7.585,
4.745,19.883,4.002,22.968,2.663,2.065
18.382,29.749,5.579,30.347,7.601,7.73,4.726,58.945,13.208,33.178,2.684,6.997,10
.503,21.103,2.786,19.707,2.118,5.473
18.638,15.245,4.264,23.116,5.779,10.266,23.262,23.771,15.063,26.058,12.46,47.19
8,4.713,25.004,3.085,27.835,1.849,1.15
17.765,8.76,11.368,15.113,4.611,10.332,24.096,13.415,26.521,2.851,3.239,4.751,3
.911,2.448,3.594,23.824,2.097,2.744
14.466,29.171,13.765,15.622,3.908,17.926,9.767,22.954,8.148,7.331,9.922,7.324,7
.292,3.557,5.342,12.708,1.798,4.629
11.467,17.845,14.484,13.258,8.846,38.365,11.993,43.354,9.556,25.307,5.976,1.965
,2.584,2.299,12.29,1.534,1.73,1.619
30.912,11.554,8.666,22.56,4.138,10.314,23.035,18.081,13.205,25.803,3.758,4.069,
4.187,6.234,15.564,5.082,2.266,10.668
12.859,34.187,3.796,13.068,4.3,10.208,8.743,9.473,9.58,2.633,7.727,1.172,3.603,
6.569,13.398,3.441,1.516,2.48
7.969,14.355,8.889,21.426,3.845,14.445,19.655,22.498,9.059,11.446,6.328,2.631,9
.525,39.245,9.06,21.587,3.065,1.53
23.955,19.832,5.717,12.575,4.234,29.456,14.749,56.542,7.742,4.643,3.491,7.427,1
0.646,8.336,5.804,6.893,2.756,2.089
15.865,23.543,8.274,28.019,4.607,11.528,31.097,21.116,9.283,7.713,3.95,10.668,6
.779,22.252,4.825,23.023,3.162,6.816
17.088,39.41,9.607,24.258,7.572,15.911,36.287,70.248,11.067,57.898,2.018,4.24,4
.936,21.233,11.99,18.028,1.761,2.054
18.313,23.507,10.892,22.267,10.121,12.457,32.343,87.711,6.926,14.48,4.617,3.759
,10.317,13.112,4.762,8.054,2.165,1.361
5.555,38.199,9.184,20.291,3.388,10.106,31.899,31.377,9.075,22.359,3.473,5.342,7
.608,1.413,7.521,16.881,1.842,2.166
15.474,17.767,9.308,20.659,5.807,36.668,15.559,13.037,14.149,11.365,3.729,7.077
,3.821,3.223,4.4,2.879,4.68,1.558
7.3,4.523,3.789,18.926,10.247,14.579,17.89,23.607,15.853,48.113,6.593,14.781,6.
382,4.711,10.953,10.34,2.331,1.11
26.421,36.724,3.486,43.648,3.918,18.407,23.245,40.075,15.498,10.711,5.019,74.13

9,9.529,3.522,7.504,15.293,2.247,1.41
17.236,15.956,3.699,26.656,12.459,16.493,15.49,6.001,6.636,6.541,2.81,6.121,5.759,18.042,9.717,26.038,3.733,3.366
10.199,12.872,12.055,9.87,6.478,13.887,17.378,10.835,11.016,15.232,3.699,14.997,13.804,21.841,5.786,25.164,1.546,5.873
25.475,7.095,5.56,23.063,6.107,12.518,6.462,38.389,11.749,9.682,2.666,7.527,10.791,6.273,6.773,4.582,2.22,4.878
16.922,17.447,7.9,19.818,7.618,31.339,16.51,39.272,15.184,12.15,2.93,4.866,5.623,6.067,8.418,7.78,3.487,5.575
23.434,64.216,4.651,13.973,6.498,11.308,44.942,3.621,5.992,26.282,9.324,11.923,5.687,2.853,15.296,20.896,1.592,1.65
22.375,16.976,8.403,10.405,3.761,1.214,10.582,18.447,15.02,2.272,3.165,7.628,6.973,1.954,7.528,23.397,1.8,2.022
8.294,25.401,15.182,19.885,4.483,8.538,10.167,4.859,12.523,3.489,4.169,7.838,12.194,23.429,8.133,21.943,4.245,2.134
6.896,38.748,6.449,57.404,4.391,9.508,3.735,7.717,14.483,50.178,8.59,12.569,6.329,12.987,7.66,19.049,2.155,3.446
24.33,14.561,5.65,12.732,5.074,7.714,12.8,42.053,17.626,8.42,2.237,6.039,5.922,13.752,6.496,4.865,1.491,2.035
8.81,16.759,6.886,6.627,3.313,14.695,11.39,23.39,19.419,18.48,3.668,1.314,10.053,6.618,11.734,26.554,1.566,1.491
11.425,15.514,6.045,16.437,3.832,8.859,29.265,68.565,7.754,43.664,4.532,11.209,8.93,6.354,5.448,2.144,1.559,2.15
11.613,48.918,2.802,9.879,5.732,3.603,21.651,25.5,8.368,15.207,2.582,11.908,3.601,4.583,10.085,12.588,1.236,1.779
17.028,26.048,4.403,34.01,8.215,8.162,13.386,44.685,5.635,18.444,5.175,5.132,3.002,1.962,8.472,10.984,3.474,2.228
15.005,36.606,4.517,11.734,4.971,14.071,25.383,17.217,10.634,29.041,2.669,10.601,11.813,4.882,4.682,3.801,1.954,1.727
7.386,19.208,4.729,15.663,6.058,24.099,5.774,11.059,7.55,10.533,3.528,10.025,8.95,2.733,6.305,3.1,2.017,7.29
19.486,3.118,8.042,19.643,4.17,1.525,26.023,11.468,15.58,6.579,1.654,5.926,4.001,4.198,12.008,3.903,1.854,1.435
23.014,38.739,4.472,17.067,5.228,9.728,12.067,17.322,9.735,12.428,3.42,7.123,4.242,2.428,8.691,8.915,1.273,6.886
18.945,37.581,4.337,15.97,5.732,26.483,26.178,18.63,25.035,9.82,5.706,4.936,3.92,2.283,11.369,9.396,2.544,1.488
15.584,29.555,8.266,22.971,2.809,8.927,18.818,14.184,13.607,33.015,9.348,5.024,7.573,3.142,4.515,15.88,1.439,1.184
12.374,46.799,4.383,21.921,6.478,14.346,20.712,3.811,10.268,32.296,2.439,2.967,5.694,2.91,11.864,2.122,1.818,4.362
58.165,4.579,7.572,28.197,4.85,9.946,27.979,43.441,8.415,5.267,3.673,4.015,1.728,6.685,5.7,39.483,1.597,7.479
17.161,25.376,9.811,34.001,7.537,11.364,16.972,15.377,11.311,3.849,2.985,8.869,5.207,2.357,9.192,22.004,3.285,1.94
8.779,39.546,7.068,12.338,3.693,10.423,40.291,35.975,12.813,16.573,2.815,5.064,5.31,5.975,11.054,17.607,1.564,75.108
18.923,20.2,6.725,3.862,3.497,22.348,12.676,20.494,8.883,17.671,2.937,1.9,9.606,24.713,7.287,5.565,1.538,3.431
9.111,17.27,9.809,22.36,5.603,30.438,21.804,29.498,7.592,29.443,3.583,3.882,9.016,1.181,9.296,2.968,1.21,1.314
20.149,22.018,4.541,13.442,4.191,10.587,37.115,35.788,4.418,6.728,1.908,11.209,7.256,9.05,9.603,3.28,3.153,5.001
10.654,25.213,21.619,26.51,5.596,26.637,29.159,10.945,38.805,18.876,2.281,11.502,10.034,33.758,9.319,4.792,3.304,12.172
28.563,27.414,6.204,13.945,4.033,11.103,10.517,13.601,9.743,2.075,5.899,8.816,8.606,2.741,8.493,14.289,2.629,3.345
10.762,36.417,5.885,25.405,5.407,32.125,37.334,17.425,14.856,10.754,2.948,6.596,11.568,10.485,2.463,14.673,2.105,7.224
7.821,23.416,9.094,46.324,3.37,9.663,13.039,13.99,17.341,6.929,5.496,8.704,3.264,2.583,2.037,23.331,2.083,1.364
11.824,33.223,3.824,21.769,5.857,12.32,18.579,78.884,17.652,20.405,8.149,9.055,9.157,10.75,1.455,1.783,2.297,4.07
10.779,22.864,10.533,13.197,7.504,15.906,36.102,39.534,7.976,6.112,4.61,3.044,3

.131,3.248,10.993,53.319,3.115,9.666
20.161,45.363,8.954,5.861,3.13,6.584,10.449,13.004,12.389,14.556,3.251,5.958,12
.948,16.748,9.881,27.202,3.223,1.487
9.7,5.202,5.386,3.294,4.384,6.827,28.32,23.655,12.166,2.092,4.422,6.59,10.471,2
.08,8.077,16.357,1.986,1.161
20.15,13.525,4.618,14.252,3.691,15.117,11.104,24.28,11.837,11.059,3.485,4.18,12
.284,2.228,7.501,7.864,1.839,2.107
24.298,30.871,5.101,31.331,3.18,8.785,14.931,22.629,25.675,8.141,3.742,10.75,8.
709,7.493,8.477,18.919,2.193,6.417
9.217,16.313,5.04,26.583,5.294,7.978,5.885,35.369,9.483,16.488,3.069,8.156,4.30
5,31.495,10.807,2.984,1.788,5.048
21.329,34.232,4.32,19.928,4.121,11.393,6.66,37.933,17.018,24.28,2.477,6.877,4.2
29,3.547,12.717,5.173,1.564,4.754
15.965,15.631,5.699,18.68,4.103,6.631,27.402,25.989,12.638,22.968,2.36,10.205,8
.488,13.419,2.008,16.342,3.179,2.448
9.857,21.169,3.461,28.56,13.394,25.413,30.38,30.086,31.886,9.998,2.174,8.801,4.
079,27.063,12.135,6.184,1.553,1.44
33.667,7.786,6.912,26.661,4.108,33.498,8.959,38.095,7.14,13.698,3.232,7.38,7.13
3,6.968,13.708,19.292,1.686,8.531
15.091,47.984,5.341,33.602,9.234,11.646,16.56,12.666,9.132,6.333,5.032,4.709,3.
509,27.77,13.167,21.267,1.854,9.866
14.788,8.697,15.331,27.122,4.936,12.159,10.442,24.142,6.947,14.9,2.718,6.801,11
.425,6.435,8.697,23.552,1.835,5.621
15.473,36.13,2.972,48.672,5.594,5.857,9.466,19.033,17.418,2.799,3.727,7.294,4.8
66,16.391,11.436,22.084,1.953,1.009
8.18,40.192,4.479,19.492,6.372,10.487,13.738,26.799,12.234,18.372,2.936,7.484,5
.144,9.188,5.09,20.548,3.213,1.85
10.614,30.255,6.835,18.12,4.876,6.564,8.202,5.306,9.553,11.216,3.69,5.84,2.074,
2.301,5.091,17.189,4.715,6.497
13.101,16.113,7.694,27.336,7.688,30.204,15.748,63.342,11.336,53.023,6.214,2.67,
10.462,2.548,8.075,7.439,2.264,94.164
24.786,11.736,11.869,16.798,4.409,12.152,11.754,23.372,14.277,3.532,4.495,4.717
,7.824,3.376,5.444,20.396,1.537,1.464
17.179,25.813,4.803,15.738,14.431,26.814,18.519,20.781,19.073,15.596,3.626,1.79
9,15.693,3.463,3.287,19.214,1.503,6.532
18.491,45.054,4.319,30.358,5.572,15.303,11.466,15.878,19.191,14.681,4.871,3.65,
9.412,3.294,11.929,18.217,2.693,1.725
9.403,24.205,4.258,15.238,5.599,13.522,11.193,23.661,4.519,11.715,1.43,10.86,8.
406,27.935,3.482,6.351,1.826,1.131
13.181,23.443,3.353,11.198,10.465,13.142,10.151,21.138,15.151,6.884,3.507,5.465
,2.96,1.302,12.957,10.647,2.004,1.322
38.17,11.035,12.749,17.608,10.741,22.919,15.976,7.542,9.092,2.112,7.183,10.585,
2.692,6.4,10.47,14.606,1.495,3.864
11.45,1.752,13.61,20.309,8.984,14.182,6.538,18.829,18.207,5.715,2.877,4.988,11.
08,16.32,13.921,2.82,2.424,2.368
13.377,16.892,15.073,13.484,2.632,12.219,8.86,4.995,37.108,13.463,2.92,7.016,11
.512,3.783,9.367,3.456,1.99,1.502
23.107,24.599,5.637,14.291,3.403,31.704,12.81,4.397,13.778,31.09,4.849,10.653,5
.719,8.344,2.783,26.583,3.58,1.675
14.096,11.294,3.927,17.525,6.568,16.988,8.305,26.476,17.668,9.994,6.085,9.623,8
.177,3.238,8.724,2.117,1.319,2.996
19.61,15.206,16.862,10.145,7.656,12.201,8.101,16.622,8.754,4.085,3.914,7.431,1.
994,5.784,2.792,20.399,2.009,1.652
7.008,23.465,4.949,8.283,8.757,16.147,6.283,27.742,4.325,22.145,4.9,8.473,16.81
9,23.784,6.862,7.526,1.535,14.138
15.301,6.741,7.26,45.323,10.937,8.71,2.732,10,14.808,16.36,2.133,4.924,2.507,4.
315,4.481,8.851,1.427,6.043
18.868,38.652,7.489,21.43,3.328,1.233,13.649,17.005,19.687,20.315,3.242,8.925,1
0.217,13.343,10.75,9.504,1.959,1.595
12.774,22.388,7.309,5.423,4.567,11.082,8.486,66.521,6.266,5.867,3.153,7.817,4.6
86,16.669,2.687,47.17,2.333,1.942
11.263,20.793,9.583,20.564,4.571,28.034,29.355,23.778,14.312,12.521,15.998,8.70
9,4.529,16.045,3.127,21.168,1.417,7.572
9.745,11.645,13.201,22.563,5.537,16.943,24.395,42.485,7.53,4.977,2.615,6.098,12

.407,12.761,4.502,6.816,1.873,2.224
21.289,27.861,3.975,25.643,18.652,15.172,6.89,27.767,16.818,21.336,2.843,2.725,
6.466,6.528,4.343,18.795,1.932,1.639
6.786,25.868,4.27,12.53,6.325,20.214,10.878,25.206,17.858,16.503,6.371,6.864,8.
008,17.773,18.965,5.161,1.587,4.537
33.546,7.11,5.302,20.204,5.294,30.134,14.787,22.235,10.277,24.381,2.161,5.182,8
.087,11.48,14.509,3.482,3.575,2.893
9.833,20.583,11.69,38.443,10.555,15.024,6.596,29.589,15.861,7.271,3.277,6.586,8
.866,20.851,7.827,20.033,2.192,1.314
20.452,14.806,5.701,10.323,3.633,10.366,9.935,4.321,7.839,3.476,3.06,11.938,2.9
6,19.322,8.654,10.591,2.407,1.631
26.168,26.112,2.923,48.965,5.08,10.646,19.344,11.4,15.799,4.641,4.553,11.207,5.
21,3.373,7.251,84.103,1.852,1.083
11.644,11.445,5.913,9.712,4.231,16.818,9.341,25.325,12.365,1.28,3.45,4.974,6.90
7,1.139,14.602,16.538,1.837,1.299
11.787,24.332,5.532,12.472,4.069,13.856,11.163,35.916,10.25,2.593,5.392,6.605,9
.47,3.502,2.974,8.605,2.323,2.141
9.645,12.92,7.374,16.434,3.958,17.742,19.982,26.452,7.404,8.883,1.536,11.082,9.
303,4.07,12.133,4.817,2.683,6.845
6.453,21.258,7.21,3.857,7.855,5.35,10.976,30.428,12.749,24.462,2.958,5.091,10.8
23,11.226,11.894,6.565,1.937,1.913
7.365,12.267,10.682,22.797,15.053,31.497,26.404,24.265,7.709,2.389,2.317,8.26,8
.326,8.057,3.539,3.199,2.257,1.036
14.271,47.08,4.762,19.399,3.741,25.39,41.96,24.805,8.032,6.516,6.004,13.856,10.
26,15.483,10.156,16.389,1.916,2.535
17.705,17.967,5.099,16.615,3.774,27.446,9.812,14.693,11.26,40.807,4.461,6.83,5.
931,11.19,4.16,6.493,1.335,1.906
12.445,15.94,6.486,40.248,3.857,35.434,16.096,31.843,16.422,32.396,4.013,3.683,
9.313,2.817,2.645,1.436,1.865,2.024
42.757,18.921,7.373,40.585,3.511,20.395,20.501,27.069,12.215,13.102,9.096,4.381
,4.066,19.549,9.269,25.792,2.736,3.295
44.4,48.929,9.463,15.848,4.973,16.434,17.445,16.642,25.451,3.016,2.378,10.821,2
.651,4.986,6.099,1.564,1.538,8.811
12.094,15.758,10.883,30.644,5.402,31.618,29.936,20.763,17.161,25.145,8.317,3.94
7,2.188,3.139,6.995,17.68,1.786,1.959
16.805,10.536,2.567,30.511,7.25,6.88,16.212,30.481,12.66,15.482,3.107,13.477,11
.488,6.511,2.7,9.211,1.696,2.146
12.701,26.565,4.399,27.314,10.289,27.808,22.222,17.174,14.005,15.123,5.125,4.53
1,17.675,6.627,3.069,13.128,2.287,1.194
4.444,5.768,12.984,33.599,6.319,16.105,4.168,44.257,24.22,18.756,2.209,15.976,2
.921,25.443,6.938,20.963,1.904,5.313
11.754,22.684,13.604,98.294,9.186,14.913,28.294,38.902,5.937,4.126,3.337,3.212,
4.312,26.003,12.409,17.683,1.599,6.963
19.644,13.113,10.738,31.089,8.138,9.534,7.645,19.906,8.464,77.346,3.251,3.638,1
0.621,4.541,10.985,16.536,4.288,3.074
28.425,56.962,4.011,46.327,6.175,32.735,15.673,17.068,10.843,23.06,3.378,1.867,
5.041,2.231,3.625,24.426,1.52,1.635
22.442,30.412,7.541,38.218,5.409,8.962,10.922,23.271,13.177,24.203,9.742,11.154
,14.613,19.549,3.401,4.723,2.215,9.074
15.201,23.702,2.324,42.787,7.328,12.544,8.505,8.116,15.864,20.846,9.723,3.602,9
.006,3.961,11.917,22.349,1.216,4.71
28.935,23.764,4.471,16.931,7.016,8.311,11.081,25.186,12.992,43.588,2.259,7.361,
8.153,18.266,15.291,8.976,2.456,2.132
16.665,19.794,11.392,10.324,3.997,9.323,15.888,24.444,8.131,2.379,2.721,10.913,
10.428,36.741,4.661,3.758,2.383,1.312
12.337,23.785,7.365,4.972,3.014,13.105,16.368,39.764,12.701,14.517,2.782,5.622,
11.262,32.471,3.959,14.474,2.153,11.052
12.091,15.42,7.074,14.83,3.323,15.14,12.484,11.058,5.262,22.409,3.749,9.786,5.3
98,17.944,4.888,23.051,1.296,1.794
19.579,15.949,5.62,34.498,5.446,26.853,25.254,60.997,14.614,14.49,9.802,9.496,1
1.124,6.733,10.975,2.039,2.633,4.92
7.284,33.09,5.702,11.881,17.218,25.281,10.816,19.664,31.473,20.358,6.845,9.94,8
.937,6.78,12.754,19.57,1.436,1.339
24.075,8.839,6.357,3.932,14.643,15.634,21.169,3.045,10.061,10.695,2.159,11.218,

2.27,3.231,12.305,36.27,2.046,3.023
14.159,26.738,9.192,17.245,10.559,19.666,15.165,22.623,6.662,5.065,1.91,1.402,4
.815,4.188,5.096,22.548,2,4.051
12.788,36.543,8.565,48.274,5.412,16.975,21.561,42.998,7.572,3.453,3.058,2.59,9.
487,1.978,10.994,9.437,3.782,1.229
13.318,7.564,3.925,23.572,4.915,15.15,5.395,21.794,21.381,10.906,5.344,5.461,4.
585,11.92,7.154,13.807,2.306,1.657
9.416,32.194,5.975,3.961,7.918,20.273,12.894,9.696,7.438,11.988,5.034,10.021,4.
231,23.312,9.693,4.806,2.748,2.822
17.482,32.511,9.934,24.224,3.995,8.313,5.779,19.643,9.608,73.262,5.157,1.303,3.
681,3.207,12.774,17.678,2.145,1.87
10.531,46.853,6.892,9.74,5.219,11.581,4.893,26.495,12.846,6.842,3.054,11.967,10
.555,28.234,4.051,21.533,2.71,11.803
40.003,28.309,4.247,47.396,5.214,11.114,19.727,5.236,15.426,22.498,2.806,4.186,
7.865,9.84,3.971,35.768,3.97,1.349
16.394,20.387,2.951,26.446,4.867,9.896,10.538,15.966,4.938,16.838,4.919,6.917,3
.879,3.971,17.189,5.315,2.67,3.03
7.927,25.513,10.42,50.151,6.377,15.712,26.361,14.905,7.634,18.027,6.141,4.755,5
.55,3.399,1.638,4.344,1.81,2.045
16.295,15.52,7.526,33.136,4.299,14.729,22.907,51.326,20.022,21.594,6.837,3.518,
9.594,2.034,9.384,4.964,1.195,3.088
12.741,15.219,2.883,14.375,9.336,16.606,22.135,25.196,8.294,43.33,5.06,1.791,3.
258,10.664,9.446,21.746,1.999,4.261
12.071,15.975,10.349,24.235,6.619,17.556,36.143,39.447,25.981,3.6,6.197,11.938,
4.83,8.259,3.624,3.222,1.62,1.517
22.572,20.922,4.717,26.676,6.621,33.791,10.239,45.792,8.611,7.499,3.509,7.798,1
0.922,35.863,7.655,7.226,2.398,1.537
19.239,31.13,7.81,6.64,3.966,9.53,20.461,25.411,22.565,7.678,4.807,4.497,8.477,
2.504,7.125,1.312,2.009,1.75
12.048,29.454,5.774,36.578,4.404,16.752,22.879,21.699,14.314,3.783,5.827,6.385,
4.359,2.082,12.276,10.349,2.374,0.884
18.195,33.673,4.544,13.677,11.434,9.38,23.821,25.248,21.26,39.467,2.613,1.575,9
.696,5.27,3.713,13.251,2.814,4.298
35.965,18.138,3.861,19.242,11.646,29.084,41.432,25.26,12.374,3.308,3.466,11,10.
022,2.701,11.181,4.551,1.457,5.305
11.908,25.247,12.626,9.965,5.139,14.486,18.871,24.095,9.449,22.696,4.123,8.125,
12.257,6.929,10.905,8.81,4.56,11.233
10.409,32.448,7.137,20.175,3.904,30.708,9.376,15.21,11.976,3.424,3.974,4.157,24
.958,18.856,4.142,20.162,1.898,4.044
23.041,15.708,5.147,18.169,5.24,4.958,19.326,26.752,7.555,5.138,6.402,2.899,6.0
91,6.867,9.722,8.874,1.903,1.347
14.929,3.616,4.987,11.733,7.575,37.779,11.537,23.418,16.789,12.371,5.459,5.05,3
.984,8.793,4.637,5.931,1.83,5.64
11.687,28.934,10.944,25.53,4.993,22.314,14.336,4.095,20.769,67.019,3.176,5.012,
3.293,3.533,10.445,4.127,2.04,2.852
7.266,20.523,4.866,21.462,5.163,15.071,21.471,10.351,10.813,8.415,3.756,4.026,2
.754,2.494,3.44,1.505,1.421,3.56
32.712,19.595,6.488,10.861,7.724,14.497,5.496,7.104,15.767,3.233,4.481,9.889,2.
621,2.19,9.238,9.878,1.349,1.711
48.73,2.379,3.103,18.075,7.464,24.218,8.241,36.41,18.636,11.784,3.51,1.923,16.8
51,19.118,7.793,3.978,1.693,1.312
10.337,16.956,9.48,22.67,4.456,13.545,25.809,11.685,8.705,2.638,3.718,4.342,14.
48,9.253,5.458,3.543,2.84,1.836
21.991,23.367,5.352,26.093,3.646,11.304,29.883,15.302,9.794,24.716,3.212,4.958,
12.463,22.01,11.553,13.714,1.442,1.461
13.023,35.393,8.209,33.797,7.252,7.43,28.057,4.601,10.507,24.701,3.29,10.645,16
.616,25.48,4.299,4.149,4.639,3.544
12.273,23.256,13.157,29.694,3.693,8.303,12.196,25.219,8.845,12.235,3.504,2.605,
8.921,3.223,6.166,5.02,1.87,1.605
15.497,20.54,11.02,18.81,5.36,24.204,18.701,22.849,17.206,54.302,5.037,3.59,11.
427,6.811,8.1,23.213,1.89,1.275
12.472,12.149,13.272,26.472,5.492,11.08,34.038,3.104,11.087,15.801,2.357,7.415,
10.439,3.218,7.564,8.291,2.07,1.411
13.2,8.609,5.376,20.508,5.164,10.76,17.465,16.736,13.969,17.369,3.971,6.3,5.413

,3.95,5.936,19.668,1.543,3.676
6.755,18.773,6.742,47.275,4.298,8.534,13.68,23.185,9.96,4.612,2.058,8.428,2.724
,7.306,12.13,8.322,3.396,4.969
26.089,25.712,6.667,57.177,5.956,9.616,21.004,47.912,5.842,22.471,6.008,2.846,4
.177,1.754,3.396,9.623,1.414,1.201
15.002,23.975,8.285,21.306,6.9,22.152,16.272,23.602,10.895,14.397,2.369,4.616,8
.249,5.78,4.34,6.821,2.05,5.087
14.109,24.671,4.518,13.462,7.212,9.645,6.316,22.955,14.746,11.166,4.371,10.538,
4.449,3.535,11.096,3.927,1.6,14.479
11.264,7.758,4.408,25.934,4.738,8.64,7.466,33.956,8.771,6.881,2.243,6.081,9.17,
7.626,8.648,7.157,3.577,4.885
11.391,2.993,4.296,24.436,4.088,8.695,17.157,15.637,9.492,3.442,6.671,5.501,11.
129,8.324,8.698,18.823,1.295,2.731
36.557,21.771,6.178,52.48,3.882,8.322,20.068,23.001,29.908,13.437,4.058,4.965,8
.647,3.968,10.112,16.462,2.36,1.298
14.251,16.078,7.048,19.966,3.443,9.048,14.126,31.93,16.601,26.804,3.793,7.235,1
0.945,28.761,15.414,24.383,1.85,1.085
8.651,14.938,10.564,38.1,4.454,23.123,12.513,36.762,16.548,5.652,4.6,3.253,10.8
67,1.97,4.01,2.367,2.336,1.348
11.274,34.432,4.284,26.899,2.46,14.891,15.952,47.167,23.613,7.722,5.28,13.772,3
.144,3.458,11.639,12.127,1.945,9.252
15.771,28.32,5.109,17.25,6.846,29.992,7.032,15.135,3.836,44.769,4.763,4.358,5.4
63,2.543,12.738,2.85,3.7,1.983
16.99,2.644,8.993,14.996,5.119,19.946,23.41,15.953,14.356,2.537,4.486,8.73,3.17
4,1.442,3.066,11.189,2.622,1.495
13.247,9.033,9.922,24.549,2.743,25.818,10.441,44.151,9.051,8.986,2.492,55.039,2
.722,3.861,9.191,3.29,1.679,0.996
14.568,20.825,6.268,33.093,2.426,11.26,19.359,61.101,7.437,10.174,6.908,11.103,
13.274,20.281,3.182,7.511,1.348,1.422
13.792,32.528,1.82,52.116,4.413,10.79,16.891,25.534,4.057,17.551,2.707,7.901,3.
549,18.786,2.706,6.978,2.704,2.247
23.952,12.205,8.185,30.075,4.927,30.018,17.119,18.173,6.033,35.444,3.924,10.176
,13.793,9.993,10.868,3.943,1.417,1.591
17.982,24.067,4.637,16.002,4.308,16.385,2.92,41.074,7.544,17.78,4.266,4.79,3.06
4,5.306,8.291,1.46,1.979,2.941
4.769,20.892,6.638,19.557,6.87,10.432,18.295,40.049,7.147,9.043,3.692,5.661,3.9
67,21.32,4.472,53.969,1.454,1.987
16.378,15.254,5.662,40.203,9.681,31.719,30.069,20.139,8.321,7.125,5.192,13.472,
9.882,3.245,4.071,4.947,2.11,1.596
11.721,20.768,7.048,50.389,8.742,36.202,13.791,45.715,9.854,5.4,6.692,3.896,9.6
59,6.867,8.148,5.195,2.091,1.5
12.826,11.66,6.449,13.513,3.067,28.323,17.832,18.48,8.65,2.209,2.834,14.881,4.8
02,39.488,9.639,11.063,2.141,4.59
5.783,9.359,5.733,5.7,8.328,9.019,24.477,25.47,18.626,5.715,3.107,10.885,9.068,
16.206,7.365,28.098,2.189,5.53
11.324,2.162,13.381,17.545,6.671,15.757,12.926,64.597,7.293,26.255,4.245,17.592
,3.418,19.998,11.033,7.026,3.32,0.857
22.466,4.123,4.593,35.274,7.561,12.915,14.665,22.589,10.148,7.383,3.82,6.603,2.
569,4.183,10.945,20.685,1.591,1.675
9.497,24.183,3.24,24.739,4.42,9.781,15.369,7.873,10.567,8.099,2.651,6.365,9.772
,5.622,5.217,11.39,2.054,1.582
103.265,18.319,10.797,28.393,7.921,10.71,15.431,18.94,8.445,0.915,4.782,2.14,13
.559,2.828,9.365,23.449,2.459,1.296
7.739,18.758,7.201,13.442,4.389,28.487,4.622,20.344,6.709,8.155,6.156,3.673,2.6
25,1.221,9.224,19.264,2.184,1.546
21.417,17.681,8.694,8.436,4.41,15.624,14.356,23.31,14.125,22.967,5.511,1.977,10
.288,43.332,4.184,6.098,5.202,2.512
11.402,59.006,7.093,14.518,5.723,14.935,14.478,15.438,15.38,14.78,3.419,3.573,3
.227,14.408,3.429,3.044,1.767,0.908
36.817,33.765,3.623,35.263,4.886,26.859,18.16,40.216,9.174,58.825,3.46,8.086,5.
975,7.995,9.157,2.508,1.759,0.933
15.339,29.725,13.374,14.208,4.066,10.095,18.021,35.306,1.743,15.153,5.011,5.571
,8.124,27.694,3.961,9.544,1.731,1.982
13.677,25.453,7.087,42.541,5.166,33.224,17.163,7.83,4.563,10.491,6.652,5.697,1.

407,23.294,2.969,8.243,1.412,1.252
6.466,18.089,5.294,15.88,5.75,26.799,12.615,2.821,12.955,12.78,4.664,5.092,3.06
2,13.559,3.293,29.346,1.89,2.743
10.668,7.41,9.033,65.358,2.189,6.727,14.616,28.938,17.658,9.486,3.142,2.828,7.2
26,2.482,2.558,4.129,1.248,1.473
24.957,23.751,8.379,55.641,3.766,11.241,12.962,18.486,8.63,22.181,7.798,7.115,1
0.435,24.556,1.624,4.474,1.808,7.112
30.06,9.019,3.349,24.472,3.467,16.25,16.136,3.081,7.355,37.265,2.541,4.495,1.75
9,1.19,1.732,16.872,3.244,1.652
9.075,21.343,8.126,28.457,3.4,22.986,34.98,21.111,15.144,26.303,4.163,4.213,2.2
74,2.458,2.491,14.913,1.252,4.631
8.591,30.052,6.169,23.654,2.508,14.163,16.482,32.349,15.839,32.512,6.964,9.7,8.
686,4.645,4.528,14.04,1.825,5.228
13.05,48.017,5.659,16.041,6.443,12.852,24.052,23.576,6.052,14.749,2.042,5.592,5
.3,24.365,4.193,5.909,3.734,5.155
16.108,19.261,5.964,20.616,4.213,16.03,34.246,20.342,17.03,24.718,2.66,7.649,10
.173,2.76,10.025,21.679,1.871,3.963
20.597,33.978,5.572,16.748,3.893,10.323,14.847,43.463,7.483,4.544,2.808,15.622,
15.284,1.466,9.095,25.449,1.952,4.6
14.012,4.181,4.544,9.746,3.257,13.708,9.691,20.85,11.209,1.536,2.894,12.326,5.4
69,2.528,3.481,3.49,1.466,3.568
12.966,32.089,10.938,28.049,4.148,39.289,11.002,28.445,11.718,1.932,3.676,3.527
,8.062,22.055,7.124,8.353,2.064,1.657
19.098,18.442,12.727,24.339,6.234,8.836,26.691,31.106,8.684,9.577,3.331,2.077,1
0.306,2.914,12.6,11.724,1.454,7.332
12.334,19.564,6.365,25.161,6.863,10.831,10.369,37.274,14.371,3.312,2.321,7.74,1
.988,14.919,10.421,11.933,1.78,1.289
10.893,7.268,14.366,24.019,10.748,11.906,14.691,39.236,13.624,36.772,4.391,8.39
9,10.597,2.648,9.911,4.373,2.761,1.836
19.323,7.843,9.137,21.343,5.781,11.399,5.235,25.424,11.496,5.411,3.979,5.816,6.
464,1.058,11.86,4.787,1.535,1.488
14.382,6.909,10.132,14.17,3.579,5.552,17.317,33.272,7.852,18.232,4.232,4.626,11
.353,6.729,10.277,3.857,2.247,1.739
21.532,13.306,8.512,19.434,12.428,13.336,22.385,22.344,5.455,3.583,3.42,6.29,8.
663,4.044,11.399,10.788,1.634,7.657
9.333,21.316,5.064,49.475,6.17,10.295,11.574,13.287,19.849,3.464,4.394,4.042,2.
591,14.328,3.861,2.201,3.955,8.733
22.077,28.452,4.273,9.472,2.926,10.098,6.666,22.176,10.042,13.056,2.917,5.331,3
.623,12.529,9.627,2.657,1.597,2.428
6.286,20.605,5.754,41.436,2.879,13.805,14.212,30.984,12.147,6.845,2.232,11.975,
8.912,5.424,5.347,19.109,6.062,1.139
26.91,21.428,5.865,4.804,6.37,5.862,13.685,34.056,20.938,5.944,6.794,8.256,7.97
6,10.917,3.275,12.348,1.831,3.682
13.345,18.187,9.637,26.81,3.289,5.366,11.071,15.95,12.33,16.58,4.049,4.909,10.5
4,2.433,9.074,17.199,1.691,2.402
9.969,11.819,10.888,8.91,5.423,9.841,7.031,8.866,13.835,4.782,2.663,4.078,9.106
,3.006,7.67,15.917,1.49,5.196
39.991,8.855,11.907,83.724,5.986,21.734,8.699,21.7,12.381,36.595,3.582,5.475,3.
355,16.014,4.972,6.498,1.517,3.069
19.587,7.38,8.225,10.112,2.458,1.426,18.818,11.876,2.596,5.7,13.556,7.322,4.356
,9.079,4.741,19.636,2.794,5.805
11.031,22.461,9.798,10.005,6.56,21.363,20.967,20.047,4.735,18.625,3.502,2.43,7.
154,11.839,2.313,7.029,2.522,9.763
22.566,13.005,4.896,9.697,5.921,24.229,10.007,6.638,7.856,6.07,5.371,17.362,1.2
88,23.131,11.453,9.587,1.653,4.704
19.997,30.604,5.828,22.655,4.112,9.439,11.795,9.268,14.091,5.488,2.425,4.562,2.
376,2.41,12.969,15.842,1.555,1.637
6.732,22.54,3.631,17.037,8.406,7.203,18.987,69.031,10.679,5.288,2.88,9.335,4.03
6,3.757,13.085,23.032,3.18,4.955
12.951,28.673,7.882,31.051,3.213,25.602,25.995,7.421,18.945,2.374,2.418,8.466,4
.498,19.584,4.935,3.357,1.504,11.066
17.002,10.308,7.523,21.045,3.559,38.559,38.184,32.948,16.236,9.2,3.526,1.189,11
.639,16.793,13.749,18.312,2.599,5.537
16.314,6.881,4.461,50.191,4.451,13.887,21.501,7.85,11.153,15.542,9.2,4.345,9.66

3,13.089,6.276,6.248,1.647,1.748
21.519,6.045,5.616,11.029,5.97,8.278,29.869,16.776,9.63,14.064,2.928,8.885,5.85
4,1.893,3.015,22.312,1.932,1.364
14.127,21.689,4.048,34.462,4.201,8.723,11.893,13.016,14.43,1.587,5.41,5.554,2.4
67,73.359,10.773,12.815,1.372,6.337
11.043,12.594,8.587,24.318,4.664,7.7,26.039,20.183,11.76,13.949,6.804,2.31,4.44
3,29.131,8.404,1.594,1.982,1.771
10.284,40.273,7.585,23.121,3.214,7.13,16.722,34.037,9.844,15.807,3.84,5.163,8.6
36,3.584,15.603,5.384,1.61,2.854
7.394,35.011,12.233,19.341,3.71,20.363,21.635,71.09,11.325,2.039,2.984,6.259,9.
244,29.099,9.7,8.439,1.276,1.141
15.579,12.716,21.025,25.76,9.59,16.184,11.305,12.024,15.447,14.105,3.505,6.739,
2.967,2.234,2.666,20.322,1.644,2.91
11.52,4.493,10.346,24.543,16.988,8.316,4.183,3.849,25.719,6.82,4.146,10.44,9.79
,3.879,8.189,17.386,2.215,7.094
9.827,13.548,16.064,23.617,2.638,14.246,19.213,30.912,12.11,20.385,3.973,3.825,
3.183,10.165,4.268,16.543,2.024,3.876
12.328,16.168,8.469,35.747,5.207,19.387,17.018,6.233,18.422,14.439,3,3.076,8.06
7,20.92,11.53,8.953,3.492,7.631
10.794,22.7,6.941,57.574,7.599,13.144,2.958,42.237,11.168,10.618,3.366,4.434,6.
704,3.265,12.026,16.206,1.37,1.344
8.84,27.359,13.906,59.827,15.875,14.101,9.808,19.372,8.507,4.808,1.7,1.938,3.68
8,47.023,2.221,4.112,2.307,1.181
34.017,16.769,8.06,13.119,5.976,14.561,26.624,16.398,13.956,7.75,10.741,5.134,3
.121,9.056,3.907,4.351,2.906,1.194
11.785,27.268,9.32,7.051,3.255,33.729,14.43,19.029,6.98,9.458,2.776,8.432,8.921
,1.509,3.127,19.899,1.726,8.244
16.475,26.396,3.937,20.432,3.015,9.123,12.265,27.951,11.845,5.507,2.778,6.73,6.
385,6.004,2.544,26.035,1.302,1.565
14.678,22.488,5.566,19.186,7.227,20.861,23.416,8.518,14.412,40.291,2.229,3.525,
11.381,2.027,10.694,5.808,6.22,7.257
11.56,49.517,4.545,14.812,4.541,14.514,37.685,9.629,10.172,14.589,3.698,2.964,1
.605,3.144,4.212,22.707,4.583,5.779
23.596,17.88,8.379,9.578,5.124,23.122,26.661,45.702,7.685,11.744,7.955,4.22,3.7
45,30.41,9.097,22.497,1.673,0.727
10.596,26.732,5.503,17.457,4.686,11.754,17.728,10.015,11.474,35.788,2.72,7.47,5
.262,2.733,13.58,2.024,2.294,1.61
11.367,22.334,3.038,22.905,5.821,17.496,13.612,29.269,5.348,1.932,1.997,5.582,2
.865,4.234,14.933,16.671,1.713,5.734
17.925,17.979,7.056,7.345,6.365,10.137,29.118,36.326,14.238,16.574,4.553,7.001,
8.003,3.049,5.29,4.504,1.438,1.626
12.978,17.436,11.6,20.275,3.556,8.711,3.989,12.727,29.565,41.277,2.666,1.476,11
.457,2.618,6.032,3.928,2.777,5.109
17.195,39.941,8.764,18.468,10.406,13.16,13.306,29.523,8.65,13.155,4.49,5.449,16
.714,17.371,7.641,13.062,2.091,2.675
12.623,19.747,9.193,23.76,2.824,16.055,22.417,6.671,10.713,10.757,2.959,4.392,3
.701,13.457,3.909,25.879,2.928,4.519
31.161,19.718,4.356,20.733,5.903,4.149,9.701,14.169,9.138,3.993,4.919,12.019,7.
248,38.298,9.615,15.688,3.389,1.628
29.114,15.089,5.761,7.37,6.061,9.299,31.655,19.481,28.185,14.407,2.791,2.617,6.
06,2.206,5.097,13.761,1.264,3.305
11.086,21.935,1.891,11.847,4.2,10.984,17.034,12.929,20.601,7.787,2.99,35.473,5.
743,19.079,12.227,38.758,2.271,1.606
12.635,49.233,6.021,34.602,2.721,2.054,19.513,15.335,10.098,45.534,3.719,5.389,
8.146,21.989,10.772,5.19,4.232,5.094
7.975,17.052,4.383,5.663,11.875,10.599,18.47,36.361,10.06,7.324,3.79,5.331,10.0
41,22.322,13.159,11.711,2.958,3.776
12.734,65.511,7.795,37.319,4.096,15.95,10.133,21.834,10.194,8.828,2.557,4.653,1
2.373,3.083,3.905,4.721,1.573,4.455
7.892,33.679,15.291,15.612,4.936,12.498,9.968,26.088,10.257,4.185,21.786,5.832,
9.027,6.508,9.837,10.768,2.768,0.658
8.568,17.223,3.492,18.482,8.513,16.097,8.991,75.398,10.465,9.027,8.207,3.372,13
.834,3.337,8.224,23.007,2.02,3.294
13.667,16.078,7.125,23.378,7.297,39.274,24.447,16.278,19.147,21.003,3.564,4.856

,5.384,10.667,8.589,20.086,2.194,11.482
18.827,31.526,5.402,8.375,2.942,14.32,6.094,40.457,6.695,24.313,4.454,3.785,12.318,14.861,14.199,10.778,1.048,1.26
15.973,14.229,6.077,19.82,4.925,8.581,10.336,23.921,12.65,3.311,3.735,9.649,8.085,16.541,11.91,25.425,2.507,1.852
32.14,7.618,5.033,8.351,8.753,14.316,25.217,27.533,6.822,20.526,3.566,2.721,6.43,15.738,3.373,19.617,1.948,1.342
24.325,21.893,3.677,3.401,3.559,11.349,16.662,11.724,9.105,27.6,5.694,1.431,8.923,4.021,8.328,13.143,1.626,6.842
9.534,16.155,13.261,31.913,2.01,14.514,29.609,14.592,14.755,19.968,5.444,7.212,9.313,5.255,7.02,3.068,1.642,8.412
28.093,16.407,6.961,95.792,5.71,19.96,6.674,15.338,9.019,5.505,3.027,10.837,4.903,5.514,2.257,24.248,2.481,4.623
13.796,19.343,19.987,10.651,7.238,11.883,23.283,22.811,13.249,16.515,3.31,8.663,9.833,11.801,15.183,7.034,2.061,5.555
12.651,29.2,6.118,18.778,7.629,7.484,15.141,21.894,22.864,4.233,5.496,5.725,7.301,23.243,5.007,1.783,1.416,6.961
18.176,6.178,7.728,39.102,5.194,8.075,9.494,21.317,14.61,5.945,1.747,12.239,5.473,4.525,9.05,13.856,1.776,1.228
21.193,21.586,6.502,26.789,7.028,29.851,27.525,29.006,11.267,9.485,7.706,8.335,3.494,10.47,6,3.493,2.669,1.487
12.297,3.857,5.32,31.475,2.371,17.113,24.516,48.276,7.317,8.339,2.358,5.658,10.419,2.603,9.552,44.981,1.732,1.051
15.076,5.36,15.599,30.206,4.513,7.556,56.94,20.806,28.898,40.299,5.303,11.274,4.636,17.139,10.352,22.892,1.292,1.482
9.1,15.906,10.999,21.241,3.127,15.451,17.523,19.159,10.183,22.632,5.918,14.466,9.702,7.026,13.564,2.299,3.076,3.341
11.008,28.071,6.933,20.677,9.072,6.133,14.331,27.902,8.347,20.82,2.979,6.07,11.188,25.336,7.322,2.196,2.233,3.103
26.589,21.856,9.67,21.672,4.148,0.852,11.087,18.222,10.797,17.796,15.573,7.259,6.949,43.301,10.728,21.252,1.608,3.407
10.579,9.463,6.837,25.673,3.683,13.134,39.814,46.511,10.238,4.755,4.484,3.306,9.124,4.115,8.605,2.82,2.537,1.767
16.011,9.626,16.615,22.035,4.643,19.058,6.996,24.451,10.719,21.851,3.911,3.727,1.77,6.657,9.114,1.845,1.865,5.283
13.359,26.54,6.312,5.039,13.287,10.171,15.046,9.186,14.296,12.755,4.5,2.099,1.427,11.307,3.315,6.123,3.241,3.925
23.597,12.17,8.661,21.582,6.441,20.251,24.936,17.934,7.289,21.781,5.207,3.6,2.972,3.218,1.934,15.953,3.309,1.713
16.883,26.646,4.618,30.457,4.556,18.387,17.506,60.433,8.35,10.977,3.546,5.242,8.574,5.809,2.964,15.559,1.05,2.871
23.336,33.093,6.582,3.449,4.397,8.031,6.543,22.625,11.412,41.491,2.568,7.292,9.397,39.163,12.348,21.372,1.415,2.452
28.282,28.476,11.701,17.462,3.353,9.582,4.302,18.94,8.041,44.603,1.953,12.557,6.648,23.395,3.646,11.195,2.53,1.684
8.859,35.635,11.897,53.532,3.435,12.071,6.123,16.64,12.523,2.385,6.951,7.002,8.682,1.602,7.569,3,3.161,1.652
8.634,5.01,6.277,39.214,4.844,21.277,9.326,2.253,11.885,7.959,5.19,3.457,16.274,19.96,18.64,7.399,2.302,6.37
21.899,39.667,4.63,5.939,4.074,17.116,32.438,5.601,13.578,9.583,2.953,10.886,1.76,23.116,3.385,11.683,4.197,1.206
11.875,19.656,9.466,20.679,7.768,10.3,24.693,5.171,17.327,2.476,7.112,10.032,6.564,3.481,2.985,21.603,1.81,1.4
46.801,21.501,5.218,119.265,5.314,14.755,27.407,27.466,7.163,21.309,4.304,2.569,6.752,4.296,6.353,11.672,3.408,3.506
17.96,5.31,4.336,19.151,2.859,20.955,3.306,4.003,6.487,26.351,2.694,3.063,2.985,3.541,3.34,24.891,4.045,5.323
12.226,21.756,3.348,19.835,7.424,33.153,21.568,31.268,6.175,5.443,4.616,3.615,3.332,9.196,9.222,17.484,2.165,4.977
28.886,3.983,5.495,2.878,4.761,2.283,31.185,16.933,9.633,3.85,5.017,0.925,9.148,22.02,7.737,6.018,1.293,3.972
13.107,26.187,7.918,20.55,6.307,16.059,7.53,19.934,3.355,7.493,2.082,7.971,5.335,10.03,4.771,35.935,1.784,3.685
9.101,16.471,21.101,32.591,3.14,42.076,9.319,21.374,10.269,2.317,9.584,12.268,4

.89,8.199,18.517,4.213,2.06,1.545
28.777,33.711,10.981,17.627,10.356,24.256,17.825,38.897,18.087,46.289,2.534,4.7
67,4.571,9.1,2.517,4.712,1.902,1.274
14.824,2.713,6.591,19.686,7.048,8.852,17.44,11.218,28.553,13.42,4.112,3.847,2.5
52,15.282,10.571,4.181,2.705,5.744
27.22,58.942,4.349,23.742,6.526,20.613,10.427,21.505,18.302,17.24,7.954,10.689,
9.349,15.782,7.342,8.531,1.826,9.403
45.001,7.282,9.673,3.91,4.939,10.751,21.187,32.672,14.28,12.78,2.778,13.48,10.3
7,8.489,9.141,14.755,2.004,3.144
11.91,26.444,11.556,4.001,4.155,15.815,6.858,1.909,6.099,9.155,2.193,4.845,4.96
1,3.559,4.127,15.517,1.569,1.558
6.359,15.526,4.34,17.858,7.018,17.835,6.825,17.728,10.457,24.645,3.355,12.358,7
.961,14.342,10.258,24.076,3.71,1.153
17.49,6.868,5.788,13.461,4.334,13.446,24.935,18.746,12.187,1.125,2.2,8.776,5.61
3,14.359,10.021,17.385,3.316,1.339
20.174,14.993,7.882,22.456,7.77,14.303,16.331,19.521,16.005,1.904,6.835,79.679,
3.556,4.739,3.419,9.86,2.922,1.375
9.807,13.501,7.064,24.645,4.465,17.702,19.636,17.291,8.212,10.629,1.731,4.614,4
.061,4.726,10.733,20.113,1.762,4.353
16.626,20.823,3.803,19.149,4.836,35.301,3.845,13.809,10.228,20.191,2.767,5.005,
16.655,28.564,15.508,27.757,1.441,3.793
12.625,18.414,10.102,13.05,13.991,13.961,8.357,30.928,7.414,11.768,2.52,4.801,1
5.677,27.155,5.198,21.195,1.274,2.018
12.673,14.822,3.126,22.246,6.047,13.241,28.517,15.712,17.803,14.246,3.621,3.249
,7.729,4.184,3.4,14.037,2.568,4.78
9.717,28.455,3.427,8.928,6.489,13.607,13.004,2.62,17.465,11.257,6.053,10.362,7.
399,11.609,7.665,1.256,1.73,2.431
11.025,22.469,4.509,25.929,8.06,12.5,7.831,23.085,4.541,31.199,4.959,3.974,12.4
24,14.385,2.483,14.331,2.842,7.493
8.255,3.511,8.731,9.76,6.403,9.594,4.093,14.622,10.516,34.497,3.57,5.902,8.222,
3.007,9.093,20.694,4.184,3.599
8.52,25.219,7.276,33.695,7.261,8.477,12.444,45.807,12.018,8.581,4.265,4.665,10.
959,3.274,4.929,16.668,1.643,2.341
11.798,26.881,6.583,13.942,4.002,8.201,12.006,35.049,21.589,46.785,3.424,8.508,
12.686,17.349,3.912,5.833,1.475,2.621
18.264,48.389,4.429,14.671,4.218,10.215,18.82,25.194,7.069,10.915,3.549,11.75,1
0.651,2.316,5.776,8.955,1.575,2.517
12.647,11.038,9.077,19.064,5.273,41.907,6.24,29.749,20.193,8.775,2.263,4.273,2.
926,1.888,13.473,5.894,2.527,1.908
18.49,10.45,5.021,25.349,6.356,13.841,4.955,26.173,7.624,3.454,2.485,8.898,7.92
5,12.052,12.151,7.885,2.348,1.548
15.38,34.845,6.646,27.479,4.361,15.838,17.042,19.632,12.438,43.052,2.722,7.045,
3.122,4.206,10.622,24.545,2.779,1.758
9.348,13.731,10.827,11.718,4.931,32.88,13.539,20.158,20.612,81.327,4.123,3.811,
4.812,20.613,10.85,5.745,1.83,2.438
8.949,23.701,5.694,25.989,6.752,24.451,18.605,10.124,9.025,2.011,3.046,6.718,11
.472,7.844,8.505,5.58,1.265,10.001
12.904,14.655,7.822,5.735,6.06,12.018,19.489,33.223,11.215,8.184,2.752,5.417,8.
382,13.35,2.473,3.548,2.011,1.231
24.396,5.306,9.599,11.275,5.163,36.093,11.142,28.102,27.855,3.903,3.004,3.27,7.
002,7.774,8.129,10.899,3.905,2.112
12.289,13.376,8.791,11.331,5.868,9.617,14.026,70.321,14.643,32.166,5.059,11.308
,6.558,3.98,5.016,6.87,1.684,1.797
16.192,22.435,7.852,19.386,4.318,32.328,16.475,15.135,5.504,4.949,6.483,6.455,1
2.079,2.034,9.273,7.512,1.654,4.474
19.64,37.509,13.611,12.035,4.609,41.199,8.652,8.363,15.401,19.842,4.417,10.778,
7.171,8.064,4.076,7.841,4.783,3.459
9.519,10.012,11.061,15.803,4.19,26.995,16.526,41.658,21.041,8.785,3.88,7.988,17
.516,18.177,8.877,4.005,4.439,12.11
20.657,25.12,3.451,19.987,7.738,25.839,33.269,43.131,16.462,5.377,4.379,8.515,2
.584,2.848,5.397,5.135,2.411,2.018
8.545,9.747,5.504,27.02,5.424,7.404,20.092,10.861,10.684,7.885,2.791,7.565,5.37
7,6.807,2.473,22.015,1.525,1.343
17.541,22.152,6.805,35.693,4.112,8.175,7.619,20.759,9.062,8.53,2.936,4.147,3.23

5,39.077,1.672,13.105,2.819,6.314
14.298,6.023,8.434,22.914,5.14,17.38,5.705,41.856,8.957,43.744,3.105,4.83,9.429,2.219,6.495,22.739,2.231,1.704
34.313,5.719,4.375,8.914,7.97,2.341,6.949,26.325,10.545,10.219,2.233,10.607,13.584,5.109,13.026,20.108,1.336,1.593
22.086,16.809,10.52,24.269,4.373,11.297,22.774,51.008,12.902,16.696,7.04,1.995,5.578,21.34,8.32,6.237,2.451,3.293
24.742,22.903,4.585,11.285,7.475,8.885,16.776,41.422,11.519,2.707,4.012,4.191,6.412,9.479,2.052,6.105,1.229,3.682
54.964,3.125,12.135,10.489,8.289,9.711,4.288,12.053,8.504,14.271,3.234,2.256,8.412,3.585,7.155,3.298,2.518,1.519
23.302,7.286,5.302,38.265,5.084,12.559,9.26,33.402,7.351,3.499,3.924,5.955,9.229,3.661,10.867,4.236,2.409,1.447
17.753,23.356,7.26,12.977,4.158,10.918,18.422,6.936,12.885,4.421,3.486,2.605,3.631,8.273,8.615,17.216,6.072,1.338
14.996,31.231,4.709,10.16,15.055,26.267,32.953,25.689,25,6.788,4.765,2.774,2.906,7.706,11.063,17.917,2.719,5.146
13.287,16.409,19.866,35.84,3.04,11.389,43.356,50.57,13.927,11.04,2.626,3.578,5.161,4.291,10.856,39.625,3.086,1.18
17.183,14.308,9.206,8.461,3.245,32.621,21.342,2.595,7.208,23.695,3.467,3.173,5.529,2.587,4.07,6.342,2.325,4.708
11.295,56.035,3.719,16.465,3.674,10.538,2.311,8.148,7.376,20.003,2.374,4.704,7.753,9.415,8.812,7.001,3.173,5.266
8.792,22.578,8.874,24.284,3.357,10.956,19.745,9.175,10.459,3.673,3.007,4.094,8.852,2.82,9.72,22.327,1.429,0.987
14.391,16.993,5.269,28.382,3.742,24.122,7.911,12.666,13.688,7.285,3.321,2.244,20.285,4.72,3.747,24.487,4.515,5.026
12.315,34.399,4.395,25.364,4.654,34.473,46.988,66.22,14.237,15.97,5.379,2.522,11.053,2.396,14.009,7.496,2.263,1.074
12.114,11.351,8.538,25.404,6.747,12.71,17.867,13.543,13.924,5.834,2.991,9.45,9.823,27.859,12.26,24.303,3.587,1.807
22.638,21.77,7.717,18.885,1.859,11.099,14.563,34.858,14.894,6.067,3.522,12.706,7.087,12.291,9.484,12.694,1.282,6.993
12.096,18.29,3.408,16.325,5.971,14.962,16.777,2.188,23.949,8.52,2.803,8.11,8.866,16.689,4.667,7.298,3.222,1.301
30.048,54.567,8.362,10.325,9.71,11.02,10.431,33.444,6.984,11.176,4.236,12.261,10.621,1.321,23.766,32.437,5.323,1.518
17.813,21.971,5.015,11.726,6.138,10.584,27.488,23.859,15.91,17.376,2.149,13.657,17.584,9.821,11.813,19.569,1.355,3.247
10.027,11.522,12.564,22.774,7.808,10.995,19.588,20.272,3.365,3.799,3.142,5.733,14.394,21.33,6.065,14.516,1.372,1.479
10.017,15.017,14.421,43.963,7.378,10.857,38.765,24.46,8.857,8.827,9.633,3.76,3.916,25.034,4.338,88.345,3.776,2.983
34.009,19.022,2.707,18.489,7.71,9.189,13.154,25.585,14.605,2.343,2.792,2.607,7.244,2.61,10.254,7.351,2.993,1.904
4.228,25.942,5.018,11.203,4.03,33.783,7.137,5.164,19.369,14.104,4.107,5.736,6.493,2.142,6.604,7.302,1.907,3.383
10.585,20.152,5.603,7.498,8.204,2.756,6,20.908,8.451,1.815,4.703,1.617,6.454,11.74,12.078,9.024,2.441,2.555
8.977,28.488,2.126,8.207,3.946,30.916,9.282,8.452,13.934,29.497,3.796,4.937,1.99,3.059,13.163,5.641,1.629,1.877
9.732,25.384,11.914,9.178,9.727,9.021,18.055,32.549,7.716,3.587,3.739,10.466,10.501,29.41,4.29,21.041,1.87,1.403
12.725,12.396,6.441,60.458,7.872,31.555,10.74,26.045,9,6.354,4.463,14.798,3.636,8.286,1.501,22.1,1.47,2.357
82.105,3.807,5.162,22.946,3.845,16.292,12.65,30.521,13.62,20.877,8.583,6.255,10.053,22.378,7.119,26.421,1.888,1.619
14.496,10.375,6.346,22.051,4.341,12.445,19.445,39.056,9.146,4.957,5.698,1.645,9.455,12.606,4.849,10.98,2.216,4.011
15.844,11.521,17.536,15.174,3.412,38.029,8.656,52.054,7.776,15.491,3.578,5.421,10.265,7.562,4.736,1.24,2.118,1.805
34.218,27.547,8.074,13.855,9.294,10.894,24.54,14.123,6.253,29.883,3.323,3.634,12.907,3.215,5.858,2.253,1.515,1.446
29.716,6.941,8.144,16.884,4.28,19.726,13.214,23.217,19.239,4.719,6.077,12.419,3

.684,1.534,8.953,1.93,4.706,1.869
12.73,18.409,4.273,19.847,5.215,8.1,7.872,50.867,9.199,19.243,3.162,9.877,13.87
4,1.223,3.74,21.192,1.612,4.662
29.764,2.857,5.113,42.93,6.611,11.676,5.935,35.13,16.986,1.673,4.53,3.818,9.389
,12.603,8.781,20.671,1.823,1.432
11.111,9.933,5.924,2.057,1.226,0.977,2.646,25.186,8.751,19.94,2.823,1.517,2.179
,3.46,3.051,4.935,1.401,1.572
6.066,7.809,2.529,4.942,2.034,8.646,8.838,27.11,4.65,17.658,4.118,1.703,5.549,6
.267,1.901,5.19,1.281,2.669
4.632,13.642,4.457,13.559,1.821,1.539,6.42,21.151,4.314,16.985,7.847,5.03,2.038
,3.798,1.165,10.842,1.071,1.964
49.153,11.102,8.885,9.584,1.452,1.598,21.271,14.411,3.893,11.798,5.889,1.749,4.
437,2.027,2.387,2.489,0.874,1.288
4.032,16.866,4.483,13.786,1.67,1.151,7.311,19.026,4.06,30.566,19.529,1.794,3.52
8,3.297,1.364,1.35,1.24,1.274
9.763,10.863,13.243,5.89,1.962,2.699,3.569,20.416,5.189,14.104,9.742,4.871,2.29
3,3.304,1.02,3.936,1.193,1.916
3.212,11.163,1.599,8.61,1.031,1.498,17.14,17.566,4.492,6.884,6.464,3.653,2.301,
1.732,4.534,3.267,0.93,2.905
5.05,3.83,8.845,3.069,1.518,3.449,16.593,20.924,3.578,17.812,3.138,4.612,4.605,
3.449,1.722,3.015,1.077,2.061
9.302,12.193,4.489,3.36,1.61,2.582,45.405,3.763,5.379,30.765,7.183,3.669,2.312,
3.556,1.327,1.647,1.32,1.106
6.389,8.292,2.472,7.436,1.692,2.984,8.008,28.109,4.112,8.53,4.043,2.296,2.538,1
3.184,1.34,0.916,1.847,3.459
4.976,18.587,3.224,4.098,3.129,7.307,28.159,16.044,2.813,8.339,6.079,4.465,1.77
1,2.877,3.024,4.23,0.726,1.536
11.14,5.525,3.899,7.714,1.102,1.236,12.47,13.809,4.862,14.892,3.479,2.973,1.576
,1.769,2.258,1.695,1.365,2.323
9.3,6.054,2.265,13.533,2.264,7.378,42.399,11.391,2.238,5.14,3.312,6.853,1.869,3
.231,0.732,4.255,1.079,1.602
4.19,9.547,17.704,8.48,2.466,1.435,6.07,39.212,3.663,16.924,6.521,1.815,5.999,3
.028,1.95,4.63,1.312,1.391
8.553,7.113,12.205,6.018,2.036,2.473,14.494,15.05,6.873,6.625,5.714,2.006,11.87
6,2.618,1.512,1.437,1.157,2.324
9.316,9.937,4.185,6.444,1.905,4.023,8.428,23.494,6.212,6.92,11.623,5.995,2.752,
5.331,1.149,4.385,2.657,2.643
4.036,12.172,9.664,4.75,1.346,6.836,8.962,8.992,5.299,3.291,14.916,3.178,2.903,
4.194,1.485,3.276,1.475,1.085
8.825,7.307,12.086,4.408,1.741,2.922,8.718,30.135,3.14,14.593,2.52,2.805,3.312,
2.701,1.003,1.067,1.002,1.919
4.809,17.459,3.433,7.367,1.27,13.108,18.303,11.898,9.249,8.128,5.323,57.226,3.1
86,4.049,1.607,2.329,0.842,1.312
7.728,7.433,6.014,5.401,1.56,1.108,6.79,18.937,7.155,11.028,9.312,2.774,2.04,3.
587,2.003,2.766,0.991,1.087
12.307,18.171,2.586,11.349,1.362,2.839,11.902,13.55,6.784,6.85,4.117,6.275,1.86
,3.34,1.3,1.225,0.833,1.571
6.575,20.214,3.986,6.563,1.453,7.805,12.219,40.926,3.435,6.856,4.793,3.312,2.44
9,4.115,2.105,3.117,0.997,1.389
11.685,9.656,6.318,9.79,2.073,4.376,13.735,13.643,7.734,97.095,13.465,8.654,3.8
37,3.594,2.474,2.816,1.078,1.808
3.155,11.278,8.028,2.401,1.34,2.717,8.79,18.1,4.999,6.111,2.794,4.288,1.182,1.7
22,1.641,6.321,1.09,1.429
6.909,7.719,9.996,11.001,1.28,2.291,7.292,6.381,4.759,8.953,2.638,3.265,2.481,3
.866,2.17,1.177,1.031,1.472
14.761,6.948,6.2,2.985,1.337,2.285,8.744,13.102,2.984,10.921,4.66,1.382,3.384,1
.617,1.501,3.443,1.054,1.923
5.249,9.51,2.396,4.773,1.368,1.735,3.464,13.363,8.133,9.102,6.837,3.709,3.813,3
.977,16.639,1.959,0.948,1.8
2.49,11.69,9.211,9.027,1.207,2.026,9.165,8.719,12.138,9.221,5.681,2.335,2.839,4
.068,1.725,86.203,1.397,1.321
5.601,18.91,3.862,6.37,2.005,10.879,5.922,38.819,3.717,32.319,2.801,3.409,3.101
,2.034,1.63,22.594,1.284,2.298
6.77,4.699,13.509,7.921,1.605,1.739,11.494,9.418,7.062,18.794,4.999,1.761,5.805

,4.03,2.692,5.342,1.126,2.456
8.191,15.781,2.42,6.243,1.557,3.12,5.328,15.3,3.85,5.307,6.668,1.763,4.541,2.75
9,1.502,3.087,1.067,2.093
14.149,8.798,3.03,12.915,1.699,1.096,10.856,10.058,3.512,24.679,6.149,3.649,2.4
73,3.41,1.256,4.516,1.12,1.848
4.142,4.416,3.337,4.402,1.102,5.048,4.648,12.248,6.36,7.713,2.827,4.393,4.359,2
.357,4.699,1.519,1.779,1.388
14.099,7.034,3.513,17.688,1.952,4.204,7.843,57.002,4.625,20.55,6.516,4.061,3.34
4,2.433,1.768,1.91,1.495,1.371
7.909,9.304,6.542,4.022,1.599,4.13,11.344,9.527,5.793,15.155,2.991,1.737,2.921,
3.265,2.826,5.31,3.923,1.673
5.937,17.211,6.662,3.966,1.411,1.925,2.689,14.508,2.755,3.621,2.418,2.346,3,3.4
54,1.1,6.075,0.824,1.507
3.886,6.411,3.549,10.146,1.683,6.154,6.273,20.826,1.897,11.903,2.791,4.631,8.33
1,2.599,1.626,2.451,1.056,1.031
5.31,7.887,7.927,9.933,1.328,7.094,19.169,14.285,7.165,11.362,4.254,3.307,2.213
,2.636,2.207,5.503,0.961,1.307
17.079,4.634,2.799,5.39,1.458,2.291,7.326,21.719,5.76,6.541,6.145,3.211,2.637,3
.868,4.734,2.986,1.094,1.384
3.351,9.619,1.502,11.653,1.8,1.596,6.859,10.247,5.408,16.075,8.564,2.221,4.966,
4.575,2.305,9.014,1.244,0.935
3.839,10.079,4.861,3.759,2.274,1.816,7.125,14.403,5.016,13.173,8.965,1.335,1.78
,4.786,1.636,3.727,1.012,1.579
4.758,26.611,3.488,6.837,1.489,7.328,6.917,24.045,4.982,6.494,3.783,3.796,2.259
,8.453,1.685,1.128,0.936,1.973
2.074,7.693,6.358,13.188,1.103,2.265,12.796,14.132,3.392,7.556,4.263,7.184,2.18
6,4.283,1.153,2.244,0.944,1.576
6.501,9.866,1.939,5.566,1.608,1.209,10.102,10.313,3.644,21.406,5.132,2.746,1.88
8,3.02,1.022,2.034,1.079,2.134
4.154,6.65,11.147,5.569,1.737,9.241,14.106,6.671,4.17,8.098,8.612,4.147,3.699,1
.19,4.471,1.017,1.533,1.479
10.223,12.232,7.244,8.326,1.633,1.905,13.587,18.97,6.794,13.617,11.218,2.76,2.1
32,2.319,1.483,5.415,1.084,0.848
2.105,16.355,5.221,12.411,1.831,3.137,10.328,11.895,4.034,11.611,7.969,1.477,2.
656,4.657,1.726,15.164,0.889,2.314
4.057,14.853,4.466,9.948,1.438,25.57,10.582,18.937,4.151,40.315,6.235,4.82,1.33
,2.86,1.052,35.613,1.068,2.66
5.895,6.439,10.798,4.827,1.581,11.248,9.164,14.598,7.565,17.374,2.928,2.519,2.6
49,3.808,1.667,1.187,1.133,1.831
6.366,15.488,13.766,8.121,1.589,1.242,10.124,13.104,6.916,4.544,4.798,3.19,4.71
,3.468,1.119,46.26,1.408,2.339
4.936,19.182,6.131,10.636,1.568,6.71,7.137,14.463,3.729,12.78,4.993,2.162,2.183
,1.181,1.377,1.305,1.242,1.527
4.63,9.263,1.225,8.85,1.394,5.072,9.827,26.426,5.773,11.216,4.806,3.144,3.074,3
.06,2.849,6.146,1.182,1.509
21.417,7.123,10.558,5.969,2.387,1.398,2.171,34.714,5.306,20.293,6.265,2.471,3.1
12,3.977,1.626,2.67,2.001,1.354
3.108,10.206,2.711,10.208,1.634,11.642,6.927,11.152,6.012,22.03,3.995,1.931,5.7
23,2.41,2.171,4.662,1.795,1.506
14.812,20.015,5.571,5.602,1.547,2.898,1.954,25.496,5.163,18.444,7.548,4.341,3.3
31,4.81,1.778,6.076,1.162,1.508
3.078,7.942,9.456,10.452,1.947,1.302,8.892,25.275,5.773,11.433,3.045,2.465,2.54
6,2.91,1.106,1.244,1.377,1.234
4.871,8.726,2.46,3.656,1.473,5.47,10.113,11.425,6.422,12.356,3.471,1.833,1.614,
2.221,0.786,1.532,1.056,1.481
4.718,5.123,2.874,5.61,1.903,1.208,7.35,13.471,5.66,4.159,5.062,2.854,4.152,4.7
07,1.75,1.155,0.954,1.572
5.019,5.902,3.651,5.576,1.78,6.644,7.599,13.394,4.496,4.779,2.473,5.224,2.9,12.
785,2.088,1.429,0.711,1.212
9.697,7.202,9.001,5.925,1.475,2.609,3.77,25.955,8.378,7.899,12.865,1.836,2.826,
3.605,0.653,16.144,1.347,1.786
3.997,8.344,2.741,7.108,1.312,3.921,5.87,1.523,4.24,8.425,6.968,18.425,3.699,5.
23,1.785,6.039,1.323,1.978
13.461,8.323,2.7,7.486,1.399,2.226,8.479,30.503,8.886,10.657,6.176,5.551,3.461,

4.045,3.784,1.88,0.678,1.489
7.364,11.511,9.296,6.467,1.334,1.466,11.384,10.076,7.105,7.493,3.584,4.293,5.23
7,2.448,0.783,5.783,1.044,1.643
95.493,7.51,8.368,4.357,2.136,2.807,1.92,14.071,2.948,6.331,5.342,41.917,1.725,
3.645,3.546,1.237,0.928,1.552
6.259,14.391,5.51,5.453,1.754,1.209,8.167,27.868,3.979,8.456,2.174,2.847,1.422,
2.381,1.415,0.876,1.205,2.952
4.468,12.186,7.814,8.844,1.991,2.17,8.399,22.693,3.199,11.7,7.479,5.1,5.98,2.46
2,1.709,2.463,1.872,2.057
4.336,7.032,1.909,5.345,1.997,2.152,7.541,15.13,7.422,16.716,4.067,1.104,3.831,
4.141,2.485,4.163,1.06,2.169
9.833,12.004,2.173,5.345,1.893,1.731,6.981,12.559,2.627,21.111,6.157,4.357,1.92
5,2.051,1.834,1.462,1.13,1.486
4.971,10.751,2.04,4.144,1.635,1.127,16.702,17.797,4.063,2.872,2.395,2.504,2.495
,4.522,1.209,4.364,1.49,3.103
7.127,13.067,1.33,8.576,1.395,5.429,6.235,21.945,2.19,11.358,2.534,4.674,2.135,
4.152,2.176,3.454,0.941,1.465
5.185,9.955,3.052,3.751,1.431,2.377,10.177,10.328,3.739,9.543,14.36,3.73,3.506,
9.163,5.186,8.623,0.995,1.387
5.359,16.142,5.909,6.677,1.556,1.602,12.774,15.679,5.885,9.754,4.127,3.714,2.17
2,4.783,1.893,3.176,1.282,1.976
5.275,12.063,8.004,6.322,1.12,12.651,12.819,25.398,3.667,8.212,5.933,2.501,5.01
8,4.142,1.781,6.555,1.182,1.465
2.95,16.29,2.886,12.123,2.173,3.449,9.86,17.39,5.639,12.203,3.321,2.024,2.201,4
.934,4.046,3.262,1.542,1.928
3.627,16.556,1.486,16.388,1.208,2.282,9.732,33.111,5.03,27.752,2.527,1.542,2.04
4,4.206,1.492,2.22,1.572,1.649
3.69,7.378,2.126,6.913,1.794,2.186,5.55,6.713,3.415,3.453,3.069,2.843,2.011,2.7
35,1.826,1.523,1.319,1.046
3.31,10.721,5.271,8.724,1.915,11.868,8.161,24.823,7.277,19.123,3.641,3.298,3.83
2,4.182,2.123,2.285,0.939,2.476
8.697,12.249,3.627,6.618,1.917,2.53,6.75,28.912,4.195,11.257,2.953,1.381,2.486,
2.165,1.136,6.611,1.525,2.061
4.173,8.368,5.24,5.499,1.492,2.402,14.163,21.21,3.728,9.485,9.475,1.877,1.737,3
.559,2.238,4.906,1.117,1.555
4.962,4.586,8.329,2.583,1.503,4.852,4.802,1.255,7.098,10.091,10.284,3.05,1.965,
3.295,1.16,1.662,1.209,2.273
3.309,7.986,2.427,4.048,2.398,1.399,8.826,25.612,6.156,11.43,4.845,4.711,1.903,
5.917,2.709,1.017,1.729,2.358
7.396,6.004,5.442,7.843,1.321,6.499,10.584,12.987,4.663,7.067,4.089,3.751,4.096
,5.327,2.741,1.737,1.074,2.155
13.985,8.749,2.804,6.186,1.474,1.585,10.852,7.463,6.31,15.318,2.831,2.927,4.06,
3.815,1.45,2.733,0.851,2.172
6.098,16.955,3.247,5.327,1.855,0.924,3.989,13.695,3.867,12.48,5.634,4.684,4.11,
4.119,2.274,3.526,1.014,1.679
5.024,8.371,6.546,3.424,1.845,1.308,12.137,7.965,3.348,8.347,5.305,4.315,4.218,
3.245,0.784,4.131,0.78,1.795
13.312,14.063,11.289,4.094,1.435,4.596,9.735,20.004,3.648,7.003,3.509,4.003,6.2
59,3.399,1.645,3.105,1.04,1.712
5.367,9.173,6.982,1.821,1.336,2.088,20,15.899,3.878,13.688,2.902,1.889,4.037,1.
843,0.679,1.295,0.902,2.185
7.54,21.066,21.564,6.236,1.537,1.354,3.341,36.559,2.417,7.959,5.173,1.399,2.583
,7.623,1.741,3.016,3.424,1.56
10.893,9.039,2.472,7.801,2.36,5.689,5.79,29.729,7.087,15.805,4.754,3.259,3.549,
2.848,1.822,1.254,0.765,1.373
3.523,7.467,4.289,2.751,1.821,7.194,14.413,12.437,4.024,10.629,11.443,2.279,2.8
13,5.812,1.985,2.337,1.016,2.509
3.685,14.787,5.84,1.824,1.275,8.319,13.416,1.767,2.978,6.681,16.176,1.962,3.377
,3.17,1.469,2.082,0.948,1.631
6.446,7.096,3.172,5.085,1.631,10.869,12.684,48.823,3.44,13.822,6.229,1.53,2.282
,4.29,1.558,6.51,61.129,1.61
16.105,6.839,9.473,5.369,1.496,1.586,7,19.382,6.251,5.947,2.963,4.179,8.519,10.
47,2.145,1.486,2.301,1.444
6.309,18.12,8.911,9.721,1.413,1.244,21.806,20.756,5.059,10.533,6.027,5.135,2.07

6,4.165,2.145,0.852,0.849,1.415
7.065,8.445,9.837,5.143,1.414,4.673,12.614,22.52,6.421,10.133,4.6,2.151,1.864,5
.816,1.361,3.844,1.036,1.588
14.802,8.841,1.671,6.304,1.362,10.437,18.652,13.6,9,14.503,3.466,4.567,1.793,3.
605,1.182,1.086,0.893,2.55
4.896,7.366,10.876,14.129,1.444,1.714,13.21,30.198,6.869,8.348,7.002,2.474,9.01
7,5.429,0.963,5.313,1.548,0.911
3.284,11.266,4.373,11.417,1.607,3.737,9.189,17.8,4.897,9.584,4.388,3.232,2.374,
13.097,1.485,3.227,1.256,1.807
6.205,6.672,6.355,10.442,1.864,7.491,10.871,23.876,3.399,9.826,3.331,2.577,5.57
8,4.48,0.897,4.425,1.164,2.209
4.349,4.758,9.965,12.929,1.845,3.641,13.515,25.626,5.443,8.898,7.186,3.183,3.02
8,4.797,1.329,1.15,1.792,1.213
5.027,8.073,4.529,8.707,1.985,2.468,4.24,2.146,5.588,12.379,1.965,2.393,1.814,3
.209,1.894,3.708,1.196,2.313
4.909,20.205,14.759,6.509,2.171,7.387,6.626,32.633,3.524,14.78,2.142,3.235,2.34
4,6.48,1.867,4.637,1.273,1.45
1.923,19.614,3.014,6.431,1.412,5.801,6.217,4.222,3.708,13.727,9.562,4.815,4.563
,5.255,2.196,2.002,1.048,1.705
15.442,5.633,6.981,3.731,1.55,4.658,11.384,10.481,3.237,14.069,3.676,2.73,5.47,
2.76,1.314,7.737,1.117,2.911
5.312,5.271,6.859,5.097,1.748,2.41,7.952,17.647,8.449,16.055,4.126,6.632,2.08,2
.77,2.022,1.972,1.005,1.99
10.111,9.36,5.558,10.02,1.804,1.4,4.351,19.894,4.11,11.89,9.675,1.013,9.53,2.85
3,1.797,1.007,1.539,1.465
4.955,19.349,6.384,4.722,1.563,2.904,13.73,11.6,5.89,11.692,2.843,2.92,1.405,3.
552,1.387,1.492,1.098,3.237
3.726,8.305,4.937,14.721,1.837,2.347,10.154,2.508,5.512,15.005,1.851,6.665,2.60
4,6.548,2.3,0.691,0.83,3.107
12.373,10.814,3.452,7.496,2.058,3.857,14.648,14.019,5.934,22.968,4.002,4.496,3.
383,3.979,1.386,5.929,0.91,2.044
4.955,8.801,2.111,4.257,1.627,5.453,7.542,17.655,8.701,6.26,2.411,3.203,2.429,2
.388,1.502,1.758,0.812,1.312
5.052,6.832,3.841,4.343,1.588,2.299,8.402,30.977,3.93,9.106,5.737,5.431,3.874,3
.231,1.94,30.948,1.065,2.1
7.37,10.107,6.938,19.122,1.753,1.361,8.173,33.392,2.338,11.228,5.961,4.466,2.57
9,6.061,2.182,1.762,1.325,3.048
6.894,7.926,8.871,6.936,1.601,8.852,11.405,9.483,5.85,11.839,5.391,3.074,3.926,
5.881,1.839,2.859,2.169,1.684
3.858,8.294,3.562,7.434,1.112,9.42,11.494,10.662,7.163,10.38,4.769,2.77,5.817,5
.733,4.066,3.174,6.852,3.026
10.573,7.708,4.685,7.361,1.853,2.97,12.59,3.69,11.38,8.42,4.949,1.878,2.995,4.2
34,1.503,2.464,1.593,3.115
20.652,25.493,10.016,6.013,2.002,2.151,4.228,16.426,4.086,17.632,5.055,10.063,3
.918,3.327,1.792,38.435,1.28,0.996
12.122,6.298,3.173,4.583,1.462,1.481,3.304,12.906,6.569,15.873,4.753,3.556,2.48
,1.895,1.44,3.497,2.134,0.993
3.678,8.372,4.789,2.026,1.686,10.086,7.244,11.82,6.877,17.934,4.099,1.311,5.353
,2.13,2.401,1.939,1.37,2.781
2.843,4.976,12.038,10.096,1.874,4.892,5.24,26.925,5.301,15.482,7.767,2.445,11.2
2,6.038,1.367,1.919,1.186,1.567
8.709,7.586,6.192,8.011,2.02,8.178,6.423,24.992,3.844,8.669,6.477,5.903,3.073,2
.836,3.693,1.268,1.093,1.946
8.774,8.764,10.124,7.707,1.948,1.088,11.771,37.039,5.961,21.032,4.75,2.884,2.91
9,3.21,1.281,2.126,1.064,1.769
17.291,6.76,16.274,6.651,1.347,1.947,8.55,35.365,4.185,12.353,4.428,2.424,1.817
,3.817,1.178,0.952,1.062,1.121
9.687,7.891,4.758,7.31,1.678,2.65,13.354,28.274,2.424,8.589,6.174,6.284,1.538,9
.471,1.341,4.801,0.806,1.137
9.177,13.836,2.769,5.731,1.799,1.125,8.65,14.581,5.606,6.829,2.523,3.251,6.73,5
.306,2.029,3.448,1.216,2.732
4.598,16.029,4.463,9.117,1.552,1.999,4.056,17.917,4.322,9.943,9.514,5.117,2.298
,5.142,1.23,2.809,1.208,1.961
8.23,9.026,6.034,5.307,1.939,14.403,14.127,14.01,3.395,5.69,3.648,2.423,4.388,4

.184,1.382,1.303,1.141,3.561
11.438,5.549,10.375,8.198,1.306,4.865,11.986,20.533,2.676,15.723,2.948,1.528,4.
717,4.849,1.386,9.254,1.429,5.727
4.116,5.66,4.197,3.041,1.673,8.137,2.325,9.919,4.414,31.512,3.758,5.735,1.605,7
.141,2.272,20.878,0.959,1.424
5.049,7.371,5.476,20.582,1.359,2.262,3.802,17.52,17.618,8.827,11.854,3.225,1.50
9,1.534,1.155,2.611,0.96,2.019
5.197,11.482,4.259,5.764,1.423,3.28,2.154,19.208,2.806,15.247,5.943,1.984,2.648
,2.876,1.682,1.415,1.067,1.445
5.149,3.286,9.301,5.442,2.069,1.676,17.307,38.473,5.694,17.519,5.19,3.569,2.356
,5.421,1.64,2.439,1.141,1.178
2.845,6.588,11.133,5.788,1.542,1.379,9.402,28.345,4.793,6.891,4.59,4.081,1.889,
7.686,2.208,3.089,1.029,1.413
6.745,11.109,4.253,7.257,1.395,4.779,3.877,46.948,6.751,4.574,7.184,3.974,3.758
,2.338,1.982,9.307,1.037,1.462
6.034,10.541,2.827,9.357,3.621,2.408,11.929,26.841,3.259,29.727,5.833,4.087,3.4
1,5.648,2.214,3.875,1.281,1.899
4.52,9.272,1.818,7.552,1.573,1.816,29.768,9.476,4.055,13.698,6.188,2.02,1.638,7
.409,1.699,4.039,0.897,1.088
9.982,13.817,8.971,9.863,1.406,8.773,6.494,15.184,5.934,10.901,7.295,3.685,2.15
,3.195,1.452,8.005,1.309,1.583
6.052,5.731,3.014,15.029,1.536,1.021,3.596,17.804,2.802,5.432,2.551,3.872,2.179
,4.188,2.203,11.925,1.311,1.802
7.457,114.45,6.748,4.029,1.236,2.587,5.736,8.065,7.072,32.079,4.178,2.939,2.243
,1.413,2.571,1.708,1.349,1.489
4.074,6.292,4.543,2.772,1.813,1.287,8.047,23.6,3.032,5.602,7.811,42.936,2.21,9.
226,1.505,4.587,1.555,2.252
13.595,10.036,2.291,8.036,1.179,2.527,8.348,39.629,6.109,10.618,5.036,4.449,3.1
8,4.151,4.835,1.586,0.984,1.896
3.275,14.229,7.212,5.521,1.367,2.327,7.416,13.162,4.668,14.96,3.788,3.637,2.508
,3.642,1.299,4.26,1.1,1.445
8.44,16.792,4.169,6.034,1.376,1.074,6.282,30.95,4.54,11.821,9.837,2.96,7.093,2.
845,2.605,4.578,0.968,1.6
4.512,7.838,3.186,7.636,1.288,8.223,8.283,15.503,8.921,21.591,9.104,1.1,4.354,8
.036,1.927,2.885,1.041,0.982
5.686,21.923,8.167,12.188,1.716,1.628,3.26,31.566,3.294,8.392,3.535,1.951,4.047
,3.657,3.751,1.514,0.932,3.719
7.254,4.298,5.051,4.161,1.332,3.653,7.18,9.372,6.548,15.613,2.791,3.066,4.572,6
.041,1.752,1.498,1.404,2.365
6.281,19.705,5.418,7.709,1.995,9.222,17.369,18.396,3.125,16.138,2.516,5.338,2.5
83,1.954,1.48,2.663,0.882,1.562
5.757,7.479,4.152,11.224,1.371,4.112,5.093,20.9,3.95,19.222,4.155,3.103,2.519,3
.459,2.586,8.311,0.966,2.702
6.882,12.676,14.862,10.289,1.018,27.378,17.976,16.398,4.105,13.972,3.468,4.576,
2.709,6.228,2.467,7.17,3.278,2.111
13.286,16.128,5.208,4.025,1.628,1.16,8.762,23.538,4.071,5.267,10.632,5.502,3.37
2,3.672,1.387,4.812,1.386,1.49
3.699,119.718,8.167,6.118,1.557,3.039,3.776,5.649,2.25,3.421,4.801,4.009,1.667,
1.45,1.536,8.744,1.014,1.643
8.549,38.387,13.984,14.486,1.362,2.355,7.208,18.168,3.795,10.612,15.573,4.681,3
.323,2.219,1.391,5.24,1.674,1.42
4.994,5.251,4.015,9.471,1.806,8.072,9.316,19.094,3.865,9.458,4.882,4.575,1.888,
2.543,1.453,50.051,1.185,2.044
3.417,6.115,7.174,8.732,1.689,3.073,4.473,17.837,10.449,10.309,7.547,2.198,1.64
8,1.718,1.972,8.426,0.967,1.321
9.417,18.043,9.869,9.162,1.591,1.835,10.464,24.915,2.368,25.738,9.952,3.842,2.4
81,8.358,1.318,6.293,2.654,0.961
14.118,9.559,10.595,7,1.273,10.062,4.034,17.46,7.696,11.171,5.873,3.772,1.575,2
.315,2.018,3.884,1.42,1.222
2.726,8.045,4.776,3.983,1.536,0.951,6.399,17.39,2.795,37.383,5.327,3.026,1.681,
2.907,1.554,6.574,1.21,2.443
3.819,24.036,15.479,7.425,1.238,1.296,13.765,11.742,3.617,13.871,7.436,5.147,2.
577,0.905,1.498,1.408,0.85,1.531
2.587,4.048,4.866,6.524,1.281,9.864,13.201,10.414,4.864,10.015,6.462,2.439,3.06

4,1.981,1.332,3.728,1.084,5.897
3.981,7.876,9.579,22.306,2.334,1.234,13.143,35.839,3.617,10.578,4.47,2.686,1.88
2,5.542,1.558,1.14,1.249,1.773
16.72,13.56,9.957,5.294,1.306,1.367,10.245,8.458,3.818,11.523,3.528,2.036,2.791
,12.885,2.712,15.052,1.016,2.882
5.849,8.084,4.035,5.445,1.385,2.841,4.234,24.612,2.361,5.4,7.023,4.142,3.203,9.
172,1.806,5.005,3.256,1.641
3.073,20.227,10.739,5.615,1.612,3.189,10.618,19.757,3.45,10.799,2.846,1.605,2.2
5,2.082,2.05,5.656,0.912,1.669
5.152,7.747,2.583,6.392,1.01,2.073,11.096,19.644,4.035,11.256,2.666,2.095,2.706
,3.985,1.734,5.298,1.018,1.784
5.436,12.45,2.309,4.576,2.177,1.145,5.415,17.678,3.871,9.586,4.944,3.082,4.861,
6.968,1.99,1.504,0.997,2.022
13.781,11.103,3.385,10.706,2.001,9.774,5.682,13.162,4.611,13.325,9.538,3.162,6.
67,2.733,1.43,10.697,1.547,2.007
7.453,13.288,3.904,11.516,1.4,1.307,7.512,21.23,3.686,6.333,3.762,3.768,3.16,3.
879,1.153,3.452,1.159,1.966
13.355,20.747,4.918,3.888,2.546,1.977,12.442,24.443,3.068,4.58,7.271,3.218,3.33
7,3.461,2.622,3.018,0.884,1.522
4.981,9.024,5.22,7.329,1.869,10.894,15.977,40.245,2.408,8.038,7.024,4.798,2.968
,3.311,4.055,1.702,1.446,1.914
3.217,8.52,3.465,7.257,1.561,3.54,5.923,33.105,5.808,76.54,2.685,5.731,3.049,2.
528,1.547,3.089,1.065,3.298
4.972,13.136,12.743,4.268,1.796,5.272,6.81,33.973,4.367,8.471,5.811,3.23,1.901,
2.393,1.433,1.361,1.638,2.077
3.539,16.991,3.211,15.153,1.227,0.993,10.021,9.851,4.793,14.709,3.428,1.863,5.4
39,5.016,1.544,2.564,1.672,2.998
3.792,9.818,3.89,7.231,1.696,7.298,10.811,29.291,3.53,17.949,5.125,5.118,1.79,2
.603,1.407,10.233,0.987,1.606
11.012,12.024,3.339,13.499,1.808,5.708,6.886,16.75,5.358,6.956,9.274,6.246,3.55
6,2.306,1.788,1.408,1.09,1.445
6.691,6.618,3.345,3.267,1.5,9.969,28.031,16.057,5.788,10.84,7.909,4.707,3.541,4
.821,1.617,1.014,0.642,1.518
8.234,6.016,5.609,12.881,1.619,2.469,8.859,15.589,3.905,22.403,9.432,1.248,1.57
2,2.19,4.107,2.993,1.898,2.163
4.042,7.919,3.915,13.782,1.161,1.772,10.193,15.579,2.683,9.155,3.867,1.148,1.83
9,4.317,1.017,1.093,1.224,2.968
44.968,6.503,2.589,7.946,1.375,1.649,12.439,9.938,6.095,14.538,5.196,2.044,1.30
5,3.026,2.652,1.715,0.971,1.682
16.558,4.332,6.633,5.907,1.332,2.246,16.012,21.367,3.269,8.655,4.286,3.75,1.42,
6.179,1.4,1.092,0.931,1.67
4.374,7.389,1.632,6.087,1.751,7.96,5.196,17.669,5.024,11.166,4.417,1.478,2.621,
2.22,3.59,3.643,1.025,1.966
8.211,3.826,2.411,6.525,1.447,2.579,4.056,15.274,4.838,20.959,4.486,4.918,3.186
,3.542,1.988,5.255,1.429,1.636
6.632,8.034,10.104,6.131,1.582,7.588,11.73,31.474,4.841,16.191,5.774,1.431,5.81
6,5.075,1.36,9.866,1.268,1.475
17.089,22.104,3.165,5.055,1.377,4.375,14.746,14.567,5.253,10.404,4.684,4.186,2.
847,6.635,1.555,1.134,0.809,2.021
4.794,20.708,4.952,29.293,1.881,1.19,7.164,57.745,6.067,4.957,5.288,1.828,3.303
,2.324,3.713,6.282,1.195,1.595
2.498,8.285,3.193,3.88,1.35,1.526,6.546,36.126,5.175,13.237,7.156,2.355,1.787,5
.412,2.467,2.92,1.328,1.084
4.229,10.568,9.166,9.047,1.481,8.267,17.215,20.741,8.883,13.641,4.675,2.059,1.6
86,4.045,1.555,6.049,1.2,1.412
6.83,17.51,8.178,26.084,2.165,1.58,5.559,16.163,5.08,21.111,3.121,2.963,5.461,5
.424,1.495,2.036,2.473,1.362
7.706,5.373,6.178,4.959,1.091,4.682,8.18,7.867,5.49,11.258,5.005,4.962,4.247,3.
435,1.397,1.145,0.977,1.409
6.747,19.049,4.987,4.638,0.998,14.635,13.217,18.483,4.363,7.862,2.241,3.905,6.5
98,2.458,1.982,61.459,0.801,1.691
5.739,13.574,4.872,3.621,1.732,2.195,8.961,14.377,7.129,14.589,7.872,6.335,3.37
6,1.591,2.313,1.594,0.979,2.521
3.938,14.321,10.487,8.704,1.427,4.991,8.177,12.297,4.98,8.52,3.862,4.318,4.661,

8.593,2.821,6.704,0.86,1.402
6.396,9.238,2.629,5.257,1.296,1.818,11.304,27.698,3.675,31.95,6.041,4.742,3.873
,4.392,1.961,2.962,39.648,1.729
4.834,7.842,2.405,14.498,2.517,1.315,4.14,20.262,4.825,8.166,5.716,2.765,2.702,
2.73,1.539,3.811,0.963,1.384
7.387,10.362,8.744,5.059,1.635,2.255,7.116,32.103,4.465,21.761,4.059,4.662,3.64
,4.725,1.273,4.009,3.202,2.22
5.809,5.175,5.958,6.909,1.487,9.556,10.489,18.554,6.831,10.825,3.024,4.373,2.78
1,3.935,3.254,2.55,1.004,1.405
6.226,35.135,10.44,11.889,1.391,1.616,9.055,7.902,2.973,12.859,3.572,2.182,2.82
7,4.602,1.768,7.521,1.119,1.553
13.535,7.427,6.25,6.789,1.358,5.183,4.927,2.469,4.759,5.959,6.089,5.91,2.553,8.
018,0.818,3.946,1.342,1.304
4.588,14.368,11.721,8.923,1.351,1.195,32.648,17.355,3.757,8.285,3.419,5.117,1.4
97,3.674,0.973,6.998,0.888,1.142
6.58,10.836,2.873,6.483,1.352,11.05,6.025,18.168,8.236,51.163,3.839,4.265,2.499
,5.371,2.512,2.843,1.154,1.715
11.999,6.948,1.955,7.231,1.441,9.957,7.814,8.079,7.57,10.901,10.14,4.464,2.211,
6.807,1.268,2.082,0.81,1.923
11.334,2.992,1.72,6.185,1.3,1.282,21.442,13.759,4.432,25.582,3.477,2.099,3.371,
3.185,1.481,0.724,1.229,1.959
5.491,4.091,11.728,8.86,1.547,3.529,3.13,28.971,10.461,17.14,3.708,2.371,2.416,
3.505,2.721,1.089,1.055,1.645
5.35,10.258,9.524,28.388,1.291,1.925,8.946,16.611,4.902,14.456,2.243,2.277,4.96
7,2.684,1.723,1.378,0.924,3.714
4.581,7.165,4.605,7.539,2.037,3.012,15.04,10.825,4.882,19.875,6.214,1.473,2.835
,2.281,2.121,1.458,1.355,2.344
9.832,9.565,8.654,3.741,1.469,5.794,10.759,12.399,3.833,6.929,8.205,1.965,2.6,9
.142,1.54,7.603,1.527,1.203
4.935,20.876,5.013,6.382,1.708,1.004,6.809,37.235,3.971,9.044,4.138,5.5,1.837,3
.805,0.966,5.083,0.977,1.534
3.73,9.25,1.924,5.062,2.116,5.188,11.403,18.763,4.813,21.371,2.778,3.489,2.812,
4.115,2.865,1.413,0.836,1.475
4.157,4.639,4.591,4.132,1.683,8.94,7.57,14.333,4.53,13.039,6.265,1.722,2.401,2.
421,1.347,1.108,1.228,1.243
3.17,9.772,4.068,5.772,2.165,2.212,10.604,10.94,3.907,11.768,2.259,4.341,5.784,
3.876,5.366,4.64,1.302,2.486
7.587,8.373,1.747,3.935,2.09,1.911,10.069,14.296,5.301,6.571,5.204,2.416,2.246,
4.628,1.636,2.823,1.073,1.739
7.341,7.819,7.253,5.633,1.514,1.163,3.743,18.645,6.837,7.408,4.261,5.672,4.331,
4.934,0.806,8.398,0.988,1.503
9.65,7.499,4.318,8.79,1.916,1.349,8.624,21.649,4.449,19.138,2.721,7.218,3.072,3
.586,2.246,1.268,0.829,3.598
3.139,11.044,10.863,7.415,1.456,2.25,12.036,18.109,5.677,9.171,2.184,4.893,2.93
7,3.218,1.457,1.866,1.337,1.412
3.057,8.732,3.914,13.821,1.668,1.605,7.393,12.022,4.444,11.77,3.424,5.005,3.445
,2.482,8.105,4.741,1.555,1.958
8.909,15.641,3.289,5.445,1.671,4.708,3.336,3.951,5.696,10.865,5.583,4.478,5.066
,4.484,1.764,1.828,0.85,1.325
10.893,4.802,2.435,4.605,1.43,6.144,10.396,13.772,5.065,12.603,3.027,3.97,2.975
,5.215,1.149,2.599,1.166,2.369
12.222,9.818,8.804,3.97,1.243,2.137,4.547,18.194,6.736,15.488,4.851,3.876,1.352
,8.506,2.882,1.19,2.137,1.673
4.753,2.938,2.03,5.001,2.451,4.45,11.401,9.428,8.083,5.555,2.959,2.656,3.379,3.
51,1.546,2.658,0.953,1.491
8.205,8.623,9.389,8.358,1.269,1.936,6.135,1.687,3.451,10.842,3.14,4.492,4.717,7
.438,2.251,7.14,0.926,3.537
4.798,12.382,3.266,4.118,1.553,8.01,7.07,12.877,3.716,7.651,3.407,3.343,5.549,2
.743,1.513,2.626,0.927,2.296
7.82,11.972,3.501,8.094,1.586,3.6,24.661,17.601,3.59,13.343,14.92,4.967,2.001,4
.179,0.77,3.267,1.573,1.604
4.08,15.532,8.138,7.539,1.664,4.363,6.234,21.583,4.651,7.945,3.058,5.356,5.458,
2.383,1.431,8.552,0.792,1.215
9.232,4.358,2.667,7.613,2.156,5.693,13.659,8.321,3.195,12.371,3.676,5.203,3.073

,4.882,1.501,5.696,1.133,1.311
9.682,4.825,5.865,2.33,1.364,7.614,12.825,1.8,9.932,5.902,6.768,3.395,10.481,7.
06,1.257,2.443,1.122,2.129
4.134,5.114,3.827,7.277,1.852,2.512,14.404,25.213,6.254,11.734,3.811,3.06,2.724
,5.003,1.698,1.761,1.026,1.757
8.258,9.881,10.329,8.328,1.376,1.145,9.502,12.715,2.701,6.742,5.493,2.707,2.496
,3.772,7.106,9.098,1.244,1.351
8.047,14.433,4.3,9.173,1.345,3.132,7.757,21.941,4.728,11.147,4.558,2.645,2.383,
3.826,3.297,1.854,0.963,1.345
43.093,8.546,5.662,5.726,1.78,1.74,11.545,8.365,7.005,19.67,5.219,2.763,27.229,
4.677,1.779,1.741,1.198,1.181
8.577,14.293,9.336,3.689,1.354,1.569,13.273,14.931,3.662,13.672,5.327,3.594,1.6
08,2.19,2.638,7.947,2.25,2.024
4.311,4.261,8.104,13.128,1.421,1.704,8.79,17.422,3.7,21.168,3.23,2.55,4.956,2.0
23,2.149,4.263,1.068,1.197
6.516,16.428,6.668,10.085,2.012,0.971,2.926,20.705,6.668,11.871,5.684,10.223,3.
528,2.587,1.738,11.773,1.517,2.062
6.755,4.334,3.938,5.044,1.549,1.915,25.104,33.224,6.101,13.419,3.389,2.805,4.20
4,2.564,4.077,1.981,1.448,1.835
8.502,19.115,11.335,4.798,1.538,2.728,6.762,5.785,5.596,3.819,3.242,2.586,1.988
,4.864,0.867,84.618,1.213,1.537
4.982,12.032,9.499,7.923,2.677,3.431,10.989,38.348,5.26,8.588,5.776,20.922,3.30
6,3.353,1.839,2.91,0.908,1.768
3.79,4.562,7.734,5.758,1.755,1.785,5.912,8.931,3.431,16.558,4.065,3.612,1.98,1.
888,1.887,2.319,1.307,2.008
4.964,15.342,6.247,7.883,1.928,1.278,12.162,3.211,8.038,9.557,7.439,2.408,2.525
,2.034,2.432,6.259,1.635,2.413
5.485,9.143,5.118,5.059,2.124,10.455,10.005,38.036,3.246,10.754,5.718,5.876,1.7
89,3.555,0.855,3.252,1.119,1.504
7.923,6.167,5.287,4.985,1.394,2.893,11.538,15.903,4.451,14.369,3.901,2.548,1.93
4,3.009,0.948,2.31,1.342,1.865
4.423,8.535,4.331,7.911,2.214,1.977,11.435,7.551,7.785,11.453,2.792,4.33,3.351,
3.105,6.206,5.531,1.562,2.511
7.362,8.275,8.059,4.296,2.971,1.655,10.616,20.949,6.248,29.761,3.152,3.145,2.84
5,2.515,1.244,1.985,2.47,3.051
4.073,2.871,6.895,3.56,1.427,1.636,10.403,22.756,4.779,12.122,4.101,2.131,33.75
6,5.736,1.555,4.466,0.914,1.356
4.912,6.626,2.025,5.549,1.406,15.182,5.601,17.103,8.387,6.927,4.133,2.382,3.714
,7.509,2.436,3.831,0.887,1.808
7.339,8.572,5.342,14.095,1.953,4.06,12.029,24.686,9.77,24.211,4.401,9.184,2.699
,1.365,0.916,3.232,1.203,1.581
6.99,10.811,4.212,8.341,1.757,1.373,8.294,21.001,2.677,13.6,4.601,2.034,1.914,3
.278,0.865,2.001,0.805,1.739
19.615,16.739,3.603,8.255,1.71,2.844,18.045,26.636,7.852,7.881,7.181,2.445,2.97
8,5.776,2.355,3.633,0.787,2.188
5.601,8.758,3.5,6.66,2.016,5.021,10.937,12.327,6.243,6.303,7.339,1.708,2.772,8.
489,1.919,1.657,1.217,2.945
7.709,11.04,16.843,3.614,1.848,1.741,10.522,9.779,3.391,18.841,3.698,4.016,1.95
9,5.693,3.85,1.728,1.297,2.158
9.619,11.951,4.506,4.132,1.756,4.839,4.995,28.332,2.876,21.31,3.515,41.968,7.85
9,2.03,1.931,7.367,0.896,1.573
7.162,10.859,5.141,12.371,1.346,2.069,30.952,16.539,4.048,7.513,3.453,1.849,4.5
28,2.375,1.94,4.684,1.061,2.236
8.394,7.906,5.919,5.209,1.607,4.544,5.143,42.825,4.099,11.194,6.13,1.791,3.949,
3.512,1.608,3.003,1.183,2.161
5.129,8.763,7.723,21.326,1.624,1.797,6.192,26.004,3.584,14.472,4.853,2.779,1.93
2,2.096,1.167,4.161,1.844,1.521
5.466,4.422,5.363,9.192,2.093,5.623,4.375,14.796,16.244,16.979,3.502,2.809,3.76
7,4.893,2.887,6.146,1.477,1.292
4.096,8.562,6.315,3.881,1.548,2.684,9.937,23.313,5.594,8.147,6.007,1.922,2.497,
4.088,1.904,7.932,0.73,2.355
6.697,14.549,7.625,5.533,1.695,1.217,10.633,25.122,3.986,73.262,8.59,2.43,1.662
,4.667,2.588,2.02,0.719,2.651
5.19,16.965,9.078,3.792,1.43,64.871,10.737,6.167,3.604,11.422,3.979,6.351,1.73,

3.223,1.841,2.311,1.103,1.659
9.933,6.949,7.204,1.921,1.499,1.072,10.457,3.774,6.87,9.912,2.357,1.136,2.788,4.406,2.362,2.033,1.423,2.275
14.162,13.945,4.67,4.051,1.372,4.488,33.435,17.185,9.64,22.409,3.975,3.951,1.599,9.415,1.522,2.987,1.1,1.663
3.939,3.406,12.072,12.669,1.4,1.9,10.565,15.085,8.994,5.361,4.218,5.384,1.725,2.914,1.282,8.071,0.908,1.246
9.306,6.642,8.134,5.64,1.124,0.968,6.211,24.837,4.103,23.818,5.043,3.069,3.331,1.509,2.049,7.295,1.065,1.448
3.562,21.926,8.394,4.338,1.983,7.795,5.75,16.702,3.683,18.451,3.95,3.613,2.136,1.466,1.732,4.351,1.481,3.057
5.843,18.108,4.785,12.896,1.648,1.573,5.952,22.351,6.095,9.989,3.491,2.543,3.991,6.551,2.82,3.51,6.923,1.562
2.917,15.838,2.134,9.619,1.321,2.875,20.604,11.761,6.574,12.521,8.471,5.245,4.202,9.196,1.367,9.328,0.978,1.521
5.929,48.592,4.781,8.816,1.89,1.197,5.322,28.543,3.011,6.533,2.555,3.13,2.674,4.08,1.481,14.469,1.079,2.319
24.649,18.48,3.772,9.106,2.372,2.447,2.243,34.645,4.073,11.163,4.442,2.086,2.383,6.272,1.791,3.104,1.247,1.508
4.686,14.367,9.852,5.946,1.408,8.424,12.603,3.556,3.068,13.68,4.21,5.04,3.206,4.058,1.097,1.204,3.496,2.251
6.837,8.089,2.548,2.96,1.608,3.618,14.678,14.123,4.53,8.977,3.89,1.147,3.692,2.635,2.012,2.105,1.102,1.487
7.373,7.863,7.514,4.148,1.348,2.021,19.657,14.821,10.452,14.001,12.748,1.642,2.572,6.978,1.951,15.132,1.787,1.846
3.889,12.103,3.641,3.707,1.941,1.006,16.307,15.077,5.877,21.524,3.516,3.692,5.053,7.026,1.518,1.939,0.855,1.376
4.007,11.045,5.914,9.996,1.579,2.542,14.391,24.771,3.226,12.846,5.891,5.91,2.443,3.995,1.207,10.239,0.806,2.743
6.109,7.558,5.113,10.978,1.648,1.632,8.973,27.429,5.243,16.671,3.255,2.495,2.18,5.821,0.694,6.719,1.193,2.668
8.156,4.933,8.028,14.308,1.384,1.74,15.74,20.246,5.087,7.055,3.798,2.151,3.61,4.675,4.643,3.065,1.109,1.524
2.381,5.745,9.774,5.873,2.412,4.475,7.765,37.144,3.706,8.459,5.19,3.886,2.673,4.209,1.644,13.502,0.964,1.432
8.764,7.634,7.62,20.023,1.464,2.469,9.649,18.994,2.903,8.931,4.013,2.823,2.282,3.083,1.578,3.693,0.855,1.873
6.047,14.498,7.044,5.571,1.686,1.679,8.529,13.172,6.243,17.984,4.771,2.622,2.566,3.754,1.364,6.376,0.898,1.707
11.2,19.459,5.541,9.462,1.508,1.665,8.048,13.776,4.933,7.787,9.043,4.492,2.177,2.278,0.918,2.365,1.026,1.906
2.51,7.675,9.447,4.885,1.464,9.715,1.546,10.589,3.443,9.06,3.365,1.564,5.95,4.597,1.802,3.831,1.195,1.896
2.422,12.177,7.592,9.527,1.975,1.317,10.406,12.452,5.536,5.885,9.354,2.793,3.336,1.302,0.734,3.984,1.66,1.265
5.869,25.759,5.087,13.663,1.729,0.958,3.271,19.299,2.988,9.145,5.254,3.313,4.138,2.759,0.967,1.631,1.08,2.378
7.215,21.497,2.812,5.037,1.44,5.527,12.361,24.872,4.061,6.112,5.296,3.357,5.52,2.751,1.265,2.399,1.311,1.205
5.196,9.078,3.76,5.202,1.398,7.99,4.747,22.107,3.825,52.449,7.884,3.307,3.277,5.21,1.497,2.045,1.093,4.013
6.314,7.257,4.028,4.254,3.684,1.368,4.093,9.442,8.842,14.925,14.199,1.721,4.99,4.726,2.64,37.3,1.082,1.808
7.974,7.828,2.478,4.874,1.222,3.481,3.051,8.771,2.735,16.503,2.517,5.013,3.084,4.954,2.357,1.098,1.086,3.751
3.146,9.441,7.666,10.16,1.444,3.236,8.264,16.826,4.006,13.428,4.749,3.157,3.444,2.51,4.873,0.916,1.024,1.275
15.438,7.578,13.346,4.144,1.732,1.559,17.255,16.567,5.322,7.459,3.88,4.132,3.643,1.929,1.551,1.249,1.145,1.632
9.332,12.059,16.748,5.853,2.432,12.052,2.914,24.043,5.511,17.037,5.496,4.254,2.087,2.432,1.632,5.377,1.649,1.594
2.893,28.789,8.758,5.498,1.419,1.173,10.214,12.657,7.758,10.341,3.391,2.243,1.889,2.23,2.129,29.701,0.923,1.475
8.151,9.654,3.735,15.757,1.385,2.154,3.304,15.975,6.789,7.35,5.72,5.809,4.276,2

.392,1.338,1.203,1.339,2.848
4.159,9.688,7.681,8.532,1.86,1.448,11.655,13.589,3.836,9.267,8.254,4.387,4.33,5
.848,0.975,3.291,1.365,1.87
3.304,8.539,4.442,3.614,1.453,3.742,8.902,7.171,3.85,23.604,8.48,4.02,2.29,1.97
8,1.715,1.134,8.657,1.484
4.895,8.465,6.956,6.067,1.359,4.235,6.889,17.122,3.964,8.631,2.042,3.866,2.546,
3.792,1.393,2.348,1.13,1.316
7.794,7.882,7.997,7.679,1.044,2.289,3.727,10.514,4.876,4.513,8.441,7.622,4.22,7
.273,1.025,2.362,1.163,1.433
5.176,10.927,10.168,8.347,1.077,2.085,13.767,18.476,4.919,9.407,3.549,1.942,3.2
3,2.682,3.42,6.709,1.004,1.114
2.586,15.702,6.05,13.509,2.293,3.426,2.73,29.95,8.079,7.565,4.039,4.904,2.541,4
.468,1.3,2.513,1.015,1.648
4.113,16.94,8.983,8.439,1.311,2.337,4.219,13.666,1.99,7.011,8.707,1.815,3.486,3
.2,2.283,1.739,1.318,2.137
14.008,4.921,14.711,5.247,1.356,3.43,4.88,35.529,4.874,24.395,7.694,4.721,2.466
,1.964,1.183,1.048,1.203,1.242
4.481,4.493,4.965,7.264,1.461,2.082,3.622,15.637,3.23,26.732,5.909,3.164,4.492,
5.139,1.089,2.965,1.293,2.314
5.549,7.337,3.223,6.153,1.261,34.577,16.413,12.245,6.237,10.757,4.836,2.254,3.1
24,2.576,1.408,6.495,1.232,2.527
38.96,5.975,4.632,9.294,1.307,3.127,18.614,12.969,5.544,22.618,3.92,3.31,7.154,
1.202,2.087,1.543,0.89,2.052
2.783,11.523,1.264,5.956,1.938,1.187,17.669,24.454,3.21,17.766,5.633,2.979,4.74
9,3.547,0.944,3.608,0.851,1.045
6.442,15.7,4.179,8.002,1.248,1.792,9.679,11.339,4.23,13.002,3.141,2.149,3.619,5
.802,1.765,6.198,1.279,2.289
6.296,7.44,5.478,9.831,1.554,1.748,11.844,12.145,4.073,8.629,7.622,2.624,3.491,
5.48,1.939,7.006,1.092,1.65
4.504,17.298,9.383,7.312,1.341,16.846,13.384,25.151,4.306,23.71,6.964,4.554,2.6
64,3.852,1.671,3.66,0.99,1.5
3.245,10.832,2.535,7.022,1.992,3.437,11.648,20.313,5.93,10.278,3.232,2.671,3.02
3,1.449,1.695,8.339,1.18,1.165
3.307,10.609,12.625,7.17,1.925,11.747,12.298,21.874,4.725,7.883,9.145,1.88,2.10
8,5.109,1.663,4.682,0.863,1.56
4.07,12.695,4.114,6.772,1.724,1.15,7.45,26.257,5.968,6.987,3.796,1.49,2.494,2.9
59,1.888,6.523,1.896,2.513
6.816,14.51,10.021,10.956,1.971,1.108,9.897,9.041,5.107,1.644,2.422,4.758,2.496
,3.632,1.652,5.18,0.914,1.547
5.002,12.538,9.469,4.139,1.38,3.586,22.765,14.26,5.177,10.492,7.253,2.463,2.075
,4.46,5.518,7.651,0.962,1.619
32.953,15.223,4.941,7.109,2.217,2.644,12.676,25.402,5.18,14.944,9.114,3.048,2.0
52,3.155,1.876,7.538,0.933,1.627
9.985,11.231,11.115,6.061,1.345,2.589,3.834,17.405,2.677,8.744,5.335,2.353,3.22
3,4.648,1.916,1.11,1.702,1.545
12.681,13.879,2.288,6.084,1.557,5.613,9.777,16.514,8.017,4.021,4.44,10.377,2.01
9,10.667,1.996,4.091,2.72,1.548
4.151,6.646,7.34,9.745,1.811,5.364,10.837,11.829,5.064,18.947,4.058,1.902,2.289
,3.302,1.974,3.588,1.2,2.308
7.547,9.81,8.303,2.984,1.472,1.064,10.485,21.15,6.201,5.804,3.086,15.342,4.846,
3.463,2.129,2.998,1.727,1.427
11.878,9.213,9.557,5.728,1.412,1.251,8.736,2.367,8.226,9.2,5.829,3.121,2.283,13
.888,1.011,3.376,1.147,1.738
5.681,8.689,12.201,14.313,1.711,4.691,2.875,19.222,2.415,14.815,2.439,4.178,2.9
76,1.531,0.988,2.248,1.546,2.098
5.529,12.68,1.44,7.339,1.047,11.884,23.076,21.279,3.095,17.452,7.947,4.069,2.36
5,3.583,1.501,4.362,1.561,2.03
20.835,19.009,9.394,7.249,1.782,7.115,10.228,14.872,3.186,8.986,4.5,6.486,1.86,
6.123,2.268,2.622,0.978,1.76
9.213,10.16,3.507,10.817,1.245,5.101,12.967,27.23,5.692,9.755,5.522,3.266,2.655
,1.976,1.02,14.337,0.867,2.011
19.728,6.051,6.245,10.312,1.995,3.64,7.132,39.103,8.572,12.191,6.804,1.705,1.39
5,4.909,4.986,1.181,1.215,1.385
4.143,6.648,8.121,9.31,2.014,13.351,15.007,18.712,3.12,4.453,6.416,2.608,3.463,

3.139,1.786,4.912,0.963,1.428
4.378,8.408,5.449,14.328,2.03,3.961,5.055,11.038,3.355,13.463,9.922,5.012,1.709,3.297,1.831,2.616,0.985,1.857
3.807,9.041,4.999,5.65,1.443,1.861,2.517,13.52,9.631,11.8,3.673,2.916,1.664,4.837,2.599,3.124,0.915,2.209
4.655,4.979,8.679,3.661,1.825,14.704,3.985,10.118,4.641,18.128,5.531,2.93,4.088,1.836,2.454,3.06,0.931,1.205
7.226,6.334,6.338,12.214,1.314,2.768,9.951,9.921,8.413,5.115,4.676,3.033,3.519,2.316,1.692,1.576,3.08,2.668
2.597,21.5,7.93,12.278,1.358,2.314,3.31,18.325,2.421,7.716,4.142,2.011,1.738,4.273,1.582,1.48,0.893,1.432
4.096,12.09,3.253,11.579,1.825,3.67,9.504,11.37,5.517,6.848,5.756,4.122,6.548,5.494,1.428,2.51,0.933,1.483
3.626,5.972,6.785,9.999,2.031,13.042,17.848,12.842,3.349,15.369,2.684,3.702,4.221,8.24,1.369,3.073,0.808,1.415
11.276,7.996,3.467,5.264,1.285,1.281,4.719,20.866,3.344,12.995,5.38,6.365,2.817,3.197,1.912,1.505,0.979,1.626
3.951,10.68,8.617,6.986,1.283,2.996,8.868,39.565,5.183,18.822,5.56,1.673,2.588,4.204,1.522,7.349,0.928,1.462
3.498,13.959,5.104,5.646,1.835,1.62,6.16,25.644,6.164,13.156,3.735,5.652,2.927,2.976,1.809,6.165,1.12,1.387
4.992,9.605,2.385,6.888,1.334,2.796,2.921,16.041,4.388,14.118,4.126,3.579,2.452,2.409,1.719,3.534,1.021,0.935
8.149,8.899,8.094,6.387,1.846,1.053,11.173,19.594,2.694,26.436,2.666,3.333,3.152,4.107,2.048,2.421,1.187,1.578
6.548,17.044,6.127,3.221,1.845,2.985,12.267,71.516,10.742,4.641,3.142,72.915,3.919,3.707,1.762,5.205,1.214,1.626
20.466,21.313,4.32,6.223,1.278,9.079,23.845,2.053,5.487,6.233,9.324,3.58,1.171,2.648,4.703,3.072,1.396,1.365
6.632,12.012,11.102,4.159,1.7,1.75,36.955,17.729,4.761,62.618,8.031,3.05,10.544,6.671,1.561,1.7,1.297,2.576
7.864,7.103,4.451,5.655,1.781,5.717,1.641,20.771,4.618,6.317,3.012,6.487,3.032,7.002,1.814,3.324,0.703,1.486
3.92,21.479,5.85,4.847,1.525,2.655,9.736,23.599,3.909,18.749,3,2.24,3.826,3.95,1.535,2.368,1.067,2.44
31.548,11.419,4.887,5.911,1.688,3.773,9.602,23.159,3.45,17.469,5.837,2.332,2.355,3.068,1.735,5.947,1.1,1.879
5.024,6.621,3.229,7.905,1.538,3.158,8.25,29.288,2.928,11.969,2.97,1.759,2.491,3.133,1.803,7.679,1.106,0.841
10.457,6.585,1.794,5.332,2.038,2.452,16.695,32.429,3.241,15.587,4.48,6.704,2.666,4.364,2.407,3.206,1.705,1.346
10.11,7.173,3.81,8.893,1.677,12.594,4.869,14.79,6.644,17.24,9.2,2.904,3.438,4.336,3.249,1.024,1.35,11.08
4.563,6.309,3.095,6.701,1.812,2.538,6.168,6.491,6.204,12.2,4.19,2.965,2.722,4.541,2.503,1.29,2.739,2.386
13.624,27.66,6.713,1.879,1.374,3.834,9.792,7.016,6.708,9.485,8.141,4.304,3.029,3.084,2.036,2.584,1.69,1.787
4.686,8.653,9.697,7.042,2.004,1.769,3.448,10.309,4.22,3.13,4.358,1.715,3.732,3.364,1.463,3.959,1.034,2.301
18.301,15.561,7.206,4.482,1.839,11.133,13.636,3.712,3.768,8.183,4.898,5.106,2.803,2.959,1.876,2.455,1.012,2.743
13.974,9.5,3.882,9.835,1.362,1.829,8.918,24.111,6.855,21.851,4.972,2.815,4.424,2.911,2.19,1.642,1.616,1.712
7.111,13.492,6.898,12.683,1.964,4.07,6.774,17.431,4.611,8.499,10.417,2.638,8.571,2.121,1.728,1.385,2.501,1.368
5.104,12.774,7.74,4.983,1.509,1.494,5.62,81.964,5.517,11.176,4.807,2.927,2.231,4.604,2.185,6.02,1.144,1.292
3.964,11.032,3.267,10.263,2.262,3.166,6.231,5.942,5.885,19.174,3.105,3.035,1.736,4.44,1.567,3.617,0.88,0.917
4.565,8.44,8.095,5.412,1.606,1.291,5.005,53.556,3.913,8.635,4.145,2.08,3.244,4.269,2.667,7.18,1.182,1.405
18.516,5.65,5.977,8.071,1.668,2.683,5.615,7.277,4.421,29.079,8.879,3.558,4.313,4.279,2.634,6.988,1.192,1.8
5.453,5.692,1.648,21.897,1.266,5.043,10.794,12.68,7.927,14.48,4.662,4.79,1.967,

8.22,1.83,1.157,1.689,1.243
2.395,9.988,5.641,5.443,1.352,8.596,6.009,12.272,12.575,5.029,4.822,2.348,4.191
,3.978,1.4,1.868,1.542,1.489
3.156,7.179,3.624,7.141,1.898,5.539,19.472,45.264,5.721,1.895,6.856,2.169,1.731
,4.183,2.132,2.059,1.08,1.737
6.693,7.713,4.731,5.699,1.667,3.5,20.482,13.576,5.669,18.527,14.683,2.699,2.409
,8.152,1.104,52.507,1.161,1.642
7.795,15.865,3.599,24.021,1.718,1.722,2.626,9.313,4.126,14.04,2.845,2.305,2.647
,5.522,1.496,3.852,1.026,2.945
4.895,18.165,11.29,3.963,1.533,2.287,7.258,23.999,7.738,9.28,6.827,4.784,2.929,
3.959,2.163,4.65,1.254,1.259
4.81,16.363,10.686,4.585,1.524,8.397,9.221,17.693,2.592,5.016,6.453,4.668,4.687
,4.169,1.615,1.238,2.229,6.571
7.896,13.152,4.052,2.833,1.718,1.248,7.526,14.198,6.08,8.982,2.557,2.928,3.388,
5.478,1.218,5.993,1.357,3.959
4.106,6.407,6.785,2.093,1.614,3.982,13.764,10.59,2.766,11.656,8.479,6.351,1.901
,2.82,1.397,1.691,1.138,1.77
3.755,17.739,12.229,4.199,1.842,1.111,16.833,33.646,2.838,19.792,2.707,2.286,2.
097,3.957,1.428,4.558,1.505,1.704
8.021,7.315,12.467,6.713,1.943,11.955,6.565,11.578,3.958,5.923,8.264,3.319,2.15
9,2.577,2.256,6.226,0.997,2.037
7.871,4.657,13.683,5.278,1.306,5.122,8.835,13.196,3.176,8.211,4.392,0.971,2.601
,3.187,1.743,1.155,1.26,1.78
8.499,9.553,14.413,4.822,1.538,5.392,6.763,24.125,3.766,5.652,9.238,2.774,4.233
,3.996,2.24,10.287,0.891,1.949
6.915,12.646,4.351,11.625,1.462,2.566,11.286,19.449,8.053,9.338,7.434,2.947,1.7
24,7.683,0.938,1.154,1.135,2.351
12.935,14.657,6.725,13.822,1.484,2.557,12.837,39.307,2.818,9.537,3.504,6.064,3.
571,4.269,1.535,1.203,0.642,2.571
3.989,6.816,2.688,11.955,1.265,4.327,10.085,26.317,7.546,13.359,3.2,5.003,9.915
,3.386,1.44,8.468,1.746,1.328
7.917,13.256,4.993,7.449,1.379,2.284,7.56,28.966,3.099,14.749,7.185,4.478,4.525
,6.224,1.428,7.86,1.089,3.523
10.307,12.904,5.62,5.102,1.492,3.44,6.494,7.506,3.401,6.28,7.012,3.081,2.336,3.
584,0.827,4.858,1.44,0.963
24.025,5.351,4.141,9.4,1.621,3.644,10.417,6.756,3.968,1.633,4.592,4.51,2.185,2.
548,1.746,3.616,0.808,1.085
4.413,9.023,9.909,7.019,1.655,2.399,10.521,13.383,2.664,7.08,10.999,3.368,3.074
,9.253,3.981,3.36,1.248,4.293
4.958,10.434,16.016,6.608,2.268,1.514,9.325,5.043,5.04,8.826,5.77,2.391,2.124,2
.866,2.175,2.138,1.538,2.437
3.758,17.47,10.35,7.872,1.684,3.386,9.716,37.592,4.814,13.42,3.201,4.79,1.748,6
.452,2.208,3.265,1.162,2.224
6.579,8.986,8.839,10.496,1.92,14.752,15.871,19.682,7.148,9.346,3.467,53.962,3.2
24,3.757,1.49,1.442,1.118,1.588
4.754,7.174,9.018,7.914,1.865,1.427,10.298,13.111,2.035,12.26,11.432,3.951,4.43
8,2.228,3.634,1.1,0.923,1.447
29.852,4.217,4.464,6.169,2.048,1.569,10.136,15.121,5.582,6.677,7.82,3.22,3.016,
2.998,1.37,4.854,1.049,1.074
6.653,12.736,6.712,10.822,1.599,2.683,22.173,41.057,2.563,29.443,5.792,6.443,2.
798,1.223,1.543,3.258,1.223,1.574
3.038,5.803,4.789,5.272,1.22,5.927,16.56,15.127,5.497,15.731,2.492,4.499,3.873,
3.784,2.194,4.645,1.198,1.478
49.647,11.163,7.297,4.423,1.629,3.125,14.051,37.454,5.736,30.069,11.352,2.462,6
.328,4.565,2.11,1.318,1.223,1.687
2.18,8.051,8.547,8.311,1.368,12.095,8.828,28.898,4.872,2.519,5.545,2.339,4.809,
2.508,1.448,6.348,1.048,2.494
4.857,6.696,6.089,7.146,1.474,1.938,15.311,1.904,4.651,8.063,4.098,2.226,3.306,
5.96,1.404,1.591,0.849,1.311
3.654,9.518,4.43,4.115,1.397,3.563,12.019,15.191,3.92,19.501,9.052,1.77,3.398,3
.132,1.203,1.585,1.202,1.753
11.238,10.124,6.716,9.681,1.927,1.221,9.23,10.783,7.027,11.049,4.159,4.311,3.26
1,3.077,1.721,3.853,1.059,1.022
8.46,13.36,4.895,18.169,2.431,5.812,20.557,13.36,4.414,23.787,2.961,2.871,1.434

,5.518,1.289,1.374,1.122,2.962
5.214,9.338,1.844,12.902,1.3,0.673,9.254,13.816,2.914,29.883,2.917,3.098,4.662,
3.426,1.509,1.192,0.788,1.755
10.643,8.442,3.669,9.934,1.302,6.872,9.578,25.537,3.291,5.7,4.725,4.776,3.671,1
3.089,1.831,5.932,1.006,1.386
3.492,5.582,2.627,4.673,1.497,4.403,15.894,4.452,3.719,16.437,3.756,4.23,9.727,
5.784,5.595,1.555,1.06,0.812
4.861,10.629,15.351,13.68,1.421,4.717,5.78,19.187,4.457,11.327,4.465,6.32,2.794
,2.583,1.35,3.492,1.13,1.491
7.546,13.713,3.214,3.811,1.166,1.556,3.062,14.996,3.318,9.424,5.357,2.268,2.931
,5.325,2.189,3.821,0.842,1.459
4.726,14.026,7.874,8.435,1.653,3.027,6.136,22.861,2.675,19.956,5.84,1.13,2.259,
4.491,1.269,3.929,1.952,2.937
3.943,6.637,4.432,7.216,2.35,7.902,12.289,9.983,3.36,15.725,2.877,4.009,2.448,2
.305,1.999,6.955,1.276,2.547
6.009,7.838,2.439,3.285,1.666,5.643,11.674,12.131,6.452,9.168,2.227,3.309,2.225
,7.151,1.807,1.487,1.219,1.635
4.925,8.197,11.346,8.288,1.76,4.544,11.883,19.917,4.516,16.39,6.129,1.859,5.554
,1.139,3.449,5.231,1.56,1.823
3.587,8.809,13.267,5.612,1.851,1.834,10.715,55.677,4.823,13.181,5.226,5.428,3.0
68,2.705,1.78,2.922,0.974,3.173
4.016,10.677,2.573,6.677,1.602,4.086,11.04,89.324,3.944,13.056,3.148,2.607,2.54
1,2.895,1.959,3.34,0.954,2.131
3.997,5.908,16.552,8.811,2.359,2.437,15.296,12.587,2.944,15.878,3.532,1.429,3.2
53,6.03,3.128,3.997,1.405,2.791
6.02,4.121,2.789,14.962,1.8,1.732,11.406,10.1,2.64,12.079,5.76,1.516,3.353,6.51
9,1.446,0.914,1.071,1.62
4.681,5.481,6.728,4.998,1.479,4.468,8.331,8.778,8.353,8.419,3.189,4.615,2.561,2
.741,1.084,3.311,1.259,1.565
7.04,8.036,4.219,5.086,1.346,13.337,19.904,33.854,3.199,14.469,6.143,2.081,2.26
1,2.428,1.784,0.966,1.133,1.519
7.331,5.087,8.194,13.449,1.376,2.254,10.559,6.574,4.885,3.151,5.115,3.691,2.363
,3.115,2.226,1.482,2.04,2.111
4.027,12.224,6.52,7.807,1.434,1.481,12.212,38.755,3.429,14.039,8.548,2.564,6.38
2,5.858,2.138,1.813,0.884,1.581
7.956,8.059,8.643,4.299,1.282,1.909,2.614,22.852,2.294,2.58,5.11,4.714,2.43,4.6
,1.786,2.682,1.307,2.572
